# Anxiety and depressive disorders in adolescent injury prevention and safety promotion: frontier and inequality mapping across 953 locations

**DOI:** 10.3389/fpubh.2026.1916777

**Published:** 2026-08-11

**Authors:** Minglu Yuan, Xuezhi Liang, Wenyi Jin, Meng Zheng, Shiyu Feng, Jianli Zhao, Shunrong Li, Liling Zhu, Luyuan Tan, Yinduo Zeng, Tongrui Shang, Yanfang Ye, Guangyao Cai, Yuanyuan Chen, Ying Wang, Yutong Lu, Dong Wang, Yuanyuan Wan, Ningning Wu, Ciqing Lin, Wei Pan, Jingxuan Zhou, You Zeng, Lukasz Szarpak, Nadia M. Hamdy, You Zuo, Claire Chenwen Zhong, Queran Lin, Xiaoliang Huang, Wen Chen

**Affiliations:** 1 Department of Medical Statistics, School of Public Health, Sun Yat-sen University, Guangzhou, China; 2 Department of Orthopaedics, Renmin Hospital of Wuhan University, Wuhan University, Wuhan, China; 3 Department of Biomedical Sciences, City University of Hong Kong, Kowloon, Hong Kong SAR, China; 4 Clinical Research Design Division, Guangdong Provincial Key Laboratory of Malignant Tumour Epigenetics and Gene Regulation, Guangdong-Hong Kong Joint Laboratory for RNA Medicine, Breast Tumor Center, Clinical Research Center, Sun Yat-Sen Memorial Hospital, Sun Yat-Sen University, Guangzhou, China; 5 Guangdong Provincial Key Laboratory of Malignant Tumour Epigenetics and Gene Regulation, Guangdong-Hong Kong Joint Laboratory for RNA Medicine, Breast Tumor Center, Sun Yat-Sen Memorial Hospital, Sun Yat-Sen University, Guangzhou, China; 6 Guangdong Provincial Key Laboratory of Malignant Tumor Epigenetics and Gene Regulation, Breast Tumor Center, Sun Yat-Sen Memorial Hospital, Sun Yat-Sen University, Guangzhou, China; 7 Breast Tumor Center, Sun Yat-Sen Memorial Hospital, Sun Yat-Sen University, Guangzhou, China; 8 Clinical Research Design Division, Clinical Research Center, Sun Yat-Sen Memorial Hospital, Sun Yat-Sen University, Guangzhou, China; 9 Department of Gynaecologic Oncology, State Key Laboratory of Oncology in South China, Guangdong Provincial Clinical Research Centre for Cancer, Sun Yat-Sen University Cancer Centre, Guangzhou, China; 10 State Key Laboratory of Oncology in South China, Sun Yat-sen University Cancer Center, Guangzhou, China; 11 National Supercomputer Center in Guangzhou, Sun Yat-Sen University, Guangzhou, China; 12 Department of Supercomputing Application Promotion, National Supercomputer Center in Guangzhou, Sun Yat-Sen University, Guangzhou, China; 13 Department of HPC Application, National Supercomputer Center in Guangzhou, Sun Yat-Sen University, Guangzhou, China; 14 School of Computing and Information Systems, The University of Melbourne, Parkville, VIC, Australia; 15 School of Computer Science and Engineering, Nanyang Technological University, Singapore, Singapore; 16 Faculty of Social Sciences, Hong Kong Baptist University, Kowloon, Hong Kong SAR, China; 17 Department of Information Studies, University College London, London, United Kingdom; 18 School of Urban Planning and Design, Peking University Shenzhen Graduate School, Shenzhen, China; 19 Department of Women and Children Health Care, Guangzhou Baiyun District Maternal and Child Health Hospital, Guangzhou, China; 20 Institute of Medical Science, Collegium Medicum, The John Paul II Catholic University of Lublin, Lubin, Poland; 21 Biochemistry Department, Faculty of Pharmacy, Ain Shams University, Cairo, Egypt; 22 Department of Neurology, Sun Yat-Sen Memorial Hospital, Sun Yat-Sen University, Guangzhou, China; 23 JC School of Public Health and Primary Care, Faculty of Medicine, The Chinese University of Hong Kong, Sha Tin, Hong Kong SAR, China; 24 Clinical Research Design Division, Guangdong Provincial Key Laboratory of Malignant Tumour Epigenetics and Gene Regulation, Guangdong-Hong Kong Joint Laboratory for RNA Medicine, Breast Tumor Center, Guangdong Provincial Clinical Research Center for Breast Diseases, Clinical Research Center, Sun Yat-Sen Memorial Hospital, Sun Yat-Sen University, Guangzhou, China; 25 WHO Collaborating Centre for Public Health Education and Training, Department of Primary Care and Public Health, School of Public Health, Faculty of Medicine, Imperial College London, London, United Kingdom; 26 The Affairs Center of Health Commission of Guangdong Province, Guangzhou, China; 27 Center for Migrant Health Policy, Sun Yat-sen University, Guangzhou, China

**Keywords:** anxiety disoders, child and adolescent, depressive disorder, health inequality, injury prevention, self-harm

## Abstract

**Purpose:**

Anxiety and depressive disorders constitute major contributors to global mental health burdens, disproportionately affecting 10-24-year-olds during critical neurodevelopmental windows. Despite their substantial disease burden, persistent treatment gaps and inequitable resource allocation remain worldwide. This study aims to systematically examine the evolving disease burden, inequalities, and achievable frontiers for anxiety and depressive disorders among 10–24 years from 1990 to 2021, providing critical epidemiological evidence to inform integrated adolescent mental health and injury prevention strategies.

**Methods:**

Employed the data from the Global Burden of Disease Study (GBD) 2021, this study analyzed anxiety and depressive indicators across 953 locations at global, regional, national, and subnational levels, with stratification by sociodemographic index (SDI) quintiles to examine socioeconomic inequalities and identify potentially achievable frontiers. Data analysis occurred from August 2024 to January 2025.

**Results:**

Key findings reveal a marginal increase in incidence rates over three decades, driven by a marked surge during the pandemic. Significant inter-regional disparities emerged, with high SDI regions such as Western Europe and High-income North America bearing disproportionately greater burdens. Notably, while some countries like Pakistan and Nigeria demonstrated relatively low burdens, subnational analyses uncovered accelerated disease trajectories and persistent health inequities, underscoring emerging challenges in resource-constrained settings. Among the top 100 affected subnational locations in 2021, Iran (31%), Brazil (27%), and Italy (19%) recorded the highest anxiety DALY rates, while the United States of America (51%), the United Kingdom (25%), and Iran (19%) dominated depressive DALYs. Socioeconomic inequalities in disease burdens intensified globally from 1990 to 2021, most pronounced in high SDI countries. In 2021, 89.2% and 92.2% of 204 countries and territories fell behind the anxiety and depressive DALYs frontiers respectively, with 99.0% and 97.1% experiencing an increasing frontier deviation between 1990 and 2021. Among 494 subnational locations, 49.2% fell behind the anxiety DALYs frontiers, and 35.8% fell behind the depressive DALYs.

**Conclusion:**

These findings emphasize the urgency of implementing targeted interventions addressing developmental-stage vulnerabilities, and integrating equity-focused strategies into mental health prevention and promotion policy frameworks, with direct implications for adolescent self-harm prevention and child safety promotion.

## Introduction

Anxiety and depressive disorders represent a leading global health burden, comprising >20% of mental health-related disability (1). Their epidemiological significance arises from both high lifetime prevalence and neurodevelopmental impacts during the critical 10–24 age window (1, 2). This age demarcation captures the neurobiological confluence of adolescence and early adulthood, characterized by prefrontal cortical maturation, hormonal flux, and identity formation processes that heighten susceptibility to psychopathology (3, 4). Beyond their direct disease burden, these disorders are also well-established risk factors for adolescent injury, including self-harm, suicidal behavior, and risk-taking behaviors that elevate unintentional injury risk. Adolescents with untreated depression face substantially increased risks of suicide attempt, while anxiety disorders are linked to engagement in hazardous behaviors. These disorders also substantially impair educational attainment and social relationship formation (5, 6), and demonstrate strong associations with long-term health risks, including metabolic syndrome, cardiovascular diseases, and premature mortality (7–9). Notably, depressive disorders encompass clinically heterogeneous subtypes: major depressive disorder (MDD) and dysthymia are classified as distinct entities in the GBD framework, differing in episode duration, severity, and associations with functional impairment, and may therefore carry different implications for injury risk and long-term health outcomes. The convergence of mental health vulnerability and injury risk during this developmental window highlights the need for integrated prevention frameworks that address both burdens simultaneously.

From 1990 to 2019, the age-standardized incidence rate (ASIR) of anxiety and depressive disorders among adolescents globally both remained relatively stable (2), with a slight decrease in depressive disorders (Estimated annual percentage change, EAPC = −0.23) (11). This relatively stable pattern was disrupted by the COVID-19 pandemic, which substantially increased the global burden of anxiety and depressive disorders (12). As these disorders are closely linked to self-harm, suicidal behavior, risk-taking, and unintentional injury during adolescence, their changing epidemiological patterns are relevant to injury prevention and underscore the need for population-specific strategies that integrate mental health promotion with injury risk reduction.

Despite substantial progress in psychiatric epidemiology, important knowledge gaps remain in understanding how mental health burdens contribute to injury risks. Evidence specific to individuals aged 10–24 years is still limited, although this life stage covers major developmental transitions during which anxiety, depression, risk-taking behaviors, self-harm, and interpersonal violence may emerge or intensify (13). Furthermore, inequalities in mental disorders across and within countries have not been adequately quantified, making it difficult to determine whether the mental health burden associated with injury risk is concentrated in particular demographic or socioeconomic groups. National estimates may obscure subnational variation in disease burden, which is especially relevant for large and heterogeneous countries where prevention resources are planned and allocated locally. In China, for example, provincial-level data have shown higher age-standardized depression incidence in more developed regions than in less-developed regions (14), suggesting that administrative-level estimates may reveal patterns that are not visible in national averages. However, current child and adolescent injury prevention frameworks rarely incorporate mental health burden data. A clearer epidemiological characterization of anxiety and depressive disorders among people aged 10–24 years, including their inequalities and spatial heterogeneity, is needed to support integrated injury prevention strategies.

This study aims to address these limitations through multilevel analysis of GBD 2021 datasets, encompassing 953 locations at regional, national, and subnational levels. We presented temporal trends in incidence, prevalence, and disability-adjusted life years (DALYs) for anxiety and depressive disorders among 10–24 years from 1990 to 2021; MDD and dysthymia were examined separately, alongside the combined depressive disorders category, to determine whether their distinct clinical profiles correspond to divergent burden trajectories and demographic distributions. By integrating these disease burden metrics with SDI stratification, we described the demographic and epidemiological changes in disease burden. Utilizing inequality analysis and frontier analysis, we systematically evaluated spatial heterogeneities in youth mental health burdens, thereby providing an evidence base for integrated mental health and injury prevention strategies, including targeted self-harm prevention, suicide prevention, and child safety promotion across varying resource settings.

## Methods

The GBD is a comprehensive epidemiological initiative designed to evaluate the impact of diseases, injuries, and risk factors at global, regional, and national levels. The dataset provides detailed breakdowns by sex, age, and cause, across 953 locations (15), encompassing 1 global entity, 7 GBD super regions (e.g., Latin America and Caribbean), 77 specific international regions (e.g., World Bank income levels), 204 countries and territories (e.g., Brazil), 20 subnational regions (e.g., Brazil North), and 652 local units (e.g., Amazonas). In addition to the global, regional, and national estimates, we also utilized GBD 2021 data sources to ensure robust local-level estimates for 18 countries and territories, including Brazil, China, Ethiopia, Indonesia, Italy, Iran, Japan, Kenya, Mexico, Nigeria, Norway, Pakistan, the Philippines, Poland, South Africa, Sweden, the United Kingdom (UK) and the United States of America (USA). GBD provides full methodological protocols and interactive data visualizations accessible via the Global Health Data Exchange (GHDx) platform http://ghdx.healthdata.org/gbd-results-tool. This study adhered to the Guidelines for Accurate and Transparent Health Estimates Reporting (GATHER) to ensure methodological rigor throughout the research process. Supplementary materials provide complete geographic hierarchies from global to local (Supplementary Table S1).

GBD uses a variety of indicators to measure population health losses, including prevalence and number of cases, years lost due to premature death (YLLs), years lived with disability (YLDs) and DALYs. DALYs are defined as the sum of YLLs and YLDs, with one DALY representing the loss of 1 year of full health (16). Given the absence of mortality data directly attributable to anxiety and depressive disorders in GBD 2021, YLL estimates for these conditions are constrained to zero. This reflects that anxiety and depressive disorders are not coded as underlying causes of death, and deaths causally related to them, including suicide, are instead attributed to the self-harm cause category. DALYs for these disorders are therefore numerically equivalent to YLDs, capturing morbidity alone rather than combined fatal and non-fatal burden. Consequently, this study primarily utilized the estimates of incidence, prevalence, and DALYs, along with their corresponding 95% uncertainty intervals (UIs), as metrics to assess anxiety and depressive burden and their relevance to injury-related outcomes.

### Case definition

Anxiety disorders are characterized by intense and persistent fear and distress, accompanied by physiological symptoms such as increased heart rate, sweating, and trembling. These disorders include panic disorder, agoraphobia, and specific phobia, as classified by DSM-IV-TR codes 300.0–300.3, 208.3, 309.21, 309.81 and ICD-10 classifications F40–F42, F43.0, F43.1, F93.0–F93.2, F93.8 (17). In contrast, the GBD stratifies depressive disorders according to DSM-IV-TR into two categories: major depressive disorders (MDDs) (DSM-IV-TR: 296.21–24, 296.31–34; ICD-10: F32.0–9, F33.0–9) and dysthymia (DSM-IV-TR: 300.4, ICD-10: F34.1), excluding mood disorders secondary to medical comorbidities or substance use (18). MDD is defined by marked depressed mood, anhedonia, and a range of physical and cognitive symptoms. It requires at least one episode of these symptoms daily for at least 2 weeks, significantly affecting functioning (19, 20). Dysthymia presents as a chronically and persistently depressed mood for most of the day, lasting at least 2 years in adults or 1 year in children and adolescents (21). Given these differences in duration, severity, and age-of-onset criteria, MDD and dysthymia may exhibit distinct epidemiological trajectories and demographic distributions, which motivated their separate examination alongside the combined depressive disorders category in this study.

### Young people

This study particularly focuses on anxiety and depressive disorders in young people aged 10–24 years. In the presentation of partial results, the 10–24 years age range is divided into three 5-year groups: early adolescence (10–14 years), late adolescence (15–19 years), and young adulthood (20–24 years) (4).

### Inequality analysis

To evaluate the inequality in the distribution of anxiety and depressive disorder burdens, we utilized the Slope Index of Inequality (SII) and the Concentration Index of Inequality (CII). The SII is calculated through regression analysis, linking the nationwide disease burden due to anxiety and depressive disorders among the population aged 10–24 years to an income-related relative social position scale, which is defined as the midpoint of the cumulative population strata ranked by gross domestic product (GDP) per capita. The SII quantifies the disparity between the most advantaged and the most disadvantaged groups. When the slope of the regression line is flat, indicating no difference in disease burden across socio-economic strata, the SII equals zero. For positive indicators, where groups are ranked from the most disadvantaged to the most advantaged, a positive SII points to a greater concentration of the burden in the most advantaged subgroup, whereas a negative SII means the burden is more prevalent in the most disadvantaged group. For negative indicators, the interpretation is reversed (22). The CII is computed by fitting a Lorenz concentration curve to the observed cumulative relative distributions of the population ranked by income and the disease burden of anxiety and depressive disorders, and numerically calculating the area under the curve (23). The CII ranges from −1 to +1, with zero value representing no inequality. Higher absolute values of the CII denote more severe inequality. Although the theoretical maximum absolute value for the CII is ±1, practical applications rarely exceed 0.5. A value of 0.2 to 0.3 is considered to represent a reasonably high level of relative inequality (22).

In this study, subnational-level analyses were restricted to 14 nations with reliable subnational SDI data, encompassing Brazil, China, Ethiopia, Indonesia, Iran, Japan, Kenya, Mexico, Norway, Pakistan, South Africa, Sweden, the UK and the USA, with a total of 494 provinces. Nigeria, the Philippines, Italy, and Poland were excluded from subnational assessments due to incomplete province-level SDI data, which precluded inequality and frontier analyses for these regions. The investigation employed World Health Organization (WHO) protocols for health equity assessment to maintain methodological uniformity, enabling cross-regional and cross-indicator comparisons (22). This standardized approach ensured data harmonization throughout spatial analyses and between measurement indicators.

### Frontier analysis

To evaluate the relationship between the burden of anxiety and depressive disorders and socio-demographic development, we employed frontier analysis, a quantitative method used to determine the lowest achievable burden of disease at different levels of the SDI (24, 25). The frontiers represent the minimum achievable values for each country or region, taking into account its current healthcare resources and socio-economic development as reflected by the SDI. The distance from the frontier, referred to as the effective difference (EF), quantifies the gap between the actual disease burden and the theoretically optimal health outcome. A larger difference indicates greater potential for improvement.

Using data from 1990 to 2021, we constructed SDI-based frontiers for the prevalence, incidence, and DALY rates of anxiety and depressive disorders. To address data uncertainty, we performed 1,000 bootstrapping by randomly sampling from all regions, countries, and subnations. The mean burden for each SDI value was then computed. A locally weighted regression (LOESS) approach, with a polynomial degree of 1 and a span of 0.2, was applied to smooth the frontier curve (24, 25). To mitigate the influence of outliers, super-efficient countries (those with DALYs significantly below the frontier) were excluded from the frontier construction. Finally, we calculated the effective difference, defined as the absolute distance between the observed values and the frontier for each SDI value. Countries or regions with lower prevalence, incidence, and DALY rates than the frontiers were assigned a distance score of zero. This methodology was applied both at the country level and within countries. Similar to the inequality analysis, subnational-level analyses were restricted to 14 nations with reliable subnational SDI data, encompassing 494 provinces. Through the above method, we were able to quantify the potential for improvement in the burden of anxiety and depressive disorders, highlighting opportunities for optimizing health outcomes within the context of socio-demographic development.

## Results

### Overview of the global burden

**Figure 1:**
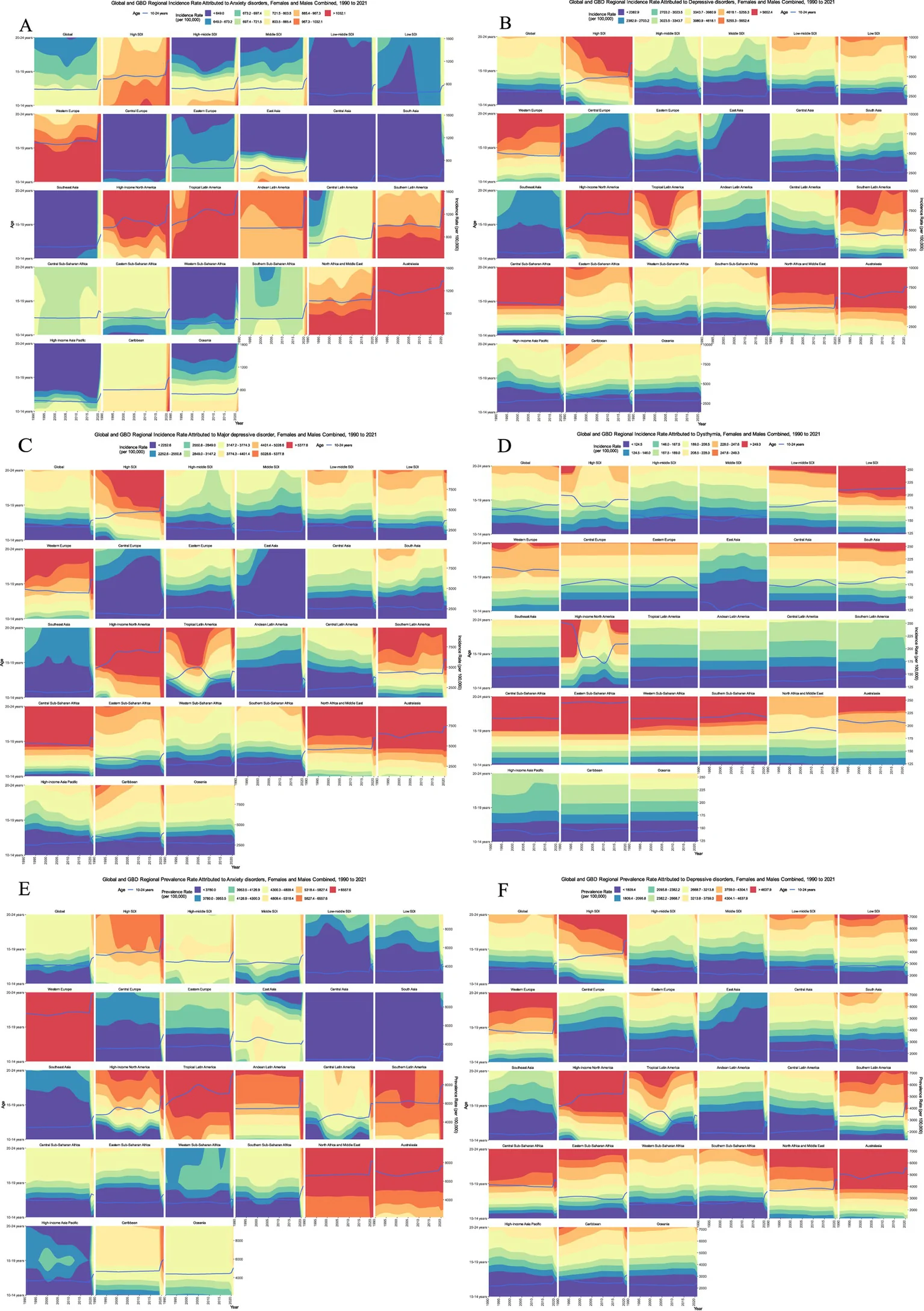
Global and GBD regional variations in the burden of anxiety and depressive disorders among young people aged 10–24 years, 1990–2021. This figure illustrates the incidence rates and prevalence rates of anxiety disorders and depressive disorders per 100,000 population globally and across the GBD regions from 1990 to 2021, combining data for males and females. The color gradient reflects the range of incidence rates or prevalence rates. The left y-axis is segmented into three age groups: 10–14 years old, 15–19 years old, and 20–24 years old, which allows for a clear comparison of data across these distinct developmental stages. **(A)** Anxiety disorders incidence. **(B)** Depressive disorders incidence. **(C)** MDD incidence. **(D)** Dysthymia incidence. **(E)** Anxiety disorders prevalence. **(F)** Depressive disorders prevalence. GBD, Global Burden of Disease; MDD, Major depressive disorder.

From 1990 to 2021, young people aged 10–24 experienced notable increases in mental health burdens, with the incidence rate of anxiety disorders rising by 24.7% (95% UI: −25.6 to 109.1) and that of depressive disorders by 26.7% (−21.6 to 104.9). The growth in depressive disorders was predominantly driven by MDD, which surged by 27.9% (−23.1 to 113.0), contrasting with the stable incidence pattern observed in dysthymia (Figures 1A–D).

By 2021, global prevalence estimates reached alarming levels, with the number of cases reaching 93,947,157 (68,842,790 to 125,013,836) for anxiety disorders and 57,488,802 (42,231,934 to 78,083,149) for depressive disorders (Table 1). Among age groups, the 10–14 group had the highest anxiety disorder incidence [925.1 (704.1 to 1,213.4)], reflecting the early onset of anxiety disorder. In contrast, depressive disorders incidence peaking in the 20–24 group [5,448.6 (3,987.8 to 7,808.0)] (Figures 1A,B). The elevated burden observed in early adolescence and young adulthood, identifying distinct developmental windows for targeted injury prevention and mental health screening.

**Table 1:** Incidence, prevalence, and DALYs rate of anxiety and depressive disorders among young people age 10–24 years globally, 1990–2021.

Disease category	Metric	Number			Rate			Change in rate		
		1990	2019	2021	1990	2019	2021	1990–2019	1990–2021	2019–2021
Anxiety disorders	Incidence	10,954,492 (7,253,757 to 15,154,940)	13,099,180 (8,687,298 to 18,143,718)	16,670,880 (11,045,571 to 23,136,222)	708.0 (468.8 to 979.5)	704.5 (467.2 to 975.8)	883.1 (585.1 to 1225.6)	−0.5% (−40.6 to 66.6)	24.7% (−25.6 to 109.1)	25.3% (−25.2 to 110.1)
	Prevalence	63,753,648 (47,253,662 to 84,038,633)	74,673,492 (54,848,339 to 99,255,273)	93,947,157 (68,842,790 to 125,013,836)	4120.6 (3054.2 to 5431.7)	4016.1 (2949.9 to 5338.2)	4976.6 (3646.8 to 6622.3)	−2.5% (−35.2 to 46.7)	20.8% (−19.9 to 82.1)	23.9% (−18.3 to 87.9)
	DALY	7,822,522 (4,876,384 to 11,645,915)	9,178,143 (5,671,541 to 13,668,048)	11,523,716 (7,139,551 to 17,187,559)	505.6 (315.2 to 752.7)	493.6 (305.0 to 735.1)	610.4 (378.2 to 910.5)	−2.4% (−47.1 to 80.2)	20.7% (−34.5 to 122.8)	23.7% (−33.1 to 128.7)
Depressive disorders	Incidence	47,739,042 (33,980,523 to 65,973,101)	56,664,428 (39,577,777 to 79,921,077)	73,809,681 (51,385,998 to 103,861,167)	3085.5 (2196.3 to 4264.0)	3047.6 (2128.6 to 4298.4)	3909.9 (2722.0 to 5501.8)	−1.2% (−38.8 to 59.7)	26.7% (−21.6 to 104.9)	28.3% (−21.5 to 109.8)
	Prevalence	38,476,683 (28,826,280 to 51,169,859)	46,544,779 (34,670,404 to 62,380,324)	57,488,802 (42,231,934 to 78,083,149)	2486.9 (1863.1 to 3307.3)	2503.3 (1864.7 to 3355.0)	3045.3 (2237.1 to 4136.3)	0.7% (−32.9 to 51.2)	22.5% (−19.3 to 85.9)	21.7% (−20.2 to 85.5)
	DALY	7,025,089 (4,440,486 to 10,480,388)	8,448,036 (5,299,323 to 12,676,544)	10,718,195 (6,669,859 to 16,195,136)	454.1 (287.0 to 677.4)	454.4 (285.0 to 681.8)	567.8 (353.3 to 857.9)	0.1% (−45.4 to 83.5)	25.0% (−32.1 to 130.5)	25.0% (−32.5 to 131.5)
MDD	Incidence	45,093,043 (31,383,990 to 63,718,689)	53,312,931 (36,410,183 to 76,595,551)	70,396,953 (47,901,653 to 100,687,703)	2914.5 (2028.4 to 4118.3)	2867.3 (1958.2 to 4119.5)	3729.1 (2537.5 to 5333.7)	−1.6% (−40.9 to 63.9)	27.9% (−23.1 to 113.0)	30.1% (−22.7 to 118.7)
	Prevalence	28,802,707 (20,020,329 to 40,620,556)	34,195,108 (23,295,247 to 49,151,273)	45,037,002 (30,674,802 to 64,522,321)	1861.6 (1294.0 to 2625.4)	1839.1 (1252.9 to 2643.5)	2385.7 (1624.9 to 3417.9)	−1.2% (−40.7 to 64.7)	28.2% (−23.0 to 113.4)	29.7% (−23.0 to 118.4)
	DALY	6,039,568 (3,698,961 to 9,293,396)	7,188,643 (4,311,308 to 11,101,662)	9,440,939 (5,668,847 to 14,519,152)	390.4 (239.1 to 600.7)	386.6 (231.9 to 597.1)	500.1 (300.3 to 769.1)	−1.0% (−48.5 to 90.4)	28.1% (−33.2 to 146.0)	29.4% (−33.1 to 150.2)
Dysthymia	Incidence	2,645,999 (1,630,857 to 3,906,225)	3,351,497 (2,072,248 to 4,944,674)	3,412,728 (2,111,894 to 5,040,907)	171.0 (105.4 to 252.5)	180.3 (111.5 to 265.9)	180.8 (111.9 to 267.0)	5.4% (−42.7 to 93.9)	5.7% (−42.5 to 94.5)	0.3% (−45.4 to 84.3)
	Prevalence	9,937,619 (6,780,909 to 13,920,335)	12,669,693 (8,633,422 to 17,808,524)	12,872,480 (8,753,348 to 18,081,877)	642.3 (438.3 to 899.7)	681.4 (464.3 to 957.8)	681.9 (463.7 to 957.8)	6.1% (−36.0 to 75.9)	6.2% (−36.0 to 76.1)	0.1% (−39.7 to 66.2)
	DALY	985,521 (570,632 to 1,542,592)	1,259,393 (727,919 to 1,996,099)	1,277,256 (740,271 to 2,023,838)	63.7 (36.9 to 99.7)	67.7 (39.1 to 107.4)	67.7 (39.2 to 107.2)	6.3% (−47.3 to 114.6)	6.2% (−47.2 to 114.1)	−0.1% (−50.6 to 102.0)

Data in parentheses are 95% uncertainty intervals. DALY = disability-adjusted life-year.

### Epidemiological trends in anxiety and depressive disorders across GBD regions

**Figure 2:**
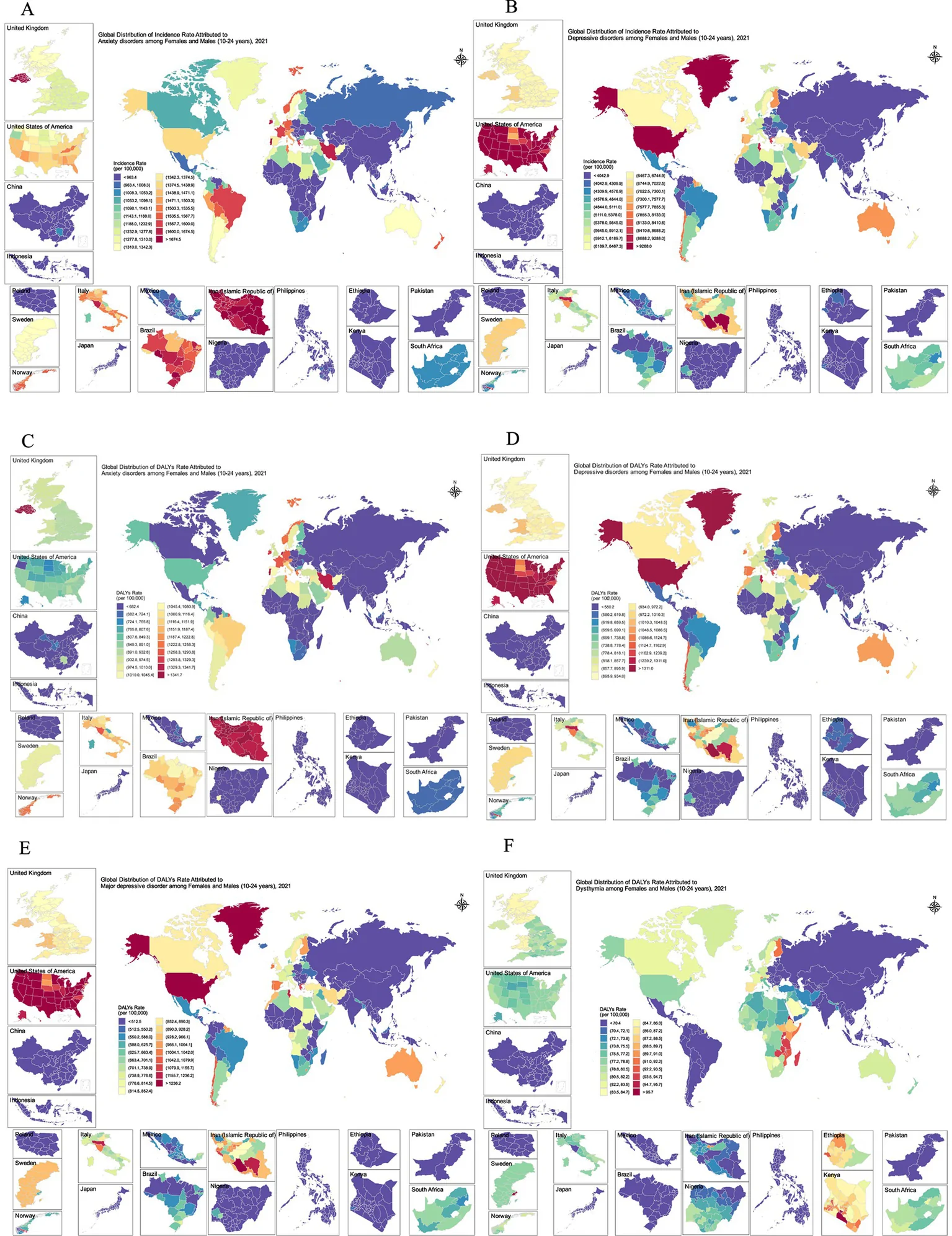
Global distribution of incidence rates and DALY rates attributed to anxiety disorders and depressive disorders among females and males (10–24 years), 2021. This figure illustrates the global burden of anxiety disorders and depressive disorders, incorporating 18 countries with robust local-level data. It presents sex-combined rates per 100,000 population for key health measures, including incidence rates and DALY rates. **(A)** Anxiety disorders incidence. **(B)** Depressive disorders incidence. **(C)** Anxiety disorders DALYs. **(D)** Depressive disorders DALYs. **(E)** MDD DALYs. **(F)** Dysthymia DALYs. All data are presented for 2021. DALYs, disability-adjusted life years; MDD, Major depressive disorder.

Across GBD regions, the annual variation in the incidence of anxiety and depressive disorders among young people aged 10–24 from 1990 to 2021 largely mirrored global temporal trends. The disease burden in most SDI regions exhibited an initial downward or stable trajectory, followed by a marked surge. By 2021, anxiety disorders exhibited the highest burden metrics in high SDI regions. Regions with the highest DALY rates included Western Europe, Tropical Latin America, Andean Latin America, and North Africa and Middle East, all exceeding 1,000 per 100,000 (Figures 1A, 2C; Supplementary Table S1).

The epidemiological pattern of depressive disorders differed slightly. There was a subtle turning point of disease burden in 2005, primarily driven by low-middle and low SDI regions. The highest DALY rates in 2021 were observed in High-income North America, with estimates of 1,387.2 (907.2 to 2,047.5). Australasia, Western Europe and North Africa and the Middle East also exhibited a substantial burden. East Asia was the only region where the incidence rate declined (Figures 1B, 2D; Supplementary Table S2). MDD demonstrated concordant temporal trajectories with depressive disorders throughout the study period, while dysthymia exhibited stable incidence rates with minimal inter-annual variation (Figures 1C,D).

### Mapping anxiety and depressive burden across national and subnational levels

During the study period, the trends in anxiety prevalence mirrored those of incidence (Figure 1E). The incidence rates increased in 203 out of 204 countries and territories, including 14 that exceeded 50% increase. Mexico experienced the largest increase in both incidence (75.8% [6.7 to 189.9]) and prevalence rates (73.3% [19.9 to 150.2]). By 2021, the highest incidence rates occurred in Portugal (1880.1 [1132.8 to 2828.3]) followed by Lebanon, Iran, and Brazil (Figure 2A; Supplementary Figures S1, S2; Supplementary Table S3). Across 652 subnational regions in 2021, Iran accounted for 31 of the top 100 highest-DALY regions, followed by Brazil (27 units) and Italy (19 units). Brazil’s DALY rates mostly driven by Rondônia, Paraná, and Santa Catarina. In contrast, Nigeria, Pakistan, and China exhibited lower DALY rate but significant subnational disparities. Pakistan exhibited 20% interprovincial variation [Khyber Pakhtunkhwa: 417.6 (242.7–662.8) vs. Balochistan: 348.8 (206.0–539.7)]. In Nigeria, Osun state constituted an extreme outlier [1,026.3 (611.5 to 1,630.1)], while others clustered between 442.8 and 460.2. China displayed pronounced heterogeneity, with Hunan province bearing the highest DALY rate [916.0 (530.2 to 1,427.2)] (Supplementary Figures S3, S4; Supplementary Tables S5–S22).

The global depression burden rose in over 95% of studied regions during 1990–2021 (Figure 1F). The USA saw the largest incidence increase (96.4% [30.7 to 195.8]) from 1990 to 2021, with Lebanon, Lesotho, Tunisia, and Ireland also showing notably rises. Greenland recorded the highest depressive DALY rate [2,114.8 (1,222.5 to 3,365.0)] in 2021 (Figure 2D). In contrast, Slovenia, Singapore, Switzerland, Cuba, China, Democratic People’s Republic of Korea, Kiribati, Maldives, Niger, Nigeria showed a declining trend. China showed the most pronounced reduction [−41.0% (−62.7 to −6.8)], achieving the lowest global incidence rates (Figure 2B; Supplementary Figures S5, S6; Supplementary Table S4). Sub-nationally, the USA accounted for 51 of the top 100 subnational regions by DALY rates, particularly in New Mexico, West Virginia, Arizona, and Arkansas. The UK and Iran accounted for 25 and 19 regions, respectively. Western Europe showed considerable variation. Italy’s burden clustered in northern regions [highest in Emilia-Romagna: 1,256.6 (720.5 to 1,939.8)], while Norway predominantly clustered in southern regions Viken and Oslo. Notably, Japan, Poland, and the Philippines maintained lower burdens despite comparable development levels MDD showed spatial patterns largely consistent with those of overall depressive disorders. (Figure 2E; Supplementary Figures S7, S8; Supplementary Tables S5–S22).

Dysthymia presented a different DALY rates distribution, with Eastern Sub-Saharan Africa showing the highest regional burden [91.9 (52.1–147.0)] in 2021. Comoros topped the list at 97.6 (52.8 to 163.5), followed by Rwanda, Eritrea, Djibouti, and United Republic of Tanzania (Figure 2F). Subnationally, Kenya contained 46 of the top 100 highest-burden regions, demonstrating significant southward concentration [Kiambu: 102.1 (59.6–160.5)]. The UK (34 regions) and Ethiopia (10 regions) followed, with urban centers like Addis Ababa and Stockholm also showed high burdens (Supplementary Figure S9; Supplementary Tables S23, S24).

**Figure 3:**
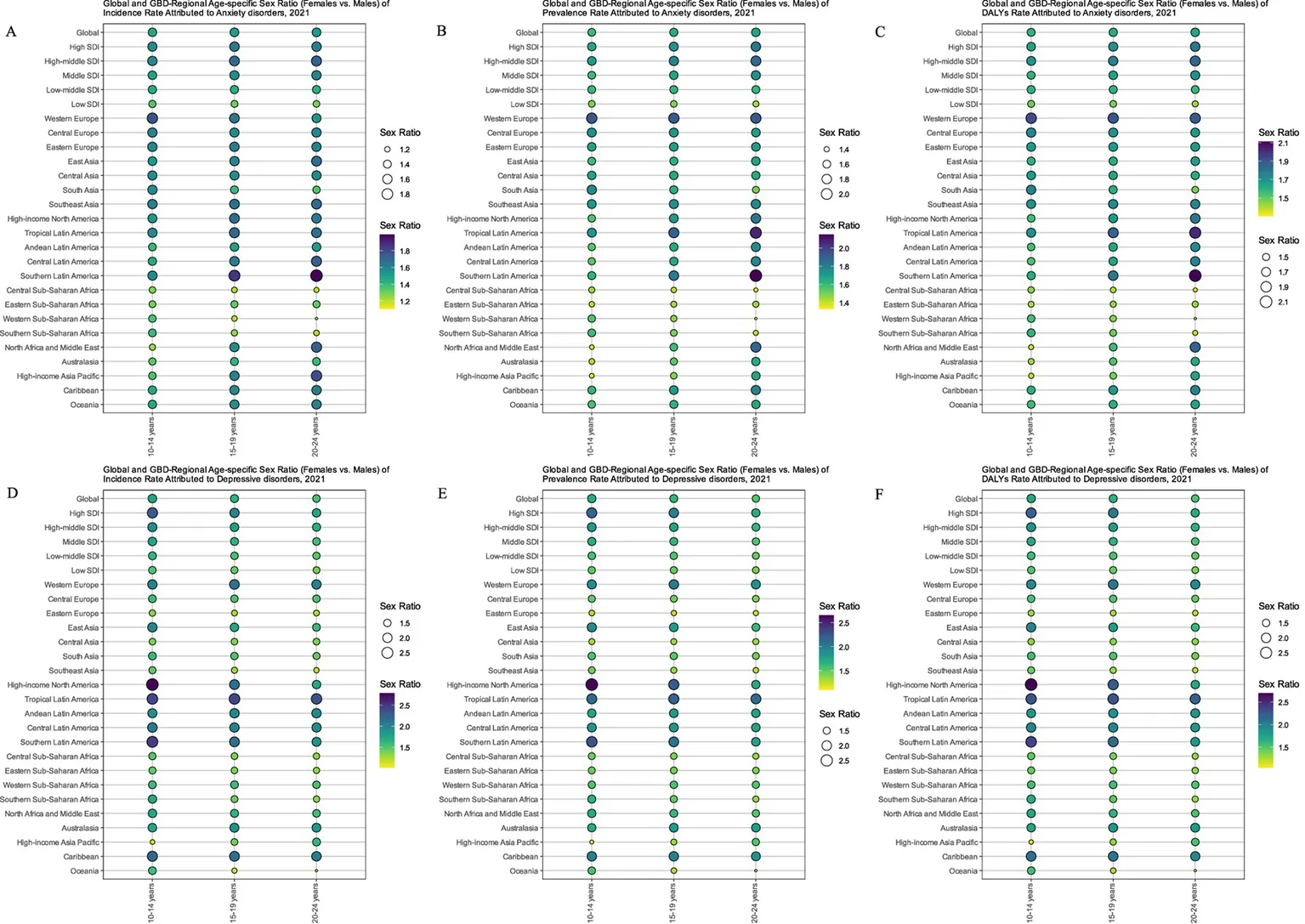
Global and GBD-regional age-specific sex ratio (females vs. males) of incidence, prevalence, and DALYs rate for anxiety disorders and depressive disorders, 2021. This figure illustrates the age-specific sex ratio (female-to-male) of anxiety disorders and depressive disorders burden across GBD regions and SDI quintiles in 2021. The horizontal axis categorizes the population into 5-year age groups, while the vertical axis represents the sex ratio, with values above 1 indicating a higher incidence in females and values below 1 indicating a higher incidence in males. **(A–C)** display the incidence, prevalence and DALYs rate attributed to anxiety disorders, respectively. **(D–F)** show the same metrics for depressive disorders. GBD, Global Burden of Disease; DALY, disability-adjusted life year; SDI, Socio-demographic Index.

During the study period, females consistently exhibited higher anxiety and depressive disorder burdens across all 21 GBD regions, with most pronounced disparities in Southern Latin America and High-income North America (Figures 3A–F).

### Inequalities change within and between countries

**Figure 4:**
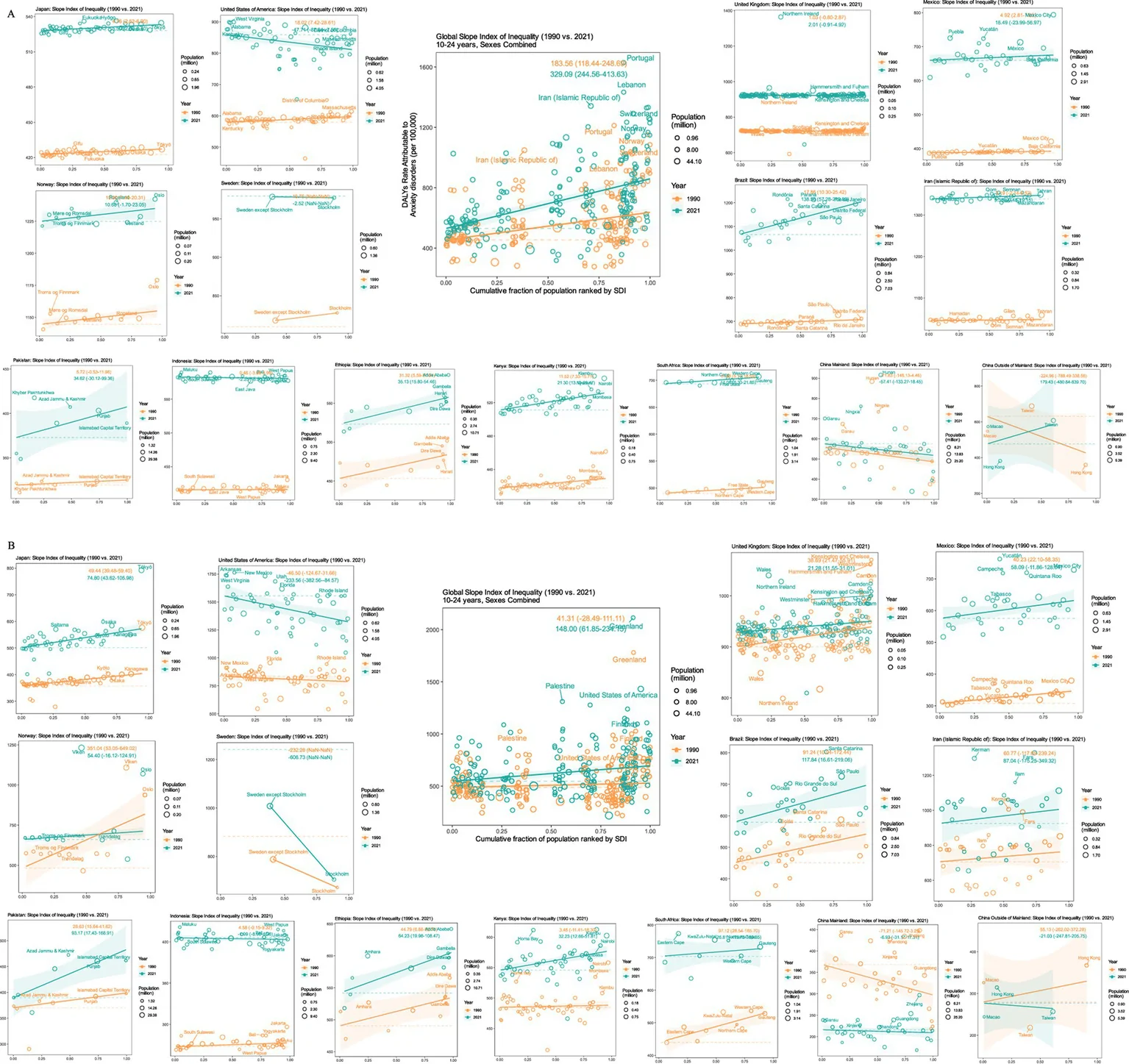
Inequalities in DALYs in anxiety disorders and depressive disorders across SDI regions (1990 and 2021). This figure presents the global SII for DALY rates attributable to anxiety disorders and depressive disorders (per 100,000 population) across all ages and sexes, ranked by SDI levels in 1990 and 2021. The cumulative fraction of the population ranked by SDI is displayed on the x-axis, while DALY rates are shown on the y-axis, highlighting inequality trends over time. **(A)** And **(B)** displayed the global inequality attributed to anxiety disorders and depressive disorders respectively, along with the inequality observed in 14 countries that have robust local-level data. DALY, disability-adjusted life year; SDI, Socio-demographic Index; SII, slope index of inequality.

We further quantified socioeconomic inequalities both between and within countries. Compared to 1990, the global absolute inequalities in anxiety burdens increased in 2021. The SII quantifying an absolute burden differential of 390.48 (289.65 to 491.31) incidence rate, 2668.65 (1970.40 to 3366.90) prevalence rate, and 329.09 (244.56 to 413.63) DALY rate per 100,000 between the highest and the lowest SDI countries. Geospatial evaluation highlighted Portugal, Lebanon, Iran, Switzerland, and Norway as principal contributors to this inequality (Figure 4A). Additionally, the gaps in anxiety burden across countries were most pronounced in high SDI countries, followed by lower-middle SDI countries (Supplementary Figure S10). Among 14 nations with subnational SDI data, China outside of mainland regions demonstrating the highest disparity in anxiety DALY rate [SII = 179.43 (−480.84 to 839.70)], succeeded by Brazil [138.56 (57.28 to 219.83)] (Supplementary Figures S11, S12).

Our revealed absolute differences of 390.48 incidence rate (289.65 to 491.31), 2668.65 prevalence rate (1970.40 to 3366.90), and 329.09 DALY rate (244.56 to 413.63) between socioeconomic extremes for depressive disorders (Supplementary Figures S13, S15). By 2021, Sweden (−4,639.40), the USA (−1,819.49), and Brazil (881.00) showed marked SII values in incidence rates. Norway achieved the most notable inequality reduction, with DALY rate SII declining from 351.04 (53.05 to 649.02) in 1990 to 54.40 (−16.12 to 124.91) in 2021. Similar downward trends occurred in the UK, Indonesia, South Africa, and China (Figure 4B). Despite these equity improvements, all studied nations except China and Norway showed increased depression burdens during the observation period (Supplementary Figures S16, S17).

### Frontier analysis

**Figure 5:**
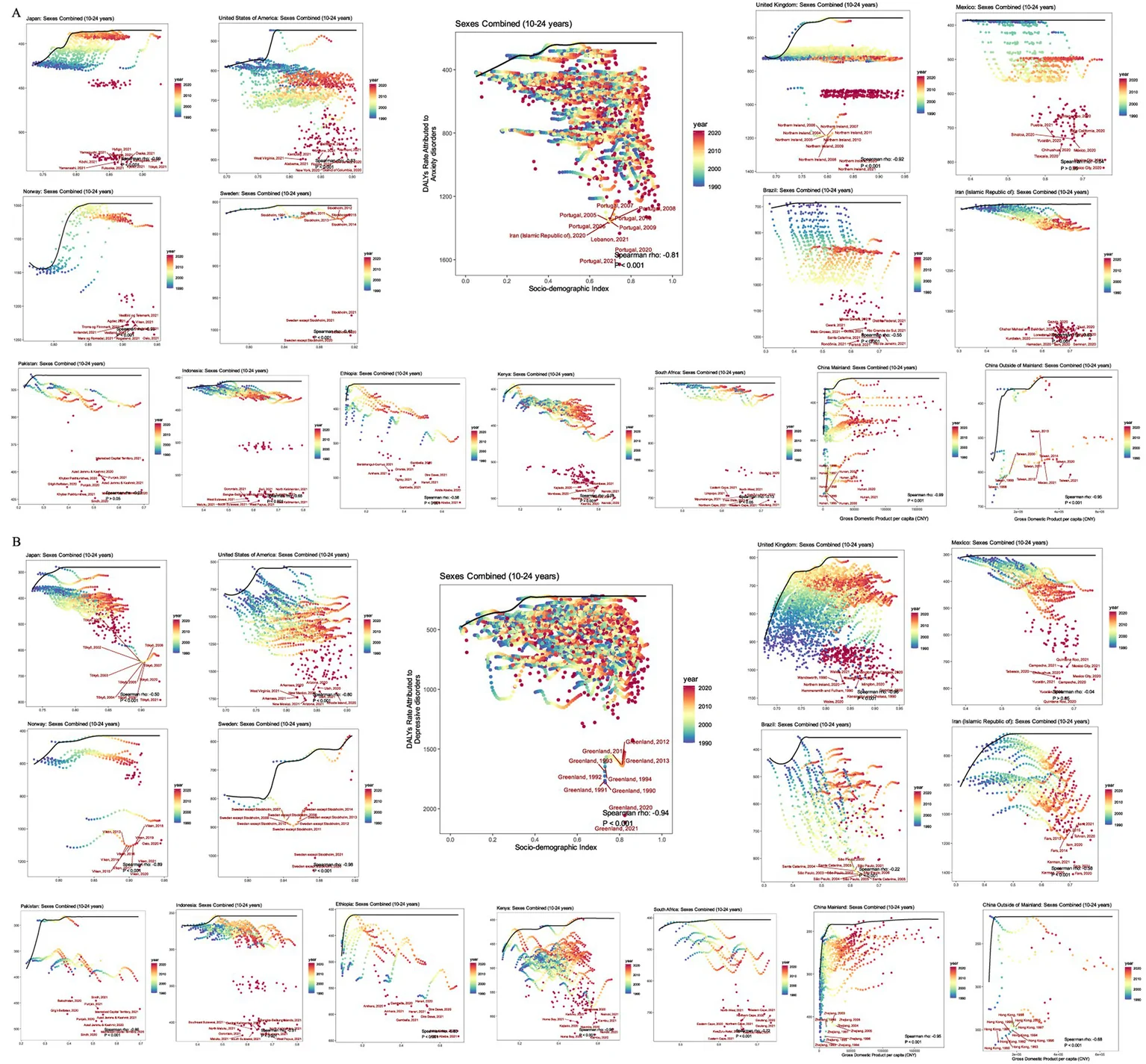
Deviation from frontier in DALYs rate attributed to anxiety disorders and depressive disorders among females and males (10–24 years), 1990–2021. This figure illustrates trends in DALYs rate due to anxiety disorders **(A)** and depressive disorders **(B)** by SDI quintiles over time (1990–2021), showing differences in disease burden reduction or aggravation trajectories among regions with varying SDI. The solid black line represents the minimum achievable disease burden for countries or regions at a given SDI level, referred to as the frontier. The origin points in the graph show the distance from the frontier over time and across SDI levels for countries and subnational regions within 14 countries with reliable specific data. The farther a point is from the solid black line, the greater the potential for improvement. DALY, disability-adjusted life year; SDI, Socio-demographic Index.

We found that over 99% of countries widened their gap for anxiety disorder from the frontier during the study period (Table 2). The 10 countries with the highest EF values for anxiety DALY rates in 2021 were Portugal, Lebanon, Iran, Netherlands, Ireland, France, Germany, Switzerland, Greece, and Norway (range: 996.953 to 1,396.556), all high SDI nations. Only 22 countries achieved the theoretical minimum burden level, primarily in low SDI regions. Among 494 subnational locations, the UK constituted 87% of the top 100 places ranked by EF attributed to DALY rates (Table 3). Iran and Norway saw significant regional EF value increases, with Semnan, Qom, Chahar Mahaal, Bakhtiari, Oslo, Rogaland, and Møre og Romsdal showing large deviations from the frontier in 2021 (Figure 5A; Supplementary Figures S18–S20).

**Table 2:** Summary of Frontier Analysis of 204 Countries from 1990 to 2021.

Metric	Frontier analysis outcome	Both	Female	Male
Anxiety disorder				
Incidence	Countries behind frontier *n* (%)	174 (85.3%)	171 (83.8%)	183 (89.7%)
	Countries with largest deviation in 2021	Portugal	Portugal	Portugal
	Number of countries with increasing deviation from frontier *n* (%)	203 (99.5%)	203 (99.5%)	203 (99.5%)
	Countries with largest decrease in deviation from 1990 to 2021	Taiwan (Province of China)	Taiwan (Province of China)	Taiwan (Province of China)
	Countries with largest increase in deviation from 1990 to 2021	Bolivia (Plurinational State of)	Brazil	Bolivia (Plurinational State of)
Prevalence	Countries behind frontier n (%)	194 (95.1%)	193 (94.6%)	196 (96.1%)
	Countries with largest deviation in 2021	Portugal	Portugal	Portugal
	Number of countries with increasing deviation from frontier *n* (%)	202 (99.0%)	202 (99.0%)	203 (99.5%)
	Countries with largest decrease in deviation from 1990 to 2021	Taiwan (Province of China)	Taiwan (Province of China)	Taiwan (Province of China)
	Countries with largest increase in deviation from 1990 to 2021	Lebanon	Brazil	Lebanon
DALY	Countries behind frontier n (%)	182 (89.2%)	182 (89.2%)	183 (89.7%)
	Countries with largest deviation in 2021	Portugal	Portugal	Portugal
	Number of countries with increasing deviation from frontier n (%)	202 (99.0%)	202 (99.0%)	203 (99.5%)
	Countries with largest decrease in deviation from 1990 to 2021	Taiwan (Province of China)	Taiwan (Province of China)	Taiwan (Province of China)
	Countries with largest increase in deviation from 1990 to 2021	Lebanon	Brazil	Lebanon
Depressive disorders				
Incidence	Countries behind frontier n (%)	197 (96.6%)	199 (97.5%)	197 (96.6%)
	Countries with largest deviation in 2021	Greenland	Greenland	Greenland
	Number of countries with increasing deviation from frontier *n* (%)	198 (97.1%)	197 (96.6%)	199 (97.5%)
	Countries with largest decrease in deviation from 1990 to 2021	Cuba	Cuba	Switzerland
	Countries with largest increase in deviation from 1990 to 2021	United States of America	United States of America	Lesotho
Prevalence	Countries behind frontier *n* (%)	195 (95.6%)	196 (96.1%)	196 (96.1%)
	Countries with largest deviation in 2021	Greenland	Greenland	Greenland
	Number of countries with increasing deviation from frontier *n* (%)	197 (96.6%)	196 (96.1%)	196 (96.1%)
	Countries with largest decrease in deviation from 1990 to 2021	Cuba	Cuba	Switzerland
	Countries with largest increase in deviation from 1990 to 2021	United States of America	United States of America	Lesotho
DALY	Countries behind frontier *n* (%)	188 (92.2%)	193 (94.6%)	189 (92.6%)
	Countries with largest deviation in 2021	Greenland	Greenland	Greenland
	Number of countries with increasing deviation from frontier *n* (%)	198 (97.1%)	196 (96.1%)	199 (97.5%)
	Countries with largest decrease in deviation from 1990 to 2021	Cuba	Cuba	Switzerland
	Countries with largest increase in deviation from 1990 to 2021	United States of America	United States of America	Lesotho
MDD				
Incidence	Countries behind frontier *n* (%)	197 (96.6%)	198 (97.1%)	195 (95.6%)
	Countries with largest deviation in 2021	Greenland	Greenland	Greenland
	Number of countries with increasing deviation from frontier *n* (%)	198 (97.1%)	197 (96.6%)	199 (97.5%)
	Countries with largest decrease in deviation from 1990 to 2021	Cuba	Cuba	Switzerland
	Countries with largest increase in deviation from 1990 to 2021	United States of America	United States of America	Lesotho
Prevalence	Countries behind frontier *n* (%)	197 (96.6%)	198 (97.1%)	196 (96.1%)
	Countries with largest deviation in 2021	Greenland	Greenland	Greenland
	Number of countries with increasing deviation from frontier *n* (%)	198 (97.1%)	197 (96.6%)	199 (97.5%)
	Countries with largest decrease in deviation from 1990 to 2021	Cuba	Cuba	Switzerland
	Countries with largest increase in deviation from 1990 to 2021	United States of America	United States of America	Lesotho
DALY	Countries behind frontier *n* (%)	189 (92.6%)	193 (94.6%)	185 (90.7%)
	Countries with largest deviation in 2021	Greenland	Greenland	Greenland
	Number of countries with increasing deviation from frontier *n* (%)	198 (97.1%)	197 (96.6%)	199 (97.5%)
	Countries with largest decrease in deviation from 1990 to 2021	Cuba	Cuba	Switzerland
	Countries with largest increase in deviation from 1990 to 2021	United States of America	United States of America	Lesotho
Dysthymia				
Incidence	Countries behind frontier n (%)	36 (17.6%)	69 (33.8%)	44 (21.6%)
	Countries with largest deviation in 2021	Comoros	Comoros	Saudi Arabia
	Number of countries with increasing deviation from frontier n (%)	154 (75.5%)	151 (74.0%)	161 (78.9%)
	Countries with largest decrease in deviation from 1990 to 2021	United States of America	United States of America	United States of America
	Countries with largest increase in deviation from 1990 to 2021	Mozambique	Mozambique	Ethiopia
Prevalence	Countries behind frontier n (%)	95 (46.6%)	96 (47.1%)	120 (58.8%)
	Countries with largest deviation in 2021	Comoros	Comoros	Saudi Arabia
	Number of countries with increasing deviation from frontier n (%)	153 (75.0%)	146 (71.6%)	156 (76.5%)
	Countries with largest decrease in deviation from 1990 to 2021	United States of America	United States of America	United States of America
	Countries with largest increase in deviation from 1990 to 2021	Mozambique	Mozambique	Ethiopia
DALY	Countries behind frontier n (%)	42 (20.6%)	69 (33.8%)	25 (12.3%)
	Countries with largest deviation in 2021	Comoros	Comoros	Saudi Arabia
	Number of countries with increasing deviation from frontier n (%)	154 (75.5%)	148 (72.5%)	157 (77.0%)
	Countries with largest decrease in deviation from 1990 to 2021	United States of America	United States of America	United States of America
	Countries with largest increase in deviation from 1990 to 2021	Mozambique	Mozambique	Ethiopia

DALY, Disability-adjusted life-year.

**Table 3:** Summary of Frontier Analysis of 494 Locations from 1990 to 2021.

Metric	Frontier analysis outcome	Both	Female	Male
Anxiety disorder				
Incidence	Locations behind frontier n (%)	263 (53.2%)	239 (48.4%)	252 (51.0%)
	Locations with largest deviation in 2021	Northern Ireland	Northern Ireland	Northern Ireland
	Number of Locations with increasing deviation from frontier n (%)	494 (100%)	494 (100%)	494 (100%)
	Locations with largest decrease in deviation from 1990 to 2021	Balochistan	Balochistan	Balochistan
	Locations with largest increase in deviation from 1990 to 2021	Northern Ireland	Rondônia	Northern Ireland
Prevalence	Locations behind frontier n (%)	289 (58.5%)	289 (58.5%)	279 (56.5%)
	Locations with largest deviation in 2021	Northern Ireland	Northern Ireland	Northern Ireland
	Number of Locations with increasing deviation from frontier n (%)	494 (100%)	494 (100%)	494 (100%)
	Locations with largest decrease in deviation from 1990 to 2021	Balochistan	Oslo	Shanghai
	Locations with largest increase in deviation from 1990 to 2021	Northern Ireland	Northern Ireland	Northern Ireland
DALY	Locations behind frontier n (%)	243 (49.2%)	230 (46.6%)	227 (46.0%)
	Locations with largest deviation in 2021	Northern Ireland	Northern Ireland	Northern Ireland
	Number of Locations with increasing deviation from frontier n (%)	494 (100%)	494 (100%)	494 (100%)
	Locations with largest decrease in deviation from 1990 to 2021	Balochistan	Oslo	Shanghai
	Locations with largest increase in deviation from 1990 to 2021	Northern Ireland	Northern Ireland	Northern Ireland
Depressive disorders				
Incidence	Locations behind frontier n (%)	389 (78.7%)	412 (83.4%)	360 (72.9%)
	Locations with largest deviation in 2021	New Mexico	Arkansas	New Mexico
	Number of Locations with increasing deviation from frontier n (%)	472 (95.5%)	473 (95.7%)	461 (93.3%)
	Locations with largest decrease in deviation from 1990 to 2021	Hubei	Hubei	Hunan
	Locations with largest increase in deviation from 1990 to 2021	New Mexico	Arkansas	West Virginia
Prevalence	Locations behind frontier n (%)	311 (63.0%)	382 (77.3%)	274 (55.5%)
	Locations with largest deviation in 2021	New Mexico	Arkansas	Fars
	Number of Locations with increasing deviation from frontier n (%)	461 (93.3%)	469 (94.9%)	431 (87.2%)
	Locations with largest decrease in deviation from 1990 to 2021	Hunan	Hubei	Westminster
	Locations with largest increase in deviation from 1990 to 2021	New Mexico	Arkansas	West Virginia
DALY	Locations behind frontier n (%)	177 (35.8%)	175 (35.4%)	184 (37.2%)
	Locations with largest deviation in 2021	New Mexico	Arkansas	Fars
	Number of Locations with increasing deviation from frontier n (%)	470 (95.1%)	469 (94.9%)	448 (90.7%)
	Locations with largest decrease in deviation from 1990 to 2021	Hubei	Hubei	Hunan
	Locations with largest increase in deviation from 1990 to 2021	New Mexico	Arkansas	West Virginia
MDD				
Incidence	Locations behind frontier n (%)	391 (79.1%)	416 (84.2%)	358 (72.5%)
	Locations with largest deviation in 2021	New Mexico	Arkansas	New Mexico
	Number of Locations with increasing deviation from frontier n (%)	473 (95.7%)	473 (95.7%)	461 (93.3%)
	Locations with largest decrease in deviation from 1990 to 2021	Hubei	Hubei	Hunan
	Locations with largest increase in deviation from 1990 to 2021	New Mexico	Arkansas	West Virginia
Prevalence	Locations behind frontier n (%)	391 (79.1%)	419 (84.8%)	355 (71.9%)
	Locations with largest deviation in 2021	New Mexico	Arkansas	New Mexico
	Number of Locations with increasing deviation from frontier n (%)	472 (95.5%)	470 (95.1%)	454 (91.9%)
	Locations with largest decrease in deviation from 1990 to 2021	Hubei	Hubei	Hunan
	Locations with largest increase in deviation from 1990 to 2021	New Mexico	Arkansas	West Virginia
DALY	Locations behind frontier n (%)	187 (37.9%)	188 (38.1%)	189 (38.3%)
	Locations with largest deviation in 2021	New Mexico	Arkansas	New Mexico
	Number of Locations with increasing deviation from frontier n (%)	471 (95.3%)	469 (94.9%)	456 (92.3%)
	Locations with largest decrease in deviation from 1990 to 2021	Hubei	Hubei	Hunan
	Locations with largest increase in deviation from 1990 to 2021	New Mexico	Arkansas	West Virginia
Dysthymia				
Incidence	Locations behind frontier n (%)	1 (0.2%)	7 (1.4%)	0 (0%)
	Locations with largest deviation in 2021	Tehran	Tehran	Dire Dawa
	Number of Locations with increasing deviation from frontier n (%)	299 (60.5%)	307 (62.1%)	300 (60.7%)
	Locations with largest decrease in deviation from 1990 to 2021	District of Columbia	District of Columbia	District of Columbia
	Locations with largest increase in deviation from 1990 to 2021	Kurdistan	Kurdistan	Harari
Prevalence	Locations behind frontier n (%)	21 (4.3%)	60 (12.1%)	26 (5.3%)
	Locations with largest deviation in 2021	Tehran	Tehran	Dire Dawa
	Number of Locations with increasing deviation from frontier n (%)	305 (61.7%)	334 (67.6%)	308 (62.3%)
	Locations with largest decrease in deviation from 1990 to 2021	District of Columbia	District of Columbia	District of Columbia
	Locations with largest increase in deviation from 1990 to 2021	Kurdistan	Kurdistan	Harari
DALY	Locations behind frontier n (%)	1 (0.2%)	1 (0.2%)	0 (0%)
	Locations with largest deviation in 2021	Tehran	Tehran	Dire Dawa
	Number of Locations with increasing deviation from frontier n (%)	305 (61.7%)	332 (67.2%)	310 (62.8%)
	Locations with largest decrease in deviation from 1990 to 2021	District of Columbia	District of Columbia	District of Columbia
	Locations with largest increase in deviation from 1990 to 2021	Kurdistan	Kurdistan	Harari

DALY, Disability-adjusted life-year.

For depressive disorders, the top 10 countries exhibiting the largest deviations in 2021 being Greenland, the USA, Palestine, Tunisia, Greece, Lebanon, Chile, Portugal, Ireland, and Finland (range: 1896.458 to 877.386) (Table 2). Subnationally, the USA had the highest representation in both the top 100 regions ranked by EF values and those ranked by EF increase, comprising 51% of these locations (Table 3). The largest discrepancies between actual and potential minimum DALY rates were observed in high SDI states of the USA such as New Mexico, Arkansas, West Virginia, Arizona, and Utah. Additionally, significant deviations from the frontier were noted in Fars and Kerman in Iran, and Viken in Norway (Figure 5B; Supplementary Figures S21–S23).

For dysthymia, 42 countries (predominantly high SDI) still did not reach the burden frontier in 2021 (Table 2). Conversely, the gap continued to widen in several low-middle SDI countries, including Comoros, Rwanda, and Eritrea (Supplementary Figures S24, S25).

## Discussion

This study, leveraging the GBD 2021 dataset, provides a comprehensive geospatial and temporal analysis of anxiety and depressive disorders among young people aged 10–24 years across 953 locations at regional, national, and subnational levels. This study has five principal findings: (1) Over the past three decades, the anxiety and depressive burdens of young people have increased significantly across over 95% of the 204 countries and territories; (2) High and high-middle SDI areas bore marked burdens in 2021, with Portugal and the USA exhibiting the heaviest incidence rates in anxiety and depressive disorders respectively, while divergent burden levels were observed in low and low-middle SDI areas; (3) Females maintained persistently greater absolute burdens; (4) Socioeconomic inequality in disease burdens has intensified globally, with pronounced impacts in high SDI countries like Norway, and also disproportionately affecting low-middle and low SDI countries (e.g., Pakistan and Nigeria); (5) Frontier analysis revealed substantial and widening frontier deviations in DALY rates, with 89.2 and 92.2% of the countries failed to achieve the anxiety and depressive frontier levels in 2021. All subnational locations experienced increasing deviation from the frontier for anxiety disorders during 1990–2021, while 95.1% of units in depressive disorders. These findings carry direct implications for adolescent injury prevention. The identified high burden and high inequality settings, spanning high SDI countries such as Portugal and the United States, middle SDI regions including Brazil and Mexico, and vulnerable subnational areas in Iran and the United Kingdom, represent priority targets for integrated mental health and safety promotion interventions. The widening frontier gaps further quantify the scale of unmet prevention potential, where addressing mental health burden could simultaneously mitigate associated injury outcomes.

### Epidemiological trends and gender disparity in burden of anxiety and depressive disorders

The burden of anxiety and depressive disorders among youth followed population-wide temporal trends (26, 27). The 2005–2010 depression burden inflection in low-middle SDI regions aligned with China’s enormous investment in controlling mental illness, supplemented by India and Sudan’s contributions (26). The COVID-19 pandemic exacerbated adolescent mental vulnerability (28), with MDD’s acute nature rendering it more responsive to pandemic-related stressors than chronic dysthymia (29), explaining its pronounced incidence increase. The post-pandemic surge documented here has potentially serious implications for adolescent injury epidemiology (30), given that population-level increases in depression and anxiety are consistently associated with elevated rates of emergency department visits for self-harm and suicide attempts in the same age group.

Globally, young females exhibited consistently higher burdens than males, reflecting both biological susceptibility and sociocultural factors (31). Estrogen fluctuations may affect limbic emotional regulation and enhance hypothalamic–pituitary–adrenal (HPA) axis responses, increasing sensitivity to stress during adolescence and early adulthood (32). Notably, the female excess varied substantially by sociocultural context, suggesting that social and cultural stressors may amplify this biological vulnerability (33). In Southern Latin America, the larger sex disparity may be related to persistent gender-based violence, unequal power relations, and sociocultural norms that normalize female self-silencing or constrain help-seeking, all linked to depression and anxiety among girls (34). In High-income North America, the wider gap may reflect a different constellation of pressures, including high exposure to social media, online harassment, appearance-based comparison, sexual violence, and rising psychological distress among adolescent girls. The US Youth Risk Behavior Survey reported that female students experienced higher levels of violence, poor mental health, and suicidal thoughts and behaviors than male students, while the US Surgeon General’s Advisory highlighted stronger associations of social media use with depressive symptoms, poor body image, and online harassment among girls than boys (35, 36). In regions with large female-to-male ratios, self-harm and injury prevention programs should integrate mental health screening with interventions addressing gender-based violence, digital safety, body image, school connectedness, and accessible counseling.

### Variations in disease burden by SDI levels and regions

Our analyses identified high and high-middle SDI regions exhibiting elevated burdens despite superior healthcare infrastructure, alongside the most pronounced intra-regional disparities. In post-industrial societies such as Western Europe and High-income North America, pressures from compulsory education and cultural emphases on achievement at school amplify performance anxiety (37), which interacts with wealth inequality to create chronic stress and intensify mental health disparities (38). Environmental factors significantly modulate disease burdens, with Greenland exhibiting extreme depression rates attributable to climatic extremes and seasonal affective disorder (39). Mediterranean regions like Italy demonstrate climate-related psychiatric impacts, where increasing flood events elevate trauma-related disorders (40). Socioeconomic and structural determinants further drive inequalities. In the USA, depression burdens concentrate in southwestern and eastern states (Arizona, Nevada, West Virginia, South Carolina), reflecting industrial decline, economic disparities, and substance use (41). Norway’s restrictive mental health services criteria on adolescents similarly create treatment gaps (42). The high anxiety and depressive burden in these high SDI settings coexists with elevated adolescent self-harm and suicide rates. In the United States, suicide is now the second leading cause of death among individuals aged 10 to 24 years, a trajectory intensified by untreated depression (43). Our subnational findings provide a spatial evidence base for targeting school-based mental health screening and safety promotion programs to the highest burden states identified here, including New Mexico, West Virginia, and Arkansas.

Notwithstanding comparable development levels, East Asian nations exhibit relatively lower mental health burdens, attributable to unique sociocultural protections. Japan’s declining birthrates have reduced absolute case numbers (44), while its “blue-space” initiatives, which integrate urban water environments such as rivers, lakes, and coastal areas into public health planning, provide ecological stress buffers that may alleviate stress among adolescents in high-density urban settings (45). Similarly, China and South Korea’s nighttime light regulations demonstrate neuroprotective effects by stabilizing circadian rhythms and attenuating cortisol responses (46).

Frontier analysis highlighted inefficiencies in mental healthcare in high and high-middle SDI countries, with Portugal, Lebanon, Greece, and Ireland demonstrating significant improvement potential. Lebanon’s compounded crises [1990 civil war (10) financial collapse (47), and Beirut explosion (48)] created exceptional youth mental health burdens, while Ireland grapples with substance abuse and bullying epidemics (49). Conversely, Cuba and China achieved notable depressive disorder reductions through targeted prevention. China’s “Healthy China 2030” initiative and three-tiered intervention strategy, incorporating digital mental health platforms for high-risk adolescents (50), proved particularly effective. These models provide adaptable frameworks for nations at comparable development stages.

Middle and low-middle SDI regions also faced distinct challenges, particularly in Andean and Tropical Latin America. Brazil’s rapid urbanization alongside traditional structures creates severe disparities, particularly in southern states (e.g., Paraná) where violence, substance use, and educational gaps converge (51). Mexico exemplifies the middle SDI paradox, where economic growth coexists with increasing mental burden. The 2017 Puebla earthquake exposed inadequate preparation in psychosocial emergency response, while subsequent pandemic overwhelmed the fragile mental healthcare system (52). South Asia and Western Sub-Saharan Africa show uneven burdens. Pakistan’s Khyber Pakhtunkhwa demonstrates a high prevalence linked to decades of sociopolitical instability and conflict (53), while Balochistan’s rural areas likely underestimate burdens due to screening limitations (Government of Balochistan (10)). Nigeria reports significant internal variations, predominantly in southwestern states like Osun and Oyo, attributed to socioeconomic conditions, insufficient mental health services, and stigma (54).

Low SDI regions exhibited substantial mental health disparities, characterized by critical shortages of mental health professionals and pervasive stigmatization that contribute to significant underreporting (38). These challenges are exacerbated by conflict and displacement (55). Notably, frontier attainment in some low SDI countries likely reflects diagnostic limitations rather than effective management, underscoring persistent systemic gaps. Future research must employ standardized assessment tools with cross-cultural validation to disentangle these measurement challenges from true epidemiological patterns.

### Implications for child injury prevention and safety promotion

Our findings have several implications for adolescent and young adult mental health promotion and injury prevention. The unequal burdens of anxiety and depressive disorders across SDI levels, regions, and subnational units suggest that injury prevention strategies should not rely only on national averages or broad age categories. Mental health indicators should be incorporated into injury surveillance, school health programs, and local priority-setting systems so that areas with overlapping burdens of psychological distress, self-harm, interpersonal violence, or unintentional injury can be identified earlier. This approach is consistent with WHO guidance, which recommends mainstreaming adolescent mental health promotion and prevention across health, education, and community platforms (56), and with the WHO-UNICEF Helping Adolescents Thrive framework, which emphasizes supportive environments, caregiver support, psychosocial interventions, and policies that reduce self-harm and other risk behaviors (57). In high-SDI settings, school-based mental health literacy, early screening, counseling pathways, and safe digital support may be particularly relevant where academic pressure, social isolation, and online exposure contribute to psychological distress. In resource-constrained settings, task-sharing, community-based psychosocial support, and integration with primary care and injury prevention programs may offer more feasible routes to scale-up interventions. For large and heterogeneous countries, subnational estimates can help direct mental health professionals, school counseling resources, and community prevention programs to the areas where young people face the greatest overlapping burden of mental disorders and injury risk (58).

## Limitations

With this study represents the most comprehensive analysis to date of anxiety and depressive disorder burdens among young people aged 10–24 years across 204 countries and 652 subnational regions, several constraints warrant careful consideration. First, GBD estimates are derived largely from secondary data sources and statistical modeling rather than direct raw data from various countries, and data availability and quality vary substantially across countries and subnational units. In low- and middle-income settings, limited mental health surveillance capacity, heterogeneous reporting protocols, delayed case ascertainment, and underdiagnosis may lead to underestimated burdens, whereas broader screening and greater service coverage in high-income settings may increase detection and potentially inflate observed differences (39). Cross-country and temporal comparisons may also be affected by cultural variation in symptom recognition, help-seeking behavior, stigma, diagnostic practices, and changes in diagnostic criteria or measurement instruments over time. Second, because YLLs are constrained to zero for these disorders in GBD, our DALY estimates reflect disability alone and are not directly comparable to DALYs for conditions where mortality contributes substantially, such as self-harm or injury. This likely understates their true population impact. Third, although 95% UIs were reported throughout the analysis, they may not fully capture measurement error, selection bias, diagnostic misclassification, or model specification uncertainty. In addition, this study was descriptive and did not apply formal trend testing methods such as EAPC estimation or joinpoint regression; therefore, percentage changes with wide uncertainty intervals should be interpreted cautiously, particularly when the intervals include both decreases and increases. Finally, the multifactorial etiology of anxiety and depressive disorders in the 10–24 age group limit causal interpretation. Observed geographic differences may reflect a mixture of true epidemiological variation and differences in detection, service access, humanitarian response, environmental exposure, socioeconomic conditions, and other unmeasured confounders.

## Conclusion

In conclusion, while modest progress has been achieved in mitigating anxiety and depressive disorders among young people aged 10–24 years, the COVID-19 pandemic has underscored the fragility of these gains. Countries with high and high-middle SDI experienced a greater burden of anxiety and depressive disorders, alongside profound health inequalities. Middle and middle-low SDI countries faced accelerating disease trajectories and persistent health inequities across subnational regions, highlighting emerging challenges for resource-limited settings. While a few low SDI countries showed the best performance in reaching the frontier level, significant disparities in mental health burden still remain. These findings emphasize the imperative for coordinated strategies that not only address mental health disparities but also leverage this evidence for child injury prevention and safety promotion. The widening frontier deviations identified here, with 89.2% and 92.2% of countries failing to achieve the anxiety and depressive DALYs frontiers, represent not only mental health treatment gaps but also substantial unrealized potential for reducing adolescent self-harm, suicide, and injury related outcomes. By identifying priority populations and settings through inequality and frontier analyses, this study provides an actionable evidence base for designing region-specific interventions that integrate mental health promotion into child safety frameworks, particularly in resource-limited settings where mental health and injury burdens converge most acutely.

## Data Availability

Publicly available datasets were analyzed in this study. This data can be found at: https://ghdx.healthdata.org/gbd-2021/sources.
