# Correction: Anxiety and depressive disorders in adolescent injury prevention and safety promotion: frontier and inequality mapping across 953 locations

**DOI:** 10.3389/fpubh.2026.1966767

**Published:** 2026-08-18

**Authors:** Frontiers Production Office

**Affiliations:** Frontiers Media SA, Lausanne, Switzerland

**Keywords:** anxiety disoders, child and adolescent, depressive disorder, health inequality, injury prevention, self-harm

In the published article, there was an error in the **Introduction** Section. A reference was cited when it should have been a year. The phrase “From 2019 to (10)” should be “From 1990 to 2019”.

A correction has been made to the Section **Introduction**, Paragraph 2:

“From 1990 to 2019, the age-standardized incidence rate (ASIR) of anxiety and depressive disorders among adolescents globally both remained relatively stable (2), with a slight decrease in depressive disorders (Estimated annual percentage change, EAPC = −0.23) (11).”

The original version of this article has been updated.

