# Supplementary material for: Anxiety and depressive disorders in adolescent injury prevention and safety promotion: frontier and inequality mapping across 953 locations
Source: Front Public Health. 2026 Aug 11;14:1916777. doi: 10.3389/fpubh.2026.1916777 (PMC13532395; doi:10.3389/fpubh.2026.1916777)
Supplement: Supplementary file 1 [file Image_1.pdf]

Supplement 2: Additional results for“ Anxiety and Depressive Disorders in Adolescent Injury Prevention and Safety Promotion: Frontier and Inequality Mapping across 953 Locations”

This supplement provides additional results for “ Anxiety and Depressive Disorders in Adolescent Injury Prevention and S afety Promotion: Frontier and Inequality Mapping across 953 Locations” .

Contents

Supplementary Figures 5

Figure S1: Incidence trends attributed to anxiety disorders among young people aged 10-24 years across GBD regions, international regions, 204 countries and territories. 5

Figure S2: DALY trends attributed to anxiety disorders among young people aged 10-24 years across GBD regions, international regions, 204 countries and territories. 41

Figure S3: DALY trends attributed to anxiety disorders among young people aged 10-24 years across subnational locations. 77

Figure S4: DALY rates map of anxiety disorders among young people aged 10-24 years across subnational locations. 161

Figure S5: Incidence trends attributed to depressive disorders among young people aged 10-24 years across GBD regions, international regions, 204 countries and territories. 162

Figure S6: DALY trends attributed to depressive disorders among young people aged 10-24 years across GBD regions, international regions, 204 countries and territories. 198

Figure S7: DALY trends attributed to depressive disorders among young people aged 10-24 years across subnational locations. 234

Figure S8: DALY rates map of depressive disorders among young people aged 10-24 years across subnational locations. 318

Figure S9: DALY rates map of dysthymia among young people aged 10-24 years across subnational locations. 319

Figure S10: Health inequality analysis of anxiety disorders in incidence, prevalence, and DALY rates from 1990 to 2021. 320

Figure S11: Health inequality analysis of anxiety disorders in incidence rates across subnational locations from 1990 to 2021. 323

Figure S12: Health inequality analysis of anxiety disorders in DALY rates across subnational locations from 1990 to 2021. 331

Figure S13: Health inequality analysis of depressive disorders in incidence, prevalence, and DALY rates from 1990 to 2021. 339

Figure S14: Health inequality analysis of MDD in incidence, prevalence, and DALY rates from 1990 to 2021. 342

Figure S15: Health inequality analysis of dysthymia in incidence, prevalence, and DALY rates from 1990 to 2021. 345

Figure S16: Health inequality analysis of depressive disorders in incidence rates across subnational locations from 1990 to 2021. 348

Figure S17: Health inequality analysis of depressive disorders in DALY rates across subnational locations from 1990 to 2021. 356

Figure S18: Deviation from frontier in incidence, prevalence, and DALY rates attributed to anxiety disorders among females and males (10-24 years) in 1990 and 2021. 364

Figure S19: Frontier analysis of anxiety disorders incidence, prevalence, and DALY rates in relation to socioeconomic development across countries. 367

Figure S20: Frontier deviation in incidence, prevalence, and DALY rates attributed to anxiety disorders across subnational locations. 370

Figure S21: Deviation from frontier in incidence, prevalence, and DALY rates attributed to depressive disorders among females and males (10-24 years) in 1990 and 2021. 415

Figure S22: Frontier analysis of depressive disorders incidence, prevalence, and DALY rates in relation to socioeconomic development across countries. 418
Figure S23: Frontier deviation in incidence, prevalence, and DALY rates attributed to depressive disorders across subnational locations. 421
Figure S24: Frontier analysis of MDD incidence, prevalence, and DALY rates in relation to socioeconomic development across countries. 466
Figure S25: Frontier analysis of dysthymia incidence, prevalence, and DALY rates in relation to socioeconomic development across countries. 469
Supplementary Tables 472
Table S1: The incidence, prevalence and DALYs rate of anxiety disorders in 1990 and 2021, and changes from 1990 to 2021 at the global level and different regions. 472
Table S2: The incidence, prevalence and DALYs rate of depressive disorders in 1990 and 2021, and changes from 1990 to 2021 at the global level and different regions. 474
Table S3: The incidence, prevalence and DALYs rate of anxiety disorders in 1990 and 2021, and changes from 1990 to 2021 at 204 countries and territories around the world. 476
Table S4: The incidence, prevalence and DALYs rate of depressive disorders in 1990 and 2021, and changes from 1990 to 2021 at 204 countries and territories around the world. 493
Table S5: The incidence, prevalence and DALYs rate of anxiety and depressive disorders in 1990 and 2021, and changes from 1990 to 2021 in Brazil. 512
Table S6: The incidence, prevalence and DALYs rate of anxiety and depressive disorders in 1990 and 2021, and changes from 1990 to 2021 in China. 517
Table S7: The incidence, prevalence and DALYs rate of anxiety and depressive disorders in 1990 and 2021, and changes from 1990 to 2021 in Ethiopia. 523
Table S8: The incidence, prevalence and DALYs rate of anxiety and depressive disorders in 1990 and 2021, and changes from 1990 to 2021 in Indonesia. 525
Table S9: The incidence, prevalence and DALYs rate of anxiety and depressive disorders in 1990 and 2021, and changes from 1990 to 2021 in Iran (Islamic Republic of). 531
Table S10: The incidence, prevalence and DALYs rate of anxiety and depressive disorders in 1990 and 2021, and changes from 1990 to 2021 in Italy. 536
Table S11: The incidence, prevalence and DALYs rate of anxiety and depressive disorders in 1990 and 2021, and changes from 1990 to 2021 in Japan. 540
Table S12: The incidence, prevalence and DALYs rate of anxiety and depressive disorders in 1990 and 2021, and changes from 1990 to 2021 in Kenya. 548
Table S13: The incidence, prevalence and DALYs rate of anxiety and depressive disorders in 1990 and 2021, and changes from 1990 to 2021 in Mexico. 556
Table S14: The incidence, prevalence and DALYs rate of anxiety and depressive disorders in 1990 and 2021, and changes from 1990 to 2021 in Nigeria. 561
Table S15: The incidence, prevalence and DALYs rate of anxiety and depressive disorders in 1990 and 2021, and changes from 1990 to 2021 in Norway. 568
Table S16: The incidence, prevalence and DALYs rate of anxiety and depressive disorders in 1990 and 2021, and changes from 1990 to 2021 in Pakistan. 570
Table S17: The incidence, prevalence and DALYs rate of anxiety and depressive disorders in 1990 and 2021, and changes from 1990 to 2021 in Philippines. 571

Table S18: The incidence, prevalence and DALYs rate of anxiety and depressive disorders in 1990 and 2021, and changes from 1990 to 2021 in Poland. 585
Table S19: The incidence, prevalence and DALYs rate of anxiety and depressive disorders in 1990 and 2021, and changes from 1990 to 2021 in South Africa. 588
Table S20: The incidence, prevalence and DALYs rate of anxiety and depressive disorders in 1990 and 2021, and changes from 1990 to 2021 in Sweden. 589
Table S21: The incidence, prevalence and DALYs rate of anxiety and depressive disorders in 1990 and 2021, and changes from 1990 to 2021 in United Kingdom. 590
Table S22: The incidence, prevalence and DALYs rate of anxiety and depressive disorders in 1990 and 2021, and changes from 1990 to 2021 in United States of America. 624
Table S23: The incidence, prevalence and DALYs rate of dysthymia in 1990 and 2021, and changes from 1990 to 2021 at the global level and different regions. 633
Table S24: The incidence, prevalence and DALYs rate of dysthymia in 1990 and 2021, and changes from 1990 to 2021 at 204 countries and territories around the world. 635

Figure S1

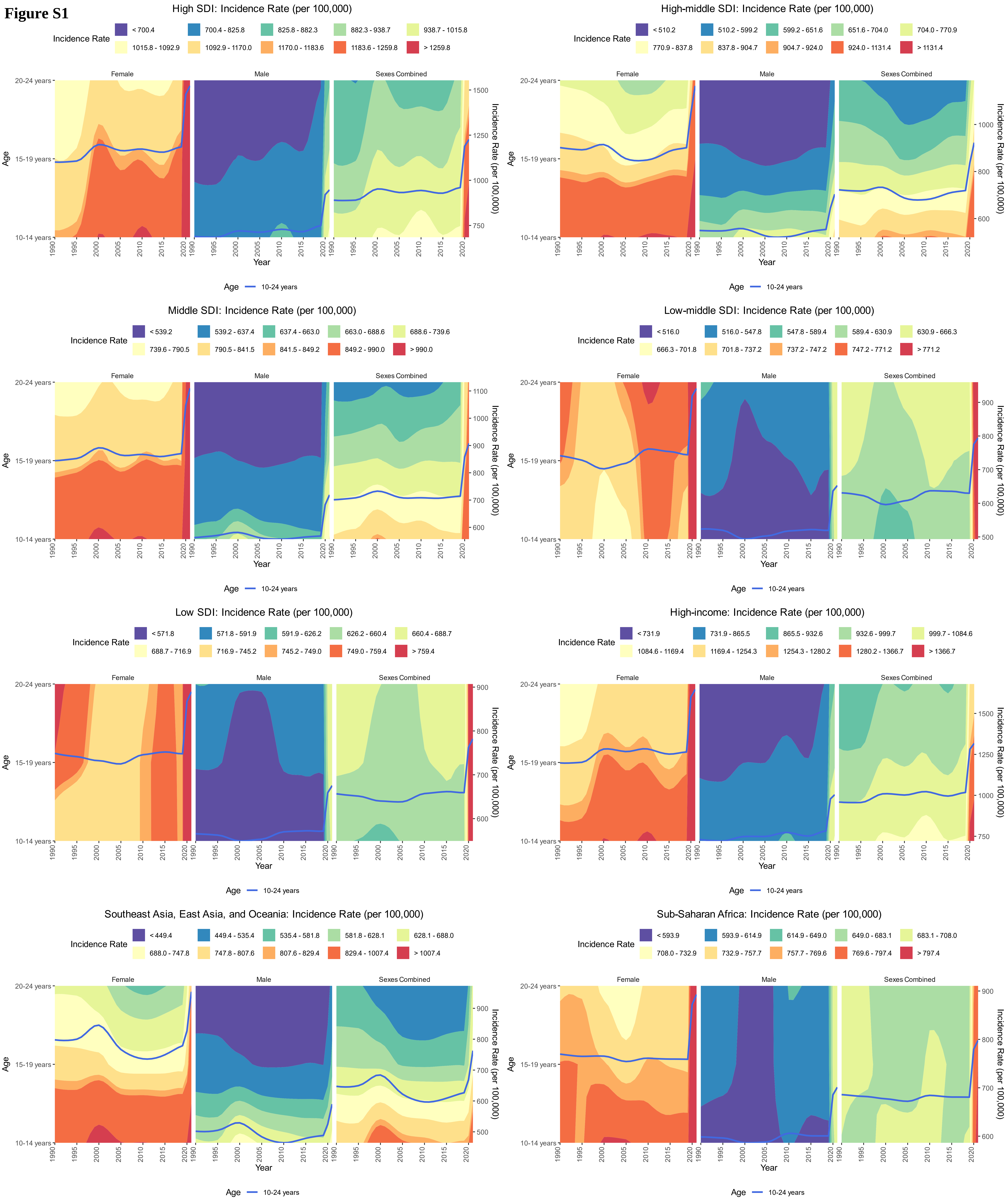
High SDI: Incidence Rate (per 100,000)
Incidence Rate
< 700.4
700.4 - 825.8
825.8 - 882.3
882.3 - 938.7
938.7 - 1015.8
1015.8 - 1092.9
1092.9 - 1170.0
1170.0 - 1183.6
1183.6 - 1259.8
> 1259.8
High-middle SDI: Incidence Rate (per 100,000)
< 510.2
510.2 - 599.2
599.2 - 651.6
651.6 - 704.0
704.0 - 770.9
770.9 - 837.8
837.8 - 904.7
904.7 - 924.0
924.0 - 1131.4
> 1131.4
Middle SDI: Incidence Rate (per 100,000)
< 539.2
539.2 - 637.4
637.4 - 663.0
663.0 - 688.6
688.6 - 739.6
739.6 - 790.5
790.5 - 841.5
841.5 - 849.2
849.2 - 990.0
> 990.0
Low-middle SDI: Incidence Rate (per 100,000)
< 516.0
516.0 - 547.8
547.8 - 589.4
589.4 - 630.9
630.9 - 666.3
666.3 - 701.8
701.8 - 737.2
737.2 - 747.2
747.2 - 771.2
> 771.2
Low SDI: Incidence Rate (per 100,000)
< 571.8
571.8 - 591.9
591.9 - 626.2
626.2 - 660.4
660.4 - 688.7
688.7 - 716.9
716.9 - 745.2
745.2 - 749.0
749.0 - 759.4
> 759.4
High-income: Incidence Rate (per 100,000)
< 731.9
731.9 - 865.5
865.5 - 932.6
932.6 - 999.7
999.7 - 1084.6
1084.6 - 1169.4
1169.4 - 1254.3
1254.3 - 1280.2
1280.2 - 1366.7
> 1366.7
Southeast Asia, East Asia, and Oceania: Incidence Rate (per 100,000)
< 449.4
449.4 - 535.4
535.4 - 581.8
581.8 - 628.1
628.1 - 688.0
688.0 - 747.8
747.8 - 807.6
807.6 - 829.4
829.4 - 1007.4
> 1007.4
Sub-Saharan Africa: Incidence Rate (per 100,000)
< 593.9
593.9 - 614.9
614.9 - 649.0
649.0 - 683.1
683.1 - 708.0
708.0 - 732.9
732.9 - 757.7
757.7 - 769.6
769.6 - 797.4
> 797.4
Female
Male
Sexes Combined
Age
20-24 years
15-19 years
10-14 years
Year
Incidence Rate (per 100,000)
Age — 10-24 years

Figure S1

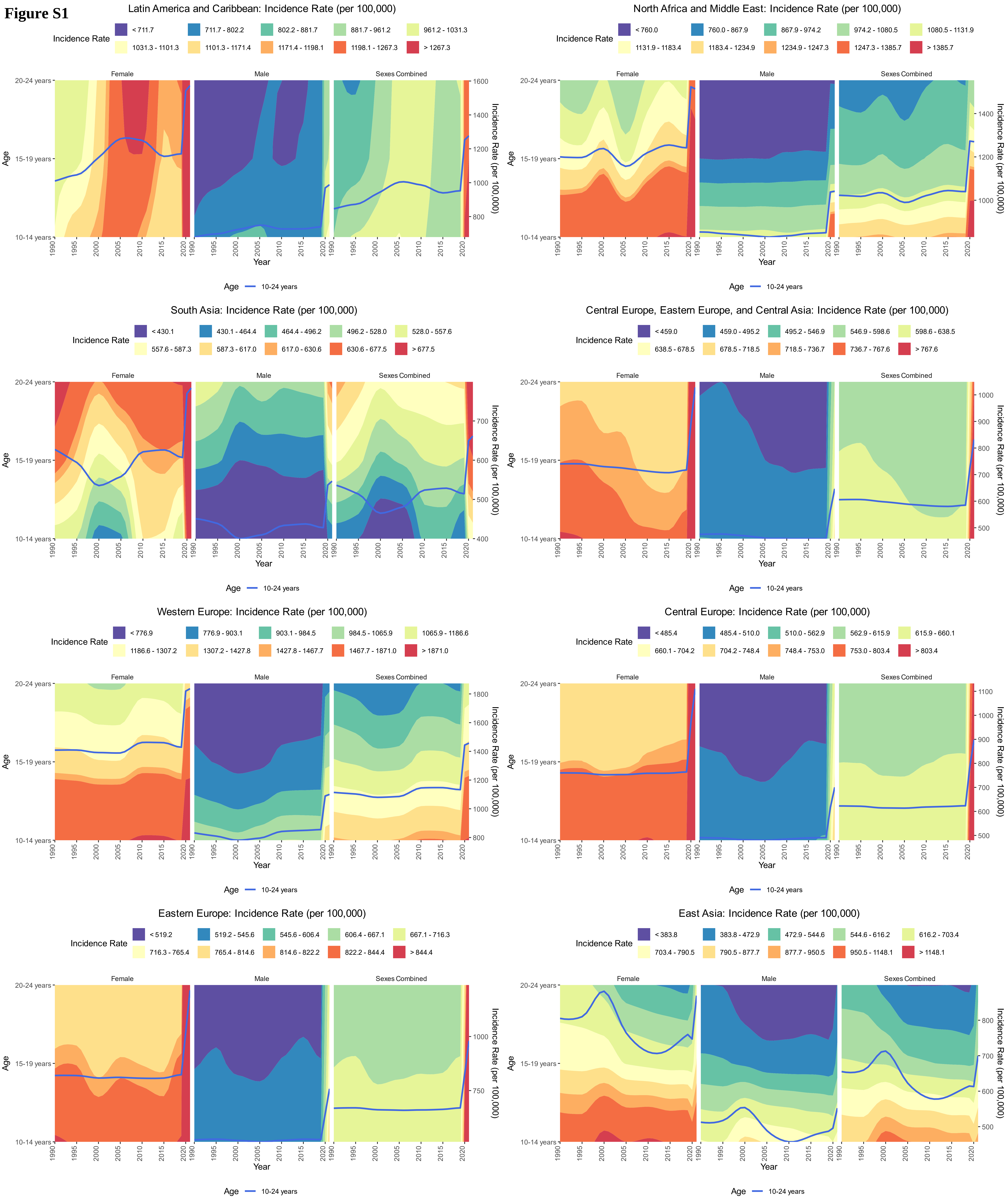
Latin America and Caribbean: Incidence Rate (per 100,000)
Incidence Rate
< 711.7
711.7 - 802.2
802.2 - 881.7
881.7 - 961.2
961.2 - 1031.3
1031.3 - 1101.3
1101.3 - 1171.4
1171.4 - 1198.1
1198.1 - 1267.3
> 1267.3
North Africa and Middle East: Incidence Rate (per 100,000)
< 760.0
760.0 - 867.9
867.9 - 974.2
974.2 - 1080.5
1080.5 - 1131.9
1131.9 - 1183.4
1183.4 - 1234.9
1234.9 - 1247.3
1247.3 - 1385.7
> 1385.7
South Asia: Incidence Rate (per 100,000)
< 430.1
430.1 - 464.4
464.4 - 496.2
496.2 - 528.0
528.0 - 557.6
557.6 - 587.3
587.3 - 617.0
617.0 - 630.6
630.6 - 677.5
> 677.5
Central Europe, Eastern Europe, and Central Asia: Incidence Rate (per 100,000)
< 459.0
459.0 - 495.2
495.2 - 546.9
546.9 - 598.6
598.6 - 638.5
638.5 - 678.5
678.5 - 718.5
718.5 - 736.7
736.7 - 767.6
> 767.6
Western Europe: Incidence Rate (per 100,000)
< 776.9
776.9 - 903.1
903.1 - 984.5
984.5 - 1065.9
1065.9 - 1186.6
1186.6 - 1307.2
1307.2 - 1427.8
1427.8 - 1467.7
1467.7 - 1871.0
> 1871.0
Central Europe: Incidence Rate (per 100,000)
< 485.4
485.4 - 510.0
510.0 - 562.9
562.9 - 615.9
615.9 - 660.1
660.1 - 704.2
704.2 - 748.4
748.4 - 753.0
753.0 - 803.4
> 803.4
Eastern Europe: Incidence Rate (per 100,000)
< 519.2
519.2 - 545.6
545.6 - 606.4
606.4 - 667.1
667.1 - 716.3
716.3 - 765.4
765.4 - 814.6
814.6 - 822.2
822.2 - 844.4
> 844.4
East Asia: Incidence Rate (per 100,000)
< 383.8
383.8 - 472.9
472.9 - 544.6
544.6 - 616.2
616.2 - 703.4
703.4 - 790.5
790.5 - 877.7
877.7 - 950.5
950.5 - 1148.1
> 1148.1
Female
Male
Sexes Combined
Age
20-24 years
15-19 years
10-14 years
Year
Incidence Rate (per 100,000)
Age
10-24 years

Figure S1

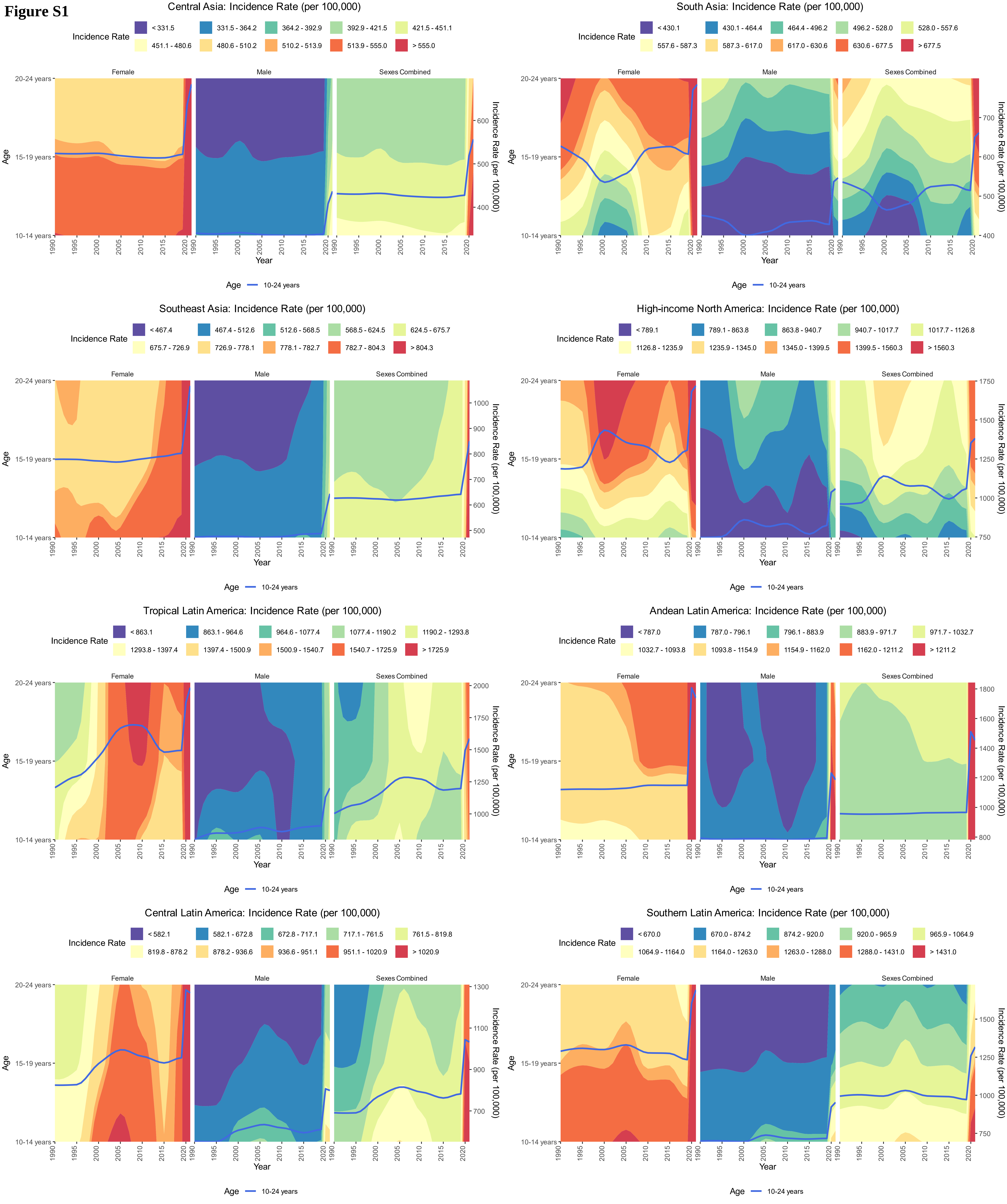
Central Asia: Incidence Rate (per 100,000)
Incidence Rate
< 331.5
331.5 - 364.2
364.2 - 392.9
392.9 - 421.5
421.5 - 451.1
451.1 - 480.6
480.6 - 510.2
510.2 - 513.9
513.9 - 555.0
> 555.0
South Asia: Incidence Rate (per 100,000)
< 430.1
430.1 - 464.4
464.4 - 496.2
496.2 - 528.0
528.0 - 557.6
557.6 - 587.3
587.3 - 617.0
617.0 - 630.6
630.6 - 677.5
> 677.5
Southeast Asia: Incidence Rate (per 100,000)
< 467.4
467.4 - 512.6
512.6 - 568.5
568.5 - 624.5
624.5 - 675.7
675.7 - 726.9
726.9 - 778.1
778.1 - 782.7
782.7 - 804.3
> 804.3
High-income North America: Incidence Rate (per 100,000)
< 789.1
789.1 - 863.8
863.8 - 940.7
940.7 - 1017.7
1017.7 - 1126.8
1126.8 - 1235.9
1235.9 - 1345.0
1345.0 - 1399.5
1399.5 - 1560.3
> 1560.3
Tropical Latin America: Incidence Rate (per 100,000)
< 863.1
863.1 - 964.6
964.6 - 1077.4
1077.4 - 1190.2
1190.2 - 1293.8
1293.8 - 1397.4
1397.4 - 1500.9
1500.9 - 1540.7
1540.7 - 1725.9
> 1725.9
Andean Latin America: Incidence Rate (per 100,000)
< 787.0
787.0 - 796.1
796.1 - 883.9
883.9 - 971.7
971.7 - 1032.7
1032.7 - 1093.8
1093.8 - 1154.9
1154.9 - 1162.0
1162.0 - 1211.2
> 1211.2
Central Latin America: Incidence Rate (per 100,000)
< 582.1
582.1 - 672.8
672.8 - 717.1
717.1 - 761.5
761.5 - 819.8
819.8 - 878.2
878.2 - 936.6
936.6 - 951.1
951.1 - 1020.9
> 1020.9
Southern Latin America: Incidence Rate (per 100,000)
< 670.0
670.0 - 874.2
874.2 - 920.0
920.0 - 965.9
965.9 - 1064.9
1064.9 - 1164.0
1164.0 - 1263.0
1263.0 - 1288.0
1288.0 - 1431.0
> 1431.0
Female
Male
Sexes Combined
Age
20-24 years
15-19 years
10-14 years
Year
Incidence Rate (per 100,000)
Age
10-24 years

Figure S1

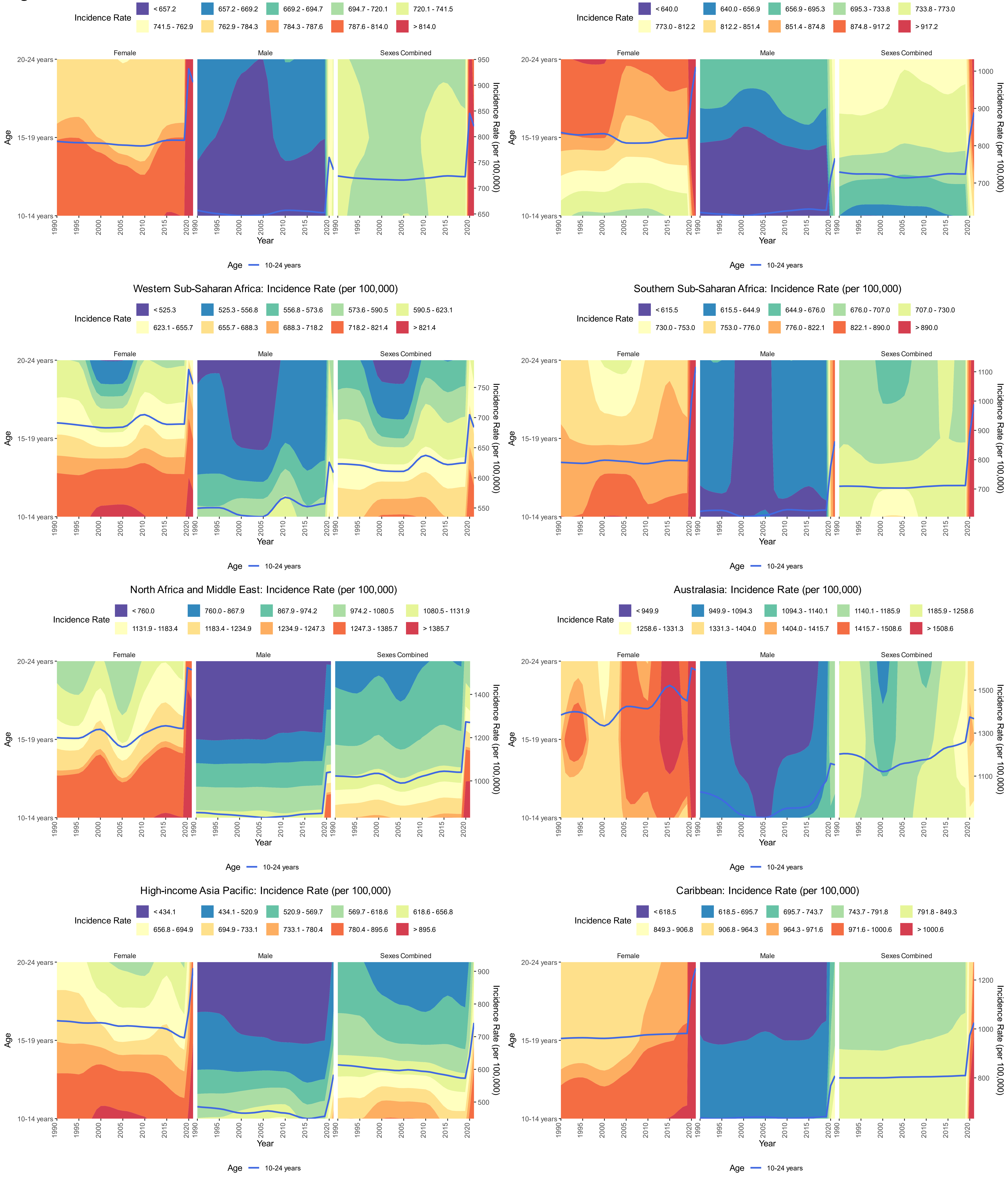
Central Sub-Saharan Africa: Incidence Rate (per 100,000)
Incidence Rate
< 657.2
657.2 - 669.2
669.2 - 694.7
694.7 - 720.1
720.1 - 741.5
741.5 - 762.9
762.9 - 784.3
784.3 - 787.6
787.6 - 814.0
> 814.0
Eastern Sub-Saharan Africa: Incidence Rate (per 100,000)
Incidence Rate
< 640.0
640.0 - 656.9
656.9 - 695.3
695.3 - 733.8
733.8 - 773.0
773.0 - 812.2
812.2 - 851.4
851.4 - 874.8
874.8 - 917.2
> 917.2
Western Sub-Saharan Africa: Incidence Rate (per 100,000)
Incidence Rate
< 525.3
525.3 - 556.8
556.8 - 573.6
573.6 - 590.5
590.5 - 623.1
623.1 - 655.7
655.7 - 688.3
688.3 - 718.2
718.2 - 821.4
> 821.4
Southern Sub-Saharan Africa: Incidence Rate (per 100,000)
Incidence Rate
< 615.5
615.5 - 644.9
644.9 - 676.0
676.0 - 707.0
707.0 - 730.0
730.0 - 753.0
753.0 - 776.0
776.0 - 822.1
822.1 - 890.0
> 890.0
North Africa and Middle East: Incidence Rate (per 100,000)
Incidence Rate
< 760.0
760.0 - 867.9
867.9 - 974.2
974.2 - 1080.5
1080.5 - 1131.9
1131.9 - 1183.4
1183.4 - 1234.9
1234.9 - 1247.3
1247.3 - 1385.7
> 1385.7
Australasia: Incidence Rate (per 100,000)
Incidence Rate
< 949.9
949.9 - 1094.3
1094.3 - 1140.1
1140.1 - 1185.9
1185.9 - 1258.6
1258.6 - 1331.3
1331.3 - 1404.0
1404.0 - 1415.7
1415.7 - 1508.6
> 1508.6
High-income Asia Pacific: Incidence Rate (per 100,000)
Incidence Rate
< 434.1
434.1 - 520.9
520.9 - 569.7
569.7 - 618.6
618.6 - 656.8
656.8 - 694.9
694.9 - 733.1
733.1 - 780.4
780.4 - 895.6
> 895.6
Caribbean: Incidence Rate (per 100,000)
Incidence Rate
< 618.5
618.5 - 695.7
695.7 - 743.7
743.7 - 791.8
791.8 - 849.3
849.3 - 906.8
906.8 - 964.3
964.3 - 971.6
971.6 - 1000.6
> 1000.6
Female
Male
Sexes Combined
Age
20-24 years
15-19 years
10-14 years
Year
Incidence Rate (per 100,000)
Age
10-24 years

Figure S1

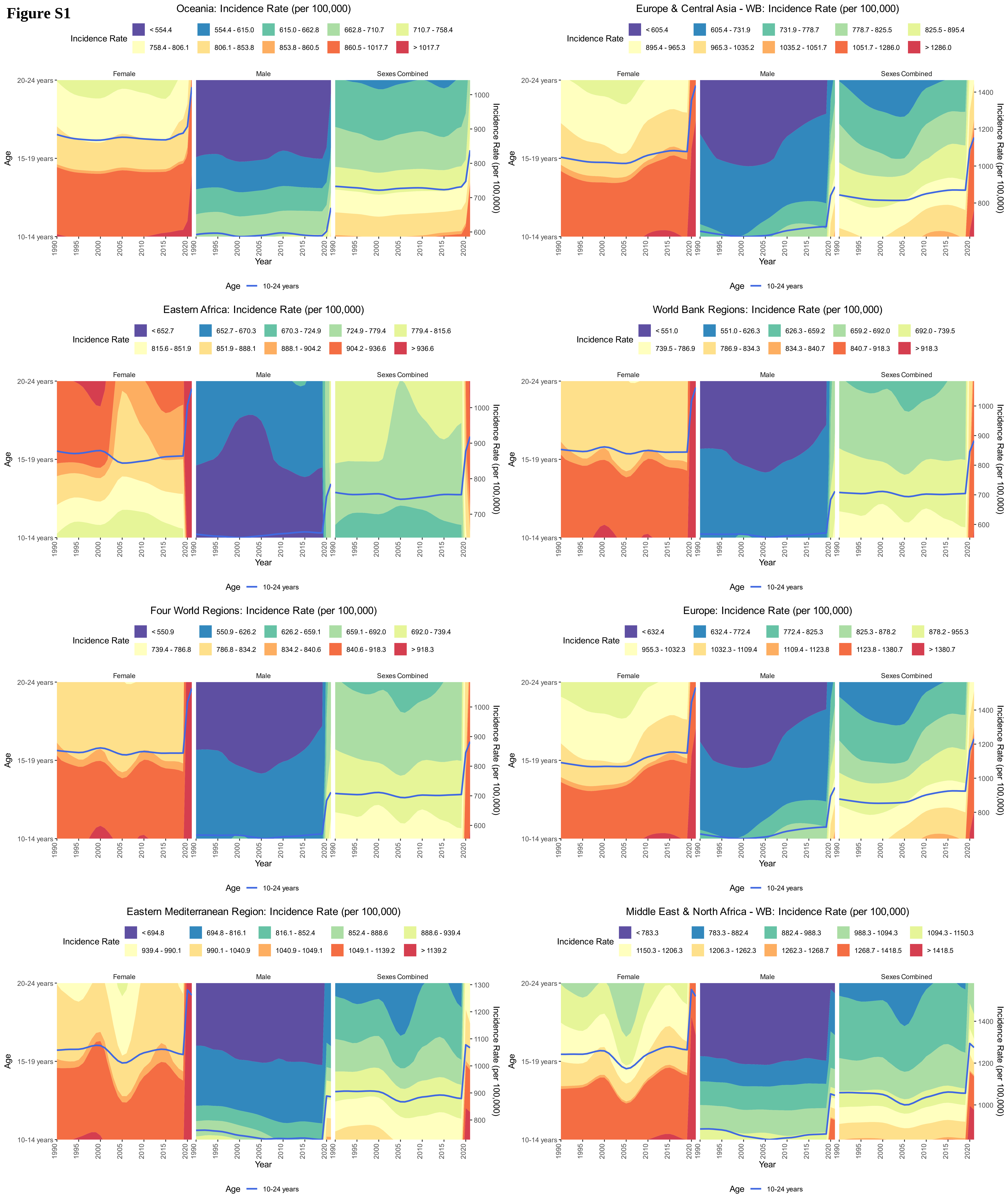
Oceania: Incidence Rate (per 100,000)
Incidence Rate
< 554.4
554.4 - 615.0
615.0 - 662.8
662.8 - 710.7
710.7 - 758.4
758.4 - 806.1
806.1 - 853.8
853.8 - 860.5
860.5 - 1017.7
> 1017.7
Europe & Central Asia - WB: Incidence Rate (per 100,000)
< 605.4
605.4 - 731.9
731.9 - 778.7
778.7 - 825.5
825.5 - 895.4
895.4 - 965.3
965.3 - 1035.2
1035.2 - 1051.7
1051.7 - 1286.0
> 1286.0
Eastern Africa: Incidence Rate (per 100,000)
< 652.7
652.7 - 670.3
670.3 - 724.9
724.9 - 779.4
779.4 - 815.6
815.6 - 851.9
851.9 - 888.1
888.1 - 904.2
904.2 - 936.6
> 936.6
World Bank Regions: Incidence Rate (per 100,000)
< 551.0
551.0 - 626.3
626.3 - 659.2
659.2 - 692.0
692.0 - 739.5
739.5 - 786.9
786.9 - 834.3
834.3 - 840.7
840.7 - 918.3
> 918.3
Four World Regions: Incidence Rate (per 100,000)
< 550.9
550.9 - 626.2
626.2 - 659.1
659.1 - 692.0
692.0 - 739.4
739.4 - 786.8
786.8 - 834.2
834.2 - 840.6
840.6 - 918.3
> 918.3
Europe: Incidence Rate (per 100,000)
< 632.4
632.4 - 772.4
772.4 - 825.3
825.3 - 878.2
878.2 - 955.3
955.3 - 1032.3
1032.3 - 1109.4
1109.4 - 1123.8
1123.8 - 1380.7
> 1380.7
Eastern Mediterranean Region: Incidence Rate (per 100,000)
< 694.8
694.8 - 816.1
816.1 - 852.4
852.4 - 888.6
888.6 - 939.4
939.4 - 990.1
990.1 - 1040.9
1040.9 - 1049.1
1049.1 - 1139.2
> 1139.2
Middle East & North Africa - WB: Incidence Rate (per 100,000)
< 783.3
783.3 - 882.4
882.4 - 988.3
988.3 - 1094.3
1094.3 - 1150.3
1150.3 - 1206.3
1206.3 - 1262.3
1262.3 - 1268.7
1268.7 - 1418.5
> 1418.5
Female
Male
Sexes Combined
20-24 years
15-19 years
10-14 years
Age
Year
Incidence Rate (per 100,000)
Age — 10-24 years

Figure S1

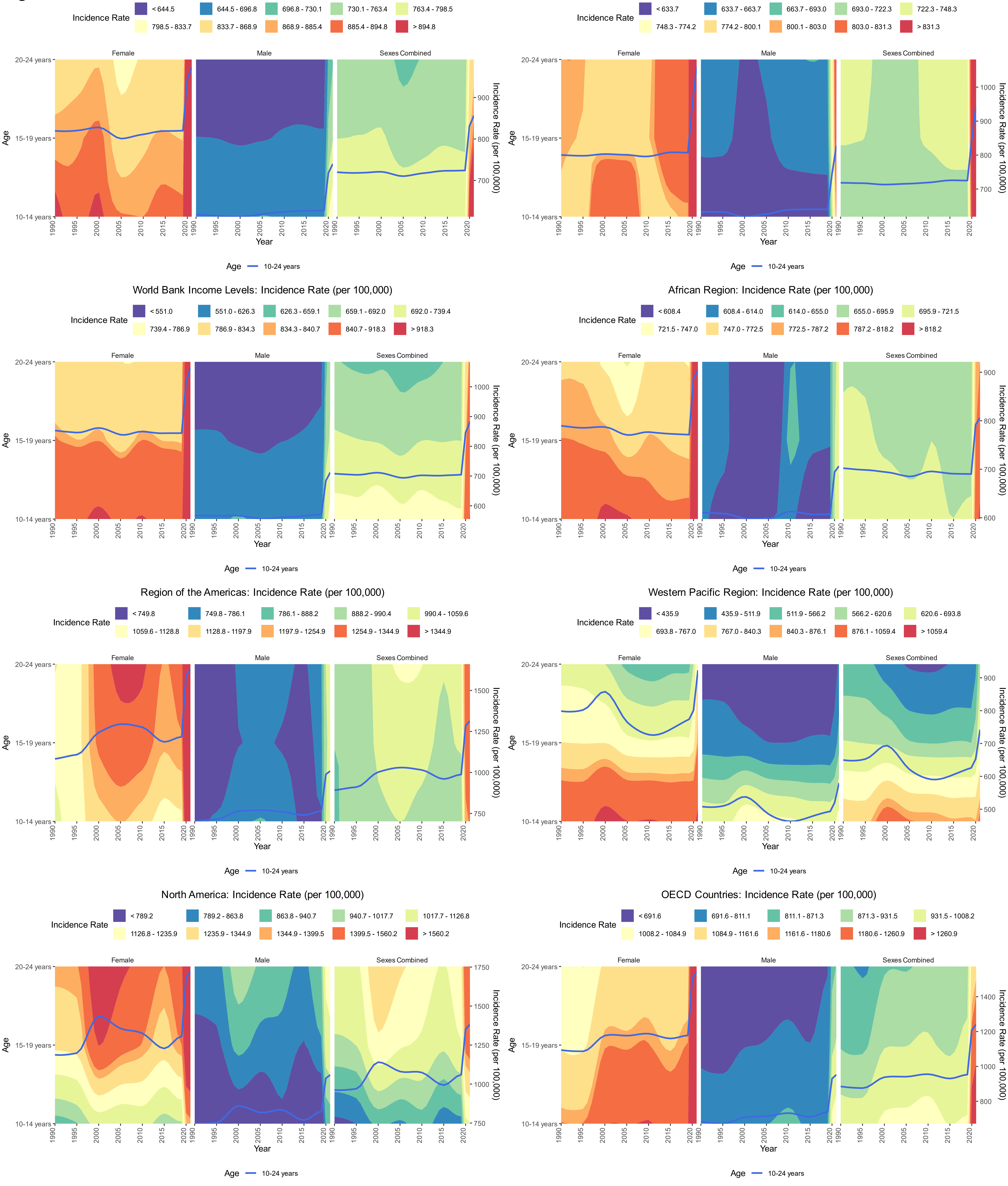
World Bank Low Income: Incidence Rate (per 100,000)
Incidence Rate
< 644.5
644.5 - 696.8
696.8 - 730.1
730.1 - 763.4
763.4 - 798.5
798.5 - 833.7
833.7 - 868.9
868.9 - 885.4
885.4 - 894.8
> 894.8
Female
Male
Sexes Combined
Age
20-24 years
15-19 years
10-14 years
Year
Incidence Rate (per 100,000)
Age
10-24 years
Southern Africa: Incidence Rate (per 100,000)
Incidence Rate
< 633.7
633.7 - 663.7
663.7 - 693.0
693.0 - 722.3
722.3 - 748.3
748.3 - 774.2
774.2 - 800.1
800.1 - 803.0
803.0 - 831.3
> 831.3
World Bank Income Levels: Incidence Rate (per 100,000)
Incidence Rate
< 551.0
551.0 - 626.3
626.3 - 659.1
659.1 - 692.0
692.0 - 739.4
739.4 - 786.9
786.9 - 834.3
834.3 - 840.7
840.7 - 918.3
> 918.3
African Region: Incidence Rate (per 100,000)
Incidence Rate
< 608.4
608.4 - 614.0
614.0 - 655.0
655.0 - 695.9
695.9 - 721.5
721.5 - 747.0
747.0 - 772.5
772.5 - 787.2
787.2 - 818.2
> 818.2
Region of the Americas: Incidence Rate (per 100,000)
Incidence Rate
< 749.8
749.8 - 786.1
786.1 - 888.2
888.2 - 990.4
990.4 - 1059.6
1059.6 - 1128.8
1128.8 - 1197.9
1197.9 - 1254.9
1254.9 - 1344.9
> 1344.9
Western Pacific Region: Incidence Rate (per 100,000)
Incidence Rate
< 435.9
435.9 - 511.9
511.9 - 566.2
566.2 - 620.6
620.6 - 693.8
693.8 - 767.0
767.0 - 840.3
840.3 - 876.1
876.1 - 1059.4
> 1059.4
North America: Incidence Rate (per 100,000)
Incidence Rate
< 789.2
789.2 - 863.8
863.8 - 940.7
940.7 - 1017.7
1017.7 - 1126.8
1126.8 - 1235.9
1235.9 - 1344.9
1344.9 - 1399.5
1399.5 - 1560.2
> 1560.2
OECD Countries: Incidence Rate (per 100,000)
Incidence Rate
< 691.6
691.6 - 811.1
811.1 - 871.3
871.3 - 931.5
931.5 - 1008.2
1008.2 - 1084.9
1084.9 - 1161.6
1161.6 - 1180.6
1180.6 - 1260.9
> 1260.9

Figure S1

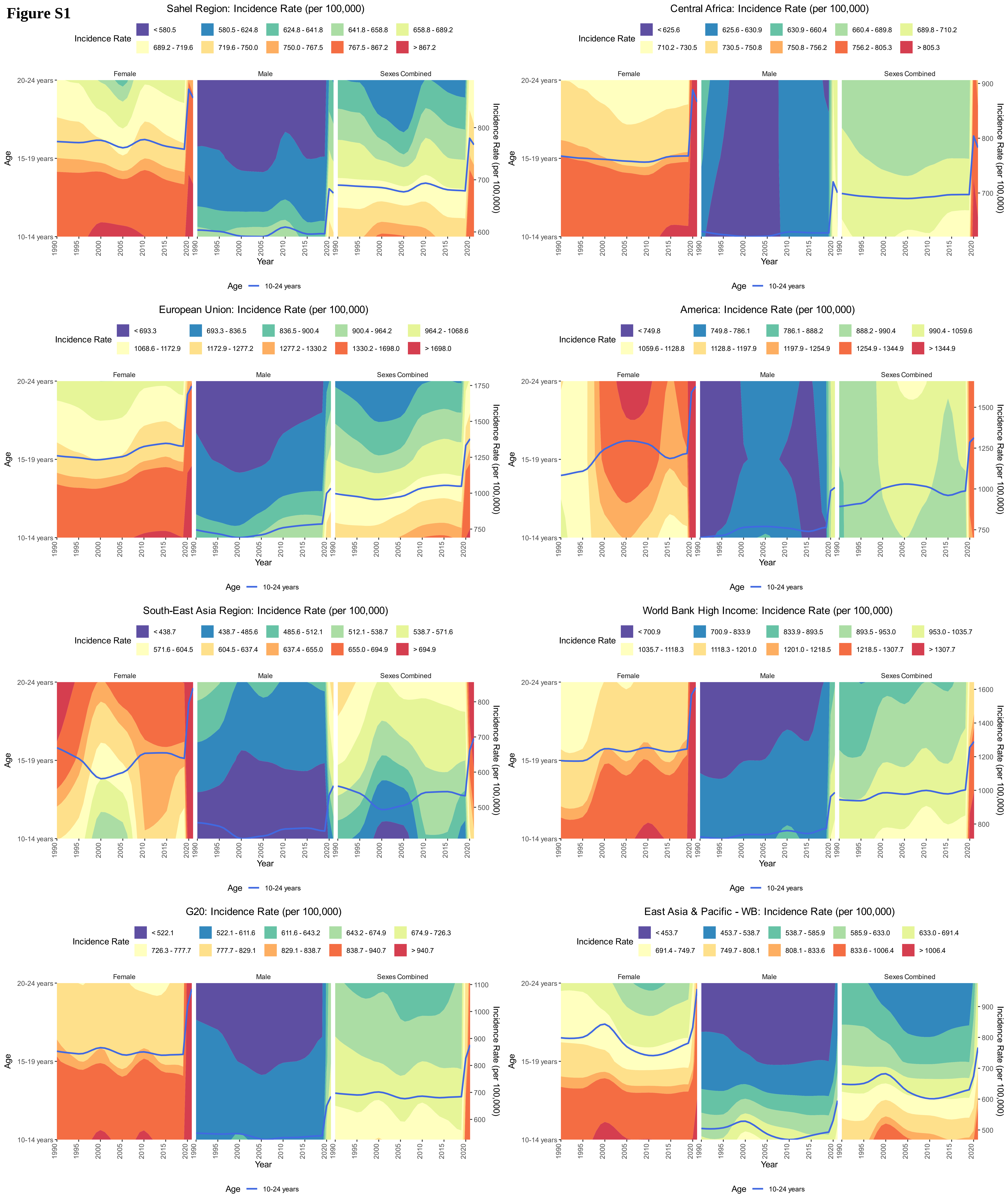

Sahel Region: Incidence Rate (per 100,000)
Incidence Rate
< 580.5
580.5 - 624.8
624.8 - 641.8
641.8 - 658.8
658.8 - 689.2
689.2 - 719.6
719.6 - 750.0
750.0 - 767.5
767.5 - 867.2
> 867.2
Female
Male
Sexes Combined
Age
Year
Incidence Rate (per 100,000)
Age — 10-24 years
Central Africa: Incidence Rate (per 100,000)
Incidence Rate
< 625.6
625.6 - 630.9
630.9 - 660.4
660.4 - 689.8
689.8 - 710.2
710.2 - 730.5
730.5 - 750.8
750.8 - 756.2
756.2 - 805.3
> 805.3
European Union: Incidence Rate (per 100,000)
Incidence Rate
< 693.3
693.3 - 836.5
836.5 - 900.4
900.4 - 964.2
964.2 - 1068.6
1068.6 - 1172.9
1172.9 - 1277.2
1277.2 - 1330.2
1330.2 - 1698.0
> 1698.0
America: Incidence Rate (per 100,000)
Incidence Rate
< 749.8
749.8 - 786.1
786.1 - 888.2
888.2 - 990.4
990.4 - 1059.6
1059.6 - 1128.8
1128.8 - 1197.9
1197.9 - 1254.9
1254.9 - 1344.9
> 1344.9
South-East Asia Region: Incidence Rate (per 100,000)
Incidence Rate
< 438.7
438.7 - 485.6
485.6 - 512.1
512.1 - 538.7
538.7 - 571.6
571.6 - 604.5
604.5 - 637.4
637.4 - 655.0
655.0 - 694.9
> 694.9
World Bank High Income: Incidence Rate (per 100,000)
Incidence Rate
< 700.9
700.9 - 833.9
833.9 - 893.5
893.5 - 953.0
953.0 - 1035.7
1035.7 - 1118.3
1118.3 - 1201.0
1201.0 - 1218.5
1218.5 - 1307.7
> 1307.7
G20: Incidence Rate (per 100,000)
Incidence Rate
< 522.1
522.1 - 611.6
611.6 - 643.2
643.2 - 674.9
674.9 - 726.3
726.3 - 777.7
777.7 - 829.1
829.1 - 838.7
838.7 - 940.7
> 940.7
East Asia & Pacific - WB: Incidence Rate (per 100,000)
Incidence Rate
< 453.7
453.7 - 538.7
538.7 - 585.9
585.9 - 633.0
633.0 - 691.4
691.4 - 749.7
749.7 - 808.1
808.1 - 833.6
833.6 - 1006.4
> 1006.4
20-24 years
15-19 years
10-14 years
1990
1995
2000
2005
2010
2015
2020

Figure S1

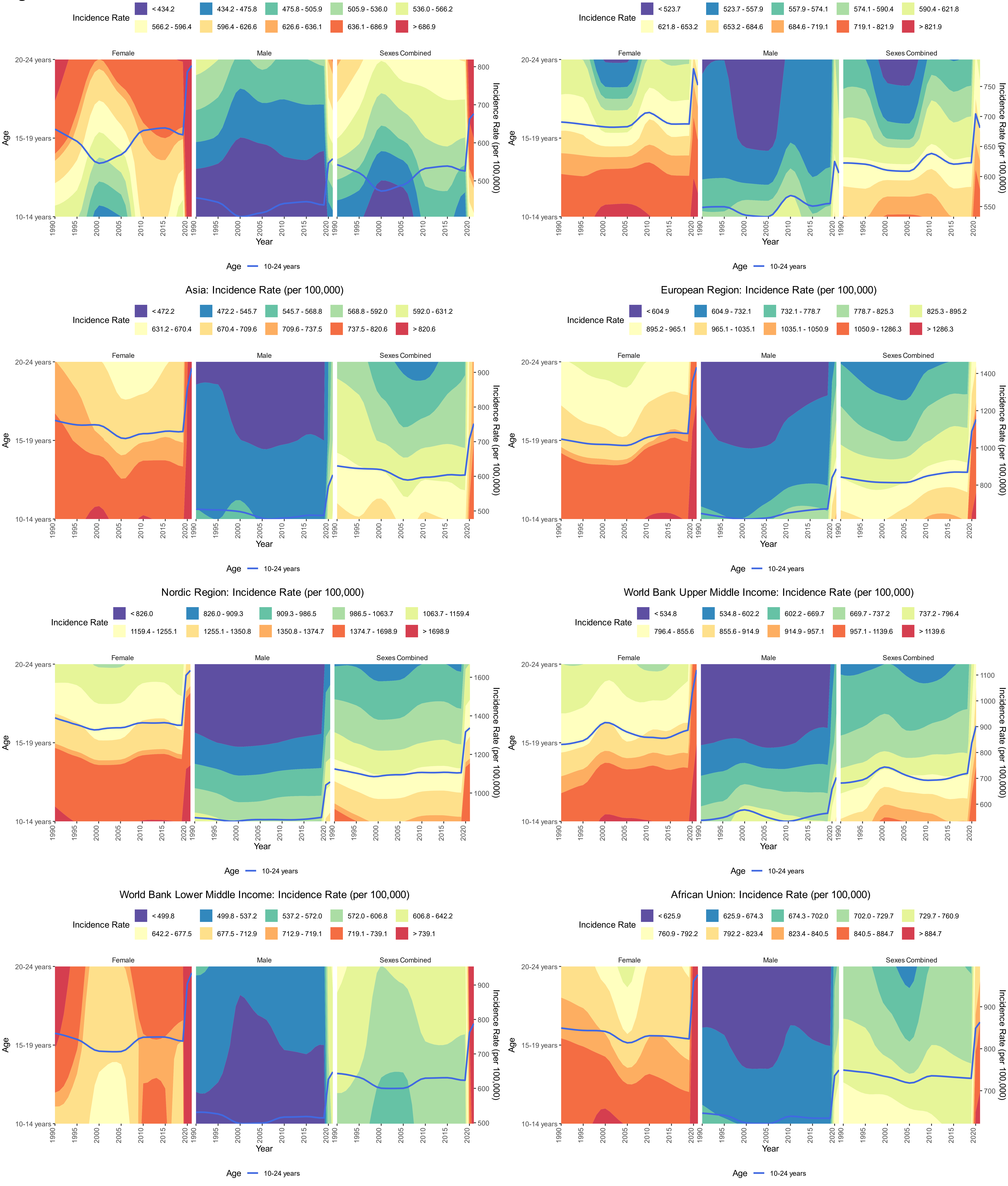
South Asia - WB: Incidence Rate (per 100,000)
Incidence Rate
< 434.2
434.2 - 475.8
475.8 - 505.9
505.9 - 536.0
536.0 - 566.2
566.2 - 596.4
596.4 - 626.6
626.6 - 636.1
636.1 - 686.9
> 686.9
Western Africa: Incidence Rate (per 100,000)
Incidence Rate
< 523.7
523.7 - 557.9
557.9 - 574.1
574.1 - 590.4
590.4 - 621.8
621.8 - 653.2
653.2 - 684.6
684.6 - 719.1
719.1 - 821.9
> 821.9
Asia: Incidence Rate (per 100,000)
Incidence Rate
< 472.2
472.2 - 545.7
545.7 - 568.8
568.8 - 592.0
592.0 - 631.2
631.2 - 670.4
670.4 - 709.6
709.6 - 737.5
737.5 - 820.6
> 820.6
European Region: Incidence Rate (per 100,000)
Incidence Rate
< 604.9
604.9 - 732.1
732.1 - 778.7
778.7 - 825.3
825.3 - 895.2
895.2 - 965.1
965.1 - 1035.1
1035.1 - 1050.9
1050.9 - 1286.3
> 1286.3
Nordic Region: Incidence Rate (per 100,000)
Incidence Rate
< 826.0
826.0 - 909.3
909.3 - 986.5
986.5 - 1063.7
1063.7 - 1159.4
1159.4 - 1255.1
1255.1 - 1350.8
1350.8 - 1374.7
1374.7 - 1698.9
> 1698.9
World Bank Upper Middle Income: Incidence Rate (per 100,000)
Incidence Rate
< 534.8
534.8 - 602.2
602.2 - 669.7
669.7 - 737.2
737.2 - 796.4
796.4 - 855.6
855.6 - 914.9
914.9 - 957.1
957.1 - 1139.6
> 1139.6
World Bank Lower Middle Income: Incidence Rate (per 100,000)
Incidence Rate
< 499.8
499.8 - 537.2
537.2 - 572.0
572.0 - 606.8
606.8 - 642.2
642.2 - 677.5
677.5 - 712.9
712.9 - 719.1
719.1 - 739.1
> 739.1
African Union: Incidence Rate (per 100,000)
Incidence Rate
< 625.9
625.9 - 674.3
674.3 - 702.0
702.0 - 729.7
729.7 - 760.9
760.9 - 792.2
792.2 - 823.4
823.4 - 840.5
840.5 - 884.7
> 884.7
Female
Male
Sexes Combined
Age
20-24 years
15-19 years
10-14 years
Year
Incidence Rate (per 100,000)
Age
10-24 years

Figure S1

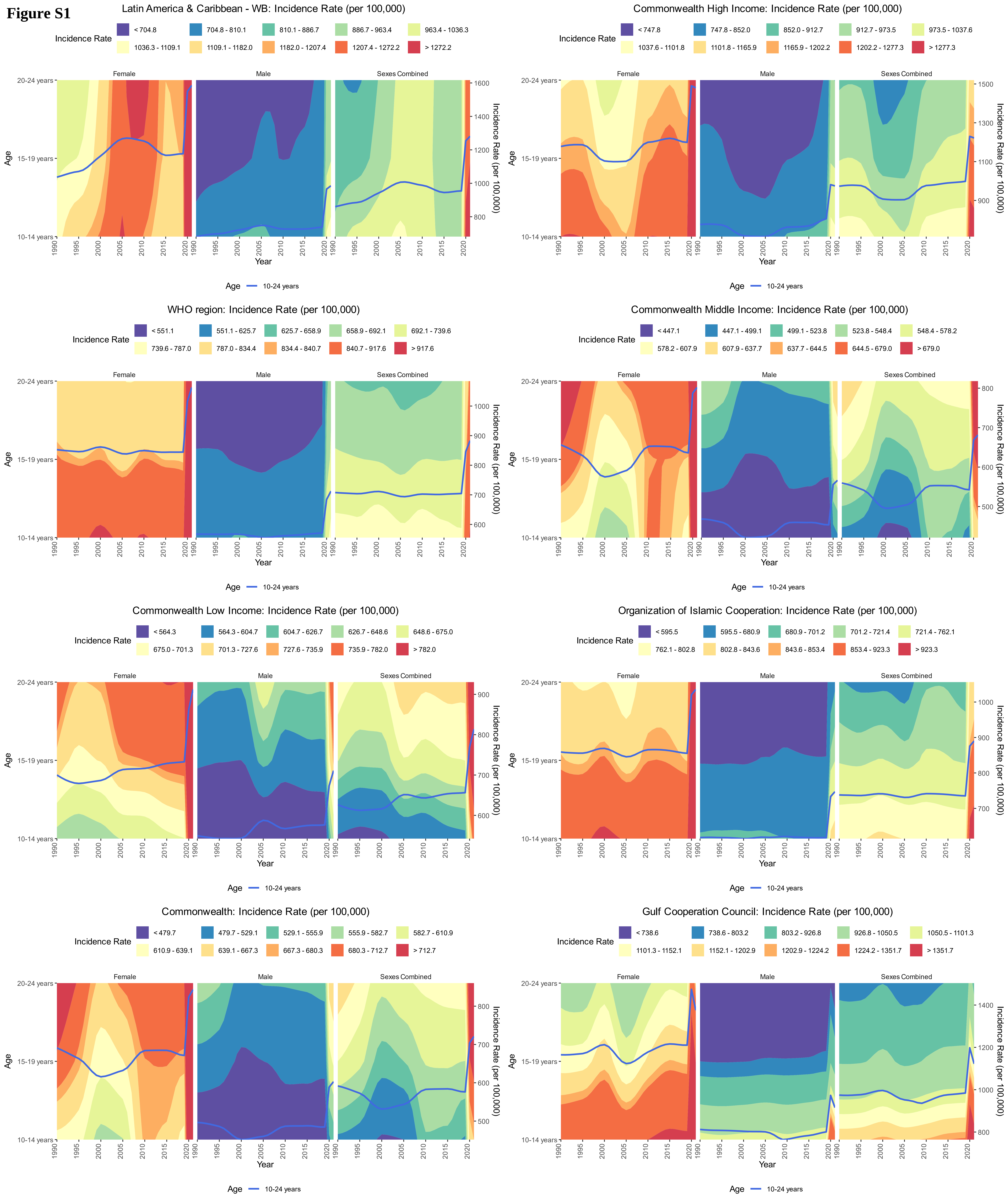

Latin America & Caribbean - WB: Incidence Rate (per 100,000)
Incidence Rate
< 704.8
704.8 - 810.1
810.1 - 886.7
886.7 - 963.4
963.4 - 1036.3
1036.3 - 1109.1
1109.1 - 1182.0
1182.0 - 1207.4
1207.4 - 1272.2
> 1272.2
Commonwealth High Income: Incidence Rate (per 100,000)
< 747.8
747.8 - 852.0
852.0 - 912.7
912.7 - 973.5
973.5 - 1037.6
1037.6 - 1101.8
1101.8 - 1165.9
1165.9 - 1202.2
1202.2 - 1277.3
> 1277.3
WHO region: Incidence Rate (per 100,000)
< 551.1
551.1 - 625.7
625.7 - 658.9
658.9 - 692.1
692.1 - 739.6
739.6 - 787.0
787.0 - 834.4
834.4 - 840.7
840.7 - 917.6
> 917.6
Commonwealth Middle Income: Incidence Rate (per 100,000)
< 447.1
447.1 - 499.1
499.1 - 523.8
523.8 - 548.4
548.4 - 578.2
578.2 - 607.9
607.9 - 637.7
637.7 - 644.5
644.5 - 679.0
> 679.0
Commonwealth Low Income: Incidence Rate (per 100,000)
< 564.3
564.3 - 604.7
604.7 - 626.7
626.7 - 648.6
648.6 - 675.0
675.0 - 701.3
701.3 - 727.6
727.6 - 735.9
735.9 - 782.0
> 782.0
Organization of Islamic Cooperation: Incidence Rate (per 100,000)
< 595.5
595.5 - 680.9
680.9 - 701.2
701.2 - 721.4
721.4 - 762.1
762.1 - 802.8
802.8 - 843.6
843.6 - 853.4
853.4 - 923.3
> 923.3
Commonwealth: Incidence Rate (per 100,000)
< 479.7
479.7 - 529.1
529.1 - 555.9
555.9 - 582.7
582.7 - 610.9
610.9 - 639.1
639.1 - 667.3
667.3 - 680.3
680.3 - 712.7
> 712.7
Gulf Cooperation Council: Incidence Rate (per 100,000)
< 738.6
738.6 - 803.2
803.2 - 926.8
926.8 - 1050.5
1050.5 - 1101.3
1101.3 - 1152.1
1152.1 - 1202.9
1202.9 - 1224.2
1224.2 - 1351.7
> 1351.7
Female
Male
Sexes Combined
Age
20-24 years
15-19 years
10-14 years
Year
Incidence Rate (per 100,000)
Age — 10-24 years

Figure S1

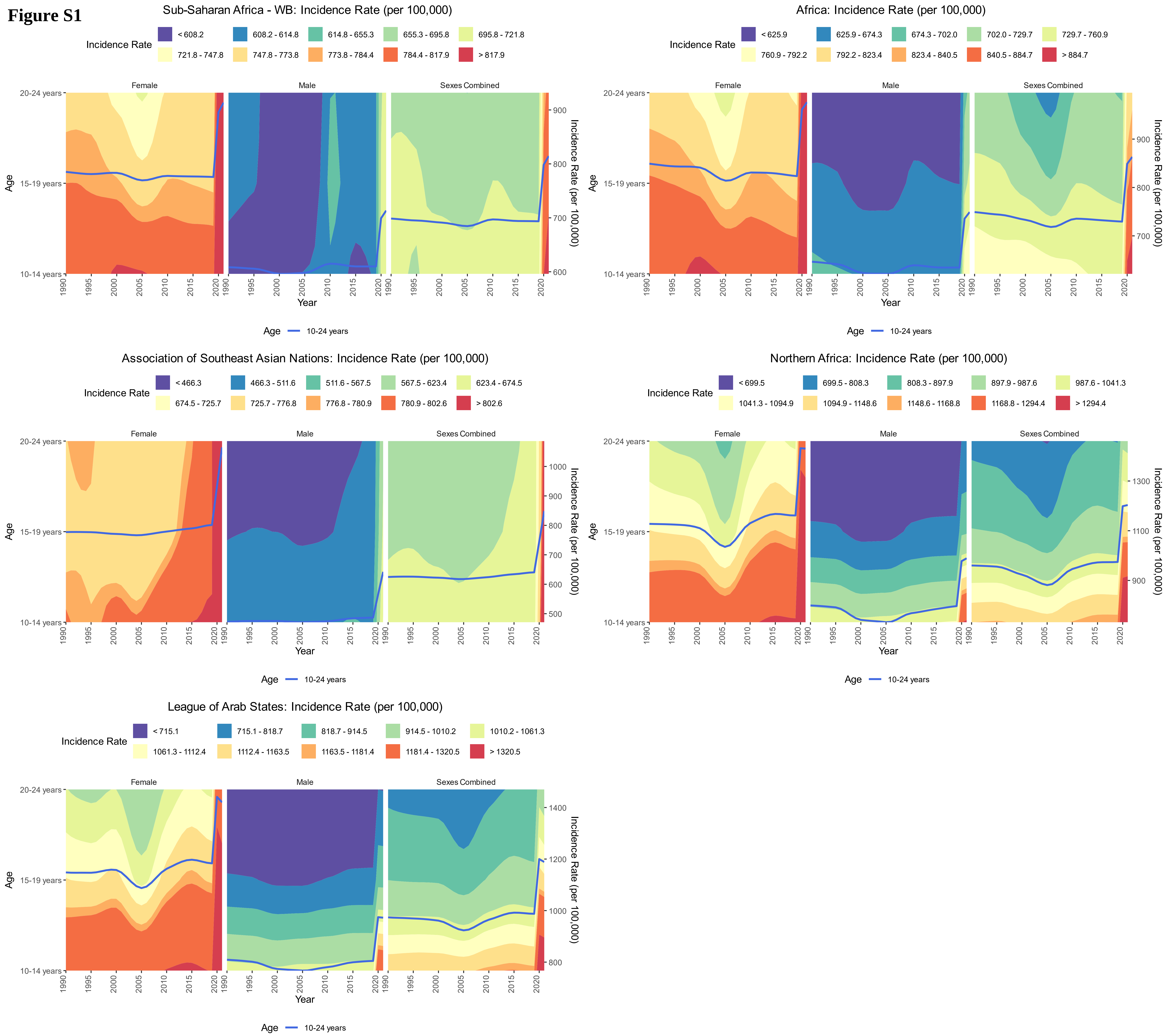

Sub-Saharan Africa - WB: Incidence Rate (per 100,000)
Incidence Rate
< 608.2
608.2 - 614.8
614.8 - 655.3
655.3 - 695.8
695.8 - 721.8
721.8 - 747.8
747.8 - 773.8
773.8 - 784.4
784.4 - 817.9
> 817.9
Female
Male
Sexes Combined
Age
Year
Incidence Rate (per 100,000)
Age
10-24 years
Africa: Incidence Rate (per 100,000)
Incidence Rate
< 625.9
625.9 - 674.3
674.3 - 702.0
702.0 - 729.7
729.7 - 760.9
760.9 - 792.2
792.2 - 823.4
823.4 - 840.5
840.5 - 884.7
> 884.7
Association of Southeast Asian Nations: Incidence Rate (per 100,000)
Incidence Rate
< 466.3
466.3 - 511.6
511.6 - 567.5
567.5 - 623.4
623.4 - 674.5
674.5 - 725.7
725.7 - 776.8
776.8 - 780.9
780.9 - 802.6
> 802.6
Northern Africa: Incidence Rate (per 100,000)
Incidence Rate
< 699.5
699.5 - 808.3
808.3 - 897.9
897.9 - 987.6
987.6 - 1041.3
1041.3 - 1094.9
1094.9 - 1148.6
1148.6 - 1168.8
1168.8 - 1294.4
> 1294.4
League of Arab States: Incidence Rate (per 100,000)
Incidence Rate
< 715.1
715.1 - 818.7
818.7 - 914.5
914.5 - 1010.2
1010.2 - 1061.3
1061.3 - 1112.4
1112.4 - 1163.5
1163.5 - 1181.4
1181.4 - 1320.5
> 1320.5
10-14 years
15-19 years
20-24 years

Figure S1

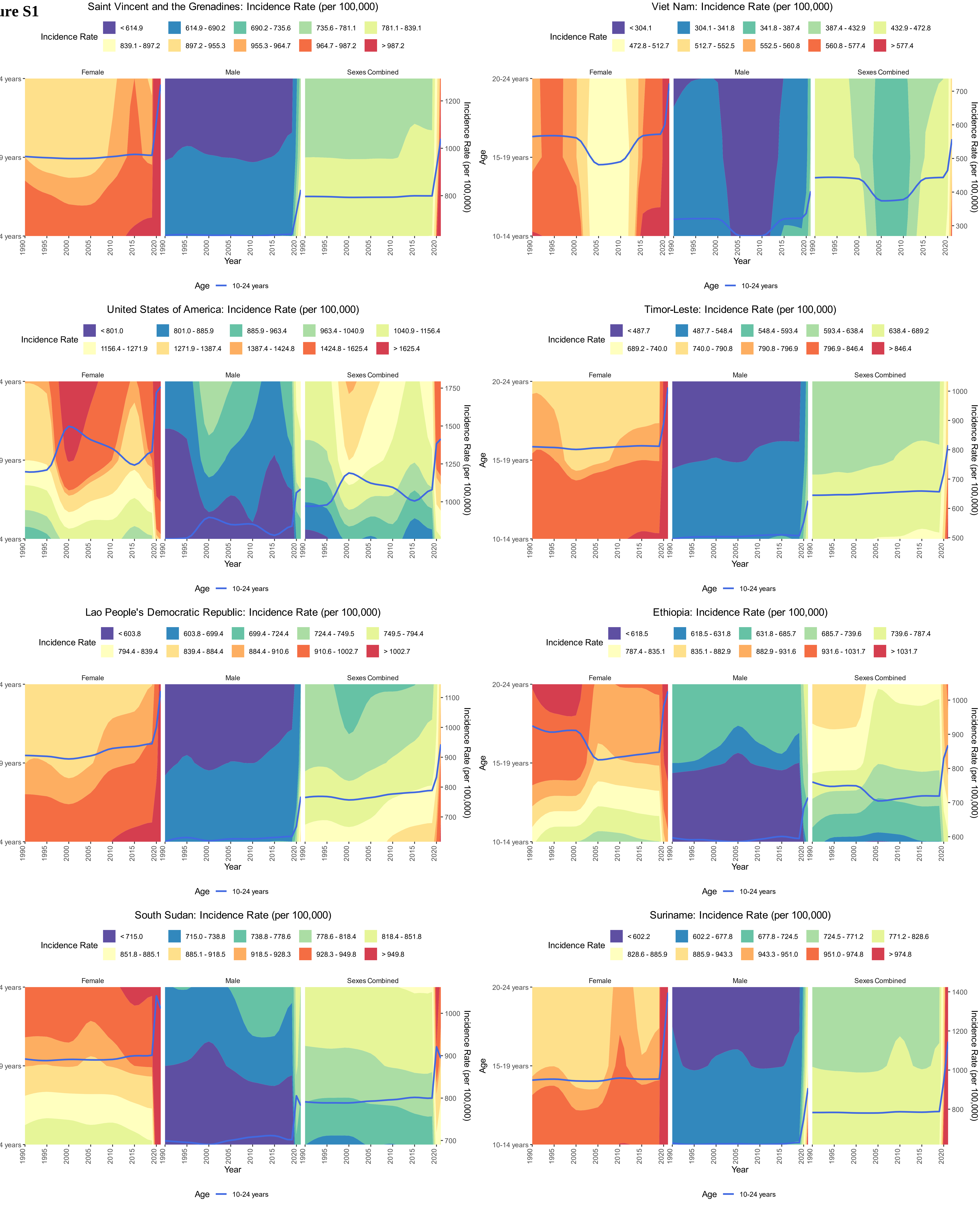

Saint Vincent and the Grenadines: Incidence Rate (per 100,000)
Incidence Rate
< 614.9
614.9 - 690.2
690.2 - 735.6
735.6 - 781.1
781.1 - 839.1
839.1 - 897.2
897.2 - 955.3
955.3 - 964.7
964.7 - 987.2
> 987.2
Viet Nam: Incidence Rate (per 100,000)
Incidence Rate
< 304.1
304.1 - 341.8
341.8 - 387.4
387.4 - 432.9
432.9 - 472.8
472.8 - 512.7
512.7 - 552.5
552.5 - 560.8
560.8 - 577.4
> 577.4
United States of America: Incidence Rate (per 100,000)
Incidence Rate
< 801.0
801.0 - 885.9
885.9 - 963.4
963.4 - 1040.9
1040.9 - 1156.4
1156.4 - 1271.9
1271.9 - 1387.4
1387.4 - 1424.8
1424.8 - 1625.4
> 1625.4
Timor-Leste: Incidence Rate (per 100,000)
Incidence Rate
< 487.7
487.7 - 548.4
548.4 - 593.4
593.4 - 638.4
638.4 - 689.2
689.2 - 740.0
740.0 - 790.8
790.8 - 796.9
796.9 - 846.4
> 846.4
Lao People's Democratic Republic: Incidence Rate (per 100,000)
Incidence Rate
< 603.8
603.8 - 699.4
699.4 - 724.4
724.4 - 749.5
749.5 - 794.4
794.4 - 839.4
839.4 - 884.4
884.4 - 910.6
910.6 - 1002.7
> 1002.7
Ethiopia: Incidence Rate (per 100,000)
Incidence Rate
< 618.5
618.5 - 631.8
631.8 - 685.7
685.7 - 739.6
739.6 - 787.4
787.4 - 835.1
835.1 - 882.9
882.9 - 931.6
931.6 - 1031.7
> 1031.7
South Sudan: Incidence Rate (per 100,000)
Incidence Rate
< 715.0
715.0 - 738.8
738.8 - 778.6
778.6 - 818.4
818.4 - 851.8
851.8 - 885.1
885.1 - 918.5
918.5 - 928.3
928.3 - 949.8
> 949.8
Suriname: Incidence Rate (per 100,000)
Incidence Rate
< 602.2
602.2 - 677.8
677.8 - 724.5
724.5 - 771.2
771.2 - 828.6
828.6 - 885.9
885.9 - 943.3
943.3 - 951.0
951.0 - 974.8
> 974.8
Female
Male
Sexes Combined
Age
20-24 years
15-19 years
10-14 years
Year
Incidence Rate (per 100,000)
Age
10-24 years

Figure S1

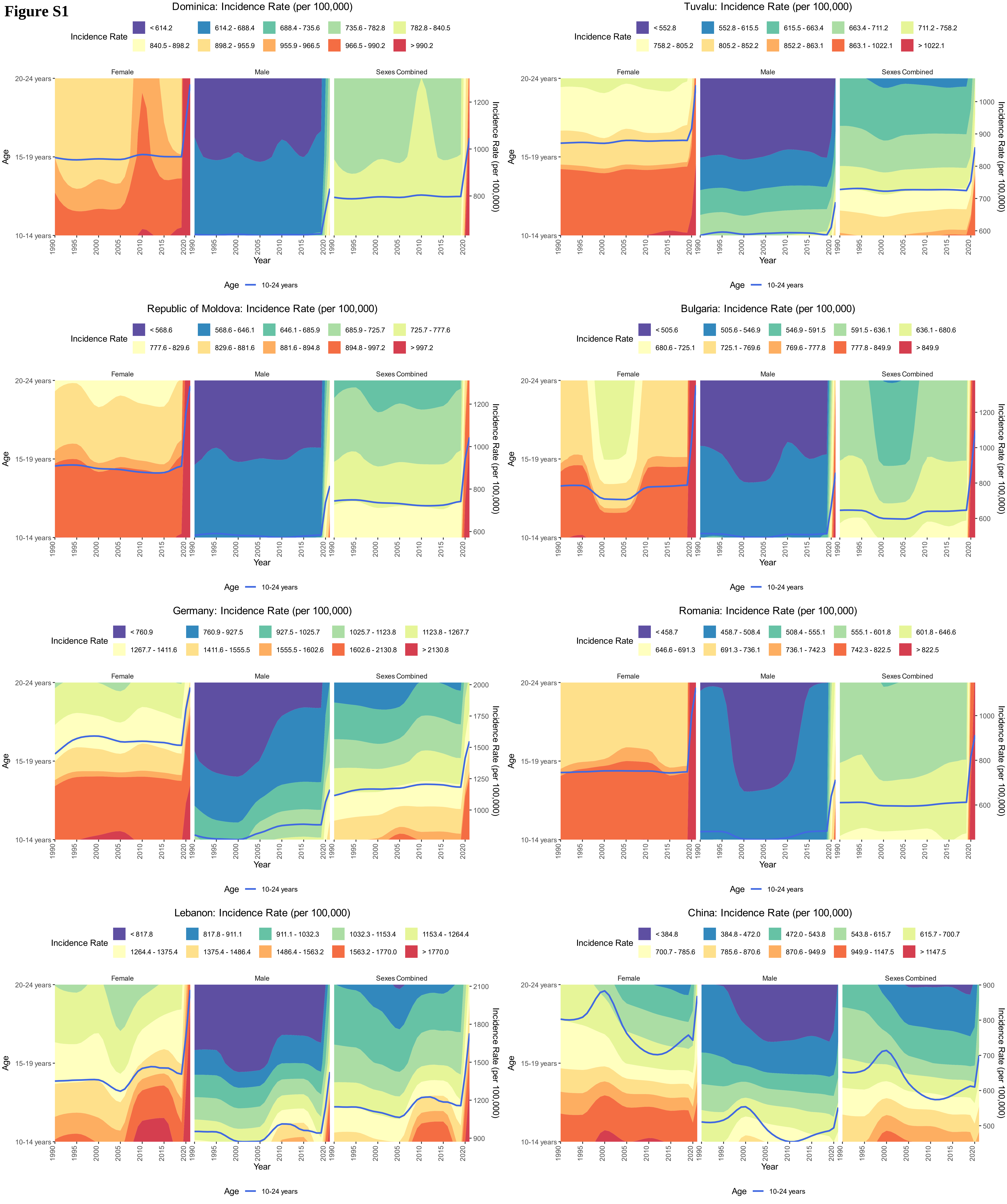
Dominica: Incidence Rate (per 100,000)
Incidence Rate
< 614.2
614.2 - 688.4
688.4 - 735.6
735.6 - 782.8
782.8 - 840.5
840.5 - 898.2
898.2 - 955.9
955.9 - 966.5
966.5 - 990.2
> 990.2
Tuvalu: Incidence Rate (per 100,000)
< 552.8
552.8 - 615.5
615.5 - 663.4
663.4 - 711.2
711.2 - 758.2
758.2 - 805.2
805.2 - 852.2
852.2 - 863.1
863.1 - 1022.1
> 1022.1
Republic of Moldova: Incidence Rate (per 100,000)
< 568.6
568.6 - 646.1
646.1 - 685.9
685.9 - 725.7
725.7 - 777.6
777.6 - 829.6
829.6 - 881.6
881.6 - 894.8
894.8 - 997.2
> 997.2
Bulgaria: Incidence Rate (per 100,000)
< 505.6
505.6 - 546.9
546.9 - 591.5
591.5 - 636.1
636.1 - 680.6
680.6 - 725.1
725.1 - 769.6
769.6 - 777.8
777.8 - 849.9
> 849.9
Germany: Incidence Rate (per 100,000)
< 760.9
760.9 - 927.5
927.5 - 1025.7
1025.7 - 1123.8
1123.8 - 1267.7
1267.7 - 1411.6
1411.6 - 1555.5
1555.5 - 1602.6
1602.6 - 2130.8
> 2130.8
Romania: Incidence Rate (per 100,000)
< 458.7
458.7 - 508.4
508.4 - 555.1
555.1 - 601.8
601.8 - 646.6
646.6 - 691.3
691.3 - 736.1
736.1 - 742.3
742.3 - 822.5
> 822.5
Lebanon: Incidence Rate (per 100,000)
< 817.8
817.8 - 911.1
911.1 - 1032.3
1032.3 - 1153.4
1153.4 - 1264.4
1264.4 - 1375.4
1375.4 - 1486.4
1486.4 - 1563.2
1563.2 - 1770.0
> 1770.0
China: Incidence Rate (per 100,000)
< 384.8
384.8 - 472.0
472.0 - 543.8
543.8 - 615.7
615.7 - 700.7
700.7 - 785.6
785.6 - 870.6
870.6 - 949.9
949.9 - 1147.5
> 1147.5
Female
Male
Sexes Combined
Age
20-24 years
15-19 years
10-14 years
Year
Incidence Rate (per 100,000)
Age
10-24 years

Figure S1

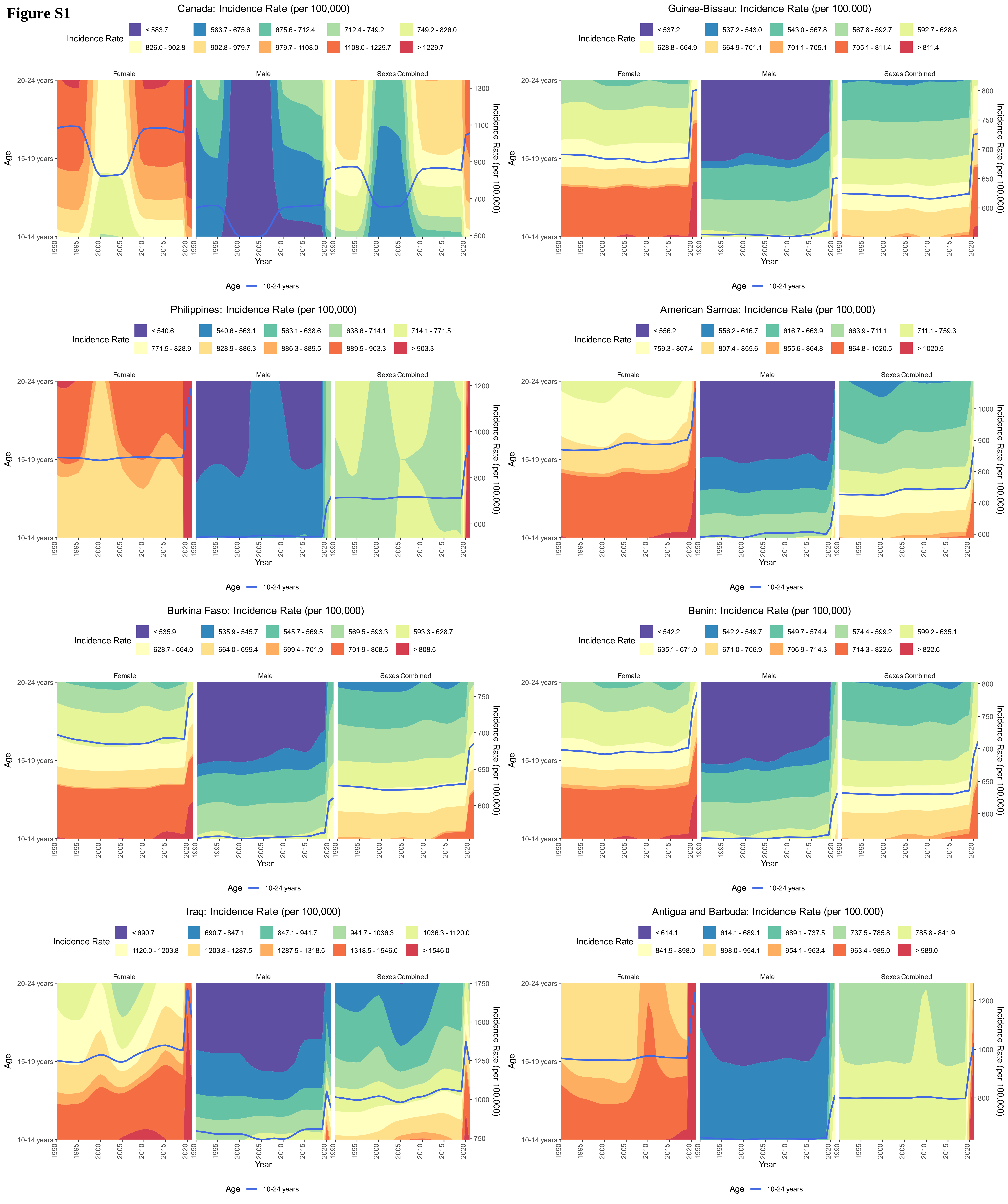

Canada: Incidence Rate (per 100,000)
Incidence Rate
< 583.7
583.7 - 675.6
675.6 - 712.4
712.4 - 749.2
749.2 - 826.0
826.0 - 902.8
902.8 - 979.7
979.7 - 1108.0
1108.0 - 1229.7
> 1229.7
Female
Male
Sexes Combined
Age
Year
Incidence Rate (per 100,000)
Age — 10-24 years
Guinea-Bissau: Incidence Rate (per 100,000)
Incidence Rate
< 537.2
537.2 - 543.0
543.0 - 567.8
567.8 - 592.7
592.7 - 628.8
628.8 - 664.9
664.9 - 701.1
701.1 - 705.1
705.1 - 811.4
> 811.4
Philippines: Incidence Rate (per 100,000)
Incidence Rate
< 540.6
540.6 - 563.1
563.1 - 638.6
638.6 - 714.1
714.1 - 771.5
771.5 - 828.9
828.9 - 886.3
886.3 - 889.5
889.5 - 903.3
> 903.3
American Samoa: Incidence Rate (per 100,000)
Incidence Rate
< 556.2
556.2 - 616.7
616.7 - 663.9
663.9 - 711.1
711.1 - 759.3
759.3 - 807.4
807.4 - 855.6
855.6 - 864.8
864.8 - 1020.5
> 1020.5
Burkina Faso: Incidence Rate (per 100,000)
Incidence Rate
< 535.9
535.9 - 545.7
545.7 - 569.5
569.5 - 593.3
593.3 - 628.7
628.7 - 664.0
664.0 - 699.4
699.4 - 701.9
701.9 - 808.5
> 808.5
Benin: Incidence Rate (per 100,000)
Incidence Rate
< 542.2
542.2 - 549.7
549.7 - 574.4
574.4 - 599.2
599.2 - 635.1
635.1 - 671.0
671.0 - 706.9
706.9 - 714.3
714.3 - 822.6
> 822.6
Iraq: Incidence Rate (per 100,000)
Incidence Rate
< 690.7
690.7 - 847.1
847.1 - 941.7
941.7 - 1036.3
1036.3 - 1120.0
1120.0 - 1203.8
1203.8 - 1287.5
1287.5 - 1318.5
1318.5 - 1546.0
> 1546.0
Antigua and Barbuda: Incidence Rate (per 100,000)
Incidence Rate
< 614.1
614.1 - 689.1
689.1 - 737.5
737.5 - 785.8
785.8 - 841.9
841.9 - 898.0
898.0 - 954.1
954.1 - 963.4
963.4 - 989.0
> 989.0
20-24 years
15-19 years
10-14 years

Figure S1

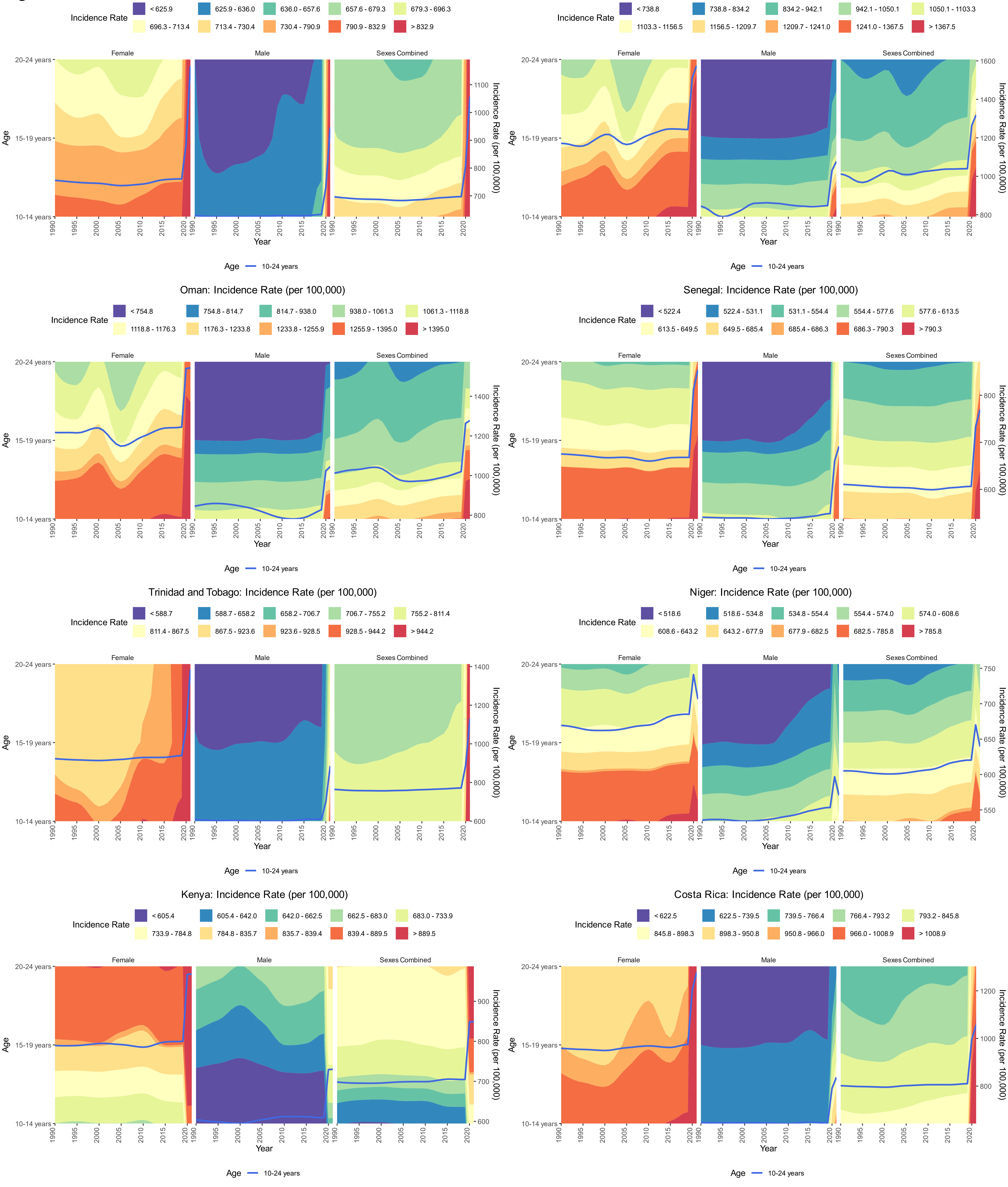
Lesotho: Incidence Rate (per 100,000)
Incidence Rate
< 625.9
625.9 - 636.0
636.0 - 657.6
657.6 - 679.3
679.3 - 696.3
696.3 - 713.4
713.4 - 730.4
730.4 - 790.9
790.9 - 832.9
> 832.9
Afghanistan: Incidence Rate (per 100,000)
< 738.8
738.8 - 834.2
834.2 - 942.1
942.1 - 1050.1
1050.1 - 1103.3
1103.3 - 1156.5
1156.5 - 1209.7
1209.7 - 1241.0
1241.0 - 1367.5
> 1367.5
Oman: Incidence Rate (per 100,000)
< 754.8
754.8 - 814.7
814.7 - 938.0
938.0 - 1061.3
1061.3 - 1118.8
1118.8 - 1176.3
1176.3 - 1233.8
1233.8 - 1255.9
1255.9 - 1395.0
> 1395.0
Senegal: Incidence Rate (per 100,000)
< 522.4
522.4 - 531.1
531.1 - 554.4
554.4 - 577.6
577.6 - 613.5
613.5 - 649.5
649.5 - 685.4
685.4 - 686.3
686.3 - 790.3
> 790.3
Trinidad and Tobago: Incidence Rate (per 100,000)
< 588.7
588.7 - 658.2
658.2 - 706.7
706.7 - 755.2
755.2 - 811.4
811.4 - 867.5
867.5 - 923.6
923.6 - 928.5
928.5 - 944.2
> 944.2
Niger: Incidence Rate (per 100,000)
< 518.6
518.6 - 534.8
534.8 - 554.4
554.4 - 574.0
574.0 - 608.6
608.6 - 643.2
643.2 - 677.9
677.9 - 682.5
682.5 - 785.8
> 785.8
Kenya: Incidence Rate (per 100,000)
< 605.4
605.4 - 642.0
642.0 - 662.5
662.5 - 683.0
683.0 - 733.9
733.9 - 784.8
784.8 - 835.7
835.7 - 839.4
839.4 - 889.5
> 889.5
Costa Rica: Incidence Rate (per 100,000)
< 622.5
622.5 - 739.5
739.5 - 766.4
766.4 - 793.2
793.2 - 845.8
845.8 - 898.3
898.3 - 950.8
950.8 - 966.0
966.0 - 1008.9
> 1008.9
Female
Male
Sexes Combined
20-24 years
15-19 years
10-14 years
Age
Year
Incidence Rate (per 100,000)
Age — 10-24 years

Figure S1

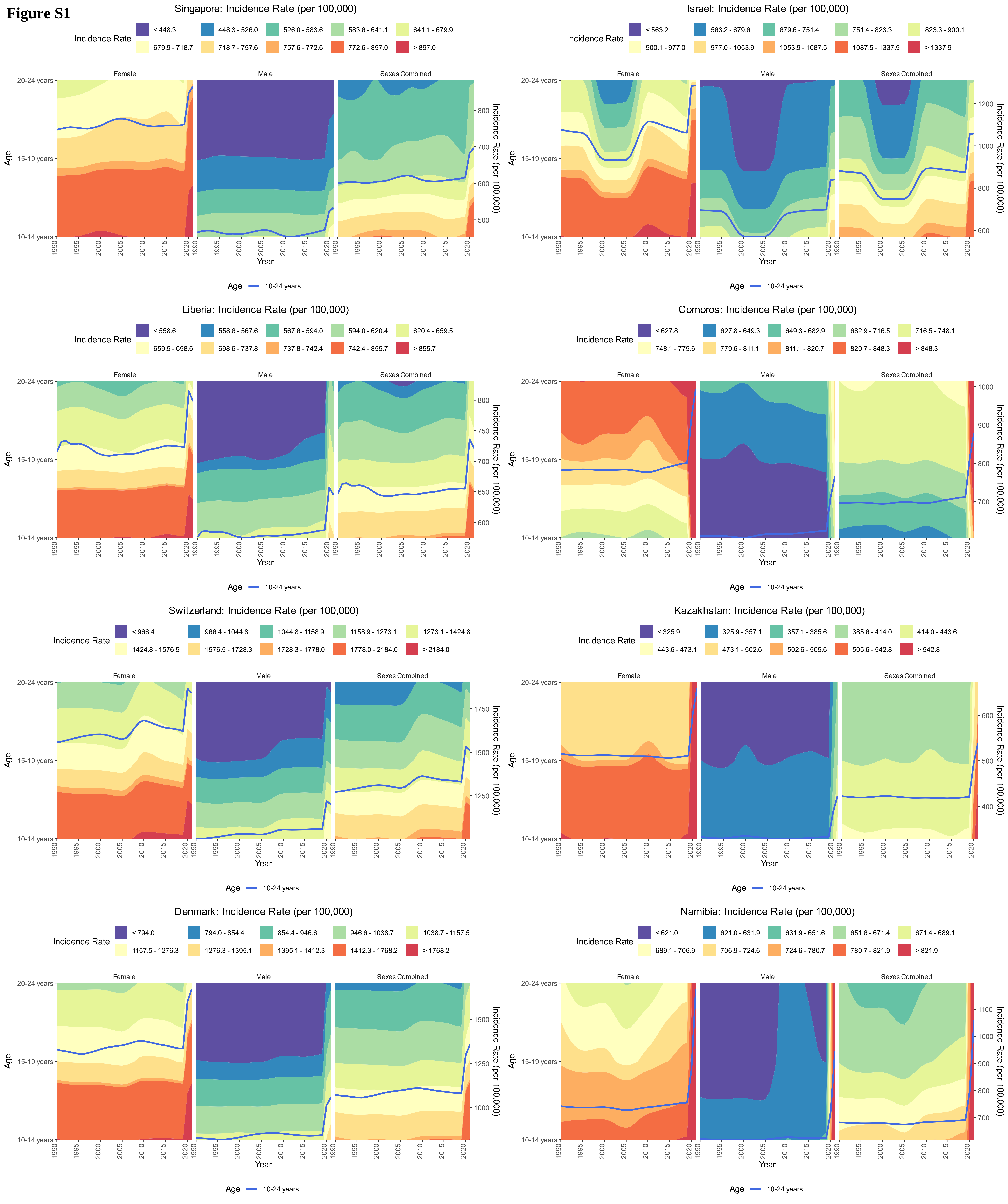
Singapore: Incidence Rate (per 100,000)
Incidence Rate
< 448.3
448.3 - 526.0
526.0 - 583.6
583.6 - 641.1
641.1 - 679.9
679.9 - 718.7
718.7 - 757.6
757.6 - 772.6
772.6 - 897.0
> 897.0
Israel: Incidence Rate (per 100,000)
< 563.2
563.2 - 679.6
679.6 - 751.4
751.4 - 823.3
823.3 - 900.1
900.1 - 977.0
977.0 - 1053.9
1053.9 - 1087.5
1087.5 - 1337.9
> 1337.9
Liberia: Incidence Rate (per 100,000)
< 558.6
558.6 - 567.6
567.6 - 594.0
594.0 - 620.4
620.4 - 659.5
659.5 - 698.6
698.6 - 737.8
737.8 - 742.4
742.4 - 855.7
> 855.7
Comoros: Incidence Rate (per 100,000)
< 627.8
627.8 - 649.3
649.3 - 682.9
682.9 - 716.5
716.5 - 748.1
748.1 - 779.6
779.6 - 811.1
811.1 - 820.7
820.7 - 848.3
> 848.3
Switzerland: Incidence Rate (per 100,000)
< 966.4
966.4 - 1044.8
1044.8 - 1158.9
1158.9 - 1273.1
1273.1 - 1424.8
1424.8 - 1576.5
1576.5 - 1728.3
1728.3 - 1778.0
1778.0 - 2184.0
> 2184.0
Kazakhstan: Incidence Rate (per 100,000)
< 325.9
325.9 - 357.1
357.1 - 385.6
385.6 - 414.0
414.0 - 443.6
443.6 - 473.1
473.1 - 502.6
502.6 - 505.6
505.6 - 542.8
> 542.8
Denmark: Incidence Rate (per 100,000)
< 794.0
794.0 - 854.4
854.4 - 946.6
946.6 - 1038.7
1038.7 - 1157.5
1157.5 - 1276.3
1276.3 - 1395.1
1395.1 - 1412.3
1412.3 - 1768.2
> 1768.2
Namibia: Incidence Rate (per 100,000)
< 621.0
621.0 - 631.9
631.9 - 651.6
651.6 - 671.4
671.4 - 689.1
689.1 - 706.9
706.9 - 724.6
724.6 - 780.7
780.7 - 821.9
> 821.9
Female
Male
Sexes Combined
20-24 years
15-19 years
10-14 years
Age
Year
Incidence Rate (per 100,000)
Age — 10-24 years

Figure S1

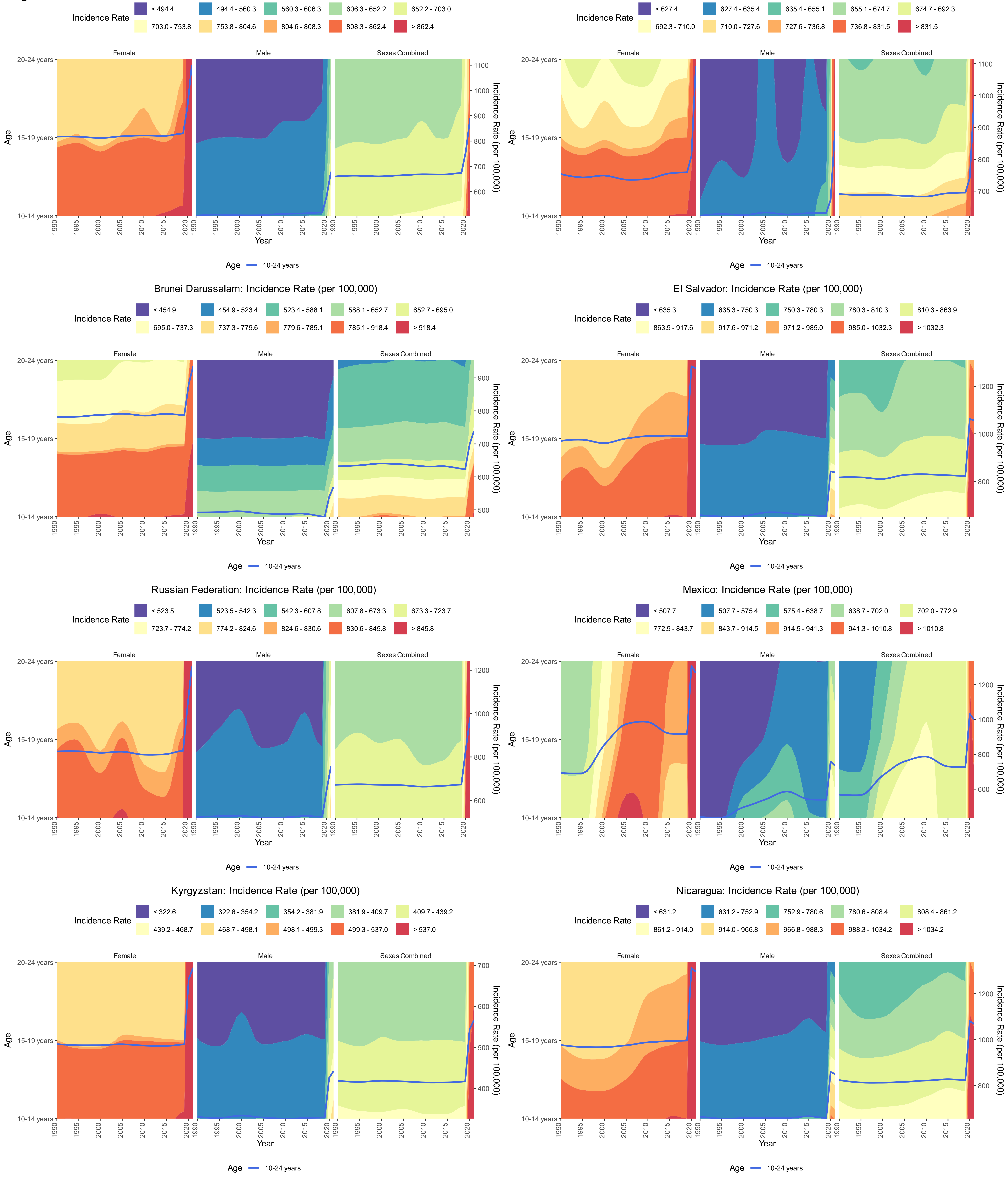

Sri Lanka: Incidence Rate (per 100,000)
Incidence Rate
< 494.4
494.4 - 560.3
560.3 - 606.3
606.3 - 652.2
652.2 - 703.0
703.0 - 753.8
753.8 - 804.6
804.6 - 808.3
808.3 - 862.4
> 862.4
Botswana: Incidence Rate (per 100,000)
Incidence Rate
< 627.4
627.4 - 635.4
635.4 - 655.1
655.1 - 674.7
674.7 - 692.3
692.3 - 710.0
710.0 - 727.6
727.6 - 736.8
736.8 - 831.5
> 831.5
Brunei Darussalam: Incidence Rate (per 100,000)
Incidence Rate
< 454.9
454.9 - 523.4
523.4 - 588.1
588.1 - 652.7
652.7 - 695.0
695.0 - 737.3
737.3 - 779.6
779.6 - 785.1
785.1 - 918.4
> 918.4
El Salvador: Incidence Rate (per 100,000)
Incidence Rate
< 635.3
635.3 - 750.3
750.3 - 780.3
780.3 - 810.3
810.3 - 863.9
863.9 - 917.6
917.6 - 971.2
971.2 - 985.0
985.0 - 1032.3
> 1032.3
Russian Federation: Incidence Rate (per 100,000)
Incidence Rate
< 523.5
523.5 - 542.3
542.3 - 607.8
607.8 - 673.3
673.3 - 723.7
723.7 - 774.2
774.2 - 824.6
824.6 - 830.6
830.6 - 845.8
> 845.8
Mexico: Incidence Rate (per 100,000)
Incidence Rate
< 507.7
507.7 - 575.4
575.4 - 638.7
638.7 - 702.0
702.0 - 772.9
772.9 - 843.7
843.7 - 914.5
914.5 - 941.3
941.3 - 1010.8
> 1010.8
Kyrgyzstan: Incidence Rate (per 100,000)
Incidence Rate
< 322.6
322.6 - 354.2
354.2 - 381.9
381.9 - 409.7
409.7 - 439.2
439.2 - 468.7
468.7 - 498.1
498.1 - 499.3
499.3 - 537.0
> 537.0
Nicaragua: Incidence Rate (per 100,000)
Incidence Rate
< 631.2
631.2 - 752.9
752.9 - 780.6
780.6 - 808.4
808.4 - 861.2
861.2 - 914.0
914.0 - 966.8
966.8 - 988.3
988.3 - 1034.2
> 1034.2
Female
Male
Sexes Combined
Age
20-24 years
15-19 years
10-14 years
Year
Incidence Rate (per 100,000)
Age — 10-24 years

Figure S1

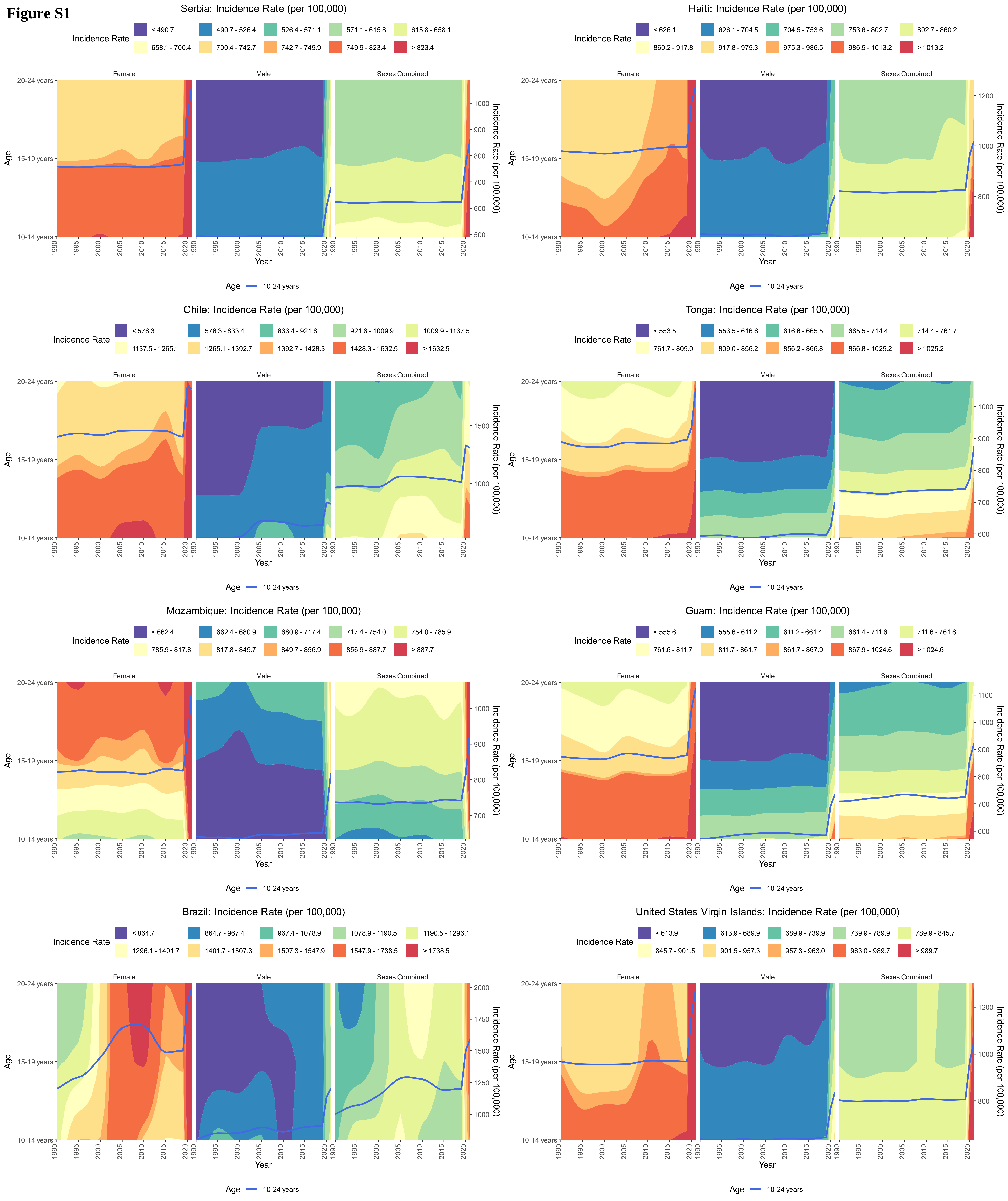

Serbia: Incidence Rate (per 100,000)
Incidence Rate
< 490.7
490.7 - 526.4
526.4 - 571.1
571.1 - 615.8
615.8 - 658.1
658.1 - 700.4
700.4 - 742.7
742.7 - 749.9
749.9 - 823.4
> 823.4
Haiti: Incidence Rate (per 100,000)
< 626.1
626.1 - 704.5
704.5 - 753.6
753.6 - 802.7
802.7 - 860.2
860.2 - 917.8
917.8 - 975.3
975.3 - 986.5
986.5 - 1013.2
> 1013.2
Chile: Incidence Rate (per 100,000)
< 576.3
576.3 - 833.4
833.4 - 921.6
921.6 - 1009.9
1009.9 - 1137.5
1137.5 - 1265.1
1265.1 - 1392.7
1392.7 - 1428.3
1428.3 - 1632.5
> 1632.5
Tonga: Incidence Rate (per 100,000)
< 553.5
553.5 - 616.6
616.6 - 665.5
665.5 - 714.4
714.4 - 761.7
761.7 - 809.0
809.0 - 856.2
856.2 - 866.8
866.8 - 1025.2
> 1025.2
Mozambique: Incidence Rate (per 100,000)
< 662.4
662.4 - 680.9
680.9 - 717.4
717.4 - 754.0
754.0 - 785.9
785.9 - 817.8
817.8 - 849.7
849.7 - 856.9
856.9 - 887.7
> 887.7
Guam: Incidence Rate (per 100,000)
< 555.6
555.6 - 611.2
611.2 - 661.4
661.4 - 711.6
711.6 - 761.6
761.6 - 811.7
811.7 - 861.7
861.7 - 867.9
867.9 - 1024.6
> 1024.6
Brazil: Incidence Rate (per 100,000)
< 864.7
864.7 - 967.4
967.4 - 1078.9
1078.9 - 1190.5
1190.5 - 1296.1
1296.1 - 1401.7
1401.7 - 1507.3
1507.3 - 1547.9
1547.9 - 1738.5
> 1738.5
United States Virgin Islands: Incidence Rate (per 100,000)
< 613.9
613.9 - 689.9
689.9 - 739.9
739.9 - 789.9
789.9 - 845.7
845.7 - 901.5
901.5 - 957.3
957.3 - 963.0
963.0 - 989.7
> 989.7
Female
Male
Sexes Combined
Age
20-24 years
15-19 years
10-14 years
Year
Incidence Rate (per 100,000)
Age — 10-24 years

Figure S1

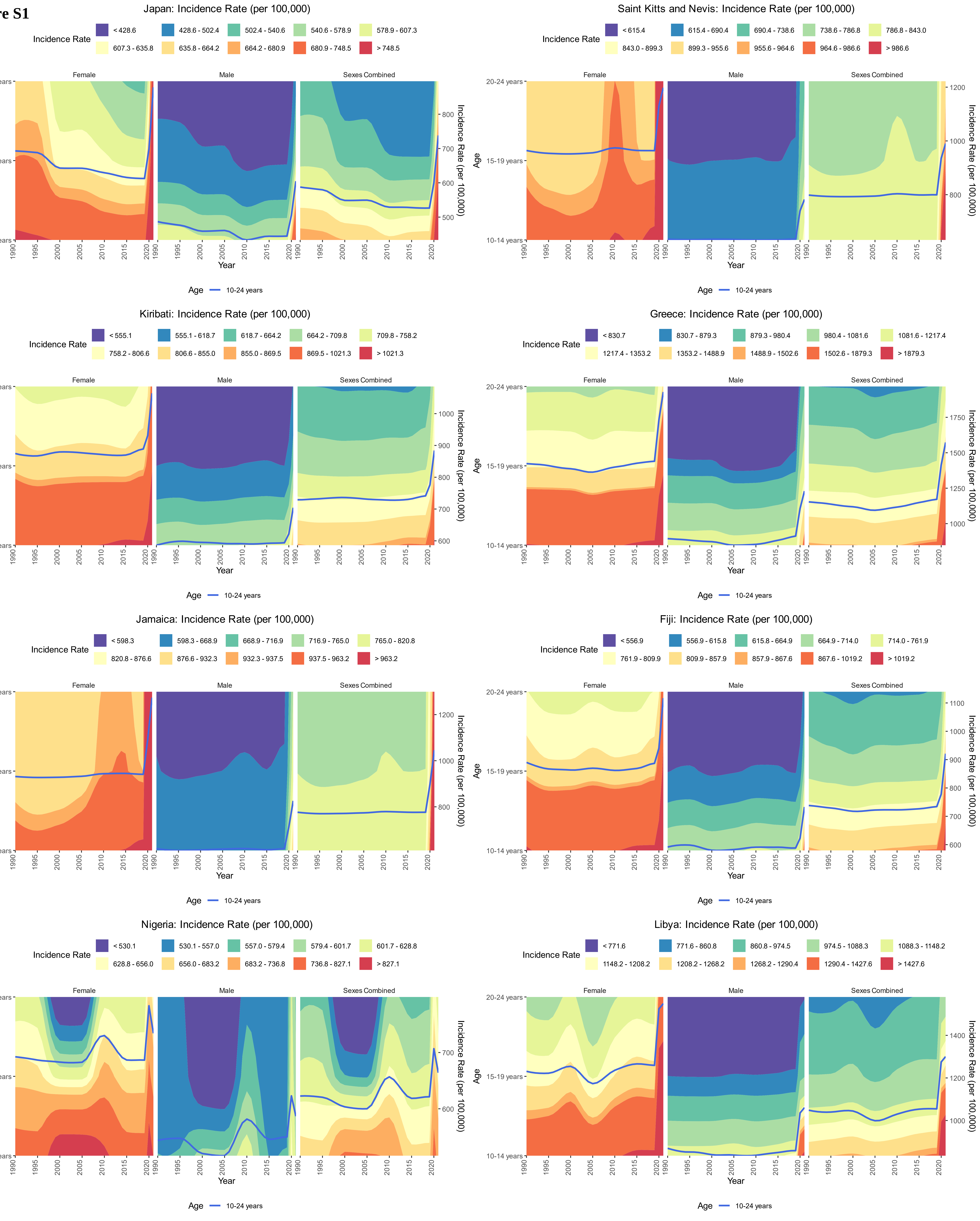

Japan: Incidence Rate (per 100,000)
Incidence Rate
< 428.6
428.6 - 502.4
502.4 - 540.6
540.6 - 578.9
578.9 - 607.3
607.3 - 635.8
635.8 - 664.2
664.2 - 680.9
680.9 - 748.5
> 748.5
Female
Male
Sexes Combined
Age
Year
Incidence Rate (per 100,000)
Age
10-24 years
Saint Kitts and Nevis: Incidence Rate (per 100,000)
< 615.4
615.4 - 690.4
690.4 - 738.6
738.6 - 786.8
786.8 - 843.0
843.0 - 899.3
899.3 - 955.6
955.6 - 964.6
964.6 - 986.6
> 986.6
Kiribati: Incidence Rate (per 100,000)
< 555.1
555.1 - 618.7
618.7 - 664.2
664.2 - 709.8
709.8 - 758.2
758.2 - 806.6
806.6 - 855.0
855.0 - 869.5
869.5 - 1021.3
> 1021.3
Greece: Incidence Rate (per 100,000)
< 830.7
830.7 - 879.3
879.3 - 980.4
980.4 - 1081.6
1081.6 - 1217.4
1217.4 - 1353.2
1353.2 - 1488.9
1488.9 - 1502.6
1502.6 - 1879.3
> 1879.3
Jamaica: Incidence Rate (per 100,000)
< 598.3
598.3 - 668.9
668.9 - 716.9
716.9 - 765.0
765.0 - 820.8
820.8 - 876.6
876.6 - 932.3
932.3 - 937.5
937.5 - 963.2
> 963.2
Fiji: Incidence Rate (per 100,000)
< 556.9
556.9 - 615.8
615.8 - 664.9
664.9 - 714.0
714.0 - 761.9
761.9 - 809.9
809.9 - 857.9
857.9 - 867.6
867.6 - 1019.2
> 1019.2
Nigeria: Incidence Rate (per 100,000)
< 530.1
530.1 - 557.0
557.0 - 579.4
579.4 - 601.7
601.7 - 628.8
628.8 - 656.0
656.0 - 683.2
683.2 - 736.8
736.8 - 827.1
> 827.1
Libya: Incidence Rate (per 100,000)
< 771.6
771.6 - 860.8
860.8 - 974.5
974.5 - 1088.3
1088.3 - 1148.2
1148.2 - 1208.2
1208.2 - 1268.2
1268.2 - 1290.4
1290.4 - 1427.6
> 1427.6
20-24 years
15-19 years
10-14 years

Figure S1

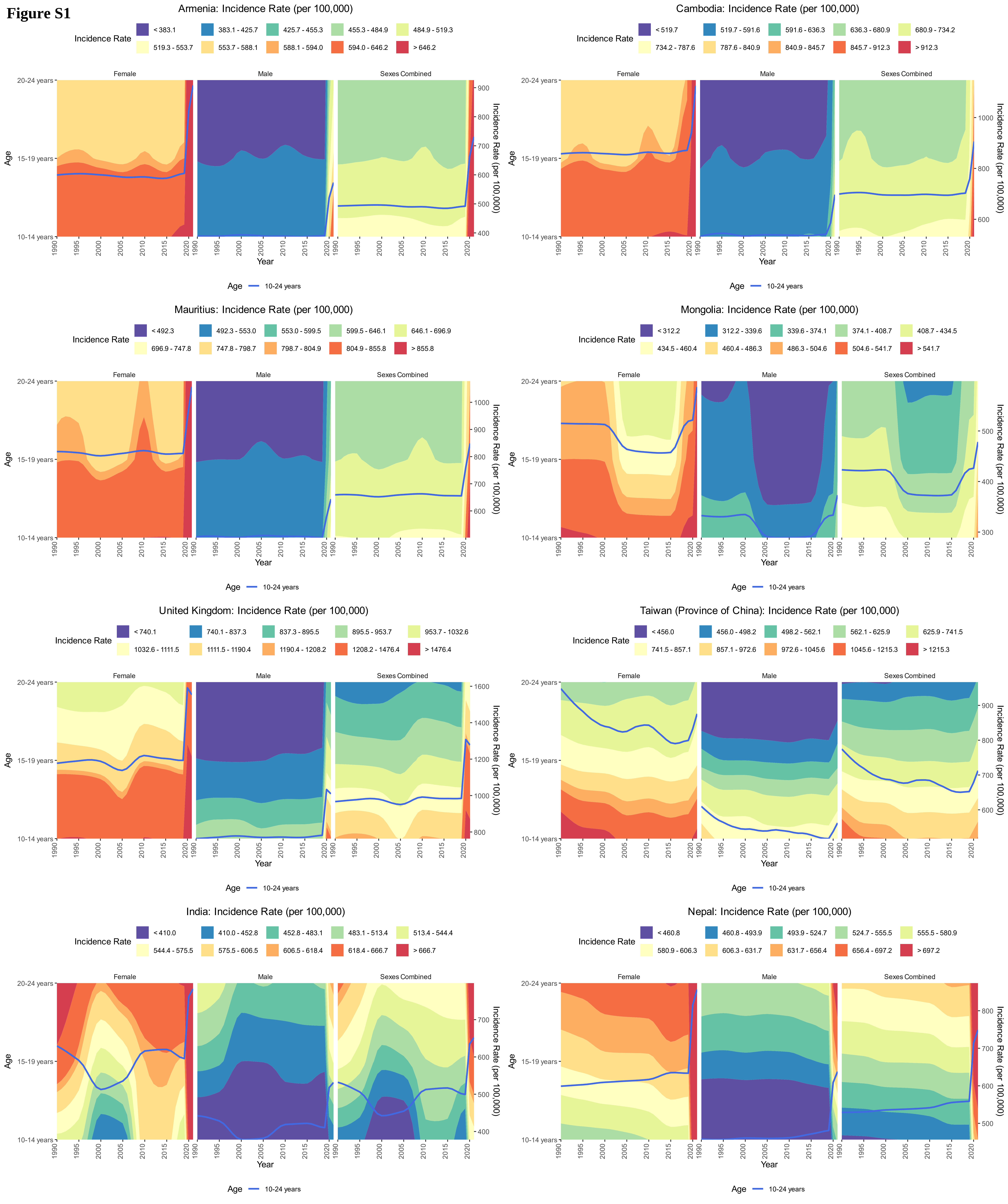
Armenia: Incidence Rate (per 100,000)
Cambodia: Incidence Rate (per 100,000)
Mauritius: Incidence Rate (per 100,000)
Mongolia: Incidence Rate (per 100,000)
United Kingdom: Incidence Rate (per 100,000)
Taiwan (Province of China): Incidence Rate (per 100,000)
India: Incidence Rate (per 100,000)
Nepal: Incidence Rate (per 100,000)
Incidence Rate
Female
Male
Sexes Combined
Age
Year
Incidence Rate (per 100,000)
10-14 years
15-19 years
20-24 years
Age — 10-24 years

Figure S1

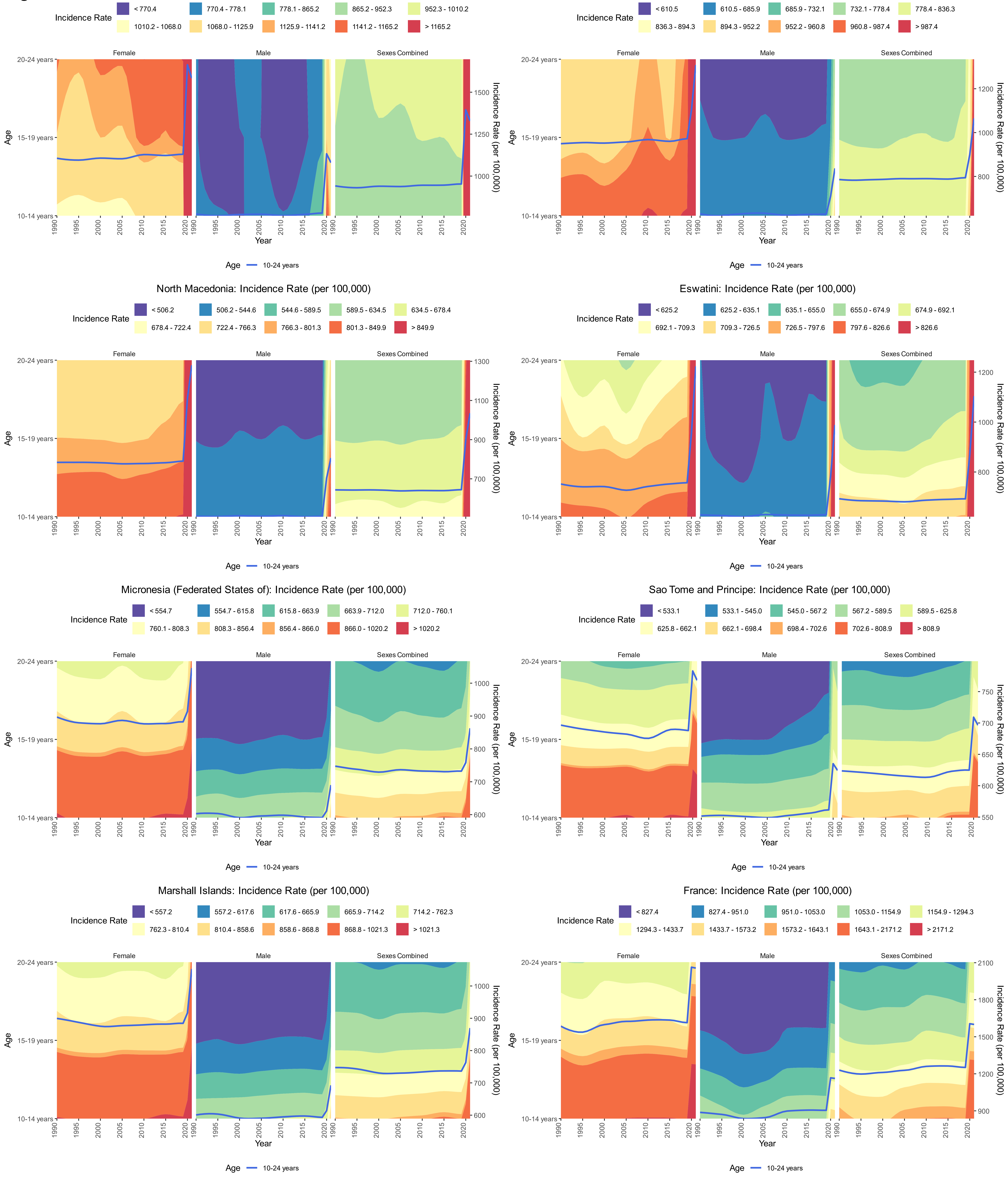
Ecuador: Incidence Rate (per 100,000)
Incidence Rate
< 770.4
770.4 - 778.1
778.1 - 865.2
865.2 - 952.3
952.3 - 1010.2
1010.2 - 1068.0
1068.0 - 1125.9
1125.9 - 1141.2
1141.2 - 1165.2
> 1165.2
Cuba: Incidence Rate (per 100,000)
< 610.5
610.5 - 685.9
685.9 - 732.1
732.1 - 778.4
778.4 - 836.3
836.3 - 894.3
894.3 - 952.2
952.2 - 960.8
960.8 - 987.4
> 987.4
North Macedonia: Incidence Rate (per 100,000)
< 506.2
506.2 - 544.6
544.6 - 589.5
589.5 - 634.5
634.5 - 678.4
678.4 - 722.4
722.4 - 766.3
766.3 - 801.3
801.3 - 849.9
> 849.9
Eswatini: Incidence Rate (per 100,000)
< 625.2
625.2 - 635.1
635.1 - 655.0
655.0 - 674.9
674.9 - 692.1
692.1 - 709.3
709.3 - 726.5
726.5 - 797.6
797.6 - 826.6
> 826.6
Micronesia (Federated States of): Incidence Rate (per 100,000)
< 554.7
554.7 - 615.8
615.8 - 663.9
663.9 - 712.0
712.0 - 760.1
760.1 - 808.3
808.3 - 856.4
856.4 - 866.0
866.0 - 1020.2
> 1020.2
Sao Tome and Principe: Incidence Rate (per 100,000)
< 533.1
533.1 - 545.0
545.0 - 567.2
567.2 - 589.5
589.5 - 625.8
625.8 - 662.1
662.1 - 698.4
698.4 - 702.6
702.6 - 808.9
> 808.9
Marshall Islands: Incidence Rate (per 100,000)
< 557.2
557.2 - 617.6
617.6 - 665.9
665.9 - 714.2
714.2 - 762.3
762.3 - 810.4
810.4 - 858.6
858.6 - 868.8
868.8 - 1021.3
> 1021.3
France: Incidence Rate (per 100,000)
< 827.4
827.4 - 951.0
951.0 - 1053.0
1053.0 - 1154.9
1154.9 - 1294.3
1294.3 - 1433.7
1433.7 - 1573.2
1573.2 - 1643.1
1643.1 - 2171.2
> 2171.2
Female
Male
Sexes Combined
Age
20-24 years
15-19 years
10-14 years
Year
Incidence Rate (per 100,000)
Age — 10-24 years

Figure S1

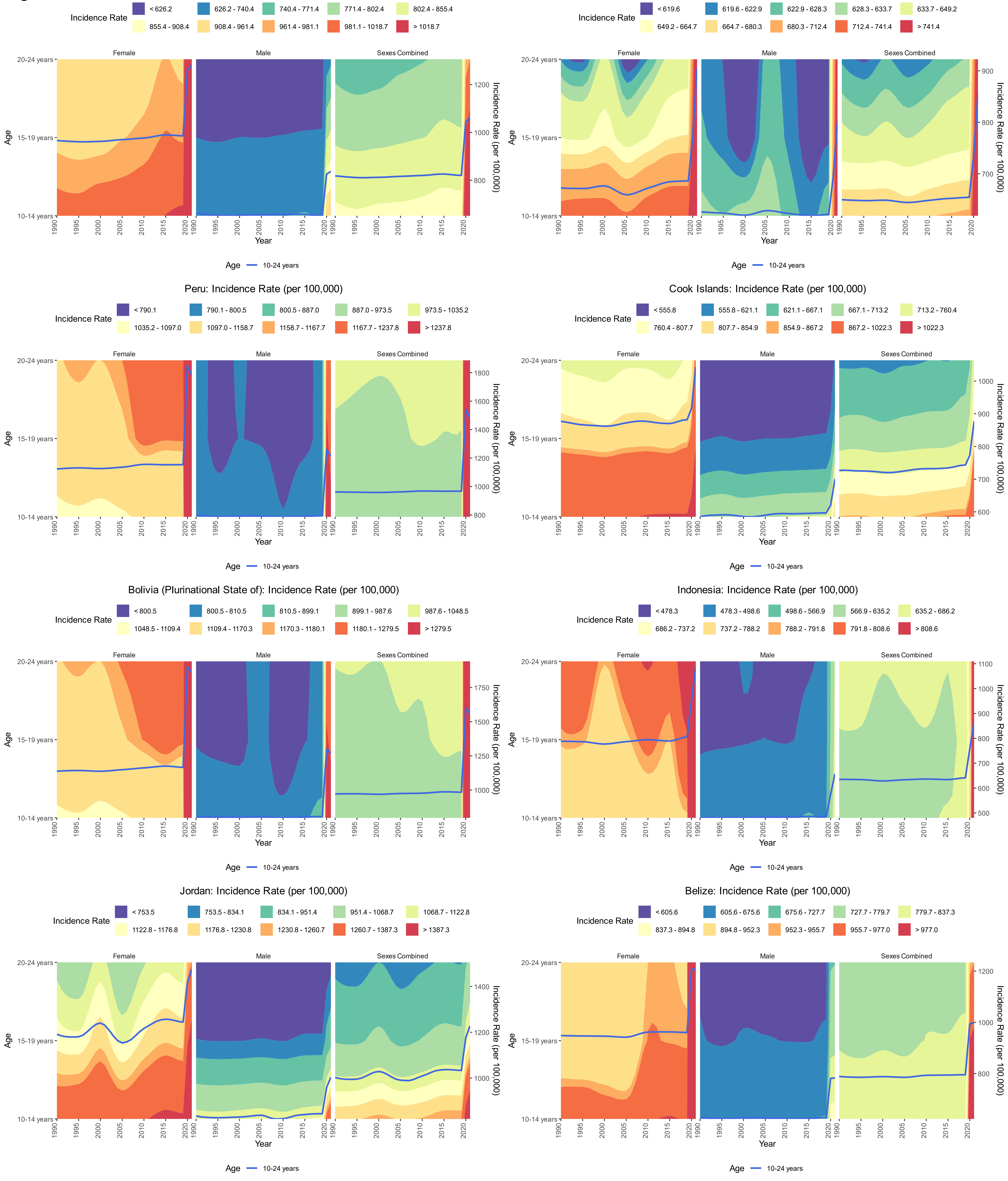
Guatemala: Incidence Rate (per 100,000)
Incidence Rate
< 626.2
626.2 - 740.4
740.4 - 771.4
771.4 - 802.4
802.4 - 855.4
855.4 - 908.4
908.4 - 961.4
961.4 - 981.1
981.1 - 1018.7
> 1018.7
Zimbabwe: Incidence Rate (per 100,000)
< 619.6
619.6 - 622.9
622.9 - 628.3
628.3 - 633.7
633.7 - 649.2
649.2 - 664.7
664.7 - 680.3
680.3 - 712.4
712.4 - 741.4
> 741.4
Peru: Incidence Rate (per 100,000)
< 790.1
790.1 - 800.5
800.5 - 887.0
887.0 - 973.5
973.5 - 1035.2
1035.2 - 1097.0
1097.0 - 1158.7
1158.7 - 1167.7
1167.7 - 1237.8
> 1237.8
Cook Islands: Incidence Rate (per 100,000)
< 555.8
555.8 - 621.1
621.1 - 667.1
667.1 - 713.2
713.2 - 760.4
760.4 - 807.7
807.7 - 854.9
854.9 - 867.2
867.2 - 1022.3
> 1022.3
Bolivia (Plurinational State of): Incidence Rate (per 100,000)
< 800.5
800.5 - 810.5
810.5 - 899.1
899.1 - 987.6
987.6 - 1048.5
1048.5 - 1109.4
1109.4 - 1170.3
1170.3 - 1180.1
1180.1 - 1279.5
> 1279.5
Indonesia: Incidence Rate (per 100,000)
< 478.3
478.3 - 498.6
498.6 - 566.9
566.9 - 635.2
635.2 - 686.2
686.2 - 737.2
737.2 - 788.2
788.2 - 791.8
791.8 - 808.6
> 808.6
Jordan: Incidence Rate (per 100,000)
< 753.5
753.5 - 834.1
834.1 - 951.4
951.4 - 1068.7
1068.7 - 1122.8
1122.8 - 1176.8
1176.8 - 1230.8
1230.8 - 1260.7
1260.7 - 1387.3
> 1387.3
Belize: Incidence Rate (per 100,000)
< 605.6
605.6 - 675.6
675.6 - 727.7
727.7 - 779.7
779.7 - 837.3
837.3 - 894.8
894.8 - 952.3
952.3 - 955.7
955.7 - 977.0
> 977.0
Female
Male
Sexes Combined
Age
20-24 years
15-19 years
10-14 years
Year
Incidence Rate (per 100,000)
Age — 10-24 years

Figure S1

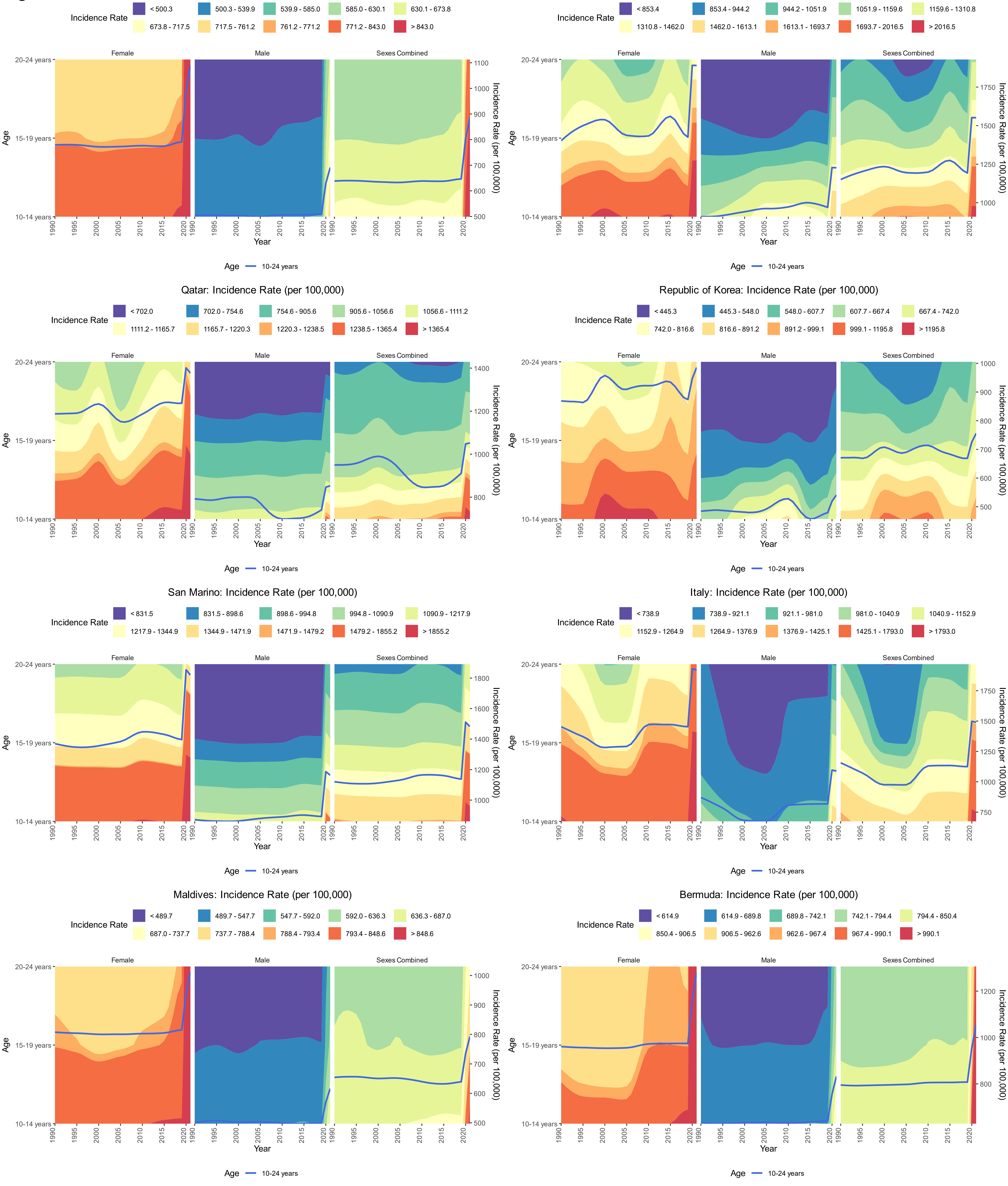
Croatia: Incidence Rate (per 100,000)
Incidence Rate
< 500.3
500.3 - 539.9
539.9 - 585.0
585.0 - 630.1
630.1 - 673.8
673.8 - 717.5
717.5 - 761.2
761.2 - 771.2
771.2 - 843.0
> 843.0
Female
Male
Sexes Combined
Age
Year
Incidence Rate (per 100,000)
Age — 10-24 years
Netherlands: Incidence Rate (per 100,000)
< 853.4
853.4 - 944.2
944.2 - 1051.9
1051.9 - 1159.6
1159.6 - 1310.8
1310.8 - 1462.0
1462.0 - 1613.1
1613.1 - 1693.7
1693.7 - 2016.5
> 2016.5
Qatar: Incidence Rate (per 100,000)
< 702.0
702.0 - 754.6
754.6 - 905.6
905.6 - 1056.6
1056.6 - 1111.2
1111.2 - 1165.7
1165.7 - 1220.3
1220.3 - 1238.5
1238.5 - 1365.4
> 1365.4
Republic of Korea: Incidence Rate (per 100,000)
< 445.3
445.3 - 548.0
548.0 - 607.7
607.7 - 667.4
667.4 - 742.0
742.0 - 816.6
816.6 - 891.2
891.2 - 999.1
999.1 - 1195.8
> 1195.8
San Marino: Incidence Rate (per 100,000)
< 831.5
831.5 - 898.6
898.6 - 994.8
994.8 - 1090.9
1090.9 - 1217.9
1217.9 - 1344.9
1344.9 - 1471.9
1471.9 - 1479.2
1479.2 - 1855.2
> 1855.2
Italy: Incidence Rate (per 100,000)
< 738.9
738.9 - 921.1
921.1 - 981.0
981.0 - 1040.9
1040.9 - 1152.9
1152.9 - 1264.9
1264.9 - 1376.9
1376.9 - 1425.1
1425.1 - 1793.0
> 1793.0
Maldives: Incidence Rate (per 100,000)
< 489.7
489.7 - 547.7
547.7 - 592.0
592.0 - 636.3
636.3 - 687.0
687.0 - 737.7
737.7 - 788.4
788.4 - 793.4
793.4 - 848.6
> 848.6
Bermuda: Incidence Rate (per 100,000)
< 614.9
614.9 - 689.8
689.8 - 742.1
742.1 - 794.4
794.4 - 850.4
850.4 - 906.5
906.5 - 962.6
962.6 - 967.4
967.4 - 990.1
> 990.1
20-24 years
15-19 years
10-14 years

Figure S1

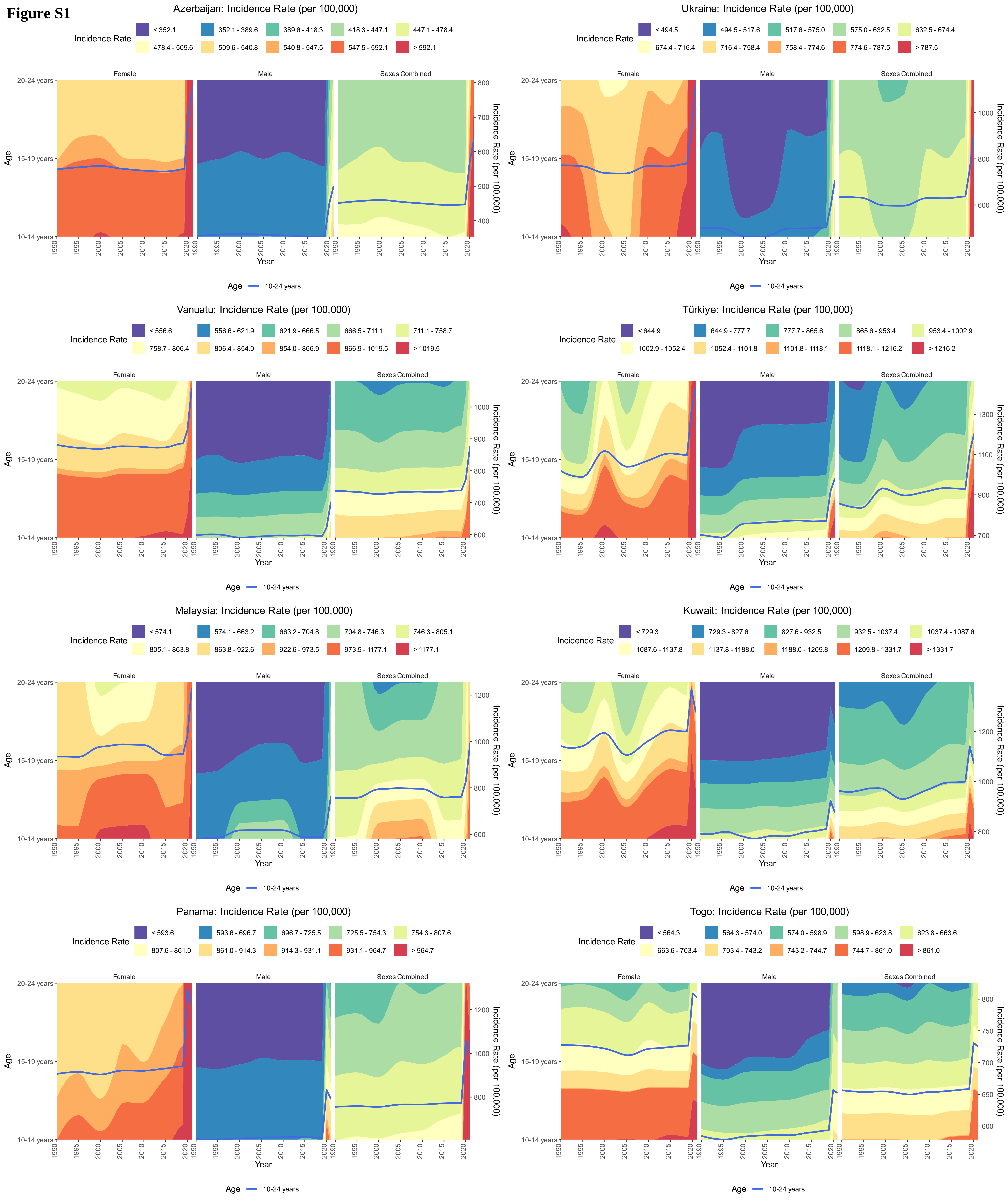

Azerbaijan: Incidence Rate (per 100,000)
Incidence Rate
< 352.1
352.1 - 389.6
389.6 - 418.3
418.3 - 447.1
447.1 - 478.4
478.4 - 509.6
509.6 - 540.8
540.8 - 547.5
547.5 - 592.1
> 592.1
Ukraine: Incidence Rate (per 100,000)
Incidence Rate
< 494.5
494.5 - 517.6
517.6 - 575.0
575.0 - 632.5
632.5 - 674.4
674.4 - 716.4
716.4 - 758.4
758.4 - 774.6
774.6 - 787.5
> 787.5
Vanuatu: Incidence Rate (per 100,000)
Incidence Rate
< 556.6
556.6 - 621.9
621.9 - 666.5
666.5 - 711.1
711.1 - 758.7
758.7 - 806.4
806.4 - 854.0
854.0 - 866.9
866.9 - 1019.5
> 1019.5
Türkiye: Incidence Rate (per 100,000)
Incidence Rate
< 644.9
644.9 - 777.7
777.7 - 865.6
865.6 - 953.4
953.4 - 1002.9
1002.9 - 1052.4
1052.4 - 1101.8
1101.8 - 1118.1
1118.1 - 1216.2
> 1216.2
Malaysia: Incidence Rate (per 100,000)
Incidence Rate
< 574.1
574.1 - 663.2
663.2 - 704.8
704.8 - 746.3
746.3 - 805.1
805.1 - 863.8
863.8 - 922.6
922.6 - 973.5
973.5 - 1177.1
> 1177.1
Kuwait: Incidence Rate (per 100,000)
Incidence Rate
< 729.3
729.3 - 827.6
827.6 - 932.5
932.5 - 1037.4
1037.4 - 1087.6
1087.6 - 1137.8
1137.8 - 1188.0
1188.0 - 1209.8
1209.8 - 1331.7
> 1331.7
Panama: Incidence Rate (per 100,000)
Incidence Rate
< 593.6
593.6 - 696.7
696.7 - 725.5
725.5 - 754.3
754.3 - 807.6
807.6 - 861.0
861.0 - 914.3
914.3 - 931.1
931.1 - 964.7
> 964.7
Togo: Incidence Rate (per 100,000)
Incidence Rate
< 564.3
564.3 - 574.0
574.0 - 598.9
598.9 - 623.8
623.8 - 663.6
663.6 - 703.4
703.4 - 743.2
743.2 - 744.7
744.7 - 861.0
> 861.0
Female
Male
Sexes Combined
Age
20-24 years
15-19 years
10-14 years
Year
Incidence Rate (per 100,000)
Age — 10-24 years

Figure S1

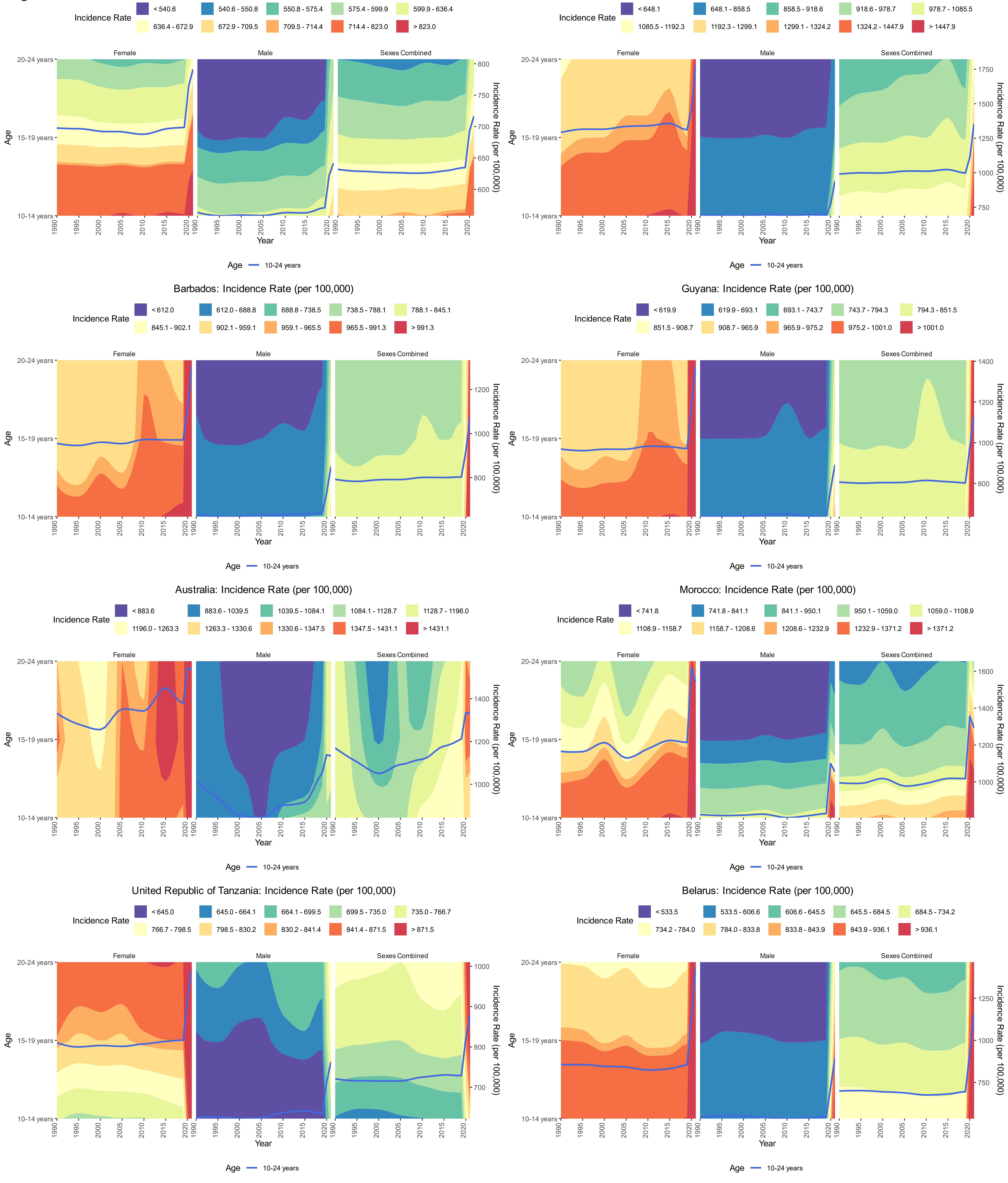

Cameroon: Incidence Rate (per 100,000)
Incidence Rate
< 540.6
540.6 - 550.8
550.8 - 575.4
575.4 - 599.9
599.9 - 636.4
636.4 - 672.9
672.9 - 709.5
709.5 - 714.4
714.4 - 823.0
> 823.0
Female
Male
Sexes Combined
Age
Year
Incidence Rate (per 100,000)
Age — 10-24 years
Uruguay: Incidence Rate (per 100,000)
Incidence Rate
< 648.1
648.1 - 858.5
858.5 - 918.6
918.6 - 978.7
978.7 - 1085.5
1085.5 - 1192.3
1192.3 - 1299.1
1299.1 - 1324.2
1324.2 - 1447.9
> 1447.9
Female
Male
Sexes Combined
Age
Year
Incidence Rate (per 100,000)
Age — 10-24 years
Barbados: Incidence Rate (per 100,000)
Incidence Rate
< 612.0
612.0 - 688.8
688.8 - 738.5
738.5 - 788.1
788.1 - 845.1
845.1 - 902.1
902.1 - 959.1
959.1 - 965.5
965.5 - 991.3
> 991.3
Female
Male
Sexes Combined
Age
Year
Incidence Rate (per 100,000)
Age — 10-24 years
Guyana: Incidence Rate (per 100,000)
Incidence Rate
< 619.9
619.9 - 693.1
693.1 - 743.7
743.7 - 794.3
794.3 - 851.5
851.5 - 908.7
908.7 - 965.9
965.9 - 975.2
975.2 - 1001.0
> 1001.0
Female
Male
Sexes Combined
Age
Year
Incidence Rate (per 100,000)
Age — 10-24 years
Australia: Incidence Rate (per 100,000)
Incidence Rate
< 883.6
883.6 - 1039.5
1039.5 - 1084.1
1084.1 - 1128.7
1128.7 - 1196.0
1196.0 - 1263.3
1263.3 - 1330.6
1330.6 - 1347.5
1347.5 - 1431.1
> 1431.1
Female
Male
Sexes Combined
Age
Year
Incidence Rate (per 100,000)
Age — 10-24 years
Morocco: Incidence Rate (per 100,000)
Incidence Rate
< 741.8
741.8 - 841.1
841.1 - 950.1
950.1 - 1059.0
1059.0 - 1108.9
1108.9 - 1158.7
1158.7 - 1208.6
1208.6 - 1232.9
1232.9 - 1371.2
> 1371.2
Female
Male
Sexes Combined
Age
Year
Incidence Rate (per 100,000)
Age — 10-24 years
United Republic of Tanzania: Incidence Rate (per 100,000)
Incidence Rate
< 645.0
645.0 - 664.1
664.1 - 699.5
699.5 - 735.0
735.0 - 766.7
766.7 - 798.5
798.5 - 830.2
830.2 - 841.4
841.4 - 871.5
> 871.5
Female
Male
Sexes Combined
Age
Year
Incidence Rate (per 100,000)
Age — 10-24 years
Belarus: Incidence Rate (per 100,000)
Incidence Rate
< 533.5
533.5 - 606.6
606.6 - 645.5
645.5 - 684.5
684.5 - 734.2
734.2 - 784.0
784.0 - 833.8
833.8 - 843.9
843.9 - 936.1
> 936.1
Female
Male
Sexes Combined
Age
Year
Incidence Rate (per 100,000)
Age — 10-24 years

Figure S1

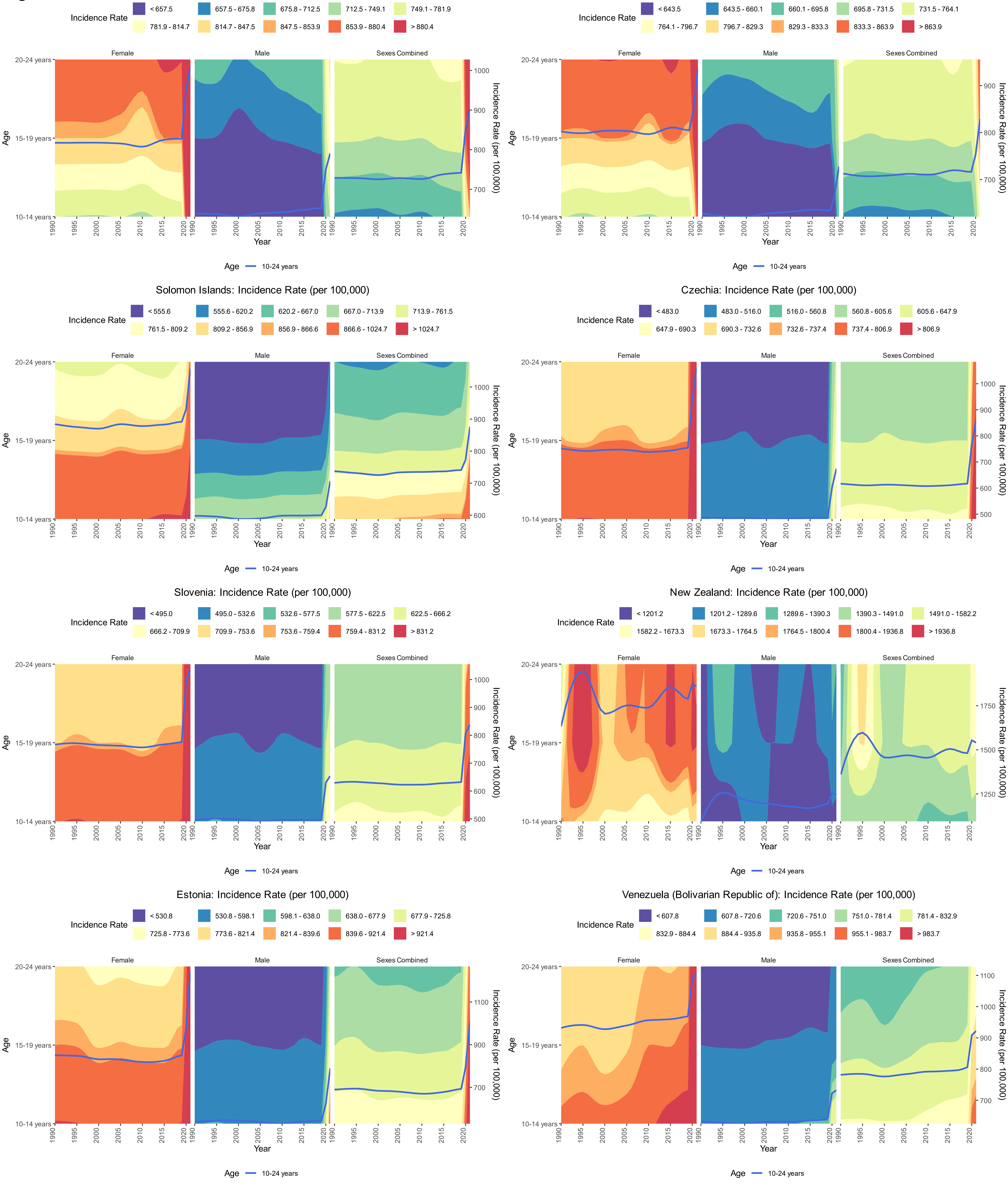
Madagascar: Incidence Rate (per 100,000)
Incidence Rate
< 657.5
657.5 - 675.8
675.8 - 712.5
712.5 - 749.1
749.1 - 781.9
781.9 - 814.7
814.7 - 847.5
847.5 - 853.9
853.9 - 880.4
> 880.4
Eritrea: Incidence Rate (per 100,000)
Incidence Rate
< 643.5
643.5 - 660.1
660.1 - 695.8
695.8 - 731.5
731.5 - 764.1
764.1 - 796.7
796.7 - 829.3
829.3 - 833.3
833.3 - 863.9
> 863.9
Solomon Islands: Incidence Rate (per 100,000)
Incidence Rate
< 555.6
555.6 - 620.2
620.2 - 667.0
667.0 - 713.9
713.9 - 761.5
761.5 - 809.2
809.2 - 856.9
856.9 - 866.6
866.6 - 1024.7
> 1024.7
Czechia: Incidence Rate (per 100,000)
Incidence Rate
< 483.0
483.0 - 516.0
516.0 - 560.8
560.8 - 605.6
605.6 - 647.9
647.9 - 690.3
690.3 - 732.6
732.6 - 737.4
737.4 - 806.9
> 806.9
Slovenia: Incidence Rate (per 100,000)
Incidence Rate
< 495.0
495.0 - 532.6
532.6 - 577.5
577.5 - 622.5
622.5 - 666.2
666.2 - 709.9
709.9 - 753.6
753.6 - 759.4
759.4 - 831.2
> 831.2
New Zealand: Incidence Rate (per 100,000)
Incidence Rate
< 1201.2
1201.2 - 1289.6
1289.6 - 1390.3
1390.3 - 1491.0
1491.0 - 1582.2
1582.2 - 1673.3
1673.3 - 1764.5
1764.5 - 1800.4
1800.4 - 1936.8
> 1936.8
Estonia: Incidence Rate (per 100,000)
Incidence Rate
< 530.8
530.8 - 598.1
598.1 - 638.0
638.0 - 677.9
677.9 - 725.8
725.8 - 773.6
773.6 - 821.4
821.4 - 839.6
839.6 - 921.4
> 921.4
Venezuela (Bolivarian Republic of): Incidence Rate (per 100,000)
Incidence Rate
< 607.8
607.8 - 720.6
720.6 - 751.0
751.0 - 781.4
781.4 - 832.9
832.9 - 884.4
884.4 - 935.8
935.8 - 955.1
955.1 - 983.7
> 983.7
Female
Male
Sexes Combined
20-24 years
15-19 years
10-14 years
Age
Year
Incidence Rate (per 100,000)
Age
10-24 years

Figure S1

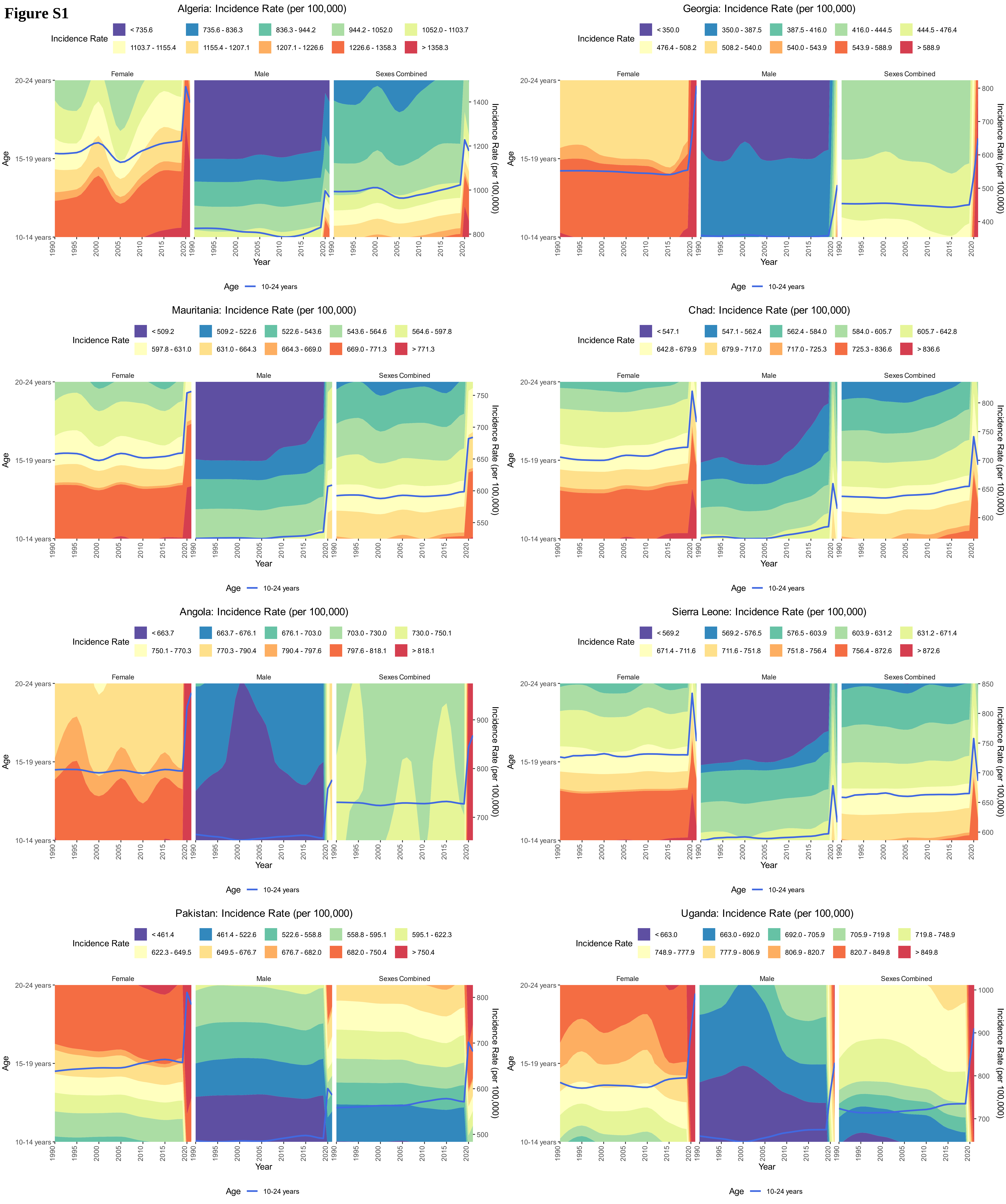
Algeria: Incidence Rate (per 100,000)
Incidence Rate
< 735.6
735.6 - 836.3
836.3 - 944.2
944.2 - 1052.0
1052.0 - 1103.7
1103.7 - 1155.4
1155.4 - 1207.1
1207.1 - 1226.6
1226.6 - 1358.3
> 1358.3
Georgia: Incidence Rate (per 100,000)
Incidence Rate
< 350.0
350.0 - 387.5
387.5 - 416.0
416.0 - 444.5
444.5 - 476.4
476.4 - 508.2
508.2 - 540.0
540.0 - 543.9
543.9 - 588.9
> 588.9
Mauritania: Incidence Rate (per 100,000)
Incidence Rate
< 509.2
509.2 - 522.6
522.6 - 543.6
543.6 - 564.6
564.6 - 597.8
597.8 - 631.0
631.0 - 664.3
664.3 - 669.0
669.0 - 771.3
> 771.3
Chad: Incidence Rate (per 100,000)
Incidence Rate
< 547.1
547.1 - 562.4
562.4 - 584.0
584.0 - 605.7
605.7 - 642.8
642.8 - 679.9
679.9 - 717.0
717.0 - 725.3
725.3 - 836.6
> 836.6
Angola: Incidence Rate (per 100,000)
Incidence Rate
< 663.7
663.7 - 676.1
676.1 - 703.0
703.0 - 730.0
730.0 - 750.1
750.1 - 770.3
770.3 - 790.4
790.4 - 797.6
797.6 - 818.1
> 818.1
Sierra Leone: Incidence Rate (per 100,000)
Incidence Rate
< 569.2
569.2 - 576.5
576.5 - 603.9
603.9 - 631.2
631.2 - 671.4
671.4 - 711.6
711.6 - 751.8
751.8 - 756.4
756.4 - 872.6
> 872.6
Pakistan: Incidence Rate (per 100,000)
Incidence Rate
< 461.4
461.4 - 522.6
522.6 - 558.8
558.8 - 595.1
595.1 - 622.3
622.3 - 649.5
649.5 - 676.7
676.7 - 682.0
682.0 - 750.4
> 750.4
Uganda: Incidence Rate (per 100,000)
Incidence Rate
< 663.0
663.0 - 692.0
692.0 - 705.9
705.9 - 719.8
719.8 - 748.9
748.9 - 777.9
777.9 - 806.9
806.9 - 820.7
820.7 - 849.8
> 849.8
Female
Male
Sexes Combined
Age
20-24 years
15-19 years
10-14 years
Year
Incidence Rate (per 100,000)
Age — 10-24 years

Figure S1

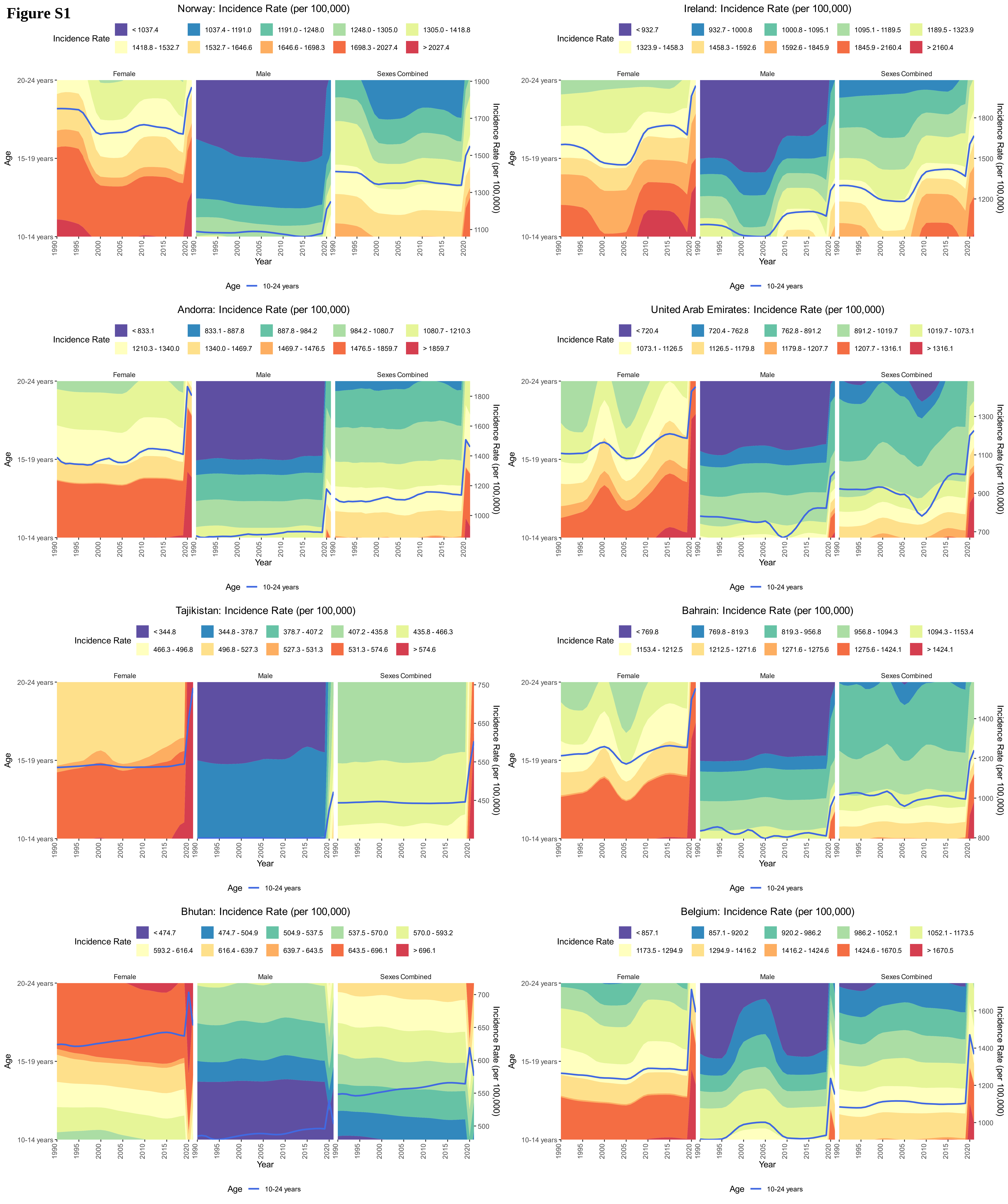

Norway: Incidence Rate (per 100,000)
Incidence Rate
< 1037.4
1037.4 - 1191.0
1191.0 - 1248.0
1248.0 - 1305.0
1305.0 - 1418.8
1418.8 - 1532.7
1532.7 - 1646.6
1646.6 - 1698.3
1698.3 - 2027.4
> 2027.4
Ireland: Incidence Rate (per 100,000)
Incidence Rate
< 932.7
932.7 - 1000.8
1000.8 - 1095.1
1095.1 - 1189.5
1189.5 - 1323.9
1323.9 - 1458.3
1458.3 - 1592.6
1592.6 - 1845.9
1845.9 - 2160.4
> 2160.4
Andorra: Incidence Rate (per 100,000)
Incidence Rate
< 833.1
833.1 - 887.8
887.8 - 984.2
984.2 - 1080.7
1080.7 - 1210.3
1210.3 - 1340.0
1340.0 - 1469.7
1469.7 - 1476.5
1476.5 - 1859.7
> 1859.7
United Arab Emirates: Incidence Rate (per 100,000)
Incidence Rate
< 720.4
720.4 - 762.8
762.8 - 891.2
891.2 - 1019.7
1019.7 - 1073.1
1073.1 - 1126.5
1126.5 - 1179.8
1179.8 - 1207.7
1207.7 - 1316.1
> 1316.1
Tajikistan: Incidence Rate (per 100,000)
Incidence Rate
< 344.8
344.8 - 378.7
378.7 - 407.2
407.2 - 435.8
435.8 - 466.3
466.3 - 496.8
496.8 - 527.3
527.3 - 531.3
531.3 - 574.6
> 574.6
Bahrain: Incidence Rate (per 100,000)
Incidence Rate
< 769.8
769.8 - 819.3
819.3 - 956.8
956.8 - 1094.3
1094.3 - 1153.4
1153.4 - 1212.5
1212.5 - 1271.6
1271.6 - 1275.6
1275.6 - 1424.1
> 1424.1
Bhutan: Incidence Rate (per 100,000)
Incidence Rate
< 474.7
474.7 - 504.9
504.9 - 537.5
537.5 - 570.0
570.0 - 593.2
593.2 - 616.4
616.4 - 639.7
639.7 - 643.5
643.5 - 696.1
> 696.1
Belgium: Incidence Rate (per 100,000)
Incidence Rate
< 857.1
857.1 - 920.2
920.2 - 986.2
986.2 - 1052.1
1052.1 - 1173.5
1173.5 - 1294.9
1294.9 - 1416.2
1416.2 - 1424.6
1424.6 - 1670.5
> 1670.5
Female
Male
Sexes Combined
Age
20-24 years
15-19 years
10-14 years
Year
Incidence Rate (per 100,000)
Age
10-24 years

Figure S1

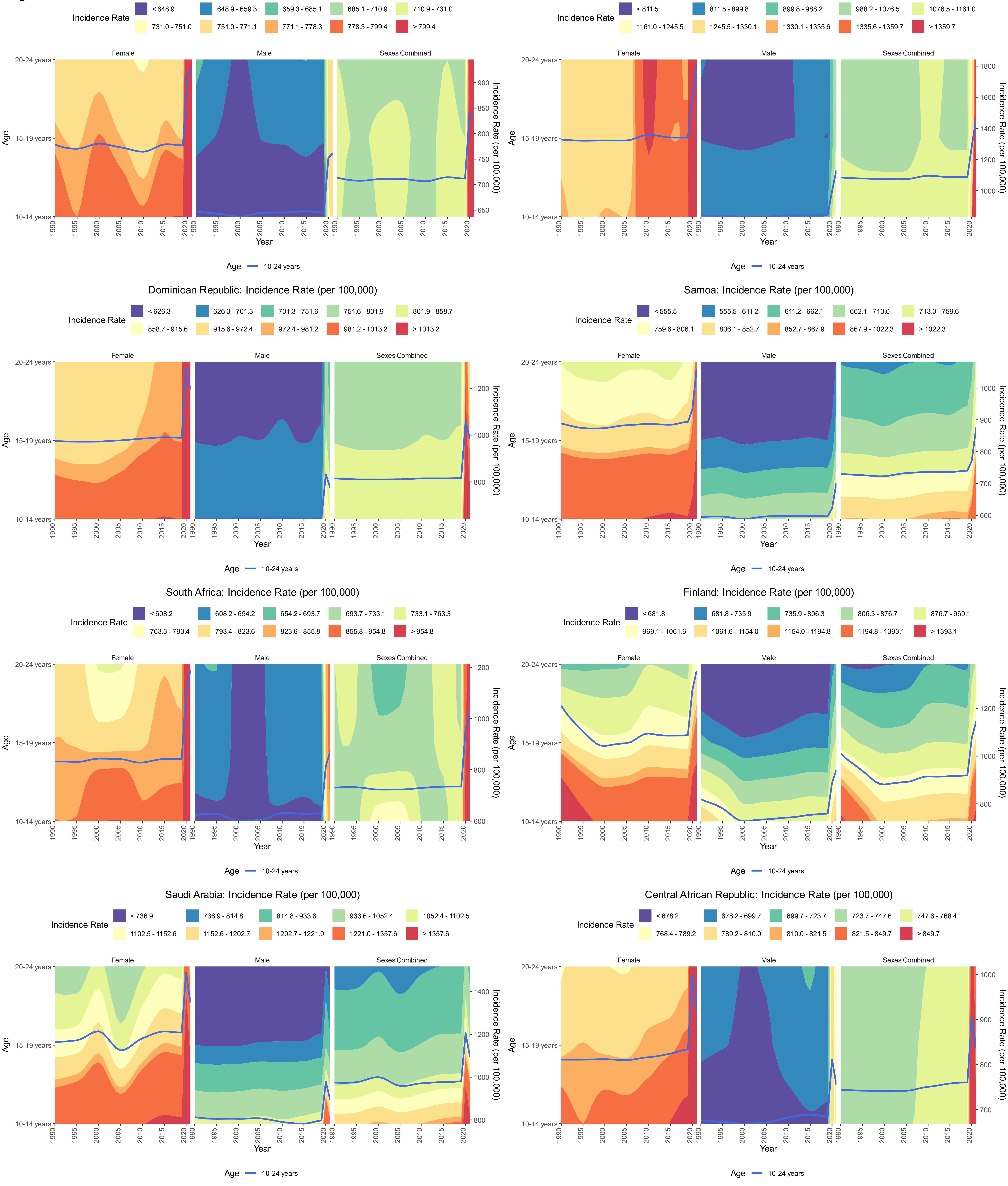
Congo: Incidence Rate (per 100,000)
Incidence Rate
< 648.9
648.9 - 659.3
659.3 - 685.1
685.1 - 710.9
710.9 - 731.0
731.0 - 751.0
751.0 - 771.1
771.1 - 778.3
778.3 - 799.4
> 799.4
Female
Male
Sexes Combined
Age
Year
Incidence Rate (per 100,000)
Age — 10-24 years
Paraguay: Incidence Rate (per 100,000)
< 811.5
811.5 - 899.8
899.8 - 988.2
988.2 - 1076.5
1076.5 - 1161.0
1161.0 - 1245.5
1245.5 - 1330.1
1330.1 - 1335.6
1335.6 - 1359.7
> 1359.7
Dominican Republic: Incidence Rate (per 100,000)
< 626.3
626.3 - 701.3
701.3 - 751.6
751.6 - 801.9
801.9 - 858.7
858.7 - 915.6
915.6 - 972.4
972.4 - 981.2
981.2 - 1013.2
> 1013.2
Samoa: Incidence Rate (per 100,000)
< 555.5
555.5 - 611.2
611.2 - 662.1
662.1 - 713.0
713.0 - 759.6
759.6 - 806.1
806.1 - 852.7
852.7 - 867.9
867.9 - 1022.3
> 1022.3
South Africa: Incidence Rate (per 100,000)
< 608.2
608.2 - 654.2
654.2 - 693.7
693.7 - 733.1
733.1 - 763.3
763.3 - 793.4
793.4 - 823.6
823.6 - 855.8
855.8 - 954.8
> 954.8
Finland: Incidence Rate (per 100,000)
< 681.8
681.8 - 735.9
735.9 - 806.3
806.3 - 876.7
876.7 - 969.1
969.1 - 1061.6
1061.6 - 1154.0
1154.0 - 1194.8
1194.8 - 1393.1
> 1393.1
Saudi Arabia: Incidence Rate (per 100,000)
< 736.9
736.9 - 814.8
814.8 - 933.6
933.6 - 1052.4
1052.4 - 1102.5
1102.5 - 1152.6
1152.6 - 1202.7
1202.7 - 1221.0
1221.0 - 1357.6
> 1357.6
Central African Republic: Incidence Rate (per 100,000)
< 678.2
678.2 - 699.7
699.7 - 723.7
723.7 - 747.6
747.6 - 768.4
768.4 - 789.2
789.2 - 810.0
810.0 - 821.5
821.5 - 849.7
> 849.7
20-24 years
15-19 years
10-14 years
1990
1995
2000
2005
2010
2015
2020

Figure S1

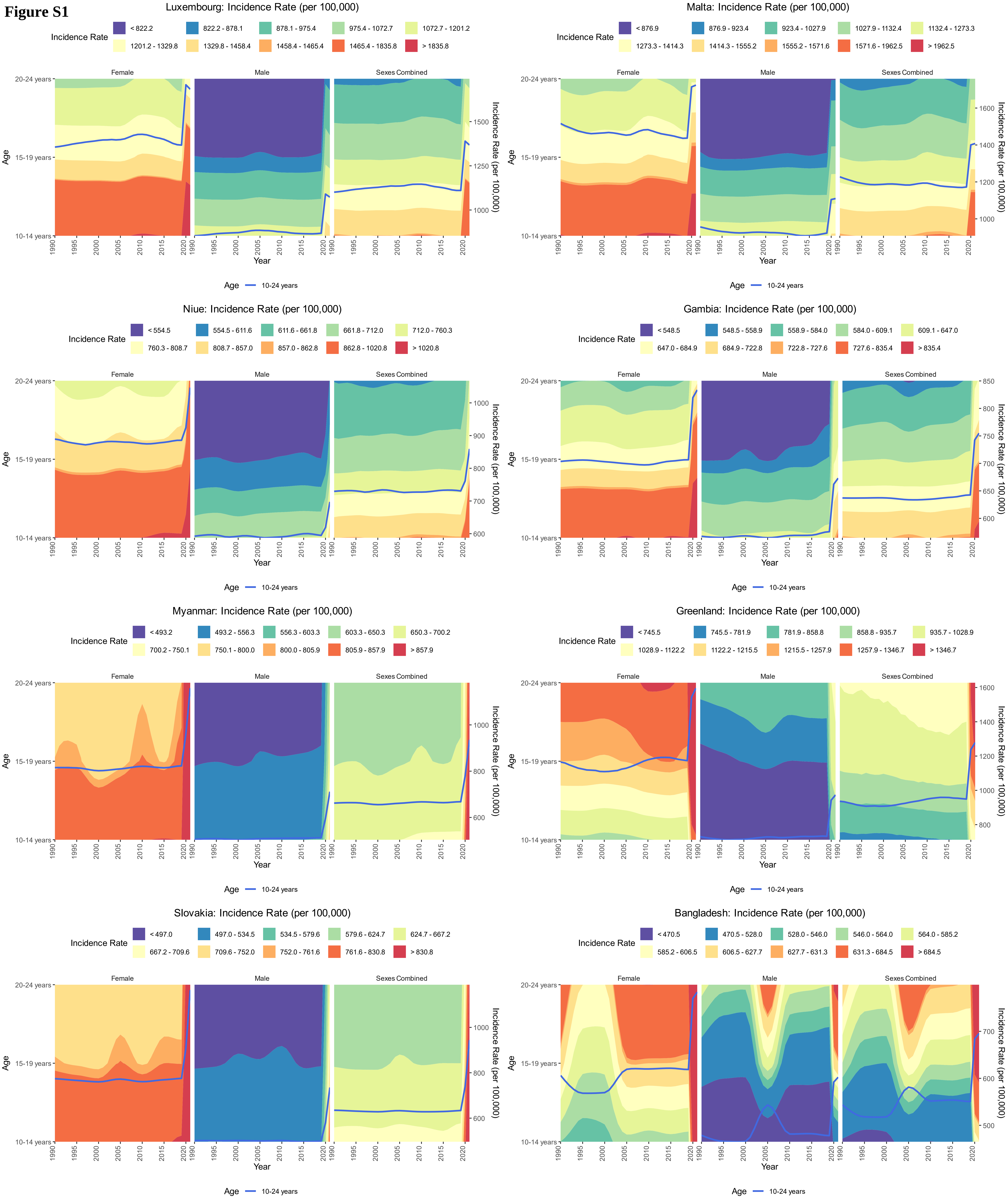

Luxembourg: Incidence Rate (per 100,000)
Incidence Rate
< 822.2
822.2 - 878.1
878.1 - 975.4
975.4 - 1072.7
1072.7 - 1201.2
1201.2 - 1329.8
1329.8 - 1458.4
1458.4 - 1465.4
1465.4 - 1835.8
> 1835.8
Malta: Incidence Rate (per 100,000)
< 876.9
876.9 - 923.4
923.4 - 1027.9
1027.9 - 1132.4
1132.4 - 1273.3
1273.3 - 1414.3
1414.3 - 1555.2
1555.2 - 1571.6
1571.6 - 1962.5
> 1962.5
Niue: Incidence Rate (per 100,000)
< 554.5
554.5 - 611.6
611.6 - 661.8
661.8 - 712.0
712.0 - 760.3
760.3 - 808.7
808.7 - 857.0
857.0 - 862.8
862.8 - 1020.8
> 1020.8
Gambia: Incidence Rate (per 100,000)
< 548.5
548.5 - 558.9
558.9 - 584.0
584.0 - 609.1
609.1 - 647.0
647.0 - 684.9
684.9 - 722.8
722.8 - 727.6
727.6 - 835.4
> 835.4
Myanmar: Incidence Rate (per 100,000)
< 493.2
493.2 - 556.3
556.3 - 603.3
603.3 - 650.3
650.3 - 700.2
700.2 - 750.1
750.1 - 800.0
800.0 - 805.9
805.9 - 857.9
> 857.9
Greenland: Incidence Rate (per 100,000)
< 745.5
745.5 - 781.9
781.9 - 858.8
858.8 - 935.7
935.7 - 1028.9
1028.9 - 1122.2
1122.2 - 1215.5
1215.5 - 1257.9
1257.9 - 1346.7
> 1346.7
Slovakia: Incidence Rate (per 100,000)
< 497.0
497.0 - 534.5
534.5 - 579.6
579.6 - 624.7
624.7 - 667.2
667.2 - 709.6
709.6 - 752.0
752.0 - 761.6
761.6 - 830.8
> 830.8
Bangladesh: Incidence Rate (per 100,000)
< 470.5
470.5 - 528.0
528.0 - 546.0
546.0 - 564.0
564.0 - 585.2
585.2 - 606.5
606.5 - 627.7
627.7 - 631.3
631.3 - 684.5
> 684.5
Female
Male
Sexes Combined
Age
20-24 years
15-19 years
10-14 years
Year
Incidence Rate (per 100,000)
Age
10-24 years

Figure S1

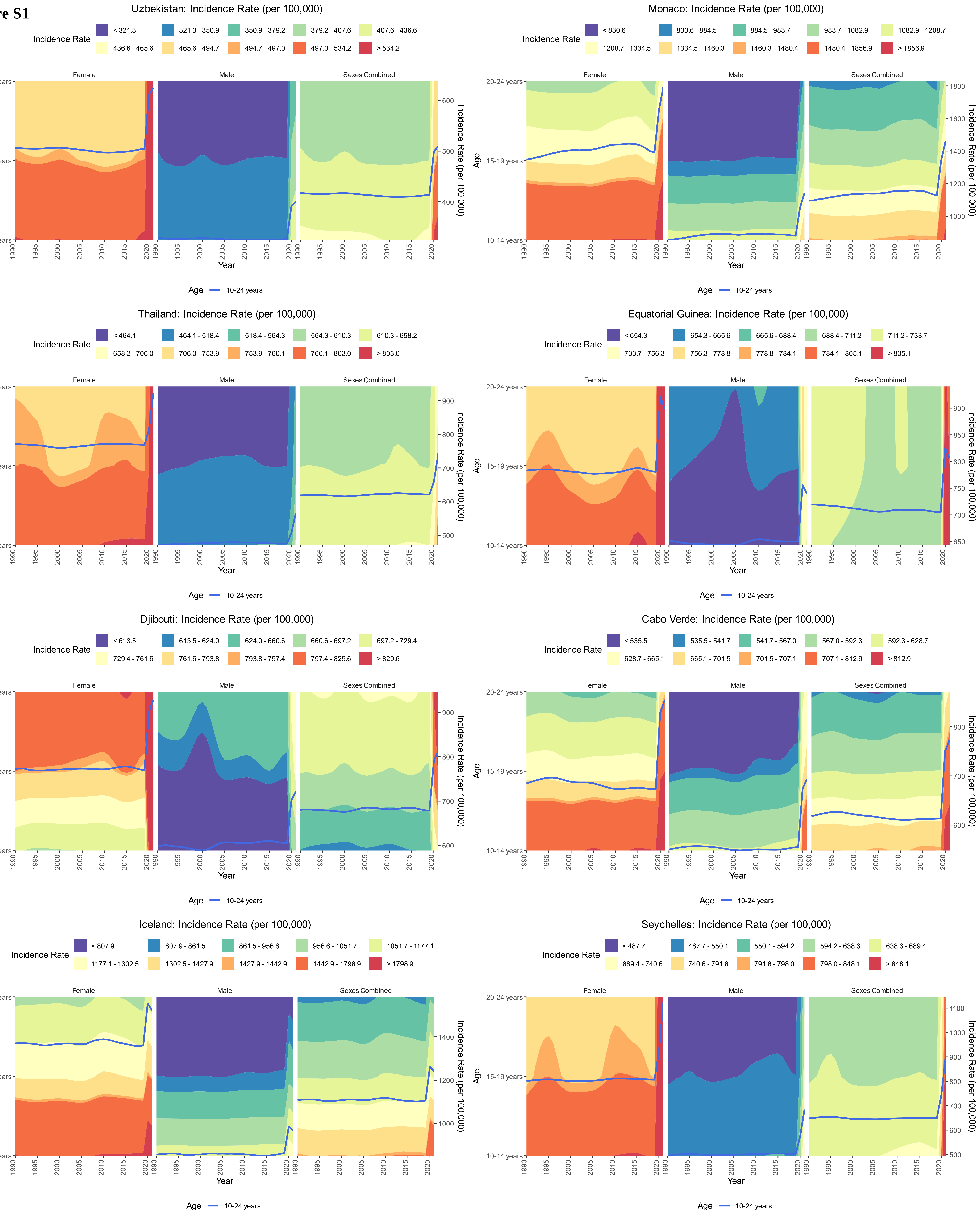
Uzbekistan: Incidence Rate (per 100,000)
Incidence Rate
< 321.3
321.3 - 350.9
350.9 - 379.2
379.2 - 407.6
407.6 - 436.6
436.6 - 465.6
465.6 - 494.7
494.7 - 497.0
497.0 - 534.2
> 534.2
Female
Male
Sexes Combined
Monaco: Incidence Rate (per 100,000)
Incidence Rate
< 830.6
830.6 - 884.5
884.5 - 983.7
983.7 - 1082.9
1082.9 - 1208.7
1208.7 - 1334.5
1334.5 - 1460.3
1460.3 - 1480.4
1480.4 - 1856.9
> 1856.9
Thailand: Incidence Rate (per 100,000)
Incidence Rate
< 464.1
464.1 - 518.4
518.4 - 564.3
564.3 - 610.3
610.3 - 658.2
658.2 - 706.0
706.0 - 753.9
753.9 - 760.1
760.1 - 803.0
> 803.0
Equatorial Guinea: Incidence Rate (per 100,000)
Incidence Rate
< 654.3
654.3 - 665.6
665.6 - 688.4
688.4 - 711.2
711.2 - 733.7
733.7 - 756.3
756.3 - 778.8
778.8 - 784.1
784.1 - 805.1
> 805.1
Djibouti: Incidence Rate (per 100,000)
Incidence Rate
< 613.5
613.5 - 624.0
624.0 - 660.6
660.6 - 697.2
697.2 - 729.4
729.4 - 761.6
761.6 - 793.8
793.8 - 797.4
797.4 - 829.6
> 829.6
Cabo Verde: Incidence Rate (per 100,000)
Incidence Rate
< 535.5
535.5 - 541.7
541.7 - 567.0
567.0 - 592.3
592.3 - 628.7
628.7 - 665.1
665.1 - 701.5
701.5 - 707.1
707.1 - 812.9
> 812.9
Iceland: Incidence Rate (per 100,000)
Incidence Rate
< 807.9
807.9 - 861.5
861.5 - 956.6
956.6 - 1051.7
1051.7 - 1177.1
1177.1 - 1302.5
1302.5 - 1427.9
1427.9 - 1442.9
1442.9 - 1798.9
> 1798.9
Seychelles: Incidence Rate (per 100,000)
Incidence Rate
< 487.7
487.7 - 550.1
550.1 - 594.2
594.2 - 638.3
638.3 - 689.4
689.4 - 740.6
740.6 - 791.8
791.8 - 798.0
798.0 - 848.1
> 848.1
Age
20-24 years
15-19 years
10-14 years
Year
1990
1995
2000
2005
2010
2015
2020
Incidence Rate (per 100,000)
Age — 10-24 years

Figure S1

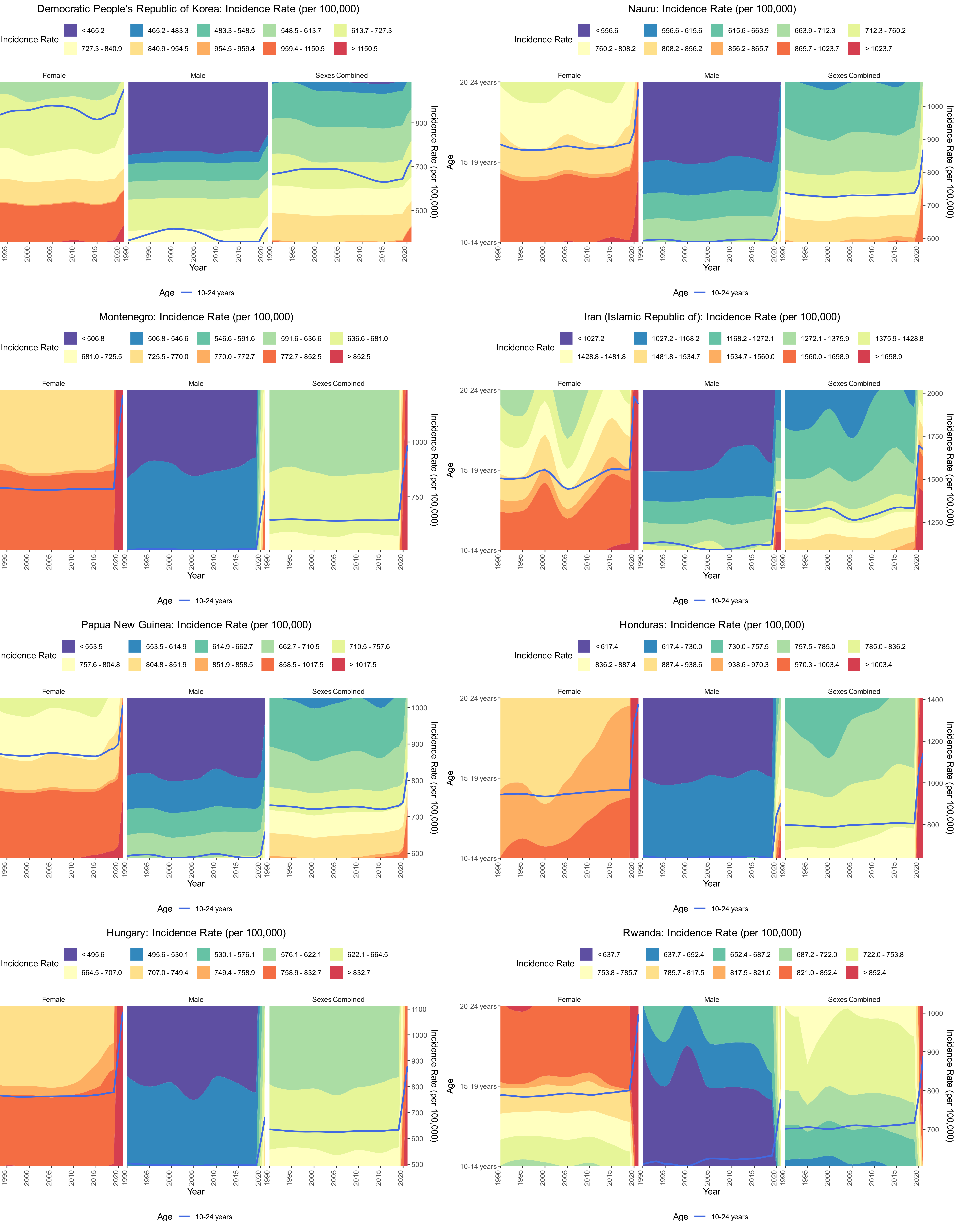

Democratic People's Republic of Korea: Incidence Rate (per 100,000)
Incidence Rate
< 465.2
465.2 - 483.3
483.3 - 548.5
548.5 - 613.7
613.7 - 727.3
727.3 - 840.9
840.9 - 954.5
954.5 - 959.4
959.4 - 1150.5
> 1150.5
Nauru: Incidence Rate (per 100,000)
Incidence Rate
< 556.6
556.6 - 615.6
615.6 - 663.9
663.9 - 712.3
712.3 - 760.2
760.2 - 808.2
808.2 - 856.2
856.2 - 865.7
865.7 - 1023.7
> 1023.7
Montenegro: Incidence Rate (per 100,000)
Incidence Rate
< 506.8
506.8 - 546.6
546.6 - 591.6
591.6 - 636.6
636.6 - 681.0
681.0 - 725.5
725.5 - 770.0
770.0 - 772.7
772.7 - 852.5
> 852.5
Iran (Islamic Republic of): Incidence Rate (per 100,000)
Incidence Rate
< 1027.2
1027.2 - 1168.2
1168.2 - 1272.1
1272.1 - 1375.9
1375.9 - 1428.8
1428.8 - 1481.8
1481.8 - 1534.7
1534.7 - 1560.0
1560.0 - 1698.9
> 1698.9
Papua New Guinea: Incidence Rate (per 100,000)
Incidence Rate
< 553.5
553.5 - 614.9
614.9 - 662.7
662.7 - 710.5
710.5 - 757.6
757.6 - 804.8
804.8 - 851.9
851.9 - 858.5
858.5 - 1017.5
> 1017.5
Honduras: Incidence Rate (per 100,000)
Incidence Rate
< 617.4
617.4 - 730.0
730.0 - 757.5
757.5 - 785.0
785.0 - 836.2
836.2 - 887.4
887.4 - 938.6
938.6 - 970.3
970.3 - 1003.4
> 1003.4
Hungary: Incidence Rate (per 100,000)
Incidence Rate
< 495.6
495.6 - 530.1
530.1 - 576.1
576.1 - 622.1
622.1 - 664.5
664.5 - 707.0
707.0 - 749.4
749.4 - 758.9
758.9 - 832.7
> 832.7
Rwanda: Incidence Rate (per 100,000)
Incidence Rate
< 637.7
637.7 - 652.4
652.4 - 687.2
687.2 - 722.0
722.0 - 753.8
753.8 - 785.7
785.7 - 817.5
817.5 - 821.0
821.0 - 852.4
> 852.4
Female
Male
Sexes Combined
20-24 years
15-19 years
10-14 years
Age
Year
Incidence Rate (per 100,000)
Age
10-24 years

Figure S1

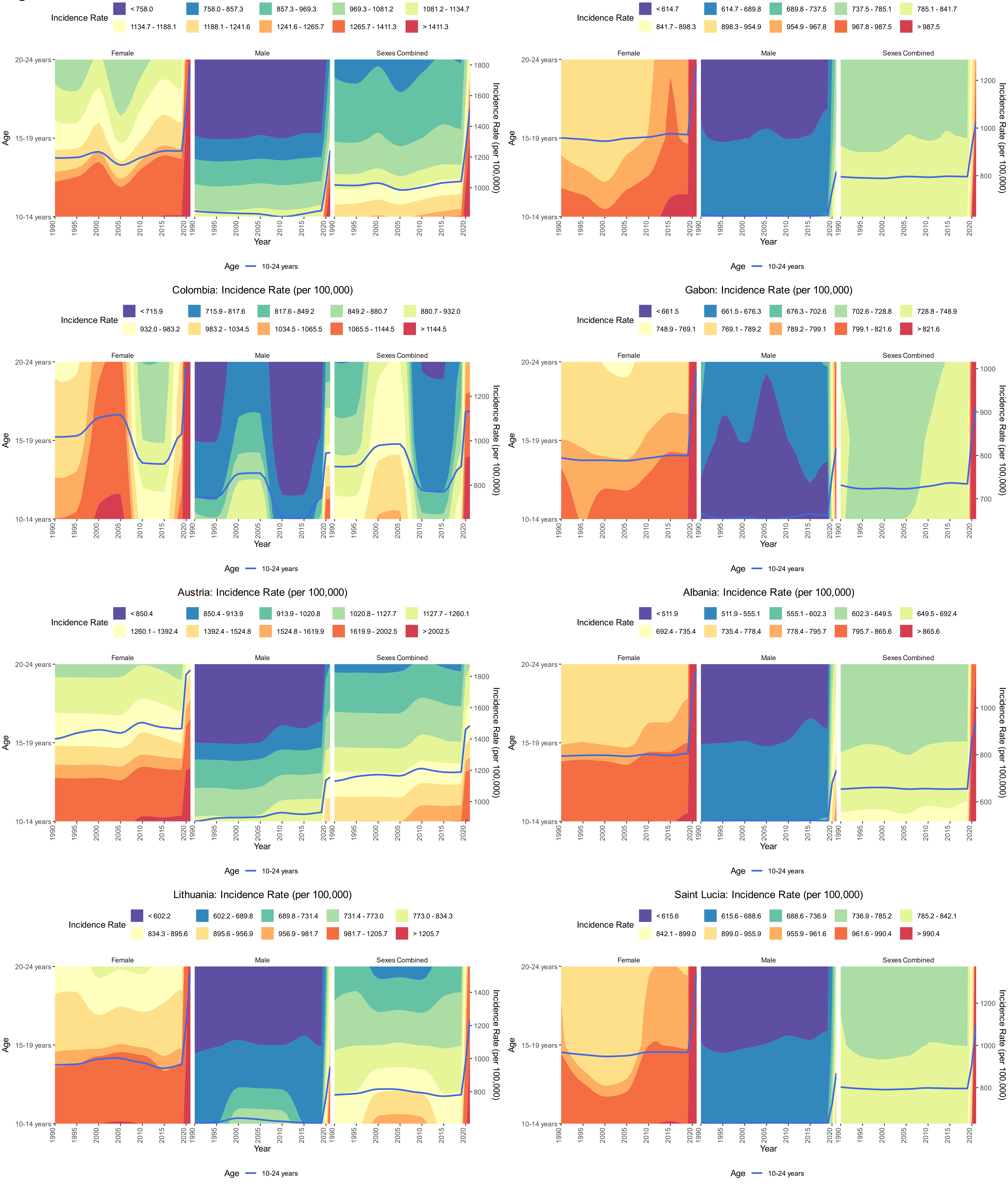
Tunisia: Incidence Rate (per 100,000)
Incidence Rate
< 758.0
758.0 - 857.3
857.3 - 969.3
969.3 - 1081.2
1081.2 - 1134.7
1134.7 - 1188.1
1188.1 - 1241.6
1241.6 - 1265.7
1265.7 - 1411.3
> 1411.3
Female
Male
Sexes Combined
Age
20-24 years
15-19 years
10-14 years
Year
Incidence Rate (per 100,000)
Age
10-24 years
Grenada: Incidence Rate (per 100,000)
Incidence Rate
< 614.7
614.7 - 689.8
689.8 - 737.5
737.5 - 785.1
785.1 - 841.7
841.7 - 898.3
898.3 - 954.9
954.9 - 967.8
967.8 - 987.5
> 987.5
Colombia: Incidence Rate (per 100,000)
Incidence Rate
< 715.9
715.9 - 817.6
817.6 - 849.2
849.2 - 880.7
880.7 - 932.0
932.0 - 983.2
983.2 - 1034.5
1034.5 - 1065.5
1065.5 - 1144.5
> 1144.5
Gabon: Incidence Rate (per 100,000)
Incidence Rate
< 661.5
661.5 - 676.3
676.3 - 702.6
702.6 - 728.8
728.8 - 748.9
748.9 - 769.1
769.1 - 789.2
789.2 - 799.1
799.1 - 821.6
> 821.6
Austria: Incidence Rate (per 100,000)
Incidence Rate
< 850.4
850.4 - 913.9
913.9 - 1020.8
1020.8 - 1127.7
1127.7 - 1260.1
1260.1 - 1392.4
1392.4 - 1524.8
1524.8 - 1619.9
1619.9 - 2002.5
> 2002.5
Albania: Incidence Rate (per 100,000)
Incidence Rate
< 511.9
511.9 - 555.1
555.1 - 602.3
602.3 - 649.5
649.5 - 692.4
692.4 - 735.4
735.4 - 778.4
778.4 - 795.7
795.7 - 865.6
> 865.6
Lithuania: Incidence Rate (per 100,000)
Incidence Rate
< 602.2
602.2 - 689.8
689.8 - 731.4
731.4 - 773.0
773.0 - 834.3
834.3 - 895.6
895.6 - 956.9
956.9 - 981.7
981.7 - 1205.7
> 1205.7
Saint Lucia: Incidence Rate (per 100,000)
Incidence Rate
< 615.6
615.6 - 688.6
688.6 - 736.9
736.9 - 785.2
785.2 - 842.1
842.1 - 899.0
899.0 - 955.9
955.9 - 961.6
961.6 - 990.4
> 990.4

Figure S1

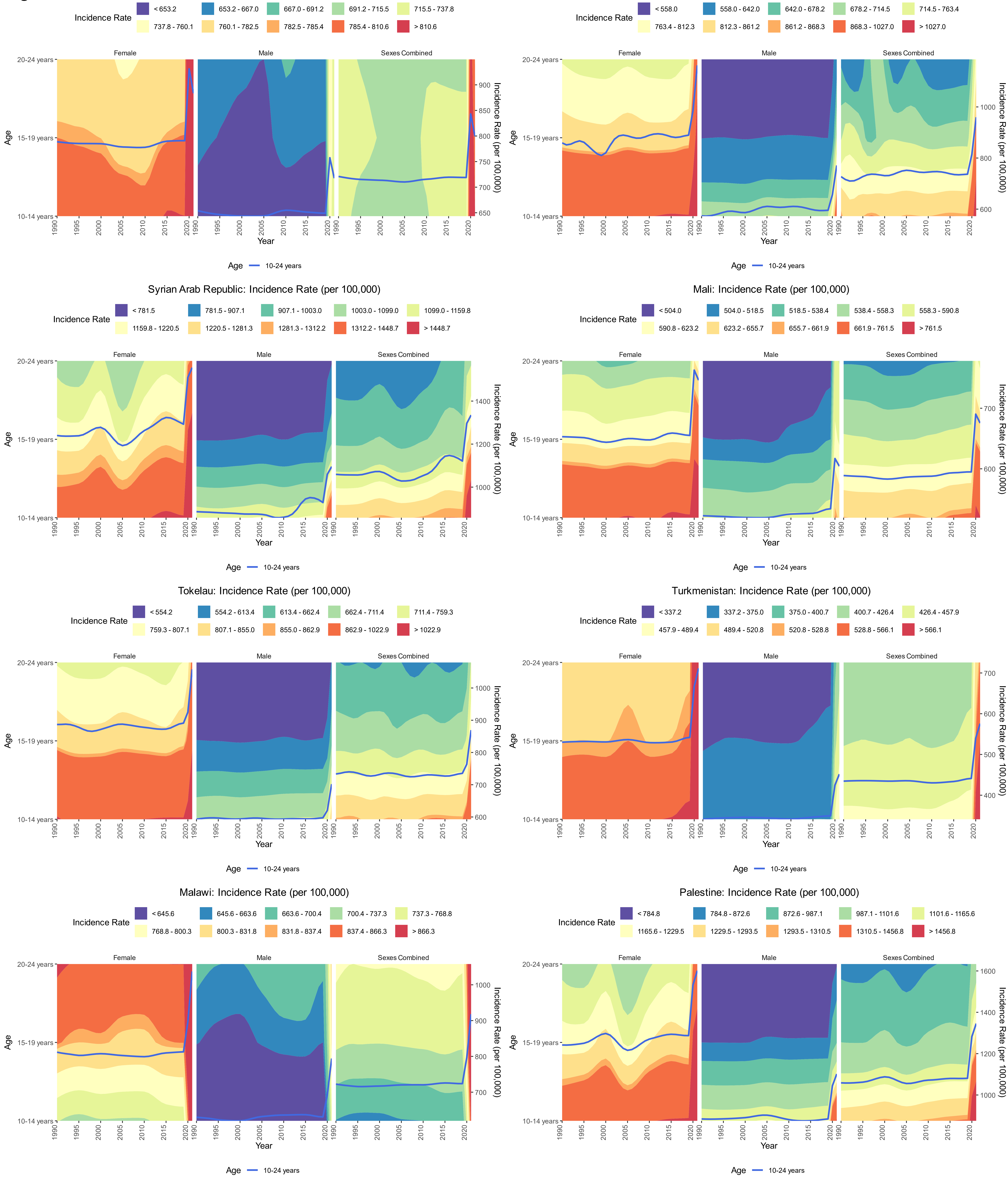

Democratic Republic of the Congo: Incidence Rate (per 100,000)
Incidence Rate
< 653.2
653.2 - 667.0
667.0 - 691.2
691.2 - 715.5
715.5 - 737.8
737.8 - 760.1
760.1 - 782.5
782.5 - 785.4
785.4 - 810.6
> 810.6
Northern Mariana Islands: Incidence Rate (per 100,000)
< 558.0
558.0 - 642.0
642.0 - 678.2
678.2 - 714.5
714.5 - 763.4
763.4 - 812.3
812.3 - 861.2
861.2 - 868.3
868.3 - 1027.0
> 1027.0
Syrian Arab Republic: Incidence Rate (per 100,000)
< 781.5
781.5 - 907.1
907.1 - 1003.0
1003.0 - 1099.0
1099.0 - 1159.8
1159.8 - 1220.5
1220.5 - 1281.3
1281.3 - 1312.2
1312.2 - 1448.7
> 1448.7
Mali: Incidence Rate (per 100,000)
< 504.0
504.0 - 518.5
518.5 - 538.4
538.4 - 558.3
558.3 - 590.8
590.8 - 623.2
623.2 - 655.7
655.7 - 661.9
661.9 - 761.5
> 761.5
Tokelau: Incidence Rate (per 100,000)
< 554.2
554.2 - 613.4
613.4 - 662.4
662.4 - 711.4
711.4 - 759.3
759.3 - 807.1
807.1 - 855.0
855.0 - 862.9
862.9 - 1022.9
> 1022.9
Turkmenistan: Incidence Rate (per 100,000)
< 337.2
337.2 - 375.0
375.0 - 400.7
400.7 - 426.4
426.4 - 457.9
457.9 - 489.4
489.4 - 520.8
520.8 - 528.8
528.8 - 566.1
> 566.1
Malawi: Incidence Rate (per 100,000)
< 645.6
645.6 - 663.6
663.6 - 700.4
700.4 - 737.3
737.3 - 768.8
768.8 - 800.3
800.3 - 831.8
831.8 - 837.4
837.4 - 866.3
> 866.3
Palestine: Incidence Rate (per 100,000)
< 784.8
784.8 - 872.6
872.6 - 987.1
987.1 - 1101.6
1101.6 - 1165.6
1165.6 - 1229.5
1229.5 - 1293.5
1293.5 - 1310.5
1310.5 - 1456.8
> 1456.8
Female
Male
Sexes Combined
Age
20-24 years
15-19 years
10-14 years
Year
Incidence Rate (per 100,000)
Age
10-24 years

Figure S1

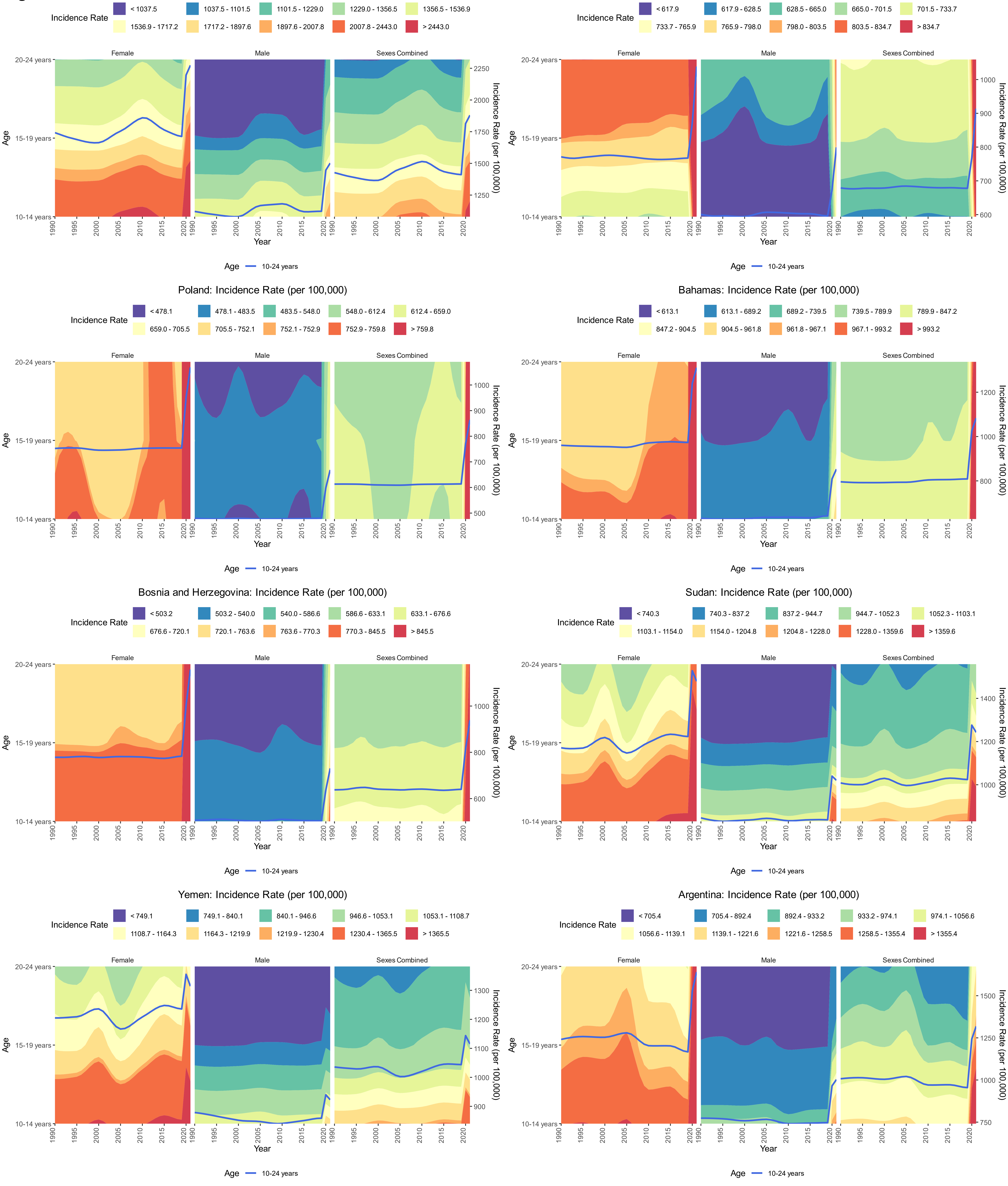
Portugal: Incidence Rate (per 100,000)
Incidence Rate
< 1037.5
1037.5 - 1101.5
1101.5 - 1229.0
1229.0 - 1356.5
1356.5 - 1536.9
1536.9 - 1717.2
1717.2 - 1897.6
1897.6 - 2007.8
2007.8 - 2443.0
> 2443.0
Female
Male
Sexes Combined
Age
Year
Incidence Rate (per 100,000)
Age 10-24 years
Somalia: Incidence Rate (per 100,000)
< 617.9
617.9 - 628.5
628.5 - 665.0
665.0 - 701.5
701.5 - 733.7
733.7 - 765.9
765.9 - 798.0
798.0 - 803.5
803.5 - 834.7
> 834.7
Poland: Incidence Rate (per 100,000)
< 478.1
478.1 - 483.5
483.5 - 548.0
548.0 - 612.4
612.4 - 659.0
659.0 - 705.5
705.5 - 752.1
752.1 - 752.9
752.9 - 759.8
> 759.8
Bahamas: Incidence Rate (per 100,000)
< 613.1
613.1 - 689.2
689.2 - 739.5
739.5 - 789.9
789.9 - 847.2
847.2 - 904.5
904.5 - 961.8
961.8 - 967.1
967.1 - 993.2
> 993.2
Bosnia and Herzegovina: Incidence Rate (per 100,000)
< 503.2
503.2 - 540.0
540.0 - 586.6
586.6 - 633.1
633.1 - 676.6
676.6 - 720.1
720.1 - 763.6
763.6 - 770.3
770.3 - 845.5
> 845.5
Sudan: Incidence Rate (per 100,000)
< 740.3
740.3 - 837.2
837.2 - 944.7
944.7 - 1052.3
1052.3 - 1103.1
1103.1 - 1154.0
1154.0 - 1204.8
1204.8 - 1228.0
1228.0 - 1359.6
> 1359.6
Yemen: Incidence Rate (per 100,000)
< 749.1
749.1 - 840.1
840.1 - 946.6
946.6 - 1053.1
1053.1 - 1108.7
1108.7 - 1164.3
1164.3 - 1219.9
1219.9 - 1230.4
1230.4 - 1365.5
> 1365.5
Argentina: Incidence Rate (per 100,000)
< 705.4
705.4 - 892.4
892.4 - 933.2
933.2 - 974.1
974.1 - 1056.6
1056.6 - 1139.1
1139.1 - 1221.6
1221.6 - 1258.5
1258.5 - 1355.4
> 1355.4
20-24 years
15-19 years
10-14 years
1990
1995
2000
2005
2010
2015
2020

Figure S1

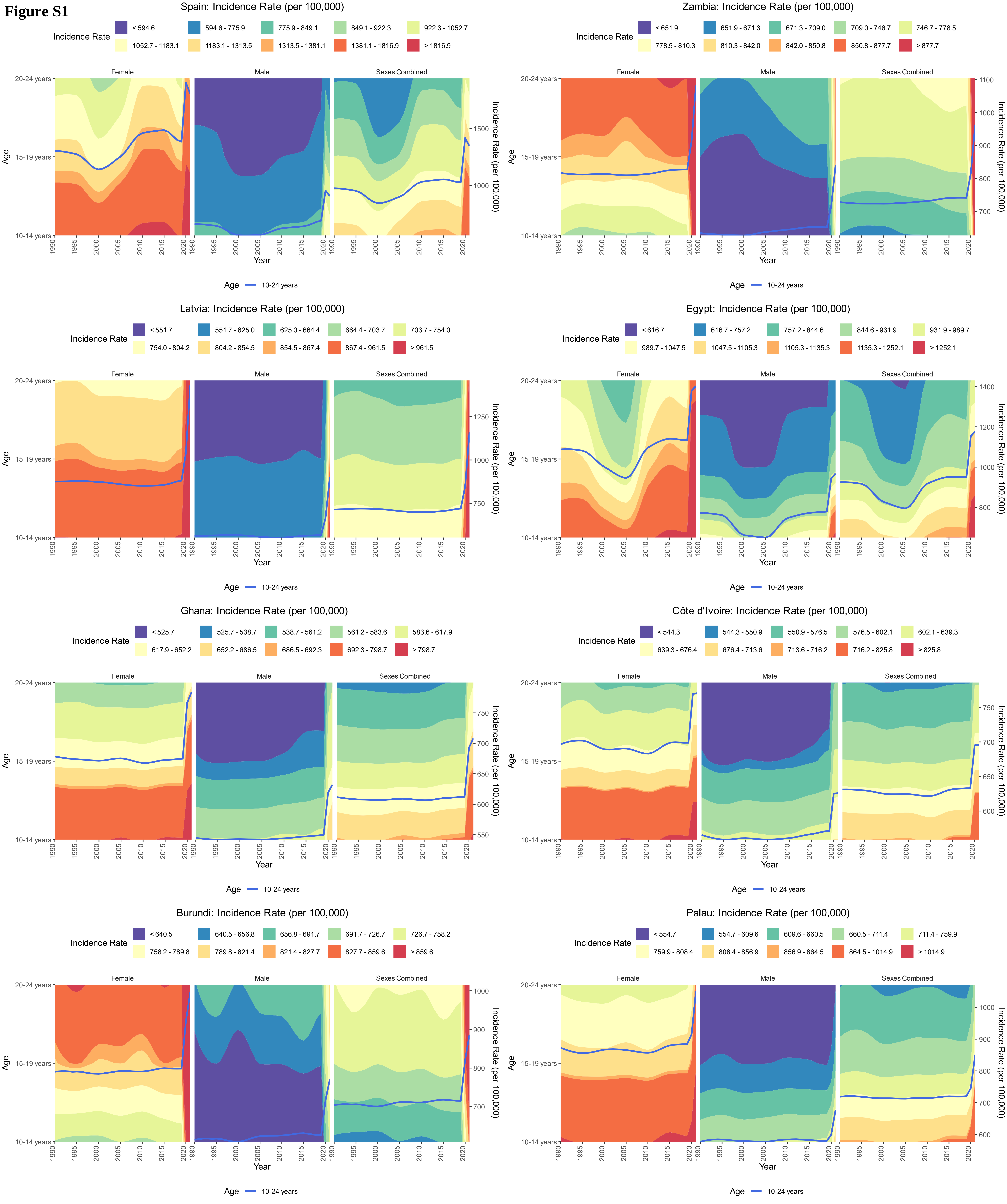

Spain: Incidence Rate (per 100,000)
Incidence Rate
< 594.6
594.6 - 775.9
775.9 - 849.1
849.1 - 922.3
922.3 - 1052.7
1052.7 - 1183.1
1183.1 - 1313.5
1313.5 - 1381.1
1381.1 - 1816.9
> 1816.9
Zambia: Incidence Rate (per 100,000)
Incidence Rate
< 651.9
651.9 - 671.3
671.3 - 709.0
709.0 - 746.7
746.7 - 778.5
778.5 - 810.3
810.3 - 842.0
842.0 - 850.8
850.8 - 877.7
> 877.7
Latvia: Incidence Rate (per 100,000)
Incidence Rate
< 551.7
551.7 - 625.0
625.0 - 664.4
664.4 - 703.7
703.7 - 754.0
754.0 - 804.2
804.2 - 854.5
854.5 - 867.4
867.4 - 961.5
> 961.5
Egypt: Incidence Rate (per 100,000)
Incidence Rate
< 616.7
616.7 - 757.2
757.2 - 844.6
844.6 - 931.9
931.9 - 989.7
989.7 - 1047.5
1047.5 - 1105.3
1105.3 - 1135.3
1135.3 - 1252.1
> 1252.1
Ghana: Incidence Rate (per 100,000)
Incidence Rate
< 525.7
525.7 - 538.7
538.7 - 561.2
561.2 - 583.6
583.6 - 617.9
617.9 - 652.2
652.2 - 686.5
686.5 - 692.3
692.3 - 798.7
> 798.7
Côte d'Ivoire: Incidence Rate (per 100,000)
Incidence Rate
< 544.3
544.3 - 550.9
550.9 - 576.5
576.5 - 602.1
602.1 - 639.3
639.3 - 676.4
676.4 - 713.6
713.6 - 716.2
716.2 - 825.8
> 825.8
Burundi: Incidence Rate (per 100,000)
Incidence Rate
< 640.5
640.5 - 656.8
656.8 - 691.7
691.7 - 726.7
726.7 - 758.2
758.2 - 789.8
789.8 - 821.4
821.4 - 827.7
827.7 - 859.6
> 859.6
Palau: Incidence Rate (per 100,000)
Incidence Rate
< 554.7
554.7 - 609.6
609.6 - 660.5
660.5 - 711.4
711.4 - 759.9
759.9 - 808.4
808.4 - 856.9
856.9 - 864.5
864.5 - 1014.9
> 1014.9
Female
Male
Sexes Combined
Age
20-24 years
15-19 years
10-14 years
Year
Incidence Rate (per 100,000)
Age — 10-24 years

Figure S1

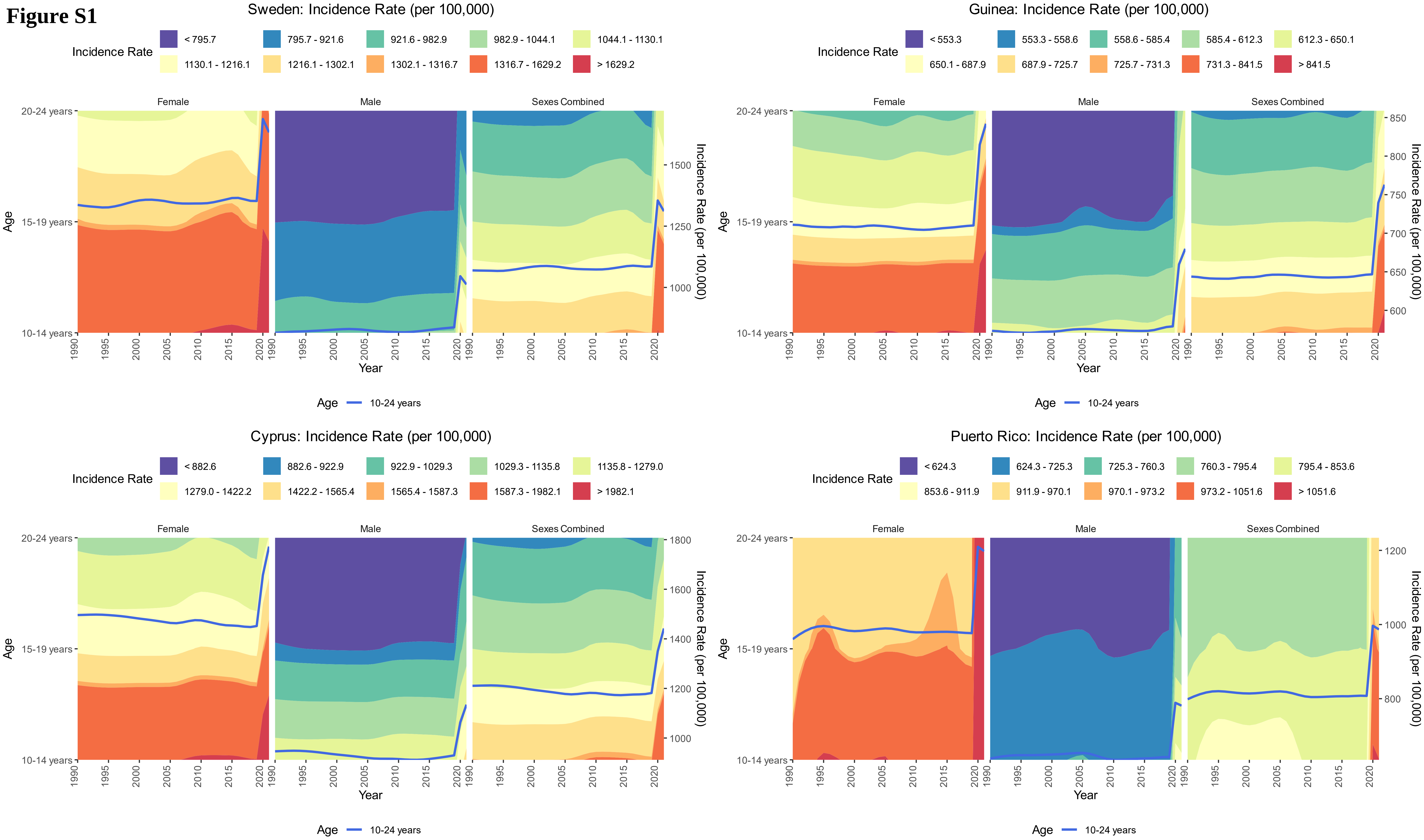

Sweden: Incidence Rate (per 100,000)
Incidence Rate
< 795.7
795.7 - 921.6
921.6 - 982.9
982.9 - 1044.1
1044.1 - 1130.1
1130.1 - 1216.1
1216.1 - 1302.1
1302.1 - 1316.7
1316.7 - 1629.2
> 1629.2
Female
Male
Sexes Combined
Age
Year
Incidence Rate (per 100,000)
10-24 years
Guinea: Incidence Rate (per 100,000)
< 553.3
553.3 - 558.6
558.6 - 585.4
585.4 - 612.3
612.3 - 650.1
650.1 - 687.9
687.9 - 725.7
725.7 - 731.3
731.3 - 841.5
> 841.5
Cyprus: Incidence Rate (per 100,000)
< 882.6
882.6 - 922.9
922.9 - 1029.3
1029.3 - 1135.8
1135.8 - 1279.0
1279.0 - 1422.2
1422.2 - 1565.4
1565.4 - 1587.3
1587.3 - 1982.1
> 1982.1
Puerto Rico: Incidence Rate (per 100,000)
< 624.3
624.3 - 725.3
725.3 - 760.3
760.3 - 795.4
795.4 - 853.6
853.6 - 911.9
911.9 - 970.1
970.1 - 973.2
973.2 - 1051.6
> 1051.6

Figure S2

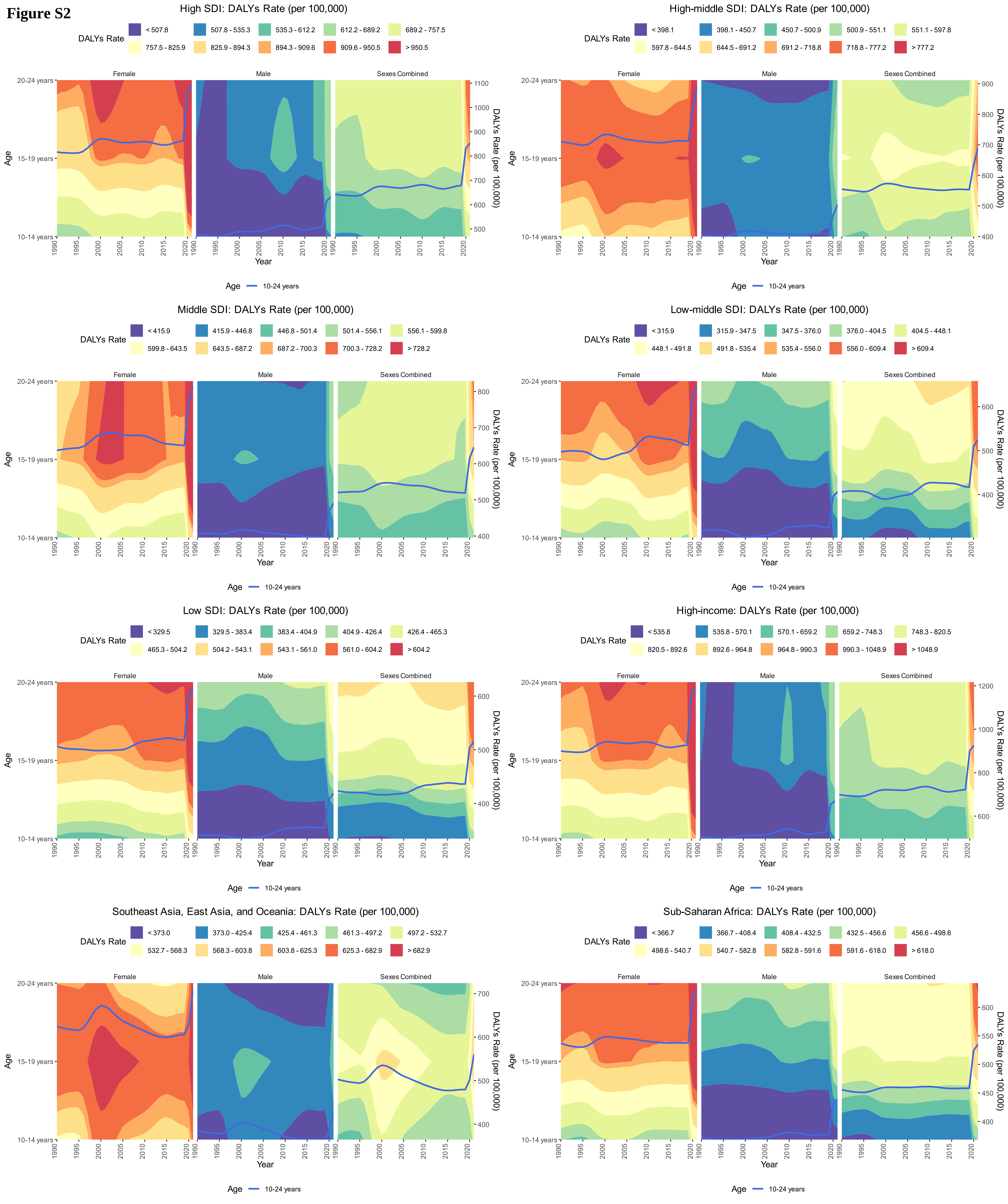
High SDI: DALYs Rate (per 100,000)
DALYs Rate
< 507.8
507.8 - 535.3
535.3 - 612.2
612.2 - 689.2
689.2 - 757.5
757.5 - 825.9
825.9 - 894.3
894.3 - 909.6
909.6 - 950.5
> 950.5
High-middle SDI: DALYs Rate (per 100,000)
DALYs Rate
< 398.1
398.1 - 450.7
450.7 - 500.9
500.9 - 551.1
551.1 - 597.8
597.8 - 644.5
644.5 - 691.2
691.2 - 718.8
718.8 - 777.2
> 777.2
Middle SDI: DALYs Rate (per 100,000)
DALYs Rate
< 415.9
415.9 - 446.8
446.8 - 501.4
501.4 - 556.1
556.1 - 599.8
599.8 - 643.5
643.5 - 687.2
687.2 - 700.3
700.3 - 728.2
> 728.2
Low-middle SDI: DALYs Rate (per 100,000)
DALYs Rate
< 315.9
315.9 - 347.5
347.5 - 376.0
376.0 - 404.5
404.5 - 448.1
448.1 - 491.8
491.8 - 535.4
535.4 - 556.0
556.0 - 609.4
> 609.4
Low SDI: DALYs Rate (per 100,000)
DALYs Rate
< 329.5
329.5 - 383.4
383.4 - 404.9
404.9 - 426.4
426.4 - 465.3
465.3 - 504.2
504.2 - 543.1
543.1 - 561.0
561.0 - 604.2
> 604.2
High-income: DALYs Rate (per 100,000)
DALYs Rate
< 535.8
535.8 - 570.1
570.1 - 659.2
659.2 - 748.3
748.3 - 820.5
820.5 - 892.6
892.6 - 964.8
964.8 - 990.3
990.3 - 1048.9
> 1048.9
Southeast Asia, East Asia, and Oceania: DALYs Rate (per 100,000)
DALYs Rate
< 373.0
373.0 - 425.4
425.4 - 461.3
461.3 - 497.2
497.2 - 532.7
532.7 - 568.3
568.3 - 603.8
603.8 - 625.3
625.3 - 682.9
> 682.9
Sub-Saharan Africa: DALYs Rate (per 100,000)
DALYs Rate
< 366.7
366.7 - 408.4
408.4 - 432.5
432.5 - 456.6
456.6 - 498.6
498.6 - 540.7
540.7 - 582.8
582.8 - 591.6
591.6 - 618.0
> 618.0
Female
Male
Sexes Combined
Age
20-24 years
15-19 years
10-14 years
Year
DALYs Rate (per 100,000)
Age — 10-24 years

Figure S2

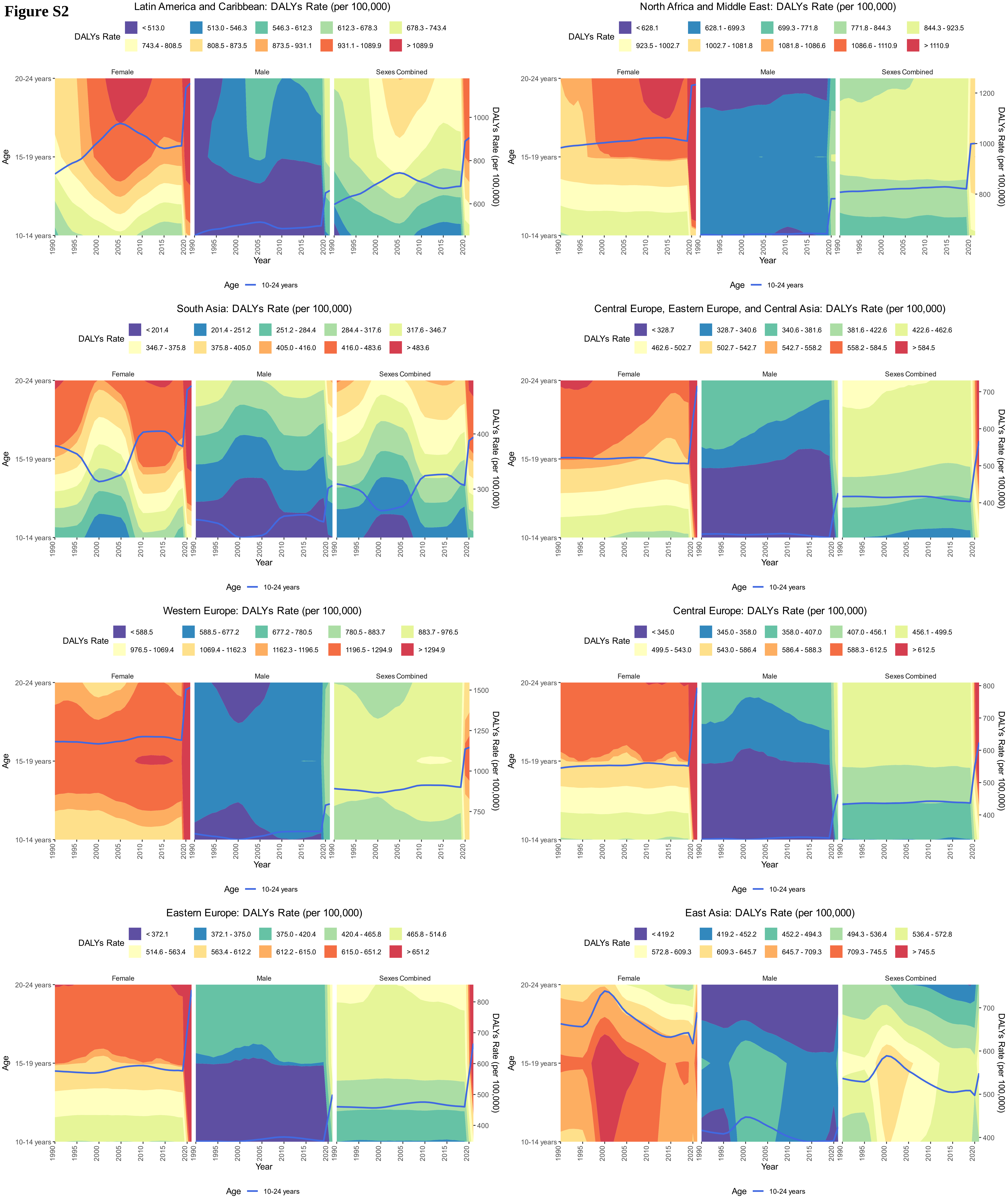

Latin America and Caribbean: DALYs Rate (per 100,000)
DALYs Rate
< 513.0
513.0 - 546.3
546.3 - 612.3
612.3 - 678.3
678.3 - 743.4
743.4 - 808.5
808.5 - 873.5
873.5 - 931.1
931.1 - 1089.9
> 1089.9
North Africa and Middle East: DALYs Rate (per 100,000)
< 628.1
628.1 - 699.3
699.3 - 771.8
771.8 - 844.3
844.3 - 923.5
923.5 - 1002.7
1002.7 - 1081.8
1081.8 - 1086.6
1086.6 - 1110.9
> 1110.9
South Asia: DALYs Rate (per 100,000)
< 201.4
201.4 - 251.2
251.2 - 284.4
284.4 - 317.6
317.6 - 346.7
346.7 - 375.8
375.8 - 405.0
405.0 - 416.0
416.0 - 483.6
> 483.6
Central Europe, Eastern Europe, and Central Asia: DALYs Rate (per 100,000)
< 328.7
328.7 - 340.6
340.6 - 381.6
381.6 - 422.6
422.6 - 462.6
462.6 - 502.7
502.7 - 542.7
542.7 - 558.2
558.2 - 584.5
> 584.5
Western Europe: DALYs Rate (per 100,000)
< 588.5
588.5 - 677.2
677.2 - 780.5
780.5 - 883.7
883.7 - 976.5
976.5 - 1069.4
1069.4 - 1162.3
1162.3 - 1196.5
1196.5 - 1294.9
> 1294.9
Central Europe: DALYs Rate (per 100,000)
< 345.0
345.0 - 358.0
358.0 - 407.0
407.0 - 456.1
456.1 - 499.5
499.5 - 543.0
543.0 - 586.4
586.4 - 588.3
588.3 - 612.5
> 612.5
Eastern Europe: DALYs Rate (per 100,000)
< 372.1
372.1 - 375.0
375.0 - 420.4
420.4 - 465.8
465.8 - 514.6
514.6 - 563.4
563.4 - 612.2
612.2 - 615.0
615.0 - 651.2
> 651.2
East Asia: DALYs Rate (per 100,000)
< 419.2
419.2 - 452.2
452.2 - 494.3
494.3 - 536.4
536.4 - 572.8
572.8 - 609.3
609.3 - 645.7
645.7 - 709.3
709.3 - 745.5
> 745.5
Female
Male
Sexes Combined
Age
20-24 years
15-19 years
10-14 years
Year
DALYs Rate (per 100,000)
Age — 10-24 years

Figure S2

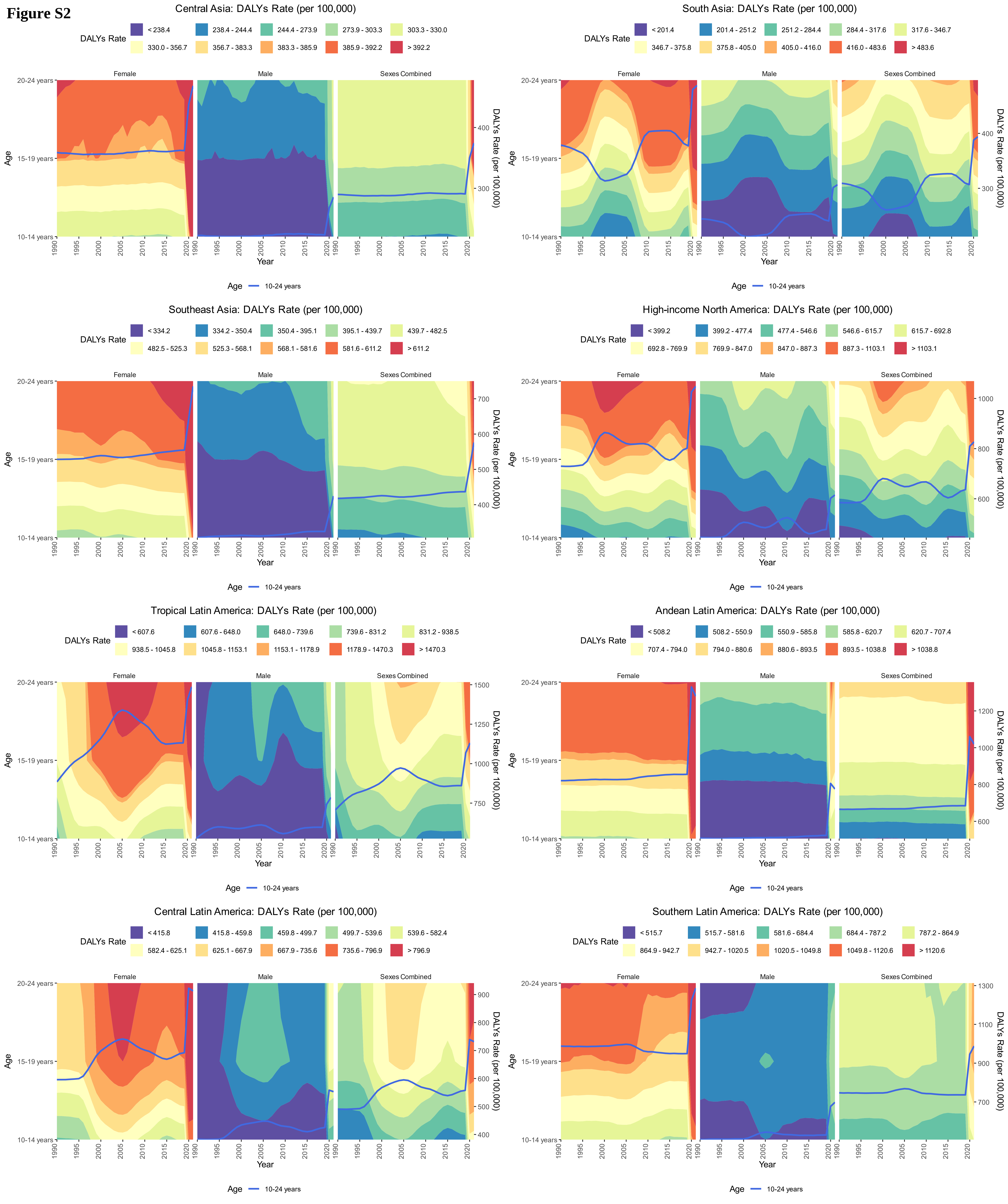

Central Asia: DALYs Rate (per 100,000)
DALYs Rate
< 238.4
238.4 - 244.4
244.4 - 273.9
273.9 - 303.3
303.3 - 330.0
330.0 - 356.7
356.7 - 383.3
383.3 - 385.9
385.9 - 392.2
> 392.2
South Asia: DALYs Rate (per 100,000)
< 201.4
201.4 - 251.2
251.2 - 284.4
284.4 - 317.6
317.6 - 346.7
346.7 - 375.8
375.8 - 405.0
405.0 - 416.0
416.0 - 483.6
> 483.6
Southeast Asia: DALYs Rate (per 100,000)
< 334.2
334.2 - 350.4
350.4 - 395.1
395.1 - 439.7
439.7 - 482.5
482.5 - 525.3
525.3 - 568.1
568.1 - 581.6
581.6 - 611.2
> 611.2
High-income North America: DALYs Rate (per 100,000)
< 399.2
399.2 - 477.4
477.4 - 546.6
546.6 - 615.7
615.7 - 692.8
692.8 - 769.9
769.9 - 847.0
847.0 - 887.3
887.3 - 1103.1
> 1103.1
Tropical Latin America: DALYs Rate (per 100,000)
< 607.6
607.6 - 648.0
648.0 - 739.6
739.6 - 831.2
831.2 - 938.5
938.5 - 1045.8
1045.8 - 1153.1
1153.1 - 1178.9
1178.9 - 1470.3
> 1470.3
Andean Latin America: DALYs Rate (per 100,000)
< 508.2
508.2 - 550.9
550.9 - 585.8
585.8 - 620.7
620.7 - 707.4
707.4 - 794.0
794.0 - 880.6
880.6 - 893.5
893.5 - 1038.8
> 1038.8
Central Latin America: DALYs Rate (per 100,000)
< 415.8
415.8 - 459.8
459.8 - 499.7
499.7 - 539.6
539.6 - 582.4
582.4 - 625.1
625.1 - 667.9
667.9 - 735.6
735.6 - 796.9
> 796.9
Southern Latin America: DALYs Rate (per 100,000)
< 515.7
515.7 - 581.6
581.6 - 684.4
684.4 - 787.2
787.2 - 864.9
864.9 - 942.7
942.7 - 1020.5
1020.5 - 1049.8
1049.8 - 1120.6
> 1120.6
Female
Male
Sexes Combined
Age
20-24 years
15-19 years
10-14 years
Year
1990
1995
2000
2005
2010
2015
2020
DALYs Rate (per 100,000)
Age
10-24 years

Figure S2

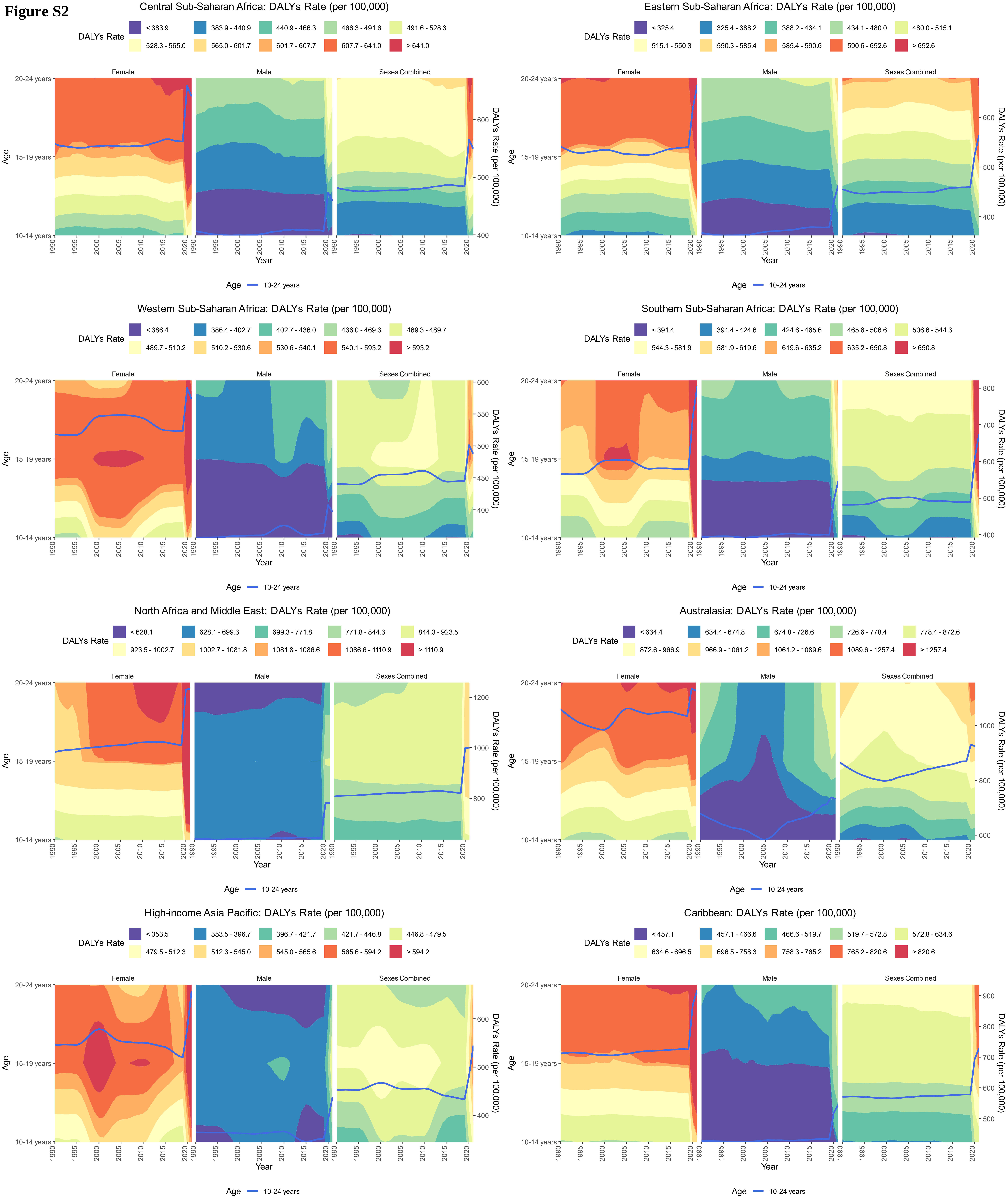

Central Sub-Saharan Africa: DALYs Rate (per 100,000)
DALYs Rate
< 383.9
383.9 - 440.9
440.9 - 466.3
466.3 - 491.6
491.6 - 528.3
528.3 - 565.0
565.0 - 601.7
601.7 - 607.7
607.7 - 641.0
> 641.0
Eastern Sub-Saharan Africa: DALYs Rate (per 100,000)
DALYs Rate
< 325.4
325.4 - 388.2
388.2 - 434.1
434.1 - 480.0
480.0 - 515.1
515.1 - 550.3
550.3 - 585.4
585.4 - 590.6
590.6 - 692.6
> 692.6
Western Sub-Saharan Africa: DALYs Rate (per 100,000)
DALYs Rate
< 386.4
386.4 - 402.7
402.7 - 436.0
436.0 - 469.3
469.3 - 489.7
489.7 - 510.2
510.2 - 530.6
530.6 - 540.1
540.1 - 593.2
> 593.2
Southern Sub-Saharan Africa: DALYs Rate (per 100,000)
DALYs Rate
< 391.4
391.4 - 424.6
424.6 - 465.6
465.6 - 506.6
506.6 - 544.3
544.3 - 581.9
581.9 - 619.6
619.6 - 635.2
635.2 - 650.8
> 650.8
North Africa and Middle East: DALYs Rate (per 100,000)
DALYs Rate
< 628.1
628.1 - 699.3
699.3 - 771.8
771.8 - 844.3
844.3 - 923.5
923.5 - 1002.7
1002.7 - 1081.8
1081.8 - 1086.6
1086.6 - 1110.9
> 1110.9
Australasia: DALYs Rate (per 100,000)
DALYs Rate
< 634.4
634.4 - 674.8
674.8 - 726.6
726.6 - 778.4
778.4 - 872.6
872.6 - 966.9
966.9 - 1061.2
1061.2 - 1089.6
1089.6 - 1257.4
> 1257.4
High-income Asia Pacific: DALYs Rate (per 100,000)
DALYs Rate
< 353.5
353.5 - 396.7
396.7 - 421.7
421.7 - 446.8
446.8 - 479.5
479.5 - 512.3
512.3 - 545.0
545.0 - 565.6
565.6 - 594.2
> 594.2
Caribbean: DALYs Rate (per 100,000)
DALYs Rate
< 457.1
457.1 - 466.6
466.6 - 519.7
519.7 - 572.8
572.8 - 634.6
634.6 - 696.5
696.5 - 758.3
758.3 - 765.2
765.2 - 820.6
> 820.6
Female
Male
Sexes Combined
Age
20-24 years
15-19 years
10-14 years
Year
DALYs Rate (per 100,000)
Age
10-24 years

Figure S2

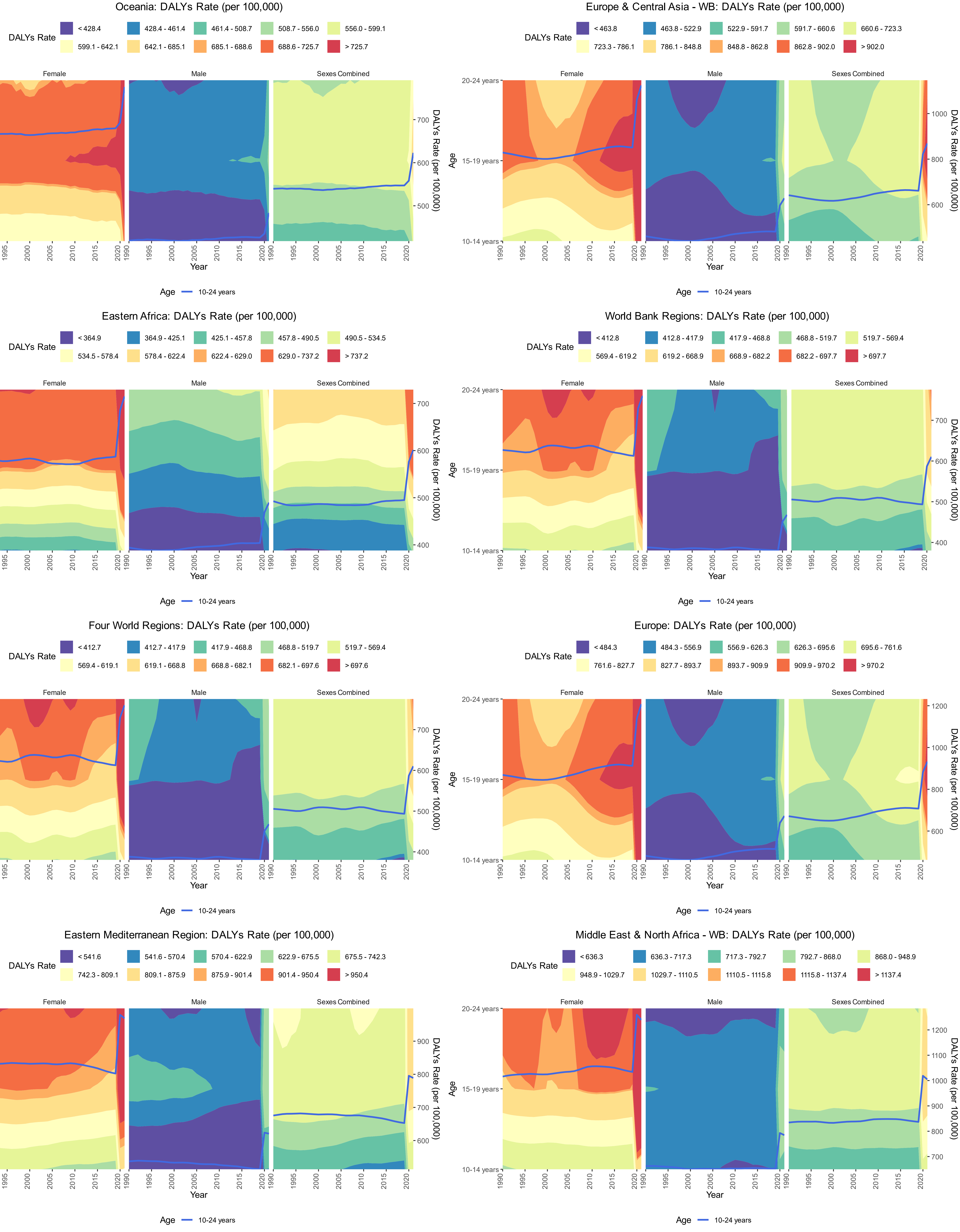
Oceania: DALYs Rate (per 100,000)
DALYs Rate
< 428.4
428.4 - 461.4
461.4 - 508.7
508.7 - 556.0
556.0 - 599.1
599.1 - 642.1
642.1 - 685.1
685.1 - 688.6
688.6 - 725.7
> 725.7
Europe & Central Asia - WB: DALYs Rate (per 100,000)
DALYs Rate
< 463.8
463.8 - 522.9
522.9 - 591.7
591.7 - 660.6
660.6 - 723.3
723.3 - 786.1
786.1 - 848.8
848.8 - 862.8
862.8 - 902.0
> 902.0
Eastern Africa: DALYs Rate (per 100,000)
DALYs Rate
< 364.9
364.9 - 425.1
425.1 - 457.8
457.8 - 490.5
490.5 - 534.5
534.5 - 578.4
578.4 - 622.4
622.4 - 629.0
629.0 - 737.2
> 737.2
World Bank Regions: DALYs Rate (per 100,000)
DALYs Rate
< 412.8
412.8 - 417.9
417.9 - 468.8
468.8 - 519.7
519.7 - 569.4
569.4 - 619.2
619.2 - 668.9
668.9 - 682.2
682.2 - 697.7
> 697.7
Four World Regions: DALYs Rate (per 100,000)
DALYs Rate
< 412.7
412.7 - 417.9
417.9 - 468.8
468.8 - 519.7
519.7 - 569.4
569.4 - 619.1
619.1 - 668.8
668.8 - 682.1
682.1 - 697.6
> 697.6
Europe: DALYs Rate (per 100,000)
DALYs Rate
< 484.3
484.3 - 556.9
556.9 - 626.3
626.3 - 695.6
695.6 - 761.6
761.6 - 827.7
827.7 - 893.7
893.7 - 909.9
909.9 - 970.2
> 970.2
Eastern Mediterranean Region: DALYs Rate (per 100,000)
DALYs Rate
< 541.6
541.6 - 570.4
570.4 - 622.9
622.9 - 675.5
675.5 - 742.3
742.3 - 809.1
809.1 - 875.9
875.9 - 901.4
901.4 - 950.4
> 950.4
Middle East & North Africa - WB: DALYs Rate (per 100,000)
DALYs Rate
< 636.3
636.3 - 717.3
717.3 - 792.7
792.7 - 868.0
868.0 - 948.9
948.9 - 1029.7
1029.7 - 1110.5
1110.5 - 1115.8
1115.8 - 1137.4
> 1137.4
Female
Male
Sexes Combined
20-24 years
15-19 years
10-14 years
Age
Year
DALYs Rate (per 100,000)
Age — 10-24 years

Figure S2

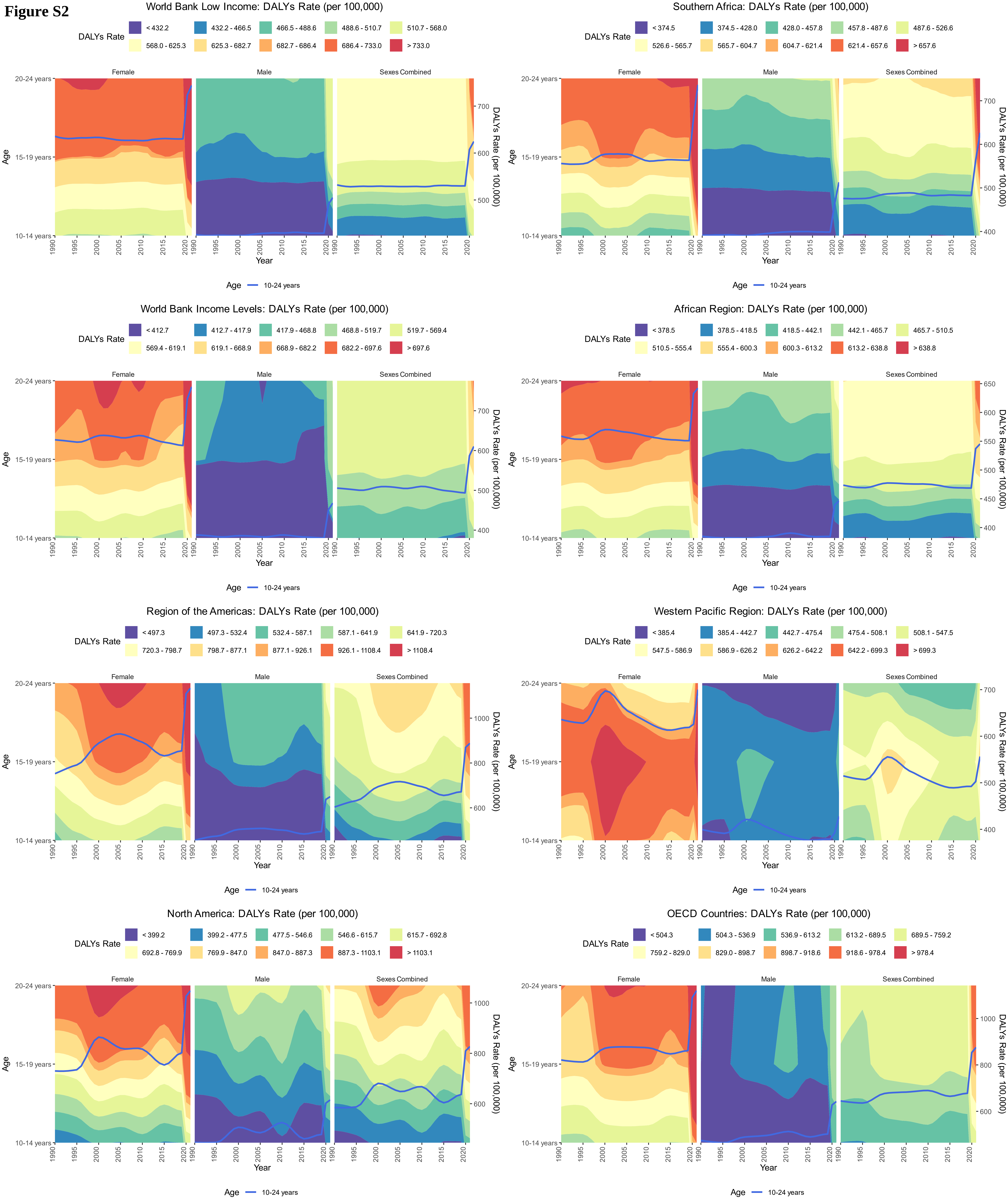
World Bank Low Income: DALYs Rate (per 100,000)
DALYs Rate
< 432.2
432.2 - 466.5
466.5 - 488.6
488.6 - 510.7
510.7 - 568.0
568.0 - 625.3
625.3 - 682.7
682.7 - 686.4
686.4 - 733.0
> 733.0
Southern Africa: DALYs Rate (per 100,000)
DALYs Rate
< 374.5
374.5 - 428.0
428.0 - 457.8
457.8 - 487.6
487.6 - 526.6
526.6 - 565.7
565.7 - 604.7
604.7 - 621.4
621.4 - 657.6
> 657.6
World Bank Income Levels: DALYs Rate (per 100,000)
DALYs Rate
< 412.7
412.7 - 417.9
417.9 - 468.8
468.8 - 519.7
519.7 - 569.4
569.4 - 619.1
619.1 - 668.9
668.9 - 682.2
682.2 - 697.6
> 697.6
African Region: DALYs Rate (per 100,000)
DALYs Rate
< 378.5
378.5 - 418.5
418.5 - 442.1
442.1 - 465.7
465.7 - 510.5
510.5 - 555.4
555.4 - 600.3
600.3 - 613.2
613.2 - 638.8
> 638.8
Region of the Americas: DALYs Rate (per 100,000)
DALYs Rate
< 497.3
497.3 - 532.4
532.4 - 587.1
587.1 - 641.9
641.9 - 720.3
720.3 - 798.7
798.7 - 877.1
877.1 - 926.1
926.1 - 1108.4
> 1108.4
Western Pacific Region: DALYs Rate (per 100,000)
DALYs Rate
< 385.4
385.4 - 442.7
442.7 - 475.4
475.4 - 508.1
508.1 - 547.5
547.5 - 586.9
586.9 - 626.2
626.2 - 642.2
642.2 - 699.3
> 699.3
North America: DALYs Rate (per 100,000)
DALYs Rate
< 399.2
399.2 - 477.5
477.5 - 546.6
546.6 - 615.7
615.7 - 692.8
692.8 - 769.9
769.9 - 847.0
847.0 - 887.3
887.3 - 1103.1
> 1103.1
OECD Countries: DALYs Rate (per 100,000)
DALYs Rate
< 504.3
504.3 - 536.9
536.9 - 613.2
613.2 - 689.5
689.5 - 759.2
759.2 - 829.0
829.0 - 898.7
898.7 - 918.6
918.6 - 978.4
> 978.4
Female
Male
Sexes Combined
Age
20-24 years
15-19 years
10-14 years
Year
DALYs Rate (per 100,000)
Age — 10-24 years

Figure S2

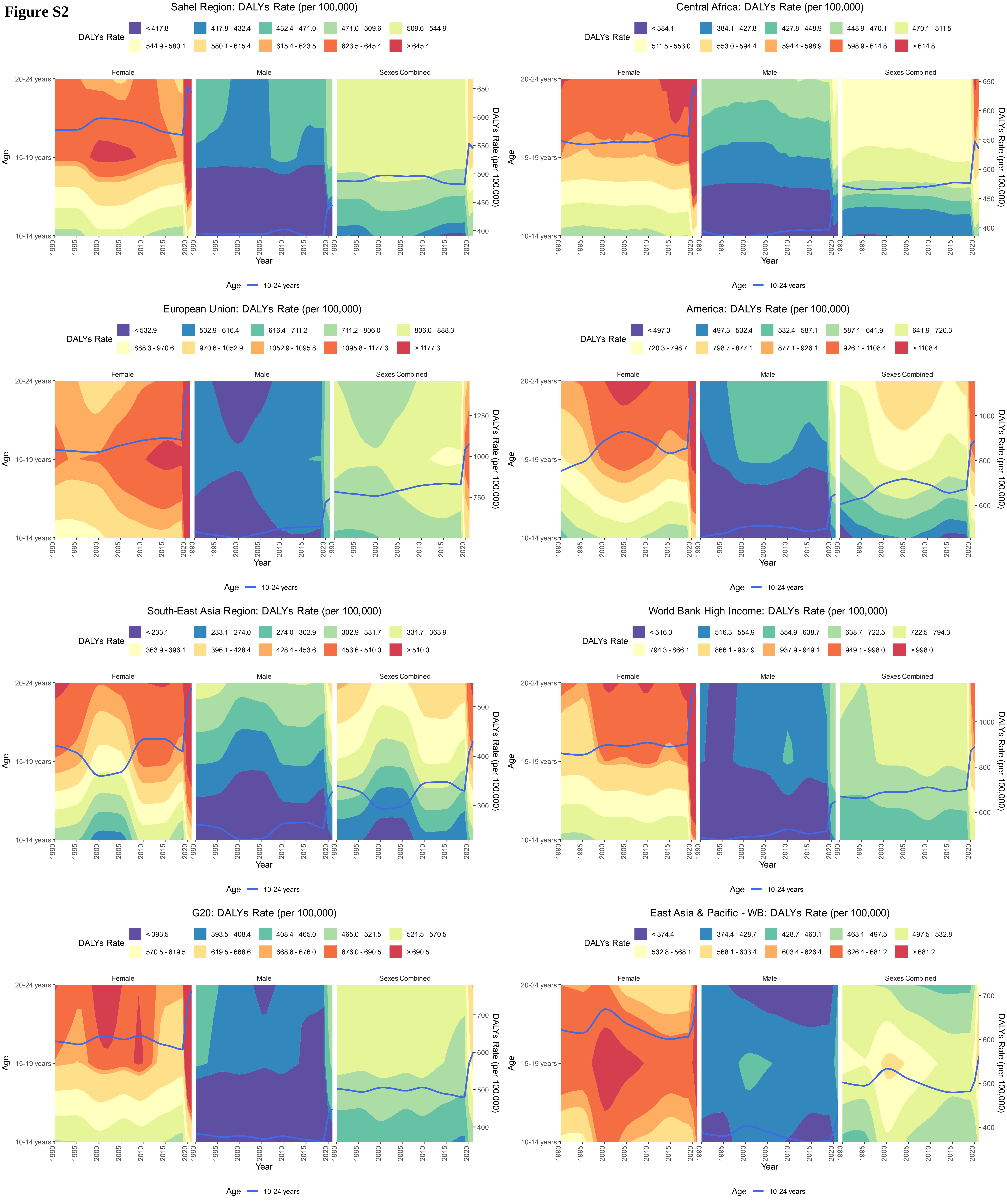
Sahel Region: DALYs Rate (per 100,000)
DALYs Rate
< 417.8
417.8 - 432.4
432.4 - 471.0
471.0 - 509.6
509.6 - 544.9
544.9 - 580.1
580.1 - 615.4
615.4 - 623.5
623.5 - 645.4
> 645.4
Female
Male
Sexes Combined
Age
20-24 years
15-19 years
10-14 years
Year
DALYs Rate (per 100,000)
Age — 10-24 years
Central Africa: DALYs Rate (per 100,000)
< 384.1
384.1 - 427.8
427.8 - 448.9
448.9 - 470.1
470.1 - 511.5
511.5 - 553.0
553.0 - 594.4
594.4 - 598.9
598.9 - 614.8
> 614.8
European Union: DALYs Rate (per 100,000)
< 532.9
532.9 - 616.4
616.4 - 711.2
711.2 - 806.0
806.0 - 888.3
888.3 - 970.6
970.6 - 1052.9
1052.9 - 1095.8
1095.8 - 1177.3
> 1177.3
America: DALYs Rate (per 100,000)
< 497.3
497.3 - 532.4
532.4 - 587.1
587.1 - 641.9
641.9 - 720.3
720.3 - 798.7
798.7 - 877.1
877.1 - 926.1
926.1 - 1108.4
> 1108.4
South-East Asia Region: DALYs Rate (per 100,000)
< 233.1
233.1 - 274.0
274.0 - 302.9
302.9 - 331.7
331.7 - 363.9
363.9 - 396.1
396.1 - 428.4
428.4 - 453.6
453.6 - 510.0
> 510.0
World Bank High Income: DALYs Rate (per 100,000)
< 516.3
516.3 - 554.9
554.9 - 638.7
638.7 - 722.5
722.5 - 794.3
794.3 - 866.1
866.1 - 937.9
937.9 - 949.1
949.1 - 998.0
> 998.0
G20: DALYs Rate (per 100,000)
< 393.5
393.5 - 408.4
408.4 - 465.0
465.0 - 521.5
521.5 - 570.5
570.5 - 619.5
619.5 - 668.6
668.6 - 676.0
676.0 - 690.5
> 690.5
East Asia & Pacific - WB: DALYs Rate (per 100,000)
< 374.4
374.4 - 428.7
428.7 - 463.1
463.1 - 497.5
497.5 - 532.8
532.8 - 568.1
568.1 - 603.4
603.4 - 626.4
626.4 - 681.2
> 681.2

Figure S2

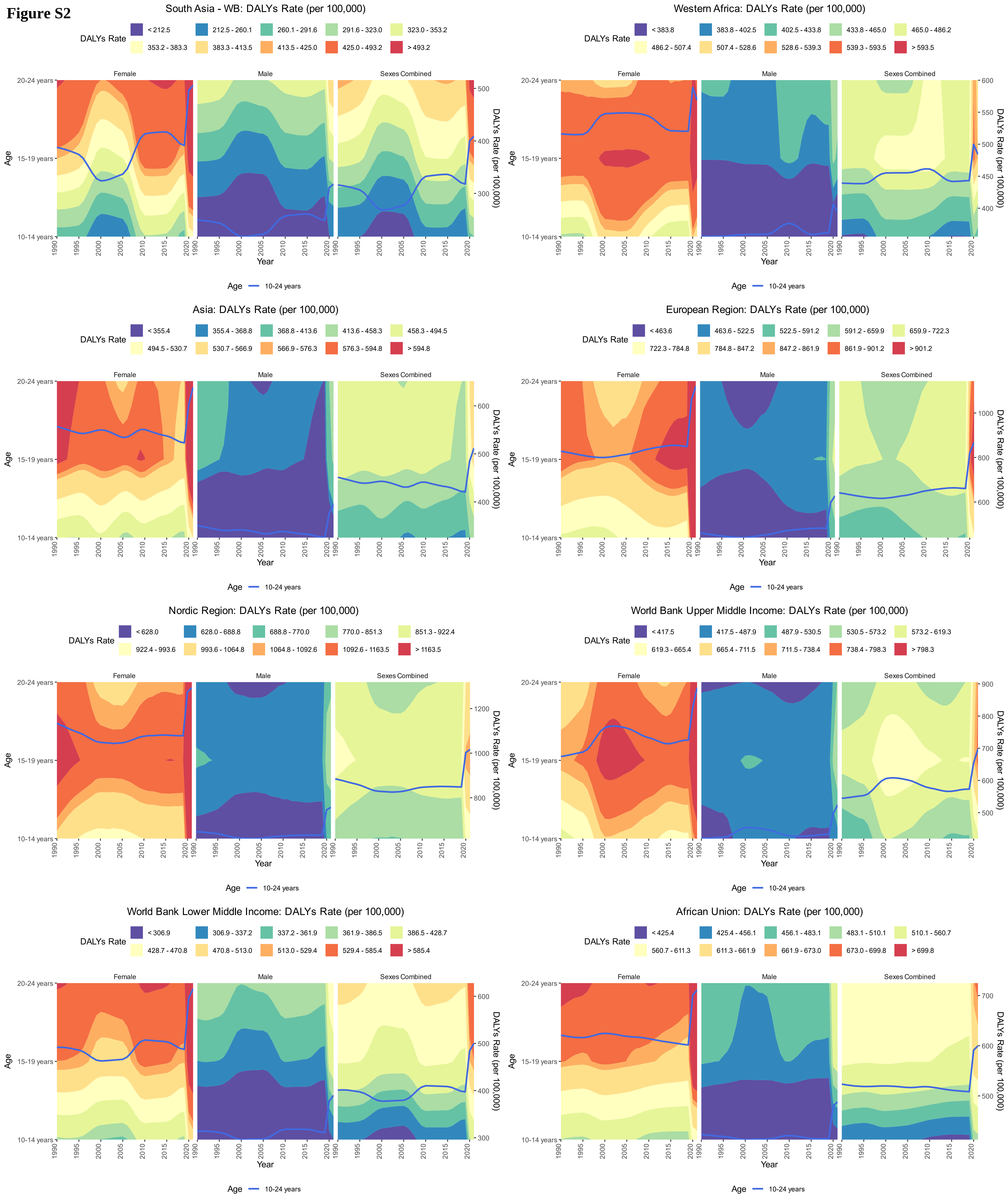
South Asia - WB: DALYs Rate (per 100,000)
DALYs Rate
< 212.5
212.5 - 260.1
260.1 - 291.6
291.6 - 323.0
323.0 - 353.2
353.2 - 383.3
383.3 - 413.5
413.5 - 425.0
425.0 - 493.2
> 493.2
Western Africa: DALYs Rate (per 100,000)
DALYs Rate
< 383.8
383.8 - 402.5
402.5 - 433.8
433.8 - 465.0
465.0 - 486.2
486.2 - 507.4
507.4 - 528.6
528.6 - 539.3
539.3 - 593.5
> 593.5
Asia: DALYs Rate (per 100,000)
DALYs Rate
< 355.4
355.4 - 368.8
368.8 - 413.6
413.6 - 458.3
458.3 - 494.5
494.5 - 530.7
530.7 - 566.9
566.9 - 576.3
576.3 - 594.8
> 594.8
European Region: DALYs Rate (per 100,000)
DALYs Rate
< 463.6
463.6 - 522.5
522.5 - 591.2
591.2 - 659.9
659.9 - 722.3
722.3 - 784.8
784.8 - 847.2
847.2 - 861.9
861.9 - 901.2
> 901.2
Nordic Region: DALYs Rate (per 100,000)
DALYs Rate
< 628.0
628.0 - 688.8
688.8 - 770.0
770.0 - 851.3
851.3 - 922.4
922.4 - 993.6
993.6 - 1064.8
1064.8 - 1092.6
1092.6 - 1163.5
> 1163.5
World Bank Upper Middle Income: DALYs Rate (per 100,000)
DALYs Rate
< 417.5
417.5 - 487.9
487.9 - 530.5
530.5 - 573.2
573.2 - 619.3
619.3 - 665.4
665.4 - 711.5
711.5 - 738.4
738.4 - 798.3
> 798.3
World Bank Lower Middle Income: DALYs Rate (per 100,000)
DALYs Rate
< 306.9
306.9 - 337.2
337.2 - 361.9
361.9 - 386.5
386.5 - 428.7
428.7 - 470.8
470.8 - 513.0
513.0 - 529.4
529.4 - 585.4
> 585.4
African Union: DALYs Rate (per 100,000)
DALYs Rate
< 425.4
425.4 - 456.1
456.1 - 483.1
483.1 - 510.1
510.1 - 560.7
560.7 - 611.3
611.3 - 661.9
661.9 - 673.0
673.0 - 699.8
> 699.8
Female
Male
Sexes Combined
Age
20-24 years
15-19 years
10-14 years
Year
DALYs Rate (per 100,000)
Age
10-24 years

Figure S2

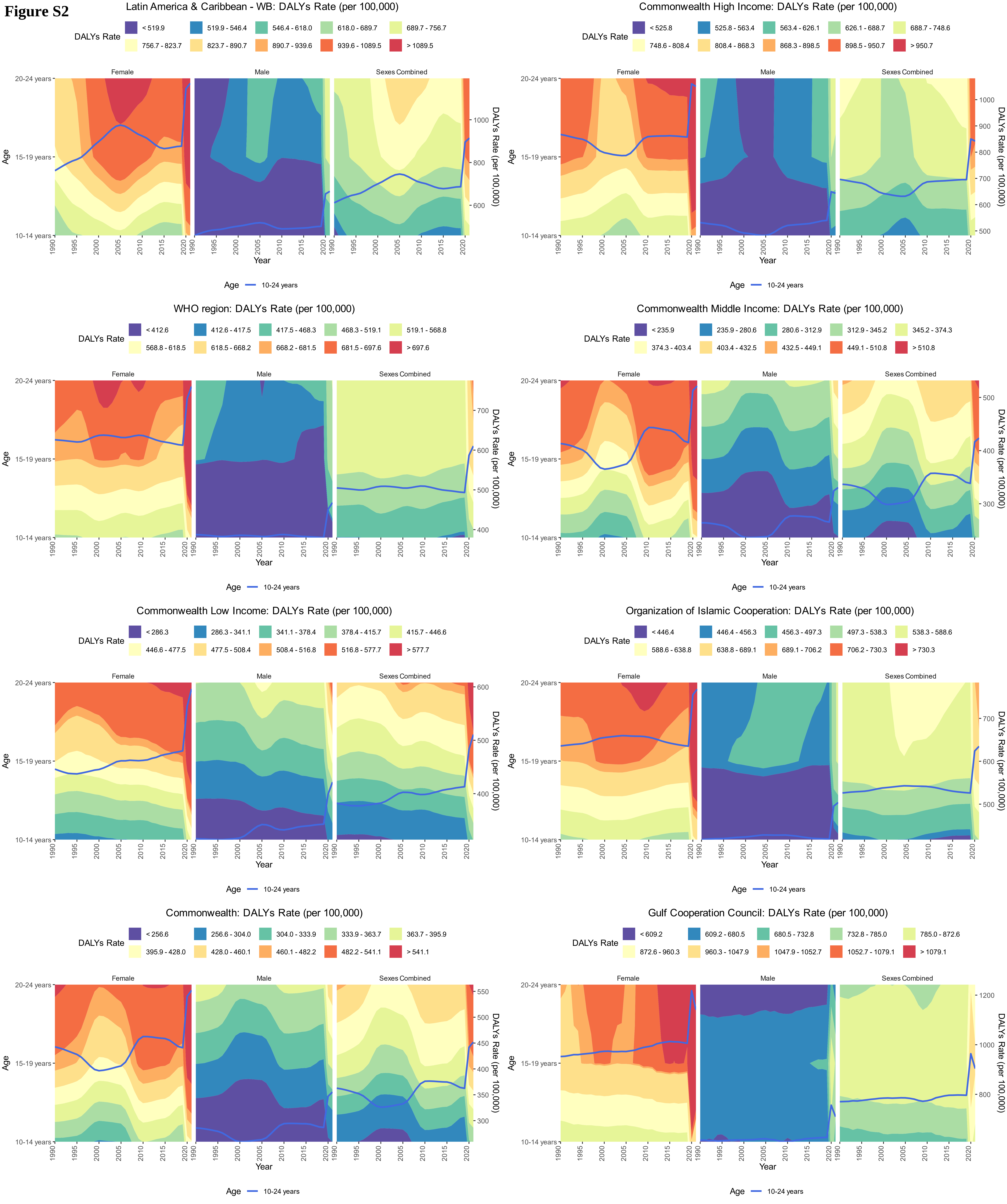

Latin America & Caribbean - WB: DALYs Rate (per 100,000)
DALYs Rate
< 519.9
519.9 - 546.4
546.4 - 618.0
618.0 - 689.7
689.7 - 756.7
756.7 - 823.7
823.7 - 890.7
890.7 - 939.6
939.6 - 1089.5
> 1089.5
Commonwealth High Income: DALYs Rate (per 100,000)
DALYs Rate
< 525.8
525.8 - 563.4
563.4 - 626.1
626.1 - 688.7
688.7 - 748.6
748.6 - 808.4
808.4 - 868.3
868.3 - 898.5
898.5 - 950.7
> 950.7
WHO region: DALYs Rate (per 100,000)
DALYs Rate
< 412.6
412.6 - 417.5
417.5 - 468.3
468.3 - 519.1
519.1 - 568.8
568.8 - 618.5
618.5 - 668.2
668.2 - 681.5
681.5 - 697.6
> 697.6
Commonwealth Middle Income: DALYs Rate (per 100,000)
DALYs Rate
< 235.9
235.9 - 280.6
280.6 - 312.9
312.9 - 345.2
345.2 - 374.3
374.3 - 403.4
403.4 - 432.5
432.5 - 449.1
449.1 - 510.8
> 510.8
Commonwealth Low Income: DALYs Rate (per 100,000)
DALYs Rate
< 286.3
286.3 - 341.1
341.1 - 378.4
378.4 - 415.7
415.7 - 446.6
446.6 - 477.5
477.5 - 508.4
508.4 - 516.8
516.8 - 577.7
> 577.7
Organization of Islamic Cooperation: DALYs Rate (per 100,000)
DALYs Rate
< 446.4
446.4 - 456.3
456.3 - 497.3
497.3 - 538.3
538.3 - 588.6
588.6 - 638.8
638.8 - 689.1
689.1 - 706.2
706.2 - 730.3
> 730.3
Commonwealth: DALYs Rate (per 100,000)
DALYs Rate
< 256.6
256.6 - 304.0
304.0 - 333.9
333.9 - 363.7
363.7 - 395.9
395.9 - 428.0
428.0 - 460.1
460.1 - 482.2
482.2 - 541.1
> 541.1
Gulf Cooperation Council: DALYs Rate (per 100,000)
DALYs Rate
< 609.2
609.2 - 680.5
680.5 - 732.8
732.8 - 785.0
785.0 - 872.6
872.6 - 960.3
960.3 - 1047.9
1047.9 - 1052.7
1052.7 - 1079.1
> 1079.1
Female
Male
Sexes Combined
Age
20-24 years
15-19 years
10-14 years
Year
1990
1995
2000
2005
2010
2015
2020
DALYs Rate (per 100,000)
Age
10-24 years

Figure S2

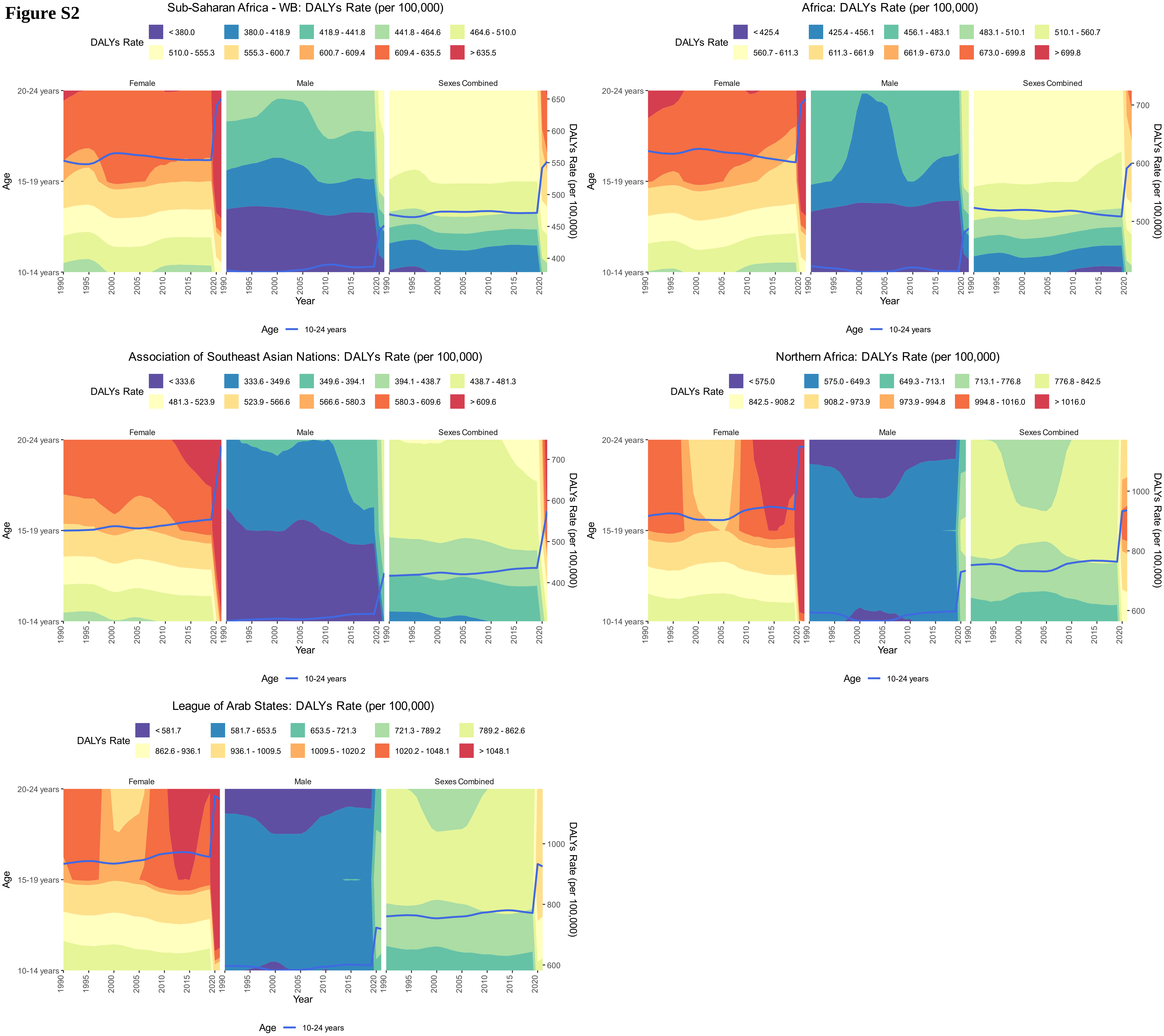
Sub-Saharan Africa - WB: DALYs Rate (per 100,000)
DALYs Rate
< 380.0
380.0 - 418.9
418.9 - 441.8
441.8 - 464.6
464.6 - 510.0
510.0 - 555.3
555.3 - 600.7
600.7 - 609.4
609.4 - 635.5
> 635.5
Female
Male
Sexes Combined
Age
Year
DALYs Rate (per 100,000)
Age 10-24 years
Africa: DALYs Rate (per 100,000)
DALYs Rate
< 425.4
425.4 - 456.1
456.1 - 483.1
483.1 - 510.1
510.1 - 560.7
560.7 - 611.3
611.3 - 661.9
661.9 - 673.0
673.0 - 699.8
> 699.8
Association of Southeast Asian Nations: DALYs Rate (per 100,000)
DALYs Rate
< 333.6
333.6 - 349.6
349.6 - 394.1
394.1 - 438.7
438.7 - 481.3
481.3 - 523.9
523.9 - 566.6
566.6 - 580.3
580.3 - 609.6
> 609.6
Northern Africa: DALYs Rate (per 100,000)
DALYs Rate
< 575.0
575.0 - 649.3
649.3 - 713.1
713.1 - 776.8
776.8 - 842.5
842.5 - 908.2
908.2 - 973.9
973.9 - 994.8
994.8 - 1016.0
> 1016.0
League of Arab States: DALYs Rate (per 100,000)
DALYs Rate
< 581.7
581.7 - 653.5
653.5 - 721.3
721.3 - 789.2
789.2 - 862.6
862.6 - 936.1
936.1 - 1009.5
1009.5 - 1020.2
1020.2 - 1048.1
> 1048.1
20-24 years
15-19 years
10-14 years

Figure S2

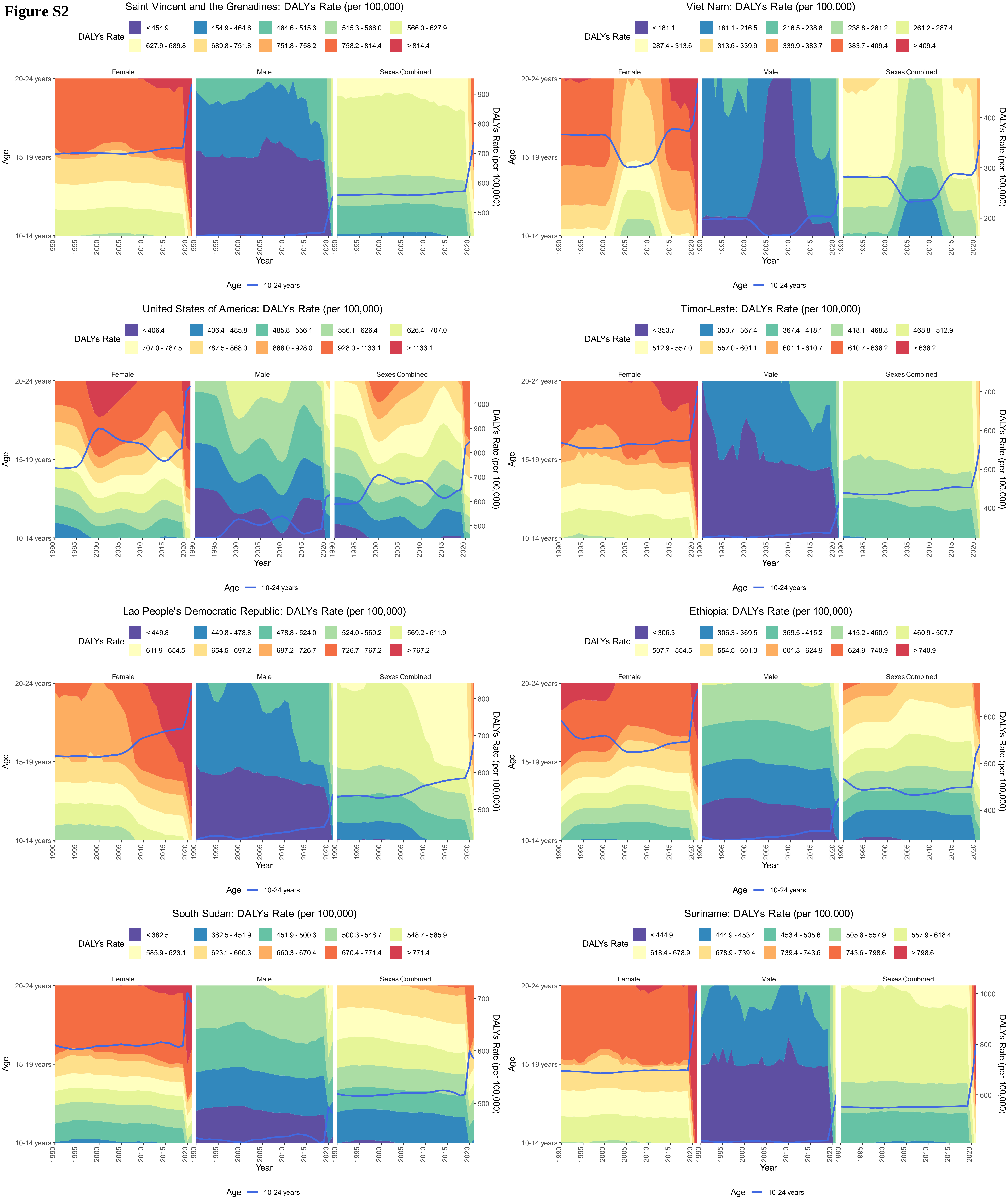

Saint Vincent and the Grenadines: DALYs Rate (per 100,000)
DALYs Rate
< 454.9
454.9 - 464.6
464.6 - 515.3
515.3 - 566.0
566.0 - 627.9
627.9 - 689.8
689.8 - 751.8
751.8 - 758.2
758.2 - 814.4
> 814.4
Viet Nam: DALYs Rate (per 100,000)
DALYs Rate
< 181.1
181.1 - 216.5
216.5 - 238.8
238.8 - 261.2
261.2 - 287.4
287.4 - 313.6
313.6 - 339.9
339.9 - 383.7
383.7 - 409.4
> 409.4
United States of America: DALYs Rate (per 100,000)
DALYs Rate
< 406.4
406.4 - 485.8
485.8 - 556.1
556.1 - 626.4
626.4 - 707.0
707.0 - 787.5
787.5 - 868.0
868.0 - 928.0
928.0 - 1133.1
> 1133.1
Timor-Leste: DALYs Rate (per 100,000)
DALYs Rate
< 353.7
353.7 - 367.4
367.4 - 418.1
418.1 - 468.8
468.8 - 512.9
512.9 - 557.0
557.0 - 601.1
601.1 - 610.7
610.7 - 636.2
> 636.2
Lao People's Democratic Republic: DALYs Rate (per 100,000)
DALYs Rate
< 449.8
449.8 - 478.8
478.8 - 524.0
524.0 - 569.2
569.2 - 611.9
611.9 - 654.5
654.5 - 697.2
697.2 - 726.7
726.7 - 767.2
> 767.2
Ethiopia: DALYs Rate (per 100,000)
DALYs Rate
< 306.3
306.3 - 369.5
369.5 - 415.2
415.2 - 460.9
460.9 - 507.7
507.7 - 554.5
554.5 - 601.3
601.3 - 624.9
624.9 - 740.9
> 740.9
South Sudan: DALYs Rate (per 100,000)
DALYs Rate
< 382.5
382.5 - 451.9
451.9 - 500.3
500.3 - 548.7
548.7 - 585.9
585.9 - 623.1
623.1 - 660.3
660.3 - 670.4
670.4 - 771.4
> 771.4
Suriname: DALYs Rate (per 100,000)
DALYs Rate
< 444.9
444.9 - 453.4
453.4 - 505.6
505.6 - 557.9
557.9 - 618.4
618.4 - 678.9
678.9 - 739.4
739.4 - 743.6
743.6 - 798.6
> 798.6
Female
Male
Sexes Combined
Age
20-24 years
15-19 years
10-14 years
Year
DALYs Rate (per 100,000)
Age — 10-24 years

Figure S2

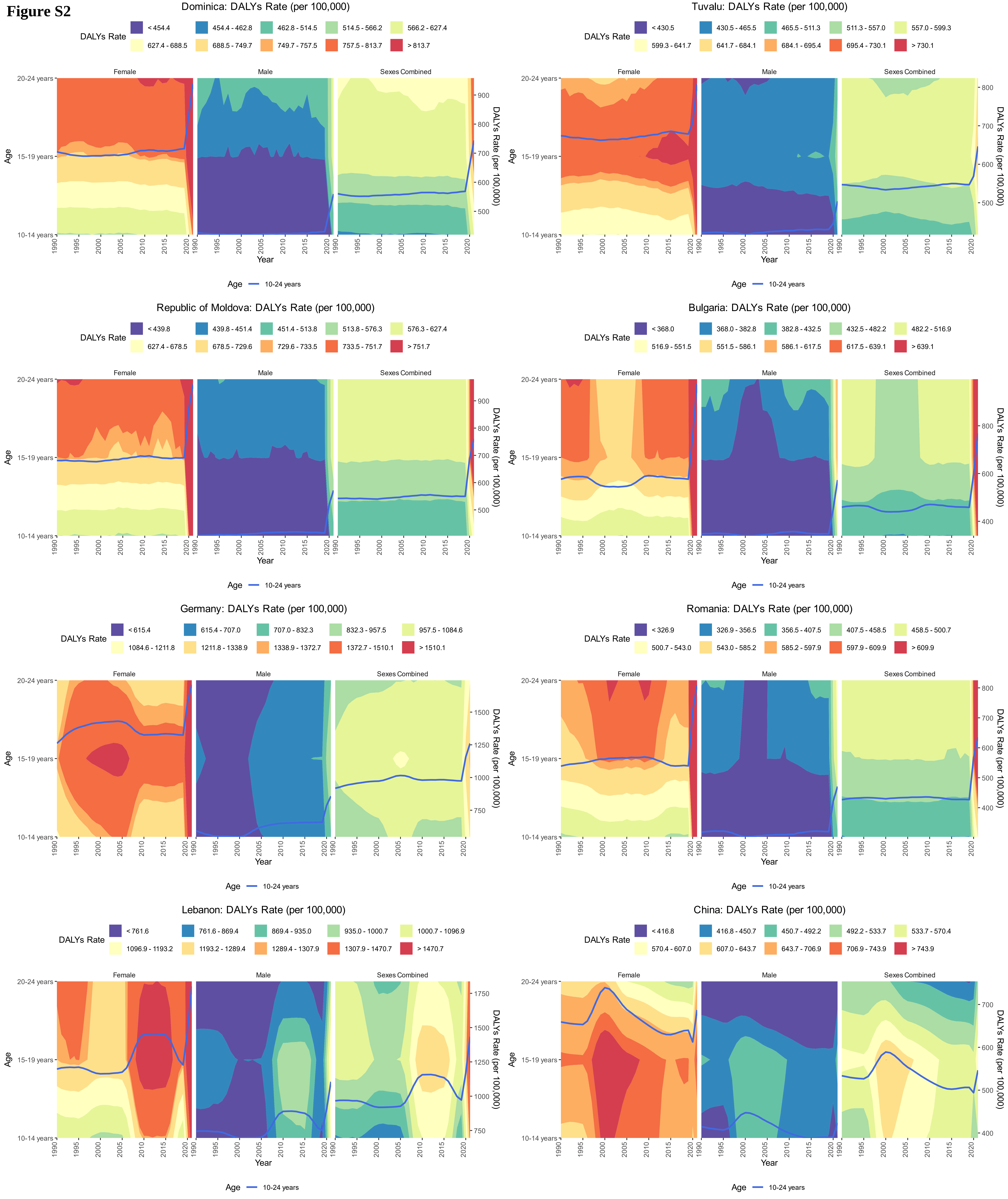

Dominica: DALYs Rate (per 100,000)
DALYs Rate
< 454.4
454.4 - 462.8
462.8 - 514.5
514.5 - 566.2
566.2 - 627.4
627.4 - 688.5
688.5 - 749.7
749.7 - 757.5
757.5 - 813.7
> 813.7
Tuvalu: DALYs Rate (per 100,000)
< 430.5
430.5 - 465.5
465.5 - 511.3
511.3 - 557.0
557.0 - 599.3
599.3 - 641.7
641.7 - 684.1
684.1 - 695.4
695.4 - 730.1
> 730.1
Republic of Moldova: DALYs Rate (per 100,000)
< 439.8
439.8 - 451.4
451.4 - 513.8
513.8 - 576.3
576.3 - 627.4
627.4 - 678.5
678.5 - 729.6
729.6 - 733.5
733.5 - 751.7
> 751.7
Bulgaria: DALYs Rate (per 100,000)
< 368.0
368.0 - 382.8
382.8 - 432.5
432.5 - 482.2
482.2 - 516.9
516.9 - 551.5
551.5 - 586.1
586.1 - 617.5
617.5 - 639.1
> 639.1
Germany: DALYs Rate (per 100,000)
< 615.4
615.4 - 707.0
707.0 - 832.3
832.3 - 957.5
957.5 - 1084.6
1084.6 - 1211.8
1211.8 - 1338.9
1338.9 - 1372.7
1372.7 - 1510.1
> 1510.1
Romania: DALYs Rate (per 100,000)
< 326.9
326.9 - 356.5
356.5 - 407.5
407.5 - 458.5
458.5 - 500.7
500.7 - 543.0
543.0 - 585.2
585.2 - 597.9
597.9 - 609.9
> 609.9
Lebanon: DALYs Rate (per 100,000)
< 761.6
761.6 - 869.4
869.4 - 935.0
935.0 - 1000.7
1000.7 - 1096.9
1096.9 - 1193.2
1193.2 - 1289.4
1289.4 - 1307.9
1307.9 - 1470.7
> 1470.7
China: DALYs Rate (per 100,000)
< 416.8
416.8 - 450.7
450.7 - 492.2
492.2 - 533.7
533.7 - 570.4
570.4 - 607.0
607.0 - 643.7
643.7 - 706.9
706.9 - 743.9
> 743.9
Female
Male
Sexes Combined
Age
20-24 years
15-19 years
10-14 years
Year
DALYs Rate (per 100,000)
Age — 10-24 years

Figure S2

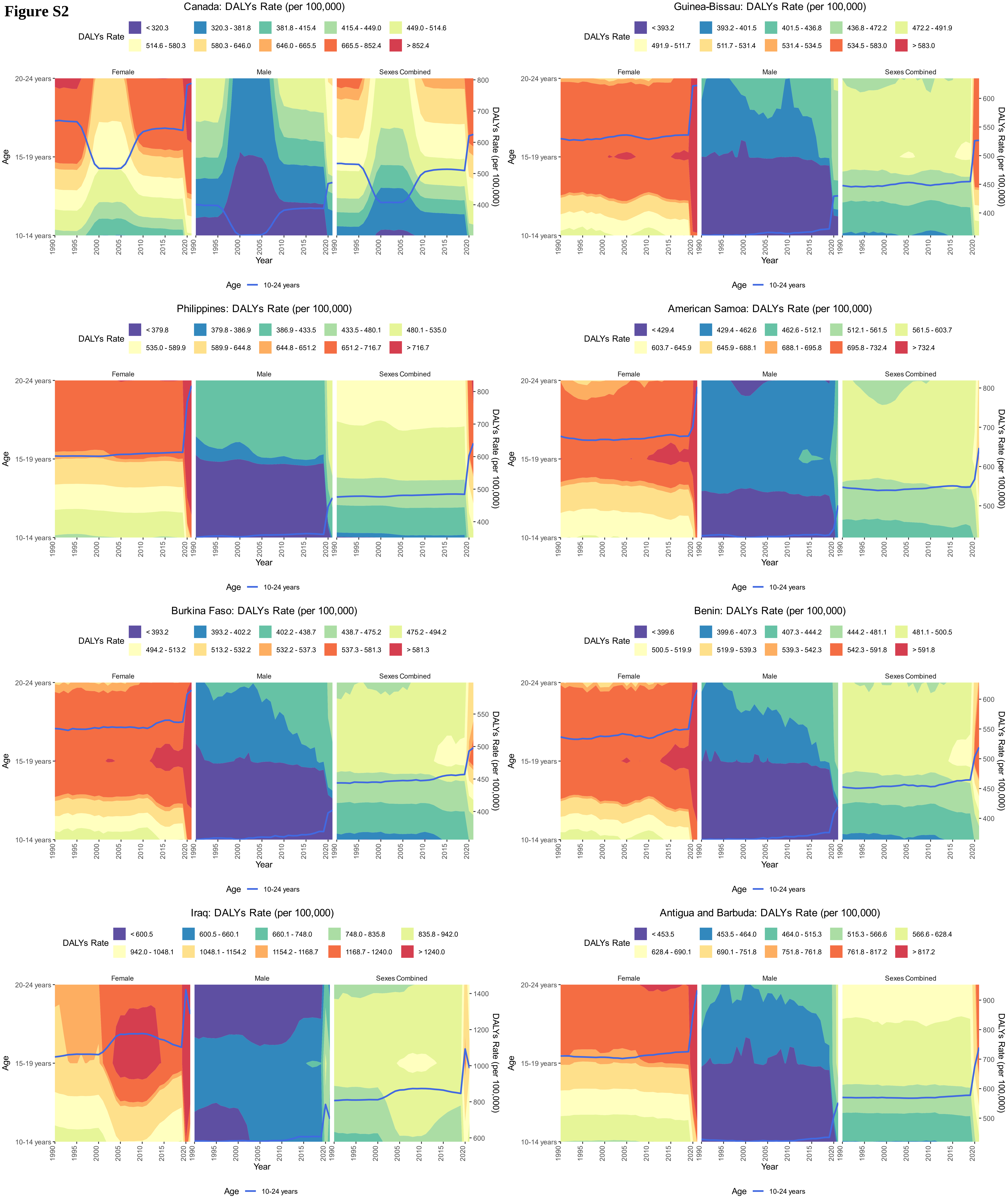

Canada: DALYs Rate (per 100,000)
DALYs Rate
< 320.3
320.3 - 381.8
381.8 - 415.4
415.4 - 449.0
449.0 - 514.6
514.6 - 580.3
580.3 - 646.0
646.0 - 665.5
665.5 - 852.4
> 852.4
Guinea-Bissau: DALYs Rate (per 100,000)
< 393.2
393.2 - 401.5
401.5 - 436.8
436.8 - 472.2
472.2 - 491.9
491.9 - 511.7
511.7 - 531.4
531.4 - 534.5
534.5 - 583.0
> 583.0
Philippines: DALYs Rate (per 100,000)
< 379.8
379.8 - 386.9
386.9 - 433.5
433.5 - 480.1
480.1 - 535.0
535.0 - 589.9
589.9 - 644.8
644.8 - 651.2
651.2 - 716.7
> 716.7
American Samoa: DALYs Rate (per 100,000)
< 429.4
429.4 - 462.6
462.6 - 512.1
512.1 - 561.5
561.5 - 603.7
603.7 - 645.9
645.9 - 688.1
688.1 - 695.8
695.8 - 732.4
> 732.4
Burkina Faso: DALYs Rate (per 100,000)
< 393.2
393.2 - 402.2
402.2 - 438.7
438.7 - 475.2
475.2 - 494.2
494.2 - 513.2
513.2 - 532.2
532.2 - 537.3
537.3 - 581.3
> 581.3
Benin: DALYs Rate (per 100,000)
< 399.6
399.6 - 407.3
407.3 - 444.2
444.2 - 481.1
481.1 - 500.5
500.5 - 519.9
519.9 - 539.3
539.3 - 542.3
542.3 - 591.8
> 591.8
Iraq: DALYs Rate (per 100,000)
< 600.5
600.5 - 660.1
660.1 - 748.0
748.0 - 835.8
835.8 - 942.0
942.0 - 1048.1
1048.1 - 1154.2
1154.2 - 1168.7
1168.7 - 1240.0
> 1240.0
Antigua and Barbuda: DALYs Rate (per 100,000)
< 453.5
453.5 - 464.0
464.0 - 515.3
515.3 - 566.6
566.6 - 628.4
628.4 - 690.1
690.1 - 751.8
751.8 - 761.8
761.8 - 817.2
> 817.2
Female
Male
Sexes Combined
Age
20-24 years
15-19 years
10-14 years
Year
DALYs Rate (per 100,000)
Age — 10-24 years

Figure S2

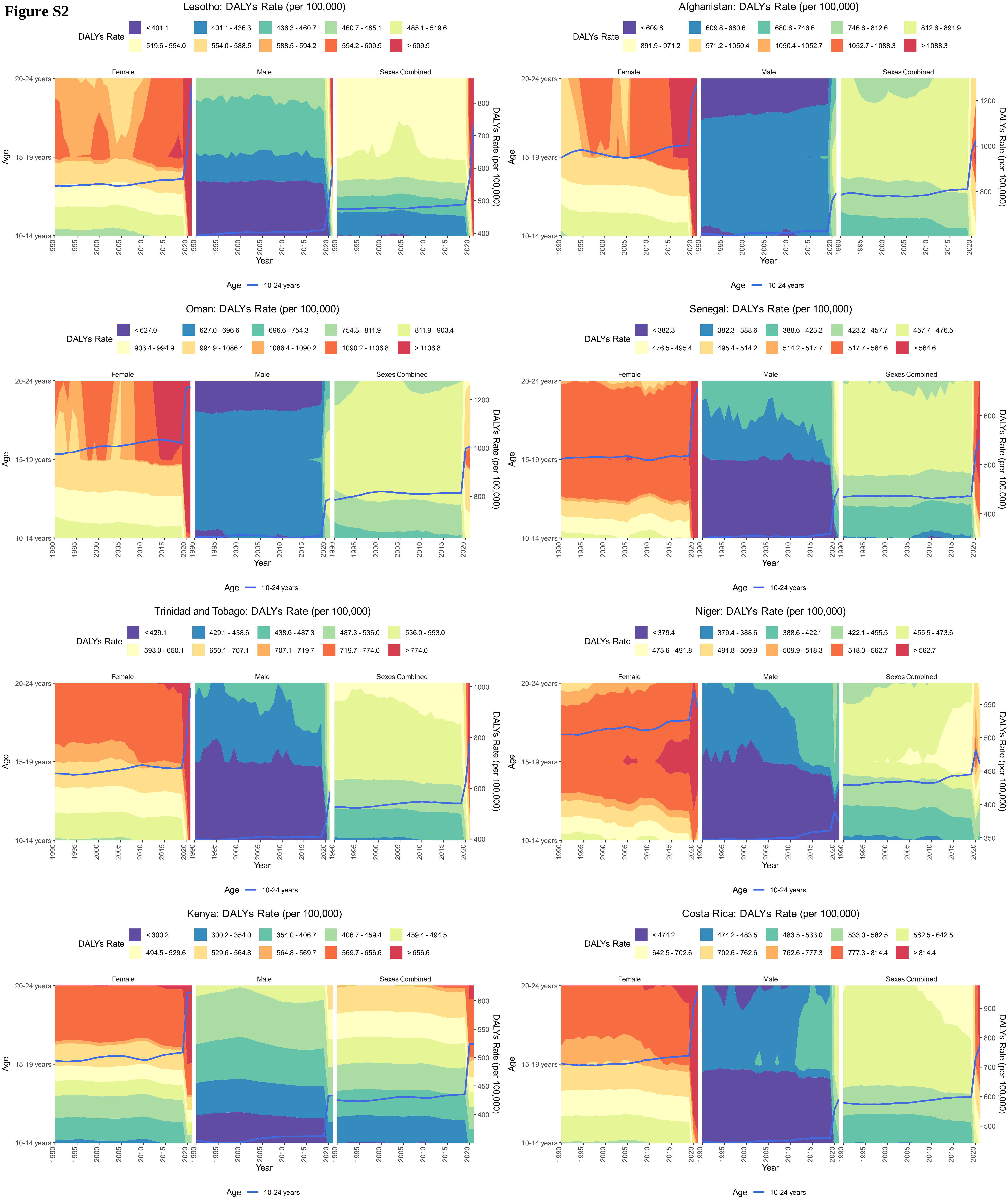
Lesotho: DALYs Rate (per 100,000)
DALYs Rate
< 401.1
401.1 - 436.3
436.3 - 460.7
460.7 - 485.1
485.1 - 519.6
519.6 - 554.0
554.0 - 588.5
588.5 - 594.2
594.2 - 609.9
> 609.9
Female
Male
Sexes Combined
Age
Year
DALYs Rate (per 100,000)
Age — 10-24 years
Afghanistan: DALYs Rate (per 100,000)
DALYs Rate
< 609.8
609.8 - 680.6
680.6 - 746.6
746.6 - 812.6
812.6 - 891.9
891.9 - 971.2
971.2 - 1050.4
1050.4 - 1052.7
1052.7 - 1088.3
> 1088.3
Oman: DALYs Rate (per 100,000)
DALYs Rate
< 627.0
627.0 - 696.6
696.6 - 754.3
754.3 - 811.9
811.9 - 903.4
903.4 - 994.9
994.9 - 1086.4
1086.4 - 1090.2
1090.2 - 1106.8
> 1106.8
Senegal: DALYs Rate (per 100,000)
DALYs Rate
< 382.3
382.3 - 388.6
388.6 - 423.2
423.2 - 457.7
457.7 - 476.5
476.5 - 495.4
495.4 - 514.2
514.2 - 517.7
517.7 - 564.6
> 564.6
Trinidad and Tobago: DALYs Rate (per 100,000)
DALYs Rate
< 429.1
429.1 - 438.6
438.6 - 487.3
487.3 - 536.0
536.0 - 593.0
593.0 - 650.1
650.1 - 707.1
707.1 - 719.7
719.7 - 774.0
> 774.0
Niger: DALYs Rate (per 100,000)
DALYs Rate
< 379.4
379.4 - 388.6
388.6 - 422.1
422.1 - 455.5
455.5 - 473.6
473.6 - 491.8
491.8 - 509.9
509.9 - 518.3
518.3 - 562.7
> 562.7
Kenya: DALYs Rate (per 100,000)
DALYs Rate
< 300.2
300.2 - 354.0
354.0 - 406.7
406.7 - 459.4
459.4 - 494.5
494.5 - 529.6
529.6 - 564.8
564.8 - 569.7
569.7 - 656.6
> 656.6
Costa Rica: DALYs Rate (per 100,000)
DALYs Rate
< 474.2
474.2 - 483.5
483.5 - 533.0
533.0 - 582.5
582.5 - 642.5
642.5 - 702.6
702.6 - 762.6
762.6 - 777.3
777.3 - 814.4
> 814.4
20-24 years
15-19 years
10-14 years
1990
1995
2000
2005
2010
2015
2020

Figure S2

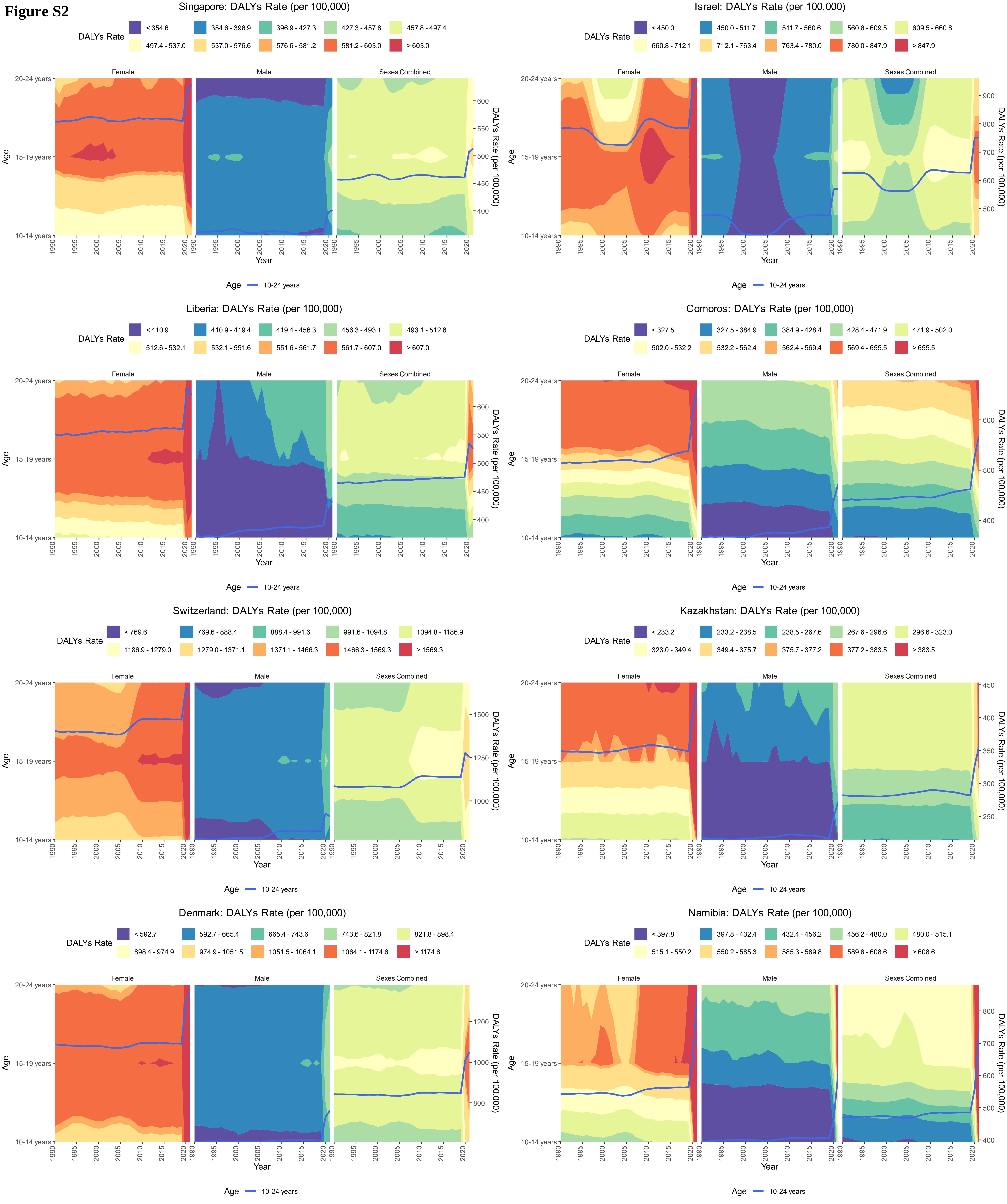

Singapore: DALYs Rate (per 100,000)
DALYs Rate
< 354.6
354.6 - 396.9
396.9 - 427.3
427.3 - 457.8
457.8 - 497.4
497.4 - 537.0
537.0 - 576.6
576.6 - 581.2
581.2 - 603.0
> 603.0
Israel: DALYs Rate (per 100,000)
DALYs Rate
< 450.0
450.0 - 511.7
511.7 - 560.6
560.6 - 609.5
609.5 - 660.8
660.8 - 712.1
712.1 - 763.4
763.4 - 780.0
780.0 - 847.9
> 847.9
Liberia: DALYs Rate (per 100,000)
DALYs Rate
< 410.9
410.9 - 419.4
419.4 - 456.3
456.3 - 493.1
493.1 - 512.6
512.6 - 532.1
532.1 - 551.6
551.6 - 561.7
561.7 - 607.0
> 607.0
Comoros: DALYs Rate (per 100,000)
DALYs Rate
< 327.5
327.5 - 384.9
384.9 - 428.4
428.4 - 471.9
471.9 - 502.0
502.0 - 532.2
532.2 - 562.4
562.4 - 569.4
569.4 - 655.5
> 655.5
Switzerland: DALYs Rate (per 100,000)
DALYs Rate
< 769.6
769.6 - 888.4
888.4 - 991.6
991.6 - 1094.8
1094.8 - 1186.9
1186.9 - 1279.0
1279.0 - 1371.1
1371.1 - 1466.3
1466.3 - 1569.3
> 1569.3
Kazakhstan: DALYs Rate (per 100,000)
DALYs Rate
< 233.2
233.2 - 238.5
238.5 - 267.6
267.6 - 296.6
296.6 - 323.0
323.0 - 349.4
349.4 - 375.7
375.7 - 377.2
377.2 - 383.5
> 383.5
Denmark: DALYs Rate (per 100,000)
DALYs Rate
< 592.7
592.7 - 665.4
665.4 - 743.6
743.6 - 821.8
821.8 - 898.4
898.4 - 974.9
974.9 - 1051.5
1051.5 - 1064.1
1064.1 - 1174.6
> 1174.6
Namibia: DALYs Rate (per 100,000)
DALYs Rate
< 397.8
397.8 - 432.4
432.4 - 456.2
456.2 - 480.0
480.0 - 515.1
515.1 - 550.2
550.2 - 585.3
585.3 - 589.8
589.8 - 608.6
> 608.6
Female
Male
Sexes Combined
20-24 years
15-19 years
10-14 years
Age
Year
DALYs Rate (per 100,000)
1990
1995
2000
2005
2010
2015
2020
Age
10-24 years

Figure S2

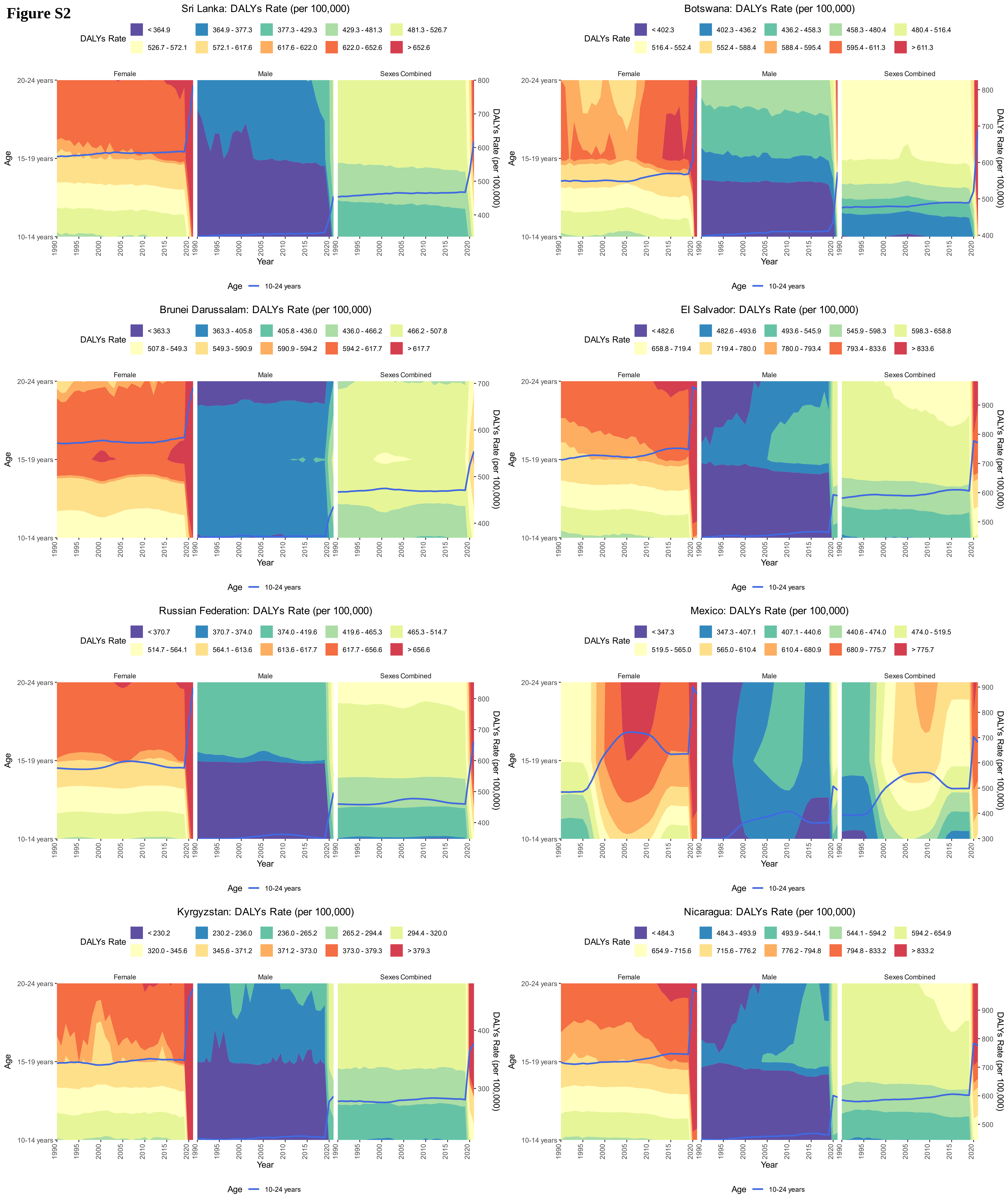

Sri Lanka: DALYs Rate (per 100,000)
DALYs Rate
< 364.9
364.9 - 377.3
377.3 - 429.3
429.3 - 481.3
481.3 - 526.7
526.7 - 572.1
572.1 - 617.6
617.6 - 622.0
622.0 - 652.6
> 652.6
Botswana: DALYs Rate (per 100,000)
< 402.3
402.3 - 436.2
436.2 - 458.3
458.3 - 480.4
480.4 - 516.4
516.4 - 552.4
552.4 - 588.4
588.4 - 595.4
595.4 - 611.3
> 611.3
Brunei Darussalam: DALYs Rate (per 100,000)
< 363.3
363.3 - 405.8
405.8 - 436.0
436.0 - 466.2
466.2 - 507.8
507.8 - 549.3
549.3 - 590.9
590.9 - 594.2
594.2 - 617.7
> 617.7
El Salvador: DALYs Rate (per 100,000)
< 482.6
482.6 - 493.6
493.6 - 545.9
545.9 - 598.3
598.3 - 658.8
658.8 - 719.4
719.4 - 780.0
780.0 - 793.4
793.4 - 833.6
> 833.6
Russian Federation: DALYs Rate (per 100,000)
< 370.7
370.7 - 374.0
374.0 - 419.6
419.6 - 465.3
465.3 - 514.7
514.7 - 564.1
564.1 - 613.6
613.6 - 617.7
617.7 - 656.6
> 656.6
Mexico: DALYs Rate (per 100,000)
< 347.3
347.3 - 407.1
407.1 - 440.6
440.6 - 474.0
474.0 - 519.5
519.5 - 565.0
565.0 - 610.4
610.4 - 680.9
680.9 - 775.7
> 775.7
Kyrgyzstan: DALYs Rate (per 100,000)
< 230.2
230.2 - 236.0
236.0 - 265.2
265.2 - 294.4
294.4 - 320.0
320.0 - 345.6
345.6 - 371.2
371.2 - 373.0
373.0 - 379.3
> 379.3
Nicaragua: DALYs Rate (per 100,000)
< 484.3
484.3 - 493.9
493.9 - 544.1
544.1 - 594.2
594.2 - 654.9
654.9 - 715.6
715.6 - 776.2
776.2 - 794.8
794.8 - 833.2
> 833.2
Female
Male
Sexes Combined
Age
20-24 years
15-19 years
10-14 years
Year
DALYs Rate (per 100,000)
Age — 10-24 years

Figure S2

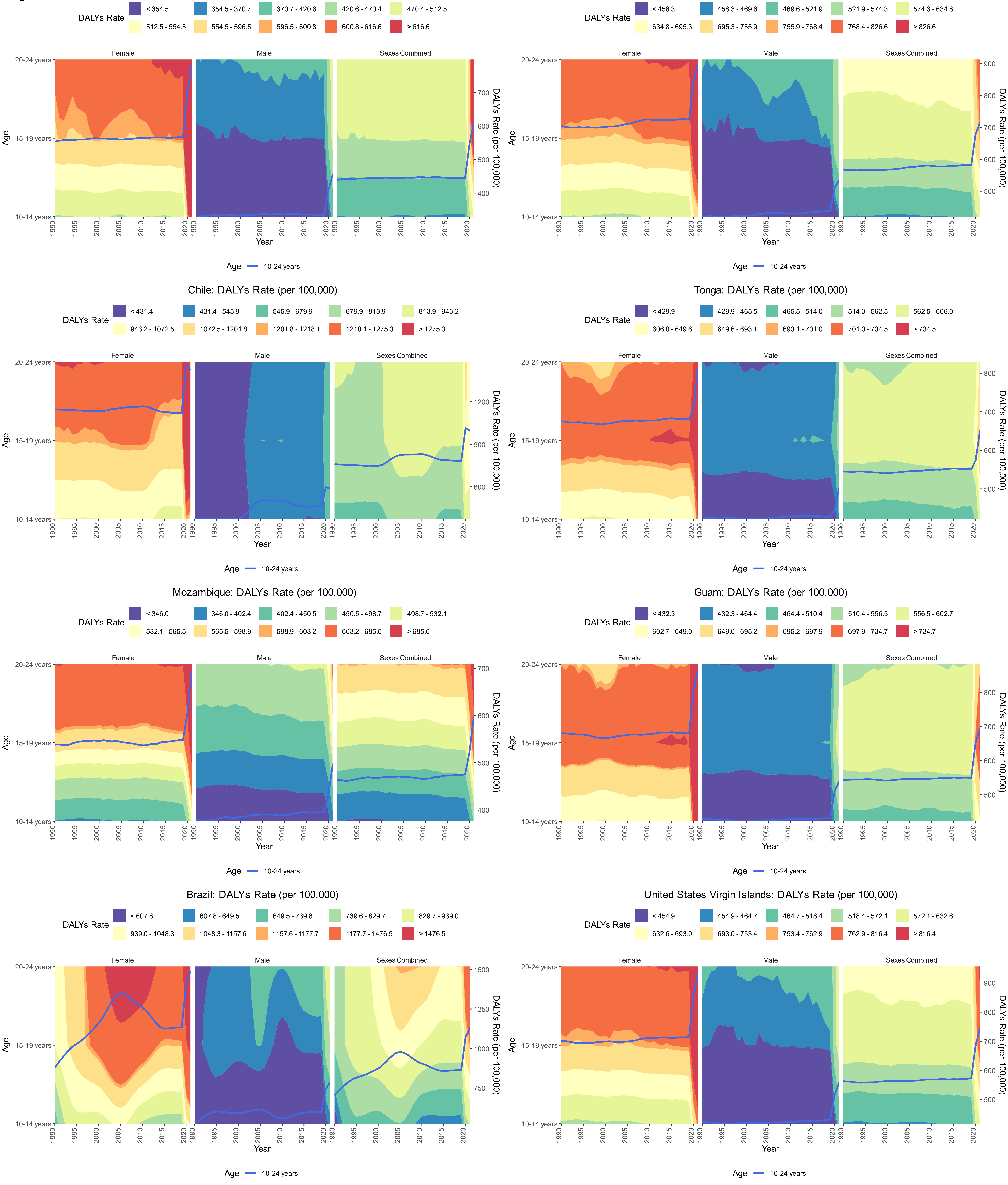

Serbia: DALYs Rate (per 100,000)
DALYs Rate
< 354.5
354.5 - 370.7
370.7 - 420.6
420.6 - 470.4
470.4 - 512.5
512.5 - 554.5
554.5 - 596.5
596.5 - 600.8
600.8 - 616.6
> 616.6
Haiti: DALYs Rate (per 100,000)
DALYs Rate
< 458.3
458.3 - 469.6
469.6 - 521.9
521.9 - 574.3
574.3 - 634.8
634.8 - 695.3
695.3 - 755.9
755.9 - 768.4
768.4 - 826.6
> 826.6
Chile: DALYs Rate (per 100,000)
DALYs Rate
< 431.4
431.4 - 545.9
545.9 - 679.9
679.9 - 813.9
813.9 - 943.2
943.2 - 1072.5
1072.5 - 1201.8
1201.8 - 1218.1
1218.1 - 1275.3
> 1275.3
Tonga: DALYs Rate (per 100,000)
DALYs Rate
< 429.9
429.9 - 465.5
465.5 - 514.0
514.0 - 562.5
562.5 - 606.0
606.0 - 649.6
649.6 - 693.1
693.1 - 701.0
701.0 - 734.5
> 734.5
Mozambique: DALYs Rate (per 100,000)
DALYs Rate
< 346.0
346.0 - 402.4
402.4 - 450.5
450.5 - 498.7
498.7 - 532.1
532.1 - 565.5
565.5 - 598.9
598.9 - 603.2
603.2 - 685.6
> 685.6
Guam: DALYs Rate (per 100,000)
DALYs Rate
< 432.3
432.3 - 464.4
464.4 - 510.4
510.4 - 556.5
556.5 - 602.7
602.7 - 649.0
649.0 - 695.2
695.2 - 697.9
697.9 - 734.7
> 734.7
Brazil: DALYs Rate (per 100,000)
DALYs Rate
< 607.8
607.8 - 649.5
649.5 - 739.6
739.6 - 829.7
829.7 - 939.0
939.0 - 1048.3
1048.3 - 1157.6
1157.6 - 1177.7
1177.7 - 1476.5
> 1476.5
United States Virgin Islands: DALYs Rate (per 100,000)
DALYs Rate
< 454.9
454.9 - 464.7
464.7 - 518.4
518.4 - 572.1
572.1 - 632.6
632.6 - 693.0
693.0 - 753.4
753.4 - 762.9
762.9 - 816.4
> 816.4
Female
Male
Sexes Combined
Age
20-24 years
15-19 years
10-14 years
Year
DALYs Rate (per 100,000)
Age — 10-24 years

Figure S2

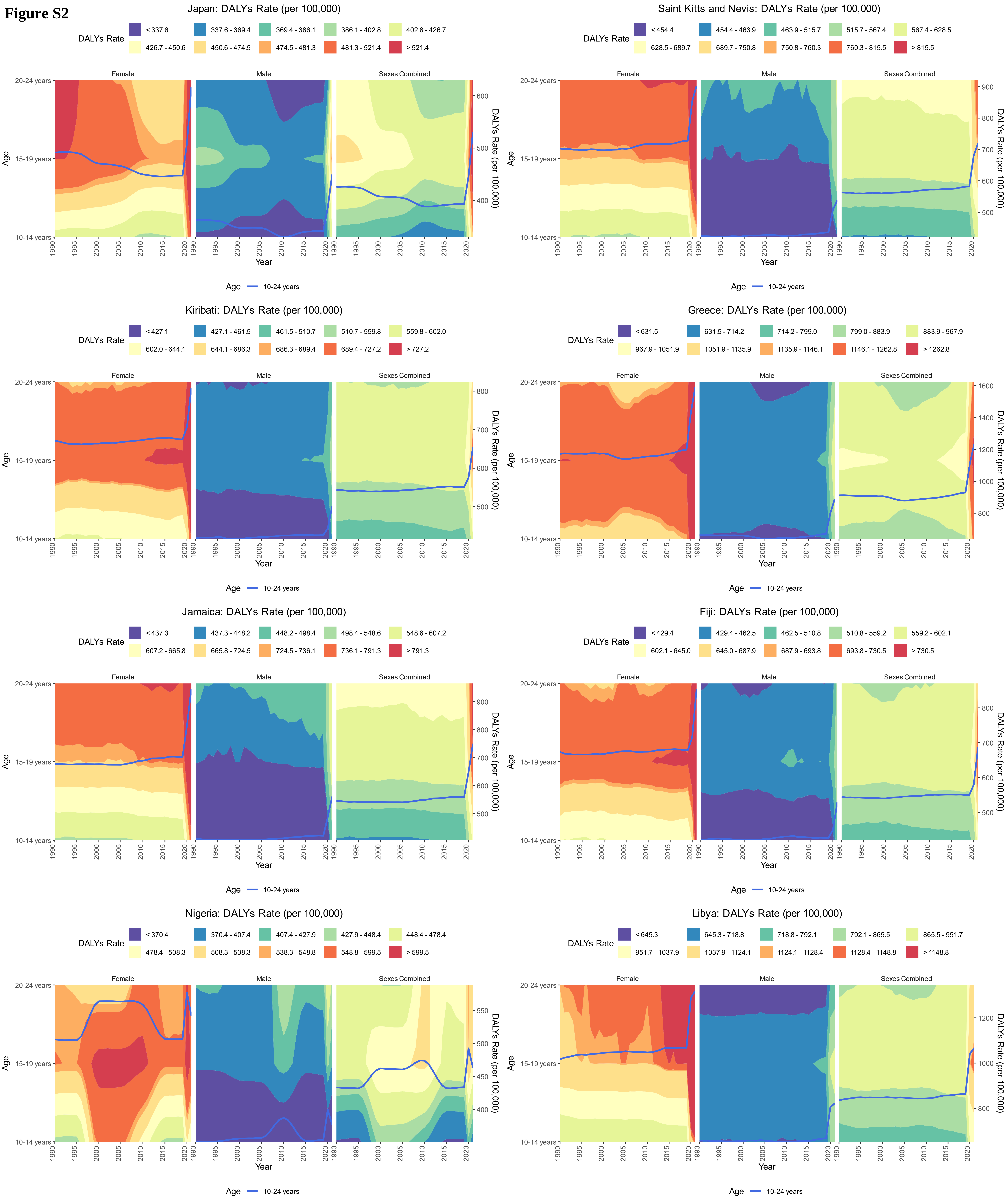

Japan: DALYs Rate (per 100,000)
DALYs Rate
< 337.6
337.6 - 369.4
369.4 - 386.1
386.1 - 402.8
402.8 - 426.7
426.7 - 450.6
450.6 - 474.5
474.5 - 481.3
481.3 - 521.4
> 521.4
Saint Kitts and Nevis: DALYs Rate (per 100,000)
< 454.4
454.4 - 463.9
463.9 - 515.7
515.7 - 567.4
567.4 - 628.5
628.5 - 689.7
689.7 - 750.8
750.8 - 760.3
760.3 - 815.5
> 815.5
Kiribati: DALYs Rate (per 100,000)
< 427.1
427.1 - 461.5
461.5 - 510.7
510.7 - 559.8
559.8 - 602.0
602.0 - 644.1
644.1 - 686.3
686.3 - 689.4
689.4 - 727.2
> 727.2
Greece: DALYs Rate (per 100,000)
< 631.5
631.5 - 714.2
714.2 - 799.0
799.0 - 883.9
883.9 - 967.9
967.9 - 1051.9
1051.9 - 1135.9
1135.9 - 1146.1
1146.1 - 1262.8
> 1262.8
Jamaica: DALYs Rate (per 100,000)
< 437.3
437.3 - 448.2
448.2 - 498.4
498.4 - 548.6
548.6 - 607.2
607.2 - 665.8
665.8 - 724.5
724.5 - 736.1
736.1 - 791.3
> 791.3
Fiji: DALYs Rate (per 100,000)
< 429.4
429.4 - 462.5
462.5 - 510.8
510.8 - 559.2
559.2 - 602.1
602.1 - 645.0
645.0 - 687.9
687.9 - 693.8
693.8 - 730.5
> 730.5
Nigeria: DALYs Rate (per 100,000)
< 370.4
370.4 - 407.4
407.4 - 427.9
427.9 - 448.4
448.4 - 478.4
478.4 - 508.3
508.3 - 538.3
538.3 - 548.8
548.8 - 599.5
> 599.5
Libya: DALYs Rate (per 100,000)
< 645.3
645.3 - 718.8
718.8 - 792.1
792.1 - 865.5
865.5 - 951.7
951.7 - 1037.9
1037.9 - 1124.1
1124.1 - 1128.4
1128.4 - 1148.8
> 1148.8
Female
Male
Sexes Combined
Age
20-24 years
15-19 years
10-14 years
Year
DALYs Rate (per 100,000)
Age — 10-24 years

Figure S2

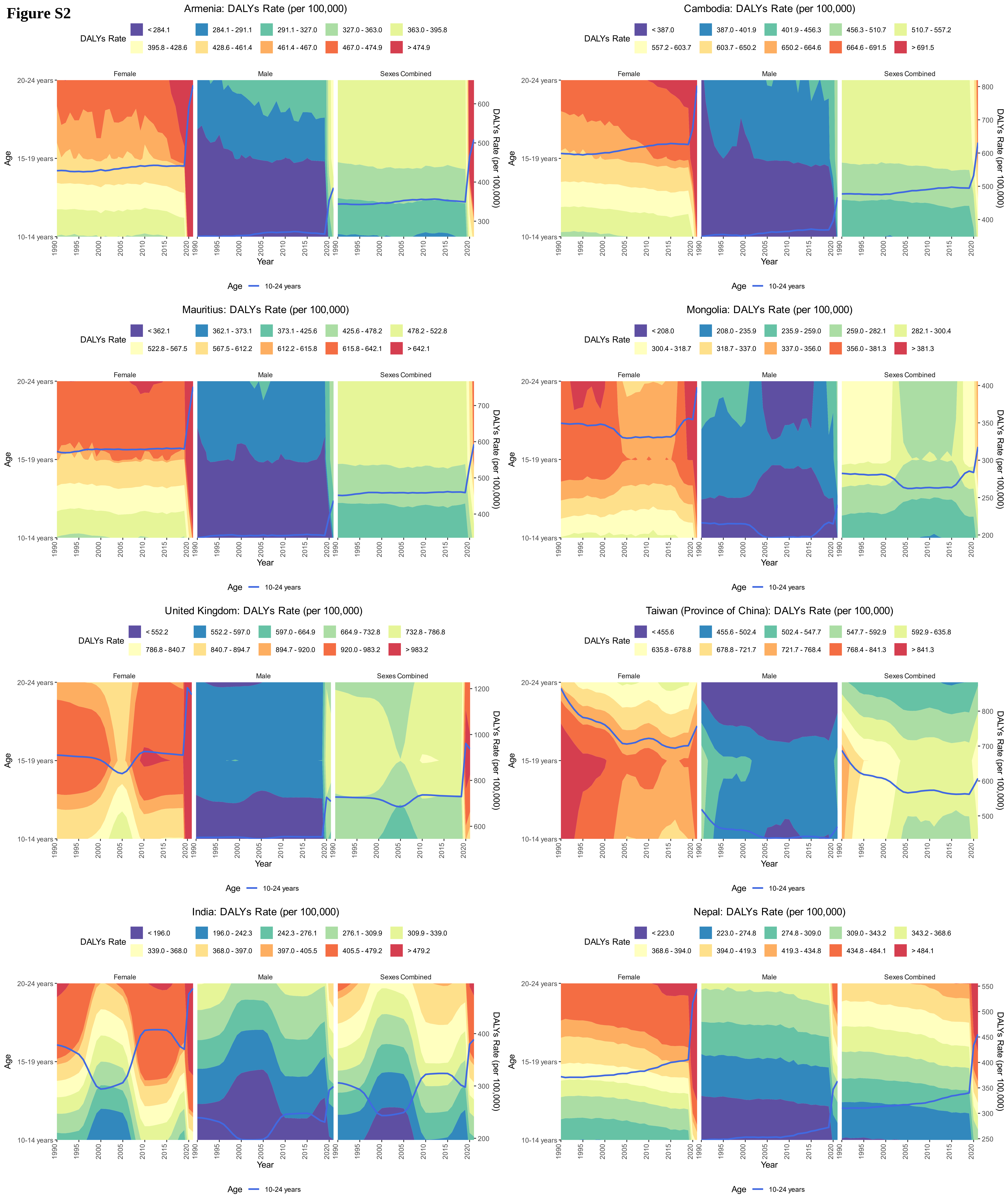

Armenia: DALYs Rate (per 100,000)
DALYs Rate
< 284.1
284.1 - 291.1
291.1 - 327.0
327.0 - 363.0
363.0 - 395.8
395.8 - 428.6
428.6 - 461.4
461.4 - 467.0
467.0 - 474.9
> 474.9
Cambodia: DALYs Rate (per 100,000)
< 387.0
387.0 - 401.9
401.9 - 456.3
456.3 - 510.7
510.7 - 557.2
557.2 - 603.7
603.7 - 650.2
650.2 - 664.6
664.6 - 691.5
> 691.5
Mauritius: DALYs Rate (per 100,000)
< 362.1
362.1 - 373.1
373.1 - 425.6
425.6 - 478.2
478.2 - 522.8
522.8 - 567.5
567.5 - 612.2
612.2 - 615.8
615.8 - 642.1
> 642.1
Mongolia: DALYs Rate (per 100,000)
< 208.0
208.0 - 235.9
235.9 - 259.0
259.0 - 282.1
282.1 - 300.4
300.4 - 318.7
318.7 - 337.0
337.0 - 356.0
356.0 - 381.3
> 381.3
United Kingdom: DALYs Rate (per 100,000)
< 552.2
552.2 - 597.0
597.0 - 664.9
664.9 - 732.8
732.8 - 786.8
786.8 - 840.7
840.7 - 894.7
894.7 - 920.0
920.0 - 983.2
> 983.2
Taiwan (Province of China): DALYs Rate (per 100,000)
< 455.6
455.6 - 502.4
502.4 - 547.7
547.7 - 592.9
592.9 - 635.8
635.8 - 678.8
678.8 - 721.7
721.7 - 768.4
768.4 - 841.3
> 841.3
India: DALYs Rate (per 100,000)
< 196.0
196.0 - 242.3
242.3 - 276.1
276.1 - 309.9
309.9 - 339.0
339.0 - 368.0
368.0 - 397.0
397.0 - 405.5
405.5 - 479.2
> 479.2
Nepal: DALYs Rate (per 100,000)
< 223.0
223.0 - 274.8
274.8 - 309.0
309.0 - 343.2
343.2 - 368.6
368.6 - 394.0
394.0 - 419.3
419.3 - 434.8
434.8 - 484.1
> 484.1
Female
Male
Sexes Combined
Age
20-24 years
15-19 years
10-14 years
Year
DALYs Rate (per 100,000)
Age — 10-24 years

Figure S2

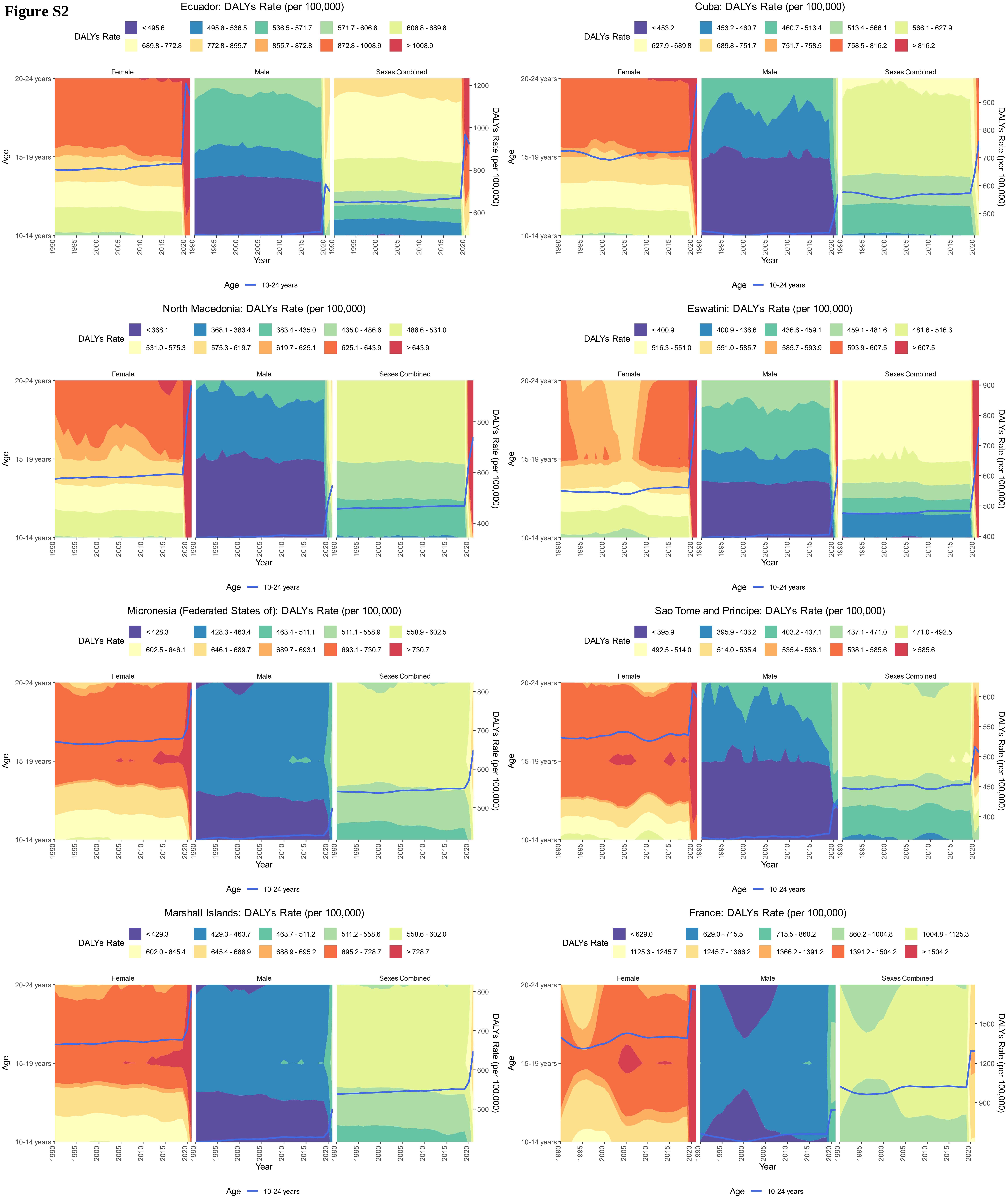

Ecuador: DALYs Rate (per 100,000)
DALYs Rate
< 495.6
495.6 - 536.5
536.5 - 571.7
571.7 - 606.8
606.8 - 689.8
689.8 - 772.8
772.8 - 855.7
855.7 - 872.8
872.8 - 1008.9
> 1008.9
Female
Male
Sexes Combined
Age
Year
DALYs Rate (per 100,000)
Age — 10-24 years
Cuba: DALYs Rate (per 100,000)
DALYs Rate
< 453.2
453.2 - 460.7
460.7 - 513.4
513.4 - 566.1
566.1 - 627.9
627.9 - 689.8
689.8 - 751.7
751.7 - 758.5
758.5 - 816.2
> 816.2
North Macedonia: DALYs Rate (per 100,000)
DALYs Rate
< 368.1
368.1 - 383.4
383.4 - 435.0
435.0 - 486.6
486.6 - 531.0
531.0 - 575.3
575.3 - 619.7
619.7 - 625.1
625.1 - 643.9
> 643.9
Eswatini: DALYs Rate (per 100,000)
DALYs Rate
< 400.9
400.9 - 436.6
436.6 - 459.1
459.1 - 481.6
481.6 - 516.3
516.3 - 551.0
551.0 - 585.7
585.7 - 593.9
593.9 - 607.5
> 607.5
Micronesia (Federated States of): DALYs Rate (per 100,000)
DALYs Rate
< 428.3
428.3 - 463.4
463.4 - 511.1
511.1 - 558.9
558.9 - 602.5
602.5 - 646.1
646.1 - 689.7
689.7 - 693.1
693.1 - 730.7
> 730.7
Sao Tome and Principe: DALYs Rate (per 100,000)
DALYs Rate
< 395.9
395.9 - 403.2
403.2 - 437.1
437.1 - 471.0
471.0 - 492.5
492.5 - 514.0
514.0 - 535.4
535.4 - 538.1
538.1 - 585.6
> 585.6
Marshall Islands: DALYs Rate (per 100,000)
DALYs Rate
< 429.3
429.3 - 463.7
463.7 - 511.2
511.2 - 558.6
558.6 - 602.0
602.0 - 645.4
645.4 - 688.9
688.9 - 695.2
695.2 - 728.7
> 728.7
France: DALYs Rate (per 100,000)
DALYs Rate
< 629.0
629.0 - 715.5
715.5 - 860.2
860.2 - 1004.8
1004.8 - 1125.3
1125.3 - 1245.7
1245.7 - 1366.2
1366.2 - 1391.2
1391.2 - 1504.2
> 1504.2
20-24 years
15-19 years
10-14 years
1990
1995
2000
2005
2010
2015
2020

Figure S2

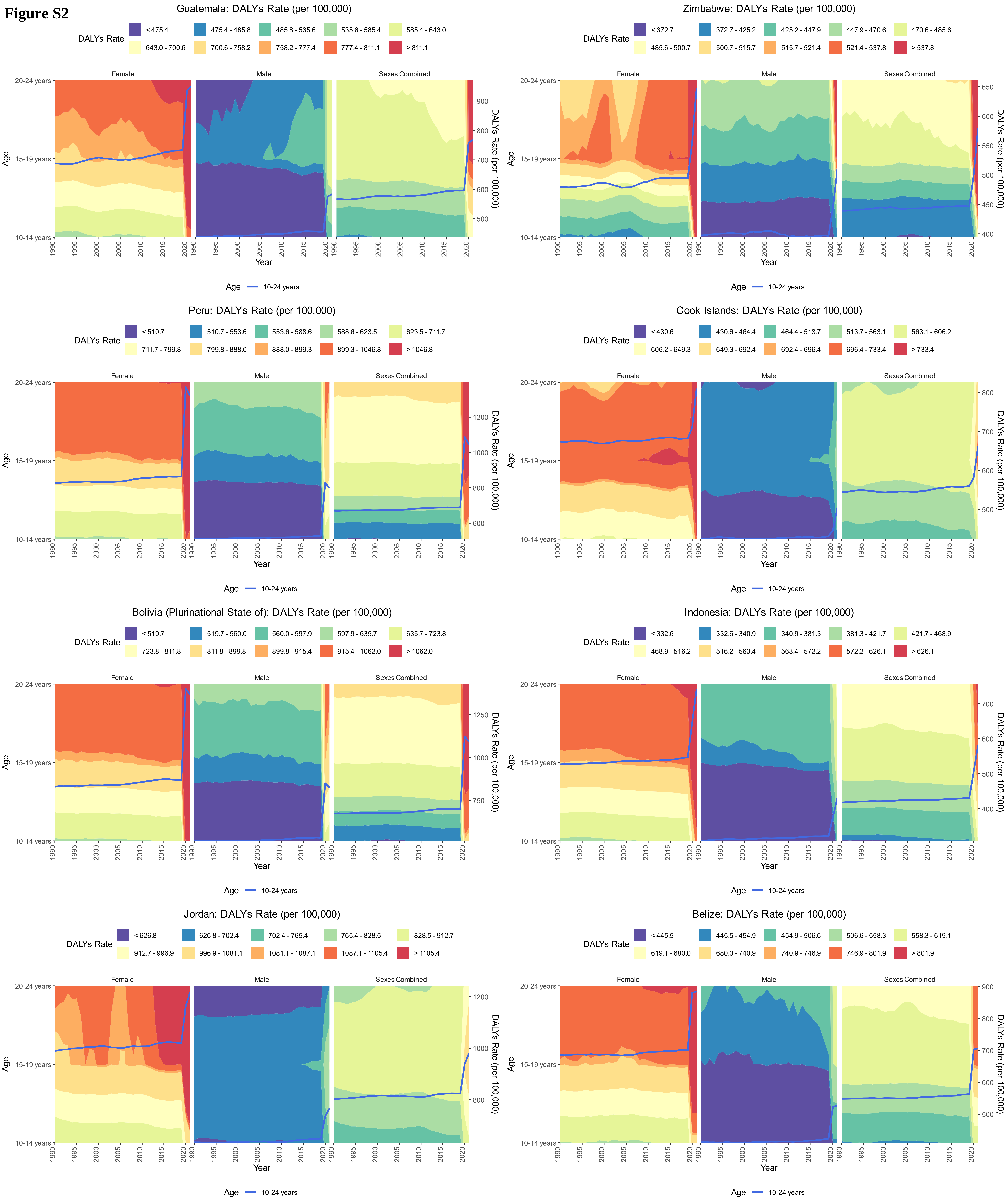

Guatemala: DALYs Rate (per 100,000)
DALYs Rate
< 475.4
475.4 - 485.8
485.8 - 535.6
535.6 - 585.4
585.4 - 643.0
643.0 - 700.6
700.6 - 758.2
758.2 - 777.4
777.4 - 811.1
> 811.1
Zimbabwe: DALYs Rate (per 100,000)
DALYs Rate
< 372.7
372.7 - 425.2
425.2 - 447.9
447.9 - 470.6
470.6 - 485.6
485.6 - 500.7
500.7 - 515.7
515.7 - 521.4
521.4 - 537.8
> 537.8
Peru: DALYs Rate (per 100,000)
DALYs Rate
< 510.7
510.7 - 553.6
553.6 - 588.6
588.6 - 623.5
623.5 - 711.7
711.7 - 799.8
799.8 - 888.0
888.0 - 899.3
899.3 - 1046.8
> 1046.8
Cook Islands: DALYs Rate (per 100,000)
DALYs Rate
< 430.6
430.6 - 464.4
464.4 - 513.7
513.7 - 563.1
563.1 - 606.2
606.2 - 649.3
649.3 - 692.4
692.4 - 696.4
696.4 - 733.4
> 733.4
Bolivia (Plurinational State of): DALYs Rate (per 100,000)
DALYs Rate
< 519.7
519.7 - 560.0
560.0 - 597.9
597.9 - 635.7
635.7 - 723.8
723.8 - 811.8
811.8 - 899.8
899.8 - 915.4
915.4 - 1062.0
> 1062.0
Indonesia: DALYs Rate (per 100,000)
DALYs Rate
< 332.6
332.6 - 340.9
340.9 - 381.3
381.3 - 421.7
421.7 - 468.9
468.9 - 516.2
516.2 - 563.4
563.4 - 572.2
572.2 - 626.1
> 626.1
Jordan: DALYs Rate (per 100,000)
DALYs Rate
< 626.8
626.8 - 702.4
702.4 - 765.4
765.4 - 828.5
828.5 - 912.7
912.7 - 996.9
996.9 - 1081.1
1081.1 - 1087.1
1087.1 - 1105.4
> 1105.4
Belize: DALYs Rate (per 100,000)
DALYs Rate
< 445.5
445.5 - 454.9
454.9 - 506.6
506.6 - 558.3
558.3 - 619.1
619.1 - 680.0
680.0 - 740.9
740.9 - 746.9
746.9 - 801.9
> 801.9
Female
Male
Sexes Combined
Age
20-24 years
15-19 years
10-14 years
Year
1990
1995
2000
2005
2010
2015
2020
DALYs Rate (per 100,000)
Age
10-24 years

Figure S2

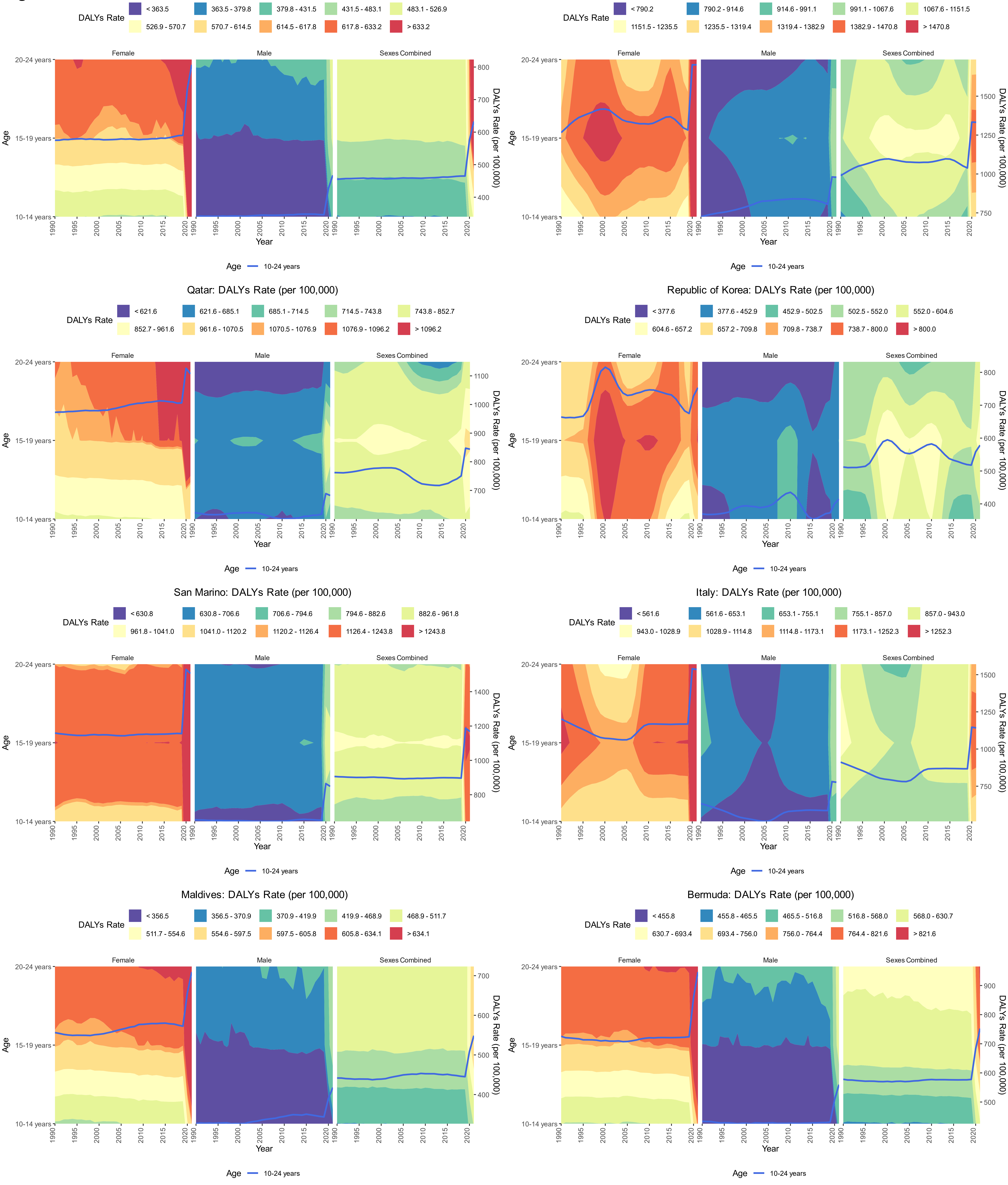

Croatia: DALYs Rate (per 100,000)
DALYs Rate
< 363.5
363.5 - 379.8
379.8 - 431.5
431.5 - 483.1
483.1 - 526.9
526.9 - 570.7
570.7 - 614.5
614.5 - 617.8
617.8 - 633.2
> 633.2
Netherlands: DALYs Rate (per 100,000)
DALYs Rate
< 790.2
790.2 - 914.6
914.6 - 991.1
991.1 - 1067.6
1067.6 - 1151.5
1151.5 - 1235.5
1235.5 - 1319.4
1319.4 - 1382.9
1382.9 - 1470.8
> 1470.8
Qatar: DALYs Rate (per 100,000)
DALYs Rate
< 621.6
621.6 - 685.1
685.1 - 714.5
714.5 - 743.8
743.8 - 852.7
852.7 - 961.6
961.6 - 1070.5
1070.5 - 1076.9
1076.9 - 1096.2
> 1096.2
Republic of Korea: DALYs Rate (per 100,000)
DALYs Rate
< 377.6
377.6 - 452.9
452.9 - 502.5
502.5 - 552.0
552.0 - 604.6
604.6 - 657.2
657.2 - 709.8
709.8 - 738.7
738.7 - 800.0
> 800.0
San Marino: DALYs Rate (per 100,000)
DALYs Rate
< 630.8
630.8 - 706.6
706.6 - 794.6
794.6 - 882.6
882.6 - 961.8
961.8 - 1041.0
1041.0 - 1120.2
1120.2 - 1126.4
1126.4 - 1243.8
> 1243.8
Italy: DALYs Rate (per 100,000)
DALYs Rate
< 561.6
561.6 - 653.1
653.1 - 755.1
755.1 - 857.0
857.0 - 943.0
943.0 - 1028.9
1028.9 - 1114.8
1114.8 - 1173.1
1173.1 - 1252.3
> 1252.3
Maldives: DALYs Rate (per 100,000)
DALYs Rate
< 356.5
356.5 - 370.9
370.9 - 419.9
419.9 - 468.9
468.9 - 511.7
511.7 - 554.6
554.6 - 597.5
597.5 - 605.8
605.8 - 634.1
> 634.1
Bermuda: DALYs Rate (per 100,000)
DALYs Rate
< 455.8
455.8 - 465.5
465.5 - 516.8
516.8 - 568.0
568.0 - 630.7
630.7 - 693.4
693.4 - 756.0
756.0 - 764.4
764.4 - 821.6
> 821.6
Female
Male
Sexes Combined
Age
20-24 years
15-19 years
10-14 years
Year
1990
1995
2000
2005
2010
2015
2020
DALYs Rate (per 100,000)
Age
10-24 years

Figure S2

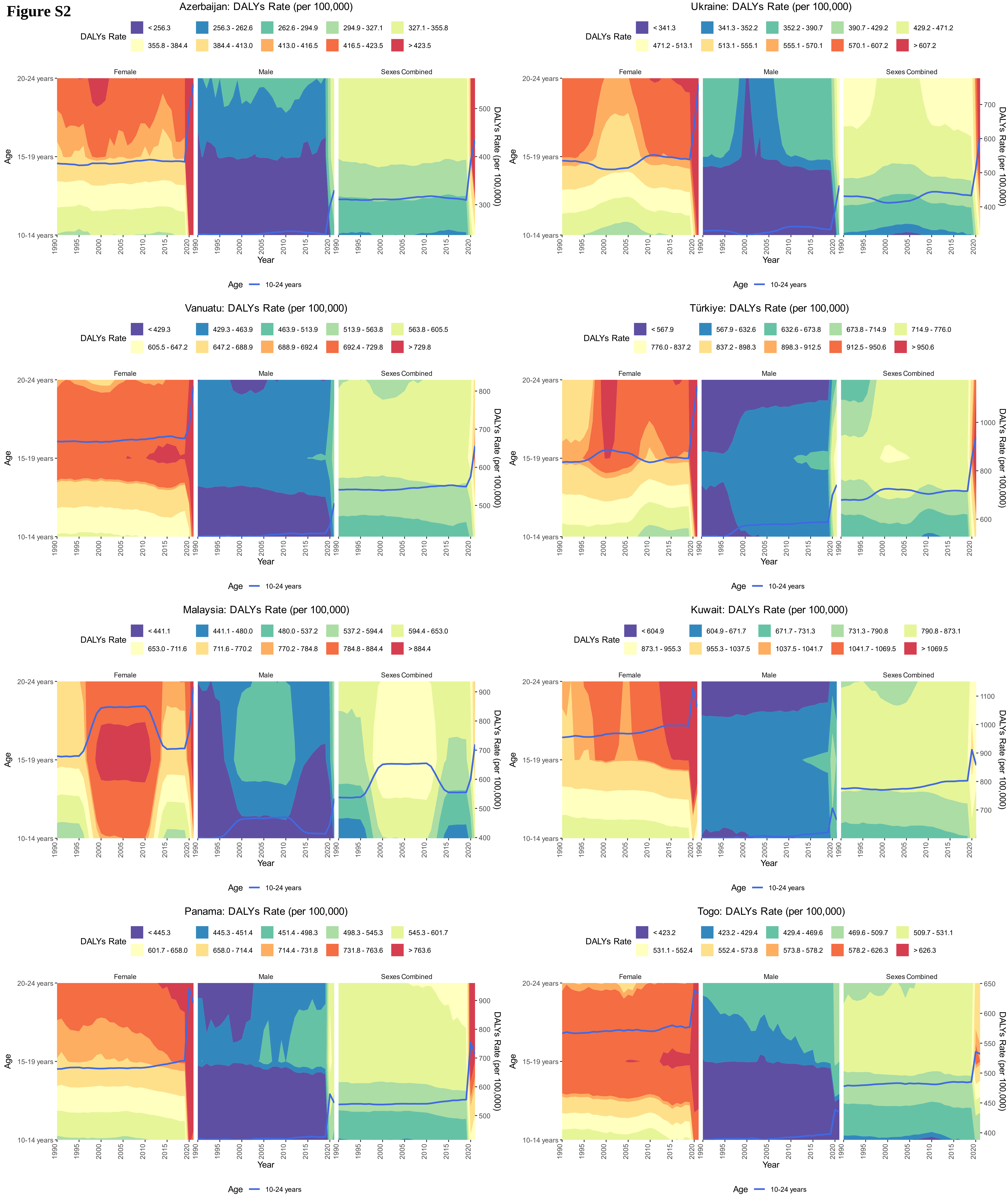
Azerbaijan: DALYs Rate (per 100,000)
DALYs Rate
< 256.3
256.3 - 262.6
262.6 - 294.9
294.9 - 327.1
327.1 - 355.8
355.8 - 384.4
384.4 - 413.0
413.0 - 416.5
416.5 - 423.5
> 423.5
Female
Male
Sexes Combined
20-24 years
15-19 years
10-14 years
Age
Year
DALYs Rate (per 100,000)
Age
10-24 years
Ukraine: DALYs Rate (per 100,000)
DALYs Rate
< 341.3
341.3 - 352.2
352.2 - 390.7
390.7 - 429.2
429.2 - 471.2
471.2 - 513.1
513.1 - 555.1
555.1 - 570.1
570.1 - 607.2
> 607.2
Vanuatu: DALYs Rate (per 100,000)
DALYs Rate
< 429.3
429.3 - 463.9
463.9 - 513.9
513.9 - 563.8
563.8 - 605.5
605.5 - 647.2
647.2 - 688.9
688.9 - 692.4
692.4 - 729.8
> 729.8
Türkiye: DALYs Rate (per 100,000)
DALYs Rate
< 567.9
567.9 - 632.6
632.6 - 673.8
673.8 - 714.9
714.9 - 776.0
776.0 - 837.2
837.2 - 898.3
898.3 - 912.5
912.5 - 950.6
> 950.6
Malaysia: DALYs Rate (per 100,000)
DALYs Rate
< 441.1
441.1 - 480.0
480.0 - 537.2
537.2 - 594.4
594.4 - 653.0
653.0 - 711.6
711.6 - 770.2
770.2 - 784.8
784.8 - 884.4
> 884.4
Kuwait: DALYs Rate (per 100,000)
DALYs Rate
< 604.9
604.9 - 671.7
671.7 - 731.3
731.3 - 790.8
790.8 - 873.1
873.1 - 955.3
955.3 - 1037.5
1037.5 - 1041.7
1041.7 - 1069.5
> 1069.5
Panama: DALYs Rate (per 100,000)
DALYs Rate
< 445.3
445.3 - 451.4
451.4 - 498.3
498.3 - 545.3
545.3 - 601.7
601.7 - 658.0
658.0 - 714.4
714.4 - 731.8
731.8 - 763.6
> 763.6
Togo: DALYs Rate (per 100,000)
DALYs Rate
< 423.2
423.2 - 429.4
429.4 - 469.6
469.6 - 509.7
509.7 - 531.1
531.1 - 552.4
552.4 - 573.8
573.8 - 578.2
578.2 - 626.3
> 626.3

Figure S2

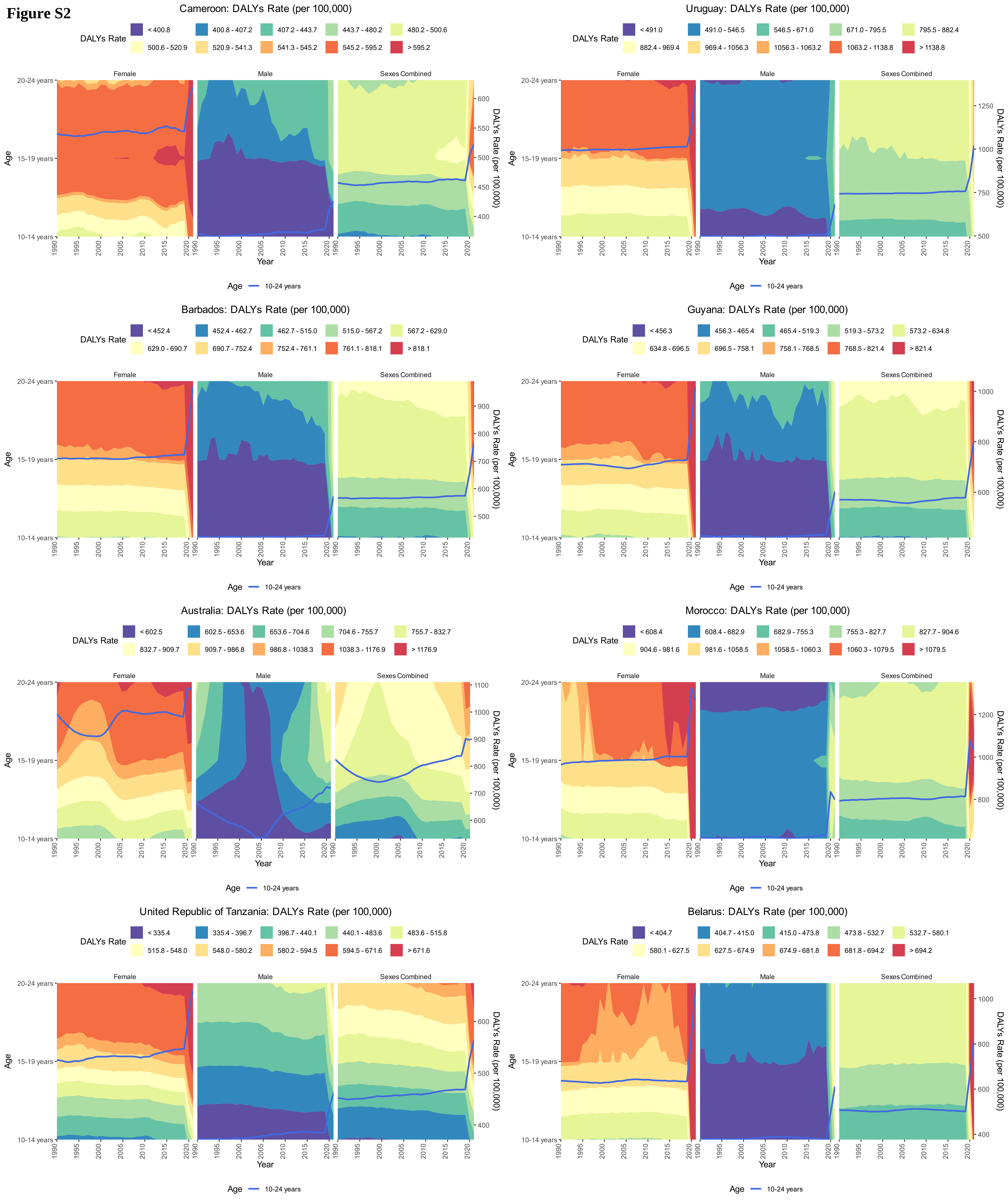
Cameroon: DALYs Rate (per 100,000)
DALYs Rate
< 400.8
400.8 - 407.2
407.2 - 443.7
443.7 - 480.2
480.2 - 500.6
500.6 - 520.9
520.9 - 541.3
541.3 - 545.2
545.2 - 595.2
> 595.2
Uruguay: DALYs Rate (per 100,000)
< 491.0
491.0 - 546.5
546.5 - 671.0
671.0 - 795.5
795.5 - 882.4
882.4 - 969.4
969.4 - 1056.3
1056.3 - 1063.2
1063.2 - 1138.8
> 1138.8
Barbados: DALYs Rate (per 100,000)
< 452.4
452.4 - 462.7
462.7 - 515.0
515.0 - 567.2
567.2 - 629.0
629.0 - 690.7
690.7 - 752.4
752.4 - 761.1
761.1 - 818.1
> 818.1
Guyana: DALYs Rate (per 100,000)
< 456.3
456.3 - 465.4
465.4 - 519.3
519.3 - 573.2
573.2 - 634.8
634.8 - 696.5
696.5 - 758.1
758.1 - 768.5
768.5 - 821.4
> 821.4
Australia: DALYs Rate (per 100,000)
< 602.5
602.5 - 653.6
653.6 - 704.6
704.6 - 755.7
755.7 - 832.7
832.7 - 909.7
909.7 - 986.8
986.8 - 1038.3
1038.3 - 1176.9
> 1176.9
Morocco: DALYs Rate (per 100,000)
< 608.4
608.4 - 682.9
682.9 - 755.3
755.3 - 827.7
827.7 - 904.6
904.6 - 981.6
981.6 - 1058.5
1058.5 - 1060.3
1060.3 - 1079.5
> 1079.5
United Republic of Tanzania: DALYs Rate (per 100,000)
< 335.4
335.4 - 396.7
396.7 - 440.1
440.1 - 483.6
483.6 - 515.8
515.8 - 548.0
548.0 - 580.2
580.2 - 594.5
594.5 - 671.6
> 671.6
Belarus: DALYs Rate (per 100,000)
< 404.7
404.7 - 415.0
415.0 - 473.8
473.8 - 532.7
532.7 - 580.1
580.1 - 627.5
627.5 - 674.9
674.9 - 681.8
681.8 - 694.2
> 694.2
Female
Male
Sexes Combined
Age
20-24 years
15-19 years
10-14 years
Year
DALYs Rate (per 100,000)
Age
10-24 years

Figure S2

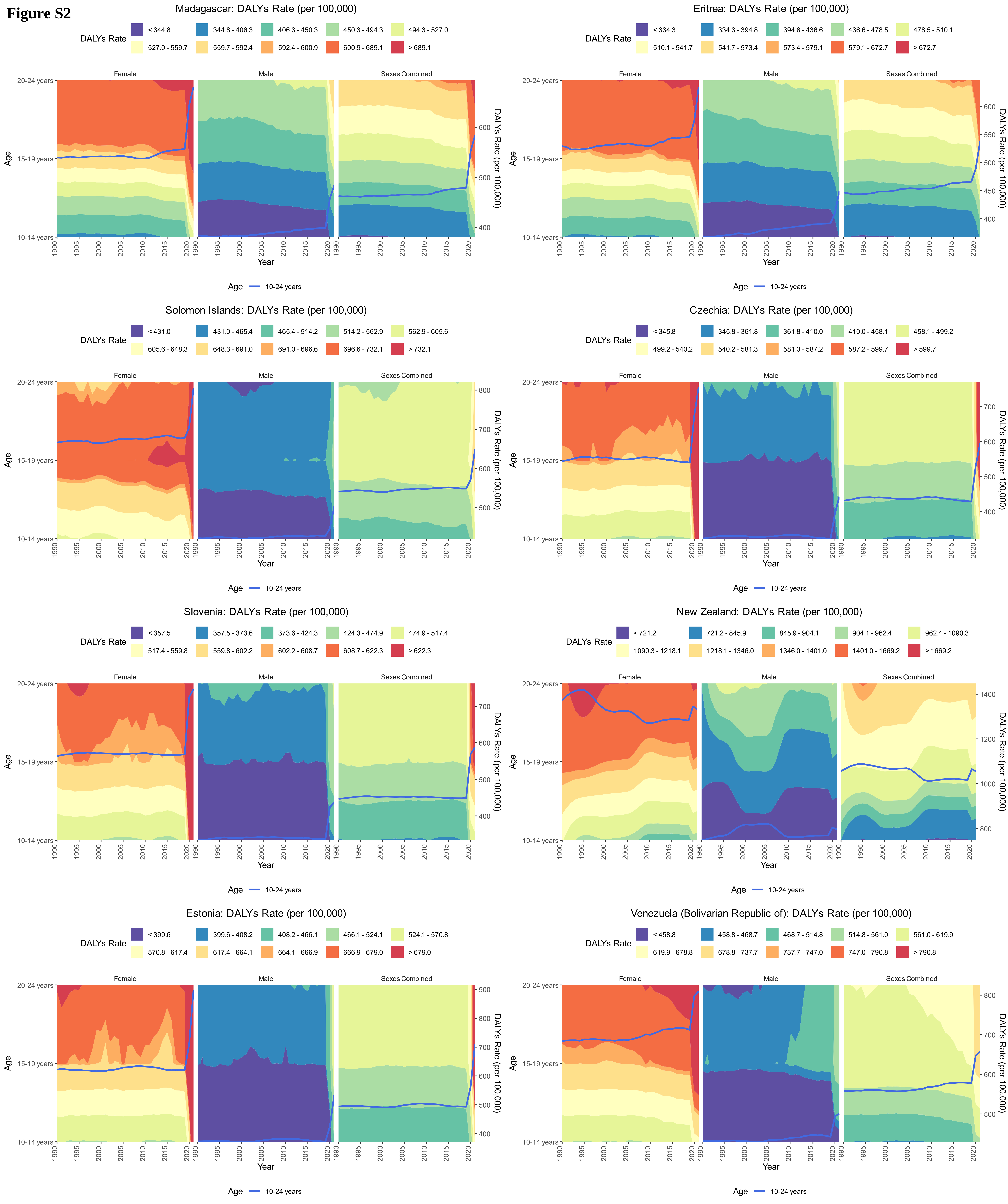

Madagascar: DALYs Rate (per 100,000)
DALYs Rate
< 344.8
344.8 - 406.3
406.3 - 450.3
450.3 - 494.3
494.3 - 527.0
527.0 - 559.7
559.7 - 592.4
592.4 - 600.9
600.9 - 689.1
> 689.1
Eritrea: DALYs Rate (per 100,000)
< 334.3
334.3 - 394.8
394.8 - 436.6
436.6 - 478.5
478.5 - 510.1
510.1 - 541.7
541.7 - 573.4
573.4 - 579.1
579.1 - 672.7
> 672.7
Solomon Islands: DALYs Rate (per 100,000)
< 431.0
431.0 - 465.4
465.4 - 514.2
514.2 - 562.9
562.9 - 605.6
605.6 - 648.3
648.3 - 691.0
691.0 - 696.6
696.6 - 732.1
> 732.1
Czechia: DALYs Rate (per 100,000)
< 345.8
345.8 - 361.8
361.8 - 410.0
410.0 - 458.1
458.1 - 499.2
499.2 - 540.2
540.2 - 581.3
581.3 - 587.2
587.2 - 599.7
> 599.7
Slovenia: DALYs Rate (per 100,000)
< 357.5
357.5 - 373.6
373.6 - 424.3
424.3 - 474.9
474.9 - 517.4
517.4 - 559.8
559.8 - 602.2
602.2 - 608.7
608.7 - 622.3
> 622.3
New Zealand: DALYs Rate (per 100,000)
< 721.2
721.2 - 845.9
845.9 - 904.1
904.1 - 962.4
962.4 - 1090.3
1090.3 - 1218.1
1218.1 - 1346.0
1346.0 - 1401.0
1401.0 - 1669.2
> 1669.2
Estonia: DALYs Rate (per 100,000)
< 399.6
399.6 - 408.2
408.2 - 466.1
466.1 - 524.1
524.1 - 570.8
570.8 - 617.4
617.4 - 664.1
664.1 - 666.9
666.9 - 679.0
> 679.0
Venezuela (Bolivarian Republic of): DALYs Rate (per 100,000)
< 458.8
458.8 - 468.7
468.7 - 514.8
514.8 - 561.0
561.0 - 619.9
619.9 - 678.8
678.8 - 737.7
737.7 - 747.0
747.0 - 790.8
> 790.8
Female
Male
Sexes Combined
Age
20-24 years
15-19 years
10-14 years
Year
DALYs Rate (per 100,000)
Age — 10-24 years

Figure S2

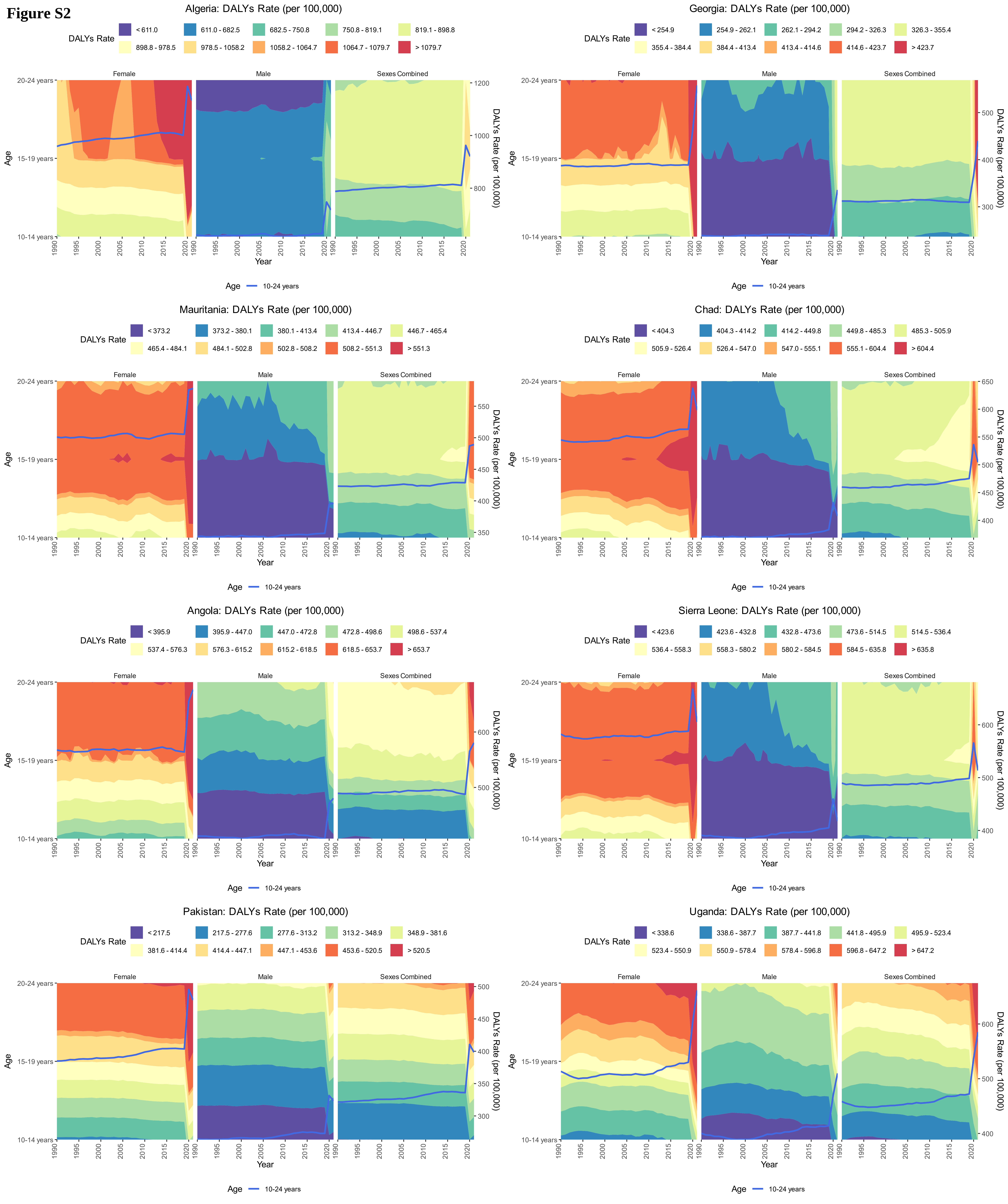
Algeria: DALYs Rate (per 100,000)
DALYs Rate
< 611.0
611.0 - 682.5
682.5 - 750.8
750.8 - 819.1
819.1 - 898.8
898.8 - 978.5
978.5 - 1058.2
1058.2 - 1064.7
1064.7 - 1079.7
> 1079.7
Georgia: DALYs Rate (per 100,000)
< 254.9
254.9 - 262.1
262.1 - 294.2
294.2 - 326.3
326.3 - 355.4
355.4 - 384.4
384.4 - 413.4
413.4 - 414.6
414.6 - 423.7
> 423.7
Mauritania: DALYs Rate (per 100,000)
< 373.2
373.2 - 380.1
380.1 - 413.4
413.4 - 446.7
446.7 - 465.4
465.4 - 484.1
484.1 - 502.8
502.8 - 508.2
508.2 - 551.3
> 551.3
Chad: DALYs Rate (per 100,000)
< 404.3
404.3 - 414.2
414.2 - 449.8
449.8 - 485.3
485.3 - 505.9
505.9 - 526.4
526.4 - 547.0
547.0 - 555.1
555.1 - 604.4
> 604.4
Angola: DALYs Rate (per 100,000)
< 395.9
395.9 - 447.0
447.0 - 472.8
472.8 - 498.6
498.6 - 537.4
537.4 - 576.3
576.3 - 615.2
615.2 - 618.5
618.5 - 653.7
> 653.7
Sierra Leone: DALYs Rate (per 100,000)
< 423.6
423.6 - 432.8
432.8 - 473.6
473.6 - 514.5
514.5 - 536.4
536.4 - 558.3
558.3 - 580.2
580.2 - 584.5
584.5 - 635.8
> 635.8
Pakistan: DALYs Rate (per 100,000)
< 217.5
217.5 - 277.6
277.6 - 313.2
313.2 - 348.9
348.9 - 381.6
381.6 - 414.4
414.4 - 447.1
447.1 - 453.6
453.6 - 520.5
> 520.5
Uganda: DALYs Rate (per 100,000)
< 338.6
338.6 - 387.7
387.7 - 441.8
441.8 - 495.9
495.9 - 523.4
523.4 - 550.9
550.9 - 578.4
578.4 - 596.8
596.8 - 647.2
> 647.2
Female
Male
Sexes Combined
20-24 years
15-19 years
10-14 years
Age
Year
DALYs Rate (per 100,000)
Age — 10-24 years

Figure S2

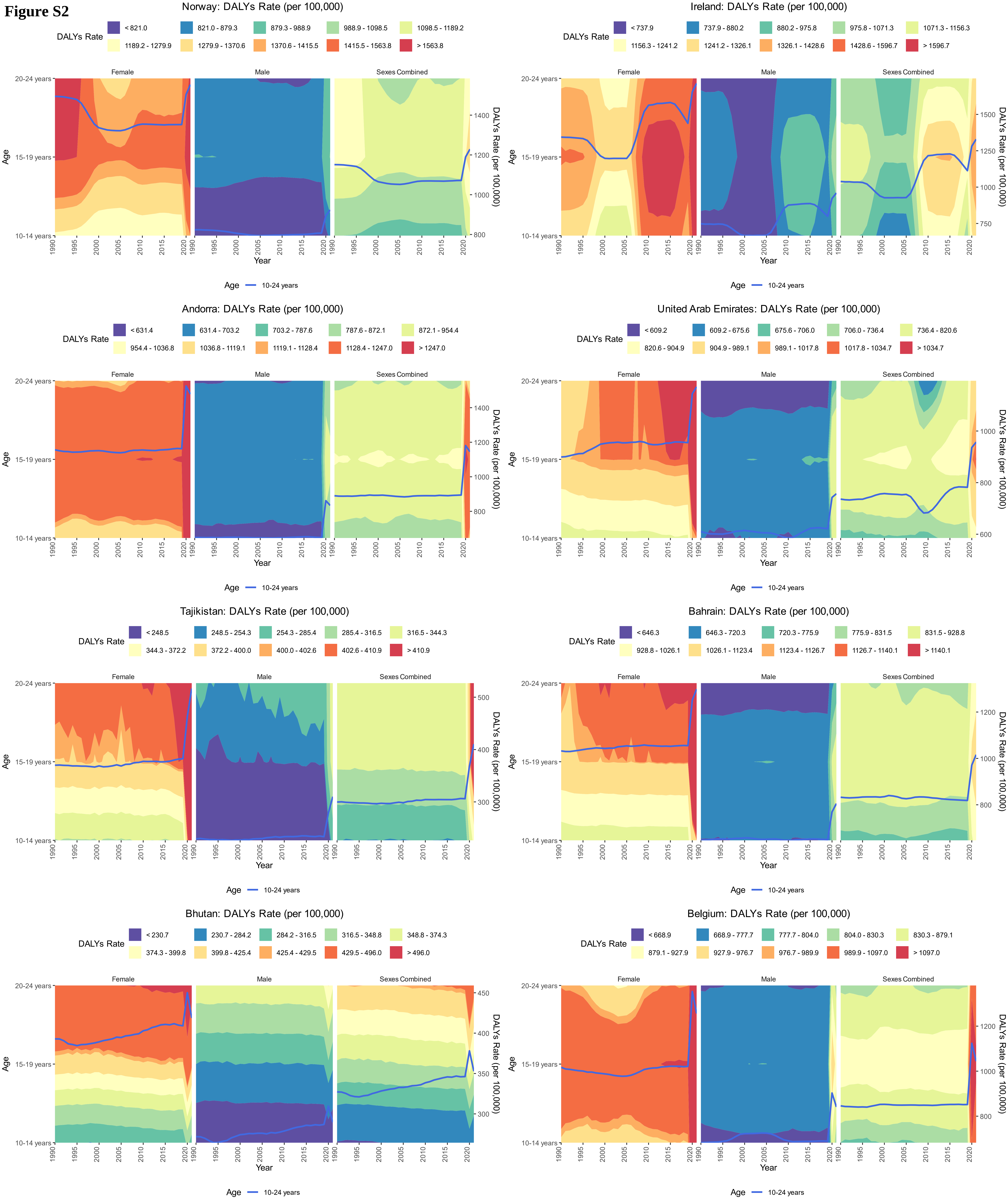

Norway: DALYs Rate (per 100,000)
DALYs Rate
< 821.0
821.0 - 879.3
879.3 - 988.9
988.9 - 1098.5
1098.5 - 1189.2
1189.2 - 1279.9
1279.9 - 1370.6
1370.6 - 1415.5
1415.5 - 1563.8
> 1563.8
Ireland: DALYs Rate (per 100,000)
< 737.9
737.9 - 880.2
880.2 - 975.8
975.8 - 1071.3
1071.3 - 1156.3
1156.3 - 1241.2
1241.2 - 1326.1
1326.1 - 1428.6
1428.6 - 1596.7
> 1596.7
Andorra: DALYs Rate (per 100,000)
< 631.4
631.4 - 703.2
703.2 - 787.6
787.6 - 872.1
872.1 - 954.4
954.4 - 1036.8
1036.8 - 1119.1
1119.1 - 1128.4
1128.4 - 1247.0
> 1247.0
United Arab Emirates: DALYs Rate (per 100,000)
< 609.2
609.2 - 675.6
675.6 - 706.0
706.0 - 736.4
736.4 - 820.6
820.6 - 904.9
904.9 - 989.1
989.1 - 1017.8
1017.8 - 1034.7
> 1034.7
Tajikistan: DALYs Rate (per 100,000)
< 248.5
248.5 - 254.3
254.3 - 285.4
285.4 - 316.5
316.5 - 344.3
344.3 - 372.2
372.2 - 400.0
400.0 - 402.6
402.6 - 410.9
> 410.9
Bahrain: DALYs Rate (per 100,000)
< 646.3
646.3 - 720.3
720.3 - 775.9
775.9 - 831.5
831.5 - 928.8
928.8 - 1026.1
1026.1 - 1123.4
1123.4 - 1126.7
1126.7 - 1140.1
> 1140.1
Bhutan: DALYs Rate (per 100,000)
< 230.7
230.7 - 284.2
284.2 - 316.5
316.5 - 348.8
348.8 - 374.3
374.3 - 399.8
399.8 - 425.4
425.4 - 429.5
429.5 - 496.0
> 496.0
Belgium: DALYs Rate (per 100,000)
< 668.9
668.9 - 777.7
777.7 - 804.0
804.0 - 830.3
830.3 - 879.1
879.1 - 927.9
927.9 - 976.7
976.7 - 989.9
989.9 - 1097.0
> 1097.0
Female
Male
Sexes Combined
Age
20-24 years
15-19 years
10-14 years
Year
DALYs Rate (per 100,000)
Age — 10-24 years

Figure S2

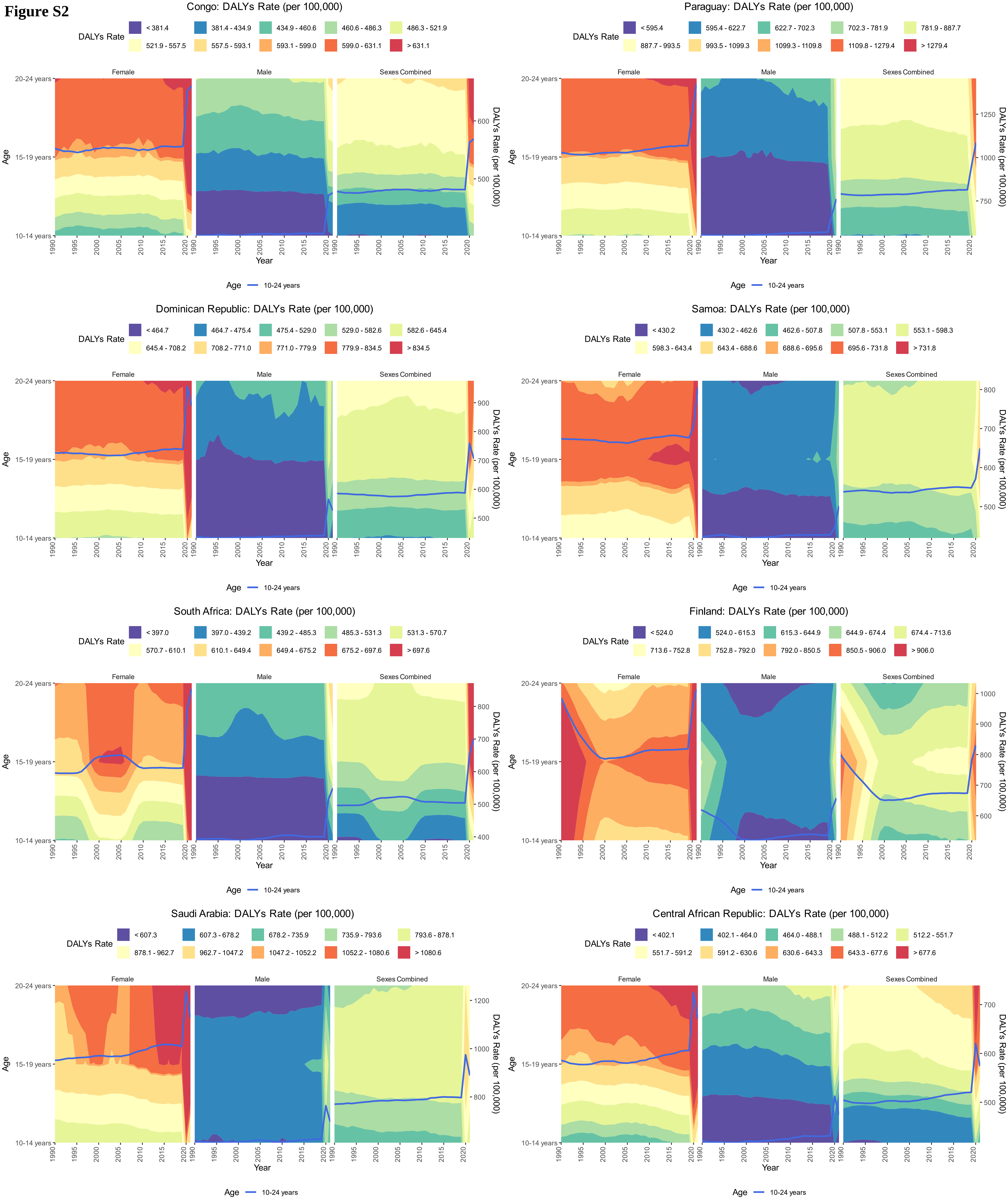
Congo: DALYs Rate (per 100,000)
DALYs Rate
< 381.4
381.4 - 434.9
434.9 - 460.6
460.6 - 486.3
486.3 - 521.9
521.9 - 557.5
557.5 - 593.1
593.1 - 599.0
599.0 - 631.1
> 631.1
Female
Male
Sexes Combined
Age
Year
DALYs Rate (per 100,000)
Age
10-24 years
Paraguay: DALYs Rate (per 100,000)
DALYs Rate
< 595.4
595.4 - 622.7
622.7 - 702.3
702.3 - 781.9
781.9 - 887.7
887.7 - 993.5
993.5 - 1099.3
1099.3 - 1109.8
1109.8 - 1279.4
> 1279.4
Dominican Republic: DALYs Rate (per 100,000)
DALYs Rate
< 464.7
464.7 - 475.4
475.4 - 529.0
529.0 - 582.6
582.6 - 645.4
645.4 - 708.2
708.2 - 771.0
771.0 - 779.9
779.9 - 834.5
> 834.5
Samoa: DALYs Rate (per 100,000)
DALYs Rate
< 430.2
430.2 - 462.6
462.6 - 507.8
507.8 - 553.1
553.1 - 598.3
598.3 - 643.4
643.4 - 688.6
688.6 - 695.6
695.6 - 731.8
> 731.8
South Africa: DALYs Rate (per 100,000)
DALYs Rate
< 397.0
397.0 - 439.2
439.2 - 485.3
485.3 - 531.3
531.3 - 570.7
570.7 - 610.1
610.1 - 649.4
649.4 - 675.2
675.2 - 697.6
> 697.6
Finland: DALYs Rate (per 100,000)
DALYs Rate
< 524.0
524.0 - 615.3
615.3 - 644.9
644.9 - 674.4
674.4 - 713.6
713.6 - 752.8
752.8 - 792.0
792.0 - 850.5
850.5 - 906.0
> 906.0
Saudi Arabia: DALYs Rate (per 100,000)
DALYs Rate
< 607.3
607.3 - 678.2
678.2 - 735.9
735.9 - 793.6
793.6 - 878.1
878.1 - 962.7
962.7 - 1047.2
1047.2 - 1052.2
1052.2 - 1080.6
> 1080.6
Central African Republic: DALYs Rate (per 100,000)
DALYs Rate
< 402.1
402.1 - 464.0
464.0 - 488.1
488.1 - 512.2
512.2 - 551.7
551.7 - 591.2
591.2 - 630.6
630.6 - 643.3
643.3 - 677.6
> 677.6
20-24 years
15-19 years
10-14 years
1990
1995
2000
2005
2010
2015
2020

Figure S2

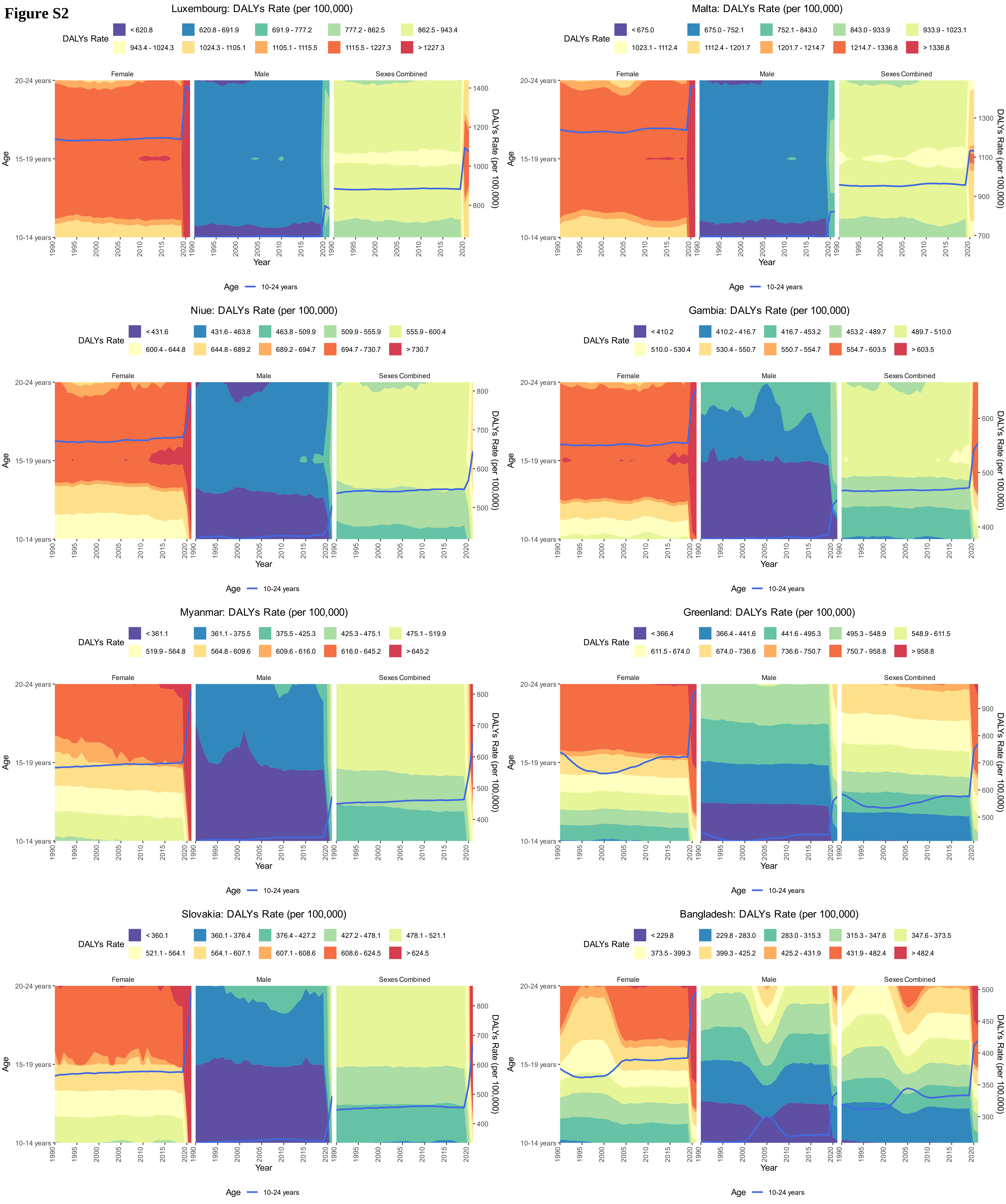

Luxembourg: DALYs Rate (per 100,000)
DALYs Rate
< 620.8
620.8 - 691.9
691.9 - 777.2
777.2 - 862.5
862.5 - 943.4
943.4 - 1024.3
1024.3 - 1105.1
1105.1 - 1115.5
1115.5 - 1227.3
> 1227.3
Malta: DALYs Rate (per 100,000)
DALYs Rate
< 675.0
675.0 - 752.1
752.1 - 843.0
843.0 - 933.9
933.9 - 1023.1
1023.1 - 1112.4
1112.4 - 1201.7
1201.7 - 1214.7
1214.7 - 1336.8
> 1336.8
Niue: DALYs Rate (per 100,000)
DALYs Rate
< 431.6
431.6 - 463.8
463.8 - 509.9
509.9 - 555.9
555.9 - 600.4
600.4 - 644.8
644.8 - 689.2
689.2 - 694.7
694.7 - 730.7
> 730.7
Gambia: DALYs Rate (per 100,000)
DALYs Rate
< 410.2
410.2 - 416.7
416.7 - 453.2
453.2 - 489.7
489.7 - 510.0
510.0 - 530.4
530.4 - 550.7
550.7 - 554.7
554.7 - 603.5
> 603.5
Myanmar: DALYs Rate (per 100,000)
DALYs Rate
< 361.1
361.1 - 375.5
375.5 - 425.3
425.3 - 475.1
475.1 - 519.9
519.9 - 564.8
564.8 - 609.6
609.6 - 616.0
616.0 - 645.2
> 645.2
Greenland: DALYs Rate (per 100,000)
DALYs Rate
< 366.4
366.4 - 441.6
441.6 - 495.3
495.3 - 548.9
548.9 - 611.5
611.5 - 674.0
674.0 - 736.6
736.6 - 750.7
750.7 - 958.8
> 958.8
Slovakia: DALYs Rate (per 100,000)
DALYs Rate
< 360.1
360.1 - 376.4
376.4 - 427.2
427.2 - 478.1
478.1 - 521.1
521.1 - 564.1
564.1 - 607.1
607.1 - 608.6
608.6 - 624.5
> 624.5
Bangladesh: DALYs Rate (per 100,000)
DALYs Rate
< 229.8
229.8 - 283.0
283.0 - 315.3
315.3 - 347.6
347.6 - 373.5
373.5 - 399.3
399.3 - 425.2
425.2 - 431.9
431.9 - 482.4
> 482.4
Female
Male
Sexes Combined
Age
20-24 years
15-19 years
10-14 years
Year
DALYs Rate (per 100,000)
Age
10-24 years

Figure S2

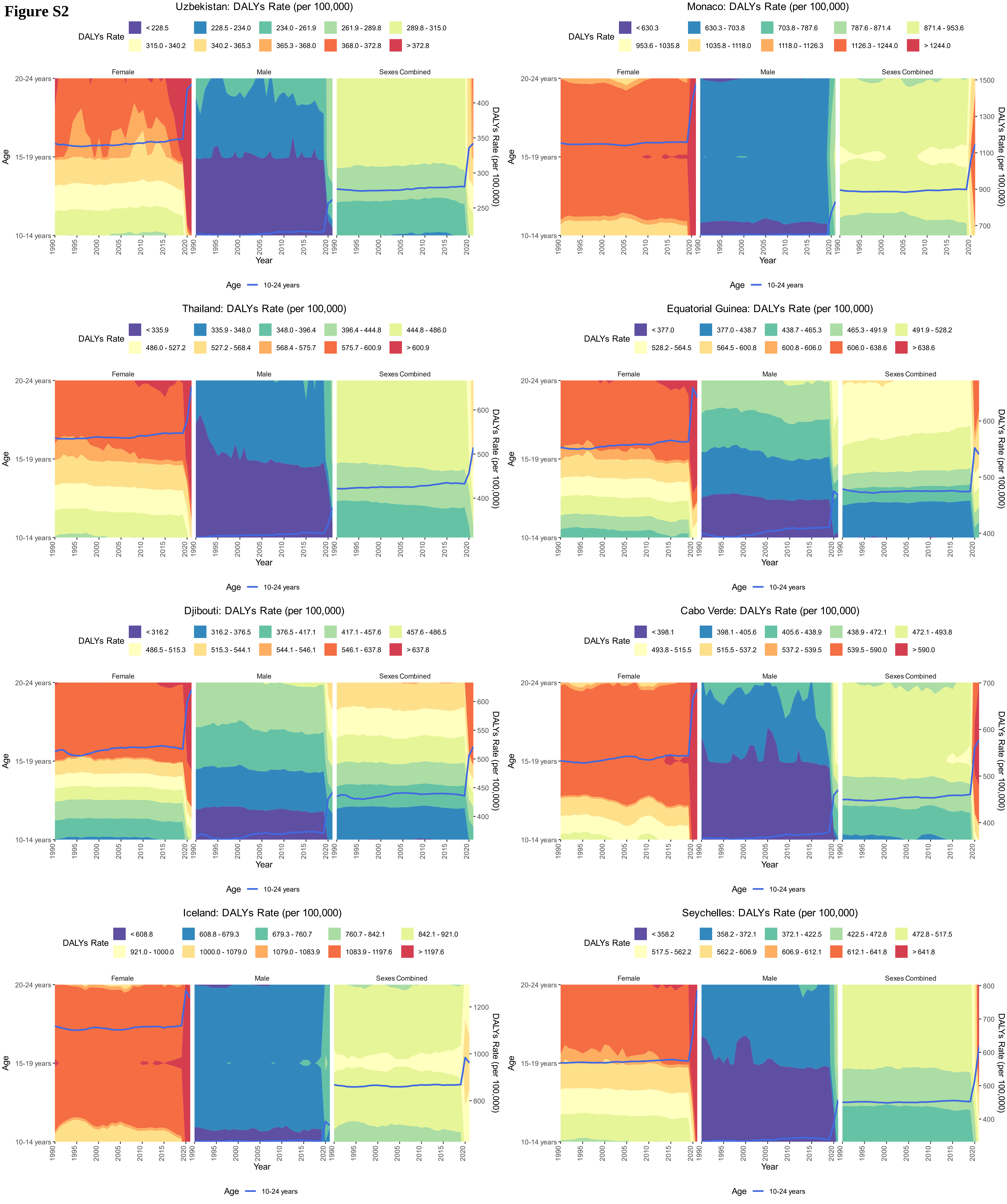

Uzbekistan: DALYs Rate (per 100,000)
DALYs Rate
< 228.5
228.5 - 234.0
234.0 - 261.9
261.9 - 289.8
289.8 - 315.0
315.0 - 340.2
340.2 - 365.3
365.3 - 368.0
368.0 - 372.8
> 372.8
Female
Male
Sexes Combined
Age
Year
DALYs Rate (per 100,000)
Age — 10-24 years
Monaco: DALYs Rate (per 100,000)
< 630.3
630.3 - 703.8
703.8 - 787.6
787.6 - 871.4
871.4 - 953.6
953.6 - 1035.8
1035.8 - 1118.0
1118.0 - 1126.3
1126.3 - 1244.0
> 1244.0
Thailand: DALYs Rate (per 100,000)
< 335.9
335.9 - 348.0
348.0 - 396.4
396.4 - 444.8
444.8 - 486.0
486.0 - 527.2
527.2 - 568.4
568.4 - 575.7
575.7 - 600.9
> 600.9
Equatorial Guinea: DALYs Rate (per 100,000)
< 377.0
377.0 - 438.7
438.7 - 465.3
465.3 - 491.9
491.9 - 528.2
528.2 - 564.5
564.5 - 600.8
600.8 - 606.0
606.0 - 638.6
> 638.6
Djibouti: DALYs Rate (per 100,000)
< 316.2
316.2 - 376.5
376.5 - 417.1
417.1 - 457.6
457.6 - 486.5
486.5 - 515.3
515.3 - 544.1
544.1 - 546.1
546.1 - 637.8
> 637.8
Cabo Verde: DALYs Rate (per 100,000)
< 398.1
398.1 - 405.6
405.6 - 438.9
438.9 - 472.1
472.1 - 493.8
493.8 - 515.5
515.5 - 537.2
537.2 - 539.5
539.5 - 590.0
> 590.0
Iceland: DALYs Rate (per 100,000)
< 608.8
608.8 - 679.3
679.3 - 760.7
760.7 - 842.1
842.1 - 921.0
921.0 - 1000.0
1000.0 - 1079.0
1079.0 - 1083.9
1083.9 - 1197.6
> 1197.6
Seychelles: DALYs Rate (per 100,000)
< 358.2
358.2 - 372.1
372.1 - 422.5
422.5 - 472.8
472.8 - 517.5
517.5 - 562.2
562.2 - 606.9
606.9 - 612.1
612.1 - 641.8
> 641.8
20-24 years
15-19 years
10-14 years
1990
1995
2000
2005
2010
2015
2020

Figure S2

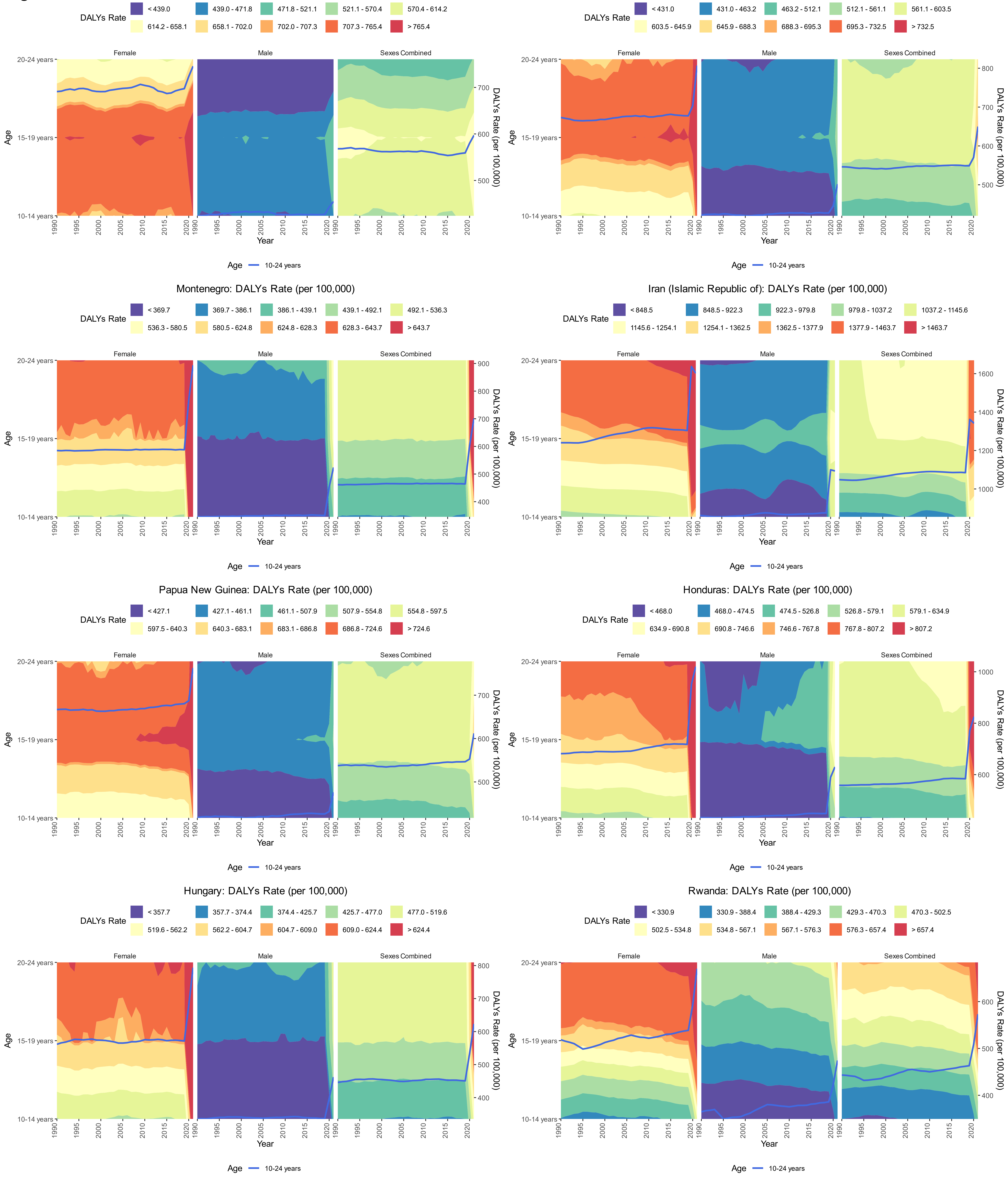
Democratic People's Republic of Korea: DALYs Rate (per 100,000)
DALYs Rate
< 439.0
439.0 - 471.8
471.8 - 521.1
521.1 - 570.4
570.4 - 614.2
614.2 - 658.1
658.1 - 702.0
702.0 - 707.3
707.3 - 765.4
> 765.4
Nauru: DALYs Rate (per 100,000)
DALYs Rate
< 431.0
431.0 - 463.2
463.2 - 512.1
512.1 - 561.1
561.1 - 603.5
603.5 - 645.9
645.9 - 688.3
688.3 - 695.3
695.3 - 732.5
> 732.5
Montenegro: DALYs Rate (per 100,000)
DALYs Rate
< 369.7
369.7 - 386.1
386.1 - 439.1
439.1 - 492.1
492.1 - 536.3
536.3 - 580.5
580.5 - 624.8
624.8 - 628.3
628.3 - 643.7
> 643.7
Iran (Islamic Republic of): DALYs Rate (per 100,000)
DALYs Rate
< 848.5
848.5 - 922.3
922.3 - 979.8
979.8 - 1037.2
1037.2 - 1145.6
1145.6 - 1254.1
1254.1 - 1362.5
1362.5 - 1377.9
1377.9 - 1463.7
> 1463.7
Papua New Guinea: DALYs Rate (per 100,000)
DALYs Rate
< 427.1
427.1 - 461.1
461.1 - 507.9
507.9 - 554.8
554.8 - 597.5
597.5 - 640.3
640.3 - 683.1
683.1 - 686.8
686.8 - 724.6
> 724.6
Honduras: DALYs Rate (per 100,000)
DALYs Rate
< 468.0
468.0 - 474.5
474.5 - 526.8
526.8 - 579.1
579.1 - 634.9
634.9 - 690.8
690.8 - 746.6
746.6 - 767.8
767.8 - 807.2
> 807.2
Hungary: DALYs Rate (per 100,000)
DALYs Rate
< 357.7
357.7 - 374.4
374.4 - 425.7
425.7 - 477.0
477.0 - 519.6
519.6 - 562.2
562.2 - 604.7
604.7 - 609.0
609.0 - 624.4
> 624.4
Rwanda: DALYs Rate (per 100,000)
DALYs Rate
< 330.9
330.9 - 388.4
388.4 - 429.3
429.3 - 470.3
470.3 - 502.5
502.5 - 534.8
534.8 - 567.1
567.1 - 576.3
576.3 - 657.4
> 657.4
Female
Male
Sexes Combined
20-24 years
15-19 years
10-14 years
Age
Year
DALYs Rate (per 100,000)
Age — 10-24 years

Figure S2

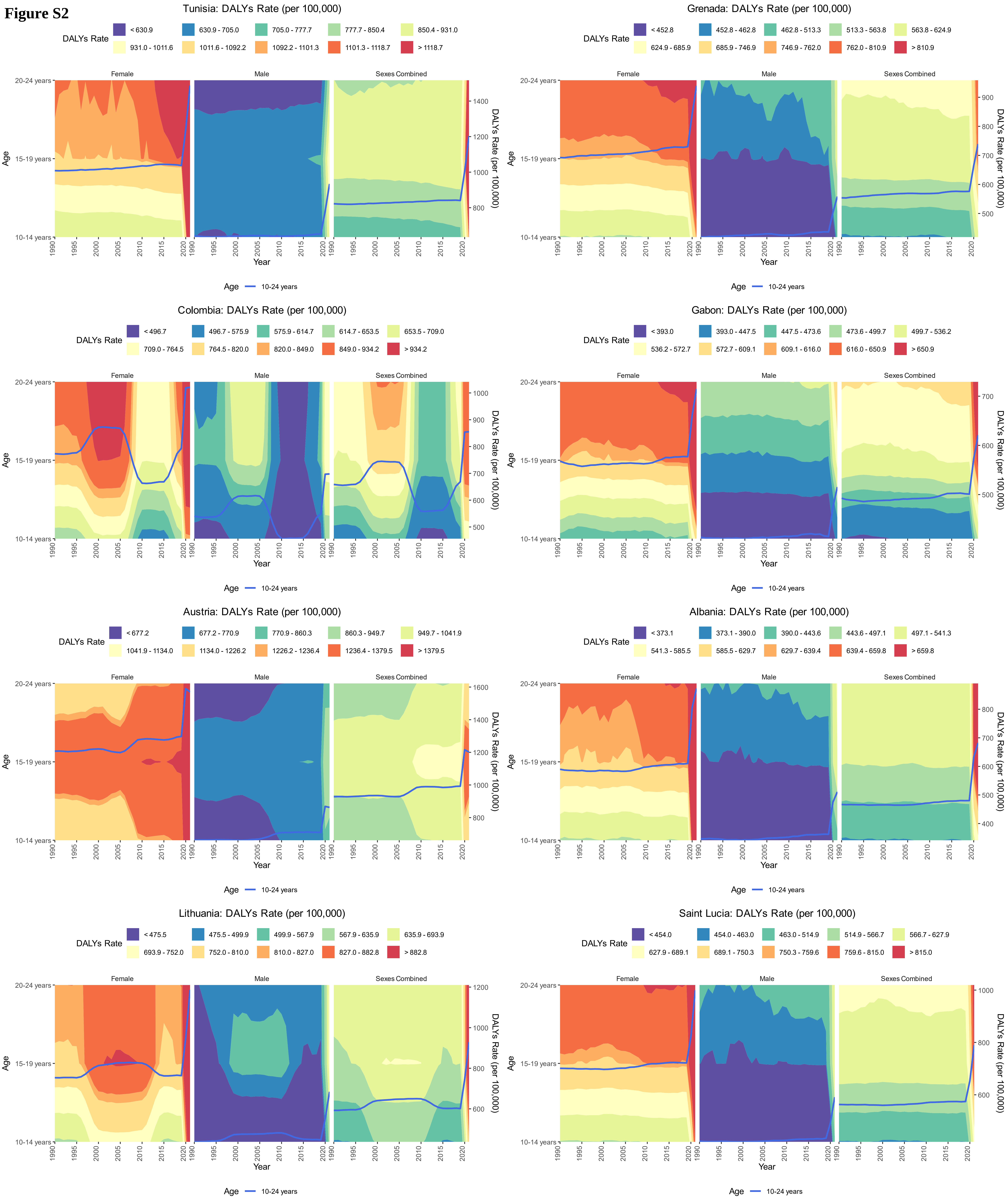

Tunisia: DALYs Rate (per 100,000)
DALYs Rate
< 630.9
630.9 - 705.0
705.0 - 777.7
777.7 - 850.4
850.4 - 931.0
931.0 - 1011.6
1011.6 - 1092.2
1092.2 - 1101.3
1101.3 - 1118.7
> 1118.7
Grenada: DALYs Rate (per 100,000)
DALYs Rate
< 452.8
452.8 - 462.8
462.8 - 513.3
513.3 - 563.8
563.8 - 624.9
624.9 - 685.9
685.9 - 746.9
746.9 - 762.0
762.0 - 810.9
> 810.9
Colombia: DALYs Rate (per 100,000)
DALYs Rate
< 496.7
496.7 - 575.9
575.9 - 614.7
614.7 - 653.5
653.5 - 709.0
709.0 - 764.5
764.5 - 820.0
820.0 - 849.0
849.0 - 934.2
> 934.2
Gabon: DALYs Rate (per 100,000)
DALYs Rate
< 393.0
393.0 - 447.5
447.5 - 473.6
473.6 - 499.7
499.7 - 536.2
536.2 - 572.7
572.7 - 609.1
609.1 - 616.0
616.0 - 650.9
> 650.9
Austria: DALYs Rate (per 100,000)
DALYs Rate
< 677.2
677.2 - 770.9
770.9 - 860.3
860.3 - 949.7
949.7 - 1041.9
1041.9 - 1134.0
1134.0 - 1226.2
1226.2 - 1236.4
1236.4 - 1379.5
> 1379.5
Albania: DALYs Rate (per 100,000)
DALYs Rate
< 373.1
373.1 - 390.0
390.0 - 443.6
443.6 - 497.1
497.1 - 541.3
541.3 - 585.5
585.5 - 629.7
629.7 - 639.4
639.4 - 659.8
> 659.8
Lithuania: DALYs Rate (per 100,000)
DALYs Rate
< 475.5
475.5 - 499.9
499.9 - 567.9
567.9 - 635.9
635.9 - 693.9
693.9 - 752.0
752.0 - 810.0
810.0 - 827.0
827.0 - 882.8
> 882.8
Saint Lucia: DALYs Rate (per 100,000)
DALYs Rate
< 454.0
454.0 - 463.0
463.0 - 514.9
514.9 - 566.7
566.7 - 627.9
627.9 - 689.1
689.1 - 750.3
750.3 - 759.6
759.6 - 815.0
> 815.0
Female
Male
Sexes Combined
Age
20-24 years
15-19 years
10-14 years
Year
DALYs Rate (per 100,000)
Age
10-24 years

Figure S2

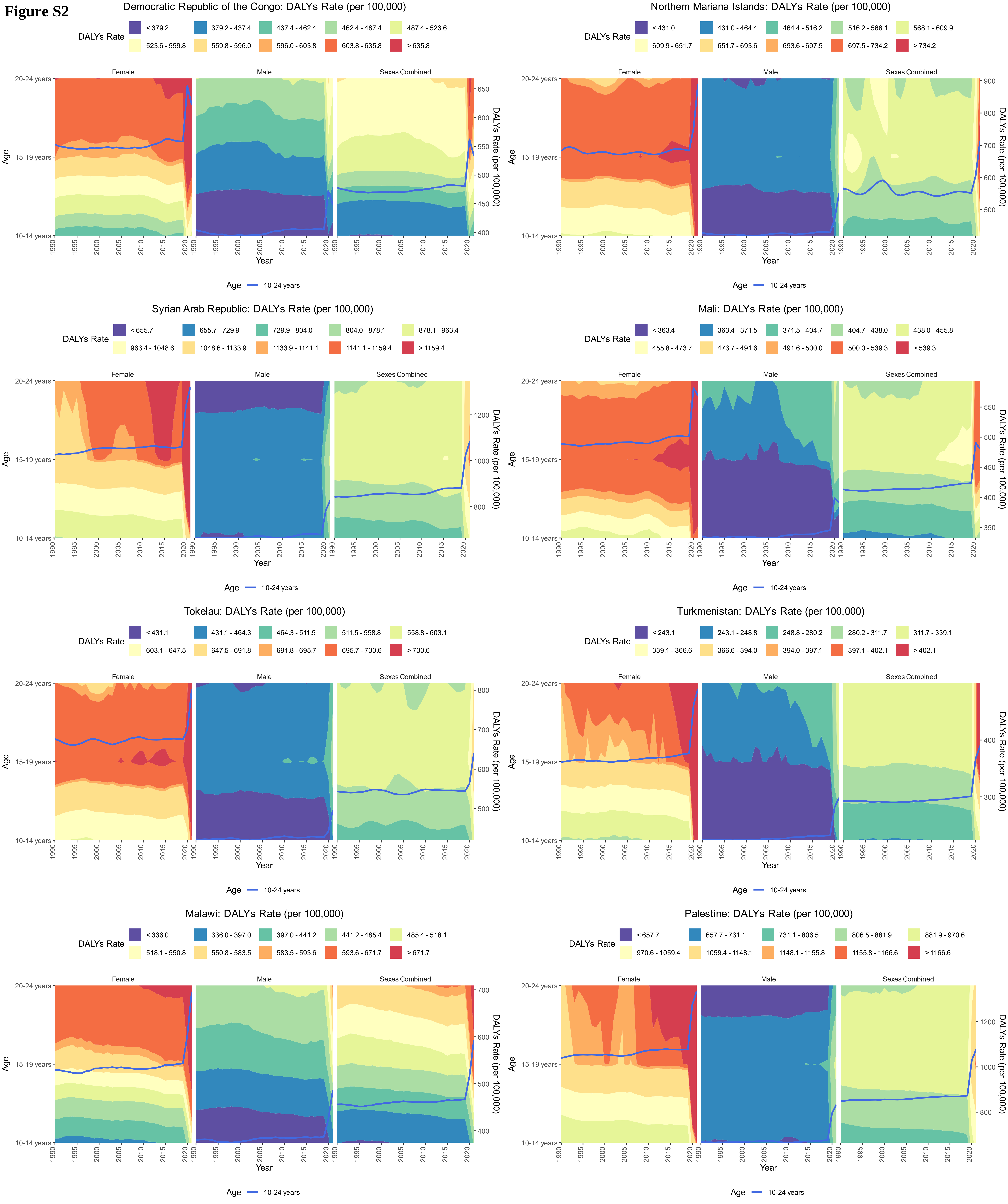
Democratic Republic of the Congo: DALYs Rate (per 100,000)
DALYs Rate
< 379.2
379.2 - 437.4
437.4 - 462.4
462.4 - 487.4
487.4 - 523.6
523.6 - 559.8
559.8 - 596.0
596.0 - 603.8
603.8 - 635.8
> 635.8
Northern Mariana Islands: DALYs Rate (per 100,000)
< 431.0
431.0 - 464.4
464.4 - 516.2
516.2 - 568.1
568.1 - 609.9
609.9 - 651.7
651.7 - 693.6
693.6 - 697.5
697.5 - 734.2
> 734.2
Syrian Arab Republic: DALYs Rate (per 100,000)
< 655.7
655.7 - 729.9
729.9 - 804.0
804.0 - 878.1
878.1 - 963.4
963.4 - 1048.6
1048.6 - 1133.9
1133.9 - 1141.1
1141.1 - 1159.4
> 1159.4
Mali: DALYs Rate (per 100,000)
< 363.4
363.4 - 371.5
371.5 - 404.7
404.7 - 438.0
438.0 - 455.8
455.8 - 473.7
473.7 - 491.6
491.6 - 500.0
500.0 - 539.3
> 539.3
Tokelau: DALYs Rate (per 100,000)
< 431.1
431.1 - 464.3
464.3 - 511.5
511.5 - 558.8
558.8 - 603.1
603.1 - 647.5
647.5 - 691.8
691.8 - 695.7
695.7 - 730.6
> 730.6
Turkmenistan: DALYs Rate (per 100,000)
< 243.1
243.1 - 248.8
248.8 - 280.2
280.2 - 311.7
311.7 - 339.1
339.1 - 366.6
366.6 - 394.0
394.0 - 397.1
397.1 - 402.1
> 402.1
Malawi: DALYs Rate (per 100,000)
< 336.0
336.0 - 397.0
397.0 - 441.2
441.2 - 485.4
485.4 - 518.1
518.1 - 550.8
550.8 - 583.5
583.5 - 593.6
593.6 - 671.7
> 671.7
Palestine: DALYs Rate (per 100,000)
< 657.7
657.7 - 731.1
731.1 - 806.5
806.5 - 881.9
881.9 - 970.6
970.6 - 1059.4
1059.4 - 1148.1
1148.1 - 1155.8
1155.8 - 1166.6
> 1166.6
Female
Male
Sexes Combined
Age
20-24 years
15-19 years
10-14 years
Year
DALYs Rate (per 100,000)
Age 10-24 years

Figure S2

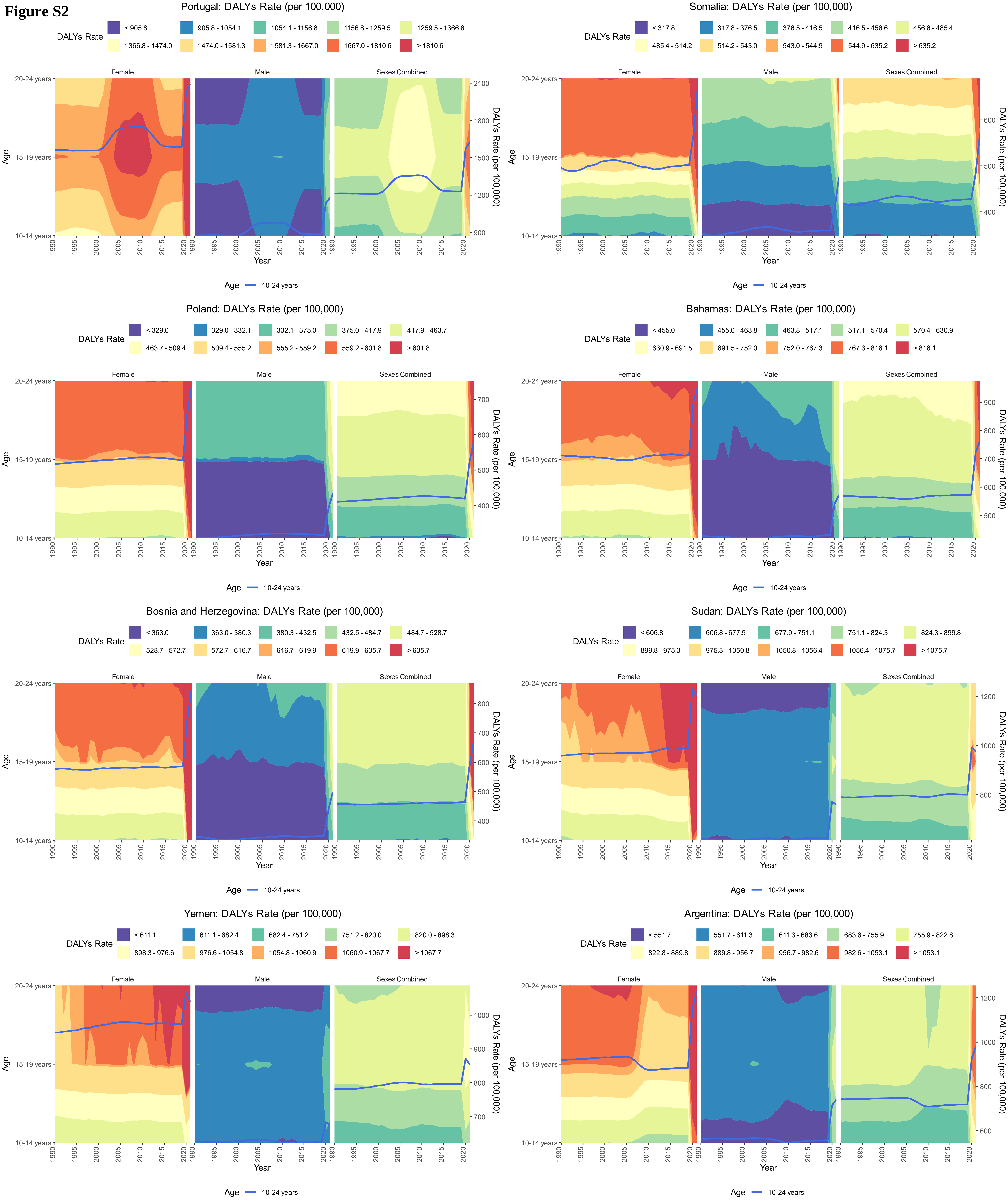
Portugal: DALYs Rate (per 100,000)
DALYs Rate
< 905.8
905.8 - 1054.1
1054.1 - 1156.8
1156.8 - 1259.5
1259.5 - 1366.8
1366.8 - 1474.0
1474.0 - 1581.3
1581.3 - 1667.0
1667.0 - 1810.6
> 1810.6
Somalia: DALYs Rate (per 100,000)
< 317.8
317.8 - 376.5
376.5 - 416.5
416.5 - 456.6
456.6 - 485.4
485.4 - 514.2
514.2 - 543.0
543.0 - 544.9
544.9 - 635.2
> 635.2
Poland: DALYs Rate (per 100,000)
< 329.0
329.0 - 332.1
332.1 - 375.0
375.0 - 417.9
417.9 - 463.7
463.7 - 509.4
509.4 - 555.2
555.2 - 559.2
559.2 - 601.8
> 601.8
Bahamas: DALYs Rate (per 100,000)
< 455.0
455.0 - 463.8
463.8 - 517.1
517.1 - 570.4
570.4 - 630.9
630.9 - 691.5
691.5 - 752.0
752.0 - 767.3
767.3 - 816.1
> 816.1
Bosnia and Herzegovina: DALYs Rate (per 100,000)
< 363.0
363.0 - 380.3
380.3 - 432.5
432.5 - 484.7
484.7 - 528.7
528.7 - 572.7
572.7 - 616.7
616.7 - 619.9
619.9 - 635.7
> 635.7
Sudan: DALYs Rate (per 100,000)
< 606.8
606.8 - 677.9
677.9 - 751.1
751.1 - 824.3
824.3 - 899.8
899.8 - 975.3
975.3 - 1050.8
1050.8 - 1056.4
1056.4 - 1075.7
> 1075.7
Yemen: DALYs Rate (per 100,000)
< 611.1
611.1 - 682.4
682.4 - 751.2
751.2 - 820.0
820.0 - 898.3
898.3 - 976.6
976.6 - 1054.8
1054.8 - 1060.9
1060.9 - 1067.7
> 1067.7
Argentina: DALYs Rate (per 100,000)
< 551.7
551.7 - 611.3
611.3 - 683.6
683.6 - 755.9
755.9 - 822.8
822.8 - 889.8
889.8 - 956.7
956.7 - 982.6
982.6 - 1053.1
> 1053.1
Female
Male
Sexes Combined
Age
20-24 years
15-19 years
10-14 years
Year
DALYs Rate (per 100,000)
Age — 10-24 years

Figure S2

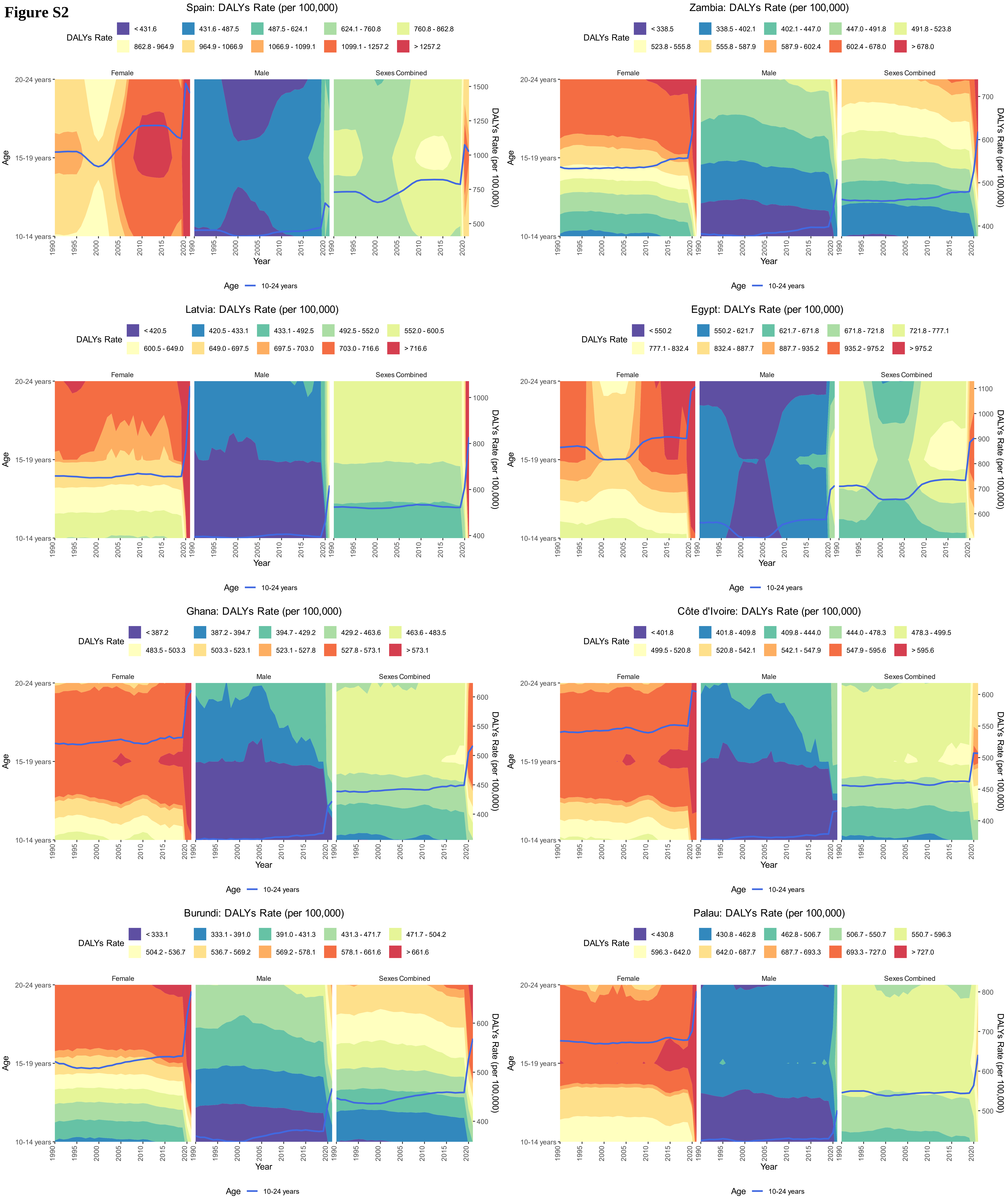

Spain: DALYs Rate (per 100,000)
DALYs Rate
< 431.6
431.6 - 487.5
487.5 - 624.1
624.1 - 760.8
760.8 - 862.8
862.8 - 964.9
964.9 - 1066.9
1066.9 - 1099.1
1099.1 - 1257.2
> 1257.2
Zambia: DALYs Rate (per 100,000)
< 338.5
338.5 - 402.1
402.1 - 447.0
447.0 - 491.8
491.8 - 523.8
523.8 - 555.8
555.8 - 587.9
587.9 - 602.4
602.4 - 678.0
> 678.0
Latvia: DALYs Rate (per 100,000)
< 420.5
420.5 - 433.1
433.1 - 492.5
492.5 - 552.0
552.0 - 600.5
600.5 - 649.0
649.0 - 697.5
697.5 - 703.0
703.0 - 716.6
> 716.6
Egypt: DALYs Rate (per 100,000)
< 550.2
550.2 - 621.7
621.7 - 671.8
671.8 - 721.8
721.8 - 777.1
777.1 - 832.4
832.4 - 887.7
887.7 - 935.2
935.2 - 975.2
> 975.2
Ghana: DALYs Rate (per 100,000)
< 387.2
387.2 - 394.7
394.7 - 429.2
429.2 - 463.6
463.6 - 483.5
483.5 - 503.3
503.3 - 523.1
523.1 - 527.8
527.8 - 573.1
> 573.1
Côte d'Ivoire: DALYs Rate (per 100,000)
< 401.8
401.8 - 409.8
409.8 - 444.0
444.0 - 478.3
478.3 - 499.5
499.5 - 520.8
520.8 - 542.1
542.1 - 547.9
547.9 - 595.6
> 595.6
Burundi: DALYs Rate (per 100,000)
< 333.1
333.1 - 391.0
391.0 - 431.3
431.3 - 471.7
471.7 - 504.2
504.2 - 536.7
536.7 - 569.2
569.2 - 578.1
578.1 - 661.6
> 661.6
Palau: DALYs Rate (per 100,000)
< 430.8
430.8 - 462.8
462.8 - 506.7
506.7 - 550.7
550.7 - 596.3
596.3 - 642.0
642.0 - 687.7
687.7 - 693.3
693.3 - 727.0
> 727.0
Female
Male
Sexes Combined
20-24 years
15-19 years
10-14 years
Age
Year
DALYs Rate (per 100,000)
Age — 10-24 years

Figure S2

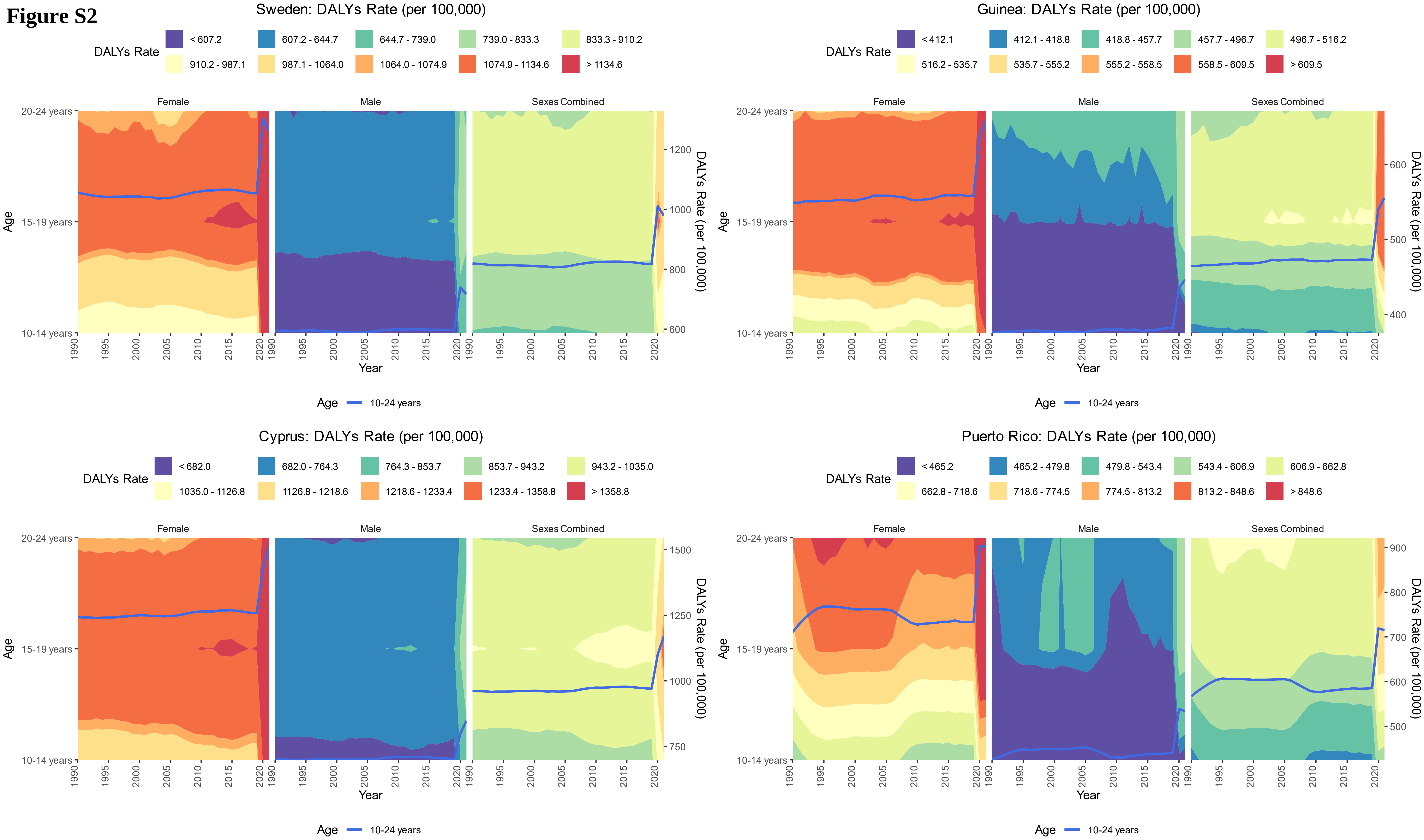

Sweden: DALYs Rate (per 100,000)
DALYs Rate
< 607.2
607.2 - 644.7
644.7 - 739.0
739.0 - 833.3
833.3 - 910.2
910.2 - 987.1
987.1 - 1064.0
1064.0 - 1074.9
1074.9 - 1134.6
> 1134.6
Female
Male
Sexes Combined
20-24 years
15-19 years
10-14 years
Age
Year
DALYs Rate (per 100,000)
Age
10-24 years
Guinea: DALYs Rate (per 100,000)
< 412.1
412.1 - 418.8
418.8 - 457.7
457.7 - 496.7
496.7 - 516.2
516.2 - 535.7
535.7 - 555.2
555.2 - 558.5
558.5 - 609.5
> 609.5
Cyprus: DALYs Rate (per 100,000)
< 682.0
682.0 - 764.3
764.3 - 853.7
853.7 - 943.2
943.2 - 1035.0
1035.0 - 1126.8
1126.8 - 1218.6
1218.6 - 1233.4
1233.4 - 1358.8
> 1358.8
Puerto Rico: DALYs Rate (per 100,000)
< 465.2
465.2 - 479.8
479.8 - 543.4
543.4 - 606.9
606.9 - 662.8
662.8 - 718.6
718.6 - 774.5
774.5 - 813.2
813.2 - 848.6
> 848.6

Figure S3

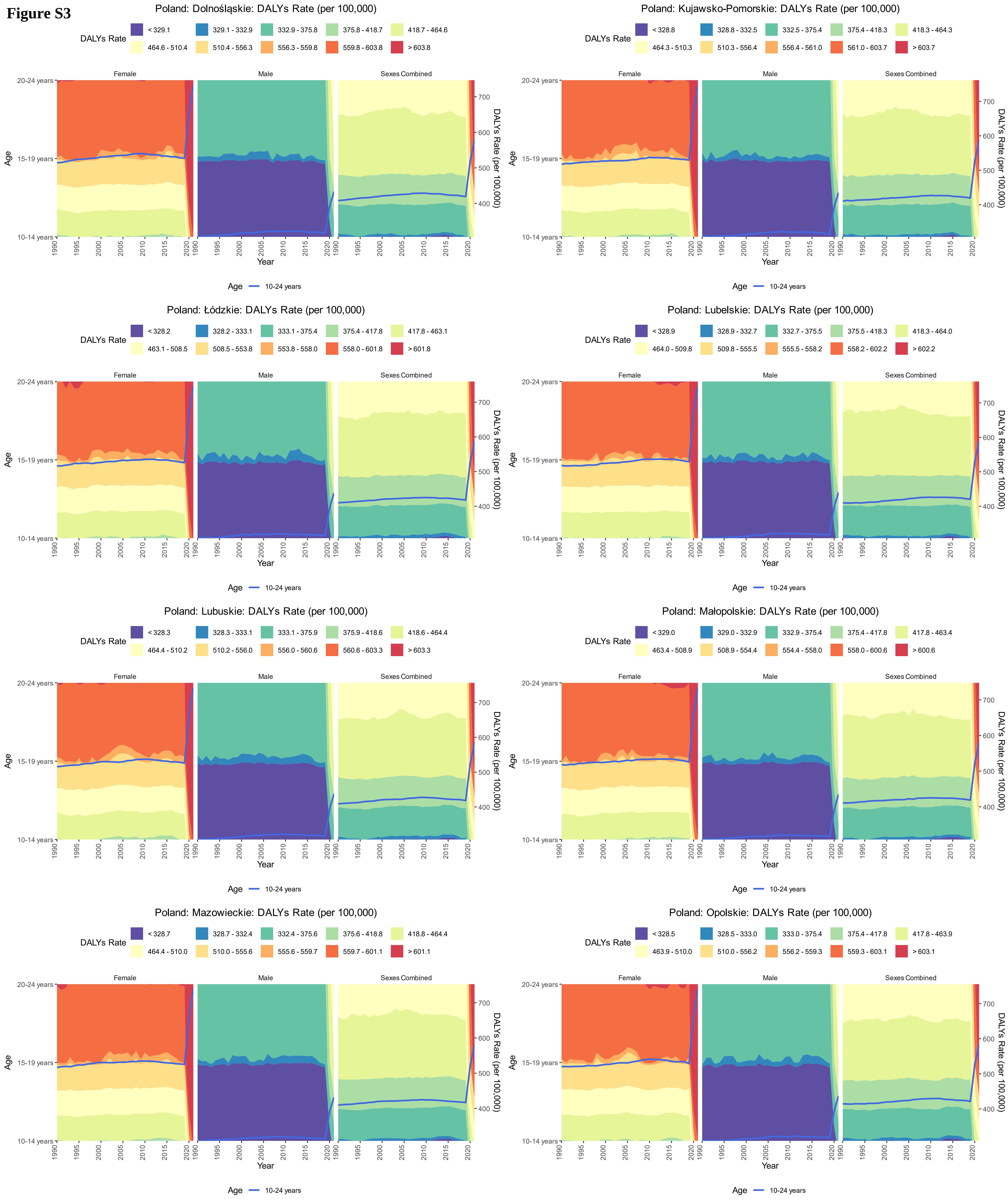

Poland: Dolnośląskie: DALYs Rate (per 100,000)
DALYs Rate
< 329.1
329.1 - 332.9
332.9 - 375.8
375.8 - 418.7
418.7 - 464.6
464.6 - 510.4
510.4 - 556.3
556.3 - 559.8
559.8 - 603.8
> 603.8
Poland: Kujawsko-Pomorskie: DALYs Rate (per 100,000)
DALYs Rate
< 328.8
328.8 - 332.5
332.5 - 375.4
375.4 - 418.3
418.3 - 464.3
464.3 - 510.3
510.3 - 556.4
556.4 - 561.0
561.0 - 603.7
> 603.7
Poland: Łódzkie: DALYs Rate (per 100,000)
DALYs Rate
< 328.2
328.2 - 333.1
333.1 - 375.4
375.4 - 417.8
417.8 - 463.1
463.1 - 508.5
508.5 - 553.8
553.8 - 558.0
558.0 - 601.8
> 601.8
Poland: Lubelskie: DALYs Rate (per 100,000)
DALYs Rate
< 328.9
328.9 - 332.7
332.7 - 375.5
375.5 - 418.3
418.3 - 464.0
464.0 - 509.8
509.8 - 555.5
555.5 - 558.2
558.2 - 602.2
> 602.2
Poland: Lubuskie: DALYs Rate (per 100,000)
DALYs Rate
< 328.3
328.3 - 333.1
333.1 - 375.9
375.9 - 418.6
418.6 - 464.4
464.4 - 510.2
510.2 - 556.0
556.0 - 560.6
560.6 - 603.3
> 603.3
Poland: Małopolskie: DALYs Rate (per 100,000)
DALYs Rate
< 329.0
329.0 - 332.9
332.9 - 375.4
375.4 - 417.8
417.8 - 463.4
463.4 - 508.9
508.9 - 554.4
554.4 - 558.0
558.0 - 600.6
> 600.6
Poland: Mazowieckie: DALYs Rate (per 100,000)
DALYs Rate
< 328.7
328.7 - 332.4
332.4 - 375.6
375.6 - 418.8
418.8 - 464.4
464.4 - 510.0
510.0 - 555.6
555.6 - 559.7
559.7 - 601.1
> 601.1
Poland: Opolskie: DALYs Rate (per 100,000)
DALYs Rate
< 328.5
328.5 - 333.0
333.0 - 375.4
375.4 - 417.8
417.8 - 463.9
463.9 - 510.0
510.0 - 556.2
556.2 - 559.3
559.3 - 603.1
> 603.1
Female
Male
Sexes Combined
Age
20-24 years
15-19 years
10-14 years
Year
1990
1995
2000
2005
2010
2015
2020
DALYs Rate (per 100,000)
400
500
600
700
Age
10-24 years

Figure S3

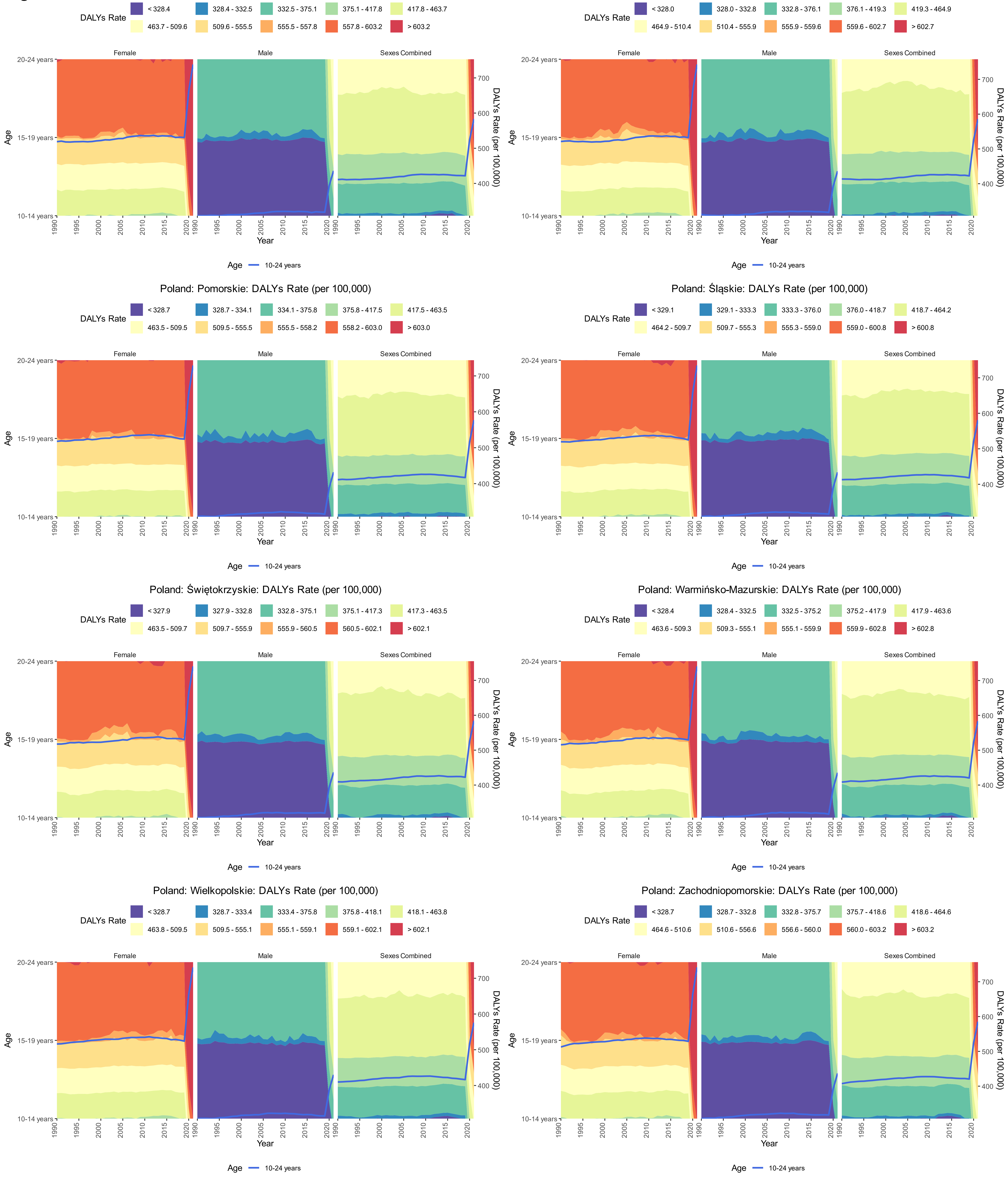

Poland: Podkarpackie: DALYs Rate (per 100,000)
DALYs Rate
< 328.4
328.4 - 332.5
332.5 - 375.1
375.1 - 417.8
417.8 - 463.7
463.7 - 509.6
509.6 - 555.5
555.5 - 557.8
557.8 - 603.2
> 603.2
Poland: Podlaskie: DALYs Rate (per 100,000)
< 328.0
328.0 - 332.8
332.8 - 376.1
376.1 - 419.3
419.3 - 464.9
464.9 - 510.4
510.4 - 555.9
555.9 - 559.6
559.6 - 602.7
> 602.7
Poland: Pomorskie: DALYs Rate (per 100,000)
< 328.7
328.7 - 334.1
334.1 - 375.8
375.8 - 417.5
417.5 - 463.5
463.5 - 509.5
509.5 - 555.5
555.5 - 558.2
558.2 - 603.0
> 603.0
Poland: Śląskie: DALYs Rate (per 100,000)
< 329.1
329.1 - 333.3
333.3 - 376.0
376.0 - 418.7
418.7 - 464.2
464.2 - 509.7
509.7 - 555.3
555.3 - 559.0
559.0 - 600.8
> 600.8
Poland: Świętokrzyskie: DALYs Rate (per 100,000)
< 327.9
327.9 - 332.8
332.8 - 375.1
375.1 - 417.3
417.3 - 463.5
463.5 - 509.7
509.7 - 555.9
555.9 - 560.5
560.5 - 602.1
> 602.1
Poland: Warmińsko-Mazurskie: DALYs Rate (per 100,000)
< 328.4
328.4 - 332.5
332.5 - 375.2
375.2 - 417.9
417.9 - 463.6
463.6 - 509.3
509.3 - 555.1
555.1 - 559.9
559.9 - 602.8
> 602.8
Poland: Wielkopolskie: DALYs Rate (per 100,000)
< 328.7
328.7 - 333.4
333.4 - 375.8
375.8 - 418.1
418.1 - 463.8
463.8 - 509.5
509.5 - 555.1
555.1 - 559.1
559.1 - 602.1
> 602.1
Poland: Zachodniopomorskie: DALYs Rate (per 100,000)
< 328.7
328.7 - 332.8
332.8 - 375.7
375.7 - 418.6
418.6 - 464.6
464.6 - 510.6
510.6 - 556.6
556.6 - 560.0
560.0 - 603.2
> 603.2
Female
Male
Sexes Combined
Age
20-24 years
15-19 years
10-14 years
Year
DALYs Rate (per 100,000)
Age — 10-24 years

Figure S3

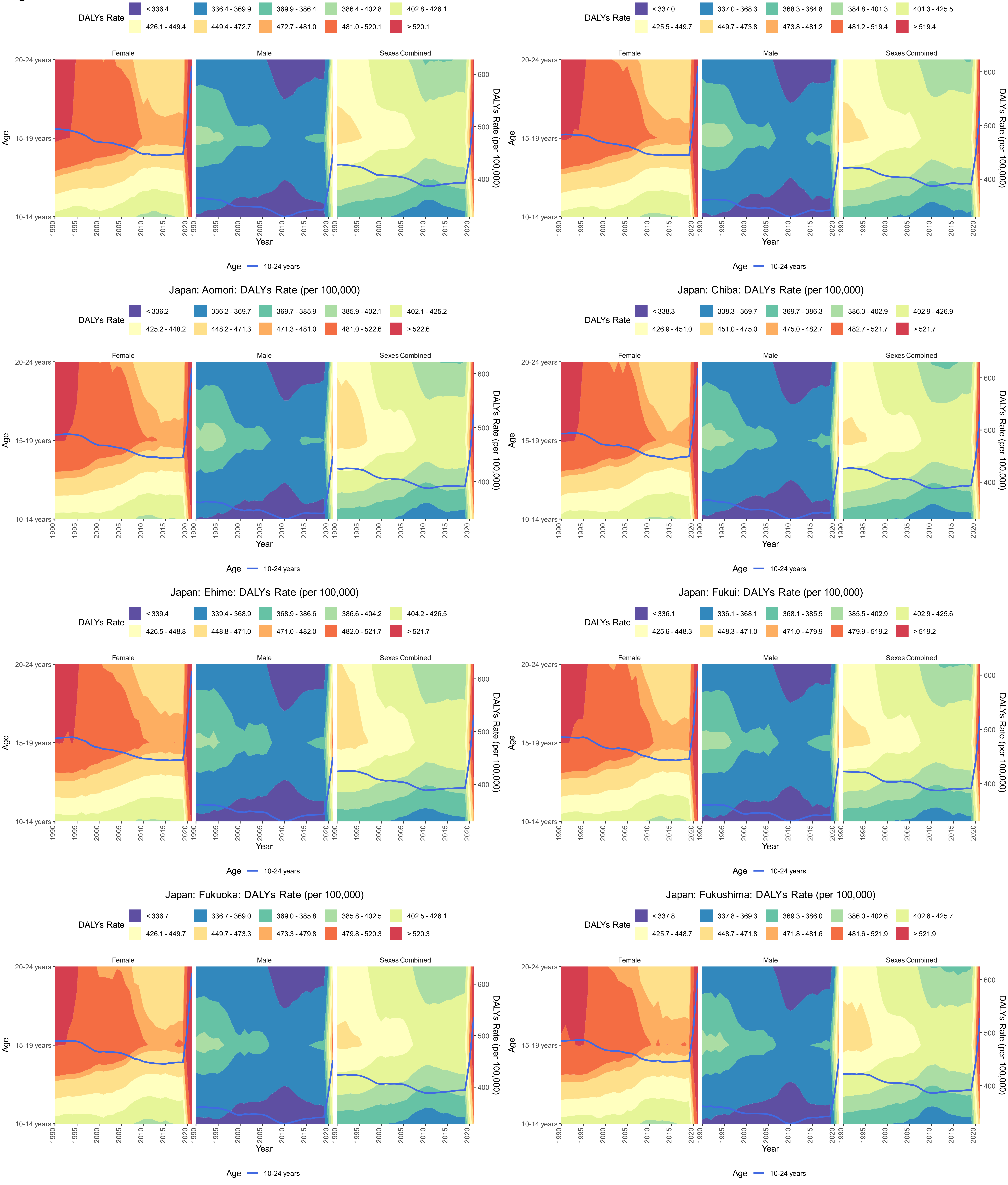
Japan: Aichi: DALYs Rate (per 100,000)
DALYs Rate
< 336.4
336.4 - 369.9
369.9 - 386.4
386.4 - 402.8
402.8 - 426.1
426.1 - 449.4
449.4 - 472.7
472.7 - 481.0
481.0 - 520.1
> 520.1
Japan: Akita: DALYs Rate (per 100,000)
< 337.0
337.0 - 368.3
368.3 - 384.8
384.8 - 401.3
401.3 - 425.5
425.5 - 449.7
449.7 - 473.8
473.8 - 481.2
481.2 - 519.4
> 519.4
Japan: Aomori: DALYs Rate (per 100,000)
< 336.2
336.2 - 369.7
369.7 - 385.9
385.9 - 402.1
402.1 - 425.2
425.2 - 448.2
448.2 - 471.3
471.3 - 481.0
481.0 - 522.6
> 522.6
Japan: Chiba: DALYs Rate (per 100,000)
< 338.3
338.3 - 369.7
369.7 - 386.3
386.3 - 402.9
402.9 - 426.9
426.9 - 451.0
451.0 - 475.0
475.0 - 482.7
482.7 - 521.7
> 521.7
Japan: Ehime: DALYs Rate (per 100,000)
< 339.4
339.4 - 368.9
368.9 - 386.6
386.6 - 404.2
404.2 - 426.5
426.5 - 448.8
448.8 - 471.0
471.0 - 482.0
482.0 - 521.7
> 521.7
Japan: Fukui: DALYs Rate (per 100,000)
< 336.1
336.1 - 368.1
368.1 - 385.5
385.5 - 402.9
402.9 - 425.6
425.6 - 448.3
448.3 - 471.0
471.0 - 479.9
479.9 - 519.2
> 519.2
Japan: Fukuoka: DALYs Rate (per 100,000)
< 336.7
336.7 - 369.0
369.0 - 385.8
385.8 - 402.5
402.5 - 426.1
426.1 - 449.7
449.7 - 473.3
473.3 - 479.8
479.8 - 520.3
> 520.3
Japan: Fukushima: DALYs Rate (per 100,000)
< 337.8
337.8 - 369.3
369.3 - 386.0
386.0 - 402.6
402.6 - 425.7
425.7 - 448.7
448.7 - 471.8
471.8 - 481.6
481.6 - 521.9
> 521.9
Female
Male
Sexes Combined
Age
20-24 years
15-19 years
10-14 years
Year
DALYs Rate (per 100,000)
Age — 10-24 years

Figure S3

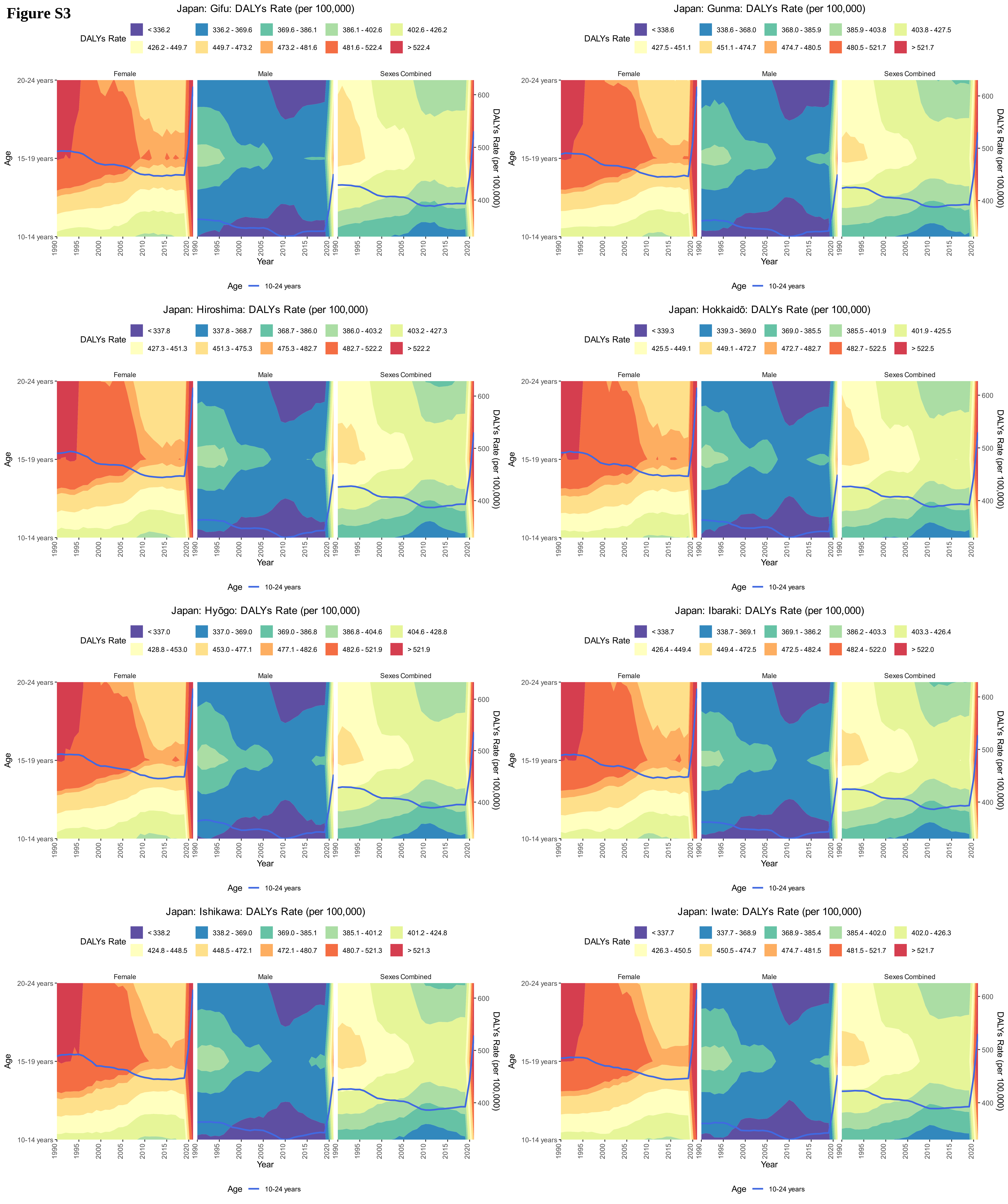

Japan: Gifu: DALYs Rate (per 100,000)
DALYs Rate
< 336.2
336.2 - 369.6
369.6 - 386.1
386.1 - 402.6
402.6 - 426.2
426.2 - 449.7
449.7 - 473.2
473.2 - 481.6
481.6 - 522.4
> 522.4
Japan: Gunma: DALYs Rate (per 100,000)
DALYs Rate
< 338.6
338.6 - 368.0
368.0 - 385.9
385.9 - 403.8
403.8 - 427.5
427.5 - 451.1
451.1 - 474.7
474.7 - 480.5
480.5 - 521.7
> 521.7
Japan: Hiroshima: DALYs Rate (per 100,000)
DALYs Rate
< 337.8
337.8 - 368.7
368.7 - 386.0
386.0 - 403.2
403.2 - 427.3
427.3 - 451.3
451.3 - 475.3
475.3 - 482.7
482.7 - 522.2
> 522.2
Japan: Hokkaidō: DALYs Rate (per 100,000)
DALYs Rate
< 339.3
339.3 - 369.0
369.0 - 385.5
385.5 - 401.9
401.9 - 425.5
425.5 - 449.1
449.1 - 472.7
472.7 - 482.7
482.7 - 522.5
> 522.5
Japan: Hyōgo: DALYs Rate (per 100,000)
DALYs Rate
< 337.0
337.0 - 369.0
369.0 - 386.8
386.8 - 404.6
404.6 - 428.8
428.8 - 453.0
453.0 - 477.1
477.1 - 482.6
482.6 - 521.9
> 521.9
Japan: Ibaraki: DALYs Rate (per 100,000)
DALYs Rate
< 338.7
338.7 - 369.1
369.1 - 386.2
386.2 - 403.3
403.3 - 426.4
426.4 - 449.4
449.4 - 472.5
472.5 - 482.4
482.4 - 522.0
> 522.0
Japan: Ishikawa: DALYs Rate (per 100,000)
DALYs Rate
< 338.2
338.2 - 369.0
369.0 - 385.1
385.1 - 401.2
401.2 - 424.8
424.8 - 448.5
448.5 - 472.1
472.1 - 480.7
480.7 - 521.3
> 521.3
Japan: Iwate: DALYs Rate (per 100,000)
DALYs Rate
< 337.7
337.7 - 368.9
368.9 - 385.4
385.4 - 402.0
402.0 - 426.3
426.3 - 450.5
450.5 - 474.7
474.7 - 481.5
481.5 - 521.7
> 521.7
Female
Male
Sexes Combined
Age
20-24 years
15-19 years
10-14 years
Year
1990
1995
2000
2005
2010
2015
2020
DALYs Rate (per 100,000)
400
500
600
Age
10-24 years

Figure S3

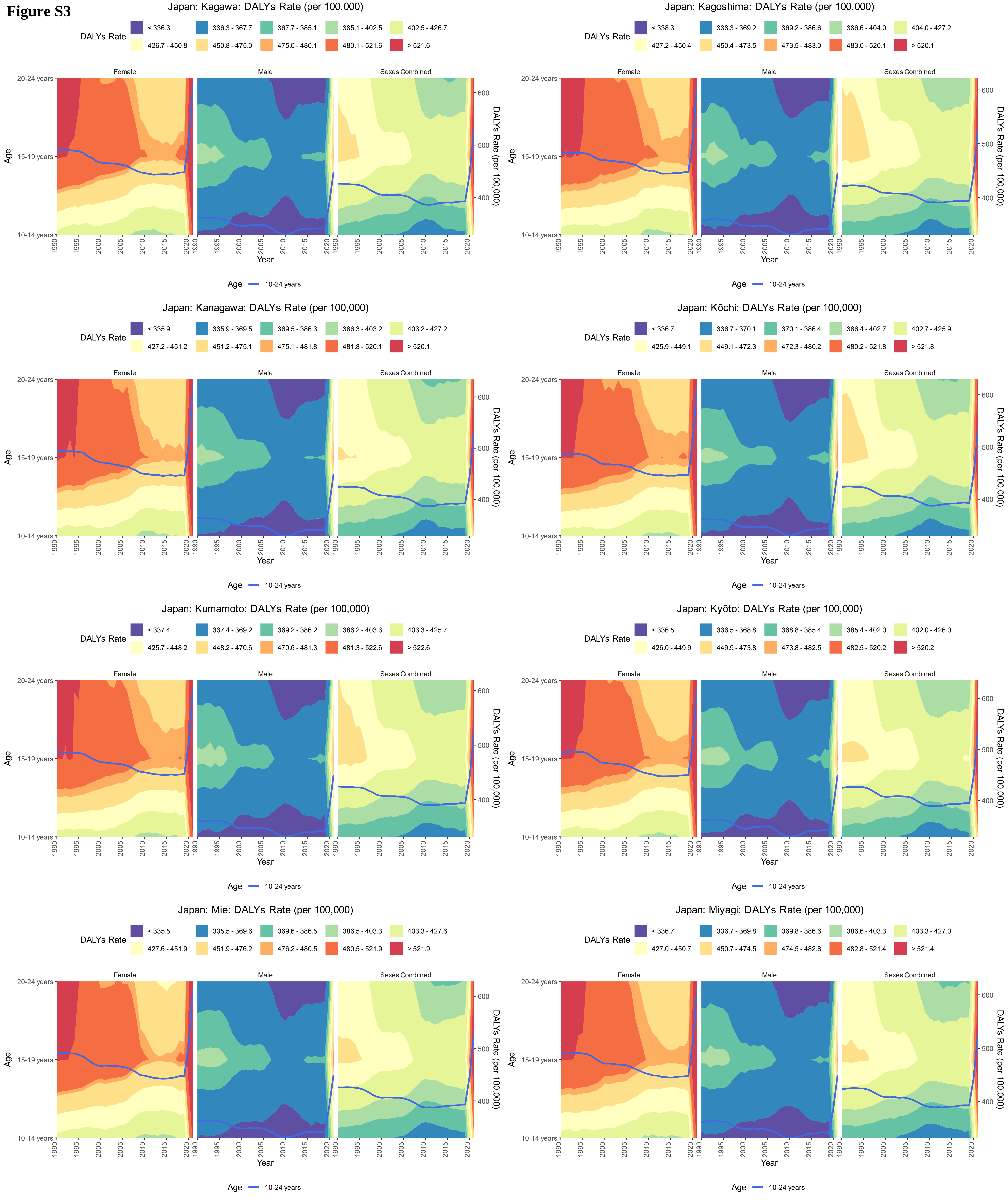
Japan: Kagawa: DALYs Rate (per 100,000)
DALYs Rate
< 336.3
336.3 - 367.7
367.7 - 385.1
385.1 - 402.5
402.5 - 426.7
426.7 - 450.8
450.8 - 475.0
475.0 - 480.1
480.1 - 521.6
> 521.6
Female
Male
Sexes Combined
20-24 years
15-19 years
10-14 years
Age
Year
DALYs Rate (per 100,000)
Age
10-24 years
Japan: Kagoshima: DALYs Rate (per 100,000)
DALYs Rate
< 338.3
338.3 - 369.2
369.2 - 386.6
386.6 - 404.0
404.0 - 427.2
427.2 - 450.4
450.4 - 473.5
473.5 - 483.0
483.0 - 520.1
> 520.1
Japan: Kanagawa: DALYs Rate (per 100,000)
DALYs Rate
< 335.9
335.9 - 369.5
369.5 - 386.3
386.3 - 403.2
403.2 - 427.2
427.2 - 451.2
451.2 - 475.1
475.1 - 481.8
481.8 - 520.1
> 520.1
Japan: Kōchi: DALYs Rate (per 100,000)
DALYs Rate
< 336.7
336.7 - 370.1
370.1 - 386.4
386.4 - 402.7
402.7 - 425.9
425.9 - 449.1
449.1 - 472.3
472.3 - 480.2
480.2 - 521.8
> 521.8
Japan: Kumamoto: DALYs Rate (per 100,000)
DALYs Rate
< 337.4
337.4 - 369.2
369.2 - 386.2
386.2 - 403.3
403.3 - 425.7
425.7 - 448.2
448.2 - 470.6
470.6 - 481.3
481.3 - 522.6
> 522.6
Japan: Kyōto: DALYs Rate (per 100,000)
DALYs Rate
< 336.5
336.5 - 368.8
368.8 - 385.4
385.4 - 402.0
402.0 - 426.0
426.0 - 449.9
449.9 - 473.8
473.8 - 482.5
482.5 - 520.2
> 520.2
Japan: Mie: DALYs Rate (per 100,000)
DALYs Rate
< 335.5
335.5 - 369.6
369.6 - 386.5
386.5 - 403.3
403.3 - 427.6
427.6 - 451.9
451.9 - 476.2
476.2 - 480.5
480.5 - 521.9
> 521.9
Japan: Miyagi: DALYs Rate (per 100,000)
DALYs Rate
< 336.7
336.7 - 369.8
369.8 - 386.6
386.6 - 403.3
403.3 - 427.0
427.0 - 450.7
450.7 - 474.5
474.5 - 482.8
482.8 - 521.4
> 521.4

Figure S3

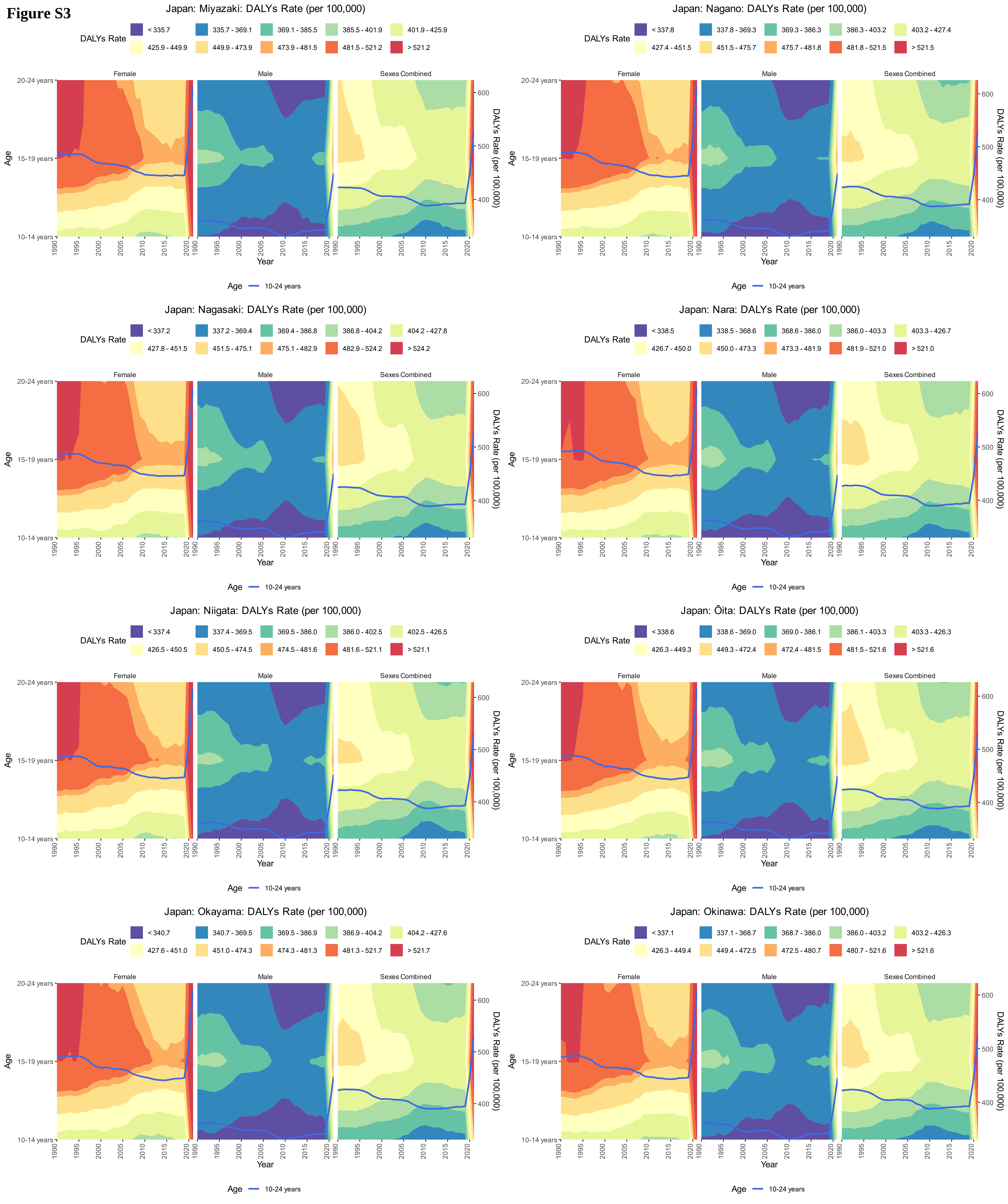

Japan: Miyazaki: DALYs Rate (per 100,000)
DALYs Rate
< 335.7
335.7 - 369.1
369.1 - 385.5
385.5 - 401.9
401.9 - 425.9
425.9 - 449.9
449.9 - 473.9
473.9 - 481.5
481.5 - 521.2
> 521.2
Japan: Nagano: DALYs Rate (per 100,000)
DALYs Rate
< 337.8
337.8 - 369.3
369.3 - 386.3
386.3 - 403.2
403.2 - 427.4
427.4 - 451.5
451.5 - 475.7
475.7 - 481.8
481.8 - 521.5
> 521.5
Japan: Nagasaki: DALYs Rate (per 100,000)
DALYs Rate
< 337.2
337.2 - 369.4
369.4 - 386.8
386.8 - 404.2
404.2 - 427.8
427.8 - 451.5
451.5 - 475.1
475.1 - 482.9
482.9 - 524.2
> 524.2
Japan: Nara: DALYs Rate (per 100,000)
DALYs Rate
< 338.5
338.5 - 368.6
368.6 - 386.0
386.0 - 403.3
403.3 - 426.7
426.7 - 450.0
450.0 - 473.3
473.3 - 481.9
481.9 - 521.0
> 521.0
Japan: Niigata: DALYs Rate (per 100,000)
DALYs Rate
< 337.4
337.4 - 369.5
369.5 - 386.0
386.0 - 402.5
402.5 - 426.5
426.5 - 450.5
450.5 - 474.5
474.5 - 481.6
481.6 - 521.1
> 521.1
Japan: Ōita: DALYs Rate (per 100,000)
DALYs Rate
< 338.6
338.6 - 369.0
369.0 - 386.1
386.1 - 403.3
403.3 - 426.3
426.3 - 449.3
449.3 - 472.4
472.4 - 481.5
481.5 - 521.6
> 521.6
Japan: Okayama: DALYs Rate (per 100,000)
DALYs Rate
< 340.7
340.7 - 369.5
369.5 - 386.9
386.9 - 404.2
404.2 - 427.6
427.6 - 451.0
451.0 - 474.3
474.3 - 481.3
481.3 - 521.7
> 521.7
Japan: Okinawa: DALYs Rate (per 100,000)
DALYs Rate
< 337.1
337.1 - 368.7
368.7 - 386.0
386.0 - 403.2
403.2 - 426.3
426.3 - 449.4
449.4 - 472.5
472.5 - 480.7
480.7 - 521.6
> 521.6
Female
Male
Sexes Combined
20-24 years
15-19 years
10-14 years
Age
1990
1995
2000
2005
2010
2015
2020
Year
400
500
600
DALYs Rate (per 100,000)
Age
10-24 years

Figure S3

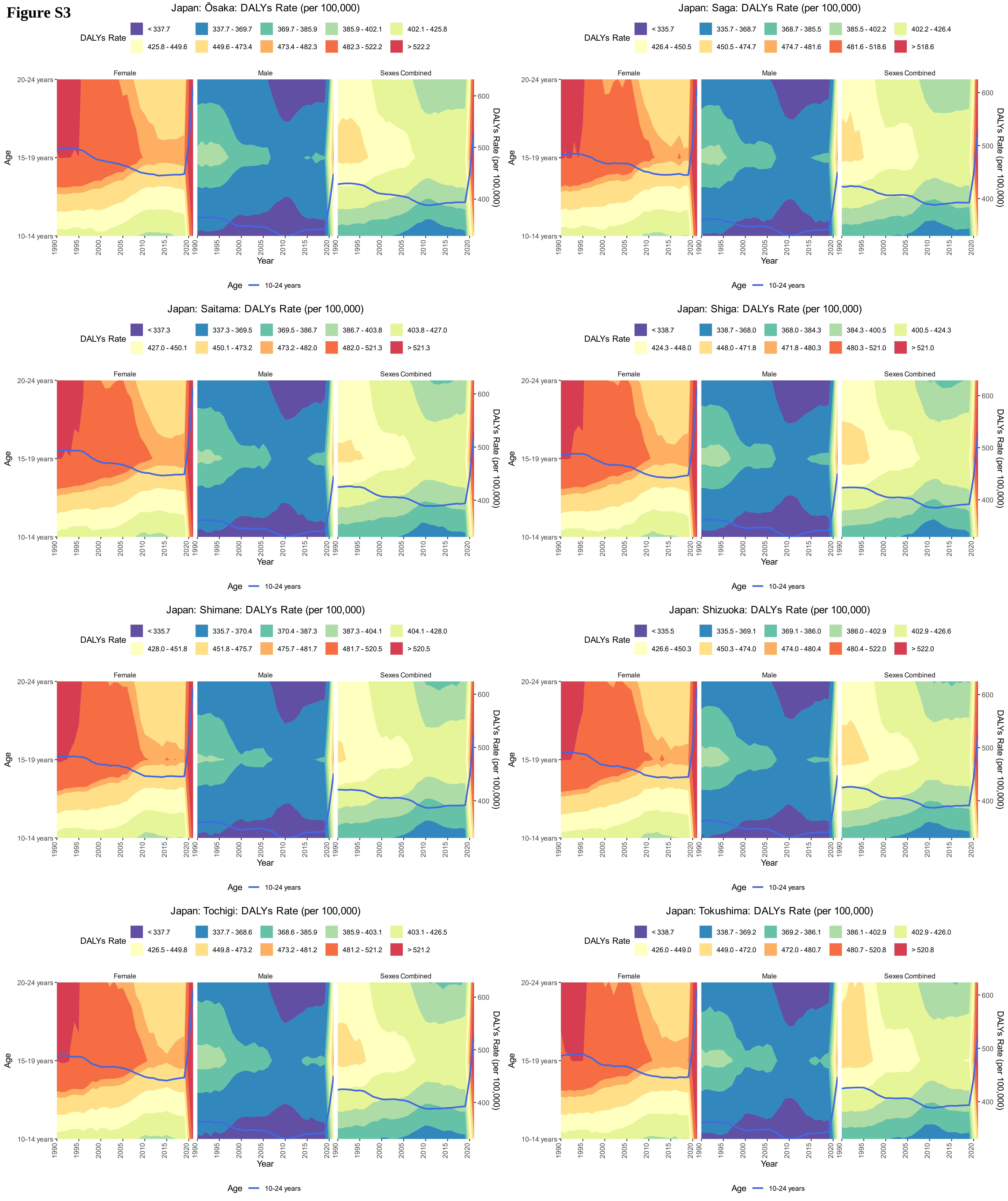
Japan: Ōsaka: DALYs Rate (per 100,000)
DALYs Rate
< 337.7
337.7 - 369.7
369.7 - 385.9
385.9 - 402.1
402.1 - 425.8
425.8 - 449.6
449.6 - 473.4
473.4 - 482.3
482.3 - 522.2
> 522.2
Japan: Saga: DALYs Rate (per 100,000)
DALYs Rate
< 335.7
335.7 - 368.7
368.7 - 385.5
385.5 - 402.2
402.2 - 426.4
426.4 - 450.5
450.5 - 474.7
474.7 - 481.6
481.6 - 518.6
> 518.6
Japan: Saitama: DALYs Rate (per 100,000)
DALYs Rate
< 337.3
337.3 - 369.5
369.5 - 386.7
386.7 - 403.8
403.8 - 427.0
427.0 - 450.1
450.1 - 473.2
473.2 - 482.0
482.0 - 521.3
> 521.3
Japan: Shiga: DALYs Rate (per 100,000)
DALYs Rate
< 338.7
338.7 - 368.0
368.0 - 384.3
384.3 - 400.5
400.5 - 424.3
424.3 - 448.0
448.0 - 471.8
471.8 - 480.3
480.3 - 521.0
> 521.0
Japan: Shimane: DALYs Rate (per 100,000)
DALYs Rate
< 335.7
335.7 - 370.4
370.4 - 387.3
387.3 - 404.1
404.1 - 428.0
428.0 - 451.8
451.8 - 475.7
475.7 - 481.7
481.7 - 520.5
> 520.5
Japan: Shizuoka: DALYs Rate (per 100,000)
DALYs Rate
< 335.5
335.5 - 369.1
369.1 - 386.0
386.0 - 402.9
402.9 - 426.6
426.6 - 450.3
450.3 - 474.0
474.0 - 480.4
480.4 - 522.0
> 522.0
Japan: Tochigi: DALYs Rate (per 100,000)
DALYs Rate
< 337.7
337.7 - 368.6
368.6 - 385.9
385.9 - 403.1
403.1 - 426.5
426.5 - 449.8
449.8 - 473.2
473.2 - 481.2
481.2 - 521.2
> 521.2
Japan: Tokushima: DALYs Rate (per 100,000)
DALYs Rate
< 338.7
338.7 - 369.2
369.2 - 386.1
386.1 - 402.9
402.9 - 426.0
426.0 - 449.0
449.0 - 472.0
472.0 - 480.7
480.7 - 520.8
> 520.8
Female
Male
Sexes Combined
Age
20-24 years
15-19 years
10-14 years
Year
DALYs Rate (per 100,000)
Age — 10-24 years

Figure S3

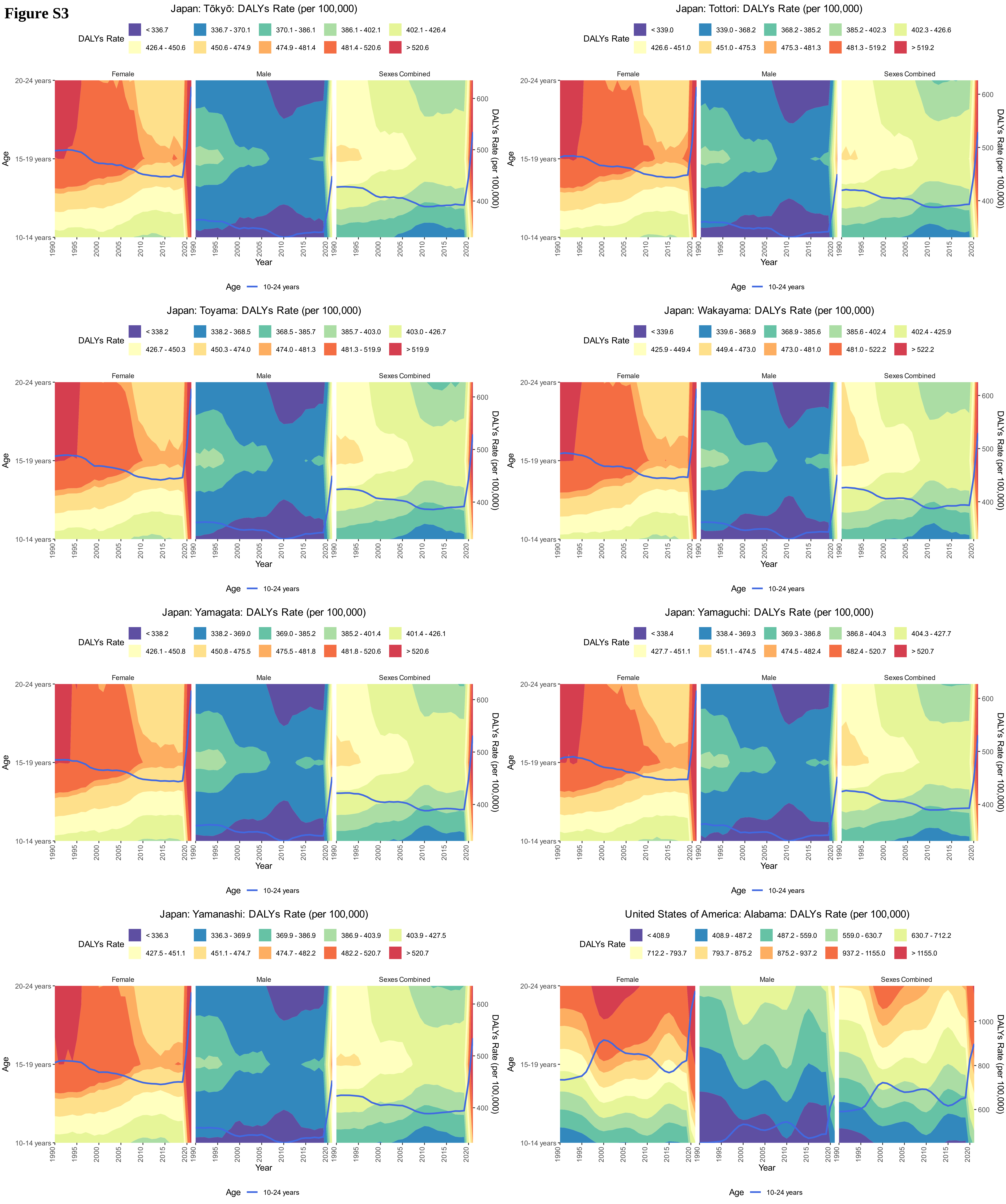

Japan: Tōkyō: DALYs Rate (per 100,000)
DALYs Rate
< 336.7
336.7 - 370.1
370.1 - 386.1
386.1 - 402.1
402.1 - 426.4
426.4 - 450.6
450.6 - 474.9
474.9 - 481.4
481.4 - 520.6
> 520.6
Japan: Tottori: DALYs Rate (per 100,000)
< 339.0
339.0 - 368.2
368.2 - 385.2
385.2 - 402.3
402.3 - 426.6
426.6 - 451.0
451.0 - 475.3
475.3 - 481.3
481.3 - 519.2
> 519.2
Japan: Toyama: DALYs Rate (per 100,000)
< 338.2
338.2 - 368.5
368.5 - 385.7
385.7 - 403.0
403.0 - 426.7
426.7 - 450.3
450.3 - 474.0
474.0 - 481.3
481.3 - 519.9
> 519.9
Japan: Wakayama: DALYs Rate (per 100,000)
< 339.6
339.6 - 368.9
368.9 - 385.6
385.6 - 402.4
402.4 - 425.9
425.9 - 449.4
449.4 - 473.0
473.0 - 481.0
481.0 - 522.2
> 522.2
Japan: Yamagata: DALYs Rate (per 100,000)
< 338.2
338.2 - 369.0
369.0 - 385.2
385.2 - 401.4
401.4 - 426.1
426.1 - 450.8
450.8 - 475.5
475.5 - 481.8
481.8 - 520.6
> 520.6
Japan: Yamaguchi: DALYs Rate (per 100,000)
< 338.4
338.4 - 369.3
369.3 - 386.8
386.8 - 404.3
404.3 - 427.7
427.7 - 451.1
451.1 - 474.5
474.5 - 482.4
482.4 - 520.7
> 520.7
Japan: Yamanashi: DALYs Rate (per 100,000)
< 336.3
336.3 - 369.9
369.9 - 386.9
386.9 - 403.9
403.9 - 427.5
427.5 - 451.1
451.1 - 474.7
474.7 - 482.2
482.2 - 520.7
> 520.7
United States of America: Alabama: DALYs Rate (per 100,000)
< 408.9
408.9 - 487.2
487.2 - 559.0
559.0 - 630.7
630.7 - 712.2
712.2 - 793.7
793.7 - 875.2
875.2 - 937.2
937.2 - 1155.0
> 1155.0
Female
Male
Sexes Combined
20-24 years
15-19 years
10-14 years
Age
Year
DALYs Rate (per 100,000)
Age — 10-24 years

Figure S3

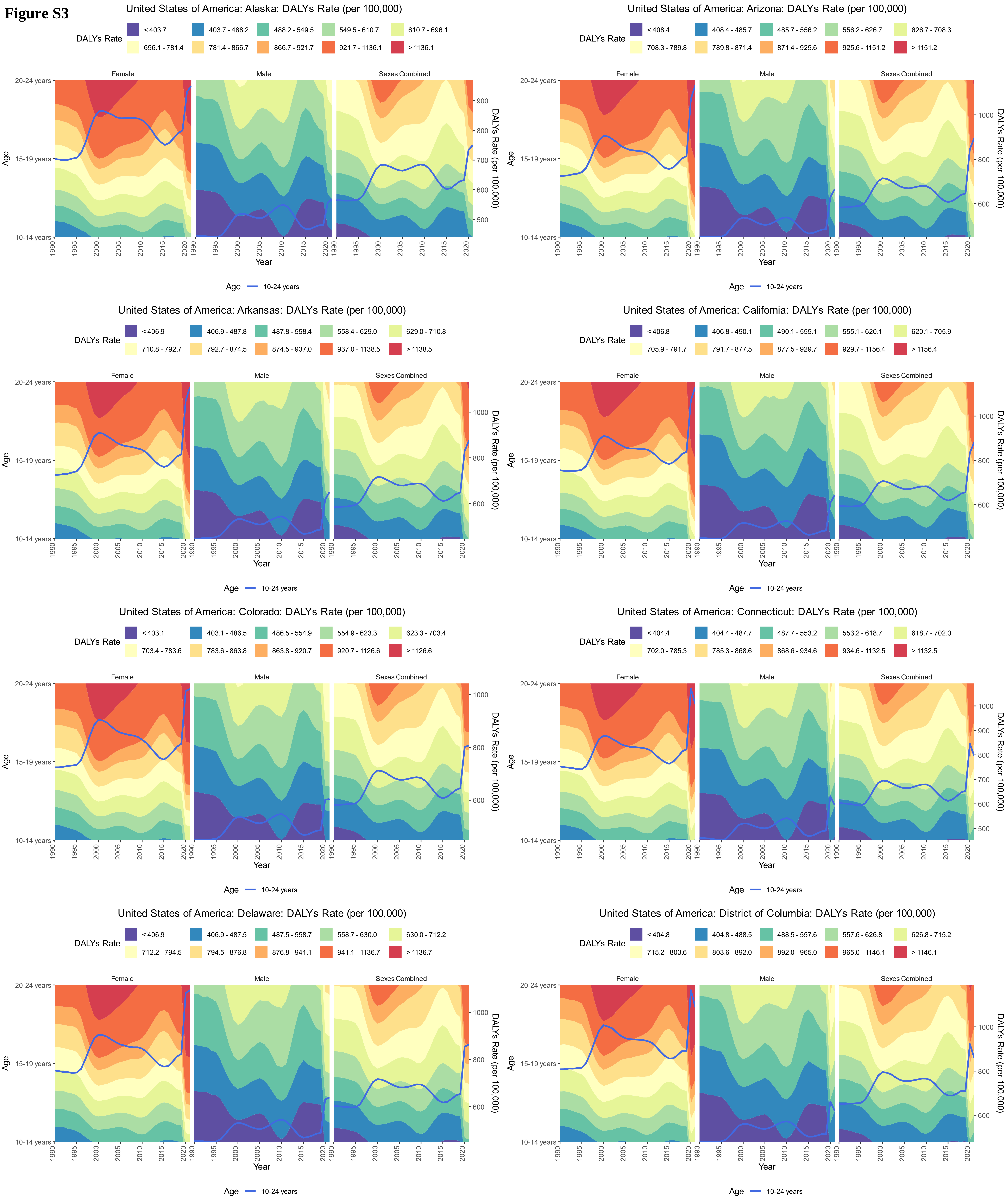
United States of America: Alaska: DALYs Rate (per 100,000)
DALYs Rate
< 403.7
403.7 - 488.2
488.2 - 549.5
549.5 - 610.7
610.7 - 696.1
696.1 - 781.4
781.4 - 866.7
866.7 - 921.7
921.7 - 1136.1
> 1136.1
United States of America: Arizona: DALYs Rate (per 100,000)
DALYs Rate
< 408.4
408.4 - 485.7
485.7 - 556.2
556.2 - 626.7
626.7 - 708.3
708.3 - 789.8
789.8 - 871.4
871.4 - 925.6
925.6 - 1151.2
> 1151.2
United States of America: Arkansas: DALYs Rate (per 100,000)
DALYs Rate
< 406.9
406.9 - 487.8
487.8 - 558.4
558.4 - 629.0
629.0 - 710.8
710.8 - 792.7
792.7 - 874.5
874.5 - 937.0
937.0 - 1138.5
> 1138.5
United States of America: California: DALYs Rate (per 100,000)
DALYs Rate
< 406.8
406.8 - 490.1
490.1 - 555.1
555.1 - 620.1
620.1 - 705.9
705.9 - 791.7
791.7 - 877.5
877.5 - 929.7
929.7 - 1156.4
> 1156.4
United States of America: Colorado: DALYs Rate (per 100,000)
DALYs Rate
< 403.1
403.1 - 486.5
486.5 - 554.9
554.9 - 623.3
623.3 - 703.4
703.4 - 783.6
783.6 - 863.8
863.8 - 920.7
920.7 - 1126.6
> 1126.6
United States of America: Connecticut: DALYs Rate (per 100,000)
DALYs Rate
< 404.4
404.4 - 487.7
487.7 - 553.2
553.2 - 618.7
618.7 - 702.0
702.0 - 785.3
785.3 - 868.6
868.6 - 934.6
934.6 - 1132.5
> 1132.5
United States of America: Delaware: DALYs Rate (per 100,000)
DALYs Rate
< 406.9
406.9 - 487.5
487.5 - 558.7
558.7 - 630.0
630.0 - 712.2
712.2 - 794.5
794.5 - 876.8
876.8 - 941.1
941.1 - 1136.7
> 1136.7
United States of America: District of Columbia: DALYs Rate (per 100,000)
DALYs Rate
< 404.8
404.8 - 488.5
488.5 - 557.6
557.6 - 626.8
626.8 - 715.2
715.2 - 803.6
803.6 - 892.0
892.0 - 965.0
965.0 - 1146.1
> 1146.1
Female
Male
Sexes Combined
20-24 years
15-19 years
10-14 years
Age
Year
DALYs Rate (per 100,000)
Age
10-24 years

Figure S3

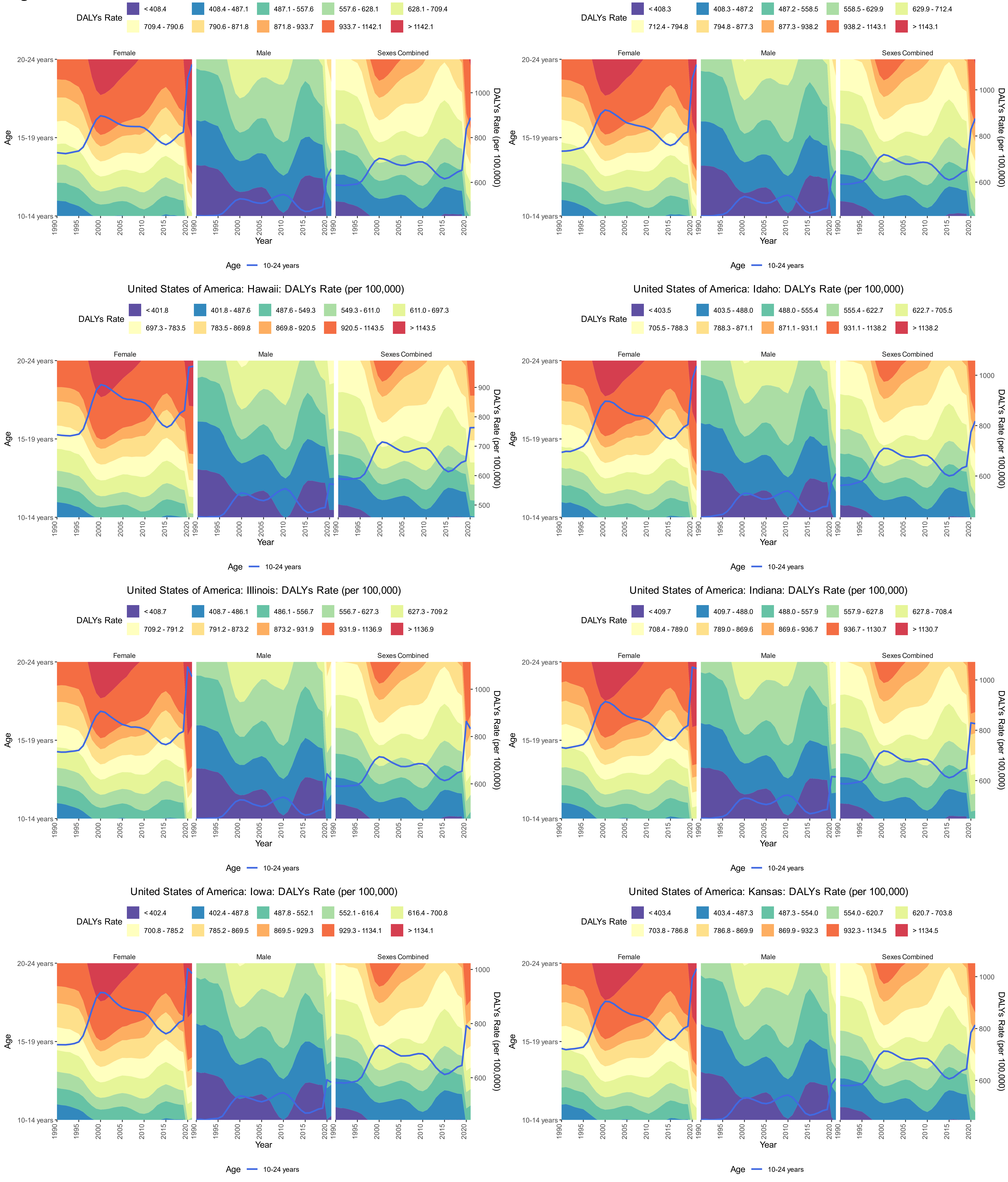
United States of America: Florida: DALYs Rate (per 100,000)
DALYs Rate
< 408.4
408.4 - 487.1
487.1 - 557.6
557.6 - 628.1
628.1 - 709.4
709.4 - 790.6
790.6 - 871.8
871.8 - 933.7
933.7 - 1142.1
> 1142.1
Female
Male
Sexes Combined
20-24 years
15-19 years
10-14 years
Age
Year
DALYs Rate (per 100,000)
Age
10-24 years
United States of America: Georgia: DALYs Rate (per 100,000)
DALYs Rate
< 408.3
408.3 - 487.2
487.2 - 558.5
558.5 - 629.9
629.9 - 712.4
712.4 - 794.8
794.8 - 877.3
877.3 - 938.2
938.2 - 1143.1
> 1143.1
United States of America: Hawaii: DALYs Rate (per 100,000)
DALYs Rate
< 401.8
401.8 - 487.6
487.6 - 549.3
549.3 - 611.0
611.0 - 697.3
697.3 - 783.5
783.5 - 869.8
869.8 - 920.5
920.5 - 1143.5
> 1143.5
United States of America: Idaho: DALYs Rate (per 100,000)
DALYs Rate
< 403.5
403.5 - 488.0
488.0 - 555.4
555.4 - 622.7
622.7 - 705.5
705.5 - 788.3
788.3 - 871.1
871.1 - 931.1
931.1 - 1138.2
> 1138.2
United States of America: Illinois: DALYs Rate (per 100,000)
DALYs Rate
< 408.7
408.7 - 486.1
486.1 - 556.7
556.7 - 627.3
627.3 - 709.2
709.2 - 791.2
791.2 - 873.2
873.2 - 931.9
931.9 - 1136.9
> 1136.9
United States of America: Indiana: DALYs Rate (per 100,000)
DALYs Rate
< 409.7
409.7 - 488.0
488.0 - 557.9
557.9 - 627.8
627.8 - 708.4
708.4 - 789.0
789.0 - 869.6
869.6 - 936.7
936.7 - 1130.7
> 1130.7
United States of America: Iowa: DALYs Rate (per 100,000)
DALYs Rate
< 402.4
402.4 - 487.8
487.8 - 552.1
552.1 - 616.4
616.4 - 700.8
700.8 - 785.2
785.2 - 869.5
869.5 - 929.3
929.3 - 1134.1
> 1134.1
United States of America: Kansas: DALYs Rate (per 100,000)
DALYs Rate
< 403.4
403.4 - 487.3
487.3 - 554.0
554.0 - 620.7
620.7 - 703.8
703.8 - 786.8
786.8 - 869.9
869.9 - 932.3
932.3 - 1134.5
> 1134.5

Figure S3

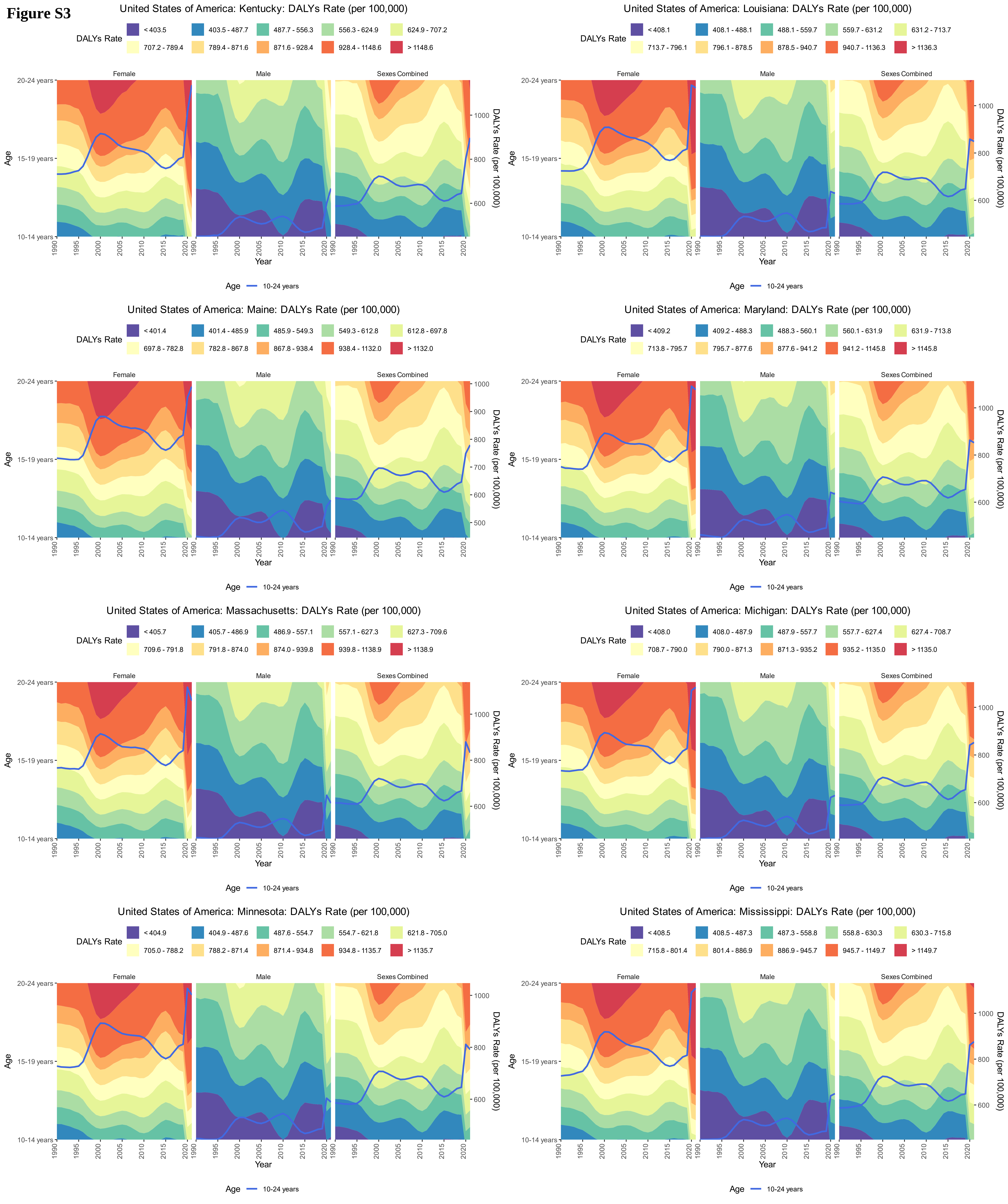
United States of America: Kentucky: DALYs Rate (per 100,000)
DALYs Rate
< 403.5
403.5 - 487.7
487.7 - 556.3
556.3 - 624.9
624.9 - 707.2
707.2 - 789.4
789.4 - 871.6
871.6 - 928.4
928.4 - 1148.6
> 1148.6
Female
Male
Sexes Combined
Age
Year
DALYs Rate (per 100,000)
Age 10-24 years
United States of America: Louisiana: DALYs Rate (per 100,000)
< 408.1
408.1 - 488.1
488.1 - 559.7
559.7 - 631.2
631.2 - 713.7
713.7 - 796.1
796.1 - 878.5
878.5 - 940.7
940.7 - 1136.3
> 1136.3
United States of America: Maine: DALYs Rate (per 100,000)
< 401.4
401.4 - 485.9
485.9 - 549.3
549.3 - 612.8
612.8 - 697.8
697.8 - 782.8
782.8 - 867.8
867.8 - 938.4
938.4 - 1132.0
> 1132.0
United States of America: Maryland: DALYs Rate (per 100,000)
< 409.2
409.2 - 488.3
488.3 - 560.1
560.1 - 631.9
631.9 - 713.8
713.8 - 795.7
795.7 - 877.6
877.6 - 941.2
941.2 - 1145.8
> 1145.8
United States of America: Massachusetts: DALYs Rate (per 100,000)
< 405.7
405.7 - 486.9
486.9 - 557.1
557.1 - 627.3
627.3 - 709.6
709.6 - 791.8
791.8 - 874.0
874.0 - 939.8
939.8 - 1138.9
> 1138.9
United States of America: Michigan: DALYs Rate (per 100,000)
< 408.0
408.0 - 487.9
487.9 - 557.7
557.7 - 627.4
627.4 - 708.7
708.7 - 790.0
790.0 - 871.3
871.3 - 935.2
935.2 - 1135.0
> 1135.0
United States of America: Minnesota: DALYs Rate (per 100,000)
< 404.9
404.9 - 487.6
487.6 - 554.7
554.7 - 621.8
621.8 - 705.0
705.0 - 788.2
788.2 - 871.4
871.4 - 934.8
934.8 - 1135.7
> 1135.7
United States of America: Mississippi: DALYs Rate (per 100,000)
< 408.5
408.5 - 487.3
487.3 - 558.8
558.8 - 630.3
630.3 - 715.8
715.8 - 801.4
801.4 - 886.9
886.9 - 945.7
945.7 - 1149.7
> 1149.7
20-24 years
15-19 years
10-14 years
1990
1995
2000
2005
2010
2015
2020

Figure S3

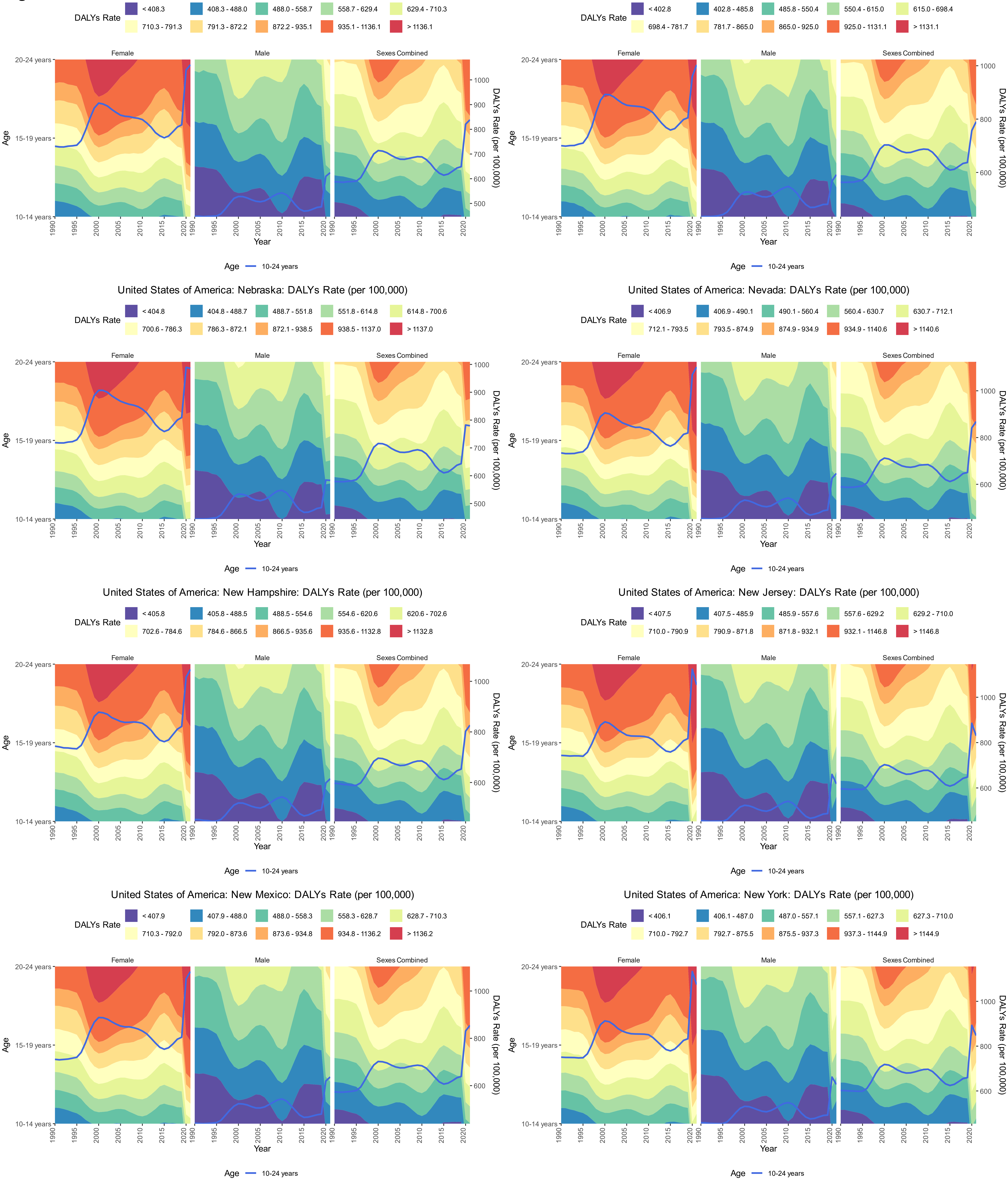
United States of America: Missouri: DALYs Rate (per 100,000)
DALYs Rate
< 408.3
408.3 - 488.0
488.0 - 558.7
558.7 - 629.4
629.4 - 710.3
710.3 - 791.3
791.3 - 872.2
872.2 - 935.1
935.1 - 1136.1
> 1136.1
Female
Male
Sexes Combined
Age
20-24 years
15-19 years
10-14 years
Year
DALYs Rate (per 100,000)
Age
10-24 years
United States of America: Montana: DALYs Rate (per 100,000)
DALYs Rate
< 402.8
402.8 - 485.8
485.8 - 550.4
550.4 - 615.0
615.0 - 698.4
698.4 - 781.7
781.7 - 865.0
865.0 - 925.0
925.0 - 1131.1
> 1131.1
United States of America: Nebraska: DALYs Rate (per 100,000)
DALYs Rate
< 404.8
404.8 - 488.7
488.7 - 551.8
551.8 - 614.8
614.8 - 700.6
700.6 - 786.3
786.3 - 872.1
872.1 - 938.5
938.5 - 1137.0
> 1137.0
United States of America: Nevada: DALYs Rate (per 100,000)
DALYs Rate
< 406.9
406.9 - 490.1
490.1 - 560.4
560.4 - 630.7
630.7 - 712.1
712.1 - 793.5
793.5 - 874.9
874.9 - 934.9
934.9 - 1140.6
> 1140.6
United States of America: New Hampshire: DALYs Rate (per 100,000)
DALYs Rate
< 405.8
405.8 - 488.5
488.5 - 554.6
554.6 - 620.6
620.6 - 702.6
702.6 - 784.6
784.6 - 866.5
866.5 - 935.6
935.6 - 1132.8
> 1132.8
United States of America: New Jersey: DALYs Rate (per 100,000)
DALYs Rate
< 407.5
407.5 - 485.9
485.9 - 557.6
557.6 - 629.2
629.2 - 710.0
710.0 - 790.9
790.9 - 871.8
871.8 - 932.1
932.1 - 1146.8
> 1146.8
United States of America: New Mexico: DALYs Rate (per 100,000)
DALYs Rate
< 407.9
407.9 - 488.0
488.0 - 558.3
558.3 - 628.7
628.7 - 710.3
710.3 - 792.0
792.0 - 873.6
873.6 - 934.8
934.8 - 1136.2
> 1136.2
United States of America: New York: DALYs Rate (per 100,000)
DALYs Rate
< 406.1
406.1 - 487.0
487.0 - 557.1
557.1 - 627.3
627.3 - 710.0
710.0 - 792.7
792.7 - 875.5
875.5 - 937.3
937.3 - 1144.9
> 1144.9

Figure S3

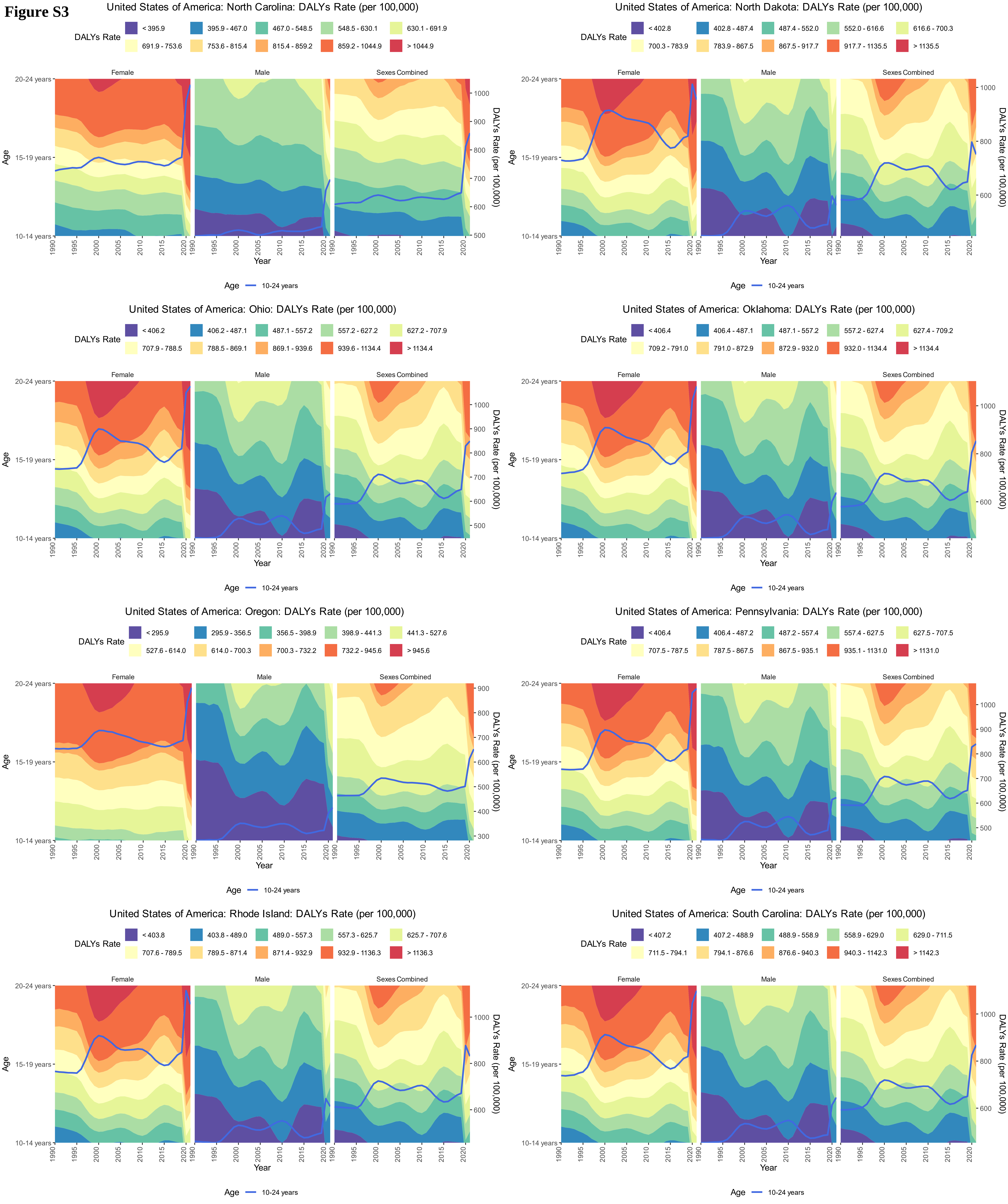
United States of America: North Carolina: DALYs Rate (per 100,000)
DALYs Rate
< 395.9
395.9 - 467.0
467.0 - 548.5
548.5 - 630.1
630.1 - 691.9
691.9 - 753.6
753.6 - 815.4
815.4 - 859.2
859.2 - 1044.9
> 1044.9
United States of America: North Dakota: DALYs Rate (per 100,000)
< 402.8
402.8 - 487.4
487.4 - 552.0
552.0 - 616.6
616.6 - 700.3
700.3 - 783.9
783.9 - 867.5
867.5 - 917.7
917.7 - 1135.5
> 1135.5
United States of America: Ohio: DALYs Rate (per 100,000)
< 406.2
406.2 - 487.1
487.1 - 557.2
557.2 - 627.2
627.2 - 707.9
707.9 - 788.5
788.5 - 869.1
869.1 - 939.6
939.6 - 1134.4
> 1134.4
United States of America: Oklahoma: DALYs Rate (per 100,000)
< 406.4
406.4 - 487.1
487.1 - 557.2
557.2 - 627.4
627.4 - 709.2
709.2 - 791.0
791.0 - 872.9
872.9 - 932.0
932.0 - 1134.4
> 1134.4
United States of America: Oregon: DALYs Rate (per 100,000)
< 295.9
295.9 - 356.5
356.5 - 398.9
398.9 - 441.3
441.3 - 527.6
527.6 - 614.0
614.0 - 700.3
700.3 - 732.2
732.2 - 945.6
> 945.6
United States of America: Pennsylvania: DALYs Rate (per 100,000)
< 406.4
406.4 - 487.2
487.2 - 557.4
557.4 - 627.5
627.5 - 707.5
707.5 - 787.5
787.5 - 867.5
867.5 - 935.1
935.1 - 1131.0
> 1131.0
United States of America: Rhode Island: DALYs Rate (per 100,000)
< 403.8
403.8 - 489.0
489.0 - 557.3
557.3 - 625.7
625.7 - 707.6
707.6 - 789.5
789.5 - 871.4
871.4 - 932.9
932.9 - 1136.3
> 1136.3
United States of America: South Carolina: DALYs Rate (per 100,000)
< 407.2
407.2 - 488.9
488.9 - 558.9
558.9 - 629.0
629.0 - 711.5
711.5 - 794.1
794.1 - 876.6
876.6 - 940.3
940.3 - 1142.3
> 1142.3
Female
Male
Sexes Combined
Age
20-24 years
15-19 years
10-14 years
Year
1990
1995
2000
2005
2010
2015
2020
DALYs Rate (per 100,000)
Age
10-24 years

Figure S3

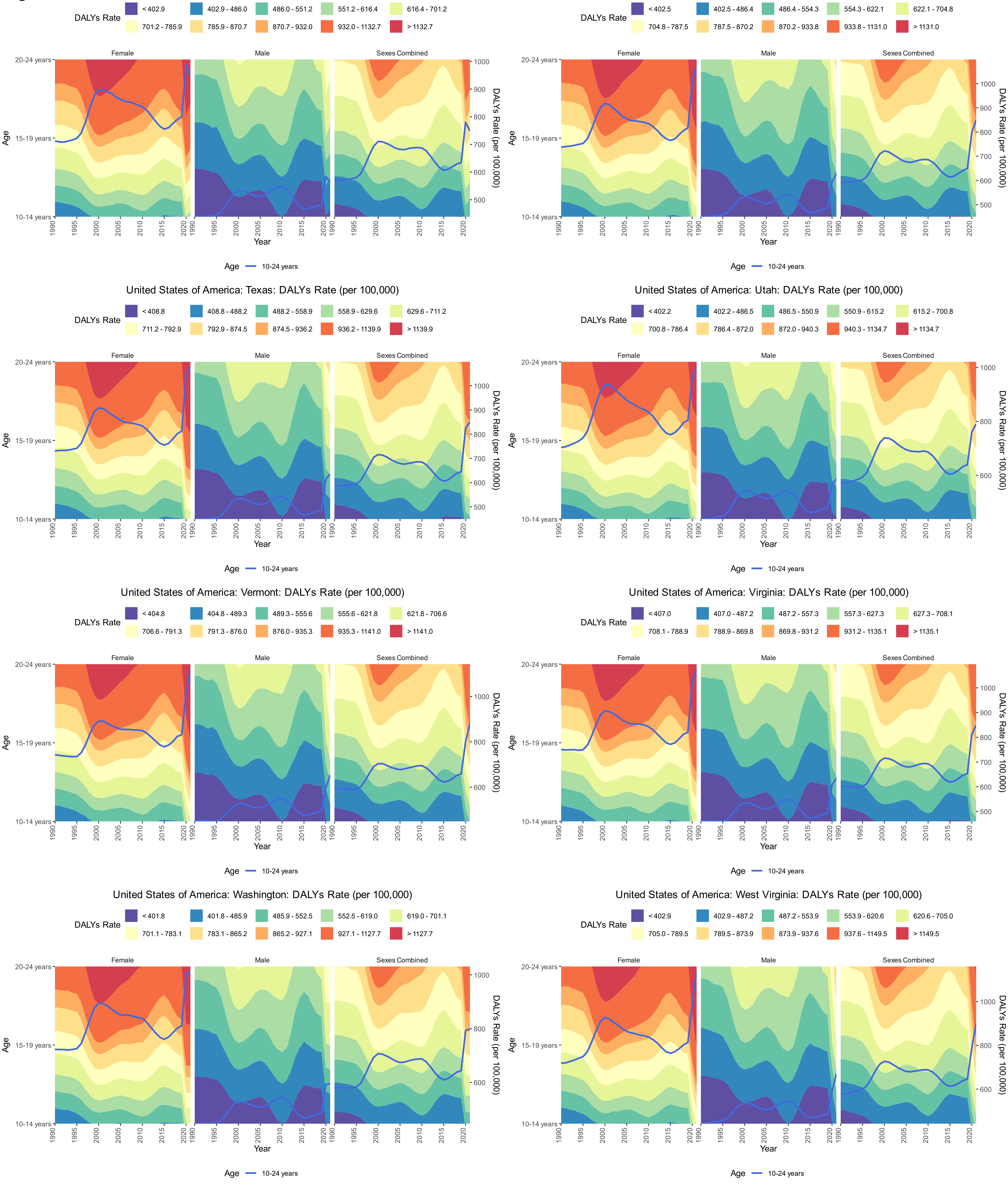
United States of America: South Dakota: DALYs Rate (per 100,000)
DALYs Rate
< 402.9
402.9 - 486.0
486.0 - 551.2
551.2 - 616.4
616.4 - 701.2
701.2 - 785.9
785.9 - 870.7
870.7 - 932.0
932.0 - 1132.7
> 1132.7
Female
Male
Sexes Combined
Age
Year
DALYs Rate (per 100,000)
Age
10-24 years
United States of America: Tennessee: DALYs Rate (per 100,000)
DALYs Rate
< 402.5
402.5 - 486.4
486.4 - 554.3
554.3 - 622.1
622.1 - 704.8
704.8 - 787.5
787.5 - 870.2
870.2 - 933.8
933.8 - 1131.0
> 1131.0
Age
10-24 years
United States of America: Texas: DALYs Rate (per 100,000)
DALYs Rate
< 408.8
408.8 - 488.2
488.2 - 558.9
558.9 - 629.6
629.6 - 711.2
711.2 - 792.9
792.9 - 874.5
874.5 - 936.2
936.2 - 1139.9
> 1139.9
Age
10-24 years
United States of America: Utah: DALYs Rate (per 100,000)
DALYs Rate
< 402.2
402.2 - 486.5
486.5 - 550.9
550.9 - 615.2
615.2 - 700.8
700.8 - 786.4
786.4 - 872.0
872.0 - 940.3
940.3 - 1134.7
> 1134.7
Age
10-24 years
United States of America: Vermont: DALYs Rate (per 100,000)
DALYs Rate
< 404.8
404.8 - 489.3
489.3 - 555.6
555.6 - 621.8
621.8 - 706.6
706.6 - 791.3
791.3 - 876.0
876.0 - 935.3
935.3 - 1141.0
> 1141.0
Age
10-24 years
United States of America: Virginia: DALYs Rate (per 100,000)
DALYs Rate
< 407.0
407.0 - 487.2
487.2 - 557.3
557.3 - 627.3
627.3 - 708.1
708.1 - 788.9
788.9 - 869.8
869.8 - 931.2
931.2 - 1135.1
> 1135.1
Age
10-24 years
United States of America: Washington: DALYs Rate (per 100,000)
DALYs Rate
< 401.8
401.8 - 485.9
485.9 - 552.5
552.5 - 619.0
619.0 - 701.1
701.1 - 783.1
783.1 - 865.2
865.2 - 927.1
927.1 - 1127.7
> 1127.7
Age
10-24 years
United States of America: West Virginia: DALYs Rate (per 100,000)
DALYs Rate
< 402.9
402.9 - 487.2
487.2 - 553.9
553.9 - 620.6
620.6 - 705.0
705.0 - 789.5
789.5 - 873.9
873.9 - 937.6
937.6 - 1149.5
> 1149.5
Age
10-24 years
20-24 years
15-19 years
10-14 years

Figure S3

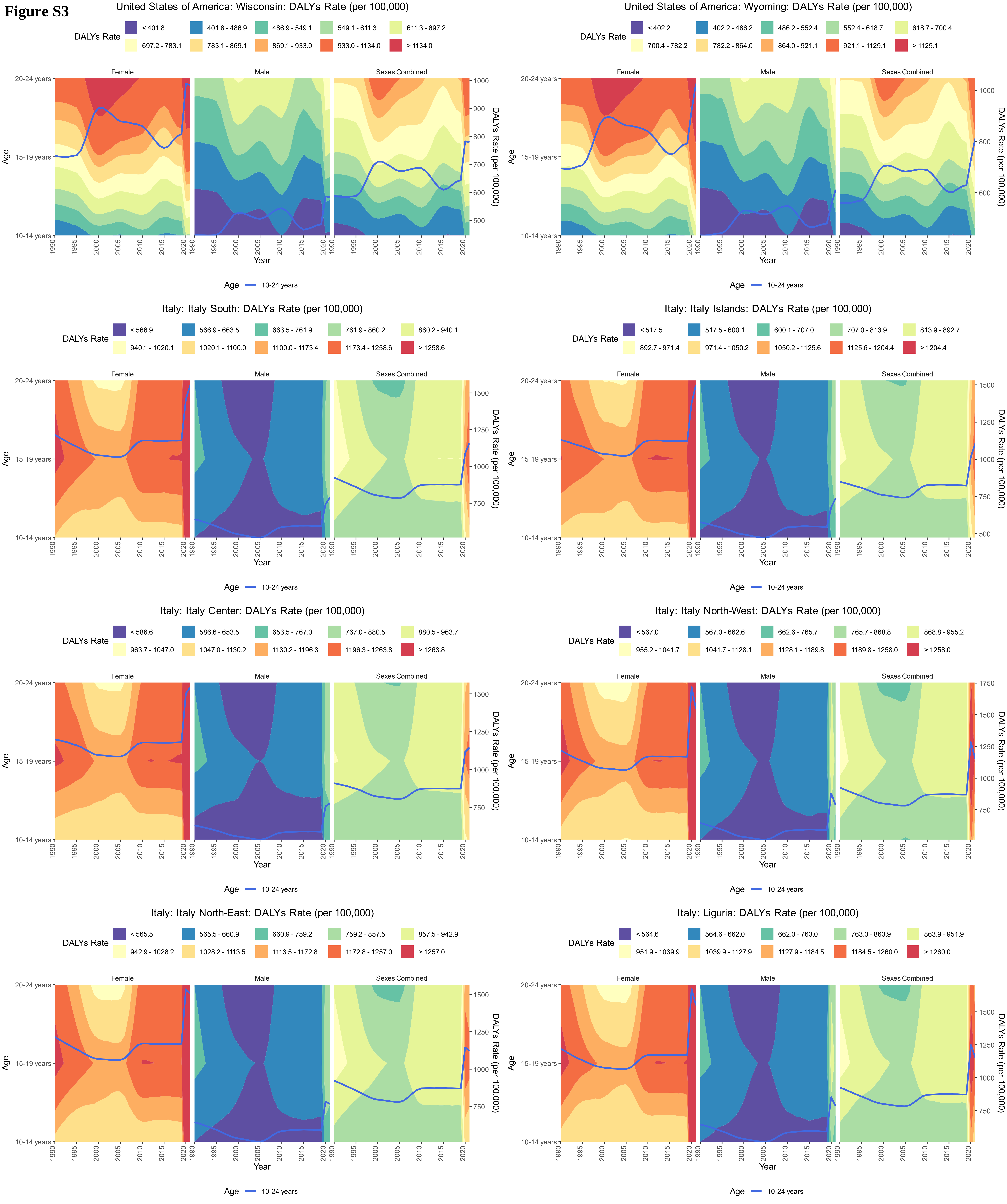
United States of America: Wisconsin: DALYs Rate (per 100,000)
DALYs Rate
< 401.8
401.8 - 486.9
486.9 - 549.1
549.1 - 611.3
611.3 - 697.2
697.2 - 783.1
783.1 - 869.1
869.1 - 933.0
933.0 - 1134.0
> 1134.0
Female
Male
Sexes Combined
Age
Year
DALYs Rate (per 100,000)
Age 10-24 years
United States of America: Wyoming: DALYs Rate (per 100,000)
< 402.2
402.2 - 486.2
486.2 - 552.4
552.4 - 618.7
618.7 - 700.4
700.4 - 782.2
782.2 - 864.0
864.0 - 921.1
921.1 - 1129.1
> 1129.1
Italy: Italy South: DALYs Rate (per 100,000)
< 566.9
566.9 - 663.5
663.5 - 761.9
761.9 - 860.2
860.2 - 940.1
940.1 - 1020.1
1020.1 - 1100.0
1100.0 - 1173.4
1173.4 - 1258.6
> 1258.6
Italy: Italy Islands: DALYs Rate (per 100,000)
< 517.5
517.5 - 600.1
600.1 - 707.0
707.0 - 813.9
813.9 - 892.7
892.7 - 971.4
971.4 - 1050.2
1050.2 - 1125.6
1125.6 - 1204.4
> 1204.4
Italy: Italy Center: DALYs Rate (per 100,000)
< 586.6
586.6 - 653.5
653.5 - 767.0
767.0 - 880.5
880.5 - 963.7
963.7 - 1047.0
1047.0 - 1130.2
1130.2 - 1196.3
1196.3 - 1263.8
> 1263.8
Italy: Italy North-West: DALYs Rate (per 100,000)
< 567.0
567.0 - 662.6
662.6 - 765.7
765.7 - 868.8
868.8 - 955.2
955.2 - 1041.7
1041.7 - 1128.1
1128.1 - 1189.8
1189.8 - 1258.0
> 1258.0
Italy: Italy North-East: DALYs Rate (per 100,000)
< 565.5
565.5 - 660.9
660.9 - 759.2
759.2 - 857.5
857.5 - 942.9
942.9 - 1028.2
1028.2 - 1113.5
1113.5 - 1172.8
1172.8 - 1257.0
> 1257.0
Italy: Liguria: DALYs Rate (per 100,000)
< 564.6
564.6 - 662.0
662.0 - 763.0
763.0 - 863.9
863.9 - 951.9
951.9 - 1039.9
1039.9 - 1127.9
1127.9 - 1184.5
1184.5 - 1260.0
> 1260.0

Figure S3

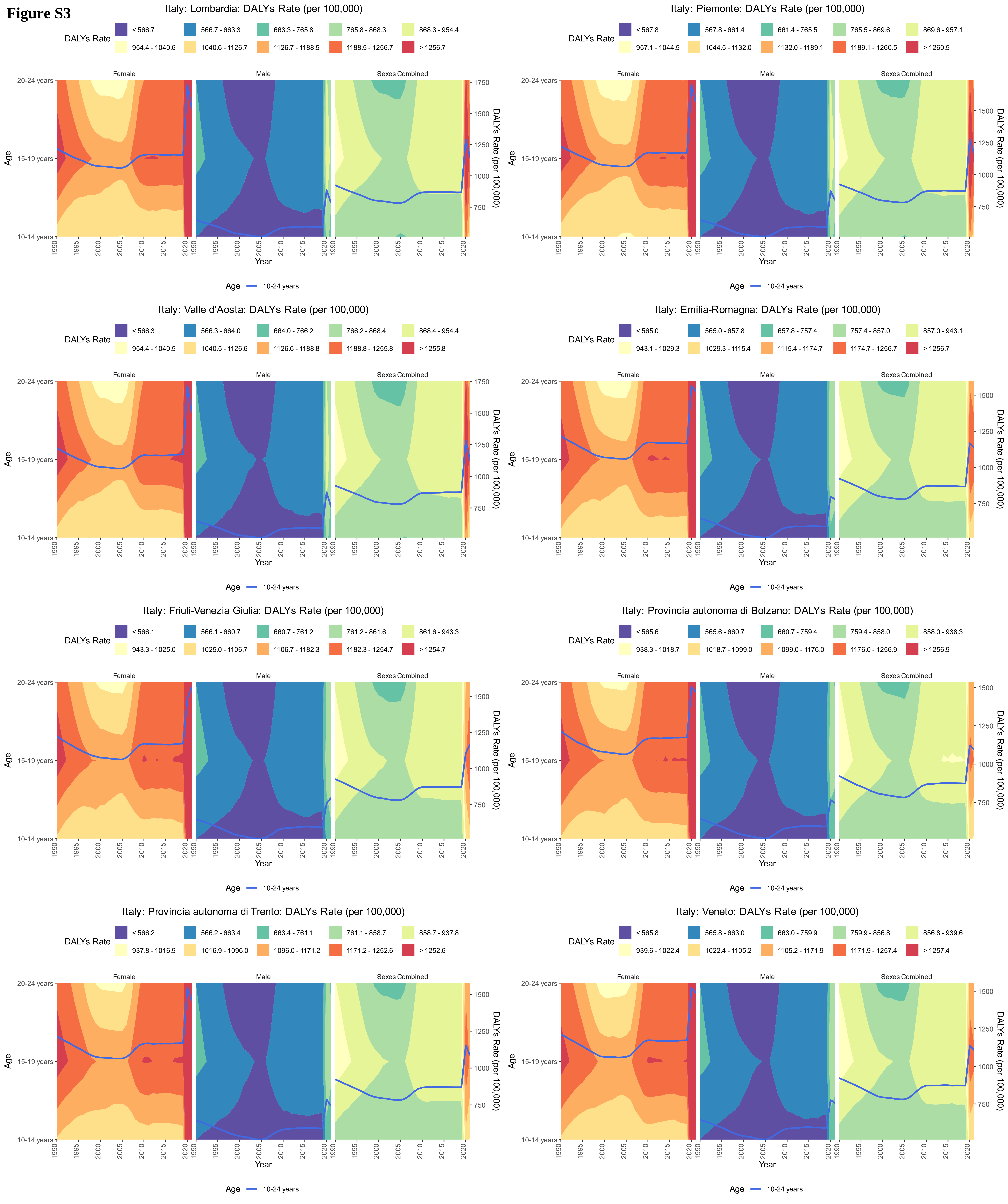
Italy: Lombardia: DALYs Rate (per 100,000)
DALYs Rate
< 566.7
566.7 - 663.3
663.3 - 765.8
765.8 - 868.3
868.3 - 954.4
954.4 - 1040.6
1040.6 - 1126.7
1126.7 - 1188.5
1188.5 - 1256.7
> 1256.7
Italy: Piemonte: DALYs Rate (per 100,000)
< 567.8
567.8 - 661.4
661.4 - 765.5
765.5 - 869.6
869.6 - 957.1
957.1 - 1044.5
1044.5 - 1132.0
1132.0 - 1189.1
1189.1 - 1260.5
> 1260.5
Italy: Valle d'Aosta: DALYs Rate (per 100,000)
< 566.3
566.3 - 664.0
664.0 - 766.2
766.2 - 868.4
868.4 - 954.4
954.4 - 1040.5
1040.5 - 1126.6
1126.6 - 1188.8
1188.8 - 1255.8
> 1255.8
Italy: Emilia-Romagna: DALYs Rate (per 100,000)
< 565.0
565.0 - 657.8
657.8 - 757.4
757.4 - 857.0
857.0 - 943.1
943.1 - 1029.3
1029.3 - 1115.4
1115.4 - 1174.7
1174.7 - 1256.7
> 1256.7
Italy: Friuli-Venezia Giulia: DALYs Rate (per 100,000)
< 566.1
566.1 - 660.7
660.7 - 761.2
761.2 - 861.6
861.6 - 943.3
943.3 - 1025.0
1025.0 - 1106.7
1106.7 - 1182.3
1182.3 - 1254.7
> 1254.7
Italy: Provincia autonoma di Bolzano: DALYs Rate (per 100,000)
< 565.6
565.6 - 660.7
660.7 - 759.4
759.4 - 858.0
858.0 - 938.3
938.3 - 1018.7
1018.7 - 1099.0
1099.0 - 1176.0
1176.0 - 1256.9
> 1256.9
Italy: Provincia autonoma di Trento: DALYs Rate (per 100,000)
< 566.2
566.2 - 663.4
663.4 - 761.1
761.1 - 858.7
858.7 - 937.8
937.8 - 1016.9
1016.9 - 1096.0
1096.0 - 1171.2
1171.2 - 1252.6
> 1252.6
Italy: Veneto: DALYs Rate (per 100,000)
< 565.8
565.8 - 663.0
663.0 - 759.9
759.9 - 856.8
856.8 - 939.6
939.6 - 1022.4
1022.4 - 1105.2
1105.2 - 1171.9
1171.9 - 1257.4
> 1257.4
Female
Male
Sexes Combined
Age
20-24 years
15-19 years
10-14 years
Year
DALYs Rate (per 100,000)
Age
10-24 years

Figure S3

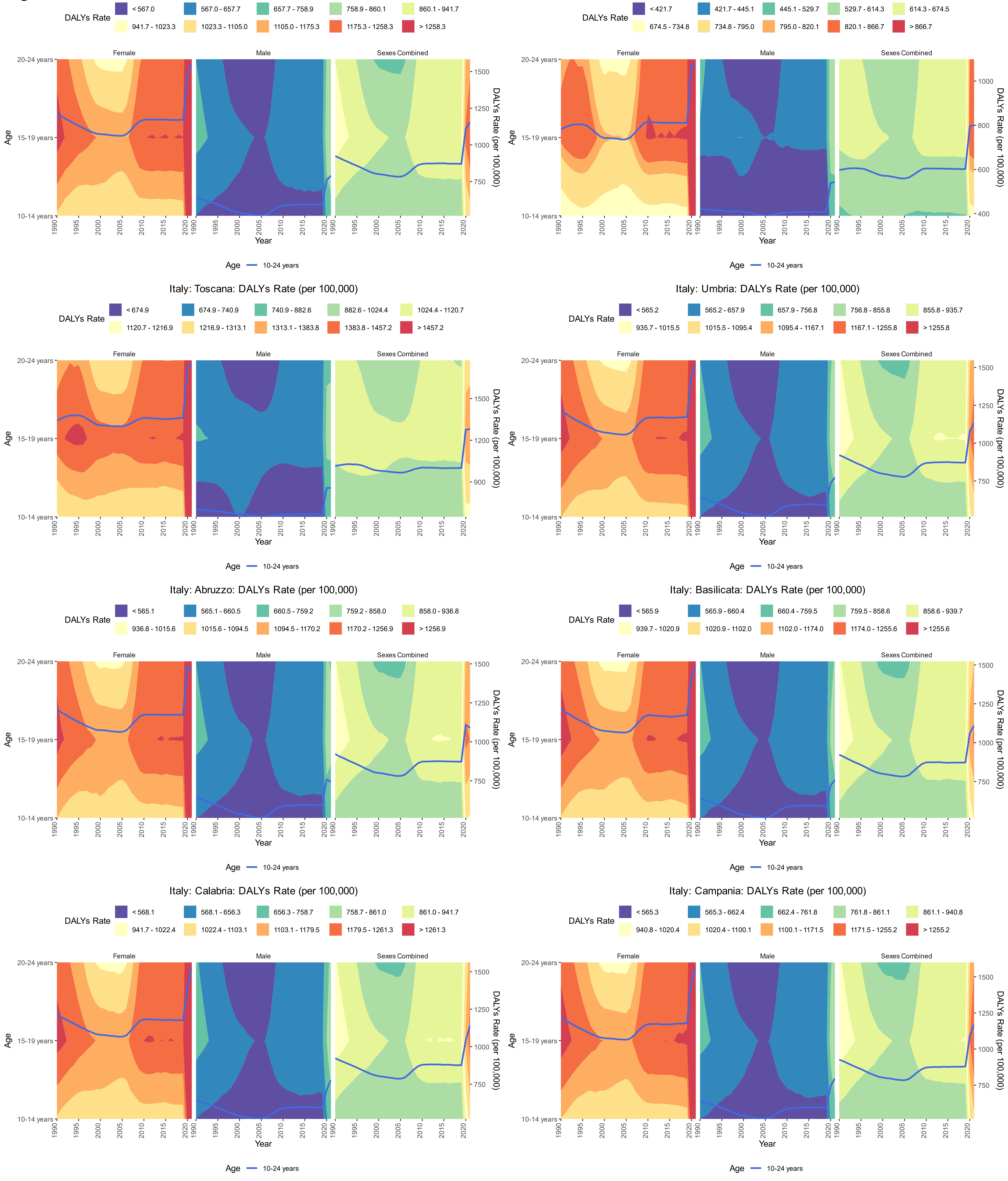

Italy: Lazio: DALYs Rate (per 100,000)
DALYs Rate
< 567.0
567.0 - 657.7
657.7 - 758.9
758.9 - 860.1
860.1 - 941.7
941.7 - 1023.3
1023.3 - 1105.0
1105.0 - 1175.3
1175.3 - 1258.3
> 1258.3
Female
Male
Sexes Combined
Age
Year
DALYs Rate (per 100,000)
Age — 10-24 years
Italy: Marche: DALYs Rate (per 100,000)
< 421.7
421.7 - 445.1
445.1 - 529.7
529.7 - 614.3
614.3 - 674.5
674.5 - 734.8
734.8 - 795.0
795.0 - 820.1
820.1 - 866.7
> 866.7
Italy: Toscana: DALYs Rate (per 100,000)
< 674.9
674.9 - 740.9
740.9 - 882.6
882.6 - 1024.4
1024.4 - 1120.7
1120.7 - 1216.9
1216.9 - 1313.1
1313.1 - 1383.8
1383.8 - 1457.2
> 1457.2
Italy: Umbria: DALYs Rate (per 100,000)
< 565.2
565.2 - 657.9
657.9 - 756.8
756.8 - 855.8
855.8 - 935.7
935.7 - 1015.5
1015.5 - 1095.4
1095.4 - 1167.1
1167.1 - 1255.8
> 1255.8
Italy: Abruzzo: DALYs Rate (per 100,000)
< 565.1
565.1 - 660.5
660.5 - 759.2
759.2 - 858.0
858.0 - 936.8
936.8 - 1015.6
1015.6 - 1094.5
1094.5 - 1170.2
1170.2 - 1256.9
> 1256.9
Italy: Basilicata: DALYs Rate (per 100,000)
< 565.9
565.9 - 660.4
660.4 - 759.5
759.5 - 858.6
858.6 - 939.7
939.7 - 1020.9
1020.9 - 1102.0
1102.0 - 1174.0
1174.0 - 1255.6
> 1255.6
Italy: Calabria: DALYs Rate (per 100,000)
< 568.1
568.1 - 656.3
656.3 - 758.7
758.7 - 861.0
861.0 - 941.7
941.7 - 1022.4
1022.4 - 1103.1
1103.1 - 1179.5
1179.5 - 1261.3
> 1261.3
Italy: Campania: DALYs Rate (per 100,000)
< 565.3
565.3 - 662.4
662.4 - 761.8
761.8 - 861.1
861.1 - 940.8
940.8 - 1020.4
1020.4 - 1100.1
1100.1 - 1171.5
1171.5 - 1255.2
> 1255.2
10-14 years
15-19 years
20-24 years

Figure S3

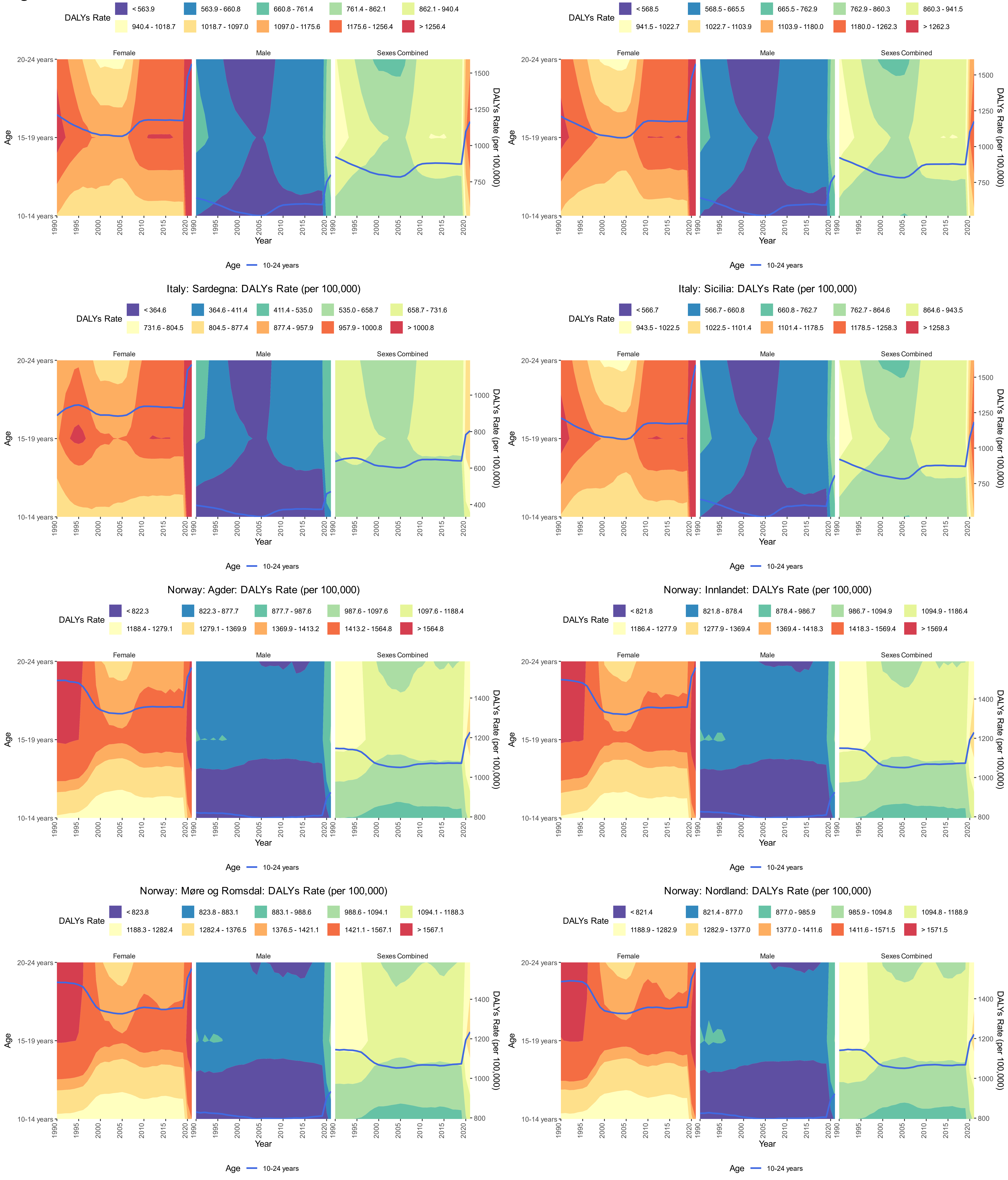

Italy: Molise: DALYs Rate (per 100,000)
DALYs Rate
< 563.9
563.9 - 660.8
660.8 - 761.4
761.4 - 862.1
862.1 - 940.4
940.4 - 1018.7
1018.7 - 1097.0
1097.0 - 1175.6
1175.6 - 1256.4
> 1256.4
Female
Male
Sexes Combined
Age
Year
DALYs Rate (per 100,000)
Age — 10-24 years
Italy: Puglia: DALYs Rate (per 100,000)
DALYs Rate
< 568.5
568.5 - 665.5
665.5 - 762.9
762.9 - 860.3
860.3 - 941.5
941.5 - 1022.7
1022.7 - 1103.9
1103.9 - 1180.0
1180.0 - 1262.3
> 1262.3
Italy: Sardegna: DALYs Rate (per 100,000)
DALYs Rate
< 364.6
364.6 - 411.4
411.4 - 535.0
535.0 - 658.7
658.7 - 731.6
731.6 - 804.5
804.5 - 877.4
877.4 - 957.9
957.9 - 1000.8
> 1000.8
Italy: Sicilia: DALYs Rate (per 100,000)
DALYs Rate
< 566.7
566.7 - 660.8
660.8 - 762.7
762.7 - 864.6
864.6 - 943.5
943.5 - 1022.5
1022.5 - 1101.4
1101.4 - 1178.5
1178.5 - 1258.3
> 1258.3
Norway: Agder: DALYs Rate (per 100,000)
DALYs Rate
< 822.3
822.3 - 877.7
877.7 - 987.6
987.6 - 1097.6
1097.6 - 1188.4
1188.4 - 1279.1
1279.1 - 1369.9
1369.9 - 1413.2
1413.2 - 1564.8
> 1564.8
Norway: Innlandet: DALYs Rate (per 100,000)
DALYs Rate
< 821.8
821.8 - 878.4
878.4 - 986.7
986.7 - 1094.9
1094.9 - 1186.4
1186.4 - 1277.9
1277.9 - 1369.4
1369.4 - 1418.3
1418.3 - 1569.4
> 1569.4
Norway: Møre og Romsdal: DALYs Rate (per 100,000)
DALYs Rate
< 823.8
823.8 - 883.1
883.1 - 988.6
988.6 - 1094.1
1094.1 - 1188.3
1188.3 - 1282.4
1282.4 - 1376.5
1376.5 - 1421.1
1421.1 - 1567.1
> 1567.1
Norway: Nordland: DALYs Rate (per 100,000)
DALYs Rate
< 821.4
821.4 - 877.0
877.0 - 985.9
985.9 - 1094.8
1094.8 - 1188.9
1188.9 - 1282.9
1282.9 - 1377.0
1377.0 - 1411.6
1411.6 - 1571.5
> 1571.5
20-24 years
15-19 years
10-14 years

Figure S3

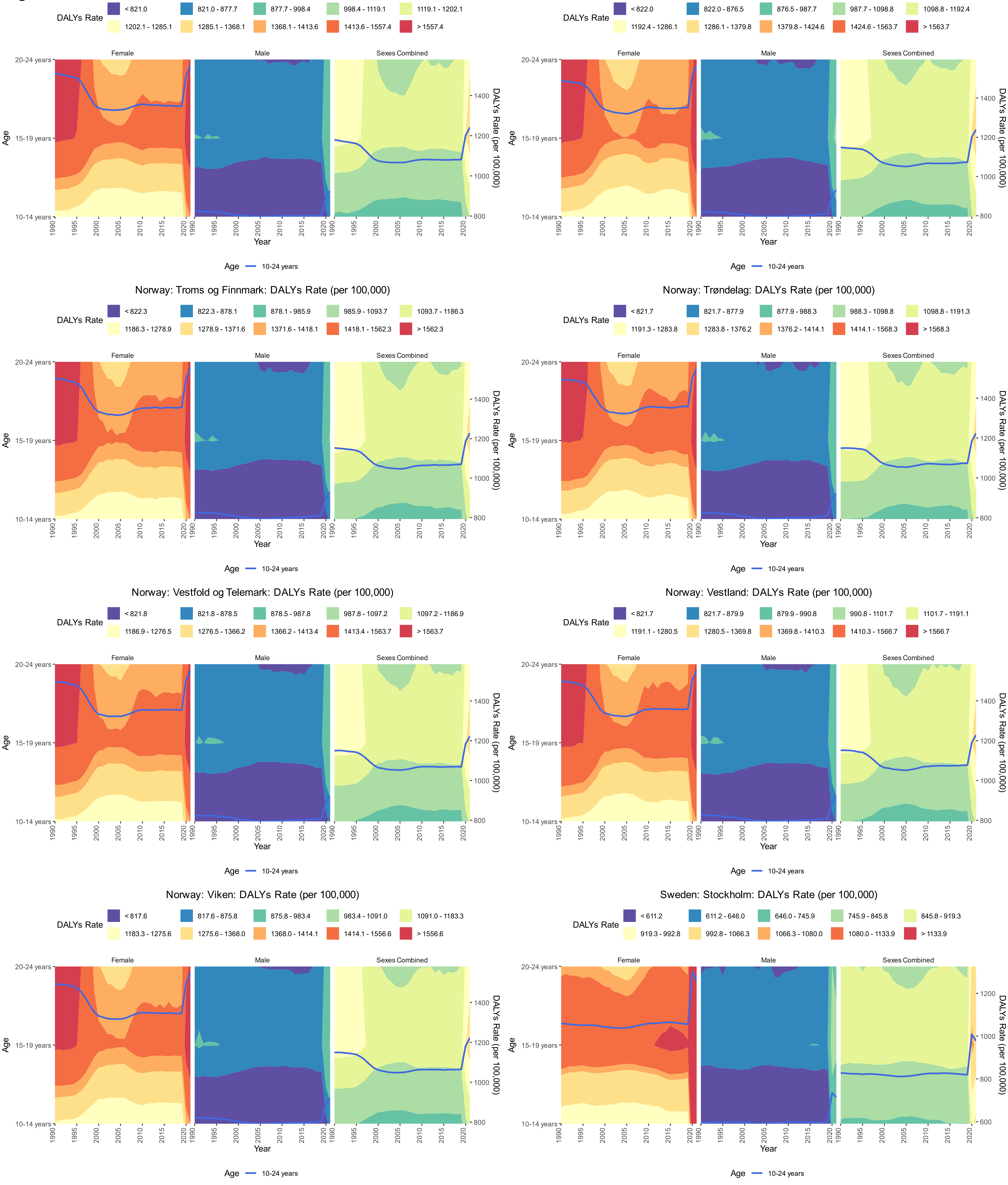
Norway: Oslo: DALYs Rate (per 100,000)
DALYs Rate
< 821.0
821.0 - 877.7
877.7 - 998.4
998.4 - 1119.1
1119.1 - 1202.1
1202.1 - 1285.1
1285.1 - 1368.1
1368.1 - 1413.6
1413.6 - 1557.4
> 1557.4
Norway: Rogaland: DALYs Rate (per 100,000)
< 822.0
822.0 - 876.5
876.5 - 987.7
987.7 - 1098.8
1098.8 - 1192.4
1192.4 - 1286.1
1286.1 - 1379.8
1379.8 - 1424.6
1424.6 - 1563.7
> 1563.7
Norway: Troms og Finnmark: DALYs Rate (per 100,000)
< 822.3
822.3 - 878.1
878.1 - 985.9
985.9 - 1093.7
1093.7 - 1186.3
1186.3 - 1278.9
1278.9 - 1371.6
1371.6 - 1418.1
1418.1 - 1562.3
> 1562.3
Norway: Trøndelag: DALYs Rate (per 100,000)
< 821.7
821.7 - 877.9
877.9 - 988.3
988.3 - 1098.8
1098.8 - 1191.3
1191.3 - 1283.8
1283.8 - 1376.2
1376.2 - 1414.1
1414.1 - 1568.3
> 1568.3
Norway: Vestfold og Telemark: DALYs Rate (per 100,000)
< 821.8
821.8 - 878.5
878.5 - 987.8
987.8 - 1097.2
1097.2 - 1186.9
1186.9 - 1276.5
1276.5 - 1366.2
1366.2 - 1413.4
1413.4 - 1563.7
> 1563.7
Norway: Vestland: DALYs Rate (per 100,000)
< 821.7
821.7 - 879.9
879.9 - 990.8
990.8 - 1101.7
1101.7 - 1191.1
1191.1 - 1280.5
1280.5 - 1369.8
1369.8 - 1410.3
1410.3 - 1566.7
> 1566.7
Norway: Viken: DALYs Rate (per 100,000)
< 817.6
817.6 - 875.8
875.8 - 983.4
983.4 - 1091.0
1091.0 - 1183.3
1183.3 - 1275.6
1275.6 - 1368.0
1368.0 - 1414.1
1414.1 - 1556.6
> 1556.6
Sweden: Stockholm: DALYs Rate (per 100,000)
< 611.2
611.2 - 646.0
646.0 - 745.9
745.9 - 845.8
845.8 - 919.3
919.3 - 992.8
992.8 - 1066.3
1066.3 - 1080.0
1080.0 - 1133.9
> 1133.9
Female
Male
Sexes Combined
Age
20-24 years
15-19 years
10-14 years
Year
DALYs Rate (per 100,000)
Age — 10-24 years

Figure S3

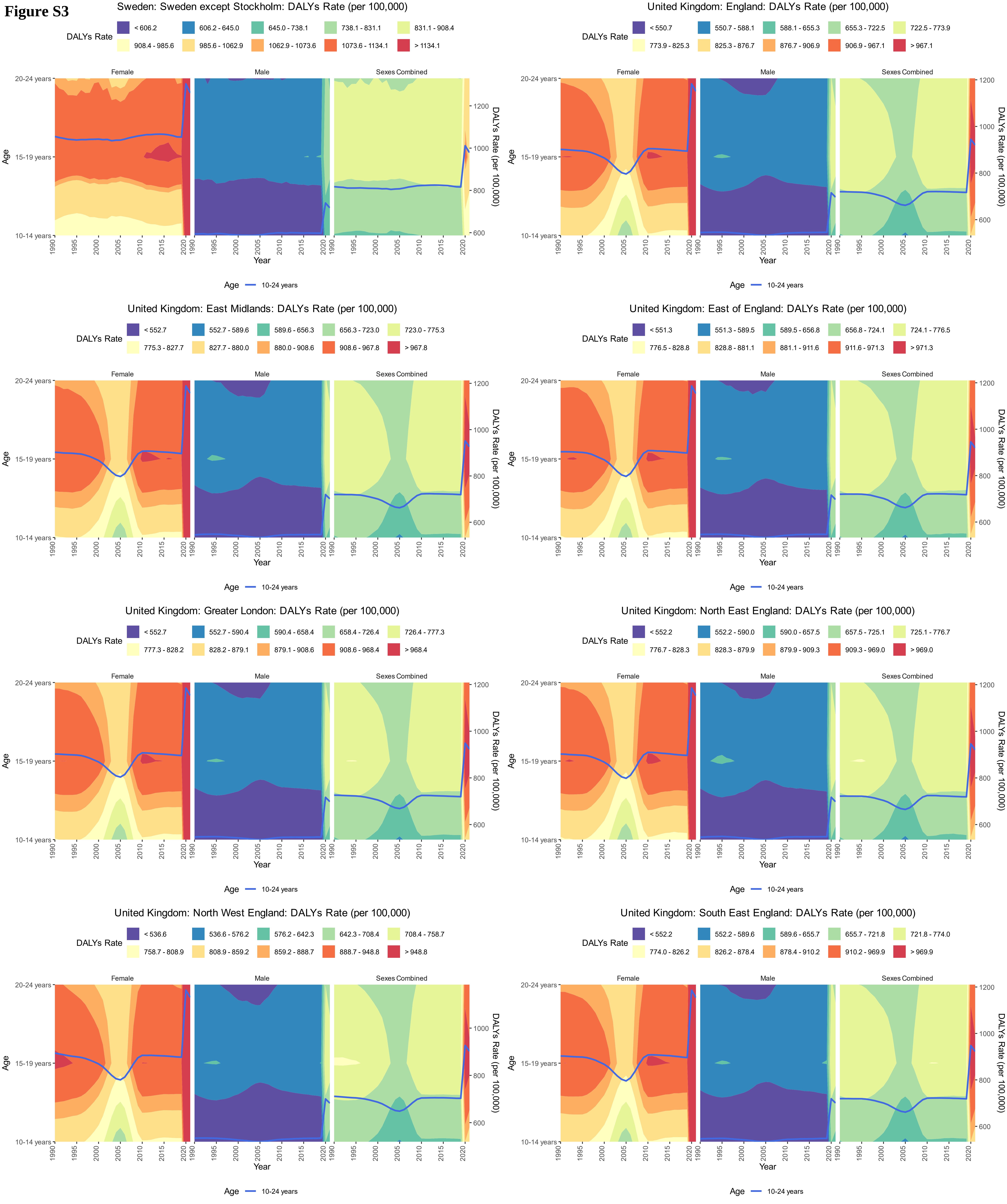
Sweden: Sweden except Stockholm: DALYs Rate (per 100,000)
DALYs Rate
< 606.2
606.2 - 645.0
645.0 - 738.1
738.1 - 831.1
831.1 - 908.4
908.4 - 985.6
985.6 - 1062.9
1062.9 - 1073.6
1073.6 - 1134.1
> 1134.1
United Kingdom: England: DALYs Rate (per 100,000)
DALYs Rate
< 550.7
550.7 - 588.1
588.1 - 655.3
655.3 - 722.5
722.5 - 773.9
773.9 - 825.3
825.3 - 876.7
876.7 - 906.9
906.9 - 967.1
> 967.1
United Kingdom: East Midlands: DALYs Rate (per 100,000)
DALYs Rate
< 552.7
552.7 - 589.6
589.6 - 656.3
656.3 - 723.0
723.0 - 775.3
775.3 - 827.7
827.7 - 880.0
880.0 - 908.6
908.6 - 967.8
> 967.8
United Kingdom: East of England: DALYs Rate (per 100,000)
DALYs Rate
< 551.3
551.3 - 589.5
589.5 - 656.8
656.8 - 724.1
724.1 - 776.5
776.5 - 828.8
828.8 - 881.1
881.1 - 911.6
911.6 - 971.3
> 971.3
United Kingdom: Greater London: DALYs Rate (per 100,000)
DALYs Rate
< 552.7
552.7 - 590.4
590.4 - 658.4
658.4 - 726.4
726.4 - 777.3
777.3 - 828.2
828.2 - 879.1
879.1 - 908.6
908.6 - 968.4
> 968.4
United Kingdom: North East England: DALYs Rate (per 100,000)
DALYs Rate
< 552.2
552.2 - 590.0
590.0 - 657.5
657.5 - 725.1
725.1 - 776.7
776.7 - 828.3
828.3 - 879.9
879.9 - 909.3
909.3 - 969.0
> 969.0
United Kingdom: North West England: DALYs Rate (per 100,000)
DALYs Rate
< 536.6
536.6 - 576.2
576.2 - 642.3
642.3 - 708.4
708.4 - 758.7
758.7 - 808.9
808.9 - 859.2
859.2 - 888.7
888.7 - 948.8
> 948.8
United Kingdom: South East England: DALYs Rate (per 100,000)
DALYs Rate
< 552.2
552.2 - 589.6
589.6 - 655.7
655.7 - 721.8
721.8 - 774.0
774.0 - 826.2
826.2 - 878.4
878.4 - 910.2
910.2 - 969.9
> 969.9
Female
Male
Sexes Combined
Age
20-24 years
15-19 years
10-14 years
Year
DALYs Rate (per 100,000)
Age — 10-24 years

Figure S3

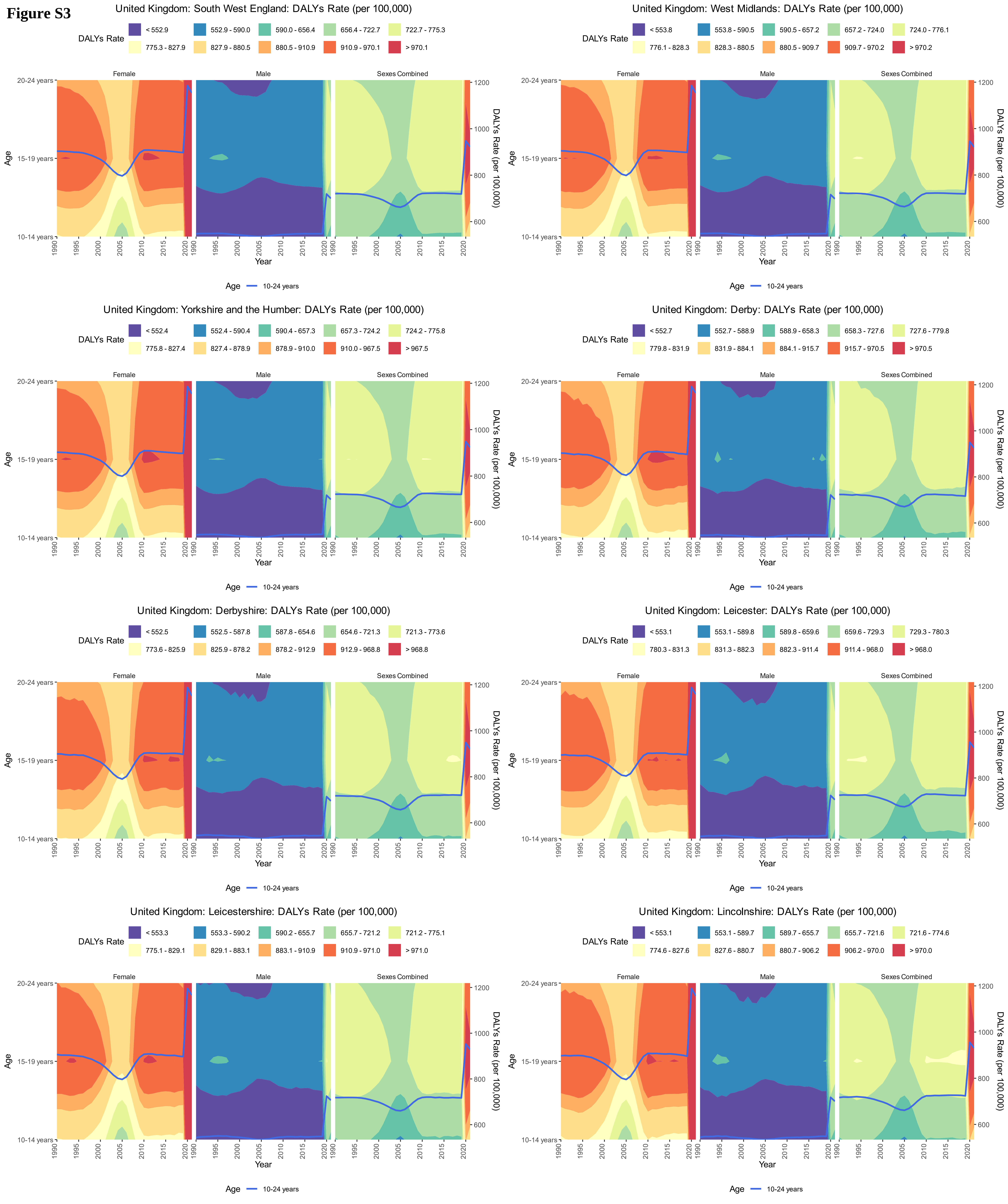
United Kingdom: South West England: DALYs Rate (per 100,000)
DALYs Rate
< 552.9
552.9 - 590.0
590.0 - 656.4
656.4 - 722.7
722.7 - 775.3
775.3 - 827.9
827.9 - 880.5
880.5 - 910.9
910.9 - 970.1
> 970.1
United Kingdom: West Midlands: DALYs Rate (per 100,000)
DALYs Rate
< 553.8
553.8 - 590.5
590.5 - 657.2
657.2 - 724.0
724.0 - 776.1
776.1 - 828.3
828.3 - 880.5
880.5 - 909.7
909.7 - 970.2
> 970.2
United Kingdom: Yorkshire and the Humber: DALYs Rate (per 100,000)
DALYs Rate
< 552.4
552.4 - 590.4
590.4 - 657.3
657.3 - 724.2
724.2 - 775.8
775.8 - 827.4
827.4 - 878.9
878.9 - 910.0
910.0 - 967.5
> 967.5
United Kingdom: Derby: DALYs Rate (per 100,000)
DALYs Rate
< 552.7
552.7 - 588.9
588.9 - 658.3
658.3 - 727.6
727.6 - 779.8
779.8 - 831.9
831.9 - 884.1
884.1 - 915.7
915.7 - 970.5
> 970.5
United Kingdom: Derbyshire: DALYs Rate (per 100,000)
DALYs Rate
< 552.5
552.5 - 587.8
587.8 - 654.6
654.6 - 721.3
721.3 - 773.6
773.6 - 825.9
825.9 - 878.2
878.2 - 912.9
912.9 - 968.8
> 968.8
United Kingdom: Leicester: DALYs Rate (per 100,000)
DALYs Rate
< 553.1
553.1 - 589.8
589.8 - 659.6
659.6 - 729.3
729.3 - 780.3
780.3 - 831.3
831.3 - 882.3
882.3 - 911.4
911.4 - 968.0
> 968.0
United Kingdom: Leicestershire: DALYs Rate (per 100,000)
DALYs Rate
< 553.3
553.3 - 590.2
590.2 - 655.7
655.7 - 721.2
721.2 - 775.1
775.1 - 829.1
829.1 - 883.1
883.1 - 910.9
910.9 - 971.0
> 971.0
United Kingdom: Lincolnshire: DALYs Rate (per 100,000)
DALYs Rate
< 553.1
553.1 - 589.7
589.7 - 655.7
655.7 - 721.6
721.6 - 774.6
774.6 - 827.6
827.6 - 880.7
880.7 - 906.2
906.2 - 970.0
> 970.0
Female
Male
Sexes Combined
Age
20-24 years
15-19 years
10-14 years
Year
1990
1995
2000
2005
2010
2015
2020
DALYs Rate (per 100,000)
600
800
1000
1200
Age
10-24 years

Figure S3

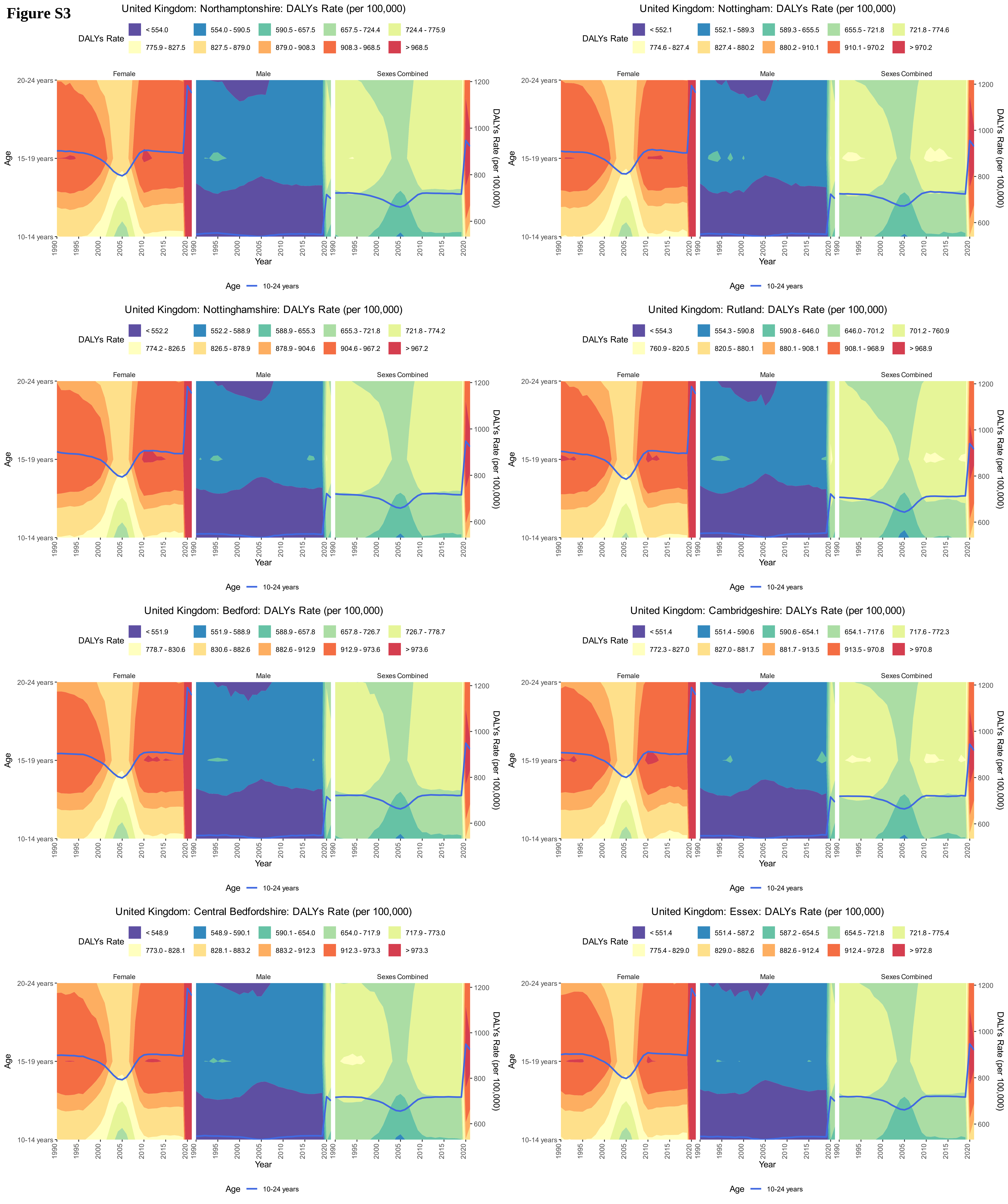
United Kingdom: Northamptonshire: DALYs Rate (per 100,000)
DALYs Rate
< 554.0
554.0 - 590.5
590.5 - 657.5
657.5 - 724.4
724.4 - 775.9
775.9 - 827.5
827.5 - 879.0
879.0 - 908.3
908.3 - 968.5
> 968.5
United Kingdom: Nottingham: DALYs Rate (per 100,000)
DALYs Rate
< 552.1
552.1 - 589.3
589.3 - 655.5
655.5 - 721.8
721.8 - 774.6
774.6 - 827.4
827.4 - 880.2
880.2 - 910.1
910.1 - 970.2
> 970.2
United Kingdom: Nottinghamshire: DALYs Rate (per 100,000)
DALYs Rate
< 552.2
552.2 - 588.9
588.9 - 655.3
655.3 - 721.8
721.8 - 774.2
774.2 - 826.5
826.5 - 878.9
878.9 - 904.6
904.6 - 967.2
> 967.2
United Kingdom: Rutland: DALYs Rate (per 100,000)
DALYs Rate
< 554.3
554.3 - 590.8
590.8 - 646.0
646.0 - 701.2
701.2 - 760.9
760.9 - 820.5
820.5 - 880.1
880.1 - 908.1
908.1 - 968.9
> 968.9
United Kingdom: Bedford: DALYs Rate (per 100,000)
DALYs Rate
< 551.9
551.9 - 588.9
588.9 - 657.8
657.8 - 726.7
726.7 - 778.7
778.7 - 830.6
830.6 - 882.6
882.6 - 912.9
912.9 - 973.6
> 973.6
United Kingdom: Cambridgeshire: DALYs Rate (per 100,000)
DALYs Rate
< 551.4
551.4 - 590.6
590.6 - 654.1
654.1 - 717.6
717.6 - 772.3
772.3 - 827.0
827.0 - 881.7
881.7 - 913.5
913.5 - 970.8
> 970.8
United Kingdom: Central Bedfordshire: DALYs Rate (per 100,000)
DALYs Rate
< 548.9
548.9 - 590.1
590.1 - 654.0
654.0 - 717.9
717.9 - 773.0
773.0 - 828.1
828.1 - 883.2
883.2 - 912.3
912.3 - 973.3
> 973.3
United Kingdom: Essex: DALYs Rate (per 100,000)
DALYs Rate
< 551.4
551.4 - 587.2
587.2 - 654.5
654.5 - 721.8
721.8 - 775.4
775.4 - 829.0
829.0 - 882.6
882.6 - 912.4
912.4 - 972.8
> 972.8
Female
Male
Sexes Combined
Age
20-24 years
15-19 years
10-14 years
Year
1990
1995
2000
2005
2010
2015
2020
DALYs Rate (per 100,000)
600
800
1000
1200
Age
10-24 years

Figure S3

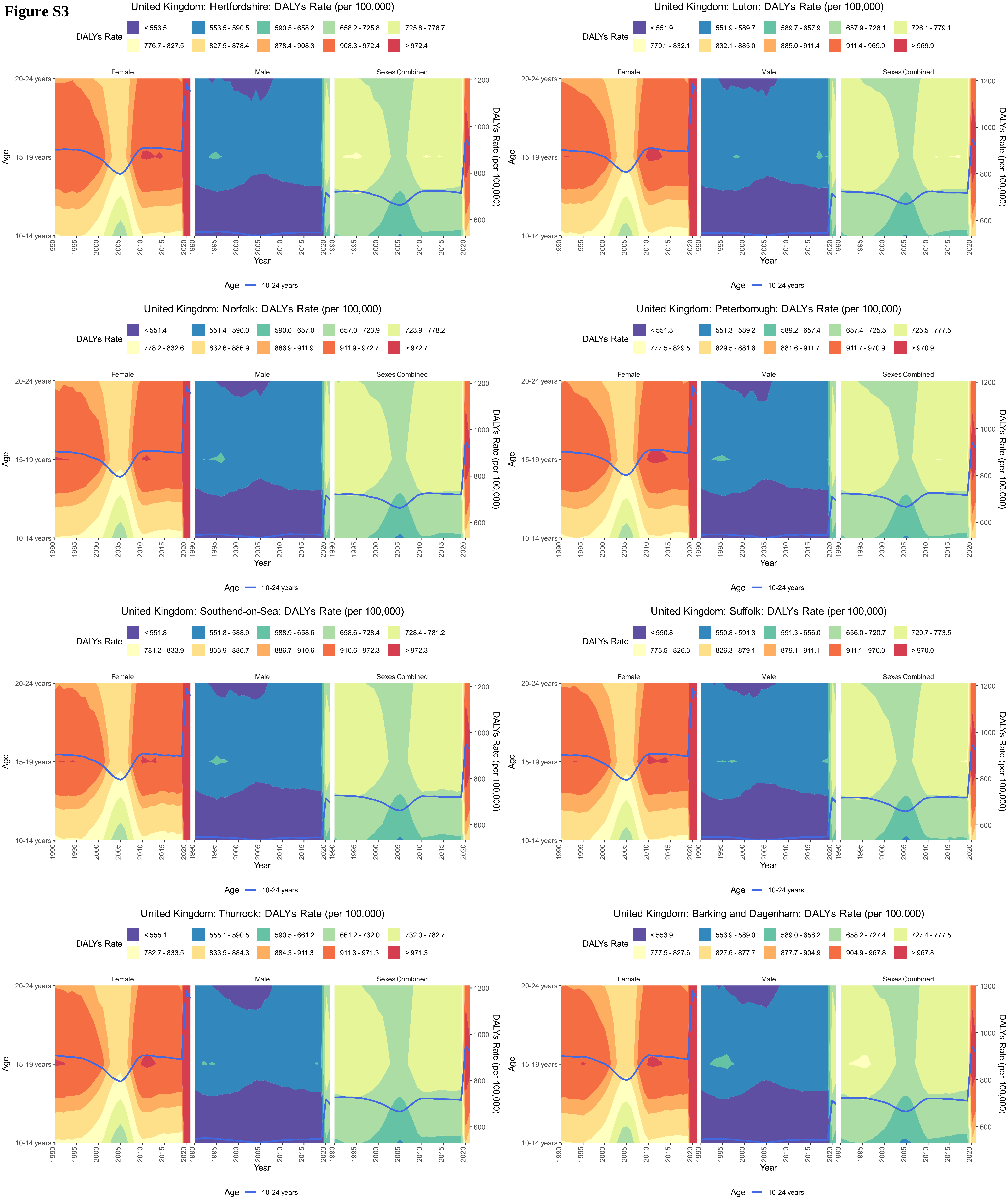
United Kingdom: Hertfordshire: DALYs Rate (per 100,000)
DALYs Rate
< 553.5
553.5 - 590.5
590.5 - 658.2
658.2 - 725.8
725.8 - 776.7
776.7 - 827.5
827.5 - 878.4
878.4 - 908.3
908.3 - 972.4
> 972.4
United Kingdom: Luton: DALYs Rate (per 100,000)
DALYs Rate
< 551.9
551.9 - 589.7
589.7 - 657.9
657.9 - 726.1
726.1 - 779.1
779.1 - 832.1
832.1 - 885.0
885.0 - 911.4
911.4 - 969.9
> 969.9
United Kingdom: Norfolk: DALYs Rate (per 100,000)
DALYs Rate
< 551.4
551.4 - 590.0
590.0 - 657.0
657.0 - 723.9
723.9 - 778.2
778.2 - 832.6
832.6 - 886.9
886.9 - 911.9
911.9 - 972.7
> 972.7
United Kingdom: Peterborough: DALYs Rate (per 100,000)
DALYs Rate
< 551.3
551.3 - 589.2
589.2 - 657.4
657.4 - 725.5
725.5 - 777.5
777.5 - 829.5
829.5 - 881.6
881.6 - 911.7
911.7 - 970.9
> 970.9
United Kingdom: Southend-on-Sea: DALYs Rate (per 100,000)
DALYs Rate
< 551.8
551.8 - 588.9
588.9 - 658.6
658.6 - 728.4
728.4 - 781.2
781.2 - 833.9
833.9 - 886.7
886.7 - 910.6
910.6 - 972.3
> 972.3
United Kingdom: Suffolk: DALYs Rate (per 100,000)
DALYs Rate
< 550.8
550.8 - 591.3
591.3 - 656.0
656.0 - 720.7
720.7 - 773.5
773.5 - 826.3
826.3 - 879.1
879.1 - 911.1
911.1 - 970.0
> 970.0
United Kingdom: Thurrock: DALYs Rate (per 100,000)
DALYs Rate
< 555.1
555.1 - 590.5
590.5 - 661.2
661.2 - 732.0
732.0 - 782.7
782.7 - 833.5
833.5 - 884.3
884.3 - 911.3
911.3 - 971.3
> 971.3
United Kingdom: Barking and Dagenham: DALYs Rate (per 100,000)
DALYs Rate
< 553.9
553.9 - 589.0
589.0 - 658.2
658.2 - 727.4
727.4 - 777.5
777.5 - 827.6
827.6 - 877.7
877.7 - 904.9
904.9 - 967.8
> 967.8
Female
Male
Sexes Combined
20-24 years
15-19 years
10-14 years
Age
Year
DALYs Rate (per 100,000)
Age — 10-24 years

Figure S3

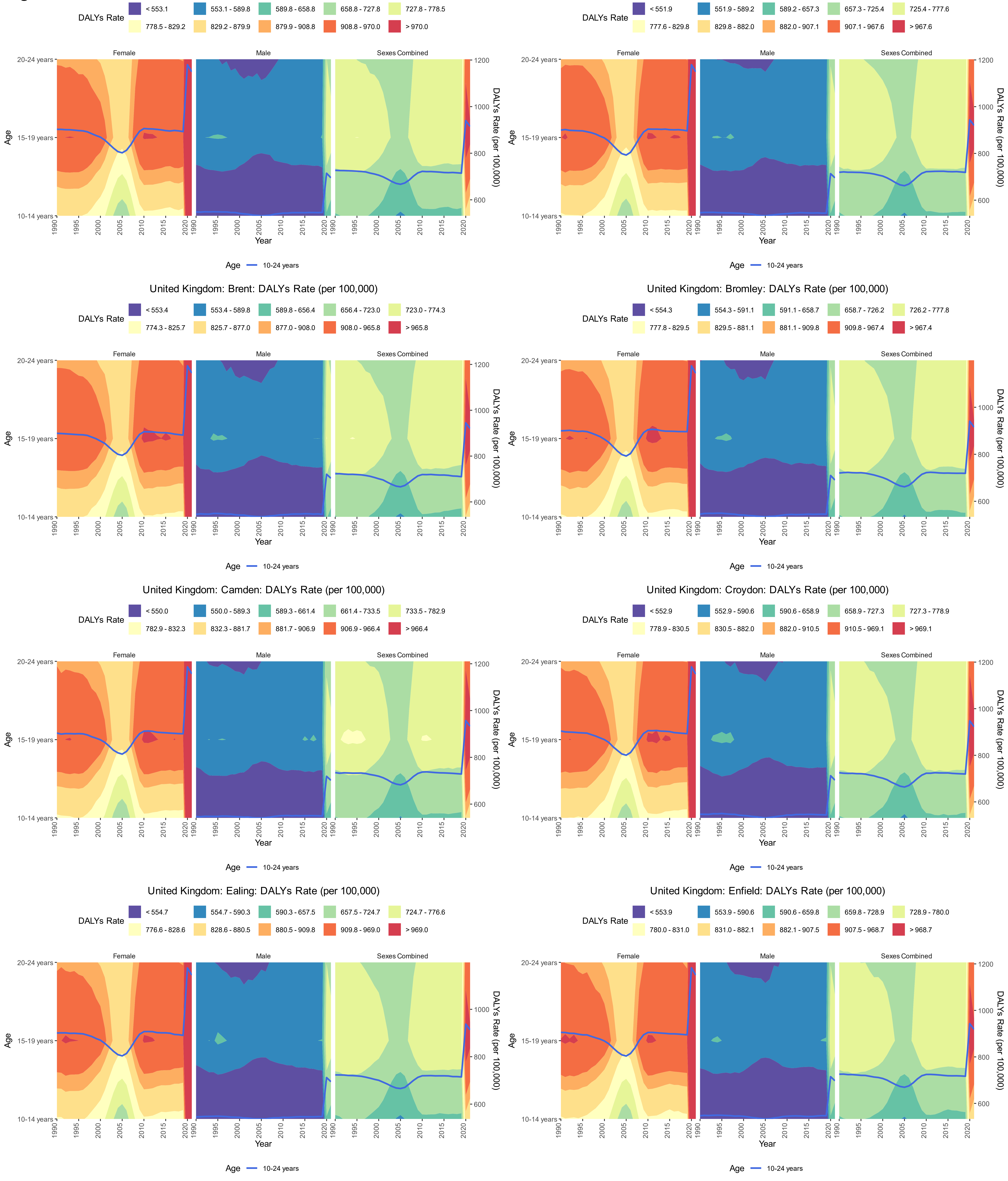

United Kingdom: Barnet: DALYs Rate (per 100,000)
DALYs Rate
< 553.1
553.1 - 589.8
589.8 - 658.8
658.8 - 727.8
727.8 - 778.5
778.5 - 829.2
829.2 - 879.9
879.9 - 908.8
908.8 - 970.0
> 970.0
United Kingdom: Bexley: DALYs Rate (per 100,000)
< 551.9
551.9 - 589.2
589.2 - 657.3
657.3 - 725.4
725.4 - 777.6
777.6 - 829.8
829.8 - 882.0
882.0 - 907.1
907.1 - 967.6
> 967.6
United Kingdom: Brent: DALYs Rate (per 100,000)
< 553.4
553.4 - 589.8
589.8 - 656.4
656.4 - 723.0
723.0 - 774.3
774.3 - 825.7
825.7 - 877.0
877.0 - 908.0
908.0 - 965.8
> 965.8
United Kingdom: Bromley: DALYs Rate (per 100,000)
< 554.3
554.3 - 591.1
591.1 - 658.7
658.7 - 726.2
726.2 - 777.8
777.8 - 829.5
829.5 - 881.1
881.1 - 909.8
909.8 - 967.4
> 967.4
United Kingdom: Camden: DALYs Rate (per 100,000)
< 550.0
550.0 - 589.3
589.3 - 661.4
661.4 - 733.5
733.5 - 782.9
782.9 - 832.3
832.3 - 881.7
881.7 - 906.9
906.9 - 966.4
> 966.4
United Kingdom: Croydon: DALYs Rate (per 100,000)
< 552.9
552.9 - 590.6
590.6 - 658.9
658.9 - 727.3
727.3 - 778.9
778.9 - 830.5
830.5 - 882.0
882.0 - 910.5
910.5 - 969.1
> 969.1
United Kingdom: Ealing: DALYs Rate (per 100,000)
< 554.7
554.7 - 590.3
590.3 - 657.5
657.5 - 724.7
724.7 - 776.6
776.6 - 828.6
828.6 - 880.5
880.5 - 909.8
909.8 - 969.0
> 969.0
United Kingdom: Enfield: DALYs Rate (per 100,000)
< 553.9
553.9 - 590.6
590.6 - 659.8
659.8 - 728.9
728.9 - 780.0
780.0 - 831.0
831.0 - 882.1
882.1 - 907.5
907.5 - 968.7
> 968.7
Female
Male
Sexes Combined
Age
20-24 years
15-19 years
10-14 years
Year
DALYs Rate (per 100,000)
Age — 10-24 years

Figure S3

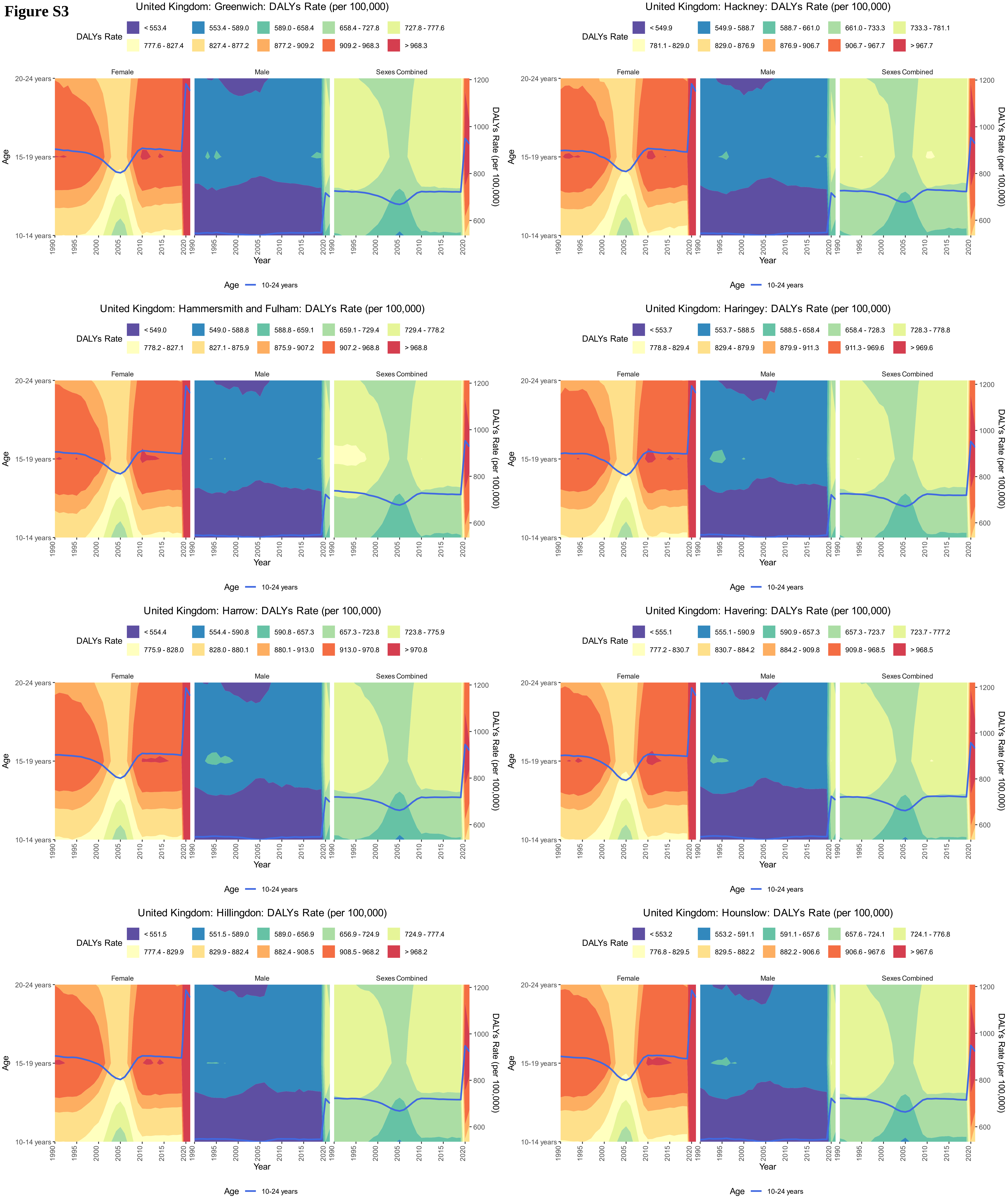
United Kingdom: Greenwich: DALYs Rate (per 100,000)
DALYs Rate
< 553.4
553.4 - 589.0
589.0 - 658.4
658.4 - 727.8
727.8 - 777.6
777.6 - 827.4
827.4 - 877.2
877.2 - 909.2
909.2 - 968.3
> 968.3
United Kingdom: Hackney: DALYs Rate (per 100,000)
DALYs Rate
< 549.9
549.9 - 588.7
588.7 - 661.0
661.0 - 733.3
733.3 - 781.1
781.1 - 829.0
829.0 - 876.9
876.9 - 906.7
906.7 - 967.7
> 967.7
United Kingdom: Hammersmith and Fulham: DALYs Rate (per 100,000)
DALYs Rate
< 549.0
549.0 - 588.8
588.8 - 659.1
659.1 - 729.4
729.4 - 778.2
778.2 - 827.1
827.1 - 875.9
875.9 - 907.2
907.2 - 968.8
> 968.8
United Kingdom: Haringey: DALYs Rate (per 100,000)
DALYs Rate
< 553.7
553.7 - 588.5
588.5 - 658.4
658.4 - 728.3
728.3 - 778.8
778.8 - 829.4
829.4 - 879.9
879.9 - 911.3
911.3 - 969.6
> 969.6
United Kingdom: Harrow: DALYs Rate (per 100,000)
DALYs Rate
< 554.4
554.4 - 590.8
590.8 - 657.3
657.3 - 723.8
723.8 - 775.9
775.9 - 828.0
828.0 - 880.1
880.1 - 913.0
913.0 - 970.8
> 970.8
United Kingdom: Havering: DALYs Rate (per 100,000)
DALYs Rate
< 555.1
555.1 - 590.9
590.9 - 657.3
657.3 - 723.7
723.7 - 777.2
777.2 - 830.7
830.7 - 884.2
884.2 - 909.8
909.8 - 968.5
> 968.5
United Kingdom: Hillingdon: DALYs Rate (per 100,000)
DALYs Rate
< 551.5
551.5 - 589.0
589.0 - 656.9
656.9 - 724.9
724.9 - 777.4
777.4 - 829.9
829.9 - 882.4
882.4 - 908.5
908.5 - 968.2
> 968.2
United Kingdom: Hounslow: DALYs Rate (per 100,000)
DALYs Rate
< 553.2
553.2 - 591.1
591.1 - 657.6
657.6 - 724.1
724.1 - 776.8
776.8 - 829.5
829.5 - 882.2
882.2 - 906.6
906.6 - 967.6
> 967.6
Female
Male
Sexes Combined
Age
20-24 years
15-19 years
10-14 years
Year
1990
1995
2000
2005
2010
2015
2020
DALYs Rate (per 100,000)
600
800
1000
1200
Age
10-24 years

Figure S3

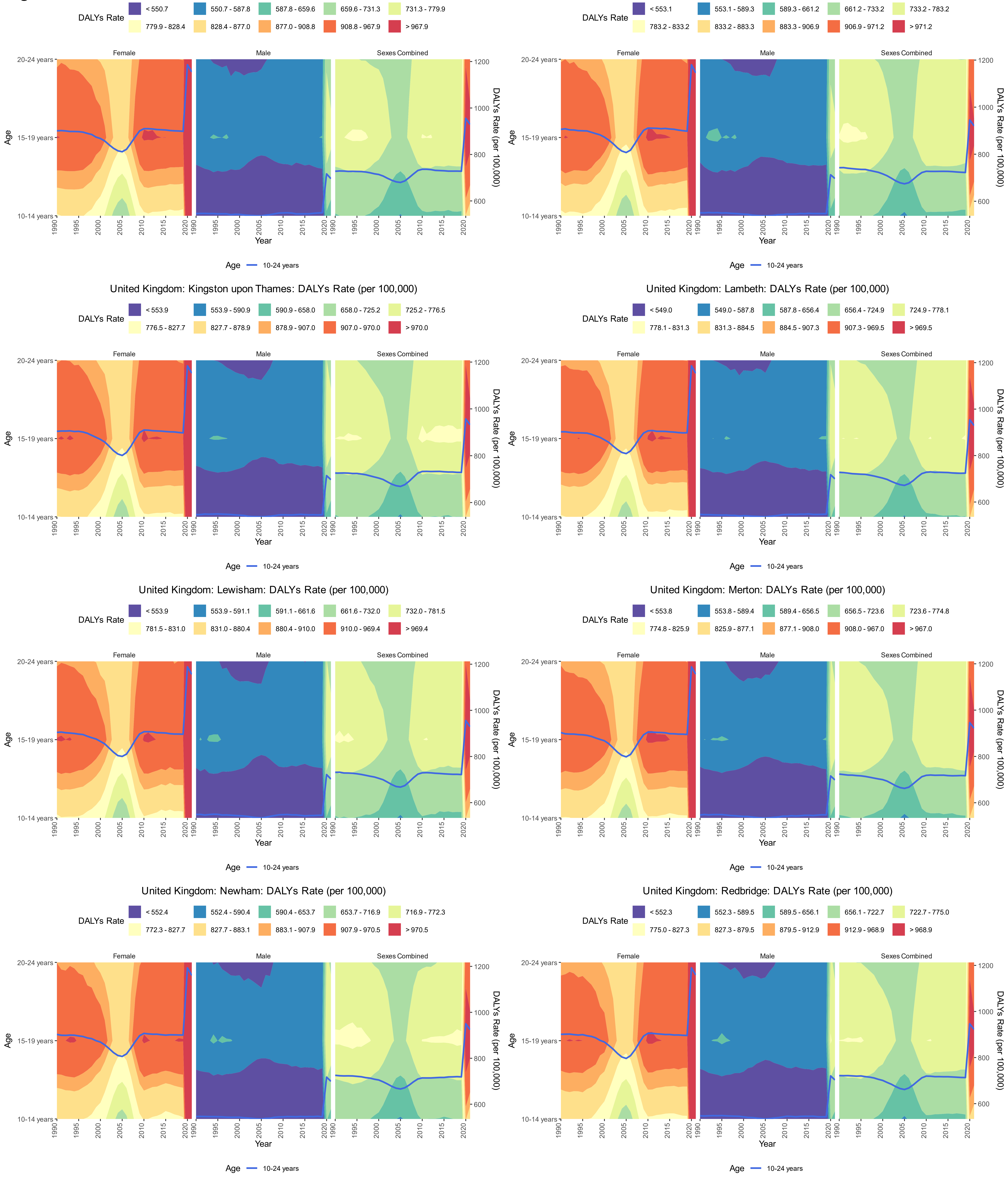
United Kingdom: Islington: DALYs Rate (per 100,000)
DALYs Rate
< 550.7
550.7 - 587.8
587.8 - 659.6
659.6 - 731.3
731.3 - 779.9
779.9 - 828.4
828.4 - 877.0
877.0 - 908.8
908.8 - 967.9
> 967.9
United Kingdom: Kensington and Chelsea: DALYs Rate (per 100,000)
< 553.1
553.1 - 589.3
589.3 - 661.2
661.2 - 733.2
733.2 - 783.2
783.2 - 833.2
833.2 - 883.3
883.3 - 906.9
906.9 - 971.2
> 971.2
United Kingdom: Kingston upon Thames: DALYs Rate (per 100,000)
< 553.9
553.9 - 590.9
590.9 - 658.0
658.0 - 725.2
725.2 - 776.5
776.5 - 827.7
827.7 - 878.9
878.9 - 907.0
907.0 - 970.0
> 970.0
United Kingdom: Lambeth: DALYs Rate (per 100,000)
< 549.0
549.0 - 587.8
587.8 - 656.4
656.4 - 724.9
724.9 - 778.1
778.1 - 831.3
831.3 - 884.5
884.5 - 907.3
907.3 - 969.5
> 969.5
United Kingdom: Lewisham: DALYs Rate (per 100,000)
< 553.9
553.9 - 591.1
591.1 - 661.6
661.6 - 732.0
732.0 - 781.5
781.5 - 831.0
831.0 - 880.4
880.4 - 910.0
910.0 - 969.4
> 969.4
United Kingdom: Merton: DALYs Rate (per 100,000)
< 553.8
553.8 - 589.4
589.4 - 656.5
656.5 - 723.6
723.6 - 774.8
774.8 - 825.9
825.9 - 877.1
877.1 - 908.0
908.0 - 967.0
> 967.0
United Kingdom: Newham: DALYs Rate (per 100,000)
< 552.4
552.4 - 590.4
590.4 - 653.7
653.7 - 716.9
716.9 - 772.3
772.3 - 827.7
827.7 - 883.1
883.1 - 907.9
907.9 - 970.5
> 970.5
United Kingdom: Redbridge: DALYs Rate (per 100,000)
< 552.3
552.3 - 589.5
589.5 - 656.1
656.1 - 722.7
722.7 - 775.0
775.0 - 827.3
827.3 - 879.5
879.5 - 912.9
912.9 - 968.9
> 968.9
Female
Male
Sexes Combined
Age
20-24 years
15-19 years
10-14 years
Year
DALYs Rate (per 100,000)
Age — 10-24 years

Figure S3

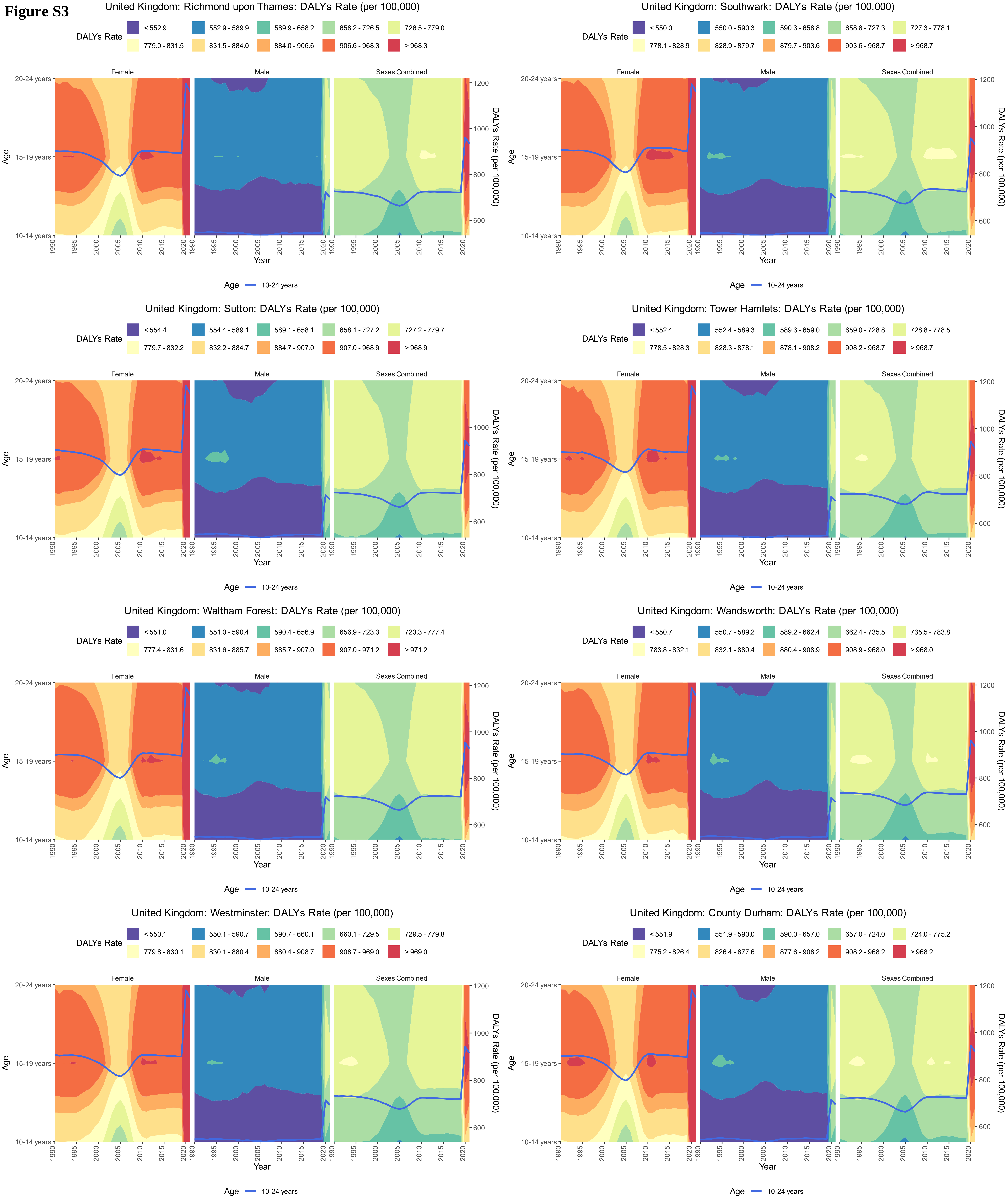

United Kingdom: Richmond upon Thames: DALYs Rate (per 100,000)
DALYs Rate
< 552.9
552.9 - 589.9
589.9 - 658.2
658.2 - 726.5
726.5 - 779.0
779.0 - 831.5
831.5 - 884.0
884.0 - 906.6
906.6 - 968.3
> 968.3
United Kingdom: Southwark: DALYs Rate (per 100,000)
< 550.0
550.0 - 590.3
590.3 - 658.8
658.8 - 727.3
727.3 - 778.1
778.1 - 828.9
828.9 - 879.7
879.7 - 903.6
903.6 - 968.7
> 968.7
United Kingdom: Sutton: DALYs Rate (per 100,000)
< 554.4
554.4 - 589.1
589.1 - 658.1
658.1 - 727.2
727.2 - 779.7
779.7 - 832.2
832.2 - 884.7
884.7 - 907.0
907.0 - 968.9
> 968.9
United Kingdom: Tower Hamlets: DALYs Rate (per 100,000)
< 552.4
552.4 - 589.3
589.3 - 659.0
659.0 - 728.8
728.8 - 778.5
778.5 - 828.3
828.3 - 878.1
878.1 - 908.2
908.2 - 968.7
> 968.7
United Kingdom: Waltham Forest: DALYs Rate (per 100,000)
< 551.0
551.0 - 590.4
590.4 - 656.9
656.9 - 723.3
723.3 - 777.4
777.4 - 831.6
831.6 - 885.7
885.7 - 907.0
907.0 - 971.2
> 971.2
United Kingdom: Wandsworth: DALYs Rate (per 100,000)
< 550.7
550.7 - 589.2
589.2 - 662.4
662.4 - 735.5
735.5 - 783.8
783.8 - 832.1
832.1 - 880.4
880.4 - 908.9
908.9 - 968.0
> 968.0
United Kingdom: Westminster: DALYs Rate (per 100,000)
< 550.1
550.1 - 590.7
590.7 - 660.1
660.1 - 729.5
729.5 - 779.8
779.8 - 830.1
830.1 - 880.4
880.4 - 908.7
908.7 - 969.0
> 969.0
United Kingdom: County Durham: DALYs Rate (per 100,000)
< 551.9
551.9 - 590.0
590.0 - 657.0
657.0 - 724.0
724.0 - 775.2
775.2 - 826.4
826.4 - 877.6
877.6 - 908.2
908.2 - 968.2
> 968.2
Female
Male
Sexes Combined
Age
20-24 years
15-19 years
10-14 years
Year
DALYs Rate (per 100,000)
Age — 10-24 years

Figure S3

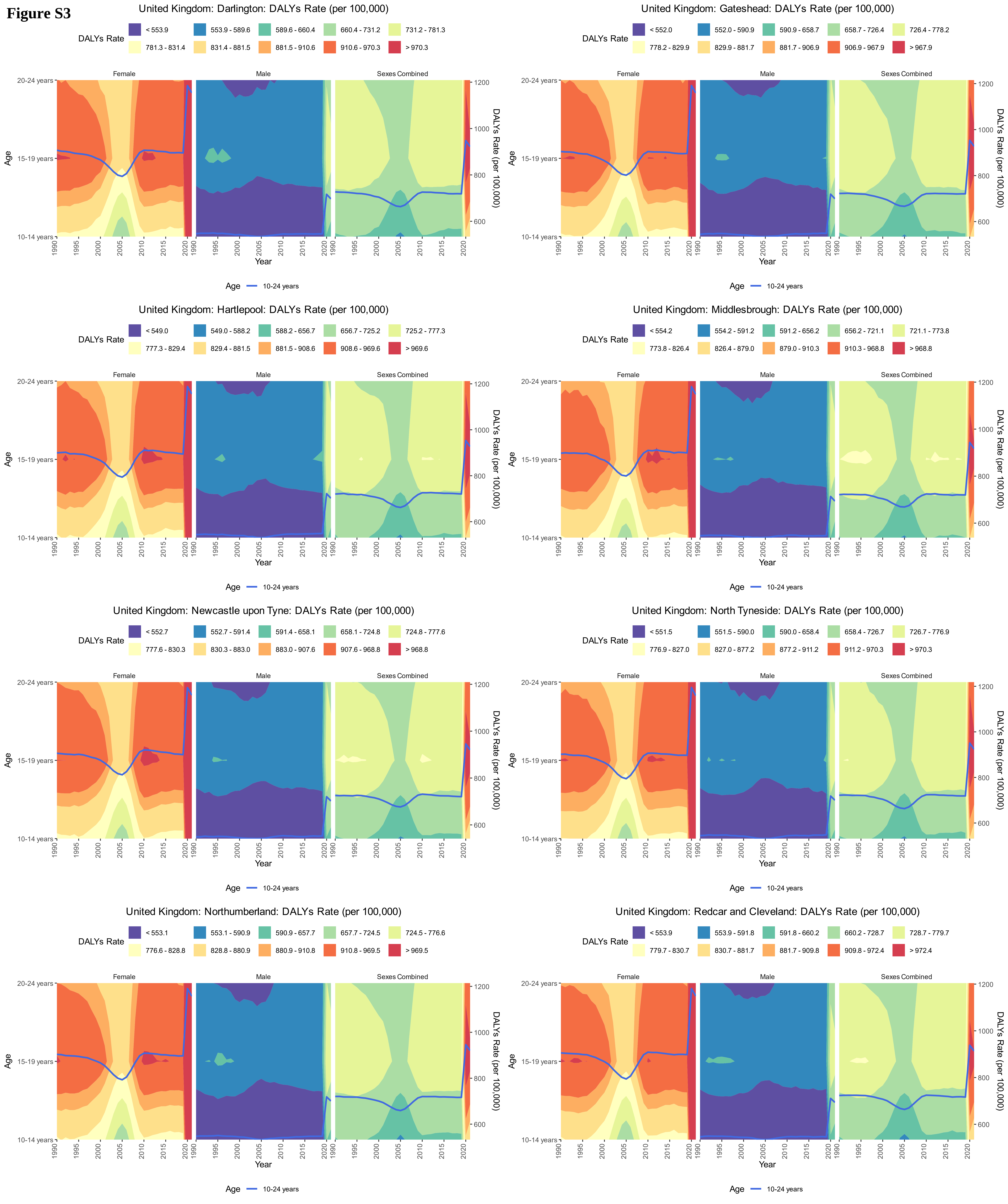
United Kingdom: Darlington: DALYs Rate (per 100,000)
DALYs Rate
< 553.9
553.9 - 589.6
589.6 - 660.4
660.4 - 731.2
731.2 - 781.3
781.3 - 831.4
831.4 - 881.5
881.5 - 910.6
910.6 - 970.3
> 970.3
United Kingdom: Gateshead: DALYs Rate (per 100,000)
DALYs Rate
< 552.0
552.0 - 590.9
590.9 - 658.7
658.7 - 726.4
726.4 - 778.2
778.2 - 829.9
829.9 - 881.7
881.7 - 906.9
906.9 - 967.9
> 967.9
United Kingdom: Hartlepool: DALYs Rate (per 100,000)
DALYs Rate
< 549.0
549.0 - 588.2
588.2 - 656.7
656.7 - 725.2
725.2 - 777.3
777.3 - 829.4
829.4 - 881.5
881.5 - 908.6
908.6 - 969.6
> 969.6
United Kingdom: Middlesbrough: DALYs Rate (per 100,000)
DALYs Rate
< 554.2
554.2 - 591.2
591.2 - 656.2
656.2 - 721.1
721.1 - 773.8
773.8 - 826.4
826.4 - 879.0
879.0 - 910.3
910.3 - 968.8
> 968.8
United Kingdom: Newcastle upon Tyne: DALYs Rate (per 100,000)
DALYs Rate
< 552.7
552.7 - 591.4
591.4 - 658.1
658.1 - 724.8
724.8 - 777.6
777.6 - 830.3
830.3 - 883.0
883.0 - 907.6
907.6 - 968.8
> 968.8
United Kingdom: North Tyneside: DALYs Rate (per 100,000)
DALYs Rate
< 551.5
551.5 - 590.0
590.0 - 658.4
658.4 - 726.7
726.7 - 776.9
776.9 - 827.0
827.0 - 877.2
877.2 - 911.2
911.2 - 970.3
> 970.3
United Kingdom: Northumberland: DALYs Rate (per 100,000)
DALYs Rate
< 553.1
553.1 - 590.9
590.9 - 657.7
657.7 - 724.5
724.5 - 776.6
776.6 - 828.8
828.8 - 880.9
880.9 - 910.8
910.8 - 969.5
> 969.5
United Kingdom: Redcar and Cleveland: DALYs Rate (per 100,000)
DALYs Rate
< 553.9
553.9 - 591.8
591.8 - 660.2
660.2 - 728.7
728.7 - 779.7
779.7 - 830.7
830.7 - 881.7
881.7 - 909.8
909.8 - 972.4
> 972.4
Female
Male
Sexes Combined
20-24 years
15-19 years
10-14 years
Age
1990
1995
2000
2005
2010
2015
2020
Year
1200
1000
800
600
DALYs Rate (per 100,000)
Age
10-24 years

Figure S3

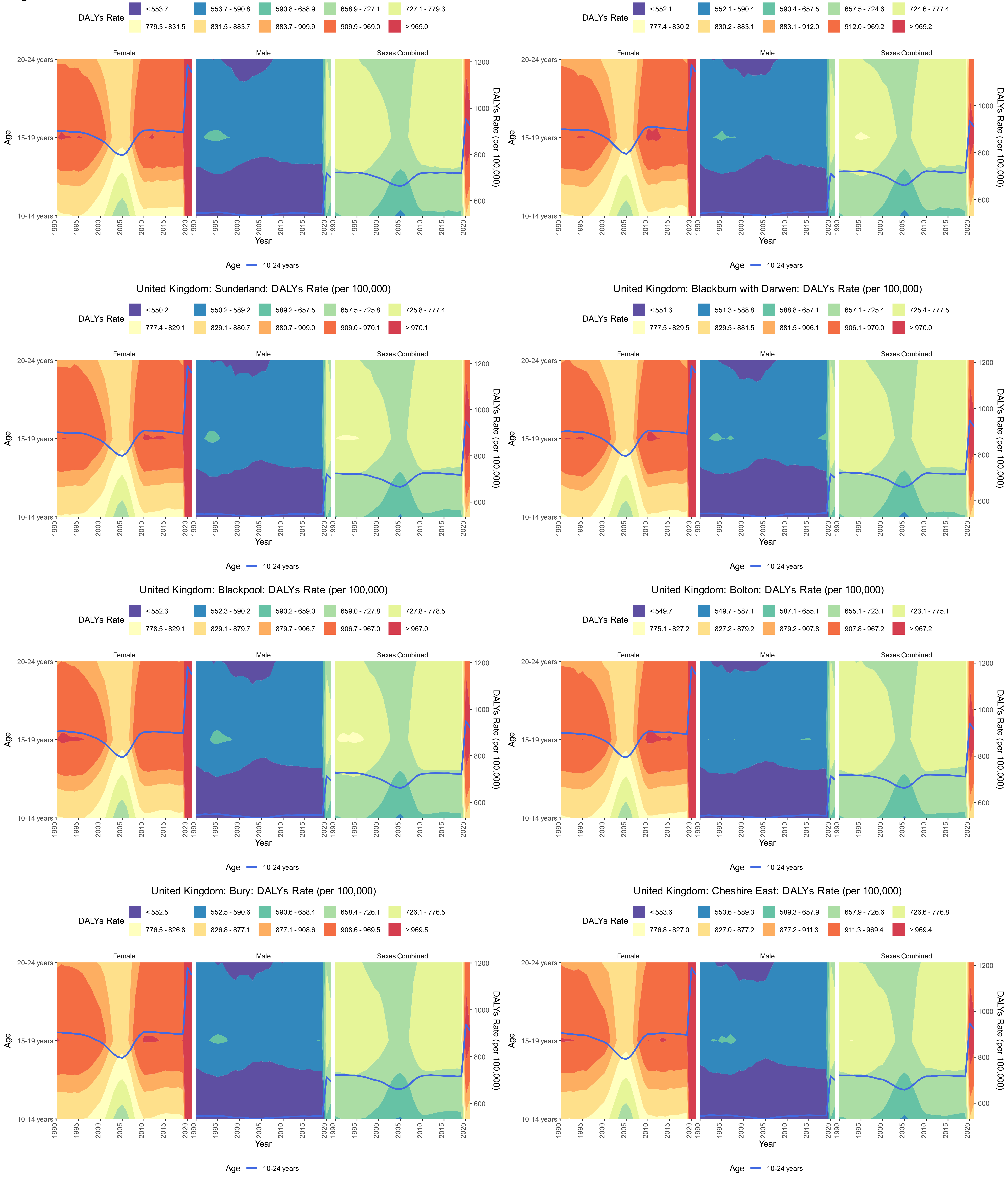

United Kingdom: South Tyneside: DALYs Rate (per 100,000)
DALYs Rate
< 553.7
553.7 - 590.8
590.8 - 658.9
658.9 - 727.1
727.1 - 779.3
779.3 - 831.5
831.5 - 883.7
883.7 - 909.9
909.9 - 969.0
> 969.0
United Kingdom: Stockton-on-Tees: DALYs Rate (per 100,000)
DALYs Rate
< 552.1
552.1 - 590.4
590.4 - 657.5
657.5 - 724.6
724.6 - 777.4
777.4 - 830.2
830.2 - 883.1
883.1 - 912.0
912.0 - 969.2
> 969.2
United Kingdom: Sunderland: DALYs Rate (per 100,000)
DALYs Rate
< 550.2
550.2 - 589.2
589.2 - 657.5
657.5 - 725.8
725.8 - 777.4
777.4 - 829.1
829.1 - 880.7
880.7 - 909.0
909.0 - 970.1
> 970.1
United Kingdom: Blackburn with Darwen: DALYs Rate (per 100,000)
DALYs Rate
< 551.3
551.3 - 588.8
588.8 - 657.1
657.1 - 725.4
725.4 - 777.5
777.5 - 829.5
829.5 - 881.5
881.5 - 906.1
906.1 - 970.0
> 970.0
United Kingdom: Blackpool: DALYs Rate (per 100,000)
DALYs Rate
< 552.3
552.3 - 590.2
590.2 - 659.0
659.0 - 727.8
727.8 - 778.5
778.5 - 829.1
829.1 - 879.7
879.7 - 906.7
906.7 - 967.0
> 967.0
United Kingdom: Bolton: DALYs Rate (per 100,000)
DALYs Rate
< 549.7
549.7 - 587.1
587.1 - 655.1
655.1 - 723.1
723.1 - 775.1
775.1 - 827.2
827.2 - 879.2
879.2 - 907.8
907.8 - 967.2
> 967.2
United Kingdom: Bury: DALYs Rate (per 100,000)
DALYs Rate
< 552.5
552.5 - 590.6
590.6 - 658.4
658.4 - 726.1
726.1 - 776.5
776.5 - 826.8
826.8 - 877.1
877.1 - 908.6
908.6 - 969.5
> 969.5
United Kingdom: Cheshire East: DALYs Rate (per 100,000)
DALYs Rate
< 553.6
553.6 - 589.3
589.3 - 657.9
657.9 - 726.6
726.6 - 776.8
776.8 - 827.0
827.0 - 877.2
877.2 - 911.3
911.3 - 969.4
> 969.4
Female
Male
Sexes Combined
Age
20-24 years
15-19 years
10-14 years
Year
1990
1995
2000
2005
2010
2015
2020
DALYs Rate (per 100,000)
600
800
1000
1200
Age
10-24 years

Figure S3

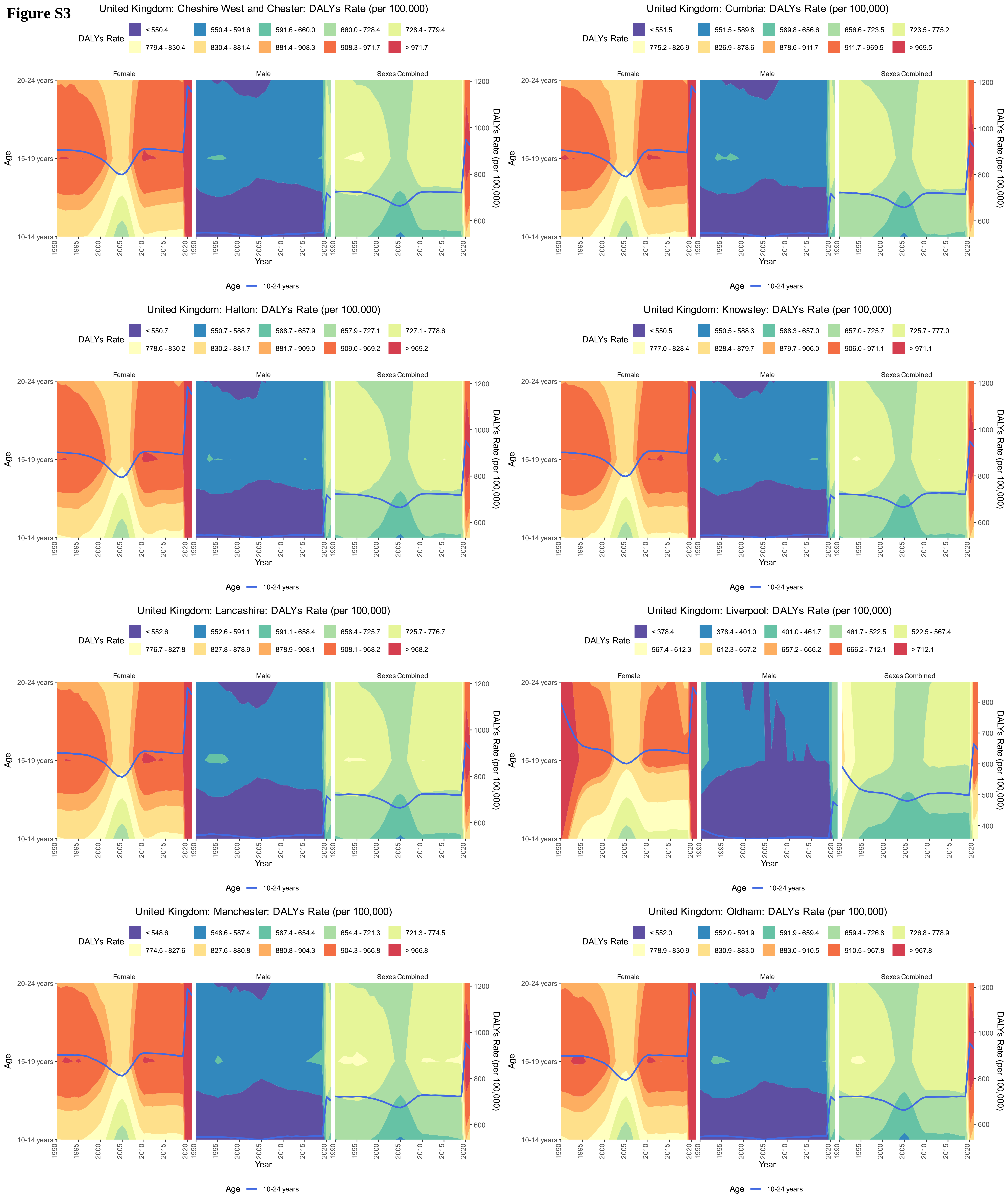

United Kingdom: Cheshire West and Chester: DALYs Rate (per 100,000)
DALYs Rate
< 550.4
550.4 - 591.6
591.6 - 660.0
660.0 - 728.4
728.4 - 779.4
779.4 - 830.4
830.4 - 881.4
881.4 - 908.3
908.3 - 971.7
> 971.7
United Kingdom: Cumbria: DALYs Rate (per 100,000)
< 551.5
551.5 - 589.8
589.8 - 656.6
656.6 - 723.5
723.5 - 775.2
775.2 - 826.9
826.9 - 878.6
878.6 - 911.7
911.7 - 969.5
> 969.5
United Kingdom: Halton: DALYs Rate (per 100,000)
< 550.7
550.7 - 588.7
588.7 - 657.9
657.9 - 727.1
727.1 - 778.6
778.6 - 830.2
830.2 - 881.7
881.7 - 909.0
909.0 - 969.2
> 969.2
United Kingdom: Knowsley: DALYs Rate (per 100,000)
< 550.5
550.5 - 588.3
588.3 - 657.0
657.0 - 725.7
725.7 - 777.0
777.0 - 828.4
828.4 - 879.7
879.7 - 906.0
906.0 - 971.1
> 971.1
United Kingdom: Lancashire: DALYs Rate (per 100,000)
< 552.6
552.6 - 591.1
591.1 - 658.4
658.4 - 725.7
725.7 - 776.7
776.7 - 827.8
827.8 - 878.9
878.9 - 908.1
908.1 - 968.2
> 968.2
United Kingdom: Liverpool: DALYs Rate (per 100,000)
< 378.4
378.4 - 401.0
401.0 - 461.7
461.7 - 522.5
522.5 - 567.4
567.4 - 612.3
612.3 - 657.2
657.2 - 666.2
666.2 - 712.1
> 712.1
United Kingdom: Manchester: DALYs Rate (per 100,000)
< 548.6
548.6 - 587.4
587.4 - 654.4
654.4 - 721.3
721.3 - 774.5
774.5 - 827.6
827.6 - 880.8
880.8 - 904.3
904.3 - 966.8
> 966.8
United Kingdom: Oldham: DALYs Rate (per 100,000)
< 552.0
552.0 - 591.9
591.9 - 659.4
659.4 - 726.8
726.8 - 778.9
778.9 - 830.9
830.9 - 883.0
883.0 - 910.5
910.5 - 967.8
> 967.8
Female
Male
Sexes Combined
Age
20-24 years
15-19 years
10-14 years
Year
DALYs Rate (per 100,000)
Age — 10-24 years

Figure S3

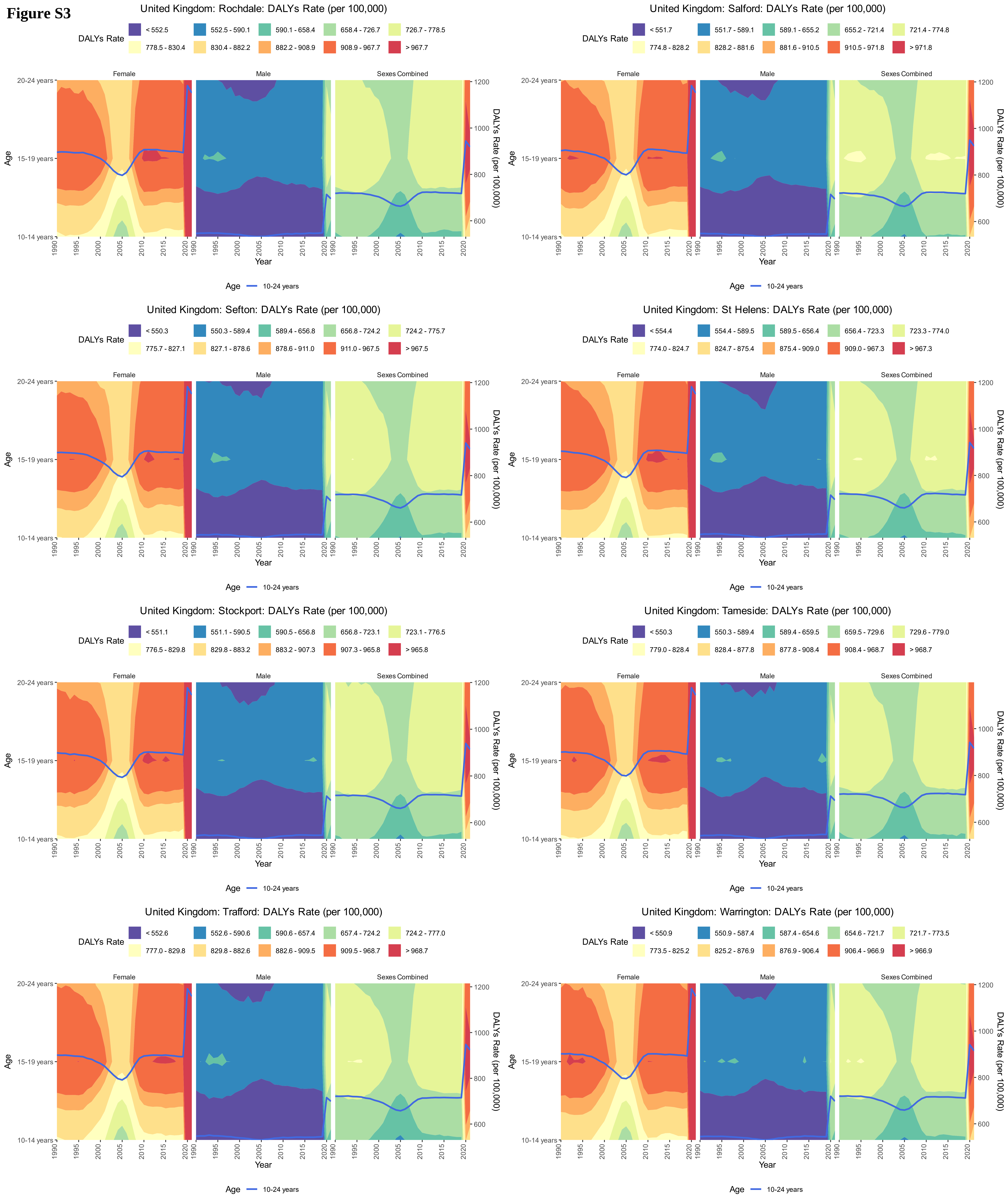

United Kingdom: Rochdale: DALYs Rate (per 100,000)
DALYs Rate
< 552.5
552.5 - 590.1
590.1 - 658.4
658.4 - 726.7
726.7 - 778.5
778.5 - 830.4
830.4 - 882.2
882.2 - 908.9
908.9 - 967.7
> 967.7
United Kingdom: Salford: DALYs Rate (per 100,000)
DALYs Rate
< 551.7
551.7 - 589.1
589.1 - 655.2
655.2 - 721.4
721.4 - 774.8
774.8 - 828.2
828.2 - 881.6
881.6 - 910.5
910.5 - 971.8
> 971.8
United Kingdom: Sefton: DALYs Rate (per 100,000)
DALYs Rate
< 550.3
550.3 - 589.4
589.4 - 656.8
656.8 - 724.2
724.2 - 775.7
775.7 - 827.1
827.1 - 878.6
878.6 - 911.0
911.0 - 967.5
> 967.5
United Kingdom: St Helens: DALYs Rate (per 100,000)
DALYs Rate
< 554.4
554.4 - 589.5
589.5 - 656.4
656.4 - 723.3
723.3 - 774.0
774.0 - 824.7
824.7 - 875.4
875.4 - 909.0
909.0 - 967.3
> 967.3
United Kingdom: Stockport: DALYs Rate (per 100,000)
DALYs Rate
< 551.1
551.1 - 590.5
590.5 - 656.8
656.8 - 723.1
723.1 - 776.5
776.5 - 829.8
829.8 - 883.2
883.2 - 907.3
907.3 - 965.8
> 965.8
United Kingdom: Tameside: DALYs Rate (per 100,000)
DALYs Rate
< 550.3
550.3 - 589.4
589.4 - 659.5
659.5 - 729.6
729.6 - 779.0
779.0 - 828.4
828.4 - 877.8
877.8 - 908.4
908.4 - 968.7
> 968.7
United Kingdom: Trafford: DALYs Rate (per 100,000)
DALYs Rate
< 552.6
552.6 - 590.6
590.6 - 657.4
657.4 - 724.2
724.2 - 777.0
777.0 - 829.8
829.8 - 882.6
882.6 - 909.5
909.5 - 968.7
> 968.7
United Kingdom: Warrington: DALYs Rate (per 100,000)
DALYs Rate
< 550.9
550.9 - 587.4
587.4 - 654.6
654.6 - 721.7
721.7 - 773.5
773.5 - 825.2
825.2 - 876.9
876.9 - 906.4
906.4 - 966.9
> 966.9
Female
Male
Sexes Combined
Age
20-24 years
15-19 years
10-14 years
Year
1990
1995
2000
2005
2010
2015
2020
DALYs Rate (per 100,000)
600
800
1000
1200
Age
10-24 years

Figure S3

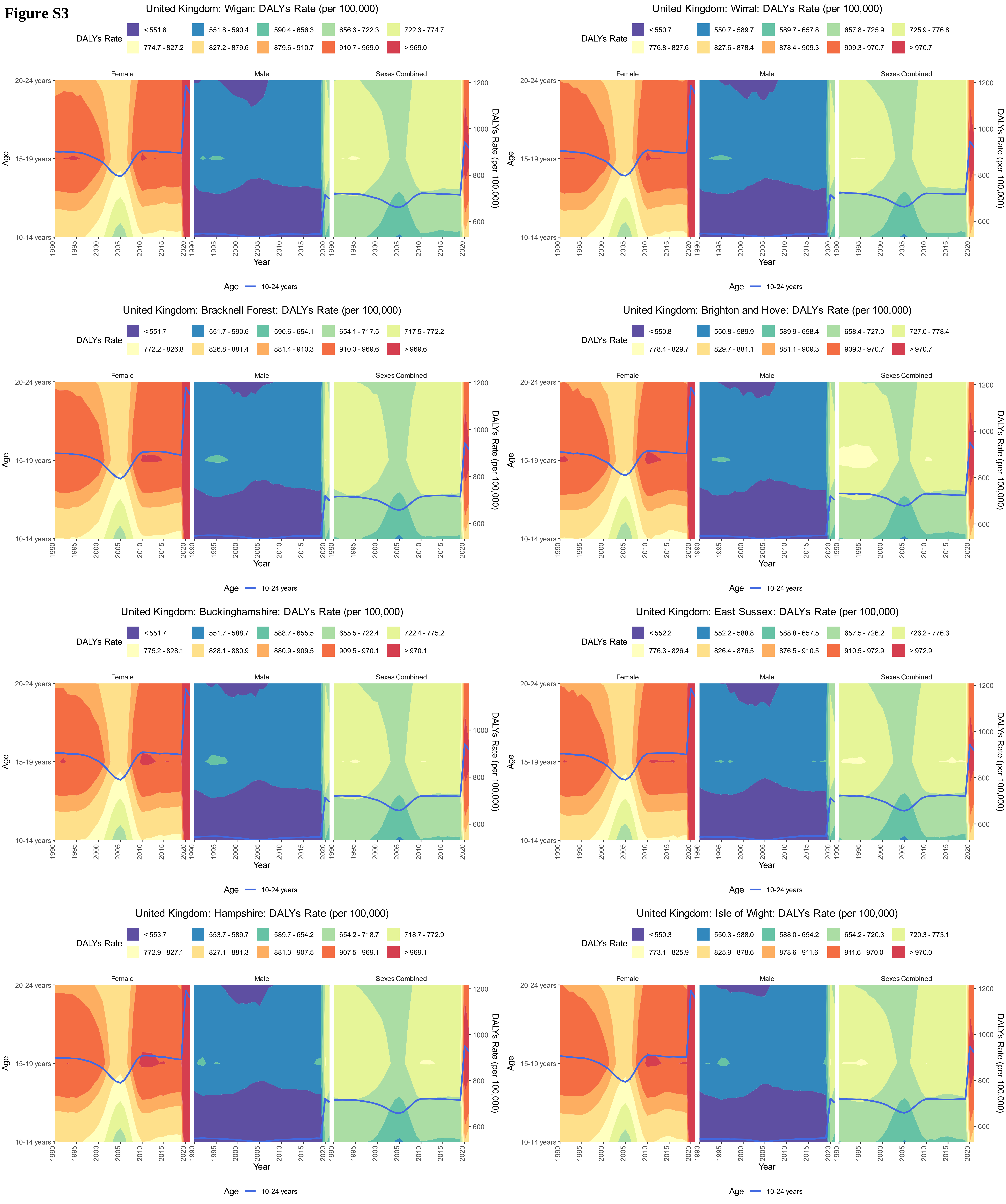

United Kingdom: Wigan: DALYs Rate (per 100,000)
DALYs Rate
< 551.8
551.8 - 590.4
590.4 - 656.3
656.3 - 722.3
722.3 - 774.7
774.7 - 827.2
827.2 - 879.6
879.6 - 910.7
910.7 - 969.0
> 969.0
United Kingdom: Wirral: DALYs Rate (per 100,000)
DALYs Rate
< 550.7
550.7 - 589.7
589.7 - 657.8
657.8 - 725.9
725.9 - 776.8
776.8 - 827.6
827.6 - 878.4
878.4 - 909.3
909.3 - 970.7
> 970.7
United Kingdom: Bracknell Forest: DALYs Rate (per 100,000)
DALYs Rate
< 551.7
551.7 - 590.6
590.6 - 654.1
654.1 - 717.5
717.5 - 772.2
772.2 - 826.8
826.8 - 881.4
881.4 - 910.3
910.3 - 969.6
> 969.6
United Kingdom: Brighton and Hove: DALYs Rate (per 100,000)
DALYs Rate
< 550.8
550.8 - 589.9
589.9 - 658.4
658.4 - 727.0
727.0 - 778.4
778.4 - 829.7
829.7 - 881.1
881.1 - 909.3
909.3 - 970.7
> 970.7
United Kingdom: Buckinghamshire: DALYs Rate (per 100,000)
DALYs Rate
< 551.7
551.7 - 588.7
588.7 - 655.5
655.5 - 722.4
722.4 - 775.2
775.2 - 828.1
828.1 - 880.9
880.9 - 909.5
909.5 - 970.1
> 970.1
United Kingdom: East Sussex: DALYs Rate (per 100,000)
DALYs Rate
< 552.2
552.2 - 588.8
588.8 - 657.5
657.5 - 726.2
726.2 - 776.3
776.3 - 826.4
826.4 - 876.5
876.5 - 910.5
910.5 - 972.9
> 972.9
United Kingdom: Hampshire: DALYs Rate (per 100,000)
DALYs Rate
< 553.7
553.7 - 589.7
589.7 - 654.2
654.2 - 718.7
718.7 - 772.9
772.9 - 827.1
827.1 - 881.3
881.3 - 907.5
907.5 - 969.1
> 969.1
United Kingdom: Isle of Wight: DALYs Rate (per 100,000)
DALYs Rate
< 550.3
550.3 - 588.0
588.0 - 654.2
654.2 - 720.3
720.3 - 773.1
773.1 - 825.9
825.9 - 878.6
878.6 - 911.6
911.6 - 970.0
> 970.0
Female
Male
Sexes Combined
Age
20-24 years
15-19 years
10-14 years
Year
DALYs Rate (per 100,000)
Age — 10-24 years

Figure S3

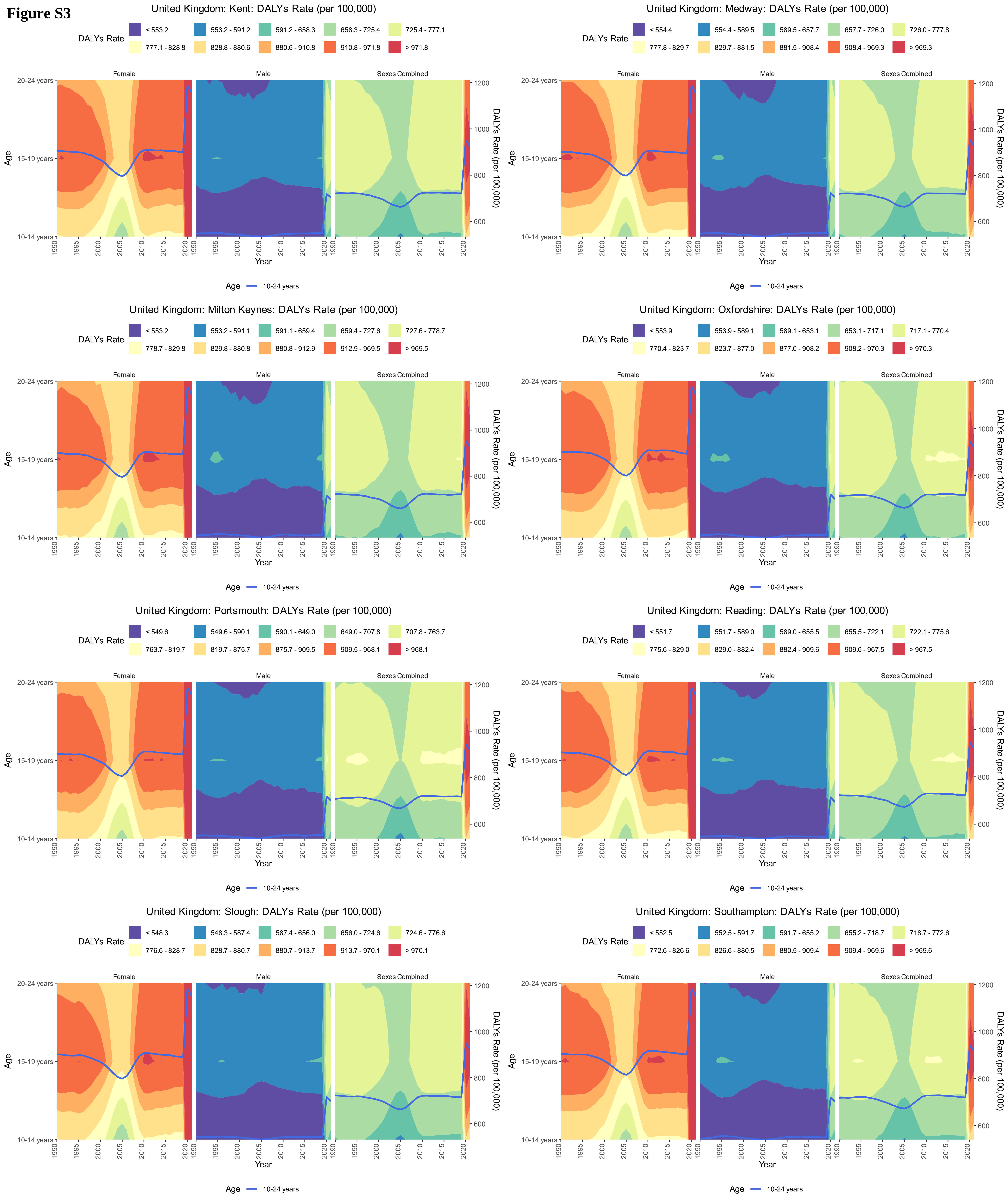
United Kingdom: Kent: DALYs Rate (per 100,000)
DALYs Rate
< 553.2
553.2 - 591.2
591.2 - 658.3
658.3 - 725.4
725.4 - 777.1
777.1 - 828.8
828.8 - 880.6
880.6 - 910.8
910.8 - 971.8
> 971.8
United Kingdom: Medway: DALYs Rate (per 100,000)
DALYs Rate
< 554.4
554.4 - 589.5
589.5 - 657.7
657.7 - 726.0
726.0 - 777.8
777.8 - 829.7
829.7 - 881.5
881.5 - 908.4
908.4 - 969.3
> 969.3
United Kingdom: Milton Keynes: DALYs Rate (per 100,000)
DALYs Rate
< 553.2
553.2 - 591.1
591.1 - 659.4
659.4 - 727.6
727.6 - 778.7
778.7 - 829.8
829.8 - 880.8
880.8 - 912.9
912.9 - 969.5
> 969.5
United Kingdom: Oxfordshire: DALYs Rate (per 100,000)
DALYs Rate
< 553.9
553.9 - 589.1
589.1 - 653.1
653.1 - 717.1
717.1 - 770.4
770.4 - 823.7
823.7 - 877.0
877.0 - 908.2
908.2 - 970.3
> 970.3
United Kingdom: Portsmouth: DALYs Rate (per 100,000)
DALYs Rate
< 549.6
549.6 - 590.1
590.1 - 649.0
649.0 - 707.8
707.8 - 763.7
763.7 - 819.7
819.7 - 875.7
875.7 - 909.5
909.5 - 968.1
> 968.1
United Kingdom: Reading: DALYs Rate (per 100,000)
DALYs Rate
< 551.7
551.7 - 589.0
589.0 - 655.5
655.5 - 722.1
722.1 - 775.6
775.6 - 829.0
829.0 - 882.4
882.4 - 909.6
909.6 - 967.5
> 967.5
United Kingdom: Slough: DALYs Rate (per 100,000)
DALYs Rate
< 548.3
548.3 - 587.4
587.4 - 656.0
656.0 - 724.6
724.6 - 776.6
776.6 - 828.7
828.7 - 880.7
880.7 - 913.7
913.7 - 970.1
> 970.1
United Kingdom: Southampton: DALYs Rate (per 100,000)
DALYs Rate
< 552.5
552.5 - 591.7
591.7 - 655.2
655.2 - 718.7
718.7 - 772.6
772.6 - 826.6
826.6 - 880.5
880.5 - 909.4
909.4 - 969.6
> 969.6
Female
Male
Sexes Combined
20-24 years
15-19 years
10-14 years
Age
1990
1995
2000
2005
2010
2015
2020
Year
600
800
1000
1200
DALYs Rate (per 100,000)
Age
10-24 years

Figure S3

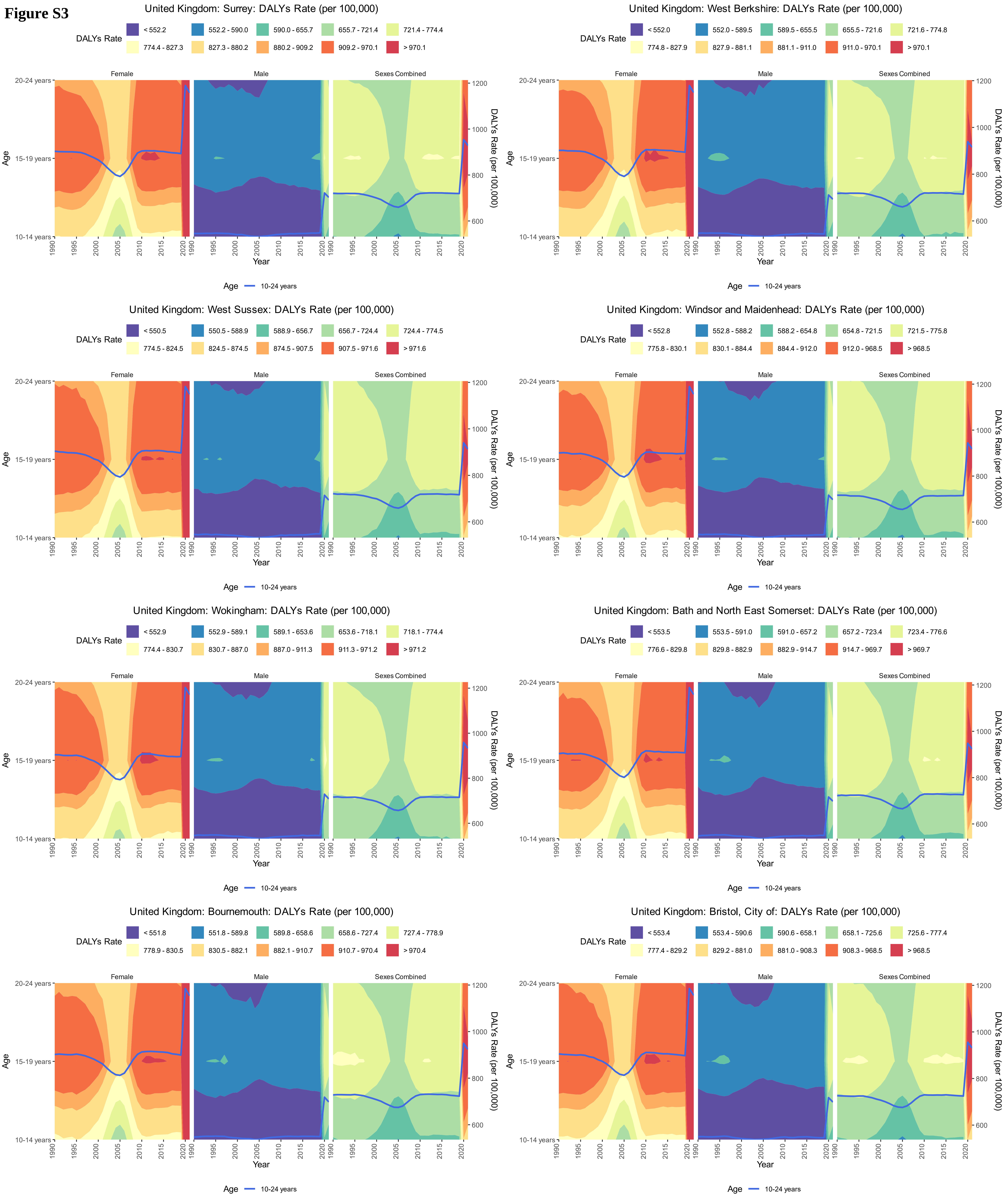

United Kingdom: Surrey: DALYs Rate (per 100,000)
DALYs Rate
< 552.2
552.2 - 590.0
590.0 - 655.7
655.7 - 721.4
721.4 - 774.4
774.4 - 827.3
827.3 - 880.2
880.2 - 909.2
909.2 - 970.1
> 970.1
United Kingdom: West Berkshire: DALYs Rate (per 100,000)
< 552.0
552.0 - 589.5
589.5 - 655.5
655.5 - 721.6
721.6 - 774.8
774.8 - 827.9
827.9 - 881.1
881.1 - 911.0
911.0 - 970.1
> 970.1
United Kingdom: West Sussex: DALYs Rate (per 100,000)
< 550.5
550.5 - 588.9
588.9 - 656.7
656.7 - 724.4
724.4 - 774.5
774.5 - 824.5
824.5 - 874.5
874.5 - 907.5
907.5 - 971.6
> 971.6
United Kingdom: Windsor and Maidenhead: DALYs Rate (per 100,000)
< 552.8
552.8 - 588.2
588.2 - 654.8
654.8 - 721.5
721.5 - 775.8
775.8 - 830.1
830.1 - 884.4
884.4 - 912.0
912.0 - 968.5
> 968.5
United Kingdom: Wokingham: DALYs Rate (per 100,000)
< 552.9
552.9 - 589.1
589.1 - 653.6
653.6 - 718.1
718.1 - 774.4
774.4 - 830.7
830.7 - 887.0
887.0 - 911.3
911.3 - 971.2
> 971.2
United Kingdom: Bath and North East Somerset: DALYs Rate (per 100,000)
< 553.5
553.5 - 591.0
591.0 - 657.2
657.2 - 723.4
723.4 - 776.6
776.6 - 829.8
829.8 - 882.9
882.9 - 914.7
914.7 - 969.7
> 969.7
United Kingdom: Bournemouth: DALYs Rate (per 100,000)
< 551.8
551.8 - 589.8
589.8 - 658.6
658.6 - 727.4
727.4 - 778.9
778.9 - 830.5
830.5 - 882.1
882.1 - 910.7
910.7 - 970.4
> 970.4
United Kingdom: Bristol, City of: DALYs Rate (per 100,000)
< 553.4
553.4 - 590.6
590.6 - 658.1
658.1 - 725.6
725.6 - 777.4
777.4 - 829.2
829.2 - 881.0
881.0 - 908.3
908.3 - 968.5
> 968.5
Female
Male
Sexes Combined
Age
20-24 years
15-19 years
10-14 years
Year
1990
1995
2000
2005
2010
2015
2020
DALYs Rate (per 100,000)
600
800
1000
1200
Age
10-24 years

Figure S3

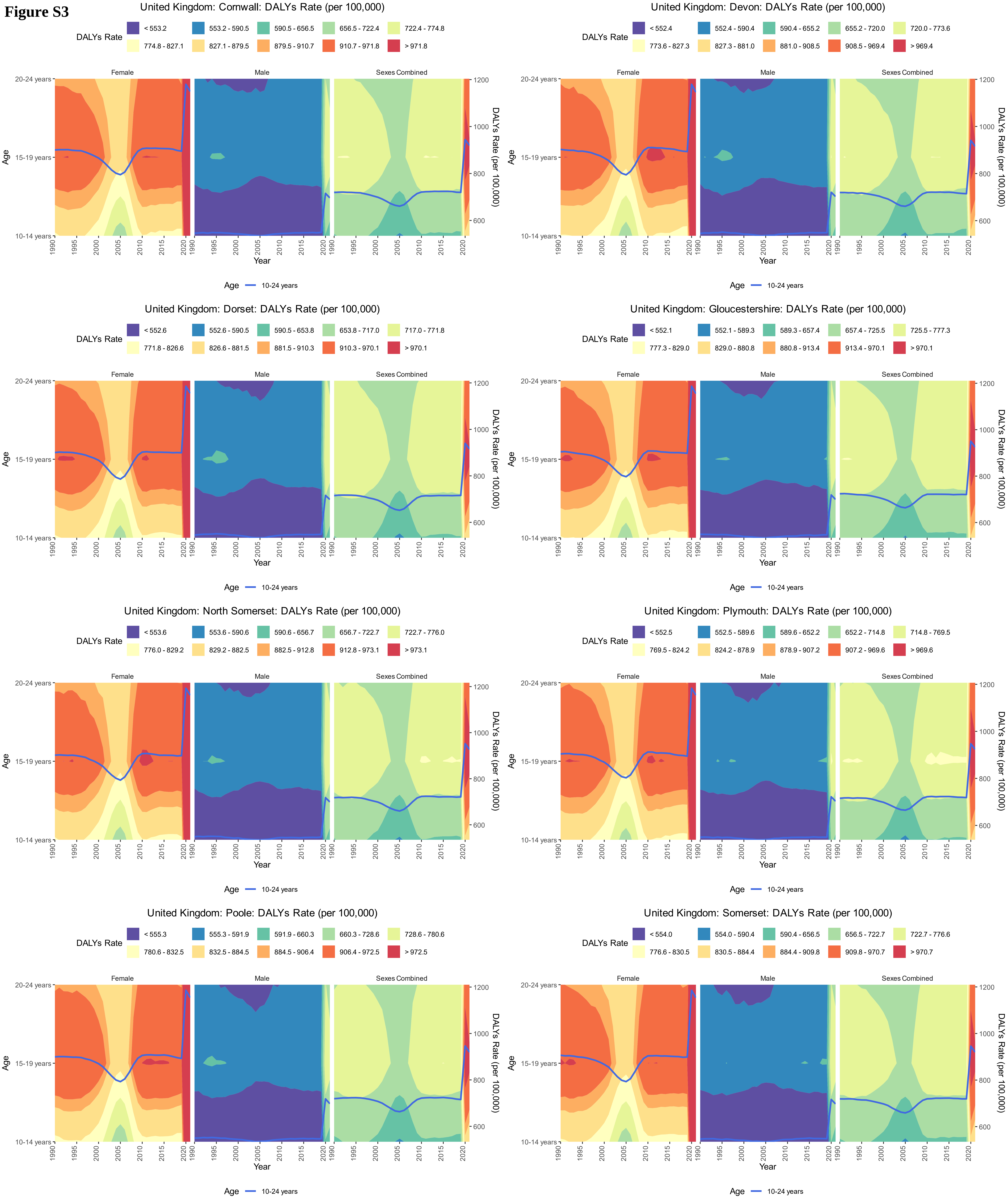

United Kingdom: Cornwall: DALYs Rate (per 100,000)
DALYs Rate
< 553.2
553.2 - 590.5
590.5 - 656.5
656.5 - 722.4
722.4 - 774.8
774.8 - 827.1
827.1 - 879.5
879.5 - 910.7
910.7 - 971.8
> 971.8
United Kingdom: Devon: DALYs Rate (per 100,000)
< 552.4
552.4 - 590.4
590.4 - 655.2
655.2 - 720.0
720.0 - 773.6
773.6 - 827.3
827.3 - 881.0
881.0 - 908.5
908.5 - 969.4
> 969.4
United Kingdom: Dorset: DALYs Rate (per 100,000)
< 552.6
552.6 - 590.5
590.5 - 653.8
653.8 - 717.0
717.0 - 771.8
771.8 - 826.6
826.6 - 881.5
881.5 - 910.3
910.3 - 970.1
> 970.1
United Kingdom: Gloucestershire: DALYs Rate (per 100,000)
< 552.1
552.1 - 589.3
589.3 - 657.4
657.4 - 725.5
725.5 - 777.3
777.3 - 829.0
829.0 - 880.8
880.8 - 913.4
913.4 - 970.1
> 970.1
United Kingdom: North Somerset: DALYs Rate (per 100,000)
< 553.6
553.6 - 590.6
590.6 - 656.7
656.7 - 722.7
722.7 - 776.0
776.0 - 829.2
829.2 - 882.5
882.5 - 912.8
912.8 - 973.1
> 973.1
United Kingdom: Plymouth: DALYs Rate (per 100,000)
< 552.5
552.5 - 589.6
589.6 - 652.2
652.2 - 714.8
714.8 - 769.5
769.5 - 824.2
824.2 - 878.9
878.9 - 907.2
907.2 - 969.6
> 969.6
United Kingdom: Poole: DALYs Rate (per 100,000)
< 555.3
555.3 - 591.9
591.9 - 660.3
660.3 - 728.6
728.6 - 780.6
780.6 - 832.5
832.5 - 884.5
884.5 - 906.4
906.4 - 972.5
> 972.5
United Kingdom: Somerset: DALYs Rate (per 100,000)
< 554.0
554.0 - 590.4
590.4 - 656.5
656.5 - 722.7
722.7 - 776.6
776.6 - 830.5
830.5 - 884.4
884.4 - 909.8
909.8 - 970.7
> 970.7
Female
Male
Sexes Combined
Age
20-24 years
15-19 years
10-14 years
Year
1990
1995
2000
2005
2010
2015
2020
DALYs Rate (per 100,000)
600
800
1000
1200
Age
10-24 years

Figure S3

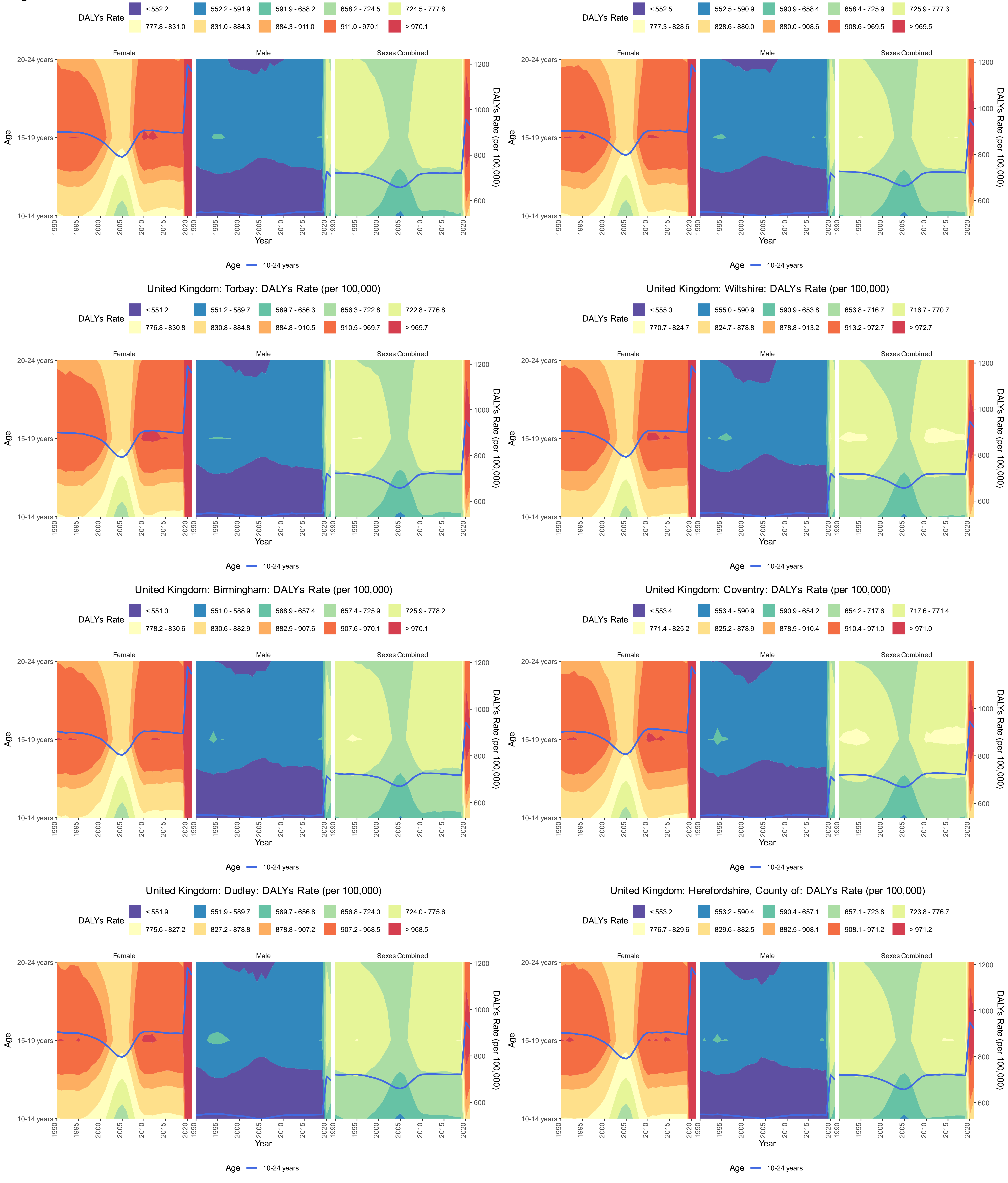
United Kingdom: South Gloucestershire: DALYs Rate (per 100,000)
DALYs Rate
< 552.2
552.2 - 591.9
591.9 - 658.2
658.2 - 724.5
724.5 - 777.8
777.8 - 831.0
831.0 - 884.3
884.3 - 911.0
911.0 - 970.1
> 970.1
Female
Male
Sexes Combined
Age
Year
DALYs Rate (per 100,000)
Age — 10-24 years
United Kingdom: Swindon: DALYs Rate (per 100,000)
DALYs Rate
< 552.5
552.5 - 590.9
590.9 - 658.4
658.4 - 725.9
725.9 - 777.3
777.3 - 828.6
828.6 - 880.0
880.0 - 908.6
908.6 - 969.5
> 969.5
United Kingdom: Torbay: DALYs Rate (per 100,000)
DALYs Rate
< 551.2
551.2 - 589.7
589.7 - 656.3
656.3 - 722.8
722.8 - 776.8
776.8 - 830.8
830.8 - 884.8
884.8 - 910.5
910.5 - 969.7
> 969.7
United Kingdom: Wiltshire: DALYs Rate (per 100,000)
DALYs Rate
< 555.0
555.0 - 590.9
590.9 - 653.8
653.8 - 716.7
716.7 - 770.7
770.7 - 824.7
824.7 - 878.8
878.8 - 913.2
913.2 - 972.7
> 972.7
United Kingdom: Birmingham: DALYs Rate (per 100,000)
DALYs Rate
< 551.0
551.0 - 588.9
588.9 - 657.4
657.4 - 725.9
725.9 - 778.2
778.2 - 830.6
830.6 - 882.9
882.9 - 907.6
907.6 - 970.1
> 970.1
United Kingdom: Coventry: DALYs Rate (per 100,000)
DALYs Rate
< 553.4
553.4 - 590.9
590.9 - 654.2
654.2 - 717.6
717.6 - 771.4
771.4 - 825.2
825.2 - 878.9
878.9 - 910.4
910.4 - 971.0
> 971.0
United Kingdom: Dudley: DALYs Rate (per 100,000)
DALYs Rate
< 551.9
551.9 - 589.7
589.7 - 656.8
656.8 - 724.0
724.0 - 775.6
775.6 - 827.2
827.2 - 878.8
878.8 - 907.2
907.2 - 968.5
> 968.5
United Kingdom: Herefordshire, County of: DALYs Rate (per 100,000)
DALYs Rate
< 553.2
553.2 - 590.4
590.4 - 657.1
657.1 - 723.8
723.8 - 776.7
776.7 - 829.6
829.6 - 882.5
882.5 - 908.1
908.1 - 971.2
> 971.2
20-24 years
15-19 years
10-14 years
1990
1995
2000
2005
2010
2015
2020
600
800
1000
1200

Figure S3

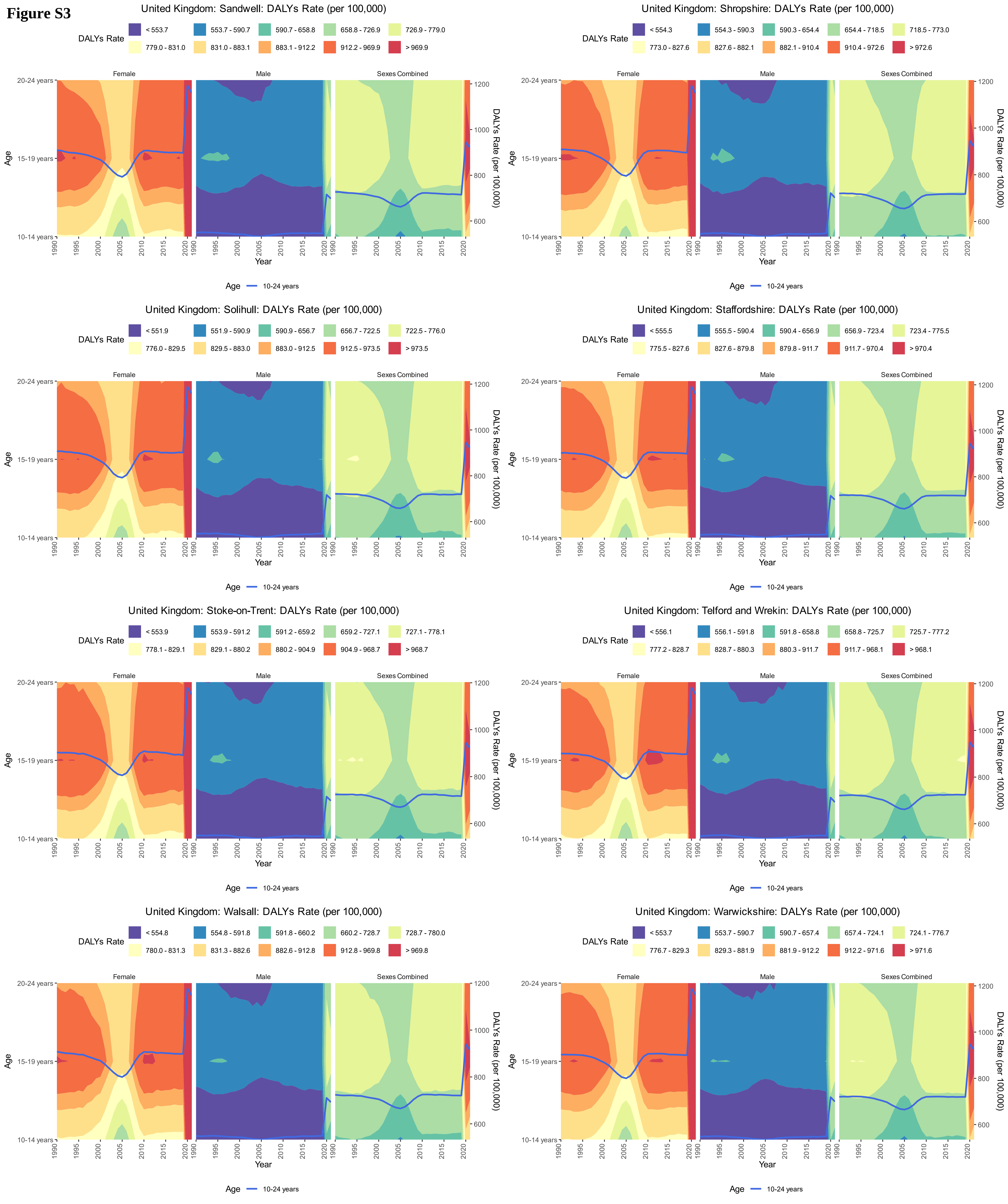

United Kingdom: Sandwell: DALYs Rate (per 100,000)
DALYs Rate
< 553.7
553.7 - 590.7
590.7 - 658.8
658.8 - 726.9
726.9 - 779.0
779.0 - 831.0
831.0 - 883.1
883.1 - 912.2
912.2 - 969.9
> 969.9
United Kingdom: Shropshire: DALYs Rate (per 100,000)
DALYs Rate
< 554.3
554.3 - 590.3
590.3 - 654.4
654.4 - 718.5
718.5 - 773.0
773.0 - 827.6
827.6 - 882.1
882.1 - 910.4
910.4 - 972.6
> 972.6
United Kingdom: Solihull: DALYs Rate (per 100,000)
DALYs Rate
< 551.9
551.9 - 590.9
590.9 - 656.7
656.7 - 722.5
722.5 - 776.0
776.0 - 829.5
829.5 - 883.0
883.0 - 912.5
912.5 - 973.5
> 973.5
United Kingdom: Staffordshire: DALYs Rate (per 100,000)
DALYs Rate
< 555.5
555.5 - 590.4
590.4 - 656.9
656.9 - 723.4
723.4 - 775.5
775.5 - 827.6
827.6 - 879.8
879.8 - 911.7
911.7 - 970.4
> 970.4
United Kingdom: Stoke-on-Trent: DALYs Rate (per 100,000)
DALYs Rate
< 553.9
553.9 - 591.2
591.2 - 659.2
659.2 - 727.1
727.1 - 778.1
778.1 - 829.1
829.1 - 880.2
880.2 - 904.9
904.9 - 968.7
> 968.7
United Kingdom: Telford and Wrekin: DALYs Rate (per 100,000)
DALYs Rate
< 556.1
556.1 - 591.8
591.8 - 658.8
658.8 - 725.7
725.7 - 777.2
777.2 - 828.7
828.7 - 880.3
880.3 - 911.7
911.7 - 968.1
> 968.1
United Kingdom: Walsall: DALYs Rate (per 100,000)
DALYs Rate
< 554.8
554.8 - 591.8
591.8 - 660.2
660.2 - 728.7
728.7 - 780.0
780.0 - 831.3
831.3 - 882.6
882.6 - 912.8
912.8 - 969.8
> 969.8
United Kingdom: Warwickshire: DALYs Rate (per 100,000)
DALYs Rate
< 553.7
553.7 - 590.7
590.7 - 657.4
657.4 - 724.1
724.1 - 776.7
776.7 - 829.3
829.3 - 881.9
881.9 - 912.2
912.2 - 971.6
> 971.6
Female
Male
Sexes Combined
Age
20-24 years
15-19 years
10-14 years
Year
1990
1995
2000
2005
2010
2015
2020
DALYs Rate (per 100,000)
600
800
1000
1200
Age
10-24 years

Figure S3

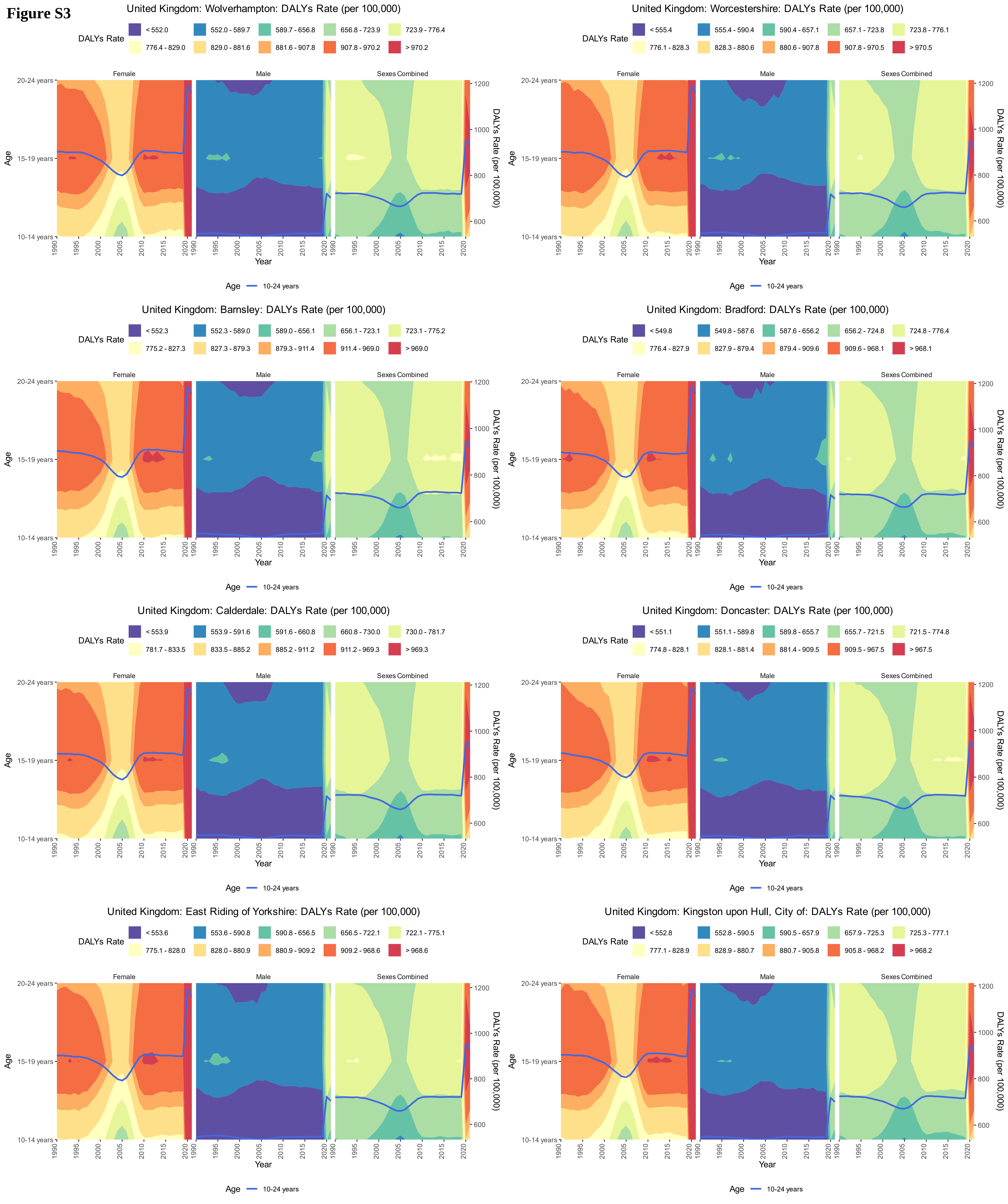
United Kingdom: Wolverhampton: DALYs Rate (per 100,000)
DALYs Rate
< 552.0 | 552.0 - 589.7 | 589.7 - 656.8 | 656.8 - 723.9 | 723.9 - 776.4
776.4 - 829.0 | 829.0 - 881.6 | 881.6 - 907.8 | 907.8 - 970.2 | > 970.2
United Kingdom: Worcestershire: DALYs Rate (per 100,000)
DALYs Rate
< 555.4 | 555.4 - 590.4 | 590.4 - 657.1 | 657.1 - 723.8 | 723.8 - 776.1
776.1 - 828.3 | 828.3 - 880.6 | 880.6 - 907.8 | 907.8 - 970.5 | > 970.5
United Kingdom: Barnsley: DALYs Rate (per 100,000)
DALYs Rate
< 552.3 | 552.3 - 589.0 | 589.0 - 656.1 | 656.1 - 723.1 | 723.1 - 775.2
775.2 - 827.3 | 827.3 - 879.3 | 879.3 - 911.4 | 911.4 - 969.0 | > 969.0
United Kingdom: Bradford: DALYs Rate (per 100,000)
DALYs Rate
< 549.8 | 549.8 - 587.6 | 587.6 - 656.2 | 656.2 - 724.8 | 724.8 - 776.4
776.4 - 827.9 | 827.9 - 879.4 | 879.4 - 909.6 | 909.6 - 968.1 | > 968.1
United Kingdom: Calderdale: DALYs Rate (per 100,000)
DALYs Rate
< 553.9 | 553.9 - 591.6 | 591.6 - 660.8 | 660.8 - 730.0 | 730.0 - 781.7
781.7 - 833.5 | 833.5 - 885.2 | 885.2 - 911.2 | 911.2 - 969.3 | > 969.3
United Kingdom: Doncaster: DALYs Rate (per 100,000)
DALYs Rate
< 551.1 | 551.1 - 589.8 | 589.8 - 655.7 | 655.7 - 721.5 | 721.5 - 774.8
774.8 - 828.1 | 828.1 - 881.4 | 881.4 - 909.5 | 909.5 - 967.5 | > 967.5
United Kingdom: East Riding of Yorkshire: DALYs Rate (per 100,000)
DALYs Rate
< 553.6 | 553.6 - 590.8 | 590.8 - 656.5 | 656.5 - 722.1 | 722.1 - 775.1
775.1 - 828.0 | 828.0 - 880.9 | 880.9 - 909.2 | 909.2 - 968.6 | > 968.6
United Kingdom: Kingston upon Hull, City of: DALYs Rate (per 100,000)
DALYs Rate
< 552.8 | 552.8 - 590.5 | 590.5 - 657.9 | 657.9 - 725.3 | 725.3 - 777.1
777.1 - 828.9 | 828.9 - 880.7 | 880.7 - 905.8 | 905.8 - 968.2 | > 968.2
Female
Male
Sexes Combined
Age
20-24 years
15-19 years
10-14 years
Year
1990
1995
2000
2005
2010
2015
2020
DALYs Rate (per 100,000)
600
800
1000
1200
Age
10-24 years

Figure S3

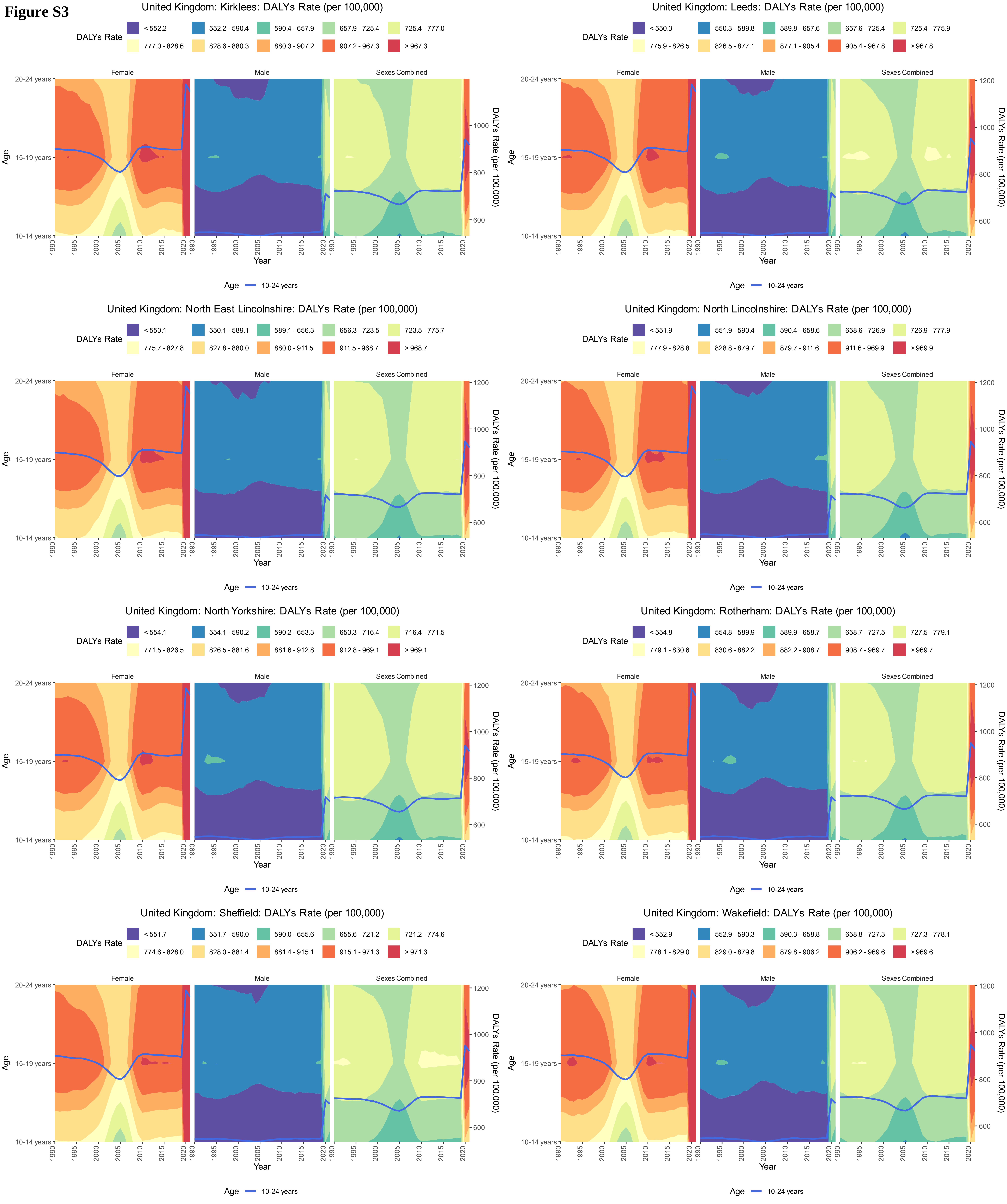
United Kingdom: Kirklees: DALYs Rate (per 100,000)
DALYs Rate
< 552.2
552.2 - 590.4
590.4 - 657.9
657.9 - 725.4
725.4 - 777.0
777.0 - 828.6
828.6 - 880.3
880.3 - 907.2
907.2 - 967.3
> 967.3
United Kingdom: Leeds: DALYs Rate (per 100,000)
DALYs Rate
< 550.3
550.3 - 589.8
589.8 - 657.6
657.6 - 725.4
725.4 - 775.9
775.9 - 826.5
826.5 - 877.1
877.1 - 905.4
905.4 - 967.8
> 967.8
United Kingdom: North East Lincolnshire: DALYs Rate (per 100,000)
DALYs Rate
< 550.1
550.1 - 589.1
589.1 - 656.3
656.3 - 723.5
723.5 - 775.7
775.7 - 827.8
827.8 - 880.0
880.0 - 911.5
911.5 - 968.7
> 968.7
United Kingdom: North Lincolnshire: DALYs Rate (per 100,000)
DALYs Rate
< 551.9
551.9 - 590.4
590.4 - 658.6
658.6 - 726.9
726.9 - 777.9
777.9 - 828.8
828.8 - 879.7
879.7 - 911.6
911.6 - 969.9
> 969.9
United Kingdom: North Yorkshire: DALYs Rate (per 100,000)
DALYs Rate
< 554.1
554.1 - 590.2
590.2 - 653.3
653.3 - 716.4
716.4 - 771.5
771.5 - 826.5
826.5 - 881.6
881.6 - 912.8
912.8 - 969.1
> 969.1
United Kingdom: Rotherham: DALYs Rate (per 100,000)
DALYs Rate
< 554.8
554.8 - 589.9
589.9 - 658.7
658.7 - 727.5
727.5 - 779.1
779.1 - 830.6
830.6 - 882.2
882.2 - 908.7
908.7 - 969.7
> 969.7
United Kingdom: Sheffield: DALYs Rate (per 100,000)
DALYs Rate
< 551.7
551.7 - 590.0
590.0 - 655.6
655.6 - 721.2
721.2 - 774.6
774.6 - 828.0
828.0 - 881.4
881.4 - 915.1
915.1 - 971.3
> 971.3
United Kingdom: Wakefield: DALYs Rate (per 100,000)
DALYs Rate
< 552.9
552.9 - 590.3
590.3 - 658.8
658.8 - 727.3
727.3 - 778.1
778.1 - 829.0
829.0 - 879.8
879.8 - 906.2
906.2 - 969.6
> 969.6
Female
Male
Sexes Combined
Age
20-24 years
15-19 years
10-14 years
Year
1990
1995
2000
2005
2010
2015
2020
DALYs Rate (per 100,000)
600
800
1000
1200
Age
10-24 years

Figure S3

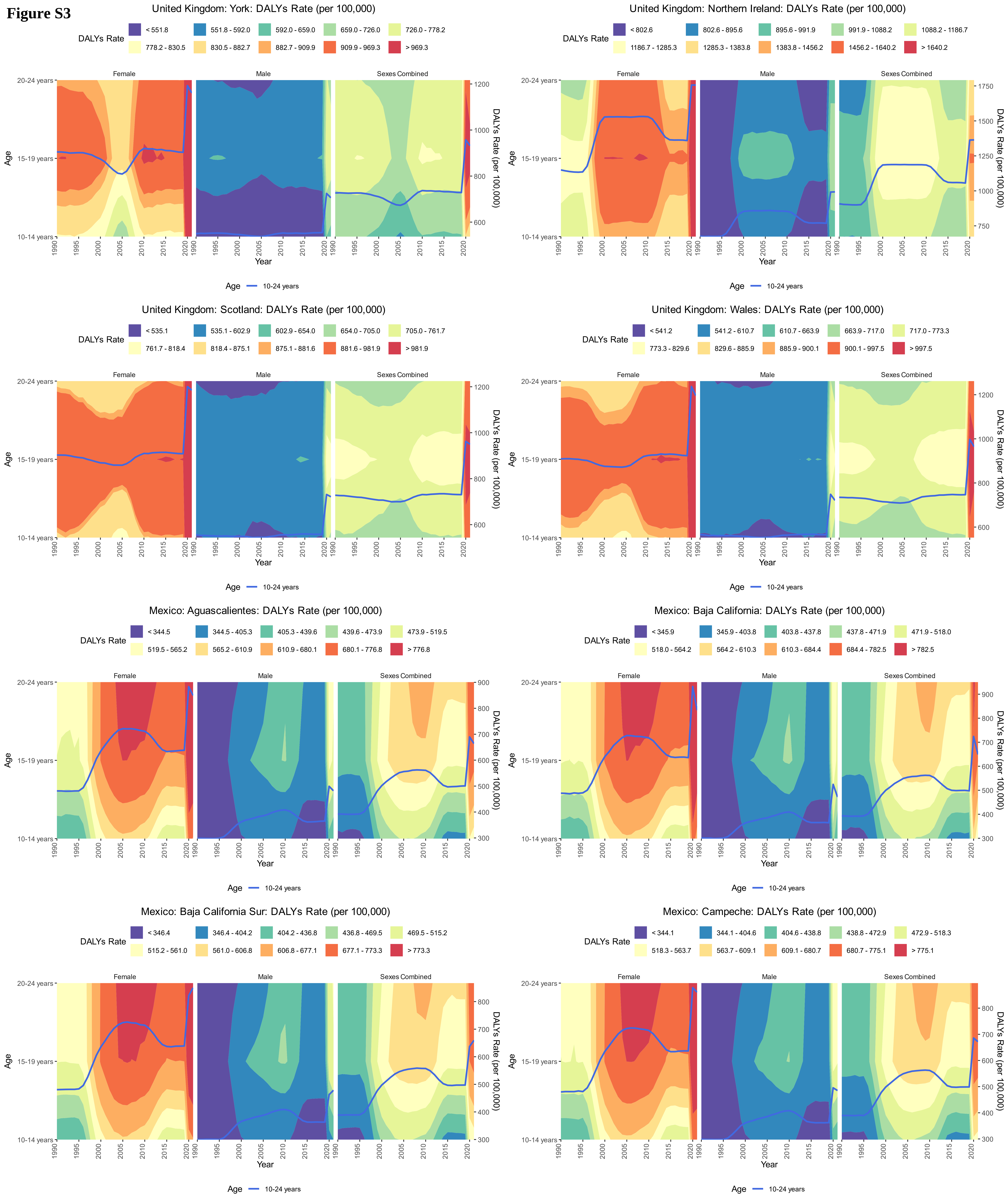
United Kingdom: York: DALYs Rate (per 100,000)
DALYs Rate
< 551.8
551.8 - 592.0
592.0 - 659.0
659.0 - 726.0
726.0 - 778.2
778.2 - 830.5
830.5 - 882.7
882.7 - 909.9
909.9 - 969.3
> 969.3
United Kingdom: Northern Ireland: DALYs Rate (per 100,000)
DALYs Rate
< 802.6
802.6 - 895.6
895.6 - 991.9
991.9 - 1088.2
1088.2 - 1186.7
1186.7 - 1285.3
1285.3 - 1383.8
1383.8 - 1456.2
1456.2 - 1640.2
> 1640.2
United Kingdom: Scotland: DALYs Rate (per 100,000)
DALYs Rate
< 535.1
535.1 - 602.9
602.9 - 654.0
654.0 - 705.0
705.0 - 761.7
761.7 - 818.4
818.4 - 875.1
875.1 - 881.6
881.6 - 981.9
> 981.9
United Kingdom: Wales: DALYs Rate (per 100,000)
DALYs Rate
< 541.2
541.2 - 610.7
610.7 - 663.9
663.9 - 717.0
717.0 - 773.3
773.3 - 829.6
829.6 - 885.9
885.9 - 900.1
900.1 - 997.5
> 997.5
Mexico: Aguascalientes: DALYs Rate (per 100,000)
DALYs Rate
< 344.5
344.5 - 405.3
405.3 - 439.6
439.6 - 473.9
473.9 - 519.5
519.5 - 565.2
565.2 - 610.9
610.9 - 680.1
680.1 - 776.8
> 776.8
Mexico: Baja California: DALYs Rate (per 100,000)
DALYs Rate
< 345.9
345.9 - 403.8
403.8 - 437.8
437.8 - 471.9
471.9 - 518.0
518.0 - 564.2
564.2 - 610.3
610.3 - 684.4
684.4 - 782.5
> 782.5
Mexico: Baja California Sur: DALYs Rate (per 100,000)
DALYs Rate
< 346.4
346.4 - 404.2
404.2 - 436.8
436.8 - 469.5
469.5 - 515.2
515.2 - 561.0
561.0 - 606.8
606.8 - 677.1
677.1 - 773.3
> 773.3
Mexico: Campeche: DALYs Rate (per 100,000)
DALYs Rate
< 344.1
344.1 - 404.6
404.6 - 438.8
438.8 - 472.9
472.9 - 518.3
518.3 - 563.7
563.7 - 609.1
609.1 - 680.7
680.7 - 775.1
> 775.1
Female
Male
Sexes Combined
Age
20-24 years
15-19 years
10-14 years
Year
DALYs Rate (per 100,000)
Age — 10-24 years

Figure S3

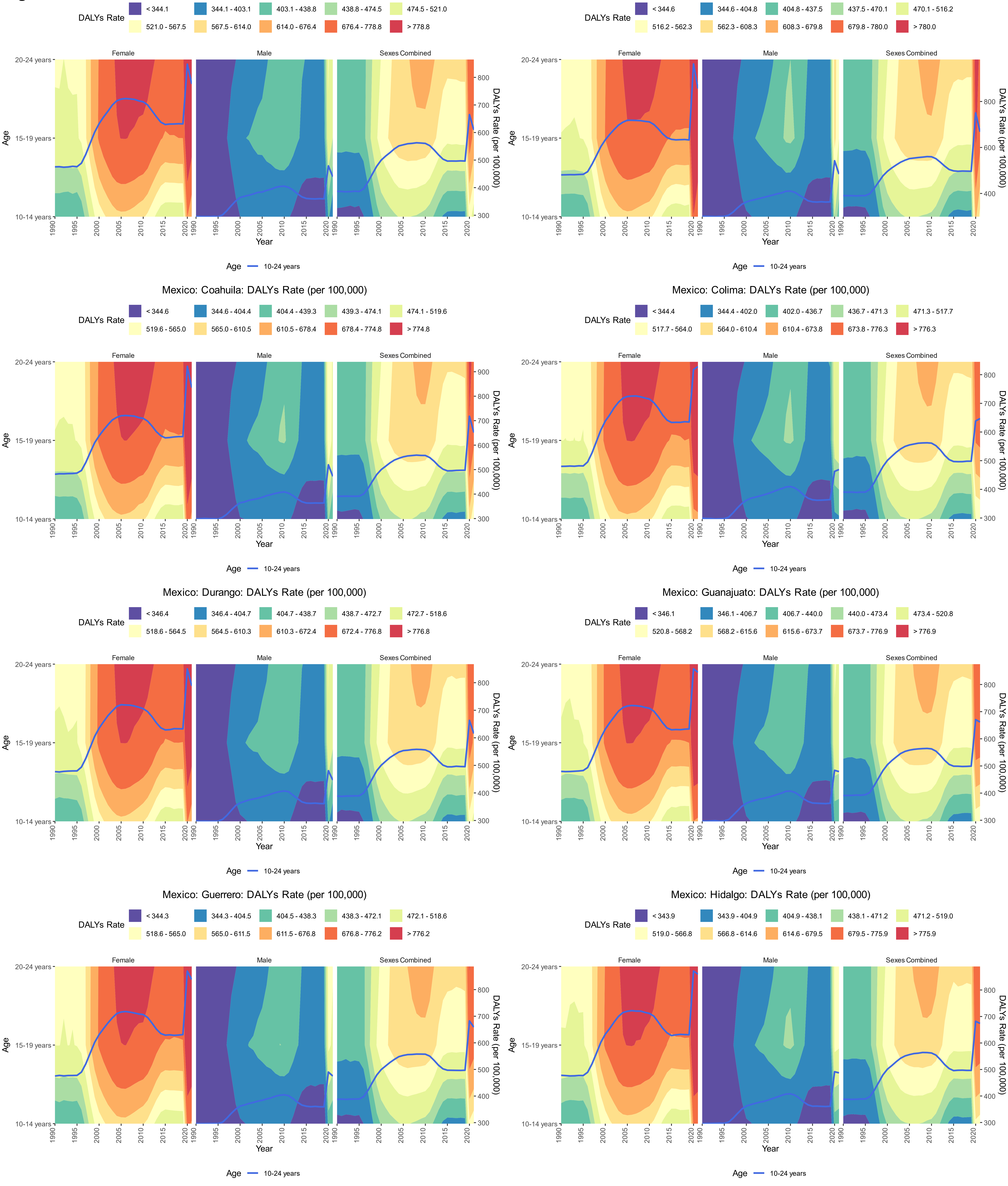

Mexico: Chiapas: DALYs Rate (per 100,000)
DALYs Rate
< 344.1
344.1 - 403.1
403.1 - 438.8
438.8 - 474.5
474.5 - 521.0
521.0 - 567.5
567.5 - 614.0
614.0 - 676.4
676.4 - 778.8
> 778.8
Mexico: Chihuahua: DALYs Rate (per 100,000)
DALYs Rate
< 344.6
344.6 - 404.8
404.8 - 437.5
437.5 - 470.1
470.1 - 516.2
516.2 - 562.3
562.3 - 608.3
608.3 - 679.8
679.8 - 780.0
> 780.0
Mexico: Coahuila: DALYs Rate (per 100,000)
DALYs Rate
< 344.6
344.6 - 404.4
404.4 - 439.3
439.3 - 474.1
474.1 - 519.6
519.6 - 565.0
565.0 - 610.5
610.5 - 678.4
678.4 - 774.8
> 774.8
Mexico: Colima: DALYs Rate (per 100,000)
DALYs Rate
< 344.4
344.4 - 402.0
402.0 - 436.7
436.7 - 471.3
471.3 - 517.7
517.7 - 564.0
564.0 - 610.4
610.4 - 673.8
673.8 - 776.3
> 776.3
Mexico: Durango: DALYs Rate (per 100,000)
DALYs Rate
< 346.4
346.4 - 404.7
404.7 - 438.7
438.7 - 472.7
472.7 - 518.6
518.6 - 564.5
564.5 - 610.3
610.3 - 672.4
672.4 - 776.8
> 776.8
Mexico: Guanajuato: DALYs Rate (per 100,000)
DALYs Rate
< 346.1
346.1 - 406.7
406.7 - 440.0
440.0 - 473.4
473.4 - 520.8
520.8 - 568.2
568.2 - 615.6
615.6 - 673.7
673.7 - 776.9
> 776.9
Mexico: Guerrero: DALYs Rate (per 100,000)
DALYs Rate
< 344.3
344.3 - 404.5
404.5 - 438.3
438.3 - 472.1
472.1 - 518.6
518.6 - 565.0
565.0 - 611.5
611.5 - 676.8
676.8 - 776.2
> 776.2
Mexico: Hidalgo: DALYs Rate (per 100,000)
DALYs Rate
< 343.9
343.9 - 404.9
404.9 - 438.1
438.1 - 471.2
471.2 - 519.0
519.0 - 566.8
566.8 - 614.6
614.6 - 679.5
679.5 - 775.9
> 775.9
Female
Male
Sexes Combined
Age
20-24 years
15-19 years
10-14 years
Year
DALYs Rate (per 100,000)
Age
10-24 years

Figure S3

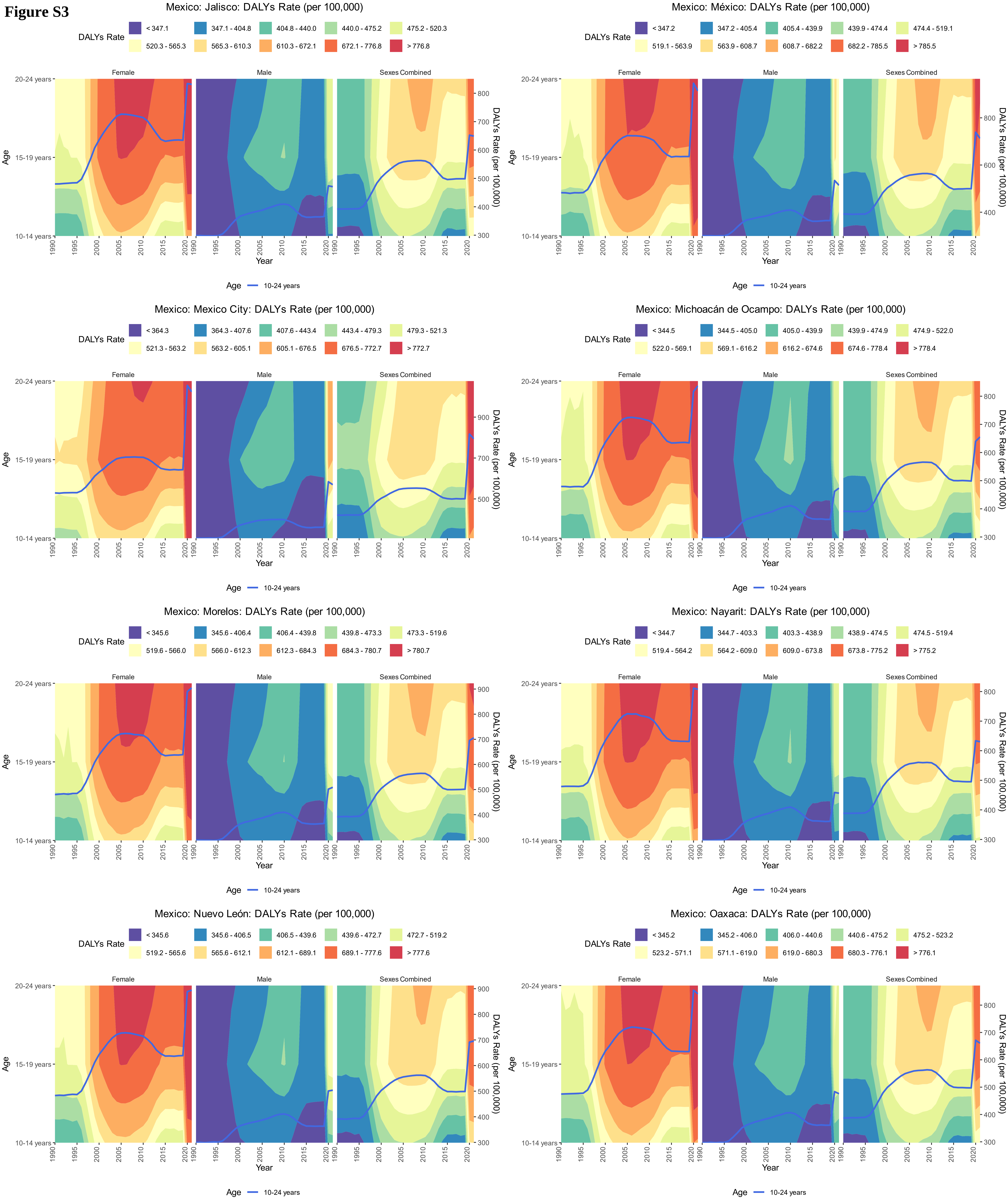
Mexico: Jalisco: DALYs Rate (per 100,000)
DALYs Rate
< 347.1
347.1 - 404.8
404.8 - 440.0
440.0 - 475.2
475.2 - 520.3
520.3 - 565.3
565.3 - 610.3
610.3 - 672.1
672.1 - 776.8
> 776.8
Mexico: México: DALYs Rate (per 100,000)
DALYs Rate
< 347.2
347.2 - 405.4
405.4 - 439.9
439.9 - 474.4
474.4 - 519.1
519.1 - 563.9
563.9 - 608.7
608.7 - 682.2
682.2 - 785.5
> 785.5
Mexico: Mexico City: DALYs Rate (per 100,000)
DALYs Rate
< 364.3
364.3 - 407.6
407.6 - 443.4
443.4 - 479.3
479.3 - 521.3
521.3 - 563.2
563.2 - 605.1
605.1 - 676.5
676.5 - 772.7
> 772.7
Mexico: Michoacán de Ocampo: DALYs Rate (per 100,000)
DALYs Rate
< 344.5
344.5 - 405.0
405.0 - 439.9
439.9 - 474.9
474.9 - 522.0
522.0 - 569.1
569.1 - 616.2
616.2 - 674.6
674.6 - 778.4
> 778.4
Mexico: Morelos: DALYs Rate (per 100,000)
DALYs Rate
< 345.6
345.6 - 406.4
406.4 - 439.8
439.8 - 473.3
473.3 - 519.6
519.6 - 566.0
566.0 - 612.3
612.3 - 684.3
684.3 - 780.7
> 780.7
Mexico: Nayarit: DALYs Rate (per 100,000)
DALYs Rate
< 344.7
344.7 - 403.3
403.3 - 438.9
438.9 - 474.5
474.5 - 519.4
519.4 - 564.2
564.2 - 609.0
609.0 - 673.8
673.8 - 775.2
> 775.2
Mexico: Nuevo León: DALYs Rate (per 100,000)
DALYs Rate
< 345.6
345.6 - 406.5
406.5 - 439.6
439.6 - 472.7
472.7 - 519.2
519.2 - 565.6
565.6 - 612.1
612.1 - 689.1
689.1 - 777.6
> 777.6
Mexico: Oaxaca: DALYs Rate (per 100,000)
DALYs Rate
< 345.2
345.2 - 406.0
406.0 - 440.6
440.6 - 475.2
475.2 - 523.2
523.2 - 571.1
571.1 - 619.0
619.0 - 680.3
680.3 - 776.1
> 776.1
Female
Male
Sexes Combined
Age
20-24 years
15-19 years
10-14 years
Year
DALYs Rate (per 100,000)
Age
10-24 years

Figure S3

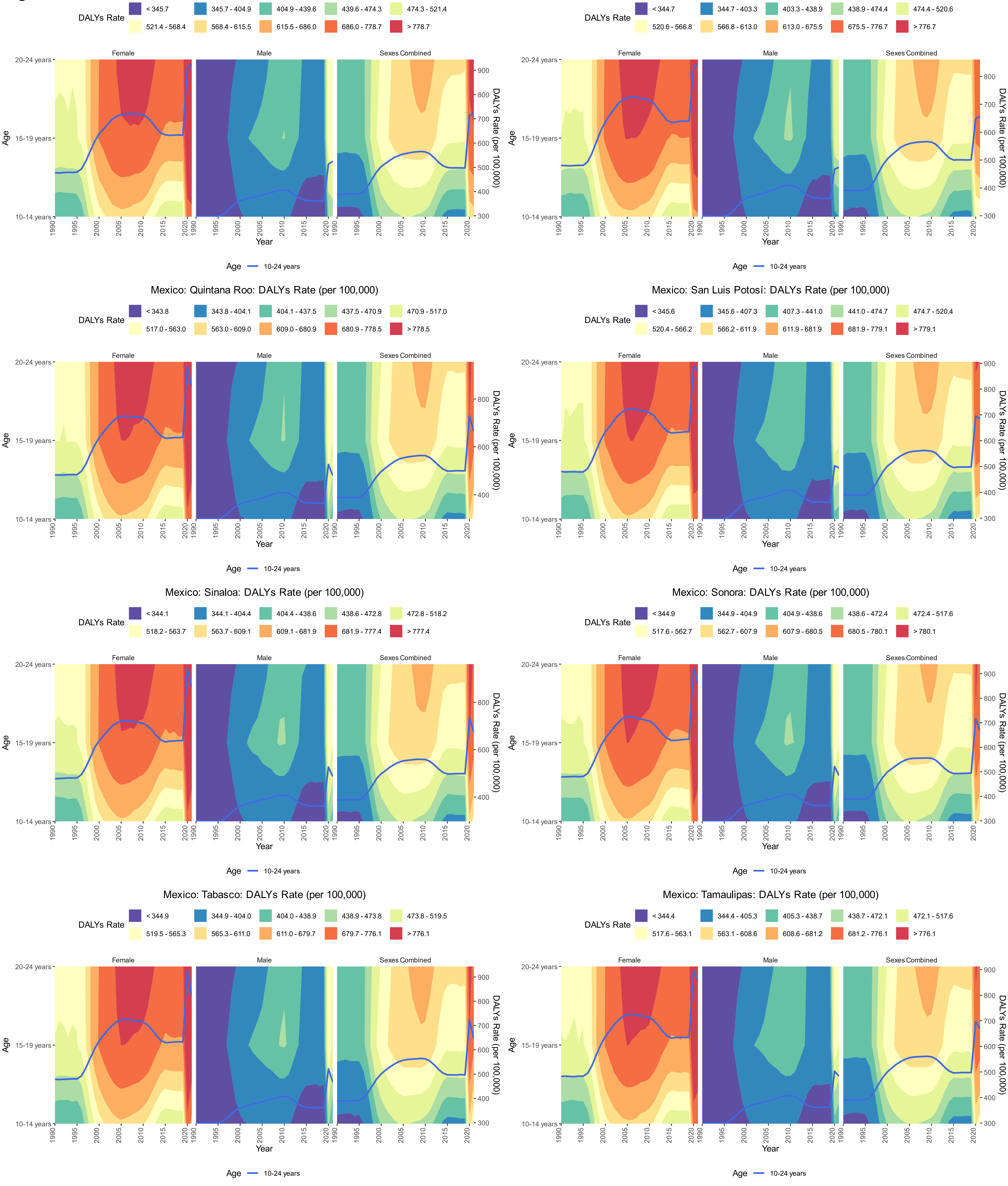
Mexico: Puebla: DALYs Rate (per 100,000)
DALYs Rate
< 345.7
345.7 - 404.9
404.9 - 439.6
439.6 - 474.3
474.3 - 521.4
521.4 - 568.4
568.4 - 615.5
615.5 - 686.0
686.0 - 778.7
> 778.7
Mexico: Querétaro: DALYs Rate (per 100,000)
< 344.7
344.7 - 403.3
403.3 - 438.9
438.9 - 474.4
474.4 - 520.6
520.6 - 566.8
566.8 - 613.0
613.0 - 675.5
675.5 - 776.7
> 776.7
Mexico: Quintana Roo: DALYs Rate (per 100,000)
< 343.8
343.8 - 404.1
404.1 - 437.5
437.5 - 470.9
470.9 - 517.0
517.0 - 563.0
563.0 - 609.0
609.0 - 680.9
680.9 - 778.5
> 778.5
Mexico: San Luis Potosí: DALYs Rate (per 100,000)
< 345.6
345.6 - 407.3
407.3 - 441.0
441.0 - 474.7
474.7 - 520.4
520.4 - 566.2
566.2 - 611.9
611.9 - 681.9
681.9 - 779.1
> 779.1
Mexico: Sinaloa: DALYs Rate (per 100,000)
< 344.1
344.1 - 404.4
404.4 - 438.6
438.6 - 472.8
472.8 - 518.2
518.2 - 563.7
563.7 - 609.1
609.1 - 681.9
681.9 - 777.4
> 777.4
Mexico: Sonora: DALYs Rate (per 100,000)
< 344.9
344.9 - 404.9
404.9 - 438.6
438.6 - 472.4
472.4 - 517.6
517.6 - 562.7
562.7 - 607.9
607.9 - 680.5
680.5 - 780.1
> 780.1
Mexico: Tabasco: DALYs Rate (per 100,000)
< 344.9
344.9 - 404.0
404.0 - 438.9
438.9 - 473.8
473.8 - 519.5
519.5 - 565.3
565.3 - 611.0
611.0 - 679.7
679.7 - 776.1
> 776.1
Mexico: Tamaulipas: DALYs Rate (per 100,000)
< 344.4
344.4 - 405.3
405.3 - 438.7
438.7 - 472.1
472.1 - 517.6
517.6 - 563.1
563.1 - 608.6
608.6 - 681.2
681.2 - 776.1
> 776.1
Female
Male
Sexes Combined
Age
10-14 years
15-19 years
20-24 years
Year
DALYs Rate (per 100,000)
Age
10-24 years

Figure S3

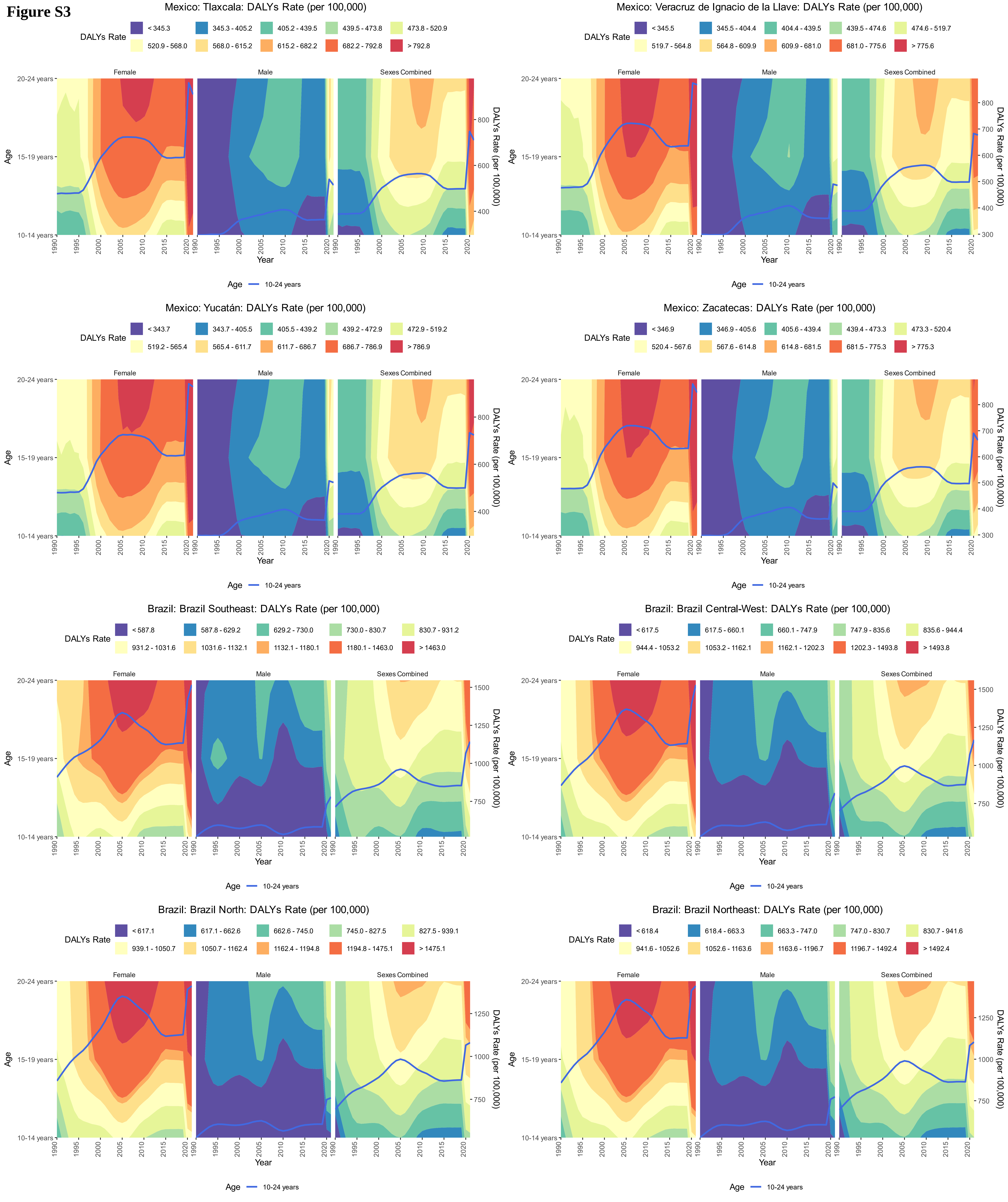
Mexico: Tlaxcala: DALYs Rate (per 100,000)
DALYs Rate
< 345.3
345.3 - 405.2
405.2 - 439.5
439.5 - 473.8
473.8 - 520.9
520.9 - 568.0
568.0 - 615.2
615.2 - 682.2
682.2 - 792.8
> 792.8
Mexico: Veracruz de Ignacio de la Llave: DALYs Rate (per 100,000)
DALYs Rate
< 345.5
345.5 - 404.4
404.4 - 439.5
439.5 - 474.6
474.6 - 519.7
519.7 - 564.8
564.8 - 609.9
609.9 - 681.0
681.0 - 775.6
> 775.6
Mexico: Yucatán: DALYs Rate (per 100,000)
DALYs Rate
< 343.7
343.7 - 405.5
405.5 - 439.2
439.2 - 472.9
472.9 - 519.2
519.2 - 565.4
565.4 - 611.7
611.7 - 686.7
686.7 - 786.9
> 786.9
Mexico: Zacatecas: DALYs Rate (per 100,000)
DALYs Rate
< 346.9
346.9 - 405.6
405.6 - 439.4
439.4 - 473.3
473.3 - 520.4
520.4 - 567.6
567.6 - 614.8
614.8 - 681.5
681.5 - 775.3
> 775.3
Brazil: Brazil Southeast: DALYs Rate (per 100,000)
DALYs Rate
< 587.8
587.8 - 629.2
629.2 - 730.0
730.0 - 830.7
830.7 - 931.2
931.2 - 1031.6
1031.6 - 1132.1
1132.1 - 1180.1
1180.1 - 1463.0
> 1463.0
Brazil: Brazil Central-West: DALYs Rate (per 100,000)
DALYs Rate
< 617.5
617.5 - 660.1
660.1 - 747.9
747.9 - 835.6
835.6 - 944.4
944.4 - 1053.2
1053.2 - 1162.1
1162.1 - 1202.3
1202.3 - 1493.8
> 1493.8
Brazil: Brazil North: DALYs Rate (per 100,000)
DALYs Rate
< 617.1
617.1 - 662.6
662.6 - 745.0
745.0 - 827.5
827.5 - 939.1
939.1 - 1050.7
1050.7 - 1162.4
1162.4 - 1194.8
1194.8 - 1475.1
> 1475.1
Brazil: Brazil Northeast: DALYs Rate (per 100,000)
DALYs Rate
< 618.4
618.4 - 663.3
663.3 - 747.0
747.0 - 830.7
830.7 - 941.6
941.6 - 1052.6
1052.6 - 1163.6
1163.6 - 1196.7
1196.7 - 1492.4
> 1492.4
Female
Male
Sexes Combined
Age
20-24 years
15-19 years
10-14 years
Year
DALYs Rate (per 100,000)
Age
10-24 years

Figure S3

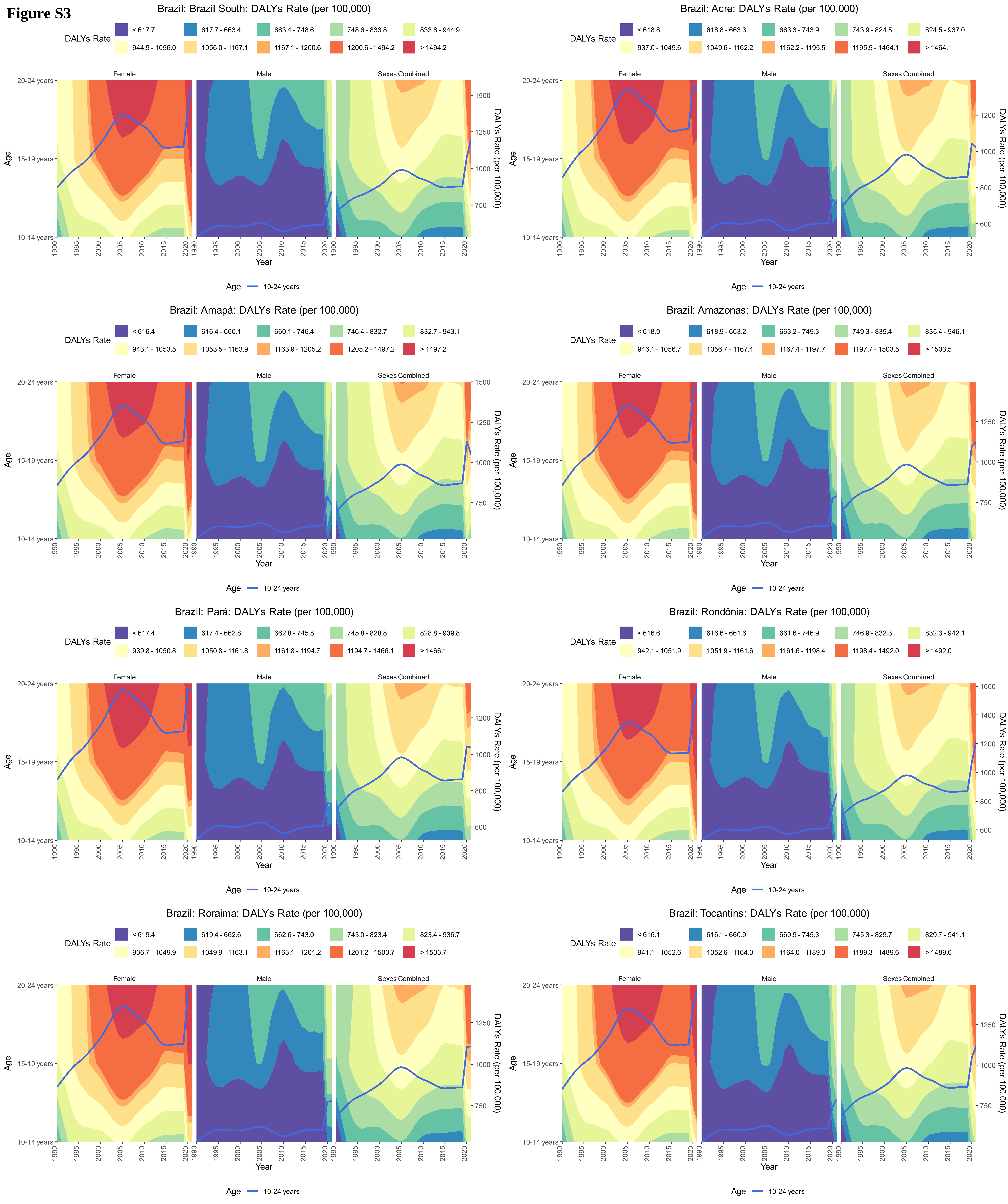
Brazil: Brazil South: DALYs Rate (per 100,000)
DALYs Rate
< 617.7
617.7 - 663.4
663.4 - 748.6
748.6 - 833.8
833.8 - 944.9
944.9 - 1056.0
1056.0 - 1167.1
1167.1 - 1200.6
1200.6 - 1494.2
> 1494.2
Female
Male
Sexes Combined
Age
20-24 years
15-19 years
10-14 years
Year
DALYs Rate (per 100,000)
Age — 10-24 years
Brazil: Acre: DALYs Rate (per 100,000)
< 618.8
618.8 - 663.3
663.3 - 743.9
743.9 - 824.5
824.5 - 937.0
937.0 - 1049.6
1049.6 - 1162.2
1162.2 - 1195.5
1195.5 - 1464.1
> 1464.1
Brazil: Amapá: DALYs Rate (per 100,000)
< 616.4
616.4 - 660.1
660.1 - 746.4
746.4 - 832.7
832.7 - 943.1
943.1 - 1053.5
1053.5 - 1163.9
1163.9 - 1205.2
1205.2 - 1497.2
> 1497.2
Brazil: Amazonas: DALYs Rate (per 100,000)
< 618.9
618.9 - 663.2
663.2 - 749.3
749.3 - 835.4
835.4 - 946.1
946.1 - 1056.7
1056.7 - 1167.4
1167.4 - 1197.7
1197.7 - 1503.5
> 1503.5
Brazil: Pará: DALYs Rate (per 100,000)
< 617.4
617.4 - 662.8
662.8 - 745.8
745.8 - 828.8
828.8 - 939.8
939.8 - 1050.8
1050.8 - 1161.8
1161.8 - 1194.7
1194.7 - 1466.1
> 1466.1
Brazil: Rondônia: DALYs Rate (per 100,000)
< 616.6
616.6 - 661.6
661.6 - 746.9
746.9 - 832.3
832.3 - 942.1
942.1 - 1051.9
1051.9 - 1161.6
1161.6 - 1198.4
1198.4 - 1492.0
> 1492.0
Brazil: Roraima: DALYs Rate (per 100,000)
< 619.4
619.4 - 662.6
662.6 - 743.0
743.0 - 823.4
823.4 - 936.7
936.7 - 1049.9
1049.9 - 1163.1
1163.1 - 1201.2
1201.2 - 1503.7
> 1503.7
Brazil: Tocantins: DALYs Rate (per 100,000)
< 616.1
616.1 - 660.9
660.9 - 745.3
745.3 - 829.7
829.7 - 941.1
941.1 - 1052.6
1052.6 - 1164.0
1164.0 - 1189.3
1189.3 - 1489.6
> 1489.6

Figure S3

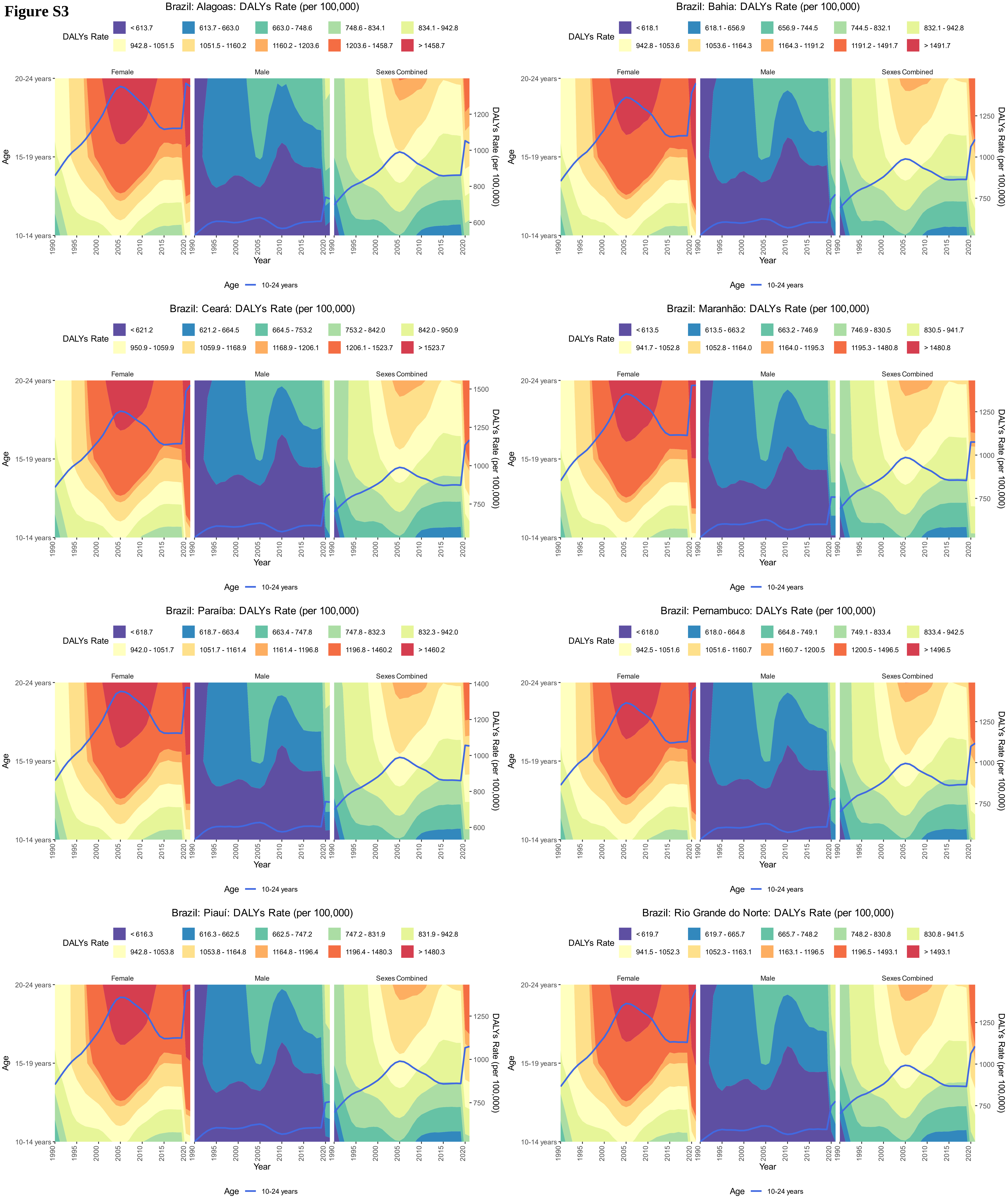

Brazil: Alagoas: DALYs Rate (per 100,000)
DALYs Rate
< 613.7
613.7 - 663.0
663.0 - 748.6
748.6 - 834.1
834.1 - 942.8
942.8 - 1051.5
1051.5 - 1160.2
1160.2 - 1203.6
1203.6 - 1458.7
> 1458.7
Brazil: Bahia: DALYs Rate (per 100,000)
< 618.1
618.1 - 656.9
656.9 - 744.5
744.5 - 832.1
832.1 - 942.8
942.8 - 1053.6
1053.6 - 1164.3
1164.3 - 1191.2
1191.2 - 1491.7
> 1491.7
Brazil: Ceará: DALYs Rate (per 100,000)
< 621.2
621.2 - 664.5
664.5 - 753.2
753.2 - 842.0
842.0 - 950.9
950.9 - 1059.9
1059.9 - 1168.9
1168.9 - 1206.1
1206.1 - 1523.7
> 1523.7
Brazil: Maranhão: DALYs Rate (per 100,000)
< 613.5
613.5 - 663.2
663.2 - 746.9
746.9 - 830.5
830.5 - 941.7
941.7 - 1052.8
1052.8 - 1164.0
1164.0 - 1195.3
1195.3 - 1480.8
> 1480.8
Brazil: Paraíba: DALYs Rate (per 100,000)
< 618.7
618.7 - 663.4
663.4 - 747.8
747.8 - 832.3
832.3 - 942.0
942.0 - 1051.7
1051.7 - 1161.4
1161.4 - 1196.8
1196.8 - 1460.2
> 1460.2
Brazil: Pernambuco: DALYs Rate (per 100,000)
< 618.0
618.0 - 664.8
664.8 - 749.1
749.1 - 833.4
833.4 - 942.5
942.5 - 1051.6
1051.6 - 1160.7
1160.7 - 1200.5
1200.5 - 1496.5
> 1496.5
Brazil: Piauí: DALYs Rate (per 100,000)
< 616.3
616.3 - 662.5
662.5 - 747.2
747.2 - 831.9
831.9 - 942.8
942.8 - 1053.8
1053.8 - 1164.8
1164.8 - 1196.4
1196.4 - 1480.3
> 1480.3
Brazil: Rio Grande do Norte: DALYs Rate (per 100,000)
< 619.7
619.7 - 665.7
665.7 - 748.2
748.2 - 830.8
830.8 - 941.5
941.5 - 1052.3
1052.3 - 1163.1
1163.1 - 1196.5
1196.5 - 1493.1
> 1493.1
Female
Male
Sexes Combined
Age
20-24 years
15-19 years
10-14 years
Year
DALYs Rate (per 100,000)
10-24 years

Figure S3

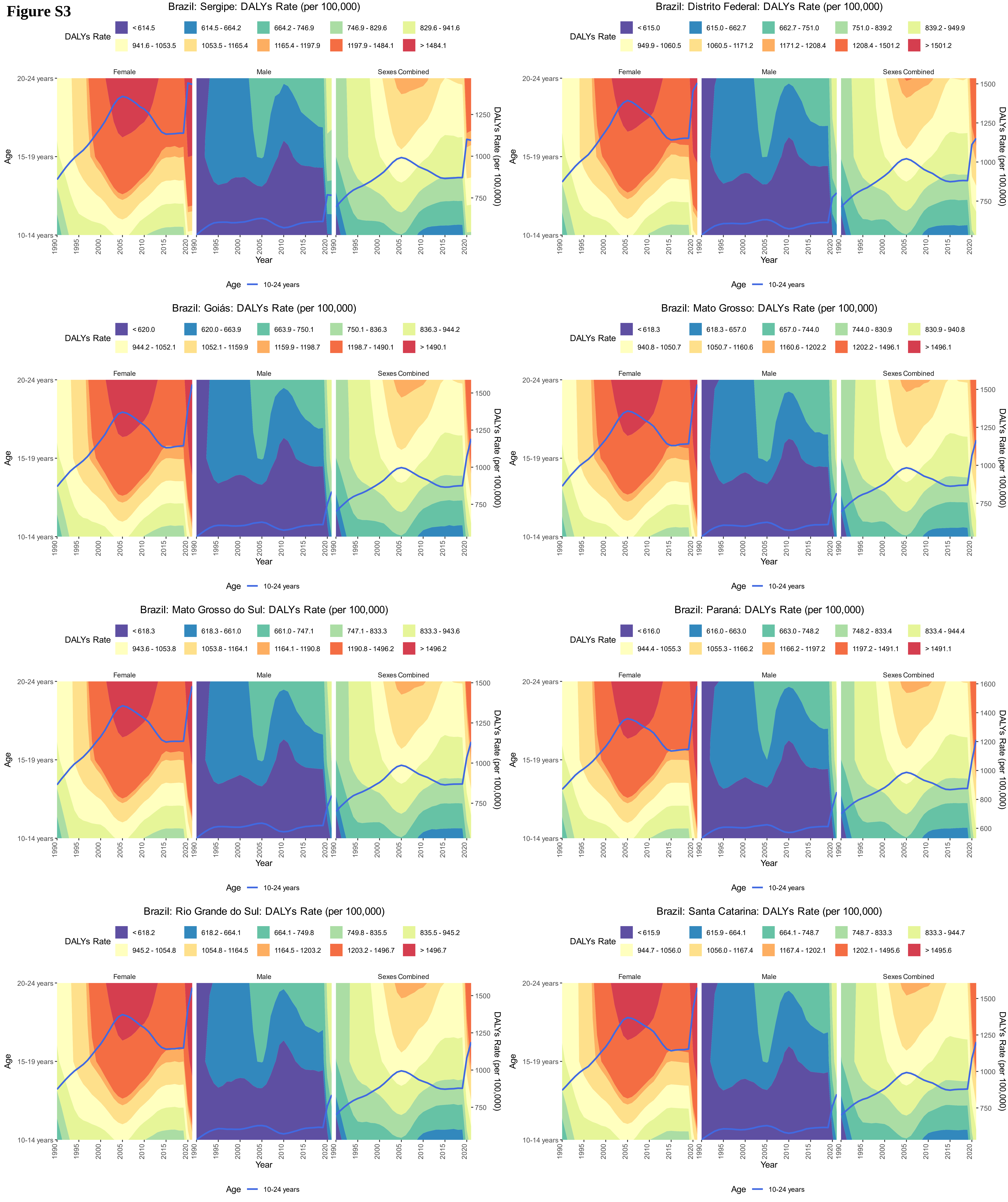

Brazil: Sergipe: DALYs Rate (per 100,000)
DALYs Rate
< 614.5
614.5 - 664.2
664.2 - 746.9
746.9 - 829.6
829.6 - 941.6
941.6 - 1053.5
1053.5 - 1165.4
1165.4 - 1197.9
1197.9 - 1484.1
> 1484.1
Brazil: Distrito Federal: DALYs Rate (per 100,000)
DALYs Rate
< 615.0
615.0 - 662.7
662.7 - 751.0
751.0 - 839.2
839.2 - 949.9
949.9 - 1060.5
1060.5 - 1171.2
1171.2 - 1208.4
1208.4 - 1501.2
> 1501.2
Brazil: Goiás: DALYs Rate (per 100,000)
DALYs Rate
< 620.0
620.0 - 663.9
663.9 - 750.1
750.1 - 836.3
836.3 - 944.2
944.2 - 1052.1
1052.1 - 1159.9
1159.9 - 1198.7
1198.7 - 1490.1
> 1490.1
Brazil: Mato Grosso: DALYs Rate (per 100,000)
DALYs Rate
< 618.3
618.3 - 657.0
657.0 - 744.0
744.0 - 830.9
830.9 - 940.8
940.8 - 1050.7
1050.7 - 1160.6
1160.6 - 1202.2
1202.2 - 1496.1
> 1496.1
Brazil: Mato Grosso do Sul: DALYs Rate (per 100,000)
DALYs Rate
< 618.3
618.3 - 661.0
661.0 - 747.1
747.1 - 833.3
833.3 - 943.6
943.6 - 1053.8
1053.8 - 1164.1
1164.1 - 1190.8
1190.8 - 1496.2
> 1496.2
Brazil: Paraná: DALYs Rate (per 100,000)
DALYs Rate
< 616.0
616.0 - 663.0
663.0 - 748.2
748.2 - 833.4
833.4 - 944.4
944.4 - 1055.3
1055.3 - 1166.2
1166.2 - 1197.2
1197.2 - 1491.1
> 1491.1
Brazil: Rio Grande do Sul: DALYs Rate (per 100,000)
DALYs Rate
< 618.2
618.2 - 664.1
664.1 - 749.8
749.8 - 835.5
835.5 - 945.2
945.2 - 1054.8
1054.8 - 1164.5
1164.5 - 1203.2
1203.2 - 1496.7
> 1496.7
Brazil: Santa Catarina: DALYs Rate (per 100,000)
DALYs Rate
< 615.9
615.9 - 664.1
664.1 - 748.7
748.7 - 833.3
833.3 - 944.7
944.7 - 1056.0
1056.0 - 1167.4
1167.4 - 1202.1
1202.1 - 1495.6
> 1495.6
Female
Male
Sexes Combined
Age
20-24 years
15-19 years
10-14 years
Year
DALYs Rate (per 100,000)
Age — 10-24 years

Figure S3

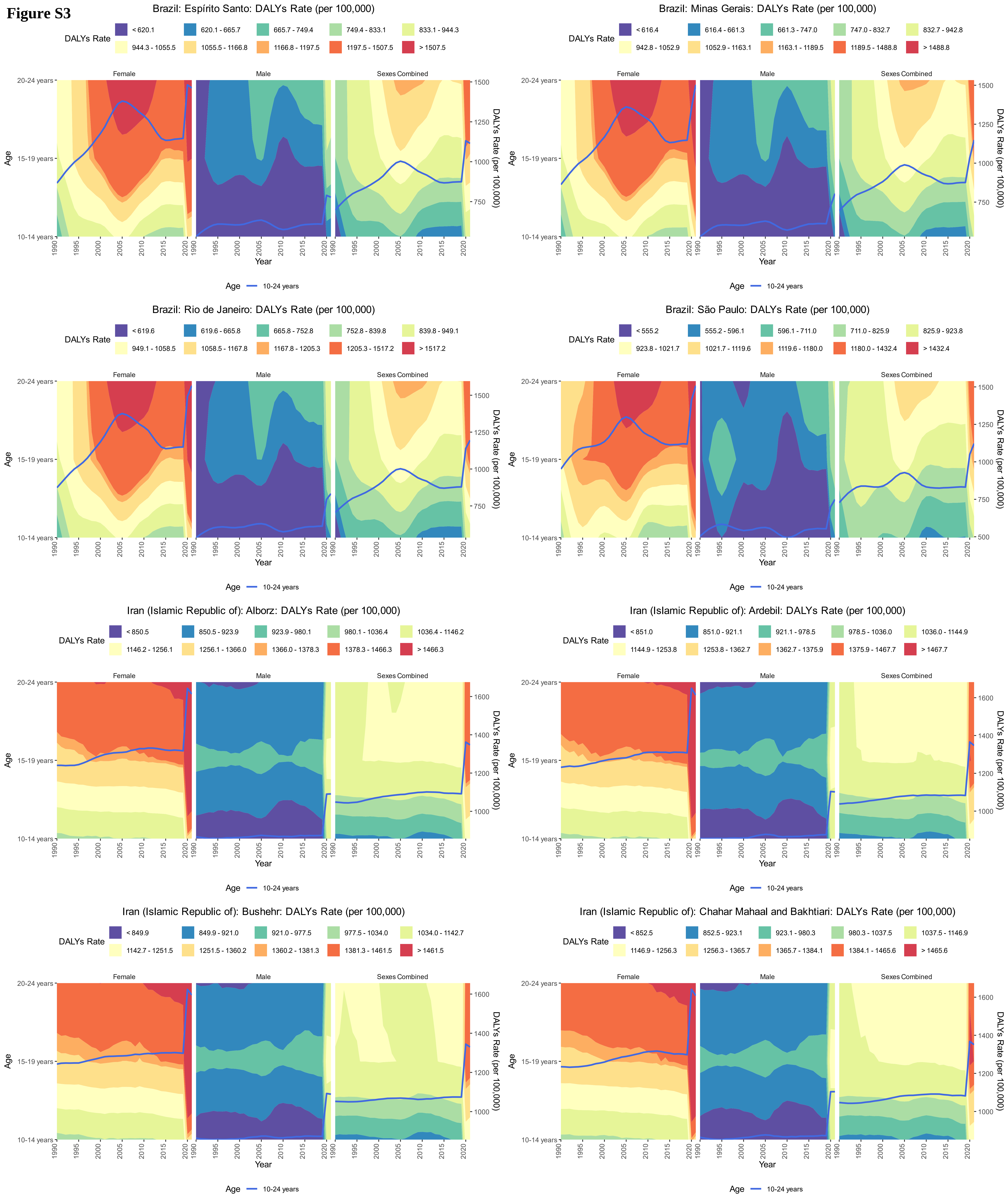

Brazil: Espírito Santo: DALYs Rate (per 100,000)
DALYs Rate
< 620.1
620.1 - 665.7
665.7 - 749.4
749.4 - 833.1
833.1 - 944.3
944.3 - 1055.5
1055.5 - 1166.8
1166.8 - 1197.5
1197.5 - 1507.5
> 1507.5
Brazil: Minas Gerais: DALYs Rate (per 100,000)
< 616.4
616.4 - 661.3
661.3 - 747.0
747.0 - 832.7
832.7 - 942.8
942.8 - 1052.9
1052.9 - 1163.1
1163.1 - 1189.5
1189.5 - 1488.8
> 1488.8
Brazil: Rio de Janeiro: DALYs Rate (per 100,000)
< 619.6
619.6 - 665.8
665.8 - 752.8
752.8 - 839.8
839.8 - 949.1
949.1 - 1058.5
1058.5 - 1167.8
1167.8 - 1205.3
1205.3 - 1517.2
> 1517.2
Brazil: São Paulo: DALYs Rate (per 100,000)
< 555.2
555.2 - 596.1
596.1 - 711.0
711.0 - 825.9
825.9 - 923.8
923.8 - 1021.7
1021.7 - 1119.6
1119.6 - 1180.0
1180.0 - 1432.4
> 1432.4
Iran (Islamic Republic of): Alborz: DALYs Rate (per 100,000)
< 850.5
850.5 - 923.9
923.9 - 980.1
980.1 - 1036.4
1036.4 - 1146.2
1146.2 - 1256.1
1256.1 - 1366.0
1366.0 - 1378.3
1378.3 - 1466.3
> 1466.3
Iran (Islamic Republic of): Ardebil: DALYs Rate (per 100,000)
< 851.0
851.0 - 921.1
921.1 - 978.5
978.5 - 1036.0
1036.0 - 1144.9
1144.9 - 1253.8
1253.8 - 1362.7
1362.7 - 1375.9
1375.9 - 1467.7
> 1467.7
Iran (Islamic Republic of): Bushehr: DALYs Rate (per 100,000)
< 849.9
849.9 - 921.0
921.0 - 977.5
977.5 - 1034.0
1034.0 - 1142.7
1142.7 - 1251.5
1251.5 - 1360.2
1360.2 - 1381.3
1381.3 - 1461.5
> 1461.5
Iran (Islamic Republic of): Chahar Mahaal and Bakhtiari: DALYs Rate (per 100,000)
< 852.5
852.5 - 923.1
923.1 - 980.3
980.3 - 1037.5
1037.5 - 1146.9
1146.9 - 1256.3
1256.3 - 1365.7
1365.7 - 1384.1
1384.1 - 1465.6
> 1465.6
Female
Male
Sexes Combined
Age
20-24 years
15-19 years
10-14 years
Year
DALYs Rate (per 100,000)
Age — 10-24 years

Figure S3

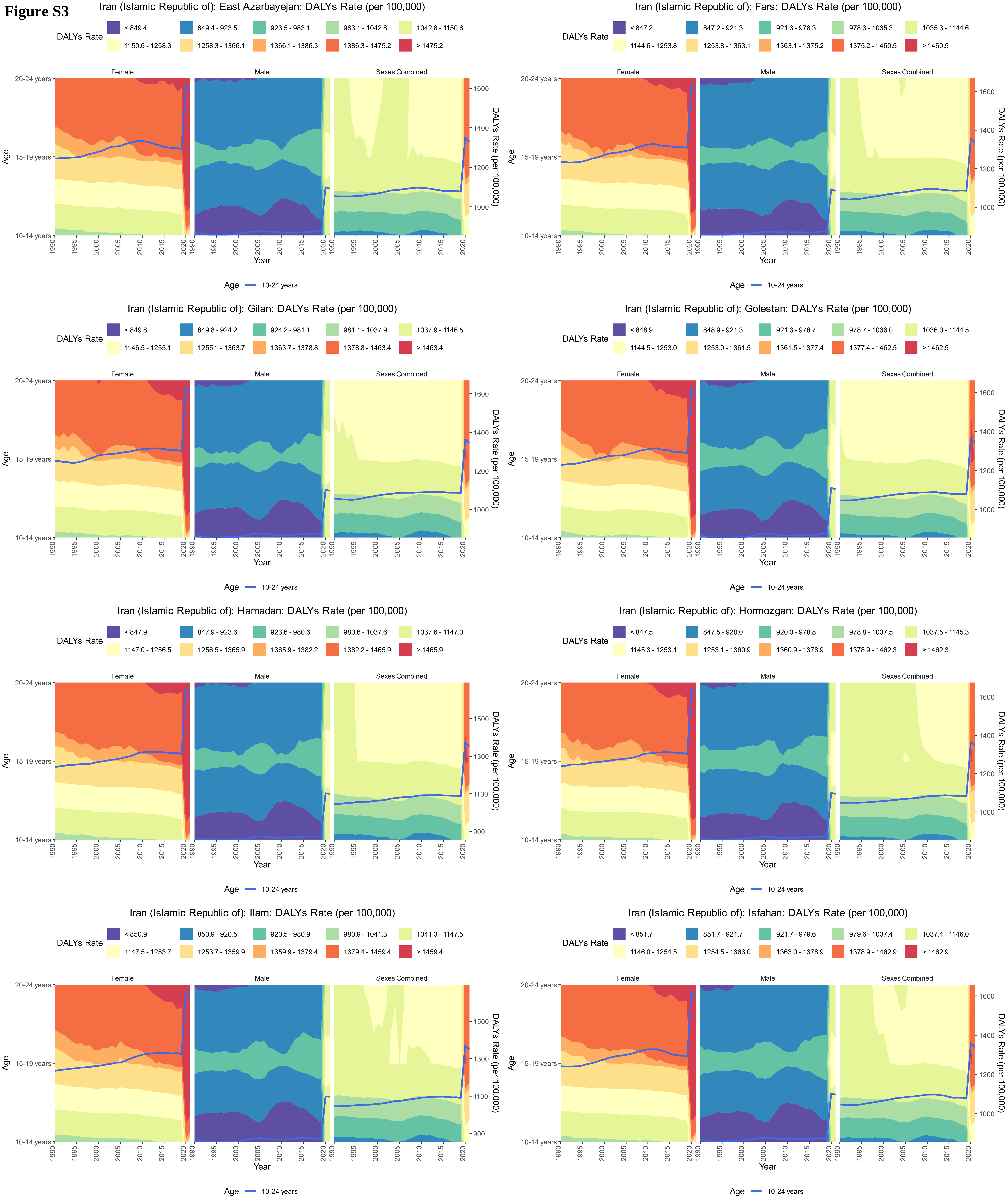

Iran (Islamic Republic of): East Azarbayejan: DALYs Rate (per 100,000)
DALYs Rate
< 849.4
849.4 - 923.5
923.5 - 983.1
983.1 - 1042.8
1042.8 - 1150.6
1150.6 - 1258.3
1258.3 - 1366.1
1366.1 - 1386.3
1386.3 - 1475.2
> 1475.2
Iran (Islamic Republic of): Fars: DALYs Rate (per 100,000)
DALYs Rate
< 847.2
847.2 - 921.3
921.3 - 978.3
978.3 - 1035.3
1035.3 - 1144.6
1144.6 - 1253.8
1253.8 - 1363.1
1363.1 - 1375.2
1375.2 - 1460.5
> 1460.5
Iran (Islamic Republic of): Gilan: DALYs Rate (per 100,000)
DALYs Rate
< 849.8
849.8 - 924.2
924.2 - 981.1
981.1 - 1037.9
1037.9 - 1146.5
1146.5 - 1255.1
1255.1 - 1363.7
1363.7 - 1378.8
1378.8 - 1463.4
> 1463.4
Iran (Islamic Republic of): Golestan: DALYs Rate (per 100,000)
DALYs Rate
< 848.9
848.9 - 921.3
921.3 - 978.7
978.7 - 1036.0
1036.0 - 1144.5
1144.5 - 1253.0
1253.0 - 1361.5
1361.5 - 1377.4
1377.4 - 1462.5
> 1462.5
Iran (Islamic Republic of): Hamadan: DALYs Rate (per 100,000)
DALYs Rate
< 847.9
847.9 - 923.6
923.6 - 980.6
980.6 - 1037.6
1037.6 - 1147.0
1147.0 - 1256.5
1256.5 - 1365.9
1365.9 - 1382.2
1382.2 - 1465.9
> 1465.9
Iran (Islamic Republic of): Hormozgan: DALYs Rate (per 100,000)
DALYs Rate
< 847.5
847.5 - 920.0
920.0 - 978.8
978.8 - 1037.5
1037.5 - 1145.3
1145.3 - 1253.1
1253.1 - 1360.9
1360.9 - 1378.9
1378.9 - 1462.3
> 1462.3
Iran (Islamic Republic of): Ilam: DALYs Rate (per 100,000)
DALYs Rate
< 850.9
850.9 - 920.5
920.5 - 980.9
980.9 - 1041.3
1041.3 - 1147.5
1147.5 - 1253.7
1253.7 - 1359.9
1359.9 - 1379.4
1379.4 - 1459.4
> 1459.4
Iran (Islamic Republic of): Isfahan: DALYs Rate (per 100,000)
DALYs Rate
< 851.7
851.7 - 921.7
921.7 - 979.6
979.6 - 1037.4
1037.4 - 1146.0
1146.0 - 1254.5
1254.5 - 1363.0
1363.0 - 1378.9
1378.9 - 1462.9
> 1462.9
Female
Male
Sexes Combined
Age
20-24 years
15-19 years
10-14 years
Year
DALYs Rate (per 100,000)
Age
10-24 years

Figure S3

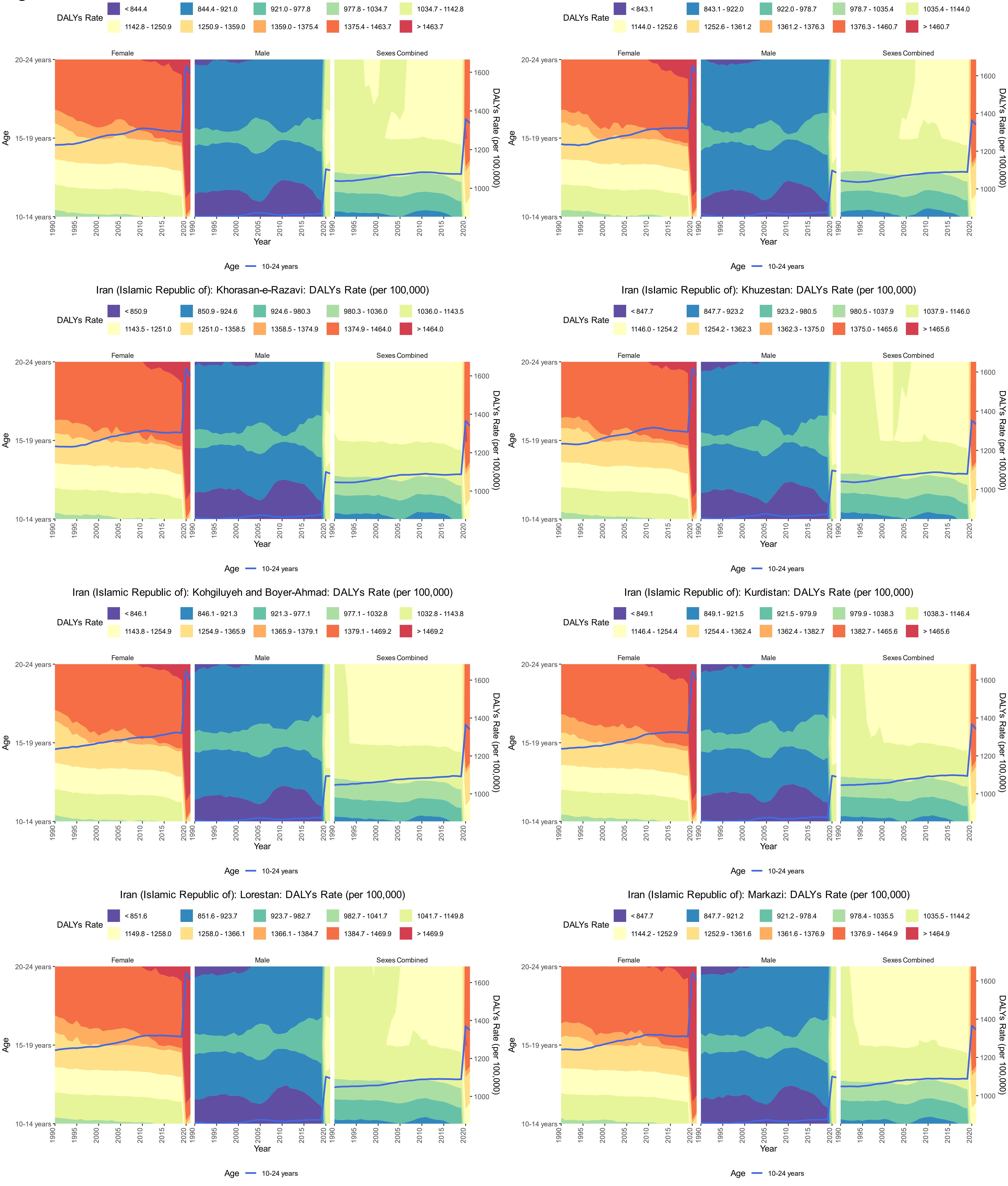
Iran (Islamic Republic of): Kerman: DALYs Rate (per 100,000)
DALYs Rate
< 844.4
844.4 - 921.0
921.0 - 977.8
977.8 - 1034.7
1034.7 - 1142.8
1142.8 - 1250.9
1250.9 - 1359.0
1359.0 - 1375.4
1375.4 - 1463.7
> 1463.7
Iran (Islamic Republic of): Kermanshah: DALYs Rate (per 100,000)
< 843.1
843.1 - 922.0
922.0 - 978.7
978.7 - 1035.4
1035.4 - 1144.0
1144.0 - 1252.6
1252.6 - 1361.2
1361.2 - 1376.3
1376.3 - 1460.7
> 1460.7
Iran (Islamic Republic of): Khorasan-e-Razavi: DALYs Rate (per 100,000)
< 850.9
850.9 - 924.6
924.6 - 980.3
980.3 - 1036.0
1036.0 - 1143.5
1143.5 - 1251.0
1251.0 - 1358.5
1358.5 - 1374.9
1374.9 - 1464.0
> 1464.0
Iran (Islamic Republic of): Khuzestan: DALYs Rate (per 100,000)
< 847.7
847.7 - 923.2
923.2 - 980.5
980.5 - 1037.9
1037.9 - 1146.0
1146.0 - 1254.2
1254.2 - 1362.3
1362.3 - 1375.0
1375.0 - 1465.6
> 1465.6
Iran (Islamic Republic of): Kohgiluyeh and Boyer-Ahmad: DALYs Rate (per 100,000)
< 846.1
846.1 - 921.3
921.3 - 977.1
977.1 - 1032.8
1032.8 - 1143.8
1143.8 - 1254.9
1254.9 - 1365.9
1365.9 - 1379.1
1379.1 - 1469.2
> 1469.2
Iran (Islamic Republic of): Kurdistan: DALYs Rate (per 100,000)
< 849.1
849.1 - 921.5
921.5 - 979.9
979.9 - 1038.3
1038.3 - 1146.4
1146.4 - 1254.4
1254.4 - 1362.4
1362.4 - 1382.7
1382.7 - 1465.6
> 1465.6
Iran (Islamic Republic of): Lorestan: DALYs Rate (per 100,000)
< 851.6
851.6 - 923.7
923.7 - 982.7
982.7 - 1041.7
1041.7 - 1149.8
1149.8 - 1258.0
1258.0 - 1366.1
1366.1 - 1384.7
1384.7 - 1469.9
> 1469.9
Iran (Islamic Republic of): Markazi: DALYs Rate (per 100,000)
< 847.7
847.7 - 921.2
921.2 - 978.4
978.4 - 1035.5
1035.5 - 1144.2
1144.2 - 1252.9
1252.9 - 1361.6
1361.6 - 1376.9
1376.9 - 1464.9
> 1464.9
Female
Male
Sexes Combined
Age
20-24 years
15-19 years
10-14 years
Year
DALYs Rate (per 100,000)
Age — 10-24 years

Figure S3

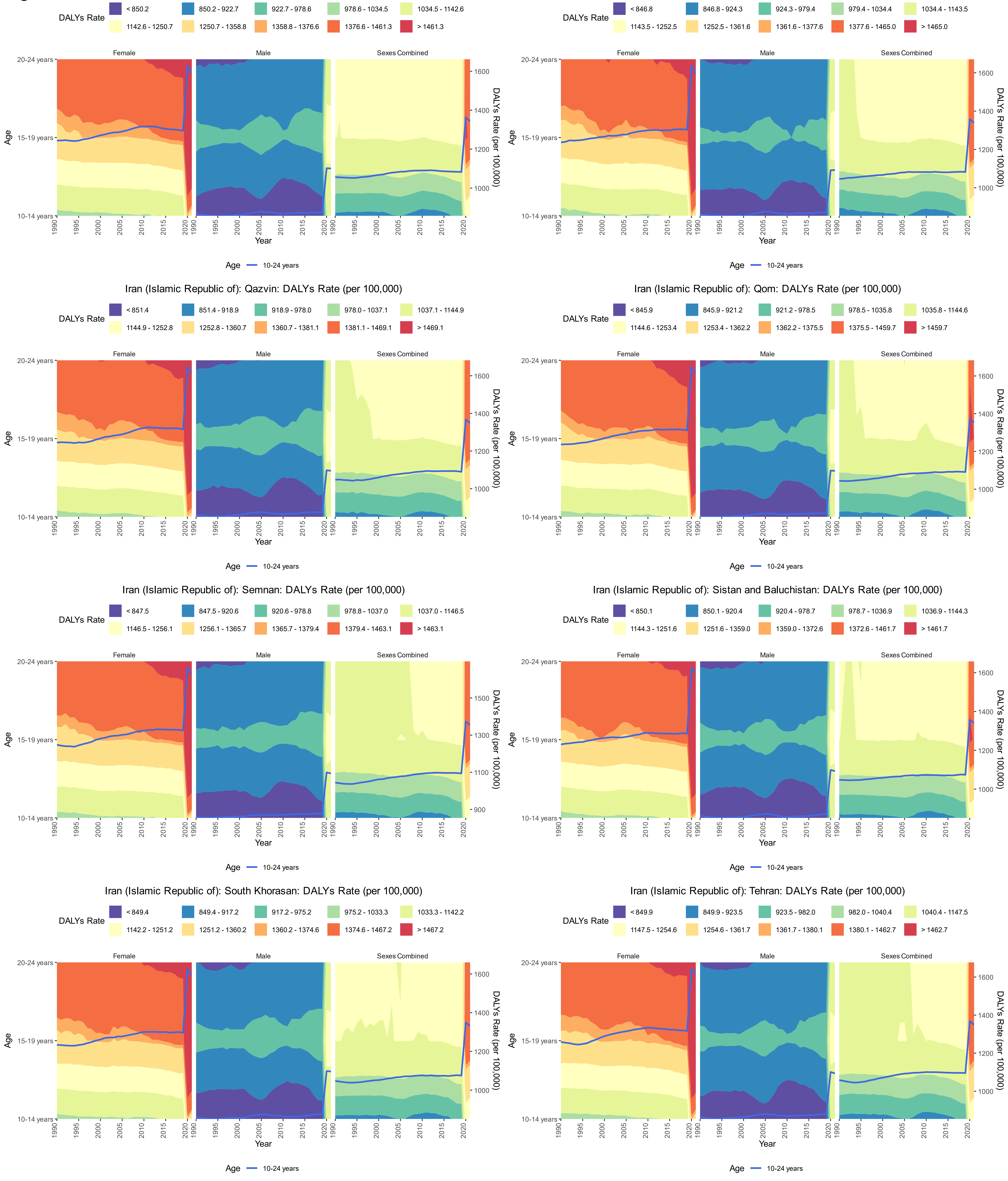
Iran (Islamic Republic of): Mazandaran: DALYs Rate (per 100,000)
DALYs Rate
< 850.2
850.2 - 922.7
922.7 - 978.6
978.6 - 1034.5
1034.5 - 1142.6
1142.6 - 1250.7
1250.7 - 1358.8
1358.8 - 1376.6
1376.6 - 1461.3
> 1461.3
Iran (Islamic Republic of): North Khorasan: DALYs Rate (per 100,000)
< 846.8
846.8 - 924.3
924.3 - 979.4
979.4 - 1034.4
1034.4 - 1143.5
1143.5 - 1252.5
1252.5 - 1361.6
1361.6 - 1377.6
1377.6 - 1465.0
> 1465.0
Iran (Islamic Republic of): Qazvin: DALYs Rate (per 100,000)
< 851.4
851.4 - 918.9
918.9 - 978.0
978.0 - 1037.1
1037.1 - 1144.9
1144.9 - 1252.8
1252.8 - 1360.7
1360.7 - 1381.1
1381.1 - 1469.1
> 1469.1
Iran (Islamic Republic of): Qom: DALYs Rate (per 100,000)
< 845.9
845.9 - 921.2
921.2 - 978.5
978.5 - 1035.8
1035.8 - 1144.6
1144.6 - 1253.4
1253.4 - 1362.2
1362.2 - 1375.5
1375.5 - 1459.7
> 1459.7
Iran (Islamic Republic of): Semnan: DALYs Rate (per 100,000)
< 847.5
847.5 - 920.6
920.6 - 978.8
978.8 - 1037.0
1037.0 - 1146.5
1146.5 - 1256.1
1256.1 - 1365.7
1365.7 - 1379.4
1379.4 - 1463.1
> 1463.1
Iran (Islamic Republic of): Sistan and Baluchistan: DALYs Rate (per 100,000)
< 850.1
850.1 - 920.4
920.4 - 978.7
978.7 - 1036.9
1036.9 - 1144.3
1144.3 - 1251.6
1251.6 - 1359.0
1359.0 - 1372.6
1372.6 - 1461.7
> 1461.7
Iran (Islamic Republic of): South Khorasan: DALYs Rate (per 100,000)
< 849.4
849.4 - 917.2
917.2 - 975.2
975.2 - 1033.3
1033.3 - 1142.2
1142.2 - 1251.2
1251.2 - 1360.2
1360.2 - 1374.6
1374.6 - 1467.2
> 1467.2
Iran (Islamic Republic of): Tehran: DALYs Rate (per 100,000)
< 849.9
849.9 - 923.5
923.5 - 982.0
982.0 - 1040.4
1040.4 - 1147.5
1147.5 - 1254.6
1254.6 - 1361.7
1361.7 - 1380.1
1380.1 - 1462.7
> 1462.7
Female
Male
Sexes Combined
Age
20-24 years
15-19 years
10-14 years
Year
DALYs Rate (per 100,000)
Age — 10-24 years

Figure S3

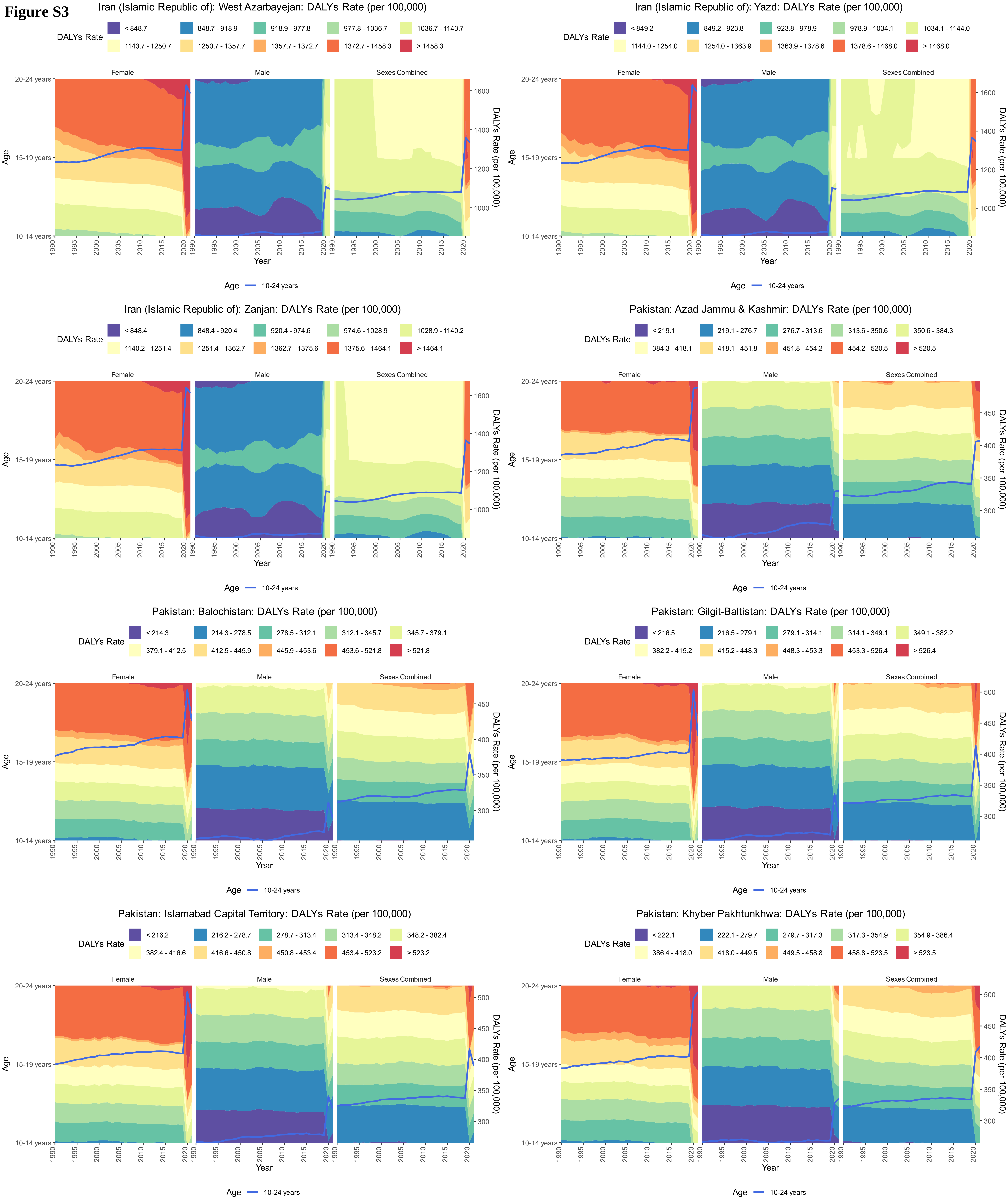

Iran (Islamic Republic of): West Azarbayejan: DALYs Rate (per 100,000)
DALYs Rate
< 848.7
848.7 - 918.9
918.9 - 977.8
977.8 - 1036.7
1036.7 - 1143.7
1143.7 - 1250.7
1250.7 - 1357.7
1357.7 - 1372.7
1372.7 - 1458.3
> 1458.3
Iran (Islamic Republic of): Yazd: DALYs Rate (per 100,000)
DALYs Rate
< 849.2
849.2 - 923.8
923.8 - 978.9
978.9 - 1034.1
1034.1 - 1144.0
1144.0 - 1254.0
1254.0 - 1363.9
1363.9 - 1378.6
1378.6 - 1468.0
> 1468.0
Iran (Islamic Republic of): Zanjan: DALYs Rate (per 100,000)
DALYs Rate
< 848.4
848.4 - 920.4
920.4 - 974.6
974.6 - 1028.9
1028.9 - 1140.2
1140.2 - 1251.4
1251.4 - 1362.7
1362.7 - 1375.6
1375.6 - 1464.1
> 1464.1
Pakistan: Azad Jammu & Kashmir: DALYs Rate (per 100,000)
DALYs Rate
< 219.1
219.1 - 276.7
276.7 - 313.6
313.6 - 350.6
350.6 - 384.3
384.3 - 418.1
418.1 - 451.8
451.8 - 454.2
454.2 - 520.5
> 520.5
Pakistan: Balochistan: DALYs Rate (per 100,000)
DALYs Rate
< 214.3
214.3 - 278.5
278.5 - 312.1
312.1 - 345.7
345.7 - 379.1
379.1 - 412.5
412.5 - 445.9
445.9 - 453.6
453.6 - 521.8
> 521.8
Pakistan: Gilgit-Baltistan: DALYs Rate (per 100,000)
DALYs Rate
< 216.5
216.5 - 279.1
279.1 - 314.1
314.1 - 349.1
349.1 - 382.2
382.2 - 415.2
415.2 - 448.3
448.3 - 453.3
453.3 - 526.4
> 526.4
Pakistan: Islamabad Capital Territory: DALYs Rate (per 100,000)
DALYs Rate
< 216.2
216.2 - 278.7
278.7 - 313.4
313.4 - 348.2
348.2 - 382.4
382.4 - 416.6
416.6 - 450.8
450.8 - 453.4
453.4 - 523.2
> 523.2
Pakistan: Khyber Pakhtunkhwa: DALYs Rate (per 100,000)
DALYs Rate
< 222.1
222.1 - 279.7
279.7 - 317.3
317.3 - 354.9
354.9 - 386.4
386.4 - 418.0
418.0 - 449.5
449.5 - 458.8
458.8 - 523.5
> 523.5
Female
Male
Sexes Combined
Age
20-24 years
15-19 years
10-14 years
Year
DALYs Rate (per 100,000)
Age
10-24 years

Figure S3

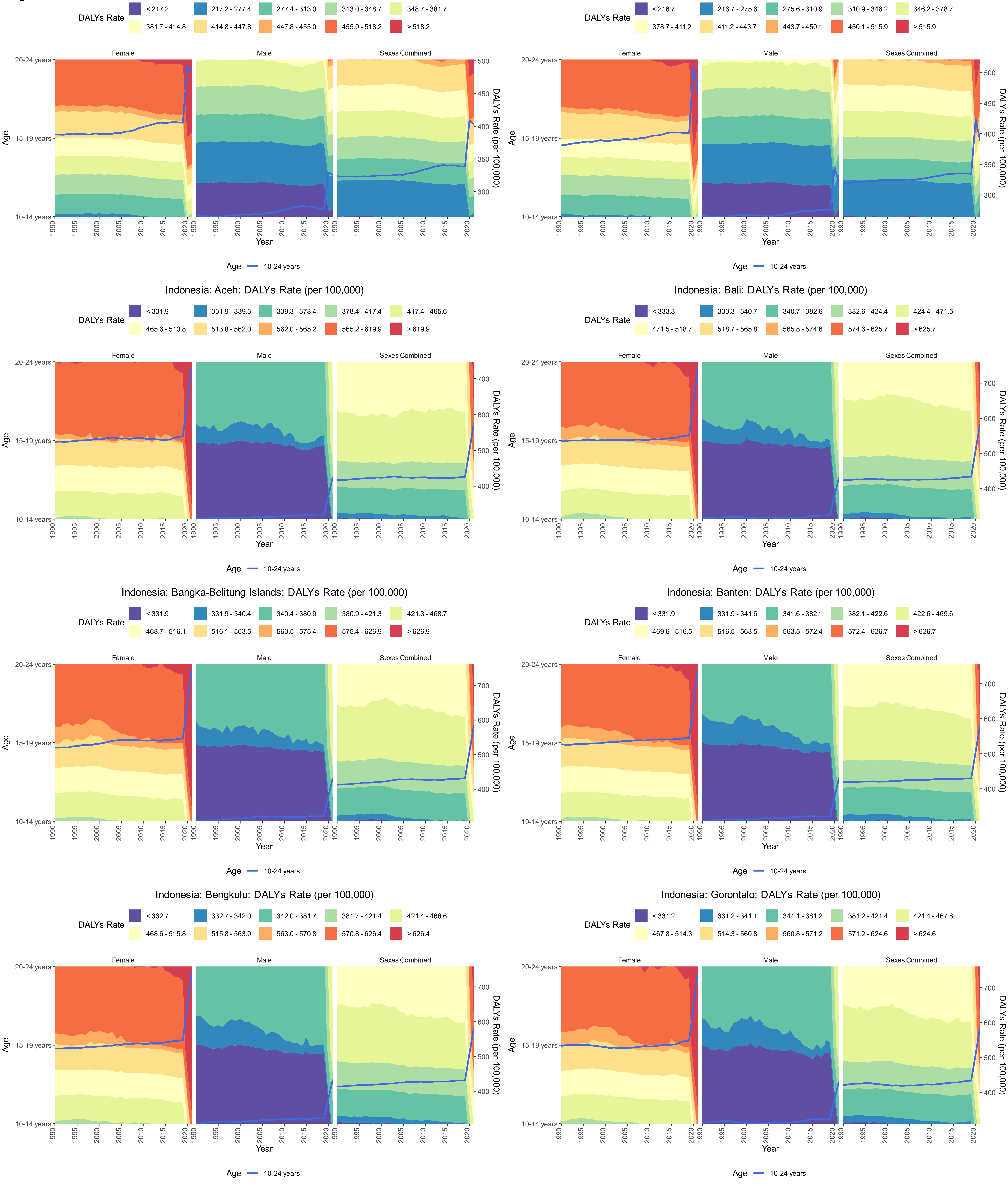
Pakistan: Punjab: DALYs Rate (per 100,000)
DALYs Rate
< 217.2
217.2 - 277.4
277.4 - 313.0
313.0 - 348.7
348.7 - 381.7
381.7 - 414.8
414.8 - 447.8
447.8 - 455.0
455.0 - 518.2
> 518.2
Female
Male
Sexes Combined
Age
Year
DALYs Rate (per 100,000)
Age
10-24 years
Pakistan: Sindh: DALYs Rate (per 100,000)
< 216.7
216.7 - 275.6
275.6 - 310.9
310.9 - 346.2
346.2 - 378.7
378.7 - 411.2
411.2 - 443.7
443.7 - 450.1
450.1 - 515.9
> 515.9
Indonesia: Aceh: DALYs Rate (per 100,000)
< 331.9
331.9 - 339.3
339.3 - 378.4
378.4 - 417.4
417.4 - 465.6
465.6 - 513.8
513.8 - 562.0
562.0 - 565.2
565.2 - 619.9
> 619.9
Indonesia: Bali: DALYs Rate (per 100,000)
< 333.3
333.3 - 340.7
340.7 - 382.6
382.6 - 424.4
424.4 - 471.5
471.5 - 518.7
518.7 - 565.8
565.8 - 574.6
574.6 - 625.7
> 625.7
Indonesia: Bangka-Belitung Islands: DALYs Rate (per 100,000)
< 331.9
331.9 - 340.4
340.4 - 380.9
380.9 - 421.3
421.3 - 468.7
468.7 - 516.1
516.1 - 563.5
563.5 - 575.4
575.4 - 626.9
> 626.9
Indonesia: Banten: DALYs Rate (per 100,000)
< 331.9
331.9 - 341.6
341.6 - 382.1
382.1 - 422.6
422.6 - 469.6
469.6 - 516.5
516.5 - 563.5
563.5 - 572.4
572.4 - 626.7
> 626.7
Indonesia: Bengkulu: DALYs Rate (per 100,000)
< 332.7
332.7 - 342.0
342.0 - 381.7
381.7 - 421.4
421.4 - 468.6
468.6 - 515.8
515.8 - 563.0
563.0 - 570.8
570.8 - 626.4
> 626.4
Indonesia: Gorontalo: DALYs Rate (per 100,000)
< 331.2
331.2 - 341.1
341.1 - 381.2
381.2 - 421.4
421.4 - 467.8
467.8 - 514.3
514.3 - 560.8
560.8 - 571.2
571.2 - 624.6
> 624.6

Figure S3

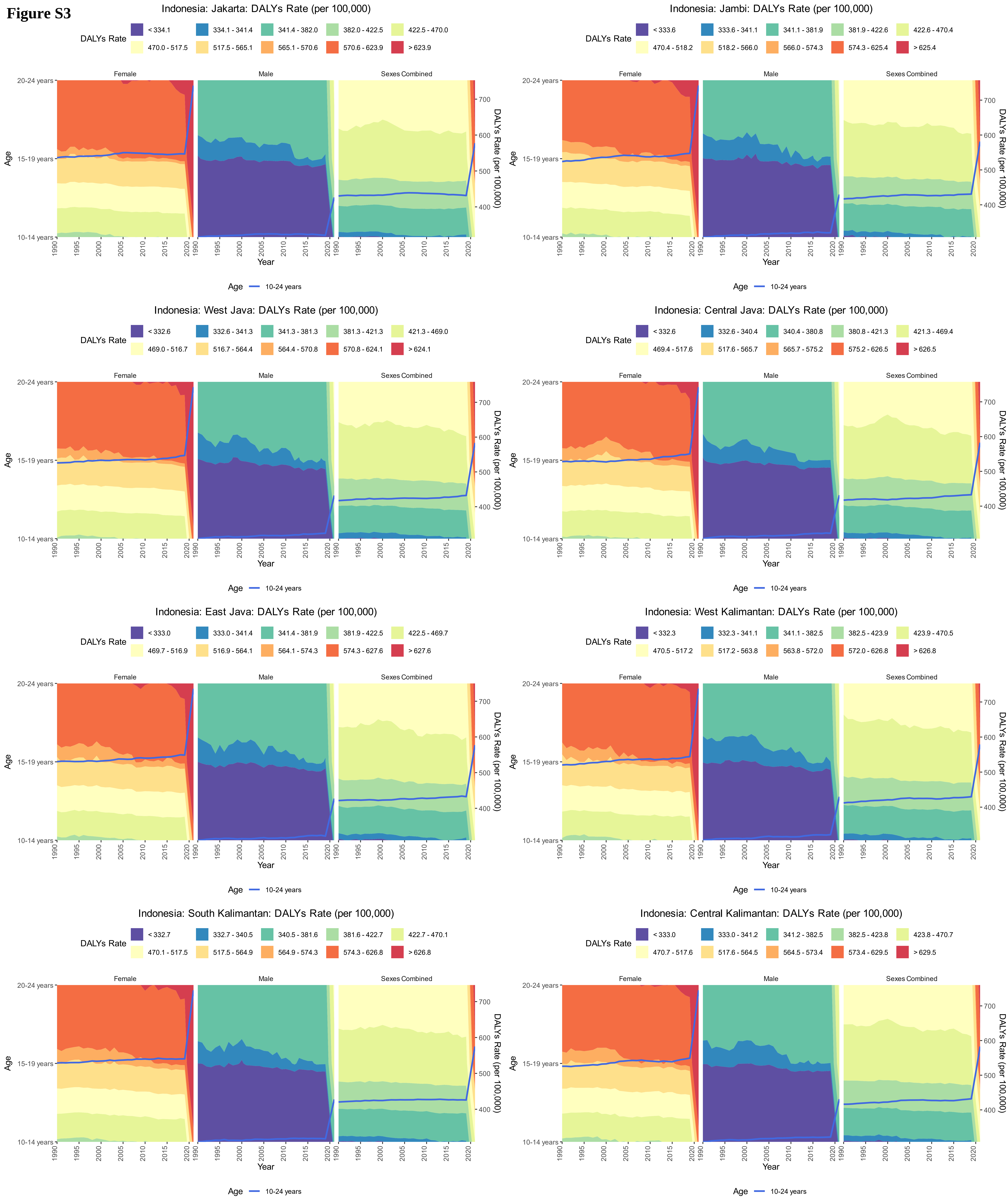
Indonesia: Jakarta: DALYs Rate (per 100,000)
DALYs Rate
< 334.1
334.1 - 341.4
341.4 - 382.0
382.0 - 422.5
422.5 - 470.0
470.0 - 517.5
517.5 - 565.1
565.1 - 570.6
570.6 - 623.9
> 623.9
Indonesia: Jambi: DALYs Rate (per 100,000)
< 333.6
333.6 - 341.1
341.1 - 381.9
381.9 - 422.6
422.6 - 470.4
470.4 - 518.2
518.2 - 566.0
566.0 - 574.3
574.3 - 625.4
> 625.4
Indonesia: West Java: DALYs Rate (per 100,000)
< 332.6
332.6 - 341.3
341.3 - 381.3
381.3 - 421.3
421.3 - 469.0
469.0 - 516.7
516.7 - 564.4
564.4 - 570.8
570.8 - 624.1
> 624.1
Indonesia: Central Java: DALYs Rate (per 100,000)
< 332.6
332.6 - 340.4
340.4 - 380.8
380.8 - 421.3
421.3 - 469.4
469.4 - 517.6
517.6 - 565.7
565.7 - 575.2
575.2 - 626.5
> 626.5
Indonesia: East Java: DALYs Rate (per 100,000)
< 333.0
333.0 - 341.4
341.4 - 381.9
381.9 - 422.5
422.5 - 469.7
469.7 - 516.9
516.9 - 564.1
564.1 - 574.3
574.3 - 627.6
> 627.6
Indonesia: West Kalimantan: DALYs Rate (per 100,000)
< 332.3
332.3 - 341.1
341.1 - 382.5
382.5 - 423.9
423.9 - 470.5
470.5 - 517.2
517.2 - 563.8
563.8 - 572.0
572.0 - 626.8
> 626.8
Indonesia: South Kalimantan: DALYs Rate (per 100,000)
< 332.7
332.7 - 340.5
340.5 - 381.6
381.6 - 422.7
422.7 - 470.1
470.1 - 517.5
517.5 - 564.9
564.9 - 574.3
574.3 - 626.8
> 626.8
Indonesia: Central Kalimantan: DALYs Rate (per 100,000)
< 333.0
333.0 - 341.2
341.2 - 382.5
382.5 - 423.8
423.8 - 470.7
470.7 - 517.6
517.6 - 564.5
564.5 - 573.4
573.4 - 629.5
> 629.5
Female
Male
Sexes Combined
Age
20-24 years
15-19 years
10-14 years
Year
DALYs Rate (per 100,000)
Age — 10-24 years

Figure S3

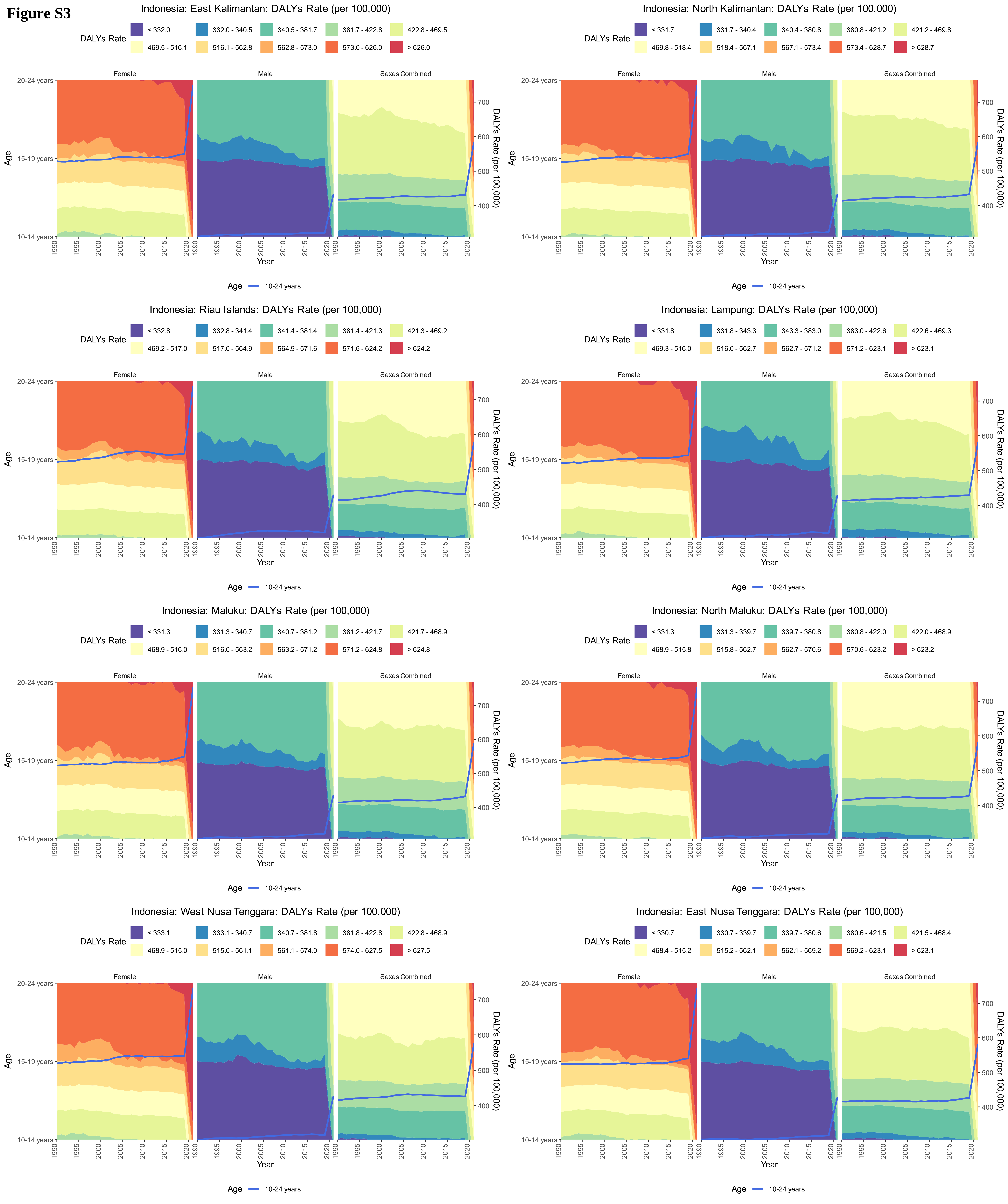

Indonesia: East Kalimantan: DALYs Rate (per 100,000)
DALYs Rate
< 332.0
332.0 - 340.5
340.5 - 381.7
381.7 - 422.8
422.8 - 469.5
469.5 - 516.1
516.1 - 562.8
562.8 - 573.0
573.0 - 626.0
> 626.0
Indonesia: North Kalimantan: DALYs Rate (per 100,000)
< 331.7
331.7 - 340.4
340.4 - 380.8
380.8 - 421.2
421.2 - 469.8
469.8 - 518.4
518.4 - 567.1
567.1 - 573.4
573.4 - 628.7
> 628.7
Indonesia: Riau Islands: DALYs Rate (per 100,000)
< 332.8
332.8 - 341.4
341.4 - 381.4
381.4 - 421.3
421.3 - 469.2
469.2 - 517.0
517.0 - 564.9
564.9 - 571.6
571.6 - 624.2
> 624.2
Indonesia: Lampung: DALYs Rate (per 100,000)
< 331.8
331.8 - 343.3
343.3 - 383.0
383.0 - 422.6
422.6 - 469.3
469.3 - 516.0
516.0 - 562.7
562.7 - 571.2
571.2 - 623.1
> 623.1
Indonesia: Maluku: DALYs Rate (per 100,000)
< 331.3
331.3 - 340.7
340.7 - 381.2
381.2 - 421.7
421.7 - 468.9
468.9 - 516.0
516.0 - 563.2
563.2 - 571.2
571.2 - 624.8
> 624.8
Indonesia: North Maluku: DALYs Rate (per 100,000)
< 331.3
331.3 - 339.7
339.7 - 380.8
380.8 - 422.0
422.0 - 468.9
468.9 - 515.8
515.8 - 562.7
562.7 - 570.6
570.6 - 623.2
> 623.2
Indonesia: West Nusa Tenggara: DALYs Rate (per 100,000)
< 333.1
333.1 - 340.7
340.7 - 381.8
381.8 - 422.8
422.8 - 468.9
468.9 - 515.0
515.0 - 561.1
561.1 - 574.0
574.0 - 627.5
> 627.5
Indonesia: East Nusa Tenggara: DALYs Rate (per 100,000)
< 330.7
330.7 - 339.7
339.7 - 380.6
380.6 - 421.5
421.5 - 468.4
468.4 - 515.2
515.2 - 562.1
562.1 - 569.2
569.2 - 623.1
> 623.1
Female
Male
Sexes Combined
Age
20-24 years
15-19 years
10-14 years
Year
1990
1995
2000
2005
2010
2015
2020
DALYs Rate (per 100,000)
400
500
600
700
Age
10-24 years

Figure S3

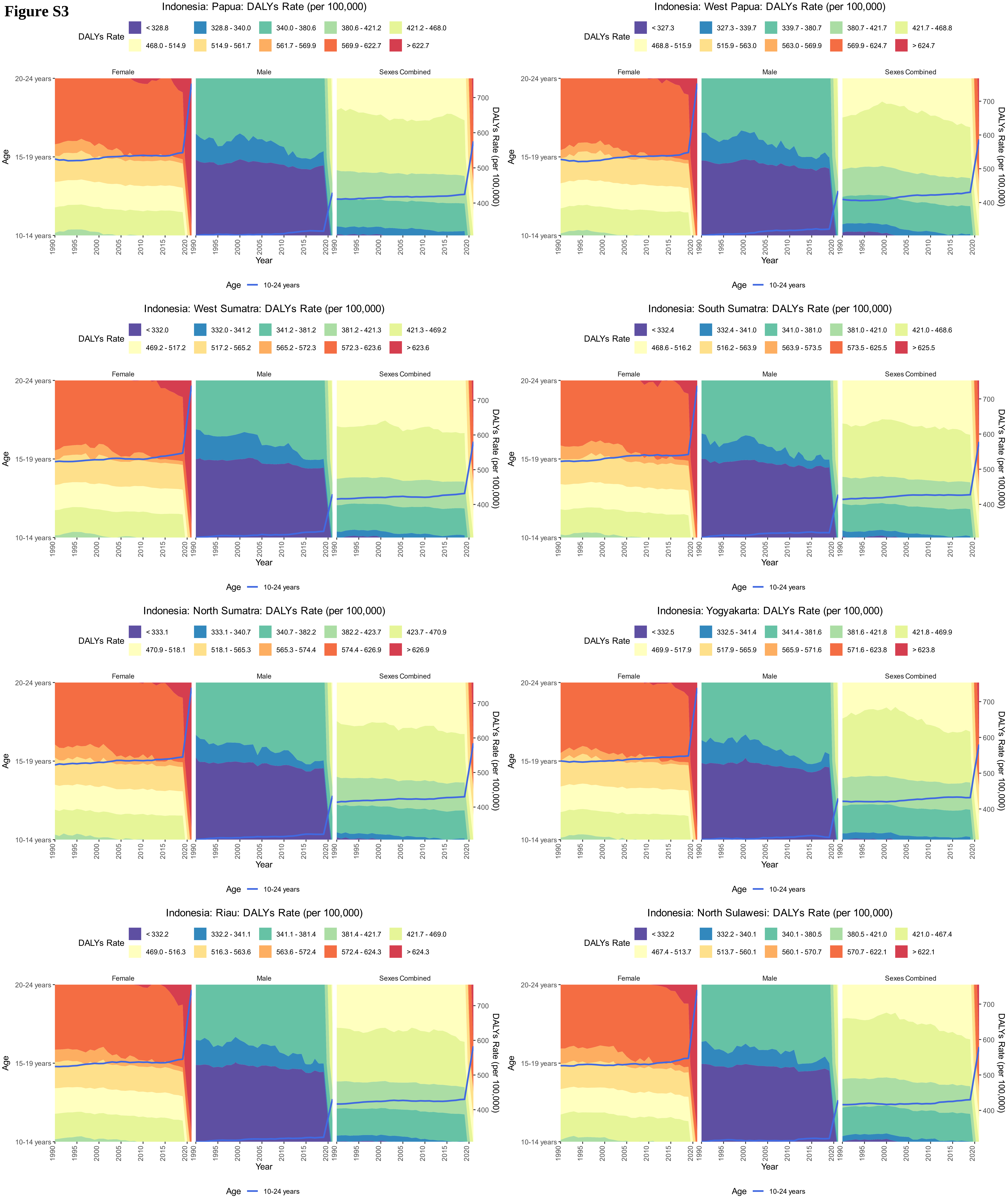
Indonesia: Papua: DALYs Rate (per 100,000)
DALYs Rate
< 328.8
328.8 - 340.0
340.0 - 380.6
380.6 - 421.2
421.2 - 468.0
468.0 - 514.9
514.9 - 561.7
561.7 - 569.9
569.9 - 622.7
> 622.7
Indonesia: West Papua: DALYs Rate (per 100,000)
< 327.3
327.3 - 339.7
339.7 - 380.7
380.7 - 421.7
421.7 - 468.8
468.8 - 515.9
515.9 - 563.0
563.0 - 569.9
569.9 - 624.7
> 624.7
Indonesia: West Sumatra: DALYs Rate (per 100,000)
< 332.0
332.0 - 341.2
341.2 - 381.2
381.2 - 421.3
421.3 - 469.2
469.2 - 517.2
517.2 - 565.2
565.2 - 572.3
572.3 - 623.6
> 623.6
Indonesia: South Sumatra: DALYs Rate (per 100,000)
< 332.4
332.4 - 341.0
341.0 - 381.0
381.0 - 421.0
421.0 - 468.6
468.6 - 516.2
516.2 - 563.9
563.9 - 573.5
573.5 - 625.5
> 625.5
Indonesia: North Sumatra: DALYs Rate (per 100,000)
< 333.1
333.1 - 340.7
340.7 - 382.2
382.2 - 423.7
423.7 - 470.9
470.9 - 518.1
518.1 - 565.3
565.3 - 574.4
574.4 - 626.9
> 626.9
Indonesia: Yogyakarta: DALYs Rate (per 100,000)
< 332.5
332.5 - 341.4
341.4 - 381.6
381.6 - 421.8
421.8 - 469.9
469.9 - 517.9
517.9 - 565.9
565.9 - 571.6
571.6 - 623.8
> 623.8
Indonesia: Riau: DALYs Rate (per 100,000)
< 332.2
332.2 - 341.1
341.1 - 381.4
381.4 - 421.7
421.7 - 469.0
469.0 - 516.3
516.3 - 563.6
563.6 - 572.4
572.4 - 624.3
> 624.3
Indonesia: North Sulawesi: DALYs Rate (per 100,000)
< 332.2
332.2 - 340.1
340.1 - 380.5
380.5 - 421.0
421.0 - 467.4
467.4 - 513.7
513.7 - 560.1
560.1 - 570.7
570.7 - 622.1
> 622.1
Female
Male
Sexes Combined
Age
20-24 years
15-19 years
10-14 years
Year
DALYs Rate (per 100,000)
Age — 10-24 years

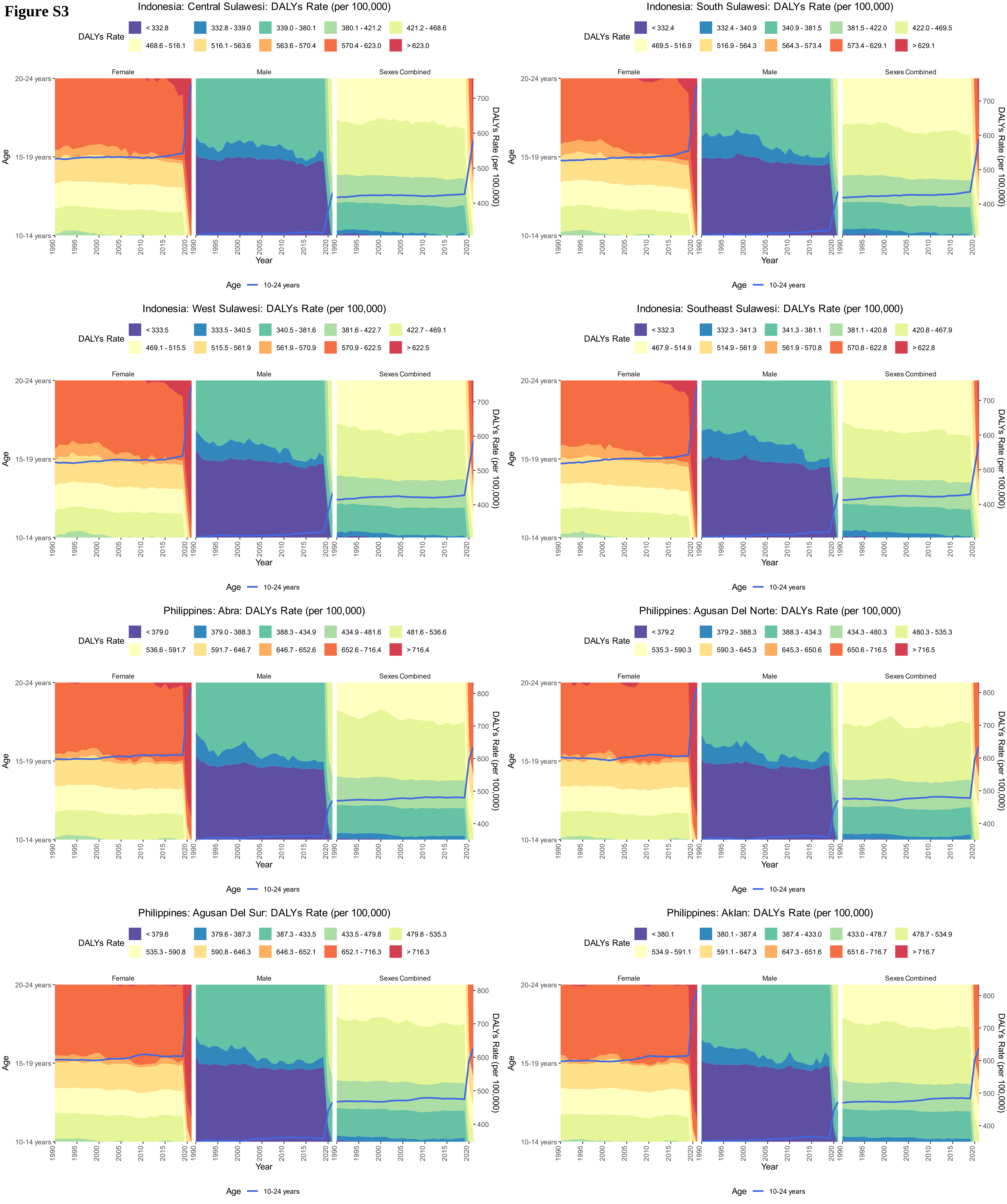
Indonesia: Central Sulawesi: DALYs Rate (per 100,000)
DALYs Rate
< 332.8
332.8 - 339.0
339.0 - 380.1
380.1 - 421.2
421.2 - 468.6
468.6 - 516.1
516.1 - 563.6
563.6 - 570.4
570.4 - 623.0
> 623.0
Indonesia: South Sulawesi: DALYs Rate (per 100,000)
DALYs Rate
< 332.4
332.4 - 340.9
340.9 - 381.5
381.5 - 422.0
422.0 - 469.5
469.5 - 516.9
516.9 - 564.3
564.3 - 573.4
573.4 - 629.1
> 629.1
Indonesia: West Sulawesi: DALYs Rate (per 100,000)
DALYs Rate
< 333.5
333.5 - 340.5
340.5 - 381.6
381.6 - 422.7
422.7 - 469.1
469.1 - 515.5
515.5 - 561.9
561.9 - 570.9
570.9 - 622.5
> 622.5
Indonesia: Southeast Sulawesi: DALYs Rate (per 100,000)
DALYs Rate
< 332.3
332.3 - 341.3
341.3 - 381.1
381.1 - 420.8
420.8 - 467.9
467.9 - 514.9
514.9 - 561.9
561.9 - 570.8
570.8 - 622.8
> 622.8
Philippines: Abra: DALYs Rate (per 100,000)
DALYs Rate
< 379.0
379.0 - 388.3
388.3 - 434.9
434.9 - 481.6
481.6 - 536.6
536.6 - 591.7
591.7 - 646.7
646.7 - 652.6
652.6 - 716.4
> 716.4
Philippines: Agusan Del Norte: DALYs Rate (per 100,000)
DALYs Rate
< 379.2
379.2 - 388.3
388.3 - 434.3
434.3 - 480.3
480.3 - 535.3
535.3 - 590.3
590.3 - 645.3
645.3 - 650.6
650.6 - 716.5
> 716.5
Philippines: Agusan Del Sur: DALYs Rate (per 100,000)
DALYs Rate
< 379.6
379.6 - 387.3
387.3 - 433.5
433.5 - 479.8
479.8 - 535.3
535.3 - 590.8
590.8 - 646.3
646.3 - 652.1
652.1 - 716.3
> 716.3
Philippines: Aklan: DALYs Rate (per 100,000)
DALYs Rate
< 380.1
380.1 - 387.4
387.4 - 433.0
433.0 - 478.7
478.7 - 534.9
534.9 - 591.1
591.1 - 647.3
647.3 - 651.6
651.6 - 716.7
> 716.7
Female
Male
Sexes Combined
Age
20-24 years
15-19 years
10-14 years
Year
DALYs Rate (per 100,000)
Age
10-24 years

Figure S3

Figure S3

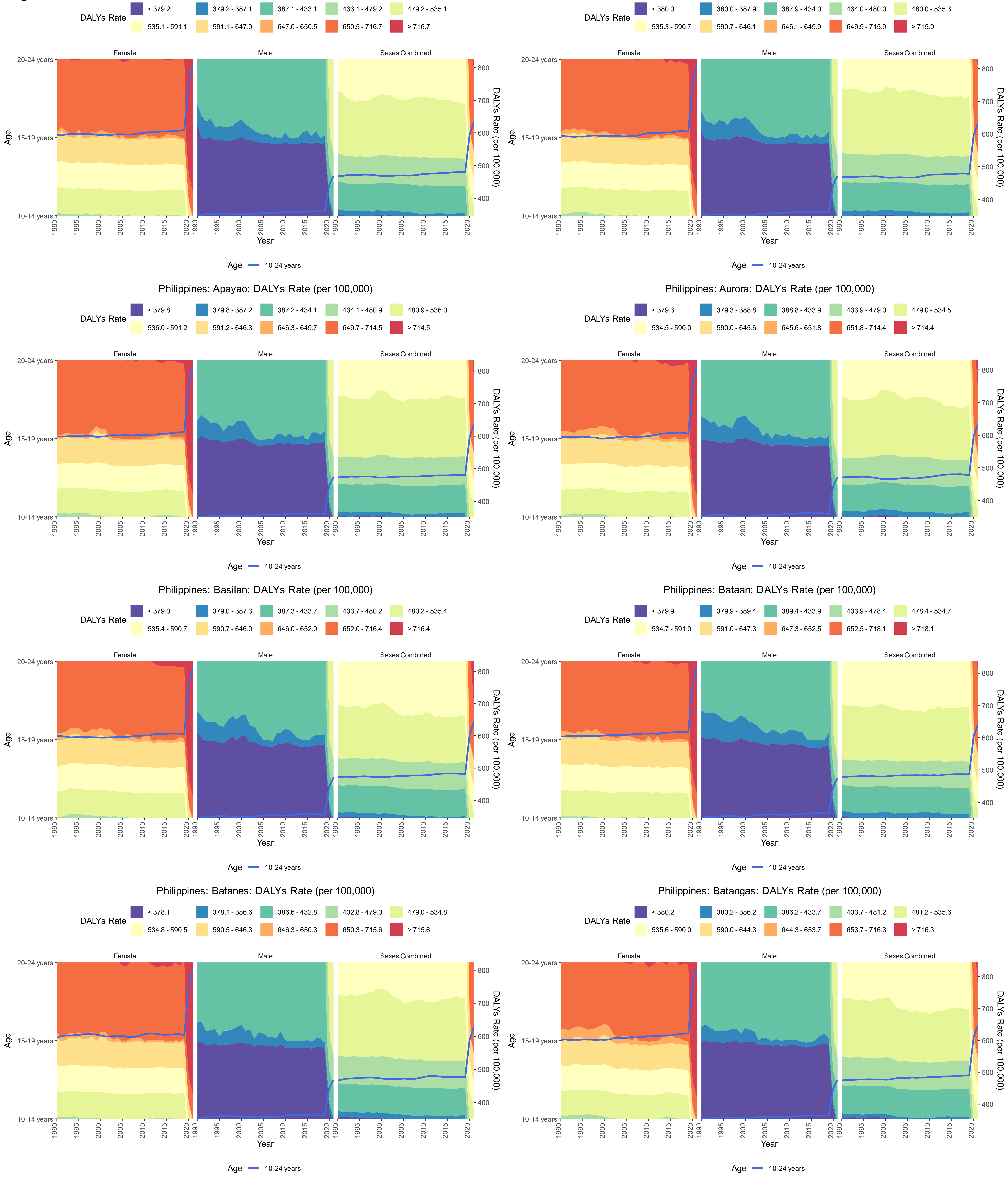

Philippines: Albay: DALYs Rate (per 100,000)
DALYs Rate
< 379.2
379.2 - 387.1
387.1 - 433.1
433.1 - 479.2
479.2 - 535.1
535.1 - 591.1
591.1 - 647.0
647.0 - 650.5
650.5 - 716.7
> 716.7
Philippines: Antique: DALYs Rate (per 100,000)
< 380.0
380.0 - 387.9
387.9 - 434.0
434.0 - 480.0
480.0 - 535.3
535.3 - 590.7
590.7 - 646.1
646.1 - 649.9
649.9 - 715.9
> 715.9
Philippines: Apayao: DALYs Rate (per 100,000)
< 379.8
379.8 - 387.2
387.2 - 434.1
434.1 - 480.9
480.9 - 536.0
536.0 - 591.2
591.2 - 646.3
646.3 - 649.7
649.7 - 714.5
> 714.5
Philippines: Aurora: DALYs Rate (per 100,000)
< 379.3
379.3 - 388.8
388.8 - 433.9
433.9 - 479.0
479.0 - 534.5
534.5 - 590.0
590.0 - 645.6
645.6 - 651.8
651.8 - 714.4
> 714.4
Philippines: Basilan: DALYs Rate (per 100,000)
< 379.0
379.0 - 387.3
387.3 - 433.7
433.7 - 480.2
480.2 - 535.4
535.4 - 590.7
590.7 - 646.0
646.0 - 652.0
652.0 - 716.4
> 716.4
Philippines: Bataan: DALYs Rate (per 100,000)
< 379.9
379.9 - 389.4
389.4 - 433.9
433.9 - 478.4
478.4 - 534.7
534.7 - 591.0
591.0 - 647.3
647.3 - 652.5
652.5 - 718.1
> 718.1
Philippines: Batanes: DALYs Rate (per 100,000)
< 378.1
378.1 - 386.6
386.6 - 432.8
432.8 - 479.0
479.0 - 534.8
534.8 - 590.5
590.5 - 646.3
646.3 - 650.3
650.3 - 715.6
> 715.6
Philippines: Batangas: DALYs Rate (per 100,000)
< 380.2
380.2 - 386.2
386.2 - 433.7
433.7 - 481.2
481.2 - 535.6
535.6 - 590.0
590.0 - 644.3
644.3 - 653.7
653.7 - 716.3
> 716.3
Female
Male
Sexes Combined
Age
20-24 years
15-19 years
10-14 years
Year
DALYs Rate (per 100,000)
Age — 10-24 years

Figure S3

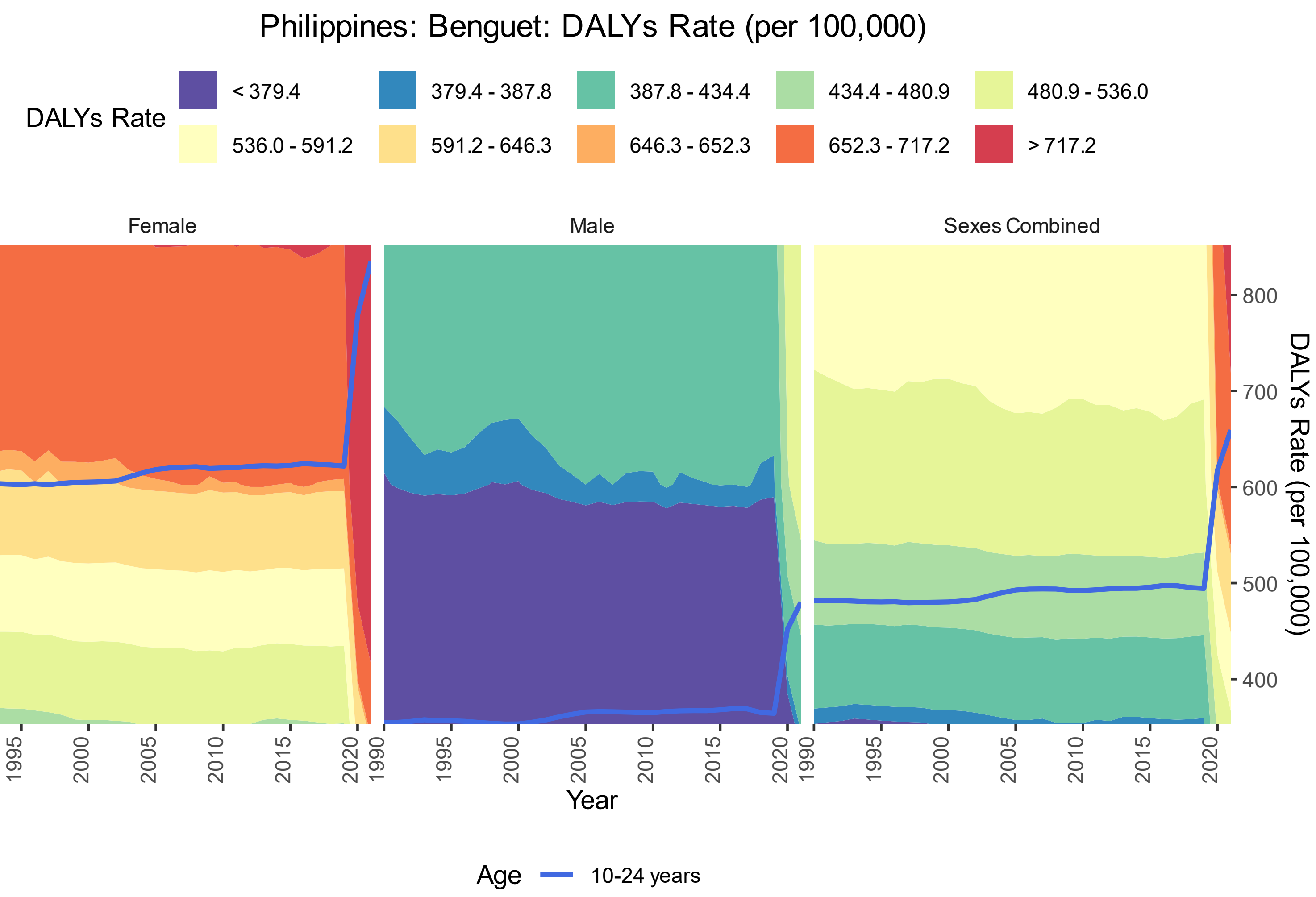
Philippines: Benguet: DALYs Rate (per 100,000)
DALYs Rate
< 379.4
379.4 - 387.8
387.8 - 434.4
434.4 - 480.9
480.9 - 536.0
536.0 - 591.2
591.2 - 646.3
646.3 - 652.3
652.3 - 717.2
> 717.2
Female
Male
Sexes Combined
Age
Year
DALYs Rate (per 100,000)
Age
10-24 years

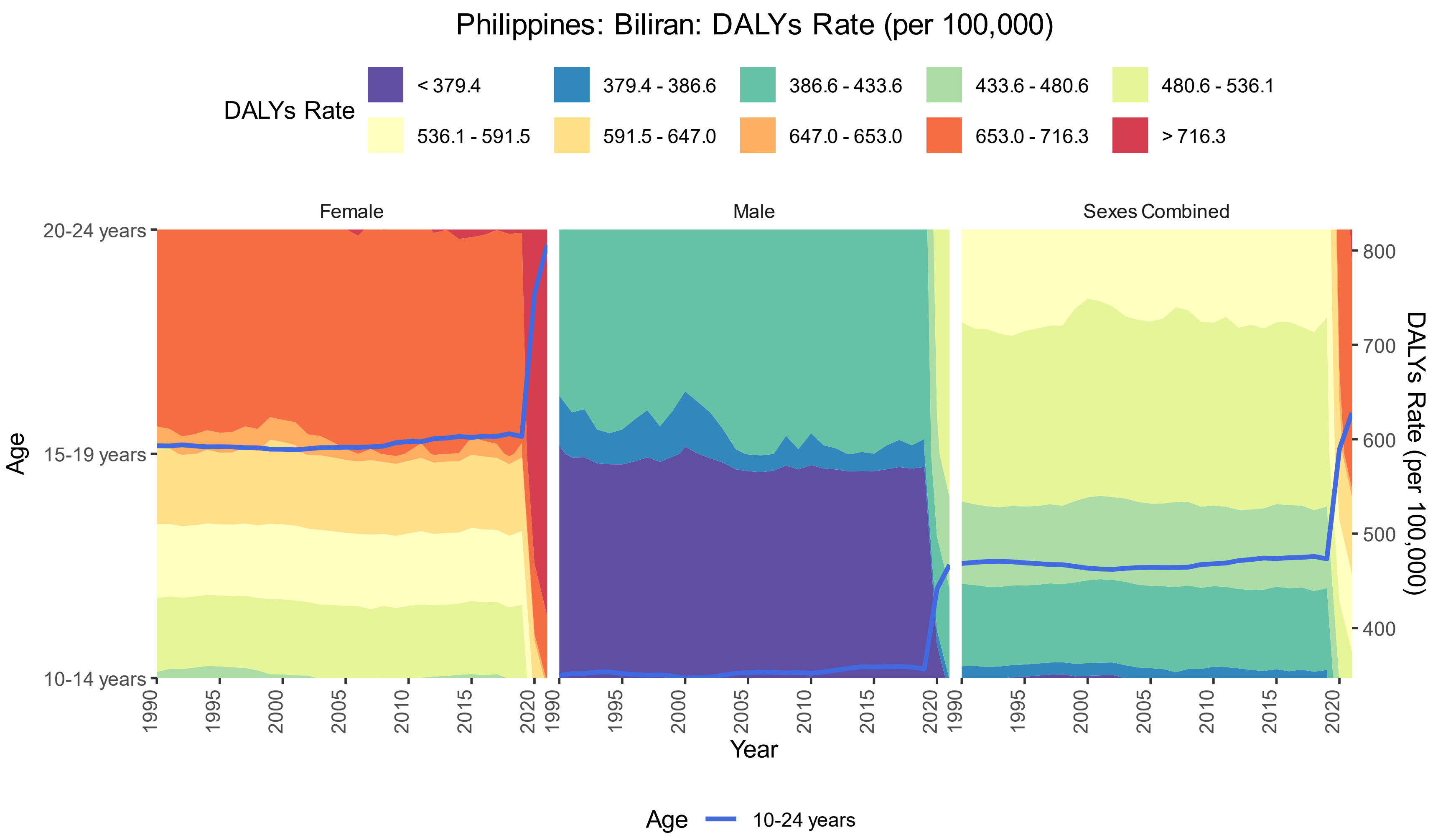
Philippines: Biliran: DALYs Rate (per 100,000)
DALYs Rate
< 379.4
379.4 - 386.6
386.6 - 433.6
433.6 - 480.6
480.6 - 536.1
536.1 - 591.5
591.5 - 647.0
647.0 - 653.0
653.0 - 716.3
> 716.3
Female
Male
Sexes Combined
Age
Year
DALYs Rate (per 100,000)
Age
10-24 years

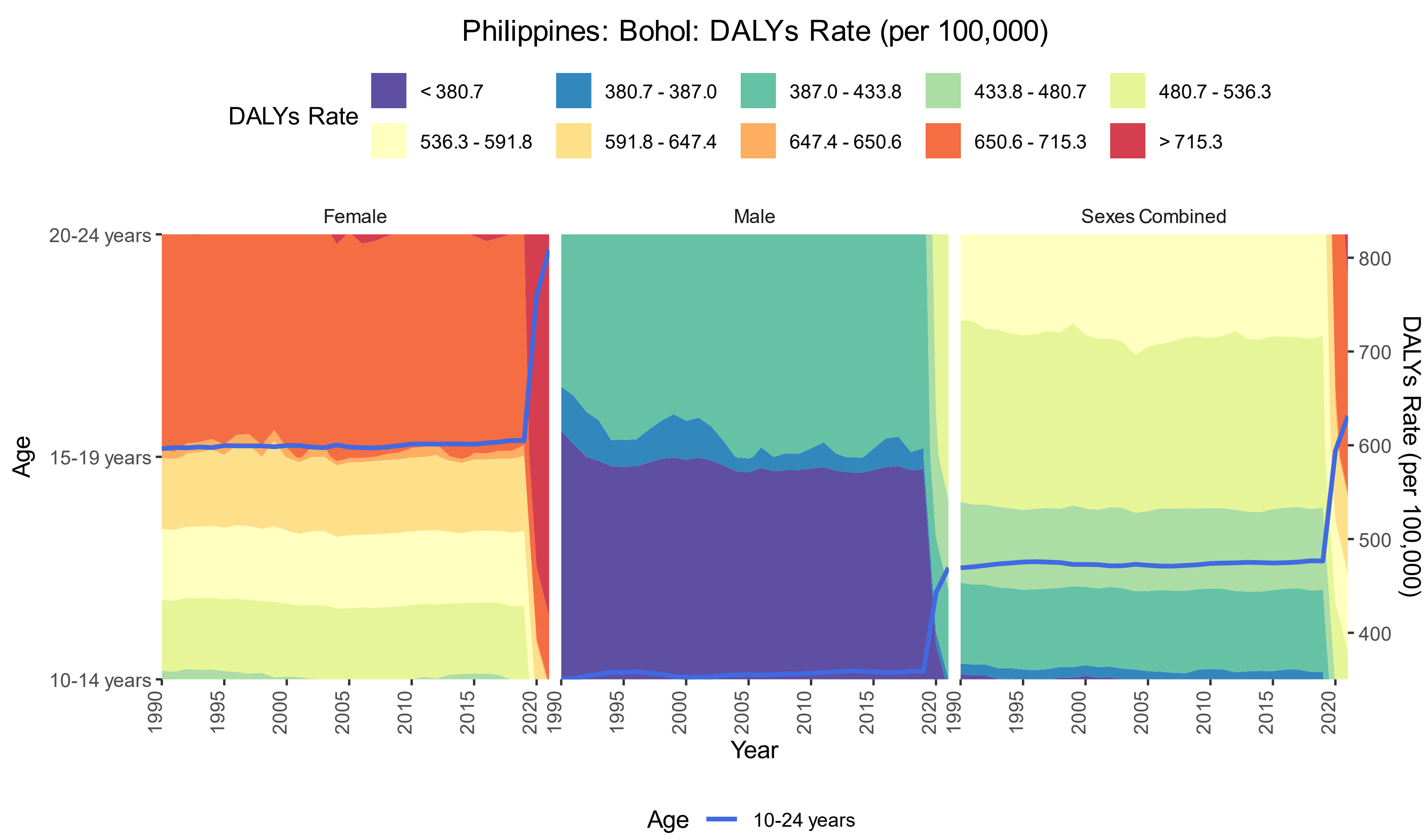
Philippines: Bohol: DALYs Rate (per 100,000)
DALYs Rate
< 380.7
380.7 - 387.0
387.0 - 433.8
433.8 - 480.7
480.7 - 536.3
536.3 - 591.8
591.8 - 647.4
647.4 - 650.6
650.6 - 715.3
> 715.3
Female
Male
Sexes Combined
Age
Year
DALYs Rate (per 100,000)
Age
10-24 years

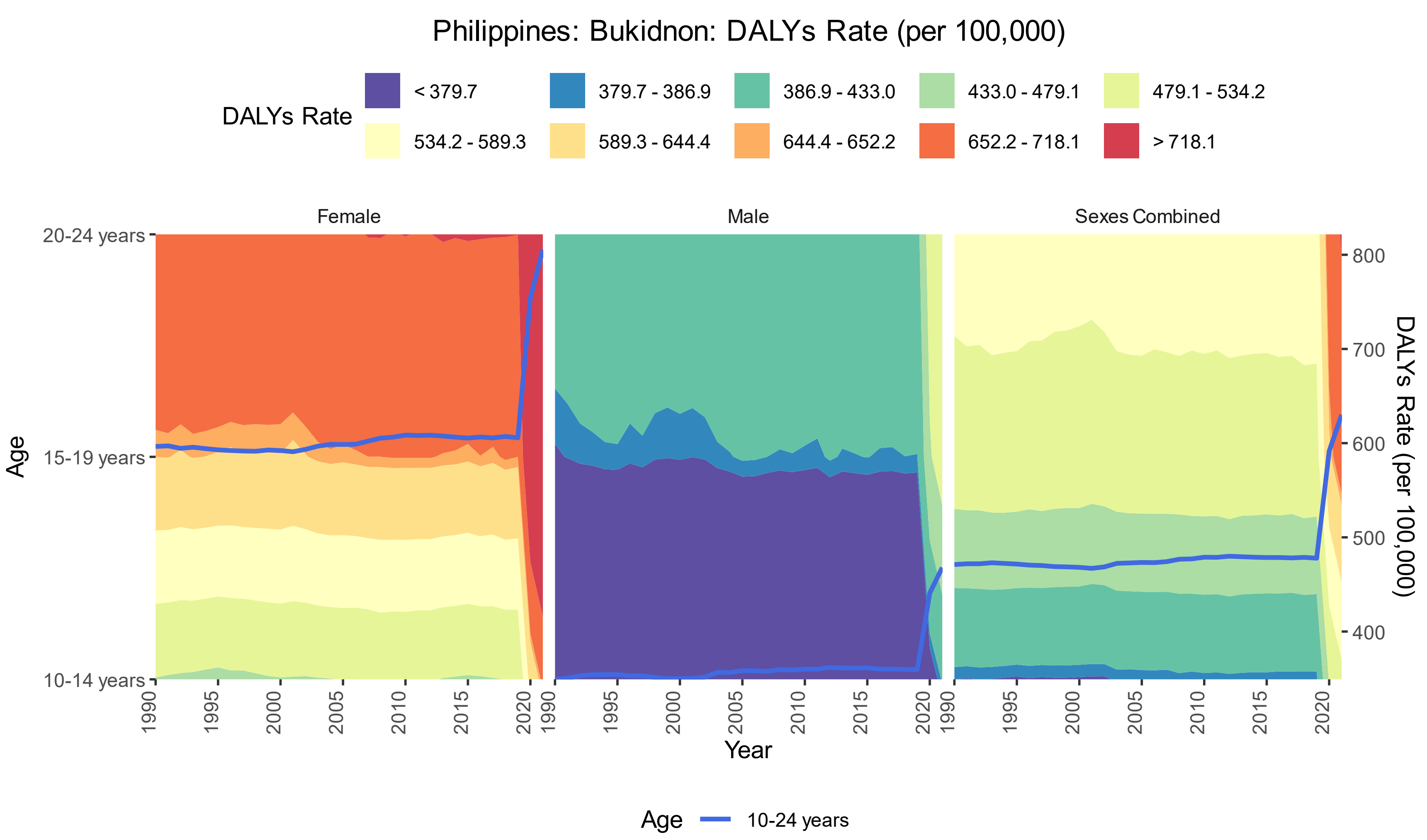
Philippines: Bukidnon: DALYs Rate (per 100,000)
DALYs Rate
< 379.7
379.7 - 386.9
386.9 - 433.0
433.0 - 479.1
479.1 - 534.2
534.2 - 589.3
589.3 - 644.4
644.4 - 652.2
652.2 - 718.1
> 718.1
Female
Male
Sexes Combined
Age
Year
DALYs Rate (per 100,000)
Age
10-24 years

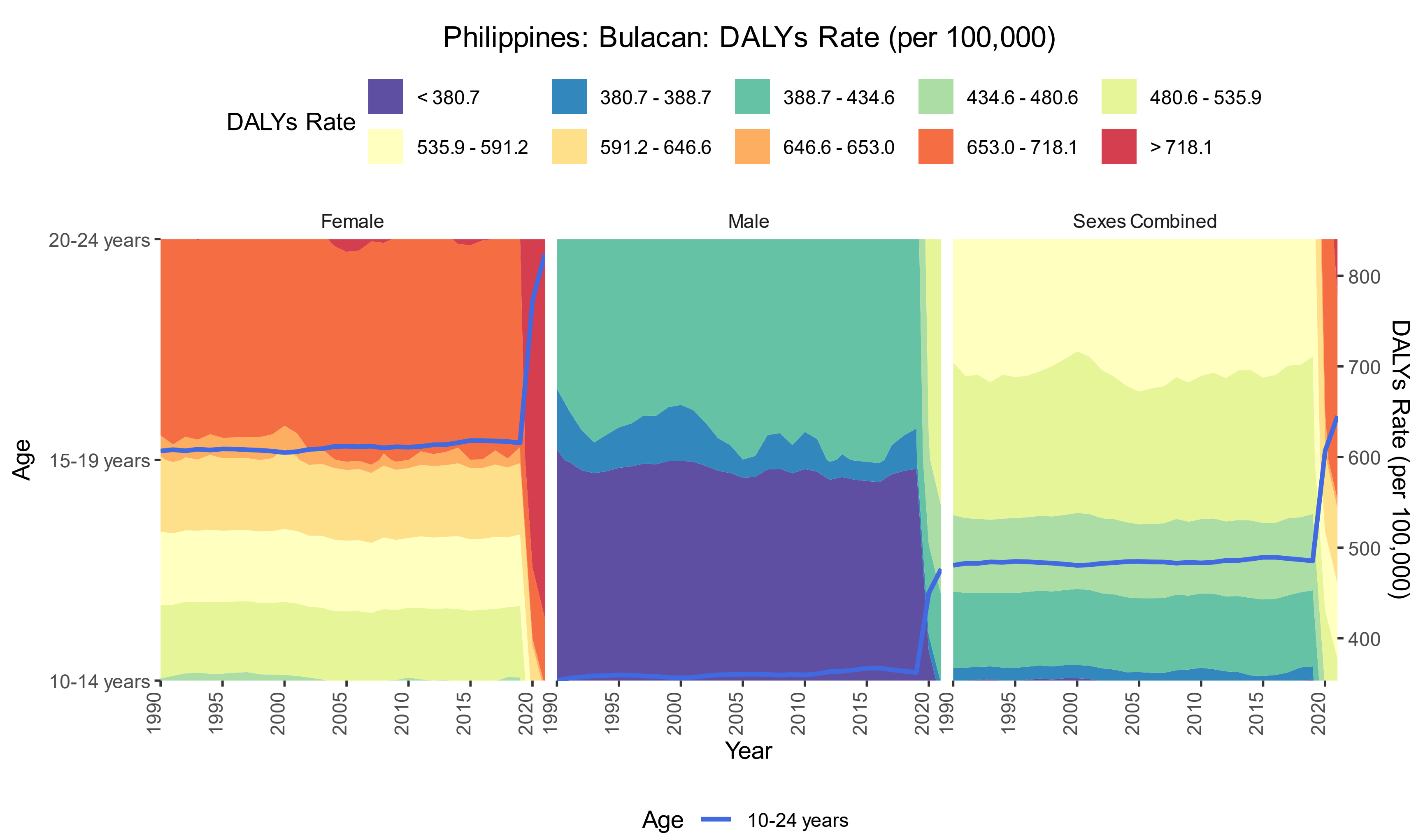
Philippines: Bulacan: DALYs Rate (per 100,000)
DALYs Rate
< 380.7
380.7 - 388.7
388.7 - 434.6
434.6 - 480.6
480.6 - 535.9
535.9 - 591.2
591.2 - 646.6
646.6 - 653.0
653.0 - 718.1
> 718.1
Female
Male
Sexes Combined
Age
Year
DALYs Rate (per 100,000)
Age
10-24 years

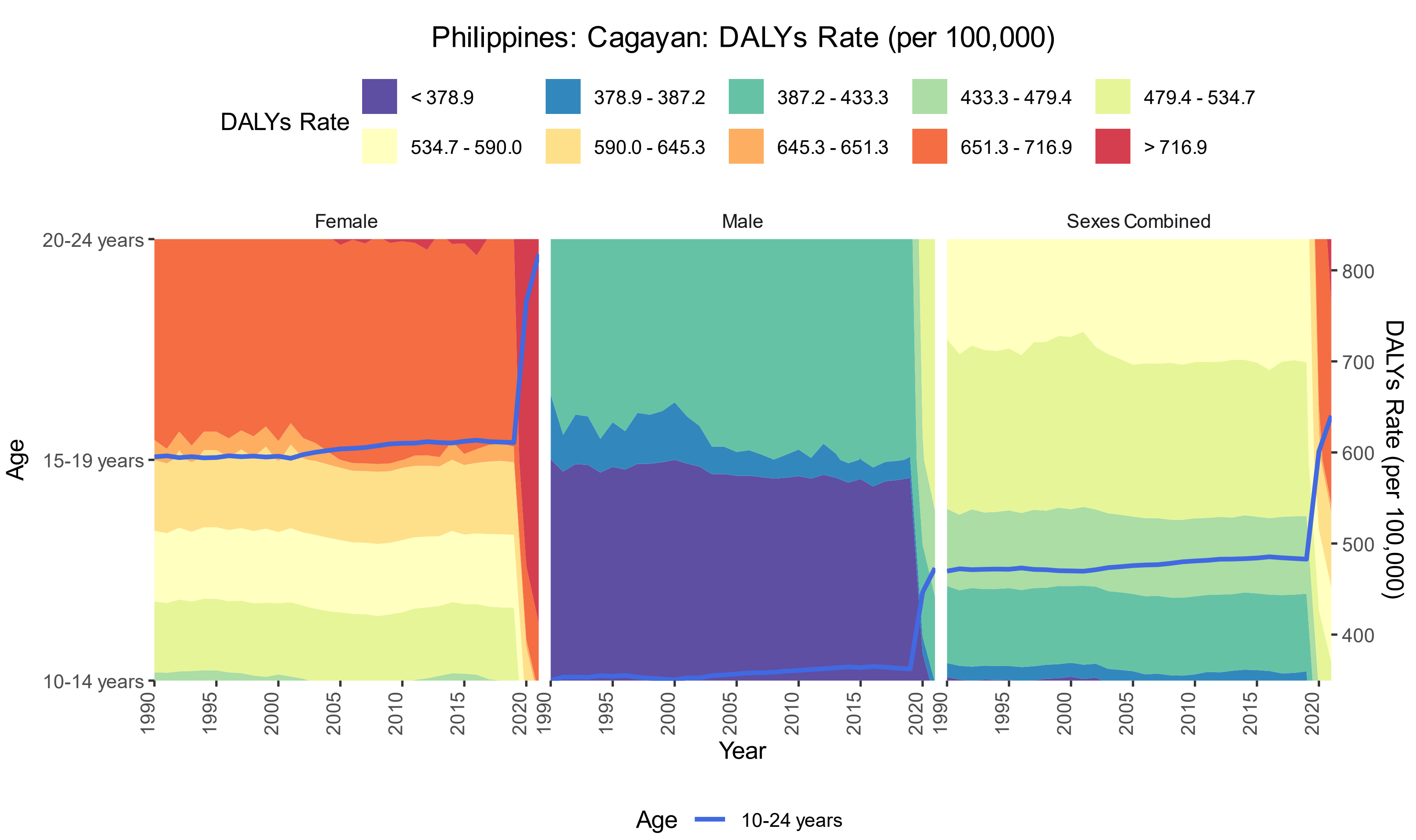
Philippines: Cagayan: DALYs Rate (per 100,000)
DALYs Rate
< 378.9
378.9 - 387.2
387.2 - 433.3
433.3 - 479.4
479.4 - 534.7
534.7 - 590.0
590.0 - 645.3
645.3 - 651.3
651.3 - 716.9
> 716.9
Female
Male
Sexes Combined
Age
Year
DALYs Rate (per 100,000)
Age
10-24 years

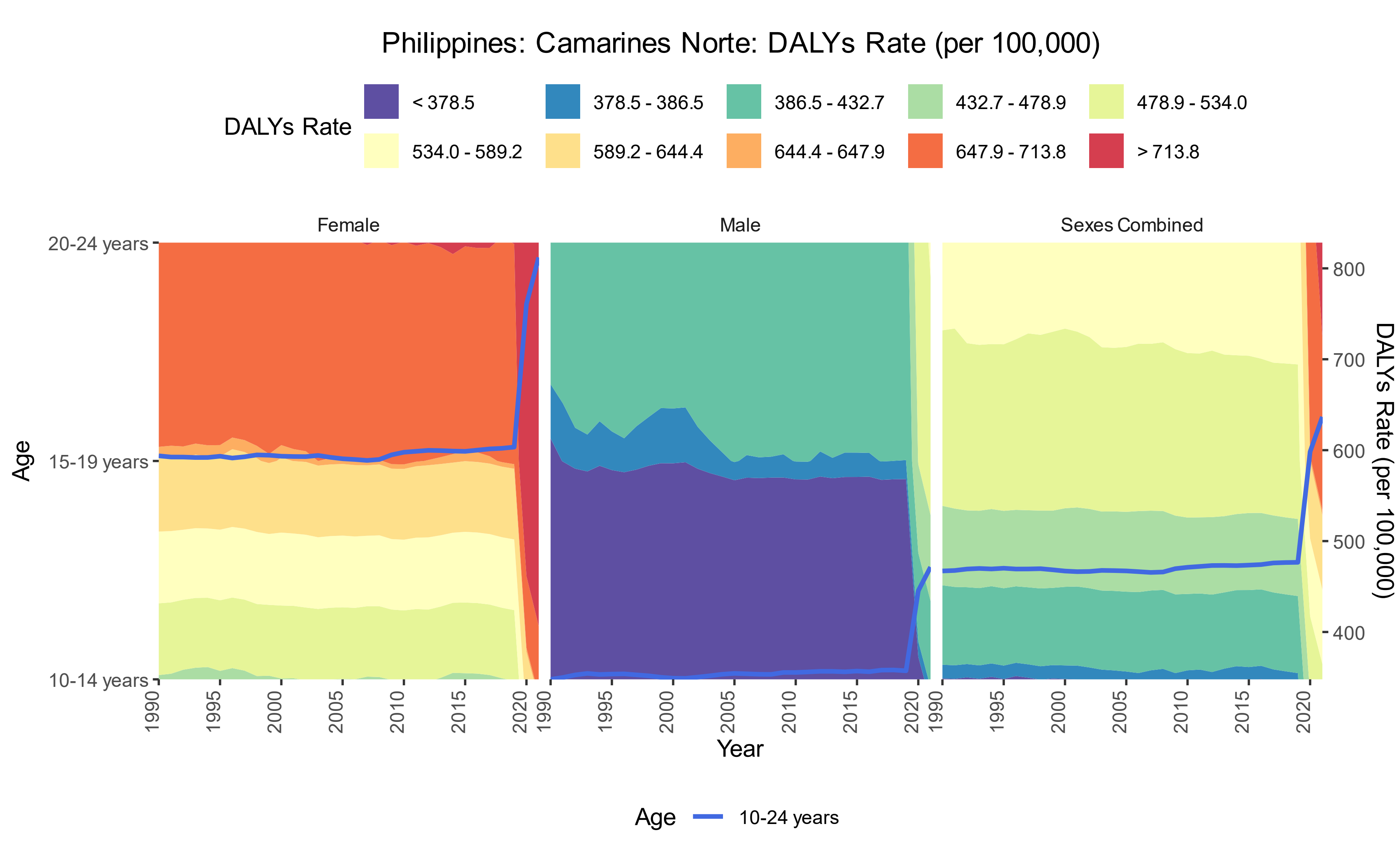
Philippines: Camarines Norte: DALYs Rate (per 100,000)
DALYs Rate
< 378.5
378.5 - 386.5
386.5 - 432.7
432.7 - 478.9
478.9 - 534.0
534.0 - 589.2
589.2 - 644.4
644.4 - 647.9
647.9 - 713.8
> 713.8
Female
Male
Sexes Combined
Age
Year
DALYs Rate (per 100,000)
Age
10-24 years

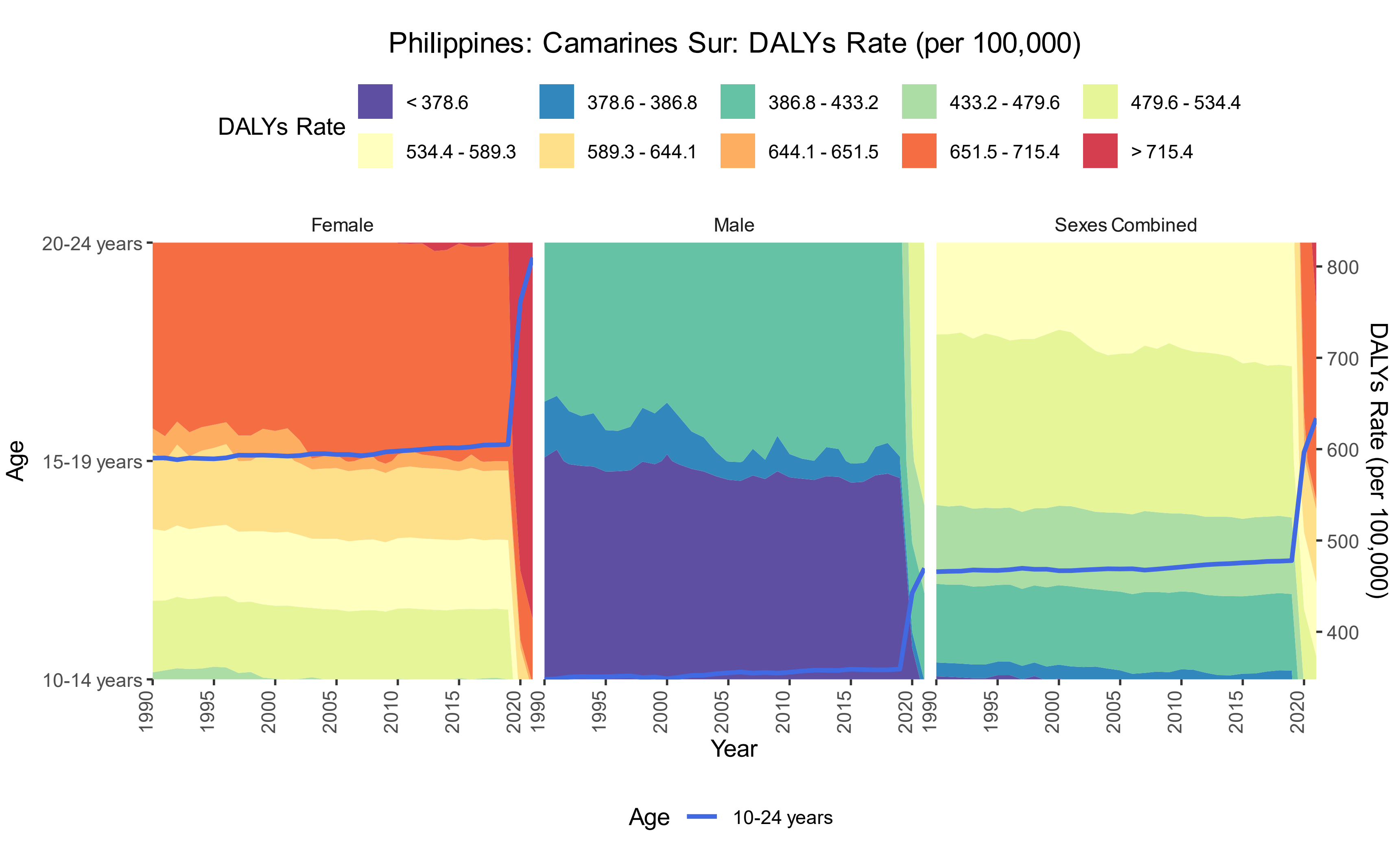
Philippines: Camarines Sur: DALYs Rate (per 100,000)
DALYs Rate
< 378.6
378.6 - 386.8
386.8 - 433.2
433.2 - 479.6
479.6 - 534.4
534.4 - 589.3
589.3 - 644.1
644.1 - 651.5
651.5 - 715.4
> 715.4
Female
Male
Sexes Combined
Age
Year
DALYs Rate (per 100,000)
Age
10-24 years

Figure S3

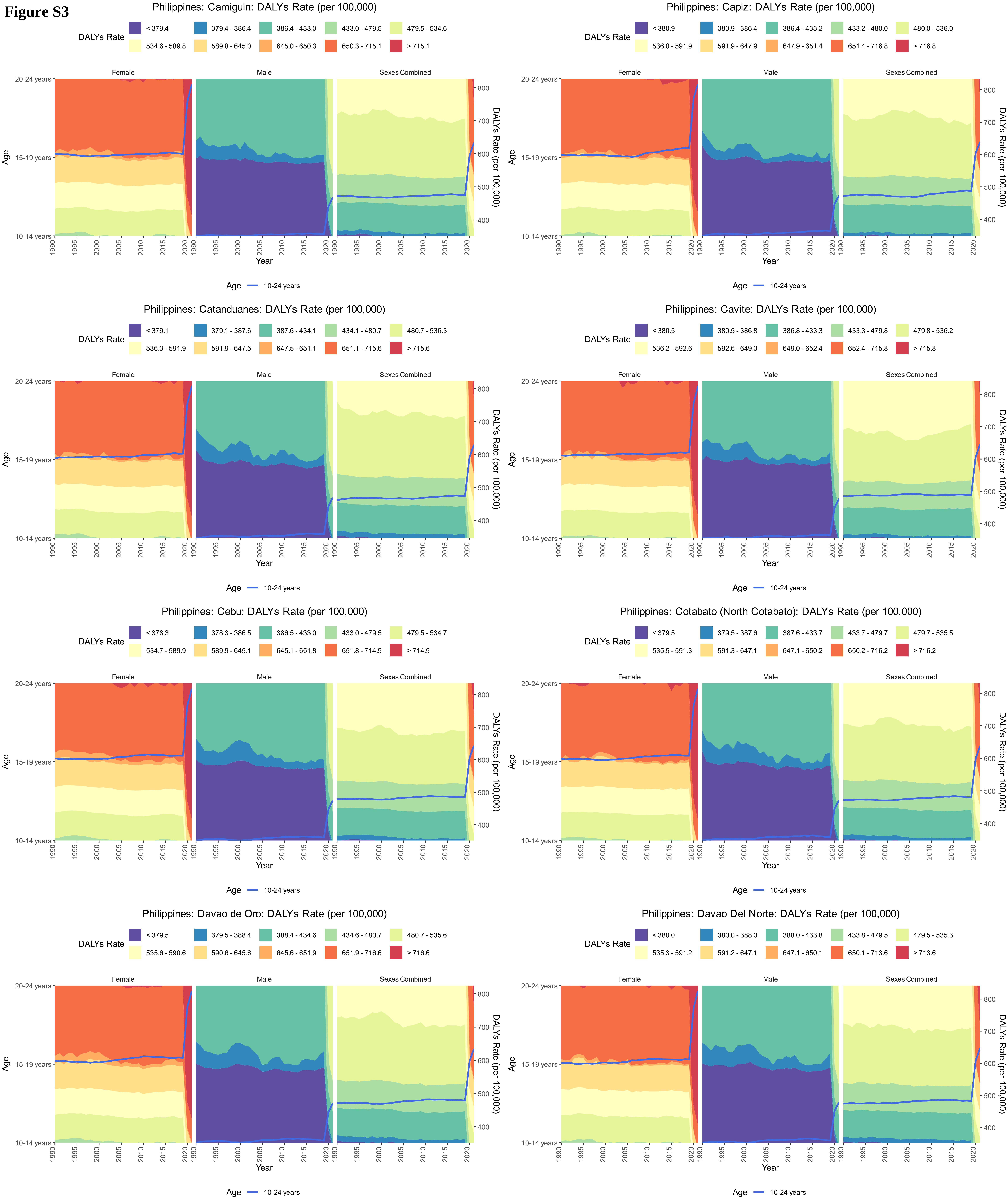
Philippines: Camiguin: DALYs Rate (per 100,000)
DALYs Rate
< 379.4
379.4 - 386.4
386.4 - 433.0
433.0 - 479.5
479.5 - 534.6
534.6 - 589.8
589.8 - 645.0
645.0 - 650.3
650.3 - 715.1
> 715.1
Philippines: Capiz: DALYs Rate (per 100,000)
DALYs Rate
< 380.9
380.9 - 386.4
386.4 - 433.2
433.2 - 480.0
480.0 - 536.0
536.0 - 591.9
591.9 - 647.9
647.9 - 651.4
651.4 - 716.8
> 716.8
Philippines: Catanduanes: DALYs Rate (per 100,000)
DALYs Rate
< 379.1
379.1 - 387.6
387.6 - 434.1
434.1 - 480.7
480.7 - 536.3
536.3 - 591.9
591.9 - 647.5
647.5 - 651.1
651.1 - 715.6
> 715.6
Philippines: Cavite: DALYs Rate (per 100,000)
DALYs Rate
< 380.5
380.5 - 386.8
386.8 - 433.3
433.3 - 479.8
479.8 - 536.2
536.2 - 592.6
592.6 - 649.0
649.0 - 652.4
652.4 - 715.8
> 715.8
Philippines: Cebu: DALYs Rate (per 100,000)
DALYs Rate
< 378.3
378.3 - 386.5
386.5 - 433.0
433.0 - 479.5
479.5 - 534.7
534.7 - 589.9
589.9 - 645.1
645.1 - 651.8
651.8 - 714.9
> 714.9
Philippines: Cotabato (North Cotabato): DALYs Rate (per 100,000)
DALYs Rate
< 379.5
379.5 - 387.6
387.6 - 433.7
433.7 - 479.7
479.7 - 535.5
535.5 - 591.3
591.3 - 647.1
647.1 - 650.2
650.2 - 716.2
> 716.2
Philippines: Davao de Oro: DALYs Rate (per 100,000)
DALYs Rate
< 379.5
379.5 - 388.4
388.4 - 434.6
434.6 - 480.7
480.7 - 535.6
535.6 - 590.6
590.6 - 645.6
645.6 - 651.9
651.9 - 716.6
> 716.6
Philippines: Davao Del Norte: DALYs Rate (per 100,000)
DALYs Rate
< 380.0
380.0 - 388.0
388.0 - 433.8
433.8 - 479.5
479.5 - 535.3
535.3 - 591.2
591.2 - 647.1
647.1 - 650.1
650.1 - 713.6
> 713.6
Female
Male
Sexes Combined
Age
20-24 years
15-19 years
10-14 years
Year
DALYs Rate (per 100,000)
Age
10-24 years

Figure S3

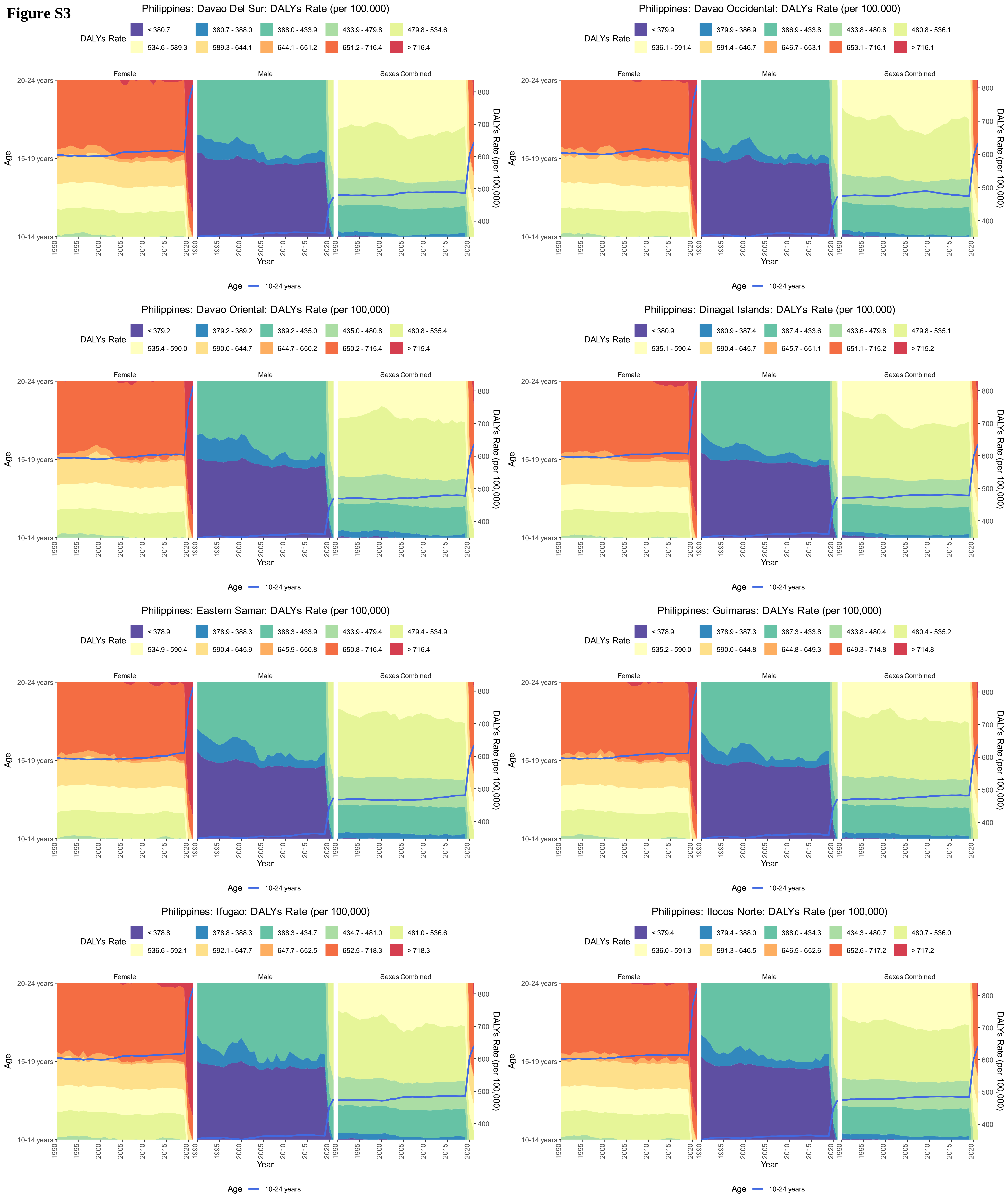
Philippines: Davao Del Sur: DALYs Rate (per 100,000)
DALYs Rate
< 380.7
380.7 - 388.0
388.0 - 433.9
433.9 - 479.8
479.8 - 534.6
534.6 - 589.3
589.3 - 644.1
644.1 - 651.2
651.2 - 716.4
> 716.4
Female
Male
Sexes Combined
Age
20-24 years
15-19 years
10-14 years
Year
DALYs Rate (per 100,000)
Age
10-24 years
Philippines: Davao Occidental: DALYs Rate (per 100,000)
< 379.9
379.9 - 386.9
386.9 - 433.8
433.8 - 480.8
480.8 - 536.1
536.1 - 591.4
591.4 - 646.7
646.7 - 653.1
653.1 - 716.1
> 716.1
Philippines: Davao Oriental: DALYs Rate (per 100,000)
< 379.2
379.2 - 389.2
389.2 - 435.0
435.0 - 480.8
480.8 - 535.4
535.4 - 590.0
590.0 - 644.7
644.7 - 650.2
650.2 - 715.4
> 715.4
Philippines: Dinagat Islands: DALYs Rate (per 100,000)
< 380.9
380.9 - 387.4
387.4 - 433.6
433.6 - 479.8
479.8 - 535.1
535.1 - 590.4
590.4 - 645.7
645.7 - 651.1
651.1 - 715.2
> 715.2
Philippines: Eastern Samar: DALYs Rate (per 100,000)
< 378.9
378.9 - 388.3
388.3 - 433.9
433.9 - 479.4
479.4 - 534.9
534.9 - 590.4
590.4 - 645.9
645.9 - 650.8
650.8 - 716.4
> 716.4
Philippines: Guimaras: DALYs Rate (per 100,000)
< 378.9
378.9 - 387.3
387.3 - 433.8
433.8 - 480.4
480.4 - 535.2
535.2 - 590.0
590.0 - 644.8
644.8 - 649.3
649.3 - 714.8
> 714.8
Philippines: Ifugao: DALYs Rate (per 100,000)
< 378.8
378.8 - 388.3
388.3 - 434.7
434.7 - 481.0
481.0 - 536.6
536.6 - 592.1
592.1 - 647.7
647.7 - 652.5
652.5 - 718.3
> 718.3
Philippines: Ilocos Norte: DALYs Rate (per 100,000)
< 379.4
379.4 - 388.0
388.0 - 434.3
434.3 - 480.7
480.7 - 536.0
536.0 - 591.3
591.3 - 646.5
646.5 - 652.6
652.6 - 717.2
> 717.2

Figure S3

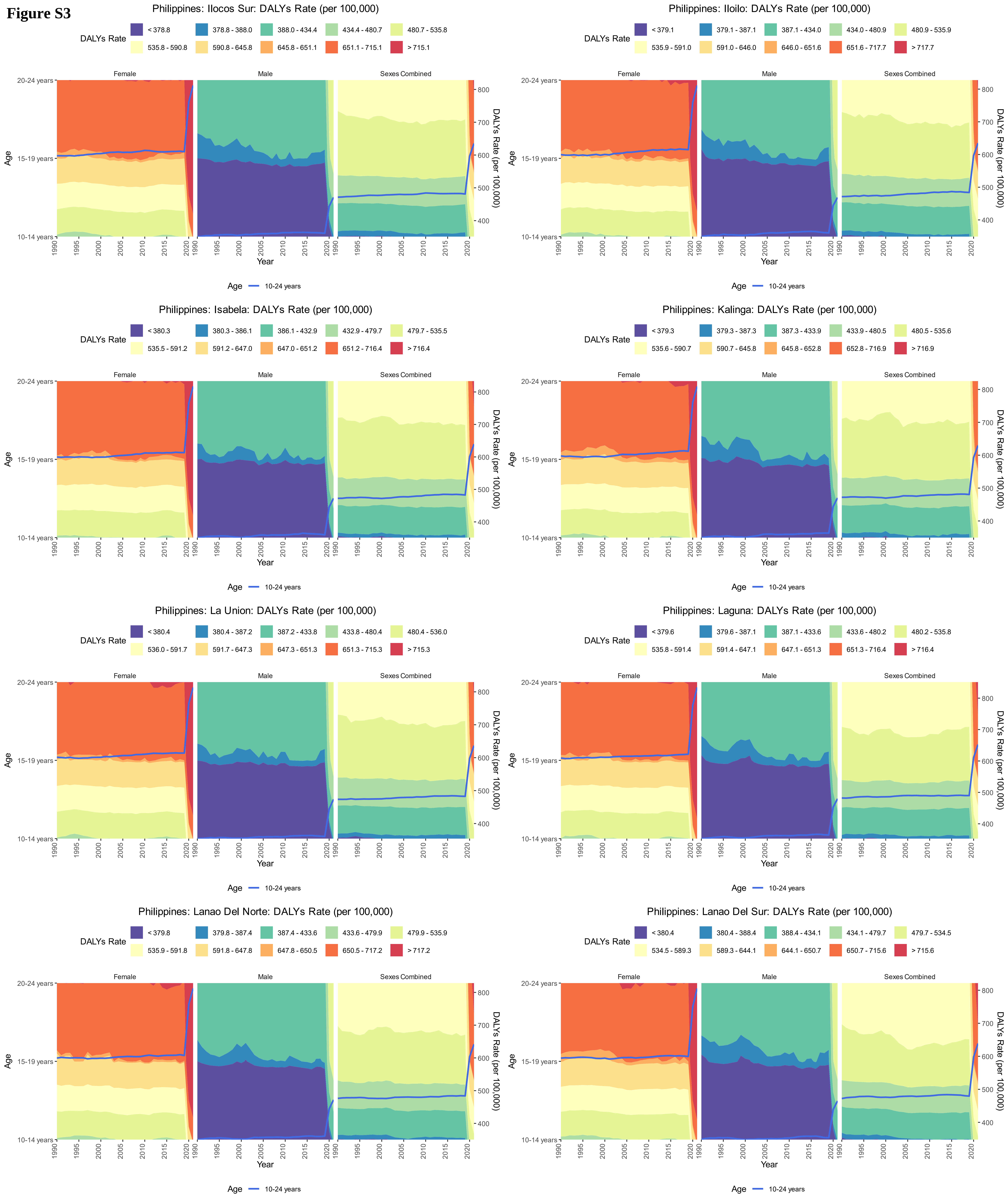

Philippines: Ilocos Sur: DALYs Rate (per 100,000)
DALYs Rate
< 378.8
378.8 - 388.0
388.0 - 434.4
434.4 - 480.7
480.7 - 535.8
535.8 - 590.8
590.8 - 645.8
645.8 - 651.1
651.1 - 715.1
> 715.1
Philippines: Iloilo: DALYs Rate (per 100,000)
< 379.1
379.1 - 387.1
387.1 - 434.0
434.0 - 480.9
480.9 - 535.9
535.9 - 591.0
591.0 - 646.0
646.0 - 651.6
651.6 - 717.7
> 717.7
Philippines: Isabela: DALYs Rate (per 100,000)
< 380.3
380.3 - 386.1
386.1 - 432.9
432.9 - 479.7
479.7 - 535.5
535.5 - 591.2
591.2 - 647.0
647.0 - 651.2
651.2 - 716.4
> 716.4
Philippines: Kalinga: DALYs Rate (per 100,000)
< 379.3
379.3 - 387.3
387.3 - 433.9
433.9 - 480.5
480.5 - 535.6
535.6 - 590.7
590.7 - 645.8
645.8 - 652.8
652.8 - 716.9
> 716.9
Philippines: La Union: DALYs Rate (per 100,000)
< 380.4
380.4 - 387.2
387.2 - 433.8
433.8 - 480.4
480.4 - 536.0
536.0 - 591.7
591.7 - 647.3
647.3 - 651.3
651.3 - 715.3
> 715.3
Philippines: Laguna: DALYs Rate (per 100,000)
< 379.6
379.6 - 387.1
387.1 - 433.6
433.6 - 480.2
480.2 - 535.8
535.8 - 591.4
591.4 - 647.1
647.1 - 651.3
651.3 - 716.4
> 716.4
Philippines: Lanao Del Norte: DALYs Rate (per 100,000)
< 379.8
379.8 - 387.4
387.4 - 433.6
433.6 - 479.9
479.9 - 535.9
535.9 - 591.8
591.8 - 647.8
647.8 - 650.5
650.5 - 717.2
> 717.2
Philippines: Lanao Del Sur: DALYs Rate (per 100,000)
< 380.4
380.4 - 388.4
388.4 - 434.1
434.1 - 479.7
479.7 - 534.5
534.5 - 589.3
589.3 - 644.1
644.1 - 650.7
650.7 - 715.6
> 715.6
Female
Male
Sexes Combined
Age
20-24 years
15-19 years
10-14 years
Year
DALYs Rate (per 100,000)
Age — 10-24 years

Figure S3

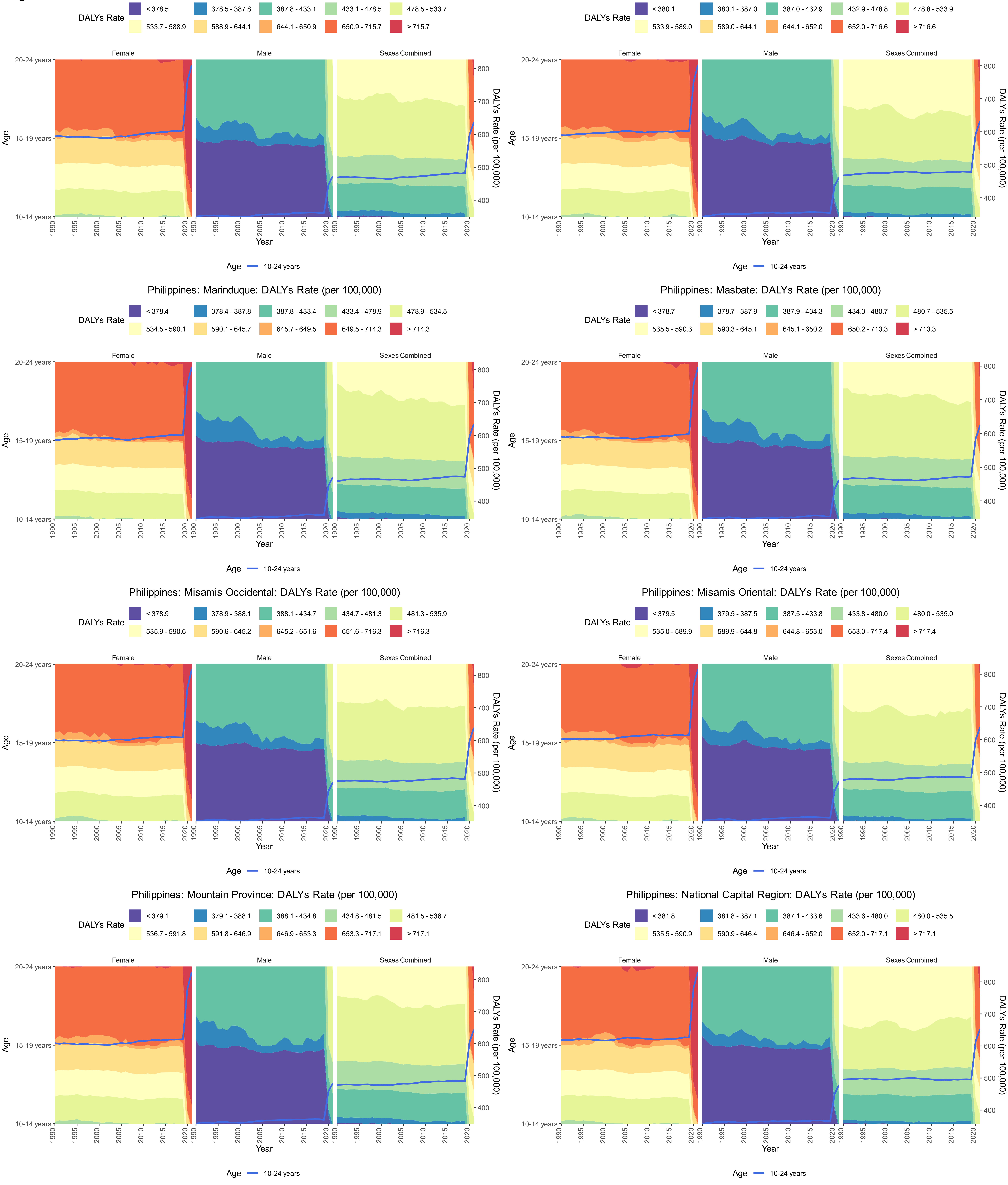

Philippines: Leyte: DALYs Rate (per 100,000)
DALYs Rate
< 378.5
378.5 - 387.8
387.8 - 433.1
433.1 - 478.5
478.5 - 533.7
533.7 - 588.9
588.9 - 644.1
644.1 - 650.9
650.9 - 715.7
> 715.7
Philippines: Maguindanao: DALYs Rate (per 100,000)
DALYs Rate
< 380.1
380.1 - 387.0
387.0 - 432.9
432.9 - 478.8
478.8 - 533.9
533.9 - 589.0
589.0 - 644.1
644.1 - 652.0
652.0 - 716.6
> 716.6
Philippines: Marinduque: DALYs Rate (per 100,000)
DALYs Rate
< 378.4
378.4 - 387.8
387.8 - 433.4
433.4 - 478.9
478.9 - 534.5
534.5 - 590.1
590.1 - 645.7
645.7 - 649.5
649.5 - 714.3
> 714.3
Philippines: Masbate: DALYs Rate (per 100,000)
DALYs Rate
< 378.7
378.7 - 387.9
387.9 - 434.3
434.3 - 480.7
480.7 - 535.5
535.5 - 590.3
590.3 - 645.1
645.1 - 650.2
650.2 - 713.3
> 713.3
Philippines: Misamis Occidental: DALYs Rate (per 100,000)
DALYs Rate
< 378.9
378.9 - 388.1
388.1 - 434.7
434.7 - 481.3
481.3 - 535.9
535.9 - 590.6
590.6 - 645.2
645.2 - 651.6
651.6 - 716.3
> 716.3
Philippines: Misamis Oriental: DALYs Rate (per 100,000)
DALYs Rate
< 379.5
379.5 - 387.5
387.5 - 433.8
433.8 - 480.0
480.0 - 535.0
535.0 - 589.9
589.9 - 644.8
644.8 - 653.0
653.0 - 717.4
> 717.4
Philippines: Mountain Province: DALYs Rate (per 100,000)
DALYs Rate
< 379.1
379.1 - 388.1
388.1 - 434.8
434.8 - 481.5
481.5 - 536.7
536.7 - 591.8
591.8 - 646.9
646.9 - 653.3
653.3 - 717.1
> 717.1
Philippines: National Capital Region: DALYs Rate (per 100,000)
DALYs Rate
< 381.8
381.8 - 387.1
387.1 - 433.6
433.6 - 480.0
480.0 - 535.5
535.5 - 590.9
590.9 - 646.4
646.4 - 652.0
652.0 - 717.1
> 717.1
Female
Male
Sexes Combined
Age
20-24 years
15-19 years
10-14 years
Year
DALYs Rate (per 100,000)
Age — 10-24 years

Figure S3

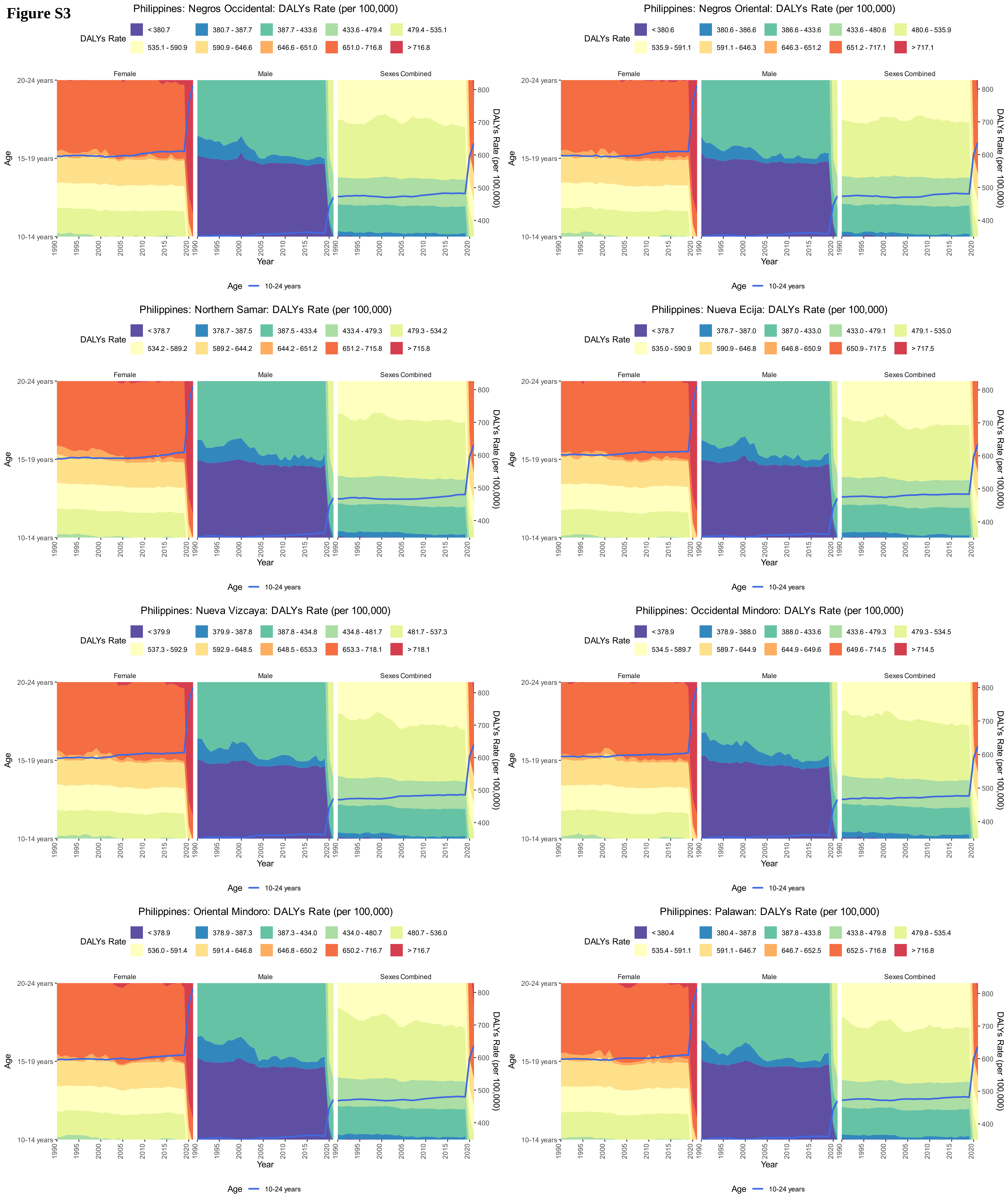
Philippines: Negros Occidental: DALYs Rate (per 100,000)
DALYs Rate
< 380.7
380.7 - 387.7
387.7 - 433.6
433.6 - 479.4
479.4 - 535.1
535.1 - 590.9
590.9 - 646.6
646.6 - 651.0
651.0 - 716.8
> 716.8
Philippines: Negros Oriental: DALYs Rate (per 100,000)
DALYs Rate
< 380.6
380.6 - 386.6
386.6 - 433.6
433.6 - 480.6
480.6 - 535.9
535.9 - 591.1
591.1 - 646.3
646.3 - 651.2
651.2 - 717.1
> 717.1
Philippines: Northern Samar: DALYs Rate (per 100,000)
DALYs Rate
< 378.7
378.7 - 387.5
387.5 - 433.4
433.4 - 479.3
479.3 - 534.2
534.2 - 589.2
589.2 - 644.2
644.2 - 651.2
651.2 - 715.8
> 715.8
Philippines: Nueva Ecija: DALYs Rate (per 100,000)
DALYs Rate
< 378.7
378.7 - 387.0
387.0 - 433.0
433.0 - 479.1
479.1 - 535.0
535.0 - 590.9
590.9 - 646.8
646.8 - 650.9
650.9 - 717.5
> 717.5
Philippines: Nueva Vizcaya: DALYs Rate (per 100,000)
DALYs Rate
< 379.9
379.9 - 387.8
387.8 - 434.8
434.8 - 481.7
481.7 - 537.3
537.3 - 592.9
592.9 - 648.5
648.5 - 653.3
653.3 - 718.1
> 718.1
Philippines: Occidental Mindoro: DALYs Rate (per 100,000)
DALYs Rate
< 378.9
378.9 - 388.0
388.0 - 433.6
433.6 - 479.3
479.3 - 534.5
534.5 - 589.7
589.7 - 644.9
644.9 - 649.6
649.6 - 714.5
> 714.5
Philippines: Oriental Mindoro: DALYs Rate (per 100,000)
DALYs Rate
< 378.9
378.9 - 387.3
387.3 - 434.0
434.0 - 480.7
480.7 - 536.0
536.0 - 591.4
591.4 - 646.8
646.8 - 650.2
650.2 - 716.7
> 716.7
Philippines: Palawan: DALYs Rate (per 100,000)
DALYs Rate
< 380.4
380.4 - 387.8
387.8 - 433.8
433.8 - 479.8
479.8 - 535.4
535.4 - 591.1
591.1 - 646.7
646.7 - 652.5
652.5 - 716.8
> 716.8
Female
Male
Sexes Combined
Age
20-24 years
15-19 years
10-14 years
Year
DALYs Rate (per 100,000)
Age — 10-24 years

Figure S3

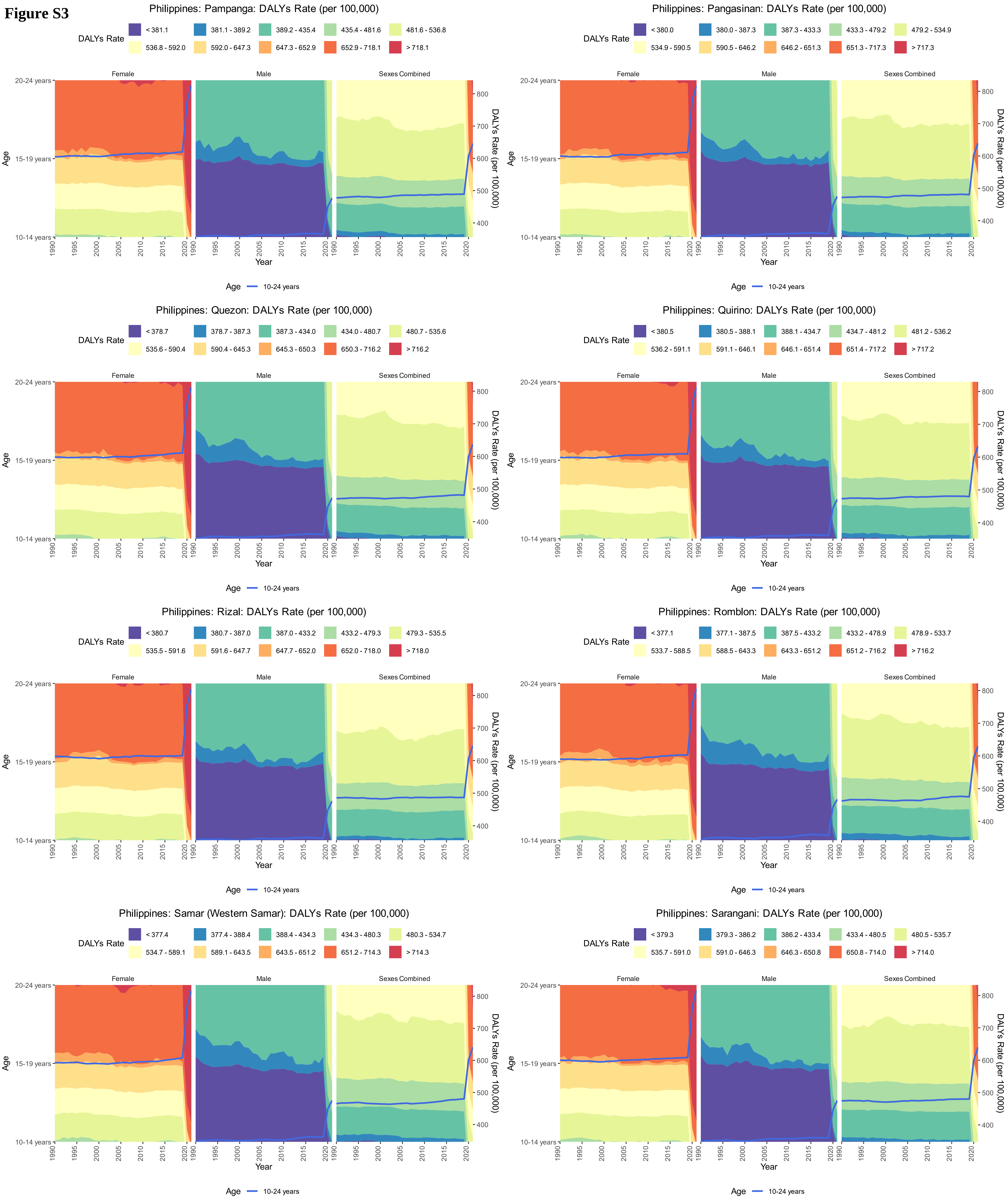
Philippines: Pampanga: DALYs Rate (per 100,000)
DALYs Rate
< 381.1
381.1 - 389.2
389.2 - 435.4
435.4 - 481.6
481.6 - 536.8
536.8 - 592.0
592.0 - 647.3
647.3 - 652.9
652.9 - 718.1
> 718.1
Female
Male
Sexes Combined
Age
Year
DALYs Rate (per 100,000)
Age — 10-24 years
Philippines: Pangasinan: DALYs Rate (per 100,000)
< 380.0
380.0 - 387.3
387.3 - 433.3
433.3 - 479.2
479.2 - 534.9
534.9 - 590.5
590.5 - 646.2
646.2 - 651.3
651.3 - 717.3
> 717.3
Philippines: Quezon: DALYs Rate (per 100,000)
< 378.7
378.7 - 387.3
387.3 - 434.0
434.0 - 480.7
480.7 - 535.6
535.6 - 590.4
590.4 - 645.3
645.3 - 650.3
650.3 - 716.2
> 716.2
Philippines: Quirino: DALYs Rate (per 100,000)
< 380.5
380.5 - 388.1
388.1 - 434.7
434.7 - 481.2
481.2 - 536.2
536.2 - 591.1
591.1 - 646.1
646.1 - 651.4
651.4 - 717.2
> 717.2
Philippines: Rizal: DALYs Rate (per 100,000)
< 380.7
380.7 - 387.0
387.0 - 433.2
433.2 - 479.3
479.3 - 535.5
535.5 - 591.6
591.6 - 647.7
647.7 - 652.0
652.0 - 718.0
> 718.0
Philippines: Romblon: DALYs Rate (per 100,000)
< 377.1
377.1 - 387.5
387.5 - 433.2
433.2 - 478.9
478.9 - 533.7
533.7 - 588.5
588.5 - 643.3
643.3 - 651.2
651.2 - 716.2
> 716.2
Philippines: Samar (Western Samar): DALYs Rate (per 100,000)
< 377.4
377.4 - 388.4
388.4 - 434.3
434.3 - 480.3
480.3 - 534.7
534.7 - 589.1
589.1 - 643.5
643.5 - 651.2
651.2 - 714.3
> 714.3
Philippines: Sarangani: DALYs Rate (per 100,000)
< 379.3
379.3 - 386.2
386.2 - 433.4
433.4 - 480.5
480.5 - 535.7
535.7 - 591.0
591.0 - 646.3
646.3 - 650.8
650.8 - 714.0
> 714.0
20-24 years
15-19 years
10-14 years
1990
1995
2000
2005
2010
2015
2020
400
500
600
700
800

Figure S3

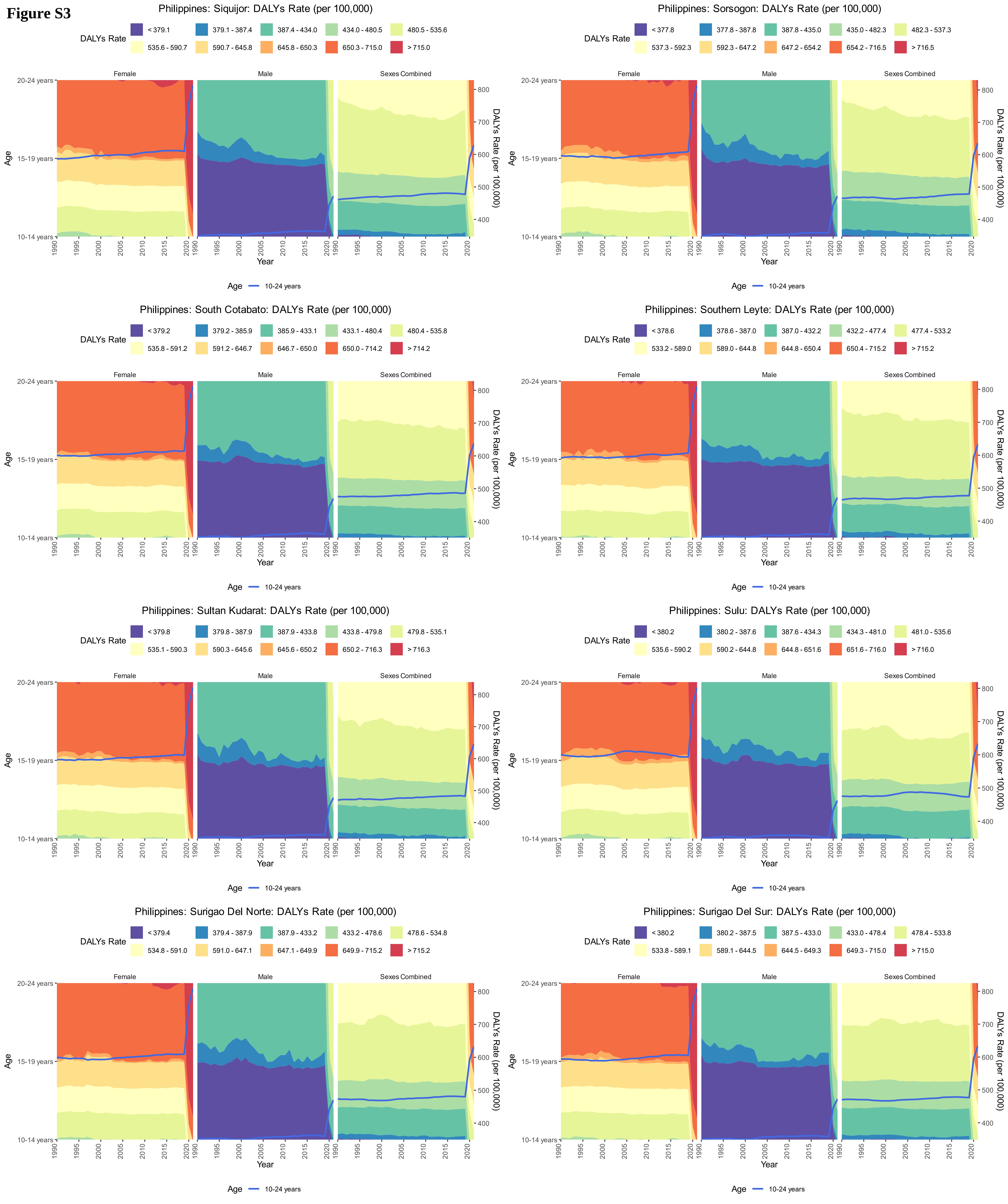

Philippines: Siquijor: DALYs Rate (per 100,000)
DALYs Rate
< 379.1
379.1 - 387.4
387.4 - 434.0
434.0 - 480.5
480.5 - 535.6
535.6 - 590.7
590.7 - 645.8
645.8 - 650.3
650.3 - 715.0
> 715.0
Female
Male
Sexes Combined
Age
Year
DALYs Rate (per 100,000)
Age
10-24 years
Philippines: Sorsogon: DALYs Rate (per 100,000)
< 377.8
377.8 - 387.8
387.8 - 435.0
435.0 - 482.3
482.3 - 537.3
537.3 - 592.3
592.3 - 647.2
647.2 - 654.2
654.2 - 716.5
> 716.5
Philippines: South Cotabato: DALYs Rate (per 100,000)
< 379.2
379.2 - 385.9
385.9 - 433.1
433.1 - 480.4
480.4 - 535.8
535.8 - 591.2
591.2 - 646.7
646.7 - 650.0
650.0 - 714.2
> 714.2
Philippines: Southern Leyte: DALYs Rate (per 100,000)
< 378.6
378.6 - 387.0
387.0 - 432.2
432.2 - 477.4
477.4 - 533.2
533.2 - 589.0
589.0 - 644.8
644.8 - 650.4
650.4 - 715.2
> 715.2
Philippines: Sultan Kudarat: DALYs Rate (per 100,000)
< 379.8
379.8 - 387.9
387.9 - 433.8
433.8 - 479.8
479.8 - 535.1
535.1 - 590.3
590.3 - 645.6
645.6 - 650.2
650.2 - 716.3
> 716.3
Philippines: Sulu: DALYs Rate (per 100,000)
< 380.2
380.2 - 387.6
387.6 - 434.3
434.3 - 481.0
481.0 - 535.6
535.6 - 590.2
590.2 - 644.8
644.8 - 651.6
651.6 - 716.0
> 716.0
Philippines: Surigao Del Norte: DALYs Rate (per 100,000)
< 379.4
379.4 - 387.9
387.9 - 433.2
433.2 - 478.6
478.6 - 534.8
534.8 - 591.0
591.0 - 647.1
647.1 - 649.9
649.9 - 715.2
> 715.2
Philippines: Surigao Del Sur: DALYs Rate (per 100,000)
< 380.2
380.2 - 387.5
387.5 - 433.0
433.0 - 478.4
478.4 - 533.8
533.8 - 589.1
589.1 - 644.5
644.5 - 649.3
649.3 - 715.0
> 715.0

Figure S3

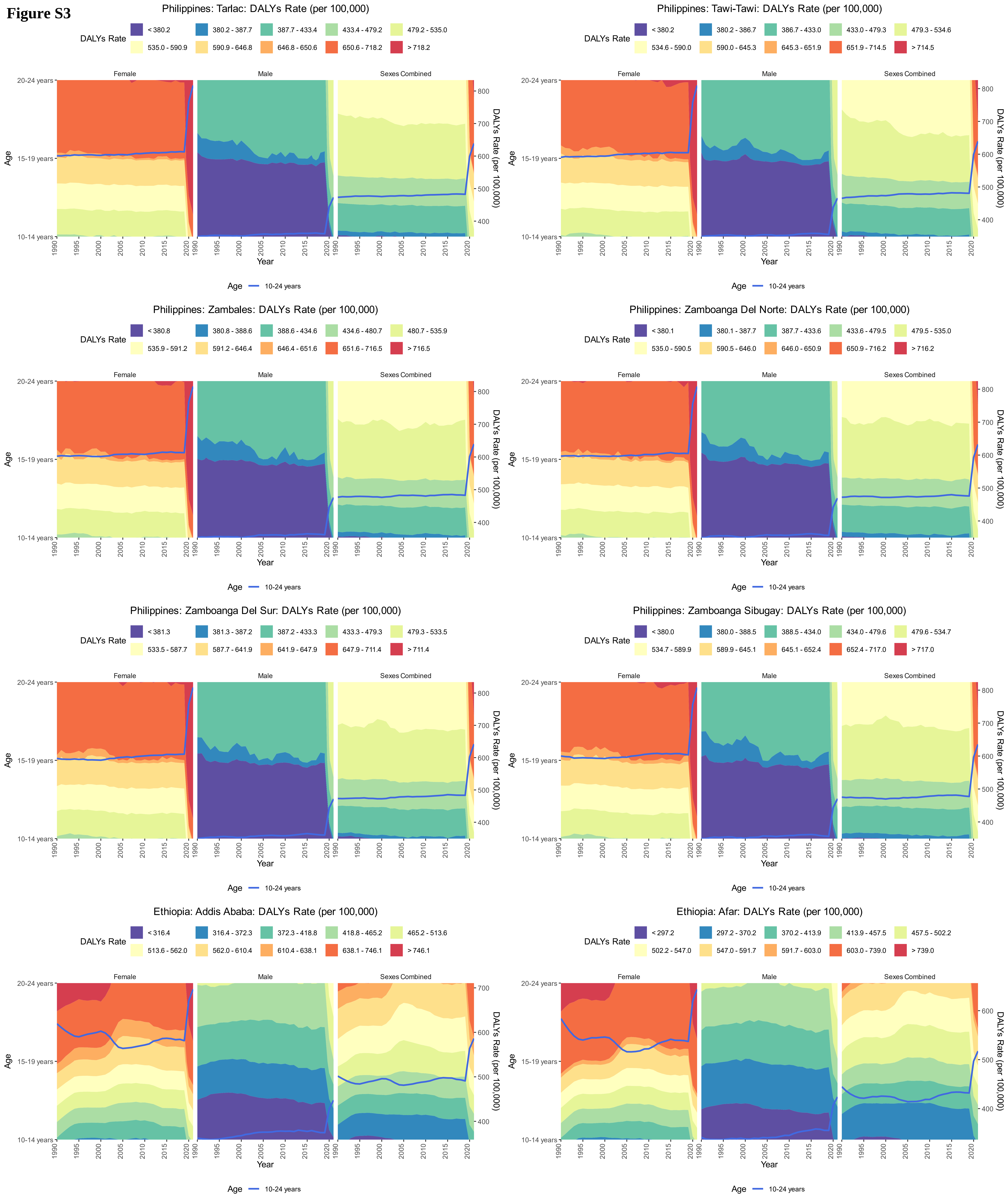

Philippines: Tarlac: DALYs Rate (per 100,000)
DALYs Rate
< 380.2
380.2 - 387.7
387.7 - 433.4
433.4 - 479.2
479.2 - 535.0
535.0 - 590.9
590.9 - 646.8
646.8 - 650.6
650.6 - 718.2
> 718.2
Philippines: Tawi-Tawi: DALYs Rate (per 100,000)
DALYs Rate
< 380.2
380.2 - 386.7
386.7 - 433.0
433.0 - 479.3
479.3 - 534.6
534.6 - 590.0
590.0 - 645.3
645.3 - 651.9
651.9 - 714.5
> 714.5
Philippines: Zambales: DALYs Rate (per 100,000)
DALYs Rate
< 380.8
380.8 - 388.6
388.6 - 434.6
434.6 - 480.7
480.7 - 535.9
535.9 - 591.2
591.2 - 646.4
646.4 - 651.6
651.6 - 716.5
> 716.5
Philippines: Zamboanga Del Norte: DALYs Rate (per 100,000)
DALYs Rate
< 380.1
380.1 - 387.7
387.7 - 433.6
433.6 - 479.5
479.5 - 535.0
535.0 - 590.5
590.5 - 646.0
646.0 - 650.9
650.9 - 716.2
> 716.2
Philippines: Zamboanga Del Sur: DALYs Rate (per 100,000)
DALYs Rate
< 381.3
381.3 - 387.2
387.2 - 433.3
433.3 - 479.3
479.3 - 533.5
533.5 - 587.7
587.7 - 641.9
641.9 - 647.9
647.9 - 711.4
> 711.4
Philippines: Zamboanga Sibugay: DALYs Rate (per 100,000)
DALYs Rate
< 380.0
380.0 - 388.5
388.5 - 434.0
434.0 - 479.6
479.6 - 534.7
534.7 - 589.9
589.9 - 645.1
645.1 - 652.4
652.4 - 717.0
> 717.0
Ethiopia: Addis Ababa: DALYs Rate (per 100,000)
DALYs Rate
< 316.4
316.4 - 372.3
372.3 - 418.8
418.8 - 465.2
465.2 - 513.6
513.6 - 562.0
562.0 - 610.4
610.4 - 638.1
638.1 - 746.1
> 746.1
Ethiopia: Afar: DALYs Rate (per 100,000)
DALYs Rate
< 297.2
297.2 - 370.2
370.2 - 413.9
413.9 - 457.5
457.5 - 502.2
502.2 - 547.0
547.0 - 591.7
591.7 - 603.0
603.0 - 739.0
> 739.0
Female
Male
Sexes Combined
Age
20-24 years
15-19 years
10-14 years
Year
DALYs Rate (per 100,000)
Age
10-24 years

Figure S3

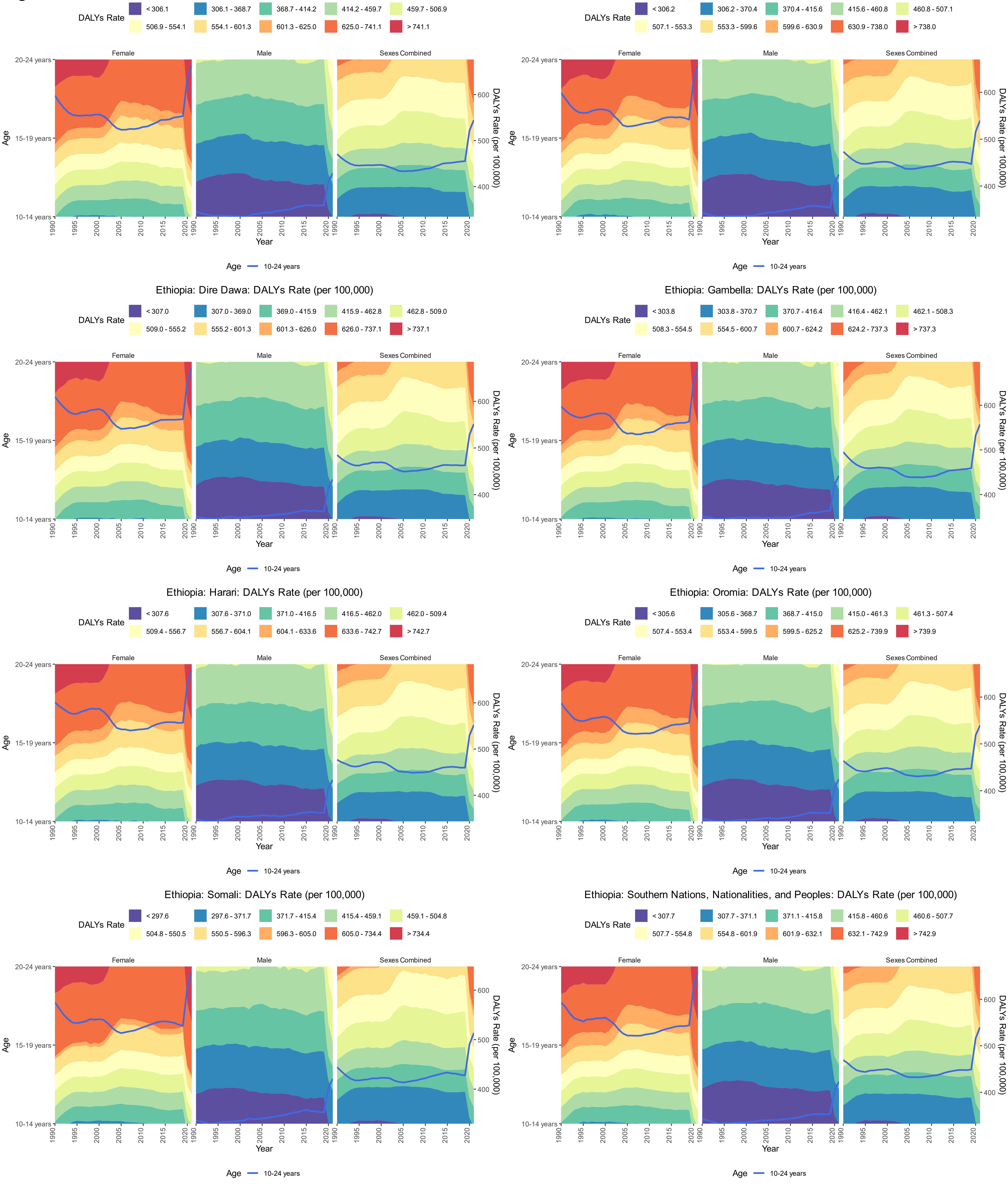
Ethiopia: Amhara: DALYs Rate (per 100,000)
DALYs Rate
< 306.1
306.1 - 368.7
368.7 - 414.2
414.2 - 459.7
459.7 - 506.9
506.9 - 554.1
554.1 - 601.3
601.3 - 625.0
625.0 - 741.1
> 741.1
Female
Male
Sexes Combined
Age
DALYs Rate (per 100,000)
Year
Age — 10-24 years
Ethiopia: Benishangul-Gumuz: DALYs Rate (per 100,000)
DALYs Rate
< 306.2
306.2 - 370.4
370.4 - 415.6
415.6 - 460.8
460.8 - 507.1
507.1 - 553.3
553.3 - 599.6
599.6 - 630.9
630.9 - 738.0
> 738.0
Ethiopia: Dire Dawa: DALYs Rate (per 100,000)
DALYs Rate
< 307.0
307.0 - 369.0
369.0 - 415.9
415.9 - 462.8
462.8 - 509.0
509.0 - 555.2
555.2 - 601.3
601.3 - 626.0
626.0 - 737.1
> 737.1
Ethiopia: Gambella: DALYs Rate (per 100,000)
DALYs Rate
< 303.8
303.8 - 370.7
370.7 - 416.4
416.4 - 462.1
462.1 - 508.3
508.3 - 554.5
554.5 - 600.7
600.7 - 624.2
624.2 - 737.3
> 737.3
Ethiopia: Harari: DALYs Rate (per 100,000)
DALYs Rate
< 307.6
307.6 - 371.0
371.0 - 416.5
416.5 - 462.0
462.0 - 509.4
509.4 - 556.7
556.7 - 604.1
604.1 - 633.6
633.6 - 742.7
> 742.7
Ethiopia: Oromia: DALYs Rate (per 100,000)
DALYs Rate
< 305.6
305.6 - 368.7
368.7 - 415.0
415.0 - 461.3
461.3 - 507.4
507.4 - 553.4
553.4 - 599.5
599.5 - 625.2
625.2 - 739.9
> 739.9
Ethiopia: Somali: DALYs Rate (per 100,000)
DALYs Rate
< 297.6
297.6 - 371.7
371.7 - 415.4
415.4 - 459.1
459.1 - 504.8
504.8 - 550.5
550.5 - 596.3
596.3 - 605.0
605.0 - 734.4
> 734.4
Ethiopia: Southern Nations, Nationalities, and Peoples: DALYs Rate (per 100,000)
DALYs Rate
< 307.7
307.7 - 371.1
371.1 - 415.8
415.8 - 460.6
460.6 - 507.7
507.7 - 554.8
554.8 - 601.9
601.9 - 632.1
632.1 - 742.9
> 742.9
20-24 years
15-19 years
10-14 years
1990
1995
2000
2005
2010
2015
2020
400
500
600

Figure S3

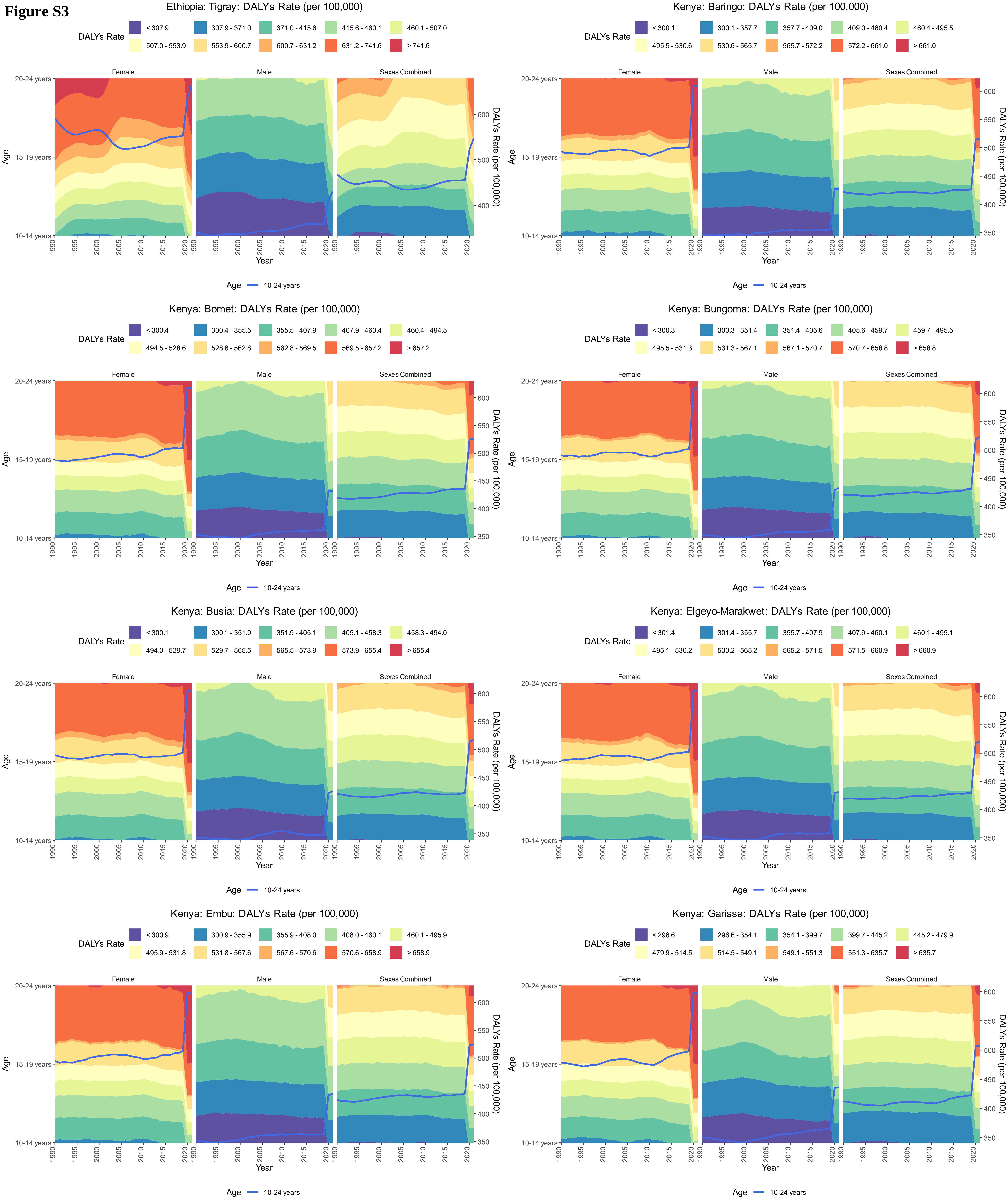

Ethiopia: Tigray: DALYs Rate (per 100,000)
DALYs Rate
< 307.9
307.9 - 371.0
371.0 - 415.6
415.6 - 460.1
460.1 - 507.0
507.0 - 553.9
553.9 - 600.7
600.7 - 631.2
631.2 - 741.6
> 741.6
Kenya: Baringo: DALYs Rate (per 100,000)
< 300.1
300.1 - 357.7
357.7 - 409.0
409.0 - 460.4
460.4 - 495.5
495.5 - 530.6
530.6 - 565.7
565.7 - 572.2
572.2 - 661.0
> 661.0
Kenya: Bomet: DALYs Rate (per 100,000)
< 300.4
300.4 - 355.5
355.5 - 407.9
407.9 - 460.4
460.4 - 494.5
494.5 - 528.6
528.6 - 562.8
562.8 - 569.5
569.5 - 657.2
> 657.2
Kenya: Bungoma: DALYs Rate (per 100,000)
< 300.3
300.3 - 351.4
351.4 - 405.6
405.6 - 459.7
459.7 - 495.5
495.5 - 531.3
531.3 - 567.1
567.1 - 570.7
570.7 - 658.8
> 658.8
Kenya: Busia: DALYs Rate (per 100,000)
< 300.1
300.1 - 351.9
351.9 - 405.1
405.1 - 458.3
458.3 - 494.0
494.0 - 529.7
529.7 - 565.5
565.5 - 573.9
573.9 - 655.4
> 655.4
Kenya: Elgeyo-Marakwet: DALYs Rate (per 100,000)
< 301.4
301.4 - 355.7
355.7 - 407.9
407.9 - 460.1
460.1 - 495.1
495.1 - 530.2
530.2 - 565.2
565.2 - 571.5
571.5 - 660.9
> 660.9
Kenya: Embu: DALYs Rate (per 100,000)
< 300.9
300.9 - 355.9
355.9 - 408.0
408.0 - 460.1
460.1 - 495.9
495.9 - 531.8
531.8 - 567.6
567.6 - 570.6
570.6 - 658.9
> 658.9
Kenya: Garissa: DALYs Rate (per 100,000)
< 296.6
296.6 - 354.1
354.1 - 399.7
399.7 - 445.2
445.2 - 479.9
479.9 - 514.5
514.5 - 549.1
549.1 - 551.3
551.3 - 635.7
> 635.7
Female
Male
Sexes Combined
20-24 years
15-19 years
10-14 years
Age
Year
DALYs Rate (per 100,000)
Age — 10-24 years

Figure S3

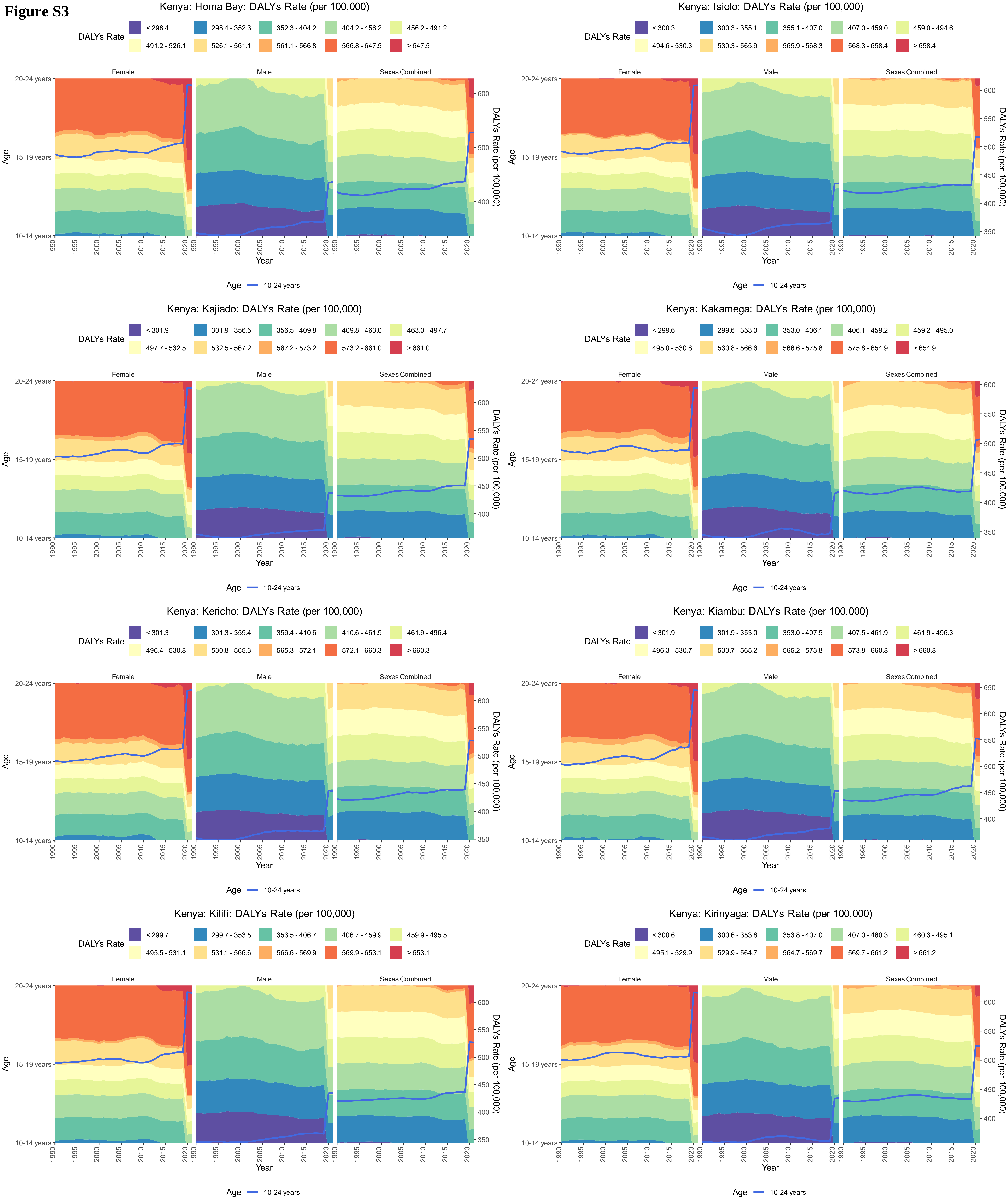

Kenya: Homa Bay: DALYs Rate (per 100,000)
DALYs Rate
< 298.4
298.4 - 352.3
352.3 - 404.2
404.2 - 456.2
456.2 - 491.2
491.2 - 526.1
526.1 - 561.1
561.1 - 566.8
566.8 - 647.5
> 647.5
Kenya: Isiolo: DALYs Rate (per 100,000)
< 300.3
300.3 - 355.1
355.1 - 407.0
407.0 - 459.0
459.0 - 494.6
494.6 - 530.3
530.3 - 565.9
565.9 - 568.3
568.3 - 658.4
> 658.4
Kenya: Kajiado: DALYs Rate (per 100,000)
< 301.9
301.9 - 356.5
356.5 - 409.8
409.8 - 463.0
463.0 - 497.7
497.7 - 532.5
532.5 - 567.2
567.2 - 573.2
573.2 - 661.0
> 661.0
Kenya: Kakamega: DALYs Rate (per 100,000)
< 299.6
299.6 - 353.0
353.0 - 406.1
406.1 - 459.2
459.2 - 495.0
495.0 - 530.8
530.8 - 566.6
566.6 - 575.8
575.8 - 654.9
> 654.9
Kenya: Kericho: DALYs Rate (per 100,000)
< 301.3
301.3 - 359.4
359.4 - 410.6
410.6 - 461.9
461.9 - 496.4
496.4 - 530.8
530.8 - 565.3
565.3 - 572.1
572.1 - 660.3
> 660.3
Kenya: Kiambu: DALYs Rate (per 100,000)
< 301.9
301.9 - 353.0
353.0 - 407.5
407.5 - 461.9
461.9 - 496.3
496.3 - 530.7
530.7 - 565.2
565.2 - 573.8
573.8 - 660.8
> 660.8
Kenya: Kilifi: DALYs Rate (per 100,000)
< 299.7
299.7 - 353.5
353.5 - 406.7
406.7 - 459.9
459.9 - 495.5
495.5 - 531.1
531.1 - 566.6
566.6 - 569.9
569.9 - 653.1
> 653.1
Kenya: Kirinyaga: DALYs Rate (per 100,000)
< 300.6
300.6 - 353.8
353.8 - 407.0
407.0 - 460.3
460.3 - 495.1
495.1 - 529.9
529.9 - 564.7
564.7 - 569.7
569.7 - 661.2
> 661.2
Female
Male
Sexes Combined
Age
20-24 years
15-19 years
10-14 years
Year
DALYs Rate (per 100,000)
Age
10-24 years

Figure S3

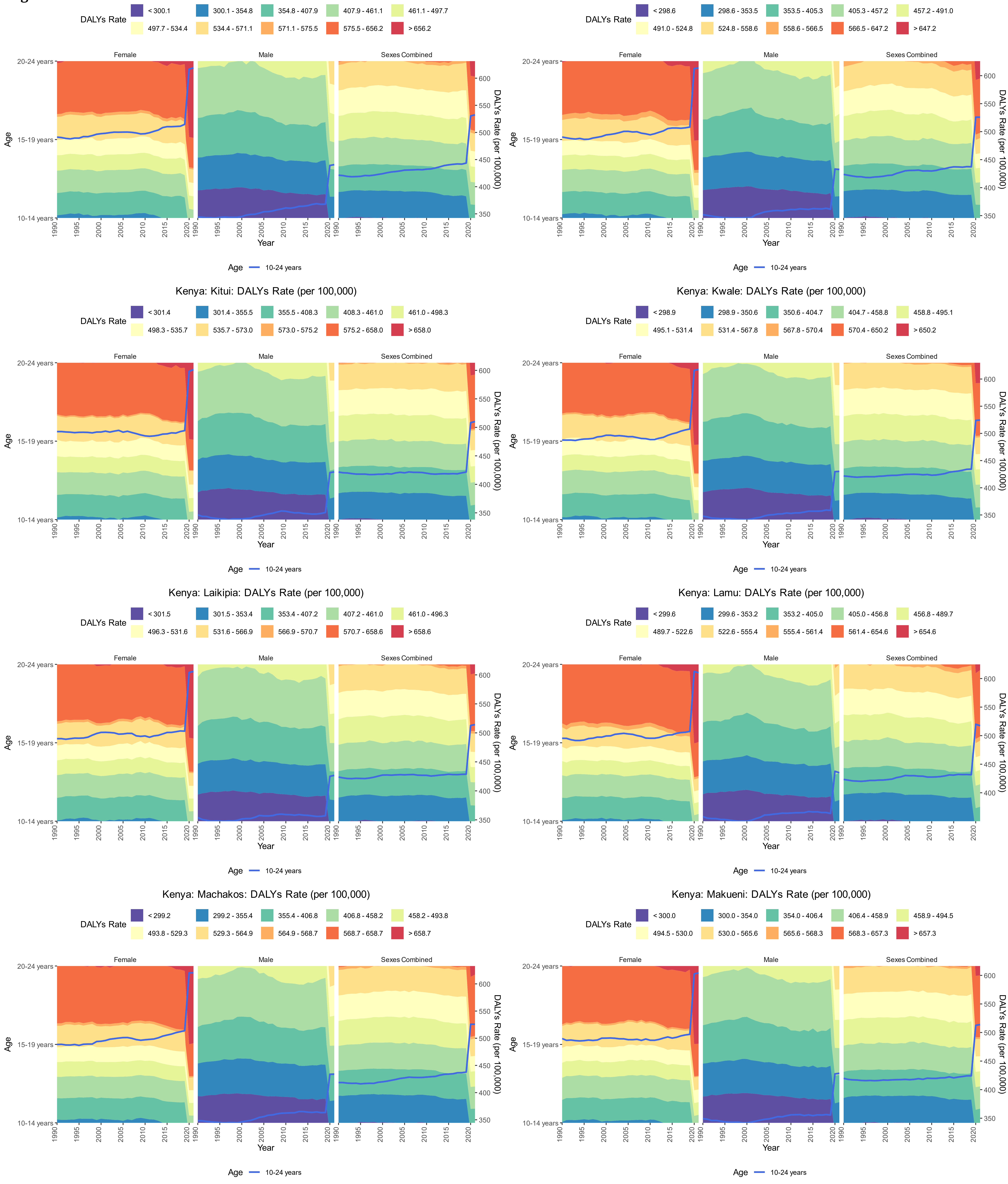
Kenya: Kisii: DALYs Rate (per 100,000)
DALYs Rate
< 300.1
300.1 - 354.8
354.8 - 407.9
407.9 - 461.1
461.1 - 497.7
497.7 - 534.4
534.4 - 571.1
571.1 - 575.5
575.5 - 656.2
> 656.2
Kenya: Kisumu: DALYs Rate (per 100,000)
DALYs Rate
< 298.6
298.6 - 353.5
353.5 - 405.3
405.3 - 457.2
457.2 - 491.0
491.0 - 524.8
524.8 - 558.6
558.6 - 566.5
566.5 - 647.2
> 647.2
Kenya: Kitui: DALYs Rate (per 100,000)
DALYs Rate
< 301.4
301.4 - 355.5
355.5 - 408.3
408.3 - 461.0
461.0 - 498.3
498.3 - 535.7
535.7 - 573.0
573.0 - 575.2
575.2 - 658.0
> 658.0
Kenya: Kwale: DALYs Rate (per 100,000)
DALYs Rate
< 298.9
298.9 - 350.6
350.6 - 404.7
404.7 - 458.8
458.8 - 495.1
495.1 - 531.4
531.4 - 567.8
567.8 - 570.4
570.4 - 650.2
> 650.2
Kenya: Laikipia: DALYs Rate (per 100,000)
DALYs Rate
< 301.5
301.5 - 353.4
353.4 - 407.2
407.2 - 461.0
461.0 - 496.3
496.3 - 531.6
531.6 - 566.9
566.9 - 570.7
570.7 - 658.6
> 658.6
Kenya: Lamu: DALYs Rate (per 100,000)
DALYs Rate
< 299.6
299.6 - 353.2
353.2 - 405.0
405.0 - 456.8
456.8 - 489.7
489.7 - 522.6
522.6 - 555.4
555.4 - 561.4
561.4 - 654.6
> 654.6
Kenya: Machakos: DALYs Rate (per 100,000)
DALYs Rate
< 299.2
299.2 - 355.4
355.4 - 406.8
406.8 - 458.2
458.2 - 493.8
493.8 - 529.3
529.3 - 564.9
564.9 - 568.7
568.7 - 658.7
> 658.7
Kenya: Makueni: DALYs Rate (per 100,000)
DALYs Rate
< 300.0
300.0 - 354.0
354.0 - 406.4
406.4 - 458.9
458.9 - 494.5
494.5 - 530.0
530.0 - 565.6
565.6 - 568.3
568.3 - 657.3
> 657.3
Female
Male
Sexes Combined
Age
20-24 years
15-19 years
10-14 years
Year
DALYs Rate (per 100,000)
10-24 years

Figure S3

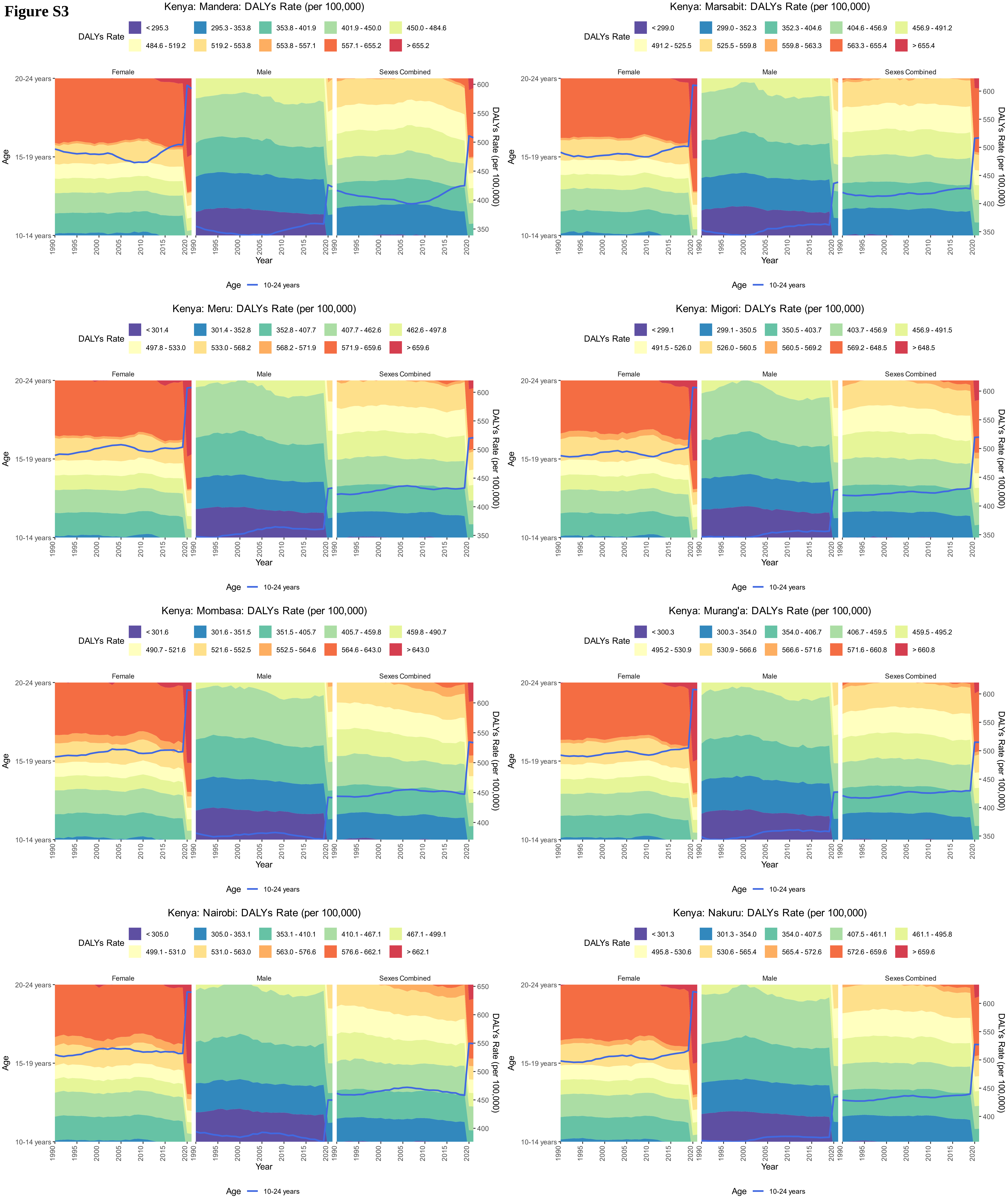
Kenya: Mandera: DALYs Rate (per 100,000)
DALYs Rate
< 295.3
295.3 - 353.8
353.8 - 401.9
401.9 - 450.0
450.0 - 484.6
484.6 - 519.2
519.2 - 553.8
553.8 - 557.1
557.1 - 655.2
> 655.2
Female
Male
Sexes Combined
Age
Year
DALYs Rate (per 100,000)
Age — 10-24 years
Kenya: Marsabit: DALYs Rate (per 100,000)
DALYs Rate
< 299.0
299.0 - 352.3
352.3 - 404.6
404.6 - 456.9
456.9 - 491.2
491.2 - 525.5
525.5 - 559.8
559.8 - 563.3
563.3 - 655.4
> 655.4
Kenya: Meru: DALYs Rate (per 100,000)
DALYs Rate
< 301.4
301.4 - 352.8
352.8 - 407.7
407.7 - 462.6
462.6 - 497.8
497.8 - 533.0
533.0 - 568.2
568.2 - 571.9
571.9 - 659.6
> 659.6
Kenya: Migori: DALYs Rate (per 100,000)
DALYs Rate
< 299.1
299.1 - 350.5
350.5 - 403.7
403.7 - 456.9
456.9 - 491.5
491.5 - 526.0
526.0 - 560.5
560.5 - 569.2
569.2 - 648.5
> 648.5
Kenya: Mombasa: DALYs Rate (per 100,000)
DALYs Rate
< 301.6
301.6 - 351.5
351.5 - 405.7
405.7 - 459.8
459.8 - 490.7
490.7 - 521.6
521.6 - 552.5
552.5 - 564.6
564.6 - 643.0
> 643.0
Kenya: Murang'a: DALYs Rate (per 100,000)
DALYs Rate
< 300.3
300.3 - 354.0
354.0 - 406.7
406.7 - 459.5
459.5 - 495.2
495.2 - 530.9
530.9 - 566.6
566.6 - 571.6
571.6 - 660.8
> 660.8
Kenya: Nairobi: DALYs Rate (per 100,000)
DALYs Rate
< 305.0
305.0 - 353.1
353.1 - 410.1
410.1 - 467.1
467.1 - 499.1
499.1 - 531.0
531.0 - 563.0
563.0 - 576.6
576.6 - 662.1
> 662.1
Kenya: Nakuru: DALYs Rate (per 100,000)
DALYs Rate
< 301.3
301.3 - 354.0
354.0 - 407.5
407.5 - 461.1
461.1 - 495.8
495.8 - 530.6
530.6 - 565.4
565.4 - 572.6
572.6 - 659.6
> 659.6
20-24 years
15-19 years
10-14 years
1990
1995
2000
2005
2010
2015
2020

Figure S3

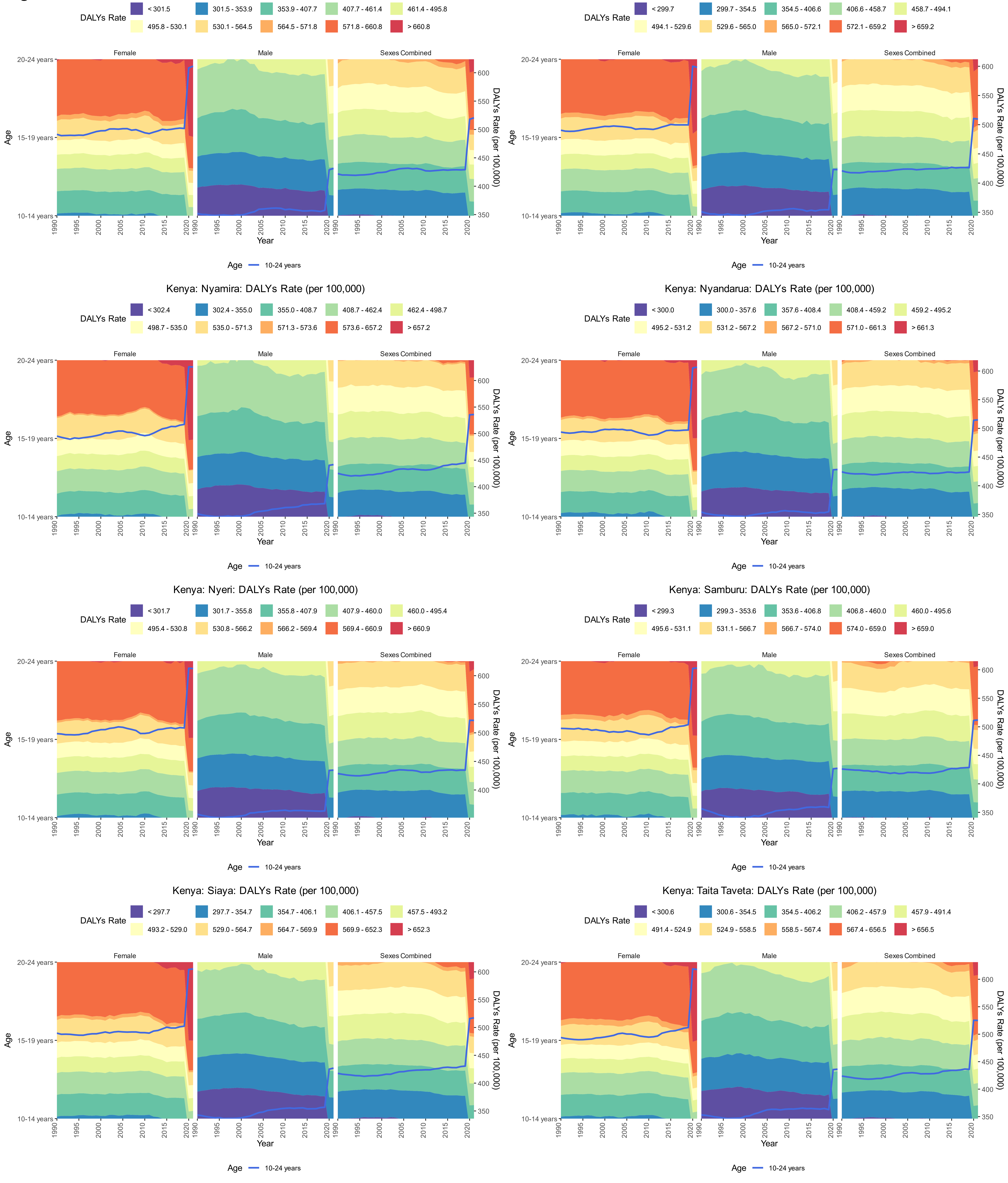
Kenya: Nandi: DALYs Rate (per 100,000)
DALYs Rate
< 301.5
301.5 - 353.9
353.9 - 407.7
407.7 - 461.4
461.4 - 495.8
495.8 - 530.1
530.1 - 564.5
564.5 - 571.8
571.8 - 660.8
> 660.8
Female
Male
Sexes Combined
Age
20-24 years
15-19 years
10-14 years
Year
DALYs Rate (per 100,000)
Age 10-24 years
Kenya: Narok: DALYs Rate (per 100,000)
< 299.7
299.7 - 354.5
354.5 - 406.6
406.6 - 458.7
458.7 - 494.1
494.1 - 529.6
529.6 - 565.0
565.0 - 572.1
572.1 - 659.2
> 659.2
Kenya: Nyamira: DALYs Rate (per 100,000)
< 302.4
302.4 - 355.0
355.0 - 408.7
408.7 - 462.4
462.4 - 498.7
498.7 - 535.0
535.0 - 571.3
571.3 - 573.6
573.6 - 657.2
> 657.2
Kenya: Nyandarua: DALYs Rate (per 100,000)
< 300.0
300.0 - 357.6
357.6 - 408.4
408.4 - 459.2
459.2 - 495.2
495.2 - 531.2
531.2 - 567.2
567.2 - 571.0
571.0 - 661.3
> 661.3
Kenya: Nyeri: DALYs Rate (per 100,000)
< 301.7
301.7 - 355.8
355.8 - 407.9
407.9 - 460.0
460.0 - 495.4
495.4 - 530.8
530.8 - 566.2
566.2 - 569.4
569.4 - 660.9
> 660.9
Kenya: Samburu: DALYs Rate (per 100,000)
< 299.3
299.3 - 353.6
353.6 - 406.8
406.8 - 460.0
460.0 - 495.6
495.6 - 531.1
531.1 - 566.7
566.7 - 574.0
574.0 - 659.0
> 659.0
Kenya: Siaya: DALYs Rate (per 100,000)
< 297.7
297.7 - 354.7
354.7 - 406.1
406.1 - 457.5
457.5 - 493.2
493.2 - 529.0
529.0 - 564.7
564.7 - 569.9
569.9 - 652.3
> 652.3
Kenya: Taita Taveta: DALYs Rate (per 100,000)
< 300.6
300.6 - 354.5
354.5 - 406.2
406.2 - 457.9
457.9 - 491.4
491.4 - 524.9
524.9 - 558.5
558.5 - 567.4
567.4 - 656.5
> 656.5

Figure S3

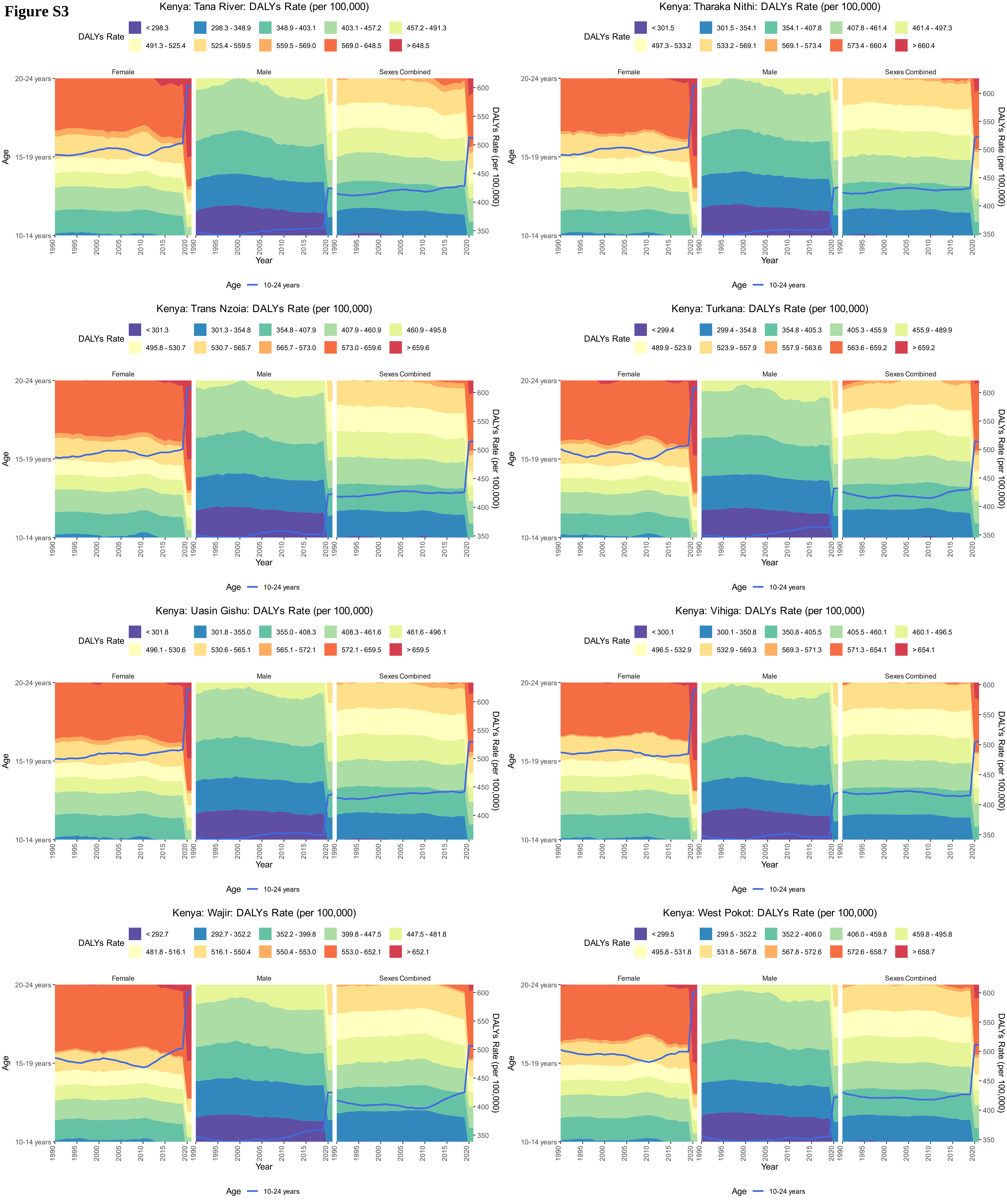
Kenya: Tana River: DALYs Rate (per 100,000)
DALYs Rate
< 298.3
298.3 - 348.9
348.9 - 403.1
403.1 - 457.2
457.2 - 491.3
491.3 - 525.4
525.4 - 559.5
559.5 - 569.0
569.0 - 648.5
> 648.5
Kenya: Tharaka Nithi: DALYs Rate (per 100,000)
DALYs Rate
< 301.5
301.5 - 354.1
354.1 - 407.8
407.8 - 461.4
461.4 - 497.3
497.3 - 533.2
533.2 - 569.1
569.1 - 573.4
573.4 - 660.4
> 660.4
Kenya: Trans Nzoia: DALYs Rate (per 100,000)
DALYs Rate
< 301.3
301.3 - 354.8
354.8 - 407.9
407.9 - 460.9
460.9 - 495.8
495.8 - 530.7
530.7 - 565.7
565.7 - 573.0
573.0 - 659.6
> 659.6
Kenya: Turkana: DALYs Rate (per 100,000)
DALYs Rate
< 299.4
299.4 - 354.8
354.8 - 405.3
405.3 - 455.9
455.9 - 489.9
489.9 - 523.9
523.9 - 557.9
557.9 - 563.6
563.6 - 659.2
> 659.2
Kenya: Uasin Gishu: DALYs Rate (per 100,000)
DALYs Rate
< 301.8
301.8 - 355.0
355.0 - 408.3
408.3 - 461.6
461.6 - 496.1
496.1 - 530.6
530.6 - 565.1
565.1 - 572.1
572.1 - 659.5
> 659.5
Kenya: Vihiga: DALYs Rate (per 100,000)
DALYs Rate
< 300.1
300.1 - 350.8
350.8 - 405.5
405.5 - 460.1
460.1 - 496.5
496.5 - 532.9
532.9 - 569.3
569.3 - 571.3
571.3 - 654.1
> 654.1
Kenya: Wajir: DALYs Rate (per 100,000)
DALYs Rate
< 292.7
292.7 - 352.2
352.2 - 399.8
399.8 - 447.5
447.5 - 481.8
481.8 - 516.1
516.1 - 550.4
550.4 - 553.0
553.0 - 652.1
> 652.1
Kenya: West Pokot: DALYs Rate (per 100,000)
DALYs Rate
< 299.5
299.5 - 352.2
352.2 - 406.0
406.0 - 459.8
459.8 - 495.8
495.8 - 531.8
531.8 - 567.8
567.8 - 572.6
572.6 - 658.7
> 658.7
Female
Male
Sexes Combined
Age
20-24 years
15-19 years
10-14 years
Year
DALYs Rate (per 100,000)
Age
10-24 years

Figure S3

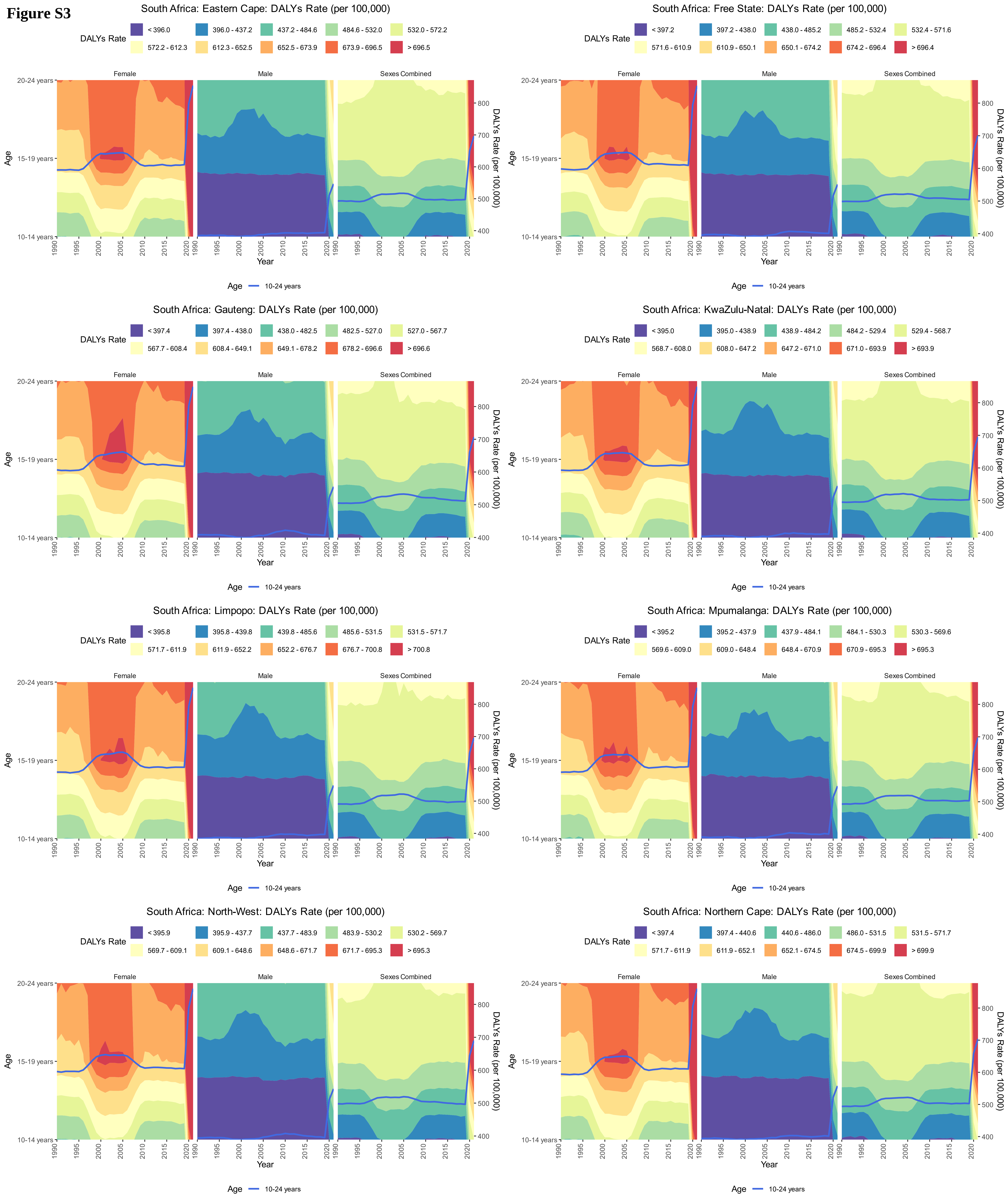
South Africa: Eastern Cape: DALYs Rate (per 100,000)
DALYs Rate
< 396.0
396.0 - 437.2
437.2 - 484.6
484.6 - 532.0
532.0 - 572.2
572.2 - 612.3
612.3 - 652.5
652.5 - 673.9
673.9 - 696.5
> 696.5
South Africa: Free State: DALYs Rate (per 100,000)
DALYs Rate
< 397.2
397.2 - 438.0
438.0 - 485.2
485.2 - 532.4
532.4 - 571.6
571.6 - 610.9
610.9 - 650.1
650.1 - 674.2
674.2 - 696.4
> 696.4
South Africa: Gauteng: DALYs Rate (per 100,000)
DALYs Rate
< 397.4
397.4 - 438.0
438.0 - 482.5
482.5 - 527.0
527.0 - 567.7
567.7 - 608.4
608.4 - 649.1
649.1 - 678.2
678.2 - 696.6
> 696.6
South Africa: KwaZulu-Natal: DALYs Rate (per 100,000)
DALYs Rate
< 395.0
395.0 - 438.9
438.9 - 484.2
484.2 - 529.4
529.4 - 568.7
568.7 - 608.0
608.0 - 647.2
647.2 - 671.0
671.0 - 693.9
> 693.9
South Africa: Limpopo: DALYs Rate (per 100,000)
DALYs Rate
< 395.8
395.8 - 439.8
439.8 - 485.6
485.6 - 531.5
531.5 - 571.7
571.7 - 611.9
611.9 - 652.2
652.2 - 676.7
676.7 - 700.8
> 700.8
South Africa: Mpumalanga: DALYs Rate (per 100,000)
DALYs Rate
< 395.2
395.2 - 437.9
437.9 - 484.1
484.1 - 530.3
530.3 - 569.6
569.6 - 609.0
609.0 - 648.4
648.4 - 670.9
670.9 - 695.3
> 695.3
South Africa: North-West: DALYs Rate (per 100,000)
DALYs Rate
< 395.9
395.9 - 437.7
437.7 - 483.9
483.9 - 530.2
530.2 - 569.7
569.7 - 609.1
609.1 - 648.6
648.6 - 671.7
671.7 - 695.3
> 695.3
South Africa: Northern Cape: DALYs Rate (per 100,000)
DALYs Rate
< 397.4
397.4 - 440.6
440.6 - 486.0
486.0 - 531.5
531.5 - 571.7
571.7 - 611.9
611.9 - 652.1
652.1 - 674.5
674.5 - 699.9
> 699.9
Female
Male
Sexes Combined
Age
20-24 years
15-19 years
10-14 years
Year
DALYs Rate (per 100,000)
Age
10-24 years

Figure S3

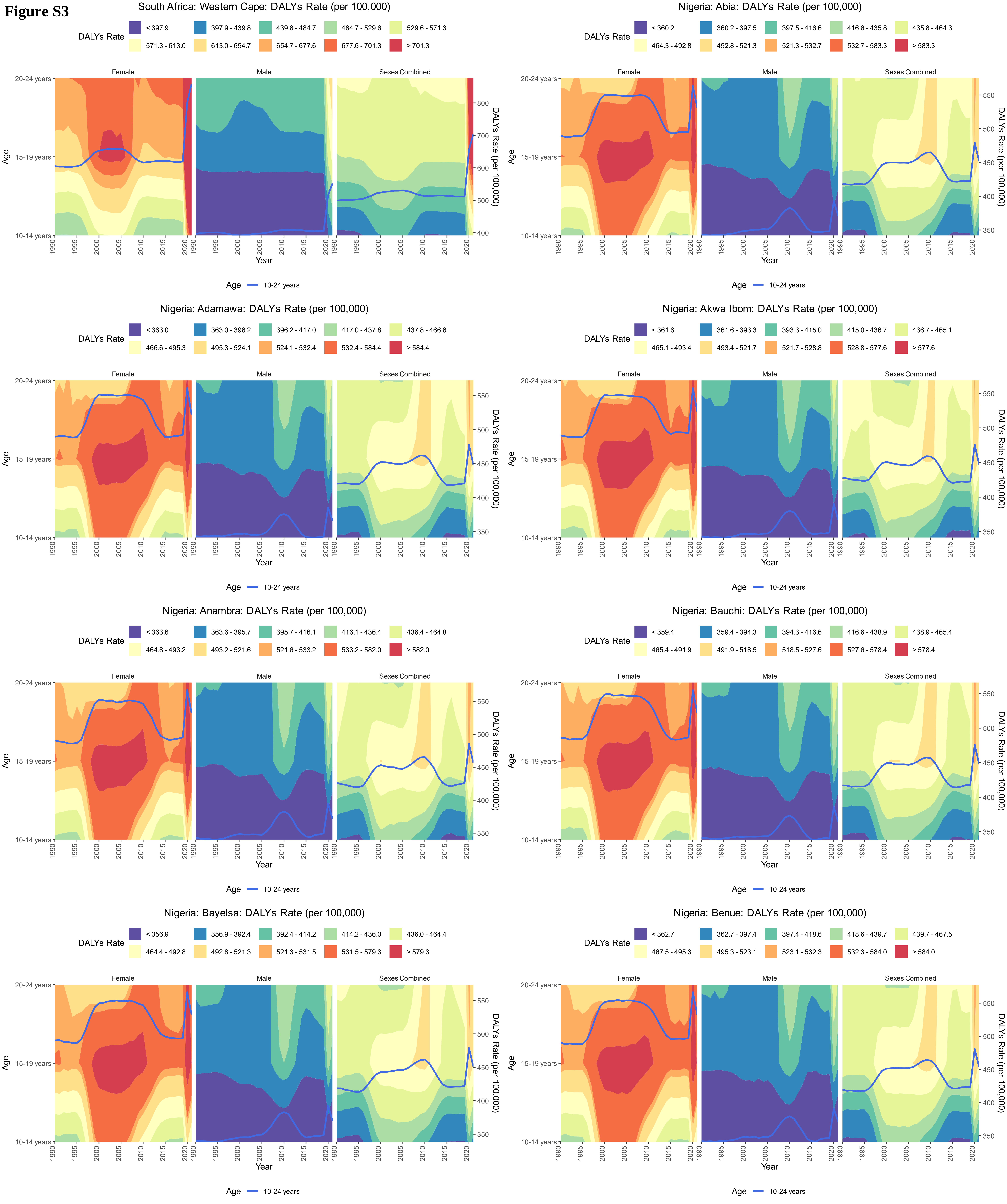

South Africa: Western Cape: DALYs Rate (per 100,000)
DALYs Rate
< 397.9
397.9 - 439.8
439.8 - 484.7
484.7 - 529.6
529.6 - 571.3
571.3 - 613.0
613.0 - 654.7
654.7 - 677.6
677.6 - 701.3
> 701.3
Nigeria: Abia: DALYs Rate (per 100,000)
DALYs Rate
< 360.2
360.2 - 397.5
397.5 - 416.6
416.6 - 435.8
435.8 - 464.3
464.3 - 492.8
492.8 - 521.3
521.3 - 532.7
532.7 - 583.3
> 583.3
Nigeria: Adamawa: DALYs Rate (per 100,000)
DALYs Rate
< 363.0
363.0 - 396.2
396.2 - 417.0
417.0 - 437.8
437.8 - 466.6
466.6 - 495.3
495.3 - 524.1
524.1 - 532.4
532.4 - 584.4
> 584.4
Nigeria: Akwa Ibom: DALYs Rate (per 100,000)
DALYs Rate
< 361.6
361.6 - 393.3
393.3 - 415.0
415.0 - 436.7
436.7 - 465.1
465.1 - 493.4
493.4 - 521.7
521.7 - 528.8
528.8 - 577.6
> 577.6
Nigeria: Anambra: DALYs Rate (per 100,000)
DALYs Rate
< 363.6
363.6 - 395.7
395.7 - 416.1
416.1 - 436.4
436.4 - 464.8
464.8 - 493.2
493.2 - 521.6
521.6 - 533.2
533.2 - 582.0
> 582.0
Nigeria: Bauchi: DALYs Rate (per 100,000)
DALYs Rate
< 359.4
359.4 - 394.3
394.3 - 416.6
416.6 - 438.9
438.9 - 465.4
465.4 - 491.9
491.9 - 518.5
518.5 - 527.6
527.6 - 578.4
> 578.4
Nigeria: Bayelsa: DALYs Rate (per 100,000)
DALYs Rate
< 356.9
356.9 - 392.4
392.4 - 414.2
414.2 - 436.0
436.0 - 464.4
464.4 - 492.8
492.8 - 521.3
521.3 - 531.5
531.5 - 579.3
> 579.3
Nigeria: Benue: DALYs Rate (per 100,000)
DALYs Rate
< 362.7
362.7 - 397.4
397.4 - 418.6
418.6 - 439.7
439.7 - 467.5
467.5 - 495.3
495.3 - 523.1
523.1 - 532.3
532.3 - 584.0
> 584.0
Female
Male
Sexes Combined
Age
20-24 years
15-19 years
10-14 years
Year
DALYs Rate (per 100,000)
Age — 10-24 years

Figure S3

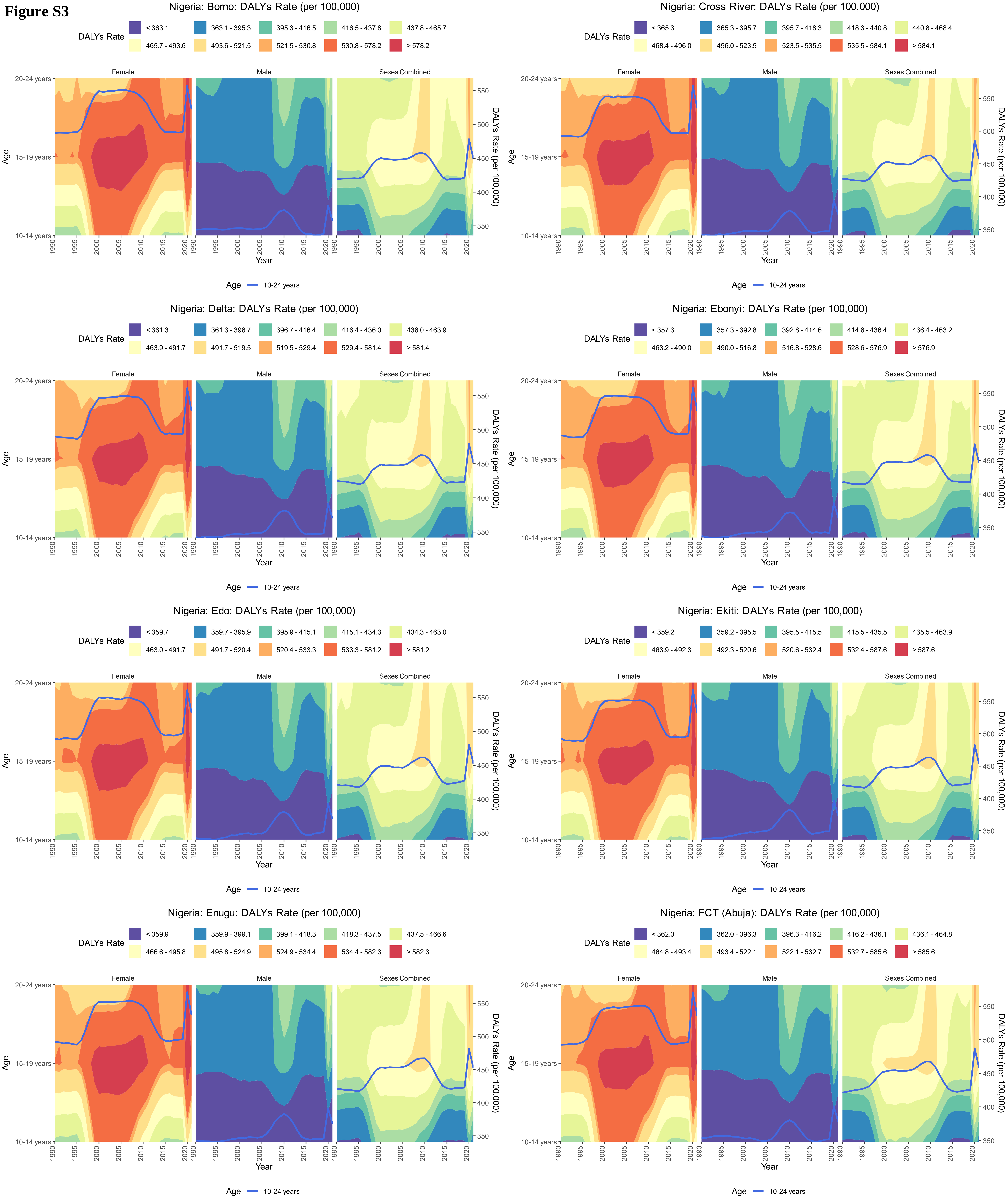

Nigeria: Borno: DALYs Rate (per 100,000)
DALYs Rate
< 363.1
363.1 - 395.3
395.3 - 416.5
416.5 - 437.8
437.8 - 465.7
465.7 - 493.6
493.6 - 521.5
521.5 - 530.8
530.8 - 578.2
> 578.2
Nigeria: Cross River: DALYs Rate (per 100,000)
< 365.3
365.3 - 395.7
395.7 - 418.3
418.3 - 440.8
440.8 - 468.4
468.4 - 496.0
496.0 - 523.5
523.5 - 535.5
535.5 - 584.1
> 584.1
Nigeria: Delta: DALYs Rate (per 100,000)
< 361.3
361.3 - 396.7
396.7 - 416.4
416.4 - 436.0
436.0 - 463.9
463.9 - 491.7
491.7 - 519.5
519.5 - 529.4
529.4 - 581.4
> 581.4
Nigeria: Ebonyi: DALYs Rate (per 100,000)
< 357.3
357.3 - 392.8
392.8 - 414.6
414.6 - 436.4
436.4 - 463.2
463.2 - 490.0
490.0 - 516.8
516.8 - 528.6
528.6 - 576.9
> 576.9
Nigeria: Edo: DALYs Rate (per 100,000)
< 359.7
359.7 - 395.9
395.9 - 415.1
415.1 - 434.3
434.3 - 463.0
463.0 - 491.7
491.7 - 520.4
520.4 - 533.3
533.3 - 581.2
> 581.2
Nigeria: Ekiti: DALYs Rate (per 100,000)
< 359.2
359.2 - 395.5
395.5 - 415.5
415.5 - 435.5
435.5 - 463.9
463.9 - 492.3
492.3 - 520.6
520.6 - 532.4
532.4 - 587.6
> 587.6
Nigeria: Enugu: DALYs Rate (per 100,000)
< 359.9
359.9 - 399.1
399.1 - 418.3
418.3 - 437.5
437.5 - 466.6
466.6 - 495.8
495.8 - 524.9
524.9 - 534.4
534.4 - 582.3
> 582.3
Nigeria: FCT (Abuja): DALYs Rate (per 100,000)
< 362.0
362.0 - 396.3
396.3 - 416.2
416.2 - 436.1
436.1 - 464.8
464.8 - 493.4
493.4 - 522.1
522.1 - 532.7
532.7 - 585.6
> 585.6
Female
Male
Sexes Combined
Age
20-24 years
15-19 years
10-14 years
Year
DALYs Rate (per 100,000)
Age
10-24 years

Figure S3

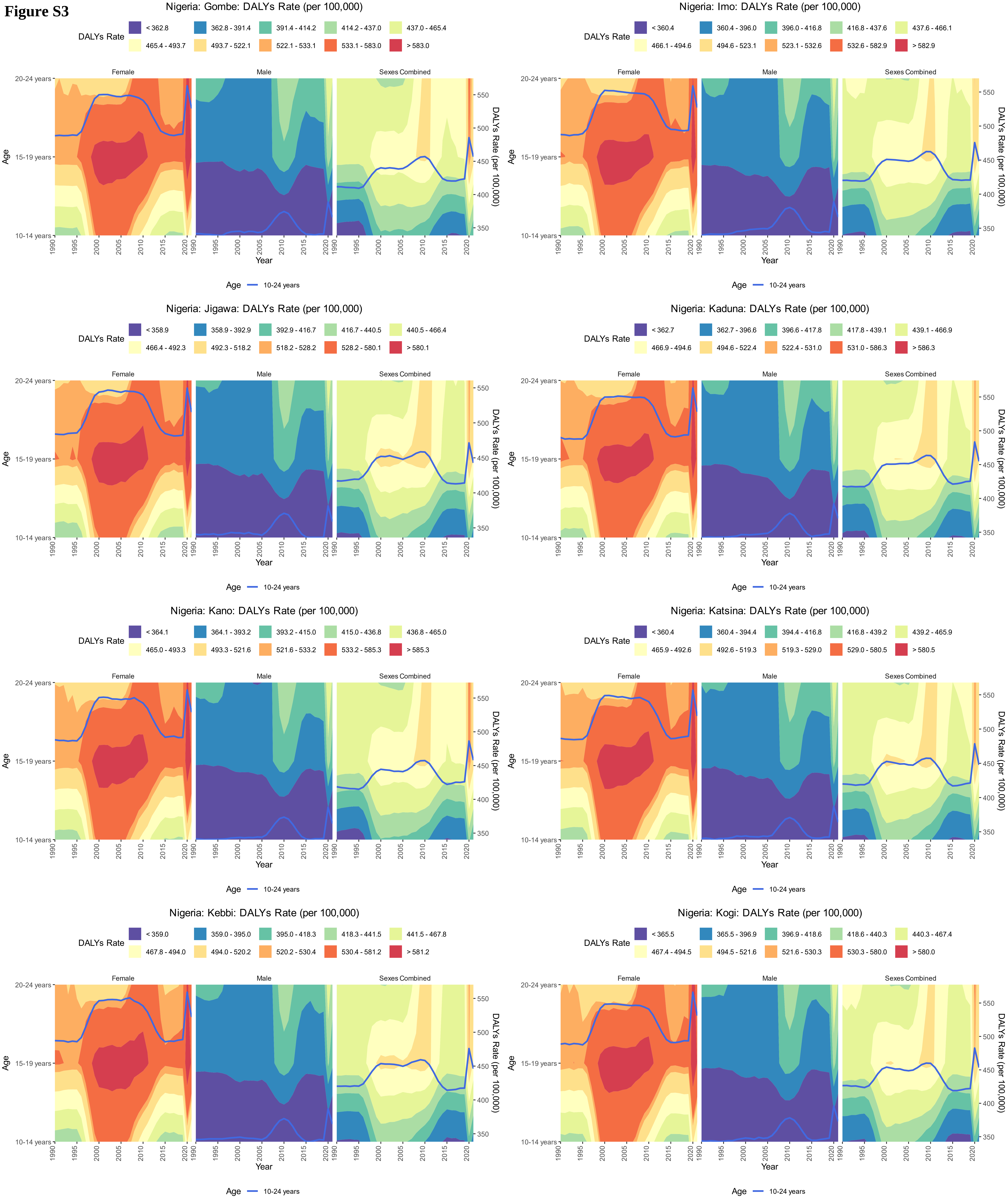

Nigeria: Gombe: DALYs Rate (per 100,000)
DALYs Rate
< 362.8
362.8 - 391.4
391.4 - 414.2
414.2 - 437.0
437.0 - 465.4
465.4 - 493.7
493.7 - 522.1
522.1 - 533.1
533.1 - 583.0
> 583.0
Female
Male
Sexes Combined
Age
Year
DALYs Rate (per 100,000)
Age — 10-24 years
Nigeria: Imo: DALYs Rate (per 100,000)
DALYs Rate
< 360.4
360.4 - 396.0
396.0 - 416.8
416.8 - 437.6
437.6 - 466.1
466.1 - 494.6
494.6 - 523.1
523.1 - 532.6
532.6 - 582.9
> 582.9
Nigeria: Jigawa: DALYs Rate (per 100,000)
DALYs Rate
< 358.9
358.9 - 392.9
392.9 - 416.7
416.7 - 440.5
440.5 - 466.4
466.4 - 492.3
492.3 - 518.2
518.2 - 528.2
528.2 - 580.1
> 580.1
Nigeria: Kaduna: DALYs Rate (per 100,000)
DALYs Rate
< 362.7
362.7 - 396.6
396.6 - 417.8
417.8 - 439.1
439.1 - 466.9
466.9 - 494.6
494.6 - 522.4
522.4 - 531.0
531.0 - 586.3
> 586.3
Nigeria: Kano: DALYs Rate (per 100,000)
DALYs Rate
< 364.1
364.1 - 393.2
393.2 - 415.0
415.0 - 436.8
436.8 - 465.0
465.0 - 493.3
493.3 - 521.6
521.6 - 533.2
533.2 - 585.3
> 585.3
Nigeria: Katsina: DALYs Rate (per 100,000)
DALYs Rate
< 360.4
360.4 - 394.4
394.4 - 416.8
416.8 - 439.2
439.2 - 465.9
465.9 - 492.6
492.6 - 519.3
519.3 - 529.0
529.0 - 580.5
> 580.5
Nigeria: Kebbi: DALYs Rate (per 100,000)
DALYs Rate
< 359.0
359.0 - 395.0
395.0 - 418.3
418.3 - 441.5
441.5 - 467.8
467.8 - 494.0
494.0 - 520.2
520.2 - 530.4
530.4 - 581.2
> 581.2
Nigeria: Kogi: DALYs Rate (per 100,000)
DALYs Rate
< 365.5
365.5 - 396.9
396.9 - 418.6
418.6 - 440.3
440.3 - 467.4
467.4 - 494.5
494.5 - 521.6
521.6 - 530.3
530.3 - 580.0
> 580.0

Figure S3

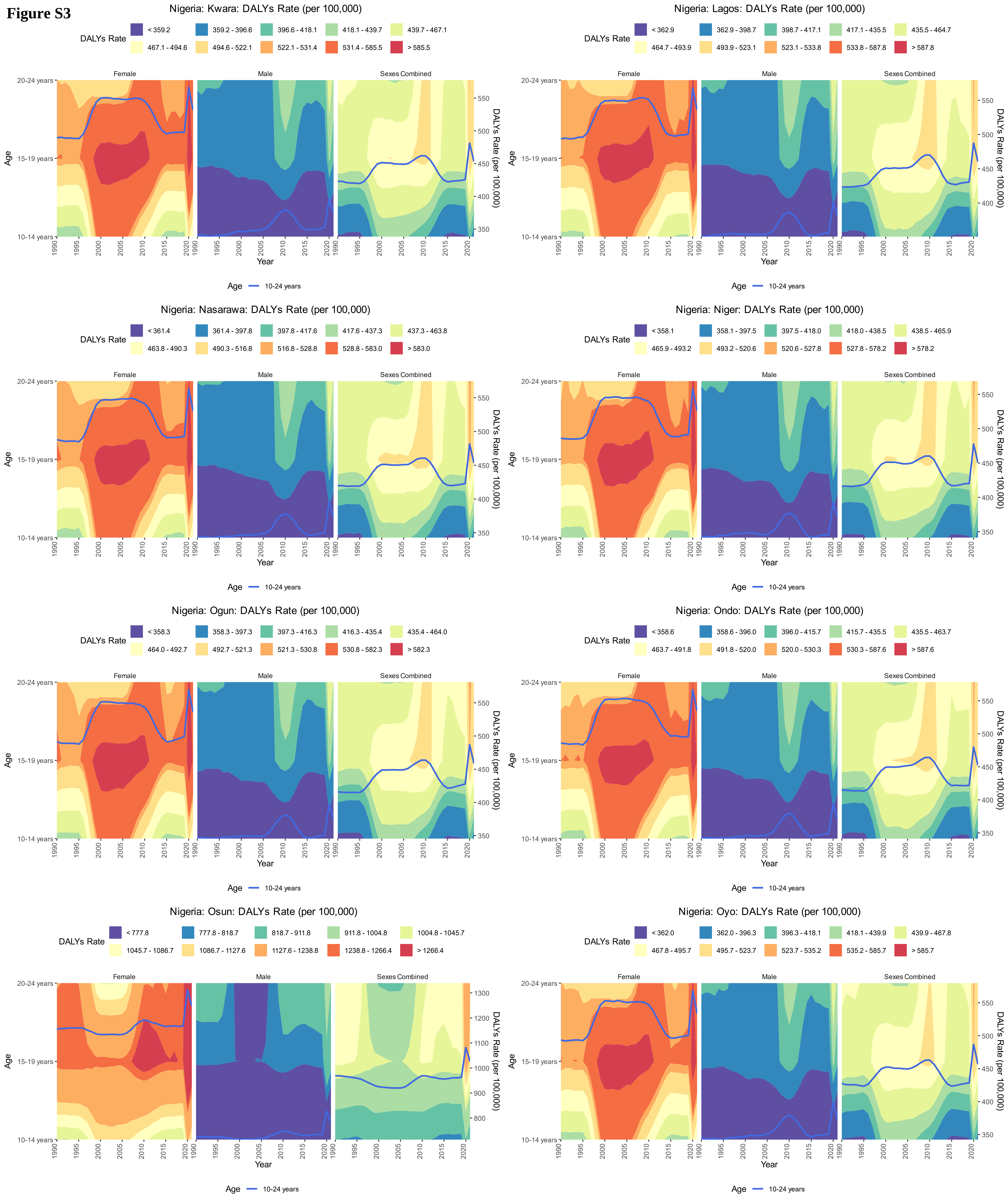
Nigeria: Kwara: DALYs Rate (per 100,000)
DALYs Rate
< 359.2
359.2 - 396.6
396.6 - 418.1
418.1 - 439.7
439.7 - 467.1
467.1 - 494.6
494.6 - 522.1
522.1 - 531.4
531.4 - 585.5
> 585.5
Female
Male
Sexes Combined
Age
20-24 years
15-19 years
10-14 years
Year
DALYs Rate (per 100,000)
Age
10-24 years
Nigeria: Lagos: DALYs Rate (per 100,000)
DALYs Rate
< 362.9
362.9 - 398.7
398.7 - 417.1
417.1 - 435.5
435.5 - 464.7
464.7 - 493.9
493.9 - 523.1
523.1 - 533.8
533.8 - 587.8
> 587.8
Nigeria: Nasarawa: DALYs Rate (per 100,000)
DALYs Rate
< 361.4
361.4 - 397.8
397.8 - 417.6
417.6 - 437.3
437.3 - 463.8
463.8 - 490.3
490.3 - 516.8
516.8 - 528.8
528.8 - 583.0
> 583.0
Nigeria: Niger: DALYs Rate (per 100,000)
DALYs Rate
< 358.1
358.1 - 397.5
397.5 - 418.0
418.0 - 438.5
438.5 - 465.9
465.9 - 493.2
493.2 - 520.6
520.6 - 527.8
527.8 - 578.2
> 578.2
Nigeria: Ogun: DALYs Rate (per 100,000)
DALYs Rate
< 358.3
358.3 - 397.3
397.3 - 416.3
416.3 - 435.4
435.4 - 464.0
464.0 - 492.7
492.7 - 521.3
521.3 - 530.8
530.8 - 582.3
> 582.3
Nigeria: Ondo: DALYs Rate (per 100,000)
DALYs Rate
< 358.6
358.6 - 396.0
396.0 - 415.7
415.7 - 435.5
435.5 - 463.7
463.7 - 491.8
491.8 - 520.0
520.0 - 530.3
530.3 - 587.6
> 587.6
Nigeria: Osun: DALYs Rate (per 100,000)
DALYs Rate
< 777.8
777.8 - 818.7
818.7 - 911.8
911.8 - 1004.8
1004.8 - 1045.7
1045.7 - 1086.7
1086.7 - 1127.6
1127.6 - 1238.8
1238.8 - 1266.4
> 1266.4
Nigeria: Oyo: DALYs Rate (per 100,000)
DALYs Rate
< 362.0
362.0 - 396.3
396.3 - 418.1
418.1 - 439.9
439.9 - 467.8
467.8 - 495.7
495.7 - 523.7
523.7 - 535.2
535.2 - 585.7
> 585.7

Figure S3

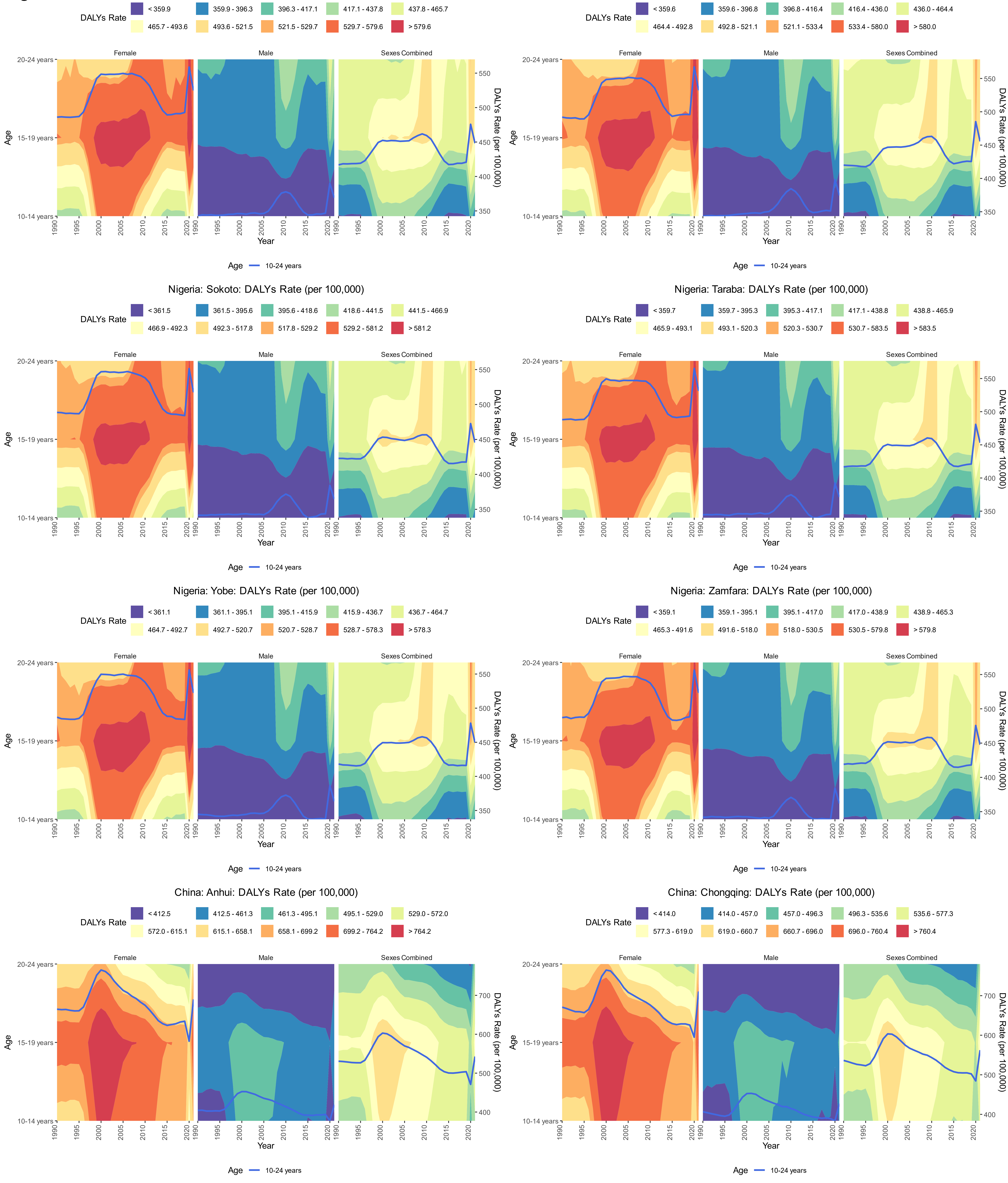
Nigeria: Plateau: DALYs Rate (per 100,000)
DALYs Rate
< 359.9
359.9 - 396.3
396.3 - 417.1
417.1 - 437.8
437.8 - 465.7
465.7 - 493.6
493.6 - 521.5
521.5 - 529.7
529.7 - 579.6
> 579.6
Nigeria: Rivers: DALYs Rate (per 100,000)
< 359.6
359.6 - 396.8
396.8 - 416.4
416.4 - 436.0
436.0 - 464.4
464.4 - 492.8
492.8 - 521.1
521.1 - 533.4
533.4 - 580.0
> 580.0
Nigeria: Sokoto: DALYs Rate (per 100,000)
< 361.5
361.5 - 395.6
395.6 - 418.6
418.6 - 441.5
441.5 - 466.9
466.9 - 492.3
492.3 - 517.8
517.8 - 529.2
529.2 - 581.2
> 581.2
Nigeria: Taraba: DALYs Rate (per 100,000)
< 359.7
359.7 - 395.3
395.3 - 417.1
417.1 - 438.8
438.8 - 465.9
465.9 - 493.1
493.1 - 520.3
520.3 - 530.7
530.7 - 583.5
> 583.5
Nigeria: Yobe: DALYs Rate (per 100,000)
< 361.1
361.1 - 395.1
395.1 - 415.9
415.9 - 436.7
436.7 - 464.7
464.7 - 492.7
492.7 - 520.7
520.7 - 528.7
528.7 - 578.3
> 578.3
Nigeria: Zamfara: DALYs Rate (per 100,000)
< 359.1
359.1 - 395.1
395.1 - 417.0
417.0 - 438.9
438.9 - 465.3
465.3 - 491.6
491.6 - 518.0
518.0 - 530.5
530.5 - 579.8
> 579.8
China: Anhui: DALYs Rate (per 100,000)
< 412.5
412.5 - 461.3
461.3 - 495.1
495.1 - 529.0
529.0 - 572.0
572.0 - 615.1
615.1 - 658.1
658.1 - 699.2
699.2 - 764.2
> 764.2
China: Chongqing: DALYs Rate (per 100,000)
< 414.0
414.0 - 457.0
457.0 - 496.3
496.3 - 535.6
535.6 - 577.3
577.3 - 619.0
619.0 - 660.7
660.7 - 696.0
696.0 - 760.4
> 760.4
Female
Male
Sexes Combined
Age
20-24 years
15-19 years
10-14 years
Year
DALYs Rate (per 100,000)
Age — 10-24 years

Figure S3

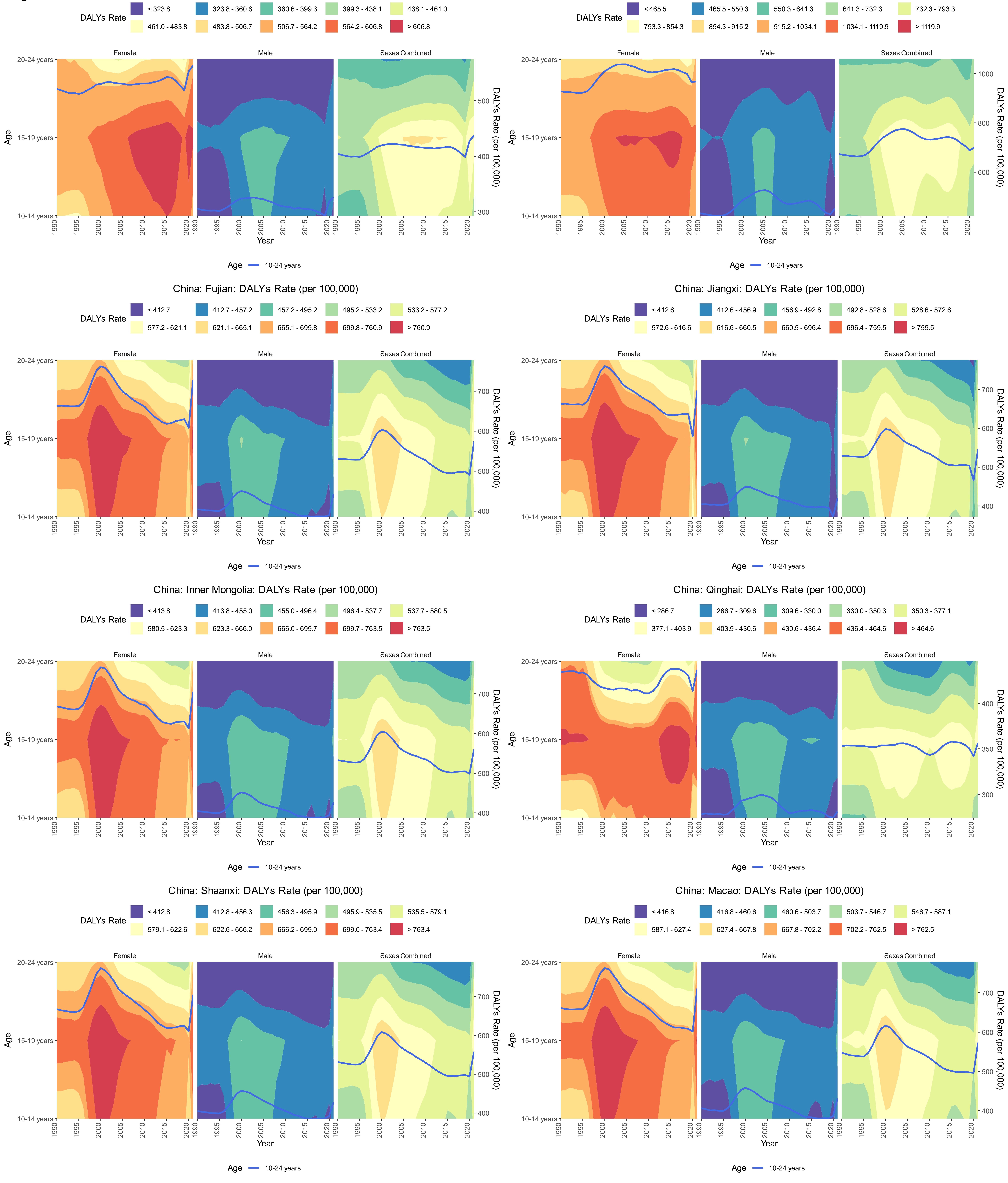
China: Beijing: DALYs Rate (per 100,000)
DALYs Rate
< 323.8
323.8 - 360.6
360.6 - 399.3
399.3 - 438.1
438.1 - 461.0
461.0 - 483.8
483.8 - 506.7
506.7 - 564.2
564.2 - 606.8
> 606.8
China: Gansu: DALYs Rate (per 100,000)
DALYs Rate
< 465.5
465.5 - 550.3
550.3 - 641.3
641.3 - 732.3
732.3 - 793.3
793.3 - 854.3
854.3 - 915.2
915.2 - 1034.1
1034.1 - 1119.9
> 1119.9
China: Fujian: DALYs Rate (per 100,000)
DALYs Rate
< 412.7
412.7 - 457.2
457.2 - 495.2
495.2 - 533.2
533.2 - 577.2
577.2 - 621.1
621.1 - 665.1
665.1 - 699.8
699.8 - 760.9
> 760.9
China: Jiangxi: DALYs Rate (per 100,000)
DALYs Rate
< 412.6
412.6 - 456.9
456.9 - 492.8
492.8 - 528.6
528.6 - 572.6
572.6 - 616.6
616.6 - 660.5
660.5 - 696.4
696.4 - 759.5
> 759.5
China: Inner Mongolia: DALYs Rate (per 100,000)
DALYs Rate
< 413.8
413.8 - 455.0
455.0 - 496.4
496.4 - 537.7
537.7 - 580.5
580.5 - 623.3
623.3 - 666.0
666.0 - 699.7
699.7 - 763.5
> 763.5
China: Qinghai: DALYs Rate (per 100,000)
DALYs Rate
< 286.7
286.7 - 309.6
309.6 - 330.0
330.0 - 350.3
350.3 - 377.1
377.1 - 403.9
403.9 - 430.6
430.6 - 436.4
436.4 - 464.6
> 464.6
China: Shaanxi: DALYs Rate (per 100,000)
DALYs Rate
< 412.8
412.8 - 456.3
456.3 - 495.9
495.9 - 535.5
535.5 - 579.1
579.1 - 622.6
622.6 - 666.2
666.2 - 699.0
699.0 - 763.4
> 763.4
China: Macao: DALYs Rate (per 100,000)
DALYs Rate
< 416.8
416.8 - 460.6
460.6 - 503.7
503.7 - 546.7
546.7 - 587.1
587.1 - 627.4
627.4 - 667.8
667.8 - 702.2
702.2 - 762.5
> 762.5
Female
Male
Sexes Combined
20-24 years
15-19 years
10-14 years
Age
Year
DALYs Rate (per 100,000)
Age
10-24 years

Figure S3

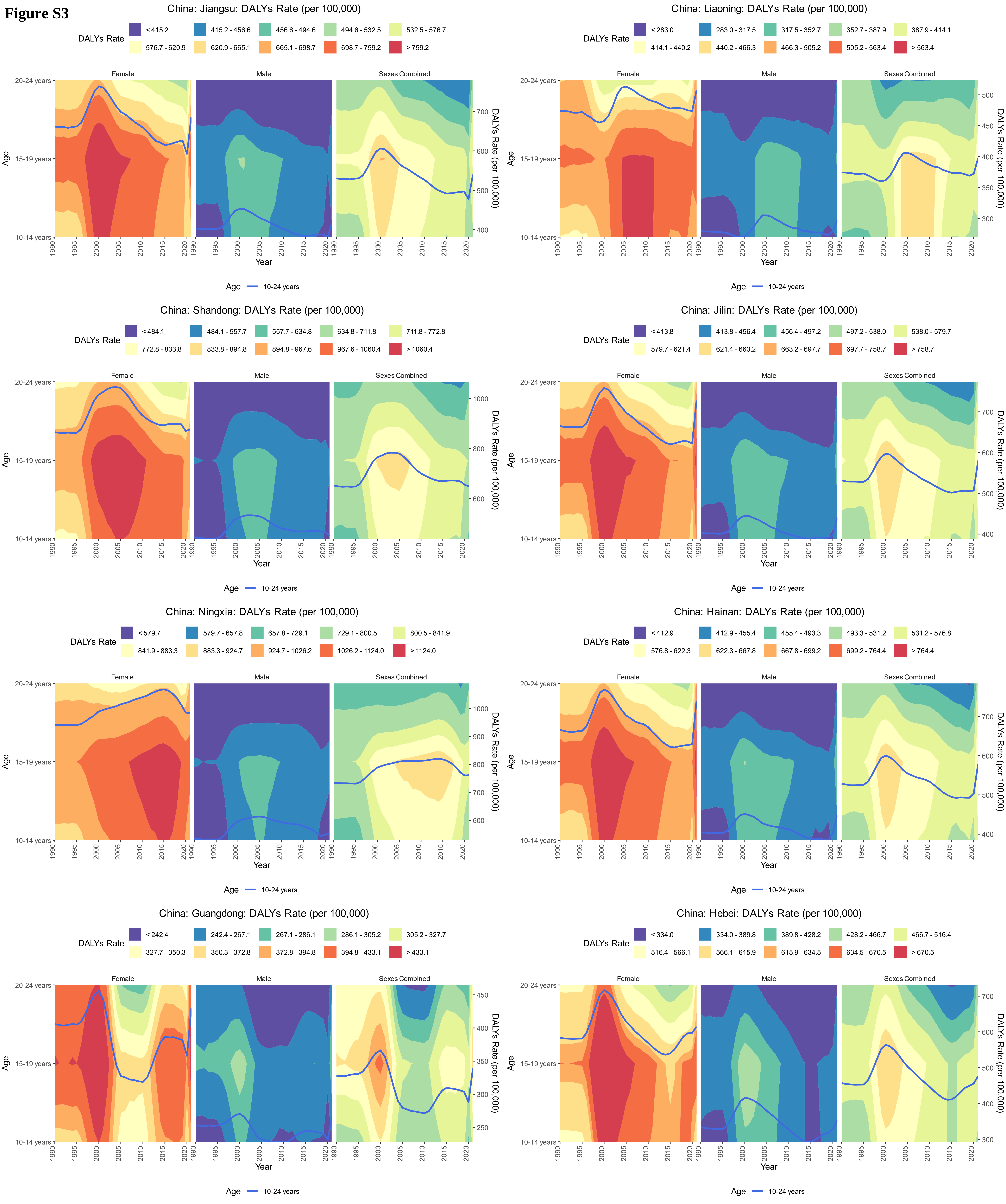
China: Jiangsu: DALYs Rate (per 100,000)
DALYs Rate
< 415.2
415.2 - 456.6
456.6 - 494.6
494.6 - 532.5
532.5 - 576.7
576.7 - 620.9
620.9 - 665.1
665.1 - 698.7
698.7 - 759.2
> 759.2
China: Liaoning: DALYs Rate (per 100,000)
DALYs Rate
< 283.0
283.0 - 317.5
317.5 - 352.7
352.7 - 387.9
387.9 - 414.1
414.1 - 440.2
440.2 - 466.3
466.3 - 505.2
505.2 - 563.4
> 563.4
China: Shandong: DALYs Rate (per 100,000)
DALYs Rate
< 484.1
484.1 - 557.7
557.7 - 634.8
634.8 - 711.8
711.8 - 772.8
772.8 - 833.8
833.8 - 894.8
894.8 - 967.6
967.6 - 1060.4
> 1060.4
China: Jilin: DALYs Rate (per 100,000)
DALYs Rate
< 413.8
413.8 - 456.4
456.4 - 497.2
497.2 - 538.0
538.0 - 579.7
579.7 - 621.4
621.4 - 663.2
663.2 - 697.7
697.7 - 758.7
> 758.7
China: Ningxia: DALYs Rate (per 100,000)
DALYs Rate
< 579.7
579.7 - 657.8
657.8 - 729.1
729.1 - 800.5
800.5 - 841.9
841.9 - 883.3
883.3 - 924.7
924.7 - 1026.2
1026.2 - 1124.0
> 1124.0
China: Hainan: DALYs Rate (per 100,000)
DALYs Rate
< 412.9
412.9 - 455.4
455.4 - 493.3
493.3 - 531.2
531.2 - 576.8
576.8 - 622.3
622.3 - 667.8
667.8 - 699.2
699.2 - 764.4
> 764.4
China: Guangdong: DALYs Rate (per 100,000)
DALYs Rate
< 242.4
242.4 - 267.1
267.1 - 286.1
286.1 - 305.2
305.2 - 327.7
327.7 - 350.3
350.3 - 372.8
372.8 - 394.8
394.8 - 433.1
> 433.1
China: Hebei: DALYs Rate (per 100,000)
DALYs Rate
< 334.0
334.0 - 389.8
389.8 - 428.2
428.2 - 466.7
466.7 - 516.4
516.4 - 566.1
566.1 - 615.9
615.9 - 634.5
634.5 - 670.5
> 670.5
Female
Male
Sexes Combined
Age
20-24 years
15-19 years
10-14 years
Year
1990
1995
2000
2005
2010
2015
2020
DALYs Rate (per 100,000)
Age — 10-24 years

Figure S3

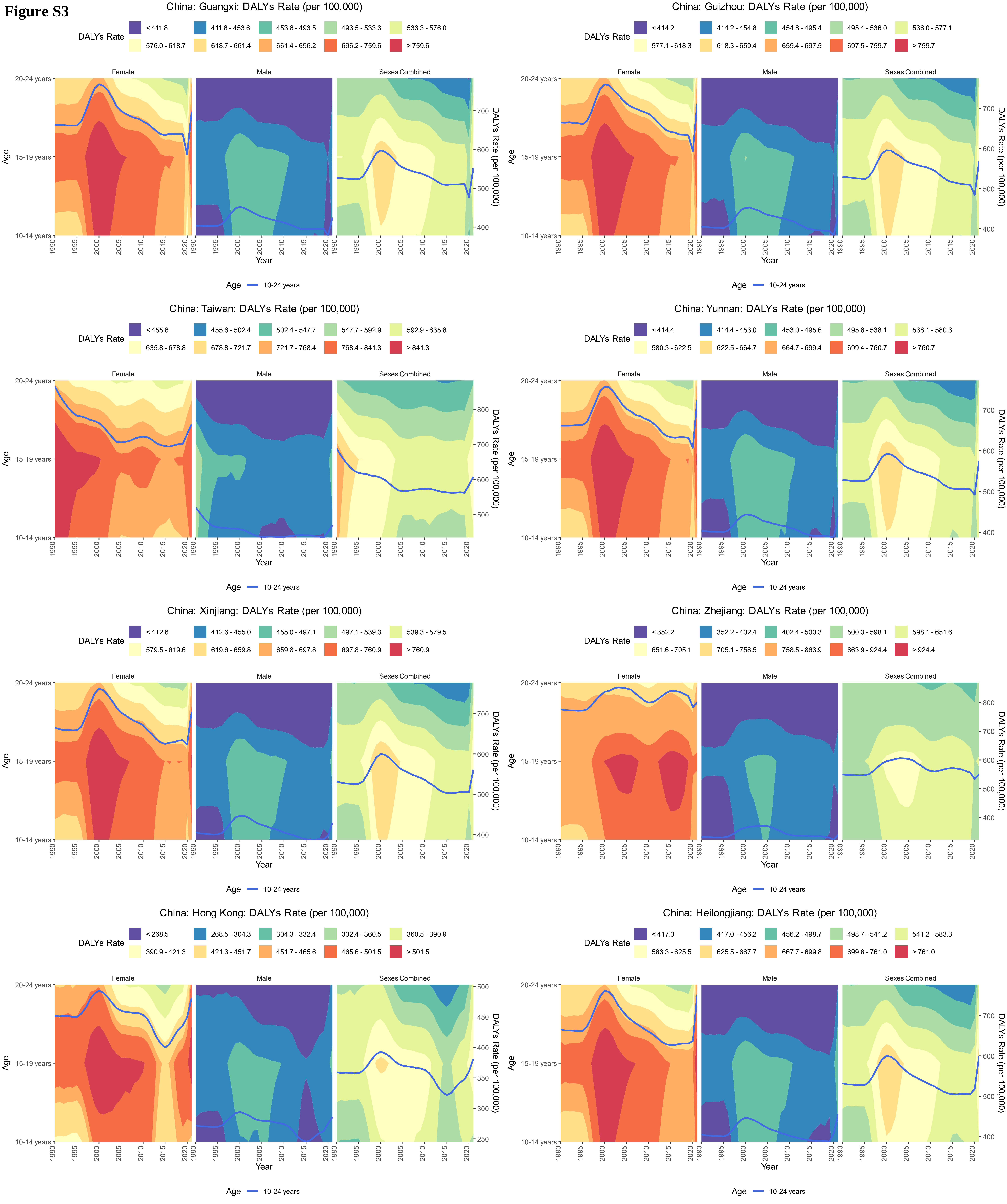
China: Guangxi: DALYs Rate (per 100,000)
DALYs Rate
< 411.8
411.8 - 453.6
453.6 - 493.5
493.5 - 533.3
533.3 - 576.0
576.0 - 618.7
618.7 - 661.4
661.4 - 696.2
696.2 - 759.6
> 759.6
China: Guizhou: DALYs Rate (per 100,000)
DALYs Rate
< 414.2
414.2 - 454.8
454.8 - 495.4
495.4 - 536.0
536.0 - 577.1
577.1 - 618.3
618.3 - 659.4
659.4 - 697.5
697.5 - 759.7
> 759.7
China: Taiwan: DALYs Rate (per 100,000)
DALYs Rate
< 455.6
455.6 - 502.4
502.4 - 547.7
547.7 - 592.9
592.9 - 635.8
635.8 - 678.8
678.8 - 721.7
721.7 - 768.4
768.4 - 841.3
> 841.3
China: Yunnan: DALYs Rate (per 100,000)
DALYs Rate
< 414.4
414.4 - 453.0
453.0 - 495.6
495.6 - 538.1
538.1 - 580.3
580.3 - 622.5
622.5 - 664.7
664.7 - 699.4
699.4 - 760.7
> 760.7
China: Xinjiang: DALYs Rate (per 100,000)
DALYs Rate
< 412.6
412.6 - 455.0
455.0 - 497.1
497.1 - 539.3
539.3 - 579.5
579.5 - 619.6
619.6 - 659.8
659.8 - 697.8
697.8 - 760.9
> 760.9
China: Zhejiang: DALYs Rate (per 100,000)
DALYs Rate
< 352.2
352.2 - 402.4
402.4 - 500.3
500.3 - 598.1
598.1 - 651.6
651.6 - 705.1
705.1 - 758.5
758.5 - 863.9
863.9 - 924.4
> 924.4
China: Hong Kong: DALYs Rate (per 100,000)
DALYs Rate
< 268.5
268.5 - 304.3
304.3 - 332.4
332.4 - 360.5
360.5 - 390.9
390.9 - 421.3
421.3 - 451.7
451.7 - 465.6
465.6 - 501.5
> 501.5
China: Heilongjiang: DALYs Rate (per 100,000)
DALYs Rate
< 417.0
417.0 - 456.2
456.2 - 498.7
498.7 - 541.2
541.2 - 583.3
583.3 - 625.5
625.5 - 667.7
667.7 - 699.8
699.8 - 761.0
> 761.0
Female
Male
Sexes Combined
20-24 years
15-19 years
10-14 years
Age
Year
DALYs Rate (per 100,000)
Age
10-24 years

Figure S3

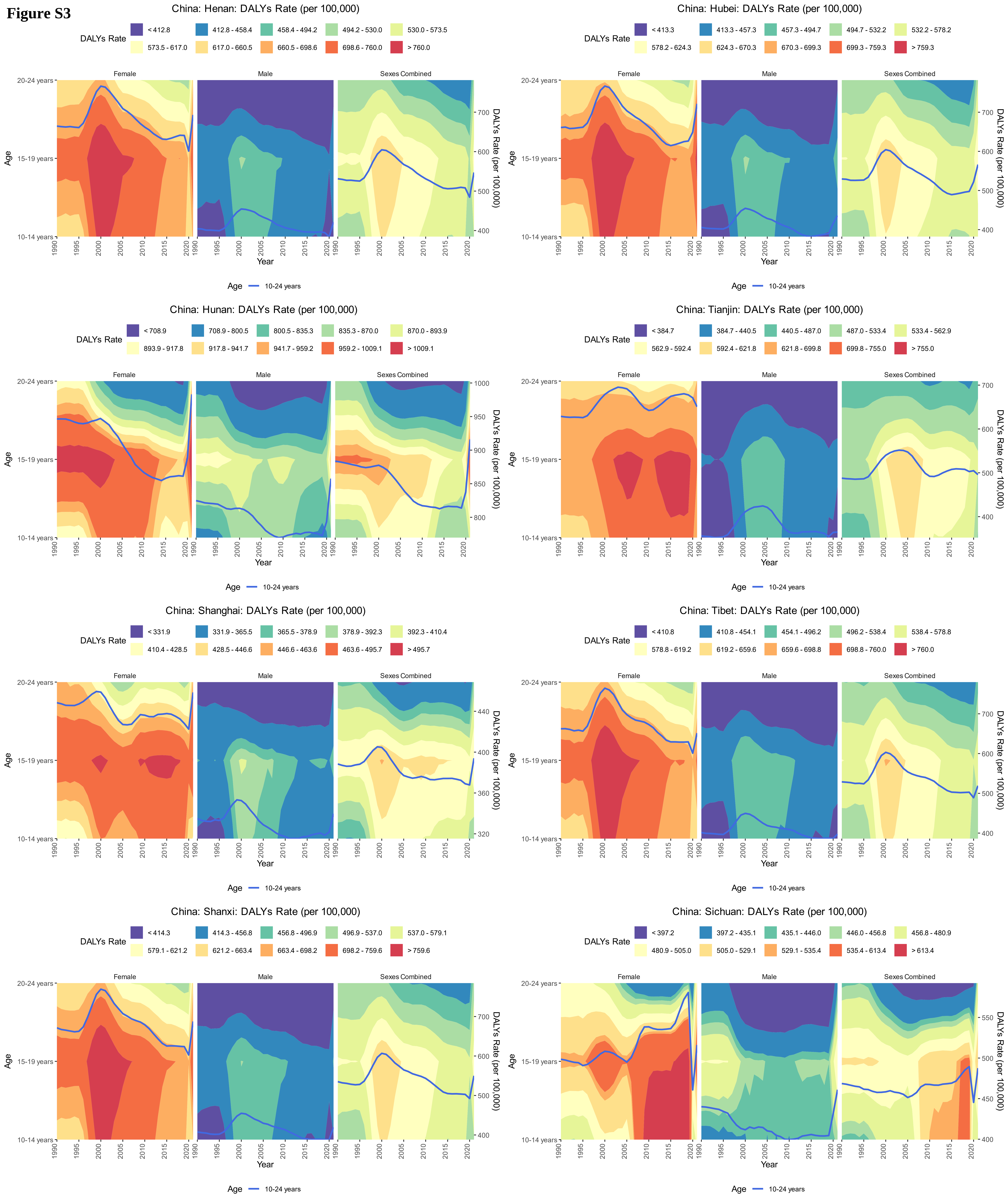

China: Henan: DALYs Rate (per 100,000)
DALYs Rate
< 412.8
412.8 - 458.4
458.4 - 494.2
494.2 - 530.0
530.0 - 573.5
573.5 - 617.0
617.0 - 660.5
660.5 - 698.6
698.6 - 760.0
> 760.0
China: Hubei: DALYs Rate (per 100,000)
DALYs Rate
< 413.3
413.3 - 457.3
457.3 - 494.7
494.7 - 532.2
532.2 - 578.2
578.2 - 624.3
624.3 - 670.3
670.3 - 699.3
699.3 - 759.3
> 759.3
China: Hunan: DALYs Rate (per 100,000)
DALYs Rate
< 708.9
708.9 - 800.5
800.5 - 835.3
835.3 - 870.0
870.0 - 893.9
893.9 - 917.8
917.8 - 941.7
941.7 - 959.2
959.2 - 1009.1
> 1009.1
China: Tianjin: DALYs Rate (per 100,000)
DALYs Rate
< 384.7
384.7 - 440.5
440.5 - 487.0
487.0 - 533.4
533.4 - 562.9
562.9 - 592.4
592.4 - 621.8
621.8 - 699.8
699.8 - 755.0
> 755.0
China: Shanghai: DALYs Rate (per 100,000)
DALYs Rate
< 331.9
331.9 - 365.5
365.5 - 378.9
378.9 - 392.3
392.3 - 410.4
410.4 - 428.5
428.5 - 446.6
446.6 - 463.6
463.6 - 495.7
> 495.7
China: Tibet: DALYs Rate (per 100,000)
DALYs Rate
< 410.8
410.8 - 454.1
454.1 - 496.2
496.2 - 538.4
538.4 - 578.8
578.8 - 619.2
619.2 - 659.6
659.6 - 698.8
698.8 - 760.0
> 760.0
China: Shanxi: DALYs Rate (per 100,000)
DALYs Rate
< 414.3
414.3 - 456.8
456.8 - 496.9
496.9 - 537.0
537.0 - 579.1
579.1 - 621.2
621.2 - 663.4
663.4 - 698.2
698.2 - 759.6
> 759.6
China: Sichuan: DALYs Rate (per 100,000)
DALYs Rate
< 397.2
397.2 - 435.1
435.1 - 446.0
446.0 - 456.8
456.8 - 480.9
480.9 - 505.0
505.0 - 529.1
529.1 - 535.4
535.4 - 613.4
> 613.4
Female
Male
Sexes Combined
Age
20-24 years
15-19 years
10-14 years
Year
DALYs Rate (per 100,000)
Age
10-24 years

Figure S4

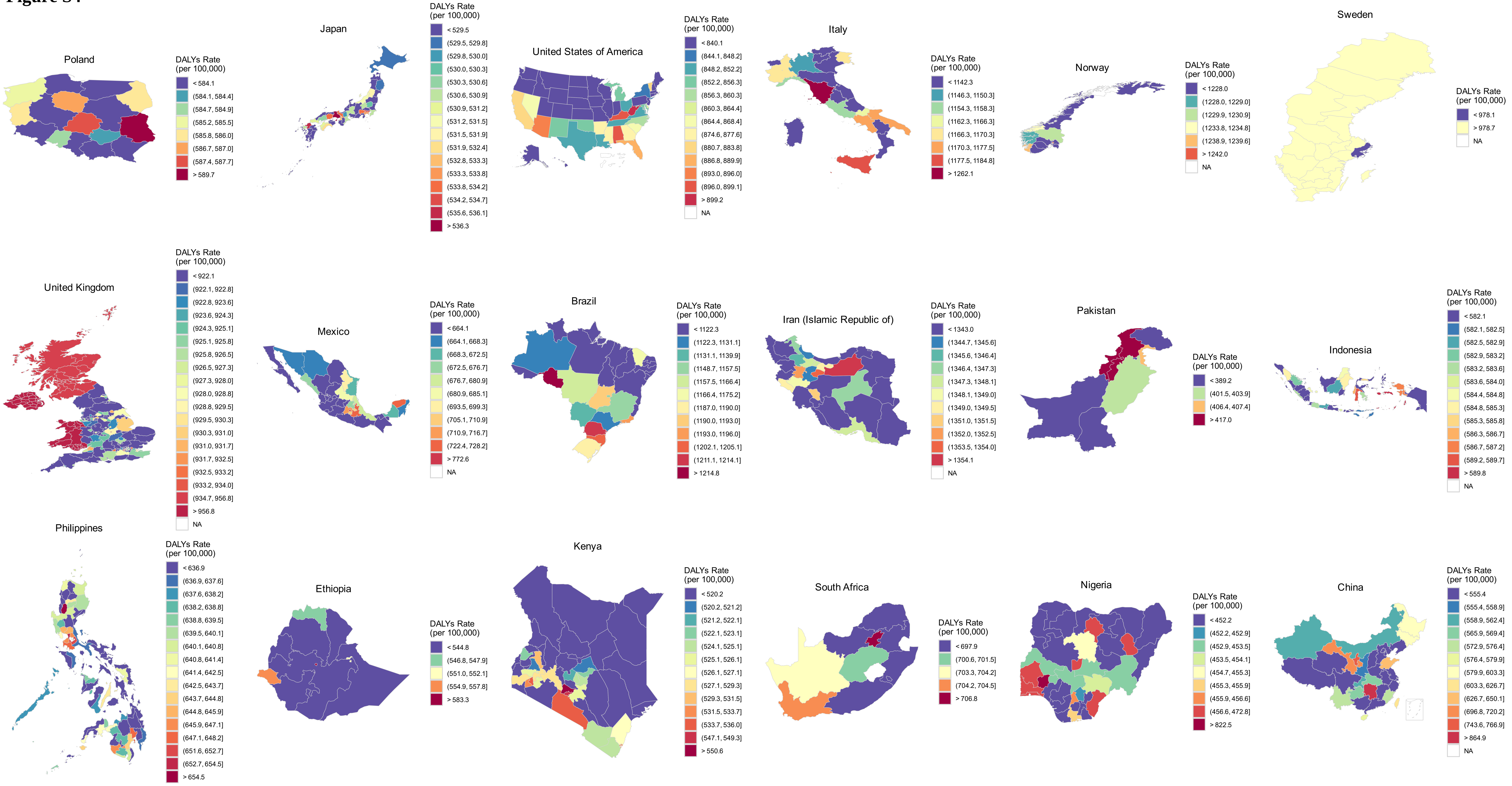
Poland
DALYs Rate (per 100,000)
< 584.1
(584.1, 584.4]
(584.7, 584.9]
(585.2, 585.5]
(585.8, 586.0]
(586.7, 587.0]
(587.4, 587.7]
> 589.7
Japan
DALYs Rate (per 100,000)
< 529.5
(529.5, 529.8]
(529.8, 530.0]
(530.0, 530.3]
(530.3, 530.6]
(530.6, 530.9]
(530.9, 531.2]
(531.2, 531.5]
(531.5, 531.9]
(531.9, 532.4]
(532.8, 533.3]
(533.3, 533.8]
(533.8, 534.2]
(534.2, 534.7]
(535.6, 536.1]
> 536.3
United States of America
DALYs Rate (per 100,000)
< 840.1
(844.1, 848.2]
(848.2, 852.2]
(852.2, 856.3]
(856.3, 860.3]
(860.3, 864.4]
(864.4, 868.4]
(874.6, 877.6]
(880.7, 883.8]
(886.8, 889.9]
(893.0, 896.0]
(896.0, 899.1]
> 899.2
NA
Italy
DALYs Rate (per 100,000)
< 1142.3
(1146.3, 1150.3]
(1154.3, 1158.3]
(1162.3, 1166.3]
(1166.3, 1170.3]
(1170.3, 1177.5]
(1177.5, 1184.8]
> 1262.1
Norway
DALYs Rate (per 100,000)
< 1228.0
(1228.0, 1229.0]
(1229.9, 1230.9]
(1233.8, 1234.8]
(1238.9, 1239.6]
> 1242.0
NA
Sweden
DALYs Rate (per 100,000)
< 978.1
> 978.7
NA
United Kingdom
DALYs Rate (per 100,000)
< 922.1
(922.1, 922.8]
(922.8, 923.6]
(923.6, 924.3]
(924.3, 925.1]
(925.1, 925.8]
(925.8, 926.5]
(926.5, 927.3]
(927.3, 928.0]
(928.0, 928.8]
(928.8, 929.5]
(929.5, 930.3]
(930.3, 931.0]
(931.0, 931.7]
(931.7, 932.5]
(932.5, 933.2]
(933.2, 934.0]
(934.7, 956.8]
> 956.8
NA
Mexico
DALYs Rate (per 100,000)
< 664.1
(664.1, 668.3]
(668.3, 672.5]
(672.5, 676.7]
(676.7, 680.9]
(680.9, 685.1]
(693.5, 699.3]
(705.1, 710.9]
(710.9, 716.7]
(722.4, 728.2]
> 772.6
NA
Brazil
DALYs Rate (per 100,000)
< 1122.3
(1122.3, 1131.1]
(1131.1, 1139.9]
(1148.7, 1157.5]
(1157.5, 1166.4]
(1166.4, 1175.2]
(1187.0, 1190.0]
(1190.0, 1193.0]
(1193.0, 1196.0]
(1202.1, 1205.1]
(1211.1, 1214.1]
> 1214.8
Iran (Islamic Republic of)
DALYs Rate (per 100,000)
< 1343.0
(1344.7, 1345.6]
(1345.6, 1346.4]
(1346.4, 1347.3]
(1347.3, 1348.1]
(1348.1, 1349.0]
(1349.0, 1349.5]
(1351.0, 1351.5]
(1352.0, 1352.5]
(1353.5, 1354.0]
> 1354.1
NA
Pakistan
DALYs Rate (per 100,000)
< 389.2
(401.5, 403.9]
(406.4, 407.4]
> 417.0
Indonesia
DALYs Rate (per 100,000)
< 582.1
(582.1, 582.5]
(582.5, 582.9]
(582.9, 583.2]
(583.2, 583.6]
(583.6, 584.0]
(584.4, 584.8]
(584.8, 585.3]
(585.3, 585.8]
(586.3, 586.7]
(586.7, 587.2]
(589.2, 589.7]
> 589.8
NA
Philippines
DALYs Rate (per 100,000)
< 636.9
(636.9, 637.6]
(637.6, 638.2]
(638.2, 638.8]
(638.8, 639.5]
(639.5, 640.1]
(640.1, 640.8]
(640.8, 641.4]
(641.4, 642.5]
(642.5, 643.7]
(643.7, 644.8]
(644.8, 645.9]
(645.9, 647.1]
(647.1, 648.2]
(651.6, 652.7]
(652.7, 654.5]
> 654.5
Ethiopia
DALYs Rate (per 100,000)
< 544.8
(546.8, 547.9]
(551.0, 552.1]
(554.9, 557.8]
> 583.3
Kenya
DALYs Rate (per 100,000)
< 520.2
(520.2, 521.2]
(521.2, 522.1]
(522.1, 523.1]
(524.1, 525.1]
(525.1, 526.1]
(526.1, 527.1]
(527.1, 529.3]
(529.3, 531.5]
(531.5, 533.7]
(533.7, 536.0]
(547.1, 549.3]
> 550.6
South Africa
DALYs Rate (per 100,000)
< 697.9
(700.6, 701.5]
(703.3, 704.2]
(704.2, 704.5]
> 706.8
Nigeria
DALYs Rate (per 100,000)
< 452.2
(452.2, 452.9]
(452.9, 453.5]
(453.5, 454.1]
(454.7, 455.3]
(455.3, 455.9]
(455.9, 456.6]
(456.6, 472.8]
> 822.5
China
DALYs Rate (per 100,000)
< 555.4
(555.4, 558.9]
(558.9, 562.4]
(565.9, 569.4]
(572.9, 576.4]
(576.4, 579.9]
(579.9, 603.3]
(603.3, 626.7]
(626.7, 650.1]
(696.8, 720.2]
(743.6, 766.9]
> 864.9
NA

Figure S5

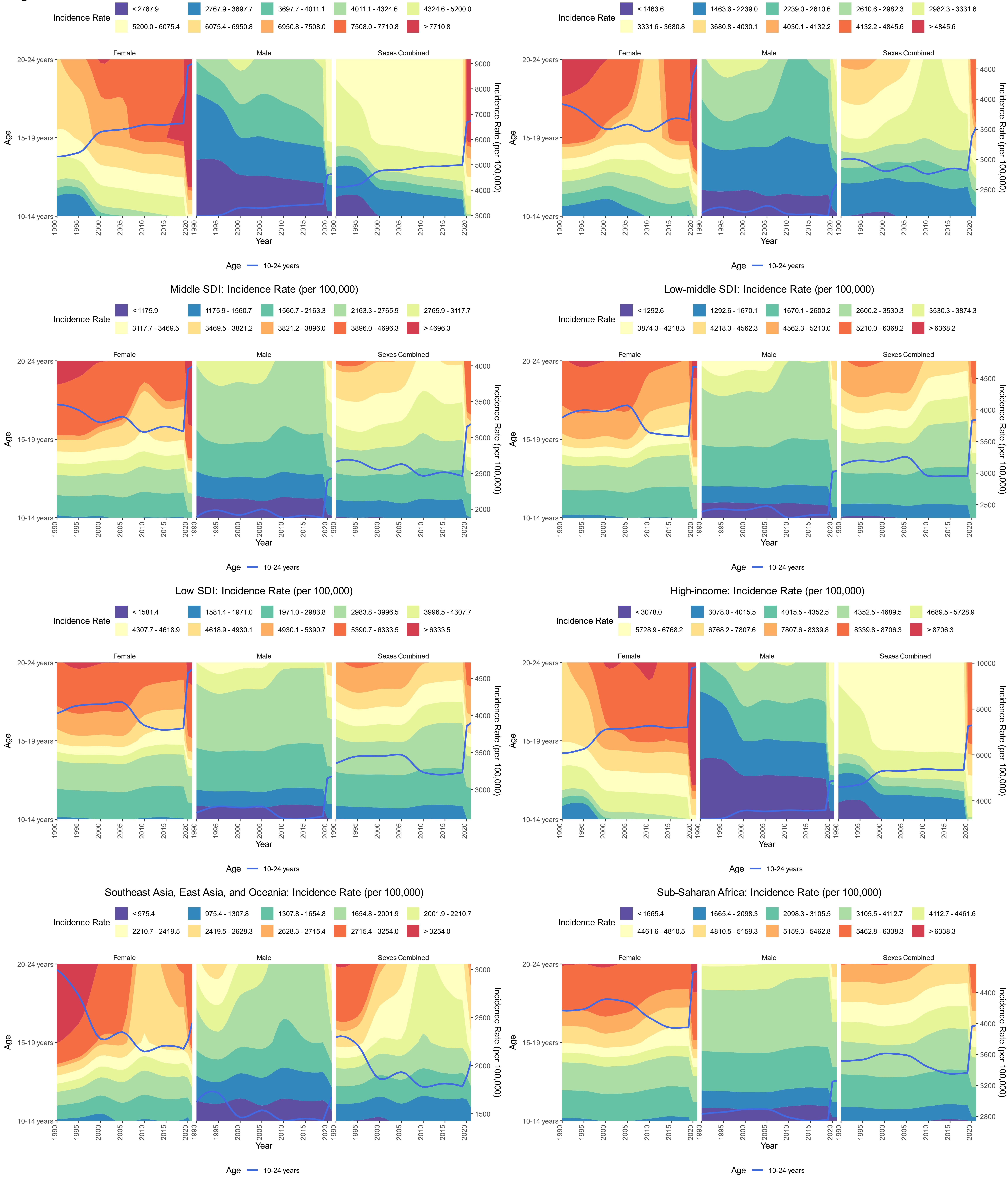

High SDI: Incidence Rate (per 100,000)
Incidence Rate
< 2767.9
2767.9 - 3697.7
3697.7 - 4011.1
4011.1 - 4324.6
4324.6 - 5200.0
5200.0 - 6075.4
6075.4 - 6950.8
6950.8 - 7508.0
7508.0 - 7710.8
> 7710.8
Female
Male
Sexes Combined
Age
Year
Incidence Rate (per 100,000)
Age — 10-24 years
High-middle SDI: Incidence Rate (per 100,000)
< 1463.6
1463.6 - 2239.0
2239.0 - 2610.6
2610.6 - 2982.3
2982.3 - 3331.6
3331.6 - 3680.8
3680.8 - 4030.1
4030.1 - 4132.2
4132.2 - 4845.6
> 4845.6
Middle SDI: Incidence Rate (per 100,000)
< 1175.9
1175.9 - 1560.7
1560.7 - 2163.3
2163.3 - 2765.9
2765.9 - 3117.7
3117.7 - 3469.5
3469.5 - 3821.2
3821.2 - 3896.0
3896.0 - 4696.3
> 4696.3
Low-middle SDI: Incidence Rate (per 100,000)
< 1292.6
1292.6 - 1670.1
1670.1 - 2600.2
2600.2 - 3530.3
3530.3 - 3874.3
3874.3 - 4218.3
4218.3 - 4562.3
4562.3 - 5210.0
5210.0 - 6368.2
> 6368.2
Low SDI: Incidence Rate (per 100,000)
< 1581.4
1581.4 - 1971.0
1971.0 - 2983.8
2983.8 - 3996.5
3996.5 - 4307.7
4307.7 - 4618.9
4618.9 - 4930.1
4930.1 - 5390.7
5390.7 - 6333.5
> 6333.5
High-income: Incidence Rate (per 100,000)
< 3078.0
3078.0 - 4015.5
4015.5 - 4352.5
4352.5 - 4689.5
4689.5 - 5728.9
5728.9 - 6768.2
6768.2 - 7807.6
7807.6 - 8339.8
8339.8 - 8706.3
> 8706.3
Southeast Asia, East Asia, and Oceania: Incidence Rate (per 100,000)
< 975.4
975.4 - 1307.8
1307.8 - 1654.8
1654.8 - 2001.9
2001.9 - 2210.7
2210.7 - 2419.5
2419.5 - 2628.3
2628.3 - 2715.4
2715.4 - 3254.0
> 3254.0
Sub-Saharan Africa: Incidence Rate (per 100,000)
< 1665.4
1665.4 - 2098.3
2098.3 - 3105.5
3105.5 - 4112.7
4112.7 - 4461.6
4461.6 - 4810.5
4810.5 - 5159.3
5159.3 - 5462.8
5462.8 - 6338.3
> 6338.3
20-24 years
15-19 years
10-14 years

Figure S5

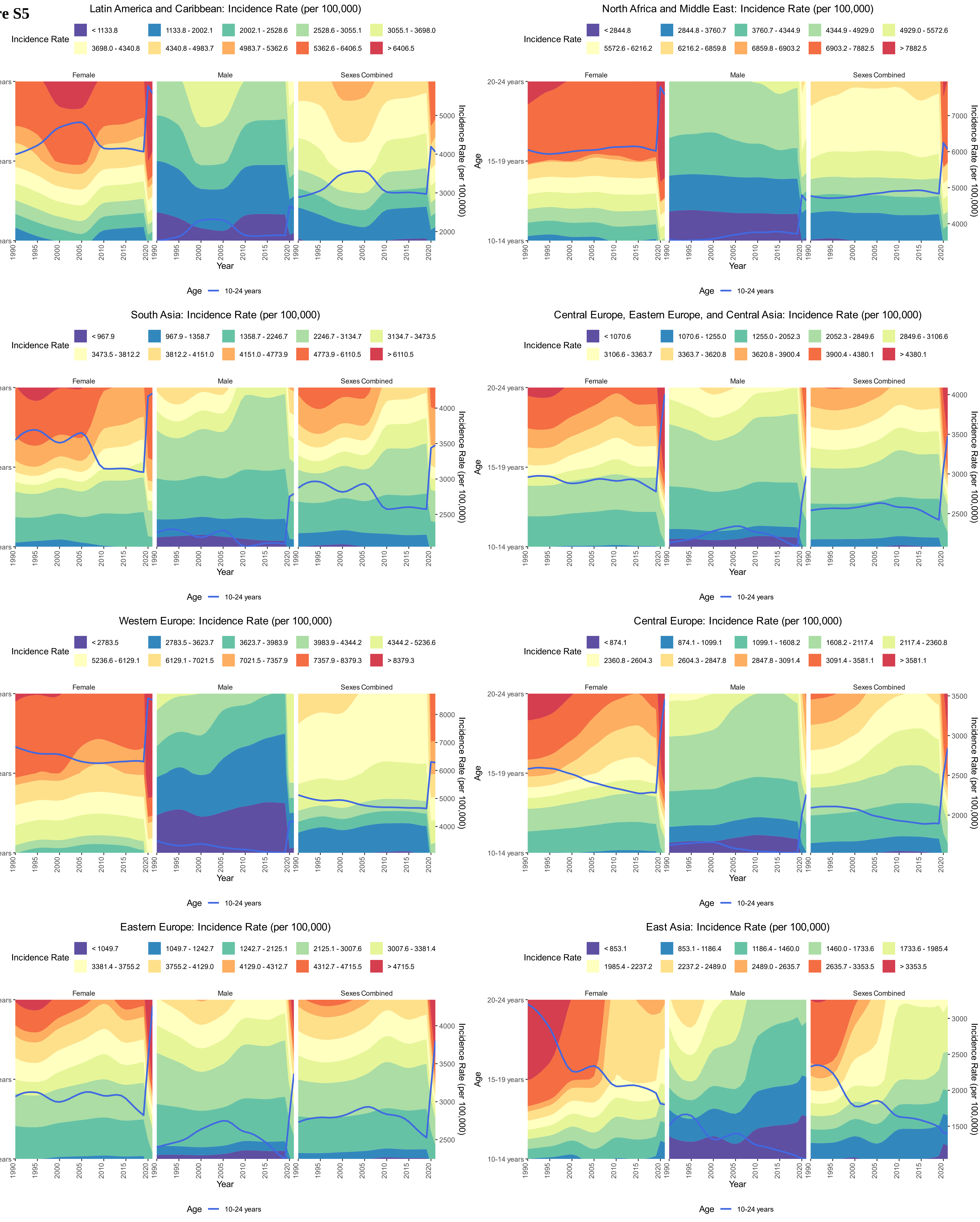

Latin America and Caribbean: Incidence Rate (per 100,000)
Incidence Rate
< 1133.8
1133.8 - 2002.1
2002.1 - 2528.6
2528.6 - 3055.1
3055.1 - 3698.0
3698.0 - 4340.8
4340.8 - 4983.7
4983.7 - 5362.6
5362.6 - 6406.5
> 6406.5
North Africa and Middle East: Incidence Rate (per 100,000)
Incidence Rate
< 2844.8
2844.8 - 3760.7
3760.7 - 4344.9
4344.9 - 4929.0
4929.0 - 5572.6
5572.6 - 6216.2
6216.2 - 6859.8
6859.8 - 6903.2
6903.2 - 7882.5
> 7882.5
South Asia: Incidence Rate (per 100,000)
Incidence Rate
< 967.9
967.9 - 1358.7
1358.7 - 2246.7
2246.7 - 3134.7
3134.7 - 3473.5
3473.5 - 3812.2
3812.2 - 4151.0
4151.0 - 4773.9
4773.9 - 6110.5
> 6110.5
Central Europe, Eastern Europe, and Central Asia: Incidence Rate (per 100,000)
Incidence Rate
< 1070.6
1070.6 - 1255.0
1255.0 - 2052.3
2052.3 - 2849.6
2849.6 - 3106.6
3106.6 - 3363.7
3363.7 - 3620.8
3620.8 - 3900.4
3900.4 - 4380.1
> 4380.1
Western Europe: Incidence Rate (per 100,000)
Incidence Rate
< 2783.5
2783.5 - 3623.7
3623.7 - 3983.9
3983.9 - 4344.2
4344.2 - 5236.6
5236.6 - 6129.1
6129.1 - 7021.5
7021.5 - 7357.9
7357.9 - 8379.3
> 8379.3
Central Europe: Incidence Rate (per 100,000)
Incidence Rate
< 874.1
874.1 - 1099.1
1099.1 - 1608.2
1608.2 - 2117.4
2117.4 - 2360.8
2360.8 - 2604.3
2604.3 - 2847.8
2847.8 - 3091.4
3091.4 - 3581.1
> 3581.1
Eastern Europe: Incidence Rate (per 100,000)
Incidence Rate
< 1049.7
1049.7 - 1242.7
1242.7 - 2125.1
2125.1 - 3007.6
3007.6 - 3381.4
3381.4 - 3755.2
3755.2 - 4129.0
4129.0 - 4312.7
4312.7 - 4715.5
> 4715.5
East Asia: Incidence Rate (per 100,000)
Incidence Rate
< 853.1
853.1 - 1186.4
1186.4 - 1460.0
1460.0 - 1733.6
1733.6 - 1985.4
1985.4 - 2237.2
2237.2 - 2489.0
2489.0 - 2635.7
2635.7 - 3353.5
> 3353.5
Female
Male
Sexes Combined
Age
20-24 years
15-19 years
10-14 years
Year
Incidence Rate (per 100,000)
Age
10-24 years

Figure S5

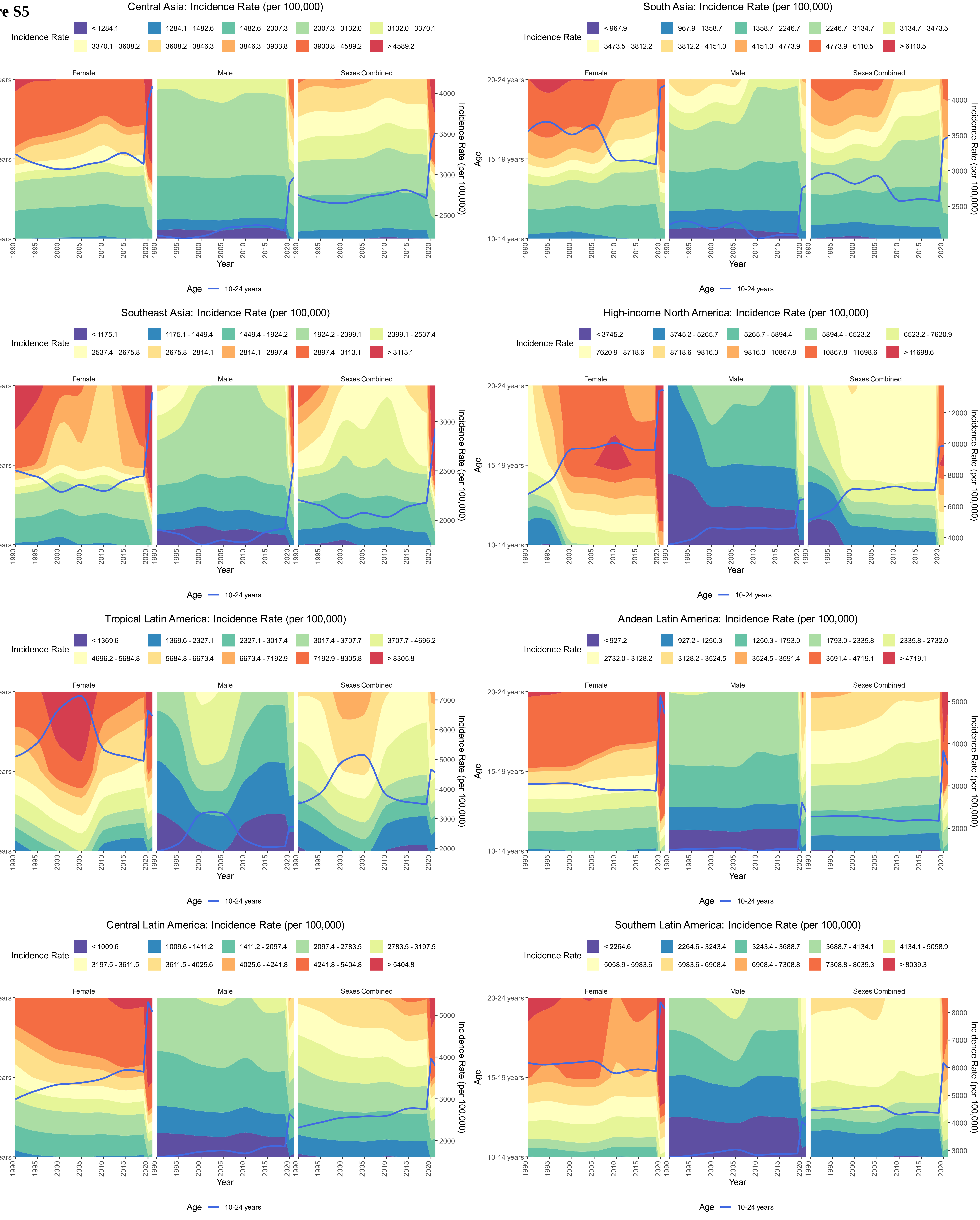

Central Asia: Incidence Rate (per 100,000)
Incidence Rate
< 1284.1
1284.1 - 1482.6
1482.6 - 2307.3
2307.3 - 3132.0
3132.0 - 3370.1
3370.1 - 3608.2
3608.2 - 3846.3
3846.3 - 3933.8
3933.8 - 4589.2
> 4589.2
South Asia: Incidence Rate (per 100,000)
< 967.9
967.9 - 1358.7
1358.7 - 2246.7
2246.7 - 3134.7
3134.7 - 3473.5
3473.5 - 3812.2
3812.2 - 4151.0
4151.0 - 4773.9
4773.9 - 6110.5
> 6110.5
Southeast Asia: Incidence Rate (per 100,000)
< 1175.1
1175.1 - 1449.4
1449.4 - 1924.2
1924.2 - 2399.1
2399.1 - 2537.4
2537.4 - 2675.8
2675.8 - 2814.1
2814.1 - 2897.4
2897.4 - 3113.1
> 3113.1
High-income North America: Incidence Rate (per 100,000)
< 3745.2
3745.2 - 5265.7
5265.7 - 5894.4
5894.4 - 6523.2
6523.2 - 7620.9
7620.9 - 8718.6
8718.6 - 9816.3
9816.3 - 10867.8
10867.8 - 11698.6
> 11698.6
Tropical Latin America: Incidence Rate (per 100,000)
< 1369.6
1369.6 - 2327.1
2327.1 - 3017.4
3017.4 - 3707.7
3707.7 - 4696.2
4696.2 - 5684.8
5684.8 - 6673.4
6673.4 - 7192.9
7192.9 - 8305.8
> 8305.8
Andean Latin America: Incidence Rate (per 100,000)
< 927.2
927.2 - 1250.3
1250.3 - 1793.0
1793.0 - 2335.8
2335.8 - 2732.0
2732.0 - 3128.2
3128.2 - 3524.5
3524.5 - 3591.4
3591.4 - 4719.1
> 4719.1
Central Latin America: Incidence Rate (per 100,000)
< 1009.6
1009.6 - 1411.2
1411.2 - 2097.4
2097.4 - 2783.5
2783.5 - 3197.5
3197.5 - 3611.5
3611.5 - 4025.6
4025.6 - 4241.8
4241.8 - 5404.8
> 5404.8
Southern Latin America: Incidence Rate (per 100,000)
< 2264.6
2264.6 - 3243.4
3243.4 - 3688.7
3688.7 - 4134.1
4134.1 - 5058.9
5058.9 - 5983.6
5983.6 - 6908.4
6908.4 - 7308.8
7308.8 - 8039.3
> 8039.3
Female
Male
Sexes Combined
Age
20-24 years
15-19 years
10-14 years
Year
Incidence Rate (per 100,000)
Age — 10-24 years

Figure S5

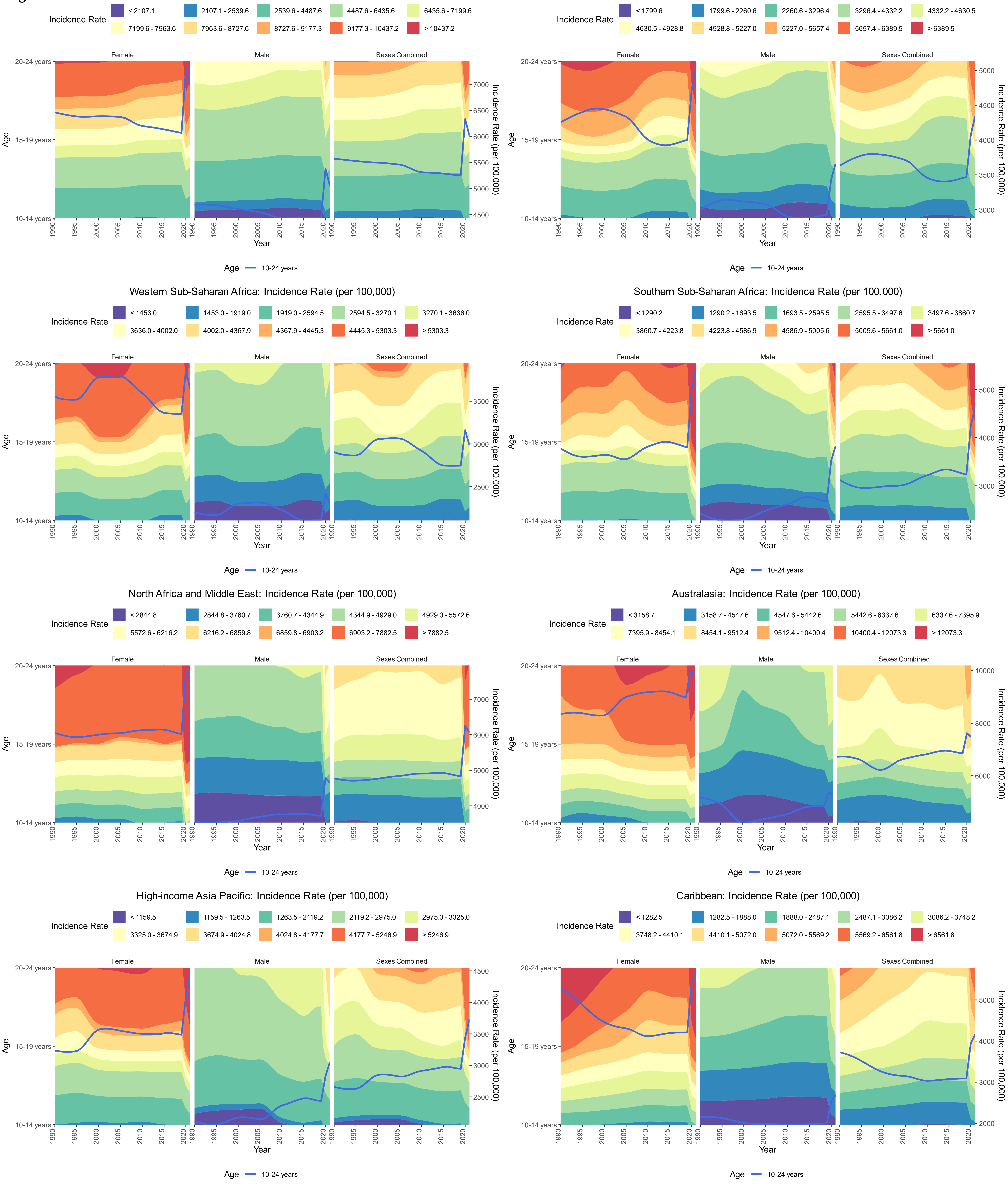

Central Sub-Saharan Africa: Incidence Rate (per 100,000)
Incidence Rate
< 2107.1
2107.1 - 2539.6
2539.6 - 4487.6
4487.6 - 6435.6
6435.6 - 7199.6
7199.6 - 7963.6
7963.6 - 8727.6
8727.6 - 9177.3
9177.3 - 10437.2
> 10437.2
Eastern Sub-Saharan Africa: Incidence Rate (per 100,000)
< 1799.6
1799.6 - 2260.6
2260.6 - 3296.4
3296.4 - 4332.2
4332.2 - 4630.5
4630.5 - 4928.8
4928.8 - 5227.0
5227.0 - 5657.4
5657.4 - 6389.5
> 6389.5
Western Sub-Saharan Africa: Incidence Rate (per 100,000)
< 1453.0
1453.0 - 1919.0
1919.0 - 2594.5
2594.5 - 3270.1
3270.1 - 3636.0
3636.0 - 4002.0
4002.0 - 4367.9
4367.9 - 4445.3
4445.3 - 5303.3
> 5303.3
Southern Sub-Saharan Africa: Incidence Rate (per 100,000)
< 1290.2
1290.2 - 1693.5
1693.5 - 2595.5
2595.5 - 3497.6
3497.6 - 3860.7
3860.7 - 4223.8
4223.8 - 4586.9
4586.9 - 5005.6
5005.6 - 5661.0
> 5661.0
North Africa and Middle East: Incidence Rate (per 100,000)
< 2844.8
2844.8 - 3760.7
3760.7 - 4344.9
4344.9 - 4929.0
4929.0 - 5572.6
5572.6 - 6216.2
6216.2 - 6859.8
6859.8 - 6903.2
6903.2 - 7882.5
> 7882.5
Australasia: Incidence Rate (per 100,000)
< 3158.7
3158.7 - 4547.6
4547.6 - 5442.6
5442.6 - 6337.6
6337.6 - 7395.9
7395.9 - 8454.1
8454.1 - 9512.4
9512.4 - 10400.4
10400.4 - 12073.3
> 12073.3
High-income Asia Pacific: Incidence Rate (per 100,000)
< 1159.5
1159.5 - 1263.5
1263.5 - 2119.2
2119.2 - 2975.0
2975.0 - 3325.0
3325.0 - 3674.9
3674.9 - 4024.8
4024.8 - 4177.7
4177.7 - 5246.9
> 5246.9
Caribbean: Incidence Rate (per 100,000)
< 1282.5
1282.5 - 1888.0
1888.0 - 2487.1
2487.1 - 3086.2
3086.2 - 3748.2
3748.2 - 4410.1
4410.1 - 5072.0
5072.0 - 5569.2
5569.2 - 6561.8
> 6561.8
Female
Male
Sexes Combined
Age
20-24 years
15-19 years
10-14 years
Year
1990
1995
2000
2005
2010
2015
2020
Incidence Rate (per 100,000)
Age
10-24 years

Figure S5

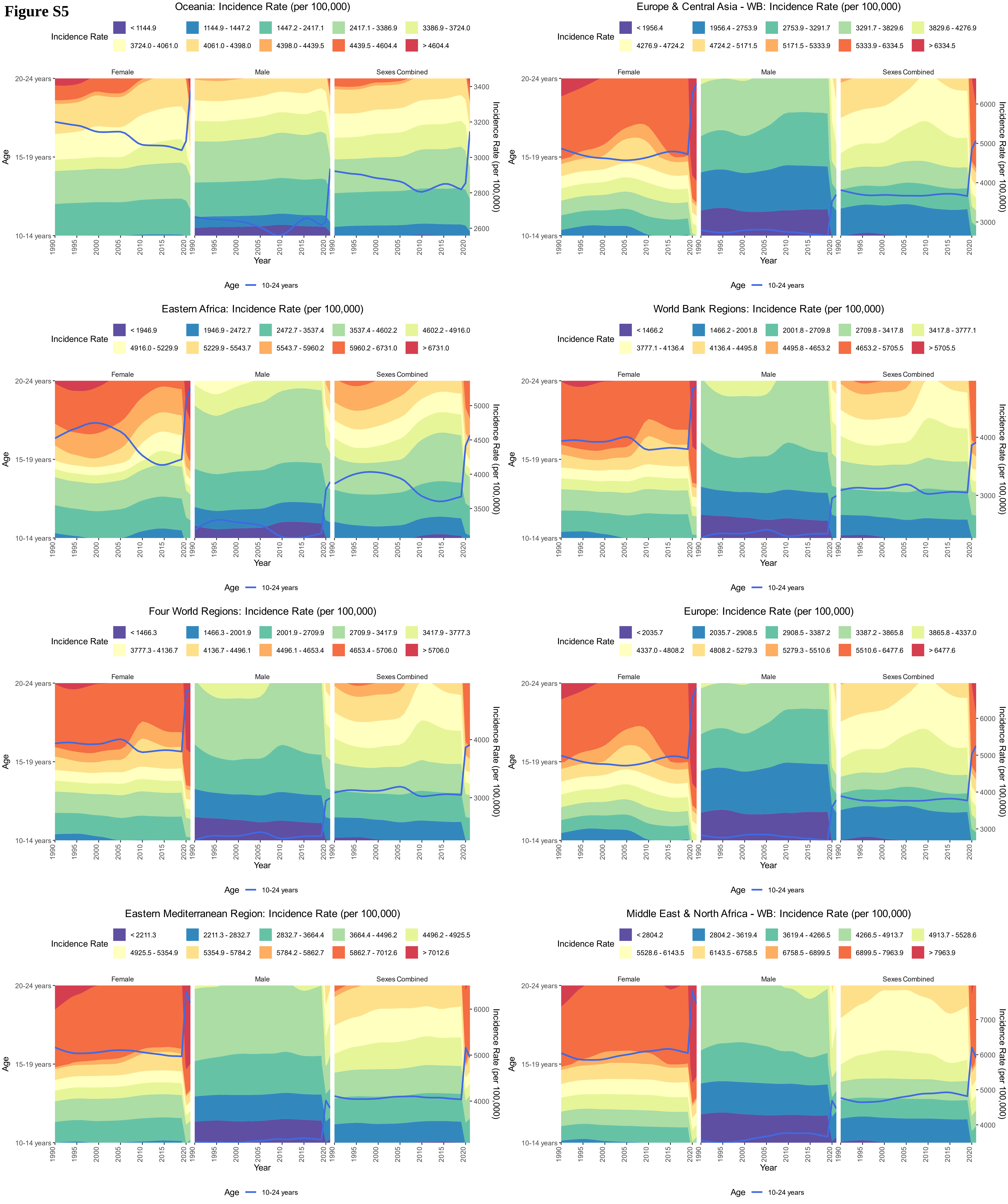

Oceania: Incidence Rate (per 100,000)
Incidence Rate
< 1144.9
1144.9 - 1447.2
1447.2 - 2417.1
2417.1 - 3386.9
3386.9 - 3724.0
3724.0 - 4061.0
4061.0 - 4398.0
4398.0 - 4439.5
4439.5 - 4604.4
> 4604.4
Female
Male
Sexes Combined
Age
20-24 years
15-19 years
10-14 years
Year
Incidence Rate (per 100,000)
Age — 10-24 years
Europe & Central Asia - WB: Incidence Rate (per 100,000)
< 1956.4
1956.4 - 2753.9
2753.9 - 3291.7
3291.7 - 3829.6
3829.6 - 4276.9
4276.9 - 4724.2
4724.2 - 5171.5
5171.5 - 5333.9
5333.9 - 6334.5
> 6334.5
Eastern Africa: Incidence Rate (per 100,000)
< 1946.9
1946.9 - 2472.7
2472.7 - 3537.4
3537.4 - 4602.2
4602.2 - 4916.0
4916.0 - 5229.9
5229.9 - 5543.7
5543.7 - 5960.2
5960.2 - 6731.0
> 6731.0
World Bank Regions: Incidence Rate (per 100,000)
< 1466.2
1466.2 - 2001.8
2001.8 - 2709.8
2709.8 - 3417.8
3417.8 - 3777.1
3777.1 - 4136.4
4136.4 - 4495.8
4495.8 - 4653.2
4653.2 - 5705.5
> 5705.5
Four World Regions: Incidence Rate (per 100,000)
< 1466.3
1466.3 - 2001.9
2001.9 - 2709.9
2709.9 - 3417.9
3417.9 - 3777.3
3777.3 - 4136.7
4136.7 - 4496.1
4496.1 - 4653.4
4653.4 - 5706.0
> 5706.0
Europe: Incidence Rate (per 100,000)
< 2035.7
2035.7 - 2908.5
2908.5 - 3387.2
3387.2 - 3865.8
3865.8 - 4337.0
4337.0 - 4808.2
4808.2 - 5279.3
5279.3 - 5510.6
5510.6 - 6477.6
> 6477.6
Eastern Mediterranean Region: Incidence Rate (per 100,000)
< 2211.3
2211.3 - 2832.7
2832.7 - 3664.4
3664.4 - 4496.2
4496.2 - 4925.5
4925.5 - 5354.9
5354.9 - 5784.2
5784.2 - 5862.7
5862.7 - 7012.6
> 7012.6
Middle East & North Africa - WB: Incidence Rate (per 100,000)
< 2804.2
2804.2 - 3619.4
3619.4 - 4266.5
4266.5 - 4913.7
4913.7 - 5528.6
5528.6 - 6143.5
6143.5 - 6758.5
6758.5 - 6899.5
6899.5 - 7963.9
> 7963.9

Figure S5

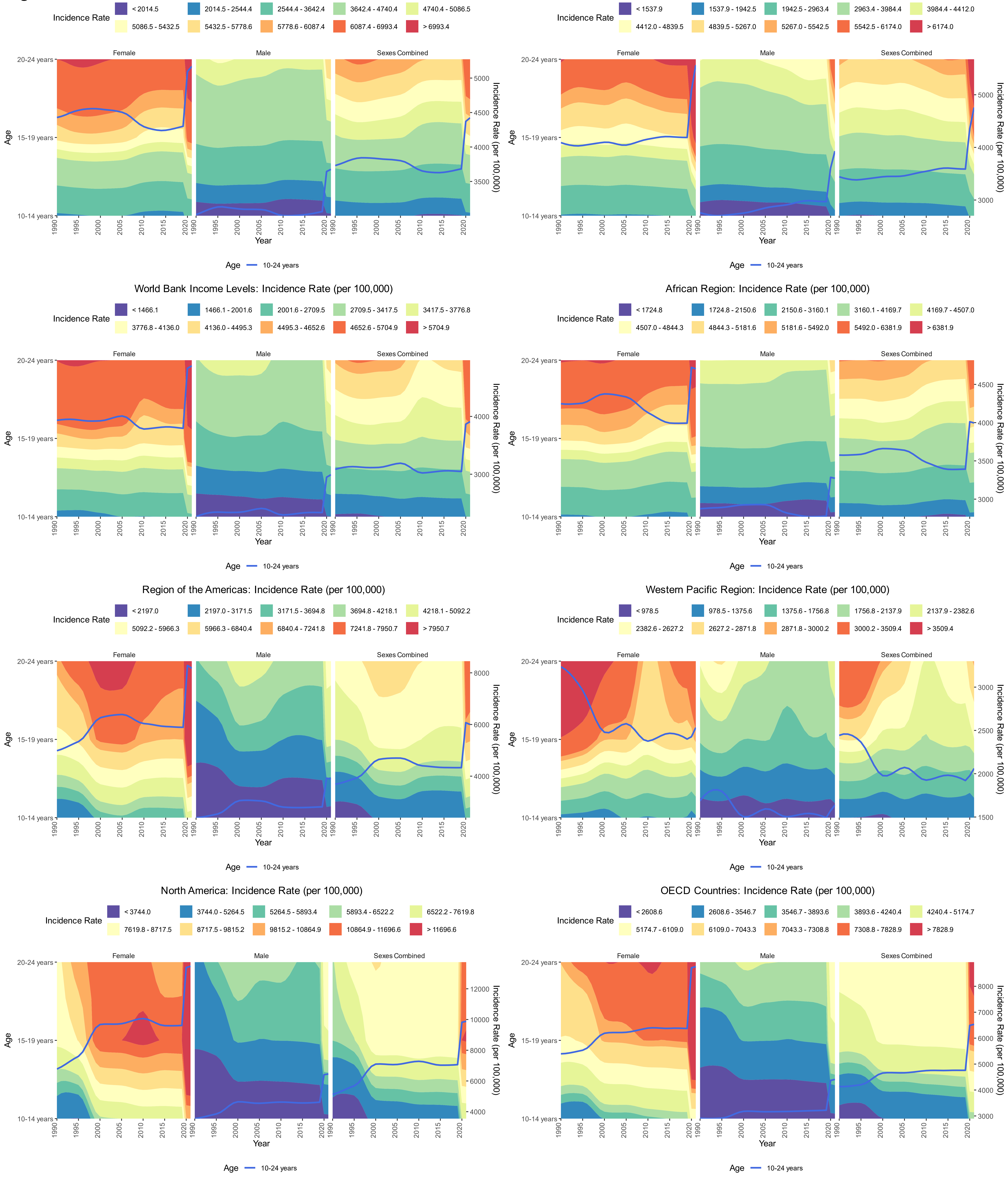
World Bank Low Income: Incidence Rate (per 100,000)
Incidence Rate
< 2014.5
2014.5 - 2544.4
2544.4 - 3642.4
3642.4 - 4740.4
4740.4 - 5086.5
5086.5 - 5432.5
5432.5 - 5778.6
5778.6 - 6087.4
6087.4 - 6993.4
> 6993.4
Southern Africa: Incidence Rate (per 100,000)
< 1537.9
1537.9 - 1942.5
1942.5 - 2963.4
2963.4 - 3984.4
3984.4 - 4412.0
4412.0 - 4839.5
4839.5 - 5267.0
5267.0 - 5542.5
5542.5 - 6174.0
> 6174.0
World Bank Income Levels: Incidence Rate (per 100,000)
< 1466.1
1466.1 - 2001.6
2001.6 - 2709.5
2709.5 - 3417.5
3417.5 - 3776.8
3776.8 - 4136.0
4136.0 - 4495.3
4495.3 - 4652.6
4652.6 - 5704.9
> 5704.9
African Region: Incidence Rate (per 100,000)
< 1724.8
1724.8 - 2150.6
2150.6 - 3160.1
3160.1 - 4169.7
4169.7 - 4507.0
4507.0 - 4844.3
4844.3 - 5181.6
5181.6 - 5492.0
5492.0 - 6381.9
> 6381.9
Region of the Americas: Incidence Rate (per 100,000)
< 2197.0
2197.0 - 3171.5
3171.5 - 3694.8
3694.8 - 4218.1
4218.1 - 5092.2
5092.2 - 5966.3
5966.3 - 6840.4
6840.4 - 7241.8
7241.8 - 7950.7
> 7950.7
Western Pacific Region: Incidence Rate (per 100,000)
< 978.5
978.5 - 1375.6
1375.6 - 1756.8
1756.8 - 2137.9
2137.9 - 2382.6
2382.6 - 2627.2
2627.2 - 2871.8
2871.8 - 3000.2
3000.2 - 3509.4
> 3509.4
North America: Incidence Rate (per 100,000)
< 3744.0
3744.0 - 5264.5
5264.5 - 5893.4
5893.4 - 6522.2
6522.2 - 7619.8
7619.8 - 8717.5
8717.5 - 9815.2
9815.2 - 10864.9
10864.9 - 11696.6
> 11696.6
OECD Countries: Incidence Rate (per 100,000)
< 2608.6
2608.6 - 3546.7
3546.7 - 3893.6
3893.6 - 4240.4
4240.4 - 5174.7
5174.7 - 6109.0
6109.0 - 7043.3
7043.3 - 7308.8
7308.8 - 7828.9
> 7828.9
Female
Male
Sexes Combined
20-24 years
15-19 years
10-14 years
Age
Year
Incidence Rate (per 100,000)
Age — 10-24 years

Figure S5

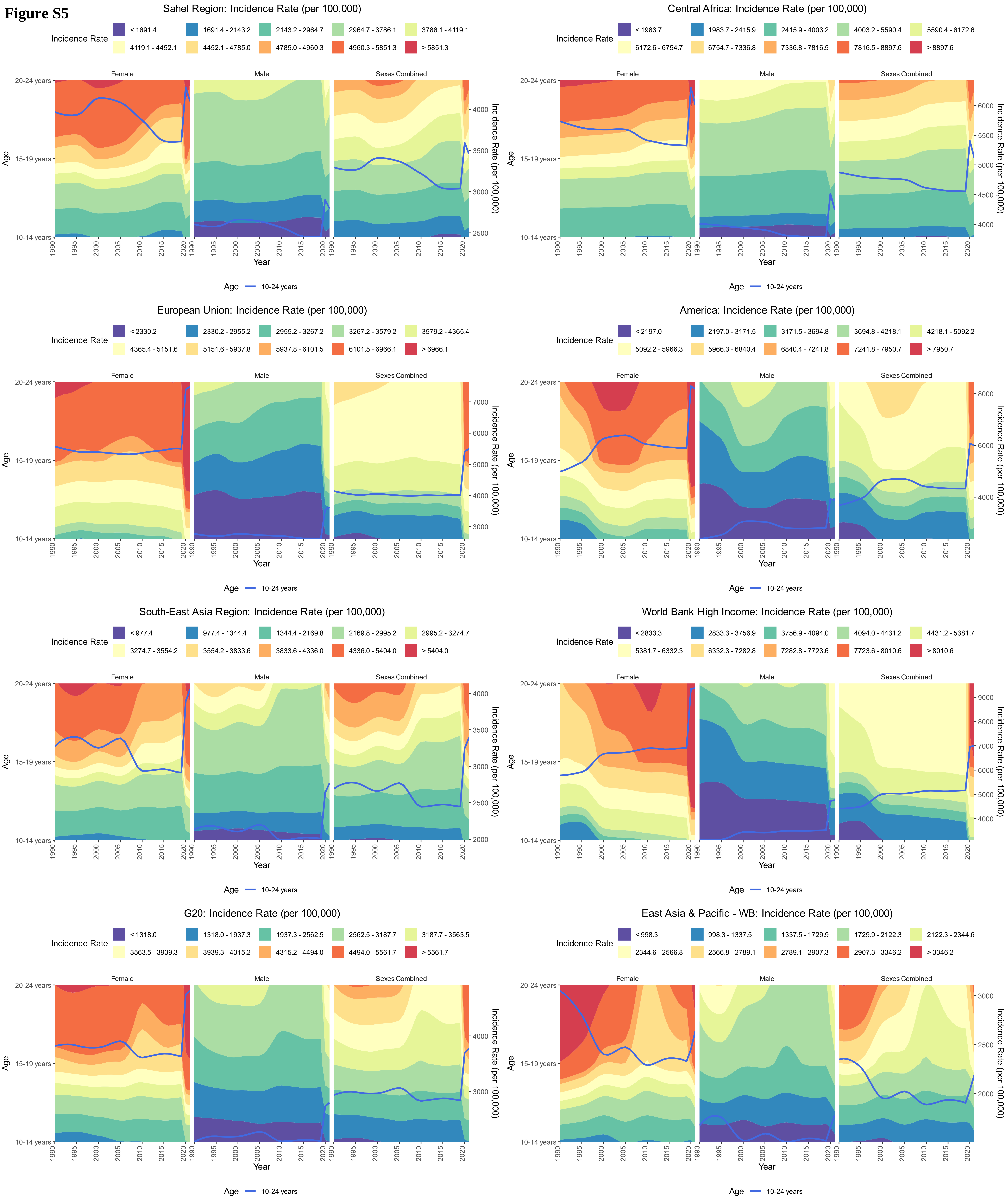
Sahel Region: Incidence Rate (per 100,000)
Incidence Rate
< 1691.4
1691.4 - 2143.2
2143.2 - 2964.7
2964.7 - 3786.1
3786.1 - 4119.1
4119.1 - 4452.1
4452.1 - 4785.0
4785.0 - 4960.3
4960.3 - 5851.3
> 5851.3
Female
Male
Sexes Combined
20-24 years
15-19 years
10-14 years
Age
Year
Incidence Rate (per 100,000)
Age
10-24 years
Central Africa: Incidence Rate (per 100,000)
Incidence Rate
< 1983.7
1983.7 - 2415.9
2415.9 - 4003.2
4003.2 - 5590.4
5590.4 - 6172.6
6172.6 - 6754.7
6754.7 - 7336.8
7336.8 - 7816.5
7816.5 - 8897.6
> 8897.6
European Union: Incidence Rate (per 100,000)
Incidence Rate
< 2330.2
2330.2 - 2955.2
2955.2 - 3267.2
3267.2 - 3579.2
3579.2 - 4365.4
4365.4 - 5151.6
5151.6 - 5937.8
5937.8 - 6101.5
6101.5 - 6966.1
> 6966.1
America: Incidence Rate (per 100,000)
Incidence Rate
< 2197.0
2197.0 - 3171.5
3171.5 - 3694.8
3694.8 - 4218.1
4218.1 - 5092.2
5092.2 - 5966.3
5966.3 - 6840.4
6840.4 - 7241.8
7241.8 - 7950.7
> 7950.7
South-East Asia Region: Incidence Rate (per 100,000)
Incidence Rate
< 977.4
977.4 - 1344.4
1344.4 - 2169.8
2169.8 - 2995.2
2995.2 - 3274.7
3274.7 - 3554.2
3554.2 - 3833.6
3833.6 - 4336.0
4336.0 - 5404.0
> 5404.0
World Bank High Income: Incidence Rate (per 100,000)
Incidence Rate
< 2833.3
2833.3 - 3756.9
3756.9 - 4094.0
4094.0 - 4431.2
4431.2 - 5381.7
5381.7 - 6332.3
6332.3 - 7282.8
7282.8 - 7723.6
7723.6 - 8010.6
> 8010.6
G20: Incidence Rate (per 100,000)
Incidence Rate
< 1318.0
1318.0 - 1937.3
1937.3 - 2562.5
2562.5 - 3187.7
3187.7 - 3563.5
3563.5 - 3939.3
3939.3 - 4315.2
4315.2 - 4494.0
4494.0 - 5561.7
> 5561.7
East Asia & Pacific - WB: Incidence Rate (per 100,000)
Incidence Rate
< 998.3
998.3 - 1337.5
1337.5 - 1729.9
1729.9 - 2122.3
2122.3 - 2344.6
2344.6 - 2566.8
2566.8 - 2789.1
2789.1 - 2907.3
2907.3 - 3346.2
> 3346.2

Figure S5

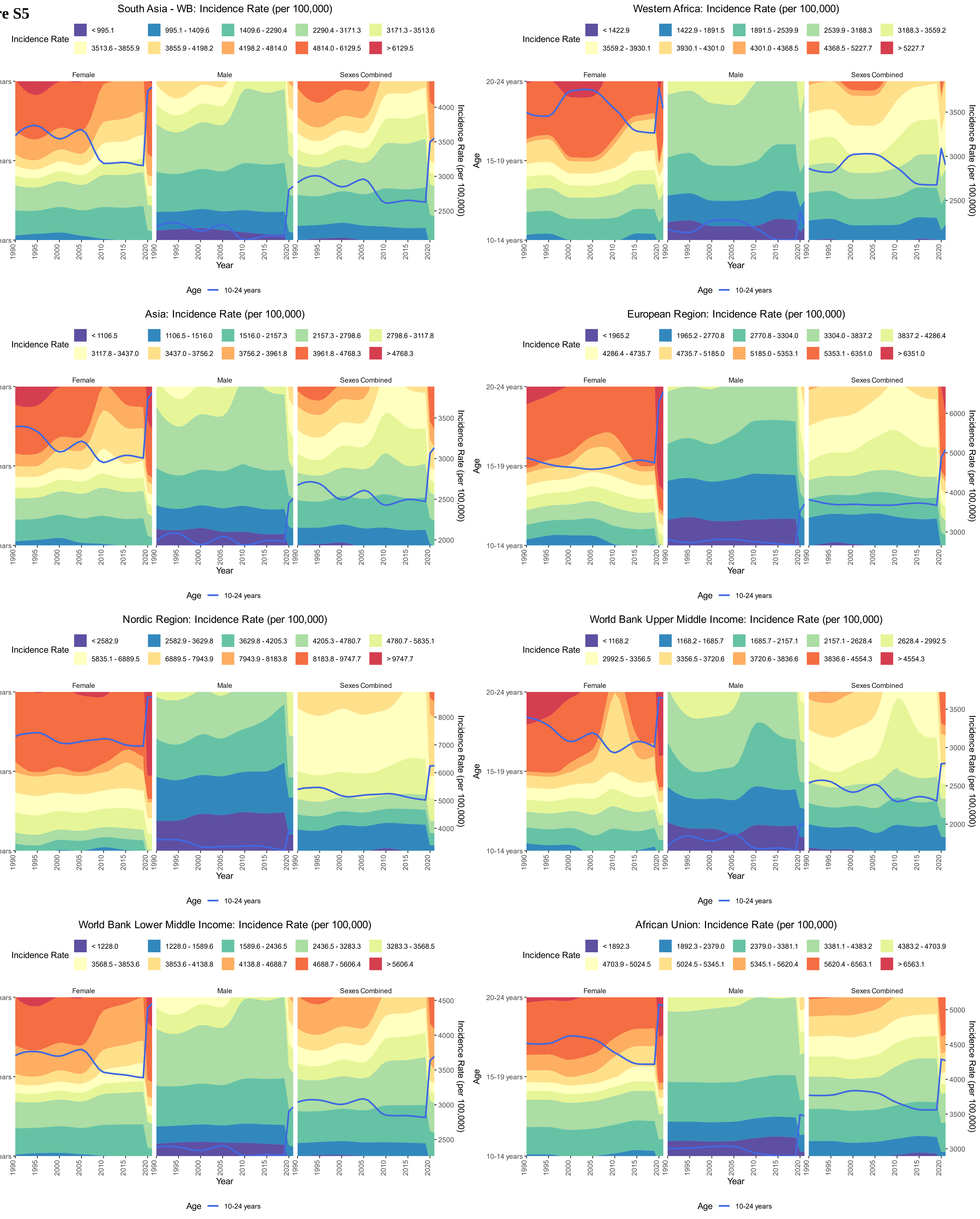

South Asia - WB: Incidence Rate (per 100,000)
Incidence Rate
< 995.1
995.1 - 1409.6
1409.6 - 2290.4
2290.4 - 3171.3
3171.3 - 3513.6
3513.6 - 3855.9
3855.9 - 4198.2
4198.2 - 4814.0
4814.0 - 6129.5
> 6129.5
Western Africa: Incidence Rate (per 100,000)
< 1422.9
1422.9 - 1891.5
1891.5 - 2539.9
2539.9 - 3188.3
3188.3 - 3559.2
3559.2 - 3930.1
3930.1 - 4301.0
4301.0 - 4368.5
4368.5 - 5227.7
> 5227.7
Asia: Incidence Rate (per 100,000)
< 1106.5
1106.5 - 1516.0
1516.0 - 2157.3
2157.3 - 2798.6
2798.6 - 3117.8
3117.8 - 3437.0
3437.0 - 3756.2
3756.2 - 3961.8
3961.8 - 4768.3
> 4768.3
European Region: Incidence Rate (per 100,000)
< 1965.2
1965.2 - 2770.8
2770.8 - 3304.0
3304.0 - 3837.2
3837.2 - 4286.4
4286.4 - 4735.7
4735.7 - 5185.0
5185.0 - 5353.1
5353.1 - 6351.0
> 6351.0
Nordic Region: Incidence Rate (per 100,000)
< 2582.9
2582.9 - 3629.8
3629.8 - 4205.3
4205.3 - 4780.7
4780.7 - 5835.1
5835.1 - 6889.5
6889.5 - 7943.9
7943.9 - 8183.8
8183.8 - 9747.7
> 9747.7
World Bank Upper Middle Income: Incidence Rate (per 100,000)
< 1168.2
1168.2 - 1685.7
1685.7 - 2157.1
2157.1 - 2628.4
2628.4 - 2992.5
2992.5 - 3356.5
3356.5 - 3720.6
3720.6 - 3836.6
3836.6 - 4554.3
> 4554.3
World Bank Lower Middle Income: Incidence Rate (per 100,000)
< 1228.0
1228.0 - 1589.6
1589.6 - 2436.5
2436.5 - 3283.3
3283.3 - 3568.5
3568.5 - 3853.6
3853.6 - 4138.8
4138.8 - 4688.7
4688.7 - 5606.4
> 5606.4
African Union: Incidence Rate (per 100,000)
< 1892.3
1892.3 - 2379.0
2379.0 - 3381.1
3381.1 - 4383.2
4383.2 - 4703.9
4703.9 - 5024.5
5024.5 - 5345.1
5345.1 - 5620.4
5620.4 - 6563.1
> 6563.1
Female
Male
Sexes Combined
Age
20-24 years
15-19 years
10-14 years
Year
Incidence Rate (per 100,000)
Age
10-24 years

Figure S5

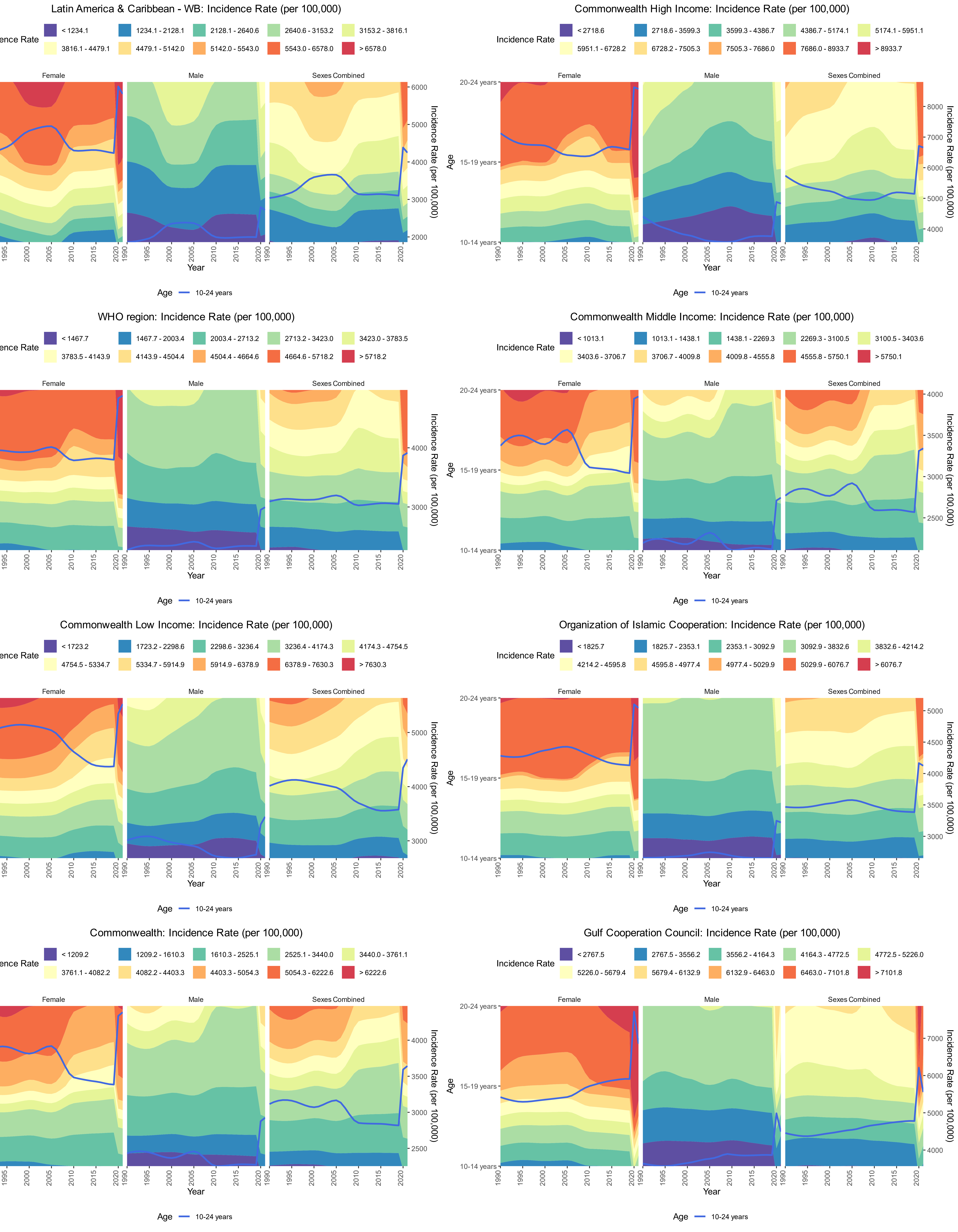
Latin America & Caribbean - WB: Incidence Rate (per 100,000)
Incidence Rate
< 1234.1
1234.1 - 2128.1
2128.1 - 2640.6
2640.6 - 3153.2
3153.2 - 3816.1
3816.1 - 4479.1
4479.1 - 5142.0
5142.0 - 5543.0
5543.0 - 6578.0
> 6578.0
Commonwealth High Income: Incidence Rate (per 100,000)
< 2718.6
2718.6 - 3599.3
3599.3 - 4386.7
4386.7 - 5174.1
5174.1 - 5951.1
5951.1 - 6728.2
6728.2 - 7505.3
7505.3 - 7686.0
7686.0 - 8933.7
> 8933.7
WHO region: Incidence Rate (per 100,000)
< 1467.7
1467.7 - 2003.4
2003.4 - 2713.2
2713.2 - 3423.0
3423.0 - 3783.5
3783.5 - 4143.9
4143.9 - 4504.4
4504.4 - 4664.6
4664.6 - 5718.2
> 5718.2
Commonwealth Middle Income: Incidence Rate (per 100,000)
< 1013.1
1013.1 - 1438.1
1438.1 - 2269.3
2269.3 - 3100.5
3100.5 - 3403.6
3403.6 - 3706.7
3706.7 - 4009.8
4009.8 - 4555.8
4555.8 - 5750.1
> 5750.1
Commonwealth Low Income: Incidence Rate (per 100,000)
< 1723.2
1723.2 - 2298.6
2298.6 - 3236.4
3236.4 - 4174.3
4174.3 - 4754.5
4754.5 - 5334.7
5334.7 - 5914.9
5914.9 - 6378.9
6378.9 - 7630.3
> 7630.3
Organization of Islamic Cooperation: Incidence Rate (per 100,000)
< 1825.7
1825.7 - 2353.1
2353.1 - 3092.9
3092.9 - 3832.6
3832.6 - 4214.2
4214.2 - 4595.8
4595.8 - 4977.4
4977.4 - 5029.9
5029.9 - 6076.7
> 6076.7
Commonwealth: Incidence Rate (per 100,000)
< 1209.2
1209.2 - 1610.3
1610.3 - 2525.1
2525.1 - 3440.0
3440.0 - 3761.1
3761.1 - 4082.2
4082.2 - 4403.3
4403.3 - 5054.3
5054.3 - 6222.6
> 6222.6
Gulf Cooperation Council: Incidence Rate (per 100,000)
< 2767.5
2767.5 - 3556.2
3556.2 - 4164.3
4164.3 - 4772.5
4772.5 - 5226.0
5226.0 - 5679.4
5679.4 - 6132.9
6132.9 - 6463.0
6463.0 - 7101.8
> 7101.8
Female
Male
Sexes Combined
Age
20-24 years
15-19 years
10-14 years
Year
Incidence Rate (per 100,000)
Age — 10-24 years

Figure S5

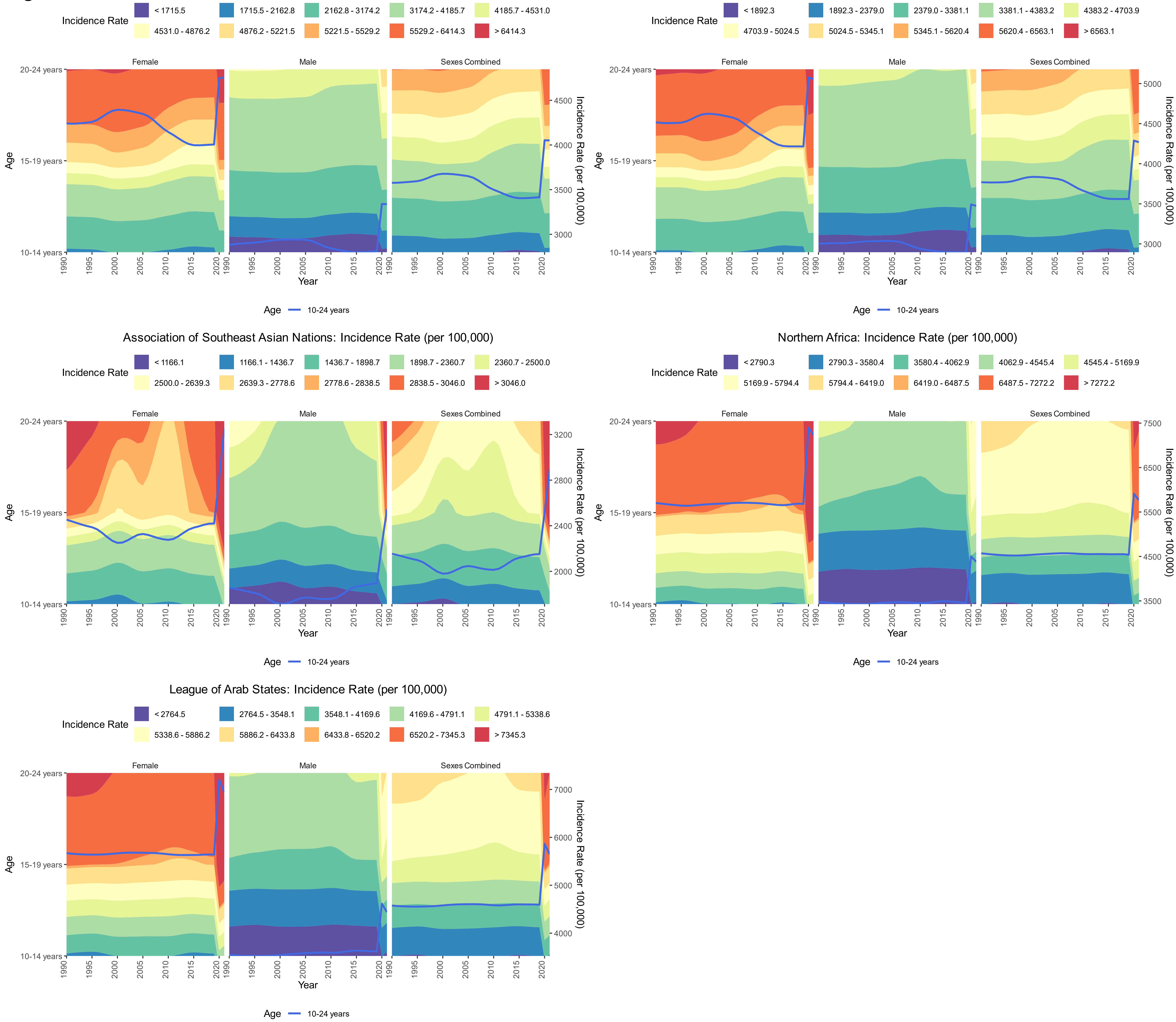

Sub-Saharan Africa - WB: Incidence Rate (per 100,000)
Incidence Rate
< 1715.5
1715.5 - 2162.8
2162.8 - 3174.2
3174.2 - 4185.7
4185.7 - 4531.0
4531.0 - 4876.2
4876.2 - 5221.5
5221.5 - 5529.2
5529.2 - 6414.3
> 6414.3
Female
Male
Sexes Combined
Age
Year
Incidence Rate (per 100,000)
Age
10-24 years
Africa: Incidence Rate (per 100,000)
Incidence Rate
< 1892.3
1892.3 - 2379.0
2379.0 - 3381.1
3381.1 - 4383.2
4383.2 - 4703.9
4703.9 - 5024.5
5024.5 - 5345.1
5345.1 - 5620.4
5620.4 - 6563.1
> 6563.1
Association of Southeast Asian Nations: Incidence Rate (per 100,000)
Incidence Rate
< 1166.1
1166.1 - 1436.7
1436.7 - 1898.7
1898.7 - 2360.7
2360.7 - 2500.0
2500.0 - 2639.3
2639.3 - 2778.6
2778.6 - 2838.5
2838.5 - 3046.0
> 3046.0
Northern Africa: Incidence Rate (per 100,000)
Incidence Rate
< 2790.3
2790.3 - 3580.4
3580.4 - 4062.9
4062.9 - 4545.4
4545.4 - 5169.9
5169.9 - 5794.4
5794.4 - 6419.0
6419.0 - 6487.5
6487.5 - 7272.2
> 7272.2
League of Arab States: Incidence Rate (per 100,000)
Incidence Rate
< 2764.5
2764.5 - 3548.1
3548.1 - 4169.6
4169.6 - 4791.1
4791.1 - 5338.6
5338.6 - 5886.2
5886.2 - 6433.8
6433.8 - 6520.2
6520.2 - 7345.3
> 7345.3
20-24 years
15-19 years
10-14 years

Figure S5

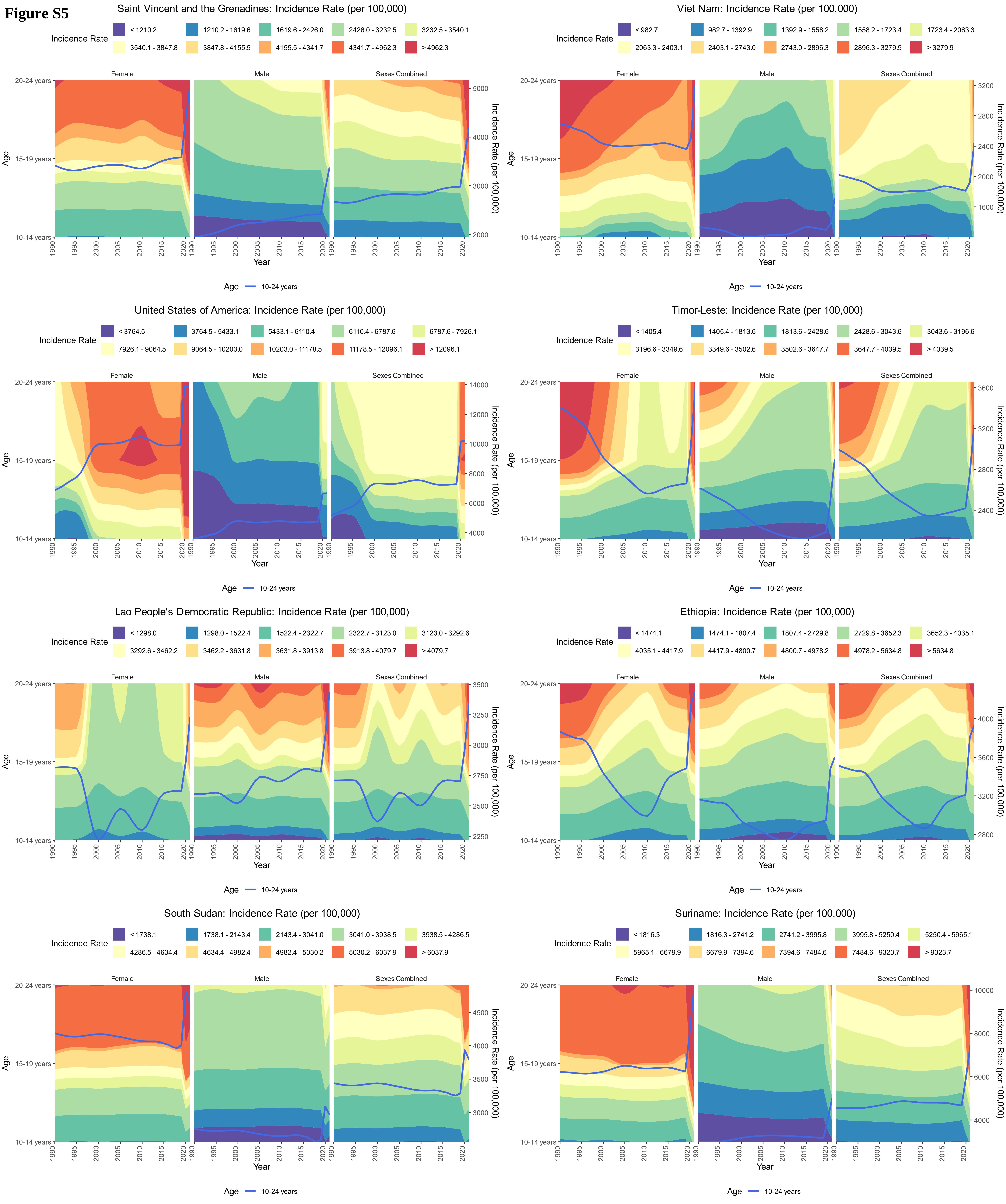

Saint Vincent and the Grenadines: Incidence Rate (per 100,000)
Incidence Rate
< 1210.2
1210.2 - 1619.6
1619.6 - 2426.0
2426.0 - 3232.5
3232.5 - 3540.1
3540.1 - 3847.8
3847.8 - 4155.5
4155.5 - 4341.7
4341.7 - 4962.3
> 4962.3
Viet Nam: Incidence Rate (per 100,000)
< 982.7
982.7 - 1392.9
1392.9 - 1558.2
1558.2 - 1723.4
1723.4 - 2063.3
2063.3 - 2403.1
2403.1 - 2743.0
2743.0 - 2896.3
2896.3 - 3279.9
> 3279.9
United States of America: Incidence Rate (per 100,000)
< 3764.5
3764.5 - 5433.1
5433.1 - 6110.4
6110.4 - 6787.6
6787.6 - 7926.1
7926.1 - 9064.5
9064.5 - 10203.0
10203.0 - 11178.5
11178.5 - 12096.1
> 12096.1
Timor-Leste: Incidence Rate (per 100,000)
< 1405.4
1405.4 - 1813.6
1813.6 - 2428.6
2428.6 - 3043.6
3043.6 - 3196.6
3196.6 - 3349.6
3349.6 - 3502.6
3502.6 - 3647.7
3647.7 - 4039.5
> 4039.5
Lao People's Democratic Republic: Incidence Rate (per 100,000)
< 1298.0
1298.0 - 1522.4
1522.4 - 2322.7
2322.7 - 3123.0
3123.0 - 3292.6
3292.6 - 3462.2
3462.2 - 3631.8
3631.8 - 3913.8
3913.8 - 4079.7
> 4079.7
Ethiopia: Incidence Rate (per 100,000)
< 1474.1
1474.1 - 1807.4
1807.4 - 2729.8
2729.8 - 3652.3
3652.3 - 4035.1
4035.1 - 4417.9
4417.9 - 4800.7
4800.7 - 4978.2
4978.2 - 5634.8
> 5634.8
South Sudan: Incidence Rate (per 100,000)
< 1738.1
1738.1 - 2143.4
2143.4 - 3041.0
3041.0 - 3938.5
3938.5 - 4286.5
4286.5 - 4634.4
4634.4 - 4982.4
4982.4 - 5030.2
5030.2 - 6037.9
> 6037.9
Suriname: Incidence Rate (per 100,000)
< 1816.3
1816.3 - 2741.2
2741.2 - 3995.8
3995.8 - 5250.4
5250.4 - 5965.1
5965.1 - 6679.9
6679.9 - 7394.6
7394.6 - 7484.6
7484.6 - 9323.7
> 9323.7
Female
Male
Sexes Combined
Age
20-24 years
15-19 years
10-14 years
Year
Incidence Rate (per 100,000)
Age — 10-24 years

Figure S5

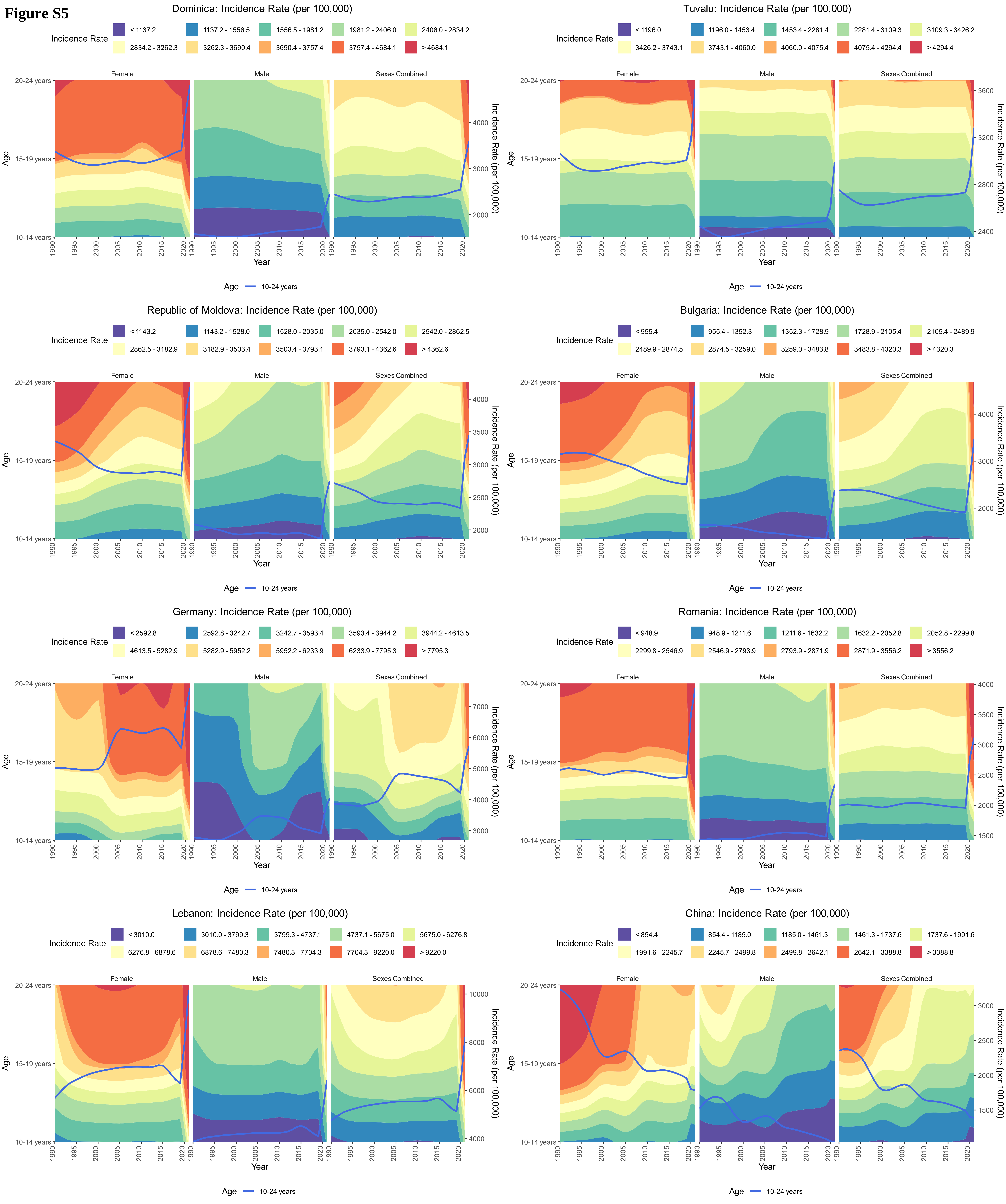
Dominica: Incidence Rate (per 100,000)
Incidence Rate
< 1137.2
1137.2 - 1556.5
1556.5 - 1981.2
1981.2 - 2406.0
2406.0 - 2834.2
2834.2 - 3262.3
3262.3 - 3690.4
3690.4 - 3757.4
3757.4 - 4684.1
> 4684.1
Tuvalu: Incidence Rate (per 100,000)
< 1196.0
1196.0 - 1453.4
1453.4 - 2281.4
2281.4 - 3109.3
3109.3 - 3426.2
3426.2 - 3743.1
3743.1 - 4060.0
4060.0 - 4075.4
4075.4 - 4294.4
> 4294.4
Republic of Moldova: Incidence Rate (per 100,000)
< 1143.2
1143.2 - 1528.0
1528.0 - 2035.0
2035.0 - 2542.0
2542.0 - 2862.5
2862.5 - 3182.9
3182.9 - 3503.4
3503.4 - 3793.1
3793.1 - 4362.6
> 4362.6
Bulgaria: Incidence Rate (per 100,000)
< 955.4
955.4 - 1352.3
1352.3 - 1728.9
1728.9 - 2105.4
2105.4 - 2489.9
2489.9 - 2874.5
2874.5 - 3259.0
3259.0 - 3483.8
3483.8 - 4320.3
> 4320.3
Germany: Incidence Rate (per 100,000)
< 2592.8
2592.8 - 3242.7
3242.7 - 3593.4
3593.4 - 3944.2
3944.2 - 4613.5
4613.5 - 5282.9
5282.9 - 5952.2
5952.2 - 6233.9
6233.9 - 7795.3
> 7795.3
Romania: Incidence Rate (per 100,000)
< 948.9
948.9 - 1211.6
1211.6 - 1632.2
1632.2 - 2052.8
2052.8 - 2299.8
2299.8 - 2546.9
2546.9 - 2793.9
2793.9 - 2871.9
2871.9 - 3556.2
> 3556.2
Lebanon: Incidence Rate (per 100,000)
< 3010.0
3010.0 - 3799.3
3799.3 - 4737.1
4737.1 - 5675.0
5675.0 - 6276.8
6276.8 - 6878.6
6878.6 - 7480.3
7480.3 - 7704.3
7704.3 - 9220.0
> 9220.0
China: Incidence Rate (per 100,000)
< 854.4
854.4 - 1185.0
1185.0 - 1461.3
1461.3 - 1737.6
1737.6 - 1991.6
1991.6 - 2245.7
2245.7 - 2499.8
2499.8 - 2642.1
2642.1 - 3388.8
> 3388.8
Female
Male
Sexes Combined
Age
20-24 years
15-19 years
10-14 years
Year
Incidence Rate (per 100,000)
Age — 10-24 years

Figure S5

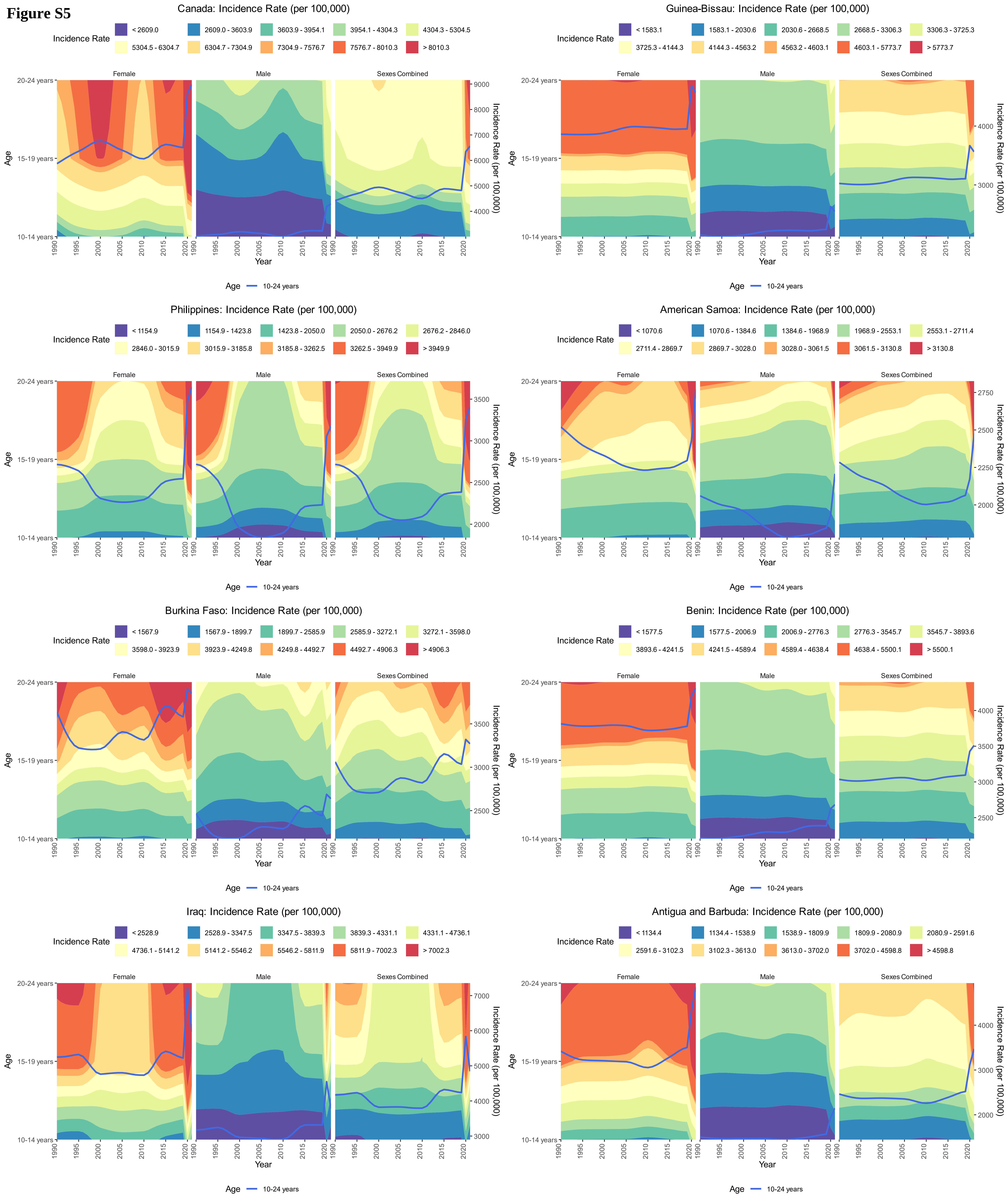
Canada: Incidence Rate (per 100,000)
Incidence Rate
< 2609.0
2609.0 - 3603.9
3603.9 - 3954.1
3954.1 - 4304.3
4304.3 - 5304.5
5304.5 - 6304.7
6304.7 - 7304.9
7304.9 - 7576.7
7576.7 - 8010.3
> 8010.3
Female
Male
Sexes Combined
Age
Year
Incidence Rate (per 100,000)
Age — 10-24 years
Guinea-Bissau: Incidence Rate (per 100,000)
< 1583.1
1583.1 - 2030.6
2030.6 - 2668.5
2668.5 - 3306.3
3306.3 - 3725.3
3725.3 - 4144.3
4144.3 - 4563.2
4563.2 - 4603.1
4603.1 - 5773.7
> 5773.7
Philippines: Incidence Rate (per 100,000)
< 1154.9
1154.9 - 1423.8
1423.8 - 2050.0
2050.0 - 2676.2
2676.2 - 2846.0
2846.0 - 3015.9
3015.9 - 3185.8
3185.8 - 3262.5
3262.5 - 3949.9
> 3949.9
American Samoa: Incidence Rate (per 100,000)
< 1070.6
1070.6 - 1384.6
1384.6 - 1968.9
1968.9 - 2553.1
2553.1 - 2711.4
2711.4 - 2869.7
2869.7 - 3028.0
3028.0 - 3061.5
3061.5 - 3130.8
> 3130.8
Burkina Faso: Incidence Rate (per 100,000)
< 1567.9
1567.9 - 1899.7
1899.7 - 2585.9
2585.9 - 3272.1
3272.1 - 3598.0
3598.0 - 3923.9
3923.9 - 4249.8
4249.8 - 4492.7
4492.7 - 4906.3
> 4906.3
Benin: Incidence Rate (per 100,000)
< 1577.5
1577.5 - 2006.9
2006.9 - 2776.3
2776.3 - 3545.7
3545.7 - 3893.6
3893.6 - 4241.5
4241.5 - 4589.4
4589.4 - 4638.4
4638.4 - 5500.1
> 5500.1
Iraq: Incidence Rate (per 100,000)
< 2528.9
2528.9 - 3347.5
3347.5 - 3839.3
3839.3 - 4331.1
4331.1 - 4736.1
4736.1 - 5141.2
5141.2 - 5546.2
5546.2 - 5811.9
5811.9 - 7002.3
> 7002.3
Antigua and Barbuda: Incidence Rate (per 100,000)
< 1134.4
1134.4 - 1538.9
1538.9 - 1809.9
1809.9 - 2080.9
2080.9 - 2591.6
2591.6 - 3102.3
3102.3 - 3613.0
3613.0 - 3702.0
3702.0 - 4598.8
> 4598.8

Figure S5

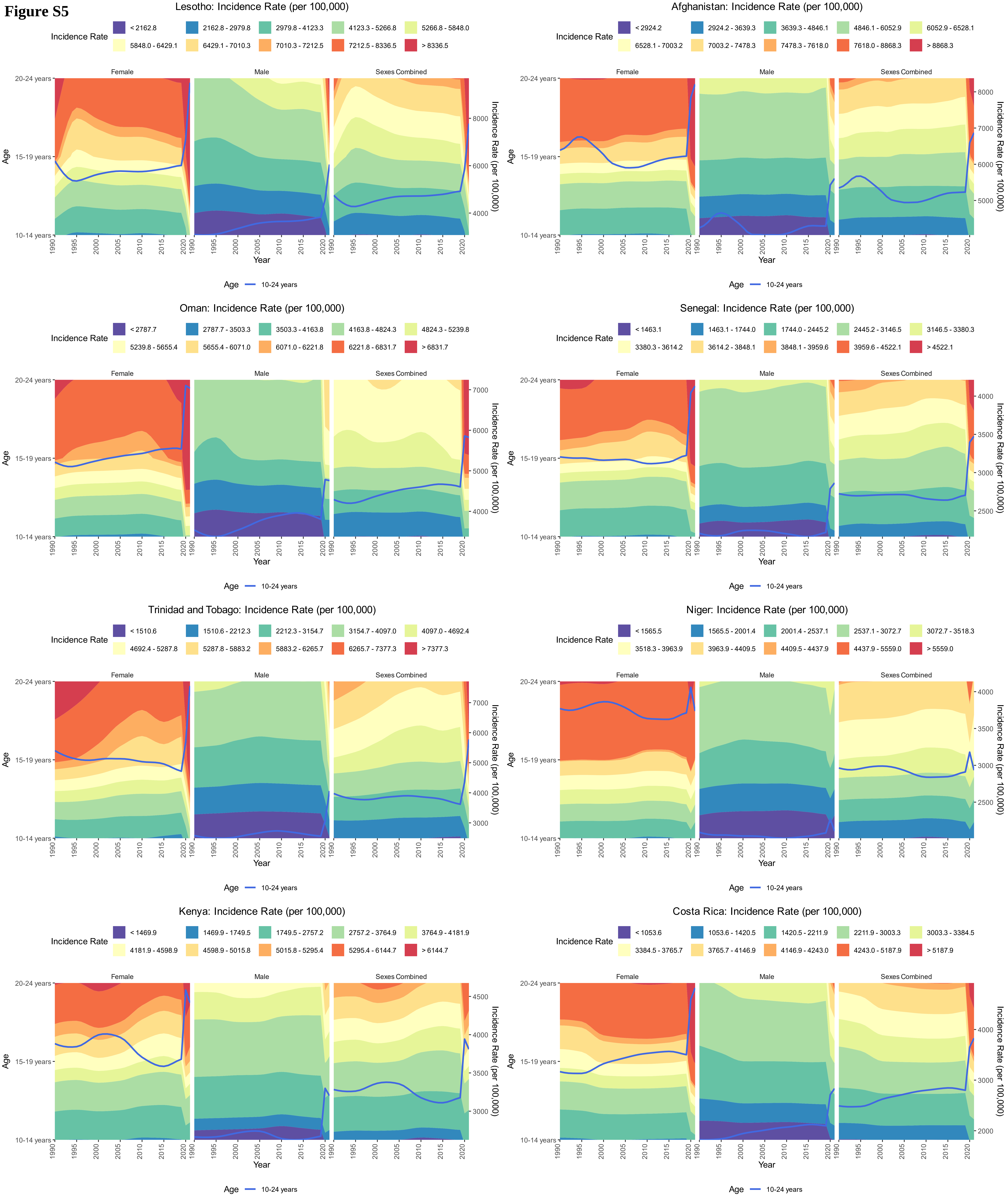
Lesotho: Incidence Rate (per 100,000)
Incidence Rate
< 2162.8
2162.8 - 2979.8
2979.8 - 4123.3
4123.3 - 5266.8
5266.8 - 5848.0
5848.0 - 6429.1
6429.1 - 7010.3
7010.3 - 7212.5
7212.5 - 8336.5
> 8336.5
Afghanistan: Incidence Rate (per 100,000)
Incidence Rate
< 2924.2
2924.2 - 3639.3
3639.3 - 4846.1
4846.1 - 6052.9
6052.9 - 6528.1
6528.1 - 7003.2
7003.2 - 7478.3
7478.3 - 7618.0
7618.0 - 8868.3
> 8868.3
Oman: Incidence Rate (per 100,000)
Incidence Rate
< 2787.7
2787.7 - 3503.3
3503.3 - 4163.8
4163.8 - 4824.3
4824.3 - 5239.8
5239.8 - 5655.4
5655.4 - 6071.0
6071.0 - 6221.8
6221.8 - 6831.7
> 6831.7
Senegal: Incidence Rate (per 100,000)
Incidence Rate
< 1463.1
1463.1 - 1744.0
1744.0 - 2445.2
2445.2 - 3146.5
3146.5 - 3380.3
3380.3 - 3614.2
3614.2 - 3848.1
3848.1 - 3959.6
3959.6 - 4522.1
> 4522.1
Trinidad and Tobago: Incidence Rate (per 100,000)
Incidence Rate
< 1510.6
1510.6 - 2212.3
2212.3 - 3154.7
3154.7 - 4097.0
4097.0 - 4692.4
4692.4 - 5287.8
5287.8 - 5883.2
5883.2 - 6265.7
6265.7 - 7377.3
> 7377.3
Niger: Incidence Rate (per 100,000)
Incidence Rate
< 1565.5
1565.5 - 2001.4
2001.4 - 2537.1
2537.1 - 3072.7
3072.7 - 3518.3
3518.3 - 3963.9
3963.9 - 4409.5
4409.5 - 4437.9
4437.9 - 5559.0
> 5559.0
Kenya: Incidence Rate (per 100,000)
Incidence Rate
< 1469.9
1469.9 - 1749.5
1749.5 - 2757.2
2757.2 - 3764.9
3764.9 - 4181.9
4181.9 - 4598.9
4598.9 - 5015.8
5015.8 - 5295.4
5295.4 - 6144.7
> 6144.7
Costa Rica: Incidence Rate (per 100,000)
Incidence Rate
< 1053.6
1053.6 - 1420.5
1420.5 - 2211.9
2211.9 - 3003.3
3003.3 - 3384.5
3384.5 - 3765.7
3765.7 - 4146.9
4146.9 - 4243.0
4243.0 - 5187.9
> 5187.9
Female
Male
Sexes Combined
Age
20-24 years
15-19 years
10-14 years
Year
Incidence Rate (per 100,000)
Age
10-24 years

Figure S5

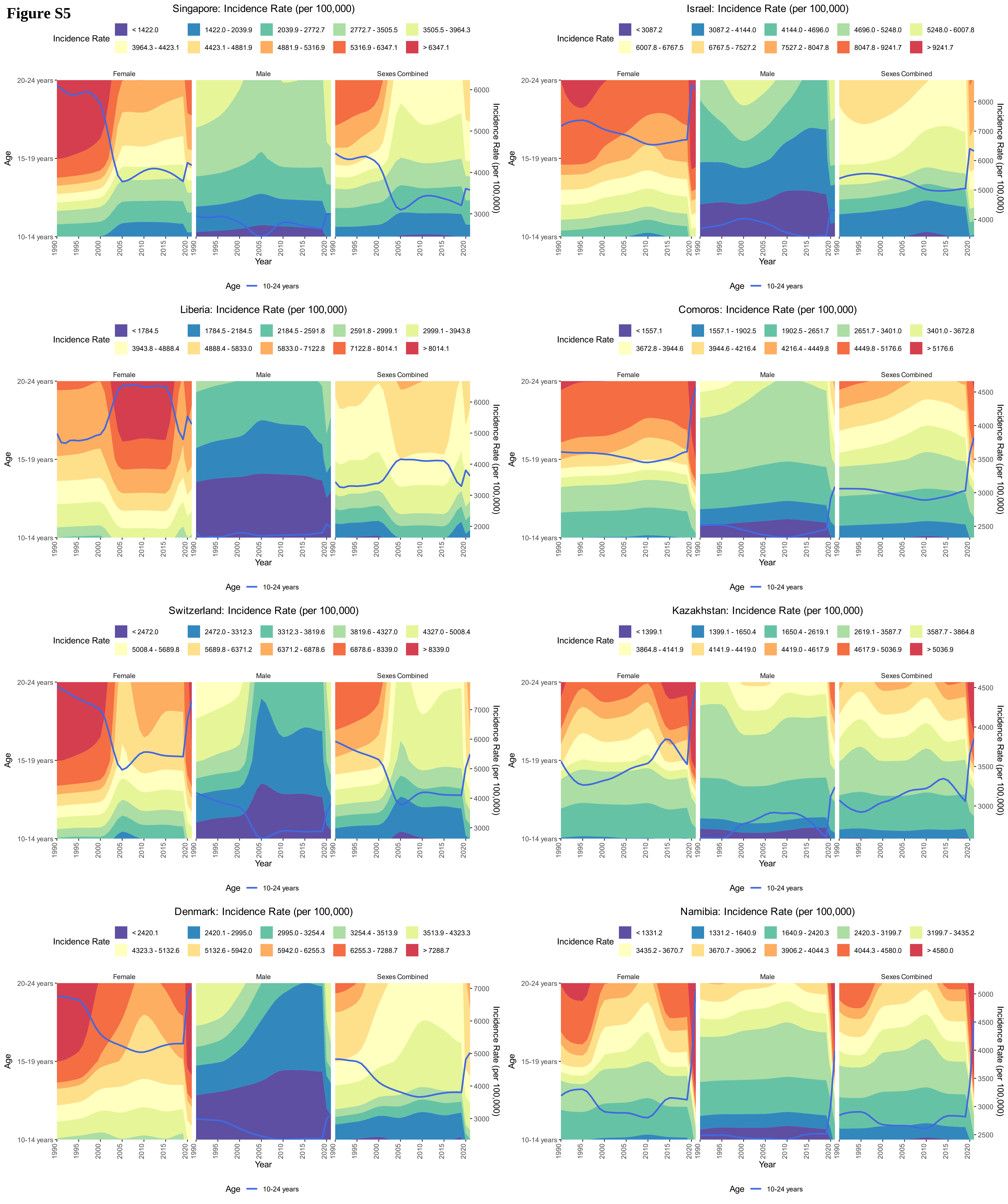
Singapore: Incidence Rate (per 100,000)
Incidence Rate
< 1422.0
1422.0 - 2039.9
2039.9 - 2772.7
2772.7 - 3505.5
3505.5 - 3964.3
3964.3 - 4423.1
4423.1 - 4881.9
4881.9 - 5316.9
5316.9 - 6347.1
> 6347.1
Israel: Incidence Rate (per 100,000)
< 3087.2
3087.2 - 4144.0
4144.0 - 4696.0
4696.0 - 5248.0
5248.0 - 6007.8
6007.8 - 6767.5
6767.5 - 7527.2
7527.2 - 8047.8
8047.8 - 9241.7
> 9241.7
Liberia: Incidence Rate (per 100,000)
< 1784.5
1784.5 - 2184.5
2184.5 - 2591.8
2591.8 - 2999.1
2999.1 - 3943.8
3943.8 - 4888.4
4888.4 - 5833.0
5833.0 - 7122.8
7122.8 - 8014.1
> 8014.1
Comoros: Incidence Rate (per 100,000)
< 1557.1
1557.1 - 1902.5
1902.5 - 2651.7
2651.7 - 3401.0
3401.0 - 3672.8
3672.8 - 3944.6
3944.6 - 4216.4
4216.4 - 4449.8
4449.8 - 5176.6
> 5176.6
Switzerland: Incidence Rate (per 100,000)
< 2472.0
2472.0 - 3312.3
3312.3 - 3819.6
3819.6 - 4327.0
4327.0 - 5008.4
5008.4 - 5689.8
5689.8 - 6371.2
6371.2 - 6878.6
6878.6 - 8339.0
> 8339.0
Kazakhstan: Incidence Rate (per 100,000)
< 1399.1
1399.1 - 1650.4
1650.4 - 2619.1
2619.1 - 3587.7
3587.7 - 3864.8
3864.8 - 4141.9
4141.9 - 4419.0
4419.0 - 4617.9
4617.9 - 5036.9
> 5036.9
Denmark: Incidence Rate (per 100,000)
< 2420.1
2420.1 - 2995.0
2995.0 - 3254.4
3254.4 - 3513.9
3513.9 - 4323.3
4323.3 - 5132.6
5132.6 - 5942.0
5942.0 - 6255.3
6255.3 - 7288.7
> 7288.7
Namibia: Incidence Rate (per 100,000)
< 1331.2
1331.2 - 1640.9
1640.9 - 2420.3
2420.3 - 3199.7
3199.7 - 3435.2
3435.2 - 3670.7
3670.7 - 3906.2
3906.2 - 4044.3
4044.3 - 4580.0
> 4580.0
Female
Male
Sexes Combined
Age
20-24 years
15-19 years
10-14 years
Year
Incidence Rate (per 100,000)
Age
10-24 years

Figure S5

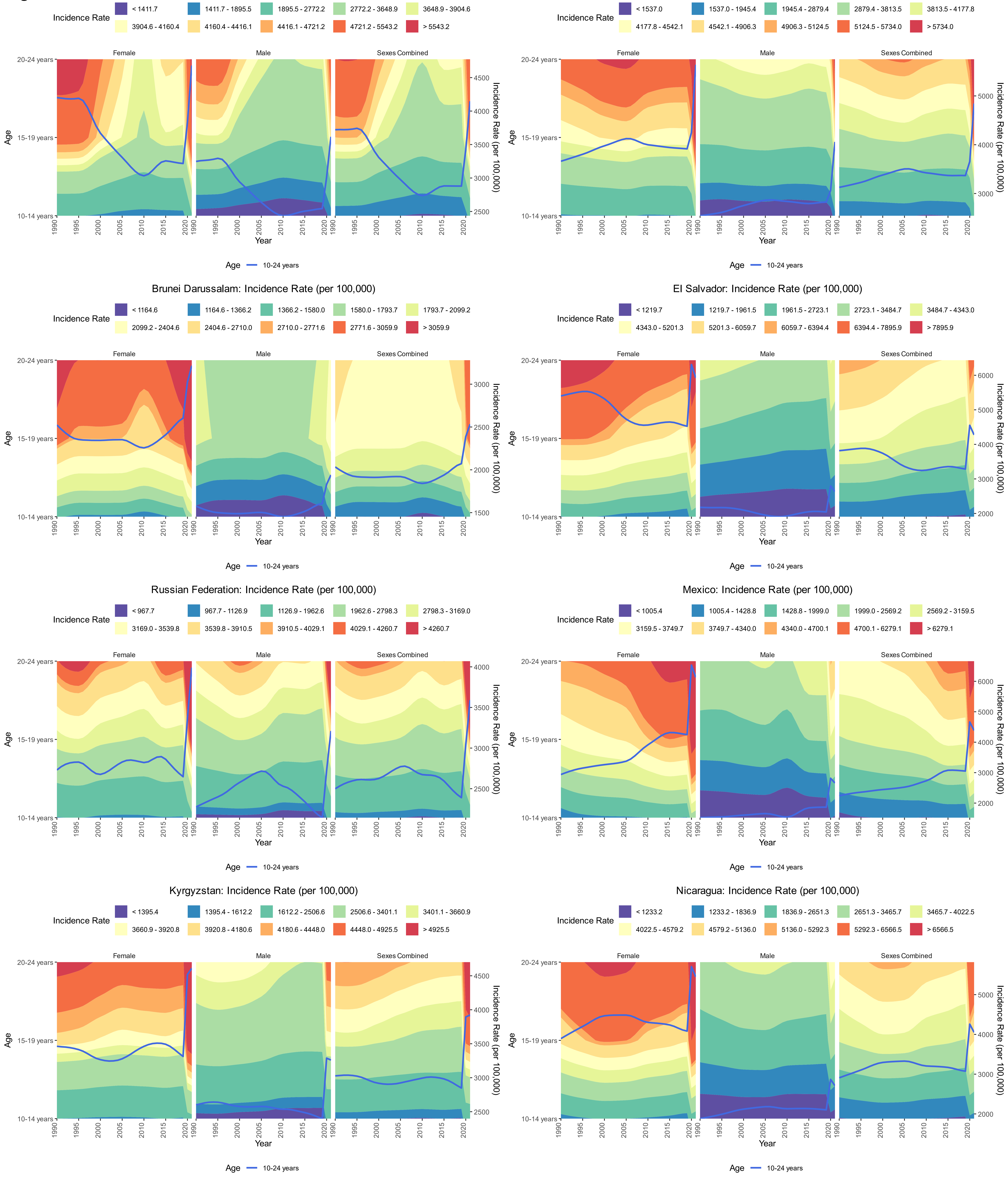

Sri Lanka: Incidence Rate (per 100,000)
Incidence Rate
< 1411.7
1411.7 - 1895.5
1895.5 - 2772.2
2772.2 - 3648.9
3648.9 - 3904.6
3904.6 - 4160.4
4160.4 - 4416.1
4416.1 - 4721.2
4721.2 - 5543.2
> 5543.2
Female
Male
Sexes Combined
Age
20-24 years
15-19 years
10-14 years
Year
Incidence Rate (per 100,000)
Age
10-24 years
Botswana: Incidence Rate (per 100,000)
Incidence Rate
< 1537.0
1537.0 - 1945.4
1945.4 - 2879.4
2879.4 - 3813.5
3813.5 - 4177.8
4177.8 - 4542.1
4542.1 - 4906.3
4906.3 - 5124.5
5124.5 - 5734.0
> 5734.0
Brunei Darussalam: Incidence Rate (per 100,000)
Incidence Rate
< 1164.6
1164.6 - 1366.2
1366.2 - 1580.0
1580.0 - 1793.7
1793.7 - 2099.2
2099.2 - 2404.6
2404.6 - 2710.0
2710.0 - 2771.6
2771.6 - 3059.9
> 3059.9
El Salvador: Incidence Rate (per 100,000)
Incidence Rate
< 1219.7
1219.7 - 1961.5
1961.5 - 2723.1
2723.1 - 3484.7
3484.7 - 4343.0
4343.0 - 5201.3
5201.3 - 6059.7
6059.7 - 6394.4
6394.4 - 7895.9
> 7895.9
Russian Federation: Incidence Rate (per 100,000)
Incidence Rate
< 967.7
967.7 - 1126.9
1126.9 - 1962.6
1962.6 - 2798.3
2798.3 - 3169.0
3169.0 - 3539.8
3539.8 - 3910.5
3910.5 - 4029.1
4029.1 - 4260.7
> 4260.7
Mexico: Incidence Rate (per 100,000)
Incidence Rate
< 1005.4
1005.4 - 1428.8
1428.8 - 1999.0
1999.0 - 2569.2
2569.2 - 3159.5
3159.5 - 3749.7
3749.7 - 4340.0
4340.0 - 4700.1
4700.1 - 6279.1
> 6279.1
Kyrgyzstan: Incidence Rate (per 100,000)
Incidence Rate
< 1395.4
1395.4 - 1612.2
1612.2 - 2506.6
2506.6 - 3401.1
3401.1 - 3660.9
3660.9 - 3920.8
3920.8 - 4180.6
4180.6 - 4448.0
4448.0 - 4925.5
> 4925.5
Nicaragua: Incidence Rate (per 100,000)
Incidence Rate
< 1233.2
1233.2 - 1836.9
1836.9 - 2651.3
2651.3 - 3465.7
3465.7 - 4022.5
4022.5 - 4579.2
4579.2 - 5136.0
5136.0 - 5292.3
5292.3 - 6566.5
> 6566.5

Figure S5

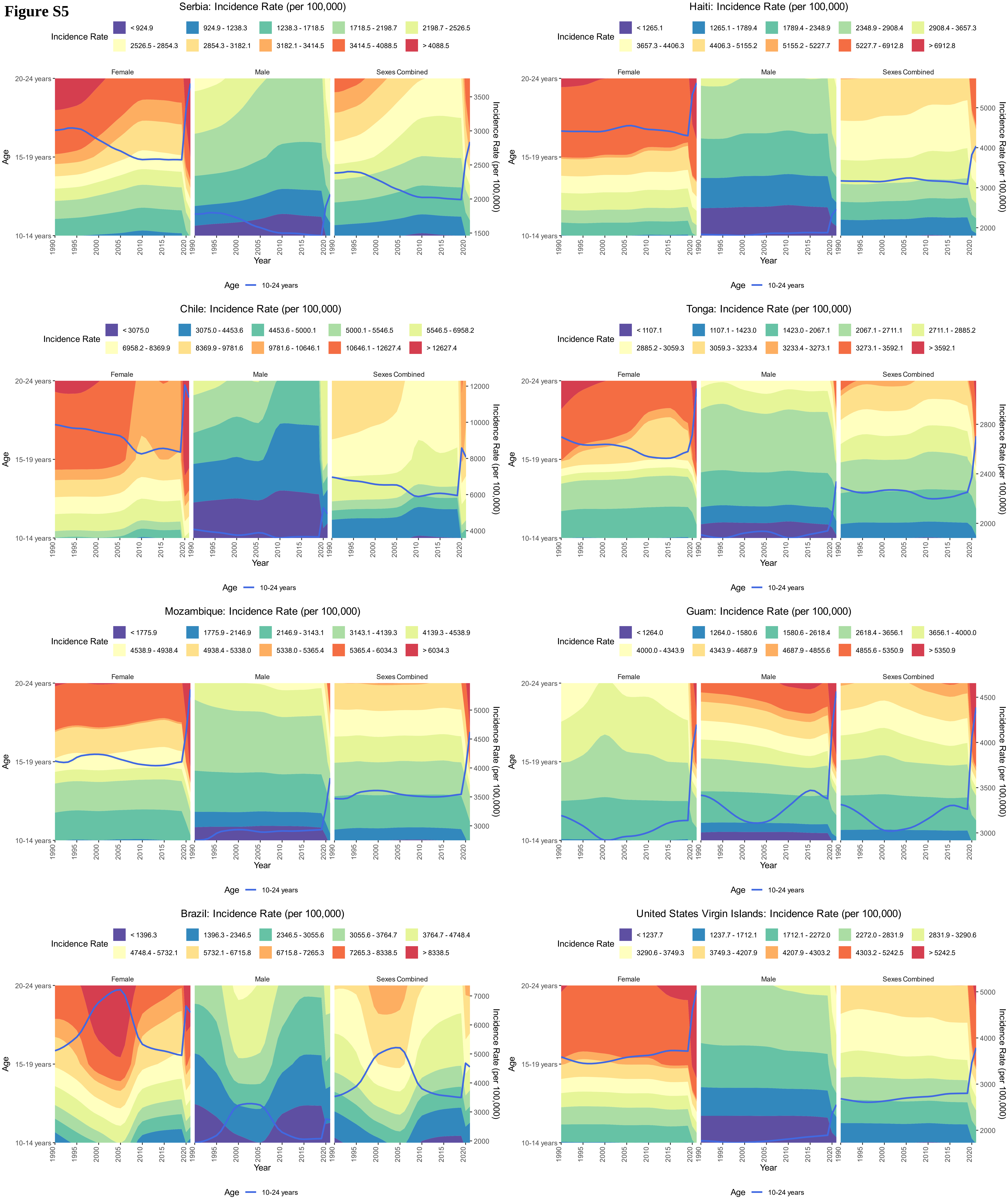

Serbia: Incidence Rate (per 100,000)
Incidence Rate
< 924.9
924.9 - 1238.3
1238.3 - 1718.5
1718.5 - 2198.7
2198.7 - 2526.5
2526.5 - 2854.3
2854.3 - 3182.1
3182.1 - 3414.5
3414.5 - 4088.5
> 4088.5
Female
Male
Sexes Combined
Age
Year
Incidence Rate (per 100,000)
Age — 10-24 years
Haiti: Incidence Rate (per 100,000)
Incidence Rate
< 1265.1
1265.1 - 1789.4
1789.4 - 2348.9
2348.9 - 2908.4
2908.4 - 3657.3
3657.3 - 4406.3
4406.3 - 5155.2
5155.2 - 5227.7
5227.7 - 6912.8
> 6912.8
Chile: Incidence Rate (per 100,000)
Incidence Rate
< 3075.0
3075.0 - 4453.6
4453.6 - 5000.1
5000.1 - 5546.5
5546.5 - 6958.2
6958.2 - 8369.9
8369.9 - 9781.6
9781.6 - 10646.1
10646.1 - 12627.4
> 12627.4
Tonga: Incidence Rate (per 100,000)
Incidence Rate
< 1107.1
1107.1 - 1423.0
1423.0 - 2067.1
2067.1 - 2711.1
2711.1 - 2885.2
2885.2 - 3059.3
3059.3 - 3233.4
3233.4 - 3273.1
3273.1 - 3592.1
> 3592.1
Mozambique: Incidence Rate (per 100,000)
Incidence Rate
< 1775.9
1775.9 - 2146.9
2146.9 - 3143.1
3143.1 - 4139.3
4139.3 - 4538.9
4538.9 - 4938.4
4938.4 - 5338.0
5338.0 - 5365.4
5365.4 - 6034.3
> 6034.3
Guam: Incidence Rate (per 100,000)
Incidence Rate
< 1264.0
1264.0 - 1580.6
1580.6 - 2618.4
2618.4 - 3656.1
3656.1 - 4000.0
4000.0 - 4343.9
4343.9 - 4687.9
4687.9 - 4855.6
4855.6 - 5350.9
> 5350.9
Brazil: Incidence Rate (per 100,000)
Incidence Rate
< 1396.3
1396.3 - 2346.5
2346.5 - 3055.6
3055.6 - 3764.7
3764.7 - 4748.4
4748.4 - 5732.1
5732.1 - 6715.8
6715.8 - 7265.3
7265.3 - 8338.5
> 8338.5
United States Virgin Islands: Incidence Rate (per 100,000)
Incidence Rate
< 1237.7
1237.7 - 1712.1
1712.1 - 2272.0
2272.0 - 2831.9
2831.9 - 3290.6
3290.6 - 3749.3
3749.3 - 4207.9
4207.9 - 4303.2
4303.2 - 5242.5
> 5242.5

Figure S5

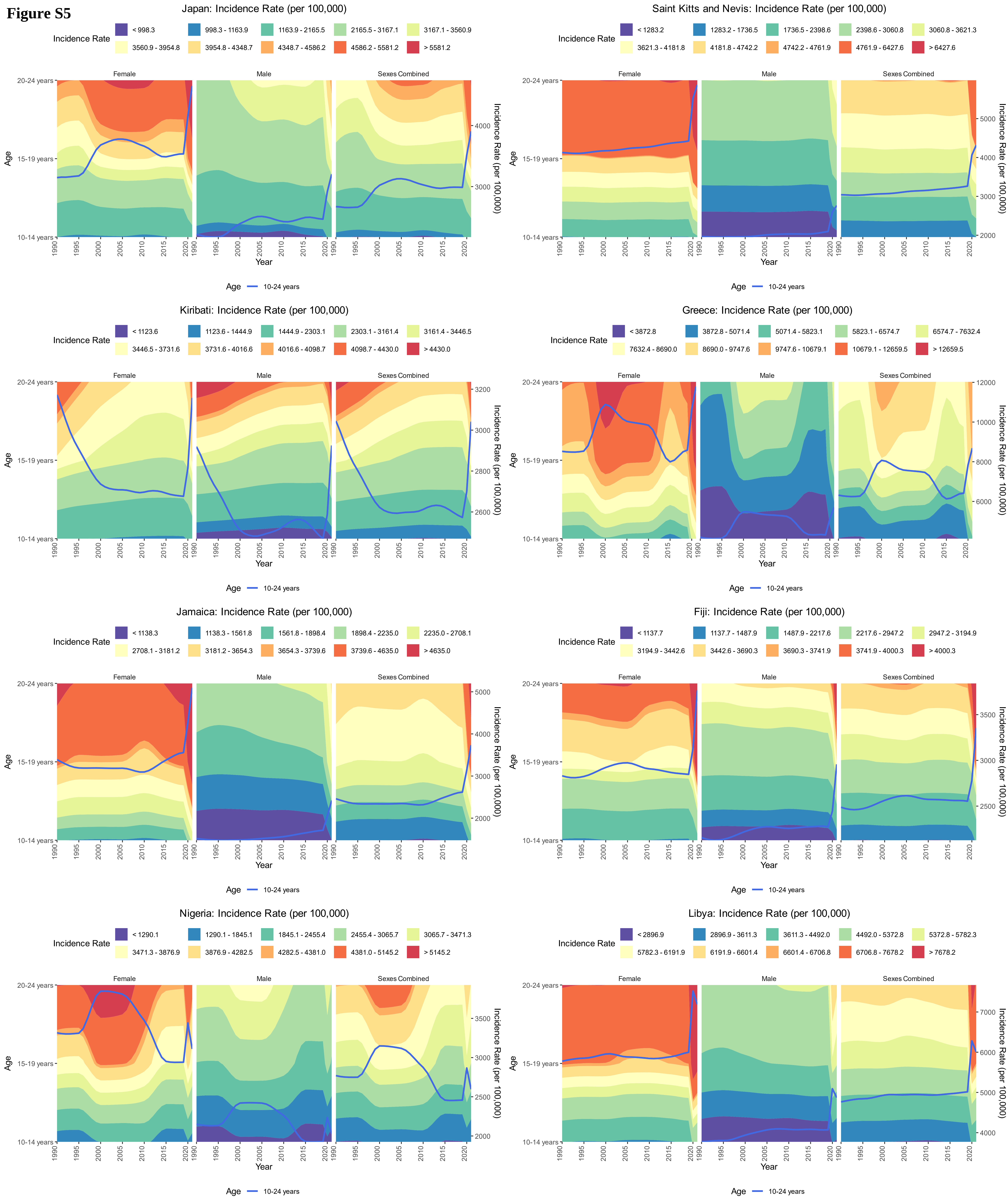

Japan: Incidence Rate (per 100,000)
Incidence Rate
< 998.3
998.3 - 1163.9
1163.9 - 2165.5
2165.5 - 3167.1
3167.1 - 3560.9
3560.9 - 3954.8
3954.8 - 4348.7
4348.7 - 4586.2
4586.2 - 5581.2
> 5581.2
Saint Kitts and Nevis: Incidence Rate (per 100,000)
Incidence Rate
< 1283.2
1283.2 - 1736.5
1736.5 - 2398.6
2398.6 - 3060.8
3060.8 - 3621.3
3621.3 - 4181.8
4181.8 - 4742.2
4742.2 - 4761.9
4761.9 - 6427.6
> 6427.6
Kiribati: Incidence Rate (per 100,000)
Incidence Rate
< 1123.6
1123.6 - 1444.9
1444.9 - 2303.1
2303.1 - 3161.4
3161.4 - 3446.5
3446.5 - 3731.6
3731.6 - 4016.6
4016.6 - 4098.7
4098.7 - 4430.0
> 4430.0
Greece: Incidence Rate (per 100,000)
Incidence Rate
< 3872.8
3872.8 - 5071.4
5071.4 - 5823.1
5823.1 - 6574.7
6574.7 - 7632.4
7632.4 - 8690.0
8690.0 - 9747.6
9747.6 - 10679.1
10679.1 - 12659.5
> 12659.5
Jamaica: Incidence Rate (per 100,000)
Incidence Rate
< 1138.3
1138.3 - 1561.8
1561.8 - 1898.4
1898.4 - 2235.0
2235.0 - 2708.1
2708.1 - 3181.2
3181.2 - 3654.3
3654.3 - 3739.6
3739.6 - 4635.0
> 4635.0
Fiji: Incidence Rate (per 100,000)
Incidence Rate
< 1137.7
1137.7 - 1487.9
1487.9 - 2217.6
2217.6 - 2947.2
2947.2 - 3194.9
3194.9 - 3442.6
3442.6 - 3690.3
3690.3 - 3741.9
3741.9 - 4000.3
> 4000.3
Nigeria: Incidence Rate (per 100,000)
Incidence Rate
< 1290.1
1290.1 - 1845.1
1845.1 - 2455.4
2455.4 - 3065.7
3065.7 - 3471.3
3471.3 - 3876.9
3876.9 - 4282.5
4282.5 - 4381.0
4381.0 - 5145.2
> 5145.2
Libya: Incidence Rate (per 100,000)
Incidence Rate
< 2896.9
2896.9 - 3611.3
3611.3 - 4492.0
4492.0 - 5372.8
5372.8 - 5782.3
5782.3 - 6191.9
6191.9 - 6601.4
6601.4 - 6706.8
6706.8 - 7678.2
> 7678.2
Female
Male
Sexes Combined
20-24 years
15-19 years
10-14 years
Age
Year
Incidence Rate (per 100,000)
Age
10-24 years

Figure S5

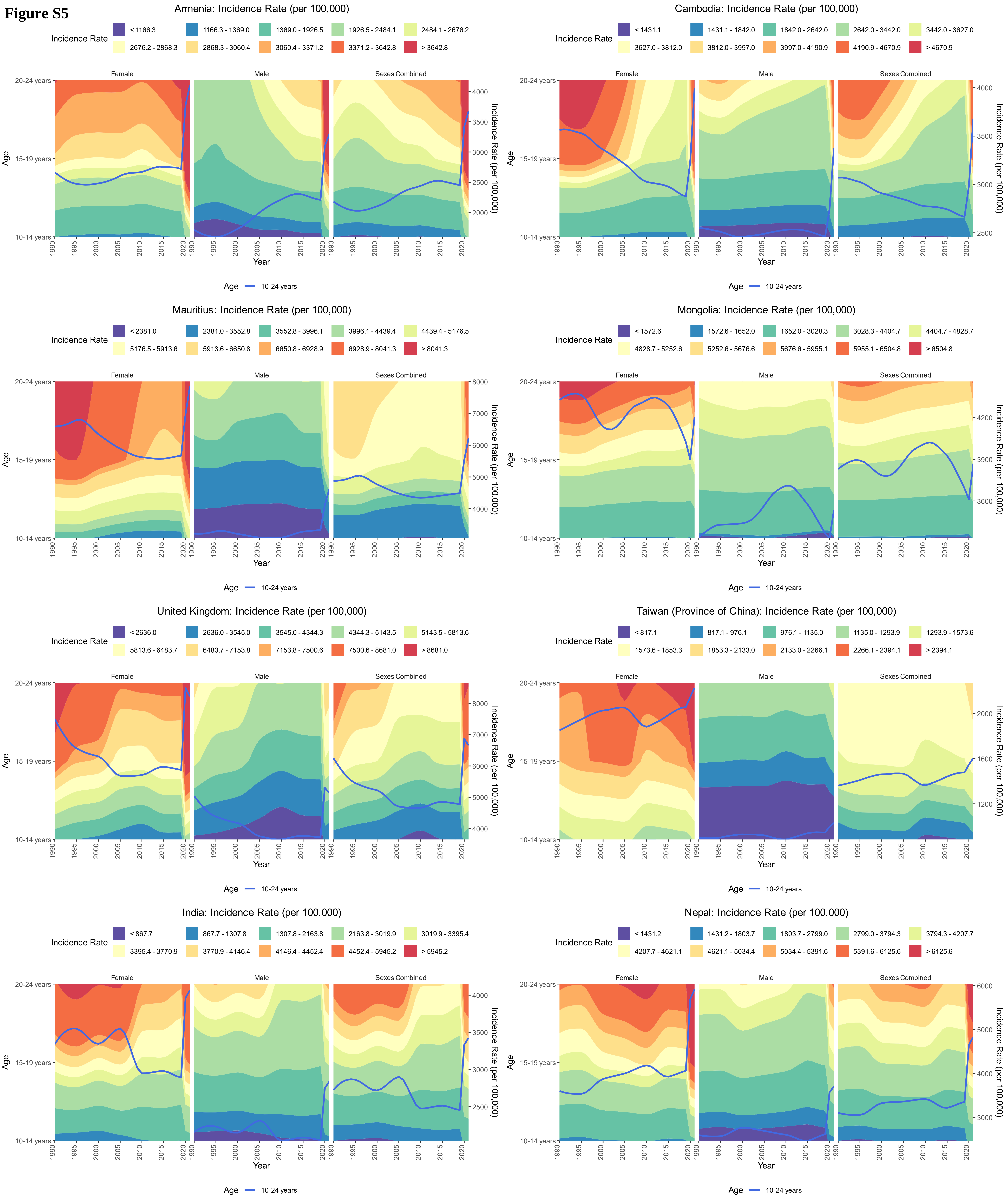

Armenia: Incidence Rate (per 100,000)
Incidence Rate
< 1166.3
1166.3 - 1369.0
1369.0 - 1926.5
1926.5 - 2484.1
2484.1 - 2676.2
2676.2 - 2868.3
2868.3 - 3060.4
3060.4 - 3371.2
3371.2 - 3642.8
> 3642.8
Cambodia: Incidence Rate (per 100,000)
< 1431.1
1431.1 - 1842.0
1842.0 - 2642.0
2642.0 - 3442.0
3442.0 - 3627.0
3627.0 - 3812.0
3812.0 - 3997.0
3997.0 - 4190.9
4190.9 - 4670.9
> 4670.9
Mauritius: Incidence Rate (per 100,000)
< 2381.0
2381.0 - 3552.8
3552.8 - 3996.1
3996.1 - 4439.4
4439.4 - 5176.5
5176.5 - 5913.6
5913.6 - 6650.8
6650.8 - 6928.9
6928.9 - 8041.3
> 8041.3
Mongolia: Incidence Rate (per 100,000)
< 1572.6
1572.6 - 1652.0
1652.0 - 3028.3
3028.3 - 4404.7
4404.7 - 4828.7
4828.7 - 5252.6
5252.6 - 5676.6
5676.6 - 5955.1
5955.1 - 6504.8
> 6504.8
United Kingdom: Incidence Rate (per 100,000)
< 2636.0
2636.0 - 3545.0
3545.0 - 4344.3
4344.3 - 5143.5
5143.5 - 5813.6
5813.6 - 6483.7
6483.7 - 7153.8
7153.8 - 7500.6
7500.6 - 8681.0
> 8681.0
Taiwan (Province of China): Incidence Rate (per 100,000)
< 817.1
817.1 - 976.1
976.1 - 1135.0
1135.0 - 1293.9
1293.9 - 1573.6
1573.6 - 1853.3
1853.3 - 2133.0
2133.0 - 2266.1
2266.1 - 2394.1
> 2394.1
India: Incidence Rate (per 100,000)
< 867.7
867.7 - 1307.8
1307.8 - 2163.8
2163.8 - 3019.9
3019.9 - 3395.4
3395.4 - 3770.9
3770.9 - 4146.4
4146.4 - 4452.4
4452.4 - 5945.2
> 5945.2
Nepal: Incidence Rate (per 100,000)
< 1431.2
1431.2 - 1803.7
1803.7 - 2799.0
2799.0 - 3794.3
3794.3 - 4207.7
4207.7 - 4621.1
4621.1 - 5034.4
5034.4 - 5391.6
5391.6 - 6125.6
> 6125.6
Female
Male
Sexes Combined
Age
20-24 years
15-19 years
10-14 years
Year
Incidence Rate (per 100,000)
Age — 10-24 years

Figure S5

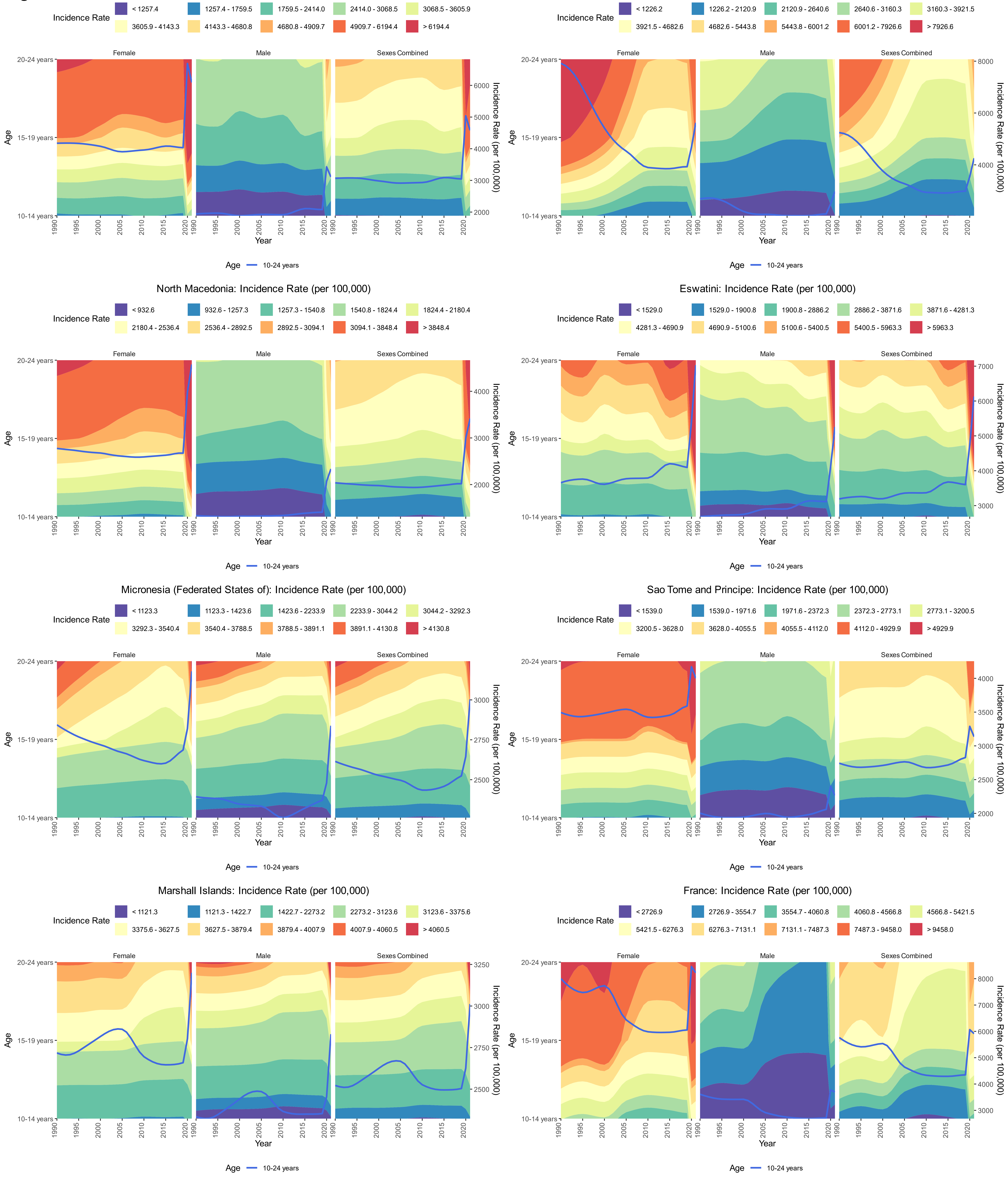

Ecuador: Incidence Rate (per 100,000)
Incidence Rate
< 1257.4
1257.4 - 1759.5
1759.5 - 2414.0
2414.0 - 3068.5
3068.5 - 3605.9
3605.9 - 4143.3
4143.3 - 4680.8
4680.8 - 4909.7
4909.7 - 6194.4
> 6194.4
Cuba: Incidence Rate (per 100,000)
< 1226.2
1226.2 - 2120.9
2120.9 - 2640.6
2640.6 - 3160.3
3160.3 - 3921.5
3921.5 - 4682.6
4682.6 - 5443.8
5443.8 - 6001.2
6001.2 - 7926.6
> 7926.6
North Macedonia: Incidence Rate (per 100,000)
< 932.6
932.6 - 1257.3
1257.3 - 1540.8
1540.8 - 1824.4
1824.4 - 2180.4
2180.4 - 2536.4
2536.4 - 2892.5
2892.5 - 3094.1
3094.1 - 3848.4
> 3848.4
Eswatini: Incidence Rate (per 100,000)
< 1529.0
1529.0 - 1900.8
1900.8 - 2886.2
2886.2 - 3871.6
3871.6 - 4281.3
4281.3 - 4690.9
4690.9 - 5100.6
5100.6 - 5400.5
5400.5 - 5963.3
> 5963.3
Micronesia (Federated States of): Incidence Rate (per 100,000)
< 1123.3
1123.3 - 1423.6
1423.6 - 2233.9
2233.9 - 3044.2
3044.2 - 3292.3
3292.3 - 3540.4
3540.4 - 3788.5
3788.5 - 3891.1
3891.1 - 4130.8
> 4130.8
Sao Tome and Principe: Incidence Rate (per 100,000)
< 1539.0
1539.0 - 1971.6
1971.6 - 2372.3
2372.3 - 2773.1
2773.1 - 3200.5
3200.5 - 3628.0
3628.0 - 4055.5
4055.5 - 4112.0
4112.0 - 4929.9
> 4929.9
Marshall Islands: Incidence Rate (per 100,000)
< 1121.3
1121.3 - 1422.7
1422.7 - 2273.2
2273.2 - 3123.6
3123.6 - 3375.6
3375.6 - 3627.5
3627.5 - 3879.4
3879.4 - 4007.9
4007.9 - 4060.5
> 4060.5
France: Incidence Rate (per 100,000)
< 2726.9
2726.9 - 3554.7
3554.7 - 4060.8
4060.8 - 4566.8
4566.8 - 5421.5
5421.5 - 6276.3
6276.3 - 7131.1
7131.1 - 7487.3
7487.3 - 9458.0
> 9458.0
Female
Male
Sexes Combined
Age
20-24 years
15-19 years
10-14 years
Year
Incidence Rate (per 100,000)
Age — 10-24 years

Figure S5

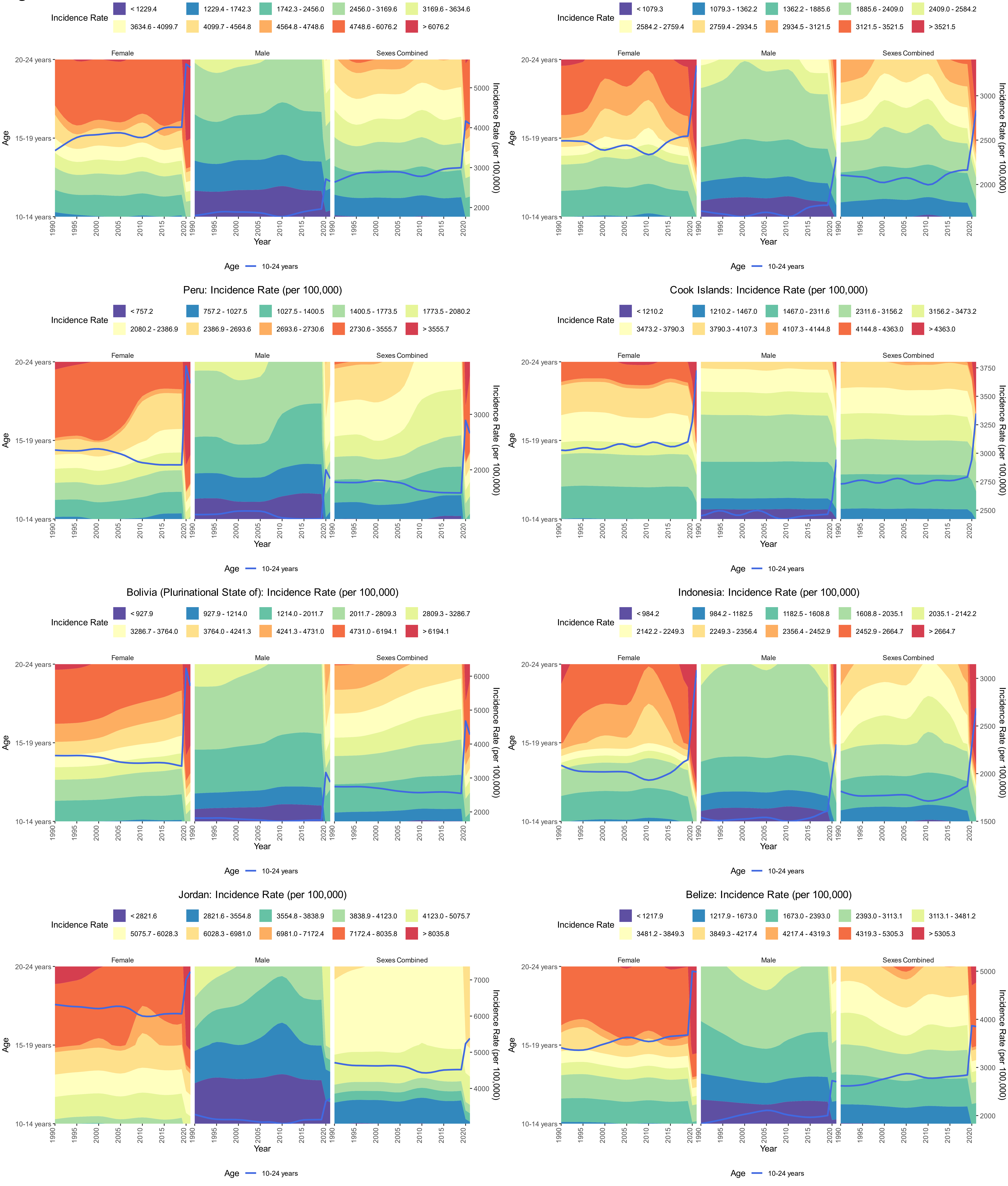
Guatemala: Incidence Rate (per 100,000)
Incidence Rate
< 1229.4
1229.4 - 1742.3
1742.3 - 2456.0
2456.0 - 3169.6
3169.6 - 3634.6
3634.6 - 4099.7
4099.7 - 4564.8
4564.8 - 4748.6
4748.6 - 6076.2
> 6076.2
Female
Male
Sexes Combined
Age
Year
Incidence Rate (per 100,000)
Age — 10-24 years
Zimbabwe: Incidence Rate (per 100,000)
< 1079.3
1079.3 - 1362.2
1362.2 - 1885.6
1885.6 - 2409.0
2409.0 - 2584.2
2584.2 - 2759.4
2759.4 - 2934.5
2934.5 - 3121.5
3121.5 - 3521.5
> 3521.5
Peru: Incidence Rate (per 100,000)
< 757.2
757.2 - 1027.5
1027.5 - 1400.5
1400.5 - 1773.5
1773.5 - 2080.2
2080.2 - 2386.9
2386.9 - 2693.6
2693.6 - 2730.6
2730.6 - 3555.7
> 3555.7
Cook Islands: Incidence Rate (per 100,000)
< 1210.2
1210.2 - 1467.0
1467.0 - 2311.6
2311.6 - 3156.2
3156.2 - 3473.2
3473.2 - 3790.3
3790.3 - 4107.3
4107.3 - 4144.8
4144.8 - 4363.0
> 4363.0
Bolivia (Plurinational State of): Incidence Rate (per 100,000)
< 927.9
927.9 - 1214.0
1214.0 - 2011.7
2011.7 - 2809.3
2809.3 - 3286.7
3286.7 - 3764.0
3764.0 - 4241.3
4241.3 - 4731.0
4731.0 - 6194.1
> 6194.1
Indonesia: Incidence Rate (per 100,000)
< 984.2
984.2 - 1182.5
1182.5 - 1608.8
1608.8 - 2035.1
2035.1 - 2142.2
2142.2 - 2249.3
2249.3 - 2356.4
2356.4 - 2452.9
2452.9 - 2664.7
> 2664.7
Jordan: Incidence Rate (per 100,000)
< 2821.6
2821.6 - 3554.8
3554.8 - 3838.9
3838.9 - 4123.0
4123.0 - 5075.7
5075.7 - 6028.3
6028.3 - 6981.0
6981.0 - 7172.4
7172.4 - 8035.8
> 8035.8
Belize: Incidence Rate (per 100,000)
< 1217.9
1217.9 - 1673.0
1673.0 - 2393.0
2393.0 - 3113.1
3113.1 - 3481.2
3481.2 - 3849.3
3849.3 - 4217.4
4217.4 - 4319.3
4319.3 - 5305.3
> 5305.3
20-24 years
15-19 years
10-14 years
1990
1995
2000
2005
2010
2015
2020

Figure S5

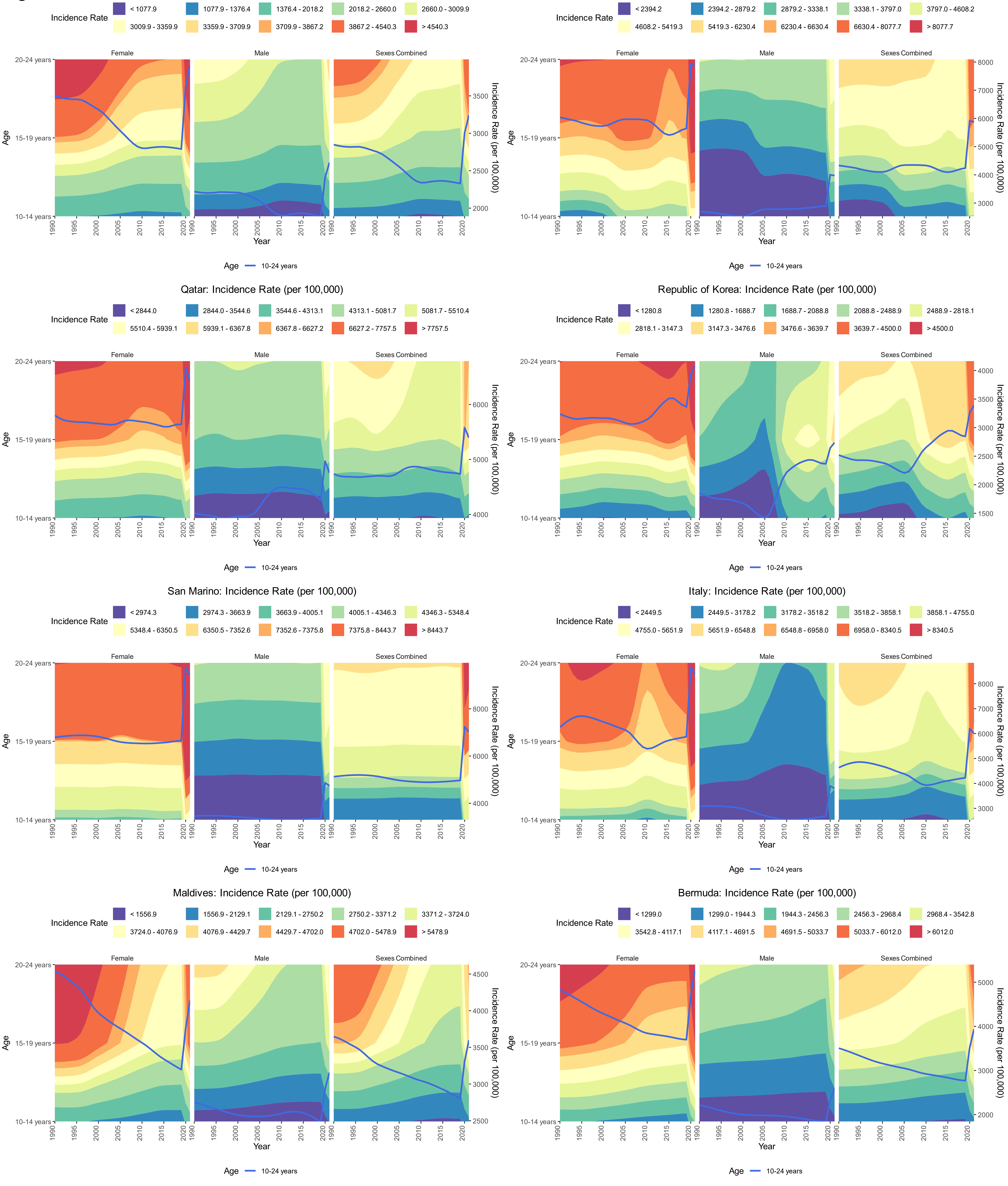

Croatia: Incidence Rate (per 100,000)
Incidence Rate
< 1077.9
1077.9 - 1376.4
1376.4 - 2018.2
2018.2 - 2660.0
2660.0 - 3009.9
3009.9 - 3359.9
3359.9 - 3709.9
3709.9 - 3867.2
3867.2 - 4540.3
> 4540.3
Netherlands: Incidence Rate (per 100,000)
Incidence Rate
< 2394.2
2394.2 - 2879.2
2879.2 - 3338.1
3338.1 - 3797.0
3797.0 - 4608.2
4608.2 - 5419.3
5419.3 - 6230.4
6230.4 - 6630.4
6630.4 - 8077.7
> 8077.7
Qatar: Incidence Rate (per 100,000)
Incidence Rate
< 2844.0
2844.0 - 3544.6
3544.6 - 4313.1
4313.1 - 5081.7
5081.7 - 5510.4
5510.4 - 5939.1
5939.1 - 6367.8
6367.8 - 6627.2
6627.2 - 7757.5
> 7757.5
Republic of Korea: Incidence Rate (per 100,000)
Incidence Rate
< 1280.8
1280.8 - 1688.7
1688.7 - 2088.8
2088.8 - 2488.9
2488.9 - 2818.1
2818.1 - 3147.3
3147.3 - 3476.6
3476.6 - 3639.7
3639.7 - 4500.0
> 4500.0
San Marino: Incidence Rate (per 100,000)
Incidence Rate
< 2974.3
2974.3 - 3663.9
3663.9 - 4005.1
4005.1 - 4346.3
4346.3 - 5348.4
5348.4 - 6350.5
6350.5 - 7352.6
7352.6 - 7375.8
7375.8 - 8443.7
> 8443.7
Italy: Incidence Rate (per 100,000)
Incidence Rate
< 2449.5
2449.5 - 3178.2
3178.2 - 3518.2
3518.2 - 3858.1
3858.1 - 4755.0
4755.0 - 5651.9
5651.9 - 6548.8
6548.8 - 6958.0
6958.0 - 8340.5
> 8340.5
Maldives: Incidence Rate (per 100,000)
Incidence Rate
< 1556.9
1556.9 - 2129.1
2129.1 - 2750.2
2750.2 - 3371.2
3371.2 - 3724.0
3724.0 - 4076.9
4076.9 - 4429.7
4429.7 - 4702.0
4702.0 - 5478.9
> 5478.9
Bermuda: Incidence Rate (per 100,000)
Incidence Rate
< 1299.0
1299.0 - 1944.3
1944.3 - 2456.3
2456.3 - 2968.4
2968.4 - 3542.8
3542.8 - 4117.1
4117.1 - 4691.5
4691.5 - 5033.7
5033.7 - 6012.0
> 6012.0
Female
Male
Sexes Combined
Age
20-24 years
15-19 years
10-14 years
Year
Incidence Rate (per 100,000)
Age
10-24 years

Figure S5

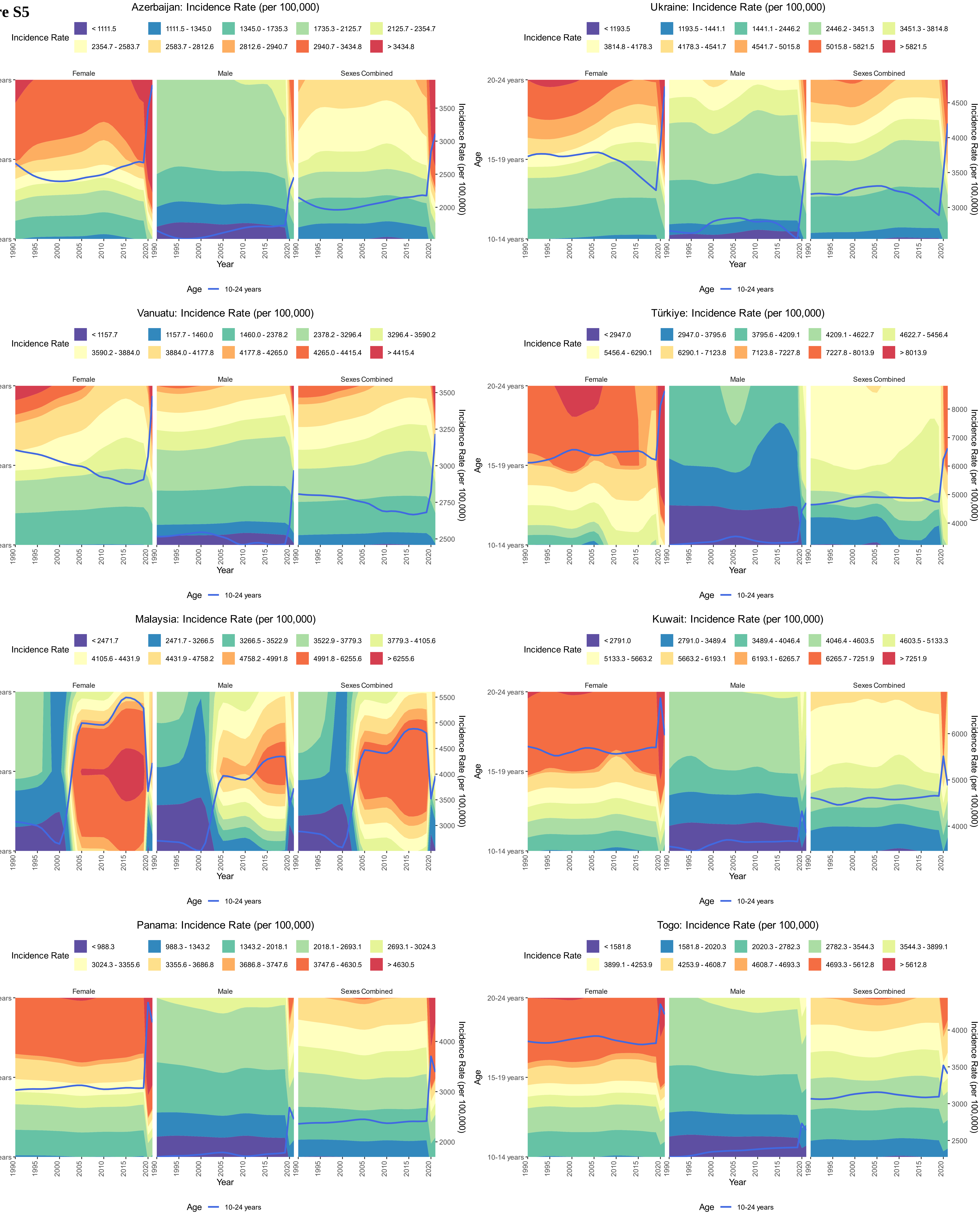

Azerbaijan: Incidence Rate (per 100,000)
Incidence Rate
< 1111.5
1111.5 - 1345.0
1345.0 - 1735.3
1735.3 - 2125.7
2125.7 - 2354.7
2354.7 - 2583.7
2583.7 - 2812.6
2812.6 - 2940.7
2940.7 - 3434.8
> 3434.8
Ukraine: Incidence Rate (per 100,000)
< 1193.5
1193.5 - 1441.1
1441.1 - 2446.2
2446.2 - 3451.3
3451.3 - 3814.8
3814.8 - 4178.3
4178.3 - 4541.7
4541.7 - 5015.8
5015.8 - 5821.5
> 5821.5
Vanuatu: Incidence Rate (per 100,000)
< 1157.7
1157.7 - 1460.0
1460.0 - 2378.2
2378.2 - 3296.4
3296.4 - 3590.2
3590.2 - 3884.0
3884.0 - 4177.8
4177.8 - 4265.0
4265.0 - 4415.4
> 4415.4
Türkiye: Incidence Rate (per 100,000)
< 2947.0
2947.0 - 3795.6
3795.6 - 4209.1
4209.1 - 4622.7
4622.7 - 5456.4
5456.4 - 6290.1
6290.1 - 7123.8
7123.8 - 7227.8
7227.8 - 8013.9
> 8013.9
Malaysia: Incidence Rate (per 100,000)
< 2471.7
2471.7 - 3266.5
3266.5 - 3522.9
3522.9 - 3779.3
3779.3 - 4105.6
4105.6 - 4431.9
4431.9 - 4758.2
4758.2 - 4991.8
4991.8 - 6255.6
> 6255.6
Kuwait: Incidence Rate (per 100,000)
< 2791.0
2791.0 - 3489.4
3489.4 - 4046.4
4046.4 - 4603.5
4603.5 - 5133.3
5133.3 - 5663.2
5663.2 - 6193.1
6193.1 - 6265.7
6265.7 - 7251.9
> 7251.9
Panama: Incidence Rate (per 100,000)
< 988.3
988.3 - 1343.2
1343.2 - 2018.1
2018.1 - 2693.1
2693.1 - 3024.3
3024.3 - 3355.6
3355.6 - 3686.8
3686.8 - 3747.6
3747.6 - 4630.5
> 4630.5
Togo: Incidence Rate (per 100,000)
< 1581.8
1581.8 - 2020.3
2020.3 - 2782.3
2782.3 - 3544.3
3544.3 - 3899.1
3899.1 - 4253.9
4253.9 - 4608.7
4608.7 - 4693.3
4693.3 - 5612.8
> 5612.8
Female
Male
Sexes Combined
Age
20-24 years
15-19 years
10-14 years
Year
Incidence Rate (per 100,000)
Age — 10-24 years

Figure S5

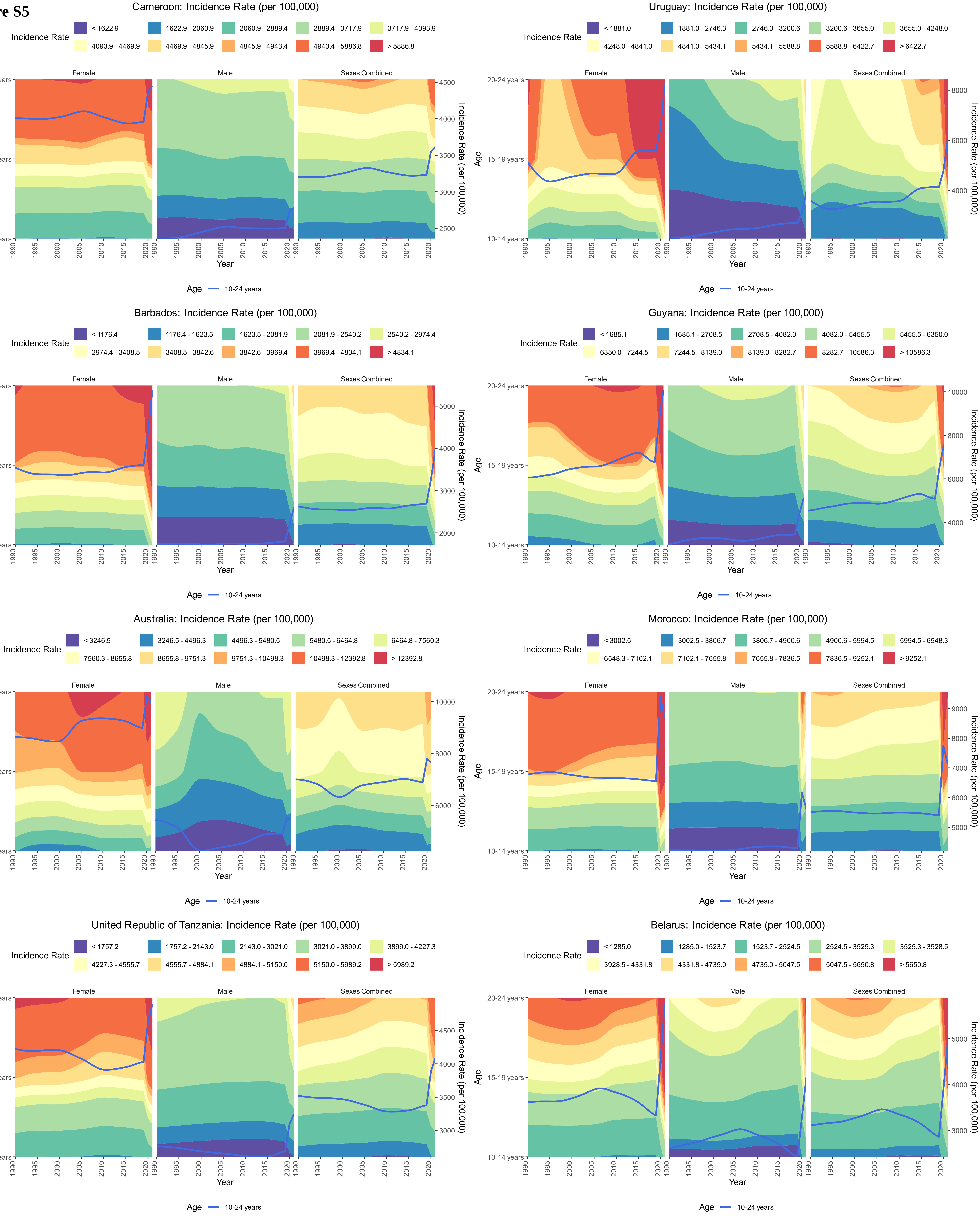

Cameroon: Incidence Rate (per 100,000)
Incidence Rate
< 1622.9
1622.9 - 2060.9
2060.9 - 2889.4
2889.4 - 3717.9
3717.9 - 4093.9
4093.9 - 4469.9
4469.9 - 4845.9
4845.9 - 4943.4
4943.4 - 5886.8
> 5886.8
Uruguay: Incidence Rate (per 100,000)
< 1881.0
1881.0 - 2746.3
2746.3 - 3200.6
3200.6 - 3655.0
3655.0 - 4248.0
4248.0 - 4841.0
4841.0 - 5434.1
5434.1 - 5588.8
5588.8 - 6422.7
> 6422.7
Barbados: Incidence Rate (per 100,000)
< 1176.4
1176.4 - 1623.5
1623.5 - 2081.9
2081.9 - 2540.2
2540.2 - 2974.4
2974.4 - 3408.5
3408.5 - 3842.6
3842.6 - 3969.4
3969.4 - 4834.1
> 4834.1
Guyana: Incidence Rate (per 100,000)
< 1685.1
1685.1 - 2708.5
2708.5 - 4082.0
4082.0 - 5455.5
5455.5 - 6350.0
6350.0 - 7244.5
7244.5 - 8139.0
8139.0 - 8282.7
8282.7 - 10586.3
> 10586.3
Australia: Incidence Rate (per 100,000)
< 3246.5
3246.5 - 4496.3
4496.3 - 5480.5
5480.5 - 6464.8
6464.8 - 7560.3
7560.3 - 8655.8
8655.8 - 9751.3
9751.3 - 10498.3
10498.3 - 12392.8
> 12392.8
Morocco: Incidence Rate (per 100,000)
< 3002.5
3002.5 - 3806.7
3806.7 - 4900.6
4900.6 - 5994.5
5994.5 - 6548.3
6548.3 - 7102.1
7102.1 - 7655.8
7655.8 - 7836.5
7836.5 - 9252.1
> 9252.1
United Republic of Tanzania: Incidence Rate (per 100,000)
< 1757.2
1757.2 - 2143.0
2143.0 - 3021.0
3021.0 - 3899.0
3899.0 - 4227.3
4227.3 - 4555.7
4555.7 - 4884.1
4884.1 - 5150.0
5150.0 - 5989.2
> 5989.2
Belarus: Incidence Rate (per 100,000)
< 1285.0
1285.0 - 1523.7
1523.7 - 2524.5
2524.5 - 3525.3
3525.3 - 3928.5
3928.5 - 4331.8
4331.8 - 4735.0
4735.0 - 5047.5
5047.5 - 5650.8
> 5650.8
Female
Male
Sexes Combined
Age
20-24 years
15-19 years
10-14 years
Year
Incidence Rate (per 100,000)
Age — 10-24 years

Figure S5

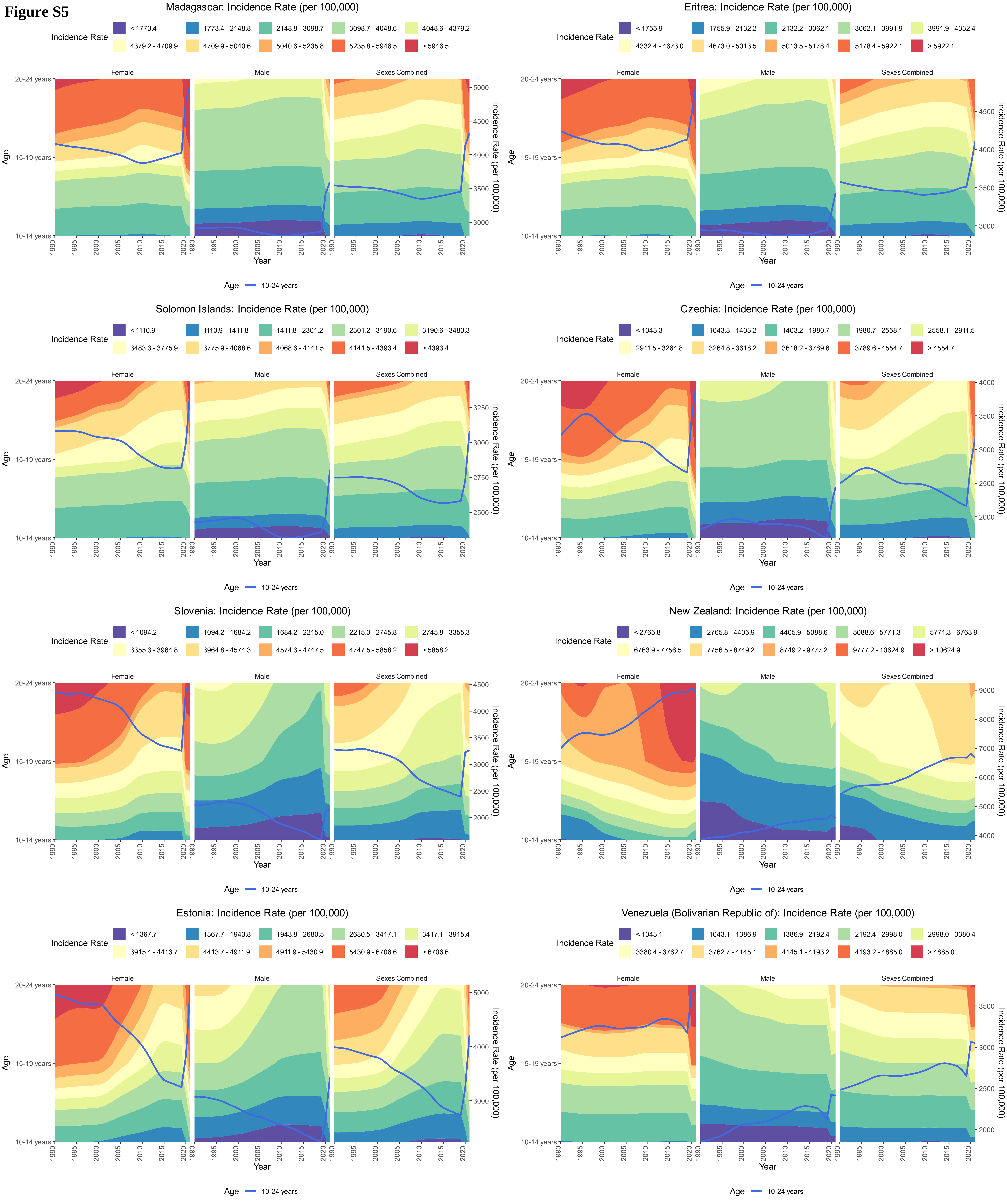

Madagascar: Incidence Rate (per 100,000)
Incidence Rate
< 1773.4
1773.4 - 2148.8
2148.8 - 3098.7
3098.7 - 4048.6
4048.6 - 4379.2
4379.2 - 4709.9
4709.9 - 5040.6
5040.6 - 5235.8
5235.8 - 5946.5
> 5946.5
Eritrea: Incidence Rate (per 100,000)
< 1755.9
1755.9 - 2132.2
2132.2 - 3062.1
3062.1 - 3991.9
3991.9 - 4332.4
4332.4 - 4673.0
4673.0 - 5013.5
5013.5 - 5178.4
5178.4 - 5922.1
> 5922.1
Solomon Islands: Incidence Rate (per 100,000)
< 1110.9
1110.9 - 1411.8
1411.8 - 2301.2
2301.2 - 3190.6
3190.6 - 3483.3
3483.3 - 3775.9
3775.9 - 4068.6
4068.6 - 4141.5
4141.5 - 4393.4
> 4393.4
Czechia: Incidence Rate (per 100,000)
< 1043.3
1043.3 - 1403.2
1403.2 - 1980.7
1980.7 - 2558.1
2558.1 - 2911.5
2911.5 - 3264.8
3264.8 - 3618.2
3618.2 - 3789.6
3789.6 - 4554.7
> 4554.7
Slovenia: Incidence Rate (per 100,000)
< 1094.2
1094.2 - 1684.2
1684.2 - 2215.0
2215.0 - 2745.8
2745.8 - 3355.3
3355.3 - 3964.8
3964.8 - 4574.3
4574.3 - 4747.5
4747.5 - 5858.2
> 5858.2
New Zealand: Incidence Rate (per 100,000)
< 2765.8
2765.8 - 4405.9
4405.9 - 5088.6
5088.6 - 5771.3
5771.3 - 6763.9
6763.9 - 7756.5
7756.5 - 8749.2
8749.2 - 9777.2
9777.2 - 10624.9
> 10624.9
Estonia: Incidence Rate (per 100,000)
< 1367.7
1367.7 - 1943.8
1943.8 - 2680.5
2680.5 - 3417.1
3417.1 - 3915.4
3915.4 - 4413.7
4413.7 - 4911.9
4911.9 - 5430.9
5430.9 - 6706.6
> 6706.6
Venezuela (Bolivarian Republic of): Incidence Rate (per 100,000)
< 1043.1
1043.1 - 1386.9
1386.9 - 2192.4
2192.4 - 2998.0
2998.0 - 3380.4
3380.4 - 3762.7
3762.7 - 4145.1
4145.1 - 4193.2
4193.2 - 4885.0
> 4885.0
Female
Male
Sexes Combined
Age
20-24 years
15-19 years
10-14 years
Year
Incidence Rate (per 100,000)
Age — 10-24 years

Figure S5

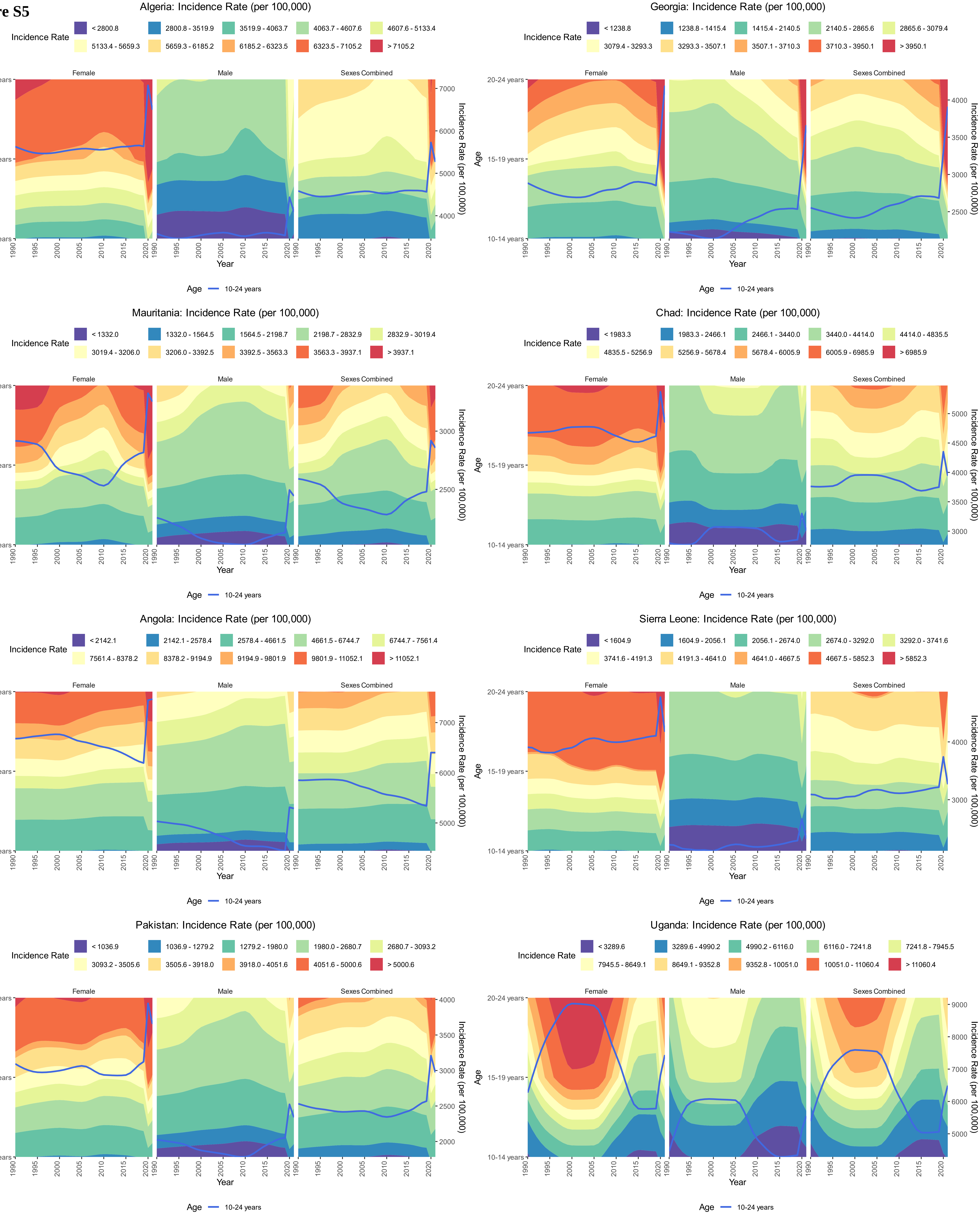

Algeria: Incidence Rate (per 100,000)
Incidence Rate
< 2800.8
2800.8 - 3519.9
3519.9 - 4063.7
4063.7 - 4607.6
4607.6 - 5133.4
5133.4 - 5659.3
5659.3 - 6185.2
6185.2 - 6323.5
6323.5 - 7105.2
> 7105.2
Georgia: Incidence Rate (per 100,000)
Incidence Rate
< 1238.8
1238.8 - 1415.4
1415.4 - 2140.5
2140.5 - 2865.6
2865.6 - 3079.4
3079.4 - 3293.3
3293.3 - 3507.1
3507.1 - 3710.3
3710.3 - 3950.1
> 3950.1
Mauritania: Incidence Rate (per 100,000)
Incidence Rate
< 1332.0
1332.0 - 1564.5
1564.5 - 2198.7
2198.7 - 2832.9
2832.9 - 3019.4
3019.4 - 3206.0
3206.0 - 3392.5
3392.5 - 3563.3
3563.3 - 3937.1
> 3937.1
Chad: Incidence Rate (per 100,000)
Incidence Rate
< 1983.3
1983.3 - 2466.1
2466.1 - 3440.0
3440.0 - 4414.0
4414.0 - 4835.5
4835.5 - 5256.9
5256.9 - 5678.4
5678.4 - 6005.9
6005.9 - 6985.9
> 6985.9
Angola: Incidence Rate (per 100,000)
Incidence Rate
< 2142.1
2142.1 - 2578.4
2578.4 - 4661.5
4661.5 - 6744.7
6744.7 - 7561.4
7561.4 - 8378.2
8378.2 - 9194.9
9194.9 - 9801.9
9801.9 - 11052.1
> 11052.1
Sierra Leone: Incidence Rate (per 100,000)
Incidence Rate
< 1604.9
1604.9 - 2056.1
2056.1 - 2674.0
2674.0 - 3292.0
3292.0 - 3741.6
3741.6 - 4191.3
4191.3 - 4641.0
4641.0 - 4667.5
4667.5 - 5852.3
> 5852.3
Pakistan: Incidence Rate (per 100,000)
Incidence Rate
< 1036.9
1036.9 - 1279.2
1279.2 - 1980.0
1980.0 - 2680.7
2680.7 - 3093.2
3093.2 - 3505.6
3505.6 - 3918.0
3918.0 - 4051.6
4051.6 - 5000.6
> 5000.6
Uganda: Incidence Rate (per 100,000)
Incidence Rate
< 3289.6
3289.6 - 4990.2
4990.2 - 6116.0
6116.0 - 7241.8
7241.8 - 7945.5
7945.5 - 8649.1
8649.1 - 9352.8
9352.8 - 10051.0
10051.0 - 11060.4
> 11060.4
Female
Male
Sexes Combined
Age
20-24 years
15-19 years
10-14 years
Year
Incidence Rate (per 100,000)
Age
10-24 years

Figure S5

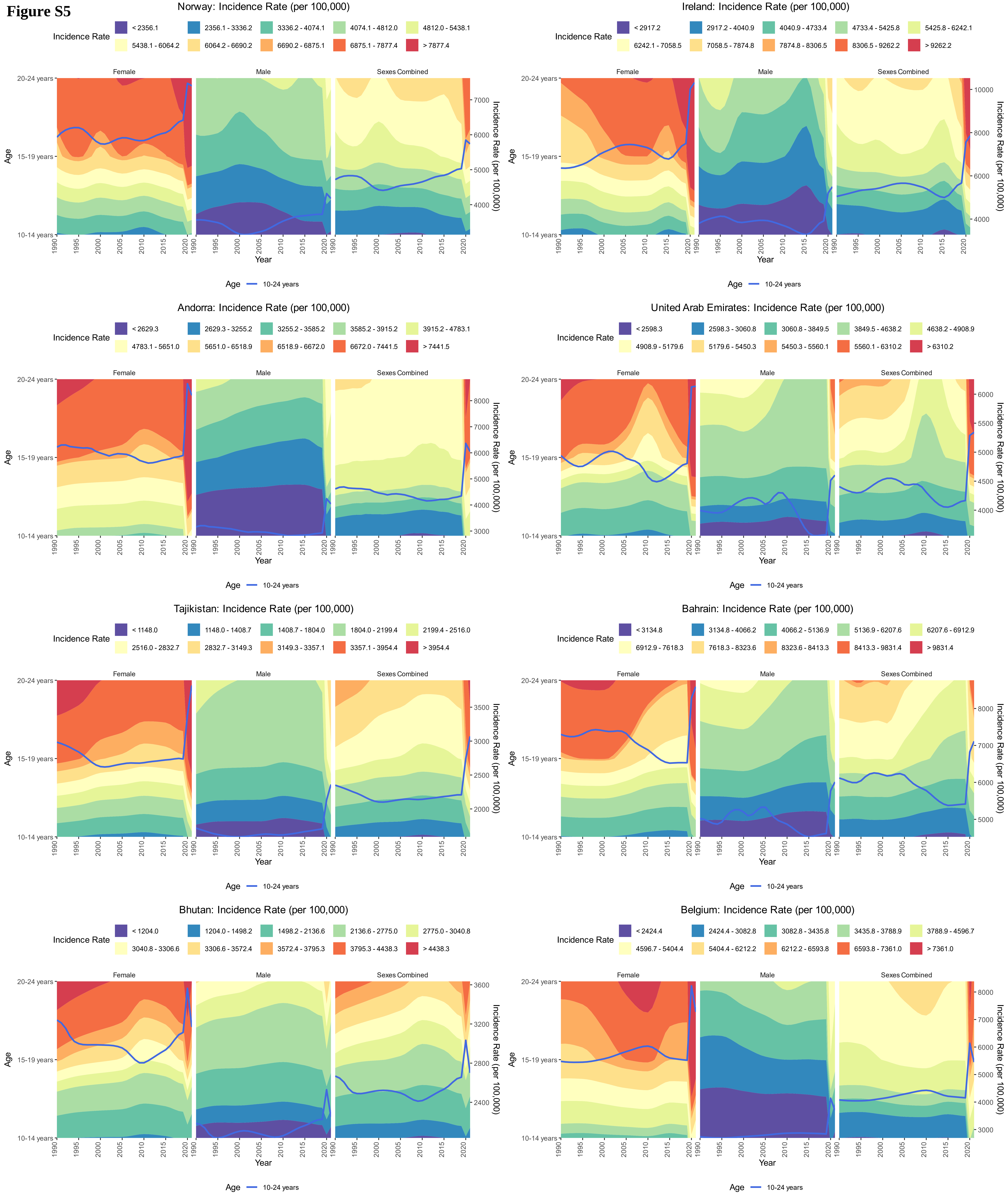

Norway: Incidence Rate (per 100,000)
Incidence Rate
< 2356.1
2356.1 - 3336.2
3336.2 - 4074.1
4074.1 - 4812.0
4812.0 - 5438.1
5438.1 - 6064.2
6064.2 - 6690.2
6690.2 - 6875.1
6875.1 - 7877.4
> 7877.4
Ireland: Incidence Rate (per 100,000)
Incidence Rate
< 2917.2
2917.2 - 4040.9
4040.9 - 4733.4
4733.4 - 5425.8
5425.8 - 6242.1
6242.1 - 7058.5
7058.5 - 7874.8
7874.8 - 8306.5
8306.5 - 9262.2
> 9262.2
Andorra: Incidence Rate (per 100,000)
Incidence Rate
< 2629.3
2629.3 - 3255.2
3255.2 - 3585.2
3585.2 - 3915.2
3915.2 - 4783.1
4783.1 - 5651.0
5651.0 - 6518.9
6518.9 - 6672.0
6672.0 - 7441.5
> 7441.5
United Arab Emirates: Incidence Rate (per 100,000)
Incidence Rate
< 2598.3
2598.3 - 3060.8
3060.8 - 3849.5
3849.5 - 4638.2
4638.2 - 4908.9
4908.9 - 5179.6
5179.6 - 5450.3
5450.3 - 5560.1
5560.1 - 6310.2
> 6310.2
Tajikistan: Incidence Rate (per 100,000)
Incidence Rate
< 1148.0
1148.0 - 1408.7
1408.7 - 1804.0
1804.0 - 2199.4
2199.4 - 2516.0
2516.0 - 2832.7
2832.7 - 3149.3
3149.3 - 3357.1
3357.1 - 3954.4
> 3954.4
Bahrain: Incidence Rate (per 100,000)
Incidence Rate
< 3134.8
3134.8 - 4066.2
4066.2 - 5136.9
5136.9 - 6207.6
6207.6 - 6912.9
6912.9 - 7618.3
7618.3 - 8323.6
8323.6 - 8413.3
8413.3 - 9831.4
> 9831.4
Bhutan: Incidence Rate (per 100,000)
Incidence Rate
< 1204.0
1204.0 - 1498.2
1498.2 - 2136.6
2136.6 - 2775.0
2775.0 - 3040.8
3040.8 - 3306.6
3306.6 - 3572.4
3572.4 - 3795.3
3795.3 - 4438.3
> 4438.3
Belgium: Incidence Rate (per 100,000)
Incidence Rate
< 2424.4
2424.4 - 3082.8
3082.8 - 3435.8
3435.8 - 3788.9
3788.9 - 4596.7
4596.7 - 5404.4
5404.4 - 6212.2
6212.2 - 6593.8
6593.8 - 7361.0
> 7361.0
Female
Male
Sexes Combined
Age
20-24 years
15-19 years
10-14 years
Year
Incidence Rate (per 100,000)
Age
10-24 years

Figure S5

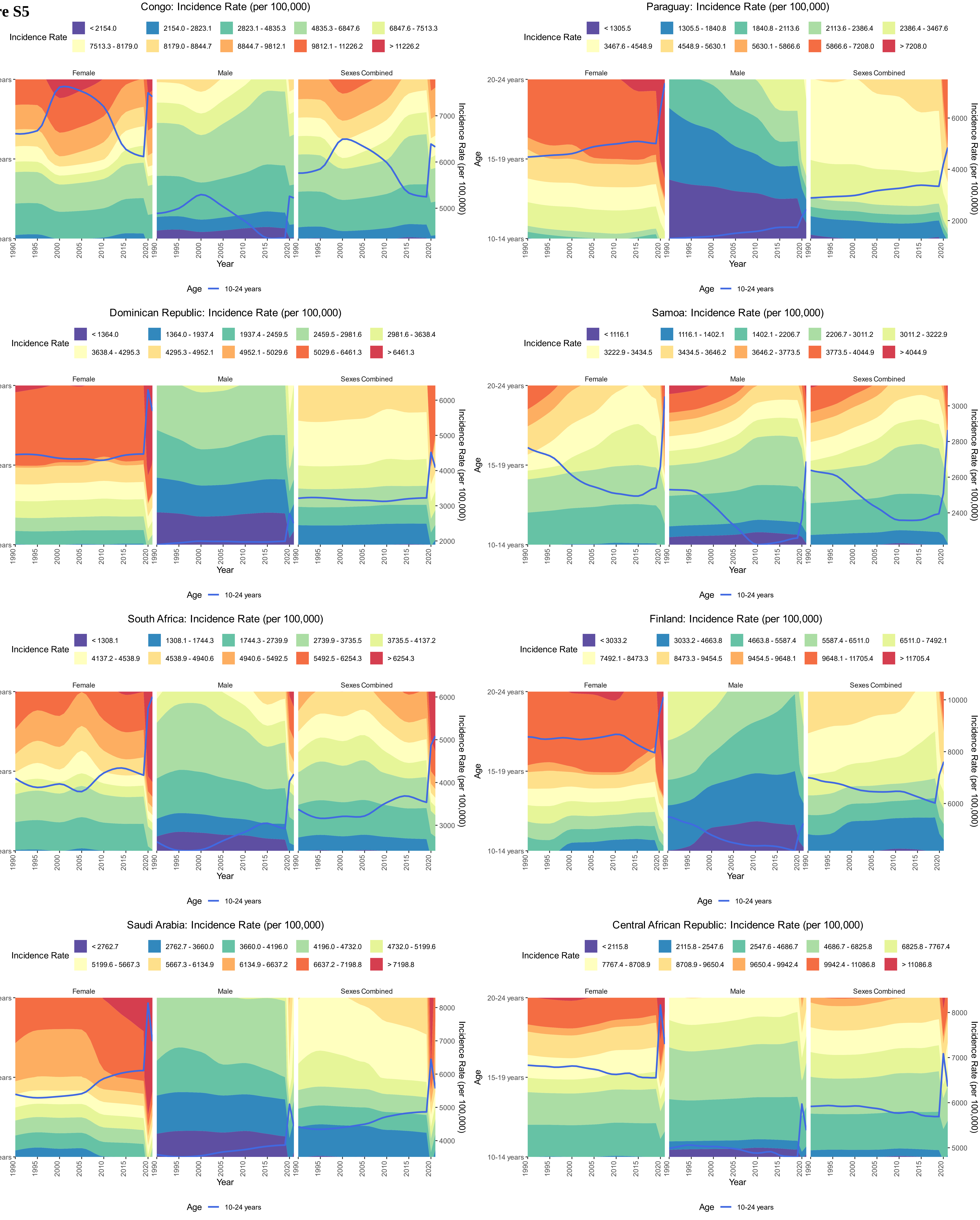

Congo: Incidence Rate (per 100,000)
Incidence Rate
< 2154.0
2154.0 - 2823.1
2823.1 - 4835.3
4835.3 - 6847.6
6847.6 - 7513.3
7513.3 - 8179.0
8179.0 - 8844.7
8844.7 - 9812.1
9812.1 - 11226.2
> 11226.2
Paraguay: Incidence Rate (per 100,000)
Incidence Rate
< 1305.5
1305.5 - 1840.8
1840.8 - 2113.6
2113.6 - 2386.4
2386.4 - 3467.6
3467.6 - 4548.9
4548.9 - 5630.1
5630.1 - 5866.6
5866.6 - 7208.0
> 7208.0
Dominican Republic: Incidence Rate (per 100,000)
Incidence Rate
< 1364.0
1364.0 - 1937.4
1937.4 - 2459.5
2459.5 - 2981.6
2981.6 - 3638.4
3638.4 - 4295.3
4295.3 - 4952.1
4952.1 - 5029.6
5029.6 - 6461.3
> 6461.3
Samoa: Incidence Rate (per 100,000)
Incidence Rate
< 1116.1
1116.1 - 1402.1
1402.1 - 2206.7
2206.7 - 3011.2
3011.2 - 3222.9
3222.9 - 3434.5
3434.5 - 3646.2
3646.2 - 3773.5
3773.5 - 4044.9
> 4044.9
South Africa: Incidence Rate (per 100,000)
Incidence Rate
< 1308.1
1308.1 - 1744.3
1744.3 - 2739.9
2739.9 - 3735.5
3735.5 - 4137.2
4137.2 - 4538.9
4538.9 - 4940.6
4940.6 - 5492.5
5492.5 - 6254.3
> 6254.3
Finland: Incidence Rate (per 100,000)
Incidence Rate
< 3033.2
3033.2 - 4663.8
4663.8 - 5587.4
5587.4 - 6511.0
6511.0 - 7492.1
7492.1 - 8473.3
8473.3 - 9454.5
9454.5 - 9648.1
9648.1 - 11705.4
> 11705.4
Saudi Arabia: Incidence Rate (per 100,000)
Incidence Rate
< 2762.7
2762.7 - 3660.0
3660.0 - 4196.0
4196.0 - 4732.0
4732.0 - 5199.6
5199.6 - 5667.3
5667.3 - 6134.9
6134.9 - 6637.2
6637.2 - 7198.8
> 7198.8
Central African Republic: Incidence Rate (per 100,000)
Incidence Rate
< 2115.8
2115.8 - 2547.6
2547.6 - 4686.7
4686.7 - 6825.8
6825.8 - 7767.4
7767.4 - 8708.9
8708.9 - 9650.4
9650.4 - 9942.4
9942.4 - 11086.8
> 11086.8
Female
Male
Sexes Combined
Age
20-24 years
15-19 years
10-14 years
Year
Incidence Rate (per 100,000)
Age
10-24 years

Figure S5

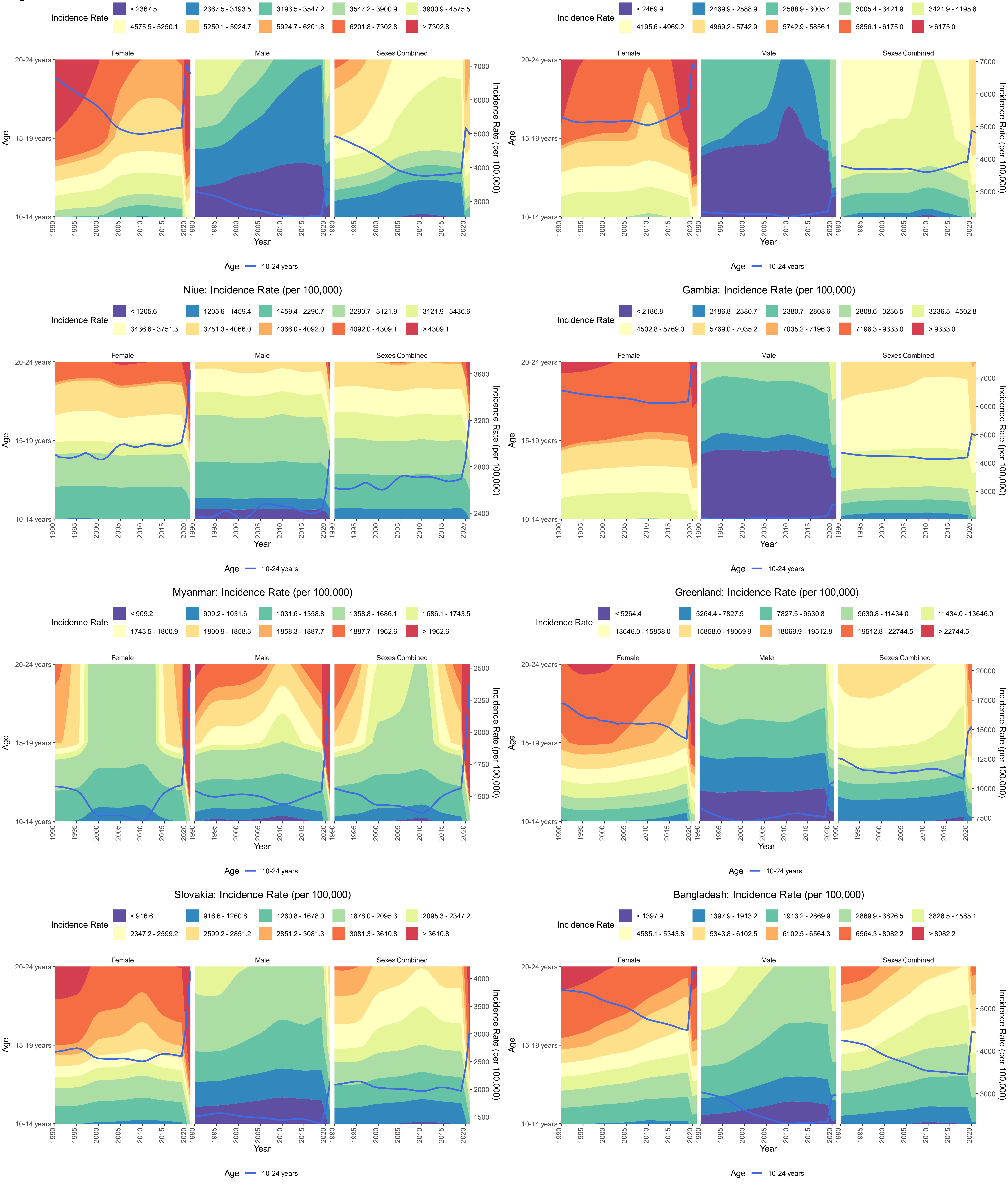

Luxembourg: Incidence Rate (per 100,000)
Incidence Rate
< 2367.5
2367.5 - 3193.5
3193.5 - 3547.2
3547.2 - 3900.9
3900.9 - 4575.5
4575.5 - 5250.1
5250.1 - 5924.7
5924.7 - 6201.8
6201.8 - 7302.8
> 7302.8
Malta: Incidence Rate (per 100,000)
< 2469.9
2469.9 - 2588.9
2588.9 - 3005.4
3005.4 - 3421.9
3421.9 - 4195.6
4195.6 - 4969.2
4969.2 - 5742.9
5742.9 - 5856.1
5856.1 - 6175.0
> 6175.0
Niue: Incidence Rate (per 100,000)
< 1205.6
1205.6 - 1459.4
1459.4 - 2290.7
2290.7 - 3121.9
3121.9 - 3436.6
3436.6 - 3751.3
3751.3 - 4066.0
4066.0 - 4092.0
4092.0 - 4309.1
> 4309.1
Gambia: Incidence Rate (per 100,000)
< 2186.8
2186.8 - 2380.7
2380.7 - 2808.6
2808.6 - 3236.5
3236.5 - 4502.8
4502.8 - 5769.0
5769.0 - 7035.2
7035.2 - 7196.3
7196.3 - 9333.0
> 9333.0
Myanmar: Incidence Rate (per 100,000)
< 909.2
909.2 - 1031.6
1031.6 - 1358.8
1358.8 - 1686.1
1686.1 - 1743.5
1743.5 - 1800.9
1800.9 - 1858.3
1858.3 - 1887.7
1887.7 - 1962.6
> 1962.6
Greenland: Incidence Rate (per 100,000)
< 5264.4
5264.4 - 7827.5
7827.5 - 9630.8
9630.8 - 11434.0
11434.0 - 13646.0
13646.0 - 15858.0
15858.0 - 18069.9
18069.9 - 19512.8
19512.8 - 22744.5
> 22744.5
Slovakia: Incidence Rate (per 100,000)
< 916.6
916.6 - 1260.8
1260.8 - 1678.0
1678.0 - 2095.3
2095.3 - 2347.2
2347.2 - 2599.2
2599.2 - 2851.2
2851.2 - 3081.3
3081.3 - 3610.8
> 3610.8
Bangladesh: Incidence Rate (per 100,000)
< 1397.9
1397.9 - 1913.2
1913.2 - 2869.9
2869.9 - 3826.5
3826.5 - 4585.1
4585.1 - 5343.8
5343.8 - 6102.5
6102.5 - 6564.3
6564.3 - 8082.2
> 8082.2
Female
Male
Sexes Combined
Age
20-24 years
15-19 years
10-14 years
Year
Incidence Rate (per 100,000)
Age — 10-24 years

Figure S5

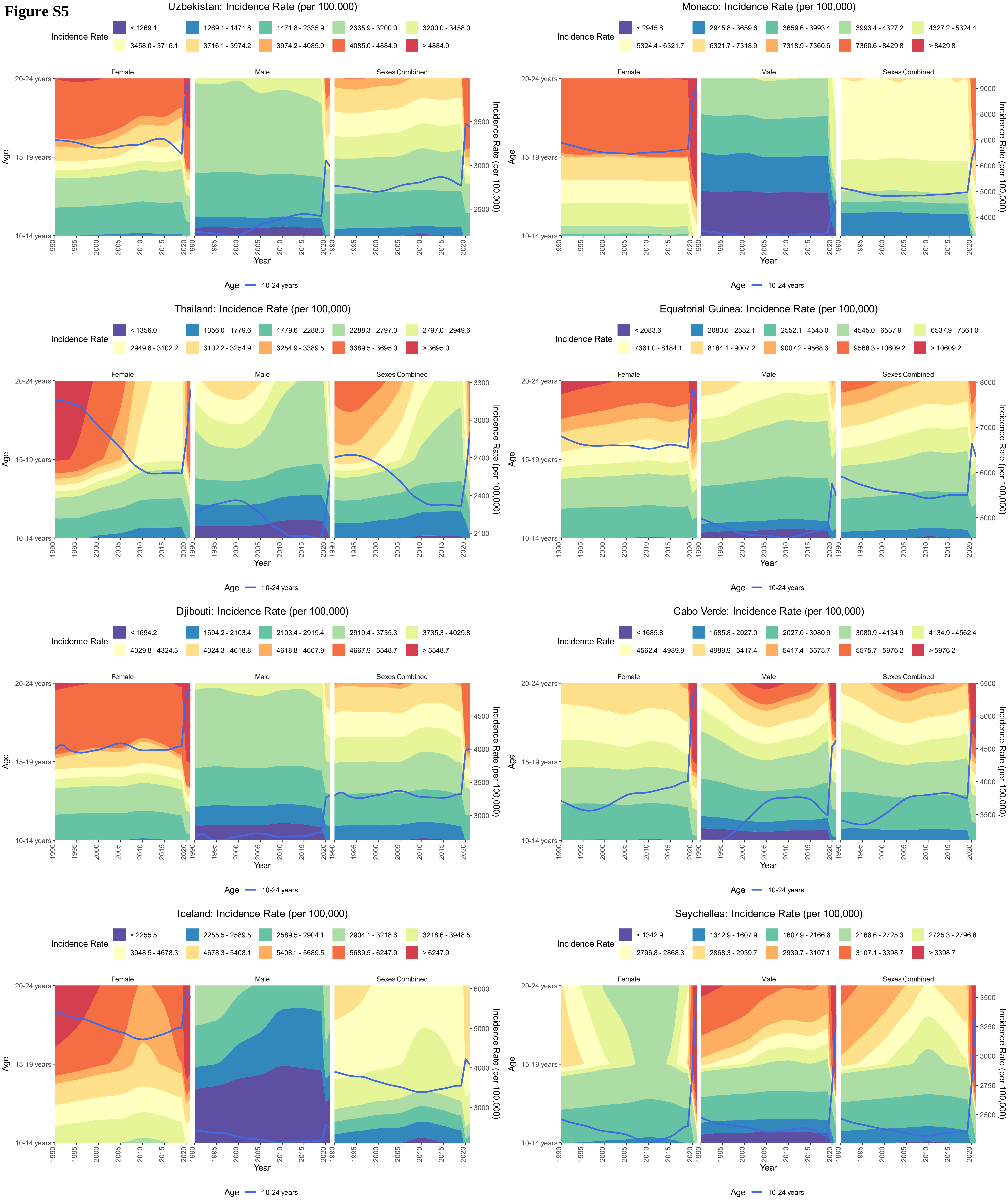

Uzbekistan: Incidence Rate (per 100,000)
Incidence Rate
< 1269.1
1269.1 - 1471.8
1471.8 - 2335.9
2335.9 - 3200.0
3200.0 - 3458.0
3458.0 - 3716.1
3716.1 - 3974.2
3974.2 - 4085.0
4085.0 - 4884.9
> 4884.9
Monaco: Incidence Rate (per 100,000)
< 2945.8
2945.8 - 3659.6
3659.6 - 3993.4
3993.4 - 4327.2
4327.2 - 5324.4
5324.4 - 6321.7
6321.7 - 7318.9
7318.9 - 7360.6
7360.6 - 8429.8
> 8429.8
Thailand: Incidence Rate (per 100,000)
< 1356.0
1356.0 - 1779.6
1779.6 - 2288.3
2288.3 - 2797.0
2797.0 - 2949.6
2949.6 - 3102.2
3102.2 - 3254.9
3254.9 - 3389.5
3389.5 - 3695.0
> 3695.0
Equatorial Guinea: Incidence Rate (per 100,000)
< 2083.6
2083.6 - 2552.1
2552.1 - 4545.0
4545.0 - 6537.9
6537.9 - 7361.0
7361.0 - 8184.1
8184.1 - 9007.2
9007.2 - 9568.3
9568.3 - 10609.2
> 10609.2
Djibouti: Incidence Rate (per 100,000)
< 1694.2
1694.2 - 2103.4
2103.4 - 2919.4
2919.4 - 3735.3
3735.3 - 4029.8
4029.8 - 4324.3
4324.3 - 4618.8
4618.8 - 4667.9
4667.9 - 5548.7
> 5548.7
Cabo Verde: Incidence Rate (per 100,000)
< 1685.8
1685.8 - 2027.0
2027.0 - 3080.9
3080.9 - 4134.9
4134.9 - 4562.4
4562.4 - 4989.9
4989.9 - 5417.4
5417.4 - 5575.7
5575.7 - 5976.2
> 5976.2
Iceland: Incidence Rate (per 100,000)
< 2255.5
2255.5 - 2589.5
2589.5 - 2904.1
2904.1 - 3218.6
3218.6 - 3948.5
3948.5 - 4678.3
4678.3 - 5408.1
5408.1 - 5689.5
5689.5 - 6247.9
> 6247.9
Seychelles: Incidence Rate (per 100,000)
< 1342.9
1342.9 - 1607.9
1607.9 - 2166.6
2166.6 - 2725.3
2725.3 - 2796.8
2796.8 - 2868.3
2868.3 - 2939.7
2939.7 - 3107.1
3107.1 - 3398.7
> 3398.7
Female
Male
Sexes Combined
Age
20-24 years
15-19 years
10-14 years
Year
Incidence Rate (per 100,000)
Age
10-24 years

Figure S5

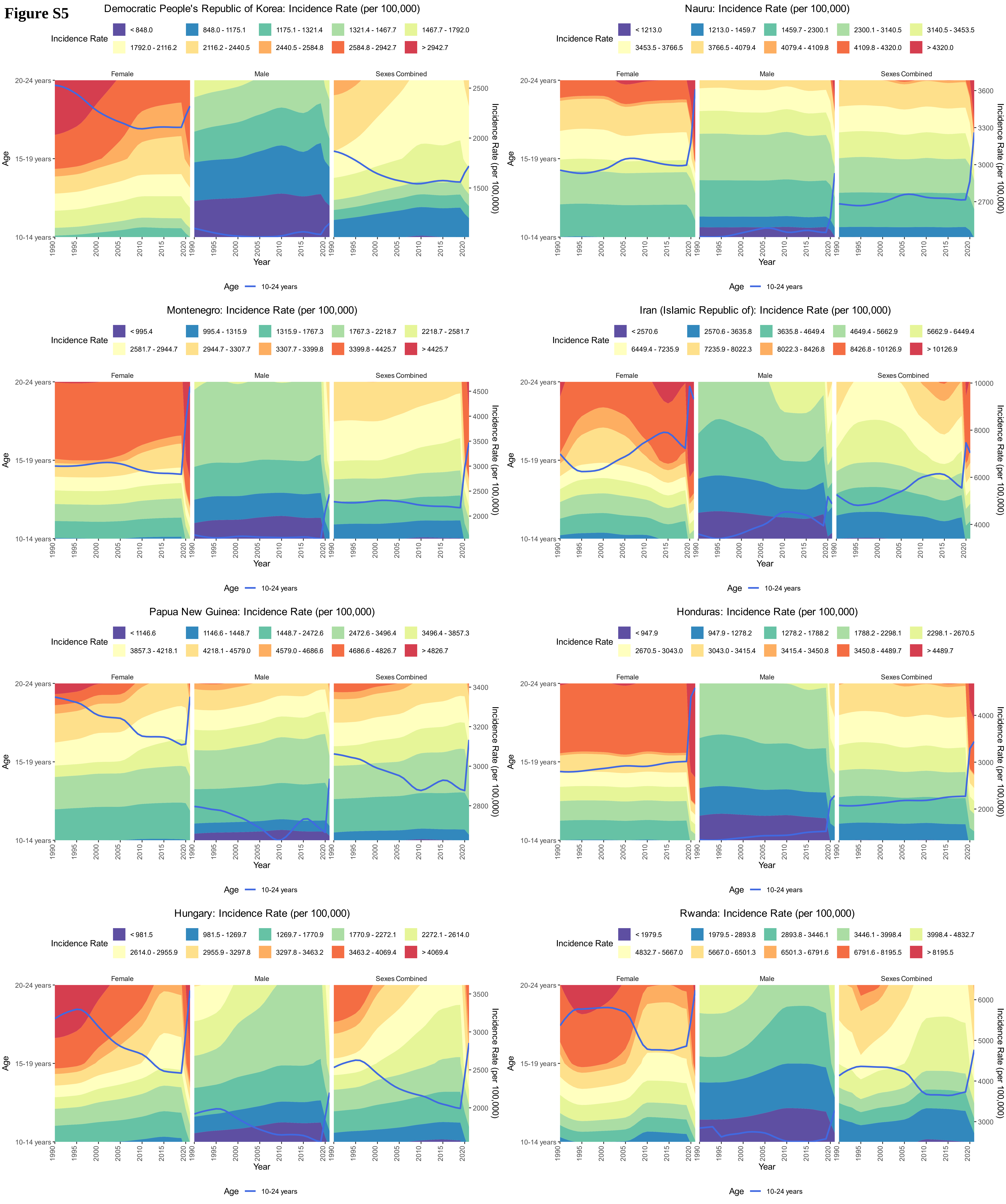
Democratic People's Republic of Korea: Incidence Rate (per 100,000)
Incidence Rate
< 848.0
848.0 - 1175.1
1175.1 - 1321.4
1321.4 - 1467.7
1467.7 - 1792.0
1792.0 - 2116.2
2116.2 - 2440.5
2440.5 - 2584.8
2584.8 - 2942.7
> 2942.7
Nauru: Incidence Rate (per 100,000)
Incidence Rate
< 1213.0
1213.0 - 1459.7
1459.7 - 2300.1
2300.1 - 3140.5
3140.5 - 3453.5
3453.5 - 3766.5
3766.5 - 4079.4
4079.4 - 4109.8
4109.8 - 4320.0
> 4320.0
Montenegro: Incidence Rate (per 100,000)
Incidence Rate
< 995.4
995.4 - 1315.9
1315.9 - 1767.3
1767.3 - 2218.7
2218.7 - 2581.7
2581.7 - 2944.7
2944.7 - 3307.7
3307.7 - 3399.8
3399.8 - 4425.7
> 4425.7
Iran (Islamic Republic of): Incidence Rate (per 100,000)
Incidence Rate
< 2570.6
2570.6 - 3635.8
3635.8 - 4649.4
4649.4 - 5662.9
5662.9 - 6449.4
6449.4 - 7235.9
7235.9 - 8022.3
8022.3 - 8426.8
8426.8 - 10126.9
> 10126.9
Papua New Guinea: Incidence Rate (per 100,000)
Incidence Rate
< 1146.6
1146.6 - 1448.7
1448.7 - 2472.6
2472.6 - 3496.4
3496.4 - 3857.3
3857.3 - 4218.1
4218.1 - 4579.0
4579.0 - 4686.6
4686.6 - 4826.7
> 4826.7
Honduras: Incidence Rate (per 100,000)
Incidence Rate
< 947.9
947.9 - 1278.2
1278.2 - 1788.2
1788.2 - 2298.1
2298.1 - 2670.5
2670.5 - 3043.0
3043.0 - 3415.4
3415.4 - 3450.8
3450.8 - 4489.7
> 4489.7
Hungary: Incidence Rate (per 100,000)
Incidence Rate
< 981.5
981.5 - 1269.7
1269.7 - 1770.9
1770.9 - 2272.1
2272.1 - 2614.0
2614.0 - 2955.9
2955.9 - 3297.8
3297.8 - 3463.2
3463.2 - 4069.4
> 4069.4
Rwanda: Incidence Rate (per 100,000)
Incidence Rate
< 1979.5
1979.5 - 2893.8
2893.8 - 3446.1
3446.1 - 3998.4
3998.4 - 4832.7
4832.7 - 5667.0
5667.0 - 6501.3
6501.3 - 6791.6
6791.6 - 8195.5
> 8195.5
Female
Male
Sexes Combined
Age
20-24 years
15-19 years
10-14 years
Year
Incidence Rate (per 100,000)
Age — 10-24 years

Figure S5

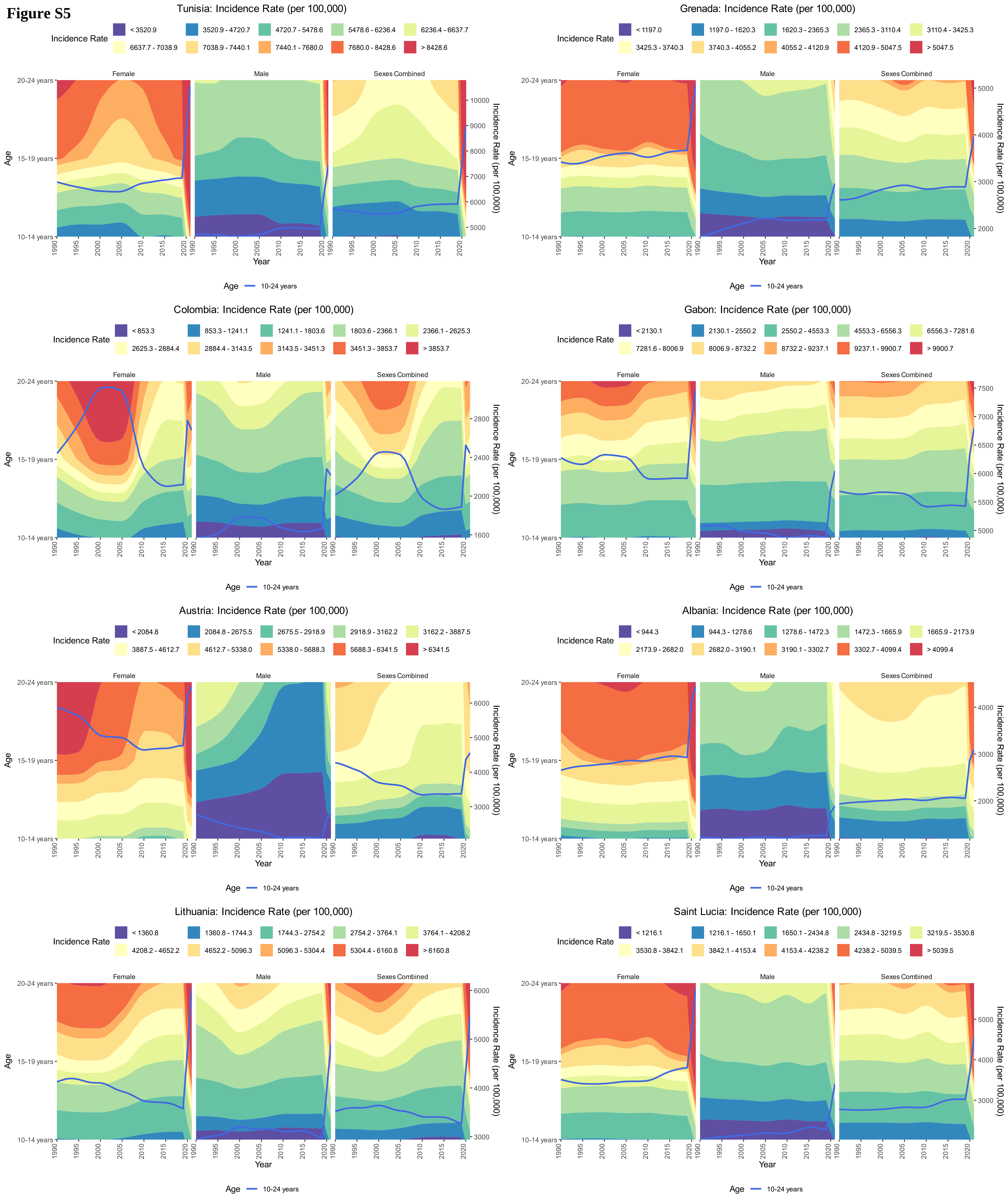

Tunisia: Incidence Rate (per 100,000)
Incidence Rate
< 3520.9
3520.9 - 4720.7
4720.7 - 5478.6
5478.6 - 6236.4
6236.4 - 6637.7
6637.7 - 7038.9
7038.9 - 7440.1
7440.1 - 7680.0
7680.0 - 8428.6
> 8428.6
Grenada: Incidence Rate (per 100,000)
Incidence Rate
< 1197.0
1197.0 - 1620.3
1620.3 - 2365.3
2365.3 - 3110.4
3110.4 - 3425.3
3425.3 - 3740.3
3740.3 - 4055.2
4055.2 - 4120.9
4120.9 - 5047.5
> 5047.5
Colombia: Incidence Rate (per 100,000)
Incidence Rate
< 853.3
853.3 - 1241.1
1241.1 - 1803.6
1803.6 - 2366.1
2366.1 - 2625.3
2625.3 - 2884.4
2884.4 - 3143.5
3143.5 - 3451.3
3451.3 - 3853.7
> 3853.7
Gabon: Incidence Rate (per 100,000)
Incidence Rate
< 2130.1
2130.1 - 2550.2
2550.2 - 4553.3
4553.3 - 6556.3
6556.3 - 7281.6
7281.6 - 8006.9
8006.9 - 8732.2
8732.2 - 9237.1
9237.1 - 9900.7
> 9900.7
Austria: Incidence Rate (per 100,000)
Incidence Rate
< 2084.8
2084.8 - 2675.5
2675.5 - 2918.9
2918.9 - 3162.2
3162.2 - 3887.5
3887.5 - 4612.7
4612.7 - 5338.0
5338.0 - 5688.3
5688.3 - 6341.5
> 6341.5
Albania: Incidence Rate (per 100,000)
Incidence Rate
< 944.3
944.3 - 1278.6
1278.6 - 1472.3
1472.3 - 1665.9
1665.9 - 2173.9
2173.9 - 2682.0
2682.0 - 3190.1
3190.1 - 3302.7
3302.7 - 4099.4
> 4099.4
Lithuania: Incidence Rate (per 100,000)
Incidence Rate
< 1360.8
1360.8 - 1744.3
1744.3 - 2754.2
2754.2 - 3764.1
3764.1 - 4208.2
4208.2 - 4652.2
4652.2 - 5096.3
5096.3 - 5304.4
5304.4 - 6160.8
> 6160.8
Saint Lucia: Incidence Rate (per 100,000)
Incidence Rate
< 1216.1
1216.1 - 1650.1
1650.1 - 2434.8
2434.8 - 3219.5
3219.5 - 3530.8
3530.8 - 3842.1
3842.1 - 4153.4
4153.4 - 4238.2
4238.2 - 5039.5
> 5039.5
Female
Male
Sexes Combined
Age
20-24 years
15-19 years
10-14 years
Year
Incidence Rate (per 100,000)
Age
10-24 years

Figure S5

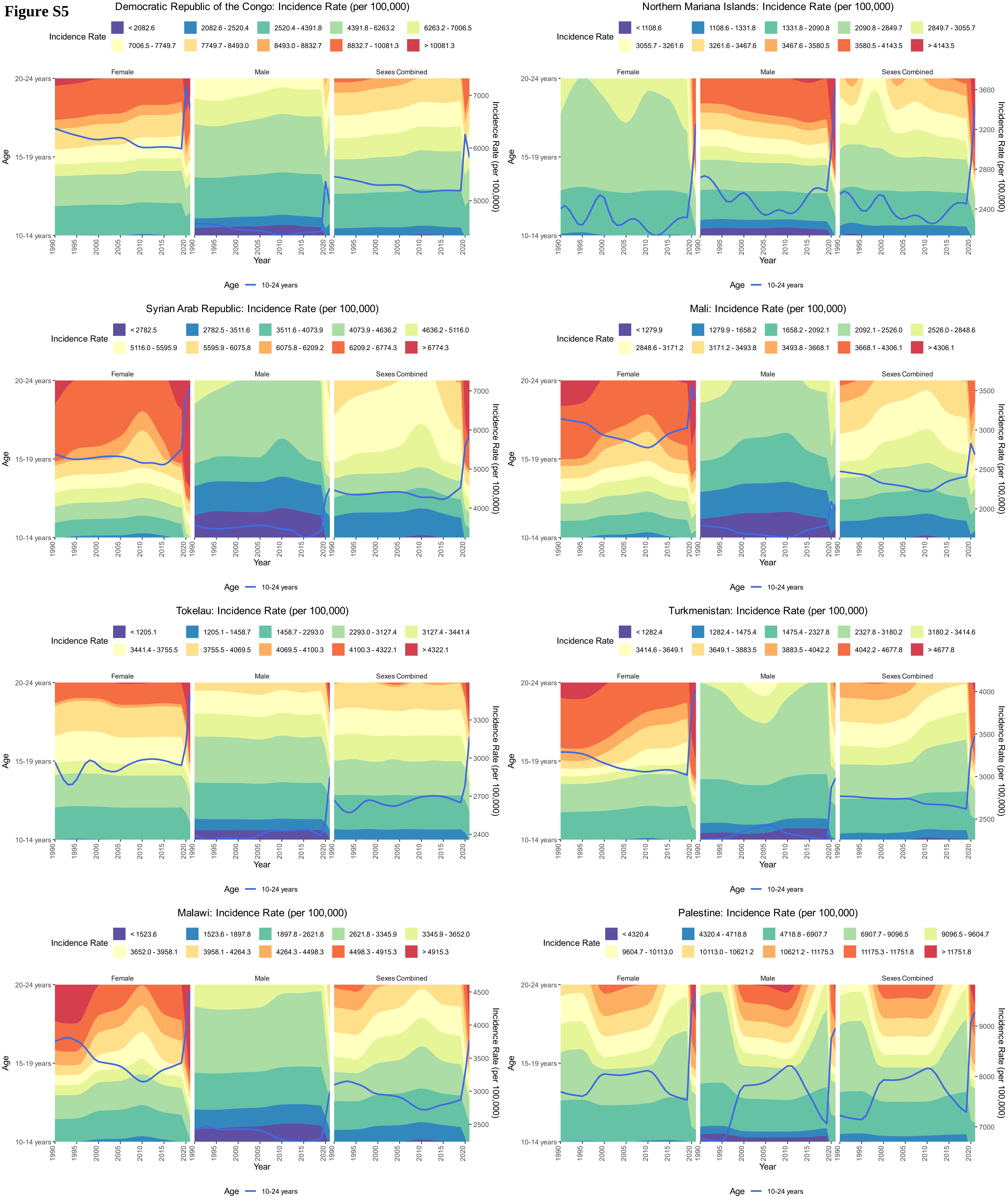
Democratic Republic of the Congo: Incidence Rate (per 100,000)
Incidence Rate
< 2082.6
2082.6 - 2520.4
2520.4 - 4391.8
4391.8 - 6263.2
6263.2 - 7006.5
7006.5 - 7749.7
7749.7 - 8493.0
8493.0 - 8832.7
8832.7 - 10081.3
> 10081.3
Northern Mariana Islands: Incidence Rate (per 100,000)
< 1108.6
1108.6 - 1331.8
1331.8 - 2090.8
2090.8 - 2849.7
2849.7 - 3055.7
3055.7 - 3261.6
3261.6 - 3467.6
3467.6 - 3580.5
3580.5 - 4143.5
> 4143.5
Syrian Arab Republic: Incidence Rate (per 100,000)
< 2782.5
2782.5 - 3511.6
3511.6 - 4073.9
4073.9 - 4636.2
4636.2 - 5116.0
5116.0 - 5595.9
5595.9 - 6075.8
6075.8 - 6209.2
6209.2 - 6774.3
> 6774.3
Mali: Incidence Rate (per 100,000)
< 1279.9
1279.9 - 1658.2
1658.2 - 2092.1
2092.1 - 2526.0
2526.0 - 2848.6
2848.6 - 3171.2
3171.2 - 3493.8
3493.8 - 3668.1
3668.1 - 4306.1
> 4306.1
Tokelau: Incidence Rate (per 100,000)
< 1205.1
1205.1 - 1458.7
1458.7 - 2293.0
2293.0 - 3127.4
3127.4 - 3441.4
3441.4 - 3755.5
3755.5 - 4069.5
4069.5 - 4100.3
4100.3 - 4322.1
> 4322.1
Turkmenistan: Incidence Rate (per 100,000)
< 1282.4
1282.4 - 1475.4
1475.4 - 2327.8
2327.8 - 3180.2
3180.2 - 3414.6
3414.6 - 3649.1
3649.1 - 3883.5
3883.5 - 4042.2
4042.2 - 4677.8
> 4677.8
Malawi: Incidence Rate (per 100,000)
< 1523.6
1523.6 - 1897.8
1897.8 - 2621.8
2621.8 - 3345.9
3345.9 - 3652.0
3652.0 - 3958.1
3958.1 - 4264.3
4264.3 - 4498.3
4498.3 - 4915.3
> 4915.3
Palestine: Incidence Rate (per 100,000)
< 4320.4
4320.4 - 4718.8
4718.8 - 6907.7
6907.7 - 9096.5
9096.5 - 9604.7
9604.7 - 10113.0
10113.0 - 10621.2
10621.2 - 11175.3
11175.3 - 11751.8
> 11751.8
Female
Male
Sexes Combined
Age
20-24 years
15-19 years
10-14 years
Year
Incidence Rate (per 100,000)
Age — 10-24 years

Figure S5

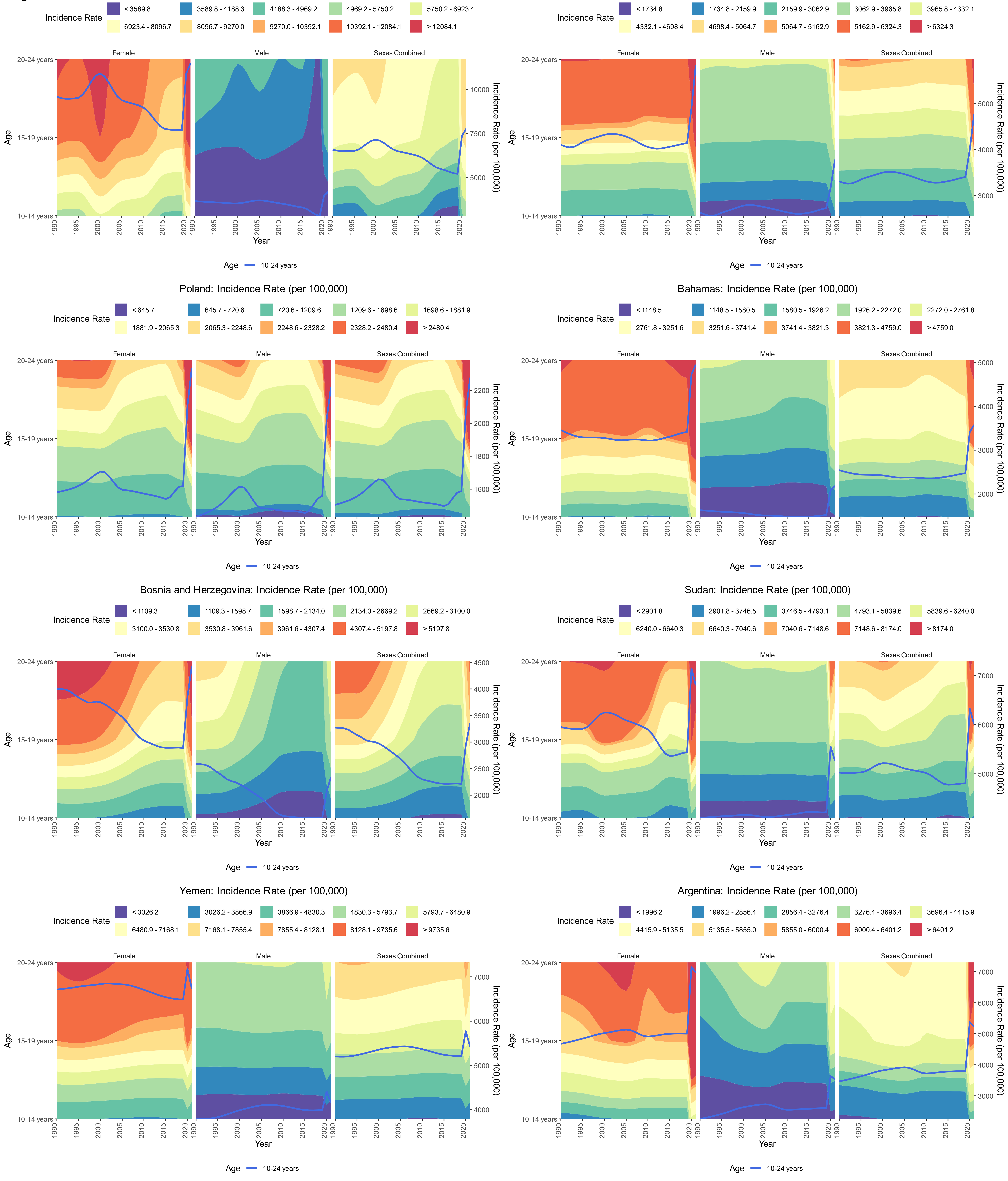

Portugal: Incidence Rate (per 100,000)
Incidence Rate
< 3589.8
3589.8 - 4188.3
4188.3 - 4969.2
4969.2 - 5750.2
5750.2 - 6923.4
6923.4 - 8096.7
8096.7 - 9270.0
9270.0 - 10392.1
10392.1 - 12084.1
> 12084.1
Female
Male
Sexes Combined
Age
20-24 years
15-19 years
10-14 years
Year
Incidence Rate (per 100,000)
Age
10-24 years
Somalia: Incidence Rate (per 100,000)
Incidence Rate
< 1734.8
1734.8 - 2159.9
2159.9 - 3062.9
3062.9 - 3965.8
3965.8 - 4332.1
4332.1 - 4698.4
4698.4 - 5064.7
5064.7 - 5162.9
5162.9 - 6324.3
> 6324.3
Poland: Incidence Rate (per 100,000)
Incidence Rate
< 645.7
645.7 - 720.6
720.6 - 1209.6
1209.6 - 1698.6
1698.6 - 1881.9
1881.9 - 2065.3
2065.3 - 2248.6
2248.6 - 2328.2
2328.2 - 2480.4
> 2480.4
Bahamas: Incidence Rate (per 100,000)
Incidence Rate
< 1148.5
1148.5 - 1580.5
1580.5 - 1926.2
1926.2 - 2272.0
2272.0 - 2761.8
2761.8 - 3251.6
3251.6 - 3741.4
3741.4 - 3821.3
3821.3 - 4759.0
> 4759.0
Bosnia and Herzegovina: Incidence Rate (per 100,000)
Incidence Rate
< 1109.3
1109.3 - 1598.7
1598.7 - 2134.0
2134.0 - 2669.2
2669.2 - 3100.0
3100.0 - 3530.8
3530.8 - 3961.6
3961.6 - 4307.4
4307.4 - 5197.8
> 5197.8
Sudan: Incidence Rate (per 100,000)
Incidence Rate
< 2901.8
2901.8 - 3746.5
3746.5 - 4793.1
4793.1 - 5839.6
5839.6 - 6240.0
6240.0 - 6640.3
6640.3 - 7040.6
7040.6 - 7148.6
7148.6 - 8174.0
> 8174.0
Yemen: Incidence Rate (per 100,000)
Incidence Rate
< 3026.2
3026.2 - 3866.9
3866.9 - 4830.3
4830.3 - 5793.7
5793.7 - 6480.9
6480.9 - 7168.1
7168.1 - 7855.4
7855.4 - 8128.1
8128.1 - 9735.6
> 9735.6
Argentina: Incidence Rate (per 100,000)
Incidence Rate
< 1996.2
1996.2 - 2856.4
2856.4 - 3276.4
3276.4 - 3696.4
3696.4 - 4415.9
4415.9 - 5135.5
5135.5 - 5855.0
5855.0 - 6000.4
6000.4 - 6401.2
> 6401.2

Figure S5

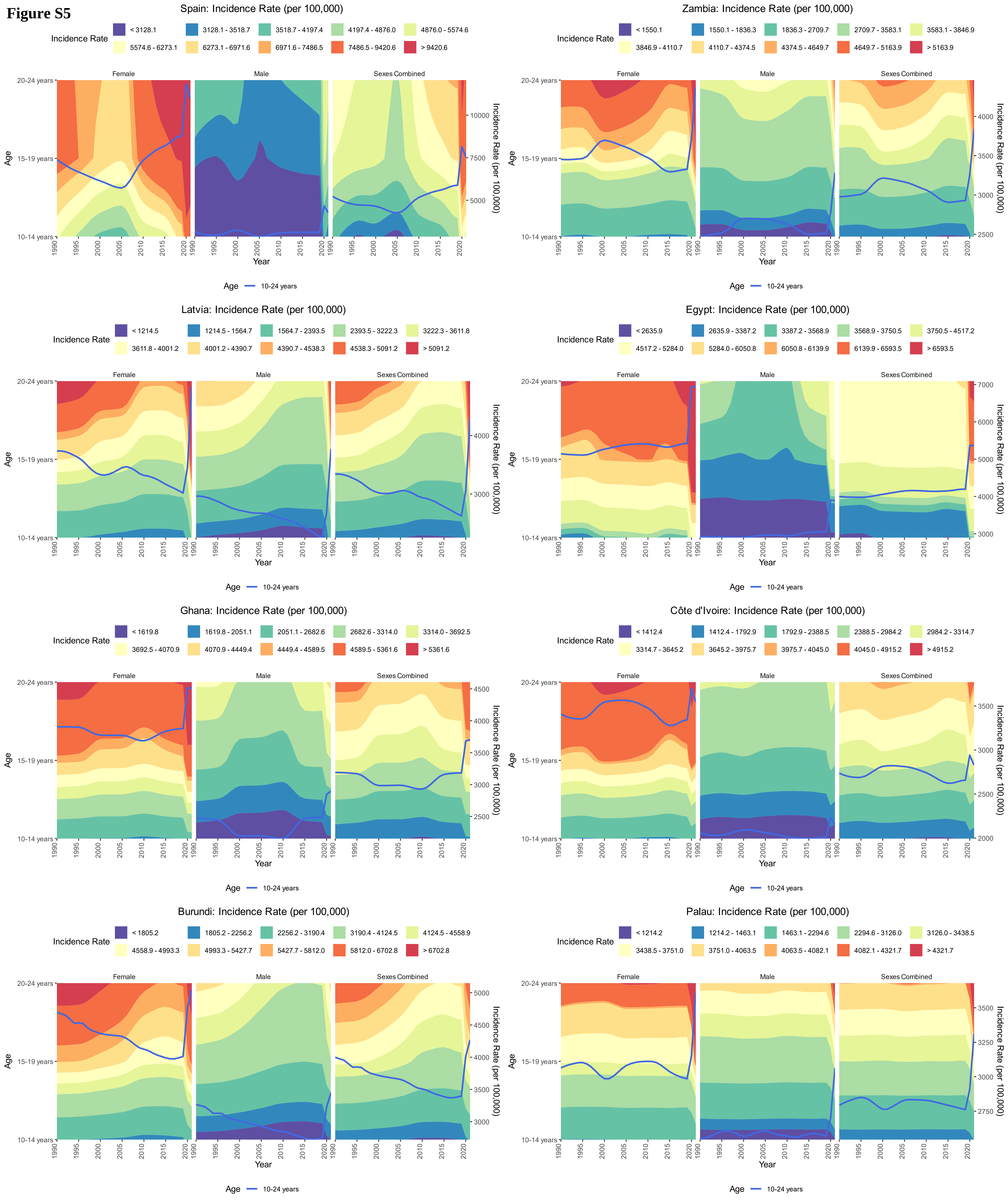
Spain: Incidence Rate (per 100,000)
Incidence Rate
< 3128.1
3128.1 - 3518.7
3518.7 - 4197.4
4197.4 - 4876.0
4876.0 - 5574.6
5574.6 - 6273.1
6273.1 - 6971.6
6971.6 - 7486.5
7486.5 - 9420.6
> 9420.6
Zambia: Incidence Rate (per 100,000)
< 1550.1
1550.1 - 1836.3
1836.3 - 2709.7
2709.7 - 3583.1
3583.1 - 3846.9
3846.9 - 4110.7
4110.7 - 4374.5
4374.5 - 4649.7
4649.7 - 5163.9
> 5163.9
Latvia: Incidence Rate (per 100,000)
< 1214.5
1214.5 - 1564.7
1564.7 - 2393.5
2393.5 - 3222.3
3222.3 - 3611.8
3611.8 - 4001.2
4001.2 - 4390.7
4390.7 - 4538.3
4538.3 - 5091.2
> 5091.2
Egypt: Incidence Rate (per 100,000)
< 2635.9
2635.9 - 3387.2
3387.2 - 3568.9
3568.9 - 3750.5
3750.5 - 4517.2
4517.2 - 5284.0
5284.0 - 6050.8
6050.8 - 6139.9
6139.9 - 6593.5
> 6593.5
Ghana: Incidence Rate (per 100,000)
< 1619.8
1619.8 - 2051.1
2051.1 - 2682.6
2682.6 - 3314.0
3314.0 - 3692.5
3692.5 - 4070.9
4070.9 - 4449.4
4449.4 - 4589.5
4589.5 - 5361.6
> 5361.6
Côte d'Ivoire: Incidence Rate (per 100,000)
< 1412.4
1412.4 - 1792.9
1792.9 - 2388.5
2388.5 - 2984.2
2984.2 - 3314.7
3314.7 - 3645.2
3645.2 - 3975.7
3975.7 - 4045.0
4045.0 - 4915.2
> 4915.2
Burundi: Incidence Rate (per 100,000)
< 1805.2
1805.2 - 2256.2
2256.2 - 3190.4
3190.4 - 4124.5
4124.5 - 4558.9
4558.9 - 4993.3
4993.3 - 5427.7
5427.7 - 5812.0
5812.0 - 6702.8
> 6702.8
Palau: Incidence Rate (per 100,000)
< 1214.2
1214.2 - 1463.1
1463.1 - 2294.6
2294.6 - 3126.0
3126.0 - 3438.5
3438.5 - 3751.0
3751.0 - 4063.5
4063.5 - 4082.1
4082.1 - 4321.7
> 4321.7
Female
Male
Sexes Combined
Age
20-24 years
15-19 years
10-14 years
Year
Incidence Rate (per 100,000)
Age — 10-24 years

Figure S5

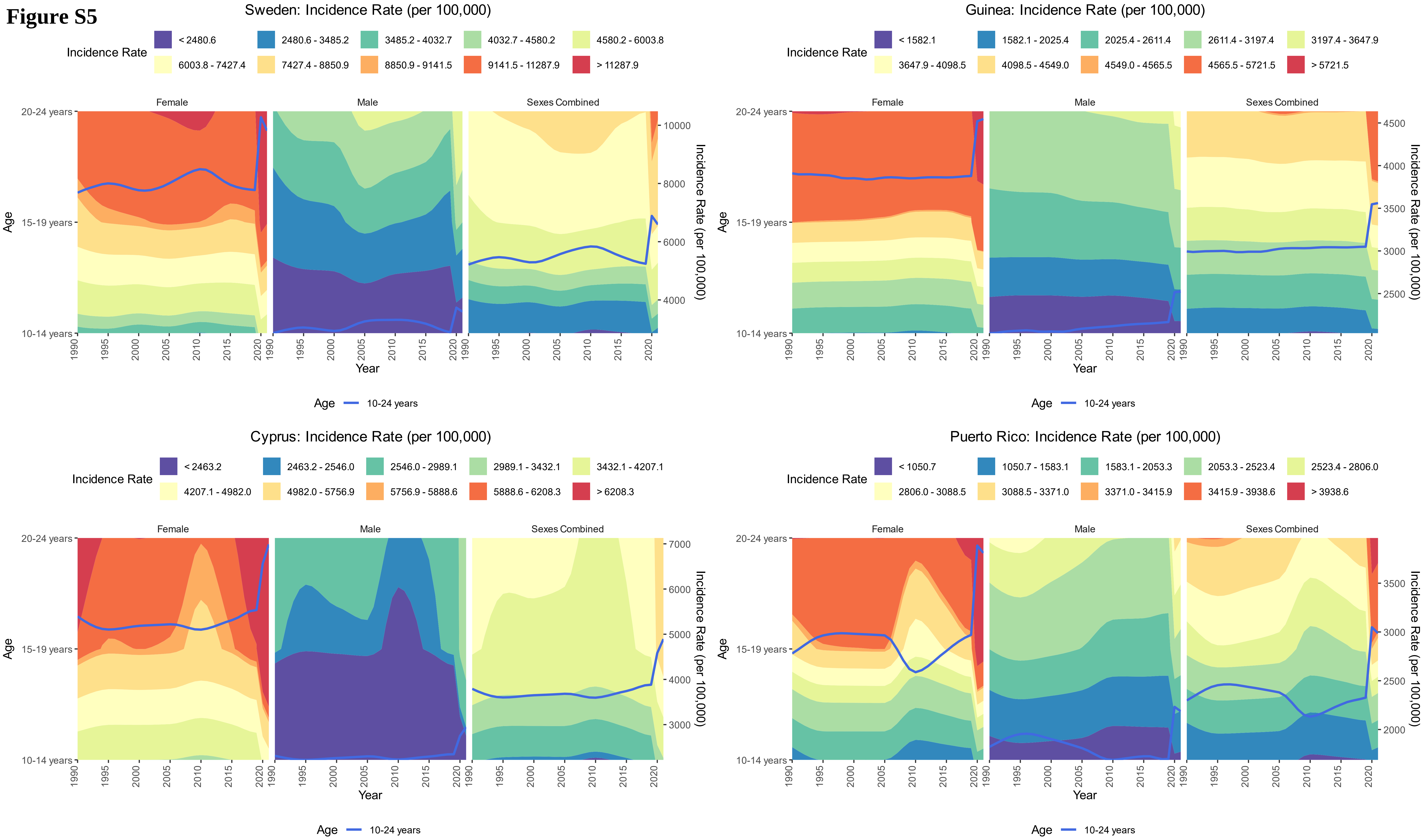
Sweden: Incidence Rate (per 100,000)
Incidence Rate
< 2480.6
2480.6 - 3485.2
3485.2 - 4032.7
4032.7 - 4580.2
4580.2 - 6003.8
6003.8 - 7427.4
7427.4 - 8850.9
8850.9 - 9141.5
9141.5 - 11287.9
> 11287.9
Guinea: Incidence Rate (per 100,000)
Incidence Rate
< 1582.1
1582.1 - 2025.4
2025.4 - 2611.4
2611.4 - 3197.4
3197.4 - 3647.9
3647.9 - 4098.5
4098.5 - 4549.0
4549.0 - 4565.5
4565.5 - 5721.5
> 5721.5
Cyprus: Incidence Rate (per 100,000)
Incidence Rate
< 2463.2
2463.2 - 2546.0
2546.0 - 2989.1
2989.1 - 3432.1
3432.1 - 4207.1
4207.1 - 4982.0
4982.0 - 5756.9
5756.9 - 5888.6
5888.6 - 6208.3
> 6208.3
Puerto Rico: Incidence Rate (per 100,000)
Incidence Rate
< 1050.7
1050.7 - 1583.1
1583.1 - 2053.3
2053.3 - 2523.4
2523.4 - 2806.0
2806.0 - 3088.5
3088.5 - 3371.0
3371.0 - 3415.9
3415.9 - 3938.6
> 3938.6
Female
Male
Sexes Combined
Age
20-24 years
15-19 years
10-14 years
Year
Incidence Rate (per 100,000)
Age
10-24 years

Figure S6

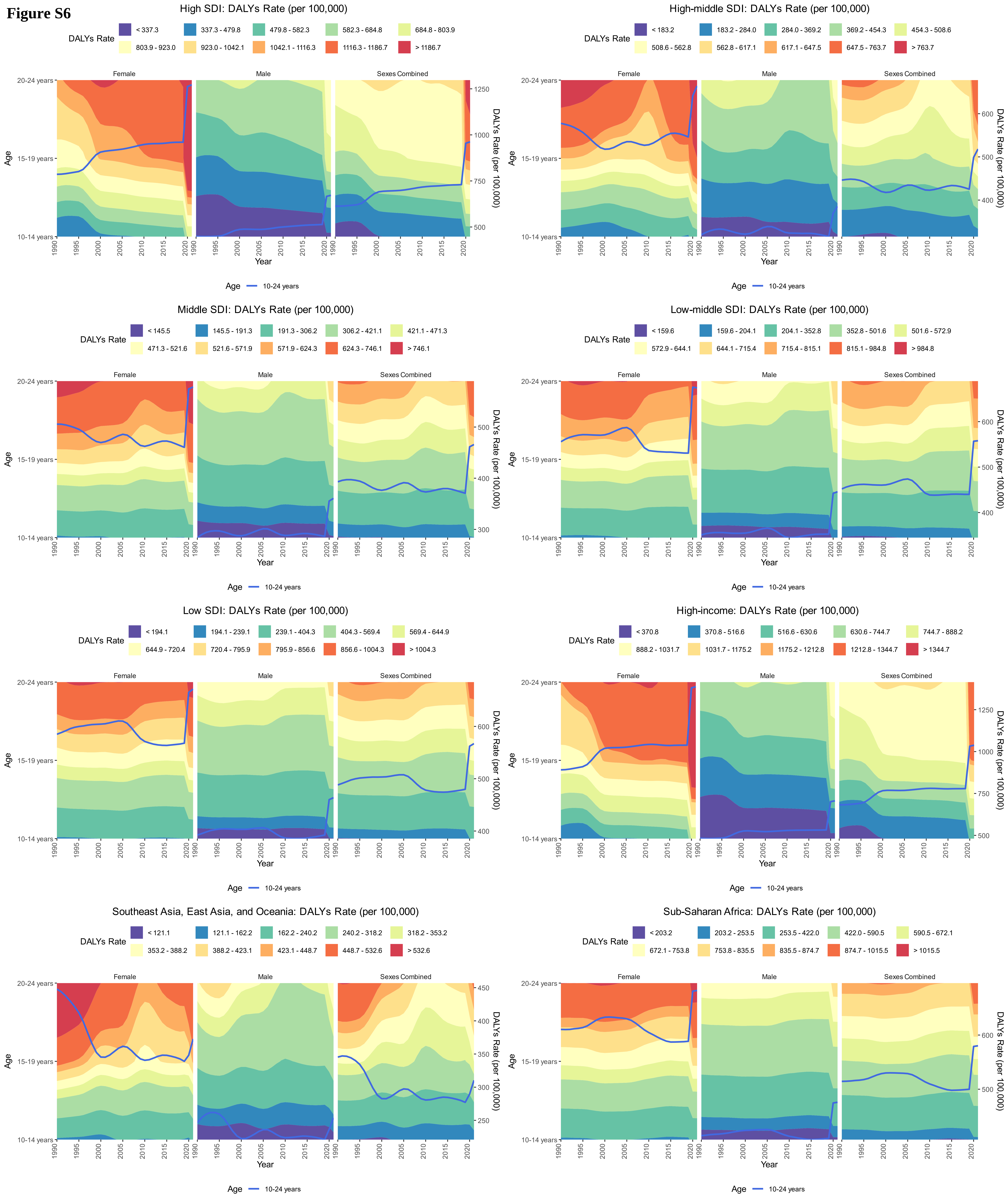

High SDI: DALYs Rate (per 100,000)
DALYs Rate
< 337.3
337.3 - 479.8
479.8 - 582.3
582.3 - 684.8
684.8 - 803.9
803.9 - 923.0
923.0 - 1042.1
1042.1 - 1116.3
1116.3 - 1186.7
> 1186.7
High-middle SDI: DALYs Rate (per 100,000)
< 183.2
183.2 - 284.0
284.0 - 369.2
369.2 - 454.3
454.3 - 508.6
508.6 - 562.8
562.8 - 617.1
617.1 - 647.5
647.5 - 763.7
> 763.7
Middle SDI: DALYs Rate (per 100,000)
< 145.5
145.5 - 191.3
191.3 - 306.2
306.2 - 421.1
421.1 - 471.3
471.3 - 521.6
521.6 - 571.9
571.9 - 624.3
624.3 - 746.1
> 746.1
Low-middle SDI: DALYs Rate (per 100,000)
< 159.6
159.6 - 204.1
204.1 - 352.8
352.8 - 501.6
501.6 - 572.9
572.9 - 644.1
644.1 - 715.4
715.4 - 815.1
815.1 - 984.8
> 984.8
Low SDI: DALYs Rate (per 100,000)
< 194.1
194.1 - 239.1
239.1 - 404.3
404.3 - 569.4
569.4 - 644.9
644.9 - 720.4
720.4 - 795.9
795.9 - 856.6
856.6 - 1004.3
> 1004.3
High-income: DALYs Rate (per 100,000)
< 370.8
370.8 - 516.6
516.6 - 630.6
630.6 - 744.7
744.7 - 888.2
888.2 - 1031.7
1031.7 - 1175.2
1175.2 - 1212.8
1212.8 - 1344.7
> 1344.7
Southeast Asia, East Asia, and Oceania: DALYs Rate (per 100,000)
< 121.1
121.1 - 162.2
162.2 - 240.2
240.2 - 318.2
318.2 - 353.2
353.2 - 388.2
388.2 - 423.1
423.1 - 448.7
448.7 - 532.6
> 532.6
Sub-Saharan Africa: DALYs Rate (per 100,000)
< 203.2
203.2 - 253.5
253.5 - 422.0
422.0 - 590.5
590.5 - 672.1
672.1 - 753.8
753.8 - 835.5
835.5 - 874.7
874.7 - 1015.5
> 1015.5
Female
Male
Sexes Combined
Age
20-24 years
15-19 years
10-14 years
Year
DALYs Rate (per 100,000)
Age — 10-24 years

Figure S6

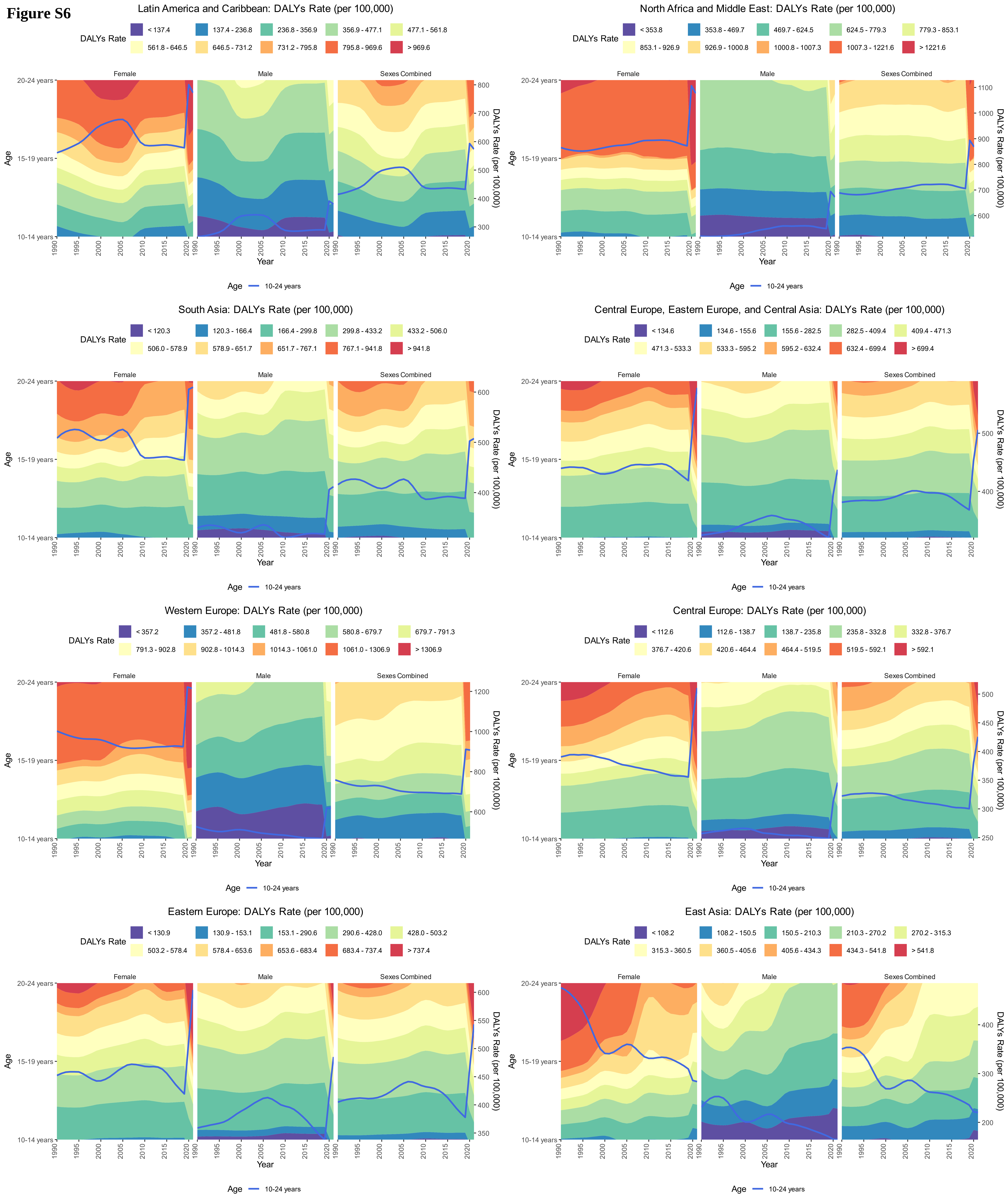

Latin America and Caribbean: DALYs Rate (per 100,000)
DALYs Rate
< 137.4
137.4 - 236.8
236.8 - 356.9
356.9 - 477.1
477.1 - 561.8
561.8 - 646.5
646.5 - 731.2
731.2 - 795.8
795.8 - 969.6
> 969.6
North Africa and Middle East: DALYs Rate (per 100,000)
DALYs Rate
< 353.8
353.8 - 469.7
469.7 - 624.5
624.5 - 779.3
779.3 - 853.1
853.1 - 926.9
926.9 - 1000.8
1000.8 - 1007.3
1007.3 - 1221.6
> 1221.6
South Asia: DALYs Rate (per 100,000)
DALYs Rate
< 120.3
120.3 - 166.4
166.4 - 299.8
299.8 - 433.2
433.2 - 506.0
506.0 - 578.9
578.9 - 651.7
651.7 - 767.1
767.1 - 941.8
> 941.8
Central Europe, Eastern Europe, and Central Asia: DALYs Rate (per 100,000)
DALYs Rate
< 134.6
134.6 - 155.6
155.6 - 282.5
282.5 - 409.4
409.4 - 471.3
471.3 - 533.3
533.3 - 595.2
595.2 - 632.4
632.4 - 699.4
> 699.4
Western Europe: DALYs Rate (per 100,000)
DALYs Rate
< 357.2
357.2 - 481.8
481.8 - 580.8
580.8 - 679.7
679.7 - 791.3
791.3 - 902.8
902.8 - 1014.3
1014.3 - 1061.0
1061.0 - 1306.9
> 1306.9
Central Europe: DALYs Rate (per 100,000)
DALYs Rate
< 112.6
112.6 - 138.7
138.7 - 235.8
235.8 - 332.8
332.8 - 376.7
376.7 - 420.6
420.6 - 464.4
464.4 - 519.5
519.5 - 592.1
> 592.1
Eastern Europe: DALYs Rate (per 100,000)
DALYs Rate
< 130.9
130.9 - 153.1
153.1 - 290.6
290.6 - 428.0
428.0 - 503.2
503.2 - 578.4
578.4 - 653.6
653.6 - 683.4
683.4 - 737.4
> 737.4
East Asia: DALYs Rate (per 100,000)
DALYs Rate
< 108.2
108.2 - 150.5
150.5 - 210.3
210.3 - 270.2
270.2 - 315.3
315.3 - 360.5
360.5 - 405.6
405.6 - 434.3
434.3 - 541.8
> 541.8
Female
Male
Sexes Combined
Age
20-24 years
15-19 years
10-14 years
Year
DALYs Rate (per 100,000)
Age
10-24 years

Figure S6

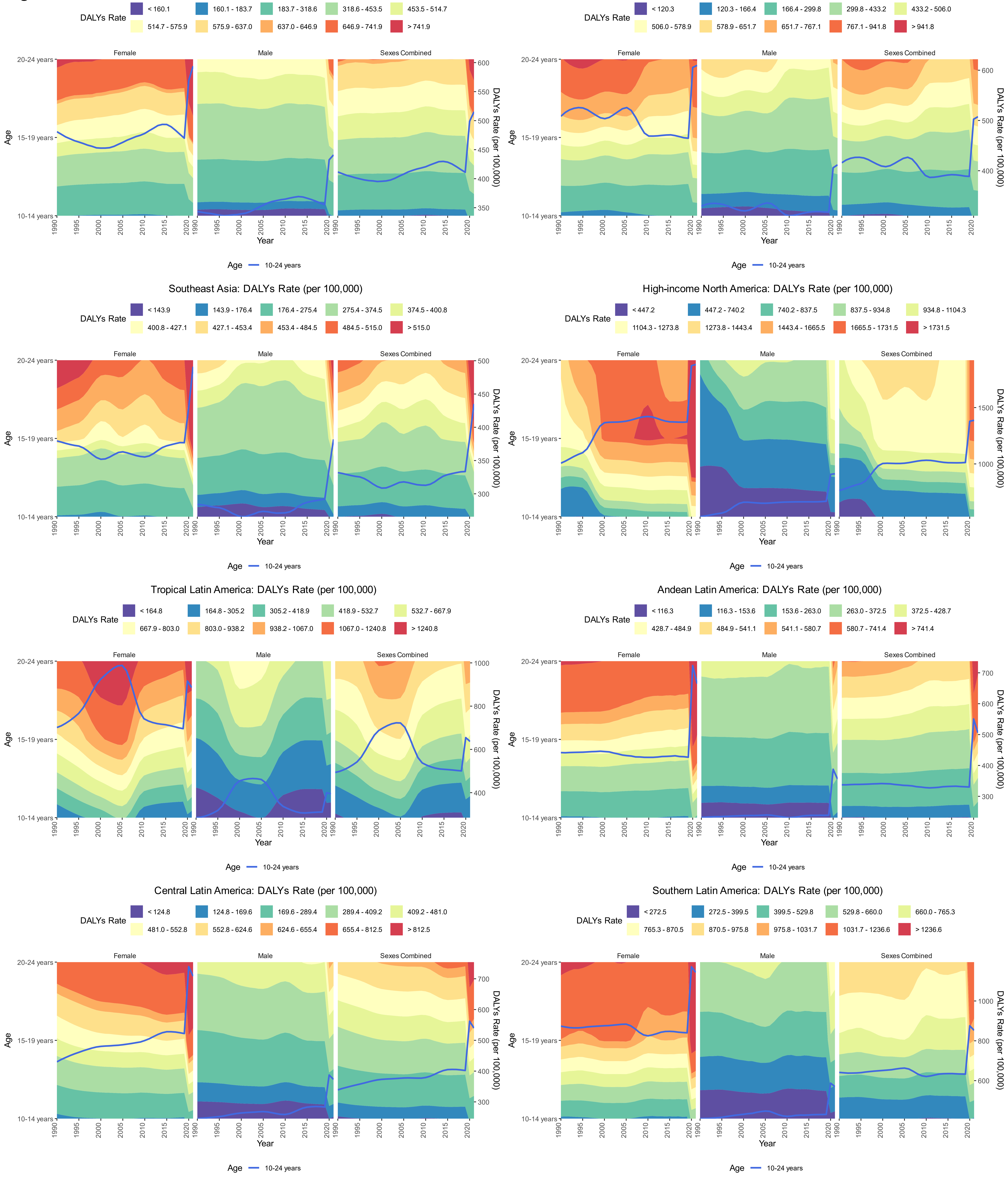

Central Asia: DALYs Rate (per 100,000)
DALYs Rate
< 160.1
160.1 - 183.7
183.7 - 318.6
318.6 - 453.5
453.5 - 514.7
514.7 - 575.9
575.9 - 637.0
637.0 - 646.9
646.9 - 741.9
> 741.9
South Asia: DALYs Rate (per 100,000)
DALYs Rate
< 120.3
120.3 - 166.4
166.4 - 299.8
299.8 - 433.2
433.2 - 506.0
506.0 - 578.9
578.9 - 651.7
651.7 - 767.1
767.1 - 941.8
> 941.8
Southeast Asia: DALYs Rate (per 100,000)
DALYs Rate
< 143.9
143.9 - 176.4
176.4 - 275.4
275.4 - 374.5
374.5 - 400.8
400.8 - 427.1
427.1 - 453.4
453.4 - 484.5
484.5 - 515.0
> 515.0
High-income North America: DALYs Rate (per 100,000)
DALYs Rate
< 447.2
447.2 - 740.2
740.2 - 837.5
837.5 - 934.8
934.8 - 1104.3
1104.3 - 1273.8
1273.8 - 1443.4
1443.4 - 1665.5
1665.5 - 1731.5
> 1731.5
Tropical Latin America: DALYs Rate (per 100,000)
DALYs Rate
< 164.8
164.8 - 305.2
305.2 - 418.9
418.9 - 532.7
532.7 - 667.9
667.9 - 803.0
803.0 - 938.2
938.2 - 1067.0
1067.0 - 1240.8
> 1240.8
Andean Latin America: DALYs Rate (per 100,000)
DALYs Rate
< 116.3
116.3 - 153.6
153.6 - 263.0
263.0 - 372.5
372.5 - 428.7
428.7 - 484.9
484.9 - 541.1
541.1 - 580.7
580.7 - 741.4
> 741.4
Central Latin America: DALYs Rate (per 100,000)
DALYs Rate
< 124.8
124.8 - 169.6
169.6 - 289.4
289.4 - 409.2
409.2 - 481.0
481.0 - 552.8
552.8 - 624.6
624.6 - 655.4
655.4 - 812.5
> 812.5
Southern Latin America: DALYs Rate (per 100,000)
DALYs Rate
< 272.5
272.5 - 399.5
399.5 - 529.8
529.8 - 660.0
660.0 - 765.3
765.3 - 870.5
870.5 - 975.8
975.8 - 1031.7
1031.7 - 1236.6
> 1236.6
Female
Male
Sexes Combined
Age
20-24 years
15-19 years
10-14 years
Year
1990
1995
2000
2005
2010
2015
2020
DALYs Rate (per 100,000)
Age
10-24 years

Figure S6

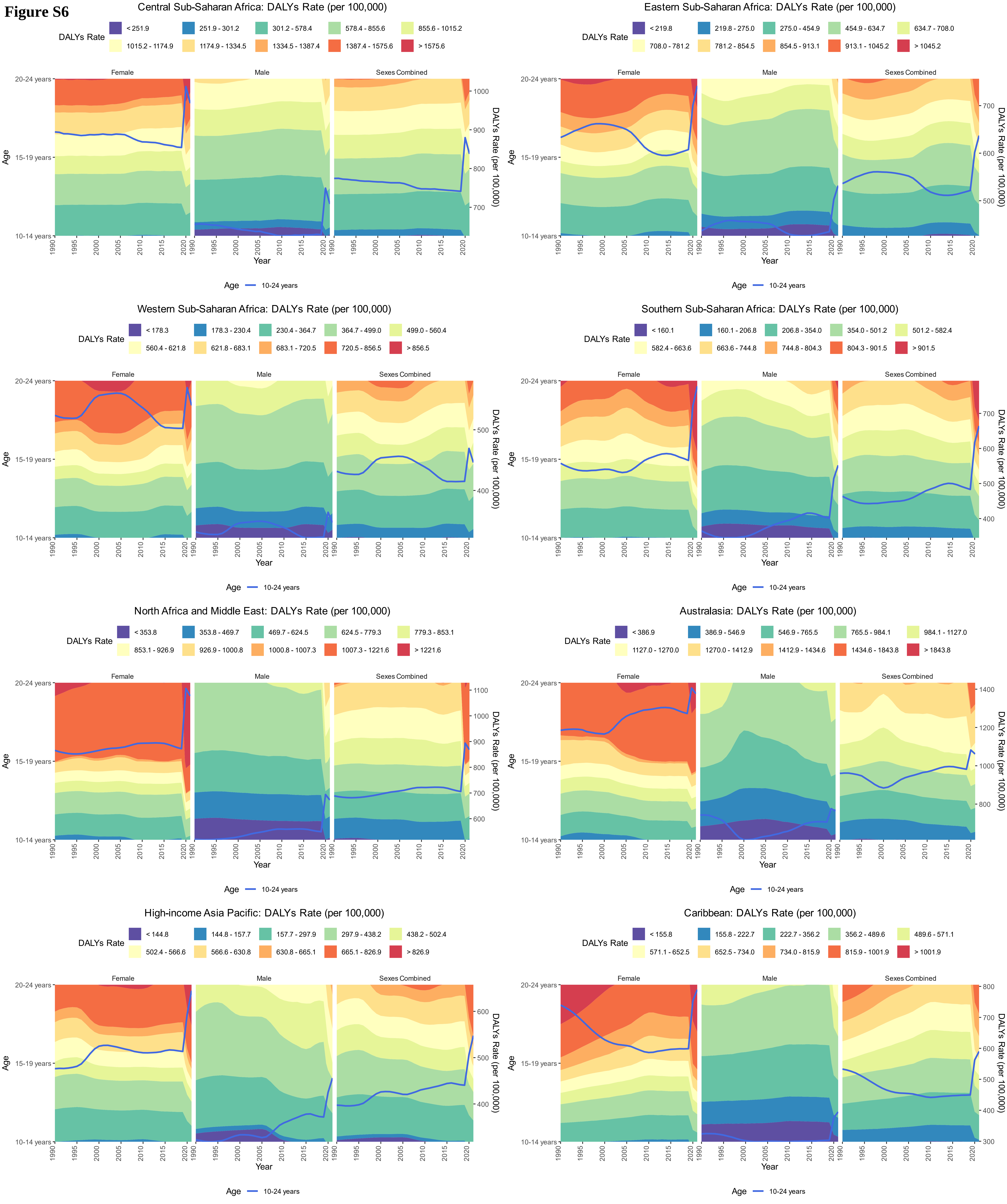
Central Sub-Saharan Africa: DALYs Rate (per 100,000)
DALYs Rate
< 251.9
251.9 - 301.2
301.2 - 578.4
578.4 - 855.6
855.6 - 1015.2
1015.2 - 1174.9
1174.9 - 1334.5
1334.5 - 1387.4
1387.4 - 1575.6
> 1575.6
Eastern Sub-Saharan Africa: DALYs Rate (per 100,000)
DALYs Rate
< 219.8
219.8 - 275.0
275.0 - 454.9
454.9 - 634.7
634.7 - 708.0
708.0 - 781.2
781.2 - 854.5
854.5 - 913.1
913.1 - 1045.2
> 1045.2
Western Sub-Saharan Africa: DALYs Rate (per 100,000)
DALYs Rate
< 178.3
178.3 - 230.4
230.4 - 364.7
364.7 - 499.0
499.0 - 560.4
560.4 - 621.8
621.8 - 683.1
683.1 - 720.5
720.5 - 856.5
> 856.5
Southern Sub-Saharan Africa: DALYs Rate (per 100,000)
DALYs Rate
< 160.1
160.1 - 206.8
206.8 - 354.0
354.0 - 501.2
501.2 - 582.4
582.4 - 663.6
663.6 - 744.8
744.8 - 804.3
804.3 - 901.5
> 901.5
North Africa and Middle East: DALYs Rate (per 100,000)
DALYs Rate
< 353.8
353.8 - 469.7
469.7 - 624.5
624.5 - 779.3
779.3 - 853.1
853.1 - 926.9
926.9 - 1000.8
1000.8 - 1007.3
1007.3 - 1221.6
> 1221.6
Australasia: DALYs Rate (per 100,000)
DALYs Rate
< 386.9
386.9 - 546.9
546.9 - 765.5
765.5 - 984.1
984.1 - 1127.0
1127.0 - 1270.0
1270.0 - 1412.9
1412.9 - 1434.6
1434.6 - 1843.8
> 1843.8
High-income Asia Pacific: DALYs Rate (per 100,000)
DALYs Rate
< 144.8
144.8 - 157.7
157.7 - 297.9
297.9 - 438.2
438.2 - 502.4
502.4 - 566.6
566.6 - 630.8
630.8 - 665.1
665.1 - 826.9
> 826.9
Caribbean: DALYs Rate (per 100,000)
DALYs Rate
< 155.8
155.8 - 222.7
222.7 - 356.2
356.2 - 489.6
489.6 - 571.1
571.1 - 652.5
652.5 - 734.0
734.0 - 815.9
815.9 - 1001.9
> 1001.9
Female
Male
Sexes Combined
Age
20-24 years
15-19 years
10-14 years
Year
DALYs Rate (per 100,000)
Age
10-24 years

Figure S6

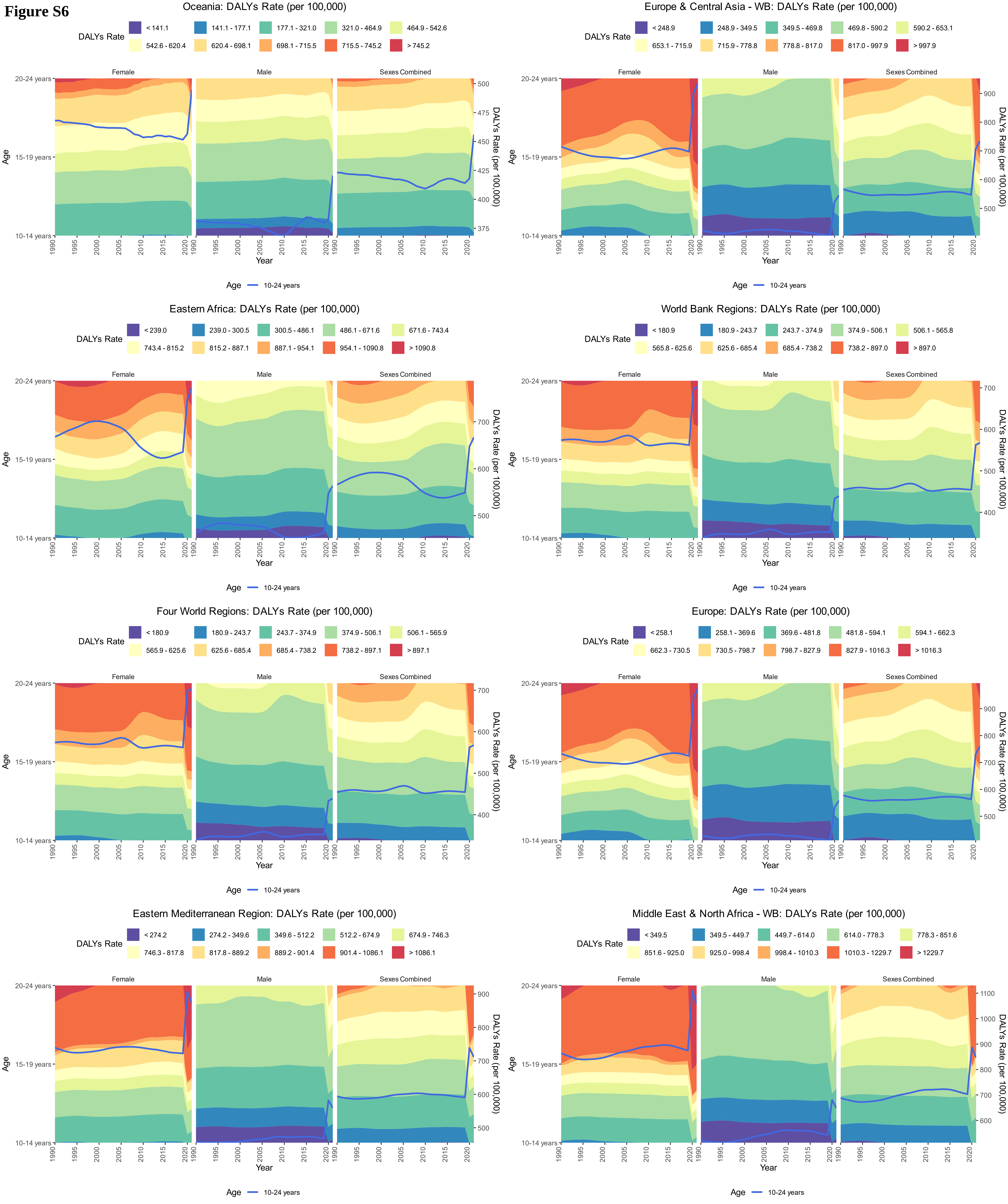

Oceania: DALYs Rate (per 100,000)
DALYs Rate
< 141.1
141.1 - 177.1
177.1 - 321.0
321.0 - 464.9
464.9 - 542.6
542.6 - 620.4
620.4 - 698.1
698.1 - 715.5
715.5 - 745.2
> 745.2
Europe & Central Asia - WB: DALYs Rate (per 100,000)
< 248.9
248.9 - 349.5
349.5 - 469.8
469.8 - 590.2
590.2 - 653.1
653.1 - 715.9
715.9 - 778.8
778.8 - 817.0
817.0 - 997.9
> 997.9
Eastern Africa: DALYs Rate (per 100,000)
< 239.0
239.0 - 300.5
300.5 - 486.1
486.1 - 671.6
671.6 - 743.4
743.4 - 815.2
815.2 - 887.1
887.1 - 954.1
954.1 - 1090.8
> 1090.8
World Bank Regions: DALYs Rate (per 100,000)
< 180.9
180.9 - 243.7
243.7 - 374.9
374.9 - 506.1
506.1 - 565.8
565.8 - 625.6
625.6 - 685.4
685.4 - 738.2
738.2 - 897.0
> 897.0
Four World Regions: DALYs Rate (per 100,000)
< 180.9
180.9 - 243.7
243.7 - 374.9
374.9 - 506.1
506.1 - 565.9
565.9 - 625.6
625.6 - 685.4
685.4 - 738.2
738.2 - 897.1
> 897.1
Europe: DALYs Rate (per 100,000)
< 258.1
258.1 - 369.6
369.6 - 481.8
481.8 - 594.1
594.1 - 662.3
662.3 - 730.5
730.5 - 798.7
798.7 - 827.9
827.9 - 1016.3
> 1016.3
Eastern Mediterranean Region: DALYs Rate (per 100,000)
< 274.2
274.2 - 349.6
349.6 - 512.2
512.2 - 674.9
674.9 - 746.3
746.3 - 817.8
817.8 - 889.2
889.2 - 901.4
901.4 - 1086.1
> 1086.1
Middle East & North Africa - WB: DALYs Rate (per 100,000)
< 349.5
349.5 - 449.7
449.7 - 614.0
614.0 - 778.3
778.3 - 851.6
851.6 - 925.0
925.0 - 998.4
998.4 - 1010.3
1010.3 - 1229.7
> 1229.7
Female
Male
Sexes Combined
Age
20-24 years
15-19 years
10-14 years
Year
DALYs Rate (per 100,000)
Age — 10-24 years

Figure S6

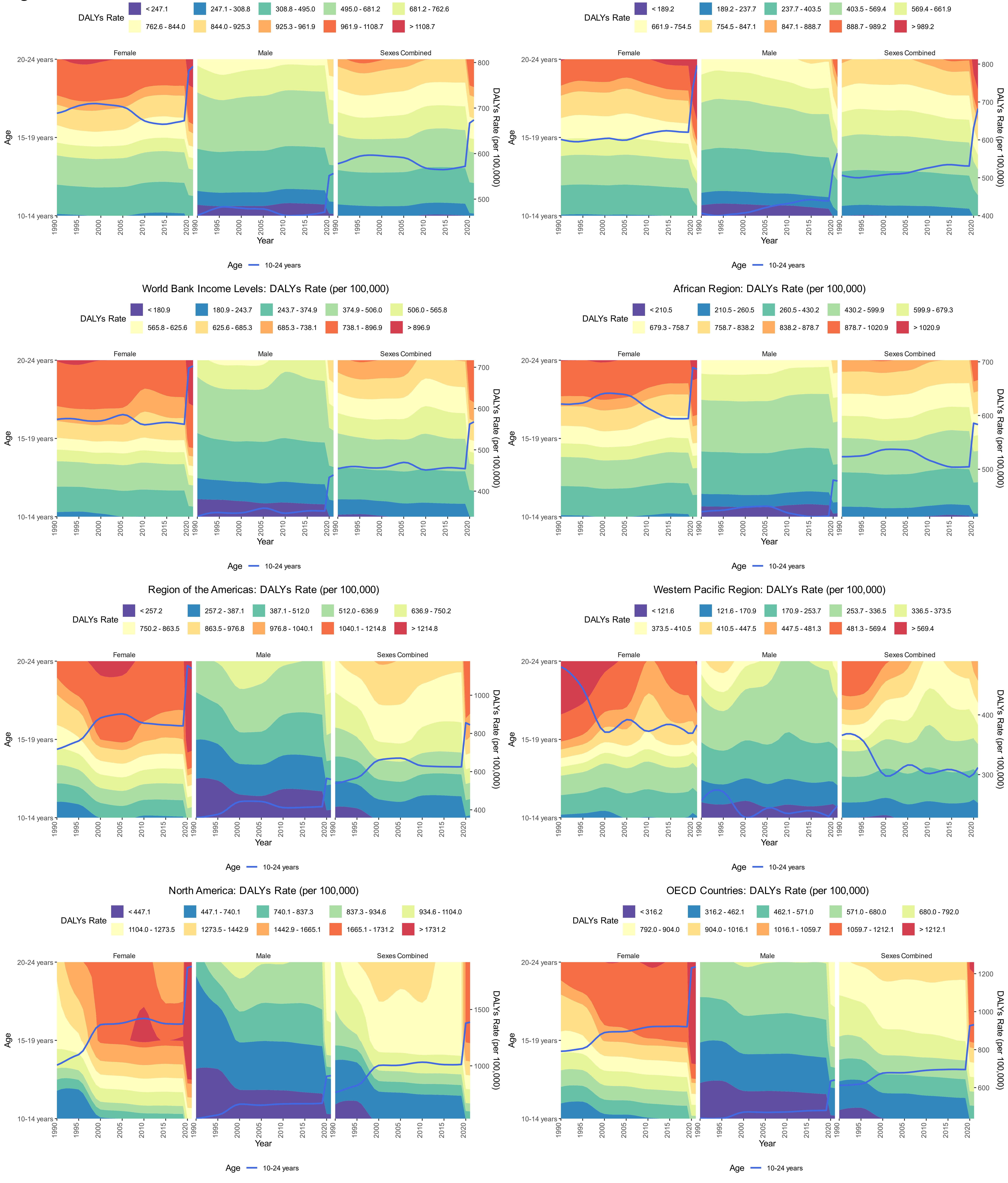
World Bank Low Income: DALYs Rate (per 100,000)
DALYs Rate
< 247.1
247.1 - 308.8
308.8 - 495.0
495.0 - 681.2
681.2 - 762.6
762.6 - 844.0
844.0 - 925.3
925.3 - 961.9
961.9 - 1108.7
> 1108.7
Southern Africa: DALYs Rate (per 100,000)
< 189.2
189.2 - 237.7
237.7 - 403.5
403.5 - 569.4
569.4 - 661.9
661.9 - 754.5
754.5 - 847.1
847.1 - 888.7
888.7 - 989.2
> 989.2
World Bank Income Levels: DALYs Rate (per 100,000)
< 180.9
180.9 - 243.7
243.7 - 374.9
374.9 - 506.0
506.0 - 565.8
565.8 - 625.6
625.6 - 685.3
685.3 - 738.1
738.1 - 896.9
> 896.9
African Region: DALYs Rate (per 100,000)
< 210.5
210.5 - 260.5
260.5 - 430.2
430.2 - 599.9
599.9 - 679.3
679.3 - 758.7
758.7 - 838.2
838.2 - 878.7
878.7 - 1020.9
> 1020.9
Region of the Americas: DALYs Rate (per 100,000)
< 257.2
257.2 - 387.1
387.1 - 512.0
512.0 - 636.9
636.9 - 750.2
750.2 - 863.5
863.5 - 976.8
976.8 - 1040.1
1040.1 - 1214.8
> 1214.8
Western Pacific Region: DALYs Rate (per 100,000)
< 121.6
121.6 - 170.9
170.9 - 253.7
253.7 - 336.5
336.5 - 373.5
373.5 - 410.5
410.5 - 447.5
447.5 - 481.3
481.3 - 569.4
> 569.4
North America: DALYs Rate (per 100,000)
< 447.1
447.1 - 740.1
740.1 - 837.3
837.3 - 934.6
934.6 - 1104.0
1104.0 - 1273.5
1273.5 - 1442.9
1442.9 - 1665.1
1665.1 - 1731.2
> 1731.2
OECD Countries: DALYs Rate (per 100,000)
< 316.2
316.2 - 462.1
462.1 - 571.0
571.0 - 680.0
680.0 - 792.0
792.0 - 904.0
904.0 - 1016.1
1016.1 - 1059.7
1059.7 - 1212.1
> 1212.1
Female
Male
Sexes Combined
Age
20-24 years
15-19 years
10-14 years
Year
DALYs Rate (per 100,000)
Age — 10-24 years

Figure S6

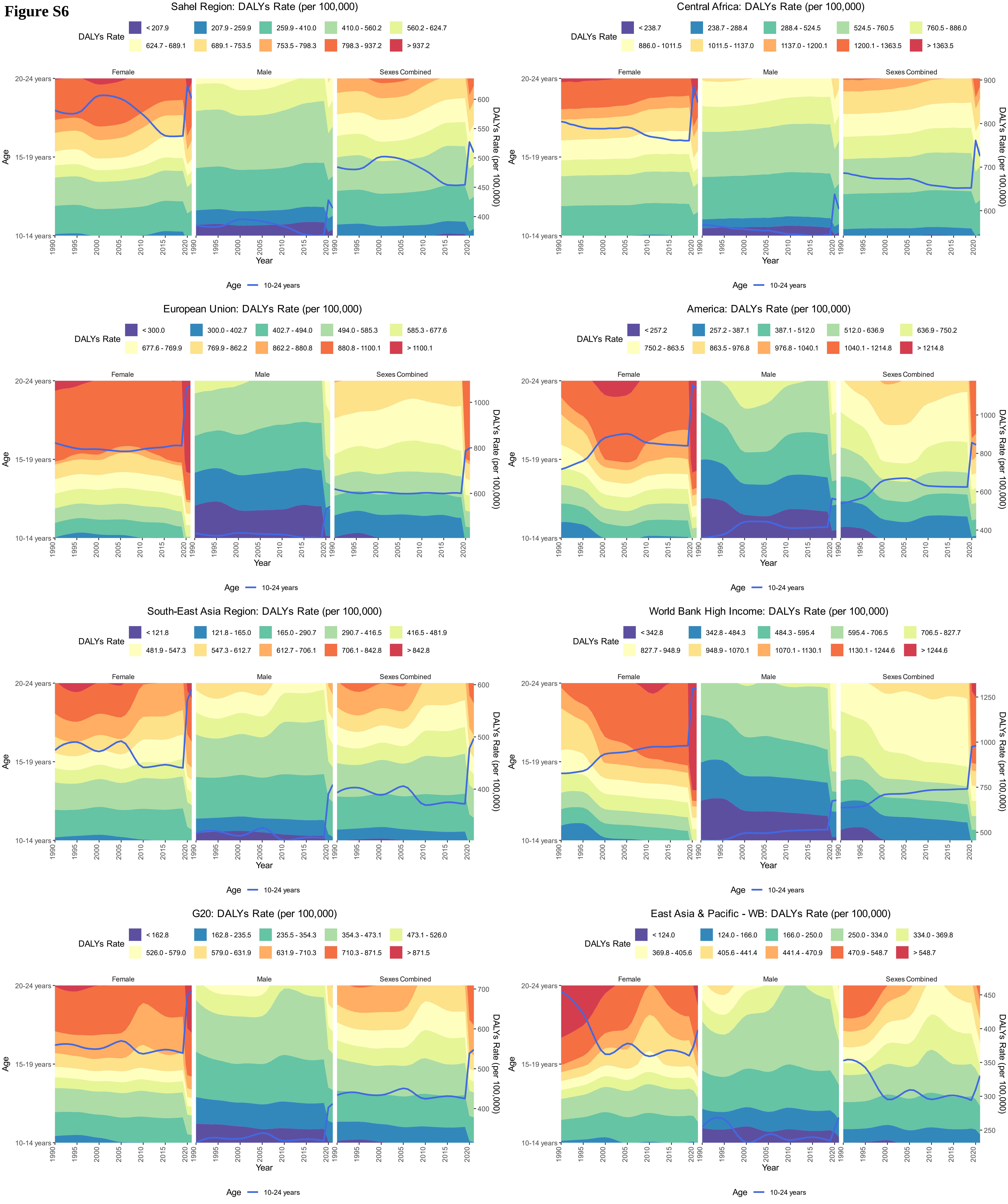
Sahel Region: DALYs Rate (per 100,000)
DALYs Rate
< 207.9
207.9 - 259.9
259.9 - 410.0
410.0 - 560.2
560.2 - 624.7
624.7 - 689.1
689.1 - 753.5
753.5 - 798.3
798.3 - 937.2
> 937.2
Central Africa: DALYs Rate (per 100,000)
DALYs Rate
< 238.7
238.7 - 288.4
288.4 - 524.5
524.5 - 760.5
760.5 - 886.0
886.0 - 1011.5
1011.5 - 1137.0
1137.0 - 1200.1
1200.1 - 1363.5
> 1363.5
European Union: DALYs Rate (per 100,000)
DALYs Rate
< 300.0
300.0 - 402.7
402.7 - 494.0
494.0 - 585.3
585.3 - 677.6
677.6 - 769.9
769.9 - 862.2
862.2 - 880.8
880.8 - 1100.1
> 1100.1
America: DALYs Rate (per 100,000)
DALYs Rate
< 257.2
257.2 - 387.1
387.1 - 512.0
512.0 - 636.9
636.9 - 750.2
750.2 - 863.5
863.5 - 976.8
976.8 - 1040.1
1040.1 - 1214.8
> 1214.8
South-East Asia Region: DALYs Rate (per 100,000)
DALYs Rate
< 121.8
121.8 - 165.0
165.0 - 290.7
290.7 - 416.5
416.5 - 481.9
481.9 - 547.3
547.3 - 612.7
612.7 - 706.1
706.1 - 842.8
> 842.8
World Bank High Income: DALYs Rate (per 100,000)
DALYs Rate
< 342.8
342.8 - 484.3
484.3 - 595.4
595.4 - 706.5
706.5 - 827.7
827.7 - 948.9
948.9 - 1070.1
1070.1 - 1130.1
1130.1 - 1244.6
> 1244.6
G20: DALYs Rate (per 100,000)
DALYs Rate
< 162.8
162.8 - 235.5
235.5 - 354.3
354.3 - 473.1
473.1 - 526.0
526.0 - 579.0
579.0 - 631.9
631.9 - 710.3
710.3 - 871.5
> 871.5
East Asia & Pacific - WB: DALYs Rate (per 100,000)
DALYs Rate
< 124.0
124.0 - 166.0
166.0 - 250.0
250.0 - 334.0
334.0 - 369.8
369.8 - 405.6
405.6 - 441.4
441.4 - 470.9
470.9 - 548.7
> 548.7
Female
Male
Sexes Combined
Age
20-24 years
15-19 years
10-14 years
Year
1990
1995
2000
2005
2010
2015
2020
DALYs Rate (per 100,000)
Age
10-24 years

Figure S6

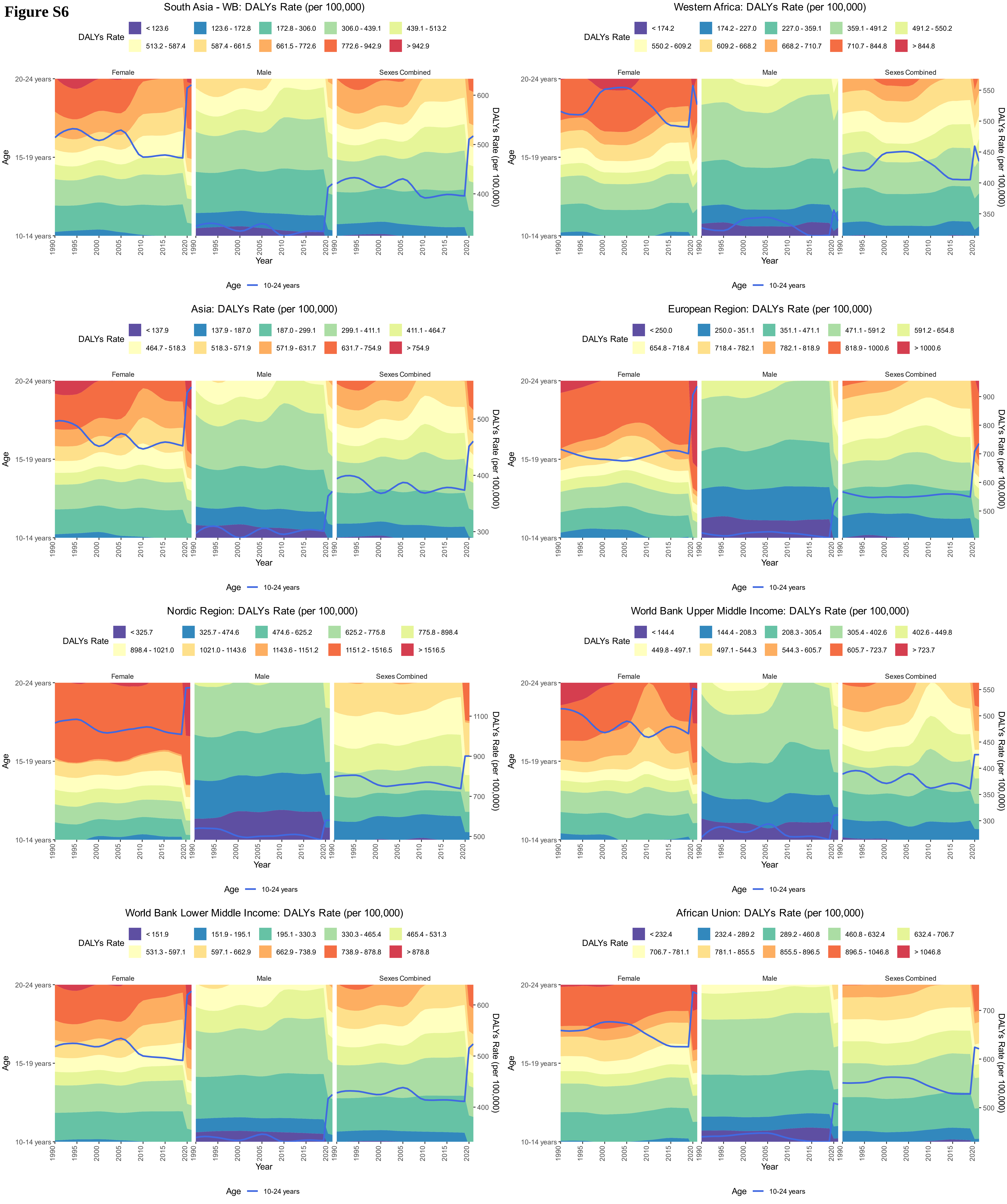
South Asia - WB: DALYs Rate (per 100,000)
DALYs Rate
< 123.6
123.6 - 172.8
172.8 - 306.0
306.0 - 439.1
439.1 - 513.2
513.2 - 587.4
587.4 - 661.5
661.5 - 772.6
772.6 - 942.9
> 942.9
Western Africa: DALYs Rate (per 100,000)
DALYs Rate
< 174.2
174.2 - 227.0
227.0 - 359.1
359.1 - 491.2
491.2 - 550.2
550.2 - 609.2
609.2 - 668.2
668.2 - 710.7
710.7 - 844.8
> 844.8
Asia: DALYs Rate (per 100,000)
DALYs Rate
< 137.9
137.9 - 187.0
187.0 - 299.1
299.1 - 411.1
411.1 - 464.7
464.7 - 518.3
518.3 - 571.9
571.9 - 631.7
631.7 - 754.9
> 754.9
European Region: DALYs Rate (per 100,000)
DALYs Rate
< 250.0
250.0 - 351.1
351.1 - 471.1
471.1 - 591.2
591.2 - 654.8
654.8 - 718.4
718.4 - 782.1
782.1 - 818.9
818.9 - 1000.6
> 1000.6
Nordic Region: DALYs Rate (per 100,000)
DALYs Rate
< 325.7
325.7 - 474.6
474.6 - 625.2
625.2 - 775.8
775.8 - 898.4
898.4 - 1021.0
1021.0 - 1143.6
1143.6 - 1151.2
1151.2 - 1516.5
> 1516.5
World Bank Upper Middle Income: DALYs Rate (per 100,000)
DALYs Rate
< 144.4
144.4 - 208.3
208.3 - 305.4
305.4 - 402.6
402.6 - 449.8
449.8 - 497.1
497.1 - 544.3
544.3 - 605.7
605.7 - 723.7
> 723.7
World Bank Lower Middle Income: DALYs Rate (per 100,000)
DALYs Rate
< 151.9
151.9 - 195.1
195.1 - 330.3
330.3 - 465.4
465.4 - 531.3
531.3 - 597.1
597.1 - 662.9
662.9 - 738.9
738.9 - 878.8
> 878.8
African Union: DALYs Rate (per 100,000)
DALYs Rate
< 232.4
232.4 - 289.2
289.2 - 460.8
460.8 - 632.4
632.4 - 706.7
706.7 - 781.1
781.1 - 855.5
855.5 - 896.5
896.5 - 1046.8
> 1046.8
Female
Male
Sexes Combined
20-24 years
15-19 years
10-14 years
Age
Year
DALYs Rate (per 100,000)
Age — 10-24 years

Figure S6

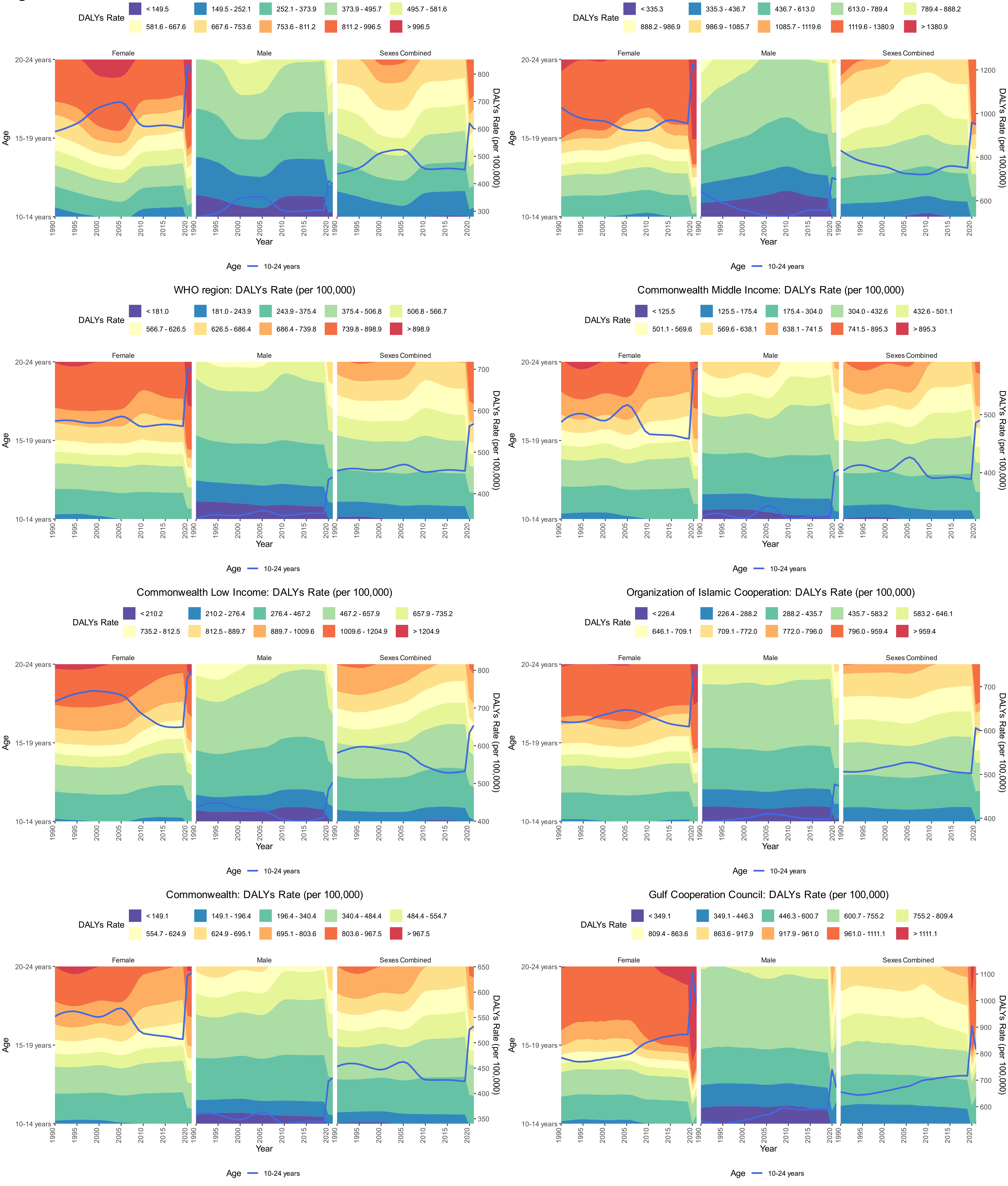

Latin America & Caribbean - WB: DALYs Rate (per 100,000)
DALYs Rate
< 149.5
149.5 - 252.1
252.1 - 373.9
373.9 - 495.7
495.7 - 581.6
581.6 - 667.6
667.6 - 753.6
753.6 - 811.2
811.2 - 996.5
> 996.5
Female
Male
Sexes Combined
Age
Year
DALYs Rate (per 100,000)
Age — 10-24 years
Commonwealth High Income: DALYs Rate (per 100,000)
< 335.3
335.3 - 436.7
436.7 - 613.0
613.0 - 789.4
789.4 - 888.2
888.2 - 986.9
986.9 - 1085.7
1085.7 - 1119.6
1119.6 - 1380.9
> 1380.9
WHO region: DALYs Rate (per 100,000)
< 181.0
181.0 - 243.9
243.9 - 375.4
375.4 - 506.8
506.8 - 566.7
566.7 - 626.5
626.5 - 686.4
686.4 - 739.8
739.8 - 898.9
> 898.9
Commonwealth Middle Income: DALYs Rate (per 100,000)
< 125.5
125.5 - 175.4
175.4 - 304.0
304.0 - 432.6
432.6 - 501.1
501.1 - 569.6
569.6 - 638.1
638.1 - 741.5
741.5 - 895.3
> 895.3
Commonwealth Low Income: DALYs Rate (per 100,000)
< 210.2
210.2 - 276.4
276.4 - 467.2
467.2 - 657.9
657.9 - 735.2
735.2 - 812.5
812.5 - 889.7
889.7 - 1009.6
1009.6 - 1204.9
> 1204.9
Organization of Islamic Cooperation: DALYs Rate (per 100,000)
< 226.4
226.4 - 288.2
288.2 - 435.7
435.7 - 583.2
583.2 - 646.1
646.1 - 709.1
709.1 - 772.0
772.0 - 796.0
796.0 - 959.4
> 959.4
Commonwealth: DALYs Rate (per 100,000)
< 149.1
149.1 - 196.4
196.4 - 340.4
340.4 - 484.4
484.4 - 554.7
554.7 - 624.9
624.9 - 695.1
695.1 - 803.6
803.6 - 967.5
> 967.5
Gulf Cooperation Council: DALYs Rate (per 100,000)
< 349.1
349.1 - 446.3
446.3 - 600.7
600.7 - 755.2
755.2 - 809.4
809.4 - 863.6
863.6 - 917.9
917.9 - 961.0
961.0 - 1111.1
> 1111.1
10-14 years
15-19 years
20-24 years
1990 1995 2000 2005 2010 2015 2020

Figure S6

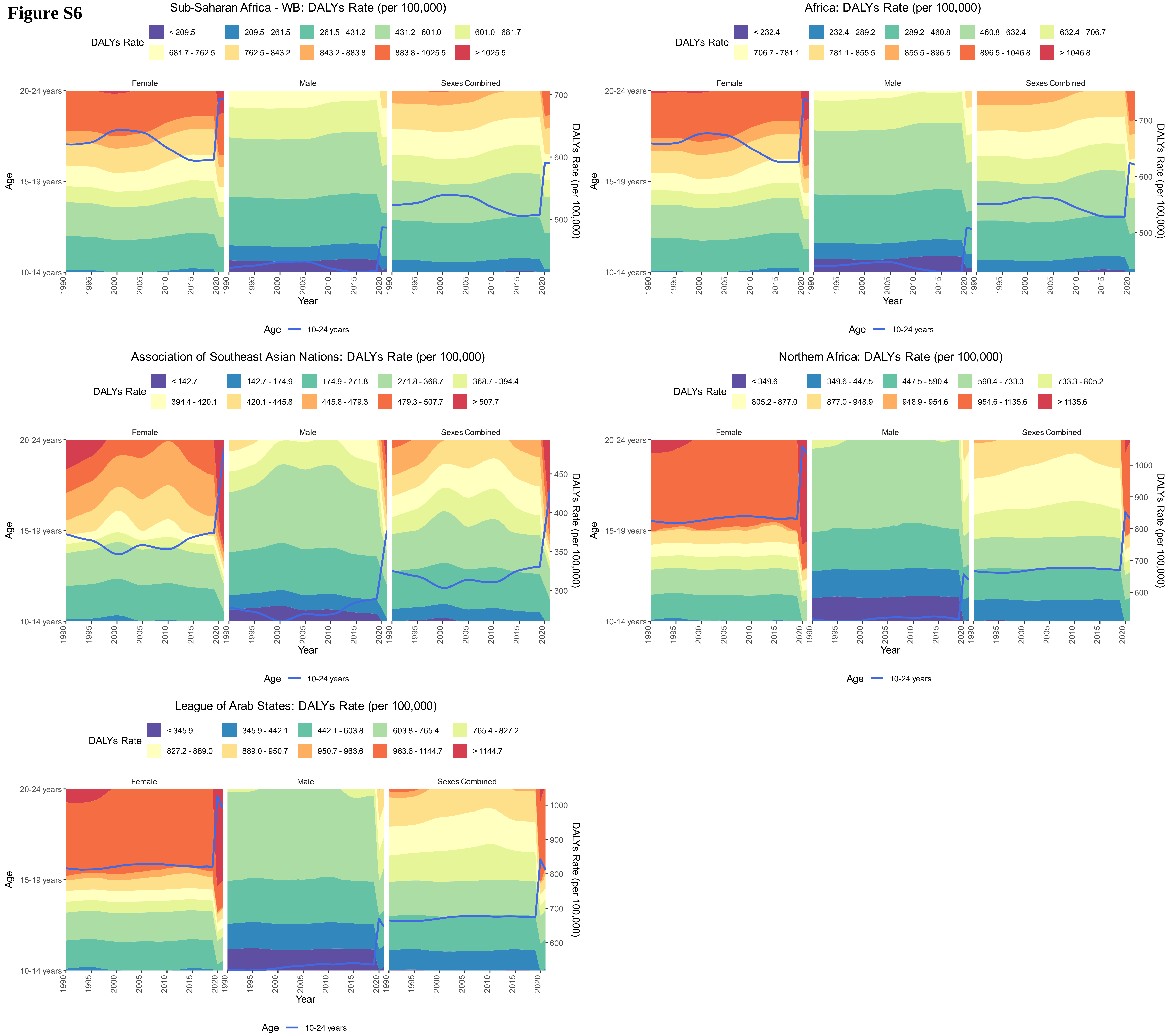
Sub-Saharan Africa - WB: DALYs Rate (per 100,000)
DALYs Rate
< 209.5
209.5 - 261.5
261.5 - 431.2
431.2 - 601.0
601.0 - 681.7
681.7 - 762.5
762.5 - 843.2
843.2 - 883.8
883.8 - 1025.5
> 1025.5
Female
Male
Sexes Combined
Age
Year
DALYs Rate (per 100,000)
Age — 10-24 years
Africa: DALYs Rate (per 100,000)
DALYs Rate
< 232.4
232.4 - 289.2
289.2 - 460.8
460.8 - 632.4
632.4 - 706.7
706.7 - 781.1
781.1 - 855.5
855.5 - 896.5
896.5 - 1046.8
> 1046.8
Association of Southeast Asian Nations: DALYs Rate (per 100,000)
DALYs Rate
< 142.7
142.7 - 174.9
174.9 - 271.8
271.8 - 368.7
368.7 - 394.4
394.4 - 420.1
420.1 - 445.8
445.8 - 479.3
479.3 - 507.7
> 507.7
Northern Africa: DALYs Rate (per 100,000)
DALYs Rate
< 349.6
349.6 - 447.5
447.5 - 590.4
590.4 - 733.3
733.3 - 805.2
805.2 - 877.0
877.0 - 948.9
948.9 - 954.6
954.6 - 1135.6
> 1135.6
League of Arab States: DALYs Rate (per 100,000)
DALYs Rate
< 345.9
345.9 - 442.1
442.1 - 603.8
603.8 - 765.4
765.4 - 827.2
827.2 - 889.0
889.0 - 950.7
950.7 - 963.6
963.6 - 1144.7
> 1144.7
20-24 years
15-19 years
10-14 years

Figure S6

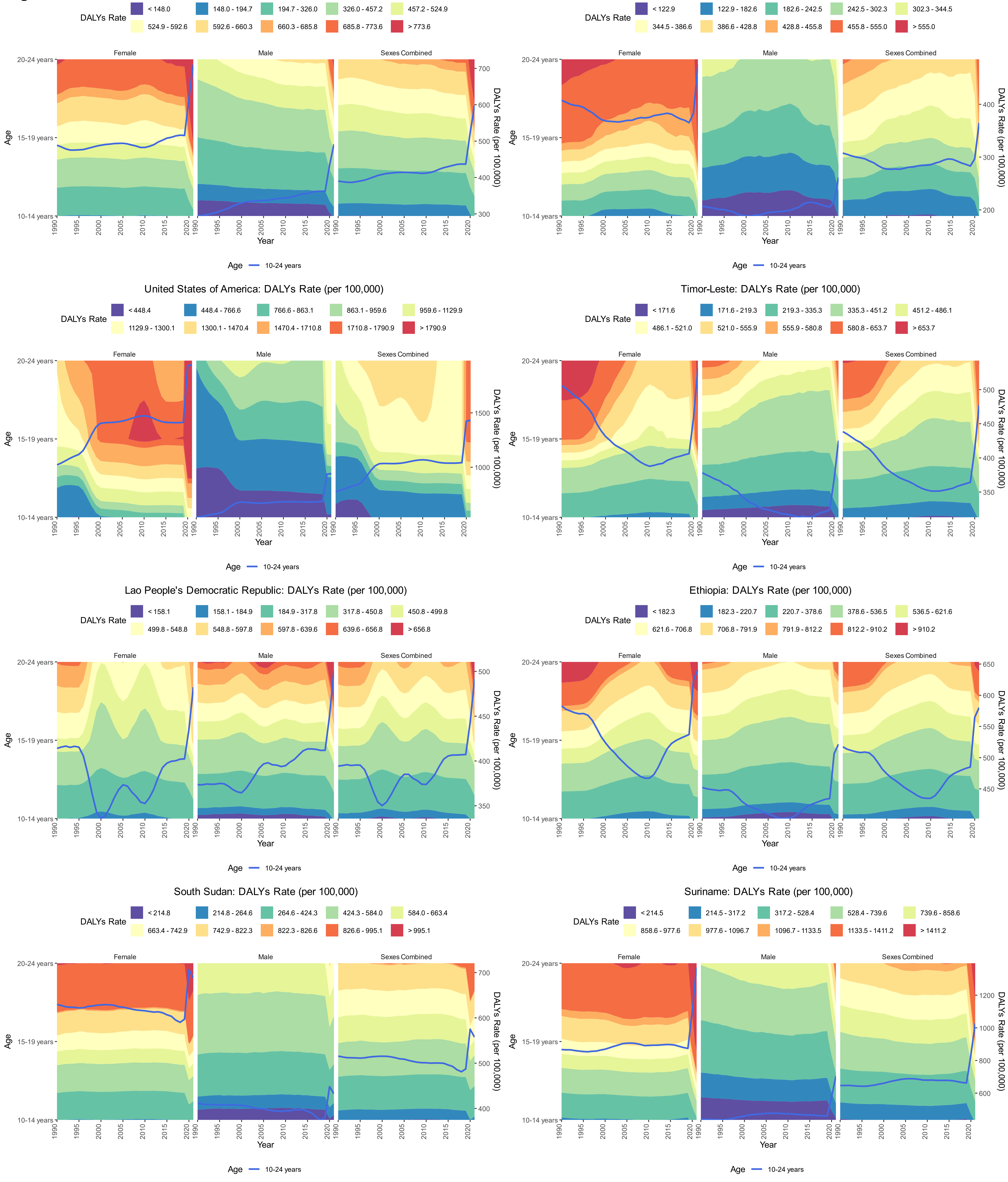
Saint Vincent and the Grenadines: DALYs Rate (per 100,000)
DALYs Rate
< 148.0
148.0 - 194.7
194.7 - 326.0
326.0 - 457.2
457.2 - 524.9
524.9 - 592.6
592.6 - 660.3
660.3 - 685.8
685.8 - 773.6
> 773.6
Female
Male
Sexes Combined
Age
Year
DALYs Rate (per 100,000)
Age 10-24 years
Viet Nam: DALYs Rate (per 100,000)
DALYs Rate
< 122.9
122.9 - 182.6
182.6 - 242.5
242.5 - 302.3
302.3 - 344.5
344.5 - 386.6
386.6 - 428.8
428.8 - 455.8
455.8 - 555.0
> 555.0
United States of America: DALYs Rate (per 100,000)
DALYs Rate
< 448.4
448.4 - 766.6
766.6 - 863.1
863.1 - 959.6
959.6 - 1129.9
1129.9 - 1300.1
1300.1 - 1470.4
1470.4 - 1710.8
1710.8 - 1790.9
> 1790.9
Timor-Leste: DALYs Rate (per 100,000)
DALYs Rate
< 171.6
171.6 - 219.3
219.3 - 335.3
335.3 - 451.2
451.2 - 486.1
486.1 - 521.0
521.0 - 555.9
555.9 - 580.8
580.8 - 653.7
> 653.7
Lao People's Democratic Republic: DALYs Rate (per 100,000)
DALYs Rate
< 158.1
158.1 - 184.9
184.9 - 317.8
317.8 - 450.8
450.8 - 499.8
499.8 - 548.8
548.8 - 597.8
597.8 - 639.6
639.6 - 656.8
> 656.8
Ethiopia: DALYs Rate (per 100,000)
DALYs Rate
< 182.3
182.3 - 220.7
220.7 - 378.6
378.6 - 536.5
536.5 - 621.6
621.6 - 706.8
706.8 - 791.9
791.9 - 812.2
812.2 - 910.2
> 910.2
South Sudan: DALYs Rate (per 100,000)
DALYs Rate
< 214.8
214.8 - 264.6
264.6 - 424.3
424.3 - 584.0
584.0 - 663.4
663.4 - 742.9
742.9 - 822.3
822.3 - 826.6
826.6 - 995.1
> 995.1
Suriname: DALYs Rate (per 100,000)
DALYs Rate
< 214.5
214.5 - 317.2
317.2 - 528.4
528.4 - 739.6
739.6 - 858.6
858.6 - 977.6
977.6 - 1096.7
1096.7 - 1133.5
1133.5 - 1411.2
> 1411.2
20-24 years
15-19 years
10-14 years
1990 1995 2000 2005 2010 2015 2020

Figure S6

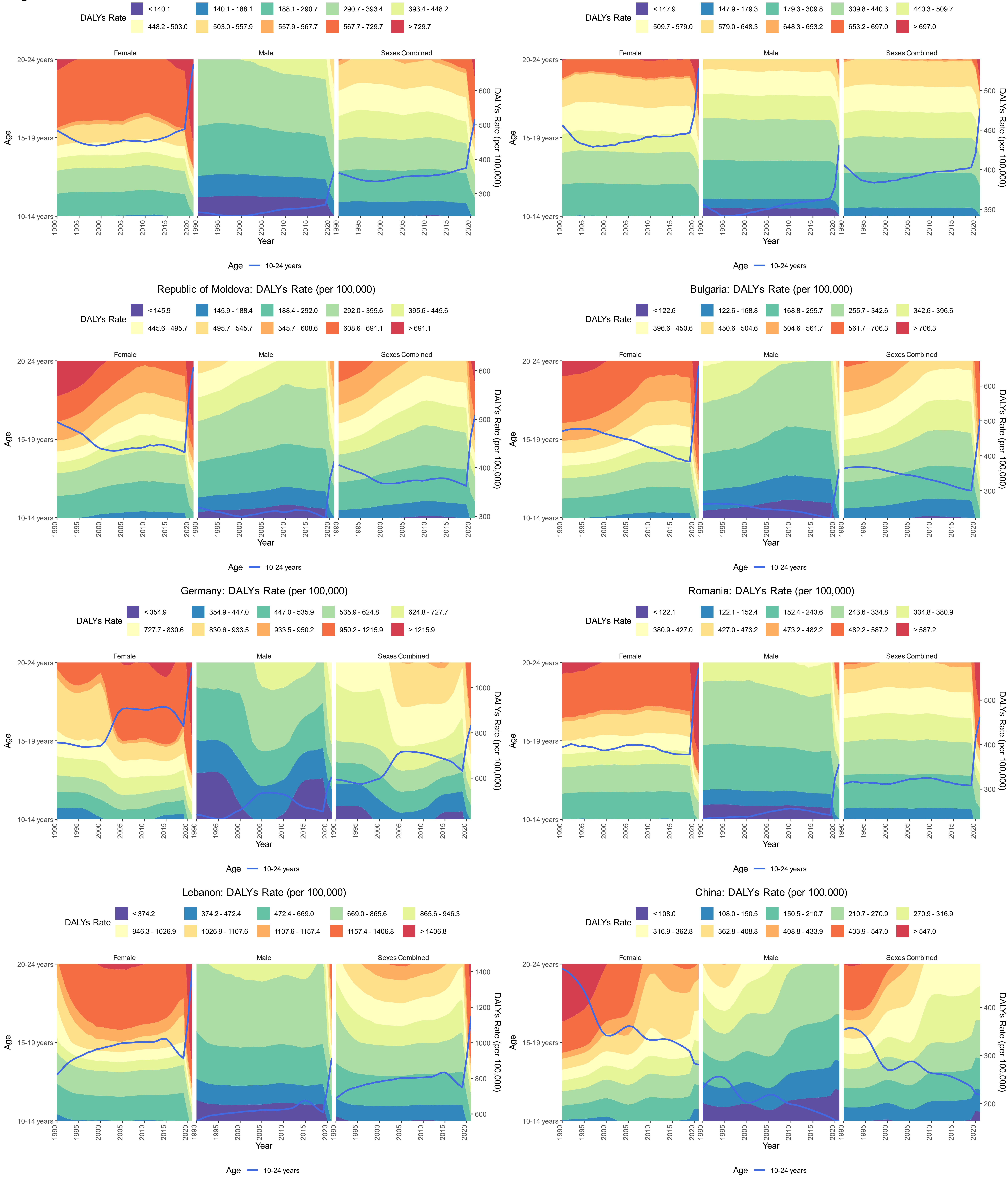

Dominica: DALYs Rate (per 100,000)
DALYs Rate
< 140.1
140.1 - 188.1
188.1 - 290.7
290.7 - 393.4
393.4 - 448.2
448.2 - 503.0
503.0 - 557.9
557.9 - 567.7
567.7 - 729.7
> 729.7
Female
Male
Sexes Combined
Age
20-24 years
15-19 years
10-14 years
Year
DALYs Rate (per 100,000)
Age
10-24 years
Tuvalu: DALYs Rate (per 100,000)
< 147.9
147.9 - 179.3
179.3 - 309.8
309.8 - 440.3
440.3 - 509.7
509.7 - 579.0
579.0 - 648.3
648.3 - 653.2
653.2 - 697.0
> 697.0
Republic of Moldova: DALYs Rate (per 100,000)
< 145.9
145.9 - 188.4
188.4 - 292.0
292.0 - 395.6
395.6 - 445.6
445.6 - 495.7
495.7 - 545.7
545.7 - 608.6
608.6 - 691.1
> 691.1
Bulgaria: DALYs Rate (per 100,000)
< 122.6
122.6 - 168.8
168.8 - 255.7
255.7 - 342.6
342.6 - 396.6
396.6 - 450.6
450.6 - 504.6
504.6 - 561.7
561.7 - 706.3
> 706.3
Germany: DALYs Rate (per 100,000)
< 354.9
354.9 - 447.0
447.0 - 535.9
535.9 - 624.8
624.8 - 727.7
727.7 - 830.6
830.6 - 933.5
933.5 - 950.2
950.2 - 1215.9
> 1215.9
Romania: DALYs Rate (per 100,000)
< 122.1
122.1 - 152.4
152.4 - 243.6
243.6 - 334.8
334.8 - 380.9
380.9 - 427.0
427.0 - 473.2
473.2 - 482.2
482.2 - 587.2
> 587.2
Lebanon: DALYs Rate (per 100,000)
< 374.2
374.2 - 472.4
472.4 - 669.0
669.0 - 865.6
865.6 - 946.3
946.3 - 1026.9
1026.9 - 1107.6
1107.6 - 1157.4
1157.4 - 1406.8
> 1406.8
China: DALYs Rate (per 100,000)
< 108.0
108.0 - 150.5
150.5 - 210.7
210.7 - 270.9
270.9 - 316.9
316.9 - 362.8
362.8 - 408.8
408.8 - 433.9
433.9 - 547.0
> 547.0

Figure S6

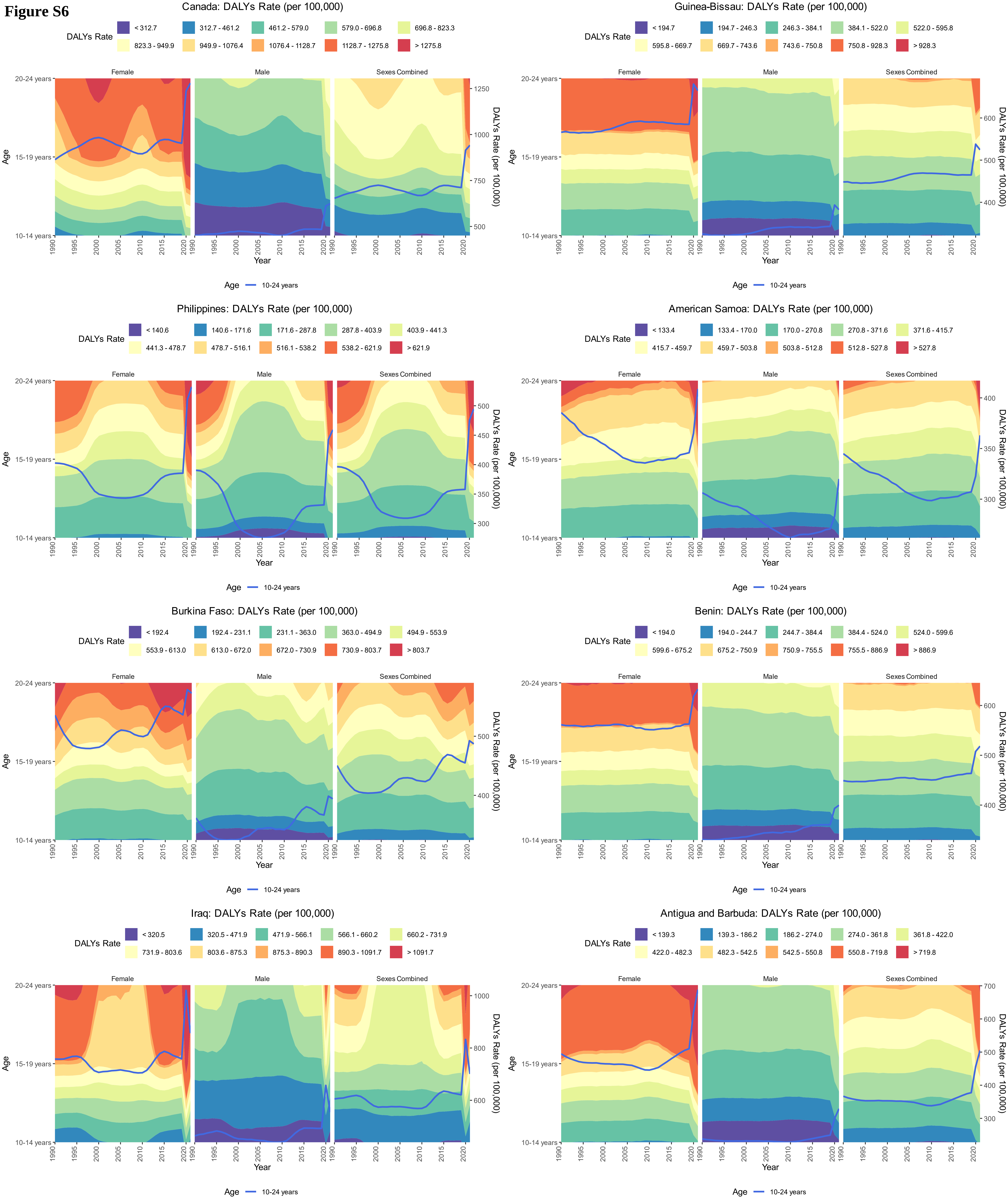
Canada: DALYs Rate (per 100,000)
DALYs Rate
< 312.7
312.7 - 461.2
461.2 - 579.0
579.0 - 696.8
696.8 - 823.3
823.3 - 949.9
949.9 - 1076.4
1076.4 - 1128.7
1128.7 - 1275.8
> 1275.8
Guinea-Bissau: DALYs Rate (per 100,000)
< 194.7
194.7 - 246.3
246.3 - 384.1
384.1 - 522.0
522.0 - 595.8
595.8 - 669.7
669.7 - 743.6
743.6 - 750.8
750.8 - 928.3
> 928.3
Philippines: DALYs Rate (per 100,000)
< 140.6
140.6 - 171.6
171.6 - 287.8
287.8 - 403.9
403.9 - 441.3
441.3 - 478.7
478.7 - 516.1
516.1 - 538.2
538.2 - 621.9
> 621.9
American Samoa: DALYs Rate (per 100,000)
< 133.4
133.4 - 170.0
170.0 - 270.8
270.8 - 371.6
371.6 - 415.7
415.7 - 459.7
459.7 - 503.8
503.8 - 512.8
512.8 - 527.8
> 527.8
Burkina Faso: DALYs Rate (per 100,000)
< 192.4
192.4 - 231.1
231.1 - 363.0
363.0 - 494.9
494.9 - 553.9
553.9 - 613.0
613.0 - 672.0
672.0 - 730.9
730.9 - 803.7
> 803.7
Benin: DALYs Rate (per 100,000)
< 194.0
194.0 - 244.7
244.7 - 384.4
384.4 - 524.0
524.0 - 599.6
599.6 - 675.2
675.2 - 750.9
750.9 - 755.5
755.5 - 886.9
> 886.9
Iraq: DALYs Rate (per 100,000)
< 320.5
320.5 - 471.9
471.9 - 566.1
566.1 - 660.2
660.2 - 731.9
731.9 - 803.6
803.6 - 875.3
875.3 - 890.3
890.3 - 1091.7
> 1091.7
Antigua and Barbuda: DALYs Rate (per 100,000)
< 139.3
139.3 - 186.2
186.2 - 274.0
274.0 - 361.8
361.8 - 422.0
422.0 - 482.3
482.3 - 542.5
542.5 - 550.8
550.8 - 719.8
> 719.8
Female
Male
Sexes Combined
Age
20-24 years
15-19 years
10-14 years
Year
DALYs Rate (per 100,000)
Age — 10-24 years

Figure S6

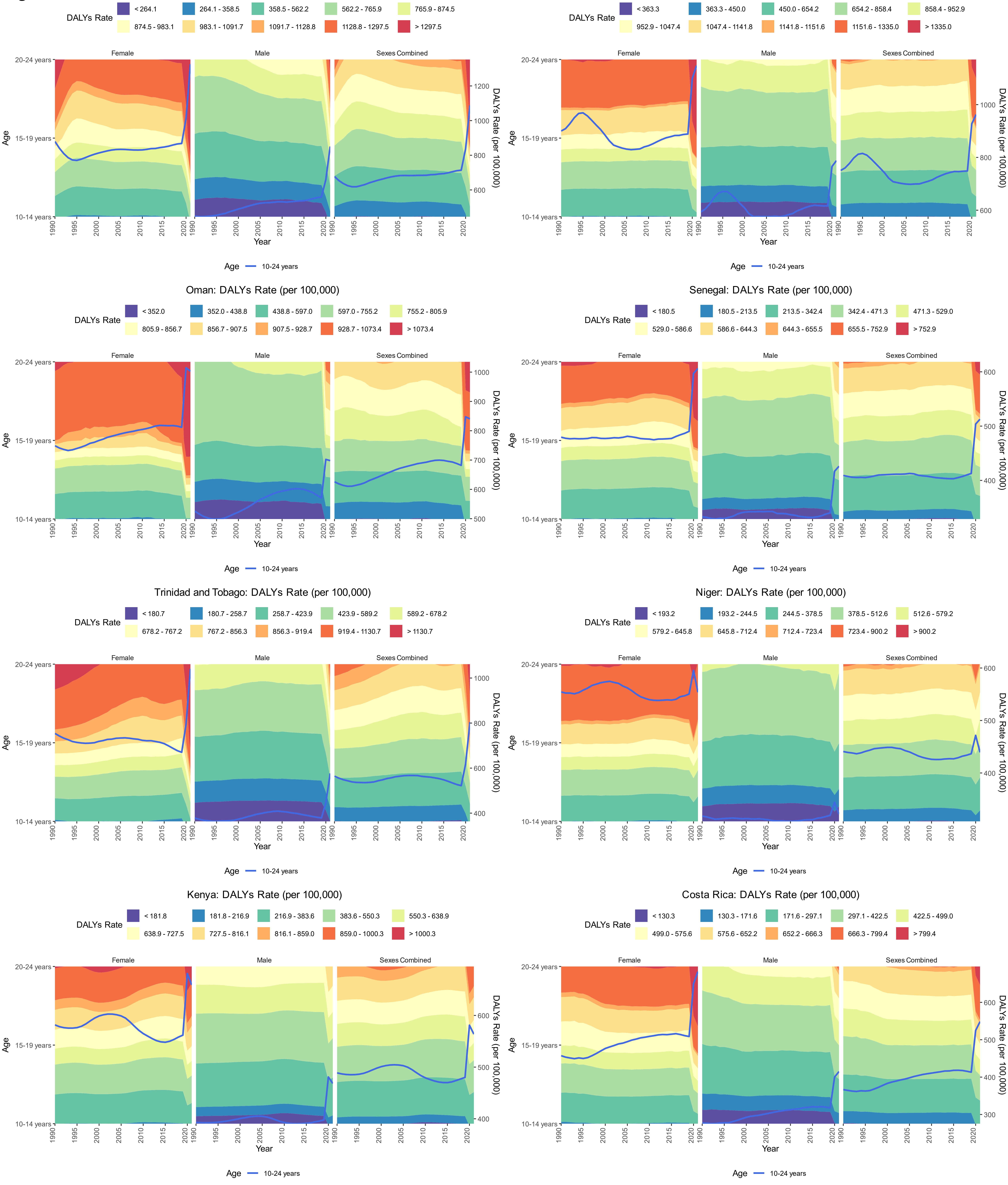
Lesotho: DALYs Rate (per 100,000)
DALYs Rate
< 264.1
264.1 - 358.5
358.5 - 562.2
562.2 - 765.9
765.9 - 874.5
874.5 - 983.1
983.1 - 1091.7
1091.7 - 1128.8
1128.8 - 1297.5
> 1297.5
Afghanistan: DALYs Rate (per 100,000)
< 363.3
363.3 - 450.0
450.0 - 654.2
654.2 - 858.4
858.4 - 952.9
952.9 - 1047.4
1047.4 - 1141.8
1141.8 - 1151.6
1151.6 - 1335.0
> 1335.0
Oman: DALYs Rate (per 100,000)
< 352.0
352.0 - 438.8
438.8 - 597.0
597.0 - 755.2
755.2 - 805.9
805.9 - 856.7
856.7 - 907.5
907.5 - 928.7
928.7 - 1073.4
> 1073.4
Senegal: DALYs Rate (per 100,000)
< 180.5
180.5 - 213.5
213.5 - 342.4
342.4 - 471.3
471.3 - 529.0
529.0 - 586.6
586.6 - 644.3
644.3 - 655.5
655.5 - 752.9
> 752.9
Trinidad and Tobago: DALYs Rate (per 100,000)
< 180.7
180.7 - 258.7
258.7 - 423.9
423.9 - 589.2
589.2 - 678.2
678.2 - 767.2
767.2 - 856.3
856.3 - 919.4
919.4 - 1130.7
> 1130.7
Niger: DALYs Rate (per 100,000)
< 193.2
193.2 - 244.5
244.5 - 378.5
378.5 - 512.6
512.6 - 579.2
579.2 - 645.8
645.8 - 712.4
712.4 - 723.4
723.4 - 900.2
> 900.2
Kenya: DALYs Rate (per 100,000)
< 181.8
181.8 - 216.9
216.9 - 383.6
383.6 - 550.3
550.3 - 638.9
638.9 - 727.5
727.5 - 816.1
816.1 - 859.0
859.0 - 1000.3
> 1000.3
Costa Rica: DALYs Rate (per 100,000)
< 130.3
130.3 - 171.6
171.6 - 297.1
297.1 - 422.5
422.5 - 499.0
499.0 - 575.6
575.6 - 652.2
652.2 - 666.3
666.3 - 799.4
> 799.4
Female
Male
Sexes Combined
Age
20-24 years
15-19 years
10-14 years
Year
1990
1995
2000
2005
2010
2015
2020
DALYs Rate (per 100,000)
Age
10-24 years

Figure S6

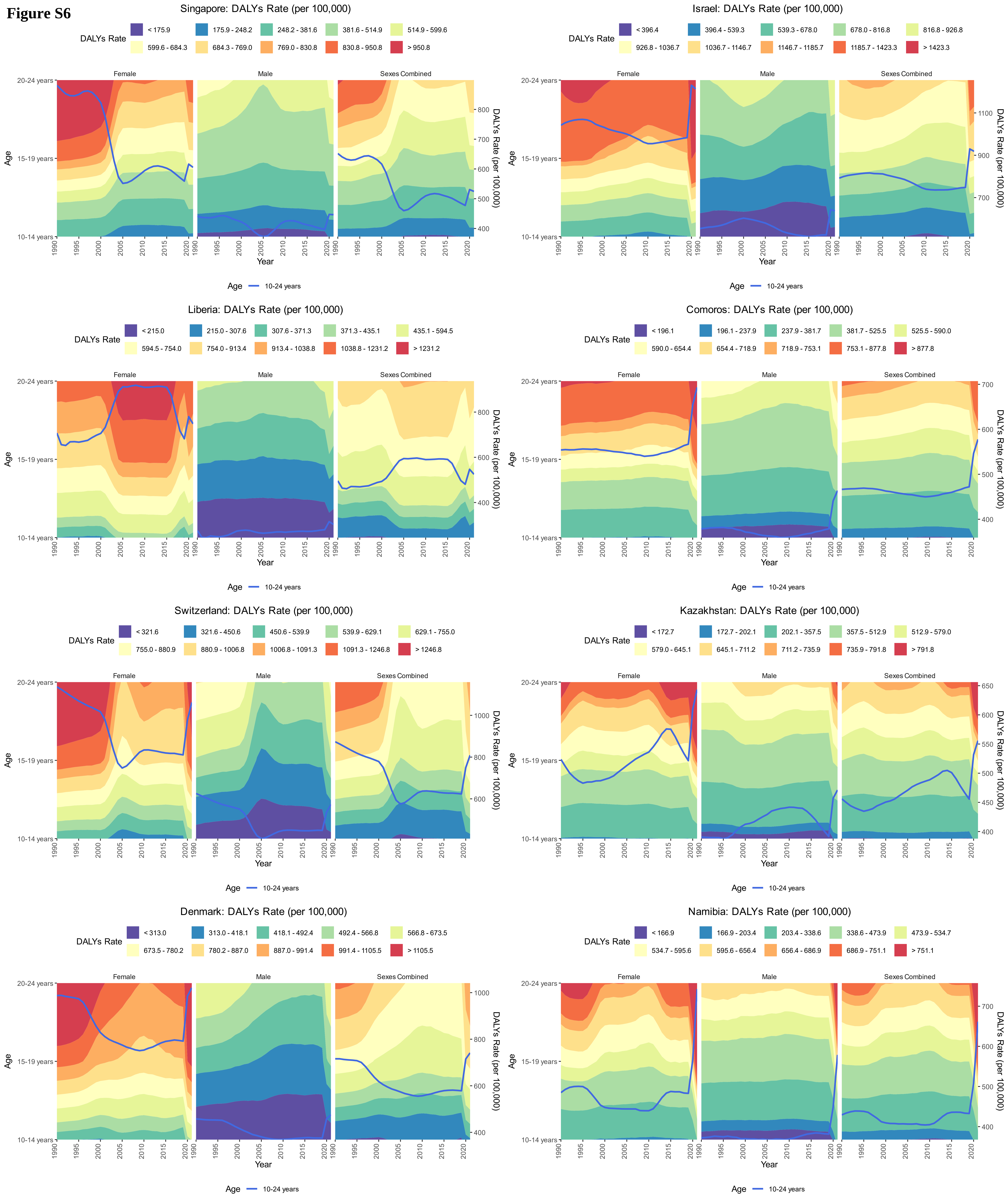

Singapore: DALYs Rate (per 100,000)
DALYs Rate
< 175.9
175.9 - 248.2
248.2 - 381.6
381.6 - 514.9
514.9 - 599.6
599.6 - 684.3
684.3 - 769.0
769.0 - 830.8
830.8 - 950.8
> 950.8
Israel: DALYs Rate (per 100,000)
DALYs Rate
< 396.4
396.4 - 539.3
539.3 - 678.0
678.0 - 816.8
816.8 - 926.8
926.8 - 1036.7
1036.7 - 1146.7
1146.7 - 1185.7
1185.7 - 1423.3
> 1423.3
Liberia: DALYs Rate (per 100,000)
DALYs Rate
< 215.0
215.0 - 307.6
307.6 - 371.3
371.3 - 435.1
435.1 - 594.5
594.5 - 754.0
754.0 - 913.4
913.4 - 1038.8
1038.8 - 1231.2
> 1231.2
Comoros: DALYs Rate (per 100,000)
DALYs Rate
< 196.1
196.1 - 237.9
237.9 - 381.7
381.7 - 525.5
525.5 - 590.0
590.0 - 654.4
654.4 - 718.9
718.9 - 753.1
753.1 - 877.8
> 877.8
Switzerland: DALYs Rate (per 100,000)
DALYs Rate
< 321.6
321.6 - 450.6
450.6 - 539.9
539.9 - 629.1
629.1 - 755.0
755.0 - 880.9
880.9 - 1006.8
1006.8 - 1091.3
1091.3 - 1246.8
> 1246.8
Kazakhstan: DALYs Rate (per 100,000)
DALYs Rate
< 172.7
172.7 - 202.1
202.1 - 357.5
357.5 - 512.9
512.9 - 579.0
579.0 - 645.1
645.1 - 711.2
711.2 - 735.9
735.9 - 791.8
> 791.8
Denmark: DALYs Rate (per 100,000)
DALYs Rate
< 313.0
313.0 - 418.1
418.1 - 492.4
492.4 - 566.8
566.8 - 673.5
673.5 - 780.2
780.2 - 887.0
887.0 - 991.4
991.4 - 1105.5
> 1105.5
Namibia: DALYs Rate (per 100,000)
DALYs Rate
< 166.9
166.9 - 203.4
203.4 - 338.6
338.6 - 473.9
473.9 - 534.7
534.7 - 595.6
595.6 - 656.4
656.4 - 686.9
686.9 - 751.1
> 751.1
Female
Male
Sexes Combined
Age
20-24 years
15-19 years
10-14 years
Year
DALYs Rate (per 100,000)
Age — 10-24 years

Figure S6

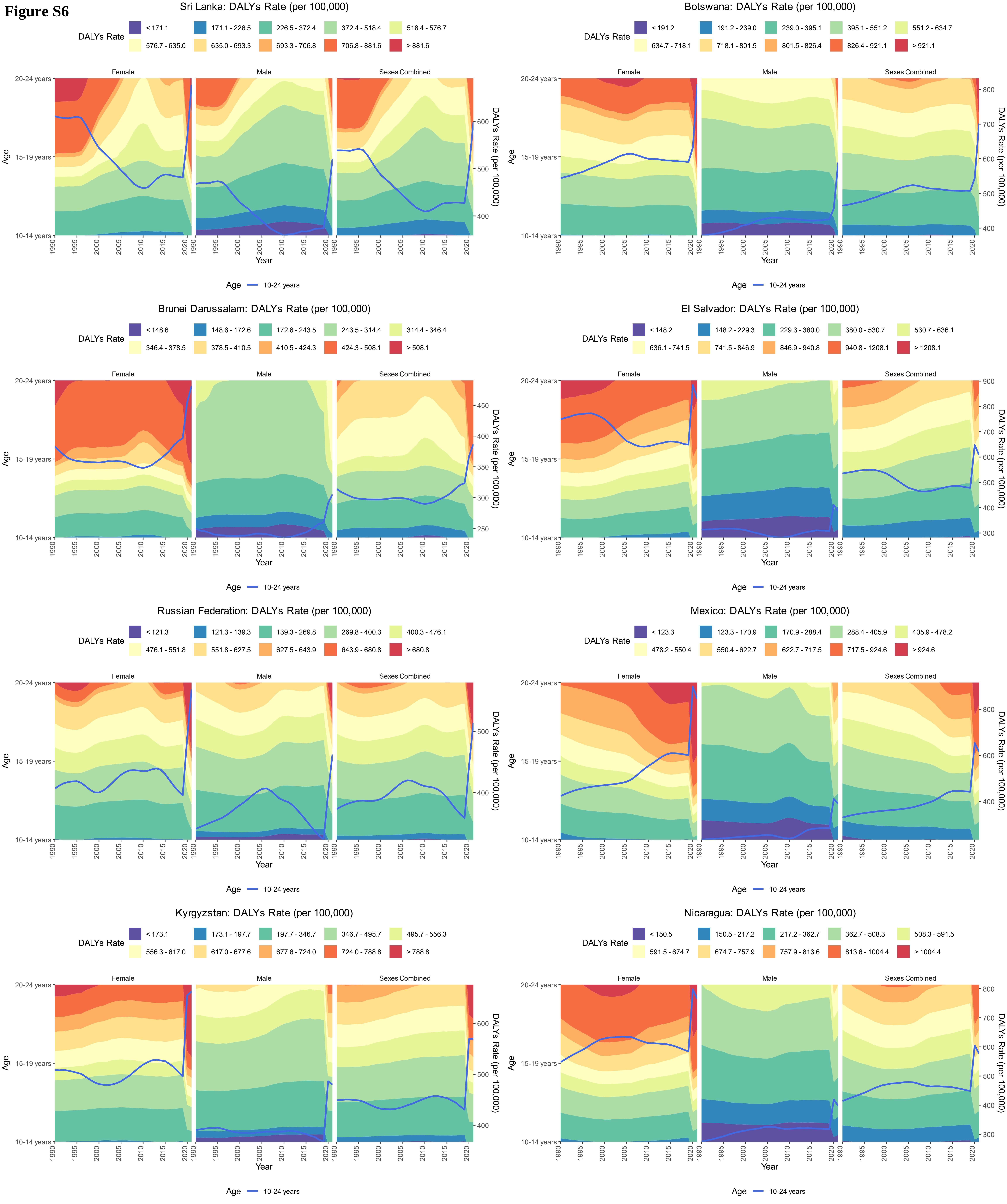

Sri Lanka: DALYs Rate (per 100,000)
DALYs Rate
< 171.1
171.1 - 226.5
226.5 - 372.4
372.4 - 518.4
518.4 - 576.7
576.7 - 635.0
635.0 - 693.3
693.3 - 706.8
706.8 - 881.6
> 881.6
Botswana: DALYs Rate (per 100,000)
< 191.2
191.2 - 239.0
239.0 - 395.1
395.1 - 551.2
551.2 - 634.7
634.7 - 718.1
718.1 - 801.5
801.5 - 826.4
826.4 - 921.1
> 921.1
Brunei Darussalam: DALYs Rate (per 100,000)
< 148.6
148.6 - 172.6
172.6 - 243.5
243.5 - 314.4
314.4 - 346.4
346.4 - 378.5
378.5 - 410.5
410.5 - 424.3
424.3 - 508.1
> 508.1
El Salvador: DALYs Rate (per 100,000)
< 148.2
148.2 - 229.3
229.3 - 380.0
380.0 - 530.7
530.7 - 636.1
636.1 - 741.5
741.5 - 846.9
846.9 - 940.8
940.8 - 1208.1
> 1208.1
Russian Federation: DALYs Rate (per 100,000)
< 121.3
121.3 - 139.3
139.3 - 269.8
269.8 - 400.3
400.3 - 476.1
476.1 - 551.8
551.8 - 627.5
627.5 - 643.9
643.9 - 680.8
> 680.8
Mexico: DALYs Rate (per 100,000)
< 123.3
123.3 - 170.9
170.9 - 288.4
288.4 - 405.9
405.9 - 478.2
478.2 - 550.4
550.4 - 622.7
622.7 - 717.5
717.5 - 924.6
> 924.6
Kyrgyzstan: DALYs Rate (per 100,000)
< 173.1
173.1 - 197.7
197.7 - 346.7
346.7 - 495.7
495.7 - 556.3
556.3 - 617.0
617.0 - 677.6
677.6 - 724.0
724.0 - 788.8
> 788.8
Nicaragua: DALYs Rate (per 100,000)
< 150.5
150.5 - 217.2
217.2 - 362.7
362.7 - 508.3
508.3 - 591.5
591.5 - 674.7
674.7 - 757.9
757.9 - 813.6
813.6 - 1004.4
> 1004.4
Female
Male
Sexes Combined
Age
20-24 years
15-19 years
10-14 years
Year
DALYs Rate (per 100,000)
Age — 10-24 years

Figure S6

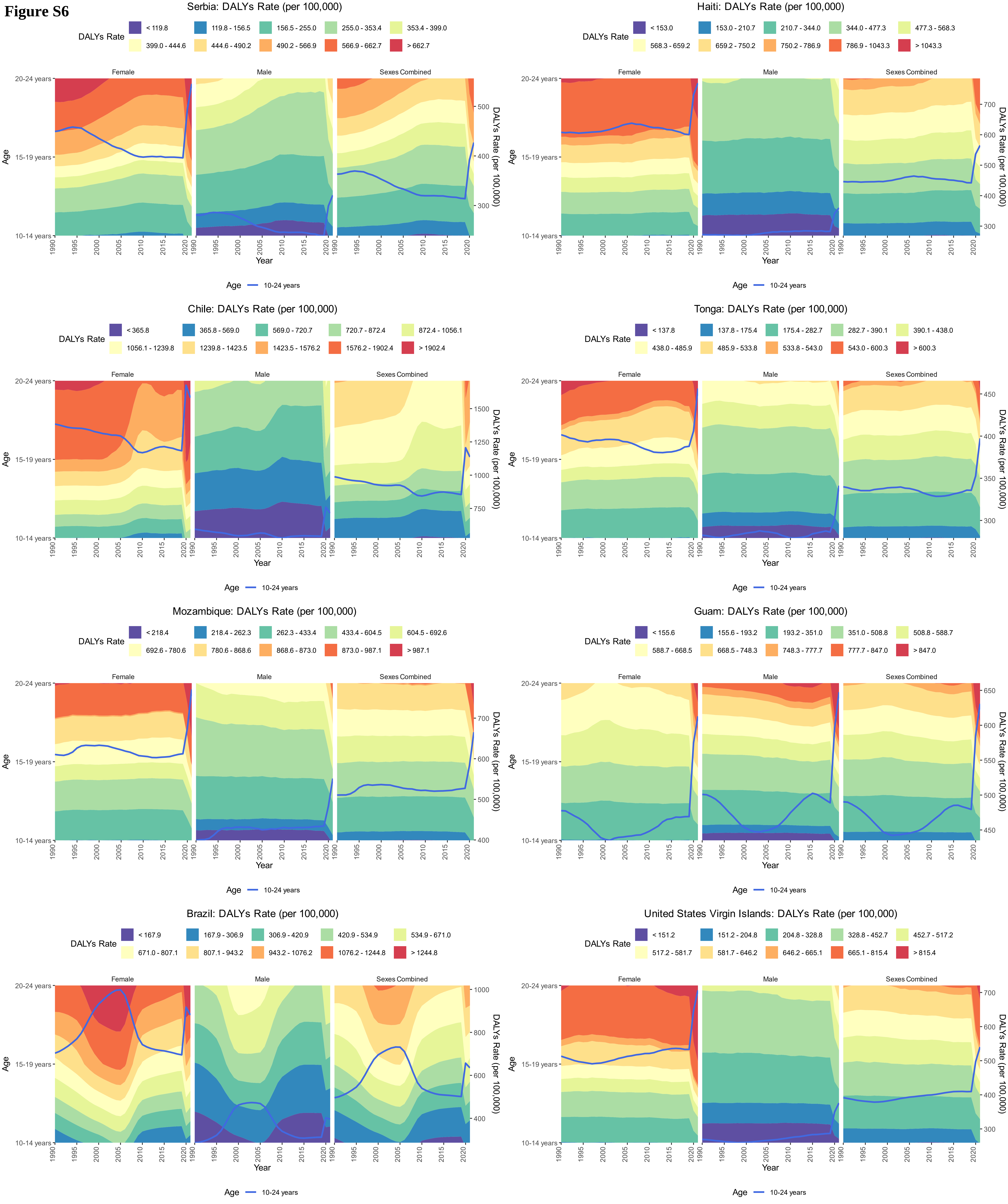
Serbia: DALYs Rate (per 100,000)
DALYs Rate
< 119.8
119.8 - 156.5
156.5 - 255.0
255.0 - 353.4
353.4 - 399.0
399.0 - 444.6
444.6 - 490.2
490.2 - 566.9
566.9 - 662.7
> 662.7
Haiti: DALYs Rate (per 100,000)
DALYs Rate
< 153.0
153.0 - 210.7
210.7 - 344.0
344.0 - 477.3
477.3 - 568.3
568.3 - 659.2
659.2 - 750.2
750.2 - 786.9
786.9 - 1043.3
> 1043.3
Chile: DALYs Rate (per 100,000)
DALYs Rate
< 365.8
365.8 - 569.0
569.0 - 720.7
720.7 - 872.4
872.4 - 1056.1
1056.1 - 1239.8
1239.8 - 1423.5
1423.5 - 1576.2
1576.2 - 1902.4
> 1902.4
Tonga: DALYs Rate (per 100,000)
DALYs Rate
< 137.8
137.8 - 175.4
175.4 - 282.7
282.7 - 390.1
390.1 - 438.0
438.0 - 485.9
485.9 - 533.8
533.8 - 543.0
543.0 - 600.3
> 600.3
Mozambique: DALYs Rate (per 100,000)
DALYs Rate
< 218.4
218.4 - 262.3
262.3 - 433.4
433.4 - 604.5
604.5 - 692.6
692.6 - 780.6
780.6 - 868.6
868.6 - 873.0
873.0 - 987.1
> 987.1
Guam: DALYs Rate (per 100,000)
DALYs Rate
< 155.6
155.6 - 193.2
193.2 - 351.0
351.0 - 508.8
508.8 - 588.7
588.7 - 668.5
668.5 - 748.3
748.3 - 777.7
777.7 - 847.0
> 847.0
Brazil: DALYs Rate (per 100,000)
DALYs Rate
< 167.9
167.9 - 306.9
306.9 - 420.9
420.9 - 534.9
534.9 - 671.0
671.0 - 807.1
807.1 - 943.2
943.2 - 1076.2
1076.2 - 1244.8
> 1244.8
United States Virgin Islands: DALYs Rate (per 100,000)
DALYs Rate
< 151.2
151.2 - 204.8
204.8 - 328.8
328.8 - 452.7
452.7 - 517.2
517.2 - 581.7
581.7 - 646.2
646.2 - 665.1
665.1 - 815.4
> 815.4
Female
Male
Sexes Combined
20-24 years
15-19 years
10-14 years
Age
Year
DALYs Rate (per 100,000)
Age
10-24 years

Figure S6

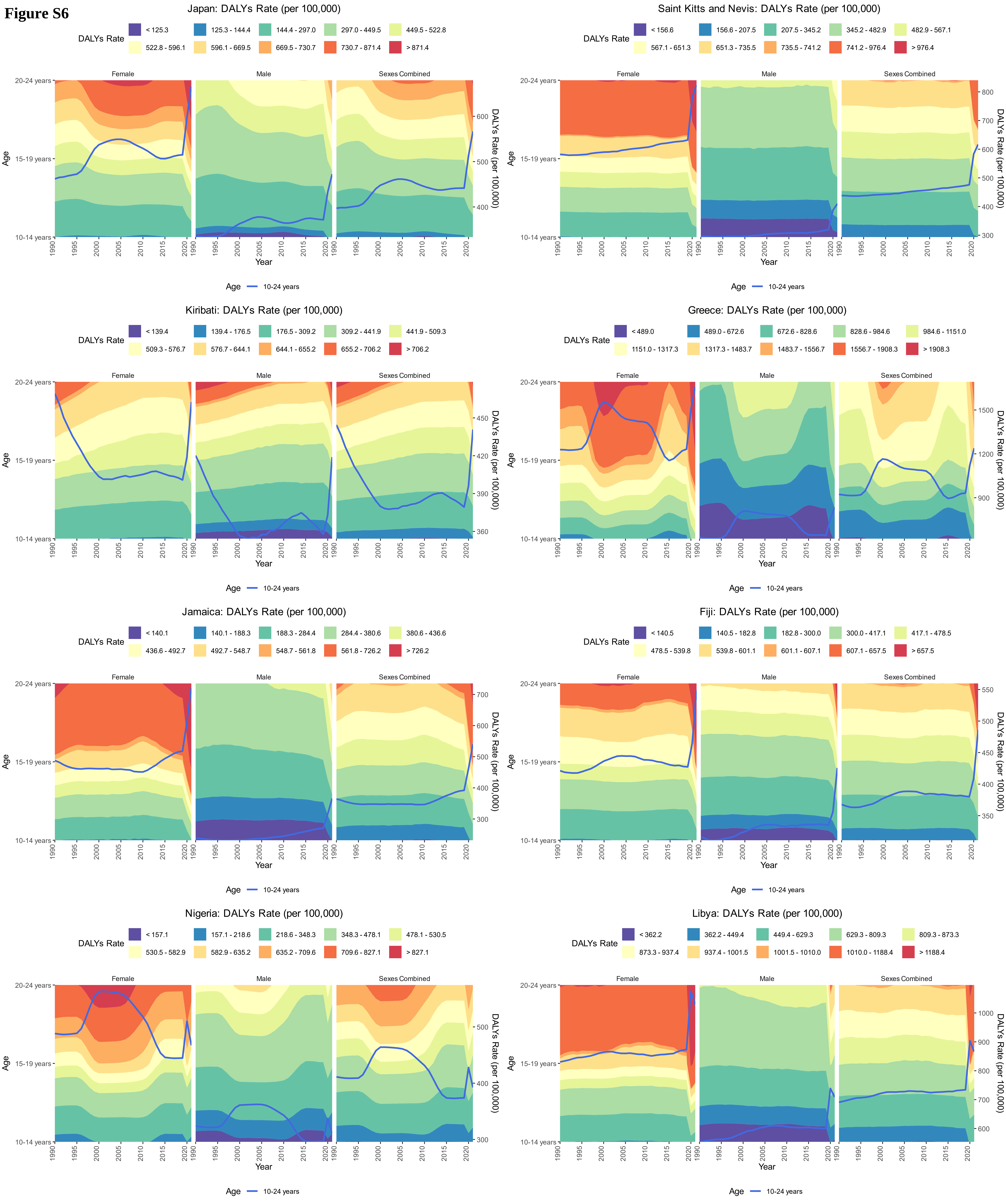

Japan: DALYs Rate (per 100,000)
DALYs Rate
< 125.3
125.3 - 144.4
144.4 - 297.0
297.0 - 449.5
449.5 - 522.8
522.8 - 596.1
596.1 - 669.5
669.5 - 730.7
730.7 - 871.4
> 871.4
Saint Kitts and Nevis: DALYs Rate (per 100,000)
DALYs Rate
< 156.6
156.6 - 207.5
207.5 - 345.2
345.2 - 482.9
482.9 - 567.1
567.1 - 651.3
651.3 - 735.5
735.5 - 741.2
741.2 - 976.4
> 976.4
Kiribati: DALYs Rate (per 100,000)
DALYs Rate
< 139.4
139.4 - 176.5
176.5 - 309.2
309.2 - 441.9
441.9 - 509.3
509.3 - 576.7
576.7 - 644.1
644.1 - 655.2
655.2 - 706.2
> 706.2
Greece: DALYs Rate (per 100,000)
DALYs Rate
< 489.0
489.0 - 672.6
672.6 - 828.6
828.6 - 984.6
984.6 - 1151.0
1151.0 - 1317.3
1317.3 - 1483.7
1483.7 - 1556.7
1556.7 - 1908.3
> 1908.3
Jamaica: DALYs Rate (per 100,000)
DALYs Rate
< 140.1
140.1 - 188.3
188.3 - 284.4
284.4 - 380.6
380.6 - 436.6
436.6 - 492.7
492.7 - 548.7
548.7 - 561.8
561.8 - 726.2
> 726.2
Fiji: DALYs Rate (per 100,000)
DALYs Rate
< 140.5
140.5 - 182.8
182.8 - 300.0
300.0 - 417.1
417.1 - 478.5
478.5 - 539.8
539.8 - 601.1
601.1 - 607.1
607.1 - 657.5
> 657.5
Nigeria: DALYs Rate (per 100,000)
DALYs Rate
< 157.1
157.1 - 218.6
218.6 - 348.3
348.3 - 478.1
478.1 - 530.5
530.5 - 582.9
582.9 - 635.2
635.2 - 709.6
709.6 - 827.1
> 827.1
Libya: DALYs Rate (per 100,000)
DALYs Rate
< 362.2
362.2 - 449.4
449.4 - 629.3
629.3 - 809.3
809.3 - 873.3
873.3 - 937.4
937.4 - 1001.5
1001.5 - 1010.0
1010.0 - 1188.4
> 1188.4
Female
Male
Sexes Combined
Age
20-24 years
15-19 years
10-14 years
Year
DALYs Rate (per 100,000)
Age
10-24 years

Figure S6

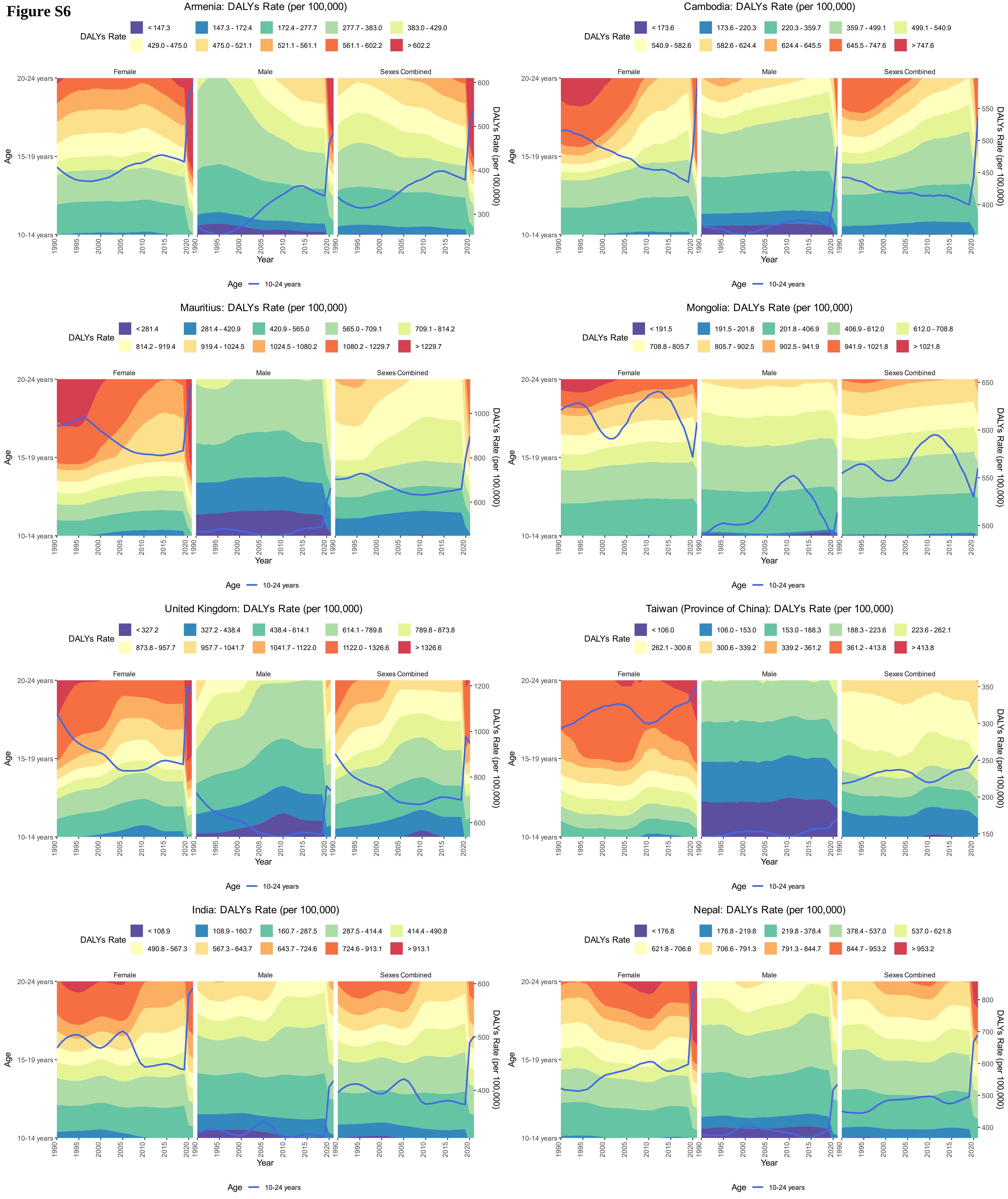

Armenia: DALYs Rate (per 100,000)
DALYs Rate
< 147.3
147.3 - 172.4
172.4 - 277.7
277.7 - 383.0
383.0 - 429.0
429.0 - 475.0
475.0 - 521.1
521.1 - 561.1
561.1 - 602.2
> 602.2
Female
Male
Sexes Combined
Age
Year
DALYs Rate (per 100,000)
Age 10-24 years
Cambodia: DALYs Rate (per 100,000)
< 173.6
173.6 - 220.3
220.3 - 359.7
359.7 - 499.1
499.1 - 540.9
540.9 - 582.6
582.6 - 624.4
624.4 - 645.5
645.5 - 747.6
> 747.6
Mauritius: DALYs Rate (per 100,000)
< 281.4
281.4 - 420.9
420.9 - 565.0
565.0 - 709.1
709.1 - 814.2
814.2 - 919.4
919.4 - 1024.5
1024.5 - 1080.2
1080.2 - 1229.7
> 1229.7
Mongolia: DALYs Rate (per 100,000)
< 191.5
191.5 - 201.8
201.8 - 406.9
406.9 - 612.0
612.0 - 708.8
708.8 - 805.7
805.7 - 902.5
902.5 - 941.9
941.9 - 1021.8
> 1021.8
United Kingdom: DALYs Rate (per 100,000)
< 327.2
327.2 - 438.4
438.4 - 614.1
614.1 - 789.8
789.8 - 873.8
873.8 - 957.7
957.7 - 1041.7
1041.7 - 1122.0
1122.0 - 1326.6
> 1326.6
Taiwan (Province of China): DALYs Rate (per 100,000)
< 106.0
106.0 - 153.0
153.0 - 188.3
188.3 - 223.6
223.6 - 262.1
262.1 - 300.6
300.6 - 339.2
339.2 - 361.2
361.2 - 413.8
> 413.8
India: DALYs Rate (per 100,000)
< 108.9
108.9 - 160.7
160.7 - 287.5
287.5 - 414.4
414.4 - 490.8
490.8 - 567.3
567.3 - 643.7
643.7 - 724.6
724.6 - 913.1
> 913.1
Nepal: DALYs Rate (per 100,000)
< 176.8
176.8 - 219.8
219.8 - 378.4
378.4 - 537.0
537.0 - 621.8
621.8 - 706.6
706.6 - 791.3
791.3 - 844.7
844.7 - 953.2
> 953.2
20-24 years
15-19 years
10-14 years

Figure S6

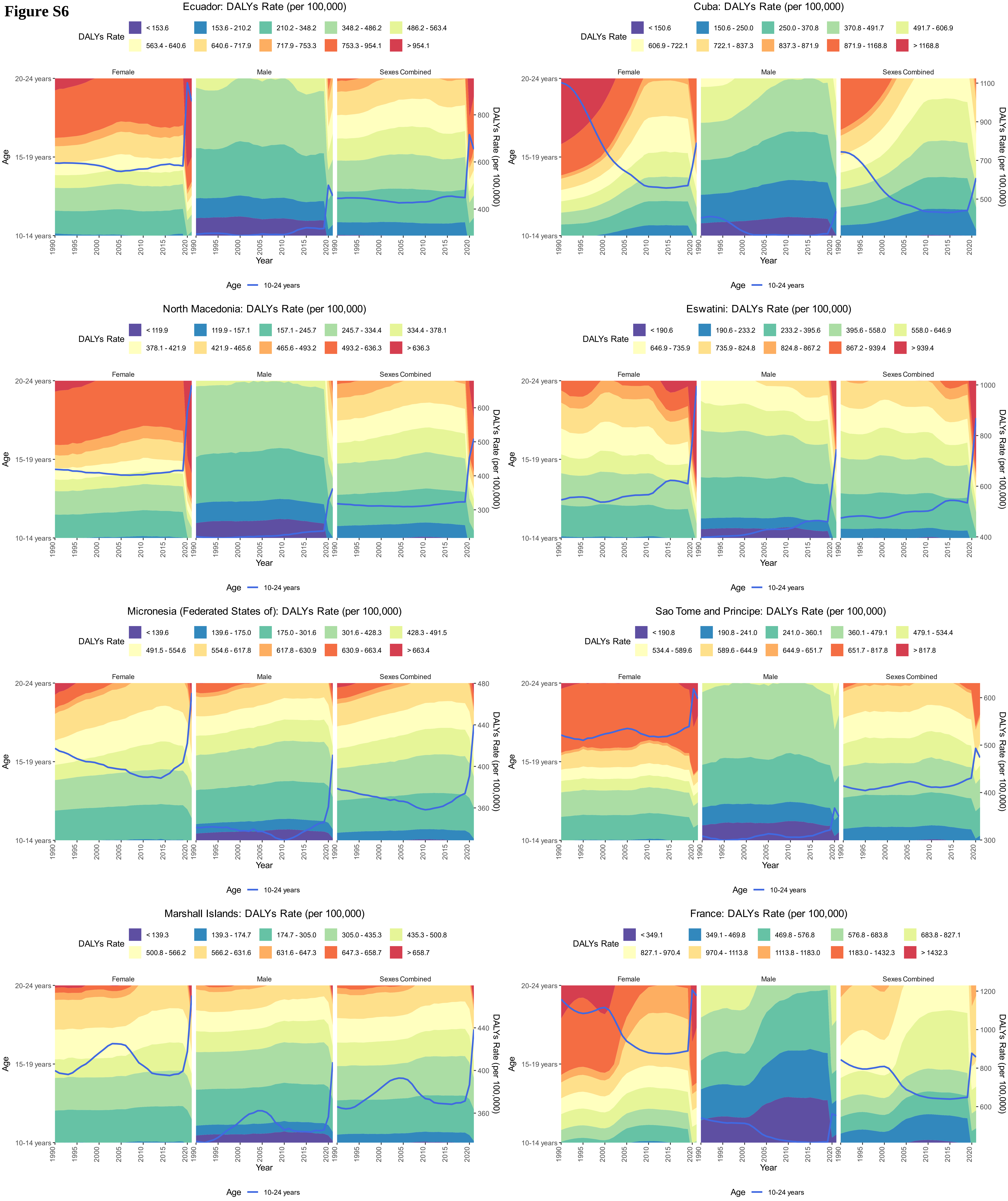

Ecuador: DALYs Rate (per 100,000)
DALYs Rate
< 153.6
153.6 - 210.2
210.2 - 348.2
348.2 - 486.2
486.2 - 563.4
563.4 - 640.6
640.6 - 717.9
717.9 - 753.3
753.3 - 954.1
> 954.1
Cuba: DALYs Rate (per 100,000)
< 150.6
150.6 - 250.0
250.0 - 370.8
370.8 - 491.7
491.7 - 606.9
606.9 - 722.1
722.1 - 837.3
837.3 - 871.9
871.9 - 1168.8
> 1168.8
North Macedonia: DALYs Rate (per 100,000)
< 119.9
119.9 - 157.1
157.1 - 245.7
245.7 - 334.4
334.4 - 378.1
378.1 - 421.9
421.9 - 465.6
465.6 - 493.2
493.2 - 636.3
> 636.3
Eswatini: DALYs Rate (per 100,000)
< 190.6
190.6 - 233.2
233.2 - 395.6
395.6 - 558.0
558.0 - 646.9
646.9 - 735.9
735.9 - 824.8
824.8 - 867.2
867.2 - 939.4
> 939.4
Micronesia (Federated States of): DALYs Rate (per 100,000)
< 139.6
139.6 - 175.0
175.0 - 301.6
301.6 - 428.3
428.3 - 491.5
491.5 - 554.6
554.6 - 617.8
617.8 - 630.9
630.9 - 663.4
> 663.4
Sao Tome and Principe: DALYs Rate (per 100,000)
< 190.8
190.8 - 241.0
241.0 - 360.1
360.1 - 479.1
479.1 - 534.4
534.4 - 589.6
589.6 - 644.9
644.9 - 651.7
651.7 - 817.8
> 817.8
Marshall Islands: DALYs Rate (per 100,000)
< 139.3
139.3 - 174.7
174.7 - 305.0
305.0 - 435.3
435.3 - 500.8
500.8 - 566.2
566.2 - 631.6
631.6 - 647.3
647.3 - 658.7
> 658.7
France: DALYs Rate (per 100,000)
< 349.1
349.1 - 469.8
469.8 - 576.8
576.8 - 683.8
683.8 - 827.1
827.1 - 970.4
970.4 - 1113.8
1113.8 - 1183.0
1183.0 - 1432.3
> 1432.3
Female
Male
Sexes Combined
Age
20-24 years
15-19 years
10-14 years
Year
DALYs Rate (per 100,000)
10-24 years

Figure S6

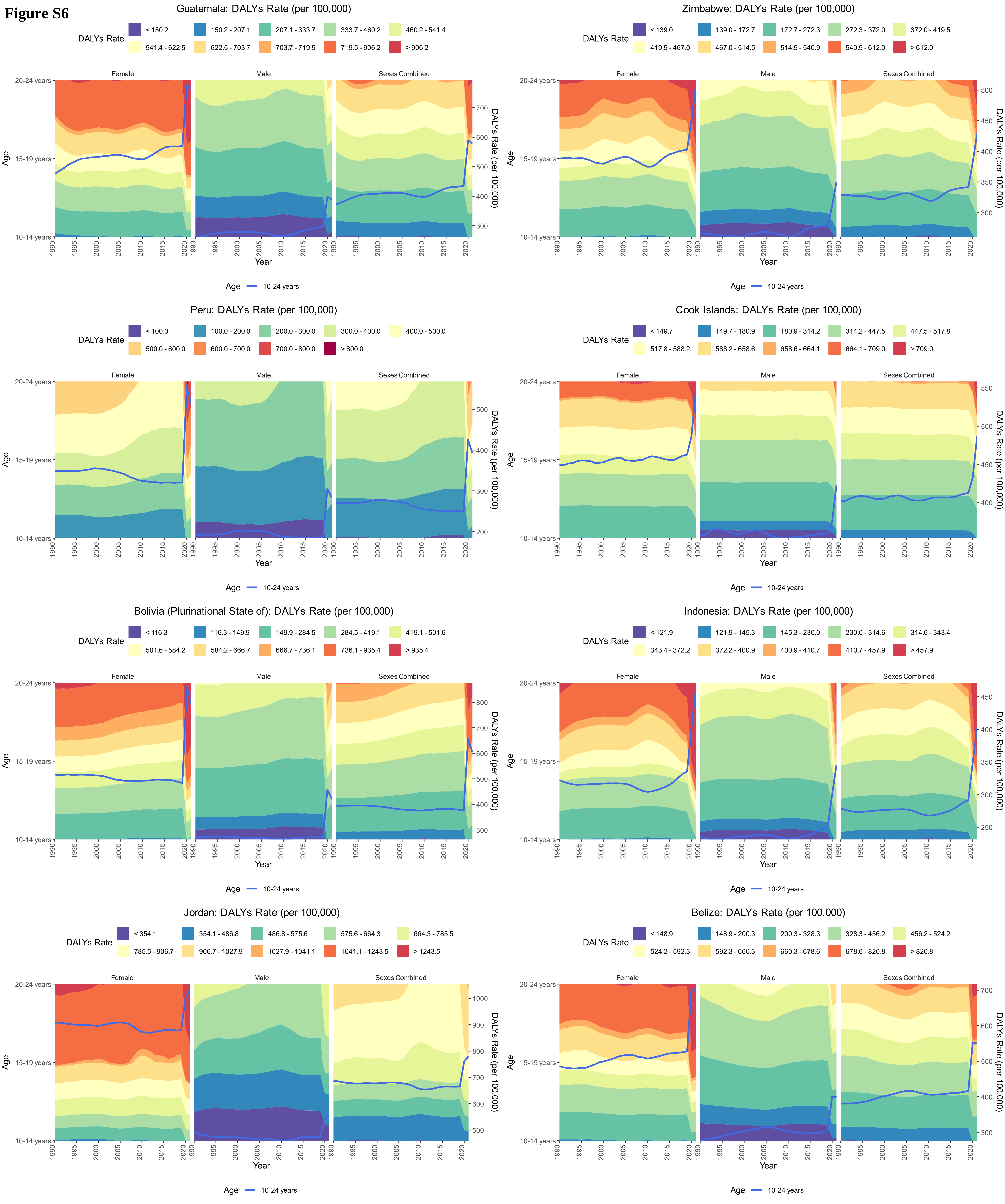
Guatemala: DALYs Rate (per 100,000)
DALYs Rate
< 150.2
150.2 - 207.1
207.1 - 333.7
333.7 - 460.2
460.2 - 541.4
541.4 - 622.5
622.5 - 703.7
703.7 - 719.5
719.5 - 906.2
> 906.2
Zimbabwe: DALYs Rate (per 100,000)
DALYs Rate
< 139.0
139.0 - 172.7
172.7 - 272.3
272.3 - 372.0
372.0 - 419.5
419.5 - 467.0
467.0 - 514.5
514.5 - 540.9
540.9 - 612.0
> 612.0
Peru: DALYs Rate (per 100,000)
DALYs Rate
< 100.0
100.0 - 200.0
200.0 - 300.0
300.0 - 400.0
400.0 - 500.0
500.0 - 600.0
600.0 - 700.0
700.0 - 800.0
> 800.0
Cook Islands: DALYs Rate (per 100,000)
DALYs Rate
< 149.7
149.7 - 180.9
180.9 - 314.2
314.2 - 447.5
447.5 - 517.8
517.8 - 588.2
588.2 - 658.6
658.6 - 664.1
664.1 - 709.0
> 709.0
Bolivia (Plurinational State of): DALYs Rate (per 100,000)
DALYs Rate
< 116.3
116.3 - 149.9
149.9 - 284.5
284.5 - 419.1
419.1 - 501.6
501.6 - 584.2
584.2 - 666.7
666.7 - 736.1
736.1 - 935.4
> 935.4
Indonesia: DALYs Rate (per 100,000)
DALYs Rate
< 121.9
121.9 - 145.3
145.3 - 230.0
230.0 - 314.6
314.6 - 343.4
343.4 - 372.2
372.2 - 400.9
400.9 - 410.7
410.7 - 457.9
> 457.9
Jordan: DALYs Rate (per 100,000)
DALYs Rate
< 354.1
354.1 - 486.8
486.8 - 575.6
575.6 - 664.3
664.3 - 785.5
785.5 - 906.7
906.7 - 1027.9
1027.9 - 1041.1
1041.1 - 1243.5
> 1243.5
Belize: DALYs Rate (per 100,000)
DALYs Rate
< 148.9
148.9 - 200.3
200.3 - 328.3
328.3 - 456.2
456.2 - 524.2
524.2 - 592.3
592.3 - 660.3
660.3 - 678.6
678.6 - 820.8
> 820.8
Female
Male
Sexes Combined
20-24 years
15-19 years
10-14 years
Age
Year
DALYs Rate (per 100,000)
1990 1995 2000 2005 2010 2015 2020
Age — 10-24 years

Figure S6

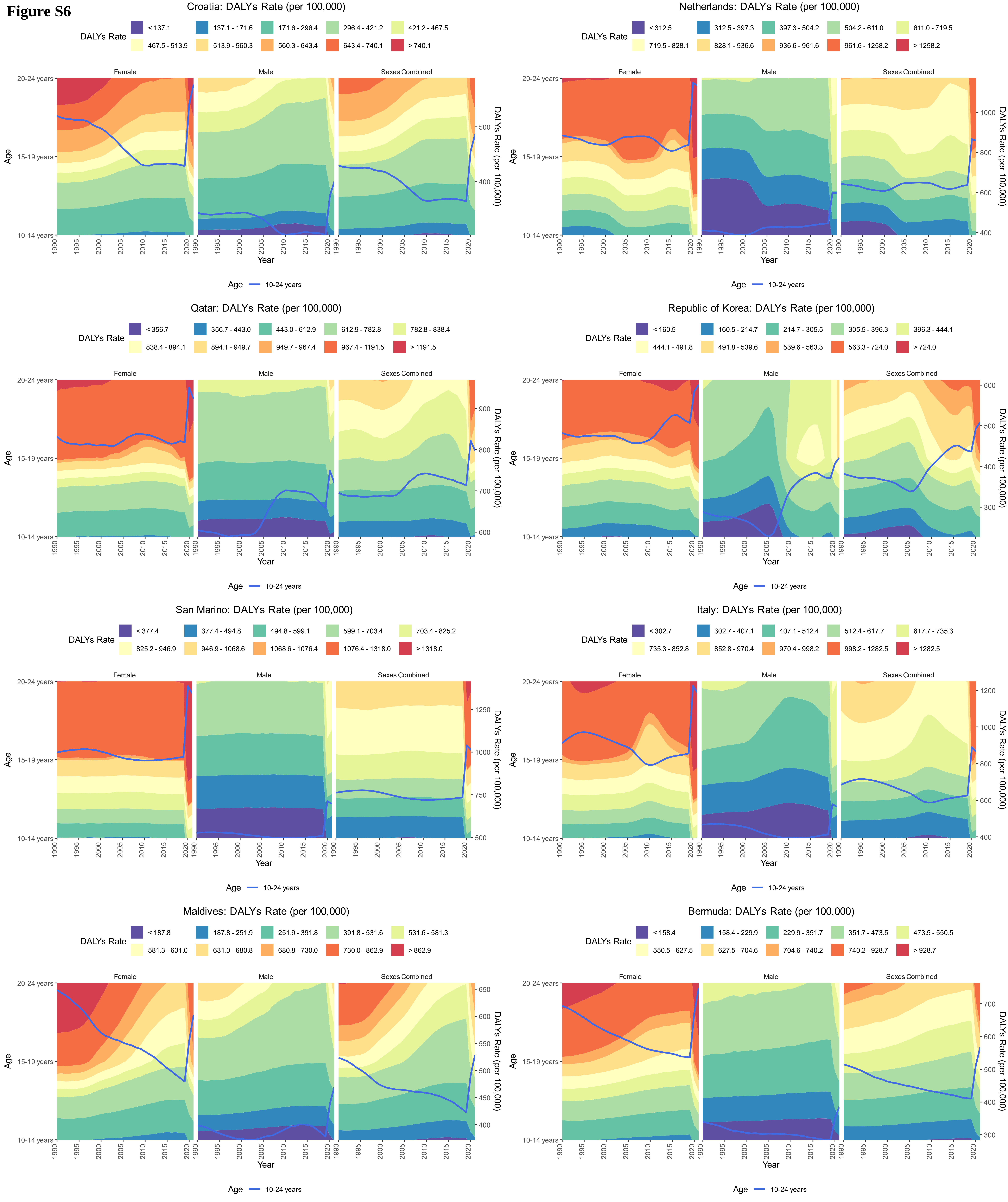
Croatia: DALYs Rate (per 100,000)
DALYs Rate
< 137.1
137.1 - 171.6
171.6 - 296.4
296.4 - 421.2
421.2 - 467.5
467.5 - 513.9
513.9 - 560.3
560.3 - 643.4
643.4 - 740.1
> 740.1
Netherlands: DALYs Rate (per 100,000)
< 312.5
312.5 - 397.3
397.3 - 504.2
504.2 - 611.0
611.0 - 719.5
719.5 - 828.1
828.1 - 936.6
936.6 - 961.6
961.6 - 1258.2
> 1258.2
Qatar: DALYs Rate (per 100,000)
< 356.7
356.7 - 443.0
443.0 - 612.9
612.9 - 782.8
782.8 - 838.4
838.4 - 894.1
894.1 - 949.7
949.7 - 967.4
967.4 - 1191.5
> 1191.5
Republic of Korea: DALYs Rate (per 100,000)
< 160.5
160.5 - 214.7
214.7 - 305.5
305.5 - 396.3
396.3 - 444.1
444.1 - 491.8
491.8 - 539.6
539.6 - 563.3
563.3 - 724.0
> 724.0
San Marino: DALYs Rate (per 100,000)
< 377.4
377.4 - 494.8
494.8 - 599.1
599.1 - 703.4
703.4 - 825.2
825.2 - 946.9
946.9 - 1068.6
1068.6 - 1076.4
1076.4 - 1318.0
> 1318.0
Italy: DALYs Rate (per 100,000)
< 302.7
302.7 - 407.1
407.1 - 512.4
512.4 - 617.7
617.7 - 735.3
735.3 - 852.8
852.8 - 970.4
970.4 - 998.2
998.2 - 1282.5
> 1282.5
Maldives: DALYs Rate (per 100,000)
< 187.8
187.8 - 251.9
251.9 - 391.8
391.8 - 531.6
531.6 - 581.3
581.3 - 631.0
631.0 - 680.8
680.8 - 730.0
730.0 - 862.9
> 862.9
Bermuda: DALYs Rate (per 100,000)
< 158.4
158.4 - 229.9
229.9 - 351.7
351.7 - 473.5
473.5 - 550.5
550.5 - 627.5
627.5 - 704.6
704.6 - 740.2
740.2 - 928.7
> 928.7
Female
Male
Sexes Combined
Age
20-24 years
15-19 years
10-14 years
Year
DALYs Rate (per 100,000)
Age
10-24 years

Figure S6

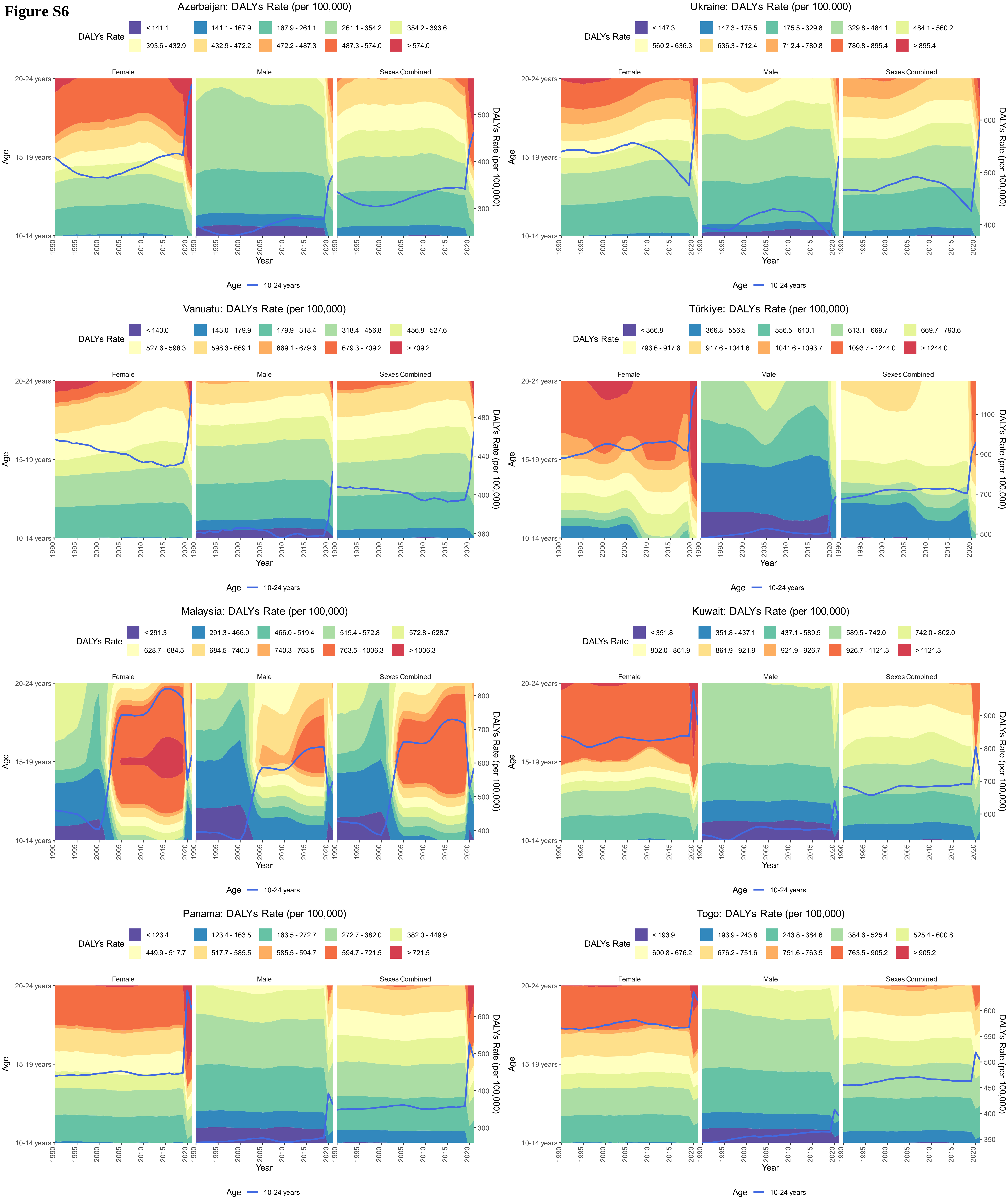

Azerbaijan: DALYs Rate (per 100,000)
DALYs Rate
< 141.1
141.1 - 167.9
167.9 - 261.1
261.1 - 354.2
354.2 - 393.6
393.6 - 432.9
432.9 - 472.2
472.2 - 487.3
487.3 - 574.0
> 574.0
Female
Male
Sexes Combined
Age
Year
DALYs Rate (per 100,000)
Age 10-24 years
Ukraine: DALYs Rate (per 100,000)
DALYs Rate
< 147.3
147.3 - 175.5
175.5 - 329.8
329.8 - 484.1
484.1 - 560.2
560.2 - 636.3
636.3 - 712.4
712.4 - 780.8
780.8 - 895.4
> 895.4
Vanuatu: DALYs Rate (per 100,000)
DALYs Rate
< 143.0
143.0 - 179.9
179.9 - 318.4
318.4 - 456.8
456.8 - 527.6
527.6 - 598.3
598.3 - 669.1
669.1 - 679.3
679.3 - 709.2
> 709.2
Türkiye: DALYs Rate (per 100,000)
DALYs Rate
< 366.8
366.8 - 556.5
556.5 - 613.1
613.1 - 669.7
669.7 - 793.6
793.6 - 917.6
917.6 - 1041.6
1041.6 - 1093.7
1093.7 - 1244.0
> 1244.0
Malaysia: DALYs Rate (per 100,000)
DALYs Rate
< 291.3
291.3 - 466.0
466.0 - 519.4
519.4 - 572.8
572.8 - 628.7
628.7 - 684.5
684.5 - 740.3
740.3 - 763.5
763.5 - 1006.3
> 1006.3
Kuwait: DALYs Rate (per 100,000)
DALYs Rate
< 351.8
351.8 - 437.1
437.1 - 589.5
589.5 - 742.0
742.0 - 802.0
802.0 - 861.9
861.9 - 921.9
921.9 - 926.7
926.7 - 1121.3
> 1121.3
Panama: DALYs Rate (per 100,000)
DALYs Rate
< 123.4
123.4 - 163.5
163.5 - 272.7
272.7 - 382.0
382.0 - 449.9
449.9 - 517.7
517.7 - 585.5
585.5 - 594.7
594.7 - 721.5
> 721.5
Togo: DALYs Rate (per 100,000)
DALYs Rate
< 193.9
193.9 - 243.8
243.8 - 384.6
384.6 - 525.4
525.4 - 600.8
600.8 - 676.2
676.2 - 751.6
751.6 - 763.5
763.5 - 905.2
> 905.2
20-24 years
15-19 years
10-14 years
1990
1995
2000
2005
2010
2015
2020

Figure S6

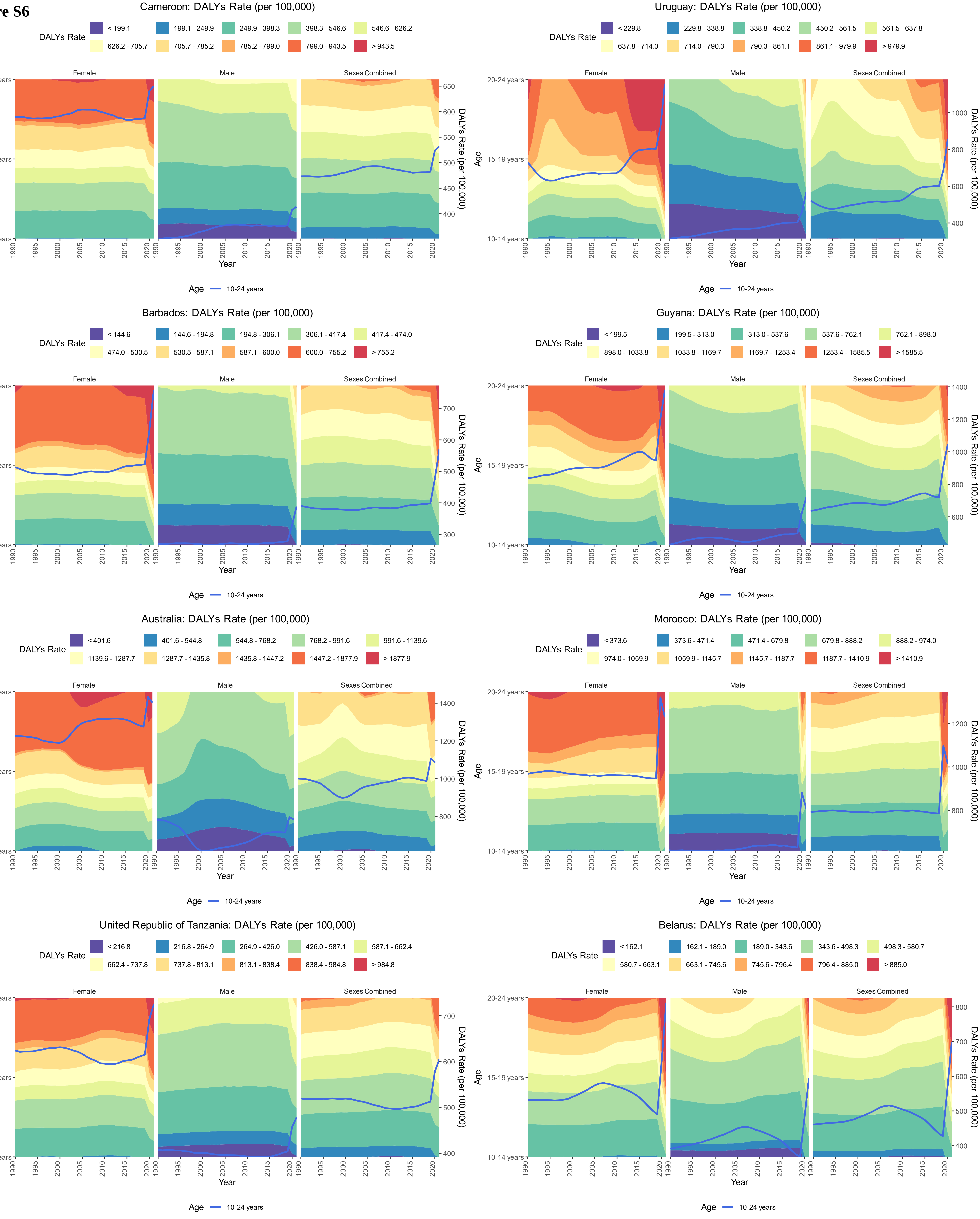
Cameroon: DALYs Rate (per 100,000)
DALYs Rate
< 199.1
199.1 - 249.9
249.9 - 398.3
398.3 - 546.6
546.6 - 626.2
626.2 - 705.7
705.7 - 785.2
785.2 - 799.0
799.0 - 943.5
> 943.5
Uruguay: DALYs Rate (per 100,000)
< 229.8
229.8 - 338.8
338.8 - 450.2
450.2 - 561.5
561.5 - 637.8
637.8 - 714.0
714.0 - 790.3
790.3 - 861.1
861.1 - 979.9
> 979.9
Barbados: DALYs Rate (per 100,000)
< 144.6
144.6 - 194.8
194.8 - 306.1
306.1 - 417.4
417.4 - 474.0
474.0 - 530.5
530.5 - 587.1
587.1 - 600.0
600.0 - 755.2
> 755.2
Guyana: DALYs Rate (per 100,000)
< 199.5
199.5 - 313.0
313.0 - 537.6
537.6 - 762.1
762.1 - 898.0
898.0 - 1033.8
1033.8 - 1169.7
1169.7 - 1253.4
1253.4 - 1585.5
> 1585.5
Australia: DALYs Rate (per 100,000)
< 401.6
401.6 - 544.8
544.8 - 768.2
768.2 - 991.6
991.6 - 1139.6
1139.6 - 1287.7
1287.7 - 1435.8
1435.8 - 1447.2
1447.2 - 1877.9
> 1877.9
Morocco: DALYs Rate (per 100,000)
< 373.6
373.6 - 471.4
471.4 - 679.8
679.8 - 888.2
888.2 - 974.0
974.0 - 1059.9
1059.9 - 1145.7
1145.7 - 1187.7
1187.7 - 1410.9
> 1410.9
United Republic of Tanzania: DALYs Rate (per 100,000)
< 216.8
216.8 - 264.9
264.9 - 426.0
426.0 - 587.1
587.1 - 662.4
662.4 - 737.8
737.8 - 813.1
813.1 - 838.4
838.4 - 984.8
> 984.8
Belarus: DALYs Rate (per 100,000)
< 162.1
162.1 - 189.0
189.0 - 343.6
343.6 - 498.3
498.3 - 580.7
580.7 - 663.1
663.1 - 745.6
745.6 - 796.4
796.4 - 885.0
> 885.0
Female
Male
Sexes Combined
Age
20-24 years
15-19 years
10-14 years
Year
DALYs Rate (per 100,000)
Age — 10-24 years

Figure S6

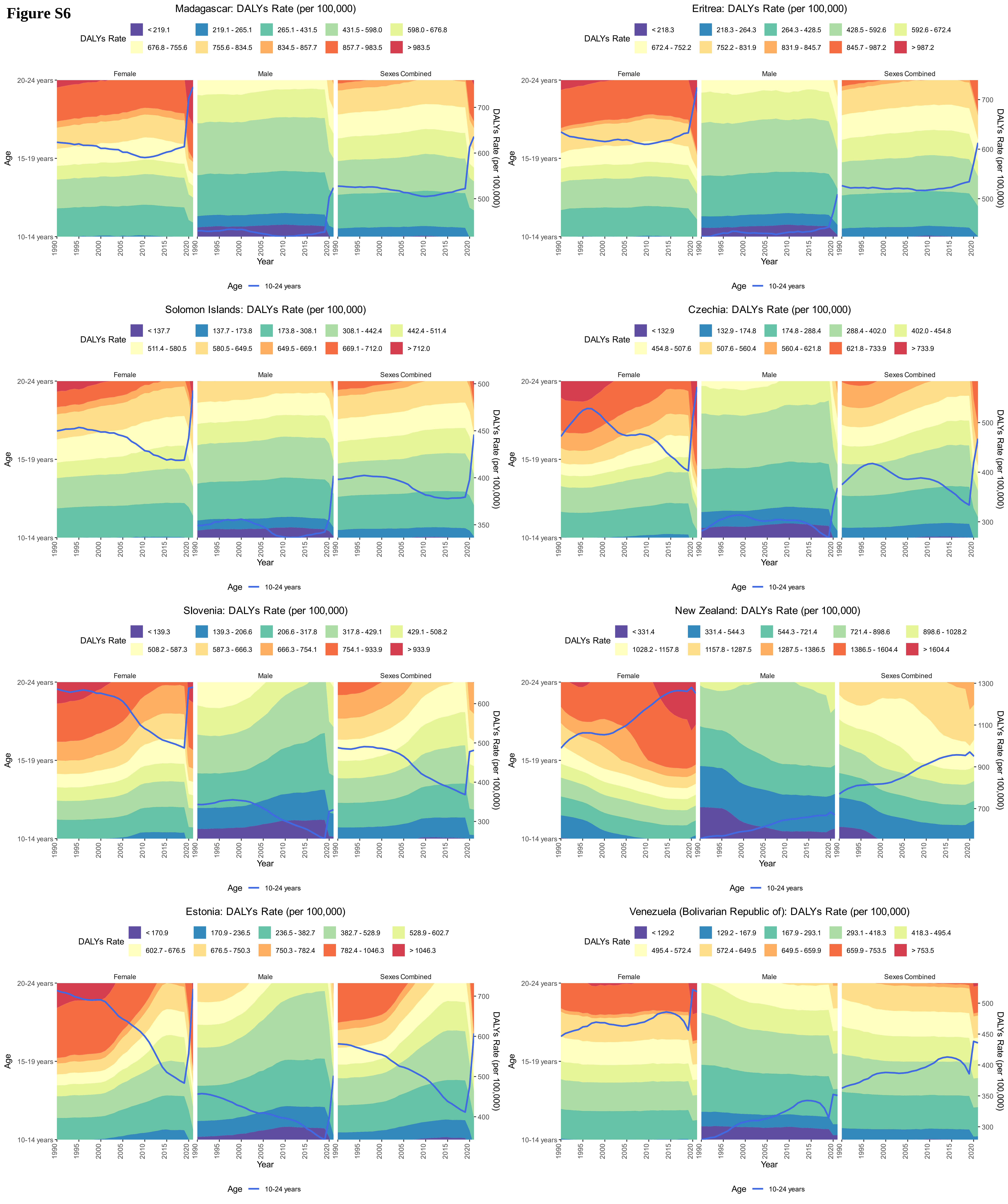

Madagascar: DALYs Rate (per 100,000)
DALYs Rate
< 219.1
219.1 - 265.1
265.1 - 431.5
431.5 - 598.0
598.0 - 676.8
676.8 - 755.6
755.6 - 834.5
834.5 - 857.7
857.7 - 983.5
> 983.5
Eritrea: DALYs Rate (per 100,000)
DALYs Rate
< 218.3
218.3 - 264.3
264.3 - 428.5
428.5 - 592.6
592.6 - 672.4
672.4 - 752.2
752.2 - 831.9
831.9 - 845.7
845.7 - 987.2
> 987.2
Solomon Islands: DALYs Rate (per 100,000)
DALYs Rate
< 137.7
137.7 - 173.8
173.8 - 308.1
308.1 - 442.4
442.4 - 511.4
511.4 - 580.5
580.5 - 649.5
649.5 - 669.1
669.1 - 712.0
> 712.0
Czechia: DALYs Rate (per 100,000)
DALYs Rate
< 132.9
132.9 - 174.8
174.8 - 288.4
288.4 - 402.0
402.0 - 454.8
454.8 - 507.6
507.6 - 560.4
560.4 - 621.8
621.8 - 733.9
> 733.9
Slovenia: DALYs Rate (per 100,000)
DALYs Rate
< 139.3
139.3 - 206.6
206.6 - 317.8
317.8 - 429.1
429.1 - 508.2
508.2 - 587.3
587.3 - 666.3
666.3 - 754.1
754.1 - 933.9
> 933.9
New Zealand: DALYs Rate (per 100,000)
DALYs Rate
< 331.4
331.4 - 544.3
544.3 - 721.4
721.4 - 898.6
898.6 - 1028.2
1028.2 - 1157.8
1157.8 - 1287.5
1287.5 - 1386.5
1386.5 - 1604.4
> 1604.4
Estonia: DALYs Rate (per 100,000)
DALYs Rate
< 170.9
170.9 - 236.5
236.5 - 382.7
382.7 - 528.9
528.9 - 602.7
602.7 - 676.5
676.5 - 750.3
750.3 - 782.4
782.4 - 1046.3
> 1046.3
Venezuela (Bolivarian Republic of): DALYs Rate (per 100,000)
DALYs Rate
< 129.2
129.2 - 167.9
167.9 - 293.1
293.1 - 418.3
418.3 - 495.4
495.4 - 572.4
572.4 - 649.5
649.5 - 659.9
659.9 - 753.5
> 753.5
Female
Male
Sexes Combined
Age
20-24 years
15-19 years
10-14 years
Year
DALYs Rate (per 100,000)
Age
10-24 years

Figure S6

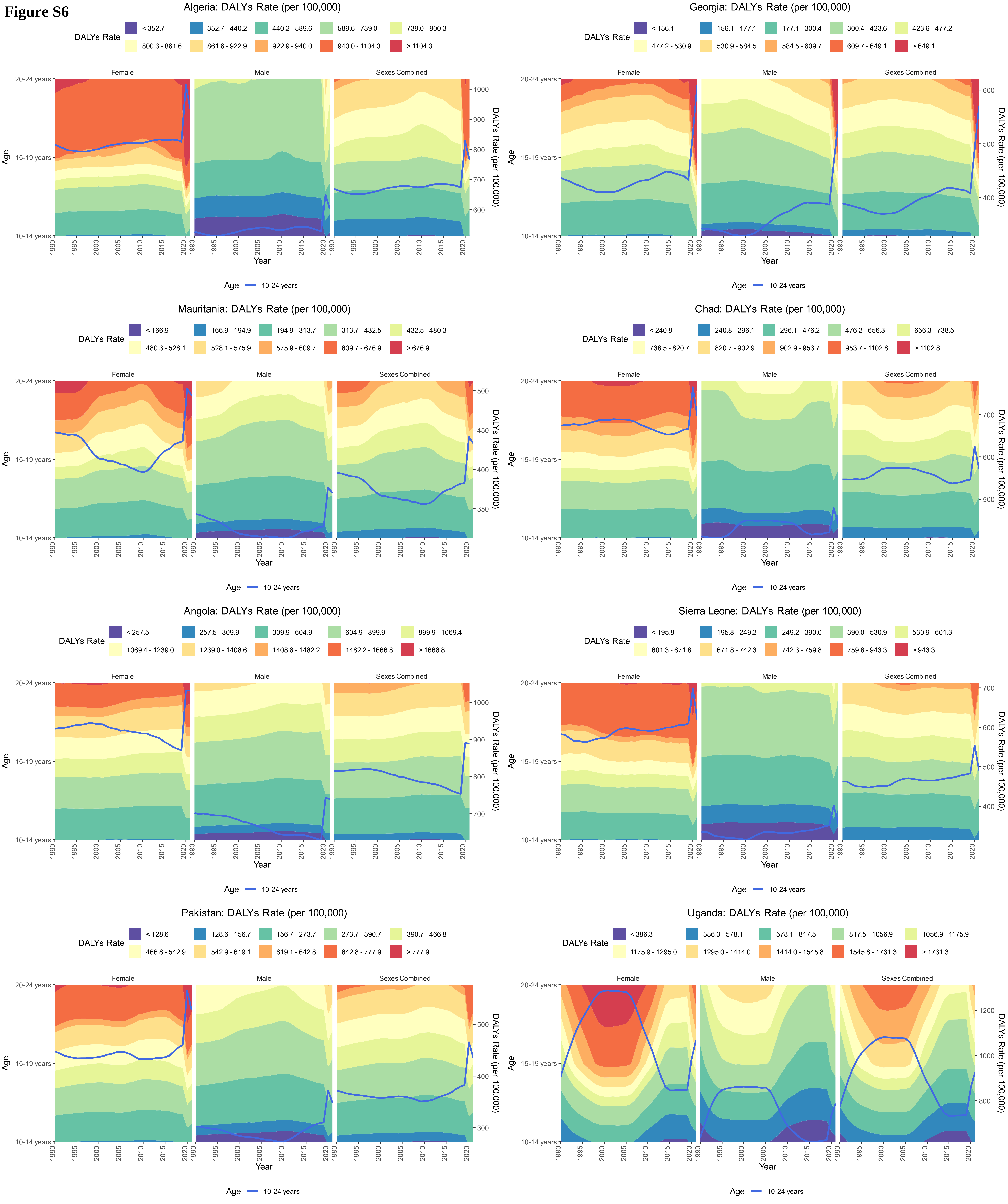

Algeria: DALYs Rate (per 100,000)
DALYs Rate
< 352.7
352.7 - 440.2
440.2 - 589.6
589.6 - 739.0
739.0 - 800.3
800.3 - 861.6
861.6 - 922.9
922.9 - 940.0
940.0 - 1104.3
> 1104.3
Georgia: DALYs Rate (per 100,000)
DALYs Rate
< 156.1
156.1 - 177.1
177.1 - 300.4
300.4 - 423.6
423.6 - 477.2
477.2 - 530.9
530.9 - 584.5
584.5 - 609.7
609.7 - 649.1
> 649.1
Mauritania: DALYs Rate (per 100,000)
DALYs Rate
< 166.9
166.9 - 194.9
194.9 - 313.7
313.7 - 432.5
432.5 - 480.3
480.3 - 528.1
528.1 - 575.9
575.9 - 609.7
609.7 - 676.9
> 676.9
Chad: DALYs Rate (per 100,000)
DALYs Rate
< 240.8
240.8 - 296.1
296.1 - 476.2
476.2 - 656.3
656.3 - 738.5
738.5 - 820.7
820.7 - 902.9
902.9 - 953.7
953.7 - 1102.8
> 1102.8
Angola: DALYs Rate (per 100,000)
DALYs Rate
< 257.5
257.5 - 309.9
309.9 - 604.9
604.9 - 899.9
899.9 - 1069.4
1069.4 - 1239.0
1239.0 - 1408.6
1408.6 - 1482.2
1482.2 - 1666.8
> 1666.8
Sierra Leone: DALYs Rate (per 100,000)
DALYs Rate
< 195.8
195.8 - 249.2
249.2 - 390.0
390.0 - 530.9
530.9 - 601.3
601.3 - 671.8
671.8 - 742.3
742.3 - 759.8
759.8 - 943.3
> 943.3
Pakistan: DALYs Rate (per 100,000)
DALYs Rate
< 128.6
128.6 - 156.7
156.7 - 273.7
273.7 - 390.7
390.7 - 466.8
466.8 - 542.9
542.9 - 619.1
619.1 - 642.8
642.8 - 777.9
> 777.9
Uganda: DALYs Rate (per 100,000)
DALYs Rate
< 386.3
386.3 - 578.1
578.1 - 817.5
817.5 - 1056.9
1056.9 - 1175.9
1175.9 - 1295.0
1295.0 - 1414.0
1414.0 - 1545.8
1545.8 - 1731.3
> 1731.3
Female
Male
Sexes Combined
Age
20-24 years
15-19 years
10-14 years
Year
DALYs Rate (per 100,000)
Age 10-24 years

Figure S6

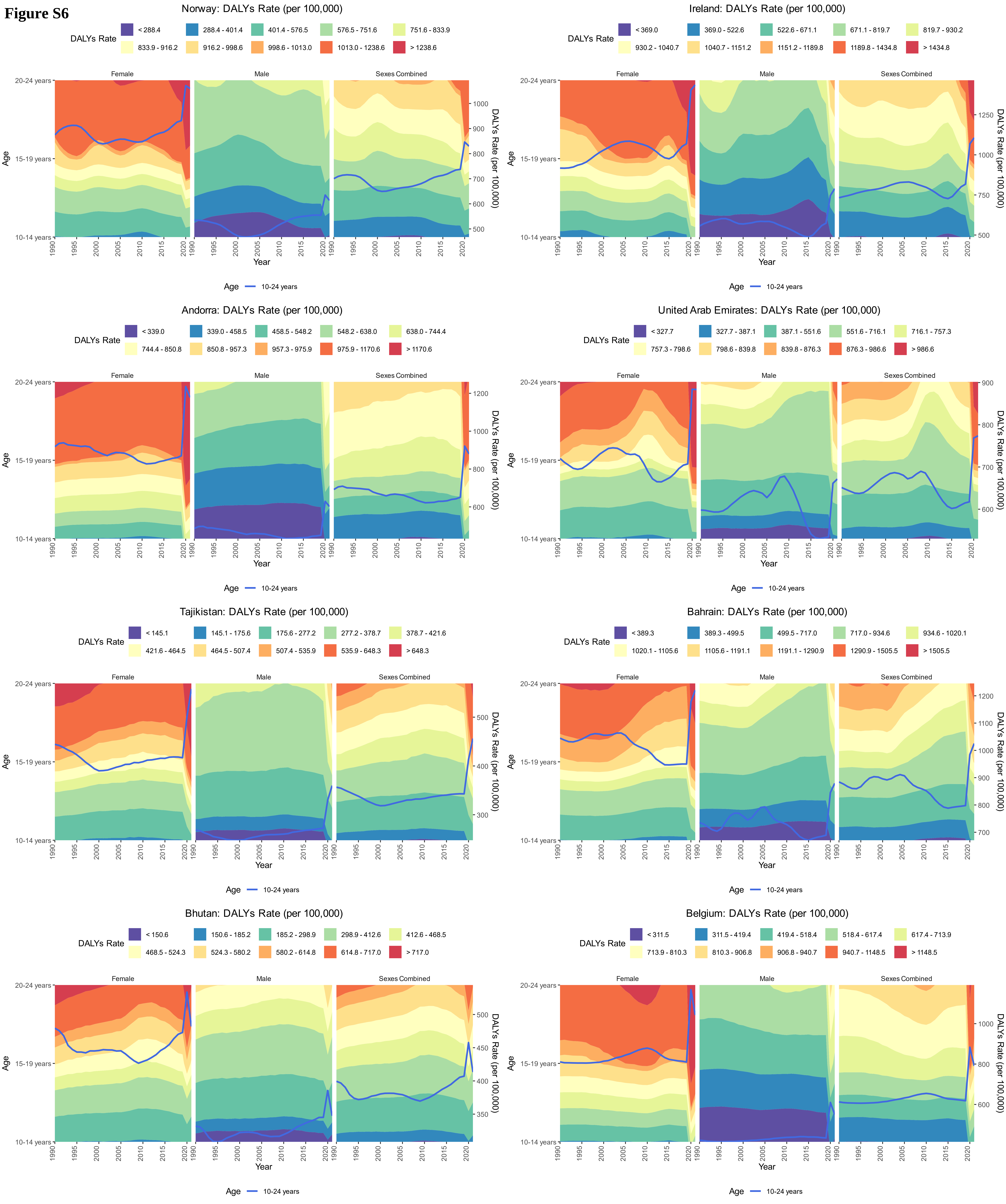

Norway: DALYs Rate (per 100,000)
DALYs Rate
< 288.4
288.4 - 401.4
401.4 - 576.5
576.5 - 751.6
751.6 - 833.9
833.9 - 916.2
916.2 - 998.6
998.6 - 1013.0
1013.0 - 1238.6
> 1238.6
Female
Male
Sexes Combined
20-24 years
15-19 years
10-14 years
Age
Year
DALYs Rate (per 100,000)
Age
10-24 years
Ireland: DALYs Rate (per 100,000)
DALYs Rate
< 369.0
369.0 - 522.6
522.6 - 671.1
671.1 - 819.7
819.7 - 930.2
930.2 - 1040.7
1040.7 - 1151.2
1151.2 - 1189.8
1189.8 - 1434.8
> 1434.8
Andorra: DALYs Rate (per 100,000)
DALYs Rate
< 339.0
339.0 - 458.5
458.5 - 548.2
548.2 - 638.0
638.0 - 744.4
744.4 - 850.8
850.8 - 957.3
957.3 - 975.9
975.9 - 1170.6
> 1170.6
United Arab Emirates: DALYs Rate (per 100,000)
DALYs Rate
< 327.7
327.7 - 387.1
387.1 - 551.6
551.6 - 716.1
716.1 - 757.3
757.3 - 798.6
798.6 - 839.8
839.8 - 876.3
876.3 - 986.6
> 986.6
Tajikistan: DALYs Rate (per 100,000)
DALYs Rate
< 145.1
145.1 - 175.6
175.6 - 277.2
277.2 - 378.7
378.7 - 421.6
421.6 - 464.5
464.5 - 507.4
507.4 - 535.9
535.9 - 648.3
> 648.3
Bahrain: DALYs Rate (per 100,000)
DALYs Rate
< 389.3
389.3 - 499.5
499.5 - 717.0
717.0 - 934.6
934.6 - 1020.1
1020.1 - 1105.6
1105.6 - 1191.1
1191.1 - 1290.9
1290.9 - 1505.5
> 1505.5
Bhutan: DALYs Rate (per 100,000)
DALYs Rate
< 150.6
150.6 - 185.2
185.2 - 298.9
298.9 - 412.6
412.6 - 468.5
468.5 - 524.3
524.3 - 580.2
580.2 - 614.8
614.8 - 717.0
> 717.0
Belgium: DALYs Rate (per 100,000)
DALYs Rate
< 311.5
311.5 - 419.4
419.4 - 518.4
518.4 - 617.4
617.4 - 713.9
713.9 - 810.3
810.3 - 906.8
906.8 - 940.7
940.7 - 1148.5
> 1148.5

Figure S6

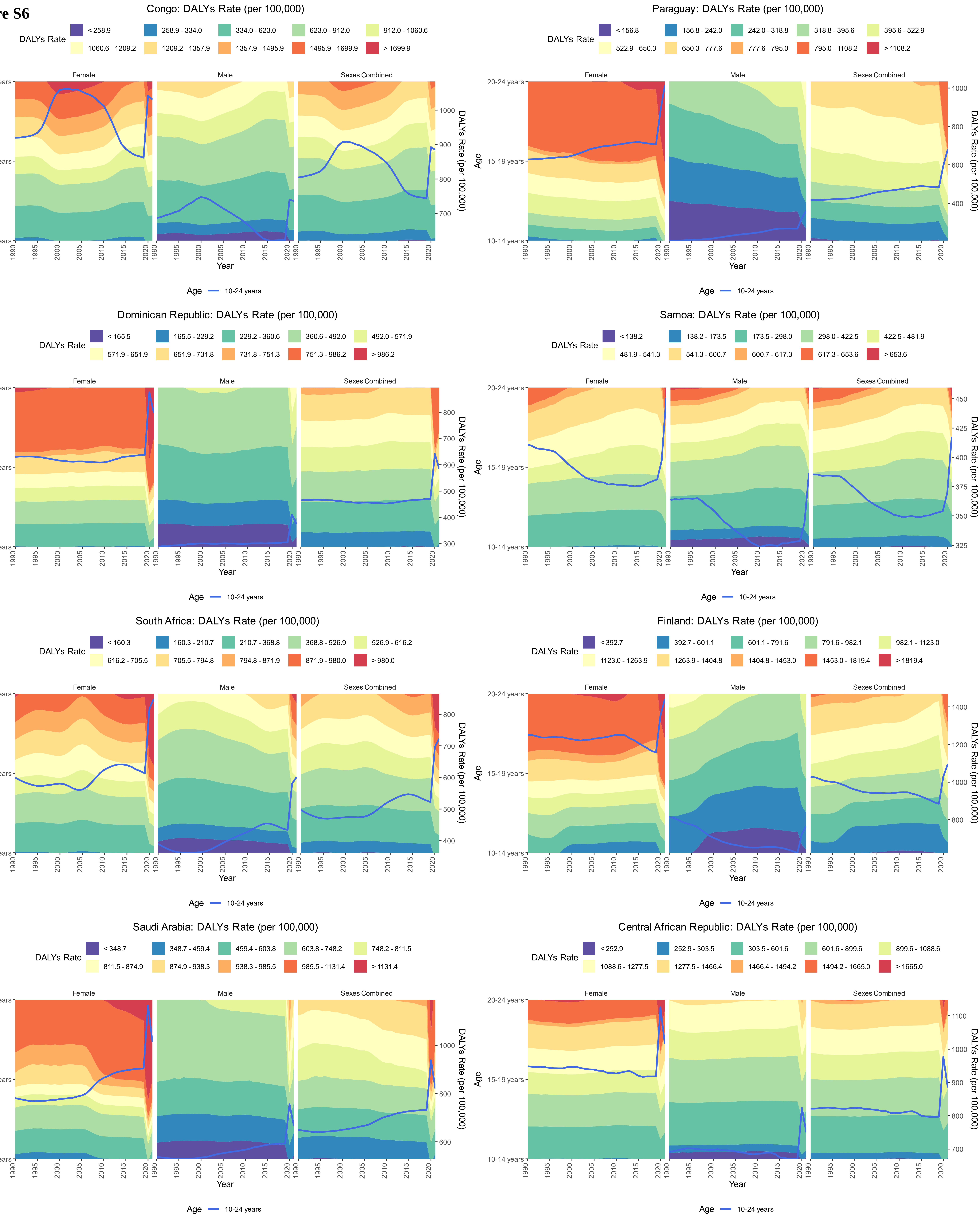
Congo: DALYs Rate (per 100,000)
DALYs Rate
< 258.9
258.9 - 334.0
334.0 - 623.0
623.0 - 912.0
912.0 - 1060.6
1060.6 - 1209.2
1209.2 - 1357.9
1357.9 - 1495.9
1495.9 - 1699.9
> 1699.9
Paraguay: DALYs Rate (per 100,000)
< 156.8
156.8 - 242.0
242.0 - 318.8
318.8 - 395.6
395.6 - 522.9
522.9 - 650.3
650.3 - 777.6
777.6 - 795.0
795.0 - 1108.2
> 1108.2
Dominican Republic: DALYs Rate (per 100,000)
< 165.5
165.5 - 229.2
229.2 - 360.6
360.6 - 492.0
492.0 - 571.9
571.9 - 651.9
651.9 - 731.8
731.8 - 751.3
751.3 - 986.2
> 986.2
Samoa: DALYs Rate (per 100,000)
< 138.2
138.2 - 173.5
173.5 - 298.0
298.0 - 422.5
422.5 - 481.9
481.9 - 541.3
541.3 - 600.7
600.7 - 617.3
617.3 - 653.6
> 653.6
South Africa: DALYs Rate (per 100,000)
< 160.3
160.3 - 210.7
210.7 - 368.8
368.8 - 526.9
526.9 - 616.2
616.2 - 705.5
705.5 - 794.8
794.8 - 871.9
871.9 - 980.0
> 980.0
Finland: DALYs Rate (per 100,000)
< 392.7
392.7 - 601.1
601.1 - 791.6
791.6 - 982.1
982.1 - 1123.0
1123.0 - 1263.9
1263.9 - 1404.8
1404.8 - 1453.0
1453.0 - 1819.4
> 1819.4
Saudi Arabia: DALYs Rate (per 100,000)
< 348.7
348.7 - 459.4
459.4 - 603.8
603.8 - 748.2
748.2 - 811.5
811.5 - 874.9
874.9 - 938.3
938.3 - 985.5
985.5 - 1131.4
> 1131.4
Central African Republic: DALYs Rate (per 100,000)
< 252.9
252.9 - 303.5
303.5 - 601.6
601.6 - 899.6
899.6 - 1088.6
1088.6 - 1277.5
1277.5 - 1466.4
1466.4 - 1494.2
1494.2 - 1665.0
> 1665.0
Female
Male
Sexes Combined
Age
20-24 years
15-19 years
10-14 years
Year
DALYs Rate (per 100,000)
Age — 10-24 years

Figure S6

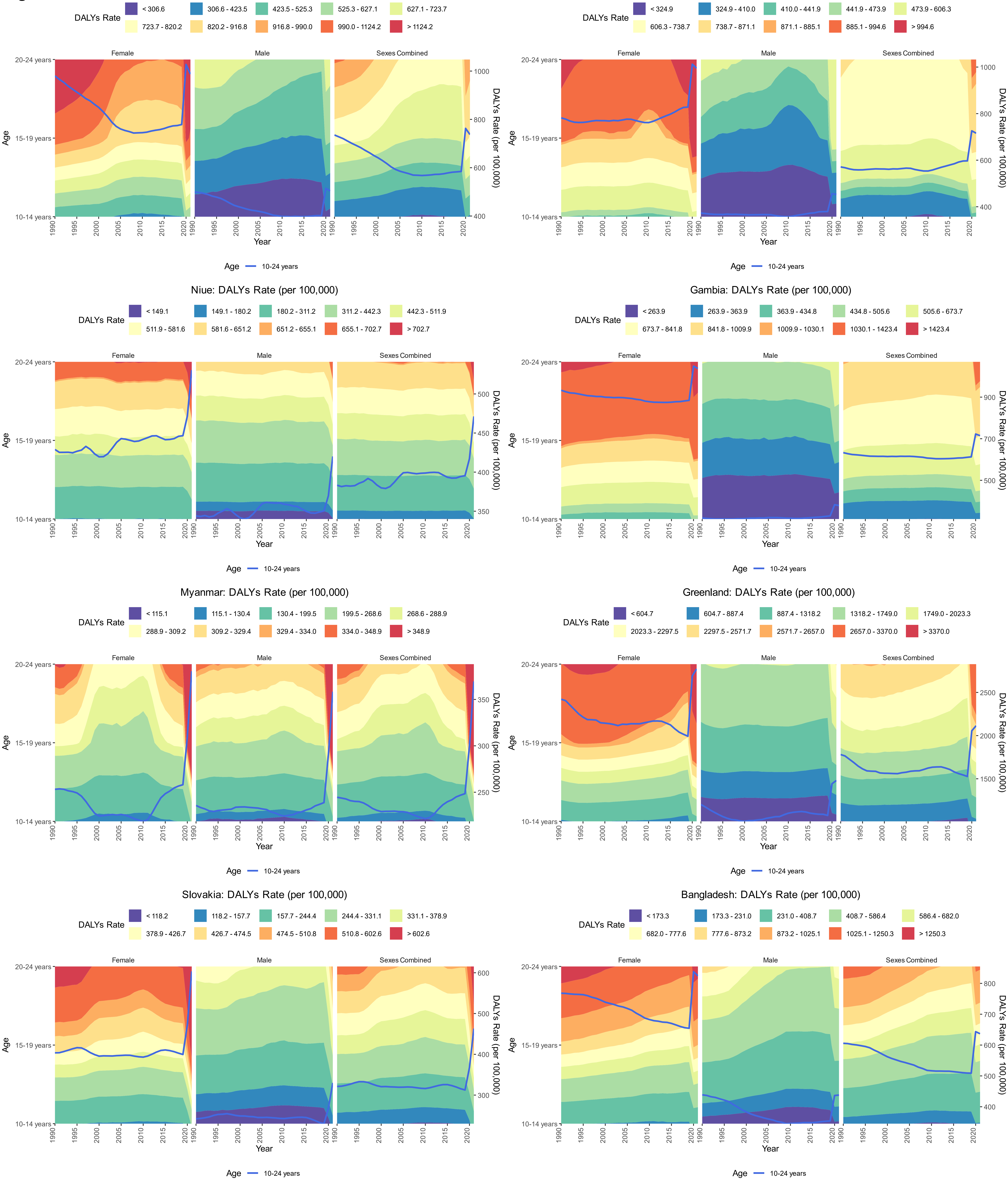
Luxembourg: DALYs Rate (per 100,000)
DALYs Rate
< 306.6
306.6 - 423.5
423.5 - 525.3
525.3 - 627.1
627.1 - 723.7
723.7 - 820.2
820.2 - 916.8
916.8 - 990.0
990.0 - 1124.2
> 1124.2
Malta: DALYs Rate (per 100,000)
< 324.9
324.9 - 410.0
410.0 - 441.9
441.9 - 473.9
473.9 - 606.3
606.3 - 738.7
738.7 - 871.1
871.1 - 885.1
885.1 - 994.6
> 994.6
Niue: DALYs Rate (per 100,000)
< 149.1
149.1 - 180.2
180.2 - 311.2
311.2 - 442.3
442.3 - 511.9
511.9 - 581.6
581.6 - 651.2
651.2 - 655.1
655.1 - 702.7
> 702.7
Gambia: DALYs Rate (per 100,000)
< 263.9
263.9 - 363.9
363.9 - 434.8
434.8 - 505.6
505.6 - 673.7
673.7 - 841.8
841.8 - 1009.9
1009.9 - 1030.1
1030.1 - 1423.4
> 1423.4
Myanmar: DALYs Rate (per 100,000)
< 115.1
115.1 - 130.4
130.4 - 199.5
199.5 - 268.6
268.6 - 288.9
288.9 - 309.2
309.2 - 329.4
329.4 - 334.0
334.0 - 348.9
> 348.9
Greenland: DALYs Rate (per 100,000)
< 604.7
604.7 - 887.4
887.4 - 1318.2
1318.2 - 1749.0
1749.0 - 2023.3
2023.3 - 2297.5
2297.5 - 2571.7
2571.7 - 2657.0
2657.0 - 3370.0
> 3370.0
Slovakia: DALYs Rate (per 100,000)
< 118.2
118.2 - 157.7
157.7 - 244.4
244.4 - 331.1
331.1 - 378.9
378.9 - 426.7
426.7 - 474.5
474.5 - 510.8
510.8 - 602.6
> 602.6
Bangladesh: DALYs Rate (per 100,000)
< 173.3
173.3 - 231.0
231.0 - 408.7
408.7 - 586.4
586.4 - 682.0
682.0 - 777.6
777.6 - 873.2
873.2 - 1025.1
1025.1 - 1250.3
> 1250.3
Female
Male
Sexes Combined
Age
20-24 years
15-19 years
10-14 years
Year
DALYs Rate (per 100,000)
Age — 10-24 years

Figure S6

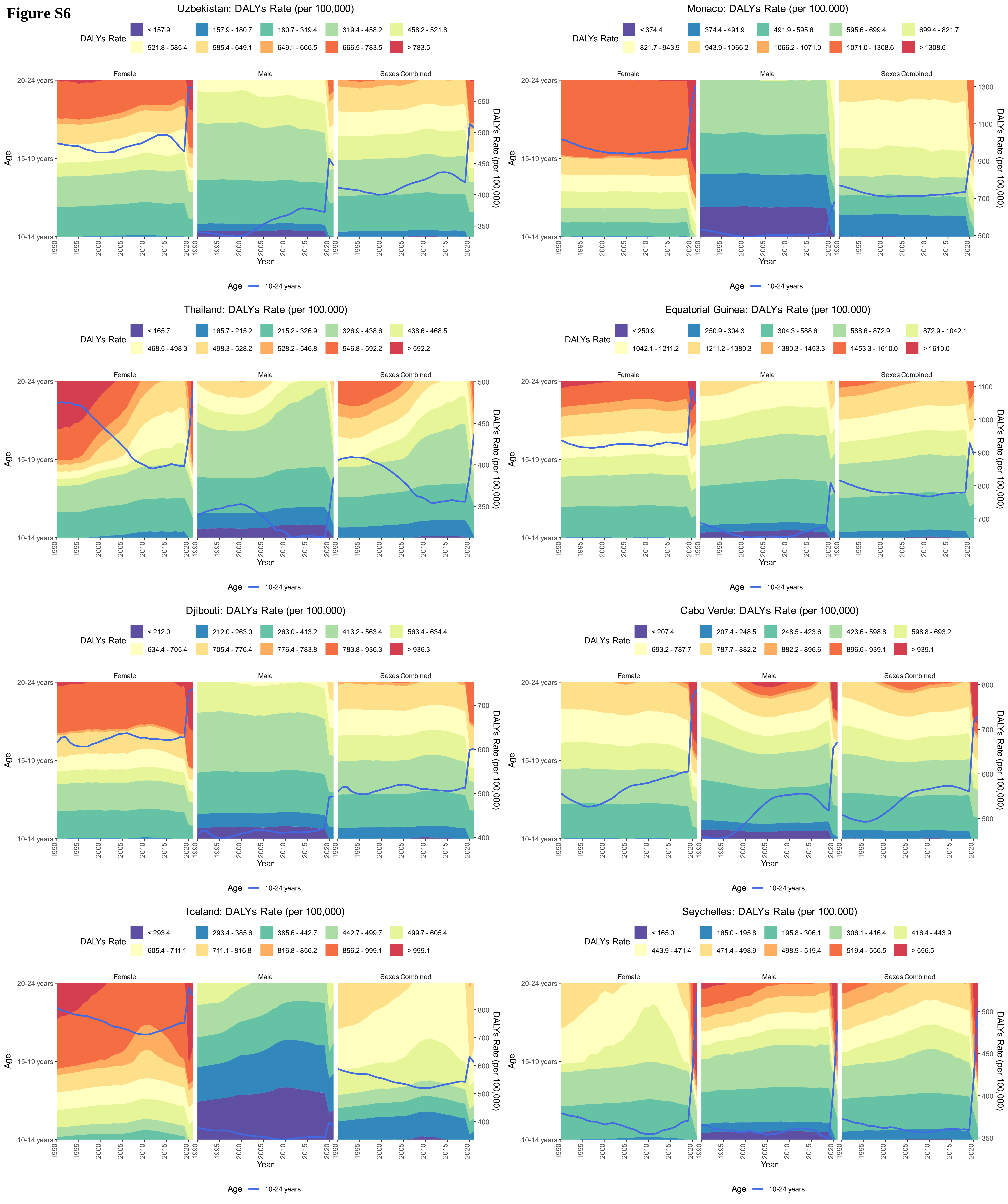

Uzbekistan: DALYs Rate (per 100,000)
DALYs Rate
< 157.9
157.9 - 180.7
180.7 - 319.4
319.4 - 458.2
458.2 - 521.8
521.8 - 585.4
585.4 - 649.1
649.1 - 666.5
666.5 - 783.5
> 783.5
Monaco: DALYs Rate (per 100,000)
< 374.4
374.4 - 491.9
491.9 - 595.6
595.6 - 699.4
699.4 - 821.7
821.7 - 943.9
943.9 - 1066.2
1066.2 - 1071.0
1071.0 - 1308.6
> 1308.6
Thailand: DALYs Rate (per 100,000)
< 165.7
165.7 - 215.2
215.2 - 326.9
326.9 - 438.6
438.6 - 468.5
468.5 - 498.3
498.3 - 528.2
528.2 - 546.8
546.8 - 592.2
> 592.2
Equatorial Guinea: DALYs Rate (per 100,000)
< 250.9
250.9 - 304.3
304.3 - 588.6
588.6 - 872.9
872.9 - 1042.1
1042.1 - 1211.2
1211.2 - 1380.3
1380.3 - 1453.3
1453.3 - 1610.0
> 1610.0
Djibouti: DALYs Rate (per 100,000)
< 212.0
212.0 - 263.0
263.0 - 413.2
413.2 - 563.4
563.4 - 634.4
634.4 - 705.4
705.4 - 776.4
776.4 - 783.8
783.8 - 936.3
> 936.3
Cabo Verde: DALYs Rate (per 100,000)
< 207.4
207.4 - 248.5
248.5 - 423.6
423.6 - 598.8
598.8 - 693.2
693.2 - 787.7
787.7 - 882.2
882.2 - 896.6
896.6 - 939.1
> 939.1
Iceland: DALYs Rate (per 100,000)
< 293.4
293.4 - 385.6
385.6 - 442.7
442.7 - 499.7
499.7 - 605.4
605.4 - 711.1
711.1 - 816.8
816.8 - 856.2
856.2 - 999.1
> 999.1
Seychelles: DALYs Rate (per 100,000)
< 165.0
165.0 - 195.8
195.8 - 306.1
306.1 - 416.4
416.4 - 443.9
443.9 - 471.4
471.4 - 498.9
498.9 - 519.4
519.4 - 556.5
> 556.5
Female
Male
Sexes Combined
Age
20-24 years
15-19 years
10-14 years
Year
DALYs Rate (per 100,000)
Age — 10-24 years

Figure S6

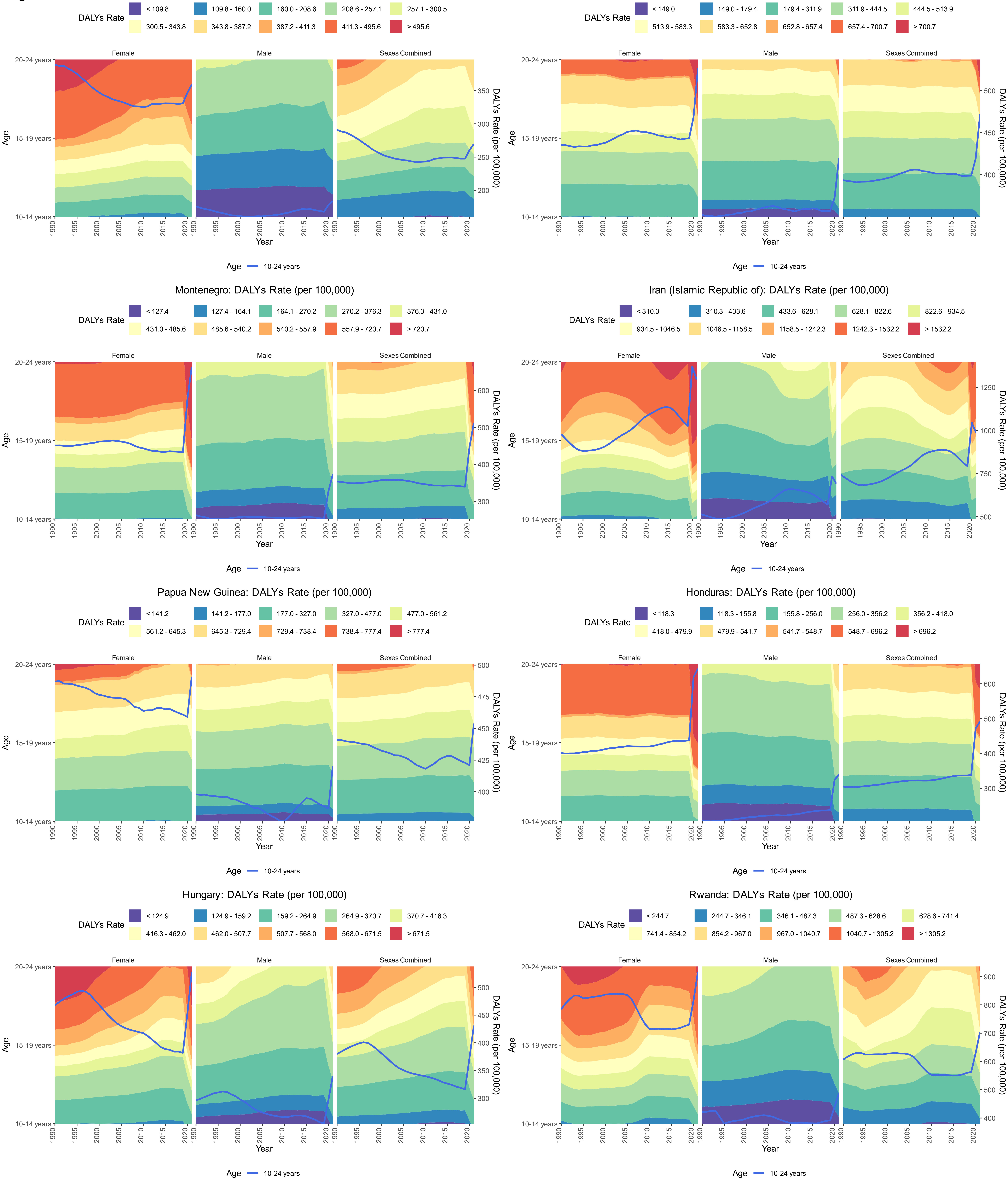

Democratic People's Republic of Korea: DALYs Rate (per 100,000)
DALYs Rate
< 109.8
109.8 - 160.0
160.0 - 208.6
208.6 - 257.1
257.1 - 300.5
300.5 - 343.8
343.8 - 387.2
387.2 - 411.3
411.3 - 495.6
> 495.6
Nauru: DALYs Rate (per 100,000)
< 149.0
149.0 - 179.4
179.4 - 311.9
311.9 - 444.5
444.5 - 513.9
513.9 - 583.3
583.3 - 652.8
652.8 - 657.4
657.4 - 700.7
> 700.7
Montenegro: DALYs Rate (per 100,000)
< 127.4
127.4 - 164.1
164.1 - 270.2
270.2 - 376.3
376.3 - 431.0
431.0 - 485.6
485.6 - 540.2
540.2 - 557.9
557.9 - 720.7
> 720.7
Iran (Islamic Republic of): DALYs Rate (per 100,000)
< 310.3
310.3 - 433.6
433.6 - 628.1
628.1 - 822.6
822.6 - 934.5
934.5 - 1046.5
1046.5 - 1158.5
1158.5 - 1242.3
1242.3 - 1532.2
> 1532.2
Papua New Guinea: DALYs Rate (per 100,000)
< 141.2
141.2 - 177.0
177.0 - 327.0
327.0 - 477.0
477.0 - 561.2
561.2 - 645.3
645.3 - 729.4
729.4 - 738.4
738.4 - 777.4
> 777.4
Honduras: DALYs Rate (per 100,000)
< 118.3
118.3 - 155.8
155.8 - 256.0
256.0 - 356.2
356.2 - 418.0
418.0 - 479.9
479.9 - 541.7
541.7 - 548.7
548.7 - 696.2
> 696.2
Hungary: DALYs Rate (per 100,000)
< 124.9
124.9 - 159.2
159.2 - 264.9
264.9 - 370.7
370.7 - 416.3
416.3 - 462.0
462.0 - 507.7
507.7 - 568.0
568.0 - 671.5
> 671.5
Rwanda: DALYs Rate (per 100,000)
< 244.7
244.7 - 346.1
346.1 - 487.3
487.3 - 628.6
628.6 - 741.4
741.4 - 854.2
854.2 - 967.0
967.0 - 1040.7
1040.7 - 1305.2
> 1305.2
Female
Male
Sexes Combined
Age
20-24 years
15-19 years
10-14 years
Year
DALYs Rate (per 100,000)
Age — 10-24 years

Figure S6

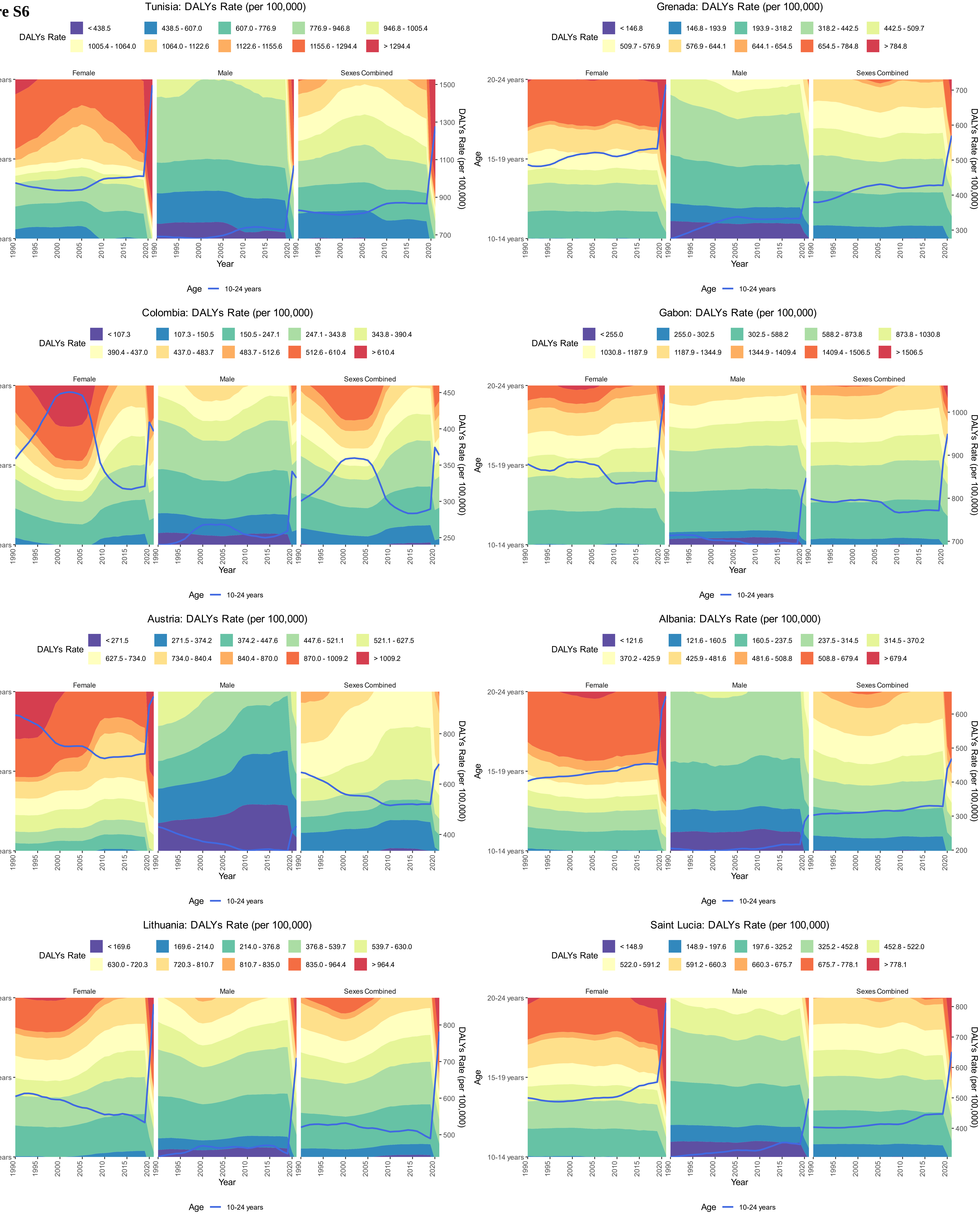
Tunisia: DALYs Rate (per 100,000)
DALYs Rate
< 438.5
438.5 - 607.0
607.0 - 776.9
776.9 - 946.8
946.8 - 1005.4
1005.4 - 1064.0
1064.0 - 1122.6
1122.6 - 1155.6
1155.6 - 1294.4
> 1294.4
Grenada: DALYs Rate (per 100,000)
DALYs Rate
< 146.8
146.8 - 193.9
193.9 - 318.2
318.2 - 442.5
442.5 - 509.7
509.7 - 576.9
576.9 - 644.1
644.1 - 654.5
654.5 - 784.8
> 784.8
Colombia: DALYs Rate (per 100,000)
DALYs Rate
< 107.3
107.3 - 150.5
150.5 - 247.1
247.1 - 343.8
343.8 - 390.4
390.4 - 437.0
437.0 - 483.7
483.7 - 512.6
512.6 - 610.4
> 610.4
Gabon: DALYs Rate (per 100,000)
DALYs Rate
< 255.0
255.0 - 302.5
302.5 - 588.2
588.2 - 873.8
873.8 - 1030.8
1030.8 - 1187.9
1187.9 - 1344.9
1344.9 - 1409.4
1409.4 - 1506.5
> 1506.5
Austria: DALYs Rate (per 100,000)
DALYs Rate
< 271.5
271.5 - 374.2
374.2 - 447.6
447.6 - 521.1
521.1 - 627.5
627.5 - 734.0
734.0 - 840.4
840.4 - 870.0
870.0 - 1009.2
> 1009.2
Albania: DALYs Rate (per 100,000)
DALYs Rate
< 121.6
121.6 - 160.5
160.5 - 237.5
237.5 - 314.5
314.5 - 370.2
370.2 - 425.9
425.9 - 481.6
481.6 - 508.8
508.8 - 679.4
> 679.4
Lithuania: DALYs Rate (per 100,000)
DALYs Rate
< 169.6
169.6 - 214.0
214.0 - 376.8
376.8 - 539.7
539.7 - 630.0
630.0 - 720.3
720.3 - 810.7
810.7 - 835.0
835.0 - 964.4
> 964.4
Saint Lucia: DALYs Rate (per 100,000)
DALYs Rate
< 148.9
148.9 - 197.6
197.6 - 325.2
325.2 - 452.8
452.8 - 522.0
522.0 - 591.2
591.2 - 660.3
660.3 - 675.7
675.7 - 778.1
> 778.1
Female
Male
Sexes Combined
Age
20-24 years
15-19 years
10-14 years
Year
1990
1995
2000
2005
2010
2015
2020
DALYs Rate (per 100,000)
Age
10-24 years

Figure S6

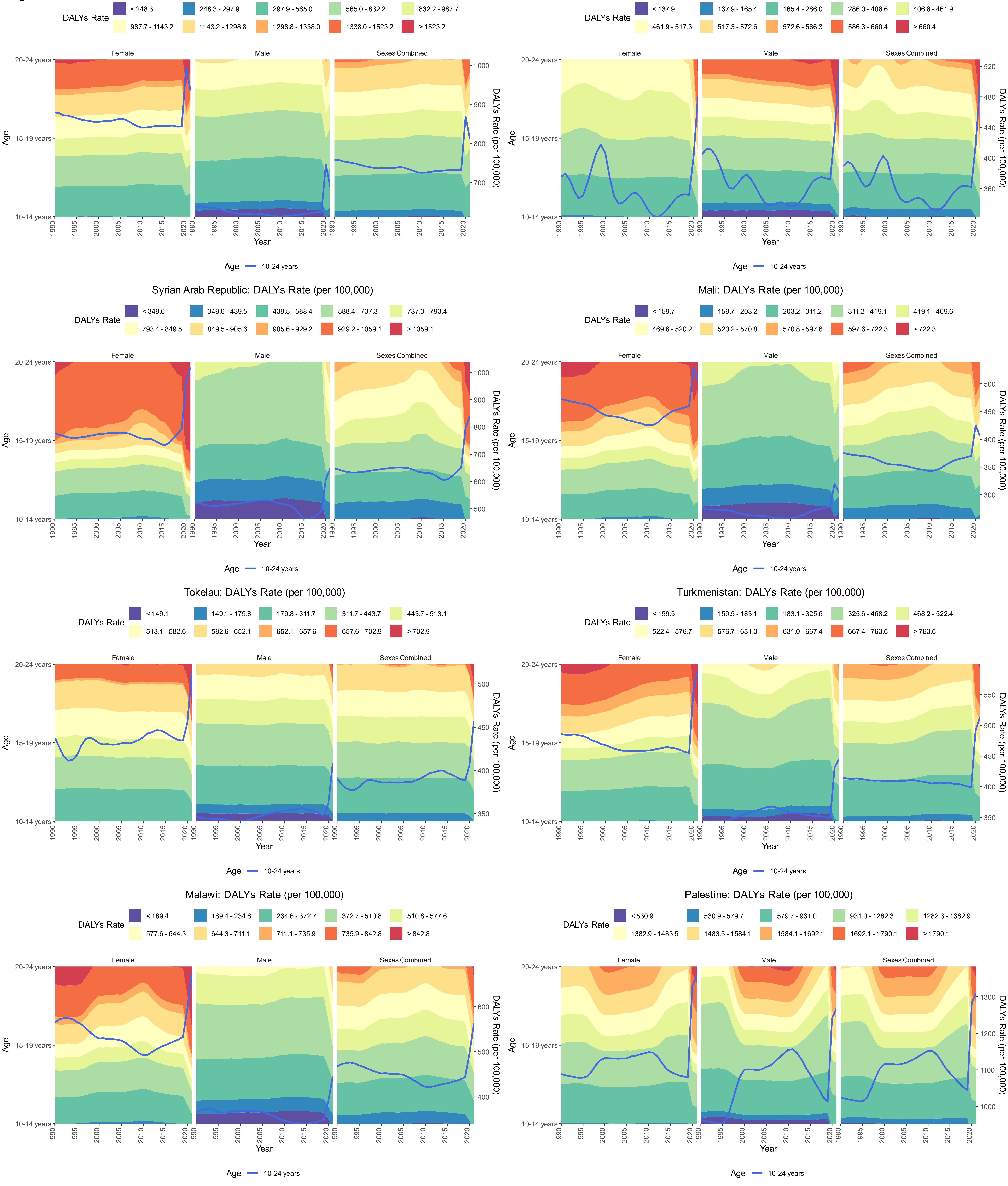
Democratic Republic of the Congo: DALYs Rate (per 100,000)
DALYs Rate
< 248.3
248.3 - 297.9
297.9 - 565.0
565.0 - 832.2
832.2 - 987.7
987.7 - 1143.2
1143.2 - 1298.8
1298.8 - 1338.0
1338.0 - 1523.2
> 1523.2
Northern Mariana Islands: DALYs Rate (per 100,000)
DALYs Rate
< 137.9
137.9 - 165.4
165.4 - 286.0
286.0 - 406.6
406.6 - 461.9
461.9 - 517.3
517.3 - 572.6
572.6 - 586.3
586.3 - 660.4
> 660.4
Syrian Arab Republic: DALYs Rate (per 100,000)
DALYs Rate
< 349.6
349.6 - 439.5
439.5 - 588.4
588.4 - 737.3
737.3 - 793.4
793.4 - 849.5
849.5 - 905.6
905.6 - 929.2
929.2 - 1059.1
> 1059.1
Mali: DALYs Rate (per 100,000)
DALYs Rate
< 159.7
159.7 - 203.2
203.2 - 311.2
311.2 - 419.1
419.1 - 469.6
469.6 - 520.2
520.2 - 570.8
570.8 - 597.6
597.6 - 722.3
> 722.3
Tokelau: DALYs Rate (per 100,000)
DALYs Rate
< 149.1
149.1 - 179.8
179.8 - 311.7
311.7 - 443.7
443.7 - 513.1
513.1 - 582.6
582.6 - 652.1
652.1 - 657.6
657.6 - 702.9
> 702.9
Turkmenistan: DALYs Rate (per 100,000)
DALYs Rate
< 159.5
159.5 - 183.1
183.1 - 325.6
325.6 - 468.2
468.2 - 522.4
522.4 - 576.7
576.7 - 631.0
631.0 - 667.4
667.4 - 763.6
> 763.6
Malawi: DALYs Rate (per 100,000)
DALYs Rate
< 189.4
189.4 - 234.6
234.6 - 372.7
372.7 - 510.8
510.8 - 577.6
577.6 - 644.3
644.3 - 711.1
711.1 - 735.9
735.9 - 842.8
> 842.8
Palestine: DALYs Rate (per 100,000)
DALYs Rate
< 530.9
530.9 - 579.7
579.7 - 931.0
931.0 - 1282.3
1282.3 - 1382.9
1382.9 - 1483.5
1483.5 - 1584.1
1584.1 - 1692.1
1692.1 - 1790.1
> 1790.1
Female
Male
Sexes Combined
20-24 years
15-19 years
10-14 years
Age
Year
DALYs Rate (per 100,000)
Age
10-24 years

Figure S6

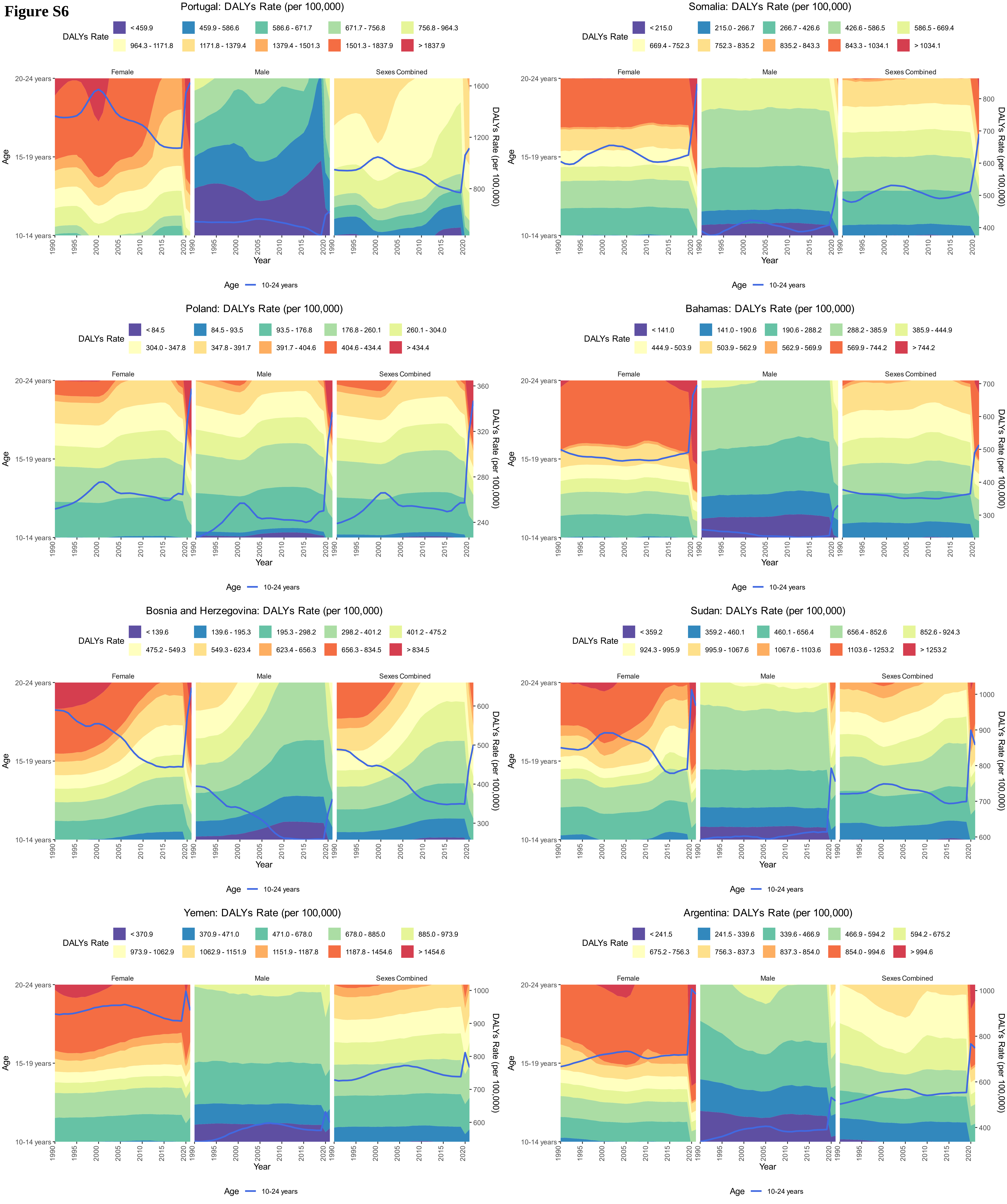

Portugal: DALYs Rate (per 100,000)
DALYs Rate
< 459.9
459.9 - 586.6
586.6 - 671.7
671.7 - 756.8
756.8 - 964.3
964.3 - 1171.8
1171.8 - 1379.4
1379.4 - 1501.3
1501.3 - 1837.9
> 1837.9
Somalia: DALYs Rate (per 100,000)
DALYs Rate
< 215.0
215.0 - 266.7
266.7 - 426.6
426.6 - 586.5
586.5 - 669.4
669.4 - 752.3
752.3 - 835.2
835.2 - 843.3
843.3 - 1034.1
> 1034.1
Poland: DALYs Rate (per 100,000)
DALYs Rate
< 84.5
84.5 - 93.5
93.5 - 176.8
176.8 - 260.1
260.1 - 304.0
304.0 - 347.8
347.8 - 391.7
391.7 - 404.6
404.6 - 434.4
> 434.4
Bahamas: DALYs Rate (per 100,000)
DALYs Rate
< 141.0
141.0 - 190.6
190.6 - 288.2
288.2 - 385.9
385.9 - 444.9
444.9 - 503.9
503.9 - 562.9
562.9 - 569.9
569.9 - 744.2
> 744.2
Bosnia and Herzegovina: DALYs Rate (per 100,000)
DALYs Rate
< 139.6
139.6 - 195.3
195.3 - 298.2
298.2 - 401.2
401.2 - 475.2
475.2 - 549.3
549.3 - 623.4
623.4 - 656.3
656.3 - 834.5
> 834.5
Sudan: DALYs Rate (per 100,000)
DALYs Rate
< 359.2
359.2 - 460.1
460.1 - 656.4
656.4 - 852.6
852.6 - 924.3
924.3 - 995.9
995.9 - 1067.6
1067.6 - 1103.6
1103.6 - 1253.2
> 1253.2
Yemen: DALYs Rate (per 100,000)
DALYs Rate
< 370.9
370.9 - 471.0
471.0 - 678.0
678.0 - 885.0
885.0 - 973.9
973.9 - 1062.9
1062.9 - 1151.9
1151.9 - 1187.8
1187.8 - 1454.6
> 1454.6
Argentina: DALYs Rate (per 100,000)
DALYs Rate
< 241.5
241.5 - 339.6
339.6 - 466.9
466.9 - 594.2
594.2 - 675.2
675.2 - 756.3
756.3 - 837.3
837.3 - 854.0
854.0 - 994.6
> 994.6
Female
Male
Sexes Combined
Age
20-24 years
15-19 years
10-14 years
Year
DALYs Rate (per 100,000)
Age
10-24 years

Figure S6

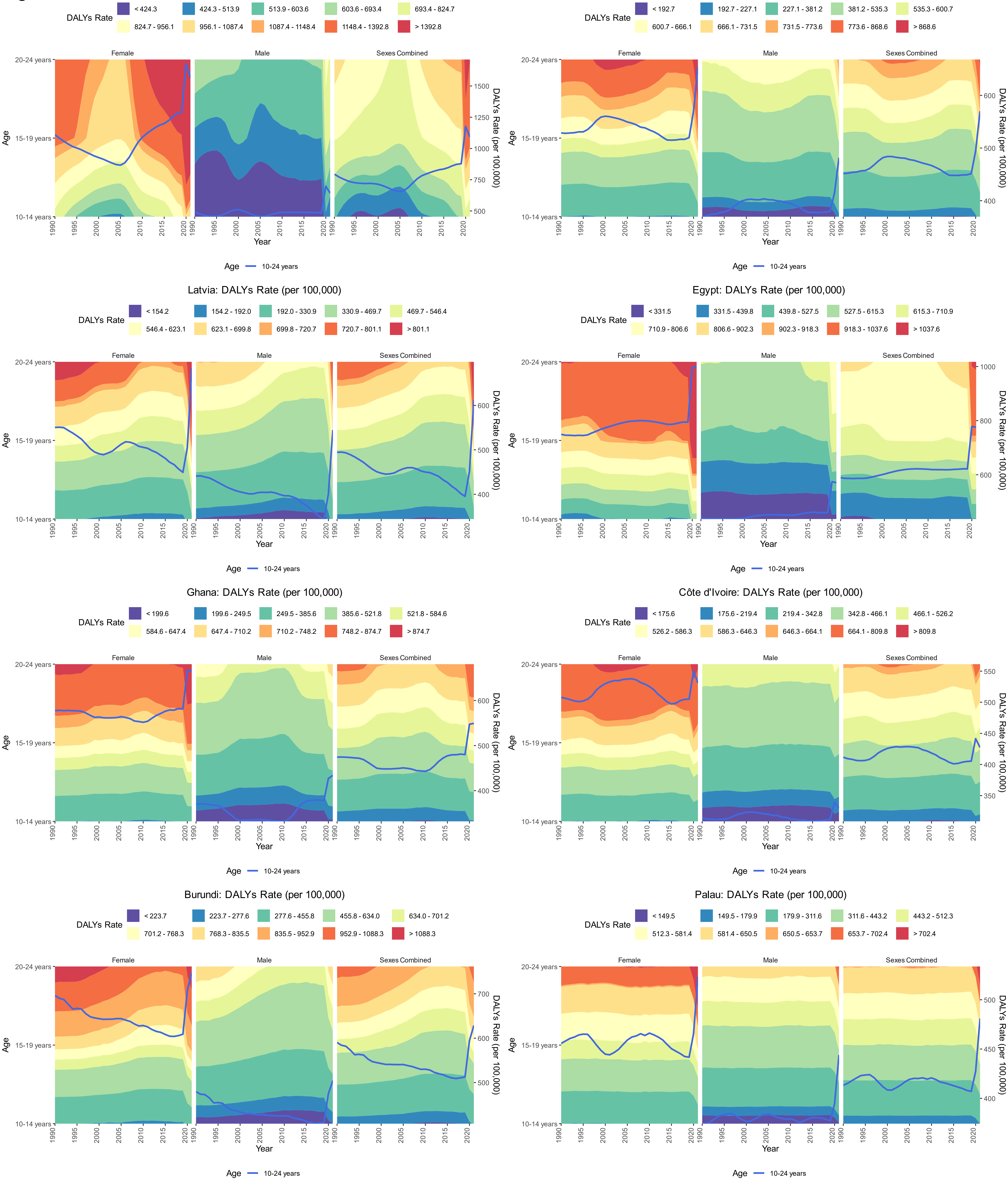

Spain: DALYs Rate (per 100,000)
DALYs Rate
< 424.3
424.3 - 513.9
513.9 - 603.6
603.6 - 693.4
693.4 - 824.7
824.7 - 956.1
956.1 - 1087.4
1087.4 - 1148.4
1148.4 - 1392.8
> 1392.8
Female
Male
Sexes Combined
20-24 years
15-19 years
10-14 years
Age
Year
DALYs Rate (per 100,000)
Age
10-24 years
Zambia: DALYs Rate (per 100,000)
< 192.7
192.7 - 227.1
227.1 - 381.2
381.2 - 535.3
535.3 - 600.7
600.7 - 666.1
666.1 - 731.5
731.5 - 773.6
773.6 - 868.6
> 868.6
Latvia: DALYs Rate (per 100,000)
< 154.2
154.2 - 192.0
192.0 - 330.9
330.9 - 469.7
469.7 - 546.4
546.4 - 623.1
623.1 - 699.8
699.8 - 720.7
720.7 - 801.1
> 801.1
Egypt: DALYs Rate (per 100,000)
< 331.5
331.5 - 439.8
439.8 - 527.5
527.5 - 615.3
615.3 - 710.9
710.9 - 806.6
806.6 - 902.3
902.3 - 918.3
918.3 - 1037.6
> 1037.6
Ghana: DALYs Rate (per 100,000)
< 199.6
199.6 - 249.5
249.5 - 385.6
385.6 - 521.8
521.8 - 584.6
584.6 - 647.4
647.4 - 710.2
710.2 - 748.2
748.2 - 874.7
> 874.7
Côte d'Ivoire: DALYs Rate (per 100,000)
< 175.6
175.6 - 219.4
219.4 - 342.8
342.8 - 466.1
466.1 - 526.2
526.2 - 586.3
586.3 - 646.3
646.3 - 664.1
664.1 - 809.8
> 809.8
Burundi: DALYs Rate (per 100,000)
< 223.7
223.7 - 277.6
277.6 - 455.8
455.8 - 634.0
634.0 - 701.2
701.2 - 768.3
768.3 - 835.5
835.5 - 952.9
952.9 - 1088.3
> 1088.3
Palau: DALYs Rate (per 100,000)
< 149.5
149.5 - 179.9
179.9 - 311.6
311.6 - 443.2
443.2 - 512.3
512.3 - 581.4
581.4 - 650.5
650.5 - 653.7
653.7 - 702.4
> 702.4

Figure S6

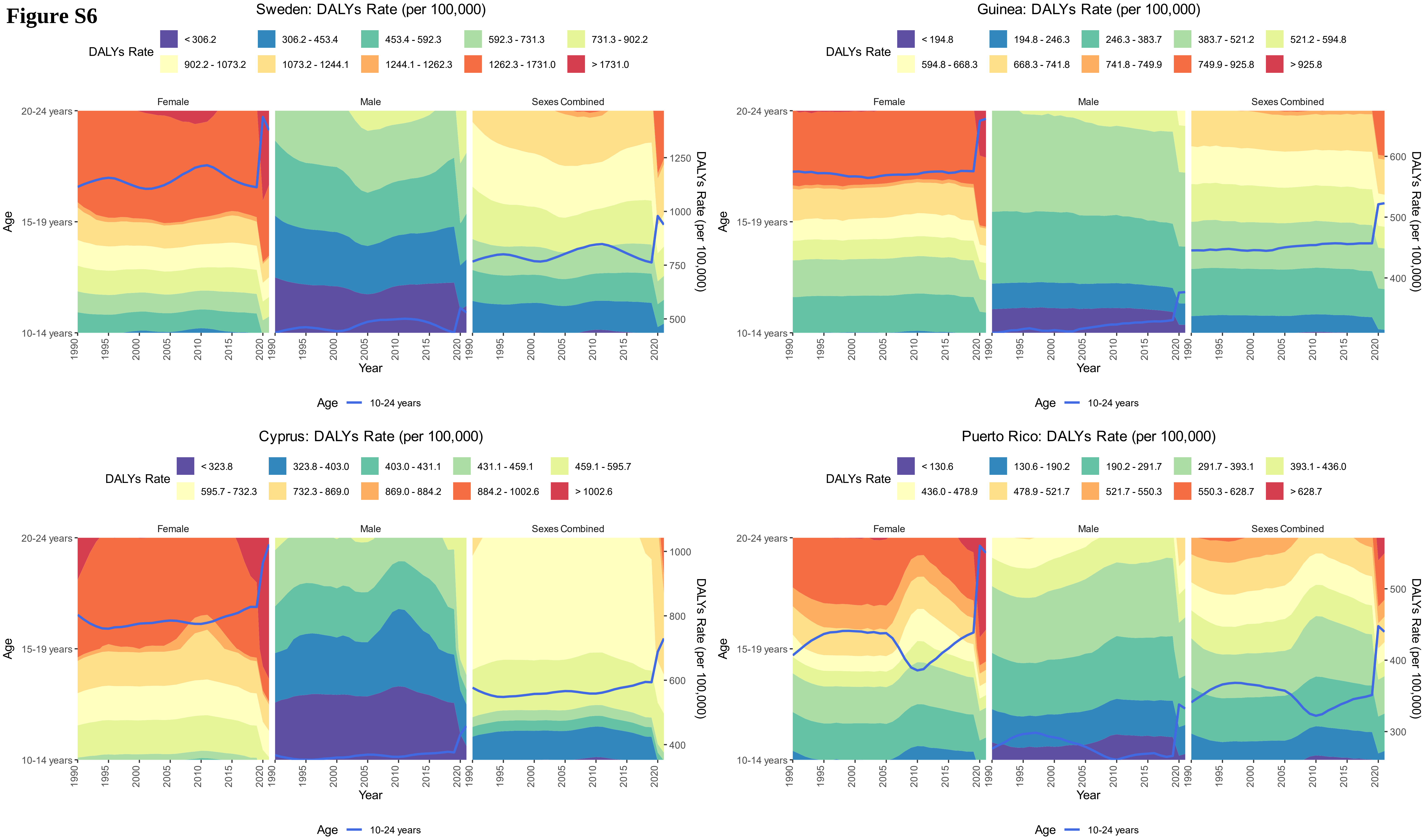
Sweden: DALYs Rate (per 100,000)
DALYs Rate
< 306.2
306.2 - 453.4
453.4 - 592.3
592.3 - 731.3
731.3 - 902.2
902.2 - 1073.2
1073.2 - 1244.1
1244.1 - 1262.3
1262.3 - 1731.0
> 1731.0
Female
Male
Sexes Combined
20-24 years
15-19 years
10-14 years
Age
1990
1995
2000
2005
2010
2015
2020
Year
1250
1000
750
500
DALYs Rate (per 100,000)
Age
10-24 years
Guinea: DALYs Rate (per 100,000)
DALYs Rate
< 194.8
194.8 - 246.3
246.3 - 383.7
383.7 - 521.2
521.2 - 594.8
594.8 - 668.3
668.3 - 741.8
741.8 - 749.9
749.9 - 925.8
> 925.8
600
500
400
Cyprus: DALYs Rate (per 100,000)
DALYs Rate
< 323.8
323.8 - 403.0
403.0 - 431.1
431.1 - 459.1
459.1 - 595.7
595.7 - 732.3
732.3 - 869.0
869.0 - 884.2
884.2 - 1002.6
> 1002.6
1000
800
600
400
Puerto Rico: DALYs Rate (per 100,000)
DALYs Rate
< 130.6
130.6 - 190.2
190.2 - 291.7
291.7 - 393.1
393.1 - 436.0
436.0 - 478.9
478.9 - 521.7
521.7 - 550.3
550.3 - 628.7
> 628.7
500
400
300

Figure S7

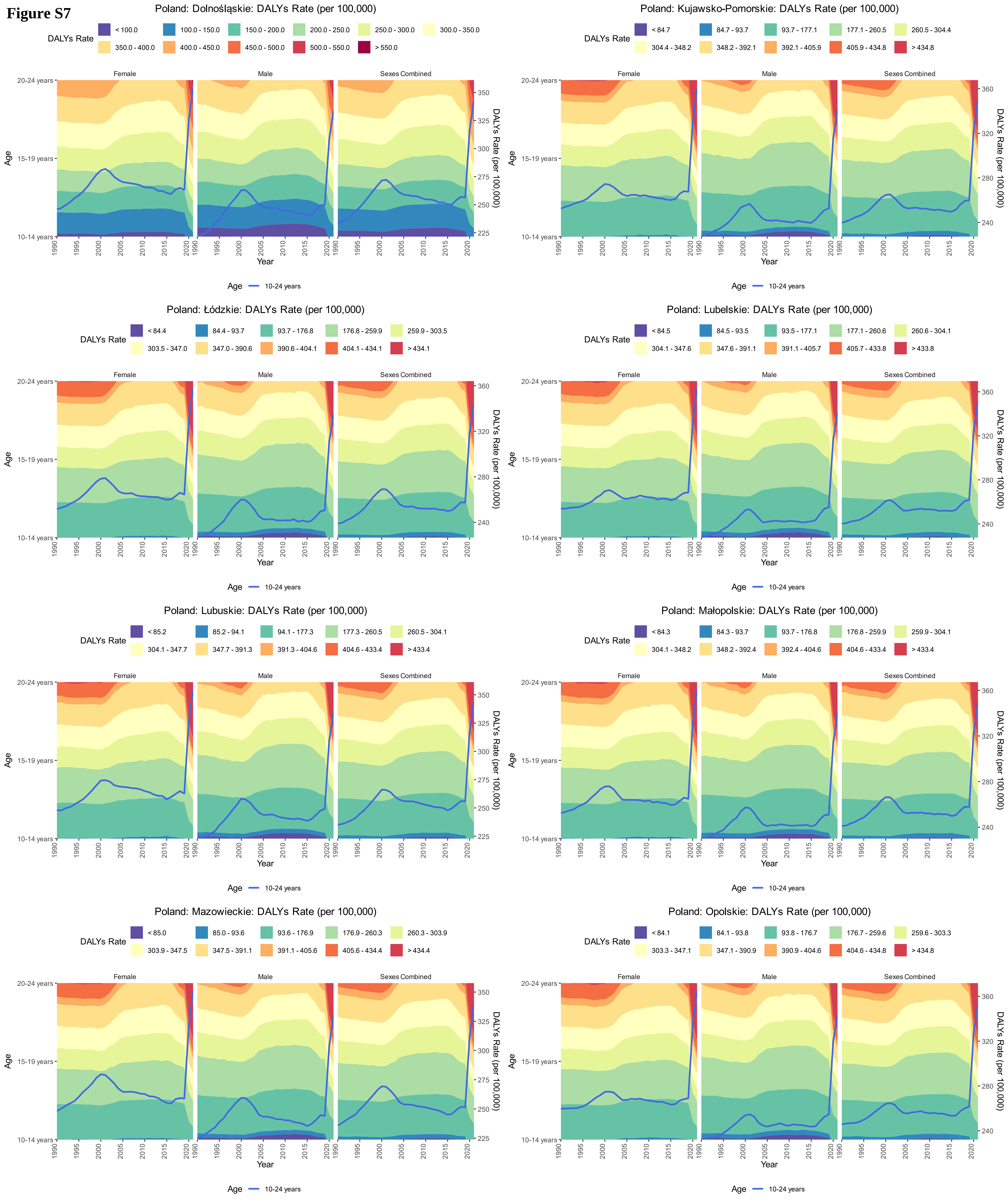
Poland: Dolnośląskie: DALYs Rate (per 100,000)
DALYs Rate
< 100.0
100.0 - 150.0
150.0 - 200.0
200.0 - 250.0
250.0 - 300.0
300.0 - 350.0
350.0 - 400.0
400.0 - 450.0
450.0 - 500.0
500.0 - 550.0
> 550.0
Poland: Kujawsko-Pomorskie: DALYs Rate (per 100,000)
< 84.7
84.7 - 93.7
93.7 - 177.1
177.1 - 260.5
260.5 - 304.4
304.4 - 348.2
348.2 - 392.1
392.1 - 405.9
405.9 - 434.8
> 434.8
Poland: Łódzkie: DALYs Rate (per 100,000)
< 84.4
84.4 - 93.7
93.7 - 176.8
176.8 - 259.9
259.9 - 303.5
303.5 - 347.0
347.0 - 390.6
390.6 - 404.1
404.1 - 434.1
> 434.1
Poland: Lubelskie: DALYs Rate (per 100,000)
< 84.5
84.5 - 93.5
93.5 - 177.1
177.1 - 260.6
260.6 - 304.1
304.1 - 347.6
347.6 - 391.1
391.1 - 405.7
405.7 - 433.8
> 433.8
Poland: Lubuskie: DALYs Rate (per 100,000)
< 85.2
85.2 - 94.1
94.1 - 177.3
177.3 - 260.5
260.5 - 304.1
304.1 - 347.7
347.7 - 391.3
391.3 - 404.6
404.6 - 433.4
> 433.4
Poland: Małopolskie: DALYs Rate (per 100,000)
< 84.3
84.3 - 93.7
93.7 - 176.8
176.8 - 259.9
259.9 - 304.1
304.1 - 348.2
348.2 - 392.4
392.4 - 404.6
404.6 - 433.4
> 433.4
Poland: Mazowieckie: DALYs Rate (per 100,000)
< 85.0
85.0 - 93.6
93.6 - 176.9
176.9 - 260.3
260.3 - 303.9
303.9 - 347.5
347.5 - 391.1
391.1 - 405.6
405.6 - 434.4
> 434.4
Poland: Opolskie: DALYs Rate (per 100,000)
< 84.1
84.1 - 93.8
93.8 - 176.7
176.7 - 259.6
259.6 - 303.3
303.3 - 347.1
347.1 - 390.9
390.9 - 404.6
404.6 - 434.8
> 434.8
Female
Male
Sexes Combined
Age
20-24 years
15-19 years
10-14 years
Year
DALYs Rate (per 100,000)
Age — 10-24 years

Figure S7

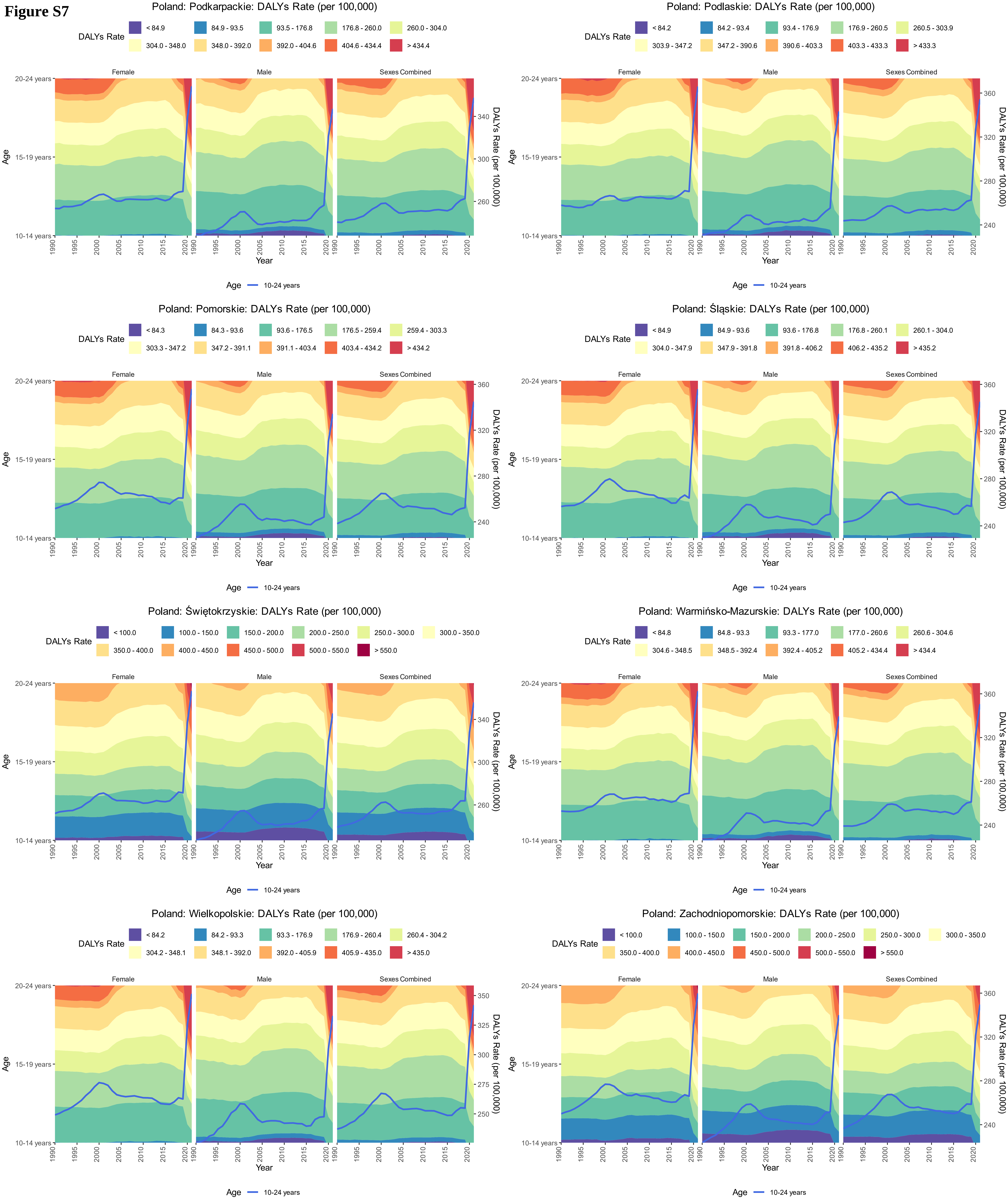
Poland: Podkarpackie: DALYs Rate (per 100,000)
DALYs Rate
< 84.9
84.9 - 93.5
93.5 - 176.8
176.8 - 260.0
260.0 - 304.0
304.0 - 348.0
348.0 - 392.0
392.0 - 404.6
404.6 - 434.4
> 434.4
Poland: Podlaskie: DALYs Rate (per 100,000)
< 84.2
84.2 - 93.4
93.4 - 176.9
176.9 - 260.5
260.5 - 303.9
303.9 - 347.2
347.2 - 390.6
390.6 - 403.3
403.3 - 433.3
> 433.3
Poland: Pomorskie: DALYs Rate (per 100,000)
< 84.3
84.3 - 93.6
93.6 - 176.5
176.5 - 259.4
259.4 - 303.3
303.3 - 347.2
347.2 - 391.1
391.1 - 403.4
403.4 - 434.2
> 434.2
Poland: Śląskie: DALYs Rate (per 100,000)
< 84.9
84.9 - 93.6
93.6 - 176.8
176.8 - 260.1
260.1 - 304.0
304.0 - 347.9
347.9 - 391.8
391.8 - 406.2
406.2 - 435.2
> 435.2
Poland: Świętokrzyskie: DALYs Rate (per 100,000)
< 100.0
100.0 - 150.0
150.0 - 200.0
200.0 - 250.0
250.0 - 300.0
300.0 - 350.0
350.0 - 400.0
400.0 - 450.0
450.0 - 500.0
500.0 - 550.0
> 550.0
Poland: Warmińsko-Mazurskie: DALYs Rate (per 100,000)
< 84.8
84.8 - 93.3
93.3 - 177.0
177.0 - 260.6
260.6 - 304.6
304.6 - 348.5
348.5 - 392.4
392.4 - 405.2
405.2 - 434.4
> 434.4
Poland: Wielkopolskie: DALYs Rate (per 100,000)
< 84.2
84.2 - 93.3
93.3 - 176.9
176.9 - 260.4
260.4 - 304.2
304.2 - 348.1
348.1 - 392.0
392.0 - 405.9
405.9 - 435.0
> 435.0
Poland: Zachodniopomorskie: DALYs Rate (per 100,000)
< 100.0
100.0 - 150.0
150.0 - 200.0
200.0 - 250.0
250.0 - 300.0
300.0 - 350.0
350.0 - 400.0
400.0 - 450.0
450.0 - 500.0
500.0 - 550.0
> 550.0
Female
Male
Sexes Combined
Age
20-24 years
15-19 years
10-14 years
Year
DALYs Rate (per 100,000)
Age — 10-24 years

Figure S7

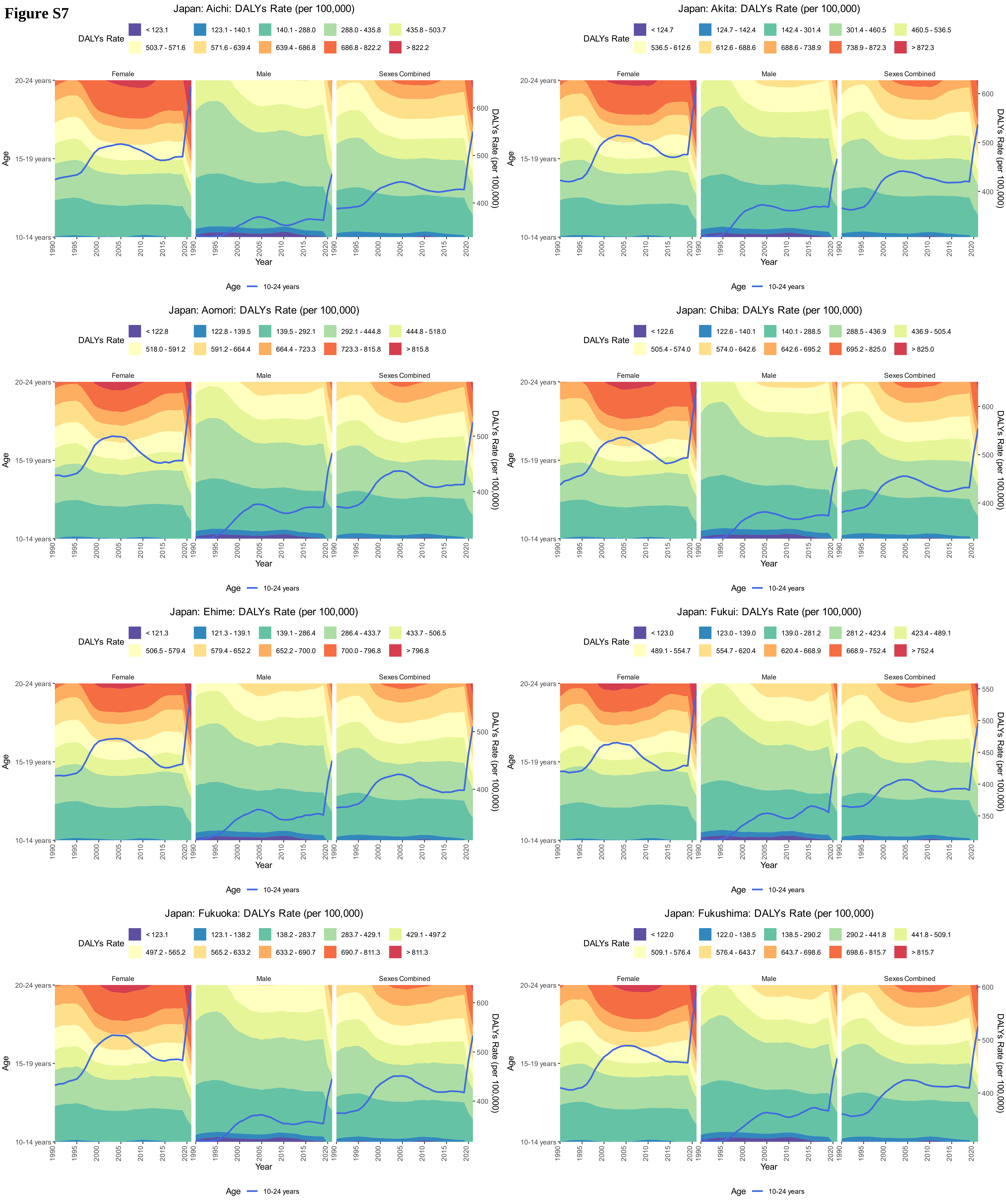

Japan: Aichi: DALYs Rate (per 100,000)
DALYs Rate
< 123.1
123.1 - 140.1
140.1 - 288.0
288.0 - 435.8
435.8 - 503.7
503.7 - 571.6
571.6 - 639.4
639.4 - 686.8
686.8 - 822.2
> 822.2
Female
Male
Sexes Combined
Age
Year
DALYs Rate (per 100,000)
Age 10-24 years
Japan: Akita: DALYs Rate (per 100,000)
< 124.7
124.7 - 142.4
142.4 - 301.4
301.4 - 460.5
460.5 - 536.5
536.5 - 612.6
612.6 - 688.6
688.6 - 738.9
738.9 - 872.3
> 872.3
Japan: Aomori: DALYs Rate (per 100,000)
< 122.8
122.8 - 139.5
139.5 - 292.1
292.1 - 444.8
444.8 - 518.0
518.0 - 591.2
591.2 - 664.4
664.4 - 723.3
723.3 - 815.8
> 815.8
Japan: Chiba: DALYs Rate (per 100,000)
< 122.6
122.6 - 140.1
140.1 - 288.5
288.5 - 436.9
436.9 - 505.4
505.4 - 574.0
574.0 - 642.6
642.6 - 695.2
695.2 - 825.0
> 825.0
Japan: Ehime: DALYs Rate (per 100,000)
< 121.3
121.3 - 139.1
139.1 - 286.4
286.4 - 433.7
433.7 - 506.5
506.5 - 579.4
579.4 - 652.2
652.2 - 700.0
700.0 - 796.8
> 796.8
Japan: Fukui: DALYs Rate (per 100,000)
< 123.0
123.0 - 139.0
139.0 - 281.2
281.2 - 423.4
423.4 - 489.1
489.1 - 554.7
554.7 - 620.4
620.4 - 668.9
668.9 - 752.4
> 752.4
Japan: Fukuoka: DALYs Rate (per 100,000)
< 123.1
123.1 - 138.2
138.2 - 283.7
283.7 - 429.1
429.1 - 497.2
497.2 - 565.2
565.2 - 633.2
633.2 - 690.7
690.7 - 811.3
> 811.3
Japan: Fukushima: DALYs Rate (per 100,000)
< 122.0
122.0 - 138.5
138.5 - 290.2
290.2 - 441.8
441.8 - 509.1
509.1 - 576.4
576.4 - 643.7
643.7 - 698.6
698.6 - 815.7
> 815.7
20-24 years
15-19 years
10-14 years
1990
1995
2000
2005
2010
2015
2020

Figure S7

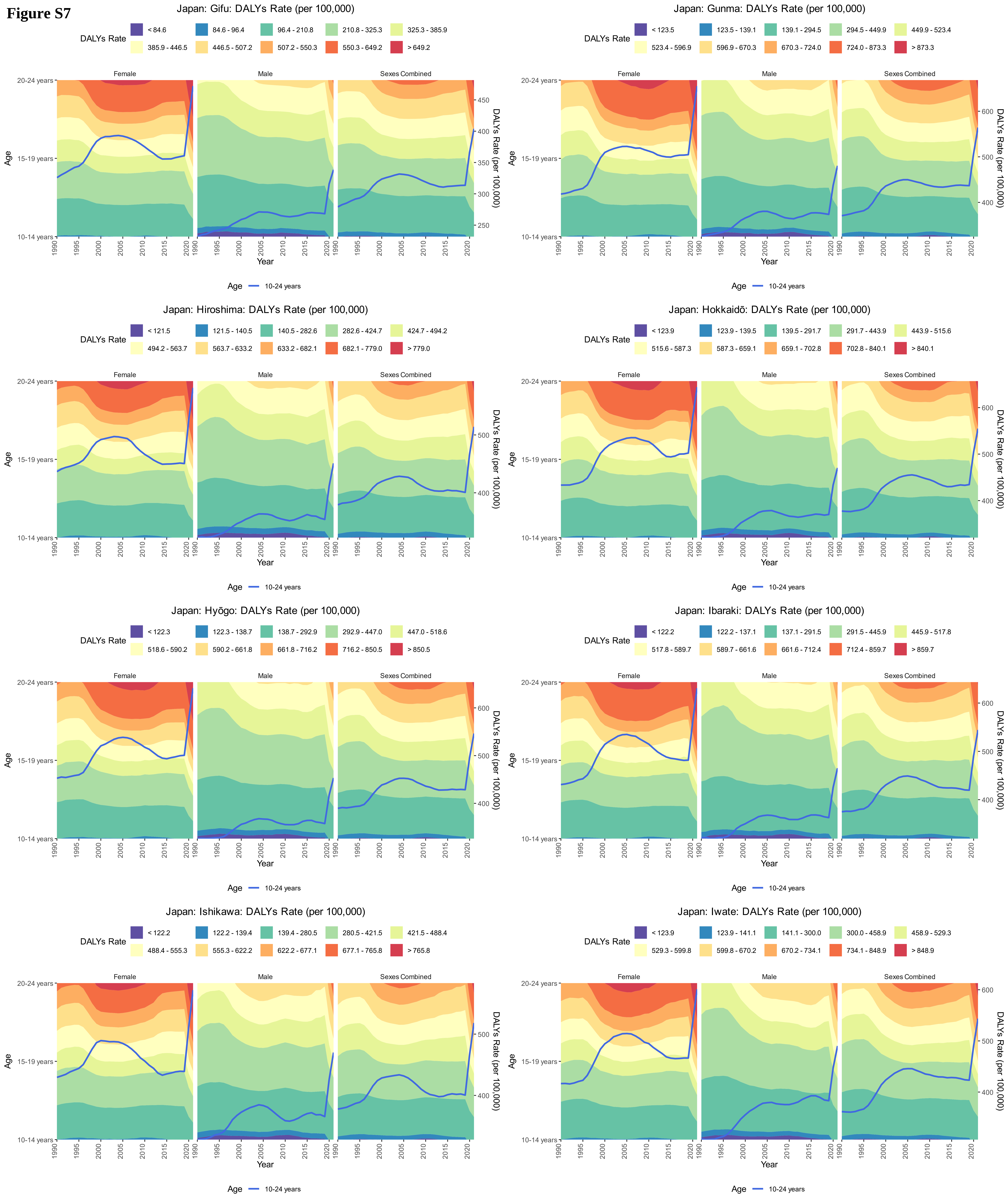

Japan: Gifu: DALYs Rate (per 100,000)
DALYs Rate
< 84.6
84.6 - 96.4
96.4 - 210.8
210.8 - 325.3
325.3 - 385.9
385.9 - 446.5
446.5 - 507.2
507.2 - 550.3
550.3 - 649.2
> 649.2
Japan: Gunma: DALYs Rate (per 100,000)
< 123.5
123.5 - 139.1
139.1 - 294.5
294.5 - 449.9
449.9 - 523.4
523.4 - 596.9
596.9 - 670.3
670.3 - 724.0
724.0 - 873.3
> 873.3
Japan: Hiroshima: DALYs Rate (per 100,000)
< 121.5
121.5 - 140.5
140.5 - 282.6
282.6 - 424.7
424.7 - 494.2
494.2 - 563.7
563.7 - 633.2
633.2 - 682.1
682.1 - 779.0
> 779.0
Japan: Hokkaidō: DALYs Rate (per 100,000)
< 123.9
123.9 - 139.5
139.5 - 291.7
291.7 - 443.9
443.9 - 515.6
515.6 - 587.3
587.3 - 659.1
659.1 - 702.8
702.8 - 840.1
> 840.1
Japan: Hyōgo: DALYs Rate (per 100,000)
< 122.3
122.3 - 138.7
138.7 - 292.9
292.9 - 447.0
447.0 - 518.6
518.6 - 590.2
590.2 - 661.8
661.8 - 716.2
716.2 - 850.5
> 850.5
Japan: Ibaraki: DALYs Rate (per 100,000)
< 122.2
122.2 - 137.1
137.1 - 291.5
291.5 - 445.9
445.9 - 517.8
517.8 - 589.7
589.7 - 661.6
661.6 - 712.4
712.4 - 859.7
> 859.7
Japan: Ishikawa: DALYs Rate (per 100,000)
< 122.2
122.2 - 139.4
139.4 - 280.5
280.5 - 421.5
421.5 - 488.4
488.4 - 555.3
555.3 - 622.2
622.2 - 677.1
677.1 - 765.8
> 765.8
Japan: Iwate: DALYs Rate (per 100,000)
< 123.9
123.9 - 141.1
141.1 - 300.0
300.0 - 458.9
458.9 - 529.3
529.3 - 599.8
599.8 - 670.2
670.2 - 734.1
734.1 - 848.9
> 848.9
Female
Male
Sexes Combined
Age
20-24 years
15-19 years
10-14 years
Year
DALYs Rate (per 100,000)
Age — 10-24 years

Figure S7

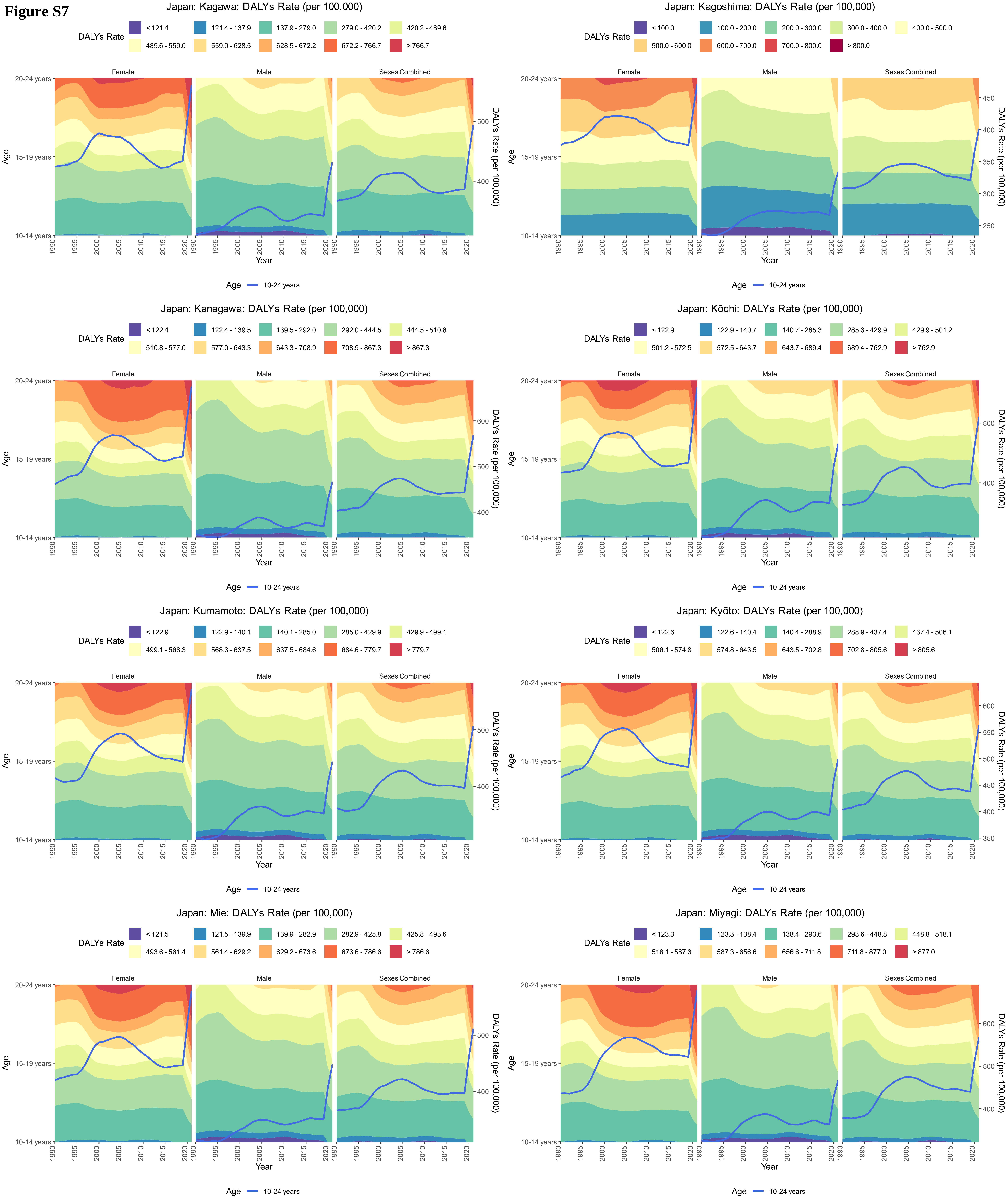

Japan: Kagawa: DALYs Rate (per 100,000)
DALYs Rate
< 121.4
121.4 - 137.9
137.9 - 279.0
279.0 - 420.2
420.2 - 489.6
489.6 - 559.0
559.0 - 628.5
628.5 - 672.2
672.2 - 766.7
> 766.7
Japan: Kagoshima: DALYs Rate (per 100,000)
DALYs Rate
< 100.0
100.0 - 200.0
200.0 - 300.0
300.0 - 400.0
400.0 - 500.0
500.0 - 600.0
600.0 - 700.0
700.0 - 800.0
> 800.0
Japan: Kanagawa: DALYs Rate (per 100,000)
DALYs Rate
< 122.4
122.4 - 139.5
139.5 - 292.0
292.0 - 444.5
444.5 - 510.8
510.8 - 577.0
577.0 - 643.3
643.3 - 708.9
708.9 - 867.3
> 867.3
Japan: Kōchi: DALYs Rate (per 100,000)
DALYs Rate
< 122.9
122.9 - 140.7
140.7 - 285.3
285.3 - 429.9
429.9 - 501.2
501.2 - 572.5
572.5 - 643.7
643.7 - 689.4
689.4 - 762.9
> 762.9
Japan: Kumamoto: DALYs Rate (per 100,000)
DALYs Rate
< 122.9
122.9 - 140.1
140.1 - 285.0
285.0 - 429.9
429.9 - 499.1
499.1 - 568.3
568.3 - 637.5
637.5 - 684.6
684.6 - 779.7
> 779.7
Japan: Kyōto: DALYs Rate (per 100,000)
DALYs Rate
< 122.6
122.6 - 140.4
140.4 - 288.9
288.9 - 437.4
437.4 - 506.1
506.1 - 574.8
574.8 - 643.5
643.5 - 702.8
702.8 - 805.6
> 805.6
Japan: Mie: DALYs Rate (per 100,000)
DALYs Rate
< 121.5
121.5 - 139.9
139.9 - 282.9
282.9 - 425.8
425.8 - 493.6
493.6 - 561.4
561.4 - 629.2
629.2 - 673.6
673.6 - 786.6
> 786.6
Japan: Miyagi: DALYs Rate (per 100,000)
DALYs Rate
< 123.3
123.3 - 138.4
138.4 - 293.6
293.6 - 448.8
448.8 - 518.1
518.1 - 587.3
587.3 - 656.6
656.6 - 711.8
711.8 - 877.0
> 877.0
Female
Male
Sexes Combined
20-24 years
15-19 years
10-14 years
Age
Year
DALYs Rate (per 100,000)
Age
10-24 years

Figure S7

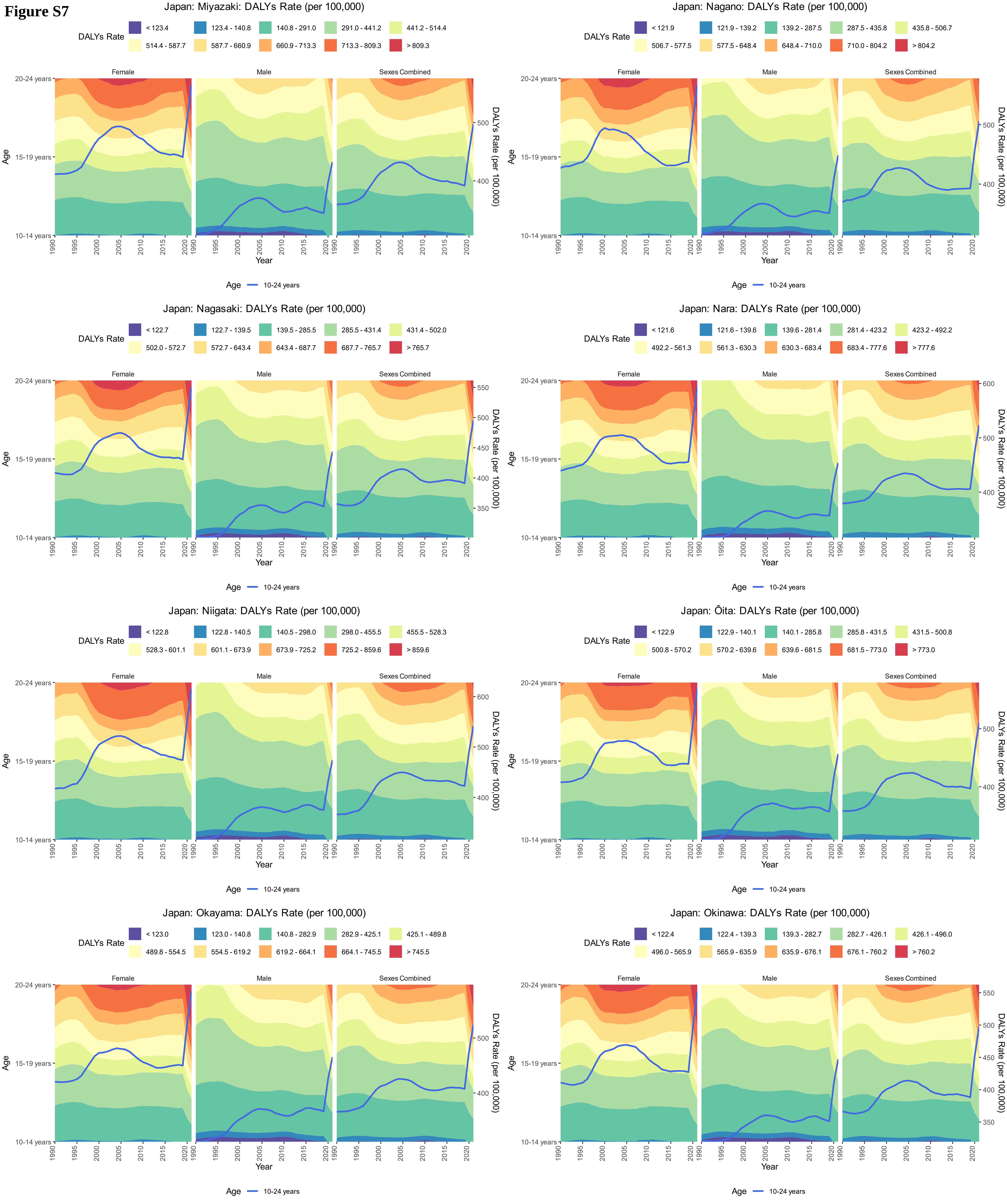
Japan: Miyazaki: DALYs Rate (per 100,000)
DALYs Rate
< 123.4
123.4 - 140.8
140.8 - 291.0
291.0 - 441.2
441.2 - 514.4
514.4 - 587.7
587.7 - 660.9
660.9 - 713.3
713.3 - 809.3
> 809.3
Female
Male
Sexes Combined
20-24 years
15-19 years
10-14 years
Age
Year
DALYs Rate (per 100,000)
Age
10-24 years
Japan: Nagano: DALYs Rate (per 100,000)
DALYs Rate
< 121.9
121.9 - 139.2
139.2 - 287.5
287.5 - 435.8
435.8 - 506.7
506.7 - 577.5
577.5 - 648.4
648.4 - 710.0
710.0 - 804.2
> 804.2
Japan: Nagasaki: DALYs Rate (per 100,000)
DALYs Rate
< 122.7
122.7 - 139.5
139.5 - 285.5
285.5 - 431.4
431.4 - 502.0
502.0 - 572.7
572.7 - 643.4
643.4 - 687.7
687.7 - 765.7
> 765.7
Japan: Nara: DALYs Rate (per 100,000)
DALYs Rate
< 121.6
121.6 - 139.6
139.6 - 281.4
281.4 - 423.2
423.2 - 492.2
492.2 - 561.3
561.3 - 630.3
630.3 - 683.4
683.4 - 777.6
> 777.6
Japan: Niigata: DALYs Rate (per 100,000)
DALYs Rate
< 122.8
122.8 - 140.5
140.5 - 298.0
298.0 - 455.5
455.5 - 528.3
528.3 - 601.1
601.1 - 673.9
673.9 - 725.2
725.2 - 859.6
> 859.6
Japan: Ōita: DALYs Rate (per 100,000)
DALYs Rate
< 122.9
122.9 - 140.1
140.1 - 285.8
285.8 - 431.5
431.5 - 500.8
500.8 - 570.2
570.2 - 639.6
639.6 - 681.5
681.5 - 773.0
> 773.0
Japan: Okayama: DALYs Rate (per 100,000)
DALYs Rate
< 123.0
123.0 - 140.8
140.8 - 282.9
282.9 - 425.1
425.1 - 489.8
489.8 - 554.5
554.5 - 619.2
619.2 - 664.1
664.1 - 745.5
> 745.5
Japan: Okinawa: DALYs Rate (per 100,000)
DALYs Rate
< 122.4
122.4 - 139.3
139.3 - 282.7
282.7 - 426.1
426.1 - 496.0
496.0 - 565.9
565.9 - 635.9
635.9 - 676.1
676.1 - 760.2
> 760.2

Figure S7

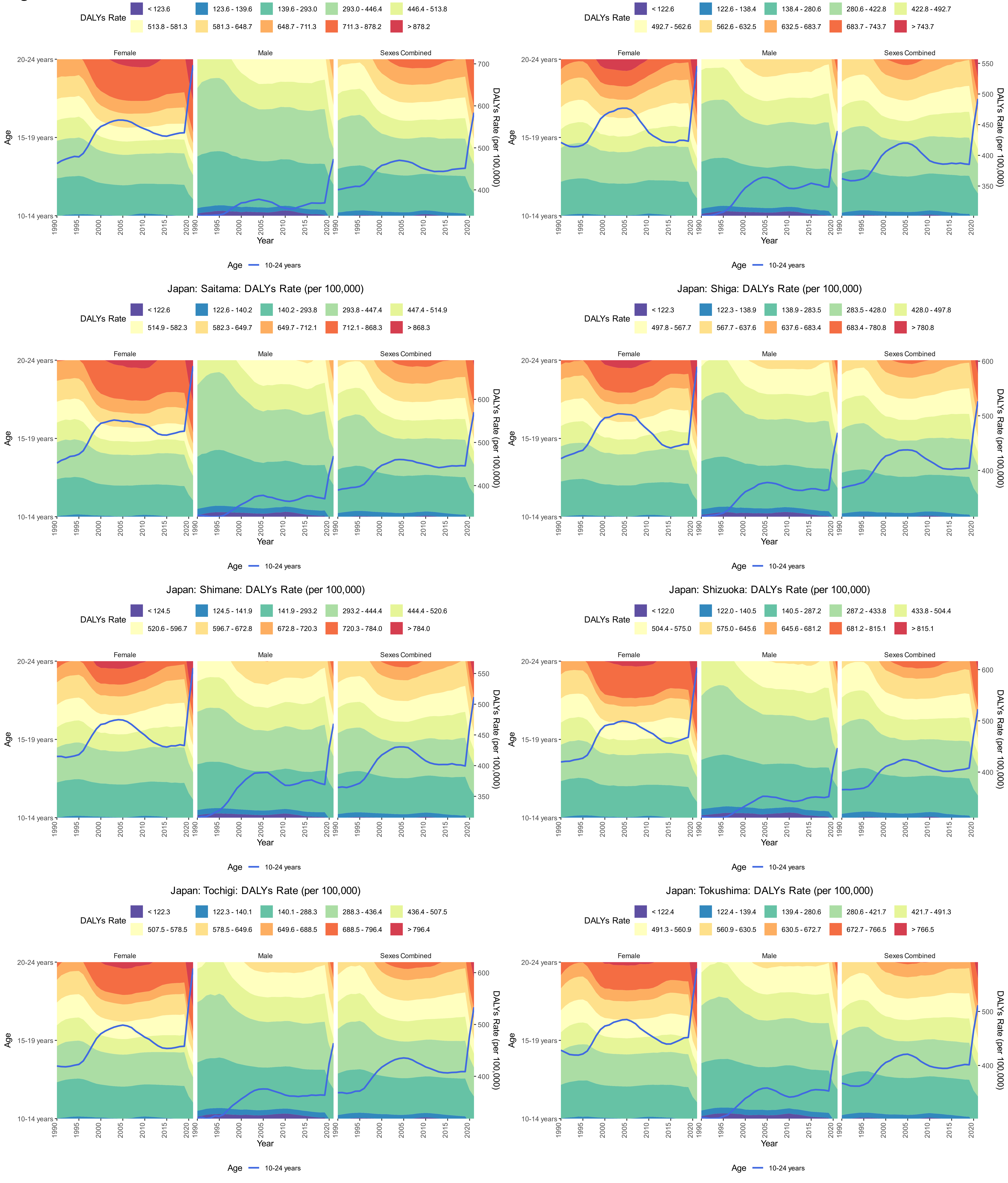
Japan: Ōsaka: DALYs Rate (per 100,000)
DALYs Rate
< 123.6
123.6 - 139.6
139.6 - 293.0
293.0 - 446.4
446.4 - 513.8
513.8 - 581.3
581.3 - 648.7
648.7 - 711.3
711.3 - 878.2
> 878.2
Female
Male
Sexes Combined
Age
Year
DALYs Rate (per 100,000)
Age — 10-24 years
Japan: Saga: DALYs Rate (per 100,000)
< 122.6
122.6 - 138.4
138.4 - 280.6
280.6 - 422.8
422.8 - 492.7
492.7 - 562.6
562.6 - 632.5
632.5 - 683.7
683.7 - 743.7
> 743.7
Japan: Saitama: DALYs Rate (per 100,000)
< 122.6
122.6 - 140.2
140.2 - 293.8
293.8 - 447.4
447.4 - 514.9
514.9 - 582.3
582.3 - 649.7
649.7 - 712.1
712.1 - 868.3
> 868.3
Japan: Shiga: DALYs Rate (per 100,000)
< 122.3
122.3 - 138.9
138.9 - 283.5
283.5 - 428.0
428.0 - 497.8
497.8 - 567.7
567.7 - 637.6
637.6 - 683.4
683.4 - 780.8
> 780.8
Japan: Shimane: DALYs Rate (per 100,000)
< 124.5
124.5 - 141.9
141.9 - 293.2
293.2 - 444.4
444.4 - 520.6
520.6 - 596.7
596.7 - 672.8
672.8 - 720.3
720.3 - 784.0
> 784.0
Japan: Shizuoka: DALYs Rate (per 100,000)
< 122.0
122.0 - 140.5
140.5 - 287.2
287.2 - 433.8
433.8 - 504.4
504.4 - 575.0
575.0 - 645.6
645.6 - 681.2
681.2 - 815.1
> 815.1
Japan: Tochigi: DALYs Rate (per 100,000)
< 122.3
122.3 - 140.1
140.1 - 288.3
288.3 - 436.4
436.4 - 507.5
507.5 - 578.5
578.5 - 649.6
649.6 - 688.5
688.5 - 796.4
> 796.4
Japan: Tokushima: DALYs Rate (per 100,000)
< 122.4
122.4 - 139.4
139.4 - 280.6
280.6 - 421.7
421.7 - 491.3
491.3 - 560.9
560.9 - 630.5
630.5 - 672.7
672.7 - 766.5
> 766.5
20-24 years
15-19 years
10-14 years
1990
1995
2000
2005
2010
2015
2020

Figure S7

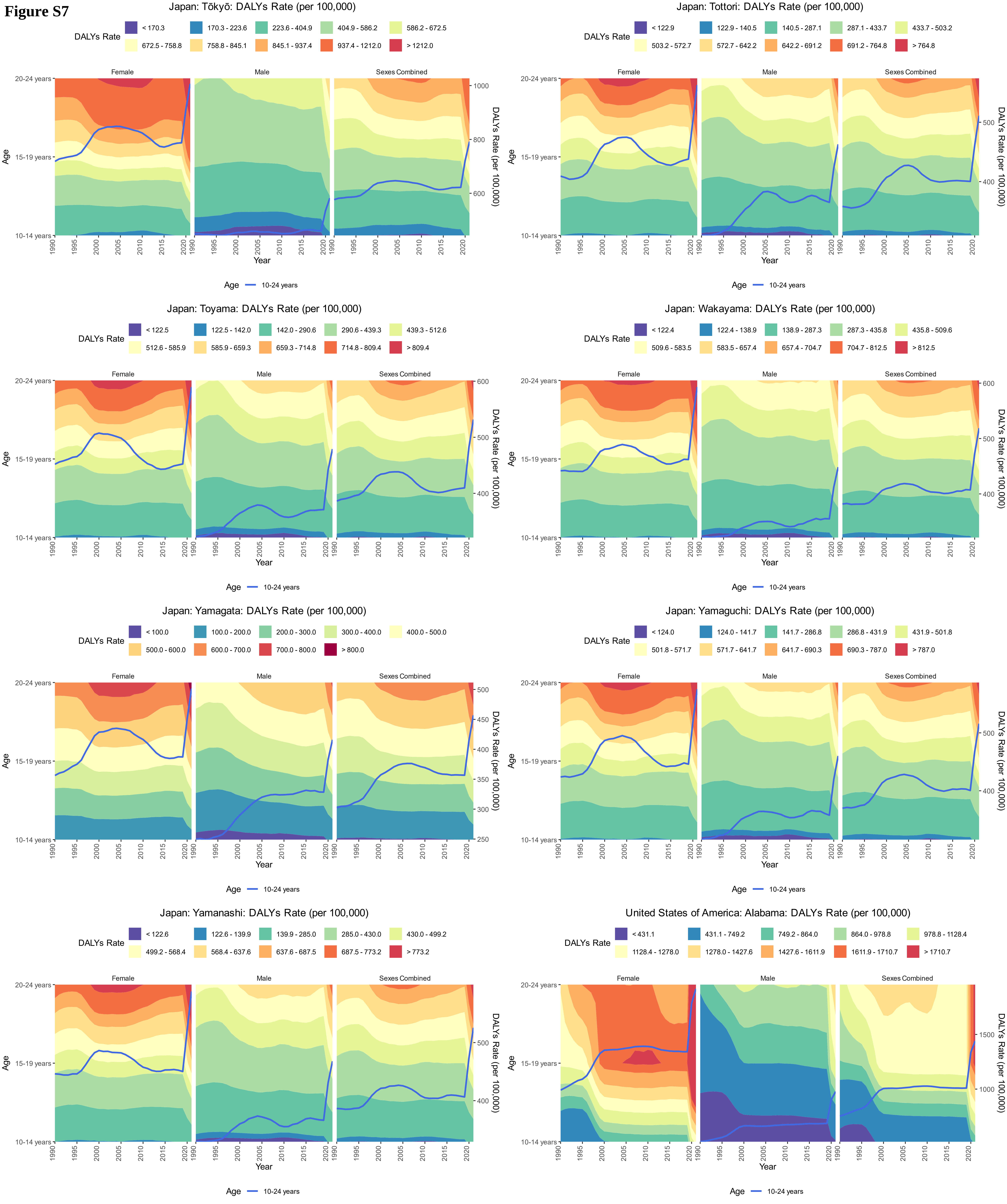

Japan: Tōkyō: DALYs Rate (per 100,000)
DALYs Rate
< 170.3
170.3 - 223.6
223.6 - 404.9
404.9 - 586.2
586.2 - 672.5
672.5 - 758.8
758.8 - 845.1
845.1 - 937.4
937.4 - 1212.0
> 1212.0
Japan: Tottori: DALYs Rate (per 100,000)
DALYs Rate
< 122.9
122.9 - 140.5
140.5 - 287.1
287.1 - 433.7
433.7 - 503.2
503.2 - 572.7
572.7 - 642.2
642.2 - 691.2
691.2 - 764.8
> 764.8
Japan: Toyama: DALYs Rate (per 100,000)
DALYs Rate
< 122.5
122.5 - 142.0
142.0 - 290.6
290.6 - 439.3
439.3 - 512.6
512.6 - 585.9
585.9 - 659.3
659.3 - 714.8
714.8 - 809.4
> 809.4
Japan: Wakayama: DALYs Rate (per 100,000)
DALYs Rate
< 122.4
122.4 - 138.9
138.9 - 287.3
287.3 - 435.8
435.8 - 509.6
509.6 - 583.5
583.5 - 657.4
657.4 - 704.7
704.7 - 812.5
> 812.5
Japan: Yamagata: DALYs Rate (per 100,000)
DALYs Rate
< 100.0
100.0 - 200.0
200.0 - 300.0
300.0 - 400.0
400.0 - 500.0
500.0 - 600.0
600.0 - 700.0
700.0 - 800.0
> 800.0
Japan: Yamaguchi: DALYs Rate (per 100,000)
DALYs Rate
< 124.0
124.0 - 141.7
141.7 - 286.8
286.8 - 431.9
431.9 - 501.8
501.8 - 571.7
571.7 - 641.7
641.7 - 690.3
690.3 - 787.0
> 787.0
Japan: Yamanashi: DALYs Rate (per 100,000)
DALYs Rate
< 122.6
122.6 - 139.9
139.9 - 285.0
285.0 - 430.0
430.0 - 499.2
499.2 - 568.4
568.4 - 637.6
637.6 - 687.5
687.5 - 773.2
> 773.2
United States of America: Alabama: DALYs Rate (per 100,000)
DALYs Rate
< 431.1
431.1 - 749.2
749.2 - 864.0
864.0 - 978.8
978.8 - 1128.4
1128.4 - 1278.0
1278.0 - 1427.6
1427.6 - 1611.9
1611.9 - 1710.7
> 1710.7
Female
Male
Sexes Combined
Age
20-24 years
15-19 years
10-14 years
Year
DALYs Rate (per 100,000)
Age — 10-24 years

Figure S7

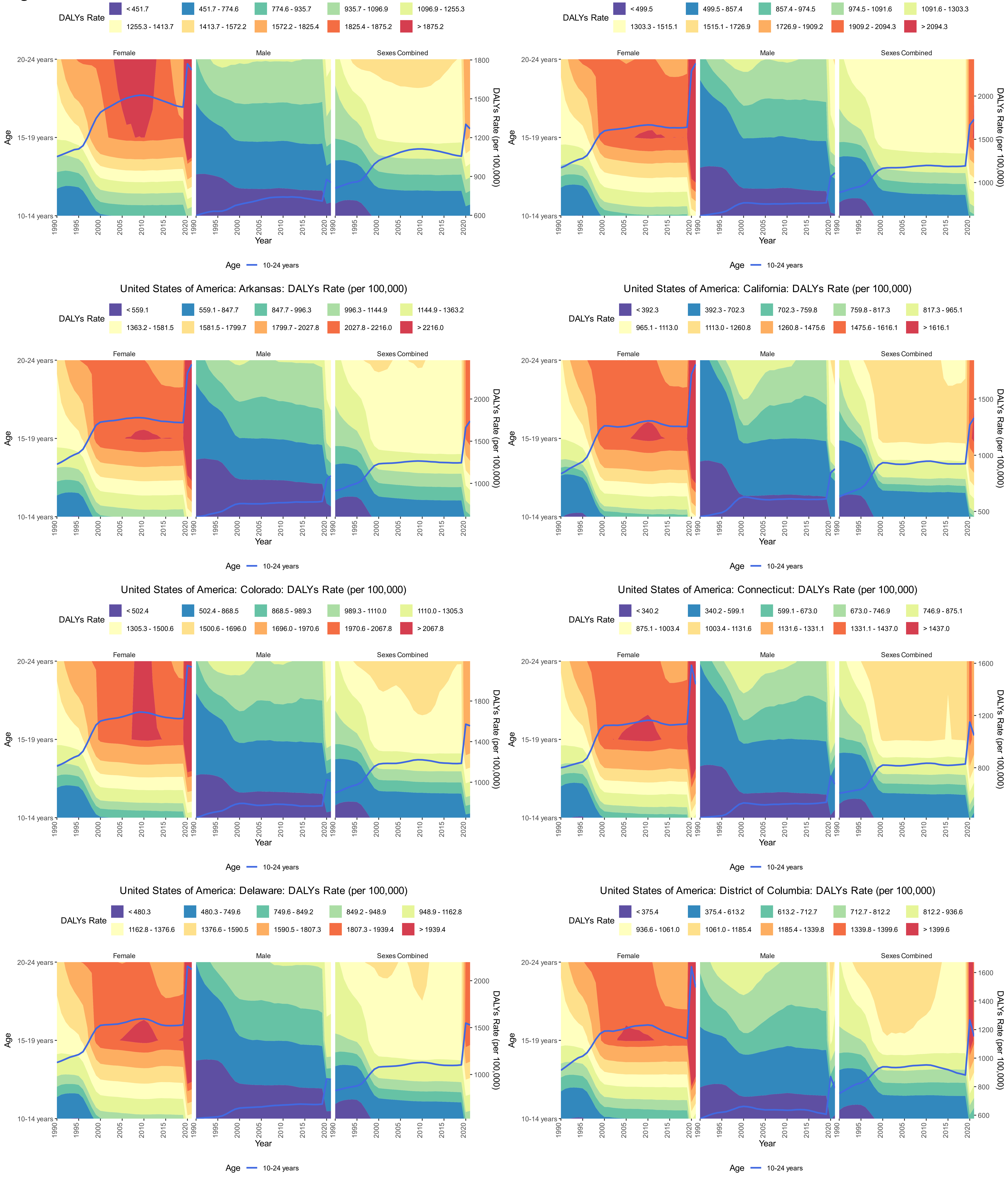
United States of America: Alaska: DALYs Rate (per 100,000)
DALYs Rate
< 451.7
451.7 - 774.6
774.6 - 935.7
935.7 - 1096.9
1096.9 - 1255.3
1255.3 - 1413.7
1413.7 - 1572.2
1572.2 - 1825.4
1825.4 - 1875.2
> 1875.2
Female
Male
Sexes Combined
Age
Year
DALYs Rate (per 100,000)
Age — 10-24 years
United States of America: Arizona: DALYs Rate (per 100,000)
< 499.5
499.5 - 857.4
857.4 - 974.5
974.5 - 1091.6
1091.6 - 1303.3
1303.3 - 1515.1
1515.1 - 1726.9
1726.9 - 1909.2
1909.2 - 2094.3
> 2094.3
United States of America: Arkansas: DALYs Rate (per 100,000)
< 559.1
559.1 - 847.7
847.7 - 996.3
996.3 - 1144.9
1144.9 - 1363.2
1363.2 - 1581.5
1581.5 - 1799.7
1799.7 - 2027.8
2027.8 - 2216.0
> 2216.0
United States of America: California: DALYs Rate (per 100,000)
< 392.3
392.3 - 702.3
702.3 - 759.8
759.8 - 817.3
817.3 - 965.1
965.1 - 1113.0
1113.0 - 1260.8
1260.8 - 1475.6
1475.6 - 1616.1
> 1616.1
United States of America: Colorado: DALYs Rate (per 100,000)
< 502.4
502.4 - 868.5
868.5 - 989.3
989.3 - 1110.0
1110.0 - 1305.3
1305.3 - 1500.6
1500.6 - 1696.0
1696.0 - 1970.6
1970.6 - 2067.8
> 2067.8
United States of America: Connecticut: DALYs Rate (per 100,000)
< 340.2
340.2 - 599.1
599.1 - 673.0
673.0 - 746.9
746.9 - 875.1
875.1 - 1003.4
1003.4 - 1131.6
1131.6 - 1331.1
1331.1 - 1437.0
> 1437.0
United States of America: Delaware: DALYs Rate (per 100,000)
< 480.3
480.3 - 749.6
749.6 - 849.2
849.2 - 948.9
948.9 - 1162.8
1162.8 - 1376.6
1376.6 - 1590.5
1590.5 - 1807.3
1807.3 - 1939.4
> 1939.4
United States of America: District of Columbia: DALYs Rate (per 100,000)
< 375.4
375.4 - 613.2
613.2 - 712.7
712.7 - 812.2
812.2 - 936.6
936.6 - 1061.0
1061.0 - 1185.4
1185.4 - 1339.8
1339.8 - 1399.6
> 1399.6
20-24 years
15-19 years
10-14 years
1990
1995
2000
2005
2010
2015
2020

Figure S7

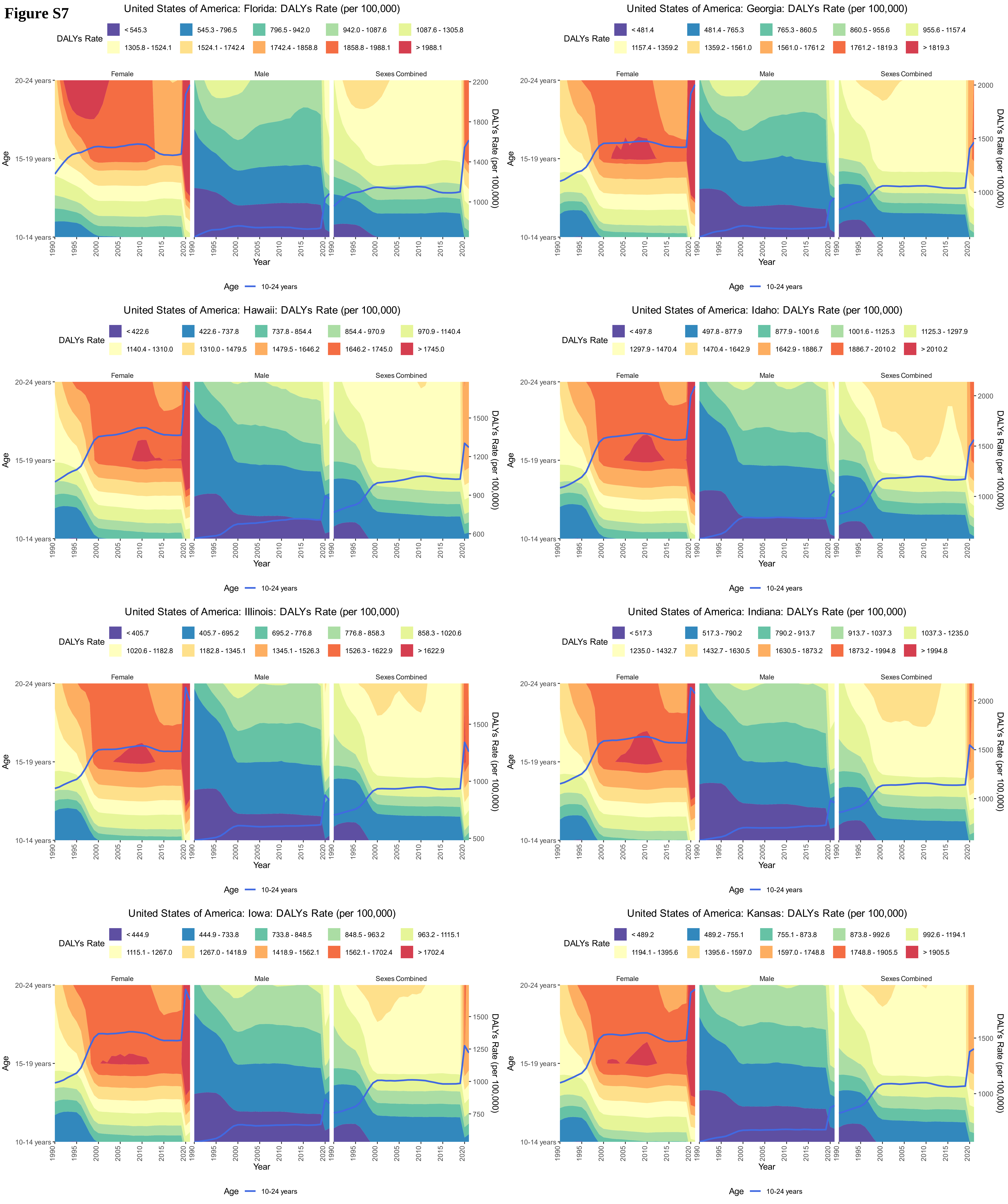
United States of America: Florida: DALYs Rate (per 100,000)
DALYs Rate
< 545.3
545.3 - 796.5
796.5 - 942.0
942.0 - 1087.6
1087.6 - 1305.8
1305.8 - 1524.1
1524.1 - 1742.4
1742.4 - 1858.8
1858.8 - 1988.1
> 1988.1
United States of America: Georgia: DALYs Rate (per 100,000)
DALYs Rate
< 481.4
481.4 - 765.3
765.3 - 860.5
860.5 - 955.6
955.6 - 1157.4
1157.4 - 1359.2
1359.2 - 1561.0
1561.0 - 1761.2
1761.2 - 1819.3
> 1819.3
United States of America: Hawaii: DALYs Rate (per 100,000)
DALYs Rate
< 422.6
422.6 - 737.8
737.8 - 854.4
854.4 - 970.9
970.9 - 1140.4
1140.4 - 1310.0
1310.0 - 1479.5
1479.5 - 1646.2
1646.2 - 1745.0
> 1745.0
United States of America: Idaho: DALYs Rate (per 100,000)
DALYs Rate
< 497.8
497.8 - 877.9
877.9 - 1001.6
1001.6 - 1125.3
1125.3 - 1297.9
1297.9 - 1470.4
1470.4 - 1642.9
1642.9 - 1886.7
1886.7 - 2010.2
> 2010.2
United States of America: Illinois: DALYs Rate (per 100,000)
DALYs Rate
< 405.7
405.7 - 695.2
695.2 - 776.8
776.8 - 858.3
858.3 - 1020.6
1020.6 - 1182.8
1182.8 - 1345.1
1345.1 - 1526.3
1526.3 - 1622.9
> 1622.9
United States of America: Indiana: DALYs Rate (per 100,000)
DALYs Rate
< 517.3
517.3 - 790.2
790.2 - 913.7
913.7 - 1037.3
1037.3 - 1235.0
1235.0 - 1432.7
1432.7 - 1630.5
1630.5 - 1873.2
1873.2 - 1994.8
> 1994.8
United States of America: Iowa: DALYs Rate (per 100,000)
DALYs Rate
< 444.9
444.9 - 733.8
733.8 - 848.5
848.5 - 963.2
963.2 - 1115.1
1115.1 - 1267.0
1267.0 - 1418.9
1418.9 - 1562.1
1562.1 - 1702.4
> 1702.4
United States of America: Kansas: DALYs Rate (per 100,000)
DALYs Rate
< 489.2
489.2 - 755.1
755.1 - 873.8
873.8 - 992.6
992.6 - 1194.1
1194.1 - 1395.6
1395.6 - 1597.0
1597.0 - 1748.8
1748.8 - 1905.5
> 1905.5
Female
Male
Sexes Combined
20-24 years
15-19 years
10-14 years
Age
Year
DALYs Rate (per 100,000)
Age
10-24 years

Figure S7

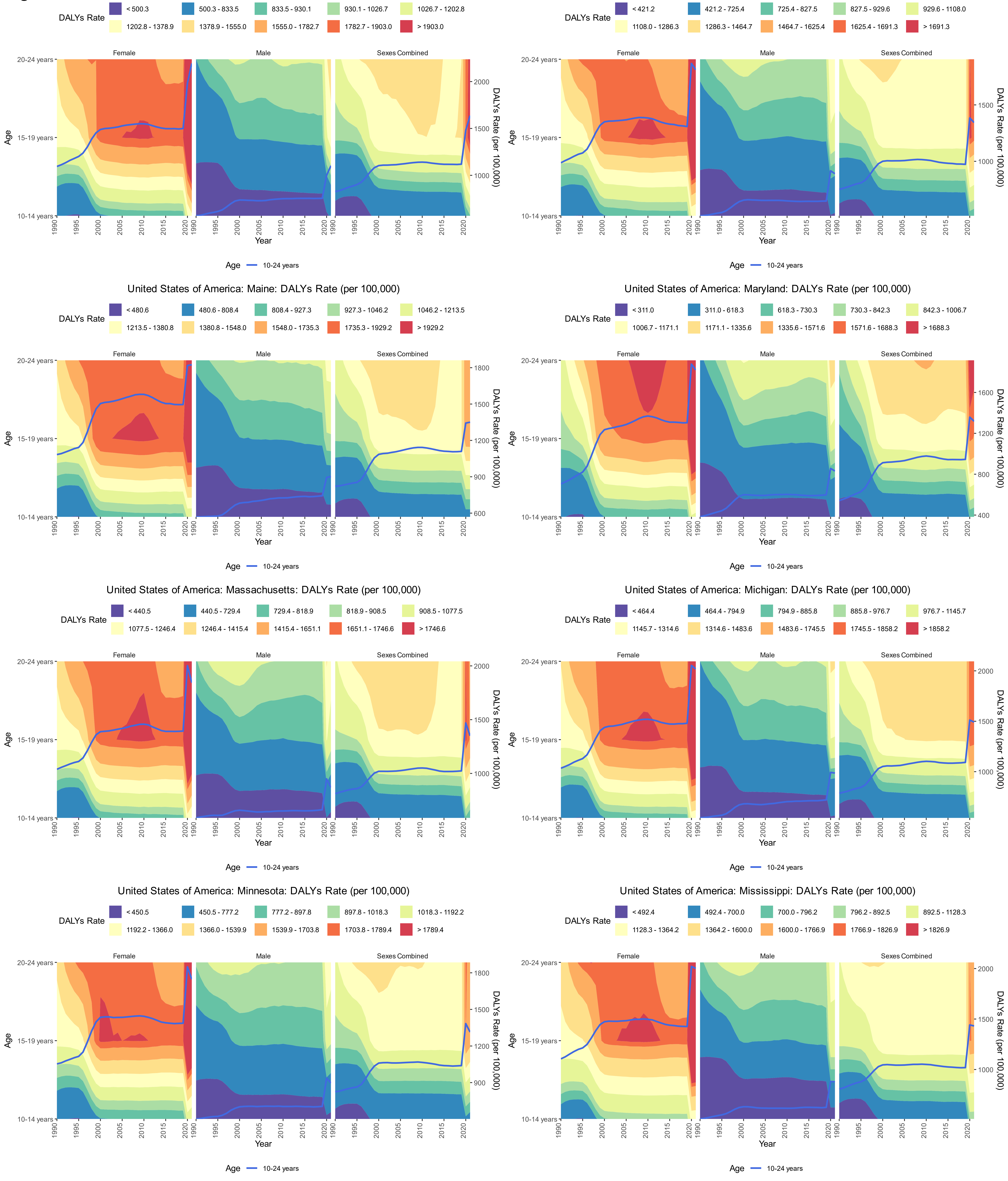

United States of America: Kentucky: DALYs Rate (per 100,000)
DALYs Rate
< 500.3
500.3 - 833.5
833.5 - 930.1
930.1 - 1026.7
1026.7 - 1202.8
1202.8 - 1378.9
1378.9 - 1555.0
1555.0 - 1782.7
1782.7 - 1903.0
> 1903.0
United States of America: Louisiana: DALYs Rate (per 100,000)
DALYs Rate
< 421.2
421.2 - 725.4
725.4 - 827.5
827.5 - 929.6
929.6 - 1108.0
1108.0 - 1286.3
1286.3 - 1464.7
1464.7 - 1625.4
1625.4 - 1691.3
> 1691.3
United States of America: Maine: DALYs Rate (per 100,000)
DALYs Rate
< 480.6
480.6 - 808.4
808.4 - 927.3
927.3 - 1046.2
1046.2 - 1213.5
1213.5 - 1380.8
1380.8 - 1548.0
1548.0 - 1735.3
1735.3 - 1929.2
> 1929.2
United States of America: Maryland: DALYs Rate (per 100,000)
DALYs Rate
< 311.0
311.0 - 618.3
618.3 - 730.3
730.3 - 842.3
842.3 - 1006.7
1006.7 - 1171.1
1171.1 - 1335.6
1335.6 - 1571.6
1571.6 - 1688.3
> 1688.3
United States of America: Massachusetts: DALYs Rate (per 100,000)
DALYs Rate
< 440.5
440.5 - 729.4
729.4 - 818.9
818.9 - 908.5
908.5 - 1077.5
1077.5 - 1246.4
1246.4 - 1415.4
1415.4 - 1651.1
1651.1 - 1746.6
> 1746.6
United States of America: Michigan: DALYs Rate (per 100,000)
DALYs Rate
< 464.4
464.4 - 794.9
794.9 - 885.8
885.8 - 976.7
976.7 - 1145.7
1145.7 - 1314.6
1314.6 - 1483.6
1483.6 - 1745.5
1745.5 - 1858.2
> 1858.2
United States of America: Minnesota: DALYs Rate (per 100,000)
DALYs Rate
< 450.5
450.5 - 777.2
777.2 - 897.8
897.8 - 1018.3
1018.3 - 1192.2
1192.2 - 1366.0
1366.0 - 1539.9
1539.9 - 1703.8
1703.8 - 1789.4
> 1789.4
United States of America: Mississippi: DALYs Rate (per 100,000)
DALYs Rate
< 492.4
492.4 - 700.0
700.0 - 796.2
796.2 - 892.5
892.5 - 1128.3
1128.3 - 1364.2
1364.2 - 1600.0
1600.0 - 1766.9
1766.9 - 1826.9
> 1826.9
Female
Male
Sexes Combined
Age
20-24 years
15-19 years
10-14 years
Year
1990
1995
2000
2005
2010
2015
2020
DALYs Rate (per 100,000)
Age
10-24 years

Figure S7

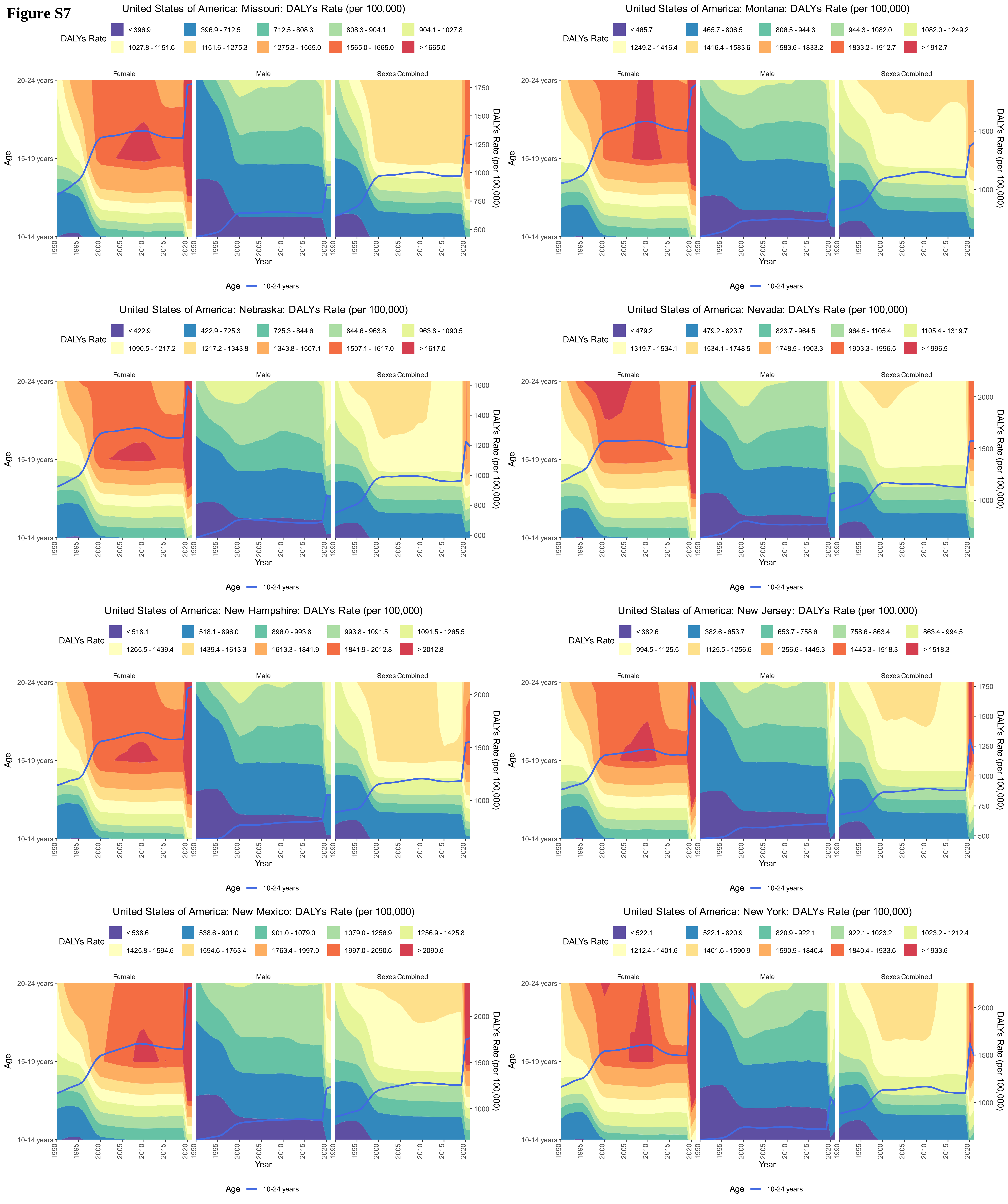
United States of America: Missouri: DALYs Rate (per 100,000)
DALYs Rate
< 396.9
396.9 - 712.5
712.5 - 808.3
808.3 - 904.1
904.1 - 1027.8
1027.8 - 1151.6
1151.6 - 1275.3
1275.3 - 1565.0
1565.0 - 1665.0
> 1665.0
Female
Male
Sexes Combined
20-24 years
15-19 years
10-14 years
Age
Year
DALYs Rate (per 100,000)
Age — 10-24 years
United States of America: Montana: DALYs Rate (per 100,000)
< 465.7
465.7 - 806.5
806.5 - 944.3
944.3 - 1082.0
1082.0 - 1249.2
1249.2 - 1416.4
1416.4 - 1583.6
1583.6 - 1833.2
1833.2 - 1912.7
> 1912.7
United States of America: Nebraska: DALYs Rate (per 100,000)
< 422.9
422.9 - 725.3
725.3 - 844.6
844.6 - 963.8
963.8 - 1090.5
1090.5 - 1217.2
1217.2 - 1343.8
1343.8 - 1507.1
1507.1 - 1617.0
> 1617.0
United States of America: Nevada: DALYs Rate (per 100,000)
< 479.2
479.2 - 823.7
823.7 - 964.5
964.5 - 1105.4
1105.4 - 1319.7
1319.7 - 1534.1
1534.1 - 1748.5
1748.5 - 1903.3
1903.3 - 1996.5
> 1996.5
United States of America: New Hampshire: DALYs Rate (per 100,000)
< 518.1
518.1 - 896.0
896.0 - 993.8
993.8 - 1091.5
1091.5 - 1265.5
1265.5 - 1439.4
1439.4 - 1613.3
1613.3 - 1841.9
1841.9 - 2012.8
> 2012.8
United States of America: New Jersey: DALYs Rate (per 100,000)
< 382.6
382.6 - 653.7
653.7 - 758.6
758.6 - 863.4
863.4 - 994.5
994.5 - 1125.5
1125.5 - 1256.6
1256.6 - 1445.3
1445.3 - 1518.3
> 1518.3
United States of America: New Mexico: DALYs Rate (per 100,000)
< 538.6
538.6 - 901.0
901.0 - 1079.0
1079.0 - 1256.9
1256.9 - 1425.8
1425.8 - 1594.6
1594.6 - 1763.4
1763.4 - 1997.0
1997.0 - 2090.6
> 2090.6
United States of America: New York: DALYs Rate (per 100,000)
< 522.1
522.1 - 820.9
820.9 - 922.1
922.1 - 1023.2
1023.2 - 1212.4
1212.4 - 1401.6
1401.6 - 1590.9
1590.9 - 1840.4
1840.4 - 1933.6
> 1933.6

Figure S7

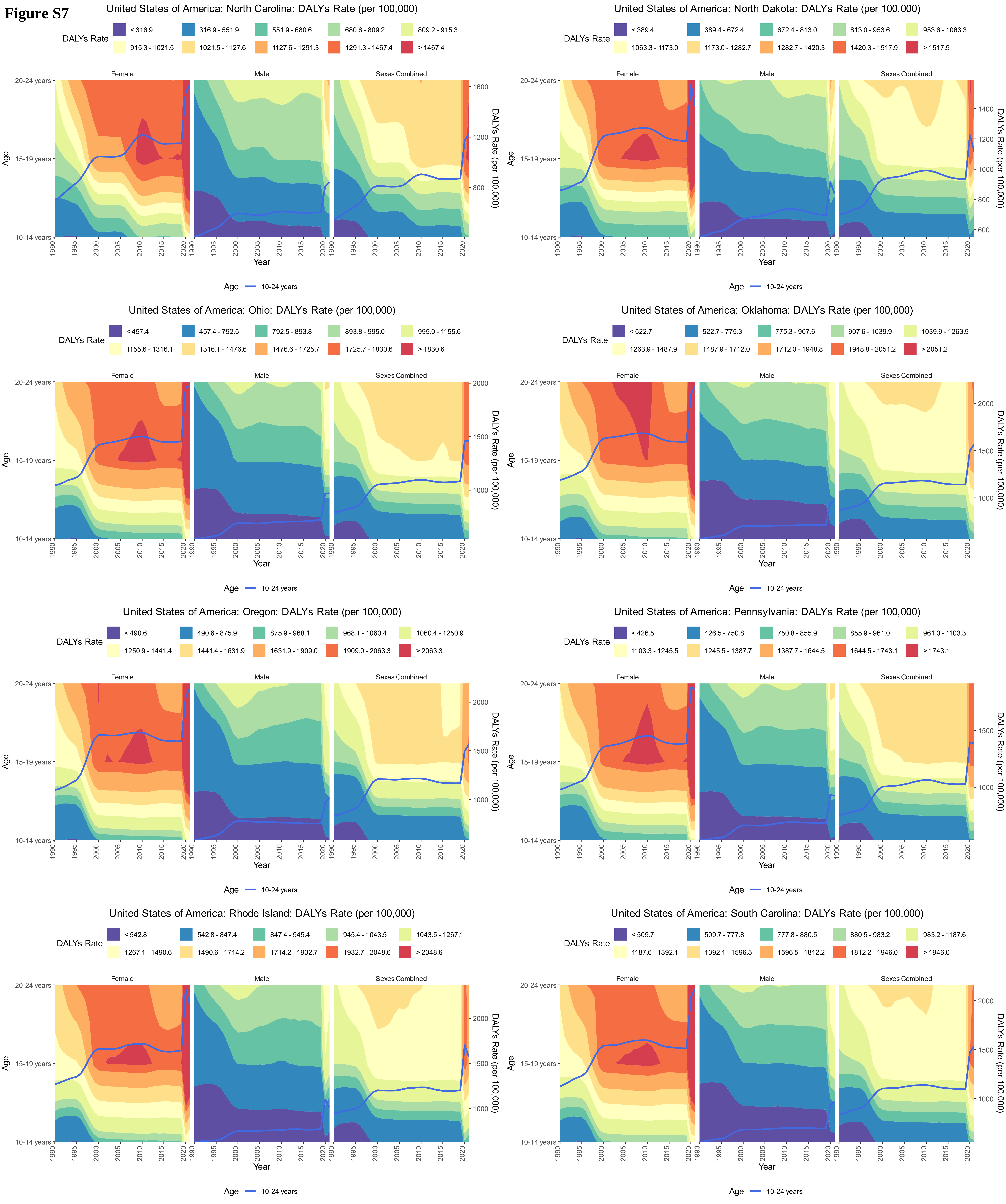

United States of America: North Carolina: DALYs Rate (per 100,000)
DALYs Rate
< 316.9
316.9 - 551.9
551.9 - 680.6
680.6 - 809.2
809.2 - 915.3
915.3 - 1021.5
1021.5 - 1127.6
1127.6 - 1291.3
1291.3 - 1467.4
> 1467.4
United States of America: North Dakota: DALYs Rate (per 100,000)
DALYs Rate
< 389.4
389.4 - 672.4
672.4 - 813.0
813.0 - 953.6
953.6 - 1063.3
1063.3 - 1173.0
1173.0 - 1282.7
1282.7 - 1420.3
1420.3 - 1517.9
> 1517.9
United States of America: Ohio: DALYs Rate (per 100,000)
DALYs Rate
< 457.4
457.4 - 792.5
792.5 - 893.8
893.8 - 995.0
995.0 - 1155.6
1155.6 - 1316.1
1316.1 - 1476.6
1476.6 - 1725.7
1725.7 - 1830.6
> 1830.6
United States of America: Oklahoma: DALYs Rate (per 100,000)
DALYs Rate
< 522.7
522.7 - 775.3
775.3 - 907.6
907.6 - 1039.9
1039.9 - 1263.9
1263.9 - 1487.9
1487.9 - 1712.0
1712.0 - 1948.8
1948.8 - 2051.2
> 2051.2
United States of America: Oregon: DALYs Rate (per 100,000)
DALYs Rate
< 490.6
490.6 - 875.9
875.9 - 968.1
968.1 - 1060.4
1060.4 - 1250.9
1250.9 - 1441.4
1441.4 - 1631.9
1631.9 - 1909.0
1909.0 - 2063.3
> 2063.3
United States of America: Pennsylvania: DALYs Rate (per 100,000)
DALYs Rate
< 426.5
426.5 - 750.8
750.8 - 855.9
855.9 - 961.0
961.0 - 1103.3
1103.3 - 1245.5
1245.5 - 1387.7
1387.7 - 1644.5
1644.5 - 1743.1
> 1743.1
United States of America: Rhode Island: DALYs Rate (per 100,000)
DALYs Rate
< 542.8
542.8 - 847.4
847.4 - 945.4
945.4 - 1043.5
1043.5 - 1267.1
1267.1 - 1490.6
1490.6 - 1714.2
1714.2 - 1932.7
1932.7 - 2048.6
> 2048.6
United States of America: South Carolina: DALYs Rate (per 100,000)
DALYs Rate
< 509.7
509.7 - 777.8
777.8 - 880.5
880.5 - 983.2
983.2 - 1187.6
1187.6 - 1392.1
1392.1 - 1596.5
1596.5 - 1812.2
1812.2 - 1946.0
> 1946.0
Female
Male
Sexes Combined
Age
20-24 years
15-19 years
10-14 years
Year
DALYs Rate (per 100,000)
Age
10-24 years

Figure S7

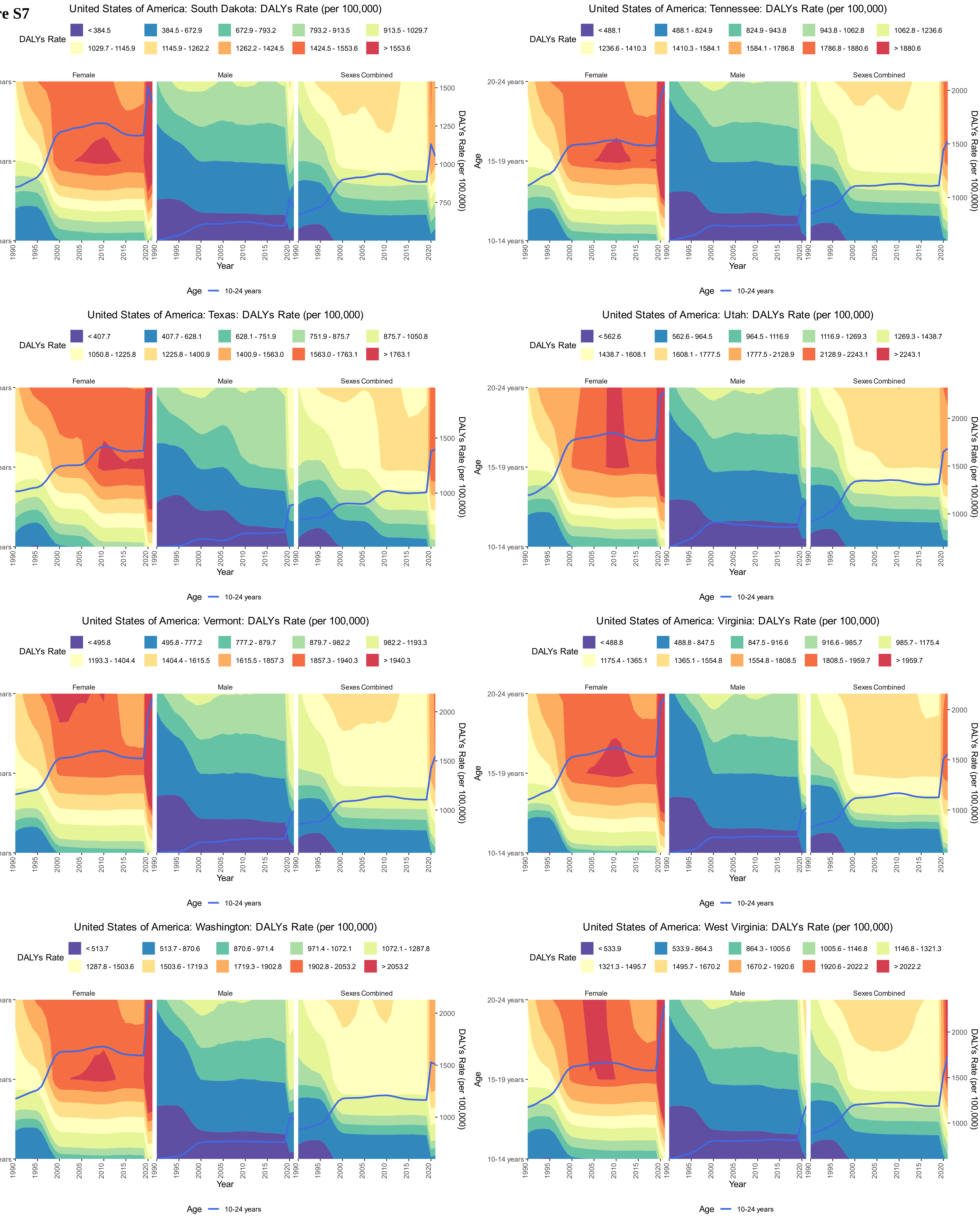

United States of America: South Dakota: DALYs Rate (per 100,000)
DALYs Rate
< 384.5
384.5 - 672.9
672.9 - 793.2
793.2 - 913.5
913.5 - 1029.7
1029.7 - 1145.9
1145.9 - 1262.2
1262.2 - 1424.5
1424.5 - 1553.6
> 1553.6
United States of America: Tennessee: DALYs Rate (per 100,000)
DALYs Rate
< 488.1
488.1 - 824.9
824.9 - 943.8
943.8 - 1062.8
1062.8 - 1236.6
1236.6 - 1410.3
1410.3 - 1584.1
1584.1 - 1786.8
1786.8 - 1880.6
> 1880.6
United States of America: Texas: DALYs Rate (per 100,000)
DALYs Rate
< 407.7
407.7 - 628.1
628.1 - 751.9
751.9 - 875.7
875.7 - 1050.8
1050.8 - 1225.8
1225.8 - 1400.9
1400.9 - 1563.0
1563.0 - 1763.1
> 1763.1
United States of America: Utah: DALYs Rate (per 100,000)
DALYs Rate
< 562.6
562.6 - 964.5
964.5 - 1116.9
1116.9 - 1269.3
1269.3 - 1438.7
1438.7 - 1608.1
1608.1 - 1777.5
1777.5 - 2128.9
2128.9 - 2243.1
> 2243.1
United States of America: Vermont: DALYs Rate (per 100,000)
DALYs Rate
< 495.8
495.8 - 777.2
777.2 - 879.7
879.7 - 982.2
982.2 - 1193.3
1193.3 - 1404.4
1404.4 - 1615.5
1615.5 - 1857.3
1857.3 - 1940.3
> 1940.3
United States of America: Virginia: DALYs Rate (per 100,000)
DALYs Rate
< 488.8
488.8 - 847.5
847.5 - 916.6
916.6 - 985.7
985.7 - 1175.4
1175.4 - 1365.1
1365.1 - 1554.8
1554.8 - 1808.5
1808.5 - 1959.7
> 1959.7
United States of America: Washington: DALYs Rate (per 100,000)
DALYs Rate
< 513.7
513.7 - 870.6
870.6 - 971.4
971.4 - 1072.1
1072.1 - 1287.8
1287.8 - 1503.6
1503.6 - 1719.3
1719.3 - 1902.8
1902.8 - 2053.2
> 2053.2
United States of America: West Virginia: DALYs Rate (per 100,000)
DALYs Rate
< 533.9
533.9 - 864.3
864.3 - 1005.6
1005.6 - 1146.8
1146.8 - 1321.3
1321.3 - 1495.7
1495.7 - 1670.2
1670.2 - 1920.6
1920.6 - 2022.2
> 2022.2
Female
Male
Sexes Combined
Age
20-24 years
15-19 years
10-14 years
Year
DALYs Rate (per 100,000)
Age
10-24 years

Figure S7

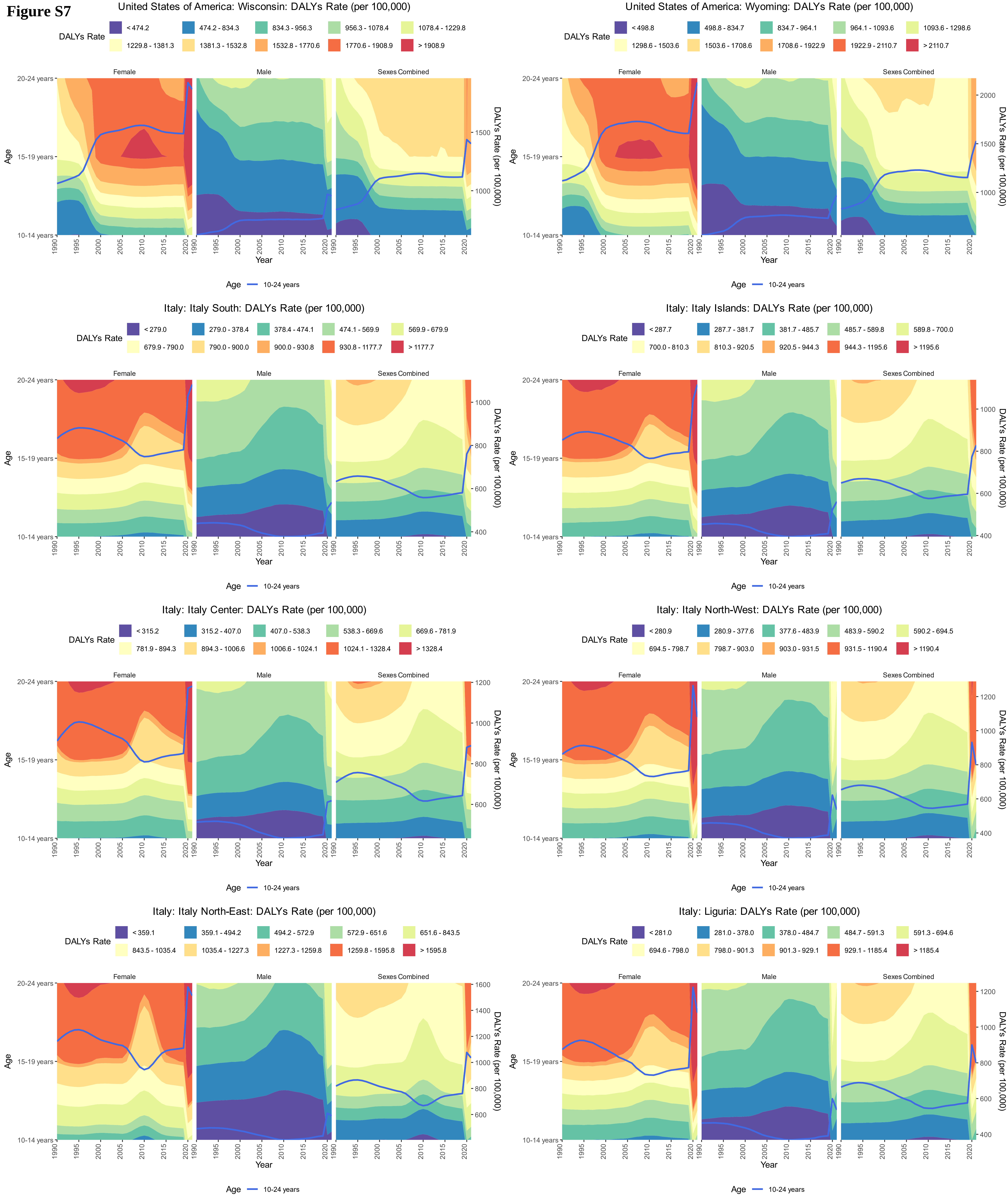
United States of America: Wisconsin: DALYs Rate (per 100,000)
DALYs Rate
< 474.2
474.2 - 834.3
834.3 - 956.3
956.3 - 1078.4
1078.4 - 1229.8
1229.8 - 1381.3
1381.3 - 1532.8
1532.8 - 1770.6
1770.6 - 1908.9
> 1908.9
United States of America: Wyoming: DALYs Rate (per 100,000)
DALYs Rate
< 498.8
498.8 - 834.7
834.7 - 964.1
964.1 - 1093.6
1093.6 - 1298.6
1298.6 - 1503.6
1503.6 - 1708.6
1708.6 - 1922.9
1922.9 - 2110.7
> 2110.7
Italy: Italy South: DALYs Rate (per 100,000)
DALYs Rate
< 279.0
279.0 - 378.4
378.4 - 474.1
474.1 - 569.9
569.9 - 679.9
679.9 - 790.0
790.0 - 900.0
900.0 - 930.8
930.8 - 1177.7
> 1177.7
Italy: Italy Islands: DALYs Rate (per 100,000)
DALYs Rate
< 287.7
287.7 - 381.7
381.7 - 485.7
485.7 - 589.8
589.8 - 700.0
700.0 - 810.3
810.3 - 920.5
920.5 - 944.3
944.3 - 1195.6
> 1195.6
Italy: Italy Center: DALYs Rate (per 100,000)
DALYs Rate
< 315.2
315.2 - 407.0
407.0 - 538.3
538.3 - 669.6
669.6 - 781.9
781.9 - 894.3
894.3 - 1006.6
1006.6 - 1024.1
1024.1 - 1328.4
> 1328.4
Italy: Italy North-West: DALYs Rate (per 100,000)
DALYs Rate
< 280.9
280.9 - 377.6
377.6 - 483.9
483.9 - 590.2
590.2 - 694.5
694.5 - 798.7
798.7 - 903.0
903.0 - 931.5
931.5 - 1190.4
> 1190.4
Italy: Italy North-East: DALYs Rate (per 100,000)
DALYs Rate
< 359.1
359.1 - 494.2
494.2 - 572.9
572.9 - 651.6
651.6 - 843.5
843.5 - 1035.4
1035.4 - 1227.3
1227.3 - 1259.8
1259.8 - 1595.8
> 1595.8
Italy: Liguria: DALYs Rate (per 100,000)
DALYs Rate
< 281.0
281.0 - 378.0
378.0 - 484.7
484.7 - 591.3
591.3 - 694.6
694.6 - 798.0
798.0 - 901.3
901.3 - 929.1
929.1 - 1185.4
> 1185.4
Female
Male
Sexes Combined
Age
20-24 years
15-19 years
10-14 years
Year
DALYs Rate (per 100,000)
Age
10-24 years

Figure S7

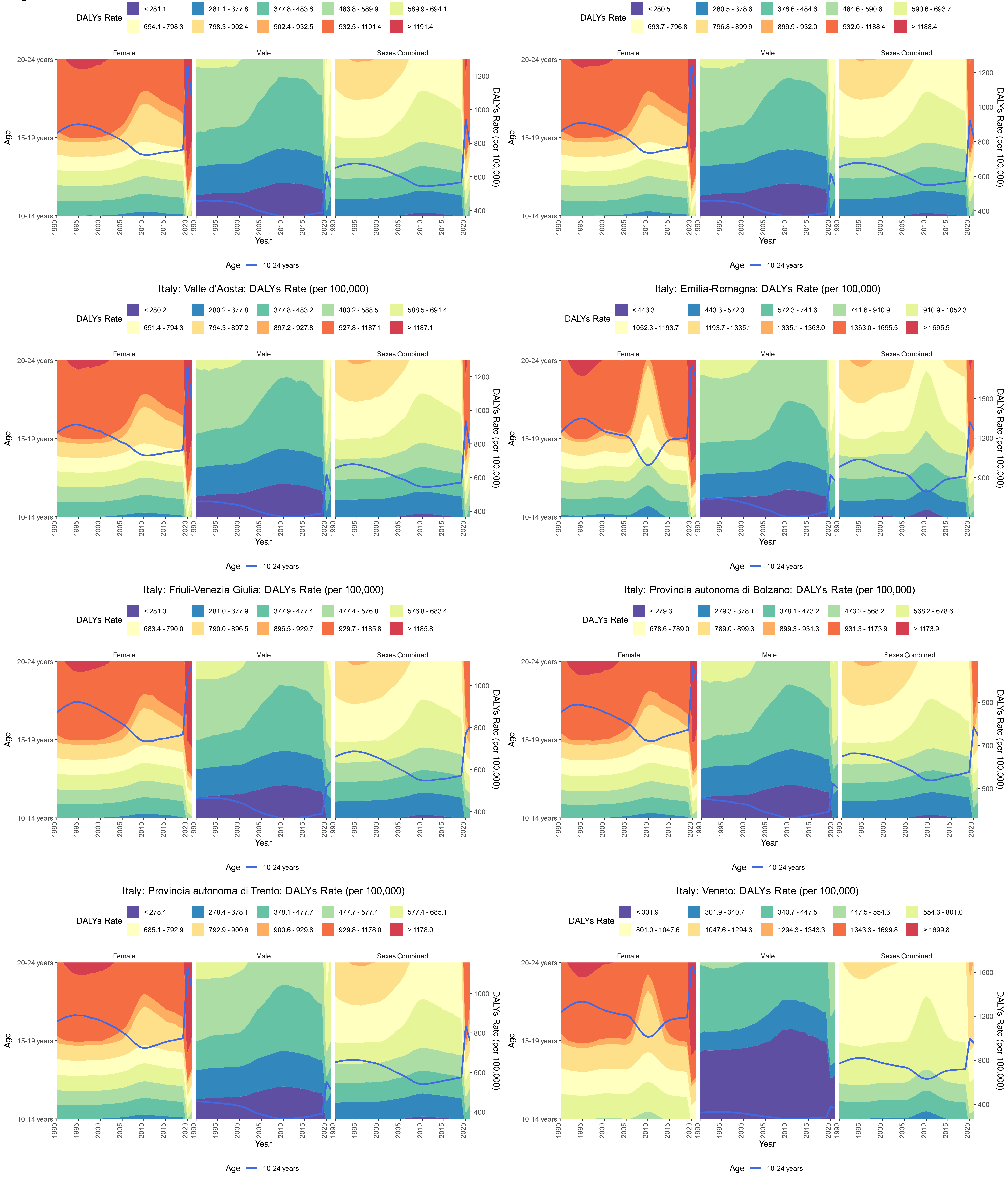

Italy: Lombardia: DALYs Rate (per 100,000)
DALYs Rate
< 281.1
281.1 - 377.8
377.8 - 483.8
483.8 - 589.9
589.9 - 694.1
694.1 - 798.3
798.3 - 902.4
902.4 - 932.5
932.5 - 1191.4
> 1191.4
Italy: Piemonte: DALYs Rate (per 100,000)
< 280.5
280.5 - 378.6
378.6 - 484.6
484.6 - 590.6
590.6 - 693.7
693.7 - 796.8
796.8 - 899.9
899.9 - 932.0
932.0 - 1188.4
> 1188.4
Italy: Valle d'Aosta: DALYs Rate (per 100,000)
< 280.2
280.2 - 377.8
377.8 - 483.2
483.2 - 588.5
588.5 - 691.4
691.4 - 794.3
794.3 - 897.2
897.2 - 927.8
927.8 - 1187.1
> 1187.1
Italy: Emilia-Romagna: DALYs Rate (per 100,000)
< 443.3
443.3 - 572.3
572.3 - 741.6
741.6 - 910.9
910.9 - 1052.3
1052.3 - 1193.7
1193.7 - 1335.1
1335.1 - 1363.0
1363.0 - 1695.5
> 1695.5
Italy: Friuli-Venezia Giulia: DALYs Rate (per 100,000)
< 281.0
281.0 - 377.9
377.9 - 477.4
477.4 - 576.8
576.8 - 683.4
683.4 - 790.0
790.0 - 896.5
896.5 - 929.7
929.7 - 1185.8
> 1185.8
Italy: Provincia autonoma di Bolzano: DALYs Rate (per 100,000)
< 279.3
279.3 - 378.1
378.1 - 473.2
473.2 - 568.2
568.2 - 678.6
678.6 - 789.0
789.0 - 899.3
899.3 - 931.3
931.3 - 1173.9
> 1173.9
Italy: Provincia autonoma di Trento: DALYs Rate (per 100,000)
< 278.4
278.4 - 378.1
378.1 - 477.7
477.7 - 577.4
577.4 - 685.1
685.1 - 792.9
792.9 - 900.6
900.6 - 929.8
929.8 - 1178.0
> 1178.0
Italy: Veneto: DALYs Rate (per 100,000)
< 301.9
301.9 - 340.7
340.7 - 447.5
447.5 - 554.3
554.3 - 801.0
801.0 - 1047.6
1047.6 - 1294.3
1294.3 - 1343.3
1343.3 - 1699.8
> 1699.8
Female
Male
Sexes Combined
20-24 years
15-19 years
10-14 years
Age
Year
DALYs Rate (per 100,000)
Age
10-24 years

Figure S7

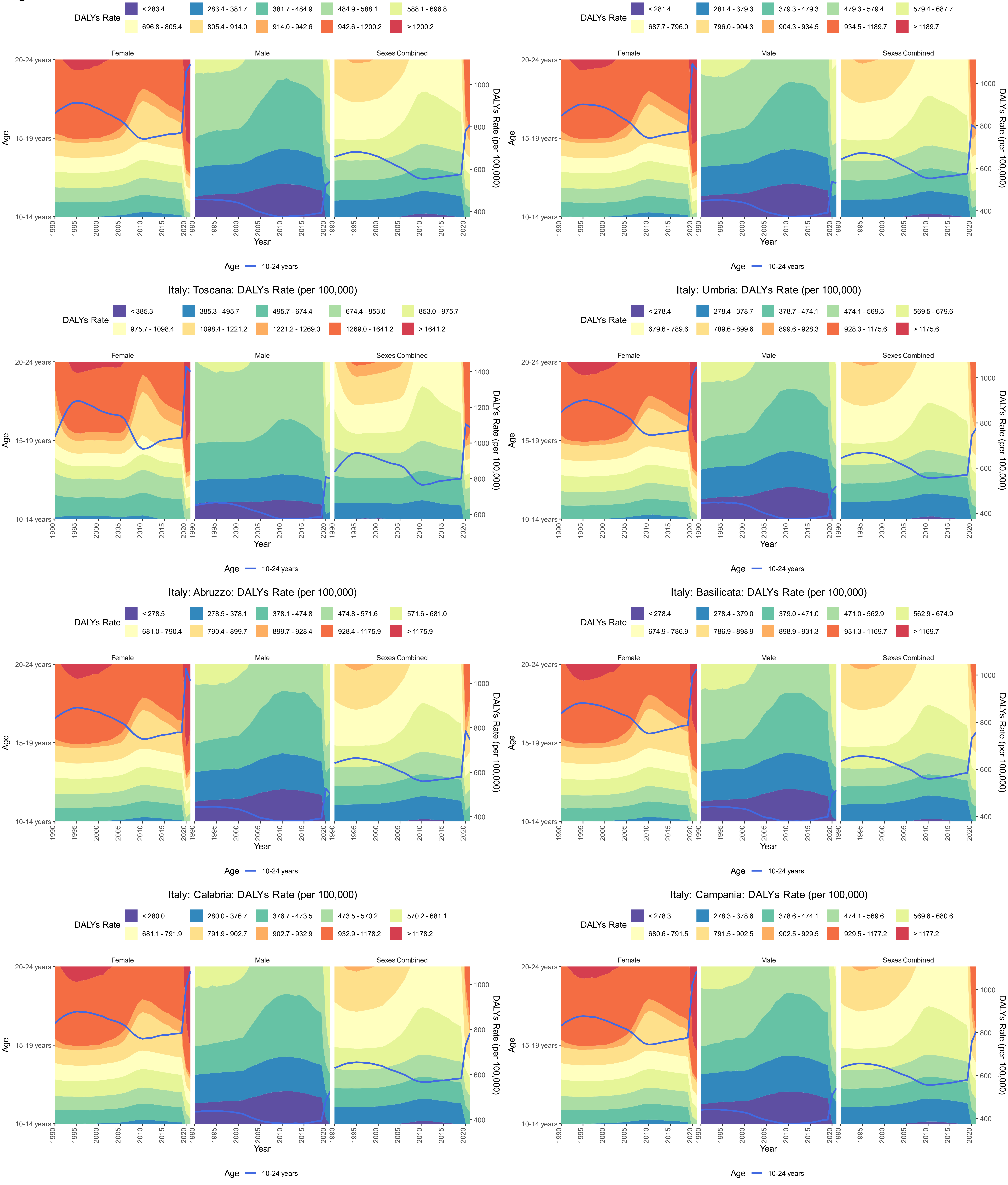
Italy: Lazio: DALYs Rate (per 100,000)
DALYs Rate
< 283.4
283.4 - 381.7
381.7 - 484.9
484.9 - 588.1
588.1 - 696.8
696.8 - 805.4
805.4 - 914.0
914.0 - 942.6
942.6 - 1200.2
> 1200.2
Italy: Marche: DALYs Rate (per 100,000)
DALYs Rate
< 281.4
281.4 - 379.3
379.3 - 479.3
479.3 - 579.4
579.4 - 687.7
687.7 - 796.0
796.0 - 904.3
904.3 - 934.5
934.5 - 1189.7
> 1189.7
Italy: Toscana: DALYs Rate (per 100,000)
DALYs Rate
< 385.3
385.3 - 495.7
495.7 - 674.4
674.4 - 853.0
853.0 - 975.7
975.7 - 1098.4
1098.4 - 1221.2
1221.2 - 1269.0
1269.0 - 1641.2
> 1641.2
Italy: Umbria: DALYs Rate (per 100,000)
DALYs Rate
< 278.4
278.4 - 378.7
378.7 - 474.1
474.1 - 569.5
569.5 - 679.6
679.6 - 789.6
789.6 - 899.6
899.6 - 928.3
928.3 - 1175.6
> 1175.6
Italy: Abruzzo: DALYs Rate (per 100,000)
DALYs Rate
< 278.5
278.5 - 378.1
378.1 - 474.8
474.8 - 571.6
571.6 - 681.0
681.0 - 790.4
790.4 - 899.7
899.7 - 928.4
928.4 - 1175.9
> 1175.9
Italy: Basilicata: DALYs Rate (per 100,000)
DALYs Rate
< 278.4
278.4 - 379.0
379.0 - 471.0
471.0 - 562.9
562.9 - 674.9
674.9 - 786.9
786.9 - 898.9
898.9 - 931.3
931.3 - 1169.7
> 1169.7
Italy: Calabria: DALYs Rate (per 100,000)
DALYs Rate
< 280.0
280.0 - 376.7
376.7 - 473.5
473.5 - 570.2
570.2 - 681.1
681.1 - 791.9
791.9 - 902.7
902.7 - 932.9
932.9 - 1178.2
> 1178.2
Italy: Campania: DALYs Rate (per 100,000)
DALYs Rate
< 278.3
278.3 - 378.6
378.6 - 474.1
474.1 - 569.6
569.6 - 680.6
680.6 - 791.5
791.5 - 902.5
902.5 - 929.5
929.5 - 1177.2
> 1177.2
Female
Male
Sexes Combined
Age
20-24 years
15-19 years
10-14 years
Year
DALYs Rate (per 100,000)
Age
10-24 years

Figure S7

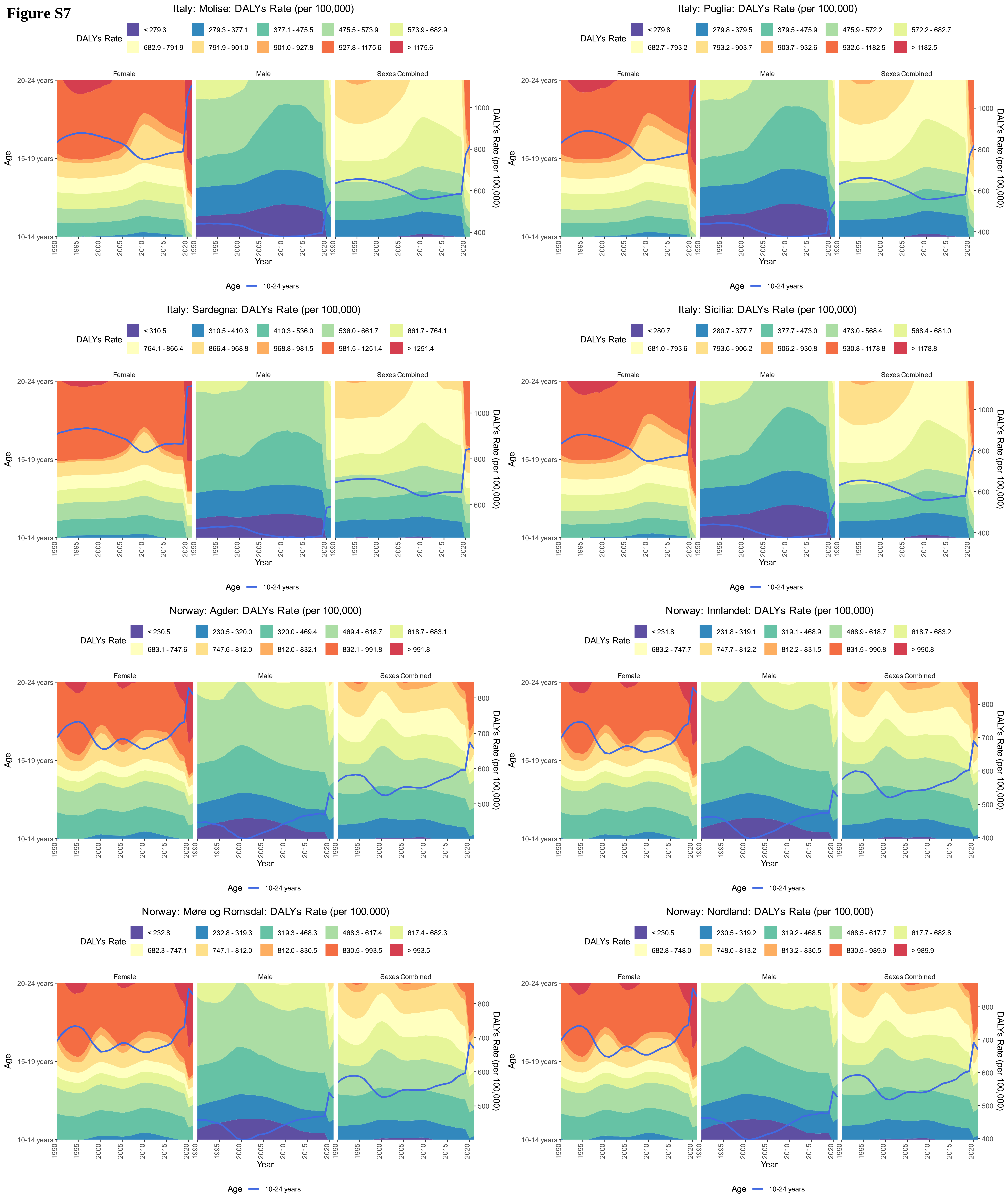

Italy: Molise: DALYs Rate (per 100,000)
DALYs Rate
< 279.3
279.3 - 377.1
377.1 - 475.5
475.5 - 573.9
573.9 - 682.9
682.9 - 791.9
791.9 - 901.0
901.0 - 927.8
927.8 - 1175.6
> 1175.6
Italy: Puglia: DALYs Rate (per 100,000)
< 279.8
279.8 - 379.5
379.5 - 475.9
475.9 - 572.2
572.2 - 682.7
682.7 - 793.2
793.2 - 903.7
903.7 - 932.6
932.6 - 1182.5
> 1182.5
Italy: Sardegna: DALYs Rate (per 100,000)
< 310.5
310.5 - 410.3
410.3 - 536.0
536.0 - 661.7
661.7 - 764.1
764.1 - 866.4
866.4 - 968.8
968.8 - 981.5
981.5 - 1251.4
> 1251.4
Italy: Sicilia: DALYs Rate (per 100,000)
< 280.7
280.7 - 377.7
377.7 - 473.0
473.0 - 568.4
568.4 - 681.0
681.0 - 793.6
793.6 - 906.2
906.2 - 930.8
930.8 - 1178.8
> 1178.8
Norway: Agder: DALYs Rate (per 100,000)
< 230.5
230.5 - 320.0
320.0 - 469.4
469.4 - 618.7
618.7 - 683.1
683.1 - 747.6
747.6 - 812.0
812.0 - 832.1
832.1 - 991.8
> 991.8
Norway: Innlandet: DALYs Rate (per 100,000)
< 231.8
231.8 - 319.1
319.1 - 468.9
468.9 - 618.7
618.7 - 683.2
683.2 - 747.7
747.7 - 812.2
812.2 - 831.5
831.5 - 990.8
> 990.8
Norway: Møre og Romsdal: DALYs Rate (per 100,000)
< 232.8
232.8 - 319.3
319.3 - 468.3
468.3 - 617.4
617.4 - 682.3
682.3 - 747.1
747.1 - 812.0
812.0 - 830.5
830.5 - 993.5
> 993.5
Norway: Nordland: DALYs Rate (per 100,000)
< 230.5
230.5 - 319.2
319.2 - 468.5
468.5 - 617.7
617.7 - 682.8
682.8 - 748.0
748.0 - 813.2
813.2 - 830.5
830.5 - 989.9
> 989.9
Female
Male
Sexes Combined
Age
20-24 years
15-19 years
10-14 years
Year
DALYs Rate (per 100,000)
Age
10-24 years

Figure S7

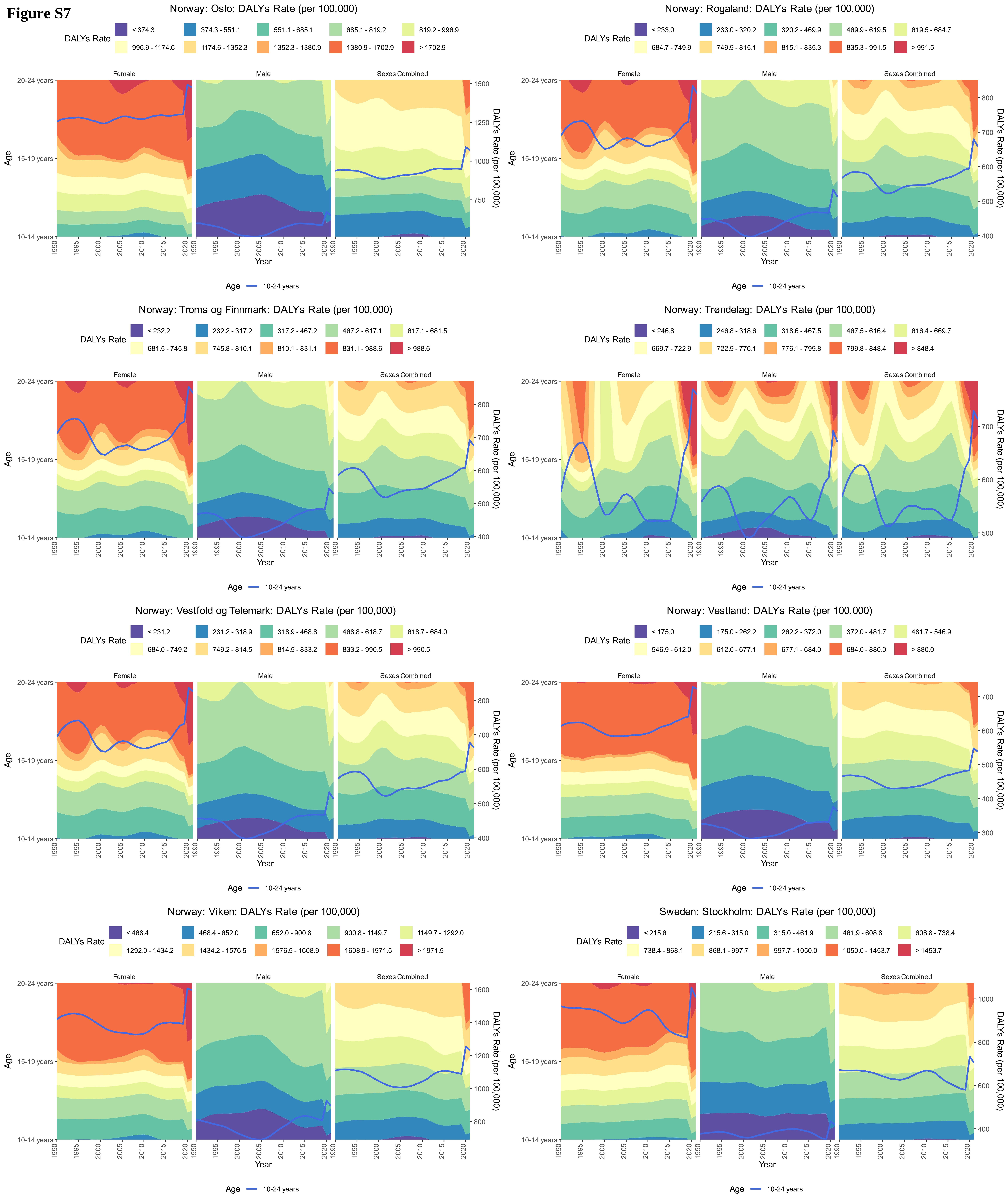

Norway: Oslo: DALYs Rate (per 100,000)
DALYs Rate
< 374.3
374.3 - 551.1
551.1 - 685.1
685.1 - 819.2
819.2 - 996.9
996.9 - 1174.6
1174.6 - 1352.3
1352.3 - 1380.9
1380.9 - 1702.9
> 1702.9
Norway: Rogaland: DALYs Rate (per 100,000)
< 233.0
233.0 - 320.2
320.2 - 469.9
469.9 - 619.5
619.5 - 684.7
684.7 - 749.9
749.9 - 815.1
815.1 - 835.3
835.3 - 991.5
> 991.5
Norway: Troms og Finnmark: DALYs Rate (per 100,000)
< 232.2
232.2 - 317.2
317.2 - 467.2
467.2 - 617.1
617.1 - 681.5
681.5 - 745.8
745.8 - 810.1
810.1 - 831.1
831.1 - 988.6
> 988.6
Norway: Trøndelag: DALYs Rate (per 100,000)
< 246.8
246.8 - 318.6
318.6 - 467.5
467.5 - 616.4
616.4 - 669.7
669.7 - 722.9
722.9 - 776.1
776.1 - 799.8
799.8 - 848.4
> 848.4
Norway: Vestfold og Telemark: DALYs Rate (per 100,000)
< 231.2
231.2 - 318.9
318.9 - 468.8
468.8 - 618.7
618.7 - 684.0
684.0 - 749.2
749.2 - 814.5
814.5 - 833.2
833.2 - 990.5
> 990.5
Norway: Vestland: DALYs Rate (per 100,000)
< 175.0
175.0 - 262.2
262.2 - 372.0
372.0 - 481.7
481.7 - 546.9
546.9 - 612.0
612.0 - 677.1
677.1 - 684.0
684.0 - 880.0
> 880.0
Norway: Viken: DALYs Rate (per 100,000)
< 468.4
468.4 - 652.0
652.0 - 900.8
900.8 - 1149.7
1149.7 - 1292.0
1292.0 - 1434.2
1434.2 - 1576.5
1576.5 - 1608.9
1608.9 - 1971.5
> 1971.5
Sweden: Stockholm: DALYs Rate (per 100,000)
< 215.6
215.6 - 315.0
315.0 - 461.9
461.9 - 608.8
608.8 - 738.4
738.4 - 868.1
868.1 - 997.7
997.7 - 1050.0
1050.0 - 1453.7
> 1453.7
Female
Male
Sexes Combined
20-24 years
15-19 years
10-14 years
Age
Year
DALYs Rate (per 100,000)
Age
10-24 years

Figure S7

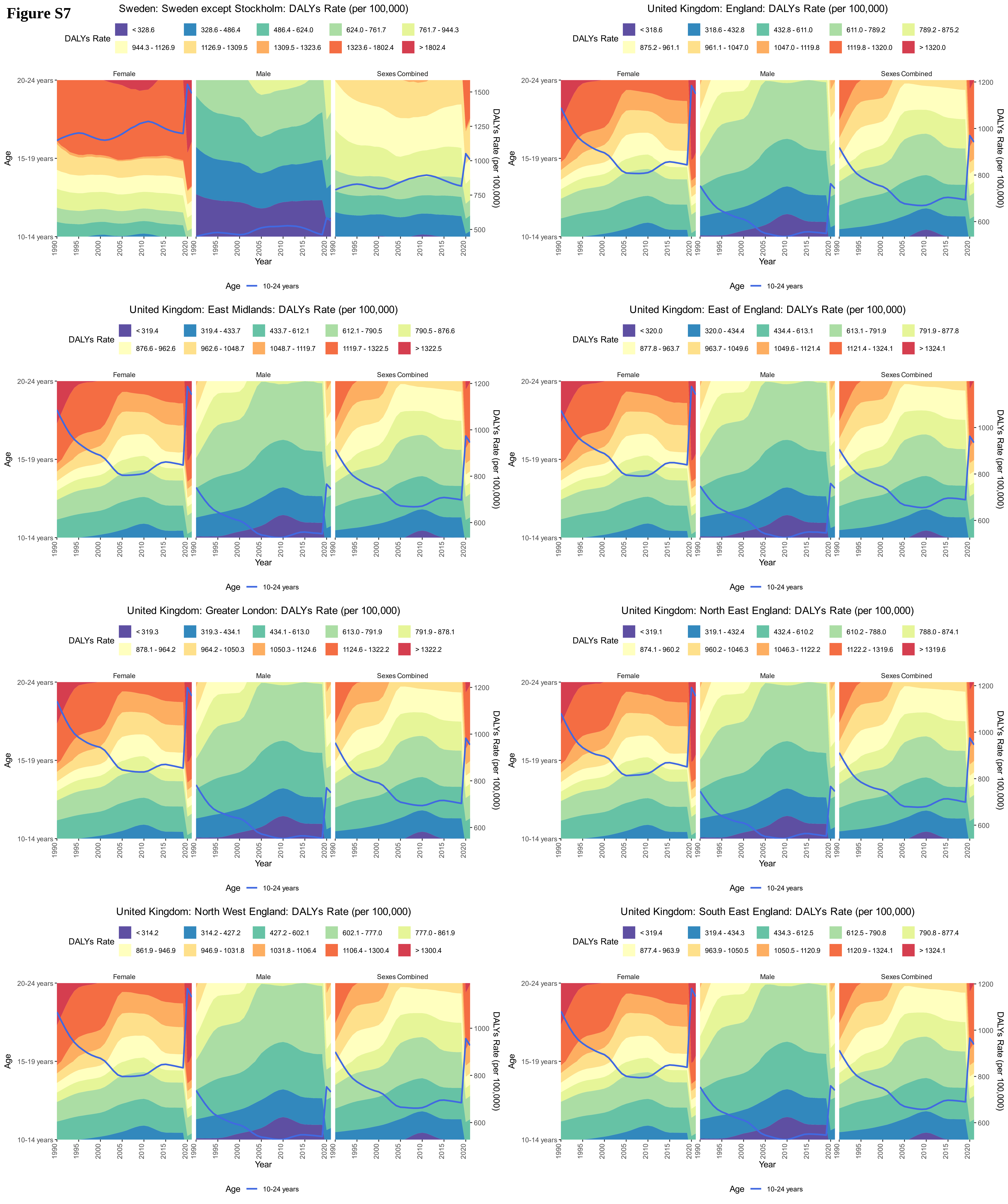

Sweden: Sweden except Stockholm: DALYs Rate (per 100,000)
DALYs Rate
< 328.6
328.6 - 486.4
486.4 - 624.0
624.0 - 761.7
761.7 - 944.3
944.3 - 1126.9
1126.9 - 1309.5
1309.5 - 1323.6
1323.6 - 1802.4
> 1802.4
Female
Male
Sexes Combined
Age
Year
DALYs Rate (per 100,000)
Age — 10-24 years
United Kingdom: England: DALYs Rate (per 100,000)
< 318.6
318.6 - 432.8
432.8 - 611.0
611.0 - 789.2
789.2 - 875.2
875.2 - 961.1
961.1 - 1047.0
1047.0 - 1119.8
1119.8 - 1320.0
> 1320.0
United Kingdom: East Midlands: DALYs Rate (per 100,000)
< 319.4
319.4 - 433.7
433.7 - 612.1
612.1 - 790.5
790.5 - 876.6
876.6 - 962.6
962.6 - 1048.7
1048.7 - 1119.7
1119.7 - 1322.5
> 1322.5
United Kingdom: East of England: DALYs Rate (per 100,000)
< 320.0
320.0 - 434.4
434.4 - 613.1
613.1 - 791.9
791.9 - 877.8
877.8 - 963.7
963.7 - 1049.6
1049.6 - 1121.4
1121.4 - 1324.1
> 1324.1
United Kingdom: Greater London: DALYs Rate (per 100,000)
< 319.3
319.3 - 434.1
434.1 - 613.0
613.0 - 791.9
791.9 - 878.1
878.1 - 964.2
964.2 - 1050.3
1050.3 - 1124.6
1124.6 - 1322.2
> 1322.2
United Kingdom: North East England: DALYs Rate (per 100,000)
< 319.1
319.1 - 432.4
432.4 - 610.2
610.2 - 788.0
788.0 - 874.1
874.1 - 960.2
960.2 - 1046.3
1046.3 - 1122.2
1122.2 - 1319.6
> 1319.6
United Kingdom: North West England: DALYs Rate (per 100,000)
< 314.2
314.2 - 427.2
427.2 - 602.1
602.1 - 777.0
777.0 - 861.9
861.9 - 946.9
946.9 - 1031.8
1031.8 - 1106.4
1106.4 - 1300.4
> 1300.4
United Kingdom: South East England: DALYs Rate (per 100,000)
< 319.4
319.4 - 434.3
434.3 - 612.5
612.5 - 790.8
790.8 - 877.4
877.4 - 963.9
963.9 - 1050.5
1050.5 - 1120.9
1120.9 - 1324.1
> 1324.1
20-24 years
15-19 years
10-14 years
1990
1995
2000
2005
2010
2015
2020

Figure S7

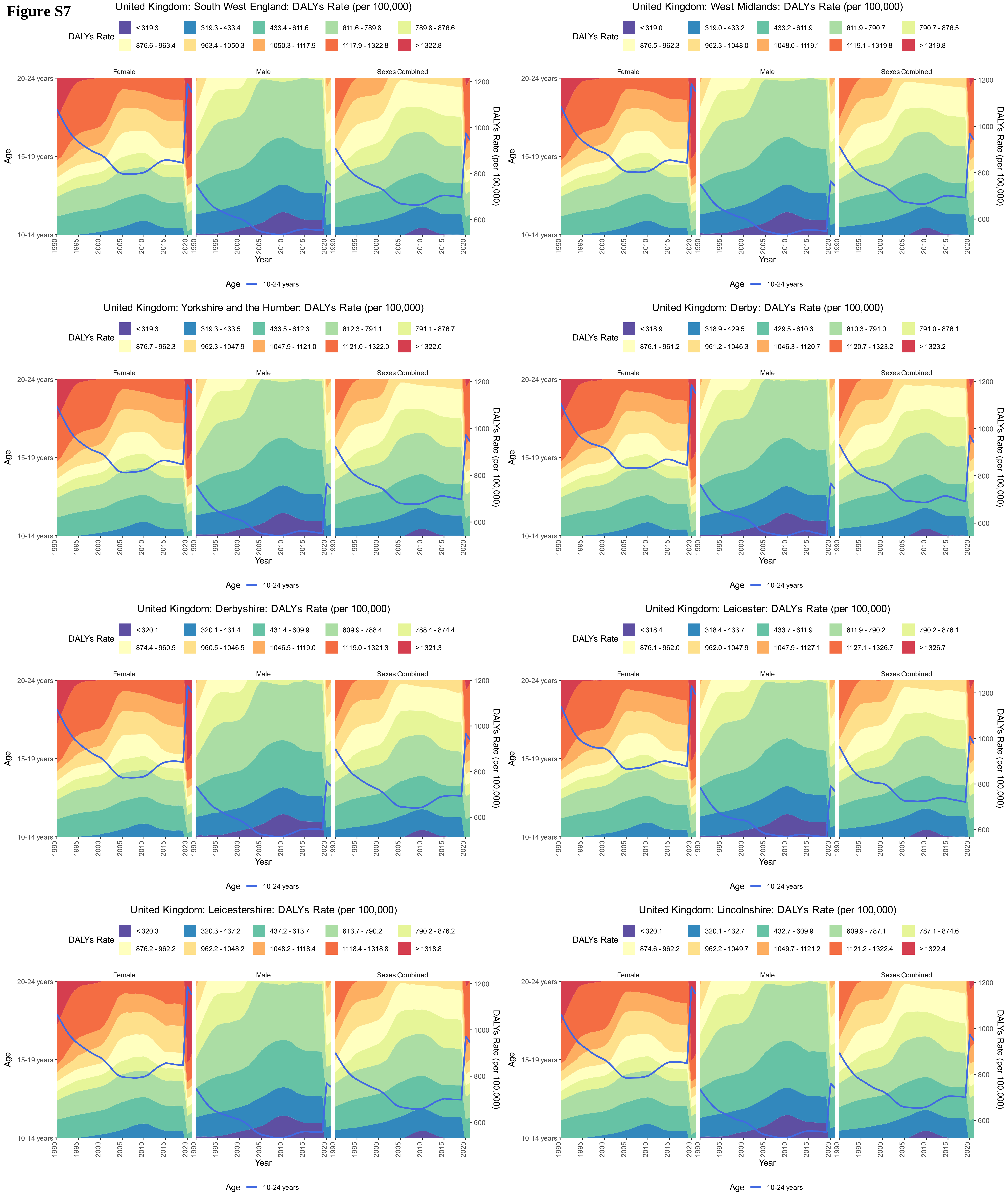

United Kingdom: South West England: DALYs Rate (per 100,000)
DALYs Rate
< 319.3
319.3 - 433.4
433.4 - 611.6
611.6 - 789.8
789.8 - 876.6
876.6 - 963.4
963.4 - 1050.3
1050.3 - 1117.9
1117.9 - 1322.8
> 1322.8
United Kingdom: West Midlands: DALYs Rate (per 100,000)
DALYs Rate
< 319.0
319.0 - 433.2
433.2 - 611.9
611.9 - 790.7
790.7 - 876.5
876.5 - 962.3
962.3 - 1048.0
1048.0 - 1119.1
1119.1 - 1319.8
> 1319.8
United Kingdom: Yorkshire and the Humber: DALYs Rate (per 100,000)
DALYs Rate
< 319.3
319.3 - 433.5
433.5 - 612.3
612.3 - 791.1
791.1 - 876.7
876.7 - 962.3
962.3 - 1047.9
1047.9 - 1121.0
1121.0 - 1322.0
> 1322.0
United Kingdom: Derby: DALYs Rate (per 100,000)
DALYs Rate
< 318.9
318.9 - 429.5
429.5 - 610.3
610.3 - 791.0
791.0 - 876.1
876.1 - 961.2
961.2 - 1046.3
1046.3 - 1120.7
1120.7 - 1323.2
> 1323.2
United Kingdom: Derbyshire: DALYs Rate (per 100,000)
DALYs Rate
< 320.1
320.1 - 431.4
431.4 - 609.9
609.9 - 788.4
788.4 - 874.4
874.4 - 960.5
960.5 - 1046.5
1046.5 - 1119.0
1119.0 - 1321.3
> 1321.3
United Kingdom: Leicester: DALYs Rate (per 100,000)
DALYs Rate
< 318.4
318.4 - 433.7
433.7 - 611.9
611.9 - 790.2
790.2 - 876.1
876.1 - 962.0
962.0 - 1047.9
1047.9 - 1127.1
1127.1 - 1326.7
> 1326.7
United Kingdom: Leicestershire: DALYs Rate (per 100,000)
DALYs Rate
< 320.3
320.3 - 437.2
437.2 - 613.7
613.7 - 790.2
790.2 - 876.2
876.2 - 962.2
962.2 - 1048.2
1048.2 - 1118.4
1118.4 - 1318.8
> 1318.8
United Kingdom: Lincolnshire: DALYs Rate (per 100,000)
DALYs Rate
< 320.1
320.1 - 432.7
432.7 - 609.9
609.9 - 787.1
787.1 - 874.6
874.6 - 962.2
962.2 - 1049.7
1049.7 - 1121.2
1121.2 - 1322.4
> 1322.4
Female
Male
Sexes Combined
Age
20-24 years
15-19 years
10-14 years
Year
1990
1995
2000
2005
2010
2015
2020
DALYs Rate (per 100,000)
600
800
1000
1200
Age
10-24 years

Figure S7

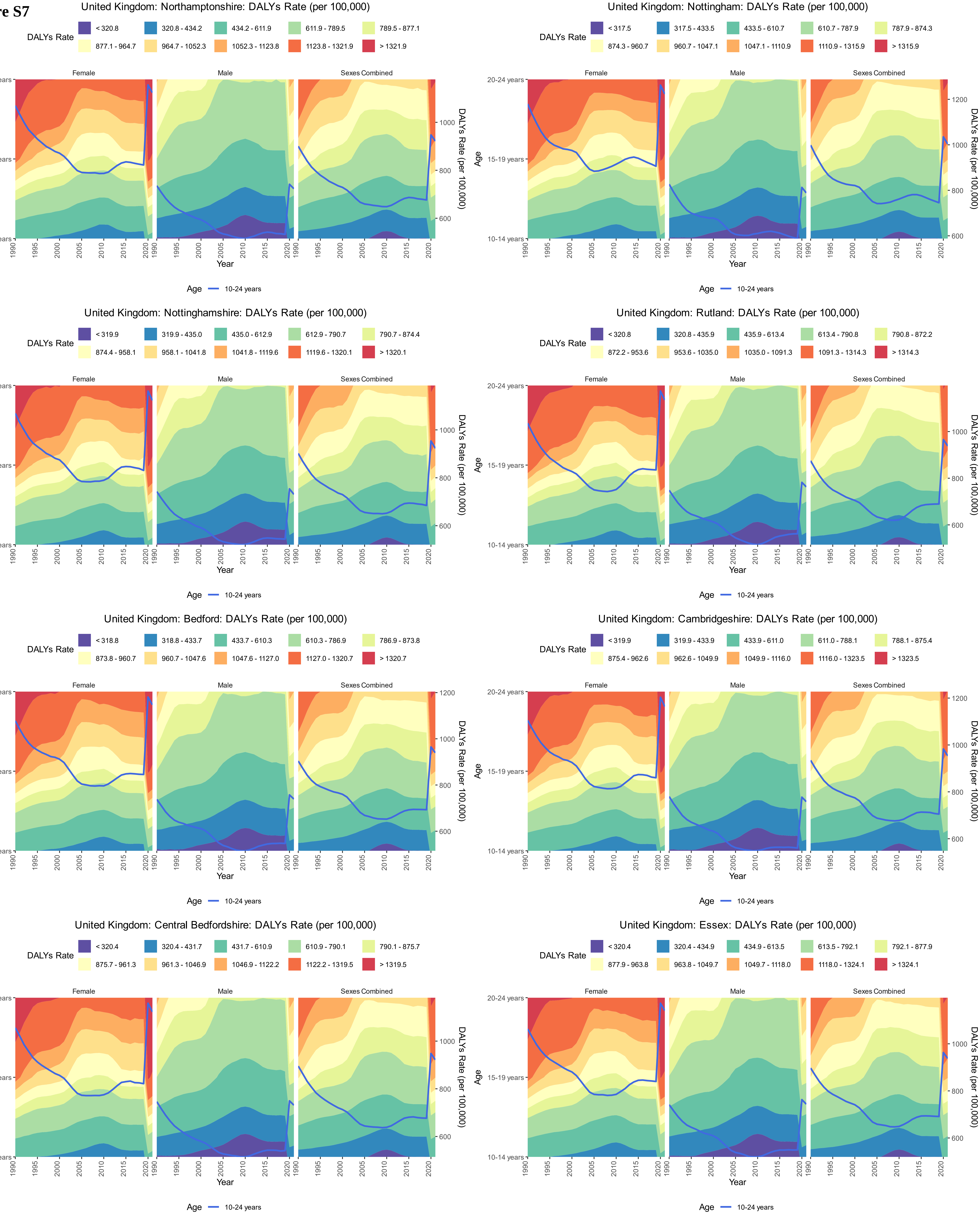

United Kingdom: Northamptonshire: DALYs Rate (per 100,000)
DALYs Rate
< 320.8
320.8 - 434.2
434.2 - 611.9
611.9 - 789.5
789.5 - 877.1
877.1 - 964.7
964.7 - 1052.3
1052.3 - 1123.8
1123.8 - 1321.9
> 1321.9
Female
Male
Sexes Combined
20-24 years
15-19 years
10-14 years
Age
Year
DALYs Rate (per 100,000)
Age
10-24 years
United Kingdom: Nottingham: DALYs Rate (per 100,000)
DALYs Rate
< 317.5
317.5 - 433.5
433.5 - 610.7
610.7 - 787.9
787.9 - 874.3
874.3 - 960.7
960.7 - 1047.1
1047.1 - 1110.9
1110.9 - 1315.9
> 1315.9
United Kingdom: Nottinghamshire: DALYs Rate (per 100,000)
DALYs Rate
< 319.9
319.9 - 435.0
435.0 - 612.9
612.9 - 790.7
790.7 - 874.4
874.4 - 958.1
958.1 - 1041.8
1041.8 - 1119.6
1119.6 - 1320.1
> 1320.1
United Kingdom: Rutland: DALYs Rate (per 100,000)
DALYs Rate
< 320.8
320.8 - 435.9
435.9 - 613.4
613.4 - 790.8
790.8 - 872.2
872.2 - 953.6
953.6 - 1035.0
1035.0 - 1091.3
1091.3 - 1314.3
> 1314.3
United Kingdom: Bedford: DALYs Rate (per 100,000)
DALYs Rate
< 318.8
318.8 - 433.7
433.7 - 610.3
610.3 - 786.9
786.9 - 873.8
873.8 - 960.7
960.7 - 1047.6
1047.6 - 1127.0
1127.0 - 1320.7
> 1320.7
United Kingdom: Cambridgeshire: DALYs Rate (per 100,000)
DALYs Rate
< 319.9
319.9 - 433.9
433.9 - 611.0
611.0 - 788.1
788.1 - 875.4
875.4 - 962.6
962.6 - 1049.9
1049.9 - 1116.0
1116.0 - 1323.5
> 1323.5
United Kingdom: Central Bedfordshire: DALYs Rate (per 100,000)
DALYs Rate
< 320.4
320.4 - 431.7
431.7 - 610.9
610.9 - 790.1
790.1 - 875.7
875.7 - 961.3
961.3 - 1046.9
1046.9 - 1122.2
1122.2 - 1319.5
> 1319.5
United Kingdom: Essex: DALYs Rate (per 100,000)
DALYs Rate
< 320.4
320.4 - 434.9
434.9 - 613.5
613.5 - 792.1
792.1 - 877.9
877.9 - 963.8
963.8 - 1049.7
1049.7 - 1118.0
1118.0 - 1324.1
> 1324.1

Figure S7

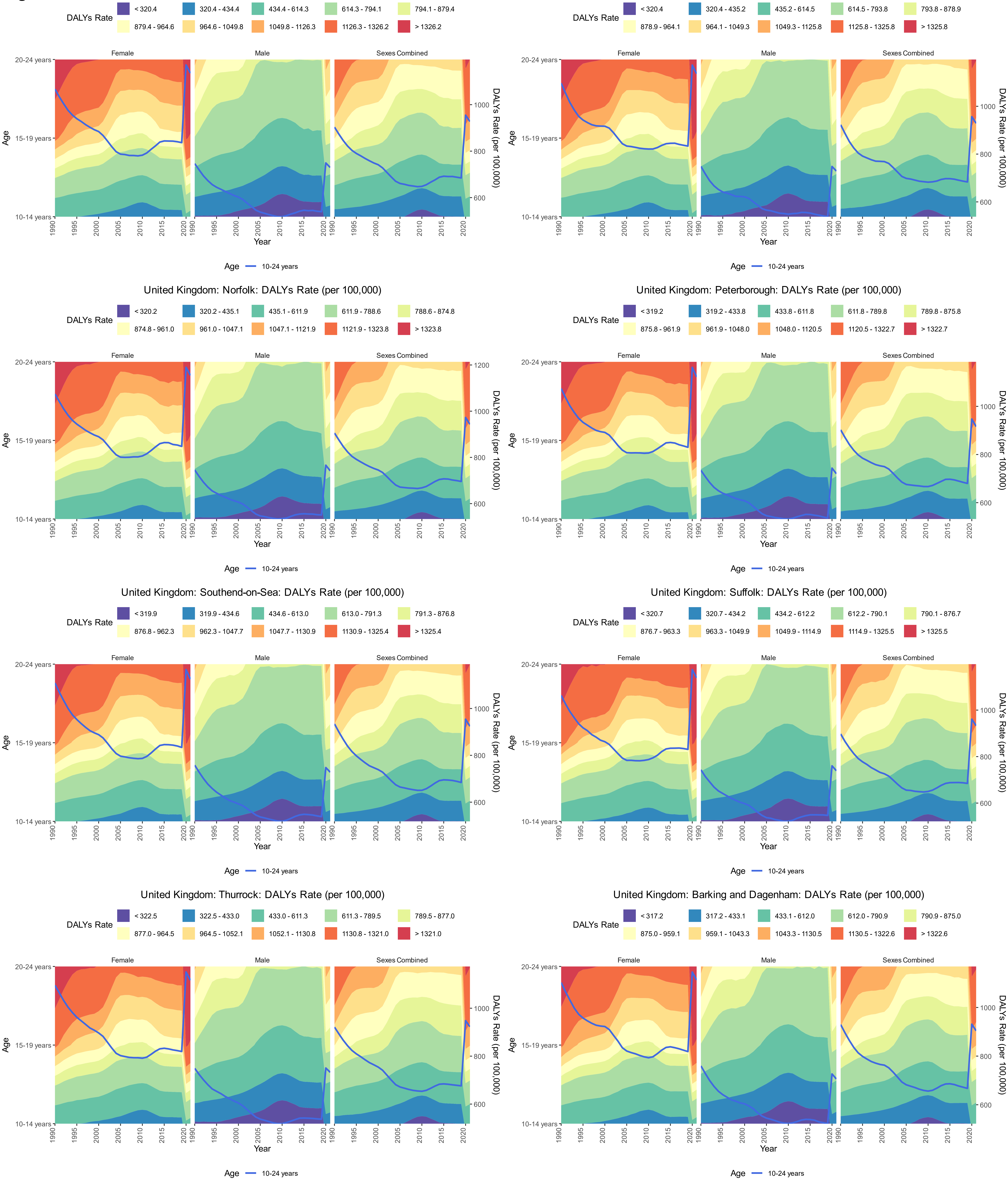
United Kingdom: Hertfordshire: DALYs Rate (per 100,000)
DALYs Rate
< 320.4
320.4 - 434.4
434.4 - 614.3
614.3 - 794.1
794.1 - 879.4
879.4 - 964.6
964.6 - 1049.8
1049.8 - 1126.3
1126.3 - 1326.2
> 1326.2
United Kingdom: Luton: DALYs Rate (per 100,000)
< 320.4
320.4 - 435.2
435.2 - 614.5
614.5 - 793.8
793.8 - 878.9
878.9 - 964.1
964.1 - 1049.3
1049.3 - 1125.8
1125.8 - 1325.8
> 1325.8
United Kingdom: Norfolk: DALYs Rate (per 100,000)
< 320.2
320.2 - 435.1
435.1 - 611.9
611.9 - 788.6
788.6 - 874.8
874.8 - 961.0
961.0 - 1047.1
1047.1 - 1121.9
1121.9 - 1323.8
> 1323.8
United Kingdom: Peterborough: DALYs Rate (per 100,000)
< 319.2
319.2 - 433.8
433.8 - 611.8
611.8 - 789.8
789.8 - 875.8
875.8 - 961.9
961.9 - 1048.0
1048.0 - 1120.5
1120.5 - 1322.7
> 1322.7
United Kingdom: Southend-on-Sea: DALYs Rate (per 100,000)
< 319.9
319.9 - 434.6
434.6 - 613.0
613.0 - 791.3
791.3 - 876.8
876.8 - 962.3
962.3 - 1047.7
1047.7 - 1130.9
1130.9 - 1325.4
> 1325.4
United Kingdom: Suffolk: DALYs Rate (per 100,000)
< 320.7
320.7 - 434.2
434.2 - 612.2
612.2 - 790.1
790.1 - 876.7
876.7 - 963.3
963.3 - 1049.9
1049.9 - 1114.9
1114.9 - 1325.5
> 1325.5
United Kingdom: Thurrock: DALYs Rate (per 100,000)
< 322.5
322.5 - 433.0
433.0 - 611.3
611.3 - 789.5
789.5 - 877.0
877.0 - 964.5
964.5 - 1052.1
1052.1 - 1130.8
1130.8 - 1321.0
> 1321.0
United Kingdom: Barking and Dagenham: DALYs Rate (per 100,000)
< 317.2
317.2 - 433.1
433.1 - 612.0
612.0 - 790.9
790.9 - 875.0
875.0 - 959.1
959.1 - 1043.3
1043.3 - 1130.5
1130.5 - 1322.6
> 1322.6
Female
Male
Sexes Combined
Age
20-24 years
15-19 years
10-14 years
Year
1990
1995
2000
2005
2010
2015
2020
DALYs Rate (per 100,000)
600
800
1000
1200
Age
10-24 years

Figure S7

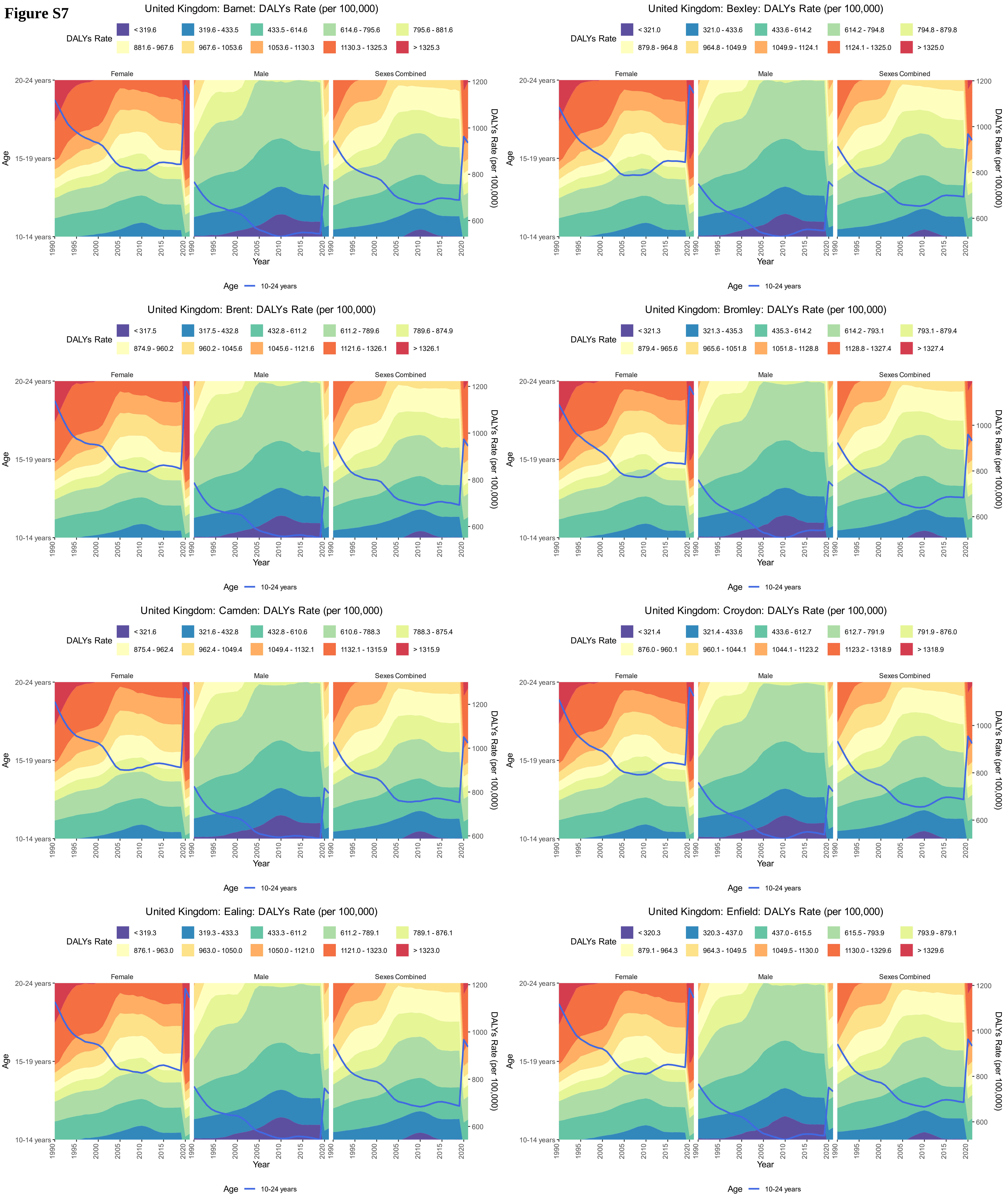
United Kingdom: Barnet: DALYs Rate (per 100,000)
DALYs Rate
< 319.6
319.6 - 433.5
433.5 - 614.6
614.6 - 795.6
795.6 - 881.6
881.6 - 967.6
967.6 - 1053.6
1053.6 - 1130.3
1130.3 - 1325.3
> 1325.3
Female
Male
Sexes Combined
Age
Year
DALYs Rate (per 100,000)
Age — 10-24 years
United Kingdom: Bexley: DALYs Rate (per 100,000)
< 321.0
321.0 - 433.6
433.6 - 614.2
614.2 - 794.8
794.8 - 879.8
879.8 - 964.8
964.8 - 1049.9
1049.9 - 1124.1
1124.1 - 1325.0
> 1325.0
United Kingdom: Brent: DALYs Rate (per 100,000)
< 317.5
317.5 - 432.8
432.8 - 611.2
611.2 - 789.6
789.6 - 874.9
874.9 - 960.2
960.2 - 1045.6
1045.6 - 1121.6
1121.6 - 1326.1
> 1326.1
United Kingdom: Bromley: DALYs Rate (per 100,000)
< 321.3
321.3 - 435.3
435.3 - 614.2
614.2 - 793.1
793.1 - 879.4
879.4 - 965.6
965.6 - 1051.8
1051.8 - 1128.8
1128.8 - 1327.4
> 1327.4
United Kingdom: Camden: DALYs Rate (per 100,000)
< 321.6
321.6 - 432.8
432.8 - 610.6
610.6 - 788.3
788.3 - 875.4
875.4 - 962.4
962.4 - 1049.4
1049.4 - 1132.1
1132.1 - 1315.9
> 1315.9
United Kingdom: Croydon: DALYs Rate (per 100,000)
< 321.4
321.4 - 433.6
433.6 - 612.7
612.7 - 791.9
791.9 - 876.0
876.0 - 960.1
960.1 - 1044.1
1044.1 - 1123.2
1123.2 - 1318.9
> 1318.9
United Kingdom: Ealing: DALYs Rate (per 100,000)
< 319.3
319.3 - 433.3
433.3 - 611.2
611.2 - 789.1
789.1 - 876.1
876.1 - 963.0
963.0 - 1050.0
1050.0 - 1121.0
1121.0 - 1323.0
> 1323.0
United Kingdom: Enfield: DALYs Rate (per 100,000)
< 320.3
320.3 - 437.0
437.0 - 615.5
615.5 - 793.9
793.9 - 879.1
879.1 - 964.3
964.3 - 1049.5
1049.5 - 1130.0
1130.0 - 1329.6
> 1329.6
20-24 years
15-19 years
10-14 years
1990
1995
2000
2005
2010
2015
2020

Figure S7

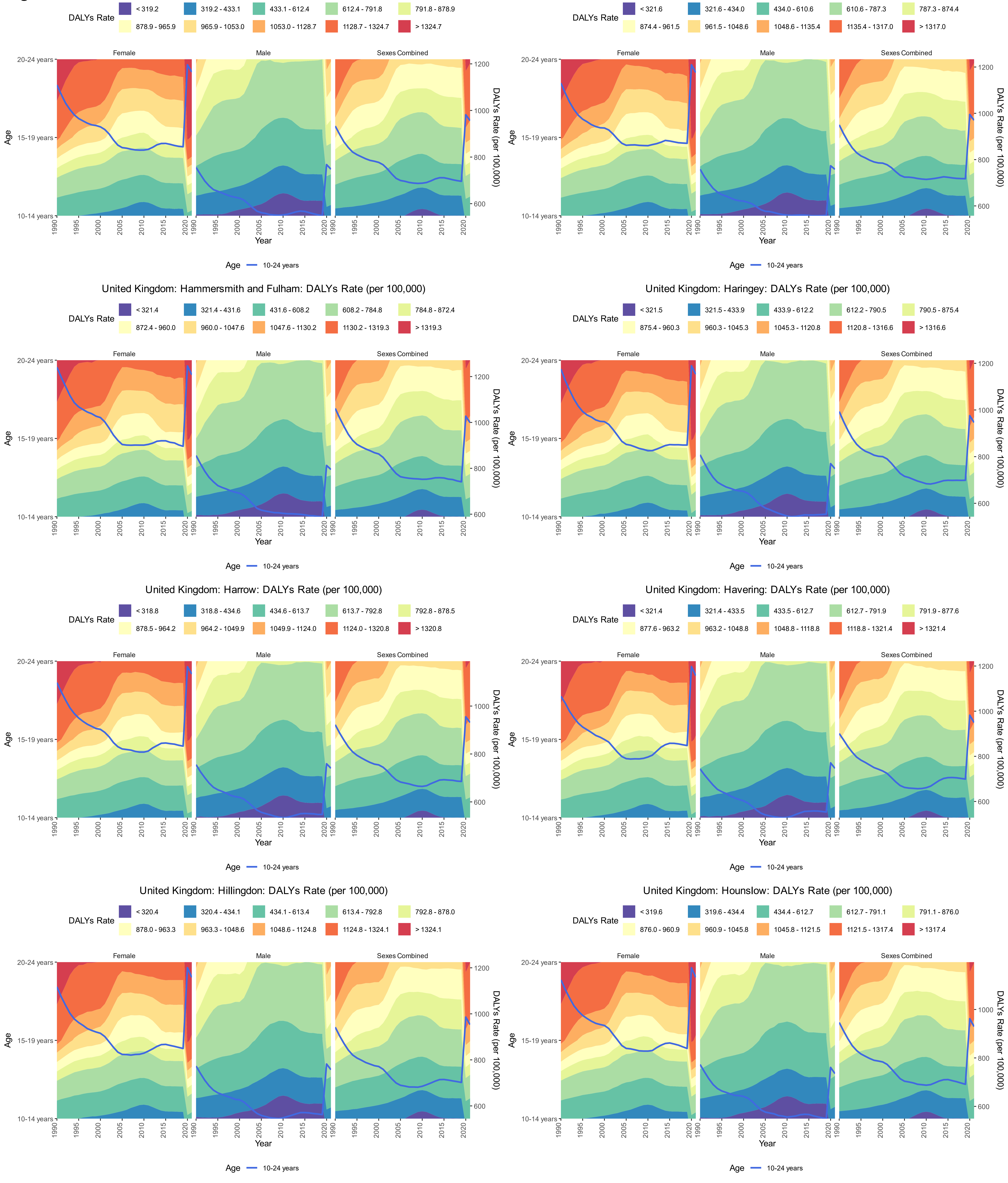

United Kingdom: Greenwich: DALYs Rate (per 100,000)
DALYs Rate
< 319.2
319.2 - 433.1
433.1 - 612.4
612.4 - 791.8
791.8 - 878.9
878.9 - 965.9
965.9 - 1053.0
1053.0 - 1128.7
1128.7 - 1324.7
> 1324.7
Female
Male
Sexes Combined
Age
Year
DALYs Rate (per 100,000)
Age — 10-24 years
United Kingdom: Hackney: DALYs Rate (per 100,000)
DALYs Rate
< 321.6
321.6 - 434.0
434.0 - 610.6
610.6 - 787.3
787.3 - 874.4
874.4 - 961.5
961.5 - 1048.6
1048.6 - 1135.4
1135.4 - 1317.0
> 1317.0
Female
Male
Sexes Combined
Age
Year
DALYs Rate (per 100,000)
Age — 10-24 years
United Kingdom: Hammersmith and Fulham: DALYs Rate (per 100,000)
DALYs Rate
< 321.4
321.4 - 431.6
431.6 - 608.2
608.2 - 784.8
784.8 - 872.4
872.4 - 960.0
960.0 - 1047.6
1047.6 - 1130.2
1130.2 - 1319.3
> 1319.3
Female
Male
Sexes Combined
Age
Year
DALYs Rate (per 100,000)
Age — 10-24 years
United Kingdom: Haringey: DALYs Rate (per 100,000)
DALYs Rate
< 321.5
321.5 - 433.9
433.9 - 612.2
612.2 - 790.5
790.5 - 875.4
875.4 - 960.3
960.3 - 1045.3
1045.3 - 1120.8
1120.8 - 1316.6
> 1316.6
Female
Male
Sexes Combined
Age
Year
DALYs Rate (per 100,000)
Age — 10-24 years
United Kingdom: Harrow: DALYs Rate (per 100,000)
DALYs Rate
< 318.8
318.8 - 434.6
434.6 - 613.7
613.7 - 792.8
792.8 - 878.5
878.5 - 964.2
964.2 - 1049.9
1049.9 - 1124.0
1124.0 - 1320.8
> 1320.8
Female
Male
Sexes Combined
Age
Year
DALYs Rate (per 100,000)
Age — 10-24 years
United Kingdom: Havering: DALYs Rate (per 100,000)
DALYs Rate
< 321.4
321.4 - 433.5
433.5 - 612.7
612.7 - 791.9
791.9 - 877.6
877.6 - 963.2
963.2 - 1048.8
1048.8 - 1118.8
1118.8 - 1321.4
> 1321.4
Female
Male
Sexes Combined
Age
Year
DALYs Rate (per 100,000)
Age — 10-24 years
United Kingdom: Hillingdon: DALYs Rate (per 100,000)
DALYs Rate
< 320.4
320.4 - 434.1
434.1 - 613.4
613.4 - 792.8
792.8 - 878.0
878.0 - 963.3
963.3 - 1048.6
1048.6 - 1124.8
1124.8 - 1324.1
> 1324.1
Female
Male
Sexes Combined
Age
Year
DALYs Rate (per 100,000)
Age — 10-24 years
United Kingdom: Hounslow: DALYs Rate (per 100,000)
DALYs Rate
< 319.6
319.6 - 434.4
434.4 - 612.7
612.7 - 791.1
791.1 - 876.0
876.0 - 960.9
960.9 - 1045.8
1045.8 - 1121.5
1121.5 - 1317.4
> 1317.4
Female
Male
Sexes Combined
Age
Year
DALYs Rate (per 100,000)
Age — 10-24 years

Figure S7

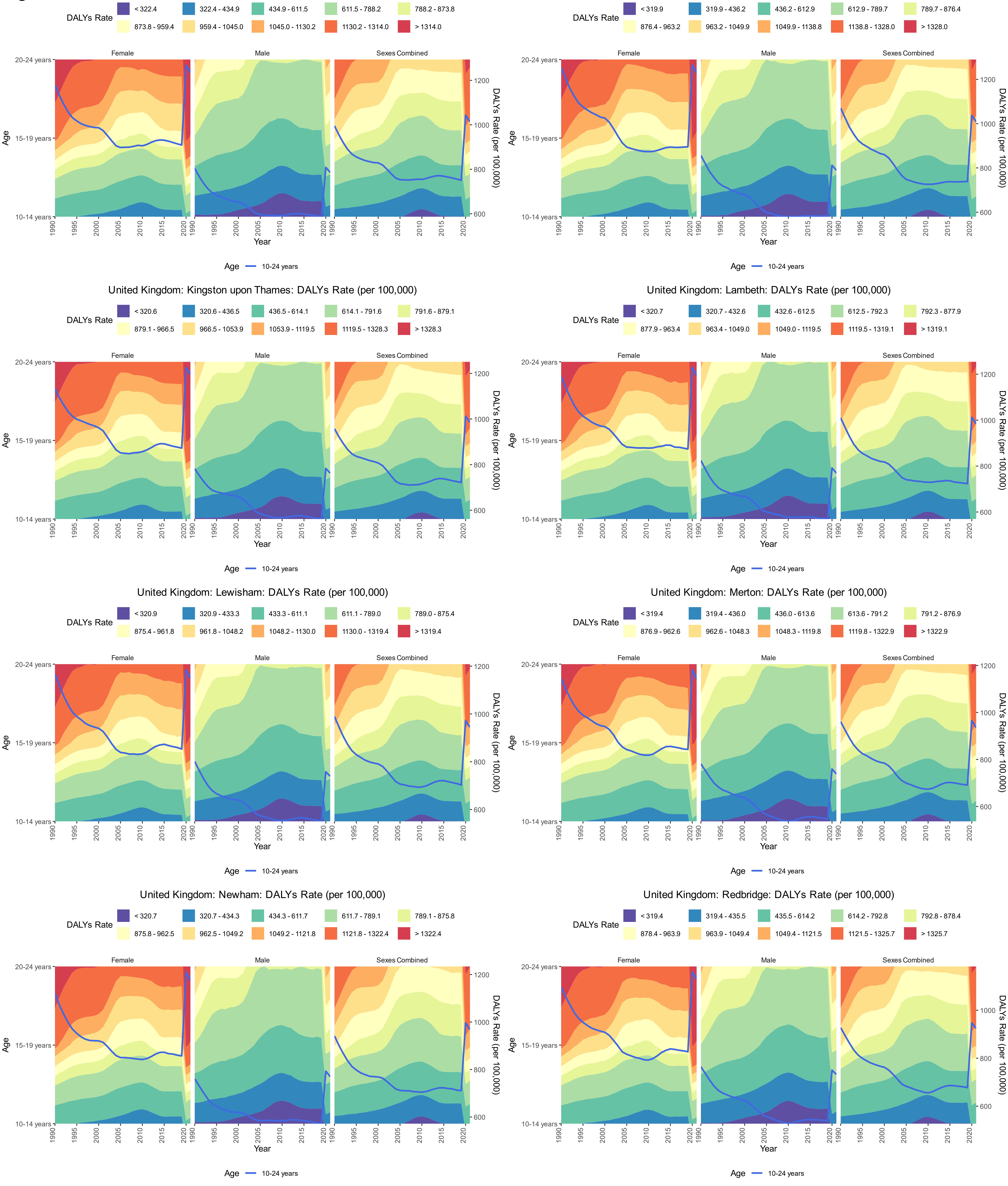

United Kingdom: Islington: DALYs Rate (per 100,000)
DALYs Rate
< 322.4
322.4 - 434.9
434.9 - 611.5
611.5 - 788.2
788.2 - 873.8
873.8 - 959.4
959.4 - 1045.0
1045.0 - 1130.2
1130.2 - 1314.0
> 1314.0
Female
Male
Sexes Combined
Age
Year
DALYs Rate (per 100,000)
Age 10-24 years
United Kingdom: Kensington and Chelsea: DALYs Rate (per 100,000)
< 319.9
319.9 - 436.2
436.2 - 612.9
612.9 - 789.7
789.7 - 876.4
876.4 - 963.2
963.2 - 1049.9
1049.9 - 1138.8
1138.8 - 1328.0
> 1328.0
United Kingdom: Kingston upon Thames: DALYs Rate (per 100,000)
< 320.6
320.6 - 436.5
436.5 - 614.1
614.1 - 791.6
791.6 - 879.1
879.1 - 966.5
966.5 - 1053.9
1053.9 - 1119.5
1119.5 - 1328.3
> 1328.3
United Kingdom: Lambeth: DALYs Rate (per 100,000)
< 320.7
320.7 - 432.6
432.6 - 612.5
612.5 - 792.3
792.3 - 877.9
877.9 - 963.4
963.4 - 1049.0
1049.0 - 1119.5
1119.5 - 1319.1
> 1319.1
United Kingdom: Lewisham: DALYs Rate (per 100,000)
< 320.9
320.9 - 433.3
433.3 - 611.1
611.1 - 789.0
789.0 - 875.4
875.4 - 961.8
961.8 - 1048.2
1048.2 - 1130.0
1130.0 - 1319.4
> 1319.4
United Kingdom: Merton: DALYs Rate (per 100,000)
< 319.4
319.4 - 436.0
436.0 - 613.6
613.6 - 791.2
791.2 - 876.9
876.9 - 962.6
962.6 - 1048.3
1048.3 - 1119.8
1119.8 - 1322.9
> 1322.9
United Kingdom: Newham: DALYs Rate (per 100,000)
< 320.7
320.7 - 434.3
434.3 - 611.7
611.7 - 789.1
789.1 - 875.8
875.8 - 962.5
962.5 - 1049.2
1049.2 - 1121.8
1121.8 - 1322.4
> 1322.4
United Kingdom: Redbridge: DALYs Rate (per 100,000)
< 319.4
319.4 - 435.5
435.5 - 614.2
614.2 - 792.8
792.8 - 878.4
878.4 - 963.9
963.9 - 1049.4
1049.4 - 1121.5
1121.5 - 1325.7
> 1325.7

Figure S7

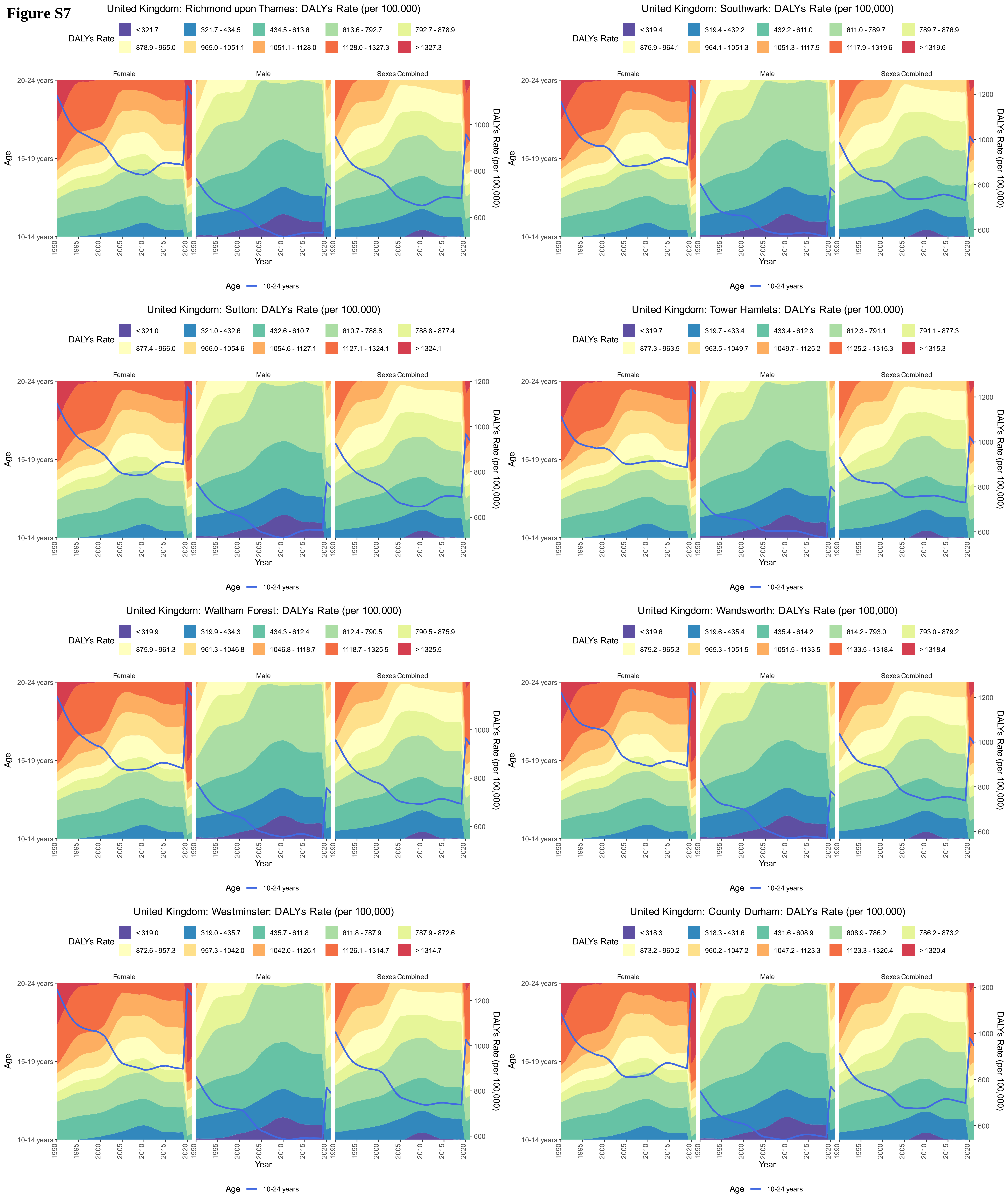
United Kingdom: Richmond upon Thames: DALYs Rate (per 100,000)
DALYs Rate
< 321.7
321.7 - 434.5
434.5 - 613.6
613.6 - 792.7
792.7 - 878.9
878.9 - 965.0
965.0 - 1051.1
1051.1 - 1128.0
1128.0 - 1327.3
> 1327.3
United Kingdom: Southwark: DALYs Rate (per 100,000)
< 319.4
319.4 - 432.2
432.2 - 611.0
611.0 - 789.7
789.7 - 876.9
876.9 - 964.1
964.1 - 1051.3
1051.3 - 1117.9
1117.9 - 1319.6
> 1319.6
United Kingdom: Sutton: DALYs Rate (per 100,000)
< 321.0
321.0 - 432.6
432.6 - 610.7
610.7 - 788.8
788.8 - 877.4
877.4 - 966.0
966.0 - 1054.6
1054.6 - 1127.1
1127.1 - 1324.1
> 1324.1
United Kingdom: Tower Hamlets: DALYs Rate (per 100,000)
< 319.7
319.7 - 433.4
433.4 - 612.3
612.3 - 791.1
791.1 - 877.3
877.3 - 963.5
963.5 - 1049.7
1049.7 - 1125.2
1125.2 - 1315.3
> 1315.3
United Kingdom: Waltham Forest: DALYs Rate (per 100,000)
< 319.9
319.9 - 434.3
434.3 - 612.4
612.4 - 790.5
790.5 - 875.9
875.9 - 961.3
961.3 - 1046.8
1046.8 - 1118.7
1118.7 - 1325.5
> 1325.5
United Kingdom: Wandsworth: DALYs Rate (per 100,000)
< 319.6
319.6 - 435.4
435.4 - 614.2
614.2 - 793.0
793.0 - 879.2
879.2 - 965.3
965.3 - 1051.5
1051.5 - 1133.5
1133.5 - 1318.4
> 1318.4
United Kingdom: Westminster: DALYs Rate (per 100,000)
< 319.0
319.0 - 435.7
435.7 - 611.8
611.8 - 787.9
787.9 - 872.6
872.6 - 957.3
957.3 - 1042.0
1042.0 - 1126.1
1126.1 - 1314.7
> 1314.7
United Kingdom: County Durham: DALYs Rate (per 100,000)
< 318.3
318.3 - 431.6
431.6 - 608.9
608.9 - 786.2
786.2 - 873.2
873.2 - 960.2
960.2 - 1047.2
1047.2 - 1123.3
1123.3 - 1320.4
> 1320.4
Female
Male
Sexes Combined
Age
20-24 years
15-19 years
10-14 years
Year
DALYs Rate (per 100,000)
Age
10-24 years

Figure S7

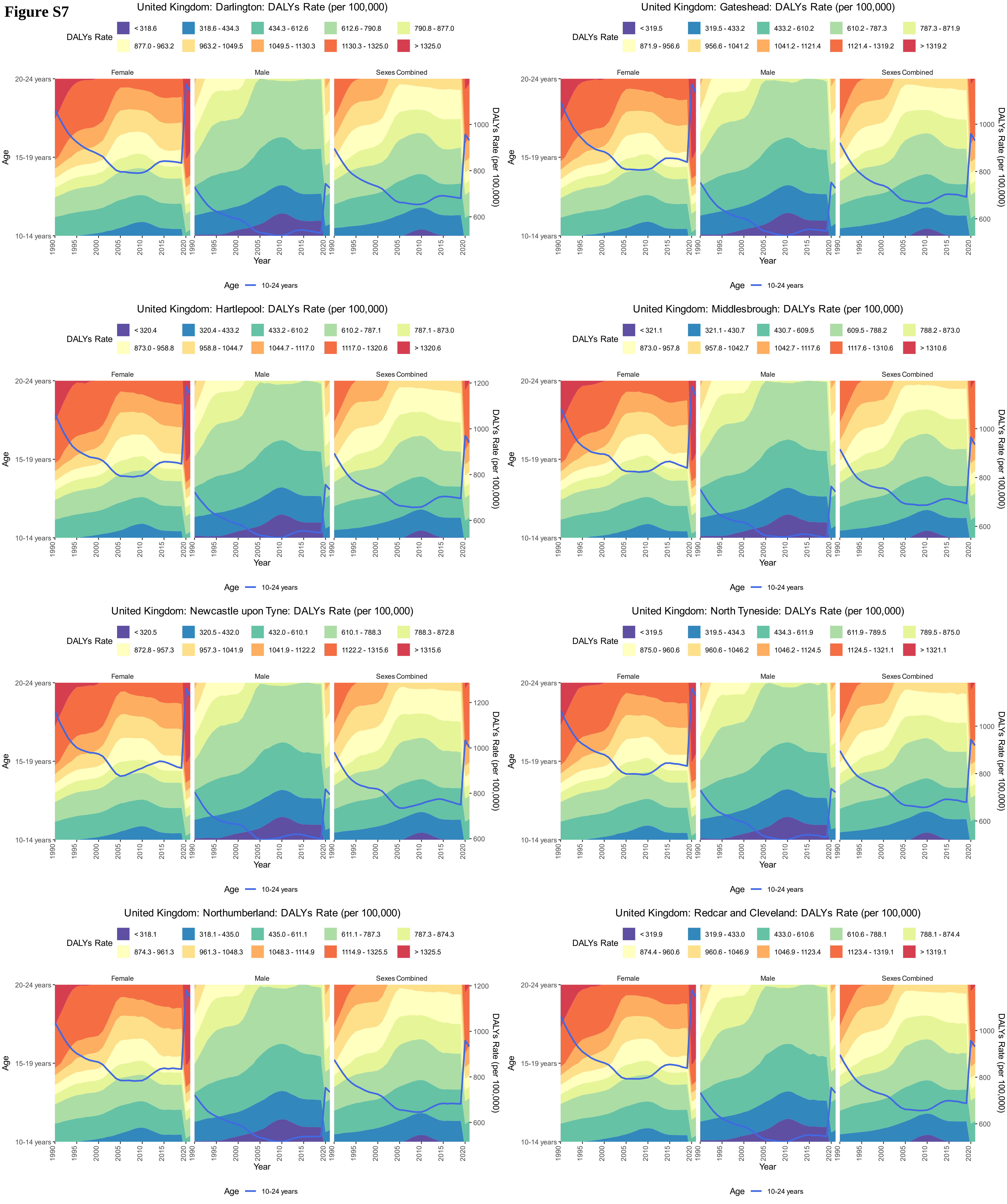
United Kingdom: Darlington: DALYs Rate (per 100,000)
DALYs Rate
< 318.6
318.6 - 434.3
434.3 - 612.6
612.6 - 790.8
790.8 - 877.0
877.0 - 963.2
963.2 - 1049.5
1049.5 - 1130.3
1130.3 - 1325.0
> 1325.0
United Kingdom: Gateshead: DALYs Rate (per 100,000)
DALYs Rate
< 319.5
319.5 - 433.2
433.2 - 610.2
610.2 - 787.3
787.3 - 871.9
871.9 - 956.6
956.6 - 1041.2
1041.2 - 1121.4
1121.4 - 1319.2
> 1319.2
United Kingdom: Hartlepool: DALYs Rate (per 100,000)
DALYs Rate
< 320.4
320.4 - 433.2
433.2 - 610.2
610.2 - 787.1
787.1 - 873.0
873.0 - 958.8
958.8 - 1044.7
1044.7 - 1117.0
1117.0 - 1320.6
> 1320.6
United Kingdom: Middlesbrough: DALYs Rate (per 100,000)
DALYs Rate
< 321.1
321.1 - 430.7
430.7 - 609.5
609.5 - 788.2
788.2 - 873.0
873.0 - 957.8
957.8 - 1042.7
1042.7 - 1117.6
1117.6 - 1310.6
> 1310.6
United Kingdom: Newcastle upon Tyne: DALYs Rate (per 100,000)
DALYs Rate
< 320.5
320.5 - 432.0
432.0 - 610.1
610.1 - 788.3
788.3 - 872.8
872.8 - 957.3
957.3 - 1041.9
1041.9 - 1122.2
1122.2 - 1315.6
> 1315.6
United Kingdom: North Tyneside: DALYs Rate (per 100,000)
DALYs Rate
< 319.5
319.5 - 434.3
434.3 - 611.9
611.9 - 789.5
789.5 - 875.0
875.0 - 960.6
960.6 - 1046.2
1046.2 - 1124.5
1124.5 - 1321.1
> 1321.1
United Kingdom: Northumberland: DALYs Rate (per 100,000)
DALYs Rate
< 318.1
318.1 - 435.0
435.0 - 611.1
611.1 - 787.3
787.3 - 874.3
874.3 - 961.3
961.3 - 1048.3
1048.3 - 1114.9
1114.9 - 1325.5
> 1325.5
United Kingdom: Redcar and Cleveland: DALYs Rate (per 100,000)
DALYs Rate
< 319.9
319.9 - 433.0
433.0 - 610.6
610.6 - 788.1
788.1 - 874.4
874.4 - 960.6
960.6 - 1046.9
1046.9 - 1123.4
1123.4 - 1319.1
> 1319.1
Female
Male
Sexes Combined
Age
20-24 years
15-19 years
10-14 years
Year
DALYs Rate (per 100,000)
Age
10-24 years

Figure S7

United Kingdom: South Tyneside: DALYs Rate (per 100,000)

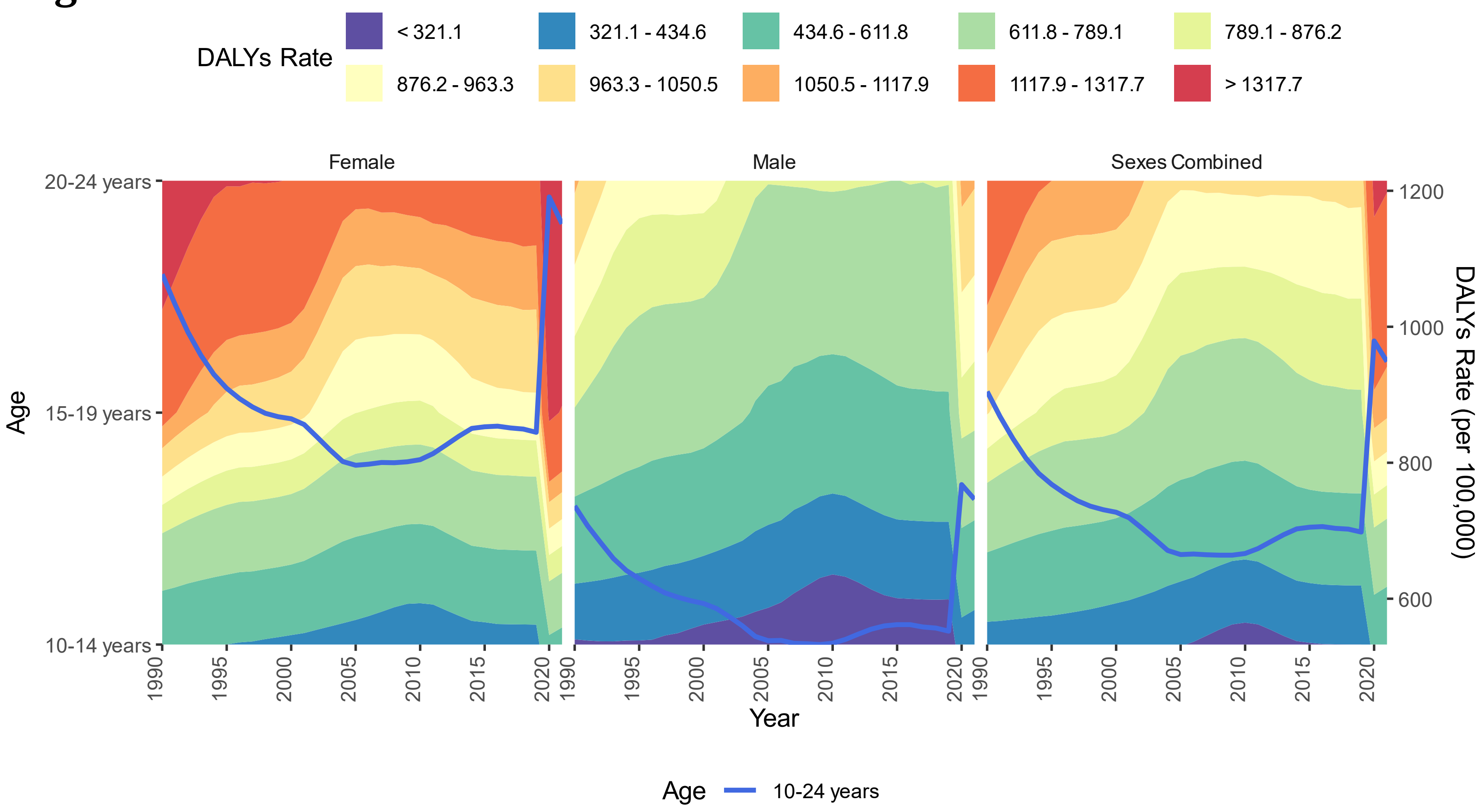

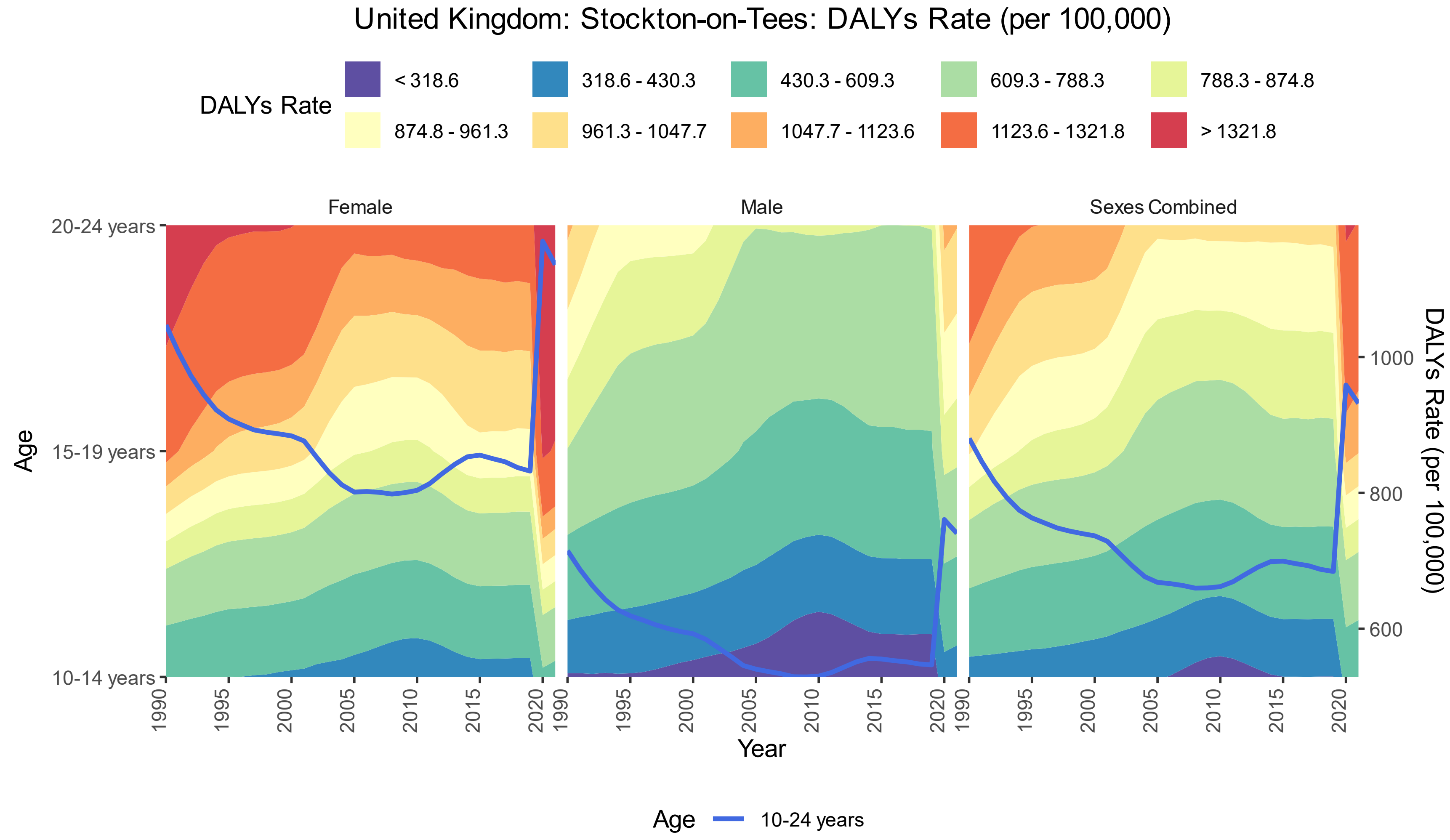

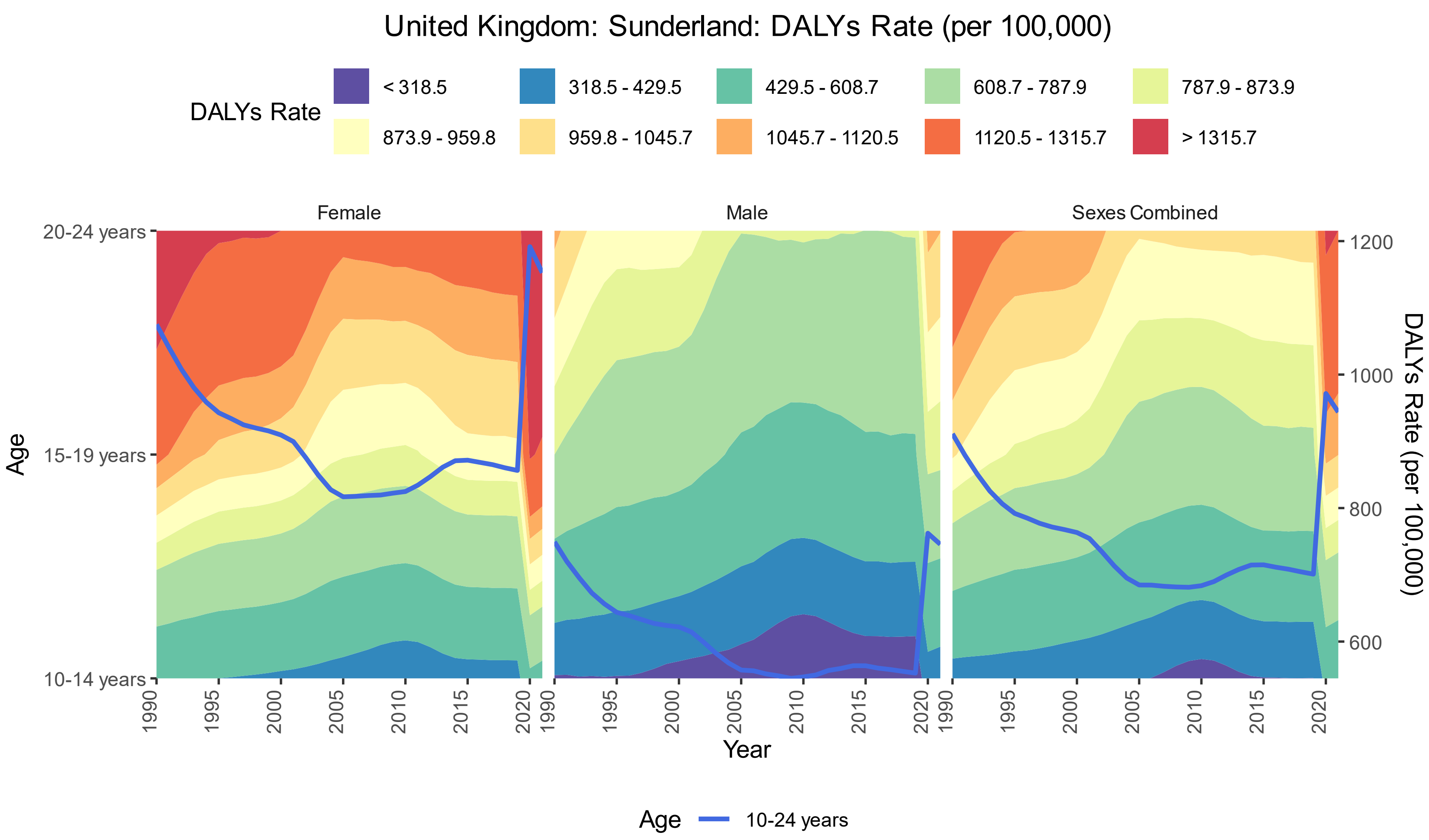

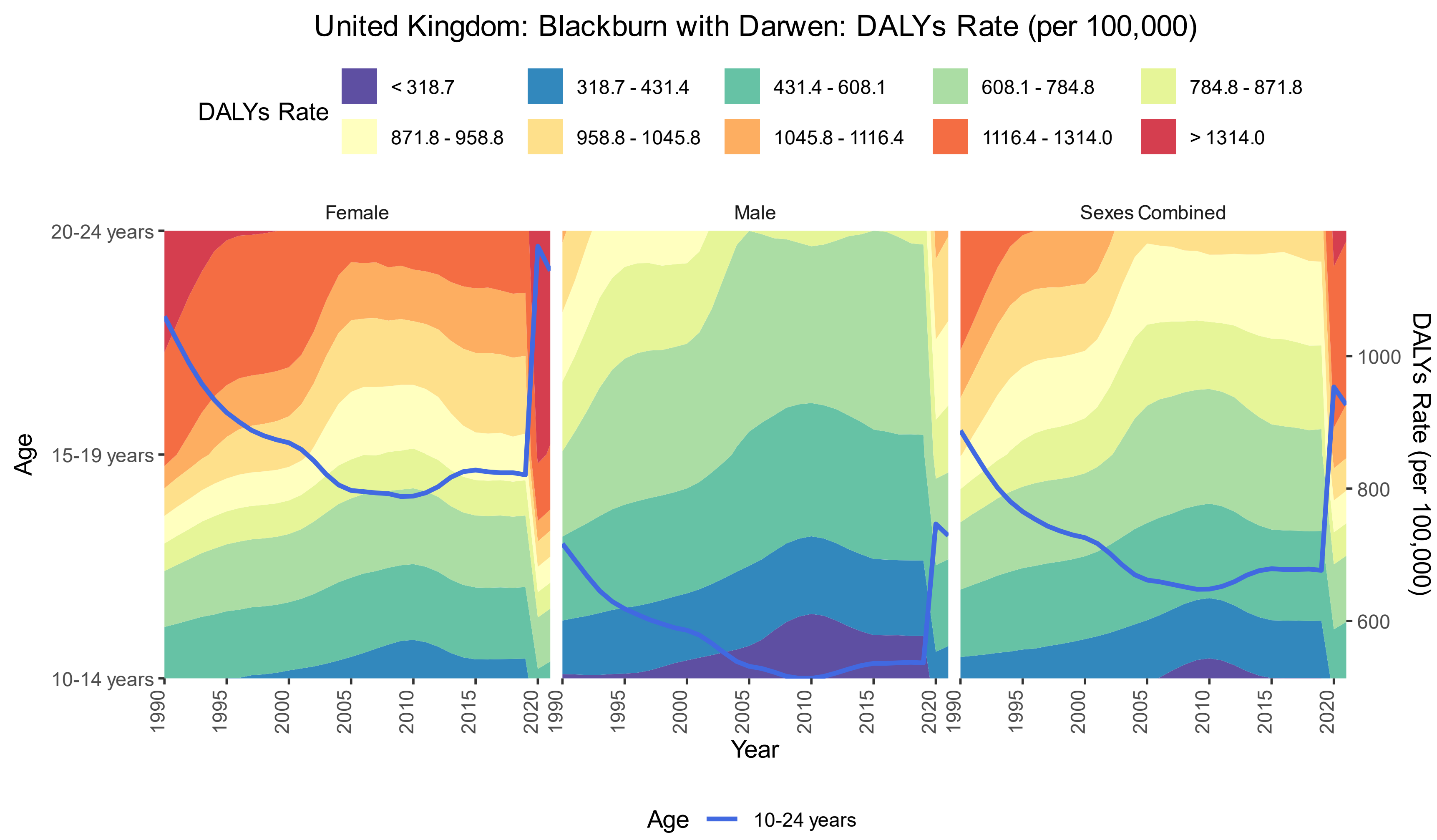

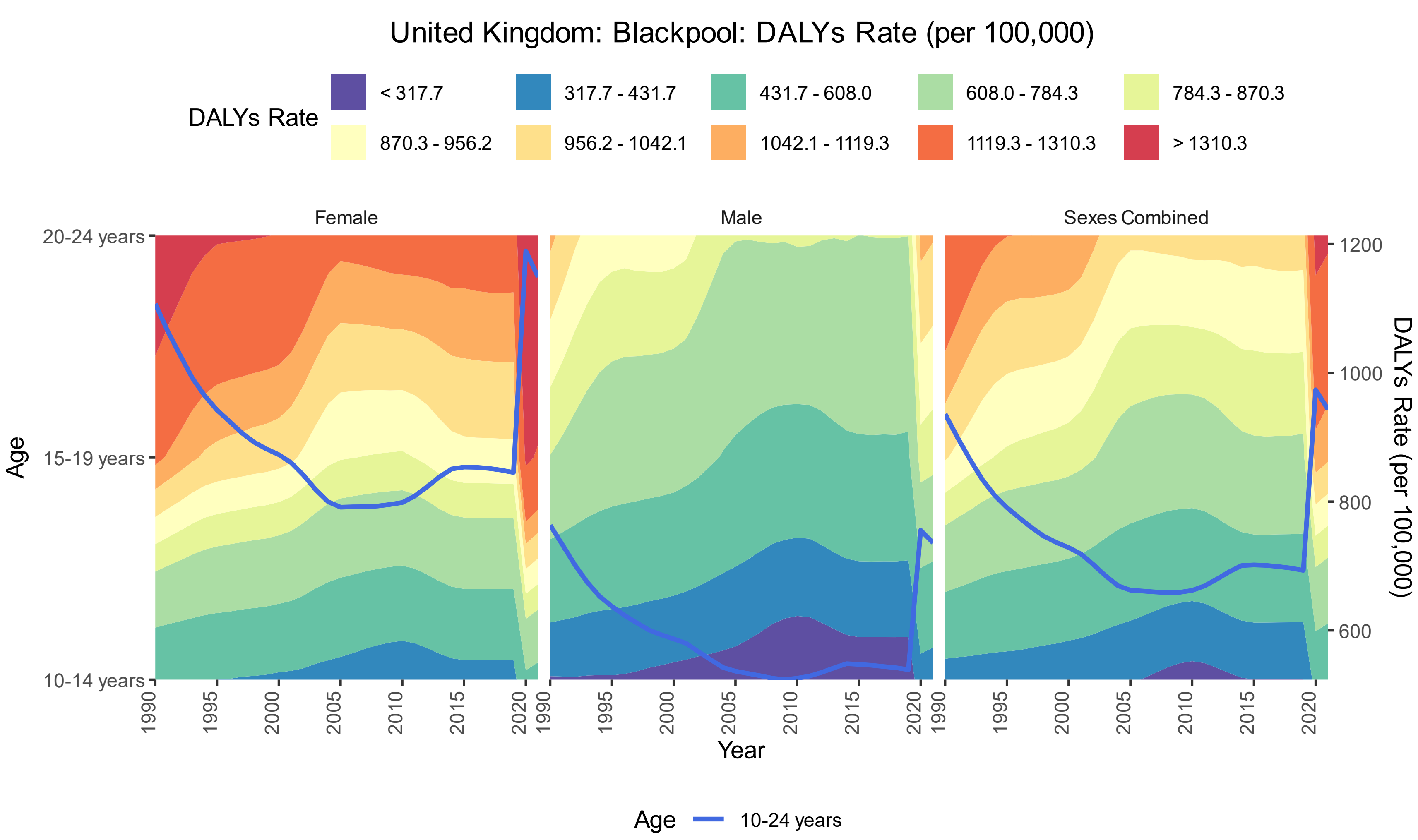

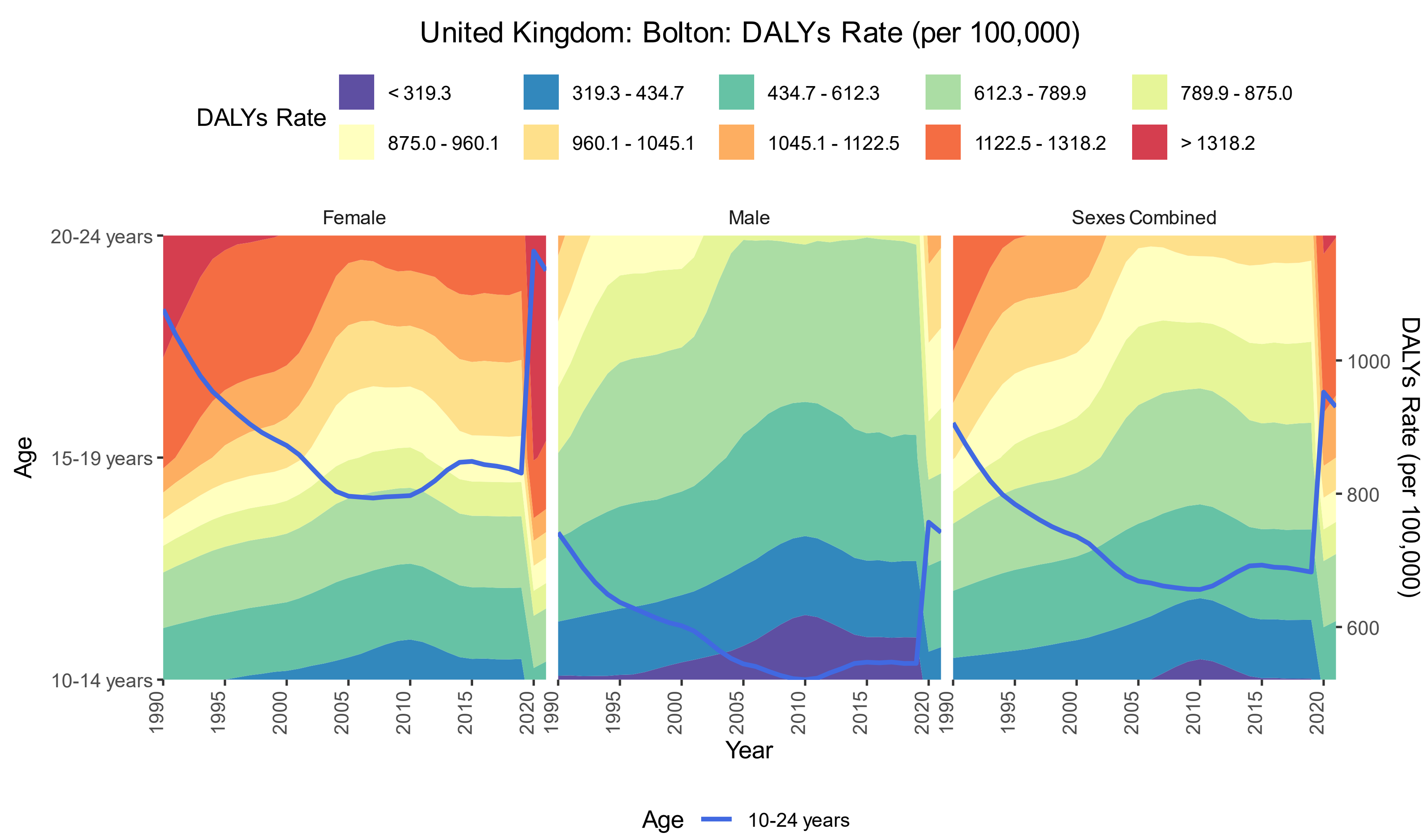

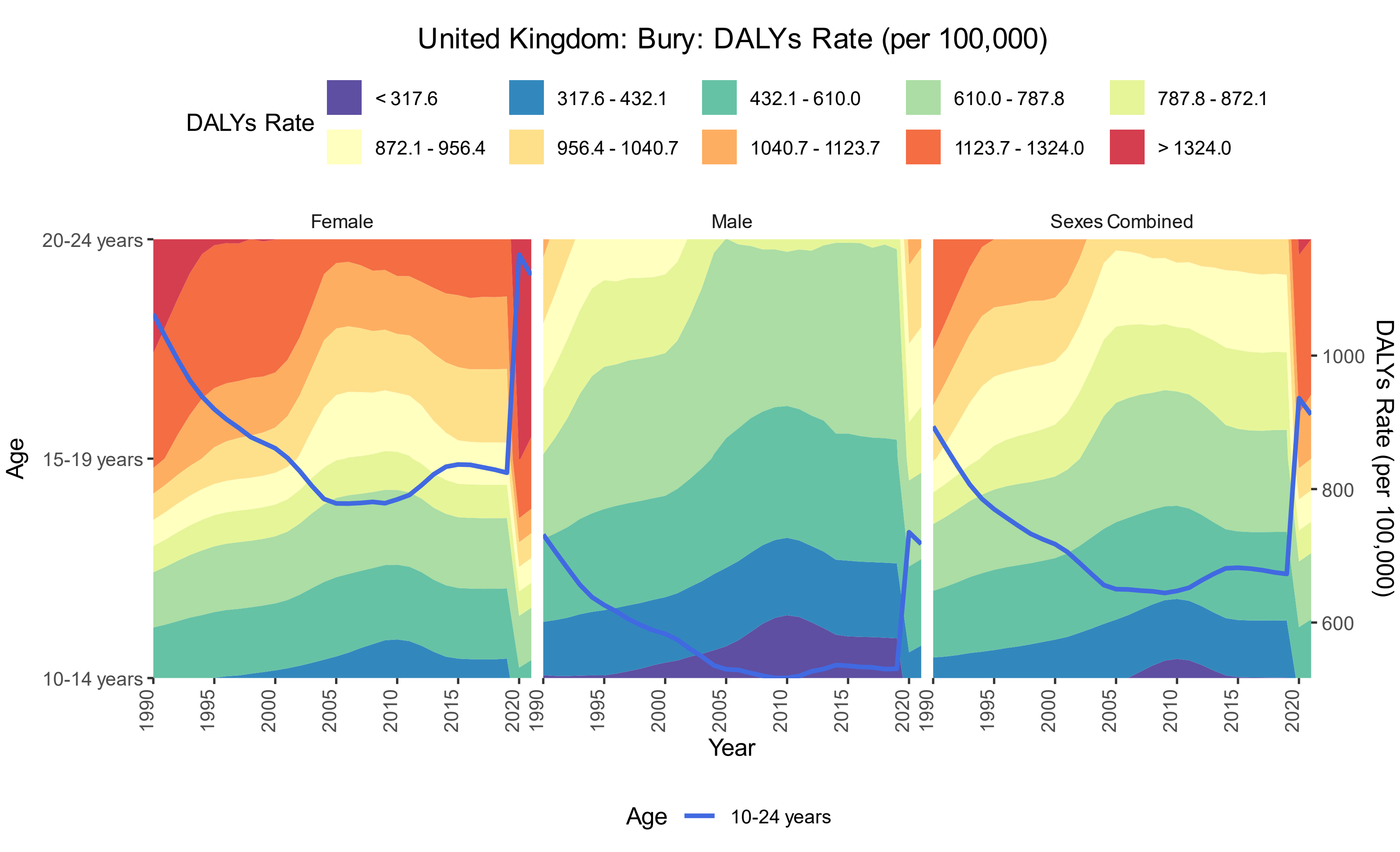

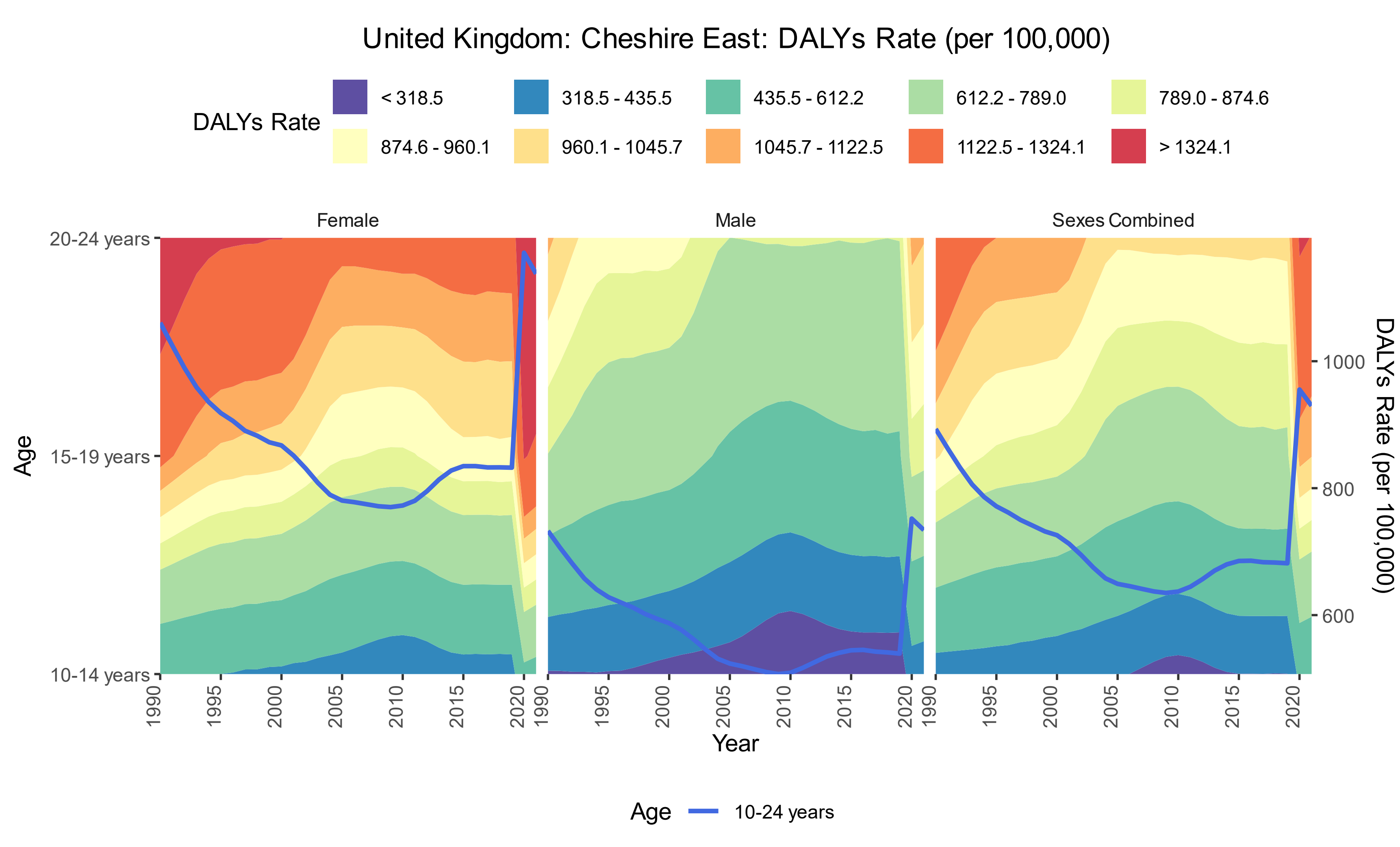

Figure S7

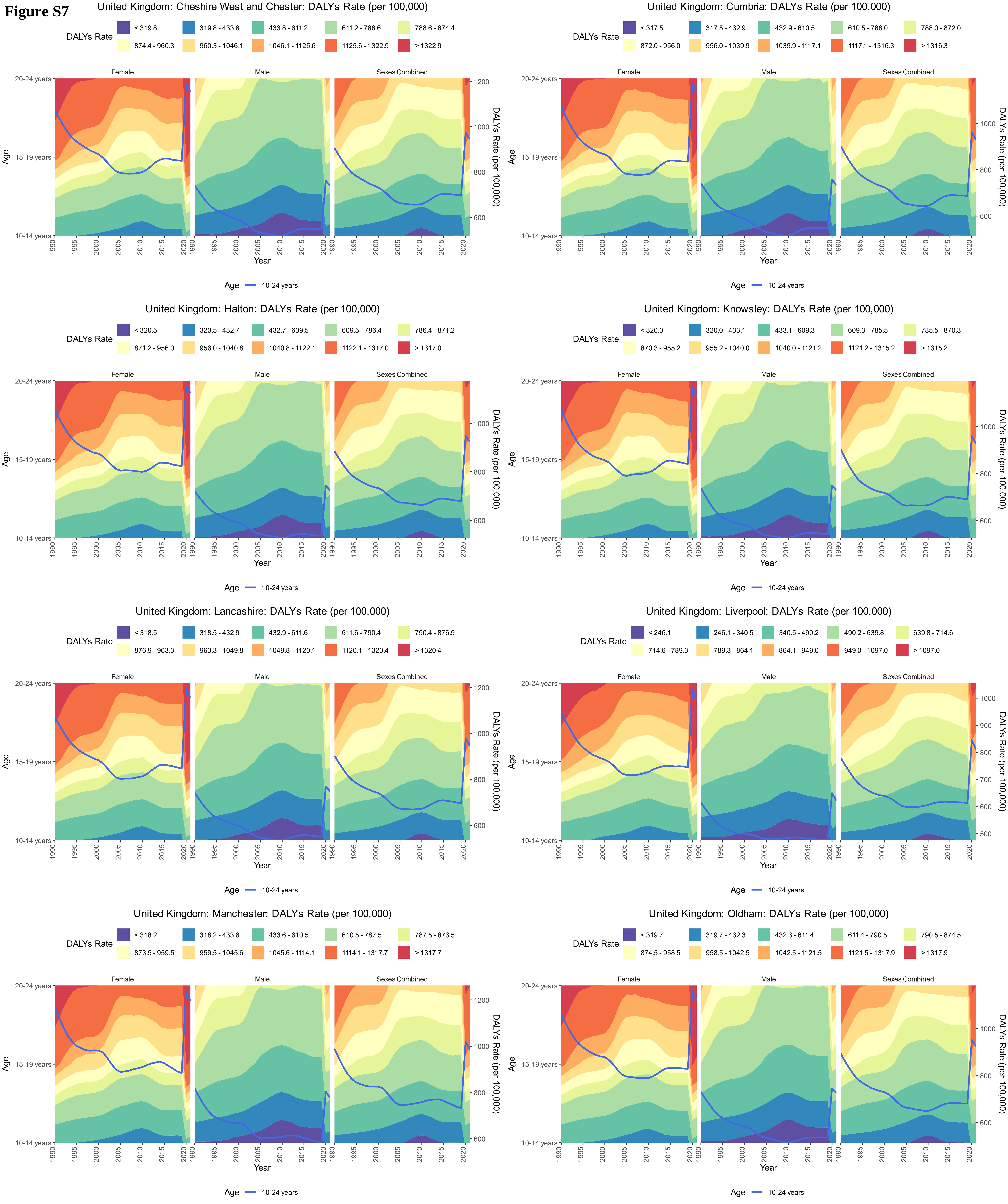
United Kingdom: Cheshire West and Chester: DALYs Rate (per 100,000)
DALYs Rate
< 319.8
319.8 - 433.8
433.8 - 611.2
611.2 - 788.6
788.6 - 874.4
874.4 - 960.3
960.3 - 1046.1
1046.1 - 1125.6
1125.6 - 1322.9
> 1322.9
United Kingdom: Cumbria: DALYs Rate (per 100,000)
DALYs Rate
< 317.5
317.5 - 432.9
432.9 - 610.5
610.5 - 788.0
788.0 - 872.0
872.0 - 956.0
956.0 - 1039.9
1039.9 - 1117.1
1117.1 - 1316.3
> 1316.3
United Kingdom: Halton: DALYs Rate (per 100,000)
DALYs Rate
< 320.5
320.5 - 432.7
432.7 - 609.5
609.5 - 786.4
786.4 - 871.2
871.2 - 956.0
956.0 - 1040.8
1040.8 - 1122.1
1122.1 - 1317.0
> 1317.0
United Kingdom: Knowsley: DALYs Rate (per 100,000)
DALYs Rate
< 320.0
320.0 - 433.1
433.1 - 609.3
609.3 - 785.5
785.5 - 870.3
870.3 - 955.2
955.2 - 1040.0
1040.0 - 1121.2
1121.2 - 1315.2
> 1315.2
United Kingdom: Lancashire: DALYs Rate (per 100,000)
DALYs Rate
< 318.5
318.5 - 432.9
432.9 - 611.6
611.6 - 790.4
790.4 - 876.9
876.9 - 963.3
963.3 - 1049.8
1049.8 - 1120.1
1120.1 - 1320.4
> 1320.4
United Kingdom: Liverpool: DALYs Rate (per 100,000)
DALYs Rate
< 246.1
246.1 - 340.5
340.5 - 490.2
490.2 - 639.8
639.8 - 714.6
714.6 - 789.3
789.3 - 864.1
864.1 - 949.0
949.0 - 1097.0
> 1097.0
United Kingdom: Manchester: DALYs Rate (per 100,000)
DALYs Rate
< 318.2
318.2 - 433.6
433.6 - 610.5
610.5 - 787.5
787.5 - 873.5
873.5 - 959.5
959.5 - 1045.6
1045.6 - 1114.1
1114.1 - 1317.7
> 1317.7
United Kingdom: Oldham: DALYs Rate (per 100,000)
DALYs Rate
< 319.7
319.7 - 432.3
432.3 - 611.4
611.4 - 790.5
790.5 - 874.5
874.5 - 958.5
958.5 - 1042.5
1042.5 - 1121.5
1121.5 - 1317.9
> 1317.9
Female
Male
Sexes Combined
Age
20-24 years
15-19 years
10-14 years
Year
DALYs Rate (per 100,000)
Age
10-24 years

Figure S7

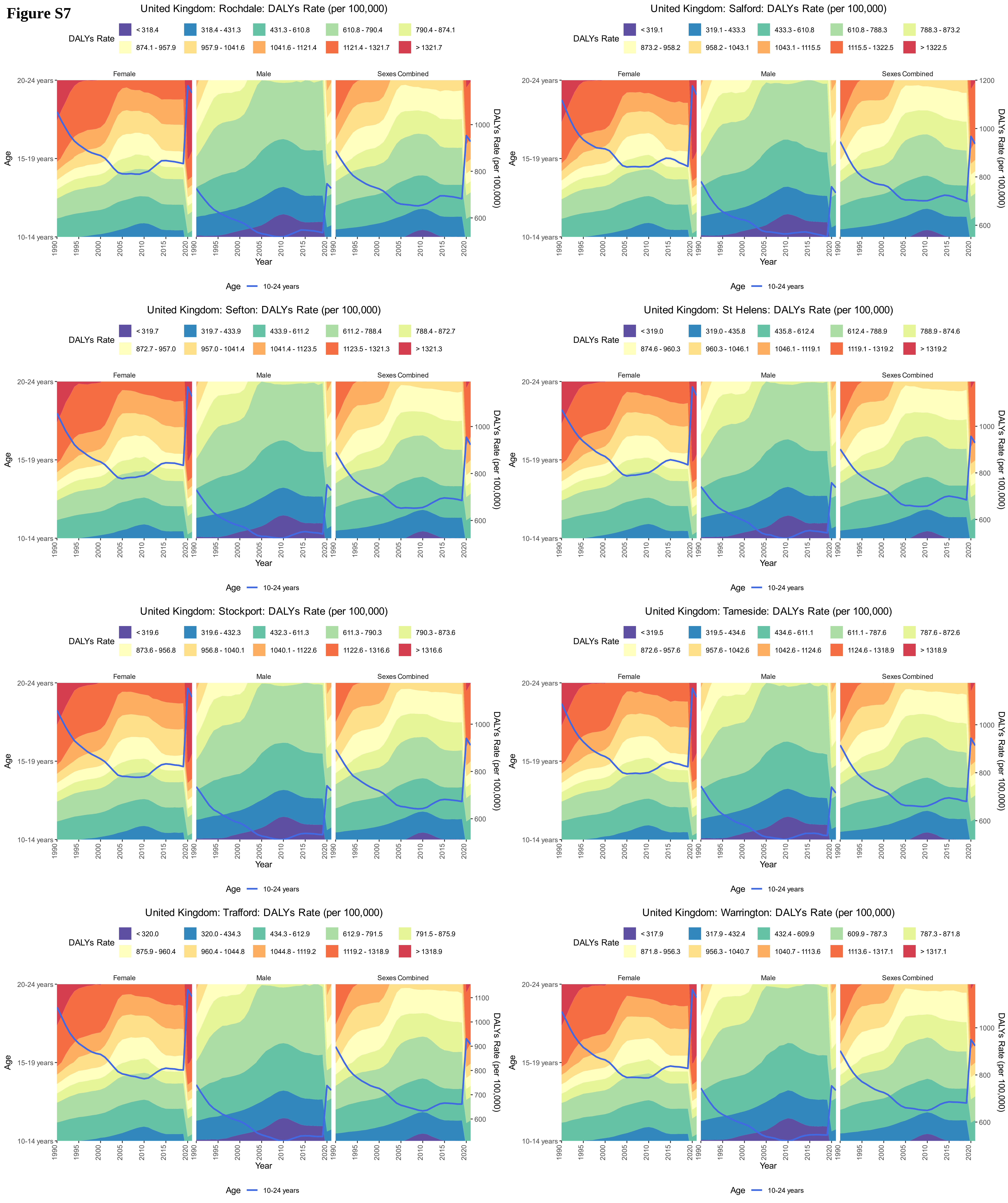

United Kingdom: Rochdale: DALYs Rate (per 100,000)
DALYs Rate
< 318.4
318.4 - 431.3
431.3 - 610.8
610.8 - 790.4
790.4 - 874.1
874.1 - 957.9
957.9 - 1041.6
1041.6 - 1121.4
1121.4 - 1321.7
> 1321.7
United Kingdom: Salford: DALYs Rate (per 100,000)
< 319.1
319.1 - 433.3
433.3 - 610.8
610.8 - 788.3
788.3 - 873.2
873.2 - 958.2
958.2 - 1043.1
1043.1 - 1115.5
1115.5 - 1322.5
> 1322.5
United Kingdom: Sefton: DALYs Rate (per 100,000)
< 319.7
319.7 - 433.9
433.9 - 611.2
611.2 - 788.4
788.4 - 872.7
872.7 - 957.0
957.0 - 1041.4
1041.4 - 1123.5
1123.5 - 1321.3
> 1321.3
United Kingdom: St Helens: DALYs Rate (per 100,000)
< 319.0
319.0 - 435.8
435.8 - 612.4
612.4 - 788.9
788.9 - 874.6
874.6 - 960.3
960.3 - 1046.1
1046.1 - 1119.1
1119.1 - 1319.2
> 1319.2
United Kingdom: Stockport: DALYs Rate (per 100,000)
< 319.6
319.6 - 432.3
432.3 - 611.3
611.3 - 790.3
790.3 - 873.6
873.6 - 956.8
956.8 - 1040.1
1040.1 - 1122.6
1122.6 - 1316.6
> 1316.6
United Kingdom: Tameside: DALYs Rate (per 100,000)
< 319.5
319.5 - 434.6
434.6 - 611.1
611.1 - 787.6
787.6 - 872.6
872.6 - 957.6
957.6 - 1042.6
1042.6 - 1124.6
1124.6 - 1318.9
> 1318.9
United Kingdom: Trafford: DALYs Rate (per 100,000)
< 320.0
320.0 - 434.3
434.3 - 612.9
612.9 - 791.5
791.5 - 875.9
875.9 - 960.4
960.4 - 1044.8
1044.8 - 1119.2
1119.2 - 1318.9
> 1318.9
United Kingdom: Warrington: DALYs Rate (per 100,000)
< 317.9
317.9 - 432.4
432.4 - 609.9
609.9 - 787.3
787.3 - 871.8
871.8 - 956.3
956.3 - 1040.7
1040.7 - 1113.6
1113.6 - 1317.1
> 1317.1
Female
Male
Sexes Combined
Age
20-24 years
15-19 years
10-14 years
Year
1990
1995
2000
2005
2010
2015
2020
DALYs Rate (per 100,000)
Age — 10-24 years

Figure S7

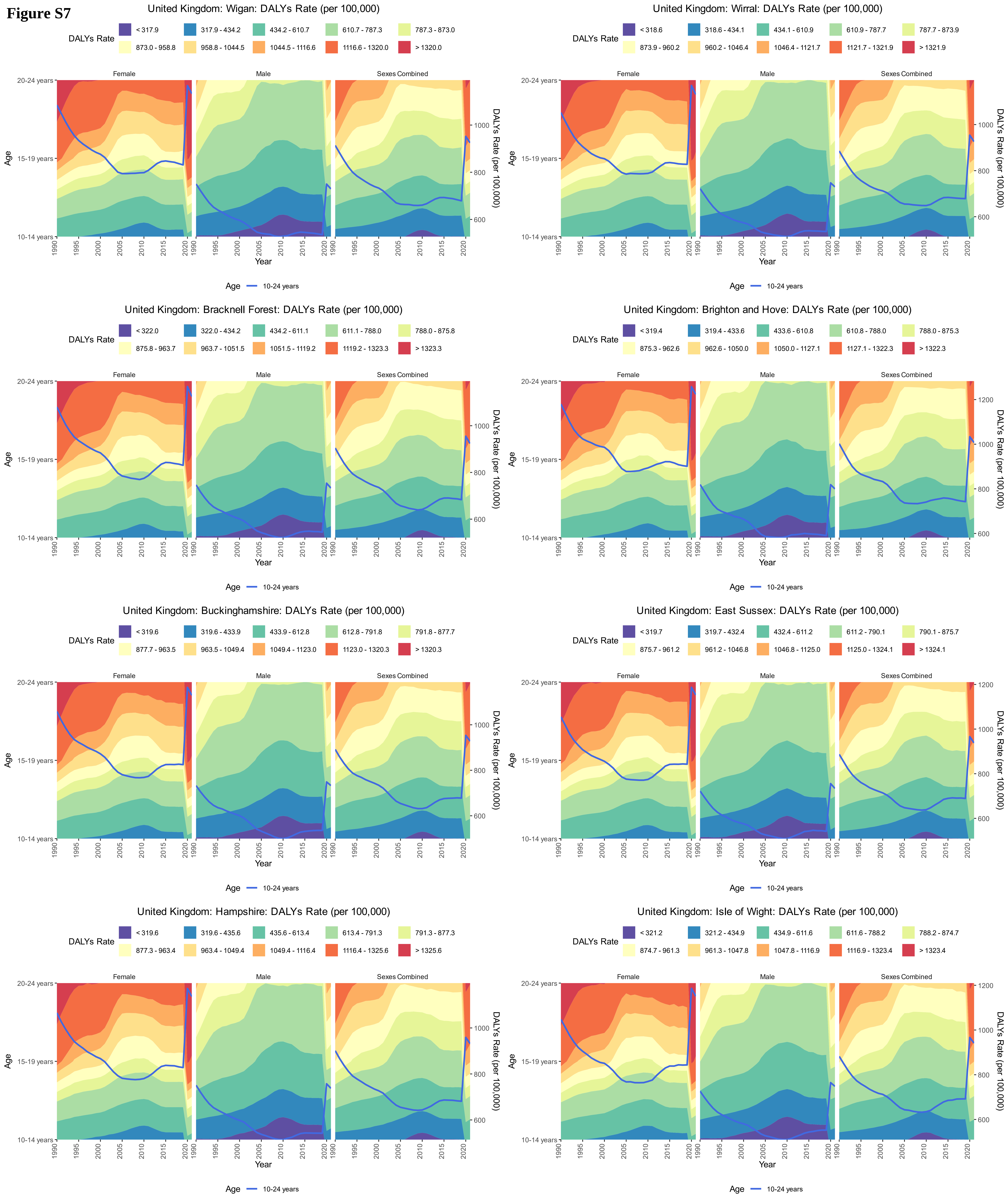

United Kingdom: Wigan: DALYs Rate (per 100,000)
DALYs Rate
< 317.9
317.9 - 434.2
434.2 - 610.7
610.7 - 787.3
787.3 - 873.0
873.0 - 958.8
958.8 - 1044.5
1044.5 - 1116.6
1116.6 - 1320.0
> 1320.0
United Kingdom: Wirral: DALYs Rate (per 100,000)
DALYs Rate
< 318.6
318.6 - 434.1
434.1 - 610.9
610.9 - 787.7
787.7 - 873.9
873.9 - 960.2
960.2 - 1046.4
1046.4 - 1121.7
1121.7 - 1321.9
> 1321.9
United Kingdom: Bracknell Forest: DALYs Rate (per 100,000)
DALYs Rate
< 322.0
322.0 - 434.2
434.2 - 611.1
611.1 - 788.0
788.0 - 875.8
875.8 - 963.7
963.7 - 1051.5
1051.5 - 1119.2
1119.2 - 1323.3
> 1323.3
United Kingdom: Brighton and Hove: DALYs Rate (per 100,000)
DALYs Rate
< 319.4
319.4 - 433.6
433.6 - 610.8
610.8 - 788.0
788.0 - 875.3
875.3 - 962.6
962.6 - 1050.0
1050.0 - 1127.1
1127.1 - 1322.3
> 1322.3
United Kingdom: Buckinghamshire: DALYs Rate (per 100,000)
DALYs Rate
< 319.6
319.6 - 433.9
433.9 - 612.8
612.8 - 791.8
791.8 - 877.7
877.7 - 963.5
963.5 - 1049.4
1049.4 - 1123.0
1123.0 - 1320.3
> 1320.3
United Kingdom: East Sussex: DALYs Rate (per 100,000)
DALYs Rate
< 319.7
319.7 - 432.4
432.4 - 611.2
611.2 - 790.1
790.1 - 875.7
875.7 - 961.2
961.2 - 1046.8
1046.8 - 1125.0
1125.0 - 1324.1
> 1324.1
United Kingdom: Hampshire: DALYs Rate (per 100,000)
DALYs Rate
< 319.6
319.6 - 435.6
435.6 - 613.4
613.4 - 791.3
791.3 - 877.3
877.3 - 963.4
963.4 - 1049.4
1049.4 - 1116.4
1116.4 - 1325.6
> 1325.6
United Kingdom: Isle of Wight: DALYs Rate (per 100,000)
DALYs Rate
< 321.2
321.2 - 434.9
434.9 - 611.6
611.6 - 788.2
788.2 - 874.7
874.7 - 961.3
961.3 - 1047.8
1047.8 - 1116.9
1116.9 - 1323.4
> 1323.4
Female
Male
Sexes Combined
Age
20-24 years
15-19 years
10-14 years
Year
DALYs Rate (per 100,000)
Age
10-24 years

Figure S7

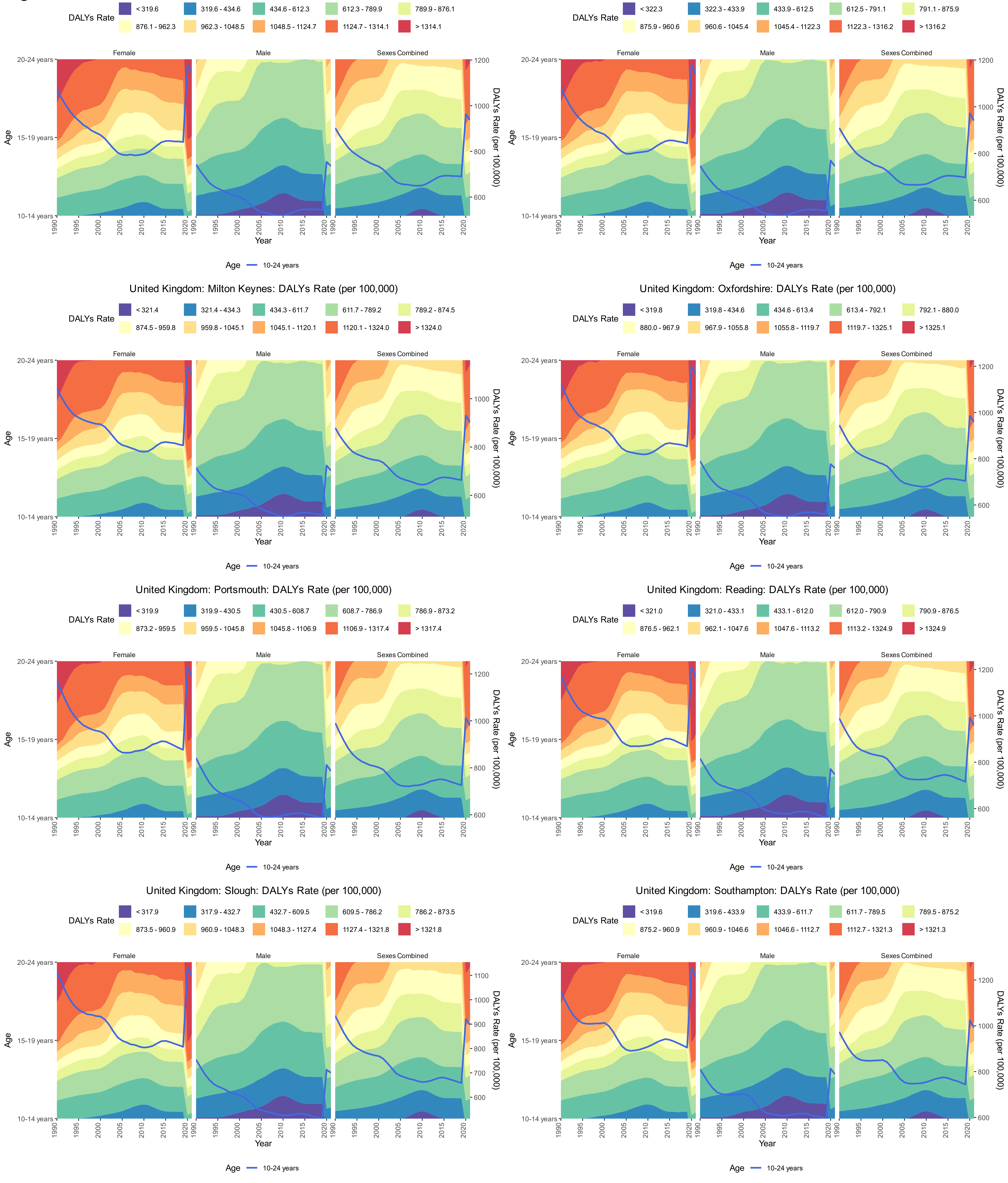

United Kingdom: Kent: DALYs Rate (per 100,000)
DALYs Rate
< 319.6
319.6 - 434.6
434.6 - 612.3
612.3 - 789.9
789.9 - 876.1
876.1 - 962.3
962.3 - 1048.5
1048.5 - 1124.7
1124.7 - 1314.1
> 1314.1
United Kingdom: Medway: DALYs Rate (per 100,000)
DALYs Rate
< 322.3
322.3 - 433.9
433.9 - 612.5
612.5 - 791.1
791.1 - 875.9
875.9 - 960.6
960.6 - 1045.4
1045.4 - 1122.3
1122.3 - 1316.2
> 1316.2
United Kingdom: Milton Keynes: DALYs Rate (per 100,000)
DALYs Rate
< 321.4
321.4 - 434.3
434.3 - 611.7
611.7 - 789.2
789.2 - 874.5
874.5 - 959.8
959.8 - 1045.1
1045.1 - 1120.1
1120.1 - 1324.0
> 1324.0
United Kingdom: Oxfordshire: DALYs Rate (per 100,000)
DALYs Rate
< 319.8
319.8 - 434.6
434.6 - 613.4
613.4 - 792.1
792.1 - 880.0
880.0 - 967.9
967.9 - 1055.8
1055.8 - 1119.7
1119.7 - 1325.1
> 1325.1
United Kingdom: Portsmouth: DALYs Rate (per 100,000)
DALYs Rate
< 319.9
319.9 - 430.5
430.5 - 608.7
608.7 - 786.9
786.9 - 873.2
873.2 - 959.5
959.5 - 1045.8
1045.8 - 1106.9
1106.9 - 1317.4
> 1317.4
United Kingdom: Reading: DALYs Rate (per 100,000)
DALYs Rate
< 321.0
321.0 - 433.1
433.1 - 612.0
612.0 - 790.9
790.9 - 876.5
876.5 - 962.1
962.1 - 1047.6
1047.6 - 1113.2
1113.2 - 1324.9
> 1324.9
United Kingdom: Slough: DALYs Rate (per 100,000)
DALYs Rate
< 317.9
317.9 - 432.7
432.7 - 609.5
609.5 - 786.2
786.2 - 873.5
873.5 - 960.9
960.9 - 1048.3
1048.3 - 1127.4
1127.4 - 1321.8
> 1321.8
United Kingdom: Southampton: DALYs Rate (per 100,000)
DALYs Rate
< 319.6
319.6 - 433.9
433.9 - 611.7
611.7 - 789.5
789.5 - 875.2
875.2 - 960.9
960.9 - 1046.6
1046.6 - 1112.7
1112.7 - 1321.3
> 1321.3
Female
Male
Sexes Combined
Age
20-24 years
15-19 years
10-14 years
Year
DALYs Rate (per 100,000)
Age
10-24 years

Figure S7

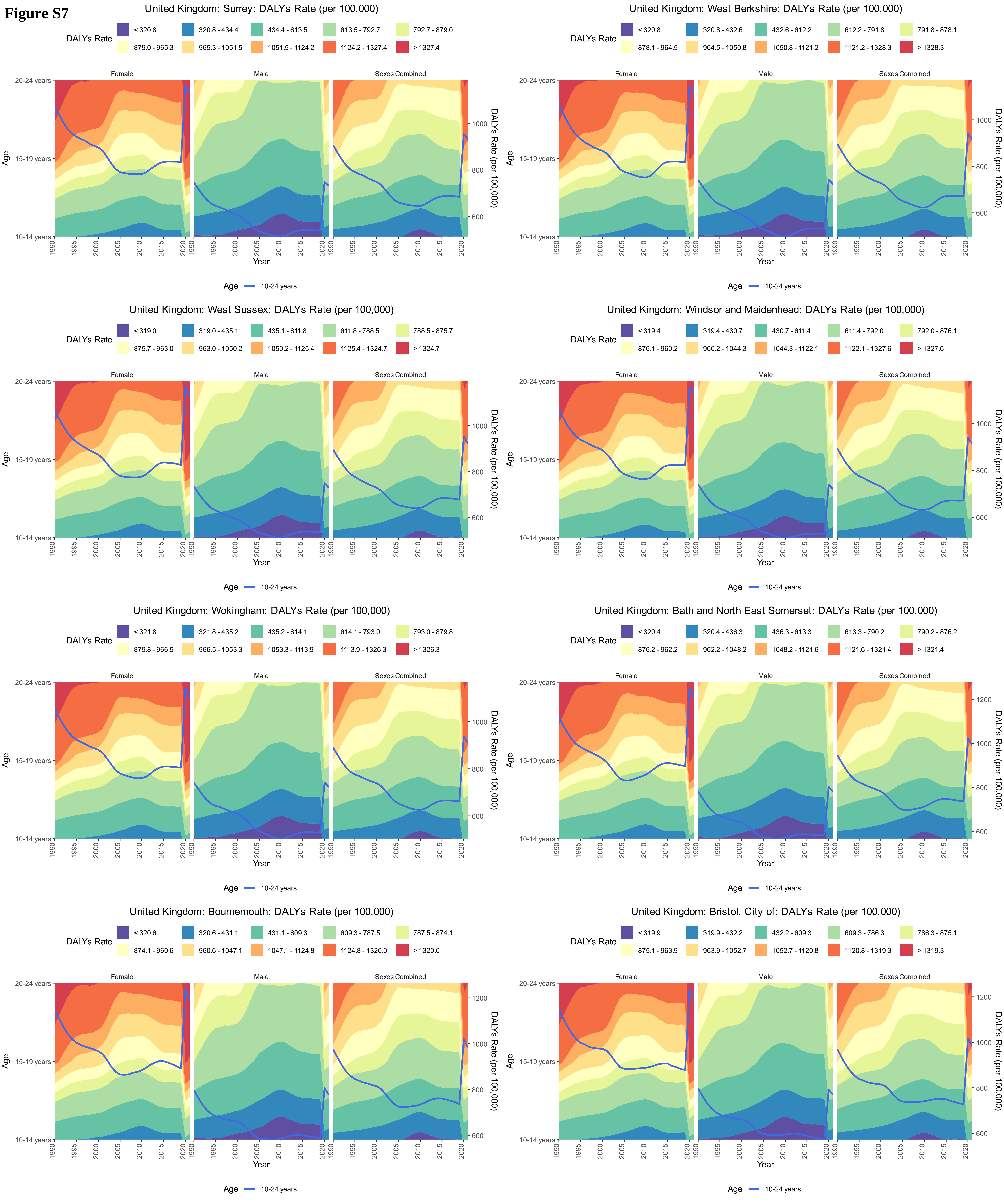

United Kingdom: Surrey: DALYs Rate (per 100,000)
DALYs Rate
< 320.8
320.8 - 434.4
434.4 - 613.5
613.5 - 792.7
792.7 - 879.0
879.0 - 965.3
965.3 - 1051.5
1051.5 - 1124.2
1124.2 - 1327.4
> 1327.4
United Kingdom: West Berkshire: DALYs Rate (per 100,000)
< 320.8
320.8 - 432.6
432.6 - 612.2
612.2 - 791.8
791.8 - 878.1
878.1 - 964.5
964.5 - 1050.8
1050.8 - 1121.2
1121.2 - 1328.3
> 1328.3
United Kingdom: West Sussex: DALYs Rate (per 100,000)
< 319.0
319.0 - 435.1
435.1 - 611.8
611.8 - 788.5
788.5 - 875.7
875.7 - 963.0
963.0 - 1050.2
1050.2 - 1125.4
1125.4 - 1324.7
> 1324.7
United Kingdom: Windsor and Maidenhead: DALYs Rate (per 100,000)
< 319.4
319.4 - 430.7
430.7 - 611.4
611.4 - 792.0
792.0 - 876.1
876.1 - 960.2
960.2 - 1044.3
1044.3 - 1122.1
1122.1 - 1327.6
> 1327.6
United Kingdom: Wokingham: DALYs Rate (per 100,000)
< 321.8
321.8 - 435.2
435.2 - 614.1
614.1 - 793.0
793.0 - 879.8
879.8 - 966.5
966.5 - 1053.3
1053.3 - 1113.9
1113.9 - 1326.3
> 1326.3
United Kingdom: Bath and North East Somerset: DALYs Rate (per 100,000)
< 320.4
320.4 - 436.3
436.3 - 613.3
613.3 - 790.2
790.2 - 876.2
876.2 - 962.2
962.2 - 1048.2
1048.2 - 1121.6
1121.6 - 1321.4
> 1321.4
United Kingdom: Bournemouth: DALYs Rate (per 100,000)
< 320.6
320.6 - 431.1
431.1 - 609.3
609.3 - 787.5
787.5 - 874.1
874.1 - 960.6
960.6 - 1047.1
1047.1 - 1124.8
1124.8 - 1320.0
> 1320.0
United Kingdom: Bristol, City of: DALYs Rate (per 100,000)
< 319.9
319.9 - 432.2
432.2 - 609.3
609.3 - 786.3
786.3 - 875.1
875.1 - 963.9
963.9 - 1052.7
1052.7 - 1120.8
1120.8 - 1319.3
> 1319.3
Female
Male
Sexes Combined
Age
20-24 years
15-19 years
10-14 years
Year
DALYs Rate (per 100,000)
Age — 10-24 years

Figure S7

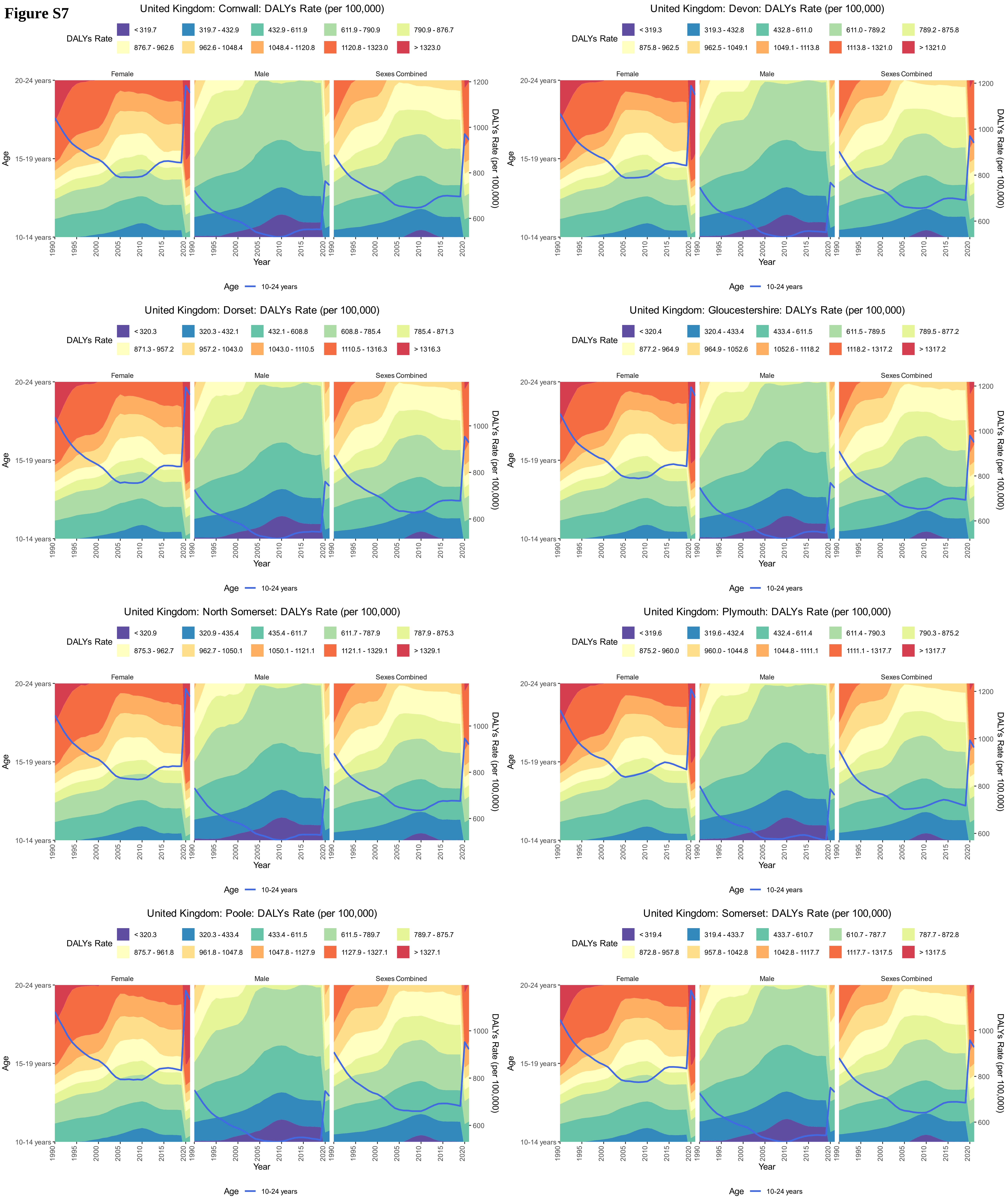

United Kingdom: Cornwall: DALYs Rate (per 100,000)
DALYs Rate
< 319.7
319.7 - 432.9
432.9 - 611.9
611.9 - 790.9
790.9 - 876.7
876.7 - 962.6
962.6 - 1048.4
1048.4 - 1120.8
1120.8 - 1323.0
> 1323.0
United Kingdom: Devon: DALYs Rate (per 100,000)
< 319.3
319.3 - 432.8
432.8 - 611.0
611.0 - 789.2
789.2 - 875.8
875.8 - 962.5
962.5 - 1049.1
1049.1 - 1113.8
1113.8 - 1321.0
> 1321.0
United Kingdom: Dorset: DALYs Rate (per 100,000)
< 320.3
320.3 - 432.1
432.1 - 608.8
608.8 - 785.4
785.4 - 871.3
871.3 - 957.2
957.2 - 1043.0
1043.0 - 1110.5
1110.5 - 1316.3
> 1316.3
United Kingdom: Gloucestershire: DALYs Rate (per 100,000)
< 320.4
320.4 - 433.4
433.4 - 611.5
611.5 - 789.5
789.5 - 877.2
877.2 - 964.9
964.9 - 1052.6
1052.6 - 1118.2
1118.2 - 1317.2
> 1317.2
United Kingdom: North Somerset: DALYs Rate (per 100,000)
< 320.9
320.9 - 435.4
435.4 - 611.7
611.7 - 787.9
787.9 - 875.3
875.3 - 962.7
962.7 - 1050.1
1050.1 - 1121.1
1121.1 - 1329.1
> 1329.1
United Kingdom: Plymouth: DALYs Rate (per 100,000)
< 319.6
319.6 - 432.4
432.4 - 611.4
611.4 - 790.3
790.3 - 875.2
875.2 - 960.0
960.0 - 1044.8
1044.8 - 1111.1
1111.1 - 1317.7
> 1317.7
United Kingdom: Poole: DALYs Rate (per 100,000)
< 320.3
320.3 - 433.4
433.4 - 611.5
611.5 - 789.7
789.7 - 875.7
875.7 - 961.8
961.8 - 1047.8
1047.8 - 1127.9
1127.9 - 1327.1
> 1327.1
United Kingdom: Somerset: DALYs Rate (per 100,000)
< 319.4
319.4 - 433.7
433.7 - 610.7
610.7 - 787.7
787.7 - 872.8
872.8 - 957.8
957.8 - 1042.8
1042.8 - 1117.7
1117.7 - 1317.5
> 1317.5
Female
Male
Sexes Combined
20-24 years
15-19 years
10-14 years
Age
Year
DALYs Rate (per 100,000)
Age
10-24 years

Figure S7

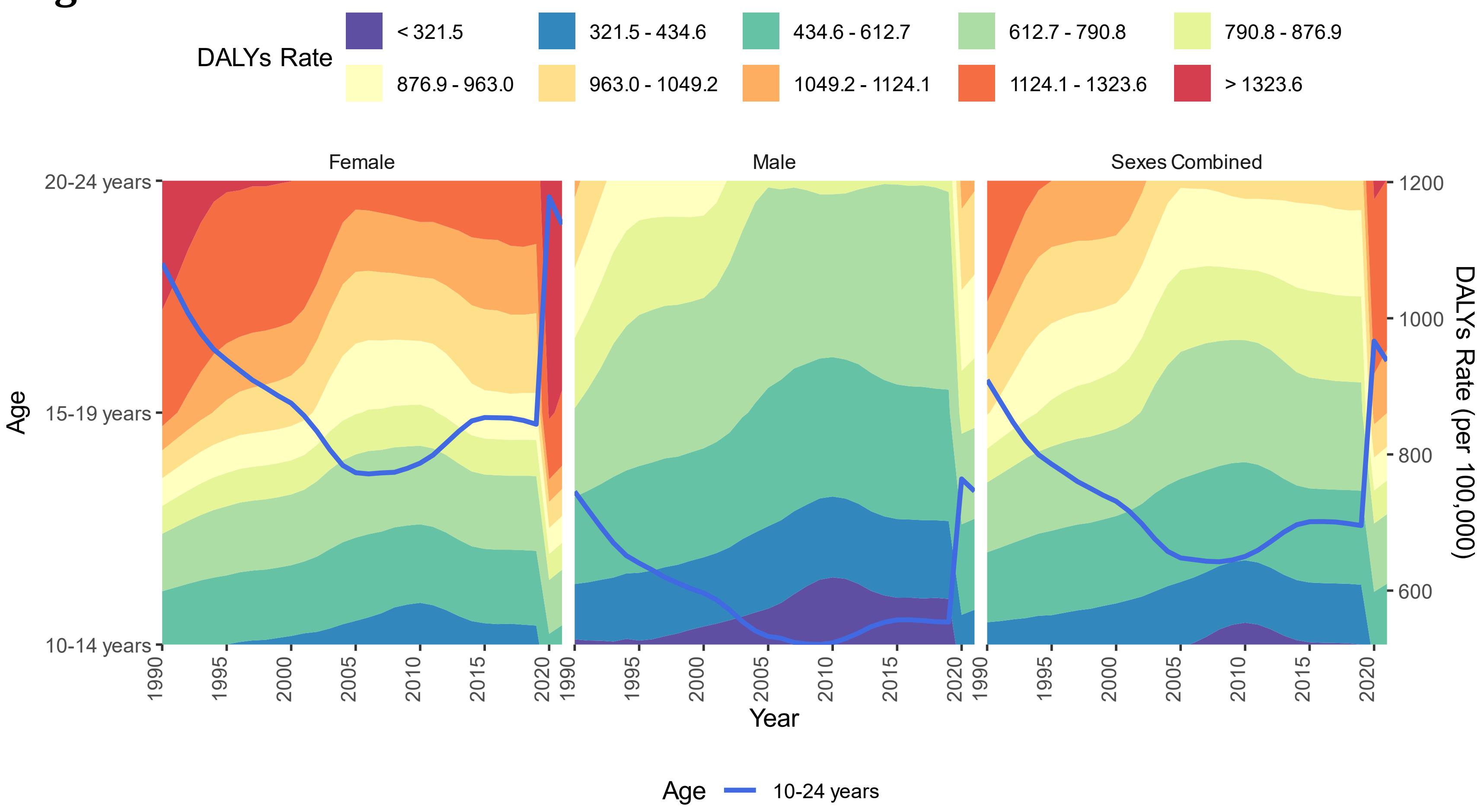
United Kingdom: South Gloucestershire: DALYs Rate (per 100,000)
DALYs Rate
< 321.5
321.5 - 434.6
434.6 - 612.7
612.7 - 790.8
790.8 - 876.9
876.9 - 963.0
963.0 - 1049.2
1049.2 - 1124.1
1124.1 - 1323.6
> 1323.6
Female
Male
Sexes Combined
Age
Year
DALYs Rate (per 100,000)
Age
10-24 years

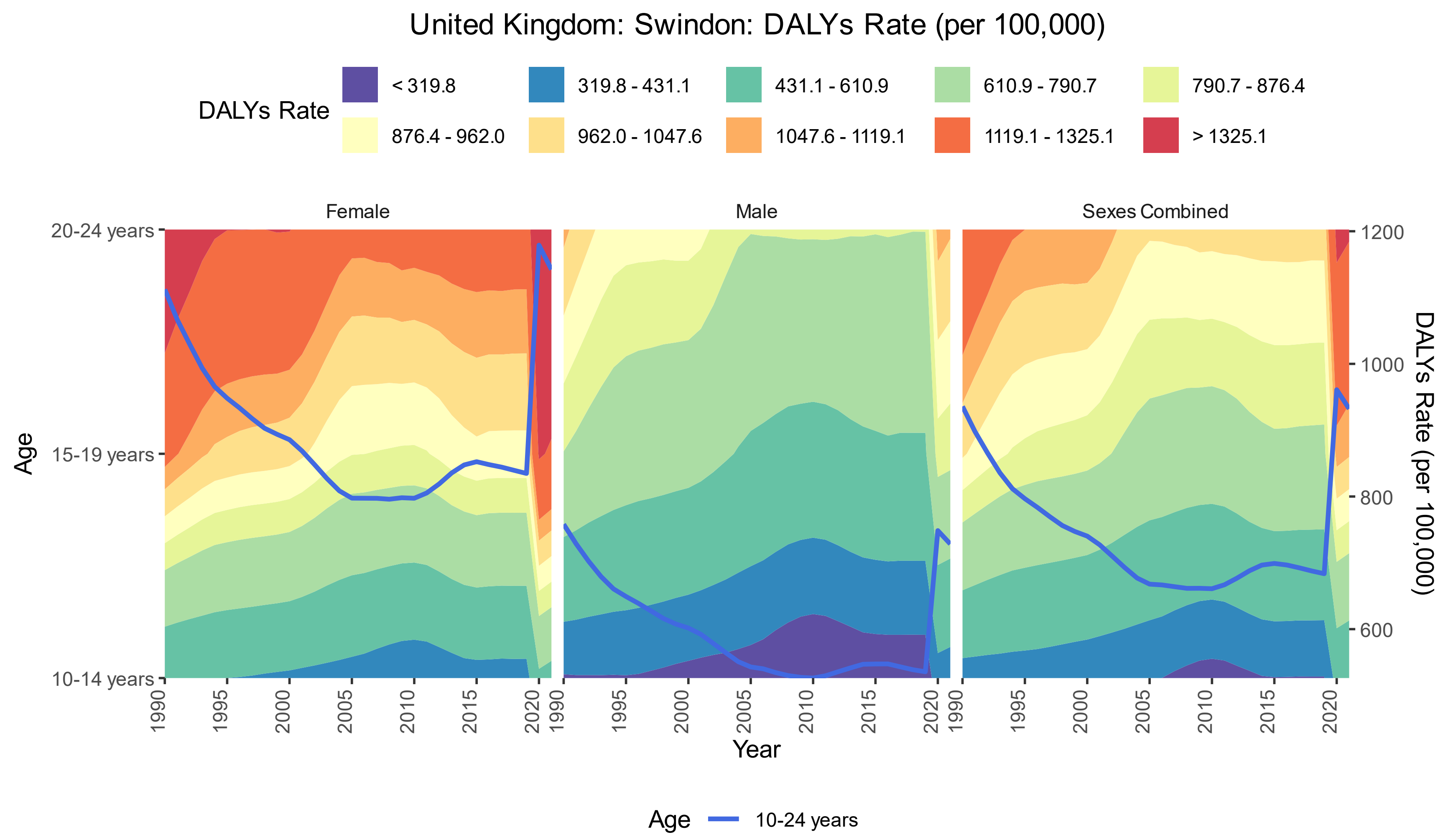
United Kingdom: Swindon: DALYs Rate (per 100,000)
DALYs Rate
< 319.8
319.8 - 431.1
431.1 - 610.9
610.9 - 790.7
790.7 - 876.4
876.4 - 962.0
962.0 - 1047.6
1047.6 - 1119.1
1119.1 - 1325.1
> 1325.1
Female
Male
Sexes Combined
Age
Year
DALYs Rate (per 100,000)
Age
10-24 years

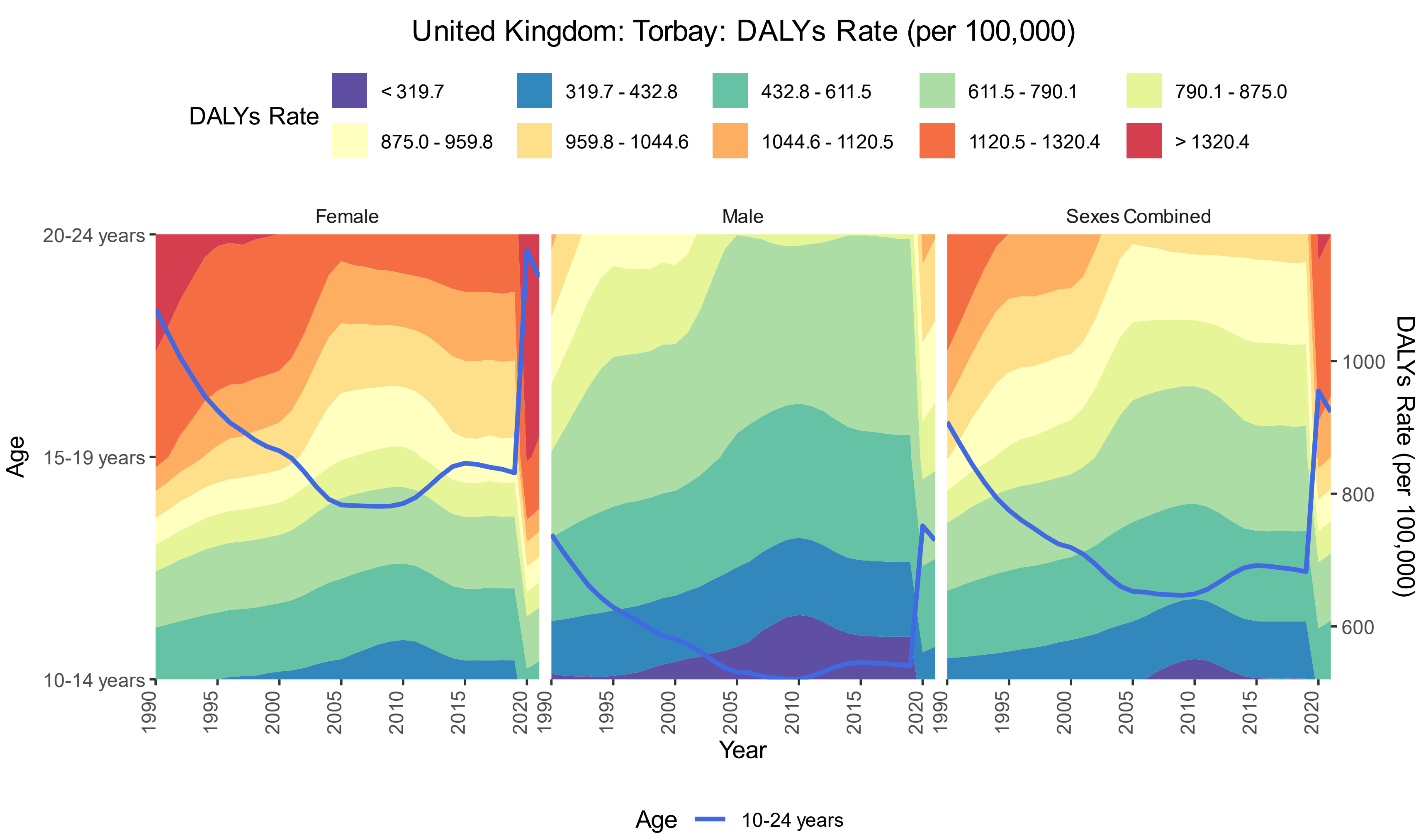
United Kingdom: Torbay: DALYs Rate (per 100,000)
DALYs Rate
< 319.7
319.7 - 432.8
432.8 - 611.5
611.5 - 790.1
790.1 - 875.0
875.0 - 959.8
959.8 - 1044.6
1044.6 - 1120.5
1120.5 - 1320.4
> 1320.4
Female
Male
Sexes Combined
Age
Year
DALYs Rate (per 100,000)
Age
10-24 years

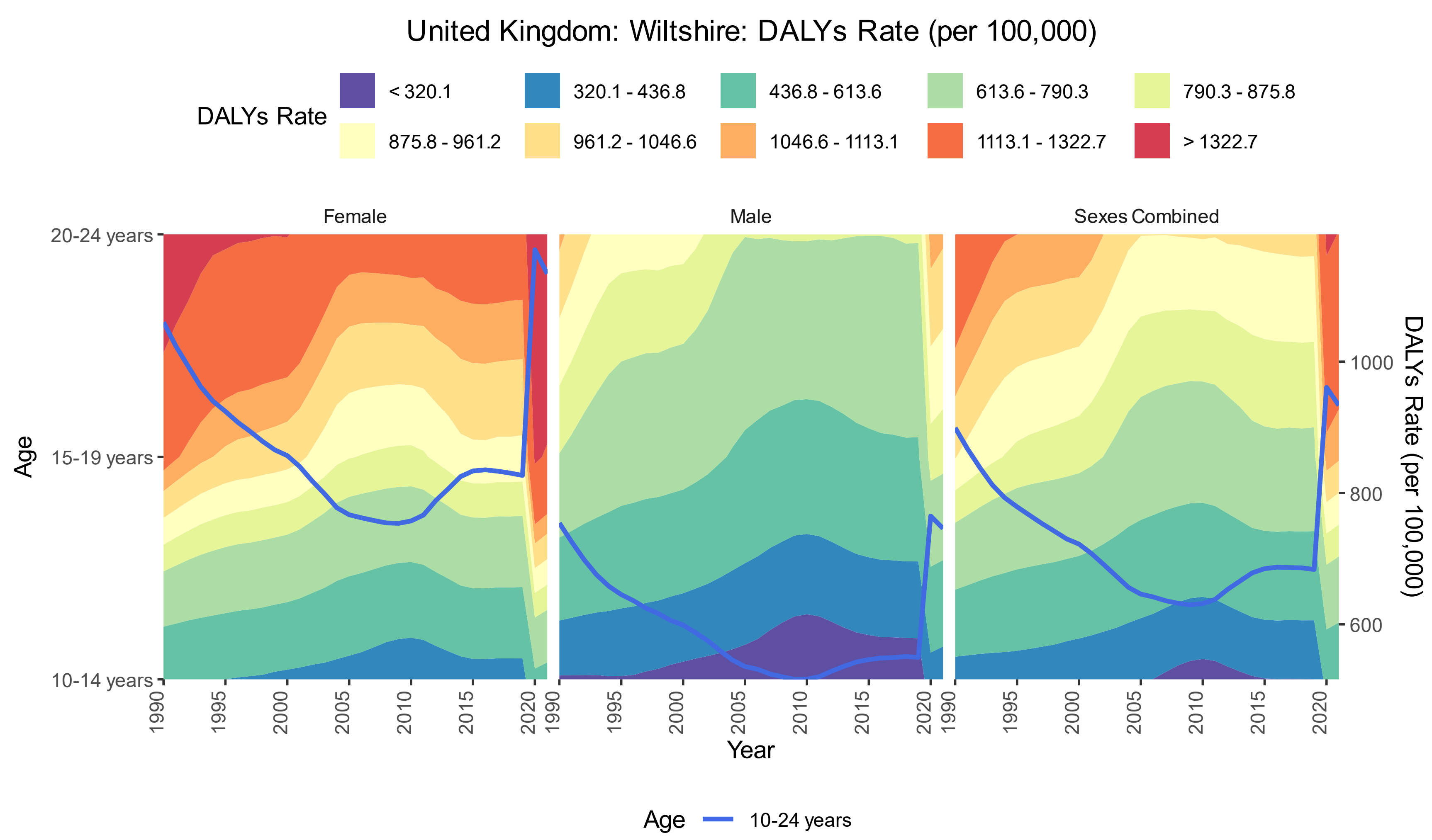
United Kingdom: Wiltshire: DALYs Rate (per 100,000)
DALYs Rate
< 320.1
320.1 - 436.8
436.8 - 613.6
613.6 - 790.3
790.3 - 875.8
875.8 - 961.2
961.2 - 1046.6
1046.6 - 1113.1
1113.1 - 1322.7
> 1322.7
Female
Male
Sexes Combined
Age
Year
DALYs Rate (per 100,000)
Age
10-24 years

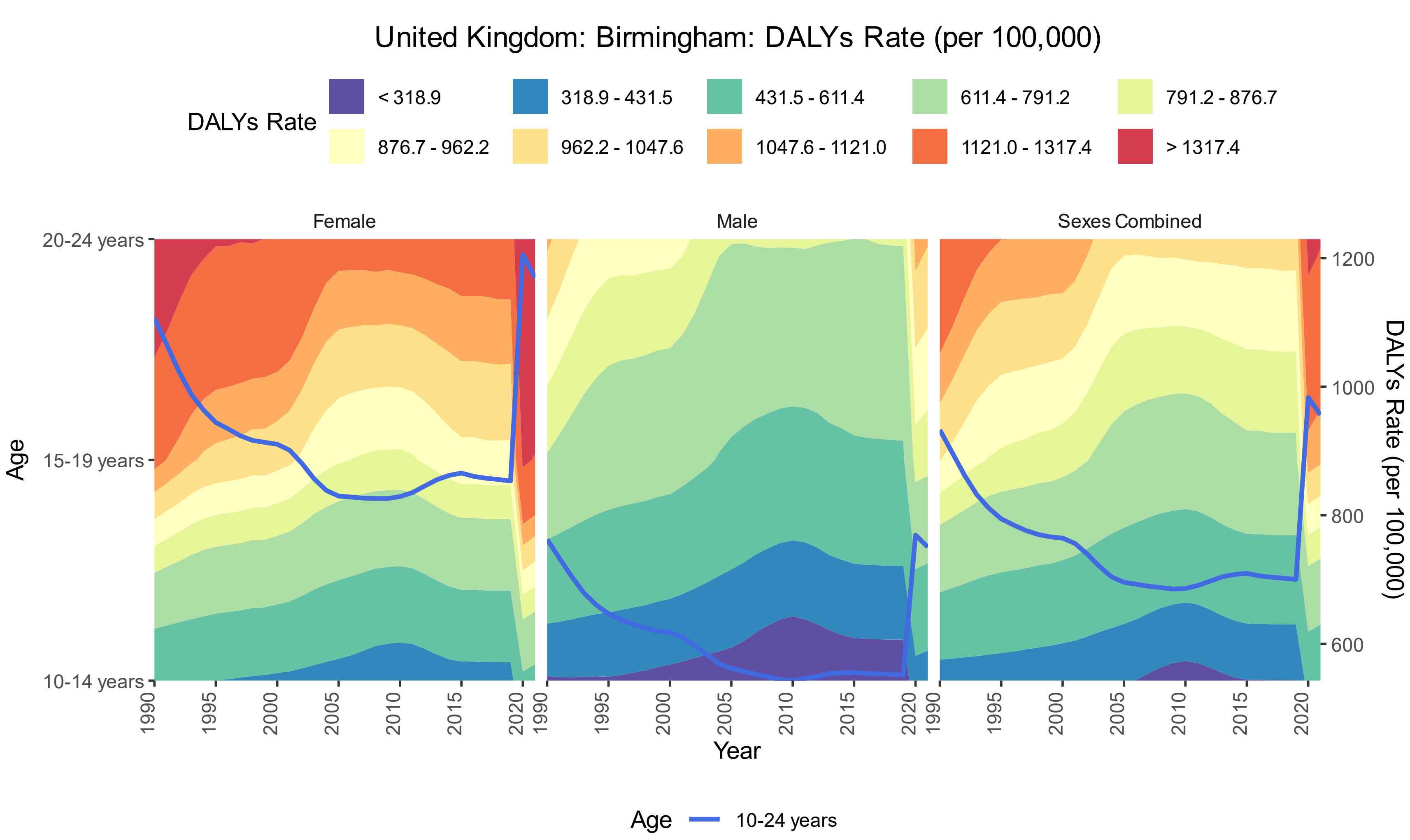
United Kingdom: Birmingham: DALYs Rate (per 100,000)
DALYs Rate
< 318.9
318.9 - 431.5
431.5 - 611.4
611.4 - 791.2
791.2 - 876.7
876.7 - 962.2
962.2 - 1047.6
1047.6 - 1121.0
1121.0 - 1317.4
> 1317.4
Female
Male
Sexes Combined
Age
Year
DALYs Rate (per 100,000)
Age
10-24 years

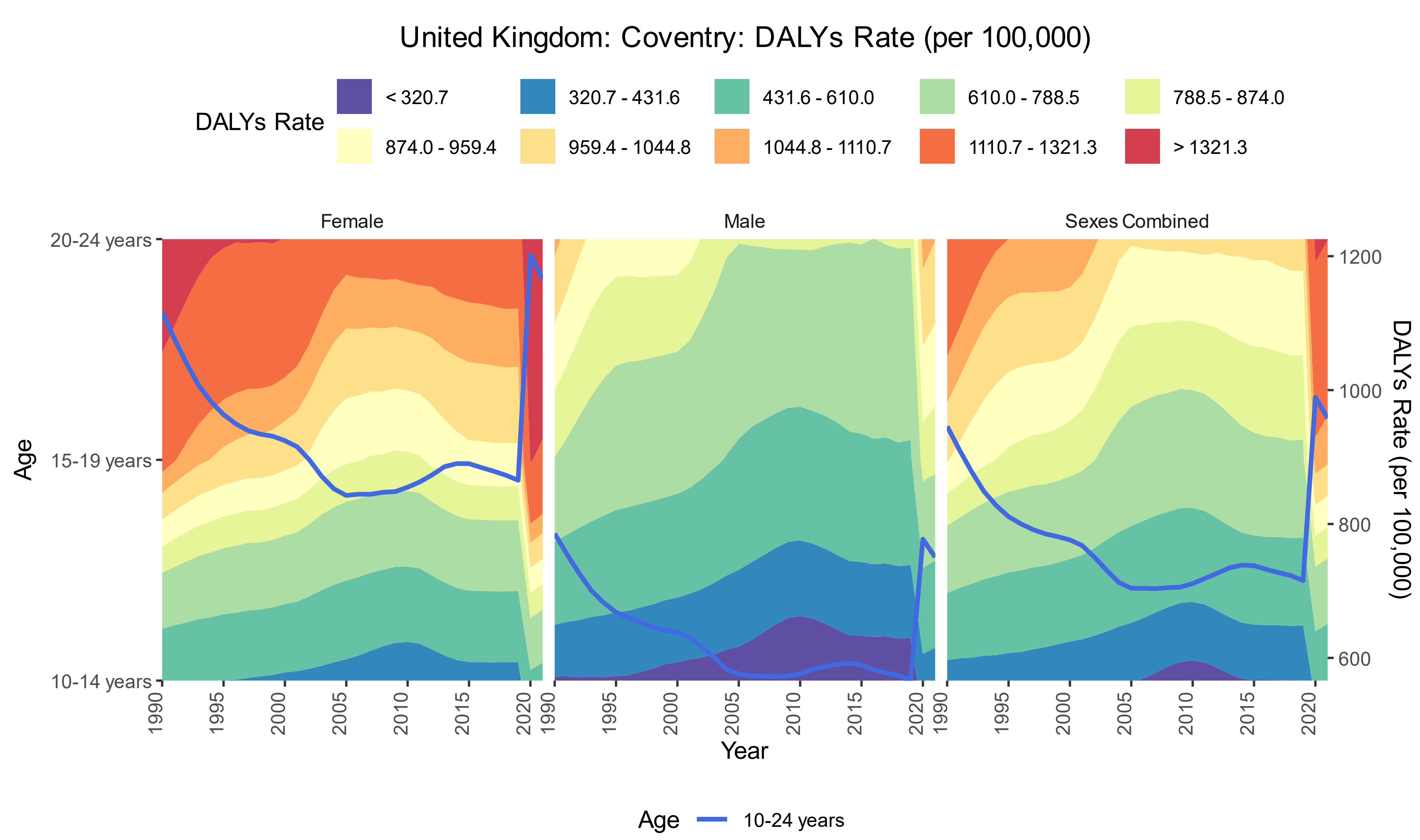
United Kingdom: Coventry: DALYs Rate (per 100,000)
DALYs Rate
< 320.7
320.7 - 431.6
431.6 - 610.0
610.0 - 788.5
788.5 - 874.0
874.0 - 959.4
959.4 - 1044.8
1044.8 - 1110.7
1110.7 - 1321.3
> 1321.3
Female
Male
Sexes Combined
Age
Year
DALYs Rate (per 100,000)
Age
10-24 years

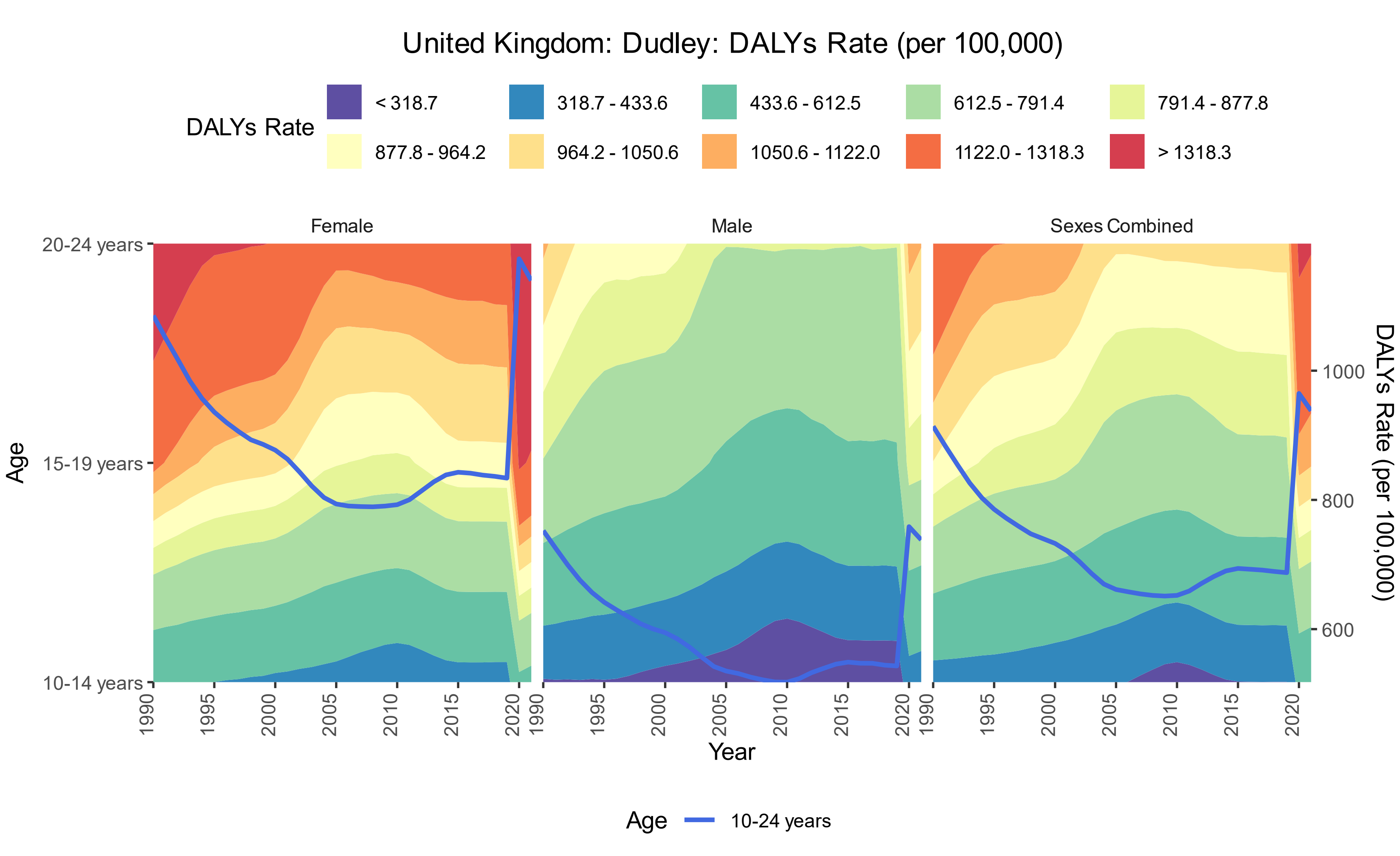
United Kingdom: Dudley: DALYs Rate (per 100,000)
DALYs Rate
< 318.7
318.7 - 433.6
433.6 - 612.5
612.5 - 791.4
791.4 - 877.8
877.8 - 964.2
964.2 - 1050.6
1050.6 - 1122.0
1122.0 - 1318.3
> 1318.3
Female
Male
Sexes Combined
Age
Year
DALYs Rate (per 100,000)
Age
10-24 years

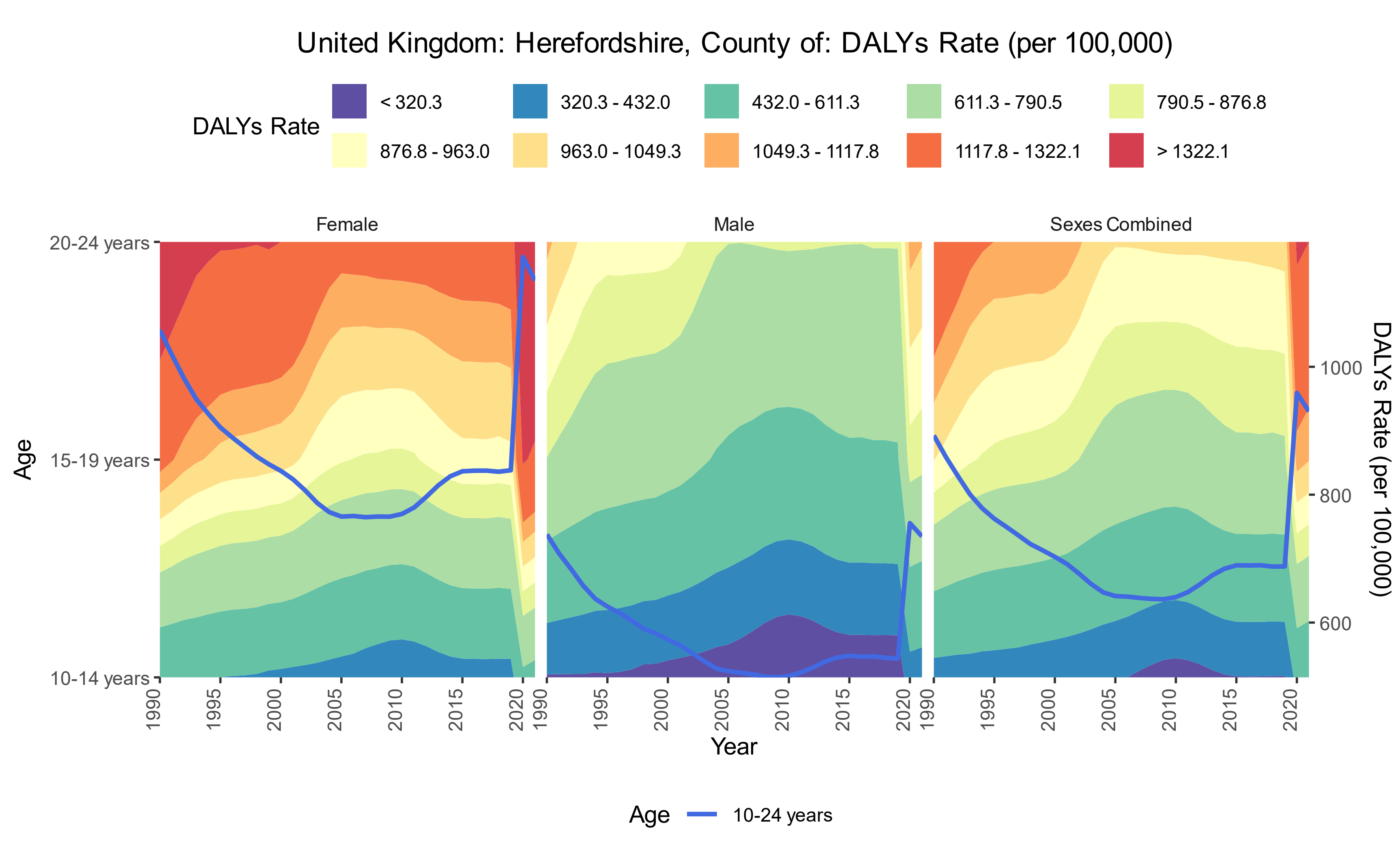
United Kingdom: Herefordshire, County of: DALYs Rate (per 100,000)
DALYs Rate
< 320.3
320.3 - 432.0
432.0 - 611.3
611.3 - 790.5
790.5 - 876.8
876.8 - 963.0
963.0 - 1049.3
1049.3 - 1117.8
1117.8 - 1322.1
> 1322.1
Female
Male
Sexes Combined
Age
Year
DALYs Rate (per 100,000)
Age
10-24 years

Figure S7

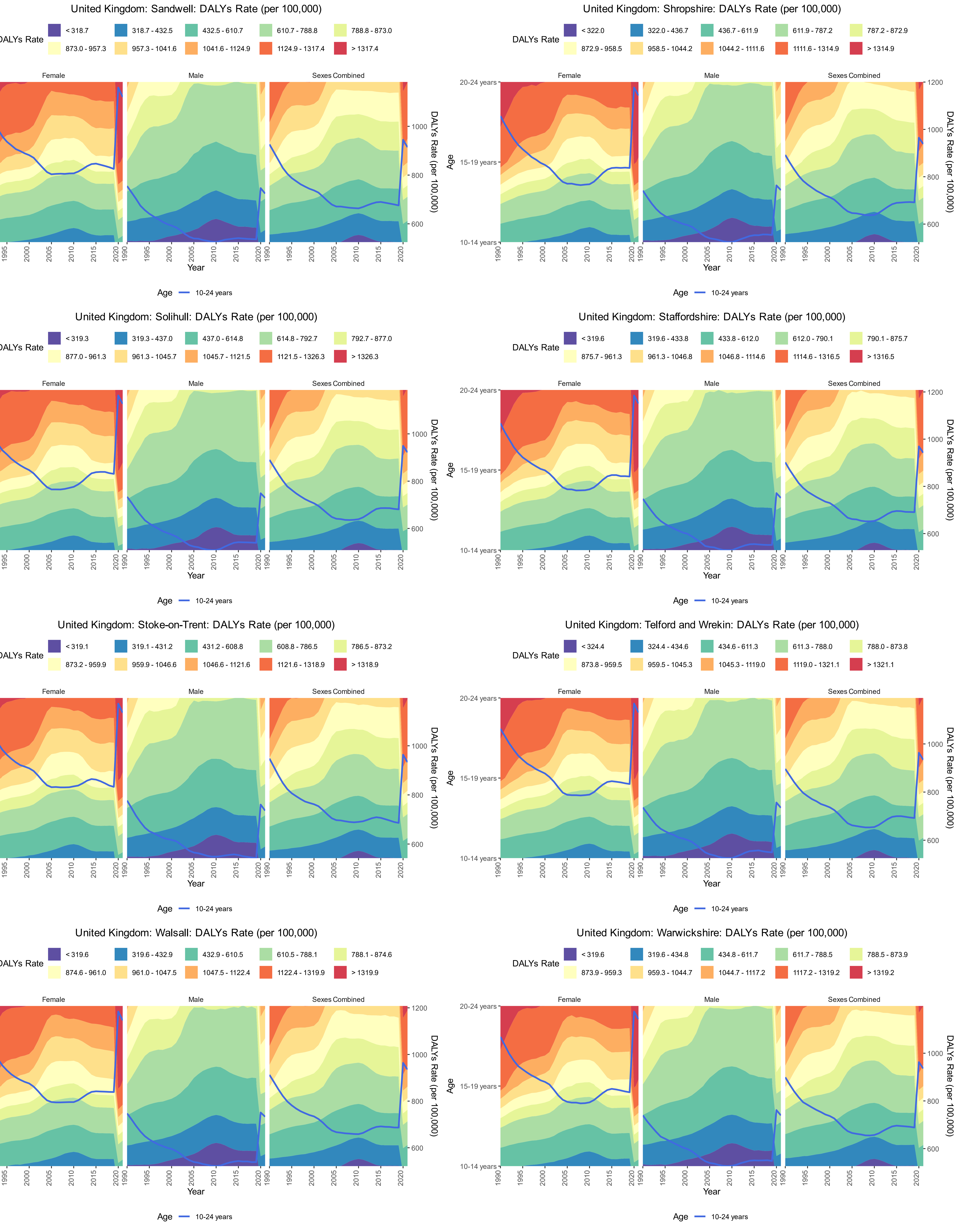
United Kingdom: Sandwell: DALYs Rate (per 100,000)
DALYs Rate
< 318.7
318.7 - 432.5
432.5 - 610.7
610.7 - 788.8
788.8 - 873.0
873.0 - 957.3
957.3 - 1041.6
1041.6 - 1124.9
1124.9 - 1317.4
> 1317.4
United Kingdom: Shropshire: DALYs Rate (per 100,000)
< 322.0
322.0 - 436.7
436.7 - 611.9
611.9 - 787.2
787.2 - 872.9
872.9 - 958.5
958.5 - 1044.2
1044.2 - 1111.6
1111.6 - 1314.9
> 1314.9
United Kingdom: Solihull: DALYs Rate (per 100,000)
< 319.3
319.3 - 437.0
437.0 - 614.8
614.8 - 792.7
792.7 - 877.0
877.0 - 961.3
961.3 - 1045.7
1045.7 - 1121.5
1121.5 - 1326.3
> 1326.3
United Kingdom: Staffordshire: DALYs Rate (per 100,000)
< 319.6
319.6 - 433.8
433.8 - 612.0
612.0 - 790.1
790.1 - 875.7
875.7 - 961.3
961.3 - 1046.8
1046.8 - 1114.6
1114.6 - 1316.5
> 1316.5
United Kingdom: Stoke-on-Trent: DALYs Rate (per 100,000)
< 319.1
319.1 - 431.2
431.2 - 608.8
608.8 - 786.5
786.5 - 873.2
873.2 - 959.9
959.9 - 1046.6
1046.6 - 1121.6
1121.6 - 1318.9
> 1318.9
United Kingdom: Telford and Wrekin: DALYs Rate (per 100,000)
< 324.4
324.4 - 434.6
434.6 - 611.3
611.3 - 788.0
788.0 - 873.8
873.8 - 959.5
959.5 - 1045.3
1045.3 - 1119.0
1119.0 - 1321.1
> 1321.1
United Kingdom: Walsall: DALYs Rate (per 100,000)
< 319.6
319.6 - 432.9
432.9 - 610.5
610.5 - 788.1
788.1 - 874.6
874.6 - 961.0
961.0 - 1047.5
1047.5 - 1122.4
1122.4 - 1319.9
> 1319.9
United Kingdom: Warwickshire: DALYs Rate (per 100,000)
< 319.6
319.6 - 434.8
434.8 - 611.7
611.7 - 788.5
788.5 - 873.9
873.9 - 959.3
959.3 - 1044.7
1044.7 - 1117.2
1117.2 - 1319.2
> 1319.2
Female
Male
Sexes Combined
Age
20-24 years
15-19 years
10-14 years
Year
DALYs Rate (per 100,000)
Age — 10-24 years

Figure S7

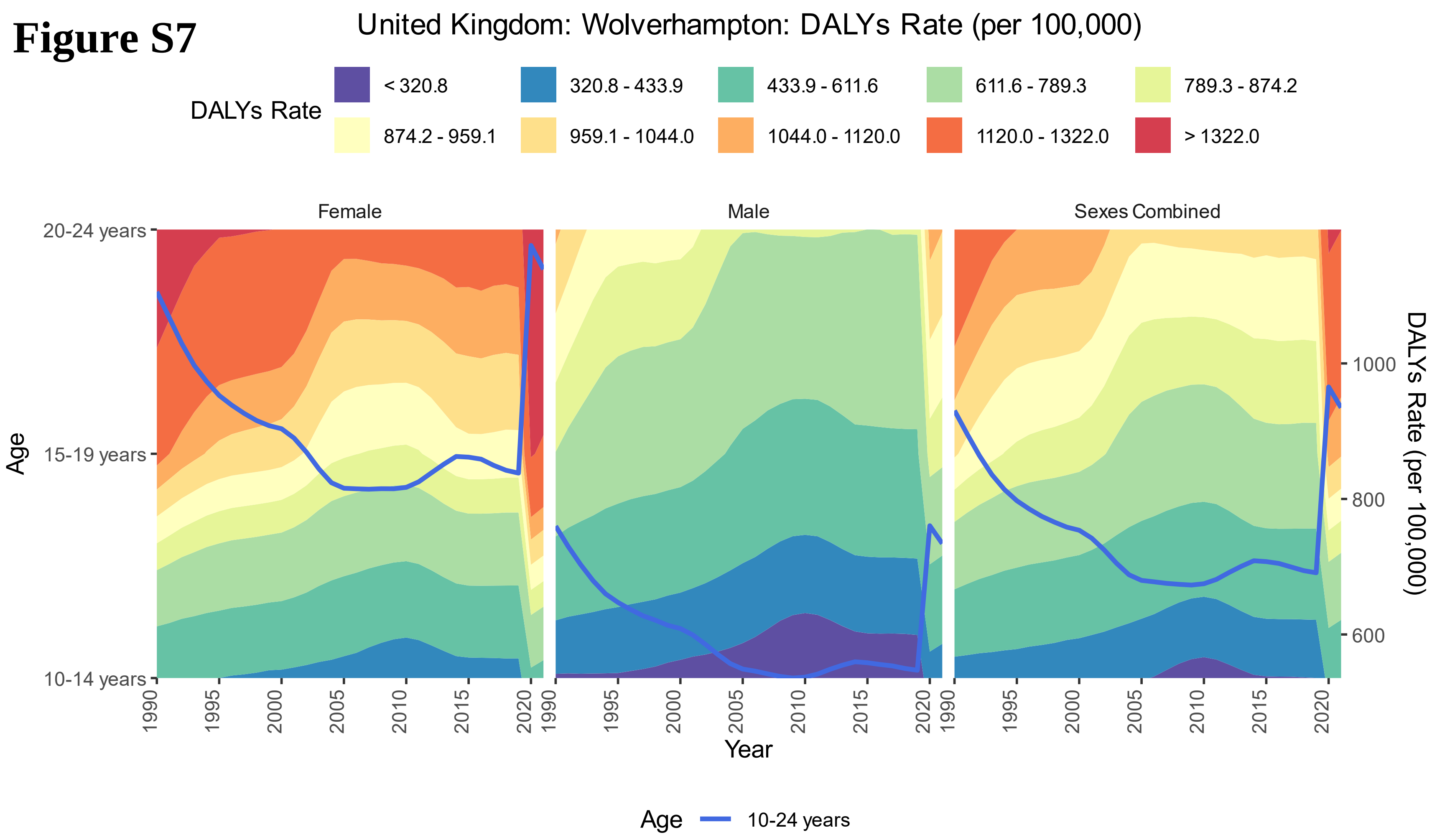
United Kingdom: Wolverhampton: DALYs Rate (per 100,000)
DALYs Rate
< 320.8
320.8 - 433.9
433.9 - 611.6
611.6 - 789.3
789.3 - 874.2
874.2 - 959.1
959.1 - 1044.0
1044.0 - 1120.0
1120.0 - 1322.0
> 1322.0
Female
Male
Sexes Combined
Age
Year
DALYs Rate (per 100,000)
Age — 10-24 years

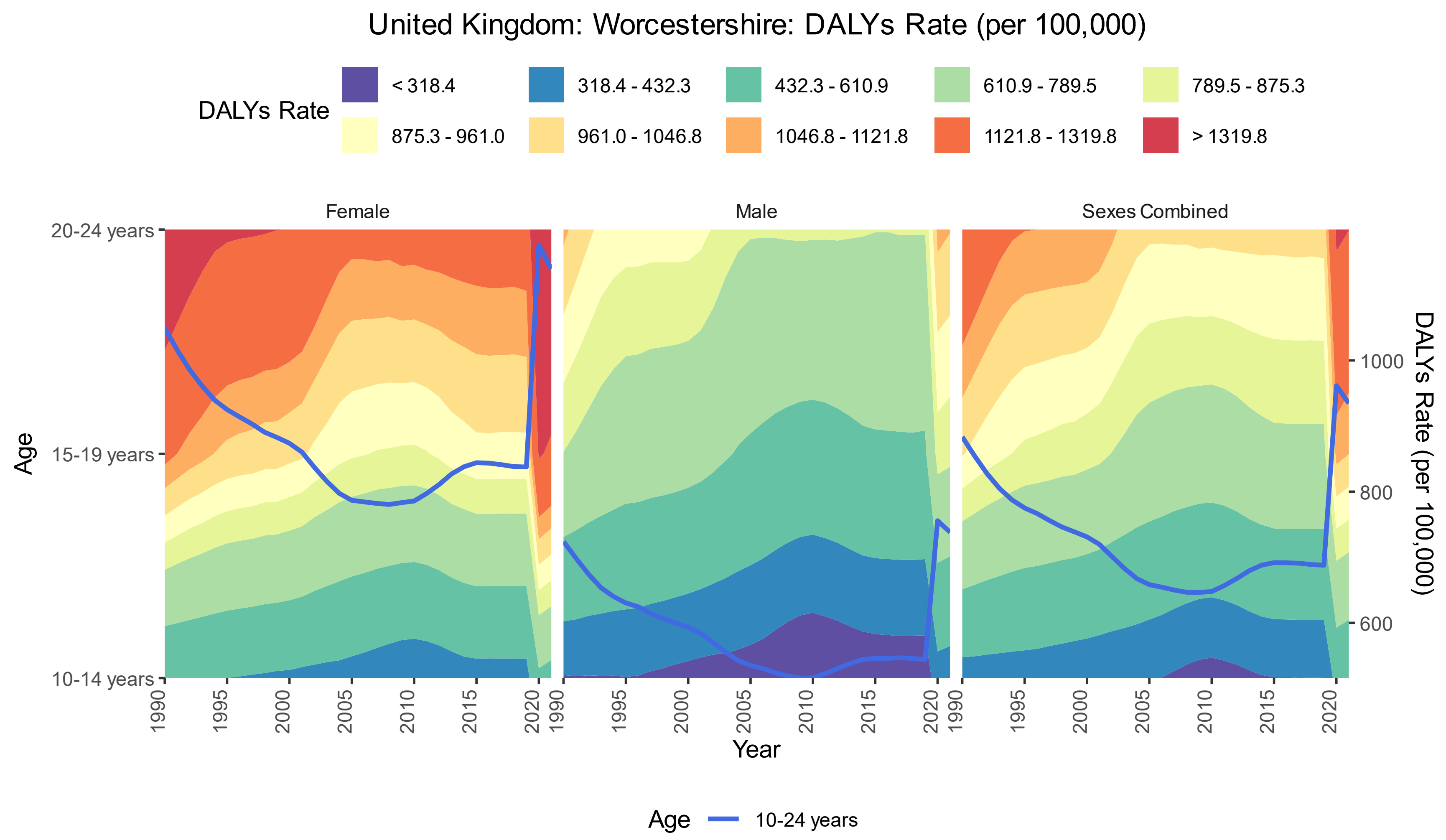
United Kingdom: Worcestershire: DALYs Rate (per 100,000)
DALYs Rate
< 318.4
318.4 - 432.3
432.3 - 610.9
610.9 - 789.5
789.5 - 875.3
875.3 - 961.0
961.0 - 1046.8
1046.8 - 1121.8
1121.8 - 1319.8
> 1319.8
Female
Male
Sexes Combined
Age
Year
DALYs Rate (per 100,000)
Age — 10-24 years

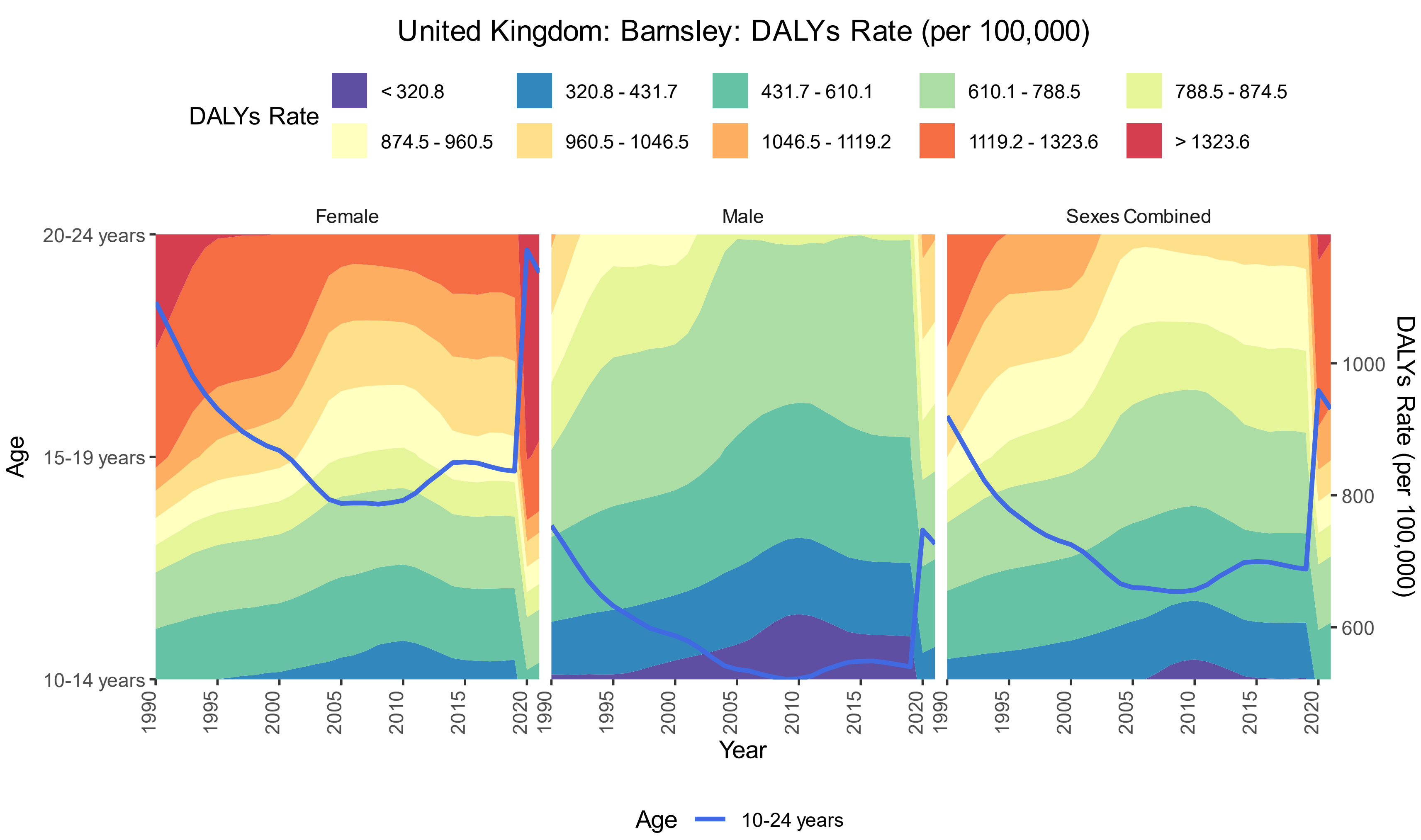
United Kingdom: Barnsley: DALYs Rate (per 100,000)
DALYs Rate
< 320.8
320.8 - 431.7
431.7 - 610.1
610.1 - 788.5
788.5 - 874.5
874.5 - 960.5
960.5 - 1046.5
1046.5 - 1119.2
1119.2 - 1323.6
> 1323.6
Female
Male
Sexes Combined
Age
Year
DALYs Rate (per 100,000)
Age — 10-24 years

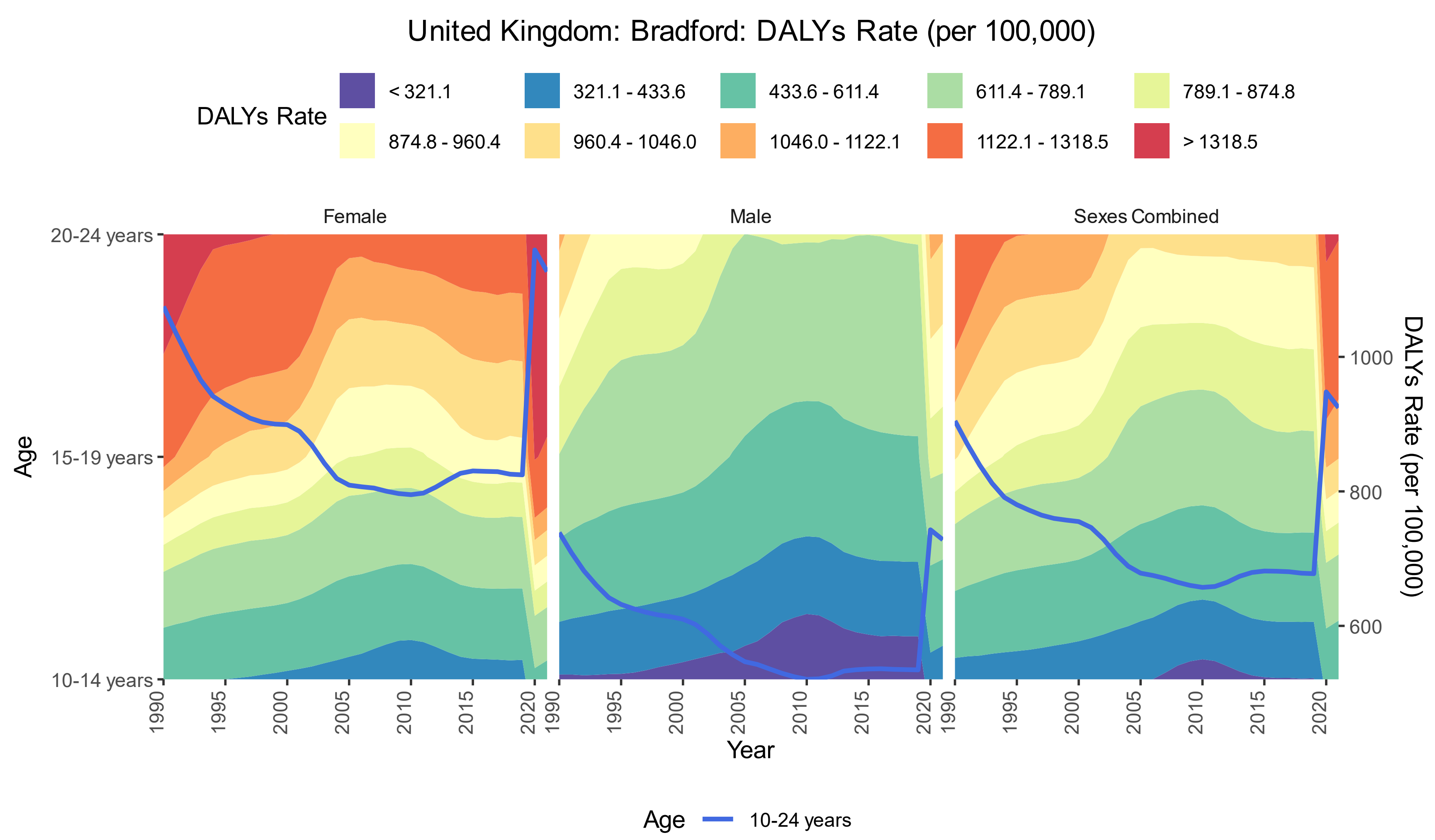
United Kingdom: Bradford: DALYs Rate (per 100,000)
DALYs Rate
< 321.1
321.1 - 433.6
433.6 - 611.4
611.4 - 789.1
789.1 - 874.8
874.8 - 960.4
960.4 - 1046.0
1046.0 - 1122.1
1122.1 - 1318.5
> 1318.5
Female
Male
Sexes Combined
Age
Year
DALYs Rate (per 100,000)
Age — 10-24 years

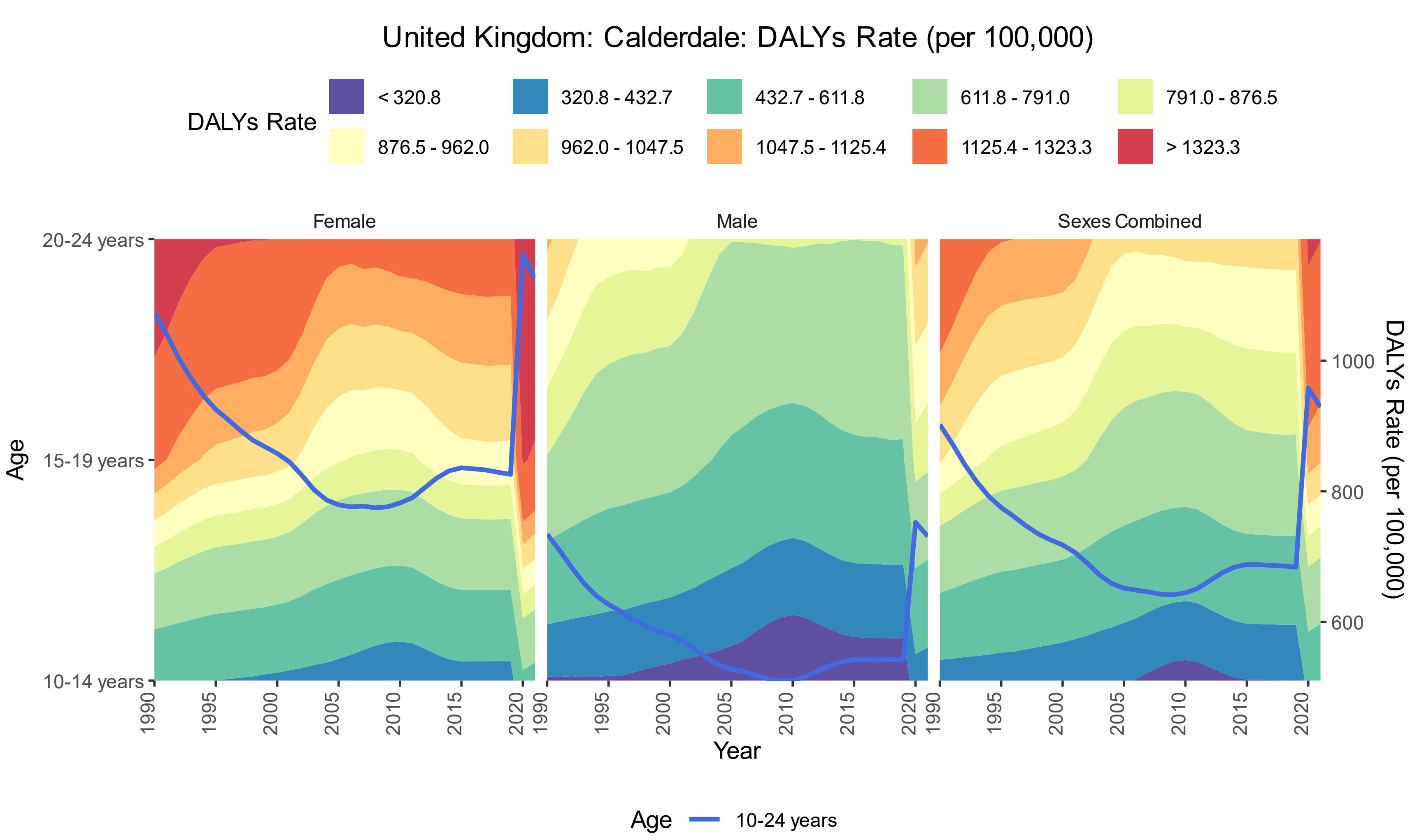
United Kingdom: Calderdale: DALYs Rate (per 100,000)
DALYs Rate
< 320.8
320.8 - 432.7
432.7 - 611.8
611.8 - 791.0
791.0 - 876.5
876.5 - 962.0
962.0 - 1047.5
1047.5 - 1125.4
1125.4 - 1323.3
> 1323.3
Female
Male
Sexes Combined
Age
Year
DALYs Rate (per 100,000)
Age — 10-24 years

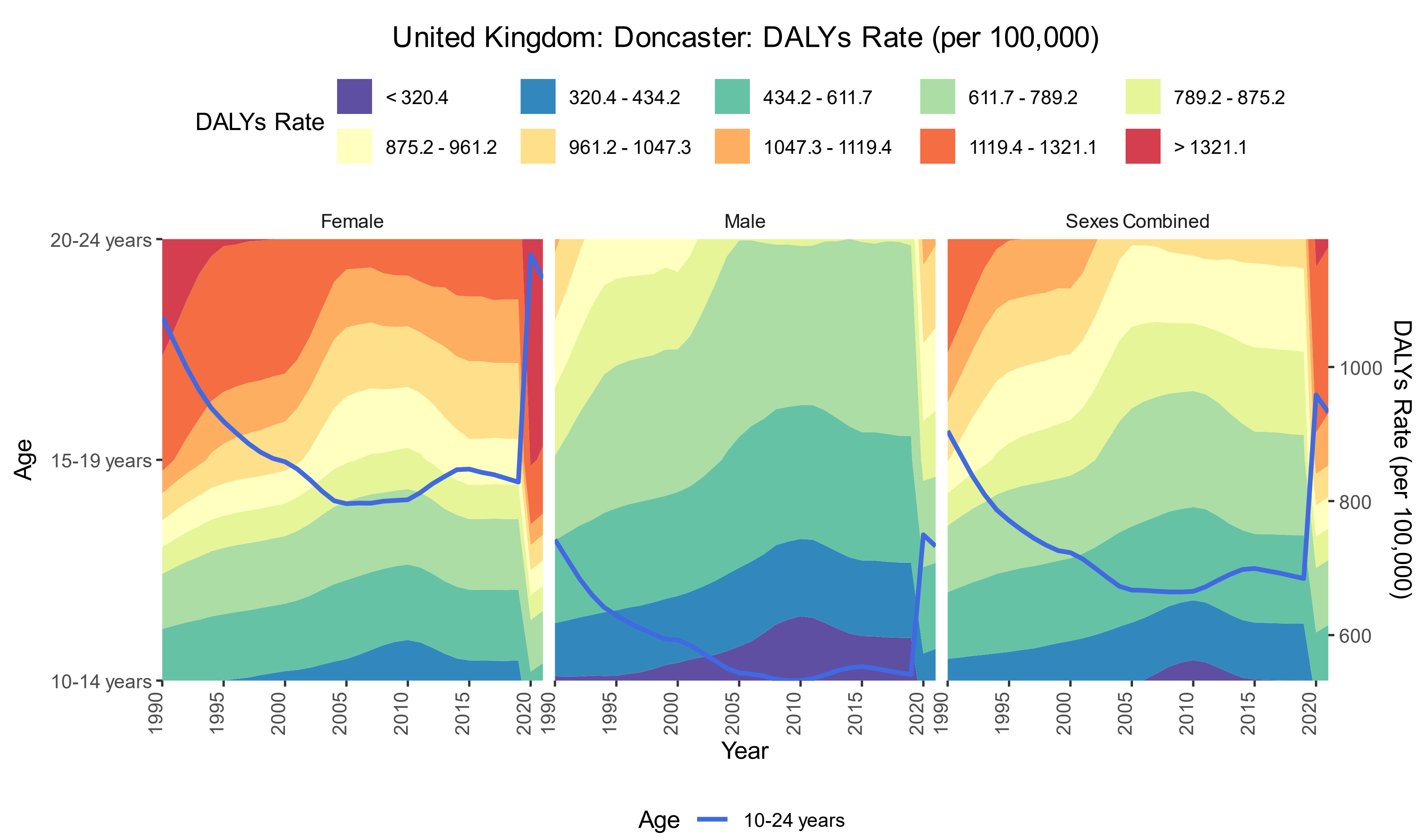
United Kingdom: Doncaster: DALYs Rate (per 100,000)
DALYs Rate
< 320.4
320.4 - 434.2
434.2 - 611.7
611.7 - 789.2
789.2 - 875.2
875.2 - 961.2
961.2 - 1047.3
1047.3 - 1119.4
1119.4 - 1321.1
> 1321.1
Female
Male
Sexes Combined
Age
Year
DALYs Rate (per 100,000)
Age — 10-24 years

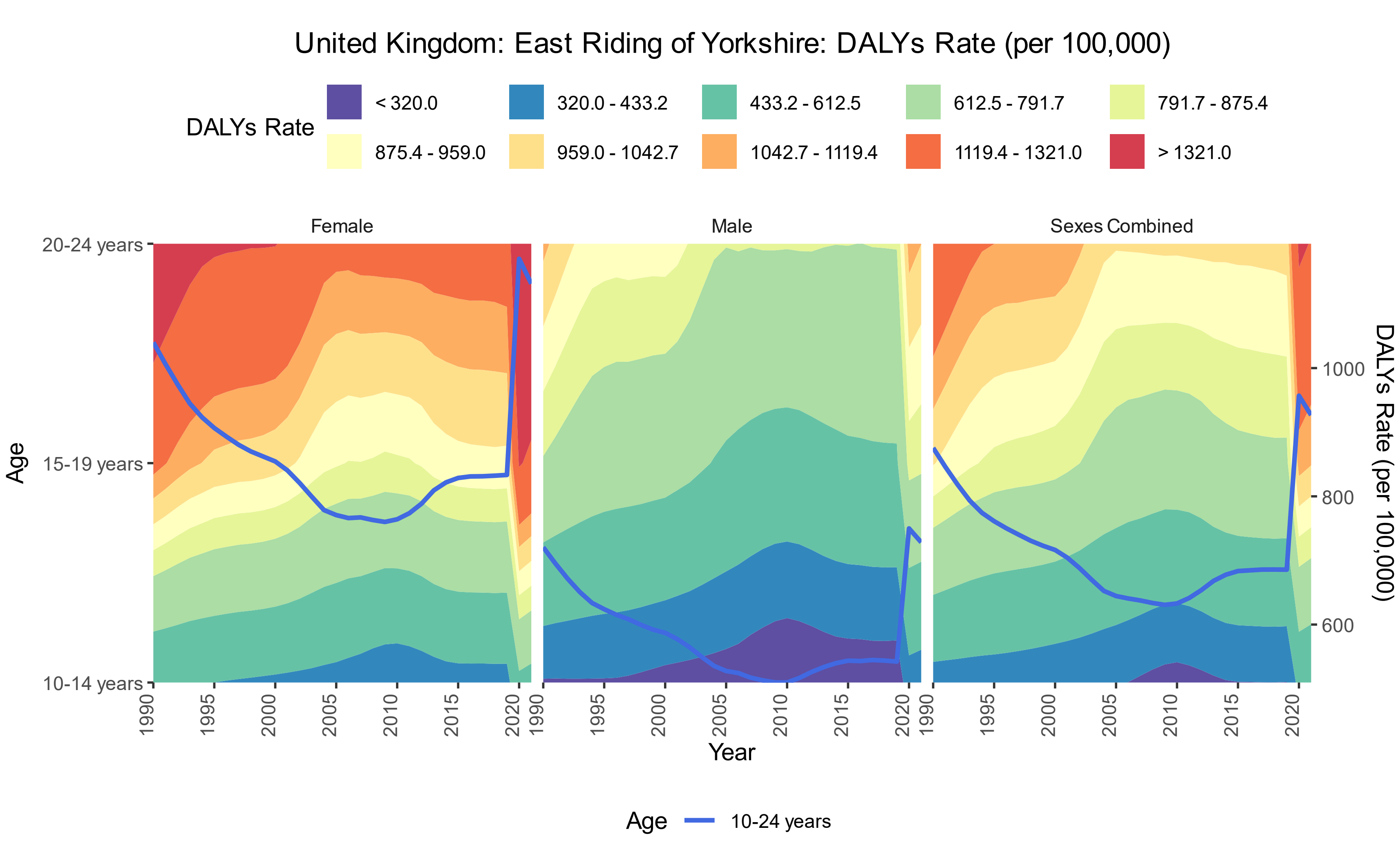
United Kingdom: East Riding of Yorkshire: DALYs Rate (per 100,000)
DALYs Rate
< 320.0
320.0 - 433.2
433.2 - 612.5
612.5 - 791.7
791.7 - 875.4
875.4 - 959.0
959.0 - 1042.7
1042.7 - 1119.4
1119.4 - 1321.0
> 1321.0
Female
Male
Sexes Combined
Age
Year
DALYs Rate (per 100,000)
Age — 10-24 years

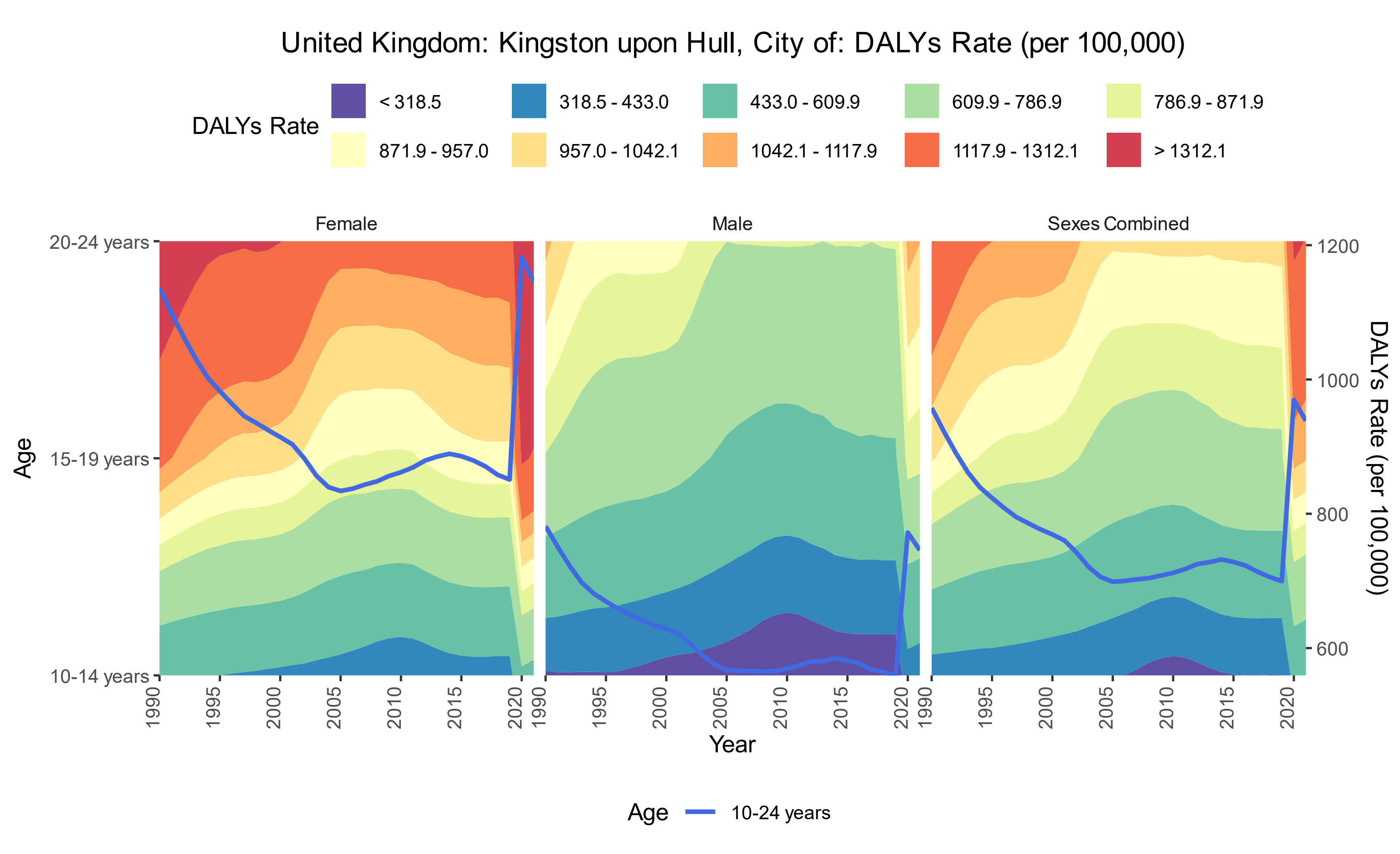
United Kingdom: Kingston upon Hull, City of: DALYs Rate (per 100,000)
DALYs Rate
< 318.5
318.5 - 433.0
433.0 - 609.9
609.9 - 786.9
786.9 - 871.9
871.9 - 957.0
957.0 - 1042.1
1042.1 - 1117.9
1117.9 - 1312.1
> 1312.1
Female
Male
Sexes Combined
Age
Year
DALYs Rate (per 100,000)
Age — 10-24 years

Figure S7

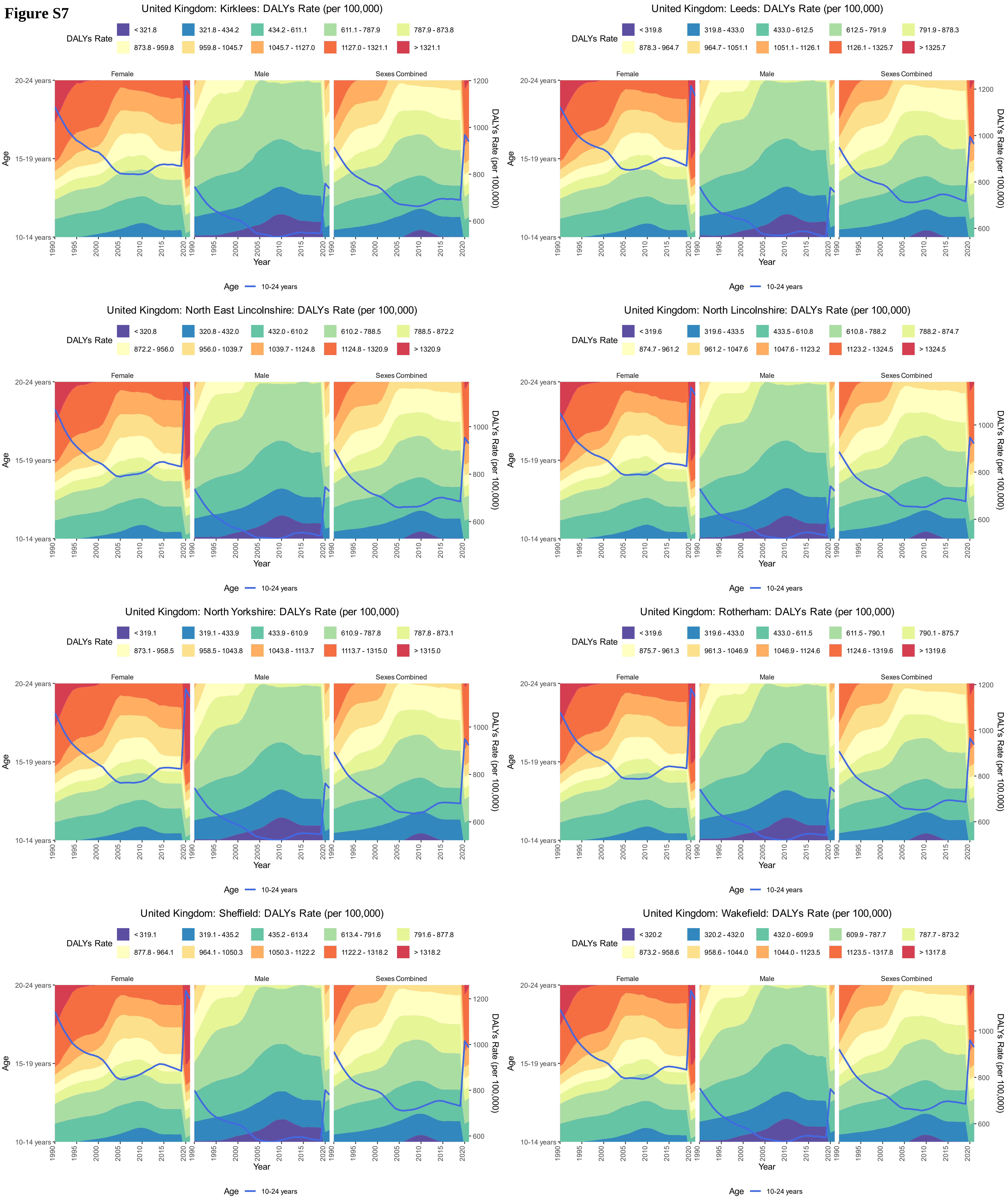
United Kingdom: Kirklees: DALYs Rate (per 100,000)
DALYs Rate
< 321.8
321.8 - 434.2
434.2 - 611.1
611.1 - 787.9
787.9 - 873.8
873.8 - 959.8
959.8 - 1045.7
1045.7 - 1127.0
1127.0 - 1321.1
> 1321.1
United Kingdom: Leeds: DALYs Rate (per 100,000)
< 319.8
319.8 - 433.0
433.0 - 612.5
612.5 - 791.9
791.9 - 878.3
878.3 - 964.7
964.7 - 1051.1
1051.1 - 1126.1
1126.1 - 1325.7
> 1325.7
United Kingdom: North East Lincolnshire: DALYs Rate (per 100,000)
< 320.8
320.8 - 432.0
432.0 - 610.2
610.2 - 788.5
788.5 - 872.2
872.2 - 956.0
956.0 - 1039.7
1039.7 - 1124.8
1124.8 - 1320.9
> 1320.9
United Kingdom: North Lincolnshire: DALYs Rate (per 100,000)
< 319.6
319.6 - 433.5
433.5 - 610.8
610.8 - 788.2
788.2 - 874.7
874.7 - 961.2
961.2 - 1047.6
1047.6 - 1123.2
1123.2 - 1324.5
> 1324.5
United Kingdom: North Yorkshire: DALYs Rate (per 100,000)
< 319.1
319.1 - 433.9
433.9 - 610.9
610.9 - 787.8
787.8 - 873.1
873.1 - 958.5
958.5 - 1043.8
1043.8 - 1113.7
1113.7 - 1315.0
> 1315.0
United Kingdom: Rotherham: DALYs Rate (per 100,000)
< 319.6
319.6 - 433.0
433.0 - 611.5
611.5 - 790.1
790.1 - 875.7
875.7 - 961.3
961.3 - 1046.9
1046.9 - 1124.6
1124.6 - 1319.6
> 1319.6
United Kingdom: Sheffield: DALYs Rate (per 100,000)
< 319.1
319.1 - 435.2
435.2 - 613.4
613.4 - 791.6
791.6 - 877.8
877.8 - 964.1
964.1 - 1050.3
1050.3 - 1122.2
1122.2 - 1318.2
> 1318.2
United Kingdom: Wakefield: DALYs Rate (per 100,000)
< 320.2
320.2 - 432.0
432.0 - 609.9
609.9 - 787.7
787.7 - 873.2
873.2 - 958.6
958.6 - 1044.0
1044.0 - 1123.5
1123.5 - 1317.8
> 1317.8
Female
Male
Sexes Combined
Age
20-24 years
15-19 years
10-14 years
Year
DALYs Rate (per 100,000)
Age — 10-24 years

Figure S7

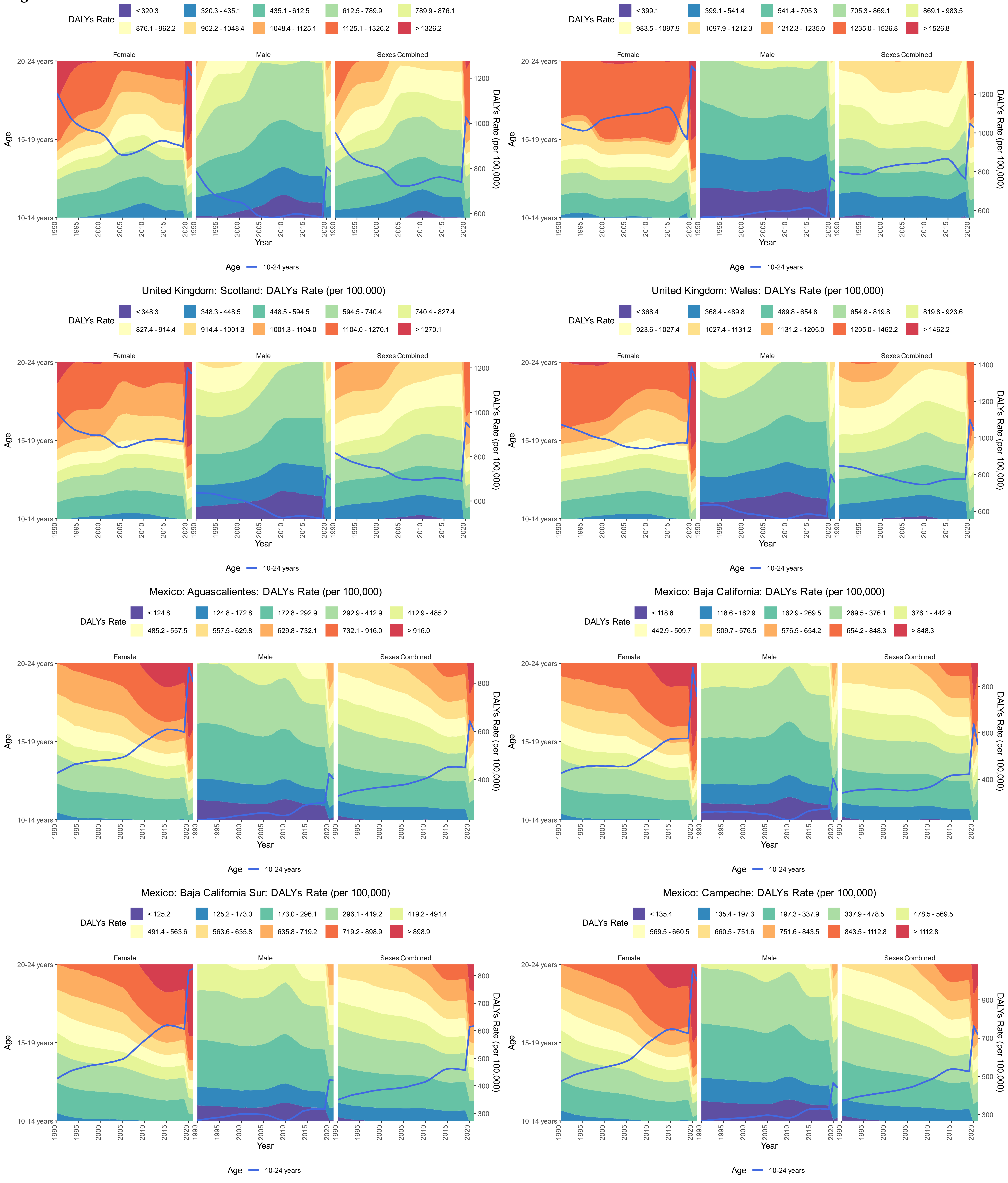
United Kingdom: York: DALYs Rate (per 100,000)
DALYs Rate
< 320.3
320.3 - 435.1
435.1 - 612.5
612.5 - 789.9
789.9 - 876.1
876.1 - 962.2
962.2 - 1048.4
1048.4 - 1125.1
1125.1 - 1326.2
> 1326.2
United Kingdom: Northern Ireland: DALYs Rate (per 100,000)
< 399.1
399.1 - 541.4
541.4 - 705.3
705.3 - 869.1
869.1 - 983.5
983.5 - 1097.9
1097.9 - 1212.3
1212.3 - 1235.0
1235.0 - 1526.8
> 1526.8
United Kingdom: Scotland: DALYs Rate (per 100,000)
< 348.3
348.3 - 448.5
448.5 - 594.5
594.5 - 740.4
740.4 - 827.4
827.4 - 914.4
914.4 - 1001.3
1001.3 - 1104.0
1104.0 - 1270.1
> 1270.1
United Kingdom: Wales: DALYs Rate (per 100,000)
< 368.4
368.4 - 489.8
489.8 - 654.8
654.8 - 819.8
819.8 - 923.6
923.6 - 1027.4
1027.4 - 1131.2
1131.2 - 1205.0
1205.0 - 1462.2
> 1462.2
Mexico: Aguascalientes: DALYs Rate (per 100,000)
< 124.8
124.8 - 172.8
172.8 - 292.9
292.9 - 412.9
412.9 - 485.2
485.2 - 557.5
557.5 - 629.8
629.8 - 732.1
732.1 - 916.0
> 916.0
Mexico: Baja California: DALYs Rate (per 100,000)
< 118.6
118.6 - 162.9
162.9 - 269.5
269.5 - 376.1
376.1 - 442.9
442.9 - 509.7
509.7 - 576.5
576.5 - 654.2
654.2 - 848.3
> 848.3
Mexico: Baja California Sur: DALYs Rate (per 100,000)
< 125.2
125.2 - 173.0
173.0 - 296.1
296.1 - 419.2
419.2 - 491.4
491.4 - 563.6
563.6 - 635.8
635.8 - 719.2
719.2 - 898.9
> 898.9
Mexico: Campeche: DALYs Rate (per 100,000)
< 135.4
135.4 - 197.3
197.3 - 337.9
337.9 - 478.5
478.5 - 569.5
569.5 - 660.5
660.5 - 751.6
751.6 - 843.5
843.5 - 1112.8
> 1112.8
Female
Male
Sexes Combined
20-24 years
15-19 years
10-14 years
Age
Year
DALYs Rate (per 100,000)
Age — 10-24 years

Figure S7

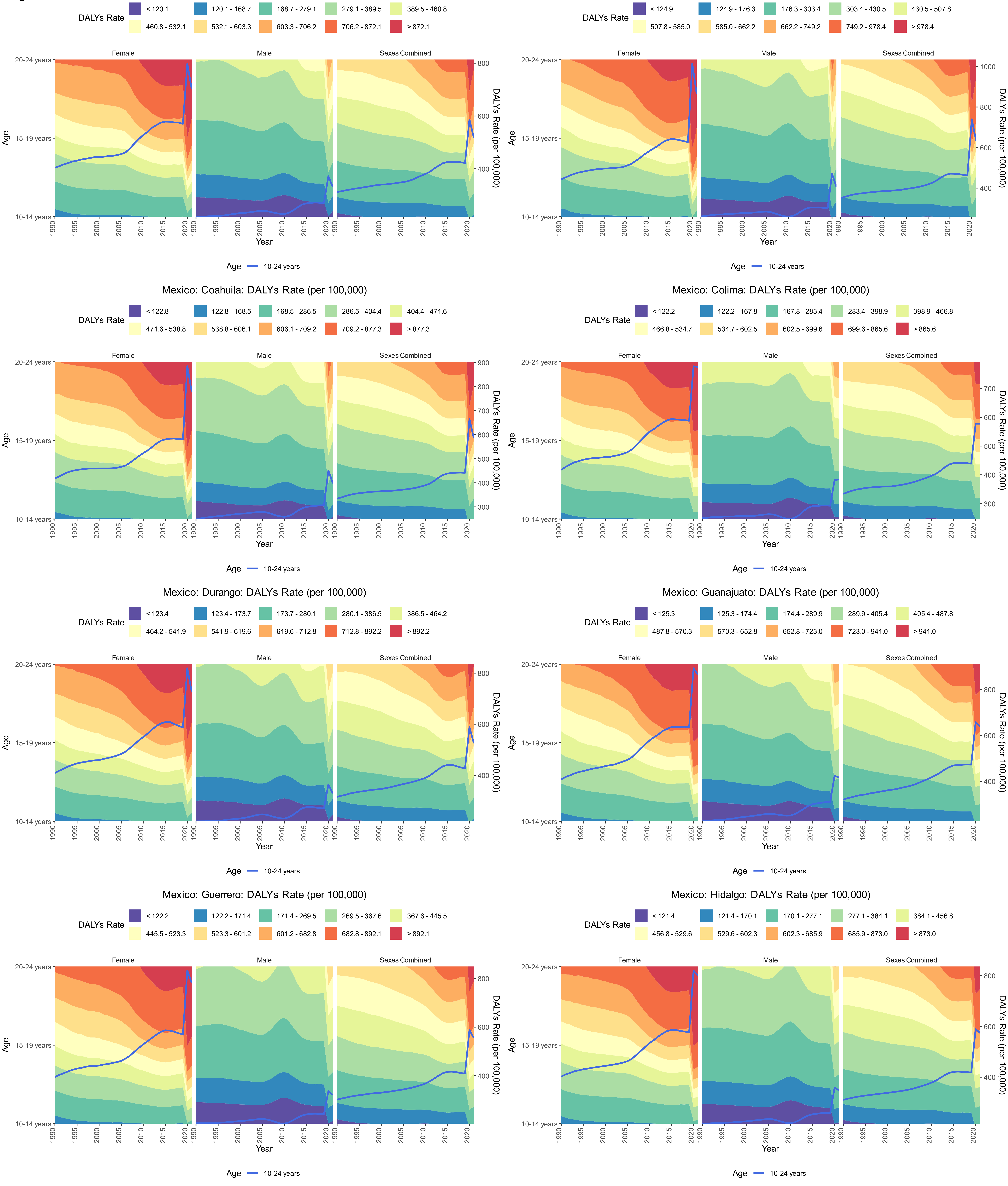
Mexico: Chiapas: DALYs Rate (per 100,000)
DALYs Rate
< 120.1
120.1 - 168.7
168.7 - 279.1
279.1 - 389.5
389.5 - 460.8
460.8 - 532.1
532.1 - 603.3
603.3 - 706.2
706.2 - 872.1
> 872.1
Mexico: Chihuahua: DALYs Rate (per 100,000)
DALYs Rate
< 124.9
124.9 - 176.3
176.3 - 303.4
303.4 - 430.5
430.5 - 507.8
507.8 - 585.0
585.0 - 662.2
662.2 - 749.2
749.2 - 978.4
> 978.4
Mexico: Coahuila: DALYs Rate (per 100,000)
DALYs Rate
< 122.8
122.8 - 168.5
168.5 - 286.5
286.5 - 404.4
404.4 - 471.6
471.6 - 538.8
538.8 - 606.1
606.1 - 709.2
709.2 - 877.3
> 877.3
Mexico: Colima: DALYs Rate (per 100,000)
DALYs Rate
< 122.2
122.2 - 167.8
167.8 - 283.4
283.4 - 398.9
398.9 - 466.8
466.8 - 534.7
534.7 - 602.5
602.5 - 699.6
699.6 - 865.6
> 865.6
Mexico: Durango: DALYs Rate (per 100,000)
DALYs Rate
< 123.4
123.4 - 173.7
173.7 - 280.1
280.1 - 386.5
386.5 - 464.2
464.2 - 541.9
541.9 - 619.6
619.6 - 712.8
712.8 - 892.2
> 892.2
Mexico: Guanajuato: DALYs Rate (per 100,000)
DALYs Rate
< 125.3
125.3 - 174.4
174.4 - 289.9
289.9 - 405.4
405.4 - 487.8
487.8 - 570.3
570.3 - 652.8
652.8 - 723.0
723.0 - 941.0
> 941.0
Mexico: Guerrero: DALYs Rate (per 100,000)
DALYs Rate
< 122.2
122.2 - 171.4
171.4 - 269.5
269.5 - 367.6
367.6 - 445.5
445.5 - 523.3
523.3 - 601.2
601.2 - 682.8
682.8 - 892.1
> 892.1
Mexico: Hidalgo: DALYs Rate (per 100,000)
DALYs Rate
< 121.4
121.4 - 170.1
170.1 - 277.1
277.1 - 384.1
384.1 - 456.8
456.8 - 529.6
529.6 - 602.3
602.3 - 685.9
685.9 - 873.0
> 873.0
Female
Male
Sexes Combined
Age
20-24 years
15-19 years
10-14 years
Year
1990
1995
2000
2005
2010
2015
2020
DALYs Rate (per 100,000)
Age
10-24 years

Figure S7

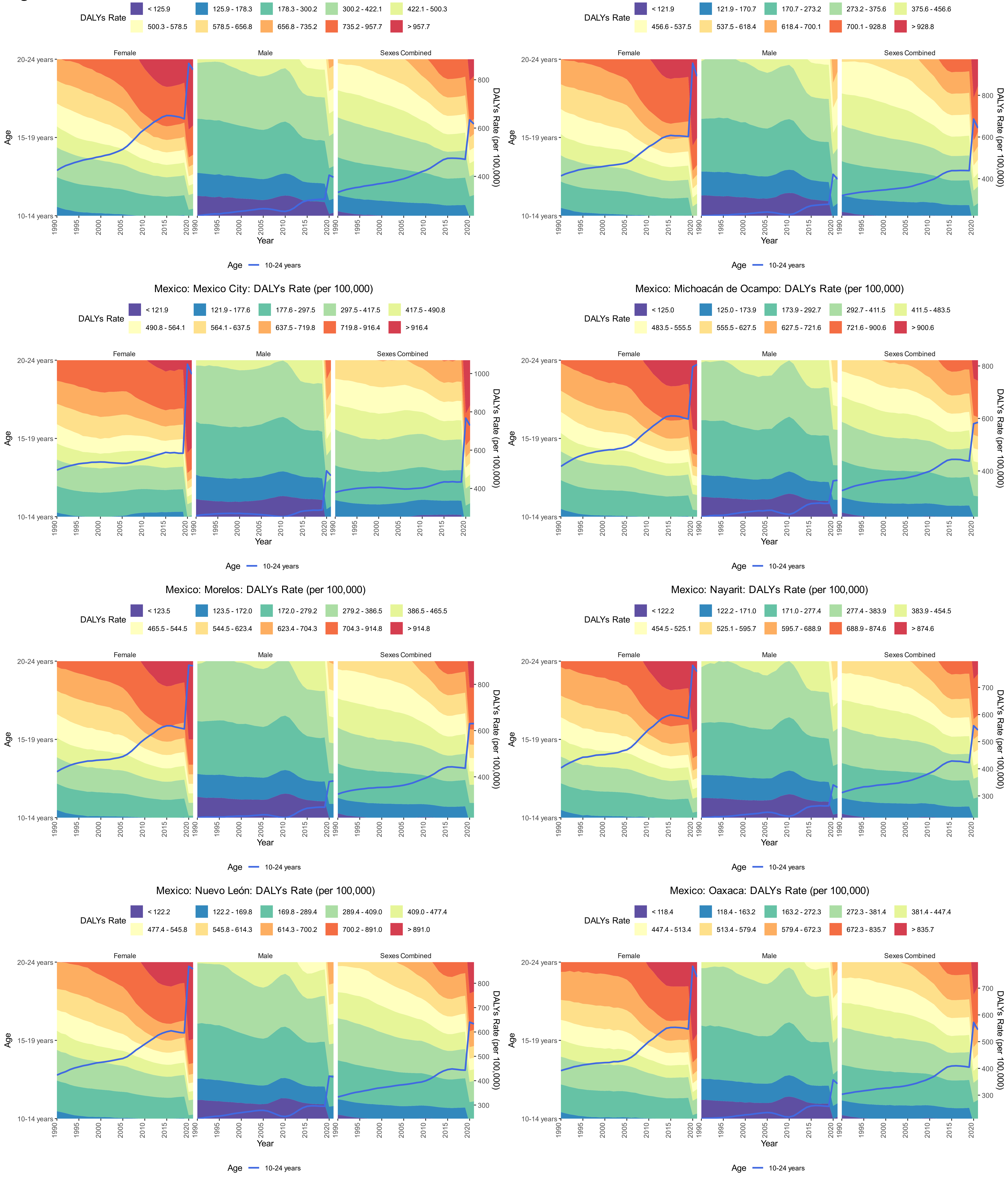
Mexico: Jalisco: DALYs Rate (per 100,000)
DALYs Rate
< 125.9
125.9 - 178.3
178.3 - 300.2
300.2 - 422.1
422.1 - 500.3
500.3 - 578.5
578.5 - 656.8
656.8 - 735.2
735.2 - 957.7
> 957.7
Female
Male
Sexes Combined
Age
Year
DALYs Rate (per 100,000)
Age — 10-24 years
Mexico: México: DALYs Rate (per 100,000)
< 121.9
121.9 - 170.7
170.7 - 273.2
273.2 - 375.6
375.6 - 456.6
456.6 - 537.5
537.5 - 618.4
618.4 - 700.1
700.1 - 928.8
> 928.8
Mexico: Mexico City: DALYs Rate (per 100,000)
< 121.9
121.9 - 177.6
177.6 - 297.5
297.5 - 417.5
417.5 - 490.8
490.8 - 564.1
564.1 - 637.5
637.5 - 719.8
719.8 - 916.4
> 916.4
Mexico: Michoacán de Ocampo: DALYs Rate (per 100,000)
< 125.0
125.0 - 173.9
173.9 - 292.7
292.7 - 411.5
411.5 - 483.5
483.5 - 555.5
555.5 - 627.5
627.5 - 721.6
721.6 - 900.6
> 900.6
Mexico: Morelos: DALYs Rate (per 100,000)
< 123.5
123.5 - 172.0
172.0 - 279.2
279.2 - 386.5
386.5 - 465.5
465.5 - 544.5
544.5 - 623.4
623.4 - 704.3
704.3 - 914.8
> 914.8
Mexico: Nayarit: DALYs Rate (per 100,000)
< 122.2
122.2 - 171.0
171.0 - 277.4
277.4 - 383.9
383.9 - 454.5
454.5 - 525.1
525.1 - 595.7
595.7 - 688.9
688.9 - 874.6
> 874.6
Mexico: Nuevo León: DALYs Rate (per 100,000)
< 122.2
122.2 - 169.8
169.8 - 289.4
289.4 - 409.0
409.0 - 477.4
477.4 - 545.8
545.8 - 614.3
614.3 - 700.2
700.2 - 891.0
> 891.0
Mexico: Oaxaca: DALYs Rate (per 100,000)
< 118.4
118.4 - 163.2
163.2 - 272.3
272.3 - 381.4
381.4 - 447.4
447.4 - 513.4
513.4 - 579.4
579.4 - 672.3
672.3 - 835.7
> 835.7
20-24 years
15-19 years
10-14 years
1990
1995
2000
2005
2010
2015
2020

Figure S7

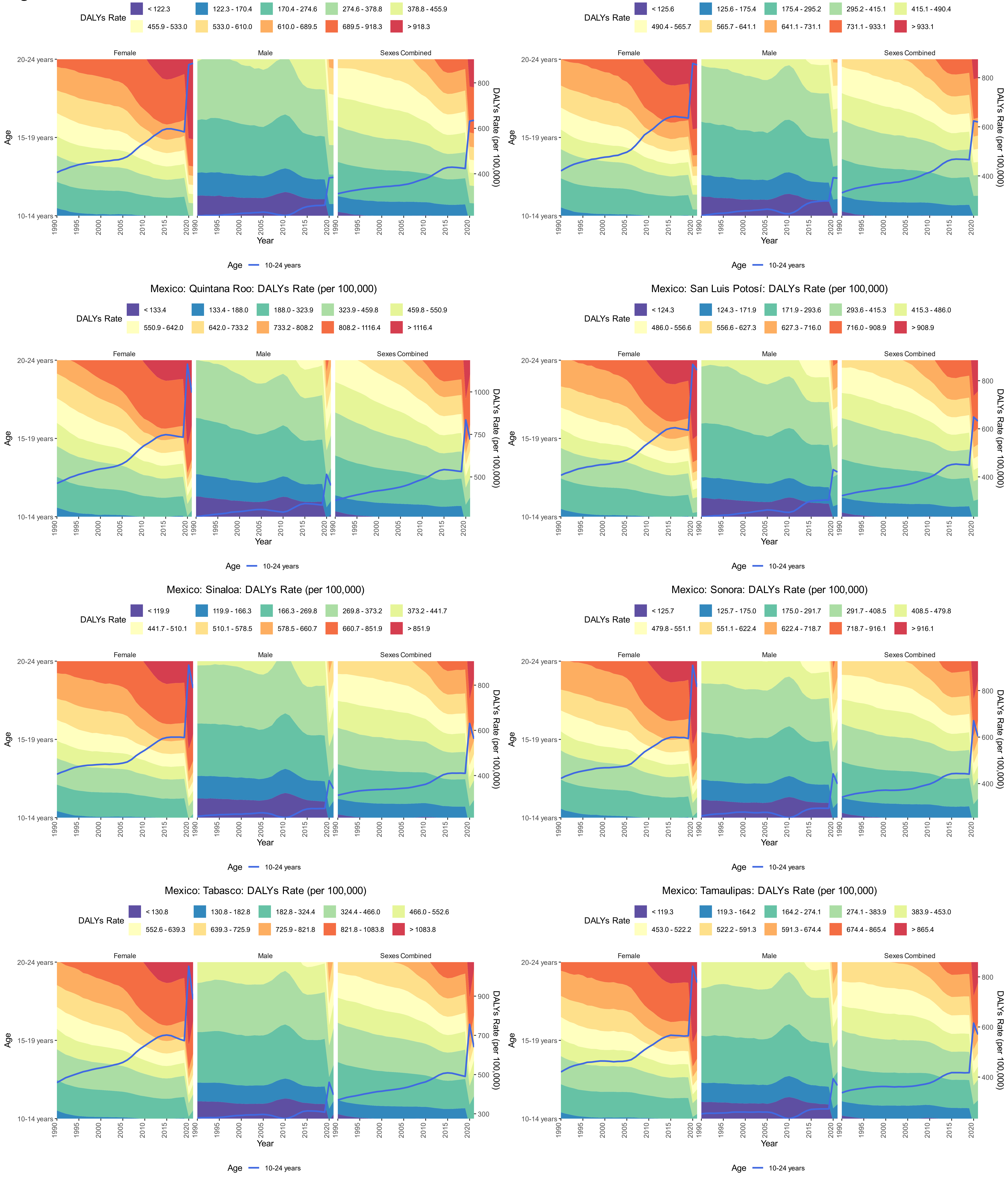

Mexico: Puebla: DALYs Rate (per 100,000)
DALYs Rate
< 122.3
122.3 - 170.4
170.4 - 274.6
274.6 - 378.8
378.8 - 455.9
455.9 - 533.0
533.0 - 610.0
610.0 - 689.5
689.5 - 918.3
> 918.3
Female
Male
Sexes Combined
20-24 years
15-19 years
10-14 years
Age
Year
DALYs Rate (per 100,000)
Age
10-24 years
Mexico: Querétaro: DALYs Rate (per 100,000)
DALYs Rate
< 125.6
125.6 - 175.4
175.4 - 295.2
295.2 - 415.1
415.1 - 490.4
490.4 - 565.7
565.7 - 641.1
641.1 - 731.1
731.1 - 933.1
> 933.1
Mexico: Quintana Roo: DALYs Rate (per 100,000)
DALYs Rate
< 133.4
133.4 - 188.0
188.0 - 323.9
323.9 - 459.8
459.8 - 550.9
550.9 - 642.0
642.0 - 733.2
733.2 - 808.2
808.2 - 1116.4
> 1116.4
Mexico: San Luis Potosí: DALYs Rate (per 100,000)
DALYs Rate
< 124.3
124.3 - 171.9
171.9 - 293.6
293.6 - 415.3
415.3 - 486.0
486.0 - 556.6
556.6 - 627.3
627.3 - 716.0
716.0 - 908.9
> 908.9
Mexico: Sinaloa: DALYs Rate (per 100,000)
DALYs Rate
< 119.9
119.9 - 166.3
166.3 - 269.8
269.8 - 373.2
373.2 - 441.7
441.7 - 510.1
510.1 - 578.5
578.5 - 660.7
660.7 - 851.9
> 851.9
Mexico: Sonora: DALYs Rate (per 100,000)
DALYs Rate
< 125.7
125.7 - 175.0
175.0 - 291.7
291.7 - 408.5
408.5 - 479.8
479.8 - 551.1
551.1 - 622.4
622.4 - 718.7
718.7 - 916.1
> 916.1
Mexico: Tabasco: DALYs Rate (per 100,000)
DALYs Rate
< 130.8
130.8 - 182.8
182.8 - 324.4
324.4 - 466.0
466.0 - 552.6
552.6 - 639.3
639.3 - 725.9
725.9 - 821.8
821.8 - 1083.8
> 1083.8
Mexico: Tamaulipas: DALYs Rate (per 100,000)
DALYs Rate
< 119.3
119.3 - 164.2
164.2 - 274.1
274.1 - 383.9
383.9 - 453.0
453.0 - 522.2
522.2 - 591.3
591.3 - 674.4
674.4 - 865.4
> 865.4

Figure S7

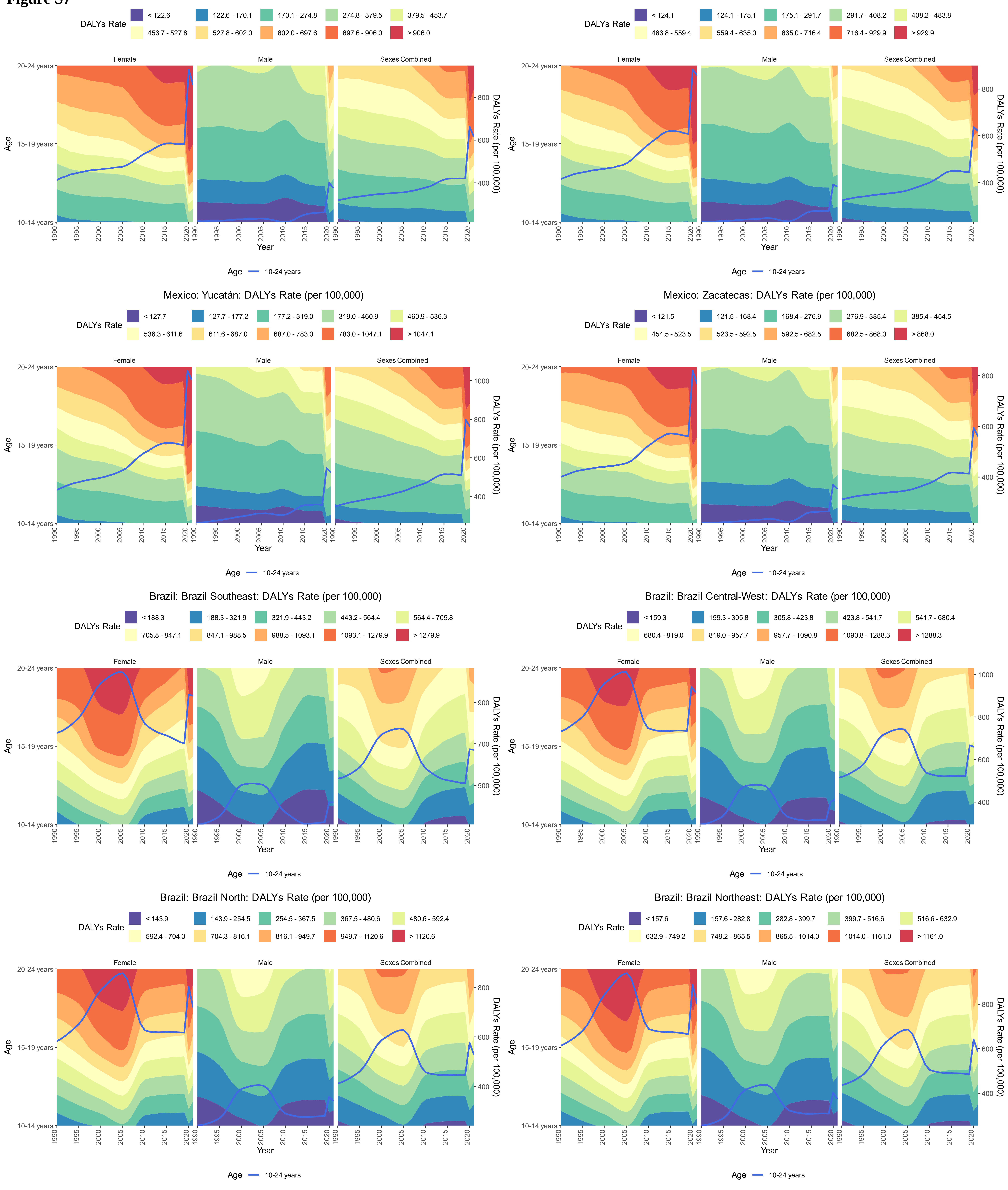

Mexico: Tlaxcala: DALYs Rate (per 100,000)
DALYs Rate
< 122.6
122.6 - 170.1
170.1 - 274.8
274.8 - 379.5
379.5 - 453.7
453.7 - 527.8
527.8 - 602.0
602.0 - 697.6
697.6 - 906.0
> 906.0
Female
Male
Sexes Combined
Mexico: Veracruz de Ignacio de la Llave: DALYs Rate (per 100,000)
DALYs Rate
< 124.1
124.1 - 175.1
175.1 - 291.7
291.7 - 408.2
408.2 - 483.8
483.8 - 559.4
559.4 - 635.0
635.0 - 716.4
716.4 - 929.9
> 929.9
Mexico: Yucatán: DALYs Rate (per 100,000)
DALYs Rate
< 127.7
127.7 - 177.2
177.2 - 319.0
319.0 - 460.9
460.9 - 536.3
536.3 - 611.6
611.6 - 687.0
687.0 - 783.0
783.0 - 1047.1
> 1047.1
Mexico: Zacatecas: DALYs Rate (per 100,000)
DALYs Rate
< 121.5
121.5 - 168.4
168.4 - 276.9
276.9 - 385.4
385.4 - 454.5
454.5 - 523.5
523.5 - 592.5
592.5 - 682.5
682.5 - 868.0
> 868.0
Brazil: Brazil Southeast: DALYs Rate (per 100,000)
DALYs Rate
< 188.3
188.3 - 321.9
321.9 - 443.2
443.2 - 564.4
564.4 - 705.8
705.8 - 847.1
847.1 - 988.5
988.5 - 1093.1
1093.1 - 1279.9
> 1279.9
Brazil: Brazil Central-West: DALYs Rate (per 100,000)
DALYs Rate
< 159.3
159.3 - 305.8
305.8 - 423.8
423.8 - 541.7
541.7 - 680.4
680.4 - 819.0
819.0 - 957.7
957.7 - 1090.8
1090.8 - 1288.3
> 1288.3
Brazil: Brazil North: DALYs Rate (per 100,000)
DALYs Rate
< 143.9
143.9 - 254.5
254.5 - 367.5
367.5 - 480.6
480.6 - 592.4
592.4 - 704.3
704.3 - 816.1
816.1 - 949.7
949.7 - 1120.6
> 1120.6
Brazil: Brazil Northeast: DALYs Rate (per 100,000)
DALYs Rate
< 157.6
157.6 - 282.8
282.8 - 399.7
399.7 - 516.6
516.6 - 632.9
632.9 - 749.2
749.2 - 865.5
865.5 - 1014.0
1014.0 - 1161.0
> 1161.0
Age
20-24 years
15-19 years
10-14 years
Year
DALYs Rate (per 100,000)
Age — 10-24 years

Figure S7

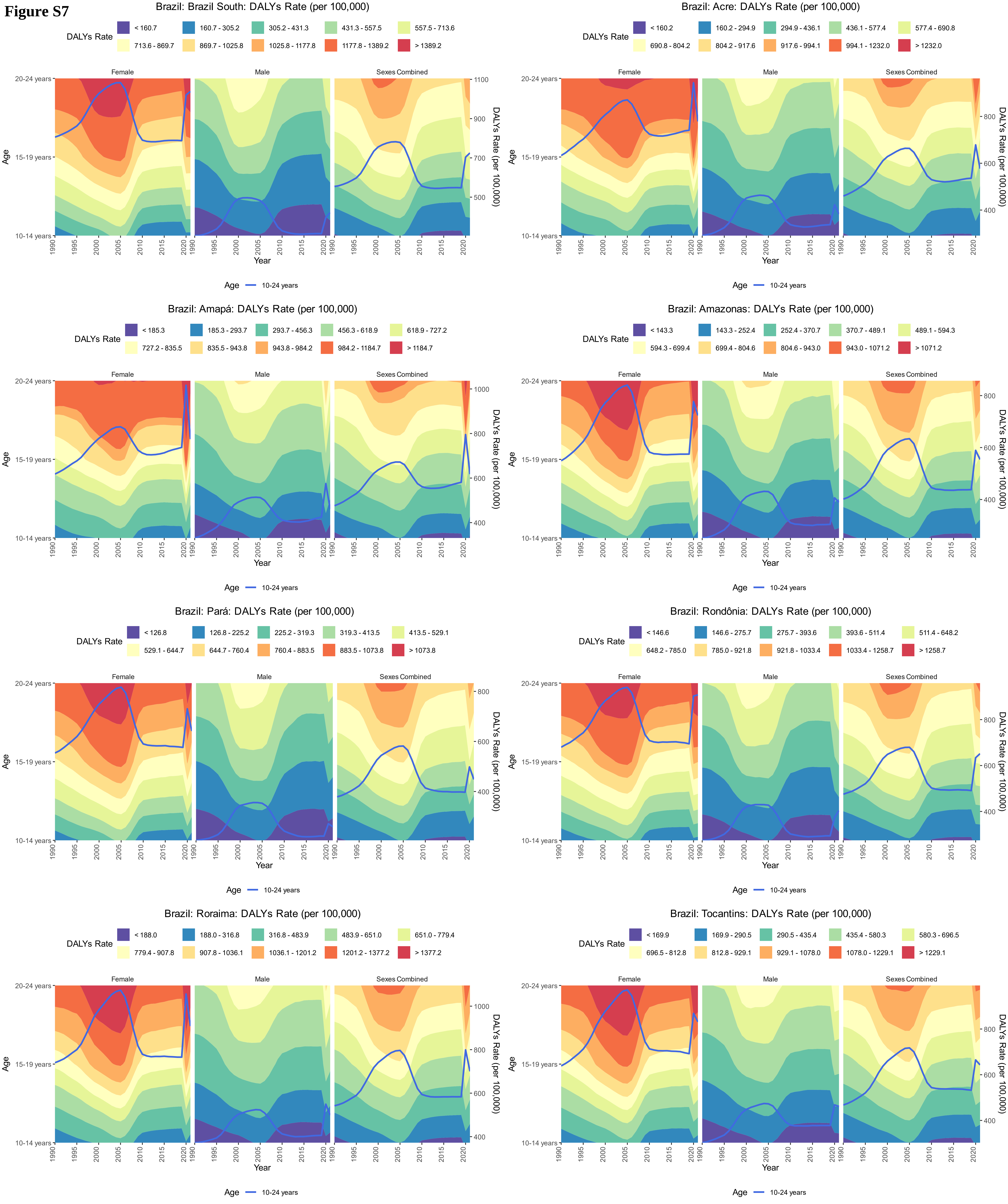

Brazil: Brazil South: DALYs Rate (per 100,000)
DALYs Rate
< 160.7
160.7 - 305.2
305.2 - 431.3
431.3 - 557.5
557.5 - 713.6
713.6 - 869.7
869.7 - 1025.8
1025.8 - 1177.8
1177.8 - 1389.2
> 1389.2
Brazil: Acre: DALYs Rate (per 100,000)
DALYs Rate
< 160.2
160.2 - 294.9
294.9 - 436.1
436.1 - 577.4
577.4 - 690.8
690.8 - 804.2
804.2 - 917.6
917.6 - 994.1
994.1 - 1232.0
> 1232.0
Brazil: Amapá: DALYs Rate (per 100,000)
DALYs Rate
< 185.3
185.3 - 293.7
293.7 - 456.3
456.3 - 618.9
618.9 - 727.2
727.2 - 835.5
835.5 - 943.8
943.8 - 984.2
984.2 - 1184.7
> 1184.7
Brazil: Amazonas: DALYs Rate (per 100,000)
DALYs Rate
< 143.3
143.3 - 252.4
252.4 - 370.7
370.7 - 489.1
489.1 - 594.3
594.3 - 699.4
699.4 - 804.6
804.6 - 943.0
943.0 - 1071.2
> 1071.2
Brazil: Pará: DALYs Rate (per 100,000)
DALYs Rate
< 126.8
126.8 - 225.2
225.2 - 319.3
319.3 - 413.5
413.5 - 529.1
529.1 - 644.7
644.7 - 760.4
760.4 - 883.5
883.5 - 1073.8
> 1073.8
Brazil: Rondônia: DALYs Rate (per 100,000)
DALYs Rate
< 146.6
146.6 - 275.7
275.7 - 393.6
393.6 - 511.4
511.4 - 648.2
648.2 - 785.0
785.0 - 921.8
921.8 - 1033.4
1033.4 - 1258.7
> 1258.7
Brazil: Roraima: DALYs Rate (per 100,000)
DALYs Rate
< 188.0
188.0 - 316.8
316.8 - 483.9
483.9 - 651.0
651.0 - 779.4
779.4 - 907.8
907.8 - 1036.1
1036.1 - 1201.2
1201.2 - 1377.2
> 1377.2
Brazil: Tocantins: DALYs Rate (per 100,000)
DALYs Rate
< 169.9
169.9 - 290.5
290.5 - 435.4
435.4 - 580.3
580.3 - 696.5
696.5 - 812.8
812.8 - 929.1
929.1 - 1078.0
1078.0 - 1229.1
> 1229.1
Female
Male
Sexes Combined
Age
20-24 years
15-19 years
10-14 years
Year
DALYs Rate (per 100,000)
Age
10-24 years

Figure S7

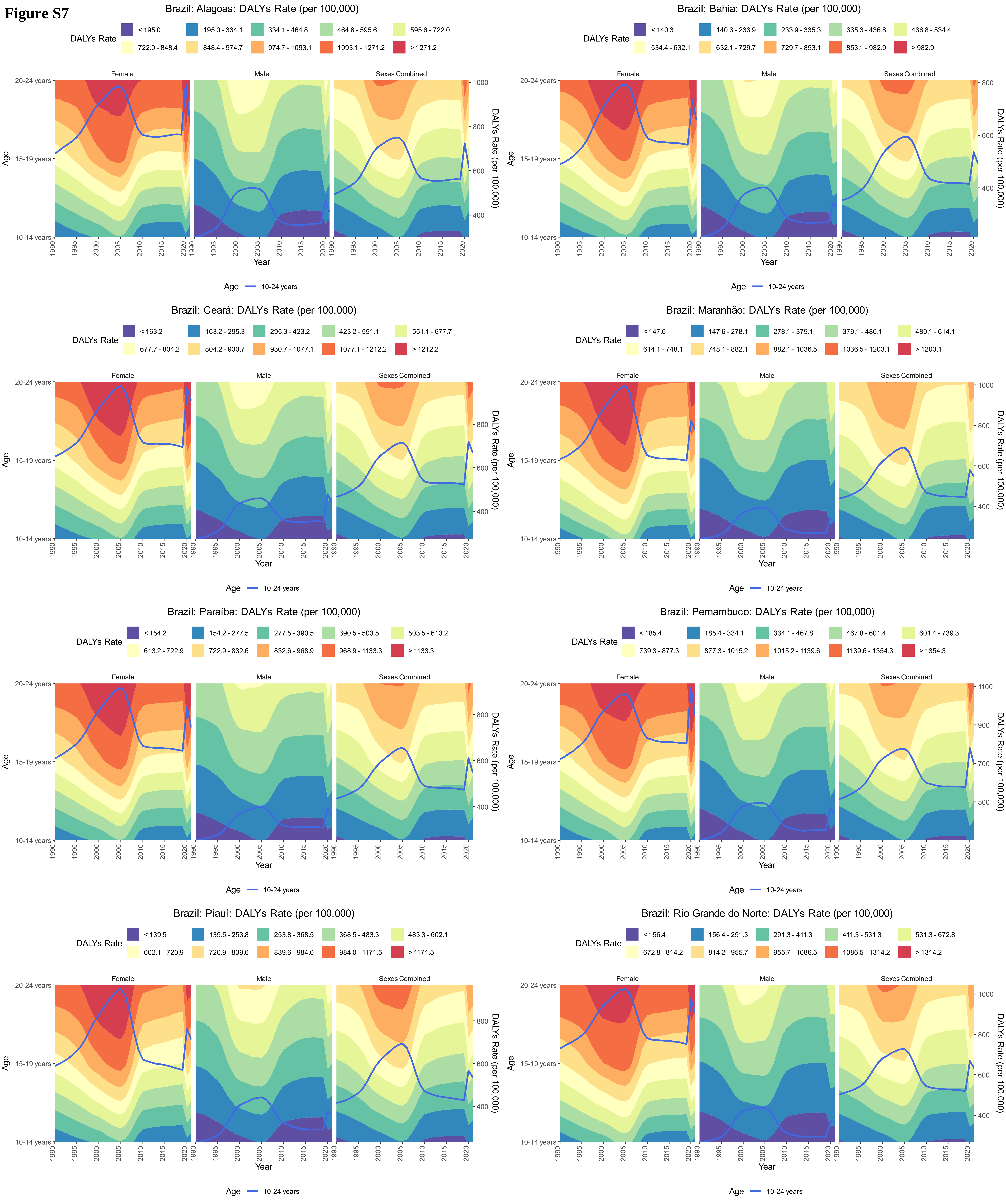

Brazil: Alagoas: DALYs Rate (per 100,000)
DALYs Rate
< 195.0
195.0 - 334.1
334.1 - 464.8
464.8 - 595.6
595.6 - 722.0
722.0 - 848.4
848.4 - 974.7
974.7 - 1093.1
1093.1 - 1271.2
> 1271.2
Brazil: Bahia: DALYs Rate (per 100,000)
< 140.3
140.3 - 233.9
233.9 - 335.3
335.3 - 436.8
436.8 - 534.4
534.4 - 632.1
632.1 - 729.7
729.7 - 853.1
853.1 - 982.9
> 982.9
Brazil: Ceará: DALYs Rate (per 100,000)
< 163.2
163.2 - 295.3
295.3 - 423.2
423.2 - 551.1
551.1 - 677.7
677.7 - 804.2
804.2 - 930.7
930.7 - 1077.1
1077.1 - 1212.2
> 1212.2
Brazil: Maranhão: DALYs Rate (per 100,000)
< 147.6
147.6 - 278.1
278.1 - 379.1
379.1 - 480.1
480.1 - 614.1
614.1 - 748.1
748.1 - 882.1
882.1 - 1036.5
1036.5 - 1203.1
> 1203.1
Brazil: Paraíba: DALYs Rate (per 100,000)
< 154.2
154.2 - 277.5
277.5 - 390.5
390.5 - 503.5
503.5 - 613.2
613.2 - 722.9
722.9 - 832.6
832.6 - 968.9
968.9 - 1133.3
> 1133.3
Brazil: Pernambuco: DALYs Rate (per 100,000)
< 185.4
185.4 - 334.1
334.1 - 467.8
467.8 - 601.4
601.4 - 739.3
739.3 - 877.3
877.3 - 1015.2
1015.2 - 1139.6
1139.6 - 1354.3
> 1354.3
Brazil: Piauí: DALYs Rate (per 100,000)
< 139.5
139.5 - 253.8
253.8 - 368.5
368.5 - 483.3
483.3 - 602.1
602.1 - 720.9
720.9 - 839.6
839.6 - 984.0
984.0 - 1171.5
> 1171.5
Brazil: Rio Grande do Norte: DALYs Rate (per 100,000)
< 156.4
156.4 - 291.3
291.3 - 411.3
411.3 - 531.3
531.3 - 672.8
672.8 - 814.2
814.2 - 955.7
955.7 - 1086.5
1086.5 - 1314.2
> 1314.2
Female
Male
Sexes Combined
Age
20-24 years
15-19 years
10-14 years
Year
DALYs Rate (per 100,000)
Age — 10-24 years

Figure S7

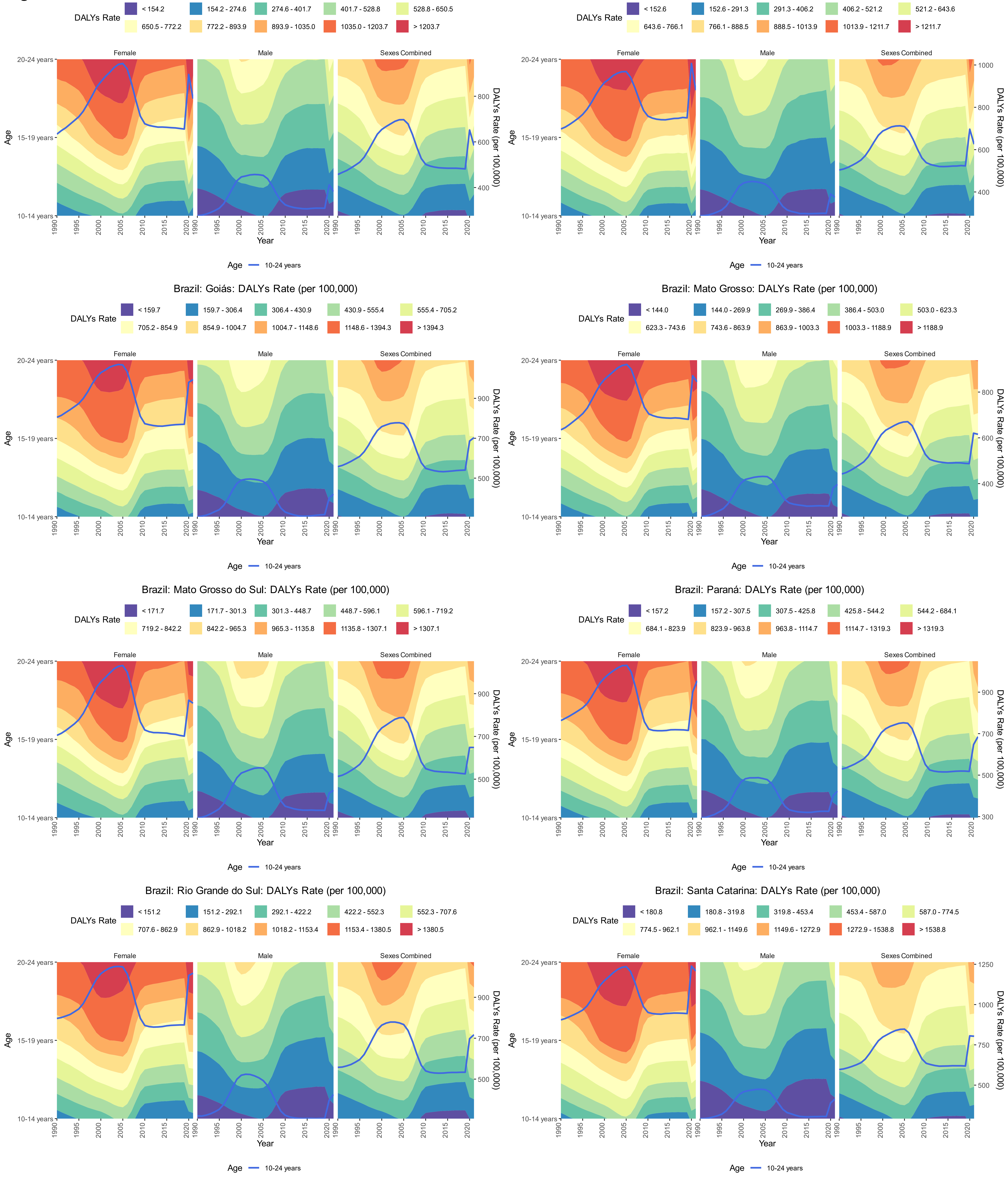
Brazil: Sergipe: DALYs Rate (per 100,000)
DALYs Rate
< 154.2
154.2 - 274.6
274.6 - 401.7
401.7 - 528.8
528.8 - 650.5
650.5 - 772.2
772.2 - 893.9
893.9 - 1035.0
1035.0 - 1203.7
> 1203.7
Brazil: Distrito Federal: DALYs Rate (per 100,000)
DALYs Rate
< 152.6
152.6 - 291.3
291.3 - 406.2
406.2 - 521.2
521.2 - 643.6
643.6 - 766.1
766.1 - 888.5
888.5 - 1013.9
1013.9 - 1211.7
> 1211.7
Brazil: Goiás: DALYs Rate (per 100,000)
DALYs Rate
< 159.7
159.7 - 306.4
306.4 - 430.9
430.9 - 555.4
555.4 - 705.2
705.2 - 854.9
854.9 - 1004.7
1004.7 - 1148.6
1148.6 - 1394.3
> 1394.3
Brazil: Mato Grosso: DALYs Rate (per 100,000)
DALYs Rate
< 144.0
144.0 - 269.9
269.9 - 386.4
386.4 - 503.0
503.0 - 623.3
623.3 - 743.6
743.6 - 863.9
863.9 - 1003.3
1003.3 - 1188.9
> 1188.9
Brazil: Mato Grosso do Sul: DALYs Rate (per 100,000)
DALYs Rate
< 171.7
171.7 - 301.3
301.3 - 448.7
448.7 - 596.1
596.1 - 719.2
719.2 - 842.2
842.2 - 965.3
965.3 - 1135.8
1135.8 - 1307.1
> 1307.1
Brazil: Paraná: DALYs Rate (per 100,000)
DALYs Rate
< 157.2
157.2 - 307.5
307.5 - 425.8
425.8 - 544.2
544.2 - 684.1
684.1 - 823.9
823.9 - 963.8
963.8 - 1114.7
1114.7 - 1319.3
> 1319.3
Brazil: Rio Grande do Sul: DALYs Rate (per 100,000)
DALYs Rate
< 151.2
151.2 - 292.1
292.1 - 422.2
422.2 - 552.3
552.3 - 707.6
707.6 - 862.9
862.9 - 1018.2
1018.2 - 1153.4
1153.4 - 1380.5
> 1380.5
Brazil: Santa Catarina: DALYs Rate (per 100,000)
DALYs Rate
< 180.8
180.8 - 319.8
319.8 - 453.4
453.4 - 587.0
587.0 - 774.5
774.5 - 962.1
962.1 - 1149.6
1149.6 - 1272.9
1272.9 - 1538.8
> 1538.8
Female
Male
Sexes Combined
Age
20-24 years
15-19 years
10-14 years
Year
DALYs Rate (per 100,000)
Age — 10-24 years

Figure S7

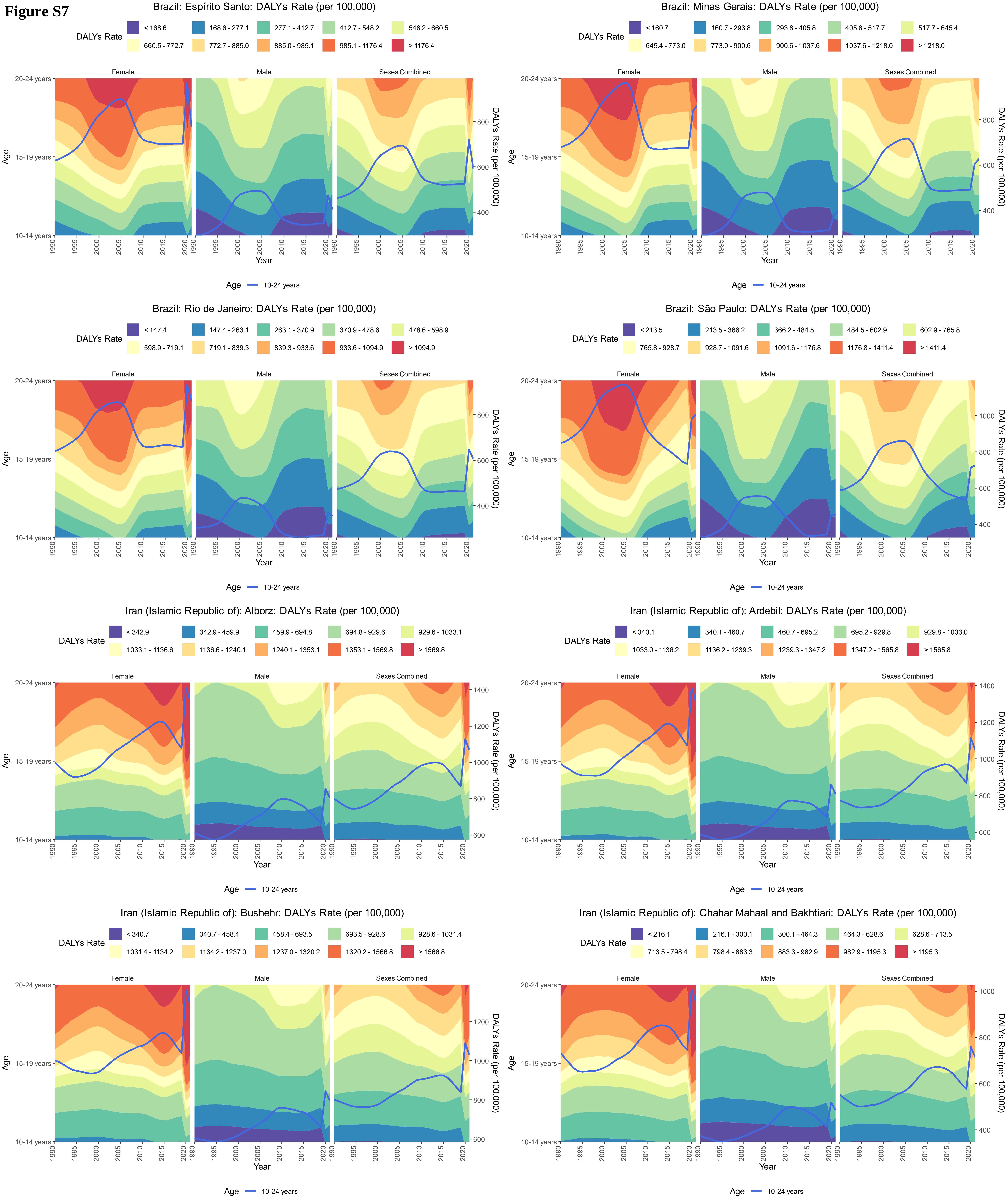

Brazil: Espírito Santo: DALYs Rate (per 100,000)
DALYs Rate
< 168.6
168.6 - 277.1
277.1 - 412.7
412.7 - 548.2
548.2 - 660.5
660.5 - 772.7
772.7 - 885.0
885.0 - 985.1
985.1 - 1176.4
> 1176.4
Female
Male
Sexes Combined
20-24 years
15-19 years
10-14 years
Age
Year
DALYs Rate (per 100,000)
Age
10-24 years
Brazil: Minas Gerais: DALYs Rate (per 100,000)
DALYs Rate
< 160.7
160.7 - 293.8
293.8 - 405.8
405.8 - 517.7
517.7 - 645.4
645.4 - 773.0
773.0 - 900.6
900.6 - 1037.6
1037.6 - 1218.0
> 1218.0
Brazil: Rio de Janeiro: DALYs Rate (per 100,000)
DALYs Rate
< 147.4
147.4 - 263.1
263.1 - 370.9
370.9 - 478.6
478.6 - 598.9
598.9 - 719.1
719.1 - 839.3
839.3 - 933.6
933.6 - 1094.9
> 1094.9
Brazil: São Paulo: DALYs Rate (per 100,000)
DALYs Rate
< 213.5
213.5 - 366.2
366.2 - 484.5
484.5 - 602.9
602.9 - 765.8
765.8 - 928.7
928.7 - 1091.6
1091.6 - 1176.8
1176.8 - 1411.4
> 1411.4
Iran (Islamic Republic of): Alborz: DALYs Rate (per 100,000)
DALYs Rate
< 342.9
342.9 - 459.9
459.9 - 694.8
694.8 - 929.6
929.6 - 1033.1
1033.1 - 1136.6
1136.6 - 1240.1
1240.1 - 1353.1
1353.1 - 1569.8
> 1569.8
Iran (Islamic Republic of): Ardebil: DALYs Rate (per 100,000)
DALYs Rate
< 340.1
340.1 - 460.7
460.7 - 695.2
695.2 - 929.8
929.8 - 1033.0
1033.0 - 1136.2
1136.2 - 1239.3
1239.3 - 1347.2
1347.2 - 1565.8
> 1565.8
Iran (Islamic Republic of): Bushehr: DALYs Rate (per 100,000)
DALYs Rate
< 340.7
340.7 - 458.4
458.4 - 693.5
693.5 - 928.6
928.6 - 1031.4
1031.4 - 1134.2
1134.2 - 1237.0
1237.0 - 1320.2
1320.2 - 1566.8
> 1566.8
Iran (Islamic Republic of): Chahar Mahaal and Bakhtiari: DALYs Rate (per 100,000)
DALYs Rate
< 216.1
216.1 - 300.1
300.1 - 464.3
464.3 - 628.6
628.6 - 713.5
713.5 - 798.4
798.4 - 883.3
883.3 - 982.9
982.9 - 1195.3
> 1195.3

Figure S7

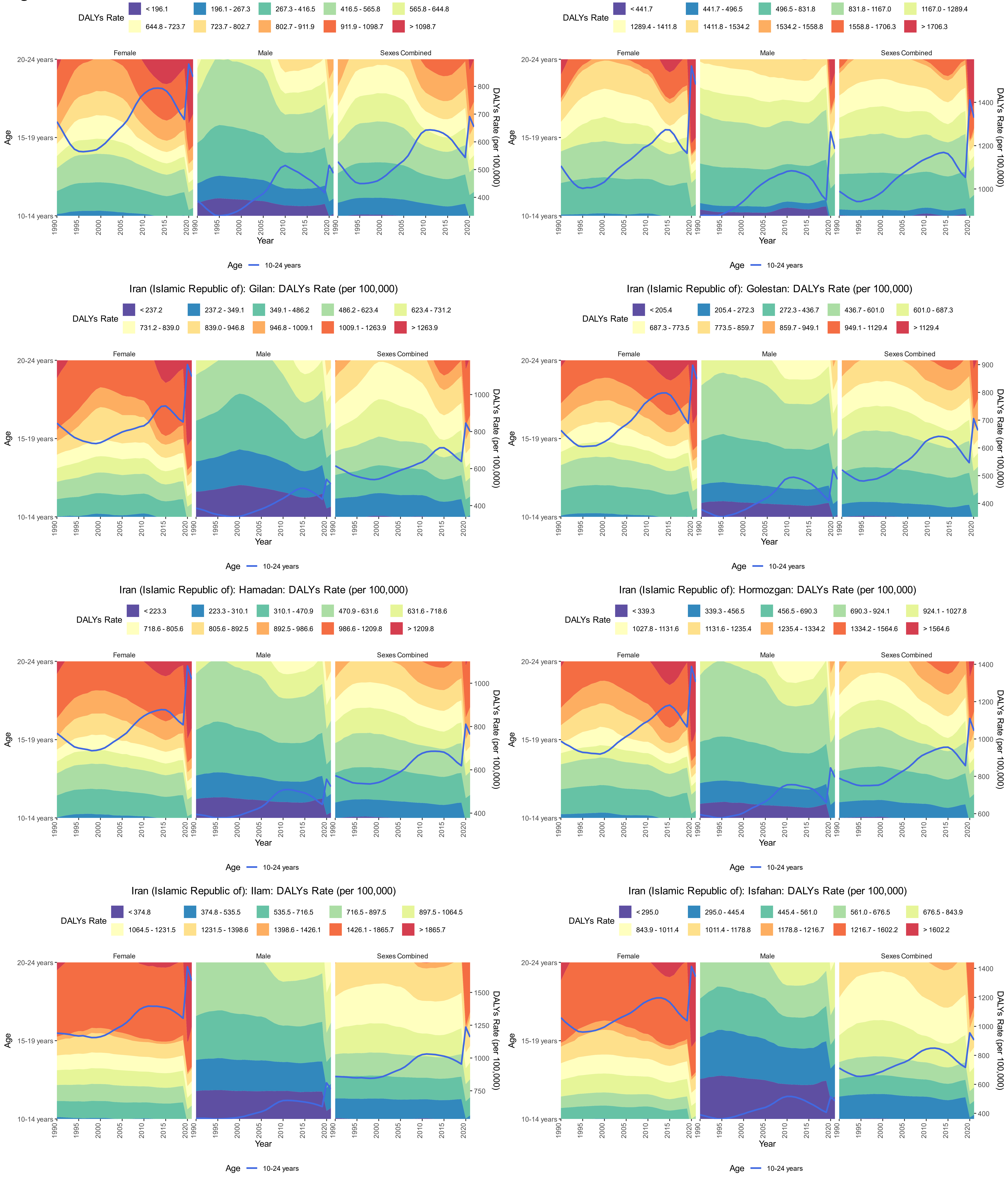

Iran (Islamic Republic of): East Azarbayejan: DALYs Rate (per 100,000)
DALYs Rate
< 196.1
196.1 - 267.3
267.3 - 416.5
416.5 - 565.8
565.8 - 644.8
644.8 - 723.7
723.7 - 802.7
802.7 - 911.9
911.9 - 1098.7
> 1098.7
Iran (Islamic Republic of): Fars: DALYs Rate (per 100,000)
DALYs Rate
< 441.7
441.7 - 496.5
496.5 - 831.8
831.8 - 1167.0
1167.0 - 1289.4
1289.4 - 1411.8
1411.8 - 1534.2
1534.2 - 1558.8
1558.8 - 1706.3
> 1706.3
Iran (Islamic Republic of): Gilan: DALYs Rate (per 100,000)
DALYs Rate
< 237.2
237.2 - 349.1
349.1 - 486.2
486.2 - 623.4
623.4 - 731.2
731.2 - 839.0
839.0 - 946.8
946.8 - 1009.1
1009.1 - 1263.9
> 1263.9
Iran (Islamic Republic of): Golestan: DALYs Rate (per 100,000)
DALYs Rate
< 205.4
205.4 - 272.3
272.3 - 436.7
436.7 - 601.0
601.0 - 687.3
687.3 - 773.5
773.5 - 859.7
859.7 - 949.1
949.1 - 1129.4
> 1129.4
Iran (Islamic Republic of): Hamadan: DALYs Rate (per 100,000)
DALYs Rate
< 223.3
223.3 - 310.1
310.1 - 470.9
470.9 - 631.6
631.6 - 718.6
718.6 - 805.6
805.6 - 892.5
892.5 - 986.6
986.6 - 1209.8
> 1209.8
Iran (Islamic Republic of): Hormozgan: DALYs Rate (per 100,000)
DALYs Rate
< 339.3
339.3 - 456.5
456.5 - 690.3
690.3 - 924.1
924.1 - 1027.8
1027.8 - 1131.6
1131.6 - 1235.4
1235.4 - 1334.2
1334.2 - 1564.6
> 1564.6
Iran (Islamic Republic of): Ilam: DALYs Rate (per 100,000)
DALYs Rate
< 374.8
374.8 - 535.5
535.5 - 716.5
716.5 - 897.5
897.5 - 1064.5
1064.5 - 1231.5
1231.5 - 1398.6
1398.6 - 1426.1
1426.1 - 1865.7
> 1865.7
Iran (Islamic Republic of): Isfahan: DALYs Rate (per 100,000)
DALYs Rate
< 295.0
295.0 - 445.4
445.4 - 561.0
561.0 - 676.5
676.5 - 843.9
843.9 - 1011.4
1011.4 - 1178.8
1178.8 - 1216.7
1216.7 - 1602.2
> 1602.2
Female
Male
Sexes Combined
Age
20-24 years
15-19 years
10-14 years
Year
DALYs Rate (per 100,000)
Age — 10-24 years

Figure S7

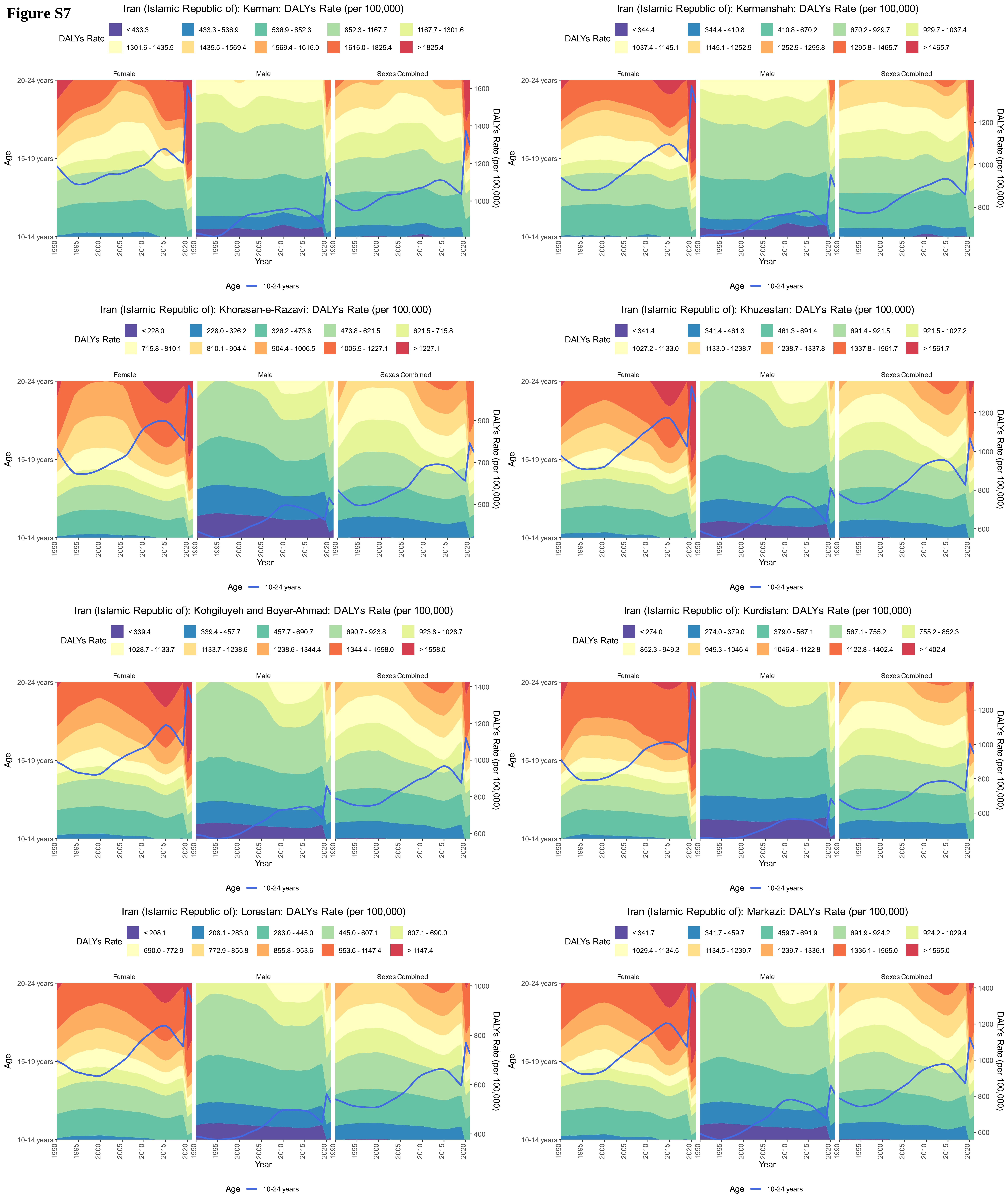

Iran (Islamic Republic of): Kerman: DALYs Rate (per 100,000)
DALYs Rate
< 433.3
433.3 - 536.9
536.9 - 852.3
852.3 - 1167.7
1167.7 - 1301.6
1301.6 - 1435.5
1435.5 - 1569.4
1569.4 - 1616.0
1616.0 - 1825.4
> 1825.4
Iran (Islamic Republic of): Kermanshah: DALYs Rate (per 100,000)
DALYs Rate
< 344.4
344.4 - 410.8
410.8 - 670.2
670.2 - 929.7
929.7 - 1037.4
1037.4 - 1145.1
1145.1 - 1252.9
1252.9 - 1295.8
1295.8 - 1465.7
> 1465.7
Iran (Islamic Republic of): Khorasan-e-Razavi: DALYs Rate (per 100,000)
DALYs Rate
< 228.0
228.0 - 326.2
326.2 - 473.8
473.8 - 621.5
621.5 - 715.8
715.8 - 810.1
810.1 - 904.4
904.4 - 1006.5
1006.5 - 1227.1
> 1227.1
Iran (Islamic Republic of): Khuzestan: DALYs Rate (per 100,000)
DALYs Rate
< 341.4
341.4 - 461.3
461.3 - 691.4
691.4 - 921.5
921.5 - 1027.2
1027.2 - 1133.0
1133.0 - 1238.7
1238.7 - 1337.8
1337.8 - 1561.7
> 1561.7
Iran (Islamic Republic of): Kohgiluyeh and Boyer-Ahmad: DALYs Rate (per 100,000)
DALYs Rate
< 339.4
339.4 - 457.7
457.7 - 690.7
690.7 - 923.8
923.8 - 1028.7
1028.7 - 1133.7
1133.7 - 1238.6
1238.6 - 1344.4
1344.4 - 1558.0
> 1558.0
Iran (Islamic Republic of): Kurdistan: DALYs Rate (per 100,000)
DALYs Rate
< 274.0
274.0 - 379.0
379.0 - 567.1
567.1 - 755.2
755.2 - 852.3
852.3 - 949.3
949.3 - 1046.4
1046.4 - 1122.8
1122.8 - 1402.4
> 1402.4
Iran (Islamic Republic of): Lorestan: DALYs Rate (per 100,000)
DALYs Rate
< 208.1
208.1 - 283.0
283.0 - 445.0
445.0 - 607.1
607.1 - 690.0
690.0 - 772.9
772.9 - 855.8
855.8 - 953.6
953.6 - 1147.4
> 1147.4
Iran (Islamic Republic of): Markazi: DALYs Rate (per 100,000)
DALYs Rate
< 341.7
341.7 - 459.7
459.7 - 691.9
691.9 - 924.2
924.2 - 1029.4
1029.4 - 1134.5
1134.5 - 1239.7
1239.7 - 1336.1
1336.1 - 1565.0
> 1565.0
Female
Male
Sexes Combined
Age
20-24 years
15-19 years
10-14 years
Year
DALYs Rate (per 100,000)
Age
10-24 years

Figure S7

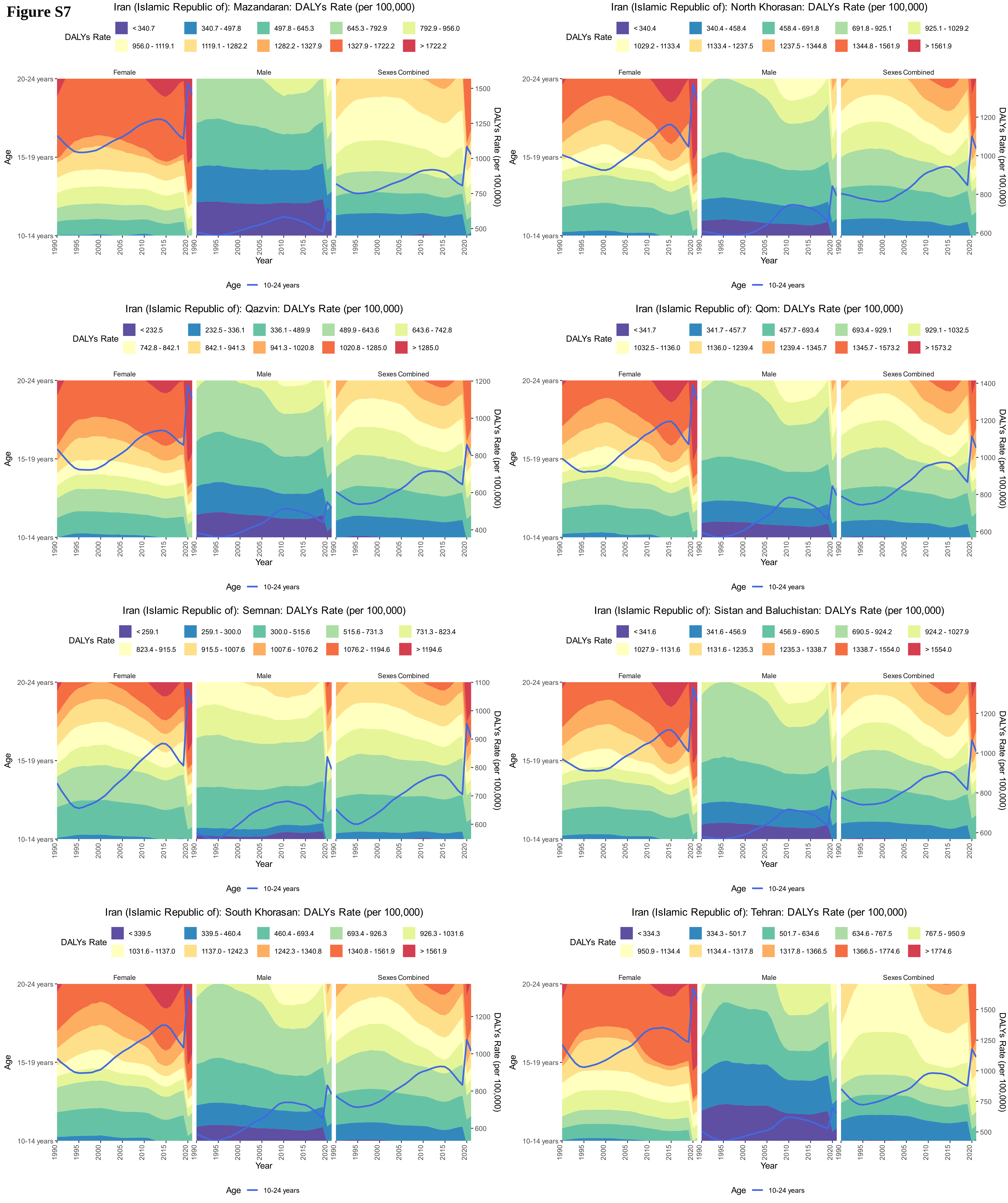
Iran (Islamic Republic of): Mazandaran: DALYs Rate (per 100,000)
DALYs Rate
< 340.7
340.7 - 497.8
497.8 - 645.3
645.3 - 792.9
792.9 - 956.0
956.0 - 1119.1
1119.1 - 1282.2
1282.2 - 1327.9
1327.9 - 1722.2
> 1722.2
Iran (Islamic Republic of): North Khorasan: DALYs Rate (per 100,000)
< 340.4
340.4 - 458.4
458.4 - 691.8
691.8 - 925.1
925.1 - 1029.2
1029.2 - 1133.4
1133.4 - 1237.5
1237.5 - 1344.8
1344.8 - 1561.9
> 1561.9
Iran (Islamic Republic of): Qazvin: DALYs Rate (per 100,000)
< 232.5
232.5 - 336.1
336.1 - 489.9
489.9 - 643.6
643.6 - 742.8
742.8 - 842.1
842.1 - 941.3
941.3 - 1020.8
1020.8 - 1285.0
> 1285.0
Iran (Islamic Republic of): Qom: DALYs Rate (per 100,000)
< 341.7
341.7 - 457.7
457.7 - 693.4
693.4 - 929.1
929.1 - 1032.5
1032.5 - 1136.0
1136.0 - 1239.4
1239.4 - 1345.7
1345.7 - 1573.2
> 1573.2
Iran (Islamic Republic of): Semnan: DALYs Rate (per 100,000)
< 259.1
259.1 - 300.0
300.0 - 515.6
515.6 - 731.3
731.3 - 823.4
823.4 - 915.5
915.5 - 1007.6
1007.6 - 1076.2
1076.2 - 1194.6
> 1194.6
Iran (Islamic Republic of): Sistan and Baluchistan: DALYs Rate (per 100,000)
< 341.6
341.6 - 456.9
456.9 - 690.5
690.5 - 924.2
924.2 - 1027.9
1027.9 - 1131.6
1131.6 - 1235.3
1235.3 - 1338.7
1338.7 - 1554.0
> 1554.0
Iran (Islamic Republic of): South Khorasan: DALYs Rate (per 100,000)
< 339.5
339.5 - 460.4
460.4 - 693.4
693.4 - 926.3
926.3 - 1031.6
1031.6 - 1137.0
1137.0 - 1242.3
1242.3 - 1340.8
1340.8 - 1561.9
> 1561.9
Iran (Islamic Republic of): Tehran: DALYs Rate (per 100,000)
< 334.3
334.3 - 501.7
501.7 - 634.6
634.6 - 767.5
767.5 - 950.9
950.9 - 1134.4
1134.4 - 1317.8
1317.8 - 1366.5
1366.5 - 1774.6
> 1774.6
Female
Male
Sexes Combined
Age
20-24 years
15-19 years
10-14 years
Year
DALYs Rate (per 100,000)
Age
10-24 years

Figure S7

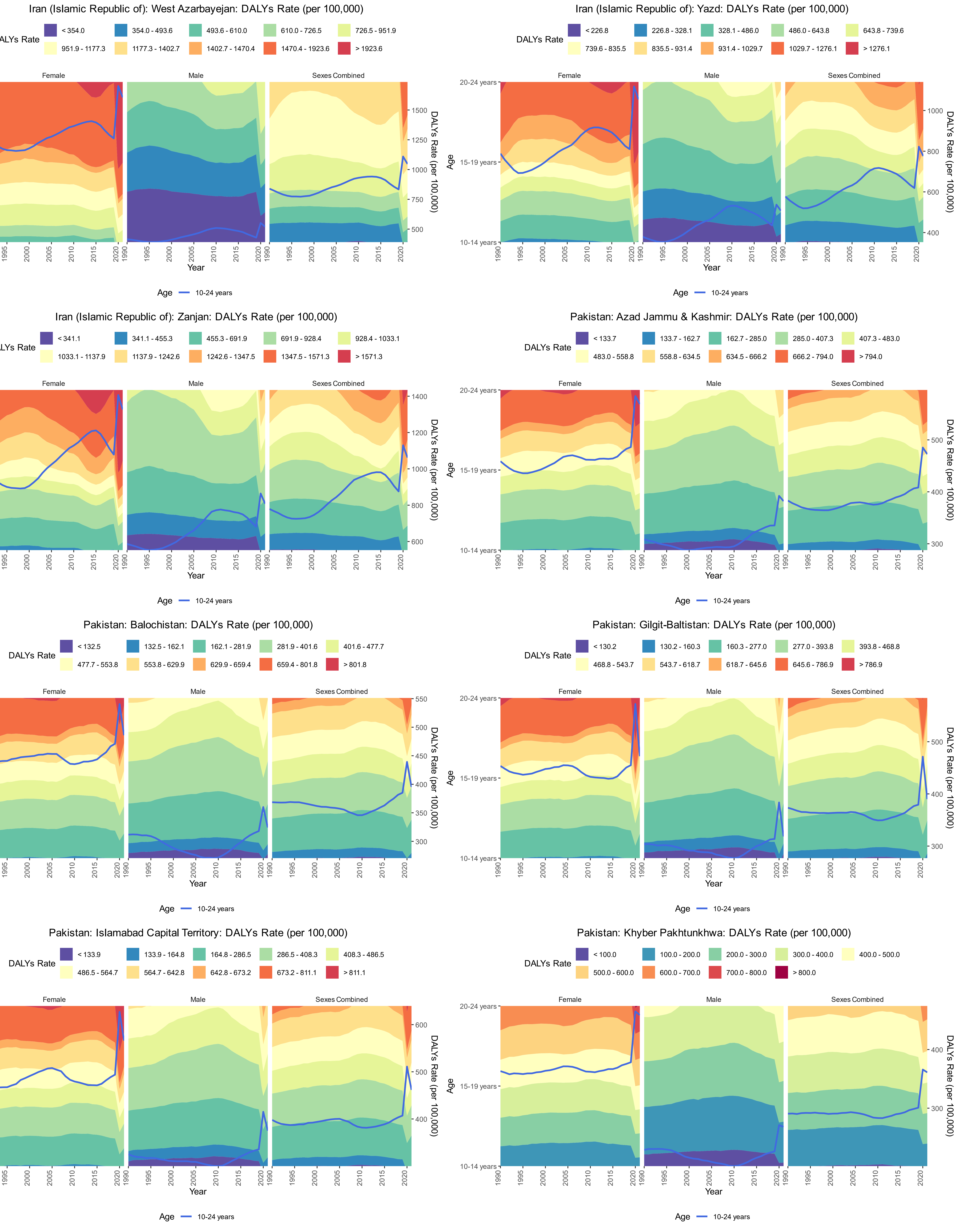

Iran (Islamic Republic of): West Azarbayejan: DALYs Rate (per 100,000)
DALYs Rate
< 354.0
354.0 - 493.6
493.6 - 610.0
610.0 - 726.5
726.5 - 951.9
951.9 - 1177.3
1177.3 - 1402.7
1402.7 - 1470.4
1470.4 - 1923.6
> 1923.6
Iran (Islamic Republic of): Yazd: DALYs Rate (per 100,000)
DALYs Rate
< 226.8
226.8 - 328.1
328.1 - 486.0
486.0 - 643.8
643.8 - 739.6
739.6 - 835.5
835.5 - 931.4
931.4 - 1029.7
1029.7 - 1276.1
> 1276.1
Iran (Islamic Republic of): Zanjan: DALYs Rate (per 100,000)
DALYs Rate
< 341.1
341.1 - 455.3
455.3 - 691.9
691.9 - 928.4
928.4 - 1033.1
1033.1 - 1137.9
1137.9 - 1242.6
1242.6 - 1347.5
1347.5 - 1571.3
> 1571.3
Pakistan: Azad Jammu & Kashmir: DALYs Rate (per 100,000)
DALYs Rate
< 133.7
133.7 - 162.7
162.7 - 285.0
285.0 - 407.3
407.3 - 483.0
483.0 - 558.8
558.8 - 634.5
634.5 - 666.2
666.2 - 794.0
> 794.0
Pakistan: Balochistan: DALYs Rate (per 100,000)
DALYs Rate
< 132.5
132.5 - 162.1
162.1 - 281.9
281.9 - 401.6
401.6 - 477.7
477.7 - 553.8
553.8 - 629.9
629.9 - 659.4
659.4 - 801.8
> 801.8
Pakistan: Gilgit-Baltistan: DALYs Rate (per 100,000)
DALYs Rate
< 130.2
130.2 - 160.3
160.3 - 277.0
277.0 - 393.8
393.8 - 468.8
468.8 - 543.7
543.7 - 618.7
618.7 - 645.6
645.6 - 786.9
> 786.9
Pakistan: Islamabad Capital Territory: DALYs Rate (per 100,000)
DALYs Rate
< 133.9
133.9 - 164.8
164.8 - 286.5
286.5 - 408.3
408.3 - 486.5
486.5 - 564.7
564.7 - 642.8
642.8 - 673.2
673.2 - 811.1
> 811.1
Pakistan: Khyber Pakhtunkhwa: DALYs Rate (per 100,000)
DALYs Rate
< 100.0
100.0 - 200.0
200.0 - 300.0
300.0 - 400.0
400.0 - 500.0
500.0 - 600.0
600.0 - 700.0
700.0 - 800.0
> 800.0
Female
Male
Sexes Combined
Age
20-24 years
15-19 years
10-14 years
Year
DALYs Rate (per 100,000)
Age — 10-24 years

Figure S7

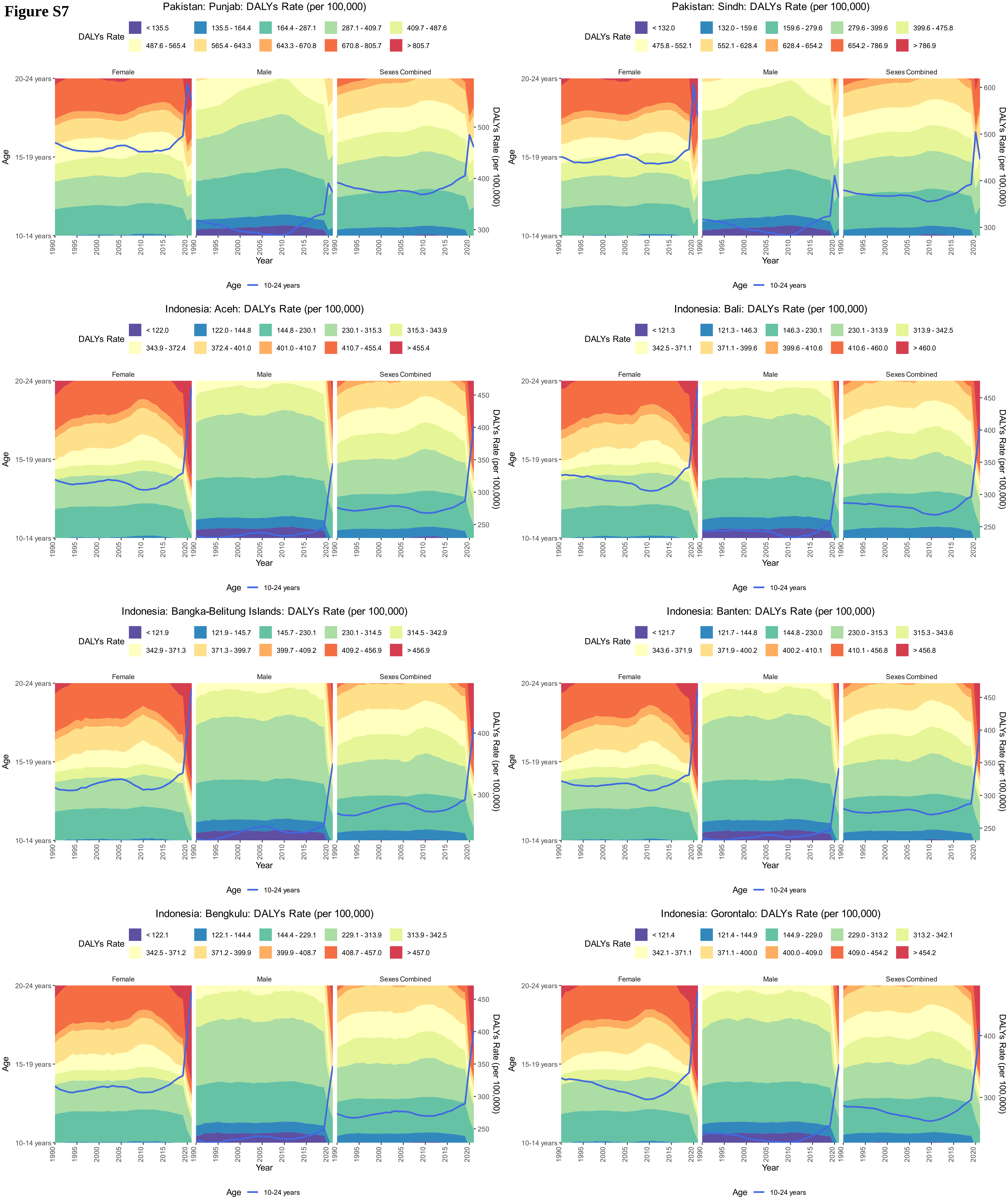

Pakistan: Punjab: DALYs Rate (per 100,000)
DALYs Rate
< 135.5
135.5 - 164.4
164.4 - 287.1
287.1 - 409.7
409.7 - 487.6
487.6 - 565.4
565.4 - 643.3
643.3 - 670.8
670.8 - 805.7
> 805.7
Pakistan: Sindh: DALYs Rate (per 100,000)
DALYs Rate
< 132.0
132.0 - 159.6
159.6 - 279.6
279.6 - 399.6
399.6 - 475.8
475.8 - 552.1
552.1 - 628.4
628.4 - 654.2
654.2 - 786.9
> 786.9
Indonesia: Aceh: DALYs Rate (per 100,000)
DALYs Rate
< 122.0
122.0 - 144.8
144.8 - 230.1
230.1 - 315.3
315.3 - 343.9
343.9 - 372.4
372.4 - 401.0
401.0 - 410.7
410.7 - 455.4
> 455.4
Indonesia: Bali: DALYs Rate (per 100,000)
DALYs Rate
< 121.3
121.3 - 146.3
146.3 - 230.1
230.1 - 313.9
313.9 - 342.5
342.5 - 371.1
371.1 - 399.6
399.6 - 410.6
410.6 - 460.0
> 460.0
Indonesia: Bangka-Belitung Islands: DALYs Rate (per 100,000)
DALYs Rate
< 121.9
121.9 - 145.7
145.7 - 230.1
230.1 - 314.5
314.5 - 342.9
342.9 - 371.3
371.3 - 399.7
399.7 - 409.2
409.2 - 456.9
> 456.9
Indonesia: Banten: DALYs Rate (per 100,000)
DALYs Rate
< 121.7
121.7 - 144.8
144.8 - 230.0
230.0 - 315.3
315.3 - 343.6
343.6 - 371.9
371.9 - 400.2
400.2 - 410.1
410.1 - 456.8
> 456.8
Indonesia: Bengkulu: DALYs Rate (per 100,000)
DALYs Rate
< 122.1
122.1 - 144.4
144.4 - 229.1
229.1 - 313.9
313.9 - 342.5
342.5 - 371.2
371.2 - 399.9
399.9 - 408.7
408.7 - 457.0
> 457.0
Indonesia: Gorontalo: DALYs Rate (per 100,000)
DALYs Rate
< 121.4
121.4 - 144.9
144.9 - 229.0
229.0 - 313.2
313.2 - 342.1
342.1 - 371.1
371.1 - 400.0
400.0 - 409.0
409.0 - 454.2
> 454.2
Female
Male
Sexes Combined
Age
20-24 years
15-19 years
10-14 years
Year
DALYs Rate (per 100,000)
Age
10-24 years

Figure S7

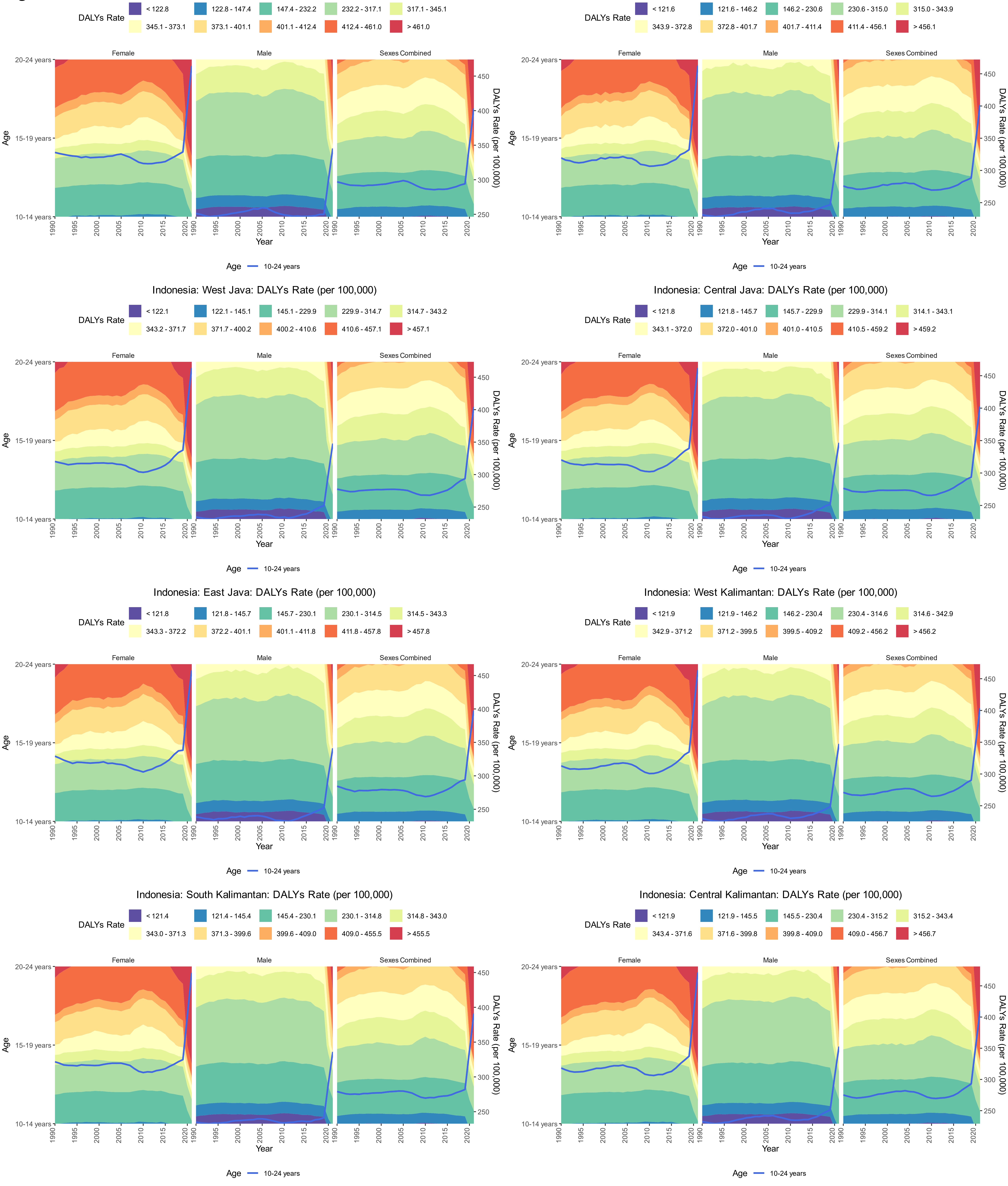

Indonesia: Jakarta: DALYs Rate (per 100,000)
DALYs Rate
< 122.8
122.8 - 147.4
147.4 - 232.2
232.2 - 317.1
317.1 - 345.1
345.1 - 373.1
373.1 - 401.1
401.1 - 412.4
412.4 - 461.0
> 461.0
Female
Male
Sexes Combined
Age
20-24 years
15-19 years
10-14 years
Year
DALYs Rate (per 100,000)
Age
10-24 years
Indonesia: Jambi: DALYs Rate (per 100,000)
DALYs Rate
< 121.6
121.6 - 146.2
146.2 - 230.6
230.6 - 315.0
315.0 - 343.9
343.9 - 372.8
372.8 - 401.7
401.7 - 411.4
411.4 - 456.1
> 456.1
Indonesia: West Java: DALYs Rate (per 100,000)
DALYs Rate
< 122.1
122.1 - 145.1
145.1 - 229.9
229.9 - 314.7
314.7 - 343.2
343.2 - 371.7
371.7 - 400.2
400.2 - 410.6
410.6 - 457.1
> 457.1
Indonesia: Central Java: DALYs Rate (per 100,000)
DALYs Rate
< 121.8
121.8 - 145.7
145.7 - 229.9
229.9 - 314.1
314.1 - 343.1
343.1 - 372.0
372.0 - 401.0
401.0 - 410.5
410.5 - 459.2
> 459.2
Indonesia: East Java: DALYs Rate (per 100,000)
DALYs Rate
< 121.8
121.8 - 145.7
145.7 - 230.1
230.1 - 314.5
314.5 - 343.3
343.3 - 372.2
372.2 - 401.1
401.1 - 411.8
411.8 - 457.8
> 457.8
Indonesia: West Kalimantan: DALYs Rate (per 100,000)
DALYs Rate
< 121.9
121.9 - 146.2
146.2 - 230.4
230.4 - 314.6
314.6 - 342.9
342.9 - 371.2
371.2 - 399.5
399.5 - 409.2
409.2 - 456.2
> 456.2
Indonesia: South Kalimantan: DALYs Rate (per 100,000)
DALYs Rate
< 121.4
121.4 - 145.4
145.4 - 230.1
230.1 - 314.8
314.8 - 343.0
343.0 - 371.3
371.3 - 399.6
399.6 - 409.0
409.0 - 455.5
> 455.5
Indonesia: Central Kalimantan: DALYs Rate (per 100,000)
DALYs Rate
< 121.9
121.9 - 145.5
145.5 - 230.4
230.4 - 315.2
315.2 - 343.4
343.4 - 371.6
371.6 - 399.8
399.8 - 409.0
409.0 - 456.7
> 456.7

Figure S7

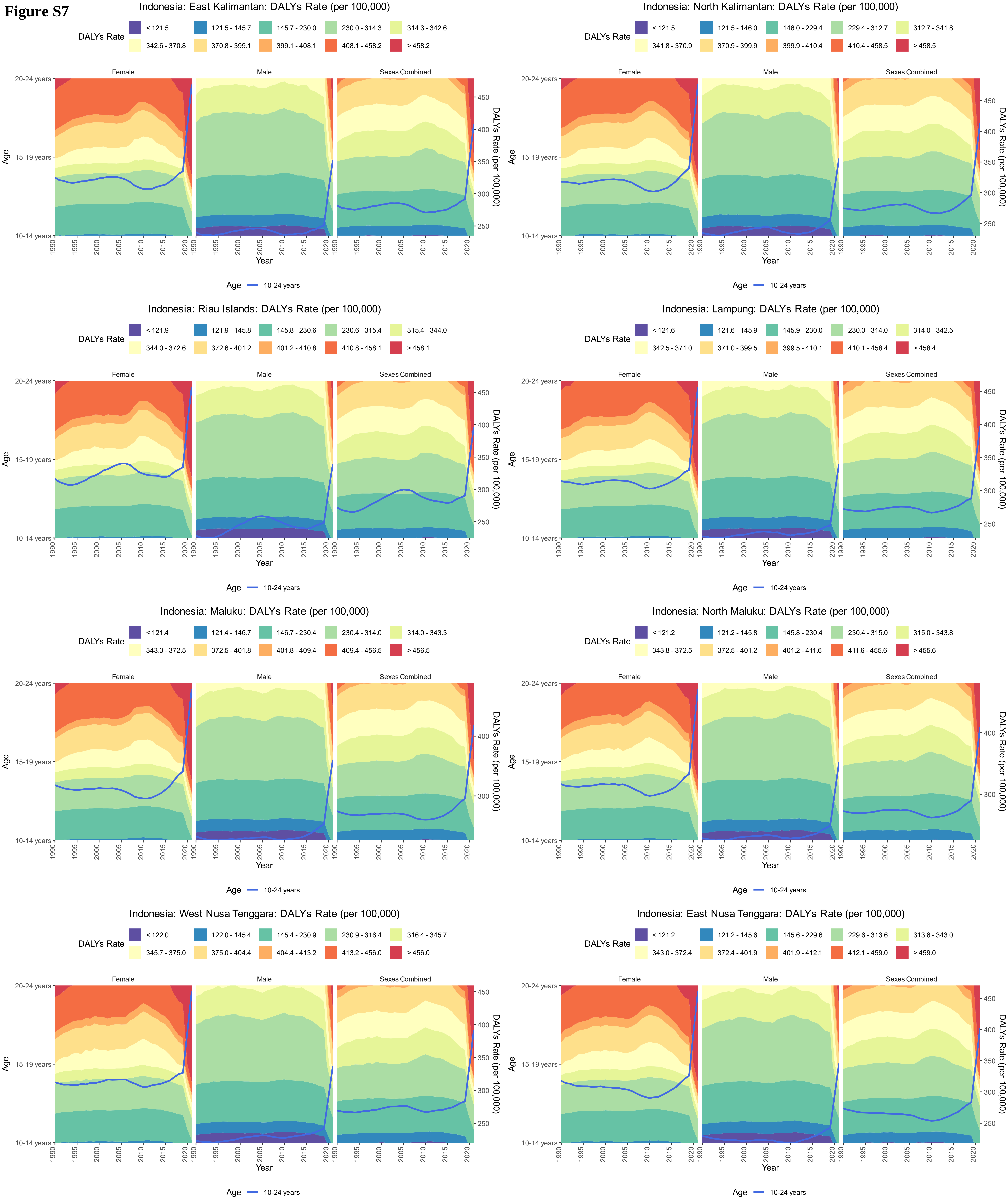

Indonesia: East Kalimantan: DALYs Rate (per 100,000)
DALYs Rate
< 121.5
121.5 - 145.7
145.7 - 230.0
230.0 - 314.3
314.3 - 342.6
342.6 - 370.8
370.8 - 399.1
399.1 - 408.1
408.1 - 458.2
> 458.2
Indonesia: North Kalimantan: DALYs Rate (per 100,000)
< 121.5
121.5 - 146.0
146.0 - 229.4
229.4 - 312.7
312.7 - 341.8
341.8 - 370.9
370.9 - 399.9
399.9 - 410.4
410.4 - 458.5
> 458.5
Indonesia: Riau Islands: DALYs Rate (per 100,000)
< 121.9
121.9 - 145.8
145.8 - 230.6
230.6 - 315.4
315.4 - 344.0
344.0 - 372.6
372.6 - 401.2
401.2 - 410.8
410.8 - 458.1
> 458.1
Indonesia: Lampung: DALYs Rate (per 100,000)
< 121.6
121.6 - 145.9
145.9 - 230.0
230.0 - 314.0
314.0 - 342.5
342.5 - 371.0
371.0 - 399.5
399.5 - 410.1
410.1 - 458.4
> 458.4
Indonesia: Maluku: DALYs Rate (per 100,000)
< 121.4
121.4 - 146.7
146.7 - 230.4
230.4 - 314.0
314.0 - 343.3
343.3 - 372.5
372.5 - 401.8
401.8 - 409.4
409.4 - 456.5
> 456.5
Indonesia: North Maluku: DALYs Rate (per 100,000)
< 121.2
121.2 - 145.8
145.8 - 230.4
230.4 - 315.0
315.0 - 343.8
343.8 - 372.5
372.5 - 401.2
401.2 - 411.6
411.6 - 455.6
> 455.6
Indonesia: West Nusa Tenggara: DALYs Rate (per 100,000)
< 122.0
122.0 - 145.4
145.4 - 230.9
230.9 - 316.4
316.4 - 345.7
345.7 - 375.0
375.0 - 404.4
404.4 - 413.2
413.2 - 456.0
> 456.0
Indonesia: East Nusa Tenggara: DALYs Rate (per 100,000)
< 121.2
121.2 - 145.6
145.6 - 229.6
229.6 - 313.6
313.6 - 343.0
343.0 - 372.4
372.4 - 401.9
401.9 - 412.1
412.1 - 459.0
> 459.0
Female
Male
Sexes Combined
Age
20-24 years
15-19 years
10-14 years
Year
DALYs Rate (per 100,000)
Age — 10-24 years

Figure S7

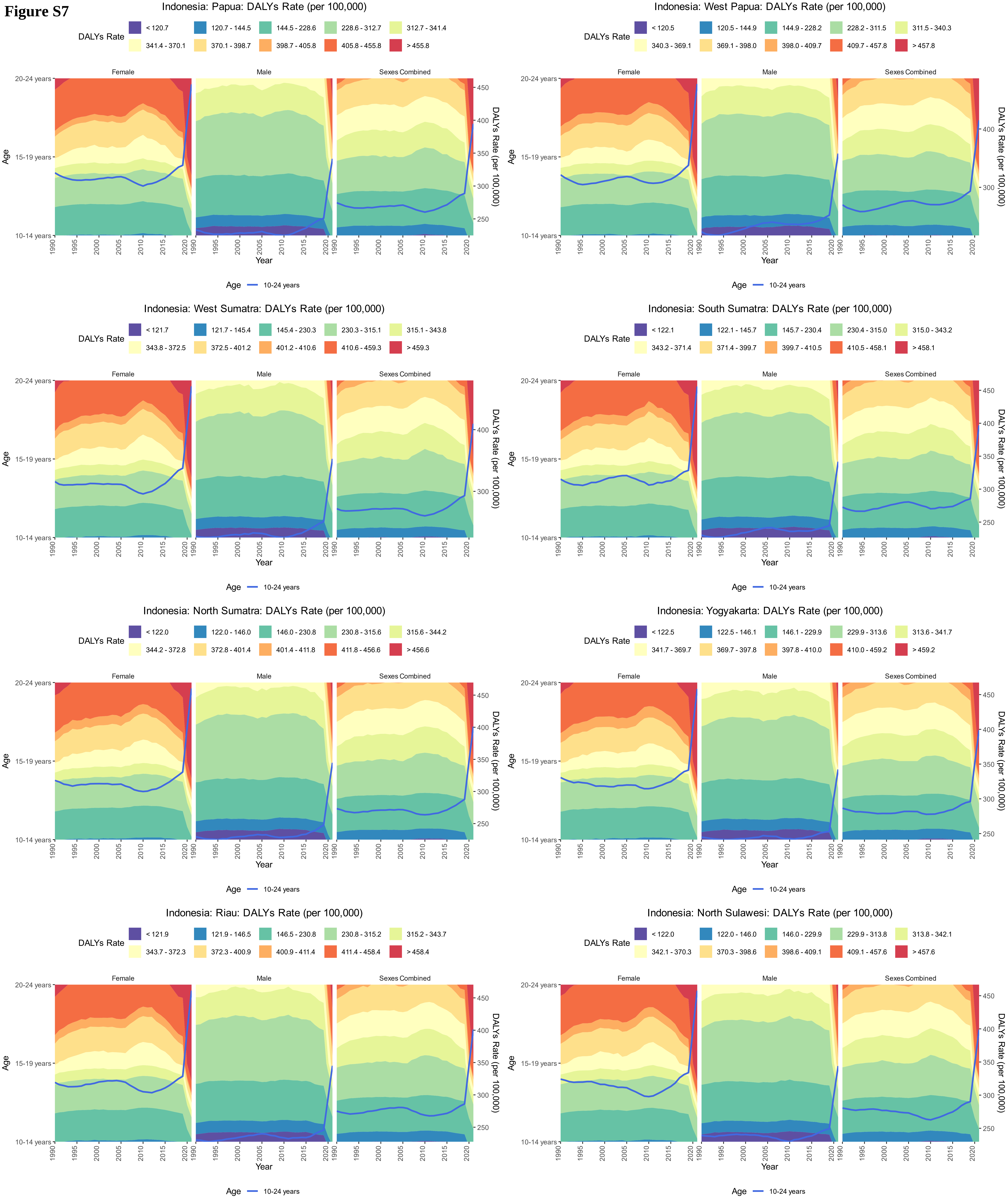
Indonesia: Papua: DALYs Rate (per 100,000)
DALYs Rate
< 120.7
120.7 - 144.5
144.5 - 228.6
228.6 - 312.7
312.7 - 341.4
341.4 - 370.1
370.1 - 398.7
398.7 - 405.8
405.8 - 455.8
> 455.8
Indonesia: West Papua: DALYs Rate (per 100,000)
DALYs Rate
< 120.5
120.5 - 144.9
144.9 - 228.2
228.2 - 311.5
311.5 - 340.3
340.3 - 369.1
369.1 - 398.0
398.0 - 409.7
409.7 - 457.8
> 457.8
Indonesia: West Sumatra: DALYs Rate (per 100,000)
DALYs Rate
< 121.7
121.7 - 145.4
145.4 - 230.3
230.3 - 315.1
315.1 - 343.8
343.8 - 372.5
372.5 - 401.2
401.2 - 410.6
410.6 - 459.3
> 459.3
Indonesia: South Sumatra: DALYs Rate (per 100,000)
DALYs Rate
< 122.1
122.1 - 145.7
145.7 - 230.4
230.4 - 315.0
315.0 - 343.2
343.2 - 371.4
371.4 - 399.7
399.7 - 410.5
410.5 - 458.1
> 458.1
Indonesia: North Sumatra: DALYs Rate (per 100,000)
DALYs Rate
< 122.0
122.0 - 146.0
146.0 - 230.8
230.8 - 315.6
315.6 - 344.2
344.2 - 372.8
372.8 - 401.4
401.4 - 411.8
411.8 - 456.6
> 456.6
Indonesia: Yogyakarta: DALYs Rate (per 100,000)
DALYs Rate
< 122.5
122.5 - 146.1
146.1 - 229.9
229.9 - 313.6
313.6 - 341.7
341.7 - 369.7
369.7 - 397.8
397.8 - 410.0
410.0 - 459.2
> 459.2
Indonesia: Riau: DALYs Rate (per 100,000)
DALYs Rate
< 121.9
121.9 - 146.5
146.5 - 230.8
230.8 - 315.2
315.2 - 343.7
343.7 - 372.3
372.3 - 400.9
400.9 - 411.4
411.4 - 458.4
> 458.4
Indonesia: North Sulawesi: DALYs Rate (per 100,000)
DALYs Rate
< 122.0
122.0 - 146.0
146.0 - 229.9
229.9 - 313.8
313.8 - 342.1
342.1 - 370.3
370.3 - 398.6
398.6 - 409.1
409.1 - 457.6
> 457.6
Female
Male
Sexes Combined
Age
20-24 years
15-19 years
10-14 years
Year
DALYs Rate (per 100,000)
Age
10-24 years

Figure S7

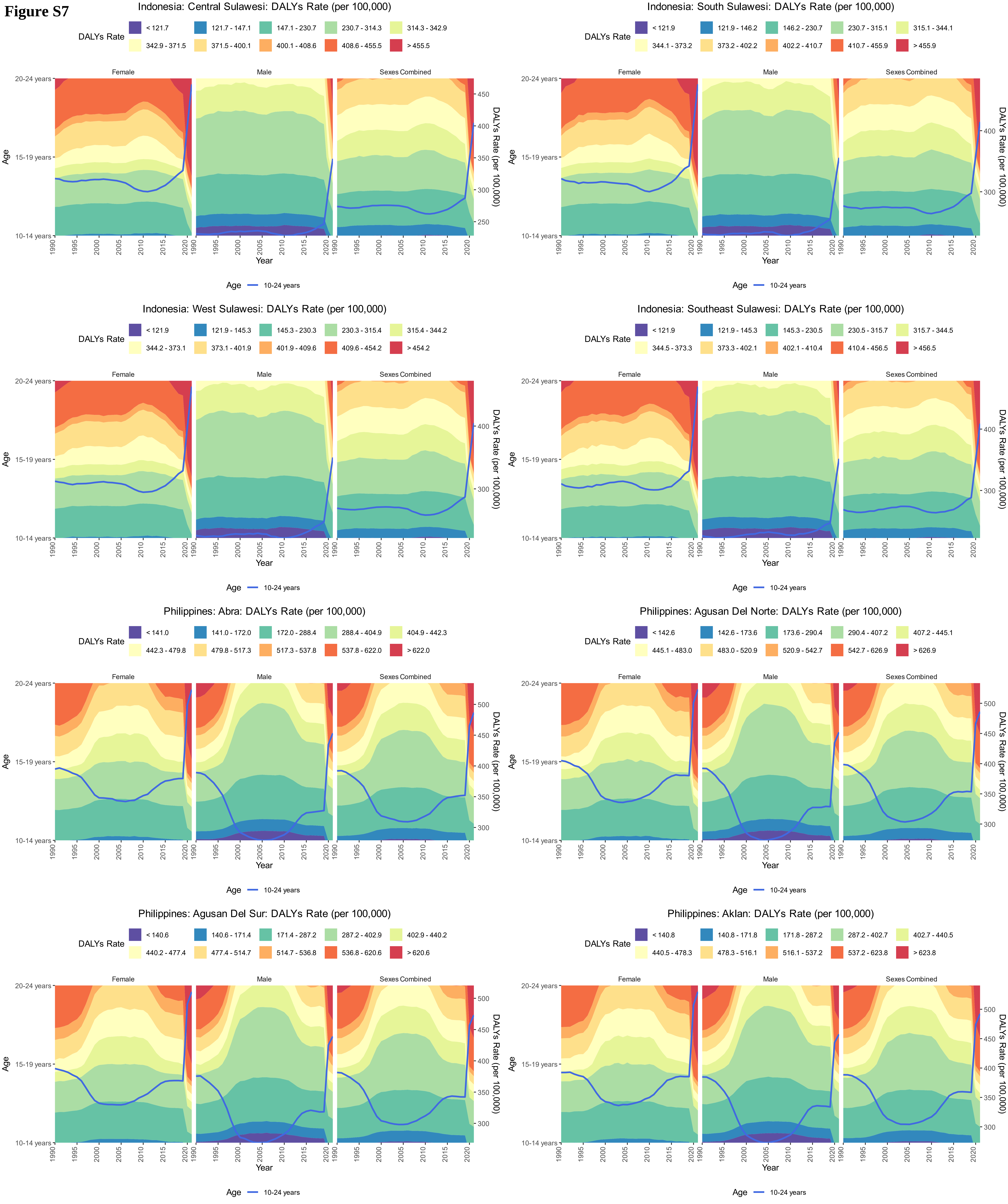

Indonesia: Central Sulawesi: DALYs Rate (per 100,000)
DALYs Rate
< 121.7
121.7 - 147.1
147.1 - 230.7
230.7 - 314.3
314.3 - 342.9
342.9 - 371.5
371.5 - 400.1
400.1 - 408.6
408.6 - 455.5
> 455.5
Indonesia: South Sulawesi: DALYs Rate (per 100,000)
< 121.9
121.9 - 146.2
146.2 - 230.7
230.7 - 315.1
315.1 - 344.1
344.1 - 373.2
373.2 - 402.2
402.2 - 410.7
410.7 - 455.9
> 455.9
Indonesia: West Sulawesi: DALYs Rate (per 100,000)
< 121.9
121.9 - 145.3
145.3 - 230.3
230.3 - 315.4
315.4 - 344.2
344.2 - 373.1
373.1 - 401.9
401.9 - 409.6
409.6 - 454.2
> 454.2
Indonesia: Southeast Sulawesi: DALYs Rate (per 100,000)
< 121.9
121.9 - 145.3
145.3 - 230.5
230.5 - 315.7
315.7 - 344.5
344.5 - 373.3
373.3 - 402.1
402.1 - 410.4
410.4 - 456.5
> 456.5
Philippines: Abra: DALYs Rate (per 100,000)
< 141.0
141.0 - 172.0
172.0 - 288.4
288.4 - 404.9
404.9 - 442.3
442.3 - 479.8
479.8 - 517.3
517.3 - 537.8
537.8 - 622.0
> 622.0
Philippines: Agusan Del Norte: DALYs Rate (per 100,000)
< 142.6
142.6 - 173.6
173.6 - 290.4
290.4 - 407.2
407.2 - 445.1
445.1 - 483.0
483.0 - 520.9
520.9 - 542.7
542.7 - 626.9
> 626.9
Philippines: Agusan Del Sur: DALYs Rate (per 100,000)
< 140.6
140.6 - 171.4
171.4 - 287.2
287.2 - 402.9
402.9 - 440.2
440.2 - 477.4
477.4 - 514.7
514.7 - 536.8
536.8 - 620.6
> 620.6
Philippines: Aklan: DALYs Rate (per 100,000)
< 140.8
140.8 - 171.8
171.8 - 287.2
287.2 - 402.7
402.7 - 440.5
440.5 - 478.3
478.3 - 516.1
516.1 - 537.2
537.2 - 623.8
> 623.8
Female
Male
Sexes Combined
Age
20-24 years
15-19 years
10-14 years
Year
DALYs Rate (per 100,000)
Age — 10-24 years

Figure S7

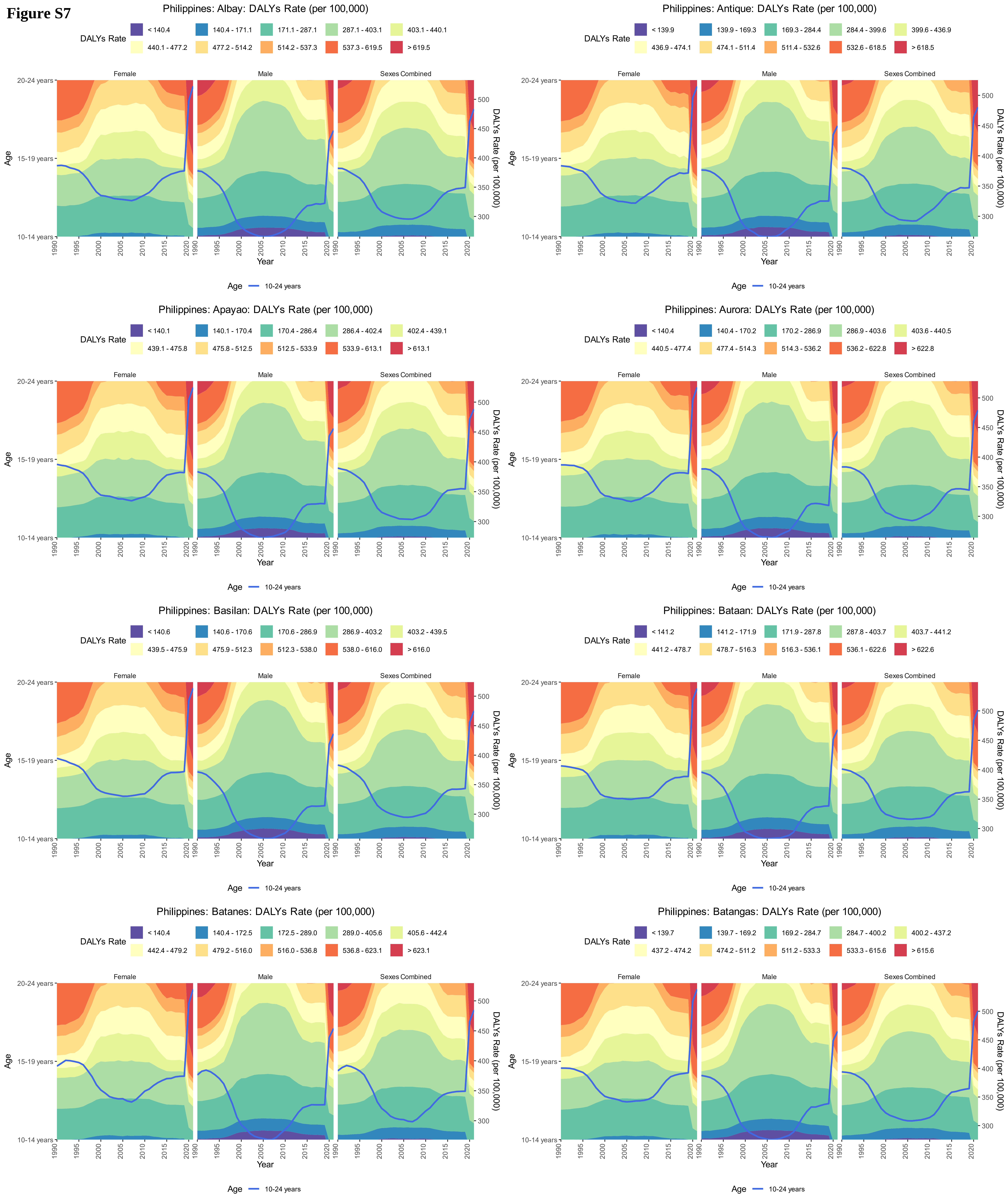

Philippines: Albay: DALYs Rate (per 100,000)
DALYs Rate
< 140.4
140.4 - 171.1
171.1 - 287.1
287.1 - 403.1
403.1 - 440.1
440.1 - 477.2
477.2 - 514.2
514.2 - 537.3
537.3 - 619.5
> 619.5
Philippines: Antique: DALYs Rate (per 100,000)
DALYs Rate
< 139.9
139.9 - 169.3
169.3 - 284.4
284.4 - 399.6
399.6 - 436.9
436.9 - 474.1
474.1 - 511.4
511.4 - 532.6
532.6 - 618.5
> 618.5
Philippines: Apayao: DALYs Rate (per 100,000)
DALYs Rate
< 140.1
140.1 - 170.4
170.4 - 286.4
286.4 - 402.4
402.4 - 439.1
439.1 - 475.8
475.8 - 512.5
512.5 - 533.9
533.9 - 613.1
> 613.1
Philippines: Aurora: DALYs Rate (per 100,000)
DALYs Rate
< 140.4
140.4 - 170.2
170.2 - 286.9
286.9 - 403.6
403.6 - 440.5
440.5 - 477.4
477.4 - 514.3
514.3 - 536.2
536.2 - 622.8
> 622.8
Philippines: Basilan: DALYs Rate (per 100,000)
DALYs Rate
< 140.6
140.6 - 170.6
170.6 - 286.9
286.9 - 403.2
403.2 - 439.5
439.5 - 475.9
475.9 - 512.3
512.3 - 538.0
538.0 - 616.0
> 616.0
Philippines: Bataan: DALYs Rate (per 100,000)
DALYs Rate
< 141.2
141.2 - 171.9
171.9 - 287.8
287.8 - 403.7
403.7 - 441.2
441.2 - 478.7
478.7 - 516.3
516.3 - 536.1
536.1 - 622.6
> 622.6
Philippines: Batanes: DALYs Rate (per 100,000)
DALYs Rate
< 140.4
140.4 - 172.5
172.5 - 289.0
289.0 - 405.6
405.6 - 442.4
442.4 - 479.2
479.2 - 516.0
516.0 - 536.8
536.8 - 623.1
> 623.1
Philippines: Batangas: DALYs Rate (per 100,000)
DALYs Rate
< 139.7
139.7 - 169.2
169.2 - 284.7
284.7 - 400.2
400.2 - 437.2
437.2 - 474.2
474.2 - 511.2
511.2 - 533.3
533.3 - 615.6
> 615.6
Female
Male
Sexes Combined
Age
20-24 years
15-19 years
10-14 years
Year
DALYs Rate (per 100,000)
Age
10-24 years

Figure S7

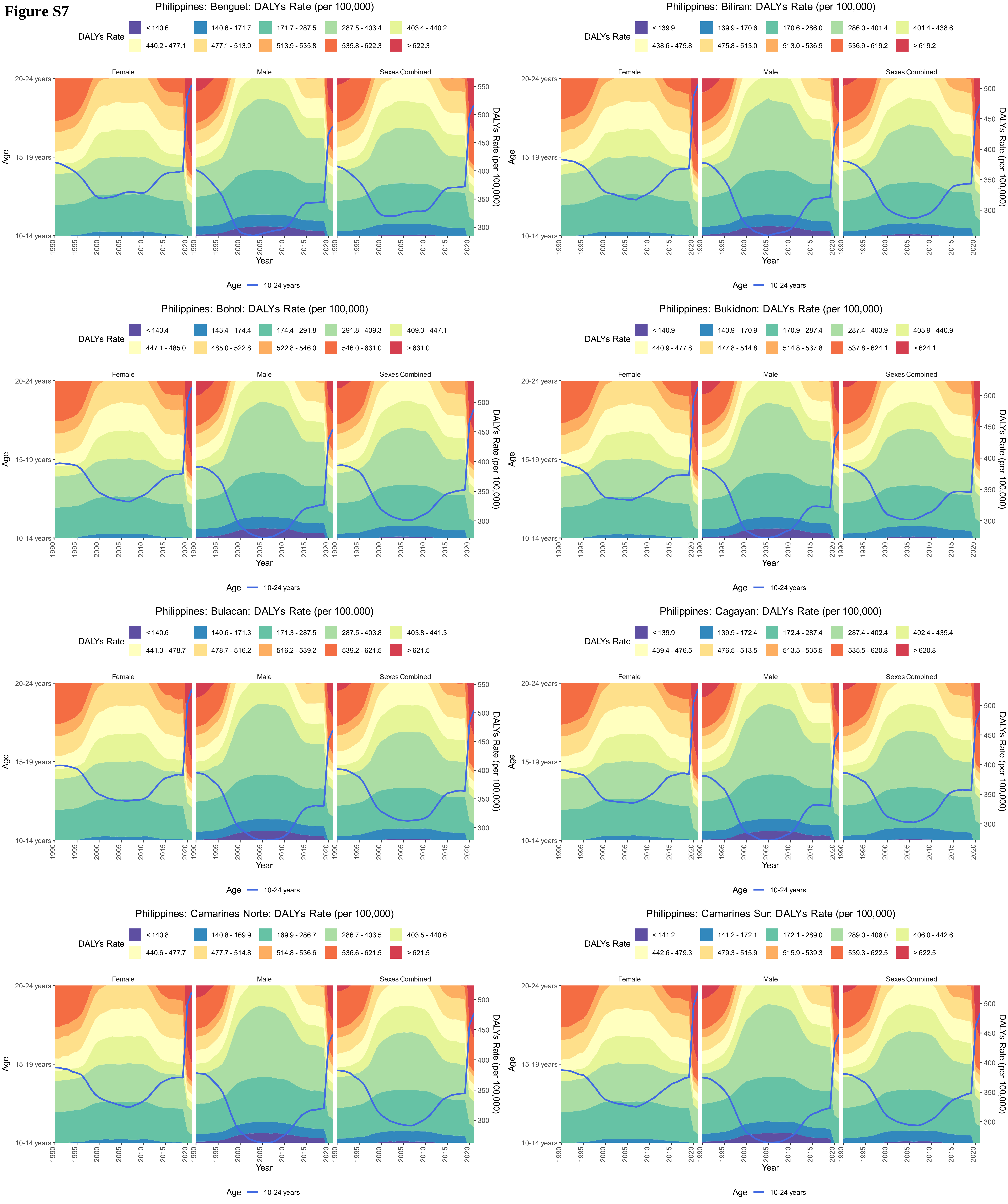
Philippines: Benguet: DALYs Rate (per 100,000)
DALYs Rate
< 140.6
140.6 - 171.7
171.7 - 287.5
287.5 - 403.4
403.4 - 440.2
440.2 - 477.1
477.1 - 513.9
513.9 - 535.8
535.8 - 622.3
> 622.3
Philippines: Biliran: DALYs Rate (per 100,000)
< 139.9
139.9 - 170.6
170.6 - 286.0
286.0 - 401.4
401.4 - 438.6
438.6 - 475.8
475.8 - 513.0
513.0 - 536.9
536.9 - 619.2
> 619.2
Philippines: Bohol: DALYs Rate (per 100,000)
< 143.4
143.4 - 174.4
174.4 - 291.8
291.8 - 409.3
409.3 - 447.1
447.1 - 485.0
485.0 - 522.8
522.8 - 546.0
546.0 - 631.0
> 631.0
Philippines: Bukidnon: DALYs Rate (per 100,000)
< 140.9
140.9 - 170.9
170.9 - 287.4
287.4 - 403.9
403.9 - 440.9
440.9 - 477.8
477.8 - 514.8
514.8 - 537.8
537.8 - 624.1
> 624.1
Philippines: Bulacan: DALYs Rate (per 100,000)
< 140.6
140.6 - 171.3
171.3 - 287.5
287.5 - 403.8
403.8 - 441.3
441.3 - 478.7
478.7 - 516.2
516.2 - 539.2
539.2 - 621.5
> 621.5
Philippines: Cagayan: DALYs Rate (per 100,000)
< 139.9
139.9 - 172.4
172.4 - 287.4
287.4 - 402.4
402.4 - 439.4
439.4 - 476.5
476.5 - 513.5
513.5 - 535.5
535.5 - 620.8
> 620.8
Philippines: Camarines Norte: DALYs Rate (per 100,000)
< 140.8
140.8 - 169.9
169.9 - 286.7
286.7 - 403.5
403.5 - 440.6
440.6 - 477.7
477.7 - 514.8
514.8 - 536.6
536.6 - 621.5
> 621.5
Philippines: Camarines Sur: DALYs Rate (per 100,000)
< 141.2
141.2 - 172.1
172.1 - 289.0
289.0 - 406.0
406.0 - 442.6
442.6 - 479.3
479.3 - 515.9
515.9 - 539.3
539.3 - 622.5
> 622.5
Female
Male
Sexes Combined
Age
20-24 years
15-19 years
10-14 years
Year
DALYs Rate (per 100,000)
Age — 10-24 years

Figure S7

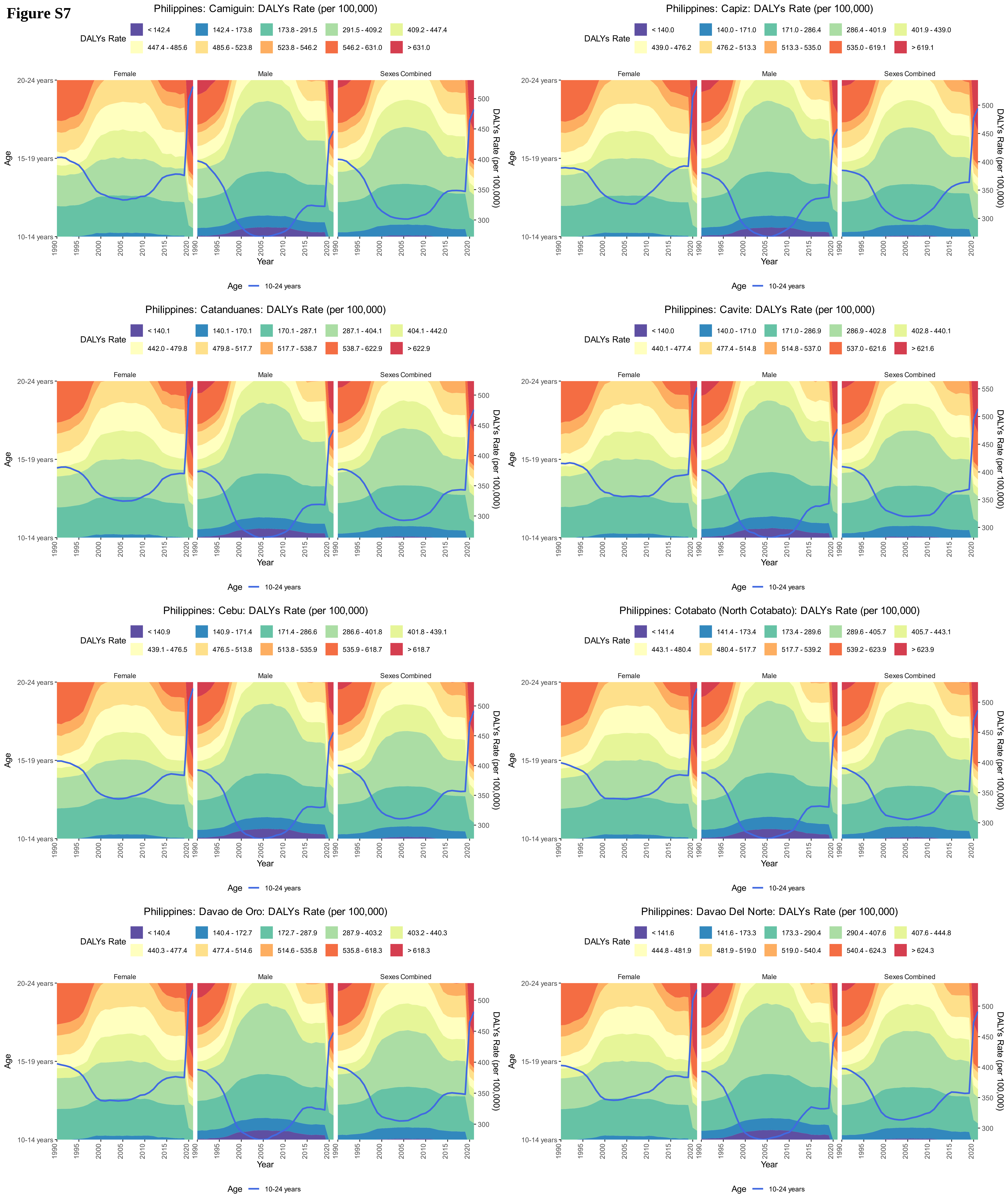
Philippines: Camiguin: DALYs Rate (per 100,000)
DALYs Rate
< 142.4
142.4 - 173.8
173.8 - 291.5
291.5 - 409.2
409.2 - 447.4
447.4 - 485.6
485.6 - 523.8
523.8 - 546.2
546.2 - 631.0
> 631.0
Philippines: Capiz: DALYs Rate (per 100,000)
< 140.0
140.0 - 171.0
171.0 - 286.4
286.4 - 401.9
401.9 - 439.0
439.0 - 476.2
476.2 - 513.3
513.3 - 535.0
535.0 - 619.1
> 619.1
Philippines: Catanduanes: DALYs Rate (per 100,000)
< 140.1
140.1 - 170.1
170.1 - 287.1
287.1 - 404.1
404.1 - 442.0
442.0 - 479.8
479.8 - 517.7
517.7 - 538.7
538.7 - 622.9
> 622.9
Philippines: Cavite: DALYs Rate (per 100,000)
< 140.0
140.0 - 171.0
171.0 - 286.9
286.9 - 402.8
402.8 - 440.1
440.1 - 477.4
477.4 - 514.8
514.8 - 537.0
537.0 - 621.6
> 621.6
Philippines: Cebu: DALYs Rate (per 100,000)
< 140.9
140.9 - 171.4
171.4 - 286.6
286.6 - 401.8
401.8 - 439.1
439.1 - 476.5
476.5 - 513.8
513.8 - 535.9
535.9 - 618.7
> 618.7
Philippines: Cotabato (North Cotabato): DALYs Rate (per 100,000)
< 141.4
141.4 - 173.4
173.4 - 289.6
289.6 - 405.7
405.7 - 443.1
443.1 - 480.4
480.4 - 517.7
517.7 - 539.2
539.2 - 623.9
> 623.9
Philippines: Davao de Oro: DALYs Rate (per 100,000)
< 140.4
140.4 - 172.7
172.7 - 287.9
287.9 - 403.2
403.2 - 440.3
440.3 - 477.4
477.4 - 514.6
514.6 - 535.8
535.8 - 618.3
> 618.3
Philippines: Davao Del Norte: DALYs Rate (per 100,000)
< 141.6
141.6 - 173.3
173.3 - 290.4
290.4 - 407.6
407.6 - 444.8
444.8 - 481.9
481.9 - 519.0
519.0 - 540.4
540.4 - 624.3
> 624.3
Female
Male
Sexes Combined
Age
20-24 years
15-19 years
10-14 years
Year
DALYs Rate (per 100,000)
Age — 10-24 years

Figure S7

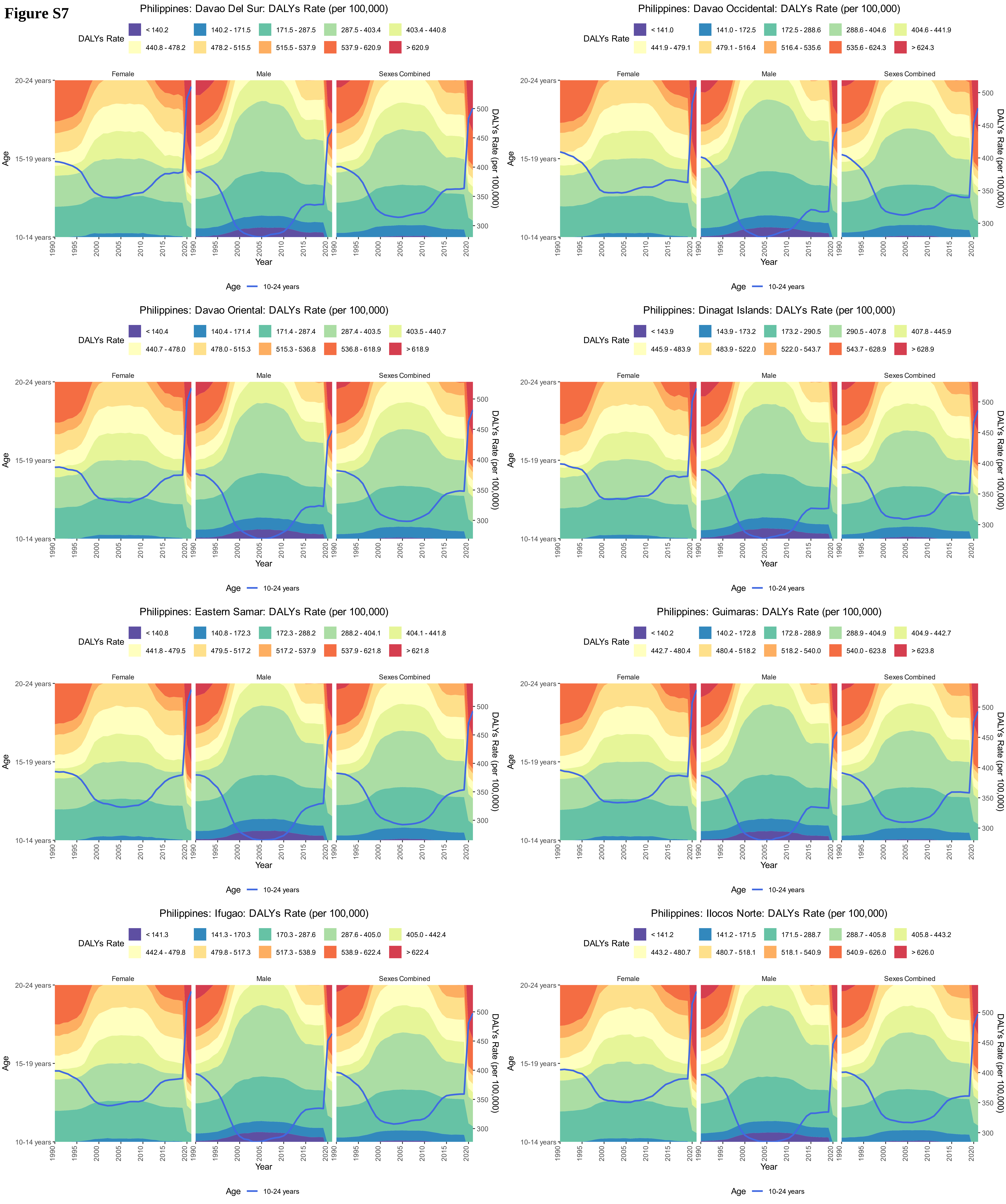

Philippines: Davao Del Sur: DALYs Rate (per 100,000)
DALYs Rate
< 140.2
140.2 - 171.5
171.5 - 287.5
287.5 - 403.4
403.4 - 440.8
440.8 - 478.2
478.2 - 515.5
515.5 - 537.9
537.9 - 620.9
> 620.9
Female
Male
Sexes Combined
Age
20-24 years
15-19 years
10-14 years
Year
DALYs Rate (per 100,000)
Age
10-24 years
Philippines: Davao Occidental: DALYs Rate (per 100,000)
< 141.0
141.0 - 172.5
172.5 - 288.6
288.6 - 404.6
404.6 - 441.9
441.9 - 479.1
479.1 - 516.4
516.4 - 535.6
535.6 - 624.3
> 624.3
Philippines: Davao Oriental: DALYs Rate (per 100,000)
< 140.4
140.4 - 171.4
171.4 - 287.4
287.4 - 403.5
403.5 - 440.7
440.7 - 478.0
478.0 - 515.3
515.3 - 536.8
536.8 - 618.9
> 618.9
Philippines: Dinagat Islands: DALYs Rate (per 100,000)
< 143.9
143.9 - 173.2
173.2 - 290.5
290.5 - 407.8
407.8 - 445.9
445.9 - 483.9
483.9 - 522.0
522.0 - 543.7
543.7 - 628.9
> 628.9
Philippines: Eastern Samar: DALYs Rate (per 100,000)
< 140.8
140.8 - 172.3
172.3 - 288.2
288.2 - 404.1
404.1 - 441.8
441.8 - 479.5
479.5 - 517.2
517.2 - 537.9
537.9 - 621.8
> 621.8
Philippines: Guimaras: DALYs Rate (per 100,000)
< 140.2
140.2 - 172.8
172.8 - 288.9
288.9 - 404.9
404.9 - 442.7
442.7 - 480.4
480.4 - 518.2
518.2 - 540.0
540.0 - 623.8
> 623.8
Philippines: Ifugao: DALYs Rate (per 100,000)
< 141.3
141.3 - 170.3
170.3 - 287.6
287.6 - 405.0
405.0 - 442.4
442.4 - 479.8
479.8 - 517.3
517.3 - 538.9
538.9 - 622.4
> 622.4
Philippines: Ilocos Norte: DALYs Rate (per 100,000)
< 141.2
141.2 - 171.5
171.5 - 288.7
288.7 - 405.8
405.8 - 443.2
443.2 - 480.7
480.7 - 518.1
518.1 - 540.9
540.9 - 626.0
> 626.0

Figure S7

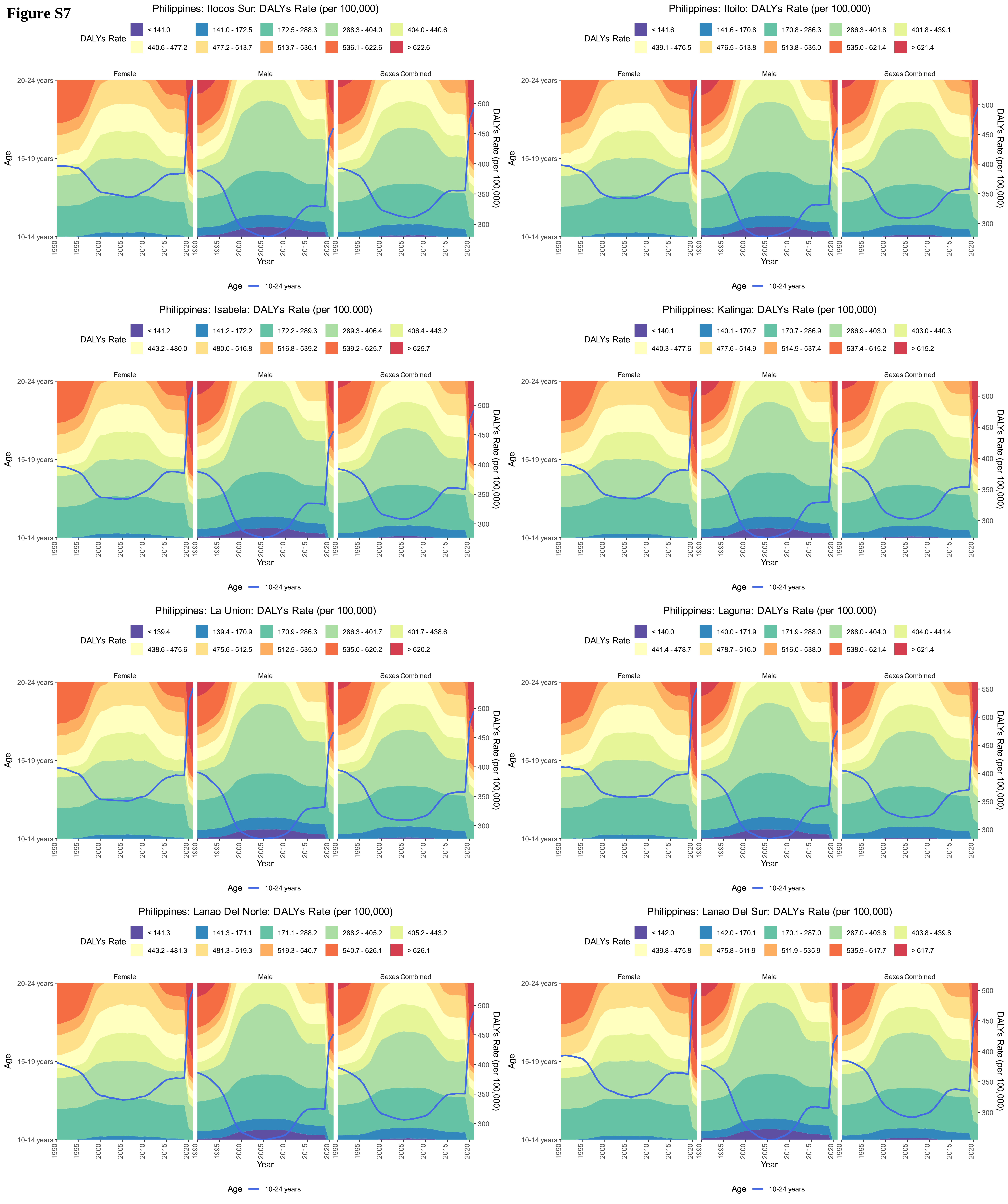
Philippines: Ilocos Sur: DALYs Rate (per 100,000)
DALYs Rate
< 141.0
141.0 - 172.5
172.5 - 288.3
288.3 - 404.0
404.0 - 440.6
440.6 - 477.2
477.2 - 513.7
513.7 - 536.1
536.1 - 622.6
> 622.6
Philippines: Iloilo: DALYs Rate (per 100,000)
< 141.6
141.6 - 170.8
170.8 - 286.3
286.3 - 401.8
401.8 - 439.1
439.1 - 476.5
476.5 - 513.8
513.8 - 535.0
535.0 - 621.4
> 621.4
Philippines: Isabela: DALYs Rate (per 100,000)
< 141.2
141.2 - 172.2
172.2 - 289.3
289.3 - 406.4
406.4 - 443.2
443.2 - 480.0
480.0 - 516.8
516.8 - 539.2
539.2 - 625.7
> 625.7
Philippines: Kalinga: DALYs Rate (per 100,000)
< 140.1
140.1 - 170.7
170.7 - 286.9
286.9 - 403.0
403.0 - 440.3
440.3 - 477.6
477.6 - 514.9
514.9 - 537.4
537.4 - 615.2
> 615.2
Philippines: La Union: DALYs Rate (per 100,000)
< 139.4
139.4 - 170.9
170.9 - 286.3
286.3 - 401.7
401.7 - 438.6
438.6 - 475.6
475.6 - 512.5
512.5 - 535.0
535.0 - 620.2
> 620.2
Philippines: Laguna: DALYs Rate (per 100,000)
< 140.0
140.0 - 171.9
171.9 - 288.0
288.0 - 404.0
404.0 - 441.4
441.4 - 478.7
478.7 - 516.0
516.0 - 538.0
538.0 - 621.4
> 621.4
Philippines: Lanao Del Norte: DALYs Rate (per 100,000)
< 141.3
141.3 - 171.1
171.1 - 288.2
288.2 - 405.2
405.2 - 443.2
443.2 - 481.3
481.3 - 519.3
519.3 - 540.7
540.7 - 626.1
> 626.1
Philippines: Lanao Del Sur: DALYs Rate (per 100,000)
< 142.0
142.0 - 170.1
170.1 - 287.0
287.0 - 403.8
403.8 - 439.8
439.8 - 475.8
475.8 - 511.9
511.9 - 535.9
535.9 - 617.7
> 617.7
Female
Male
Sexes Combined
Age
20-24 years
15-19 years
10-14 years
Year
1990
1995
2000
2005
2010
2015
2020
DALYs Rate (per 100,000)
Age
10-24 years

Figure S7

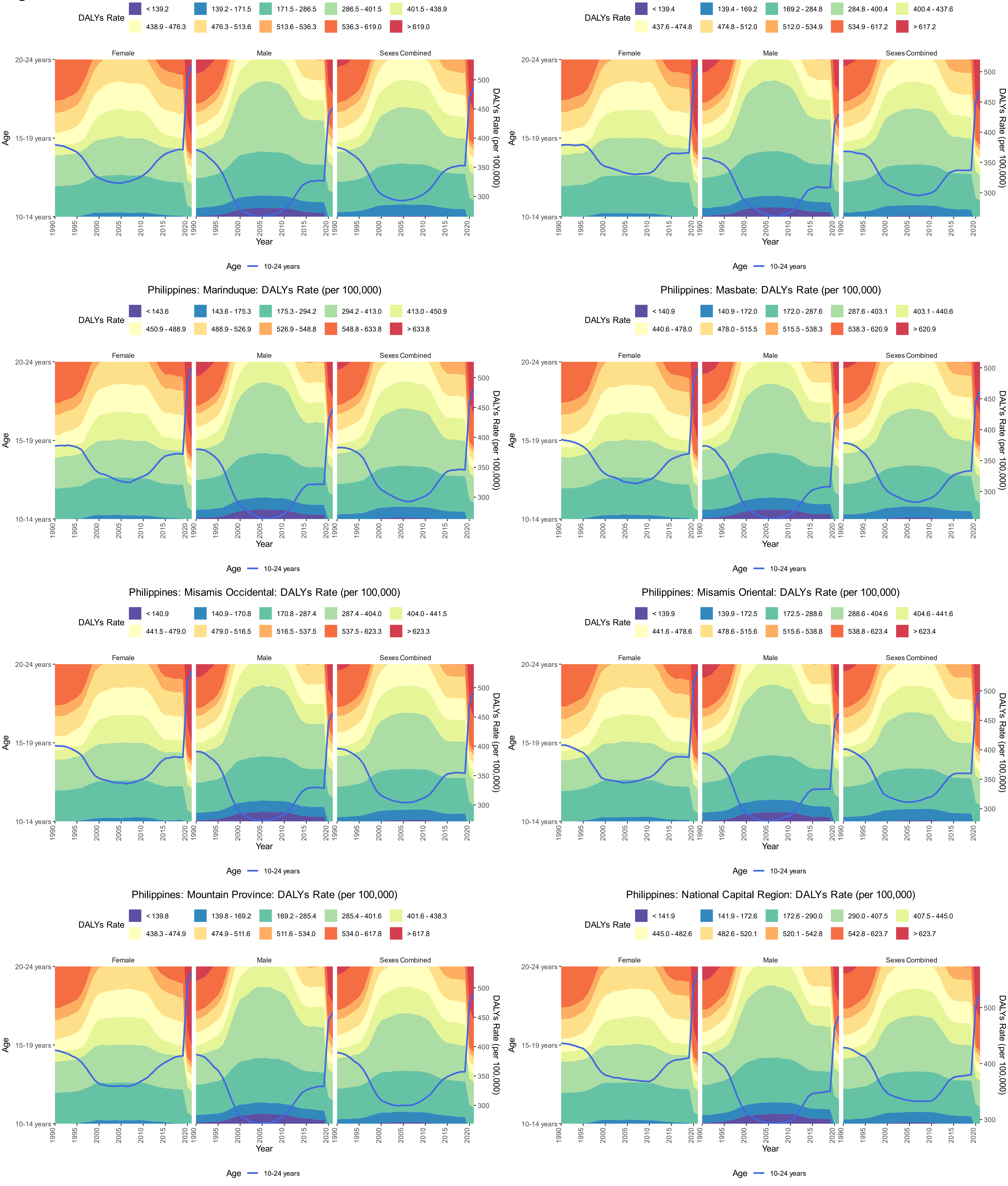

Philippines: Leyte: DALYs Rate (per 100,000)
DALYs Rate
< 139.2
139.2 - 171.5
171.5 - 286.5
286.5 - 401.5
401.5 - 438.9
438.9 - 476.3
476.3 - 513.6
513.6 - 536.3
536.3 - 619.0
> 619.0
Philippines: Maguindanao: DALYs Rate (per 100,000)
DALYs Rate
< 139.4
139.4 - 169.2
169.2 - 284.8
284.8 - 400.4
400.4 - 437.6
437.6 - 474.8
474.8 - 512.0
512.0 - 534.9
534.9 - 617.2
> 617.2
Philippines: Marinduque: DALYs Rate (per 100,000)
DALYs Rate
< 143.6
143.6 - 175.3
175.3 - 294.2
294.2 - 413.0
413.0 - 450.9
450.9 - 488.9
488.9 - 526.9
526.9 - 548.8
548.8 - 633.8
> 633.8
Philippines: Masbate: DALYs Rate (per 100,000)
DALYs Rate
< 140.9
140.9 - 172.0
172.0 - 287.6
287.6 - 403.1
403.1 - 440.6
440.6 - 478.0
478.0 - 515.5
515.5 - 538.3
538.3 - 620.9
> 620.9
Philippines: Misamis Occidental: DALYs Rate (per 100,000)
DALYs Rate
< 140.9
140.9 - 170.8
170.8 - 287.4
287.4 - 404.0
404.0 - 441.5
441.5 - 479.0
479.0 - 516.5
516.5 - 537.5
537.5 - 623.3
> 623.3
Philippines: Misamis Oriental: DALYs Rate (per 100,000)
DALYs Rate
< 139.9
139.9 - 172.5
172.5 - 288.6
288.6 - 404.6
404.6 - 441.6
441.6 - 478.6
478.6 - 515.6
515.6 - 538.8
538.8 - 623.4
> 623.4
Philippines: Mountain Province: DALYs Rate (per 100,000)
DALYs Rate
< 139.8
139.8 - 169.2
169.2 - 285.4
285.4 - 401.6
401.6 - 438.3
438.3 - 474.9
474.9 - 511.6
511.6 - 534.0
534.0 - 617.8
> 617.8
Philippines: National Capital Region: DALYs Rate (per 100,000)
DALYs Rate
< 141.9
141.9 - 172.6
172.6 - 290.0
290.0 - 407.5
407.5 - 445.0
445.0 - 482.6
482.6 - 520.1
520.1 - 542.8
542.8 - 623.7
> 623.7
Female
Male
Sexes Combined
Age
20-24 years
15-19 years
10-14 years
Year
1990
1995
2000
2005
2010
2015
2020
DALYs Rate (per 100,000)
Age
10-24 years

Figure S7

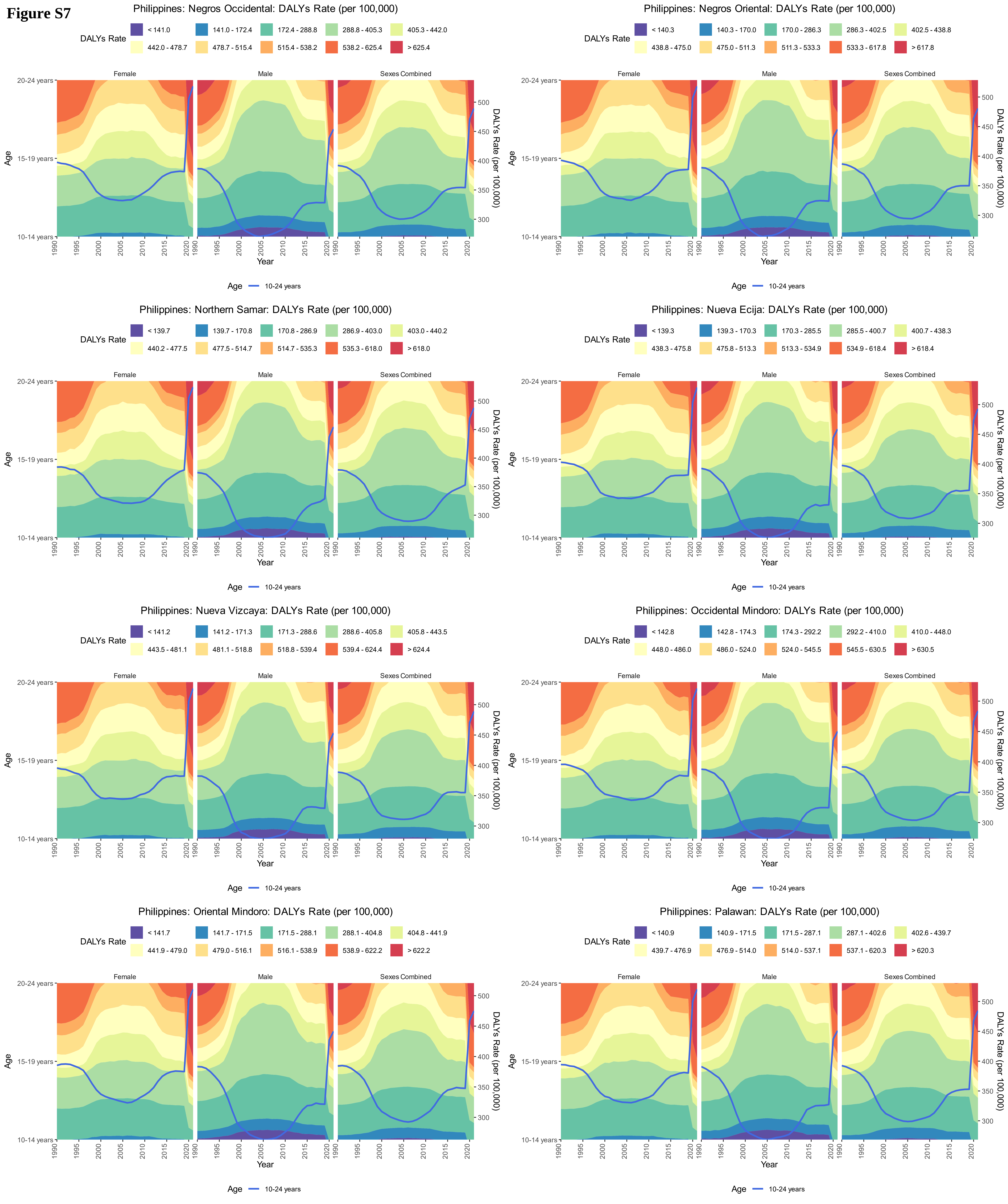

Philippines: Negros Occidental: DALYs Rate (per 100,000)
DALYs Rate
< 141.0
141.0 - 172.4
172.4 - 288.8
288.8 - 405.3
405.3 - 442.0
442.0 - 478.7
478.7 - 515.4
515.4 - 538.2
538.2 - 625.4
> 625.4
Philippines: Negros Oriental: DALYs Rate (per 100,000)
DALYs Rate
< 140.3
140.3 - 170.0
170.0 - 286.3
286.3 - 402.5
402.5 - 438.8
438.8 - 475.0
475.0 - 511.3
511.3 - 533.3
533.3 - 617.8
> 617.8
Philippines: Northern Samar: DALYs Rate (per 100,000)
DALYs Rate
< 139.7
139.7 - 170.8
170.8 - 286.9
286.9 - 403.0
403.0 - 440.2
440.2 - 477.5
477.5 - 514.7
514.7 - 535.3
535.3 - 618.0
> 618.0
Philippines: Nueva Ecija: DALYs Rate (per 100,000)
DALYs Rate
< 139.3
139.3 - 170.3
170.3 - 285.5
285.5 - 400.7
400.7 - 438.3
438.3 - 475.8
475.8 - 513.3
513.3 - 534.9
534.9 - 618.4
> 618.4
Philippines: Nueva Vizcaya: DALYs Rate (per 100,000)
DALYs Rate
< 141.2
141.2 - 171.3
171.3 - 288.6
288.6 - 405.8
405.8 - 443.5
443.5 - 481.1
481.1 - 518.8
518.8 - 539.4
539.4 - 624.4
> 624.4
Philippines: Occidental Mindoro: DALYs Rate (per 100,000)
DALYs Rate
< 142.8
142.8 - 174.3
174.3 - 292.2
292.2 - 410.0
410.0 - 448.0
448.0 - 486.0
486.0 - 524.0
524.0 - 545.5
545.5 - 630.5
> 630.5
Philippines: Oriental Mindoro: DALYs Rate (per 100,000)
DALYs Rate
< 141.7
141.7 - 171.5
171.5 - 288.1
288.1 - 404.8
404.8 - 441.9
441.9 - 479.0
479.0 - 516.1
516.1 - 538.9
538.9 - 622.2
> 622.2
Philippines: Palawan: DALYs Rate (per 100,000)
DALYs Rate
< 140.9
140.9 - 171.5
171.5 - 287.1
287.1 - 402.6
402.6 - 439.7
439.7 - 476.9
476.9 - 514.0
514.0 - 537.1
537.1 - 620.3
> 620.3
Female
Male
Sexes Combined
Age
20-24 years
15-19 years
10-14 years
Year
DALYs Rate (per 100,000)
Age
10-24 years

Figure S7

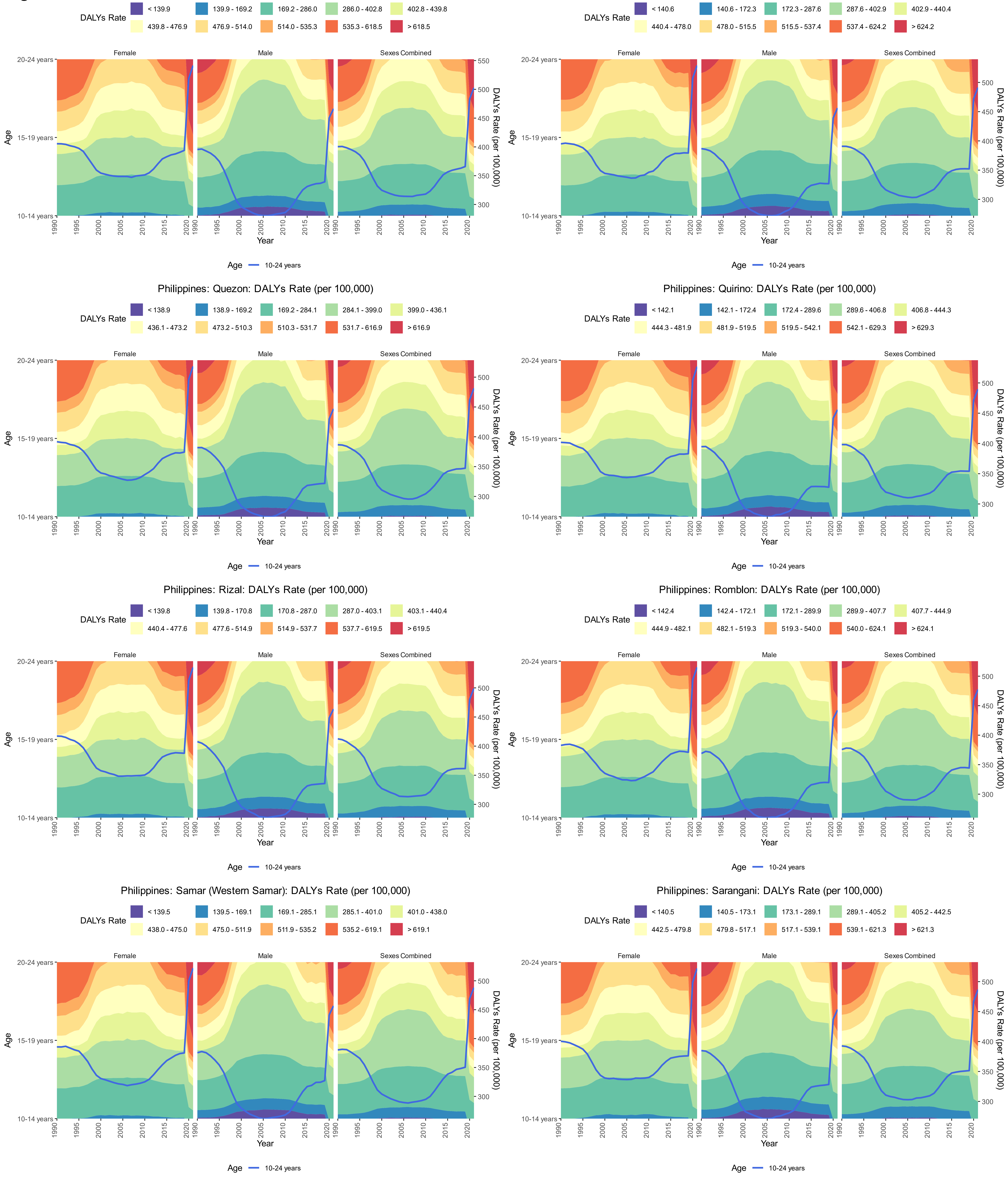

Philippines: Pampanga: DALYs Rate (per 100,000)
DALYs Rate
< 139.9
139.9 - 169.2
169.2 - 286.0
286.0 - 402.8
402.8 - 439.8
439.8 - 476.9
476.9 - 514.0
514.0 - 535.3
535.3 - 618.5
> 618.5
Philippines: Pangasinan: DALYs Rate (per 100,000)
DALYs Rate
< 140.6
140.6 - 172.3
172.3 - 287.6
287.6 - 402.9
402.9 - 440.4
440.4 - 478.0
478.0 - 515.5
515.5 - 537.4
537.4 - 624.2
> 624.2
Philippines: Quezon: DALYs Rate (per 100,000)
DALYs Rate
< 138.9
138.9 - 169.2
169.2 - 284.1
284.1 - 399.0
399.0 - 436.1
436.1 - 473.2
473.2 - 510.3
510.3 - 531.7
531.7 - 616.9
> 616.9
Philippines: Quirino: DALYs Rate (per 100,000)
DALYs Rate
< 142.1
142.1 - 172.4
172.4 - 289.6
289.6 - 406.8
406.8 - 444.3
444.3 - 481.9
481.9 - 519.5
519.5 - 542.1
542.1 - 629.3
> 629.3
Philippines: Rizal: DALYs Rate (per 100,000)
DALYs Rate
< 139.8
139.8 - 170.8
170.8 - 287.0
287.0 - 403.1
403.1 - 440.4
440.4 - 477.6
477.6 - 514.9
514.9 - 537.7
537.7 - 619.5
> 619.5
Philippines: Romblon: DALYs Rate (per 100,000)
DALYs Rate
< 142.4
142.4 - 172.1
172.1 - 289.9
289.9 - 407.7
407.7 - 444.9
444.9 - 482.1
482.1 - 519.3
519.3 - 540.0
540.0 - 624.1
> 624.1
Philippines: Samar (Western Samar): DALYs Rate (per 100,000)
DALYs Rate
< 139.5
139.5 - 169.1
169.1 - 285.1
285.1 - 401.0
401.0 - 438.0
438.0 - 475.0
475.0 - 511.9
511.9 - 535.2
535.2 - 619.1
> 619.1
Philippines: Sarangani: DALYs Rate (per 100,000)
DALYs Rate
< 140.5
140.5 - 173.1
173.1 - 289.1
289.1 - 405.2
405.2 - 442.5
442.5 - 479.8
479.8 - 517.1
517.1 - 539.1
539.1 - 621.3
> 621.3
Female
Male
Sexes Combined
Age
20-24 years
15-19 years
10-14 years
Year
DALYs Rate (per 100,000)
Age
10-24 years

Figure S7

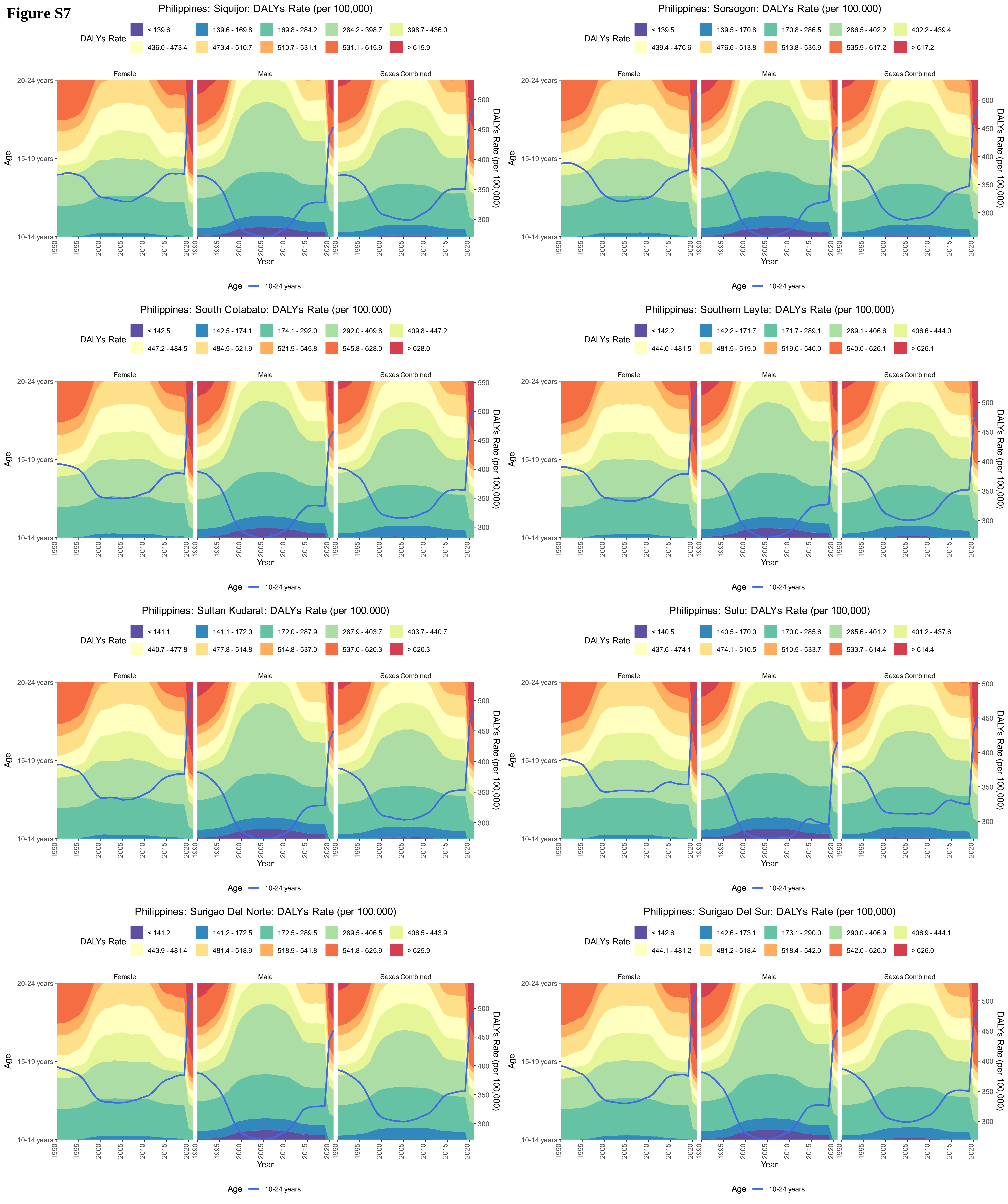

Philippines: Siquijor: DALYs Rate (per 100,000)
DALYs Rate
< 139.6
139.6 - 169.8
169.8 - 284.2
284.2 - 398.7
398.7 - 436.0
436.0 - 473.4
473.4 - 510.7
510.7 - 531.1
531.1 - 615.9
> 615.9
Philippines: Sorsogon: DALYs Rate (per 100,000)
< 139.5
139.5 - 170.8
170.8 - 286.5
286.5 - 402.2
402.2 - 439.4
439.4 - 476.6
476.6 - 513.8
513.8 - 535.9
535.9 - 617.2
> 617.2
Philippines: South Cotabato: DALYs Rate (per 100,000)
< 142.5
142.5 - 174.1
174.1 - 292.0
292.0 - 409.8
409.8 - 447.2
447.2 - 484.5
484.5 - 521.9
521.9 - 545.8
545.8 - 628.0
> 628.0
Philippines: Southern Leyte: DALYs Rate (per 100,000)
< 142.2
142.2 - 171.7
171.7 - 289.1
289.1 - 406.6
406.6 - 444.0
444.0 - 481.5
481.5 - 519.0
519.0 - 540.0
540.0 - 626.1
> 626.1
Philippines: Sultan Kudarat: DALYs Rate (per 100,000)
< 141.1
141.1 - 172.0
172.0 - 287.9
287.9 - 403.7
403.7 - 440.7
440.7 - 477.8
477.8 - 514.8
514.8 - 537.0
537.0 - 620.3
> 620.3
Philippines: Sulu: DALYs Rate (per 100,000)
< 140.5
140.5 - 170.0
170.0 - 285.6
285.6 - 401.2
401.2 - 437.6
437.6 - 474.1
474.1 - 510.5
510.5 - 533.7
533.7 - 614.4
> 614.4
Philippines: Surigao Del Norte: DALYs Rate (per 100,000)
< 141.2
141.2 - 172.5
172.5 - 289.5
289.5 - 406.5
406.5 - 443.9
443.9 - 481.4
481.4 - 518.9
518.9 - 541.8
541.8 - 625.9
> 625.9
Philippines: Surigao Del Sur: DALYs Rate (per 100,000)
< 142.6
142.6 - 173.1
173.1 - 290.0
290.0 - 406.9
406.9 - 444.1
444.1 - 481.2
481.2 - 518.4
518.4 - 542.0
542.0 - 626.0
> 626.0
Female
Male
Sexes Combined
Age
20-24 years
15-19 years
10-14 years
Year
DALYs Rate (per 100,000)
Age — 10-24 years

Figure S7

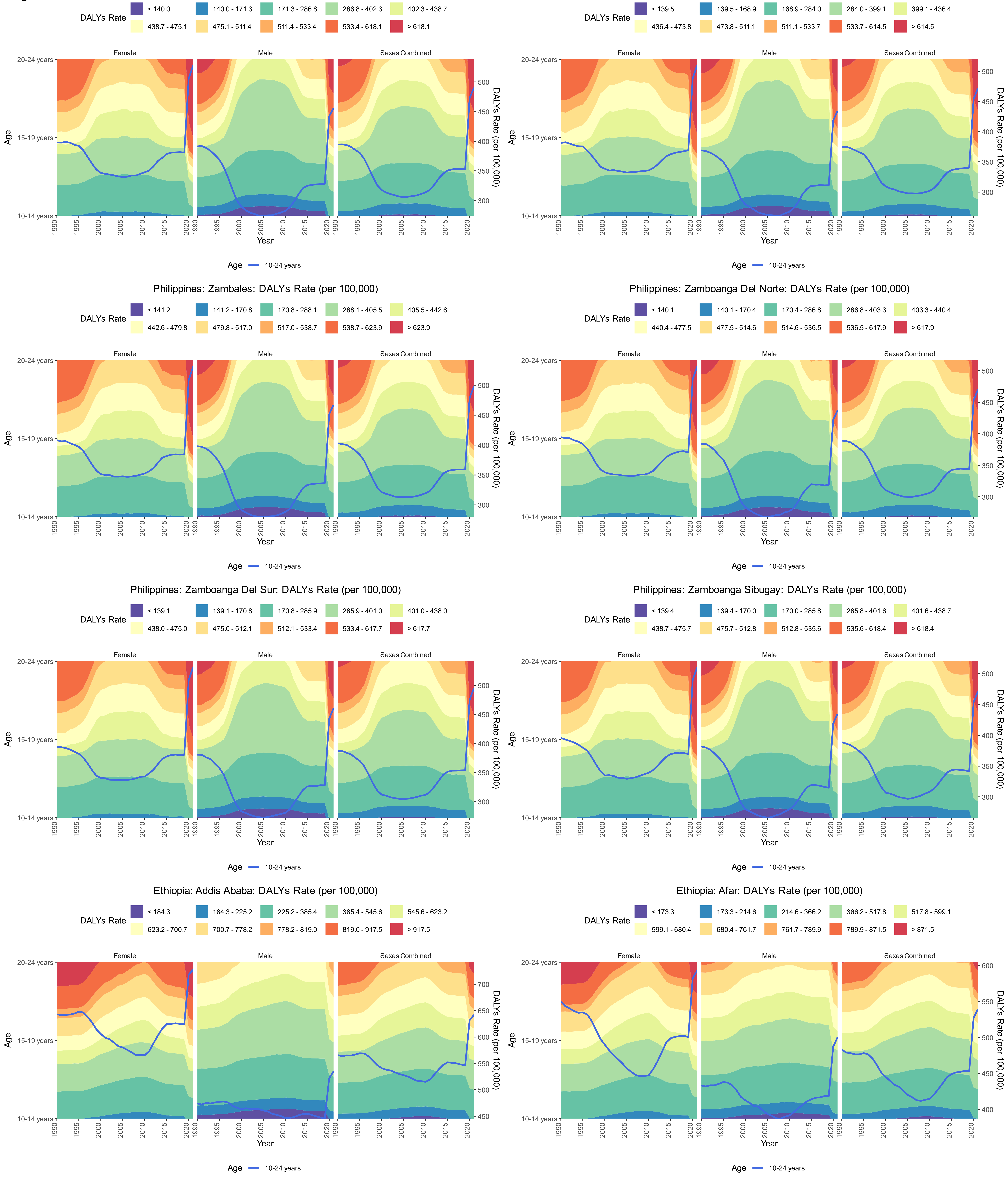
Philippines: Tarlac: DALYs Rate (per 100,000)
DALYs Rate
< 140.0
140.0 - 171.3
171.3 - 286.8
286.8 - 402.3
402.3 - 438.7
438.7 - 475.1
475.1 - 511.4
511.4 - 533.4
533.4 - 618.1
> 618.1
Philippines: Tawi-Tawi: DALYs Rate (per 100,000)
DALYs Rate
< 139.5
139.5 - 168.9
168.9 - 284.0
284.0 - 399.1
399.1 - 436.4
436.4 - 473.8
473.8 - 511.1
511.1 - 533.7
533.7 - 614.5
> 614.5
Philippines: Zambales: DALYs Rate (per 100,000)
DALYs Rate
< 141.2
141.2 - 170.8
170.8 - 288.1
288.1 - 405.5
405.5 - 442.6
442.6 - 479.8
479.8 - 517.0
517.0 - 538.7
538.7 - 623.9
> 623.9
Philippines: Zamboanga Del Norte: DALYs Rate (per 100,000)
DALYs Rate
< 140.1
140.1 - 170.4
170.4 - 286.8
286.8 - 403.3
403.3 - 440.4
440.4 - 477.5
477.5 - 514.6
514.6 - 536.5
536.5 - 617.9
> 617.9
Philippines: Zamboanga Del Sur: DALYs Rate (per 100,000)
DALYs Rate
< 139.1
139.1 - 170.8
170.8 - 285.9
285.9 - 401.0
401.0 - 438.0
438.0 - 475.0
475.0 - 512.1
512.1 - 533.4
533.4 - 617.7
> 617.7
Philippines: Zamboanga Sibugay: DALYs Rate (per 100,000)
DALYs Rate
< 139.4
139.4 - 170.0
170.0 - 285.8
285.8 - 401.6
401.6 - 438.7
438.7 - 475.7
475.7 - 512.8
512.8 - 535.6
535.6 - 618.4
> 618.4
Ethiopia: Addis Ababa: DALYs Rate (per 100,000)
DALYs Rate
< 184.3
184.3 - 225.2
225.2 - 385.4
385.4 - 545.6
545.6 - 623.2
623.2 - 700.7
700.7 - 778.2
778.2 - 819.0
819.0 - 917.5
> 917.5
Ethiopia: Afar: DALYs Rate (per 100,000)
DALYs Rate
< 173.3
173.3 - 214.6
214.6 - 366.2
366.2 - 517.8
517.8 - 599.1
599.1 - 680.4
680.4 - 761.7
761.7 - 789.9
789.9 - 871.5
> 871.5
Female
Male
Sexes Combined
Age
20-24 years
15-19 years
10-14 years
Year
DALYs Rate (per 100,000)
Age
10-24 years

Figure S7

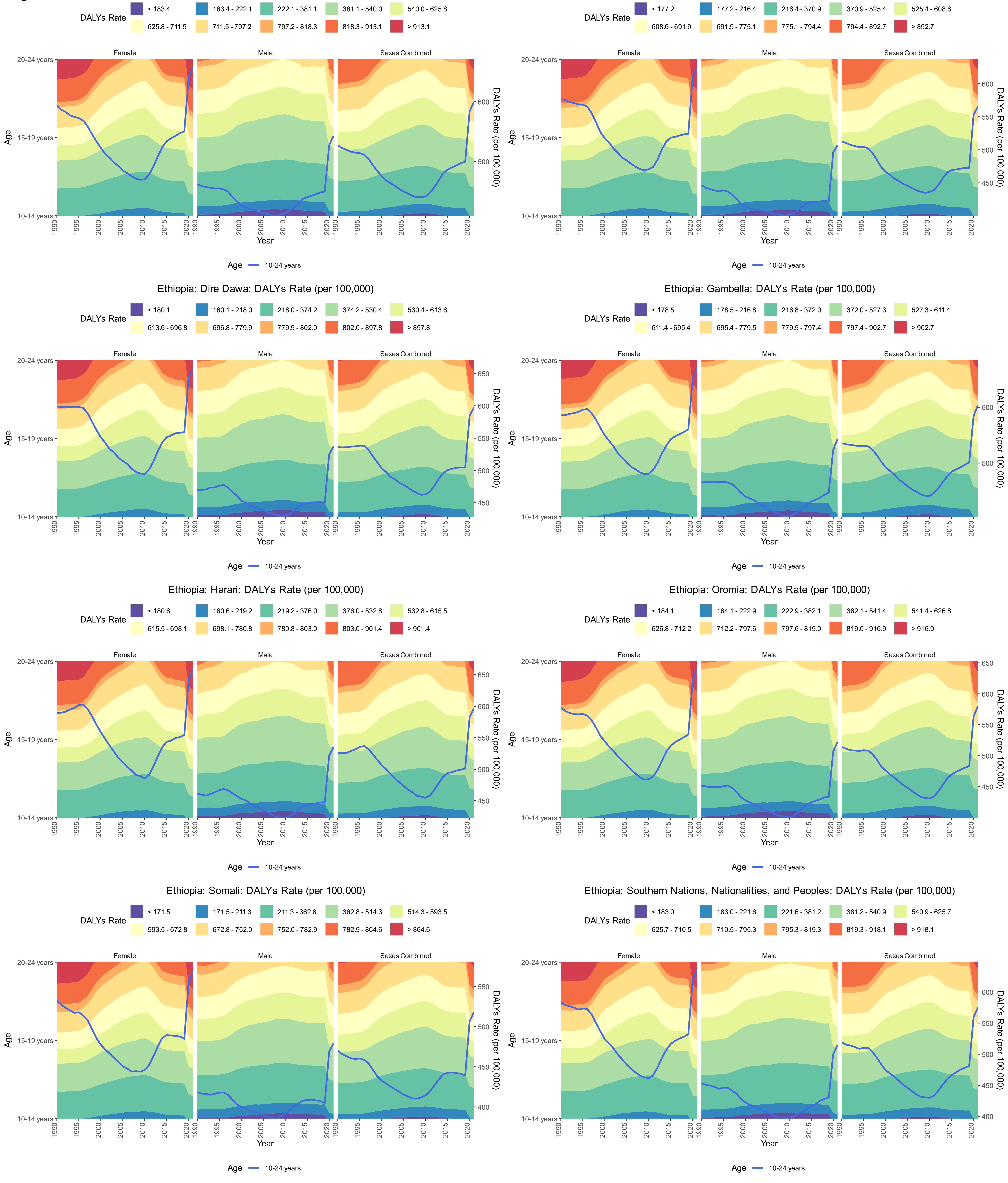

Ethiopia: Amhara: DALYs Rate (per 100,000)
DALYs Rate
< 183.4
183.4 - 222.1
222.1 - 381.1
381.1 - 540.0
540.0 - 625.8
625.8 - 711.5
711.5 - 797.2
797.2 - 818.3
818.3 - 913.1
> 913.1
Ethiopia: Benishangul-Gumuz: DALYs Rate (per 100,000)
DALYs Rate
< 177.2
177.2 - 216.4
216.4 - 370.9
370.9 - 525.4
525.4 - 608.6
608.6 - 691.9
691.9 - 775.1
775.1 - 794.4
794.4 - 892.7
> 892.7
Ethiopia: Dire Dawa: DALYs Rate (per 100,000)
DALYs Rate
< 180.1
180.1 - 218.0
218.0 - 374.2
374.2 - 530.4
530.4 - 613.6
613.6 - 696.8
696.8 - 779.9
779.9 - 802.0
802.0 - 897.8
> 897.8
Ethiopia: Gambella: DALYs Rate (per 100,000)
DALYs Rate
< 178.5
178.5 - 216.8
216.8 - 372.0
372.0 - 527.3
527.3 - 611.4
611.4 - 695.4
695.4 - 779.5
779.5 - 797.4
797.4 - 902.7
> 902.7
Ethiopia: Harari: DALYs Rate (per 100,000)
DALYs Rate
< 180.6
180.6 - 219.2
219.2 - 376.0
376.0 - 532.8
532.8 - 615.5
615.5 - 698.1
698.1 - 780.8
780.8 - 803.0
803.0 - 901.4
> 901.4
Ethiopia: Oromia: DALYs Rate (per 100,000)
DALYs Rate
< 184.1
184.1 - 222.9
222.9 - 382.1
382.1 - 541.4
541.4 - 626.8
626.8 - 712.2
712.2 - 797.6
797.6 - 819.0
819.0 - 916.9
> 916.9
Ethiopia: Somali: DALYs Rate (per 100,000)
DALYs Rate
< 171.5
171.5 - 211.3
211.3 - 362.8
362.8 - 514.3
514.3 - 593.5
593.5 - 672.8
672.8 - 752.0
752.0 - 782.9
782.9 - 864.6
> 864.6
Ethiopia: Southern Nations, Nationalities, and Peoples: DALYs Rate (per 100,000)
DALYs Rate
< 183.0
183.0 - 221.6
221.6 - 381.2
381.2 - 540.9
540.9 - 625.7
625.7 - 710.5
710.5 - 795.3
795.3 - 819.3
819.3 - 918.1
> 918.1
Female
Male
Sexes Combined
Age
20-24 years
15-19 years
10-14 years
Year
1990
1995
2000
2005
2010
2015
2020
DALYs Rate (per 100,000)
Age
10-24 years

Figure S7

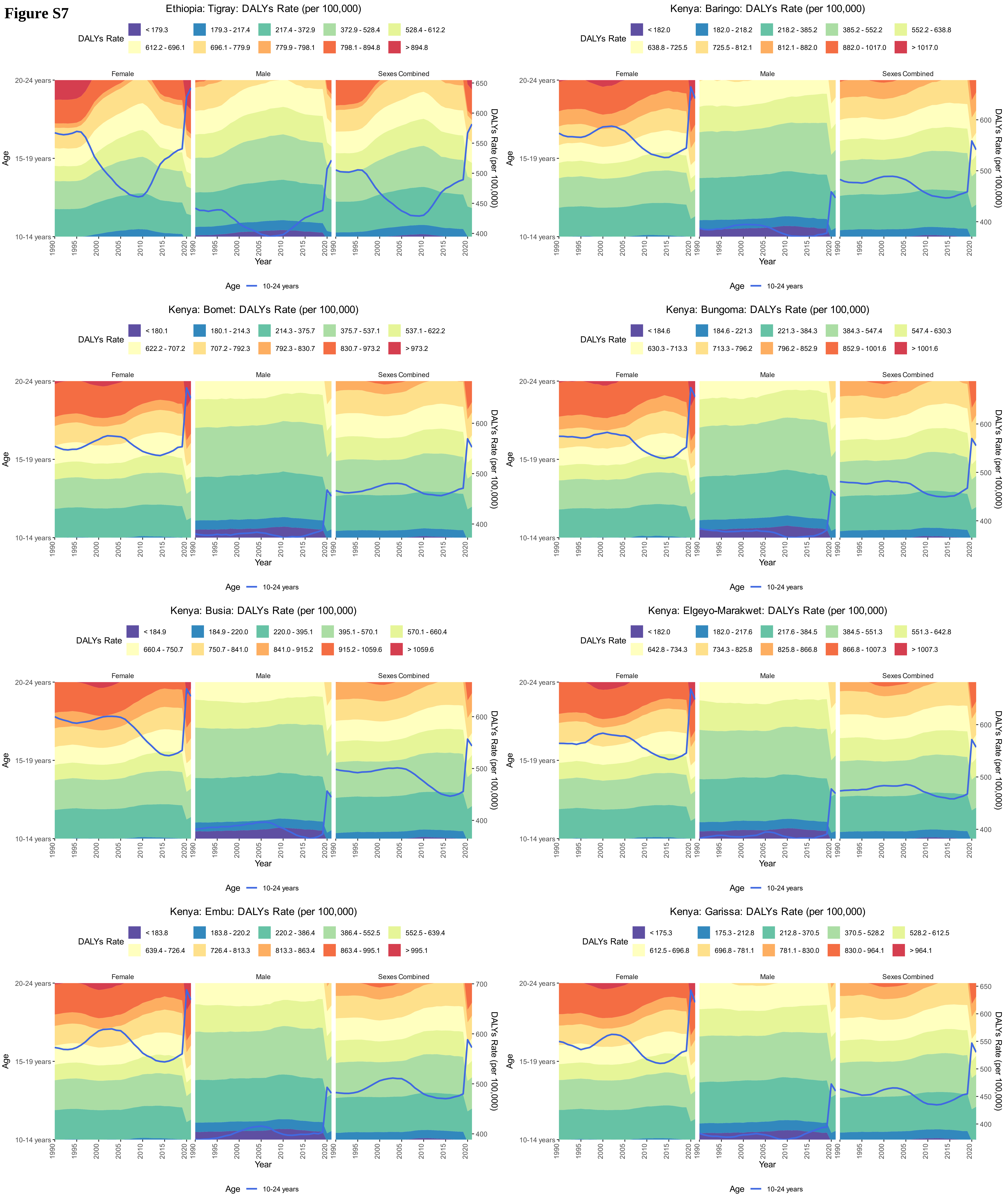
Ethiopia: Tigray: DALYs Rate (per 100,000)
DALYs Rate
< 179.3
179.3 - 217.4
217.4 - 372.9
372.9 - 528.4
528.4 - 612.2
612.2 - 696.1
696.1 - 779.9
779.9 - 798.1
798.1 - 894.8
> 894.8
Female
Male
Sexes Combined
Age
Year
DALYs Rate (per 100,000)
Age — 10-24 years
Kenya: Baringo: DALYs Rate (per 100,000)
< 182.0
182.0 - 218.2
218.2 - 385.2
385.2 - 552.2
552.2 - 638.8
638.8 - 725.5
725.5 - 812.1
812.1 - 882.0
882.0 - 1017.0
> 1017.0
Kenya: Bomet: DALYs Rate (per 100,000)
< 180.1
180.1 - 214.3
214.3 - 375.7
375.7 - 537.1
537.1 - 622.2
622.2 - 707.2
707.2 - 792.3
792.3 - 830.7
830.7 - 973.2
> 973.2
Kenya: Bungoma: DALYs Rate (per 100,000)
< 184.6
184.6 - 221.3
221.3 - 384.3
384.3 - 547.4
547.4 - 630.3
630.3 - 713.3
713.3 - 796.2
796.2 - 852.9
852.9 - 1001.6
> 1001.6
Kenya: Busia: DALYs Rate (per 100,000)
< 184.9
184.9 - 220.0
220.0 - 395.1
395.1 - 570.1
570.1 - 660.4
660.4 - 750.7
750.7 - 841.0
841.0 - 915.2
915.2 - 1059.6
> 1059.6
Kenya: Elgeyo-Marakwet: DALYs Rate (per 100,000)
< 182.0
182.0 - 217.6
217.6 - 384.5
384.5 - 551.3
551.3 - 642.8
642.8 - 734.3
734.3 - 825.8
825.8 - 866.8
866.8 - 1007.3
> 1007.3
Kenya: Embu: DALYs Rate (per 100,000)
< 183.8
183.8 - 220.2
220.2 - 386.4
386.4 - 552.5
552.5 - 639.4
639.4 - 726.4
726.4 - 813.3
813.3 - 863.4
863.4 - 995.1
> 995.1
Kenya: Garissa: DALYs Rate (per 100,000)
< 175.3
175.3 - 212.8
212.8 - 370.5
370.5 - 528.2
528.2 - 612.5
612.5 - 696.8
696.8 - 781.1
781.1 - 830.0
830.0 - 964.1
> 964.1
20-24 years
15-19 years
10-14 years
1990
1995
2000
2005
2010
2015
2020

Figure S7

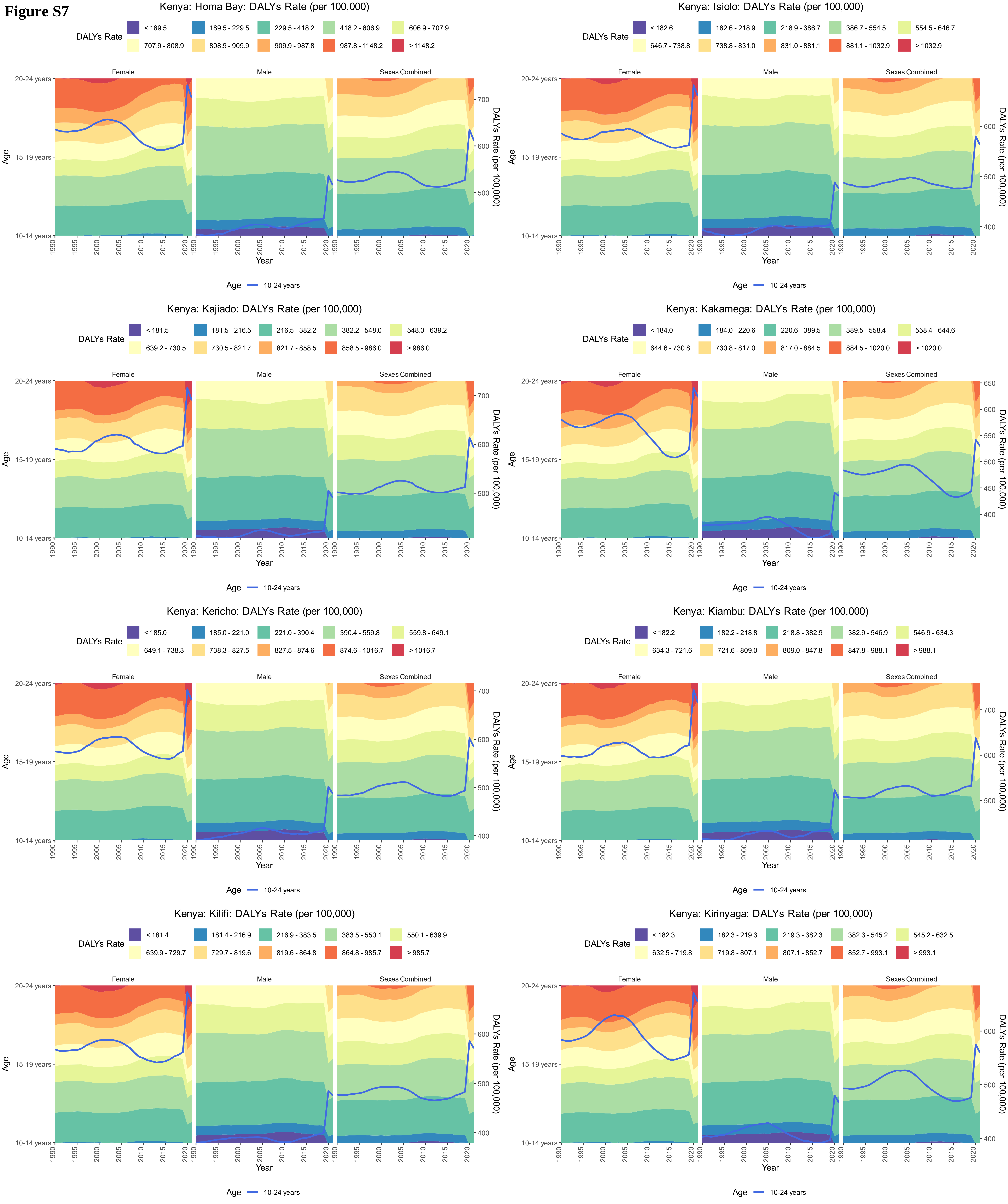
Kenya: Homa Bay: DALYs Rate (per 100,000)
DALYs Rate
< 189.5
189.5 - 229.5
229.5 - 418.2
418.2 - 606.9
606.9 - 707.9
707.9 - 808.9
808.9 - 909.9
909.9 - 987.8
987.8 - 1148.2
> 1148.2
Kenya: Isiolo: DALYs Rate (per 100,000)
DALYs Rate
< 182.6
182.6 - 218.9
218.9 - 386.7
386.7 - 554.5
554.5 - 646.7
646.7 - 738.8
738.8 - 831.0
831.0 - 881.1
881.1 - 1032.9
> 1032.9
Kenya: Kajiado: DALYs Rate (per 100,000)
DALYs Rate
< 181.5
181.5 - 216.5
216.5 - 382.2
382.2 - 548.0
548.0 - 639.2
639.2 - 730.5
730.5 - 821.7
821.7 - 858.5
858.5 - 986.0
> 986.0
Kenya: Kakamega: DALYs Rate (per 100,000)
DALYs Rate
< 184.0
184.0 - 220.6
220.6 - 389.5
389.5 - 558.4
558.4 - 644.6
644.6 - 730.8
730.8 - 817.0
817.0 - 884.5
884.5 - 1020.0
> 1020.0
Kenya: Kericho: DALYs Rate (per 100,000)
DALYs Rate
< 185.0
185.0 - 221.0
221.0 - 390.4
390.4 - 559.8
559.8 - 649.1
649.1 - 738.3
738.3 - 827.5
827.5 - 874.6
874.6 - 1016.7
> 1016.7
Kenya: Kiambu: DALYs Rate (per 100,000)
DALYs Rate
< 182.2
182.2 - 218.8
218.8 - 382.9
382.9 - 546.9
546.9 - 634.3
634.3 - 721.6
721.6 - 809.0
809.0 - 847.8
847.8 - 988.1
> 988.1
Kenya: Kilifi: DALYs Rate (per 100,000)
DALYs Rate
< 181.4
181.4 - 216.9
216.9 - 383.5
383.5 - 550.1
550.1 - 639.9
639.9 - 729.7
729.7 - 819.6
819.6 - 864.8
864.8 - 985.7
> 985.7
Kenya: Kirinyaga: DALYs Rate (per 100,000)
DALYs Rate
< 182.3
182.3 - 219.3
219.3 - 382.3
382.3 - 545.2
545.2 - 632.5
632.5 - 719.8
719.8 - 807.1
807.1 - 852.7
852.7 - 993.1
> 993.1
Female
Male
Sexes Combined
Age
20-24 years
15-19 years
10-14 years
Year
DALYs Rate (per 100,000)
Age
10-24 years

Figure S7

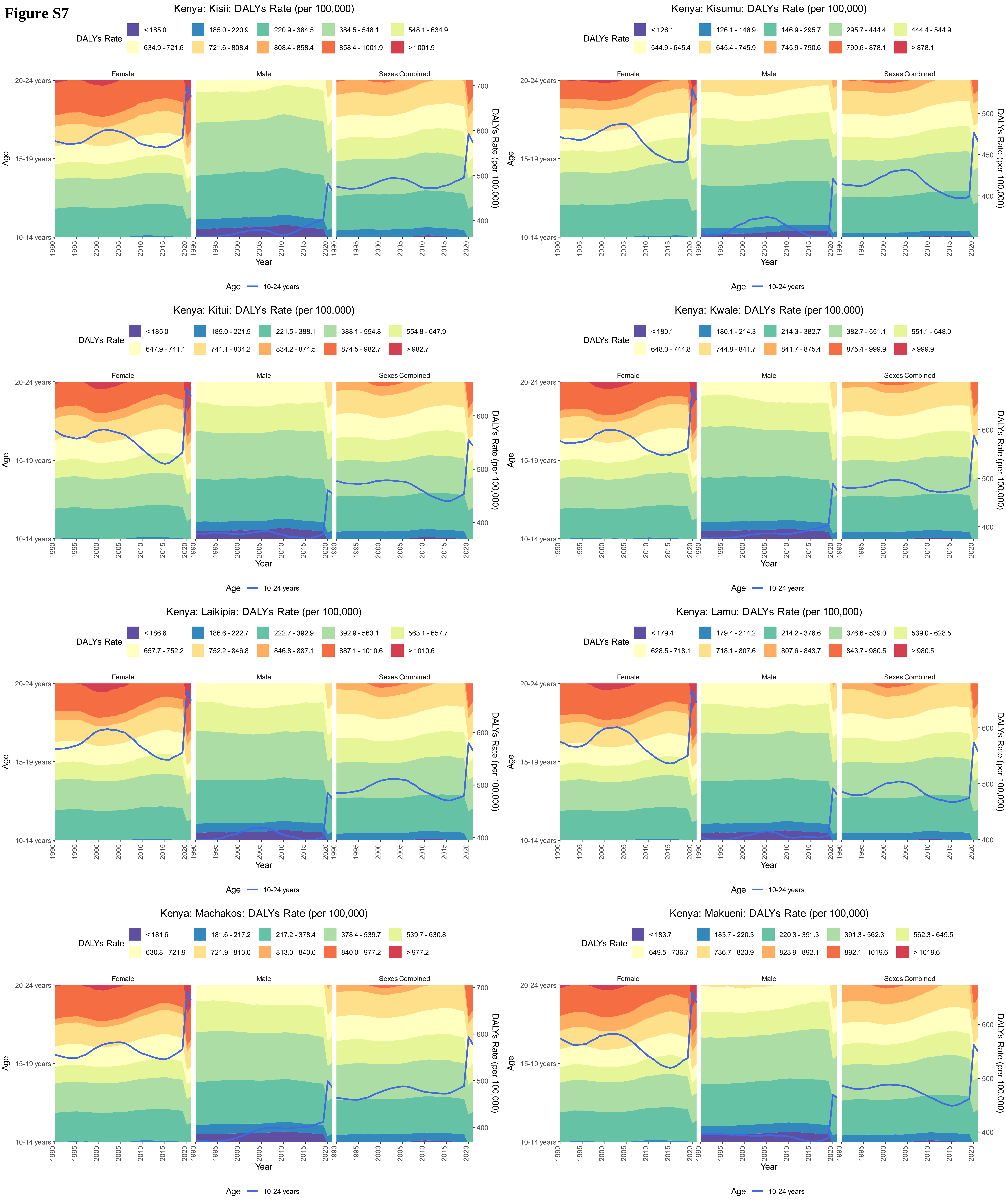
Kenya: Kisii: DALYs Rate (per 100,000)
DALYs Rate
< 185.0
185.0 - 220.9
220.9 - 384.5
384.5 - 548.1
548.1 - 634.9
634.9 - 721.6
721.6 - 808.4
808.4 - 858.4
858.4 - 1001.9
> 1001.9
Female
Male
Sexes Combined
Age
Year
DALYs Rate (per 100,000)
Age — 10-24 years
Kenya: Kisumu: DALYs Rate (per 100,000)
DALYs Rate
< 126.1
126.1 - 146.9
146.9 - 295.7
295.7 - 444.4
444.4 - 544.9
544.9 - 645.4
645.4 - 745.9
745.9 - 790.6
790.6 - 878.1
> 878.1
Female
Male
Sexes Combined
Age
Year
DALYs Rate (per 100,000)
Age — 10-24 years
Kenya: Kitui: DALYs Rate (per 100,000)
DALYs Rate
< 185.0
185.0 - 221.5
221.5 - 388.1
388.1 - 554.8
554.8 - 647.9
647.9 - 741.1
741.1 - 834.2
834.2 - 874.5
874.5 - 982.7
> 982.7
Female
Male
Sexes Combined
Age
Year
DALYs Rate (per 100,000)
Age — 10-24 years
Kenya: Kwale: DALYs Rate (per 100,000)
DALYs Rate
< 180.1
180.1 - 214.3
214.3 - 382.7
382.7 - 551.1
551.1 - 648.0
648.0 - 744.8
744.8 - 841.7
841.7 - 875.4
875.4 - 999.9
> 999.9
Female
Male
Sexes Combined
Age
Year
DALYs Rate (per 100,000)
Age — 10-24 years
Kenya: Laikipia: DALYs Rate (per 100,000)
DALYs Rate
< 186.6
186.6 - 222.7
222.7 - 392.9
392.9 - 563.1
563.1 - 657.7
657.7 - 752.2
752.2 - 846.8
846.8 - 887.1
887.1 - 1010.6
> 1010.6
Female
Male
Sexes Combined
Age
Year
DALYs Rate (per 100,000)
Age — 10-24 years
Kenya: Lamu: DALYs Rate (per 100,000)
DALYs Rate
< 179.4
179.4 - 214.2
214.2 - 376.6
376.6 - 539.0
539.0 - 628.5
628.5 - 718.1
718.1 - 807.6
807.6 - 843.7
843.7 - 980.5
> 980.5
Female
Male
Sexes Combined
Age
Year
DALYs Rate (per 100,000)
Age — 10-24 years
Kenya: Machakos: DALYs Rate (per 100,000)
DALYs Rate
< 181.6
181.6 - 217.2
217.2 - 378.4
378.4 - 539.7
539.7 - 630.8
630.8 - 721.9
721.9 - 813.0
813.0 - 840.0
840.0 - 977.2
> 977.2
Female
Male
Sexes Combined
Age
Year
DALYs Rate (per 100,000)
Age — 10-24 years
Kenya: Makueni: DALYs Rate (per 100,000)
DALYs Rate
< 183.7
183.7 - 220.3
220.3 - 391.3
391.3 - 562.3
562.3 - 649.5
649.5 - 736.7
736.7 - 823.9
823.9 - 892.1
892.1 - 1019.6
> 1019.6
Female
Male
Sexes Combined
Age
Year
DALYs Rate (per 100,000)
Age — 10-24 years

Figure S7

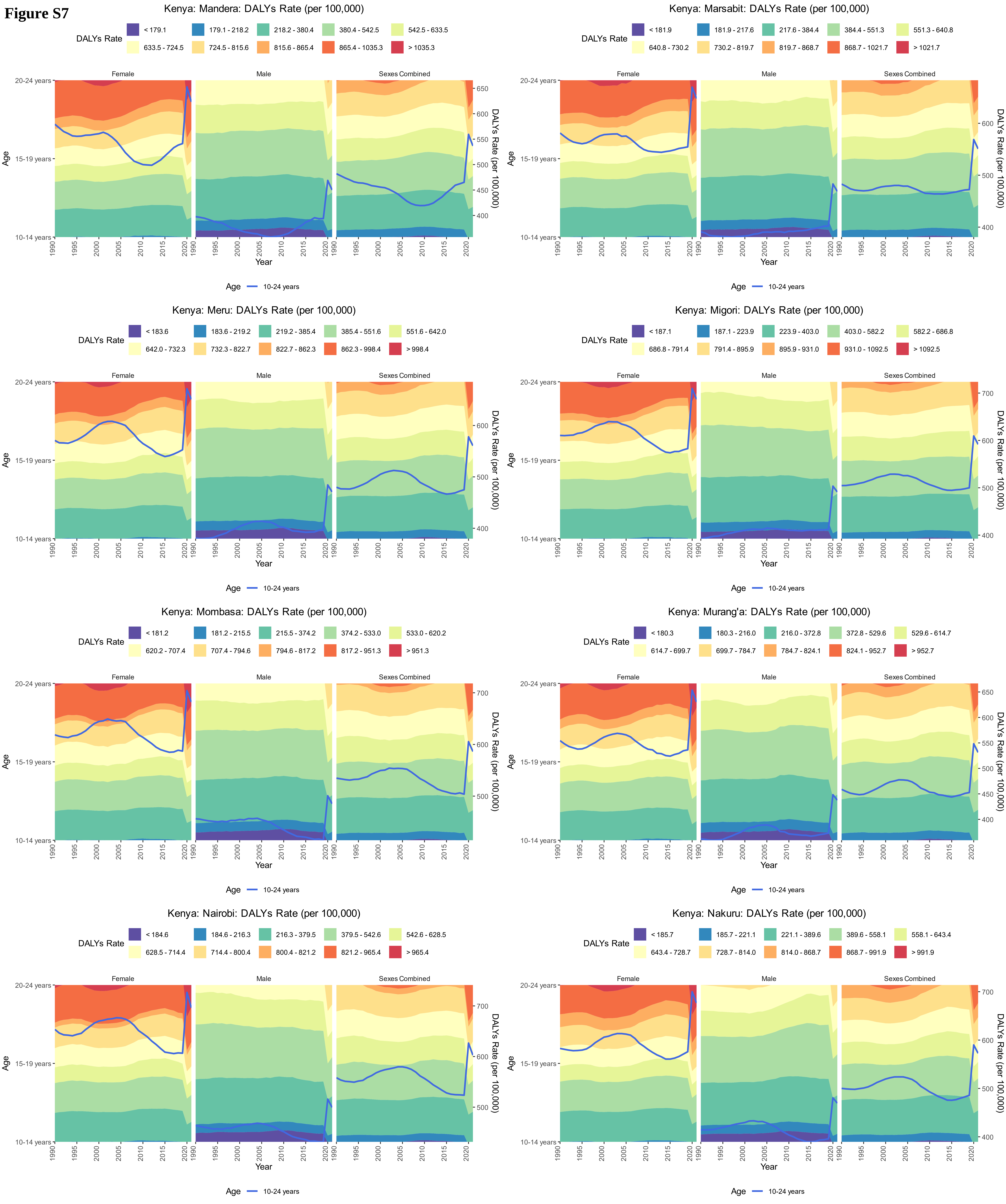
Kenya: Mandera: DALYs Rate (per 100,000)
DALYs Rate
< 179.1
179.1 - 218.2
218.2 - 380.4
380.4 - 542.5
542.5 - 633.5
633.5 - 724.5
724.5 - 815.6
815.6 - 865.4
865.4 - 1035.3
> 1035.3
Kenya: Marsabit: DALYs Rate (per 100,000)
< 181.9
181.9 - 217.6
217.6 - 384.4
384.4 - 551.3
551.3 - 640.8
640.8 - 730.2
730.2 - 819.7
819.7 - 868.7
868.7 - 1021.7
> 1021.7
Kenya: Meru: DALYs Rate (per 100,000)
< 183.6
183.6 - 219.2
219.2 - 385.4
385.4 - 551.6
551.6 - 642.0
642.0 - 732.3
732.3 - 822.7
822.7 - 862.3
862.3 - 998.4
> 998.4
Kenya: Migori: DALYs Rate (per 100,000)
< 187.1
187.1 - 223.9
223.9 - 403.0
403.0 - 582.2
582.2 - 686.8
686.8 - 791.4
791.4 - 895.9
895.9 - 931.0
931.0 - 1092.5
> 1092.5
Kenya: Mombasa: DALYs Rate (per 100,000)
< 181.2
181.2 - 215.5
215.5 - 374.2
374.2 - 533.0
533.0 - 620.2
620.2 - 707.4
707.4 - 794.6
794.6 - 817.2
817.2 - 951.3
> 951.3
Kenya: Murang'a: DALYs Rate (per 100,000)
< 180.3
180.3 - 216.0
216.0 - 372.8
372.8 - 529.6
529.6 - 614.7
614.7 - 699.7
699.7 - 784.7
784.7 - 824.1
824.1 - 952.7
> 952.7
Kenya: Nairobi: DALYs Rate (per 100,000)
< 184.6
184.6 - 216.3
216.3 - 379.5
379.5 - 542.6
542.6 - 628.5
628.5 - 714.4
714.4 - 800.4
800.4 - 821.2
821.2 - 965.4
> 965.4
Kenya: Nakuru: DALYs Rate (per 100,000)
< 185.7
185.7 - 221.1
221.1 - 389.6
389.6 - 558.1
558.1 - 643.4
643.4 - 728.7
728.7 - 814.0
814.0 - 868.7
868.7 - 991.9
> 991.9
Female
Male
Sexes Combined
Age
20-24 years
15-19 years
10-14 years
Year
DALYs Rate (per 100,000)
Age — 10-24 years

Figure S7

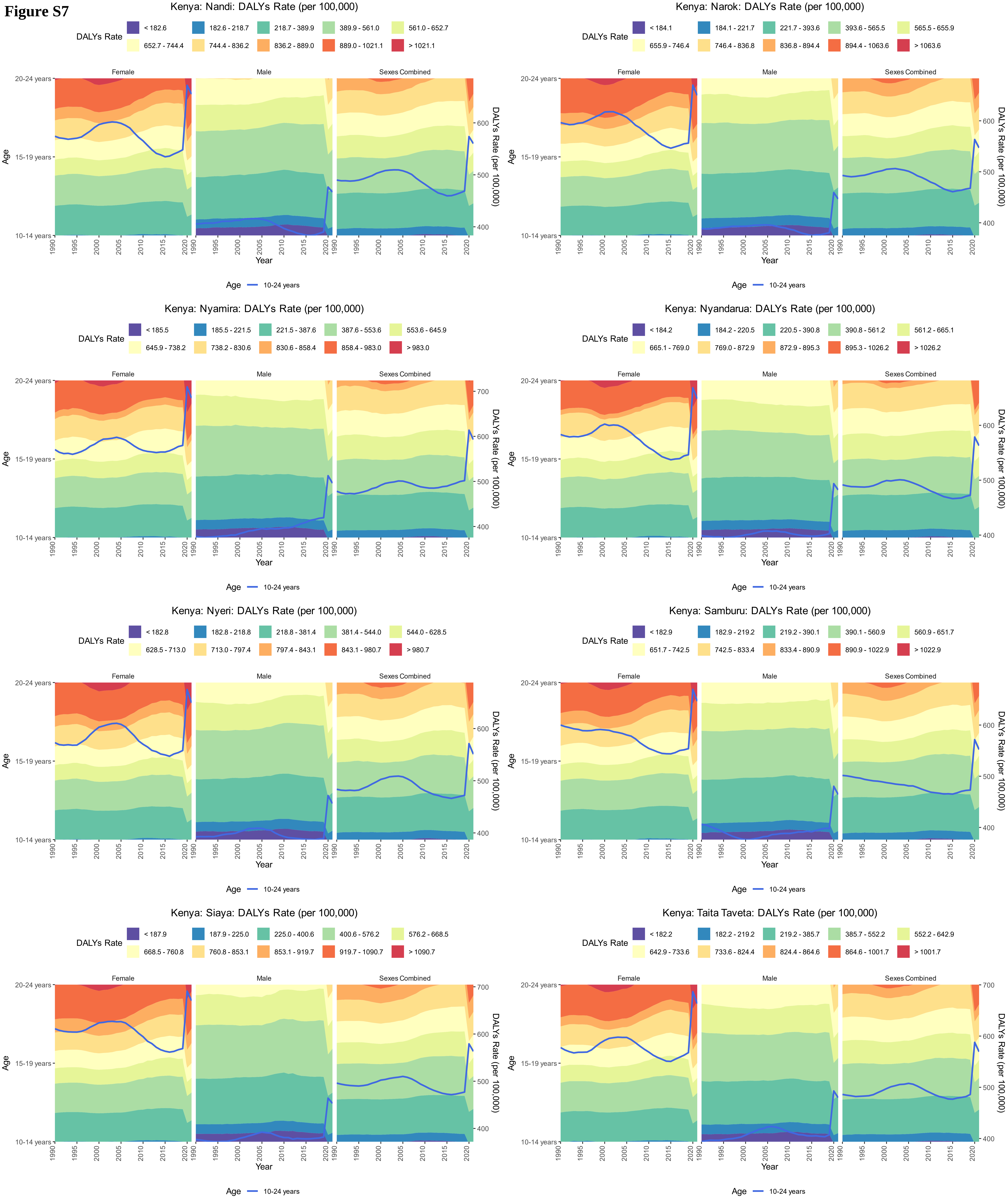

Kenya: Nandi: DALYs Rate (per 100,000)
DALYs Rate
< 182.6
182.6 - 218.7
218.7 - 389.9
389.9 - 561.0
561.0 - 652.7
652.7 - 744.4
744.4 - 836.2
836.2 - 889.0
889.0 - 1021.1
> 1021.1
Kenya: Narok: DALYs Rate (per 100,000)
< 184.1
184.1 - 221.7
221.7 - 393.6
393.6 - 565.5
565.5 - 655.9
655.9 - 746.4
746.4 - 836.8
836.8 - 894.4
894.4 - 1063.6
> 1063.6
Kenya: Nyamira: DALYs Rate (per 100,000)
< 185.5
185.5 - 221.5
221.5 - 387.6
387.6 - 553.6
553.6 - 645.9
645.9 - 738.2
738.2 - 830.6
830.6 - 858.4
858.4 - 983.0
> 983.0
Kenya: Nyandarua: DALYs Rate (per 100,000)
< 184.2
184.2 - 220.5
220.5 - 390.8
390.8 - 561.2
561.2 - 665.1
665.1 - 769.0
769.0 - 872.9
872.9 - 895.3
895.3 - 1026.2
> 1026.2
Kenya: Nyeri: DALYs Rate (per 100,000)
< 182.8
182.8 - 218.8
218.8 - 381.4
381.4 - 544.0
544.0 - 628.5
628.5 - 713.0
713.0 - 797.4
797.4 - 843.1
843.1 - 980.7
> 980.7
Kenya: Samburu: DALYs Rate (per 100,000)
< 182.9
182.9 - 219.2
219.2 - 390.1
390.1 - 560.9
560.9 - 651.7
651.7 - 742.5
742.5 - 833.4
833.4 - 890.9
890.9 - 1022.9
> 1022.9
Kenya: Siaya: DALYs Rate (per 100,000)
< 187.9
187.9 - 225.0
225.0 - 400.6
400.6 - 576.2
576.2 - 668.5
668.5 - 760.8
760.8 - 853.1
853.1 - 919.7
919.7 - 1090.7
> 1090.7
Kenya: Taita Taveta: DALYs Rate (per 100,000)
< 182.2
182.2 - 219.2
219.2 - 385.7
385.7 - 552.2
552.2 - 642.9
642.9 - 733.6
733.6 - 824.4
824.4 - 864.6
864.6 - 1001.7
> 1001.7
Female
Male
Sexes Combined
Age
20-24 years
15-19 years
10-14 years
Year
DALYs Rate (per 100,000)
Age — 10-24 years

Figure S7

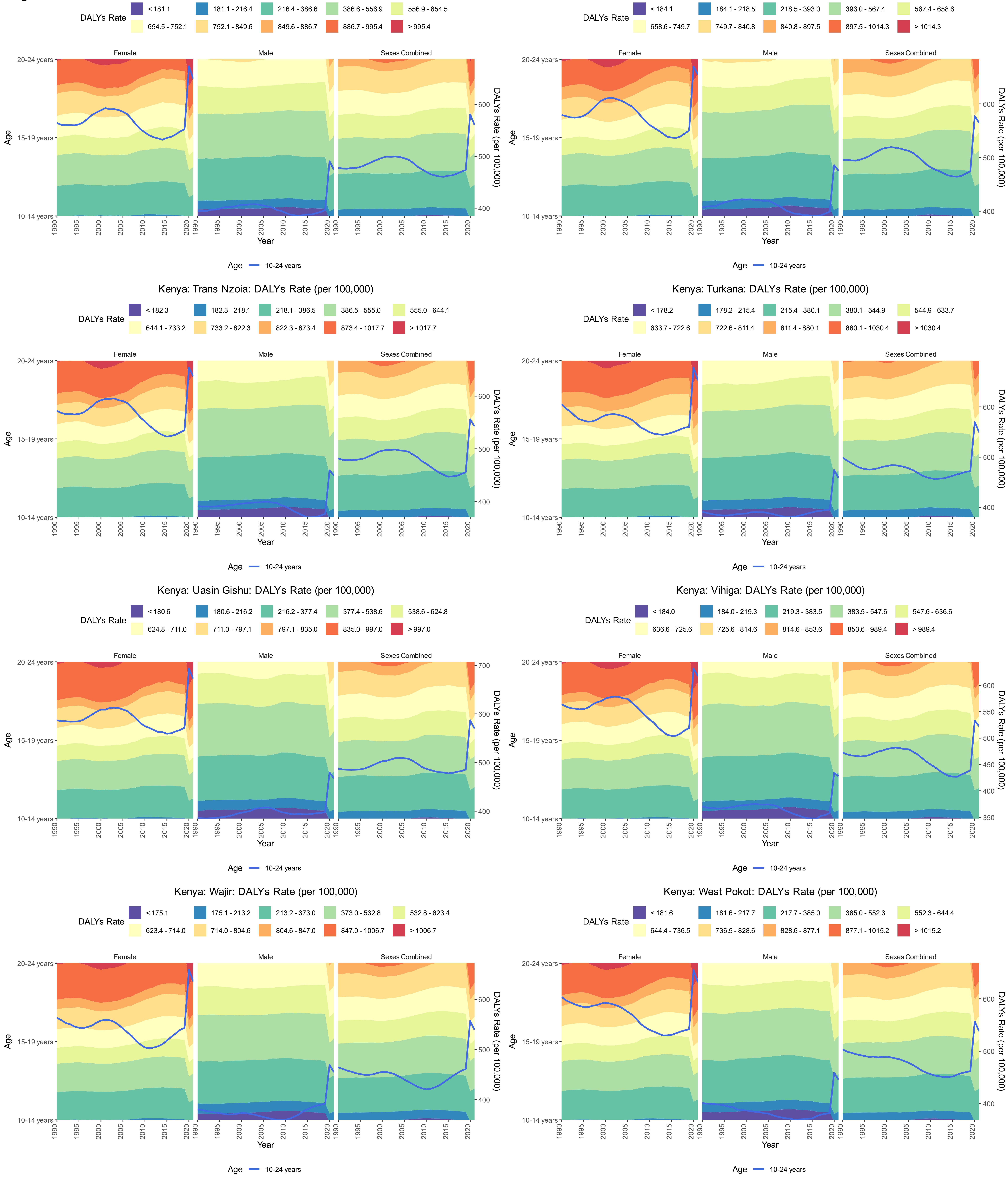
Kenya: Tana River: DALYs Rate (per 100,000)
DALYs Rate
< 181.1
181.1 - 216.4
216.4 - 386.6
386.6 - 556.9
556.9 - 654.5
654.5 - 752.1
752.1 - 849.6
849.6 - 886.7
886.7 - 995.4
> 995.4
Kenya: Tharaka Nithi: DALYs Rate (per 100,000)
< 184.1
184.1 - 218.5
218.5 - 393.0
393.0 - 567.4
567.4 - 658.6
658.6 - 749.7
749.7 - 840.8
840.8 - 897.5
897.5 - 1014.3
> 1014.3
Kenya: Trans Nzoia: DALYs Rate (per 100,000)
< 182.3
182.3 - 218.1
218.1 - 386.5
386.5 - 555.0
555.0 - 644.1
644.1 - 733.2
733.2 - 822.3
822.3 - 873.4
873.4 - 1017.7
> 1017.7
Kenya: Turkana: DALYs Rate (per 100,000)
< 178.2
178.2 - 215.4
215.4 - 380.1
380.1 - 544.9
544.9 - 633.7
633.7 - 722.6
722.6 - 811.4
811.4 - 880.1
880.1 - 1030.4
> 1030.4
Kenya: Uasin Gishu: DALYs Rate (per 100,000)
< 180.6
180.6 - 216.2
216.2 - 377.4
377.4 - 538.6
538.6 - 624.8
624.8 - 711.0
711.0 - 797.1
797.1 - 835.0
835.0 - 997.0
> 997.0
Kenya: Vihiga: DALYs Rate (per 100,000)
< 184.0
184.0 - 219.3
219.3 - 383.5
383.5 - 547.6
547.6 - 636.6
636.6 - 725.6
725.6 - 814.6
814.6 - 853.6
853.6 - 989.4
> 989.4
Kenya: Wajir: DALYs Rate (per 100,000)
< 175.1
175.1 - 213.2
213.2 - 373.0
373.0 - 532.8
532.8 - 623.4
623.4 - 714.0
714.0 - 804.6
804.6 - 847.0
847.0 - 1006.7
> 1006.7
Kenya: West Pokot: DALYs Rate (per 100,000)
< 181.6
181.6 - 217.7
217.7 - 385.0
385.0 - 552.3
552.3 - 644.4
644.4 - 736.5
736.5 - 828.6
828.6 - 877.1
877.1 - 1015.2
> 1015.2
Female
Male
Sexes Combined
20-24 years
15-19 years
10-14 years
Age
Year
DALYs Rate (per 100,000)
Age — 10-24 years

Figure S7

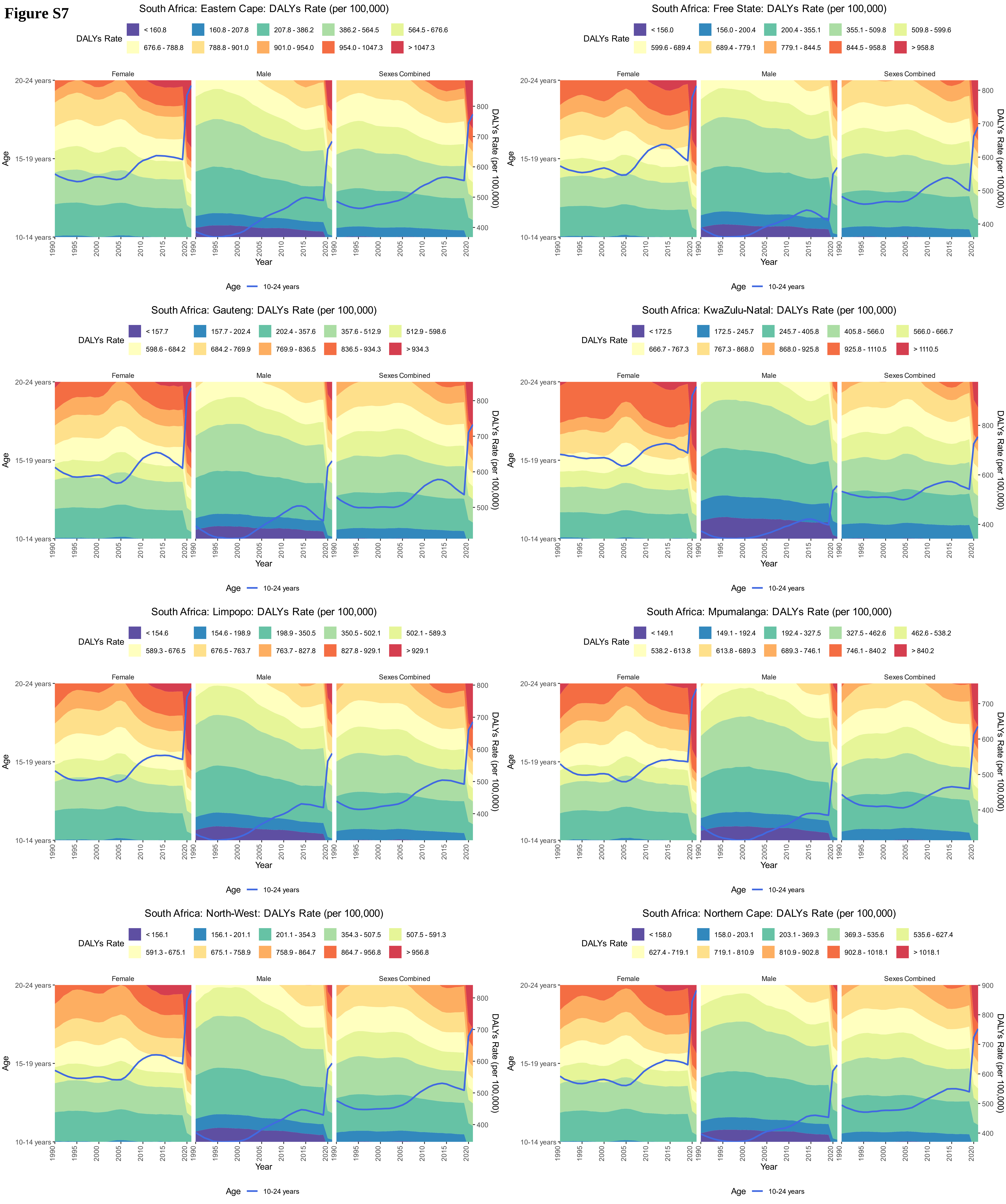
South Africa: Eastern Cape: DALYs Rate (per 100,000)
DALYs Rate
< 160.8
160.8 - 207.8
207.8 - 386.2
386.2 - 564.5
564.5 - 676.6
676.6 - 788.8
788.8 - 901.0
901.0 - 954.0
954.0 - 1047.3
> 1047.3
South Africa: Free State: DALYs Rate (per 100,000)
DALYs Rate
< 156.0
156.0 - 200.4
200.4 - 355.1
355.1 - 509.8
509.8 - 599.6
599.6 - 689.4
689.4 - 779.1
779.1 - 844.5
844.5 - 958.8
> 958.8
South Africa: Gauteng: DALYs Rate (per 100,000)
DALYs Rate
< 157.7
157.7 - 202.4
202.4 - 357.6
357.6 - 512.9
512.9 - 598.6
598.6 - 684.2
684.2 - 769.9
769.9 - 836.5
836.5 - 934.3
> 934.3
South Africa: KwaZulu-Natal: DALYs Rate (per 100,000)
DALYs Rate
< 172.5
172.5 - 245.7
245.7 - 405.8
405.8 - 566.0
566.0 - 666.7
666.7 - 767.3
767.3 - 868.0
868.0 - 925.8
925.8 - 1110.5
> 1110.5
South Africa: Limpopo: DALYs Rate (per 100,000)
DALYs Rate
< 154.6
154.6 - 198.9
198.9 - 350.5
350.5 - 502.1
502.1 - 589.3
589.3 - 676.5
676.5 - 763.7
763.7 - 827.8
827.8 - 929.1
> 929.1
South Africa: Mpumalanga: DALYs Rate (per 100,000)
DALYs Rate
< 149.1
149.1 - 192.4
192.4 - 327.5
327.5 - 462.6
462.6 - 538.2
538.2 - 613.8
613.8 - 689.3
689.3 - 746.1
746.1 - 840.2
> 840.2
South Africa: North-West: DALYs Rate (per 100,000)
DALYs Rate
< 156.1
156.1 - 201.1
201.1 - 354.3
354.3 - 507.5
507.5 - 591.3
591.3 - 675.1
675.1 - 758.9
758.9 - 864.7
864.7 - 956.8
> 956.8
South Africa: Northern Cape: DALYs Rate (per 100,000)
DALYs Rate
< 158.0
158.0 - 203.1
203.1 - 369.3
369.3 - 535.6
535.6 - 627.4
627.4 - 719.1
719.1 - 810.9
810.9 - 902.8
902.8 - 1018.1
> 1018.1
Female
Male
Sexes Combined
Age
20-24 years
15-19 years
10-14 years
Year
1990
1995
2000
2005
2010
2015
2020
DALYs Rate (per 100,000)
Age
10-24 years

Figure S7

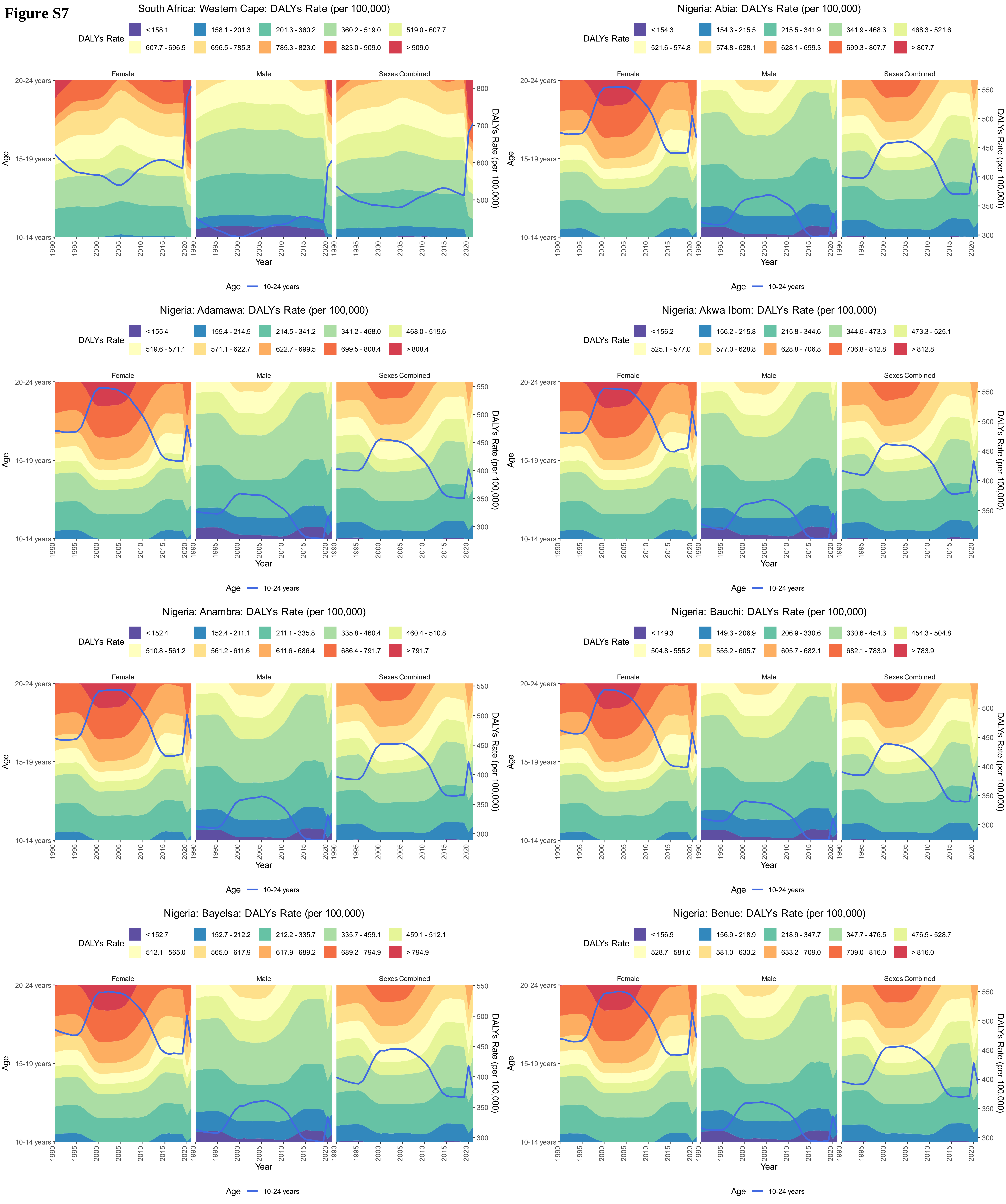
South Africa: Western Cape: DALYs Rate (per 100,000)
DALYs Rate
< 158.1
158.1 - 201.3
201.3 - 360.2
360.2 - 519.0
519.0 - 607.7
607.7 - 696.5
696.5 - 785.3
785.3 - 823.0
823.0 - 909.0
> 909.0
Nigeria: Abia: DALYs Rate (per 100,000)
< 154.3
154.3 - 215.5
215.5 - 341.9
341.9 - 468.3
468.3 - 521.6
521.6 - 574.8
574.8 - 628.1
628.1 - 699.3
699.3 - 807.7
> 807.7
Nigeria: Adamawa: DALYs Rate (per 100,000)
< 155.4
155.4 - 214.5
214.5 - 341.2
341.2 - 468.0
468.0 - 519.6
519.6 - 571.1
571.1 - 622.7
622.7 - 699.5
699.5 - 808.4
> 808.4
Nigeria: Akwa Ibom: DALYs Rate (per 100,000)
< 156.2
156.2 - 215.8
215.8 - 344.6
344.6 - 473.3
473.3 - 525.1
525.1 - 577.0
577.0 - 628.8
628.8 - 706.8
706.8 - 812.8
> 812.8
Nigeria: Anambra: DALYs Rate (per 100,000)
< 152.4
152.4 - 211.1
211.1 - 335.8
335.8 - 460.4
460.4 - 510.8
510.8 - 561.2
561.2 - 611.6
611.6 - 686.4
686.4 - 791.7
> 791.7
Nigeria: Bauchi: DALYs Rate (per 100,000)
< 149.3
149.3 - 206.9
206.9 - 330.6
330.6 - 454.3
454.3 - 504.8
504.8 - 555.2
555.2 - 605.7
605.7 - 682.1
682.1 - 783.9
> 783.9
Nigeria: Bayelsa: DALYs Rate (per 100,000)
< 152.7
152.7 - 212.2
212.2 - 335.7
335.7 - 459.1
459.1 - 512.1
512.1 - 565.0
565.0 - 617.9
617.9 - 689.2
689.2 - 794.9
> 794.9
Nigeria: Benue: DALYs Rate (per 100,000)
< 156.9
156.9 - 218.9
218.9 - 347.7
347.7 - 476.5
476.5 - 528.7
528.7 - 581.0
581.0 - 633.2
633.2 - 709.0
709.0 - 816.0
> 816.0
Female
Male
Sexes Combined
Age
20-24 years
15-19 years
10-14 years
Year
DALYs Rate (per 100,000)
Age — 10-24 years

Figure S7

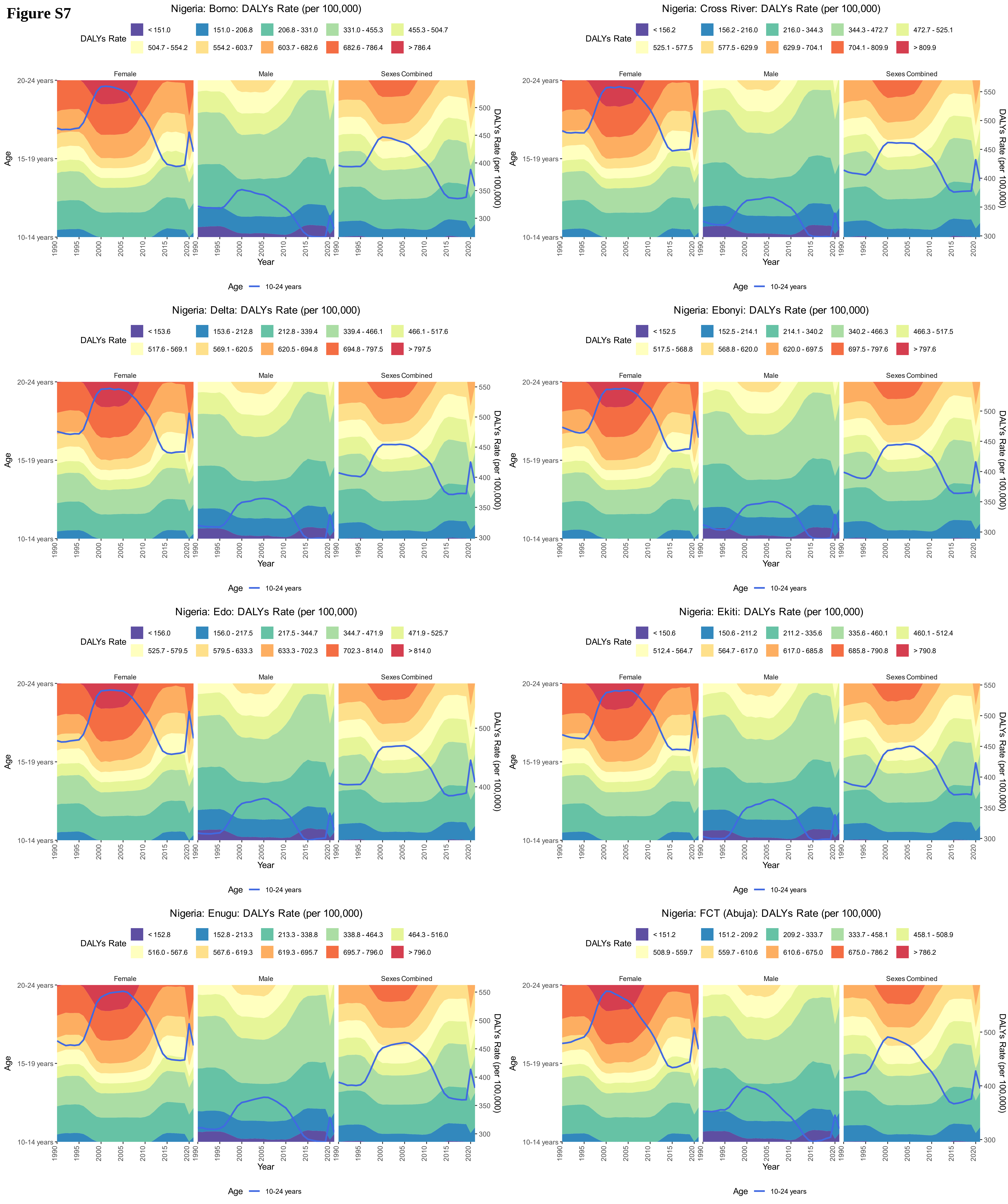
Nigeria: Borno: DALYs Rate (per 100,000)
DALYs Rate
< 151.0
151.0 - 206.8
206.8 - 331.0
331.0 - 455.3
455.3 - 504.7
504.7 - 554.2
554.2 - 603.7
603.7 - 682.6
682.6 - 786.4
> 786.4
Nigeria: Cross River: DALYs Rate (per 100,000)
< 156.2
156.2 - 216.0
216.0 - 344.3
344.3 - 472.7
472.7 - 525.1
525.1 - 577.5
577.5 - 629.9
629.9 - 704.1
704.1 - 809.9
> 809.9
Nigeria: Delta: DALYs Rate (per 100,000)
< 153.6
153.6 - 212.8
212.8 - 339.4
339.4 - 466.1
466.1 - 517.6
517.6 - 569.1
569.1 - 620.5
620.5 - 694.8
694.8 - 797.5
> 797.5
Nigeria: Ebonyi: DALYs Rate (per 100,000)
< 152.5
152.5 - 214.1
214.1 - 340.2
340.2 - 466.3
466.3 - 517.5
517.5 - 568.8
568.8 - 620.0
620.0 - 697.5
697.5 - 797.6
> 797.6
Nigeria: Edo: DALYs Rate (per 100,000)
< 156.0
156.0 - 217.5
217.5 - 344.7
344.7 - 471.9
471.9 - 525.7
525.7 - 579.5
579.5 - 633.3
633.3 - 702.3
702.3 - 814.0
> 814.0
Nigeria: Ekiti: DALYs Rate (per 100,000)
< 150.6
150.6 - 211.2
211.2 - 335.6
335.6 - 460.1
460.1 - 512.4
512.4 - 564.7
564.7 - 617.0
617.0 - 685.8
685.8 - 790.8
> 790.8
Nigeria: Enugu: DALYs Rate (per 100,000)
< 152.8
152.8 - 213.3
213.3 - 338.8
338.8 - 464.3
464.3 - 516.0
516.0 - 567.6
567.6 - 619.3
619.3 - 695.7
695.7 - 796.0
> 796.0
Nigeria: FCT (Abuja): DALYs Rate (per 100,000)
< 151.2
151.2 - 209.2
209.2 - 333.7
333.7 - 458.1
458.1 - 508.9
508.9 - 559.7
559.7 - 610.6
610.6 - 675.0
675.0 - 786.2
> 786.2
Female
Male
Sexes Combined
Age
20-24 years
15-19 years
10-14 years
Year
1990
1995
2000
2005
2010
2015
2020
DALYs Rate (per 100,000)
300
350
400
450
500
550
Age
10-24 years

Figure S7

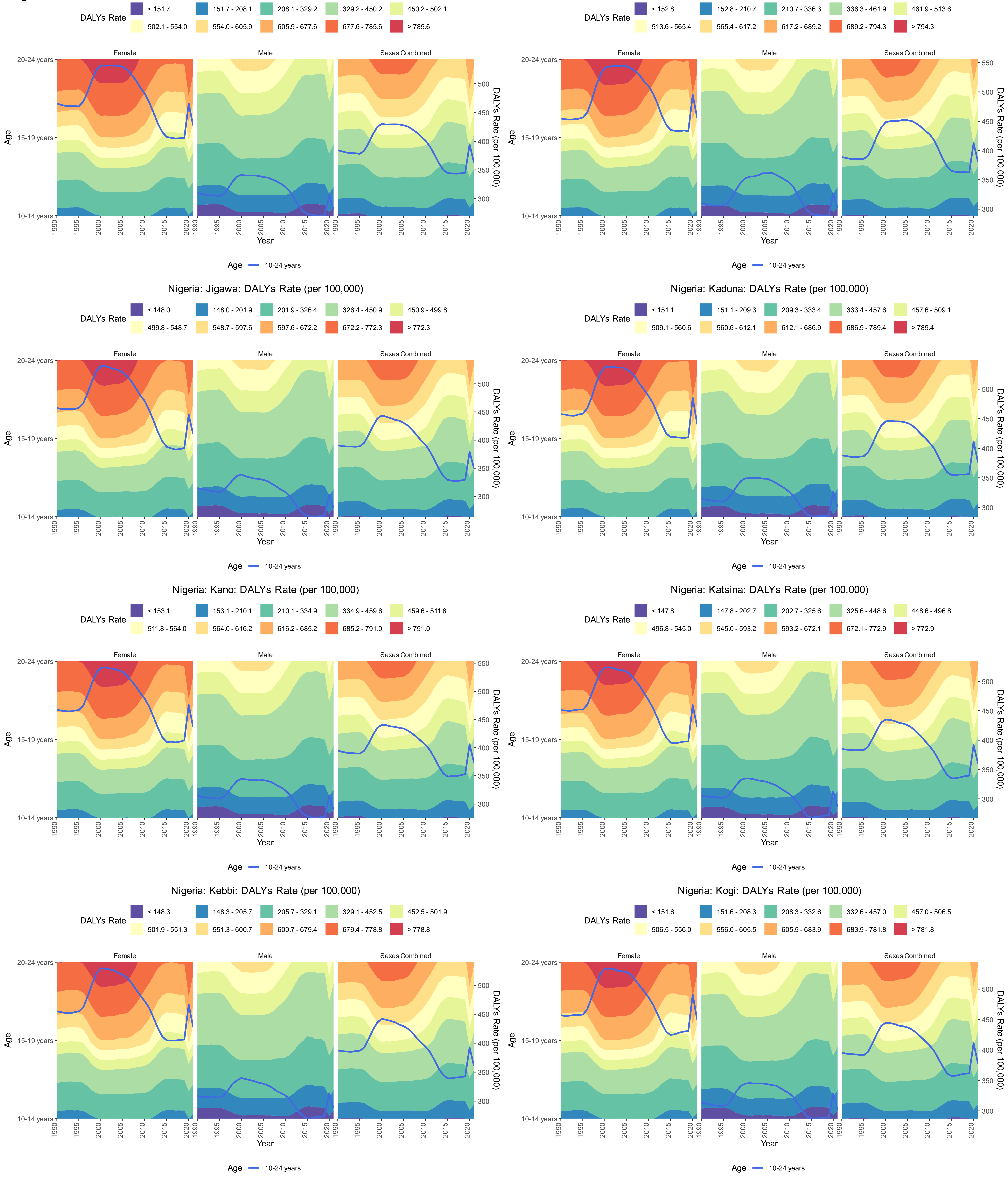

Nigeria: Gombe: DALYs Rate (per 100,000)
DALYs Rate
< 151.7
151.7 - 208.1
208.1 - 329.2
329.2 - 450.2
450.2 - 502.1
502.1 - 554.0
554.0 - 605.9
605.9 - 677.6
677.6 - 785.6
> 785.6
Nigeria: Imo: DALYs Rate (per 100,000)
< 152.8
152.8 - 210.7
210.7 - 336.3
336.3 - 461.9
461.9 - 513.6
513.6 - 565.4
565.4 - 617.2
617.2 - 689.2
689.2 - 794.3
> 794.3
Nigeria: Jigawa: DALYs Rate (per 100,000)
< 148.0
148.0 - 201.9
201.9 - 326.4
326.4 - 450.9
450.9 - 499.8
499.8 - 548.7
548.7 - 597.6
597.6 - 672.2
672.2 - 772.3
> 772.3
Nigeria: Kaduna: DALYs Rate (per 100,000)
< 151.1
151.1 - 209.3
209.3 - 333.4
333.4 - 457.6
457.6 - 509.1
509.1 - 560.6
560.6 - 612.1
612.1 - 686.9
686.9 - 789.4
> 789.4
Nigeria: Kano: DALYs Rate (per 100,000)
< 153.1
153.1 - 210.1
210.1 - 334.9
334.9 - 459.6
459.6 - 511.8
511.8 - 564.0
564.0 - 616.2
616.2 - 685.2
685.2 - 791.0
> 791.0
Nigeria: Katsina: DALYs Rate (per 100,000)
< 147.8
147.8 - 202.7
202.7 - 325.6
325.6 - 448.6
448.6 - 496.8
496.8 - 545.0
545.0 - 593.2
593.2 - 672.1
672.1 - 772.9
> 772.9
Nigeria: Kebbi: DALYs Rate (per 100,000)
< 148.3
148.3 - 205.7
205.7 - 329.1
329.1 - 452.5
452.5 - 501.9
501.9 - 551.3
551.3 - 600.7
600.7 - 679.4
679.4 - 778.8
> 778.8
Nigeria: Kogi: DALYs Rate (per 100,000)
< 151.6
151.6 - 208.3
208.3 - 332.6
332.6 - 457.0
457.0 - 506.5
506.5 - 556.0
556.0 - 605.5
605.5 - 683.9
683.9 - 781.8
> 781.8
Female
Male
Sexes Combined
20-24 years
15-19 years
10-14 years
Age
Year
DALYs Rate (per 100,000)
Age — 10-24 years

Figure S7

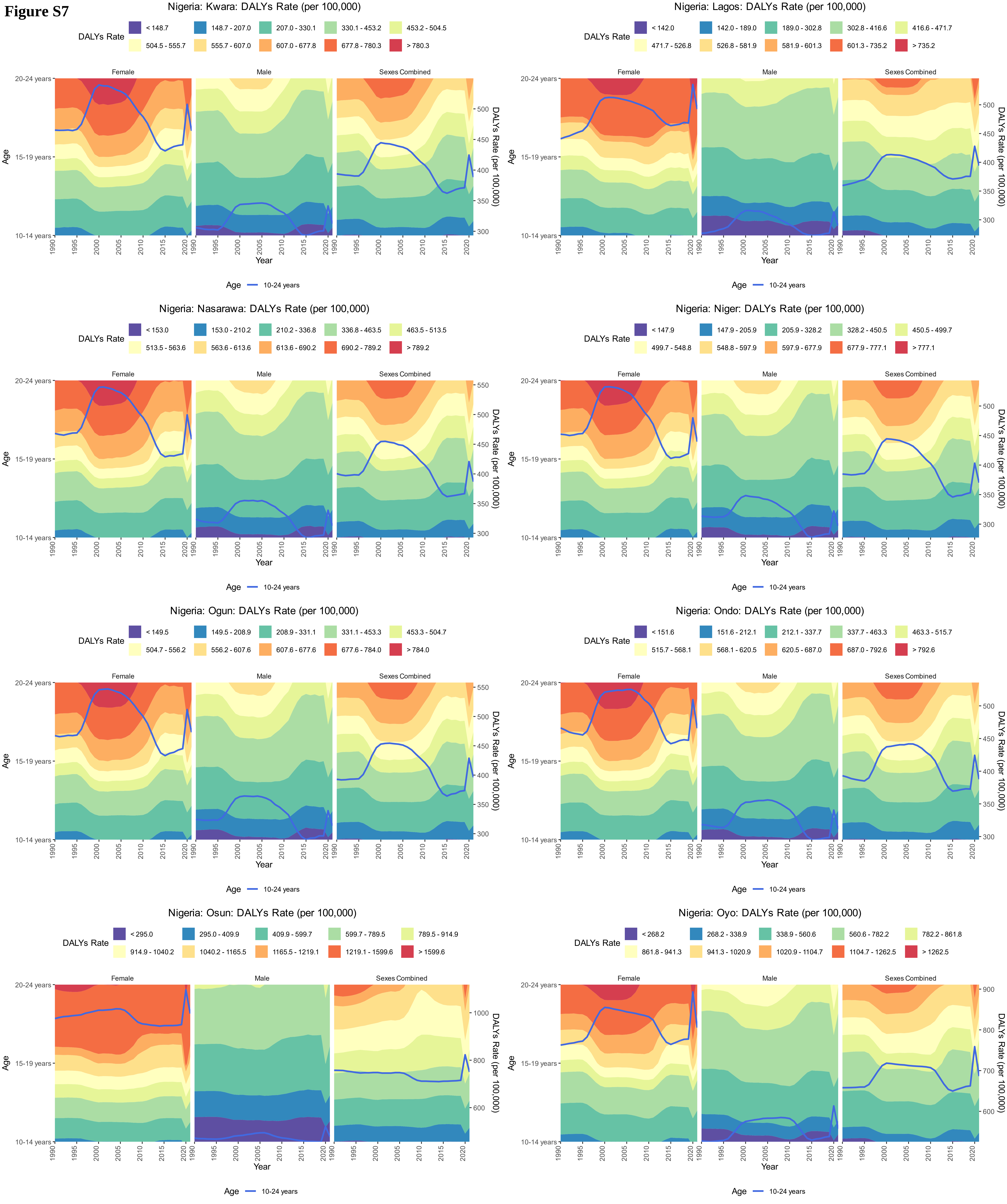
Nigeria: Kwara: DALYs Rate (per 100,000)
DALYs Rate
< 148.7
148.7 - 207.0
207.0 - 330.1
330.1 - 453.2
453.2 - 504.5
504.5 - 555.7
555.7 - 607.0
607.0 - 677.8
677.8 - 780.3
> 780.3
Nigeria: Lagos: DALYs Rate (per 100,000)
DALYs Rate
< 142.0
142.0 - 189.0
189.0 - 302.8
302.8 - 416.6
416.6 - 471.7
471.7 - 526.8
526.8 - 581.9
581.9 - 601.3
601.3 - 735.2
> 735.2
Nigeria: Nasarawa: DALYs Rate (per 100,000)
DALYs Rate
< 153.0
153.0 - 210.2
210.2 - 336.8
336.8 - 463.5
463.5 - 513.5
513.5 - 563.6
563.6 - 613.6
613.6 - 690.2
690.2 - 789.2
> 789.2
Nigeria: Niger: DALYs Rate (per 100,000)
DALYs Rate
< 147.9
147.9 - 205.9
205.9 - 328.2
328.2 - 450.5
450.5 - 499.7
499.7 - 548.8
548.8 - 597.9
597.9 - 677.9
677.9 - 777.1
> 777.1
Nigeria: Ogun: DALYs Rate (per 100,000)
DALYs Rate
< 149.5
149.5 - 208.9
208.9 - 331.1
331.1 - 453.3
453.3 - 504.7
504.7 - 556.2
556.2 - 607.6
607.6 - 677.6
677.6 - 784.0
> 784.0
Nigeria: Ondo: DALYs Rate (per 100,000)
DALYs Rate
< 151.6
151.6 - 212.1
212.1 - 337.7
337.7 - 463.3
463.3 - 515.7
515.7 - 568.1
568.1 - 620.5
620.5 - 687.0
687.0 - 792.6
> 792.6
Nigeria: Osun: DALYs Rate (per 100,000)
DALYs Rate
< 295.0
295.0 - 409.9
409.9 - 599.7
599.7 - 789.5
789.5 - 914.9
914.9 - 1040.2
1040.2 - 1165.5
1165.5 - 1219.1
1219.1 - 1599.6
> 1599.6
Nigeria: Oyo: DALYs Rate (per 100,000)
DALYs Rate
< 268.2
268.2 - 338.9
338.9 - 560.6
560.6 - 782.2
782.2 - 861.8
861.8 - 941.3
941.3 - 1020.9
1020.9 - 1104.7
1104.7 - 1262.5
> 1262.5
Female
Male
Sexes Combined
20-24 years
15-19 years
10-14 years
Age
Year
DALYs Rate (per 100,000)
Age
10-24 years

Figure S7

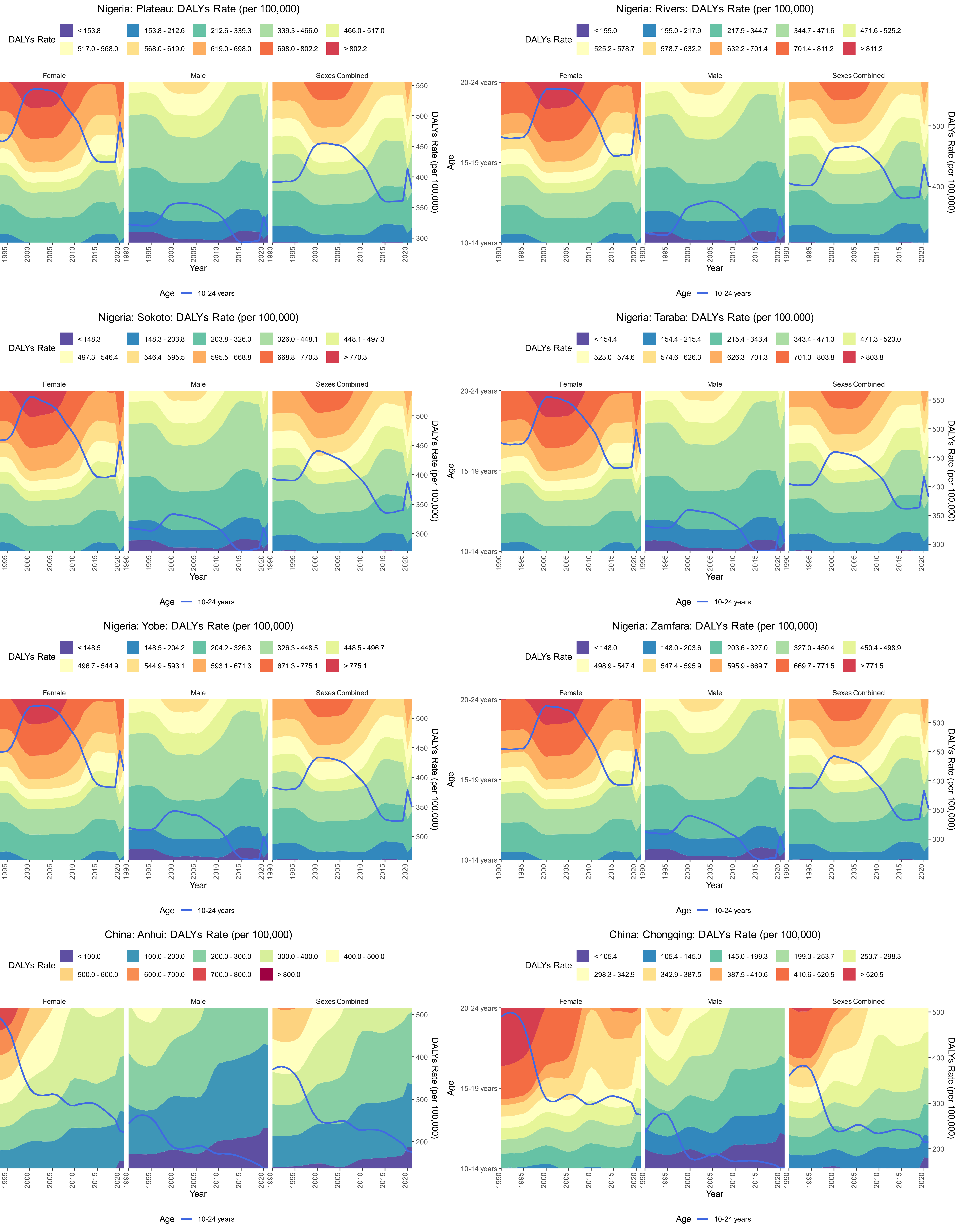
Nigeria: Plateau: DALYs Rate (per 100,000)
DALYs Rate
< 153.8
153.8 - 212.6
212.6 - 339.3
339.3 - 466.0
466.0 - 517.0
517.0 - 568.0
568.0 - 619.0
619.0 - 698.0
698.0 - 802.2
> 802.2
Female
Male
Sexes Combined
Age
Year
DALYs Rate (per 100,000)
Age
10-24 years
Nigeria: Rivers: DALYs Rate (per 100,000)
DALYs Rate
< 155.0
155.0 - 217.9
217.9 - 344.7
344.7 - 471.6
471.6 - 525.2
525.2 - 578.7
578.7 - 632.2
632.2 - 701.4
701.4 - 811.2
> 811.2
Nigeria: Sokoto: DALYs Rate (per 100,000)
DALYs Rate
< 148.3
148.3 - 203.8
203.8 - 326.0
326.0 - 448.1
448.1 - 497.3
497.3 - 546.4
546.4 - 595.5
595.5 - 668.8
668.8 - 770.3
> 770.3
Nigeria: Taraba: DALYs Rate (per 100,000)
DALYs Rate
< 154.4
154.4 - 215.4
215.4 - 343.4
343.4 - 471.3
471.3 - 523.0
523.0 - 574.6
574.6 - 626.3
626.3 - 701.3
701.3 - 803.8
> 803.8
Nigeria: Yobe: DALYs Rate (per 100,000)
DALYs Rate
< 148.5
148.5 - 204.2
204.2 - 326.3
326.3 - 448.5
448.5 - 496.7
496.7 - 544.9
544.9 - 593.1
593.1 - 671.3
671.3 - 775.1
> 775.1
Nigeria: Zamfara: DALYs Rate (per 100,000)
DALYs Rate
< 148.0
148.0 - 203.6
203.6 - 327.0
327.0 - 450.4
450.4 - 498.9
498.9 - 547.4
547.4 - 595.9
595.9 - 669.7
669.7 - 771.5
> 771.5
China: Anhui: DALYs Rate (per 100,000)
DALYs Rate
< 100.0
100.0 - 200.0
200.0 - 300.0
300.0 - 400.0
400.0 - 500.0
500.0 - 600.0
600.0 - 700.0
700.0 - 800.0
> 800.0
China: Chongqing: DALYs Rate (per 100,000)
DALYs Rate
< 105.4
105.4 - 145.0
145.0 - 199.3
199.3 - 253.7
253.7 - 298.3
298.3 - 342.9
342.9 - 387.5
387.5 - 410.6
410.6 - 520.5
> 520.5
20-24 years
15-19 years
10-14 years
1990
1995
2000
2005
2010
2015
2020

Figure S7

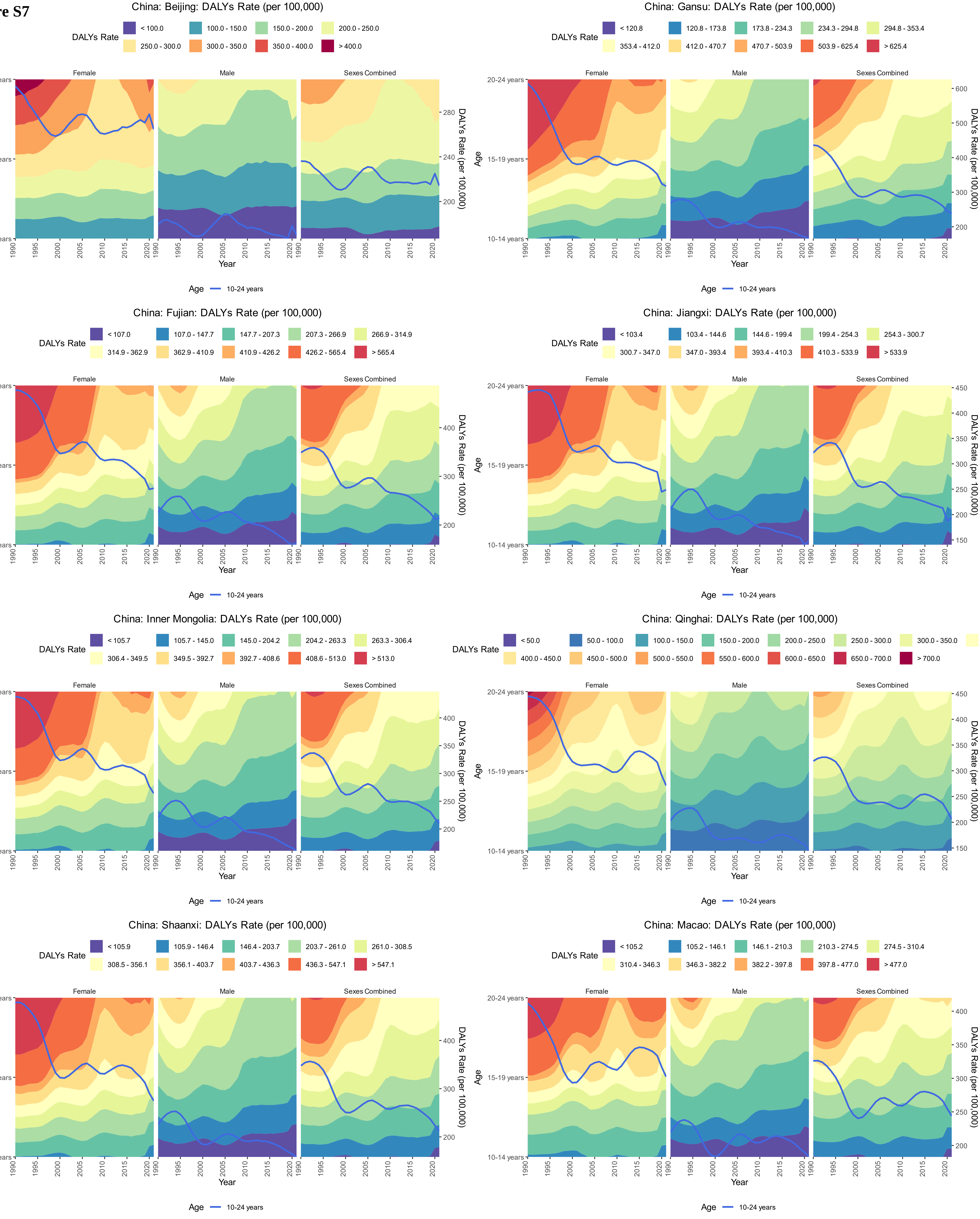
China: Beijing: DALYs Rate (per 100,000)
DALYs Rate
< 100.0
100.0 - 150.0
150.0 - 200.0
200.0 - 250.0
250.0 - 300.0
300.0 - 350.0
350.0 - 400.0
> 400.0
Female
Male
Sexes Combined
20-24 years
15-19 years
10-14 years
Age
Year
DALYs Rate (per 100,000)
Age
10-24 years
China: Gansu: DALYs Rate (per 100,000)
< 120.8
120.8 - 173.8
173.8 - 234.3
234.3 - 294.8
294.8 - 353.4
353.4 - 412.0
412.0 - 470.7
470.7 - 503.9
503.9 - 625.4
> 625.4
China: Fujian: DALYs Rate (per 100,000)
< 107.0
107.0 - 147.7
147.7 - 207.3
207.3 - 266.9
266.9 - 314.9
314.9 - 362.9
362.9 - 410.9
410.9 - 426.2
426.2 - 565.4
> 565.4
China: Jiangxi: DALYs Rate (per 100,000)
< 103.4
103.4 - 144.6
144.6 - 199.4
199.4 - 254.3
254.3 - 300.7
300.7 - 347.0
347.0 - 393.4
393.4 - 410.3
410.3 - 533.9
> 533.9
China: Inner Mongolia: DALYs Rate (per 100,000)
< 105.7
105.7 - 145.0
145.0 - 204.2
204.2 - 263.3
263.3 - 306.4
306.4 - 349.5
349.5 - 392.7
392.7 - 408.6
408.6 - 513.0
> 513.0
China: Qinghai: DALYs Rate (per 100,000)
< 50.0
50.0 - 100.0
100.0 - 150.0
150.0 - 200.0
200.0 - 250.0
250.0 - 300.0
300.0 - 350.0
400.0 - 450.0
450.0 - 500.0
500.0 - 550.0
550.0 - 600.0
600.0 - 650.0
650.0 - 700.0
> 700.0
China: Shaanxi: DALYs Rate (per 100,000)
< 105.9
105.9 - 146.4
146.4 - 203.7
203.7 - 261.0
261.0 - 308.5
308.5 - 356.1
356.1 - 403.7
403.7 - 436.3
436.3 - 547.1
> 547.1
China: Macao: DALYs Rate (per 100,000)
< 105.2
105.2 - 146.1
146.1 - 210.3
210.3 - 274.5
274.5 - 310.4
310.4 - 346.3
346.3 - 382.2
382.2 - 397.8
397.8 - 477.0
> 477.0

Figure S7

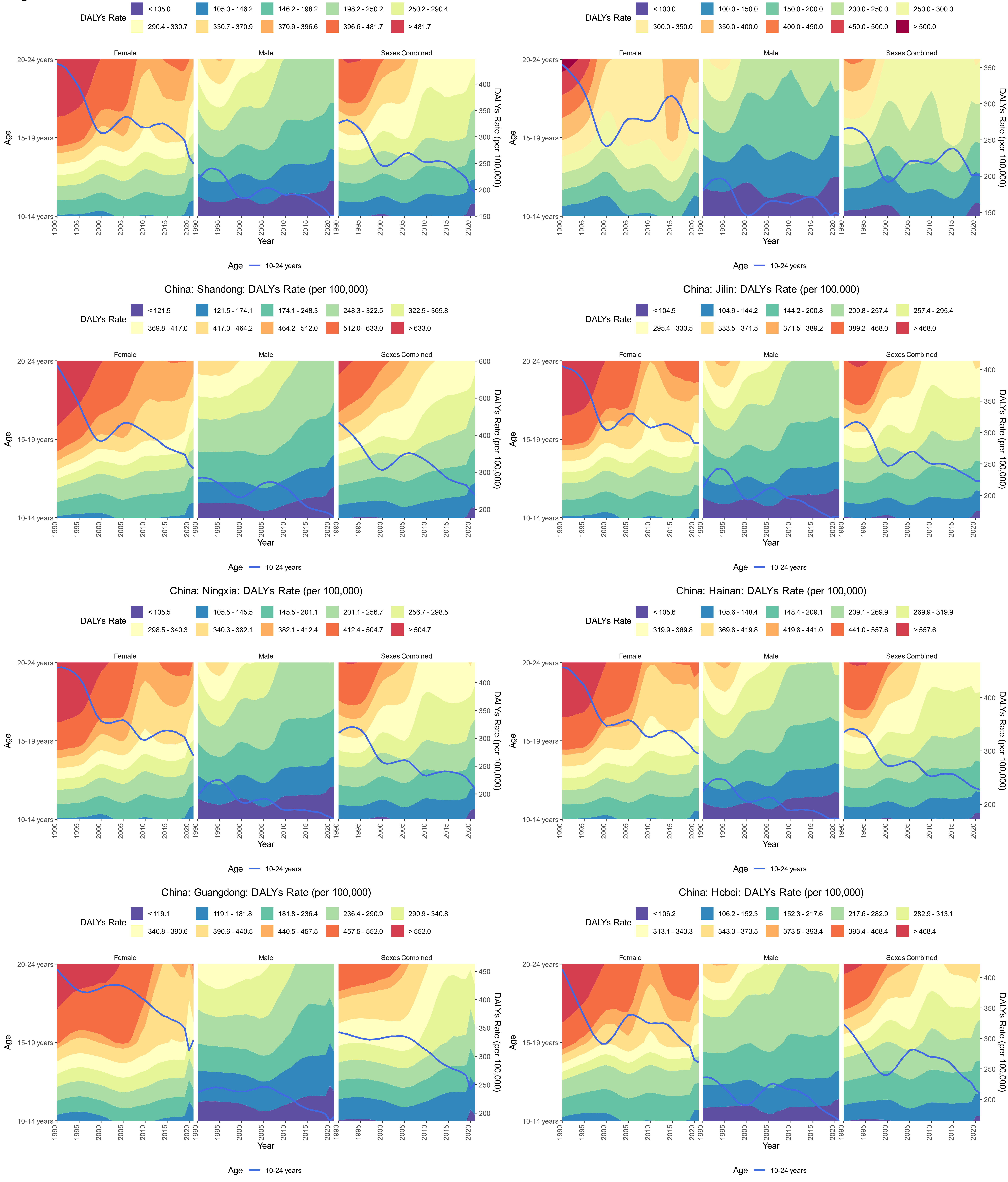

China: Jiangsu: DALYs Rate (per 100,000)
DALYs Rate
< 105.0
105.0 - 146.2
146.2 - 198.2
198.2 - 250.2
250.2 - 290.4
290.4 - 330.7
330.7 - 370.9
370.9 - 396.6
396.6 - 481.7
> 481.7
China: Liaoning: DALYs Rate (per 100,000)
DALYs Rate
< 100.0
100.0 - 150.0
150.0 - 200.0
200.0 - 250.0
250.0 - 300.0
300.0 - 350.0
350.0 - 400.0
400.0 - 450.0
450.0 - 500.0
> 500.0
China: Shandong: DALYs Rate (per 100,000)
DALYs Rate
< 121.5
121.5 - 174.1
174.1 - 248.3
248.3 - 322.5
322.5 - 369.8
369.8 - 417.0
417.0 - 464.2
464.2 - 512.0
512.0 - 633.0
> 633.0
China: Jilin: DALYs Rate (per 100,000)
DALYs Rate
< 104.9
104.9 - 144.2
144.2 - 200.8
200.8 - 257.4
257.4 - 295.4
295.4 - 333.5
333.5 - 371.5
371.5 - 389.2
389.2 - 468.0
> 468.0
China: Ningxia: DALYs Rate (per 100,000)
DALYs Rate
< 105.5
105.5 - 145.5
145.5 - 201.1
201.1 - 256.7
256.7 - 298.5
298.5 - 340.3
340.3 - 382.1
382.1 - 412.4
412.4 - 504.7
> 504.7
China: Hainan: DALYs Rate (per 100,000)
DALYs Rate
< 105.6
105.6 - 148.4
148.4 - 209.1
209.1 - 269.9
269.9 - 319.9
319.9 - 369.8
369.8 - 419.8
419.8 - 441.0
441.0 - 557.6
> 557.6
China: Guangdong: DALYs Rate (per 100,000)
DALYs Rate
< 119.1
119.1 - 181.8
181.8 - 236.4
236.4 - 290.9
290.9 - 340.8
340.8 - 390.6
390.6 - 440.5
440.5 - 457.5
457.5 - 552.0
> 552.0
China: Hebei: DALYs Rate (per 100,000)
DALYs Rate
< 106.2
106.2 - 152.3
152.3 - 217.6
217.6 - 282.9
282.9 - 313.1
313.1 - 343.3
343.3 - 373.5
373.5 - 393.4
393.4 - 468.4
> 468.4
Female
Male
Sexes Combined
Age
20-24 years
15-19 years
10-14 years
Year
1990
1995
2000
2005
2010
2015
2020
DALYs Rate (per 100,000)
Age
10-24 years

Figure S7

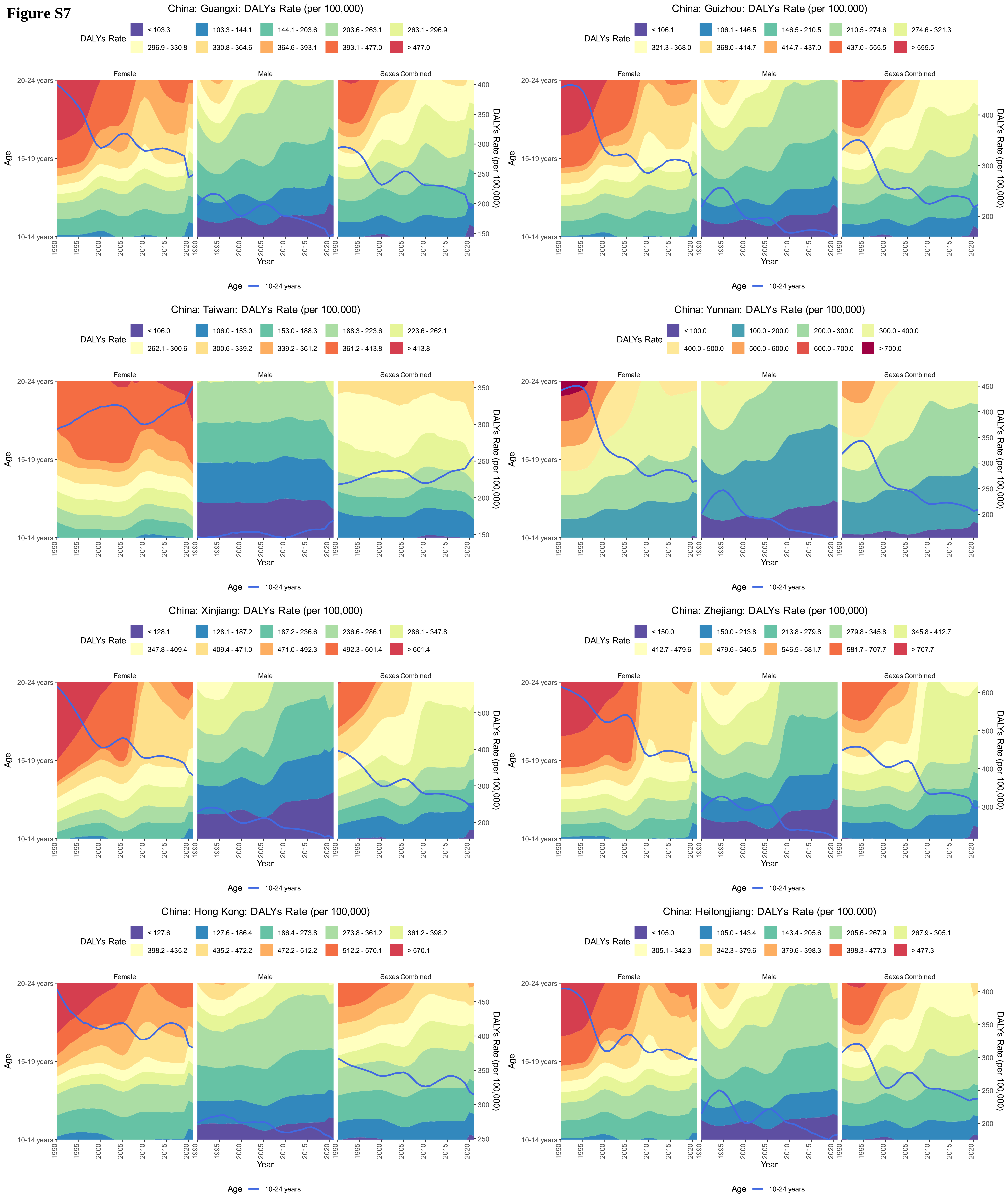
China: Guangxi: DALYs Rate (per 100,000)
DALYs Rate
< 103.3
103.3 - 144.1
144.1 - 203.6
203.6 - 263.1
263.1 - 296.9
296.9 - 330.8
330.8 - 364.6
364.6 - 393.1
393.1 - 477.0
> 477.0
China: Guizhou: DALYs Rate (per 100,000)
DALYs Rate
< 106.1
106.1 - 146.5
146.5 - 210.5
210.5 - 274.6
274.6 - 321.3
321.3 - 368.0
368.0 - 414.7
414.7 - 437.0
437.0 - 555.5
> 555.5
China: Taiwan: DALYs Rate (per 100,000)
DALYs Rate
< 106.0
106.0 - 153.0
153.0 - 188.3
188.3 - 223.6
223.6 - 262.1
262.1 - 300.6
300.6 - 339.2
339.2 - 361.2
361.2 - 413.8
> 413.8
China: Yunnan: DALYs Rate (per 100,000)
DALYs Rate
< 100.0
100.0 - 200.0
200.0 - 300.0
300.0 - 400.0
400.0 - 500.0
500.0 - 600.0
600.0 - 700.0
> 700.0
China: Xinjiang: DALYs Rate (per 100,000)
DALYs Rate
< 128.1
128.1 - 187.2
187.2 - 236.6
236.6 - 286.1
286.1 - 347.8
347.8 - 409.4
409.4 - 471.0
471.0 - 492.3
492.3 - 601.4
> 601.4
China: Zhejiang: DALYs Rate (per 100,000)
DALYs Rate
< 150.0
150.0 - 213.8
213.8 - 279.8
279.8 - 345.8
345.8 - 412.7
412.7 - 479.6
479.6 - 546.5
546.5 - 581.7
581.7 - 707.7
> 707.7
China: Hong Kong: DALYs Rate (per 100,000)
DALYs Rate
< 127.6
127.6 - 186.4
186.4 - 273.8
273.8 - 361.2
361.2 - 398.2
398.2 - 435.2
435.2 - 472.2
472.2 - 512.2
512.2 - 570.1
> 570.1
China: Heilongjiang: DALYs Rate (per 100,000)
DALYs Rate
< 105.0
105.0 - 143.4
143.4 - 205.6
205.6 - 267.9
267.9 - 305.1
305.1 - 342.3
342.3 - 379.6
379.6 - 398.3
398.3 - 477.3
> 477.3
Female
Male
Sexes Combined
Age
20-24 years
15-19 years
10-14 years
Year
DALYs Rate (per 100,000)
Age
10-24 years

Figure S7

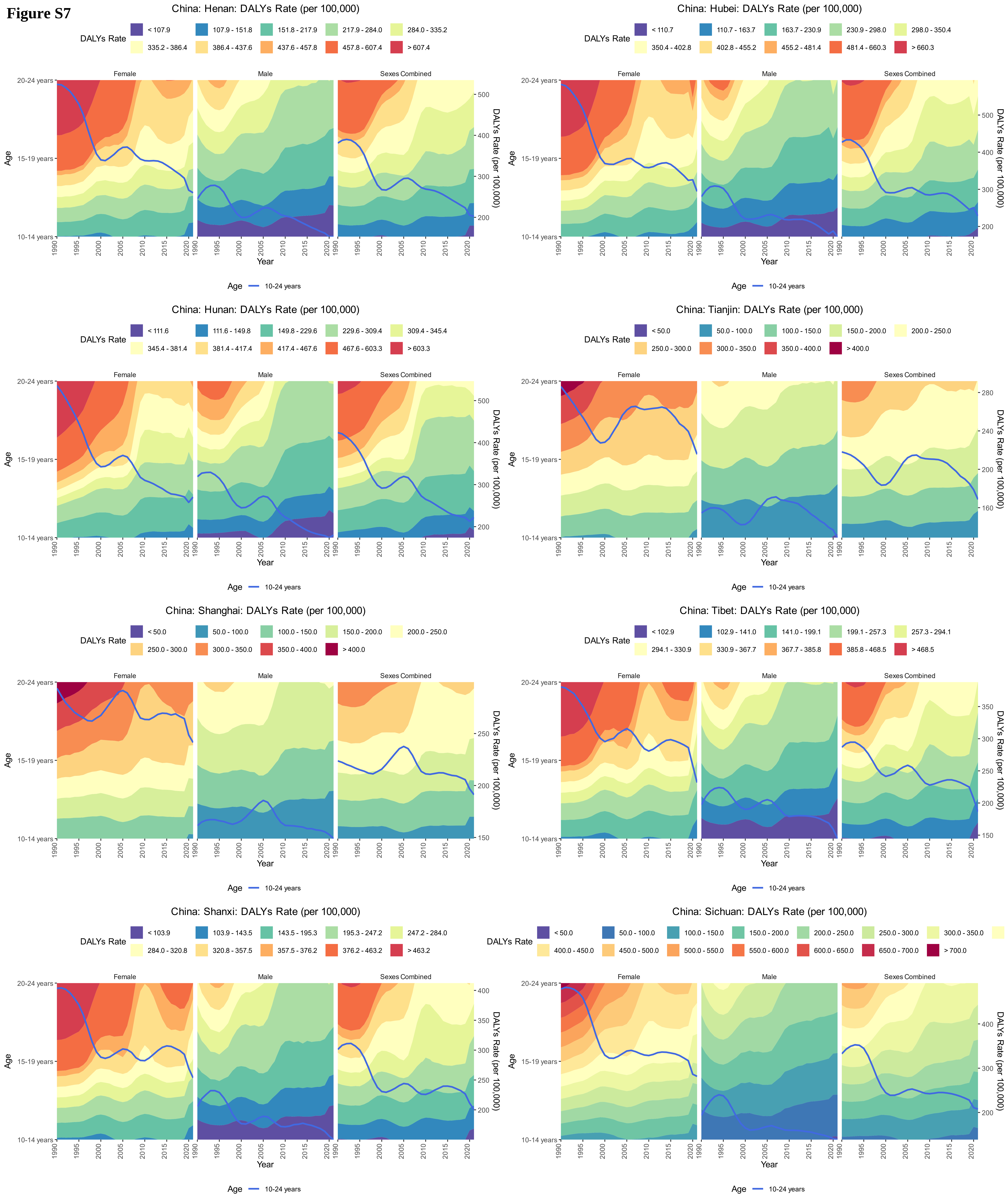
China: Henan: DALYs Rate (per 100,000)
DALYs Rate
< 107.9
107.9 - 151.8
151.8 - 217.9
217.9 - 284.0
284.0 - 335.2
335.2 - 386.4
386.4 - 437.6
437.6 - 457.8
457.8 - 607.4
> 607.4
Female
Male
Sexes Combined
20-24 years
15-19 years
10-14 years
Age
Year
DALYs Rate (per 100,000)
Age
10-24 years
China: Hubei: DALYs Rate (per 100,000)
DALYs Rate
< 110.7
110.7 - 163.7
163.7 - 230.9
230.9 - 298.0
298.0 - 350.4
350.4 - 402.8
402.8 - 455.2
455.2 - 481.4
481.4 - 660.3
> 660.3
China: Hunan: DALYs Rate (per 100,000)
DALYs Rate
< 111.6
111.6 - 149.8
149.8 - 229.6
229.6 - 309.4
309.4 - 345.4
345.4 - 381.4
381.4 - 417.4
417.4 - 467.6
467.6 - 603.3
> 603.3
China: Tianjin: DALYs Rate (per 100,000)
DALYs Rate
< 50.0
50.0 - 100.0
100.0 - 150.0
150.0 - 200.0
200.0 - 250.0
250.0 - 300.0
300.0 - 350.0
350.0 - 400.0
> 400.0
China: Shanghai: DALYs Rate (per 100,000)
DALYs Rate
< 50.0
50.0 - 100.0
100.0 - 150.0
150.0 - 200.0
200.0 - 250.0
250.0 - 300.0
300.0 - 350.0
350.0 - 400.0
> 400.0
China: Tibet: DALYs Rate (per 100,000)
DALYs Rate
< 102.9
102.9 - 141.0
141.0 - 199.1
199.1 - 257.3
257.3 - 294.1
294.1 - 330.9
330.9 - 367.7
367.7 - 385.8
385.8 - 468.5
> 468.5
China: Shanxi: DALYs Rate (per 100,000)
DALYs Rate
< 103.9
103.9 - 143.5
143.5 - 195.3
195.3 - 247.2
247.2 - 284.0
284.0 - 320.8
320.8 - 357.5
357.5 - 376.2
376.2 - 463.2
> 463.2
China: Sichuan: DALYs Rate (per 100,000)
DALYs Rate
< 50.0
50.0 - 100.0
100.0 - 150.0
150.0 - 200.0
200.0 - 250.0
250.0 - 300.0
300.0 - 350.0
400.0 - 450.0
450.0 - 500.0
500.0 - 550.0
550.0 - 600.0
600.0 - 650.0
650.0 - 700.0
> 700.0

Figure S8

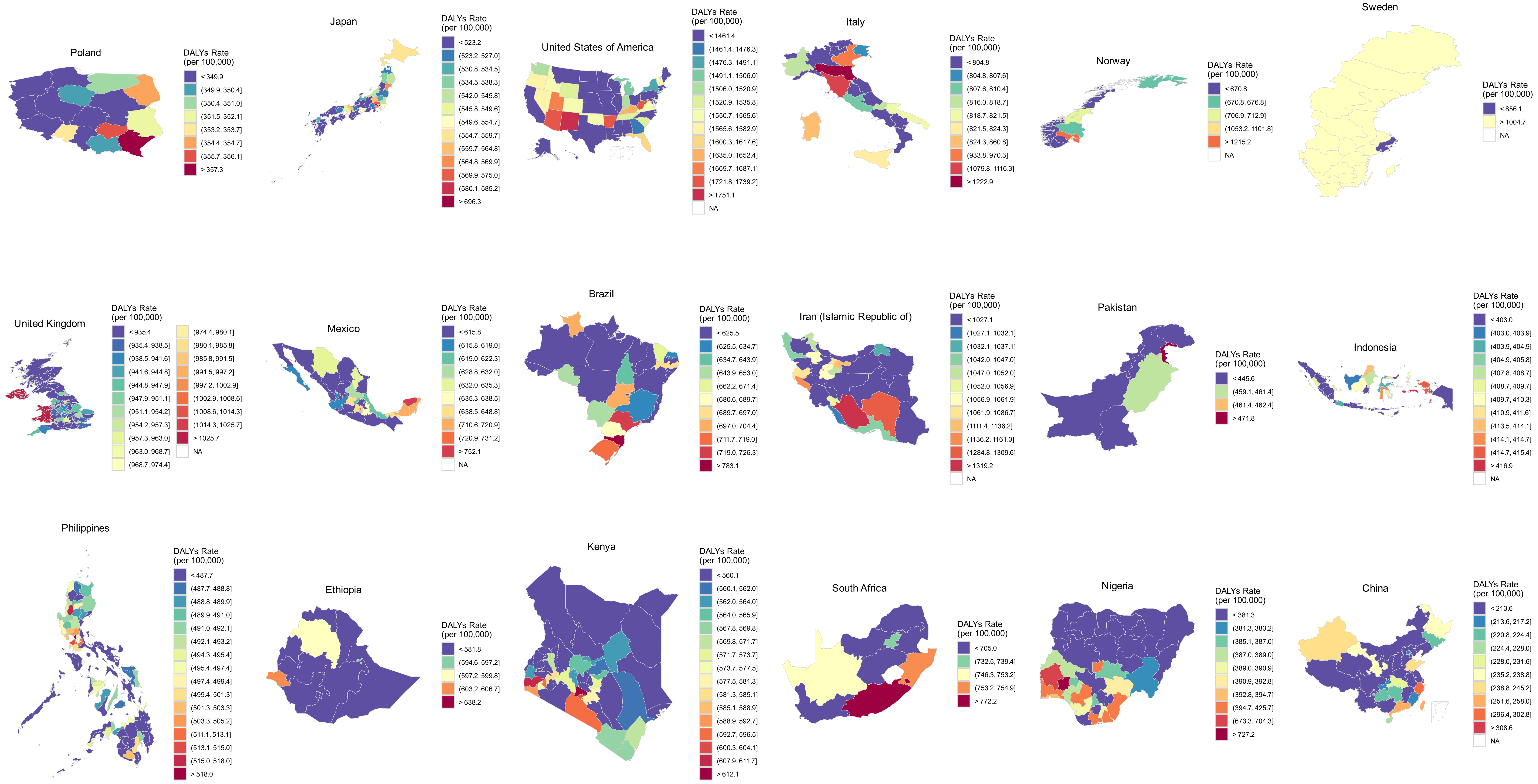
Poland
DALYs Rate (per 100,000)
< 349.9
(349.9, 350.4]
(350.4, 351.0]
(351.5, 352.1]
(353.2, 353.7]
(354.4, 354.7]
(355.7, 356.1]
> 357.3
Japan
DALYs Rate (per 100,000)
< 523.2
(523.2, 527.0]
(530.8, 534.5]
(534.5, 538.3]
(542.0, 545.8]
(545.8, 549.6]
(549.6, 554.7]
(554.7, 559.7]
(559.7, 564.8]
(564.8, 569.9]
(569.9, 575.0]
(580.1, 585.2]
> 696.3
United States of America
DALYs Rate (per 100,000)
< 1461.4
(1461.4, 1476.3]
(1476.3, 1491.1]
(1491.1, 1506.0]
(1506.0, 1520.9]
(1520.9, 1535.8]
(1550.7, 1565.6]
(1565.6, 1582.9]
(1600.3, 1617.6]
(1635.0, 1652.4]
(1669.7, 1687.1]
(1721.8, 1739.2]
> 1751.1
NA
Italy
DALYs Rate (per 100,000)
< 804.8
(804.8, 807.6]
(807.6, 810.4]
(816.0, 818.7]
(818.7, 821.5]
(821.5, 824.3]
(824.3, 860.8]
(933.8, 970.3]
(1079.8, 1116.3]
> 1222.9
Norway
DALYs Rate (per 100,000)
< 670.8
(670.8, 676.8]
(706.9, 712.9]
(1053.2, 1101.8]
> 1215.2
NA
Sweden
DALYs Rate (per 100,000)
< 856.1
> 1004.7
NA
United Kingdom
DALYs Rate (per 100,000)
< 935.4
(935.4, 938.5]
(938.5, 941.6]
(941.6, 944.8]
(944.8, 947.9]
(947.9, 951.1]
(951.1, 954.2]
(954.2, 957.3]
(957.3, 963.0]
(963.0, 968.7]
(968.7, 974.4]
(974.4, 980.1]
(980.1, 985.8]
(985.8, 991.5]
(991.5, 997.2]
(997.2, 1002.9]
(1002.9, 1008.6]
(1008.6, 1014.3]
(1014.3, 1025.7]
> 1025.7
NA
Mexico
DALYs Rate (per 100,000)
< 615.8
(615.8, 619.0]
(619.0, 622.3]
(628.8, 632.0]
(632.0, 635.3]
(635.3, 638.5]
(638.5, 648.8]
(710.6, 720.9]
(720.9, 731.2]
> 752.1
NA
Brazil
DALYs Rate (per 100,000)
< 625.5
(625.5, 634.7]
(634.7, 643.9]
(643.9, 653.0]
(662.2, 671.4]
(680.6, 689.7]
(689.7, 697.0]
(697.0, 704.4]
(711.7, 719.0]
(719.0, 726.3]
> 783.1
Iran (Islamic Republic of)
DALYs Rate (per 100,000)
< 1027.1
(1027.1, 1032.1]
(1032.1, 1037.1]
(1042.0, 1047.0]
(1047.0, 1052.0]
(1052.0, 1056.9]
(1056.9, 1061.9]
(1061.9, 1086.7]
(1111.4, 1136.2]
(1136.2, 1161.0]
(1284.8, 1309.6]
> 1319.2
NA
Pakistan
DALYs Rate (per 100,000)
< 445.6
(459.1, 461.4]
(461.4, 462.4]
> 471.8
Indonesia
DALYs Rate (per 100,000)
< 403.0
(403.0, 403.9]
(403.9, 404.9]
(404.9, 405.8]
(407.8, 408.7]
(408.7, 409.7]
(409.7, 410.3]
(410.9, 411.6]
(413.5, 414.1]
(414.1, 414.7]
(414.7, 415.4]
> 416.9
NA
Philippines
DALYs Rate (per 100,000)
< 487.7
(487.7, 488.8]
(488.8, 489.9]
(489.9, 491.0]
(491.0, 492.1]
(492.1, 493.2]
(494.3, 495.4]
(495.4, 497.4]
(497.4, 499.4]
(499.4, 501.3]
(501.3, 503.3]
(503.3, 505.2]
(511.1, 513.1]
(513.1, 515.0]
(515.0, 518.0]
> 518.0
Ethiopia
DALYs Rate (per 100,000)
< 581.8
(594.6, 597.2]
(597.2, 599.8]
(603.2, 606.7]
> 638.2
Kenya
DALYs Rate (per 100,000)
< 560.1
(560.1, 562.0]
(562.0, 564.0]
(564.0, 565.9]
(567.8, 569.8]
(569.8, 571.7]
(571.7, 573.7]
(573.7, 577.5]
(577.5, 581.3]
(581.3, 585.1]
(585.1, 588.9]
(588.9, 592.7]
(592.7, 596.5]
(600.3, 604.1]
(607.9, 611.7]
> 612.1
South Africa
DALYs Rate (per 100,000)
< 705.0
(732.5, 739.4]
(746.3, 753.2]
(753.2, 754.9]
> 772.2
Nigeria
DALYs Rate (per 100,000)
< 381.3
(381.3, 383.2]
(385.1, 387.0]
(387.0, 389.0]
(389.0, 390.9]
(390.9, 392.8]
(392.8, 394.7]
(394.7, 425.7]
(673.3, 704.3]
> 727.2
China
DALYs Rate (per 100,000)
< 213.6
(213.6, 217.2]
(220.8, 224.4]
(224.4, 228.0]
(228.0, 231.6]
(235.2, 238.8]
(238.8, 245.2]
(251.6, 258.0]
(296.4, 302.8]
> 308.6
NA

Figure S9

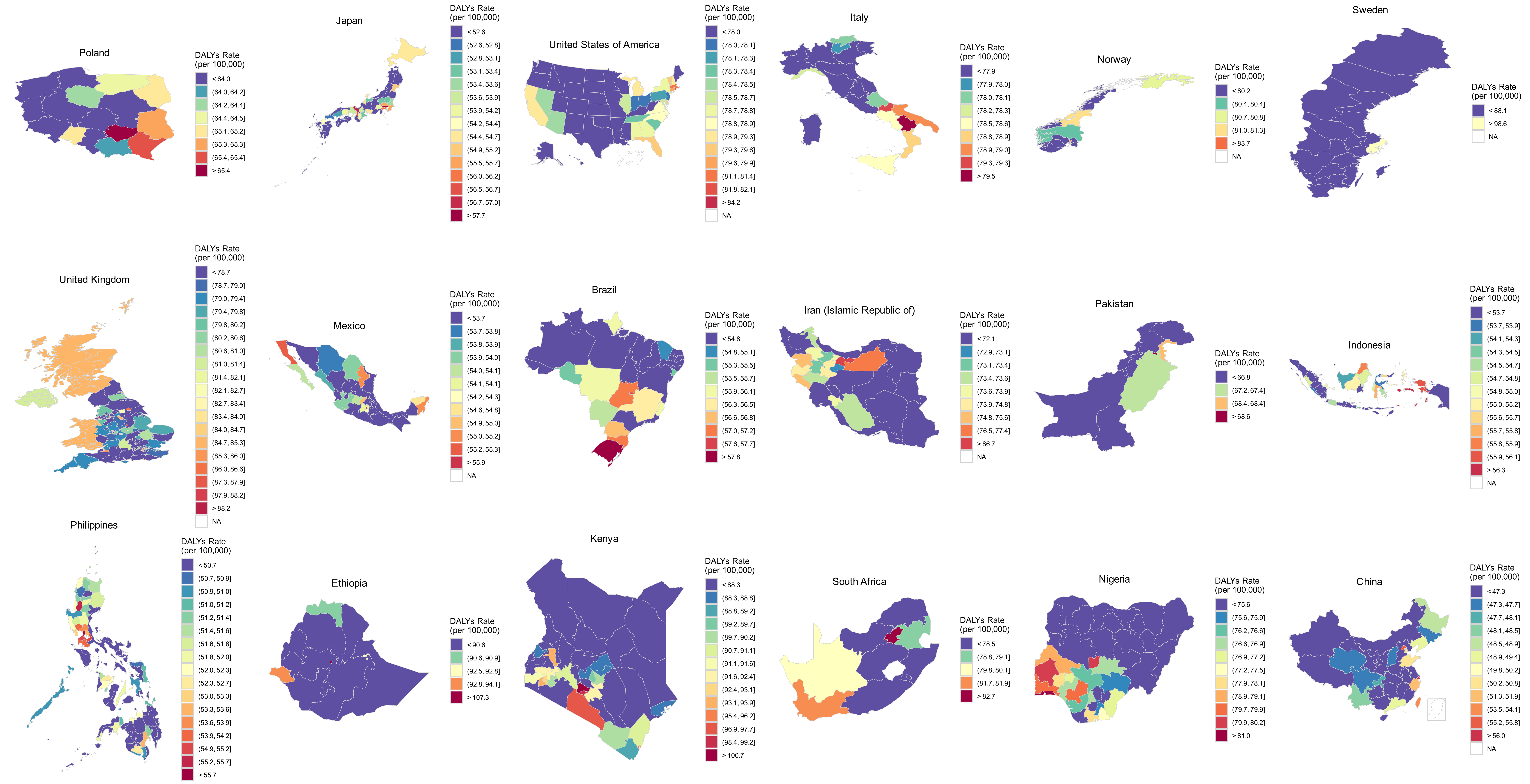

Poland
DALYs Rate (per 100,000)
< 64.0
(64.0, 64.2]
(64.2, 64.4]
(64.4, 64.5]
(65.1, 65.2]
(65.3, 65.3]
(65.4, 65.4]
> 65.4
Japan
DALYs Rate (per 100,000)
< 52.6
(52.6, 52.8]
(52.8, 53.1]
(53.1, 53.4]
(53.4, 53.6]
(53.6, 53.9]
(53.9, 54.2]
(54.2, 54.4]
(54.4, 54.7]
(54.9, 55.2]
(55.5, 55.7]
(56.0, 56.2]
(56.5, 56.7]
(56.7, 57.0]
> 57.7
United States of America
DALYs Rate (per 100,000)
< 78.0
(78.0, 78.1]
(78.1, 78.3]
(78.3, 78.4]
(78.4, 78.5]
(78.5, 78.7]
(78.7, 78.8]
(78.8, 78.9]
(78.9, 79.3]
(79.3, 79.6]
(79.6, 79.9]
(81.1, 81.4]
(81.8, 82.1]
> 84.2
NA
Italy
DALYs Rate (per 100,000)
< 77.9
(77.9, 78.0]
(78.0, 78.1]
(78.2, 78.3]
(78.5, 78.6]
(78.8, 78.9]
(78.9, 79.0]
(79.3, 79.3]
> 79.5
Norway
DALYs Rate (per 100,000)
< 80.2
(80.4, 80.4]
(80.7, 80.8]
(81.0, 81.3]
> 83.7
NA
Sweden
DALYs Rate (per 100,000)
< 88.1
> 98.6
NA
United Kingdom
DALYs Rate (per 100,000)
< 78.7
(78.7, 79.0]
(79.0, 79.4]
(79.4, 79.8]
(79.8, 80.2]
(80.2, 80.6]
(80.6, 81.0]
(81.0, 81.4]
(81.4, 82.1]
(82.1, 82.7]
(82.7, 83.4]
(83.4, 84.0]
(84.0, 84.7]
(84.7, 85.3]
(85.3, 86.0]
(86.0, 86.6]
(87.3, 87.9]
(87.9, 88.2]
> 88.2
NA
Mexico
DALYs Rate (per 100,000)
< 53.7
(53.7, 53.8]
(53.8, 53.9]
(53.9, 54.0]
(54.0, 54.1]
(54.1, 54.1]
(54.2, 54.3]
(54.6, 54.8]
(54.9, 55.0]
(55.0, 55.2]
(55.2, 55.3]
> 55.9
NA
Brazil
DALYs Rate (per 100,000)
< 54.8
(54.8, 55.1]
(55.3, 55.5]
(55.5, 55.7]
(55.9, 56.1]
(56.3, 56.5]
(56.6, 56.8]
(57.0, 57.2]
(57.6, 57.7]
> 57.8
Iran (Islamic Republic of)
DALYs Rate (per 100,000)
< 72.1
(72.9, 73.1]
(73.1, 73.4]
(73.4, 73.6]
(73.6, 73.9]
(73.9, 74.8]
(74.8, 75.6]
(76.5, 77.4]
> 86.7
NA
Pakistan
DALYs Rate (per 100,000)
< 66.8
(67.2, 67.4]
(68.4, 68.4]
> 68.6
Indonesia
DALYs Rate (per 100,000)
< 53.7
(53.7, 53.9]
(54.1, 54.3]
(54.3, 54.5]
(54.5, 54.7]
(54.7, 54.8]
(54.8, 55.0]
(55.0, 55.2]
(55.6, 55.7]
(55.7, 55.8]
(55.8, 55.9]
(55.9, 56.1]
> 56.3
NA
Philippines
DALYs Rate (per 100,000)
< 50.7
(50.7, 50.9]
(50.9, 51.0]
(51.0, 51.2]
(51.2, 51.4]
(51.4, 51.6]
(51.6, 51.8]
(51.8, 52.0]
(52.0, 52.3]
(52.3, 52.7]
(53.0, 53.3]
(53.3, 53.6]
(53.6, 53.9]
(53.9, 54.2]
(54.9, 55.2]
(55.2, 55.7]
> 55.7
Ethiopia
DALYs Rate (per 100,000)
< 90.6
(90.6, 90.9]
(92.5, 92.8]
(92.8, 94.1]
> 107.3
Kenya
DALYs Rate (per 100,000)
< 88.3
(88.3, 88.8]
(88.8, 89.2]
(89.2, 89.7]
(89.7, 90.2]
(90.7, 91.1]
(91.1, 91.6]
(91.6, 92.4]
(92.4, 93.1]
(93.1, 93.9]
(95.4, 96.2]
(96.9, 97.7]
(98.4, 99.2]
> 100.7
South Africa
DALYs Rate (per 100,000)
< 78.5
(78.8, 79.1]
(79.8, 80.1]
(81.7, 81.9]
> 82.7
Nigeria
DALYs Rate (per 100,000)
< 75.6
(75.6, 75.9]
(76.2, 76.6]
(76.6, 76.9]
(76.9, 77.2]
(77.2, 77.5]
(77.9, 78.1]
(78.9, 79.1]
(79.7, 79.9]
(79.9, 80.2]
> 81.0
China
DALYs Rate (per 100,000)
< 47.3
(47.3, 47.7]
(47.7, 48.1]
(48.1, 48.5]
(48.5, 48.9]
(48.9, 49.4]
(49.8, 50.2]
(50.2, 50.8]
(51.3, 51.9]
(53.5, 54.1]
(55.2, 55.8]
> 56.0
NA

Figure S10

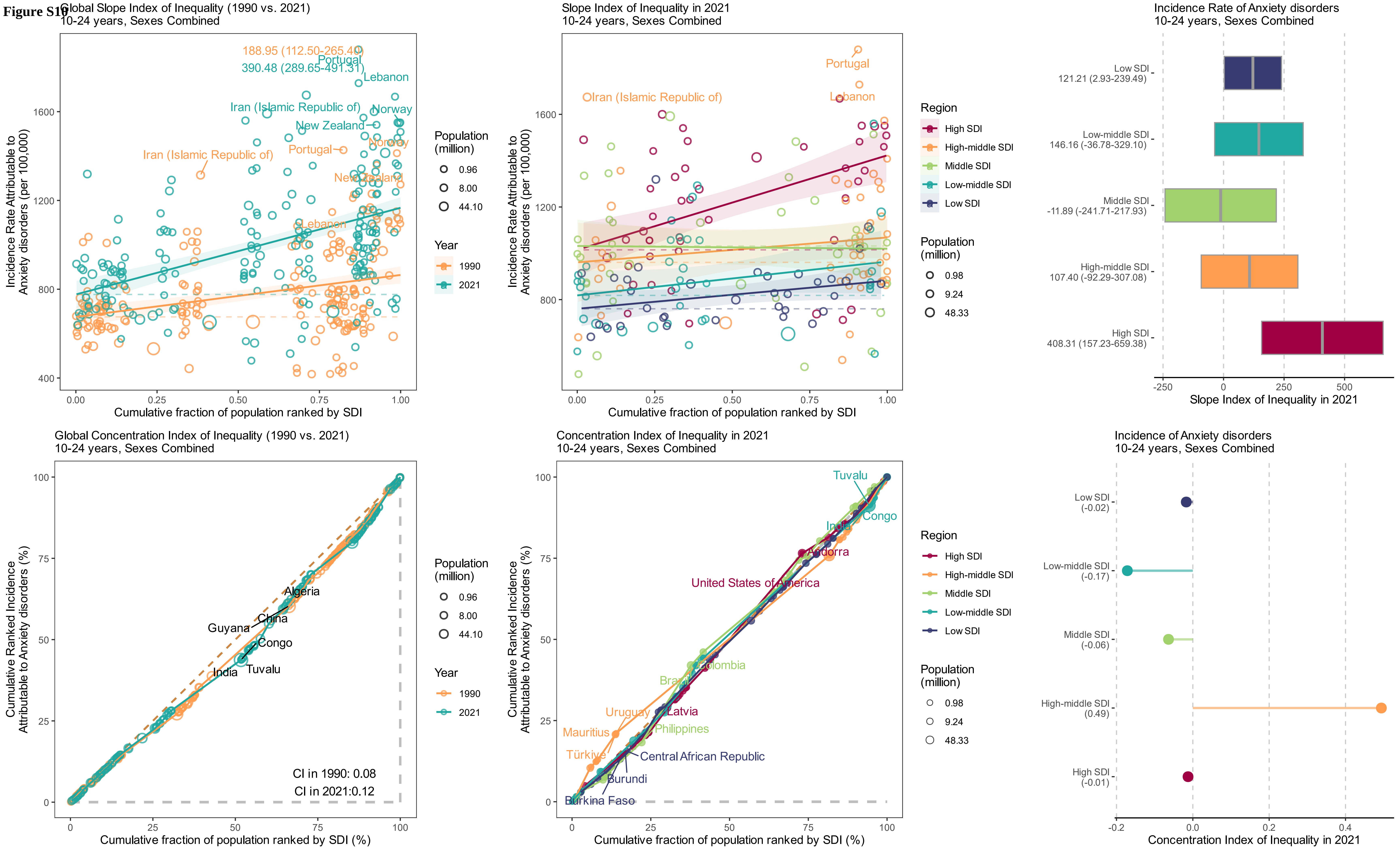
Global Slope Index of Inequality (1990 vs. 2021)
10-24 years, Sexes Combined
188.95 (112.50-265.40)
390.48 (289.65-491.31)
Portugal
Lebanon
Iran (Islamic Republic of)
Norway
New Zealand
Incidence Rate Attributable to Anxiety disorders (per 100,000)
Cumulative fraction of population ranked by SDI
Population (million)
0.96
8.00
44.10
Year
1990
2021
Slope Index of Inequality in 2021
10-24 years, Sexes Combined
Region
High SDI
High-middle SDI
Middle SDI
Low-middle SDI
Low SDI
Population (million)
0.98
9.24
48.33
Incidence Rate of Anxiety disorders
10-24 years, Sexes Combined
Low SDI 121.21 (2.93-239.49)
Low-middle SDI 146.16 (-36.78-329.10)
Middle SDI -11.89 (-241.71-217.93)
High-middle SDI 107.40 (-92.29-307.08)
High SDI 408.31 (157.23-659.38)
Slope Index of Inequality in 2021
Global Concentration Index of Inequality (1990 vs. 2021)
10-24 years, Sexes Combined
Cumulative Ranked Incidence Attributable to Anxiety disorders (%)
Cumulative fraction of population ranked by SDI (%)
Algeria
China
Guyana
Congo
India
Tuvalu
CI in 1990: 0.08
CI in 2021:0.12
Concentration Index of Inequality in 2021
10-24 years, Sexes Combined
Tuvalu
Congo
India
Andorra
United States of America
Colombia
Brazil
Uruguay
Latvia
Mauritius
Philippines
Türkiye
Central African Republic
Burundi
Burkina Faso
Incidence of Anxiety disorders
10-24 years, Sexes Combined
Low SDI (-0.02)
Low-middle SDI (-0.17)
Middle SDI (-0.06)
High-middle SDI (0.49)
High SDI (-0.01)
Concentration Index of Inequality in 2021

Figure S10

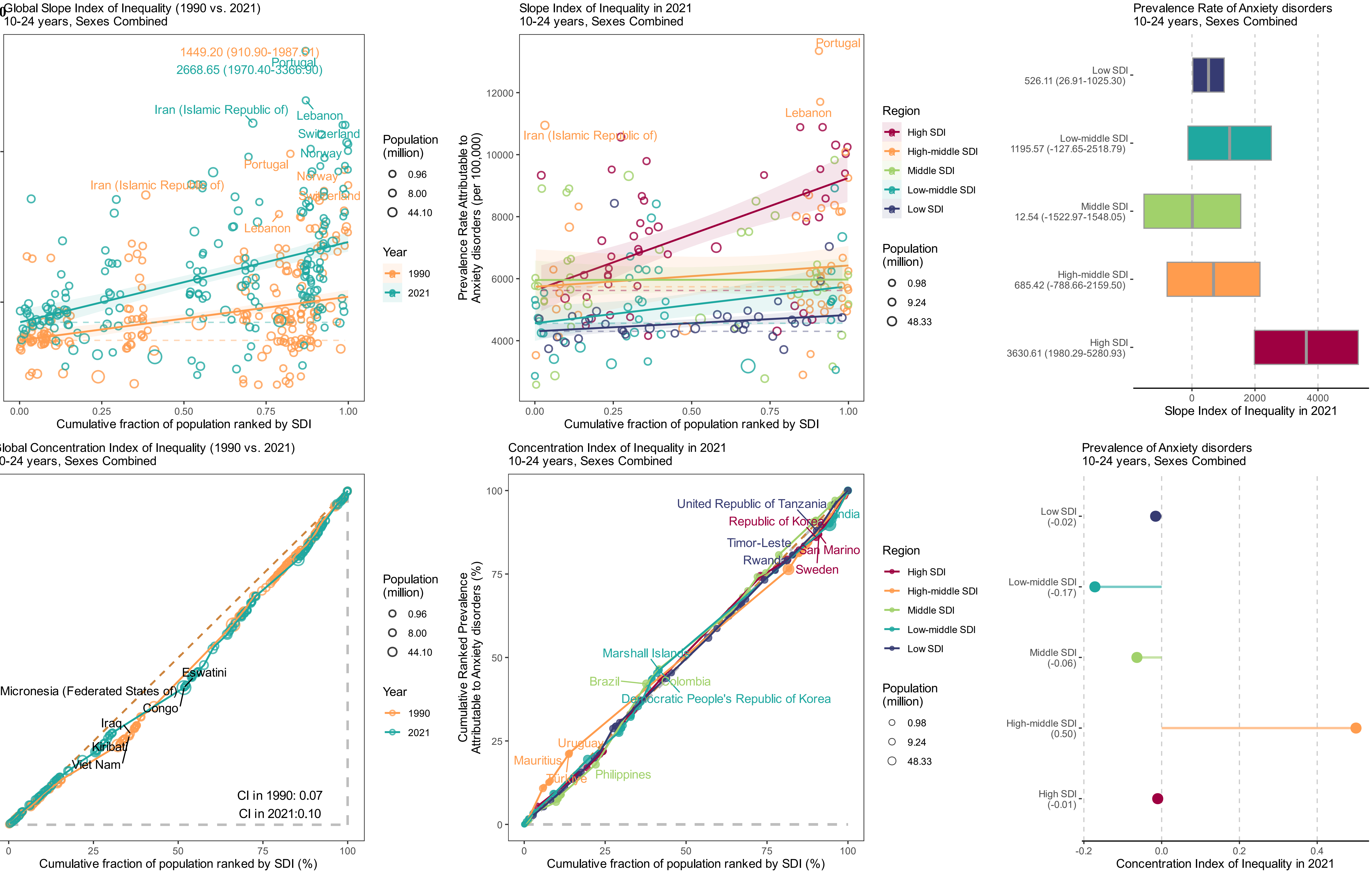
Global Slope Index of Inequality (1990 vs. 2021)
10-24 years, Sexes Combined
1449.20 (910.90-1987.51)
2668.65 (1970.40-3366.90)
Prevalence Rate Attributable to Anxiety disorders (per 100,000)
Cumulative fraction of population ranked by SDI
Population (million)
0.96
8.00
44.10
Year
1990
2021
Slope Index of Inequality in 2021
10-24 years, Sexes Combined
Region
High SDI
High-middle SDI
Middle SDI
Low-middle SDI
Low SDI
Population (million)
0.98
9.24
48.33
Prevalence Rate of Anxiety disorders
10-24 years, Sexes Combined
Low SDI 526.11 (26.91-1025.30)
Low-middle SDI 1195.57 (-127.65-2518.79)
Middle SDI 12.54 (-1522.97-1548.05)
High-middle SDI 685.42 (-788.66-2159.50)
High SDI 3630.61 (1980.29-5280.93)
Slope Index of Inequality in 2021
Global Concentration Index of Inequality (1990 vs. 2021)
10-24 years, Sexes Combined
Cumulative Ranked Prevalence Attributable to Anxiety disorders (%)
Cumulative fraction of population ranked by SDI (%)
CI in 1990: 0.07
CI in 2021:0.10
Concentration Index of Inequality in 2021
10-24 years, Sexes Combined
Prevalence of Anxiety disorders
10-24 years, Sexes Combined
Low SDI (-0.02)
Low-middle SDI (-0.17)
Middle SDI (-0.06)
High-middle SDI (0.50)
High SDI (-0.01)
Concentration Index of Inequality in 2021

Figure S10

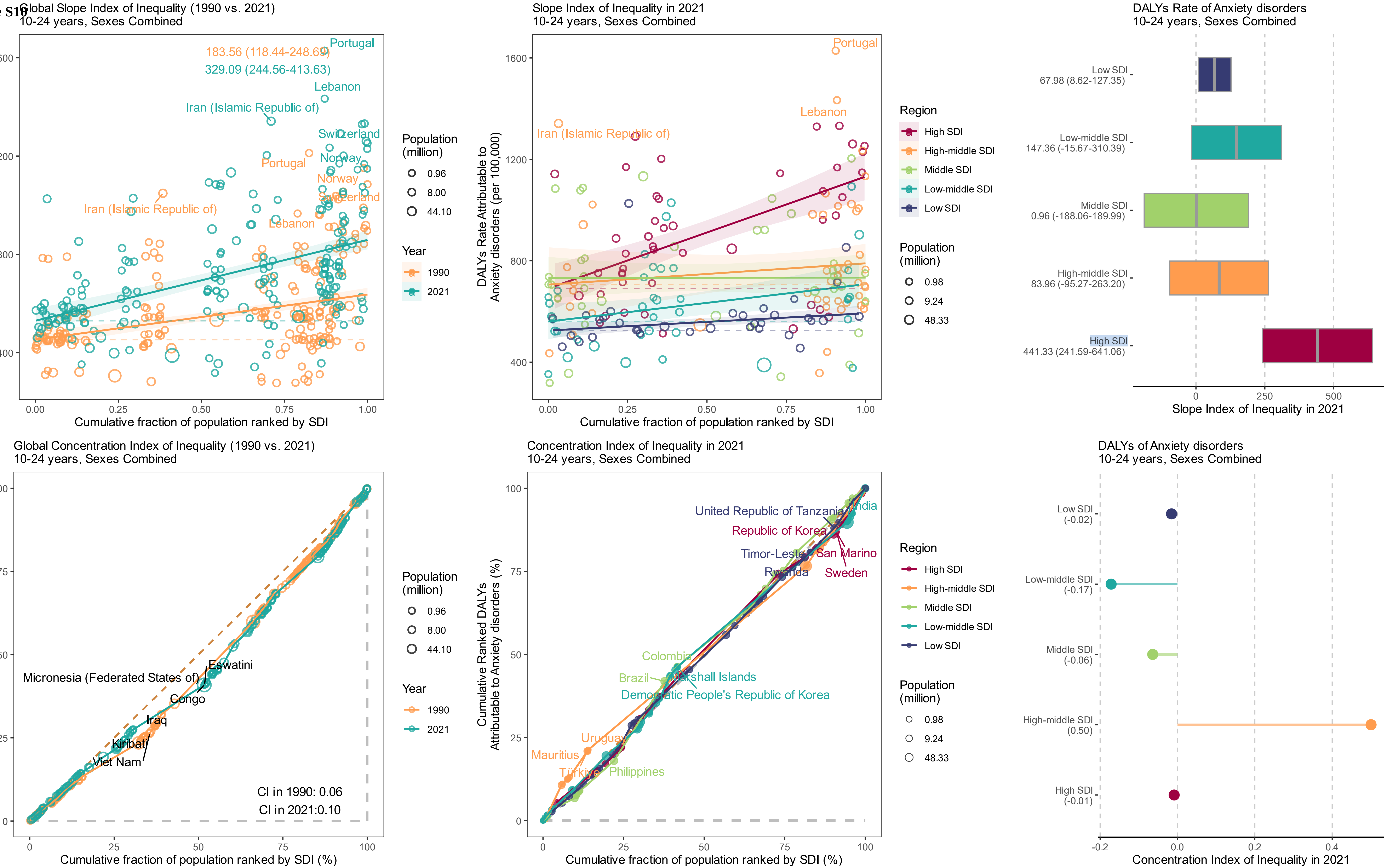
Global Slope Index of Inequality (1990 vs. 2021)
10-24 years, Sexes Combined
183.56 (118.44-248.69)
329.09 (244.56-413.63)
Portugal
Lebanon
Iran (Islamic Republic of)
Switzerland
Norway
Portugal
Norway
Switzerland
Iran (Islamic Republic of)
Lebanon
DALYs Rate Attributable to Anxiety disorders (per 100,000)
Cumulative fraction of population ranked by SDI
Population (million)
0.96
8.00
44.10
Year
1990
2021
Slope Index of Inequality in 2021
10-24 years, Sexes Combined
Portugal
Lebanon
Iran (Islamic Republic of)
DALYs Rate Attributable to Anxiety disorders (per 100,000)
Cumulative fraction of population ranked by SDI
Region
High SDI
High-middle SDI
Middle SDI
Low-middle SDI
Low SDI
Population (million)
0.98
9.24
48.33
DALYs Rate of Anxiety disorders
10-24 years, Sexes Combined
Low SDI
67.98 (8.62-127.35)
Low-middle SDI
147.36 (-15.67-310.39)
Middle SDI
0.96 (-188.06-189.99)
High-middle SDI
83.96 (-95.27-263.20)
High SDI
441.33 (241.59-641.06)
Slope Index of Inequality in 2021
Global Concentration Index of Inequality (1990 vs. 2021)
10-24 years, Sexes Combined
Micronesia (Federated States of)
Eswatini
Congo
Iraq
Kiribati
Viet Nam
CI in 1990: 0.06
CI in 2021:0.10
Cumulative Ranked DALYs Attributable to Anxiety disorders (%)
Cumulative fraction of population ranked by SDI (%)
Population (million)
0.96
8.00
44.10
Year
1990
2021
Concentration Index of Inequality in 2021
10-24 years, Sexes Combined
United Republic of Tanzania
India
Republic of Korea
Timor-Leste
San Marino
Rwanda
Sweden
Colombia
Brazil
Marshall Islands
Democratic People's Republic of Korea
Uruguay
Mauritius
Türkiye
Philippines
Cumulative Ranked DALYs Attributable to Anxiety disorders (%)
Cumulative fraction of population ranked by SDI (%)
Region
High SDI
High-middle SDI
Middle SDI
Low-middle SDI
Low SDI
Population (million)
0.98
9.24
48.33
DALYs of Anxiety disorders
10-24 years, Sexes Combined
Low SDI (-0.02)
Low-middle SDI (-0.17)
Middle SDI (-0.06)
High-middle SDI (0.50)
High SDI (-0.01)
Concentration Index of Inequality in 2021

Figure S11

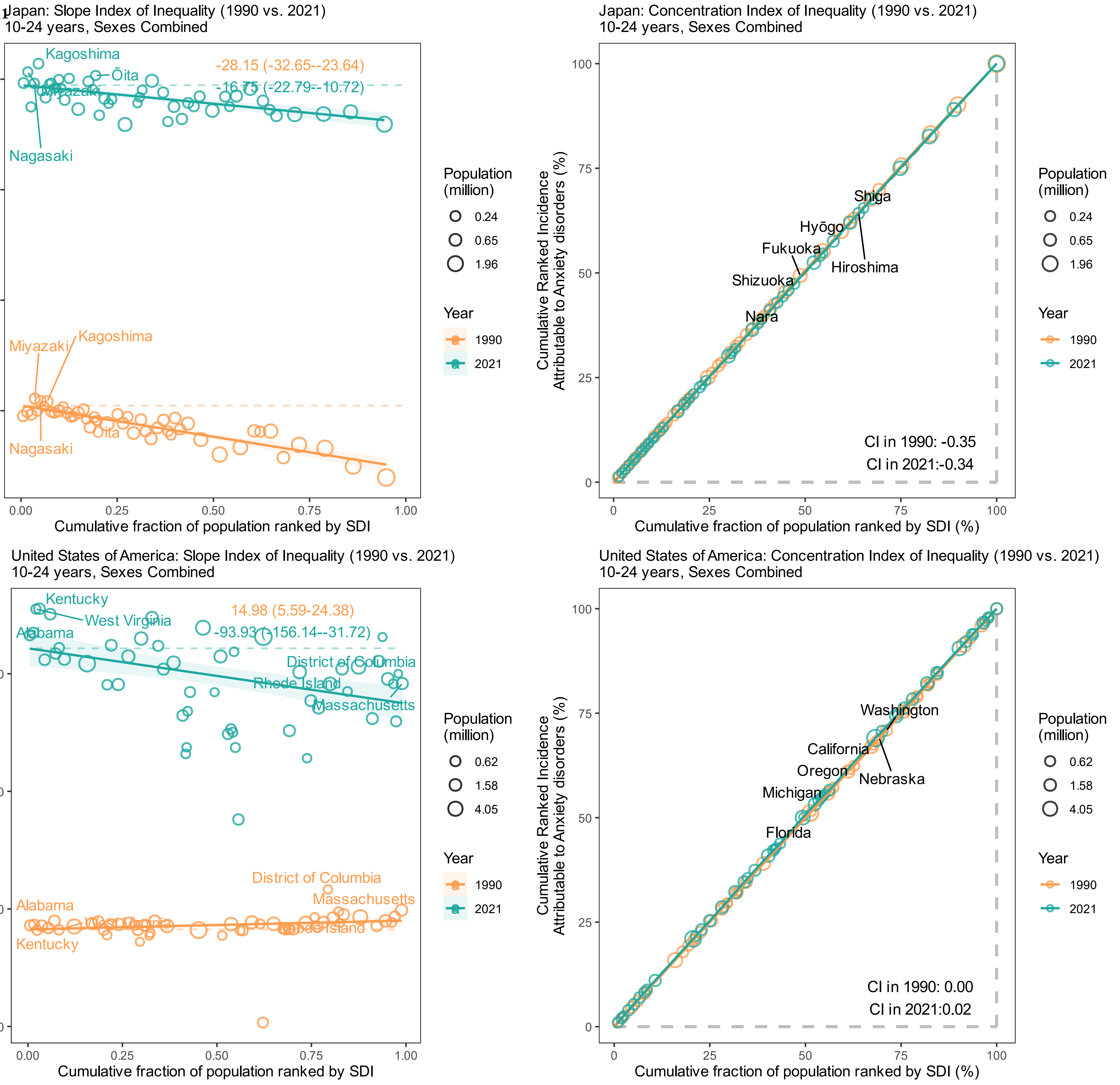
Japan: Slope Index of Inequality (1990 vs. 2021)
10-24 years, Sexes Combined
Kagoshima
Ōita
Miyazaki
Nagasaki
-28.15 (-32.65--23.64)
-16.75 (-22.79--10.72)
Incidence Rate Attributable to Anxiety disorders (per 100,000)
Cumulative fraction of population ranked by SDI
Population (million)
0.24
0.65
1.96
Year
1990
2021
Japan: Concentration Index of Inequality (1990 vs. 2021)
10-24 years, Sexes Combined
Shiga
Hyōgo
Fukuoka
Hiroshima
Shizuoka
Nara
CI in 1990: -0.35
CI in 2021:-0.34
Cumulative Ranked Incidence Attributable to Anxiety disorders (%)
Cumulative fraction of population ranked by SDI (%)
United States of America: Slope Index of Inequality (1990 vs. 2021)
10-24 years, Sexes Combined
Kentucky
West Virginia
Alabama
14.98 (5.59-24.38)
-93.93 (-156.14--31.72)
District of Columbia
Rhode Island
Massachusetts
Population (million)
0.62
1.58
4.05
United States of America: Concentration Index of Inequality (1990 vs. 2021)
10-24 years, Sexes Combined
Washington
California
Oregon
Nebraska
Michigan
Florida
CI in 1990: 0.00
CI in 2021:0.02

Figure S11

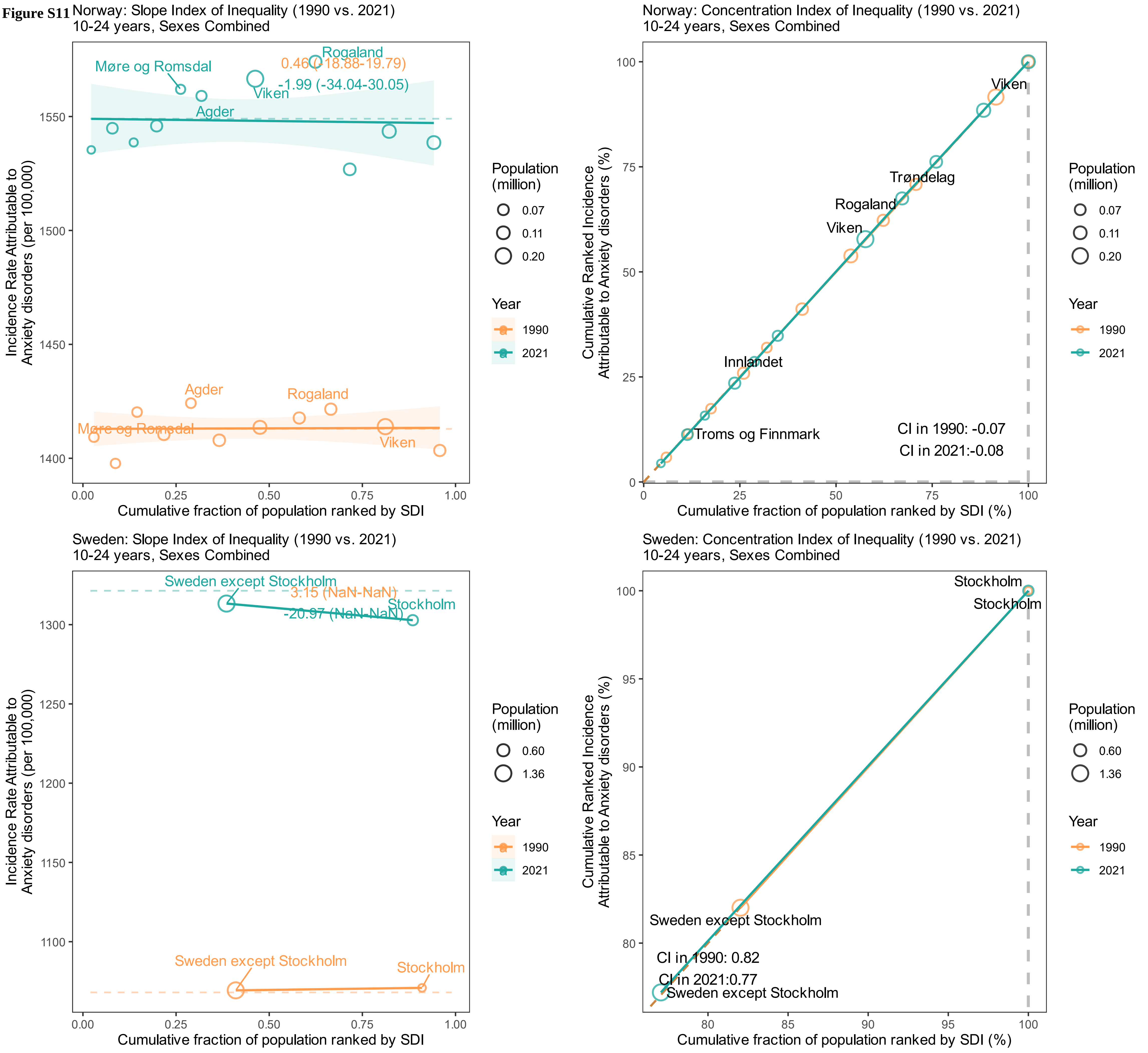
Norway: Slope Index of Inequality (1990 vs. 2021)
10-24 years, Sexes Combined
Møre og Romsdal
Rogaland
0.46 (-18.88-19.79)
-1.99 (-34.04-30.05)
Viken
Agder
Agder
Rogaland
Møre og Romsdal
Viken
Incidence Rate Attributable to Anxiety disorders (per 100,000)
Cumulative fraction of population ranked by SDI
Norway: Concentration Index of Inequality (1990 vs. 2021)
10-24 years, Sexes Combined
Viken
Trøndelag
Rogaland
Viken
Innlandet
Troms og Finnmark
CI in 1990: -0.07
CI in 2021:-0.08
Cumulative Ranked Incidence Attributable to Anxiety disorders (%)
Cumulative fraction of population ranked by SDI (%)
Population (million)
0.07
0.11
0.20
Year
1990
2021
Sweden: Slope Index of Inequality (1990 vs. 2021)
10-24 years, Sexes Combined
Sweden except Stockholm
3.15 (NaN-NaN)
-20.97 (NaN-NaN)
Stockholm
Sweden except Stockholm
Stockholm
Incidence Rate Attributable to Anxiety disorders (per 100,000)
Cumulative fraction of population ranked by SDI
Sweden: Concentration Index of Inequality (1990 vs. 2021)
10-24 years, Sexes Combined
Stockholm
Stockholm
Sweden except Stockholm
CI in 1990: 0.82
CI in 2021:0.77
Sweden except Stockholm
Cumulative Ranked Incidence Attributable to Anxiety disorders (%)
Cumulative fraction of population ranked by SDI (%)
Population (million)
0.60
1.36
Year
1990
2021

Figure S11

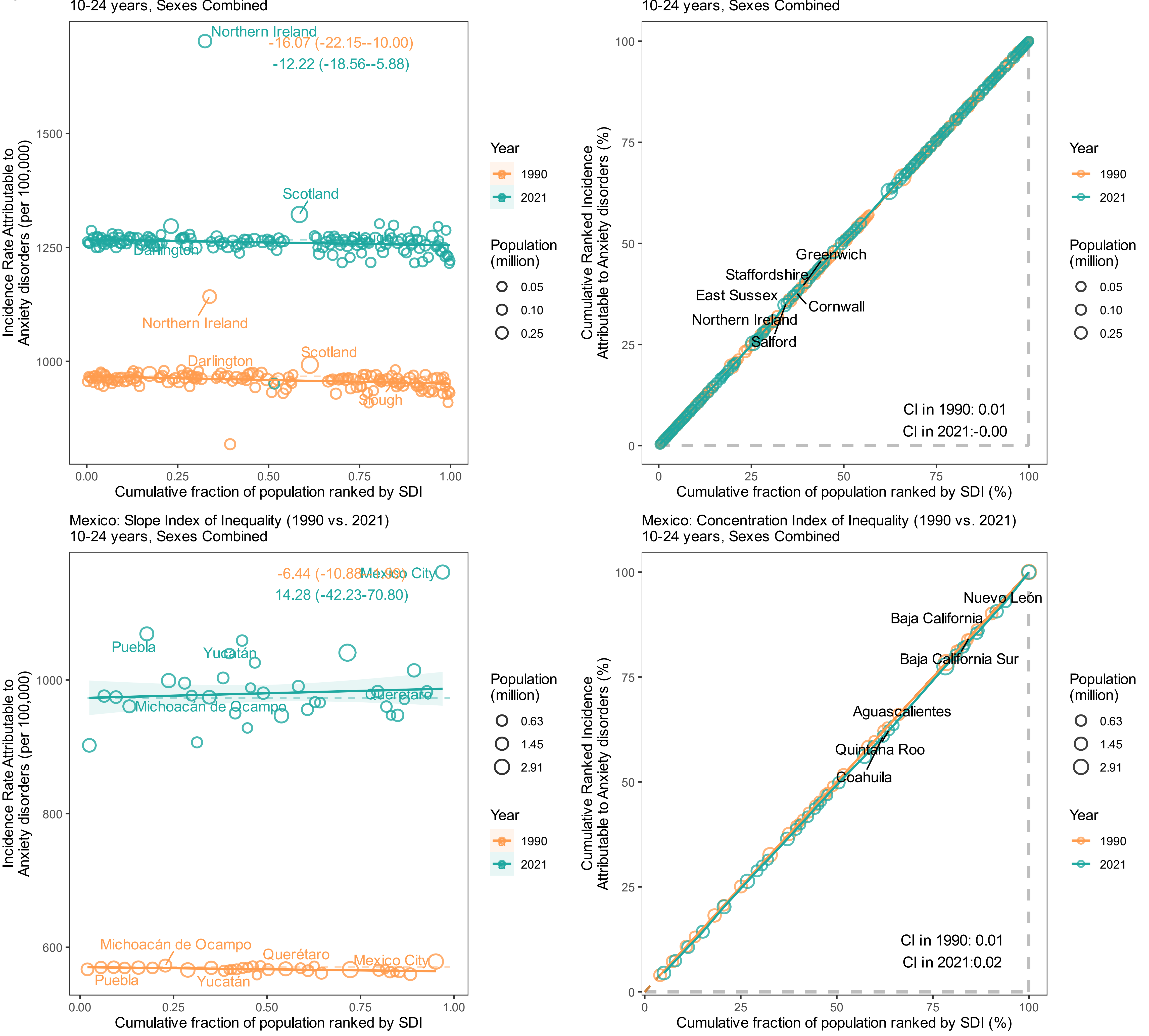

United Kingdom: Slope Index of Inequality (1990 vs. 2021)
10-24 years, Sexes Combined
Incidence Rate Attributable to Anxiety disorders (per 100,000)
Cumulative fraction of population ranked by SDI
-16.07 (-22.15--10.00)
-12.22 (-18.56--5.88)
Northern Ireland
Scotland
Darlington
Slough
Year
1990
2021
Population (million)
0.05
0.10
0.25
United Kingdom: Concentration Index of Inequality (1990 vs. 2021)
10-24 years, Sexes Combined
Cumulative Ranked Incidence Attributable to Anxiety disorders (%)
Cumulative fraction of population ranked by SDI (%)
Greenwich
Staffordshire
East Sussex
Cornwall
Northern Ireland
Salford
CI in 1990: 0.01
CI in 2021:-0.00
Mexico: Slope Index of Inequality (1990 vs. 2021)
10-24 years, Sexes Combined
-6.44 (-10.88--1.99)
14.28 (-42.23-70.80)
Mexico City
Puebla
Yucatán
Querétaro
Michoacán de Ocampo
Population (million)
0.63
1.45
2.91
Mexico: Concentration Index of Inequality (1990 vs. 2021)
10-24 years, Sexes Combined
Nuevo León
Baja California
Baja California Sur
Aguascalientes
Quintana Roo
Coahuila
CI in 1990: 0.01
CI in 2021:0.02

Figure S11

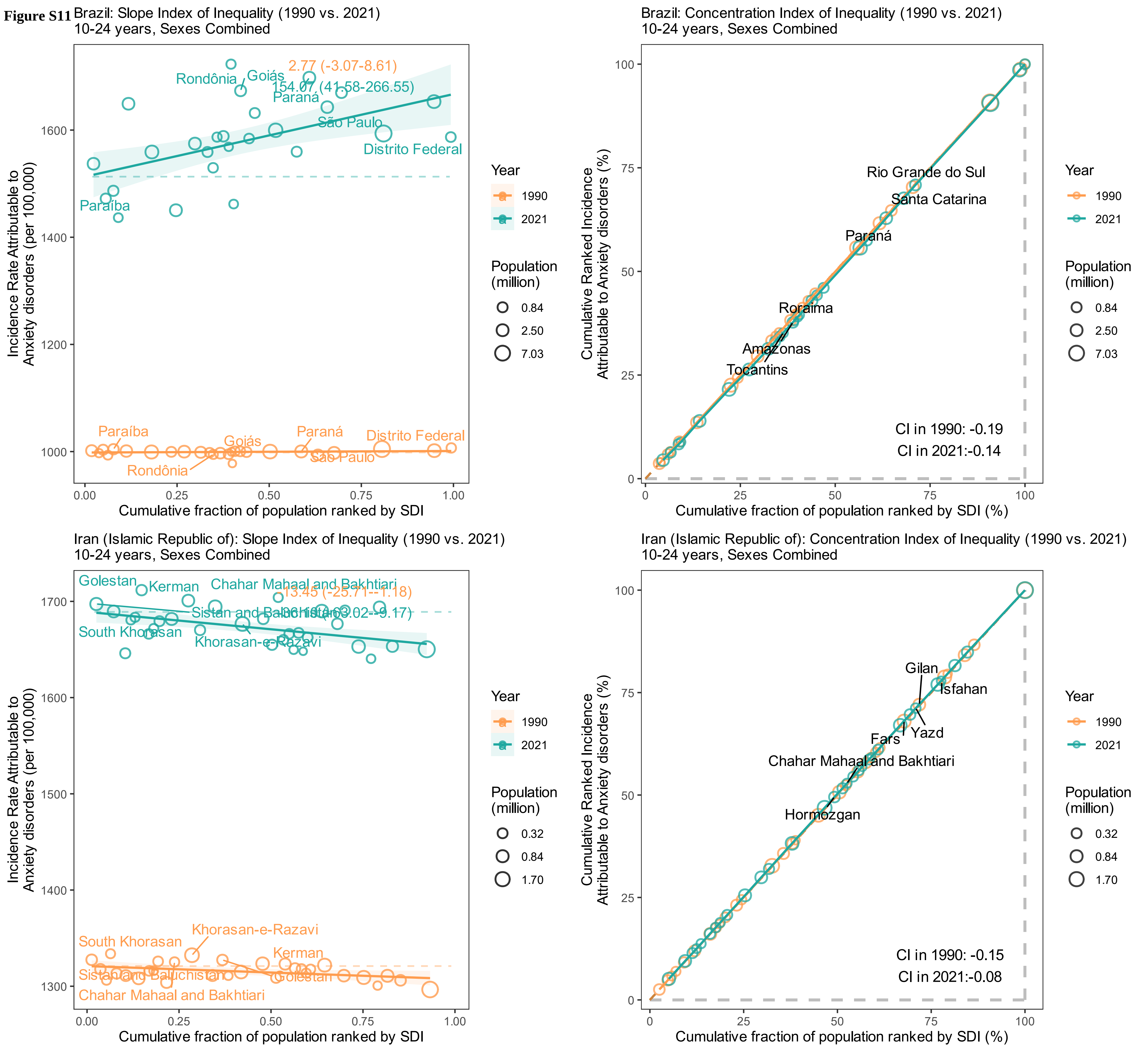

Brazil: Slope Index of Inequality (1990 vs. 2021)
10-24 years, Sexes Combined
Incidence Rate Attributable to Anxiety disorders (per 100,000)
Cumulative fraction of population ranked by SDI
2.77 (-3.07-8.61)
154.07 (41.58-266.55)
Rondônia
Goiás
Paraná
São Paulo
Distrito Federal
Paraíba
Year
1990
2021
Population (million)
0.84
2.50
7.03
Brazil: Concentration Index of Inequality (1990 vs. 2021)
10-24 years, Sexes Combined
Cumulative Ranked Incidence Attributable to Anxiety disorders (%)
Cumulative fraction of population ranked by SDI (%)
Rio Grande do Sul
Santa Catarina
Paraná
Roraima
Amazonas
Tocantins
CI in 1990: -0.19
CI in 2021:-0.14
Iran (Islamic Republic of): Slope Index of Inequality (1990 vs. 2021)
10-24 years, Sexes Combined
Golestan
Kerman
Chahar Mahaal and Bakhtiari
-13.45 (-25.71--1.18)
Sistan and Baluchistan
South Khorasan
Khorasan-e-Razavi
Population (million)
0.32
0.84
1.70
Iran (Islamic Republic of): Concentration Index of Inequality (1990 vs. 2021)
10-24 years, Sexes Combined
Gilan
Isfahan
Yazd
Fars
Hormozgan
CI in 1990: -0.15
CI in 2021:-0.08

Figure S11

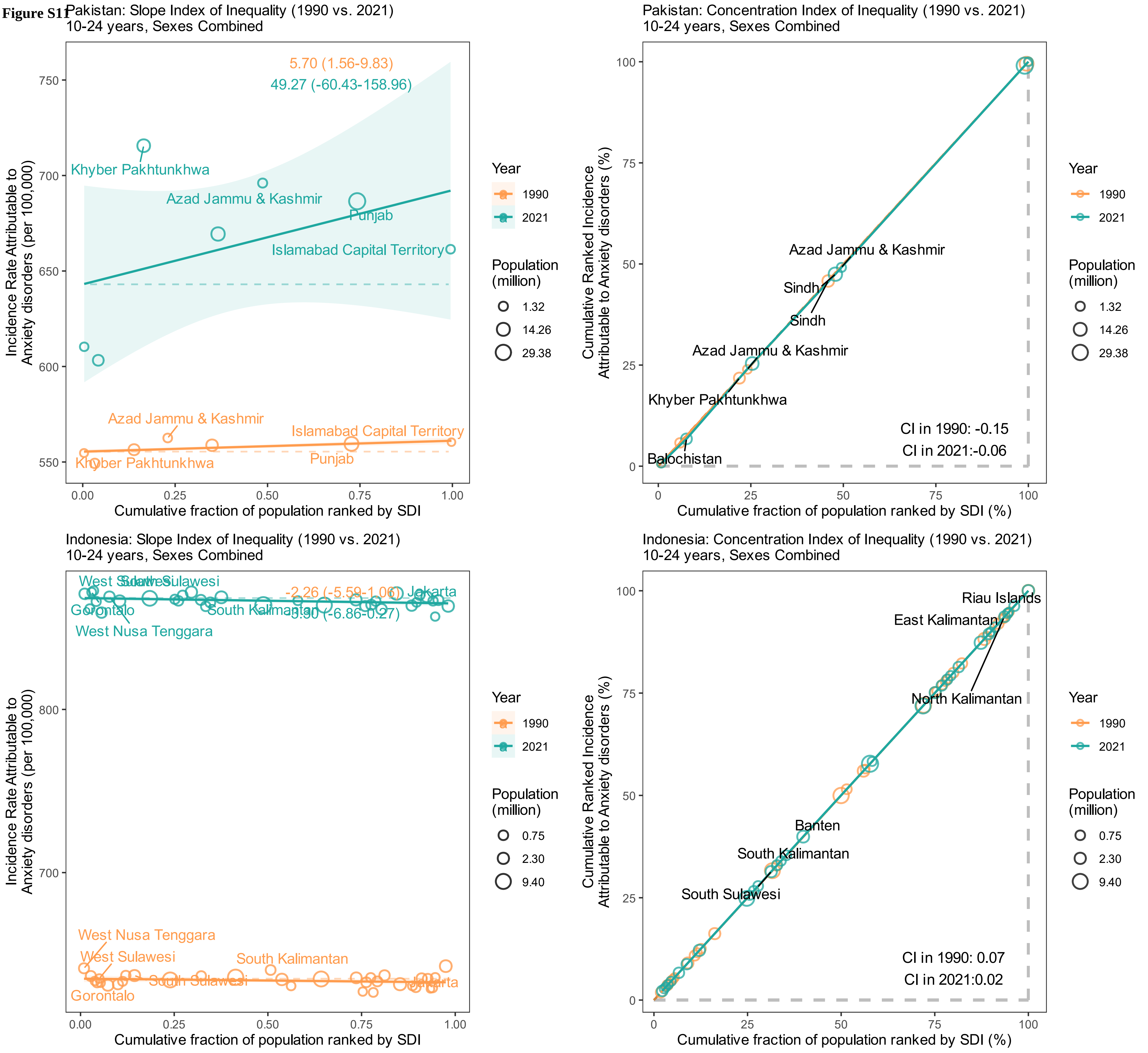

Pakistan: Slope Index of Inequality (1990 vs. 2021)
10-24 years, Sexes Combined
5.70 (1.56-9.83)
49.27 (-60.43-158.96)
Khyber Pakhtunkhwa
Azad Jammu & Kashmir
Punjab
Islamabad Capital Territory
Azad Jammu & Kashmir
Islamabad Capital Territory
Punjab
Khyber Pakhtunkhwa
Incidence Rate Attributable to Anxiety disorders (per 100,000)
Cumulative fraction of population ranked by SDI
Year
1990
2021
Population (million)
1.32
14.26
29.38
Pakistan: Concentration Index of Inequality (1990 vs. 2021)
10-24 years, Sexes Combined
Azad Jammu & Kashmir
Sindh
Sindh
Azad Jammu & Kashmir
Khyber Pakhtunkhwa
Balochistan
CI in 1990: -0.15
CI in 2021:-0.06
Cumulative Ranked Incidence Attributable to Anxiety disorders (%)
Cumulative fraction of population ranked by SDI (%)
Indonesia: Slope Index of Inequality (1990 vs. 2021)
10-24 years, Sexes Combined
West Sulawesi
South Sulawesi
-2.26 (-5.59-1.06)
Jakarta
Gorontalo
South Kalimantan
-3.30 (-6.86-0.27)
West Nusa Tenggara
West Nusa Tenggara
West Sulawesi
South Kalimantan
South Sulawesi
Jakarta
Gorontalo
Population (million)
0.75
2.30
9.40
Indonesia: Concentration Index of Inequality (1990 vs. 2021)
10-24 years, Sexes Combined
Riau Islands
East Kalimantan
North Kalimantan
Banten
South Kalimantan
South Sulawesi
CI in 1990: 0.07
CI in 2021:0.02

Figure S11

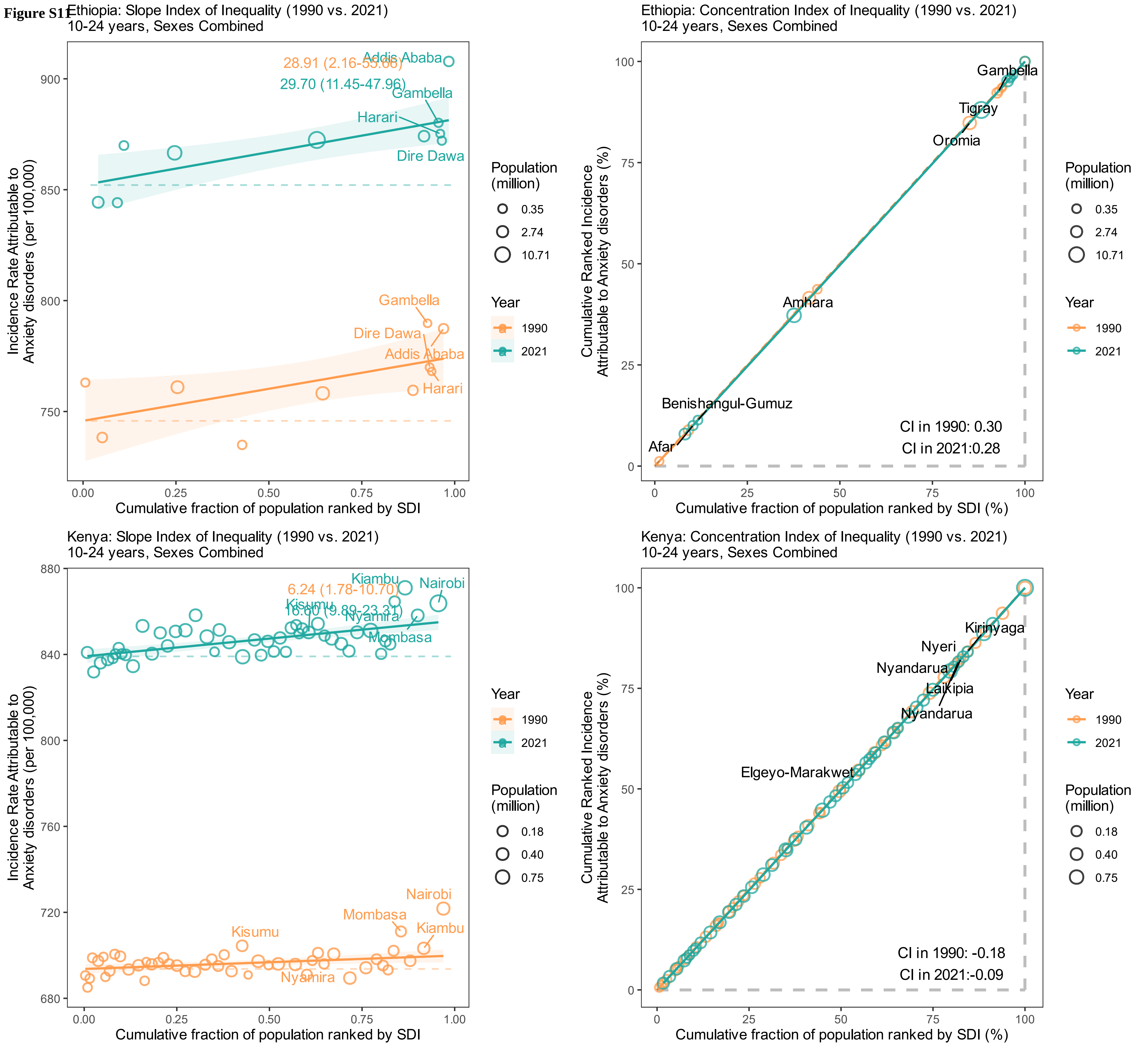

Ethiopia: Slope Index of Inequality (1990 vs. 2021)
10-24 years, Sexes Combined
28.91 (2.16-55.66)
29.70 (11.45-47.96)
Addis Ababa
Gambella
Harari
Dire Dawa
Incidence Rate Attributable to Anxiety disorders (per 100,000)
Cumulative fraction of population ranked by SDI
Population (million)
0.35
2.74
10.71
Year
1990
2021
Ethiopia: Concentration Index of Inequality (1990 vs. 2021)
10-24 years, Sexes Combined
Gambella
Tigray
Oromia
Amhara
Benishangul-Gumuz
Afar
CI in 1990: 0.30
CI in 2021:0.28
Cumulative Ranked Incidence Attributable to Anxiety disorders (%)
Cumulative fraction of population ranked by SDI (%)
Kenya: Slope Index of Inequality (1990 vs. 2021)
10-24 years, Sexes Combined
6.24 (1.78-10.70)
16.60 (9.89-23.31)
Kiambu
Nairobi
Kisumu
Nyamira
Mombasa
Incidence Rate Attributable to Anxiety disorders (per 100,000)
Cumulative fraction of population ranked by SDI
Population (million)
0.18
0.40
0.75
Kenya: Concentration Index of Inequality (1990 vs. 2021)
10-24 years, Sexes Combined
Kirinyaga
Nyeri
Nyandarua
Laikipia
Elgeyo-Marakwet
CI in 1990: -0.18
CI in 2021:-0.09
Cumulative Ranked Incidence Attributable to Anxiety disorders (%)
Cumulative fraction of population ranked by SDI (%)

Figure S11

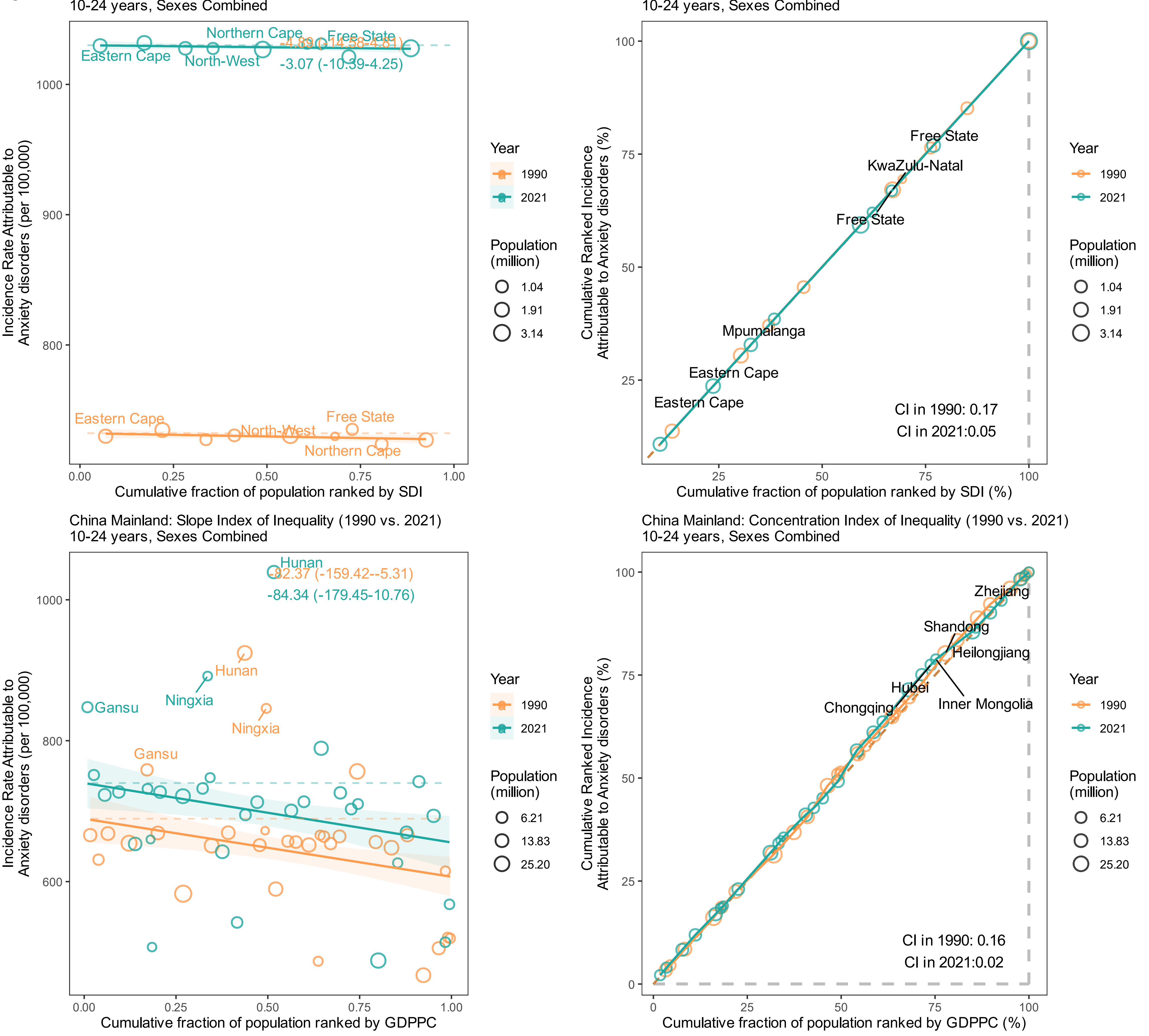

South Africa: Slope Index of Inequality (1990 vs. 2021)
10-24 years, Sexes Combined
Incidence Rate Attributable to Anxiety disorders (per 100,000)
Cumulative fraction of population ranked by SDI
Northern Cape
Free State
Eastern Cape
North-West
-4.89 (-14.58-4.81)
-3.07 (-10.39-4.25)
Eastern Cape
North-West
Free State
Northern Cape
Year
1990
2021
Population (million)
1.04
1.91
3.14
South Africa: Concentration Index of Inequality (1990 vs. 2021)
10-24 years, Sexes Combined
Cumulative Ranked Incidence Attributable to Anxiety disorders (%)
Cumulative fraction of population ranked by SDI (%)
Free State
KwaZulu-Natal
Free State
Mpumalanga
Eastern Cape
Eastern Cape
CI in 1990: 0.17
CI in 2021:0.05
China Mainland: Slope Index of Inequality (1990 vs. 2021)
10-24 years, Sexes Combined
Incidence Rate Attributable to Anxiety disorders (per 100,000)
Cumulative fraction of population ranked by GDPPC
Hunan
-82.37 (-159.42--5.31)
-84.34 (-179.45-10.76)
Hunan
Ningxia
Gansu
Ningxia
Gansu
Year
1990
2021
Population (million)
6.21
13.83
25.20
China Mainland: Concentration Index of Inequality (1990 vs. 2021)
10-24 years, Sexes Combined
Cumulative Ranked Incidence Attributable to Anxiety disorders (%)
Cumulative fraction of population ranked by GDPPC (%)
Zhejiang
Shandong
Heilongjiang
Hubei
Chongqing
Inner Mongolia
CI in 1990: 0.16
CI in 2021:0.02

Figure S11

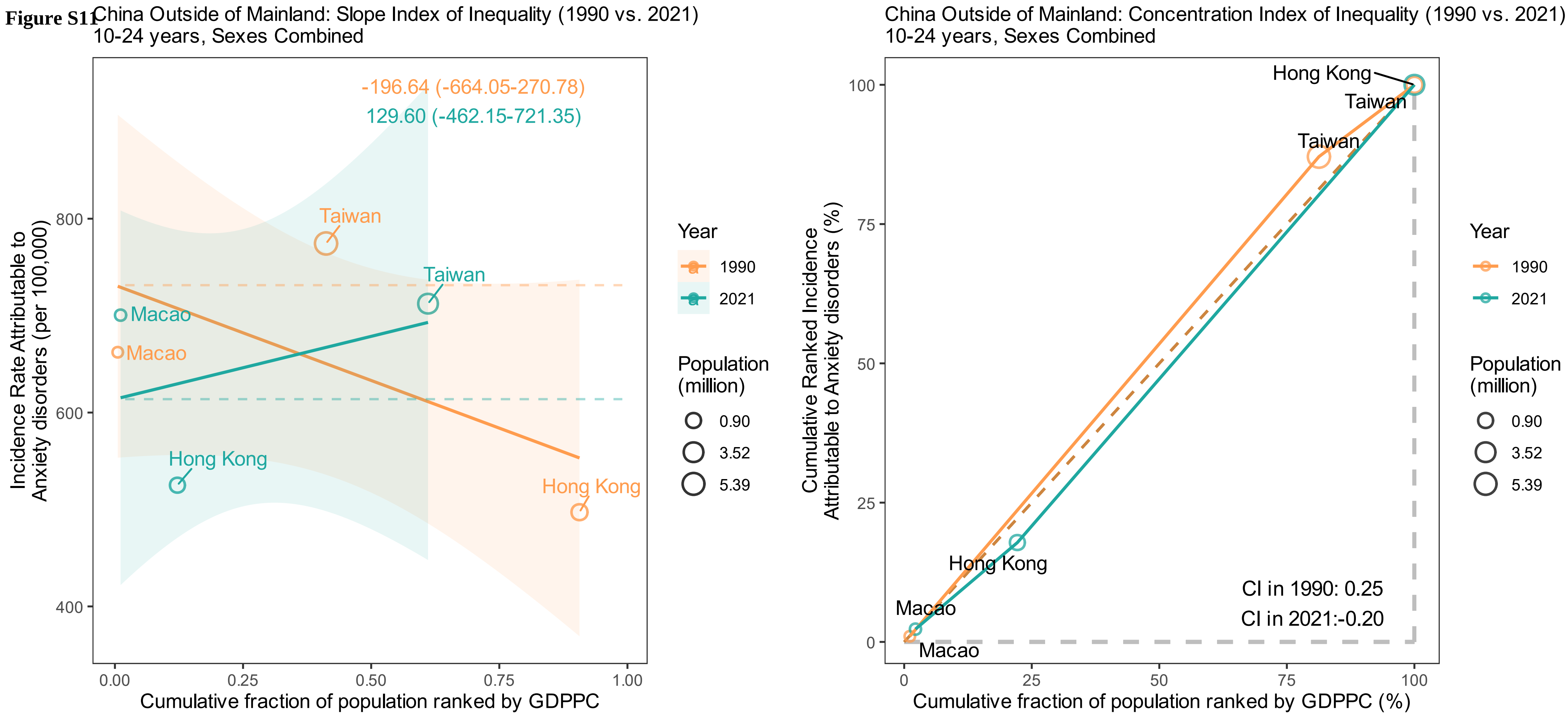
China Outside of Mainland: Slope Index of Inequality (1990 vs. 2021)
10-24 years, Sexes Combined
-196.64 (-664.05-270.78)
129.60 (-462.15-721.35)
Taiwan
Taiwan
Macao
Macao
Hong Kong
Hong Kong
Incidence Rate Attributable to Anxiety disorders (per 100,000)
Cumulative fraction of population ranked by GDPPC
Year
1990
2021
Population (million)
0.90
3.52
5.39
China Outside of Mainland: Concentration Index of Inequality (1990 vs. 2021)
10-24 years, Sexes Combined
Hong Kong
Taiwan
Taiwan
Hong Kong
Macao
Macao
CI in 1990: 0.25
CI in 2021:-0.20
Cumulative Ranked Incidence Attributable to Anxiety disorders (%)
Cumulative fraction of population ranked by GDPPC (%)

Figure S12

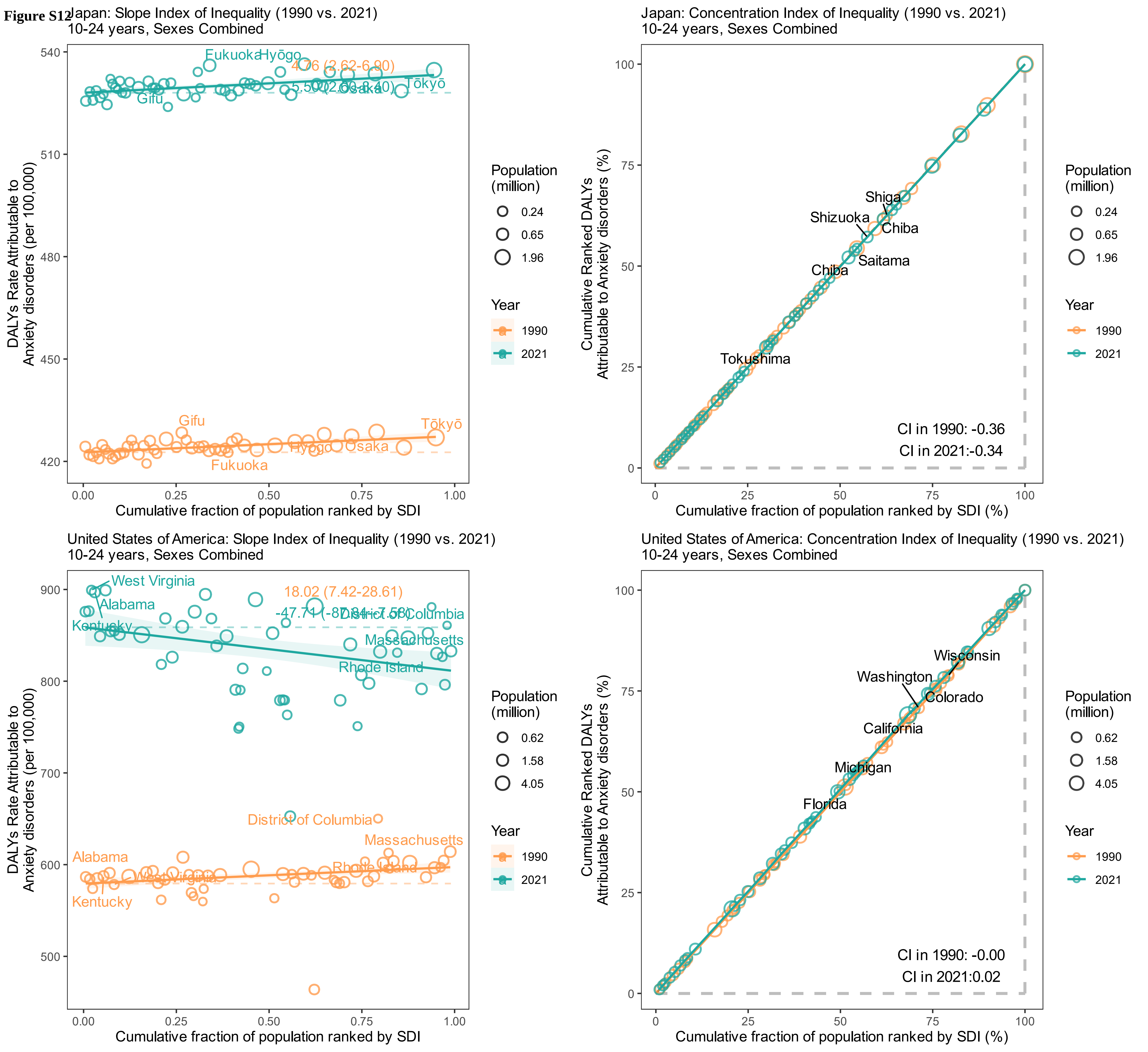
Japan: Slope Index of Inequality (1990 vs. 2021)
10-24 years, Sexes Combined
DALYs Rate Attributable to Anxiety disorders (per 100,000)
Cumulative fraction of population ranked by SDI
4.76 (2.62-6.90)
5.50 (2.60-8.40)
Fukuoka
Hyōgo
Tōkyō
Gifu
Ōsaka
Japan: Concentration Index of Inequality (1990 vs. 2021)
10-24 years, Sexes Combined
Cumulative Ranked DALYs Attributable to Anxiety disorders (%)
Cumulative fraction of population ranked by SDI (%)
Shiga
Shizuoka
Chiba
Saitama
Tokushima
CI in 1990: -0.36
CI in 2021:-0.34
Population (million)
0.24
0.65
1.96
Year
1990
2021
United States of America: Slope Index of Inequality (1990 vs. 2021)
10-24 years, Sexes Combined
DALYs Rate Attributable to Anxiety disorders (per 100,000)
Cumulative fraction of population ranked by SDI
18.02 (7.42-28.61)
-47.71 (-87.84--7.58)
West Virginia
Alabama
Kentucky
District of Columbia
Massachusetts
Rhode Island
United States of America: Concentration Index of Inequality (1990 vs. 2021)
10-24 years, Sexes Combined
Cumulative Ranked DALYs Attributable to Anxiety disorders (%)
Cumulative fraction of population ranked by SDI (%)
Wisconsin
Washington
Colorado
California
Michigan
Florida
CI in 1990: -0.00
CI in 2021:0.02
Population (million)
0.62
1.58
4.05
Year
1990
2021

Figure S12

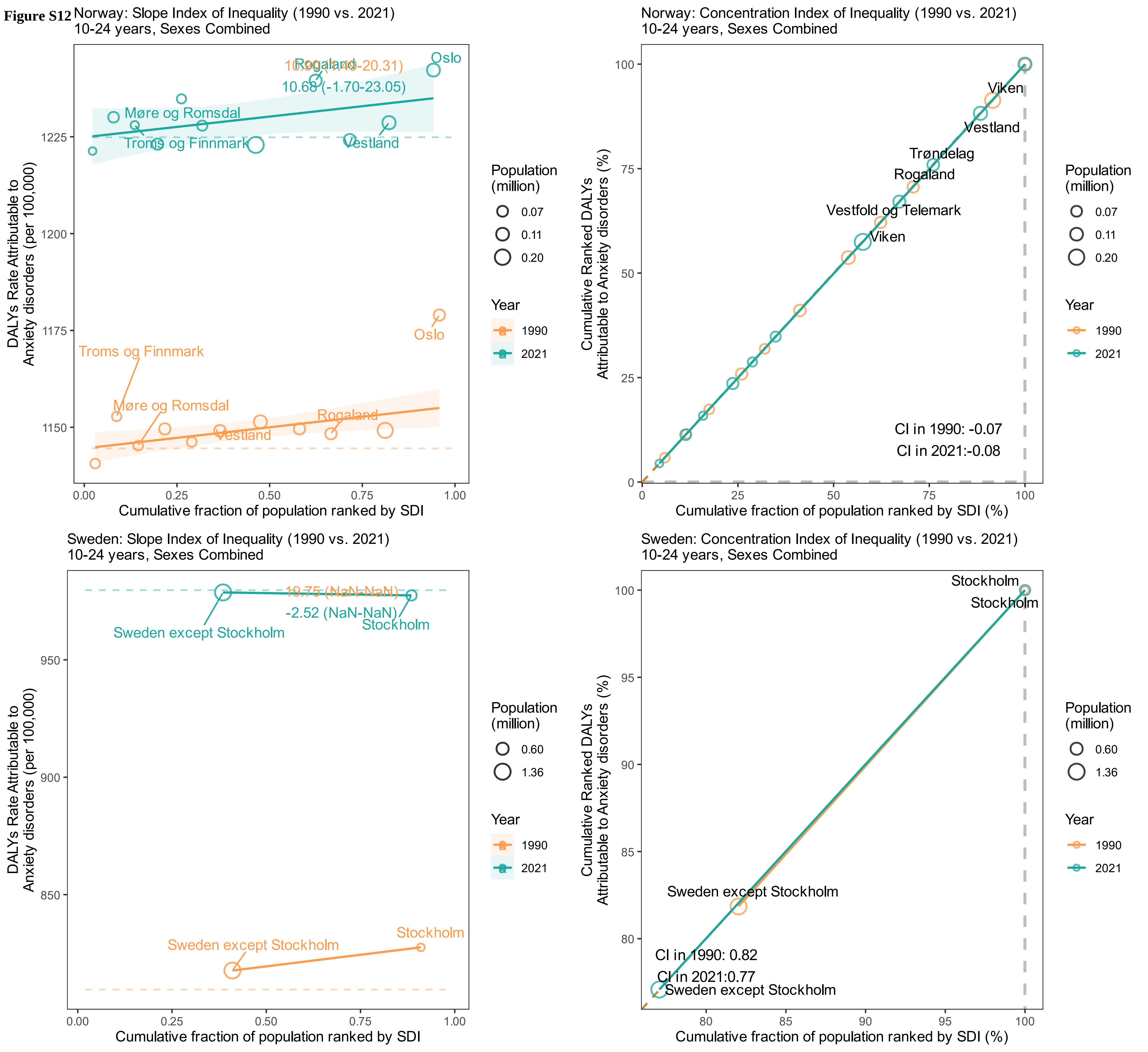

Norway: Slope Index of Inequality (1990 vs. 2021)
10-24 years, Sexes Combined
DALYs Rate Attributable to Anxiety disorders (per 100,000)
Cumulative fraction of population ranked by SDI
10.90 (1.49-20.31)
10.68 (-1.70-23.05)
Oslo
Rogaland
Møre og Romsdal
Troms og Finnmark
Vestland
Population (million)
0.07
0.11
0.20
Year
1990
2021
Norway: Concentration Index of Inequality (1990 vs. 2021)
10-24 years, Sexes Combined
Cumulative Ranked DALYs Attributable to Anxiety disorders (%)
Cumulative fraction of population ranked by SDI (%)
Viken
Vestland
Trøndelag
Rogaland
Vestfold og Telemark
CI in 1990: -0.07
CI in 2021:-0.08
Sweden: Slope Index of Inequality (1990 vs. 2021)
10-24 years, Sexes Combined
19.75 (NaN-NaN)
-2.52 (NaN-NaN)
Sweden except Stockholm
Stockholm
0.60
1.36
Sweden: Concentration Index of Inequality (1990 vs. 2021)
10-24 years, Sexes Combined
CI in 1990: 0.82
CI in 2021:0.77

Figure S12

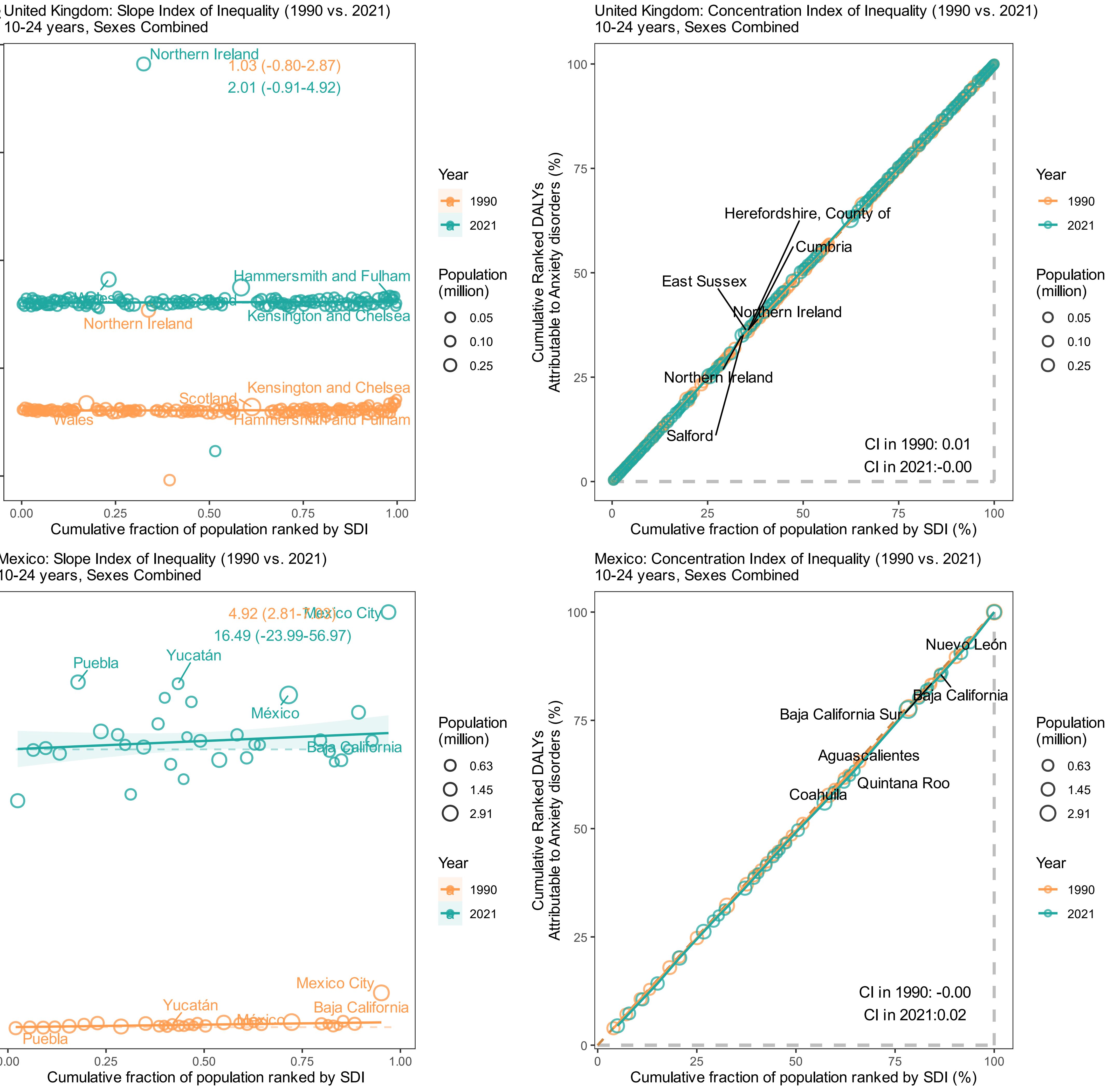
United Kingdom: Slope Index of Inequality (1990 vs. 2021)
10-24 years, Sexes Combined
DALYs Rate Attributable to Anxiety disorders (per 100,000)
Cumulative fraction of population ranked by SDI
1.03 (-0.80-2.87)
2.01 (-0.91-4.92)
Northern Ireland
Hammersmith and Fulham
Kensington and Chelsea
Scotland
Wales
Year
1990
2021
Population (million)
0.05
0.10
0.25
United Kingdom: Concentration Index of Inequality (1990 vs. 2021)
10-24 years, Sexes Combined
Cumulative Ranked DALYs Attributable to Anxiety disorders (%)
Cumulative fraction of population ranked by SDI (%)
Herefordshire, County of
Cumbria
East Sussex
Northern Ireland
Salford
CI in 1990: 0.01
CI in 2021:-0.00
Mexico: Slope Index of Inequality (1990 vs. 2021)
10-24 years, Sexes Combined
DALYs Rate Attributable to Anxiety disorders (per 100,000)
Cumulative fraction of population ranked by SDI
16.49 (-23.99-56.97)
Mexico City
Puebla
Yucatán
México
Baja California
Population (million)
0.63
1.45
2.91
Mexico: Concentration Index of Inequality (1990 vs. 2021)
10-24 years, Sexes Combined
Cumulative Ranked DALYs Attributable to Anxiety disorders (%)
Cumulative fraction of population ranked by SDI (%)
Nuevo León
Baja California
Baja California Sur
Aguascalientes
Quintana Roo
Coahuila
CI in 1990: -0.00
CI in 2021:0.02

Figure S12

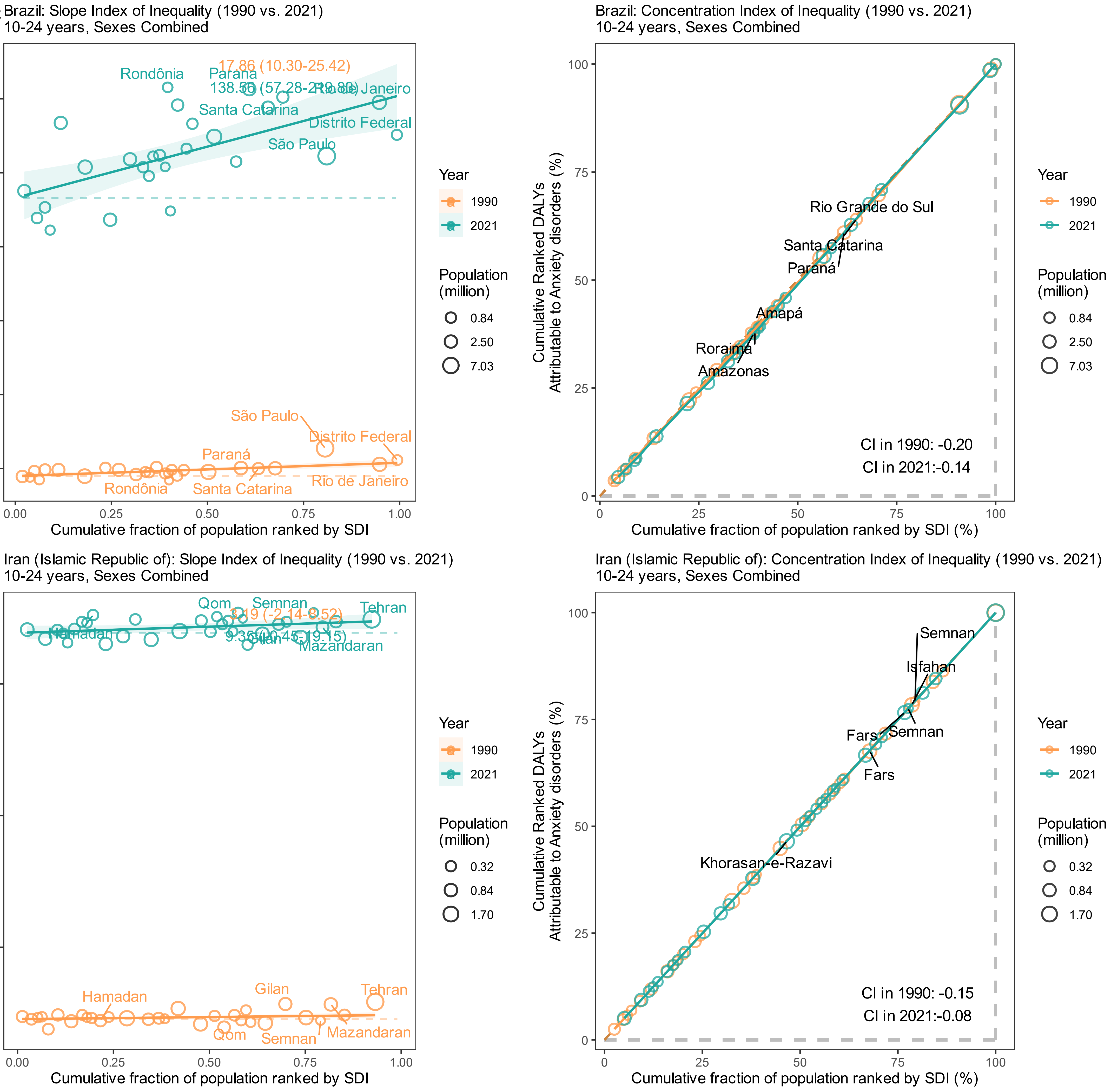
Brazil: Slope Index of Inequality (1990 vs. 2021)
10-24 years, Sexes Combined
17.86 (10.30-25.42)
Rondônia
Parana
Rio de Janeiro
Santa Catarina
Distrito Federal
São Paulo
São Paulo
Distrito Federal
Paraná
Rondônia
Santa Catarina
Rio de Janeiro
DALYs Rate Attributable to Anxiety disorders (per 100,000)
Cumulative fraction of population ranked by SDI
Year
1990
2021
Population (million)
0.84
2.50
7.03
Brazil: Concentration Index of Inequality (1990 vs. 2021)
10-24 years, Sexes Combined
Rio Grande do Sul
Santa Catarina
Paraná
Amapá
Roraima
Amazonas
CI in 1990: -0.20
CI in 2021:-0.14
Cumulative Ranked DALYs Attributable to Anxiety disorders (%)
Cumulative fraction of population ranked by SDI (%)
Iran (Islamic Republic of): Slope Index of Inequality (1990 vs. 2021)
10-24 years, Sexes Combined
Qom
Semnan
Tehran
Hamadan
Mazandaran
Hamadan
Gilan
Tehran
Qom
Semnan
Mazandaran
Population (million)
0.32
0.84
1.70
Iran (Islamic Republic of): Concentration Index of Inequality (1990 vs. 2021)
10-24 years, Sexes Combined
Semnan
Isfahan
Fars
Semnan
Fars
Khorasan-e-Razavi
CI in 1990: -0.15
CI in 2021:-0.08

Figure S12

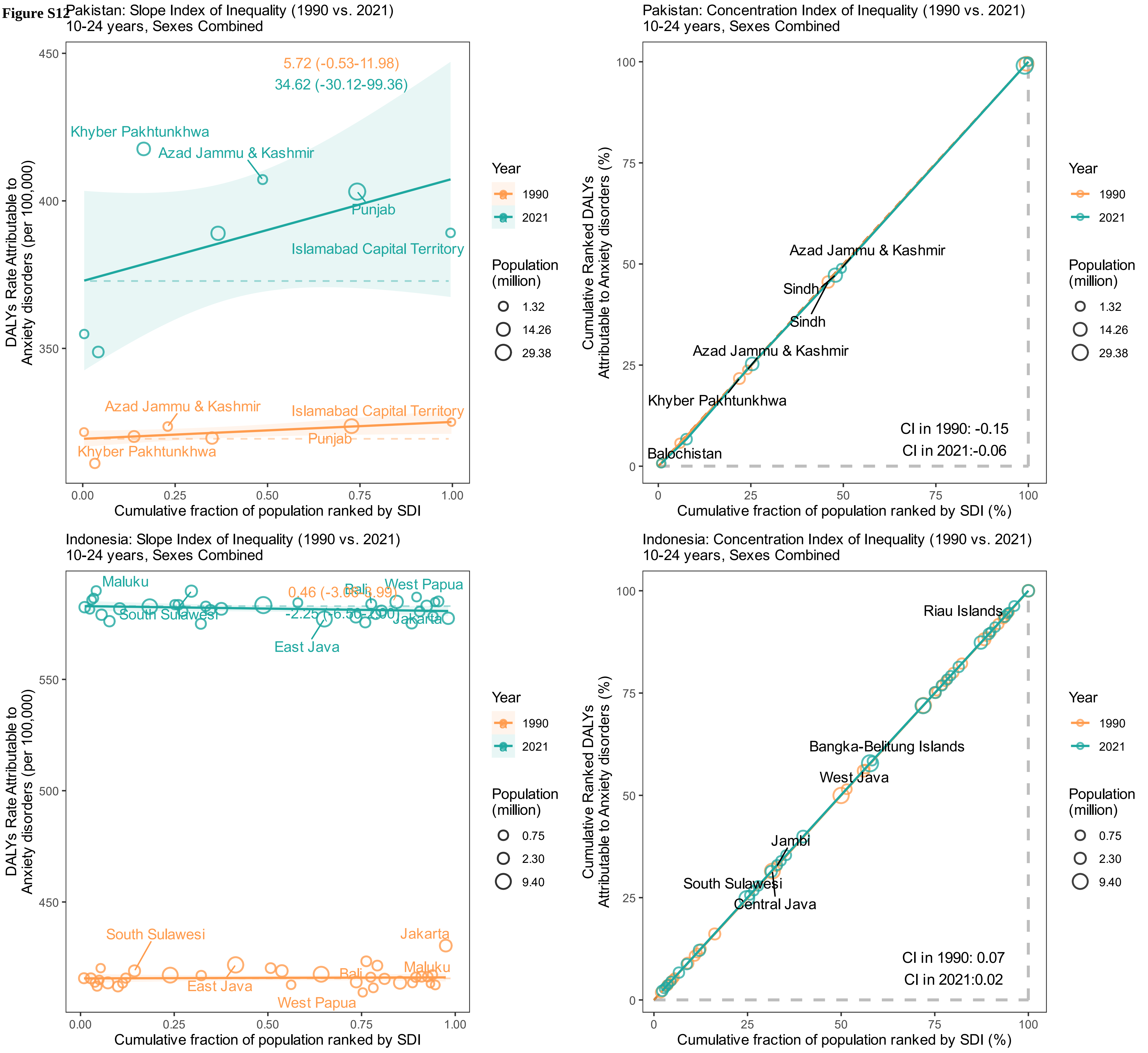

Pakistan: Slope Index of Inequality (1990 vs. 2021)
10-24 years, Sexes Combined
5.72 (-0.53-11.98)
34.62 (-30.12-99.36)
Khyber Pakhtunkhwa
Azad Jammu & Kashmir
Punjab
Islamabad Capital Territory
Azad Jammu & Kashmir
Islamabad Capital Territory
Punjab
Khyber Pakhtunkhwa
DALYs Rate Attributable to Anxiety disorders (per 100,000)
Cumulative fraction of population ranked by SDI
Year
1990
2021
Population (million)
1.32
14.26
29.38
Pakistan: Concentration Index of Inequality (1990 vs. 2021)
10-24 years, Sexes Combined
Azad Jammu & Kashmir
Sindh
Sindh
Azad Jammu & Kashmir
Khyber Pakhtunkhwa
Balochistan
CI in 1990: -0.15
CI in 2021:-0.06
Cumulative Ranked DALYs Attributable to Anxiety disorders (%)
Cumulative fraction of population ranked by SDI (%)
Indonesia: Slope Index of Inequality (1990 vs. 2021)
10-24 years, Sexes Combined
Maluku
Bali
West Papua
0.46 (-3.08-3.99)
-2.25 (-6.50-2.00)
South Sulawesi
Jakarta
East Java
South Sulawesi
Jakarta
Maluku
Bali
East Java
West Papua
DALYs Rate Attributable to Anxiety disorders (per 100,000)
Cumulative fraction of population ranked by SDI
Population (million)
0.75
2.30
9.40
Indonesia: Concentration Index of Inequality (1990 vs. 2021)
10-24 years, Sexes Combined
Riau Islands
Bangka-Belitung Islands
West Java
Jambi
South Sulawesi
Central Java
CI in 1990: 0.07
CI in 2021:0.02
Cumulative Ranked DALYs Attributable to Anxiety disorders (%)
Cumulative fraction of population ranked by SDI (%)

Figure S12

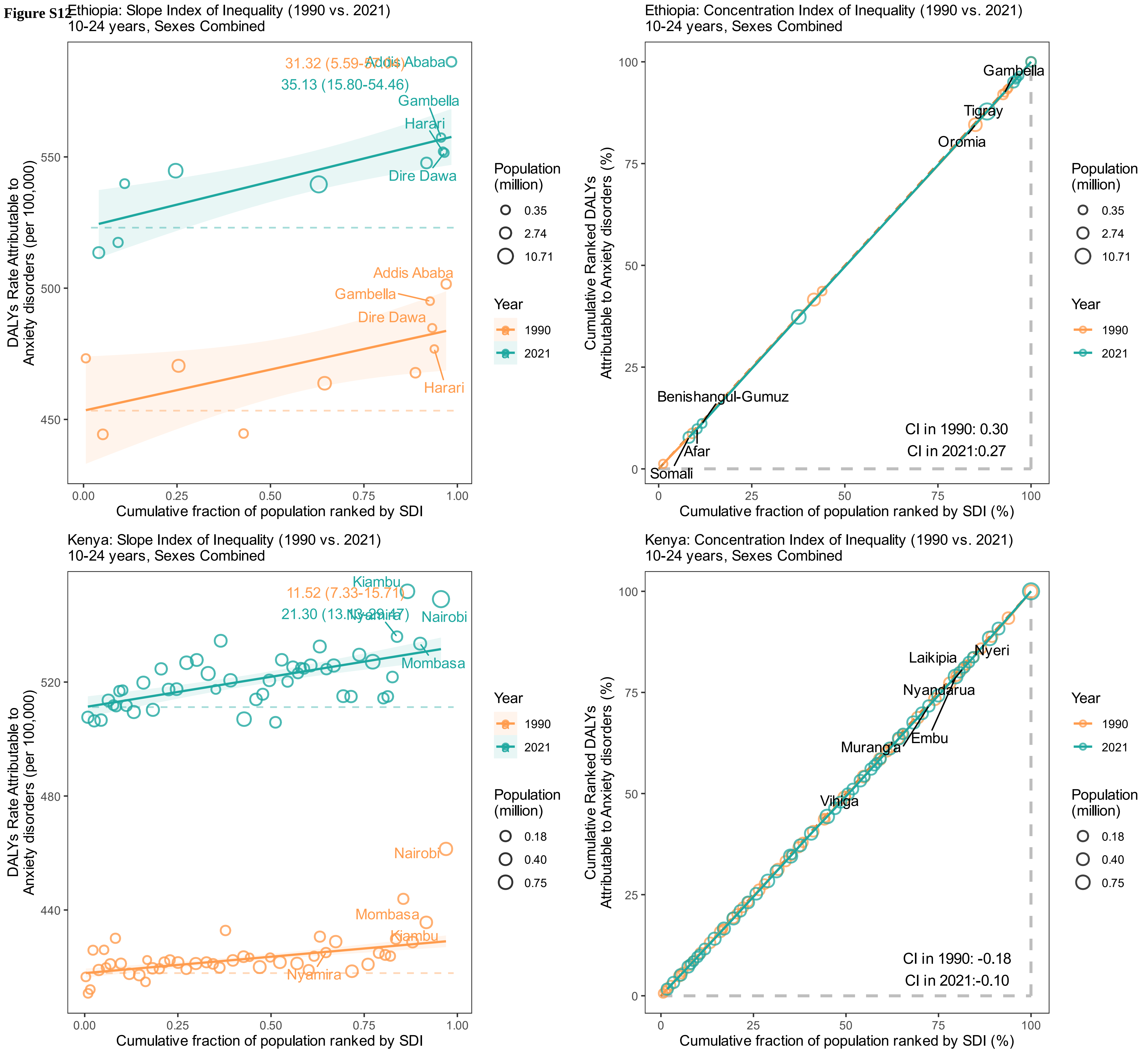

Ethiopia: Slope Index of Inequality (1990 vs. 2021)
10-24 years, Sexes Combined
31.32 (5.59-57.04)
35.13 (15.80-54.46)
Addis Ababa
Gambella
Harari
Dire Dawa
DALYs Rate Attributable to Anxiety disorders (per 100,000)
Cumulative fraction of population ranked by SDI
Population (million)
0.35
2.74
10.71
Year
1990
2021
Ethiopia: Concentration Index of Inequality (1990 vs. 2021)
10-24 years, Sexes Combined
Gambella
Tigray
Oromia
Benishangul-Gumuz
Afar
Somali
CI in 1990: 0.30
CI in 2021:0.27
Cumulative Ranked DALYs Attributable to Anxiety disorders (%)
Cumulative fraction of population ranked by SDI (%)
Kenya: Slope Index of Inequality (1990 vs. 2021)
10-24 years, Sexes Combined
11.52 (7.33-15.71)
21.30 (13.13-29.47)
Kiambu
Nyamira
Nairobi
Mombasa
DALYs Rate Attributable to Anxiety disorders (per 100,000)
Cumulative fraction of population ranked by SDI
Year
1990
2021
Population (million)
0.18
0.40
0.75
Kenya: Concentration Index of Inequality (1990 vs. 2021)
10-24 years, Sexes Combined
Laikipia
Nyeri
Nyandarua
Embu
Murang'a
Vihiga
CI in 1990: -0.18
CI in 2021:-0.10
Cumulative Ranked DALYs Attributable to Anxiety disorders (%)
Cumulative fraction of population ranked by SDI (%)

Figure S12

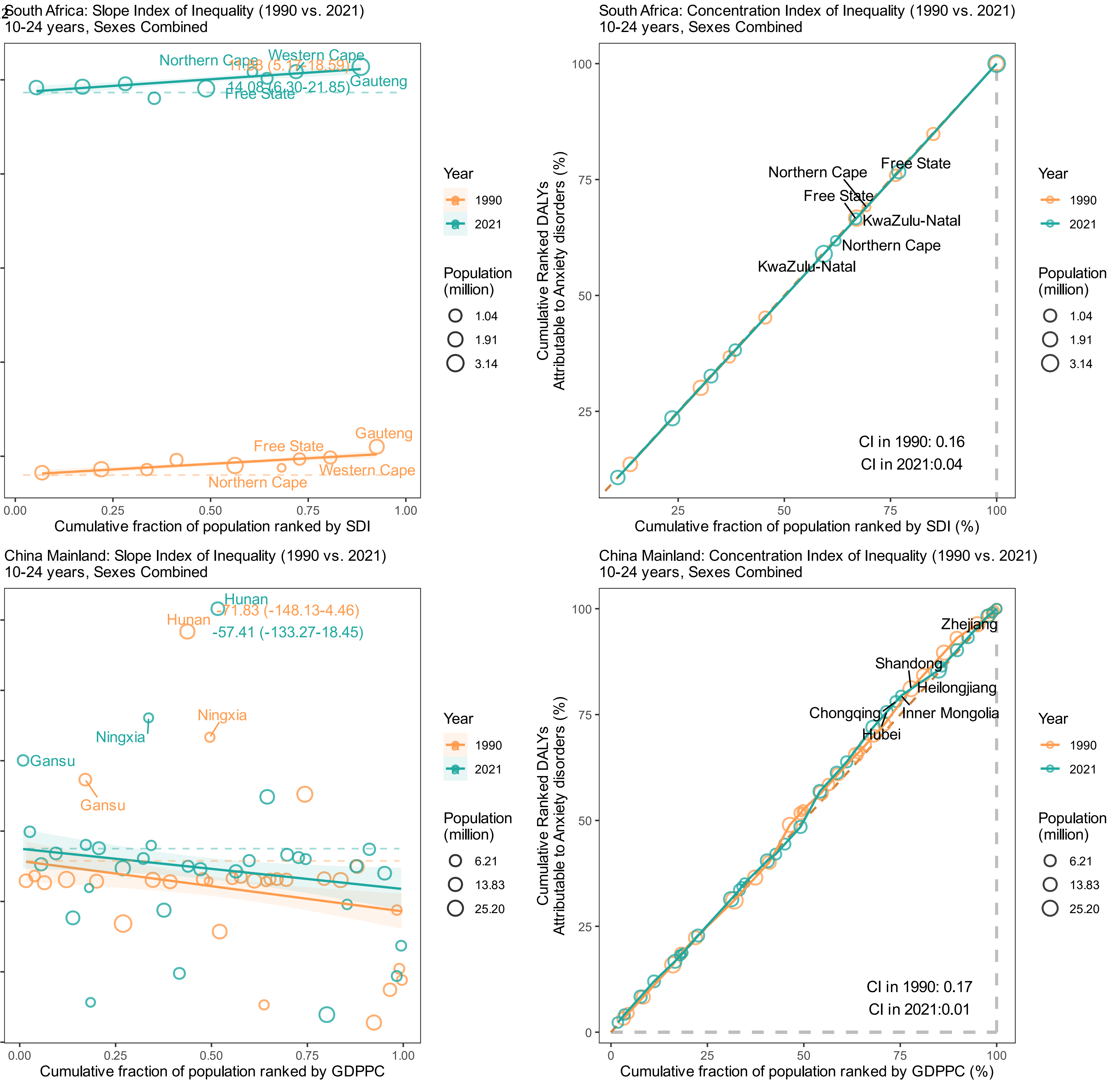
South Africa: Slope Index of Inequality (1990 vs. 2021)
10-24 years, Sexes Combined
DALYs Rate Attributable to Anxiety disorders (per 100,000)
Cumulative fraction of population ranked by SDI
Northern Cape
Western Cape
11.88 (5.17-18.59)
14.08 (6.30-21.85)
Gauteng
Free State
Free State
Western Cape
Northern Cape
Year
1990
2021
Population (million)
1.04
1.91
3.14
South Africa: Concentration Index of Inequality (1990 vs. 2021)
10-24 years, Sexes Combined
Cumulative Ranked DALYs Attributable to Anxiety disorders (%)
Cumulative fraction of population ranked by SDI (%)
Free State
Northern Cape
Free State
KwaZulu-Natal
Northern Cape
KwaZulu-Natal
CI in 1990: 0.16
CI in 2021:0.04
China Mainland: Slope Index of Inequality (1990 vs. 2021)
10-24 years, Sexes Combined
DALYs Rate Attributable to Anxiety disorders (per 100,000)
Cumulative fraction of population ranked by GDPPC
Hunan
-71.83 (-148.13-4.46)
Hunan
-57.41 (-133.27-18.45)
Ningxia
Ningxia
Gansu
Gansu
Population (million)
6.21
13.83
25.20
China Mainland: Concentration Index of Inequality (1990 vs. 2021)
10-24 years, Sexes Combined
Cumulative Ranked DALYs Attributable to Anxiety disorders (%)
Cumulative fraction of population ranked by GDPPC (%)
Zhejiang
Shandong
Heilongjiang
Chongqing
Inner Mongolia
Hubei
CI in 1990: 0.17
CI in 2021:0.01

Figure S12

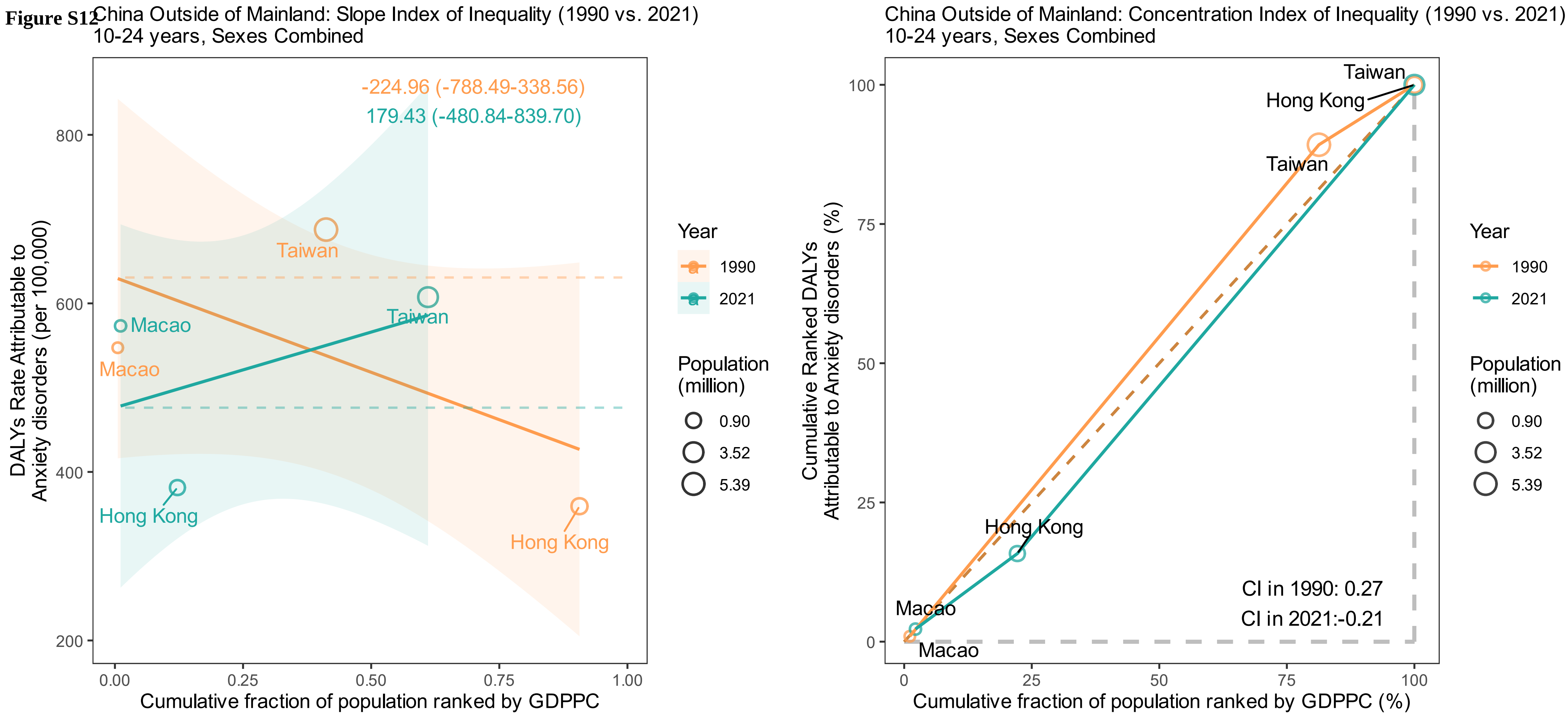

China Outside of Mainland: Slope Index of Inequality (1990 vs. 2021)
10-24 years, Sexes Combined
-224.96 (-788.49-338.56)
179.43 (-480.84-839.70)
Taiwan
Taiwan
Macao
Macao
Hong Kong
Hong Kong
DALYs Rate Attributable to Anxiety disorders (per 100,000)
Cumulative fraction of population ranked by GDPPC
Year
1990
2021
Population (million)
0.90
3.52
5.39
China Outside of Mainland: Concentration Index of Inequality (1990 vs. 2021)
10-24 years, Sexes Combined
Taiwan
Hong Kong
Taiwan
Hong Kong
Macao
Macao
CI in 1990: 0.27
CI in 2021:-0.21
Cumulative Ranked DALYs Attributable to Anxiety disorders (%)
Cumulative fraction of population ranked by GDPPC (%)

Figure S13

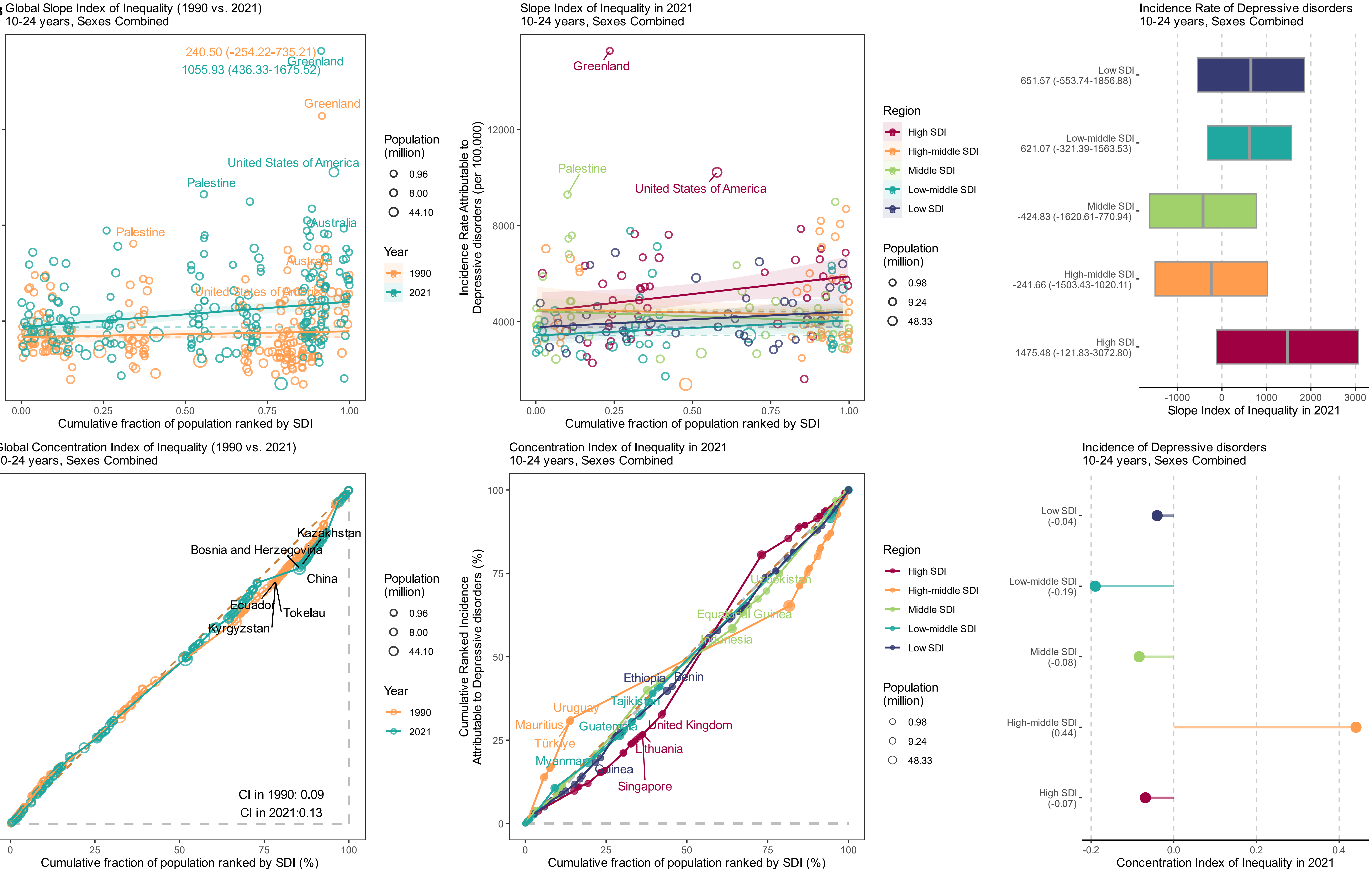
Global Slope Index of Inequality (1990 vs. 2021)
10-24 years, Sexes Combined
240.50 (-254.22-735.21)
1055.93 (436.33-1675.52)
Incidence Rate Attributable to Depressive disorders (per 100,000)
Cumulative fraction of population ranked by SDI
Population (million)
0.96
8.00
44.10
Year
1990
2021
Slope Index of Inequality in 2021
10-24 years, Sexes Combined
Region
High SDI
High-middle SDI
Middle SDI
Low-middle SDI
Low SDI
0.98
9.24
48.33
Incidence Rate of Depressive disorders
10-24 years, Sexes Combined
Low SDI 651.57 (-553.74-1856.88)
Low-middle SDI 621.07 (-321.39-1563.53)
Middle SDI -424.83 (-1620.61-770.94)
High-middle SDI -241.66 (-1503.43-1020.11)
High SDI 1475.48 (-121.83-3072.80)
Slope Index of Inequality in 2021
Global Concentration Index of Inequality (1990 vs. 2021)
10-24 years, Sexes Combined
Cumulative Ranked Incidence Attributable to Depressive disorders (%)
Cumulative fraction of population ranked by SDI (%)
CI in 1990: 0.09
CI in 2021:0.13
Concentration Index of Inequality in 2021
10-24 years, Sexes Combined
Incidence of Depressive disorders
10-24 years, Sexes Combined
Low SDI (-0.04)
Low-middle SDI (-0.19)
Middle SDI (-0.08)
High-middle SDI (0.44)
High SDI (-0.07)
Concentration Index of Inequality in 2021

Figure S13

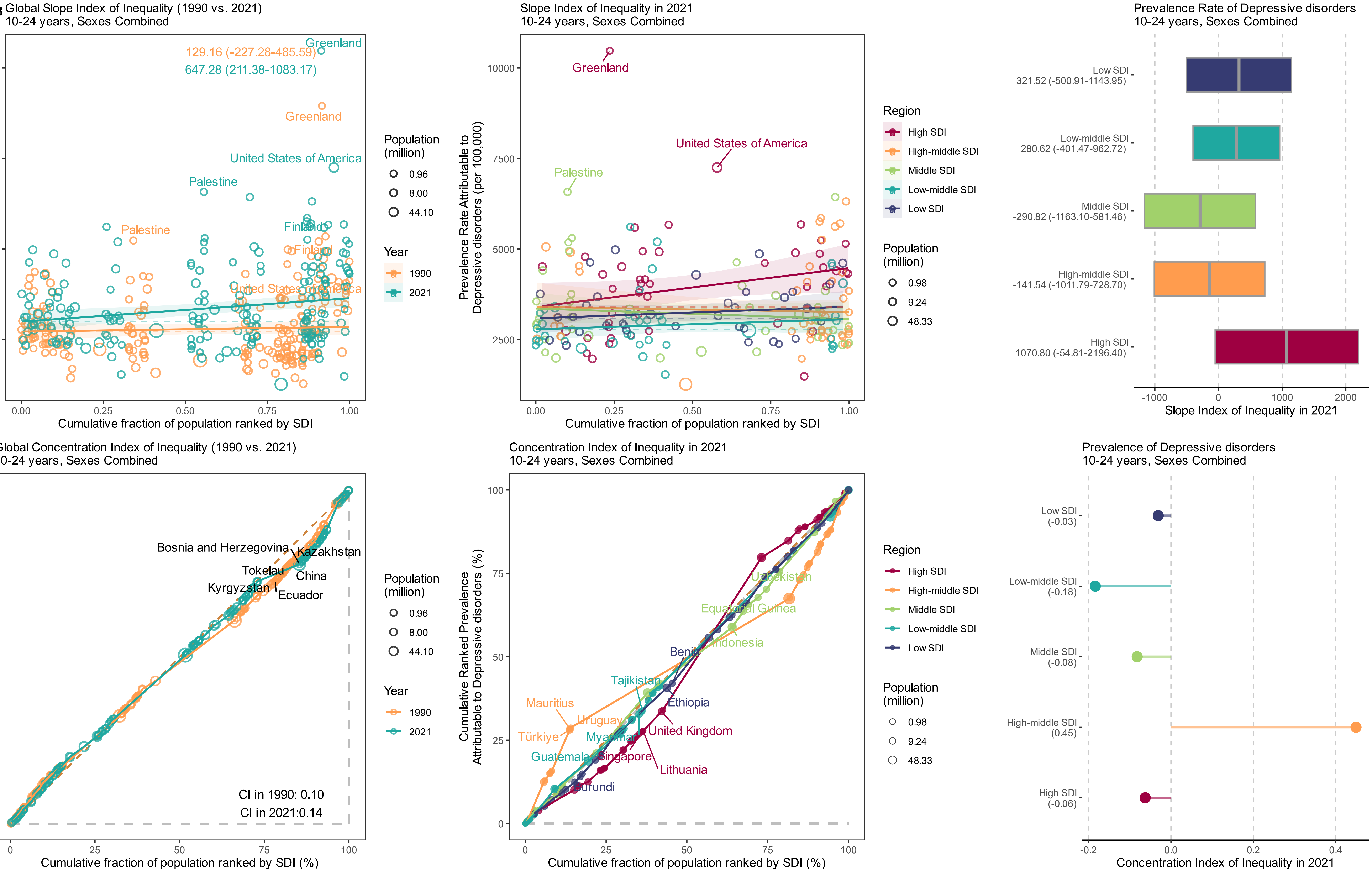

Global Slope Index of Inequality (1990 vs. 2021)
10-24 years, Sexes Combined
129.16 (-227.28-485.59)
647.28 (211.38-1083.17)
Greenland
United States of America
Palestine
Finland
Prevalence Rate Attributable to Depressive disorders (per 100,000)
Cumulative fraction of population ranked by SDI
Population (million)
0.96
8.00
44.10
Year
1990
2021
Slope Index of Inequality in 2021
10-24 years, Sexes Combined
Region
High SDI
High-middle SDI
Middle SDI
Low-middle SDI
Low SDI
Population (million)
0.98
9.24
48.33
Prevalence Rate of Depressive disorders
10-24 years, Sexes Combined
Low SDI
321.52 (-500.91-1143.95)
Low-middle SDI
280.62 (-401.47-962.72)
Middle SDI
-290.82 (-1163.10-581.46)
High-middle SDI
-141.54 (-1011.79-728.70)
High SDI
1070.80 (-54.81-2196.40)
Slope Index of Inequality in 2021
Global Concentration Index of Inequality (1990 vs. 2021)
10-24 years, Sexes Combined
Bosnia and Herzegovina
Kazakhstan
Tokelau
China
Kyrgyzstan
Ecuador
CI in 1990: 0.10
CI in 2021:0.14
Cumulative Ranked Prevalence Attributable to Depressive disorders (%)
Cumulative fraction of population ranked by SDI (%)
Concentration Index of Inequality in 2021
10-24 years, Sexes Combined
Uzbekistan
Equatorial Guinea
Indonesia
Benin
Tajikistan
Ethiopia
Mauritius
Uruguay
Türkiye
Myanmar
United Kingdom
Guatemala
Singapore
Lithuania
Burundi
Prevalence of Depressive disorders
10-24 years, Sexes Combined
Low SDI (-0.03)
Low-middle SDI (-0.18)
Middle SDI (-0.08)
High-middle SDI (0.45)
High SDI (-0.06)
Concentration Index of Inequality in 2021

Figure S13

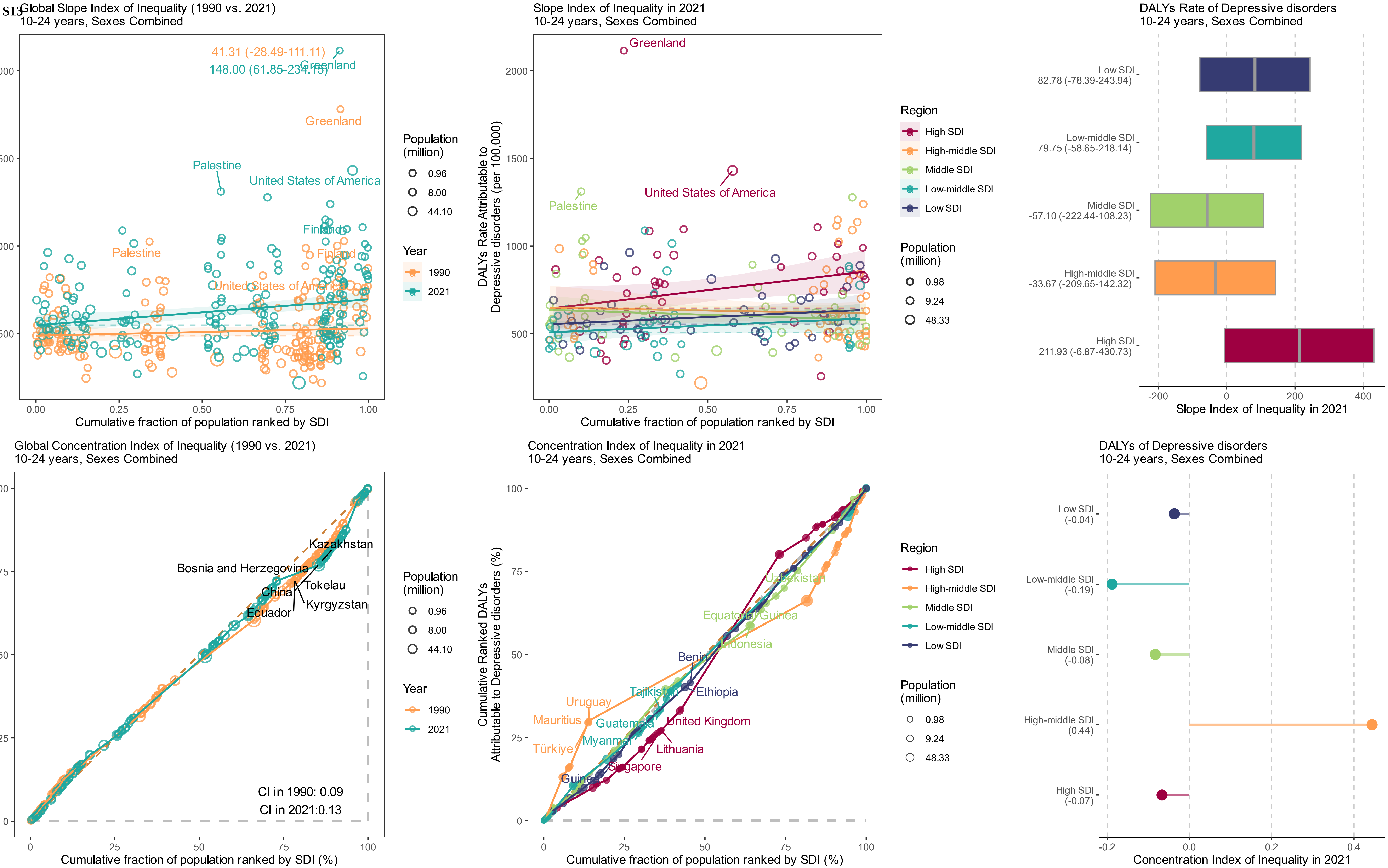

Global Slope Index of Inequality (1990 vs. 2021)
10-24 years, Sexes Combined
41.31 (-28.49-111.11)
148.00 (61.85-234.15)
Greenland
Greenland
Palestine
United States of America
Finland
Palestine
Finland
United States of America
DALYs Rate Attributable to Depressive disorders (per 100,000)
Cumulative fraction of population ranked by SDI
Population (million)
0.96
8.00
44.10
Year
1990
2021
Slope Index of Inequality in 2021
10-24 years, Sexes Combined
Greenland
United States of America
Palestine
DALYs Rate Attributable to Depressive disorders (per 100,000)
Cumulative fraction of population ranked by SDI
Region
High SDI
High-middle SDI
Middle SDI
Low-middle SDI
Low SDI
Population (million)
0.98
9.24
48.33
DALYs Rate of Depressive disorders
10-24 years, Sexes Combined
Low SDI
82.78 (-78.39-243.94)
Low-middle SDI
79.75 (-58.65-218.14)
Middle SDI
-57.10 (-222.44-108.23)
High-middle SDI
-33.67 (-209.65-142.32)
High SDI
211.93 (-6.87-430.73)
Slope Index of Inequality in 2021
Global Concentration Index of Inequality (1990 vs. 2021)
10-24 years, Sexes Combined
Kazakhstan
Bosnia and Herzegovina
Tokelau
China
Kyrgyzstan
Ecuador
CI in 1990: 0.09
CI in 2021:0.13
Cumulative Ranked DALYs Attributable to Depressive disorders (%)
Cumulative fraction of population ranked by SDI (%)
Population (million)
0.96
8.00
44.10
Year
1990
2021
Concentration Index of Inequality in 2021
10-24 years, Sexes Combined
Uzbekistan
Equatorial Guinea
Indonesia
Benin
Ethiopia
Tajikistan
Uruguay
Mauritius
Guatemala
United Kingdom
Myanmar
Türkiye
Lithuania
Singapore
Guinea
Cumulative Ranked DALYs Attributable to Depressive disorders (%)
Cumulative fraction of population ranked by SDI (%)
Region
High SDI
High-middle SDI
Middle SDI
Low-middle SDI
Low SDI
Population (million)
0.98
9.24
48.33
DALYs of Depressive disorders
10-24 years, Sexes Combined
Low SDI
(-0.04)
Low-middle SDI
(-0.19)
Middle SDI
(-0.08)
High-middle SDI
(0.44)
High SDI
(-0.07)
Concentration Index of Inequality in 2021

Figure S14

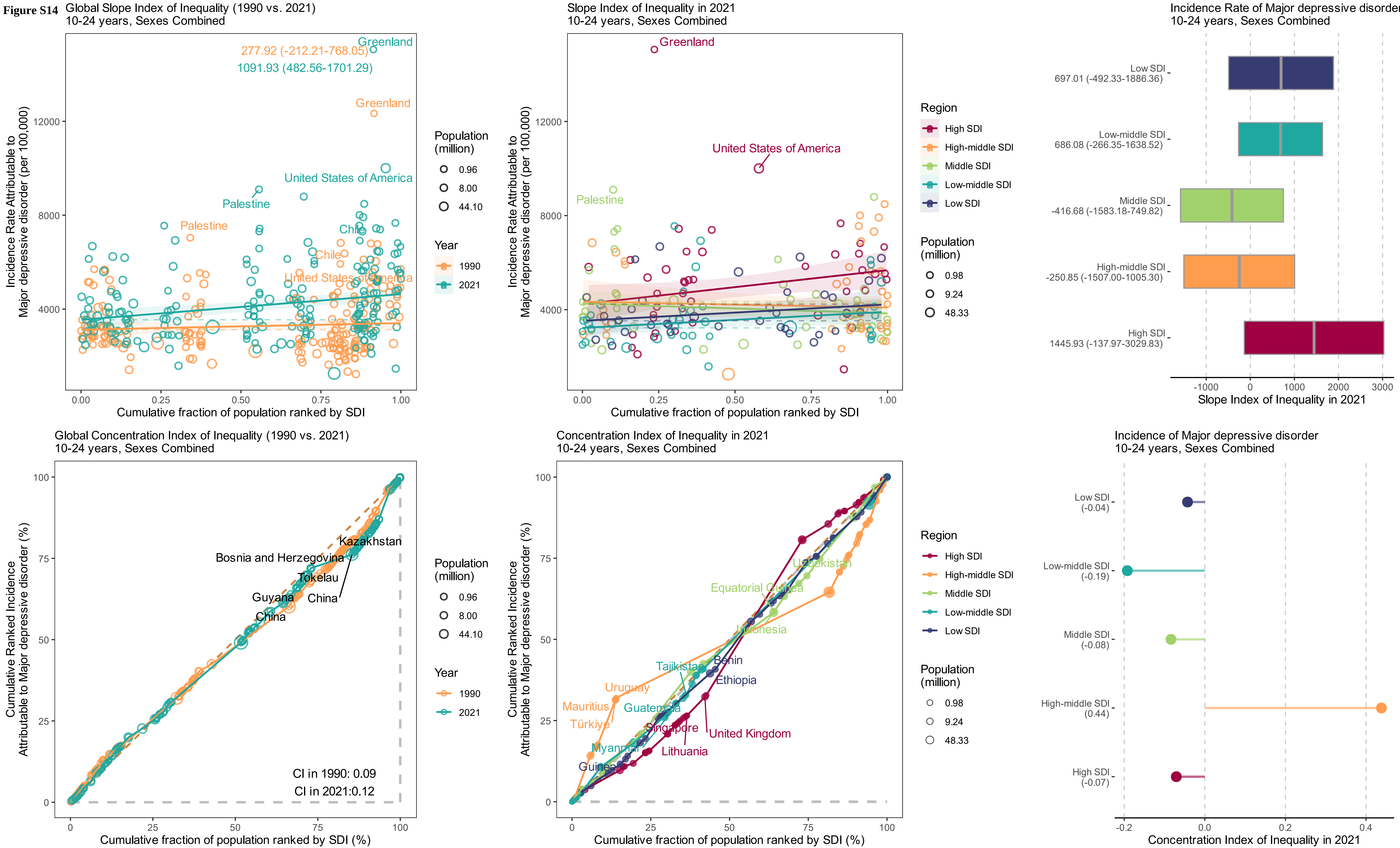
Global Slope Index of Inequality (1990 vs. 2021)
10-24 years, Sexes Combined
277.92 (-212.21-768.05)
1091.93 (482.56-1701.29)
Incidence Rate Attributable to Major depressive disorder (per 100,000)
Cumulative fraction of population ranked by SDI
Slope Index of Inequality in 2021
10-24 years, Sexes Combined
Incidence Rate of Major depressive disorder
10-24 years, Sexes Combined
Low SDI
697.01 (-492.33-1886.36)
Low-middle SDI
686.08 (-266.35-1638.52)
Middle SDI
-416.68 (-1583.18-749.82)
High-middle SDI
-250.85 (-1507.00-1005.30)
High SDI
1445.93 (-137.97-3029.83)
Slope Index of Inequality in 2021
Global Concentration Index of Inequality (1990 vs. 2021)
10-24 years, Sexes Combined
Cumulative Ranked Incidence Attributable to Major depressive disorder (%)
Cumulative fraction of population ranked by SDI (%)
CI in 1990: 0.09
CI in 2021:0.12
Concentration Index of Inequality in 2021
10-24 years, Sexes Combined
Incidence of Major depressive disorder
10-24 years, Sexes Combined
Low SDI (-0.04)
Low-middle SDI (-0.19)
Middle SDI (-0.08)
High-middle SDI (0.44)
High SDI (-0.07)
Concentration Index of Inequality in 2021
Population (million)
Year
1990
2021
Region
High SDI
High-middle SDI
Middle SDI
Low-middle SDI
Low SDI

Figure S14

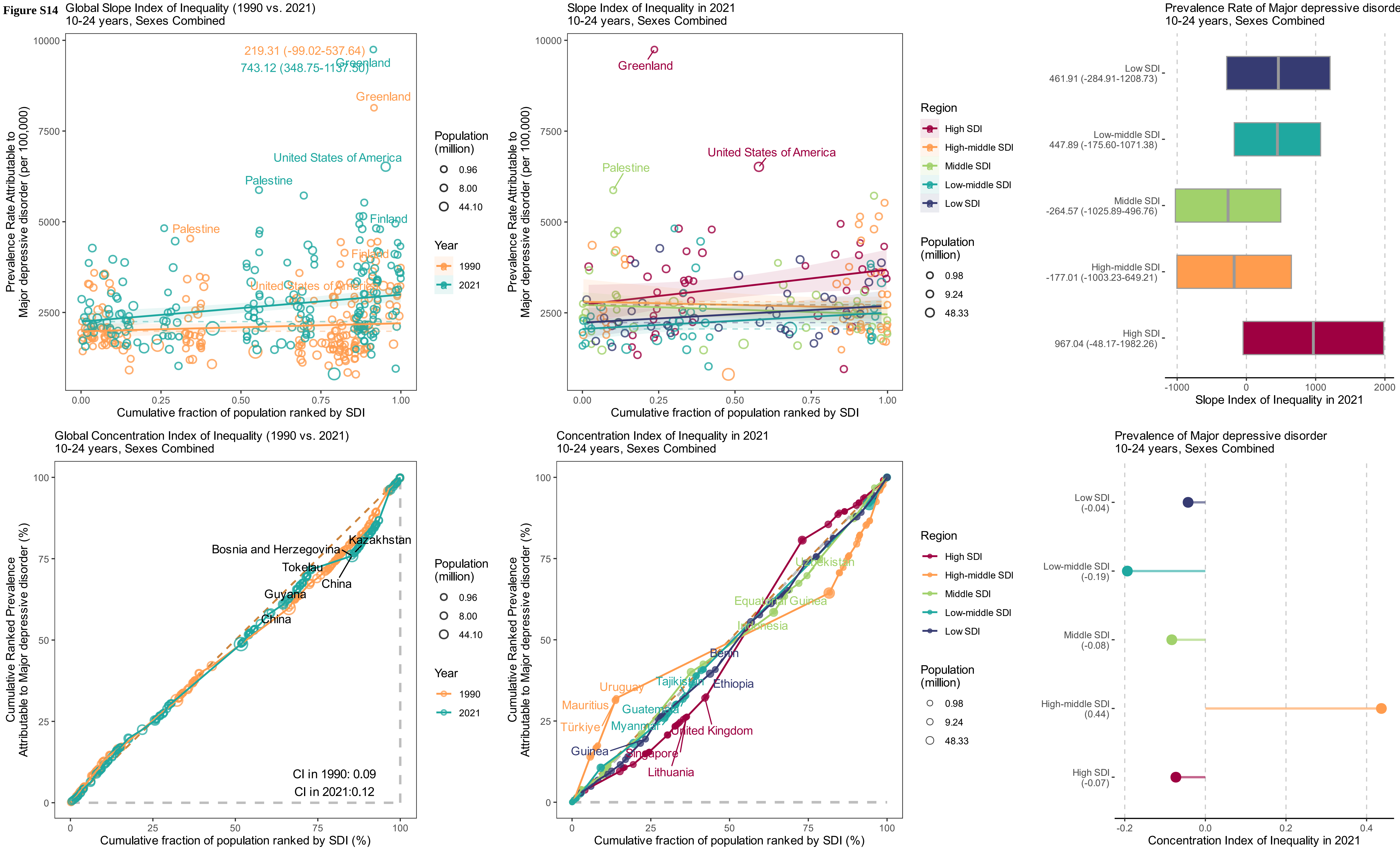

Global Slope Index of Inequality (1990 vs. 2021)
10-24 years, Sexes Combined
219.31 (-99.02-537.64)
743.12 (348.75-1137.50)
Greenland
United States of America
Palestine
Finland
Population (million)
0.96
8.00
44.10
Year
1990
2021
Prevalence Rate Attributable to Major depressive disorder (per 100,000)
Cumulative fraction of population ranked by SDI
Slope Index of Inequality in 2021
10-24 years, Sexes Combined
Region
High SDI
High-middle SDI
Middle SDI
Low-middle SDI
Low SDI
0.98
9.24
48.33
Prevalence Rate of Major depressive disorder
Low SDI 461.91 (-284.91-1208.73)
Low-middle SDI 447.89 (-175.60-1071.38)
Middle SDI -264.57 (-1025.89-496.76)
High-middle SDI -177.01 (-1003.23-649.21)
High SDI 967.04 (-48.17-1982.26)
Slope Index of Inequality in 2021
Global Concentration Index of Inequality (1990 vs. 2021)
Bosnia and Herzegovina
Kazakhstan
Tokelau
China
Guyana
CI in 1990: 0.09
CI in 2021:0.12
Cumulative Ranked Prevalence Attributable to Major depressive disorder (%)
Cumulative fraction of population ranked by SDI (%)
Concentration Index of Inequality in 2021
Uzbekistan
Equatorial Guinea
Indonesia
Benin
Ethiopia
Uruguay
Tajikistan
Mauritius
Guatemala
Türkiye
Myanmar
United Kingdom
Guinea
Singapore
Lithuania
Prevalence of Major depressive disorder
Low SDI (-0.04)
Low-middle SDI (-0.19)
Middle SDI (-0.08)
High-middle SDI (0.44)
High SDI (-0.07)
Concentration Index of Inequality in 2021

Figure S14

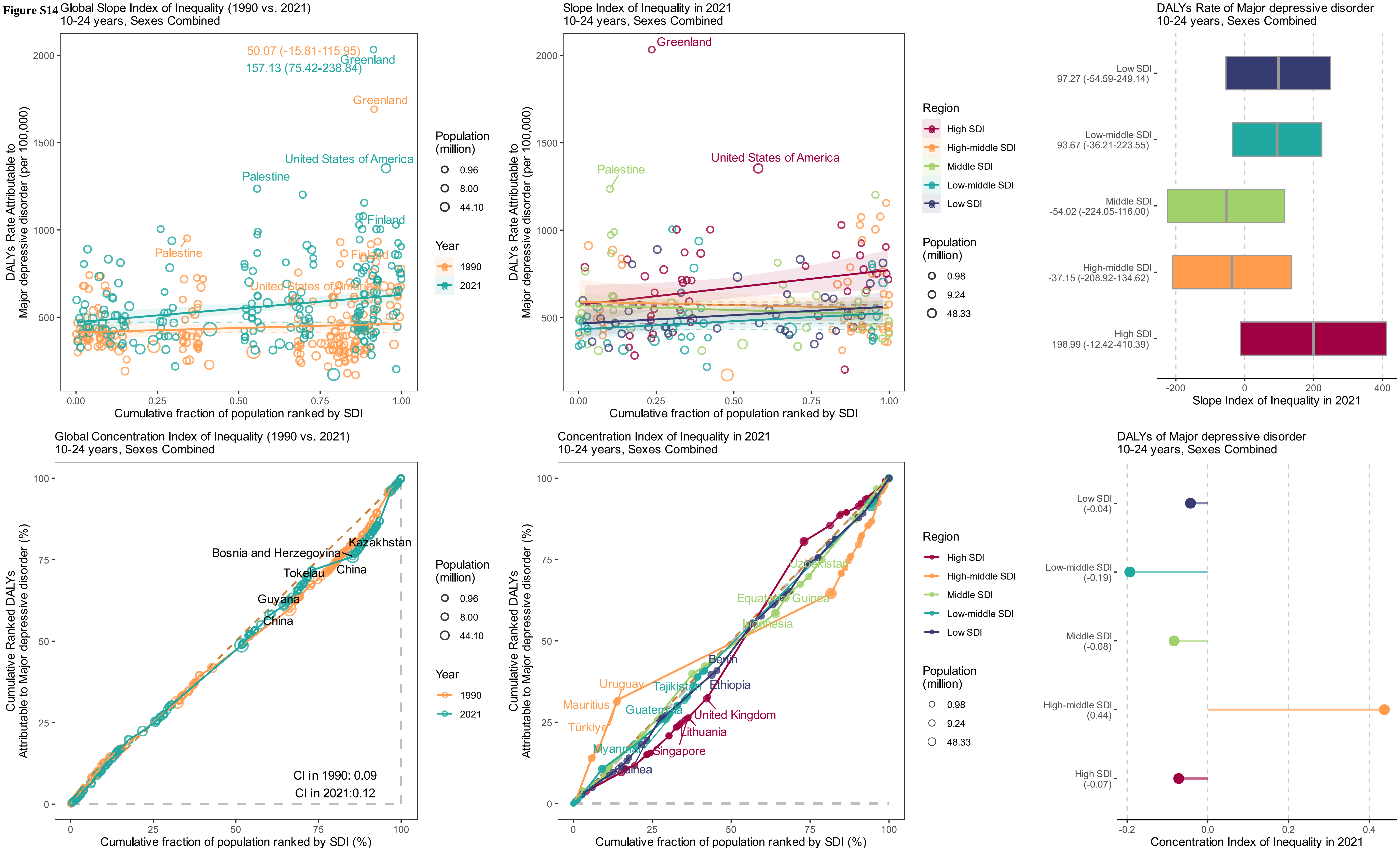
Global Slope Index of Inequality (1990 vs. 2021)
10-24 years, Sexes Combined
50.07 (-15.81-115.95)
157.13 (75.42-238.84)
Greenland
United States of America
Palestine
Finland
DALYs Rate Attributable to Major depressive disorder (per 100,000)
Cumulative fraction of population ranked by SDI
Population (million)
0.96
8.00
44.10
Year
1990
2021
Slope Index of Inequality in 2021
10-24 years, Sexes Combined
Region
High SDI
High-middle SDI
Middle SDI
Low-middle SDI
Low SDI
Population (million)
0.98
9.24
48.33
DALYs Rate of Major depressive disorder
10-24 years, Sexes Combined
Low SDI
97.27 (-54.59-249.14)
Low-middle SDI
93.67 (-36.21-223.55)
Middle SDI
-54.02 (-224.05-116.00)
High-middle SDI
-37.15 (-208.92-134.62)
High SDI
198.99 (-12.42-410.39)
Slope Index of Inequality in 2021
Global Concentration Index of Inequality (1990 vs. 2021)
10-24 years, Sexes Combined
Bosnia and Herzegovina
Kazakhstan
China
Tokelau
Guyana
CI in 1990: 0.09
CI in 2021:0.12
Cumulative Ranked DALYs Attributable to Major depressive disorder (%)
Cumulative fraction of population ranked by SDI (%)
Concentration Index of Inequality in 2021
10-24 years, Sexes Combined
Uzbekistan
Equatorial Guinea
Indonesia
Benin
Ethiopia
Uruguay
Tajikistan
Mauritius
Guatemala
United Kingdom
Türkiye
Lithuania
Myanmar
Singapore
Guinea
DALYs of Major depressive disorder
10-24 years, Sexes Combined
Low SDI
(-0.04)
Low-middle SDI
(-0.19)
Middle SDI
(-0.08)
High-middle SDI
(0.44)
High SDI
(-0.07)
Concentration Index of Inequality in 2021

Figure S15

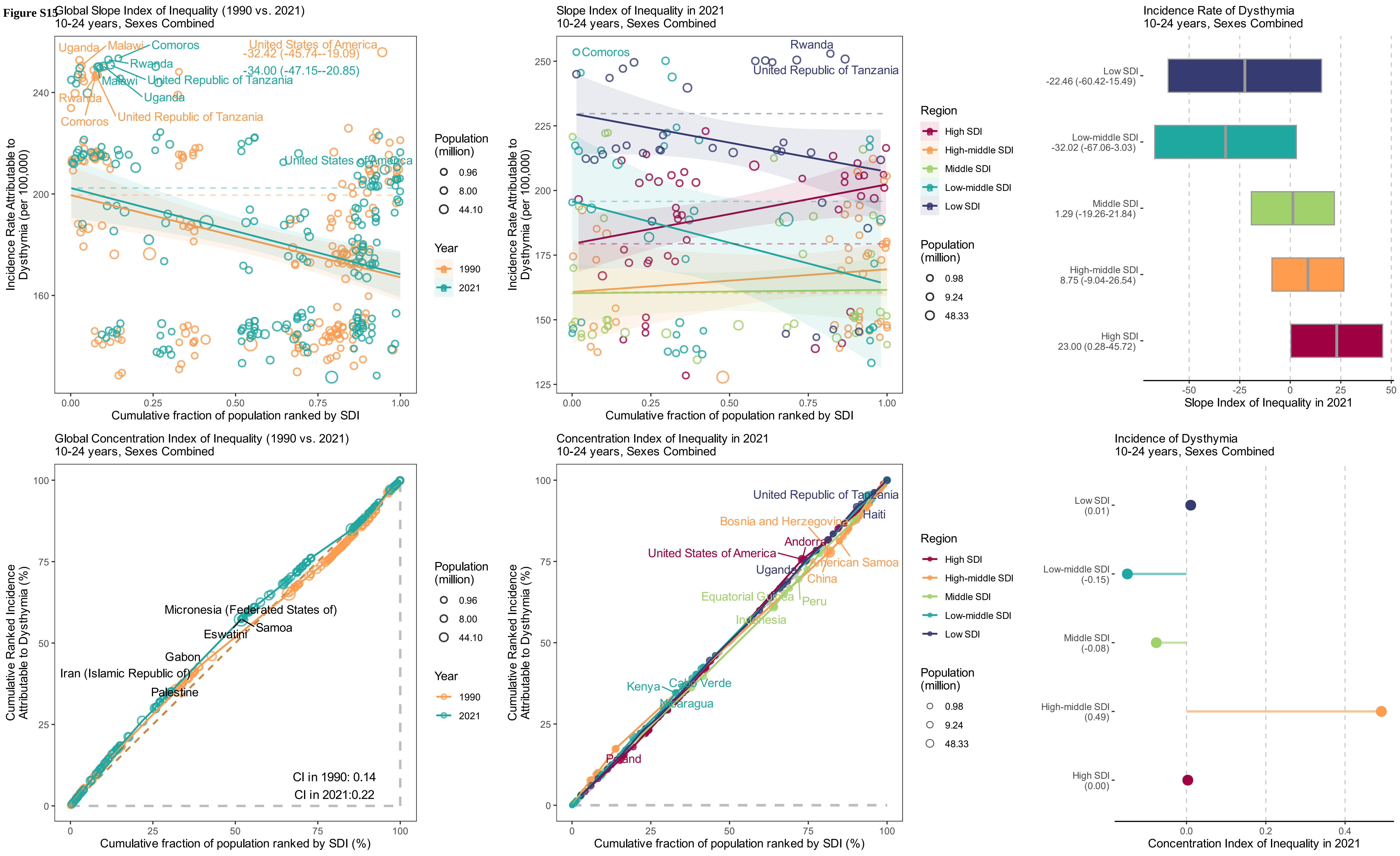
Global Slope Index of Inequality (1990 vs. 2021)
10-24 years, Sexes Combined
Incidence Rate Attributable to Dysthymia (per 100,000)
Cumulative fraction of population ranked by SDI
United States of America
-32.42 (-45.74--19.09)
-34.00 (-47.15--20.85)
Population (million)
0.96
8.00
44.10
Year
1990
2021
Slope Index of Inequality in 2021
10-24 years, Sexes Combined
Region
High SDI
High-middle SDI
Middle SDI
Low-middle SDI
Low SDI
0.98
9.24
48.33
Incidence Rate of Dysthymia
10-24 years, Sexes Combined
Low SDI -22.46 (-60.42-15.49)
Low-middle SDI -32.02 (-67.06-3.03)
Middle SDI 1.29 (-19.26-21.84)
High-middle SDI 8.75 (-9.04-26.54)
High SDI 23.00 (0.28-45.72)
Slope Index of Inequality in 2021
Global Concentration Index of Inequality (1990 vs. 2021)
10-24 years, Sexes Combined
Cumulative Ranked Incidence Attributable to Dysthymia (%)
Cumulative fraction of population ranked by SDI (%)
CI in 1990: 0.14
CI in 2021:0.22
Concentration Index of Inequality in 2021
10-24 years, Sexes Combined
Incidence of Dysthymia
10-24 years, Sexes Combined
Low SDI (0.01)
Low-middle SDI (-0.15)
Middle SDI (-0.08)
High-middle SDI (0.49)
High SDI (0.00)
Concentration Index of Inequality in 2021

Figure S15

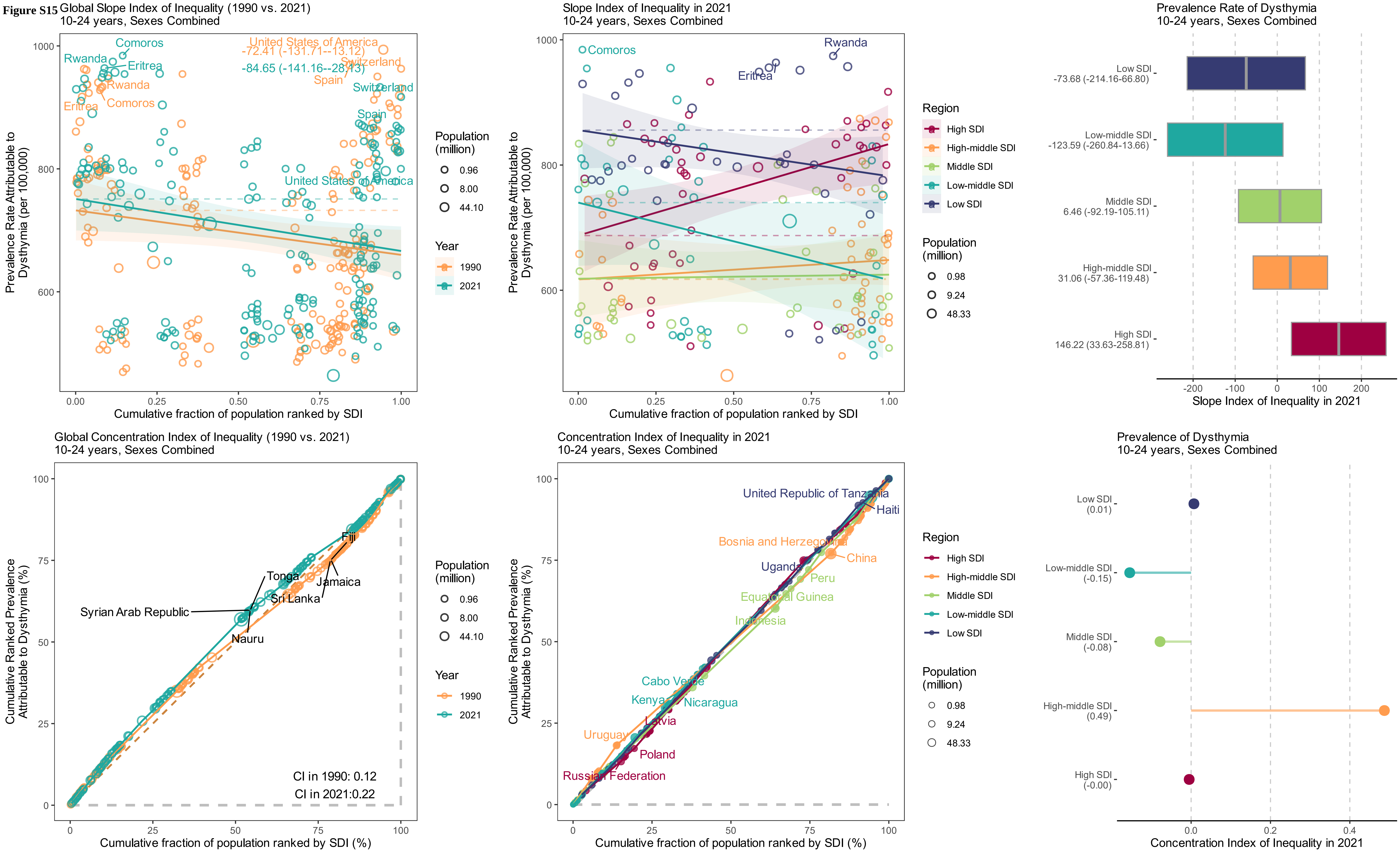
Global Slope Index of Inequality (1990 vs. 2021)
10-24 years, Sexes Combined
Prevalence Rate Attributable to Dysthymia (per 100,000)
Cumulative fraction of population ranked by SDI
United States of America
-72.41 (-131.71--13.12)
-84.65 (-141.16--28.13)
Comoros
Rwanda
Eritrea
Switzerland
Spain
Population (million)
0.96
8.00
44.10
Year
1990
2021
Slope Index of Inequality in 2021
10-24 years, Sexes Combined
Region
High SDI
High-middle SDI
Middle SDI
Low-middle SDI
Low SDI
Population (million)
0.98
9.24
48.33
Prevalence Rate of Dysthymia
10-24 years, Sexes Combined
Low SDI
-73.68 (-214.16-66.80)
Low-middle SDI
-123.59 (-260.84-13.66)
Middle SDI
6.46 (-92.19-105.11)
High-middle SDI
31.06 (-57.36-119.48)
High SDI
146.22 (33.63-258.81)
Slope Index of Inequality in 2021
Global Concentration Index of Inequality (1990 vs. 2021)
10-24 years, Sexes Combined
Cumulative Ranked Prevalence Attributable to Dysthymia (%)
Cumulative fraction of population ranked by SDI (%)
Fiji
Tonga
Jamaica
Sri Lanka
Syrian Arab Republic
Nauru
CI in 1990: 0.12
CI in 2021:0.22
Concentration Index of Inequality in 2021
10-24 years, Sexes Combined
United Republic of Tanzania
Haiti
Bosnia and Herzegovina
China
Uganda
Peru
Equatorial Guinea
Indonesia
Cabo Verde
Kenya
Nicaragua
Latvia
Uruguay
Poland
Russian Federation
Prevalence of Dysthymia
10-24 years, Sexes Combined
Low SDI
(0.01)
Low-middle SDI
(-0.15)
Middle SDI
(-0.08)
High-middle SDI
(0.49)
High SDI
(-0.00)
Concentration Index of Inequality in 2021

Figure S15

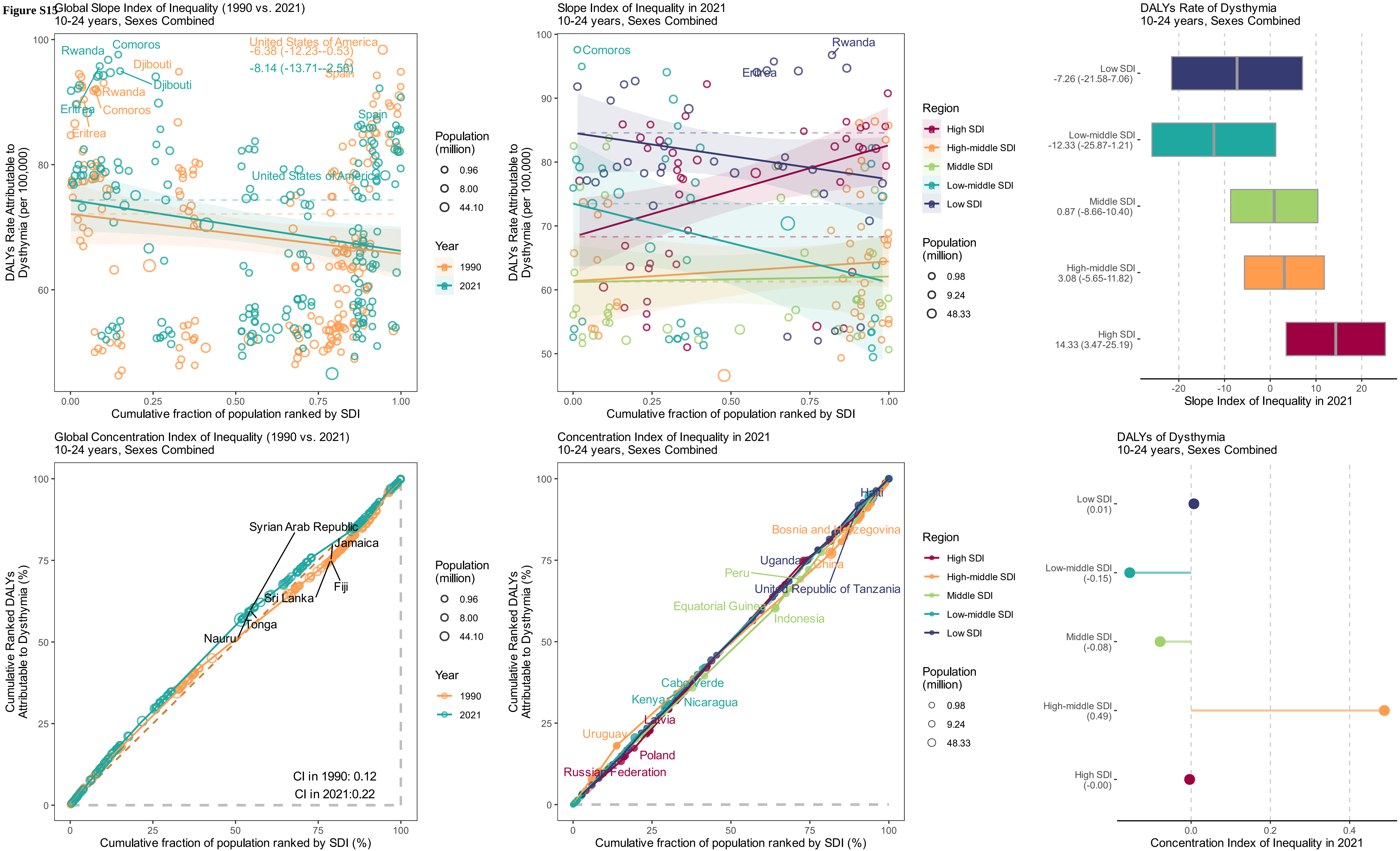
Global Slope Index of Inequality (1990 vs. 2021)
10-24 years, Sexes Combined
DALYs Rate Attributable to Dysthymia (per 100,000)
Cumulative fraction of population ranked by SDI
United States of America
-6.38 (-12.23--0.53)
-8.14 (-13.71--2.56)
Rwanda
Comoros
Djibouti
Eritrea
Spain
Population (million)
0.96
8.00
44.10
Year
1990
2021
Slope Index of Inequality in 2021
10-24 years, Sexes Combined
Region
High SDI
High-middle SDI
Middle SDI
Low-middle SDI
Low SDI
Population (million)
0.98
9.24
48.33
DALYs Rate of Dysthymia
10-24 years, Sexes Combined
Low SDI
-7.26 (-21.58-7.06)
Low-middle SDI
-12.33 (-25.87-1.21)
Middle SDI
0.87 (-8.66-10.40)
High-middle SDI
3.08 (-5.65-11.82)
High SDI
14.33 (3.47-25.19)
Slope Index of Inequality in 2021
Global Concentration Index of Inequality (1990 vs. 2021)
10-24 years, Sexes Combined
Cumulative Ranked DALYs Attributable to Dysthymia (%)
Cumulative fraction of population ranked by SDI (%)
Syrian Arab Republic
Jamaica
Fiji
Sri Lanka
Tonga
Nauru
CI in 1990: 0.12
CI in 2021:0.22
Concentration Index of Inequality in 2021
10-24 years, Sexes Combined
Haiti
Bosnia and Herzegovina
Uganda
China
Peru
United Republic of Tanzania
Equatorial Guinea
Indonesia
Cabo Verde
Kenya
Nicaragua
Latvia
Uruguay
Poland
Russian Federation
DALYs of Dysthymia
10-24 years, Sexes Combined
Low SDI
(0.01)
Low-middle SDI
(-0.15)
Middle SDI
(-0.08)
High-middle SDI
(0.49)
High SDI
(-0.00)
Concentration Index of Inequality in 2021

Figure S16

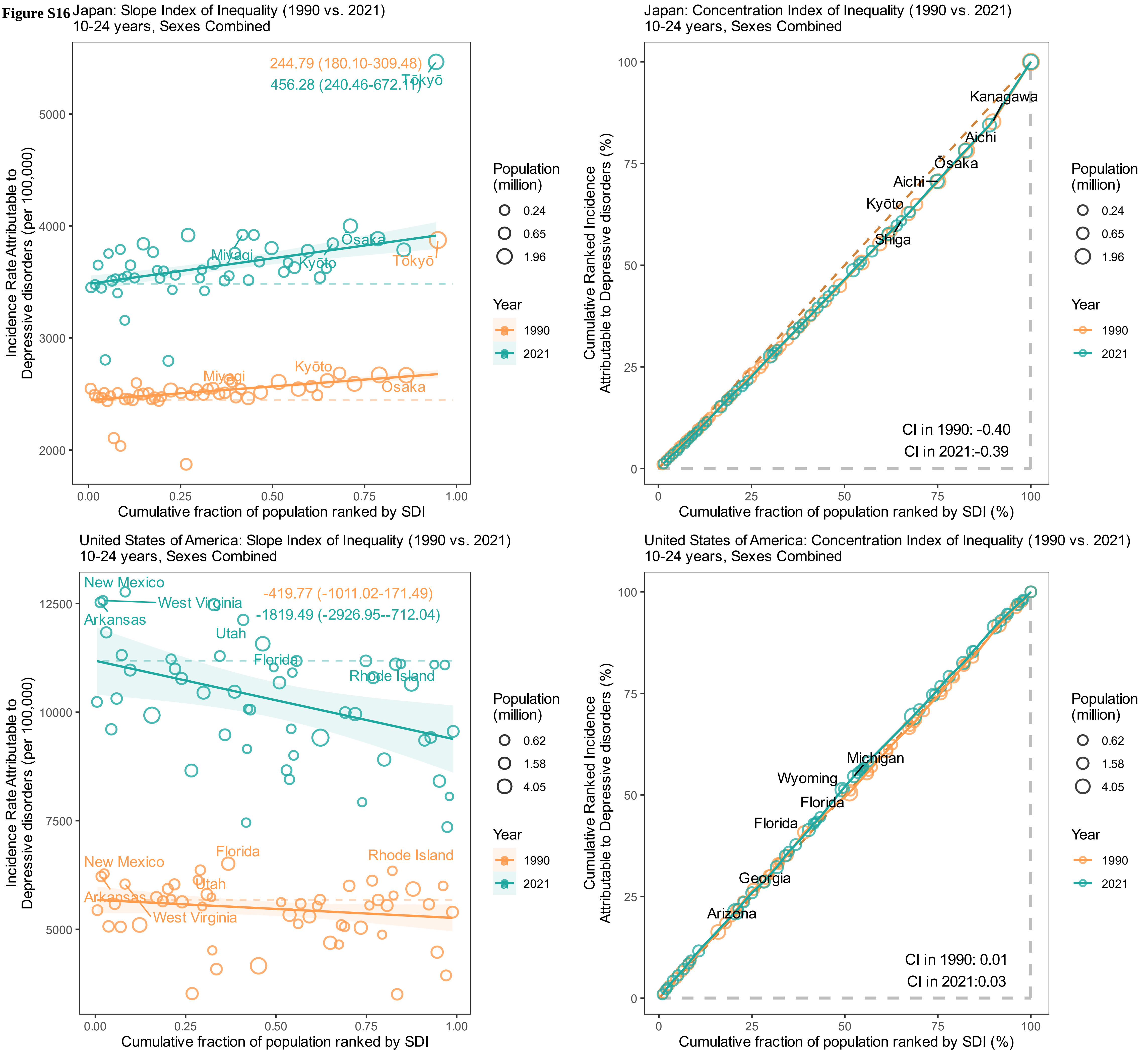

Japan: Slope Index of Inequality (1990 vs. 2021)
10-24 years, Sexes Combined
244.79 (180.10-309.48)
456.28 (240.46-672.11)
Tōkyō
Miyagi
Ōsaka
Kyōto
Tokyō
Miyagi
Kyōto
Osaka
Incidence Rate Attributable to Depressive disorders (per 100,000)
Cumulative fraction of population ranked by SDI
Population (million)
0.24
0.65
1.96
Year
1990
2021
Japan: Concentration Index of Inequality (1990 vs. 2021)
10-24 years, Sexes Combined
Kanagawa
Aichi
Ōsaka
Aichi
Kyōto
Shiga
CI in 1990: -0.40
CI in 2021:-0.39
Cumulative Ranked Incidence Attributable to Depressive disorders (%)
Cumulative fraction of population ranked by SDI (%)
United States of America: Slope Index of Inequality (1990 vs. 2021)
10-24 years, Sexes Combined
New Mexico
West Virginia
Arkansas
Utah
Florida
Rhode Island
-419.77 (-1011.02-171.49)
-1819.49 (-2926.95--712.04)
New Mexico
Florida
Rhode Island
Utah
Arkansas
West Virginia
Incidence Rate Attributable to Depressive disorders (per 100,000)
Cumulative fraction of population ranked by SDI
Population (million)
0.62
1.58
4.05
Year
1990
2021
United States of America: Concentration Index of Inequality (1990 vs. 2021)
10-24 years, Sexes Combined
Michigan
Wyoming
Florida
Florida
Georgia
Arizona
CI in 1990: 0.01
CI in 2021:0.03
Cumulative Ranked Incidence Attributable to Depressive disorders (%)
Cumulative fraction of population ranked by SDI (%)

Figure S16

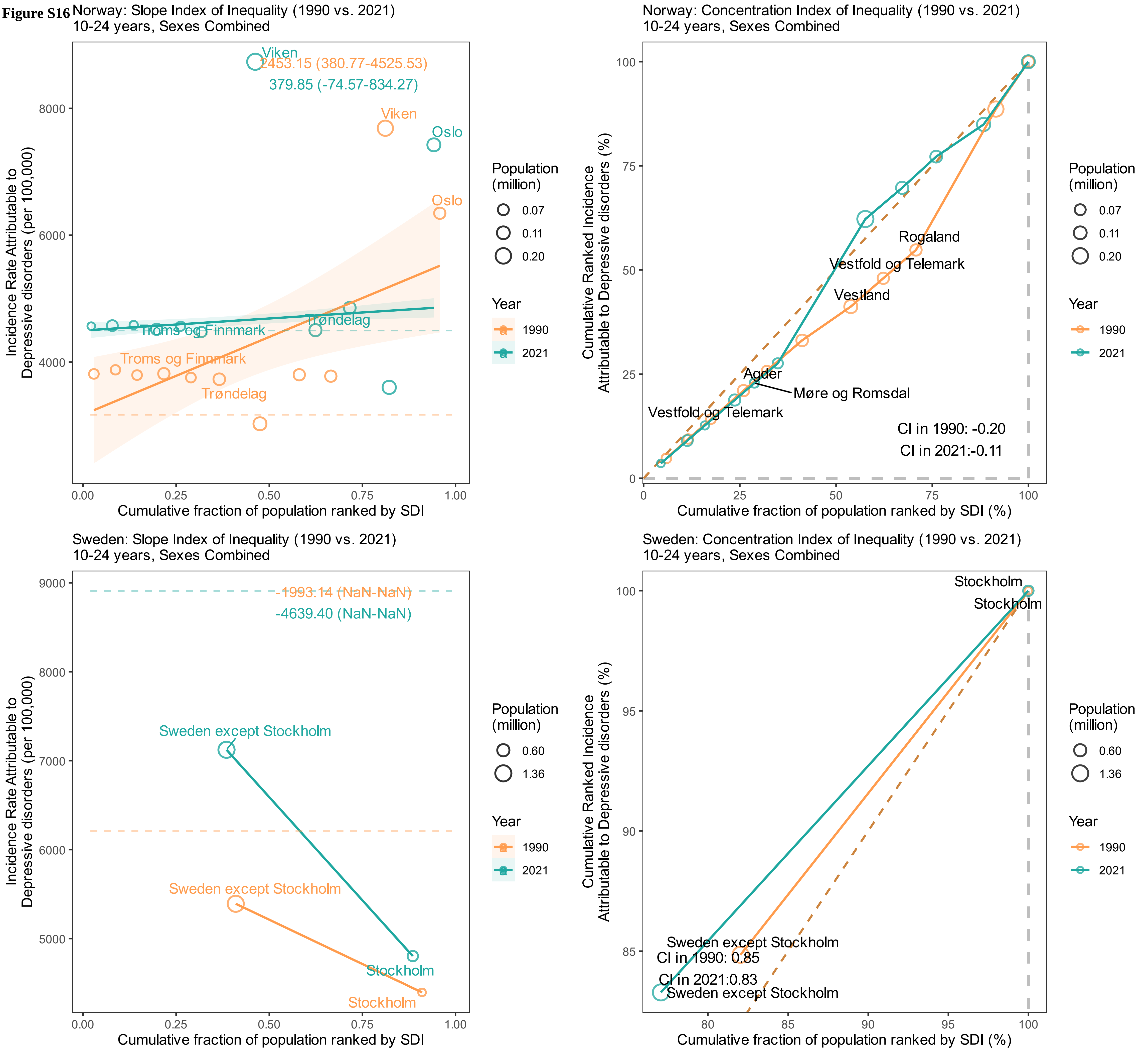
Norway: Slope Index of Inequality (1990 vs. 2021)
10-24 years, Sexes Combined
2453.15 (380.77-4525.53)
379.85 (-74.57-834.27)
Viken
Oslo
Troms og Finnmark
Trøndelag
Incidence Rate Attributable to Depressive disorders (per 100,000)
Cumulative fraction of population ranked by SDI
Population (million)
0.07
0.11
0.20
Year
1990
2021
Norway: Concentration Index of Inequality (1990 vs. 2021)
10-24 years, Sexes Combined
Rogaland
Vestfold og Telemark
Vestland
Agder
Møre og Romsdal
CI in 1990: -0.20
CI in 2021:-0.11
Cumulative Ranked Incidence Attributable to Depressive disorders (%)
Cumulative fraction of population ranked by SDI (%)
Sweden: Slope Index of Inequality (1990 vs. 2021)
10-24 years, Sexes Combined
-1993.14 (NaN-NaN)
-4639.40 (NaN-NaN)
Sweden except Stockholm
Stockholm
Population (million)
0.60
1.36
Sweden: Concentration Index of Inequality (1990 vs. 2021)
10-24 years, Sexes Combined
CI in 1990: 0.85
CI in 2021:0.83

Figure S16

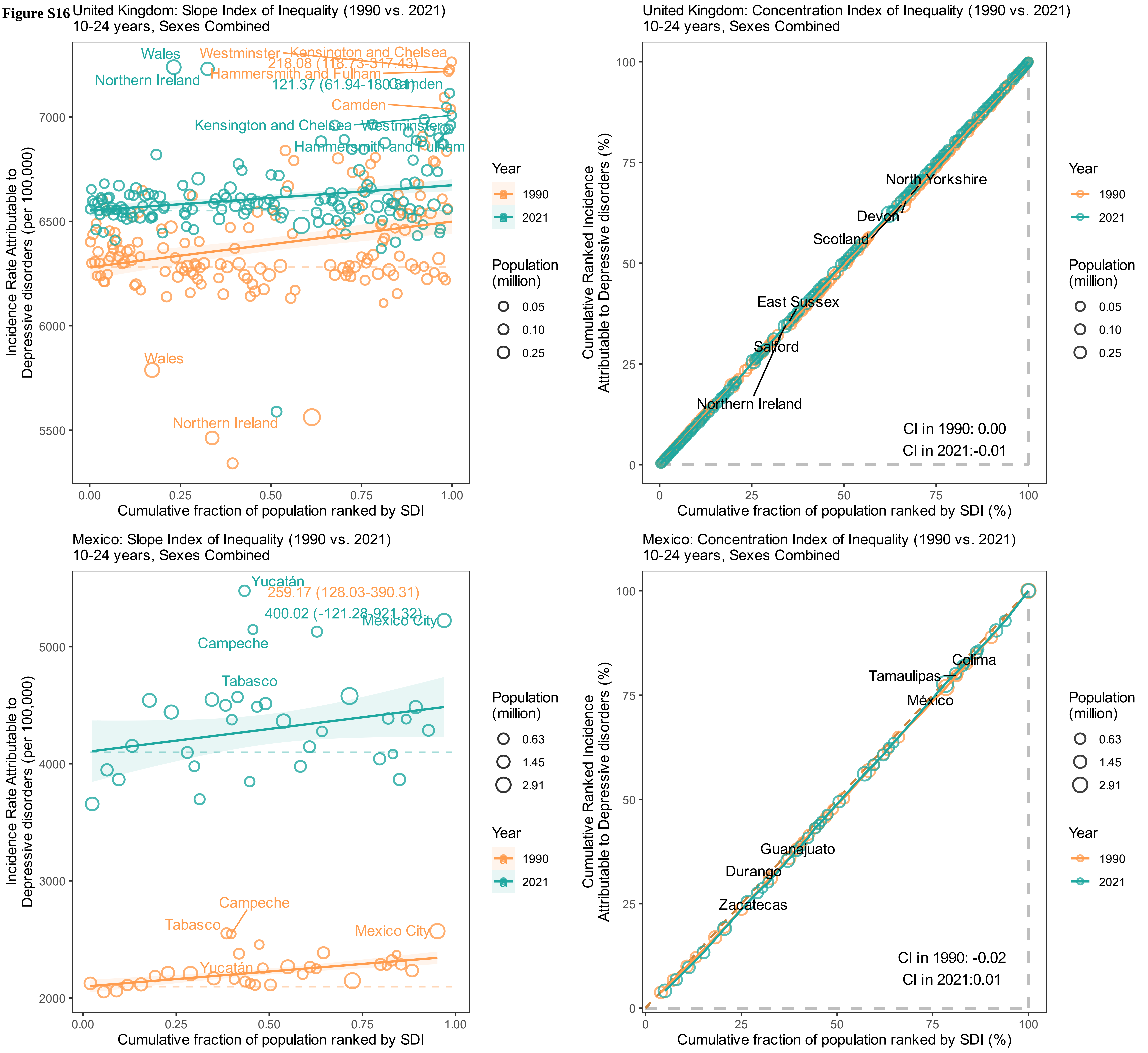
United Kingdom: Slope Index of Inequality (1990 vs. 2021)
10-24 years, Sexes Combined
Incidence Rate Attributable to Depressive disorders (per 100,000)
Cumulative fraction of population ranked by SDI
218.08 (118.73-317.43)
121.37 (61.94-180.81)
Wales
Northern Ireland
Westminster
Kensington and Chelsea
Hammersmith and Fulham
Camden
Year
1990
2021
Population (million)
0.05
0.10
0.25
United Kingdom: Concentration Index of Inequality (1990 vs. 2021)
10-24 years, Sexes Combined
Cumulative Ranked Incidence Attributable to Depressive disorders (%)
Cumulative fraction of population ranked by SDI (%)
North Yorkshire
Devon
Scotland
East Sussex
Salford
Northern Ireland
CI in 1990: 0.00
CI in 2021:-0.01
Mexico: Slope Index of Inequality (1990 vs. 2021)
10-24 years, Sexes Combined
259.17 (128.03-390.31)
400.02 (-121.28-921.32)
Yucatán
Mexico City
Campeche
Tabasco
Population (million)
0.63
1.45
2.91
Mexico: Concentration Index of Inequality (1990 vs. 2021)
10-24 years, Sexes Combined
Colima
Tamaulipas
México
Guanajuato
Durango
Zacatecas
CI in 1990: -0.02
CI in 2021:0.01

Figure S16

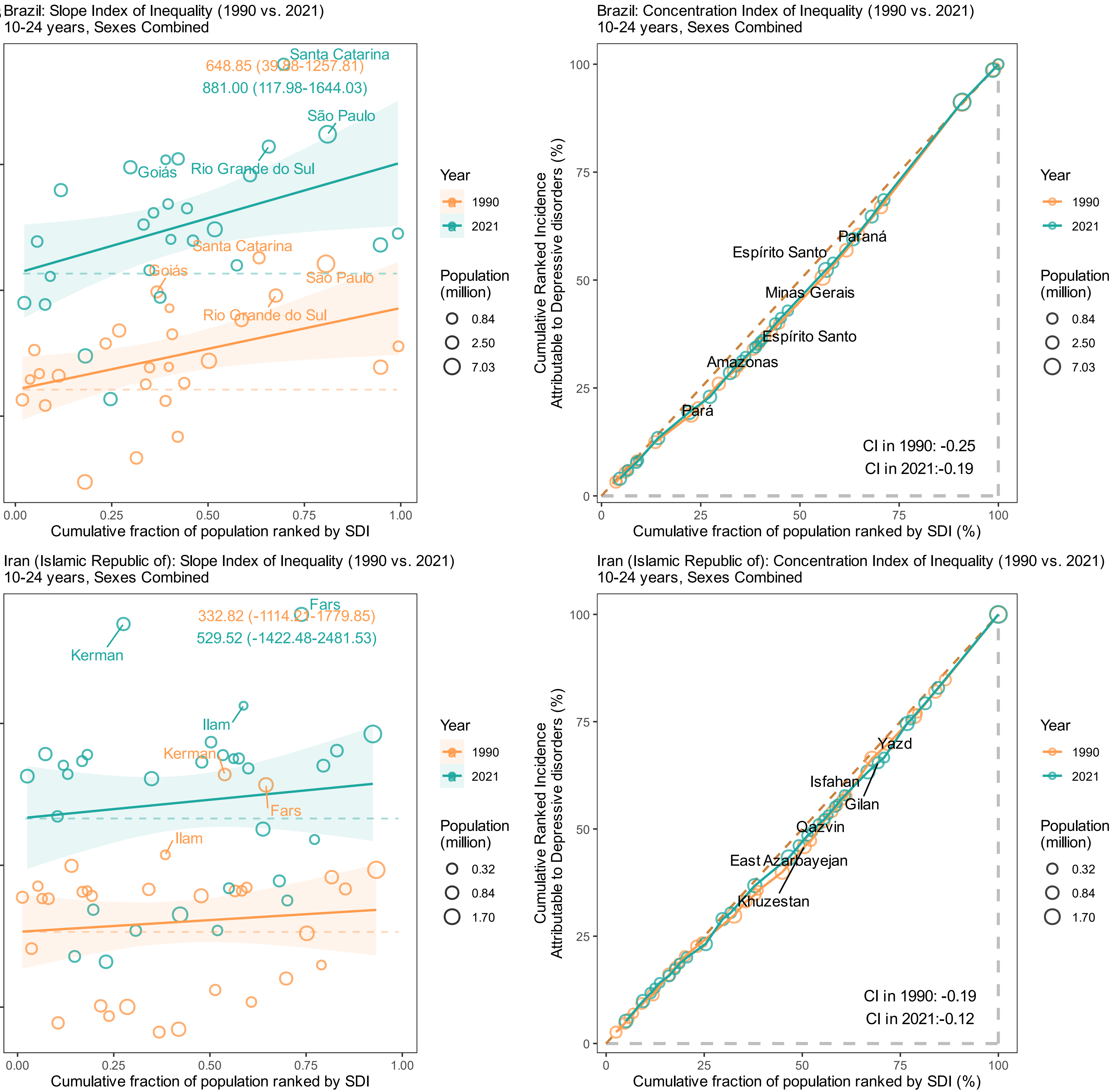

Brazil: Slope Index of Inequality (1990 vs. 2021)
10-24 years, Sexes Combined
648.85 (39.88-1257.81)
881.00 (117.98-1644.03)
Santa Catarina
São Paulo
Goiás
Rio Grande do Sul
Incidence Rate Attributable to Depressive disorders (per 100,000)
Cumulative fraction of population ranked by SDI
Year
1990
2021
Population (million)
0.84
2.50
7.03
Brazil: Concentration Index of Inequality (1990 vs. 2021)
10-24 years, Sexes Combined
Paraná
Espírito Santo
Minas Gerais
Amazonas
Pará
CI in 1990: -0.25
CI in 2021:-0.19
Cumulative Ranked Incidence Attributable to Depressive disorders (%)
Cumulative fraction of population ranked by SDI (%)
Iran (Islamic Republic of): Slope Index of Inequality (1990 vs. 2021)
10-24 years, Sexes Combined
332.82 (-1114.21-1779.85)
529.52 (-1422.48-2481.53)
Fars
Kerman
Ilam
Population (million)
0.32
0.84
1.70
Iran (Islamic Republic of): Concentration Index of Inequality (1990 vs. 2021)
10-24 years, Sexes Combined
Yazd
Isfahan
Gilan
Qazvin
East Azarbayejan
Khuzestan
CI in 1990: -0.19
CI in 2021:-0.12

Figure S16

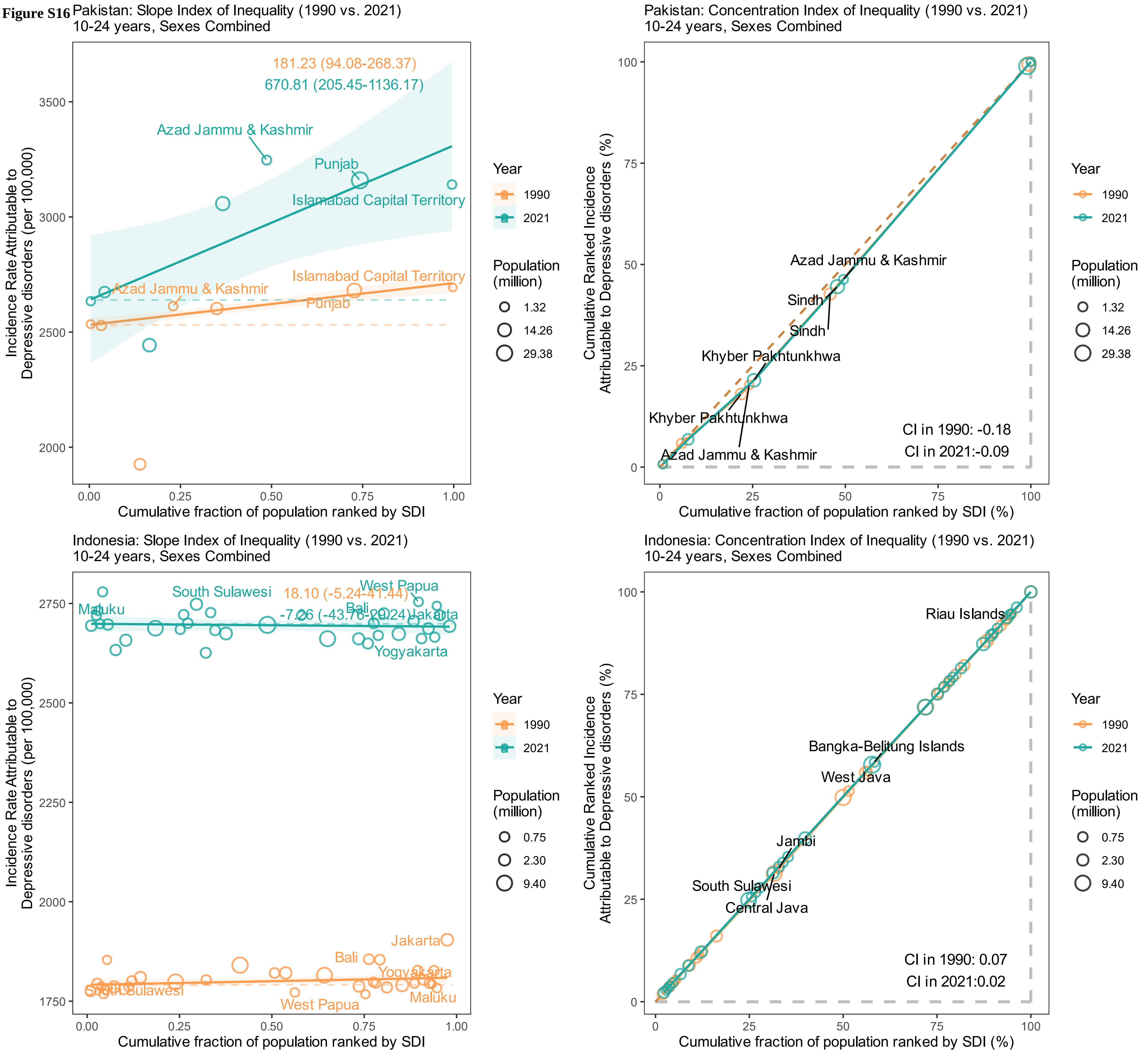
Pakistan: Slope Index of Inequality (1990 vs. 2021)
10-24 years, Sexes Combined
181.23 (94.08-268.37)
670.81 (205.45-1136.17)
Azad Jammu & Kashmir
Punjab
Islamabad Capital Territory
Azad Jammu & Kashmir
Islamabad Capital Territory
Punjab
Incidence Rate Attributable to Depressive disorders (per 100,000)
Cumulative fraction of population ranked by SDI
Year
1990
2021
Population (million)
1.32
14.26
29.38
Pakistan: Concentration Index of Inequality (1990 vs. 2021)
10-24 years, Sexes Combined
Azad Jammu & Kashmir
Sindh
Sindh
Khyber Pakhtunkhwa
Khyber Pakhtunkhwa
Azad Jammu & Kashmir
CI in 1990: -0.18
CI in 2021:-0.09
Cumulative Ranked Incidence Attributable to Depressive disorders (%)
Cumulative fraction of population ranked by SDI (%)
Indonesia: Slope Index of Inequality (1990 vs. 2021)
10-24 years, Sexes Combined
South Sulawesi
18.10 (-5.24-41.44)
West Papua
Maluku
Bali
-7.26 (-43.76-29.24)
Jakarta
Yogyakarta
Jakarta
Bali
Yogyakarta
South Sulawesi
West Papua
Maluku
Incidence Rate Attributable to Depressive disorders (per 100,000)
Cumulative fraction of population ranked by SDI
Year
1990
2021
Population (million)
0.75
2.30
9.40
Indonesia: Concentration Index of Inequality (1990 vs. 2021)
10-24 years, Sexes Combined
Riau Islands
Bangka-Belitung Islands
West Java
Jambi
South Sulawesi
Central Java
CI in 1990: 0.07
CI in 2021:0.02
Cumulative Ranked Incidence Attributable to Depressive disorders (%)
Cumulative fraction of population ranked by SDI (%)

Figure S16

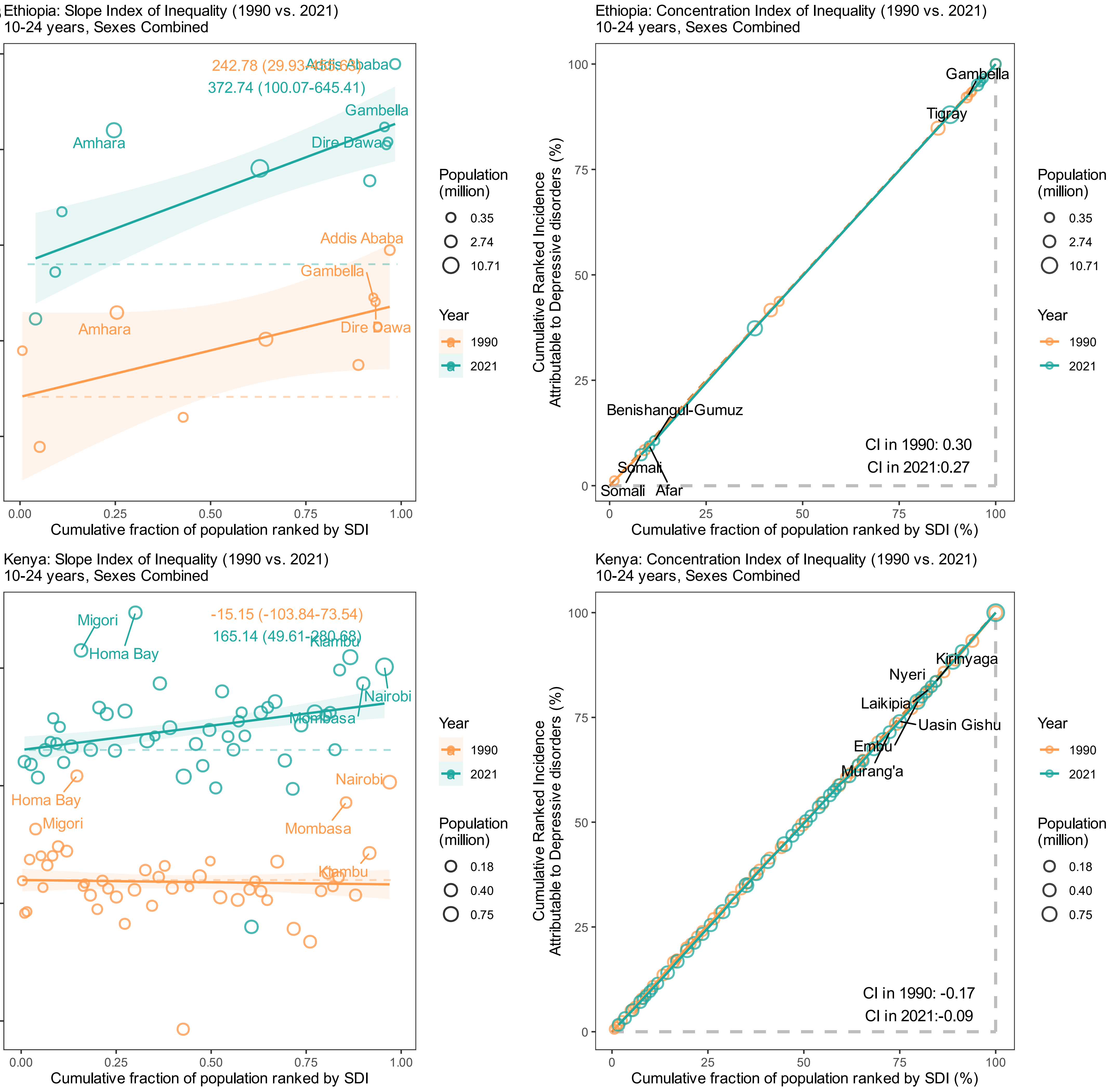

Ethiopia: Slope Index of Inequality (1990 vs. 2021)
10-24 years, Sexes Combined
242.78 (29.93-455.63)
372.74 (100.07-645.41)
Addis Ababa
Gambella
Amhara
Dire Dawa
Incidence Rate Attributable to Depressive disorders (per 100,000)
Cumulative fraction of population ranked by SDI
Population (million)
0.35
2.74
10.71
Year
1990
2021
Ethiopia: Concentration Index of Inequality (1990 vs. 2021)
10-24 years, Sexes Combined
Gambella
Tigray
Benishangul-Gumuz
Somali
Afar
CI in 1990: 0.30
CI in 2021:0.27
Cumulative Ranked Incidence Attributable to Depressive disorders (%)
Cumulative fraction of population ranked by SDI (%)
Kenya: Slope Index of Inequality (1990 vs. 2021)
10-24 years, Sexes Combined
-15.15 (-103.84-73.54)
165.14 (49.61-280.68)
Migori
Homa Bay
Kiambu
Nairobi
Mombasa
Incidence Rate Attributable to Depressive disorders (per 100,000)
Cumulative fraction of population ranked by SDI
Year
1990
2021
Population (million)
0.18
0.40
0.75
Kenya: Concentration Index of Inequality (1990 vs. 2021)
10-24 years, Sexes Combined
Kirinyaga
Nyeri
Laikipia
Uasin Gishu
Embu
Murang'a
CI in 1990: -0.17
CI in 2021:-0.09
Cumulative Ranked Incidence Attributable to Depressive disorders (%)
Cumulative fraction of population ranked by SDI (%)

Figure S16

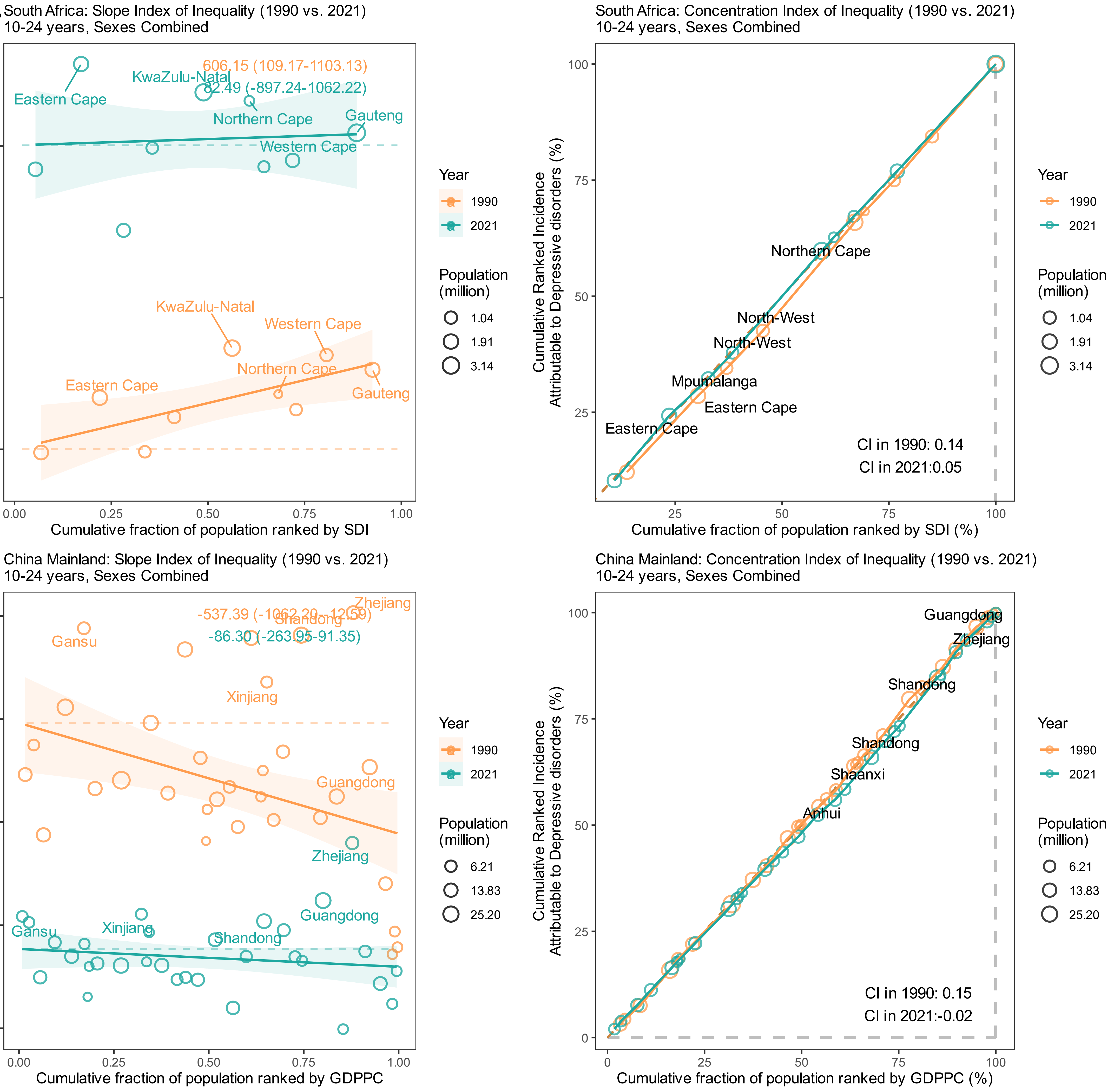
South Africa: Slope Index of Inequality (1990 vs. 2021)
10-24 years, Sexes Combined
606.15 (109.17-1103.13)
82.49 (-897.24-1062.22)
Eastern Cape
KwaZulu-Natal
Northern Cape
Gauteng
Western Cape
KwaZulu-Natal
Western Cape
Northern Cape
Eastern Cape
Gauteng
Incidence Rate Attributable to Depressive disorders (per 100,000)
Cumulative fraction of population ranked by SDI
Year
1990
2021
Population (million)
1.04
1.91
3.14
South Africa: Concentration Index of Inequality (1990 vs. 2021)
10-24 years, Sexes Combined
Northern Cape
North-West
North-West
Mpumalanga
Eastern Cape
Eastern Cape
CI in 1990: 0.14
CI in 2021:0.05
Cumulative Ranked Incidence Attributable to Depressive disorders (%)
Cumulative fraction of population ranked by SDI (%)
China Mainland: Slope Index of Inequality (1990 vs. 2021)
10-24 years, Sexes Combined
-537.39 (-1062.20--12.59)
-86.30 (-263.95-91.35)
Zhejiang
Shandong
Gansu
Xinjiang
Guangdong
Zhejiang
Guangdong
Gansu
Xinjiang
Shandong
Incidence Rate Attributable to Depressive disorders (per 100,000)
Cumulative fraction of population ranked by GDPPC
Population (million)
6.21
13.83
25.20
China Mainland: Concentration Index of Inequality (1990 vs. 2021)
10-24 years, Sexes Combined
Guangdong
Zhejiang
Shandong
Shandong
Shaanxi
Anhui
CI in 1990: 0.15
CI in 2021:-0.02
Cumulative Ranked Incidence Attributable to Depressive disorders (%)
Cumulative fraction of population ranked by GDPPC (%)

Figure S16

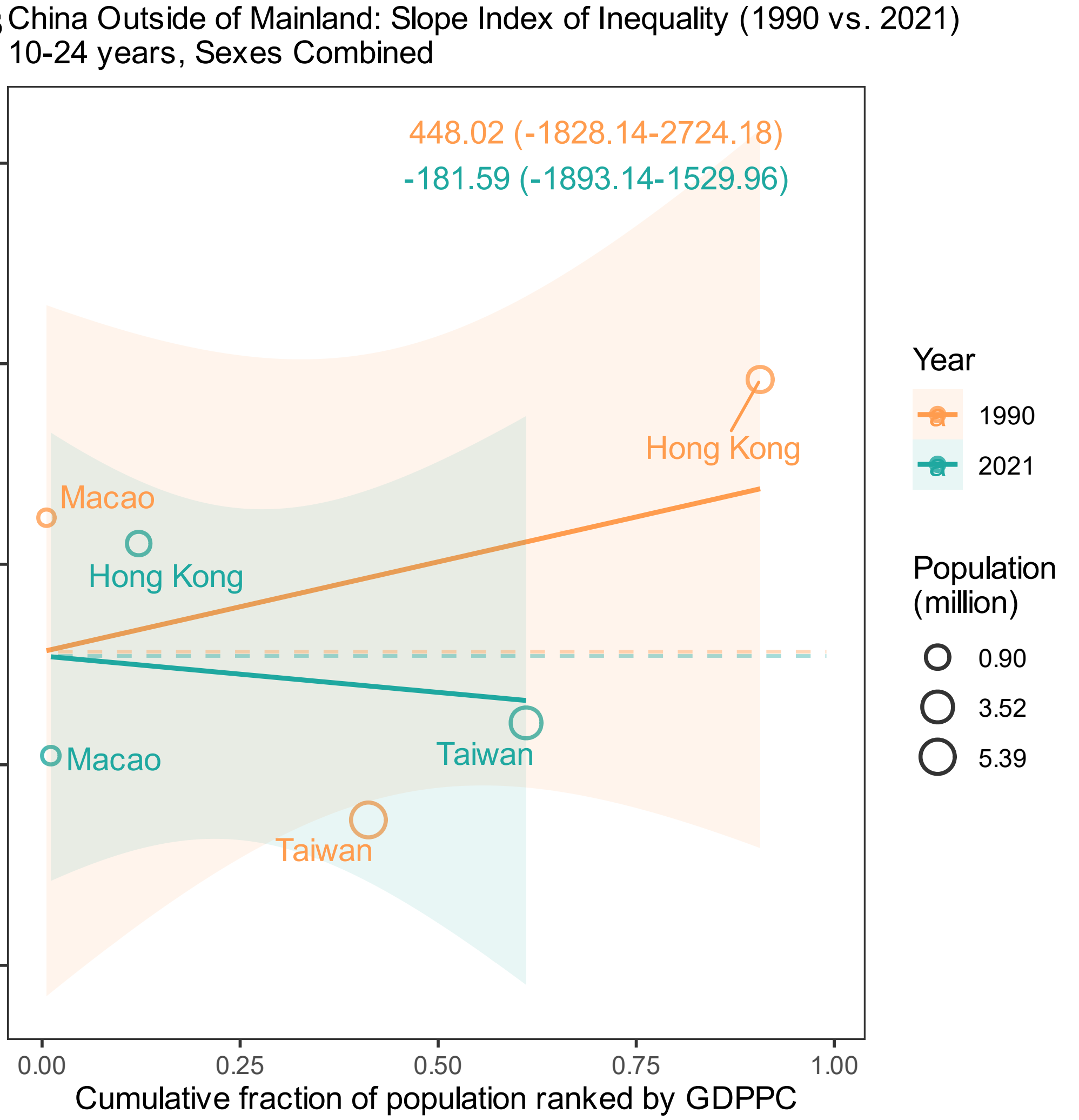
China Outside of Mainland: Slope Index of Inequality (1990 vs. 2021)
10-24 years, Sexes Combined
448.02 (-1828.14-2724.18)
-181.59 (-1893.14-1529.96)
Macao
Hong Kong
Hong Kong
Macao
Taiwan
Taiwan
Incidence Rate Attributable to Depressive disorders (per 100,000)
Cumulative fraction of population ranked by GDPPC
Year
1990
2021
Population (million)
0.90
3.52
5.39

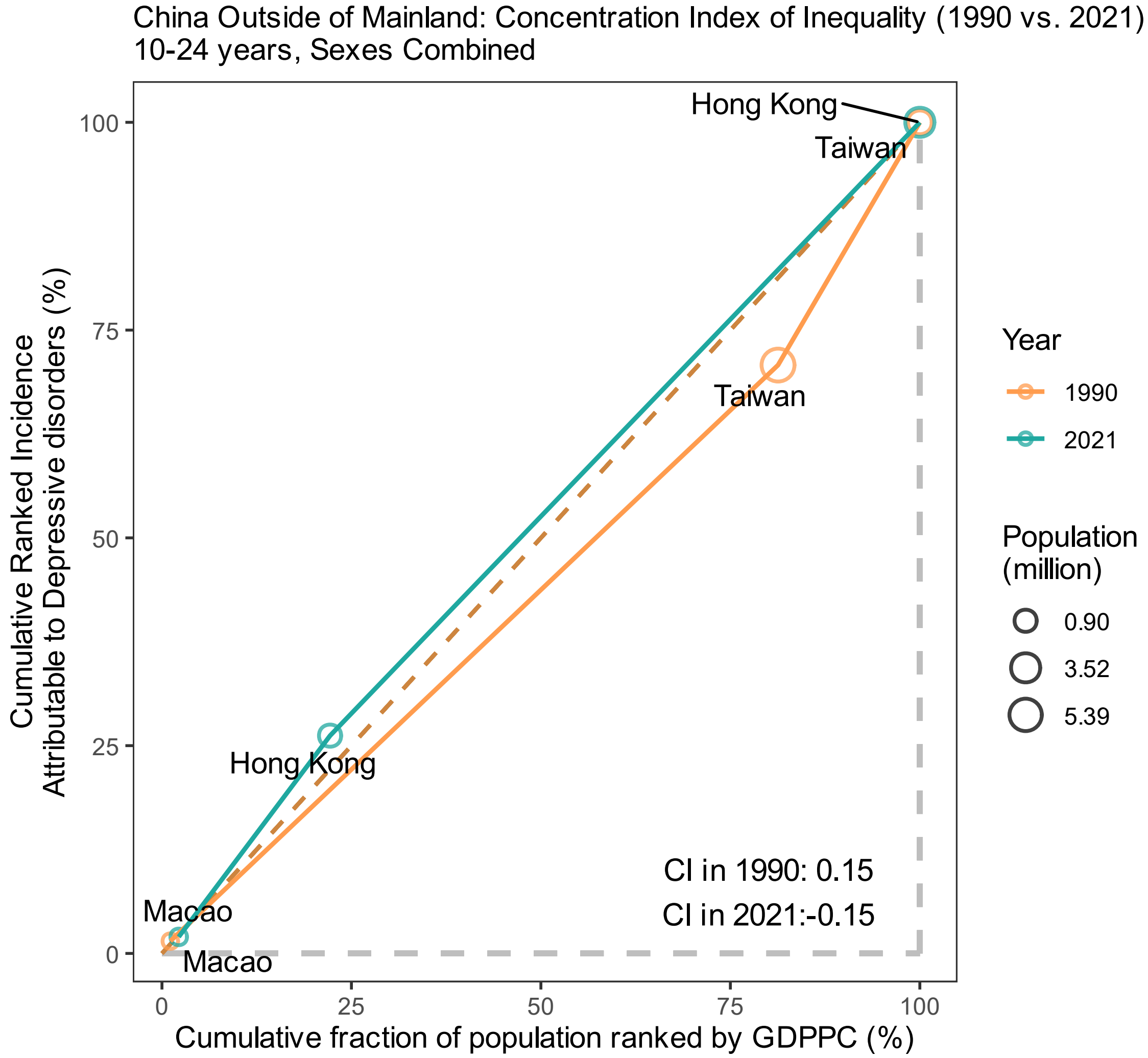
China Outside of Mainland: Concentration Index of Inequality (1990 vs. 2021)
10-24 years, Sexes Combined
Hong Kong
Taiwan
Taiwan
Hong Kong
Macao
Macao
CI in 1990: 0.15
CI in 2021:-0.15
Cumulative Ranked Incidence Attributable to Depressive disorders (%)
Cumulative fraction of population ranked by GDPPC (%)
Year
1990
2021
Population (million)
0.90
3.52
5.39

Figure S17

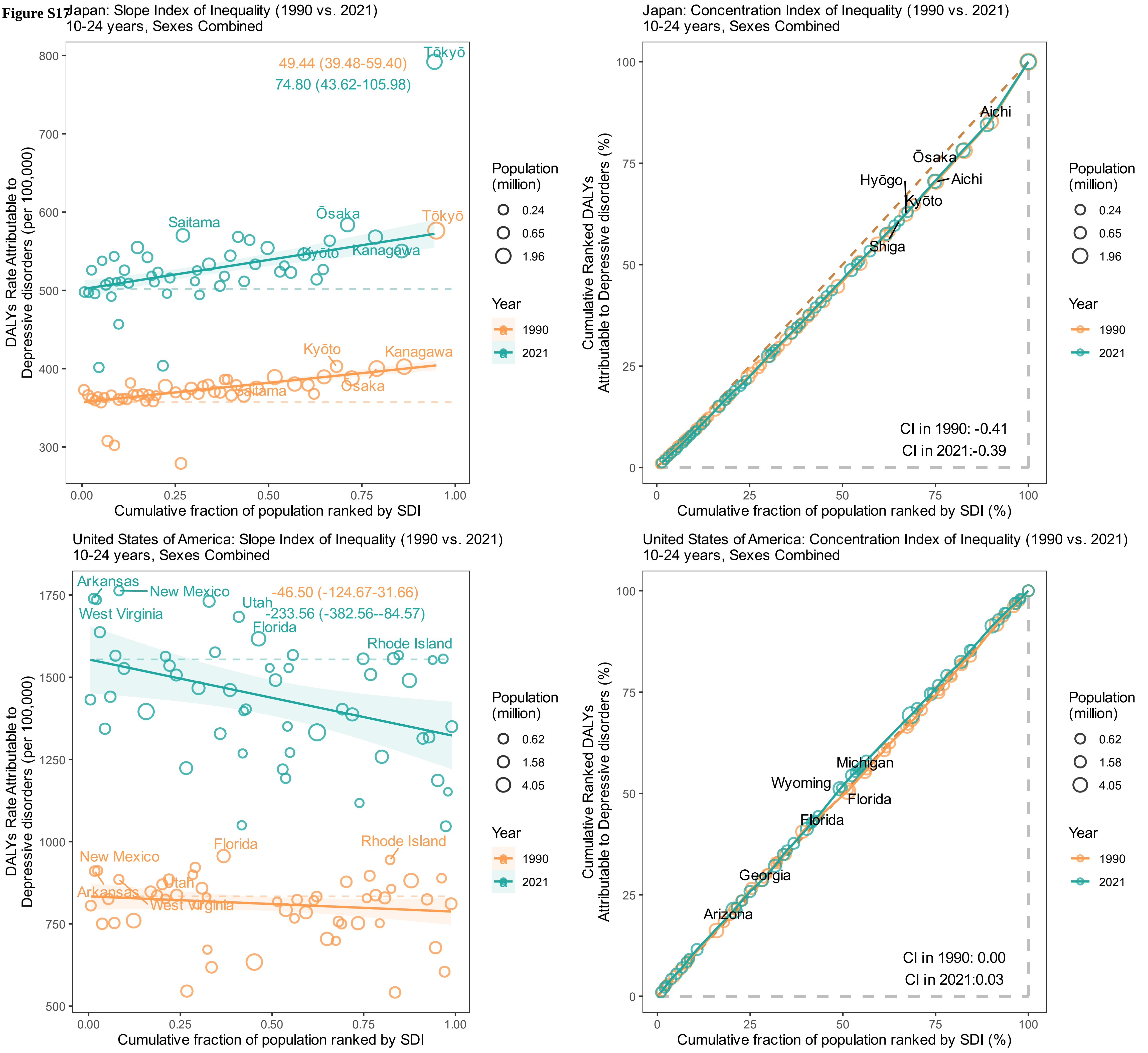
Japan: Slope Index of Inequality (1990 vs. 2021)
10-24 years, Sexes Combined
49.44 (39.48-59.40)
74.80 (43.62-105.98)
DALYs Rate Attributable to Depressive disorders (per 100,000)
Cumulative fraction of population ranked by SDI
Tōkyō
Saitama
Ōsaka
Kyōto
Kanagawa
Population (million)
0.24
0.65
1.96
Year
1990
2021
Japan: Concentration Index of Inequality (1990 vs. 2021)
10-24 years, Sexes Combined
Cumulative Ranked DALYs Attributable to Depressive disorders (%)
Cumulative fraction of population ranked by SDI (%)
Aichi
Hyōgo
Shiga
CI in 1990: -0.41
CI in 2021:-0.39
United States of America: Slope Index of Inequality (1990 vs. 2021)
10-24 years, Sexes Combined
-46.50 (-124.67-31.66)
-233.56 (-382.56--84.57)
Arkansas
New Mexico
West Virginia
Utah
Florida
Rhode Island
0.62
1.58
4.05
United States of America: Concentration Index of Inequality (1990 vs. 2021)
10-24 years, Sexes Combined
Michigan
Wyoming
Georgia
Arizona
CI in 1990: 0.00
CI in 2021:0.03

Figure S17

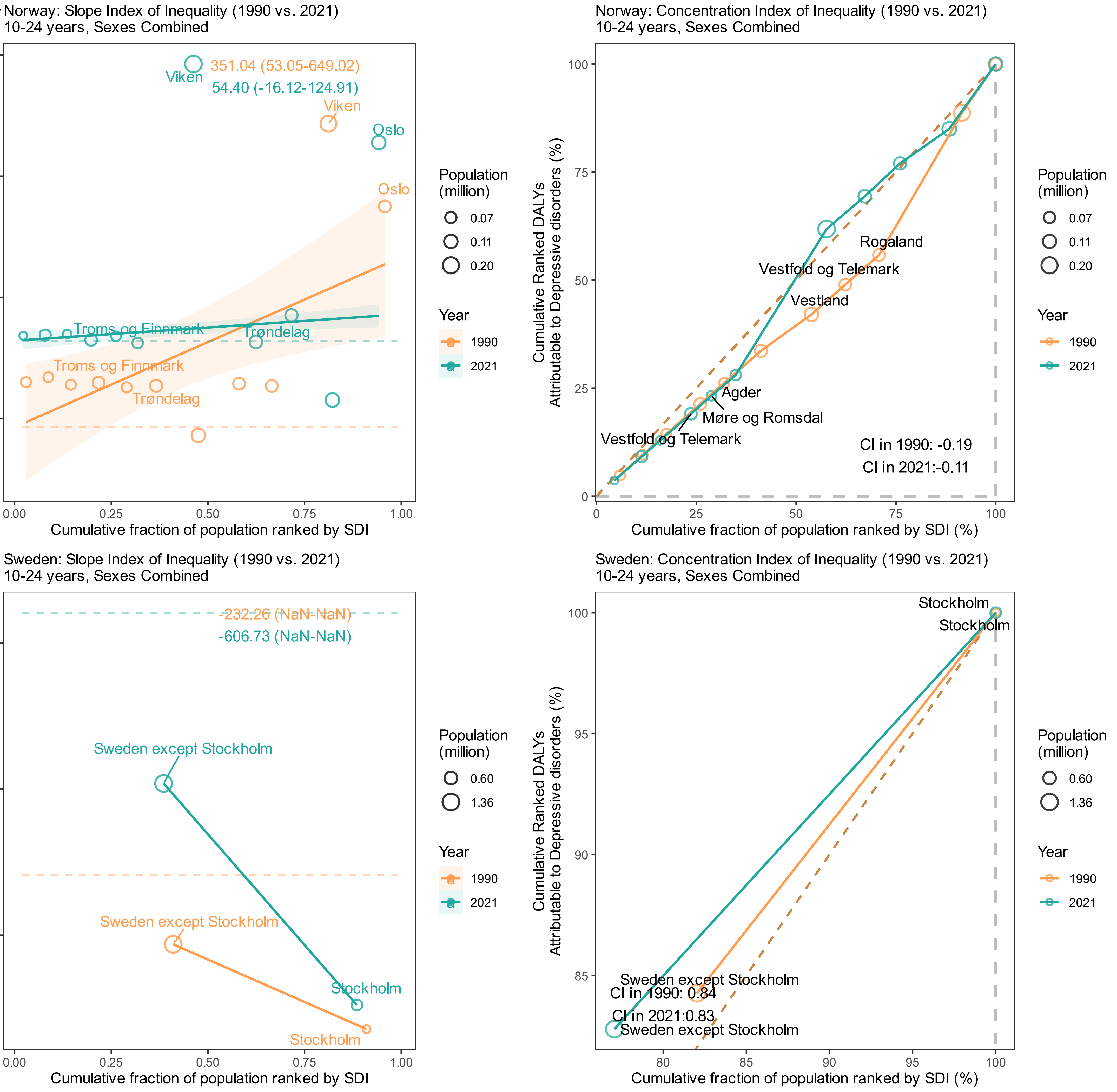

Norway: Slope Index of Inequality (1990 vs. 2021)
10-24 years, Sexes Combined
351.04 (53.05-649.02)
54.40 (-16.12-124.91)
Viken
Viken
Oslo
Oslo
Troms og Finnmark
Trøndelag
Troms og Finnmark
Trøndelag
DALYs Rate Attributable to Depressive disorders (per 100,000)
Cumulative fraction of population ranked by SDI
Population (million)
0.07
0.11
0.20
Year
1990
2021
Norway: Concentration Index of Inequality (1990 vs. 2021)
10-24 years, Sexes Combined
Rogaland
Vestfold og Telemark
Vestland
Agder
Møre og Romsdal
Vestfold og Telemark
CI in 1990: -0.19
CI in 2021:-0.11
Cumulative Ranked DALYs Attributable to Depressive disorders (%)
Cumulative fraction of population ranked by SDI (%)
Population (million)
0.07
0.11
0.20
Year
1990
2021
Sweden: Slope Index of Inequality (1990 vs. 2021)
10-24 years, Sexes Combined
-232.26 (NaN-NaN)
-606.73 (NaN-NaN)
Sweden except Stockholm
Sweden except Stockholm
Stockholm
Stockholm
DALYs Rate Attributable to Depressive disorders (per 100,000)
Cumulative fraction of population ranked by SDI
Population (million)
0.60
1.36
Year
1990
2021
Sweden: Concentration Index of Inequality (1990 vs. 2021)
10-24 years, Sexes Combined
Stockholm
Stockholm
Sweden except Stockholm
CI in 1990: 0.84
CI in 2021:0.83
Sweden except Stockholm
Cumulative Ranked DALYs Attributable to Depressive disorders (%)
Cumulative fraction of population ranked by SDI (%)
Population (million)
0.60
1.36
Year
1990
2021

Figure S17

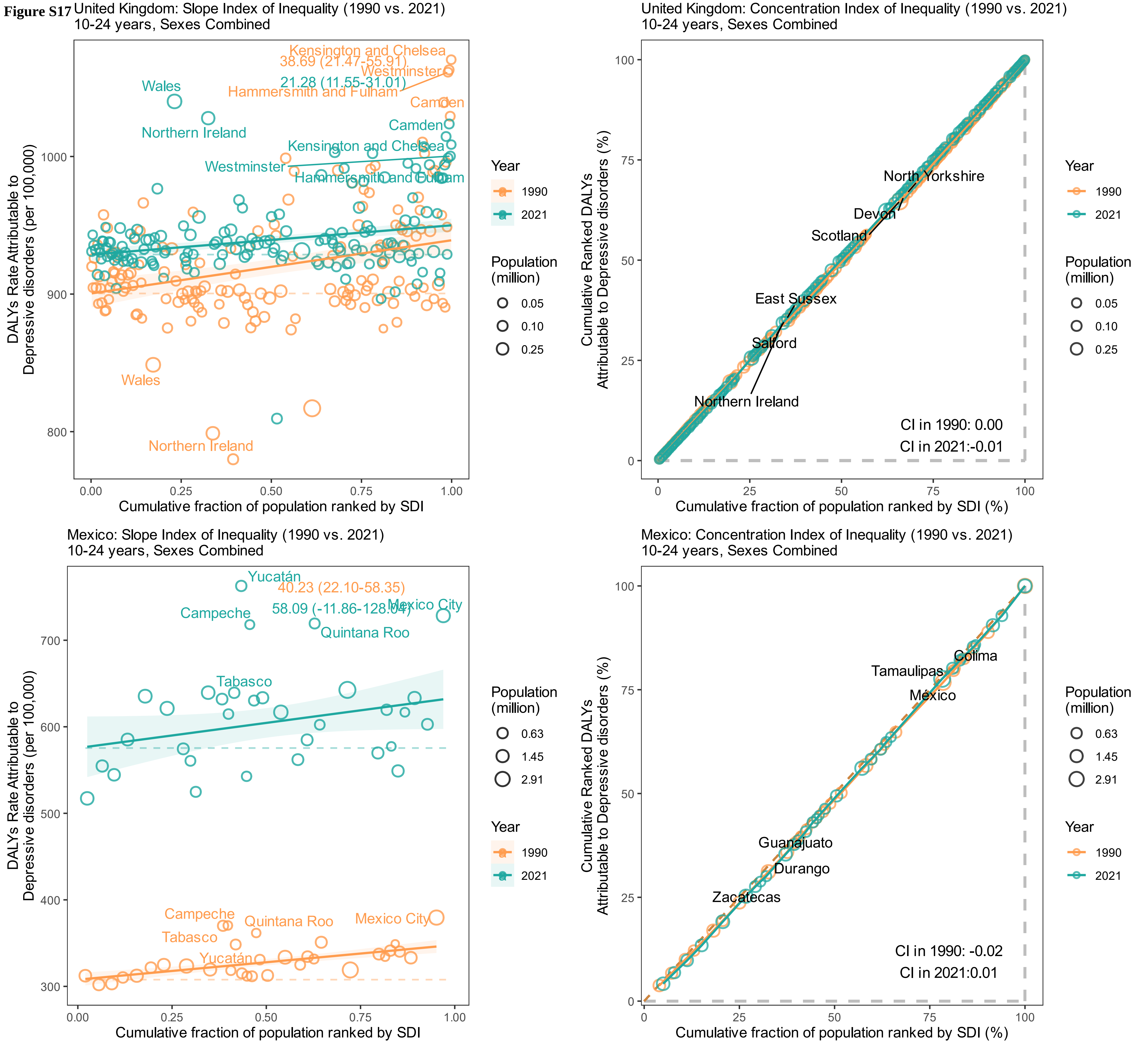

United Kingdom: Slope Index of Inequality (1990 vs. 2021)
10-24 years, Sexes Combined
Kensington and Chelsea
38.69 (21.47-55.91)
Westminster
21.28 (11.55-31.01)
Hammersmith and Fulham
Wales
Camden
Northern Ireland
Camden
Kensington and Chelsea
Westminster
Hammersmith and Fulham
Wales
Northern Ireland
DALYs Rate Attributable to Depressive disorders (per 100,000)
Cumulative fraction of population ranked by SDI
800
900
1000
0.00
0.25
0.50
0.75
1.00
Year
1990
2021
Population (million)
0.05
0.10
0.25
United Kingdom: Concentration Index of Inequality (1990 vs. 2021)
10-24 years, Sexes Combined
North Yorkshire
Devon
Scotland
East Sussex
Salford
Northern Ireland
CI in 1990: 0.00
CI in 2021:-0.01
Cumulative Ranked DALYs Attributable to Depressive disorders (%)
Cumulative fraction of population ranked by SDI (%)
0
25
50
75
100
Year
1990
2021
Population (million)
0.05
0.10
0.25
Mexico: Slope Index of Inequality (1990 vs. 2021)
10-24 years, Sexes Combined
Yucatán
40.23 (22.10-58.35)
Campeche
58.09 (-11.86-128.04)
Mexico City
Quintana Roo
Tabasco
Campeche
Quintana Roo
Mexico City
Tabasco
Yucatán
DALYs Rate Attributable to Depressive disorders (per 100,000)
Cumulative fraction of population ranked by SDI
300
400
500
600
700
0.00
0.25
0.50
0.75
1.00
Population (million)
0.63
1.45
2.91
Year
1990
2021
Mexico: Concentration Index of Inequality (1990 vs. 2021)
10-24 years, Sexes Combined
Colima
Tamaulipas
México
Guanajuato
Durango
Zacatecas
CI in 1990: -0.02
CI in 2021:0.01
Cumulative Ranked DALYs Attributable to Depressive disorders (%)
Cumulative fraction of population ranked by SDI (%)
0
25
50
75
100
Population (million)
0.63
1.45
2.91
Year
1990
2021

Figure S17

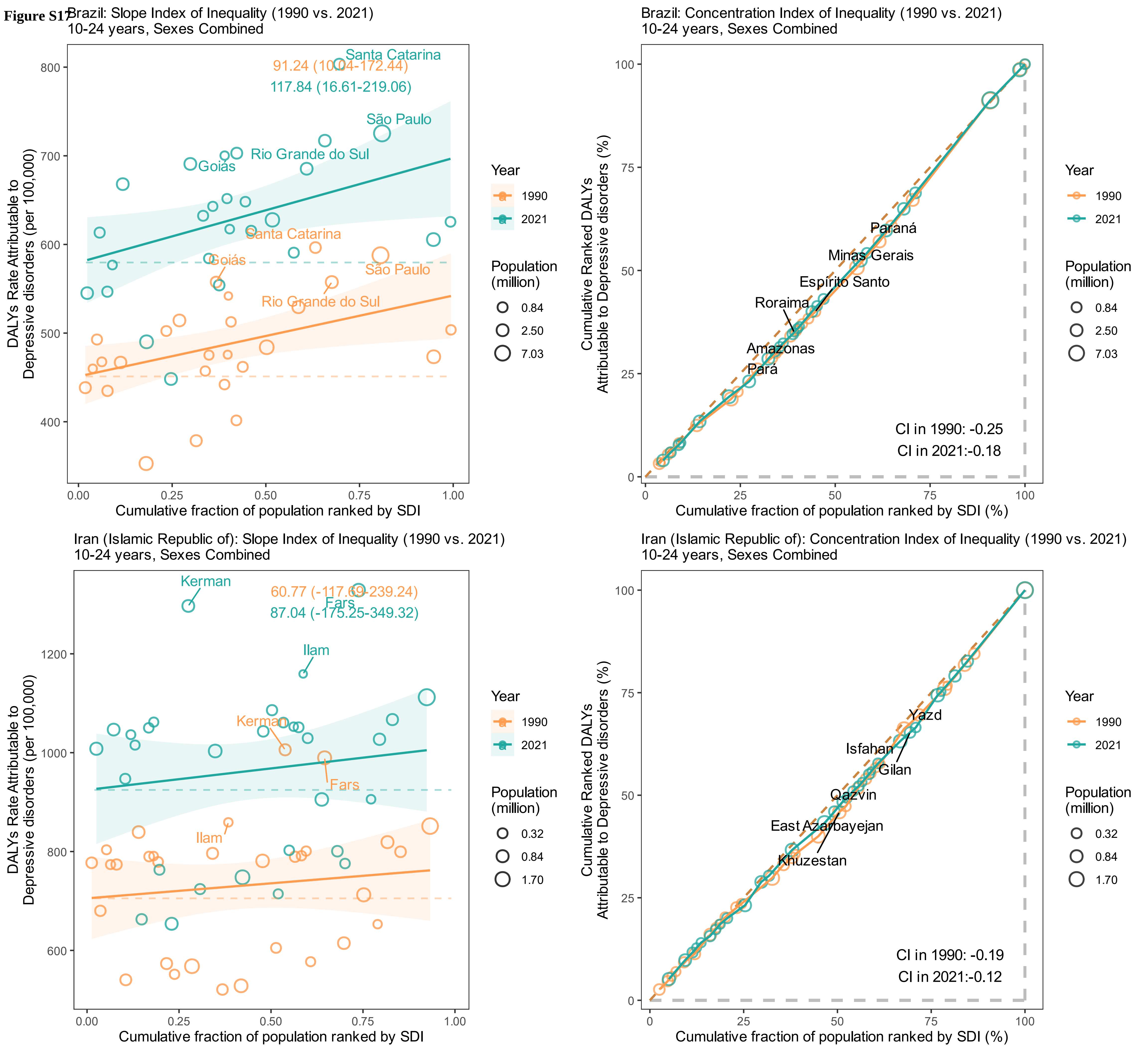

Brazil: Slope Index of Inequality (1990 vs. 2021)
10-24 years, Sexes Combined
91.24 (10.04-172.44)
117.84 (16.61-219.06)
Santa Catarina
São Paulo
Rio Grande do Sul
Goiás
DALYs Rate Attributable to Depressive disorders (per 100,000)
Cumulative fraction of population ranked by SDI
Year
1990
2021
Population (million)
0.84
2.50
7.03
Brazil: Concentration Index of Inequality (1990 vs. 2021)
10-24 years, Sexes Combined
Paraná
Minas Gerais
Espírito Santo
Roraima
Amazonas
Pará
CI in 1990: -0.25
CI in 2021:-0.18
Cumulative Ranked DALYs Attributable to Depressive disorders (%)
Cumulative fraction of population ranked by SDI (%)
Iran (Islamic Republic of): Slope Index of Inequality (1990 vs. 2021)
10-24 years, Sexes Combined
Kerman
60.77 (-117.69-239.24)
87.04 (-175.25-349.32)
Fars
Ilam
Population (million)
0.32
0.84
1.70
Iran (Islamic Republic of): Concentration Index of Inequality (1990 vs. 2021)
10-24 years, Sexes Combined
Yazd
Isfahan
Gilan
Qazvin
East Azarbayejan
Khuzestan
CI in 1990: -0.19
CI in 2021:-0.12

Figure S17

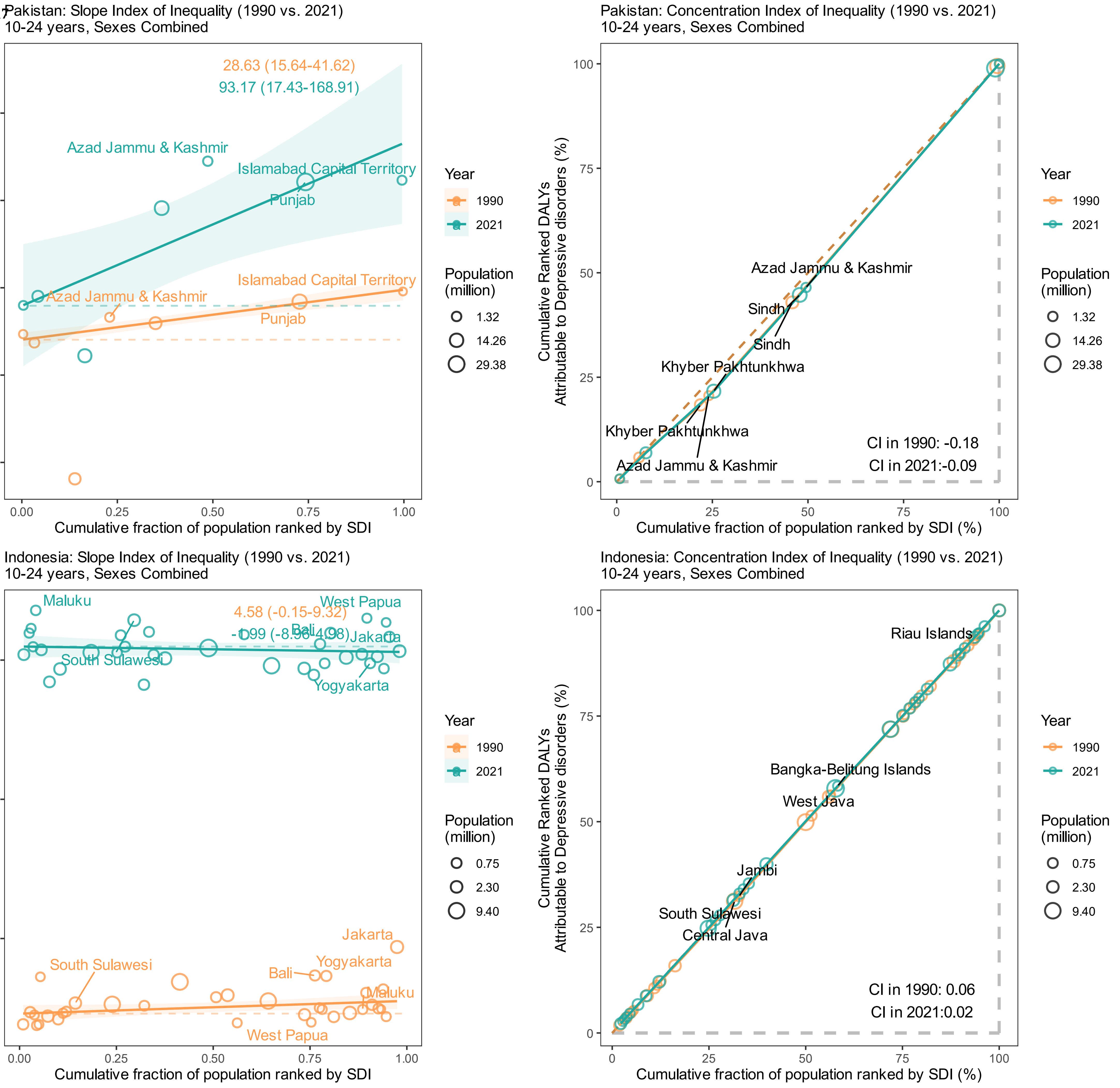

Pakistan: Slope Index of Inequality (1990 vs. 2021)
10-24 years, Sexes Combined
28.63 (15.64-41.62)
93.17 (17.43-168.91)
Azad Jammu & Kashmir
Islamabad Capital Territory
Punjab
Islamabad Capital Territory
Azad Jammu & Kashmir
Punjab
DALYs Rate Attributable to Depressive disorders (per 100,000)
Cumulative fraction of population ranked by SDI
Year
1990
2021
Population (million)
1.32
14.26
29.38
Pakistan: Concentration Index of Inequality (1990 vs. 2021)
10-24 years, Sexes Combined
Azad Jammu & Kashmir
Sindh
Sindh
Khyber Pakhtunkhwa
Khyber Pakhtunkhwa
Azad Jammu & Kashmir
CI in 1990: -0.18
CI in 2021:-0.09
Cumulative Ranked DALYs Attributable to Depressive disorders (%)
Cumulative fraction of population ranked by SDI (%)
Indonesia: Slope Index of Inequality (1990 vs. 2021)
10-24 years, Sexes Combined
Maluku
West Papua
4.58 (-0.15-9.32)
-1.99 (-8.96-4.98)
Bali
Jakarta
South Sulawesi
Yogyakarta
Jakarta
South Sulawesi
Yogyakarta
Bali
Maluku
West Papua
DALYs Rate Attributable to Depressive disorders (per 100,000)
Cumulative fraction of population ranked by SDI
Population (million)
0.75
2.30
9.40
Indonesia: Concentration Index of Inequality (1990 vs. 2021)
10-24 years, Sexes Combined
Riau Islands
Bangka-Belitung Islands
West Java
Jambi
South Sulawesi
Central Java
CI in 1990: 0.06
CI in 2021:0.02
Cumulative Ranked DALYs Attributable to Depressive disorders (%)
Cumulative fraction of population ranked by SDI (%)

Figure S17

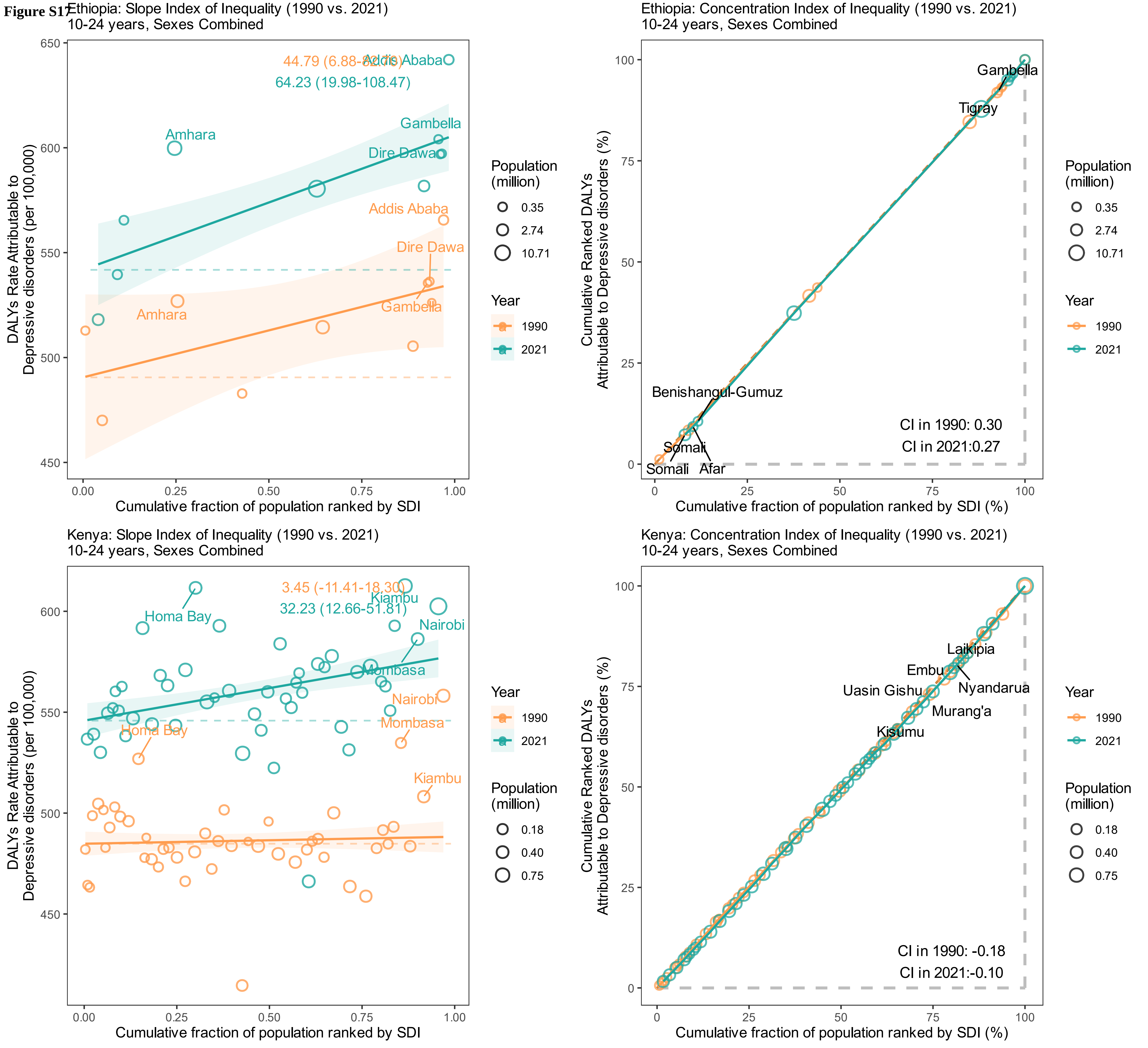

Ethiopia: Slope Index of Inequality (1990 vs. 2021)
10-24 years, Sexes Combined
44.79 (6.88-82.70)
64.23 (19.98-108.47)
Addis Ababa
Amhara
Gambella
Dire Dawa
Addis Ababa
Dire Dawa
Amhara
Gambella
DALYs Rate Attributable to Depressive disorders (per 100,000)
Cumulative fraction of population ranked by SDI
Population (million)
0.35
2.74
10.71
Year
1990
2021
Ethiopia: Concentration Index of Inequality (1990 vs. 2021)
10-24 years, Sexes Combined
Gambella
Tigray
Benishangul-Gumuz
Somali
Somali
Afar
CI in 1990: 0.30
CI in 2021:0.27
Cumulative Ranked DALYs Attributable to Depressive disorders (%)
Cumulative fraction of population ranked by SDI (%)
Population (million)
0.35
2.74
10.71
Year
1990
2021
Kenya: Slope Index of Inequality (1990 vs. 2021)
10-24 years, Sexes Combined
3.45 (-11.41-18.30)
32.23 (12.66-51.81)
Homa Bay
Kiambu
Nairobi
Mombasa
Nairobi
Homa Bay
Mombasa
Kiambu
DALYs Rate Attributable to Depressive disorders (per 100,000)
Cumulative fraction of population ranked by SDI
Year
1990
2021
Population (million)
0.18
0.40
0.75
Kenya: Concentration Index of Inequality (1990 vs. 2021)
10-24 years, Sexes Combined
Laikipia
Embu
Nyandarua
Uasin Gishu
Murang'a
Kisumu
CI in 1990: -0.18
CI in 2021:-0.10
Cumulative Ranked DALYs Attributable to Depressive disorders (%)
Cumulative fraction of population ranked by SDI (%)
Year
1990
2021
Population (million)
0.18
0.40
0.75

Figure S17

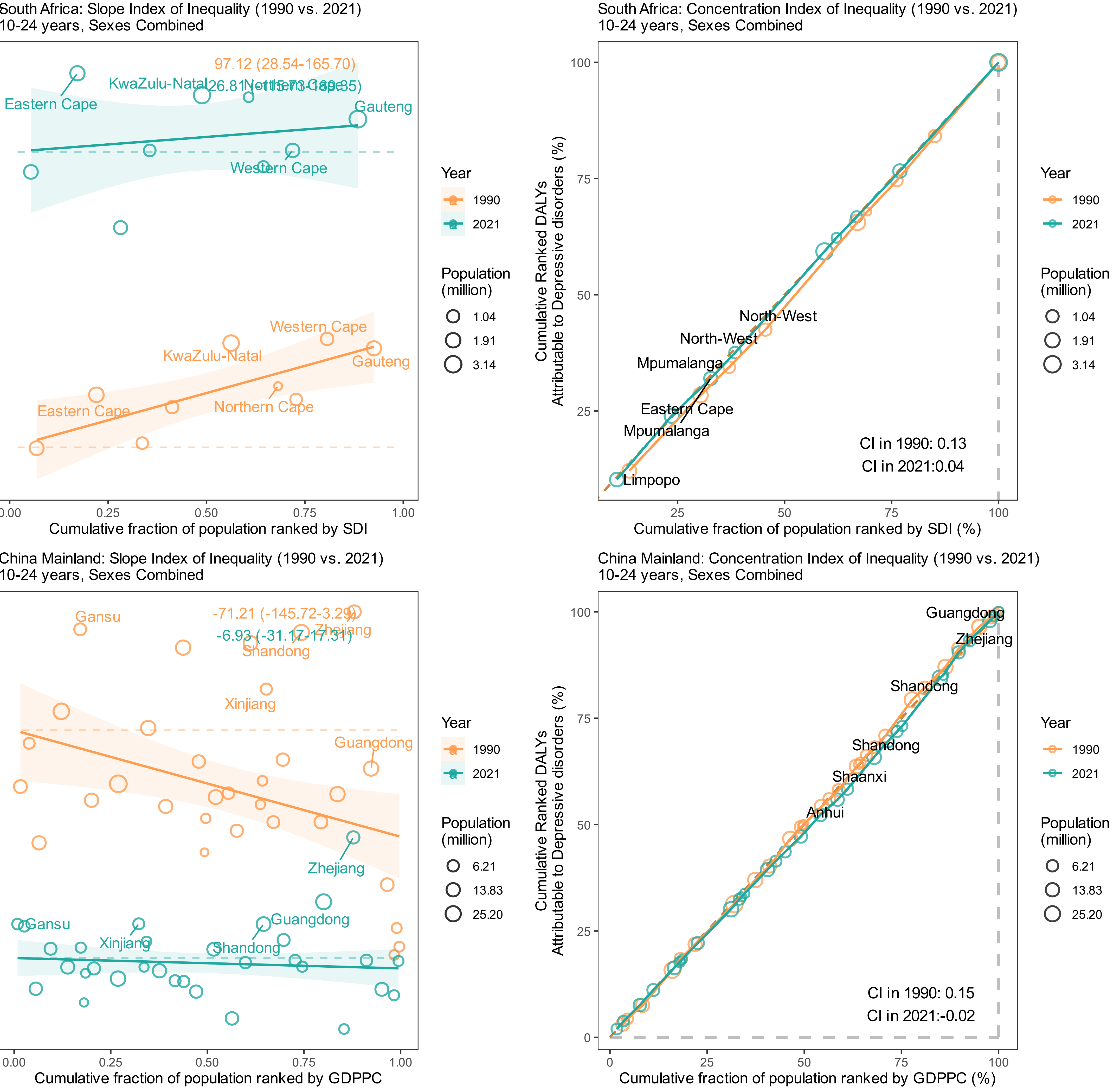
South Africa: Slope Index of Inequality (1990 vs. 2021)
10-24 years, Sexes Combined
DALYs Rate Attributable to Depressive disorders (per 100,000)
Cumulative fraction of population ranked by SDI
97.12 (28.54-165.70)
Eastern Cape
KwaZulu-Natal
Gauteng
Western Cape
Northern Cape
South Africa: Concentration Index of Inequality (1990 vs. 2021)
10-24 years, Sexes Combined
Cumulative Ranked DALYs Attributable to Depressive disorders (%)
Cumulative fraction of population ranked by SDI (%)
North-West
Mpumalanga
Eastern Cape
Limpopo
CI in 1990: 0.13
CI in 2021:0.04
Year
1990
2021
Population (million)
1.04
1.91
3.14
China Mainland: Slope Index of Inequality (1990 vs. 2021)
10-24 years, Sexes Combined
DALYs Rate Attributable to Depressive disorders (per 100,000)
Cumulative fraction of population ranked by GDPPC
-71.21 (-145.72-3.29)
-6.93 (-31.17-17.31)
Gansu
Zhejiang
Shandong
Xinjiang
Guangdong
China Mainland: Concentration Index of Inequality (1990 vs. 2021)
10-24 years, Sexes Combined
Cumulative Ranked DALYs Attributable to Depressive disorders (%)
Cumulative fraction of population ranked by GDPPC (%)
Guangdong
Zhejiang
Shandong
Shaanxi
Anhui
CI in 1990: 0.15
CI in 2021:-0.02
Year
1990
2021
Population (million)
6.21
13.83
25.20

Figure S17

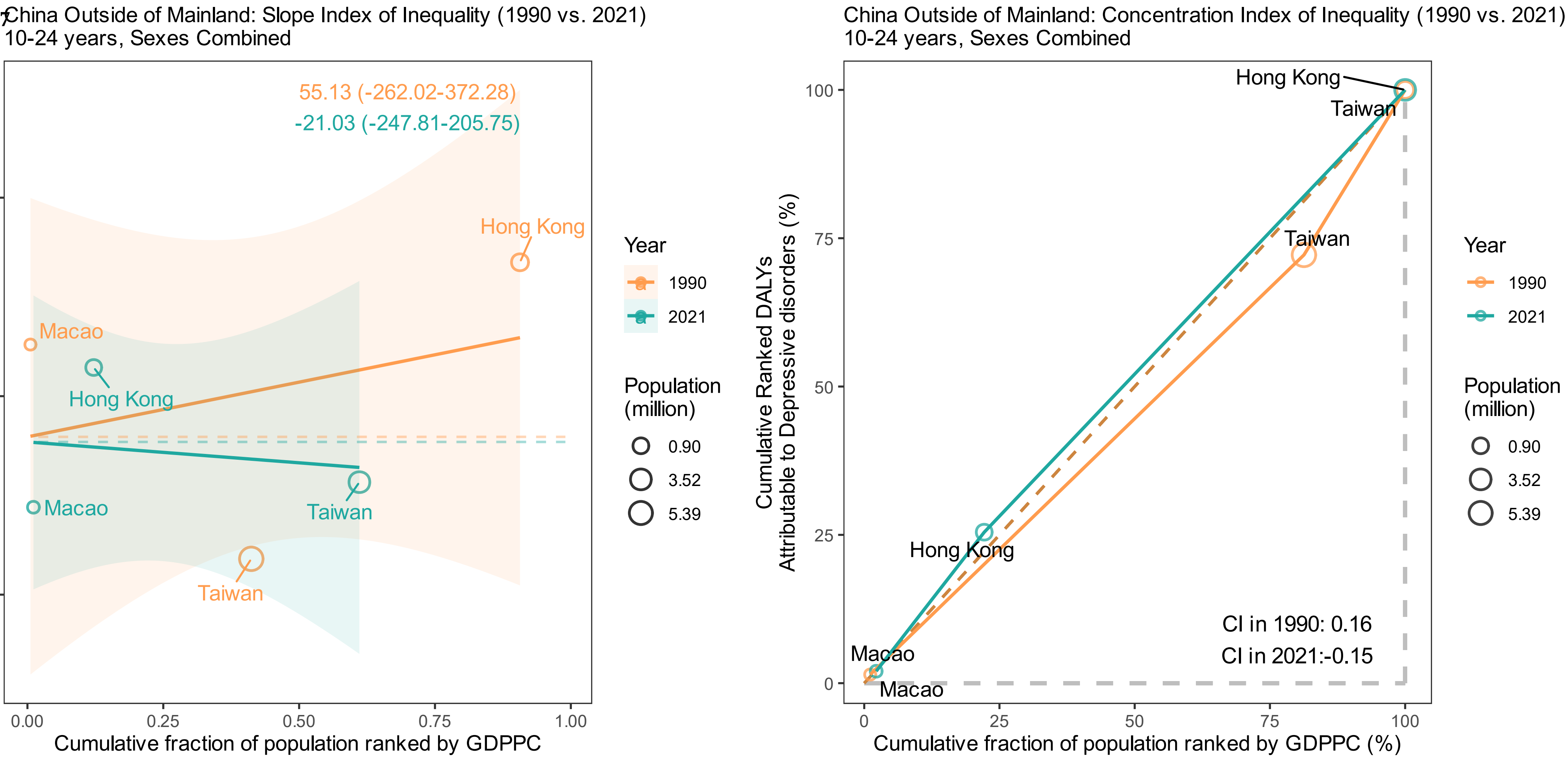
China Outside of Mainland: Slope Index of Inequality (1990 vs. 2021)
10-24 years, Sexes Combined
55.13 (-262.02-372.28)
-21.03 (-247.81-205.75)
Hong Kong
Macao
Hong Kong
Macao
Taiwan
Taiwan
DALYs Rate Attributable to Depressive disorders (per 100,000)
Cumulative fraction of population ranked by GDPPC
0.00
0.25
0.50
0.75
1.00
200
300
400
Year
1990
2021
Population (million)
0.90
3.52
5.39
China Outside of Mainland: Concentration Index of Inequality (1990 vs. 2021)
10-24 years, Sexes Combined
Hong Kong
Taiwan
Taiwan
Hong Kong
Macao
Macao
CI in 1990: 0.16
CI in 2021:-0.15
Cumulative Ranked DALYs Attributable to Depressive disorders (%)
Cumulative fraction of population ranked by GDPPC (%)
0
25
50
75
100
Year
1990
2021
Population (million)
0.90
3.52
5.39

Figure S18

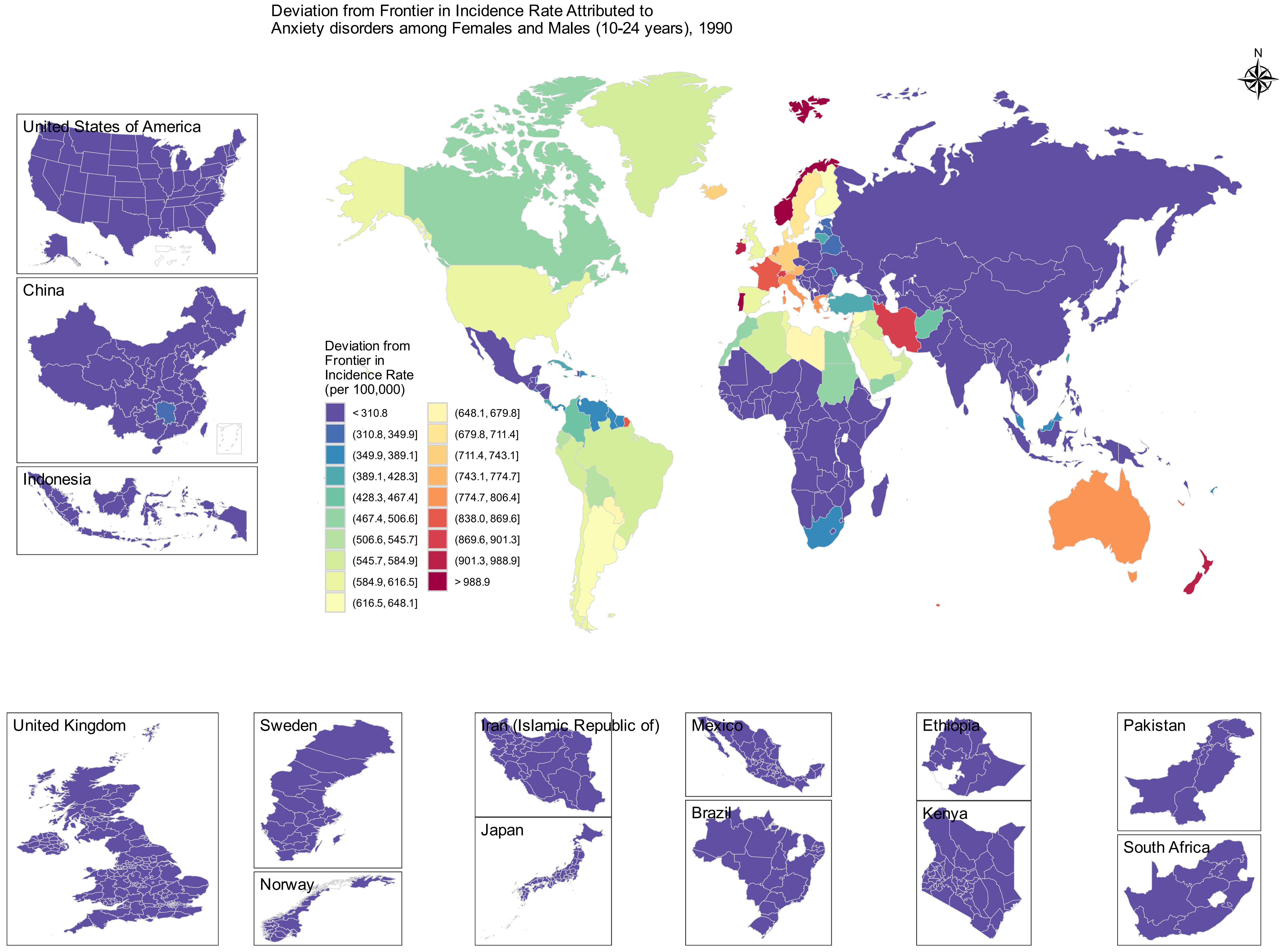

Deviation from Frontier in Incidence Rate Attributed to
Anxiety disorders among Females and Males (10-24 years), 1990
N
United States of America
China
Indonesia
Deviation from
Frontier in
Incidence Rate
(per 100,000)
< 310.8
(310.8, 349.9]
(349.9, 389.1]
(389.1, 428.3]
(428.3, 467.4]
(467.4, 506.6]
(506.6, 545.7]
(545.7, 584.9]
(584.9, 616.5]
(616.5, 648.1]
(648.1, 679.8]
(679.8, 711.4]
(711.4, 743.1]
(743.1, 774.7]
(774.7, 806.4]
(838.0, 869.6]
(869.6, 901.3]
(901.3, 988.9]
> 988.9
United Kingdom
Sweden
Norway
Iran (Islamic Republic of)
Japan
Mexico
Brazil
Ethiopia
Kenya
Pakistan
South Africa

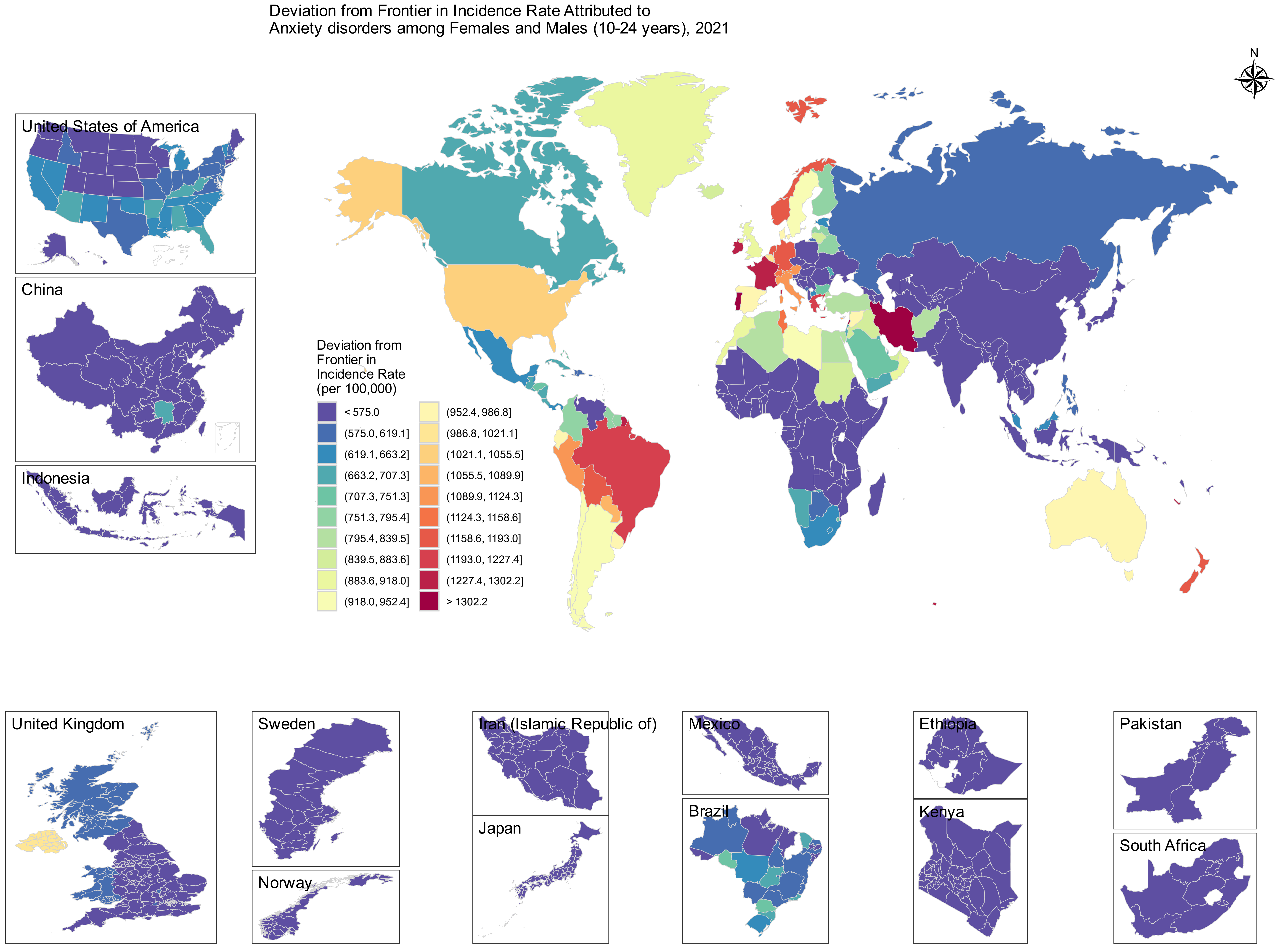

Deviation from Frontier in Incidence Rate Attributed to
Anxiety disorders among Females and Males (10-24 years), 2021
N
United States of America
China
Indonesia
Deviation from
Frontier in
Incidence Rate
(per 100,000)
< 575.0
(575.0, 619.1]
(619.1, 663.2]
(663.2, 707.3]
(707.3, 751.3]
(751.3, 795.4]
(795.4, 839.5]
(839.5, 883.6]
(883.6, 918.0]
(918.0, 952.4]
(952.4, 986.8]
(986.8, 1021.1]
(1021.1, 1055.5]
(1055.5, 1089.9]
(1089.9, 1124.3]
(1124.3, 1158.6]
(1158.6, 1193.0]
(1193.0, 1227.4]
(1227.4, 1302.2]
> 1302.2
United Kingdom
Sweden
Norway
Iran (Islamic Republic of)
Japan
Mexico
Brazil
Ethiopia
Kenya
Pakistan
South Africa

Figure S18

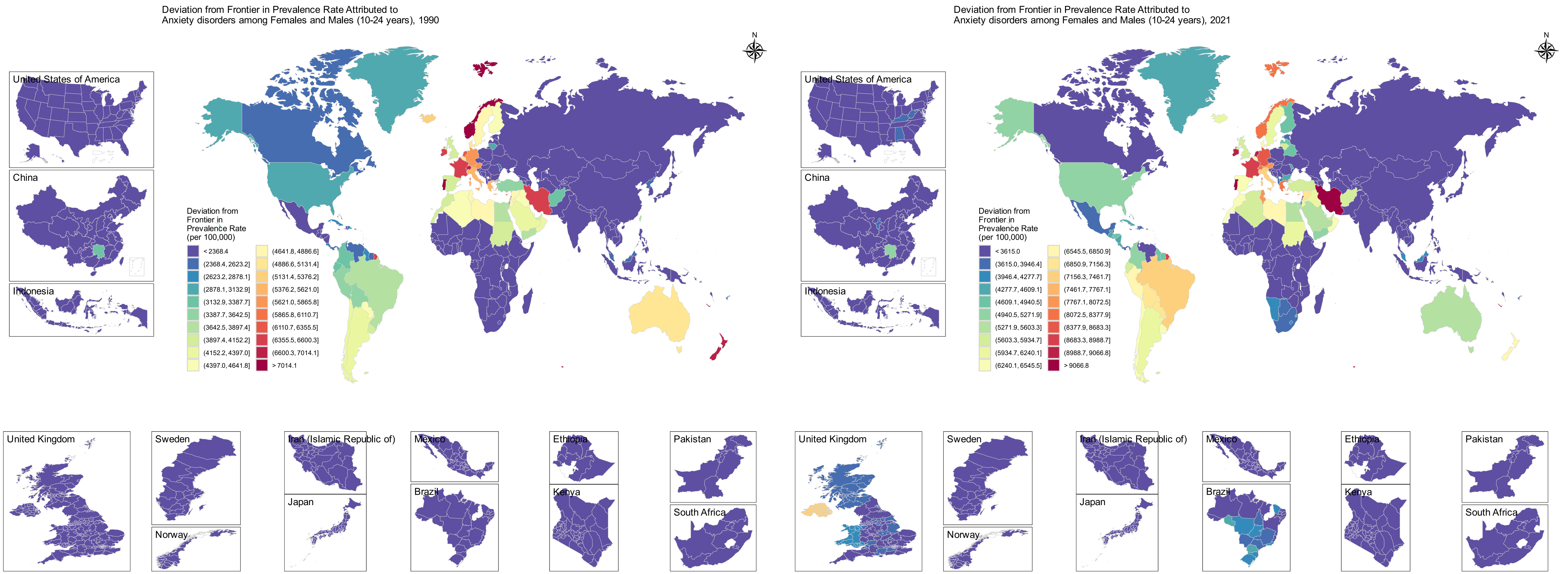

Deviation from Frontier in Prevalence Rate Attributed to
Anxiety disorders among Females and Males (10-24 years), 1990
N
United States of America
China
Indonesia
Deviation from Frontier in Prevalence Rate (per 100,000)
< 2368.4
(2368.4, 2623.2]
(2623.2, 2878.1]
(2878.1, 3132.9]
(3132.9, 3387.7]
(3387.7, 3642.5]
(3642.5, 3897.4]
(3897.4, 4152.2]
(4152.2, 4397.0]
(4397.0, 4641.8]
(4641.8, 4886.6]
(4886.6, 5131.4]
(5131.4, 5376.2]
(5376.2, 5621.0]
(5621.0, 5865.8]
(5865.8, 6110.7]
(6110.7, 6355.5]
(6355.5, 6600.3]
(6600.3, 7014.1]
> 7014.1
United Kingdom
Sweden
Norway
Iran (Islamic Republic of)
Japan
Mexico
Brazil
Ethiopia
Kenya
Pakistan
South Africa
Deviation from Frontier in Prevalence Rate Attributed to
Anxiety disorders among Females and Males (10-24 years), 2021
N
United States of America
China
Indonesia
Deviation from Frontier in Prevalence Rate (per 100,000)
< 3615.0
(3615.0, 3946.4]
(3946.4, 4277.7]
(4277.7, 4609.1]
(4609.1, 4940.5]
(4940.5, 5271.9]
(5271.9, 5603.3]
(5603.3, 5934.7]
(5934.7, 6240.1]
(6240.1, 6545.5]
(6545.5, 6850.9]
(6850.9, 7156.3]
(7156.3, 7461.7]
(7461.7, 7767.1]
(7767.1, 8072.5]
(8072.5, 8377.9]
(8377.9, 8683.3]
(8683.3, 8988.7]
(8988.7, 9066.8]
> 9066.8
United Kingdom
Sweden
Norway
Iran (Islamic Republic of)
Japan
Mexico
Brazil
Ethiopia
Kenya
Pakistan
South Africa

Figure S18

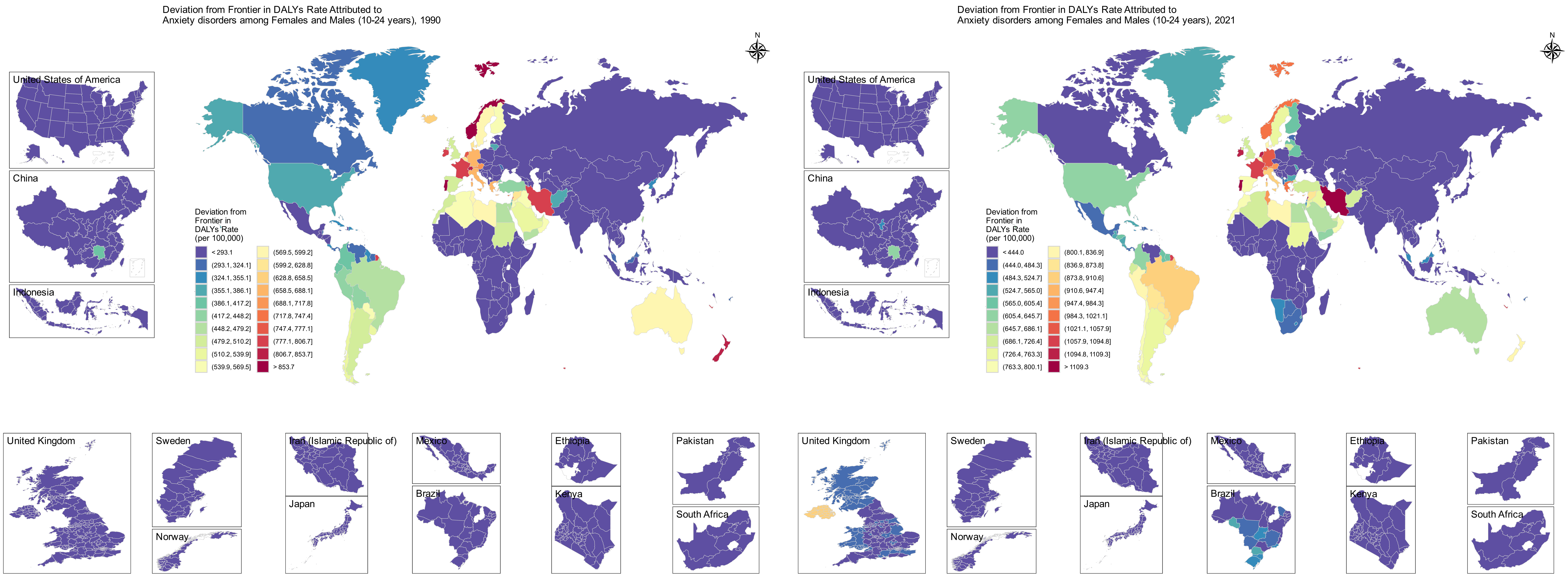
Deviation from Frontier in DALYs Rate Attributed to
Anxiety disorders among Females and Males (10-24 years), 1990
N
United States of America
China
Indonesia
Deviation from
Frontier in
DALYs Rate
(per 100,000)
< 293.1
(293.1, 324.1]
(324.1, 355.1]
(355.1, 386.1]
(386.1, 417.2]
(417.2, 448.2]
(448.2, 479.2]
(479.2, 510.2]
(510.2, 539.9]
(539.9, 569.5]
(569.5, 599.2]
(599.2, 628.8]
(628.8, 658.5]
(658.5, 688.1]
(688.1, 717.8]
(717.8, 747.4]
(747.4, 777.1]
(777.1, 806.7]
(806.7, 853.7]
> 853.7
United Kingdom
Sweden
Norway
Iran (Islamic Republic of)
Japan
Mexico
Brazil
Ethiopia
Kenya
Pakistan
South Africa
Deviation from Frontier in DALYs Rate Attributed to
Anxiety disorders among Females and Males (10-24 years), 2021
N
United States of America
China
Indonesia
Deviation from
Frontier in
DALYs Rate
(per 100,000)
< 444.0
(444.0, 484.3]
(484.3, 524.7]
(524.7, 565.0]
(565.0, 605.4]
(605.4, 645.7]
(645.7, 686.1]
(686.1, 726.4]
(726.4, 763.3]
(763.3, 800.1]
(800.1, 836.9]
(836.9, 873.8]
(873.8, 910.6]
(910.6, 947.4]
(947.4, 984.3]
(984.3, 1021.1]
(1021.1, 1057.9]
(1057.9, 1094.8]
(1094.8, 1109.3]
> 1109.3
United Kingdom
Sweden
Norway
Iran (Islamic Republic of)
Japan
Mexico
Brazil
Ethiopia
Kenya
Pakistan
South Africa

Figure S19

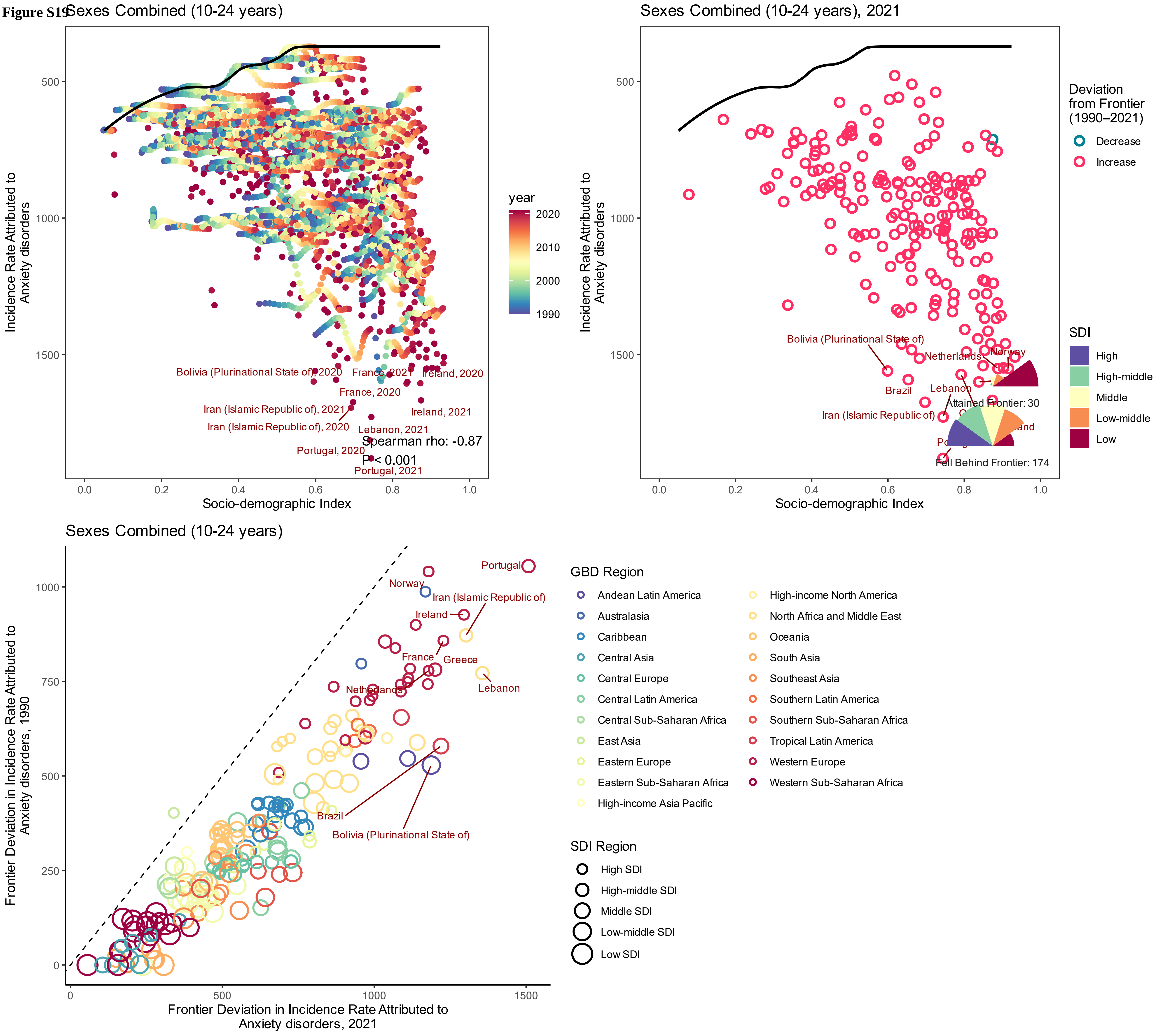

Sexes Combined (10-24 years)
Incidence Rate Attributed to Anxiety disorders
Socio-demographic Index
year
2020
2010
2000
1990
Bolivia (Plurinational State of), 2020
France, 2021
Ireland, 2020
France, 2020
Iran (Islamic Republic of), 2021
Ireland, 2021
Iran (Islamic Republic of), 2020
Lebanon, 2021
Spearman rho: -0.87
Portugal, 2020
P < 0.001
Portugal, 2021
Sexes Combined (10-24 years), 2021
Deviation from Frontier (1990–2021)
Decrease
Increase
Bolivia (Plurinational State of)
Netherlands
Norway
Brazil
Lebanon
Attained Frontier: 30
Iran (Islamic Republic of)
Fell Behind Frontier: 174
SDI
High
High-middle
Middle
Low-middle
Low
Sexes Combined (10-24 years)
Frontier Deviation in Incidence Rate Attributed to Anxiety disorders, 1990
Frontier Deviation in Incidence Rate Attributed to Anxiety disorders, 2021
Portugal
Norway
Iran (Islamic Republic of)
Ireland
France
Greece
Netherlands
Lebanon
Brazil
Bolivia (Plurinational State of)
GBD Region
Andean Latin America
Australasia
Caribbean
Central Asia
Central Europe
Central Latin America
Central Sub-Saharan Africa
East Asia
Eastern Europe
Eastern Sub-Saharan Africa
High-income Asia Pacific
High-income North America
North Africa and Middle East
Oceania
South Asia
Southeast Asia
Southern Latin America
Southern Sub-Saharan Africa
Tropical Latin America
Western Europe
Western Sub-Saharan Africa
SDI Region
High SDI
High-middle SDI
Middle SDI
Low-middle SDI
Low SDI

Figure S19

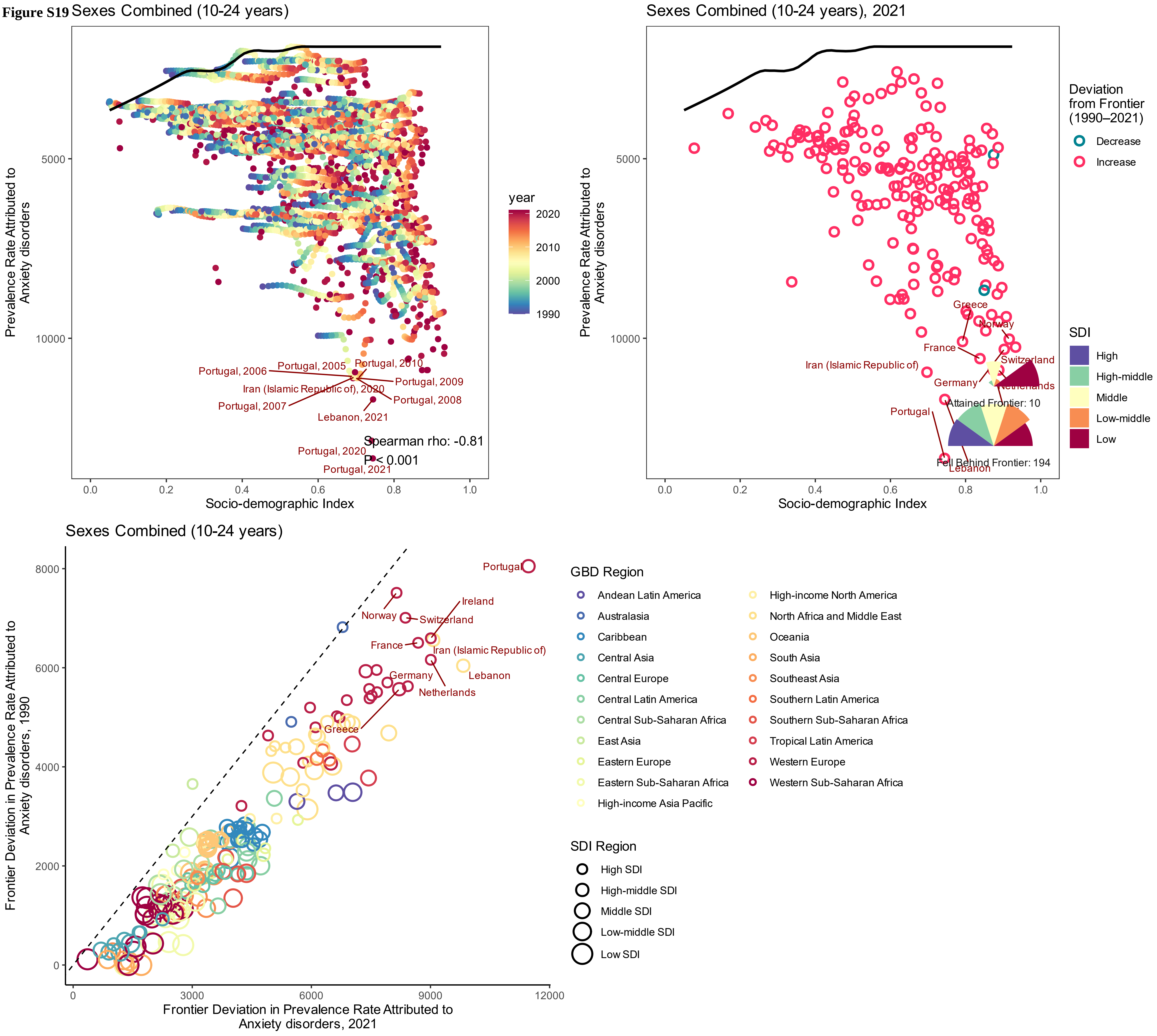

Sexes Combined (10-24 years)
Prevalence Rate Attributed to Anxiety disorders
Socio-demographic Index
year
Spearman rho: -0.81
P < 0.001
Portugal, 2006
Portugal, 2005
Portugal, 2010
Portugal, 2009
Iran (Islamic Republic of), 2020
Portugal, 2007
Portugal, 2008
Lebanon, 2021
Portugal, 2020
Portugal, 2021
Sexes Combined (10-24 years), 2021
Deviation from Frontier (1990–2021)
Decrease
Increase
SDI
High
High-middle
Middle
Low-middle
Low
Greece
Norway
France
Switzerland
Iran (Islamic Republic of)
Germany
Netherlands
Attained Frontier: 10
Portugal
Fell Behind Frontier: 194
Lebanon
Sexes Combined (10-24 years)
Frontier Deviation in Prevalence Rate Attributed to Anxiety disorders, 1990
Frontier Deviation in Prevalence Rate Attributed to Anxiety disorders, 2021
Portugal
Ireland
Norway
Switzerland
France
Iran (Islamic Republic of)
Lebanon
Germany
Netherlands
Greece
GBD Region
Andean Latin America
Australasia
Caribbean
Central Asia
Central Europe
Central Latin America
Central Sub-Saharan Africa
East Asia
Eastern Europe
Eastern Sub-Saharan Africa
High-income Asia Pacific
High-income North America
North Africa and Middle East
Oceania
South Asia
Southeast Asia
Southern Latin America
Southern Sub-Saharan Africa
Tropical Latin America
Western Europe
Western Sub-Saharan Africa
SDI Region
High SDI
High-middle SDI
Middle SDI
Low-middle SDI
Low SDI

Figure S19

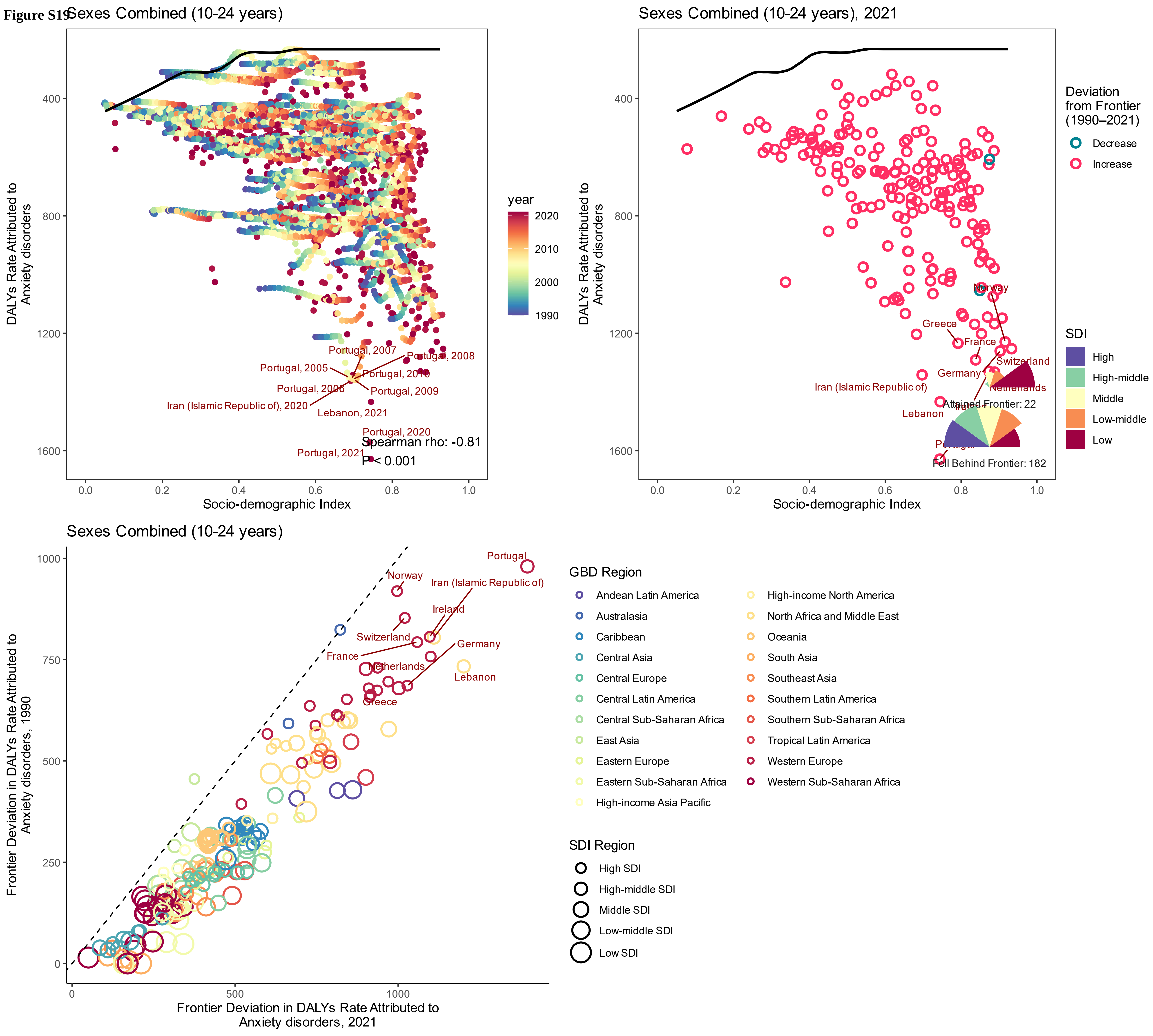
Sexes Combined (10-24 years)
DALYs Rate Attributed to Anxiety disorders
Socio-demographic Index
year
Portugal, 2007
Portugal, 2008
Portugal, 2005
Portugal, 2010
Portugal, 2006
Portugal, 2009
Iran (Islamic Republic of), 2020
Lebanon, 2021
Portugal, 2020
Portugal, 2021
Spearman rho: -0.81
P < 0.001
Sexes Combined (10-24 years), 2021
Deviation from Frontier (1990–2021)
Decrease
Increase
SDI
High
High-middle
Middle
Low-middle
Low
Norway
Greece
France
Switzerland
Germany
Iran (Islamic Republic of)
Netherlands
Attained Frontier: 22
Lebanon
Fell Behind Frontier: 182
Frontier Deviation in DALYs Rate Attributed to Anxiety disorders, 1990
Frontier Deviation in DALYs Rate Attributed to Anxiety disorders, 2021
Portugal
Ireland
GBD Region
Andean Latin America
Australasia
Caribbean
Central Asia
Central Europe
Central Latin America
Central Sub-Saharan Africa
East Asia
Eastern Europe
Eastern Sub-Saharan Africa
High-income Asia Pacific
High-income North America
North Africa and Middle East
Oceania
South Asia
Southeast Asia
Southern Latin America
Southern Sub-Saharan Africa
Tropical Latin America
Western Europe
Western Sub-Saharan Africa
SDI Region
High SDI
High-middle SDI
Middle SDI
Low-middle SDI
Low SDI

Figure S20

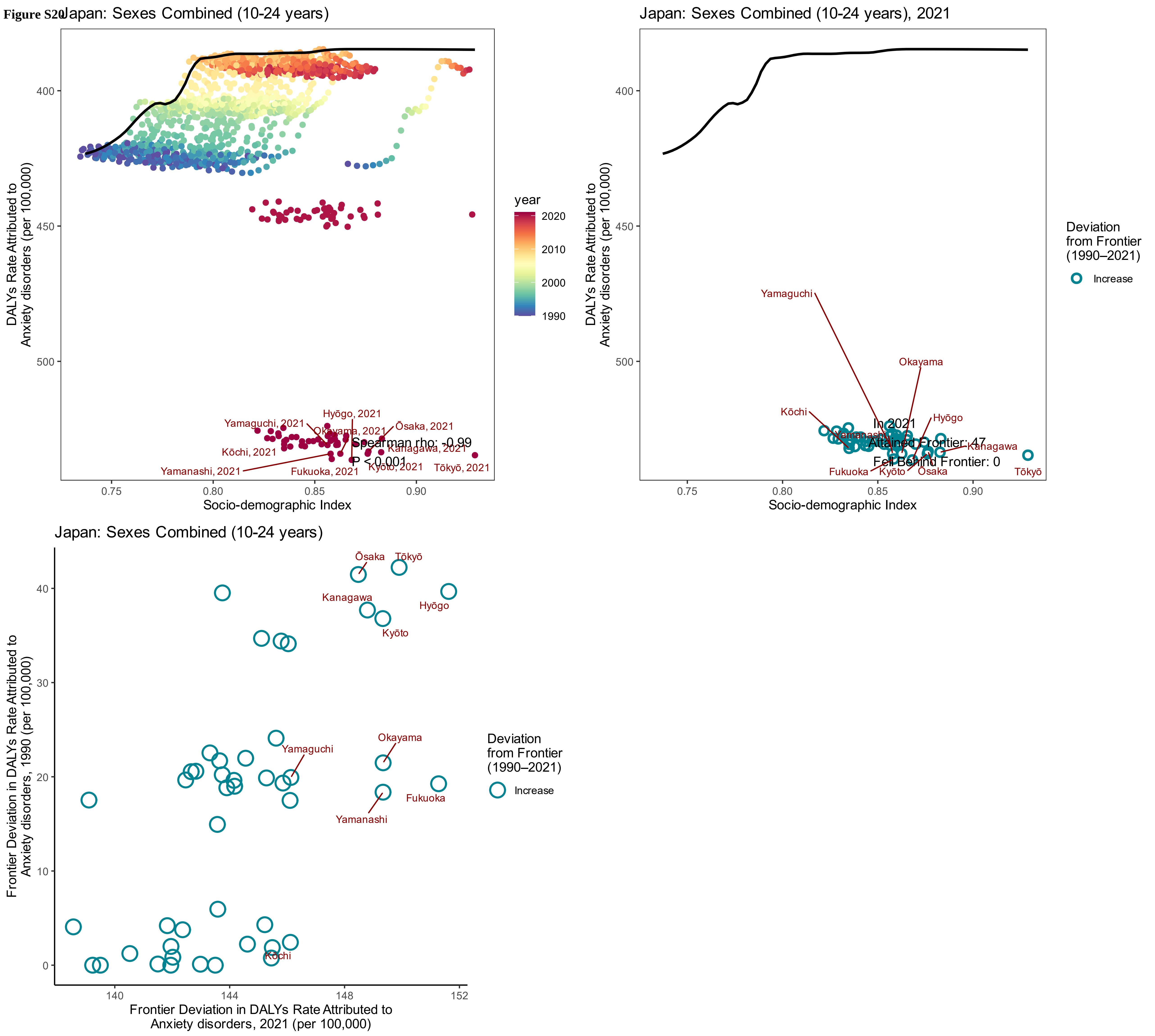

Japan: Sexes Combined (10-24 years)
DALYs Rate Attributed to Anxiety disorders (per 100,000)
Socio-demographic Index
year
2020
2010
2000
1990
Spearman rho: -0.99
P < 0.001
Yamaguchi, 2021
Hyōgo, 2021
Okayama, 2021
Ōsaka, 2021
Kōchi, 2021
Kanagawa, 2021
Yamanashi, 2021
Fukuoka, 2021
Kyōto, 2021
Tōkyō, 2021
Japan: Sexes Combined (10-24 years), 2021
In 2021
Attained Frontier: 47
Fell Behind Frontier: 0
Yamaguchi
Okayama
Kōchi
Hyōgo
Yamanashi
Kanagawa
Fukuoka
Kyōto
Ōsaka
Tōkyō
Deviation from Frontier (1990–2021)
Increase
Japan: Sexes Combined (10-24 years)
Frontier Deviation in DALYs Rate Attributed to Anxiety disorders, 1990 (per 100,000)
Frontier Deviation in DALYs Rate Attributed to Anxiety disorders, 2021 (per 100,000)
Ōsaka
Tōkyō
Kanagawa
Hyōgo
Kyōto
Okayama
Yamaguchi
Fukuoka
Yamanashi
Kōchi
Deviation from Frontier (1990–2021)
Increase

Figure S20

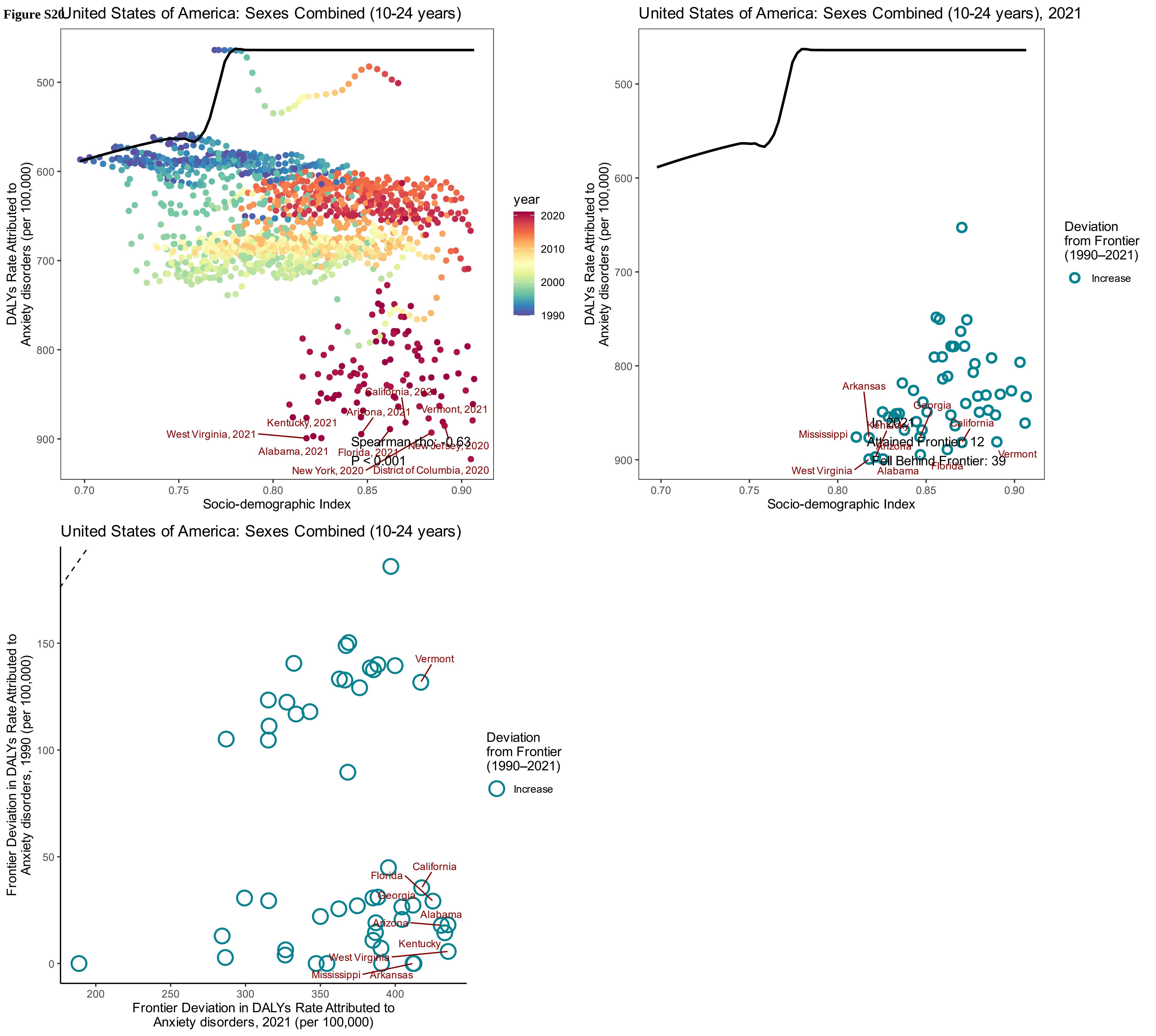
United States of America: Sexes Combined (10-24 years)
DALYs Rate Attributed to Anxiety disorders (per 100,000)
Socio-demographic Index
year
Spearman rho: -0.63
P < 0.001
California, 2021
Arizona, 2021
Vermont, 2021
Kentucky, 2021
West Virginia, 2021
Alabama, 2021
Florida, 2021
New Jersey, 2020
New York, 2020
District of Columbia, 2020
United States of America: Sexes Combined (10-24 years), 2021
Deviation from Frontier (1990–2021)
Increase
In 2021
Attained Frontier: 12
Fell Behind Frontier: 39
Arkansas
Georgia
Mississippi
Kentucky
California
Arizona
Vermont
West Virginia
Alabama
Florida
United States of America: Sexes Combined (10-24 years)
Frontier Deviation in DALYs Rate Attributed to Anxiety disorders, 1990 (per 100,000)
Frontier Deviation in DALYs Rate Attributed to Anxiety disorders, 2021 (per 100,000)
Deviation from Frontier (1990–2021)
Increase
Vermont
California
Florida
Georgia
Alabama
Arizona
Kentucky
West Virginia
Mississippi
Arkansas

Figure S20

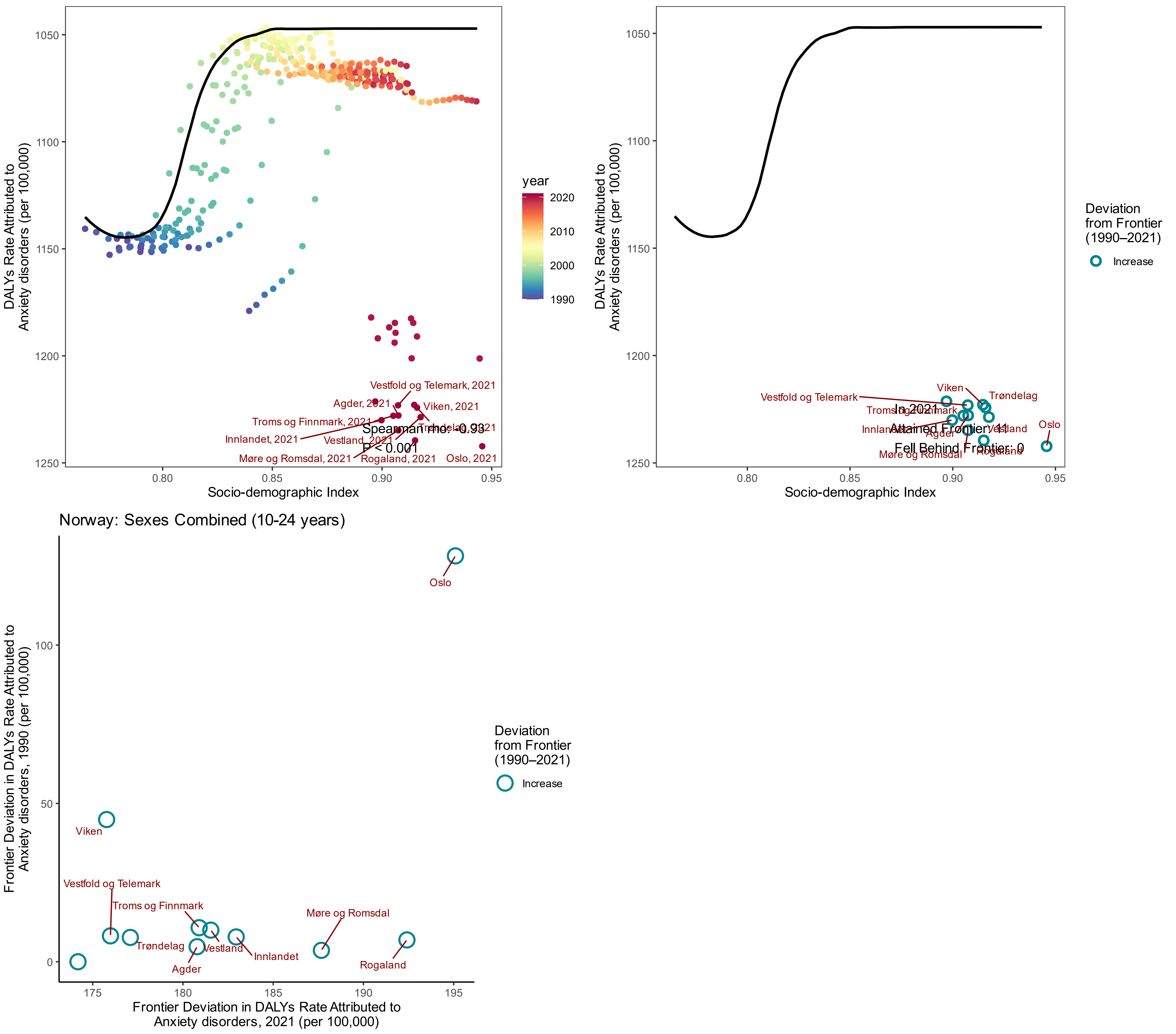

Norway: Sexes Combined (10-24 years)
DALYs Rate Attributed to Anxiety disorders (per 100,000)
Socio-demographic Index
year
2020
2010
2000
1990
Vestfold og Telemark, 2021
Agder, 2021
Viken, 2021
Troms og Finnmark, 2021
Trøndelag, 2021
Innlandet, 2021
Vestland, 2021
Spearman rho: -0.93
P < 0.001
Møre og Romsdal, 2021
Rogaland, 2021
Oslo, 2021
Norway: Sexes Combined (10-24 years), 2021
Deviation from Frontier (1990–2021)
Increase
Viken
Trøndelag
Vestfold og Telemark
Troms og Finnmark
In 2021
Innlandet
Attained Frontier: 11
Vestland
Oslo
Agder
Fell Behind Frontier: 0
Møre og Romsdal
Rogaland
Norway: Sexes Combined (10-24 years)
Frontier Deviation in DALYs Rate Attributed to Anxiety disorders, 1990 (per 100,000)
Frontier Deviation in DALYs Rate Attributed to Anxiety disorders, 2021 (per 100,000)
Oslo
Viken
Vestfold og Telemark
Troms og Finnmark
Møre og Romsdal
Trøndelag
Vestland
Innlandet
Agder
Rogaland
Deviation from Frontier (1990–2021)
Increase

Figure S20

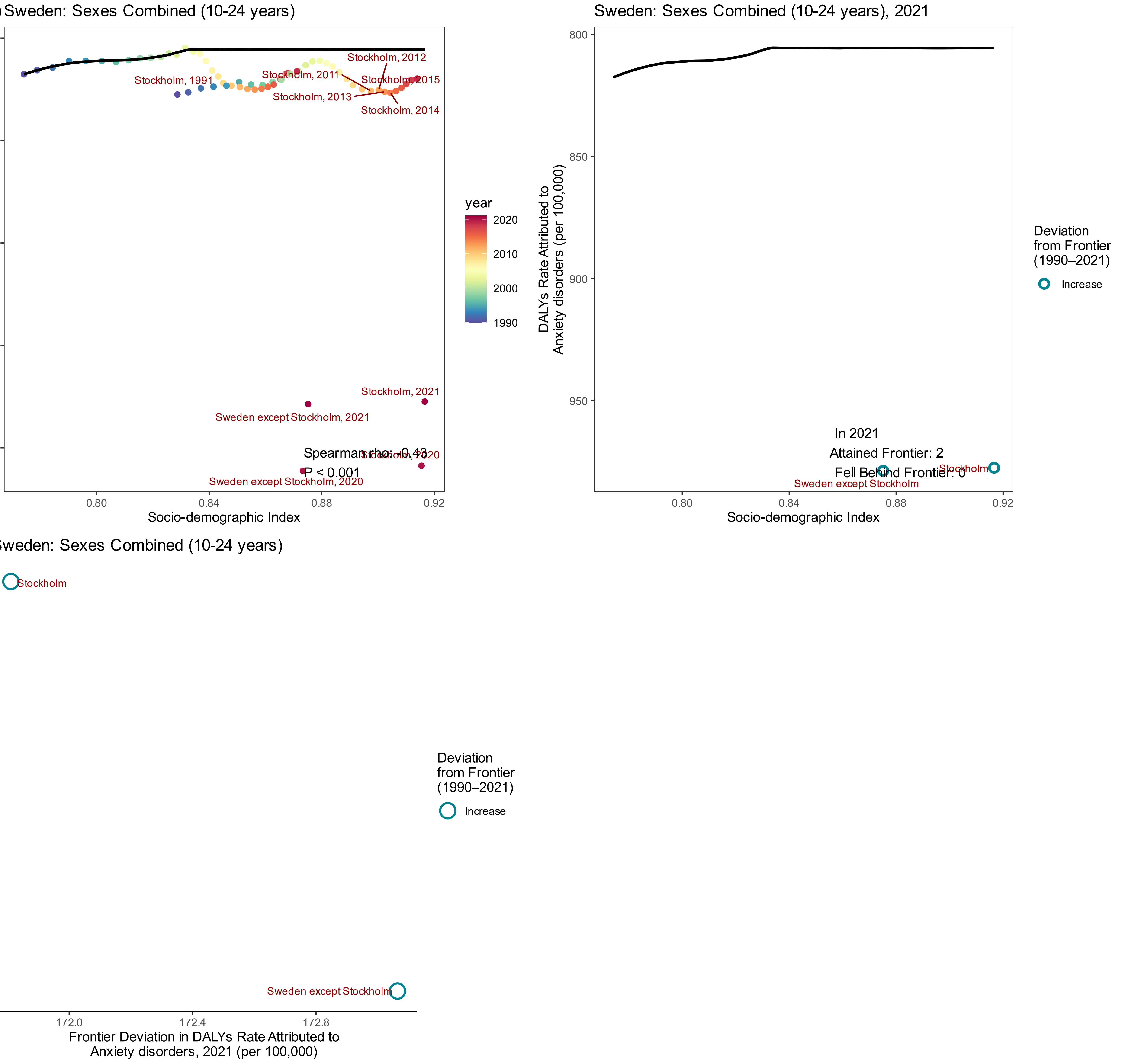
Sweden: Sexes Combined (10-24 years)
DALYs Rate Attributed to Anxiety disorders (per 100,000)
Socio-demographic Index
Stockholm, 1991
Stockholm, 2011
Stockholm, 2012
Stockholm, 2013
Stockholm, 2014
Stockholm, 2015
Stockholm, 2021
Sweden except Stockholm, 2021
Stockholm, 2020
Sweden except Stockholm, 2020
Spearman rho: -0.43
P < 0.001
year
Sweden: Sexes Combined (10-24 years), 2021
In 2021
Attained Frontier: 2
Fell Behind Frontier: 0
Stockholm
Sweden except Stockholm
Deviation from Frontier (1990–2021)
Increase
Frontier Deviation in DALYs Rate Attributed to Anxiety disorders, 1990 (per 100,000)
Frontier Deviation in DALYs Rate Attributed to Anxiety disorders, 2021 (per 100,000)

Figure S20

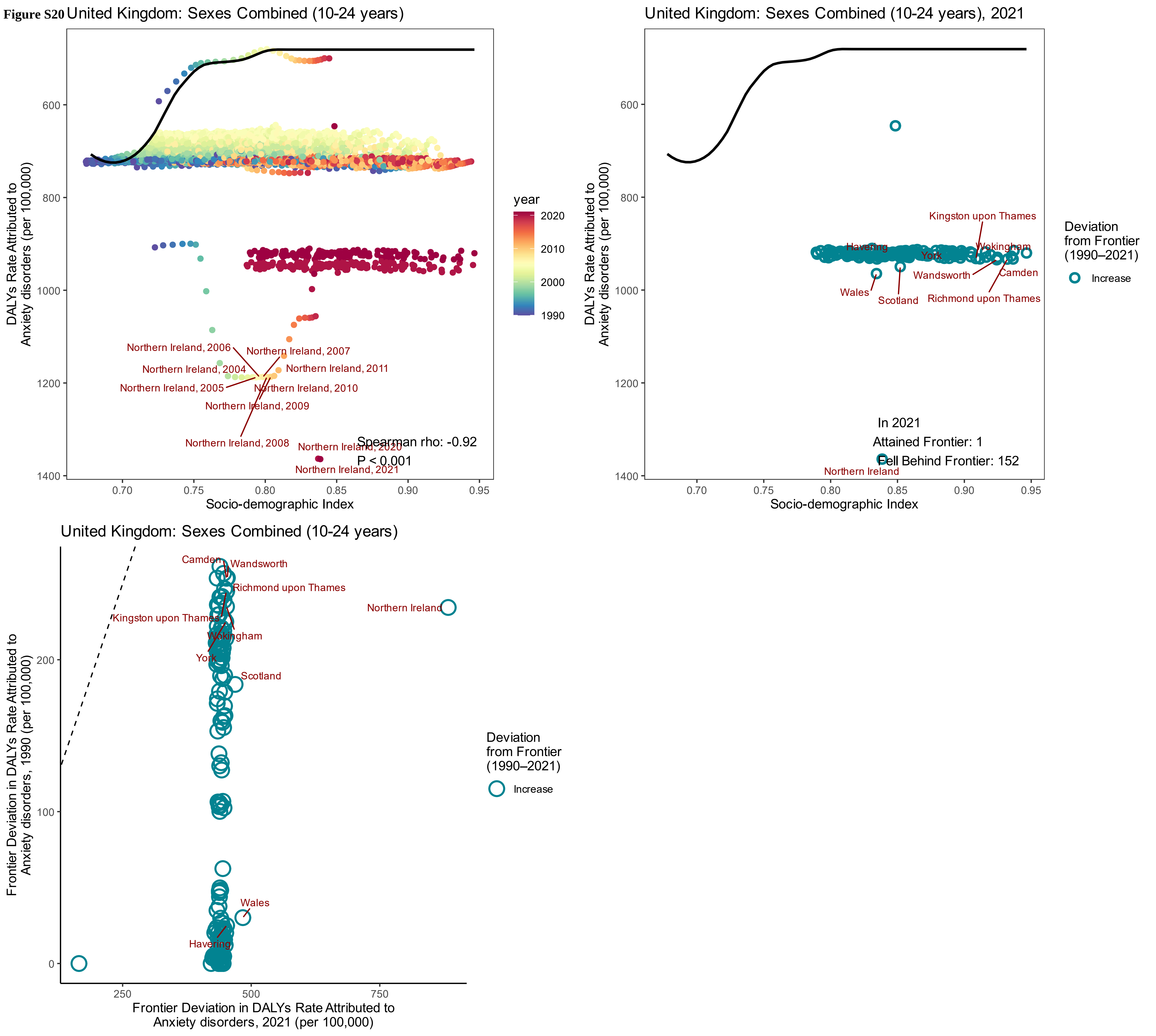
United Kingdom: Sexes Combined (10-24 years)
DALYs Rate Attributed to Anxiety disorders (per 100,000)
Socio-demographic Index
year
2020
2010
2000
1990
Northern Ireland, 2006
Northern Ireland, 2007
Northern Ireland, 2004
Northern Ireland, 2011
Northern Ireland, 2005
Northern Ireland, 2010
Northern Ireland, 2009
Northern Ireland, 2008
Northern Ireland, 2020
Northern Ireland, 2021
Spearman rho: -0.92
P < 0.001
United Kingdom: Sexes Combined (10-24 years), 2021
Kingston upon Thames
Havering
Wokingham
York
Wandsworth
Camden
Wales
Scotland
Richmond upon Thames
In 2021
Attained Frontier: 1
Fell Behind Frontier: 152
Northern Ireland
Deviation from Frontier (1990–2021)
Increase
United Kingdom: Sexes Combined (10-24 years)
Frontier Deviation in DALYs Rate Attributed to Anxiety disorders, 1990 (per 100,000)
Frontier Deviation in DALYs Rate Attributed to Anxiety disorders, 2021 (per 100,000)
Deviation from Frontier (1990–2021)
Increase

Figure S20

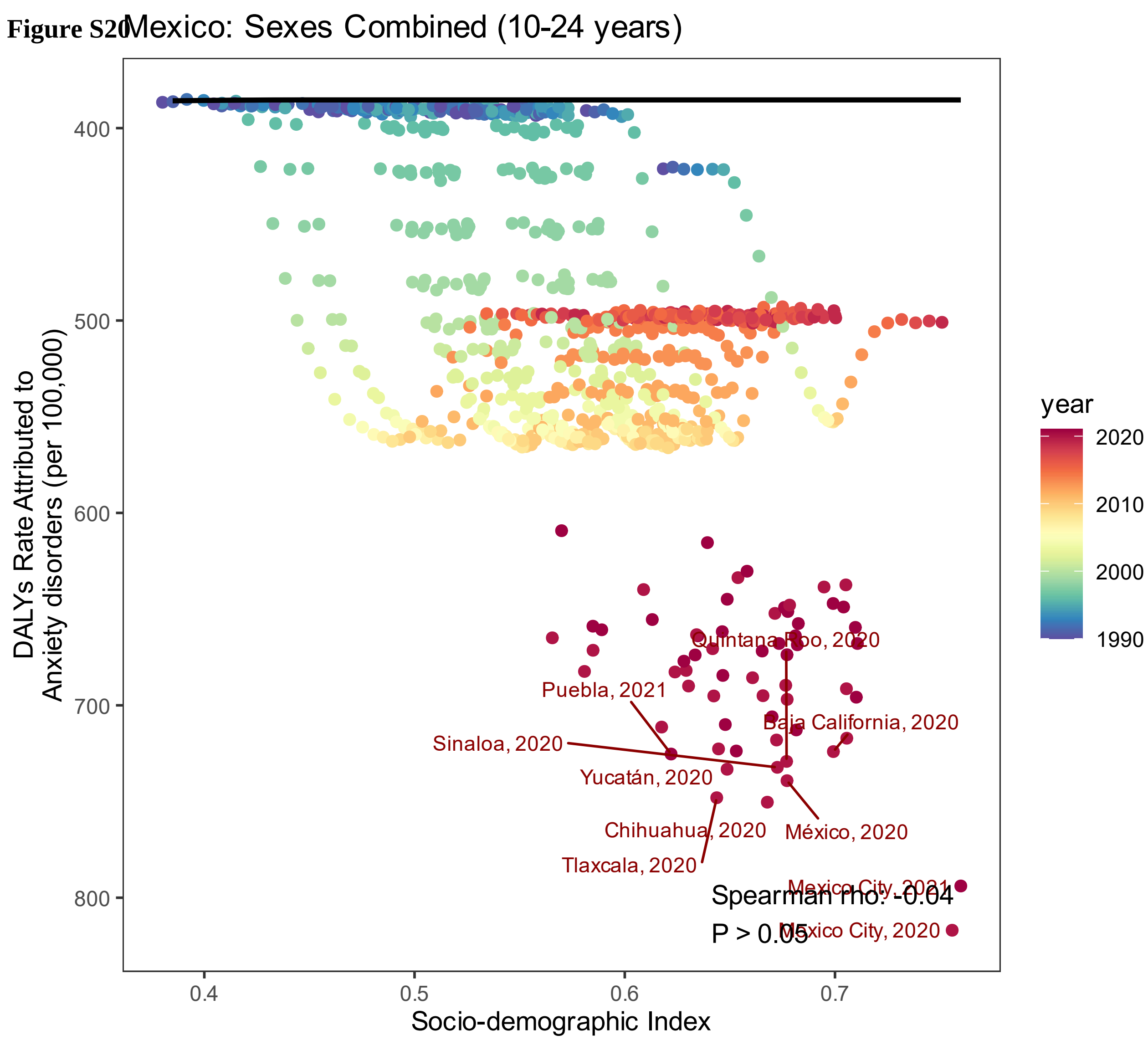

Mexico: Sexes Combined (10-24 years)
DALYs Rate Attributed to Anxiety disorders (per 100,000)
Socio-demographic Index
year
2020
2010
2000
1990
Quintana Roo, 2020
Puebla, 2021
Baja California, 2020
Sinaloa, 2020
Yucatán, 2020
Chihuahua, 2020
México, 2020
Tlaxcala, 2020
Mexico City, 2021
Mexico City, 2020
Spearman rho: -0.04
P > 0.05

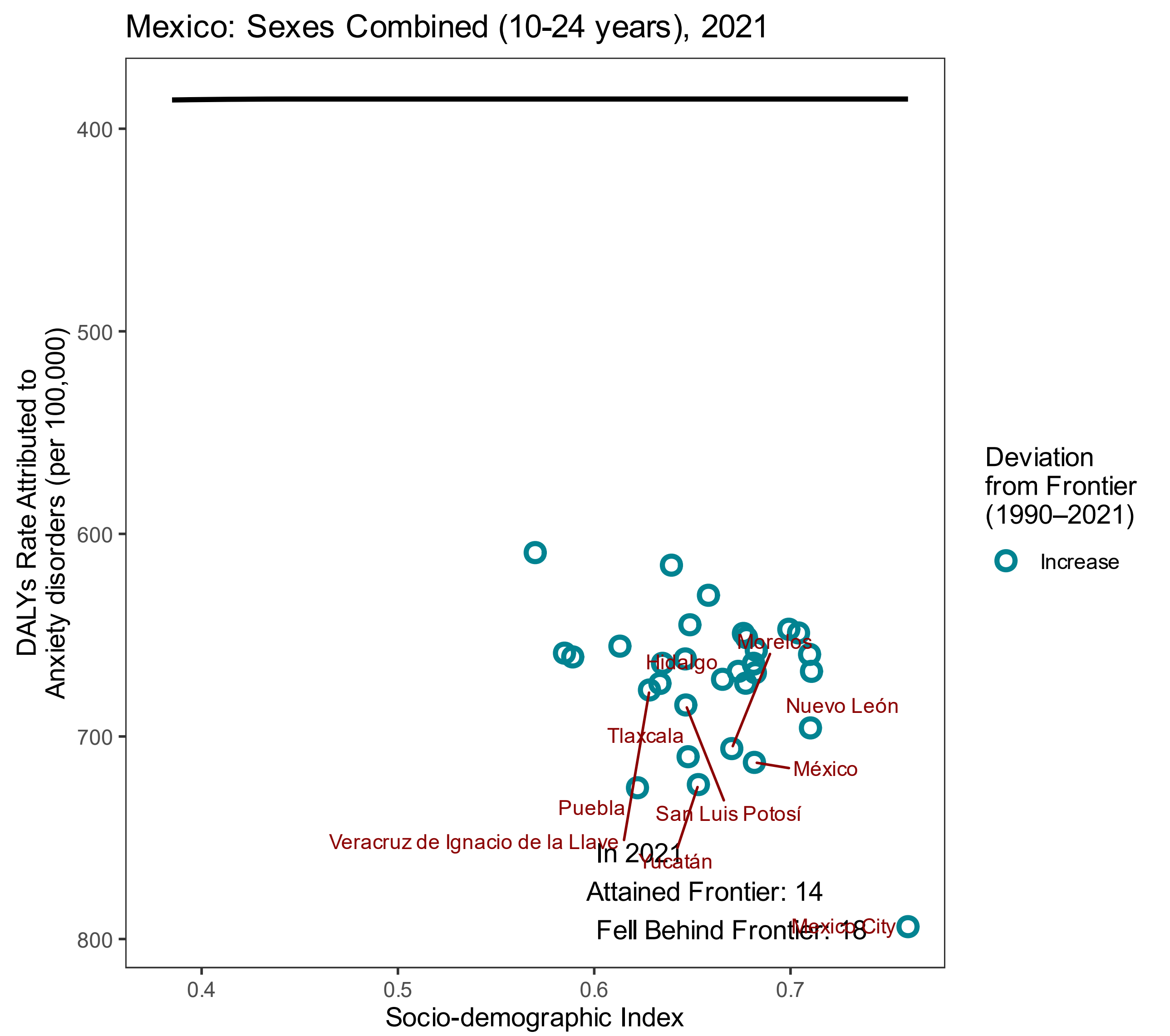

Mexico: Sexes Combined (10-24 years), 2021
DALYs Rate Attributed to Anxiety disorders (per 100,000)
Socio-demographic Index
Deviation from Frontier (1990–2021)
Increase
Hidalgo
Morelos
Nuevo León
Tlaxcala
México
Puebla
San Luis Potosí
Veracruz de Ignacio de la Llave
Yucatán
Mexico City
In 2021
Attained Frontier: 14
Fell Behind Frontier: 18

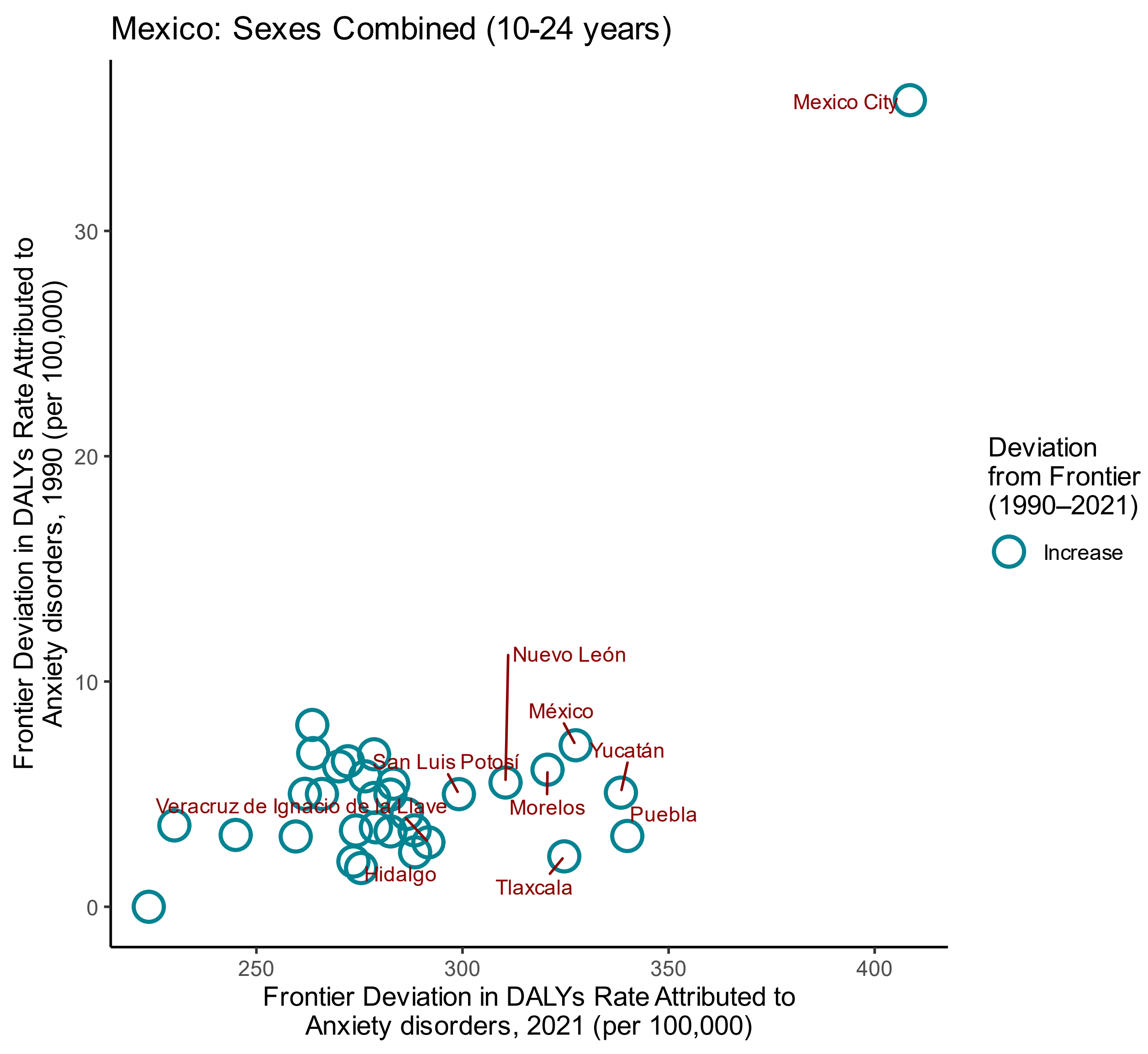

Mexico: Sexes Combined (10-24 years)
Frontier Deviation in DALYs Rate Attributed to Anxiety disorders, 1990 (per 100,000)
Frontier Deviation in DALYs Rate Attributed to Anxiety disorders, 2021 (per 100,000)
Deviation from Frontier (1990–2021)
Increase
Mexico City
Nuevo León
México
Yucatán
San Luis Potosí
Veracruz de Ignacio de la Llave
Morelos
Puebla
Hidalgo
Tlaxcala

Figure S20

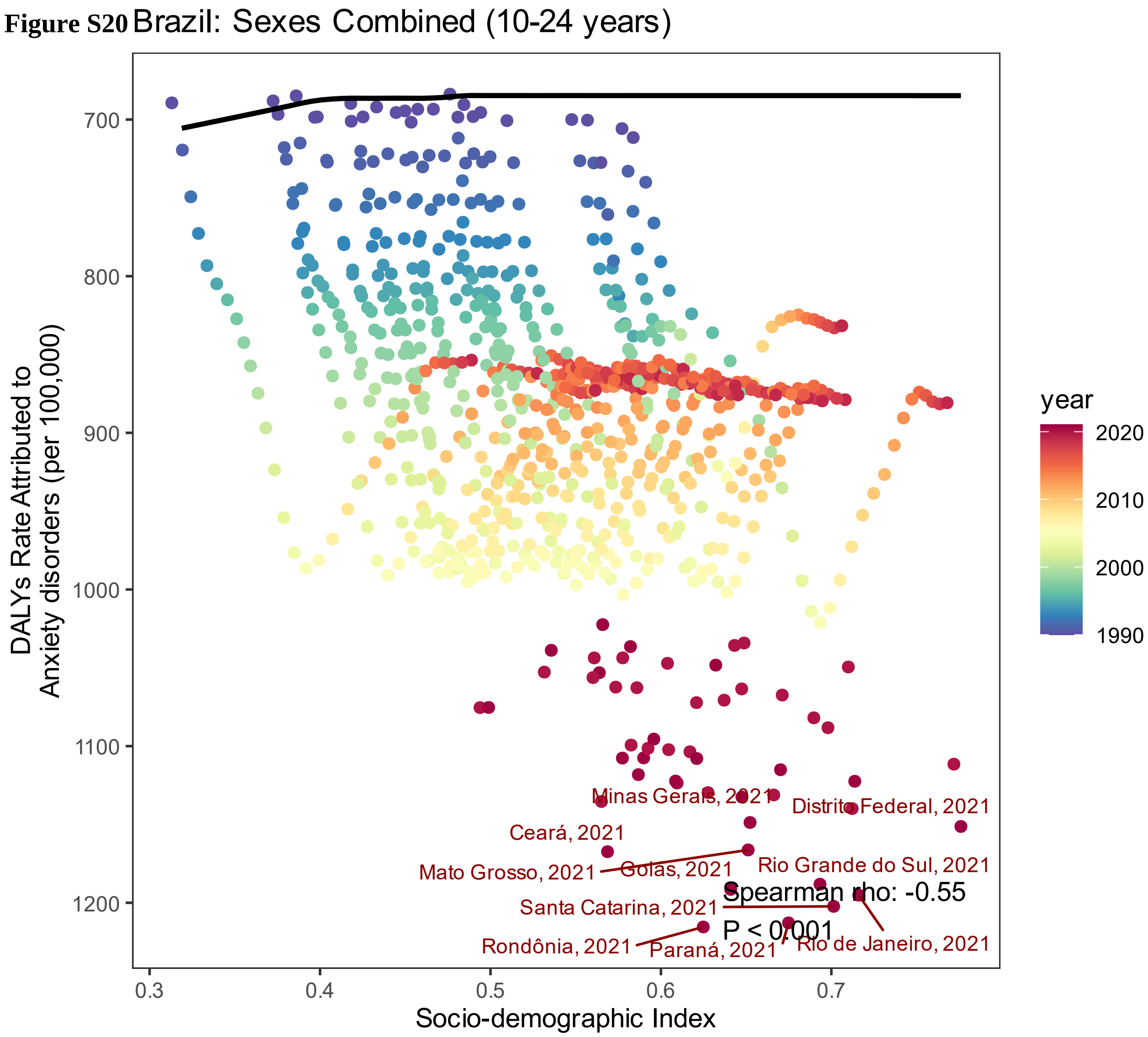

Brazil: Sexes Combined (10-24 years)
DALYs Rate Attributed to Anxiety disorders (per 100,000)
Socio-demographic Index
year
2020
2010
2000
1990
Minas Gerais, 2021
Distrito Federal, 2021
Ceará, 2021
Mato Grosso, 2021
Goiás, 2021
Rio Grande do Sul, 2021
Spearman rho: -0.55
Santa Catarina, 2021
P < 0.001
Rondônia, 2021
Paraná, 2021
Rio de Janeiro, 2021

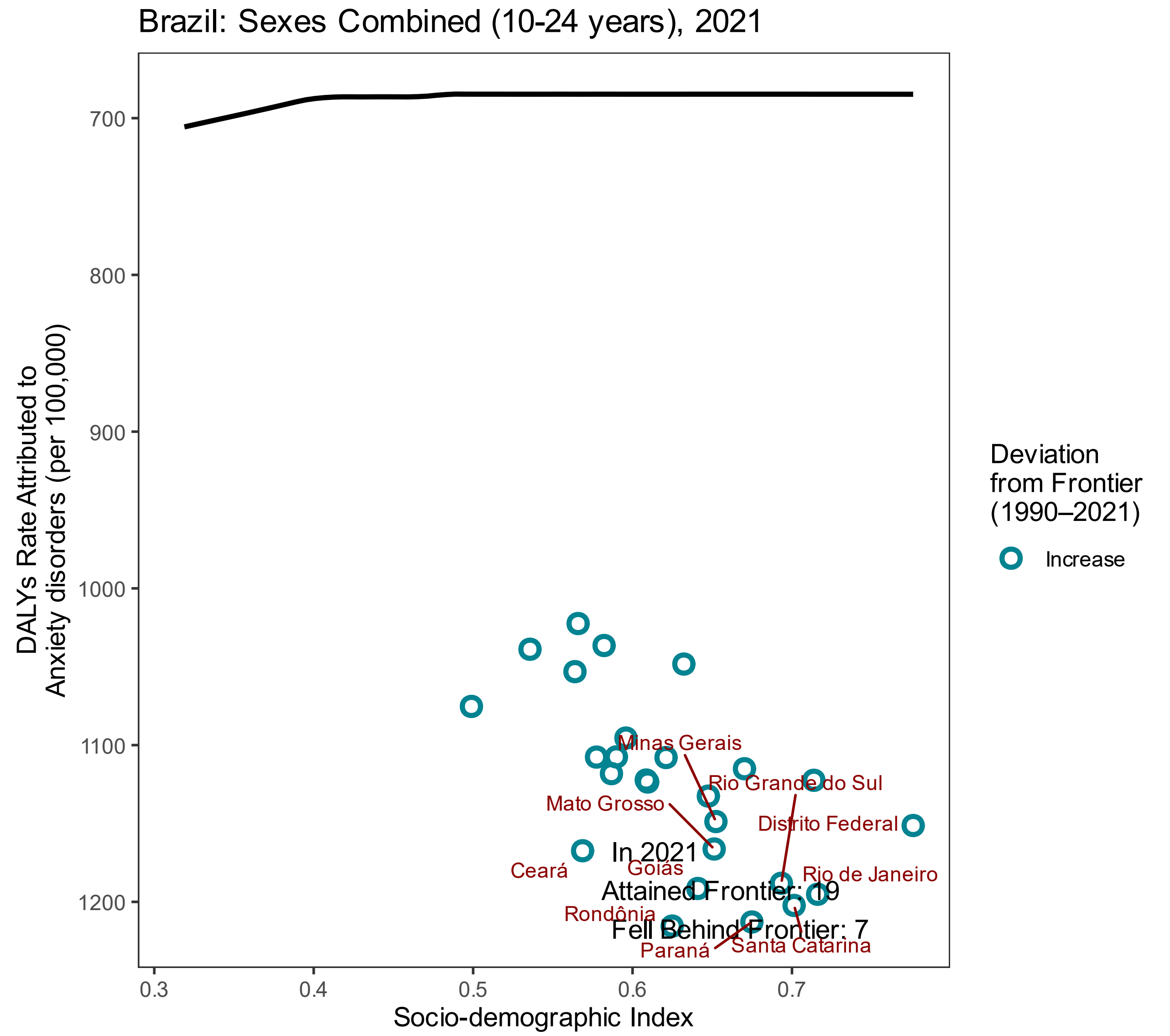

Brazil: Sexes Combined (10-24 years), 2021
DALYs Rate Attributed to Anxiety disorders (per 100,000)
Socio-demographic Index
Deviation from Frontier (1990–2021)
Increase
Minas Gerais
Rio Grande do Sul
Mato Grosso
Distrito Federal
In 2021
Ceará
Goiás
Rio de Janeiro
Attained Frontier: 19
Rondônia
Fell Behind Frontier: 7
Paraná
Santa Catarina

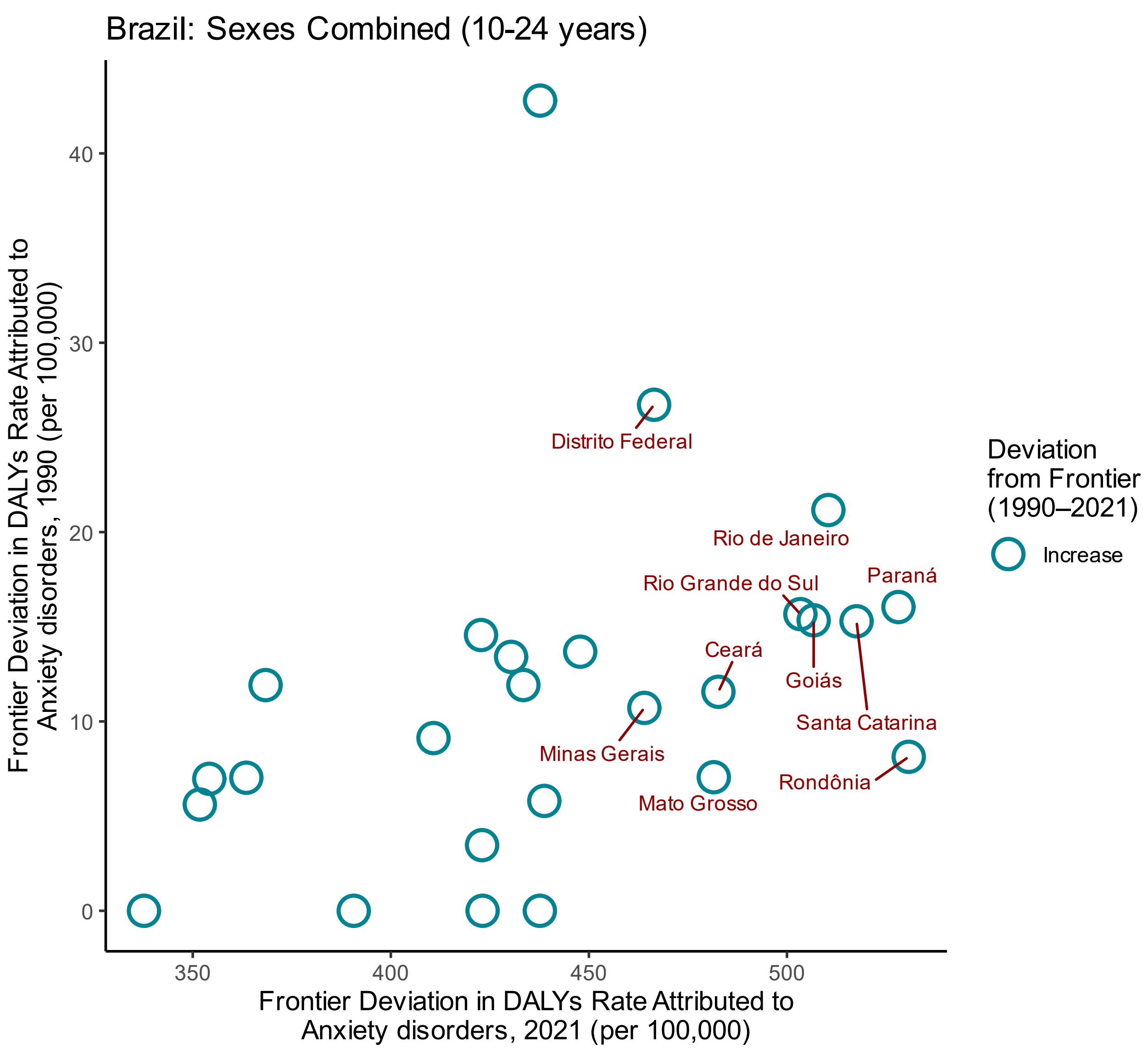

Brazil: Sexes Combined (10-24 years)
Frontier Deviation in DALYs Rate Attributed to Anxiety disorders, 1990 (per 100,000)
Frontier Deviation in DALYs Rate Attributed to Anxiety disorders, 2021 (per 100,000)
Deviation from Frontier (1990–2021)
Increase
Distrito Federal
Rio de Janeiro
Paraná
Rio Grande do Sul
Ceará
Goiás
Santa Catarina
Minas Gerais
Rondônia
Mato Grosso

Figure S20

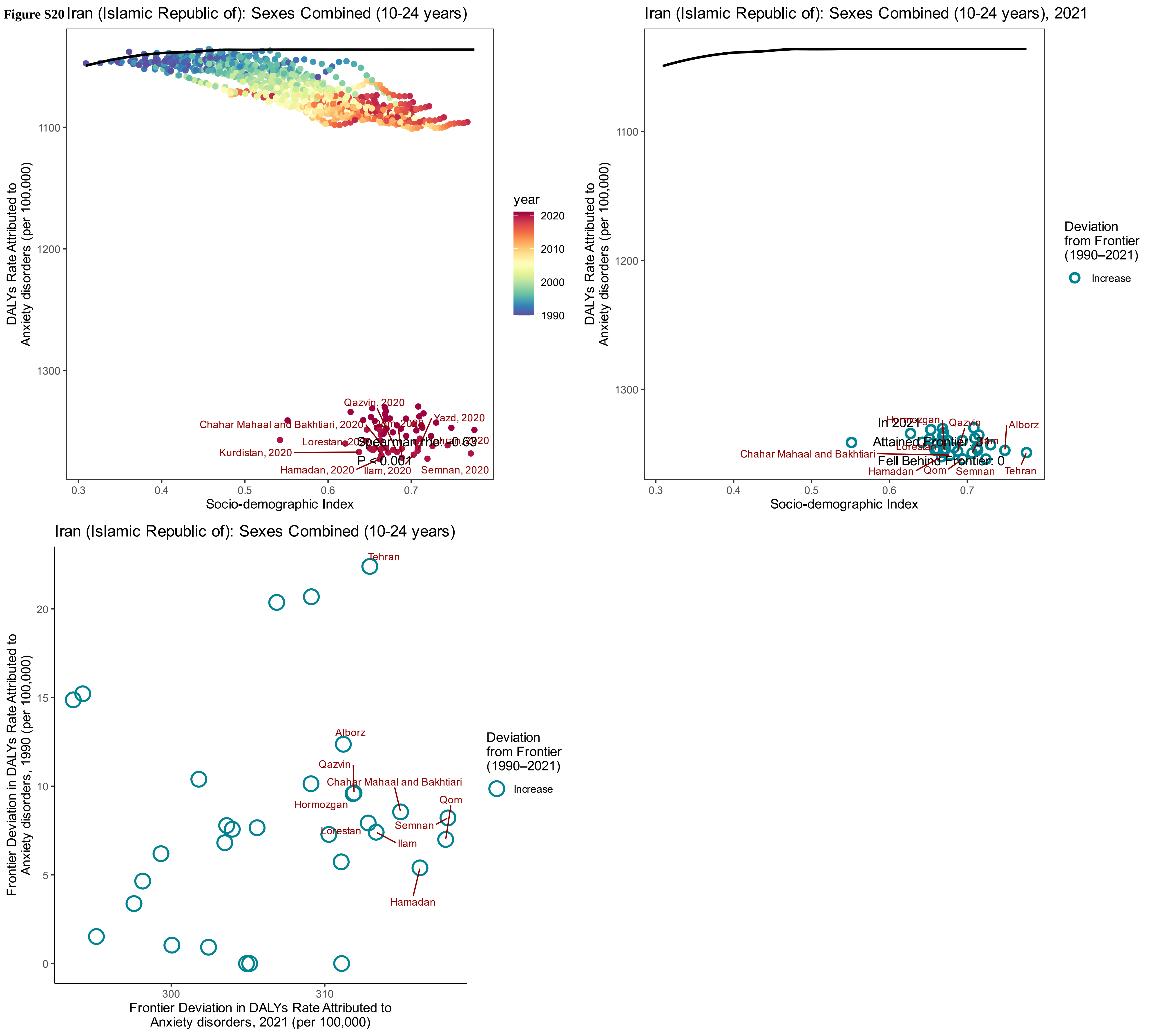

Iran (Islamic Republic of): Sexes Combined (10-24 years)
DALYs Rate Attributed to Anxiety disorders (per 100,000)
Socio-demographic Index
year
2020
2010
2000
1990
Qazvin, 2020
Yazd, 2020
Chahar Mahaal and Bakhtiari, 2020
Lorestan, 2020
Kurdistan, 2020
Hamadan, 2020
Ilam, 2020
Semnan, 2020
Spearman rho: -0.63
P < 0.001
Iran (Islamic Republic of): Sexes Combined (10-24 years), 2021
Deviation from Frontier (1990–2021)
Increase
In 2021
Attained Frontier: 31
Fell Behind Frontier: 0
Hormozgan
Qazvin
Alborz
Lorestan
Chahar Mahaal and Bakhtiari
Hamadan
Qom
Semnan
Tehran
Frontier Deviation in DALYs Rate Attributed to Anxiety disorders, 1990 (per 100,000)
Frontier Deviation in DALYs Rate Attributed to Anxiety disorders, 2021 (per 100,000)
Ilam

Figure S20

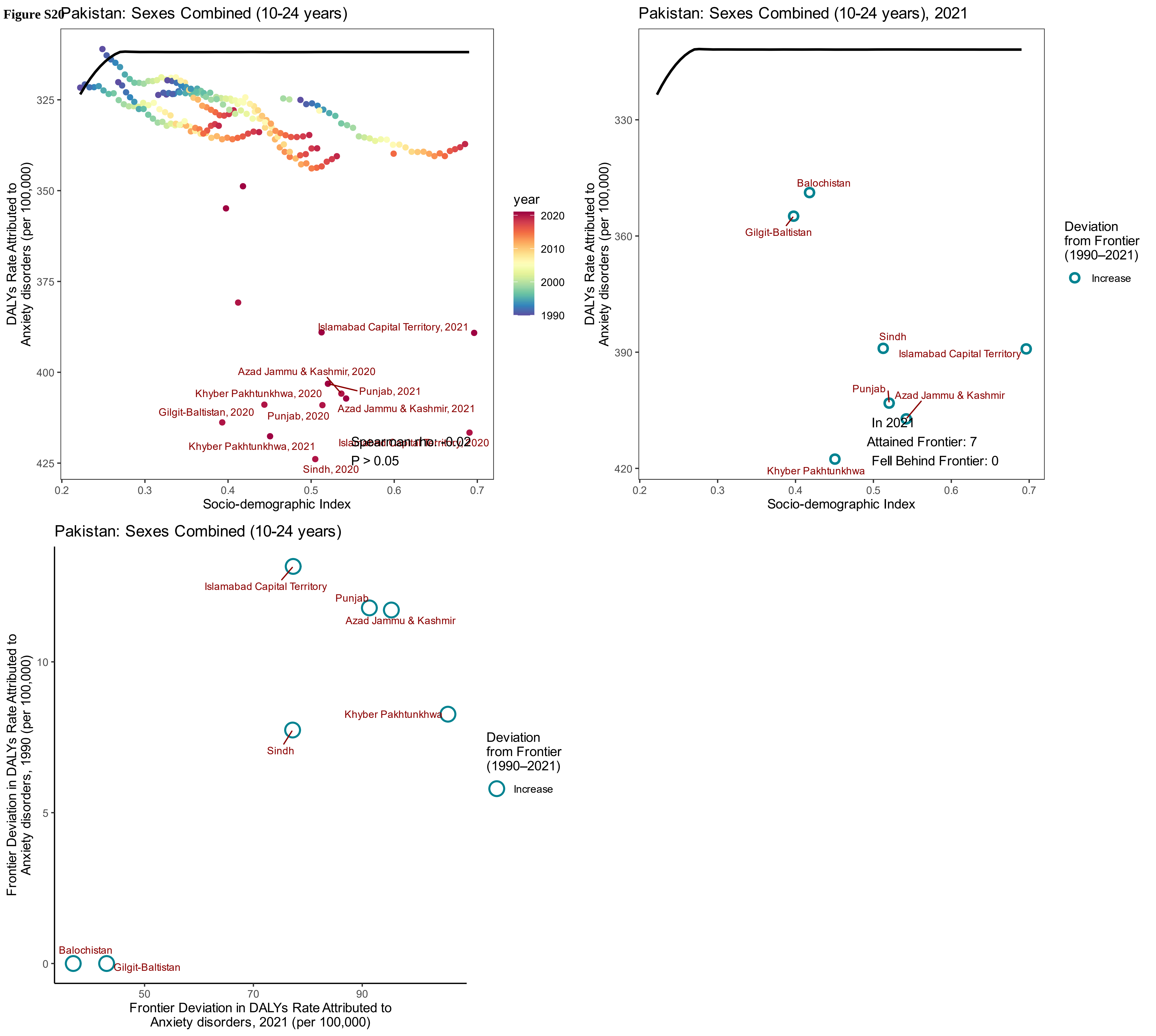

Pakistan: Sexes Combined (10-24 years)
DALYs Rate Attributed to Anxiety disorders (per 100,000)
Socio-demographic Index
year
Islamabad Capital Territory, 2021
Azad Jammu & Kashmir, 2020
Punjab, 2021
Khyber Pakhtunkhwa, 2020
Azad Jammu & Kashmir, 2021
Gilgit-Baltistan, 2020
Punjab, 2020
Khyber Pakhtunkhwa, 2021
Islamabad Capital Territory, 2020
Spearman rho: -0.02
P > 0.05
Sindh, 2020
Pakistan: Sexes Combined (10-24 years), 2021
Balochistan
Gilgit-Baltistan
Sindh
Islamabad Capital Territory
Punjab
Azad Jammu & Kashmir
In 2021
Attained Frontier: 7
Fell Behind Frontier: 0
Khyber Pakhtunkhwa
Deviation from Frontier (1990–2021)
Increase
Pakistan: Sexes Combined (10-24 years)
Frontier Deviation in DALYs Rate Attributed to Anxiety disorders, 1990 (per 100,000)
Frontier Deviation in DALYs Rate Attributed to Anxiety disorders, 2021 (per 100,000)
Islamabad Capital Territory
Punjab
Azad Jammu & Kashmir
Khyber Pakhtunkhwa
Sindh
Balochistan
Gilgit-Baltistan
Deviation from Frontier (1990–2021)
Increase

Figure S20

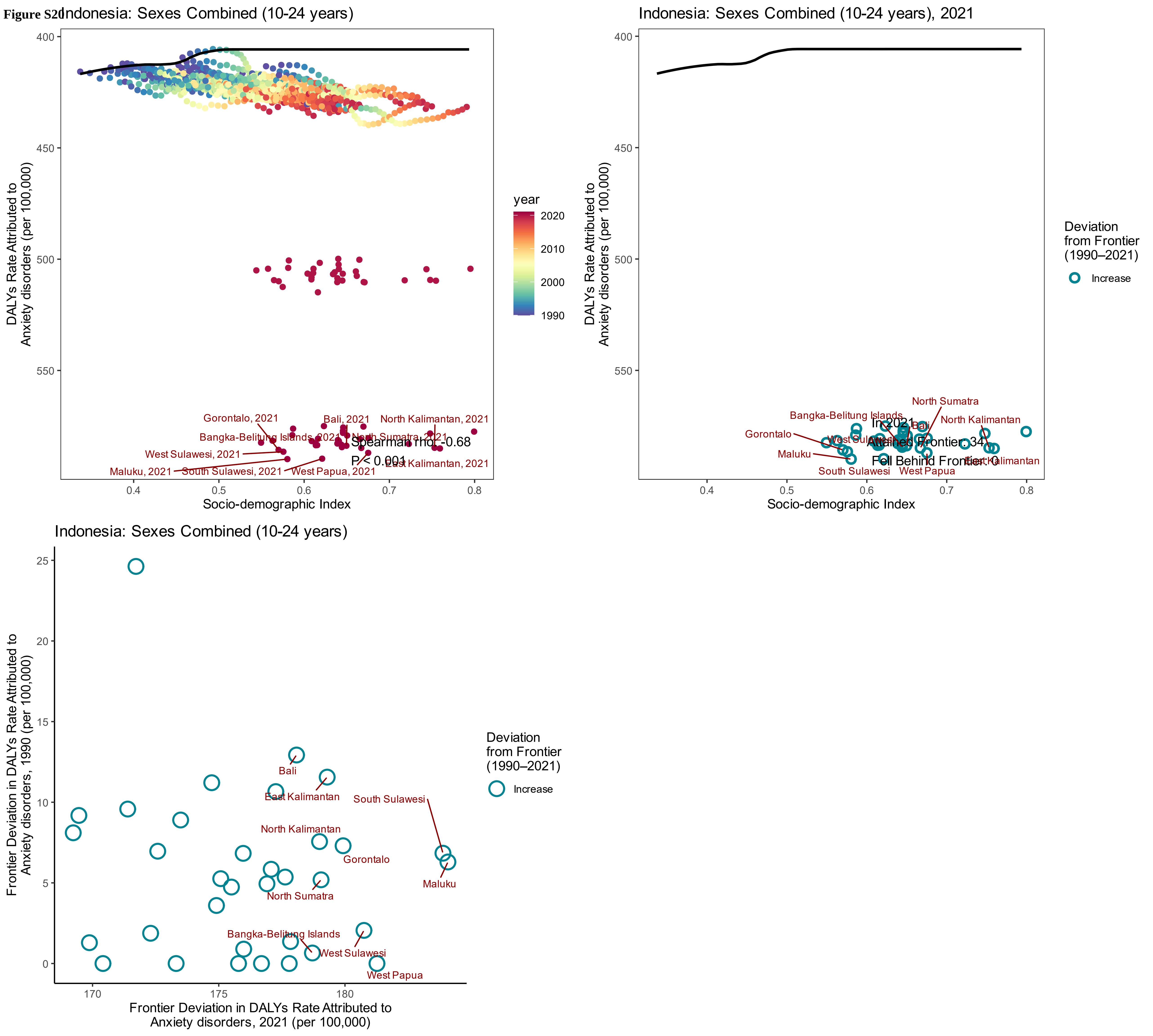

Indonesia: Sexes Combined (10-24 years)
DALYs Rate Attributed to Anxiety disorders (per 100,000)
Socio-demographic Index
year
2020
2010
2000
1990
Gorontalo, 2021
Bali, 2021
North Kalimantan, 2021
Bangka-Belitung Islands, 2021
North Sumatra, 2021
Spearman rho: -0.68
P < 0.001
West Sulawesi, 2021
East Kalimantan, 2021
Maluku, 2021
South Sulawesi, 2021
West Papua, 2021
Indonesia: Sexes Combined (10-24 years), 2021
In 2021
Attained Frontier: 34
Fell Behind Frontier: 0
North Sumatra
Bangka-Belitung Islands
North Kalimantan
Gorontalo
Bali
West Sulawesi
Maluku
East Kalimantan
South Sulawesi
West Papua
Deviation from Frontier (1990–2021)
Increase
Indonesia: Sexes Combined (10-24 years)
Frontier Deviation in DALYs Rate Attributed to Anxiety disorders, 1990 (per 100,000)
Frontier Deviation in DALYs Rate Attributed to Anxiety disorders, 2021 (per 100,000)
Bali
East Kalimantan
South Sulawesi
North Kalimantan
Gorontalo
Maluku
North Sumatra
Bangka-Belitung Islands
West Sulawesi
West Papua
Deviation from Frontier (1990–2021)
Increase

Figure S20

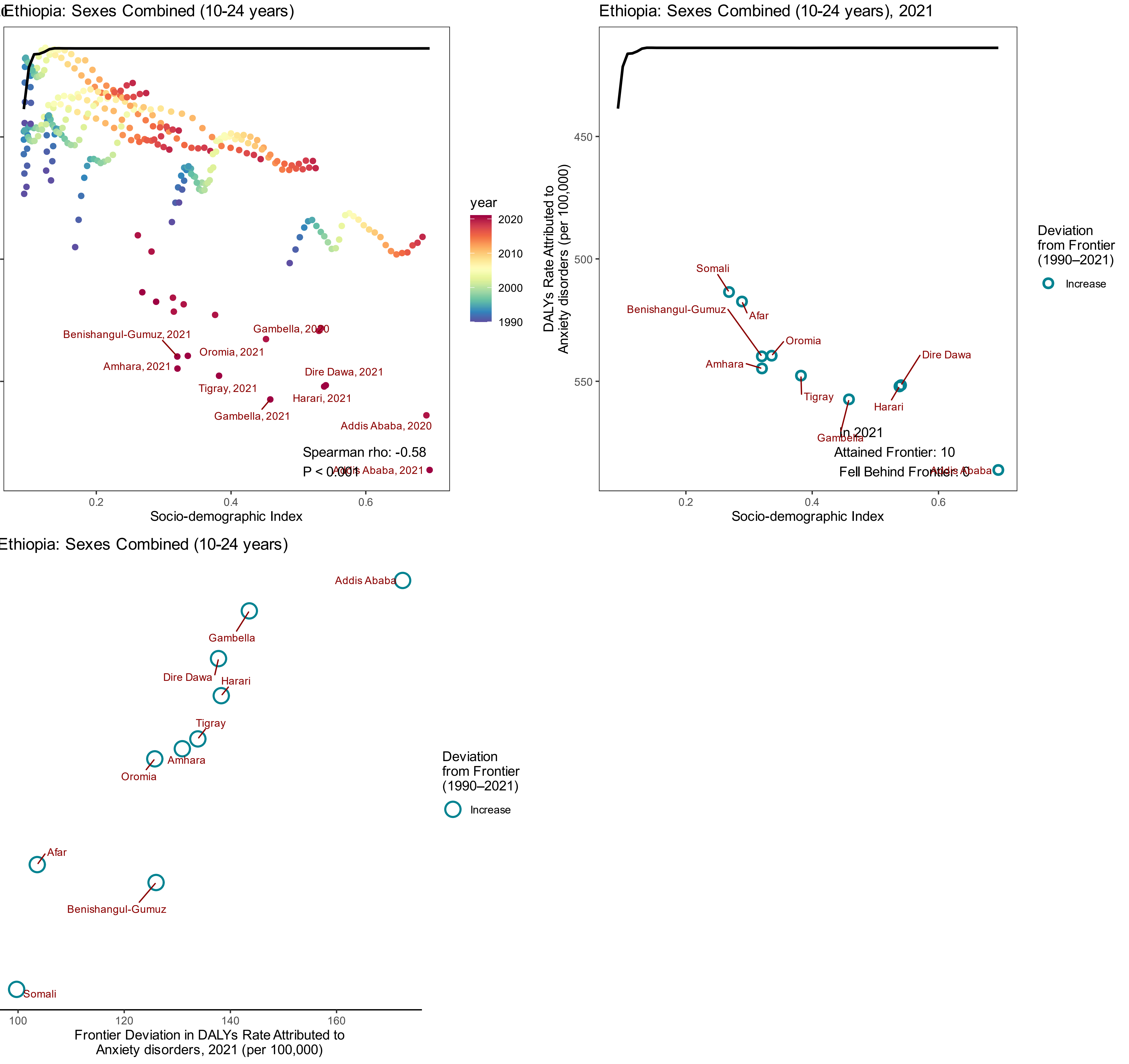

Ethiopia: Sexes Combined (10-24 years)
DALYs Rate Attributed to Anxiety disorders (per 100,000)
Socio-demographic Index
year
2020
2010
2000
1990
Benishangul-Gumuz, 2021
Amhara, 2021
Oromia, 2021
Tigray, 2021
Gambella, 2020
Gambella, 2021
Dire Dawa, 2021
Harari, 2021
Addis Ababa, 2020
Addis Ababa, 2021
Spearman rho: -0.58
P < 0.001
Ethiopia: Sexes Combined (10-24 years), 2021
Somali
Afar
Benishangul-Gumuz
Oromia
Amhara
Tigray
Dire Dawa
Harari
Gambella
Addis Ababa
In 2021
Attained Frontier: 10
Fell Behind Frontier: 0
Deviation from Frontier (1990–2021)
Increase
Ethiopia: Sexes Combined (10-24 years)
Frontier Deviation in DALYs Rate Attributed to Anxiety disorders, 1990 (per 100,000)
Frontier Deviation in DALYs Rate Attributed to Anxiety disorders, 2021 (per 100,000)
Deviation from Frontier (1990–2021)
Increase

Figure S20

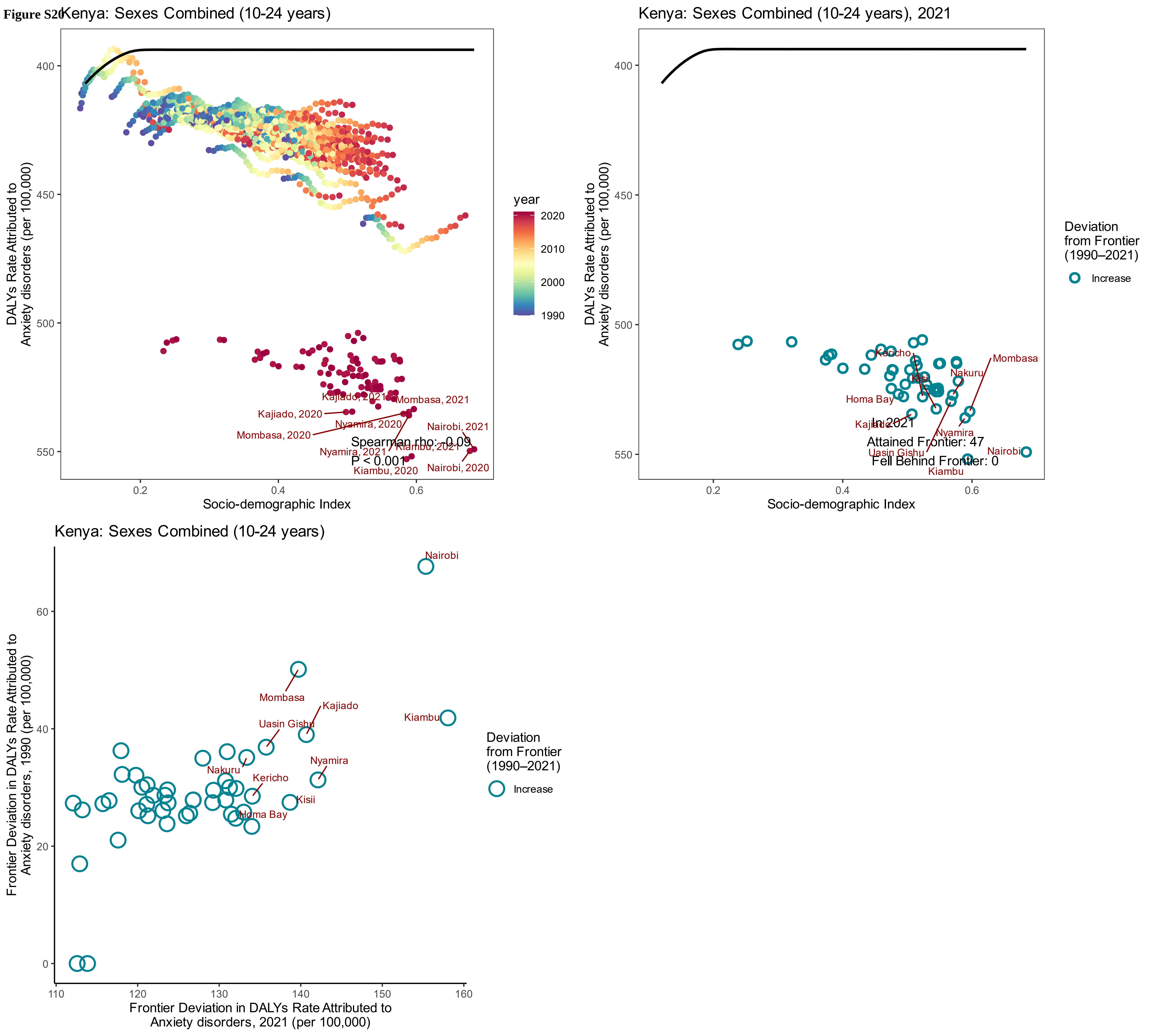
Kenya: Sexes Combined (10-24 years)
DALYs Rate Attributed to Anxiety disorders (per 100,000)
Socio-demographic Index
year
2020
2010
2000
1990
Kajiado, 2021
Mombasa, 2021
Kajiado, 2020
Nyamira, 2020
Nairobi, 2021
Mombasa, 2020
Spearman rho: -0.09
Nyamira, 2021
Kiambu, 2021
P < 0.001
Kiambu, 2020
Nairobi, 2020
Kenya: Sexes Combined (10-24 years), 2021
Deviation from Frontier (1990–2021)
Increase
Kericho
Mombasa
Nakuru
Kisii
Homa Bay
Kajiado
In 2021
Nyamira
Attained Frontier: 47
Uasin Gishu
Nairobi
Fell Behind Frontier: 0
Kiambu
Kenya: Sexes Combined (10-24 years)
Frontier Deviation in DALYs Rate Attributed to Anxiety disorders, 1990 (per 100,000)
Frontier Deviation in DALYs Rate Attributed to Anxiety disorders, 2021 (per 100,000)
Nairobi
Mombasa
Kajiado
Uasin Gishu
Kiambu
Nyamira
Nakuru
Kericho
Kisii
Homa Bay
Deviation from Frontier (1990–2021)
Increase

Figure S20

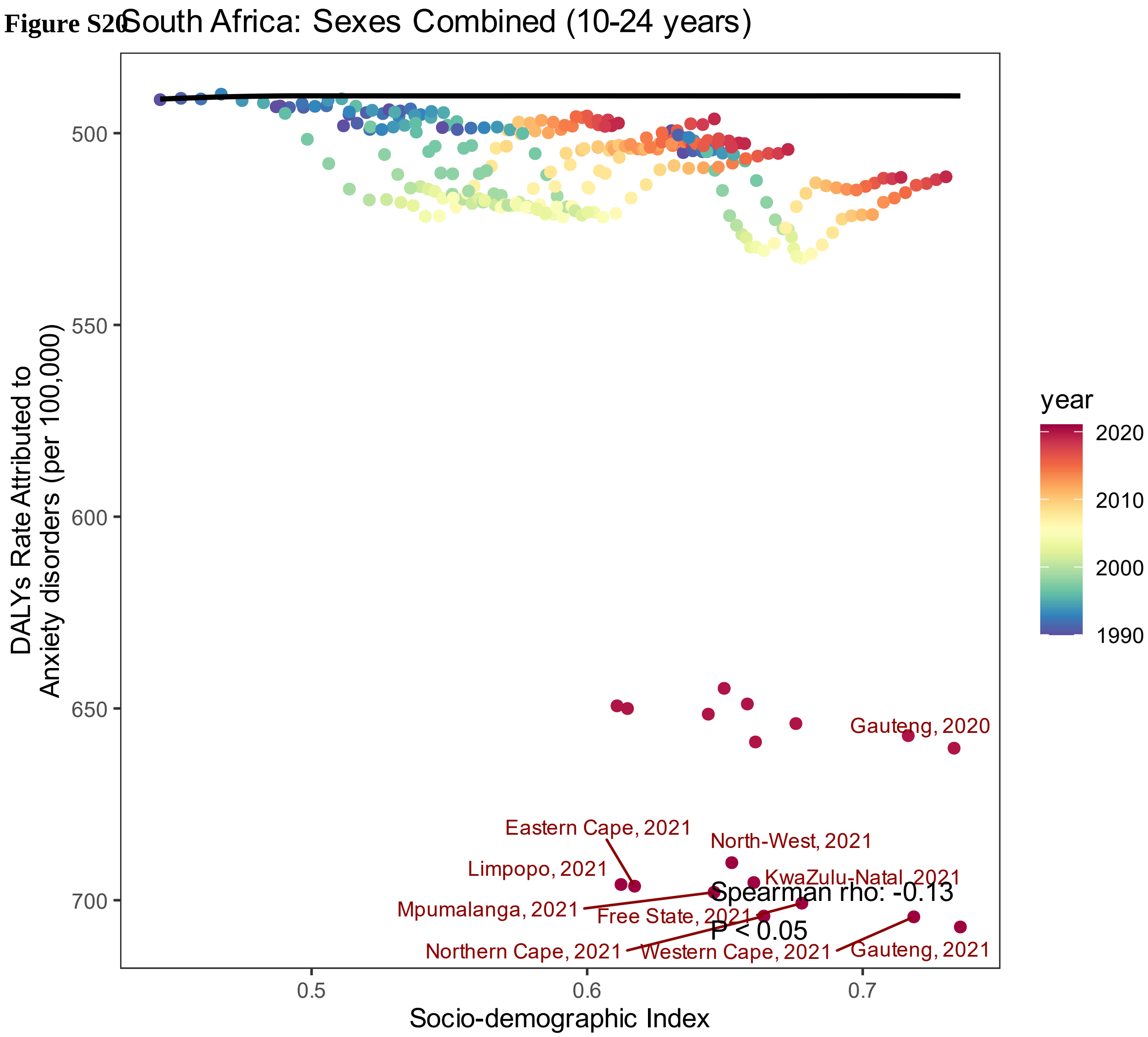
South Africa: Sexes Combined (10-24 years)
DALYs Rate Attributed to Anxiety disorders (per 100,000)
Socio-demographic Index
year
Gauteng, 2020
Eastern Cape, 2021
North-West, 2021
Limpopo, 2021
KwaZulu-Natal, 2021
Spearman rho: -0.13
Mpumalanga, 2021
Free State, 2021
P < 0.05
Northern Cape, 2021
Western Cape, 2021
Gauteng, 2021

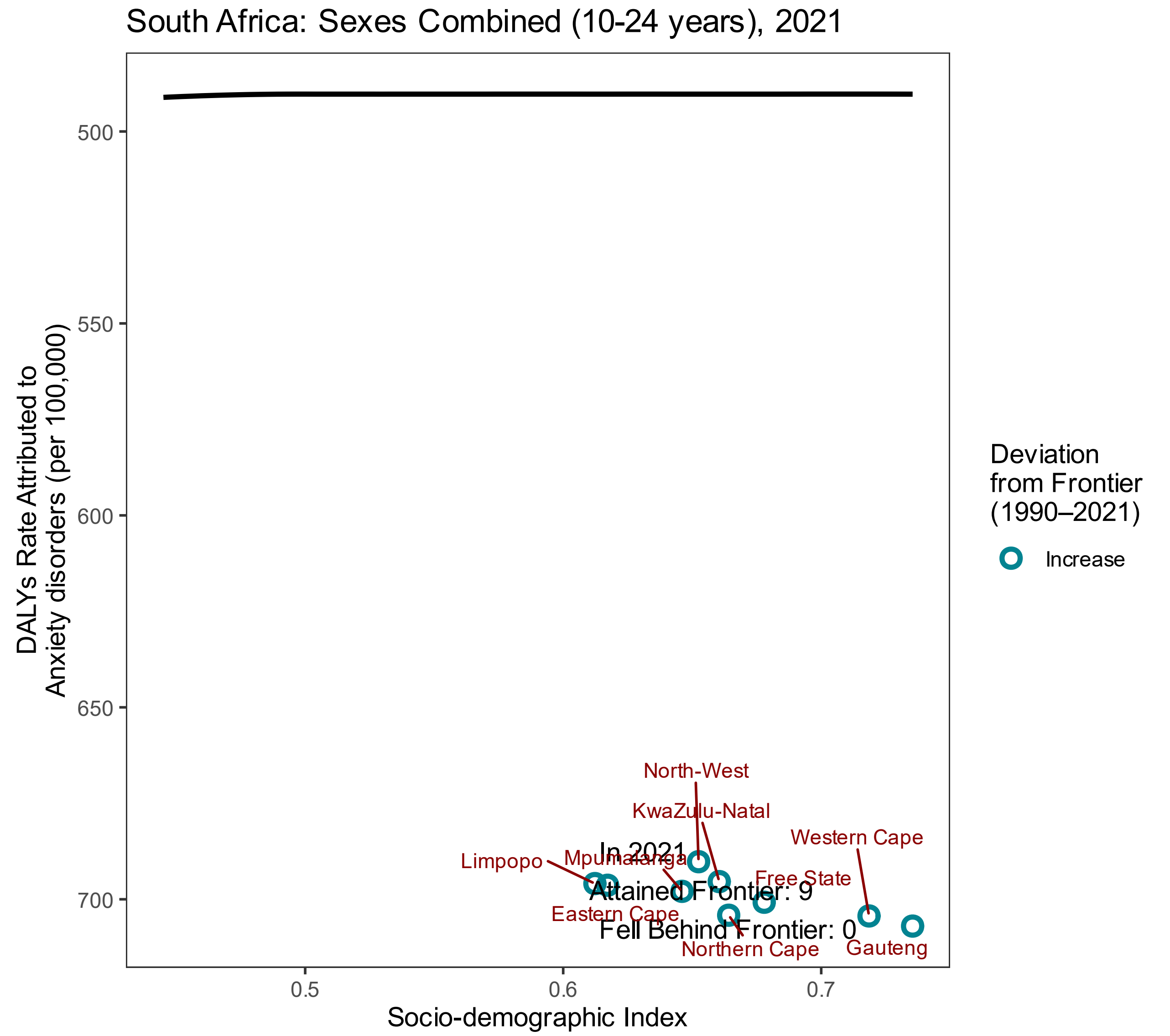
South Africa: Sexes Combined (10-24 years), 2021
DALYs Rate Attributed to Anxiety disorders (per 100,000)
Socio-demographic Index
Deviation from Frontier (1990–2021)
Increase
North-West
KwaZulu-Natal
Western Cape
Limpopo
Mpumalanga
In 2021
Free State
Attained Frontier: 9
Eastern Cape
Fell Behind Frontier: 0
Northern Cape
Gauteng

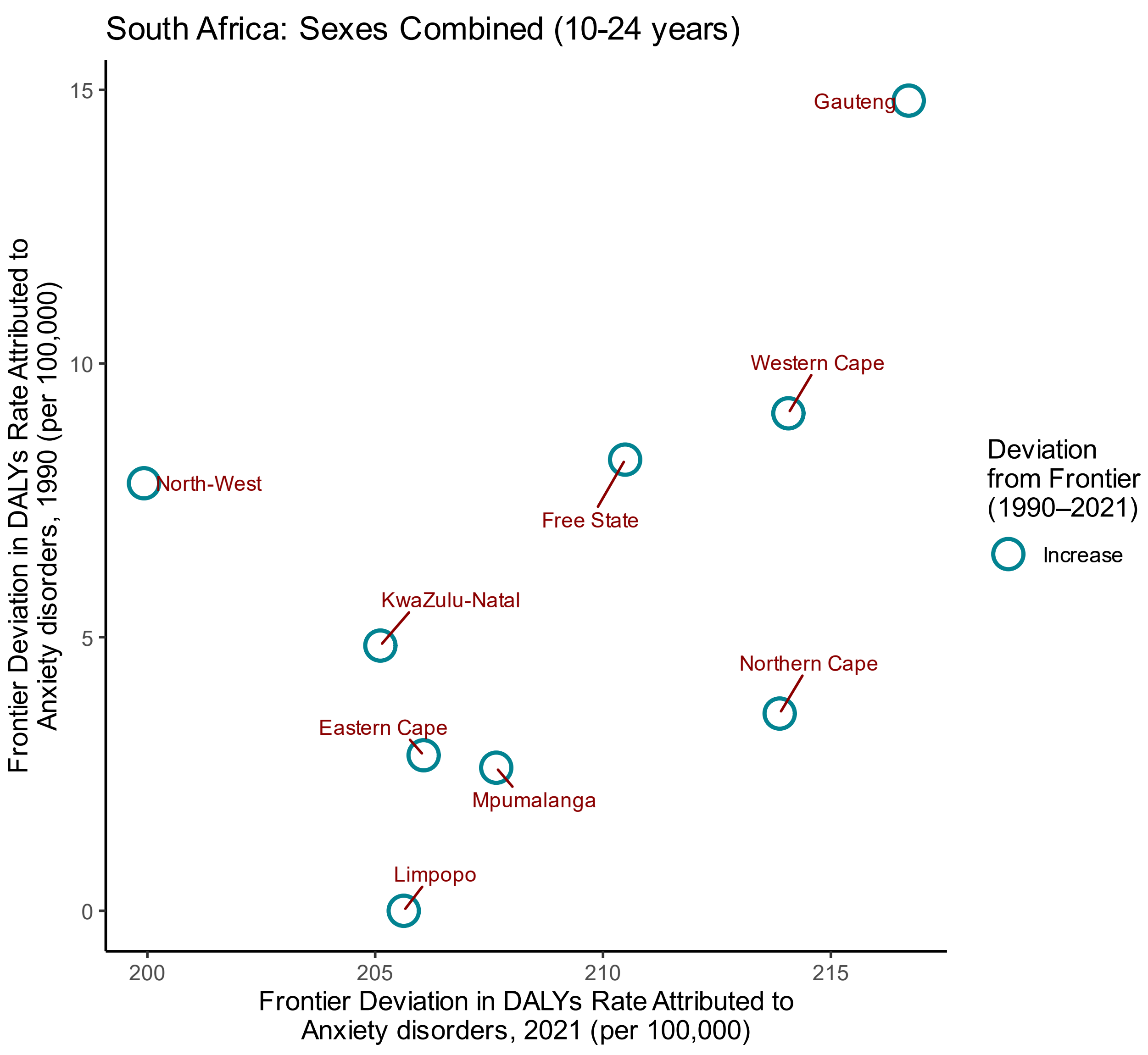
South Africa: Sexes Combined (10-24 years)
Frontier Deviation in DALYs Rate Attributed to Anxiety disorders, 1990 (per 100,000)
Frontier Deviation in DALYs Rate Attributed to Anxiety disorders, 2021 (per 100,000)
Gauteng
Western Cape
North-West
Free State
KwaZulu-Natal
Northern Cape
Eastern Cape
Mpumalanga
Limpopo
Deviation from Frontier (1990–2021)
Increase

Figure S20

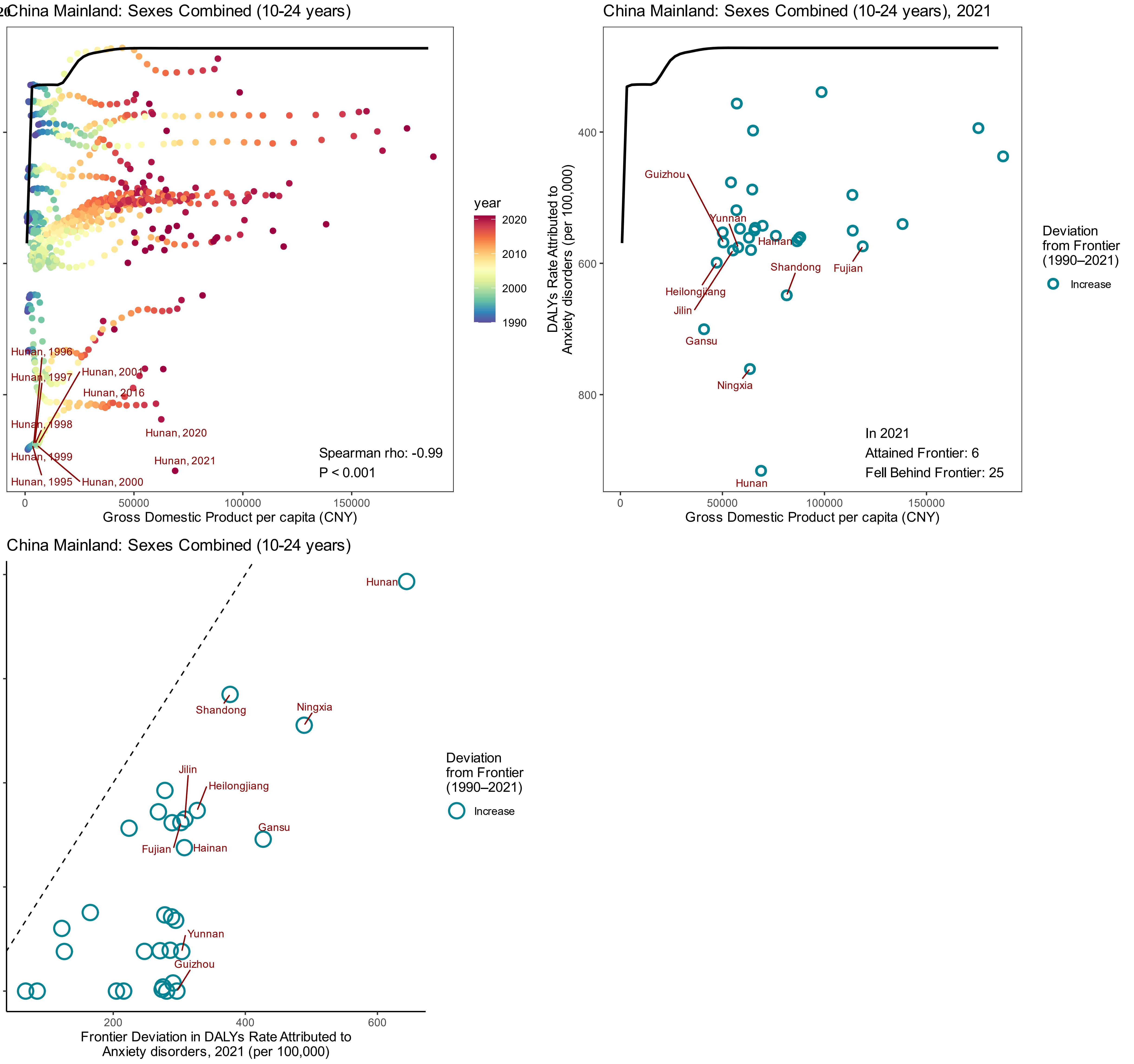
China Mainland: Sexes Combined (10-24 years)
DALYs Rate Attributed to Anxiety disorders (per 100,000)
Gross Domestic Product per capita (CNY)
year
2020
2010
2000
1990
Hunan, 1996
Hunan, 1997
Hunan, 2001
Hunan, 2016
Hunan, 1998
Hunan, 2020
Hunan, 1999
Hunan, 2021
Hunan, 1995
Hunan, 2000
Spearman rho: -0.99
P < 0.001
China Mainland: Sexes Combined (10-24 years), 2021
Guizhou
Yunnan
Hainan
Shandong
Fujian
Heilongjiang
Jilin
Gansu
Ningxia
Hunan
In 2021
Attained Frontier: 6
Fell Behind Frontier: 25
Deviation from Frontier (1990–2021)
Increase
Frontier Deviation in DALYs Rate Attributed to Anxiety disorders, 1990 (per 100,000)
Frontier Deviation in DALYs Rate Attributed to Anxiety disorders, 2021 (per 100,000)

Figure S20

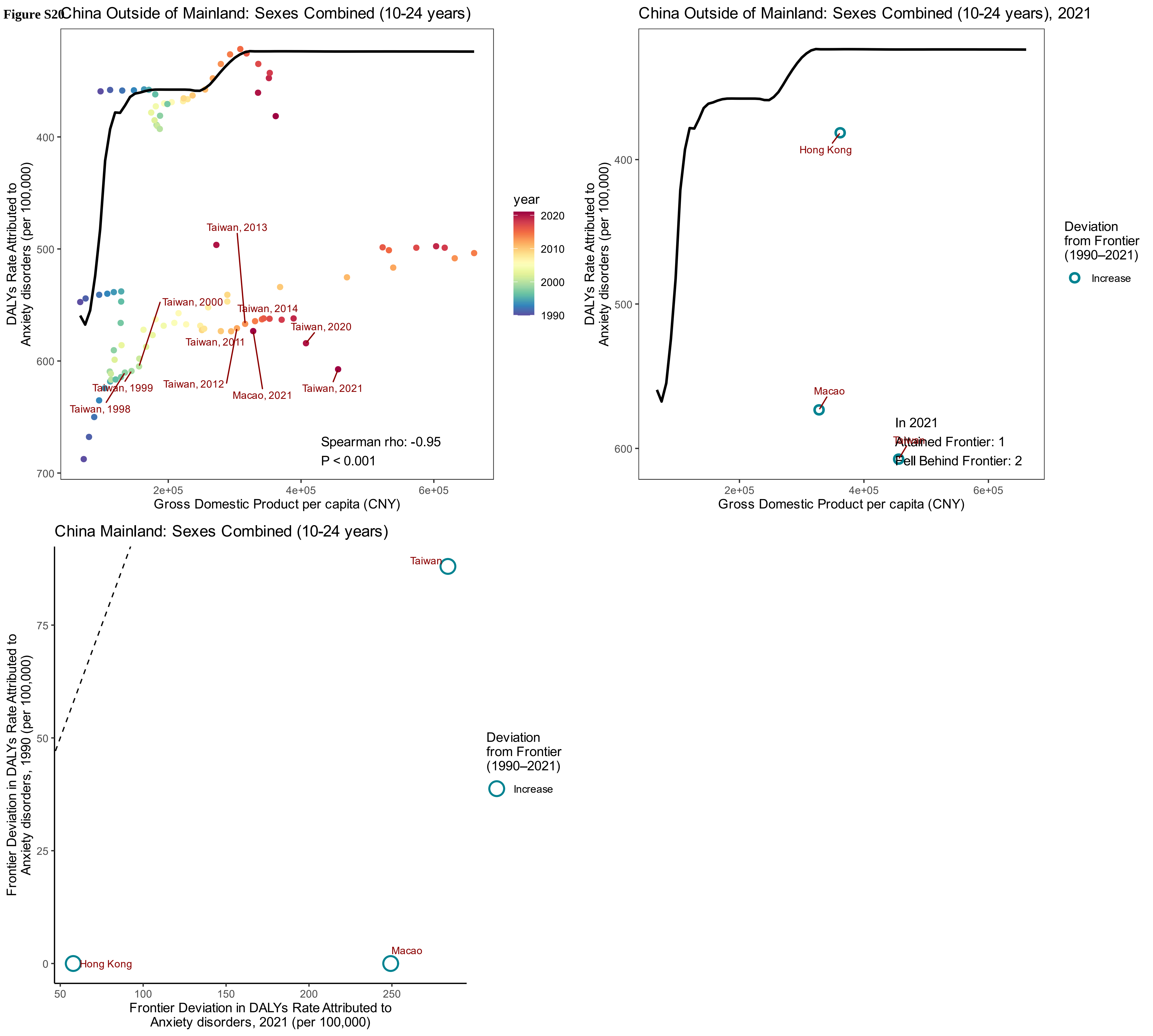

China Outside of Mainland: Sexes Combined (10-24 years)
DALYs Rate Attributed to Anxiety disorders (per 100,000)
Gross Domestic Product per capita (CNY)
year
2020
2010
2000
1990
Taiwan, 2013
Taiwan, 2000
Taiwan, 2014
Taiwan, 2020
Taiwan, 2011
Taiwan, 2012
Taiwan, 1999
Taiwan, 1998
Macao, 2021
Taiwan, 2021
Spearman rho: -0.95
P < 0.001
China Outside of Mainland: Sexes Combined (10-24 years), 2021
Hong Kong
Macao
Taiwan
In 2021
Attained Frontier: 1
Fell Behind Frontier: 2
Deviation from Frontier (1990–2021)
Increase
China Mainland: Sexes Combined (10-24 years)
Frontier Deviation in DALYs Rate Attributed to Anxiety disorders, 1990 (per 100,000)
Frontier Deviation in DALYs Rate Attributed to Anxiety disorders, 2021 (per 100,000)
Taiwan
Macao
Hong Kong
Deviation from Frontier (1990–2021)
Increase

Figure S20

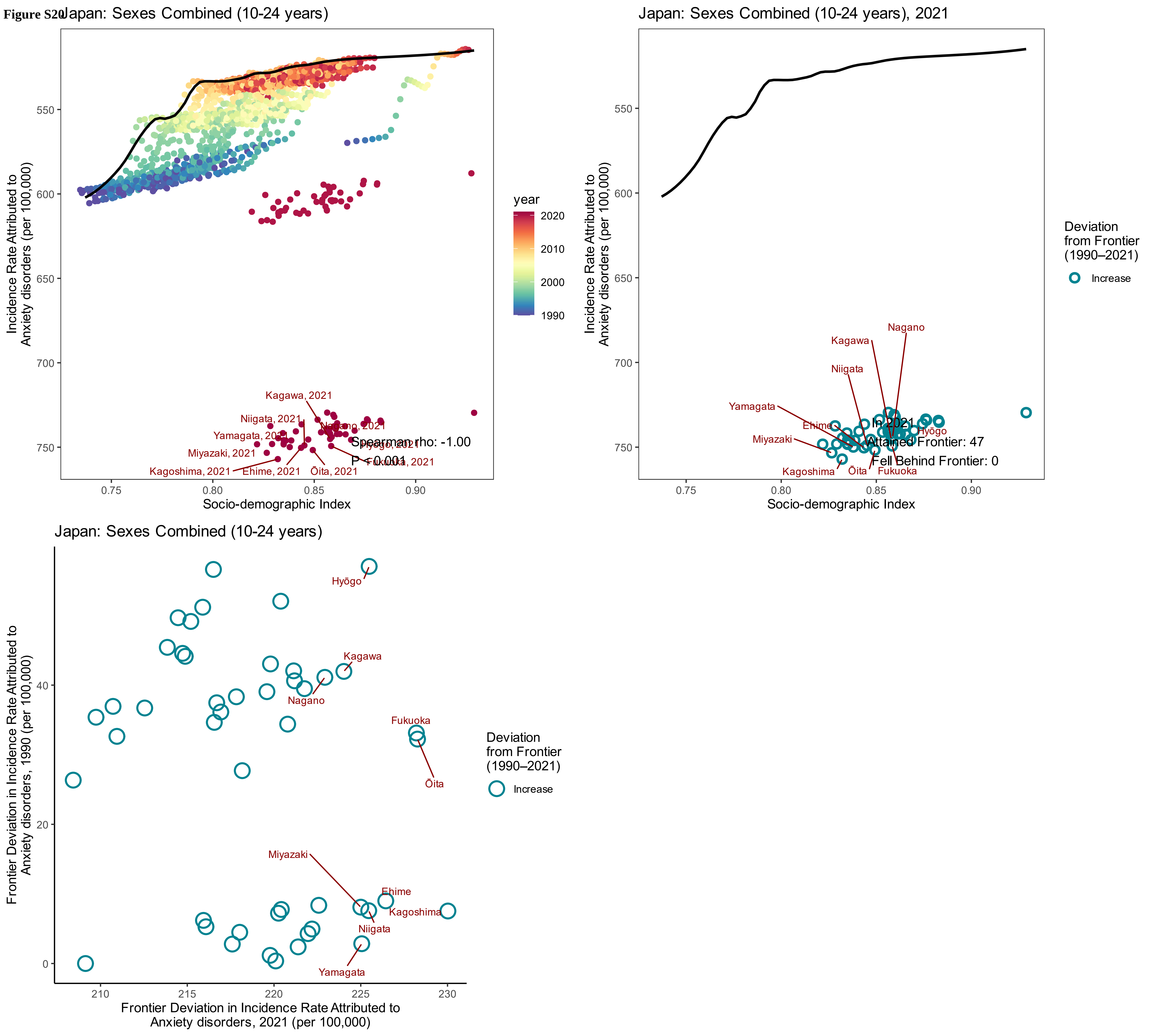
Japan: Sexes Combined (10-24 years)
Incidence Rate Attributed to Anxiety disorders (per 100,000)
Socio-demographic Index
year
2020
2010
2000
1990
Kagawa, 2021
Niigata, 2021
Nagano, 2021
Yamagata, 2021
Hyōgo, 2021
Miyazaki, 2021
Kagoshima, 2021
Ehime, 2021
Ōita, 2021
Fukuoka, 2021
Spearman rho: -1.00
P < 0.001
Japan: Sexes Combined (10-24 years), 2021
Nagano
Kagawa
Niigata
Yamagata
Ehime
Miyazaki
Kagoshima
Ōita
Fukuoka
Hyōgo
In 2021
Attained Frontier: 47
Fell Behind Frontier: 0
Deviation from Frontier (1990–2021)
Increase
Japan: Sexes Combined (10-24 years)
Frontier Deviation in Incidence Rate Attributed to Anxiety disorders, 1990 (per 100,000)
Frontier Deviation in Incidence Rate Attributed to Anxiety disorders, 2021 (per 100,000)
Hyōgo
Kagawa
Nagano
Fukuoka
Ōita
Miyazaki
Ehime
Kagoshima
Niigata
Yamagata
Deviation from Frontier (1990–2021)
Increase

Figure S20

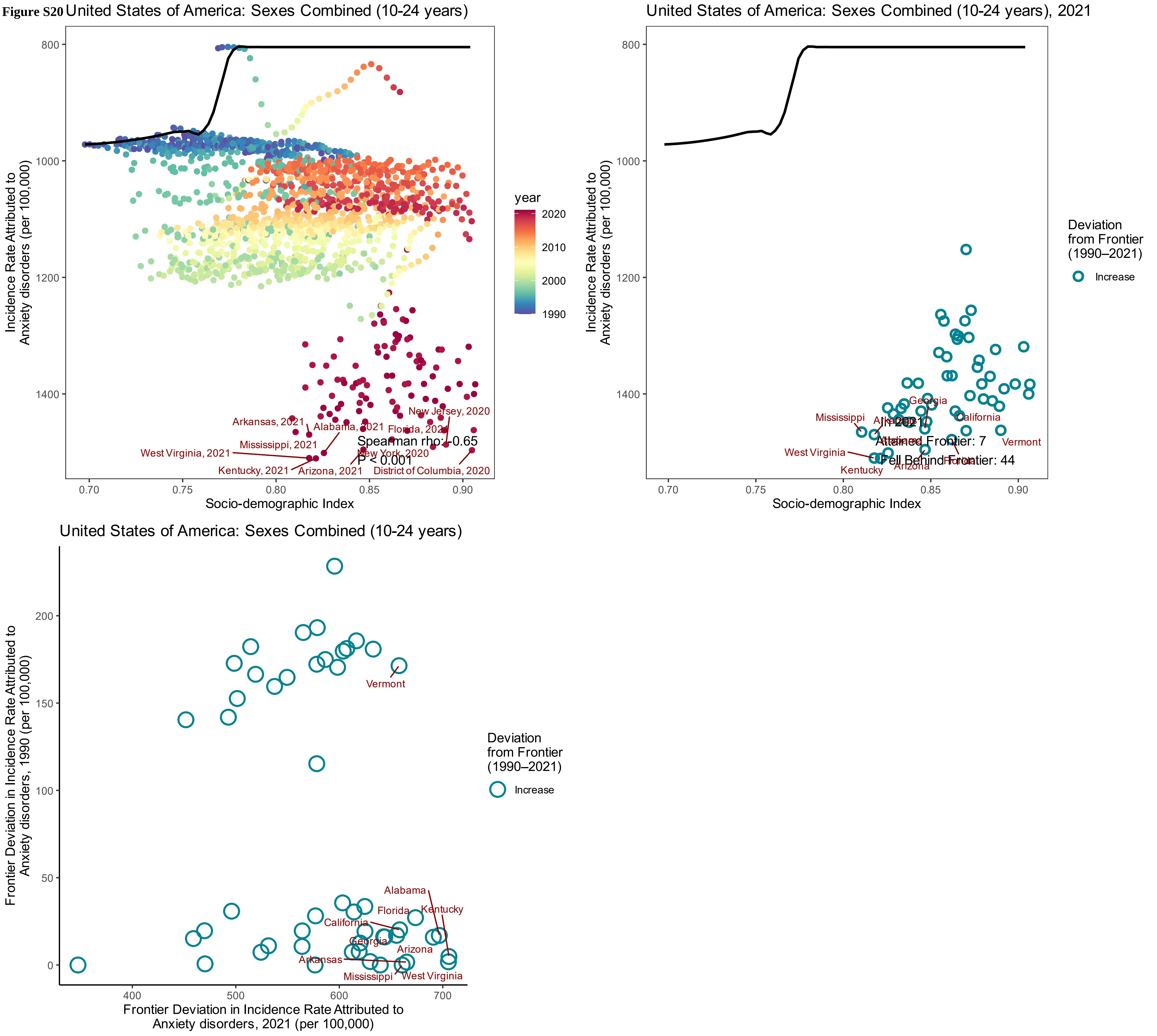
United States of America: Sexes Combined (10-24 years)
Incidence Rate Attributed to Anxiety disorders (per 100,000)
Socio-demographic Index
year
Spearman rho: -0.65
P < 0.001
Arkansas, 2021
Alabama, 2021
Florida, 2021
New Jersey, 2020
Mississippi, 2021
West Virginia, 2021
New York, 2020
Kentucky, 2021
Arizona, 2021
District of Columbia, 2020
United States of America: Sexes Combined (10-24 years), 2021
Deviation from Frontier (1990–2021)
Increase
In 2021,
Attained Frontier: 7
Fell Behind Frontier: 44
Mississippi
Georgia
California
Vermont
West Virginia
Kentucky
Arizona
Frontier Deviation in Incidence Rate Attributed to Anxiety disorders, 1990 (per 100,000)
Frontier Deviation in Incidence Rate Attributed to Anxiety disorders, 2021 (per 100,000)
Alabama
Florida
California
Georgia
Arizona
Arkansas
Mississippi
West Virginia

Figure S20

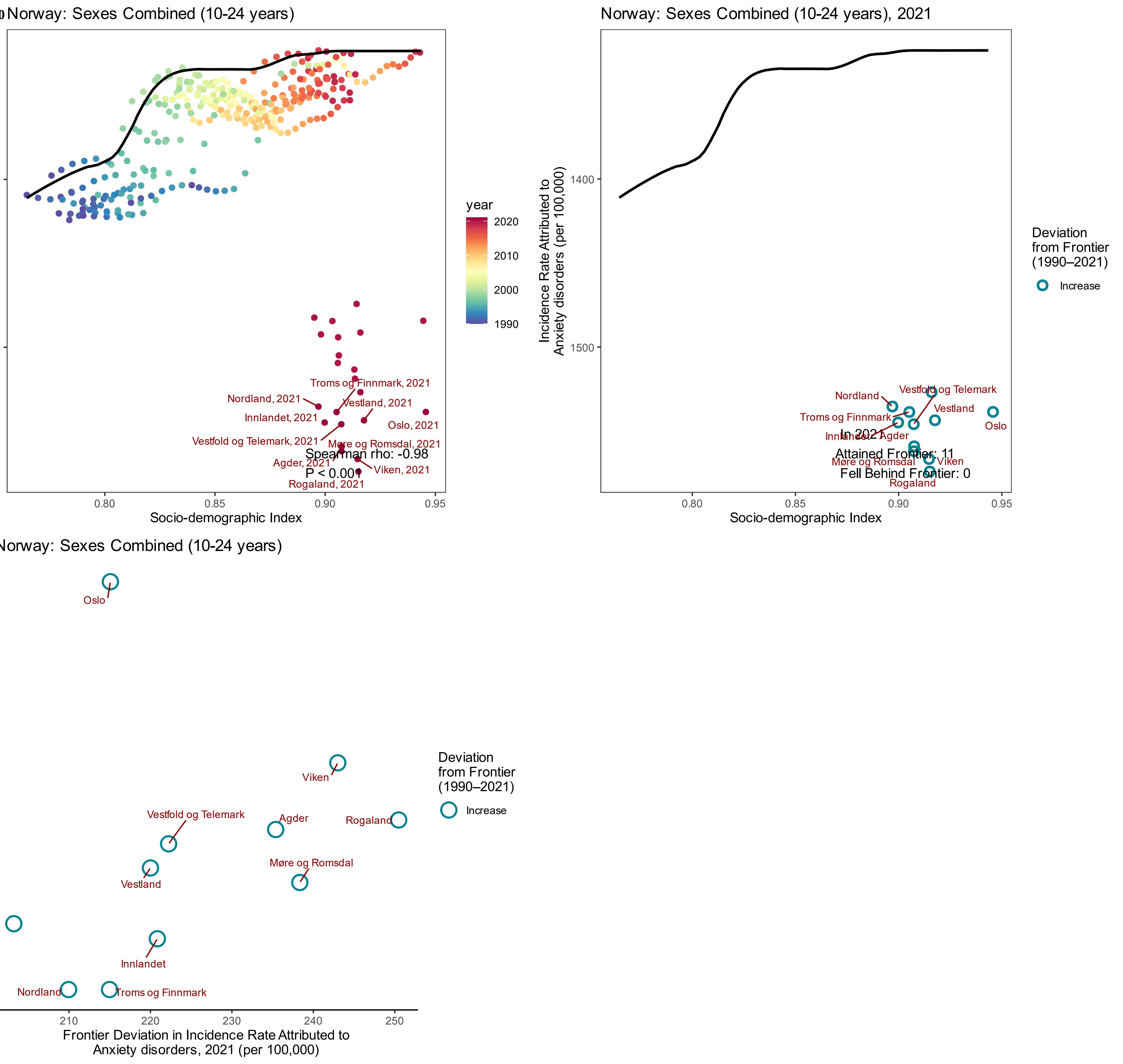

Norway: Sexes Combined (10-24 years)
Incidence Rate Attributed to Anxiety disorders (per 100,000)
Socio-demographic Index
year
2020
2010
2000
1990
Troms og Finnmark, 2021
Nordland, 2021
Vestland, 2021
Innlandet, 2021
Oslo, 2021
Vestfold og Telemark, 2021
Møre og Romsdal, 2021
Spearman rho: -0.98
Agder, 2021
P < 0.001
Viken, 2021
Rogaland, 2021
Norway: Sexes Combined (10-24 years), 2021
Deviation from Frontier (1990–2021)
Increase
In 2021
Attained Frontier: 11
Fell Behind Frontier: 0
Frontier Deviation in Incidence Rate Attributed to Anxiety disorders, 1990 (per 100,000)
Frontier Deviation in Incidence Rate Attributed to Anxiety disorders, 2021 (per 100,000)

Figure S20

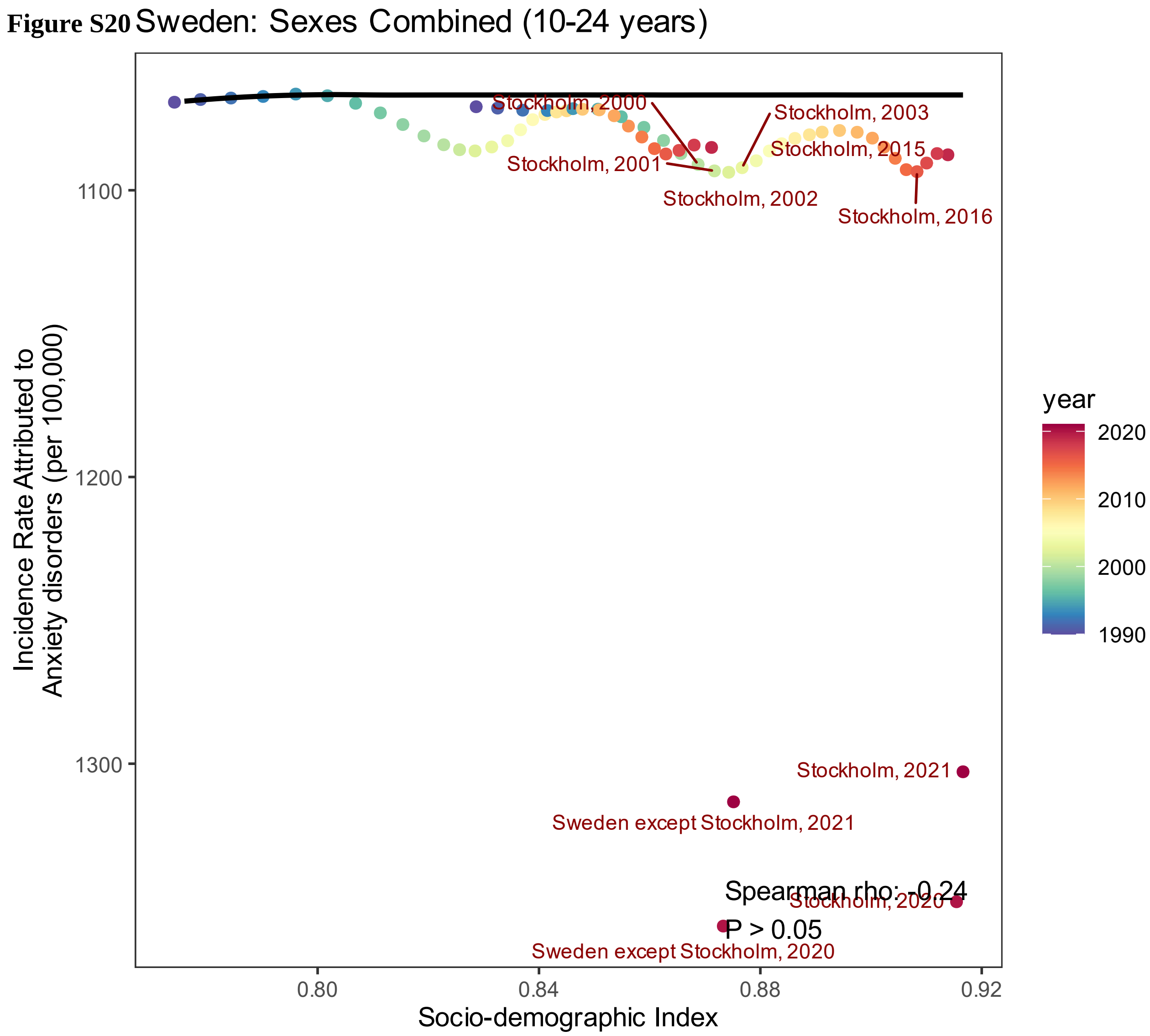

Sweden: Sexes Combined (10-24 years)
Incidence Rate Attributed to Anxiety disorders (per 100,000)
Socio-demographic Index
year
2020
2010
2000
1990
Stockholm, 2000
Stockholm, 2003
Stockholm, 2015
Stockholm, 2001
Stockholm, 2002
Stockholm, 2016
Stockholm, 2021
Sweden except Stockholm, 2021
Spearman rho: -0.24
P > 0.05
Stockholm, 2020
Sweden except Stockholm, 2020

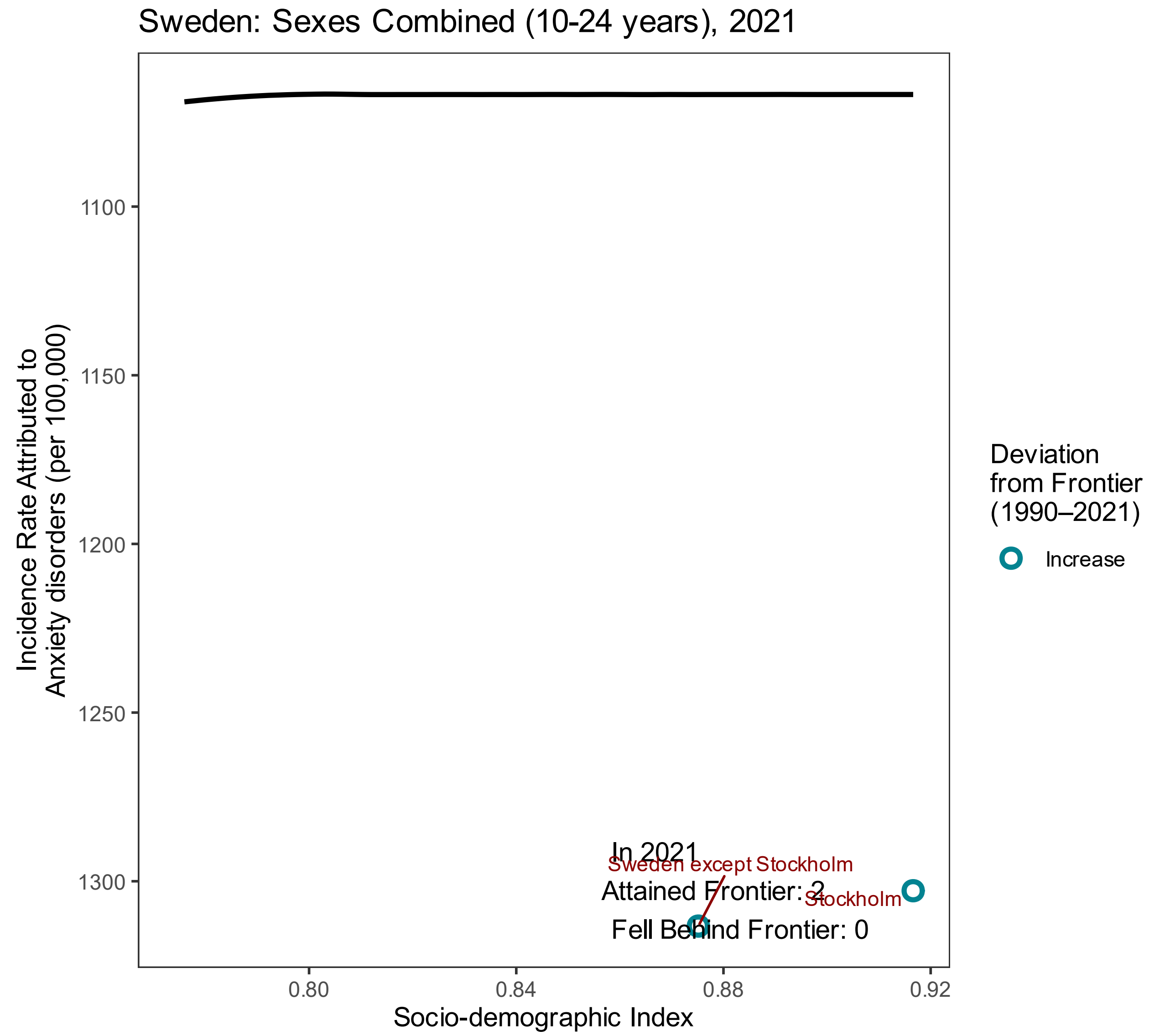

Sweden: Sexes Combined (10-24 years), 2021
Incidence Rate Attributed to Anxiety disorders (per 100,000)
Socio-demographic Index
Deviation from Frontier (1990–2021)
Increase
In 2021
Sweden except Stockholm
Attained Frontier: 2
Stockholm
Fell Behind Frontier: 0

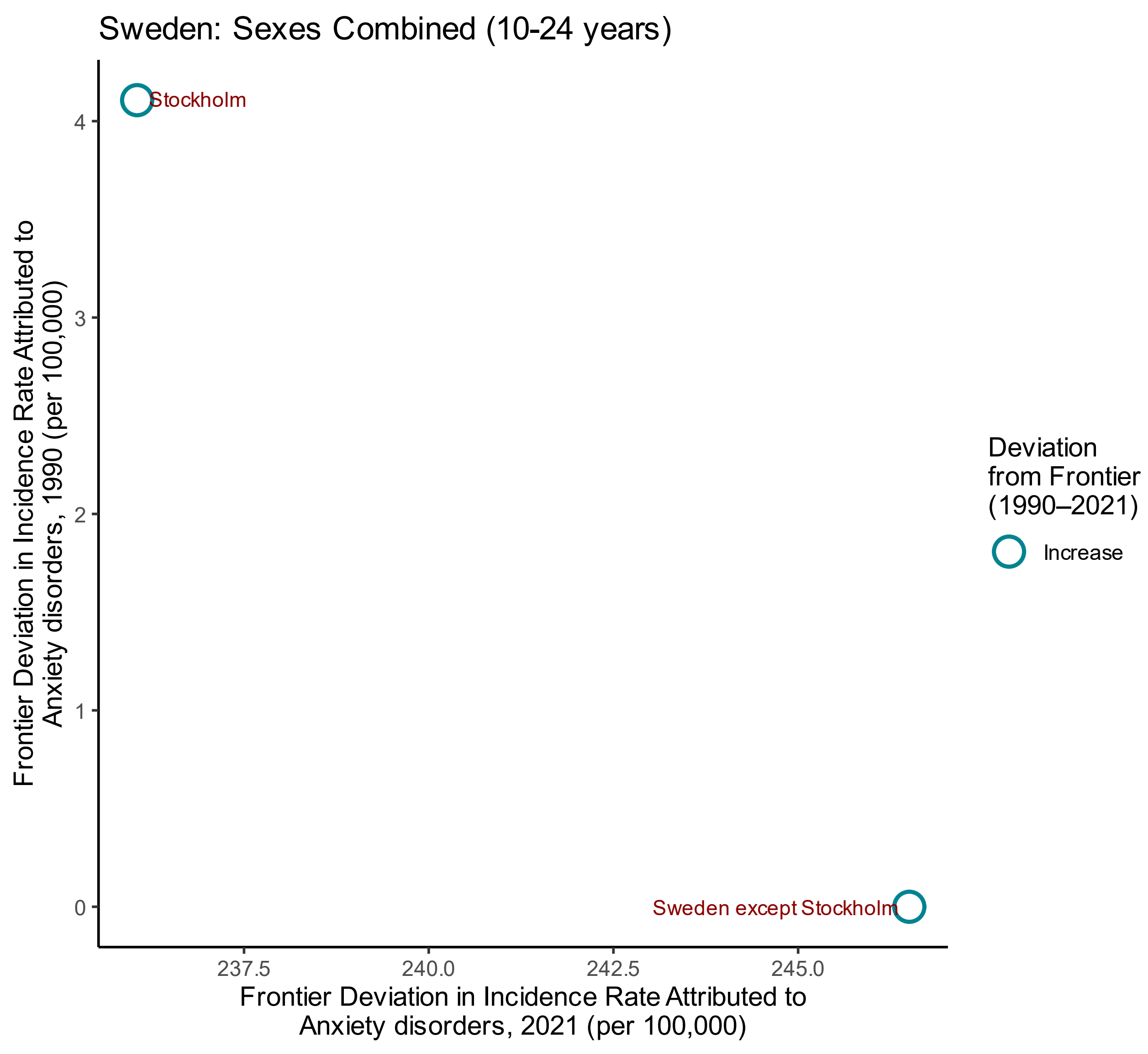

Sweden: Sexes Combined (10-24 years)
Frontier Deviation in Incidence Rate Attributed to Anxiety disorders, 1990 (per 100,000)
Frontier Deviation in Incidence Rate Attributed to Anxiety disorders, 2021 (per 100,000)
Stockholm
Sweden except Stockholm
Deviation from Frontier (1990–2021)
Increase

Figure S20

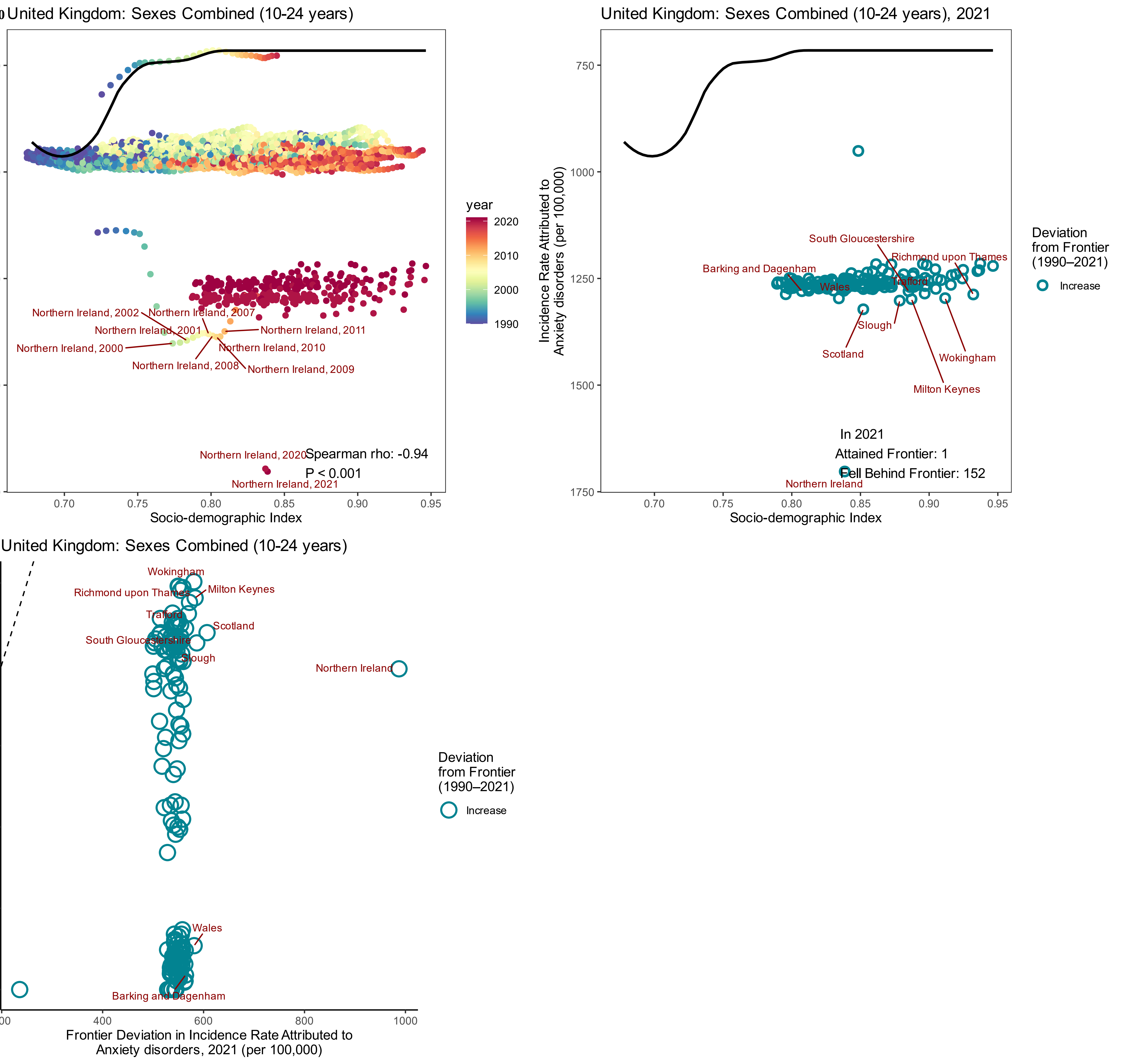
United Kingdom: Sexes Combined (10-24 years)
Incidence Rate Attributed to Anxiety disorders (per 100,000)
Socio-demographic Index
year
Northern Ireland, 2002
Northern Ireland, 2007
Northern Ireland, 2001
Northern Ireland, 2011
Northern Ireland, 2000
Northern Ireland, 2010
Northern Ireland, 2008
Northern Ireland, 2009
Northern Ireland, 2020
Northern Ireland, 2021
Spearman rho: -0.94
P < 0.001
United Kingdom: Sexes Combined (10-24 years), 2021
South Gloucestershire
Richmond upon Thames
Barking and Dagenham
Wales
Trafford
Slough
Scotland
Wokingham
Milton Keynes
In 2021
Attained Frontier: 1
Fell Behind Frontier: 152
Northern Ireland
Deviation from Frontier (1990–2021)
Increase
United Kingdom: Sexes Combined (10-24 years)
Frontier Deviation in Incidence Rate Attributed to Anxiety disorders, 1990 (per 100,000)
Frontier Deviation in Incidence Rate Attributed to Anxiety disorders, 2021 (per 100,000)
Wokingham
Richmond upon Thames
Milton Keynes
Trafford
Scotland
South Gloucestershire
Slough
Northern Ireland
Wales
Barking and Dagenham
Deviation from Frontier (1990–2021)
Increase

Figure S20

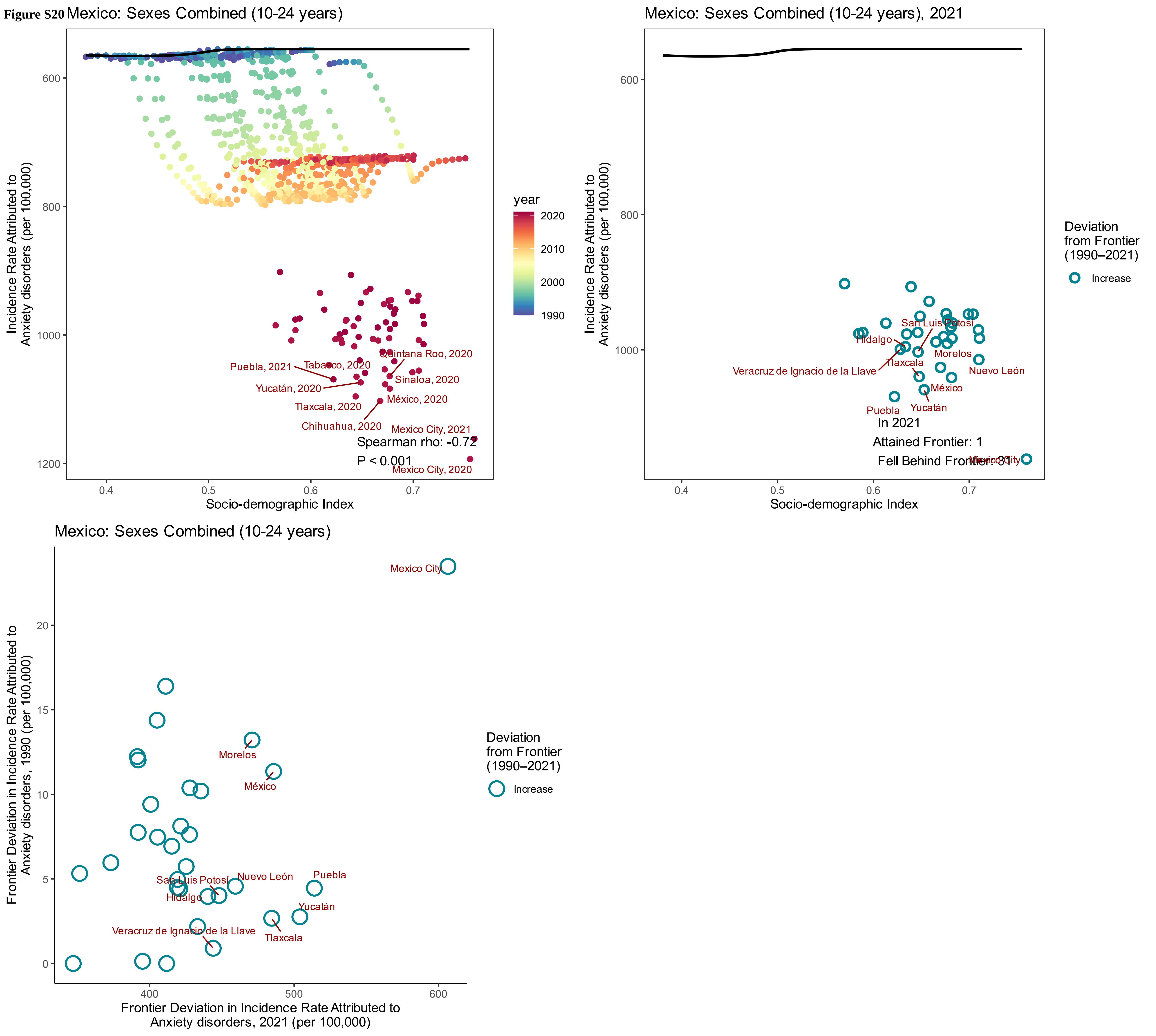
Mexico: Sexes Combined (10-24 years)
Incidence Rate Attributed to Anxiety disorders (per 100,000)
Socio-demographic Index
year
2020
2010
2000
1990
Puebla, 2021
Tabasco, 2020
Quintana Roo, 2020
Sinaloa, 2020
Yucatán, 2020
Tlaxcala, 2020
México, 2020
Chihuahua, 2020
Mexico City, 2021
Mexico City, 2020
Spearman rho: -0.72
P < 0.001
Mexico: Sexes Combined (10-24 years), 2021
San Luis Potosí
Hidalgo
Morelos
Tlaxcala
Veracruz de Ignacio de la Llave
Nuevo León
México
Puebla
Yucatán
Mexico City
In 2021
Attained Frontier: 1
Fell Behind Frontier: 31
Deviation from Frontier (1990–2021)
Increase
Mexico: Sexes Combined (10-24 years)
Frontier Deviation in Incidence Rate Attributed to Anxiety disorders, 1990 (per 100,000)
Frontier Deviation in Incidence Rate Attributed to Anxiety disorders, 2021 (per 100,000)

Figure S20

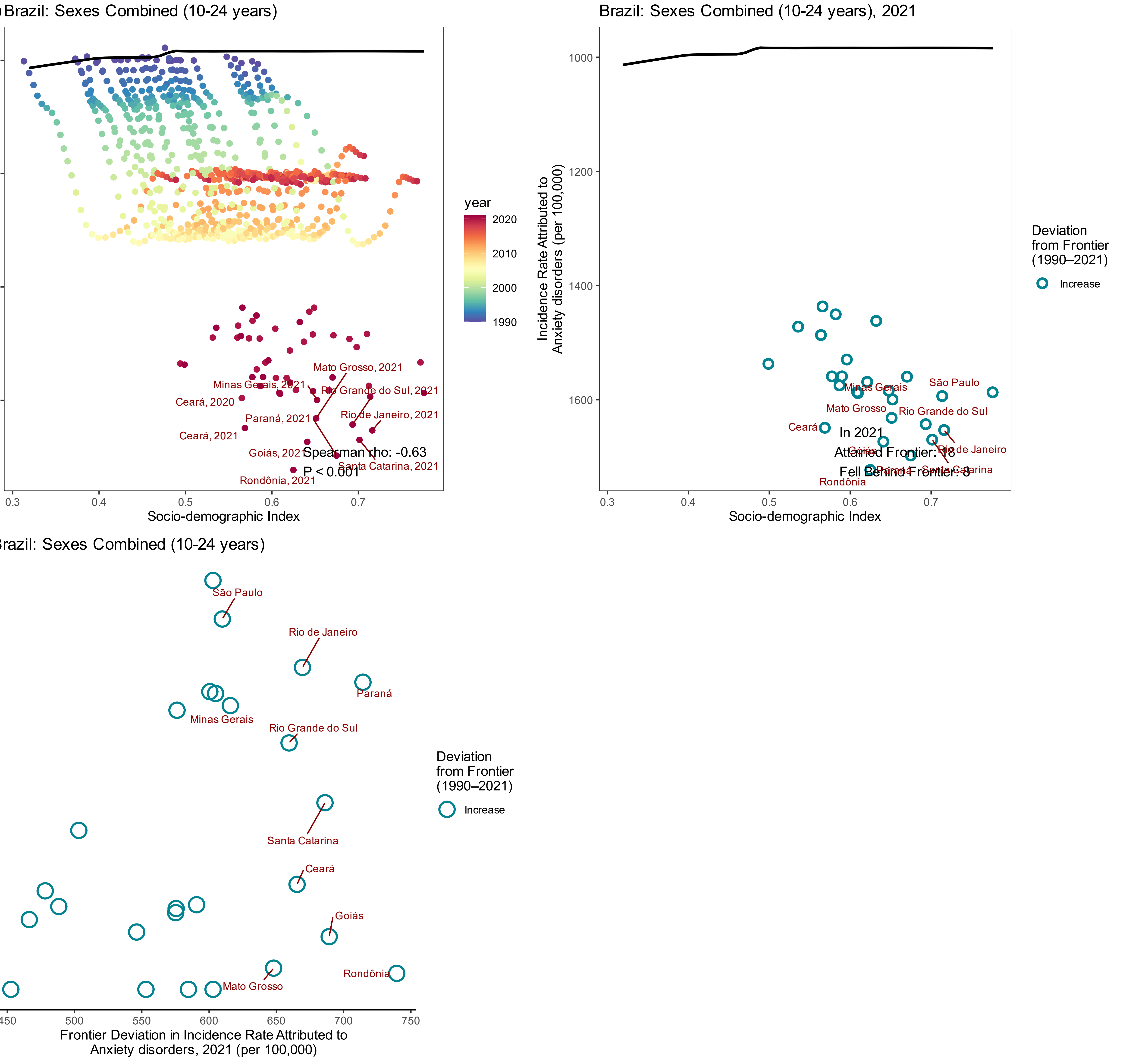

Brazil: Sexes Combined (10-24 years)
Incidence Rate Attributed to Anxiety disorders (per 100,000)
Socio-demographic Index
year
Mato Grosso, 2021
Minas Gerais, 2021
Rio Grande do Sul, 2021
Ceará, 2020
Paraná, 2021
Rio de Janeiro, 2021
Ceará, 2021
Goiás, 2021
Spearman rho: -0.63
P < 0.001
Santa Catarina, 2021
Rondônia, 2021
Brazil: Sexes Combined (10-24 years), 2021
Deviation from Frontier (1990–2021)
Increase
São Paulo
Minas Gerais
Mato Grosso
Rio Grande do Sul
Ceará
In 2021
Attained Frontier: 18
Fell Behind Frontier: 8
Rio de Janeiro
Goiás
Paraná
Santa Catarina
Rondônia
Brazil: Sexes Combined (10-24 years)
Frontier Deviation in Incidence Rate Attributed to Anxiety disorders, 1990 (per 100,000)
Frontier Deviation in Incidence Rate Attributed to Anxiety disorders, 2021 (per 100,000)

Figure S20

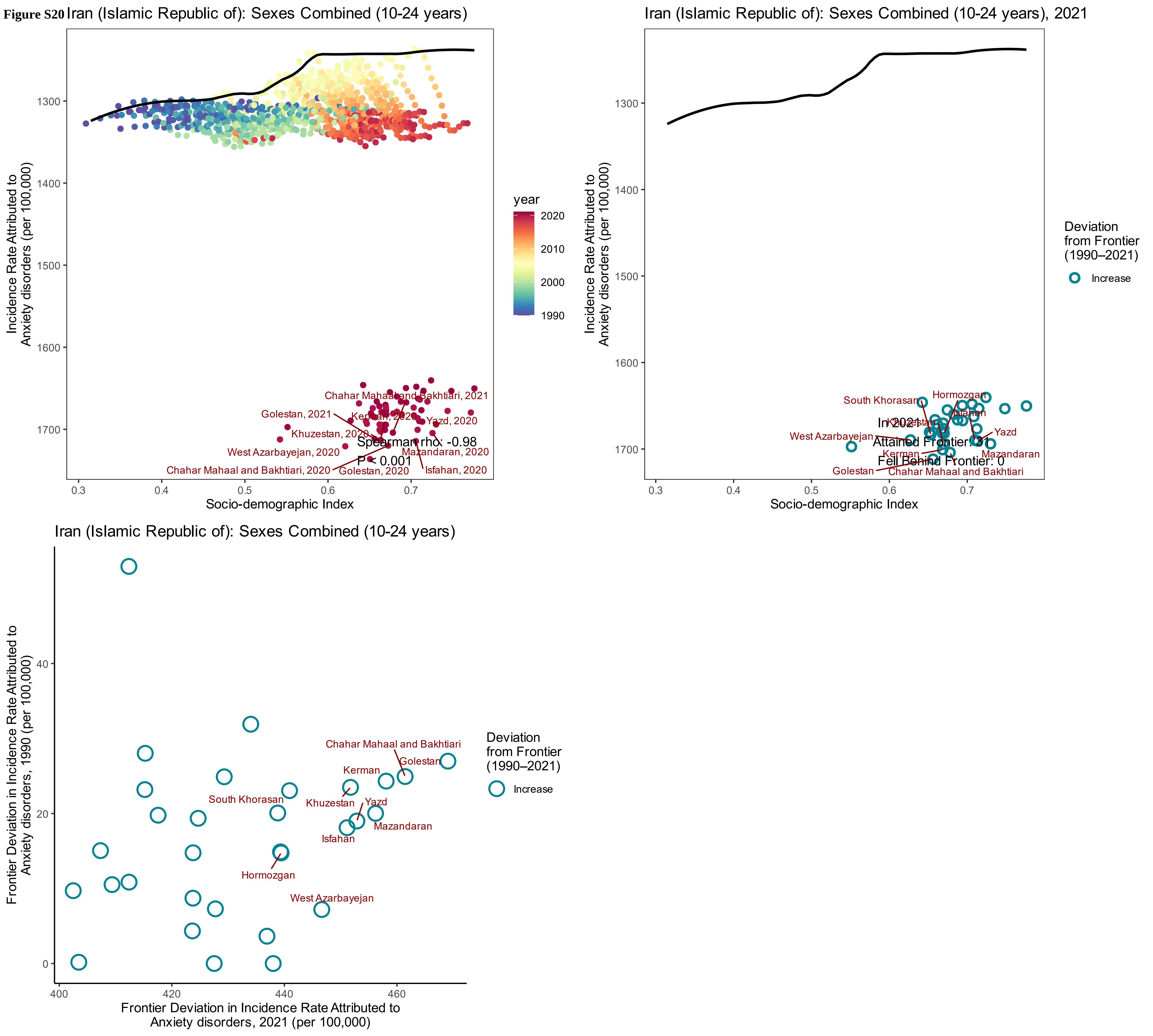

Iran (Islamic Republic of): Sexes Combined (10-24 years)
Incidence Rate Attributed to Anxiety disorders (per 100,000)
Socio-demographic Index
year
2020
2010
2000
1990
Chahar Mahaal and Bakhtiari, 2021
Golestan, 2021
Kerman, 2020
Yazd, 2020
Khuzestan, 2020
Spearman rho: -0.98
West Azarbayejan, 2020
Mazandaran, 2020
P < 0.001
Chahar Mahaal and Bakhtiari, 2020
Golestan, 2020
Isfahan, 2020
Iran (Islamic Republic of): Sexes Combined (10-24 years), 2021
Deviation from Frontier (1990–2021)
Increase
South Khorasan
Hormozgan
Isfahan
Khuzestan
In 2021
Yazd
West Azarbayejan
Attained Frontier: 31
Kerman
Mazandaran
Fell Behind Frontier: 0
Golestan
Chahar Mahaal and Bakhtiari
Frontier Deviation in Incidence Rate Attributed to Anxiety disorders, 1990 (per 100,000)
Frontier Deviation in Incidence Rate Attributed to Anxiety disorders, 2021 (per 100,000)
Chahar Mahaal and Bakhtiari
Golestan
Kerman
South Khorasan
Khuzestan
Yazd
Mazandaran
Isfahan
Hormozgan
West Azarbayejan

Figure S20

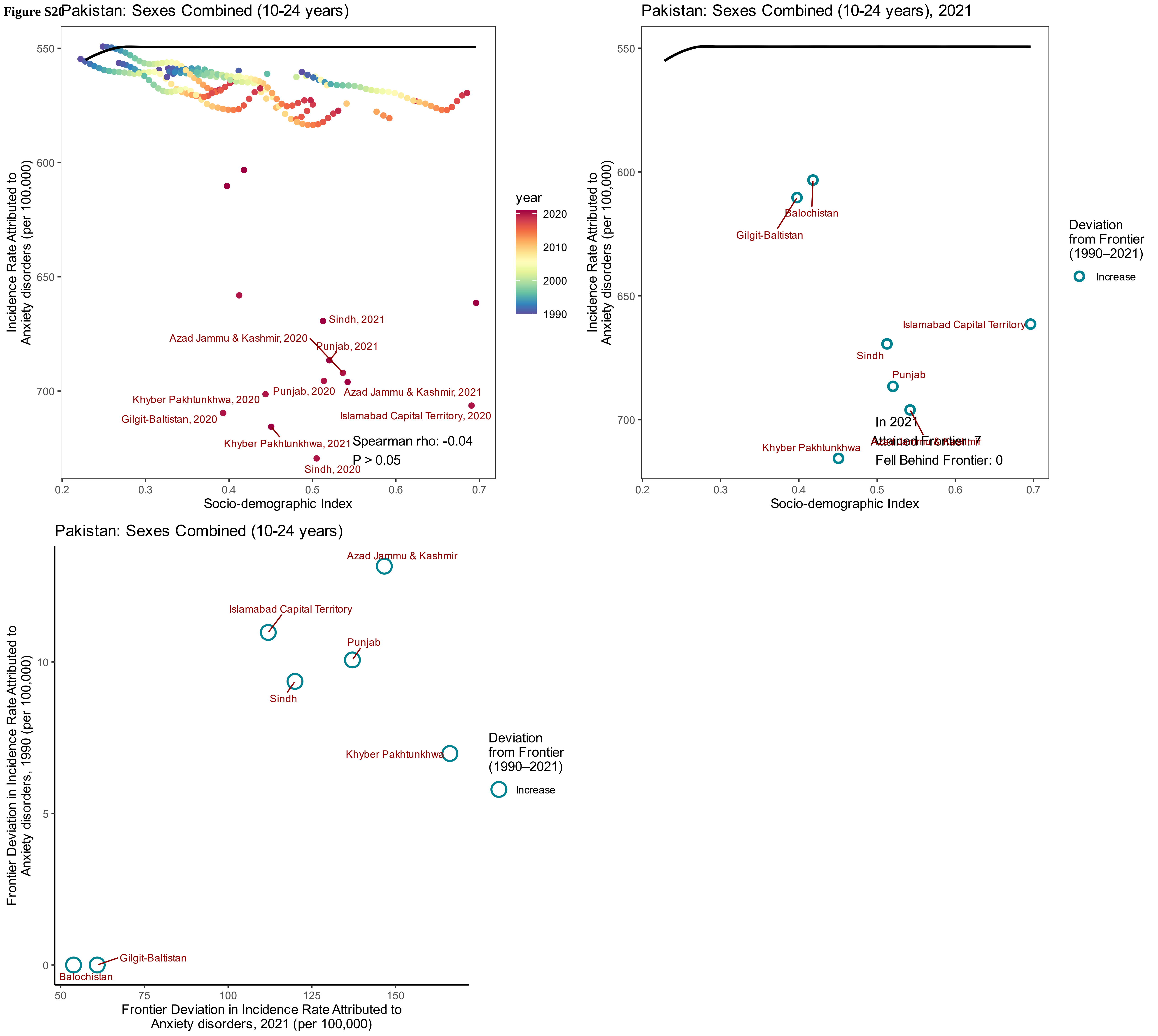

Pakistan: Sexes Combined (10-24 years)
Incidence Rate Attributed to Anxiety disorders (per 100,000)
Socio-demographic Index
year
2020
2010
2000
1990
Sindh, 2021
Azad Jammu & Kashmir, 2020
Punjab, 2021
Punjab, 2020
Azad Jammu & Kashmir, 2021
Khyber Pakhtunkhwa, 2020
Gilgit-Baltistan, 2020
Islamabad Capital Territory, 2020
Khyber Pakhtunkhwa, 2021
Spearman rho: -0.04
P > 0.05
Sindh, 2020
Pakistan: Sexes Combined (10-24 years), 2021
Balochistan
Gilgit-Baltistan
Islamabad Capital Territory
Sindh
Punjab
In 2021
Attained Frontier: 7
Azad Jammu & Kashmir
Fell Behind Frontier: 0
Khyber Pakhtunkhwa
Deviation from Frontier (1990–2021)
Increase
Pakistan: Sexes Combined (10-24 years)
Frontier Deviation in Incidence Rate Attributed to Anxiety disorders, 1990 (per 100,000)
Frontier Deviation in Incidence Rate Attributed to Anxiety disorders, 2021 (per 100,000)
Azad Jammu & Kashmir
Islamabad Capital Territory
Punjab
Sindh
Khyber Pakhtunkhwa
Gilgit-Baltistan
Balochistan
Deviation from Frontier (1990–2021)
Increase

Figure S20

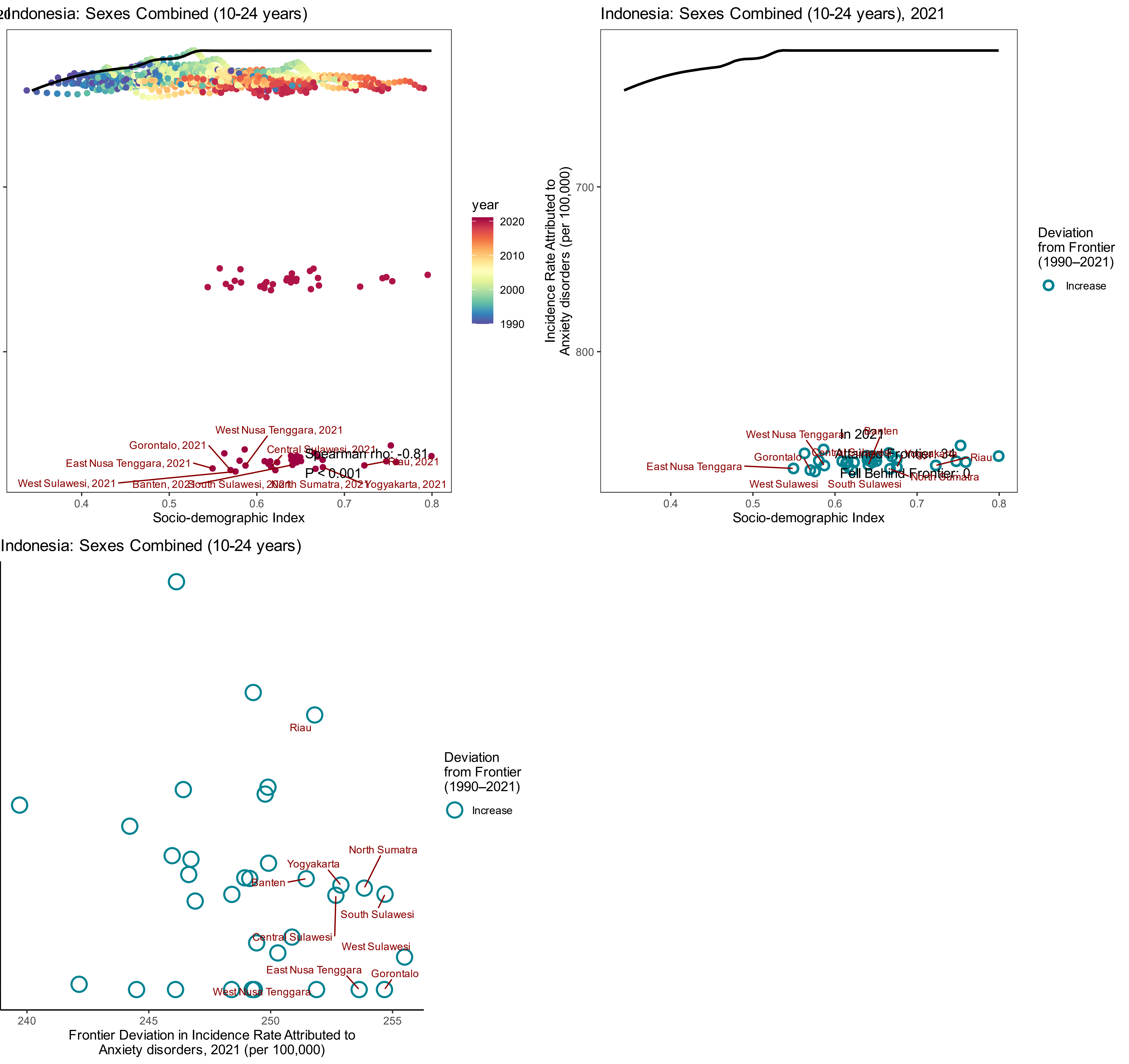
Indonesia: Sexes Combined (10-24 years)
Incidence Rate Attributed to Anxiety disorders (per 100,000)
Socio-demographic Index
year
2020
2010
2000
1990
West Nusa Tenggara, 2021
Gorontalo, 2021
Central Sulawesi, 2021
East Nusa Tenggara, 2021
Spearman rho: -0.81
P < 0.001
Riau, 2021
West Sulawesi, 2021
Banten, 2021
South Sulawesi, 2021
North Sumatra, 2021
Yogyakarta, 2021
Indonesia: Sexes Combined (10-24 years), 2021
In 2021
Attained Frontier: 34
Fell Behind Frontier: 0
Deviation from Frontier (1990–2021)
Increase
Frontier Deviation in Incidence Rate Attributed to Anxiety disorders, 1990 (per 100,000)
Frontier Deviation in Incidence Rate Attributed to Anxiety disorders, 2021 (per 100,000)
Riau
North Sumatra
Yogyakarta
Banten
South Sulawesi
Central Sulawesi
West Sulawesi
East Nusa Tenggara
Gorontalo
West Nusa Tenggara

Figure S20

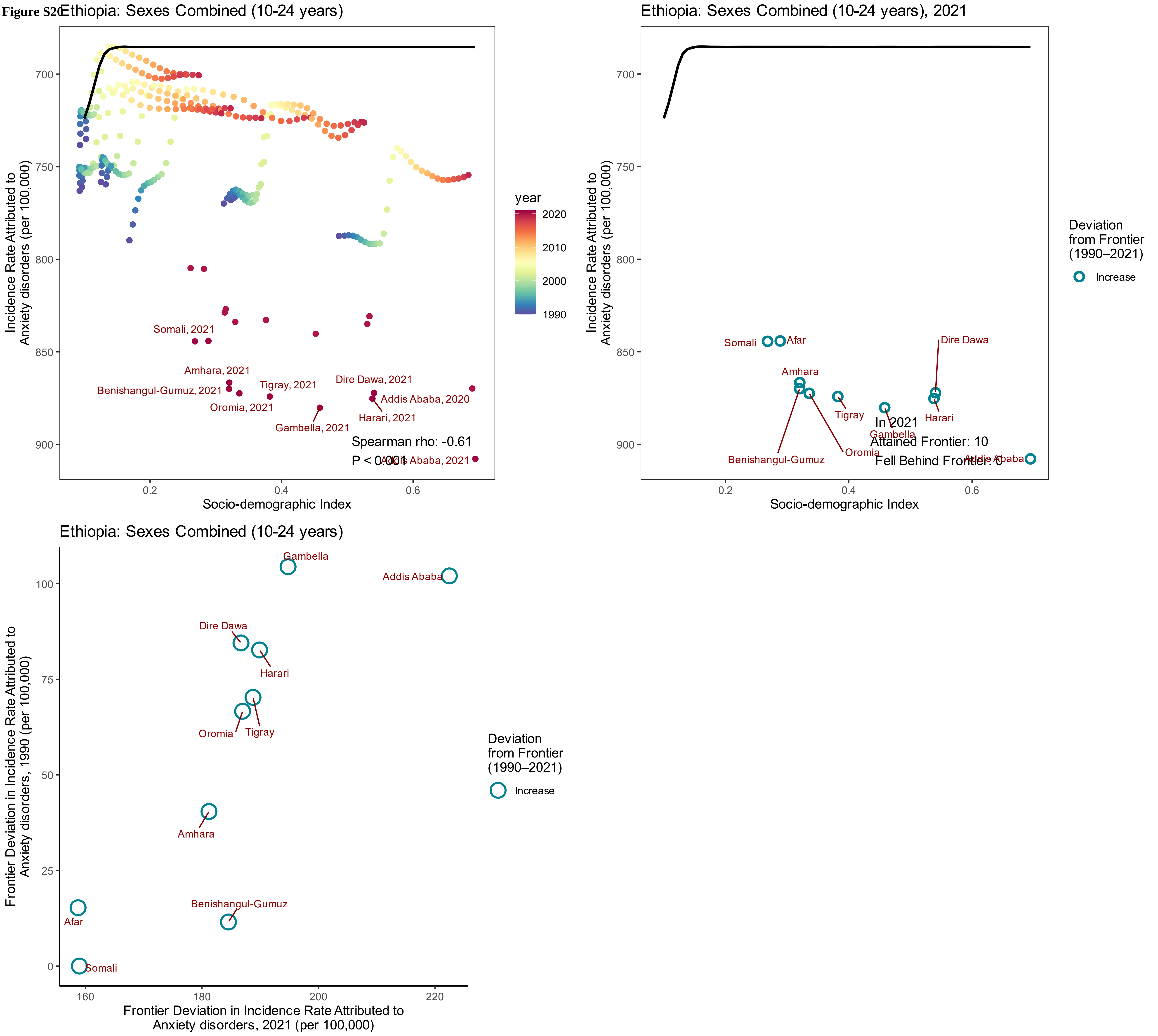
Ethiopia: Sexes Combined (10-24 years)
Incidence Rate Attributed to Anxiety disorders (per 100,000)
Socio-demographic Index
year
2020
2010
2000
1990
Somali, 2021
Amhara, 2021
Benishangul-Gumuz, 2021
Tigray, 2021
Dire Dawa, 2021
Addis Ababa, 2020
Oromia, 2021
Harari, 2021
Gambella, 2021
Spearman rho: -0.61
P < 0.001
Addis Ababa, 2021
Ethiopia: Sexes Combined (10-24 years), 2021
Somali
Afar
Dire Dawa
Amhara
Tigray
Harari
Gambella
Benishangul-Gumuz
Oromia
In 2021
Attained Frontier: 10
Fell Behind Frontier: 0
Addis Ababa
Deviation from Frontier (1990–2021)
Increase
Ethiopia: Sexes Combined (10-24 years)
Frontier Deviation in Incidence Rate Attributed to Anxiety disorders, 1990 (per 100,000)
Frontier Deviation in Incidence Rate Attributed to Anxiety disorders, 2021 (per 100,000)
Gambella
Addis Ababa
Dire Dawa
Harari
Oromia
Tigray
Amhara
Benishangul-Gumuz
Afar
Somali
Deviation from Frontier (1990–2021)
Increase

Figure S20

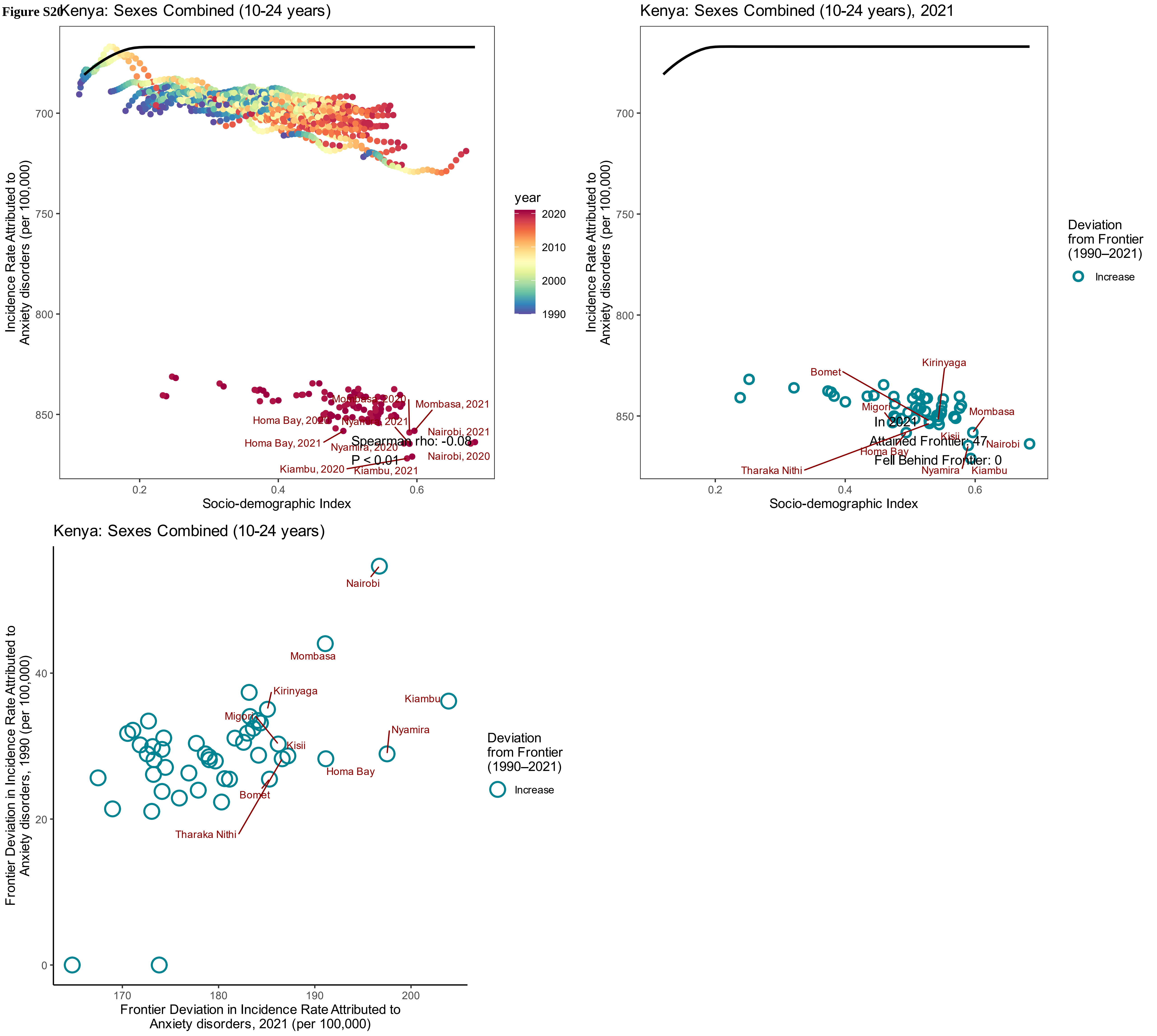

Kenya: Sexes Combined (10-24 years)
Incidence Rate Attributed to Anxiety disorders (per 100,000)
Socio-demographic Index
year
2020
2010
2000
1990
Mombasa, 2020
Mombasa, 2021
Homa Bay, 2020
Nyamira, 2021
Nairobi, 2021
Homa Bay, 2021
Spearman rho: -0.08
Nyamira, 2020
P < 0.01
Nairobi, 2020
Kiambu, 2020
Kiambu, 2021
Kenya: Sexes Combined (10-24 years), 2021
Deviation from Frontier (1990–2021)
Increase
Kirinyaga
Bomet
Migori
Mombasa
In 2021
Kisii
Attained Frontier: 47
Homa Bay
Nairobi
Fell Behind Frontier: 0
Tharaka Nithi
Nyamira
Kiambu
Kenya: Sexes Combined (10-24 years)
Frontier Deviation in Incidence Rate Attributed to Anxiety disorders, 1990 (per 100,000)
Frontier Deviation in Incidence Rate Attributed to Anxiety disorders, 2021 (per 100,000)
Nairobi
Mombasa
Kirinyaga
Kiambu
Migori
Kisii
Nyamira
Homa Bay
Bomet
Tharaka Nithi
Deviation from Frontier (1990–2021)
Increase

Figure S20

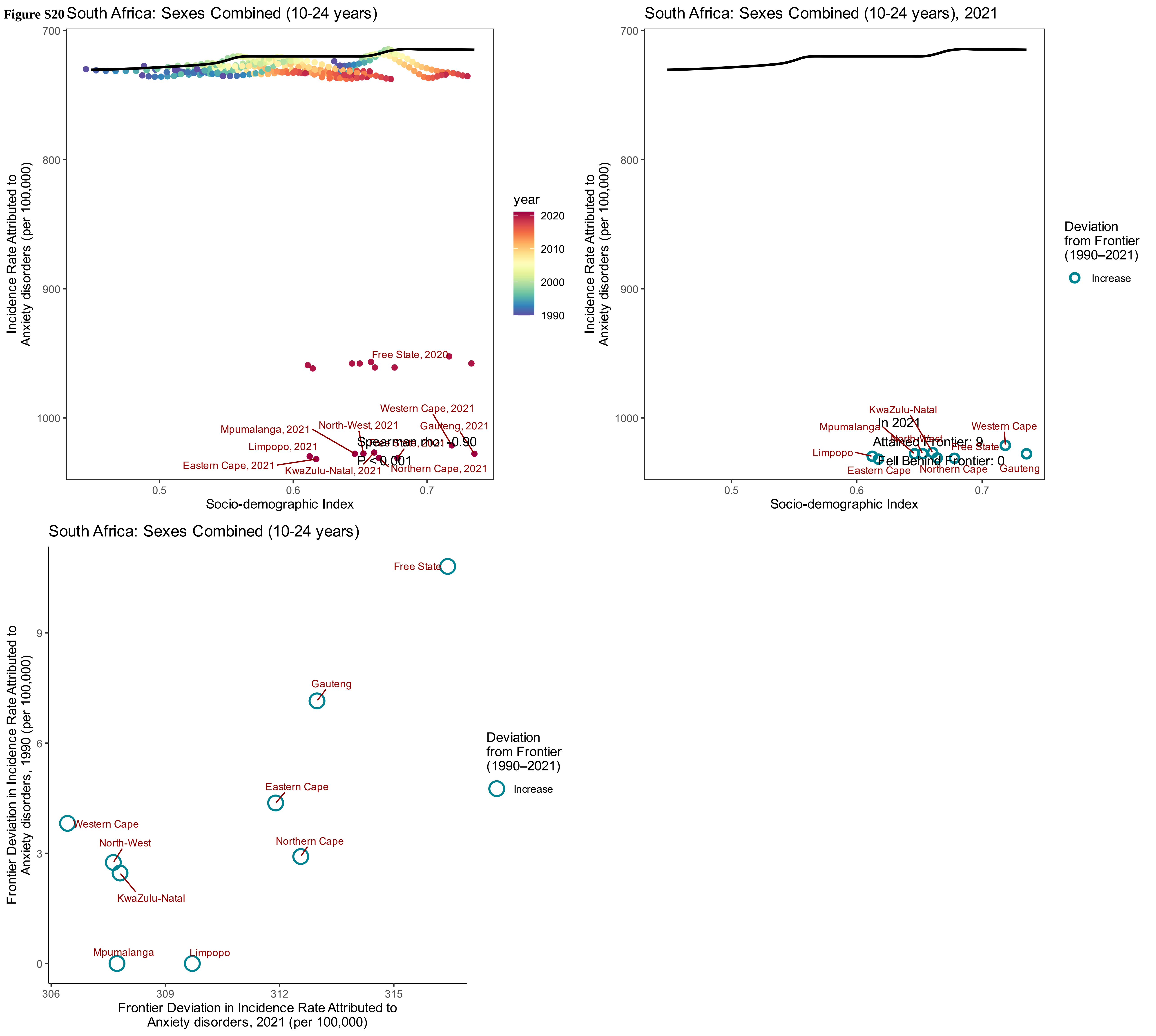

South Africa: Sexes Combined (10-24 years)
Incidence Rate Attributed to Anxiety disorders (per 100,000)
Socio-demographic Index
year
2020
2010
2000
1990
Free State, 2020
Western Cape, 2021
Mpumalanga, 2021
North-West, 2021
Gauteng, 2021
Limpopo, 2021
Spearman rho: -0.90
P < 0.001
Eastern Cape, 2021
KwaZulu-Natal, 2021
Northern Cape, 2021
South Africa: Sexes Combined (10-24 years), 2021
Deviation from Frontier (1990–2021)
Increase
KwaZulu-Natal
Mpumalanga
In 2021
Attained Frontier: 9
Fell Behind Frontier: 0
Western Cape
Free State
Limpopo
Eastern Cape
Northern Cape
Gauteng
Frontier Deviation in Incidence Rate Attributed to Anxiety disorders, 1990 (per 100,000)
Frontier Deviation in Incidence Rate Attributed to Anxiety disorders, 2021 (per 100,000)
Free State
Gauteng
Eastern Cape
Western Cape
North-West
Northern Cape
KwaZulu-Natal
Mpumalanga
Limpopo

Figure S20

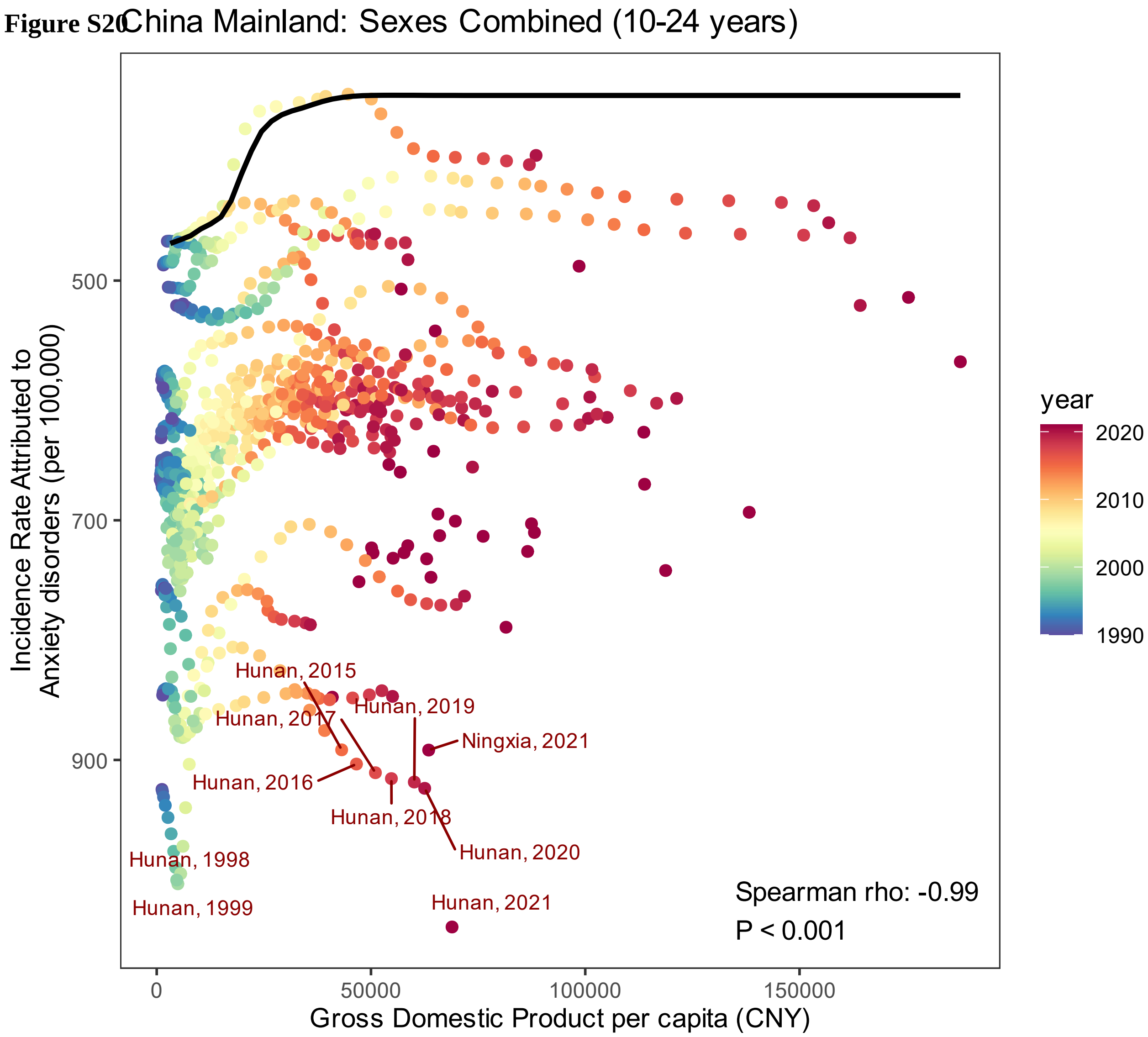

China Mainland: Sexes Combined (10-24 years)
Incidence Rate Attributed to Anxiety disorders (per 100,000)
Gross Domestic Product per capita (CNY)
year
2020
2010
2000
1990
Hunan, 2015
Hunan, 2017
Hunan, 2019
Ningxia, 2021
Hunan, 2016
Hunan, 2018
Hunan, 2020
Hunan, 1998
Hunan, 1999
Hunan, 2021
Spearman rho: -0.99
P < 0.001

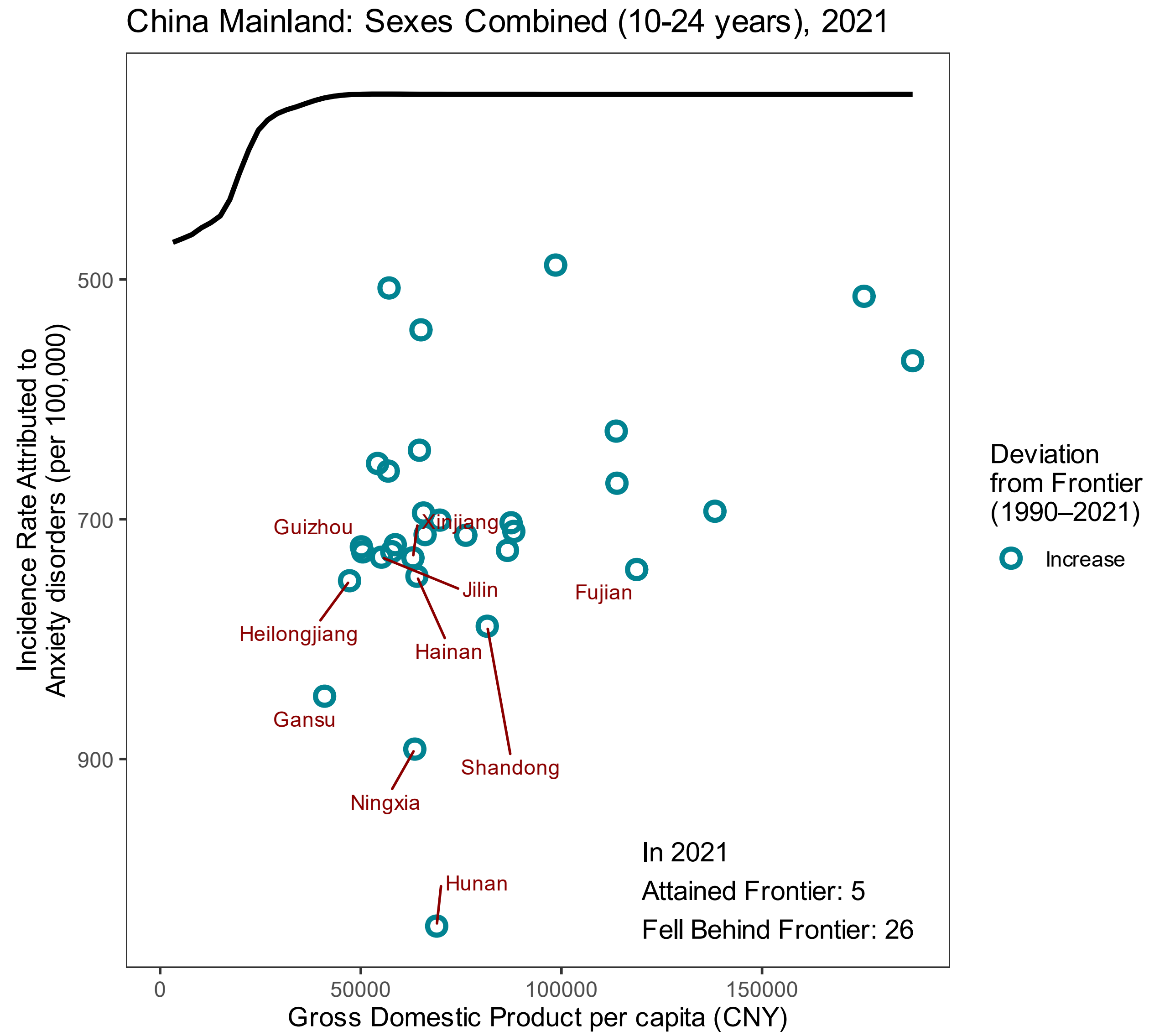

China Mainland: Sexes Combined (10-24 years), 2021
Incidence Rate Attributed to Anxiety disorders (per 100,000)
Gross Domestic Product per capita (CNY)
Guizhou
Xinjiang
Jilin
Fujian
Heilongjiang
Hainan
Gansu
Shandong
Ningxia
Hunan
In 2021
Attained Frontier: 5
Fell Behind Frontier: 26
Deviation from Frontier (1990–2021)
Increase

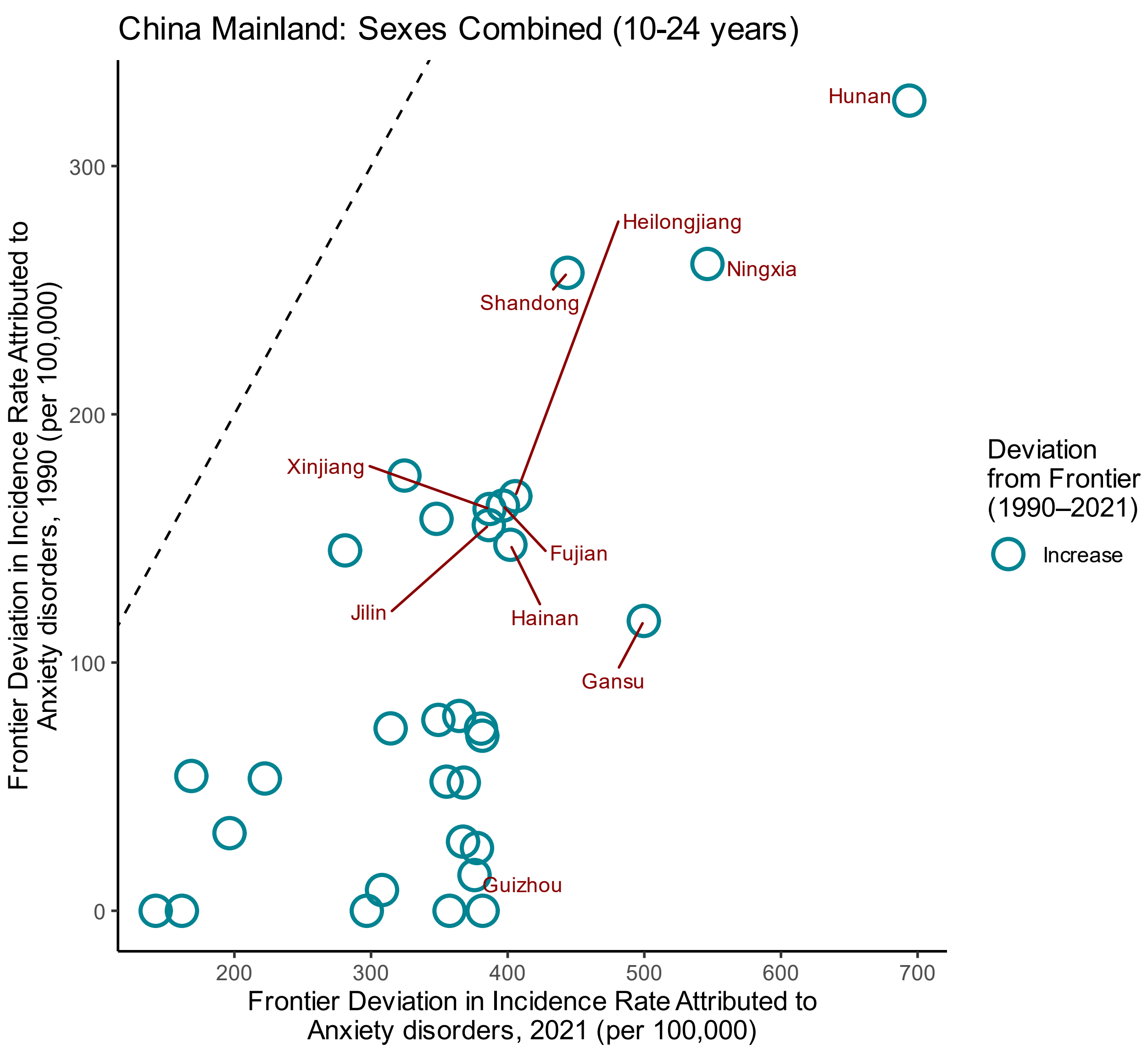

China Mainland: Sexes Combined (10-24 years)
Frontier Deviation in Incidence Rate Attributed to Anxiety disorders, 1990 (per 100,000)
Frontier Deviation in Incidence Rate Attributed to Anxiety disorders, 2021 (per 100,000)
Hunan
Heilongjiang
Shandong
Ningxia
Xinjiang
Fujian
Jilin
Hainan
Gansu
Guizhou
Deviation from Frontier (1990–2021)
Increase

Figure S20

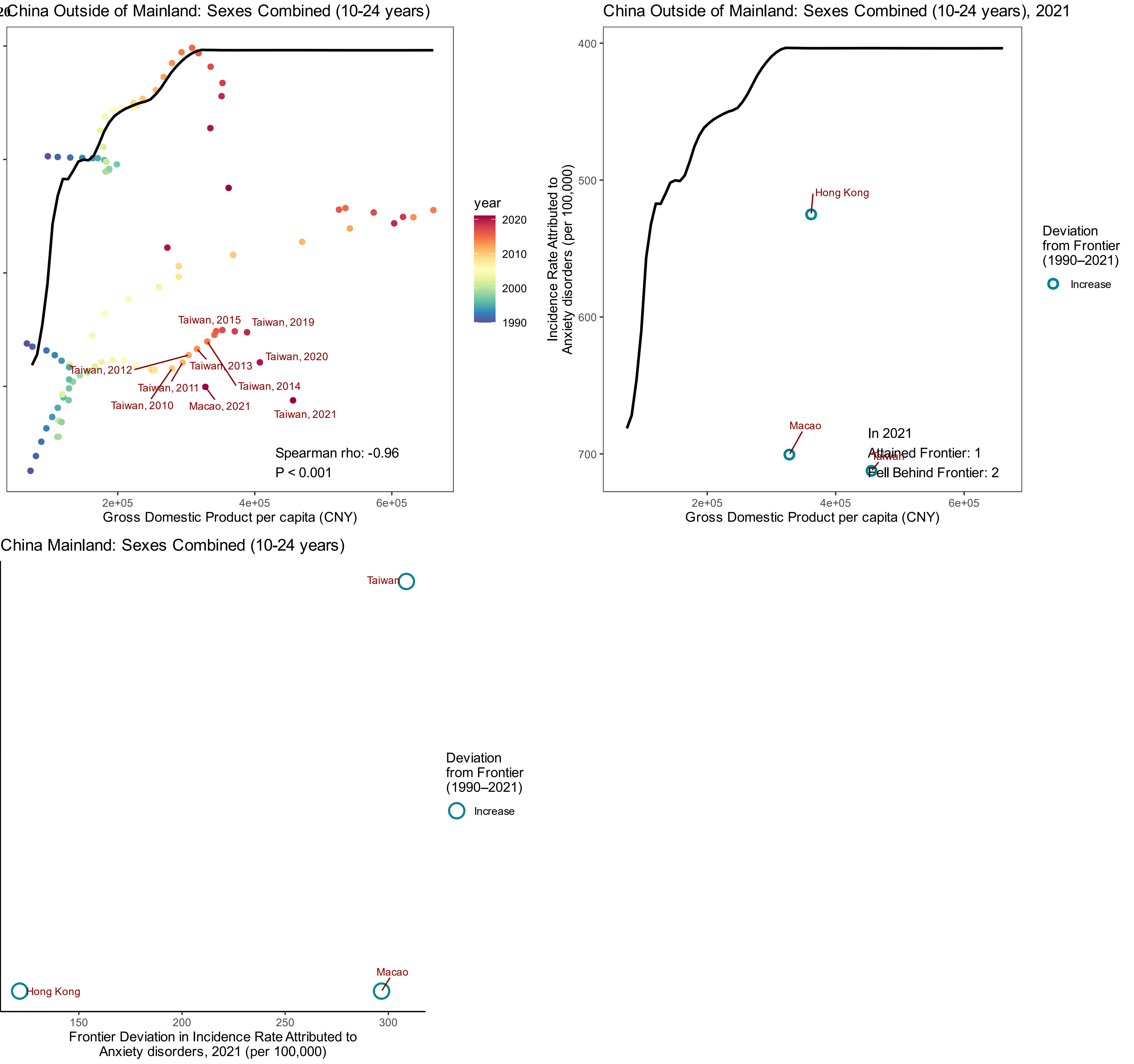

China Outside of Mainland: Sexes Combined (10-24 years)
Incidence Rate Attributed to Anxiety disorders (per 100,000)
Gross Domestic Product per capita (CNY)
year
2020
2010
2000
1990
Taiwan, 2015
Taiwan, 2019
Taiwan, 2020
Taiwan, 2012
Taiwan, 2013
Taiwan, 2011
Taiwan, 2014
Taiwan, 2010
Macao, 2021
Taiwan, 2021
Spearman rho: -0.96
P < 0.001
China Outside of Mainland: Sexes Combined (10-24 years), 2021
Hong Kong
Macao
Taiwan
In 2021
Attained Frontier: 1
Fell Behind Frontier: 2
Deviation from Frontier (1990–2021)
Increase
China Mainland: Sexes Combined (10-24 years)
Frontier Deviation in Incidence Rate Attributed to Anxiety disorders, 1990 (per 100,000)
Frontier Deviation in Incidence Rate Attributed to Anxiety disorders, 2021 (per 100,000)
Taiwan
Hong Kong
Macao
Deviation from Frontier (1990–2021)
Increase

Figure S20

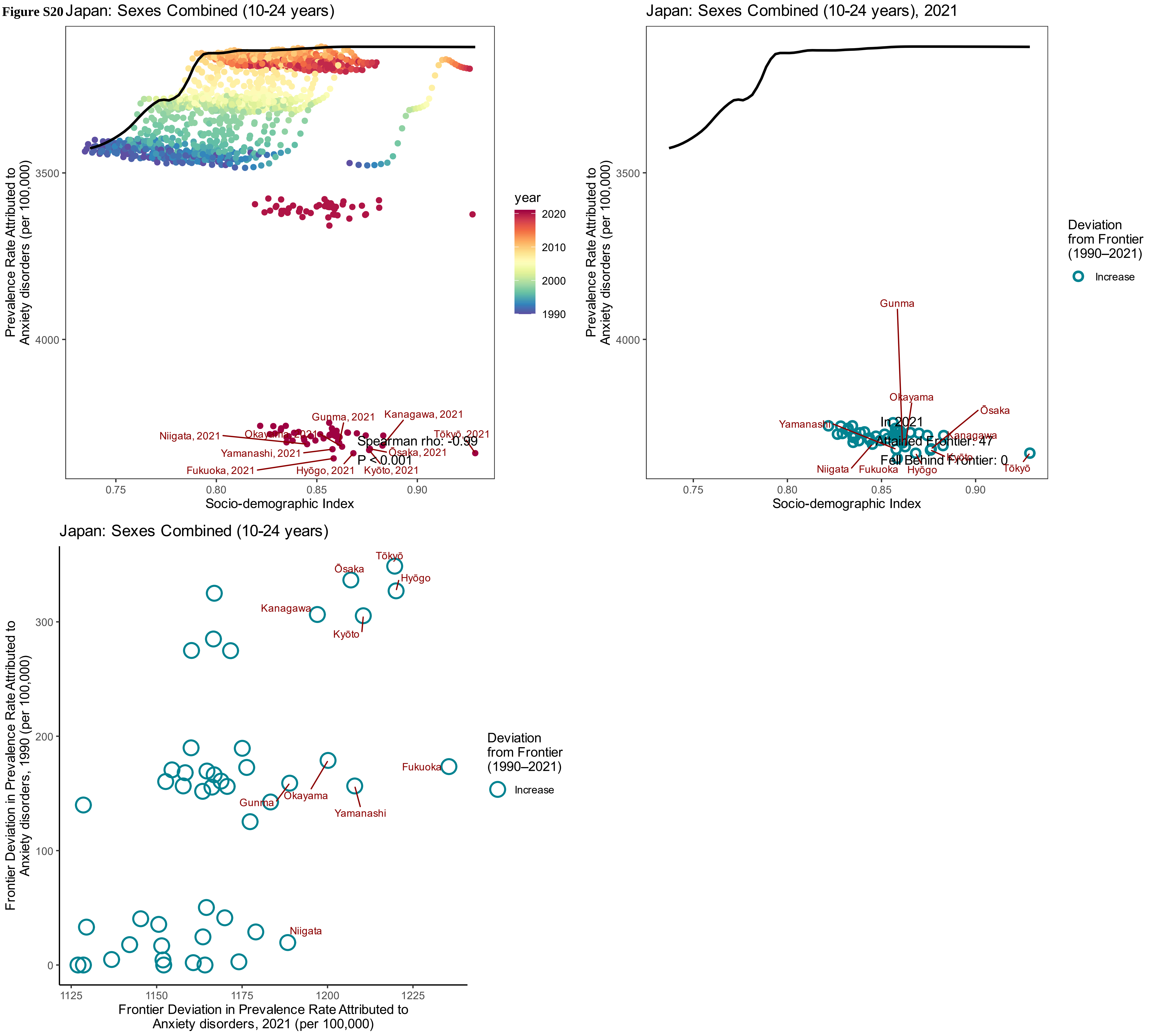

Japan: Sexes Combined (10-24 years)
Prevalence Rate Attributed to Anxiety disorders (per 100,000)
Socio-demographic Index
year
2020
2010
2000
1990
Gunma, 2021
Kanagawa, 2021
Niigata, 2021
Okayama, 2021
Tōkyō, 2021
Spearman rho: -0.99
Yamanashi, 2021
Ōsaka, 2021
P < 0.001
Fukuoka, 2021
Hyōgo, 2021
Kyōto, 2021
Japan: Sexes Combined (10-24 years), 2021
Gunma
Okayama
Ōsaka
Yamanashi
In 2021
Attained Frontier: 47
Kanagawa
Kyōto
Fell Behind Frontier: 0
Niigata
Fukuoka
Hyōgo
Tōkyō
Deviation from Frontier (1990–2021)
Increase
Japan: Sexes Combined (10-24 years)
Frontier Deviation in Prevalence Rate Attributed to Anxiety disorders, 1990 (per 100,000)
Frontier Deviation in Prevalence Rate Attributed to Anxiety disorders, 2021 (per 100,000)
Tōkyō
Ōsaka
Hyōgo
Kanagawa
Kyōto
Fukuoka
Gunma
Okayama
Yamanashi
Niigata
Deviation from Frontier (1990–2021)
Increase

Figure S20

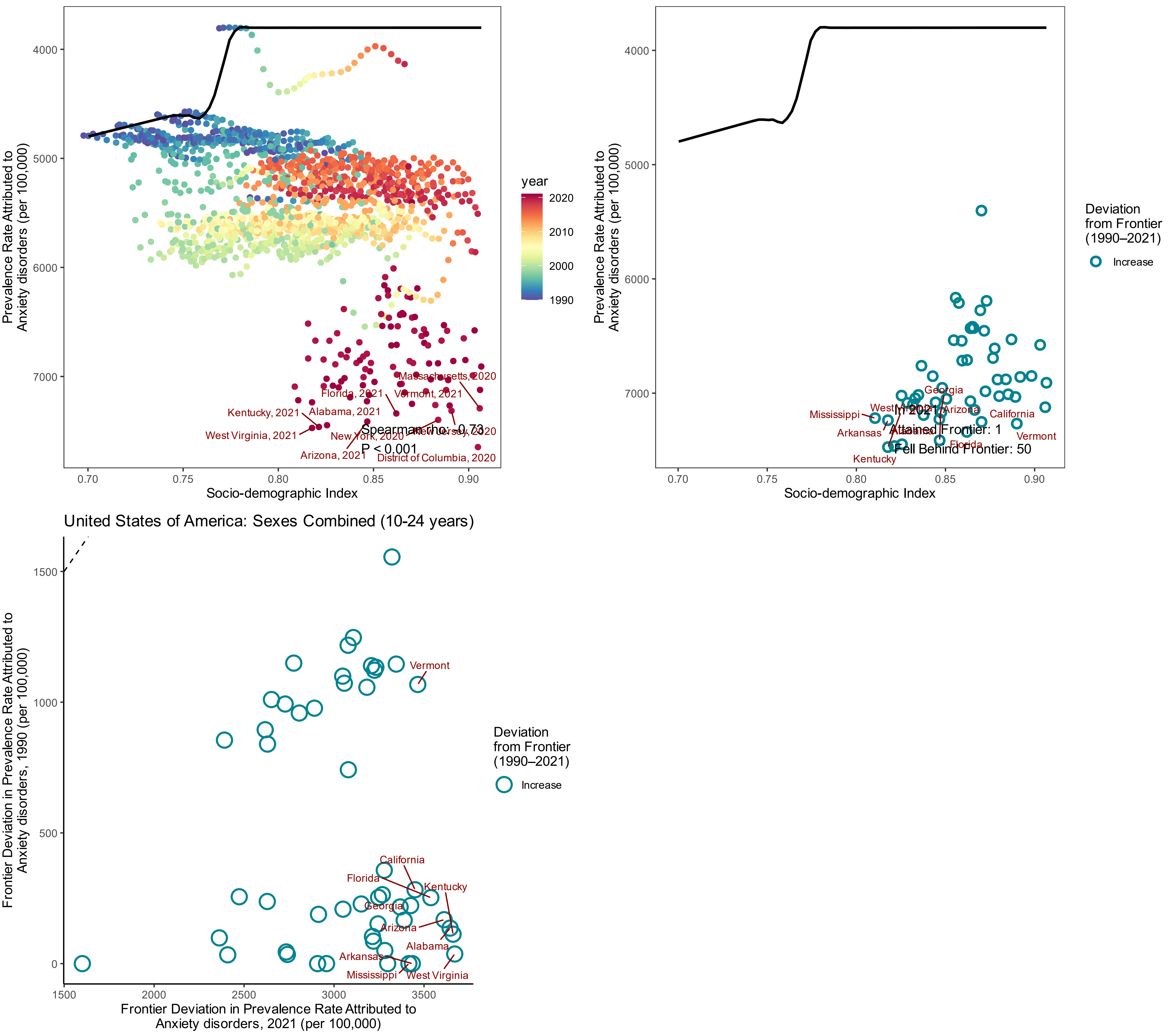

United States of America: Sexes Combined (10-24 years)
Prevalence Rate Attributed to Anxiety disorders (per 100,000)
Socio-demographic Index
year
2020
2010
2000
1990
Massachusetts, 2020
Florida, 2021
Vermont, 2021
Kentucky, 2021
Alabama, 2021
West Virginia, 2021
New York, 2020
New Jersey, 2020
Arizona, 2021
District of Columbia, 2020
Spearman rho: -0.73
P < 0.001
United States of America: Sexes Combined (10-24 years), 2021
Deviation from Frontier (1990–2021)
Increase
Mississippi
Arkansas
Georgia
Arizona
California
Vermont
Florida
Kentucky
In 2021
Attained Frontier: 1
Fell Behind Frontier: 50
United States of America: Sexes Combined (10-24 years)
Frontier Deviation in Prevalence Rate Attributed to Anxiety disorders, 1990 (per 100,000)
Frontier Deviation in Prevalence Rate Attributed to Anxiety disorders, 2021 (per 100,000)
Deviation from Frontier (1990–2021)
Increase
Vermont
California
Florida
Kentucky
Georgia
Arizona
Alabama
Arkansas
Mississippi
West Virginia

Figure S20

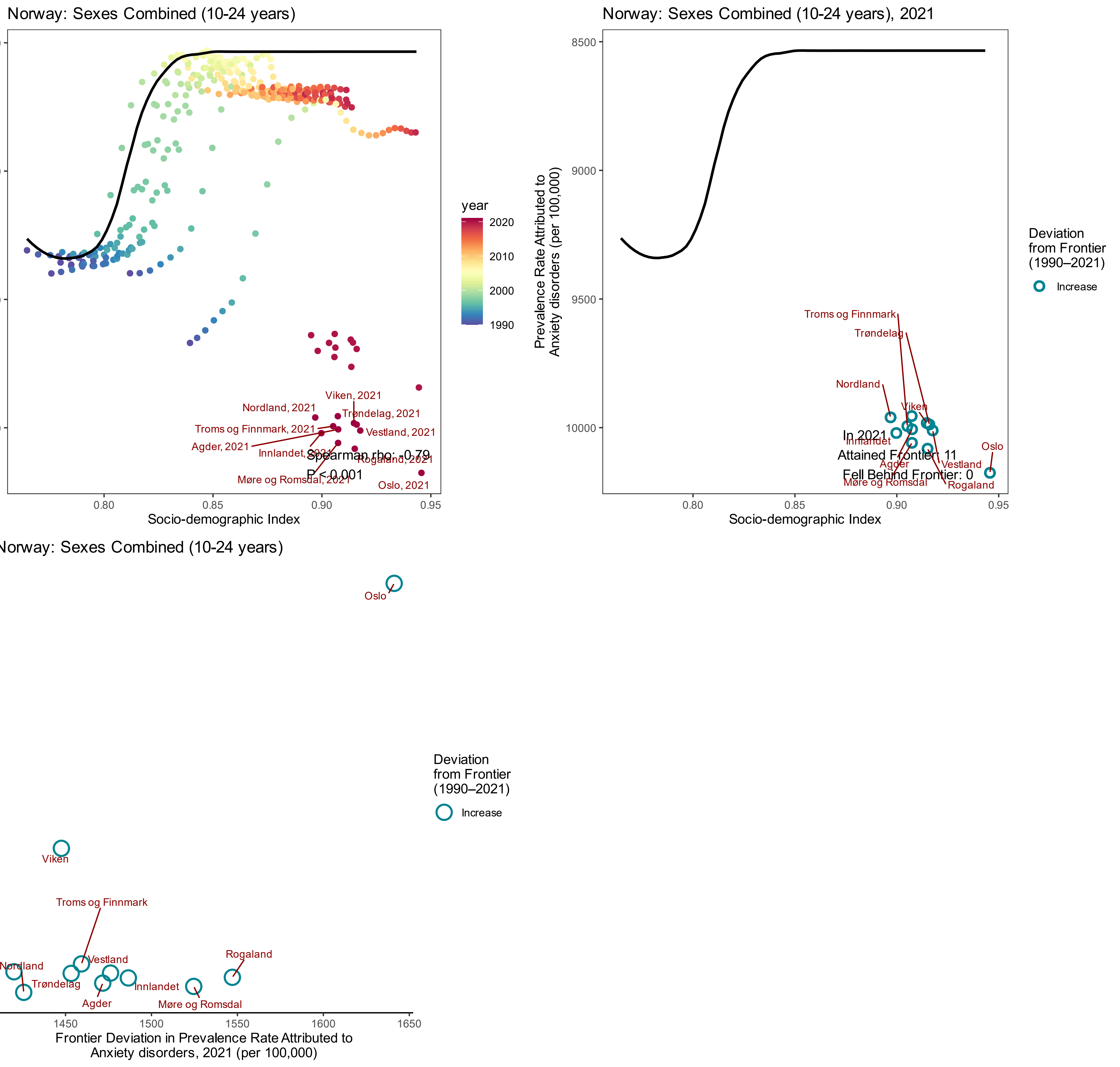
Norway: Sexes Combined (10-24 years)
Prevalence Rate Attributed to Anxiety disorders (per 100,000)
Socio-demographic Index
year
Nordland, 2021
Viken, 2021
Trøndelag, 2021
Troms og Finnmark, 2021
Vestland, 2021
Agder, 2021
Innlandet, 2021
Rogaland, 2021
Møre og Romsdal, 2021
Oslo, 2021
Spearman rho: -0.79
P < 0.001
Norway: Sexes Combined (10-24 years), 2021
Troms og Finnmark
Trøndelag
Nordland
Viken
Innlandet
Oslo
Agder
Vestland
Møre og Romsdal
Rogaland
In 2021
Attained Frontier: 11
Fell Behind Frontier: 0
Deviation from Frontier (1990–2021)
Increase
Frontier Deviation in Prevalence Rate Attributed to Anxiety disorders, 1990 (per 100,000)
Frontier Deviation in Prevalence Rate Attributed to Anxiety disorders, 2021 (per 100,000)

Figure S20

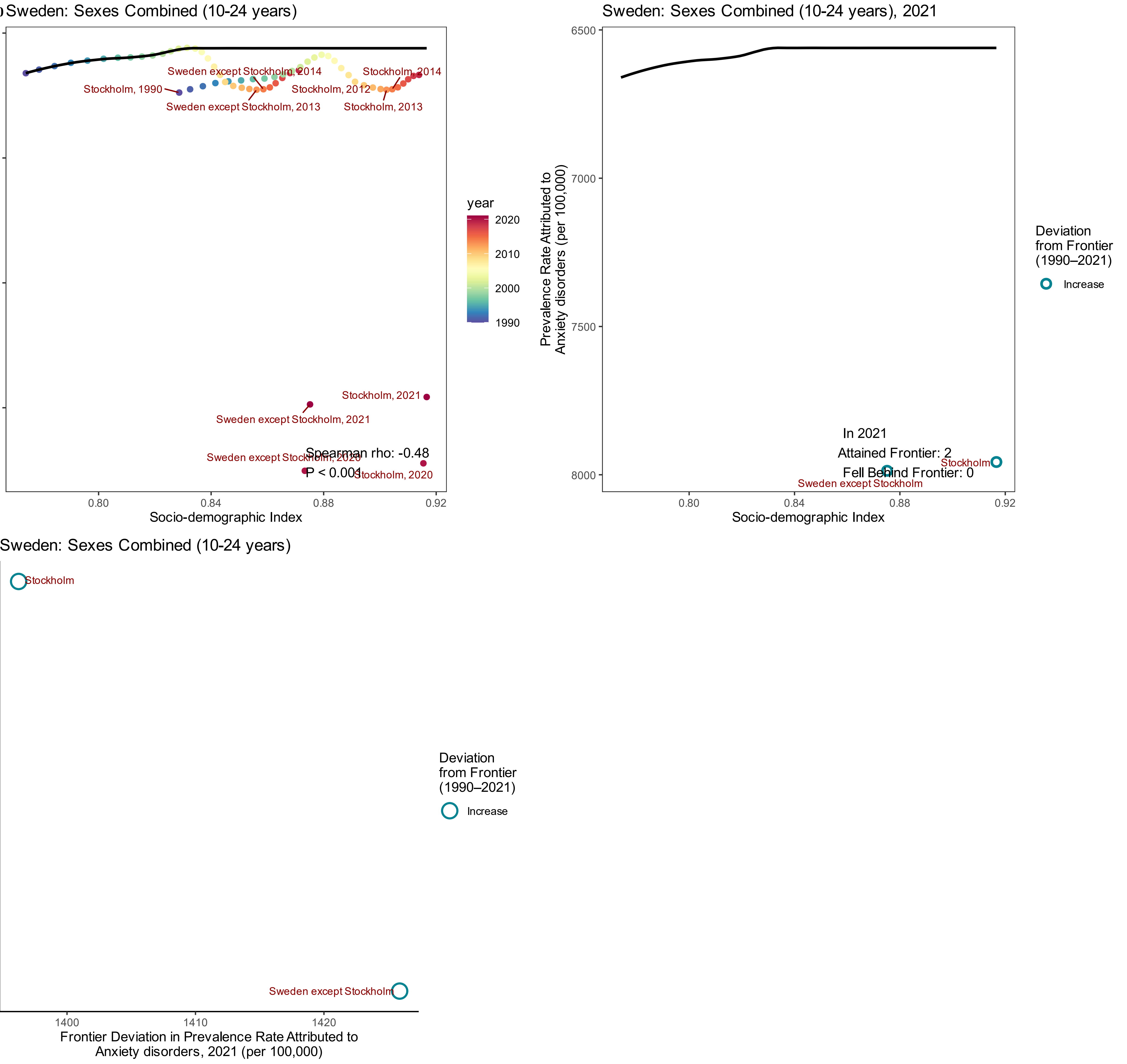

Sweden: Sexes Combined (10-24 years)
Prevalence Rate Attributed to Anxiety disorders (per 100,000)
6500
7000
7500
8000
Socio-demographic Index
0.80
0.84
0.88
0.92
Sweden except Stockholm, 2014
Stockholm, 2014
Stockholm, 1990
Stockholm, 2012
Sweden except Stockholm, 2013
Stockholm, 2013
Stockholm, 2021
Sweden except Stockholm, 2021
Spearman rho: -0.48
Sweden except Stockholm, 2020
P < 0.001
Stockholm, 2020
year
2020
2010
2000
1990
Sweden: Sexes Combined (10-24 years), 2021
In 2021
Attained Frontier: 2
Fell Behind Frontier: 0
Stockholm
Sweden except Stockholm
Deviation from Frontier (1990–2021)
Increase
Sweden: Sexes Combined (10-24 years)
Frontier Deviation in Prevalence Rate Attributed to Anxiety disorders, 1990 (per 100,000)
150
100
50
0
Frontier Deviation in Prevalence Rate Attributed to Anxiety disorders, 2021 (per 100,000)
1400
1410
1420
Stockholm
Sweden except Stockholm
Deviation from Frontier (1990–2021)
Increase

Figure S20

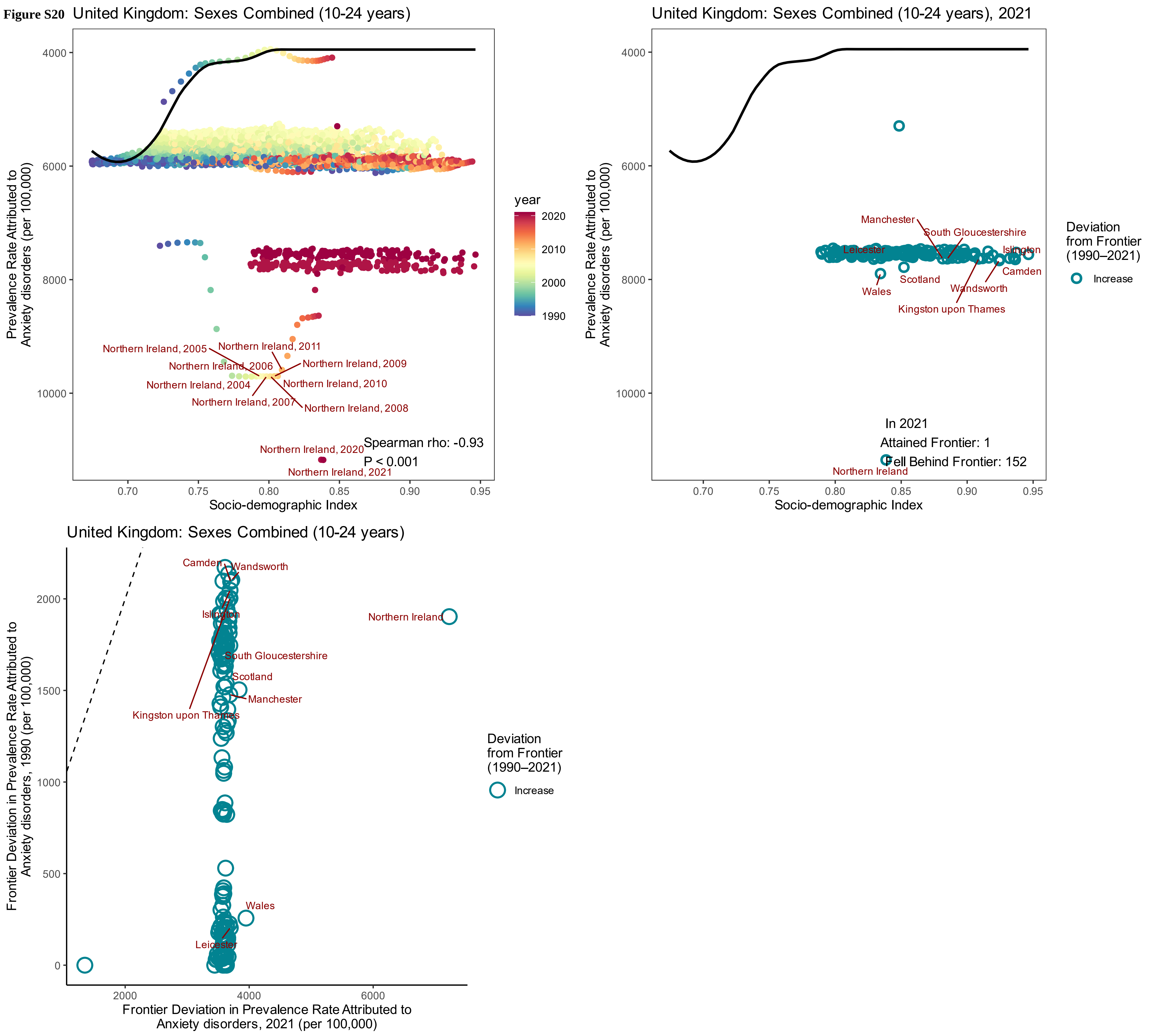
United Kingdom: Sexes Combined (10-24 years)
Prevalence Rate Attributed to Anxiety disorders (per 100,000)
Socio-demographic Index
year
2020
2010
2000
1990
Northern Ireland, 2005
Northern Ireland, 2011
Northern Ireland, 2006
Northern Ireland, 2009
Northern Ireland, 2004
Northern Ireland, 2010
Northern Ireland, 2007
Northern Ireland, 2008
Northern Ireland, 2020
Northern Ireland, 2021
Spearman rho: -0.93
P < 0.001
United Kingdom: Sexes Combined (10-24 years), 2021
Manchester
South Gloucestershire
Leicester
Islington
Camden
Scotland
Wales
Wandsworth
Kingston upon Thames
In 2021
Attained Frontier: 1
Fell Behind Frontier: 152
Northern Ireland
Deviation from Frontier (1990–2021)
Increase
United Kingdom: Sexes Combined (10-24 years)
Frontier Deviation in Prevalence Rate Attributed to Anxiety disorders, 1990 (per 100,000)
Frontier Deviation in Prevalence Rate Attributed to Anxiety disorders, 2021 (per 100,000)
Camden
Wandsworth
Islington
Northern Ireland
South Gloucestershire
Scotland
Manchester
Kingston upon Thames
Wales
Leicester
Deviation from Frontier (1990–2021)
Increase

Figure S20

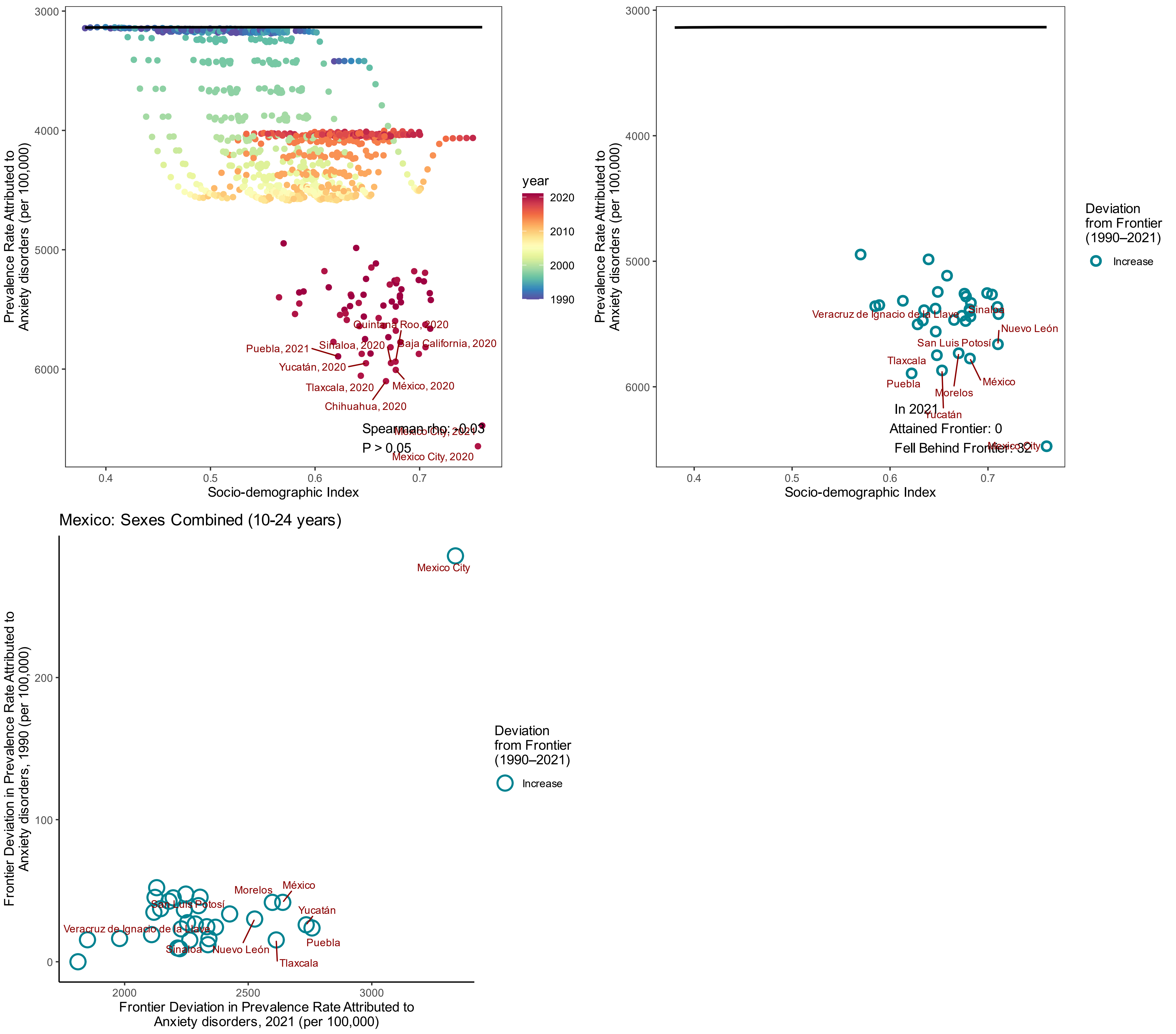

Mexico: Sexes Combined (10-24 years)
Prevalence Rate Attributed to Anxiety disorders (per 100,000)
Socio-demographic Index
year
2020
2010
2000
1990
Quintana Roo, 2020
Puebla, 2021
Sinaloa, 2020
Baja California, 2020
Yucatán, 2020
Tlaxcala, 2020
México, 2020
Chihuahua, 2020
Spearman rho: -0.03
P > 0.05
Mexico City, 2021
Mexico City, 2020
Mexico: Sexes Combined (10-24 years), 2021
Deviation from Frontier (1990–2021)
Increase
Veracruz de Ignacio de la Llave
Sinaloa
Nuevo León
San Luis Potosí
Tlaxcala
Puebla
Morelos
México
Yucatán
In 2021
Attained Frontier: 0
Fell Behind Frontier: 32
Mexico City
Mexico: Sexes Combined (10-24 years)
Frontier Deviation in Prevalence Rate Attributed to Anxiety disorders, 1990 (per 100,000)
Frontier Deviation in Prevalence Rate Attributed to Anxiety disorders, 2021 (per 100,000)
Deviation from Frontier (1990–2021)
Increase

Figure S20

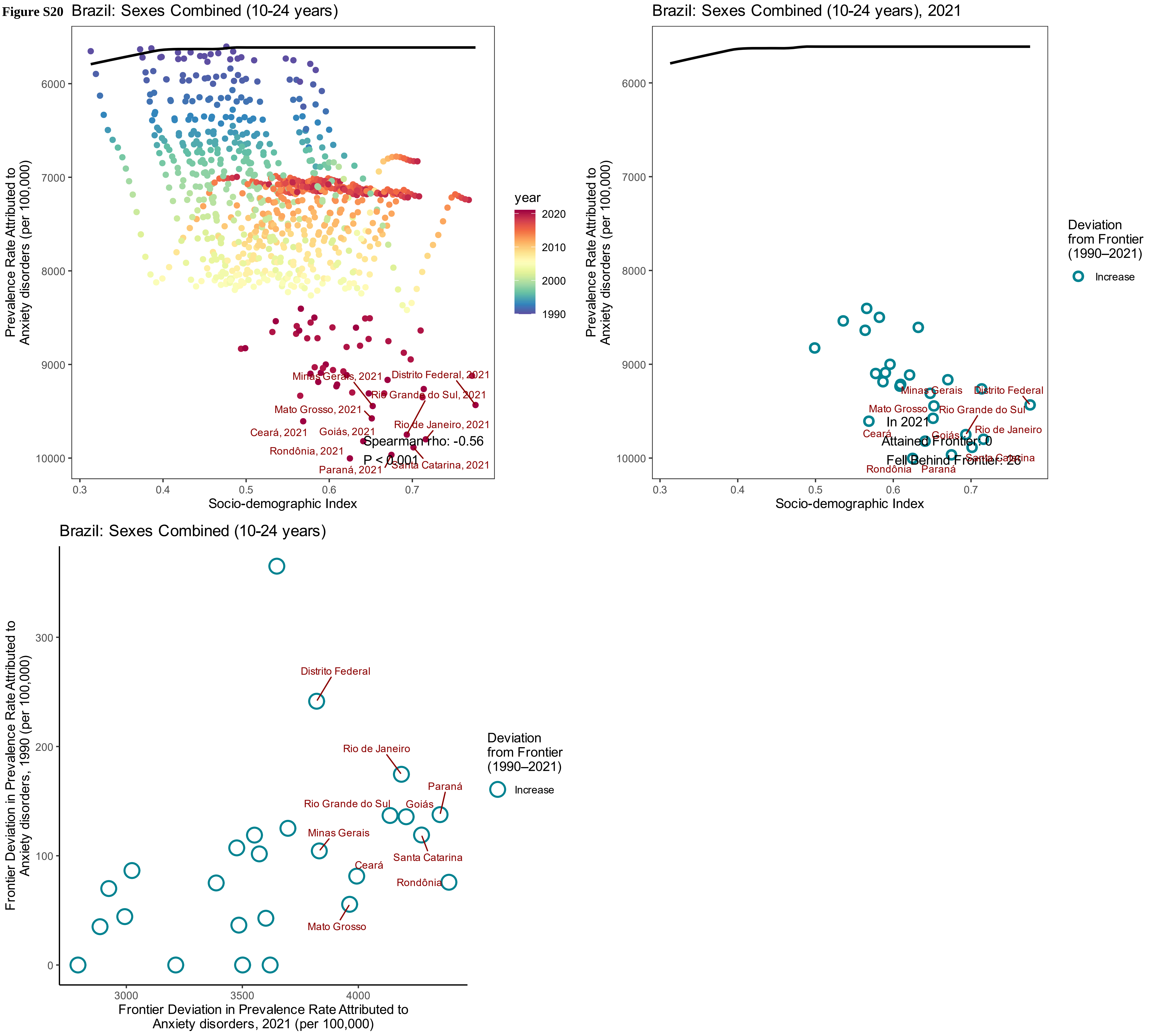

Brazil: Sexes Combined (10-24 years)
Prevalence Rate Attributed to Anxiety disorders (per 100,000)
Socio-demographic Index
year
2020
2010
2000
1990
Minas Gerais, 2021
Distrito Federal, 2021
Rio Grande do Sul, 2021
Mato Grosso, 2021
Rio de Janeiro, 2021
Ceará, 2021
Goiás, 2021
Spearman rho: -0.56
Rondônia, 2021
P < 0.001
Paraná, 2021
Santa Catarina, 2021
Brazil: Sexes Combined (10-24 years), 2021
Deviation from Frontier (1990–2021)
Increase
Minas Gerais
Distrito Federal
Mato Grosso
Rio Grande do Sul
In 2021
Ceará
Goiás
Rio de Janeiro
Attained Frontier: 0
Fell Behind Frontier: 26
Santa Catarina
Rondônia
Paraná
Brazil: Sexes Combined (10-24 years)
Frontier Deviation in Prevalence Rate Attributed to Anxiety disorders, 1990 (per 100,000)
Frontier Deviation in Prevalence Rate Attributed to Anxiety disorders, 2021 (per 100,000)
Distrito Federal
Rio de Janeiro
Paraná
Rio Grande do Sul
Goiás
Minas Gerais
Santa Catarina
Ceará
Rondônia
Mato Grosso

Figure S20

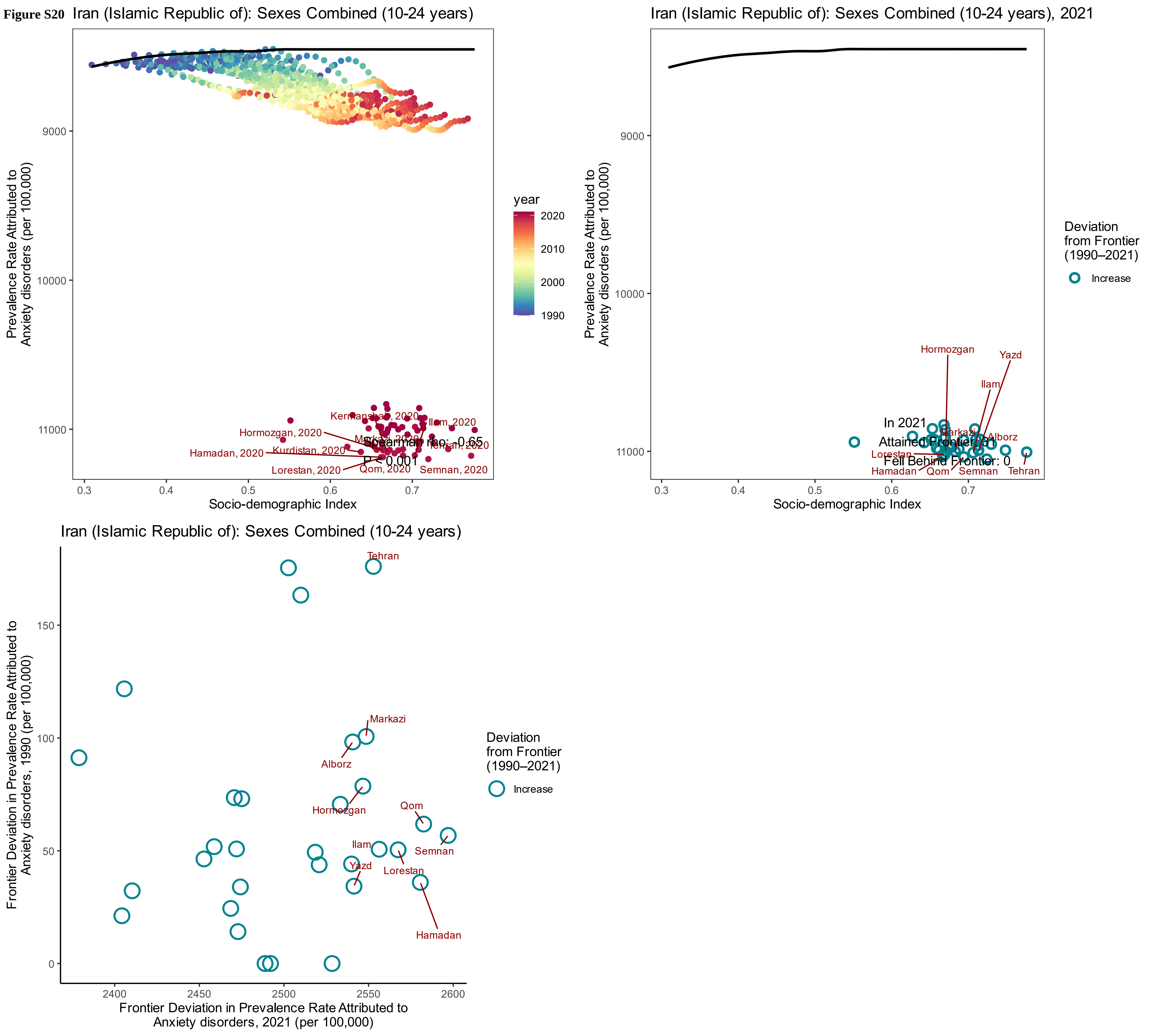

Iran (Islamic Republic of): Sexes Combined (10-24 years)
Prevalence Rate Attributed to Anxiety disorders (per 100,000)
Socio-demographic Index
year
Spearman rho: -0.65
P < 0.001
Kermanshah, 2020
Ilam, 2020
Hormozgan, 2020
Markazi, 2020
Tehran, 2020
Kurdistan, 2020
Hamadan, 2020
Lorestan, 2020
Qom, 2020
Semnan, 2020
Iran (Islamic Republic of): Sexes Combined (10-24 years), 2021
In 2021
Attained Frontier: 31
Fell Behind Frontier: 0
Hormozgan
Yazd
Ilam
Markazi
Alborz
Lorestan
Hamadan
Qom
Semnan
Tehran
Deviation from Frontier (1990–2021)
Increase
Frontier Deviation in Prevalence Rate Attributed to Anxiety disorders, 1990 (per 100,000)
Frontier Deviation in Prevalence Rate Attributed to Anxiety disorders, 2021 (per 100,000)

Figure S20

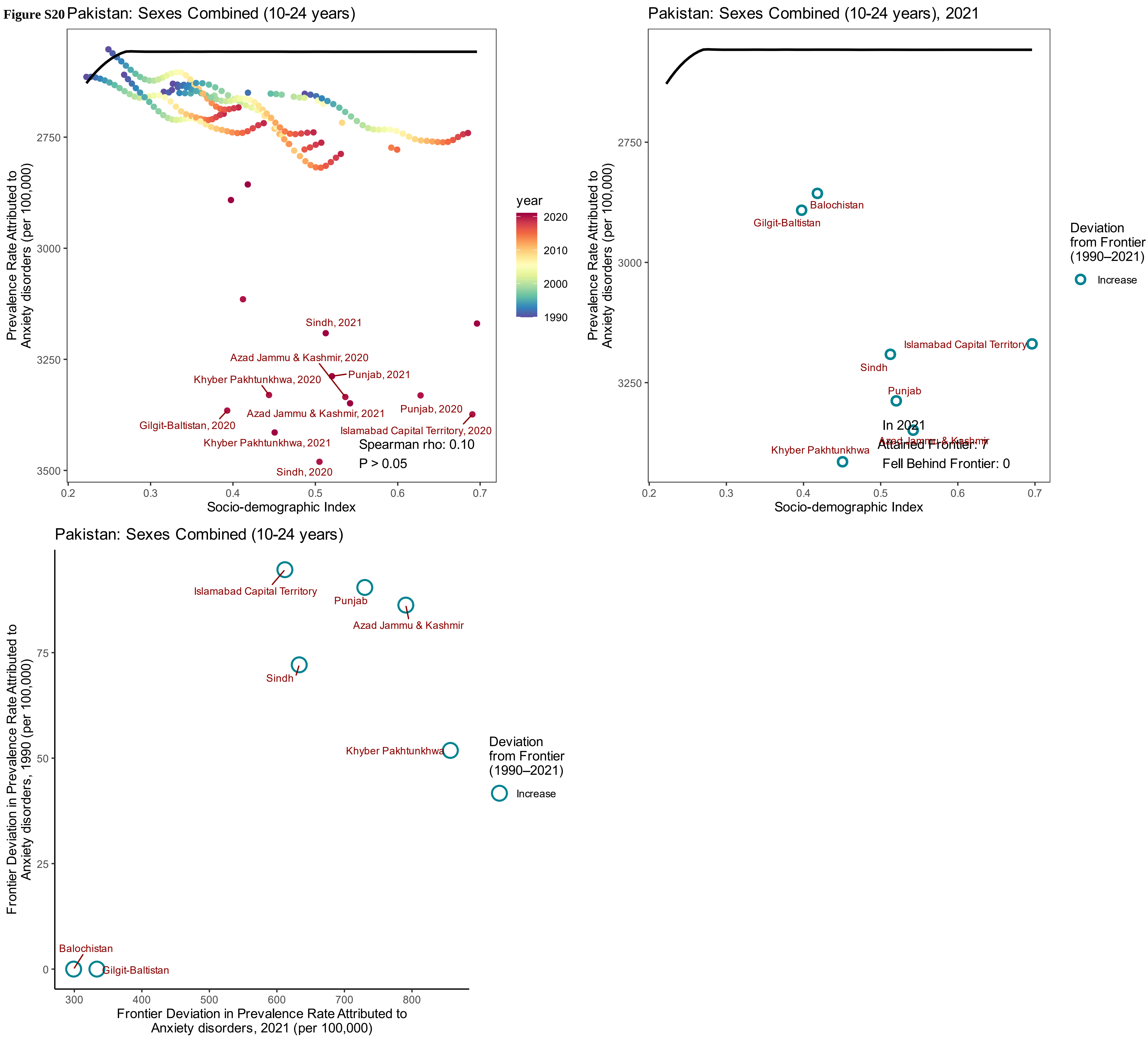
Pakistan: Sexes Combined (10-24 years)
Prevalence Rate Attributed to Anxiety disorders (per 100,000)
Socio-demographic Index
year
2020
2010
2000
1990
Sindh, 2021
Azad Jammu & Kashmir, 2020
Punjab, 2021
Khyber Pakhtunkhwa, 2020
Azad Jammu & Kashmir, 2021
Punjab, 2020
Gilgit-Baltistan, 2020
Islamabad Capital Territory, 2020
Khyber Pakhtunkhwa, 2021
Spearman rho: 0.10
P > 0.05
Sindh, 2020
Pakistan: Sexes Combined (10-24 years), 2021
Balochistan
Gilgit-Baltistan
Islamabad Capital Territory
Sindh
Punjab
In 2021
Attained Frontier: 7
Azad Jammu & Kashmir
Khyber Pakhtunkhwa
Fell Behind Frontier: 0
Deviation from Frontier (1990–2021)
Increase
Pakistan: Sexes Combined (10-24 years)
Frontier Deviation in Prevalence Rate Attributed to Anxiety disorders, 1990 (per 100,000)
Frontier Deviation in Prevalence Rate Attributed to Anxiety disorders, 2021 (per 100,000)
Islamabad Capital Territory
Punjab
Azad Jammu & Kashmir
Sindh
Khyber Pakhtunkhwa
Balochistan
Gilgit-Baltistan
Deviation from Frontier (1990–2021)
Increase

Figure S20

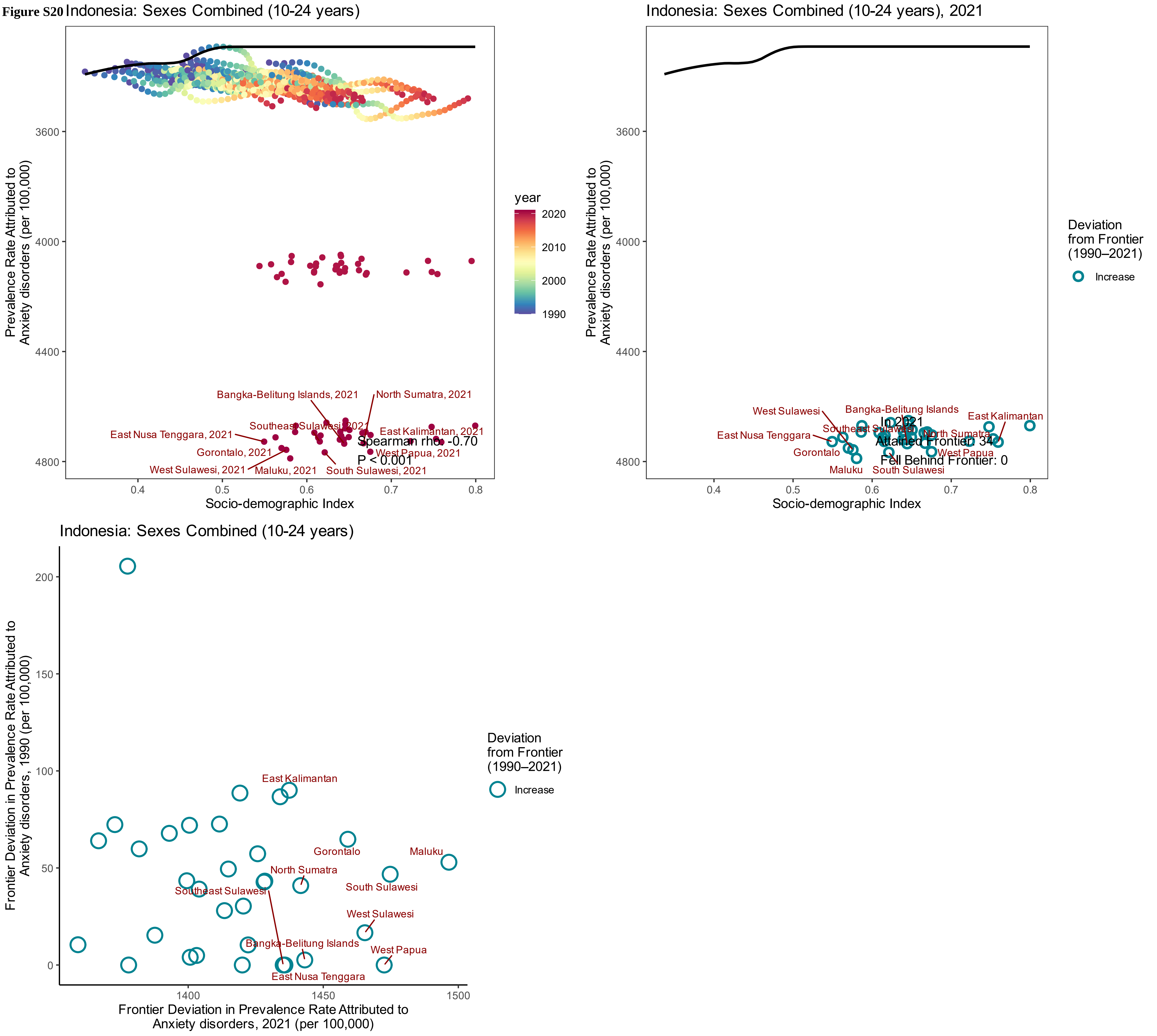

Indonesia: Sexes Combined (10-24 years)
Prevalence Rate Attributed to Anxiety disorders (per 100,000)
Socio-demographic Index
year
2020
2010
2000
1990
Bangka-Belitung Islands, 2021
North Sumatra, 2021
Southeast Sulawesi, 2021
East Kalimantan, 2021
East Nusa Tenggara, 2021
Spearman rho: -0.70
P < 0.001
Gorontalo, 2021
West Papua, 2021
West Sulawesi, 2021
Maluku, 2021
South Sulawesi, 2021
Indonesia: Sexes Combined (10-24 years), 2021
In 2021
Attained Frontier: 34
Fell Behind Frontier: 0
West Sulawesi
Bangka-Belitung Islands
East Kalimantan
Southeast Sulawesi
North Sumatra
East Nusa Tenggara
Gorontalo
West Papua
Maluku
South Sulawesi
Deviation from Frontier (1990–2021)
Increase
Frontier Deviation in Prevalence Rate Attributed to Anxiety disorders, 1990 (per 100,000)
Frontier Deviation in Prevalence Rate Attributed to Anxiety disorders, 2021 (per 100,000)

Figure S20

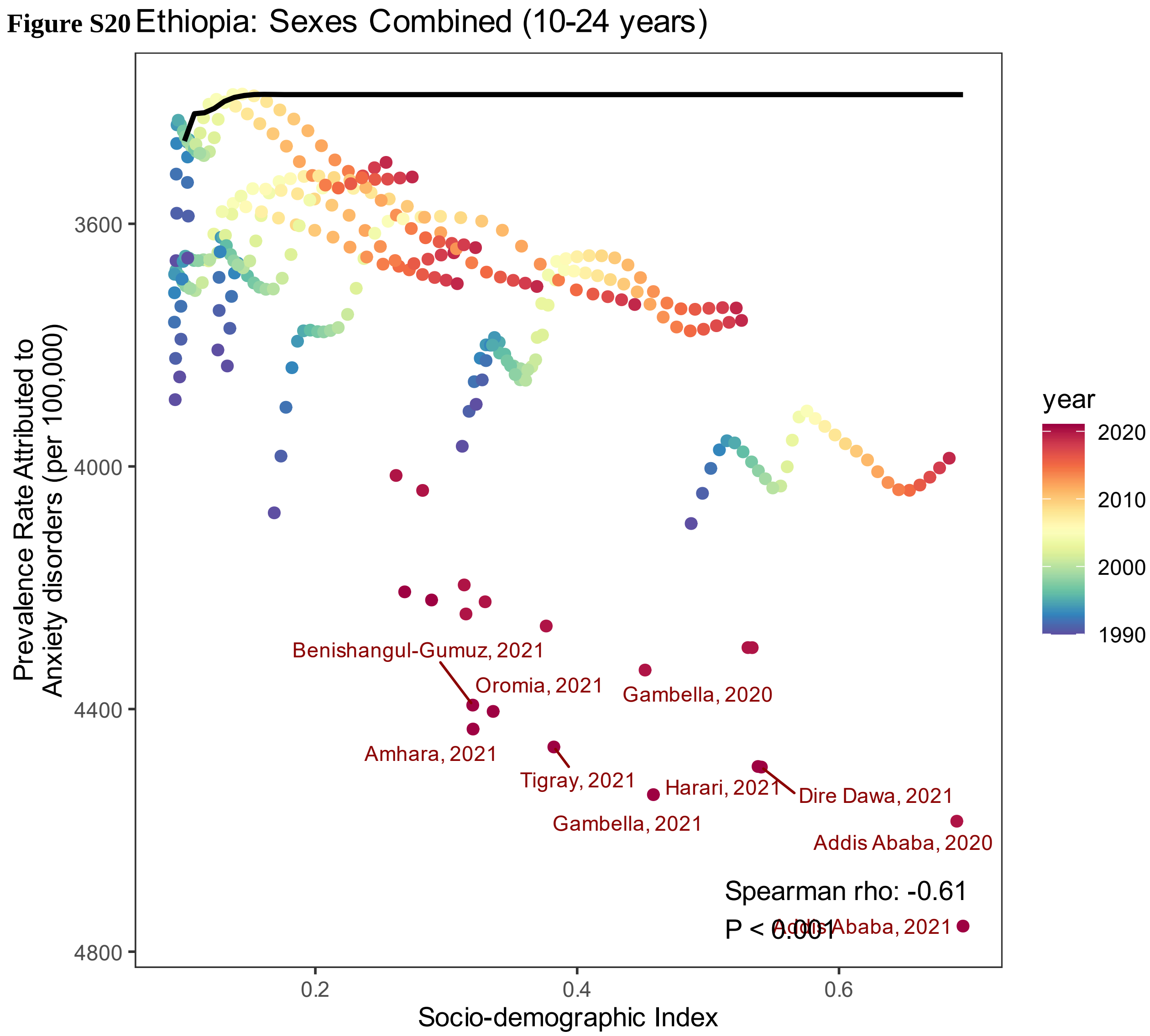

Ethiopia: Sexes Combined (10-24 years)
Prevalence Rate Attributed to Anxiety disorders (per 100,000)
Socio-demographic Index
year
2020
2010
2000
1990
Benishangul-Gumuz, 2021
Oromia, 2021
Gambella, 2020
Amhara, 2021
Tigray, 2021
Harari, 2021
Dire Dawa, 2021
Gambella, 2021
Addis Ababa, 2020
Spearman rho: -0.61
P < 0.001
Addis Ababa, 2021

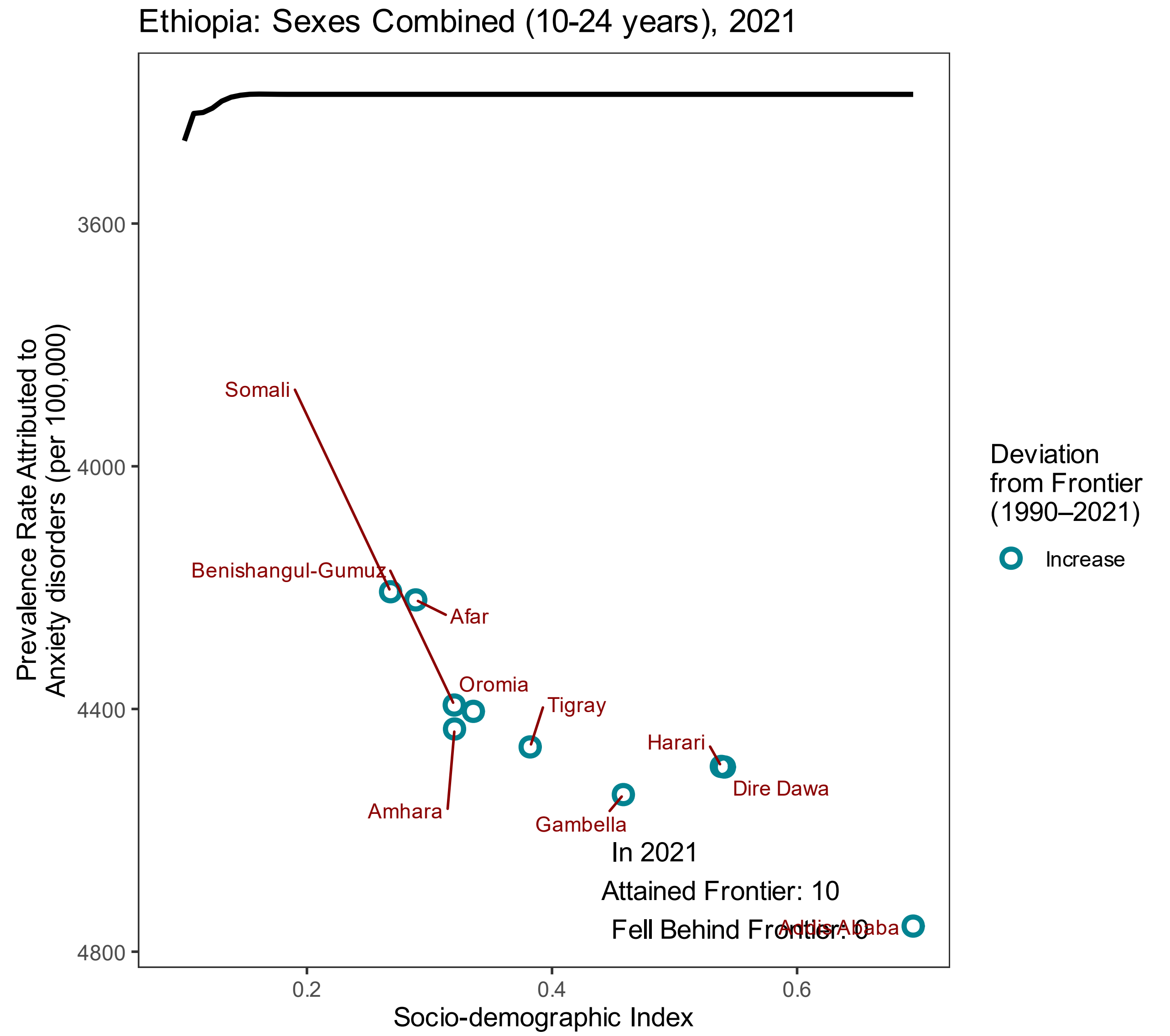

Ethiopia: Sexes Combined (10-24 years), 2021
Prevalence Rate Attributed to Anxiety disorders (per 100,000)
Socio-demographic Index
Somali
Benishangul-Gumuz
Afar
Oromia
Tigray
Harari
Dire Dawa
Amhara
Gambella
In 2021
Attained Frontier: 10
Fell Behind Frontier: 0
Addis Ababa
Deviation from Frontier (1990–2021)
Increase

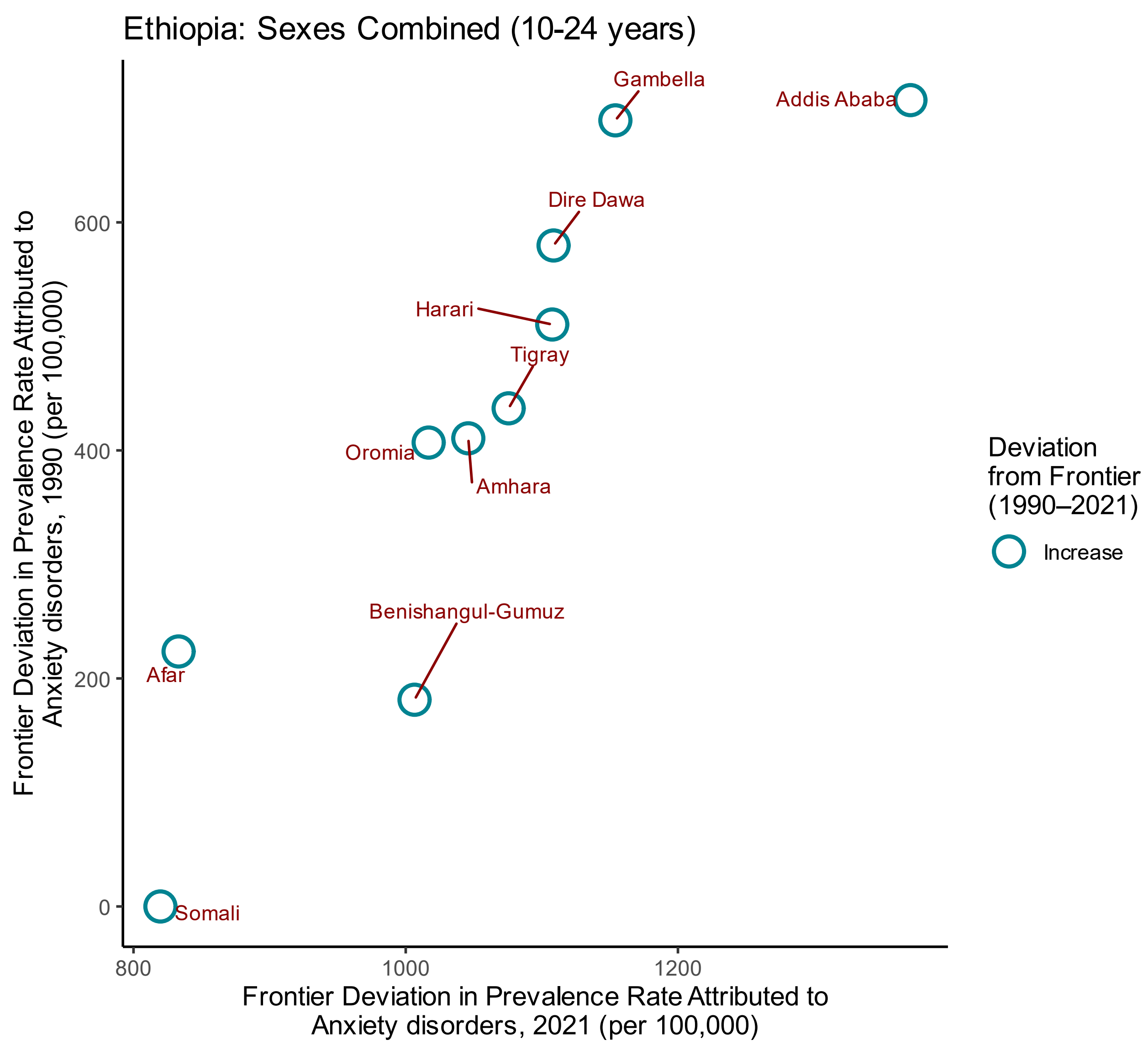

Ethiopia: Sexes Combined (10-24 years)
Frontier Deviation in Prevalence Rate Attributed to Anxiety disorders, 1990 (per 100,000)
Frontier Deviation in Prevalence Rate Attributed to Anxiety disorders, 2021 (per 100,000)
Gambella
Addis Ababa
Dire Dawa
Harari
Tigray
Oromia
Amhara
Benishangul-Gumuz
Afar
Somali
Deviation from Frontier (1990–2021)
Increase

Figure S20

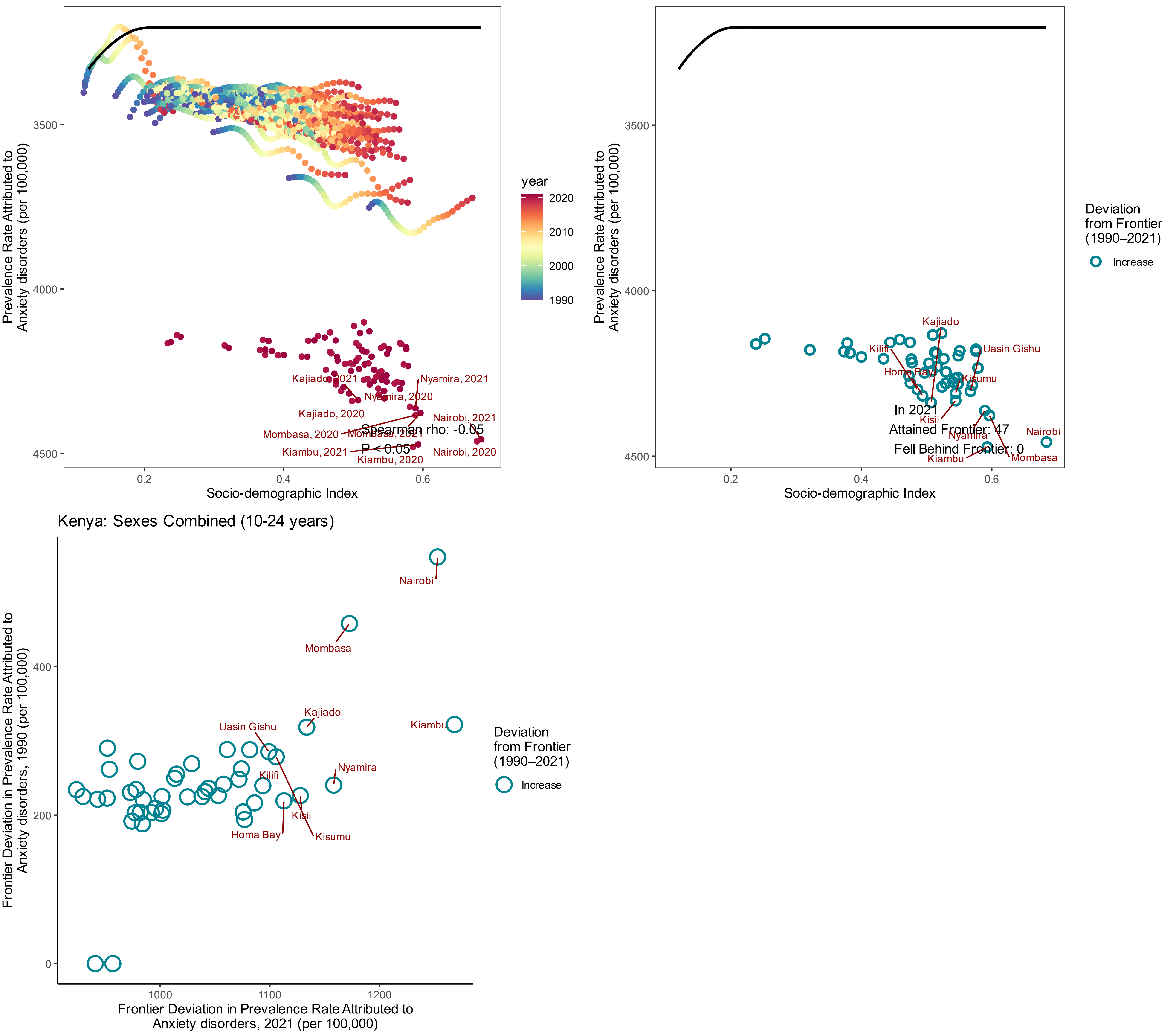
Kenya: Sexes Combined (10-24 years)
Prevalence Rate Attributed to Anxiety disorders (per 100,000)
Socio-demographic Index
year
2020
2010
2000
1990
Kajiado, 2021
Nyamira, 2021
Nyamira, 2020
Kajiado, 2020
Nairobi, 2021
Mombasa, 2020
Mombasa, 2021
Spearman rho: -0.05
P < 0.05
Kiambu, 2021
Kiambu, 2020
Nairobi, 2020
Kenya: Sexes Combined (10-24 years), 2021
Kajiado
Kilifi
Uasin Gishu
Homa Bay
Kisumu
In 2021
Kisii
Attained Frontier: 47
Nyamira
Nairobi
Fell Behind Frontier: 0
Kiambu
Mombasa
Deviation from Frontier (1990–2021)
Increase
Kenya: Sexes Combined (10-24 years)
Frontier Deviation in Prevalence Rate Attributed to Anxiety disorders, 1990 (per 100,000)
Frontier Deviation in Prevalence Rate Attributed to Anxiety disorders, 2021 (per 100,000)
Nairobi
Mombasa
Kajiado
Uasin Gishu
Kiambu
Nyamira
Kilifi
Kisii
Homa Bay
Kisumu
Deviation from Frontier (1990–2021)
Increase

Figure S20

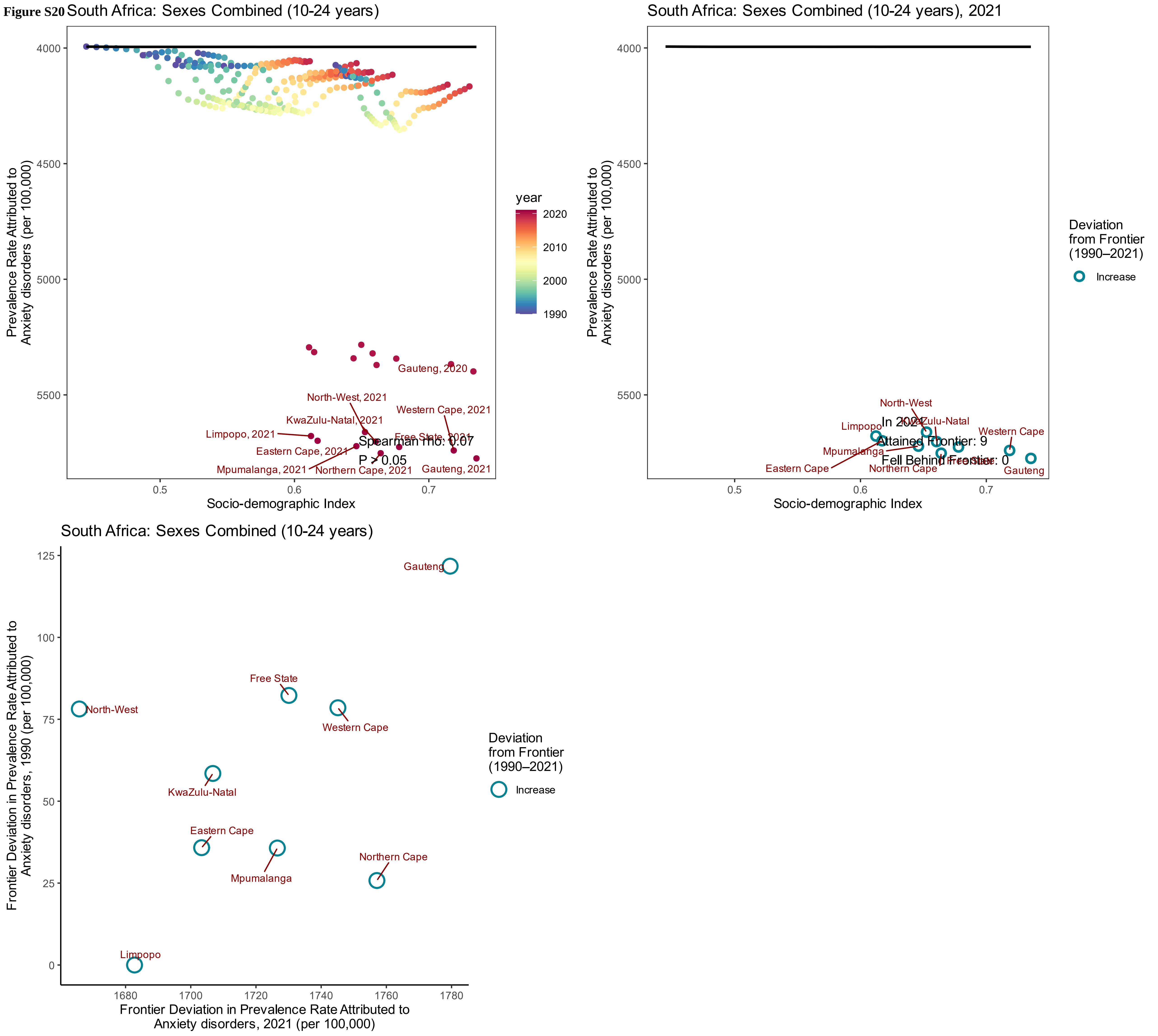
South Africa: Sexes Combined (10-24 years)
Prevalence Rate Attributed to Anxiety disorders (per 100,000)
Socio-demographic Index
year
2020
2010
2000
1990
Gauteng, 2020
North-West, 2021
Western Cape, 2021
KwaZulu-Natal, 2021
Limpopo, 2021
Free State, 2021
Spearman rho: 0.07
Eastern Cape, 2021
P > 0.05
Mpumalanga, 2021
Northern Cape, 2021
Gauteng, 2021
South Africa: Sexes Combined (10-24 years), 2021
North-West
Limpopo
In 2021
KwaZulu-Natal
Western Cape
Attained Frontier: 9
Mpumalanga
Fell Behind Frontier: 0
Free State
Eastern Cape
Northern Cape
Gauteng
Deviation from Frontier (1990–2021)
Increase
South Africa: Sexes Combined (10-24 years)
Frontier Deviation in Prevalence Rate Attributed to Anxiety disorders, 1990 (per 100,000)
Frontier Deviation in Prevalence Rate Attributed to Anxiety disorders, 2021 (per 100,000)
Gauteng
Free State
North-West
Western Cape
KwaZulu-Natal
Eastern Cape
Mpumalanga
Northern Cape
Limpopo
Deviation from Frontier (1990–2021)
Increase

Figure S20

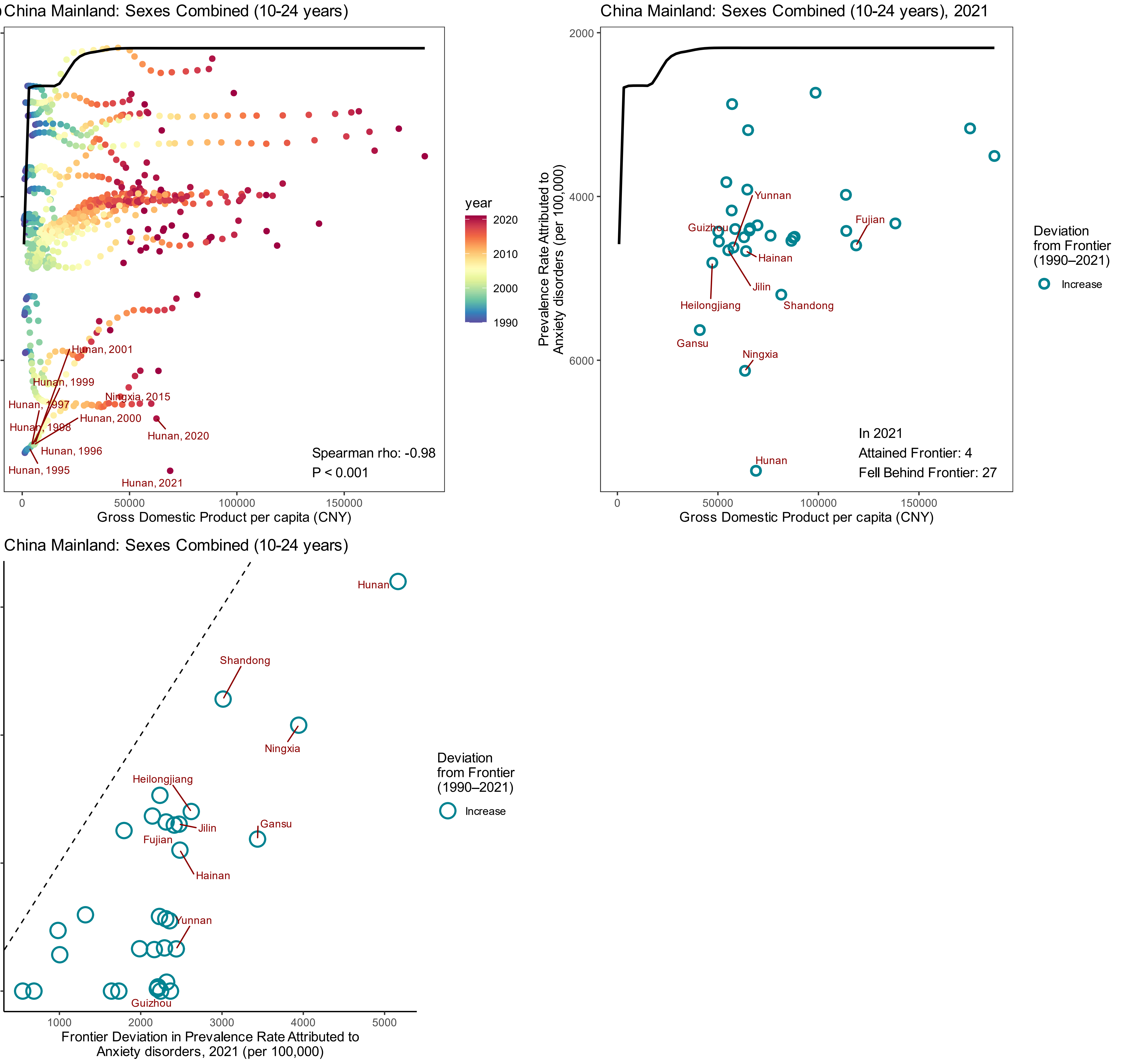
China Mainland: Sexes Combined (10-24 years)
Prevalence Rate Attributed to Anxiety disorders (per 100,000)
Gross Domestic Product per capita (CNY)
year
2020
2010
2000
1990
Hunan, 2001
Hunan, 1999
Hunan, 1997
Hunan, 1998
Hunan, 2000
Ningxia, 2015
Hunan, 2020
Hunan, 1996
Hunan, 1995
Hunan, 2021
Spearman rho: -0.98
P < 0.001
China Mainland: Sexes Combined (10-24 years), 2021
Yunnan
Guizhou
Fujian
Hainan
Jilin
Heilongjiang
Shandong
Gansu
Ningxia
Hunan
In 2021
Attained Frontier: 4
Fell Behind Frontier: 27
Deviation from Frontier (1990–2021)
Increase
China Mainland: Sexes Combined (10-24 years)
Frontier Deviation in Prevalence Rate Attributed to Anxiety disorders, 1990 (per 100,000)
Frontier Deviation in Prevalence Rate Attributed to Anxiety disorders, 2021 (per 100,000)
Hunan
Shandong
Ningxia
Heilongjiang
Gansu
Jilin
Fujian
Hainan
Yunnan
Guizhou
Deviation from Frontier (1990–2021)
Increase

Figure S20

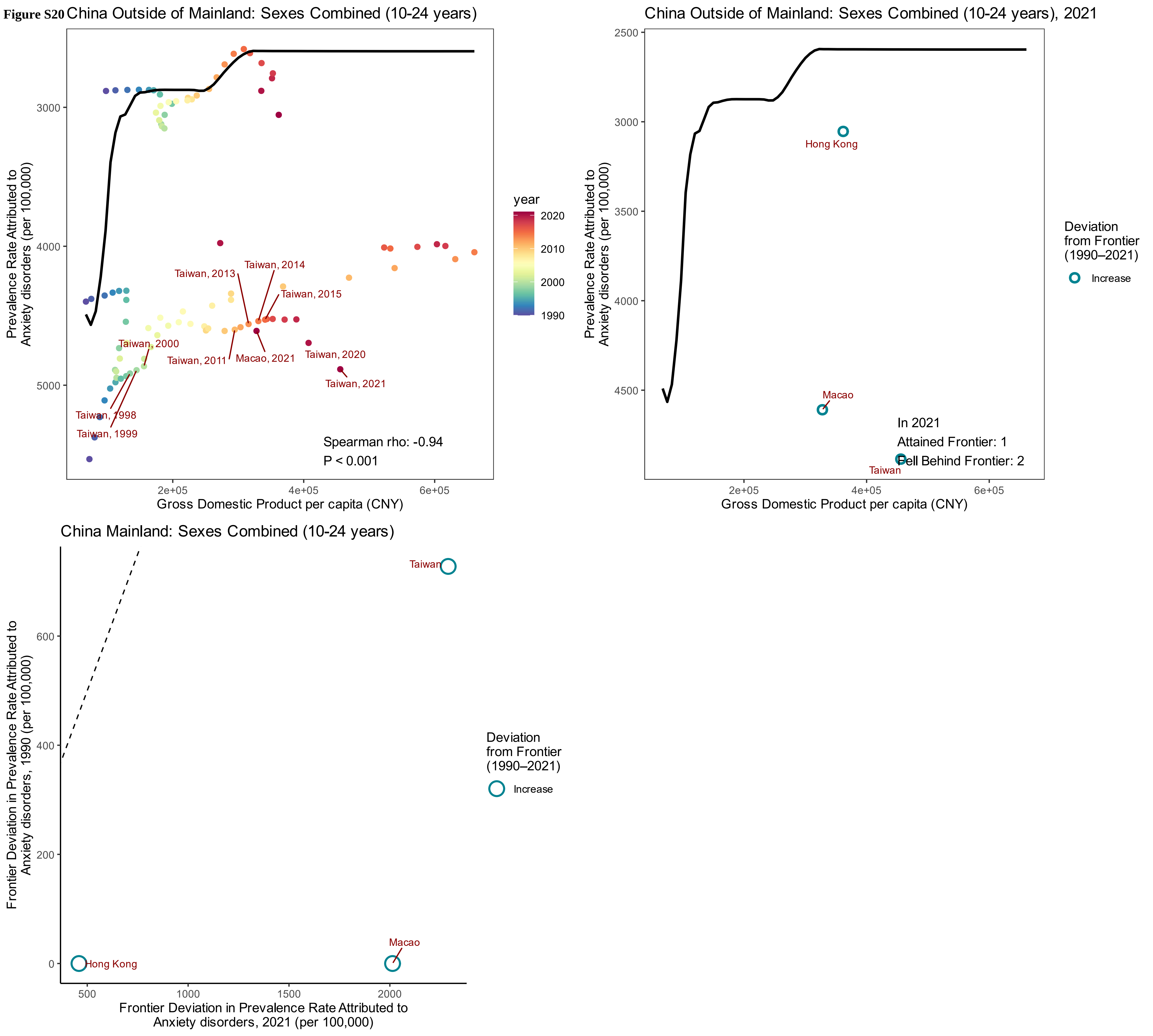

China Outside of Mainland: Sexes Combined (10-24 years)
Prevalence Rate Attributed to Anxiety disorders (per 100,000)
Gross Domestic Product per capita (CNY)
year
Taiwan, 2013
Taiwan, 2014
Taiwan, 2015
Taiwan, 2000
Taiwan, 2011
Macao, 2021
Taiwan, 2020
Taiwan, 2021
Taiwan, 1998
Taiwan, 1999
Spearman rho: -0.94
P < 0.001
China Outside of Mainland: Sexes Combined (10-24 years), 2021
Hong Kong
Macao
Taiwan
In 2021
Attained Frontier: 1
Fell Behind Frontier: 2
Deviation from Frontier (1990–2021)
Increase
China Mainland: Sexes Combined (10-24 years)
Frontier Deviation in Prevalence Rate Attributed to Anxiety disorders, 1990 (per 100,000)
Frontier Deviation in Prevalence Rate Attributed to Anxiety disorders, 2021 (per 100,000)
Taiwan
Macao
Hong Kong
Deviation from Frontier (1990–2021)
Increase

Figure S21

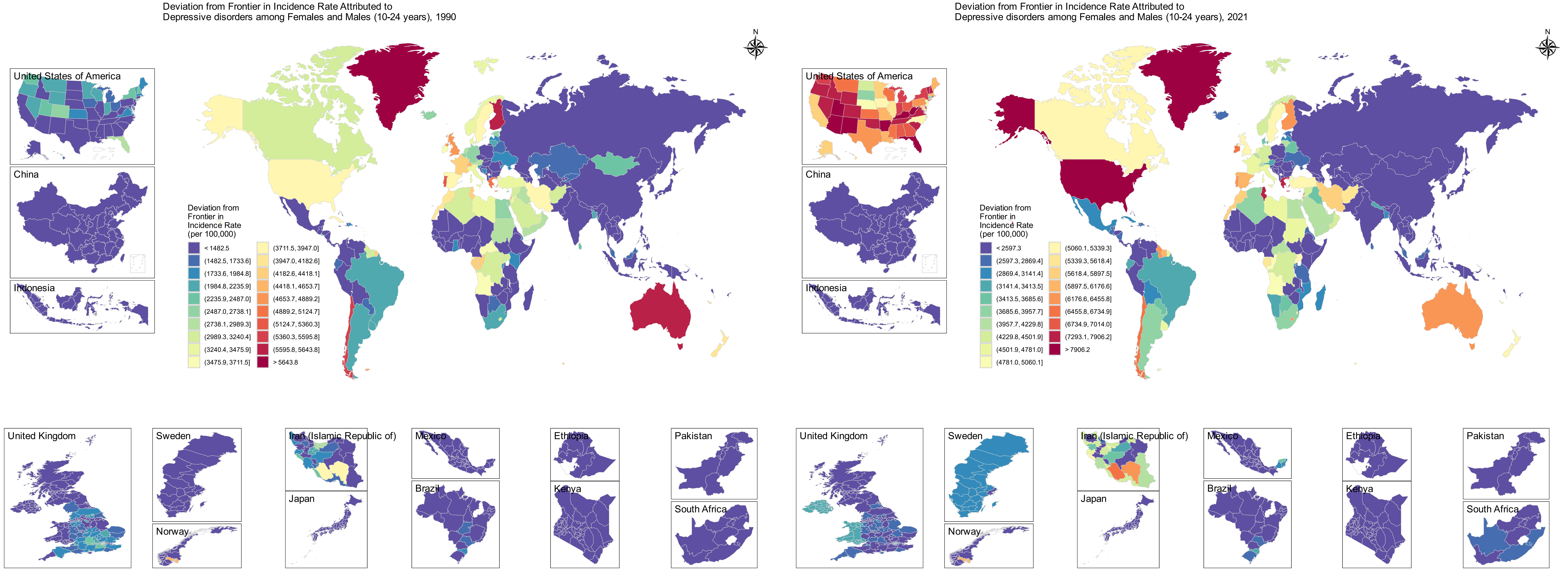

Deviation from Frontier in Incidence Rate Attributed to
Depressive disorders among Females and Males (10-24 years), 1990
N
United States of America
China
Indonesia
Deviation from
Frontier in
Incidence Rate
(per 100,000)
< 1482.5
(1482.5, 1733.6]
(1733.6, 1984.8]
(1984.8, 2235.9]
(2235.9, 2487.0]
(2487.0, 2738.1]
(2738.1, 2989.3]
(2989.3, 3240.4]
(3240.4, 3475.9]
(3475.9, 3711.5]
(3711.5, 3947.0]
(3947.0, 4182.6]
(4182.6, 4418.1]
(4418.1, 4653.7]
(4653.7, 4889.2]
(4889.2, 5124.7]
(5124.7, 5360.3]
(5360.3, 5595.8]
(5595.8, 5643.8]
> 5643.8
United Kingdom
Sweden
Norway
Iran (Islamic Republic of)
Japan
Mexico
Brazil
Ethiopia
Kenya
Pakistan
South Africa
Deviation from Frontier in Incidence Rate Attributed to
Depressive disorders among Females and Males (10-24 years), 2021
N
United States of America
China
Indonesia
Deviation from
Frontier in
Incidence Rate
(per 100,000)
< 2597.3
(2597.3, 2869.4]
(2869.4, 3141.4]
(3141.4, 3413.5]
(3413.5, 3685.6]
(3685.6, 3957.7]
(3957.7, 4229.8]
(4229.8, 4501.9]
(4501.9, 4781.0]
(4781.0, 5060.1]
(5060.1, 5339.3]
(5339.3, 5618.4]
(5618.4, 5897.5]
(5897.5, 6176.6]
(6176.6, 6455.8]
(6455.8, 6734.9]
(6734.9, 7014.0]
(7293.1, 7906.2]
> 7906.2
United Kingdom
Sweden
Norway
Iran (Islamic Republic of)
Japan
Mexico
Brazil
Ethiopia
Kenya
Pakistan
South Africa

Figure S21

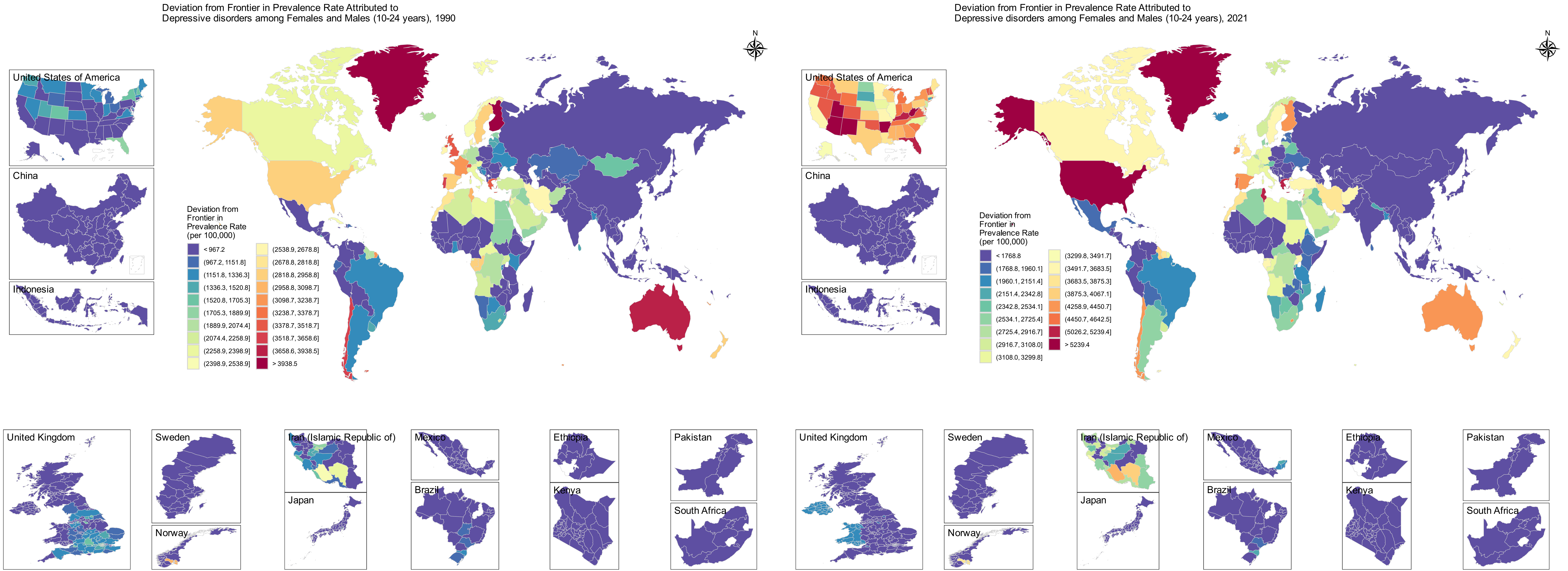

Deviation from Frontier in Prevalence Rate Attributed to
Depressive disorders among Females and Males (10-24 years), 1990
N
United States of America
China
Indonesia
Deviation from
Frontier in
Prevalence Rate
(per 100,000)
< 967.2
(967.2, 1151.8]
(1151.8, 1336.3]
(1336.3, 1520.8]
(1520.8, 1705.3]
(1705.3, 1889.9]
(1889.9, 2074.4]
(2074.4, 2258.9]
(2258.9, 2398.9]
(2398.9, 2538.9]
(2538.9, 2678.8]
(2678.8, 2818.8]
(2818.8, 2958.8]
(2958.8, 3098.7]
(3098.7, 3238.7]
(3238.7, 3378.7]
(3378.7, 3518.7]
(3518.7, 3658.6]
(3658.6, 3938.5]
> 3938.5
United Kingdom
Sweden
Norway
Iran (Islamic Republic of)
Japan
Mexico
Brazil
Ethiopia
Kenya
Pakistan
South Africa
Deviation from Frontier in Prevalence Rate Attributed to
Depressive disorders among Females and Males (10-24 years), 2021
N
United States of America
China
Indonesia
Deviation from
Frontier in
Prevalence Rate
(per 100,000)
< 1768.8
(1768.8, 1960.1]
(1960.1, 2151.4]
(2151.4, 2342.8]
(2342.8, 2534.1]
(2534.1, 2725.4]
(2725.4, 2916.7]
(2916.7, 3108.0]
(3108.0, 3299.8]
(3299.8, 3491.7]
(3491.7, 3683.5]
(3683.5, 3875.3]
(3875.3, 4067.1]
(4258.9, 4450.7]
(4450.7, 4642.5]
(5026.2, 5239.4]
> 5239.4
United Kingdom
Sweden
Norway
Iran (Islamic Republic of)
Japan
Mexico
Brazil
Ethiopia
Kenya
Pakistan
South Africa

Figure S21

Deviation from Frontier in DALYs Rate Attributed to
Depressive disorders among Females and Males (10-24 years), 1990

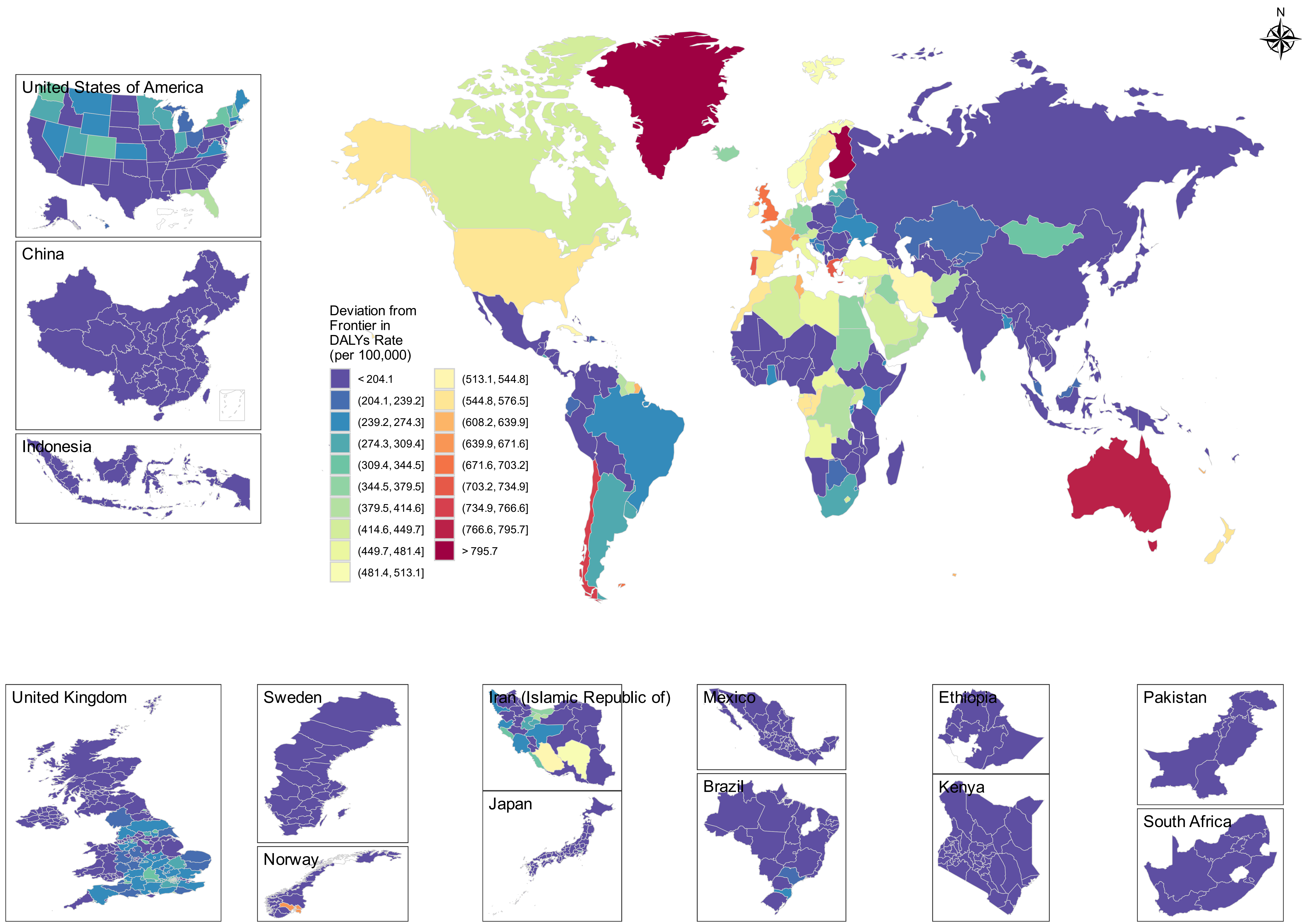

Deviation from Frontier in DALYs Rate Attributed to
Depressive disorders among Females and Males (10-24 years), 2021

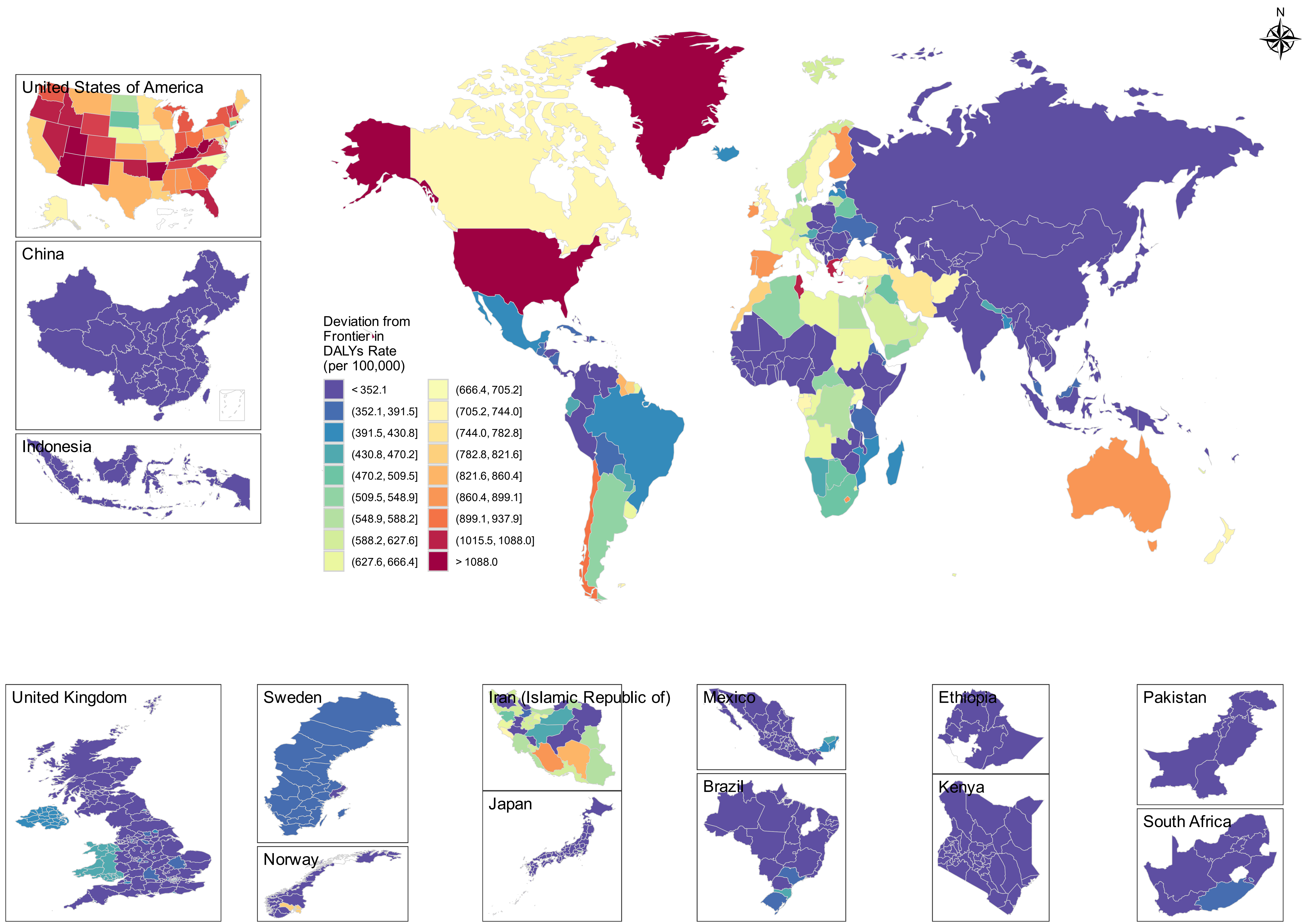

Figure S22

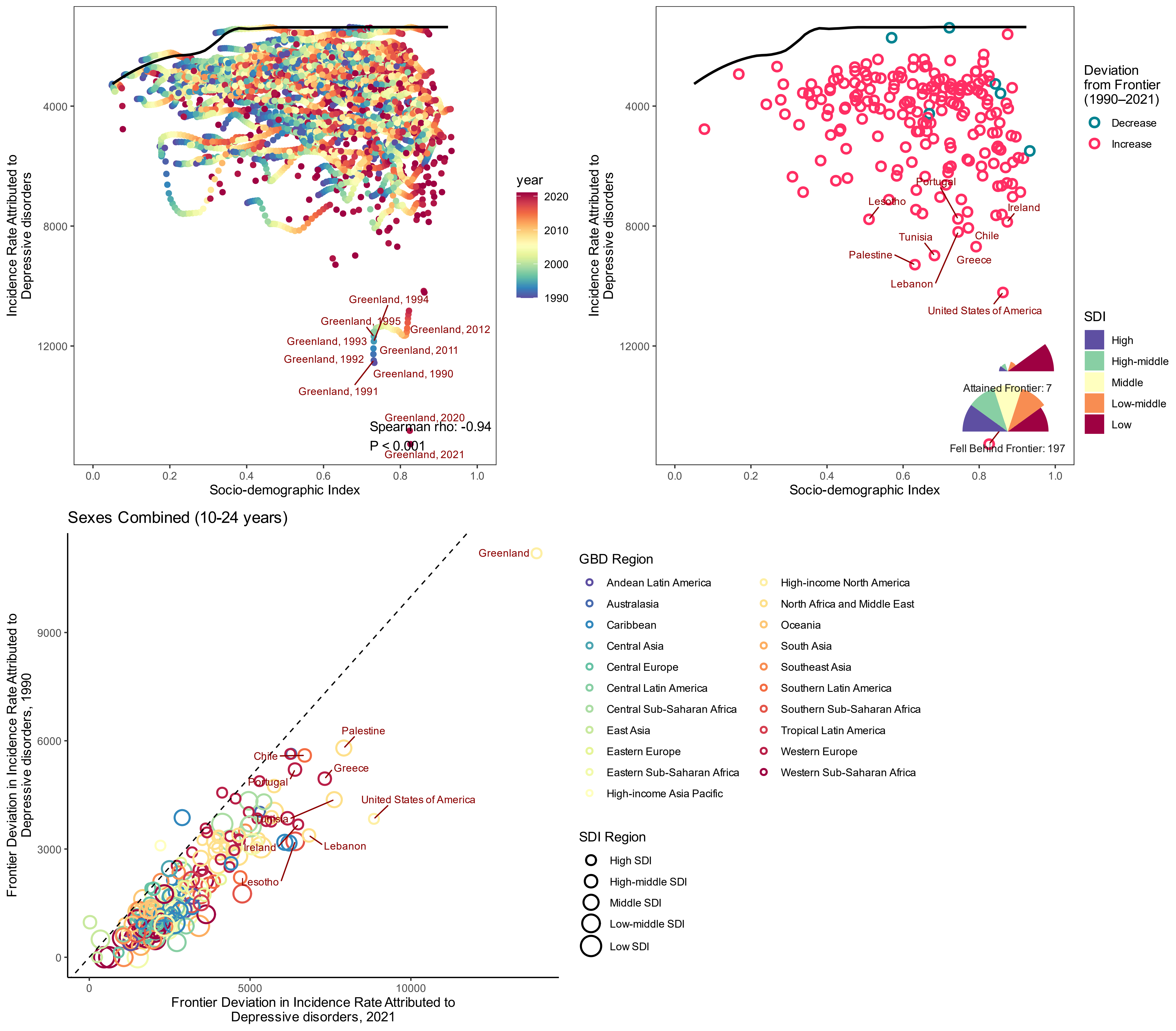
Sexes Combined (10-24 years)
Incidence Rate Attributed to Depressive disorders
Socio-demographic Index
year
2020
2010
2000
1990
Greenland, 1994
Greenland, 1995
Greenland, 2012
Greenland, 1993
Greenland, 2011
Greenland, 1992
Greenland, 1990
Greenland, 1991
Greenland, 2020
Spearman rho: -0.94
P < 0.001
Greenland, 2021
Sexes Combined (10-24 years), 2021
Deviation from Frontier (1990–2021)
Decrease
Increase
SDI
High
High-middle
Middle
Low-middle
Low
Portugal
Lesotho
Ireland
Tunisia
Chile
Palestine
Greece
Lebanon
United States of America
Attained Frontier: 7
Fell Behind Frontier: 197
Sexes Combined (10-24 years)
Greenland
Palestine
Chile
Greece
Portugal
United States of America
Tunisia
Ireland
Lebanon
Lesotho
Frontier Deviation in Incidence Rate Attributed to Depressive disorders, 1990
Frontier Deviation in Incidence Rate Attributed to Depressive disorders, 2021
GBD Region
Andean Latin America
Australasia
Caribbean
Central Asia
Central Europe
Central Latin America
Central Sub-Saharan Africa
East Asia
Eastern Europe
Eastern Sub-Saharan Africa
High-income Asia Pacific
High-income North America
North Africa and Middle East
Oceania
South Asia
Southeast Asia
Southern Latin America
Southern Sub-Saharan Africa
Tropical Latin America
Western Europe
Western Sub-Saharan Africa
SDI Region
High SDI
High-middle SDI
Middle SDI
Low-middle SDI
Low SDI

Figure S22

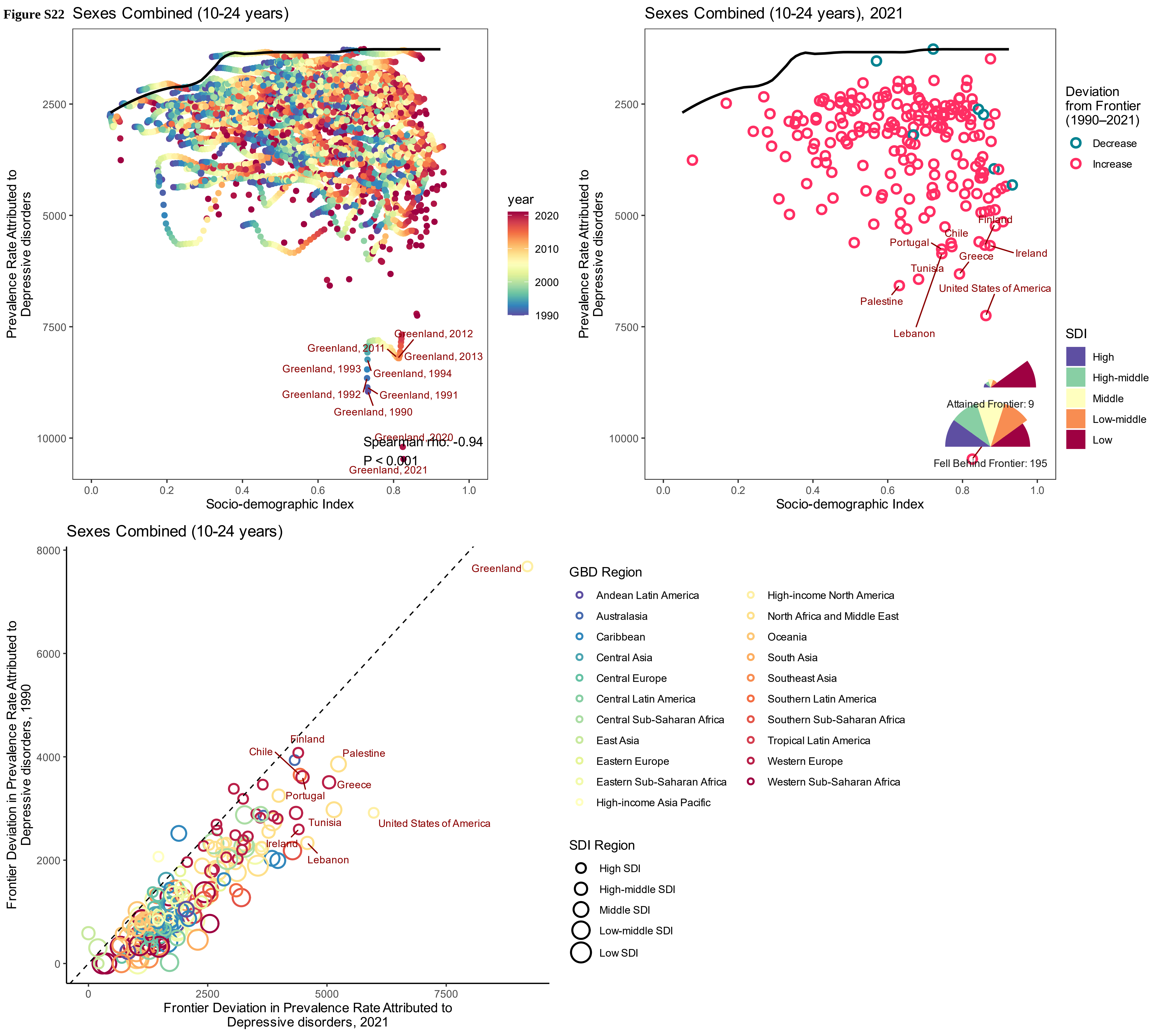

Sexes Combined (10-24 years)
Prevalence Rate Attributed to Depressive disorders
Socio-demographic Index
year
2020
2010
2000
1990
Greenland, 2012
Greenland, 2011
Greenland, 2013
Greenland, 1993
Greenland, 1994
Greenland, 1992
Greenland, 1991
Greenland, 1990
Greenland, 2020
Greenland, 2021
Spearman rho: -0.94
P < 0.001
Sexes Combined (10-24 years), 2021
Deviation from Frontier (1990–2021)
Decrease
Increase
Finland
Chile
Portugal
Ireland
Greece
Tunisia
United States of America
Palestine
Lebanon
SDI
High
High-middle
Middle
Low-middle
Low
Attained Frontier: 9
Fell Behind Frontier: 195
Sexes Combined (10-24 years)
Frontier Deviation in Prevalence Rate Attributed to Depressive disorders, 1990
Frontier Deviation in Prevalence Rate Attributed to Depressive disorders, 2021
Greenland
GBD Region
Andean Latin America
Australasia
Caribbean
Central Asia
Central Europe
Central Latin America
Central Sub-Saharan Africa
East Asia
Eastern Europe
Eastern Sub-Saharan Africa
High-income Asia Pacific
High-income North America
North Africa and Middle East
Oceania
South Asia
Southeast Asia
Southern Latin America
Southern Sub-Saharan Africa
Tropical Latin America
Western Europe
Western Sub-Saharan Africa
SDI Region
High SDI
High-middle SDI
Middle SDI
Low-middle SDI
Low SDI

Figure S22

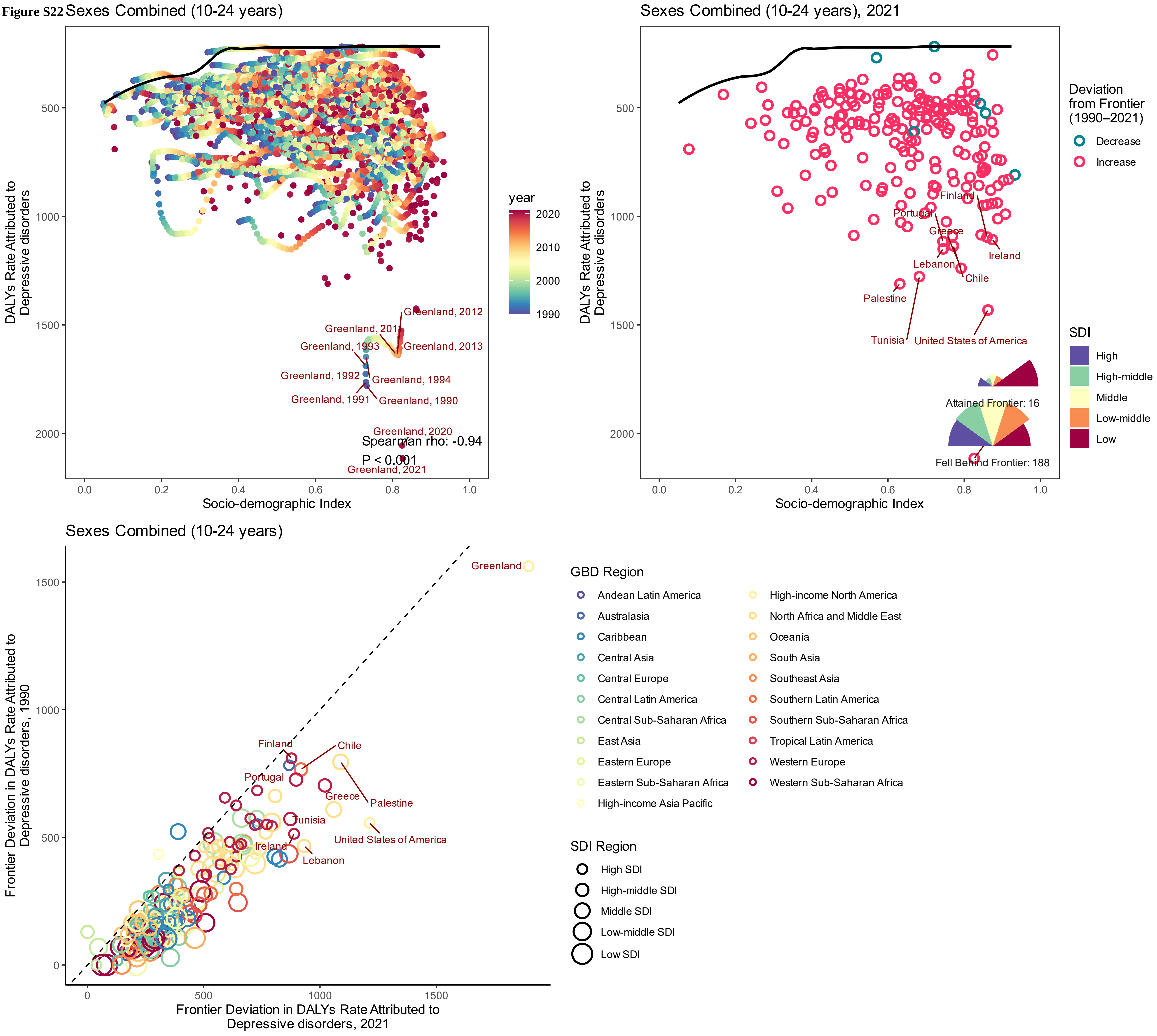

Sexes Combined (10-24 years)
DALYs Rate Attributed to Depressive disorders
Socio-demographic Index
year
2020
2010
2000
1990
Greenland, 2012
Greenland, 2011
Greenland, 1993
Greenland, 2013
Greenland, 1992
Greenland, 1994
Greenland, 1991
Greenland, 1990
Greenland, 2020
Spearman rho: -0.94
P < 0.001
Greenland, 2021
Sexes Combined (10-24 years), 2021
Deviation from Frontier (1990–2021)
Decrease
Increase
Finland
Portugal
Greece
Ireland
Lebanon
Chile
Palestine
Tunisia
United States of America
Attained Frontier: 16
Fell Behind Frontier: 188
SDI
High
High-middle
Middle
Low-middle
Low
Sexes Combined (10-24 years)
Frontier Deviation in DALYs Rate Attributed to Depressive disorders, 1990
Frontier Deviation in DALYs Rate Attributed to Depressive disorders, 2021
Greenland
Finland
Chile
Portugal
Greece
Palestine
Tunisia
Ireland
United States of America
Lebanon
GBD Region
Andean Latin America
Australasia
Caribbean
Central Asia
Central Europe
Central Latin America
Central Sub-Saharan Africa
East Asia
Eastern Europe
Eastern Sub-Saharan Africa
High-income Asia Pacific
High-income North America
North Africa and Middle East
Oceania
South Asia
Southeast Asia
Southern Latin America
Southern Sub-Saharan Africa
Tropical Latin America
Western Europe
Western Sub-Saharan Africa
SDI Region
High SDI
High-middle SDI
Middle SDI
Low-middle SDI
Low SDI

Figure S23

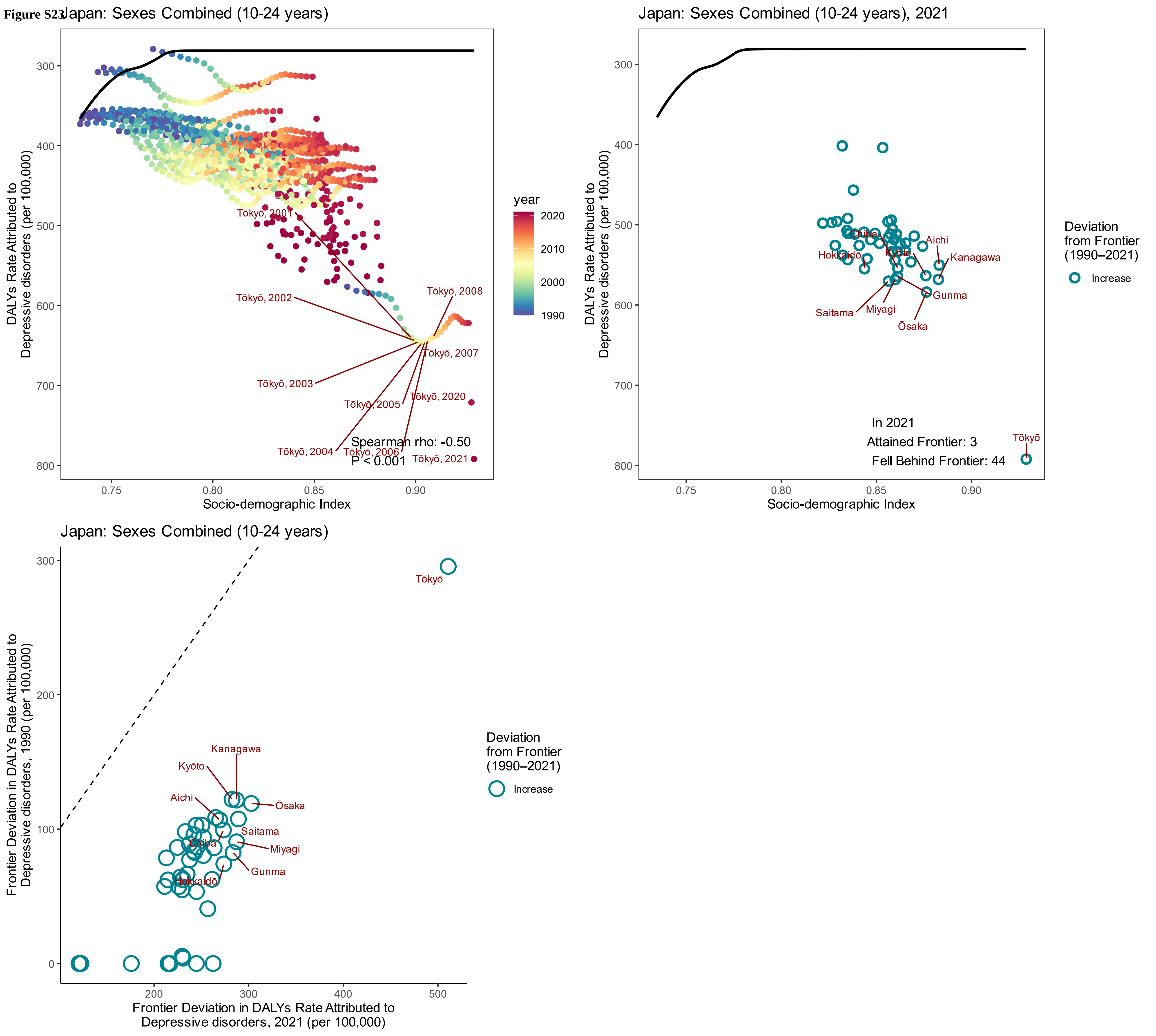

Japan: Sexes Combined (10-24 years)
DALYs Rate Attributed to Depressive disorders (per 100,000)
Socio-demographic Index
year
2020
2010
2000
1990
Tōkyō, 2001
Tōkyō, 2002
Tōkyō, 2003
Tōkyō, 2004
Tōkyō, 2005
Tōkyō, 2006
Tōkyō, 2007
Tōkyō, 2008
Tōkyō, 2020
Tōkyō, 2021
Spearman rho: -0.50
P < 0.001
Japan: Sexes Combined (10-24 years), 2021
Chiba
Hokkaidō
Kyōto
Aichi
Kanagawa
Gunma
Saitama
Miyagi
Ōsaka
Tōkyō
In 2021
Attained Frontier: 3
Fell Behind Frontier: 44
Deviation from Frontier (1990–2021)
Increase
Japan: Sexes Combined (10-24 years)
Frontier Deviation in DALYs Rate Attributed to Depressive disorders, 1990 (per 100,000)
Frontier Deviation in DALYs Rate Attributed to Depressive disorders, 2021 (per 100,000)
Tōkyō
Kanagawa
Kyōto
Aichi
Ōsaka
Saitama
Chiba
Miyagi
Gunma
Hokkaidō
Deviation from Frontier (1990–2021)
Increase

Figure S23

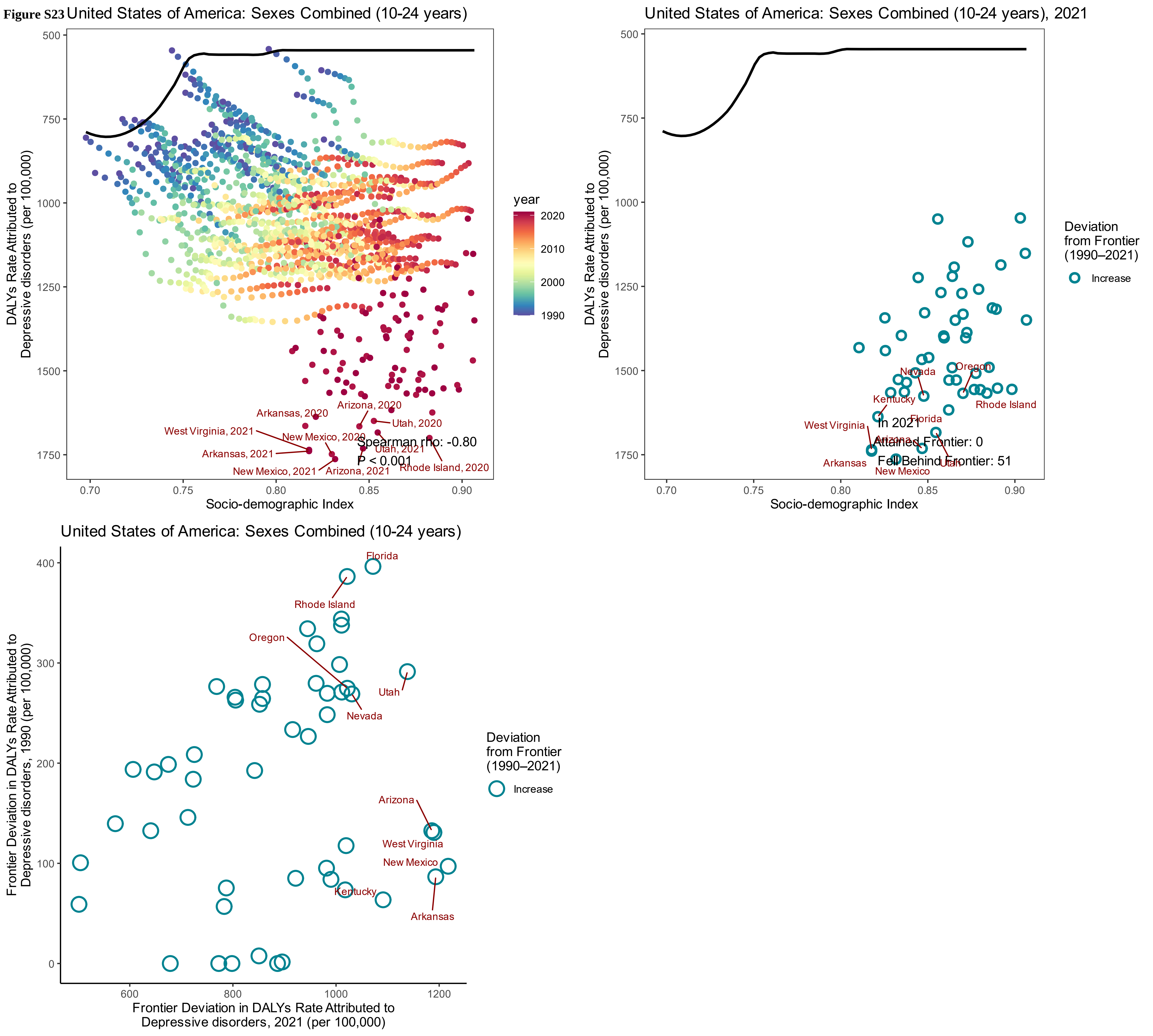

United States of America: Sexes Combined (10-24 years)
DALYs Rate Attributed to Depressive disorders (per 100,000)
Socio-demographic Index
year
2020
2010
2000
1990
Arkansas, 2020
Arizona, 2020
Utah, 2020
West Virginia, 2021
New Mexico, 2020
Spearman rho: -0.80
P < 0.001
Arkansas, 2021
Utah, 2021
New Mexico, 2021
Arizona, 2021
Rhode Island, 2020
United States of America: Sexes Combined (10-24 years), 2021
Deviation from Frontier (1990–2021)
Increase
Nevada
Oregon
Kentucky
Rhode Island
West Virginia
In 2021
Florida
Attained Frontier: 0
Arizona
Fell Behind Frontier: 51
Arkansas
New Mexico
Utah
United States of America: Sexes Combined (10-24 years)
Frontier Deviation in DALYs Rate Attributed to Depressive disorders, 1990 (per 100,000)
Frontier Deviation in DALYs Rate Attributed to Depressive disorders, 2021 (per 100,000)
Florida
Rhode Island
Oregon
Utah
Nevada
Arizona
West Virginia
New Mexico
Kentucky
Arkansas

Figure S23

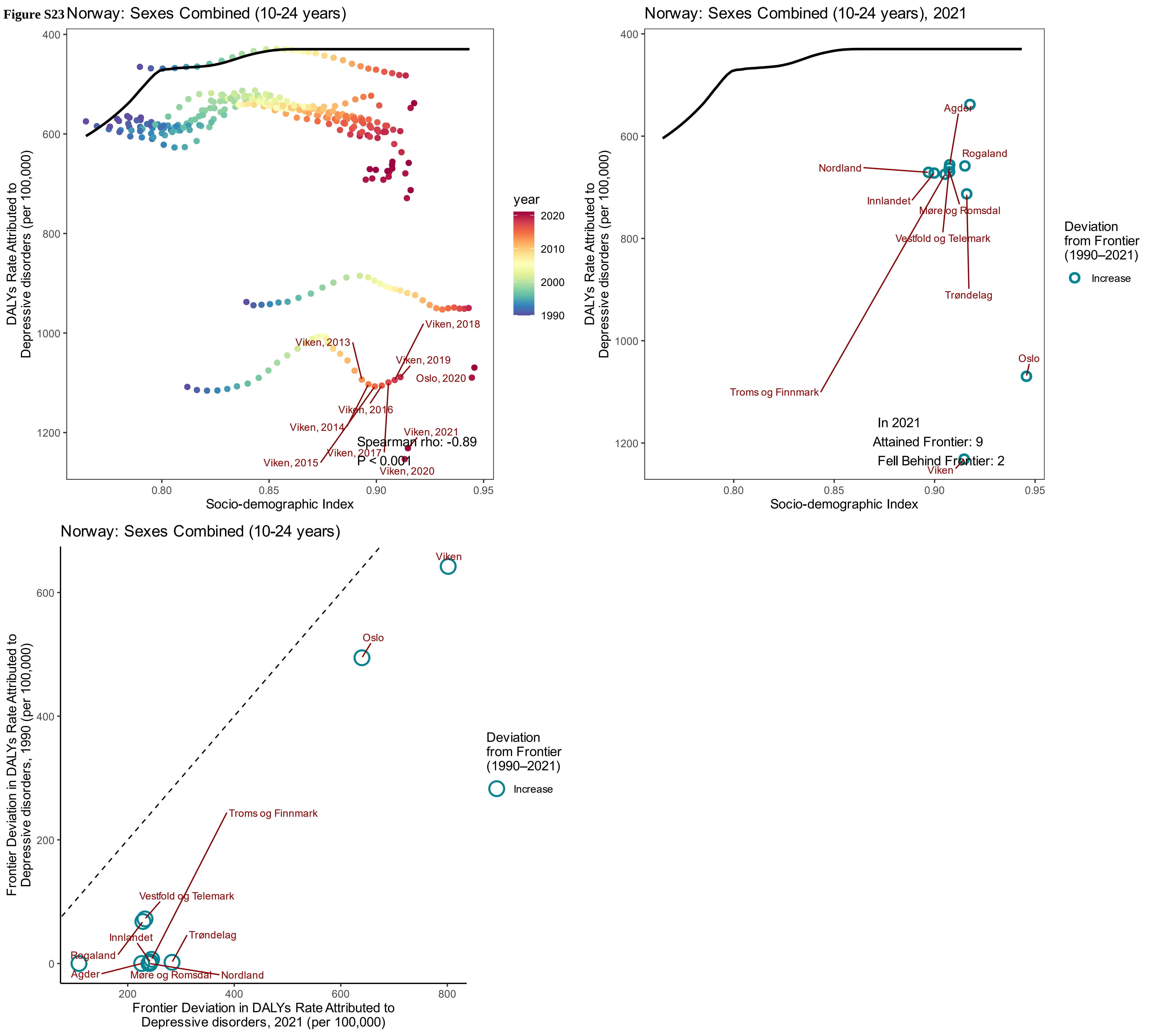

Norway: Sexes Combined (10-24 years)
DALYs Rate Attributed to Depressive disorders (per 100,000)
Socio-demographic Index
year
2020
2010
2000
1990
Viken, 2018
Viken, 2013
Viken, 2019
Oslo, 2020
Viken, 2016
Viken, 2014
Viken, 2021
Spearman rho: -0.89
Viken, 2017
P < 0.001
Viken, 2015
Viken, 2020
Norway: Sexes Combined (10-24 years), 2021
Agder
Rogaland
Nordland
Innlandet
Møre og Romsdal
Vestfold og Telemark
Trøndelag
Oslo
Troms og Finnmark
In 2021
Attained Frontier: 9
Fell Behind Frontier: 2
Viken
Deviation from Frontier (1990–2021)
Increase
Norway: Sexes Combined (10-24 years)
Frontier Deviation in DALYs Rate Attributed to Depressive disorders, 1990 (per 100,000)
Frontier Deviation in DALYs Rate Attributed to Depressive disorders, 2021 (per 100,000)
Viken
Oslo
Troms og Finnmark
Vestfold og Telemark
Trøndelag
Innlandet
Rogaland
Agder
Møre og Romsdal
Nordland
Deviation from Frontier (1990–2021)
Increase

Figure S23

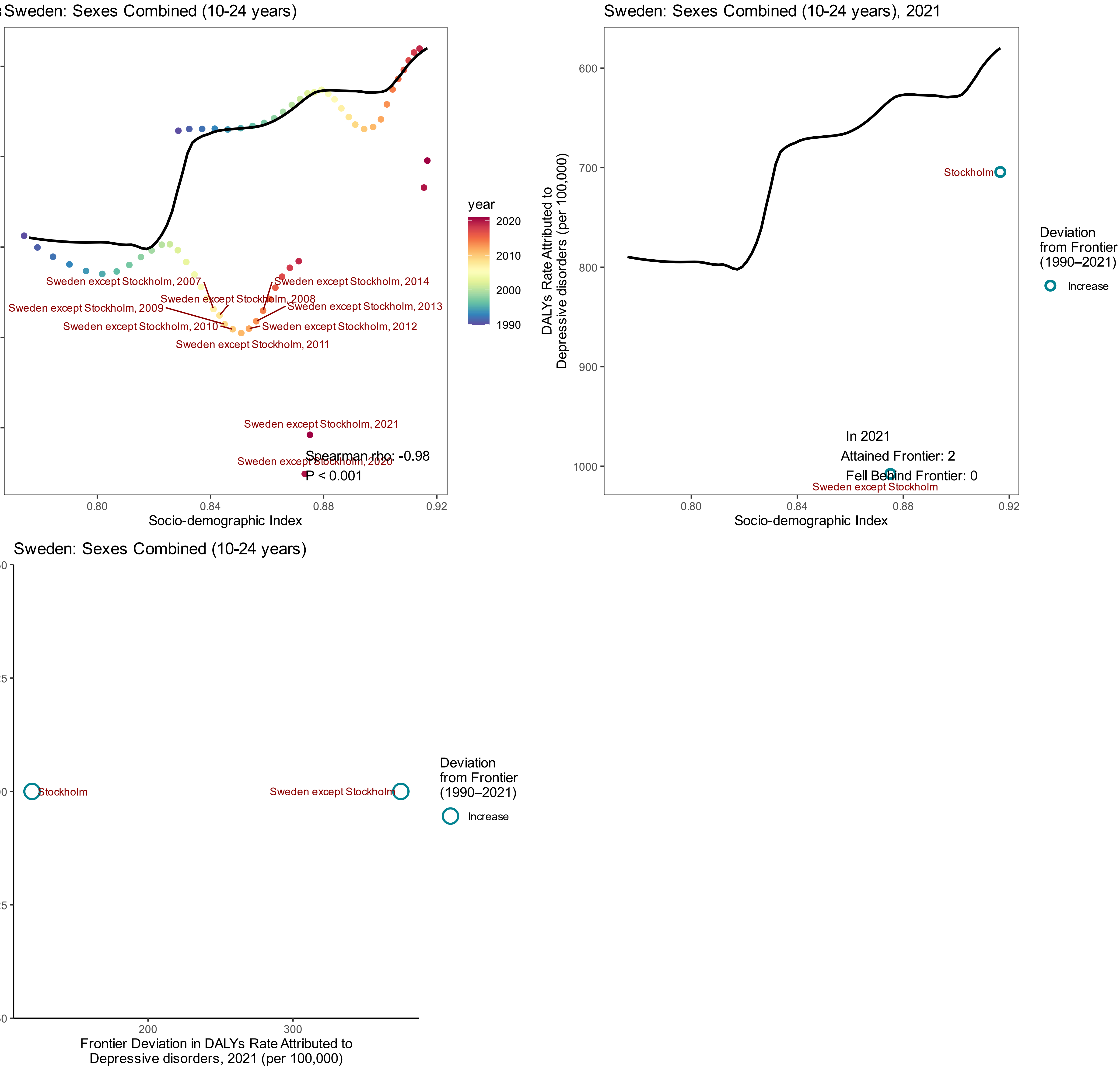

Sweden: Sexes Combined (10-24 years)
DALYs Rate Attributed to Depressive disorders (per 100,000)
Socio-demographic Index
year
2020
2010
2000
1990
Sweden except Stockholm, 2007
Sweden except Stockholm, 2008
Sweden except Stockholm, 2009
Sweden except Stockholm, 2010
Sweden except Stockholm, 2011
Sweden except Stockholm, 2012
Sweden except Stockholm, 2013
Sweden except Stockholm, 2014
Sweden except Stockholm, 2021
Sweden except Stockholm, 2020
Spearman rho: -0.98
P < 0.001
Sweden: Sexes Combined (10-24 years), 2021
Stockholm
Sweden except Stockholm
In 2021
Attained Frontier: 2
Fell Behind Frontier: 0
Deviation from Frontier (1990–2021)
Increase
Frontier Deviation in DALYs Rate Attributed to Depressive disorders, 1990 (per 100,000)
Frontier Deviation in DALYs Rate Attributed to Depressive disorders, 2021 (per 100,000)

Figure S23

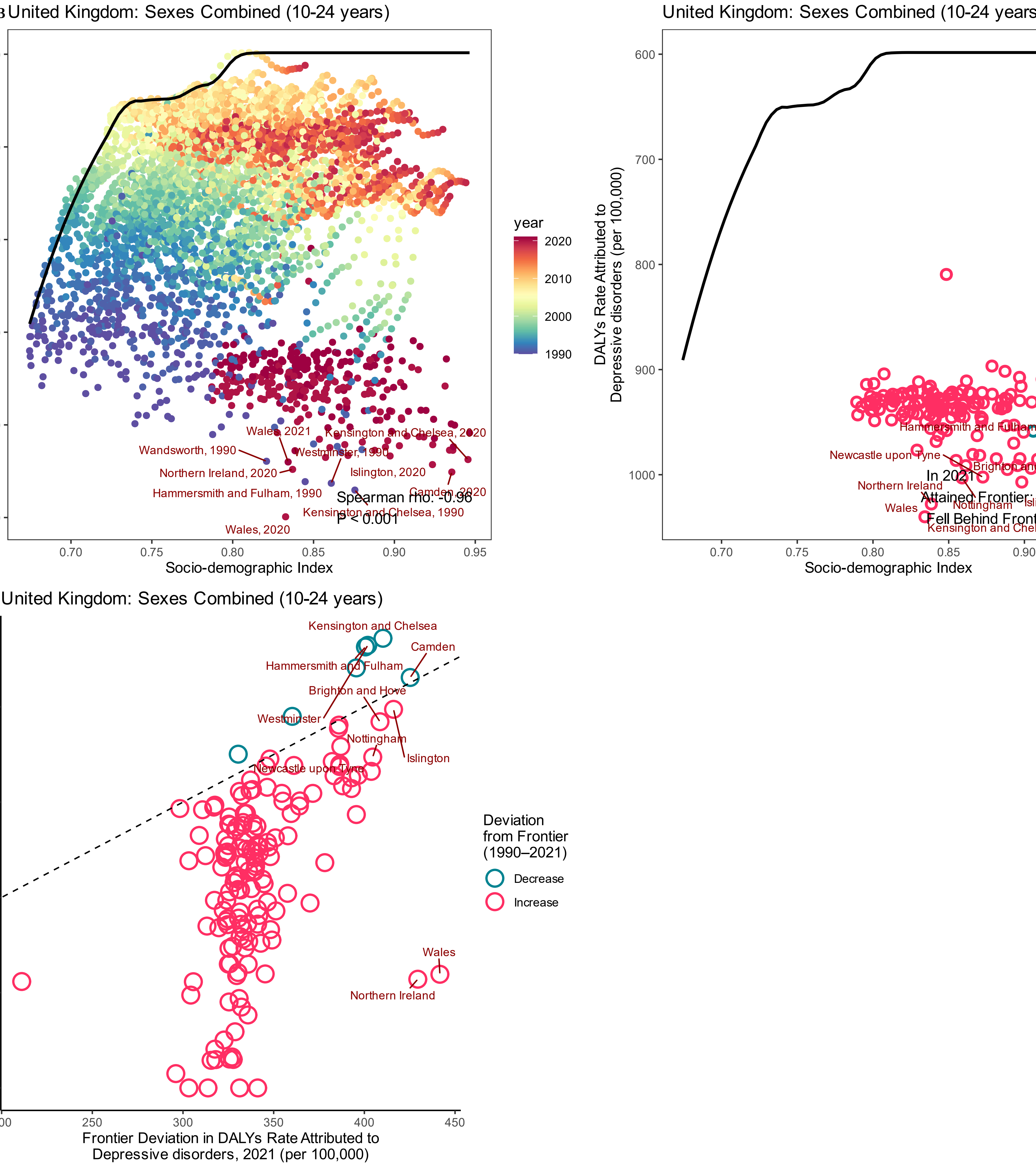

United Kingdom: Sexes Combined (10-24 years)
DALYs Rate Attributed to Depressive disorders (per 100,000)
Socio-demographic Index
year
Wales, 2021
Kensington and Chelsea, 2020
Wandsworth, 1990
Westminster, 1990
Northern Ireland, 2020
Islington, 2020
Hammersmith and Fulham, 1990
Camden, 2020
Spearman rho: -0.96
P < 0.001
Kensington and Chelsea, 1990
Wales, 2020
United Kingdom: Sexes Combined (10-24 years), 2021
Deviation from Frontier (1990–2021)
Decrease
Increase
Hammersmith and Fulham
Westminster
Newcastle upon Tyne
Brighton and Hove
In 2021
Northern Ireland
Attained Frontier: 152
Wales
Nottingham
Islington
Fell Behind Frontier: 1
Kensington and Chelsea
Camden
Frontier Deviation in DALYs Rate Attributed to Depressive disorders, 1990 (per 100,000)
Frontier Deviation in DALYs Rate Attributed to Depressive disorders, 2021 (per 100,000)

Figure S23

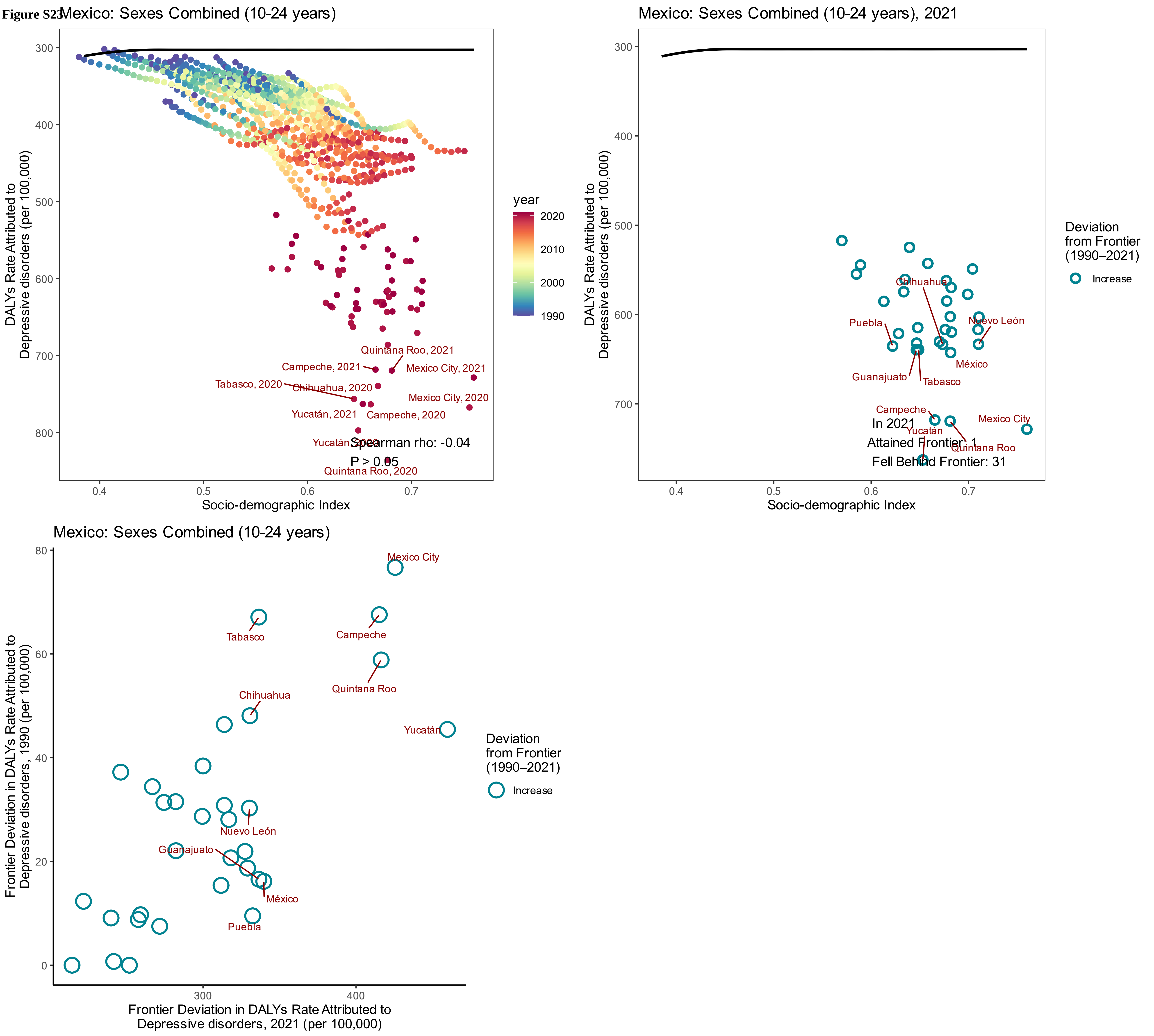

Mexico: Sexes Combined (10-24 years)
DALYs Rate Attributed to Depressive disorders (per 100,000)
Socio-demographic Index
year
2020
2010
2000
1990
Quintana Roo, 2021
Campeche, 2021
Mexico City, 2021
Tabasco, 2020
Chihuahua, 2020
Mexico City, 2020
Yucatán, 2021
Campeche, 2020
Yucatán, 2020
Spearman rho: -0.04
P > 0.05
Quintana Roo, 2020
Mexico: Sexes Combined (10-24 years), 2021
Deviation from Frontier (1990–2021)
Increase
Chihuahua
Puebla
Nuevo León
México
Guanajuato
Tabasco
Campeche
In 2021
Yucatán
Mexico City
Attained Frontier: 1
Quintana Roo
Fell Behind Frontier: 31
Mexico: Sexes Combined (10-24 years)
Frontier Deviation in DALYs Rate Attributed to Depressive disorders, 1990 (per 100,000)
Frontier Deviation in DALYs Rate Attributed to Depressive disorders, 2021 (per 100,000)

Figure S23

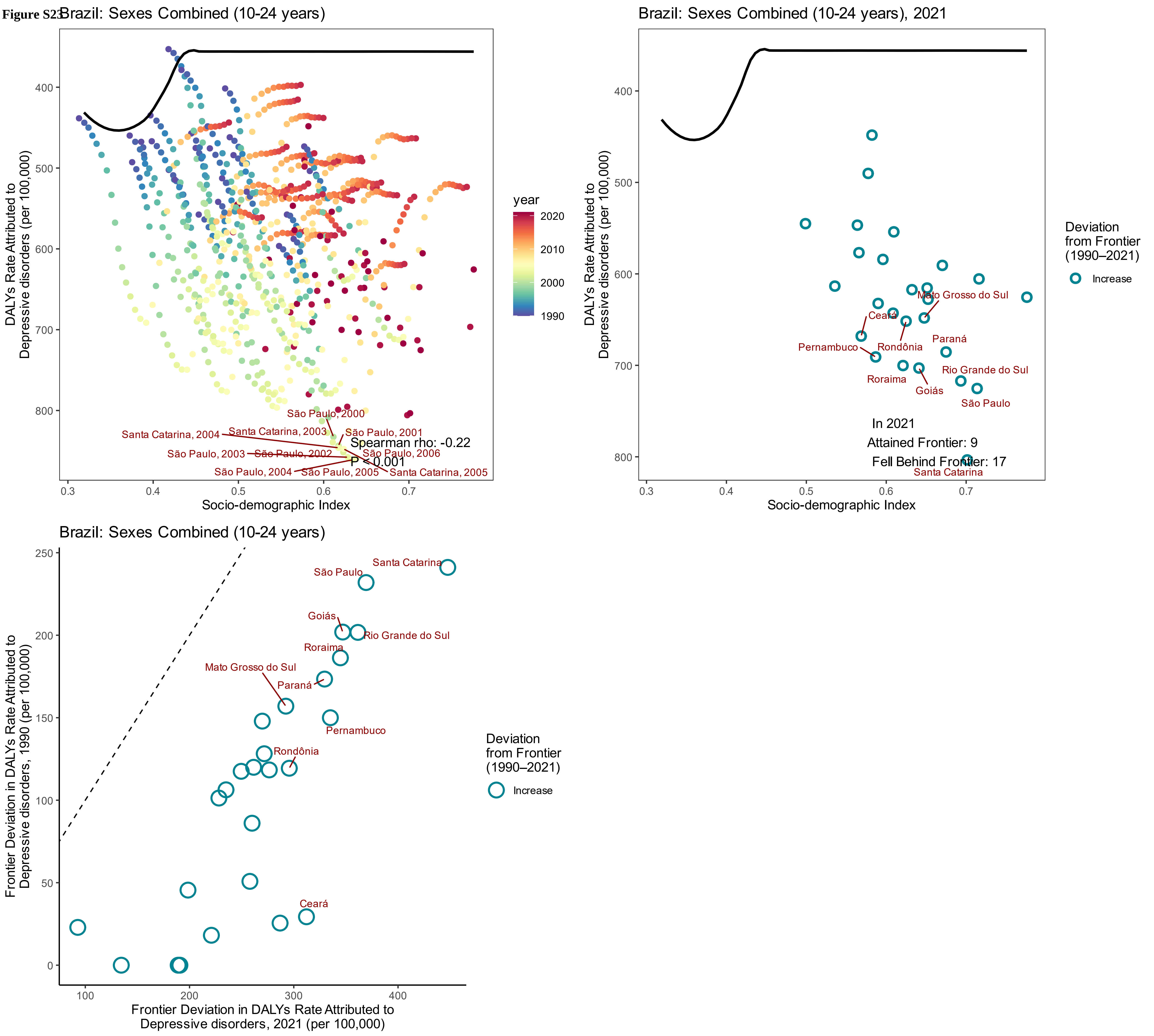
Brazil: Sexes Combined (10-24 years)
DALYs Rate Attributed to Depressive disorders (per 100,000)
Socio-demographic Index
year
2020
2010
2000
1990
São Paulo, 2000
Santa Catarina, 2004
Santa Catarina, 2003
São Paulo, 2001
Spearman rho: -0.22
São Paulo, 2003
São Paulo, 2002
São Paulo, 2006
P < 0.001
São Paulo, 2004
São Paulo, 2005
Santa Catarina, 2005
Brazil: Sexes Combined (10-24 years), 2021
Mato Grosso do Sul
Ceará
Pernambuco
Rondônia
Paraná
Roraima
Goiás
Rio Grande do Sul
São Paulo
In 2021
Attained Frontier: 9
Fell Behind Frontier: 17
Santa Catarina
Deviation from Frontier (1990–2021)
Increase
Brazil: Sexes Combined (10-24 years)
Frontier Deviation in DALYs Rate Attributed to Depressive disorders, 1990 (per 100,000)
Frontier Deviation in DALYs Rate Attributed to Depressive disorders, 2021 (per 100,000)
Santa Catarina
São Paulo
Goiás
Rio Grande do Sul
Roraima
Mato Grosso do Sul
Paraná
Pernambuco
Rondônia
Ceará
Deviation from Frontier (1990–2021)
Increase

Figure S23

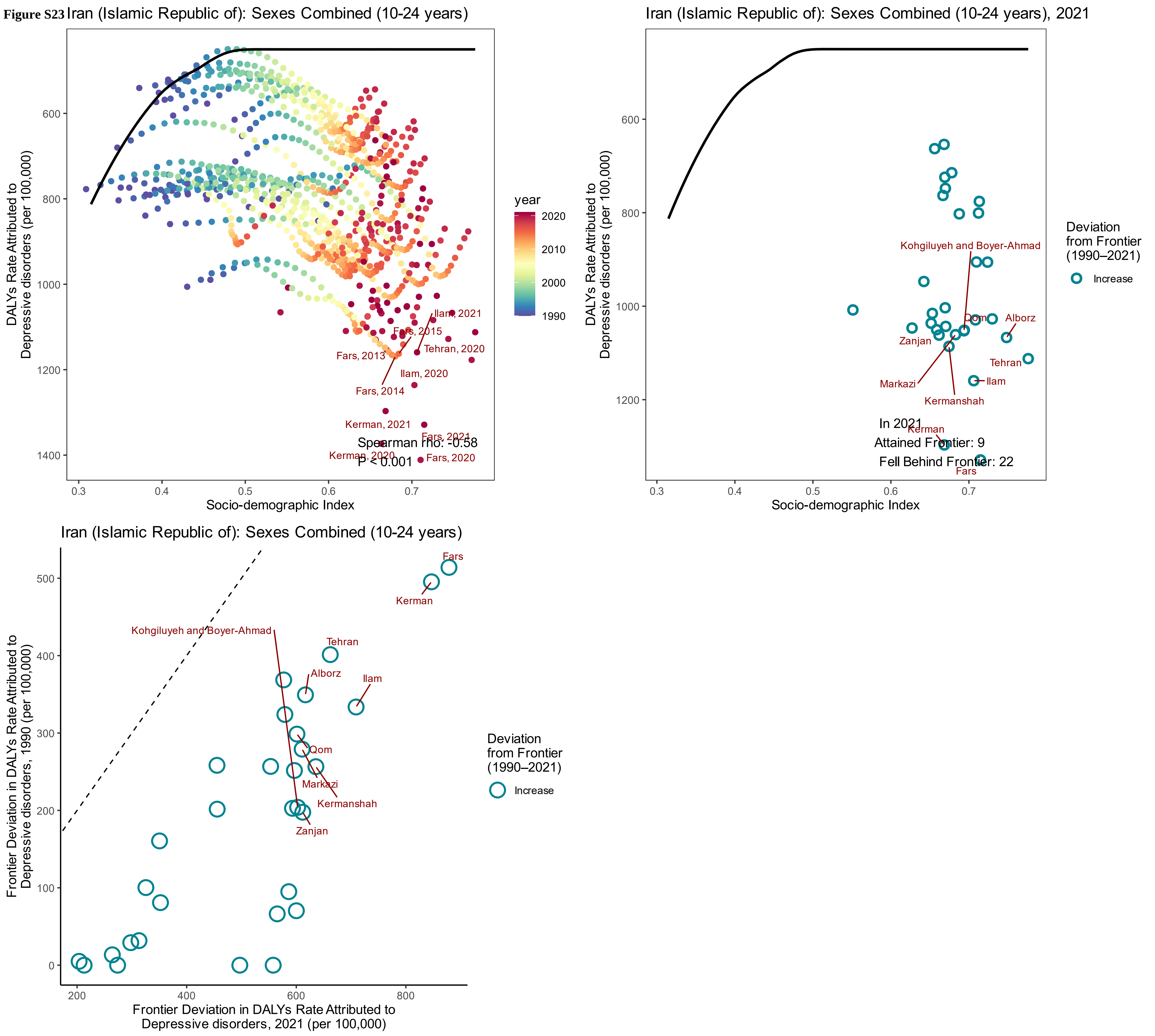

Iran (Islamic Republic of): Sexes Combined (10-24 years)
DALYs Rate Attributed to Depressive disorders (per 100,000)
Socio-demographic Index
year
Spearman rho: -0.58
P < 0.001
Ilam, 2021
Fars, 2015
Tehran, 2020
Fars, 2013
Ilam, 2020
Fars, 2014
Kerman, 2021
Fars, 2021
Kerman, 2020
Fars, 2020
Iran (Islamic Republic of): Sexes Combined (10-24 years), 2021
Kohgiluyeh and Boyer-Ahmad
Qom
Alborz
Zanjan
Tehran
Markazi
Ilam
Kermanshah
In 2021
Kerman
Attained Frontier: 9
Fell Behind Frontier: 22
Fars
Deviation from Frontier (1990–2021)
Increase
Frontier Deviation in DALYs Rate Attributed to Depressive disorders, 1990 (per 100,000)
Frontier Deviation in DALYs Rate Attributed to Depressive disorders, 2021 (per 100,000)

Figure S23

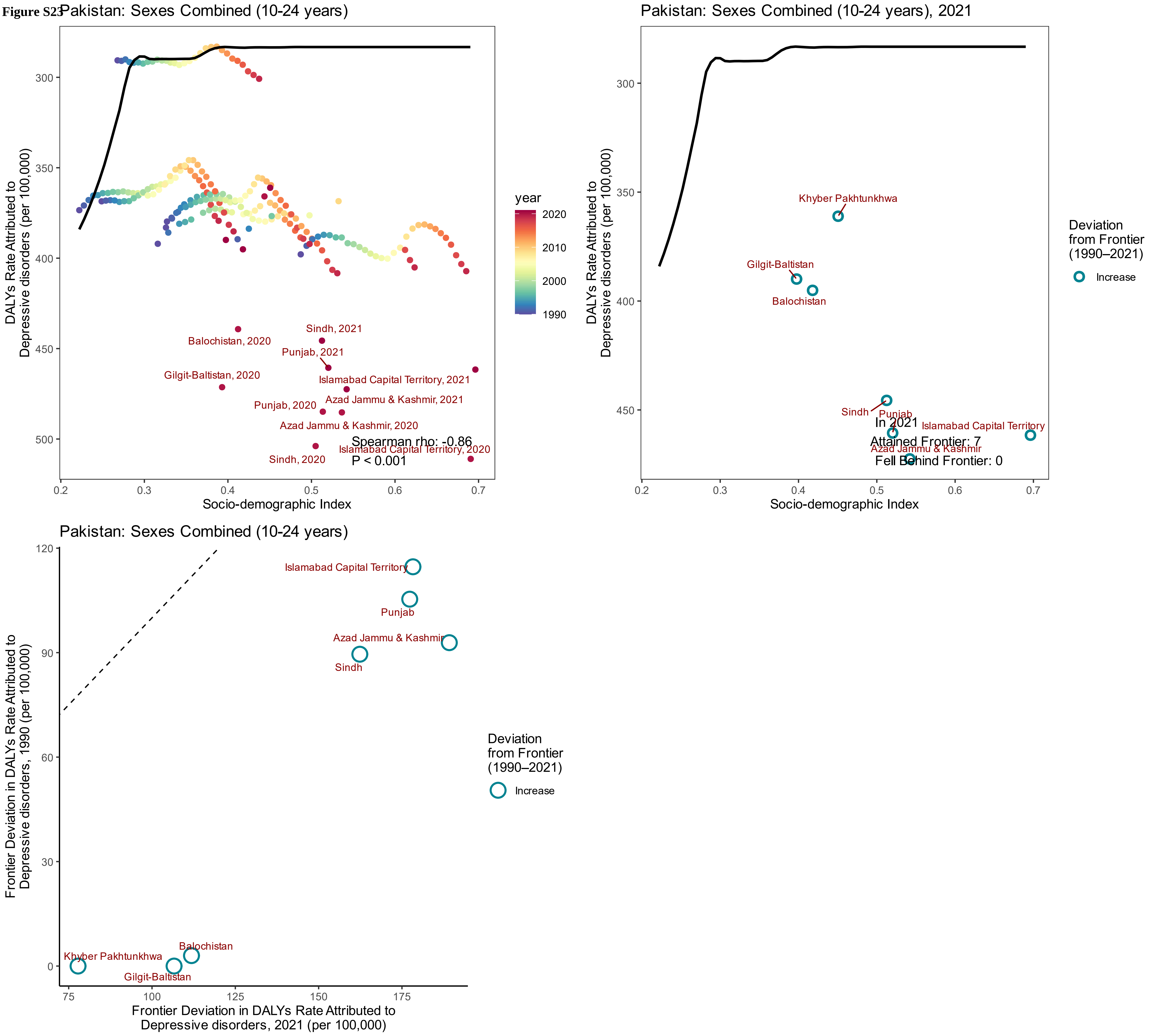

Pakistan: Sexes Combined (10-24 years)
DALYs Rate Attributed to Depressive disorders (per 100,000)
Socio-demographic Index
year
Balochistan, 2020
Sindh, 2021
Punjab, 2021
Gilgit-Baltistan, 2020
Islamabad Capital Territory, 2021
Punjab, 2020
Azad Jammu & Kashmir, 2021
Azad Jammu & Kashmir, 2020
Spearman rho: -0.86
P < 0.001
Sindh, 2020
Islamabad Capital Territory, 2020
Pakistan: Sexes Combined (10-24 years), 2021
Khyber Pakhtunkhwa
Gilgit-Baltistan
Balochistan
Sindh
Punjab
In 2021
Attained Frontier: 7
Fell Behind Frontier: 0
Islamabad Capital Territory
Azad Jammu & Kashmir
Deviation from Frontier (1990–2021)
Increase
Pakistan: Sexes Combined (10-24 years)
Frontier Deviation in DALYs Rate Attributed to Depressive disorders, 1990 (per 100,000)
Frontier Deviation in DALYs Rate Attributed to Depressive disorders, 2021 (per 100,000)
Islamabad Capital Territory
Punjab
Azad Jammu & Kashmir
Sindh
Khyber Pakhtunkhwa
Balochistan
Gilgit-Baltistan
Deviation from Frontier (1990–2021)
Increase

Figure S23

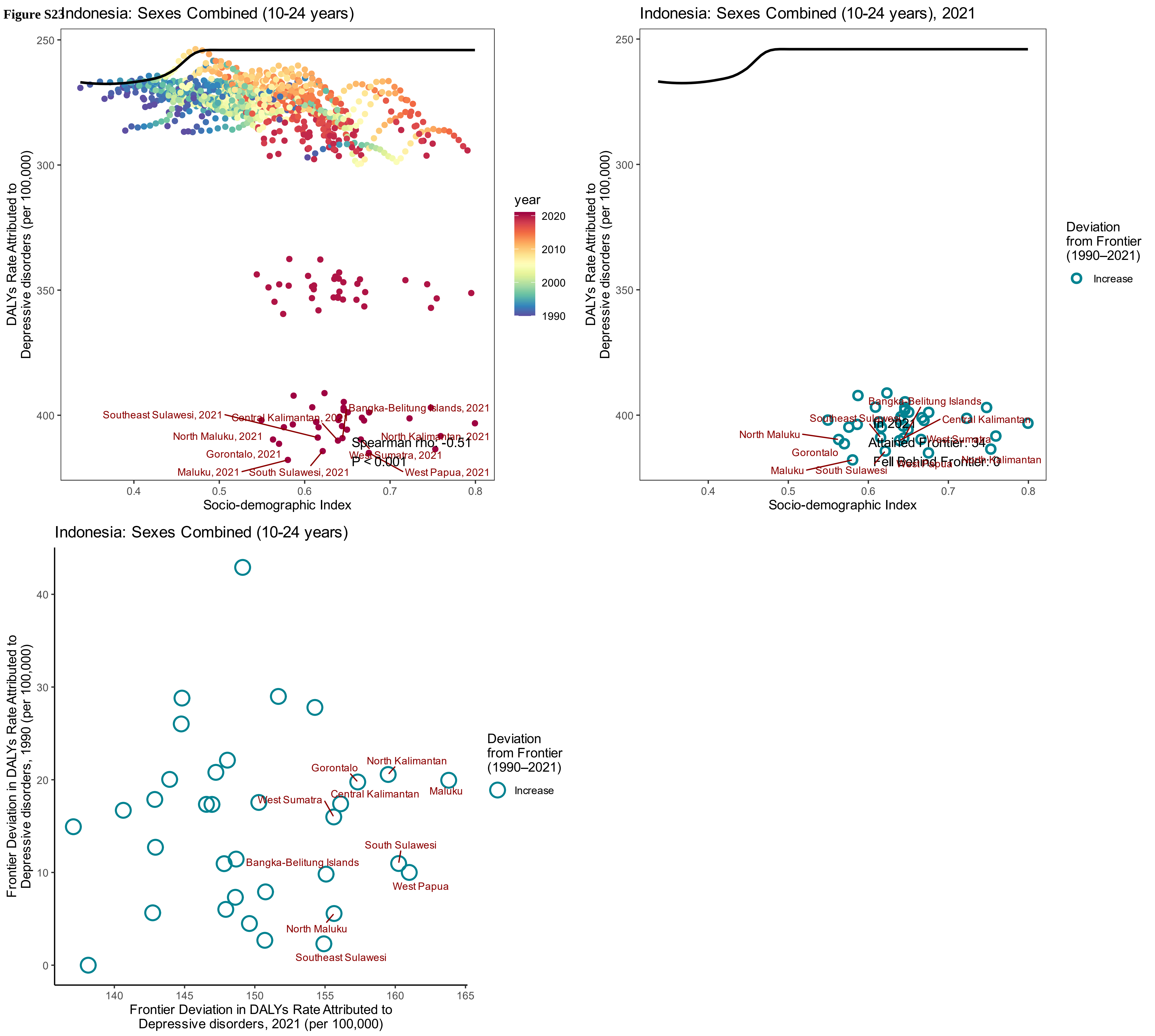
Indonesia: Sexes Combined (10-24 years)
DALYs Rate Attributed to Depressive disorders (per 100,000)
Socio-demographic Index
year
2020
2010
2000
1990
Southeast Sulawesi, 2021
Central Kalimantan, 2021
Bangka-Belitung Islands, 2021
North Maluku, 2021
North Kalimantan, 2021
Gorontalo, 2021
West Sumatra, 2021
Maluku, 2021
South Sulawesi, 2021
West Papua, 2021
Spearman rho: -0.51
P < 0.001
Indonesia: Sexes Combined (10-24 years), 2021
Deviation from Frontier (1990–2021)
Increase
Bangka-Belitung Islands
Southeast Sulawesi
Central Kalimantan
North Maluku
West Sumatra
Gorontalo
North Kalimantan
West Papua
Maluku
South Sulawesi
In 2021
Attained Frontier: 34
Fell Behind Frontier: 0
Indonesia: Sexes Combined (10-24 years)
Frontier Deviation in DALYs Rate Attributed to Depressive disorders, 1990 (per 100,000)
Frontier Deviation in DALYs Rate Attributed to Depressive disorders, 2021 (per 100,000)
Gorontalo
North Kalimantan
Central Kalimantan
Maluku
West Sumatra
South Sulawesi
Bangka-Belitung Islands
West Papua
North Maluku
Southeast Sulawesi
Deviation from Frontier (1990–2021)
Increase

Figure S23

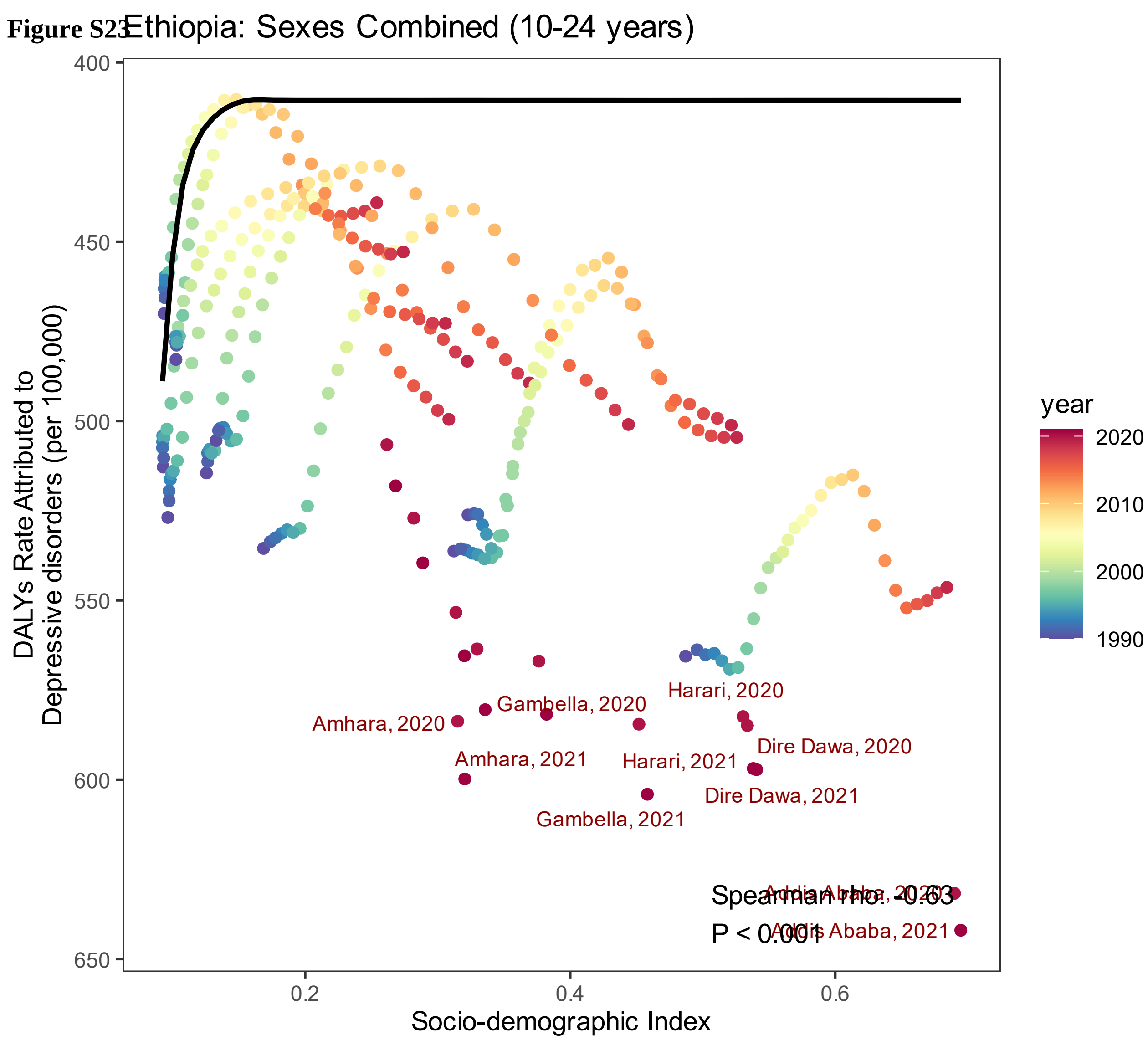
Ethiopia: Sexes Combined (10-24 years)
DALYs Rate Attributed to Depressive disorders (per 100,000)
Socio-demographic Index
year
2020
2010
2000
1990
Amhara, 2020
Gambella, 2020
Harari, 2020
Amhara, 2021
Harari, 2021
Dire Dawa, 2020
Dire Dawa, 2021
Gambella, 2021
Spearman rho: -0.63
P < 0.001
Addis Ababa, 2020
Addis Ababa, 2021

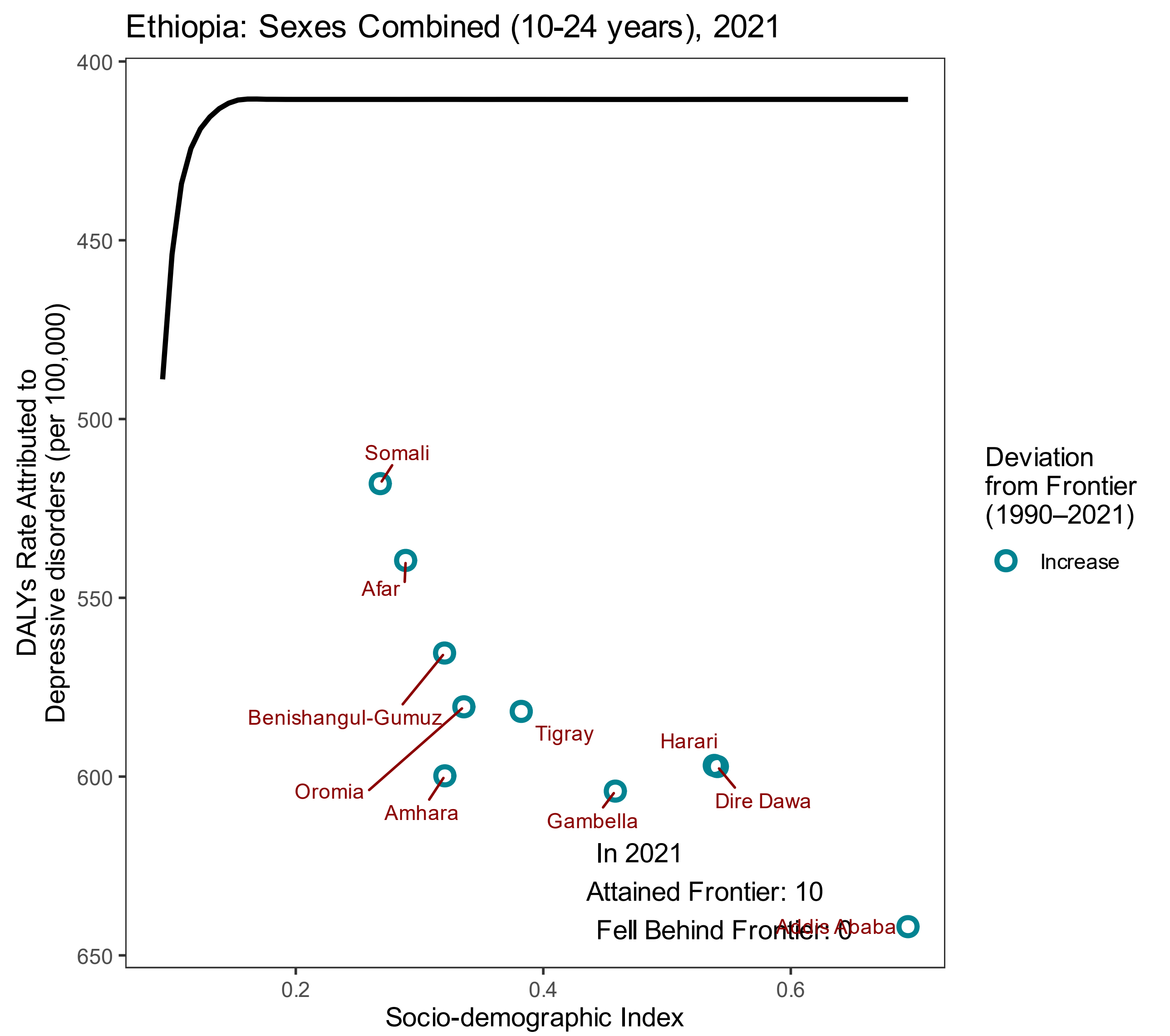
Ethiopia: Sexes Combined (10-24 years), 2021
DALYs Rate Attributed to Depressive disorders (per 100,000)
Socio-demographic Index
Somali
Afar
Benishangul-Gumuz
Tigray
Harari
Oromia
Amhara
Gambella
Dire Dawa
Addis Ababa
In 2021
Attained Frontier: 10
Fell Behind Frontier: 0
Deviation from Frontier (1990–2021)
Increase

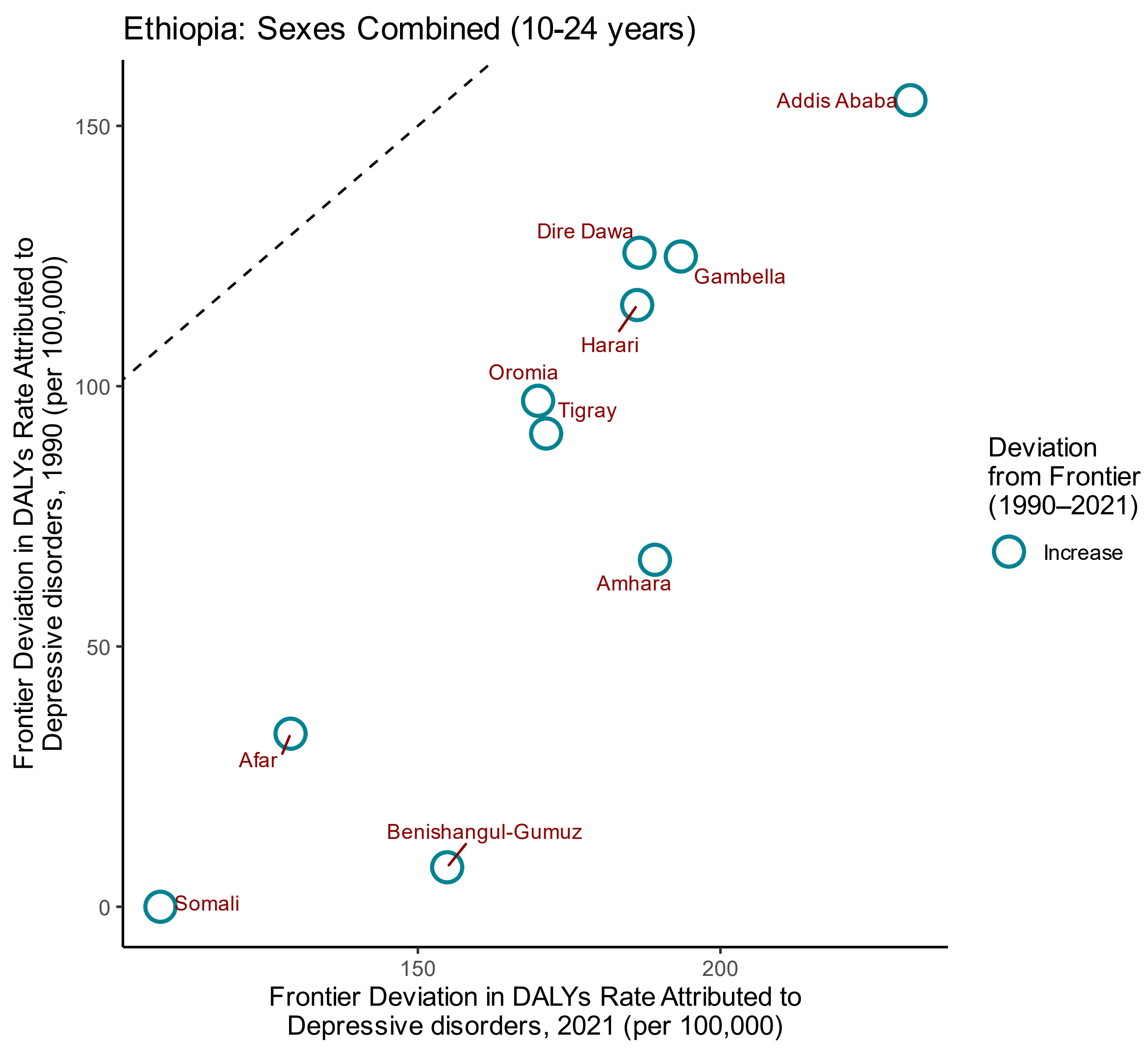
Ethiopia: Sexes Combined (10-24 years)
Frontier Deviation in DALYs Rate Attributed to Depressive disorders, 1990 (per 100,000)
Frontier Deviation in DALYs Rate Attributed to Depressive disorders, 2021 (per 100,000)
Addis Ababa
Dire Dawa
Gambella
Harari
Oromia
Tigray
Amhara
Afar
Benishangul-Gumuz
Somali
Deviation from Frontier (1990–2021)
Increase

Figure S23

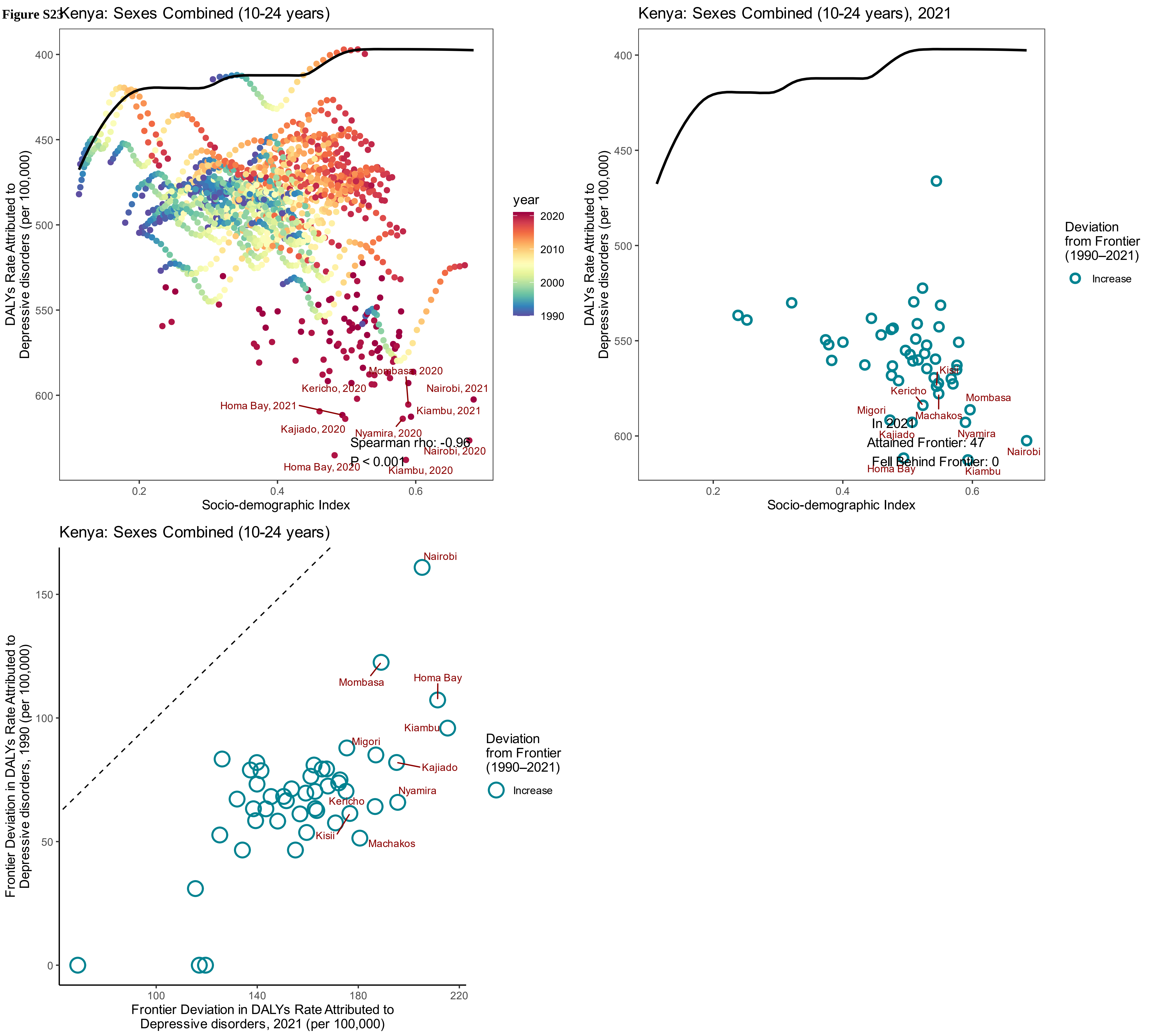

Kenya: Sexes Combined (10-24 years)
DALYs Rate Attributed to Depressive disorders (per 100,000)
Socio-demographic Index
year
Mombasa, 2020
Kericho, 2020
Nairobi, 2021
Homa Bay, 2021
Kiambu, 2021
Kajiado, 2020
Nyamira, 2020
Spearman rho: -0.96
P < 0.001
Nairobi, 2020
Homa Bay, 2020
Kiambu, 2020
Kenya: Sexes Combined (10-24 years), 2021
Kisii
Kericho
Mombasa
Migori
Machakos
In 2021
Kajiado
Nyamira
Attained Frontier: 47
Nairobi
Fell Behind Frontier: 0
Homa Bay
Kiambu
Deviation from Frontier (1990–2021)
Increase
Kenya: Sexes Combined (10-24 years)
Frontier Deviation in DALYs Rate Attributed to Depressive disorders, 1990 (per 100,000)
Frontier Deviation in DALYs Rate Attributed to Depressive disorders, 2021 (per 100,000)
Nairobi
Mombasa
Homa Bay
Kiambu
Migori
Kajiado
Nyamira
Kericho
Kisii
Machakos
Deviation from Frontier (1990–2021)
Increase

Figure S23

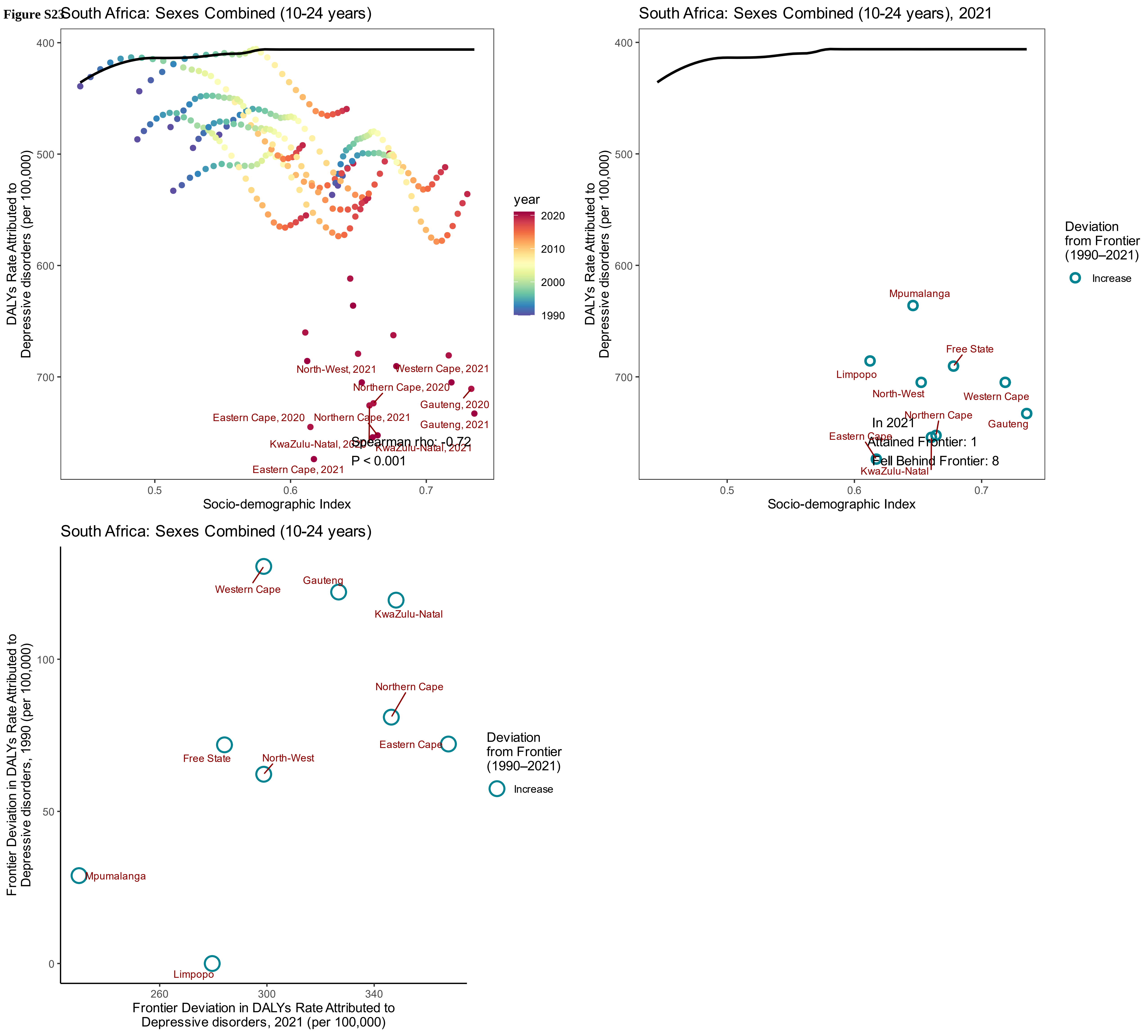

South Africa: Sexes Combined (10-24 years)
DALYs Rate Attributed to Depressive disorders (per 100,000)
Socio-demographic Index
year
2020
2010
2000
1990
North-West, 2021
Western Cape, 2021
Northern Cape, 2020
Gauteng, 2020
Eastern Cape, 2020
Northern Cape, 2021
Gauteng, 2021
KwaZulu-Natal, 2020
KwaZulu-Natal, 2021
Eastern Cape, 2021
Spearman rho: -0.72
P < 0.001
South Africa: Sexes Combined (10-24 years), 2021
Mpumalanga
Free State
Limpopo
North-West
Western Cape
Northern Cape
Gauteng
Eastern Cape
KwaZulu-Natal
In 2021
Attained Frontier: 1
Fell Behind Frontier: 8
Deviation from Frontier (1990–2021)
Increase
South Africa: Sexes Combined (10-24 years)
Frontier Deviation in DALYs Rate Attributed to Depressive disorders, 1990 (per 100,000)
Frontier Deviation in DALYs Rate Attributed to Depressive disorders, 2021 (per 100,000)
Western Cape
Gauteng
KwaZulu-Natal
Northern Cape
Free State
North-West
Eastern Cape
Mpumalanga
Limpopo
Deviation from Frontier (1990–2021)
Increase

Figure S23

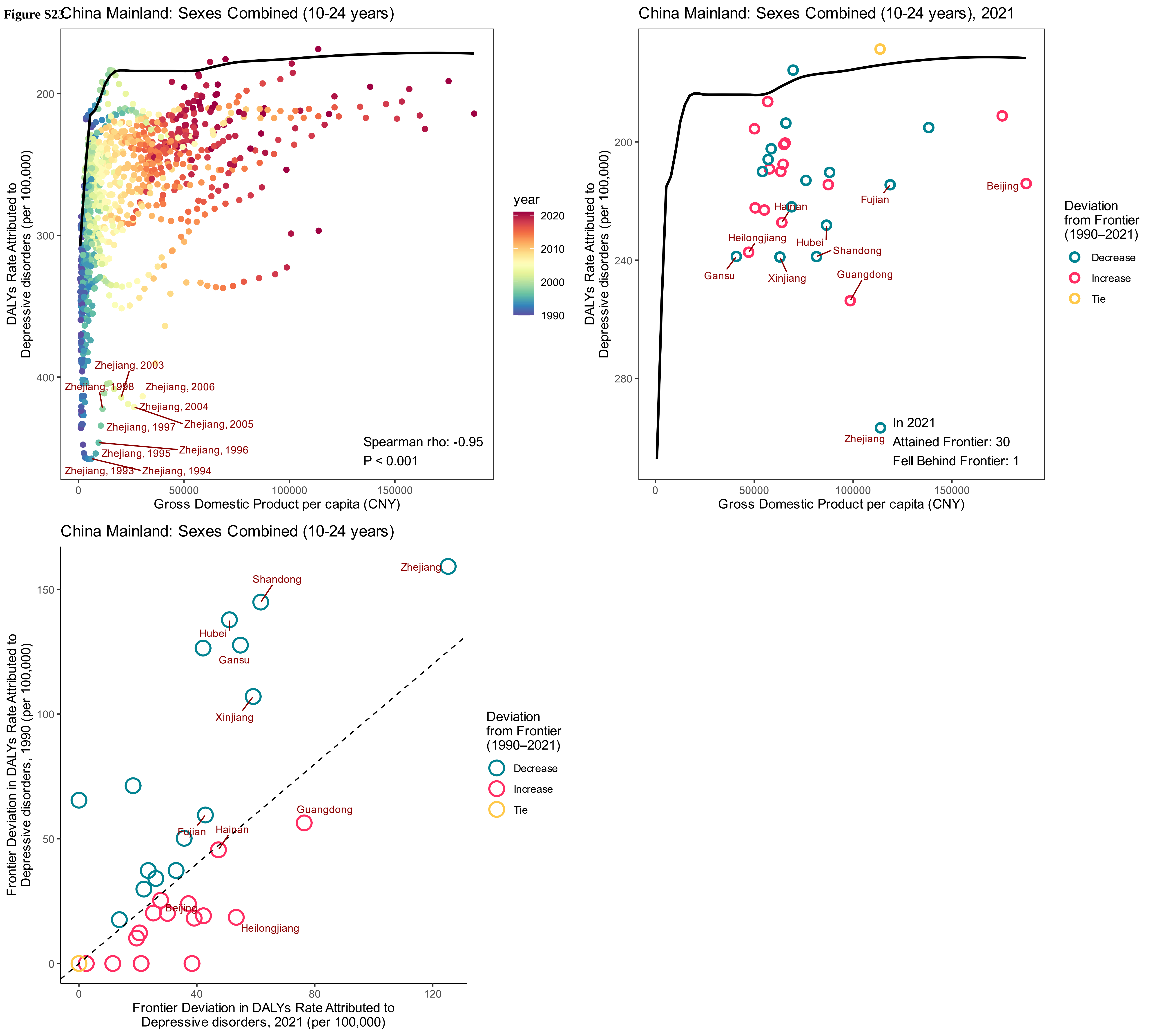

China Mainland: Sexes Combined (10-24 years)
DALYs Rate Attributed to Depressive disorders (per 100,000)
Gross Domestic Product per capita (CNY)
year
2020
2010
2000
1990
Zhejiang, 2003
Zhejiang, 1998
Zhejiang, 2006
Zhejiang, 2004
Zhejiang, 1997
Zhejiang, 2005
Zhejiang, 1996
Zhejiang, 1995
Zhejiang, 1993
Zhejiang, 1994
Spearman rho: -0.95
P < 0.001
China Mainland: Sexes Combined (10-24 years), 2021
Beijing
Fujian
Hainan
Heilongjiang
Hubei
Shandong
Gansu
Xinjiang
Guangdong
Zhejiang
In 2021
Attained Frontier: 30
Fell Behind Frontier: 1
Deviation from Frontier (1990–2021)
Decrease
Increase
Tie
China Mainland: Sexes Combined (10-24 years)
Frontier Deviation in DALYs Rate Attributed to Depressive disorders, 1990 (per 100,000)
Frontier Deviation in DALYs Rate Attributed to Depressive disorders, 2021 (per 100,000)
Zhejiang
Shandong
Hubei
Gansu
Xinjiang
Guangdong
Fujian
Hainan
Beijing
Heilongjiang
Deviation from Frontier (1990–2021)
Decrease
Increase
Tie

Figure S23

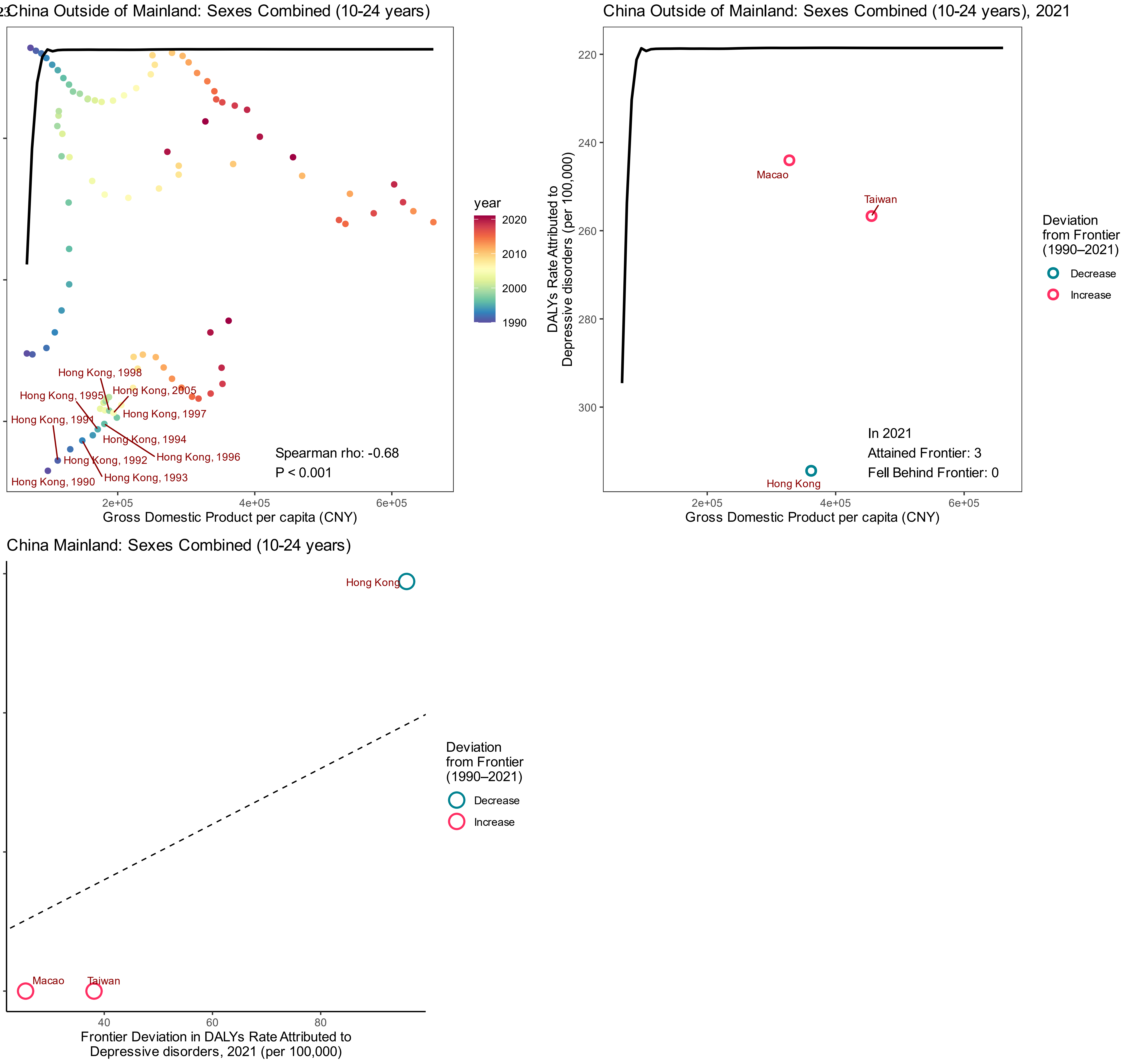

China Outside of Mainland: Sexes Combined (10-24 years)
DALYs Rate Attributed to Depressive disorders (per 100,000)
Gross Domestic Product per capita (CNY)
year
2020
2010
2000
1990
Hong Kong, 1998
Hong Kong, 2005
Hong Kong, 1995
Hong Kong, 1997
Hong Kong, 1991
Hong Kong, 1994
Hong Kong, 1996
Hong Kong, 1992
Hong Kong, 1993
Hong Kong, 1990
Spearman rho: -0.68
P < 0.001
China Outside of Mainland: Sexes Combined (10-24 years), 2021
Macao
Taiwan
Hong Kong
In 2021
Attained Frontier: 3
Fell Behind Frontier: 0
Deviation from Frontier (1990–2021)
Decrease
Increase
China Mainland: Sexes Combined (10-24 years)
Frontier Deviation in DALYs Rate Attributed to Depressive disorders, 1990 (per 100,000)
Frontier Deviation in DALYs Rate Attributed to Depressive disorders, 2021 (per 100,000)
Hong Kong
Macao
Taiwan
Deviation from Frontier (1990–2021)
Decrease
Increase

Figure S23

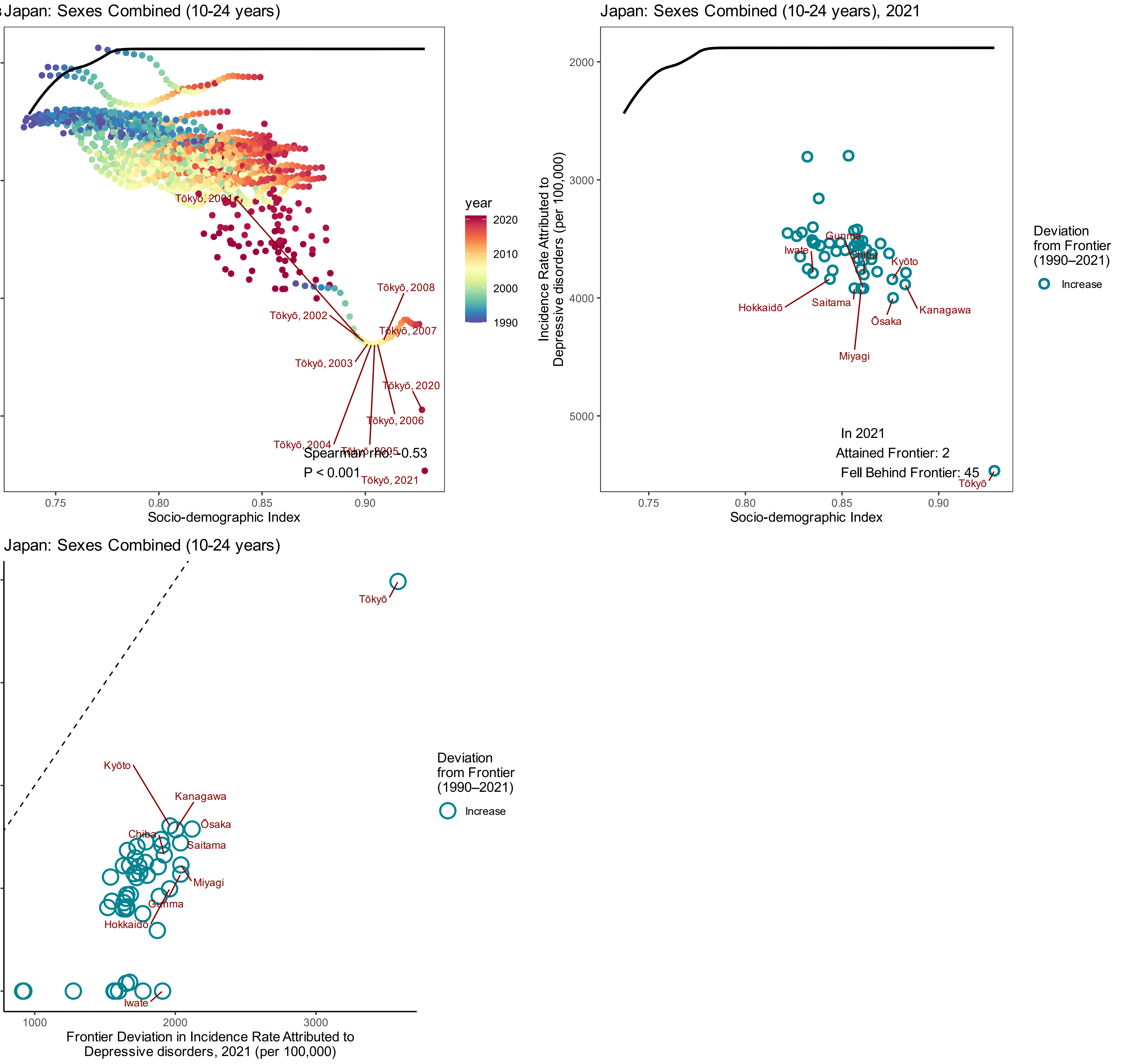

Japan: Sexes Combined (10-24 years)
Incidence Rate Attributed to Depressive disorders (per 100,000)
Socio-demographic Index
year
2020
2010
2000
1990
Tōkyō, 2001
Tōkyō, 2002
Tōkyō, 2003
Tōkyō, 2004
Tōkyō, 2005
Tōkyō, 2006
Tōkyō, 2007
Tōkyō, 2008
Tōkyō, 2020
Tōkyō, 2021
Spearman rho: -0.53
P < 0.001
Japan: Sexes Combined (10-24 years), 2021
Gunma
Iwate
Chiba
Kyōto
Hokkaidō
Saitama
Ōsaka
Kanagawa
Miyagi
Tōkyō
In 2021
Attained Frontier: 2
Fell Behind Frontier: 45
Deviation from Frontier (1990–2021)
Increase
Japan: Sexes Combined (10-24 years)
Frontier Deviation in Incidence Rate Attributed to Depressive disorders, 1990 (per 100,000)
Frontier Deviation in Incidence Rate Attributed to Depressive disorders, 2021 (per 100,000)

Figure S23

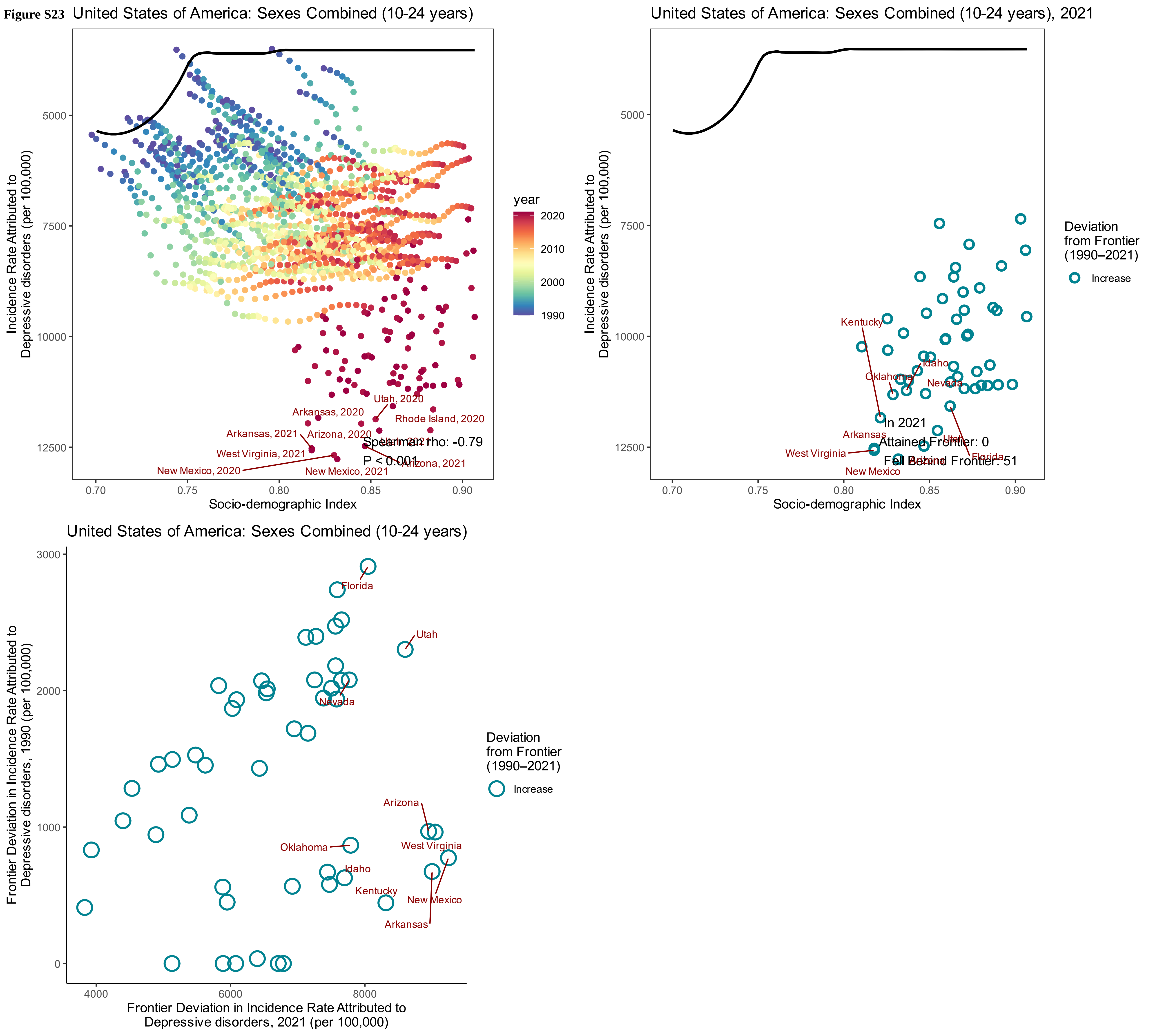

United States of America: Sexes Combined (10-24 years)
Incidence Rate Attributed to Depressive disorders (per 100,000)
Socio-demographic Index
year
Utah, 2020
Arkansas, 2020
Rhode Island, 2020
Arkansas, 2021
Arizona, 2020
Spearman rho: -0.79
P < 0.001
West Virginia, 2021
New Mexico, 2020
New Mexico, 2021
Arizona, 2021
United States of America: Sexes Combined (10-24 years), 2021
Kentucky
Idaho
Oklahoma
Nevada
In 2021
Arkansas
Utah
Attained Frontier: 0
West Virginia
Florida
Fell Behind Frontier: 51
New Mexico
Deviation from Frontier (1990–2021)
Increase
Frontier Deviation in Incidence Rate Attributed to Depressive disorders, 1990 (per 100,000)
Frontier Deviation in Incidence Rate Attributed to Depressive disorders, 2021 (per 100,000)

Figure S23

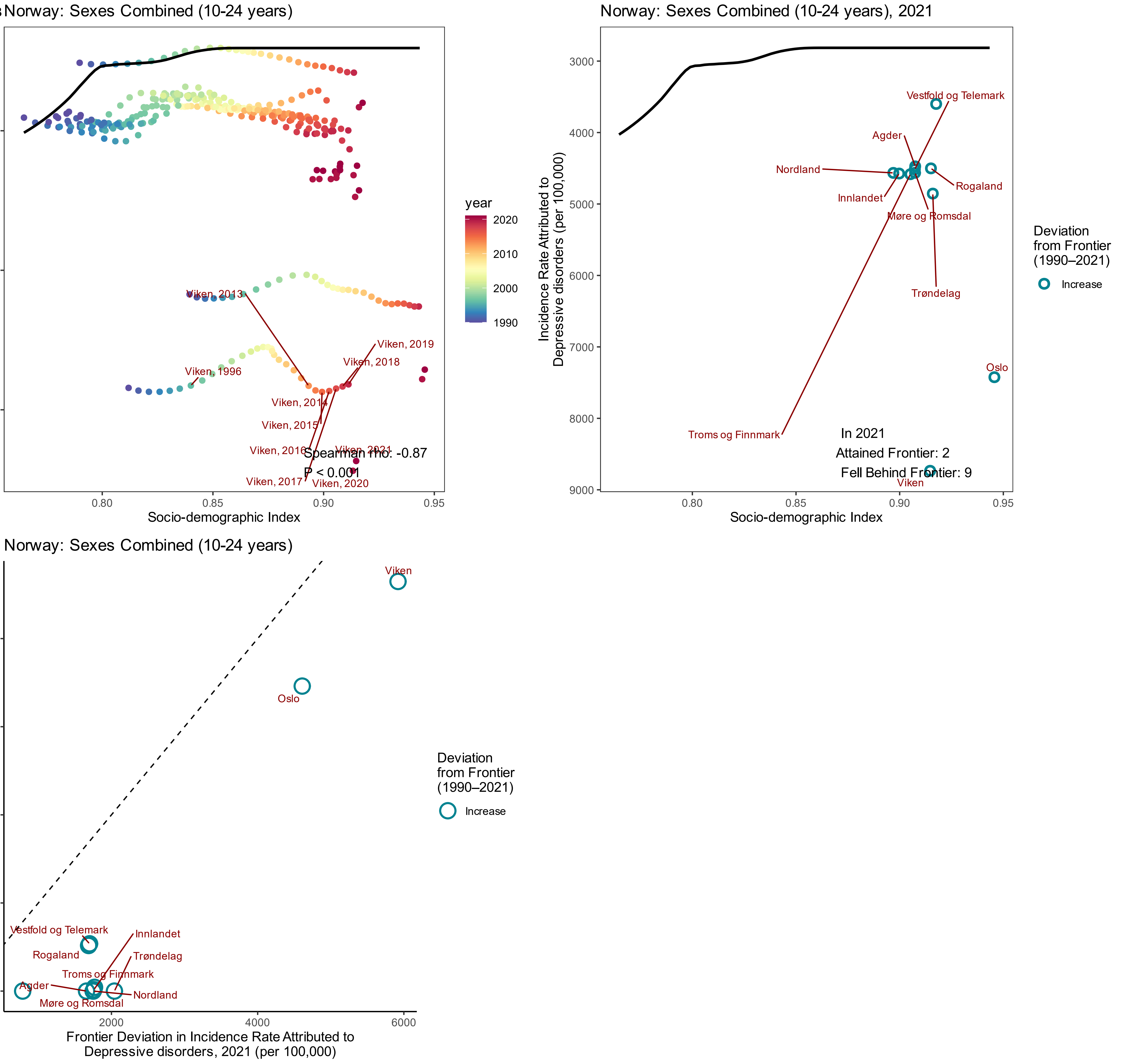

Norway: Sexes Combined (10-24 years)
Incidence Rate Attributed to Depressive disorders (per 100,000)
Socio-demographic Index
year
Viken, 1996
Viken, 2013
Viken, 2014
Viken, 2015
Viken, 2016
Viken, 2017
Viken, 2018
Viken, 2019
Viken, 2020
Viken, 2021
Spearman rho: -0.87
P < 0.001
Norway: Sexes Combined (10-24 years), 2021
Vestfold og Telemark
Agder
Nordland
Innlandet
Rogaland
Møre og Romsdal
Trøndelag
Oslo
Troms og Finnmark
Viken
In 2021
Attained Frontier: 2
Fell Behind Frontier: 9
Deviation from Frontier (1990–2021)
Increase
Frontier Deviation in Incidence Rate Attributed to Depressive disorders, 1990 (per 100,000)
Frontier Deviation in Incidence Rate Attributed to Depressive disorders, 2021 (per 100,000)

Figure S23

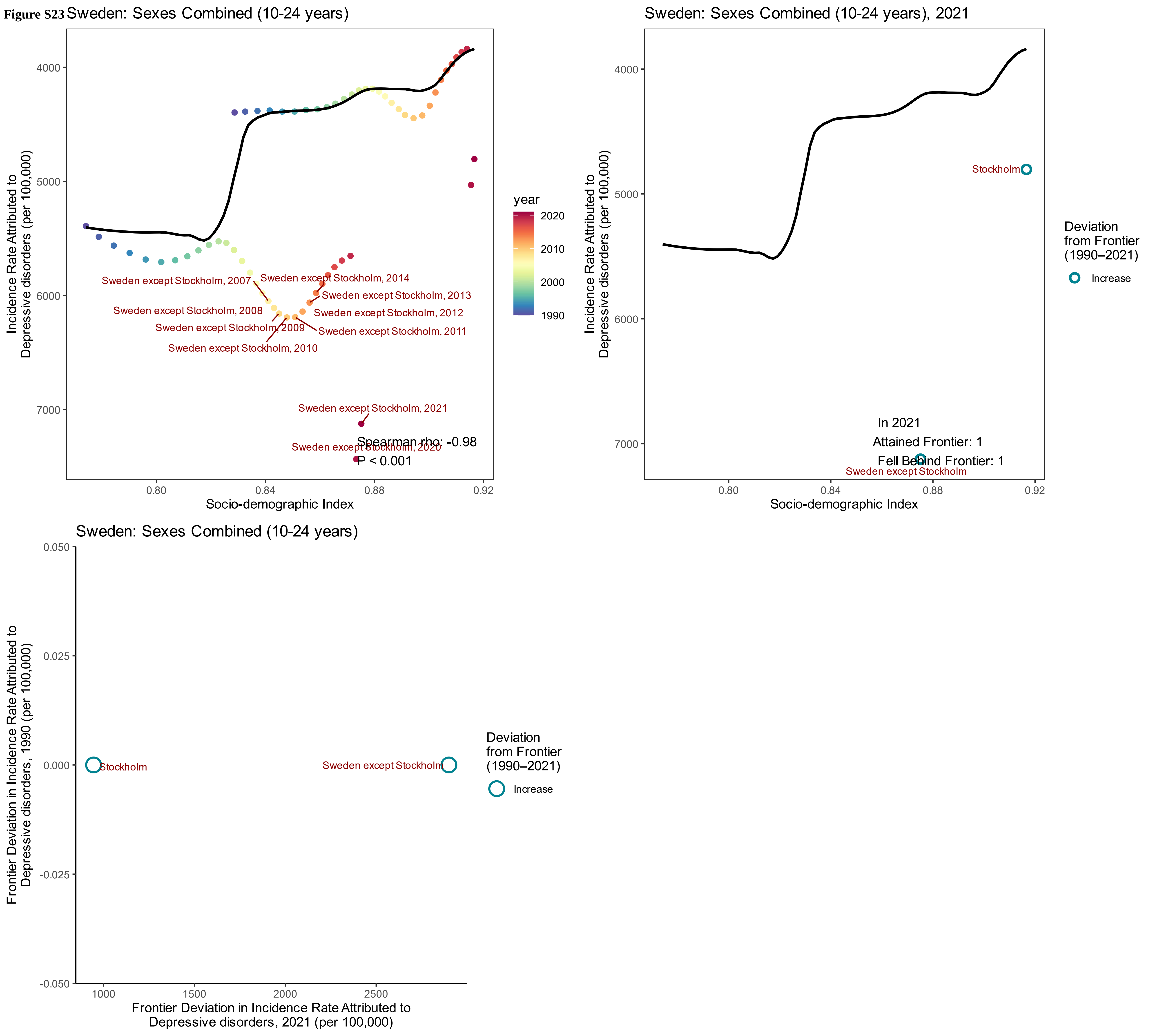

Sweden: Sexes Combined (10-24 years)
Incidence Rate Attributed to Depressive disorders (per 100,000)
Socio-demographic Index
year
2020
2010
2000
1990
Sweden except Stockholm, 2007
Sweden except Stockholm, 2008
Sweden except Stockholm, 2009
Sweden except Stockholm, 2010
Sweden except Stockholm, 2011
Sweden except Stockholm, 2012
Sweden except Stockholm, 2013
Sweden except Stockholm, 2014
Sweden except Stockholm, 2021
Sweden except Stockholm, 2020
Spearman rho: -0.98
P < 0.001
Sweden: Sexes Combined (10-24 years), 2021
Stockholm
In 2021
Attained Frontier: 1
Fell Behind Frontier: 1
Sweden except Stockholm
Deviation from Frontier (1990–2021)
Increase
Sweden: Sexes Combined (10-24 years)
Frontier Deviation in Incidence Rate Attributed to Depressive disorders, 1990 (per 100,000)
Frontier Deviation in Incidence Rate Attributed to Depressive disorders, 2021 (per 100,000)
Stockholm
Sweden except Stockholm
Deviation from Frontier (1990–2021)
Increase

Figure S23

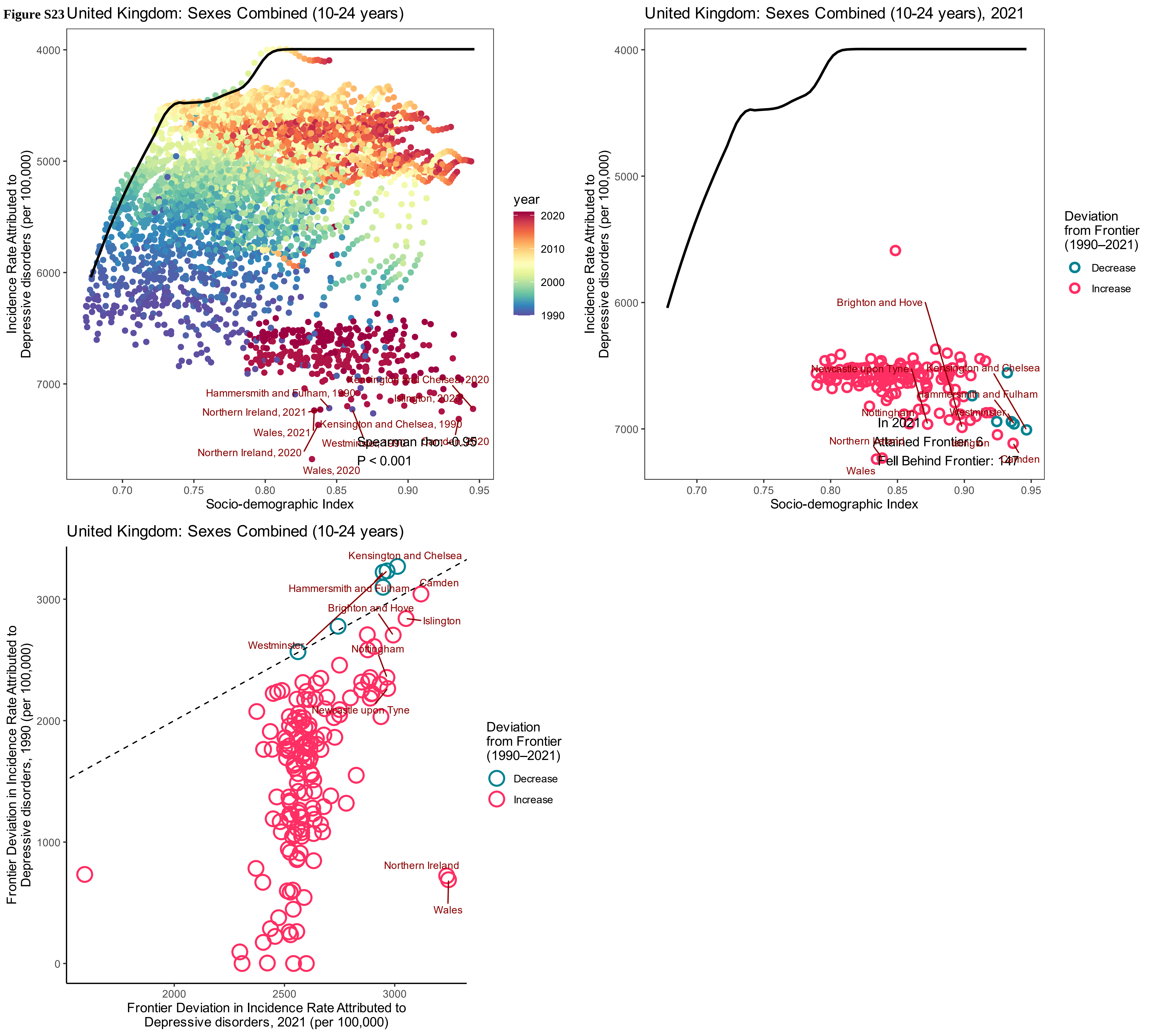

United Kingdom: Sexes Combined (10-24 years)
Incidence Rate Attributed to Depressive disorders (per 100,000)
Socio-demographic Index
year
2020
2010
2000
1990
Kensington and Chelsea, 2020
Hammersmith and Fulham, 1990
Islington, 2020
Northern Ireland, 2021
Kensington and Chelsea, 1990
Wales, 2021
Northern Ireland, 2020
Wales, 2020
Spearman rho: -0.95
P < 0.001
United Kingdom: Sexes Combined (10-24 years), 2021
Brighton and Hove
Newcastle upon Tyne
Kensington and Chelsea
Hammersmith and Fulham
Nottingham
Westminster
In 2021
Attained Frontier: 6
Fell Behind Frontier: 147
Camden
Wales
Deviation from Frontier (1990–2021)
Decrease
Increase
United Kingdom: Sexes Combined (10-24 years)
Frontier Deviation in Incidence Rate Attributed to Depressive disorders, 1990 (per 100,000)
Frontier Deviation in Incidence Rate Attributed to Depressive disorders, 2021 (per 100,000)
Kensington and Chelsea
Hammersmith and Fulham
Camden
Brighton and Hove
Islington
Westminster
Nottingham
Newcastle upon Tyne
Northern Ireland
Wales

Figure S23

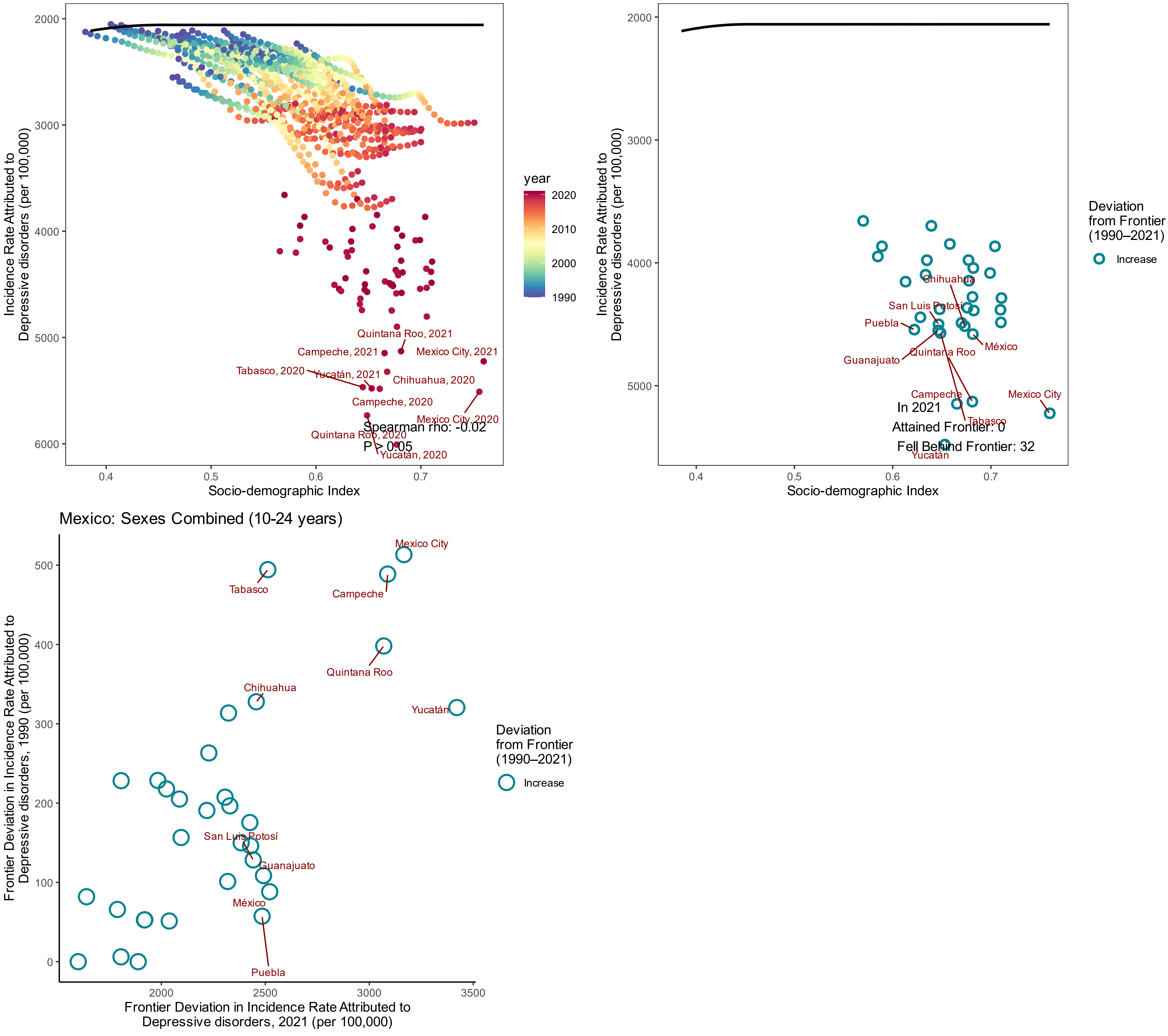

Mexico: Sexes Combined (10-24 years)
Incidence Rate Attributed to Depressive disorders (per 100,000)
Socio-demographic Index
year
2020
2010
2000
1990
Quintana Roo, 2021
Campeche, 2021
Mexico City, 2021
Tabasco, 2020
Yucatán, 2021
Chihuahua, 2020
Campeche, 2020
Mexico City, 2020
Spearman rho: -0.02
P > 0.05
Quintana Roo, 2020
Yucatán, 2020
Mexico: Sexes Combined (10-24 years), 2021
Incidence Rate Attributed to Depressive disorders (per 100,000)
Socio-demographic Index
Deviation from Frontier (1990–2021)
Increase
Chihuahua
San Luis Potosí
Puebla
México
Guanajuato
Quintana Roo
Campeche
Mexico City
In 2021
Attained Frontier: 0
Tabasco
Fell Behind Frontier: 32
Yucatán
Mexico: Sexes Combined (10-24 years)
Frontier Deviation in Incidence Rate Attributed to Depressive disorders, 1990 (per 100,000)
Frontier Deviation in Incidence Rate Attributed to Depressive disorders, 2021 (per 100,000)
Mexico City
Tabasco
Campeche
Quintana Roo
Chihuahua
Yucatán
Deviation from Frontier (1990–2021)
Increase
San Luis Potosí
Guanajuato
México
Puebla

Figure S23

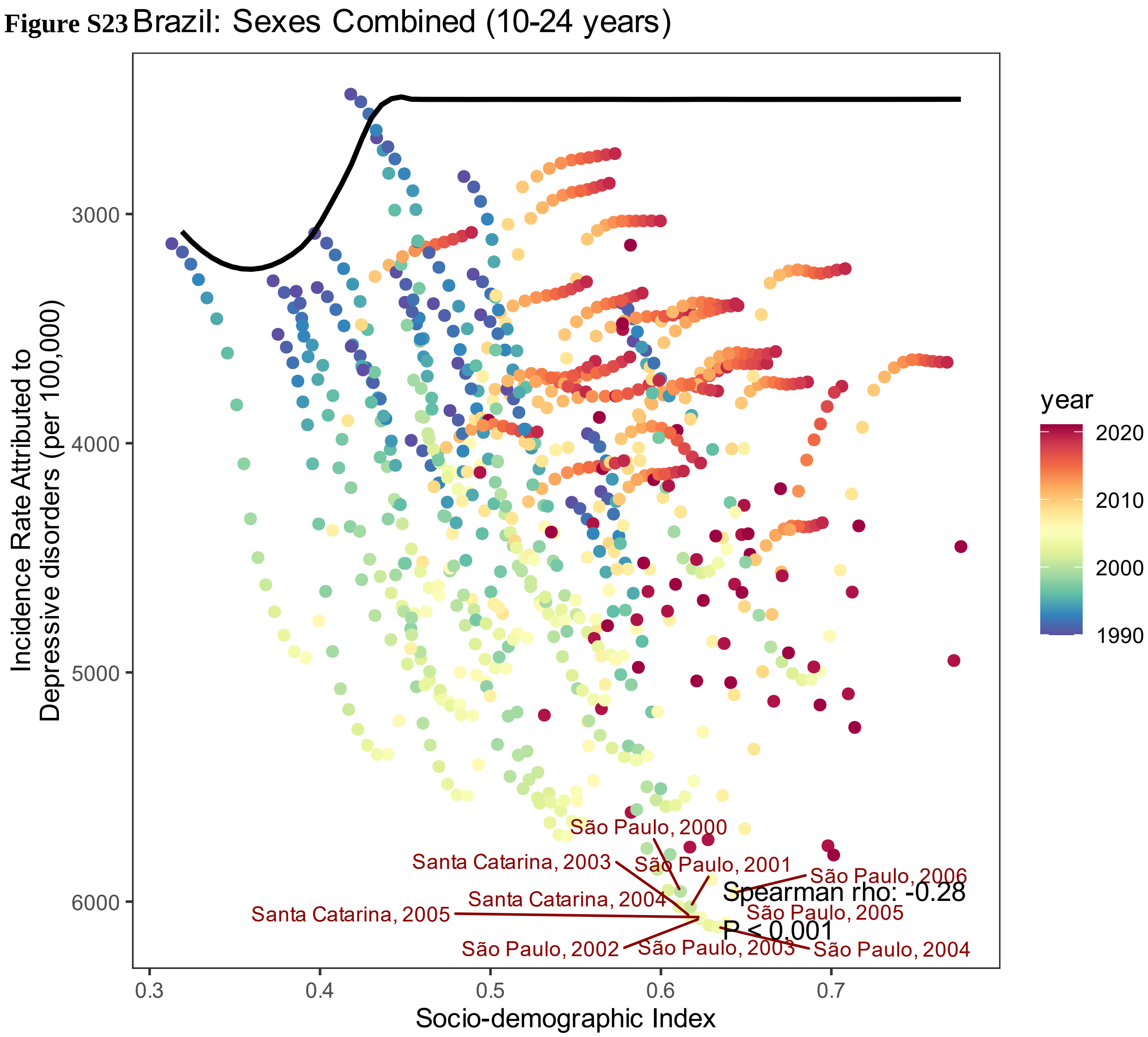
Brazil: Sexes Combined (10-24 years)
Incidence Rate Attributed to Depressive disorders (per 100,000)
Socio-demographic Index
year
2020
2010
2000
1990
São Paulo, 2000
Santa Catarina, 2003
São Paulo, 2001
São Paulo, 2006
Santa Catarina, 2004
Spearman rho: -0.28
Santa Catarina, 2005
São Paulo, 2005
P < 0.001
São Paulo, 2002
São Paulo, 2003
São Paulo, 2004

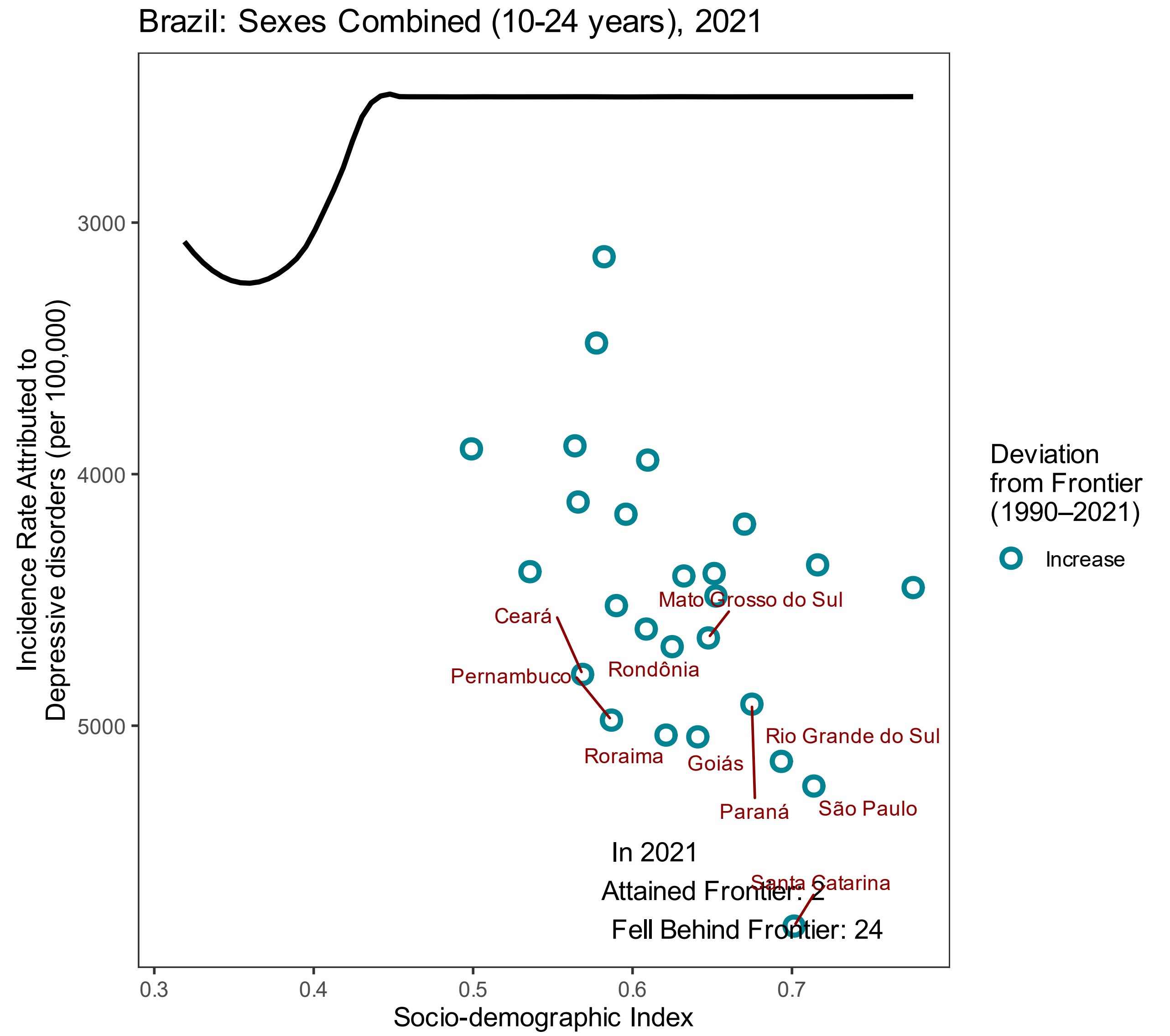
Brazil: Sexes Combined (10-24 years), 2021
Incidence Rate Attributed to Depressive disorders (per 100,000)
Socio-demographic Index
Deviation from Frontier (1990–2021)
Increase
Ceará
Mato Grosso do Sul
Pernambuco
Rondônia
Roraima
Goiás
Rio Grande do Sul
Paraná
São Paulo
In 2021
Attained Frontier: 2
Santa Catarina
Fell Behind Frontier: 24

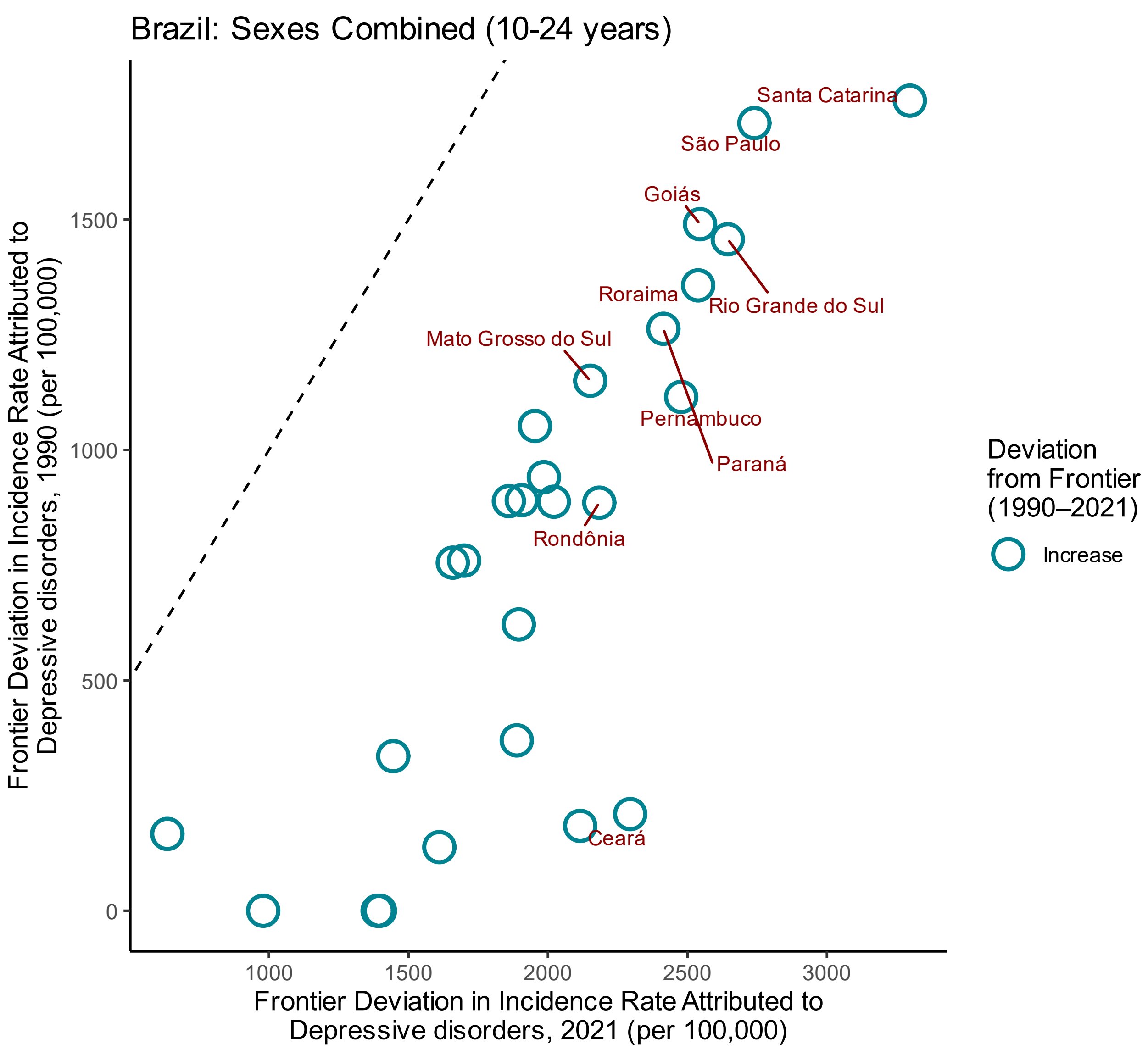
Brazil: Sexes Combined (10-24 years)
Frontier Deviation in Incidence Rate Attributed to Depressive disorders, 1990 (per 100,000)
Frontier Deviation in Incidence Rate Attributed to Depressive disorders, 2021 (per 100,000)
Santa Catarina
São Paulo
Goiás
Roraima
Rio Grande do Sul
Mato Grosso do Sul
Pernambuco
Paraná
Rondônia
Ceará
Deviation from Frontier (1990–2021)
Increase

Figure S23

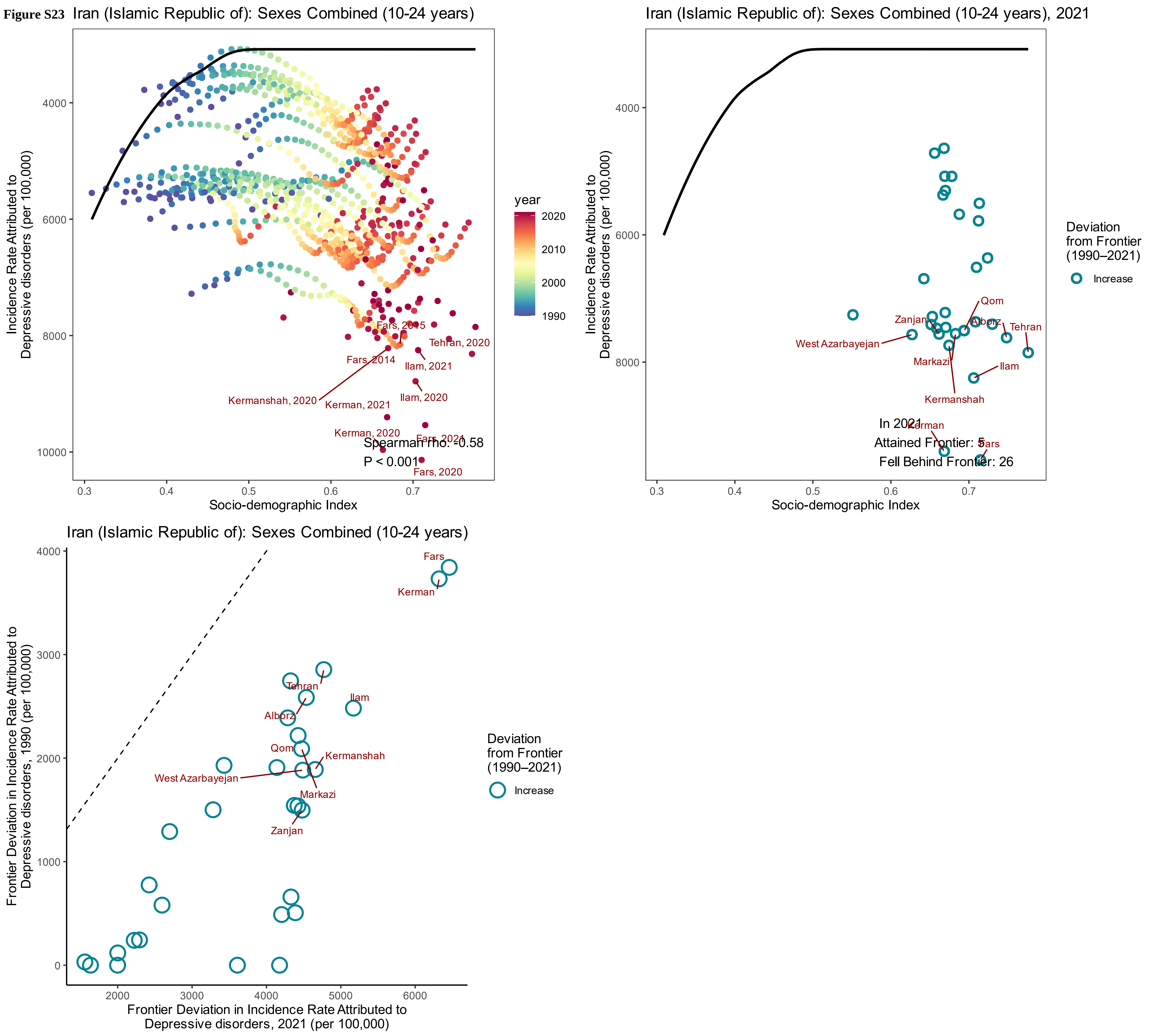

Iran (Islamic Republic of): Sexes Combined (10-24 years)
Incidence Rate Attributed to Depressive disorders (per 100,000)
Socio-demographic Index
year
2020
2010
2000
1990
Fars, 2015
Tehran, 2020
Fars, 2014
Ilam, 2021
Kermanshah, 2020
Ilam, 2020
Kerman, 2021
Kerman, 2020
Fars, 2021
Fars, 2020
Spearman rho: -0.58
P < 0.001
Iran (Islamic Republic of): Sexes Combined (10-24 years), 2021
Qom
Zanjan
Alborz
Tehran
West Azarbayejan
Markazi
Ilam
Kermanshah
In 2021
Kerman
Attained Frontier: 5
Fars
Fell Behind Frontier: 26
Deviation from Frontier (1990–2021)
Increase
Frontier Deviation in Incidence Rate Attributed to Depressive disorders, 1990 (per 100,000)
Frontier Deviation in Incidence Rate Attributed to Depressive disorders, 2021 (per 100,000)

Figure S23

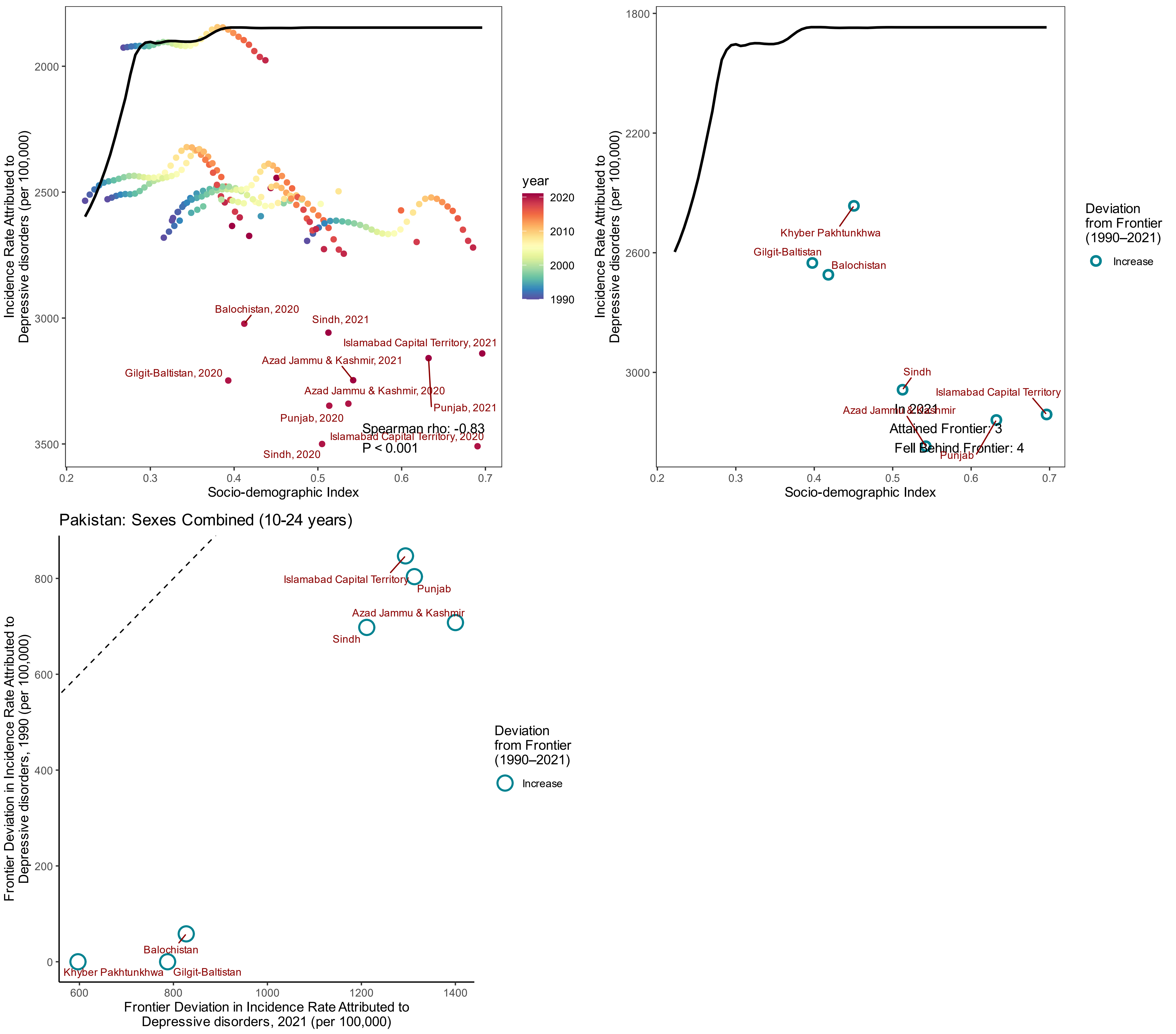
Pakistan: Sexes Combined (10-24 years)
Incidence Rate Attributed to Depressive disorders (per 100,000)
Socio-demographic Index
year
2020
2010
2000
1990
Balochistan, 2020
Sindh, 2021
Islamabad Capital Territory, 2021
Azad Jammu & Kashmir, 2021
Gilgit-Baltistan, 2020
Azad Jammu & Kashmir, 2020
Punjab, 2021
Punjab, 2020
Spearman rho: -0.83
P < 0.001
Islamabad Capital Territory, 2020
Sindh, 2020
Pakistan: Sexes Combined (10-24 years), 2021
Khyber Pakhtunkhwa
Gilgit-Baltistan
Balochistan
Sindh
Islamabad Capital Territory
Azad Jammu & Kashmir
In 2021
Attained Frontier: 3
Fell Behind Frontier: 4
Punjab
Deviation from Frontier (1990–2021)
Increase
Pakistan: Sexes Combined (10-24 years)
Frontier Deviation in Incidence Rate Attributed to Depressive disorders, 1990 (per 100,000)
Frontier Deviation in Incidence Rate Attributed to Depressive disorders, 2021 (per 100,000)
Islamabad Capital Territory
Punjab
Azad Jammu & Kashmir
Sindh
Balochistan
Khyber Pakhtunkhwa
Gilgit-Baltistan
Deviation from Frontier (1990–2021)
Increase

Figure S23

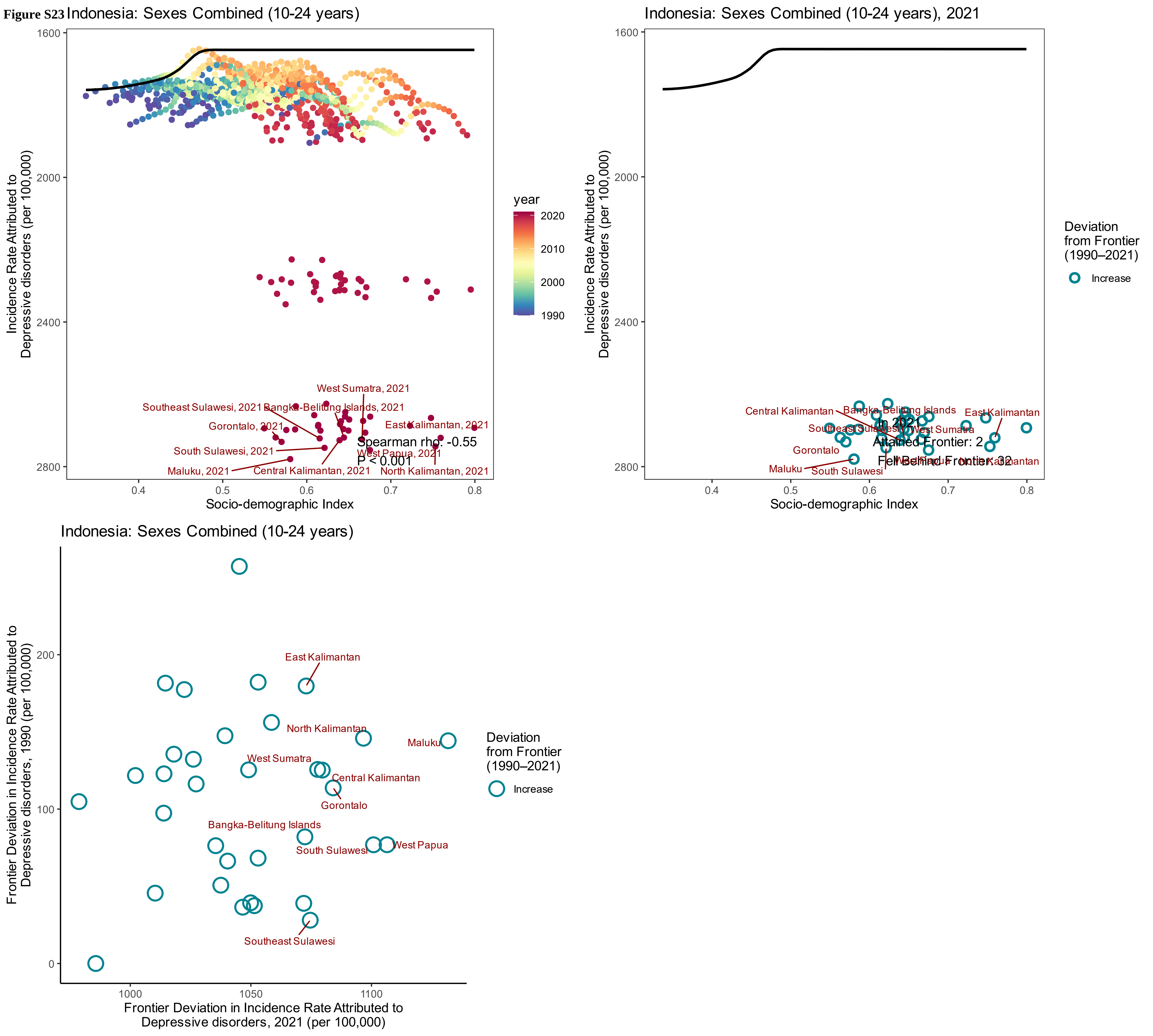

Indonesia: Sexes Combined (10-24 years)
Incidence Rate Attributed to Depressive disorders (per 100,000)
Socio-demographic Index
year
Spearman rho: -0.55
P < 0.001
West Sumatra, 2021
Southeast Sulawesi, 2021
Bangka-Belitung Islands, 2021
Gorontalo, 2021
East Kalimantan, 2021
South Sulawesi, 2021
West Papua, 2021
Maluku, 2021
Central Kalimantan, 2021
North Kalimantan, 2021
Indonesia: Sexes Combined (10-24 years), 2021
Deviation from Frontier (1990–2021)
Increase
In 2021
Attained Frontier: 2
Fell Behind Frontier: 32
Frontier Deviation in Incidence Rate Attributed to Depressive disorders, 1990 (per 100,000)
Frontier Deviation in Incidence Rate Attributed to Depressive disorders, 2021 (per 100,000)

Figure S23

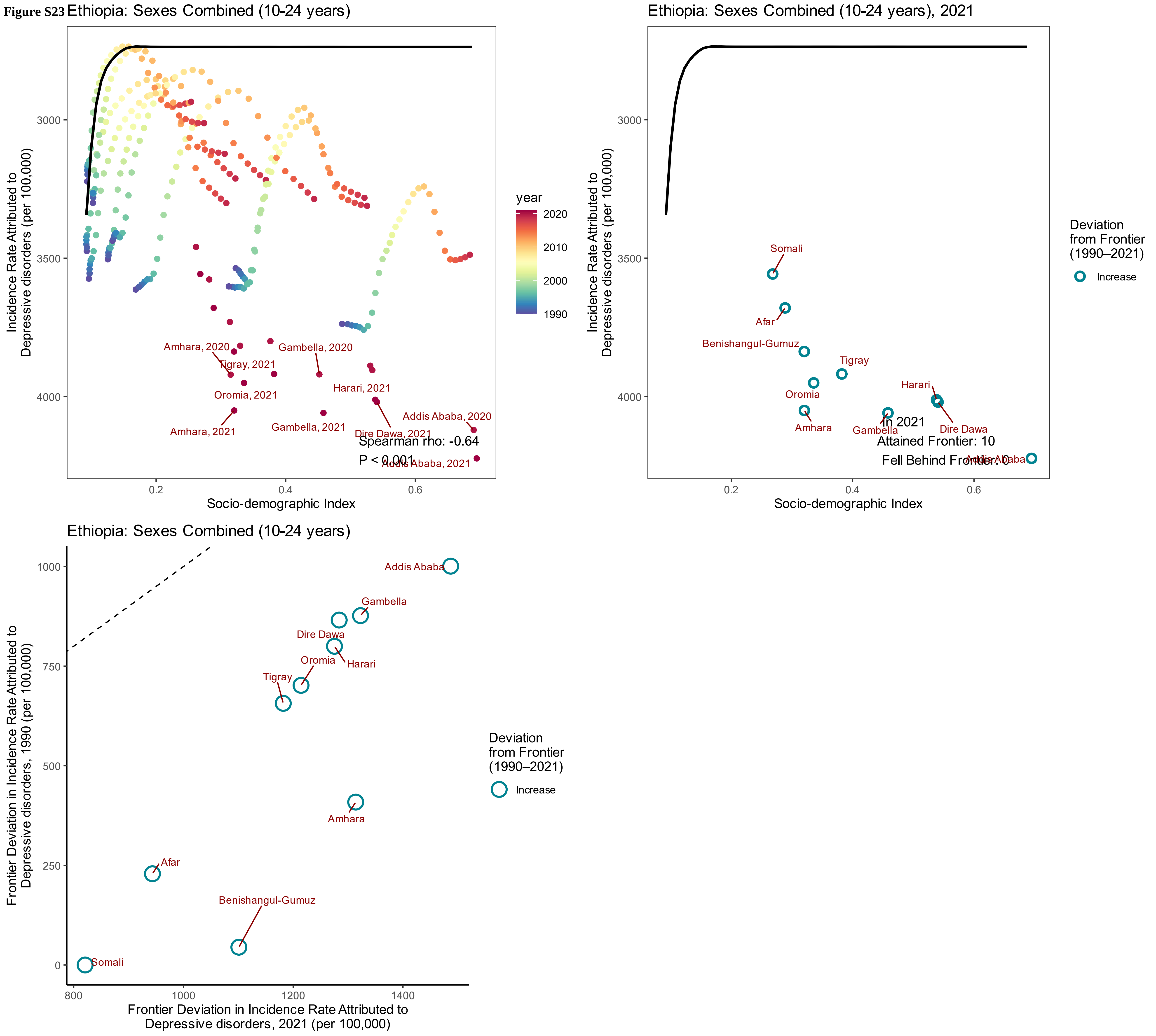
Ethiopia: Sexes Combined (10-24 years)
Incidence Rate Attributed to Depressive disorders (per 100,000)
Socio-demographic Index
year
2020
2010
2000
1990
Amhara, 2020
Gambella, 2020
Tigray, 2021
Harari, 2021
Oromia, 2021
Addis Ababa, 2020
Amhara, 2021
Gambella, 2021
Dire Dawa, 2021
Spearman rho: -0.64
P < 0.001
Addis Ababa, 2021
Ethiopia: Sexes Combined (10-24 years), 2021
Somali
Afar
Benishangul-Gumuz
Tigray
Oromia
Harari
Amhara
Gambella
Dire Dawa
In 2021
Attained Frontier: 10
Fell Behind Frontier: 0
Addis Ababa
Deviation from Frontier (1990–2021)
Increase
Ethiopia: Sexes Combined (10-24 years)
Frontier Deviation in Incidence Rate Attributed to Depressive disorders, 1990 (per 100,000)
Frontier Deviation in Incidence Rate Attributed to Depressive disorders, 2021 (per 100,000)
Addis Ababa
Gambella
Dire Dawa
Harari
Oromia
Tigray
Amhara
Afar
Benishangul-Gumuz
Somali
Deviation from Frontier (1990–2021)
Increase

Figure S23

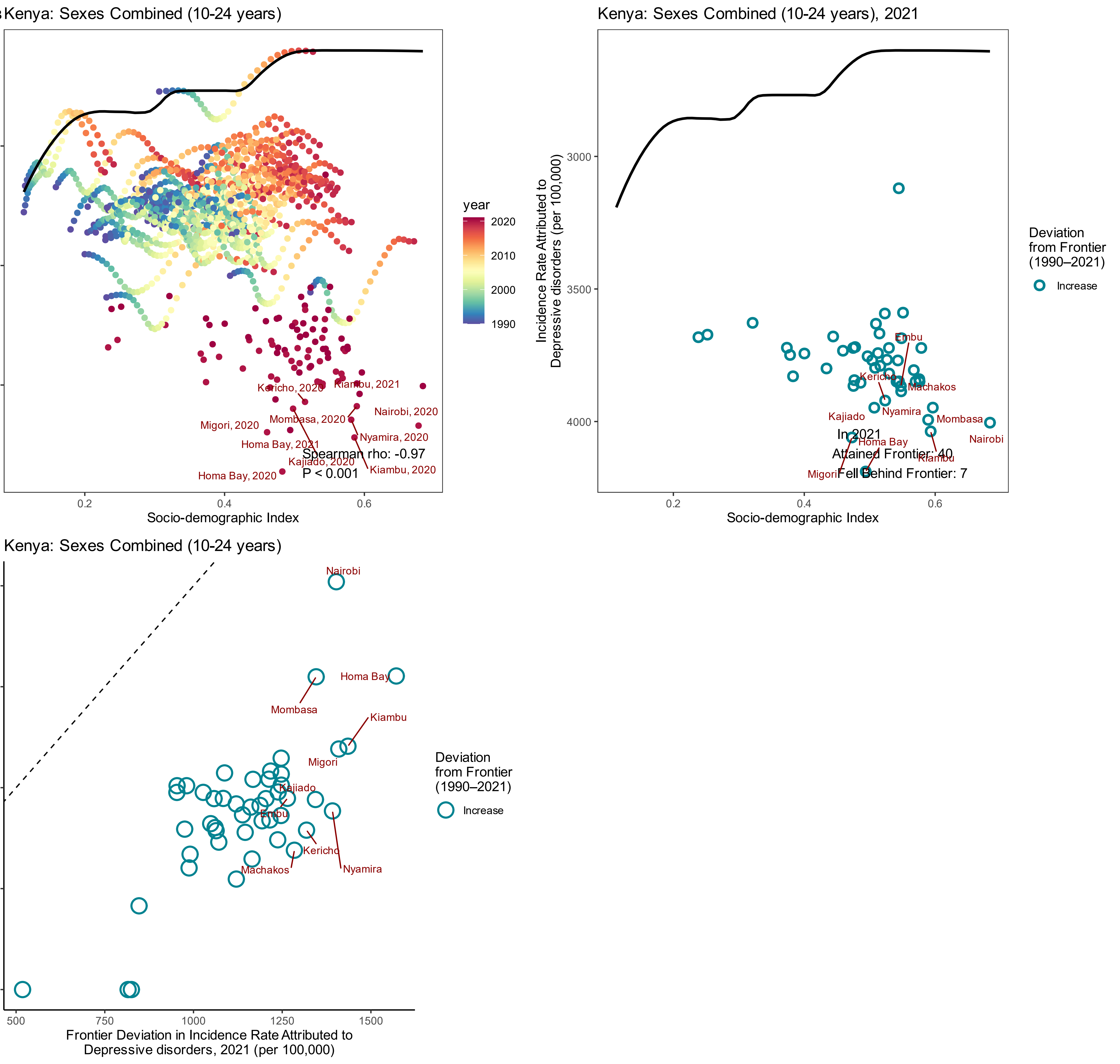

Kenya: Sexes Combined (10-24 years)
Incidence Rate Attributed to Depressive disorders (per 100,000)
Socio-demographic Index
year
2020
2010
2000
1990
Kericho, 2020
Kiambu, 2021
Nairobi, 2020
Migori, 2020
Mombasa, 2020
Nyamira, 2020
Homa Bay, 2021
Kajiado, 2020
Homa Bay, 2020
Kiambu, 2020
Spearman rho: -0.97
P < 0.001
Kenya: Sexes Combined (10-24 years), 2021
Deviation from Frontier (1990–2021)
Increase
Embu
Kericho
Machakos
Kajiado
Nyamira
Mombasa
Nairobi
In 2021
Homa Bay
Attained Frontier: 40
Kiambu
Migori
Fell Behind Frontier: 7
Kenya: Sexes Combined (10-24 years)
Frontier Deviation in Incidence Rate Attributed to Depressive disorders, 1990 (per 100,000)
Frontier Deviation in Incidence Rate Attributed to Depressive disorders, 2021 (per 100,000)
Nairobi
Homa Bay
Mombasa
Kiambu
Migori
Kajiado
Embu
Kericho
Machakos
Nyamira
Deviation from Frontier (1990–2021)
Increase

Figure S23

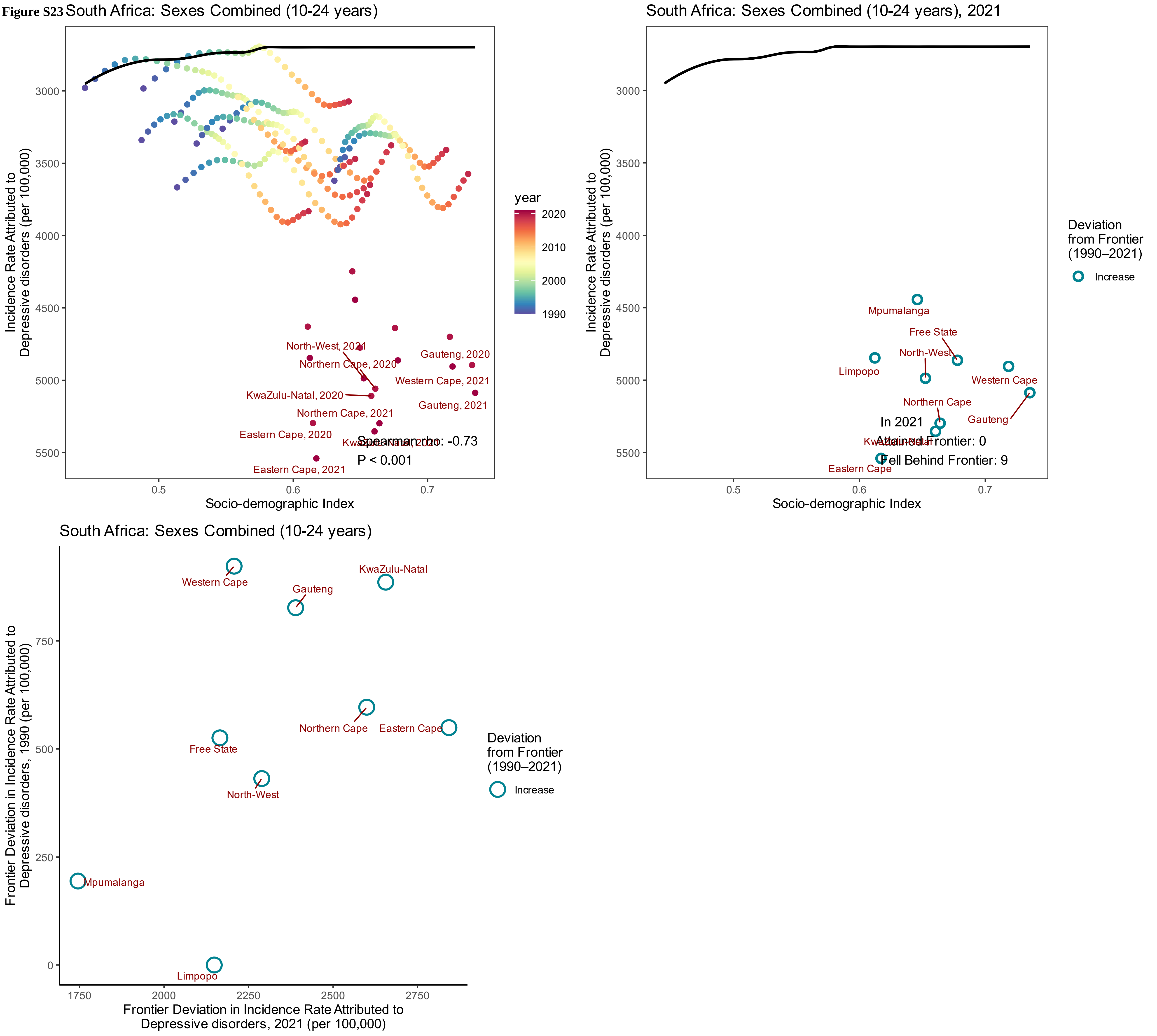
South Africa: Sexes Combined (10-24 years)
Incidence Rate Attributed to Depressive disorders (per 100,000)
Socio-demographic Index
year
2020
2010
2000
1990
North-West, 2021
Northern Cape, 2020
Gauteng, 2020
Western Cape, 2021
KwaZulu-Natal, 2020
Gauteng, 2021
Northern Cape, 2021
Eastern Cape, 2020
KwaZulu-Natal, 2021
Spearman rho: -0.73
P < 0.001
Eastern Cape, 2021
South Africa: Sexes Combined (10-24 years), 2021
Mpumalanga
Free State
North-West
Limpopo
Western Cape
Northern Cape
Gauteng
In 2021
Attained Frontier: 0
Fell Behind Frontier: 9
Eastern Cape
Deviation from Frontier (1990–2021)
Increase
South Africa: Sexes Combined (10-24 years)
Frontier Deviation in Incidence Rate Attributed to Depressive disorders, 1990 (per 100,000)
Frontier Deviation in Incidence Rate Attributed to Depressive disorders, 2021 (per 100,000)
Western Cape
KwaZulu-Natal
Gauteng
Northern Cape
Eastern Cape
Free State
North-West
Mpumalanga
Limpopo
Deviation from Frontier (1990–2021)
Increase

Figure S23

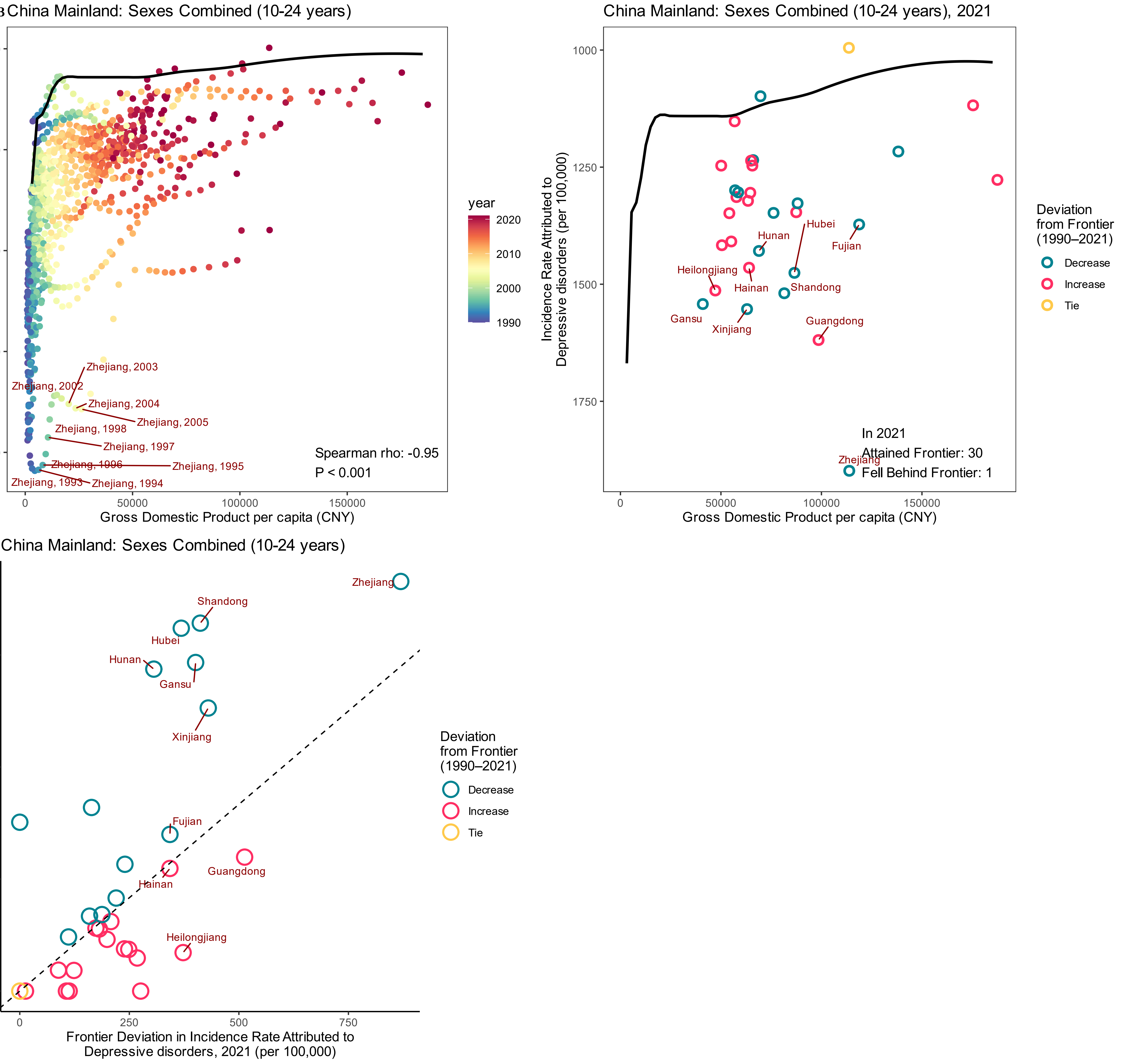

China Mainland: Sexes Combined (10-24 years)
Incidence Rate Attributed to Depressive disorders (per 100,000)
Gross Domestic Product per capita (CNY)
year
Spearman rho: -0.95
P < 0.001
Zhejiang, 2003
Zhejiang, 2002
Zhejiang, 2004
Zhejiang, 2005
Zhejiang, 1998
Zhejiang, 1997
Zhejiang, 1996
Zhejiang, 1995
Zhejiang, 1993
Zhejiang, 1994
China Mainland: Sexes Combined (10-24 years), 2021
Deviation from Frontier (1990–2021)
Decrease
Increase
Tie
In 2021
Attained Frontier: 30
Fell Behind Frontier: 1
Hubei
Hunan
Fujian
Heilongjiang
Hainan
Shandong
Gansu
Xinjiang
Guangdong
Zhejiang
Frontier Deviation in Incidence Rate Attributed to Depressive disorders, 1990 (per 100,000)
Frontier Deviation in Incidence Rate Attributed to Depressive disorders, 2021 (per 100,000)

Figure S23

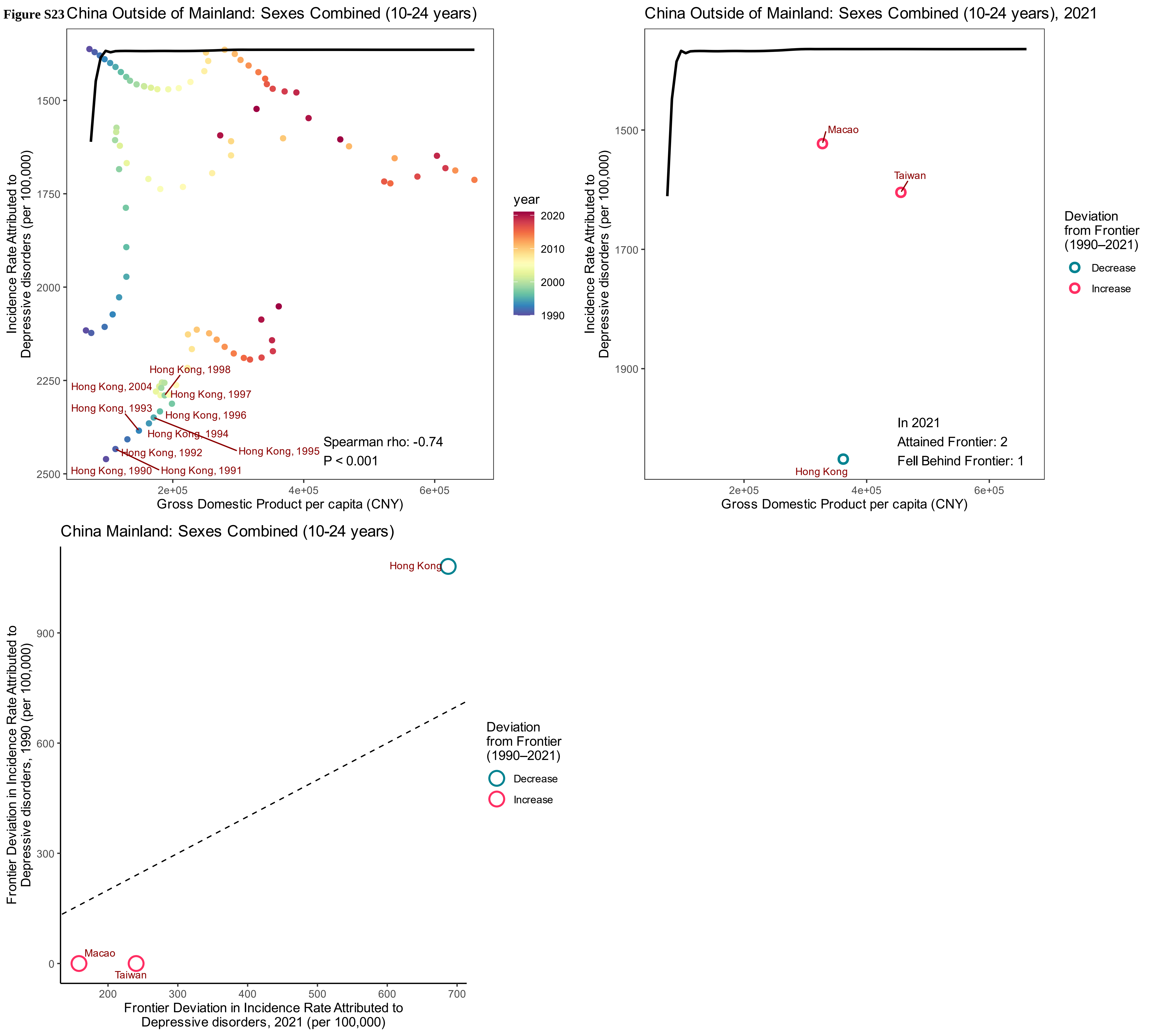
China Outside of Mainland: Sexes Combined (10-24 years)
Incidence Rate Attributed to Depressive disorders (per 100,000)
Gross Domestic Product per capita (CNY)
year
2020
2010
2000
1990
Hong Kong, 1998
Hong Kong, 2004
Hong Kong, 1997
Hong Kong, 1993
Hong Kong, 1996
Hong Kong, 1994
Hong Kong, 1995
Hong Kong, 1992
Hong Kong, 1990
Hong Kong, 1991
Spearman rho: -0.74
P < 0.001
China Outside of Mainland: Sexes Combined (10-24 years), 2021
Macao
Taiwan
Hong Kong
In 2021
Attained Frontier: 2
Fell Behind Frontier: 1
Deviation from Frontier (1990–2021)
Decrease
Increase
China Mainland: Sexes Combined (10-24 years)
Frontier Deviation in Incidence Rate Attributed to Depressive disorders, 1990 (per 100,000)
Frontier Deviation in Incidence Rate Attributed to Depressive disorders, 2021 (per 100,000)
Hong Kong
Macao
Taiwan
Deviation from Frontier (1990–2021)
Decrease
Increase

Figure S23

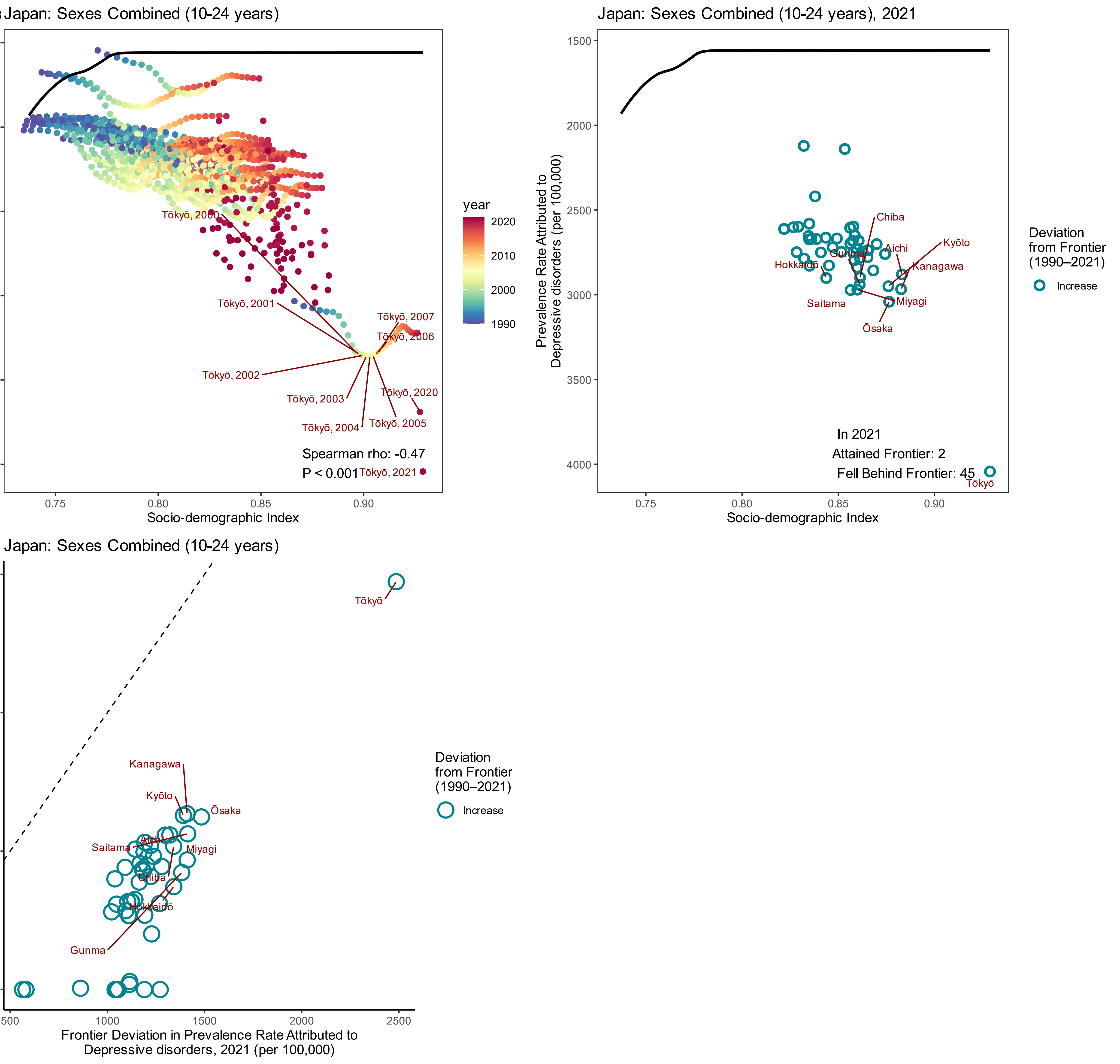
Japan: Sexes Combined (10-24 years)
Prevalence Rate Attributed to Depressive disorders (per 100,000)
Socio-demographic Index
year
2020
2010
2000
1990
Tōkyō, 2000
Tōkyō, 2001
Tōkyō, 2002
Tōkyō, 2003
Tōkyō, 2004
Tōkyō, 2005
Tōkyō, 2006
Tōkyō, 2007
Tōkyō, 2020
Tōkyō, 2021
Spearman rho: -0.47
P < 0.001
Japan: Sexes Combined (10-24 years), 2021
Chiba
Kyōto
Aichi
Kanagawa
Gunma
Hokkaidō
Saitama
Miyagi
Ōsaka
Tōkyō
In 2021
Attained Frontier: 2
Fell Behind Frontier: 45
Deviation from Frontier (1990–2021)
Increase
Japan: Sexes Combined (10-24 years)
Frontier Deviation in Prevalence Rate Attributed to Depressive disorders, 1990 (per 100,000)
Frontier Deviation in Prevalence Rate Attributed to Depressive disorders, 2021 (per 100,000)
Tōkyō
Kanagawa
Kyōto
Ōsaka
Saitama
Aichi
Miyagi
Chiba
Hokkaidō
Gunma
Deviation from Frontier (1990–2021)
Increase

Figure S23

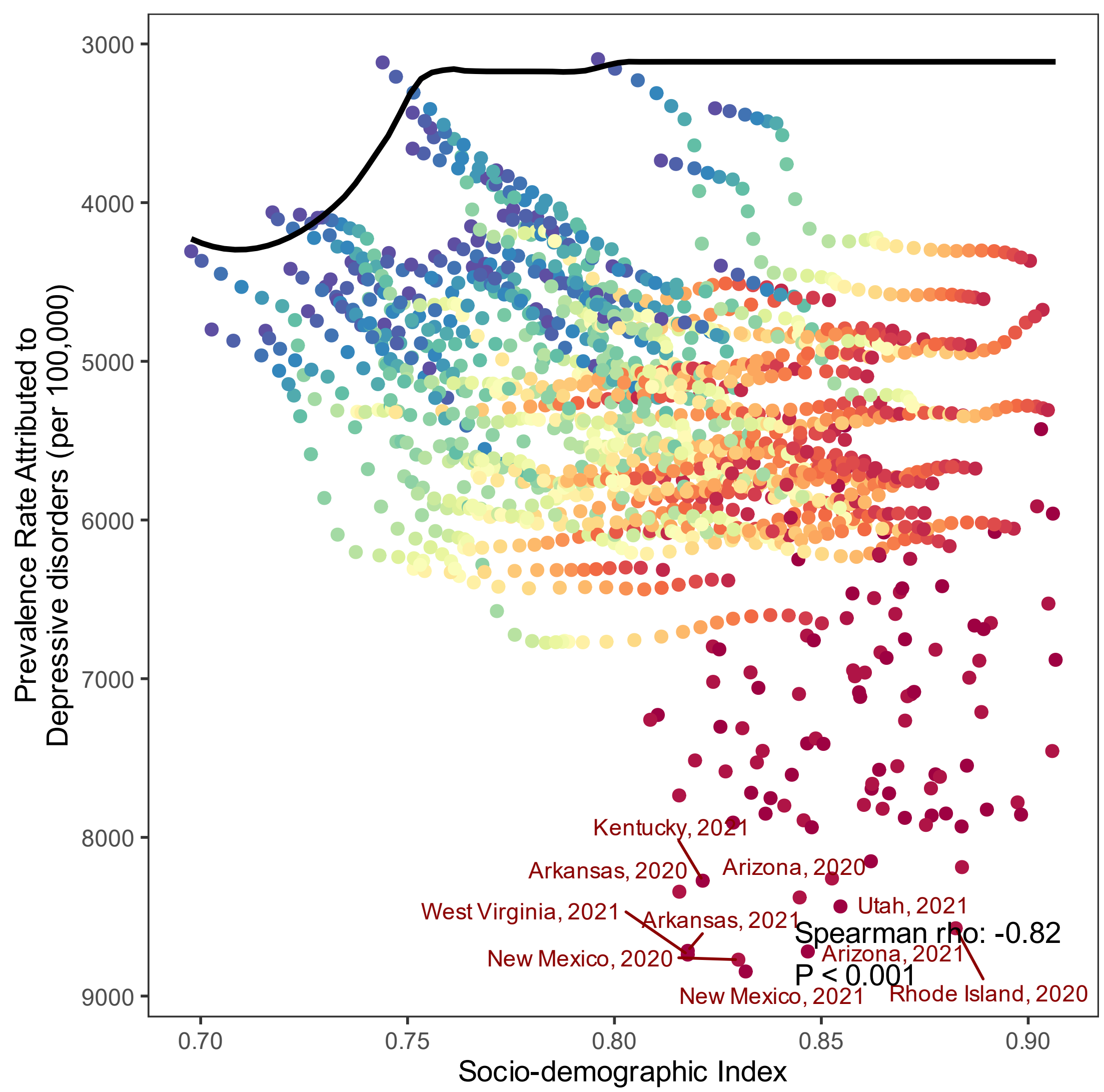

United States of America: Sexes Combined (10-24 years)
Prevalence Rate Attributed to Depressive disorders (per 100,000)
Socio-demographic Index
year
2020
2010
2000
1990
Kentucky, 2021
Arkansas, 2020
Arizona, 2020
Utah, 2021
West Virginia, 2021
Arkansas, 2021
Spearman rho: -0.82
P < 0.001
New Mexico, 2020
Arizona, 2021
New Mexico, 2021
Rhode Island, 2020

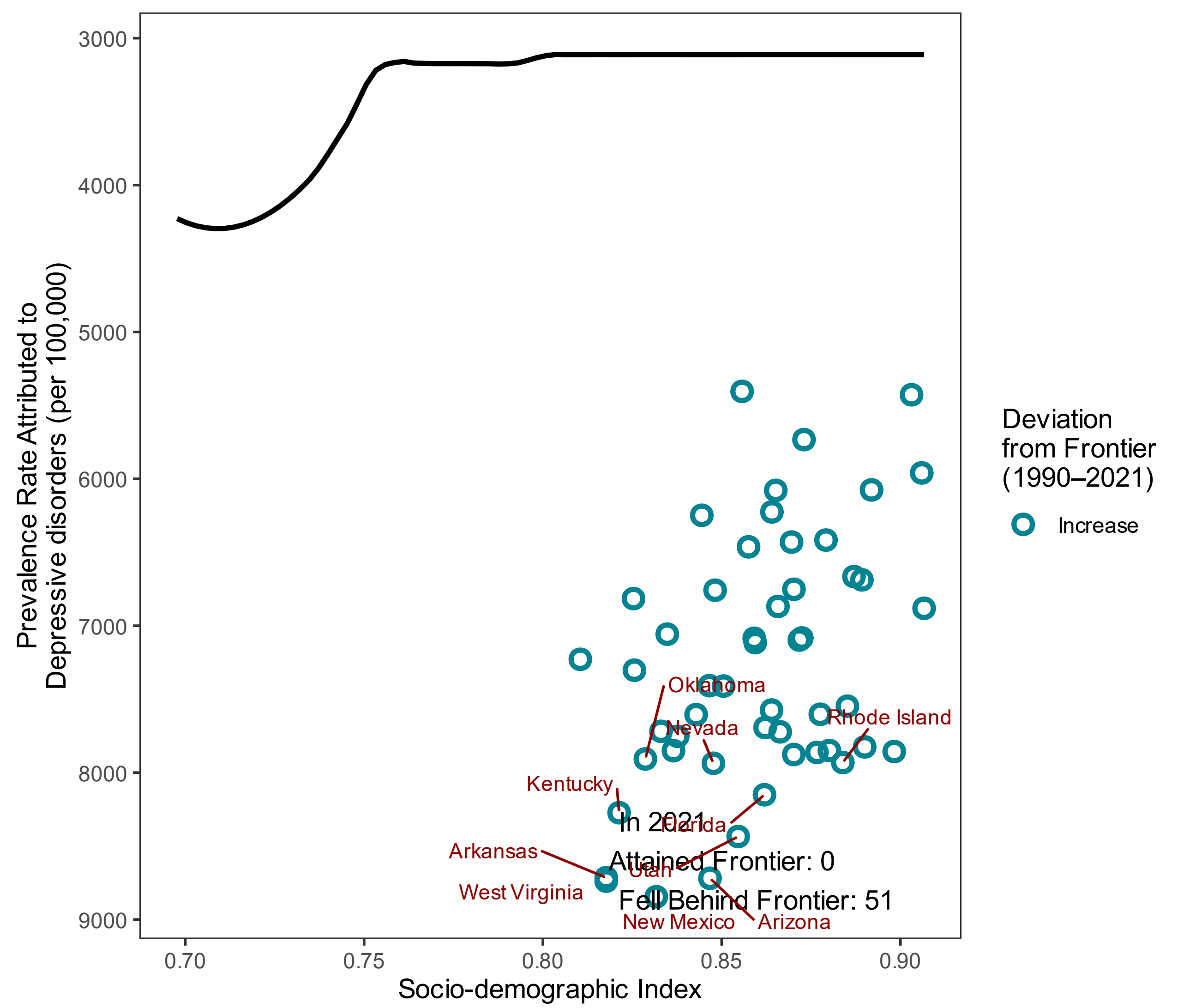

United States of America: Sexes Combined (10-24 years), 2021
Prevalence Rate Attributed to Depressive disorders (per 100,000)
Socio-demographic Index
Deviation from Frontier (1990–2021)
Increase
Oklahoma
Nevada
Rhode Island
Kentucky
In 2021
Florida
Arkansas
Utah
Attained Frontier: 0
West Virginia
Fell Behind Frontier: 51
New Mexico
Arizona

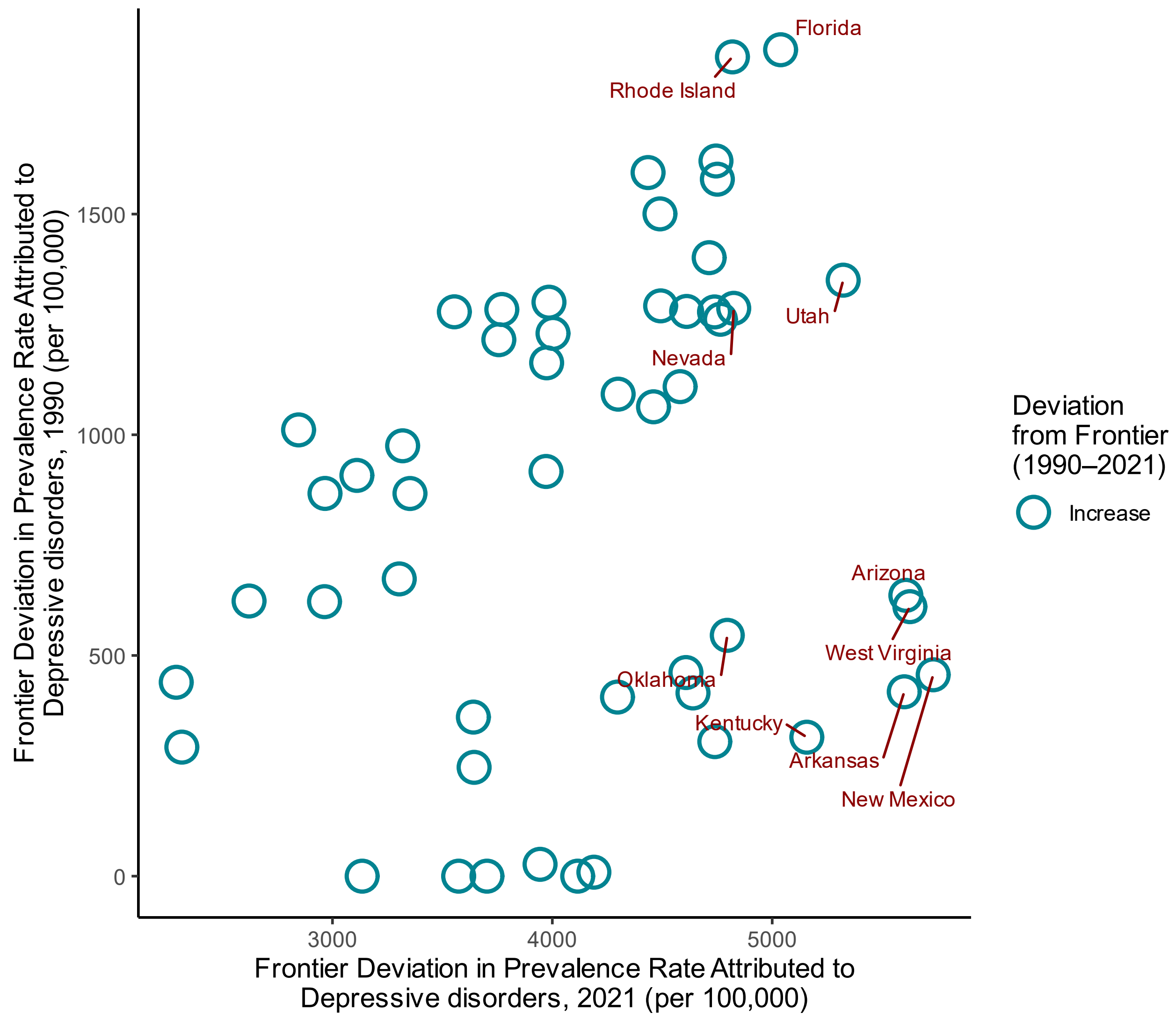

United States of America: Sexes Combined (10-24 years)
Frontier Deviation in Prevalence Rate Attributed to Depressive disorders, 1990 (per 100,000)
Frontier Deviation in Prevalence Rate Attributed to Depressive disorders, 2021 (per 100,000)
Deviation from Frontier (1990–2021)
Increase
Florida
Rhode Island
Utah
Nevada
Arizona
West Virginia
Oklahoma
Kentucky
Arkansas
New Mexico

Figure S23

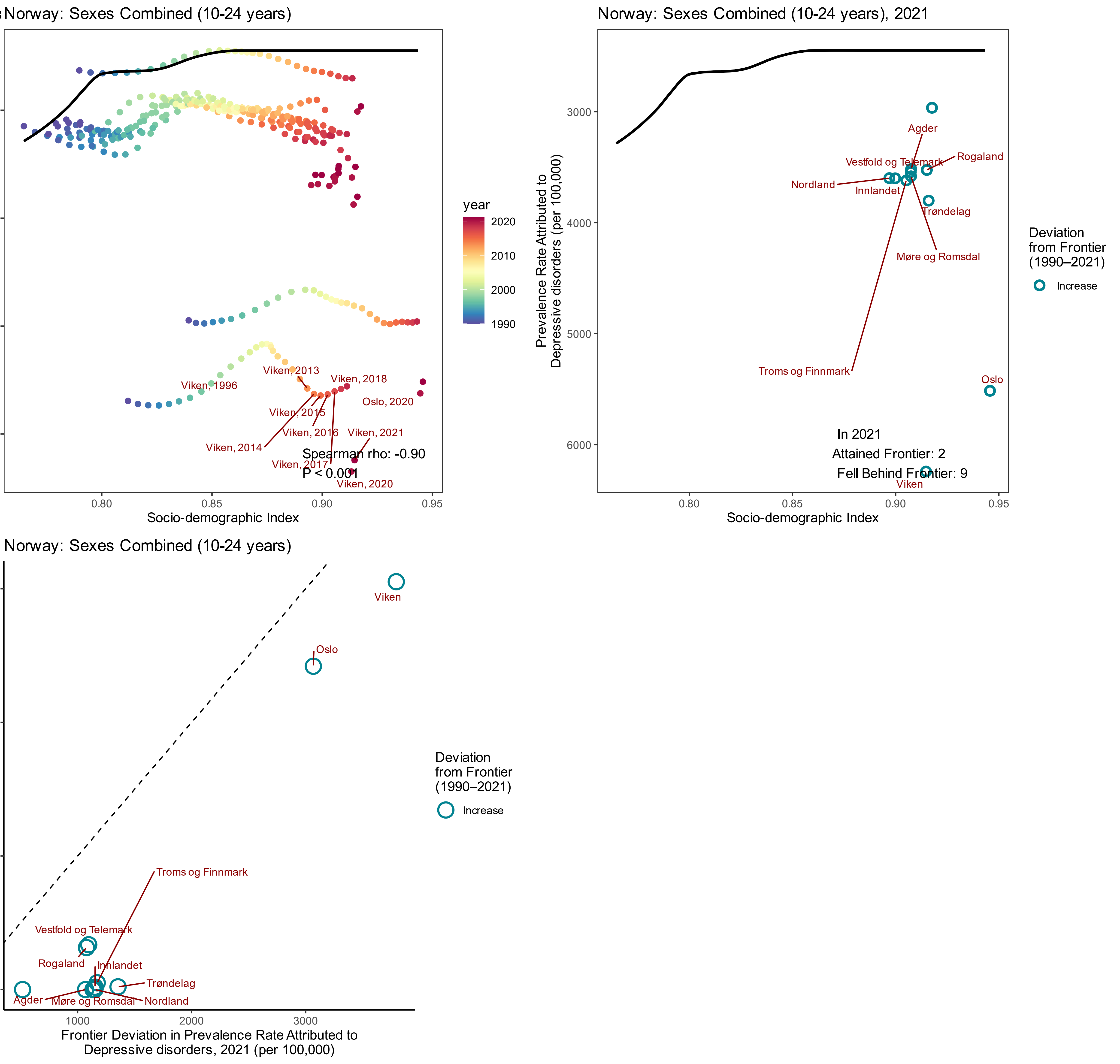

Norway: Sexes Combined (10-24 years)
Prevalence Rate Attributed to Depressive disorders (per 100,000)
Socio-demographic Index
year
2020
2010
2000
1990
Viken, 2013
Viken, 2018
Viken, 1996
Oslo, 2020
Viken, 2015
Viken, 2016
Viken, 2021
Viken, 2014
Spearman rho: -0.90
Viken, 2017
P < 0.001
Viken, 2020
Norway: Sexes Combined (10-24 years), 2021
Agder
Vestfold og Telemark
Rogaland
Nordland
Innlandet
Trøndelag
Møre og Romsdal
Troms og Finnmark
Oslo
In 2021
Attained Frontier: 2
Fell Behind Frontier: 9
Viken
Deviation from Frontier (1990–2021)
Increase
Norway: Sexes Combined (10-24 years)
Frontier Deviation in Prevalence Rate Attributed to Depressive disorders, 1990 (per 100,000)
Frontier Deviation in Prevalence Rate Attributed to Depressive disorders, 2021 (per 100,000)
Viken
Oslo
Troms og Finnmark
Vestfold og Telemark
Rogaland
Innlandet
Trøndelag
Agder
Møre og Romsdal
Nordland
Deviation from Frontier (1990–2021)
Increase

Figure S23

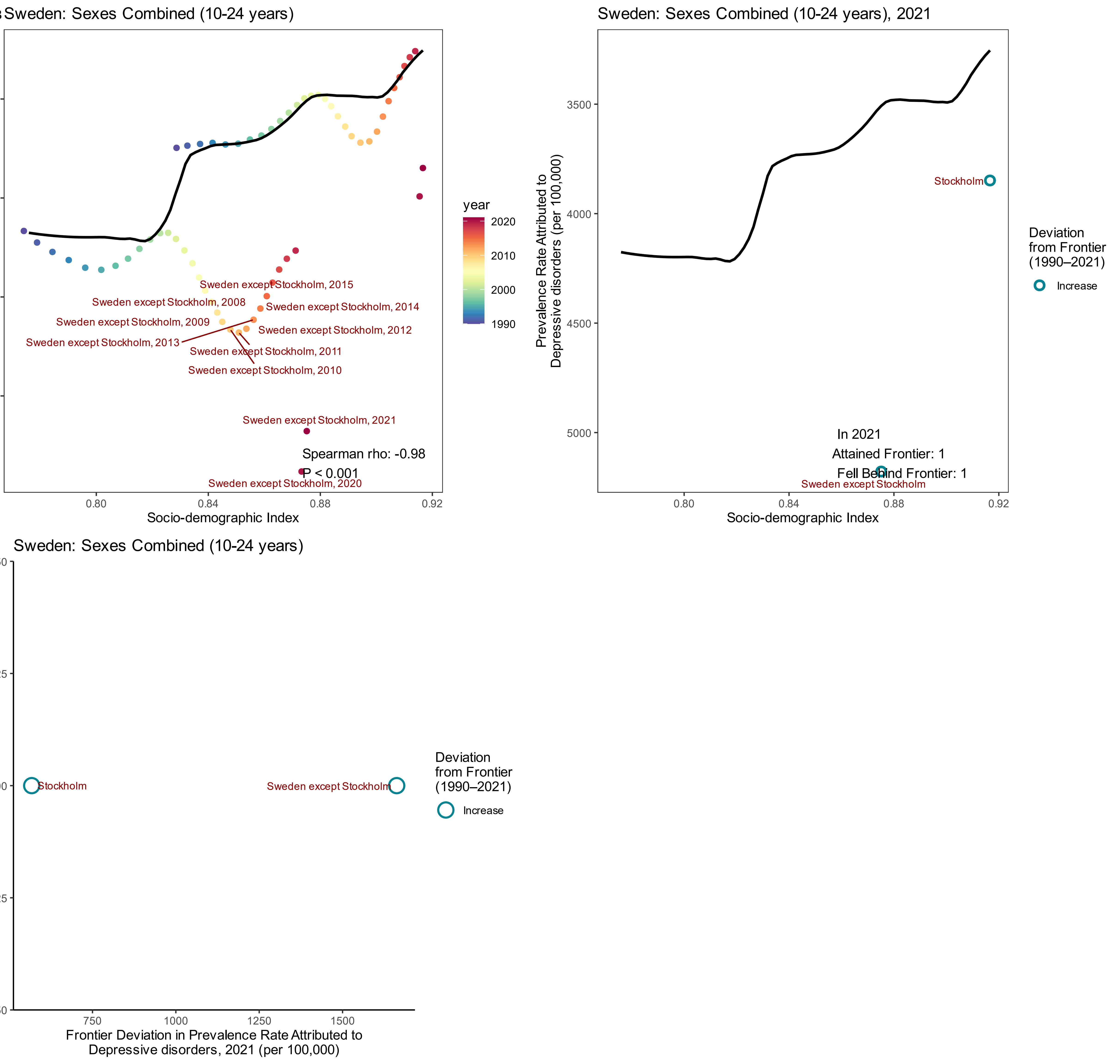
Sweden: Sexes Combined (10-24 years)
Prevalence Rate Attributed to Depressive disorders (per 100,000)
Socio-demographic Index
year
2020
2010
2000
1990
Sweden except Stockholm, 2015
Sweden except Stockholm, 2008
Sweden except Stockholm, 2014
Sweden except Stockholm, 2009
Sweden except Stockholm, 2012
Sweden except Stockholm, 2013
Sweden except Stockholm, 2011
Sweden except Stockholm, 2010
Sweden except Stockholm, 2021
Spearman rho: -0.98
P < 0.001
Sweden except Stockholm, 2020
Sweden: Sexes Combined (10-24 years), 2021
Stockholm
Deviation from Frontier (1990–2021)
Increase
In 2021
Attained Frontier: 1
Fell Behind Frontier: 1
Sweden except Stockholm
Sweden: Sexes Combined (10-24 years)
Frontier Deviation in Prevalence Rate Attributed to Depressive disorders, 1990 (per 100,000)
Frontier Deviation in Prevalence Rate Attributed to Depressive disorders, 2021 (per 100,000)
Stockholm
Sweden except Stockholm
Deviation from Frontier (1990–2021)
Increase

Figure S23

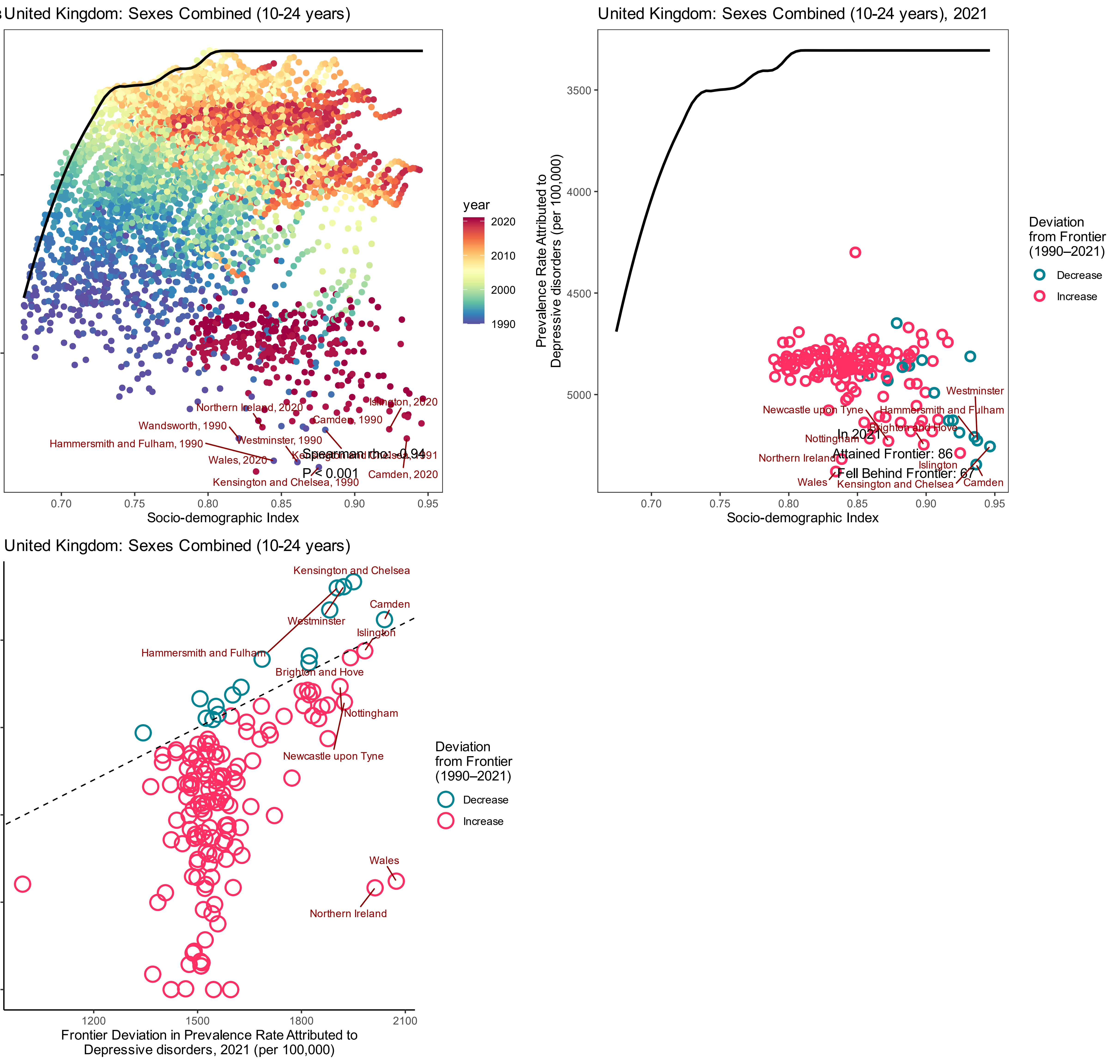
United Kingdom: Sexes Combined (10-24 years)
Prevalence Rate Attributed to Depressive disorders (per 100,000)
Socio-demographic Index
year
2020
2010
2000
1990
Northern Ireland, 2020
Islington, 2020
Camden, 1990
Wandsworth, 1990
Westminster, 1990
Hammersmith and Fulham, 1990
Wales, 2020
Kensington and Chelsea, 1991
Spearman rho: -0.94
P < 0.001
Camden, 2020
Kensington and Chelsea, 1990
United Kingdom: Sexes Combined (10-24 years), 2021
Deviation from Frontier (1990–2021)
Decrease
Increase
Westminster
Newcastle upon Tyne
Hammersmith and Fulham
Brighton and Hove
Nottingham
In 2021
Northern Ireland
Attained Frontier: 86
Islington
Fell Behind Frontier: 67
Wales
Kensington and Chelsea
Camden
United Kingdom: Sexes Combined (10-24 years)
Frontier Deviation in Prevalence Rate Attributed to Depressive disorders, 1990 (per 100,000)
Frontier Deviation in Prevalence Rate Attributed to Depressive disorders, 2021 (per 100,000)
Kensington and Chelsea
Camden
Westminster
Islington
Hammersmith and Fulham
Brighton and Hove
Nottingham
Newcastle upon Tyne
Wales
Northern Ireland

Figure S23

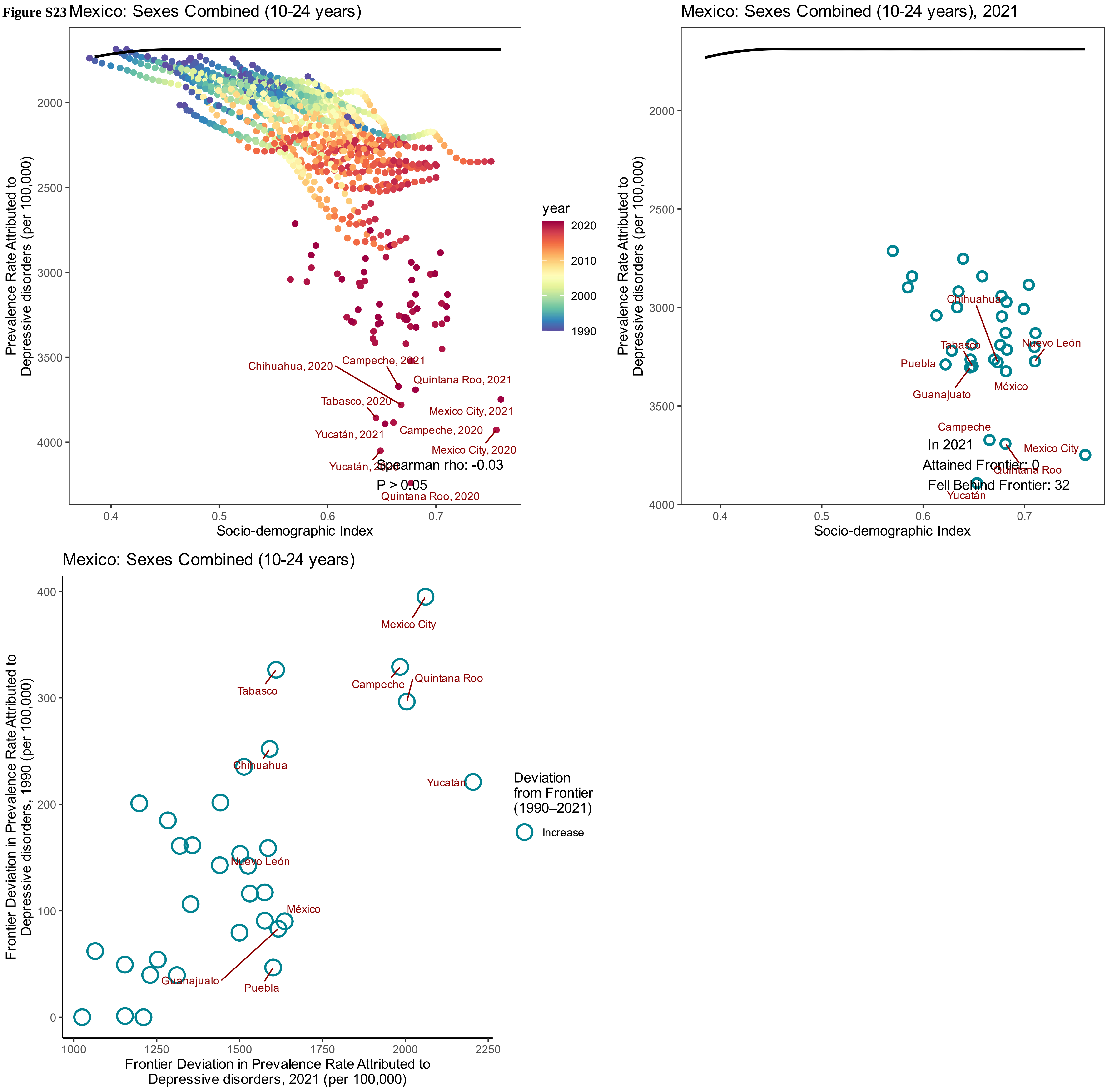

Mexico: Sexes Combined (10-24 years)
Prevalence Rate Attributed to Depressive disorders (per 100,000)
Socio-demographic Index
year
Spearman rho: -0.03
P > 0.05
Chihuahua, 2020
Campeche, 2021
Quintana Roo, 2021
Tabasco, 2020
Mexico City, 2021
Yucatán, 2021
Campeche, 2020
Mexico City, 2020
Quintana Roo, 2020
Mexico: Sexes Combined (10-24 years), 2021
Deviation from Frontier (1990–2021)
Increase
In 2021
Attained Frontier: 0
Fell Behind Frontier: 32
Chihuahua
Tabasco
Nuevo León
Puebla
Guanajuato
México
Campeche
Mexico City
Quintana Roo
Yucatán
Mexico: Sexes Combined (10-24 years)
Frontier Deviation in Prevalence Rate Attributed to Depressive disorders, 1990 (per 100,000)
Frontier Deviation in Prevalence Rate Attributed to Depressive disorders, 2021 (per 100,000)

Figure S23

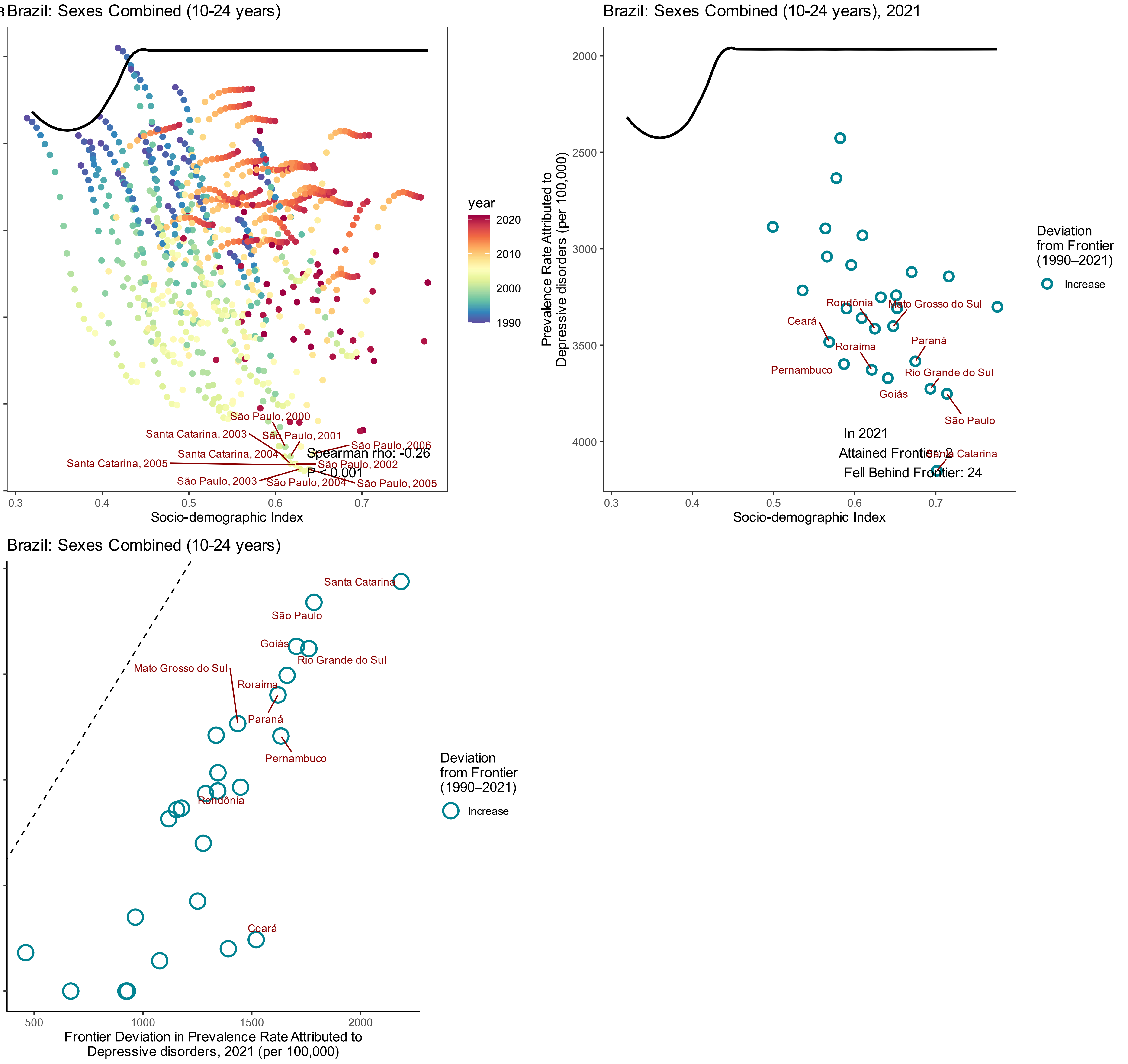
Brazil: Sexes Combined (10-24 years)
Prevalence Rate Attributed to Depressive disorders (per 100,000)
Socio-demographic Index
year
2020
2010
2000
1990
Spearman rho: -0.26
P < 0.001
São Paulo, 2000
São Paulo, 2001
Santa Catarina, 2003
Santa Catarina, 2004
Santa Catarina, 2005
São Paulo, 2006
São Paulo, 2002
São Paulo, 2003
São Paulo, 2004
São Paulo, 2005
Brazil: Sexes Combined (10-24 years), 2021
Deviation from Frontier (1990–2021)
Increase
Rondônia
Mato Grosso do Sul
Ceará
Roraima
Paraná
Pernambuco
Rio Grande do Sul
Goiás
São Paulo
Santa Catarina
In 2021
Attained Frontier: 2
Fell Behind Frontier: 24
Brazil: Sexes Combined (10-24 years)
Frontier Deviation in Prevalence Rate Attributed to Depressive disorders, 1990 (per 100,000)
Frontier Deviation in Prevalence Rate Attributed to Depressive disorders, 2021 (per 100,000)
Santa Catarina
São Paulo
Goiás
Rio Grande do Sul
Mato Grosso do Sul
Roraima
Paraná
Pernambuco
Rondônia
Ceará
Deviation from Frontier (1990–2021)
Increase

Figure S23

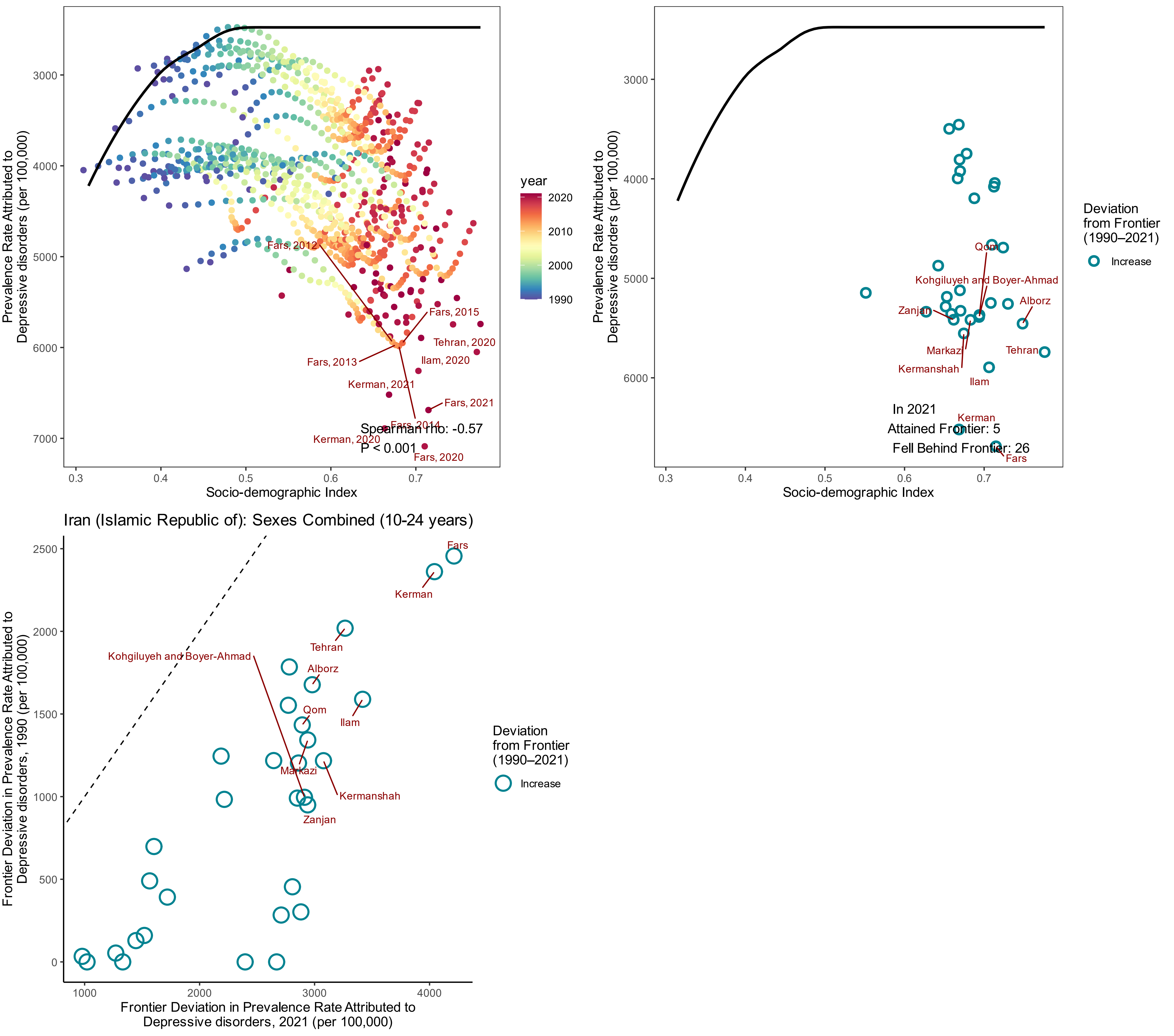

Iran (Islamic Republic of): Sexes Combined (10-24 years)
Prevalence Rate Attributed to Depressive disorders (per 100,000)
Socio-demographic Index
year
2020
2010
2000
1990
Fars, 2012
Fars, 2015
Fars, 2013
Tehran, 2020
Ilam, 2020
Kerman, 2021
Fars, 2021
Fars, 2014
Kerman, 2020
Fars, 2020
Spearman rho: -0.57
P < 0.001
Iran (Islamic Republic of): Sexes Combined (10-24 years), 2021
Prevalence Rate Attributed to Depressive disorders (per 100,000)
Socio-demographic Index
Deviation from Frontier (1990–2021)
Increase
Qom
Kohgiluyeh and Boyer-Ahmad
Zanjan
Alborz
Markazi
Tehran
Kermanshah
Ilam
Kerman
Fars
In 2021
Attained Frontier: 5
Fell Behind Frontier: 26
Iran (Islamic Republic of): Sexes Combined (10-24 years)
Frontier Deviation in Prevalence Rate Attributed to Depressive disorders, 1990 (per 100,000)
Frontier Deviation in Prevalence Rate Attributed to Depressive disorders, 2021 (per 100,000)
Fars
Kerman
Tehran
Kohgiluyeh and Boyer-Ahmad
Alborz
Qom
Ilam
Markazi
Kermanshah
Zanjan
Deviation from Frontier (1990–2021)
Increase

Figure S23

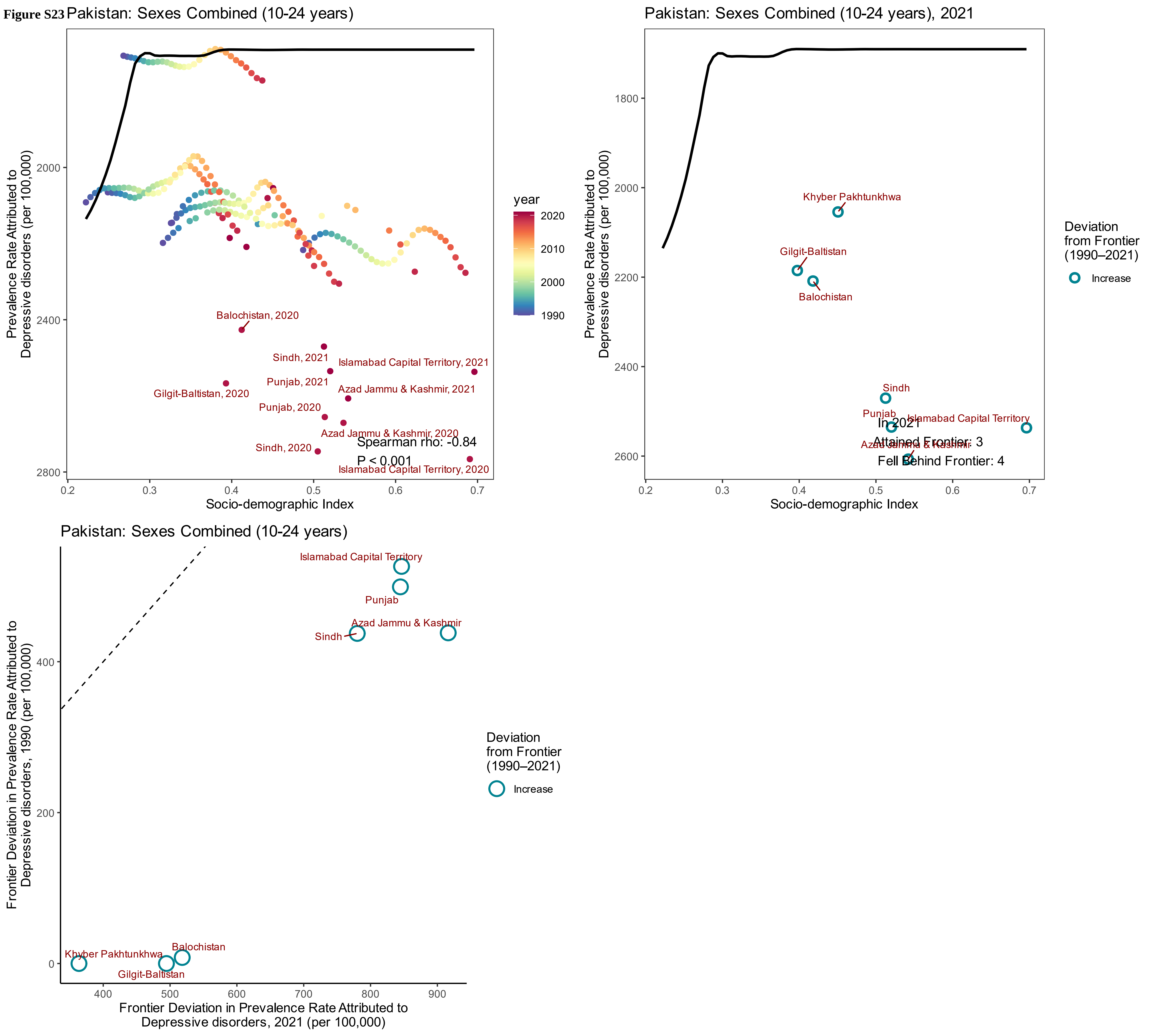
Pakistan: Sexes Combined (10-24 years)
Prevalence Rate Attributed to Depressive disorders (per 100,000)
Socio-demographic Index
year
2020
2010
2000
1990
Balochistan, 2020
Sindh, 2021
Islamabad Capital Territory, 2021
Punjab, 2021
Gilgit-Baltistan, 2020
Azad Jammu & Kashmir, 2021
Punjab, 2020
Azad Jammu & Kashmir, 2020
Sindh, 2020
Islamabad Capital Territory, 2020
Spearman rho: -0.84
P < 0.001
Pakistan: Sexes Combined (10-24 years), 2021
Khyber Pakhtunkhwa
Gilgit-Baltistan
Balochistan
Sindh
Punjab
Islamabad Capital Territory
Azad Jammu & Kashmir
In 2021
Attained Frontier: 3
Fell Behind Frontier: 4
Deviation from Frontier (1990–2021)
Increase
Pakistan: Sexes Combined (10-24 years)
Frontier Deviation in Prevalence Rate Attributed to Depressive disorders, 1990 (per 100,000)
Frontier Deviation in Prevalence Rate Attributed to Depressive disorders, 2021 (per 100,000)
Islamabad Capital Territory
Punjab
Azad Jammu & Kashmir
Sindh
Khyber Pakhtunkhwa
Balochistan
Gilgit-Baltistan
Deviation from Frontier (1990–2021)
Increase

Figure S23

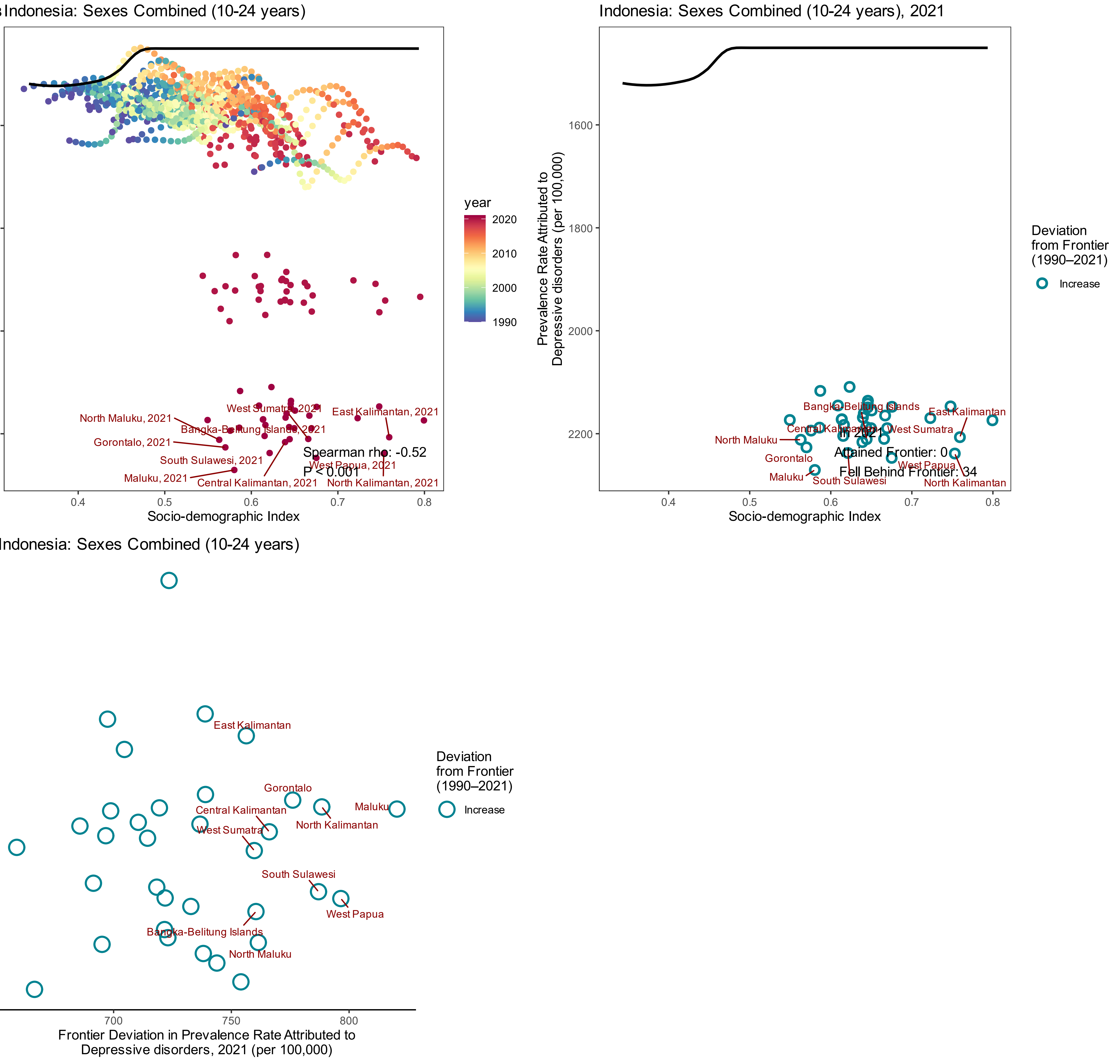

Indonesia: Sexes Combined (10-24 years)
Prevalence Rate Attributed to Depressive disorders (per 100,000)
Socio-demographic Index
year
2020
2010
2000
1990
North Maluku, 2021
Gorontalo, 2021
Bangka-Belitung Islands, 2021
West Sumatra, 2021
East Kalimantan, 2021
South Sulawesi, 2021
Maluku, 2021
Central Kalimantan, 2021
West Papua, 2021
North Kalimantan, 2021
Spearman rho: -0.52
P < 0.001
Indonesia: Sexes Combined (10-24 years), 2021
Bangka-Belitung Islands
East Kalimantan
Central Kalimantan
West Sumatra
North Maluku
Gorontalo
Maluku
South Sulawesi
West Papua
North Kalimantan
In 2021
Attained Frontier: 0
Fell Behind Frontier: 34
Deviation from Frontier (1990–2021)
Increase
Indonesia: Sexes Combined (10-24 years)
Frontier Deviation in Prevalence Rate Attributed to Depressive disorders, 1990 (per 100,000)
Frontier Deviation in Prevalence Rate Attributed to Depressive disorders, 2021 (per 100,000)
East Kalimantan
Gorontalo
Central Kalimantan
Maluku
North Kalimantan
West Sumatra
South Sulawesi
West Papua
Bangka-Belitung Islands
North Maluku
Deviation from Frontier (1990–2021)
Increase

Figure S23

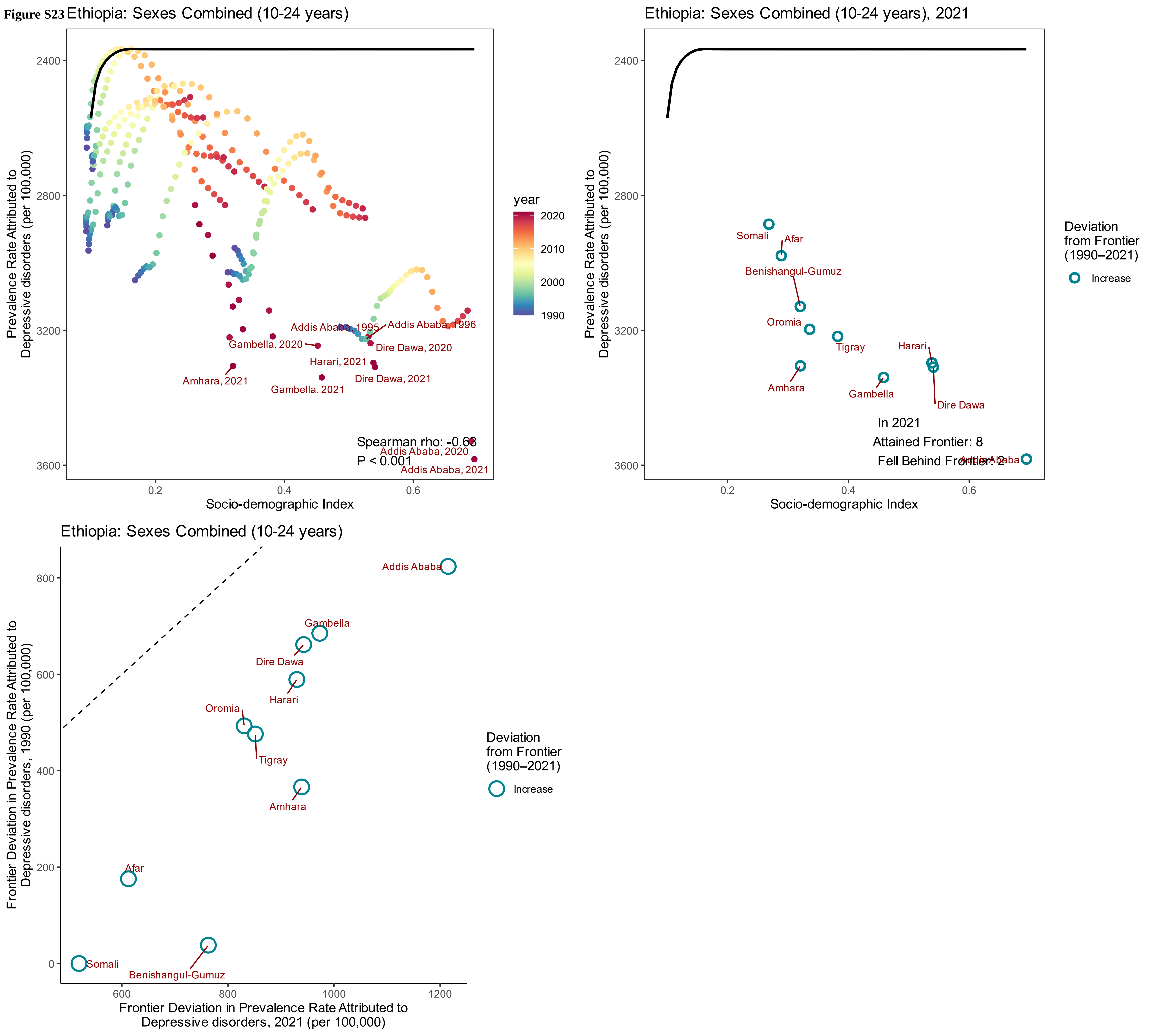

Ethiopia: Sexes Combined (10-24 years)
Prevalence Rate Attributed to Depressive disorders (per 100,000)
Socio-demographic Index
year
2020
2010
2000
1990
Addis Ababa, 1995
Addis Ababa, 1996
Gambella, 2020
Dire Dawa, 2020
Harari, 2021
Dire Dawa, 2021
Amhara, 2021
Gambella, 2021
Spearman rho: -0.63
P < 0.001
Addis Ababa, 2020
Addis Ababa, 2021
Ethiopia: Sexes Combined (10-24 years), 2021
Somali
Afar
Benishangul-Gumuz
Oromia
Tigray
Harari
Amhara
Gambella
Dire Dawa
In 2021
Attained Frontier: 8
Fell Behind Frontier: 2
Addis Ababa
Deviation from Frontier (1990–2021)
Increase
Ethiopia: Sexes Combined (10-24 years)
Frontier Deviation in Prevalence Rate Attributed to Depressive disorders, 1990 (per 100,000)
Frontier Deviation in Prevalence Rate Attributed to Depressive disorders, 2021 (per 100,000)
Addis Ababa
Gambella
Dire Dawa
Harari
Oromia
Tigray
Amhara
Afar
Somali
Benishangul-Gumuz
Deviation from Frontier (1990–2021)
Increase

Figure S23

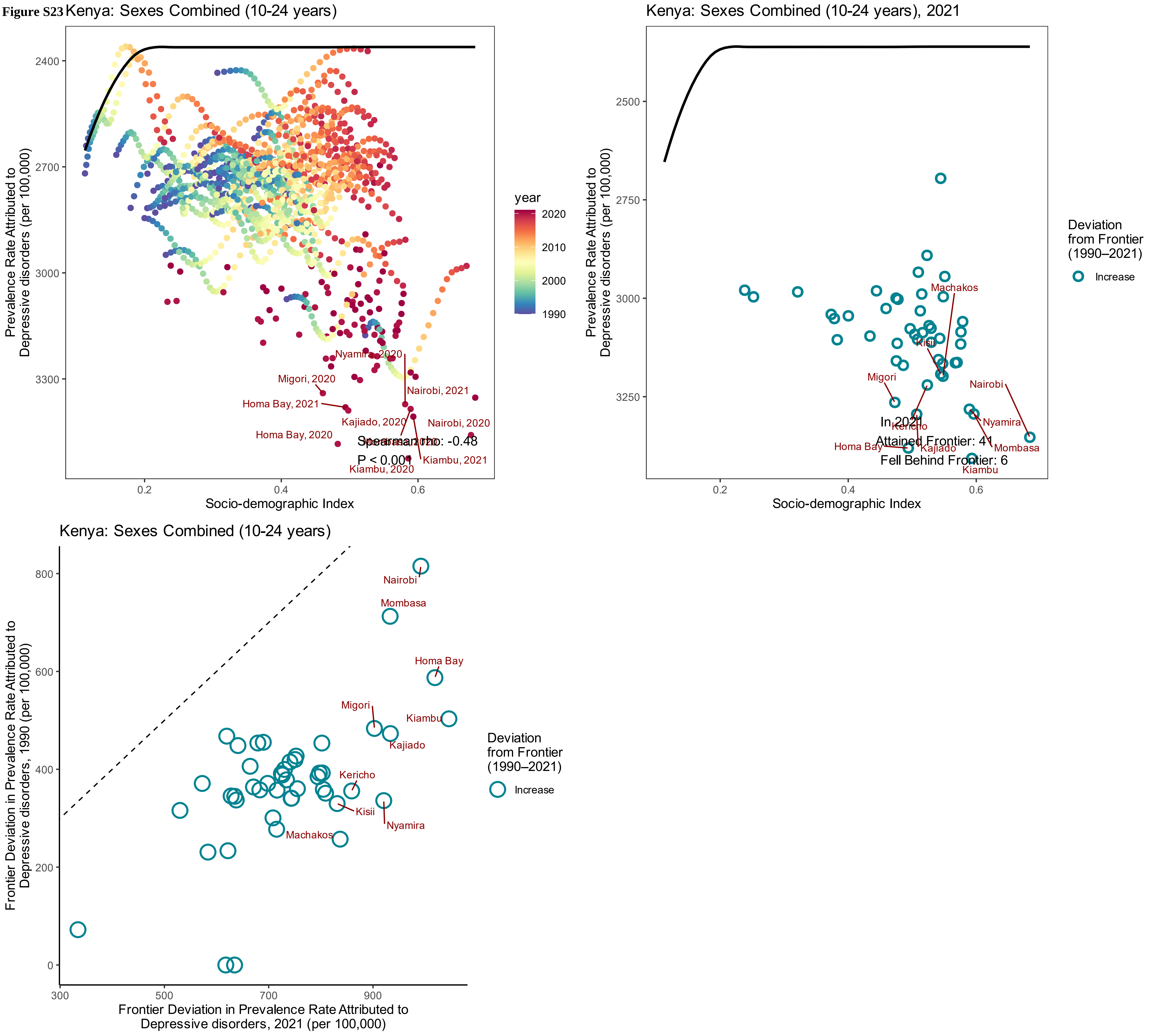

Kenya: Sexes Combined (10-24 years)
Prevalence Rate Attributed to Depressive disorders (per 100,000)
Socio-demographic Index
year
2020
2010
2000
1990
Nyamira, 2020
Migori, 2020
Nairobi, 2021
Homa Bay, 2021
Kajiado, 2020
Nairobi, 2020
Homa Bay, 2020
Spearman rho: -0.48
P < 0.001
Kiambu, 2021
Kiambu, 2020
Kenya: Sexes Combined (10-24 years), 2021
Machakos
Kisii
Migori
Nairobi
In 2021
Kericho
Nyamira
Attained Frontier: 41
Homa Bay
Kajiado
Mombasa
Fell Behind Frontier: 6
Kiambu
Deviation from Frontier (1990–2021)
Increase
Kenya: Sexes Combined (10-24 years)
Nairobi
Mombasa
Homa Bay
Migori
Kiambu
Kajiado
Kericho
Kisii
Nyamira
Machakos
Frontier Deviation in Prevalence Rate Attributed to Depressive disorders, 1990 (per 100,000)
Frontier Deviation in Prevalence Rate Attributed to Depressive disorders, 2021 (per 100,000)
Deviation from Frontier (1990–2021)
Increase

Figure S23

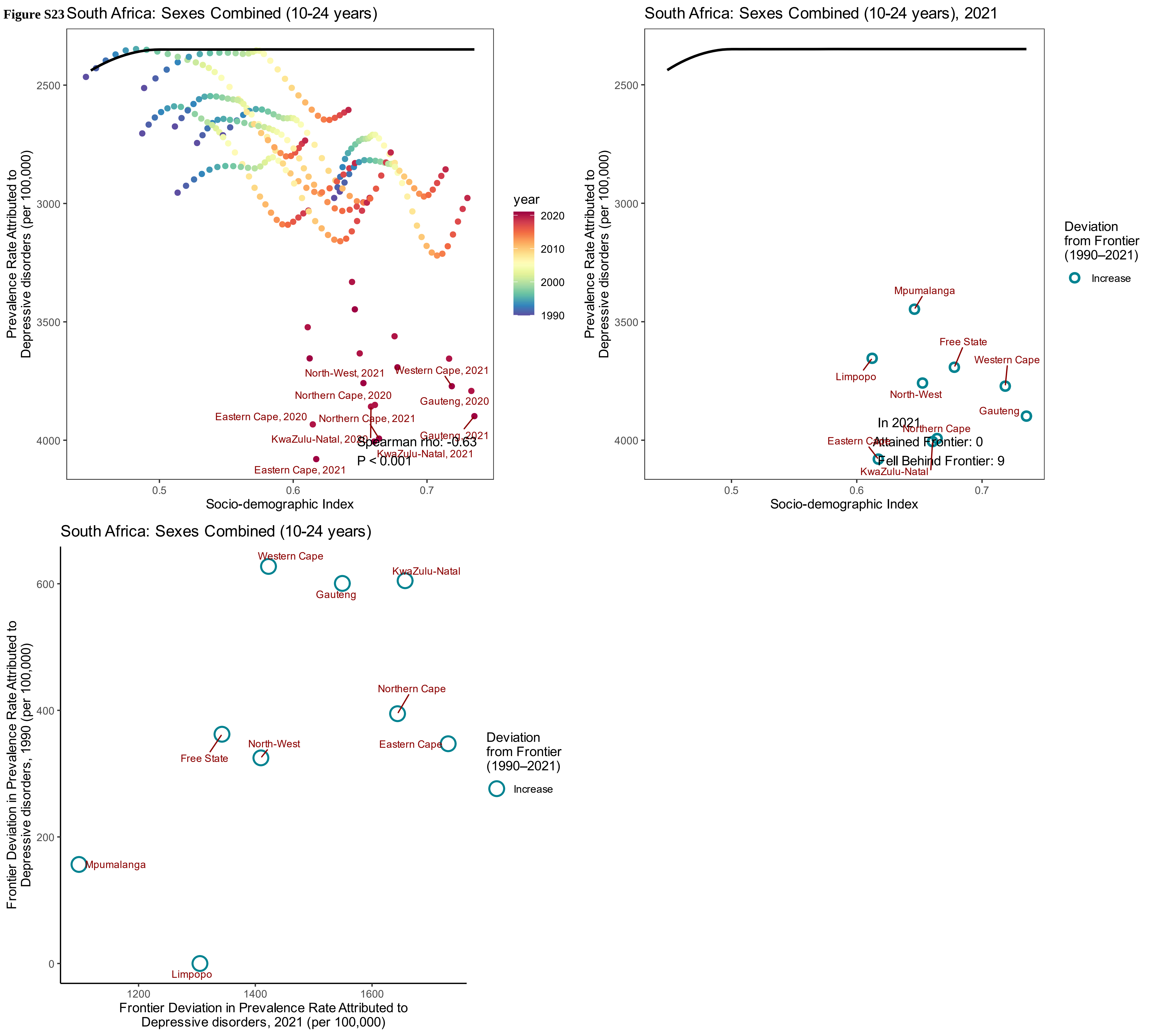
South Africa: Sexes Combined (10-24 years)
Prevalence Rate Attributed to Depressive disorders (per 100,000)
Socio-demographic Index
year
2020
2010
2000
1990
North-West, 2021
Western Cape, 2021
Northern Cape, 2020
Gauteng, 2020
Eastern Cape, 2020
Northern Cape, 2021
KwaZulu-Natal, 2020
Gauteng, 2021
Spearman rho: -0.63
P < 0.001
KwaZulu-Natal, 2021
Eastern Cape, 2021
South Africa: Sexes Combined (10-24 years), 2021
Mpumalanga
Free State
Western Cape
Limpopo
North-West
Gauteng
In 2021
Northern Cape
Eastern Cape
Attained Frontier: 0
Fell Behind Frontier: 9
KwaZulu-Natal
Deviation from Frontier (1990–2021)
Increase
South Africa: Sexes Combined (10-24 years)
Frontier Deviation in Prevalence Rate Attributed to Depressive disorders, 1990 (per 100,000)
Frontier Deviation in Prevalence Rate Attributed to Depressive disorders, 2021 (per 100,000)
Western Cape
KwaZulu-Natal
Gauteng
Northern Cape
North-West
Free State
Eastern Cape
Mpumalanga
Limpopo
Deviation from Frontier (1990–2021)
Increase

Figure S23

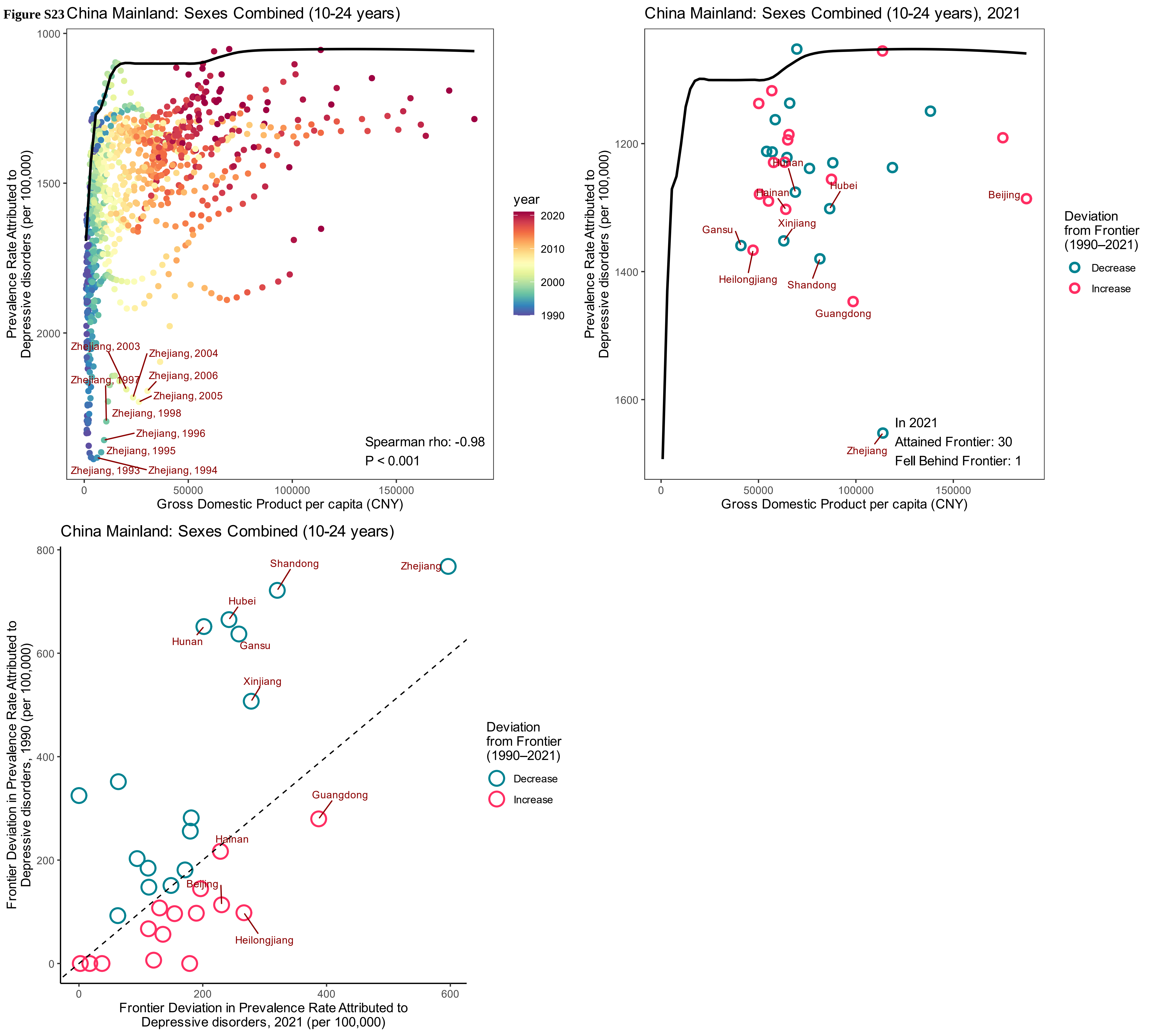

China Mainland: Sexes Combined (10-24 years)
Prevalence Rate Attributed to Depressive disorders (per 100,000)
Gross Domestic Product per capita (CNY)
year
Spearman rho: -0.98
P < 0.001
Zhejiang, 2003
Zhejiang, 2004
Zhejiang, 1997
Zhejiang, 2006
Zhejiang, 2005
Zhejiang, 1998
Zhejiang, 1996
Zhejiang, 1995
Zhejiang, 1993
Zhejiang, 1994
China Mainland: Sexes Combined (10-24 years), 2021
Deviation from Frontier (1990–2021)
Decrease
Increase
Hunan
Hubei
Hainan
Beijing
Xinjiang
Gansu
Heilongjiang
Shandong
Guangdong
Zhejiang
In 2021
Attained Frontier: 30
Fell Behind Frontier: 1
China Mainland: Sexes Combined (10-24 years)
Frontier Deviation in Prevalence Rate Attributed to Depressive disorders, 1990 (per 100,000)
Frontier Deviation in Prevalence Rate Attributed to Depressive disorders, 2021 (per 100,000)

Figure S23

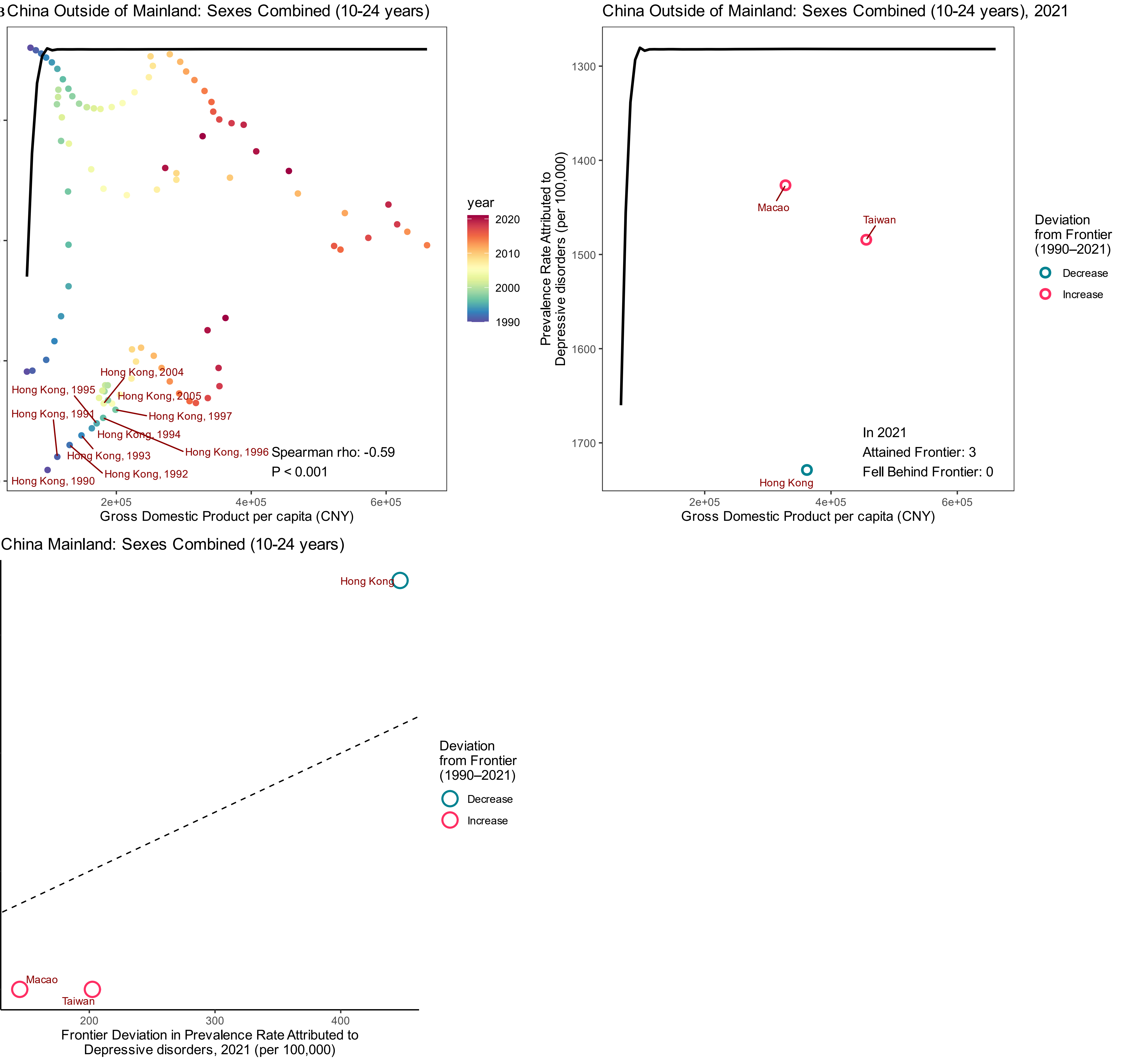

China Outside of Mainland: Sexes Combined (10-24 years)
Prevalence Rate Attributed to Depressive disorders (per 100,000)
Gross Domestic Product per capita (CNY)
year
Hong Kong, 2004
Hong Kong, 1995
Hong Kong, 2005
Hong Kong, 1991
Hong Kong, 1997
Hong Kong, 1994
Hong Kong, 1993
Hong Kong, 1996
Hong Kong, 1992
Hong Kong, 1990
Spearman rho: -0.59
P < 0.001
China Outside of Mainland: Sexes Combined (10-24 years), 2021
Macao
Taiwan
Hong Kong
In 2021
Attained Frontier: 3
Fell Behind Frontier: 0
Deviation from Frontier (1990–2021)
Decrease
Increase
China Mainland: Sexes Combined (10-24 years)
Frontier Deviation in Prevalence Rate Attributed to Depressive disorders, 1990 (per 100,000)
Frontier Deviation in Prevalence Rate Attributed to Depressive disorders, 2021 (per 100,000)

Figure S24

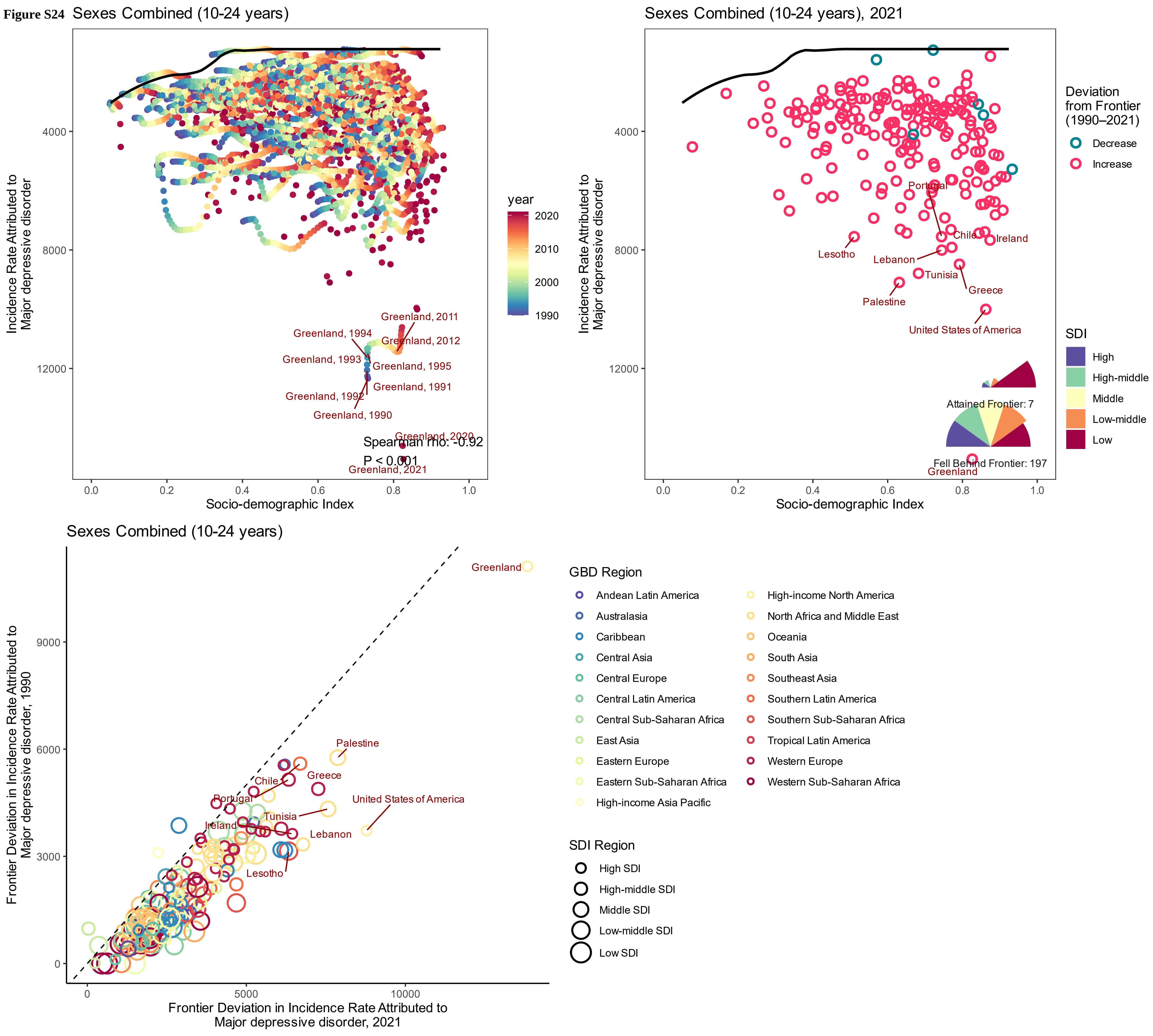

Sexes Combined (10-24 years)
Incidence Rate Attributed to Major depressive disorder
Socio-demographic Index
year
Greenland, 2011
Greenland, 1994
Greenland, 2012
Greenland, 1993
Greenland, 1995
Greenland, 1991
Greenland, 1992
Greenland, 1990
Greenland, 2020
Spearman rho: -0.92
P < 0.001
Greenland, 2021
Sexes Combined (10-24 years), 2021
Deviation from Frontier (1990–2021)
Decrease
Increase
Portugal
Chile
Ireland
Lesotho
Lebanon
Tunisia
Greece
Palestine
United States of America
SDI
High
High-middle
Middle
Low-middle
Low
Attained Frontier: 7
Fell Behind Frontier: 197
Greenland
Frontier Deviation in Incidence Rate Attributed to Major depressive disorder, 1990
Frontier Deviation in Incidence Rate Attributed to Major depressive disorder, 2021
GBD Region
Andean Latin America
Australasia
Caribbean
Central Asia
Central Europe
Central Latin America
Central Sub-Saharan Africa
East Asia
Eastern Europe
Eastern Sub-Saharan Africa
High-income Asia Pacific
High-income North America
North Africa and Middle East
Oceania
South Asia
Southeast Asia
Southern Latin America
Southern Sub-Saharan Africa
Tropical Latin America
Western Europe
Western Sub-Saharan Africa
SDI Region
High SDI
High-middle SDI
Middle SDI
Low-middle SDI
Low SDI

Figure S24

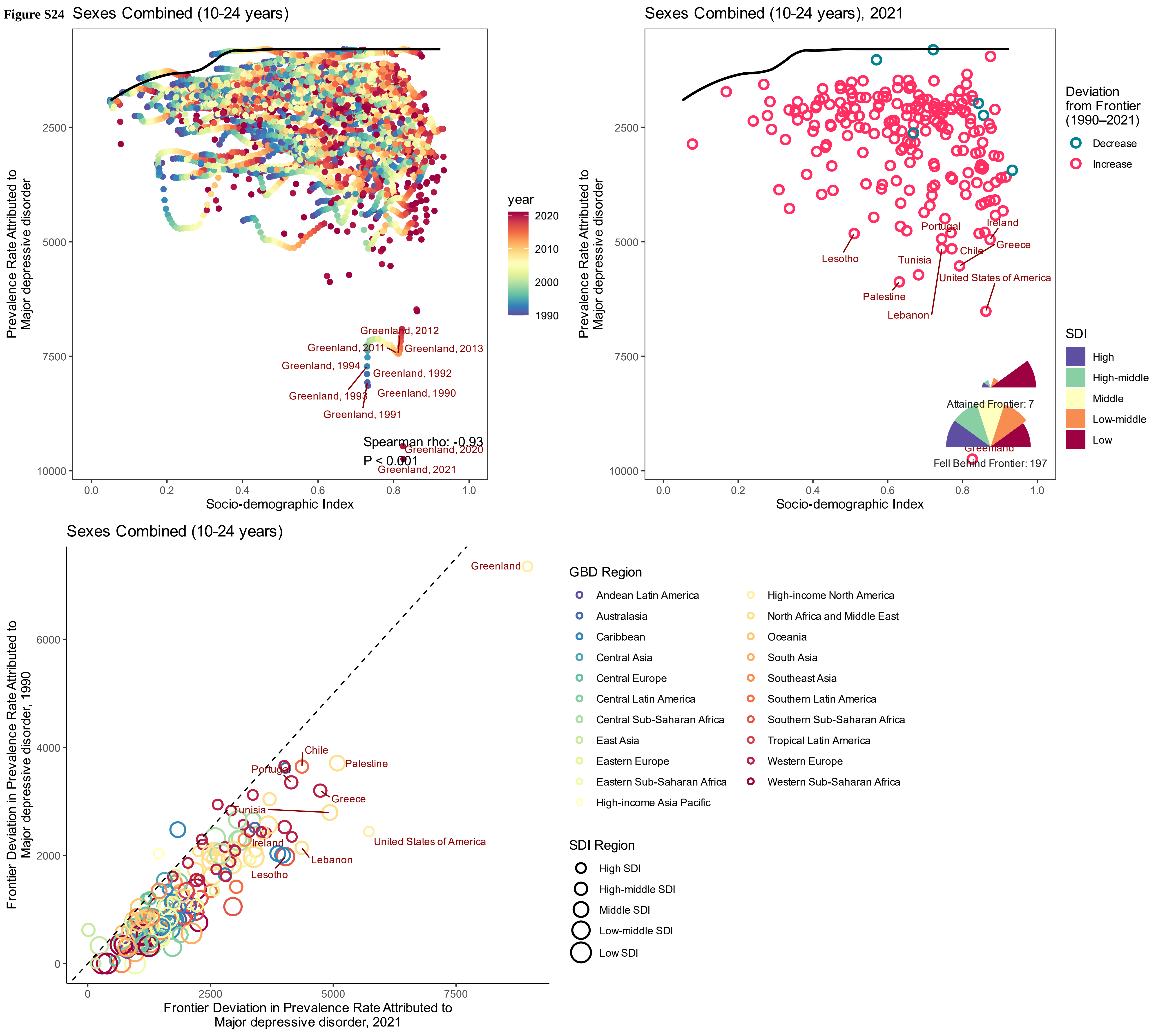
Sexes Combined (10-24 years)
Prevalence Rate Attributed to Major depressive disorder
Socio-demographic Index
year
2020
2010
2000
1990
Greenland, 2012
Greenland, 2011
Greenland, 2013
Greenland, 1994
Greenland, 1992
Greenland, 1993
Greenland, 1990
Greenland, 1991
Spearman rho: -0.93
P < 0.001
Greenland, 2020
Greenland, 2021
Sexes Combined (10-24 years), 2021
Deviation from Frontier (1990–2021)
Decrease
Increase
Portugal
Ireland
Greece
Chile
Lesotho
Tunisia
United States of America
Palestine
Lebanon
SDI
High
High-middle
Middle
Low-middle
Low
Attained Frontier: 7
Greenland
Fell Behind Frontier: 197
Sexes Combined (10-24 years)
Frontier Deviation in Prevalence Rate Attributed to Major depressive disorder, 1990
Frontier Deviation in Prevalence Rate Attributed to Major depressive disorder, 2021
Greenland
Chile
Palestine
Portugal
Greece
Tunisia
Ireland
United States of America
Lebanon
Lesotho
GBD Region
Andean Latin America
Australasia
Caribbean
Central Asia
Central Europe
Central Latin America
Central Sub-Saharan Africa
East Asia
Eastern Europe
Eastern Sub-Saharan Africa
High-income Asia Pacific
High-income North America
North Africa and Middle East
Oceania
South Asia
Southeast Asia
Southern Latin America
Southern Sub-Saharan Africa
Tropical Latin America
Western Europe
Western Sub-Saharan Africa
SDI Region
High SDI
High-middle SDI
Middle SDI
Low-middle SDI
Low SDI

Figure S24

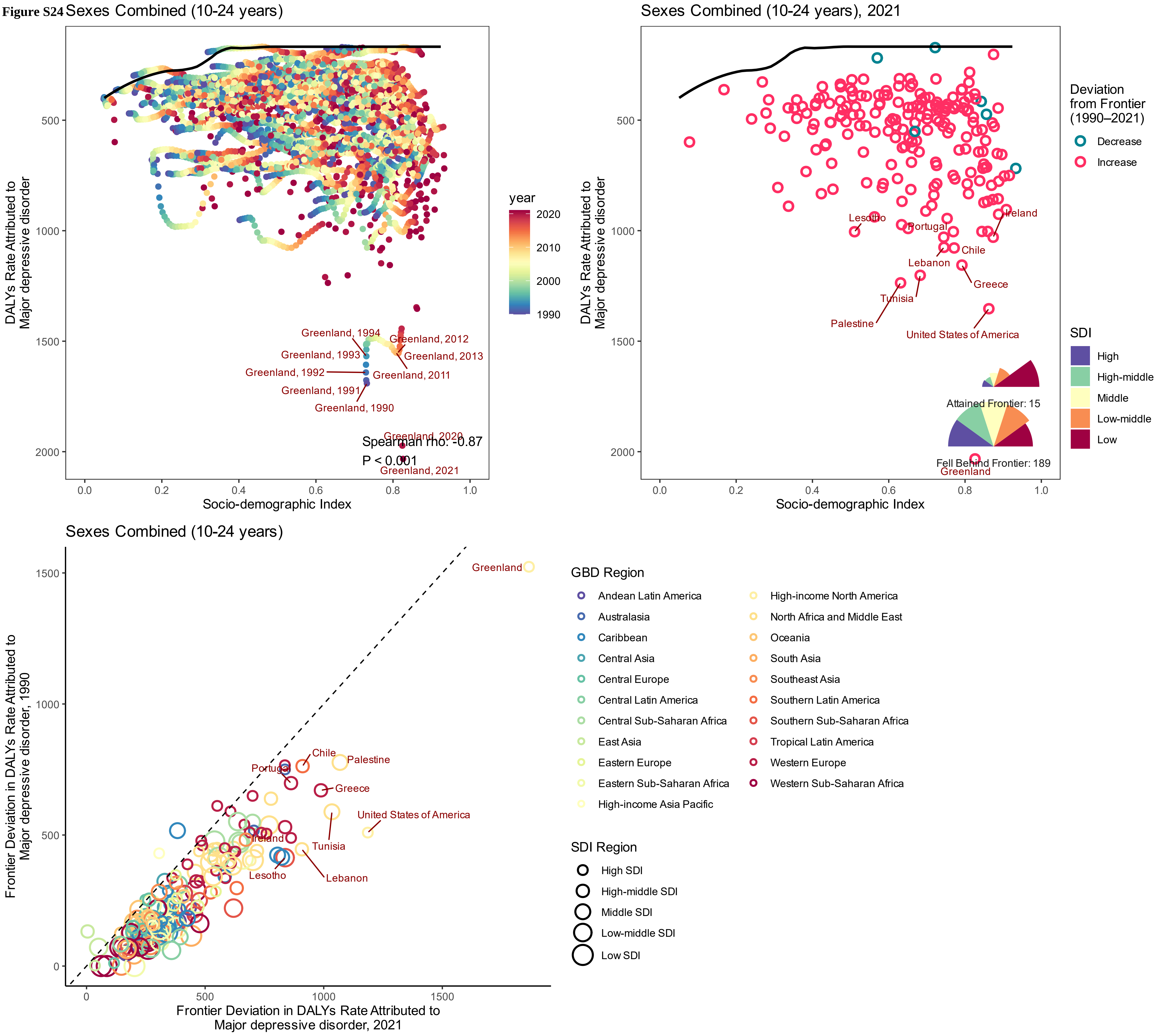

Sexes Combined (10-24 years)
DALYs Rate Attributed to Major depressive disorder
Socio-demographic Index
year
2020
2010
2000
1990
Greenland, 1994
Greenland, 1993
Greenland, 1992
Greenland, 1991
Greenland, 1990
Greenland, 2012
Greenland, 2013
Greenland, 2011
Greenland, 2020
Greenland, 2021
Spearman rho: -0.87
P < 0.001
Sexes Combined (10-24 years), 2021
Deviation from Frontier (1990–2021)
Decrease
Increase
Lesotho
Portugal
Ireland
Chile
Lebanon
Greece
Tunisia
Palestine
United States of America
Greenland
Attained Frontier: 15
Fell Behind Frontier: 189
SDI
High
High-middle
Middle
Low-middle
Low
Sexes Combined (10-24 years)
Frontier Deviation in DALYs Rate Attributed to Major depressive disorder, 1990
Frontier Deviation in DALYs Rate Attributed to Major depressive disorder, 2021
GBD Region
Andean Latin America
Australasia
Caribbean
Central Asia
Central Europe
Central Latin America
Central Sub-Saharan Africa
East Asia
Eastern Europe
Eastern Sub-Saharan Africa
High-income Asia Pacific
High-income North America
North Africa and Middle East
Oceania
South Asia
Southeast Asia
Southern Latin America
Southern Sub-Saharan Africa
Tropical Latin America
Western Europe
Western Sub-Saharan Africa
SDI Region
High SDI
High-middle SDI
Middle SDI
Low-middle SDI
Low SDI

Figure S25

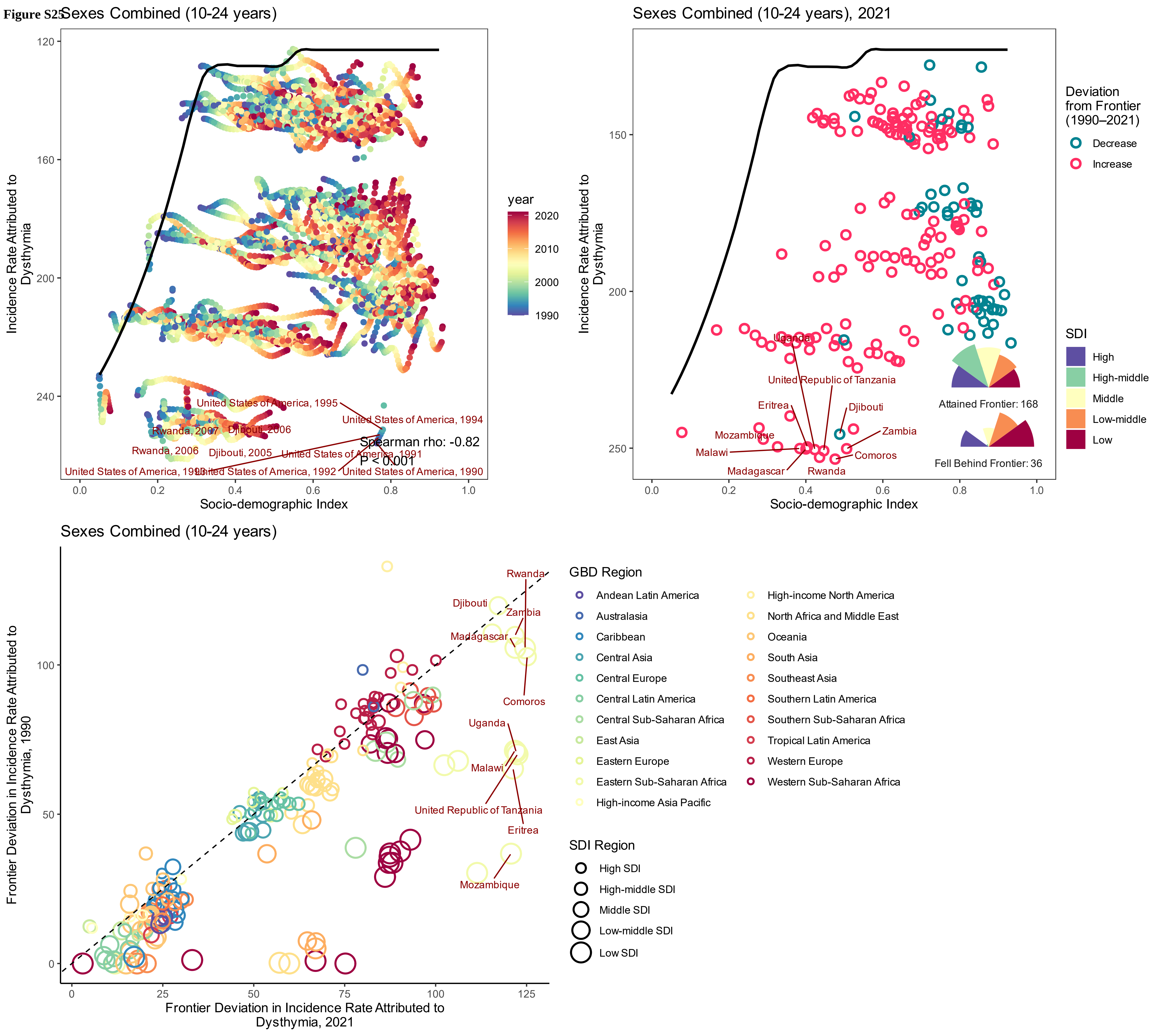

Sexes Combined (10-24 years)
Incidence Rate Attributed to Dysthymia
Socio-demographic Index
year
2020
2010
2000
1990
United States of America, 1995
United States of America, 1994
Rwanda, 2007
Djibouti, 2006
Rwanda, 2006
Djibouti, 2005
United States of America, 1991
United States of America, 1993
United States of America, 1992
United States of America, 1990
Spearman rho: -0.82
P < 0.001
Sexes Combined (10-24 years), 2021
Deviation from Frontier (1990–2021)
Decrease
Increase
SDI
High
High-middle
Middle
Low-middle
Low
Attained Frontier: 168
Fell Behind Frontier: 36
Uganda
United Republic of Tanzania
Eritrea
Djibouti
Mozambique
Zambia
Malawi
Comoros
Madagascar
Rwanda
Sexes Combined (10-24 years)
Frontier Deviation in Incidence Rate Attributed to Dysthymia, 1990
Frontier Deviation in Incidence Rate Attributed to Dysthymia, 2021
GBD Region
Andean Latin America
Australasia
Caribbean
Central Asia
Central Europe
Central Latin America
Central Sub-Saharan Africa
East Asia
Eastern Europe
Eastern Sub-Saharan Africa
High-income Asia Pacific
High-income North America
North Africa and Middle East
Oceania
South Asia
Southeast Asia
Southern Latin America
Southern Sub-Saharan Africa
Tropical Latin America
Western Europe
Western Sub-Saharan Africa
SDI Region
High SDI
High-middle SDI
Middle SDI
Low-middle SDI
Low SDI

Figure S25

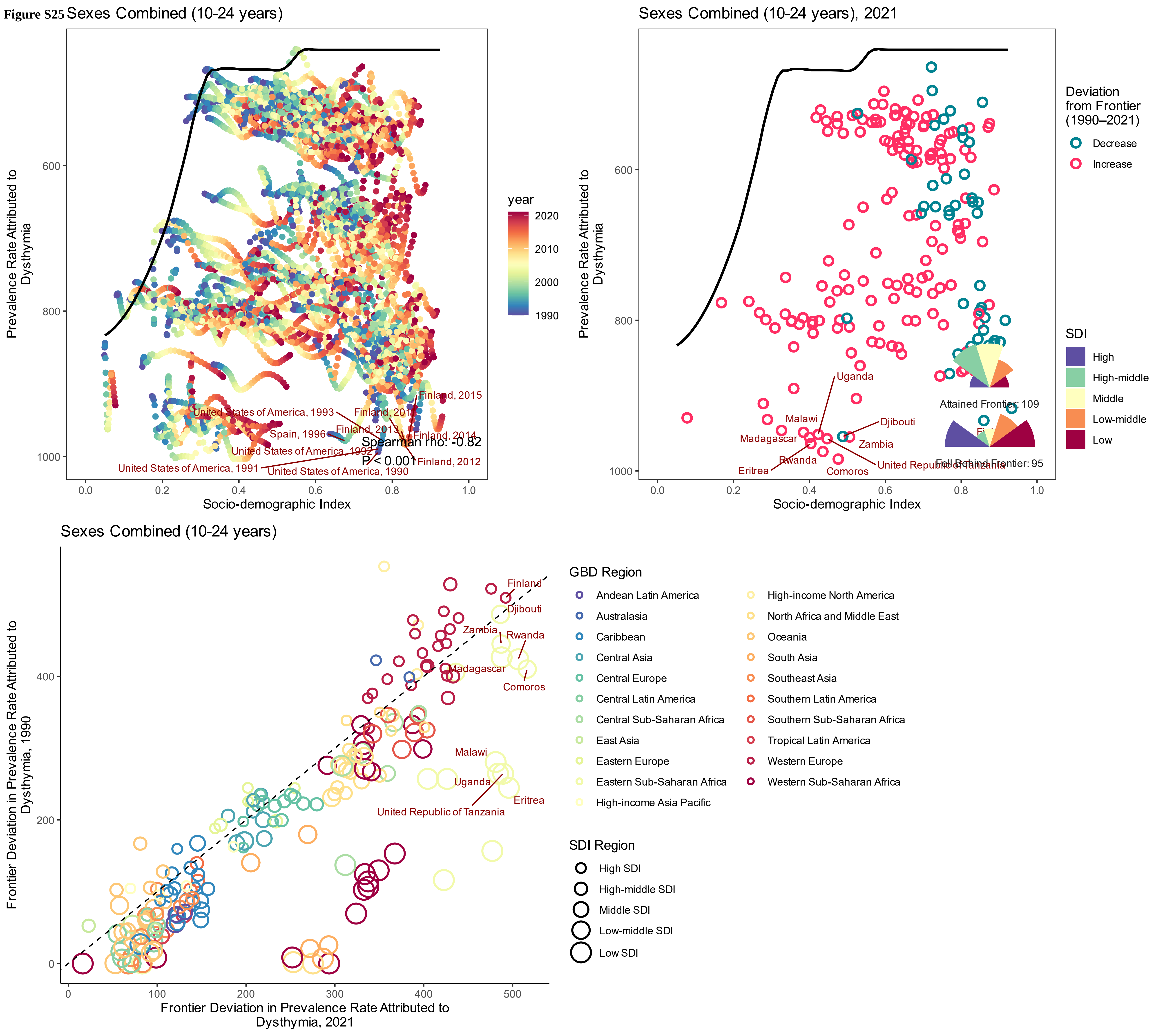

Sexes Combined (10-24 years)
Prevalence Rate Attributed to Dysthymia
Socio-demographic Index
year
2020
2010
2000
1990
Spearman rho: -0.82
P < 0.001
Finland, 2015
Finland, 2016
Finland, 2013
Finland, 2014
Finland, 2012
United States of America, 1993
Spain, 1996
United States of America, 1992
United States of America, 1991
United States of America, 1990
Sexes Combined (10-24 years), 2021
Deviation from Frontier (1990–2021)
Decrease
Increase
SDI
High
High-middle
Middle
Low-middle
Low
Attained Frontier: 109
Fell Behind Frontier: 95
Uganda
Malawi
Djibouti
Madagascar
Zambia
Rwanda
Eritrea
Comoros
United Republic of Tanzania
Sexes Combined (10-24 years)
Frontier Deviation in Prevalence Rate Attributed to Dysthymia, 1990
Frontier Deviation in Prevalence Rate Attributed to Dysthymia, 2021
Finland
Djibouti
Zambia
Rwanda
Madagascar
Comoros
Malawi
Uganda
Eritrea
United Republic of Tanzania
GBD Region
Andean Latin America
Australasia
Caribbean
Central Asia
Central Europe
Central Latin America
Central Sub-Saharan Africa
East Asia
Eastern Europe
Eastern Sub-Saharan Africa
High-income Asia Pacific
High-income North America
North Africa and Middle East
Oceania
South Asia
Southeast Asia
Southern Latin America
Southern Sub-Saharan Africa
Tropical Latin America
Western Europe
Western Sub-Saharan Africa
SDI Region
High SDI
High-middle SDI
Middle SDI
Low-middle SDI
Low SDI

Figure S25

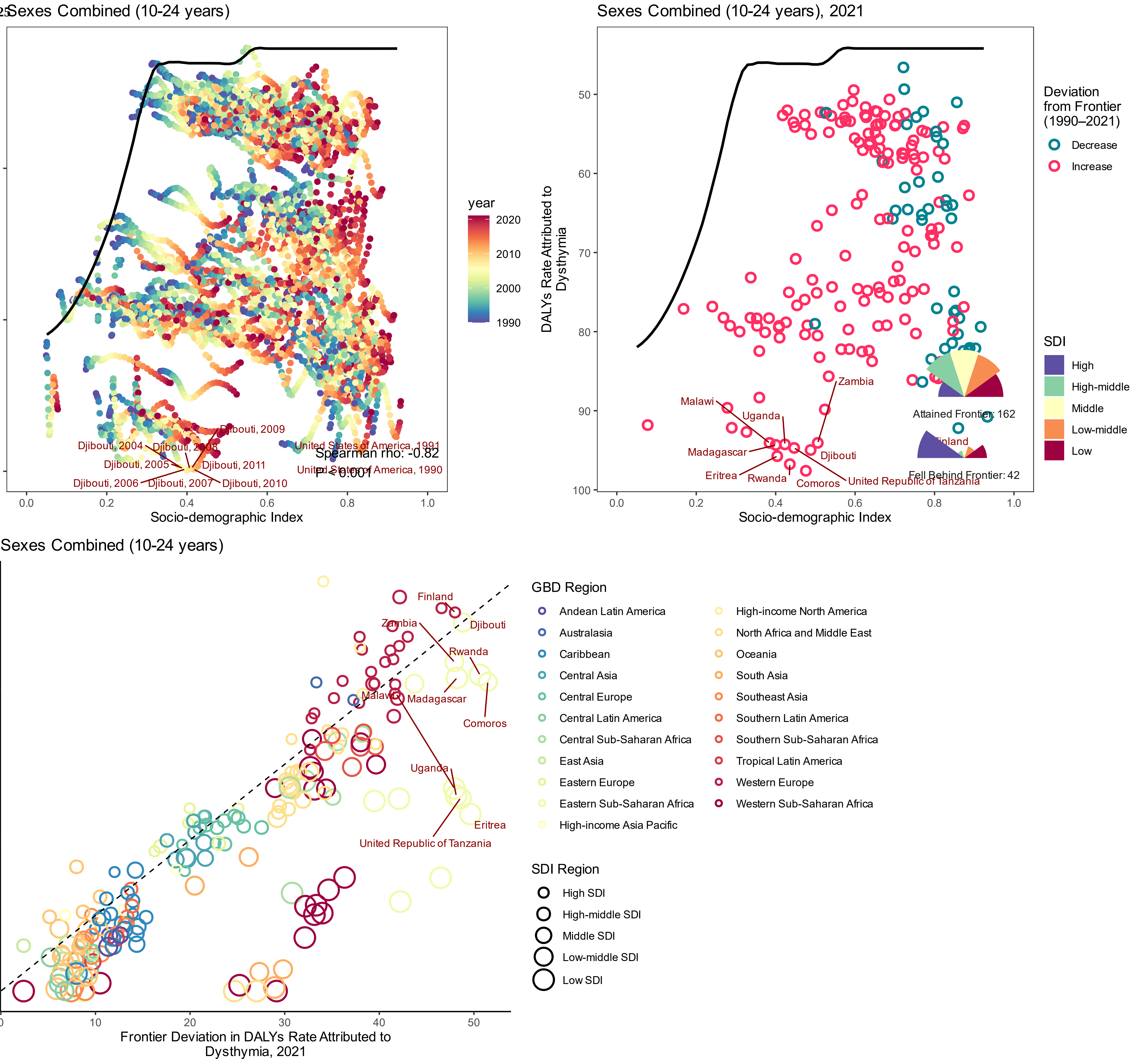
Sexes Combined (10-24 years)
DALYs Rate Attributed to Dysthymia
Socio-demographic Index
year
2020
2010
2000
1990
Djibouti, 2004
Djibouti, 2005
Djibouti, 2006
Djibouti, 2007
Djibouti, 2008
Djibouti, 2009
Djibouti, 2010
Djibouti, 2011
United States of America, 1991
United States of America, 1990
Spearman rho: -0.82
P < 0.001
Sexes Combined (10-24 years), 2021
Deviation from Frontier (1990–2021)
Decrease
Increase
SDI
High
High-middle
Middle
Low-middle
Low
Attained Frontier: 162
Fell Behind Frontier: 42
Finland
Zambia
Malawi
Uganda
Madagascar
Djibouti
Eritrea
Rwanda
Comoros
United Republic of Tanzania
Frontier Deviation in DALYs Rate Attributed to Dysthymia, 1990
Frontier Deviation in DALYs Rate Attributed to Dysthymia, 2021
GBD Region
Andean Latin America
Australasia
Caribbean
Central Asia
Central Europe
Central Latin America
Central Sub-Saharan Africa
East Asia
Eastern Europe
Eastern Sub-Saharan Africa
High-income Asia Pacific
High-income North America
North Africa and Middle East
Oceania
South Asia
Southeast Asia
Southern Latin America
Southern Sub-Saharan Africa
Tropical Latin America
Western Europe
Western Sub-Saharan Africa
SDI Region
High SDI
High-middle SDI
Middle SDI
Low-middle SDI
Low SDI

Table S1 The incidence, prevalence and DALYs rate of anxiety disorders in 1990 and 2021, and changes from 1990 to 2021 at the global level and different regions.

Location	Incidence (95% UI)			Prevalence (95% UI)			DALYs (95% UI)		
	1990	2021	Percentage change	1990	2021	Percentage change	1990	2021	Percentage change
Global	708.0 (468.8 to 979.5)	883.1 (585.1 to 1225.6)	24.7% (-25.6 to 109.1)	4120.6 (3054.2 to 5431.7)	4976.6 (3646.8 to 6622.3)	20.8% (-19.9 to 82.1)	505.6 (315.2 to 752.7)	610.4 (378.2 to 910.5)	20.7% (-34.5 to 122.8)
High SDI	889.9 (597.1 to 1224.0)	1225.8 (816.9 to 1680.4)	37.7% (-16.7 to 128.0)	5241.3 (3895.5 to 6930.3)	6994.4 (5163.7 to 9272.3)	33.4% (-11.1 to 100.5)	642.7 (398.8 to 954.3)	853.7 (529.5 to 1268.3)	32.8% (-27.9 to 144.6)
High-middle SDI	701.1 (463.8 to 965.7)	908.0 (603.0 to 1253.2)	29.5% (-22.4 to 116.1)	4495.7 (3389.3 to 5832.7)	5589.8 (4065.6 to 7454.3)	24.3% (-17.1 to 86.8)	554.7 (350.0 to 816.2)	689.8 (425.3 to 1034.7)	24.3% (-32.3 to 128.9)
Middle SDI	722.2 (479.2 to 999.4)	922.8 (599.0 to 1295.3)	27.8% (-24.6 to 116.9)	4224.4 (3173.6 to 5498.7)	5249.9 (3912.4 to 6854.9)	24.3% (-15.7 to 83.4)	520.1 (326.9 to 768.9)	646.3 (402.6 to 955.4)	24.3% (-31.9 to 127.0)
Low-middle SDI	630.9 (417.5 to 879.4)	794.3 (523.6 to 1103.2)	25.9% (-25.2 to 112.0)	3331.4 (2415.0 to 4479.4)	4276.5 (3104.3 to 5753.6)	28.4% (-16.7 to 97.7)	406.1 (251.5 to 606.7)	523.5 (321.5 to 780.8)	28.9% (-30.5 to 139.4)
Low SDI	656.6 (426.9 to 924.3)	781.9 (506.7 to 1107.0)	19.1% (-30.9 to 105.2)	3493.7 (2485.3 to 4810.9)	4223.7 (2981.3 to 5831.1)	20.9% (-24.1 to 92.8)	423.4 (259.2 to 639.8)	515.8 (312.0 to 786.7)	21.8% (-35.7 to 131.1)
North Africa and Middle East	1022.8 (685.1 to 1431.5)	1270.1 (810.4 to 1823.3)	24.2% (-27.9 to 114.4)	6589.0 (4796.9 to 8807.5)	8163.7 (5685.5 to 11370.6)	23.9% (-21.7 to 96.5)	807.1 (499.0 to 1203.0)	1000.4 (604.0 to 1529.7)	23.9% (-34.3 to 134.0)
South Asia	535.9 (352.9 to 741.1)	661.4 (437.4 to 907.6)	23.4% (-26.3 to 106.5)	2543.9 (1862.4 to 3363.6)	3223.1 (2380.1 to 4244.9)	26.7% (-15.8 to 90.7)	309.2 (191.6 to 458.4)	394.0 (242.3 to 577.1)	27.4% (-30.7 to 134.2)
Western Europe	1114.2 (722.9 to 1585.3)	1457.4 (947.5 to 2035.5)	30.8% (-23.9 to 124.7)	7281.6 (5252.1 to 9777.4)	9368.6 (6754.1 to 12672.8)	28.7% (-16.9 to 99.4)	891.4 (543.4 to 1338.6)	1145.8 (701.1 to 1698.2)	28.5% (-31.2 to 140.0)
Central Europe	622.7 (396.2 to 896.4)	899.4 (569.0 to 1296.1)	44.4% (-18.6 to 156.5)	3507.5 (2461.2 to 4863.8)	5063.2 (3577.4 to 7041.4)	44.4% (-10.3 to 132.1)	432.2 (260.9 to 666.4)	624.2 (374.9 to 942.0)	44.4% (-24.6 to 176.6)
Eastern Europe	668.3 (447.3 to 921.4)	980.4 (660.2 to 1348.9)	46.7% (-11.3 to 142.4)	3737.8 (2806.6 to 4836.7)	5390.5 (4045.0 to 7081.3)	44.2% (-2.0 to 112.3)	460.4 (289.9 to 684.4)	665.2 (417.3 to 986.7)	44.5% (-20.8 to 163.7)

East Asia	655.2 (429.5 to 908.8)	700.2 (471.1 to 964.1)	6.9% (-35.9 to 78.2)	4327.2 (3306.1 to 5599.1)	4403.7 (3304.3 to 5775.9)	1.8% (-30.5 to 49.0)	536.5 (341.1 to 785.4)	548.6 (340.7 to 816.6)	2.3% (-43.8 to 86.3)
Central Asia	431.3 (266.7 to 636.1)	556.5 (338.2 to 830.1)	29.0% (-30.5 to 139.6)	2359.9 (1588.5 to 3384.0)	3038.4 (2037.7 to 4461.9)	28.8% (-24.9 to 120.8)	290.0 (168.6 to 455.0)	374.4 (216.6 to 583.5)	29.1% (-35.5 to 158.4)
Southeast Asia	626.2 (406.1 to 874.3)	850.3 (550.0 to 1187.2)	35.8% (-20.7 to 132.4)	3395.8 (2469.4 to 4556.3)	4670.1 (3407.0 to 6246.9)	37.5% (-10.3 to 110.6)	417.6 (257.3 to 627.5)	576.1 (360.5 to 866.8)	37.9% (-25.7 to 156.0)
High-income North America	961.8 (661.0 to 1294.1)	1381.9 (916.6 to 1872.4)	43.7% (-11.7 to 133.9)	4786.7 (3637.1 to 6102.8)	6831.5 (5098.1 to 8800.3)	42.7% (-1.7 to 107.4)	584.9 (371.1 to 850.0)	827.0 (529.7 to 1213.8)	41.4% (-20.8 to 152.5)
Tropical Latin America	1002.9 (673.6 to 1375.2)	1586.8 (1030.7 to 2185.7)	58.2% (-5.4 to 164.8)	5789.5 (4444.7 to 7405.2)	9300.1 (7038.0 to 12005.1)	60.6% (11.4 to 131.7)	705.4 (448.0 to 1024.4)	1131.4 (709.3 to 1655.8)	60.4% (-10.7 to 188.3)
Andean Latin America	957.9 (586.3 to 1411.7)	1453.5 (854.5 to 2213.8)	51.7% (-20.2 to 188.9)	5428.3 (3697.2 to 7754.8)	8300.4 (5448.9 to 12122.7)	52.9% (-11.0 to 162.8)	665.7 (397.7 to 1028.2)	1019.6 (589.5 to 1614.9)	53.2% (-22.9 to 204.8)
Central Latin America	691.1 (446.2 to 973.9)	1032.7 (665.5 to 1448.8)	49.4% (-13.4 to 157.7)	3992.0 (2852.7 to 5368.5)	5947.9 (4263.5 to 8129.1)	49.0% (-4.7 to 133.2)	490.6 (301.7 to 740.9)	731.8 (447.6 to 1110.7)	49.2% (-20.7 to 181.0)
Southern Latin America	994.3 (651.9 to 1404.9)	1317.6 (792.2 to 1984.3)	32.5% (-27.2 to 141.6)	6087.7 (4416.9 to 8215.1)	8067.0 (5170.8 to 11646.7)	32.5% (-20.2 to 121.1)	747.1 (463.0 to 1123.5)	988.3 (552.7 to 1539.7)	32.3% (-32.7 to 160.8)
Central Sub-Saharan Africa	723.7 (447.3 to 1075.6)	820.1 (495.6 to 1254.3)	13.3% (-39.9 to 113.8)	3993.4 (2705.1 to 5726.7)	4508.4 (2958.5 to 6718.8)	12.9% (-35.0 to 96.3)	481.8 (284.4 to 747.3)	549.0 (316.6 to 885.6)	13.9% (-43.4 to 129.7)
Eastern Sub-Saharan Africa	729.4 (472.9 to 1025.0)	889.4 (565.9 to 1268.9)	21.9% (-29.9 to 112.3)	3749.9 (2694.8 to 5123.9)	4609.3 (3276.7 to 6346.0)	22.9% (-22.1 to 94.0)	455.3 (278.7 to 688.6)	564.0 (341.4 to 862.3)	23.9% (-34.7 to 135.2)
Western Sub-Saharan Africa	623.2 (405.5 to 873.7)	683.7 (448.8 to 947.6)	9.7% (-35.4 to 86.2)	3631.3 (2607.9 to 4919.3)	3981.7 (2860.4 to 5431.9)	9.7% (-29.8 to 71.5)	440.7 (269.5 to 666.4)	487.2 (295.4 to 739.2)	10.5% (-41.6 to 109.4)
Southern Sub-Saharan Africa	709.3 (465.0 to 985.8)	989.3 (642.1 to 1381.7)	39.5% (-17.9 to 137.4)	3936.6 (2902.5 to 5195.1)	5514.8 (4088.9 to 7272.0)	40.1% (-6.6 to 110.0)	482.2 (300.3 to 715.8)	674.7 (420.1 to 991.5)	39.9% (-23.6 to 155.9)

Australasia	1202.4 (747.5 to 1744.8)	1366.9 (831.7 to 1991.1)	13.7% (-37.7 to 107.6)	7119.9 (5048.4 to 9812.2)	7599.1 (5090.3 to 10945.7)	6.7% (-35.6 to 77.3)	865.2 (521.7 to 1328.2)	923.9 (539.2 to 1444.5)	6.8% (-45.5 to 109.7)
High-income Asia Pacific	613.8 (401.3 to 846.6)	742.3 (483.8 to 1037.6)	20.9% (-28.6 to 105.2)	3673.7 (2697.5 to 4900.9)	4414.9 (3216.7 to 5909.7)	20.2% (-21.2 to 83.3)	453.0 (280.7 to 684.0)	544.7 (337.7 to 825.7)	20.2% (-35.6 to 124.5)
Caribbean	799.8 (490.4 to 1179.7)	1025.7 (626.2 to 1547.6)	28.3% (-31.3 to 139.4)	4672.6 (3163.0 to 6631.9)	5977.6 (3921.3 to 8703.9)	27.9% (-25.5 to 119.6)	571.1 (334.0 to 892.9)	729.1 (425.0 to 1153.9)	27.7% (-36.2 to 155.7)
Oceania	732.9 (456.6 to 1076.1)	838.1 (498.1 to 1285.8)	14.4% (-39.4 to 116.3)	4403.1 (2949.1 to 6331.7)	5056.2 (3250.0 to 7641.4)	14.8% (-35.1 to 103.8)	539.5 (318.1 to 841.3)	623.0 (339.4 to 1020.6)	15.5% (-44.4 to 140.7)

UI, uncertainly interval; DALYs, disability-adjusted life-years.

Table S2 The incidence, prevalence and DALYs rate of depressive disorders in 1990 and 2021, and changes from 1990 to 2021 at the global level and different regions.

**Location**	**Incidence (95% UI)**			**Prevalence (95% UI)**			**DALYs (95% UI)**		
	**1990**	**2021**	**Percentage change**	**1990**	**2021**	**Percentage change**	**1990**	**2021**	**Percentage change**
Global	3085.5 (2196.3 to 4264.0)	3909.9 (2722.0 to 5501.8)	27.2% (-29.6 to 130.2)	2486.9 (1863.1 to 3307.3)	3045.3 (2237.1 to 4136.3)	22.5% (-19.3 to 85.9)	454.1 (287.0 to 677.4)	567.8 (353.3 to 857.9)	25.0% (-32.1 to 130.5)
High SDI	4122.8 (3035.7 to 5465.9)	6741.9 (4880.6 to 9050.4)	11.8% (-34.6 to 91.0)	3315.6 (2545.6 to 4269.6)	4967.5 (3742.7 to 6571.8)	49.8% (2.5 to 119.1)	613.2 (397.3 to 904.4)	962.2 (620.3 to 1435.4)	56.9% (-12.2 to 180.9)
High-middle SDI	2995.9 (2177.8 to 4052.9)	3531.7 (2404.5 to 4988.1)	11.8% (-34.6 to 91.0)	2419.8 (1834.3 to 3151.3)	2755.8 (1988.6 to 3757.1)	13.9% (-25.0 to 73.2)	446.2 (284.8 to 661.7)	518.2 (316.1 to 785.7)	16.1% (-37.3 to 115.1)
Middle SDI	2662.0 (1884.6 to 3683.7)	3187.2 (2215.2 to 4488.3)	11.8% (-34.6 to 91.0)	2150.6 (1606.7 to 2847.7)	2509.0 (1848.2 to 3403.9)	16.7% (-23.0 to 76.8)	393.0 (248.2 to 587.9)	465.8 (288.9 to 703.2)	18.5% (-35.8 to 118.9)
Low-middle SDI	3121.7 (2168.6 to 4438.5)	3844.5 (2624.5 to 5480.1)	11.8% (-34.6 to 91.0)	2492.6 (1828.4 to 3357.0)	3013.0 (2191.1 to 4110.6)	20.9% (-21.6 to 86.4)	452.0 (282.6 to 684.6)	558.0 (345.2 to 845.4)	23.4% (-33.8 to 130.4)
Low SDI	3354.2 (2259.5 to 4835.8)	3897.6 (2565.4 to 5628.1)	11.8% (-34.6 to 91.0)	2739.5 (1985.8 to 3746.6)	3107.8 (2225.3 to 4258.5)	13.4% (-27.6 to 77.9)	488.1 (296.1 to 741.6)	567.1 (343.1 to 871.3)	16.2% (-39.2 to 122.2)

North Africa and Middle East	4773.1 (3211.1 to 6849.8)	6064.4 (3901.0 to 8913.3)	11.8% (-34.6 to 91.0)	3643.5 (2583.2 to 5057.0)	4503.4 (3089.6 to 6373.5)	23.6% (-24.3 to 101.8)	688.8 (416.2 to 1058.8)	868.4 (503.3 to 1369.2)	26.1% (-36.1 to 148.8)
South Asia	2871.7 (2044.4 to 4032.3)	3476.3 (2432.5 to 4861.0)	11.8% (-34.6 to 91.0)	2324.5 (1744.6 to 3090.6)	2775.3 (2059.5 to 3746.1)	19.4% (-20.8 to 80.0)	415.8 (262.1 to 625.6)	507.5 (317.6 to 767.0)	22.0% (-33.9 to 125.5)
Western Europe	5124.4 (3818.8 to 6763.6)	6281.6 (4189.3 to 8917.0)	11.8% (-34.6 to 91.0)	4054.1 (3159.7 to 5189.0)	4739.4 (3350.1 to 6594.0)	16.9% (-23.0 to 77.8)	758.9 (490.9 to 1113.6)	907.6 (551.2 to 1394.7)	19.6% (-35.4 to 122.1)
Central Europe	2091.6 (1447.4 to 2928.1)	2842.5 (1908.7 to 4110.9)	45.8% (-19.0 to 162.6)	1856.9 (1360.2 to 2487.4)	2352.4 (1696.1 to 3210.3)	26.7% (-18.0 to 96.0)	322.4 (201.7 to 483.6)	425.8 (256.1 to 651.7)	32.1% (-30.0 to 149.3)
Eastern Europe	2729.1 (1870.6 to 3860.6)	3801.9 (2538.9 to 5346.8)	27.2% (-29.6 to 130.2)	2240.3 (1664.8 to 3021.3)	2879.0 (2071.4 to 3932.7)	28.5% (-16.8 to 98.5)	404.8 (250.8 to 610.0)	543.2 (335.4 to 828.7)	34.2% (-28.4 to 151.6)
East Asia	2329.8 (1685.9 to 3198.1)	1398.0 (988.4 to 1927.9)	27.2% (-29.6 to 130.2)	1915.9 (1450.8 to 2477.5)	1274.6 (960.1 to 1662.1)	-33.5% (-54.5 to -2.8)	350.4 (224.5 to 520.3)	220.2 (137.7 to 327.8)	-37.2% (-65.4 to 14.3)
Central Asia	2748.2 (1853.8 to 3891.0)	3511.2 (2311.3 to 5167.5)	45.8% (-19.0 to 162.6)	2282.0 (1650.6 to 3103.9)	2764.9 (1926.7 to 3837.2)	21.2% (-23.8 to 92.8)	411.6 (254.0 to 624.3)	515.2 (312.2 to 799.1)	25.2% (-34.3 to 138.6)
Southeast Asia	2204.2 (1503.5 to 3121.0)	2932.9 (1940.1 to 4202.7)	11.8% (-34.6 to 91.0)	1840.0 (1341.4 to 2484.9)	2329.3 (1667.7 to 3197.7)	26.6% (-18.8 to 97.6)	331.8 (204.1 to 504.2)	435.2 (263.5 to 663.7)	31.2% (-30.8 to 148.9)
High-income North America	5128.6 (3734.6 to 6849.7)	9884.4 (7369.7 to 12933.5)	11.8% (-34.6 to 91.0)	4125.3 (3134.0 to 5333.1)	7036.5 (5433.8 to 9033.4)	70.6% (18.6 to 145.2)	763.7 (491.3 to 1135.4)	1387.2 (907.2 to 2047.5)	81.6% (2.0 to 223.3)
Tropical Latin America	3513.7 (2500.1 to 4850.6)	4562.7 (3261.1 to 6288.9)	11.8% (-34.6 to 91.0)	2624.1 (1950.9 to 3495.0)	3333.4 (2476.7 to 4505.0)	27.0% (-16.0 to 92.1)	493.6 (314.4 to 737.1)	636.6 (403.8 to 959.6)	29.0% (-29.3 to 135.5)
Andean Latin America	2276.2 (1537.7 to 3263.0)	3501.9 (2266.4 to 5179.3)	#N/A	1878.9 (1345.9 to 2556.5)	2690.9 (1854.4 to 3811.0)	43.2% (-11.4 to 132.1)	337.4 (202.4 to 516.9)	505.5 (297.3 to 795.2)	49.8% (-23.6 to 194.3)
Central Latin America	2311.9 (1609.1 to 3231.6)	3798.1 (2657.6 to 5346.2)	45.8% (-19.0 to 162.6)	1870.3 (1383.6 to 2514.8)	2830.3 (2053.3 to 3866.8)	51.3% (-1.7 to 133.2)	339.0 (210.6 to 513.9)	539.6 (334.3 to 822.7)	59.2% (-15.0 to 198.3)

Southern Latin America	4468.0 (3210.9 to 6023.5)	5998.1 (4103.0 to 8362.6)	11.8% (-34.6 to 91.0)	3325.7 (2486.4 to 4394.6)	4347.4 (3101.5 to 5863.0)	30.7% (-14.6 to 100.4)	641.3 (405.7 to 966.2)	852.5 (523.8 to 1291.4)	32.9% (-28.5 to 147.3)
Central Sub-Saharan Africa	5573.9 (3590.2 to 8254.8)	6014.4 (3750.6 to 9113.0)	45.8% (-19.0 to 162.6)	4135.4 (2849.3 to 5857.7)	4401.6 (2958.3 to 6367.0)	6.4% (-36.9 to 79.5)	774.5 (458.1 to 1216.7)	837.7 (480.8 to 1328.5)	8.2% (-46.2 to 117.5)
Eastern Sub-Saharan Africa	3634.5 (2447.8 to 5207.6)	4346.9 (2862.0 to 6310.3)	27.2% (-29.6 to 130.2)	3029.8 (2216.4 to 4107.6)	3496.6 (2506.1 to 4786.8)	15.4% (-25.9 to 79.9)	536.1 (328.0 to 810.9)	637.0 (381.0 to 978.4)	18.8% (-37.8 to 127.2)
Western Sub-Saharan Africa	2904.3 (1958.4 to 4165.6)	2983.2 (1991.7 to 4355.7)	11.8% (-34.6 to 91.0)	2469.5 (1788.5 to 3333.0)	2521.9 (1792.8 to 3440.1)	2.1% (-34.7 to 59.7)	431.4 (263.0 to 653.8)	446.0 (271.1 to 684.9)	3.4% (-45.6 to 96.8)
Southern Sub-Saharan Africa	3120.8 (2199.4 to 4307.5)	4628.1 (3196.8 to 6450.7)	11.8% (-34.6 to 91.0)	2612.2 (1966.9 to 3425.1)	3569.7 (2611.0 to 4758.3)	36.7% (-8.9 to 105.1)	463.4 (291.3 to 689.9)	664.2 (411.9 to 994.0)	43.3% (-22.1 to 164.0)
Australasia	6725.0 (4765.4 to 9048.4)	7472.4 (4970.2 to 10684.7)	#N/A	5018.1 (3711.7 to 6573.6)	5476.5 (3893.2 to 7734.1)	9.1% (-30.2 to 70.9)	960.1 (610.1 to 1423.3)	1061.4 (649.5 to 1646.9)	10.5% (-40.8 to 107.0)
High-income Asia Pacific	2658.0 (1926.6 to 3575.0)	3739.0 (2639.6 to 5071.2)	11.8% (-34.6 to 91.0)	2163.8 (1671.2 to 2769.5)	2875.7 (2161.0 to 3761.3)	32.9% (-8.4 to 93.1)	397.3 (254.5 to 588.0)	547.6 (344.7 to 818.8)	37.8% (-24.0 to 150.2)
Caribbean	3726.7 (2526.5 to 5273.8)	4159.9 (2710.6 to 6258.5)	45.8% (-19.0 to 162.6)	2831.8 (2031.0 to 3839.1)	3108.2 (2142.7 to 4485.0)	9.8% (-32.5 to 79.0)	533.0 (325.8 to 819.6)	590.0 (350.1 to 922.3)	10.7% (-43.0 to 115.2)
Oceania	2922.4 (1941.5 to 4307.6)	3147.2 (1994.4 to 4795.2)	11.8% (-34.6 to 91.0)	2279.0 (1605.5 to 3193.2)	2432.8 (1670.9 to 3474.4)	6.7% (-35.2 to 75.8)	423.2 (256.3 to 658.6)	455.9 (258.5 to 731.2)	7.7% (-46.4 to 117.0)

UI, uncertainly interval; DALYs, disability-adjusted life-years.

Table S3 The incidence, prevalence and DALYs rate of anxiety disorders in 1990 and 2021, and changes from 1990 to 2021 at 204 countries and territories around the world.

**Location**	**Incidence (95% UI)**			**Prevalence (95% UI)**			**DALYs (95% UI)**		
	**1990**	**2021**	**Percentage change**	**1990**	**2021**	**Percentage change**	**1990**	**2021**	**Percentage change**
Saint Vincent and the Grenadines	796.7 (491.4 to 1190.4)	1040.6 (602.6 to 1634.0)	30.6% (-32.8 to 154.3)	4559.5 (3089.7 to 6547.8)	6047.1 (3792.4 to 9112.4)	32.6% (-25.5 to 136.6)	558.5 (320.3 to 868.0)	739.5 (412.5 to 1203.5)	32.4% (-36.0 to 173.8)

Viet Nam	442.6 (278.3 to 649.1)	557.6 (320.5 to 858.0)	26.0% (-34.1 to 141.7)	2289.2 (1547.0 to 3309.5)	2872.0 (1745.0 to 4363.2)	25.5% (-30.9 to 128.0)	282.5 (161.0 to 441.1)	355.8 (191.7 to 600.3)	25.9% (-41.0 to 169.6)
United States of America	972.3 (654.7 to 1320.4)	1414.3 (944.8 to 1917.4)	45.5% (-11.2 to 138.3)	4833.3 (3626.6 to 6225.3)	7002.0 (5269.1 to 8961.2)	44.9% (-0.4 to 110.7)	590.5 (372.9 to 859.5)	847.1 (541.4 to 1241.0)	43.5% (-19.9 to 156.8)
Timor-Leste	644.9 (396.0 to 944.6)	816.0 (473.2 to 1248.5)	26.5% (-33.8 to 142.7)	3595.9 (2430.3 to 5152.6)	4549.4 (2813.3 to 6800.5)	26.5% (-29.1 to 126.4)	439.4 (256.1 to 683.2)	562.0 (299.8 to 914.3)	27.9% (-39.0 to 168.9)
Lao People's Democratic Republic	765.1 (482.8 to 1126.2)	943.4 (547.2 to 1470.4)	23.3% (-35.6 to 136.9)	4349.8 (2926.5 to 6175.5)	5543.5 (3439.5 to 8367.0)	27.4% (-28.7 to 128.2)	533.5 (309.9 to 839.5)	682.3 (365.4 to 1087.4)	27.9% (-38.6 to 166.8)
Ethiopia	760.2 (512.6 to 1038.4)	868.0 (571.6 to 1188.6)	14.2% (-31.0 to 89.0)	3834.1 (2895.4 to 4946.1)	4404.9 (3271.8 to 5789.3)	14.9% (-22.1 to 69.5)	467.5 (295.1 to 682.8)	540.5 (337.6 to 816.7)	15.6% (-36.8 to 111.8)
South Sudan	791.3 (481.4 to 1170.2)	894.0 (510.1 to 1409.6)	13.0% (-42.4 to 121.9)	4269.5 (2868.8 to 6131.8)	4789.0 (2962.2 to 7221.8)	12.2% (-37.5 to 101.9)	518.1 (299.0 to 807.7)	584.3 (317.9 to 959.0)	12.8% (-46.1 to 136.6)
Suriname	783.0 (471.8 to 1168.4)	1145.7 (651.7 to 1802.7)	46.3% (-25.8 to 189.4)	4534.8 (3039.6 to 6498.5)	6576.5 (3998.9 to 9949.0)	45.0% (-19.9 to 163.1)	553.3 (322.5 to 886.1)	801.4 (430.9 to 1299.6)	44.8% (-31.1 to 204.9)
Dominica	794.1 (483.0 to 1169.7)	1047.0 (595.3 to 1639.5)	31.8% (-32.6 to 158.3)	4567.4 (3053.9 to 6528.8)	6088.5 (3781.1 to 9243.6)	33.3% (-25.8 to 140.3)	560.6 (325.3 to 875.8)	743.8 (405.5 to 1230.1)	32.7% (-36.7 to 179.2)
Tuvalu	727.8 (454.3 to 1063.3)	858.8 (508.4 to 1348.3)	18.0% (-38.1 to 125.4)	4437.3 (2998.0 to 6355.7)	5240.9 (3297.1 to 8129.5)	18.1% (-34.4 to 113.0)	547.1 (319.9 to 850.4)	646.4 (354.3 to 1053.7)	18.1% (-43.0 to 145.6)
Republic of Moldova	743.9 (461.8 to 1109.0)	1043.9 (593.9 to 1646.1)	40.3% (-28.3 to 175.5)	4389.7 (2938.7 to 6236.3)	6173.5 (3893.5 to 9401.2)	40.6% (-21.2 to 151.6)	542.1 (313.2 to 855.7)	760.5 (425.2 to 1244.9)	40.3% (-32.4 to 191.3)
Bulgaria	645.9 (394.6 to 958.3)	1098.5 (636.2 to 1723.6)	70.1% (-12.5 to 231.6)	3728.9 (2503.8 to 5344.4)	6252.1 (3821.5 to 9446.7)	67.7% (-7.1 to 203.2)	459.0 (264.0 to 718.0)	768.2 (423.8 to 1268.6)	67.4% (-19.9 to 250.5)
Germany	1115.1 (706.6 to 1628.1)	1548.6 (923.9 to 2369.3)	38.9% (-25.8 to 160.4)	7504.9 (5275.8 to 10491.9)	10307.8 (6755.4 to 14991.9)	37.3% (-18.8 to 132.8)	918.0 (547.3 to 1410.8)	1260.6 (702.6 to 2023.3)	37.3% (-32.2 to 179.3)

Romania	610.6 (375.9 to 920.6)	913.3 (520.3 to 1430.6)	49.6% (-23.7 to 193.8)	3474.8 (2336.3 to 5015.5)	5151.8 (3184.4 to 7741.1)	48.3% (-17.4 to 166.9)	426.6 (244.5 to 682.6)	635.0 (339.6 to 1040.1)	48.8% (-29.9 to 216.7)
Lebanon	1147.5 (721.3 to 1677.9)	1728.5 (1038.9 to 2614.6)	50.6% (-19.1 to 180.6)	7926.6 (5403.4 to 11206.6)	11703.6 (7424.5 to 17134.1)	47.6% (-15.1 to 157.2)	966.3 (573.1 to 1502.5)	1433.3 (784.9 to 2254.7)	48.3% (-27.0 to 202.1)
China	652.9 (426.9 to 906.1)	699.7 (470.3 to 963.2)	7.2% (-35.8 to 79.0)	4304.3 (3283.9 to 5565.9)	4387.1 (3300.7 to 5739.4)	1.9% (-30.2 to 49.0)	533.6 (339.7 to 779.7)	546.6 (340.1 to 809.9)	2.4% (-43.5 to 85.9)
Canada	862.2 (631.3 to 1109.1)	1056.6 (600.5 to 1611.7)	22.5% (-30.4 to 116.6)	4344.1 (3548.4 to 5251.6)	5123.9 (3187.9 to 7767.2)	18.0% (-27.0 to 91.9)	532.2 (350.5 to 751.3)	625.2 (355.0 to 997.0)	17.5% (-37.9 to 123.0)
Guinea-Bissau	624.8 (395.1 to 906.6)	727.1 (418.2 to 1148.3)	16.4% (-39.4 to 124.0)	3678.1 (2464.2 to 5256.3)	4309.5 (2681.8 to 6641.6)	17.2% (-35.1 to 112.0)	447.7 (260.3 to 693.9)	526.7 (291.7 to 867.4)	17.6% (-43.2 to 144.4)
Philippines	713.7 (478.0 to 982.8)	951.3 (635.1 to 1310.2)	33.3% (-19.6 to 121.0)	3873.4 (2933.1 to 4971.5)	5207.1 (3945.0 to 6705.5)	34.4% (-7.2 to 94.7)	475.9 (302.0 to 700.4)	641.2 (408.8 to 945.8)	34.7% (-25.1 to 142.4)
American Samoa	726.8 (455.4 to 1086.6)	879.6 (502.4 to 1365.5)	21.0% (-37.5 to 135.0)	4433.8 (3004.9 to 6325.2)	5233.7 (3207.0 to 8090.9)	18.0% (-34.7 to 113.6)	547.3 (316.8 to 867.1)	647.5 (360.6 to 1072.7)	18.3% (-43.4 to 147.4)
Burkina Faso	627.7 (402.9 to 940.5)	685.7 (406.8 to 1065.2)	9.2% (-42.4 to 107.5)	3658.9 (2470.9 to 5268.6)	4067.3 (2531.2 to 6259.8)	11.2% (-38.4 to 101.0)	444.0 (258.7 to 694.9)	498.1 (272.9 to 797.7)	12.2% (-45.6 to 131.6)
Benin	632.2 (398.2 to 937.8)	711.1 (409.3 to 1112.4)	12.5% (-41.7 to 117.7)	3727.1 (2476.1 to 5333.8)	4236.5 (2611.8 to 6491.6)	13.7% (-37.3 to 106.6)	452.7 (262.3 to 712.9)	519.3 (276.8 to 859.8)	14.7% (-46.0 to 144.2)
Iraq	1014.7 (651.8 to 1482.5)	1228.3 (731.2 to 1914.3)	21.1% (-35.6 to 128.4)	6604.4 (4477.9 to 9298.1)	8032.4 (5096.9 to 12243.3)	21.6% (-31.3 to 115.7)	806.8 (471.1 to 1253.5)	985.9 (547.3 to 1602.0)	22.2% (-40.6 to 152.0)
Antigua and Barbuda	801.3 (488.6 to 1194.4)	1026.0 (591.7 to 1648.3)	28.0% (-35.0 to 152.8)	4650.3 (3124.2 to 6658.7)	6034.1 (3778.6 to 9225.3)	29.8% (-27.7 to 133.5)	570.1 (329.9 to 902.2)	739.7 (398.3 to 1205.2)	29.8% (-38.3 to 173.5)
Lesotho	696.4 (438.8 to 1037.8)	1057.1 (608.5 to 1637.1)	51.8% (-21.1 to 192.7)	3869.3 (2606.5 to 5664.2)	6027.3 (3765.1 to 9196.4)	55.8% (-13.7 to 181.7)	473.6 (272.2 to 747.7)	734.5 (413.8 to 1202.0)	55.1% (-25.2 to 221.7)

Afghanistan	1012.1 (642.6 to 1465.9)	1319.0 (787.7 to 2028.7)	30.3% (-30.3 to 144.2)	6521.2 (4445.3 to 9195.6)	8430.3 (5404.9 to 12688.5)	29.3% (-26.1 to 126.9)	785.3 (466.7 to 1212.8)	1026.0 (594.2 to 1644.1)	30.7% (-34.6 to 161.4)
Oman	1012.0 (661.8 to 1477.2)	1277.8 (766.5 to 1935.8)	26.3% (-31.5 to 133.4)	6384.0 (4321.1 to 8991.6)	8173.6 (5028.7 to 12225.6)	28.0% (-28.0 to 128.0)	784.8 (455.3 to 1211.5)	1006.6 (555.8 to 1612.8)	28.3% (-37.4 to 163.3)
Senegal	610.5 (383.7 to 897.9)	769.9 (455.4 to 1192.0)	26.1% (-33.5 to 139.6)	3582.5 (2412.0 to 5158.6)	4516.0 (2786.9 to 6939.1)	26.1% (-30.4 to 128.8)	434.3 (252.4 to 685.1)	551.5 (295.1 to 934.3)	27.0% (-40.7 to 172.6)
Trinidad and Tobago	764.3 (471.0 to 1128.9)	1131.1 (654.7 to 1774.1)	48.0% (-23.6 to 187.3)	4310.3 (2897.4 to 6185.0)	6416.6 (4043.1 to 9842.8)	48.9% (-17.0 to 167.7)	527.5 (304.8 to 834.0)	787.2 (439.7 to 1293.5)	49.2% (-28.3 to 210.6)
Niger	605.2 (385.9 to 885.1)	639.4 (384.6 to 997.3)	5.7% (-43.7 to 98.9)	3522.5 (2378.4 to 5073.3)	3740.6 (2354.7 to 5654.0)	6.2% (-40.4 to 89.7)	429.1 (249.7 to 675.6)	460.4 (240.0 to 758.7)	7.3% (-49.8 to 130.0)
Kenya	698.1 (471.4 to 953.4)	849.1 (569.4 to 1159.8)	21.6% (-25.9 to 99.7)	3466.1 (2617.4 to 4454.8)	4267.3 (3155.8 to 5511.9)	23.1% (-16.0 to 80.5)	425.6 (266.8 to 627.4)	524.0 (331.1 to 767.5)	23.1% (-32.0 to 122.8)
Costa Rica	801.7 (499.5 to 1182.4)	1057.1 (608.7 to 1652.8)	31.9% (-31.8 to 155.5)	4705.7 (3190.4 to 6664.7)	6293.9 (3998.9 to 9489.4)	33.8% (-24.1 to 136.5)	579.5 (337.0 to 911.6)	774.6 (416.7 to 1269.1)	33.7% (-36.5 to 182.1)
Singapore	600.6 (372.2 to 885.2)	696.9 (410.0 to 1116.0)	16.0% (-40.1 to 125.2)	3707.4 (2500.3 to 5313.6)	4155.1 (2589.0 to 6388.1)	12.1% (-37.8 to 102.3)	457.0 (263.1 to 733.4)	513.2 (278.8 to 848.1)	12.3% (-47.0 to 138.1)
Israel	881.2 (569.4 to 1297.0)	1058.4 (616.4 to 1638.9)	20.1% (-36.6 to 128.1)	5089.8 (3480.3 to 7251.1)	6115.6 (3864.2 to 9226.9)	20.2% (-32.0 to 112.7)	626.0 (361.5 to 990.3)	751.5 (420.4 to 1229.9)	20.1% (-42.2 to 149.5)
Liberia	646.9 (410.0 to 948.6)	721.3 (422.1 to 1131.4)	11.5% (-41.6 to 113.6)	3894.9 (2612.7 to 5598.6)	4345.1 (2701.6 to 6608.0)	11.6% (-38.0 to 101.2)	465.6 (274.8 to 743.2)	525.5 (286.6 to 887.8)	12.9% (-46.7 to 139.8)
Comoros	695.7 (428.5 to 1022.3)	879.5 (505.5 to 1393.7)	26.4% (-35.1 to 147.1)	3603.9 (2443.9 to 5128.1)	4631.8 (2916.3 to 7215.9)	28.5% (-28.3 to 131.0)	440.6 (255.7 to 689.6)	568.0 (312.0 to 948.4)	28.9% (-38.6 to 171.7)
Switzerland	1271.8 (799.8 to 1860.9)	1508.8 (882.1 to 2345.6)	18.6% (-37.8 to 126.7)	8886.7 (6035.0 to 12592.3)	10246.9 (6493.7 to 15164.4)	15.3% (-34.1 to 102.4)	1085.3 (634.0 to 1666.6)	1252.8 (695.7 to 1972.9)	15.4% (-43.0 to 133.8)

Kazakhstan	422.5 (260.9 to 629.5)	539.3 (308.3 to 835.9)	27.6% (-34.2 to 148.2)	2293.4 (1532.0 to 3292.3)	2897.5 (1801.1 to 4348.3)	26.3% (-29.4 to 126.7)	282.0 (161.7 to 449.3)	357.0 (201.5 to 570.1)	26.6% (-38.5 to 160.8)
Denmark	1071.9 (677.7 to 1583.8)	1357.2 (808.1 to 2079.0)	26.6% (-32.7 to 138.7)	6872.3 (4697.7 to 9676.1)	8586.5 (5480.6 to 12672.1)	24.9% (-28.1 to 117.6)	842.5 (494.3 to 1297.0)	1050.8 (599.5 to 1704.4)	24.7% (-38.4 to 152.8)
Namibia	682.1 (419.8 to 1012.5)	1059.9 (629.8 to 1630.6)	55.4% (-18.4 to 195.9)	3835.1 (2583.9 to 5515.2)	6023.4 (3822.0 to 9004.6)	57.1% (-11.2 to 178.2)	469.5 (272.9 to 736.0)	736.3 (409.8 to 1182.6)	56.8% (-23.7 to 222.6)
Sri Lanka	660.1 (404.2 to 977.9)	889.5 (506.3 to 1373.8)	34.7% (-30.6 to 162.3)	3694.5 (2492.9 to 5298.9)	5016.8 (3096.9 to 7560.8)	35.8% (-24.3 to 144.1)	453.4 (262.9 to 714.4)	619.3 (338.2 to 1037.9)	36.6% (-35.3 to 189.6)
Botswana	690.7 (426.6 to 1029.1)	990.3 (578.5 to 1576.5)	43.4% (-26.3 to 179.6)	3890.5 (2620.6 to 5556.6)	5649.4 (3518.8 to 8602.0)	45.2% (-19.0 to 160.9)	475.6 (276.4 to 760.9)	691.3 (378.2 to 1146.5)	45.4% (-31.0 to 207.0)
Brunei Darussalam	632.2 (396.3 to 933.9)	739.8 (422.9 to 1151.6)	17.0% (-39.4 to 126.7)	3789.2 (2554.9 to 5416.8)	4485.7 (2753.4 to 6804.3)	18.4% (-34.3 to 113.6)	466.9 (274.4 to 728.0)	554.4 (306.1 to 919.8)	18.7% (-42.9 to 147.6)
El Salvador	817.1 (511.5 to 1202.4)	1056.3 (603.3 to 1656.1)	29.3% (-33.3 to 151.2)	4769.8 (3224.7 to 6778.3)	6280.3 (3880.9 to 9508.4)	31.7% (-26.5 to 136.1)	581.1 (336.2 to 908.7)	770.8 (421.6 to 1234.0)	32.6% (-35.9 to 174.5)
Russian Federation	671.7 (449.7 to 920.9)	981.7 (663.6 to 1336.6)	46.1% (-11.0 to 139.7)	3733.9 (2839.8 to 4761.7)	5367.6 (4077.8 to 6873.5)	43.8% (-0.1 to 106.9)	459.7 (290.5 to 679.9)	662.4 (416.8 to 970.1)	44.1% (-20.4 to 160.7)
Mexico	568.2 (381.9 to 775.3)	998.8 (666.1 to 1367.8)	75.8% (6.7 to 189.9)	3190.5 (2422.9 to 4106.5)	5527.6 (4183.5 to 7061.0)	73.3% (19.9 to 150.2)	393.1 (249.8 to 577.4)	680.8 (426.8 to 994.5)	73.2% (-3.9 to 212.4)
Kyrgyzstan	418.3 (260.4 to 629.7)	565.8 (331.1 to 889.6)	35.3% (-30.1 to 162.5)	2260.3 (1515.3 to 3252.4)	3058.3 (1907.8 to 4681.7)	35.3% (-24.9 to 144.5)	277.8 (159.4 to 443.3)	377.3 (197.4 to 617.9)	35.8% (-36.6 to 192.2)
Nicaragua	823.8 (520.7 to 1214.9)	1068.4 (632.6 to 1652.9)	29.7% (-31.5 to 146.0)	4785.7 (3267.6 to 6853.6)	6311.2 (3996.6 to 9361.6)	31.9% (-24.9 to 132.0)	585.2 (340.4 to 923.3)	775.6 (430.4 to 1270.2)	32.5% (-36.2 to 175.5)
Serbia	622.9 (378.7 to 925.7)	862.4 (485.7 to 1331.2)	38.5% (-29.2 to 171.5)	3562.5 (2389.8 to 5108.5)	4947.5 (3029.7 to 7480.6)	38.9% (-23.1 to 151.1)	439.2 (253.3 to 697.4)	610.4 (335.8 to 1029.0)	39.0% (-34.4 to 195.0)

Haiti	820.4 (511.3 to 1202.2)	1020.2 (603.2 to 1600.2)	24.3% (-34.9 to 137.9)	4702.1 (3153.9 to 6718.9)	5922.9 (3650.8 to 9067.1)	26.0% (-30.3 to 128.1)	567.2 (325.3 to 887.9)	715.0 (397.3 to 1169.8)	26.1% (-39.4 to 162.2)
Chile	964.8 (676.1 to 1305.7)	1308.8 (757.0 to 2018.3)	35.7% (-24.7 to 144.7)	6206.4 (4989.0 to 7575.5)	8160.7 (5077.6 to 12159.4)	31.5% (-18.4 to 113.1)	759.1 (502.5 to 1074.6)	995.5 (543.9 to 1592.5)	31.1% (-31.8 to 152.6)
Tonga	736.4 (468.3 to 1079.0)	874.7 (509.8 to 1350.4)	18.8% (-37.4 to 126.0)	4397.0 (2972.5 to 6281.6)	5291.9 (3293.5 to 8207.9)	20.4% (-33.2 to 117.4)	544.8 (318.4 to 853.4)	653.3 (358.3 to 1069.3)	19.9% (-42.3 to 149.8)
Mozambique	736.8 (454.1 to 1077.9)	940.0 (547.6 to 1467.1)	27.6% (-33.6 to 145.8)	3842.2 (2579.8 to 5543.9)	4950.5 (3080.5 to 7563.3)	28.8% (-28.5 to 133.0)	462.9 (265.0 to 732.3)	599.8 (327.6 to 982.5)	29.6% (-38.4 to 172.6)
Guam	709.4 (437.8 to 1045.1)	922.3 (547.9 to 1413.2)	30.0% (-31.3 to 146.5)	4393.8 (2955.2 to 6276.8)	5634.6 (3564.4 to 8526.9)	28.2% (-27.9 to 128.5)	543.3 (314.2 to 856.2)	695.8 (381.6 to 1127.3)	28.1% (-38.5 to 166.7)
Brazil	1000.7 (673.4 to 1372.2)	1591.9 (1035.8 to 2196.9)	59.1% (-4.8 to 166.0)	5771.9 (4440.7 to 7370.0)	9316.3 (7065.4 to 12001.2)	61.4% (12.2 to 132.2)	703.2 (447.1 to 1018.2)	1133.2 (714.1 to 1661.3)	61.2% (-10.0 to 189.0)
United States Virgin Islands	803.1 (499.1 to 1203.2)	1055.8 (614.8 to 1659.7)	31.5% (-32.1 to 155.1)	4577.5 (3093.4 to 6514.0)	6077.2 (3862.5 to 9106.3)	32.8% (-24.7 to 134.5)	562.6 (328.6 to 893.2)	746.9 (411.3 to 1216.0)	32.8% (-36.2 to 176.3)
Japan	587.5 (392.4 to 803.1)	738.2 (498.1 to 1013.9)	25.6% (-23.8 to 107.0)	3448.6 (2614.0 to 4433.0)	4306.1 (3237.3 to 5598.1)	24.9% (-14.3 to 82.0)	425.2 (269.4 to 628.2)	531.1 (337.9 to 785.0)	24.9% (-30.8 to 125.5)
Saint Kitts and Nevis	798.4 (492.1 to 1185.0)	990.3 (557.5 to 1565.7)	24.0% (-37.0 to 145.0)	4595.1 (3086.8 to 6570.8)	5880.3 (3659.5 to 8977.6)	28.0% (-28.8 to 130.5)	563.0 (325.8 to 888.5)	720.1 (395.5 to 1199.6)	27.9% (-39.2 to 169.7)
Kiribati	729.1 (455.9 to 1083.9)	884.2 (513.5 to 1390.8)	21.3% (-37.2 to 134.9)	4443.7 (2986.2 to 6436.4)	5328.3 (3283.5 to 8144.9)	19.9% (-33.8 to 117.8)	543.8 (317.1 to 850.4)	654.3 (357.2 to 1031.2)	20.3% (-41.4 to 147.0)
Greece	1153.4 (733.1 to 1677.4)	1572.9 (961.8 to 2441.2)	36.4% (-26.7 to 154.3)	7444.8 (5073.0 to 10543.5)	10091.3 (6568.8 to 15109.4)	35.5% (-22.0 to 136.1)	911.7 (544.2 to 1412.0)	1234.1 (692.9 to 1980.1)	35.4% (-33.1 to 174.5)
Jamaica	776.5 (479.9 to 1146.5)	1046.0 (604.6 to 1640.5)	34.7% (-30.4 to 161.3)	4432.4 (2998.5 to 6268.1)	6123.0 (3759.5 to 9257.0)	38.1% (-22.7 to 147.5)	544.3 (323.8 to 846.8)	750.2 (406.2 to 1245.8)	37.8% (-34.0 to 189.1)

Fiji	738.1 (464.9 to 1090.3)	921.1 (536.8 to 1423.1)	24.8% (-34.6 to 138.4)	4432.4 (2959.7 to 6358.0)	5576.6 (3395.8 to 8495.8)	25.8% (-30.8 to 129.1)	544.8 (320.6 to 850.2)	687.7 (375.2 to 1142.4)	26.2% (-39.6 to 164.7)
Nigeria	622.7 (421.9 to 849.7)	664.0 (450.6 to 917.7)	6.6% (-34.9 to 74.9)	3574.0 (2724.2 to 4592.2)	3790.1 (2876.6 to 4931.9)	6.0% (-26.8 to 53.8)	433.3 (274.8 to 631.5)	463.2 (292.2 to 679.7)	6.9% (-40.5 to 92.4)
Libya	1048.4 (668.2 to 1526.8)	1300.2 (780.8 to 2018.2)	24.0% (-33.8 to 132.9)	6798.5 (4648.6 to 9715.0)	8693.1 (5469.3 to 12911.6)	27.9% (-27.3 to 125.7)	835.0 (485.4 to 1277.8)	1065.5 (583.4 to 1732.3)	27.6% (-38.2 to 164.4)
Armenia	492.8 (298.2 to 725.2)	731.1 (409.9 to 1174.8)	48.4% (-25.5 to 196.2)	2803.5 (1897.6 to 3994.2)	4129.0 (2503.6 to 6291.2)	47.3% (-18.4 to 166.2)	343.8 (200.6 to 545.3)	509.0 (271.3 to 848.9)	48.1% (-30.5 to 216.2)
Cambodia	699.2 (438.2 to 1038.4)	906.0 (514.8 to 1389.2)	29.6% (-32.8 to 150.4)	3913.1 (2629.9 to 5611.2)	5142.6 (3184.9 to 7867.4)	31.4% (-27.2 to 137.5)	477.0 (278.1 to 749.8)	631.5 (343.9 to 1035.0)	32.4% (-36.6 to 177.2)
Mauritius	659.2 (406.8 to 977.6)	848.4 (491.2 to 1341.3)	28.7% (-33.8 to 150.8)	3681.2 (2479.5 to 5260.9)	4836.8 (3035.2 to 7367.3)	31.4% (-26.5 to 135.6)	451.9 (262.7 to 709.2)	593.4 (327.7 to 967.6)	31.3% (-36.7 to 172.6)
Mongolia	423.1 (261.4 to 625.1)	478.3 (272.7 to 743.7)	13.0% (-41.8 to 119.9)	2299.8 (1534.4 to 3330.7)	2580.5 (1554.8 to 3954.0)	12.2% (-38.9 to 106.3)	282.4 (163.5 to 451.3)	317.9 (172.3 to 533.9)	12.6% (-47.1 to 140.5)
United Kingdom	966.9 (640.8 to 1327.2)	1277.1 (860.0 to 1741.5)	32.1% (-20.0 to 117.9)	5959.2 (4497.2 to 7723.4)	7671.1 (5715.5 to 9916.4)	28.7% (-12.1 to 88.7)	727.6 (461.8 to 1065.2)	937.3 (590.7 to 1368.7)	28.8% (-28.3 to 131.6)
Taiwan (Province of China)	774.5 (513.6 to 1084.8)	712.3 (418.0 to 1124.8)	-8.0% (-50.3 to 71.1)	5531.9 (3874.5 to 7402.0)	4885.2 (2986.5 to 7420.2)	-11.7% (-49.3 to 54.1)	687.6 (413.5 to 1051.7)	607.4 (331.3 to 1030.4)	-11.7% (-57.6 to 84.4)
India	531.9 (350.9 to 733.1)	651.8 (432.7 to 892.8)	22.5% (-26.5 to 104.2)	2521.0 (1857.3 to 3313.9)	3183.8 (2345.2 to 4186.2)	26.3% (-15.8 to 89.4)	306.1 (190.4 to 451.3)	388.8 (240.2 to 564.7)	27.0% (-30.4 to 131.6)
Nepal	529.2 (321.5 to 790.3)	748.9 (424.6 to 1195.6)	41.5% (-28.6 to 181.2)	2552.7 (1709.5 to 3692.2)	3713.2 (2284.1 to 5874.2)	45.5% (-20.8 to 167.7)	310.5 (179.4 to 494.3)	455.4 (242.4 to 760.8)	46.7% (-31.5 to 214.7)
Ecuador	941.4 (579.3 to 1394.1)	1328.5 (753.2 to 2075.6)	41.1% (-27.7 to 176.2)	5298.9 (3611.7 to 7578.4)	7514.5 (4690.6 to 11471.9)	41.8% (-20.7 to 154.0)	651.1 (383.2 to 998.7)	921.5 (508.3 to 1512.6)	41.5% (-31.4 to 192.7)

Cuba	785.9 (470.6 to 1160.5)	1064.8 (611.3 to 1678.7)	35.5% (-31.0 to 166.8)	4707.2 (3210.0 to 6658.9)	6203.6 (3887.4 to 9392.5)	31.8% (-25.7 to 134.0)	577.1 (335.3 to 899.8)	761.5 (425.7 to 1247.3)	32.0% (-36.1 to 172.6)
North Macedonia	643.6 (393.8 to 951.5)	1034.9 (593.1 to 1618.2)	60.8% (-17.4 to 213.9)	3705.3 (2499.0 to 5275.5)	6021.6 (3721.2 to 9067.9)	62.5% (-9.1 to 191.2)	456.6 (263.6 to 710.4)	740.3 (407.2 to 1208.8)	62.1% (-22.0 to 237.5)
Eswatini	692.8 (427.8 to 1023.5)	1104.1 (640.8 to 1714.4)	59.4% (-17.2 to 207.5)	3881.9 (2622.4 to 5580.9)	6242.0 (3849.2 to 9636.6)	60.8% (-11.2 to 191.6)	476.6 (273.5 to 757.7)	762.0 (432.5 to 1241.6)	59.9% (-22.6 to 230.6)
Micronesia (Federated States of)	747.5 (476.7 to 1101.4)	862.8 (506.1 to 1344.7)	15.4% (-39.3 to 120.1)	4400.8 (2955.7 to 6310.9)	5265.4 (3306.7 to 8092.4)	19.6% (-33.4 to 115.6)	542.3 (318.8 to 856.0)	649.4 (355.0 to 1038.5)	19.8% (-41.9 to 147.3)
Sao Tome and Principe	623.6 (393.8 to 908.0)	696.4 (403.4 to 1070.3)	11.7% (-41.2 to 112.7)	3649.8 (2453.2 to 5241.6)	4133.9 (2588.3 to 6286.7)	13.3% (-36.8 to 103.5)	448.0 (263.1 to 703.2)	507.0 (281.2 to 822.7)	13.2% (-45.1 to 133.4)
Marshall Islands	747.4 (477.2 to 1082.3)	869.8 (505.5 to 1363.5)	16.4% (-38.8 to 121.8)	4376.5 (2940.3 to 6201.1)	5264.3 (3258.3 to 7978.2)	20.3% (-32.9 to 116.0)	538.6 (312.7 to 839.3)	649.2 (358.3 to 1073.5)	20.5% (-42.1 to 151.7)
France	1229.8 (785.5 to 1769.0)	1600.0 (955.8 to 2438.7)	30.1% (-29.9 to 142.1)	8381.5 (5702.0 to 11737.7)	10566.7 (6718.6 to 15575.7)	26.1% (-27.5 to 119.9)	1025.5 (604.6 to 1588.0)	1290.8 (715.4 to 2056.3)	25.9% (-38.1 to 156.5)
Guatemala	818.4 (516.2 to 1204.2)	1060.4 (602.7 to 1669.6)	29.6% (-33.2 to 152.0)	4663.1 (3122.2 to 6633.1)	6282.4 (3890.1 to 9303.1)	34.7% (-24.2 to 140.0)	566.7 (325.7 to 869.6)	769.0 (429.0 to 1263.9)	35.7% (-34.3 to 180.9)
Zimbabwe	649.7 (402.2 to 967.8)	854.2 (492.6 to 1344.3)	31.5% (-32.4 to 156.1)	3583.4 (2426.9 to 5116.7)	4728.9 (2883.0 to 7258.9)	32.0% (-27.0 to 138.9)	439.3 (253.2 to 695.9)	580.1 (314.6 to 951.6)	32.0% (-37.3 to 178.5)
Peru	961.8 (581.8 to 1426.3)	1482.3 (829.3 to 2373.8)	54.1% (-22.7 to 208.4)	5471.9 (3686.8 to 7791.0)	8499.0 (5187.4 to 12851.8)	55.3% (-13.7 to 180.0)	670.5 (389.5 to 1039.7)	1045.8 (568.6 to 1706.4)	56.0% (-25.1 to 226.0)
Cook Islands	726.7 (460.1 to 1069.9)	877.2 (505.3 to 1394.5)	20.7% (-37.6 to 134.2)	4394.5 (3015.6 to 6289.3)	5367.6 (3377.5 to 8081.1)	22.1% (-31.0 to 116.5)	545.1 (313.7 to 859.1)	662.1 (366.3 to 1099.1)	21.5% (-42.0 to 154.9)
Bolivia (Plurinational State of)	971.3 (595.0 to 1443.0)	1559.9 (918.7 to 2426.3)	60.6% (-16.4 to 209.1)	5485.8 (3703.6 to 7880.1)	8918.3 (5677.4 to 13457.6)	62.6% (-8.2 to 188.4)	672.5 (391.1 to 1047.3)	1092.7 (623.2 to 1779.0)	62.5% (-20.4 to 232.1)

Indonesia	635.0 (423.0 to 868.6)	866.2 (574.4 to 1198.2)	36.4% (-18.0 to 127.1)	3393.6 (2566.2 to 4376.0)	4704.4 (3546.2 to 6138.5)	38.6% (-5.1 to 102.5)	418.2 (262.3 to 621.0)	581.4 (364.2 to 872.0)	39.0% (-24.2 to 155.4)
Jordan	1002.4 (635.8 to 1477.5)	1228.0 (737.0 to 1875.0)	22.5% (-34.4 to 129.4)	6532.8 (4453.2 to 9293.3)	7974.4 (4995.5 to 11864.4)	22.1% (-30.8 to 115.9)	802.2 (468.1 to 1245.8)	980.9 (520.7 to 1612.7)	22.3% (-42.0 to 158.6)
Belize	788.8 (487.6 to 1161.6)	997.6 (577.7 to 1545.8)	26.5% (-34.2 to 143.8)	4471.9 (3027.6 to 6354.6)	5766.6 (3590.2 to 8727.4)	29.0% (-27.7 to 130.5)	548.4 (320.6 to 872.8)	706.3 (388.2 to 1148.0)	28.8% (-38.1 to 168.2)
Croatia	638.4 (391.5 to 948.6)	885.8 (506.7 to 1397.7)	38.7% (-29.1 to 172.1)	3695.1 (2484.7 to 5327.9)	5128.1 (3181.9 to 7827.3)	38.8% (-23.0 to 150.9)	455.1 (264.5 to 715.3)	632.8 (347.7 to 1051.6)	39.0% (-33.6 to 192.2)
Netherlands	1150.4 (718.9 to 1674.7)	1551.1 (905.7 to 2380.6)	34.8% (-28.9 to 156.2)	8039.3 (5647.9 to 10967.7)	10885.6 (6845.9 to 15975.0)	35.4% (-20.7 to 131.9)	989.9 (590.1 to 1512.3)	1332.3 (729.3 to 2138.3)	34.6% (-34.0 to 175.0)
Qatar	949.9 (596.2 to 1404.2)	1052.4 (615.8 to 1638.5)	10.8% (-42.1 to 112.3)	6196.9 (4163.8 to 8755.7)	6864.9 (4301.4 to 10315.7)	10.8% (-37.6 to 97.2)	762.2 (439.8 to 1183.5)	844.0 (472.6 to 1342.0)	10.7% (-45.7 to 126.3)
Republic of Korea	670.8 (417.9 to 992.3)	755.8 (445.7 to 1170.6)	12.7% (-40.9 to 115.5)	4152.1 (2812.9 to 5906.9)	4686.2 (2900.1 to 7104.4)	12.9% (-36.9 to 102.3)	512.0 (299.7 to 810.6)	578.4 (312.9 to 964.7)	13.0% (-46.5 to 139.3)
San Marino	1120.4 (707.1 to 1655.5)	1482.0 (892.5 to 2278.3)	32.3% (-29.5 to 148.6)	7385.3 (5065.4 to 10398.6)	9532.1 (6126.8 to 13804.5)	29.1% (-24.8 to 122.0)	906.3 (529.2 to 1409.1)	1167.9 (646.8 to 1882.2)	28.9% (-37.2 to 164.9)
Italy	1156.2 (762.5 to 1591.2)	1490.3 (998.2 to 2041.9)	28.9% (-22.4 to 114.0)	7449.9 (5696.6 to 9573.4)	9334.1 (7206.2 to 11828.3)	25.3% (-12.1 to 78.5)	911.6 (583.0 to 1322.2)	1142.2 (731.4 to 1653.7)	25.3% (-29.2 to 121.9)
Maldives	654.4 (402.4 to 962.3)	793.4 (450.4 to 1265.5)	21.2% (-38.3 to 138.9)	3603.7 (2427.5 to 5172.3)	4456.0 (2752.3 to 6980.3)	23.7% (-32.0 to 125.3)	441.7 (253.6 to 688.1)	548.6 (296.7 to 891.3)	24.2% (-40.6 to 160.4)
Bermuda	795.1 (476.2 to 1209.9)	1055.6 (616.8 to 1676.0)	32.8% (-32.7 to 161.8)	4685.1 (3171.3 to 6657.1)	6117.7 (3834.2 to 9204.1)	30.6% (-26.4 to 132.3)	578.1 (337.0 to 913.7)	752.3 (401.9 to 1214.3)	30.1% (-37.9 to 173.4)
Azerbaijan	451.4 (274.2 to 670.8)	637.4 (367.5 to 1028.1)	41.2% (-28.5 to 179.5)	2532.6 (1691.8 to 3664.8)	3531.6 (2169.7 to 5498.3)	39.4% (-23.8 to 155.9)	310.9 (176.7 to 495.0)	434.8 (237.1 to 717.1)	39.8% (-34.0 to 196.3)

Ukraine	633.4 (418.6 to 873.3)	908.0 (556.6 to 1319.3)	43.3% (-18.6 to 153.3)	3499.3 (2653.1 to 4519.3)	4929.8 (3389.4 to 6956.6)	40.9% (-9.7 to 120.2)	431.1 (271.5 to 643.1)	608.2 (357.1 to 952.8)	41.1% (-26.4 to 171.3)
Vanuatu	737.1 (466.0 to 1075.9)	878.0 (509.1 to 1372.9)	19.1% (-37.7 to 128.4)	4395.3 (2962.7 to 6235.2)	5316.7 (3345.5 to 8171.9)	21.0% (-32.4 to 116.8)	541.1 (311.2 to 844.9)	656.5 (351.7 to 1079.8)	21.3% (-42.6 to 157.2)
Türkiye	857.3 (621.9 to 1119.2)	1203.5 (701.8 to 1845.4)	40.4% (-20.0 to 146.8)	5522.5 (4569.2 to 6587.3)	7662.3 (4744.1 to 11475.9)	38.7% (-13.5 to 123.7)	679.2 (445.5 to 947.5)	942.3 (498.8 to 1503.0)	38.7% (-28.7 to 170.4)
Malaysia	756.6 (464.7 to 1132.4)	991.9 (555.8 to 1568.0)	31.1% (-33.8 to 160.4)	4366.9 (2927.3 to 6328.5)	5846.5 (3678.1 to 8935.9)	33.9% (-25.5 to 141.2)	537.7 (308.4 to 843.1)	720.1 (395.7 to 1175.4)	33.9% (-35.8 to 179.7)
Kuwait	962.9 (602.7 to 1418.2)	1069.8 (640.4 to 1614.0)	11.1% (-40.6 to 107.8)	6295.6 (4275.8 to 8898.1)	6956.6 (4396.0 to 10416.5)	10.5% (-37.2 to 95.1)	775.3 (447.5 to 1197.9)	856.8 (469.6 to 1405.9)	10.5% (-46.9 to 130.7)
Panama	755.3 (462.9 to 1135.0)	1001.7 (571.5 to 1556.9)	32.6% (-32.1 to 159.9)	4380.0 (2947.3 to 6278.9)	5813.9 (3557.6 to 8901.7)	32.7% (-26.7 to 140.8)	538.6 (309.0 to 844.0)	713.0 (384.5 to 1131.1)	32.4% (-36.4 to 175.3)
Togo	656.4 (416.6 to 967.0)	725.3 (416.8 to 1146.5)	10.5% (-42.8 to 114.0)	3934.2 (2635.1 to 5643.9)	4354.1 (2684.0 to 6649.3)	10.7% (-38.8 to 100.5)	477.9 (274.3 to 756.4)	532.1 (288.8 to 867.9)	11.4% (-47.0 to 134.3)
Cameroon	631.8 (394.6 to 919.3)	716.7 (423.3 to 1110.7)	13.4% (-40.2 to 115.5)	3758.7 (2519.9 to 5371.3)	4254.3 (2633.9 to 6504.4)	13.2% (-37.2 to 104.5)	457.1 (266.5 to 709.4)	522.3 (284.9 to 859.9)	14.3% (-45.2 to 138.9)
Uruguay	990.5 (621.3 to 1458.5)	1356.7 (791.9 to 2115.7)	37.0% (-28.5 to 162.9)	6026.8 (4090.5 to 8579.2)	8326.1 (5315.9 to 12236.8)	38.2% (-20.7 to 141.3)	742.9 (436.4 to 1152.9)	1021.7 (568.0 to 1622.4)	37.5% (-32.4 to 179.9)
Barbados	792.5 (481.8 to 1186.0)	1074.6 (623.7 to 1668.3)	35.6% (-30.1 to 163.2)	4622.6 (3111.5 to 6497.7)	6273.3 (3945.5 to 9481.3)	35.7% (-23.5 to 141.0)	566.8 (328.3 to 882.5)	768.6 (417.0 to 1260.6)	35.6% (-35.2 to 184.8)
Guyana	806.5 (498.5 to 1207.9)	1133.0 (657.4 to 1790.7)	40.5% (-27.8 to 174.0)	4677.6 (3156.4 to 6639.5)	6640.9 (4119.9 to 10030.9)	42.0% (-20.5 to 154.1)	569.8 (329.4 to 899.4)	809.6 (432.0 to 1354.0)	42.1% (-33.4 to 204.2)
Australia	1169.2 (712.6 to 1720.6)	1329.6 (779.8 to 2002.0)	13.7% (-40.1 to 115.9)	6785.7 (4667.9 to 9570.9)	7370.8 (4625.0 to 10872.2)	8.6% (-37.8 to 90.2)	824.8 (482.1 to 1291.4)	895.9 (500.4 to 1433.1)	8.6% (-46.9 to 122.3)

Morocco	995.0 (624.0 to 1459.9)	1292.1 (769.5 to 1968.8)	29.9% (-30.8 to 144.2)	6474.4 (4405.6 to 9054.5)	8410.5 (5334.3 to 12473.6)	29.9% (-25.5 to 127.2)	792.5 (461.2 to 1234.2)	1028.6 (577.7 to 1679.5)	29.8% (-36.9 to 167.0)
United Republic of Tanzania	720.6 (446.5 to 1064.9)	879.7 (499.1 to 1392.9)	22.1% (-37.7 to 139.8)	3758.9 (2552.7 to 5354.9)	4616.8 (2832.5 to 7018.2)	22.8% (-31.5 to 120.8)	451.7 (265.8 to 712.8)	563.9 (308.5 to 969.6)	24.8% (-41.3 to 166.5)
Belarus	699.2 (429.3 to 1038.6)	1159.4 (669.3 to 1784.3)	65.8% (-14.0 to 220.2)	4102.8 (2754.9 to 5870.9)	6672.7 (4146.7 to 10067.8)	62.6% (-9.1 to 192.0)	506.8 (292.2 to 795.7)	823.4 (452.0 to 1362.5)	62.5% (-22.5 to 241.5)
Madagascar	728.9 (448.8 to 1063.6)	900.7 (507.7 to 1398.1)	23.6% (-36.4 to 141.0)	3811.3 (2574.8 to 5448.5)	4773.4 (2905.8 to 7280.7)	25.2% (-30.7 to 126.7)	462.4 (263.6 to 715.4)	583.6 (309.8 to 973.9)	26.2% (-40.8 to 170.0)
Eritrea	712.7 (436.2 to 1041.5)	828.6 (470.7 to 1289.1)	16.3% (-40.2 to 126.6)	3707.9 (2475.9 to 5255.0)	4380.1 (2668.2 to 6621.7)	18.1% (-34.6 to 113.5)	446.8 (260.2 to 697.7)	538.2 (288.9 to 893.6)	20.5% (-43.0 to 155.0)
Solomon Islands	737.3 (467.5 to 1077.4)	876.1 (509.5 to 1388.5)	18.8% (-38.1 to 128.8)	4387.5 (2935.8 to 6232.5)	5270.7 (3252.5 to 8028.0)	20.1% (-33.3 to 116.8)	540.9 (314.8 to 840.8)	649.0 (356.2 to 1077.7)	20.0% (-42.6 to 151.4)
Czechia	616.5 (378.0 to 925.4)	863.4 (493.4 to 1345.3)	40.0% (-28.3 to 174.4)	3494.3 (2361.7 to 4972.8)	4825.7 (2952.9 to 7288.1)	38.1% (-23.1 to 148.3)	431.0 (248.1 to 679.7)	595.2 (325.3 to 974.0)	38.1% (-34.1 to 189.6)
Slovenia	629.2 (382.9 to 923.8)	838.3 (490.0 to 1328.9)	33.2% (-31.3 to 159.1)	3632.9 (2429.3 to 5178.8)	4761.9 (2932.8 to 7253.2)	31.1% (-27.4 to 137.0)	446.8 (262.2 to 704.7)	587.5 (306.4 to 980.5)	31.5% (-38.6 to 183.1)
New Zealand	1359.4 (888.5 to 1886.3)	1540.7 (989.2 to 2207.2)	13.3% (-34.4 to 95.8)	8697.7 (6745.1 to 11110.2)	8664.4 (6239.4 to 11522.2)	-0.4% (-32.9 to 48.0)	1055.8 (674.6 to 1519.7)	1054.8 (639.0 to 1588.7)	-0.1% (-45.6 to 84.0)
Estonia	689.9 (427.0 to 1033.5)	1009.4 (575.4 to 1612.7)	46.3% (-25.7 to 189.2)	4012.5 (2677.0 to 5767.2)	5767.0 (3580.9 to 8796.7)	43.7% (-20.3 to 160.1)	494.0 (286.7 to 782.0)	711.3 (385.3 to 1174.7)	44.0% (-31.7 to 204.2)
Venezuela (Bolivarian Republic of)	781.4 (485.4 to 1145.9)	922.0 (540.8 to 1433.9)	18.0% (-38.3 to 126.0)	4536.2 (3050.1 to 6408.6)	5342.8 (3372.7 to 8200.3)	17.8% (-34.0 to 110.6)	557.7 (324.7 to 869.1)	657.0 (367.7 to 1082.1)	17.8% (-43.0 to 143.9)
Algeria	993.4 (633.4 to 1449.8)	1177.4 (704.4 to 1834.8)	18.5% (-37.0 to 123.4)	6402.9 (4349.6 to 9091.1)	7497.2 (4696.5 to 11351.7)	17.1% (-34.1 to 108.6)	787.6 (465.5 to 1223.3)	920.6 (505.9 to 1495.7)	16.9% (-43.3 to 141.7)

Georgia	454.0 (278.6 to 689.5)	649.6 (378.5 to 1042.6)	43.1% (-27.3 to 182.4)	2533.3 (1679.5 to 3626.9)	3567.9 (2216.6 to 5504.1)	40.8% (-22.3 to 156.1)	312.2 (179.3 to 491.1)	440.1 (229.6 to 744.3)	41.0% (-34.9 to 206.5)
Mauritania	592.3 (373.6 to 866.6)	684.0 (397.4 to 1075.0)	15.5% (-39.8 to 122.1)	3462.8 (2329.0 to 4984.9)	3981.8 (2501.2 to 6133.7)	15.0% (-36.1 to 107.6)	423.3 (243.0 to 659.8)	489.3 (264.6 to 819.4)	15.6% (-45.5 to 145.6)
Chad	637.2 (400.5 to 937.4)	692.0 (407.5 to 1094.6)	8.6% (-43.4 to 108.9)	3779.5 (2526.2 to 5446.0)	4117.4 (2545.2 to 6274.1)	8.9% (-39.7 to 97.5)	459.9 (269.2 to 726.2)	504.4 (267.3 to 832.0)	9.7% (-48.3 to 133.2)
Angola	730.8 (453.5 to 1090.8)	868.2 (508.3 to 1344.6)	18.8% (-38.0 to 128.3)	4037.0 (2703.9 to 5872.4)	4744.6 (2973.4 to 7205.3)	17.5% (-34.6 to 111.7)	489.5 (281.6 to 771.1)	580.6 (316.6 to 937.6)	18.6% (-43.2 to 147.5)
Sierra Leone	658.7 (412.8 to 975.8)	686.2 (395.2 to 1088.2)	4.2% (-46.4 to 103.0)	4025.8 (2702.6 to 5712.3)	4200.8 (2608.9 to 6366.4)	4.3% (-41.7 to 87.2)	489.7 (283.3 to 763.2)	513.5 (280.9 to 855.9)	4.9% (-50.1 to 121.1)
Pakistan	558.4 (375.4 to 768.0)	681.5 (439.1 to 945.6)	22.0% (-27.5 to 105.5)	2632.6 (1964.1 to 3417.6)	3255.9 (2309.4 to 4390.9)	23.7% (-19.0 to 89.3)	321.6 (200.7 to 474.1)	398.4 (241.1 to 607.6)	23.9% (-33.8 to 131.9)
Uganda	723.9 (433.8 to 1082.3)	910.7 (534.7 to 1417.8)	25.8% (-35.2 to 144.4)	3780.9 (2541.1 to 5442.2)	4769.8 (2973.4 to 7288.2)	26.2% (-29.9 to 127.7)	458.4 (263.3 to 715.2)	585.3 (318.8 to 944.3)	27.7% (-38.6 to 165.9)
Norway	1412.9 (933.2 to 1966.6)	1551.2 (1038.5 to 2123.3)	9.8% (-34.1 to 82.8)	9391.3 (7232.1 to 12047.7)	10023.7 (7550.0 to 13060.6)	6.7% (-26.4 to 54.8)	1151.3 (734.2 to 1701.3)	1229.1 (780.7 to 1802.0)	6.8% (-40.6 to 91.9)
Ireland	1298.5 (837.0 to 1894.3)	1667.6 (1014.7 to 2551.3)	28.4% (-30.5 to 137.7)	8473.3 (5765.3 to 12015.9)	10886.7 (6915.5 to 16350.7)	28.5% (-27.0 to 126.7)	1038.6 (614.1 to 1605.0)	1329.3 (748.9 to 2185.2)	28.0% (-37.4 to 162.7)
Andorra	1114.1 (700.2 to 1638.4)	1459.7 (880.0 to 2205.5)	31.0% (-29.6 to 144.1)	7258.5 (4903.1 to 10232.2)	9341.7 (5981.0 to 13679.3)	28.7% (-25.8 to 123.9)	890.1 (528.6 to 1396.6)	1144.9 (624.9 to 1861.2)	28.6% (-37.9 to 167.2)
United Arab Emirates	923.7 (593.1 to 1338.0)	1227.0 (724.9 to 1917.1)	32.8% (-29.5 to 151.0)	5985.0 (4201.8 to 8296.6)	7790.4 (4910.6 to 11801.5)	30.2% (-25.1 to 126.7)	736.1 (441.0 to 1122.8)	956.9 (528.2 to 1554.0)	30.0% (-36.2 to 165.7)
Tajikistan	443.8 (274.4 to 659.3)	603.7 (342.3 to 978.7)	36.0% (-31.4 to 170.4)	2433.0 (1634.4 to 3495.8)	3329.6 (2046.2 to 5245.1)	36.9% (-25.1 to 150.6)	299.7 (170.8 to 474.1)	410.4 (221.1 to 679.1)	36.9% (-35.5 to 191.6)

Bahrain	1016.9 (639.6 to 1489.8)	1241.5 (743.6 to 1885.0)	22.1% (-34.6 to 128.4)	6781.0 (4614.7 to 9517.8)	8277.3 (5231.2 to 12160.6)	22.1% (-29.9 to 113.2)	832.5 (485.9 to 1290.1)	1016.0 (546.1 to 1657.0)	22.0% (-41.6 to 155.7)
Bhutan	548.4 (330.2 to 813.5)	576.3 (318.0 to 895.5)	5.1% (-47.0 to 108.9)	2665.4 (1785.1 to 3836.4)	2864.2 (1733.8 to 4348.1)	7.5% (-40.9 to 95.9)	327.2 (184.9 to 515.6)	352.7 (189.0 to 596.6)	7.8% (-49.9 to 133.0)
Belgium	1084.1 (687.7 to 1604.3)	1366.7 (817.4 to 2076.6)	26.1% (-32.6 to 136.2)	6899.0 (4686.4 to 9750.9)	8518.0 (5422.4 to 12815.2)	23.5% (-29.8 to 117.7)	846.0 (494.3 to 1321.5)	1044.3 (581.8 to 1684.4)	23.4% (-39.9 to 153.5)
Congo	713.7 (441.6 to 1061.3)	844.8 (488.0 to 1311.7)	18.4% (-38.7 to 129.1)	3930.0 (2655.7 to 5671.9)	4663.6 (2926.8 to 7111.3)	18.7% (-33.8 to 113.2)	478.2 (280.1 to 742.9)	568.8 (320.8 to 948.3)	18.9% (-42.4 to 146.1)
Paraguay	1084.7 (678.1 to 1581.6)	1461.4 (843.8 to 2240.2)	34.7% (-29.3 to 157.4)	6458.1 (4372.8 to 9210.3)	8905.6 (5626.7 to 13232.7)	37.9% (-21.7 to 143.3)	790.8 (463.5 to 1235.2)	1087.7 (612.9 to 1789.7)	37.5% (-33.1 to 183.3)
Dominican Republic	815.1 (499.9 to 1210.5)	982.7 (558.3 to 1559.6)	20.6% (-38.7 to 138.0)	4777.0 (3240.4 to 6789.5)	5758.5 (3545.4 to 8755.2)	20.5% (-32.7 to 116.4)	585.3 (335.4 to 915.1)	705.8 (387.4 to 1166.1)	20.6% (-42.5 to 153.4)
Samoa	729.3 (455.7 to 1075.4)	875.9 (520.9 to 1371.6)	20.1% (-37.0 to 129.3)	4355.3 (2897.5 to 6263.3)	5262.7 (3331.6 to 8043.9)	20.8% (-32.6 to 117.0)	537.9 (311.1 to 839.7)	649.4 (356.9 to 1073.4)	20.7% (-42.2 to 152.9)
South Africa	730.1 (484.3 to 1012.7)	1027.9 (675.3 to 1409.4)	40.8% (-15.9 to 135.7)	4053.8 (3078.3 to 5191.2)	5721.5 (4312.5 to 7475.8)	41.1% (-3.1 to 105.8)	496.4 (313.1 to 730.6)	699.6 (437.7 to 1025.7)	40.9% (-22.2 to 155.4)
Finland	1010.7 (669.2 to 1410.1)	1144.8 (662.5 to 1782.4)	13.3% (-38.7 to 110.4)	6509.4 (4606.8 to 8812.1)	6787.2 (4088.6 to 10319.3)	4.3% (-40.6 to 83.2)	798.9 (482.9 to 1223.5)	831.3 (449.6 to 1318.2)	4.1% (-48.8 to 112.1)
Saudi Arabia	975.0 (618.8 to 1443.6)	1095.0 (629.8 to 1691.1)	12.3% (-41.3 to 115.8)	6272.2 (4272.2 to 8957.9)	7225.2 (4538.4 to 10946.0)	15.2% (-35.2 to 105.1)	769.8 (450.0 to 1196.5)	888.5 (482.2 to 1472.0)	15.4% (-44.9 to 142.4)
Central African Republic	744.3 (457.0 to 1102.2)	837.4 (478.0 to 1327.8)	12.5% (-42.6 to 121.3)	4168.7 (2794.5 to 5961.1)	4704.9 (2880.8 to 7171.1)	12.9% (-37.6 to 104.6)	504.1 (292.6 to 784.6)	573.9 (313.1 to 948.9)	13.8% (-45.6 to 139.2)
Luxembourg	1100.9 (690.7 to 1624.2)	1367.6 (810.9 to 2120.8)	24.2% (-34.5 to 136.5)	7224.2 (4986.8 to 10210.5)	8770.3 (5634.9 to 12946.3)	21.4% (-29.8 to 110.6)	884.1 (516.7 to 1377.2)	1075.1 (605.8 to 1743.2)	21.6% (-40.6 to 148.8)

Malta	1227.6 (790.3 to 1768.6)	1408.1 (844.6 to 2205.1)	14.7% (-38.7 to 115.1)	7807.5 (5370.1 to 11017.5)	9247.1 (5945.0 to 13828.7)	18.4% (-31.9 to 106.6)	959.2 (563.4 to 1493.6)	1133.4 (641.8 to 1843.8)	18.2% (-42.1 to 141.3)
Niue	729.5 (465.9 to 1064.7)	859.4 (493.1 to 1349.2)	17.8% (-38.5 to 126.2)	4341.6 (2920.5 to 6225.5)	5230.9 (3261.5 to 7993.2)	20.5% (-33.0 to 117.1)	536.8 (318.6 to 849.2)	645.6 (343.0 to 1054.7)	20.3% (-42.8 to 153.6)
Gambia	637.1 (401.3 to 935.9)	754.9 (433.8 to 1180.8)	18.5% (-38.5 to 128.9)	3821.1 (2551.2 to 5454.7)	4523.4 (2805.3 to 6820.0)	18.4% (-34.0 to 112.8)	466.8 (270.2 to 740.7)	553.4 (298.1 to 889.6)	18.6% (-43.4 to 148.3)
Myanmar	661.9 (413.9 to 980.4)	935.5 (543.8 to 1469.2)	41.3% (-26.7 to 173.3)	3688.8 (2493.3 to 5309.2)	5252.1 (3290.3 to 7899.9)	42.4% (-20.1 to 154.3)	449.8 (262.5 to 704.7)	645.2 (342.6 to 1053.2)	43.4% (-31.9 to 203.1)
Greenland	936.3 (558.0 to 1384.1)	1279.0 (706.7 to 1965.3)	36.6% (-30.9 to 170.7)	4827.2 (3235.4 to 6941.7)	6330.2 (3849.3 to 9540.5)	31.1% (-27.5 to 137.8)	584.6 (339.7 to 919.0)	769.3 (405.5 to 1286.2)	31.6% (-38.5 to 182.6)
Slovakia	635.0 (390.3 to 941.5)	944.3 (549.0 to 1462.7)	48.7% (-22.7 to 187.1)	3621.1 (2447.4 to 5170.5)	5411.1 (3389.4 to 8218.2)	49.4% (-16.3 to 167.3)	446.5 (260.3 to 694.7)	667.9 (364.6 to 1112.8)	49.6% (-28.7 to 214.7)
Bangladesh	543.4 (325.2 to 809.6)	695.6 (392.6 to 1111.8)	28.0% (-35.8 to 155.8)	2619.9 (1752.0 to 3826.6)	3413.4 (2105.4 to 5229.7)	30.3% (-28.4 to 137.9)	319.3 (184.2 to 508.5)	419.1 (220.4 to 694.5)	31.2% (-38.8 to 182.2)
Uzbekistan	417.5 (255.8 to 621.3)	509.8 (285.0 to 795.4)	22.1% (-38.0 to 141.0)	2257.3 (1518.6 to 3235.3)	2776.7 (1703.3 to 4254.6)	23.0% (-32.1 to 123.1)	277.1 (157.1 to 432.3)	342.2 (179.6 to 582.9)	23.5% (-43.1 to 169.2)
Monaco	1095.2 (672.1 to 1627.6)	1460.0 (864.5 to 2240.8)	33.3% (-30.1 to 154.2)	7315.0 (4975.6 to 10267.7)	9399.6 (5919.0 to 14151.4)	28.5% (-27.1 to 127.0)	895.7 (523.6 to 1384.3)	1148.5 (618.1 to 1818.6)	28.2% (-37.7 to 164.6)
Thailand	618.8 (375.8 to 921.8)	743.7 (411.1 to 1191.5)	20.2% (-40.1 to 141.6)	3426.4 (2293.9 to 4886.1)	4175.1 (2557.0 to 6519.8)	21.9% (-33.1 to 122.4)	422.0 (240.9 to 655.1)	515.3 (284.1 to 866.9)	22.1% (-42.0 to 158.3)
Equatorial Guinea	719.6 (447.7 to 1061.9)	805.9 (465.4 to 1274.8)	12.0% (-42.3 to 117.9)	3966.6 (2663.3 to 5672.5)	4417.8 (2767.9 to 6791.0)	11.4% (-38.1 to 100.7)	478.8 (275.4 to 743.5)	540.4 (301.3 to 901.3)	12.9% (-45.9 to 135.9)
Djibouti	680.2 (410.9 to 1002.2)	813.3 (466.2 to 1288.7)	19.6% (-39.1 to 135.2)	3537.8 (2412.0 to 5118.1)	4232.3 (2581.8 to 6461.1)	19.6% (-33.8 to 116.7)	434.5 (248.2 to 683.1)	521.1 (281.1 to 868.5)	19.9% (-43.6 to 155.7)

Cabo Verde	617.7 (389.9 to 893.0)	773.2 (454.6 to 1201.3)	25.2% (-33.8 to 137.6)	3664.8 (2469.4 to 5231.2)	4692.1 (2945.9 to 7097.6)	28.0% (-28.1 to 128.7)	449.5 (259.2 to 699.9)	577.3 (317.6 to 958.2)	28.4% (-38.6 to 169.4)
Iceland	1108.2 (704.6 to 1605.7)	1238.6 (728.1 to 1933.9)	11.8% (-41.0 to 112.2)	7074.0 (4834.6 to 10109.9)	7844.5 (4831.4 to 11637.3)	10.9% (-37.5 to 97.2)	868.1 (506.4 to 1347.9)	961.3 (524.8 to 1537.9)	10.7% (-46.2 to 128.5)
Seychelles	647.7 (395.3 to 952.0)	896.9 (512.0 to 1393.5)	38.5% (-28.7 to 169.7)	3643.8 (2456.1 to 5167.2)	5000.3 (3111.0 to 7565.2)	37.2% (-23.1 to 145.5)	450.4 (260.0 to 713.6)	617.6 (336.8 to 1042.7)	37.1% (-35.5 to 192.6)
Democratic People's Republic of Korea	683.1 (443.0 to 998.9)	715.4 (432.1 to 1111.1)	4.7% (-43.8 to 95.7)	4579.3 (3118.5 to 6516.0)	4805.7 (3016.1 to 7273.4)	4.9% (-40.9 to 86.7)	568.2 (330.1 to 901.3)	597.1 (335.1 to 966.6)	5.1% (-49.0 to 117.1)
Nauru	736.9 (458.0 to 1089.0)	868.3 (511.3 to 1337.7)	17.8% (-38.1 to 124.7)	4427.3 (2946.0 to 6372.8)	5257.9 (3256.3 to 8049.4)	18.8% (-34.4 to 115.7)	546.3 (318.2 to 859.5)	649.9 (362.0 to 1080.1)	19.0% (-42.9 to 148.4)
Montenegro	644.5 (397.3 to 966.1)	985.6 (569.0 to 1573.6)	52.9% (-22.0 to 200.5)	3741.0 (2508.3 to 5306.3)	5704.9 (3497.6 to 8613.0)	52.5% (-15.2 to 174.6)	461.6 (268.3 to 728.8)	701.4 (379.6 to 1164.7)	52.0% (-28.1 to 222.2)
Iran (Islamic Republic of)	1314.2 (897.4 to 1789.6)	1674.5 (1140.5 to 2275.9)	27.4% (-21.4 to 106.5)	8559.0 (6602.1 to 10882.9)	10946.2 (8348.5 to 13944.8)	27.9% (-10.3 to 82.3)	1048.4 (672.3 to 1521.1)	1341.7 (845.3 to 1953.8)	28.0% (-28.2 to 128.4)
Papua New Guinea	731.9 (454.6 to 1082.1)	823.9 (479.4 to 1291.8)	12.6% (-41.6 to 117.5)	4399.7 (2928.4 to 6329.4)	4968.2 (3088.2 to 7594.4)	12.9% (-37.5 to 104.6)	537.6 (312.1 to 842.8)	612.0 (321.2 to 1026.6)	13.9% (-46.9 to 145.2)
Honduras	797.7 (499.3 to 1179.1)	1141.9 (656.0 to 1825.6)	43.1% (-26.6 to 179.9)	4545.7 (3035.0 to 6433.5)	6718.4 (4200.2 to 10270.6)	47.8% (-17.6 to 165.5)	558.4 (326.4 to 873.0)	825.5 (460.7 to 1391.0)	47.8% (-29.2 to 210.0)
Hungary	634.9 (391.5 to 939.2)	881.1 (500.5 to 1398.9)	38.8% (-29.3 to 173.3)	3612.6 (2432.9 to 5230.6)	5060.8 (3144.4 to 7687.1)	40.1% (-22.1 to 152.8)	445.7 (257.9 to 723.7)	623.3 (337.5 to 1015.7)	39.9% (-33.9 to 196.0)
Rwanda	701.6 (423.0 to 1023.2)	889.3 (507.4 to 1415.6)	26.7% (-35.5 to 150.0)	3634.8 (2428.9 to 5184.7)	4673.2 (2920.1 to 7062.4)	28.6% (-28.1 to 130.6)	443.2 (256.2 to 696.2)	573.2 (311.8 to 969.4)	29.3% (-39.1 to 175.4)
Tunisia	1018.7 (640.1 to 1504.6)	1513.6 (901.8 to 2342.7)	48.6% (-21.5 to 182.0)	6680.0 (4537.0 to 9423.9)	9827.5 (6162.5 to 14931.6)	47.1% (-17.1 to 161.6)	822.2 (477.6 to 1278.7)	1203.6 (671.4 to 1906.3)	46.4% (-28.2 to 198.6)

Grenada	795.7 (493.5 to 1180.5)	1028.7 (581.4 to 1633.1)	29.3% (-34.2 to 154.8)	4523.7 (3048.8 to 6371.2)	6040.3 (3815.1 to 9146.0)	33.5% (-24.6 to 137.0)	553.8 (319.9 to 858.4)	739.1 (405.9 to 1212.2)	33.5% (-35.8 to 178.2)
Colombia	884.4 (551.5 to 1326.7)	1132.6 (648.5 to 1740.2)	28.1% (-33.7 to 148.0)	5361.1 (3617.7 to 7628.9)	6944.7 (4335.0 to 10575.1)	29.5% (-27.6 to 132.2)	658.6 (382.6 to 1033.5)	855.4 (458.6 to 1443.8)	29.9% (-39.1 to 177.8)
Gabon	731.5 (455.7 to 1087.6)	912.3 (524.2 to 1448.1)	24.7% (-36.0 to 143.9)	4060.9 (2747.3 to 5776.3)	5110.9 (3153.3 to 7800.0)	25.9% (-29.9 to 126.3)	492.6 (285.4 to 763.2)	620.9 (347.3 to 1029.8)	26.0% (-39.2 to 161.8)
Austria	1130.4 (705.4 to 1652.0)	1484.5 (881.1 to 2253.0)	31.3% (-30.0 to 147.0)	7579.1 (5203.5 to 10672.3)	9792.1 (6236.5 to 14554.7)	29.2% (-25.8 to 125.7)	927.9 (545.7 to 1448.3)	1202.1 (665.2 to 1968.2)	29.5% (-37.3 to 168.2)
Albania	653.4 (400.2 to 975.9)	937.4 (536.6 to 1479.8)	43.5% (-26.8 to 182.0)	3790.8 (2532.8 to 5446.7)	5539.1 (3510.8 to 8550.7)	46.1% (-18.7 to 163.4)	466.7 (269.9 to 734.4)	682.4 (373.3 to 1116.3)	46.2% (-30.0 to 206.0)
Lithuania	780.7 (477.1 to 1157.8)	1231.3 (716.3 to 1895.6)	57.7% (-18.0 to 203.9)	4806.6 (3240.8 to 6812.1)	7537.7 (4697.2 to 11444.9)	56.8% (-12.2 to 180.7)	592.5 (339.1 to 913.9)	928.8 (514.2 to 1542.8)	56.8% (-24.9 to 227.9)
Saint Lucia	803.0 (496.7 to 1205.4)	1101.9 (633.6 to 1719.6)	37.2% (-29.4 to 167.6)	4604.7 (3114.0 to 6549.0)	6463.6 (3992.3 to 9632.5)	40.4% (-21.1 to 150.3)	563.0 (328.8 to 894.1)	789.9 (425.3 to 1282.5)	40.3% (-33.1 to 194.8)
Democratic Republic of the Congo	720.9 (442.9 to 1078.3)	799.2 (466.3 to 1248.4)	10.9% (-42.7 to 114.9)	3972.4 (2688.2 to 5696.3)	4394.3 (2734.8 to 6795.4)	10.6% (-38.7 to 99.8)	478.2 (282.6 to 739.0)	533.9 (290.7 to 892.3)	11.7% (-46.6 to 134.2)
Northern Mariana Islands	727.1 (444.2 to 1093.6)	959.7 (559.9 to 1492.2)	32.0% (-31.8 to 155.8)	4568.9 (3053.0 to 6453.5)	5776.0 (3580.7 to 8843.4)	26.4% (-29.7 to 127.8)	565.0 (325.1 to 882.6)	713.6 (383.4 to 1175.2)	26.3% (-40.2 to 167.6)
Syrian Arab Republic	1059.4 (678.3 to 1530.3)	1335.0 (791.0 to 2083.8)	26.0% (-33.1 to 137.7)	6865.0 (4653.6 to 9687.0)	8906.1 (5591.4 to 13424.4)	29.7% (-26.7 to 130.1)	842.9 (506.5 to 1303.9)	1084.5 (602.2 to 1736.4)	28.7% (-36.6 to 161.8)
Mali	588.9 (370.0 to 875.1)	675.5 (394.7 to 1074.1)	14.7% (-40.7 to 122.5)	3396.6 (2280.8 to 4873.5)	3936.0 (2397.1 to 6048.3)	15.9% (-36.3 to 111.0)	413.1 (238.0 to 650.9)	480.3 (271.1 to 822.5)	16.3% (-44.8 to 145.3)
Tokelau	733.3 (466.0 to 1072.2)	869.5 (514.7 to 1322.0)	18.6% (-36.6 to 122.4)	4401.0 (2949.4 to 6265.3)	5182.1 (3281.6 to 7643.4)	17.7% (-33.0 to 107.5)	543.6 (315.1 to 865.8)	640.5 (350.5 to 1053.9)	17.8% (-43.9 to 147.7)

Turkmenistan	434.5 (266.0 to 640.6)	575.8 (333.2 to 908.1)	32.5% (-31.8 to 158.2)	2378.1 (1596.3 to 3418.6)	3167.2 (1971.5 to 4859.5)	33.2% (-26.2 to 140.8)	292.5 (168.2 to 466.9)	390.0 (203.5 to 649.9)	33.3% (-38.3 to 189.1)
Malawi	722.5 (441.0 to 1069.2)	918.2 (525.5 to 1441.0)	27.1% (-34.9 to 148.8)	3784.7 (2557.3 to 5423.1)	4845.6 (3014.4 to 7329.4)	28.0% (-28.4 to 129.6)	456.3 (266.5 to 712.5)	593.0 (318.9 to 963.8)	30.0% (-37.8 to 172.5)
Palestine	1058.7 (689.8 to 1548.8)	1345.5 (794.3 to 2091.7)	27.1% (-32.4 to 139.4)	6944.5 (4708.4 to 9886.6)	8783.1 (5416.7 to 13028.8)	26.5% (-28.8 to 125.2)	849.7 (503.1 to 1319.7)	1077.3 (582.0 to 1797.6)	26.8% (-39.6 to 166.9)
Portugal	1427.0 (911.9 to 2075.8)	1880.1 (1132.8 to 2828.3)	31.7% (-28.5 to 143.4)	9928.0 (6846.8 to 13926.8)	13346.2 (8523.2 to 19460.0)	34.4% (-22.0 to 132.2)	1212.3 (706.1 to 1838.1)	1628.8 (900.3 to 2623.1)	34.3% (-34.2 to 175.4)
Somalia	678.9 (416.0 to 1001.0)	913.8 (518.7 to 1428.1)	34.6% (-31.0 to 163.4)	3444.6 (2315.1 to 4911.9)	4705.8 (2857.1 to 7149.2)	36.6% (-24.4 to 147.4)	418.6 (245.2 to 655.9)	572.4 (307.6 to 931.8)	36.7% (-34.6 to 186.9)
Poland	613.1 (412.9 to 845.1)	863.9 (572.6 to 1188.8)	40.9% (-15.0 to 133.9)	3322.9 (2513.8 to 4276.4)	4718.4 (3540.7 to 6110.7)	42.0% (-2.6 to 107.0)	410.3 (256.9 to 610.1)	582.2 (361.2 to 860.1)	41.9% (-22.6 to 160.2)
Bahamas	795.8 (487.1 to 1183.6)	1083.3 (607.1 to 1669.0)	36.1% (-30.4 to 167.0)	4630.9 (3109.7 to 6666.7)	6247.8 (3944.2 to 9349.7)	34.9% (-24.0 to 139.9)	569.4 (332.1 to 901.3)	765.4 (424.2 to 1207.5)	34.4% (-34.4 to 175.6)
Bosnia and Herzegovina	637.8 (391.8 to 954.0)	938.9 (529.7 to 1497.3)	47.2% (-25.6 to 192.3)	3705.8 (2500.6 to 5258.5)	5458.1 (3458.9 to 8307.7)	47.3% (-17.0 to 162.2)	457.2 (264.2 to 715.3)	671.6 (359.6 to 1098.0)	46.9% (-30.3 to 210.5)
Sudan	1008.3 (646.8 to 1472.6)	1242.8 (757.1 to 1943.5)	23.2% (-34.0 to 130.5)	6467.8 (4394.4 to 9127.0)	7958.0 (5101.0 to 12235.1)	23.0% (-30.4 to 118.1)	790.2 (463.2 to 1220.1)	974.5 (553.4 to 1630.6)	23.3% (-40.1 to 154.8)
Yemen	1035.7 (671.9 to 1494.1)	1115.1 (681.2 to 1712.0)	7.7% (-41.4 to 98.4)	6444.9 (4335.8 to 9150.9)	7035.9 (4409.6 to 10517.2)	9.2% (-38.4 to 94.0)	781.8 (450.1 to 1217.5)	852.7 (472.8 to 1375.9)	9.1% (-47.2 to 125.2)
Argentina	1007.4 (634.6 to 1482.2)	1318.2 (766.7 to 2022.9)	30.9% (-31.2 to 149.2)	6041.8 (4084.3 to 8651.6)	8015.1 (4963.3 to 11984.0)	32.7% (-25.6 to 137.2)	742.3 (436.2 to 1155.0)	983.4 (531.5 to 1570.5)	32.5% (-35.8 to 174.1)
Spain	974.1 (627.0 to 1417.4)	1342.9 (816.1 to 2025.5)	37.9% (-24.9 to 153.8)	5945.4 (4147.0 to 8336.9)	8368.0 (5393.1 to 12220.6)	40.7% (-17.7 to 141.5)	729.6 (426.4 to 1116.2)	1023.7 (575.5 to 1619.8)	40.3% (-30.5 to 183.0)

Zambia	727.6 (443.2 to 1079.7)	963.4 (540.0 to 1525.2)	32.4% (-33.1 to 163.0)	3813.5 (2574.0 to 5490.2)	5083.3 (3098.9 to 7809.7)	33.3% (-26.6 to 142.5)	461.7 (265.7 to 732.1)	618.9 (332.5 to 1016.3)	34.1% (-36.6 to 184.3)
Latvia	713.8 (437.0 to 1061.8)	1159.3 (651.6 to 1855.9)	62.4% (-18.2 to 223.5)	4234.5 (2850.8 to 6013.0)	6717.2 (4053.8 to 10140.3)	58.6% (-12.0 to 186.6)	523.6 (302.6 to 825.5)	828.2 (452.3 to 1349.8)	58.2% (-24.3 to 230.8)
Egypt	924.4 (581.6 to 1354.0)	1177.5 (700.2 to 1844.2)	27.4% (-32.9 to 142.3)	5795.4 (3962.3 to 8192.4)	7344.8 (4592.3 to 10980.9)	26.7% (-28.2 to 124.0)	709.6 (415.5 to 1108.5)	902.7 (488.5 to 1479.3)	27.2% (-39.1 to 166.5)
Ghana	611.6 (384.9 to 914.3)	708.2 (412.9 to 1112.3)	15.8% (-39.9 to 123.6)	3607.0 (2388.5 to 5200.7)	4207.7 (2587.4 to 6441.6)	16.7% (-35.9 to 112.9)	438.9 (252.4 to 692.7)	516.7 (271.9 to 826.3)	17.7% (-44.2 to 148.9)
Côte d'Ivoire	631.3 (391.9 to 925.1)	695.9 (411.2 to 1103.5)	10.2% (-42.6 to 112.2)	3763.1 (2533.4 to 5354.3)	4138.3 (2657.8 to 6305.8)	10.0% (-37.8 to 94.9)	456.0 (262.2 to 718.4)	506.8 (282.1 to 849.4)	11.1% (-47.1 to 133.7)
Burundi	704.2 (427.6 to 1022.9)	886.6 (511.9 to 1388.1)	25.9% (-35.0 to 144.2)	3660.2 (2477.4 to 5243.4)	4621.5 (2841.6 to 6978.6)	26.3% (-29.7 to 127.2)	447.5 (255.5 to 703.7)	568.4 (302.2 to 939.3)	27.0% (-40.4 to 171.8)
Palau	719.0 (446.5 to 1065.4)	851.1 (499.8 to 1308.1)	18.4% (-37.9 to 126.0)	4414.4 (2963.5 to 6310.5)	5179.1 (3187.0 to 7841.8)	17.3% (-34.8 to 111.6)	545.3 (316.3 to 854.8)	641.2 (344.2 to 1062.1)	17.6% (-44.4 to 149.2)
Sweden	1069.6 (716.9 to 1491.8)	1310.9 (839.1 to 1901.4)	22.6% (-29.0 to 112.3)	6674.5 (5103.3 to 8540.5)	7979.8 (5705.9 to 10807.9)	19.6% (-20.6 to 80.1)	819.5 (520.0 to 1194.2)	978.4 (591.2 to 1459.6)	19.4% (-35.1 to 119.8)
Guinea	643.8 (411.2 to 944.0)	763.0 (444.6 to 1175.0)	18.5% (-37.4 to 125.1)	3813.5 (2547.9 to 5431.0)	4539.2 (2759.4 to 6848.0)	19.0% (-34.1 to 115.6)	464.4 (271.2 to 737.5)	555.1 (311.4 to 900.2)	19.5% (-42.0 to 146.8)
Cyprus	1210.5 (772.3 to 1774.9)	1441.9 (850.1 to 2177.7)	19.1% (-36.3 to 123.1)	7832.9 (5352.1 to 11097.8)	9520.3 (5934.7 to 14165.4)	21.5% (-31.1 to 114.8)	962.2 (562.7 to 1500.1)	1169.4 (643.1 to 1878.2)	21.5% (-40.9 to 150.4)
Puerto Rico	798.5 (489.2 to 1172.0)	986.8 (566.4 to 1557.6)	23.6% (-36.6 to 141.5)	4618.5 (3096.0 to 6609.2)	5815.5 (3588.7 to 8864.6)	25.9% (-30.2 to 127.7)	567.1 (326.8 to 883.6)	715.2 (386.1 to 1164.7)	26.1% (-39.7 to 164.6)

UI, uncertainly interval; DALYs, disability-adjusted life-years.

Table S4 The incidence, prevalence and DALYs rate of depressive disorders in 1990 and 2021, and changes from 1990 to 2021 at 204 countries and territories around the world.

Location	Incidence (95% UI)	Prevalence (95% UI)	DALYs (95% UI)

	1990	2021	Percentage change	1990	2021	Percentage change	1990	2021	Percentage change
Saint Vincent and the Grenadines	2674.1 (1715.6 to 3949.9)	4195.7 (2592.6 to 6522.1)	56.9% (-15.4 to 191.7)	2129.0 (1492.6 to 2968.2)	3127.7 (2084.2 to 4631.0)	46.9% (-13.2 to 149.2)	390.2 (229.8 to 609.5)	598.0 (329.8 to 967.0)	53.3% (-25.5 to 216.0)
Viet Nam	2019.6 (1319.4 to 2882.4)	2435.9 (1468.9 to 3772.7)	20.6% (-34.7 to 123.0)	1727.5 (1201.7 to 2368.7)	1992.1 (1320.1 to 2897.6)	15.3% (-31.3 to 94.1)	307.6 (181.3 to 479.9)	365.1 (198.3 to 599.4)	18.7% (-43.1 to 148.1)
United States of America	5201.2 (3790.0 to 6950.7)	10214.9 (7638.9 to 13371.9)	96.4% (30.7 to 195.8)	4186.4 (3187.2 to 5405.7)	7249.4 (5621.1 to 9279.8)	73.2% (20.8 to 148.1)	775.1 (499.5 to 1151.5)	1431.5 (941.4 to 2107.4)	84.7% (4.1 to 227.5)
Timor-Leste	2990.2 (1960.4 to 4394.0)	3237.7 (1976.9 to 4954.3)	8.3% (-41.1 to 99.7)	2368.6 (1636.4 to 3294.7)	2522.0 (1649.3 to 3695.0)	6.5% (-37.5 to 81.8)	438.5 (255.8 to 677.0)	477.6 (264.0 to 787.1)	8.9% (-47.4 to 126.5)
Lao People's Democratic Republic	2709.4 (1751.9 to 4042.8)	3336.8 (2035.3 to 5261.9)	23.2% (-34.5 to 132.0)	2134.6 (1477.7 to 2993.4)	2604.0 (1719.3 to 3802.2)	22.0% (-28.1 to 107.5)	394.3 (236.9 to 615.1)	491.6 (276.6 to 800.6)	24.7% (-38.7 to 154.5)
Ethiopia	3513.3 (2411.7 to 4984.4)	3935.3 (2644.1 to 5671.3)	12.0% (-33.6 to 89.0)	2911.4 (2167.9 to 3890.9)	3202.6 (2346.0 to 4350.1)	10.0% (-27.9 to 67.8)	517.4 (323.3 to 779.2)	580.2 (359.2 to 891.9)	12.1% (-40.1 to 109.9)
South Sudan	3438.4 (2196.0 to 5051.7)	3790.9 (2324.0 to 5867.4)	10.3% (-40.6 to 105.2)	2943.0 (2094.0 to 4062.9)	3126.4 (2103.6 to 4446.1)	6.2% (-35.4 to 75.1)	515.6 (297.5 to 814.3)	557.5 (312.9 to 905.6)	8.1% (-47.7 to 123.7)
Suriname	4558.4 (3058.4 to 6496.0)	7453.1 (4825.8 to 11354.4)	63.5% (-7.4 to 189.2)	3371.3 (2364.8 to 4686.1)	5183.9 (3463.9 to 7536.1)	53.8% (-8.2 to 158.1)	646.9 (392.7 to 999.6)	1027.8 (600.7 to 1688.9)	58.9% (-20.5 to 218.3)
Dominica	2441.8 (1558.8 to 3645.5)	3594.5 (2179.4 to 5537.0)	47.2% (-21.3 to 176.0)	1996.6 (1409.8 to 2795.8)	2738.9 (1828.4 to 4028.3)	37.2% (-18.6 to 131.6)	360.7 (210.7 to 564.1)	516.4 (286.1 to 852.1)	43.2% (-31.0 to 198.0)
Tuvalu	2751.2 (1685.8 to 4341.3)	3283.1 (1909.1 to 5303.7)	19.3% (-40.2 to 138.4)	2196.2 (1466.5 to 3214.2)	2524.2 (1632.0 to 3836.3)	14.9% (-35.4 to 104.6)	406.1 (224.4 to 659.8)	477.2 (257.2 to 811.5)	17.5% (-46.3 to 157.1)
Republic of Moldova	2716.6 (1831.9 to 3887.1)	3444.5 (2126.8 to 5313.9)	26.8% (-29.8 to 129.6)	2243.3 (1612.3 to 3064.8)	2743.4 (1820.9 to 3969.1)	22.3% (-26.2 to 102.8)	405.9 (241.6 to 630.0)	507.8 (285.4 to 836.0)	25.1% (-38.9 to 157.1)

Bulgaria	2373.4 (1605.5 to 3395.0)	3459.7 (2150.9 to 5307.6)	45.8% (-19.0 to 162.6)	2060.0 (1502.2 to 2800.0)	2735.7 (1845.8 to 3906.1)	32.8% (-18.4 to 116.6)	363.6 (222.2 to 556.4)	506.5 (283.8 to 811.7)	39.3% (-30.5 to 180.0)
Germany	3867.7 (2864.7 to 5117.5)	5722.2 (3460.7 to 8838.4)	47.9% (-14.4 to 156.6)	3295.9 (2545.6 to 4228.2)	4398.9 (2888.0 to 6398.0)	33.5% (-16.5 to 113.7)	593.7 (382.7 to 864.3)	835.1 (473.3 to 1346.6)	40.7% (-27.3 to 172.9)
Romania	1991.4 (1329.2 to 2826.4)	3115.7 (1912.4 to 4769.8)	56.5% (-13.5 to 183.4)	1820.2 (1298.3 to 2462.7)	2527.1 (1723.0 to 3626.8)	38.8% (-15.0 to 127.2)	311.8 (187.1 to 482.9)	461.9 (268.1 to 746.6)	48.1% (-25.9 to 196.4)
Lebanon	4748.7 (3148.8 to 6884.9)	8196.7 (4849.0 to 12863.2)	72.6% (-7.4 to 222.4)	3667.9 (2580.2 to 5147.1)	5858.4 (3728.8 to 8823.9)	59.7% (-7.8 to 177.2)	687.9 (410.8 to 1078.8)	1150.3 (632.4 to 1884.6)	67.2% (-19.1 to 246.4)
China	2352.1 (1702.8 to 3229.6)	1387.4 (983.2 to 1913.0)	-41.0% (-62.7 to -6.8)	1930.2 (1461.1 to 2494.4)	1265.4 (955.5 to 1646.3)	-34.4% (-55.1 to -4.3)	353.4 (226.6 to 524.5)	218.5 (136.9 to 325.2)	-38.2% (-65.9 to 12.3)
Canada	4421.5 (3158.9 to 6088.8)	6562.3 (4113.3 to 9953.9)	48.4% (-14.1 to 157.1)	3533.7 (2641.7 to 4656.8)	4897.1 (3286.3 to 7093.6)	38.6% (-13.8 to 123.2)	653.5 (413.0 to 973.6)	941.8 (539.1 to 1527.8)	44.1% (-26.6 to 183.2)
Guinea-Bissau	3025.4 (1905.4 to 4518.0)	3562.1 (2156.4 to 5542.0)	17.7% (-37.6 to 122.5)	2543.5 (1769.0 to 3543.3)	2899.8 (1928.0 to 4189.3)	14.0% (-32.1 to 91.9)	448.3 (261.6 to 704.1)	524.4 (291.5 to 859.8)	17.0% (-43.5 to 142.5)
Philippines	2717.6 (1837.9 to 3836.6)	3404.2 (2287.0 to 4833.7)	25.3% (-25.5 to 110.7)	2140.0 (1555.1 to 2876.6)	2605.9 (1876.6 to 3571.2)	21.8% (-21.7 to 89.4)	396.5 (243.5 to 600.4)	494.7 (301.0 to 762.2)	24.8% (-34.2 to 136.9)
American Samoa	2284.6 (1515.7 to 3313.6)	2480.4 (1529.7 to 3775.9)	8.6% (-40.2 to 97.5)	1908.8 (1346.6 to 2615.6)	1971.2 (1337.0 to 2849.7)	3.3% (-37.5 to 70.9)	344.9 (204.6 to 534.4)	363.0 (206.2 to 573.9)	5.3% (-47.5 to 111.4)
Burkina Faso	3062.2 (1972.2 to 4552.5)	3269.8 (2019.5 to 5116.1)	6.8% (-42.7 to 99.2)	2544.5 (1796.5 to 3575.3)	2718.3 (1845.5 to 3989.3)	6.8% (-36.1 to 79.1)	450.4 (267.9 to 698.4)	487.0 (268.3 to 789.1)	8.1% (-47.3 to 122.5)
Benin	3036.0 (1958.7 to 4527.5)	3511.6 (2097.4 to 5488.6)	15.7% (-38.8 to 119.0)	2555.5 (1770.5 to 3565.7)	2874.0 (1915.7 to 4175.6)	12.5% (-33.2 to 89.7)	449.1 (258.0 to 706.9)	518.3 (291.6 to 845.1)	15.4% (-44.2 to 139.0)

Iraq	4170.4 (2689.6 to 6069.0)	4843.5 (2968.8 to 7396.7)	16.1% (-36.8 to 114.2)	3263.1 (2256.7 to 4576.6)	3719.2 (2468.5 to 5398.3)	14.0% (-32.5 to 92.8)	605.2 (348.6 to 947.7)	700.6 (399.0 to 1160.2)	15.8% (-44.0 to 139.4)
Antigua and Barbuda	2463.2 (1567.0 to 3750.6)	3471.2 (2089.7 to 5532.5)	40.9% (-26.5 to 171.0)	2033.0 (1390.0 to 2913.0)	2697.9 (1794.0 to 4046.2)	32.7% (-23.1 to 129.5)	366.6 (210.5 to 580.0)	505.3 (279.5 to 837.8)	37.8% (-34.4 to 189.6)
Lesotho	4743.5 (3046.7 to 7059.1)	7775.9 (4843.3 to 11846.1)	63.9% (-10.8 to 201.6)	3608.3 (2478.0 to 5044.4)	5612.1 (3730.1 to 8051.5)	55.5% (-7.5 to 161.8)	677.2 (394.0 to 1063.5)	1088.0 (608.6 to 1712.1)	60.7% (-21.0 to 227.1)
Afghanistan	5342.7 (3330.4 to 7963.2)	6870.3 (4216.4 to 10569.4)	28.6% (-31.4 to 141.3)	3990.7 (2684.1 to 5687.4)	4977.9 (3239.5 to 7294.6)	24.7% (-28.0 to 116.1)	750.6 (423.5 to 1172.7)	962.6 (531.0 to 1557.3)	28.2% (-38.5 to 167.7)
Oman	4290.2 (2657.8 to 6354.4)	5823.0 (3378.8 to 9074.6)	35.7% (-29.6 to 162.3)	3314.0 (2265.7 to 4704.4)	4352.3 (2738.4 to 6465.8)	31.3% (-25.2 to 131.4)	625.8 (355.1 to 995.0)	840.5 (447.5 to 1386.2)	34.3% (-37.2 to 187.8)
Senegal	2724.4 (1755.2 to 4033.2)	3474.9 (2131.3 to 5421.6)	27.5% (-31.6 to 138.5)	2365.6 (1661.0 to 3264.7)	2856.0 (1936.0 to 4107.4)	20.7% (-27.0 to 100.0)	409.0 (243.0 to 632.3)	512.9 (281.3 to 839.7)	25.4% (-39.2 to 159.3)
Trinidad and Tobago	3975.1 (2698.7 to 5801.0)	5776.8 (3636.9 to 8859.1)	45.3% (-19.1 to 161.9)	2963.5 (2127.6 to 4093.1)	4111.1 (2750.3 to 6063.5)	38.7% (-17.0 to 132.1)	564.1 (347.1 to 870.4)	804.5 (451.7 to 1318.9)	42.6% (-29.5 to 189.7)
Niger	2963.5 (1890.8 to 4444.0)	2933.4 (1735.0 to 4494.0)	-1.0% (-47.6 to 87.5)	2501.4 (1743.0 to 3509.3)	2482.0 (1632.7 to 3531.9)	-0.8% (-40.9 to 66.8)	440.9 (256.6 to 696.5)	439.2 (246.0 to 711.2)	-0.4% (-51.7 to 105.5)
Kenya	3282.5 (2317.9 to 4550.0)	3809.0 (2689.8 to 5317.8)	16.0% (-27.8 to 86.6)	2762.9 (2087.9 to 3629.4)	3127.5 (2346.9 to 4133.4)	13.2% (-23.4 to 67.5)	488.8 (306.7 to 730.7)	563.8 (354.9 to 843.3)	15.3% (-37.1 to 111.5)
Costa Rica	2491.2 (1637.6 to 3653.0)	3827.8 (2395.6 to 5890.6)	53.7% (-15.7 to 180.9)	1999.3 (1406.0 to 2796.1)	2876.1 (1946.0 to 4283.8)	43.9% (-14.6 to 142.9)	366.6 (218.3 to 573.0)	548.6 (304.5 to 895.3)	49.6% (-27.2 to 208.6)
Singapore	4460.8 (3394.6 to 5922.6)	3575.8 (2340.5 to 5223.7)	-19.8% (-50.7 to 30.6)	3351.2 (2640.3 to 4326.9)	2736.5 (1905.0 to 3864.3)	-18.3% (-46.9 to 25.6)	651.8 (414.1 to 955.9)	524.6 (305.2 to 832.7)	-19.5% (-58.1 to 55.1)

Israel	5389.7 (3498.2 to 7717.0)	6324.1 (3779.5 to 9839.0)	17.3% (-37.0 to 118.7)	4176.5 (2901.6 to 5786.1)	4787.7 (3140.5 to 7041.7)	14.6% (-32.5 to 95.3)	792.1 (467.9 to 1233.0)	918.6 (513.9 to 1493.6)	16.0% (-43.3 to 137.9)
Liberia	3429.7 (2179.9 to 5079.9)	3623.3 (2131.0 to 5692.0)	5.6% (-44.7 to 102.4)	2822.4 (1967.9 to 3920.2)	2944.4 (1959.4 to 4228.3)	4.3% (-37.6 to 74.9)	495.9 (286.5 to 770.2)	524.9 (291.6 to 871.3)	5.9% (-49.1 to 120.9)
Comoros	3059.3 (1962.8 to 4553.4)	3825.0 (2310.1 to 6031.6)	25.0% (-33.8 to 136.8)	2687.5 (1896.7 to 3675.3)	3231.0 (2210.0 to 4736.2)	20.2% (-27.3 to 99.3)	466.4 (275.9 to 730.6)	577.6 (312.7 to 954.2)	23.8% (-40.8 to 159.8)
Switzerland	5925.8 (4006.8 to 8339.5)	5495.0 (3459.0 to 8257.5)	-7.3% (-47.5 to 64.1)	4651.5 (3338.8 to 6256.1)	4316.9 (2897.9 to 6187.0)	-7.2% (-43.3 to 52.1)	873.4 (538.8 to 1325.4)	809.2 (466.4 to 1264.2)	-7.4% (-52.5 to 81.0)
Kazakhstan	3073.9 (2084.6 to 4403.2)	3850.7 (2423.9 to 5918.3)	25.3% (-30.0 to 124.7)	2486.9 (1811.7 to 3349.7)	2939.3 (1999.0 to 4148.2)	18.2% (-26.7 to 90.8)	455.9 (277.6 to 689.7)	555.9 (325.5 to 896.9)	21.9% (-38.1 to 140.7)
Denmark	4825.2 (3226.6 to 6896.8)	5023.6 (3056.4 to 7601.5)	4.1% (-42.5 to 88.9)	3847.5 (2743.4 to 5306.2)	3966.9 (2603.8 to 5772.2)	3.1% (-38.5 to 73.2)	715.9 (433.4 to 1103.6)	741.3 (419.2 to 1195.0)	3.6% (-48.5 to 109.0)
Namibia	2843.3 (1852.6 to 4122.4)	4550.0 (2749.6 to 6931.7)	60.0% (-13.1 to 195.2)	2462.3 (1734.2 to 3376.8)	3552.9 (2389.8 to 5121.4)	44.3% (-12.9 to 139.4)	429.3 (254.5 to 662.8)	661.5 (361.7 to 1062.3)	54.1% (-24.8 to 216.7)
Sri Lanka	3721.2 (2564.1 to 5222.8)	4147.1 (2639.0 to 6230.2)	11.4% (-36.2 to 95.0)	2822.8 (2046.3 to 3817.5)	3102.0 (2116.5 to 4383.7)	9.9% (-31.9 to 77.7)	538.8 (330.7 to 826.4)	598.8 (350.1 to 987.7)	11.1% (-44.2 to 121.9)
Botswana	3126.4 (2024.1 to 4616.5)	4844.5 (2979.2 to 7490.0)	55.0% (-16.3 to 187.7)	2616.1 (1831.5 to 3604.4)	3756.5 (2473.3 to 5420.5)	43.6% (-14.4 to 141.4)	464.5 (271.3 to 726.0)	701.7 (385.0 to 1117.5)	51.1% (-26.5 to 210.6)
Brunei Darussalam	2033.4 (1311.7 to 2997.8)	2533.1 (1537.6 to 3964.0)	24.6% (-33.4 to 133.6)	1767.8 (1237.8 to 2440.6)	2121.0 (1440.8 to 3129.7)	20.0% (-28.2 to 100.9)	314.2 (184.0 to 492.9)	386.8 (212.5 to 634.0)	23.1% (-40.8 to 156.4)
El Salvador	3817.4 (2576.6 to 5472.6)	4274.4 (2700.9 to 6575.1)	12.0% (-37.5 to 101.0)	2818.3 (1982.1 to 3905.2)	3158.1 (2115.6 to 4673.2)	12.1% (-33.4 to 89.1)	534.8 (330.2 to 820.8)	608.0 (342.6 to 993.5)	13.7% (-43.5 to 129.6)

Russian Federation	2499.3 (1691.3 to 3532.4)	3591.1 (2396.4 to 5108.2)	43.7% (-14.8 to 142.4)	2090.1 (1548.5 to 2800.9)	2738.8 (1987.6 to 3741.9)	31.0% (-14.8 to 101.6)	373.2 (231.6 to 567.4)	514.0 (316.9 to 782.4)	37.7% (-26.6 to 158.7)
Mexico	2239.2 (1607.6 to 3052.0)	4384.7 (3117.9 to 6082.7)	95.8% (23.8 to 209.9)	1828.9 (1387.0 to 2410.3)	3194.9 (2361.1 to 4292.6)	74.7% (16.6 to 161.8)	330.1 (206.2 to 494.8)	616.2 (388.3 to 939.8)	86.7% (0.9 to 245.8)
Kyrgyzstan	3027.3 (2025.1 to 4360.2)	3923.2 (2427.9 to 5942.0)	29.6% (-28.0 to 134.1)	2448.4 (1749.5 to 3365.3)	3014.3 (2031.9 to 4343.2)	23.1% (-25.4 to 103.6)	448.9 (268.4 to 694.3)	569.6 (306.8 to 937.4)	26.9% (-39.0 to 165.0)
Nicaragua	2908.1 (1948.5 to 4217.0)	4049.8 (2531.1 to 6040.5)	39.3% (-22.0 to 149.1)	2230.0 (1576.8 to 3103.7)	3004.8 (2017.2 to 4309.2)	34.7% (-18.8 to 124.1)	413.8 (247.2 to 647.9)	577.3 (335.7 to 918.8)	39.5% (-30.1 to 178.5)
Serbia	2380.7 (1606.6 to 3388.7)	2838.1 (1753.1 to 4354.4)	19.2% (-33.7 to 114.9)	2058.0 (1473.3 to 2798.8)	2362.2 (1649.0 to 3349.1)	14.8% (-28.6 to 84.9)	363.2 (221.2 to 559.4)	427.1 (241.8 to 694.1)	17.6% (-41.6 to 137.5)
Haiti	3170.6 (2038.9 to 4747.5)	4042.9 (2477.9 to 6262.9)	27.5% (-31.6 to 138.2)	2424.8 (1670.2 to 3438.1)	3004.3 (1990.3 to 4447.7)	23.9% (-27.6 to 112.6)	446.0 (261.5 to 711.7)	564.9 (315.1 to 919.0)	26.7% (-38.8 to 162.3)
Chile	6966.8 (5364.8 to 9013.2)	8063.8 (4983.1 to 12208.8)	15.7% (-30.8 to 94.3)	4985.5 (3925.5 to 6350.7)	5702.3 (3662.9 to 8399.2)	14.4% (-29.0 to 84.8)	988.0 (650.0 to 1460.0)	1136.2 (624.1 to 1779.3)	15.0% (-40.4 to 122.9)
Tonga	2288.8 (1523.0 to 3336.7)	2704.1 (1679.6 to 4231.0)	18.1% (-35.5 to 117.1)	1863.9 (1314.4 to 2556.0)	2144.4 (1443.5 to 3084.0)	15.0% (-30.4 to 90.6)	339.9 (199.3 to 527.2)	397.3 (229.5 to 643.8)	16.9% (-42.2 to 136.4)
Mozambique	3471.9 (2219.4 to 5174.4)	4623.3 (2732.5 to 7313.3)	33.2% (-30.4 to 155.5)	2930.1 (2048.5 to 4090.5)	3679.2 (2403.7 to 5335.0)	25.6% (-25.8 to 113.1)	510.6 (293.9 to 801.2)	665.5 (362.5 to 1090.0)	30.3% (-37.8 to 173.7)
Guam	3313.8 (2255.8 to 4690.8)	4388.7 (2761.4 to 6726.7)	32.4% (-25.5 to 135.9)	2603.2 (1853.5 to 3573.0)	3251.8 (2176.3 to 4669.7)	24.9% (-24.4 to 107.0)	490.3 (293.2 to 759.1)	630.8 (358.0 to 1019.1)	28.7% (-36.3 to 160.3)
Brazil	3530.4 (2515.6 to 4870.6)	4550.9 (3260.8 to 6269.2)	28.9% (-18.7 to 104.1)	2633.7 (1959.5 to 3503.3)	3324.7 (2472.6 to 4483.9)	26.2% (-16.3 to 90.6)	495.7 (316.2 to 740.3)	634.8 (402.1 to 958.3)	28.1% (-29.8 to 133.9)

United States Virgin Islands	2682.9 (1744.3 to 3970.2)	3788.3 (2343.4 to 5851.5)	41.2% (-23.4 to 160.9)	2127.9 (1493.8 to 2985.6)	2841.3 (1910.8 to 4087.0)	33.5% (-19.9 to 122.9)	391.6 (231.2 to 617.5)	540.3 (302.2 to 863.8)	38.0% (-32.4 to 181.9)
Japan	2671.7 (1939.3 to 3574.1)	3906.7 (2824.7 to 5330.0)	46.2% (-5.5 to 126.3)	2151.7 (1648.0 to 2759.9)	2953.2 (2246.6 to 3839.9)	37.3% (-5.0 to 98.4)	397.2 (256.8 to 588.2)	566.8 (359.5 to 836.4)	42.7% (-20.5 to 156.4)
Saint Kitts and Nevis	3041.5 (1847.6 to 4791.8)	4317.2 (2482.2 to 7135.4)	41.9% (-30.0 to 188.5)	2365.0 (1566.2 to 3486.3)	3236.7 (2015.0 to 5067.4)	36.9% (-25.6 to 152.3)	438.1 (244.4 to 716.9)	616.8 (330.3 to 1031.8)	40.8% (-35.3 to 206.7)
Kiribati	3044.7 (2052.6 to 4364.6)	3041.4 (1917.3 to 4706.7)	-0.1% (-44.4 to 79.9)	2395.5 (1695.8 to 3264.3)	2359.1 (1585.0 to 3442.9)	-1.5% (-40.7 to 63.9)	444.5 (268.3 to 684.0)	440.9 (252.3 to 723.3)	-0.8% (-50.8 to 100.7)
Greece	6315.6 (3944.7 to 9527.4)	8688.2 (5209.5 to 13466.2)	37.6% (-27.7 to 161.8)	4805.4 (3216.3 to 6960.8)	6315.0 (4027.9 to 9446.3)	31.4% (-25.8 to 133.1)	922.8 (519.5 to 1468.6)	1239.2 (667.9 to 2025.7)	34.3% (-36.9 to 185.7)
Jamaica	2459.8 (1563.9 to 3734.9)	3733.8 (2250.2 to 5890.4)	51.8% (-20.3 to 189.9)	2011.6 (1385.6 to 2849.1)	2869.6 (1898.1 to 4271.2)	42.7% (-16.9 to 145.5)	364.1 (210.7 to 575.2)	540.3 (302.0 to 908.7)	48.4% (-29.2 to 211.9)
Fiji	2487.5 (1661.6 to 3684.2)	3353.1 (2111.4 to 5071.2)	34.8% (-25.1 to 143.2)	2012.6 (1432.2 to 2772.1)	2563.6 (1718.7 to 3670.4)	27.4% (-22.9 to 110.8)	367.7 (220.2 to 567.1)	486.0 (279.3 to 797.9)	32.2% (-34.5 to 167.4)
Nigeria	2769.8 (1888.0 to 3940.8)	2595.4 (1782.1 to 3650.2)	-6.3% (-43.6 to 55.6)	2372.3 (1770.0 to 3146.6)	2259.5 (1683.8 to 2982.8)	-4.8% (-36.2 to 42.3)	411.2 (256.9 to 619.2)	392.3 (245.5 to 590.2)	-4.6% (-48.4 to 76.3)
Libya	4765.0 (3022.7 to 7091.6)	6007.0 (3661.2 to 9279.8)	26.1% (-32.6 to 136.1)	3640.7 (2473.7 to 5196.2)	4519.7 (2955.1 to 6649.8)	24.1% (-28.1 to 114.5)	690.8 (391.8 to 1102.0)	865.8 (487.9 to 1426.7)	25.3% (-40.1 to 162.3)
Armenia	2179.7 (1411.3 to 3160.9)	3676.3 (2206.2 to 5613.9)	68.7% (-8.9 to 213.1)	1940.3 (1353.6 to 2666.8)	2870.1 (1897.0 to 4129.6)	47.9% (-11.6 to 147.9)	338.6 (197.6 to 523.3)	538.2 (296.2 to 880.9)	58.9% (-23.2 to 230.2)
Cambodia	3070.1 (1972.1 to 4571.4)	3679.2 (2278.7 to 5665.7)	19.8% (-35.2 to 121.9)	2378.1 (1632.4 to 3363.8)	2807.5 (1869.3 to 4135.2)	18.1% (-30.7 to 101.6)	442.5 (253.0 to 699.7)	537.3 (300.3 to 875.1)	21.4% (-41.6 to 152.9)

Mauritius	4878.5 (3203.7 to 7063.4)	6215.0 (3856.8 to 9436.6)	27.4% (-29.7 to 131.4)	3605.9 (2471.2 to 5046.8)	4539.6 (2897.5 to 6625.0)	25.9% (-27.0 to 117.7)	703.6 (419.9 to 1095.7)	896.6 (499.5 to 1490.1)	27.4% (-38.3 to 163.8)
Mongolia	3829.5 (2585.6 to 5543.8)	3864.1 (2483.7 to 5763.2)	0.9% (-42.6 to 77.8)	2948.4 (2093.1 to 4102.8)	2964.7 (2013.4 to 4242.2)	0.6% (-38.9 to 65.9)	554.6 (329.7 to 875.9)	560.3 (322.0 to 901.3)	1.0% (-50.0 to 104.5)
United Kingdom	6246.6 (4289.7 to 8785.7)	6656.8 (4542.6 to 9476.4)	6.6% (-35.9 to 77.2)	4731.8 (3453.3 to 6466.5)	4924.3 (3536.1 to 6778.6)	4.1% (-33.5 to 62.9)	902.1 (560.1 to 1363.6)	947.8 (584.8 to 1449.5)	5.1% (-43.9 to 97.3)
Taiwan (Province of China)	1362.5 (902.3 to 1987.1)	1604.5 (996.6 to 2464.2)	17.8% (-35.3 to 114.8)	1279.4 (906.7 to 1752.2)	1484.3 (1016.9 to 2093.0)	16.0% (-28.6 to 88.7)	218.0 (130.1 to 337.6)	256.7 (141.4 to 417.9)	17.7% (-42.7 to 142.4)
India	2726.8 (1957.5 to 3788.8)	3426.9 (2415.6 to 4788.4)	25.7% (-21.5 to 101.3)	2227.6 (1681.0 to 2942.9)	2749.8 (2048.9 to 3704.5)	23.4% (-17.6 to 85.1)	395.5 (250.4 to 594.9)	501.6 (313.4 to 758.6)	26.8% (-31.2 to 134.1)
Nepal	3101.2 (2044.9 to 4503.7)	4829.0 (2934.7 to 7524.3)	55.7% (-15.8 to 188.5)	2509.8 (1781.5 to 3458.4)	3661.1 (2402.4 to 5433.1)	45.9% (-13.7 to 146.8)	450.3 (268.5 to 693.0)	690.1 (385.2 to 1126.4)	53.2% (-24.9 to 213.4)
Ecuador	3066.0 (2044.9 to 4464.3)	4580.1 (2797.6 to 6909.3)	49.4% (-17.7 to 171.8)	2386.0 (1684.2 to 3292.6)	3385.2 (2214.4 to 4880.2)	41.9% (-15.5 to 138.7)	445.7 (266.0 to 698.5)	653.6 (364.0 to 1068.4)	46.7% (-28.6 to 202.2)
Cuba	5243.3 (3675.9 to 7214.3)	4253.9 (2696.9 to 6670.1)	-18.9% (-53.7 to 42.5)	3853.3 (2807.5 to 5151.9)	3192.7 (2126.7 to 4784.1)	-17.1% (-49.9 to 37.4)	744.4 (457.8 to 1136.2)	609.3 (353.9 to 1003.8)	-18.2% (-58.9 to 63.5)
North Macedonia	2037.5 (1368.6 to 2970.0)	3416.1 (2101.0 to 5220.0)	67.7% (-7.7 to 205.5)	1847.8 (1313.9 to 2501.9)	2774.0 (1880.6 to 4013.3)	50.1% (-8.7 to 147.4)	318.0 (191.8 to 488.1)	509.3 (284.6 to 825.8)	60.1% (-21.0 to 225.4)
Eswatini	3191.0 (2060.0 to 4674.6)	6129.6 (3730.7 to 9348.8)	92.1% (4.1 to 255.6)	2651.8 (1852.9 to 3677.8)	4539.2 (3002.3 to 6563.3)	71.2% (1.9 to 188.0)	474.2 (271.3 to 743.4)	869.4 (477.3 to 1431.8)	83.3% (-12.5 to 284.7)
Micronesia (Federated States of)	2614.6 (1725.6	3003.7 (1892.0	14.9% (-36.5 to 108.2)	2039.3 (1422.8	2351.5 (1599.1	15.3% (-30.7 to 92.2)	378.7 (222.0	440.2 (249.0	16.3% (-43.4 to 138.8)

	to 3873.1)	to 4565.2)		to 2831.5)	to 3417.1)		to 594.0)	to 721.2)	
Sao Tome and Principe	2743.5 (1725.8 to 4085.4)	3135.6 (1873.9 to 4840.6)	14.3% (-39.5 to 116.5)	2356.1 (1609.8 to 3291.5)	2650.5 (1760.0 to 3792.5)	12.5% (-33.2 to 89.4)	414.0 (234.9 to 641.9)	473.7 (263.8 to 765.4)	14.4% (-44.7 to 136.9)
Marshall Islands	2522.1 (1668.5 to 3678.9)	3012.4 (1872.2 to 4630.5)	19.4% (-34.4 to 117.9)	1980.6 (1383.3 to 2751.3)	2351.2 (1589.7 to 3432.0)	18.7% (-29.0 to 98.9)	366.0 (220.4 to 569.1)	438.6 (250.5 to 716.0)	19.8% (-40.7 to 142.7)
France	5763.9 (4533.6 to 7190.4)	5912.1 (3653.4 to 8956.3)	2.6% (-37.7 to 69.6)	4457.1 (3596.4 to 5506.6)	4511.5 (2965.2 to 6590.5)	1.2% (-35.5 to 59.0)	843.8 (551.7 to 1214.5)	857.6 (477.3 to 1374.2)	1.6% (-47.3 to 96.5)
Guatemala	2636.1 (1698.9 to 3969.0)	4093.3 (2471.0 to 6482.7)	55.3% (-18.2 to 195.3)	2029.8 (1403.8 to 2863.6)	3034.5 (1959.3 to 4614.1)	49.5% (-14.4 to 161.6)	372.0 (213.2 to 587.8)	578.4 (321.5 to 950.6)	55.5% (-25.6 to 225.1)
Zimbabwe	2104.2 (1357.6 to 3096.8)	2836.1 (1705.1 to 4498.4)	34.8% (-28.6 to 155.5)	1970.1 (1376.2 to 2726.5)	2449.7 (1675.3 to 3559.0)	24.3% (-25.0 to 106.5)	328.7 (191.9 to 511.3)	429.5 (248.1 to 693.5)	30.7% (-35.4 to 164.6)
Peru	1777.7 (1193.6 to 2544.3)	2657.1 (1628.0 to 4113.3)	49.5% (-17.7 to 172.1)	1567.3 (1128.6 to 2120.3)	2158.9 (1443.1 to 3124.0)	37.7% (-16.2 to 126.9)	270.8 (157.4 to 423.6)	392.1 (226.4 to 635.1)	44.8% (-28.7 to 194.2)
Cook Islands	2730.5 (1648.1 to 4299.0)	3349.3 (1908.6 to 5417.1)	22.7% (-39.3 to 148.1)	2161.7 (1433.1 to 3140.9)	2579.1 (1636.3 to 3917.6)	19.3% (-33.4 to 114.3)	401.8 (221.3 to 652.9)	487.2 (259.9 to 838.8)	21.3% (-45.1 to 167.9)
Bolivia (Plurinational State of)	2744.3 (1816.9 to 4066.3)	4276.5 (2669.9 to 6628.5)	55.8% (-14.9 to 186.0)	2149.7 (1514.8 to 3013.7)	3156.0 (2106.4 to 4639.8)	46.8% (-12.9 to 148.0)	395.4 (229.7 to 610.5)	604.3 (333.3 to 992.1)	52.8% (-26.2 to 217.9)
Indonesia	1814.9 (1232.0 to 2573.3)	2686.0 (1795.8 to 3859.3)	48.0% (-12.5 to 150.5)	1580.0 (1163.1 to 2095.5)	2172.9 (1567.4 to 2937.3)	37.5% (-10.3 to 110.9)	278.5 (172.1 to 416.2)	402.2 (247.0 to 614.3)	44.4% (-22.9 to 170.7)
Jordan	4712.3 (2936.6 to 7109.1)	5383.7 (3280.8 to 8311.3)	14.2% (-39.5 to 116.1)	3635.9 (2486.9 to 5259.8)	4088.5 (2658.9 to 5977.5)	12.4% (-35.0 to 94.6)	687.2 (387.7 to 1087.9)	780.7 (439.9 to 1299.5)	13.6% (-45.9 to 138.9)
Belize	2618.7 (1683.9 to 3950.9)	3850.7 (2391.9 to 5918.0)	47.0% (-20.7 to 172.9)	2081.7 (1453.8 to 2944.2)	2906.3 (1938.7 to 4237.3)	39.6% (-17.3 to 136.0)	381.1 (223.8 to 602.3)	550.0 (307.9 to 874.8)	44.3% (-29.3 to 195.0)

Croatia	2848.3 (1939.4 to 4154.7)	3242.7 (2030.0 to 4965.7)	13.8% (-36.7 to 105.4)	2376.1 (1697.0 to 3223.0)	2649.2 (1783.3 to 3783.2)	11.5% (-31.9 to 83.1)	429.6 (259.5 to 652.9)	486.3 (271.1 to 778.6)	13.2% (-43.7 to 128.1)
Netherlands	4333.0 (3377.6 to 5552.7)	5872.8 (3594.1 to 8883.1)	35.5% (-18.9 to 126.8)	3471.3 (2772.8 to 4326.3)	4489.3 (2926.3 to 6493.1)	29.3% (-17.9 to 104.1)	642.8 (421.3 to 922.2)	858.6 (477.5 to 1406.1)	33.6% (-31.2 to 160.1)
Qatar	4735.6 (2992.5 to 7024.8)	5393.4 (3215.5 to 8753.0)	13.9% (-41.0 to 120.5)	3674.5 (2527.8 to 5235.6)	4159.5 (2714.6 to 6402.0)	13.2% (-35.5 to 99.2)	695.4 (403.4 to 1110.9)	797.5 (429.2 to 1343.4)	14.7% (-46.4 to 145.9)
Republic of Korea	2519.4 (1828.7 to 3435.7)	3388.6 (2178.0 to 4953.4)	34.5% (-19.6 to 126.0)	2117.6 (1617.2 to 2770.0)	2722.4 (1901.7 to 3741.9)	28.6% (-16.4 to 98.0)	382.1 (239.3 to 568.6)	508.3 (298.1 to 805.4)	33.0% (-31.1 to 157.2)
San Marino	5124.2 (3148.9 to 7943.8)	7022.7 (3977.3 to 11275.5)	37.0% (-31.5 to 175.1)	4070.6 (2719.6 to 5993.3)	5236.5 (3266.4 to 8030.8)	28.6% (-29.2 to 134.2)	762.1 (424.7 to 1247.2)	1011.3 (526.3 to 1649.0)	32.7% (-39.1 to 189.5)
Italy	4633.9 (3155.6 to 6546.0)	6010.1 (4040.0 to 8518.4)	29.7% (-22.6 to 117.5)	3671.5 (2679.0 to 4961.8)	4512.9 (3232.1 to 6175.8)	22.9% (-21.1 to 91.7)	684.5 (423.1 to 1042.2)	865.3 (526.9 to 1319.2)	26.4% (-33.1 to 139.1)
Maldives	3646.3 (2402.4 to 5328.6)	3599.6 (2192.5 to 5483.7)	-1.3% (-46.1 to 81.4)	2748.6 (1914.1 to 3869.2)	2781.4 (1821.1 to 4068.0)	1.2% (-40.6 to 72.6)	523.6 (310.1 to 809.8)	528.8 (296.1 to 854.9)	1.0% (-50.4 to 106.0)
Bermuda	3503.9 (2377.3 to 5092.7)	3937.2 (2344.1 to 6103.2)	12.4% (-38.8 to 107.0)	2749.3 (1951.2 to 3815.7)	2966.9 (1928.4 to 4395.7)	7.9% (-36.5 to 83.6)	514.8 (305.3 to 793.3)	566.4 (310.1 to 936.0)	10.0% (-46.9 to 128.7)
Azerbaijan	2150.5 (1398.8 to 3179.6)	3112.6 (1903.3 to 4719.2)	44.7% (-21.2 to 166.5)	1935.7 (1355.8 to 2683.0)	2525.2 (1697.4 to 3654.1)	30.5% (-21.8 to 118.1)	335.3 (199.6 to 519.7)	463.2 (258.8 to 766.6)	38.2% (-32.8 to 185.0)
Ukraine	3191.1 (2169.6 to 4585.9)	4202.6 (2623.9 to 6184.0)	31.7% (-25.3 to 132.7)	2528.1 (1862.9 to 3431.4)	3131.9 (2137.1 to 4407.9)	23.9% (-22.8 to 99.3)	466.2 (284.6 to 699.9)	597.1 (349.6 to 959.1)	28.1% (-34.7 to 151.9)
Vanuatu	2806.8 (1853.8 to 4067.0)	3217.8 (1952.2 to 4968.5)	14.6% (-37.7 to 111.5)	2194.7 (1539.6 to 3044.9)	2476.5 (1633.6 to 3623.0)	12.8% (-33.1 to 90.9)	408.3 (241.5 to 635.7)	465.3 (259.1 to 748.8)	13.9% (-44.2 to 133.0)

Türkiye	4644.6 (3419.5 to 6216.1)	6625.5 (3904.1 to 10139.0)	42.6% (-18.5 to 150.3)	3563.9 (2733.8 to 4617.4)	4914.2 (3117.9 to 7259.2)	37.9% (-15.8 to 126.6)	676.3 (426.2 to 992.1)	959.4 (519.9 to 1549.0)	41.9% (-28.6 to 182.9)
Malaysia	2885.6 (1845.8 to 4268.6)	3965.2 (2364.5 to 6110.0)	37.4% (-26.9 to 158.8)	2294.6 (1573.9 to 3241.1)	3038.9 (2004.5 to 4452.4)	32.4% (-22.4 to 126.5)	427.7 (245.9 to 676.7)	583.8 (316.5 to 973.1)	36.5% (-35.6 to 190.1)
Kuwait	4619.6 (2919.3 to 6970.3)	4880.8 (2871.4 to 7625.8)	5.7% (-44.9 to 103.3)	3630.6 (2490.7 to 5211.1)	3801.3 (2463.6 to 5684.4)	4.7% (-39.9 to 82.8)	683.7 (387.0 to 1089.2)	720.2 (393.6 to 1193.8)	5.3% (-50.4 to 123.8)
Panama	2358.7 (1551.8 to 3489.0)	3390.0 (2104.1 to 5326.1)	43.7% (-22.3 to 166.2)	1918.7 (1365.4 to 2687.3)	2582.9 (1689.1 to 3888.6)	34.6% (-21.2 to 130.3)	348.6 (206.4 to 548.0)	487.5 (268.2 to 808.5)	39.9% (-32.9 to 192.0)
Togo	3067.1 (1966.4 to 4600.9)	3405.6 (2035.0 to 5264.0)	11.0% (-41.1 to 110.1)	2578.4 (1782.1 to 3598.7)	2805.8 (1859.3 to 4064.3)	8.8% (-35.5 to 83.9)	455.0 (258.2 to 708.3)	504.0 (274.1 to 813.0)	10.8% (-47.0 to 131.4)
Cameroon	3202.8 (2063.7 to 4746.0)	3615.6 (2171.9 to 5752.3)	12.9% (-40.5 to 114.7)	2673.8 (1866.5 to 3711.2)	2934.0 (1940.8 to 4237.5)	9.7% (-34.7 to 84.6)	473.3 (270.7 to 738.8)	531.9 (292.5 to 865.3)	12.4% (-46.0 to 134.2)
Uruguay	3584.3 (2326.3 to 5249.3)	6058.8 (3801.7 to 9258.9)	69.0% (-7.1 to 208.0)	2753.1 (1922.0 to 3851.7)	4375.2 (2875.6 to 6471.9)	58.9% (-6.8 to 171.6)	520.5 (308.5 to 813.7)	858.5 (487.1 to 1417.6)	64.9% (-19.5 to 238.6)
Barbados	2627.8 (1697.3 to 3906.2)	3969.4 (2414.8 to 6174.0)	51.1% (-19.2 to 183.0)	2150.5 (1513.4 to 2991.5)	3005.3 (1982.2 to 4440.8)	39.8% (-17.6 to 137.4)	390.4 (231.3 to 615.4)	569.9 (318.8 to 964.4)	46.0% (-30.0 to 205.4)
Guyana	4542.3 (3067.6 to 6525.1)	7583.6 (4862.3 to 11721.0)	67.0% (-6.4 to 198.7)	3328.8 (2331.6 to 4567.9)	5304.3 (3533.0 to 7770.9)	59.3% (-5.0 to 168.0)	636.8 (385.9 to 997.1)	1047.5 (593.6 to 1705.4)	64.5% (-18.8 to 233.9)
Australia	7005.9 (4966.4 to 9411.8)	7644.0 (4905.8 to 11194.6)	9.1% (-35.1 to 83.9)	5205.6 (3821.8 to 6802.3)	5592.0 (3854.0 to 7981.9)	7.4% (-32.3 to 70.9)	1000.4 (636.8 to 1489.3)	1085.2 (649.2 to 1724.8)	8.5% (-43.1 to 107.4)
Morocco	5509.4 (3529.3 to 8160.9)	7120.7 (4238.3 to	29.2% (-31.2 to 143.5)	4141.7 (2797.5 to 5839.7)	5196.9 (3298.4 to 7792.1)	25.5% (-28.7 to 121.3)	793.0 (454.5 to	1014.4 (563.2 to	27.9% (-38.9 to 168.0)

		10963.2)					1242.8)	1685.7)	
United Republic of Tanzania	3521.2 (2254.7 to 5242.5)	4092.2 (2444.0 to 6287.3)	16.2% (-38.1 to 119.0)	2991.1 (2113.7 to 4134.2)	3367.6 (2290.2 to 4822.3)	12.6% (-31.6 to 85.6)	519.6 (309.5 to 801.7)	604.9 (342.7 to 969.0)	16.4% (-42.2 to 134.6)
Belarus	3106.4 (2095.7 to 4466.0)	4943.0 (3106.4 to 7543.3)	59.1% (-11.1 to 185.7)	2519.8 (1795.7 to 3431.5)	3635.9 (2425.1 to 5307.4)	44.3% (-13.2 to 140.2)	460.6 (275.9 to 710.8)	699.8 (393.1 to 1092.9)	51.9% (-24.0 to 203.6)
Madagascar	3547.2 (2282.6 to 5270.1)	4322.2 (2599.2 to 6762.4)	21.9% (-35.3 to 130.0)	2999.6 (2119.7 to 4157.3)	3509.2 (2381.1 to 5032.6)	17.0% (-29.1 to 93.3)	527.6 (306.8 to 822.5)	636.2 (345.1 to 1047.8)	20.6% (-42.5 to 153.4)
Eritrea	3578.7 (2279.5 to 5348.8)	4104.6 (2502.7 to 6424.4)	14.7% (-39.0 to 116.2)	3009.8 (2106.0 to 4173.7)	3388.8 (2269.3 to 4951.1)	12.6% (-32.9 to 89.2)	526.3 (306.1 to 831.4)	612.9 (335.4 to 972.0)	16.5% (-43.6 to 140.5)
Solomon Islands	2748.6 (1811.9 to 4047.3)	3080.8 (1885.5 to 4805.4)	12.1% (-39.4 to 108.2)	2146.5 (1496.6 to 3014.8)	2376.9 (1576.5 to 3471.9)	10.7% (-34.5 to 87.7)	398.3 (234.0 to 628.9)	446.1 (253.8 to 713.2)	12.0% (-44.9 to 127.6)
Czechia	2495.9 (1675.9 to 3587.0)	3174.6 (1960.7 to 4846.0)	27.2% (-29.6 to 130.2)	2105.1 (1507.5 to 2855.6)	2536.4 (1692.8 to 3647.8)	20.5% (-26.9 to 98.9)	374.7 (223.5 to 572.9)	467.7 (260.5 to 747.7)	24.8% (-38.3 to 153.2)
Slovenia	3278.5 (2249.4 to 4688.7)	3261.8 (2052.7 to 4982.8)	-0.5% (-44.1 to 77.3)	2652.5 (1932.5 to 3580.5)	2617.1 (1800.3 to 3754.9)	-1.3% (-38.9 to 59.7)	487.9 (296.5 to 755.2)	481.2 (277.8 to 781.6)	-1.4% (-50.7 to 97.7)
New Zealand	5398.8 (3572.1 to 7757.5)	6672.0 (4445.5 to 9808.9)	23.6% (-28.5 to 114.0)	4132.7 (2954.3 to 5692.8)	4937.4 (3465.6 to 6806.5)	19.5% (-25.0 to 90.4)	769.8 (466.2 to 1187.2)	950.4 (573.4 to 1475.3)	23.5% (-36.0 to 138.5)
Estonia	3989.7 (2724.0 to 5747.1)	4218.6 (2582.2 to 6553.7)	5.7% (-41.6 to 91.9)	3084.6 (2211.7 to 4177.3)	3196.2 (2117.4 to 4661.1)	3.6% (-37.5 to 71.9)	581.4 (353.0 to 906.1)	607.3 (349.8 to 971.5)	4.5% (-47.6 to 108.4)
Venezuela (Bolivarian Republic of)	2482.1 (1625.0 to 3640.2)	3050.9 (1905.5 to 4659.1)	22.9% (-32.4 to 124.1)	1985.0 (1404.0 to 2750.9)	2320.2 (1535.7 to 3322.2)	16.9% (-29.8 to 95.1)	362.4 (216.7 to 563.2)	435.8 (242.3 to 701.8)	20.3% (-40.9 to 145.5)
Algeria	4584.1 (2865.1 to 6901.4)	5266.9 (3087.2 to 8409.7)	14.9% (-40.9 to 123.9)	3547.7 (2410.2 to 5059.6)	3983.8 (2585.1 to 6068.2)	12.3% (-36.1 to 97.8)	669.0 (381.4 to	765.0 (410.2 to	14.3% (-46.9 to 147.2)

							1062.0)	1297.1)	
Georgia	2552.2 (1672.7 to 3735.2)	3911.4 (2351.2 to 5929.1)	53.3% (-16.8 to 183.1)	2181.6 (1539.8 to 2998.1)	3022.8 (1958.7 to 4360.1)	38.6% (-17.7 to 133.7)	389.2 (231.8 to 604.7)	570.9 (325.2 to 921.3)	46.7% (-27.4 to 196.4)
Mauritania	2590.7 (1652.4 to 3832.3)	2856.2 (1710.1 to 4594.6)	10.2% (-42.3 to 111.5)	2291.4 (1600.6 to 3167.1)	2454.9 (1644.5 to 3546.2)	7.1% (-35.8 to 79.1)	395.8 (229.6 to 617.5)	433.1 (237.3 to 719.2)	9.4% (-47.8 to 130.2)
Chad	3761.8 (2365.6 to 5705.2)	3940.8 (2354.7 to 6188.4)	4.8% (-45.3 to 100.9)	3013.7 (2069.8 to 4253.5)	3113.3 (2038.3 to 4618.6)	3.3% (-40.0 to 78.2)	546.9 (315.3 to 871.3)	571.6 (325.2 to 944.2)	4.5% (-49.6 to 117.2)
Angola	5851.0 (3799.6 to 8679.7)	6401.3 (3905.4 to 9965.3)	9.4% (-41.3 to 104.3)	4298.5 (2942.2 to 6137.5)	4610.7 (3058.9 to 6856.1)	7.3% (-37.6 to 84.8)	814.9 (475.5 to 1276.1)	889.1 (486.1 to 1431.2)	9.1% (-47.2 to 125.9)
Sierra Leone	3091.6 (1958.9 to 4607.8)	3266.6 (1976.9 to 5080.9)	5.7% (-43.9 to 99.4)	2642.6 (1840.3 to 3661.3)	2756.0 (1833.1 to 3956.5)	4.3% (-37.6 to 74.8)	462.9 (266.4 to 726.4)	490.6 (274.8 to 808.2)	6.0% (-49.0 to 120.2)
Pakistan	2532.1 (1707.0 to 3662.1)	2973.3 (1972.3 to 4410.5)	17.4% (-32.3 to 103.7)	2098.9 (1549.0 to 2816.5)	2411.4 (1745.5 to 3318.3)	14.9% (-25.7 to 77.6)	371.6 (230.3 to 567.6)	434.7 (261.8 to 679.9)	17.0% (-39.0 to 124.7)
Uganda	5392.6 (3484.0 to 7935.5)	6489.8 (3866.5 to 9984.8)	20.3% (-35.7 to 125.8)	4194.3 (2908.9 to 5836.0)	4866.7 (3162.8 to 7075.3)	16.0% (-31.8 to 97.8)	773.8 (445.8 to 1218.0)	927.1 (512.2 to 1498.0)	19.8% (-42.3 to 148.7)
Norway	4718.6 (3250.0 to 6640.0)	5739.6 (3876.4 to 8240.4)	21.6% (-27.3 to 103.8)	3755.0 (2768.6 to 5045.3)	4347.8 (3101.6 to 5968.3)	15.8% (-25.5 to 80.1)	700.4 (433.5 to 1054.6)	829.7 (502.7 to 1267.5)	18.5% (-37.2 to 123.7)
Ireland	5046.8 (3418.1 to 7072.9)	7861.4 (4985.0 to 11917.6)	55.8% (-11.7 to 175.4)	3873.8 (2762.5 to 5267.5)	5678.4 (3762.9 to 8306.2)	46.6% (-11.9 to 144.4)	732.5 (450.8 to 1110.7)	1106.6 (634.4 to 1780.0)	51.1% (-23.7 to 199.7)
Andorra	4616.2 (3034.3 to 6800.1)	6046.9 (3636.6 to 9414.9)	31.0% (-29.8 to 145.0)	3729.7 (2588.9 to 5243.9)	4611.6 (2980.7 to 6820.0)	23.6% (-28.2 to 113.5)	691.9 (406.2 to 1071.2)	879.6 (486.6 to 1430.0)	27.1% (-38.2 to 162.0)

United Arab Emirates	4406.2 (2809.7 to 6652.6)	5342.7 (3198.1 to 8318.9)	21.3% (-36.0 to 130.4)	3482.6 (2410.5 to 5025.9)	4044.4 (2569.6 to 6029.1)	16.1% (-33.8 to 104.2)	651.3 (383.1 to 1041.8)	773.9 (422.6 to 1287.5)	18.8% (-43.6 to 151.0)
Tajikistan	2349.7 (1535.0 to 3449.5)	3070.1 (1871.6 to 4692.2)	30.7% (-29.0 to 141.1)	2018.0 (1424.3 to 2791.3)	2487.4 (1670.8 to 3556.6)	23.3% (-25.5 to 104.5)	356.4 (207.3 to 551.4)	456.1 (259.2 to 749.0)	28.0% (-37.5 to 162.2)
Bahrain	6117.6 (3935.3 to 9105.5)	7113.1 (4303.4 to 10889.6)	16.3% (-37.6 to 117.0)	4583.3 (3100.6 to 6448.2)	5257.5 (3403.6 to 7842.1)	14.7% (-34.0 to 99.9)	884.3 (519.6 to 1388.0)	1025.2 (567.9 to 1666.2)	15.9% (-43.8 to 139.5)
Bhutan	2667.9 (1736.2 to 3980.2)	2700.2 (1649.7 to 4158.2)	1.2% (-45.4 to 88.3)	2255.0 (1590.8 to 3167.1)	2338.0 (1610.5 to 3349.3)	3.7% (-37.0 to 70.8)	399.4 (236.8 to 610.8)	413.2 (232.1 to 685.3)	3.5% (-49.5 to 112.4)
Belgium	4079.1 (3154.3 to 5289.8)	5449.7 (3361.9 to 8262.9)	33.6% (-20.2 to 124.7)	3325.9 (2634.7 to 4186.7)	4159.6 (2774.8 to 6002.1)	25.1% (-20.0 to 95.9)	612.4 (399.0 to 887.4)	790.7 (437.1 to 1263.0)	29.1% (-33.2 to 150.8)
Congo	5759.4 (3736.8 to 8551.9)	6326.9 (3782.1 to 9884.0)	9.9% (-41.7 to 107.5)	4250.3 (2930.5 to 6025.4)	4610.5 (2963.7 to 6842.0)	8.5% (-37.5 to 88.7)	804.6 (469.9 to 1257.2)	884.7 (485.9 to 1449.7)	10.0% (-47.1 to 129.0)
Paraguay	2883.9 (1881.8 to 4270.6)	4850.4 (3084.5 to 7414.9)	68.2% (-7.3 to 205.3)	2261.5 (1584.0 to 3164.0)	3547.0 (2353.9 to 5191.9)	56.8% (-7.1 to 165.2)	416.4 (244.1 to 651.4)	680.8 (387.9 to 1111.9)	63.5% (-20.0 to 234.2)
Dominican Republic	3213.6 (2032.3 to 4885.1)	4081.1 (2467.3 to 6403.1)	27.0% (-33.2 to 141.8)	2494.9 (1718.1 to 3575.0)	3068.6 (2004.3 to 4506.1)	23.0% (-28.5 to 112.0)	464.6 (266.1 to 738.6)	583.9 (321.2 to 958.2)	25.7% (-40.2 to 164.2)
Samoa	2638.5 (1736.6 to 3798.1)	2864.9 (1735.5 to 4460.8)	8.6% (-41.2 to 100.9)	2078.1 (1462.9 to 2865.2)	2235.4 (1492.0 to 3236.8)	7.6% (-35.5 to 79.8)	385.4 (230.0 to 590.7)	417.6 (238.1 to 687.3)	8.4% (-46.5 to 120.4)
South Africa	3373.5 (2442.8 to 4592.9)	5087.4 (3564.1 to 7026.4)	50.8% (-4.8 to 139.0)	2772.1 (2108.5 to 3576.5)	3848.8 (2855.2 to 5084.2)	38.8% (-5.9 to 104.9)	496.8 (317.4 to 736.0)	722.8 (450.3 to 1084.6)	45.5% (-20.4 to 166.0)
Finland	7000.0 (4728.2	7613.8 (4838.8 to	8.8% (-37.9 to 90.6)	5348.7 (3795.4	5668.3 (3790.2	6.0% (-35.4 to 74.4)	1028.1 (636.7 to	1095.7 (643.6 to	6.6% (-45.1 to 107.4)

	to 9637.9)	11478.7)		to 7214.6)	to 8068.0)		1561.2)	1731.2)	
Saudi Arabia	4415.8 (2778.2 to 6656.3)	5567.2 (3481.1 to 8530.5)	26.1% (-32.1 to 134.4)	3479.7 (2370.6 to 4917.7)	4299.5 (2916.4 to 6307.5)	23.6% (-27.0 to 109.4)	649.7 (375.4 to 1008.3)	820.4 (465.8 to 1324.0)	26.3% (-38.1 to 157.9)
Central African Republic	5916.7 (3824.0 to 8780.9)	6356.6 (3868.0 to 9968.4)	7.4% (-42.7 to 101.9)	4366.2 (3016.5 to 6210.7)	4629.6 (2998.2 to 6923.3)	6.0% (-38.9 to 84.5)	821.2 (485.7 to 1301.4)	884.7 (497.6 to 1444.8)	7.7% (-47.6 to 121.6)
Luxembourg	4935.1 (3326.0 to 6948.9)	4983.4 (3084.3 to 7517.3)	1.0% (-43.4 to 80.3)	3961.1 (2831.9 to 5349.9)	3953.4 (2597.0 to 5666.2)	-0.2% (-39.6 to 65.2)	735.4 (442.2 to 1122.7)	737.3 (416.9 to 1169.1)	0.3% (-49.7 to 100.5)
Malta	3792.0 (2405.1 to 5603.3)	4803.3 (2879.2 to 7475.9)	26.7% (-32.9 to 139.5)	3122.5 (2142.2 to 4390.2)	3843.9 (2521.4 to 5564.3)	23.1% (-27.6 to 109.7)	571.1 (329.0 to 896.6)	715.9 (400.9 to 1172.4)	25.4% (-39.5 to 160.0)
Niue	2619.5 (1604.3 to 4012.8)	3260.2 (1892.7 to 5214.9)	24.5% (-36.9 to 146.0)	2062.3 (1364.9 to 2952.7)	2495.4 (1607.7 to 3727.7)	21.0% (-31.3 to 113.4)	383.2 (213.9 to 615.1)	471.3 (251.6 to 768.9)	23.0% (-42.6 to 164.0)
Gambia	4366.0 (2764.6 to 6484.1)	4963.6 (3000.4 to 7734.4)	13.7% (-39.7 to 114.6)	3423.2 (2361.3 to 4829.4)	3813.3 (2479.0 to 5660.1)	11.4% (-35.4 to 92.6)	633.0 (358.9 to 1008.7)	714.8 (386.8 to 1173.9)	12.9% (-46.8 to 139.8)
Myanmar	1559.9 (1001.8 to 2312.1)	2444.8 (1459.0 to 3841.2)	56.7% (-17.2 to 197.5)	1441.5 (1003.2 to 1973.1)	2025.5 (1354.5 to 2936.7)	40.5% (-15.8 to 134.9)	244.5 (142.3 to 378.6)	369.4 (202.2 to 599.6)	51.1% (-27.0 to 213.3)
Greenland	12560.9 (8807.8 to 17667.0)	15273.5 (9909.2 to 22437.8)	21.6% (-28.9 to 108.4)	8955.0 (6355.1 to 12317.7)	10472.4 (6946.2 to 15004.7)	16.9% (-29.6 to 94.7)	1780.6 (1102.0 to 2740.9)	2114.8 (1222.5 to 3365.0)	18.8% (-39.7 to 134.3)
Slovakia	2081.4 (1387.4 to 2989.6)	3112.9 (1942.6 to 4813.5)	49.6% (-17.4 to 171.6)	1845.2 (1322.2 to 2497.7)	2535.6 (1721.3 to 3636.2)	37.4% (-15.8 to 125.0)	321.2 (188.2 to 492.4)	463.1 (264.0 to 747.8)	44.2% (-28.7 to 191.6)
Bangladesh	4250.5 (2868.2 to 6220.8)	4427.9 (2786.5 to 6666.2)	4.2% (-41.7 to 86.6)	3239.1 (2300.8 to 4511.2)	3393.3 (2293.1 to 4827.3)	4.8% (-36.4 to 72.8)	605.7 (369.9 to 924.4)	636.8 (359.9 to 1016.0)	5.1% (-47.2 to 110.0)

Uzbekistan	2763.6 (1835.1 to 3960.9)	3438.6 (2167.5 to 5245.3)	24.4% (-30.6 to 123.7)	2283.0 (1624.9 to 3098.9)	2737.3 (1861.7 to 3966.2)	19.9% (-27.0 to 97.5)	411.4 (249.5 to 627.0)	507.4 (292.4 to 816.0)	23.3% (-37.9 to 145.7)
Monaco	5137.2 (3147.1 to 7965.5)	6868.6 (4038.2 to 10870.3)	33.7% (-31.8 to 162.4)	4124.9 (2726.9 to 6026.3)	5143.3 (3254.4 to 7775.7)	24.7% (-30.5 to 124.3)	768.8 (434.4 to 1257.4)	990.1 (513.7 to 1681.6)	28.8% (-41.7 to 185.5)
Thailand	2702.0 (1773.9 to 3906.0)	2905.7 (1794.4 to 4336.7)	7.5% (-40.3 to 94.4)	2205.0 (1551.2 to 3016.5)	2357.3 (1565.5 to 3439.8)	6.9% (-36.1 to 79.3)	405.5 (236.7 to 627.5)	437.7 (240.4 to 712.4)	7.9% (-47.8 to 123.8)
Equatorial Guinea	5915.5 (3841.3 to 8804.5)	6348.6 (3986.0 to 9883.9)	7.3% (-41.7 to 97.9)	4338.9 (2988.9 to 6252.0)	4631.5 (3086.0 to 6896.7)	6.7% (-37.9 to 83.7)	816.4 (477.0 to 1285.4)	893.2 (507.8 to 1506.0)	9.4% (-47.3 to 127.7)
Djibouti	3298.1 (2128.9 to 4896.6)	4013.4 (2409.0 to 6225.8)	21.7% (-35.2 to 128.9)	2873.6 (2028.4 to 3967.4)	3329.0 (2259.1 to 4824.1)	15.8% (-30.0 to 92.1)	504.1 (293.3 to 783.6)	602.1 (332.1 to 978.9)	19.4% (-42.2 to 147.4)
Cabo Verde	3411.7 (2210.0 to 5061.9)	5006.5 (3060.0 to 7794.2)	46.7% (-21.2 to 174.0)	2815.2 (1955.3 to 3894.1)	3878.6 (2573.5 to 5673.5)	37.8% (-18.3 to 132.8)	508.5 (292.5 to 785.2)	730.4 (403.4 to 1185.4)	43.6% (-30.5 to 197.1)
Iceland	3903.3 (2569.6 to 5559.2)	4081.9 (2481.4 to 6278.1)	4.6% (-42.8 to 91.7)	3232.6 (2266.3 to 4438.2)	3339.8 (2238.9 to 4808.1)	3.3% (-37.8 to 71.9)	588.8 (346.7 to 905.7)	612.1 (337.8 to 976.1)	3.9% (-48.9 to 112.2)
Seychelles	2466.7 (1614.9 to 3590.2)	3437.4 (2028.6 to 5250.5)	39.4% (-25.1 to 159.9)	2038.9 (1422.0 to 2815.5)	2660.5 (1713.8 to 3852.6)	30.5% (-23.2 to 122.0)	372.5 (220.2 to 578.5)	505.6 (269.8 to 826.8)	35.8% (-35.1 to 185.0)
Democratic People's Republic of Korea	1868.7 (1247.8 to 2725.7)	1720.5 (1093.6 to 2611.1)	-7.9% (-48.5 to 65.1)	1636.1 (1153.3 to 2252.6)	1533.2 (1051.3 to 2149.0)	-6.3% (-42.4 to 52.4)	290.9 (173.2 to 452.4)	269.8 (158.8 to 426.4)	-7.2% (-53.1 to 83.4)
Nauru	2683.0 (1620.7 to 4191.7)	3261.5 (1900.8 to 5349.4)	21.6% (-39.5 to 144.4)	2125.6 (1418.8 to 3103.3)	2497.5 (1586.1 to 3825.9)	17.5% (-34.7 to 111.8)	393.6 (218.7 to 638.7)	471.6 (251.5 to 818.9)	19.8% (-45.7 to 165.3)
Montenegro	2288.8 (1537.7 to 3270.9)	3474.0 (2104.9 to 5281.9)	51.8% (-16.2 to 175.4)	2011.7 (1434.1 to 2741.9)	2781.9 (1849.8 to 3970.9)	38.3% (-16.2 to 128.7)	353.4 (212.5 to 533.1)	512.6 (283.8 to 809.7)	45.0% (-27.6 to 191.5)

Iran (Islamic Republic of)	5263.9 (3462.6 to 7699.7)	7033.8 (4696.5 to 10253.0)	33.6% (-23.1 to 132.1)	3894.8 (2767.2 to 5415.4)	5058.3 (3532.4 to 7090.4)	29.9% (-19.6 to 109.8)	742.5 (450.0 to 1142.6)	985.1 (597.9 to 1533.5)	32.7% (-31.1 to 155.8)
Papua New Guinea	3062.1 (2031.6 to 4551.9)	3133.7 (1949.7 to 4821.5)	2.3% (-44.1 to 87.7)	2366.8 (1655.2 to 3317.1)	2425.5 (1632.1 to 3517.1)	2.5% (-38.7 to 71.7)	440.7 (265.0 to 693.4)	453.9 (250.7 to 735.8)	3.0% (-49.8 to 112.0)
Honduras	2078.7 (1327.0 to 3106.0)	3441.1 (2044.0 to 5373.8)	65.5% (-12.9 to 215.3)	1690.1 (1172.0 to 2404.3)	2607.0 (1687.7 to 3873.9)	54.3% (-10.9 to 167.5)	304.5 (175.8 to 484.3)	491.6 (265.6 to 807.9)	61.5% (-23.5 to 241.7)
Hungary	2531.2 (1728.5 to 3564.3)	2854.7 (1765.2 to 4300.0)	12.8% (-36.4 to 100.2)	2126.7 (1555.5 to 2831.5)	2385.8 (1641.0 to 3355.5)	12.2% (-29.6 to 79.2)	380.0 (231.5 to 579.9)	431.1 (240.7 to 683.3)	13.5% (-43.3 to 127.4)
Rwanda	4129.2 (2660.9 to 6132.7)	4771.2 (2944.0 to 7429.4)	15.5% (-37.8 to 115.1)	3360.6 (2334.0 to 4717.1)	3817.1 (2586.6 to 5588.9)	13.6% (-32.3 to 90.9)	606.8 (355.6 to 959.2)	704.8 (398.0 to 1161.6)	16.2% (-43.8 to 139.9)
Tunisia	5740.4 (3575.9 to 8539.4)	8982.6 (5539.7 to 14019.2)	56.5% (-16.8 to 194.5)	4311.7 (2880.2 to 6147.8)	6433.8 (4099.0 to 9601.3)	49.2% (-15.4 to 164.0)	831.2 (462.2 to 1306.2)	1277.7 (713.4 to 2144.6)	53.7% (-27.4 to 226.1)
Grenada	2611.1 (1686.8 to 3899.9)	3960.9 (2444.4 to 6191.9)	51.7% (-18.6 to 183.1)	2081.7 (1459.3 to 2931.6)	3014.3 (1978.8 to 4414.4)	44.8% (-14.8 to 146.6)	379.8 (223.3 to 599.4)	570.0 (314.9 to 923.4)	50.1% (-27.3 to 210.2)
Colombia	2012.0 (1350.1 to 2891.9)	2440.4 (1548.0 to 3666.1)	21.3% (-31.6 to 115.5)	1680.6 (1193.7 to 2323.1)	1990.4 (1368.7 to 2776.2)	18.4% (-26.9 to 91.9)	300.3 (177.3 to 467.1)	363.6 (204.5 to 567.3)	21.1% (-39.7 to 143.6)
Gabon	5688.1 (3676.8 to 8567.1)	6801.4 (4151.4 to 10445.3)	19.6% (-35.8 to 122.8)	4229.8 (2902.3 to 6117.2)	4945.6 (3206.3 to 7234.6)	16.9% (-32.4 to 102.4)	798.5 (454.1 to 1270.0)	949.8 (513.1 to 1587.5)	18.9% (-44.3 to 154.6)
Austria	4275.1 (2886.4 to 5880.8)	4560.7 (2829.5 to 6982.9)	6.7% (-39.8 to 89.6)	3545.5 (2570.0 to 4748.2)	3682.4 (2478.8 to 5286.6)	3.9% (-36.1 to 69.1)	646.6 (387.1 to 987.1)	680.1 (379.2 to 1098.5)	5.2% (-48.1 to 113.7)
Albania	1930.6 (1282.3 to 2810.2)	3100.5 (1940.3 to 4938.0)	60.6% (-12.7 to 196.2)	1780.8 (1275.8 to 2417.5)	2593.4 (1755.5 to 3826.3)	45.6% (-11.9 to 141.2)	302.6 (182.9 to 462.4)	469.0 (267.2 to 768.9)	55.0% (-23.1 to 213.2)

Lithuania	3520.5 (2382.9 to 4992.3)	5460.5 (3445.8 to 8342.7)	55.1% (-12.8 to 176.6)	2805.5 (2023.1 to 3823.4)	4038.3 (2777.4 to 5864.3)	43.9% (-11.8 to 135.6)	520.4 (316.5 to 807.3)	781.7 (452.7 to 1277.6)	50.2% (-25.0 to 201.5)
Saint Lucia	2775.0 (1815.7 to 4018.1)	4563.3 (2857.1 to 6965.7)	64.4% (-9.3 to 198.8)	2203.0 (1549.2 to 3109.9)	3399.1 (2266.4 to 4985.2)	54.3% (-8.6 to 161.0)	403.6 (237.7 to 631.9)	651.2 (362.2 to 1070.3)	61.3% (-21.9 to 234.0)
Democratic Republic of the Congo	5456.8 (3511.5 to 8043.4)	5817.9 (3580.1 to 8967.9)	6.6% (-42.3 to 97.6)	4063.2 (2782.0 to 5732.3)	4287.4 (2804.7 to 6307.1)	5.5% (-38.6 to 81.7)	757.4 (442.3 to 1196.4)	810.9 (453.4 to 1309.0)	7.1% (-48.0 to 120.5)
Northern Mariana Islands	2549.0 (1689.1 to 3799.1)	3455.5 (2159.6 to 5243.9)	35.6% (-25.4 to 146.5)	2150.2 (1538.7 to 2960.9)	2621.5 (1758.6 to 3841.1)	21.9% (-26.8 to 103.3)	389.2 (230.2 to 601.1)	500.0 (281.6 to 805.2)	28.5% (-36.6 to 160.9)
Syrian Arab Republic	4458.3 (2813.1 to 6678.1)	5870.1 (3460.5 to 9132.5)	31.7% (-31.1 to 152.3)	3439.5 (2335.7 to 4914.0)	4405.6 (2825.2 to 6484.6)	28.1% (-26.5 to 123.7)	647.7 (369.1 to 1027.8)	840.5 (464.5 to 1394.9)	29.8% (-38.5 to 173.7)
Mali	2475.4 (1579.2 to 3713.5)	2682.9 (1634.3 to 4165.7)	8.4% (-42.2 to 103.7)	2200.1 (1544.1 to 3048.7)	2337.7 (1587.0 to 3363.6)	6.3% (-35.8 to 76.1)	375.6 (220.4 to 575.9)	405.5 (227.9 to 675.4)	8.0% (-47.6 to 122.8)
Tokelau	2668.6 (1619.4 to 4136.7)	3168.0 (1800.0 to 5155.5)	18.7% (-41.2 to 140.3)	2106.3 (1401.6 to 3061.5)	2414.6 (1499.2 to 3654.0)	14.6% (-36.5 to 107.4)	390.1 (218.9 to 637.2)	457.6 (239.2 to 764.2)	17.3% (-46.4 to 157.0)
Turkmenistan	2769.1 (1844.2 to 3992.2)	3478.6 (2166.1 to 5397.4)	25.6% (-30.9 to 128.9)	2289.7 (1614.1 to 3141.6)	2760.8 (1878.6 to 3976.9)	20.6% (-26.8 to 99.1)	414.3 (252.4 to 638.9)	513.0 (294.5 to 832.6)	23.8% (-38.1 to 148.7)
Malawi	3096.2 (2011.6 to 4542.0)	3775.2 (2275.7 to 5792.6)	21.9% (-34.3 to 126.7)	2745.5 (1956.1 to 3743.5)	3155.1 (2148.1 to 4521.9)	14.9% (-29.8 to 88.4)	467.5 (275.8 to 727.5)	562.8 (324.5 to 893.6)	20.4% (-39.9 to 141.4)
Palestine	7228.1 (4513.4 to 10775.5)	9288.0 (5726.7 to 14467.2)	28.5% (-31.6 to 141.7)	5234.2 (3478.3 to 7596.1)	6576.2 (4257.4 to 9836.4)	25.6% (-28.9 to 121.9)	1024.7 (588.4 to 1633.2)	1311.0 (735.5 to 2174.8)	27.9% (-38.9 to 168.2)
Portugal	6574.9 (4291.0 to 9342.9)	7762.1 (4805.6 to	18.1% (-35.0 to 115.1)	4940.7 (3410.9 to 6770.4)	5757.6 (3781.9 to 8462.0)	16.5% (-31.3 to 98.2)	948.4 (566.3 to	1115.6 (609.0 to	17.6% (-42.8 to 142.6)

		11917.4)					1480.6)	1798.8)	
Somalia	3307.7 (2092.6 to 4900.0)	4768.9 (2828.6 to 7372.2)	44.2% (-23.8 to 173.6)	2772.9 (1941.4 to 3862.8)	3759.8 (2507.2 to 5487.2)	35.6% (-19.4 to 128.5)	488.4 (288.0 to 764.0)	690.9 (379.1 to 1112.8)	41.4% (-31.3 to 191.9)
Poland	1504.6 (1031.6 to 2102.3)	2276.6 (1543.2 to 3254.9)	51.3% (-9.3 to 152.5)	1442.2 (1084.1 to 1873.3)	1969.1 (1450.8 to 2632.9)	36.5% (-8.6 to 104.1)	239.0 (150.4 to 357.7)	347.1 (214.7 to 526.8)	45.3% (-21.7 to 169.5)
Bahamas	2541.2 (1634.1 to 3827.4)	3574.1 (2162.2 to 5594.7)	40.6% (-25.5 to 166.2)	2084.4 (1441.3 to 2940.2)	2721.9 (1803.9 to 4038.1)	30.6% (-23.6 to 123.5)	377.7 (220.3 to 593.6)	513.8 (276.6 to 848.2)	36.1% (-35.4 to 187.3)
Bosnia and Herzegovina	3271.7 (2221.7 to 4696.6)	3360.4 (2070.5 to 5101.4)	2.7% (-42.9 to 84.9)	2662.5 (1926.4 to 3632.2)	2728.0 (1847.2 to 3927.9)	2.5% (-37.4 to 68.0)	488.9 (297.1 to 748.6)	501.0 (279.3 to 813.5)	2.5% (-49.4 to 108.0)
Sudan	5025.9 (3193.6 to 7464.3)	6009.2 (3600.5 to 9337.7)	19.6% (-36.7 to 126.4)	3806.6 (2568.9 to 5457.1)	4450.7 (2853.8 to 6599.7)	16.9% (-33.2 to 105.2)	720.9 (415.1 to 1144.9)	857.7 (477.3 to 1408.8)	19.0% (-43.0 to 148.6)
Yemen	5200.8 (3242.8 to 7792.8)	5422.1 (3214.1 to 8513.7)	4.3% (-45.7 to 100.4)	3837.2 (2568.0 to 5484.0)	4034.3 (2625.3 to 5994.0)	5.1% (-39.7 to 83.6)	728.6 (410.9 to 1155.6)	766.2 (414.5 to 1299.8)	5.2% (-51.1 to 126.8)
Argentina	3468.0 (2267.0 to 4989.9)	5235.8 (3524.6 to 7435.9)	51.0% (-11.7 to 158.2)	2660.2 (1844.7 to 3658.0)	3848.2 (2722.3 to 5279.8)	44.7% (-9.7 to 131.5)	502.4 (293.2 to 782.9)	748.0 (442.1 to 1156.6)	48.9% (-24.5 to 193.7)
Spain	5224.4 (4199.9 to 6493.3)	7530.1 (4752.4 to 11344.2)	44.1% (-10.9 to 134.4)	4248.2 (3518.8 to 5131.9)	5621.1 (3764.5 to 8106.8)	32.3% (-13.3 to 102.8)	793.9 (532.4 to 1122.8)	1090.9 (628.8 to 1806.9)	37.4% (-27.7 to 161.6)
Zambia	2977.6 (1905.7 to 4385.5)	3840.6 (2364.5 to 5968.2)	29.0% (-30.6 to 140.1)	2653.6 (1875.1 to 3619.1)	3201.8 (2194.2 to 4585.7)	20.7% (-26.2 to 97.8)	451.7 (268.3 to 704.9)	570.1 (322.3 to 932.5)	26.2% (-38.1 to 158.2)
Latvia	3341.6 (2263.5 to 4855.3)	4288.9 (2580.3 to 6593.0)	28.4% (-29.8 to 135.0)	2686.9 (1893.9 to 3713.3)	3223.7 (2122.9 to 4741.5)	20.0% (-29.0 to 103.0)	494.4 (294.4 to 764.7)	612.7 (344.2 to 985.8)	23.9% (-38.8 to 151.6)

Egypt	3996.6 (2497.2 to 6008.1)	5367.5 (3197.4 to 8401.8)	34.3% (-29.8 to 157.4)	3170.5 (2157.8 to 4546.2)	4055.5 (2619.0 to 6020.9)	27.9% (-26.6 to 123.7)	588.9 (332.6 to 937.8)	775.7 (422.0 to 1274.8)	31.7% (-37.9 to 179.6)
Ghana	3189.2 (2030.3 to 4718.9)	3703.0 (2265.3 to 5915.7)	16.1% (-38.6 to 120.1)	2670.3 (1863.5 to 3718.1)	3026.7 (2032.0 to 4437.1)	13.3% (-32.6 to 90.8)	474.3 (283.1 to 744.4)	549.3 (307.3 to 901.2)	15.8% (-43.6 to 138.4)
Côte d'Ivoire	2736.4 (1765.0 to 4039.4)	2826.6 (1720.2 to 4400.7)	3.3% (-44.6 to 93.2)	2396.3 (1684.9 to 3303.0)	2441.7 (1664.1 to 3457.3)	1.9% (-37.8 to 67.0)	411.6 (244.8 to 638.1)	427.2 (241.0 to 680.3)	3.8% (-48.5 to 109.4)
Burundi	3997.9 (2579.8 to 5930.3)	4268.4 (2585.8 to 6647.4)	6.8% (-43.0 to 100.3)	3280.9 (2305.2 to 4566.5)	3445.8 (2276.8 to 4976.1)	5.0% (-37.4 to 76.5)	590.2 (348.0 to 910.5)	628.6 (339.5 to 1029.6)	6.5% (-48.8 to 122.2)
Palau	2793.2 (1702.9 to 4325.9)	3315.6 (1882.0 to 5285.4)	18.7% (-40.5 to 137.5)	2226.2 (1471.5 to 3209.7)	2541.3 (1623.2 to 3854.2)	14.2% (-36.0 to 104.0)	413.2 (227.1 to 667.2)	480.9 (264.3 to 818.9)	16.4% (-46.4 to 152.9)
Sweden	5213.2 (3719.1 to 7025.9)	6591.4 (4338.4 to 9355.5)	26.4% (-23.3 to 108.6)	4091.2 (3052.0 to 5299.1)	4872.9 (3386.4 to 6645.0)	19.1% (-22.9 to 84.2)	766.6 (482.6 to 1159.0)	938.1 (558.7 to 1460.8)	22.4% (-35.9 to 133.9)
Guinea	2993.7 (1900.6 to 4498.8)	3560.6 (2113.2 to 5552.9)	18.9% (-37.6 to 127.3)	2529.8 (1758.9 to 3536.2)	2889.6 (1919.4 to 4179.4)	14.2% (-32.1 to 92.6)	445.3 (258.2 to 691.7)	522.9 (288.7 to 865.0)	17.4% (-43.6 to 145.3)
Cyprus	3791.4 (2390.3 to 5669.0)	4893.9 (2897.2 to 7624.5)	29.1% (-32.3 to 147.0)	3160.0 (2185.4 to 4456.3)	3901.4 (2550.3 to 5712.0)	23.5% (-27.7 to 111.4)	576.8 (329.8 to 910.5)	729.7 (404.4 to 1183.9)	26.5% (-39.2 to 163.7)
Puerto Rico	2298.2 (1541.1 to 3337.9)	2987.4 (1834.1 to 4630.3)	30.0% (-28.9 to 138.2)	1904.5 (1365.1 to 2604.5)	2385.1 (1599.4 to 3488.5)	25.2% (-24.6 to 108.1)	341.0 (203.6 to 526.1)	439.8 (245.4 to 703.8)	29.0% (-36.3 to 161.6)

UI, uncertainly interval; DALYs, disability-adjusted life-years.

Table S5 The incidence, prevalence and DALYs rate of anxiety and depressive disorders in 1990 and 2021, and changes from 1990 to 2021 in Brazil.

**Location**	**Incidence (95% UI)**			**Prevalence (95% UI)**			**DALYs (95% UI)**		
	**1990**	**2021**	**Percentage change**	**1990**	**2021**	**Percentage change**	**1990**	**2021**	**Percentage change**
Anxiety disorders									

Acre	997.1 (667.7 to 1363.2)	1436.6 (870.0 to 2098.5)	44.1% (-18.2 to 153.9)	5632.8 (4231.7 to 7294.5)	8404.5 (5707.7 to 11663.5)	49.2% (-4.5 to 134.0)	688.1 (430.3 to 1025.3)	1022.4 (612.9 to 1580.6)	48.6% (-21.5 to 181.5)
Amapá	999.5 (675.6 to 1392.9)	1461.9 (891.4 to 2211.1)	46.3% (-17.9 to 161.3)	5674.6 (4285.0 to 7386.9)	8606.6 (5925.7 to 12099.9)	51.7% (-2.9 to 137.8)	693.4 (437.0 to 1028.7)	1048.3 (632.2 to 1582.8)	51.2% (-18.9 to 182.0)
Amazonas	1000.5 (655.3 to 1386.5)	1588.4 (983.6 to 2437.3)	58.8% (-11.9 to 186.5)	5656.3 (4259.7 to 7353.4)	9214.4 (6227.7 to 12947.3)	62.9% (3.4 to 157.1)	690.5 (436.2 to 1032.8)	1123.5 (663.9 to 1734.9)	62.7% (-14.4 to 210.1)
Pará	998.7 (660.7 to 1395.2)	1450.4 (888.9 to 2165.6)	45.2% (-18.8 to 160.3)	5665.4 (4265.8 to 7391.1)	8499.2 (5783.2 to 11778.6)	50.0% (-4.0 to 135.2)	692.0 (432.6 to 1022.7)	1036.5 (597.8 to 1625.2)	49.8% (-22.5 to 190.4)
Rondônia	994.7 (666.6 to 1391.3)	1723.0 (1052.8 to 2551.2)	73.2% (-2.6 to 208.7)	5706.0 (4310.3 to 7421.4)	10003.6 (6831.9 to 14008.6)	75.3% (12.1 to 175.1)	694.5 (437.1 to 1022.7)	1215.5 (726.6 to 1907.7)	75.0% (-7.8 to 233.0)
Roraima	977.8 (643.8 to 1346.2)	1568.7 (980.6 to 2336.9)	60.4% (-9.2 to 184.3)	5603.6 (4256.0 to 7223.0)	9114.2 (6237.8 to 12596.3)	62.6% (5.1 to 152.5)	683.8 (431.8 to 996.9)	1107.9 (671.7 to 1698.9)	62.0% (-12.9 to 202.1)
Tocantins	993.3 (660.6 to 1380.1)	1586.9 (962.4 to 2372.6)	59.8% (-10.7 to 186.2)	5622.6 (4263.1 to 7322.5)	9233.4 (6222.6 to 13040.2)	64.2% (4.1 to 159.6)	684.9 (430.8 to 1002.9)	1122.3 (664.4 to 1778.4)	63.9% (-14.2 to 214.2)
Alagoas	1003.5 (648.8 to 1396.9)	1472.0 (913.7 to 2211.7)	46.7% (-18.2 to 163.6)	5720.4 (4348.2 to 7456.0)	8537.8 (5915.5 to 11945.0)	49.3% (-3.8 to 132.7)	696.7 (440.8 to 1029.9)	1038.8 (625.5 to 1558.3)	49.1% (-19.7 to 176.9)
Bahia	999.3 (662.7 to 1398.2)	1559.1 (948.1 to 2318.0)	56.0% (-12.9 to 180.0)	5666.7 (4266.0 to 7348.3)	9098.6 (6325.8 to 12371.5)	60.6% (4.4 to 147.2)	689.8 (439.0 to 1022.0)	1107.6 (644.9 to 1666.1)	60.6% (-14.7 to 203.3)
Ceará	1001.0 (662.7 to 1375.5)	1649.1 (988.8 to 2443.6)	64.7% (-7.7 to 194.5)	5714.2 (4341.0 to 7395.6)	9607.0 (6597.1 to 13326.1)	68.1% (8.5 to 161.4)	698.3 (434.3 to 1026.8)	1167.4 (704.1 to 1785.7)	67.2% (-10.9 to 213.9)
Maranhão	1001.7 (653.2 to 1389.4)	1537.2 (949.6 to 2258.9)	53.5% (-13.5 to 172.8)	5652.9 (4293.6 to 7375.9)	8826.2 (6179.6 to 12275.7)	56.1% (1.1 to 141.7)	689.4 (432.6 to 1017.7)	1075.3 (646.8 to 1651.5)	56.0% (-16.9 to 193.4)
Paraíba	1004.4 (663.7 to 1390.3)	1486.7 (894.2 to 2235.6)	48.0% (-17.7 to 166.6)	5719.0 (4339.7 to 7407.7)	8637.7 (5823.6 to 12195.7)	51.0% (-4.1 to 138.1)	698.6 (441.5 to 1024.0)	1053.1 (615.5 to 1614.7)	50.8% (-20.4 to 186.0)

Pernambuco	999.9 (671.3 to 1391.7)	1574.8 (962.8 to 2305.1)	57.5% (-10.8 to 178.8)	5732.3 (4327.3 to 7505.7)	9186.5 (6368.5 to 12756.0)	60.3% (3.1 to 149.6)	698.3 (433.7 to 1027.4)	1118.2 (677.0 to 1740.8)	60.1% (-15.1 to 202.6)
Piauí	1001.3 (662.0 to 1364.0)	1524.6 (938.8 to 2229.7)	52.3% (-13.4 to 168.1)	5683.2 (4311.4 to 7380.5)	8821.9 (6047.9 to 12124.7)	55.2% (0.3 to 140.9)	693.1 (434.2 to 1023.4)	1074.7 (649.8 to 1676.4)	55.1% (-17.8 to 193.2)
Rio Grande do Norte	999.5 (662.3 to 1403.1)	1559.1 (933.9 to 2268.7)	56.0% (-12.7 to 179.6)	5737.4 (4310.5 to 7470.5)	9089.3 (6240.6 to 12707.1)	58.4% (1.3 to 148.3)	701.1 (436.9 to 1026.3)	1107.5 (636.5 to 1715.5)	58.0% (-17.8 to 204.5)
Sergipe	998.1 (655.5 to 1391.0)	1529.6 (915.0 to 2266.0)	53.3% (-15.0 to 176.8)	5704.5 (4299.0 to 7408.7)	9000.4 (6226.9 to 12526.3)	57.8% (1.5 to 145.7)	695.5 (428.1 to 1026.6)	1095.5 (649.2 to 1708.5)	57.5% (-17.6 to 201.8)
Distrito Federal	1006.8 (666.9 to 1400.0)	1587.0 (979.0 to 2392.2)	57.6% (-11.8 to 182.2)	5854.2 (4414.1 to 7644.5)	9433.3 (6473.4 to 13182.5)	61.1% (3.1 to 152.5)	711.5 (448.6 to 1066.0)	1151.3 (672.8 to 1798.7)	61.8% (-15.8 to 211.6)
Goiás	997.1 (645.5 to 1387.9)	1673.4 (1032.2 to 2570.0)	67.8% (-7.5 to 205.1)	5765.7 (4311.7 to 7503.6)	9819.7 (6722.6 to 13779.5)	70.3% (8.5 to 168.0)	701.7 (442.2 to 1031.3)	1191.5 (710.7 to 1840.2)	69.8% (-10.0 to 221.2)
Mato Grosso	995.1 (658.1 to 1393.4)	1631.8 (1004.6 to 2471.2)	64.0% (-8.7 to 195.3)	5685.3 (4272.1 to 7422.3)	9576.1 (6582.9 to 13414.6)	68.4% (7.6 to 164.3)	693.4 (437.9 to 1038.3)	1166.3 (673.4 to 1799.6)	68.2% (-12.4 to 223.8)
Mato Grosso do Sul	1000.7 (656.3 to 1388.7)	1584.2 (941.1 to 2350.3)	58.3% (-12.3 to 186.3)	5740.0 (4355.0 to 7436.0)	9309.5 (6488.7 to 13031.3)	62.2% (4.8 to 151.9)	698.4 (452.3 to 1027.1)	1132.5 (663.7 to 1730.0)	62.1% (-13.6 to 205.2)
Paraná	1000.3 (663.4 to 1392.4)	1697.7 (1036.0 to 2528.7)	69.7% (-5.0 to 203.8)	5750.3 (4329.5 to 7538.2)	9965.7 (6754.9 to 13795.2)	73.3% (10.5 to 172.3)	700.7 (445.2 to 1023.6)	1212.8 (716.1 to 1856.6)	73.1% (-7.9 to 225.9)
Rio Grande do Sul	997.5 (646.9 to 1383.5)	1642.8 (992.4 to 2527.4)	64.7% (-9.7 to 200.9)	5749.8 (4380.2 to 7443.1)	9749.6 (6739.3 to 13799.1)	69.6% (8.9 to 164.6)	700.4 (441.2 to 1034.6)	1188.1 (704.4 to 1822.6)	69.6% (-10.1 to 221.2)
Santa Catarina	993.8 (648.0 to 1381.8)	1670.0 (999.8 to 2430.7)	68.0% (-6.2 to 201.9)	5731.8 (4333.4 to 7455.8)	9885.9 (6879.7 to 13714.8)	72.5% (11.4 to 167.5)	700.0 (440.1 to 1029.6)	1202.3 (702.4 to 1921.4)	71.8% (-11.1 to 232.6)
Espírito Santo	999.2 (655.4 to 1388.5)	1559.7 (952.5 to 2320.9)	56.1% (-12.8 to 180.0)	5732.4 (4305.8 to 7451.9)	9164.9 (6294.8 to 12549.4)	59.9% (3.1 to 148.3)	698.1 (445.2 to 1027.0)	1115.1 (666.2 to 1734.2)	59.7% (-15.2 to 201.5)

Minas Gerais	1000.0 (648.8 to 1399.3)	1599.6 (961.8 to 2442.7)	60.0% (-12.6 to 193.1)	5717.8 (4305.0 to 7398.5)	9444.2 (6438.7 to 13230.8)	65.2% (5.6 to 159.1)	695.5 (435.7 to 1006.7)	1148.8 (669.6 to 1762.0)	65.2% (-12.8 to 213.5)
Rio de Janeiro	1001.8 (652.2 to 1392.1)	1653.1 (995.9 to 2456.7)	65.0% (-8.5 to 198.2)	5788.5 (4377.4 to 7498.3)	9799.6 (6729.8 to 13433.6)	69.3% (9.5 to 162.3)	705.8 (445.2 to 1030.6)	1195.1 (701.2 to 1804.7)	69.3% (-9.8 to 218.6)
São Paulo	1004.8 (679.4 to 1364.8)	1593.6 (948.9 to 2321.3)	58.6% (-9.8 to 179.6)	5979.1 (4670.4 to 7526.6)	9262.3 (6471.4 to 12866.3)	54.9% (2.1 to 135.4)	727.5 (465.3 to 1049.5)	1122.4 (651.7 to 1723.8)	54.3% (-18.2 to 191.6)
Depressive disorders									
Acre	3291.3 (2317.9 to 4589.5)	4110.7 (2679.8 to 5986.8)	24.9% (-26.3 to 112.1)	2454.5 (1813.0 to 3255.8)	3040.6 (2123.2 to 4226.1)	23.9% (-21.1 to 95.0)	459.9 (281.4 to 701.1)	576.8 (340.2 to 928.6)	25.4% (-36.1 to 146.7)
Amapá	3391.1 (2402.4 to 4746.5)	4404.8 (2950.0 to 6423.2)	29.9% (-22.4 to 117.8)	2526.3 (1874.7 to 3399.3)	3251.7 (2315.3 to 4452.1)	28.7% (-17.0 to 100.0)	475.7 (291.6 to 717.8)	617.3 (369.9 to 958.9)	29.8% (-32.3 to 148.9)
Amazonas	2836.2 (1907.5 to 4115.9)	3944.1 (2580.8 to 5839.3)	39.1% (-20.3 to 142.8)	2176.4 (1576.4 to 2970.5)	2930.8 (2063.7 to 4161.8)	34.7% (-15.8 to 115.7)	401.5 (239.1 to 623.4)	554.1 (324.0 to 877.3)	38.0% (-30.4 to 173.8)
Pará	2667.8 (1767.9 to 4006.3)	3135.7 (1994.9 to 4689.2)	17.5% (-34.6 to 111.4)	2073.6 (1496.3 to 2902.5)	2426.9 (1685.4 to 3469.1)	17.0% (-28.0 to 90.5)	378.6 (222.7 to 592.4)	448.3 (257.4 to 719.9)	18.4% (-41.4 to 139.7)
Rondônia	3385.2 (2290.3 to 4809.6)	4685.8 (3031.9 to 6831.2)	38.4% (-19.8 to 139.3)	2543.4 (1811.3 to 3422.4)	3414.6 (2337.0 to 4771.0)	34.3% (-16.6 to 116.6)	475.2 (296.8 to 741.0)	651.7 (371.6 to 1023.0)	37.2% (-30.4 to 170.9)
Roraima	3857.4 (2728.9 to 5405.2)	5037.4 (3386.0 to 7311.7)	30.6% (-21.8 to 118.5)	2861.5 (2098.0 to 3845.5)	3627.8 (2554.1 to 5075.5)	26.8% (-19.7 to 100.4)	541.9 (335.9 to 818.9)	700.2 (410.1 to 1122.2)	29.2% (-33.8 to 152.9)
Tocantins	3337.3 (2309.9 to 4700.9)	4615.7 (3095.0 to 6741.7)	38.3% (-18.0 to 133.7)	2491.0 (1832.8 to 3361.1)	3359.4 (2366.9 to 4687.8)	34.9% (-14.5 to 112.9)	467.6 (286.8 to 715.8)	643.0 (374.5 to 1009.6)	37.5% (-29.6 to 168.8)
Alagoas	3524.9 (2491.3 to 4886.4)	4387.7 (2906.0 to 6392.3)	24.5% (-25.8 to 109.4)	2626.8 (1957.2 to 3489.0)	3216.2 (2257.0 to 4549.9)	22.4% (-22.3 to 93.1)	492.8 (314.0 to 747.8)	613.4 (359.1 to 980.8)	24.5% (-35.8 to 142.1)
Bahia	2477.0 (1626.4 to 3683.0)	3478.5 (2214.3 to 5317.1)	40.4% (-22.6 to 154.6)	1949.7 (1412.6 to 2706.8)	2634.1 (1825.1 to 3729.8)	35.1% (-16.4 to 118.7)	352.9 (208.5 to 550.9)	490.2 (285.2 to 772.6)	38.9% (-30.2 to 176.7)

Ceará	3320.8 (2304.7 to 4623.1)	4796.0 (3154.8 to 7085.8)	44.4% (-15.2 to 146.7)	2499.2 (1822.7 to 3322.9)	3483.5 (2454.3 to 4893.4)	39.4% (-11.7 to 120.3)	466.9 (285.8 to 716.3)	668.0 (387.0 to 1033.6)	43.1% (-26.6 to 179.1)
Maranhão	3129.3 (2170.8 to 4443.5)	3899.5 (2540.5 to 5600.2)	24.6% (-26.6 to 112.1)	2354.5 (1736.9 to 3197.6)	2887.0 (2004.2 to 4004.2)	22.6% (-22.6 to 94.5)	438.5 (269.7 to 662.8)	545.1 (322.2 to 857.8)	24.3% (-35.7 to 140.8)
Paraíba	3084.6 (2130.1 to 4364.4)	3887.4 (2615.4 to 5611.2)	26.0% (-25.1 to 112.1)	2344.6 (1713.6 to 3158.4)	2894.9 (2076.2 to 4007.6)	23.5% (-21.0 to 92.9)	434.8 (267.3 to 665.6)	546.7 (322.4 to 864.4)	25.7% (-35.5 to 145.2)
Pernambuco	3680.2 (2603.5 to 5075.2)	4977.8 (3254.2 to 7237.5)	35.3% (-19.7 to 128.1)	2732.0 (2043.4 to 3669.5)	3598.7 (2520.5 to 5070.4)	31.7% (-16.5 to 108.1)	514.3 (320.5 to 778.1)	690.8 (421.7 to 1081.0)	34.3% (-29.3 to 155.5)
Piauí	2945.5 (1972.7 to 4302.3)	3816.1 (2478.4 to 5762.9)	29.6% (-26.8 to 129.3)	2244.4 (1596.6 to 3151.4)	2839.8 (1972.0 to 4054.0)	26.5% (-22.6 to 107.0)	415.1 (249.3 to 645.7)	534.8 (309.0 to 853.4)	28.8% (-35.5 to 157.2)
Rio Grande do Norte	3577.4 (2494.0 to 4909.0)	4522.6 (2963.2 to 6572.2)	26.4% (-25.0 to 113.7)	2673.7 (1962.8 to 3548.5)	3309.9 (2312.0 to 4623.6)	23.8% (-21.5 to 95.6)	502.3 (312.5 to 751.5)	632.3 (371.8 to 987.2)	25.9% (-34.5 to 142.4)
Sergipe	3253.6 (2227.6 to 4585.6)	4159.3 (2705.8 to 6204.6)	27.8% (-26.2 to 121.7)	2456.4 (1777.5 to 3346.6)	3084.4 (2128.5 to 4345.2)	25.6% (-21.9 to 102.2)	457.2 (277.6 to 707.6)	584.2 (334.3 to 938.9)	27.8% (-36.1 to 156.0)
Distrito Federal	3554.4 (2462.0 to 5004.6)	4451.0 (2917.8 to 6597.2)	25.2% (-27.0 to 115.2)	2693.3 (1980.8 to 3624.8)	3301.7 (2274.9 to 4642.9)	22.6% (-23.2 to 96.0)	503.7 (312.3 to 773.1)	625.5 (369.2 to 984.9)	24.2% (-36.0 to 141.2)
Goiás	3987.5 (2784.2 to 5552.7)	5044.4 (3303.1 to 7494.9)	26.5% (-26.0 to 116.5)	2946.5 (2177.2 to 3956.6)	3671.1 (2537.4 to 5231.6)	24.6% (-22.1 to 99.4)	557.6 (351.2 to 840.7)	703.0 (407.5 to 1120.0)	26.1% (-35.3 to 146.3)
Mato Grosso	3120.9 (2140.8 to 4452.7)	4395.0 (2911.1 to 6381.7)	40.8% (-17.4 to 140.0)	2386.2 (1739.1 to 3258.8)	3241.8 (2263.8 to 4529.5)	35.9% (-14.6 to 116.6)	442.0 (268.3 to 678.7)	615.5 (351.8 to 992.4)	39.2% (-30.3 to 179.3)
Mato Grosso do Sul	3650.9 (2500.7 to 5225.6)	4651.0 (3090.7 to 6820.4)	27.4% (-25.6 to 118.0)	2725.3 (1986.0 to 3711.4)	3401.8 (2405.3 to 4839.5)	24.8% (-21.7 to 99.5)	512.7 (315.6 to 784.8)	648.2 (379.5 to 1022.2)	26.4% (-35.2 to 146.8)
Paraná	3764.3 (2623.6 to 5198.4)	4914.5 (3274.9 to 7184.5)	30.6% (-22.3 to 119.9)	2806.7 (2079.2 to 3754.1)	3583.6 (2504.2 to 5064.1)	27.7% (-19.4 to 102.5)	529.3 (324.8 to 803.0)	685.4 (410.9 to 1083.9)	29.5% (-33.0 to 150.2)

Rio Grande do Sul	3958.3 (2818.5 to 5417.0)	5141.8 (3461.9 to 7479.8)	29.9% (-21.6 to 115.7)	2937.7 (2189.9 to 3899.1)	3726.5 (2601.0 to 5257.5)	26.8% (-19.5 to 100.1)	557.5 (347.5 to 833.0)	717.1 (413.4 to 1141.8)	28.6% (-34.1 to 152.0)
Santa Catarina	4257.9 (2956.3 to 5939.0)	5796.6 (3776.8 to 8384.5)	36.1% (-19.7 to 131.2)	3131.2 (2275.4 to 4274.3)	4151.3 (2848.5 to 5842.0)	32.6% (-17.7 to 113.9)	596.5 (372.5 to 907.2)	803.4 (477.9 to 1260.1)	34.7% (-29.9 to 159.1)
Espírito Santo	3262.8 (2247.6 to 4566.3)	4198.6 (2716.4 to 6031.6)	28.7% (-24.4 to 119.4)	2480.1 (1820.7 to 3336.9)	3121.3 (2173.8 to 4281.6)	25.9% (-20.0 to 98.3)	461.9 (286.4 to 693.3)	590.7 (348.9 to 961.0)	27.9% (-34.6 to 150.6)
Minas Gerais	3439.5 (2401.0 to 4764.5)	4484.6 (3045.5 to 6363.7)	30.4% (-20.9 to 114.9)	2586.1 (1893.7 to 3459.7)	3308.3 (2388.4 to 4510.7)	27.9% (-17.1 to 97.7)	484.0 (298.3 to 731.9)	627.7 (387.1 to 943.9)	29.7% (-30.7 to 142.5)
Rio de Janeiro	3388.9 (2327.7 to 4719.0)	4360.7 (2837.8 to 6524.9)	28.7% (-25.4 to 122.7)	2485.1 (1796.9 to 3383.8)	3143.7 (2176.8 to 4494.7)	26.5% (-21.7 to 104.7)	473.3 (290.2 to 723.7)	605.5 (357.7 to 967.5)	27.9% (-34.6 to 150.4)
São Paulo	4210.7 (3081.9 to 5785.9)	5239.9 (3520.6 to 7789.9)	24.4% (-24.8 to 106.5)	3069.7 (2331.8 to 4015.5)	3752.4 (2657.3 to 5348.9)	22.2% (-21.3 to 90.4)	587.8 (378.6 to 882.4)	725.3 (431.9 to 1146.3)	23.4% (-35.2 to 135.7)

UI, uncertainly interval; DALYs, disability-adjusted life-years.

Table S6 The incidence, prevalence and DALYs rate of anxiety and depressive disorders in 1990 and 2021, and changes from 1990 to 2021 in China.

**Location**	**Incidence (95% UI)**			**Prevalence (95% UI)**			**DALYs (95% UI)**		
	**1990**	**2021**	**Percentage change**	**1990**	**2021**	**Percentage change**	**1990**	**2021**	**Percentage change**
Anxiety disorders									
Anhui	651.1 (427.1 to 900.3)	700.7 (448.1 to 1043.2)	7.6% (-38.6 to 89.1)	4281.5 (3252.0 to 5553.9)	4352.3 (2973.2 to 6151.6)	1.7% (-35.1 to 59.5)	530.7 (333.4 to 781.0)	542.8 (308.6 to 857.5)	2.3% (-47.4 to 99.4)
Chongqing	631.2 (406.8 to 882.5)	702.9 (432.7 to 1050.9)	11.4% (-38.1 to 100.7)	4335.7 (3319.8 to 5577.8)	4503.8 (3047.3 to 6378.3)	3.9% (-33.7 to 62.8)	536.2 (332.5 to 791.3)	562.4 (322.4 to 867.4)	4.9% (-45.6 to 102.5)
Beijing	520.8 (325.4 to 747.0)	567.8 (324.8 to 887.3)	9.0% (-43.2 to 109.7)	3250.6 (2318.0 to 4457.2)	3505.3 (2252.6 to 5134.5)	7.8% (-36.1 to 82.4)	404.4 (243.3 to 618.6)	437.1 (237.6 to 715.8)	8.1% (-47.5 to 123.2)
Gansu	758.6 (490.3 to 1080.8)	847.6 (525.5 to 1252.6)	11.7% (-37.6 to 100.6)	5444.4 (4129.0 to 7029.6)	5631.4 (3816.6 to 7910.7)	3.4% (-34.0 to 62.4)	673.1 (425.1 to 997.6)	700.4 (399.3 to 1107.8)	4.1% (-46.5 to 102.9)

Fujian	664.2 (442.7 to 925.7)	741.9 (457.7 to 1092.8)	11.7% (-36.8 to 98.1)	4279.8 (3244.1 to 5584.4)	4597.5 (3164.1 to 6544.1)	7.4% (-31.6 to 69.0)	530.8 (331.9 to 786.2)	574.2 (333.3 to 904.3)	8.2% (-44.0 to 109.5)
Jiangxi	669.1 (443.3 to 937.9)	712.8 (449.9 to 1095.9)	6.5% (-40.5 to 91.0)	4271.7 (3217.6 to 5591.3)	4389.6 (3023.8 to 6316.4)	2.8% (-34.9 to 62.8)	528.8 (334.4 to 779.7)	545.9 (322.6 to 863.4)	3.2% (-46.0 to 98.1)
Inner Mongolia	657.3 (435.9 to 901.0)	710.2 (445.4 to 1069.5)	8.0% (-38.9 to 91.2)	4289.9 (3260.3 to 5574.4)	4493.0 (3101.7 to 6420.0)	4.7% (-33.2 to 64.4)	532.8 (335.2 to 790.7)	560.2 (321.7 to 895.3)	5.1% (-46.1 to 105.5)
Qinghai	487.0 (302.6 to 719.3)	507.1 (292.6 to 786.7)	4.1% (-45.9 to 100.9)	2848.8 (1939.1 to 4011.2)	2871.7 (1790.9 to 4370.4)	0.8% (-43.2 to 79.3)	353.0 (203.6 to 549.0)	356.6 (191.0 to 571.3)	1.0% (-51.5 to 111.1)
Shaanxi	651.8 (426.6 to 909.9)	713.4 (448.6 to 1057.9)	9.4% (-38.1 to 93.9)	4284.0 (3225.5 to 5548.5)	4478.3 (3060.3 to 6286.5)	4.5% (-33.2 to 64.1)	531.5 (334.2 to 779.6)	558.0 (332.0 to 891.0)	5.0% (-45.2 to 101.7)
Macao	662.2 (429.2 to 923.6)	700.5 (421.1 to 1069.7)	5.8% (-42.1 to 93.6)	4398.2 (3350.6 to 5724.6)	4609.3 (3081.0 to 6587.1)	4.8% (-34.0 to 66.5)	547.4 (339.7 to 809.0)	573.3 (327.7 to 902.9)	4.7% (-46.2 to 104.5)
Jiangsu	648.2 (416.9 to 905.9)	693.3 (426.5 to 1041.1)	7.0% (-40.7 to 93.4)	4271.6 (3228.2 to 5533.7)	4329.1 (2934.8 to 6153.4)	1.3% (-35.7 to 60.2)	530.4 (334.6 to 772.6)	540.2 (304.7 to 852.7)	1.8% (-47.5 to 97.7)
Liaoning	505.5 (311.9 to 735.9)	542.0 (311.0 to 852.0)	7.2% (-44.6 to 108.4)	3012.6 (2078.9 to 4250.8)	3189.8 (2008.5 to 4799.8)	5.9% (-39.7 to 86.2)	374.5 (219.9 to 586.8)	397.8 (211.5 to 650.9)	6.2% (-49.5 to 124.0)
Shandong	756.4 (489.8 to 1065.8)	789.2 (489.1 to 1171.5)	4.3% (-41.7 to 87.3)	5252.2 (3997.6 to 6745.1)	5198.9 (3578.5 to 7346.6)	-1.0% (-36.4 to 54.4)	652.4 (411.3 to 964.4)	648.7 (377.2 to 1036.2)	-0.6% (-48.7 to 93.0)
Jilin	653.9 (424.1 to 904.2)	731.7 (450.2 to 1081.0)	11.9% (-37.2 to 99.9)	4284.5 (3231.3 to 5561.7)	4657.0 (3154.2 to 6499.8)	8.7% (-30.6 to 70.8)	531.8 (338.8 to 773.9)	580.4 (337.7 to 930.7)	9.1% (-43.1 to 110.0)
Ningxia	845.9 (559.6 to 1191.2)	891.7 (550.0 to 1316.0)	5.4% (-40.7 to 87.9)	5908.9 (4457.4 to 7662.6)	6128.7 (4137.1 to 8615.2)	3.7% (-34.1 to 63.5)	733.4 (464.5 to 1067.0)	761.0 (441.8 to 1166.4)	3.8% (-45.2 to 97.0)
Hainan	665.9 (439.6 to 928.5)	747.6 (465.9 to 1112.6)	12.3% (-36.7 to 99.7)	4251.5 (3218.3 to 5552.1)	4667.5 (3227.2 to 6530.8)	9.8% (-29.5 to 71.4)	528.3 (330.7 to 783.9)	579.6 (338.7 to 888.3)	9.7% (-42.4 to 109.6)

Guangdong	467.2 (284.6 to 717.2)	488.0 (280.0 to 777.1)	4.5% (-47.3 to 107.5)	2647.6 (1713.5 to 4012.3)	2733.2 (1639.0 to 4372.3)	3.2% (-46.0 to 97.9)	327.9 (183.0 to 532.4)	339.2 (177.9 to 573.7)	3.4% (-52.9 to 127.7)
Hebei	589.5 (376.4 to 829.3)	653.5 (402.0 to 993.4)	10.9% (-39.1 to 102.1)	3687.9 (2713.7 to 4858.8)	3825.3 (2516.8 to 5554.0)	3.7% (-36.3 to 69.4)	457.2 (279.7 to 685.7)	476.7 (264.3 to 773.8)	4.3% (-48.2 to 110.5)
Guangxi	668.2 (448.8 to 926.4)	723.0 (454.9 to 1090.3)	8.2% (-38.7 to 91.3)	4248.7 (3208.2 to 5507.9)	4429.0 (2969.7 to 6434.4)	4.2% (-34.8 to 66.9)	526.7 (335.8 to 788.1)	552.8 (322.4 to 873.1)	5.0% (-45.5 to 102.6)
Guizhou	666.0 (445.8 to 915.0)	727.3 (457.3 to 1071.6)	9.2% (-37.4 to 91.0)	4278.2 (3231.1 to 5516.9)	4550.6 (3134.4 to 6472.2)	6.4% (-32.0 to 66.8)	529.4 (329.4 to 783.8)	568.3 (332.1 to 897.2)	7.3% (-44.4 to 107.6)
Taiwan	774.5 (513.6 to 1084.8)	712.3 (418.0 to 1124.8)	-8.0% (-50.3 to 71.1)	5531.9 (3874.5 to 7402.0)	4885.2 (2986.5 to 7420.2)	-11.7% (-49.3 to 54.1)	687.6 (413.5 to 1051.7)	607.4 (331.3 to 1030.4)	-11.7% (-57.6 to 84.4)
Yunnan	669.4 (445.9 to 921.8)	727.1 (451.5 to 1083.1)	8.6% (-38.5 to 92.1)	4266.7 (3232.7 to 5545.4)	4622.7 (3161.9 to 6408.1)	8.3% (-30.3 to 69.1)	528.0 (329.5 to 778.3)	575.6 (336.6 to 901.6)	9.0% (-43.2 to 109.7)
Xinjiang	664.0 (446.2 to 926.7)	732.2 (467.5 to 1097.5)	10.3% (-37.1 to 93.7)	4295.7 (3291.7 to 5586.6)	4497.6 (3085.8 to 6384.9)	4.7% (-33.1 to 64.1)	531.7 (334.4 to 783.9)	561.0 (329.1 to 885.6)	5.5% (-45.0 to 103.2)
Zhejiang	665.9 (431.2 to 933.5)	670.0 (412.1 to 1029.0)	0.6% (-44.7 to 83.5)	4433.7 (3329.7 to 5805.6)	4420.7 (3009.8 to 6357.1)	-0.3% (-37.2 to 58.7)	549.7 (344.3 to 808.2)	550.2 (313.2 to 837.6)	0.1% (-47.7 to 92.2)
Hong Kong	497.2 (310.1 to 720.8)	525.1 (308.5 to 835.9)	5.6% (-45.0 to 103.4)	2882.1 (1943.8 to 4136.9)	3054.0 (1929.2 to 4691.0)	6.0% (-40.8 to 90.3)	359.3 (205.2 to 563.0)	381.4 (201.7 to 655.6)	6.2% (-51.1 to 131.2)
Heilongjiang	656.1 (434.9 to 909.1)	751.3 (469.8 to 1132.5)	14.5% (-35.5 to 103.7)	4287.6 (3244.3 to 5551.3)	4809.3 (3298.2 to 6798.6)	12.2% (-28.3 to 75.9)	532.4 (331.6 to 786.8)	599.0 (343.1 to 943.5)	12.5% (-42.1 to 119.3)
Henan	654.5 (428.4 to 899.0)	721.2 (442.8 to 1063.5)	10.2% (-37.9 to 96.0)	4286.7 (3252.5 to 5556.3)	4397.3 (2968.9 to 6252.0)	2.6% (-35.0 to 62.2)	531.2 (330.5 to 780.4)	547.2 (324.5 to 849.6)	3.0% (-45.8 to 96.4)
Hubei	652.0 (432.3 to 908.7)	726.0 (461.0 to 1101.6)	11.3% (-37.1 to 97.8)	4274.2 (3229.4 to 5532.7)	4541.4 (3112.4 to 6491.9)	6.3% (-32.4 to 67.5)	529.4 (328.7 to 783.1)	566.3 (330.1 to 901.9)	7.0% (-44.9 to 108.1)

Hunan	924.6 (599.6 to 1316.0)	1039.4 (650.8 to 1536.8)	12.4% (-37.0 to 100.6)	7129.7 (5378.3 to 9261.1)	7349.9 (4846.0 to 10253.2)	3.1% (-35.0 to 63.7)	883.6 (557.3 to 1295.9)	916.0 (530.2 to 1427.2)	3.7% (-45.9 to 99.2)
Tianjin	615.0 (401.8 to 849.8)	626.5 (382.6 to 932.8)	1.9% (-43.1 to 82.7)	3929.0 (2943.8 to 5132.0)	3979.7 (2670.9 to 5664.5)	1.3% (-36.3 to 61.5)	487.7 (299.7 to 726.4)	495.8 (285.3 to 778.3)	1.6% (-47.8 to 98.5)
Shanghai	519.5 (328.8 to 757.3)	514.0 (300.2 to 801.8)	-1.1% (-48.0 to 89.0)	3120.6 (2193.2 to 4320.4)	3168.7 (1991.6 to 4853.9)	1.5% (-41.8 to 77.9)	388.3 (227.6 to 591.0)	394.0 (213.6 to 655.1)	1.5% (-51.4 to 112.4)
Tibet	672.3 (449.3 to 922.6)	659.8 (407.1 to 986.4)	-1.9% (-44.5 to 73.6)	4277.6 (3243.4 to 5564.9)	4171.3 (2796.7 to 5987.4)	-2.5% (-38.7 to 55.2)	528.8 (329.9 to 793.0)	519.1 (297.1 to 798.2)	-1.8% (-49.2 to 90.0)
Shanxi	656.2 (428.2 to 902.3)	694.8 (418.0 to 1035.9)	5.9% (-41.1 to 90.7)	4309.4 (3253.6 to 5573.7)	4415.5 (2971.5 to 6366.4)	2.5% (-35.6 to 63.1)	535.0 (339.2 to 788.8)	550.0 (320.2 to 866.7)	2.8% (-46.4 to 98.0)
Sichuan	582.9 (370.2 to 821.4)	642.4 (390.0 to 979.4)	10.2% (-40.0 to 102.8)	3783.0 (2777.3 to 5054.9)	3915.9 (2565.0 to 5650.3)	3.5% (-36.8 to 70.0)	468.6 (286.5 to 713.8)	487.6 (279.7 to 781.4)	4.1% (-47.5 to 106.9)
Depressive disorders									
Anhui	2481.1 (1743.6 to 3482.7)	1098.6 (698.3 to 1626.4)	-55.7% (-74.3 to -23.5)	2003.6 (1479.6 to 2708.6)	1052.2 (765.9 to 1420.6)	-47.5% (-65.7 to -19.4)	370.0 (228.1 to 564.5)	175.7 (101.3 to 275.4)	-52.5% (-75.7 to -6.9)
Chongqing	2373.9 (1704.6 to 3258.0)	1346.3 (902.4 to 1933.9)	-43.3% (-65.6 to -6.3)	1976.4 (1507.3 to 2570.7)	1255.7 (915.0 to 1676.6)	-36.5% (-57.5 to -4.9)	359.7 (225.4 to 530.5)	214.5 (124.4 to 332.0)	-40.4% (-68.9 to 14.4)
Beijing	1468.7 (1018.5 to 2109.6)	1277.2 (843.0 to 1899.8)	-13.0% (-49.5 to 50.1)	1394.4 (1045.1 to 1856.0)	1286.0 (928.3 to 1760.8)	-7.8% (-39.9 to 41.8)	236.2 (147.2 to 356.6)	214.1 (123.4 to 340.4)	-9.3% (-53.7 to 77.8)
Gansu	2940.4 (2131.0 to 3990.2)	1542.3 (1032.2 to 2253.7)	-47.5% (-68.2 to -13.5)	2334.5 (1770.1 to 3051.8)	1359.3 (992.6 to 1815.3)	-41.8% (-61.1 to -12.7)	436.0 (274.7 to 652.0)	238.8 (140.5 to 375.0)	-45.2% (-71.5 to 5.4)
Fujian	2341.5 (1688.5 to 3246.8)	1372.4 (896.5 to 1992.6)	-41.4% (-65.0 to -1.7)	1894.6 (1429.7 to 2475.5)	1237.4 (908.0 to 1687.7)	-34.7% (-56.8 to -1.2)	348.8 (219.4 to 524.4)	214.5 (126.0 to 342.7)	-38.5% (-68.3 to 19.5)
Jiangxi	2162.6 (1558.2 to 2968.0)	1235.0 (807.0 to 1809.6)	-42.9% (-65.8 to -4.3)	1762.7 (1331.7 to 2302.8)	1136.8 (831.8 to 1539.3)	-35.5% (-57.2 to -2.6)	321.6 (202.7 to 491.4)	193.6 (115.6 to 299.9)	-39.8% (-68.5 to 14.9)

Inner Mongolia	2170.6 (1554.7 to 2939.9)	1327.2 (888.7 to 1905.4)	-38.9% (-62.8 to 0.7)	1794.6 (1365.5 to 2320.2)	1230.0 (912.0 to 1636.2)	-31.5% (-53.7 to 1.6)	326.4 (202.6 to 484.4)	210.3 (131.0 to 322.6)	-35.6% (-65.4 to 19.9)
Qinghai	2122.3 (1525.8 to 2927.9)	1299.4 (869.5 to 1911.4)	-38.8% (-63.3 to 2.3)	1760.1 (1330.4 to 2297.9)	1213.0 (895.5 to 1634.5)	-31.1% (-54.0 to 3.3)	318.8 (200.4 to 477.5)	205.9 (123.8 to 324.7)	-35.4% (-66.1 to 23.4)
Shaanxi	2311.7 (1652.6 to 3136.4)	1347.7 (886.5 to 1955.8)	-41.7% (-64.9 to -3.0)	1897.8 (1429.5 to 2429.6)	1238.7 (912.1 to 1653.2)	-34.7% (-56.1 to -2.7)	347.5 (215.1 to 519.1)	213.0 (123.8 to 340.1)	-38.7% (-68.6 to 19.9)
Macao	2116.0 (1528.8 to 2908.7)	1522.8 (1007.1 to 2187.8)	-28.0% (-56.5 to 19.3)	1818.0 (1378.4 to 2384.7)	1426.5 (1043.5 to 1895.8)	-21.5% (-47.5 to 17.4)	326.0 (201.3 to 493.6)	244.0 (145.7 to 369.0)	-25.1% (-60.5 to 42.0)
Jiangsu	2124.0 (1525.2 to 2884.5)	1216.6 (811.7 to 1763.2)	-42.7% (-65.3 to -5.3)	1811.4 (1373.8 to 2366.9)	1149.3 (854.2 to 1543.4)	-36.6% (-57.4 to -5.4)	325.8 (207.5 to 490.1)	195.1 (115.9 to 306.1)	-40.1% (-68.6 to 14.5)
Liaoning	1701.2 (1201.9 to 2371.8)	1236.2 (801.8 to 1802.8)	-27.3% (-57.2 to 23.5)	1512.2 (1141.4 to 1980.6)	1194.2 (874.1 to 1592.0)	-21.0% (-47.3 to 18.4)	265.2 (164.7 to 409.7)	200.9 (118.1 to 315.4)	-24.2% (-61.1 to 47.4)
Shandong	2906.9 (2116.3 to 3992.4)	1519.3 (1017.5 to 2201.9)	-47.7% (-68.3 to -13.8)	2333.7 (1778.6 to 3067.2)	1380.0 (1005.6 to 1856.7)	-40.9% (-60.7 to -10.9)	433.9 (276.2 to 646.7)	238.8 (140.2 to 377.6)	-45.0% (-71.3 to 6.0)
Jilin	2010.2 (1452.1 to 2756.5)	1408.7 (929.1 to 2035.0)	-29.9% (-57.7 to 16.4)	1709.9 (1303.4 to 2224.2)	1289.8 (937.7 to 1724.3)	-24.6% (-49.6 to 13.1)	307.0 (192.7 to 461.1)	223.1 (134.2 to 351.8)	-27.3% (-61.9 to 38.9)
Ningxia	2061.1 (1468.5 to 2795.9)	1322.0 (883.8 to 1918.2)	-35.9% (-61.3 to 6.4)	1710.4 (1287.8 to 2227.6)	1229.1 (905.2 to 1627.2)	-28.1% (-51.8 to 7.1)	309.5 (191.2 to 467.1)	210.0 (126.1 to 324.4)	-32.2% (-64.4 to 29.4)
Hainan	2249.7 (1622.8 to 3095.5)	1464.7 (971.8 to 2149.6)	-34.9% (-60.9 to 8.6)	1829.5 (1395.6 to 2361.3)	1302.6 (954.9 to 1759.2)	-28.8% (-52.4 to 6.7)	334.6 (210.9 to 505.7)	227.2 (136.5 to 353.4)	-32.1% (-64.2 to 29.1)
Guangdong	2266.6 (1620.4 to 3077.4)	1619.2 (1061.7 to 2350.7)	-28.6% (-57.1 to 19.0)	1872.0 (1423.1 to 2443.8)	1446.8 (1042.1 to 1975.5)	-22.7% (-49.1 to 17.7)	342.8 (213.9 to 512.3)	253.7 (147.2 to 389.1)	-26.0% (-61.3 to 42.1)
Hebei	2110.2 (1523.3 to 2874.7)	1348.4 (887.8 to 1946.3)	-36.1% (-61.4 to 5.8)	1800.0 (1369.7 to 2349.7)	1212.1 (879.2 to 1641.5)	-32.7% (-55.4 to 1.8)	323.8 (201.2 to 493.2)	210.0 (120.2 to 330.9)	-35.1% (-66.9 to 27.5)

Guangxi	1937.9 (1384.3 to 2634.8)	1246.8 (824.4 to 1814.2)	-35.7% (-61.3 to 7.1)	1622.8 (1229.7 to 2116.7)	1137.3 (830.2 to 1522.1)	-29.9% (-53.3 to 5.3)	293.1 (183.3 to 451.5)	195.5 (115.8 to 306.8)	-33.3% (-65.5 to 29.0)
Guizhou	2230.5 (1604.5 to 3077.1)	1416.7 (959.5 to 2055.2)	-36.5% (-61.5 to 5.0)	1805.3 (1377.8 to 2356.8)	1279.0 (954.1 to 1708.7)	-29.2% (-52.2 to 5.0)	330.8 (203.8 to 501.1)	222.3 (136.9 to 349.3)	-32.8% (-64.7 to 27.9)
Taiwan	1362.5 (902.3 to 1987.1)	1604.5 (996.6 to 2464.2)	17.8% (-35.3 to 114.8)	1279.4 (906.7 to 1752.2)	1484.3 (1016.9 to 2093.0)	16.0% (-28.6 to 88.7)	218.0 (130.1 to 337.6)	256.7 (141.4 to 417.9)	17.7% (-42.7 to 142.4)
Yunnan	2140.5 (1456.7 to 3062.0)	1313.9 (842.9 to 1980.3)	-38.6% (-65.1 to 8.2)	1746.0 (1269.7 to 2370.9)	1229.4 (880.3 to 1699.1)	-29.6% (-55.1 to 10.5)	317.5 (186.8 to 491.2)	209.1 (121.4 to 333.2)	-34.1% (-67.1 to 31.8)
Xinjiang	2679.4 (1913.4 to 3687.3)	1553.1 (1033.5 to 2243.4)	-42.0% (-65.1 to -3.5)	2119.5 (1576.1 to 2813.0)	1352.0 (974.0 to 1836.8)	-36.2% (-58.4 to -2.2)	396.0 (250.0 to 597.9)	238.9 (138.1 to 373.3)	-39.7% (-68.8 to 16.9)
Zhejiang	3016.1 (2162.4 to 4159.9)	1898.4 (1261.6 to 2769.1)	-37.1% (-62.3 to 5.2)	2376.6 (1768.2 to 3147.6)	1652.3 (1213.3 to 2251.7)	-30.5% (-54.3 to 5.8)	447.7 (283.7 to 673.5)	296.8 (174.7 to 467.7)	-33.7% (-65.5 to 27.7)
Hong Kong	2460.6 (1752.1 to 3325.7)	2051.6 (1366.7 to 3002.6)	-16.6% (-49.7 to 38.5)	1981.6 (1496.4 to 2572.0)	1728.8 (1260.5 to 2339.0)	-12.8% (-42.1 to 31.6)	367.4 (230.5 to 552.5)	314.4 (181.7 to 483.7)	-14.4% (-55.5 to 65.0)
Heilongjiang	2021.3 (1445.5 to 2776.6)	1513.5 (1019.2 to 2166.1)	-25.1% (-54.5 to 23.4)	1706.5 (1289.8 to 2240.8)	1366.5 (1001.1 to 1820.4)	-19.9% (-46.5 to 20.0)	307.1 (191.8 to 458.8)	237.3 (141.5 to 367.9)	-22.7% (-59.3 to 47.2)
Henan	2557.1 (1837.8 to 3586.1)	1303.9 (870.3 to 1846.5)	-49.0% (-69.1 to -15.7)	2050.0 (1555.6 to 2691.6)	1162.6 (852.2 to 1561.9)	-43.3% (-62.2 to -14.8)	381.1 (234.4 to 572.3)	202.3 (121.3 to 318.9)	-46.9% (-72.4 to 2.1)
Hubei	2892.9 (2091.4 to 3962.6)	1475.7 (982.1 to 2183.3)	-49.0% (-69.3 to -15.0)	2277.2 (1728.3 to 2978.8)	1301.6 (956.1 to 1748.9)	-42.8% (-61.8 to -14.3)	426.8 (269.7 to 640.6)	228.2 (137.1 to 351.1)	-46.5% (-71.7 to 0.9)
Hunan	2837.4 (2038.0 to 3905.4)	1429.0 (956.2 to 2102.8)	-49.6% (-69.8 to -15.9)	2293.2 (1728.5 to 3017.7)	1275.8 (941.0 to 1721.4)	-44.4% (-63.0 to -16.3)	423.8 (265.3 to 644.6)	222.0 (133.8 to 353.8)	-47.6% (-72.8 to 0.9)
Tianjin	1358.9 (921.2 to 1998.7)	995.2 (628.7 to 1498.5)	-26.8% (-59.0 to 31.1)	1292.1 (951.5 to 1751.4)	1055.2 (768.6 to 1426.3)	-18.3% (-46.9 to 25.6)	218.1 (133.4 to 335.8)	168.6 (96.2 to 268.1)	-22.7% (-61.1 to 53.7)

Shanghai	1392.7 (941.1 to 2032.8)	1118.0 (718.1 to 1660.6)	-19.7% (-54.4 to 41.4)	1318.9 (973.0 to 1771.9)	1190.8 (869.6 to 1605.5)	-9.7% (-40.9 to 38.1)	223.7 (135.5 to 344.5)	191.2 (111.4 to 293.5)	-14.5% (-56.1 to 66.5)
Tibet	1908.0 (1369.6 to 2619.2)	1152.2 (771.4 to 1663.8)	-39.6% (-63.5 to 0.0)	1605.5 (1215.6 to 2103.7)	1117.3 (826.4 to 1506.5)	-30.4% (-53.5 to 4.3)	286.8 (177.8 to 431.5)	186.4 (113.1 to 285.7)	-35.0% (-65.6 to 22.8)
Shanxi	1976.3 (1412.9 to 2680.9)	1246.8 (819.1 to 1816.4)	-36.9% (-62.1 to 5.1)	1679.6 (1278.4 to 2178.2)	1185.8 (868.4 to 1569.1)	-29.4% (-52.5 to 5.0)	301.2 (189.8 to 453.7)	200.4 (120.0 to 314.2)	-33.5% (-65.1 to 27.1)
Sichuan	2203.0 (1576.2 to 3137.0)	1304.7 (851.3 to 1949.4)	-40.8% (-65.4 to 1.7)	1840.0 (1384.4 to 2474.4)	1221.7 (883.1 to 1658.4)	-33.6% (-56.6 to 1.6)	332.6 (210.2 to 510.9)	207.6 (118.9 to 325.7)	-37.6% (-68.0 to 22.1)

UI, uncertainly interval; DALYs, disability-adjusted life-years.

Table S7 The incidence, prevalence and DALYs rate of anxiety and depressive disorders in 1990 and 2021, and changes from 1990 to 2021 in Ethiopia.

**Location**	**Incidence (95% UI)**			**Prevalence (95% UI)**			**DALYs (95% UI)**		
	**1990**	**2021**	**Percentage change**	**1990**	**2021**	**Percentage change**	**1990**	**2021**	**Percentage change**
Anxiety disorders									
Addis Ababa	787.4 (525.9 to 1099.0)	907.8 (545.4 to 1342.3)	15.3% (-35.5 to 106.5)	4094.2 (3082.6 to 5257.8)	4757.8 (3229.8 to 6708.2)	16.2% (-25.9 to 82.7)	501.6 (317.7 to 728.3)	586.2 (347.6 to 906.3)	16.9% (-37.9 to 120.5)
Afar	735.0 (504.8 to 1003.2)	844.2 (518.1 to 1250.7)	14.9% (-34.1 to 100.8)	3656.5 (2753.9 to 4705.7)	4220.2 (2808.4 to 5973.0)	15.4% (-27.2 to 83.0)	444.6 (271.6 to 660.7)	517.4 (294.4 to 809.0)	16.4% (-40.5 to 128.2)
Amhara	761.0 (506.7 to 1044.2)	866.6 (530.4 to 1286.1)	13.9% (-35.6 to 101.9)	3852.4 (2891.1 to 4978.2)	4433.0 (2961.3 to 6278.7)	15.1% (-27.5 to 82.8)	470.4 (291.6 to 696.0)	544.8 (316.9 to 835.7)	15.8% (-39.4 to 122.1)
Benishangul-Gumuz	763.0 (521.6 to 1039.1)	869.9 (526.4 to 1287.5)	14.0% (-34.9 to 100.7)	3889.9 (2931.8 to 5074.2)	4393.5 (2992.8 to 6231.6)	12.9% (-28.4 to 78.5)	473.3 (294.0 to 698.1)	539.8 (316.2 to 831.8)	14.1% (-40.2 to 118.3)
Dire Dawa	769.9 (523.1 to 1050.4)	872.1 (547.8 to 1277.4)	13.3% (-34.5 to 96.2)	3966.8 (2994.7 to 5166.4)	4495.6 (3019.9 to 6325.8)	13.3% (-28.2 to 79.2)	484.8 (301.5 to 714.5)	551.6 (324.5 to 887.0)	13.8% (-41.3 to 121.2)
Gambella	789.8 (534.4 to 1092.0)	880.2 (531.7 to 1326.3)	11.4% (-37.4 to 99.1)	4076.5 (3088.1 to 5272.1)	4541.1 (3101.4 to 6523.6)	11.4% (-29.4 to 76.0)	495.1 (310.2 to 727.2)	557.4 (322.4 to 867.0)	12.6% (-41.3 to 116.7)

Harari	768.1 (525.7 to 1055.4)	875.3 (534.9 to 1308.2)	14.0% (-35.2 to 100.9)	3897.6 (2916.5 to 5041.9)	4494.7 (3053.2 to 6375.3)	15.3% (-26.9 to 82.3)	476.8 (296.1 to 697.2)	552.1 (318.2 to 866.3)	15.8% (-40.0 to 124.3)
Oromia	758.2 (507.8 to 1038.8)	872.4 (530.3 to 1295.4)	15.1% (-34.9 to 103.8)	3808.0 (2856.9 to 4923.8)	4403.9 (2972.8 to 6225.1)	15.6% (-26.7 to 82.8)	463.8 (288.1 to 682.3)	539.5 (316.8 to 861.5)	16.3% (-39.8 to 125.6)
Somali	738.3 (497.7 to 1010.8)	844.3 (528.8 to 1226.6)	14.4% (-34.0 to 98.6)	3660.7 (2734.7 to 4756.4)	4206.8 (2808.4 to 5899.3)	14.9% (-27.5 to 82.5)	444.3 (275.1 to 653.4)	513.5 (297.1 to 820.8)	15.6% (-40.6 to 125.9)
Southern Nations, Nationalities, and Peoples	764.2 (510.5 to 1057.3)	863.5 (526.2 to 1274.8)	13.0% (-36.3 to 100.6)	3848.8 (2873.1 to 5009.6)	4392.7 (2954.8 to 6201.5)	14.1% (-27.9 to 81.3)	469.4 (289.0 to 697.8)	540.3 (315.8 to 850.9)	15.1% (-40.5 to 123.5)
Tigray	759.5 (512.5 to 1038.9)	874.1 (550.2 to 1279.5)	15.1% (-33.6 to 100.0)	3834.4 (2866.6 to 5021.5)	4462.5 (3036.5 to 6321.3)	16.4% (-26.4 to 84.8)	467.8 (288.2 to 697.8)	547.7 (319.9 to 858.3)	17.1% (-39.4 to 127.1)
Depressive disorders									
Addis Ababa	3737.3 (2563.7 to 5284.1)	4223.4 (2705.8 to 6222.0)	13.0% (-34.8 to 96.3)	3190.4 (2386.0 to 4231.5)	3581.9 (2554.9 to 5003.8)	12.3% (-27.8 to 74.9)	565.6 (353.2 to 866.2)	642.0 (381.1 to 1019.4)	13.5% (-41.4 to 120.3)
Afar	3300.0 (2238.5 to 4674.5)	3679.8 (2375.8 to 5593.7)	11.5% (-36.6 to 96.5)	2721.8 (2025.1 to 3670.4)	2978.8 (2112.9 to 4215.4)	9.4% (-30.6 to 72.9)	482.8 (296.6 to 731.1)	539.5 (324.5 to 870.0)	11.7% (-42.5 to 117.3)
Amhara	3573.9 (2454.6 to 5085.0)	4050.3 (2573.8 to 6059.2)	13.3% (-35.4 to 99.3)	2963.6 (2202.5 to 4003.8)	3305.5 (2334.5 to 4606.7)	11.5% (-29.0 to 75.4)	526.9 (327.8 to 802.8)	599.8 (350.6 to 931.3)	13.8% (-41.0 to 120.0)
Benishangul-Gumuz	3474.2 (2365.1 to 4892.9)	3837.3 (2451.7 to 5757.2)	10.5% (-36.9 to 93.9)	2906.1 (2170.2 to 3876.1)	3129.5 (2227.4 to 4426.0)	7.7% (-31.3 to 69.1)	512.8 (323.6 to 781.6)	565.4 (338.5 to 900.4)	10.3% (-42.7 to 112.6)
Dire Dawa	3602.0 (2467.4 to 5111.5)	4020.1 (2590.6 to 5944.2)	11.6% (-35.7 to 94.0)	3028.2 (2260.8 to 4041.2)	3309.3 (2355.7 to 4632.5)	9.3% (-30.0 to 71.0)	536.3 (328.5 to 818.5)	597.2 (357.1 to 961.6)	11.4% (-42.9 to 117.5)
Gambella	3613.2 (2466.9 to 5134.8)	4059.1 (2640.1 to 6150.5)	12.3% (-35.7 to 96.8)	3051.6 (2279.5 to 4100.0)	3339.7 (2374.3 to 4651.8)	9.4% (-29.9 to 71.0)	535.5 (329.6 to 813.1)	604.1 (360.1 to 956.4)	12.8% (-41.7 to 118.5)
Harari	3536.3 (2422.8 to 5005.3)	4011.5 (2594.5 to 6031.0)	13.4% (-34.9 to 98.3)	2955.6 (2201.4 to 3941.6)	3296.4 (2345.7 to 4554.1)	11.5% (-28.2 to 73.5)	526.2 (328.3 to 793.2)	596.9 (357.2 to 937.4)	13.4% (-40.7 to 117.4)

Oromia	3504.1 (2409.6 to 4978.8)	3950.7 (2533.5 to 5938.4)	12.7% (-35.5 to 97.7)	2888.7 (2163.5 to 3882.7)	3197.0 (2256.3 to 4426.8)	10.7% (-29.1 to 73.0)	514.5 (319.9 to 783.8)	580.5 (338.8 to 938.3)	12.8% (-42.6 to 122.4)
Somali	3222.6 (2190.8 to 4563.5)	3557.2 (2308.6 to 5290.4)	10.4% (-36.4 to 91.9)	2658.4 (1977.9 to 3568.4)	2885.5 (2080.1 to 4036.1)	8.5% (-30.2 to 69.2)	470.0 (287.8 to 720.8)	518.1 (316.6 to 813.6)	10.2% (-42.6 to 111.5)
Southern Nations, Nationalities, and Peoples	3525.8 (2373.7 to 5015.2)	3902.2 (2516.6 to 5733.4)	10.7% (-36.3 to 92.7)	2919.4 (2172.4 to 3911.1)	3177.9 (2283.8 to 4365.1)	8.9% (-29.5 to 68.4)	519.1 (312.4 to 792.1)	575.0 (337.9 to 900.9)	10.8% (-43.3 to 116.8)
Tigray	3436.9 (2333.0 to 4937.6)	3918.4 (2524.7 to 5836.4)	14.0% (-34.8 to 100.1)	2858.6 (2143.9 to 3859.4)	3218.1 (2274.7 to 4453.4)	12.6% (-27.9 to 75.9)	505.4 (313.1 to 781.7)	581.8 (342.2 to 930.7)	15.1% (-41.3 to 125.9)

UI, uncertainly interval; DALYs, disability-adjusted life-years.

Table S8 The incidence, prevalence and DALYs rate of anxiety and depressive disorders in 1990 and 2021, and changes from 1990 to 2021 in Indonesia.

**Location**	**Incidence (95% UI)**			**Prevalence (95% UI)**			**DALYs (95% UI)**		
	**1990**	**2021**	**Percentage change**	**1990**	**2021**	**Percentage change**	**1990**	**2021**	**Percentage change**
Anxiety disorders									
Aceh	635.2 (426.8 to 880.3)	863.7 (526.5 to 1295.1)	36.0% (-23.5 to 142.1)	3379.9 (2528.9 to 4452.7)	4692.5 (3190.1 to 6654.6)	38.8% (-12.4 to 121.0)	416.5 (257.3 to 620.3)	575.2 (338.5 to 895.6)	38.1% (-28.0 to 165.6)
Bali	632.8 (418.8 to 878.6)	864.1 (524.2 to 1260.3)	36.6% (-23.1 to 143.0)	3422.6 (2576.5 to 4447.8)	4711.1 (3223.0 to 6686.8)	37.6% (-12.5 to 117.0)	423.3 (259.6 to 628.2)	583.8 (335.8 to 891.9)	37.9% (-28.3 to 166.0)
Bangka-Belitung Islands	630.5 (424.5 to 864.8)	866.7 (539.1 to 1296.0)	37.5% (-21.8 to 141.8)	3351.1 (2521.6 to 4346.4)	4735.0 (3201.8 to 6641.7)	41.3% (-10.2 to 122.7)	412.8 (254.9 to 613.6)	584.5 (349.2 to 910.8)	41.6% (-25.7 to 170.3)
Banten	634.5 (418.2 to 877.7)	868.7 (516.7 to 1303.3)	36.9% (-24.1 to 147.5)	3399.0 (2553.0 to 4418.5)	4706.7 (3142.0 to 6668.5)	38.5% (-12.9 to 120.6)	419.0 (256.4 to 616.1)	581.7 (328.6 to 913.1)	38.8% (-29.1 to 172.8)
Bengkulu	633.3 (420.4 to 890.0)	867.5 (514.3 to 1280.9)	37.0% (-24.1 to 147.5)	3360.0 (2517.2 to 4368.8)	4714.1 (3205.5 to 6751.7)	40.3% (-11.5 to 122.8)	413.5 (259.3 to 621.6)	583.6 (337.8 to 928.0)	41.1% (-27.6 to 175.6)
Gorontalo	632.4 (418.6 to 886.4)	871.9 (509.8 to 1331.5)	37.9% (-24.8 to 153.5)	3421.4 (2584.7 to 4500.4)	4751.0 (3194.7 to 6761.0)	38.9% (-12.6 to 121.2)	420.3 (264.7 to 623.6)	585.7 (340.3 to 924.6)	39.4% (-27.8 to 169.8)

Jakarta	642.7 (425.3 to 901.9)	863.4 (530.3 to 1281.8)	34.3% (-24.7 to 140.4)	3497.4 (2602.8 to 4585.6)	4669.5 (3185.5 to 6713.5)	33.5% (-16.2 to 113.3)	430.4 (269.3 to 632.7)	577.5 (333.1 to 908.3)	34.2% (-30.5 to 160.0)
Jambi	636.7 (426.6 to 888.4)	865.6 (534.1 to 1280.7)	36.0% (-23.2 to 141.1)	3393.0 (2532.7 to 4416.6)	4691.4 (3213.4 to 6639.2)	38.3% (-12.2 to 118.6)	416.9 (259.9 to 622.6)	581.2 (332.9 to 935.0)	39.4% (-29.1 to 174.9)
West Java	634.8 (421.4 to 877.4)	863.9 (531.1 to 1292.0)	36.1% (-23.5 to 142.5)	3391.7 (2571.0 to 4444.5)	4720.0 (3190.7 to 6729.6)	39.2% (-12.1 to 121.0)	417.5 (261.7 to 619.4)	583.4 (342.5 to 911.6)	39.7% (-27.1 to 168.3)
Central Java	634.2 (424.7 to 874.2)	868.1 (525.7 to 1284.4)	36.9% (-22.8 to 143.0)	3379.8 (2556.9 to 4416.4)	4712.4 (3165.1 to 6726.0)	39.4% (-12.3 to 122.0)	417.1 (260.2 to 617.2)	582.7 (335.9 to 908.7)	39.7% (-27.6 to 170.3)
East Java	635.8 (409.8 to 881.6)	863.9 (533.2 to 1293.6)	35.9% (-24.3 to 144.4)	3421.8 (2535.7 to 4455.8)	4664.8 (3152.3 to 6765.5)	36.3% (-14.9 to 118.9)	421.7 (260.9 to 637.6)	577.1 (331.9 to 932.4)	36.8% (-30.8 to 171.3)
West Kalimantan	631.5 (424.3 to 869.8)	859.4 (525.8 to 1280.1)	36.1% (-23.0 to 140.9)	3353.3 (2506.2 to 4409.0)	4692.6 (3189.8 to 6596.9)	39.9% (-11.5 to 121.7)	412.0 (253.0 to 610.0)	579.1 (343.7 to 897.7)	40.5% (-26.4 to 168.6)
South Kalimantan	640.3 (428.8 to 903.8)	867.1 (530.4 to 1283.7)	35.4% (-24.0 to 141.9)	3413.6 (2558.4 to 4498.4)	4658.7 (3130.6 to 6612.8)	36.5% (-14.2 to 118.1)	420.3 (257.3 to 619.5)	575.0 (328.7 to 881.7)	36.8% (-29.2 to 165.0)
Central Kalimantan	635.6 (430.8 to 885.6)	863.2 (512.8 to 1306.4)	35.8% (-24.5 to 145.2)	3377.2 (2548.3 to 4391.3)	4720.3 (3213.3 to 6680.4)	39.8% (-11.2 to 120.3)	416.2 (261.3 to 623.7)	582.8 (340.0 to 933.5)	40.0% (-28.0 to 173.3)
East Kalimantan	629.4 (418.2 to 878.8)	867.0 (522.9 to 1305.1)	37.7% (-23.5 to 148.3)	3382.0 (2540.7 to 4404.3)	4729.4 (3214.0 to 6686.6)	39.8% (-11.2 to 121.0)	417.3 (257.2 to 623.5)	585.0 (344.7 to 911.1)	40.2% (-27.0 to 169.9)
North Kalimantan	628.7 (410.0 to 877.7)	856.9 (513.1 to 1284.5)	36.3% (-24.9 to 147.9)	3349.2 (2516.6 to 4354.6)	4717.6 (3248.8 to 6748.4)	40.9% (-10.5 to 122.4)	413.3 (254.9 to 621.0)	584.7 (338.5 to 911.0)	41.5% (-27.0 to 174.8)
Riau Islands	635.8 (428.5 to 876.5)	866.5 (535.0 to 1302.1)	36.3% (-22.8 to 141.1)	3351.7 (2528.1 to 4349.1)	4673.8 (3150.9 to 6725.5)	39.4% (-12.3 to 122.0)	412.7 (255.2 to 613.0)	578.3 (339.6 to 915.6)	40.1% (-27.5 to 171.5)
Lampung	631.0 (429.5 to 872.2)	866.5 (531.0 to 1282.5)	37.3% (-21.8 to 141.6)	3354.9 (2520.4 to 4372.6)	4695.0 (3186.4 to 6638.4)	39.9% (-11.2 to 121.4)	413.7 (259.2 to 625.7)	581.7 (325.3 to 922.4)	40.6% (-28.9 to 179.0)

Maluku	630.6 (422.4 to 869.7)	866.2 (525.1 to 1284.6)	37.4% (-22.6 to 144.0)	3361.3 (2511.8 to 4418.2)	4788.6 (3274.2 to 6785.5)	42.5% (-9.9 to 125.9)	413.7 (257.3 to 621.1)	589.8 (342.1 to 918.3)	42.6% (-26.2 to 176.5)
North Maluku	633.6 (428.9 to 882.5)	861.7 (530.5 to 1281.6)	36.0% (-23.0 to 140.7)	3369.7 (2527.3 to 4372.2)	4712.0 (3138.9 to 6633.4)	39.8% (-11.8 to 122.3)	413.8 (255.2 to 618.0)	581.5 (343.2 to 918.3)	40.5% (-27.2 to 172.0)
West Nusa Tenggara	641.3 (435.1 to 870.5)	869.1 (542.7 to 1285.8)	35.5% (-21.9 to 135.6)	3382.4 (2541.8 to 4410.7)	4669.9 (3172.4 to 6674.8)	38.1% (-12.9 to 119.2)	415.7 (258.5 to 619.7)	576.2 (337.2 to 893.3)	38.6% (-27.7 to 166.5)
East Nusa Tenggara	636.6 (422.4 to 881.2)	870.9 (531.1 to 1322.2)	36.8% (-23.7 to 145.6)	3378.8 (2537.9 to 4445.9)	4727.7 (3147.5 to 6677.2)	39.9% (-12.2 to 123.5)	415.7 (253.1 to 615.1)	582.4 (330.0 to 911.6)	40.1% (-28.5 to 175.1)
Papua	626.6 (419.8 to 868.7)	863.4 (519.1 to 1285.1)	37.8% (-22.7 to 146.2)	3341.0 (2517.5 to 4374.0)	4651.2 (3082.7 to 6656.8)	39.2% (-13.1 to 123.4)	411.4 (254.7 to 615.1)	575.6 (333.7 to 930.9)	39.9% (-28.8 to 175.9)
West Papua	627.1 (416.5 to 875.2)	865.6 (508.9 to 1308.2)	38.0% (-24.1 to 151.7)	3324.5 (2474.3 to 4307.3)	4764.4 (3225.7 to 6756.8)	43.3% (-9.4 to 127.4)	409.4 (255.5 to 605.9)	587.0 (343.2 to 917.9)	43.4% (-25.3 to 176.1)
West Sumatra	637.0 (423.0 to 891.0)	861.5 (519.9 to 1280.8)	35.2% (-24.7 to 143.1)	3371.1 (2535.0 to 4388.8)	4696.1 (3169.7 to 6718.1)	39.3% (-12.3 to 121.8)	415.5 (258.0 to 625.7)	580.8 (327.5 to 909.8)	39.8% (-28.8 to 175.3)
South Sumatra	635.5 (418.6 to 875.6)	867.2 (526.5 to 1295.0)	36.5% (-23.7 to 144.4)	3364.3 (2506.4 to 4381.6)	4679.6 (3180.9 to 6613.9)	39.1% (-12.0 to 120.6)	414.1 (257.5 to 616.0)	578.1 (340.4 to 896.0)	39.6% (-26.9 to 167.3)
North Sumatra	631.6 (421.3 to 873.8)	871.1 (546.0 to 1320.8)	37.9% (-22.2 to 145.0)	3360.1 (2520.7 to 4406.3)	4733.6 (3235.3 to 6694.0)	40.9% (-10.6 to 123.1)	413.7 (255.6 to 616.6)	584.8 (345.9 to 909.3)	41.3% (-26.2 to 171.1)
Yogyakarta	633.3 (413.5 to 885.5)	870.1 (515.6 to 1291.5)	37.4% (-24.4 to 150.0)	3404.3 (2552.4 to 4458.6)	4703.5 (3177.2 to 6709.9)	38.2% (-13.1 to 120.2)	421.4 (262.0 to 628.6)	580.5 (330.7 to 907.8)	37.7% (-29.3 to 169.0)
Riau	635.0 (420.1 to 886.9)	869.0 (532.3 to 1276.6)	36.9% (-22.9 to 143.8)	3378.6 (2553.9 to 4405.6)	4726.0 (3121.5 to 6626.8)	39.9% (-11.9 to 122.5)	416.4 (259.8 to 617.4)	583.0 (331.9 to 920.7)	40.0% (-28.2 to 174.1)
North Sulawesi	629.7 (420.2 to 868.9)	866.4 (527.4 to 1281.6)	37.6% (-22.4 to 144.4)	3376.7 (2537.0 to 4377.2)	4685.1 (3194.4 to 6594.1)	38.7% (-11.5 to 118.3)	416.2 (254.8 to 615.0)	579.2 (340.8 to 916.5)	39.2% (-28.1 to 170.1)

Central Sulawesi	637.0 (419.8 to 892.0)	869.9 (515.8 to 1331.4)	36.6% (-25.2 to 150.1)	3377.8 (2541.7 to 4386.2)	4705.4 (3097.9 to 6680.5)	39.3% (-12.8 to 122.8)	415.8 (256.9 to 619.0)	580.7 (334.2 to 901.4)	39.7% (-27.8 to 170.9)
South Sulawesi	636.9 (421.0 to 890.2)	871.9 (530.0 to 1288.8)	36.9% (-23.4 to 145.2)	3396.4 (2543.9 to 4425.0)	4766.7 (3242.8 to 6734.0)	40.3% (-11.0 to 122.0)	419.0 (259.5 to 629.7)	589.6 (337.8 to 944.7)	40.7% (-28.6 to 178.0)
West Sulawesi	635.1 (425.1 to 868.7)	872.7 (533.8 to 1309.1)	37.4% (-22.3 to 143.7)	3372.1 (2536.1 to 4384.2)	4757.4 (3192.4 to 6770.7)	41.1% (-11.2 to 124.6)	415.0 (258.1 to 616.8)	586.5 (345.4 to 900.0)	41.3% (-25.7 to 169.5)
Southeast Sulawesi	632.5 (424.0 to 875.7)	866.6 (523.3 to 1310.5)	37.0% (-23.4 to 145.9)	3347.0 (2496.4 to 4350.5)	4727.1 (3228.9 to 6771.6)	41.2% (-10.8 to 124.3)	412.2 (258.0 to 613.2)	583.5 (323.1 to 931.2)	41.6% (-28.4 to 180.8)
Depressive disorders									
Aceh	1803.8 (1229.1 to 2563.0)	2706.0 (1662.9 to 4123.1)	50.0% (-16.2 to 168.9)	1564.6 (1154.7 to 2066.4)	2189.3 (1471.2 to 3154.6)	39.9% (-13.1 to 126.2)	276.2 (168.3 to 421.7)	402.1 (234.3 to 644.0)	45.6% (-26.1 to 187.6)
Bali	1855.6 (1260.5 to 2635.2)	2700.3 (1672.9 to 4055.5)	45.5% (-18.2 to 159.5)	1628.7 (1202.8 to 2163.9)	2189.2 (1496.8 to 3084.3)	34.4% (-15.6 to 114.2)	286.7 (170.8 to 437.0)	405.8 (231.5 to 646.9)	41.5% (-29.1 to 183.0)
Bangka-Belitung Islands	1771.9 (1205.5 to 2502.7)	2719.6 (1703.0 to 4089.5)	53.5% (-13.2 to 172.1)	1526.7 (1132.0 to 2019.6)	2210.6 (1537.6 to 3142.6)	44.8% (-8.5 to 129.3)	269.7 (163.5 to 416.4)	409.1 (241.0 to 675.3)	51.7% (-24.0 to 203.5)
Banten	1821.0 (1232.8 to 2565.9)	2674.5 (1677.4 to 4013.0)	46.9% (-16.9 to 160.1)	1590.1 (1174.2 to 2126.5)	2160.8 (1484.2 to 3044.6)	35.9% (-14.8 to 116.9)	279.6 (171.0 to 431.6)	400.6 (232.5 to 642.1)	43.3% (-27.6 to 184.1)
Bengkulu	1786.1 (1209.8 to 2521.9)	2684.7 (1688.0 to 4020.8)	50.3% (-14.8 to 166.0)	1547.4 (1143.0 to 2047.8)	2171.9 (1517.7 to 3059.0)	40.4% (-11.1 to 121.8)	273.2 (165.3 to 411.9)	402.7 (235.3 to 658.2)	47.4% (-25.7 to 193.2)
Gorontalo	1853.8 (1247.7 to 2596.3)	2731.5 (1704.1 to 4178.7)	47.4% (-17.3 to 163.2)	1629.4 (1200.5 to 2161.4)	2226.5 (1528.8 to 3179.2)	36.6% (-14.4 to 118.4)	286.2 (172.2 to 441.6)	411.4 (239.2 to 655.7)	43.7% (-27.6 to 185.2)
Jakarta	1904.4 (1300.7 to 2678.8)	2692.7 (1710.0 to 4095.2)	41.4% (-19.8 to 149.7)	1689.9 (1248.8 to 2261.0)	2174.0 (1513.9 to 3110.8)	28.6% (-19.3 to 105.3)	297.0 (180.9 to 446.0)	403.2 (238.1 to 654.5)	35.8% (-30.9 to 167.5)
Jambi	1803.6 (1220.5 to 2543.6)	2682.6 (1669.8 to 3995.8)	48.7% (-15.9 to 163.6)	1569.5 (1152.5 to 2087.3)	2168.5 (1477.8 to 3069.2)	38.2% (-13.6 to 121.3)	275.9 (169.0 to 422.1)	401.9 (225.0 to 645.4)	45.7% (-27.4 to 193.1)

West Java	1815.2 (1228.8 to 2580.3)	2696.2 (1699.0 to 3993.0)	48.5% (-15.6 to 161.8)	1577.7 (1154.2 to 2102.2)	2187.0 (1527.2 to 3026.2)	38.6% (-11.9 to 118.5)	277.6 (168.8 to 424.4)	404.3 (233.5 to 640.6)	45.7% (-26.1 to 187.6)
Central Java	1798.5 (1215.0 to 2559.2)	2687.9 (1675.5 to 4095.8)	49.4% (-16.5 to 168.0)	1563.1 (1150.3 to 2071.2)	2172.3 (1487.7 to 3098.2)	39.0% (-13.0 to 122.3)	276.4 (169.1 to 427.2)	402.7 (230.9 to 641.9)	45.7% (-26.6 to 189.9)
East Java	1840.5 (1250.3 to 2599.3)	2661.4 (1660.4 to 4055.8)	44.6% (-18.7 to 157.8)	1612.9 (1182.5 to 2141.1)	2149.0 (1471.9 to 3094.5)	33.2% (-17.0 to 114.3)	284.4 (173.2 to 430.3)	398.0 (228.1 to 639.8)	39.9% (-29.5 to 178.3)
West Kalimantan	1779.8 (1192.4 to 2514.0)	2697.1 (1745.6 to 4082.8)	51.5% (-13.8 to 166.9)	1539.5 (1132.7 to 2056.8)	2188.2 (1530.0 to 3071.0)	42.1% (-10.1 to 125.2)	271.0 (162.9 to 416.6)	403.7 (239.8 to 634.7)	49.0% (-23.7 to 191.1)
South Kalimantan	1820.9 (1222.6 to 2584.7)	2626.1 (1692.2 to 3954.2)	44.2% (-17.9 to 154.0)	1589.1 (1166.9 to 2106.6)	2109.1 (1483.0 to 2977.1)	32.7% (-15.9 to 110.0)	278.9 (171.6 to 429.2)	391.2 (227.3 to 618.1)	40.2% (-28.5 to 175.3)
Central Kalimantan	1798.0 (1219.2 to 2539.3)	2727.0 (1710.1 to 4023.9)	51.7% (-13.6 to 167.0)	1560.1 (1151.7 to 2085.7)	2216.3 (1536.3 to 3055.7)	42.1% (-9.7 to 124.0)	275.1 (168.0 to 416.0)	410.1 (238.4 to 660.0)	49.1% (-24.4 to 195.0)
East Kalimantan	1827.1 (1234.0 to 2584.3)	2720.3 (1713.8 to 4116.7)	48.9% (-16.1 to 164.6)	1598.9 (1178.3 to 2130.5)	2206.7 (1525.0 to 3131.2)	38.0% (-13.4 to 120.1)	281.8 (169.6 to 421.5)	408.3 (234.0 to 652.4)	44.9% (-26.8 to 187.6)
North Kalimantan	1793.0 (1208.6 to 2528.1)	2744.1 (1724.8 to 4061.9)	53.0% (-13.0 to 169.8)	1557.3 (1149.0 to 2076.8)	2238.5 (1576.2 to 3096.0)	43.7% (-8.1 to 125.3)	274.6 (165.7 to 425.1)	413.5 (240.8 to 659.3)	50.6% (-24.1 to 198.7)
Riau Islands	1782.8 (1203.4 to 2556.9)	2665.4 (1666.0 to 4027.6)	49.5% (-16.3 to 167.7)	1540.2 (1135.0 to 2050.8)	2146.9 (1478.9 to 3000.4)	39.4% (-12.1 to 121.4)	271.9 (164.4 to 415.5)	396.9 (229.7 to 632.2)	46.0% (-26.2 to 188.9)
Lampung	1785.8 (1217.1 to 2532.5)	2657.6 (1645.9 to 3965.3)	48.8% (-16.0 to 164.4)	1544.9 (1143.1 to 2042.7)	2145.2 (1475.1 to 3004.4)	38.9% (-12.2 to 119.9)	272.1 (165.3 to 420.5)	396.8 (225.7 to 643.4)	45.8% (-27.5 to 194.3)
Maluku	1794.7 (1212.9 to 2560.0)	2779.2 (1728.4 to 4178.7)	54.9% (-13.1 to 176.7)	1557.5 (1149.0 to 2083.2)	2270.5 (1563.0 to 3223.6)	45.8% (-8.7 to 133.1)	274.5 (166.5 to 422.3)	417.8 (241.5 to 678.2)	52.2% (-23.7 to 204.5)
North Maluku	1786.7 (1203.2 to 2553.1)	2719.3 (1721.4 to 4125.1)	52.2% (-14.4 to 171.4)	1546.9 (1144.8 to 2054.7)	2211.7 (1529.0 to 3192.8)	43.0% (-10.4 to 128.7)	272.5 (167.6 to 416.5)	409.7 (232.7 to 669.7)	50.3% (-25.1 to 202.6)

West Nusa Tenggara	1774.9 (1193.6 to 2551.9)	2633.0 (1642.5 to 3954.8)	48.3% (-16.9 to 165.5)	1528.8 (1127.2 to 2047.1)	2116.8 (1477.6 to 2985.3)	38.5% (-12.7 to 120.0)	269.2 (163.6 to 411.2)	392.2 (224.8 to 622.3)	45.7% (-26.4 to 188.9)
East Nusa Tenggara	1794.0 (1214.2 to 2556.1)	2694.1 (1743.3 to 4034.1)	50.2% (-14.1 to 163.1)	1553.0 (1137.7 to 2069.2)	2173.3 (1515.1 to 3082.9)	39.9% (-12.0 to 123.0)	273.5 (165.9 to 419.6)	402.0 (232.5 to 644.4)	46.9% (-25.9 to 192.1)
Papua	1794.7 (1214.7 to 2563.6)	2649.4 (1696.3 to 3999.5)	47.6% (-16.3 to 160.8)	1564.2 (1152.3 to 2087.8)	2136.0 (1496.6 to 3009.5)	36.6% (-13.6 to 116.4)	274.5 (167.4 to 419.0)	394.7 (236.2 to 635.3)	43.8% (-26.4 to 181.0)
West Papua	1767.3 (1197.4 to 2493.5)	2754.2 (1719.6 to 4149.1)	55.8% (-12.2 to 177.1)	1534.4 (1128.6 to 2032.2)	2247.0 (1558.1 to 3173.9)	46.4% (-7.7 to 132.7)	269.9 (160.5 to 418.8)	415.1 (242.3 to 687.1)	53.8% (-23.9 to 210.9)
West Sumatra	1784.5 (1212.7 to 2532.9)	2724.8 (1740.0 to 4122.2)	52.7% (-13.3 to 169.8)	1538.4 (1132.9 to 2030.1)	2210.1 (1543.7 to 3174.9)	43.7% (-9.5 to 128.4)	271.9 (165.3 to 411.8)	409.7 (235.9 to 656.2)	50.7% (-23.9 to 199.3)
South Sumatra	1787.5 (1200.8 to 2549.1)	2661.1 (1685.5 to 4011.5)	48.9% (-16.1 to 164.6)	1543.6 (1135.7 to 2053.9)	2141.6 (1512.3 to 3036.1)	38.7% (-12.1 to 119.6)	272.6 (168.3 to 414.2)	397.0 (234.0 to 644.2)	45.6% (-25.9 to 186.9)
North Sumatra	1790.3 (1216.1 to 2555.9)	2673.5 (1694.7 to 3974.0)	49.3% (-15.0 to 162.9)	1546.1 (1145.3 to 2073.3)	2164.7 (1485.0 to 3086.6)	40.0% (-12.5 to 124.2)	273.3 (167.1 to 420.8)	401.0 (231.4 to 627.6)	46.7% (-25.3 to 188.4)
Yogyakarta	1854.3 (1260.4 to 2638.5)	2661.8 (1666.4 to 4000.8)	43.5% (-19.0 to 155.1)	1626.5 (1214.3 to 2168.2)	2147.7 (1473.7 to 3064.9)	32.0% (-17.0 to 110.6)	286.6 (173.2 to 438.6)	398.9 (227.7 to 629.2)	39.2% (-29.7 to 176.2)
Riau	1794.9 (1197.1 to 2575.6)	2686.8 (1682.4 to 4075.9)	49.7% (-16.5 to 169.0)	1556.6 (1141.6 to 2082.0)	2169.6 (1504.8 to 3042.8)	39.4% (-12.2 to 121.8)	274.8 (164.9 to 420.2)	401.3 (232.5 to 635.0)	46.0% (-26.2 to 188.7)
North Sulawesi	1827.3 (1243.2 to 2599.7)	2669.7 (1688.1 to 4041.0)	46.1% (-17.5 to 159.3)	1592.4 (1180.1 to 2126.8)	2154.9 (1493.9 to 3031.2)	35.3% (-14.6 to 114.8)	280.5 (170.1 to 422.6)	398.8 (231.7 to 623.1)	42.2% (-27.0 to 177.3)
Central Sulawesi	1801.2 (1207.1 to 2581.4)	2700.3 (1695.7 to 4092.1)	49.9% (-16.1 to 168.7)	1561.0 (1148.0 to 2067.8)	2183.0 (1511.4 to 3079.4)	39.8% (-11.9 to 122.2)	273.8 (167.8 to 415.5)	404.8 (235.2 to 656.9)	47.9% (-25.3 to 193.3)
South Sulawesi	1809.7 (1231.4 to 2597.7)	2748.0 (1719.3 to 4161.4)	51.9% (-14.8 to 171.4)	1569.5 (1155.2 to 2093.0)	2237.5 (1548.1 to 3157.3)	42.6% (-10.4 to 127.2)	276.8 (168.1 to 422.2)	414.3 (229.3 to 673.8)	49.7% (-26.2 to 204.8)

West Sulawesi	1778.4 (1202.4 to 2530.1)	2698.7 (1680.9 to 4031.4)	51.8% (-14.5 to 170.1)	1533.9 (1134.0 to 2044.9)	2194.0 (1502.9 to 3067.0)	43.0% (-10.0 to 127.5)	269.2 (165.1 to 410.6)	404.8 (235.9 to 645.6)	50.4% (-23.4 to 195.9)
Southeast Sulawesi	1768.6 (1190.9 to 2512.1)	2721.9 (1722.2 to 4160.3)	53.9% (-13.6 to 174.9)	1522.9 (1120.6 to 2031.4)	2204.3 (1516.3 to 3174.2)	44.7% (-9.8 to 132.5)	268.8 (164.4 to 409.4)	408.9 (235.8 to 648.3)	52.1% (-22.7 to 200.1)

UI, uncertainly interval; DALYs, disability-adjusted life-years.

Table S9 The incidence, prevalence and DALYs rate of anxiety and depressive disorders in 1990 and 2021, and changes from 1990 to 2021 in Iran (Islamic Republic of).

Location	Incidence (95% UI)			Prevalence (95% UI)			DALYs (95% UI)		
	1990	2021	Percentage change	1990	2021	Percentage change	1990	2021	Percentage change
Anxiety disorders									
Alborz	1306.1 (891.5 to 1807.5)	1653.3 (1034.2 to 2422.2)	26.6% (-27.2 to 120.5)	8550.5 (6537.3 to 10993.1)	10992.8 (7702.6 to 15186.8)	28.6% (-15.9 to 97.3)	1048.5 (659.0 to 1529.8)	1347.3 (819.2 to 2082.4)	28.5% (-31.2 to 140.5)
Ardebil	1313.3 (893.2 to 1821.4)	1666.2 (1043.7 to 2459.8)	26.9% (-27.4 to 122.0)	8488.2 (6527.5 to 10874.4)	10980.9 (7722.2 to 15120.9)	29.4% (-15.0 to 97.3)	1037.8 (666.4 to 1510.6)	1347.2 (799.1 to 2067.7)	29.8% (-30.6 to 143.6)
Bushehr	1313.5 (888.8 to 1804.4)	1662.5 (1032.2 to 2467.7)	26.6% (-27.7 to 121.9)	8595.1 (6510.2 to 11034.9)	10857.9 (7603.2 to 15073.7)	26.3% (-17.8 to 94.7)	1052.2 (661.9 to 1532.7)	1329.7 (804.1 to 2094.7)	26.4% (-33.0 to 139.0)
Chahar Mahaal and Bakhtiari	1325.2 (898.4 to 1821.3)	1704.1 (1079.1 to 2504.7)	28.6% (-25.8 to 123.2)	8531.7 (6491.4 to 10869.5)	10973.3 (7602.5 to 15176.8)	28.6% (-16.2 to 97.9)	1047.0 (668.8 to 1528.4)	1351.0 (791.8 to 2116.0)	29.0% (-32.1 to 145.8)
East Azarbayejan	1314.8 (892.6 to 1821.4)	1681.7 (1059.2 to 2475.3)	27.9% (-26.5 to 123.1)	8572.6 (6571.2 to 11003.9)	10831.6 (7540.5 to 15204.9)	26.4% (-18.0 to 95.1)	1053.5 (684.0 to 1547.6)	1330.4 (771.0 to 1972.2)	26.3% (-32.1 to 135.5)
Fars	1321.9 (897.1 to 1808.3)	1653.1 (1050.1 to 2436.6)	25.1% (-27.7 to 116.7)	8539.6 (6534.7 to 10943.6)	10923.1 (7644.0 to 15187.2)	27.9% (-16.5 to 96.4)	1042.4 (658.8 to 1519.7)	1335.4 (798.7 to 2062.6)	28.1% (-31.8 to 141.1)
Gilan	1311.0 (881.4 to 1830.0)	1676.7 (1024.2 to 2431.3)	27.9% (-27.4 to 125.7)	8628.7 (6587.6 to 11088.4)	10962.4 (7576.1 to 15136.6)	27.0% (-17.3 to 95.9)	1056.9 (666.7 to 1551.1)	1345.2 (778.5 to 2056.7)	27.3% (-33.0 to 142.5)
Golestan	1327.3 (895.9 to 1784.4)	1711.7 (1077.3 to 2552.0)	29.0% (-25.5 to 123.9)	8521.8 (6502.4 to 11010.0)	10926.8 (7623.7 to 15222.7)	28.2% (-16.8 to 98.2)	1046.1 (667.7 to 1534.0)	1341.7 (800.2 to 2083.6)	28.3% (-31.9 to 142.0)

Hamadan	1304.0 (880.5 to 1791.4)	1679.4 (1034.4 to 2486.9)	28.8% (-26.7 to 126.4)	8526.5 (6477.2 to 10954.3)	11033.2 (7566.9 to 15348.3)	29.4% (-16.5 to 100.9)	1044.2 (657.3 to 1516.6)	1352.3 (800.8 to 2078.3)	29.5% (-31.2 to 144.2)
Hormozgan	1316.0 (891.3 to 1799.1)	1681.9 (1027.1 to 2478.3)	27.8% (-27.0 to 124.4)	8570.7 (6546.3 to 11027.7)	10999.1 (7672.0 to 15303.5)	28.3% (-16.5 to 97.8)	1048.7 (672.9 to 1531.8)	1347.9 (780.2 to 2036.4)	28.5% (-31.6 to 142.3)
Ilam	1310.9 (897.6 to 1792.8)	1648.1 (1034.5 to 2457.3)	25.7% (-27.5 to 118.7)	8532.1 (6496.9 to 10896.3)	11008.5 (7705.1 to 15243.3)	29.0% (-15.7 to 98.0)	1045.7 (666.5 to 1520.1)	1349.5 (796.4 to 2083.1)	29.0% (-31.4 to 143.6)
Isfahan	1309.1 (886.3 to 1795.6)	1689.9 (1065.7 to 2441.3)	29.1% (-25.1 to 123.0)	8515.9 (6525.0 to 10991.9)	10924.4 (7686.0 to 14678.2)	28.3% (-15.2 to 94.3)	1046.6 (667.9 to 1529.8)	1337.9 (800.2 to 2011.1)	27.8% (-31.0 to 137.2)
Kerman	1323.1 (894.2 to 1811.8)	1700.8 (1053.1 to 2469.8)	28.5% (-26.1 to 124.0)	8504.5 (6522.5 to 10855.1)	10920.8 (7576.7 to 15000.4)	28.4% (-15.9 to 96.5)	1039.2 (654.1 to 1517.6)	1336.2 (799.1 to 2063.2)	28.6% (-31.6 to 142.3)
Kermanshah	1311.2 (875.7 to 1800.8)	1654.9 (1031.1 to 2413.8)	26.2% (-27.7 to 120.9)	8537.5 (6483.5 to 10921.3)	10971.1 (7726.0 to 15146.9)	28.5% (-15.9 to 96.7)	1045.4 (658.8 to 1519.1)	1339.6 (808.9 to 2085.6)	28.1% (-31.7 to 141.0)
Khorasan-e-Razavi	1332.3 (904.3 to 1808.5)	1676.6 (1058.5 to 2539.6)	25.8% (-27.8 to 119.8)	8539.8 (6573.5 to 11007.8)	10911.1 (7729.6 to 15212.3)	27.8% (-16.3 to 95.7)	1046.1 (666.4 to 1527.3)	1340.1 (775.9 to 2073.8)	28.1% (-32.7 to 144.3)
Khuzestan	1323.4 (898.2 to 1831.0)	1694.4 (1057.4 to 2450.8)	28.0% (-26.2 to 122.6)	8513.6 (6485.9 to 11028.9)	10862.6 (7657.8 to 15066.5)	27.6% (-16.9 to 96.1)	1041.6 (654.1 to 1544.5)	1333.7 (805.7 to 2022.0)	28.0% (-31.5 to 139.3)
Kohgiluyeh and Boyer-Ahmad	1316.0 (893.7 to 1798.9)	1649.8 (1023.6 to 2422.0)	25.4% (-27.9 to 118.3)	8565.8 (6540.7 to 10984.6)	10927.7 (7785.6 to 14980.9)	27.6% (-15.8 to 93.6)	1046.8 (661.4 to 1521.9)	1339.7 (784.3 to 2059.4)	28.0% (-32.3 to 142.5)
Kurdistan	1317.7 (899.6 to 1824.5)	1646.1 (1019.0 to 2416.0)	24.9% (-28.4 to 118.4)	8536.2 (6542.9 to 11085.7)	10944.5 (7564.9 to 15240.5)	28.2% (-17.0 to 98.7)	1045.4 (661.8 to 1552.1)	1341.0 (787.8 to 2067.4)	28.3% (-32.4 to 144.1)
Lorestan	1311.1 (892.8 to 1799.8)	1670.2 (1037.3 to 2449.9)	27.4% (-26.7 to 121.9)	8555.7 (6571.3 to 11056.9)	11019.9 (7652.9 to 15263.1)	28.8% (-16.1 to 98.5)	1048.6 (664.8 to 1549.5)	1349.0 (810.7 to 2039.0)	28.6% (-30.9 to 139.7)
Markazi	1318.6 (913.5 to 1811.6)	1660.0 (1038.1 to 2420.1)	25.9% (-26.9 to 116.9)	8580.7 (6560.3 to 10989.3)	11000.7 (7631.6 to 15039.5)	28.2% (-16.1 to 96.4)	1048.4 (659.4 to 1538.2)	1345.3 (820.9 to 2067.8)	28.3% (-31.1 to 139.3)

Mazandaran	1310.8 (865.9 to 1804.8)	1694.0 (1051.4 to 2519.5)	29.2% (-27.0 to 129.1)	8640.7 (6568.9 to 11064.4)	10955.1 (7538.2 to 15175.0)	26.8% (-17.8 to 96.0)	1056.6 (671.9 to 1535.6)	1343.0 (786.3 to 2167.0)	27.1% (-33.8 to 144.6)
North Khorasan	1306.2 (882.8 to 1791.3)	1680.6 (1030.1 to 2443.8)	28.7% (-26.3 to 125.1)	8554.6 (6556.0 to 10976.0)	10925.0 (7566.8 to 14880.4)	27.7% (-16.3 to 95.5)	1046.6 (655.3 to 1508.9)	1338.5 (798.3 to 2042.0)	27.9% (-31.6 to 139.7)
Qazvin	1308.7 (882.3 to 1794.7)	1666.3 (1001.9 to 2430.9)	27.3% (-27.7 to 124.5)	8552.1 (6563.5 to 10863.9)	10985.6 (7506.0 to 15190.7)	28.5% (-16.5 to 97.8)	1047.8 (660.1 to 1529.7)	1348.0 (801.5 to 2088.6)	28.6% (-31.8 to 143.4)
Qom	1318.1 (898.8 to 1818.6)	1667.2 (1029.6 to 2454.4)	26.5% (-27.6 to 121.3)	8535.1 (6491.5 to 11071.0)	11035.0 (7605.5 to 15310.1)	29.3% (-16.5 to 100.9)	1044.3 (657.7 to 1524.1)	1354.0 (799.1 to 2059.8)	29.7% (-31.0 to 144.1)
Semnan	1300.7 (875.2 to 1783.8)	1640.4 (1017.9 to 2438.6)	26.1% (-28.1 to 121.7)	8522.0 (6542.7 to 10825.9)	11049.2 (7677.9 to 14982.7)	29.7% (-14.4 to 97.0)	1044.4 (661.0 to 1527.0)	1354.1 (833.8 to 2020.9)	29.7% (-29.2 to 137.5)
Sistan and Baluchistan	1327.5 (910.0 to 1827.1)	1697.3 (1076.5 to 2442.4)	27.9% (-25.3 to 119.4)	8556.3 (6513.5 to 10960.2)	10941.2 (7613.7 to 15372.0)	27.9% (-17.2 to 97.8)	1047.4 (660.8 to 1529.7)	1341.2 (813.6 to 2086.9)	28.1% (-31.7 to 140.8)
South Khorasan	1333.8 (900.9 to 1829.8)	1683.5 (1041.0 to 2440.4)	26.2% (-27.5 to 120.1)	8562.1 (6516.3 to 11039.8)	10856.7 (7485.0 to 15082.8)	26.8% (-17.9 to 96.5)	1047.2 (657.8 to 1526.3)	1331.3 (777.7 to 2035.7)	27.1% (-32.8 to 140.9)
Tehran	1296.6 (868.3 to 1783.6)	1650.2 (1023.9 to 2498.1)	27.3% (-28.1 to 125.7)	8628.6 (6623.3 to 11009.7)	11004.9 (7588.3 to 15132.0)	27.5% (-16.7 to 95.6)	1058.5 (676.3 to 1568.1)	1349.1 (783.6 to 2103.9)	27.4% (-33.3 to 144.4)
West Azarbayejan	1308.2 (885.5 to 1802.9)	1689.5 (1065.3 to 2498.8)	29.1% (-25.9 to 125.5)	8541.7 (6522.6 to 10972.7)	10904.8 (7730.4 to 14920.3)	27.7% (-15.8 to 94.1)	1043.8 (663.3 to 1523.0)	1334.3 (794.4 to 2060.2)	27.8% (-32.0 to 140.6)
Yazd	1317.8 (896.4 to 1813.4)	1690.7 (1059.8 to 2479.5)	28.3% (-26.2 to 123.3)	8507.5 (6520.7 to 10979.8)	10993.5 (7634.1 to 15128.7)	29.2% (-15.7 to 98.8)	1043.1 (656.0 to 1533.4)	1347.2 (800.8 to 2056.6)	29.2% (-31.3 to 143.2)
Zanjan	1325.8 (883.4 to 1831.3)	1671.8 (1039.8 to 2463.3)	26.1% (-28.3 to 122.2)	8535.6 (6498.4 to 10967.3)	10992.1 (7688.4 to 15194.5)	28.8% (-15.9 to 98.1)	1046.3 (663.0 to 1534.5)	1346.4 (817.2 to 2062.2)	28.7% (-30.8 to 139.9)
Depressive disorders									
Alborz	5666.8 (3799.2 to 8076.5)	7616.8 (4694.9 to 11493.7)	34.4% (-25.1 to 141.8)	4153.2 (2958.5 to 5689.7)	5455.6 (3586.4 to 7882.9)	31.4% (-21.3 to 119.6)	799.5 (491.3 to 1226.8)	1067.0 (629.5 to 1698.4)	33.5% (-31.7 to 161.0)

Ardebil	5528.7 (3702.5 to 7886.8)	7468.6 (4744.2 to 11193.0)	35.1% (-23.6 to 139.4)	4026.0 (2877.2 to 5559.5)	5357.0 (3615.0 to 7702.5)	33.1% (-19.3 to 119.8)	773.9 (466.2 to 1204.2)	1050.2 (585.4 to 1687.2)	35.7% (-33.1 to 176.3)
Bushehr	5686.9 (3807.5 to 8075.2)	7367.1 (4617.4 to 10957.3)	29.5% (-26.8 to 129.9)	4167.2 (2990.8 to 5747.0)	5247.8 (3461.6 to 7508.2)	25.9% (-24.1 to 109.3)	801.0 (481.0 to 1211.6)	1029.3 (590.4 to 1634.0)	28.5% (-35.1 to 155.0)
Chahar Mahaal and Bakhtiari	3873.2 (2367.8 to 6067.8)	5080.9 (2879.7 to 8231.4)	31.2% (-35.0 to 165.5)	2968.4 (1960.0 to 4311.8)	3747.0 (2332.3 to 5761.0)	26.2% (-30.6 to 130.1)	551.7 (304.2 to 885.3)	714.4 (375.9 to 1187.8)	29.5% (-40.6 to 182.4)
East Azarbayejan	3688.6 (2118.7 to 5875.0)	4639.4 (2596.8 to 7713.7)	25.8% (-40.0 to 163.9)	2872.5 (1859.0 to 4276.2)	3455.6 (2155.7 to 5429.2)	20.3% (-35.2 to 123.7)	528.4 (280.9 to 872.4)	653.9 (345.5 to 1109.3)	23.7% (-44.6 to 176.8)
Fars	7133.8 (4822.8 to 10128.1)	9537.8 (6054.1 to 14025.4)	33.7% (-23.5 to 134.1)	5060.9 (3603.6 to 6988.4)	6688.5 (4512.6 to 9616.6)	32.2% (-19.9 to 118.5)	989.6 (600.6 to 1519.4)	1328.8 (782.9 to 2079.2)	34.3% (-31.2 to 162.3)
Gilan	4403.4 (2762.1 to 6730.6)	5779.4 (3459.1 to 9312.3)	31.2% (-32.3 to 155.1)	3193.7 (2120.7 to 4670.9)	4079.1 (2632.8 to 6263.8)	27.7% (-28.7 to 129.1)	614.8 (348.2 to 981.9)	800.7 (425.8 to 1336.9)	30.2% (-39.5 to 181.2)
Golestan	3647.8 (2168.1 to 5755.5)	4715.1 (2622.9 to 7921.1)	29.3% (-38.0 to 170.1)	2822.5 (1878.2 to 4156.4)	3497.6 (2166.1 to 5501.0)	23.9% (-32.8 to 129.1)	520.9 (281.0 to 861.6)	662.9 (337.6 to 1171.0)	27.3% (-44.6 to 193.3)
Hamadan	4017.8 (2497.4 to 6186.3)	5373.8 (3139.0 to 8437.1)	33.8% (-31.3 to 160.6)	3090.3 (2089.3 to 4451.8)	3996.5 (2568.1 to 5962.9)	29.3% (-26.4 to 127.6)	573.3 (324.5 to 927.1)	763.1 (408.2 to 1282.3)	33.1% (-38.4 to 188.1)
Hormozgan	5625.5 (3787.5 to 8031.8)	7455.4 (4766.9 to 11273.9)	32.5% (-25.0 to 134.7)	4114.5 (2961.3 to 5677.4)	5326.2 (3597.6 to 7749.5)	29.4% (-21.7 to 114.5)	789.9 (483.0 to 1211.3)	1043.2 (607.9 to 1642.9)	32.1% (-32.6 to 158.9)
Ilam	6147.3 (4087.4 to 8866.3)	8249.9 (5173.4 to 12423.3)	34.2% (-25.0 to 140.7)	4437.3 (3151.3 to 6128.6)	5894.5 (3934.6 to 8622.9)	32.8% (-20.5 to 122.6)	859.1 (513.8 to 1317.6)	1159.3 (650.9 to 1876.1)	34.9% (-33.4 to 174.2)
Isfahan	5041.0 (3334.0 to 7405.2)	6510.4 (4053.8 to 9915.3)	29.1% (-28.9 to 135.3)	3741.2 (2642.5 to 5266.0)	4662.9 (3105.3 to 6774.2)	24.6% (-25.8 to 109.7)	712.4 (425.8 to 1119.6)	905.4 (518.6 to 1447.7)	27.1% (-36.9 to 156.1)
Kerman	7280.8 (4924.0 to 10355.2)	9402.2 (5822.8 to 13965.5)	29.1% (-27.2 to 129.8)	5134.5 (3628.7 to 7051.2)	6519.3 (4372.7 to 9371.0)	27.0% (-23.3 to 110.6)	1005.9 (616.0 to 1545.7)	1296.8 (768.4 to 2117.1)	28.9% (-34.7 to 155.1)

Kermanshah	5660.6 (3755.1 to 8184.4)	7736.5 (4833.4 to 11816.5)	36.7% (-24.3 to 147.4)	4136.2 (2928.5 to 5667.7)	5553.8 (3697.1 to 8150.3)	34.3% (-19.8 to 125.2)	796.3 (485.0 to 1215.9)	1086.0 (645.2 to 1740.5)	36.4% (-30.3 to 167.0)
Khorasan-e-Razavi	4003.2 (2506.2 to 6155.2)	5303.6 (3034.8 to 8432.3)	32.5% (-32.8 to 161.8)	3048.6 (2061.4 to 4370.0)	3922.2 (2470.8 to 5891.9)	28.7% (-27.5 to 128.8)	568.0 (322.3 to 914.5)	747.9 (398.8 to 1227.3)	31.7% (-38.5 to 181.9)
Khuzestan	5569.6 (3754.4 to 7915.1)	7221.6 (4574.8 to 10597.6)	29.7% (-25.9 to 127.4)	4063.0 (2914.0 to 5585.4)	5120.7 (3415.5 to 7215.1)	26.0% (-23.1 to 107.0)	781.1 (481.7 to 1193.8)	1003.4 (589.0 to 1640.2)	28.5% (-35.0 to 154.5)
Kohgiluyeh and Boyer-Ahmad	5641.4 (3788.0 to 7964.8)	7503.4 (4729.4 to 11442.8)	33.0% (-25.3 to 137.4)	4126.6 (2950.9 to 5692.3)	5388.3 (3640.3 to 7856.8)	30.6% (-21.2 to 116.9)	790.9 (478.8 to 1217.9)	1052.6 (609.4 to 1687.2)	33.1% (-33.0 to 164.5)
Kurdistan	4824.7 (3140.3 to 7039.6)	6689.5 (4079.7 to 10297.4)	38.7% (-24.9 to 156.4)	3588.2 (2485.2 to 4995.1)	4872.8 (3191.1 to 7282.1)	35.8% (-20.9 to 133.6)	680.1 (408.0 to 1047.0)	947.1 (529.9 to 1571.5)	39.3% (-32.1 to 186.4)
Lorestan	3778.1 (2282.1 to 5950.4)	5079.5 (2928.1 to 8277.1)	34.4% (-33.4 to 171.4)	2928.2 (1952.1 to 4272.2)	3807.1 (2441.0 to 5821.0)	30.0% (-27.3 to 133.0)	540.5 (295.4 to 893.3)	724.0 (388.5 to 1225.5)	34.0% (-39.2 to 195.3)
Markazi	5638.6 (3814.5 to 8023.2)	7553.3 (4704.0 to 11541.3)	34.0% (-25.2 to 140.4)	4117.4 (2972.3 to 5672.8)	5417.3 (3598.3 to 7905.0)	31.6% (-20.9 to 119.1)	789.4 (482.1 to 1215.2)	1060.9 (601.5 to 1697.1)	34.4% (-32.7 to 169.3)
Mazandaran	5832.6 (3837.4 to 8340.8)	7404.6 (4662.6 to 10862.5)	27.0% (-28.2 to 124.6)	4261.9 (3006.4 to 5842.1)	5256.7 (3525.3 to 7442.7)	23.3% (-25.0 to 103.4)	819.0 (492.5 to 1271.0)	1027.1 (596.1 to 1664.2)	25.4% (-37.3 to 151.2)
North Khorasan	5705.5 (3840.7 to 8121.7)	7410.0 (4749.3 to 11040.3)	29.9% (-26.0 to 128.3)	4184.5 (2997.7 to 5772.1)	5283.9 (3580.7 to 7646.5)	26.3% (-23.4 to 108.7)	803.6 (487.2 to 1240.8)	1036.4 (601.2 to 1606.8)	29.0% (-34.2 to 153.1)
Qazvin	4241.8 (2655.0 to 6403.1)	5676.5 (3385.9 to 8850.7)	33.8% (-29.9 to 155.8)	3234.2 (2214.1 to 4635.5)	4195.6 (2747.7 to 6206.1)	29.7% (-24.9 to 124.5)	605.1 (342.9 to 966.9)	802.4 (436.2 to 1325.5)	32.6% (-37.7 to 182.1)
Qom	5640.2 (3783.5 to 8037.9)	7506.2 (4696.6 to 11288.1)	33.1% (-25.3 to 137.8)	4124.6 (2962.2 to 5712.1)	5369.0 (3578.2 to 7702.9)	30.2% (-21.4 to 116.0)	791.5 (485.6 to 1211.3)	1051.4 (607.0 to 1693.0)	32.8% (-33.0 to 164.2)
Semnan	4593.6 (2969.1 to 6717.1)	6363.5 (3919.8 to 9736.0)	38.5% (-24.6 to 155.2)	3466.6 (2410.2 to 4903.0)	4691.2 (3077.1 to 6920.3)	35.3% (-20.9 to 132.0)	653.1 (382.5 to 1019.7)	905.8 (507.3 to 1490.5)	38.7% (-32.7 to 186.7)

Sistan and Baluchistan	5549.1 (3711.5 to 7934.9)	7256.8 (4557.1 to 11136.3)	30.8% (-27.2 to 135.7)	4047.7 (2908.5 to 5595.1)	5146.1 (3464.3 to 7448.8)	27.1% (-23.1 to 110.7)	777.3 (469.3 to 1207.3)	1008.0 (589.3 to 1652.7)	29.7% (-35.2 to 159.9)
South Khorasan	5531.6 (3690.4 to 7872.8)	7284.4 (4516.3 to 11078.5)	31.7% (-26.8 to 137.4)	4021.9 (2875.7 to 5524.1)	5185.8 (3420.4 to 7584.3)	28.9% (-22.9 to 115.9)	773.7 (466.9 to 1198.9)	1015.3 (575.8 to 1583.8)	31.2% (-34.0 to 160.9)
Tehran	5935.5 (3933.4 to 8609.2)	7851.5 (4944.3 to 11604.8)	32.3% (-25.6 to 135.3)	4495.2 (3204.8 to 6200.4)	5741.4 (3916.3 to 8089.4)	27.7% (-21.5 to 108.2)	851.5 (511.0 to 1308.3)	1112.2 (647.1 to 1790.9)	30.6% (-34.4 to 160.1)
West Azarbayejan	5992.6 (4002.8 to 8664.3)	7568.5 (4742.1 to 11368.0)	26.3% (-29.3 to 126.2)	4354.7 (3082.8 to 6046.3)	5336.2 (3572.0 to 7780.9)	22.5% (-26.7 to 105.2)	839.4 (510.6 to 1284.9)	1046.7 (594.3 to 1648.0)	24.7% (-37.0 to 147.6)
Yazd	4072.0 (2517.0 to 6297.4)	5503.4 (3313.6 to 8686.3)	35.2% (-30.1 to 161.8)	3100.1 (2091.3 to 4507.4)	4042.0 (2637.2 to 6098.0)	30.4% (-25.8 to 129.5)	577.1 (327.8 to 925.2)	775.8 (422.6 to 1324.9)	34.4% (-37.6 to 190.1)
Zanjan	5568.5 (3738.4 to 7885.1)	7560.0 (4711.2 to 11346.2)	35.8% (-23.7 to 142.2)	4059.7 (2875.1 to 5628.3)	5416.3 (3618.2 to 7785.2)	33.4% (-19.7 to 122.1)	779.0 (467.9 to 1195.7)	1061.9 (600.9 to 1679.8)	36.3% (-31.7 to 172.5)

UI, uncertainly interval; DALYs, disability-adjusted life-years.

Table S10 The incidence, prevalence and DALYs rate of anxiety and depressive disorders in 1990 and 2021, and changes from 1990 to 2021 in Italy.

Location	Incidence (95% UI)			Prevalence (95% UI)			DALYs (95% UI)		
	1990	2021	Percentage change	1990	2021	Percentage change	1990	2021	Percentage change
Anxiety disorders									
Liguria	1149.5 (750.3 to 1622.9)	1496.9 (933.6 to 2212.7)	30.2% (-26.8 to 132.4)	7585.3 (5751.0 to 9793.4)	9443.1 (6606.7 to 12967.7)	24.5% (-18.8 to 91.3)	925.5 (579.1 to 1359.4)	1155.1 (710.5 to 1747.5)	24.8% (-32.5 to 131.1)
Lombardia	1157.9 (747.7 to 1613.1)	1498.7 (927.7 to 2220.7)	29.4% (-27.5 to 131.6)	7559.2 (5718.4 to 9686.0)	9347.3 (6430.9 to 13046.2)	23.7% (-20.3 to 92.1)	924.9 (595.5 to 1350.7)	1146.7 (666.9 to 1791.5)	24.0% (-34.8 to 136.1)
Piemonte	1153.1 (739.9 to 1593.5)	1534.0 (923.4 to 2286.5)	33.0% (-26.5 to 141.5)	7553.6 (5789.3 to 9710.2)	9557.7 (6578.3 to 13031.1)	26.5% (-17.3 to 94.3)	925.1 (584.1 to 1357.5)	1170.3 (692.5 to 1783.8)	26.5% (-32.7 to 138.5)
Valle d'Aosta	1155.2 (753.5 to 1587.9)	1465.3 (899.7 to 2112.8)	26.8% (-27.9 to 123.6)	7579.3 (5793.6 to 9753.6)	9168.2 (6286.1 to 12759.3)	21.0% (-21.8 to 87.6)	926.6 (590.0 to 1358.1)	1122.5 (661.8 to 1701.3)	21.2% (-35.3 to 127.4)

Emilia-Romagna	1155.6 (735.2 to 1619.3)	1489.7 (942.8 to 2165.3)	28.9% (-27.0 to 127.9)	7562.3 (5747.6 to 9819.2)	9308.1 (6528.5 to 12802.5)	23.1% (-19.8 to 89.2)	922.1 (587.7 to 1331.9)	1138.1 (685.4 to 1753.3)	23.4% (-33.7 to 130.2)
Friuli-Venezia Giulia	1159.9 (754.1 to 1610.0)	1528.5 (939.4 to 2259.5)	31.8% (-26.1 to 135.7)	7582.9 (5792.1 to 9749.2)	9544.6 (6523.9 to 13293.9)	25.9% (-18.8 to 95.6)	926.4 (584.5 to 1346.3)	1167.0 (710.6 to 1789.4)	26.0% (-32.1 to 134.3)
Provincia autonoma di Bolzano	1162.4 (770.1 to 1608.3)	1426.6 (867.2 to 2109.1)	22.7% (-31.1 to 119.0)	7553.2 (5736.7 to 9737.1)	8972.4 (6244.4 to 12535.2)	18.8% (-23.1 to 84.1)	922.3 (583.5 to 1369.0)	1095.3 (663.7 to 1694.6)	18.8% (-36.7 to 123.3)
Provincia autonoma di Trento	1168.6 (762.8 to 1630.6)	1409.8 (875.9 to 2108.6)	20.6% (-32.4 to 115.9)	7538.8 (5757.0 to 9692.5)	8873.1 (6063.2 to 12263.5)	17.7% (-23.8 to 82.3)	922.5 (591.0 to 1334.7)	1086.5 (640.1 to 1675.9)	17.8% (-37.3 to 121.8)
Veneto	1161.1 (754.3 to 1645.9)	1452.8 (917.3 to 2128.0)	25.1% (-29.2 to 121.2)	7541.4 (5702.6 to 9752.2)	9097.9 (6309.9 to 12571.5)	20.6% (-21.9 to 86.7)	921.9 (581.3 to 1350.2)	1109.2 (687.2 to 1661.7)	20.3% (-34.3 to 120.5)
Lazio	1160.9 (758.4 to 1632.6)	1517.4 (962.6 to 2229.2)	30.7% (-25.7 to 130.2)	7545.5 (5734.7 to 9755.7)	9462.1 (6679.6 to 13172.2)	25.4% (-18.3 to 93.0)	923.5 (595.1 to 1358.5)	1157.6 (687.5 to 1782.0)	25.3% (-33.2 to 135.7)
Marche	858.4 (528.5 to 1271.2)	1160.7 (665.2 to 1765.5)	35.2% (-29.6 to 160.3)	4865.2 (3350.1 to 6875.9)	6525.5 (4206.3 to 9621.9)	34.1% (-22.4 to 132.2)	597.4 (350.8 to 933.3)	800.2 (453.9 to 1285.0)	34.0% (-34.1 to 172.4)
Toscana	1232.4 (826.8 to 1693.9)	1613.2 (1000.6 to 2390.0)	30.9% (-25.5 to 130.3)	8292.1 (6584.6 to 10372.5)	10489.8 (7215.6 to 14270.6)	26.5% (-15.9 to 90.4)	1014.0 (659.8 to 1460.6)	1281.5 (764.4 to 1909.4)	26.4% (-31.0 to 131.9)
Umbria	1169.9 (757.0 to 1615.7)	1475.3 (896.2 to 2162.0)	26.1% (-29.4 to 125.9)	7530.6 (5735.5 to 9652.7)	9244.6 (6277.5 to 12689.8)	22.8% (-20.5 to 90.1)	921.0 (588.1 to 1332.7)	1131.2 (673.9 to 1723.1)	22.8% (-34.0 to 129.0)
Abruzzo	1171.9 (770.1 to 1607.4)	1416.5 (871.3 to 2101.3)	20.9% (-31.9 to 115.0)	7525.5 (5680.7 to 9747.2)	8883.5 (6166.5 to 12284.3)	18.0% (-23.6 to 82.8)	922.0 (581.5 to 1345.7)	1088.6 (648.5 to 1661.0)	18.1% (-37.0 to 121.8)
Basilicata	1173.2 (773.5 to 1610.3)	1425.2 (875.0 to 2157.5)	21.5% (-32.0 to 117.3)	7529.5 (5645.3 to 9773.5)	9060.4 (6283.1 to 12794.9)	20.3% (-23.0 to 88.6)	922.6 (587.9 to 1361.5)	1111.0 (648.7 to 1735.7)	20.4% (-36.9 to 130.5)
Calabria	1184.5 (790.1 to 1663.5)	1479.6 (917.2 to 2157.4)	24.9% (-29.0 to 120.4)	7517.1 (5757.9 to 9683.7)	9317.6 (6584.9 to 12864.1)	24.0% (-18.7 to 89.4)	922.5 (584.5 to 1338.2)	1142.3 (668.6 to 1740.7)	23.8% (-34.2 to 133.4)

Campania	1183.8 (771.3 to 1633.7)	1519.8 (940.3 to 2223.3)	28.4% (-27.4 to 127.3)	7575.9 (5846.1 to 9773.2)	9576.9 (6632.7 to 13323.0)	26.4% (-17.8 to 94.7)	926.4 (592.4 to 1346.6)	1170.4 (691.1 to 1794.8)	26.3% (-32.6 to 137.6)
Molise	1182.2 (768.3 to 1657.7)	1508.7 (922.7 to 2249.7)	27.6% (-29.1 to 130.3)	7542.7 (5720.4 to 9690.4)	9529.7 (6750.1 to 13227.5)	26.3% (-17.4 to 93.7)	922.0 (576.3 to 1340.0)	1165.2 (692.1 to 1795.8)	26.4% (-33.0 to 138.7)
Puglia	1189.3 (776.8 to 1642.1)	1514.9 (942.3 to 2217.5)	27.4% (-27.7 to 124.9)	7532.2 (5736.2 to 9758.6)	9590.9 (6633.3 to 13585.5)	27.3% (-18.3 to 98.8)	923.1 (586.3 to 1358.8)	1176.0 (691.7 to 1852.1)	27.4% (-33.3 to 144.0)
Sardegna	891.8 (562.2 to 1280.5)	1136.0 (668.4 to 1749.6)	27.4% (-32.3 to 140.4)	5195.5 (3633.1 to 7185.8)	6561.3 (4262.8 to 9511.0)	26.3% (-25.4 to 114.3)	636.3 (381.8 to 987.7)	804.6 (452.1 to 1279.3)	26.5% (-37.2 to 155.0)
Sicilia	1181.2 (790.1 to 1652.9)	1535.3 (950.5 to 2264.9)	30.0% (-26.4 to 130.4)	7520.3 (5726.1 to 9718.1)	9688.2 (6776.1 to 13309.1)	28.8% (-15.9 to 97.8)	921.3 (590.1 to 1358.9)	1184.4 (741.7 to 1822.3)	28.6% (-30.1 to 136.4)
Depressive disorders									
Liguria	4452.1 (3021.7 to 6345.7)	5519.4 (3497.7 to 8089.9)	24.0% (-29.0 to 116.9)	3602.4 (2643.4 to 4905.4)	4202.9 (2872.8 to 5906.4)	16.7% (-27.4 to 87.9)	664.1 (409.8 to 1007.6)	799.1 (457.6 to 1271.4)	20.3% (-39.0 to 137.8)
Lombardia	4388.6 (2971.6 to 6261.0)	5475.7 (3474.1 to 8136.1)	24.8% (-29.0 to 119.7)	3537.8 (2571.6 to 4804.2)	4155.0 (2846.4 to 5893.9)	17.4% (-27.2 to 90.1)	652.6 (403.5 to 1003.1)	789.8 (459.9 to 1232.8)	21.0% (-37.8 to 135.8)
Piemonte	4379.6 (2957.8 to 6182.9)	5662.4 (3602.1 to 8424.7)	29.3% (-26.3 to 127.2)	3533.1 (2569.9 to 4787.7)	4285.0 (2929.5 to 6098.9)	21.3% (-25.0 to 96.6)	652.2 (398.1 to 1011.6)	818.7 (465.6 to 1304.4)	25.5% (-37.1 to 150.9)
Valle d'Aosta	4406.3 (2968.0 to 6237.0)	5357.2 (3376.2 to 8082.5)	21.6% (-31.4 to 116.2)	3561.1 (2602.4 to 4849.2)	4082.5 (2791.2 to 5851.3)	14.6% (-29.3 to 86.2)	656.4 (409.9 to 1006.7)	775.7 (446.2 to 1239.1)	18.2% (-40.0 to 133.4)
Emilia-Romagna	6729.4 (4561.4 to 9516.7)	8870.9 (5641.6 to 13165.6)	31.8% (-24.7 to 131.3)	5086.6 (3641.6 to 6926.6)	6371.8 (4250.9 to 9123.4)	25.3% (-24.0 to 106.7)	979.1 (595.0 to 1488.1)	1256.6 (720.5 to 1939.8)	28.3% (-34.3 to 150.9)
Friuli-Venezia Giulia	4424.1 (2995.0 to 6330.6)	5588.7 (3537.4 to 8402.0)	26.3% (-28.6 to 124.0)	3572.4 (2622.8 to 4834.4)	4234.9 (2920.1 to 6078.1)	18.5% (-26.5 to 91.4)	660.7 (407.0 to 999.5)	807.3 (477.0 to 1305.9)	22.2% (-37.6 to 140.0)
Provincia autonoma di Bolzano	4374.2 (2956.9 to 6199.4)	5135.1 (3247.5 to 7629.5)	17.4% (-33.2 to 106.8)	3523.4 (2554.8 to 4768.5)	3959.5 (2733.3 to 5561.8)	12.4% (-29.9 to 80.4)	648.2 (394.8 to 1003.8)	746.2 (427.6 to 1193.8)	15.1% (-42.2 to 129.9)

Provincia autonoma di Trento	4376.6 (2957.8 to 6251.5)	5250.7 (3323.5 to 7697.4)	20.0% (-31.5 to 110.6)	3524.5 (2561.4 to 4797.9)	4030.0 (2757.5 to 5602.2)	14.3% (-28.6 to 83.5)	651.1 (405.9 to 997.1)	761.2 (437.7 to 1204.0)	16.9% (-40.4 to 130.0)
Veneto	5246.6 (3558.4 to 7523.6)	6692.5 (4242.8 to 10034.7)	27.6% (-27.8 to 125.8)	4093.9 (2948.2 to 5554.9)	4951.4 (3334.6 to 7038.2)	20.9% (-25.9 to 97.8)	769.3 (461.8 to 1179.2)	956.3 (547.9 to 1524.2)	24.3% (-37.6 to 147.8)
Lazio	4424.4 (2973.1 to 6306.8)	5617.7 (3519.6 to 8383.5)	27.0% (-28.4 to 125.7)	3546.8 (2592.0 to 4819.7)	4249.5 (2898.3 to 6012.2)	19.8% (-25.7 to 93.8)	657.8 (401.3 to 1001.0)	809.9 (468.0 to 1255.2)	23.1% (-36.9 to 140.1)
Marche	4309.9 (2932.9 to 6045.3)	5421.3 (3460.9 to 8108.8)	25.8% (-28.0 to 120.4)	3459.4 (2535.5 to 4660.3)	4130.4 (2850.7 to 5922.4)	19.4% (-25.8 to 92.5)	641.5 (400.9 to 999.4)	787.5 (456.2 to 1245.7)	22.8% (-37.5 to 141.5)
Toscana	5766.7 (3980.5 to 8013.3)	7687.9 (4923.9 to 11414.4)	33.3% (-22.9 to 130.9)	4361.9 (3176.4 to 5844.1)	5526.5 (3730.5 to 7880.8)	26.7% (-21.7 to 105.4)	838.1 (520.9 to 1270.0)	1088.5 (629.0 to 1722.9)	29.9% (-33.5 to 154.3)
Umbria	4330.1 (2939.8 to 6144.8)	5353.7 (3395.4 to 8016.5)	23.6% (-29.7 to 118.2)	3474.7 (2547.7 to 4684.9)	4082.8 (2786.5 to 5783.9)	17.5% (-27.0 to 89.4)	642.9 (396.7 to 995.1)	774.9 (460.1 to 1236.1)	20.5% (-38.4 to 135.6)
Abruzzo	4311.4 (2908.8 to 6072.1)	5116.9 (3179.1 to 7638.6)	18.7% (-33.0 to 110.8)	3453.2 (2526.8 to 4682.7)	3944.7 (2661.6 to 5613.1)	14.2% (-29.6 to 85.7)	640.1 (391.2 to 977.4)	747.4 (425.8 to 1203.3)	16.8% (-41.4 to 133.4)
Basilicata	4282.3 (2879.3 to 6072.2)	5190.5 (3317.7 to 7716.8)	21.2% (-30.8 to 112.8)	3421.1 (2495.8 to 4612.4)	4017.7 (2800.8 to 5722.5)	17.4% (-26.6 to 88.4)	633.9 (390.6 to 973.3)	756.9 (442.0 to 1206.2)	19.4% (-39.1 to 134.8)
Calabria	4255.6 (2876.4 to 6046.9)	5393.0 (3405.2 to 8191.1)	26.7% (-28.7 to 125.7)	3389.1 (2443.5 to 4552.4)	4131.2 (2808.7 to 5960.0)	21.9% (-25.2 to 98.9)	628.5 (384.9 to 955.8)	782.9 (461.0 to 1268.9)	24.6% (-36.7 to 145.8)
Campania	4292.9 (2902.0 to 6029.3)	5552.2 (3538.4 to 8228.5)	29.3% (-25.9 to 126.3)	3424.8 (2499.4 to 4604.4)	4236.8 (2914.9 to 5977.0)	23.7% (-22.7 to 98.6)	633.8 (393.4 to 966.1)	804.8 (467.2 to 1291.7)	27.0% (-35.4 to 150.2)
Molise	4292.4 (2897.7 to 6152.8)	5646.7 (3585.4 to 8353.4)	31.5% (-25.2 to 131.8)	3433.7 (2518.9 to 4633.5)	4307.7 (2952.4 to 6111.8)	25.5% (-22.0 to 102.0)	634.9 (389.3 to 971.8)	817.8 (477.0 to 1301.7)	28.8% (-34.4 to 153.4)
Puglia	4268.1 (2879.2 to 6053.2)	5664.2 (3573.4 to 8374.2)	32.7% (-24.5 to 133.6)	3401.3 (2464.7 to 4599.3)	4312.2 (2934.4 to 6034.8)	26.8% (-21.2 to 104.4)	631.2 (378.2 to 968.8)	821.2 (466.0 to 1318.1)	30.1% (-35.2 to 161.7)

Sardegna	4745.8 (3390.4 to 6564.4)	5828.5 (3719.6 to 8797.6)	22.8% (-28.3 to 111.6)	3723.7 (2789.4 to 4936.4)	4407.4 (3009.2 to 6277.4)	18.4% (-25.5 to 88.5)	698.5 (438.9 to 1038.7)	843.7 (480.2 to 1334.1)	20.8% (-38.1 to 136.1)
Sicilia	4269.9 (2896.3 to 6052.5)	5688.3 (3538.0 to 8709.1)	33.2% (-25.5 to 138.6)	3405.2 (2489.9 to 4663.1)	4321.7 (2939.2 to 6318.2)	26.9% (-22.6 to 108.3)	632.3 (388.9 to 980.7)	824.3 (481.5 to 1317.7)	30.4% (-33.9 to 157.2)

UI, uncertainly interval; DALYs, disability-adjusted life-years.

Table S11 The incidence, prevalence and DALYs rate of anxiety and depressive disorders in 1990 and 2021, and changes from 1990 to 2021 in Japan.

Location	Incidence (95% UI)			Prevalence (95% UI)			DALYs (95% UI)		
	1990	2021	Percentage change	1990	2021	Percentage change	1990	2021	Percentage change
Anxiety disorders									
Aichi	584.6 (382.6 to 809.9)	735.3 (445.6 to 1093.8)	25.8% (-29.9 to 126.3)	3466.0 (2612.8 to 4482.0)	4288.6 (2988.0 to 6023.3)	23.7% (-20.3 to 92.7)	427.3 (266.4 to 633.0)	528.5 (312.1 to 847.5)	23.7% (-36.0 to 139.8)
Akita	599.4 (405.1 to 820.7)	744.6 (446.9 to 1098.5)	24.2% (-29.7 to 120.0)	3407.8 (2556.0 to 4426.5)	4266.9 (2842.8 to 6054.1)	25.2% (-21.3 to 99.7)	420.8 (258.8 to 631.3)	527.6 (303.7 to 838.5)	25.4% (-36.1 to 146.6)
Aomori	597.6 (399.0 to 817.3)	737.5 (462.4 to 1098.6)	23.4% (-29.7 to 116.9)	3435.4 (2592.2 to 4459.9)	4260.3 (2907.0 to 6060.1)	24.0% (-21.2 to 95.6)	424.3 (264.3 to 630.7)	525.8 (302.9 to 801.7)	23.9% (-35.3 to 138.1)
Chiba	583.4 (386.8 to 797.0)	735.8 (434.1 to 1086.1)	26.1% (-29.4 to 126.1)	3449.2 (2614.4 to 4444.6)	4293.4 (2861.7 to 6064.1)	24.5% (-21.2 to 96.8)	425.8 (264.5 to 634.8)	530.8 (300.6 to 826.5)	24.7% (-36.1 to 143.6)
Ehime	600.6 (402.1 to 828.3)	750.4 (461.4 to 1130.7)	24.9% (-29.6 to 122.1)	3443.3 (2605.6 to 4439.6)	4296.0 (2943.3 to 6086.8)	24.8% (-20.3 to 95.7)	424.6 (266.1 to 635.9)	531.1 (315.0 to 824.0)	25.1% (-34.3 to 138.9)
Fukui	595.5 (400.8 to 806.6)	738.9 (457.5 to 1084.9)	24.1% (-28.7 to 116.2)	3422.2 (2582.8 to 4470.9)	4250.2 (2900.5 to 5940.5)	24.2% (-20.7 to 95.2)	422.3 (267.5 to 631.2)	523.9 (310.3 to 807.7)	24.1% (-34.5 to 135.8)
Fukuoka	586.9 (386.3 to 806.1)	749.3 (461.5 to 1111.6)	27.7% (-28.0 to 126.9)	3444.6 (2583.5 to 4467.0)	4357.2 (2952.7 to 6225.6)	26.5% (-20.1 to 100.8)	423.4 (264.5 to 628.1)	536.1 (319.9 to 828.1)	26.6% (-33.1 to 140.3)
Fukushima	599.9 (409.3 to 825.2)	740.6 (452.1 to 1098.2)	23.5% (-29.6 to 117.3)	3419.3 (2590.5 to 4456.0)	4277.7 (2929.7 to 6073.2)	25.1% (-20.4 to 96.9)	422.2 (267.7 to 624.3)	528.0 (306.3 to 821.9)	25.0% (-34.7 to 140.2)

Gifu	594.3 (397.2 to 809.2)	741.2 (449.5 to 1105.1)	24.7% (-29.5 to 121.2)	3468.2 (2628.0 to 4467.5)	4297.1 (2968.6 to 6187.4)	23.9% (-21.1 to 94.7)	428.4 (271.7 to 635.2)	530.6 (308.9 to 807.0)	23.9% (-34.6 to 135.0)
Gunma	592.2 (401.0 to 812.5)	737.8 (456.2 to 1085.2)	24.6% (-28.7 to 117.9)	3437.6 (2584.2 to 4452.9)	4310.6 (2890.4 to 6091.9)	25.4% (-20.8 to 98.9)	423.6 (264.5 to 623.8)	530.6 (304.6 to 817.8)	25.3% (-34.8 to 141.2)
Hiroshima	590.8 (395.0 to 815.1)	740.2 (445.3 to 1087.3)	25.3% (-29.4 to 122.6)	3450.6 (2619.4 to 4481.0)	4281.9 (2915.5 to 6130.4)	24.1% (-21.4 to 96.1)	426.0 (270.5 to 636.4)	530.6 (306.8 to 833.7)	24.5% (-35.4 to 141.0)
Hokkaidō	594.1 (393.5 to 827.5)	736.5 (453.8 to 1089.3)	24.0% (-30.2 to 120.7)	3454.1 (2615.6 to 4461.1)	4302.3 (2924.2 to 6262.5)	24.6% (-21.6 to 98.0)	426.6 (269.7 to 637.8)	529.5 (314.9 to 845.0)	24.1% (-35.4 to 139.1)
Hyōgo	590.8 (381.7 to 832.9)	745.6 (443.7 to 1113.1)	26.2% (-30.9 to 131.3)	3470.4 (2628.2 to 4519.1)	4341.7 (2945.5 to 6145.9)	25.1% (-20.5 to 97.3)	427.9 (268.1 to 639.8)	536.4 (308.2 to 850.6)	25.3% (-35.7 to 145.1)
Ibaraki	589.9 (403.6 to 801.1)	737.6 (444.8 to 1088.7)	25.0% (-28.6 to 120.0)	3439.0 (2608.5 to 4466.3)	4274.3 (2907.0 to 6124.1)	24.3% (-21.3 to 96.6)	424.0 (267.2 to 627.9)	527.3 (310.8 to 841.1)	24.4% (-35.4 to 140.3)
Ishikawa	590.7 (401.1 to 809.2)	730.8 (436.2 to 1098.7)	23.7% (-30.5 to 121.1)	3439.4 (2618.8 to 4441.4)	4290.6 (2946.9 to 6236.4)	24.7% (-21.0 to 97.0)	424.5 (268.2 to 631.6)	528.5 (313.3 to 842.0)	24.5% (-35.2 to 139.8)
Iwate	598.2 (411.2 to 818.7)	745.8 (463.5 to 1119.4)	24.7% (-28.5 to 118.1)	3413.7 (2571.6 to 4446.2)	4294.1 (2854.2 to 6138.3)	25.8% (-21.2 to 101.1)	421.5 (264.6 to 628.9)	529.5 (311.8 to 858.8)	25.6% (-35.4 to 145.2)
Kagawa	597.2 (399.5 to 820.2)	745.1 (452.7 to 1093.9)	24.8% (-29.3 to 120.5)	3449.2 (2607.8 to 4440.2)	4276.3 (2873.1 to 6219.9)	24.0% (-22.3 to 98.0)	426.3 (263.8 to 640.1)	529.3 (299.2 to 847.3)	24.2% (-37.3 to 146.7)
Kagoshima	604.2 (407.0 to 820.7)	757.1 (465.9 to 1139.6)	25.3% (-28.8 to 121.1)	3424.0 (2567.3 to 4464.5)	4272.2 (2892.4 to 6132.3)	24.8% (-21.5 to 98.8)	422.3 (264.4 to 631.6)	526.6 (311.5 to 829.4)	24.7% (-35.1 to 140.2)
Kanagawa	574.8 (369.3 to 799.9)	734.3 (440.6 to 1089.4)	27.7% (-29.5 to 132.1)	3438.9 (2585.2 to 4449.3)	4318.7 (2914.6 to 6076.4)	25.6% (-20.2 to 98.2)	424.1 (263.6 to 628.4)	533.6 (303.9 to 827.9)	25.8% (-35.1 to 144.5)
Kōchi	602.0 (408.0 to 840.2)	747.9 (464.0 to 1111.9)	24.2% (-29.5 to 119.4)	3435.7 (2612.3 to 4487.2)	4308.9 (2969.8 to 6043.1)	25.4% (-19.5 to 96.1)	423.5 (262.6 to 633.4)	532.1 (312.1 to 828.4)	25.7% (-34.6 to 142.0)

Kumamoto	599.1 (403.4 to 833.4)	741.7 (455.8 to 1092.6)	23.8% (-29.9 to 118.9)	3435.2 (2557.6 to 4472.9)	4259.4 (2901.3 to 5972.3)	24.0% (-21.3 to 95.7)	424.3 (266.1 to 646.5)	524.6 (305.4 to 808.3)	23.6% (-35.7 to 138.3)
Kyōto	578.8 (377.5 to 804.7)	733.5 (437.7 to 1107.2)	26.7% (-30.3 to 130.8)	3446.4 (2601.3 to 4463.3)	4332.1 (2961.5 to 6149.8)	25.7% (-20.0 to 97.8)	424.6 (265.9 to 628.8)	534.1 (324.8 to 818.7)	25.8% (-32.8 to 135.5)
Mie	592.5 (393.0 to 811.5)	739.3 (446.7 to 1108.1)	24.8% (-30.0 to 123.1)	3451.8 (2614.7 to 4479.3)	4305.0 (2963.2 to 6092.9)	24.7% (-20.3 to 95.5)	426.0 (266.9 to 629.2)	530.9 (309.7 to 820.2)	24.6% (-34.7 to 138.5)
Miyagi	587.3 (387.7 to 810.0)	732.0 (443.6 to 1092.0)	24.6% (-30.3 to 123.2)	3430.4 (2591.0 to 4470.9)	4285.2 (2956.4 to 6125.9)	24.9% (-20.5 to 96.8)	423.1 (261.0 to 634.7)	528.7 (312.2 to 841.6)	25.0% (-35.6 to 143.2)
Miyazaki	605.5 (416.4 to 826.4)	753.3 (478.5 to 1108.9)	24.4% (-27.6 to 114.1)	3421.0 (2566.1 to 4437.2)	4284.4 (2912.8 to 6117.7)	25.2% (-20.8 to 98.4)	422.6 (265.4 to 631.9)	528.4 (313.2 to 820.7)	25.0% (-34.4 to 138.7)
Nagano	595.7 (402.8 to 815.3)	744.0 (464.1 to 1109.7)	24.9% (-28.6 to 118.7)	3433.2 (2572.9 to 4459.8)	4288.0 (2889.1 to 6079.0)	24.9% (-21.1 to 98.2)	423.2 (260.6 to 628.9)	529.0 (302.4 to 834.0)	25.0% (-36.1 to 145.0)
Nagasaki	604.3 (400.2 to 838.1)	748.1 (455.6 to 1102.2)	23.8% (-30.4 to 120.7)	3442.5 (2611.3 to 4535.0)	4282.3 (2887.2 to 6055.3)	24.4% (-21.4 to 97.3)	424.8 (266.9 to 633.6)	528.6 (311.4 to 837.8)	24.4% (-35.4 to 140.1)
Nara	591.6 (386.2 to 821.1)	733.7 (452.5 to 1097.9)	24.0% (-30.7 to 122.5)	3465.2 (2614.1 to 4482.3)	4284.7 (2882.2 to 6049.1)	23.6% (-21.6 to 95.5)	426.7 (269.1 to 633.1)	528.8 (304.7 to 833.4)	23.9% (-35.9 to 140.6)
Niigata	599.1 (400.7 to 822.1)	748.9 (458.3 to 1114.6)	25.0% (-29.3 to 121.3)	3421.6 (2599.6 to 4436.8)	4314.4 (2902.5 to 6018.1)	26.1% (-19.5 to 98.1)	422.1 (263.4 to 633.5)	531.4 (315.0 to 837.7)	25.9% (-34.5 to 142.7)
Ōita	597.1 (409.9 to 816.2)	751.7 (460.7 to 1135.1)	25.9% (-28.3 to 122.2)	3434.2 (2600.8 to 4460.9)	4303.4 (2939.2 to 6123.9)	25.3% (-20.3 to 97.5)	423.4 (262.2 to 626.1)	529.5 (311.8 to 828.2)	25.0% (-34.8 to 140.7)
Okayama	596.7 (394.4 to 829.9)	742.1 (453.0 to 1111.9)	24.4% (-30.6 to 123.3)	3456.6 (2607.6 to 4479.2)	4321.9 (2888.9 to 6208.6)	25.0% (-21.5 to 99.4)	425.7 (264.9 to 632.0)	534.1 (316.3 to 862.1)	25.5% (-35.3 to 143.9)
Okinawa	599.4 (400.6 to 824.1)	748.3 (461.2 to 1129.4)	24.8% (-29.6 to 121.7)	3428.2 (2594.6 to 4456.3)	4259.4 (2924.6 to 6115.8)	24.2% (-21.1 to 96.2)	422.0 (260.2 to 618.1)	525.6 (320.2 to 846.3)	24.6% (-34.8 to 138.9)

Ōsaka	583.0 (378.0 to 815.4)	734.1 (451.9 to 1102.2)	25.9% (-30.0 to 127.2)	3476.3 (2624.9 to 4482.0)	4328.5 (2923.0 to 6112.8)	24.5% (-20.9 to 96.3)	428.6 (269.7 to 643.2)	533.3 (319.3 to 828.3)	24.4% (-34.4 to 136.6)
Saga	601.7 (413.7 to 826.2)	748.3 (461.8 to 1096.7)	24.4% (-28.3 to 116.3)	3427.1 (2614.3 to 4419.3)	4293.5 (2866.7 to 6126.5)	25.3% (-20.9 to 98.7)	422.5 (264.2 to 633.5)	530.6 (304.4 to 832.2)	25.6% (-35.4 to 144.8)
Saitama	580.2 (388.0 to 820.1)	729.5 (443.9 to 1101.8)	25.7% (-30.2 to 126.8)	3440.7 (2575.3 to 4447.3)	4286.4 (2887.6 to 6184.8)	24.6% (-21.8 to 98.9)	424.7 (265.3 to 629.0)	527.6 (297.5 to 824.7)	24.2% (-36.3 to 142.9)
Shiga	590.5 (397.0 to 816.6)	736.2 (452.2 to 1099.0)	24.7% (-29.6 to 120.9)	3429.2 (2588.9 to 4431.7)	4288.3 (2902.2 to 6120.7)	25.1% (-20.9 to 97.9)	423.1 (264.9 to 628.2)	529.9 (313.1 to 831.0)	25.2% (-34.5 to 140.2)
Shimane	599.8 (415.9 to 807.5)	746.0 (467.7 to 1102.7)	24.4% (-27.5 to 113.9)	3400.7 (2565.9 to 4405.3)	4280.3 (2881.4 to 6011.6)	25.9% (-20.0 to 98.5)	420.7 (259.1 to 630.7)	528.0 (306.5 to 809.6)	25.5% (-34.7 to 141.6)
Shizuoka	594.1 (398.8 to 819.1)	742.4 (450.2 to 1111.1)	25.0% (-29.7 to 122.5)	3443.5 (2606.5 to 4475.5)	4280.0 (2874.5 to 6047.2)	24.3% (-21.3 to 96.8)	424.7 (268.8 to 621.2)	527.5 (308.3 to 830.5)	24.2% (-35.1 to 138.2)
Tochigi	595.9 (401.0 to 824.5)	742.2 (446.1 to 1094.9)	24.6% (-29.8 to 121.4)	3434.7 (2581.4 to 4462.0)	4292.4 (2937.5 to 6052.1)	25.0% (-20.3 to 96.7)	424.2 (266.0 to 637.4)	530.1 (304.2 to 846.6)	25.0% (-36.2 to 145.5)
Tokushima	597.4 (409.6 to 807.8)	739.2 (456.1 to 1102.3)	23.7% (-28.8 to 116.1)	3442.6 (2597.0 to 4450.9)	4267.1 (2944.7 to 5995.6)	24.0% (-20.4 to 93.7)	424.5 (264.3 to 632.0)	526.6 (305.6 to 817.2)	24.1% (-35.5 to 139.3)
Tōkyō	569.8 (360.3 to 797.2)	729.6 (447.1 to 1098.5)	28.1% (-29.5 to 133.2)	3470.4 (2608.9 to 4488.2)	4341.4 (2967.4 to 6224.9)	25.1% (-20.7 to 97.9)	427.0 (263.7 to 627.8)	534.7 (307.2 to 830.8)	25.2% (-35.2 to 142.4)
Tottori	596.1 (411.1 to 803.6)	746.1 (460.1 to 1116.9)	25.2% (-27.9 to 118.3)	3402.8 (2566.3 to 4407.1)	4290.7 (2907.7 to 6118.0)	26.1% (-20.2 to 99.7)	419.4 (261.2 to 618.6)	529.0 (315.0 to 816.9)	26.1% (-33.4 to 139.4)
Toyama	590.8 (406.0 to 799.6)	737.6 (446.6 to 1101.3)	24.8% (-28.7 to 119.5)	3434.1 (2588.5 to 4437.2)	4279.6 (2905.1 to 6044.9)	24.6% (-20.7 to 96.2)	423.9 (262.7 to 636.7)	528.9 (304.7 to 833.5)	24.8% (-36.0 to 144.0)
Wakayama	597.2 (398.5 to 816.6)	745.4 (455.3 to 1096.0)	24.8% (-29.1 to 120.2)	3452.3 (2612.1 to 4431.7)	4290.6 (2975.5 to 6102.9)	24.3% (-20.2 to 94.0)	426.2 (266.4 to 636.5)	529.5 (313.3 to 833.2)	24.2% (-35.3 to 139.3)

Yamagata	599.0 (402.9 to 814.1)	749.9 (462.2 to 1124.6)	25.2% (-28.7 to 120.6)	3405.2 (2552.4 to 4437.1)	4304.0 (2987.2 to 6046.4)	26.4% (-19.1 to 97.8)	421.5 (261.7 to 618.7)	531.5 (318.4 to 823.8)	26.1% (-33.3 to 139.0)
Yamaguchi	598.4 (405.7 to 824.7)	740.9 (466.3 to 1098.5)	23.8% (-29.0 to 116.3)	3445.2 (2613.8 to 4483.5)	4288.5 (2871.3 to 6011.2)	24.5% (-21.0 to 96.7)	424.2 (264.4 to 643.6)	530.9 (303.0 to 830.3)	25.1% (-35.9 to 145.1)
Yamanashi	589.0 (395.4 to 833.9)	741.9 (456.3 to 1091.8)	26.0% (-29.0 to 124.0)	3434.2 (2610.1 to 4465.8)	4329.6 (2963.1 to 6184.6)	26.1% (-19.9 to 98.8)	422.6 (267.4 to 624.6)	534.1 (304.0 to 845.2)	26.4% (-35.0 to 146.2)
Depressive disorders									
Aichi	2590.8 (1852.0 to 3536.2)	3786.9 (2570.8 to 5563.4)	46.2% (-11.7 to 142.3)	2116.7 (1613.9 to 2730.6)	2880.3 (2091.9 to 4026.6)	36.1% (-10.4 to 107.0)	387.8 (244.1 to 580.3)	550.4 (330.5 to 860.9)	41.9% (-25.3 to 170.1)
Akita	2510.5 (1816.9 to 3399.8)	3754.0 (2471.5 to 5410.3)	49.5% (-9.2 to 146.8)	1977.2 (1504.4 to 2555.4)	2786.5 (1978.0 to 3810.6)	40.9% (-7.4 to 114.8)	366.3 (233.8 to 552.8)	537.9 (318.3 to 845.3)	46.9% (-23.2 to 181.6)
Aomori	2547.2 (1810.2 to 3474.9)	3651.0 (2454.0 to 5250.8)	43.3% (-13.1 to 136.9)	2019.1 (1534.6 to 2602.0)	2749.0 (1972.1 to 3788.2)	36.2% (-10.4 to 107.1)	372.9 (236.1 to 553.8)	525.8 (309.3 to 819.6)	41.0% (-26.1 to 169.3)
Chiba	2543.9 (1838.1 to 3423.1)	3802.4 (2584.9 to 5427.1)	49.5% (-7.9 to 142.9)	2076.5 (1596.7 to 2660.9)	2898.7 (2088.2 to 3938.1)	39.6% (-7.0 to 109.7)	380.5 (244.5 to 567.1)	554.3 (338.1 to 840.1)	45.7% (-21.3 to 169.7)
Ehime	2508.1 (1802.5 to 3421.8)	3535.9 (2360.8 to 5099.5)	41.0% (-14.6 to 133.1)	1997.0 (1519.4 to 2588.4)	2663.3 (1891.3 to 3698.2)	33.4% (-12.9 to 104.6)	367.8 (230.1 to 549.3)	509.3 (301.3 to 809.3)	38.4% (-28.2 to 167.5)
Fukui	2475.1 (1779.3 to 3409.5)	3430.4 (2309.3 to 4877.8)	38.6% (-15.5 to 127.6)	1987.3 (1505.5 to 2569.8)	2605.1 (1876.1 to 3538.3)	31.1% (-13.4 to 98.8)	365.5 (228.9 to 550.3)	496.0 (297.9 to 784.7)	35.7% (-29.1 to 160.4)
Fukuoka	2515.0 (1818.0 to 3400.3)	3668.1 (2465.2 to 5256.0)	45.8% (-10.8 to 138.7)	2042.8 (1559.6 to 2641.6)	2796.6 (2009.6 to 3845.1)	36.9% (-9.8 to 107.9)	375.5 (235.5 to 564.0)	533.3 (325.3 to 853.6)	42.0% (-25.6 to 171.7)
Fukushima	2454.4 (1780.1 to 3299.3)	3649.8 (2480.5 to 5216.6)	48.7% (-8.3 to 141.4)	1958.9 (1507.6 to 2514.8)	2750.4 (1975.3 to 3758.3)	40.4% (-6.7 to 111.7)	360.7 (226.7 to 535.1)	525.8 (316.4 to 812.8)	45.8% (-22.6 to 175.4)
Gifu	1872.3 (1274.7 to 2651.8)	2795.4 (1773.3 to 4172.5)	49.3% (-14.9 to 162.7)	1545.0 (1119.3 to 2077.2)	2139.9 (1481.0 to 3021.2)	38.5% (-13.5 to 122.2)	279.1 (169.2 to 430.1)	403.9 (235.2 to 650.2)	44.7% (-27.0 to 187.4)

Gunma	2502.1 (1792.6 to 3432.6)	3919.9 (2664.6 to 5782.1)	56.7% (-5.5 to 160.2)	2015.7 (1534.3 to 2613.3)	2940.1 (2125.4 to 4082.0)	45.9% (-4.2 to 122.4)	370.8 (228.7 to 551.4)	564.4 (345.1 to 863.4)	52.2% (-18.9 to 185.8)
Hiroshima	2566.5 (1869.4 to 3481.4)	3540.6 (2376.8 to 5065.9)	38.0% (-15.4 to 125.3)	2065.0 (1593.0 to 2663.4)	2701.0 (1966.2 to 3755.6)	30.8% (-13.3 to 97.7)	379.2 (239.9 to 560.2)	514.1 (311.7 to 801.5)	35.6% (-27.9 to 155.6)
Hokkaidō	2535.2 (1832.8 to 3462.5)	3840.7 (2573.4 to 5496.2)	51.5% (-7.7 to 149.0)	2050.3 (1568.5 to 2655.7)	2901.3 (2096.9 to 3983.5)	41.5% (-6.5 to 114.4)	377.5 (239.5 to 568.9)	554.8 (331.3 to 858.4)	47.0% (-22.4 to 179.1)
Hyōgo	2619.4 (1875.3 to 3544.5)	3778.5 (2562.8 to 5459.1)	44.2% (-12.0 to 136.8)	2115.3 (1611.8 to 2709.1)	2856.3 (2067.9 to 3916.3)	35.0% (-10.5 to 103.8)	389.6 (246.9 to 589.4)	546.3 (331.4 to 822.8)	40.2% (-24.8 to 161.8)
Ibaraki	2537.3 (1858.2 to 3399.9)	3758.5 (2560.3 to 5326.5)	48.1% (-7.9 to 138.4)	2039.3 (1553.4 to 2638.4)	2839.0 (2068.4 to 3891.9)	39.2% (-7.8 to 110.6)	375.0 (233.8 to 551.2)	544.4 (334.0 to 864.3)	45.2% (-23.2 to 174.9)
Ishikawa	2544.9 (1854.8 to 3383.6)	3555.0 (2388.9 to 5047.3)	39.7% (-13.4 to 125.6)	2051.5 (1572.8 to 2614.6)	2725.9 (1920.6 to 3710.8)	32.9% (-12.1 to 101.4)	377.7 (240.5 to 566.5)	518.3 (305.4 to 797.9)	37.2% (-27.7 to 161.2)
Iwate	2468.4 (1761.9 to 3340.9)	3790.4 (2556.2 to 5390.9)	53.6% (-6.0 to 151.4)	1962.5 (1493.6 to 2520.4)	2829.3 (2018.8 to 3846.3)	44.2% (-4.7 to 118.4)	361.8 (228.0 to 545.2)	543.5 (337.1 to 856.4)	50.2% (-20.3 to 183.2)
Kagawa	2489.5 (1803.0 to 3360.0)	3420.8 (2302.4 to 4934.7)	37.4% (-15.9 to 124.9)	1992.8 (1539.1 to 2554.5)	2597.5 (1873.8 to 3546.6)	30.3% (-13.1 to 95.8)	366.8 (231.1 to 550.4)	494.2 (298.5 to 748.6)	34.7% (-28.0 to 152.5)
Kagoshima	2105.8 (1467.9 to 2923.5)	2804.2 (1831.6 to 4153.6)	33.2% (-22.0 to 127.8)	1675.6 (1229.3 to 2217.6)	2121.6 (1496.5 to 2948.1)	26.6% (-19.1 to 98.6)	307.8 (190.7 to 464.5)	401.7 (241.4 to 636.4)	30.5% (-32.1 to 151.1)
Kanagawa	2667.2 (1952.9 to 3592.1)	3886.6 (2644.6 to 5507.6)	45.7% (-9.6 to 135.2)	2192.9 (1682.0 to 2847.9)	2967.3 (2133.3 to 4070.8)	35.3% (-10.7 to 105.4)	402.7 (250.8 to 604.1)	568.2 (339.2 to 866.7)	41.1% (-25.5 to 167.3)
Kōchi	2483.2 (1764.4 to 3375.4)	3532.1 (2358.4 to 5142.3)	42.2% (-14.4 to 136.7)	1973.2 (1492.7 to 2535.3)	2673.6 (1922.1 to 3680.8)	35.5% (-10.8 to 106.1)	363.4 (226.3 to 539.8)	510.6 (299.9 to 803.9)	40.5% (-27.0 to 171.2)
Kumamoto	2445.9 (1750.8 to 3354.1)	3510.1 (2318.9 to 5005.7)	43.5% (-13.3 to 137.9)	1964.4 (1497.0 to 2547.3)	2653.2 (1889.3 to 3594.0)	35.1% (-11.0 to 105.2)	361.3 (229.0 to 536.9)	507.1 (303.8 to 781.4)	40.3% (-25.4 to 164.7)

Kyōto	2685.2 (1924.5 to 3643.3)	3843.3 (2603.6 to 5441.2)	43.1% (-12.0 to 133.2)	2188.3 (1672.2 to 2814.5)	2949.7 (2126.7 to 3992.4)	34.8% (-10.4 to 103.1)	403.1 (250.4 to 596.3)	563.5 (342.9 to 869.0)	39.8% (-25.7 to 162.9)
Mie	2467.7 (1778.6 to 3349.3)	3516.7 (2396.3 to 5009.7)	42.5% (-12.3 to 132.1)	1997.8 (1519.6 to 2587.8)	2680.6 (1990.0 to 3674.2)	34.2% (-10.5 to 101.5)	366.0 (227.3 to 550.1)	511.6 (305.7 to 814.5)	39.8% (-27.5 to 169.9)
Miyagi	2551.9 (1866.9 to 3411.6)	3921.2 (2633.1 to 5630.5)	53.7% (-5.1 to 149.5)	2063.7 (1585.7 to 2636.4)	2970.0 (2121.0 to 4141.0)	43.9% (-5.2 to 119.2)	379.3 (236.6 to 562.1)	568.5 (345.4 to 877.4)	49.9% (-20.3 to 181.9)
Miyazaki	2468.6 (1787.9 to 3383.2)	3477.5 (2373.3 to 5114.1)	40.9% (-14.5 to 132.5)	1943.5 (1483.9 to 2503.1)	2602.0 (1888.3 to 3629.9)	33.9% (-11.7 to 103.4)	359.3 (227.1 to 546.6)	497.3 (301.2 to 766.0)	38.4% (-26.7 to 161.6)
Nagano	2510.2 (1802.7 to 3428.0)	3512.2 (2330.0 to 5068.5)	39.9% (-15.5 to 132.0)	2009.2 (1519.1 to 2594.4)	2650.4 (1877.9 to 3680.1)	31.9% (-14.0 to 102.7)	369.8 (231.7 to 545.8)	505.6 (301.3 to 804.9)	36.7% (-28.6 to 162.5)
Nagasaki	2437.0 (1757.8 to 3331.2)	3445.7 (2328.3 to 4910.4)	41.4% (-13.4 to 131.4)	1940.4 (1486.2 to 2491.5)	2598.6 (1875.6 to 3528.6)	33.9% (-10.9 to 101.5)	357.0 (223.1 to 538.1)	495.8 (294.2 to 760.8)	38.9% (-27.0 to 164.3)
Nara	2544.5 (1837.3 to 3461.7)	3597.1 (2426.3 to 5080.5)	41.4% (-13.0 to 130.4)	2066.5 (1582.2 to 2684.8)	2746.6 (1967.0 to 3737.9)	32.9% (-12.3 to 101.6)	379.1 (242.5 to 573.8)	523.2 (300.8 to 791.4)	38.0% (-27.6 to 163.9)
Niigata	2499.9 (1778.5 to 3416.6)	3767.1 (2499.6 to 5479.1)	50.7% (-9.6 to 151.6)	1989.8 (1502.9 to 2576.3)	2827.6 (2003.7 to 3961.1)	42.1% (-7.7 to 119.4)	366.2 (232.4 to 552.9)	542.4 (318.2 to 853.8)	48.1% (-23.0 to 185.9)
Ōita	2437.6 (1743.5 to 3331.2)	3532.4 (2365.7 to 5049.1)	44.9% (-11.9 to 139.0)	1946.3 (1479.6 to 2524.8)	2667.8 (1908.0 to 3635.6)	37.1% (-9.8 to 108.7)	358.1 (227.6 to 538.4)	510.7 (300.2 to 796.4)	42.6% (-25.4 to 173.3)
Okayama	2471.1 (1778.8 to 3335.3)	3589.4 (2395.3 to 5148.8)	45.3% (-11.4 to 138.6)	1996.5 (1524.0 to 2556.4)	2744.4 (1966.8 to 3705.3)	37.5% (-8.6 to 107.0)	366.1 (229.9 to 548.6)	523.9 (315.4 to 804.8)	43.1% (-24.2 to 170.0)
Okinawa	2493.4 (1781.3 to 3384.7)	3451.3 (2281.6 to 4992.0)	38.4% (-16.5 to 129.8)	1989.3 (1516.8 to 2574.8)	2611.8 (1868.6 to 3597.5)	31.3% (-13.7 to 100.0)	366.2 (232.5 to 550.3)	498.0 (299.5 to 775.6)	36.0% (-28.1 to 157.9)
Ōsaka	2670.0 (1922.2 to 3568.6)	4000.1 (2720.5 to 5864.1)	49.8% (-8.4 to 145.2)	2181.7 (1667.7 to 2789.3)	3041.1 (2177.8 to 4235.5)	39.4% (-8.2 to 112.2)	400.3 (253.7 to 600.2)	583.9 (346.0 to 913.6)	45.9% (-23.6 to 179.1)

Saga	2457.2 (1757.6 to 3342.5)	3400.8 (2270.3 to 4929.5)	38.4% (-16.4 to 129.4)	1965.0 (1493.3 to 2539.9)	2580.9 (1858.1 to 3578.4)	31.3% (-13.8 to 100.2)	362.0 (229.8 to 547.7)	492.1 (290.4 to 786.4)	35.9% (-29.7 to 163.3)
Saitama	2607.4 (1893.9 to 3553.9)	3918.1 (2614.2 to 5627.5)	50.3% (-8.4 to 147.0)	2124.3 (1625.0 to 2745.4)	2972.2 (2143.6 to 4088.2)	39.9% (-7.6 to 112.1)	389.4 (245.4 to 590.6)	570.3 (341.3 to 904.3)	46.5% (-23.7 to 181.7)
Shiga	2489.4 (1791.7 to 3391.9)	3623.0 (2435.9 to 5222.2)	45.5% (-11.5 to 139.7)	2007.8 (1541.8 to 2607.0)	2759.7 (1980.3 to 3803.1)	37.5% (-9.5 to 108.9)	367.9 (229.6 to 550.3)	526.7 (317.3 to 829.3)	43.2% (-24.9 to 173.5)
Shimane	2513.5 (1793.8 to 3418.2)	3557.4 (2383.4 to 5124.5)	41.5% (-14.2 to 133.8)	1969.4 (1497.8 to 2518.7)	2671.2 (1913.7 to 3671.3)	35.6% (-10.4 to 105.7)	364.4 (227.4 to 548.9)	511.7 (305.1 to 799.6)	40.4% (-26.6 to 168.9)
Shizuoka	2461.9 (1781.1 to 3329.2)	3627.9 (2451.9 to 5259.8)	47.4% (-9.9 to 141.5)	1995.2 (1530.5 to 2564.7)	2737.3 (1949.7 to 3797.8)	37.2% (-9.8 to 109.1)	365.6 (231.6 to 553.4)	522.9 (313.2 to 818.0)	43.0% (-24.9 to 172.9)
Tochigi	2495.2 (1778.3 to 3407.0)	3681.3 (2435.5 to 5368.9)	47.5% (-11.5 to 146.3)	2004.3 (1524.8 to 2582.1)	2781.9 (1980.8 to 3816.2)	38.8% (-8.7 to 111.3)	368.4 (228.3 to 542.7)	533.4 (325.4 to 836.7)	44.8% (-23.3 to 173.8)
Tokushima	2497.2 (1781.3 to 3415.5)	3532.0 (2359.4 to 5070.4)	41.4% (-14.4 to 134.2)	1998.7 (1517.4 to 2607.7)	2681.8 (1928.5 to 3712.5)	34.2% (-12.3 to 105.5)	367.1 (232.1 to 553.5)	511.7 (307.0 to 792.1)	39.4% (-26.4 to 164.1)
Tōkyō	3874.3 (2804.7 to 5222.8)	5465.2 (3680.9 to 7940.5)	41.1% (-13.9 to 131.2)	3032.4 (2305.3 to 3967.6)	4043.1 (2864.7 to 5654.1)	33.3% (-13.5 to 106.0)	576.4 (367.6 to 858.1)	792.0 (471.8 to 1265.8)	37.4% (-28.2 to 164.0)
Tottori	2447.5 (1743.8 to 3280.3)	3537.2 (2373.7 to 5032.5)	44.5% (-11.5 to 136.5)	1946.0 (1483.9 to 2514.4)	2670.6 (1921.2 to 3685.1)	37.2% (-9.7 to 108.6)	358.3 (223.8 to 532.3)	511.0 (309.2 to 797.2)	42.6% (-24.6 to 170.1)
Toyama	2628.5 (1903.8 to 3537.6)	3673.6 (2462.7 to 5238.6)	39.8% (-14.2 to 127.9)	2087.3 (1601.1 to 2704.3)	2778.8 (2002.5 to 3784.7)	33.1% (-11.8 to 101.2)	386.2 (244.7 to 582.9)	531.6 (316.1 to 824.4)	37.6% (-27.6 to 162.3)
Wakayama	2598.1 (1883.4 to 3518.0)	3605.3 (2403.9 to 5163.5)	38.8% (-15.2 to 127.5)	2067.9 (1580.3 to 2653.0)	2719.6 (1925.3 to 3774.8)	31.5% (-13.8 to 101.3)	381.8 (241.6 to 577.2)	518.6 (308.1 to 802.0)	35.8% (-28.5 to 158.8)
Yamagata	2035.7 (1366.6 to 2879.4)	3157.5 (1996.2 to 4808.6)	55.1% (-12.8 to 176.7)	1683.0 (1232.4 to 2236.9)	2419.8 (1672.9 to 3478.4)	43.8% (-10.2 to 130.6)	302.3 (181.7 to 462.4)	456.9 (250.0 to 751.5)	51.1% (-26.5 to 212.0)

Yamaguchi	2507.8 (1819.2 to 3424.5)	3561.7 (2456.8 to 5217.9)	42.0% (-13.2 to 132.6)	2004.2 (1539.2 to 2593.4)	2699.4 (1976.1 to 3713.4)	34.7% (-10.6 to 103.1)	369.9 (234.0 to 560.5)	515.9 (307.1 to 819.1)	39.5% (-27.5 to 169.2)
Yamanashi	2603.2 (1862.3 to 3563.3)	3609.2 (2415.5 to 5225.5)	38.6% (-16.3 to 129.9)	2096.8 (1597.4 to 2728.0)	2750.5 (1976.3 to 3771.9)	31.2% (-13.8 to 99.8)	386.5 (240.1 to 575.6)	525.5 (304.5 to 844.8)	36.0% (-30.5 to 166.8)

UI, uncertainly interval; DALYs, disability-adjusted life-years.

Table S12 The incidence, prevalence and DALYs rate of anxiety and depressive disorders in 1990 and 2021, and changes from 1990 to 2021 in Kenya.

Location	Incidence (95% UI)			Prevalence (95% UI)			DALYs (95% UI)		
	1990	2021	Percentage change	1990	2021	Percentage change	1990	2021	Percentage change
Anxiety disorders									
Baringo	696.0 (469.9 to 954.1)	839.7 (517.0 to 1225.8)	20.6% (-30.9 to 111.0)	3425.5 (2562.9 to 4423.7)	4189.3 (2828.0 to 5910.7)	22.3% (-22.5 to 93.3)	422.4 (264.1 to 635.4)	515.7 (301.4 to 826.7)	22.1% (-37.4 to 138.5)
Bomet	692.5 (475.9 to 948.2)	852.4 (520.1 to 1298.7)	23.1% (-30.3 to 118.3)	3408.7 (2555.2 to 4433.8)	4280.2 (2925.4 to 6050.7)	25.6% (-20.2 to 98.2)	419.2 (254.7 to 619.8)	525.3 (309.9 to 813.6)	25.3% (-34.7 to 140.5)
Bungoma	692.5 (470.7 to 946.2)	848.2 (524.6 to 1237.0)	22.5% (-29.5 to 113.0)	3440.6 (2582.8 to 4468.8)	4248.6 (2901.8 to 6038.3)	23.5% (-21.7 to 95.0)	421.2 (260.2 to 620.8)	523.0 (301.8 to 827.7)	24.2% (-36.1 to 142.2)
Busia	699.5 (480.7 to 959.3)	850.7 (527.6 to 1245.1)	21.6% (-29.8 to 110.9)	3454.0 (2578.9 to 4494.1)	4217.6 (2850.9 to 5903.2)	22.1% (-22.5 to 93.1)	421.2 (264.3 to 624.6)	517.5 (306.8 to 802.1)	22.9% (-35.3 to 134.2)
Elgeyo-Marakwet	696.7 (476.1 to 956.4)	841.2 (515.9 to 1264.9)	20.8% (-31.4 to 113.1)	3406.5 (2558.5 to 4378.5)	4205.9 (2869.8 to 5861.0)	23.5% (-20.8 to 93.0)	419.4 (262.6 to 618.6)	520.2 (301.7 to 815.2)	24.0% (-35.6 to 139.5)
Embu	698.2 (467.4 to 945.3)	848.8 (512.6 to 1246.8)	21.6% (-30.8 to 114.3)	3446.1 (2590.2 to 4506.4)	4262.5 (2826.5 to 6055.7)	23.7% (-22.6 to 98.1)	424.9 (263.7 to 626.4)	524.5 (299.7 to 828.0)	23.4% (-36.6 to 141.2)
Garissa	689.2 (463.8 to 936.6)	836.1 (517.3 to 1253.4)	21.3% (-30.9 to 113.4)	3412.3 (2575.2 to 4413.5)	4179.0 (2825.6 to 5988.2)	22.5% (-22.7 to 94.2)	412.1 (259.9 to 608.8)	506.6 (299.9 to 805.4)	22.9% (-35.9 to 136.4)
Homa Bay	695.4 (482.1 to 956.9)	858.2 (537.3 to 1263.5)	23.4% (-28.5 to 113.4)	3423.8 (2573.0 to 4422.0)	4317.6 (2911.2 to 6097.3)	26.1% (-20.0 to 99.3)	417.2 (262.7 to 614.1)	527.7 (301.6 to 830.3)	26.5% (-34.7 to 145.5)

Isiolo	697.0 (473.9 to 958.1)	840.2 (522.1 to 1236.5)	20.5% (-30.8 to 110.4)	3429.6 (2574.1 to 4408.4)	4206.1 (2877.5 to 6068.5)	22.6% (-22.4 to 94.1)	422.5 (262.9 to 624.6)	517.1 (304.5 to 782.2)	22.4% (-35.1 to 131.3)
Kajiado	700.3 (461.3 to 961.0)	851.4 (511.1 to 1252.5)	21.6% (-31.8 to 117.2)	3522.8 (2617.2 to 4564.9)	4338.3 (2852.7 to 6164.7)	23.2% (-23.2 to 97.9)	432.8 (263.9 to 647.1)	534.5 (299.6 to 843.1)	23.5% (-37.6 to 145.2)
Kakamega	697.3 (474.4 to 949.7)	838.9 (511.5 to 1236.6)	20.3% (-31.2 to 111.0)	3429.7 (2554.1 to 4461.2)	4134.4 (2789.9 to 5793.6)	20.5% (-23.6 to 91.0)	420.0 (260.9 to 619.4)	507.0 (296.5 to 793.0)	20.7% (-37.2 to 132.5)
Kericho	692.6 (467.9 to 943.3)	847.7 (526.8 to 1246.8)	22.4% (-29.7 to 113.3)	3421.1 (2568.0 to 4433.6)	4290.6 (2944.3 to 6128.7)	25.4% (-20.4 to 97.9)	422.3 (259.2 to 618.3)	527.8 (307.9 to 844.4)	25.0% (-35.7 to 143.7)
Kiambu	703.3 (473.7 to 969.4)	871.0 (530.6 to 1315.6)	23.9% (-30.3 to 120.8)	3526.4 (2622.7 to 4592.8)	4472.7 (2997.3 to 6371.5)	26.8% (-20.5 to 102.7)	435.7 (271.2 to 647.2)	551.8 (313.1 to 844.7)	26.7% (-34.4 to 145.1)
Kilifi	695.8 (473.9 to 941.8)	851.3 (526.1 to 1281.3)	22.3% (-30.0 to 114.7)	3444.1 (2566.5 to 4448.0)	4298.2 (2915.7 to 6199.1)	24.8% (-21.6 to 99.0)	419.5 (256.4 to 622.1)	526.8 (304.8 to 799.3)	25.6% (-34.4 to 140.7)
Kirinyaga	702.1 (463.7 to 978.7)	852.1 (527.5 to 1260.4)	21.4% (-31.5 to 115.8)	3492.7 (2596.4 to 4534.9)	4265.6 (2897.6 to 6117.2)	22.1% (-23.2 to 94.6)	429.9 (265.5 to 639.6)	524.7 (296.6 to 824.1)	22.1% (-37.7 to 140.1)
Kisii	695.7 (464.3 to 961.0)	854.3 (527.5 to 1254.1)	22.8% (-30.3 to 116.6)	3430.9 (2568.8 to 4440.2)	4332.4 (2924.9 to 6135.8)	26.3% (-20.1 to 100.0)	421.2 (258.2 to 634.3)	532.5 (304.8 to 834.2)	26.4% (-35.4 to 148.3)
Kisumu	704.4 (466.0 to 975.6)	850.3 (515.5 to 1237.7)	20.7% (-31.9 to 114.5)	3482.9 (2598.5 to 4498.4)	4310.2 (2925.7 to 6138.6)	23.8% (-21.8 to 96.1)	423.6 (264.2 to 627.2)	525.8 (304.7 to 829.9)	24.1% (-35.9 to 141.0)
Kitui	695.2 (476.9 to 966.2)	840.3 (527.5 to 1253.1)	20.9% (-30.8 to 111.4)	3427.4 (2557.2 to 4424.3)	4156.5 (2837.8 to 5951.5)	21.3% (-23.3 to 92.1)	421.6 (261.8 to 634.2)	510.2 (294.5 to 792.8)	21.0% (-37.5 to 135.2)
Kwale	698.9 (468.9 to 960.5)	850.0 (532.8 to 1242.1)	21.6% (-30.1 to 112.3)	3466.5 (2571.6 to 4509.2)	4279.0 (2909.7 to 6014.5)	23.4% (-21.8 to 95.3)	421.7 (262.3 to 624.2)	524.6 (310.2 to 821.0)	24.4% (-35.0 to 138.9)
Laikipia	693.2 (466.0 to 944.3)	840.3 (518.7 to 1237.0)	21.2% (-30.7 to 112.2)	3435.0 (2591.0 to 4449.4)	4177.4 (2836.6 to 5924.9)	21.6% (-22.8 to 92.0)	423.8 (265.1 to 633.3)	514.3 (293.4 to 815.9)	21.4% (-38.0 to 138.2)

Lamu	690.9 (448.4 to 938.5)	841.2 (524.5 to 1242.3)	21.8% (-30.9 to 115.2)	3459.7 (2588.4 to 4488.6)	4219.6 (2873.3 to 5848.0)	22.0% (-22.0 to 91.2)	423.4 (263.1 to 624.1)	517.4 (297.3 to 785.1)	22.2% (-36.0 to 134.2)
Machakos	689.4 (468.7 to 942.0)	847.4 (525.7 to 1274.7)	22.9% (-29.8 to 116.0)	3398.6 (2548.4 to 4445.4)	4281.7 (2893.0 to 6090.6)	26.0% (-20.7 to 100.4)	418.5 (257.2 to 621.4)	525.8 (309.4 to 818.4)	25.6% (-34.6 to 141.7)
Makueni	695.1 (470.9 to 967.3)	846.8 (538.3 to 1235.9)	21.8% (-29.6 to 111.3)	3408.4 (2540.1 to 4433.4)	4186.4 (2850.6 to 6021.3)	22.8% (-22.7 to 95.6)	419.8 (261.5 to 623.9)	513.9 (308.0 to 801.4)	22.4% (-35.6 to 133.1)
Mandera	690.6 (470.8 to 962.2)	840.9 (533.3 to 1248.6)	21.8% (-30.1 to 112.6)	3402.3 (2548.4 to 4386.6)	4161.5 (2848.4 to 5771.1)	22.3% (-21.4 to 91.1)	416.5 (258.1 to 615.2)	507.7 (301.0 to 794.0)	21.9% (-36.2 to 133.7)
Marsabit	690.0 (469.8 to 943.4)	843.0 (512.5 to 1284.1)	22.2% (-31.1 to 117.6)	3413.4 (2544.6 to 4416.0)	4200.4 (2824.2 to 5949.4)	23.1% (-22.4 to 95.5)	419.8 (256.0 to 630.5)	516.8 (288.3 to 800.6)	23.1% (-37.5 to 143.2)
Meru	696.0 (469.9 to 947.4)	845.7 (525.1 to 1244.5)	21.5% (-30.2 to 111.9)	3429.4 (2573.4 to 4469.8)	4242.8 (2871.8 to 6008.1)	23.7% (-21.8 to 96.1)	421.7 (259.0 to 617.5)	520.6 (303.4 to 794.9)	23.5% (-35.2 to 135.7)
Migori	697.4 (471.3 to 974.2)	853.3 (524.7 to 1286.2)	22.4% (-31.2 to 117.8)	3431.0 (2565.9 to 4472.2)	4257.6 (2844.4 to 6142.6)	24.1% (-22.6 to 99.3)	418.9 (256.6 to 624.3)	519.8 (297.9 to 815.0)	24.1% (-36.4 to 142.8)
Mombasa	711.1 (471.8 to 994.5)	858.2 (529.9 to 1293.5)	20.7% (-32.5 to 116.4)	3662.5 (2749.4 to 4731.5)	4377.0 (2896.9 to 6216.3)	19.5% (-25.0 to 90.6)	443.9 (276.4 to 659.7)	533.5 (314.0 to 838.3)	20.2% (-37.5 to 131.5)
Murang'a	694.2 (470.3 to 958.6)	841.6 (516.4 to 1278.0)	21.2% (-31.7 to 115.7)	3407.5 (2571.1 to 4377.3)	4181.8 (2760.7 to 6113.6)	22.7% (-23.8 to 97.9)	420.9 (263.1 to 624.5)	514.9 (298.2 to 832.1)	22.3% (-37.5 to 139.8)
Nairobi	721.8 (476.1 to 1008.2)	863.8 (529.0 to 1310.4)	19.7% (-33.6 to 116.0)	3751.9 (2815.3 to 4793.2)	4457.4 (2949.2 to 6391.7)	18.8% (-25.6 to 89.8)	461.4 (284.0 to 685.5)	549.1 (313.8 to 874.6)	19.0% (-39.4 to 134.5)
Nakuru	700.6 (469.8 to 971.3)	851.2 (518.0 to 1271.9)	21.5% (-31.7 to 116.4)	3492.5 (2598.9 to 4538.6)	4286.2 (2839.8 to 6062.4)	22.7% (-23.1 to 96.4)	428.9 (269.9 to 637.5)	527.1 (303.2 to 837.9)	22.9% (-36.8 to 139.7)
Nandi	695.6 (470.3 to 954.4)	846.1 (525.6 to 1256.1)	21.6% (-30.5 to 113.2)	3429.1 (2573.4 to 4433.2)	4229.7 (2900.2 to 5947.7)	23.3% (-21.2 to 93.6)	421.6 (259.6 to 625.8)	520.6 (296.6 to 804.6)	23.5% (-36.4 to 140.2)

Narok	692.8 (472.2 to 946.1)	834.6 (523.6 to 1264.0)	20.5% (-31.1 to 111.2)	3425.8 (2554.8 to 4434.7)	4147.7 (2809.5 to 5933.5)	21.1% (-23.7 to 92.5)	421.0 (258.7 to 639.4)	509.5 (291.5 to 805.1)	21.0% (-38.5 to 139.0)
Nyamira	696.0 (471.6 to 937.7)	864.6 (531.8 to 1287.2)	24.2% (-28.8 to 117.4)	3445.0 (2581.4 to 4455.4)	4362.6 (2946.0 to 6236.6)	26.6% (-20.2 to 101.3)	425.1 (263.2 to 630.7)	535.9 (304.0 to 858.9)	26.1% (-36.0 to 149.1)
Nyandarua	695.2 (464.8 to 952.7)	846.1 (521.8 to 1269.1)	21.7% (-31.1 to 115.4)	3438.9 (2547.1 to 4485.1)	4182.7 (2901.6 to 5976.6)	21.6% (-23.0 to 92.4)	424.2 (260.1 to 622.4)	514.9 (301.6 to 809.5)	21.4% (-37.0 to 134.5)
Nyeri	697.5 (472.9 to 958.0)	844.7 (508.6 to 1246.0)	21.1% (-31.3 to 114.3)	3473.9 (2597.7 to 4500.0)	4233.5 (2824.0 to 6018.0)	21.9% (-23.5 to 94.4)	428.8 (268.3 to 641.9)	521.8 (300.5 to 826.6)	21.7% (-37.5 to 137.8)
Samburu	699.3 (465.3 to 959.9)	838.2 (515.3 to 1245.9)	19.9% (-32.2 to 112.3)	3466.3 (2586.5 to 4514.7)	4158.2 (2805.0 to 5867.2)	20.0% (-24.2 to 90.5)	426.0 (268.9 to 624.1)	511.9 (297.4 to 800.6)	20.2% (-37.2 to 130.8)
Siaya	693.4 (465.4 to 941.4)	844.0 (523.4 to 1246.6)	21.7% (-30.3 to 112.9)	3411.1 (2550.1 to 4404.1)	4207.1 (2908.1 to 5918.1)	23.3% (-21.0 to 93.3)	417.6 (260.0 to 624.4)	517.4 (303.9 to 810.7)	23.9% (-35.6 to 139.2)
Taita Taveta	697.6 (464.7 to 960.0)	849.7 (532.3 to 1266.6)	21.8% (-30.8 to 114.8)	3452.8 (2571.0 to 4493.1)	4276.6 (2917.4 to 6055.9)	23.9% (-21.5 to 96.3)	423.8 (259.1 to 626.1)	525.0 (308.5 to 805.6)	23.9% (-35.1 to 136.8)
Tana River	688.2 (472.8 to 931.3)	840.1 (510.8 to 1256.2)	22.1% (-30.2 to 114.4)	3392.7 (2522.6 to 4366.3)	4188.6 (2783.1 to 6043.7)	23.5% (-23.0 to 98.1)	414.8 (256.4 to 611.0)	511.4 (293.8 to 816.3)	23.3% (-36.9 to 141.8)
Tharaka Nithi	695.4 (477.1 to 948.4)	853.7 (524.1 to 1264.5)	22.8% (-29.6 to 114.6)	3435.9 (2578.7 to 4407.7)	4245.4 (2880.5 to 5943.2)	23.6% (-21.1 to 93.7)	423.3 (263.1 to 627.9)	523.1 (307.8 to 830.1)	23.6% (-36.0 to 139.1)
Trans Nzoia	691.0 (467.2 to 945.2)	845.0 (520.1 to 1269.4)	22.3% (-30.5 to 115.9)	3408.0 (2560.2 to 4419.9)	4196.6 (2820.5 to 5901.2)	23.1% (-22.0 to 94.8)	418.9 (263.9 to 623.1)	515.1 (297.2 to 803.3)	22.9% (-36.2 to 137.5)
Turkana	698.8 (461.6 to 948.5)	837.6 (515.9 to 1254.3)	19.9% (-32.3 to 112.3)	3477.0 (2578.9 to 4505.5)	4184.2 (2818.9 to 5998.8)	20.3% (-24.5 to 92.3)	425.9 (262.8 to 629.9)	513.6 (301.9 to 802.1)	20.6% (-37.2 to 132.3)
Uasin Gishu	701.1 (470.1 to 974.3)	850.3 (526.1 to 1280.3)	21.3% (-31.7 to 115.7)	3489.8 (2629.7 to 4481.6)	4303.7 (2917.9 to 6146.4)	23.3% (-21.8 to 94.7)	430.6 (268.0 to 629.8)	529.6 (308.1 to 858.8)	23.0% (-36.9 to 140.3)

Vihiga	698.2 (473.4 to 966.9)	841.4 (536.7 to 1227.2)	20.5% (-30.2 to 108.4)	3438.8 (2569.2 to 4442.3)	4128.3 (2845.1 to 5882.9)	20.1% (-23.5 to 89.2)	421.1 (261.4 to 622.3)	505.9 (305.2 to 821.4)	20.1% (-37.7 to 132.1)
Wajir	685.0 (466.6 to 934.9)	831.8 (529.9 to 1234.6)	21.4% (-29.7 to 110.1)	3370.8 (2519.9 to 4370.2)	4145.4 (2799.0 to 5908.8)	23.0% (-22.4 to 95.4)	410.7 (253.0 to 606.9)	506.4 (285.7 to 768.8)	23.3% (-36.1 to 138.5)
West Pokot	700.5 (468.5 to 971.6)	839.8 (515.5 to 1243.5)	19.9% (-32.3 to 112.8)	3494.7 (2614.0 to 4531.5)	4156.4 (2793.8 to 5955.0)	18.9% (-25.3 to 89.9)	430.1 (266.8 to 645.5)	511.7 (296.0 to 804.3)	19.0% (-38.8 to 131.8)
Depressive disorders									
Baringo	3250.1 (2297.5 to 4541.5)	3667.9 (2401.3 to 5357.0)	12.9% (-33.3 to 91.5)	2707.2 (2060.7 to 3558.6)	2989.2 (2148.5 to 4080.2)	10.4% (-27.5 to 68.6)	482.9 (302.2 to 728.4)	541.0 (314.2 to 846.3)	12.0% (-42.1 to 117.4)
Bomet	3130.4 (2177.9 to 4384.8)	3722.5 (2472.5 to 5419.4)	18.9% (-29.6 to 101.2)	2639.2 (1976.8 to 3491.9)	3076.2 (2255.6 to 4171.1)	16.6% (-23.1 to 76.7)	466.2 (287.7 to 708.1)	552.3 (336.3 to 865.0)	18.5% (-38.0 to 126.6)
Bungoma	3246.0 (2297.9 to 4486.6)	3753.7 (2421.8 to 5507.1)	15.6% (-31.8 to 96.5)	2719.0 (2052.2 to 3589.7)	3077.4 (2197.7 to 4232.4)	13.2% (-26.4 to 74.4)	480.8 (303.3 to 724.1)	555.1 (331.9 to 870.7)	15.5% (-39.4 to 120.7)
Busia	3393.4 (2390.3 to 4757.0)	3719.2 (2481.7 to 5398.2)	9.6% (-34.6 to 84.1)	2810.2 (2122.6 to 3668.1)	3002.6 (2193.6 to 4072.6)	6.8% (-29.2 to 61.4)	498.3 (307.6 to 742.9)	543.4 (324.2 to 863.9)	9.1% (-43.4 to 110.5)
Elgeyo-Marakwet	3180.0 (2228.1 to 4428.9)	3766.9 (2465.9 to 5597.2)	18.5% (-30.6 to 102.6)	2662.5 (2016.7 to 3491.1)	3069.5 (2196.4 to 4223.1)	15.3% (-24.8 to 76.9)	473.3 (292.6 to 706.7)	556.7 (336.4 to 868.5)	17.6% (-38.2 to 123.8)
Embu	3242.0 (2297.0 to 4513.6)	3866.5 (2555.9 to 5537.4)	19.3% (-28.5 to 99.3)	2721.0 (2043.5 to 3590.6)	3165.9 (2310.3 to 4305.4)	16.4% (-23.3 to 76.8)	482.6 (304.0 to 713.4)	572.3 (354.6 to 884.4)	18.6% (-36.3 to 120.7)
Garissa	3171.9 (2243.4 to 4369.5)	3627.8 (2396.8 to 5339.4)	14.4% (-32.1 to 93.0)	2666.0 (2023.0 to 3497.6)	2983.8 (2140.4 to 4096.6)	11.9% (-26.8 to 71.4)	463.2 (285.4 to 692.1)	530.1 (312.7 to 832.5)	14.4% (-40.6 to 121.0)
Homa Bay	3633.4 (2565.4 to 5063.2)	4188.6 (2776.9 to 5984.3)	15.3% (-30.8 to 92.4)	2949.1 (2227.3 to 3847.0)	3380.4 (2417.8 to 4594.0)	14.6% (-24.8 to 75.0)	526.9 (330.2 to 797.9)	611.6 (369.6 to 925.8)	16.1% (-38.2 to 118.2)
Isiolo	3267.5 (2314.2 to 4512.7)	3799.8 (2509.2 to 5582.7)	16.3% (-30.9 to 96.2)	2740.3 (2074.4 to 3605.0)	3095.9 (2240.0 to 4259.9)	13.0% (-26.0 to 72.9)	487.9 (300.6 to 738.7)	562.7 (343.2 to 911.0)	15.3% (-40.3 to 123.0)

Kajiado	3327.5 (2362.2 to 4656.9)	3947.8 (2642.2 to 5711.4)	18.6% (-28.9 to 98.3)	2834.7 (2143.1 to 3695.8)	3294.4 (2416.6 to 4452.4)	16.2% (-22.7 to 75.1)	501.6 (310.1 to 756.2)	592.8 (358.9 to 937.1)	18.2% (-38.3 to 126.5)
Kakamega	3291.6 (2302.9 to 4587.0)	3631.2 (2387.4 to 5340.4)	10.3% (-35.0 to 87.7)	2732.4 (2061.9 to 3590.0)	2933.8 (2132.7 to 4023.1)	7.4% (-29.5 to 63.7)	483.7 (302.5 to 727.6)	529.6 (311.3 to 821.2)	9.5% (-42.8 to 110.2)
Kericho	3251.6 (2304.8 to 4543.9)	3920.9 (2566.7 to 5917.6)	20.6% (-29.5 to 106.6)	2717.3 (2058.2 to 3533.0)	3220.3 (2317.6 to 4503.9)	18.5% (-22.7 to 81.9)	483.8 (300.1 to 727.1)	583.9 (345.4 to 908.7)	20.7% (-37.1 to 131.7)
Kiambu	3371.0 (2394.1 to 4730.3)	4037.2 (2711.3 to 5952.7)	19.8% (-28.7 to 101.6)	2864.8 (2166.1 to 3760.6)	3406.7 (2498.6 to 4646.5)	18.9% (-21.4 to 80.1)	508.1 (317.7 to 768.0)	612.6 (370.0 to 978.8)	20.5% (-37.2 to 132.0)
Kilifi	3227.7 (2266.9 to 4503.8)	3853.8 (2518.3 to 5612.4)	19.4% (-29.5 to 102.7)	2712.9 (2043.6 to 3536.5)	3170.4 (2303.1 to 4373.5)	16.9% (-23.3 to 78.4)	477.1 (296.1 to 721.2)	571.0 (347.8 to 904.3)	19.7% (-37.4 to 128.9)
Kirinyaga	3289.0 (2311.5 to 4582.7)	3769.5 (2410.3 to 5504.9)	14.6% (-33.0 to 96.3)	2776.8 (2103.2 to 3657.5)	3101.9 (2216.0 to 4283.2)	11.7% (-27.4 to 72.0)	493.2 (301.2 to 740.6)	559.7 (327.7 to 870.9)	13.5% (-41.3 to 119.6)
Kisii	3211.7 (2258.3 to 4515.4)	3848.4 (2518.4 to 5809.0)	19.8% (-30.4 to 106.6)	2691.6 (2027.7 to 3551.2)	3192.4 (2324.1 to 4425.8)	18.6% (-22.5 to 81.9)	475.7 (296.4 to 713.4)	573.9 (340.8 to 903.6)	20.6% (-37.2 to 132.1)
Kisumu	2772.1 (1743.8 to 4203.6)	3120.1 (1922.1 to 4916.6)	12.6% (-40.6 to 113.3)	2434.0 (1743.5 to 3380.6)	2695.5 (1885.1 to 3857.9)	10.7% (-31.8 to 79.8)	414.6 (240.4 to 658.9)	466.2 (259.4 to 775.1)	12.4% (-46.3 to 135.6)
Kitui	3221.7 (2282.8 to 4458.3)	3722.4 (2451.8 to 5556.2)	15.5% (-31.8 to 96.2)	2698.8 (2041.7 to 3545.6)	2999.6 (2178.7 to 4194.1)	11.1% (-27.6 to 70.8)	478.0 (298.9 to 718.3)	544.1 (327.4 to 859.1)	13.8% (-40.4 to 117.9)
Kwale	3276.9 (2331.2 to 4589.1)	3865.7 (2546.0 to 5570.5)	18.0% (-29.6 to 98.2)	2754.0 (2094.2 to 3599.6)	3159.0 (2263.7 to 4293.2)	14.7% (-24.6 to 74.7)	482.2 (303.3 to 719.4)	568.2 (339.6 to 880.0)	17.8% (-37.8 to 123.7)
Laikipia	3257.6 (2300.7 to 4523.0)	3840.3 (2521.6 to 5682.1)	17.9% (-30.5 to 100.4)	2721.9 (2053.8 to 3549.7)	3116.1 (2258.6 to 4280.4)	14.5% (-24.8 to 74.6)	484.7 (297.5 to 733.1)	565.2 (339.2 to 871.4)	16.6% (-38.9 to 122.8)
Lamu	3254.6 (2314.0 to 4540.5)	3768.9 (2475.1 to 5493.9)	15.8% (-31.3 to 95.6)	2761.5 (2100.2 to 3587.6)	3091.5 (2228.4 to 4198.6)	11.9% (-26.0 to 69.8)	485.9 (295.3 to 731.0)	557.2 (335.3 to 891.1)	14.7% (-40.9 to 122.3)

Machakos	3113.4 (2176.5 to 4349.7)	3886.1 (2566.2 to 5661.5)	24.8% (-26.1 to 111.1)	2619.0 (1972.5 to 3455.8)	3198.0 (2294.3 to 4360.5)	22.1% (-20.2 to 87.0)	463.6 (285.8 to 692.4)	577.8 (349.6 to 910.8)	24.6% (-34.8 to 138.3)
Makueni	3289.5 (2339.3 to 4571.4)	3741.9 (2420.8 to 5545.1)	13.8% (-33.1 to 93.8)	2725.3 (2052.2 to 3566.6)	3031.9 (2173.4 to 4185.9)	11.3% (-27.5 to 71.0)	486.1 (306.3 to 733.4)	549.1 (322.2 to 829.4)	13.0% (-40.4 to 114.1)
Mandera	3276.8 (2299.5 to 4523.1)	3681.9 (2381.5 to 5470.6)	12.4% (-34.2 to 92.1)	2718.6 (2054.9 to 3573.1)	2979.5 (2143.5 to 4166.1)	9.6% (-28.9 to 69.2)	482.0 (301.6 to 726.7)	536.7 (321.0 to 858.8)	11.3% (-42.3 to 115.5)
Marsabit	3253.9 (2309.8 to 4573.1)	3743.5 (2480.0 to 5476.0)	15.0% (-31.8 to 94.4)	2719.4 (2061.1 to 3580.2)	3044.6 (2191.2 to 4130.6)	12.0% (-26.4 to 70.5)	482.9 (299.0 to 723.2)	550.8 (332.1 to 870.8)	14.1% (-40.4 to 118.5)
Meru	3220.8 (2266.8 to 4475.5)	3797.5 (2514.9 to 5573.5)	17.9% (-30.1 to 99.4)	2702.3 (2035.2 to 3547.1)	3104.2 (2256.5 to 4265.1)	14.9% (-24.6 to 75.3)	479.8 (301.1 to 714.7)	560.6 (335.5 to 871.3)	16.8% (-38.4 to 122.0)
Migori	3452.6 (2448.6 to 4824.9)	4060.2 (2688.7 to 6161.7)	17.6% (-31.1 to 101.1)	2845.1 (2150.2 to 3721.2)	3264.5 (2358.3 to 4535.0)	14.7% (-25.1 to 76.1)	504.6 (315.6 to 751.2)	591.7 (354.3 to 948.2)	17.2% (-39.0 to 126.0)
Mombasa	3542.8 (2519.0 to 4905.4)	3947.5 (2629.9 to 5903.1)	11.4% (-34.0 to 88.4)	3074.4 (2332.8 to 4009.2)	3293.9 (2404.8 to 4486.8)	7.1% (-29.0 to 62.0)	534.7 (338.3 to 806.4)	586.2 (355.4 to 925.9)	9.6% (-42.3 to 108.8)
Murang'a	3069.6 (2144.5 to 4229.0)	3589.4 (2341.3 to 5414.9)	16.9% (-31.7 to 100.5)	2592.4 (1959.5 to 3422.3)	2944.6 (2126.8 to 4054.4)	13.6% (-25.8 to 74.1)	458.8 (283.1 to 689.6)	531.3 (311.0 to 834.8)	15.8% (-40.2 to 124.7)
Nairobi	3612.1 (2546.0 to 5090.5)	4004.3 (2668.6 to 5860.4)	10.9% (-34.3 to 87.3)	3176.3 (2408.8 to 4238.9)	3353.0 (2436.6 to 4596.9)	5.6% (-30.9 to 61.5)	558.2 (353.9 to 845.2)	602.5 (361.3 to 932.1)	7.9% (-43.0 to 104.6)
Nakuru	3341.9 (2364.4 to 4590.1)	3849.0 (2508.1 to 5739.4)	15.2% (-32.1 to 95.7)	2815.3 (2123.3 to 3663.7)	3163.0 (2247.5 to 4351.8)	12.4% (-26.8 to 72.6)	500.1 (312.8 to 750.0)	572.7 (327.5 to 897.1)	14.5% (-41.2 to 123.4)
Nandi	3313.0 (2367.9 to 4636.4)	3789.8 (2458.4 to 5607.6)	14.4% (-32.7 to 94.8)	2750.0 (2082.3 to 3597.8)	3087.3 (2212.1 to 4245.9)	12.3% (-26.6 to 72.1)	489.9 (306.4 to 745.8)	560.1 (334.8 to 859.9)	14.3% (-39.9 to 117.7)
Narok	3329.7 (2347.6 to 4595.7)	3733.0 (2434.0 to 5577.0)	12.1% (-34.2 to 91.1)	2767.8 (2096.7 to 3613.3)	3026.0 (2169.0 to 4154.9)	9.3% (-28.4 to 67.3)	492.9 (312.3 to 746.9)	546.9 (325.5 to 870.0)	11.0% (-42.3 to 114.0)

Nyamira	3210.9 (2264.2 to 4435.8)	3993.6 (2619.1 to 5908.5)	24.4% (-26.6 to 111.2)	2697.8 (2036.3 to 3548.7)	3281.8 (2372.7 to 4528.7)	21.6% (-20.5 to 86.6)	478.1 (299.6 to 717.9)	592.8 (354.6 to 923.3)	24.0% (-34.8 to 136.4)
Nyandarua	3302.0 (2335.7 to 4593.5)	3849.1 (2483.5 to 5631.5)	16.6% (-31.5 to 98.6)	2753.1 (2076.9 to 3654.9)	3085.5 (2216.7 to 4238.2)	12.1% (-27.0 to 72.3)	491.6 (309.3 to 737.1)	562.9 (334.0 to 885.1)	14.5% (-40.2 to 120.0)
Nyeri	3228.2 (2282.0 to 4488.6)	3722.4 (2445.3 to 5541.4)	15.3% (-32.2 to 96.4)	2732.9 (2068.5 to 3622.6)	3059.3 (2199.8 to 4210.1)	11.9% (-27.0 to 72.1)	483.6 (299.6 to 729.3)	550.8 (323.3 to 863.3)	13.9% (-41.0 to 120.5)
Samburu	3361.7 (2385.5 to 4697.2)	3748.9 (2488.1 to 5509.0)	11.5% (-33.8 to 88.4)	2816.8 (2131.9 to 3675.0)	3051.6 (2235.7 to 4215.0)	8.3% (-28.6 to 64.8)	501.6 (312.2 to 756.1)	552.1 (333.9 to 862.3)	10.1% (-42.2 to 109.6)
Siaya	3377.6 (2397.1 to 4757.8)	3843.8 (2555.9 to 5632.2)	13.8% (-32.5 to 92.2)	2788.6 (2117.1 to 3692.4)	3114.5 (2266.1 to 4256.3)	11.7% (-26.5 to 70.0)	496.0 (306.6 to 749.9)	563.2 (333.9 to 878.3)	13.6% (-41.0 to 118.6)
Taita Taveta	3275.1 (2322.3 to 4613.7)	3849.2 (2566.3 to 5641.4)	17.5% (-30.2 to 98.2)	2746.8 (2064.9 to 3600.1)	3155.7 (2299.0 to 4317.3)	14.9% (-24.4 to 74.9)	486.1 (304.7 to 719.1)	569.4 (345.4 to 892.7)	17.2% (-38.0 to 121.8)
Tana River	3257.8 (2304.1 to 4472.2)	3829.3 (2465.7 to 5500.7)	17.5% (-30.2 to 98.1)	2702.9 (2039.3 to 3539.1)	3105.3 (2208.5 to 4217.3)	14.9% (-24.8 to 76.0)	477.9 (297.6 to 720.8)	560.3 (327.2 to 865.8)	17.3% (-39.0 to 125.8)
Tharaka Nithi	3343.7 (2351.6 to 4574.8)	3819.2 (2520.0 to 5691.8)	14.2% (-32.4 to 93.4)	2781.8 (2101.8 to 3623.0)	3111.7 (2250.4 to 4277.0)	11.9% (-26.5 to 70.7)	495.8 (306.8 to 748.0)	564.6 (329.0 to 889.2)	13.9% (-41.4 to 122.0)
Trans Nzoia	3247.1 (2315.6 to 4483.0)	3685.8 (2360.6 to 5451.0)	13.5% (-33.2 to 93.4)	2706.5 (2055.8 to 3538.9)	2996.0 (2134.2 to 4116.6)	10.7% (-27.7 to 69.8)	481.9 (305.6 to 725.3)	542.7 (326.2 to 866.1)	12.6% (-41.2 to 116.0)
Turkana	3349.1 (2340.8 to 4670.2)	3721.6 (2412.8 to 5466.5)	11.1% (-34.9 to 90.2)	2816.2 (2117.4 to 3730.5)	3040.8 (2184.5 to 4196.4)	8.0% (-29.9 to 66.5)	498.7 (307.4 to 754.7)	549.5 (329.4 to 859.0)	10.2% (-42.6 to 111.6)
Uasin Gishu	3241.3 (2271.7 to 4524.1)	3806.1 (2481.4 to 5699.8)	17.4% (-31.6 to 101.9)	2754.4 (2085.4 to 3611.2)	3163.9 (2285.1 to 4381.4)	14.9% (-25.0 to 76.1)	487.2 (299.5 to 737.0)	569.9 (335.9 to 892.6)	17.0% (-39.5 to 126.4)
Vihiga	3191.9 (2215.1 to 4548.2)	3592.5 (2296.6 to 5304.6)	12.6% (-35.1 to 95.9)	2677.9 (2011.1 to 3566.8)	2891.0 (2071.4 to 3970.2)	8.0% (-29.9 to 66.6)	472.3 (292.0 to 719.2)	522.4 (303.9 to 821.4)	10.6% (-43.2 to 115.9)

Wajir	3166.5 (2243.5 to 4394.3)	3672.4 (2415.0 to 5318.7)	16.0% (-30.9 to 95.2)	2640.3 (1989.3 to 3423.9)	2996.4 (2200.0 to 4067.3)	13.5% (-24.6 to 71.0)	464.3 (287.6 to 698.1)	539.1 (321.6 to 836.9)	16.1% (-39.3 to 121.9)
West Pokot	3361.6 (2378.0 to 4719.5)	3679.2 (2412.2 to 5372.8)	9.4% (-35.3 to 85.7)	2829.4 (2124.1 to 3709.7)	2981.1 (2143.3 to 4096.6)	5.4% (-31.2 to 61.8)	503.0 (314.5 to 755.4)	538.2 (326.3 to 824.3)	7.0% (-43.2 to 101.6)

UI, uncertainly interval; DALYs, disability-adjusted life-years.

Table S13 The incidence, prevalence and DALYs rate of anxiety and depressive disorders in 1990 and 2021, and changes from 1990 to 2021 in Mexico.

Location	Incidence (95% UI)			Prevalence (95% UI)			DALYs (95% UI)		
	**1990**	**2021**	**Percentage change**	**1990**	**2021**	**Percentage change**	**1990**	**2021**	**Percentage change**
Anxiety disorders									
Aguascalientes	571.7 (388.9 to 783.2)	966.2 (605.0 to 1434.1)	69.0% (-2.9 to 194.6)	3182.9 (2399.9 to 4160.6)	5382.8 (3688.0 to 7641.4)	69.1% (7.5 to 167.0)	392.1 (246.1 to 571.8)	663.9 (391.4 to 1042.7)	69.3% (-11.2 to 223.9)
Baja California	562.8 (369.9 to 778.7)	947.2 (566.7 to 1402.0)	68.3% (-6.3 to 202.8)	3187.4 (2391.7 to 4122.3)	5264.9 (3566.1 to 7382.4)	65.2% (5.1 to 160.1)	393.4 (247.0 to 585.1)	648.9 (378.4 to 1049.6)	64.9% (-15.4 to 222.5)
Baja California Sur	562.0 (376.1 to 773.0)	970.4 (599.8 to 1452.1)	72.7% (-2.2 to 205.5)	3158.4 (2397.8 to 4141.9)	5363.9 (3592.3 to 7729.7)	69.8% (6.3 to 171.5)	388.7 (244.6 to 575.2)	659.5 (373.1 to 1044.8)	69.6% (-13.2 to 232.1)
Campeche	566.2 (377.4 to 777.8)	988.3 (606.5 to 1453.3)	74.5% (-1.1 to 208.3)	3159.9 (2371.3 to 4100.5)	5467.7 (3739.9 to 7852.2)	73.0% (9.5 to 174.1)	389.5 (241.8 to 585.5)	671.8 (389.1 to 1047.6)	72.5% (-10.9 to 235.2)
Chiapas	566.9 (377.4 to 786.7)	902.2 (555.2 to 1333.8)	59.2% (-10.2 to 182.5)	3143.3 (2346.1 to 4098.6)	4946.4 (3318.2 to 6945.8)	57.4% (-0.6 to 150.0)	386.6 (237.4 to 577.0)	609.2 (350.3 to 937.6)	57.6% (-18.4 to 205.3)
Chihuahua	561.2 (371.4 to 783.7)	980.4 (607.4 to 1442.9)	74.7% (-1.3 to 209.8)	3174.7 (2374.0 to 4119.3)	5434.0 (3680.2 to 7821.8)	71.2% (7.6 to 173.0)	390.3 (242.9 to 578.6)	667.9 (391.3 to 1040.9)	71.1% (-10.8 to 229.0)
Coahuila	564.8 (376.8 to 783.9)	955.8 (584.9 to 1400.3)	69.2% (-4.3 to 199.8)	3172.6 (2390.2 to 4133.3)	5280.7 (3587.9 to 7476.9)	66.4% (5.5 to 163.0)	390.3 (244.8 to 572.8)	651.2 (364.7 to 1005.9)	66.8% (-14.0 to 224.2)
Colima	567.1 (383.9 to 783.4)	947.1 (571.5 to 1410.8)	67.0% (-5.8 to 196.9)	3170.1 (2379.6 to 4146.7)	5253.6 (3644.6 to 7455.7)	65.7% (5.6 to 160.7)	390.4 (242.1 to 580.2)	647.1 (376.6 to 1016.9)	65.8% (-14.3 to 221.4)

Durango	569.1 (384.8 to 778.8)	906.6 (554.7 to 1329.3)	59.3% (-9.0 to 179.1)	3150.7 (2344.7 to 4101.6)	4984.9 (3393.6 to 6952.9)	58.2% (0.6 to 149.3)	388.9 (236.8 to 573.6)	615.4 (355.1 to 989.5)	58.2% (-19.5 to 211.9)
Guanajuato	569.2 (381.7 to 781.4)	974.2 (605.3 to 1455.0)	71.2% (-2.7 to 201.8)	3171.7 (2379.3 to 4139.5)	5377.1 (3647.8 to 7623.7)	69.5% (7.3 to 168.8)	391.1 (242.4 to 580.6)	661.7 (386.0 to 1028.4)	69.2% (-12.0 to 226.3)
Guerrero	569.7 (385.2 to 787.2)	975.9 (611.2 to 1458.2)	71.3% (-2.4 to 201.0)	3144.8 (2328.9 to 4074.5)	5358.1 (3596.8 to 7503.1)	70.4% (7.6 to 170.6)	387.4 (238.8 to 576.4)	658.9 (388.8 to 1013.0)	70.1% (-10.9 to 224.7)
Hidalgo	569.0 (384.7 to 789.2)	995.3 (593.9 to 1458.8)	74.9% (-1.3 to 210.8)	3147.2 (2345.1 to 4102.0)	5472.1 (3641.3 to 7721.0)	73.9% (9.1 to 177.5)	387.8 (239.8 to 575.0)	673.8 (379.6 to 1036.0)	73.8% (-10.6 to 238.4)
Jalisco	567.7 (381.7 to 777.3)	946.5 (600.0 to 1398.0)	66.7% (-4.0 to 190.3)	3180.5 (2393.3 to 4156.5)	5258.3 (3606.5 to 7314.3)	65.3% (5.8 to 158.9)	392.2 (243.2 to 582.5)	649.1 (380.5 to 987.8)	65.5% (-12.9 to 214.9)
México	566.5 (378.8 to 786.7)	1040.9 (640.8 to 1546.8)	83.8% (3.6 to 226.4)	3177.1 (2393.0 to 4140.7)	5774.9 (3945.3 to 8189.2)	81.8% (15.5 to 186.9)	392.5 (243.7 to 589.1)	712.8 (430.0 to 1115.6)	81.6% (-4.7 to 246.3)
Mexico City	578.5 (377.9 to 801.7)	1161.6 (704.3 to 1763.9)	100.8% (11.0 to 263.8)	3420.8 (2640.1 to 4457.5)	6473.7 (4416.2 to 9167.4)	89.2% (21.0 to 196.3)	421.1 (264.6 to 624.2)	793.9 (467.1 to 1220.7)	88.5% (-0.7 to 259.1)
Michoacán de Ocampo	572.5 (384.1 to 792.6)	960.6 (583.8 to 1408.0)	67.8% (-5.1 to 197.0)	3177.8 (2370.0 to 4167.4)	5314.9 (3639.7 to 7512.8)	67.2% (5.9 to 164.7)	391.6 (245.6 to 579.8)	655.4 (382.9 to 1039.3)	67.4% (-13.3 to 224.1)
Morelos	568.6 (379.5 to 774.8)	1026.0 (634.6 to 1513.3)	80.5% (2.9 to 216.9)	3177.0 (2387.8 to 4139.7)	5732.8 (3904.8 to 8055.9)	80.4% (14.8 to 184.5)	391.4 (244.8 to 577.9)	706.0 (408.9 to 1110.2)	80.4% (-6.6 to 249.3)
Nayarit	569.7 (385.2 to 786.3)	928.2 (575.4 to 1392.6)	62.9% (-7.5 to 187.4)	3151.6 (2365.6 to 4097.0)	5114.9 (3473.4 to 7220.6)	62.3% (3.1 to 156.4)	388.5 (239.1 to 573.0)	630.3 (369.7 to 994.0)	62.2% (-15.9 to 213.7)
Nuevo León	559.6 (370.8 to 776.0)	1014.4 (608.4 to 1509.8)	81.3% (1.0 to 225.6)	3165.3 (2383.6 to 4124.3)	5661.3 (3871.7 to 8034.1)	78.9% (13.7 to 182.3)	390.9 (243.3 to 585.6)	695.8 (409.7 to 1055.8)	78.0% (-6.3 to 238.0)
Oaxaca	570.1 (386.7 to 783.5)	974.4 (596.6 to 1469.3)	70.9% (-3.3 to 203.0)	3144.9 (2360.9 to 4116.2)	5349.5 (3655.2 to 7531.8)	70.1% (8.1 to 168.7)	387.1 (241.3 to 573.5)	660.7 (393.7 to 1035.4)	70.7% (-10.5 to 226.7)

Puebla	569.5 (382.0 to 788.4)	1069.1 (667.7 to 1574.0)	87.7% (7.1 to 229.8)	3159.0 (2377.5 to 4129.4)	5893.4 (4013.6 to 8287.5)	86.6% (18.6 to 194.4)	388.5 (237.5 to 567.1)	725.3 (418.9 to 1099.4)	86.7% (-2.1 to 256.9)
Querétaro	571.9 (381.0 to 784.6)	960.2 (594.1 to 1402.6)	67.9% (-4.2 to 194.9)	3180.2 (2393.4 to 4142.7)	5331.7 (3680.5 to 7547.0)	67.7% (7.0 to 163.6)	391.8 (244.3 to 582.2)	657.5 (384.8 to 1039.5)	67.8% (-13.1 to 224.7)
Quintana Roo	558.1 (372.0 to 761.2)	966.8 (578.1 to 1432.4)	73.2% (-2.5 to 208.7)	3150.5 (2372.0 to 4093.7)	5398.7 (3695.5 to 7545.2)	71.4% (9.7 to 168.8)	388.9 (240.1 to 577.1)	664.3 (383.2 to 1046.3)	70.8% (-12.1 to 232.9)
San Luis Potosí	569.0 (382.3 to 786.7)	1003.1 (617.4 to 1491.7)	76.3% (-0.2 to 211.9)	3168.9 (2389.3 to 4117.6)	5560.2 (3779.1 to 7787.6)	75.5% (12.0 to 175.8)	390.4 (241.5 to 579.8)	684.5 (402.2 to 1079.0)	75.3% (-9.1 to 239.1)
Sinaloa	566.4 (381.6 to 775.3)	990.6 (604.4 to 1454.0)	74.9% (-0.4 to 207.4)	3151.5 (2346.6 to 4100.2)	5476.1 (3651.6 to 7691.3)	73.8% (9.4 to 176.6)	388.7 (240.2 to 568.5)	673.7 (388.3 to 1038.2)	73.3% (-9.7 to 233.3)
Sonora	562.7 (374.4 to 770.1)	982.7 (597.1 to 1470.8)	74.6% (-1.7 to 210.9)	3161.8 (2374.8 to 4143.5)	5421.1 (3645.7 to 7506.7)	71.5% (9.0 to 170.6)	388.7 (244.4 to 575.5)	667.9 (383.6 to 1035.8)	71.8% (-10.7 to 231.6)
Tabasco	564.7 (378.4 to 787.7)	950.2 (579.9 to 1414.3)	68.3% (-5.5 to 199.9)	3154.4 (2384.2 to 4088.3)	5244.3 (3545.7 to 7511.4)	66.3% (5.0 to 163.7)	388.5 (239.7 to 573.9)	644.9 (364.6 to 1024.3)	66.0% (-15.6 to 227.3)
Tamaulipas	565.5 (375.4 to 805.7)	982.9 (614.1 to 1481.1)	73.8% (-2.8 to 211.7)	3180.7 (2395.4 to 4158.0)	5440.4 (3679.9 to 7561.3)	71.0% (9.0 to 169.5)	390.8 (239.4 to 582.7)	668.5 (398.8 to 1032.9)	71.1% (-10.4 to 226.7)
Tlaxcala	566.7 (367.2 to 784.8)	1039.6 (635.9 to 1553.8)	83.4% (2.1 to 230.6)	3150.6 (2333.5 to 4160.6)	5748.5 (3894.3 to 8061.4)	82.5% (14.9 to 190.3)	387.6 (240.0 to 576.2)	710.0 (403.2 to 1108.8)	83.2% (-6.1 to 258.0)
Veracruz de Ignacio de la Llave	565.9 (374.4 to 778.8)	999.1 (604.3 to 1494.5)	76.5% (-1.2 to 215.9)	3159.5 (2362.1 to 4118.6)	5502.7 (3668.4 to 7826.5)	74.2% (9.2 to 178.5)	388.2 (240.4 to 577.0)	677.1 (388.7 to 1089.5)	74.4% (-11.2 to 244.0)
Yucatán	566.8 (382.1 to 786.0)	1059.1 (636.9 to 1573.0)	86.9% (5.1 to 233.1)	3161.3 (2374.8 to 4129.7)	5869.8 (3955.6 to 8244.4)	85.7% (17.7 to 194.1)	390.4 (239.6 to 579.0)	723.8 (418.2 to 1108.0)	85.4% (-3.5 to 257.0)
Zacatecas	571.3 (382.3 to 792.5)	976.6 (607.6 to 1465.5)	70.9% (-3.5 to 203.3)	3162.8 (2357.6 to 4114.0)	5389.7 (3678.6 to 7723.7)	70.4% (7.6 to 170.9)	390.2 (237.3 to 581.2)	663.9 (391.7 to 1036.7)	70.2% (-11.7 to 228.3)
Depressive disorders									

Aguascalientes	2249.4 (1593.0 to 3133.7)	4276.9 (2809.2 to 6183.2)	90.1% (13.2 to 220.3)	1831.1 (1372.4 to 2426.9)	3128.8 (2198.9 to 4408.6)	70.9% (9.1 to 168.1)	331.6 (204.3 to 506.6)	602.4 (356.1 to 957.8)	81.6% (-6.7 to 254.1)
Baja California	2287.0 (1627.1 to 3157.0)	3865.4 (2593.2 to 5713.0)	69.0% (0.9 to 183.6)	1889.0 (1415.8 to 2479.8)	2884.9 (2082.7 to 4065.8)	52.7% (-1.3 to 136.6)	340.2 (208.0 to 505.9)	549.0 (318.0 to 857.8)	61.4% (-16.8 to 214.2)
Baja California Sur	2372.2 (1703.4 to 3272.2)	4382.1 (2916.7 to 6356.9)	84.7% (11.1 to 207.6)	1923.7 (1462.7 to 2517.8)	3201.7 (2263.6 to 4511.9)	66.4% (7.5 to 158.2)	349.2 (217.6 to 522.0)	616.8 (370.7 to 985.7)	76.6% (-8.1 to 240.5)
Campeche	2547.8 (1834.2 to 3533.6)	5147.2 (3465.1 to 7450.9)	102.0% (22.1 to 234.9)	2017.0 (1519.7 to 2662.8)	3673.0 (2610.7 to 5154.1)	82.1% (17.2 to 183.2)	370.4 (232.5 to 554.6)	718.1 (434.5 to 1106.7)	93.9% (2.8 to 265.4)
Chiapas	2124.8 (1503.0 to 2933.4)	3658.6 (2450.7 to 5326.8)	72.2% (3.2 to 187.9)	1738.6 (1312.6 to 2291.1)	2713.2 (1937.9 to 3807.2)	56.1% (0.7 to 142.0)	312.3 (194.5 to 475.6)	517.3 (302.6 to 813.6)	65.6% (-14.6 to 221.9)
Chihuahua	2386.6 (1683.2 to 3277.1)	4515.2 (3060.2 to 6572.6)	89.2% (14.1 to 214.2)	1940.0 (1457.9 to 2539.8)	3278.8 (2355.5 to 4596.4)	69.0% (9.4 to 161.4)	351.0 (221.9 to 530.4)	633.5 (376.5 to 1009.6)	80.5% (-6.3 to 248.4)
Coahuila	2263.9 (1599.5 to 3130.3)	4145.6 (2733.2 to 6087.4)	83.1% (8.6 to 209.4)	1849.7 (1390.6 to 2439.0)	3045.5 (2125.2 to 4293.5)	64.7% (5.2 to 158.2)	334.4 (207.3 to 503.7)	584.9 (338.9 to 939.1)	74.9% (-10.9 to 244.0)
Colima	2276.9 (1634.6 to 3165.8)	4082.9 (2758.8 to 5877.5)	79.3% (8.7 to 196.3)	1848.9 (1395.3 to 2431.3)	3007.8 (2172.6 to 4172.9)	62.7% (6.0 to 150.1)	334.2 (208.4 to 502.2)	577.3 (355.5 to 891.5)	72.8% (-8.0 to 224.7)
Durango	2140.7 (1487.1 to 2952.4)	3698.8 (2419.2 to 5500.5)	72.8% (1.2 to 195.7)	1750.2 (1303.5 to 2304.7)	2753.0 (1940.6 to 3798.0)	57.3% (1.3 to 144.8)	315.1 (194.1 to 480.8)	524.8 (305.2 to 812.5)	66.6% (-14.2 to 223.3)
Guanajuato	2167.8 (1520.3 to 2978.1)	4549.7 (2943.8 to 6620.0)	109.9% (23.9 to 256.0)	1771.7 (1335.8 to 2342.3)	3305.0 (2305.2 to 4660.4)	86.5% (19.2 to 192.6)	319.5 (197.7 to 480.7)	639.5 (387.7 to 1016.2)	100.2% (4.4 to 283.9)
Guerrero	2052.4 (1435.8 to 2858.4)	3947.9 (2585.5 to 5757.7)	92.4% (13.5 to 226.8)	1685.0 (1270.4 to 2238.4)	2898.3 (2035.1 to 4056.2)	72.0% (10.2 to 169.0)	302.0 (184.3 to 456.9)	554.7 (320.9 to 853.0)	83.7% (-5.3 to 256.3)
Hidalgo	2110.6 (1478.0 to 2895.0)	4096.5 (2653.2 to 6056.3)	94.1% (14.1 to 230.6)	1727.5 (1301.2 to 2266.0)	2999.2 (2076.8 to 4224.1)	73.6% (10.9 to 172.4)	310.3 (190.5 to 468.4)	574.5 (343.1 to 925.5)	85.1% (-4.8 to 261.0)

Jalisco	2266.6 (1636.8 to 3156.9)	4365.3 (2837.0 to 6383.3)	92.6% (14.4 to 224.5)	1841.7 (1404.2 to 2452.1)	3190.3 (2233.8 to 4509.3)	73.2% (10.9 to 171.2)	333.7 (205.1 to 504.9)	616.8 (366.1 to 989.5)	84.9% (-5.1 to 260.8)
México	2147.4 (1523.0 to 2975.6)	4580.0 (3032.8 to 6673.2)	113.3% (27.2 to 258.6)	1778.6 (1351.3 to 2361.1)	3324.4 (2370.4 to 4702.0)	86.9% (20.3 to 190.8)	319.0 (196.3 to 487.0)	642.6 (375.6 to 1022.3)	101.5% (2.9 to 295.3)
Mexico City	2572.3 (1872.6 to 3540.0)	5224.4 (3426.0 to 7703.1)	103.1% (21.6 to 240.0)	2083.2 (1594.2 to 2756.3)	3748.8 (2605.8 to 5343.5)	80.0% (14.9 to 182.8)	379.5 (235.0 to 575.9)	728.4 (427.3 to 1176.6)	91.9% (-2.1 to 277.2)
Michoacán de Ocampo	2214.7 (1567.2 to 3067.0)	4153.2 (2698.4 to 6101.9)	87.5% (10.6 to 218.3)	1794.5 (1353.3 to 2362.7)	3040.4 (2120.0 to 4284.0)	69.4% (8.4 to 165.3)	324.9 (198.5 to 487.4)	585.3 (335.8 to 934.5)	80.1% (-8.6 to 256.1)
Morelos	2204.6 (1562.3 to 3043.9)	4488.4 (2990.0 to 6585.9)	103.6% (21.4 to 242.0)	1805.8 (1361.7 to 2404.5)	3263.9 (2259.5 to 4529.3)	80.7% (15.4 to 183.4)	324.9 (201.7 to 494.1)	630.3 (363.9 to 985.7)	94.0% (-0.4 to 278.6)
Nayarit	2124.3 (1505.8 to 2933.6)	3846.9 (2512.9 to 5568.0)	81.1% (7.7 to 204.9)	1737.7 (1321.9 to 2296.1)	2842.0 (1984.8 to 3927.1)	63.6% (5.6 to 153.6)	311.9 (194.9 to 468.8)	542.7 (319.3 to 855.1)	74.0% (-9.8 to 236.7)
Nuevo León	2234.1 (1584.2 to 3069.1)	4484.8 (2998.8 to 6609.8)	100.7% (19.9 to 236.7)	1846.9 (1397.1 to 2450.6)	3273.9 (2307.5 to 4574.6)	77.3% (13.9 to 176.2)	333.1 (204.7 to 506.5)	633.3 (384.8 to 981.7)	90.1% (-0.2 to 262.5)
Oaxaca	2061.6 (1463.3 to 2845.4)	3865.4 (2555.9 to 5641.1)	87.5% (11.8 to 215.0)	1686.7 (1273.0 to 2231.6)	2842.3 (1983.8 to 3989.3)	68.5% (7.8 to 163.7)	303.1 (187.4 to 453.3)	544.5 (313.4 to 857.1)	79.6% (-7.9 to 251.1)
Puebla	2116.2 (1494.9 to 2941.9)	4543.2 (3021.0 to 6659.4)	114.7% (27.7 to 261.8)	1735.2 (1307.7 to 2294.6)	3289.6 (2320.8 to 4660.0)	89.6% (21.3 to 196.6)	312.3 (193.5 to 474.7)	635.3 (375.8 to 1014.3)	103.4% (4.6 to 296.4)
Querétaro	2254.6 (1592.6 to 3113.2)	4387.9 (2891.6 to 6426.6)	94.6% (15.5 to 228.4)	1830.8 (1381.6 to 2439.0)	3214.2 (2266.8 to 4516.7)	75.6% (12.3 to 174.7)	331.0 (210.2 to 495.3)	619.7 (362.8 to 991.8)	87.2% (-3.2 to 263.4)
Quintana Roo	2456.6 (1745.5 to 3426.3)	5129.0 (3436.8 to 7694.3)	108.8% (23.4 to 253.8)	1984.6 (1505.3 to 2663.6)	3692.1 (2597.9 to 5243.2)	86.0% (18.4 to 192.5)	361.7 (225.9 to 539.6)	719.4 (432.1 to 1097.3)	98.9% (5.6 to 274.7)
San Luis Potosí	2186.9 (1547.8 to 3057.7)	4500.0 (2954.8 to 6561.0)	105.8% (21.9 to 248.3)	1778.8 (1327.4 to 2352.3)	3264.4 (2300.9 to 4536.6)	83.5% (17.7 to 186.5)	321.7 (197.6 to 477.6)	632.0 (367.9 to 994.3)	96.5% (1.3 to 282.0)

Sinaloa	2111.3 (1474.3 to 2916.9)	3977.9 (2578.4 to 5933.5)	88.4% (10.1 to 223.1)	1742.7 (1319.7 to 2295.7)	2941.6 (2051.1 to 4187.9)	68.8% (7.7 to 165.2)	312.7 (189.7 to 469.1)	562.0 (339.5 to 910.5)	79.8% (-7.5 to 249.8)
Sonora	2322.1 (1666.5 to 3205.7)	4286.3 (2836.6 to 6394.3)	84.6% (9.7 to 210.8)	1889.7 (1416.9 to 2493.4)	3130.6 (2208.6 to 4496.3)	65.7% (5.4 to 160.8)	341.3 (213.0 to 511.8)	602.8 (348.9 to 975.2)	76.6% (-10.0 to 247.9)
Tabasco	2553.0 (1834.0 to 3530.4)	4571.1 (3041.8 to 6650.9)	79.1% (7.5 to 198.8)	2014.6 (1526.9 to 2661.7)	3298.7 (2339.4 to 4580.3)	63.7% (5.8 to 153.6)	370.0 (225.9 to 563.3)	639.3 (375.2 to 998.8)	72.8% (-11.2 to 236.0)
Tamaulipas	2287.1 (1630.5 to 3123.4)	4042.0 (2685.0 to 5902.7)	76.7% (6.0 to 194.9)	1872.8 (1413.5 to 2442.6)	2971.8 (2109.1 to 4155.6)	58.7% (2.8 to 145.2)	337.3 (207.4 to 512.9)	569.7 (333.4 to 907.3)	68.9% (-13.6 to 231.1)
Tlaxcala	2160.0 (1517.5 to 3000.5)	4377.5 (2900.0 to 6390.4)	102.7% (20.4 to 242.0)	1767.6 (1326.0 to 2299.7)	3188.0 (2264.3 to 4431.4)	80.4% (16.9 to 178.6)	318.4 (193.7 to 485.0)	614.7 (356.2 to 975.6)	93.1% (-1.9 to 280.7)
Veracruz de Ignacio de la Llave	2208.5 (1585.2 to 3007.5)	4442.8 (2941.7 to 6468.3)	101.2% (21.2 to 234.3)	1804.8 (1359.5 to 2358.9)	3219.6 (2262.8 to 4522.1)	78.4% (14.9 to 177.6)	323.6 (200.6 to 482.1)	621.3 (372.4 to 995.9)	92.0% (-0.4 to 271.3)
Yucatán	2379.0 (1682.6 to 3274.9)	5478.9 (3644.4 to 8050.2)	130.3% (37.2 to 287.1)	1909.6 (1434.1 to 2517.3)	3892.3 (2738.6 to 5489.6)	103.8% (30.5 to 218.7)	348.4 (216.0 to 517.1)	762.8 (467.6 to 1191.6)	118.9% (16.0 to 313.3)
Zacatecas	2111.5 (1484.9 to 2918.5)	3978.7 (2636.9 to 5815.6)	88.4% (12.1 to 217.6)	1728.2 (1294.4 to 2292.6)	2918.5 (2072.8 to 4046.6)	68.9% (8.8 to 162.6)	311.7 (192.9 to 469.5)	560.6 (325.2 to 894.8)	79.9% (-8.1 to 253.1)

UI, uncertainly interval; DALYs, disability-adjusted life-years.

Table S14 The incidence, prevalence and DALYs rate of anxiety and depressive disorders in 1990 and 2021, and changes from 1990 to 2021 in Nigeria.

**Location**	**Incidence (95% UI)**			**Prevalence (95% UI)**			**DALYs (95% UI)**		
	**1990**	**2021**	**Percentage change**	**1990**	**2021**	**Percentage change**	**1990**	**2021**	**Percentage change**
Anxiety disorders									
Abia	607.7 (404.9 to 827.0)	650.2 (400.8 to 971.4)	7.0% (-39.3 to 88.9)	3441.8 (2596.2 to 4501.9)	3685.3 (2493.6 to 5246.1)	7.1% (-32.4 to 69.9)	418.5 (259.9 to 617.2)	451.9 (258.8 to 729.6)	8.0% (-45.0 to 112.6)
Adamawa	608.7 (409.5 to 825.6)	656.9 (404.0 to 987.7)	7.9% (-38.7 to 90.4)	3465.9 (2626.1 to 4501.4)	3652.9 (2491.9 to 5300.2)	5.4% (-33.5 to 67.3)	420.6 (264.5 to 631.4)	448.0 (262.5 to 723.7)	6.5% (-45.3 to 108.3)

Akwa Ibom	615.7 (409.5 to 843.3)	643.6 (394.6 to 974.5)	4.5% (-41.2 to 86.4)	3537.6 (2673.9 to 4583.7)	3671.5 (2472.2 to 5351.8)	3.8% (-35.1 to 66.1)	428.0 (271.2 to 637.2)	447.3 (258.6 to 689.9)	4.5% (-45.4 to 100.4)
Anambra	615.4 (403.7 to 846.0)	657.0 (400.4 to 1002.2)	6.8% (-40.7 to 92.3)	3501.4 (2646.0 to 4599.9)	3735.2 (2518.4 to 5406.1)	6.7% (-33.3 to 70.8)	426.3 (266.1 to 620.5)	456.2 (256.2 to 709.5)	7.0% (-44.8 to 108.0)
Bauchi	612.0 (402.0 to 830.5)	661.5 (415.5 to 984.9)	8.1% (-38.5 to 90.3)	3457.2 (2605.1 to 4506.2)	3688.0 (2526.7 to 5271.7)	6.7% (-32.4 to 68.6)	417.0 (259.2 to 617.5)	448.2 (269.4 to 724.8)	7.5% (-44.2 to 107.7)
Bayelsa	611.6 (416.6 to 854.1)	652.4 (397.8 to 983.1)	6.7% (-39.9 to 90.0)	3461.0 (2588.4 to 4528.4)	3679.3 (2504.1 to 5297.8)	6.3% (-33.2 to 69.6)	418.6 (262.8 to 617.2)	450.7 (262.7 to 717.5)	7.7% (-44.3 to 108.8)
Benue	614.3 (423.4 to 846.2)	651.6 (415.1 to 980.7)	6.1% (-38.8 to 84.1)	3443.1 (2599.4 to 4502.5)	3696.1 (2519.3 to 5251.6)	7.3% (-32.0 to 69.7)	419.5 (258.4 to 620.7)	453.6 (257.8 to 720.2)	8.1% (-44.9 to 113.0)
Borno	606.6 (407.8 to 824.7)	662.3 (414.9 to 988.3)	9.2% (-37.5 to 90.9)	3457.3 (2605.5 to 4494.2)	3681.8 (2531.2 to 5368.7)	6.5% (-32.9 to 69.3)	419.3 (261.1 to 623.9)	449.1 (266.4 to 700.9)	7.1% (-43.9 to 105.0)
Cross River	614.4 (414.9 to 857.6)	657.6 (412.8 to 959.8)	7.0% (-38.6 to 87.1)	3517.8 (2637.5 to 4584.2)	3737.1 (2582.2 to 5259.0)	6.2% (-32.1 to 66.7)	426.8 (263.8 to 629.5)	457.9 (265.4 to 715.4)	7.3% (-44.4 to 107.6)
Delta	615.4 (409.5 to 847.4)	649.1 (403.8 to 971.2)	5.5% (-40.4 to 86.9)	3509.1 (2640.3 to 4554.8)	3699.8 (2490.9 to 5252.7)	5.4% (-33.4 to 67.3)	425.1 (263.8 to 621.8)	451.5 (257.8 to 695.3)	6.2% (-44.8 to 104.9)
Ebonyi	614.0 (403.9 to 832.7)	651.0 (397.6 to 966.9)	6.0% (-40.1 to 88.1)	3469.1 (2611.8 to 4521.2)	3675.6 (2490.9 to 5228.6)	6.0% (-33.0 to 67.9)	418.2 (262.5 to 618.2)	446.2 (257.5 to 705.1)	6.7% (-44.9 to 107.3)
Edo	613.8 (410.6 to 848.6)	644.6 (388.2 to 956.4)	5.0% (-41.0 to 87.2)	3475.0 (2604.5 to 4520.1)	3708.9 (2528.0 to 5227.9)	6.7% (-32.2 to 68.5)	421.5 (265.1 to 622.6)	452.2 (259.1 to 699.4)	7.3% (-44.2 to 107.0)
Ekiti	610.4 (415.3 to 846.5)	656.6 (400.8 to 1016.1)	7.6% (-39.9 to 93.5)	3460.9 (2593.3 to 4538.2)	3713.4 (2508.1 to 5378.4)	7.3% (-32.9 to 72.1)	420.8 (259.7 to 619.6)	453.3 (261.7 to 730.9)	7.7% (-45.0 to 111.7)
Enugu	615.7 (410.8 to 862.5)	656.8 (396.0 to 963.9)	6.7% (-40.2 to 90.8)	3458.4 (2625.4 to 4516.5)	3695.9 (2474.3 to 5316.8)	6.9% (-33.0 to 70.5)	420.9 (268.4 to 614.7)	452.4 (262.9 to 716.2)	7.5% (-43.9 to 106.3)

FCT (Abuja)	599.1 (391.4 to 824.5)	650.1 (405.8 to 994.4)	8.5% (-39.4 to 94.8)	3468.3 (2590.0 to 4536.4)	3741.4 (2536.8 to 5468.1)	7.9% (-32.8 to 73.5)	421.4 (262.4 to 618.9)	458.1 (272.7 to 724.2)	8.7% (-43.1 to 108.4)
Gombe	604.0 (401.4 to 837.3)	667.2 (410.7 to 985.1)	10.5% (-37.6 to 96.0)	3396.2 (2583.0 to 4435.5)	3739.9 (2541.4 to 5354.2)	10.1% (-30.3 to 74.4)	411.7 (260.2 to 604.3)	456.9 (261.7 to 710.3)	11.0% (-42.2 to 113.7)
Imo	614.1 (419.7 to 836.1)	644.7 (391.2 to 959.8)	5.0% (-40.1 to 85.1)	3462.7 (2607.2 to 4502.6)	3659.7 (2442.0 to 5240.7)	5.7% (-33.7 to 68.8)	420.2 (257.5 to 623.0)	448.3 (257.0 to 729.6)	6.7% (-46.1 to 111.9)
Jigawa	612.9 (401.5 to 832.8)	656.8 (417.5 to 961.0)	7.2% (-38.3 to 86.5)	3478.4 (2622.9 to 4534.5)	3644.7 (2472.3 to 5188.2)	4.8% (-33.7 to 66.0)	417.2 (258.2 to 610.0)	442.8 (257.0 to 695.2)	6.1% (-44.9 to 105.1)
Kaduna	609.3 (411.3 to 836.3)	658.2 (401.3 to 1009.3)	8.0% (-39.4 to 93.5)	3432.6 (2576.0 to 4483.5)	3716.9 (2486.4 to 5387.1)	8.3% (-32.5 to 74.0)	418.0 (256.2 to 627.1)	454.7 (257.5 to 711.8)	8.8% (-44.6 to 114.2)
Kano	608.1 (403.3 to 835.4)	664.8 (408.3 to 990.6)	9.3% (-38.4 to 94.2)	3462.8 (2615.6 to 4486.7)	3736.0 (2518.3 to 5420.1)	7.9% (-32.3 to 72.1)	418.6 (258.4 to 618.2)	457.9 (261.2 to 720.0)	9.4% (-43.9 to 114.0)
Katsina	611.8 (411.7 to 849.4)	654.9 (405.9 to 963.7)	7.1% (-39.1 to 88.5)	3482.7 (2620.7 to 4504.2)	3664.0 (2450.8 to 5223.5)	5.2% (-33.8 to 67.3)	419.9 (263.8 to 622.6)	447.7 (259.3 to 700.5)	6.6% (-44.6 to 105.9)
Kebbi	612.9 (415.4 to 846.7)	656.6 (412.3 to 994.6)	7.1% (-39.1 to 88.7)	3479.4 (2616.9 to 4550.1)	3661.5 (2519.9 to 5299.4)	5.2% (-33.6 to 67.1)	420.3 (258.8 to 618.9)	446.2 (259.7 to 711.0)	6.2% (-45.4 to 106.9)
Kogi	617.5 (415.3 to 846.8)	654.6 (403.9 to 994.3)	6.0% (-40.1 to 88.2)	3520.6 (2628.5 to 4564.5)	3712.6 (2490.6 to 5345.9)	5.5% (-34.0 to 68.7)	426.4 (265.0 to 625.3)	453.4 (261.5 to 727.9)	6.3% (-45.5 to 107.8)
Kwara	615.0 (414.8 to 856.6)	651.7 (391.1 to 972.2)	6.0% (-40.6 to 89.5)	3485.2 (2637.9 to 4540.2)	3701.9 (2474.3 to 5203.6)	6.2% (-32.8 to 68.3)	422.7 (261.7 to 629.2)	453.0 (262.8 to 727.3)	7.2% (-45.2 to 110.3)
Lagos	606.0 (403.0 to 851.5)	646.3 (389.3 to 961.3)	6.6% (-40.7 to 92.1)	3468.3 (2632.1 to 4516.1)	3739.6 (2510.6 to 5341.4)	7.8% (-32.0 to 71.2)	423.1 (266.1 to 619.5)	460.2 (263.1 to 744.9)	8.8% (-44.3 to 112.6)
Nasarawa	608.8 (403.5 to 834.3)	652.0 (405.5 to 956.8)	7.1% (-38.9 to 88.3)	3472.3 (2631.5 to 4485.9)	3710.3 (2506.2 to 5280.3)	6.9% (-32.3 to 68.7)	419.8 (258.2 to 613.2)	453.1 (261.5 to 705.7)	8.0% (-44.0 to 108.6)

Niger	606.0 (403.0 to 831.6)	654.0 (406.3 to 973.0)	7.9% (-38.8 to 90.7)	3438.4 (2560.8 to 4514.9)	3696.1 (2505.6 to 5245.1)	7.5% (-32.3 to 71.4)	416.3 (259.4 to 616.3)	450.2 (259.5 to 698.8)	8.1% (-43.9 to 108.8)
Ogun	604.2 (406.0 to 842.4)	652.3 (398.0 to 971.7)	8.0% (-39.3 to 92.3)	3424.7 (2573.1 to 4452.4)	3753.9 (2602.5 to 5412.1)	9.6% (-30.4 to 73.1)	415.9 (257.5 to 610.9)	458.5 (270.4 to 720.5)	10.3% (-42.5 to 111.9)
Ondo	609.1 (410.4 to 833.4)	648.0 (397.2 to 968.6)	6.4% (-39.6 to 87.9)	3430.1 (2593.8 to 4441.6)	3695.5 (2518.8 to 5208.4)	7.7% (-31.3 to 69.2)	414.8 (262.3 to 603.5)	452.2 (263.9 to 711.4)	9.0% (-43.0 to 108.8)
Osun	1115.7 (744.4 to 1541.9)	1160.6 (692.8 to 1740.0)	4.0% (-42.0 to 87.0)	7990.1 (6087.1 to 10349.3)	8402.9 (5775.2 to 11934.5)	5.2% (-32.7 to 64.8)	969.1 (606.3 to 1440.3)	1026.3 (611.5 to 1630.1)	5.9% (-44.8 to 103.6)
Oyo	615.2 (402.6 to 836.2)	650.7 (387.0 to 959.1)	5.8% (-40.8 to 89.3)	3507.6 (2643.1 to 4568.8)	3737.5 (2540.1 to 5278.3)	6.6% (-32.3 to 68.2)	427.4 (264.7 to 630.0)	456.7 (268.5 to 724.5)	6.8% (-44.6 to 106.5)
Plateau	607.3 (406.1 to 836.4)	646.8 (393.5 to 973.1)	6.5% (-40.1 to 90.0)	3442.9 (2605.2 to 4469.5)	3653.7 (2482.1 to 5239.0)	6.1% (-32.9 to 68.1)	416.8 (257.0 to 607.8)	448.2 (253.9 to 705.9)	7.5% (-44.9 to 110.2)
Rivers	611.3 (418.9 to 843.7)	654.0 (383.7 to 968.3)	7.0% (-39.9 to 91.2)	3454.1 (2607.9 to 4512.8)	3734.7 (2523.6 to 5278.4)	8.1% (-31.5 to 71.1)	420.0 (264.6 to 621.2)	455.5 (272.7 to 701.6)	8.5% (-42.4 to 104.7)
Sokoto	612.3 (410.3 to 854.4)	653.8 (402.9 to 992.4)	6.8% (-40.2 to 91.0)	3510.7 (2643.3 to 4578.9)	3670.6 (2495.3 to 5322.7)	4.6% (-34.4 to 66.9)	422.9 (267.7 to 620.0)	445.6 (265.4 to 707.4)	5.4% (-44.7 to 101.3)
Taraba	603.7 (405.4 to 820.9)	657.5 (413.4 to 970.2)	8.9% (-37.4 to 89.8)	3436.3 (2590.2 to 4462.4)	3701.3 (2474.6 to 5226.8)	7.7% (-32.0 to 71.0)	416.8 (262.0 to 610.6)	453.2 (265.6 to 713.3)	8.7% (-43.2 to 108.7)
Yobe	609.4 (410.5 to 839.2)	664.4 (419.7 to 982.7)	9.0% (-37.5 to 90.4)	3450.4 (2580.8 to 4508.6)	3686.4 (2506.3 to 5297.7)	6.8% (-32.8 to 70.3)	418.1 (262.7 to 622.2)	449.5 (254.1 to 703.8)	7.5% (-44.8 to 110.1)
Zamfara	610.2 (409.8 to 849.9)	660.8 (417.9 to 988.9)	8.3% (-38.4 to 90.8)	3477.3 (2616.4 to 4535.6)	3681.0 (2511.8 to 5217.2)	5.9% (-32.8 to 67.2)	419.0 (262.6 to 619.6)	447.6 (254.6 to 707.9)	6.8% (-45.2 to 108.7)
Depressive disorders									
Abia	2697.2 (1847.4 to 3861.0)	2558.7 (1643.6 to 3820.7)	-5.1% (-45.7 to 66.1)	2319.5 (1740.4 to 3062.9)	2242.6 (1611.9 to 3096.5)	-3.3% (-37.2 to 49.0)	401.9 (251.0 to 618.1)	389.4 (225.0 to 623.3)	-3.1% (-50.8 to 91.2)

Adamawa	2703.5 (1865.3 to 3868.8)	2462.0 (1587.3 to 3692.8)	-8.9% (-47.8 to 59.3)	2328.3 (1747.8 to 3106.4)	2136.7 (1541.8 to 2954.2)	-8.2% (-40.4 to 41.6)	403.0 (247.8 to 613.4)	371.4 (216.7 to 584.2)	-7.9% (-52.7 to 79.8)
Akwa Ibom	2806.5 (1907.2 to 3952.1)	2604.6 (1680.6 to 3861.6)	-7.2% (-46.5 to 61.3)	2413.0 (1792.2 to 3177.6)	2289.7 (1637.7 to 3093.5)	-5.1% (-38.0 to 45.4)	416.7 (255.2 to 642.0)	396.5 (235.3 to 634.9)	-4.9% (-51.5 to 86.5)
Anambra	2667.1 (1836.4 to 3778.1)	2544.6 (1626.5 to 3828.0)	-4.6% (-45.5 to 67.3)	2301.8 (1720.1 to 3063.4)	2240.5 (1602.2 to 3094.2)	-2.7% (-37.1 to 50.8)	396.8 (241.4 to 604.5)	386.8 (226.4 to 603.9)	-2.5% (-50.0 to 90.0)
Bauchi	2630.9 (1798.9 to 3764.4)	2384.8 (1516.1 to 3556.6)	-9.4% (-48.4 to 59.5)	2279.3 (1717.5 to 3018.1)	2078.2 (1462.7 to 2855.7)	-8.8% (-41.1 to 41.4)	390.5 (241.5 to 601.4)	357.7 (205.5 to 560.9)	-8.4% (-53.3 to 80.1)
Bayelsa	2679.1 (1825.8 to 3837.1)	2515.7 (1593.6 to 3762.6)	-6.1% (-46.7 to 65.9)	2313.5 (1716.7 to 3081.6)	2206.2 (1574.6 to 3019.9)	-4.6% (-38.3 to 47.6)	399.1 (249.1 to 610.0)	381.3 (219.5 to 596.9)	-4.5% (-51.0 to 86.9)
Benue	2675.5 (1813.3 to 3807.6)	2577.4 (1641.5 to 3893.2)	-3.7% (-45.4 to 70.6)	2282.1 (1703.4 to 3048.6)	2253.6 (1629.9 to 3094.4)	-1.2% (-35.8 to 52.1)	396.2 (240.5 to 602.6)	391.4 (232.1 to 610.5)	-1.2% (-49.0 to 91.7)
Borno	2641.5 (1784.2 to 3746.3)	2386.1 (1543.4 to 3550.0)	-9.7% (-48.2 to 57.8)	2296.1 (1716.3 to 3022.3)	2070.4 (1481.6 to 2857.2)	-9.8% (-41.5 to 39.3)	395.5 (244.3 to 598.4)	358.0 (208.1 to 556.1)	-9.5% (-53.2 to 75.5)
Cross River	2779.5 (1897.5 to 3941.6)	2601.4 (1671.0 to 3905.4)	-6.4% (-46.5 to 64.2)	2388.7 (1769.2 to 3159.5)	2281.8 (1645.8 to 3151.4)	-4.5% (-38.1 to 47.7)	414.0 (253.4 to 633.6)	394.9 (237.3 to 618.7)	-4.6% (-50.6 to 84.2)
Delta	2738.8 (1884.8 to 3896.3)	2565.5 (1643.2 to 3904.9)	-6.3% (-46.8 to 65.1)	2362.8 (1775.5 to 3150.0)	2256.4 (1612.3 to 3199.9)	-4.5% (-38.9 to 49.5)	407.2 (249.5 to 618.1)	389.9 (236.5 to 614.5)	-4.3% (-50.2 to 84.2)
Ebonyi	2700.4 (1836.4 to 3829.3)	2528.9 (1606.0 to 3703.7)	-6.4% (-46.2 to 63.4)	2323.7 (1728.2 to 3102.1)	2214.0 (1599.9 to 2991.0)	-4.7% (-37.7 to 45.8)	398.8 (244.5 to 609.5)	380.3 (225.3 to 588.5)	-4.6% (-50.6 to 84.2)
Edo	2734.0 (1864.5 to 3896.1)	2668.0 (1748.5 to 3970.0)	-2.4% (-43.6 to 69.2)	2342.2 (1739.8 to 3113.7)	2358.3 (1705.4 to 3259.9)	0.7% (-34.7 to 55.5)	406.1 (250.4 to 612.9)	407.5 (243.0 to 649.5)	0.4% (-48.2 to 94.7)
Ekiti	2637.1 (1783.0 to 3768.1)	2532.6 (1639.3 to 3732.7)	-4.0% (-44.7 to 67.2)	2274.2 (1692.9 to 3017.6)	2240.3 (1626.5 to 3064.9)	-1.5% (-35.6 to 51.0)	392.7 (240.9 to 593.9)	386.6 (233.8 to 599.8)	-1.6% (-48.4 to 88.0)

Enugu	2621.2 (1792.4 to 3762.7)	2517.0 (1609.5 to 3779.4)	-4.0% (-45.4 to 69.2)	2257.0 (1696.3 to 2970.8)	2200.8 (1586.6 to 3056.1)	-2.5% (-36.6 to 50.3)	390.9 (240.8 to 600.6)	380.9 (224.2 to 611.6)	-2.5% (-50.3 to 91.7)
FCT (Abuja)	2743.0 (1874.9 to 3897.7)	2570.5 (1657.3 to 3821.1)	-6.3% (-46.1 to 63.3)	2408.9 (1809.8 to 3202.6)	2296.7 (1653.8 to 3134.0)	-4.7% (-37.8 to 46.4)	414.4 (252.8 to 622.7)	394.9 (233.1 to 625.3)	-4.7% (-50.9 to 85.3)
Gombe	2575.9 (1763.7 to 3703.7)	2403.4 (1575.0 to 3548.2)	-6.7% (-46.0 to 61.3)	2233.3 (1667.8 to 2955.6)	2097.6 (1524.8 to 2854.7)	-6.1% (-38.4 to 43.3)	384.3 (236.2 to 582.7)	362.5 (218.8 to 561.7)	-5.7% (-50.6 to 80.2)
Imo	2619.7 (1773.7 to 3717.0)	2500.1 (1629.9 to 3745.4)	-4.6% (-45.2 to 66.6)	2252.6 (1678.6 to 2958.1)	2202.5 (1606.0 to 2995.7)	-2.2% (-35.7 to 48.8)	389.0 (239.1 to 586.2)	380.6 (229.2 to 593.2)	-2.1% (-48.8 to 87.3)
Jigawa	2624.1 (1791.5 to 3699.6)	2335.2 (1504.4 to 3493.9)	-11.0% (-48.9 to 55.4)	2287.9 (1709.8 to 3012.6)	2032.1 (1455.7 to 2746.9)	-11.2% (-41.9 to 36.0)	390.5 (240.1 to 594.2)	349.6 (205.7 to 535.3)	-10.5% (-53.4 to 72.2)
Kaduna	2596.3 (1780.3 to 3700.3)	2476.4 (1612.1 to 3708.9)	-4.6% (-45.1 to 66.2)	2247.0 (1681.5 to 2999.7)	2177.3 (1591.9 to 2987.8)	-3.1% (-36.6 to 48.2)	387.9 (237.4 to 589.0)	376.0 (227.6 to 599.1)	-3.1% (-49.8 to 87.5)
Kano	2653.4 (1811.3 to 3773.5)	2472.5 (1586.7 to 3740.1)	-6.8% (-47.0 to 64.1)	2295.5 (1720.3 to 3030.5)	2153.6 (1540.6 to 2969.0)	-6.2% (-39.1 to 44.9)	394.8 (243.9 to 596.8)	373.4 (220.6 to 581.6)	-5.4% (-50.9 to 82.2)
Katsina	2589.1 (1785.2 to 3657.1)	2384.5 (1542.1 to 3585.9)	-7.9% (-47.0 to 60.6)	2255.8 (1683.4 to 2994.6)	2087.6 (1508.0 to 2881.4)	-7.5% (-39.9 to 42.8)	384.9 (235.4 to 576.1)	360.1 (216.1 to 572.2)	-6.4% (-51.5 to 80.6)
Kebbi	2606.9 (1798.6 to 3639.3)	2393.6 (1581.9 to 3585.8)	-8.2% (-46.5 to 57.8)	2263.7 (1691.9 to 2974.6)	2098.0 (1539.6 to 2872.3)	-7.3% (-39.0 to 41.0)	387.2 (238.5 to 583.4)	360.3 (212.1 to 565.4)	-6.9% (-51.9 to 80.2)
Kogi	2655.4 (1818.4 to 3775.2)	2472.5 (1585.1 to 3675.1)	-6.9% (-46.6 to 62.7)	2302.2 (1706.8 to 3036.9)	2191.7 (1590.4 to 2993.1)	-4.8% (-37.8 to 45.8)	395.5 (242.3 to 596.3)	377.4 (228.8 to 587.7)	-4.6% (-50.0 to 82.3)
Kwara	2631.7 (1790.7 to 3742.8)	2525.8 (1622.7 to 3757.9)	-4.0% (-45.0 to 67.9)	2290.0 (1716.8 to 3018.8)	2259.0 (1642.3 to 3098.4)	-1.4% (-35.4 to 50.9)	393.7 (242.6 to 590.5)	388.4 (229.1 to 619.3)	-1.4% (-49.2 to 92.3)
Lagos	2327.8 (1536.5 to 3404.8)	2538.4 (1702.6 to 3597.0)	9.0% (-36.4 to 87.2)	2131.7 (1566.2 to 2798.6)	2293.4 (1690.6 to 3034.8)	7.6% (-28.4 to 61.8)	359.6 (212.6 to 552.2)	394.1 (239.0 to 611.1)	9.6% (-43.5 to 112.4)

Nasarawa	2695.2 (1859.9 to 3837.8)	2545.9 (1616.4 to 3826.2)	-5.5% (-46.2 to 66.2)	2328.4 (1732.4 to 3103.9)	2249.9 (1622.4 to 3144.0)	-3.4% (-37.7 to 50.2)	400.4 (248.3 to 614.2)	386.9 (234.0 to 608.0)	-3.4% (-49.7 to 85.8)
Niger	2589.1 (1778.6 to 3650.7)	2445.0 (1579.3 to 3638.3)	-5.6% (-45.5 to 64.1)	2247.9 (1681.4 to 2968.4)	2165.6 (1555.8 to 2957.5)	-3.7% (-37.2 to 47.9)	384.8 (241.1 to 585.9)	370.1 (217.9 to 566.3)	-3.8% (-49.7 to 83.8)
Ogun	2617.1 (1799.3 to 3675.6)	2579.3 (1660.8 to 3829.3)	-1.4% (-43.1 to 71.1)	2280.6 (1716.0 to 3014.3)	2303.1 (1687.0 to 3153.6)	1.0% (-33.5 to 53.7)	392.8 (245.1 to 593.7)	396.2 (230.9 to 626.0)	0.9% (-48.1 to 96.4)
Ondo	2646.2 (1812.6 to 3779.7)	2542.4 (1657.7 to 3782.2)	-3.9% (-44.6 to 67.0)	2283.5 (1710.9 to 3025.2)	2247.1 (1627.7 to 3105.2)	-1.6% (-35.9 to 51.4)	393.1 (241.7 to 600.2)	387.7 (232.7 to 605.5)	-1.4% (-48.7 to 90.0)
Osun	5382.9 (3685.5 to 7659.6)	5224.2 (3276.8 to 7767.2)	-2.9% (-44.9 to 71.3)	4031.5 (2929.5 to 5455.5)	3981.7 (2718.6 to 5669.5)	-1.2% (-39.0 to 60.1)	757.5 (459.4 to 1153.3)	750.2 (435.9 to 1193.0)	-1.0% (-49.7 to 95.2)
Oyo	4588.3 (3124.5 to 6537.0)	4711.2 (3000.8 to 6935.8)	2.7% (-41.1 to 79.5)	3546.8 (2594.2 to 4816.8)	3671.1 (2538.6 to 5153.5)	3.5% (-35.2 to 65.6)	658.0 (407.6 to 1002.5)	686.4 (394.6 to 1095.9)	4.3% (-47.1 to 106.1)
Plateau	2637.4 (1806.6 to 3728.5)	2501.5 (1595.3 to 3717.1)	-5.2% (-45.6 to 65.8)	2269.5 (1685.6 to 3028.3)	2196.4 (1590.1 to 3020.8)	-3.2% (-37.1 to 49.2)	392.3 (242.2 to 599.4)	380.5 (224.5 to 602.7)	-3.0% (-50.1 to 88.9)
Rivers	2719.4 (1871.6 to 3867.4)	2631.7 (1675.2 to 3946.4)	-3.2% (-44.8 to 70.1)	2335.0 (1749.8 to 3117.4)	2314.9 (1672.5 to 3155.6)	-0.9% (-35.3 to 52.1)	405.3 (252.2 to 620.3)	400.6 (238.8 to 628.8)	-1.2% (-48.7 to 90.5)
Sokoto	2648.2 (1821.3 to 3740.1)	2367.2 (1498.8 to 3484.4)	-10.6% (-48.6 to 55.9)	2311.4 (1725.4 to 3042.0)	2081.4 (1490.3 to 2809.7)	-10.0% (-41.0 to 37.8)	394.1 (242.9 to 597.9)	355.6 (204.8 to 550.0)	-9.8% (-53.5 to 75.6)
Taraba	2713.7 (1853.4 to 3866.9)	2527.8 (1628.8 to 3712.9)	-6.9% (-46.3 to 61.9)	2339.1 (1756.3 to 3118.9)	2211.1 (1579.5 to 3036.0)	-5.5% (-38.7 to 46.1)	404.4 (255.3 to 613.6)	382.2 (232.6 to 600.4)	-5.5% (-50.2 to 79.6)
Yobe	2570.3 (1765.9 to 3651.2)	2338.5 (1520.7 to 3522.5)	-9.0% (-47.7 to 58.7)	2233.8 (1666.6 to 2963.6)	2027.9 (1464.4 to 2828.1)	-9.2% (-41.3 to 40.6)	383.1 (232.8 to 588.1)	350.2 (205.1 to 563.9)	-8.6% (-53.7 to 80.8)
Zamfara	2610.2 (1786.6 to 3678.4)	2354.8 (1532.8 to 3503.4)	-9.8% (-47.8 to 56.2)	2271.7 (1706.0 to 3015.7)	2060.2 (1483.8 to 2791.8)	-9.3% (-40.6 to 38.6)	387.9 (243.6 to 589.8)	352.4 (211.4 to 541.4)	-9.2% (-52.1 to 72.5)

UI, uncertainly interval; DALYs, disability-adjusted life-years.

Table S15 The incidence, prevalence and DALYs rate of anxiety and depressive disorders in 1990 and 2021, and changes from 1990 to 2021 in Norway.

Location	Incidence (95% UI)			Prevalence (95% UI)			DALYs (95% UI)		
	1990	2021	Percentage change	1990	2021	Percentage change	1990	2021	Percentage change
Anxiety disorders									
Agder	1424.2 (943.5 to 1991.2)	1559.1 (972.0 to 2268.8)	9.5% (-37.7 to 92.6)	9338.7 (7120.2 to 12053.5)	10006.3 (6822.9 to 13829.9)	7.1% (-30.9 to 66.3)	1146.2 (719.5 to 1697.2)	1227.9 (739.1 to 1909.9)	7.1% (-43.3 to 102.8)
Innlandet	1410.5 (927.4 to 1978.9)	1544.9 (947.4 to 2317.8)	9.5% (-39.0 to 97.3)	9356.1 (7141.5 to 12065.5)	10020.9 (6916.8 to 14070.3)	7.1% (-30.9 to 66.4)	1149.6 (727.1 to 1705.7)	1230.1 (709.8 to 1884.2)	7.0% (-43.9 to 104.6)
Møre og Romsdal	1420.3 (958.1 to 1992.4)	1562.0 (969.8 to 2312.6)	10.0% (-37.7 to 94.5)	9331.8 (7172.6 to 11999.9)	10059.0 (6862.7 to 13808.2)	7.8% (-30.0 to 66.2)	1145.3 (732.7 to 1695.8)	1234.8 (744.5 to 1903.9)	7.8% (-42.4 to 102.4)
Nordland	1409.3 (944.8 to 1959.5)	1535.4 (946.2 to 2246.4)	8.9% (-38.1 to 92.2)	9308.6 (7124.3 to 11925.2)	9960.0 (6771.4 to 13717.8)	7.0% (-30.7 to 65.6)	1140.7 (727.0 to 1661.6)	1221.3 (735.3 to 1948.1)	7.1% (-43.4 to 103.4)
Oslo	1403.4 (887.9 to 1998.8)	1538.6 (960.5 to 2272.3)	9.6% (-39.0 to 97.4)	9669.5 (7357.7 to 12400.5)	10175.7 (7086.6 to 14196.3)	5.2% (-31.6 to 62.5)	1179.0 (740.0 to 1726.7)	1242.2 (767.8 to 1963.3)	5.4% (-43.8 to 97.9)
Rogaland	1421.6 (943.0 to 1962.8)	1574.0 (984.6 to 2363.6)	10.7% (-37.4 to 96.4)	9354.0 (7186.2 to 11980.3)	10081.8 (7046.0 to 14099.7)	7.8% (-29.8 to 65.7)	1148.4 (729.2 to 1676.5)	1239.5 (737.9 to 1934.3)	7.9% (-42.8 to 104.3)
Troms og Finnmark	1397.8 (910.2 to 1969.8)	1538.6 (950.4 to 2311.7)	10.1% (-38.8 to 98.5)	9398.7 (7176.2 to 12119.0)	9993.9 (6826.2 to 14050.7)	6.3% (-31.8 to 66.0)	1152.8 (730.3 to 1691.7)	1228.0 (701.3 to 1902.5)	6.5% (-44.5 to 104.9)
Trøndelag	1407.9 (928.1 to 1968.8)	1526.8 (930.8 to 2242.5)	8.4% (-39.1 to 93.8)	9364.5 (7164.9 to 12104.7)	9987.7 (7063.4 to 13660.5)	6.7% (-29.9 to 62.5)	1149.1 (734.1 to 1694.1)	1224.2 (741.1 to 1899.4)	6.5% (-43.1 to 100.0)
Vestfold og Telemark	1417.7 (934.3 to 1987.2)	1545.9 (958.3 to 2283.1)	9.0% (-38.6 to 94.0)	9369.2 (7146.2 to 12089.8)	9954.9 (6854.4 to 13813.9)	6.3% (-31.2 to 64.6)	1149.6 (730.5 to 1699.8)	1223.1 (716.6 to 1878.5)	6.4% (-43.8 to 102.0)
Vestland	1413.6 (929.8 to 1983.0)	1543.5 (968.5 to 2224.7)	9.2% (-37.5 to 91.3)	9365.3 (7142.3 to 12086.2)	10010.9 (6899.3 to 13764.7)	6.9% (-30.6 to 65.1)	1151.4 (729.5 to 1701.3)	1228.6 (738.6 to 1883.6)	6.7% (-43.0 to 100.2)

Viken	1413.9 (928.4 to 1984.7)	1566.6 (993.1 to 2279.4)	10.8% (-36.7 to 94.0)	9398.1 (7170.0 to 12125.3)	9982.1 (6898.9 to 14013.6)	6.2% (-31.5 to 65.0)	1149.2 (727.7 to 1702.0)	1222.9 (725.4 to 1876.8)	6.4% (-43.6 to 101.4)
Depressive disorders									
Agder	3754.1 (2545.3 to 5311.9)	4473.3 (2883.2 to 6548.5)	19.2% (-31.1 to 106.7)	3087.5 (2274.8 to 4160.9)	3517.3 (2465.5 to 4911.1)	13.9% (-27.8 to 80.1)	564.2 (344.0 to 871.5)	655.9 (385.8 to 1047.9)	16.2% (-41.0 to 128.9)
Innlandet	3815.7 (2603.6 to 5461.4)	4573.7 (2864.3 to 6958.7)	19.9% (-32.8 to 114.2)	3149.4 (2328.0 to 4252.8)	3600.6 (2472.8 to 5134.7)	14.3% (-28.8 to 83.8)	573.9 (352.5 to 886.5)	672.2 (389.8 to 1075.5)	17.1% (-40.7 to 132.0)
Møre og Romsdal	3793.2 (2603.7 to 5386.6)	4563.5 (2892.3 to 6833.1)	20.3% (-31.5 to 111.7)	3122.2 (2312.3 to 4202.2)	3582.7 (2483.0 to 5050.7)	14.8% (-27.8 to 82.8)	569.7 (347.2 to 887.4)	669.5 (380.8 to 1062.0)	17.5% (-41.1 to 134.6)
Nordland	3811.7 (2583.3 to 5392.8)	4564.7 (2876.0 to 6756.5)	19.8% (-31.8 to 110.8)	3148.0 (2320.0 to 4249.4)	3599.1 (2485.2 to 5006.7)	14.3% (-28.0 to 81.8)	574.7 (354.6 to 879.1)	670.8 (389.9 to 1085.5)	16.7% (-41.0 to 131.3)
Oslo	6345.5 (4529.3 to 8698.2)	7424.2 (4753.1 to 11033.0)	17.0% (-31.1 to 99.3)	4947.0 (3702.3 to 6609.1)	5515.1 (3810.8 to 7922.5)	11.5% (-29.9 to 77.7)	937.6 (591.7 to 1389.2)	1069.5 (620.7 to 1726.6)	14.1% (-41.4 to 122.7)
Rogaland	3776.2 (2561.1 to 5385.2)	4501.4 (2808.9 to 6796.4)	19.2% (-33.1 to 112.7)	3112.2 (2289.5 to 4197.4)	3525.3 (2408.0 to 5034.9)	13.3% (-29.7 to 82.7)	567.1 (346.9 to 881.0)	658.2 (379.0 to 1061.0)	16.1% (-41.8 to 131.9)
Troms og Finnmark	3876.2 (2634.1 to 5517.9)	4583.2 (2887.7 to 6846.7)	18.2% (-33.0 to 109.1)	3220.8 (2367.3 to 4322.7)	3619.3 (2505.6 to 5032.5)	12.4% (-29.1 to 78.3)	585.3 (358.1 to 900.7)	674.6 (393.6 to 1079.6)	15.2% (-41.5 to 127.5)
Trøndelag	3727.8 (2556.7 to 5237.9)	4854.5 (3053.1 to 7192.9)	30.2% (-25.5 to 128.2)	3117.6 (2298.8 to 4166.5)	3802.0 (2640.5 to 5338.0)	22.0% (-23.1 to 93.6)	567.4 (345.0 to 868.3)	712.9 (410.5 to 1151.6)	25.7% (-36.9 to 151.1)
Vestfold og Telemark	3798.4 (2594.7 to 5401.4)	4514.8 (2885.3 to 6886.7)	18.9% (-32.7 to 110.3)	3132.9 (2319.3 to 4237.6)	3544.8 (2450.8 to 5053.5)	13.1% (-29.4 to 81.6)	571.9 (349.5 to 867.1)	662.2 (388.4 to 1021.7)	15.8% (-40.1 to 123.9)
Vestland	3025.7 (1996.8 to 4420.6)	3599.7 (2163.8 to 5648.0)	19.0% (-36.2 to 122.3)	2631.1 (1911.4 to 3605.0)	2965.0 (2025.7 to 4223.8)	12.7% (-30.6 to 83.3)	465.2 (280.3 to 719.4)	538.2 (301.7 to 900.5)	15.7% (-43.7 to 138.6)
Viken	7685.8 (5266.4 to 10915.5)	8734.1 (5547.6 to 13008.6)	13.6% (-35.1 to 99.5)	5692.3 (4045.8 to 7848.3)	6242.5 (4160.7 to 8937.7)	9.7% (-33.8 to 82.0)	1108.3 (668.1 to 1677.1)	1231.4 (710.2 to 1915.6)	11.1% (-43.3 to 117.6)

UI, uncertainly interval; DALYs, disability-adjusted life-years.

Table S16 The incidence, prevalence and DALYs rate of anxiety and depressive disorders in 1990 and 2021, and changes from 1990 to 2021 in Pakistan.

**Location**	**Incidence (95% UI)**			**Prevalence (95% UI)**			**DALYs (95% UI)**		
	**1990**	**2021**	**Percentage change**	**1990**	**2021**	**Percentage change**	**1990**	**2021**	**Percentage change**
Anxiety disorders									
Azad Jammu & Kashmir	562.6 (379.1 to 769.8)	696.0 (432.6 to 1044.8)	23.7% (-29.6 to 117.7)	2643.9 (1962.9 to 3452.5)	3348.9 (2266.6 to 4719.5)	26.7% (-20.0 to 101.4)	323.5 (199.1 to 479.1)	407.2 (236.4 to 635.6)	25.9% (-34.9 to 143.9)
Balochistan	549.3 (370.7 to 752.6)	603.3 (377.5 to 903.8)	9.8% (-37.3 to 92.6)	2552.4 (1895.6 to 3333.2)	2856.4 (1910.8 to 4082.2)	11.9% (-30.1 to 79.4)	311.0 (193.2 to 455.1)	348.8 (206.0 to 539.7)	12.1% (-41.0 to 113.7)
Gilgit-Baltistan	554.7 (373.0 to 767.7)	610.3 (379.3 to 917.6)	10.0% (-37.7 to 94.8)	2614.4 (1934.0 to 3439.4)	2891.5 (1969.2 to 4104.0)	10.6% (-30.5 to 76.3)	321.6 (196.6 to 479.4)	354.9 (213.7 to 548.6)	10.3% (-42.0 to 110.2)
Islamabad Capital Territory	560.4 (372.1 to 771.0)	661.4 (406.7 to 992.0)	18.0% (-33.6 to 110.1)	2652.3 (1968.4 to 3465.9)	3169.4 (2113.6 to 4547.1)	19.5% (-25.5 to 92.2)	325.0 (201.8 to 482.7)	389.2 (218.8 to 614.0)	19.7% (-39.0 to 135.8)
Khyber Pakhtunkhwa	556.5 (375.7 to 769.8)	715.6 (438.6 to 1059.4)	28.6% (-27.1 to 127.1)	2609.5 (1931.9 to 3436.5)	3414.6 (2259.2 to 4880.4)	30.9% (-18.9 to 111.5)	320.1 (199.3 to 472.7)	417.6 (242.7 to 662.8)	30.4% (-32.7 to 153.6)
Punjab	559.5 (372.1 to 780.3)	686.5 (422.3 to 1007.7)	22.7% (-30.6 to 117.7)	2647.9 (1972.9 to 3446.5)	3287.9 (2203.3 to 4724.1)	24.2% (-22.4 to 99.1)	323.7 (200.3 to 479.3)	403.2 (232.6 to 650.2)	24.6% (-36.5 to 145.1)
Sindh	558.8 (378.8 to 770.4)	669.4 (410.2 to 975.1)	19.8% (-31.5 to 109.9)	2629.8 (1961.2 to 3423.6)	3191.1 (2157.3 to 4516.3)	21.3% (-23.3 to 92.8)	319.6 (196.2 to 471.8)	389.0 (231.1 to 616.7)	21.7% (-36.8 to 135.1)
Depressive disorders									
Azad Jammu & Kashmir	2612.8 (1785.4 to 3704.3)	3246.8 (2035.1 to 4809.0)	24.3% (-29.3 to 118.9)	2146.1 (1580.2 to 2868.3)	2606.8 (1826.5 to 3595.9)	21.5% (-22.5 to 90.8)	383.1 (238.2 to 593.6)	472.5 (277.5 to 748.9)	23.3% (-36.9 to 141.1)
Balochistan	2528.1 (1712.8 to 3589.9)	2673.7 (1728.2 to 4027.6)	5.8% (-39.6 to 85.5)	2065.2 (1523.6 to 2766.0)	2208.7 (1563.7 to 3106.6)	6.9% (-32.1 to 68.6)	368.6 (222.9 to 563.8)	395.1 (236.2 to 642.5)	7.2% (-45.6 to 111.1)
Gilgit-Baltistan	2534.1 (1734.7 to 3610.9)	2634.2 (1677.7 to 3888.7)	3.9% (-40.4 to 81.6)	2091.5 (1545.4 to 2794.1)	2185.2 (1552.5 to 2995.3)	4.5% (-32.7 to 62.4)	373.5 (230.8 to 560.6)	389.9 (228.0 to 612.5)	4.4% (-46.1 to 102.7)

Islamabad Capital Territory	2693.5 (1833.3 to 3876.5)	3140.6 (1997.0 to 4728.4)	16.6% (-34.0 to 106.5)	2216.8 (1643.0 to 2980.5)	2536.8 (1796.2 to 3627.2)	14.4% (-27.8 to 81.7)	397.9 (247.6 to 602.5)	461.6 (267.9 to 729.7)	16.0% (-40.5 to 126.6)
Khyber Pakhtunkhwa	1926.0 (1186.0 to 2985.7)	2443.3 (1423.6 to 3917.9)	26.9% (-35.8 to 151.0)	1706.3 (1189.0 to 2411.6)	2054.2 (1366.3 to 2956.2)	20.4% (-28.4 to 102.6)	290.6 (160.7 to 467.6)	361.1 (196.2 to 600.6)	24.2% (-42.3 to 167.7)
Punjab	2680.8 (1819.5 to 3802.0)	3159.1 (2011.4 to 4706.0)	17.8% (-32.8 to 107.1)	2198.2 (1629.0 to 2964.1)	2535.1 (1785.5 to 3583.9)	15.3% (-27.1 to 82.9)	392.0 (243.9 to 603.6)	460.6 (266.5 to 727.2)	17.5% (-40.0 to 130.6)
Sindh	2602.4 (1766.9 to 3737.1)	3057.9 (1972.5 to 4527.9)	17.5% (-32.6 to 105.3)	2145.2 (1583.4 to 2861.3)	2470.5 (1762.1 to 3421.3)	15.2% (-26.0 to 79.7)	379.7 (234.0 to 571.9)	445.6 (264.8 to 713.2)	17.3% (-39.5 to 128.2)

UI, uncertainly interval; DALYs, disability-adjusted life-years.

Table S17 The incidence, prevalence and DALYs rate of anxiety and depressive disorders in 1990 and 2021, and changes from 1990 to 2021 in Philippines.

Location	Incidence (95% UI)			Prevalence (95% UI)			DALYs (95% UI)		
	1990	2021	Percentage change	1990	2021	Percentage change	1990	2021	Percentage change
Anxiety disorders									
Abra	705.9 (471.7 to 984.9)	941.7 (579.0 to 1356.3)	33.4% (-23.9 to 134.4)	3818.2 (2876.9 to 4949.4)	5154.4 (3471.9 to 7145.1)	35.0% (-13.8 to 112.0)	470.2 (291.0 to 701.9)	635.0 (373.1 to 1008.5)	35.1% (-30.3 to 162.2)
Agusan Del Norte	718.9 (479.5 to 989.0)	951.1 (577.2 to 1423.2)	32.3% (-25.6 to 135.8)	3872.7 (2917.6 to 5007.3)	5156.1 (3547.0 to 7274.6)	33.1% (-14.8 to 108.6)	476.1 (298.6 to 703.2)	636.1 (368.7 to 1023.6)	33.6% (-31.4 to 160.8)
Agusan Del Sur	712.3 (484.6 to 991.4)	943.9 (564.5 to 1393.4)	32.5% (-25.3 to 135.7)	3802.1 (2885.7 to 4954.7)	5094.9 (3428.0 to 7264.5)	34.0% (-15.4 to 112.7)	467.0 (290.6 to 696.1)	626.4 (363.3 to 967.4)	34.1% (-30.2 to 158.7)
Aklan	708.7 (473.3 to 982.1)	950.8 (579.3 to 1385.9)	34.2% (-24.0 to 137.5)	3819.7 (2861.0 to 4970.5)	5190.1 (3551.2 to 7430.0)	35.9% (-14.1 to 115.3)	470.2 (292.3 to 695.1)	639.1 (366.4 to 1011.1)	35.9% (-30.2 to 165.5)
Albay	711.6 (481.9 to 987.0)	945.9 (581.4 to 1372.4)	32.9% (-24.0 to 133.1)	3809.5 (2860.9 to 4963.8)	5158.4 (3501.6 to 7355.2)	35.4% (-14.5 to 114.8)	467.2 (294.9 to 683.4)	633.5 (375.2 to 1009.2)	35.6% (-29.1 to 160.3)
Antique	709.5 (480.2 to 959.2)	946.6 (588.8 to 1411.0)	33.4% (-23.4 to 132.9)	3800.7 (2868.2 to 4931.9)	5140.1 (3455.8 to 7277.6)	35.2% (-14.5 to 114.3)	467.6 (294.7 to 699.3)	633.1 (367.5 to 998.9)	35.4% (-30.0 to 162.6)

Apayao	711.2 (483.9 to 998.8)	949.3 (578.0 to 1391.4)	33.5% (-24.5 to 136.2)	3850.2 (2894.2 to 4994.4)	5166.8 (3474.2 to 7287.5)	34.2% (-15.0 to 112.4)	473.4 (296.8 to 703.1)	636.4 (368.7 to 993.0)	34.4% (-30.2 to 159.4)
Aurora	711.6 (476.4 to 993.7)	957.8 (603.1 to 1425.0)	34.6% (-23.5 to 137.4)	3823.5 (2857.6 to 5015.2)	5149.6 (3530.4 to 7168.4)	34.7% (-14.1 to 111.6)	470.5 (292.3 to 701.4)	635.3 (373.4 to 1012.6)	35.0% (-30.3 to 162.4)
Basilan	716.7 (476.7 to 997.3)	969.8 (608.8 to 1427.2)	35.3% (-22.9 to 138.0)	3847.7 (2901.0 to 5011.0)	5227.5 (3567.9 to 7442.0)	35.9% (-13.8 to 114.6)	472.6 (298.2 to 695.5)	643.9 (373.6 to 1013.1)	36.3% (-29.1 to 162.9)
Bataan	715.5 (472.1 to 998.6)	950.3 (568.1 to 1413.7)	32.8% (-26.3 to 139.8)	3891.8 (2934.7 to 5063.4)	5214.8 (3492.4 to 7418.0)	34.0% (-15.6 to 113.2)	477.8 (300.2 to 711.3)	641.5 (375.6 to 1013.6)	34.3% (-30.3 to 159.4)
Batanes	706.2 (480.1 to 992.9)	942.0 (565.0 to 1393.9)	33.4% (-25.1 to 138.0)	3781.7 (2840.0 to 4960.0)	5100.8 (3461.7 to 7261.4)	34.9% (-14.9 to 114.4)	465.6 (290.6 to 696.5)	628.6 (369.8 to 1011.4)	35.0% (-30.6 to 163.2)
Batangas	709.3 (475.5 to 986.9)	950.9 (582.4 to 1415.6)	34.1% (-24.5 to 138.4)	3854.0 (2894.3 to 5046.7)	5244.3 (3626.1 to 7397.0)	36.1% (-13.2 to 113.8)	474.1 (300.5 to 705.2)	647.4 (376.1 to 1011.2)	36.6% (-28.9 to 162.8)
Benguet	715.5 (472.0 to 1008.2)	960.4 (578.5 to 1422.0)	34.2% (-25.5 to 142.3)	3915.9 (2983.2 to 5097.4)	5348.7 (3621.4 to 7470.1)	36.6% (-12.7 to 114.1)	481.6 (298.9 to 712.5)	659.5 (382.6 to 1033.6)	36.9% (-29.1 to 164.9)
Biliran	715.5 (483.7 to 984.6)	953.0 (592.2 to 1448.6)	33.2% (-24.6 to 135.7)	3812.6 (2866.0 to 4977.9)	5104.5 (3407.6 to 7317.1)	33.9% (-16.2 to 114.3)	468.4 (291.2 to 696.2)	628.0 (367.2 to 994.2)	34.1% (-30.7 to 159.8)
Bohol	711.3 (476.3 to 984.7)	942.7 (579.0 to 1409.1)	32.5% (-25.3 to 135.5)	3818.5 (2865.9 to 4919.8)	5123.1 (3549.4 to 7269.5)	34.2% (-14.1 to 110.2)	469.3 (291.5 to 697.9)	631.5 (364.5 to 1009.6)	34.6% (-31.1 to 163.8)
Bukidnon	708.4 (468.2 to 987.1)	946.3 (583.6 to 1388.8)	33.6% (-24.5 to 137.1)	3831.5 (2886.1 to 4928.5)	5126.0 (3514.9 to 7285.9)	33.8% (-14.6 to 110.2)	471.1 (296.6 to 702.7)	630.1 (376.1 to 963.7)	33.8% (-29.0 to 152.3)
Bulacan	718.0 (476.5 to 986.1)	954.3 (565.4 to 1443.7)	32.9% (-26.4 to 140.5)	3908.9 (2933.9 to 5105.8)	5241.1 (3554.8 to 7496.2)	34.1% (-15.5 to 113.2)	480.5 (295.2 to 708.8)	645.1 (375.8 to 1016.7)	34.2% (-30.7 to 160.6)
Cagayan	712.5 (472.1 to 988.2)	952.1 (595.8 to 1427.7)	33.6% (-24.6 to 137.3)	3823.5 (2866.9 to 4963.1)	5196.1 (3544.4 to 7407.2)	35.9% (-13.9 to 115.0)	469.7 (294.2 to 696.1)	640.4 (380.3 to 1006.7)	36.3% (-28.6 to 161.0)

Camarines Norte	710.9 (481.2 to 960.9)	962.4 (581.4 to 1438.8)	35.4% (-23.1 to 139.6)	3807.7 (2873.2 to 4940.5)	5176.4 (3479.2 to 7370.7)	35.9% (-14.2 to 115.8)	467.1 (291.1 to 695.4)	636.6 (364.5 to 1005.2)	36.3% (-30.1 to 166.6)
Camarines Sur	711.5 (473.6 to 984.9)	950.7 (599.4 to 1453.9)	33.6% (-24.8 to 137.8)	3795.4 (2845.3 to 4929.1)	5144.4 (3510.0 to 7369.9)	35.5% (-14.3 to 114.9)	465.8 (290.5 to 698.0)	633.9 (382.5 to 987.9)	36.1% (-28.3 to 158.6)
Camiguin	713.0 (479.2 to 988.0)	950.1 (588.6 to 1380.5)	33.3% (-23.8 to 133.6)	3837.7 (2909.6 to 5006.7)	5141.9 (3548.5 to 7112.7)	34.0% (-13.6 to 108.2)	472.4 (291.0 to 707.9)	633.9 (370.2 to 978.8)	34.2% (-30.2 to 158.5)
Capiz	711.8 (478.4 to 1001.2)	948.5 (575.3 to 1437.2)	33.2% (-25.9 to 139.8)	3844.5 (2890.9 to 5019.7)	5186.4 (3480.6 to 7414.5)	34.9% (-15.3 to 115.4)	472.3 (293.8 to 707.5)	638.8 (366.7 to 1008.9)	35.2% (-30.7 to 164.6)
Catanduanes	709.0 (476.2 to 982.5)	950.7 (579.7 to 1395.1)	34.1% (-24.1 to 137.2)	3762.5 (2830.3 to 4893.3)	5112.6 (3523.3 to 7304.5)	35.9% (-13.6 to 114.3)	461.3 (286.6 to 681.5)	629.9 (350.4 to 1011.1)	36.6% (-31.0 to 171.0)
Cavite	716.8 (476.2 to 1002.0)	948.2 (571.4 to 1416.7)	32.3% (-26.4 to 138.1)	3940.1 (2972.4 to 5120.9)	5257.0 (3517.7 to 7500.6)	33.4% (-16.1 to 112.5)	485.4 (302.7 to 719.6)	646.1 (389.9 to 1021.6)	33.1% (-30.1 to 154.0)
Cebu	709.9 (465.2 to 986.3)	955.6 (584.1 to 1419.4)	34.6% (-24.7 to 141.4)	3898.0 (2927.7 to 5052.7)	5227.3 (3547.4 to 7378.5)	34.1% (-14.8 to 111.6)	479.1 (301.3 to 707.3)	643.5 (385.3 to 1008.3)	34.3% (-29.2 to 155.5)
Cotabato (North Cotabato)	713.6 (470.4 to 1003.5)	953.8 (588.1 to 1389.7)	33.7% (-24.5 to 137.1)	3845.2 (2901.0 to 5002.9)	5181.3 (3519.2 to 7357.3)	34.7% (-14.6 to 113.0)	472.3 (295.7 to 695.5)	638.6 (357.2 to 996.1)	35.2% (-30.7 to 164.4)
Davao de Oro	708.8 (476.9 to 988.7)	952.5 (573.8 to 1430.9)	34.4% (-24.9 to 141.1)	3825.9 (2893.9 to 4981.9)	5152.8 (3470.5 to 7234.7)	34.7% (-14.5 to 112.5)	470.8 (294.2 to 704.8)	634.4 (369.0 to 986.5)	34.7% (-30.0 to 160.1)
Davao Del Norte	713.6 (477.7 to 1001.7)	965.6 (595.8 to 1436.0)	35.3% (-23.9 to 140.9)	3868.0 (2901.3 to 4986.4)	5255.3 (3497.2 to 7397.0)	35.9% (-14.2 to 115.5)	474.4 (293.6 to 704.3)	647.6 (366.3 to 1006.7)	36.5% (-30.0 to 166.7)
Davao Del Sur	716.0 (472.6 to 984.8)	952.3 (591.8 to 1438.0)	33.0% (-25.3 to 137.0)	3918.8 (2977.8 to 5087.2)	5239.7 (3581.2 to 7377.4)	33.7% (-14.5 to 109.5)	480.9 (299.4 to 711.8)	644.2 (384.1 to 1011.3)	34.0% (-29.8 to 156.5)
Davao Occidental	707.2 (465.2 to 987.7)	956.9 (594.0 to 1451.3)	35.3% (-24.5 to 143.2)	3854.5 (2907.9 to 5010.8)	5141.6 (3543.6 to 7225.2)	33.4% (-14.5 to 109.0)	474.1 (295.3 to 702.1)	634.5 (376.3 to 994.9)	33.8% (-30.0 to 156.8)

Davao Oriental	717.1 (481.8 to 987.1)	954.4 (577.1 to 1411.9)	33.1% (-24.8 to 136.0)	3824.1 (2861.3 to 4989.4)	5172.3 (3499.3 to 7423.2)	35.3% (-15.0 to 115.8)	470.5 (293.7 to 694.7)	637.1 (369.7 to 1008.8)	35.4% (-30.0 to 162.9)
Dinagat Islands	705.0 (473.9 to 969.8)	952.8 (576.8 to 1419.6)	35.1% (-23.8 to 140.1)	3812.2 (2875.8 to 5002.9)	5164.6 (3516.9 to 7335.2)	35.5% (-14.2 to 114.6)	469.0 (289.3 to 691.2)	636.4 (380.8 to 986.6)	35.7% (-28.5 to 157.8)
Eastern Samar	709.7 (473.5 to 987.0)	941.3 (568.6 to 1403.9)	32.6% (-25.8 to 137.5)	3798.8 (2870.0 to 4945.0)	5147.3 (3495.1 to 7293.2)	35.5% (-14.0 to 113.9)	467.0 (298.0 to 681.6)	634.8 (373.9 to 997.1)	35.9% (-28.4 to 158.8)
Guimaras	707.2 (471.4 to 980.3)	955.2 (581.8 to 1431.3)	35.1% (-24.3 to 141.3)	3814.2 (2862.5 to 4946.4)	5187.7 (3554.4 to 7317.5)	36.0% (-13.3 to 114.0)	469.4 (295.6 to 695.6)	638.4 (372.7 to 993.0)	36.0% (-28.9 to 160.7)
Ifugao	707.4 (473.8 to 986.7)	953.1 (590.6 to 1422.9)	34.7% (-24.0 to 139.4)	3836.1 (2906.7 to 5014.4)	5189.1 (3459.9 to 7432.5)	35.3% (-15.2 to 116.0)	471.8 (291.6 to 704.8)	640.1 (369.5 to 1013.8)	35.7% (-30.5 to 165.2)
Ilocos Norte	710.2 (468.0 to 989.9)	952.3 (576.0 to 1424.3)	34.1% (-25.5 to 141.7)	3852.7 (2912.1 to 5035.6)	5206.8 (3477.5 to 7350.8)	35.1% (-14.8 to 114.8)	474.3 (301.0 to 705.3)	641.1 (361.6 to 994.7)	35.2% (-30.2 to 162.5)
Ilocos Sur	705.4 (465.6 to 981.9)	940.1 (575.7 to 1396.5)	33.3% (-25.3 to 138.3)	3831.7 (2883.3 to 4985.9)	5151.4 (3500.2 to 7239.3)	34.4% (-14.4 to 111.9)	471.5 (291.9 to 704.7)	635.2 (365.6 to 967.2)	34.7% (-29.8 to 159.1)
Iloilo	712.0 (472.2 to 992.4)	939.8 (559.8 to 1405.2)	32.0% (-26.8 to 138.4)	3840.2 (2901.2 to 4995.2)	5151.5 (3505.5 to 7262.4)	34.1% (-14.6 to 111.2)	471.3 (297.3 to 700.3)	634.9 (363.4 to 984.1)	34.7% (-30.1 to 160.4)
Isabela	714.8 (475.1 to 994.2)	954.7 (580.3 to 1429.2)	33.6% (-25.3 to 139.4)	3843.5 (2873.7 to 4961.8)	5191.6 (3447.6 to 7438.0)	35.1% (-15.5 to 116.1)	472.5 (295.6 to 699.4)	639.6 (379.7 to 986.3)	35.3% (-28.5 to 156.9)
Kalinga	718.1 (481.3 to 986.4)	934.4 (572.4 to 1358.9)	30.1% (-25.8 to 128.7)	3844.2 (2894.5 to 4975.7)	5101.9 (3440.2 to 7226.6)	32.7% (-16.0 to 110.1)	472.0 (295.2 to 708.3)	629.6 (356.1 to 979.0)	33.4% (-31.6 to 160.6)
La Union	707.1 (469.1 to 975.6)	943.4 (575.0 to 1399.7)	33.4% (-25.0 to 137.6)	3843.4 (2895.6 to 4998.4)	5170.3 (3474.3 to 7267.9)	34.5% (-14.8 to 112.7)	473.6 (295.6 to 704.4)	636.7 (366.9 to 988.4)	34.4% (-30.3 to 159.9)
Laguna	718.1 (477.7 to 998.7)	959.1 (597.6 to 1454.4)	33.5% (-25.1 to 138.4)	3924.9 (2960.4 to 5078.7)	5289.9 (3571.8 to 7632.9)	34.8% (-15.2 to 114.4)	482.1 (304.2 to 713.3)	652.4 (385.2 to 1013.9)	35.3% (-28.8 to 157.9)

Lanao Del Norte	716.1 (474.6 to 975.1)	950.5 (586.7 to 1400.1)	32.7% (-24.6 to 133.9)	3874.8 (2937.5 to 5025.0)	5195.7 (3485.7 to 7425.3)	34.1% (-15.5 to 112.8)	476.4 (300.9 to 711.6)	641.6 (359.9 to 1009.6)	34.7% (-31.2 to 164.4)
Lanao Del Sur	715.3 (483.0 to 997.1)	968.8 (598.4 to 1442.0)	35.4% (-23.4 to 139.8)	3849.3 (2915.1 to 5008.8)	5187.4 (3569.7 to 7352.8)	34.8% (-13.9 to 111.6)	472.0 (294.8 to 695.9)	638.8 (363.3 to 983.3)	35.3% (-29.8 to 161.6)
Leyte	712.5 (471.4 to 972.0)	951.2 (593.8 to 1412.8)	33.5% (-24.1 to 135.3)	3812.5 (2879.5 to 4908.4)	5176.8 (3540.3 to 7318.3)	35.8% (-13.2 to 113.0)	468.6 (293.9 to 695.3)	636.0 (379.1 to 974.9)	35.7% (-28.1 to 156.6)
Maguindanao	710.9 (474.4 to 983.8)	961.2 (574.6 to 1436.9)	35.2% (-24.5 to 142.8)	3811.5 (2863.9 to 4946.7)	5152.9 (3506.2 to 7337.7)	35.2% (-14.4 to 113.9)	468.1 (289.5 to 698.0)	632.4 (362.2 to 983.6)	35.1% (-30.4 to 162.9)
Marinduque	708.9 (478.3 to 965.4)	955.2 (589.7 to 1420.2)	34.7% (-23.0 to 136.4)	3762.7 (2847.4 to 4880.3)	5140.5 (3441.1 to 7261.8)	36.6% (-13.6 to 116.3)	460.5 (286.8 to 677.6)	634.8 (358.4 to 1019.9)	37.9% (-29.9 to 171.6)
Masbate	709.5 (469.6 to 983.3)	945.8 (587.4 to 1398.9)	33.3% (-24.6 to 136.2)	3794.6 (2811.6 to 4909.3)	5059.9 (3415.2 to 7246.8)	33.3% (-16.3 to 112.8)	465.4 (289.4 to 692.3)	624.0 (360.5 to 1000.4)	34.1% (-31.4 to 163.0)
Misamis Occidental	712.6 (474.5 to 989.0)	953.4 (587.4 to 1431.2)	33.8% (-24.9 to 138.6)	3872.8 (2924.8 to 5015.0)	5189.6 (3537.0 to 7263.3)	34.0% (-14.3 to 110.0)	475.4 (298.0 to 709.5)	638.9 (368.9 to 994.9)	34.4% (-30.3 to 159.7)
Misamis Oriental	713.1 (472.8 to 987.4)	946.4 (581.7 to 1434.0)	32.7% (-25.8 to 137.7)	3888.0 (2939.1 to 5019.7)	5182.0 (3543.5 to 7367.1)	33.3% (-15.1 to 109.7)	476.9 (301.8 to 702.2)	638.2 (361.9 to 987.2)	33.8% (-30.6 to 158.4)
Mountain Province	709.6 (477.9 to 984.5)	957.6 (594.7 to 1473.1)	34.9% (-24.2 to 140.9)	3827.8 (2864.0 to 4974.9)	5218.8 (3505.8 to 7587.8)	36.3% (-15.0 to 118.8)	470.8 (293.9 to 706.6)	643.6 (378.9 to 1014.2)	36.7% (-29.1 to 164.5)
National Capital Region	722.4 (470.6 to 1006.4)	950.0 (557.6 to 1456.2)	31.5% (-28.5 to 142.5)	4032.7 (3037.0 to 5247.1)	5313.0 (3533.2 to 7587.4)	31.7% (-17.4 to 110.5)	495.4 (306.3 to 732.2)	653.3 (392.2 to 1026.3)	31.9% (-30.8 to 151.9)
Negros Occidental	716.0 (471.4 to 995.5)	951.0 (600.9 to 1398.0)	32.8% (-24.3 to 133.3)	3850.9 (2911.5 to 4965.8)	5155.3 (3534.4 to 7201.3)	33.9% (-13.9 to 108.8)	473.0 (292.8 to 697.0)	636.4 (364.3 to 998.0)	34.5% (-30.7 to 162.2)
Negros Oriental	717.9 (481.7 to 985.4)	947.4 (576.8 to 1420.0)	32.0% (-25.5 to 134.5)	3852.8 (2890.2 to 4973.2)	5168.3 (3486.6 to 7376.9)	34.1% (-15.3 to 112.9)	473.1 (298.3 to 695.6)	637.8 (363.4 to 1022.3)	34.8% (-30.8 to 163.3)

Northern Samar	708.4 (474.7 to 982.7)	942.0 (586.2 to 1432.8)	33.0% (-25.2 to 136.7)	3798.7 (2868.7 to 4944.8)	5152.9 (3545.1 to 7384.8)	35.6% (-13.9 to 114.1)	466.2 (288.4 to 688.3)	632.3 (366.1 to 998.1)	35.6% (-30.1 to 163.8)
Nueva Ecija	711.6 (467.7 to 990.3)	943.2 (577.7 to 1384.8)	32.5% (-25.4 to 136.3)	3859.7 (2895.5 to 5005.4)	5159.9 (3518.1 to 7219.3)	33.7% (-14.7 to 110.2)	475.6 (292.3 to 698.7)	636.6 (365.5 to 999.4)	33.9% (-31.1 to 160.7)
Nueva Vizcaya	704.5 (466.0 to 979.8)	949.6 (575.7 to 1425.4)	34.8% (-24.9 to 142.5)	3815.4 (2868.0 to 4967.2)	5195.2 (3460.0 to 7481.0)	36.2% (-15.0 to 118.3)	470.1 (290.5 to 711.4)	640.2 (368.2 to 996.3)	36.2% (-30.0 to 165.5)
Occidental Mindoro	709.5 (473.8 to 985.1)	944.3 (580.4 to 1400.1)	33.1% (-24.9 to 136.5)	3792.4 (2851.6 to 4932.5)	5077.4 (3477.5 to 7247.1)	33.9% (-15.1 to 111.6)	466.2 (290.2 to 683.6)	625.9 (372.1 to 1012.9)	34.3% (-30.5 to 160.1)
Oriental Mindoro	708.7 (474.4 to 980.2)	954.5 (601.3 to 1435.3)	34.7% (-23.6 to 137.9)	3803.8 (2870.3 to 4930.2)	5178.0 (3558.9 to 7364.9)	36.1% (-13.2 to 114.0)	467.6 (288.3 to 696.0)	637.4 (371.5 to 976.6)	36.3% (-28.8 to 161.5)
Palawan	709.5 (477.3 to 984.0)	952.1 (585.4 to 1410.1)	34.2% (-24.0 to 137.3)	3847.4 (2882.1 to 4996.7)	5184.2 (3536.9 to 7318.4)	34.7% (-14.3 to 112.6)	472.5 (295.0 to 695.5)	638.1 (379.5 to 985.8)	35.1% (-28.6 to 156.3)
Pampanga	713.3 (472.1 to 996.5)	946.4 (569.6 to 1430.7)	32.7% (-26.6 to 140.1)	3880.9 (2914.6 to 5075.5)	5248.7 (3509.0 to 7422.3)	35.2% (-14.9 to 115.4)	477.7 (301.0 to 698.4)	645.9 (383.8 to 1046.0)	35.2% (-29.8 to 160.8)
Pangasinan	711.2 (480.7 to 988.0)	952.1 (577.0 to 1409.6)	33.9% (-24.5 to 137.5)	3837.8 (2926.1 to 4998.6)	5191.8 (3501.8 to 7322.5)	35.3% (-14.0 to 113.3)	471.9 (294.4 to 705.7)	640.4 (376.8 to 1013.0)	35.7% (-29.7 to 162.4)
Quezon	711.7 (474.7 to 988.5)	950.9 (579.2 to 1411.7)	33.6% (-24.9 to 138.1)	3828.9 (2904.5 to 5035.1)	5167.4 (3527.9 to 7321.9)	35.0% (-14.3 to 113.2)	469.7 (290.5 to 702.5)	637.4 (375.6 to 1000.9)	35.7% (-29.6 to 162.0)
Quirino	713.7 (480.1 to 966.6)	943.6 (585.4 to 1420.3)	32.2% (-24.6 to 132.5)	3857.7 (2912.0 to 5001.3)	5135.7 (3487.9 to 7381.3)	33.1% (-15.9 to 111.2)	473.3 (297.6 to 708.4)	633.9 (366.9 to 1013.7)	33.9% (-31.3 to 161.8)
Rizal	715.4 (462.0 to 997.0)	952.0 (584.1 to 1441.6)	33.1% (-26.4 to 141.4)	3945.4 (2965.5 to 5099.1)	5255.3 (3614.9 to 7550.4)	33.2% (-15.4 to 110.2)	485.8 (303.3 to 710.5)	646.6 (378.9 to 1008.0)	33.1% (-30.3 to 154.8)
Romblon	709.9 (482.8 to 980.7)	954.7 (582.0 to 1429.9)	34.5% (-23.9 to 138.4)	3775.9 (2820.3 to 4916.7)	5123.6 (3476.4 to 7232.0)	35.7% (-14.1 to 115.0)	462.2 (286.2 to 690.8)	630.4 (365.8 to 971.4)	36.4% (-29.1 to 162.8)

Samar (Western Samar)	708.2 (471.7 to 983.4)	953.5 (587.6 to 1422.5)	34.6% (-24.2 to 139.8)	3792.2 (2862.4 to 4954.6)	5206.2 (3501.1 to 7481.6)	37.3% (-13.9 to 119.1)	465.0 (291.4 to 682.2)	641.1 (380.5 to 1025.5)	37.9% (-28.2 to 165.6)
Sarangani	712.5 (484.7 to 984.4)	960.0 (589.7 to 1425.2)	34.7% (-23.3 to 137.1)	3857.6 (2925.0 to 5021.0)	5208.5 (3555.7 to 7411.8)	35.0% (-14.2 to 112.8)	474.1 (292.8 to 700.1)	640.3 (367.5 to 997.9)	35.1% (-30.3 to 162.2)
Siquijor	706.6 (474.3 to 969.7)	938.6 (563.1 to 1431.5)	32.8% (-25.9 to 139.4)	3755.3 (2827.4 to 4925.8)	5111.5 (3505.9 to 7334.2)	36.1% (-13.9 to 116.0)	460.7 (291.0 to 681.1)	629.4 (369.3 to 970.3)	36.6% (-28.1 to 160.1)
Sorsogon	706.5 (474.5 to 982.5)	946.2 (586.2 to 1413.4)	33.9% (-24.4 to 137.5)	3785.5 (2830.6 to 4884.3)	5153.3 (3506.3 to 7366.7)	36.1% (-13.9 to 115.7)	465.1 (293.0 to 687.3)	636.6 (372.6 to 1016.2)	36.9% (-29.1 to 165.1)
South Cotabato	712.1 (472.4 to 994.3)	942.3 (568.8 to 1394.7)	32.3% (-26.1 to 137.5)	3871.1 (2927.2 to 5035.6)	5183.0 (3476.4 to 7394.9)	33.9% (-15.7 to 112.9)	476.4 (301.3 to 704.1)	638.3 (364.2 to 1002.9)	34.0% (-30.8 to 160.0)
Southern Leyte	704.6 (472.0 to 981.5)	952.7 (602.3 to 1405.4)	35.2% (-22.7 to 137.0)	3786.6 (2860.9 to 4933.3)	5163.1 (3450.6 to 7312.3)	36.4% (-14.1 to 116.7)	464.5 (288.1 to 689.3)	633.5 (365.1 to 988.0)	36.4% (-29.5 to 164.5)
Sultan Kudarat	712.4 (469.0 to 990.3)	964.1 (580.7 to 1444.1)	35.3% (-24.9 to 144.1)	3831.2 (2897.0 to 4938.4)	5230.4 (3524.2 to 7434.8)	36.5% (-13.5 to 115.7)	470.3 (288.3 to 693.7)	646.0 (383.2 to 1026.2)	37.4% (-28.8 to 165.8)
Sulu	718.6 (484.5 to 995.4)	958.7 (599.0 to 1424.6)	33.4% (-24.1 to 134.8)	3867.6 (2921.2 to 4999.8)	5142.8 (3508.7 to 7321.5)	33.0% (-15.5 to 109.7)	475.4 (294.6 to 698.6)	633.8 (367.1 to 1012.1)	33.3% (-31.4 to 160.2)
Surigao Del Norte	711.2 (475.2 to 992.5)	944.0 (574.0 to 1389.6)	32.7% (-25.4 to 136.6)	3851.8 (2878.1 to 5026.8)	5153.1 (3502.8 to 7217.7)	33.8% (-15.0 to 111.3)	473.3 (297.7 to 707.5)	633.3 (377.7 to 967.2)	33.8% (-29.1 to 152.5)
Surigao Del Sur	718.8 (483.4 to 986.2)	951.6 (590.8 to 1462.7)	32.4% (-25.4 to 135.7)	3834.3 (2887.8 to 4962.3)	5138.5 (3500.9 to 7438.6)	34.0% (-15.5 to 113.0)	470.4 (296.8 to 700.7)	634.1 (357.7 to 1006.0)	34.8% (-31.2 to 164.7)
Tarlac	711.2 (480.5 to 1001.7)	947.5 (581.9 to 1426.3)	33.2% (-25.3 to 138.0)	3849.2 (2892.7 to 5048.4)	5189.1 (3471.0 to 7344.6)	34.8% (-15.2 to 114.9)	473.2 (294.7 to 703.4)	638.8 (380.0 to 989.7)	35.0% (-29.0 to 157.2)
Tawi-Tawi	707.5 (474.6 to 984.7)	955.6 (568.6 to 1419.2)	35.1% (-24.5 to 142.4)	3792.3 (2865.8 to 4939.9)	5176.7 (3484.6 to 7381.6)	36.5% (-13.9 to 116.9)	465.3 (288.4 to 677.8)	639.3 (359.8 to 1017.6)	37.4% (-29.8 to 169.6)

Zambales	713.6 (478.7 to 993.0)	947.8 (566.2 to 1395.1)	32.8% (-25.5 to 137.1)	3884.7 (2925.2 to 5034.9)	5195.5 (3586.1 to 7424.9)	33.7% (-14.9 to 110.5)	477.2 (297.4 to 709.7)	639.6 (373.2 to 994.3)	34.0% (-30.2 to 158.1)
Zamboanga Del Norte	706.5 (465.9 to 982.1)	956.5 (600.1 to 1442.4)	35.4% (-23.8 to 141.3)	3843.5 (2890.7 to 4984.1)	5128.6 (3487.7 to 7315.1)	33.4% (-15.5 to 111.2)	472.0 (296.8 to 691.9)	632.4 (363.7 to 987.1)	34.0% (-30.3 to 158.5)
Zamboanga Del Sur	716.6 (483.5 to 1003.3)	953.3 (585.9 to 1423.2)	33.0% (-25.1 to 136.5)	3863.3 (2903.2 to 5062.5)	5204.6 (3553.7 to 7463.7)	34.7% (-15.0 to 114.1)	473.5 (295.5 to 703.1)	641.9 (371.7 to 1011.2)	35.6% (-30.0 to 163.1)
Zamboanga Sibugay	712.5 (482.0 to 978.8)	958.9 (590.1 to 1424.0)	34.6% (-23.4 to 136.7)	3861.3 (2914.1 to 4997.2)	5163.0 (3475.4 to 7334.8)	33.7% (-15.4 to 111.7)	475.3 (300.0 to 707.2)	635.3 (374.7 to 988.8)	33.7% (-29.8 to 155.3)
Depressive disorders									
Abra	2696.2 (1830.4 to 3812.1)	3359.4 (2128.3 to 5003.7)	24.6% (-29.0 to 119.3)	2111.4 (1520.7 to 2872.1)	2556.5 (1752.5 to 3606.0)	21.1% (-25.0 to 95.9)	392.0 (237.5 to 607.6)	486.8 (278.4 to 778.4)	24.2% (-37.8 to 148.4)
Agusan Del Norte	2740.0 (1840.2 to 3926.4)	3356.3 (2099.3 to 5046.0)	22.5% (-31.3 to 119.0)	2148.1 (1553.3 to 2913.3)	2550.8 (1718.5 to 3680.1)	18.7% (-27.5 to 94.9)	398.6 (241.0 to 605.8)	486.2 (275.7 to 773.0)	22.0% (-38.7 to 143.6)
Agusan Del Sur	2635.6 (1784.1 to 3740.5)	3287.0 (2046.8 to 4826.8)	24.7% (-29.2 to 120.1)	2056.4 (1487.2 to 2785.9)	2482.3 (1695.4 to 3502.5)	20.7% (-25.2 to 95.2)	381.4 (230.5 to 582.4)	473.2 (263.3 to 764.4)	24.1% (-38.7 to 151.7)
Aklan	2678.7 (1801.1 to 3826.8)	3379.7 (2156.3 to 5039.8)	26.2% (-28.3 to 122.6)	2095.1 (1522.0 to 2856.9)	2583.3 (1781.1 to 3686.3)	23.3% (-23.7 to 99.6)	388.5 (237.5 to 597.0)	492.3 (289.8 to 794.0)	26.7% (-35.7 to 150.0)
Albay	2644.4 (1779.5 to 3727.2)	3342.3 (2105.3 to 4979.9)	26.4% (-28.3 to 123.4)	2063.7 (1505.9 to 2794.0)	2546.0 (1746.0 to 3621.2)	23.4% (-23.5 to 99.4)	382.2 (227.8 to 589.1)	483.5 (271.9 to 755.9)	26.5% (-36.8 to 153.0)
Antique	2620.0 (1763.9 to 3706.4)	3332.7 (2064.1 to 5041.5)	27.2% (-28.9 to 127.9)	2048.2 (1486.7 to 2784.8)	2527.5 (1703.9 to 3620.7)	23.4% (-24.4 to 101.9)	379.9 (228.8 to 582.9)	480.6 (272.0 to 757.7)	26.5% (-36.5 to 152.3)
Apayao	2674.1 (1797.0 to 3812.4)	3365.0 (2085.0 to 5162.7)	25.8% (-30.2 to 127.3)	2103.4 (1520.6 to 2848.0)	2572.2 (1736.8 to 3668.8)	22.3% (-25.0 to 99.6)	389.5 (235.5 to 602.6)	488.4 (279.8 to 803.0)	25.4% (-37.9 to 154.2)
Aurora	2645.7 (1782.6 to 3809.1)	3317.5 (2091.7 to 4887.4)	25.4% (-28.9 to 121.6)	2069.9 (1501.8 to 2806.2)	2512.6 (1719.9 to 3587.5)	21.4% (-25.0 to 97.1)	383.3 (231.5 to 590.4)	479.2 (276.8 to 761.1)	25.0% (-36.9 to 148.0)

Basilan	2642.5 (1761.0 to 3754.3)	3295.1 (2019.0 to 5003.8)	24.7% (-31.0 to 125.7)	2071.9 (1509.8 to 2808.0)	2501.1 (1680.3 to 3522.4)	20.7% (-25.5 to 96.0)	383.2 (228.2 to 594.3)	475.2 (273.9 to 761.9)	24.0% (-38.2 to 148.7)
Bataan	2739.4 (1852.7 to 3863.6)	3439.6 (2222.7 to 5104.0)	25.6% (-27.7 to 118.6)	2167.0 (1578.8 to 2930.8)	2642.3 (1824.4 to 3684.6)	21.9% (-23.5 to 94.8)	401.2 (237.0 to 616.0)	501.7 (294.9 to 812.7)	25.1% (-37.3 to 149.9)
Batanes	2648.0 (1809.8 to 3704.5)	3354.5 (2108.1 to 5099.8)	26.7% (-28.2 to 123.7)	2058.6 (1498.8 to 2775.3)	2545.6 (1740.0 to 3675.5)	23.7% (-23.8 to 100.9)	383.3 (234.7 to 584.4)	484.8 (275.7 to 767.7)	26.5% (-36.1 to 151.2)
Batangas	2695.2 (1820.7 to 3806.9)	3421.6 (2156.4 to 5171.6)	27.0% (-28.4 to 125.4)	2124.6 (1549.2 to 2892.4)	2640.9 (1821.3 to 3783.8)	24.3% (-23.1 to 101.4)	393.9 (237.0 to 603.0)	500.6 (283.9 to 812.5)	27.1% (-36.9 to 156.7)
Benguet	2773.1 (1871.7 to 3956.4)	3531.4 (2238.4 to 5396.5)	27.3% (-28.5 to 127.5)	2203.0 (1610.5 to 2982.7)	2733.8 (1889.3 to 3883.2)	24.1% (-22.7 to 99.7)	408.1 (243.4 to 637.6)	516.7 (293.4 to 851.7)	26.6% (-38.0 to 159.1)
Biliran	2624.9 (1768.8 to 3751.0)	3284.2 (2073.6 to 4894.9)	25.1% (-29.2 to 121.4)	2045.2 (1489.0 to 2769.4)	2483.2 (1697.7 to 3542.9)	21.4% (-25.0 to 96.8)	380.1 (231.9 to 584.5)	473.2 (273.2 to 753.2)	24.5% (-37.0 to 146.6)
Bohol	2713.0 (1838.5 to 3850.3)	3390.5 (2097.8 to 5070.3)	25.0% (-29.7 to 122.7)	2112.7 (1520.8 to 2856.0)	2566.5 (1746.7 to 3612.6)	21.5% (-24.8 to 96.7)	393.1 (238.7 to 609.5)	488.2 (280.2 to 759.9)	24.2% (-37.0 to 145.2)
Bukidnon	2675.8 (1806.7 to 3799.5)	3310.4 (2081.0 to 5029.4)	23.7% (-30.5 to 120.7)	2095.5 (1515.5 to 2833.0)	2505.0 (1706.8 to 3625.3)	19.5% (-26.8 to 95.4)	388.9 (237.7 to 584.0)	476.8 (278.2 to 769.4)	22.6% (-37.6 to 141.6)
Bulacan	2737.6 (1841.2 to 3878.8)	3453.9 (2217.9 to 5152.0)	26.2% (-27.9 to 121.3)	2166.2 (1568.4 to 2932.4)	2658.7 (1814.6 to 3824.1)	22.7% (-24.6 to 100.0)	401.9 (241.5 to 607.4)	504.3 (287.7 to 823.8)	25.5% (-37.5 to 152.7)
Cagayan	2655.3 (1792.2 to 3778.8)	3373.4 (2080.7 to 5136.9)	27.0% (-29.3 to 128.6)	2078.6 (1498.9 to 2819.8)	2579.7 (1748.7 to 3731.3)	24.1% (-24.2 to 103.7)	385.6 (234.1 to 588.2)	490.0 (276.8 to 813.1)	27.1% (-37.4 to 158.8)
Camarines Norte	2644.8 (1797.7 to 3749.5)	3308.6 (2047.9 to 4982.7)	25.1% (-29.8 to 123.1)	2063.5 (1487.2 to 2802.1)	2505.3 (1701.4 to 3594.2)	21.4% (-25.6 to 98.6)	382.5 (235.1 to 585.9)	476.8 (270.9 to 763.2)	24.6% (-37.4 to 148.6)
Camarines Sur	2638.3 (1768.5 to 3735.7)	3341.1 (2103.1 to 5069.8)	26.6% (-28.9 to 126.2)	2052.8 (1484.7 to 2789.3)	2533.6 (1735.2 to 3647.3)	23.4% (-24.1 to 101.3)	380.8 (230.0 to 577.4)	482.7 (269.3 to 769.6)	26.8% (-36.8 to 154.9)

Camiguin	2748.1 (1844.7 to 3939.3)	3350.7 (2131.7 to 4974.3)	21.9% (-30.8 to 115.3)	2150.5 (1564.8 to 2930.9)	2532.1 (1742.6 to 3564.4)	17.7% (-26.7 to 89.6)	400.0 (241.5 to 619.2)	482.2 (279.0 to 767.3)	20.5% (-39.3 to 139.4)
Capiz	2652.8 (1788.8 to 3754.8)	3392.0 (2154.3 to 5167.8)	27.9% (-27.9 to 127.4)	2078.8 (1502.1 to 2785.4)	2604.0 (1772.7 to 3700.7)	25.3% (-22.5 to 102.9)	384.9 (230.1 to 587.3)	494.4 (283.3 to 804.3)	28.5% (-36.0 to 158.8)
Catanduanes	2611.1 (1745.8 to 3740.7)	3311.4 (2077.5 to 5028.3)	26.8% (-29.2 to 127.7)	2025.1 (1469.1 to 2750.1)	2502.0 (1710.2 to 3518.7)	23.5% (-23.3 to 99.4)	376.3 (228.4 to 568.3)	475.9 (269.3 to 766.5)	26.5% (-36.7 to 153.2)
Cavite	2778.8 (1882.4 to 3966.8)	3505.3 (2216.2 to 5272.0)	26.1% (-28.7 to 123.8)	2208.8 (1612.0 to 3036.6)	2711.2 (1818.2 to 3830.9)	22.7% (-24.7 to 100.6)	409.8 (247.9 to 629.9)	514.0 (291.6 to 826.5)	25.4% (-37.4 to 152.4)
Cebu	2735.2 (1846.3 to 3882.4)	3392.3 (2123.0 to 5153.6)	24.0% (-30.5 to 121.6)	2161.6 (1564.9 to 2916.9)	2595.6 (1752.3 to 3739.9)	20.1% (-26.4 to 96.3)	400.1 (240.3 to 614.0)	492.0 (285.0 to 788.8)	23.0% (-38.1 to 144.7)
Cotabato (North Cotabato)	2688.4 (1819.2 to 3812.7)	3364.3 (2127.4 to 5029.0)	25.1% (-29.0 to 121.1)	2103.3 (1510.8 to 2843.3)	2557.3 (1767.0 to 3585.4)	21.6% (-24.2 to 95.5)	391.1 (235.9 to 603.4)	486.6 (283.9 to 777.6)	24.4% (-37.2 to 146.6)
Davao de Oro	2693.4 (1824.6 to 3804.4)	3338.9 (2083.2 to 5101.1)	24.0% (-30.5 to 121.4)	2115.5 (1539.5 to 2876.6)	2537.9 (1729.7 to 3624.5)	20.0% (-26.1 to 95.1)	392.7 (234.7 to 604.0)	481.6 (280.9 to 771.4)	22.6% (-38.3 to 143.8)
Davao Del Norte	2728.8 (1838.4 to 3911.0)	3391.9 (2168.4 to 5020.6)	24.3% (-29.1 to 118.3)	2142.1 (1566.3 to 2876.5)	2586.9 (1769.5 to 3649.4)	20.8% (-24.7 to 94.1)	397.8 (243.7 to 610.1)	491.3 (286.2 to 785.9)	23.5% (-37.3 to 143.8)
Davao Del Sur	2737.5 (1841.8 to 3883.3)	3436.2 (2162.7 to 5225.7)	25.5% (-29.5 to 124.2)	2164.7 (1577.5 to 2935.6)	2643.7 (1792.4 to 3827.1)	22.1% (-25.1 to 99.5)	400.7 (242.7 to 621.1)	500.7 (279.5 to 831.6)	25.0% (-39.1 to 157.2)
Davao Occidental	2759.6 (1874.4 to 3931.8)	3311.9 (2033.1 to 5070.7)	20.0% (-33.3 to 116.1)	2180.9 (1576.3 to 2947.2)	2494.6 (1689.6 to 3560.2)	14.4% (-29.7 to 86.4)	405.1 (247.3 to 618.7)	476.4 (271.0 to 765.0)	17.6% (-41.0 to 134.9)
Davao Oriental	2632.9 (1775.3 to 3743.6)	3337.7 (2058.4 to 4956.5)	26.8% (-28.7 to 126.2)	2057.6 (1485.9 to 2812.1)	2535.3 (1705.8 to 3571.7)	23.2% (-24.3 to 101.0)	382.1 (231.8 to 582.2)	482.1 (269.9 to 761.2)	26.2% (-36.8 to 152.4)
Dinagat Islands	2711.3 (1816.1 to 3847.6)	3361.7 (2080.2 to 5040.2)	24.0% (-30.6 to 122.0)	2116.8 (1528.8 to 2902.9)	2549.2 (1751.8 to 3682.2)	20.4% (-26.2 to 96.9)	394.0 (236.5 to 611.2)	486.1 (279.3 to 790.0)	23.4% (-38.7 to 148.7)

Eastern Samar	2646.2 (1776.9 to 3787.5)	3392.2 (2117.0 to 5122.9)	28.2% (-28.3 to 129.8)	2063.1 (1494.0 to 2798.5)	2584.1 (1784.0 to 3643.5)	25.3% (-22.0 to 101.6)	382.6 (231.6 to 589.8)	492.1 (287.1 to 789.2)	28.6% (-35.1 to 155.0)
Guimaras	2688.3 (1821.5 to 3856.3)	3408.6 (2129.1 to 5170.7)	26.8% (-29.1 to 127.2)	2103.2 (1533.9 to 2870.9)	2598.2 (1745.6 to 3775.9)	23.5% (-24.8 to 103.1)	391.3 (243.3 to 601.7)	492.9 (283.2 to 808.3)	26.0% (-36.9 to 152.3)
Ifugao	2720.2 (1831.2 to 3832.3)	3418.9 (2138.5 to 5131.6)	25.7% (-29.1 to 123.3)	2137.7 (1551.3 to 2886.1)	2611.9 (1759.4 to 3750.7)	22.2% (-25.1 to 99.5)	396.3 (238.1 to 600.7)	497.6 (284.1 to 801.1)	25.6% (-37.1 to 151.6)
Ilocos Norte	2740.3 (1862.7 to 3891.8)	3425.8 (2151.2 to 5232.9)	25.0% (-29.8 to 123.1)	2154.8 (1561.5 to 2922.3)	2621.7 (1790.9 to 3759.7)	21.7% (-25.1 to 98.1)	400.4 (241.8 to 613.5)	497.3 (283.3 to 776.1)	24.2% (-37.2 to 145.6)
Ilocos Sur	2688.1 (1805.0 to 3797.0)	3382.9 (2132.1 to 5120.5)	25.8% (-29.1 to 124.1)	2108.0 (1518.6 to 2852.6)	2585.1 (1763.3 to 3733.1)	22.6% (-24.9 to 100.5)	392.1 (237.6 to 595.0)	492.4 (287.2 to 811.4)	25.6% (-37.0 to 151.1)
Iloilo	2697.5 (1811.7 to 3820.4)	3405.6 (2144.6 to 5038.8)	26.2% (-28.3 to 122.6)	2116.4 (1530.3 to 2863.2)	2606.5 (1800.9 to 3687.5)	23.2% (-23.4 to 98.2)	393.5 (237.0 to 608.5)	495.5 (289.0 to 780.0)	25.9% (-36.1 to 148.7)
Isabela	2697.1 (1822.6 to 3828.1)	3389.8 (2119.8 to 5069.7)	25.7% (-29.1 to 123.3)	2112.8 (1537.9 to 2851.0)	2588.2 (1772.6 to 3679.2)	22.5% (-24.0 to 97.9)	392.6 (231.9 to 598.1)	492.0 (278.5 to 781.8)	25.3% (-37.5 to 151.5)
Kalinga	2653.7 (1786.7 to 3782.6)	3302.8 (2068.6 to 4954.7)	24.5% (-29.9 to 121.8)	2081.3 (1512.2 to 2802.6)	2521.6 (1708.3 to 3637.6)	21.2% (-25.5 to 97.4)	385.4 (230.7 to 591.5)	479.0 (275.2 to 757.3)	24.3% (-37.5 to 146.9)
La Union	2700.7 (1808.8 to 3879.3)	3394.5 (2152.9 to 5109.6)	25.7% (-29.2 to 123.7)	2124.5 (1542.1 to 2912.8)	2602.6 (1777.8 to 3702.6)	22.5% (-24.5 to 99.3)	394.8 (236.5 to 600.9)	495.1 (288.2 to 803.2)	25.4% (-37.0 to 150.2)
Laguna	2757.2 (1839.0 to 3936.2)	3502.2 (2252.3 to 5305.0)	27.0% (-28.2 to 125.3)	2185.0 (1586.1 to 2996.8)	2705.4 (1870.3 to 3824.9)	23.8% (-23.1 to 99.9)	404.7 (244.8 to 620.6)	512.7 (299.3 to 834.8)	26.7% (-36.3 to 152.6)
Lanao Del Norte	2702.9 (1814.6 to 3843.1)	3389.3 (2130.5 to 5158.0)	25.4% (-29.7 to 124.5)	2126.5 (1537.0 to 2867.3)	2573.4 (1765.1 to 3674.6)	21.0% (-25.2 to 96.2)	394.7 (236.7 to 598.9)	489.1 (280.5 to 775.4)	23.9% (-37.4 to 145.9)
Lanao Del Sur	2655.9 (1810.1 to 3774.1)	3239.7 (2025.7 to 4958.7)	22.0% (-31.6 to 117.9)	2080.1 (1508.1 to 2808.3)	2443.2 (1681.1 to 3593.4)	17.5% (-28.0 to 92.0)	384.8 (230.1 to 586.5)	464.9 (256.3 to 735.0)	20.8% (-40.1 to 144.4)

Leyte	2648.7 (1801.4 to 3803.4)	3373.9 (2154.8 to 5135.2)	27.4% (-28.1 to 126.3)	2069.5 (1496.8 to 2823.0)	2573.9 (1782.8 to 3722.1)	24.4% (-23.4 to 102.5)	384.0 (236.5 to 586.7)	487.3 (289.1 to 790.7)	26.9% (-35.3 to 149.5)
Maguindanao	2549.1 (1711.8 to 3602.4)	3269.2 (2008.3 to 4971.6)	28.2% (-28.6 to 130.7)	1985.6 (1443.3 to 2687.2)	2475.3 (1661.2 to 3553.1)	24.7% (-23.6 to 103.8)	368.0 (222.3 to 561.0)	470.2 (265.4 to 752.3)	27.8% (-36.2 to 156.5)
Marinduque	2659.8 (1800.9 to 3779.4)	3352.1 (2079.0 to 5097.3)	26.0% (-29.6 to 125.9)	2051.3 (1491.7 to 2770.1)	2516.5 (1721.3 to 3615.2)	22.7% (-24.3 to 99.3)	382.6 (231.7 to 587.8)	480.8 (281.6 to 767.2)	25.7% (-36.3 to 147.9)
Masbate	2627.8 (1771.1 to 3749.8)	3229.0 (2040.7 to 4887.2)	22.9% (-30.8 to 119.0)	2047.0 (1494.1 to 2791.4)	2426.3 (1642.8 to 3448.9)	18.5% (-27.0 to 92.8)	378.7 (226.5 to 573.2)	462.6 (261.1 to 761.7)	22.2% (-39.8 to 148.4)
Misamis Occidental	2717.1 (1816.9 to 3865.8)	3392.8 (2099.4 to 5136.2)	24.9% (-30.5 to 124.7)	2137.9 (1552.3 to 2881.1)	2586.6 (1741.0 to 3733.2)	21.0% (-25.9 to 97.7)	395.9 (238.5 to 610.0)	492.0 (273.0 to 795.1)	24.3% (-38.9 to 153.4)
Misamis Oriental	2740.5 (1850.0 to 3904.2)	3428.3 (2128.8 to 5172.9)	25.1% (-30.0 to 123.9)	2163.0 (1573.2 to 2920.9)	2620.6 (1776.1 to 3730.9)	21.2% (-25.3 to 96.8)	401.1 (240.4 to 618.3)	497.1 (278.9 to 806.9)	23.9% (-38.9 to 152.4)
Mountain Province	2669.3 (1795.2 to 3784.5)	3378.4 (2129.0 to 5062.3)	26.6% (-28.5 to 124.5)	2096.0 (1522.7 to 2844.5)	2580.7 (1748.7 to 3695.5)	23.1% (-24.4 to 100.9)	390.1 (233.7 to 592.4)	490.6 (281.4 to 789.7)	25.8% (-36.9 to 151.5)
National Capital Region	2896.2 (1949.6 to 4109.5)	3558.2 (2241.1 to 5275.4)	22.9% (-30.3 to 116.9)	2325.1 (1681.6 to 3141.4)	2764.4 (1896.3 to 3859.4)	18.9% (-25.8 to 90.9)	428.4 (254.2 to 660.5)	523.6 (305.8 to 860.0)	22.2% (-39.2 to 146.0)
Negros Occidental	2696.7 (1802.7 to 3827.9)	3384.4 (2142.2 to 5062.4)	25.5% (-29.0 to 122.3)	2111.1 (1530.1 to 2870.1)	2575.5 (1780.9 to 3672.2)	22.0% (-24.4 to 97.2)	391.8 (233.1 to 600.1)	489.3 (289.5 to 771.7)	24.9% (-36.4 to 145.2)
Negros Oriental	2654.8 (1780.2 to 3767.2)	3319.0 (2024.6 to 5140.3)	25.0% (-31.1 to 127.2)	2085.5 (1513.5 to 2816.7)	2529.5 (1722.2 to 3731.6)	21.3% (-26.0 to 99.1)	386.8 (233.0 to 590.3)	480.7 (279.0 to 791.5)	24.3% (-38.0 to 150.0)
Northern Samar	2624.0 (1765.3 to 3709.7)	3368.7 (2106.5 to 4994.5)	28.4% (-27.3 to 127.3)	2045.3 (1478.8 to 2748.4)	2568.4 (1745.8 to 3601.8)	25.6% (-22.0 to 102.6)	379.3 (228.3 to 577.0)	488.0 (273.3 to 768.4)	28.6% (-35.5 to 157.5)
Nueva Ecija	2718.9 (1830.6 to 3852.8)	3386.3 (2066.7 to 5191.6)	24.5% (-31.0 to 125.1)	2146.2 (1548.3 to 2930.9)	2593.0 (1744.3 to 3737.6)	20.8% (-26.5 to 98.9)	397.5 (240.5 to 598.1)	492.9 (281.8 to 785.7)	24.0% (-37.4 to 146.2)

Nueva Vizcaya	2667.2 (1796.2 to 3797.6)	3373.4 (2081.5 to 5101.6)	26.5% (-29.5 to 127.3)	2090.1 (1513.9 to 2829.7)	2573.4 (1756.3 to 3716.0)	23.1% (-24.4 to 101.0)	388.8 (233.8 to 602.8)	489.0 (276.9 to 775.1)	25.8% (-37.2 to 152.1)
Occidental Mindoro	2705.5 (1833.6 to 3861.2)	3366.8 (2083.5 to 5146.1)	24.4% (-30.7 to 123.8)	2105.1 (1516.6 to 2861.6)	2540.2 (1735.3 to 3721.9)	20.7% (-26.6 to 98.6)	391.6 (236.9 to 596.3)	484.0 (276.1 to 819.3)	23.6% (-39.4 to 152.9)
Oriental Mindoro	2661.6 (1796.8 to 3782.3)	3296.9 (2070.0 to 4922.4)	23.9% (-30.0 to 119.7)	2075.1 (1505.4 to 2811.3)	2501.4 (1713.6 to 3538.6)	20.5% (-25.2 to 94.8)	384.4 (234.8 to 588.9)	475.0 (270.1 to 770.3)	23.6% (-38.3 to 148.3)
Palawan	2688.4 (1801.2 to 3832.3)	3341.6 (2131.7 to 5059.6)	24.3% (-29.9 to 120.7)	2112.9 (1529.6 to 2870.5)	2551.0 (1773.5 to 3691.0)	20.7% (-25.5 to 96.1)	392.5 (235.6 to 595.2)	484.7 (280.1 to 777.0)	23.5% (-37.7 to 145.5)
Pampanga	2732.1 (1850.0 to 3893.3)	3436.2 (2162.2 to 5276.6)	25.8% (-29.7 to 125.4)	2160.1 (1563.8 to 2936.4)	2654.1 (1836.7 to 3799.6)	22.9% (-24.0 to 98.9)	400.4 (239.3 to 612.6)	502.8 (290.4 to 814.7)	25.6% (-37.2 to 151.6)
Pangasinan	2686.7 (1814.0 to 3818.3)	3387.7 (2144.5 to 5161.2)	26.1% (-29.1 to 124.7)	2105.1 (1519.4 to 2843.9)	2580.9 (1745.1 to 3675.3)	22.6% (-24.6 to 99.8)	390.8 (238.4 to 601.4)	491.4 (287.4 to 804.0)	25.7% (-36.8 to 150.8)
Quezon	2664.7 (1794.5 to 3792.0)	3325.6 (2083.6 to 4974.5)	24.8% (-29.6 to 122.0)	2093.5 (1524.5 to 2857.4)	2533.8 (1740.5 to 3584.4)	21.0% (-24.9 to 95.5)	386.7 (234.6 to 605.0)	481.3 (279.1 to 785.6)	24.5% (-38.0 to 150.1)
Quirino	2740.8 (1846.7 to 3911.2)	3377.0 (2121.8 to 5146.2)	23.2% (-31.0 to 120.7)	2149.6 (1555.0 to 2896.4)	2566.8 (1745.7 to 3674.0)	19.4% (-26.5 to 94.3)	398.2 (239.0 to 615.8)	489.6 (276.8 to 805.3)	23.0% (-39.6 to 150.9)
Rizal	2803.3 (1889.9 to 3999.0)	3421.6 (2161.4 to 5123.4)	22.1% (-31.0 to 116.4)	2228.8 (1617.5 to 3041.6)	2634.4 (1831.2 to 3761.4)	18.2% (-26.7 to 90.9)	412.5 (249.7 to 628.8)	499.1 (289.5 to 802.2)	21.0% (-38.9 to 140.2)
Romblon	2613.4 (1744.5 to 3717.9)	3322.7 (2057.0 to 5058.4)	27.1% (-29.4 to 129.3)	2026.2 (1467.8 to 2753.0)	2504.2 (1689.3 to 3593.8)	23.6% (-24.4 to 102.4)	375.6 (224.7 to 573.6)	477.4 (268.6 to 780.2)	27.1% (-37.4 to 158.6)
Samar (Western Samar)	2636.9 (1781.9 to 3765.9)	3372.6 (2151.4 to 5081.3)	27.9% (-27.6 to 126.2)	2056.6 (1490.2 to 2766.9)	2570.2 (1759.0 to 3653.5)	25.0% (-22.6 to 102.1)	380.4 (230.7 to 591.1)	488.2 (283.9 to 789.5)	28.3% (-35.6 to 156.0)
Sarangani	2692.4 (1799.5 to 3825.9)	3362.5 (2124.6 to 4936.7)	24.9% (-28.9 to 119.9)	2115.2 (1520.1 to 2887.0)	2564.3 (1756.9 to 3595.2)	21.2% (-24.9 to 96.1)	392.7 (237.4 to 604.7)	486.7 (285.4 to 771.2)	23.9% (-37.0 to 144.2)

Siquijor	2578.8 (1732.0 to 3656.9)	3351.8 (2096.0 to 5069.3)	30.0% (-27.1 to 132.3)	2008.0 (1448.5 to 2740.1)	2559.3 (1743.7 to 3650.6)	27.5% (-21.7 to 107.9)	373.0 (223.9 to 568.4)	485.5 (279.7 to 766.8)	30.2% (-34.2 to 157.5)
Sorsogon	2645.6 (1796.5 to 3728.5)	3368.1 (2072.7 to 5064.7)	27.3% (-28.4 to 126.9)	2060.6 (1499.5 to 2749.6)	2559.7 (1723.2 to 3712.8)	24.2% (-23.6 to 102.6)	382.9 (234.0 to 584.0)	487.4 (281.3 to 776.2)	27.3% (-35.5 to 151.6)
South Cotabato	2762.4 (1872.1 to 3940.5)	3458.5 (2184.5 to 5181.4)	25.2% (-29.1 to 121.8)	2167.0 (1573.6 to 2934.2)	2643.2 (1834.4 to 3754.4)	22.0% (-24.0 to 96.2)	402.5 (245.1 to 618.4)	502.6 (290.5 to 785.8)	24.9% (-36.4 to 145.2)
Southern Leyte	2673.0 (1776.5 to 3828.4)	3374.8 (2103.8 to 5092.7)	26.3% (-29.6 to 127.0)	2078.6 (1496.1 to 2844.1)	2564.5 (1757.7 to 3703.3)	23.4% (-24.5 to 102.1)	386.8 (232.8 to 598.8)	487.5 (279.1 to 781.5)	26.0% (-37.0 to 152.4)
Sultan Kudarat	2676.8 (1792.2 to 3779.5)	3340.2 (2124.1 to 5066.6)	24.8% (-29.6 to 121.8)	2095.1 (1520.4 to 2830.2)	2544.5 (1762.3 to 3676.4)	21.5% (-24.9 to 96.9)	388.8 (236.3 to 594.7)	483.8 (275.3 to 798.4)	24.5% (-38.4 to 152.1)
Sulu	2624.1 (1769.9 to 3720.8)	3186.5 (1995.6 to 4778.1)	21.4% (-31.5 to 115.9)	2046.2 (1486.7 to 2754.1)	2397.0 (1650.8 to 3439.8)	17.1% (-27.5 to 89.5)	379.2 (231.5 to 578.8)	454.1 (263.3 to 707.6)	19.7% (-38.7 to 133.9)
Surigao Del Norte	2707.7 (1815.2 to 3861.3)	3421.8 (2146.9 to 5091.8)	26.4% (-28.7 to 124.3)	2117.0 (1521.6 to 2858.3)	2603.0 (1783.4 to 3714.1)	23.0% (-24.1 to 99.8)	392.8 (239.6 to 597.0)	495.8 (285.6 to 799.9)	26.2% (-36.4 to 151.1)
Surigao Del Sur	2669.7 (1786.4 to 3777.9)	3355.0 (2100.3 to 4970.2)	25.7% (-28.9 to 122.6)	2079.6 (1504.5 to 2825.7)	2539.3 (1734.4 to 3567.4)	22.1% (-24.3 to 97.1)	386.9 (234.1 to 592.4)	484.4 (278.5 to 763.8)	25.2% (-36.6 to 147.5)
Tarlac	2704.6 (1813.8 to 3892.4)	3373.8 (2131.9 to 5138.2)	24.7% (-30.2 to 123.6)	2128.3 (1554.0 to 2899.4)	2580.2 (1732.8 to 3751.5)	21.2% (-26.1 to 99.1)	394.5 (238.2 to 603.7)	490.4 (284.0 to 792.4)	24.3% (-37.5 to 147.9)
Tawi-Tawi	2594.6 (1757.0 to 3653.6)	3278.4 (2055.9 to 4886.4)	26.4% (-28.3 to 123.2)	2021.3 (1470.5 to 2712.6)	2493.4 (1714.4 to 3560.8)	23.4% (-23.4 to 99.0)	374.9 (226.0 to 578.1)	472.3 (278.0 to 749.6)	26.0% (-36.0 to 148.3)
Zambales	2755.7 (1867.6 to 3926.6)	3426.7 (2131.4 to 5146.1)	24.3% (-30.1 to 121.7)	2172.3 (1588.0 to 2949.8)	2620.4 (1784.4 to 3718.8)	20.6% (-25.4 to 95.3)	403.0 (244.3 to 626.2)	498.0 (289.2 to 802.6)	23.6% (-38.0 to 146.4)
Zamboanga Del Norte	2678.9 (1810.8 to 3813.9)	3268.9 (2089.5 to 4964.7)	22.0% (-31.0 to 116.4)	2103.4 (1529.4 to 2845.9)	2476.8 (1696.7 to 3542.0)	17.8% (-27.2 to 90.9)	389.1 (234.1 to 595.7)	470.6 (271.4 to 744.1)	20.9% (-38.9 to 139.5)

Zamboanga Del Sur	2668.4 (1817.4 to 3766.2)	3415.6 (2113.1 to 5193.8)	28.0% (-28.1 to 128.3)	2099.5 (1521.1 to 2825.8)	2610.2 (1780.5 to 3748.7)	24.3% (-23.4 to 102.2)	387.3 (235.7 to 602.2)	495.0 (284.9 to 801.9)	27.8% (-36.1 to 156.4)
Zamboanga Sibugay	2666.9 (1778.3 to 3821.7)	3269.3 (2048.6 to 4882.4)	22.6% (-31.1 to 118.7)	2095.4 (1512.8 to 2817.4)	2477.7 (1702.4 to 3493.5)	18.2% (-26.4 to 90.4)	388.7 (232.5 to 600.9)	471.6 (266.2 to 754.6)	21.3% (-39.8 to 144.9)

UI, uncertainly interval; DALYs, disability-adjusted life-years.

Table S18 The incidence, prevalence and DALYs rate of anxiety and depressive disorders in 1990 and 2021, and changes from 1990 to 2021 in Poland.

Location	Incidence (95% UI)			Prevalence (95% UI)			DALYs (95% UI)		
	1990	2021	Percentage change	1990	2021	Percentage change	1990	2021	Percentage change
Anxiety disorders									
Dolnośląskie	614.1 (406.9 to 852.2)	862.9 (511.2 to 1295.9)	40.5% (-22.2 to 154.4)	3297.3 (2468.4 to 4305.2)	4694.1 (3086.5 to 6734.8)	42.4% (-11.6 to 129.5)	407.9 (249.1 to 603.1)	579.1 (329.3 to 909.7)	42.0% (-27.5 to 178.7)
Kujawsko-Pomorskie	615.1 (404.0 to 858.9)	866.5 (522.2 to 1295.6)	40.9% (-22.0 to 154.8)	3329.7 (2488.6 to 4376.7)	4748.9 (3217.9 to 6753.0)	42.6% (-10.2 to 127.4)	410.5 (258.5 to 605.7)	587.0 (342.3 to 935.6)	43.0% (-26.0 to 177.1)
Łódzkie	614.8 (413.1 to 859.9)	868.0 (531.9 to 1261.6)	41.2% (-19.8 to 149.4)	3329.0 (2485.8 to 4362.3)	4755.7 (3202.9 to 6634.5)	42.9% (-9.6 to 126.3)	410.0 (252.7 to 613.6)	587.6 (335.0 to 928.2)	43.3% (-27.0 to 181.8)
Lubelskie	612.1 (408.3 to 847.9)	869.9 (537.0 to 1298.6)	42.1% (-19.9 to 152.6)	3321.0 (2495.3 to 4325.7)	4779.4 (3231.3 to 6732.9)	43.9% (-8.7 to 127.6)	410.0 (251.8 to 605.9)	590.1 (338.4 to 927.1)	43.9% (-26.1 to 181.0)
Lubuskie	614.3 (414.9 to 846.8)	866.1 (528.6 to 1324.8)	41.0% (-21.0 to 152.2)	3307.8 (2508.5 to 4305.7)	4736.0 (3200.9 to 6816.1)	43.2% (-9.8 to 127.5)	409.1 (254.7 to 604.5)	586.0 (333.6 to 943.0)	43.2% (-27.2 to 182.3)
Małopolskie	609.8 (410.2 to 837.2)	865.3 (524.0 to 1274.1)	41.9% (-19.6 to 150.7)	3322.7 (2493.8 to 4355.8)	4718.9 (3176.5 to 6626.3)	42.0% (-10.2 to 125.4)	411.0 (254.4 to 612.0)	580.4 (329.1 to 909.2)	41.2% (-27.7 to 176.8)
Mazowieckie	615.7 (413.2 to 846.3)	867.2 (530.8 to 1265.7)	40.9% (-19.8 to 147.8)	3315.3 (2499.0 to 4308.6)	4713.6 (3194.0 to 6647.0)	42.2% (-9.7 to 124.3)	409.3 (252.7 to 610.1)	581.1 (334.8 to 895.1)	42.0% (-26.4 to 174.8)
Opolskie	615.9 (415.7 to 831.9)	867.4 (523.0 to 1318.6)	40.8% (-20.6 to 150.9)	3354.2 (2521.8 to 4378.8)	4753.6 (3173.4 to 6870.3)	41.7% (-11.6 to 127.4)	414.6 (257.1 to 613.4)	584.9 (335.4 to 945.6)	41.1% (-28.3 to 178.2)

Podkarpackie	614.8 (410.5 to 850.3)	858.0 (527.5 to 1269.3)	39.6% (-21.1 to 147.4)	3331.6 (2515.9 to 4363.0)	4732.0 (3204.0 to 6756.5)	42.0% (-10.4 to 125.6)	410.9 (253.3 to 617.9)	583.3 (333.0 to 906.6)	42.0% (-27.2 to 177.7)
Podlaskie	615.4 (406.6 to 842.7)	862.2 (520.6 to 1280.3)	40.1% (-21.4 to 149.9)	3349.4 (2510.7 to 4365.3)	4751.1 (3210.6 to 6843.5)	41.8% (-11.0 to 126.6)	413.6 (258.1 to 612.8)	585.8 (337.0 to 925.1)	41.6% (-27.1 to 175.9)
Pomorskie	612.3 (408.3 to 852.7)	859.5 (533.5 to 1259.7)	40.4% (-20.2 to 147.6)	3322.7 (2511.3 to 4330.2)	4671.8 (3207.6 to 6504.8)	40.6% (-9.8 to 119.8)	410.9 (249.8 to 614.9)	576.9 (333.2 to 886.9)	40.4% (-27.5 to 171.9)
Śląskie	611.0 (408.4 to 851.7)	861.7 (530.0 to 1290.3)	41.0% (-20.8 to 151.6)	3347.0 (2508.0 to 4364.2)	4698.3 (3159.9 to 6596.1)	40.4% (-11.1 to 122.5)	413.0 (260.6 to 619.0)	580.9 (332.9 to 927.7)	40.7% (-28.1 to 175.7)
Świętokrzyskie	609.7 (411.6 to 840.3)	861.6 (515.9 to 1294.7)	41.3% (-20.8 to 152.9)	3306.7 (2459.2 to 4306.1)	4742.0 (3246.7 to 6734.1)	43.4% (-9.2 to 127.4)	409.4 (250.2 to 616.4)	584.4 (343.2 to 922.3)	42.8% (-26.5 to 178.0)
Warmińsko-Mazurskie	612.6 (412.3 to 853.2)	862.1 (533.1 to 1300.5)	40.7% (-20.7 to 150.3)	3315.9 (2495.5 to 4292.7)	4729.3 (3214.4 to 6699.7)	42.6% (-9.4 to 125.0)	408.6 (248.0 to 606.2)	583.9 (343.3 to 904.6)	42.9% (-25.7 to 175.1)
Wielkopolskie	612.0 (407.9 to 840.6)	860.1 (521.3 to 1286.1)	40.5% (-21.0 to 150.5)	3313.9 (2490.7 to 4289.0)	4671.4 (3158.4 to 6588.4)	41.0% (-10.5 to 122.5)	409.8 (254.5 to 607.0)	575.8 (331.2 to 921.2)	40.5% (-28.1 to 175.8)
Zachodniopomorskie	612.3 (414.7 to 833.6)	863.9 (531.3 to 1291.7)	41.1% (-19.6 to 148.2)	3302.6 (2460.5 to 4287.7)	4725.9 (3197.9 to 6683.3)	43.1% (-9.5 to 127.0)	407.2 (254.0 to 607.9)	585.3 (333.5 to 920.0)	43.7% (-26.3 to 181.4)
Depressive disorders									
Dolnośląskie	1474.6 (1010.2 to 2064.3)	2251.5 (1448.0 to 3292.3)	52.7% (-11.3 to 163.3)	1407.5 (1065.5 to 1833.6)	1948.7 (1391.3 to 2638.3)	38.4% (-9.0 to 111.0)	233.4 (143.9 to 357.3)	343.5 (204.9 to 539.0)	47.2% (-23.8 to 184.7)
Kujawsko-Pomorskie	1510.6 (1037.9 to 2100.2)	2298.5 (1463.3 to 3458.1)	52.2% (-12.6 to 165.5)	1447.7 (1086.1 to 1882.6)	1987.5 (1420.0 to 2737.0)	37.3% (-10.5 to 111.0)	239.6 (148.8 to 360.1)	350.4 (207.6 to 546.8)	46.2% (-23.7 to 180.9)
Łódzkie	1502.8 (1027.0 to 2108.3)	2272.8 (1430.1 to 3435.1)	51.2% (-14.1 to 166.8)	1440.2 (1079.8 to 1887.9)	1967.3 (1414.5 to 2740.3)	36.6% (-11.4 to 110.9)	238.8 (146.5 to 364.3)	345.6 (208.0 to 547.0)	44.7% (-25.1 to 180.0)
Lubelskie	1511.3 (1043.1 to 2122.5)	2299.7 (1471.7 to 3427.7)	52.2% (-12.4 to 164.8)	1451.8 (1088.2 to 1893.6)	2000.0 (1406.2 to 2779.0)	37.8% (-11.1 to 113.7)	240.3 (146.5 to 363.2)	351.7 (208.8 to 544.9)	46.3% (-24.0 to 181.9)

Lubuskie	1484.7 (1019.8 to 2072.9)	2257.8 (1419.3 to 3447.1)	52.1% (-13.6 to 168.3)	1417.9 (1064.5 to 1856.1)	1953.6 (1392.1 to 2727.8)	37.8% (-11.0 to 113.4)	235.2 (147.6 to 358.6)	344.0 (208.9 to 549.3)	46.3% (-23.8 to 180.8)
Małopolskie	1518.8 (1043.0 to 2164.9)	2300.2 (1488.5 to 3478.5)	51.4% (-13.4 to 165.6)	1458.1 (1092.0 to 1909.0)	1988.7 (1411.6 to 2771.3)	36.4% (-12.0 to 111.6)	241.0 (150.3 to 366.8)	350.1 (205.2 to 549.9)	45.3% (-25.0 to 181.8)
Mazowieckie	1489.1 (1011.8 to 2093.4)	2240.7 (1445.7 to 3325.5)	50.5% (-13.3 to 161.6)	1423.6 (1073.9 to 1852.9)	1928.1 (1378.1 to 2608.9)	35.4% (-10.9 to 106.4)	235.7 (145.6 to 355.0)	340.2 (200.4 to 547.6)	44.3% (-26.1 to 182.4)
Opolskie	1541.5 (1047.6 to 2173.7)	2312.7 (1480.6 to 3403.3)	50.0% (-13.6 to 161.0)	1485.5 (1113.3 to 1937.5)	2010.2 (1418.0 to 2733.2)	35.3% (-11.9 to 108.2)	245.7 (153.9 to 379.2)	353.7 (212.0 to 552.8)	43.9% (-25.1 to 176.8)
Podkarpackie	1511.8 (1036.2 to 2126.9)	2336.0 (1506.8 to 3551.0)	54.5% (-11.7 to 170.9)	1451.1 (1085.6 to 1887.3)	2028.1 (1443.5 to 2867.1)	39.8% (-9.9 to 117.1)	240.5 (147.1 to 364.6)	357.5 (207.2 to 562.7)	48.7% (-24.0 to 191.3)
Podlaskie	1530.2 (1054.4 to 2138.9)	2320.8 (1506.5 to 3511.6)	51.7% (-12.6 to 163.8)	1474.0 (1110.7 to 1923.2)	2016.7 (1435.6 to 2830.4)	36.8% (-11.5 to 111.6)	243.7 (150.6 to 363.3)	354.4 (211.2 to 560.8)	45.4% (-24.4 to 180.1)
Pomorskie	1503.4 (1034.7 to 2120.2)	2266.1 (1474.9 to 3399.2)	50.7% (-13.0 to 161.9)	1439.5 (1082.0 to 1887.4)	1954.1 (1407.0 to 2676.4)	35.7% (-11.2 to 107.8)	238.4 (147.5 to 359.9)	344.9 (205.4 to 563.7)	44.6% (-26.0 to 183.6)
Śląskie	1523.6 (1044.7 to 2164.4)	2266.5 (1419.0 to 3484.0)	48.8% (-16.5 to 165.2)	1465.9 (1104.6 to 1934.9)	1956.9 (1394.1 to 2780.0)	33.5% (-14.3 to 108.3)	242.9 (149.0 to 373.0)	345.3 (199.1 to 552.1)	42.2% (-28.1 to 181.8)
Świętokrzyskie	1506.8 (1027.9 to 2129.0)	2317.3 (1482.6 to 3537.6)	53.8% (-12.8 to 171.7)	1443.0 (1086.9 to 1880.7)	2014.6 (1438.7 to 2820.9)	39.6% (-9.5 to 115.6)	239.1 (146.3 to 362.8)	355.9 (209.8 to 570.5)	48.9% (-23.9 to 191.9)
Warmińsko-Mazurskie	1504.0 (1036.9 to 2095.7)	2287.2 (1451.7 to 3470.2)	52.1% (-13.0 to 166.2)	1442.6 (1084.6 to 1866.9)	1984.4 (1424.3 to 2762.5)	37.6% (-10.3 to 111.2)	239.1 (147.9 to 358.3)	350.7 (206.8 to 555.4)	46.7% (-24.2 to 184.7)
Wielkopolskie	1491.7 (1021.2 to 2107.8)	2243.6 (1419.0 to 3344.7)	50.4% (-14.2 to 164.2)	1427.6 (1073.0 to 1867.1)	1938.1 (1383.3 to 2654.9)	35.8% (-11.4 to 108.6)	237.1 (145.5 to 361.2)	341.9 (197.6 to 547.5)	44.2% (-27.0 to 185.5)
Zachodniopomorskie	1490.6 (1019.8 to 2116.2)	2286.4 (1457.7 to 3512.7)	53.4% (-13.4 to 172.2)	1423.9 (1068.0 to 1865.4)	1981.0 (1404.4 to 2756.2)	39.1% (-10.2 to 115.8)	236.4 (144.7 to 358.2)	349.7 (206.5 to 545.8)	47.9% (-23.6 to 186.1)

UI, uncertainly interval; DALYs, disability-adjusted life-years.

Table S19 The incidence, prevalence and DALYs rate of anxiety and depressive disorders in 1990 and 2021, and changes from 1990 to 2021 in South Africa.

Location	Incidence (95% UI)			Prevalence (95% UI)			DALYs (95% UI)		
	1990	2021	Percentage change	1990	2021	Percentage change	1990	2021	Percentage change
Anxiety disorders									
Eastern Cape	734.7 (488.7 to 1015.0)	1031.9 (632.6 to 1554.5)	40.4% (-21.2 to 150.7)	4031.0 (3020.4 to 5226.7)	5698.4 (3780.2 to 8198.0)	41.4% (-11.8 to 126.8)	493.1 (310.3 to 716.2)	696.3 (391.1 to 1106.7)	41.2% (-27.4 to 175.1)
Free State	735.2 (482.1 to 1021.5)	1031.2 (645.0 to 1540.5)	40.3% (-21.0 to 149.6)	4077.4 (3049.2 to 5323.0)	5725.1 (3840.7 to 8136.2)	40.4% (-11.8 to 123.9)	498.5 (309.0 to 745.4)	700.8 (424.3 to 1134.4)	40.6% (-27.1 to 172.0)
Gauteng	727.1 (467.6 to 1010.9)	1027.8 (629.3 to 1544.8)	41.3% (-21.7 to 156.0)	4117.0 (3105.7 to 5325.8)	5774.6 (3876.5 to 8207.6)	40.3% (-11.4 to 122.5)	505.1 (320.9 to 754.3)	707.0 (421.7 to 1094.4)	40.0% (-26.0 to 165.6)
KwaZulu-Natal	730.4 (478.6 to 1030.6)	1026.8 (631.3 to 1530.7)	40.6% (-21.7 to 152.8)	4053.6 (3055.9 to 5255.5)	5701.9 (3874.4 to 7997.5)	40.7% (-10.3 to 121.1)	495.1 (311.5 to 732.8)	695.4 (403.8 to 1084.6)	40.5% (-26.8 to 170.3)
Limpopo	730.0 (489.7 to 1008.3)	1029.7 (641.9 to 1561.9)	41.1% (-20.4 to 150.2)	3993.6 (2992.7 to 5184.4)	5677.8 (3873.5 to 8026.5)	42.2% (-9.6 to 124.4)	491.3 (308.3 to 734.3)	695.9 (404.9 to 1118.0)	41.7% (-27.3 to 176.9)
Mpumalanga	727.4 (480.9 to 1004.1)	1027.7 (617.1 to 1522.3)	41.3% (-21.0 to 153.1)	4030.9 (3037.3 to 5275.4)	5721.6 (3899.2 to 8136.6)	41.9% (-10.1 to 124.8)	492.9 (308.1 to 717.2)	697.9 (423.6 to 1105.1)	41.6% (-25.1 to 168.3)
North-West	730.6 (477.2 to 1005.1)	1027.6 (630.5 to 1513.6)	40.6% (-20.8 to 150.5)	4073.1 (3091.2 to 5271.9)	5660.9 (3831.3 to 7981.1)	39.0% (-11.5 to 118.7)	498.1 (311.9 to 736.5)	690.2 (411.3 to 1093.9)	38.6% (-27.6 to 165.9)
Northern Cape	729.8 (479.9 to 1012.1)	1031.0 (636.8 to 1542.7)	41.3% (-20.8 to 152.5)	4020.9 (3039.4 to 5254.1)	5752.2 (3968.1 to 8102.7)	43.1% (-8.5 to 124.5)	493.9 (304.9 to 731.7)	704.1 (412.0 to 1096.8)	42.6% (-25.9 to 175.0)
Western Cape	723.8 (473.3 to 1006.6)	1021.2 (636.3 to 1521.4)	41.1% (-20.6 to 151.4)	4073.5 (3041.7 to 5320.6)	5740.2 (3961.1 to 8055.7)	40.9% (-10.1 to 121.4)	499.4 (312.7 to 731.3)	704.3 (411.3 to 1104.4)	41.1% (-26.4 to 171.2)
Depressive disorders									
Eastern Cape	3340.0 (2368.5 to 4604.3)	5540.4 (3692.7 to 7939.5)	65.9% (0.0 to 175.6)	2704.0 (2037.9 to 3534.6)	4079.8 (2902.9 to 5574.9)	50.9% (-1.5 to 131.6)	486.8 (300.6 to 727.3)	773.8 (462.7 to 1209.3)	59.0% (-16.8 to 204.1)

Free State	3261.3 (2319.0 to 4561.9)	4862.9 (3209.0 to 7103.5)	49.1% (-11.5 to 151.8)	2711.9 (2034.5 to 3511.3)	3692.1 (2622.5 to 5112.7)	36.1% (-11.5 to 109.7)	482.6 (299.1 to 727.1)	690.4 (409.0 to 1064.3)	43.1% (-25.2 to 173.5)
Gauteng	3524.6 (2527.5 to 4811.5)	5087.0 (3301.0 to 7532.3)	44.3% (-14.2 to 143.5)	2949.9 (2231.4 to 3817.0)	3898.3 (2737.1 to 5354.4)	32.2% (-13.8 to 103.0)	528.2 (329.5 to 787.1)	732.9 (420.4 to 1145.3)	38.7% (-28.5 to 169.7)
KwaZulu-Natal	3666.9 (2658.0 to 5001.1)	5353.8 (3485.4 to 7780.4)	46.0% (-12.2 to 143.4)	2954.3 (2253.5 to 3812.0)	4005.9 (2810.1 to 5544.4)	35.6% (-11.5 to 108.4)	532.9 (340.8 to 790.6)	754.3 (446.0 to 1184.2)	41.5% (-25.6 to 170.2)
Limpopo	2978.3 (2105.4 to 4082.5)	4846.4 (3158.4 to 7023.5)	62.7% (-3.2 to 173.9)	2465.3 (1870.6 to 3209.1)	3654.4 (2580.7 to 5043.4)	48.2% (-3.5 to 127.8)	439.1 (273.5 to 666.0)	685.8 (408.8 to 1058.9)	56.2% (-18.2 to 198.3)
Mpumalanga	2983.1 (2119.3 to 4087.1)	4443.6 (2885.3 to 6566.2)	49.0% (-11.7 to 152.0)	2512.2 (1897.2 to 3292.3)	3447.2 (2456.8 to 4781.8)	37.2% (-11.0 to 111.7)	443.6 (280.7 to 668.5)	636.0 (378.5 to 997.8)	43.4% (-25.0 to 174.8)
North-West	3212.1 (2303.5 to 4443.3)	4987.0 (3266.6 to 7259.2)	55.3% (-7.4 to 160.6)	2674.7 (2028.0 to 3453.2)	3758.8 (2671.9 to 5138.9)	40.5% (-7.7 to 114.3)	475.7 (298.0 to 713.7)	705.0 (413.6 to 1106.3)	48.2% (-23.1 to 186.0)
Northern Cape	3363.7 (2402.8 to 4674.0)	5297.6 (3509.8 to 7730.5)	57.5% (-6.0 to 164.4)	2744.5 (2077.8 to 3612.7)	3993.1 (2872.2 to 5484.2)	45.5% (-4.9 to 123.0)	494.5 (307.4 to 751.8)	752.5 (452.4 to 1177.1)	52.2% (-20.6 to 191.6)
Western Cape	3621.1 (2604.6 to 4924.8)	4905.0 (3302.9 to 7038.2)	35.5% (-17.4 to 122.5)	2976.7 (2284.8 to 3829.6)	3771.9 (2712.5 to 5160.9)	26.7% (-16.0 to 91.3)	536.6 (340.9 to 798.1)	704.9 (416.0 to 1087.7)	31.4% (-30.7 to 149.4)

UI, uncertainly interval; DALYs, disability-adjusted life-years.

Table S20 The incidence, prevalence and DALYs rate of anxiety and depressive disorders in 1990 and 2021, and changes from 1990 to 2021 in Sweden.

Location	Incidence (95% UI)			Prevalence (95% UI)			DALYs (95% UI)		
	1990	2021	Percentage change	1990	2021	Percentage change	1990	2021	Percentage change
Anxiety disorders									
Stockholm	1070.9 (697.3 to 1493.6)	1302.8 (817.3 to 1908.1)	21.7% (-31.0 to 114.9)	6738.2 (5158.8 to 8694.7)	7957.0 (5439.8 to 11271.8)	18.1% (-24.4 to 84.6)	827.6 (522.7 to 1232.9)	977.5 (585.1 to 1509.3)	18.1% (-37.4 to 123.4)
Sweden except Stockholm	1069.3 (719.7 to 1499.7)	1313.3 (822.1 to 1946.9)	22.8% (-30.2 to 116.9)	6660.6 (5087.5 to 8539.4)	7986.6 (5499.7 to 11157.1)	19.9% (-22.4 to 85.8)	817.7 (516.0 to 1192.4)	978.7 (567.4 to 1495.2)	19.7% (-36.8 to 127.4)
Depressive disorders									

Stockholm	4395.7 (3065.1 to 6165.2)	4803.8 (3066.9 to 7138.4)	9.3% (-36.9 to 89.4)	3746.7 (2797.5 to 4917.2)	3848.6 (2670.0 to 5325.3)	2.7% (-34.2 to 60.5)	671.3 (413.7 to 1015.3)	704.4 (418.3 to 1143.7)	4.9% (-46.4 to 106.0)
Sweden except Stockholm	5392.3 (3843.7 to 7276.9)	7123.5 (4675.6 to 10158.8)	32.1% (-20.0 to 118.5)	4166.6 (3105.4 to 5401.1)	5177.8 (3594.7 to 7188.7)	24.3% (-20.0 to 93.6)	787.4 (497.4 to 1193.8)	1007.7 (591.7 to 1591.0)	28.0% (-33.7 to 147.6)

UI, uncertainly interval; DALYs, disability-adjusted life-years.

Table S21 The incidence, prevalence and DALYs rate of anxiety and depressive disorders in 1990 and 2021, and changes from 1990 to 2021 in United Kingdom.

Location	Incidence (95% UI)			Prevalence (95% UI)			DALYs (95% UI)		
	1990	2021	Percentage change	1990	2021	Percentage change	1990	2021	Percentage change
Anxiety disorders									
England	956.7 (637.8 to 1309.3)	1257.1 (855.5 to 1704.0)	31.4% (-19.7 to 114.8)	5898.6 (4526.1 to 7521.8)	7525.8 (5701.3 to 9587.9)	27.6% (-10.9 to 82.9)	719.9 (461.4 to 1055.3)	919.7 (587.2 to 1332.9)	27.8% (-28.3 to 127.3)
East Midlands	957.8 (633.5 to 1315.2)	1263.7 (854.7 to 1719.9)	31.9% (-19.9 to 117.3)	5892.4 (4529.2 to 7534.5)	7570.0 (5702.6 to 9825.1)	28.5% (-11.3 to 86.0)	719.8 (460.1 to 1060.1)	926.3 (577.7 to 1364.7)	28.7% (-28.8 to 132.9)
East of England	960.5 (641.2 to 1321.4)	1260.8 (842.6 to 1740.8)	31.3% (-20.9 to 117.9)	5890.3 (4509.2 to 7547.2)	7520.1 (5641.2 to 9806.4)	27.7% (-12.3 to 85.8)	719.7 (462.7 to 1056.3)	919.4 (576.7 to 1326.5)	27.7% (-28.5 to 128.4)
Greater London	948.8 (626.6 to 1307.6)	1256.9 (851.2 to 1706.5)	32.5% (-19.6 to 118.5)	5958.9 (4561.2 to 7600.8)	7560.3 (5766.0 to 9615.1)	26.9% (-11.3 to 81.4)	725.8 (465.8 to 1062.6)	923.2 (593.4 to 1349.9)	27.2% (-28.5 to 126.2)
North East England	963.9 (645.5 to 1334.9)	1254.9 (839.1 to 1731.6)	30.2% (-21.6 to 116.2)	5916.0 (4539.3 to 7563.5)	7540.9 (5680.5 to 9808.7)	27.5% (-12.1 to 84.8)	721.9 (462.1 to 1052.8)	920.5 (586.6 to 1364.7)	27.5% (-28.8 to 128.7)
North West England	952.4 (635.4 to 1306.7)	1239.1 (830.4 to 1696.0)	30.1% (-21.3 to 114.9)	5833.9 (4466.8 to 7454.4)	7387.2 (5609.9 to 9480.7)	26.6% (-11.9 to 82.1)	711.4 (452.4 to 1048.8)	902.2 (582.6 to 1323.3)	26.8% (-29.1 to 126.5)

South East England	957.7 (639.5 to 1312.7)	1263.0 (843.2 to 1728.2)	31.9% (-20.2 to 117.9)	5884.8 (4528.4 to 7517.0)	7543.5 (5637.8 to 9814.9)	28.2% (-11.7 to 86.3)	719.1 (458.3 to 1049.7)	922.5 (584.6 to 1362.7)	28.3% (-28.6 to 130.7)
South West England	960.0 (643.4 to 1318.2)	1258.2 (856.4 to 1704.1)	31.1% (-19.8 to 114.0)	5900.9 (4530.8 to 7522.1)	7546.2 (5788.6 to 9685.6)	27.9% (-10.6 to 82.9)	720.5 (460.9 to 1053.3)	922.4 (581.6 to 1346.6)	28.0% (-28.5 to 129.5)
West Midlands	960.5 (641.1 to 1308.0)	1258.3 (850.1 to 1705.0)	31.0% (-20.0 to 114.3)	5912.5 (4528.6 to 7570.7)	7531.3 (5597.5 to 9667.5)	27.4% (-12.2 to 84.9)	721.8 (462.0 to 1056.0)	920.3 (577.6 to 1330.4)	27.5% (-28.7 to 128.1)
Yorkshire and the Humber	956.6 (634.7 to 1319.0)	1259.6 (837.6 to 1750.3)	31.7% (-21.2 to 120.2)	5914.9 (4524.3 to 7547.1)	7554.1 (5714.5 to 9778.8)	27.7% (-11.6 to 84.6)	721.6 (461.3 to 1057.0)	923.3 (588.8 to 1366.4)	28.0% (-28.7 to 129.8)
Derby	957.9 (620.3 to 1311.6)	1268.0 (776.0 to 1869.5)	32.4% (-25.6 to 136.4)	5934.0 (4532.3 to 7669.7)	7557.9 (5153.5 to 10585.0)	27.4% (-18.2 to 98.9)	723.7 (455.6 to 1061.8)	926.0 (550.8 to 1424.9)	27.9% (-32.1 to 141.9)
Derbyshire	954.5 (629.3 to 1309.1)	1264.8 (784.7 to 1873.1)	32.5% (-25.0 to 134.5)	5864.0 (4483.8 to 7482.5)	7527.1 (5221.9 to 10536.6)	28.4% (-16.7 to 98.2)	717.3 (456.3 to 1066.5)	921.5 (544.0 to 1447.2)	28.5% (-32.7 to 145.8)
Leicester	945.1 (617.7 to 1325.6)	1252.0 (772.1 to 1857.2)	32.5% (-25.9 to 137.2)	5956.2 (4540.6 to 7648.3)	7643.4 (5300.0 to 10935.9)	28.3% (-17.7 to 100.4)	724.9 (462.4 to 1078.6)	931.7 (561.6 to 1432.1)	28.5% (-31.4 to 141.2)
Leicestershire	961.5 (637.1 to 1330.1)	1263.9 (775.4 to 1857.9)	31.5% (-25.8 to 133.2)	5875.2 (4449.2 to 7582.0)	7578.8 (5125.0 to 10592.8)	29.0% (-17.5 to 102.3)	718.3 (457.1 to 1052.2)	929.5 (534.3 to 1465.6)	29.4% (-32.8 to 149.5)
Lincolnshire	962.7 (641.7 to 1347.1)	1270.0 (792.8 to 1877.5)	31.9% (-25.2 to 133.4)	5862.1 (4464.3 to 7622.0)	7603.3 (5224.4 to 10568.7)	29.7% (-16.5 to 101.9)	716.4 (447.5 to 1060.7)	930.6 (548.0 to 1422.4)	29.9% (-31.5 to 146.6)
Northamptonshire	968.5 (637.8 to 1322.8)	1273.7 (810.0 to 1859.3)	31.5% (-24.2 to 128.7)	5906.1 (4511.5 to 7653.1)	7550.2 (5207.3 to	27.8% (-17.4 to 98.4)	721.3 (449.4 to	922.1 (545.5 to	27.8% (-32.1 to 141.2)

					10512.5)		1057.3)	1401.7)	
Nottingham	929.9 (580.2 to 1295.9)	1233.2 (752.9 to 1816.0)	32.6% (-26.6 to 140.1)	5951.9 (4563.8 to 7663.7)	7611.4 (5316.7 to 10522.2)	27.9% (-16.5 to 96.4)	725.0 (461.0 to 1062.0)	930.0 (550.0 to 1442.9)	28.3% (-32.1 to 143.0)
Nottinghamshire	964.2 (640.5 to 1335.6)	1269.7 (808.6 to 1871.5)	31.7% (-24.5 to 130.0)	5881.6 (4471.7 to 7571.5)	7534.1 (5357.1 to 10792.2)	28.1% (-17.1 to 98.5)	719.4 (461.3 to 1066.4)	923.4 (535.8 to 1418.0)	28.3% (-32.4 to 144.4)
Rutland	953.1 (634.0 to 1317.9)	1249.8 (787.0 to 1843.9)	31.1% (-25.1 to 130.4)	5775.8 (4400.6 to 7509.6)	7490.8 (5195.7 to 10539.0)	29.7% (-16.5 to 102.1)	707.7 (446.8 to 1041.9)	915.8 (545.8 to 1393.2)	29.4% (-30.9 to 142.9)
Bedford	963.6 (642.8 to 1338.6)	1255.3 (783.8 to 1826.8)	30.3% (-25.5 to 128.3)	5893.3 (4473.1 to 7595.1)	7564.9 (5232.8 to 10567.1)	28.4% (-17.0 to 99.3)	720.4 (457.0 to 1051.5)	923.2 (549.8 to 1438.3)	28.2% (-32.1 to 142.4)
Cambridgeshire	946.9 (618.0 to 1334.7)	1244.1 (750.0 to 1860.0)	31.4% (-27.5 to 138.9)	5887.1 (4532.5 to 7625.3)	7500.4 (5136.7 to 10552.2)	27.4% (-18.1 to 98.4)	719.3 (458.8 to 1045.8)	917.1 (526.2 to 1428.7)	27.5% (-33.3 to 144.0)
Central Bedfordshire	963.6 (644.0 to 1353.0)	1272.1 (818.7 to 1859.8)	32.0% (-23.8 to 129.2)	5860.7 (4447.2 to 7520.2)	7555.3 (5234.7 to 10706.3)	28.9% (-17.1 to 100.9)	716.1 (458.1 to 1047.1)	925.8 (569.0 to 1444.4)	29.3% (-30.5 to 141.0)
Essex	960.0 (645.1 to 1334.3)	1263.1 (776.4 to 1903.0)	31.6% (-26.0 to 134.3)	5872.9 (4457.4 to 7635.4)	7536.1 (5212.9 to 10742.9)	28.3% (-18.0 to 101.2)	717.8 (458.0 to 1054.8)	919.7 (541.3 to 1437.2)	28.1% (-32.5 to 144.1)
Hertfordshire	962.6 (637.0 to 1339.6)	1261.1 (798.7 to 1856.4)	31.0% (-25.2 to 129.9)	5891.8 (4489.7 to 7645.9)	7482.8 (5228.1 to 10344.5)	27.0% (-17.4 to 95.8)	719.8 (458.9 to 1045.1)	917.7 (547.5 to 1413.9)	27.5% (-31.9 to 139.2)
Luton	961.0 (623.3 to 1356.0)	1264.1 (781.2 to 1861.9)	31.5% (-26.3 to 135.6)	5924.7 (4481.4 to 7648.8)	7531.1 (5228.3 to 10459.8)	27.1% (-17.7 to 97.0)	723.4 (449.2 to 1066.3)	921.7 (537.1 to 1407.2)	27.4% (-33.1 to 143.2)

Norfolk	961.9 (645.7 to 1323.0)	1255.2 (781.8 to 1812.7)	30.5% (-24.9 to 127.3)	5907.3 (4484.9 to 7648.6)	7523.5 (5116.5 to 10360.2)	27.4% (-17.9 to 98.3)	721.4 (461.9 to 1065.9)	919.2 (529.2 to 1406.5)	27.4% (-33.0 to 142.9)
Peterborough	966.6 (632.5 to 1360.2)	1269.9 (796.2 to 1847.7)	31.4% (-25.3 to 131.7)	5909.7 (4473.2 to 7625.2)	7492.5 (5191.1 to 10451.7)	26.8% (-18.1 to 97.0)	722.1 (453.5 to 1063.5)	914.6 (551.6 to 1411.9)	26.7% (-32.6 to 138.4)
Southend-on-Sea	964.0 (627.9 to 1352.7)	1274.2 (801.6 to 1866.2)	32.2% (-25.0 to 133.5)	5951.0 (4511.0 to 7663.2)	7550.9 (5165.8 to 10639.2)	26.9% (-18.7 to 98.5)	726.4 (463.7 to 1058.0)	924.9 (543.4 to 1442.3)	27.3% (-32.8 to 141.8)
Suffolk	962.3 (641.7 to 1342.1)	1264.5 (797.3 to 1877.2)	31.4% (-25.3 to 131.6)	5870.7 (4482.6 to 7564.2)	7533.4 (5319.3 to 10425.1)	28.3% (-16.0 to 96.5)	717.9 (458.4 to 1056.4)	919.1 (544.7 to 1376.1)	28.0% (-31.1 to 138.5)
Thurrock	970.1 (633.3 to 1324.1)	1273.7 (802.6 to 1873.0)	31.3% (-25.0 to 130.4)	5957.9 (4530.9 to 7672.2)	7544.9 (5216.0 to 10612.1)	26.6% (-18.4 to 96.9)	726.9 (463.9 to 1078.3)	923.9 (545.7 to 1417.3)	27.1% (-32.6 to 140.2)
Barking and Dagenham	956.5 (634.7 to 1333.3)	1279.8 (816.8 to 1865.8)	33.8% (-23.0 to 132.9)	5930.5 (4515.8 to 7669.2)	7491.8 (5233.8 to 10518.6)	26.3% (-18.3 to 95.8)	721.9 (459.4 to 1049.0)	916.4 (565.1 to 1414.9)	26.9% (-31.3 to 135.0)
Barnet	955.7 (632.7 to 1314.6)	1270.2 (801.3 to 1882.5)	32.9% (-24.2 to 133.6)	5942.9 (4535.7 to 7679.0)	7505.8 (5082.1 to 10559.7)	26.3% (-19.3 to 98.1)	726.5 (463.9 to 1061.1)	915.0 (558.8 to 1389.7)	25.9% (-31.7 to 132.6)
Bexley	956.4 (627.1 to 1333.7)	1260.0 (788.4 to 1856.1)	31.7% (-25.4 to 133.1)	5896.7 (4468.8 to 7626.1)	7534.1 (5232.0 to 10435.4)	27.8% (-17.2 to 97.7)	719.3 (463.6 to 1063.1)	919.9 (555.5 to 1442.4)	27.9% (-31.9 to 140.9)
Brent	945.1 (631.6 to 1343.5)	1255.4 (781.0 to 1833.7)	32.8% (-24.7 to 134.7)	5934.6 (4546.6 to 7656.7)	7528.6 (5175.4 to 10776.3)	26.9% (-18.9 to 98.6)	722.9 (459.3 to 1043.3)	920.6 (539.0 to 1456.3)	27.4% (-33.2 to 143.0)
Bromley	959.0 (641.6 to 1325.4)	1260.6 (788.6 to 1876.3)	31.5% (-25.3 to 131.8)	5906.5 (4505.8 to 7611.1)	7486.6 (5142.3 to	26.8% (-18.3 to 97.1)	720.3 (453.9 to	915.3 (529.4 to	27.1% (-33.1 to 141.9)

					10482.1)		1050.4)	1399.4)	
Camden	934.8 (585.7 to 1329.6)	1230.7 (730.2 to 1835.3)	31.6% (-28.8 to 144.0)	6042.8 (4604.8 to 7739.5)	7648.3 (5274.8 to 10649.0)	26.6% (-18.0 to 95.8)	734.6 (466.3 to 1081.5)	933.2 (544.8 to 1415.4)	27.0% (-32.6 to 140.0)
Croydon	959.4 (633.2 to 1342.2)	1274.8 (795.4 to 1865.6)	32.9% (-24.6 to 134.6)	5947.6 (4522.7 to 7645.4)	7553.1 (5184.3 to 10737.5)	27.0% (-18.7 to 98.8)	725.1 (459.5 to 1073.8)	922.8 (541.4 to 1389.2)	27.3% (-32.2 to 139.5)
Ealing	949.7 (631.8 to 1312.6)	1256.7 (774.6 to 1860.7)	32.3% (-25.2 to 134.7)	5925.2 (4499.6 to 7653.2)	7470.3 (5008.0 to 10467.3)	26.1% (-19.8 to 98.5)	723.2 (462.3 to 1056.4)	913.8 (546.9 to 1470.0)	26.3% (-33.7 to 141.3)
Enfield	954.3 (644.6 to 1330.5)	1255.6 (763.8 to 1813.9)	31.6% (-25.2 to 131.8)	5948.4 (4519.2 to 7593.1)	7508.4 (5270.1 to 10645.8)	26.2% (-18.2 to 95.2)	725.6 (459.5 to 1060.8)	918.3 (545.8 to 1451.4)	26.6% (-33.4 to 141.4)
Greenwich	968.9 (648.4 to 1353.1)	1262.9 (771.8 to 1882.2)	30.3% (-26.9 to 132.6)	5956.2 (4554.9 to 7673.6)	7567.9 (5160.9 to 10617.2)	27.1% (-18.4 to 98.1)	725.9 (465.2 to 1065.2)	925.8 (549.2 to 1437.4)	27.5% (-32.3 to 141.1)
Hackney	953.7 (614.6 to 1337.1)	1254.9 (761.4 to 1866.8)	31.6% (-27.2 to 138.5)	5967.7 (4517.0 to 7719.6)	7625.3 (5148.7 to 10805.3)	27.8% (-18.9 to 101.7)	726.2 (463.9 to 1067.8)	927.6 (529.9 to 1392.4)	27.7% (-32.4 to 142.2)
Hammersmith and Fulham	926.6 (565.6 to 1320.1)	1232.8 (737.0 to 1802.6)	33.0% (-27.8 to 145.4)	6084.0 (4611.9 to 7826.8)	7613.6 (5235.9 to 10733.1)	25.1% (-19.6 to 95.2)	738.0 (473.2 to 1078.3)	927.1 (548.8 to 1450.2)	25.6% (-33.5 to 138.1)
Haringey	935.6 (618.1 to 1316.1)	1259.6 (768.7 to 1862.7)	34.6% (-24.7 to 141.4)	5960.3 (4543.1 to 7685.1)	7585.1 (5197.1 to 10706.7)	27.3% (-18.4 to 98.9)	725.4 (455.3 to 1062.0)	926.5 (552.5 to 1423.0)	27.7% (-32.1 to 141.0)
Harrow	954.7 (638.5 to 1322.2)	1263.1 (779.8 to 1834.8)	32.3% (-24.5 to 132.6)	5883.2 (4481.3 to 7592.9)	7522.5 (5203.7 to 10548.4)	27.9% (-17.5 to 98.6)	718.9 (459.9 to 1047.0)	918.5 (552.5 to 1430.4)	27.8% (-31.8 to 139.9)

Havering	962.8 (631.6 to 1340.0)	1268.0 (788.8 to 1876.1)	31.7% (-25.7 to 134.0)	5899.2 (4497.4 to 7617.7)	7628.2 (5299.2 to 10698.9)	29.3% (-16.4 to 100.5)	720.0 (451.1 to 1066.5)	933.1 (552.3 to 1469.3)	29.6% (-32.3 to 148.7)
Hillingdon	952.7 (624.3 to 1326.8)	1258.2 (764.8 to 1870.6)	32.1% (-26.4 to 137.5)	5924.7 (4510.9 to 7663.3)	7558.1 (5122.9 to 10703.4)	27.6% (-18.8 to 100.7)	722.4 (454.8 to 1065.8)	922.9 (541.0 to 1455.2)	27.8% (-33.4 to 145.9)
Hounslow	952.8 (621.6 to 1335.1)	1267.8 (790.2 to 1869.0)	33.1% (-25.0 to 136.6)	5921.0 (4526.4 to 7655.7)	7542.5 (5209.6 to 10598.6)	27.4% (-17.9 to 98.0)	721.7 (460.9 to 1076.4)	922.3 (555.8 to 1450.2)	27.8% (-32.4 to 142.2)
Islington	943.6 (591.3 to 1322.5)	1229.8 (737.9 to 1844.7)	30.3% (-29.0 to 140.0)	5994.6 (4568.6 to 7711.7)	7640.3 (5150.8 to 10798.6)	27.5% (-18.8 to 100.2)	727.7 (457.5 to 1057.6)	929.4 (559.5 to 1433.1)	27.7% (-31.8 to 139.9)
Kensington and Chelsea	931.0 (570.5 to 1336.4)	1220.6 (739.6 to 1848.9)	31.1% (-29.6 to 144.0)	6120.1 (4621.7 to 7883.6)	7558.8 (5307.9 to 10616.2)	23.5% (-20.0 to 91.4)	742.5 (466.8 to 1082.7)	920.2 (558.5 to 1429.4)	23.9% (-33.8 to 132.9)
Kingston upon Thames	947.0 (607.5 to 1330.5)	1253.9 (771.7 to 1841.1)	32.4% (-26.0 to 137.4)	5951.3 (4538.1 to 7692.4)	7638.4 (5308.2 to 10621.4)	28.3% (-16.8 to 98.6)	725.6 (452.2 to 1069.6)	932.2 (552.5 to 1463.1)	28.5% (-32.7 to 146.0)
Lambeth	933.8 (591.7 to 1318.6)	1242.4 (758.4 to 1830.0)	33.0% (-26.4 to 140.8)	5990.2 (4568.0 to 7733.0)	7620.9 (5083.7 to 10493.2)	27.2% (-18.5 to 99.0)	727.9 (464.5 to 1071.9)	929.1 (548.6 to 1429.8)	27.6% (-32.3 to 141.1)
Lewisham	951.7 (598.2 to 1321.6)	1274.0 (784.4 to 1875.9)	33.9% (-25.4 to 140.8)	6011.4 (4543.1 to 7799.6)	7603.1 (5164.8 to 10638.9)	26.5% (-19.3 to 98.5)	731.5 (464.7 to 1070.7)	929.2 (550.1 to 1484.8)	27.0% (-33.6 to 143.5)
Merton	943.8 (605.9 to 1306.0)	1269.0 (766.5 to 1869.4)	34.5% (-25.3 to 142.6)	5934.4 (4508.1 to 7655.1)	7541.9 (5181.1 to 10475.9)	27.1% (-17.9 to 97.4)	723.8 (457.9 to 1045.2)	921.7 (560.9 to 1398.8)	27.3% (-30.9 to 135.3)
Newham	961.1 (623.2 to 1333.2)	1251.1 (776.9 to 1834.6)	30.2% (-26.5 to 130.9)	5959.7 (4517.6 to 7671.3)	7563.3 (5255.4 to	26.9% (-18.4 to 97.7)	725.7 (463.1 to	925.0 (528.1 to	27.5% (-34.5 to 148.5)

					10724.1)		1072.7)	1492.7)	
Redbridge	956.1 (620.7 to 1337.0)	1270.3 (803.3 to 1865.7)	32.9% (-24.6 to 134.5)	5918.6 (4509.2 to 7650.7)	7528.0 (5207.2 to 10419.4)	27.2% (-17.5 to 96.8)	721.7 (448.0 to 1052.2)	922.0 (538.9 to 1440.6)	27.7% (-33.3 to 145.2)
Richmond upon Thames	953.4 (628.6 to 1335.9)	1287.5 (799.5 to 1898.8)	35.0% (-23.8 to 139.8)	5951.1 (4513.9 to 7678.1)	7628.3 (5302.1 to 10640.2)	28.2% (-17.0 to 98.8)	726.2 (459.4 to 1063.6)	933.4 (549.1 to 1470.5)	28.5% (-32.7 to 146.1)
Southwark	937.0 (591.6 to 1321.2)	1240.2 (777.3 to 1848.0)	32.4% (-26.4 to 138.0)	5979.7 (4583.5 to 7802.7)	7572.2 (5264.9 to 10367.9)	26.6% (-17.5 to 94.8)	726.5 (456.7 to 1069.0)	924.0 (545.5 to 1400.6)	27.2% (-32.3 to 139.6)
Sutton	959.4 (645.1 to 1332.2)	1267.6 (783.3 to 1859.2)	32.1% (-24.9 to 132.7)	5921.3 (4477.7 to 7658.7)	7522.5 (5122.4 to 10486.9)	27.0% (-18.6 to 98.7)	722.8 (462.4 to 1061.9)	919.4 (538.1 to 1422.5)	27.2% (-32.8 to 141.7)
Tower Hamlets	962.2 (630.7 to 1344.7)	1228.3 (759.8 to 1821.3)	27.7% (-28.3 to 127.8)	5951.1 (4502.7 to 7723.4)	7559.2 (5329.6 to 10546.4)	27.0% (-17.6 to 96.2)	724.3 (451.9 to 1076.2)	917.8 (548.9 to 1425.5)	26.7% (-33.2 to 141.0)
Waltham Forest	944.4 (615.0 to 1343.3)	1260.6 (792.6 to 1837.2)	33.5% (-24.5 to 136.0)	5935.7 (4507.7 to 7613.4)	7583.3 (5231.9 to 10595.0)	27.8% (-17.5 to 98.1)	722.1 (461.6 to 1069.1)	928.1 (541.1 to 1417.5)	28.5% (-32.0 to 143.6)
Wandsworth	929.5 (586.7 to 1339.7)	1250.1 (752.6 to 1839.7)	34.5% (-26.5 to 146.2)	6051.7 (4597.1 to 7761.2)	7667.6 (5299.3 to 10692.7)	26.7% (-18.0 to 96.2)	734.9 (469.2 to 1079.0)	934.8 (550.7 to 1430.0)	27.2% (-32.4 to 139.7)
Westminster	909.5 (574.3 to 1304.1)	1214.9 (732.4 to 1812.2)	33.6% (-27.2 to 145.6)	6046.5 (4614.0 to 7762.2)	7523.5 (5141.2 to 10506.5)	24.4% (-19.8 to 93.6)	734.8 (466.2 to 1086.4)	914.9 (552.3 to 1410.7)	24.5% (-33.5 to 133.7)
County Durham	957.5 (646.5 to 1341.2)	1250.0 (773.8 to 1825.4)	30.6% (-25.6 to 129.9)	5901.0 (4488.0 to 7589.2)	7526.2 (5232.8 to 10512.7)	27.5% (-17.3 to 97.4)	719.1 (454.4 to 1052.7)	918.3 (546.0 to 1400.2)	27.7% (-31.9 to 140.1)

Darlington	982.3 (648.6 to 1335.0)	1273.5 (816.7 to 1879.4)	29.7% (-25.2 to 125.2)	5950.8 (4522.1 to 7672.0)	7545.2 (5156.0 to 10536.0)	26.8% (-18.5 to 97.6)	726.8 (457.3 to 1082.4)	922.1 (551.0 to 1422.1)	26.9% (-32.9 to 140.3)
Gateshead	961.9 (644.5 to 1336.5)	1278.2 (800.0 to 1872.2)	32.9% (-24.0 to 133.1)	5930.1 (4517.9 to 7668.3)	7601.6 (5204.8 to 10629.9)	28.2% (-17.6 to 99.7)	722.3 (458.8 to 1055.6)	927.3 (549.7 to 1437.3)	28.4% (-32.0 to 142.8)
Hartlepool	968.4 (636.4 to 1346.1)	1273.0 (772.0 to 1882.5)	31.5% (-26.6 to 135.8)	5918.7 (4510.7 to 7650.0)	7592.9 (5217.6 to 10635.3)	28.3% (-17.4 to 99.7)	720.4 (455.3 to 1050.0)	927.2 (559.4 to 1459.0)	28.7% (-31.7 to 143.2)
Middlesbrough	961.8 (642.6 to 1317.6)	1257.1 (765.6 to 1836.7)	30.7% (-25.7 to 130.4)	5922.5 (4518.9 to 7650.0)	7519.7 (5182.1 to 10613.7)	27.0% (-18.4 to 98.0)	721.4 (465.2 to 1053.3)	917.0 (549.5 to 1441.3)	27.1% (-32.4 to 139.8)
Newcastle upon Tyne	940.0 (604.1 to 1327.6)	1216.9 (757.6 to 1807.4)	29.5% (-27.7 to 132.3)	5954.0 (4520.2 to 7655.3)	7557.3 (5186.3 to 10672.3)	26.9% (-18.6 to 98.4)	725.3 (465.3 to 1053.6)	920.6 (550.7 to 1426.0)	26.9% (-32.1 to 138.2)
North Tyneside	969.6 (655.1 to 1343.0)	1269.7 (802.0 to 1901.0)	30.9% (-25.3 to 130.1)	5904.6 (4481.9 to 7639.1)	7557.6 (5224.6 to 10572.7)	28.0% (-17.5 to 99.2)	721.8 (454.9 to 1059.6)	923.2 (553.0 to 1384.2)	27.9% (-31.2 to 137.8)
Northumberland	979.6 (652.6 to 1342.4)	1257.6 (797.2 to 1863.6)	28.4% (-26.4 to 124.6)	5895.4 (4467.2 to 7597.5)	7529.1 (5162.9 to 10609.0)	27.7% (-18.1 to 99.6)	720.6 (455.6 to 1067.5)	920.6 (562.5 to 1428.2)	27.7% (-31.7 to 139.4)
Redcar and Cleveland	972.0 (651.2 to 1321.9)	1248.8 (764.5 to 1843.7)	28.5% (-26.8 to 125.9)	5917.7 (4505.2 to 7655.1)	7450.3 (5165.3 to 10474.9)	25.9% (-18.8 to 95.8)	723.2 (456.1 to 1055.3)	913.4 (536.4 to 1472.0)	26.3% (-34.5 to 144.1)
South Tyneside	966.8 (625.5 to 1356.4)	1272.4 (799.5 to 1858.6)	31.6% (-25.4 to 132.5)	5906.7 (4499.4 to 7560.6)	7608.0 (5259.7 to 10857.0)	28.8% (-17.3 to 100.9)	722.1 (459.5 to 1058.9)	926.7 (548.6 to 1410.6)	28.3% (-31.5 to 140.9)
Stockton-on-Tees	975.1 (648.6 to 1342.2)	1259.1 (770.1 to 1851.9)	29.1% (-27.0 to 128.7)	5893.7 (4467.4 to 7638.7)	7455.3 (5068.0 to	26.5% (-18.5 to 97.1)	719.6 (459.4 to	910.2 (543.8 to	26.5% (-32.7 to 138.5)

					10253.9)		1060.2)	1408.6)	
Sunderland	963.6 (620.1 to 1353.4)	1257.7 (761.8 to 1853.1)	30.5% (-27.6 to 136.0)	5921.7 (4476.9 to 7665.8)	7571.1 (5220.4 to 10737.9)	27.9% (-18.3 to 100.4)	723.8 (451.2 to 1068.3)	924.4 (545.7 to 1457.9)	27.7% (-33.4 to 145.6)
Blackburn with Darwen	977.0 (643.1 to 1347.2)	1272.8 (792.5 to 1860.4)	30.3% (-25.8 to 129.4)	5915.2 (4500.6 to 7619.4)	7552.6 (5186.3 to 10583.6)	27.7% (-17.8 to 98.7)	721.2 (452.3 to 1066.4)	923.3 (553.5 to 1413.3)	28.0% (-31.9 to 140.9)
Blackpool	953.4 (619.1 to 1310.1)	1258.8 (790.5 to 1856.2)	32.0% (-25.1 to 133.1)	5968.2 (4508.3 to 7682.3)	7552.2 (5137.6 to 10761.0)	26.5% (-19.6 to 99.4)	726.2 (464.0 to 1059.7)	922.7 (542.2 to 1475.2)	27.1% (-33.6 to 143.5)
Bolton	966.5 (631.9 to 1329.0)	1258.1 (787.7 to 1907.6)	30.2% (-27.0 to 132.4)	5894.1 (4442.1 to 7658.4)	7489.4 (5114.2 to 10742.3)	27.1% (-19.6 to 101.3)	718.9 (454.0 to 1051.2)	915.1 (539.8 to 1443.1)	27.3% (-33.3 to 143.6)
Bury	969.0 (646.6 to 1339.2)	1259.1 (790.9 to 1853.5)	29.9% (-25.7 to 128.0)	5909.0 (4503.3 to 7645.8)	7466.3 (5148.8 to 10486.2)	26.4% (-18.7 to 96.7)	721.5 (459.2 to 1074.8)	912.2 (544.4 to 1421.0)	26.4% (-33.2 to 139.9)
Cheshire East	968.9 (645.0 to 1335.3)	1264.7 (795.9 to 1891.7)	30.5% (-25.9 to 130.2)	5903.5 (4521.1 to 7622.4)	7544.6 (5108.3 to 10791.0)	27.8% (-18.9 to 101.5)	720.9 (454.0 to 1066.7)	922.7 (563.9 to 1474.2)	28.0% (-32.5 to 143.5)
Cheshire West and Chester	971.3 (649.0 to 1354.2)	1257.6 (790.8 to 1869.4)	29.5% (-26.4 to 128.5)	5938.3 (4511.6 to 7681.9)	7572.8 (5228.5 to 10733.4)	27.5% (-18.3 to 99.3)	724.0 (455.9 to 1064.0)	923.5 (554.7 to 1421.6)	27.6% (-32.0 to 139.9)
Cumbria	967.1 (646.0 to 1348.2)	1267.4 (764.1 to 1872.8)	31.1% (-26.6 to 134.3)	5918.1 (4497.9 to 7653.3)	7536.9 (5190.3 to 10674.0)	27.4% (-18.4 to 99.1)	722.1 (453.5 to 1063.8)	920.1 (540.8 to 1446.3)	27.4% (-33.5 to 144.6)
Halton	974.5 (660.8 to 1343.2)	1272.1 (792.5 to 1861.9)	30.5% (-25.1 to 127.9)	5911.4 (4464.2 to 7653.9)	7586.7 (5257.4 to 10451.5)	28.3% (-16.9 to 98.6)	722.2 (455.0 to 1061.5)	925.1 (558.7 to 1426.4)	28.1% (-31.6 to 140.5)

Knowsley	968.6 (643.6 to 1341.1)	1258.2 (767.5 to 1845.2)	29.9% (-26.7 to 130.6)	5931.8 (4520.7 to 7675.7)	7569.1 (5303.1 to 10633.0)	27.6% (-17.4 to 97.7)	721.6 (460.3 to 1063.3)	925.8 (556.6 to 1441.2)	28.3% (-31.8 to 141.9)
Lancashire	964.5 (631.1 to 1356.2)	1251.6 (768.5 to 1832.2)	29.8% (-27.1 to 131.6)	5902.4 (4481.0 to 7613.7)	7517.1 (5195.9 to 10415.8)	27.4% (-17.5 to 97.3)	721.4 (456.8 to 1071.8)	918.5 (548.0 to 1430.8)	27.3% (-32.8 to 142.0)
Liverpool	818.1 (529.9 to 1159.7)	951.0 (542.9 to 1506.2)	16.2% (-38.6 to 121.5)	4866.8 (3534.2 to 6513.9)	5297.7 (3340.0 to 8073.7)	8.9% (-36.3 to 86.1)	592.4 (362.7 to 893.0)	646.2 (357.0 to 1060.5)	9.1% (-46.2 to 121.6)
Manchester	929.1 (599.0 to 1314.4)	1240.1 (759.6 to 1859.2)	33.5% (-26.3 to 142.3)	5940.9 (4512.0 to 7660.3)	7635.2 (5297.4 to 10862.1)	28.5% (-17.5 to 100.6)	722.5 (461.5 to 1063.8)	929.6 (575.0 to 1459.0)	28.7% (-30.9 to 140.4)
Oldham	966.2 (641.4 to 1356.0)	1274.3 (785.6 to 1883.4)	31.9% (-25.8 to 134.9)	5901.1 (4489.0 to 7664.9)	7608.2 (5263.1 to 10793.2)	28.9% (-17.4 to 101.6)	719.7 (456.2 to 1066.4)	930.5 (571.1 to 1424.0)	29.3% (-30.4 to 140.2)
Rochdale	973.8 (644.8 to 1348.2)	1262.2 (802.1 to 1869.0)	29.6% (-25.9 to 127.3)	5893.9 (4461.2 to 7656.7)	7532.5 (5139.1 to 10557.1)	27.8% (-18.2 to 100.4)	718.2 (451.1 to 1062.6)	918.6 (539.5 to 1397.2)	27.9% (-32.3 to 142.2)
Salford	955.6 (619.4 to 1340.6)	1262.4 (792.4 to 1815.7)	32.1% (-24.8 to 131.9)	5938.5 (4521.0 to 7631.5)	7550.8 (5229.2 to 10680.6)	27.2% (-18.1 to 97.9)	721.7 (461.6 to 1069.3)	924.3 (549.0 to 1472.1)	28.1% (-32.9 to 145.4)
Sefton	965.3 (647.5 to 1320.1)	1262.3 (786.9 to 1818.1)	30.8% (-24.5 to 127.1)	5886.0 (4509.2 to 7603.9)	7489.7 (5113.6 to 10096.4)	27.2% (-16.9 to 95.6)	718.3 (449.2 to 1056.8)	914.4 (564.1 to 1398.2)	27.3% (-31.4 to 136.6)
St Helens	964.1 (631.4 to 1312.8)	1263.4 (816.7 to 1823.9)	31.1% (-23.6 to 125.2)	5903.0 (4512.5 to 7626.2)	7492.9 (5235.8 to 10311.1)	26.9% (-17.1 to 94.8)	720.5 (447.7 to 1067.0)	915.9 (547.8 to 1404.5)	27.1% (-32.7 to 140.2)
Stockport	963.9 (650.8 to 1317.9)	1267.6 (786.3 to 1867.5)	31.5% (-24.6 to 129.9)	5867.2 (4506.7 to 7604.6)	7488.4 (5139.0 to	27.6% (-17.4 to 97.6)	716.7 (451.5 to	914.0 (532.5 to	27.5% (-32.4 to 141.3)

					10373.0)		1057.8)	1375.9)	
Tameside	959.5 (631.7 to 1326.2)	1267.5 (798.5 to 1864.2)	32.1% (-24.7 to 132.0)	5914.8 (4505.3 to 7625.5)	7483.6 (5106.4 to 10511.3)	26.5% (-18.9 to 97.8)	719.9 (452.8 to 1063.3)	915.7 (562.0 to 1411.5)	27.2% (-31.8 to 137.3)
Trafford	969.8 (636.3 to 1356.6)	1285.4 (797.7 to 1913.0)	32.5% (-25.6 to 136.6)	5905.7 (4470.8 to 7623.9)	7559.7 (5184.5 to 10721.1)	28.0% (-18.2 to 100.8)	721.3 (459.7 to 1058.2)	923.9 (539.9 to 1416.7)	28.1% (-32.2 to 142.7)
Warrington	962.6 (643.2 to 1330.4)	1251.3 (778.1 to 1830.6)	30.0% (-25.8 to 128.4)	5901.2 (4484.0 to 7670.8)	7485.4 (5120.8 to 10464.5)	26.8% (-18.6 to 98.3)	718.9 (460.1 to 1051.2)	914.8 (552.1 to 1470.4)	27.2% (-32.9 to 142.0)
Wigan	956.4 (645.3 to 1332.2)	1261.3 (787.2 to 1874.5)	31.9% (-25.1 to 132.6)	5906.1 (4480.8 to 7662.1)	7496.0 (5177.4 to 10380.7)	26.9% (-18.0 to 97.0)	719.3 (463.2 to 1049.8)	916.7 (553.3 to 1410.5)	27.5% (-31.4 to 137.3)
Wirral	978.8 (649.2 to 1344.1)	1265.6 (789.8 to 1851.5)	29.3% (-26.1 to 126.9)	5924.8 (4490.4 to 7635.5)	7543.0 (5196.5 to 10486.3)	27.3% (-17.8 to 97.7)	722.8 (454.0 to 1064.1)	920.2 (540.7 to 1419.0)	27.3% (-32.9 to 142.3)
Bracknell Forest	956.3 (636.3 to 1310.5)	1265.9 (788.8 to 1851.9)	32.4% (-24.3 to 132.1)	5842.7 (4437.4 to 7581.9)	7484.4 (5081.7 to 10448.2)	28.1% (-18.0 to 100.6)	713.1 (453.9 to 1063.9)	915.6 (553.1 to 1417.1)	28.4% (-31.6 to 141.8)
Brighton and Hove	938.0 (593.9 to 1325.2)	1218.7 (741.5 to 1790.3)	29.9% (-28.1 to 135.3)	6006.6 (4553.6 to 7805.1)	7601.3 (5151.8 to 10704.8)	26.5% (-19.4 to 99.1)	731.9 (469.6 to 1081.3)	924.2 (562.5 to 1425.8)	26.3% (-32.2 to 135.8)
Buckinghamshire	970.1 (641.6 to 1322.0)	1269.8 (789.9 to 1877.4)	30.9% (-25.5 to 130.6)	5888.0 (4496.6 to 7628.7)	7498.3 (5152.7 to 10516.1)	27.3% (-18.1 to 98.4)	719.8 (444.6 to 1055.8)	916.1 (560.5 to 1390.3)	27.3% (-31.7 to 137.4)
East Sussex	973.9 (644.4 to 1356.0)	1249.6 (751.3 to 1841.9)	28.3% (-28.4 to 130.2)	5905.8 (4501.2 to 7672.4)	7496.2 (5173.2 to 10683.8)	26.9% (-18.8 to 98.9)	722.1 (462.1 to 1071.5)	916.4 (529.6 to 1402.2)	26.9% (-33.2 to 141.8)

Hampshire	959.6 (647.5 to 1330.5)	1269.5 (801.8 to 1848.7)	32.3% (-23.7 to 130.0)	5858.8 (4515.0 to 7539.4)	7562.6 (5132.0 to 10830.1)	29.1% (-17.8 to 102.9)	716.1 (452.3 to 1043.6)	926.5 (543.0 to 1478.3)	29.4% (-32.6 to 149.1)
Isle of Wight	977.0 (652.3 to 1340.1)	1258.2 (772.7 to 1836.8)	28.8% (-26.7 to 126.6)	5888.4 (4481.2 to 7600.5)	7538.9 (5212.2 to 10527.4)	28.0% (-17.2 to 98.7)	719.2 (455.5 to 1042.6)	920.0 (542.0 to 1426.8)	27.9% (-32.3 to 142.3)
Kent	960.8 (640.4 to 1340.1)	1266.6 (766.3 to 1904.9)	31.8% (-26.6 to 136.9)	5897.4 (4501.1 to 7694.3)	7553.8 (5076.8 to 10624.9)	28.1% (-18.7 to 102.1)	720.6 (454.8 to 1075.8)	925.2 (545.6 to 1483.7)	28.4% (-33.6 to 148.9)
Medway	960.3 (623.2 to 1349.3)	1272.3 (781.6 to 1884.1)	32.5% (-26.1 to 138.1)	5890.9 (4455.8 to 7605.1)	7601.2 (5194.8 to 10612.7)	29.0% (-17.2 to 101.5)	720.1 (456.9 to 1062.3)	929.2 (542.7 to 1418.2)	29.0% (-31.8 to 144.7)
Milton Keynes	981.7 (654.1 to 1349.1)	1298.5 (806.4 to 1973.8)	32.3% (-25.5 to 135.1)	5901.7 (4497.3 to 7556.5)	7568.2 (5134.9 to 10757.9)	28.2% (-18.2 to 101.1)	721.3 (452.1 to 1072.7)	926.2 (538.3 to 1434.7)	28.4% (-33.0 to 146.8)
Oxfordshire	933.3 (604.1 to 1296.6)	1254.3 (785.0 to 1847.3)	34.4% (-24.1 to 138.6)	5844.7 (4473.1 to 7529.0)	7535.8 (5217.7 to 10566.0)	28.9% (-16.6 to 99.7)	715.0 (457.7 to 1038.4)	919.3 (543.1 to 1424.3)	28.6% (-31.6 to 142.7)
Portsmouth	908.7 (587.1 to 1262.5)	1228.1 (753.7 to 1855.9)	35.1% (-25.1 to 144.7)	5820.1 (4483.5 to 7526.9)	7522.4 (5210.1 to 10632.3)	29.2% (-16.6 to 100.8)	708.4 (449.4 to 1032.8)	918.7 (541.8 to 1459.1)	29.7% (-32.1 to 148.2)
Reading	932.7 (595.6 to 1315.1)	1251.0 (774.3 to 1840.2)	34.1% (-25.1 to 140.4)	5935.5 (4541.5 to 7665.5)	7535.1 (5162.9 to 10379.7)	26.9% (-17.7 to 96.3)	722.4 (457.5 to 1070.5)	920.2 (554.9 to 1449.7)	27.4% (-32.7 to 141.8)
Slough	954.0 (615.9 to 1336.3)	1301.9 (825.0 to 1925.2)	36.5% (-22.8 to 141.6)	5934.3 (4519.5 to 7678.1)	7553.1 (5180.6 to 10709.4)	27.3% (-18.6 to 99.5)	723.0 (457.7 to 1072.5)	923.6 (548.1 to 1472.2)	27.7% (-33.4 to 145.7)
Southampton	931.0 (597.9 to 1299.6)	1216.5 (734.8 to 1784.9)	30.7% (-27.4 to 135.6)	5897.3 (4493.9 to 7611.6)	7513.7 (5097.5 to	27.4% (-19.1 to 100.9)	718.7 (450.8 to	917.7 (526.1 to	27.7% (-33.3 to 145.2)

					10760.2)		1066.9)	1400.7)	
Surrey	962.8 (637.6 to 1331.8)	1274.4 (787.5 to 1878.2)	32.4% (-25.1 to 134.5)	5889.8 (4501.9 to 7653.8)	7590.5 (5176.5 to 10769.2)	28.9% (-17.8 to 102.4)	720.2 (449.6 to 1048.3)	928.2 (543.4 to 1453.4)	28.9% (-32.6 to 147.2)
West Berkshire	963.6 (651.7 to 1333.2)	1266.0 (798.8 to 1880.8)	31.4% (-24.8 to 130.1)	5865.3 (4516.6 to 7545.2)	7469.9 (5194.7 to 10440.4)	27.4% (-17.2 to 96.3)	717.5 (456.3 to 1048.4)	915.2 (549.7 to 1404.3)	27.6% (-31.7 to 138.7)
West Sussex	969.6 (650.4 to 1324.3)	1253.4 (777.7 to 1834.8)	29.3% (-26.0 to 126.1)	5890.4 (4432.3 to 7591.4)	7461.3 (5122.8 to 10398.0)	26.7% (-18.6 to 97.7)	721.2 (460.5 to 1053.9)	912.8 (545.0 to 1430.3)	26.6% (-32.9 to 139.6)
Windsor and Maidenhead	963.4 (648.3 to 1326.1)	1265.0 (778.1 to 1837.3)	31.3% (-24.9 to 130.2)	5863.2 (4447.7 to 7593.2)	7503.3 (4973.6 to 10728.5)	28.0% (-19.7 to 104.4)	716.7 (454.7 to 1051.3)	917.8 (527.6 to 1397.3)	28.1% (-32.6 to 144.0)
Wokingham	966.2 (649.5 to 1318.6)	1296.2 (814.8 to 1877.6)	34.2% (-22.4 to 132.4)	5855.4 (4407.7 to 7545.2)	7630.6 (5194.3 to 10682.2)	30.3% (-16.7 to 104.2)	716.1 (455.4 to 1044.5)	932.9 (556.6 to 1448.8)	30.3% (-30.8 to 145.7)
Bath and North East Somerset	946.6 (614.6 to 1329.0)	1215.9 (755.3 to 1806.7)	28.5% (-28.1 to 129.8)	5925.6 (4503.9 to 7665.4)	7537.7 (5243.9 to 10526.6)	27.2% (-17.7 to 97.3)	721.9 (459.6 to 1053.6)	923.5 (540.5 to 1458.3)	27.9% (-33.0 to 144.9)
Bournemouth	943.7 (621.0 to 1282.7)	1237.4 (753.7 to 1840.1)	31.1% (-26.1 to 133.1)	5983.3 (4578.8 to 7718.8)	7563.4 (5132.3 to 10681.8)	26.4% (-19.2 to 98.0)	728.2 (458.0 to 1076.3)	923.7 (530.8 to 1418.5)	26.9% (-33.8 to 143.5)
Bristol, City of	945.9 (604.3 to 1337.4)	1248.7 (770.2 to 1824.8)	32.0% (-26.2 to 136.4)	5951.4 (4512.7 to 7666.4)	7614.5 (5291.6 to 10740.2)	27.9% (-17.6 to 99.0)	726.1 (456.7 to 1067.2)	931.1 (549.0 to 1466.0)	28.2% (-32.9 to 145.7)
Cornwall	973.8 (661.1 to 1315.0)	1257.7 (784.7 to 1869.1)	29.2% (-25.5 to 124.6)	5883.2 (4477.8 to 7556.1)	7516.6 (5176.7 to 10540.5)	27.8% (-17.6 to 98.5)	719.1 (451.1 to 1045.9)	919.2 (532.1 to 1407.4)	27.8% (-32.7 to 143.4)

Devon	956.0 (638.9 to 1345.9)	1250.7 (752.9 to 1812.8)	30.8% (-26.4 to 133.3)	5891.3 (4479.9 to 7578.0)	7476.4 (5116.9 to 10350.1)	26.9% (-18.0 to 96.9)	719.6 (453.2 to 1068.5)	915.3 (544.2 to 1397.8)	27.2% (-32.5 to 140.1)
Dorset	965.3 (654.3 to 1335.6)	1261.8 (800.6 to 1875.7)	30.7% (-25.0 to 128.2)	5854.5 (4463.9 to 7537.6)	7496.0 (5107.8 to 10620.3)	28.0% (-18.2 to 100.6)	714.8 (452.3 to 1052.3)	916.1 (541.3 to 1392.0)	28.2% (-31.8 to 141.5)
Gloucestershire	968.2 (636.0 to 1334.4)	1270.0 (779.5 to 1876.7)	31.2% (-26.2 to 133.5)	5931.4 (4499.5 to 7635.5)	7585.6 (5175.6 to 10556.7)	27.9% (-17.7 to 99.2)	724.1 (456.9 to 1060.8)	924.8 (532.3 to 1395.5)	27.7% (-32.5 to 142.2)
North Somerset	971.0 (655.3 to 1344.9)	1275.1 (803.5 to 1875.4)	31.3% (-24.6 to 129.4)	5875.2 (4468.3 to 7590.4)	7578.0 (5251.6 to 10579.8)	29.0% (-16.6 to 100.1)	717.6 (458.1 to 1054.7)	927.3 (547.7 to 1410.9)	29.2% (-31.1 to 142.8)
Plymouth	931.9 (596.1 to 1310.8)	1237.4 (762.4 to 1801.4)	32.8% (-25.6 to 137.1)	5872.9 (4481.8 to 7581.3)	7537.5 (5206.1 to 10482.1)	28.3% (-16.9 to 98.8)	715.4 (452.4 to 1037.4)	923.4 (532.6 to 1471.1)	29.1% (-32.9 to 149.0)
Poole	958.7 (637.3 to 1334.4)	1262.7 (782.8 to 1805.9)	31.7% (-24.4 to 130.0)	5893.4 (4474.7 to 7593.8)	7510.1 (5213.5 to 10230.8)	27.4% (-16.8 to 95.6)	719.2 (460.1 to 1045.3)	919.2 (556.0 to 1429.9)	27.8% (-31.5 to 139.2)
Somerset	975.0 (662.4 to 1347.1)	1259.0 (791.5 to 1844.3)	29.1% (-25.7 to 124.7)	5883.1 (4500.9 to 7573.8)	7533.0 (5286.7 to 10478.2)	28.0% (-16.5 to 96.9)	719.2 (453.7 to 1060.0)	919.8 (554.2 to 1376.4)	27.9% (-31.1 to 137.3)
South Gloucestershire	962.9 (636.2 to 1330.2)	1279.9 (800.9 to 1878.8)	32.9% (-24.3 to 133.8)	5900.6 (4505.7 to 7596.8)	7636.7 (5218.4 to 10853.5)	29.4% (-17.3 to 102.6)	720.9 (451.3 to 1057.6)	931.6 (555.6 to 1415.4)	29.2% (-31.0 to 142.7)
Swindon	958.0 (643.2 to 1326.2)	1270.8 (781.6 to 1910.2)	32.6% (-25.3 to 135.7)	5938.9 (4543.2 to 7642.4)	7573.5 (5140.6 to 10564.2)	27.5% (-18.0 to 98.6)	726.1 (462.1 to 1077.1)	924.6 (553.8 to 1428.4)	27.3% (-32.3 to 140.4)
Torbay	967.5 (643.6 to 1316.1)	1272.4 (799.4 to 1928.0)	31.5% (-25.3 to 132.0)	5917.0 (4469.8 to 7708.2)	7577.4 (5246.3 to	28.1% (-18.2 to 101.3)	721.9 (453.7 to	926.3 (548.3 to	28.3% (-32.3 to 143.7)

					10796.2)		1050.1)	1442.6)	
Wiltshire	958.9 (636.3 to 1344.3)	1268.1 (786.2 to 1852.2)	32.2% (-25.0 to 133.6)	5870.4 (4501.0 to 7603.5)	7540.0 (5186.4 to 10541.9)	28.4% (-17.1 to 99.5)	717.6 (456.0 to 1061.9)	921.4 (550.6 to 1405.6)	28.4% (-31.4 to 140.9)
Birmingham	955.4 (633.4 to 1338.4)	1249.6 (753.4 to 1842.1)	30.8% (-27.0 to 134.8)	5934.2 (4520.2 to 7683.0)	7520.8 (5160.7 to 10516.4)	26.7% (-18.5 to 97.4)	724.6 (463.4 to 1070.6)	917.8 (543.6 to 1383.4)	26.7% (-32.1 to 137.2)
Coventry	942.7 (628.1 to 1308.4)	1243.9 (781.5 to 1827.7)	31.9% (-24.7 to 131.6)	5909.5 (4522.1 to 7608.5)	7513.8 (5220.4 to 10425.8)	27.1% (-17.3 to 96.0)	719.7 (462.4 to 1057.8)	919.0 (554.8 to 1454.3)	27.7% (-32.3 to 141.6)
Dudley	955.2 (639.9 to 1321.8)	1256.0 (785.8 to 1860.4)	31.5% (-25.1 to 131.4)	5885.6 (4485.5 to 7592.2)	7527.0 (5192.5 to 10492.0)	27.9% (-17.4 to 98.3)	719.5 (456.8 to 1054.0)	919.9 (540.4 to 1387.8)	27.9% (-31.8 to 140.0)
Herefordshire, County of	962.5 (651.4 to 1330.0)	1265.7 (788.5 to 1889.3)	31.5% (-25.1 to 131.4)	5890.1 (4505.1 to 7577.4)	7539.8 (5199.0 to 10527.2)	28.0% (-17.2 to 98.2)	721.2 (456.9 to 1074.4)	921.7 (536.8 to 1448.4)	27.8% (-33.6 to 146.6)
Sandwell	964.1 (621.5 to 1372.0)	1271.0 (791.6 to 1901.0)	31.8% (-26.7 to 137.5)	5963.8 (4566.6 to 7752.4)	7520.3 (5176.2 to 10751.4)	26.1% (-19.5 to 97.9)	727.1 (459.2 to 1059.1)	919.9 (545.0 to 1445.2)	26.5% (-33.4 to 140.9)
Shropshire	964.7 (644.1 to 1328.9)	1256.2 (774.3 to 1855.2)	30.2% (-26.2 to 130.1)	5887.9 (4443.3 to 7592.4)	7518.8 (5195.6 to 10377.4)	27.7% (-17.4 to 97.8)	720.0 (461.8 to 1053.4)	919.5 (529.8 to 1438.9)	27.7% (-33.2 to 144.5)
Solihull	971.2 (651.4 to 1330.9)	1262.2 (782.7 to 1843.3)	30.0% (-25.6 to 127.5)	5889.3 (4487.4 to 7675.4)	7500.7 (5221.1 to 10645.3)	27.4% (-18.2 to 98.9)	721.1 (455.1 to 1051.6)	920.8 (545.1 to 1475.0)	27.7% (-33.4 to 145.3)
Staffordshire	965.1 (642.0 to 1328.7)	1254.1 (783.4 to 1861.0)	29.9% (-26.2 to 129.1)	5897.6 (4512.0 to 7565.5)	7524.9 (5223.9 to 10449.2)	27.6% (-17.0 to 96.5)	719.6 (452.9 to 1051.1)	919.2 (548.6 to 1376.3)	27.7% (-31.2 to 137.5)

Stoke-on-Trent	945.2 (609.6 to 1307.9)	1263.2 (790.4 to 1868.7)	33.6% (-24.6 to 137.5)	5942.6 (4561.5 to 7643.6)	7540.4 (5171.2 to 10777.8)	26.9% (-18.8 to 98.4)	723.8 (464.9 to 1068.9)	921.0 (545.9 to 1429.3)	27.2% (-32.6 to 140.7)
Telford and Wrekin	970.2 (636.1 to 1338.6)	1266.8 (772.7 to 1873.1)	30.6% (-26.8 to 133.3)	5906.3 (4498.0 to 7608.7)	7553.7 (5180.0 to 10655.0)	27.9% (-18.0 to 99.8)	720.9 (458.4 to 1047.9)	925.2 (551.2 to 1400.0)	28.3% (-31.0 to 139.2)
Walsall	968.5 (636.3 to 1351.7)	1259.3 (772.0 to 1844.1)	30.0% (-26.8 to 131.5)	5928.8 (4512.0 to 7626.7)	7488.3 (5071.1 to 10455.8)	26.3% (-19.0 to 97.4)	723.8 (458.2 to 1073.7)	915.2 (539.5 to 1436.2)	26.4% (-33.8 to 142.1)
Warwickshire	961.7 (634.6 to 1319.9)	1263.9 (794.6 to 1895.4)	31.4% (-25.6 to 132.4)	5896.0 (4531.1 to 7587.8)	7559.0 (5181.9 to 10605.6)	28.2% (-17.3 to 99.2)	719.9 (459.2 to 1046.2)	924.4 (548.3 to 1438.5)	28.4% (-31.8 to 142.6)
Wolverhampton	951.5 (638.1 to 1337.0)	1278.7 (801.5 to 1920.7)	34.4% (-24.2 to 138.8)	5919.2 (4504.5 to 7609.6)	7608.0 (5172.3 to 10839.1)	28.5% (-18.1 to 101.9)	721.8 (458.1 to 1061.6)	927.7 (562.7 to 1465.2)	28.5% (-31.9 to 143.2)
Worcestershire	975.1 (644.6 to 1360.3)	1266.6 (788.7 to 1882.4)	29.9% (-26.7 to 130.9)	5893.7 (4462.6 to 7606.6)	7552.7 (5181.4 to 10780.9)	28.1% (-18.4 to 101.6)	720.3 (457.2 to 1055.9)	922.9 (528.0 to 1441.6)	28.1% (-33.4 to 147.0)
Barnsley	955.7 (635.0 to 1309.2)	1262.5 (783.5 to 1864.8)	32.1% (-24.9 to 132.9)	5926.5 (4531.5 to 7697.5)	7514.0 (5138.4 to 10421.7)	26.8% (-18.2 to 97.1)	722.8 (458.5 to 1063.2)	918.5 (532.7 to 1446.3)	27.1% (-33.9 to 144.6)
Bradford	964.2 (638.0 to 1332.2)	1264.8 (791.1 to 1886.1)	31.2% (-25.7 to 132.4)	5899.5 (4441.8 to 7611.6)	7544.9 (5143.3 to 10726.1)	27.9% (-18.7 to 101.6)	720.2 (456.8 to 1057.0)	924.7 (537.6 to 1470.6)	28.4% (-33.3 to 147.9)
Calderdale	968.9 (635.3 to 1349.2)	1279.0 (790.5 to 1878.2)	32.0% (-25.5 to 134.4)	5920.4 (4493.3 to 7711.9)	7585.1 (5295.5 to 10485.5)	28.1% (-16.9 to 98.0)	722.2 (450.6 to 1069.2)	928.3 (562.3 to 1447.0)	28.5% (-31.9 to 143.1)
Doncaster	963.2 (641.1 to 1329.7)	1287.1 (800.4 to 1915.5)	33.6% (-24.3 to 136.5)	5902.6 (4475.3 to 7648.4)	7611.4 (5300.0 to	28.9% (-16.9 to 100.7)	720.4 (452.8 to	929.5 (557.0 to	29.0% (-31.8 to 145.0)

					10700.5)		1064.0)	1448.2)	
East Riding of Yorkshire	972.1 (643.3 to 1359.0)	1270.9 (795.9 to 1902.9)	30.7% (-26.3 to 132.6)	5874.2 (4462.7 to 7583.9)	7586.1 (5242.9 to 10777.1)	29.1% (-17.2 to 101.8)	718.5 (452.4 to 1051.0)	928.2 (540.4 to 1444.7)	29.2% (-32.3 to 147.4)
Kingston upon Hull, City of	949.0 (611.3 to 1327.6)	1250.2 (754.5 to 1824.0)	31.7% (-26.7 to 137.2)	5951.9 (4513.4 to 7715.2)	7522.3 (5252.2 to 10337.9)	26.4% (-17.7 to 94.6)	724.1 (461.3 to 1064.5)	919.0 (537.9 to 1408.0)	26.9% (-32.8 to 140.3)
Kirklees	964.9 (643.0 to 1330.4)	1255.1 (781.6 to 1848.7)	30.1% (-25.9 to 129.0)	5914.8 (4522.3 to 7629.8)	7503.1 (5171.4 to 10617.8)	26.9% (-18.5 to 97.9)	721.1 (453.9 to 1069.2)	916.6 (543.1 to 1428.6)	27.1% (-33.2 to 142.4)
Leeds	944.2 (618.4 to 1322.9)	1256.0 (781.6 to 1848.3)	33.0% (-24.9 to 136.1)	5932.3 (4515.3 to 7634.8)	7579.6 (5225.3 to 10542.9)	27.8% (-17.4 to 98.0)	723.5 (458.0 to 1064.6)	925.2 (547.7 to 1444.7)	27.9% (-32.7 to 143.4)
North East Lincolnshire	961.9 (632.3 to 1350.3)	1265.2 (782.5 to 1878.8)	31.5% (-26.2 to 135.0)	5906.9 (4459.4 to 7641.3)	7540.8 (5162.5 to 10574.8)	27.7% (-18.2 to 99.8)	720.6 (461.0 to 1057.4)	920.5 (542.5 to 1428.9)	27.8% (-32.4 to 142.2)
North Lincolnshire	978.8 (647.8 to 1329.1)	1271.4 (798.1 to 1860.3)	29.9% (-25.4 to 126.8)	5911.9 (4492.5 to 7623.0)	7522.9 (5209.1 to 10398.2)	27.3% (-17.4 to 96.8)	721.0 (454.9 to 1063.5)	918.5 (533.3 to 1403.8)	27.4% (-33.0 to 142.7)
North Yorkshire	960.1 (642.7 to 1336.1)	1262.3 (780.1 to 1884.5)	31.5% (-25.9 to 133.7)	5863.2 (4417.0 to 7613.7)	7487.5 (5076.3 to 10619.3)	27.7% (-19.0 to 101.8)	715.6 (451.7 to 1057.4)	915.0 (544.6 to 1426.2)	27.9% (-32.6 to 142.9)
Rotherham	960.9 (642.6 to 1315.4)	1266.3 (789.2 to 1902.9)	31.8% (-25.2 to 132.5)	5900.1 (4474.8 to 7636.9)	7570.6 (5246.8 to 10587.9)	28.3% (-17.2 to 99.6)	720.7 (459.9 to 1053.6)	924.7 (552.3 to 1435.6)	28.3% (-31.7 to 141.8)
Sheffield	939.7 (597.3 to 1335.6)	1236.0 (741.4 to 1829.7)	31.5% (-28.0 to 141.0)	5942.9 (4504.6 to 7660.1)	7591.2 (5205.6 to 10690.5)	27.7% (-18.1 to 99.6)	725.2 (461.9 to 1072.3)	927.8 (562.1 to 1441.5)	27.9% (-31.8 to 140.7)

Wakefield	958.5 (620.3 to 1321.1)	1264.7 (779.1 to 1866.5)	31.9% (-25.9 to 135.4)	5929.4 (4546.8 to 7682.4)	7536.3 (5114.6 to 10521.3)	27.1% (-18.4 to 98.5)	722.6 (462.8 to 1057.9)	921.2 (542.5 to 1418.2)	27.5% (-32.3 to 140.8)
York	944.0 (608.3 to 1306.3)	1237.9 (737.1 to 1862.8)	31.1% (-28.1 to 139.4)	5953.6 (4527.5 to 7685.6)	7622.9 (5209.1 to 10575.6)	28.0% (-17.5 to 99.1)	726.5 (465.1 to 1052.2)	931.6 (542.3 to 1430.3)	28.2% (-32.0 to 142.2)
Northern Ireland	1142.0 (735.1 to 1650.5)	1702.6 (1049.8 to 2564.5)	49.1% (-18.1 to 171.8)	7403.4 (5162.2 to 10308.1)	11174.8 (7258.9 to 16384.6)	50.9% (-11.5 to 158.1)	907.5 (543.0 to 1379.4)	1364.2 (760.6 to 2149.7)	50.3% (-24.9 to 202.0)
Scotland	992.3 (626.5 to 1443.7)	1322.3 (788.5 to 2028.1)	33.3% (-28.9 to 150.2)	5957.7 (4091.1 to 8375.6)	7784.8 (5009.1 to 11802.7)	30.7% (-25.3 to 128.9)	728.0 (426.4 to 1139.8)	949.6 (532.1 to 1566.5)	30.4% (-36.8 to 170.1)
Wales	972.5 (614.4 to 1414.9)	1296.7 (774.8 to 1985.6)	33.3% (-28.7 to 150.0)	6008.0 (4146.0 to 8453.0)	7897.1 (4983.6 to 11815.1)	31.4% (-24.9 to 130.4)	734.9 (432.3 to 1126.5)	964.6 (540.6 to 1584.3)	31.3% (-35.9 to 169.8)
Depressive disorders									
England	6379.7 (4327.3 to 9039.8)	6620.9 (4513.3 to 9395.1)	3.8% (-37.9 to 73.5)	4809.5 (3480.4 to 6630.5)	4890.0 (3497.2 to 6716.1)	1.7% (-35.4 to 60.2)	918.6 (565.7 to 1410.3)	941.4 (579.8 to 1444.1)	2.5% (-45.9 to 94.0)
East Midlands	6358.1 (4303.1 to 9017.1)	6645.0 (4473.7 to 9507.9)	4.5% (-38.0 to 76.4)	4783.0 (3465.1 to 6609.5)	4909.2 (3473.5 to 6772.7)	2.6% (-35.2 to 62.6)	915.0 (563.0 to 1404.8)	945.2 (581.0 to 1444.7)	3.3% (-45.4 to 95.6)
East of England	6302.1 (4284.5 to 8892.3)	6576.8 (4444.5 to 9423.4)	4.4% (-37.8 to 75.3)	4734.8 (3418.4 to 6499.1)	4846.9 (3449.2 to 6752.0)	2.4% (-35.4 to 62.3)	906.5 (560.2 to 1391.2)	934.8 (572.3 to 1446.0)	3.1% (-45.7 to 96.3)
Greater London	6644.6 (4502.8 to 9385.5)	6698.8 (4537.9 to 9581.7)	0.8% (-40.0 to 69.4)	5056.8 (3649.9 to 6993.0)	4958.2 (3528.1 to 6858.6)	-2.0% (-38.1 to 55.4)	963.2 (595.8 to 1475.6)	953.9 (581.9 to 1467.8)	-1.0% (-47.8 to 88.2)
North East England	6343.1 (4282.1	6646.2 (4489.9	4.8% (-37.6 to 75.9)	4769.5 (3458.6	4915.8 (3490.1	3.1% (-34.7 to 62.7)	911.7 (562.1 to	945.5 (576.2 to	3.7% (-45.6 to 97.9)

	to 8968.8)	to 9419.7)		to 6581.0)	to 6757.6)		1402.2)	1462.3)	
North West England	6260.8 (4244.7 to 8932.2)	6523.5 (4351.1 to 9265.1)	4.2% (-38.4 to 76.3)	4719.0 (3399.0 to 6491.9)	4825.0 (3423.2 to 6568.9)	2.2% (-35.1 to 61.1)	899.7 (553.7 to 1372.9)	927.0 (572.2 to 1424.5)	3.0% (-45.5 to 94.8)
South East England	6347.7 (4304.6 to 8967.2)	6606.9 (4479.5 to 9430.0)	4.1% (-38.0 to 74.7)	4774.8 (3449.1 to 6566.9)	4869.4 (3475.2 to 6744.6)	2.0% (-35.4 to 61.1)	913.2 (560.6 to 1408.1)	938.0 (571.7 to 1433.7)	2.7% (-46.0 to 95.5)
South West England	6338.8 (4308.3 to 8990.3)	6642.8 (4494.8 to 9494.4)	4.8% (-37.6 to 76.2)	4767.6 (3452.1 to 6557.3)	4907.0 (3496.1 to 6810.0)	2.9% (-34.9 to 62.8)	911.3 (561.2 to 1385.5)	945.4 (574.5 to 1442.5)	3.7% (-45.2 to 96.6)
West Midlands	6357.9 (4312.5 to 9000.2)	6617.5 (4496.8 to 9583.0)	4.1% (-38.3 to 75.5)	4786.0 (3469.1 to 6565.3)	4883.2 (3484.0 to 6780.1)	2.0% (-35.4 to 61.2)	914.0 (566.0 to 1408.0)	940.7 (581.6 to 1467.0)	2.9% (-45.9 to 95.9)
Yorkshire and the Humber	6409.3 (4339.8 to 9088.9)	6637.4 (4536.1 to 9387.1)	3.6% (-38.0 to 72.9)	4833.2 (3499.0 to 6677.8)	4903.5 (3491.3 to 6726.2)	1.5% (-35.7 to 60.1)	923.0 (568.9 to 1407.5)	943.6 (575.9 to 1456.0)	2.2% (-46.1 to 94.3)
Derby	6468.9 (4352.8 to 9181.1)	6603.8 (4224.9 to 10072.4)	2.1% (-42.4 to 81.4)	4886.2 (3501.4 to 6759.3)	4874.9 (3332.8 to 7198.5)	-0.2% (-39.8 to 65.8)	934.6 (564.3 to 1432.0)	939.2 (562.2 to 1508.4)	0.5% (-48.7 to 97.2)
Derbyshire	6277.5 (4242.4 to 8912.6)	6630.8 (4167.9 to 9997.6)	5.6% (-40.5 to 87.9)	4703.7 (3391.0 to 6478.9)	4883.7 (3294.8 to 7012.6)	3.8% (-36.8 to 71.0)	900.1 (553.3 to 1382.9)	939.5 (553.2 to 1496.6)	4.4% (-46.7 to 104.4)
Leicester	6647.7 (4518.5 to 9454.9)	6820.3 (4319.7 to 10260.4)	2.6% (-41.9 to 81.6)	5066.3 (3638.0 to 7019.4)	5077.3 (3450.4 to 7294.7)	0.2% (-39.0 to 65.0)	966.2 (596.6 to 1495.2)	976.6 (565.4 to 1604.8)	1.1% (-49.4 to 102.6)
Leicestershire	6277.0 (4232.2 to 8995.1)	6640.9 (4265.2 to 10030.2)	5.8% (-40.0 to 87.1)	4704.0 (3389.0 to 6485.8)	4909.6 (3382.2 to 7104.0)	4.4% (-36.2 to 70.9)	901.5 (552.7 to 1385.9)	945.6 (551.6 to 1505.6)	4.9% (-46.6 to 106.4)

Lincolnshire	6235.5 (4203.0 to 8919.0)	6658.3 (4185.7 to 9816.8)	6.8% (-39.4 to 88.5)	4666.8 (3384.5 to 6437.3)	4914.0 (3272.1 to 7008.1)	5.3% (-36.0 to 73.6)	894.3 (548.4 to 1379.7)	946.7 (541.4 to 1510.5)	5.9% (-46.7 to 110.7)
Northamptonshire	6274.6 (4239.2 to 8920.6)	6510.8 (4110.1 to 9624.6)	3.8% (-40.9 to 82.6)	4705.1 (3386.4 to 6444.7)	4778.5 (3190.1 to 6858.3)	1.6% (-38.4 to 67.7)	900.4 (549.0 to 1394.7)	922.0 (536.3 to 1479.5)	2.4% (-48.3 to 103.1)
Nottingham	6839.6 (4627.7 to 9682.7)	6959.4 (4441.7 to 10378.7)	1.8% (-41.9 to 78.7)	5243.6 (3766.0 to 7232.8)	5216.9 (3517.2 to 7475.5)	-0.5% (-39.5 to 63.9)	998.7 (616.1 to 1517.9)	1003.1 (580.5 to 1613.6)	0.4% (-49.1 to 98.6)
Nottinghamshire	6286.0 (4256.4 to 8961.8)	6518.9 (4235.1 to 9622.3)	3.7% (-40.2 to 80.2)	4712.8 (3391.9 to 6488.9)	4799.1 (3269.4 to 6851.0)	1.8% (-37.6 to 66.6)	901.8 (548.0 to 1389.1)	923.0 (542.5 to 1483.5)	2.4% (-48.2 to 102.2)
Rutland	6108.7 (4136.6 to 8642.0)	6595.3 (4216.1 to 9807.1)	8.0% (-38.2 to 89.1)	4550.6 (3270.2 to 6208.4)	4863.3 (3303.7 to 6905.0)	6.9% (-34.4 to 74.3)	874.9 (531.2 to 1352.3)	938.1 (543.7 to 1496.0)	7.2% (-45.9 to 112.6)
Bedford	6289.7 (4264.4 to 8881.6)	6597.2 (4204.0 to 9976.2)	4.9% (-40.5 to 85.3)	4719.9 (3420.2 to 6496.2)	4870.1 (3320.8 to 7029.6)	3.2% (-37.0 to 69.3)	903.8 (554.1 to 1388.2)	939.3 (531.4 to 1506.4)	3.9% (-48.0 to 108.2)
Cambridgeshire	6459.3 (4363.0 to 9126.2)	6678.1 (4251.6 to 9949.3)	3.4% (-41.1 to 81.7)	4882.9 (3513.0 to 6731.3)	4946.9 (3384.9 to 7001.2)	1.3% (-37.6 to 65.0)	934.6 (570.9 to 1435.4)	953.5 (562.8 to 1514.5)	2.0% (-47.9 to 99.7)
Central Bedfordshire	6240.4 (4220.8 to 8835.0)	6494.9 (4151.2 to 9621.1)	4.1% (-40.4 to 82.2)	4674.1 (3359.0 to 6433.1)	4769.8 (3236.1 to 6757.5)	2.0% (-37.4 to 66.7)	895.3 (549.9 to 1379.7)	921.4 (531.8 to 1474.1)	2.9% (-48.0 to 104.1)
Essex	6243.6 (4212.8 to 8884.8)	6588.1 (4136.3 to 9928.0)	5.5% (-40.6 to 88.0)	4681.7 (3385.6 to 6405.6)	4860.4 (3272.7 to 7086.0)	3.8% (-37.1 to 71.5)	897.1 (547.8 to 1389.6)	937.9 (531.0 to 1549.7)	4.5% (-48.5 to 112.8)
Hertfordshire	6291.2 (4279.3 to 8879.5)	6533.3 (4121.0 to 9919.3)	3.8% (-41.4 to 84.2)	4721.3 (3406.8 to 6497.9)	4802.3 (3272.6 to 6857.7)	1.7% (-37.7 to 66.2)	903.4 (557.3 to	927.2 (544.9 to	2.6% (-47.4 to 100.8)

							1383.3)	1474.4)	
Luton	6403.0 (4368.0 to 9135.0)	6557.6 (4126.9 to 9982.5)	2.4% (-42.4 to 82.5)	4834.6 (3479.7 to 6681.2)	4822.2 (3235.8 to 6936.3)	-0.3% (-39.6 to 65.0)	922.7 (569.6 to 1417.7)	928.7 (545.4 to 1502.8)	0.7% (-48.9 to 98.7)
Norfolk	6298.3 (4307.2 to 8921.9)	6627.1 (4225.9 to 9845.5)	5.2% (-39.7 to 84.2)	4728.9 (3412.7 to 6484.2)	4897.9 (3342.5 to 7046.8)	3.6% (-36.6 to 69.7)	905.5 (557.0 to 1386.4)	943.1 (545.5 to 1516.3)	4.2% (-47.3 to 106.7)
Peterborough	6278.1 (4208.3 to 8962.2)	6460.1 (4107.4 to 9770.5)	2.9% (-42.0 to 82.9)	4713.5 (3389.9 to 6481.4)	4744.7 (3242.1 to 6924.5)	0.7% (-38.8 to 66.1)	902.4 (546.7 to 1369.5)	915.7 (540.1 to 1515.9)	1.5% (-49.0 to 102.5)
Southend-on-Sea	6464.2 (4421.1 to 9163.4)	6514.8 (4244.3 to 9791.1)	0.8% (-42.0 to 75.5)	4885.6 (3545.2 to 6702.8)	4792.6 (3290.7 to 6920.5)	-1.9% (-39.8 to 60.3)	934.3 (570.7 to 1436.5)	924.1 (549.5 to 1485.5)	-1.1% (-49.6 to 94.0)
Suffolk	6242.9 (4239.2 to 8804.5)	6574.0 (4183.2 to 9993.0)	5.3% (-40.4 to 86.3)	4683.4 (3384.5 to 6403.9)	4837.8 (3304.6 to 6988.1)	3.3% (-36.8 to 69.3)	897.4 (559.3 to 1373.8)	932.7 (540.7 to 1470.1)	3.9% (-46.7 to 103.4)
Thurrock	6396.2 (4341.4 to 9097.5)	6519.7 (4047.8 to 9726.6)	1.9% (-42.6 to 81.2)	4821.3 (3503.0 to 6681.0)	4780.7 (3235.3 to 6817.7)	-0.8% (-39.4 to 62.5)	921.2 (567.2 to 1419.0)	922.6 (526.8 to 1450.6)	0.1% (-49.2 to 97.9)
Barking and Dagenham	6454.8 (4360.1 to 9193.6)	6410.2 (4041.7 to 9549.8)	-0.7% (-43.7 to 75.7)	4878.9 (3493.6 to 6776.4)	4692.4 (3180.0 to 6713.3)	-3.8% (-41.5 to 58.4)	929.7 (569.5 to 1430.0)	904.1 (523.1 to 1418.7)	-2.8% (-50.4 to 90.9)
Barnet	6537.6 (4443.0 to 9254.6)	6585.9 (4130.0 to 9941.8)	0.7% (-43.2 to 79.0)	4950.2 (3562.9 to 6773.1)	4849.1 (3280.8 to 7030.5)	-2.0% (-40.5 to 61.5)	945.4 (586.8 to 1463.2)	935.4 (544.3 to 1488.2)	-1.1% (-49.6 to 94.8)
Bexley	6369.9 (4332.7 to 9011.7)	6610.1 (4261.3 to 10120.0)	3.8% (-41.1 to 83.3)	4790.4 (3453.8 to 6566.8)	4877.1 (3346.9 to 7041.8)	1.8% (-37.7 to 66.6)	914.5 (555.0 to 1426.3)	940.4 (544.1 to 1500.4)	2.8% (-48.3 to 104.6)

Brent	6645.0 (4496.1 to 9457.6)	6635.1 (4205.1 to 9861.3)	-0.2% (-43.2 to 75.8)	5059.4 (3641.5 to 7035.1)	4902.1 (3296.2 to 7095.6)	-3.1% (-41.5 to 60.9)	963.8 (594.3 to 1470.7)	944.8 (539.4 to 1542.3)	-2.0% (-51.0 to 96.5)
Bromley	6418.9 (4361.3 to 9161.7)	6575.6 (4178.3 to 9904.0)	2.4% (-42.0 to 81.4)	4829.6 (3483.1 to 6665.9)	4833.7 (3262.6 to 6905.6)	0.1% (-39.0 to 64.5)	924.2 (571.4 to 1431.7)	931.5 (538.6 to 1489.3)	0.8% (-49.0 to 99.6)
Camden	7037.4 (4760.0 to 9964.0)	7113.9 (4562.0 to 10793.7)	1.1% (-42.6 to 78.6)	5422.8 (3940.5 to 7483.7)	5344.7 (3651.0 to 7734.4)	-1.4% (-39.8 to 61.8)	1029.3 (630.7 to 1607.0)	1023.6 (604.3 to 1674.7)	-0.5% (-49.9 to 97.8)
Croydon	6484.3 (4404.0 to 9188.4)	6542.2 (4148.4 to 9730.2)	0.9% (-42.5 to 77.4)	4905.5 (3538.1 to 6757.1)	4806.5 (3263.9 to 6891.2)	-2.0% (-40.2 to 60.7)	934.4 (570.3 to 1454.6)	923.8 (544.9 to 1472.6)	-1.1% (-49.8 to 94.9)
Ealing	6554.9 (4462.1 to 9310.9)	6607.5 (4108.0 to 9792.5)	0.8% (-42.9 to 78.5)	4986.5 (3555.8 to 6842.8)	4864.9 (3302.9 to 6900.6)	-2.4% (-40.3 to 59.7)	948.7 (580.9 to 1451.6)	936.7 (544.0 to 1496.6)	-1.3% (-49.9 to 95.0)
Enfield	6527.1 (4405.5 to 9323.9)	6581.1 (4132.6 to 9808.1)	0.8% (-43.0 to 78.9)	4931.6 (3544.9 to 6814.0)	4843.7 (3251.1 to 6951.2)	-1.8% (-40.5 to 62.4)	942.6 (575.8 to 1431.8)	933.4 (528.5 to 1459.2)	-1.0% (-49.8 to 95.8)
Greenwich	6470.5 (4379.4 to 9161.6)	6723.0 (4278.5 to 10067.4)	3.9% (-40.9 to 83.1)	4888.1 (3561.5 to 6723.5)	4964.2 (3384.8 to 7220.0)	1.6% (-38.1 to 66.8)	933.1 (577.4 to 1423.8)	956.3 (551.6 to 1534.6)	2.5% (-48.1 to 102.7)
Hackney	6578.8 (4466.5 to 9348.4)	6794.0 (4416.6 to 10223.1)	3.3% (-40.9 to 80.7)	4998.7 (3590.0 to 6932.3)	5054.9 (3494.0 to 7291.8)	1.1% (-38.1 to 65.7)	950.3 (589.2 to 1464.2)	970.0 (573.9 to 1577.6)	2.1% (-48.1 to 101.2)
Hammersmith and Fulham	7217.4 (4956.5 to 10327.2)	6942.7 (4337.7 to 10451.9)	-3.8% (-45.8 to 71.0)	5603.7 (4013.9 to 7773.8)	5208.5 (3457.5 to 7541.7)	-7.1% (-44.2 to 55.3)	1061.3 (662.0 to 1634.1)	999.0 (570.7 to 1619.0)	-5.9% (-52.7 to 87.9)
Haringey	6806.5 (4575.2 to 9603.5)	6659.5 (4301.8 to 9880.1)	-2.2% (-43.8 to 70.8)	5216.8 (3722.1 to 7178.8)	4930.9 (3383.8 to 6965.5)	-5.5% (-41.8 to 53.7)	993.3 (603.4 to	946.3 (568.6 to	-4.7% (-50.9 to 85.1)

							1511.7)	1502.9)	
Harrow	6403.1 (4357.1 to 9044.9)	6559.7 (4138.1 to 9671.1)	2.4% (-41.4 to 79.7)	4824.6 (3478.0 to 6557.3)	4826.8 (3258.5 to 6965.3)	0.0% (-39.0 to 64.4)	922.0 (558.9 to 1433.7)	931.3 (533.4 to 1484.6)	1.0% (-49.4 to 101.6)
Havering	6288.5 (4234.8 to 9027.5)	6673.2 (4211.6 to 9852.3)	6.1% (-39.8 to 87.6)	4713.6 (3421.9 to 6513.2)	4928.4 (3352.8 to 7022.6)	4.6% (-35.9 to 70.8)	900.9 (560.1 to 1403.5)	949.6 (555.8 to 1507.2)	5.4% (-46.2 to 106.8)
Hillingdon	6515.1 (4436.5 to 9194.3)	6687.9 (4206.5 to 9943.1)	2.7% (-41.6 to 80.8)	4934.3 (3552.7 to 6788.8)	4946.6 (3331.4 to 7122.5)	0.2% (-39.1 to 65.5)	942.1 (577.6 to 1471.9)	952.8 (555.3 to 1548.5)	1.1% (-49.2 to 101.9)
Hounslow	6540.0 (4488.3 to 9216.8)	6550.8 (4129.5 to 9598.3)	0.2% (-42.4 to 74.7)	4963.9 (3580.9 to 6857.4)	4830.2 (3273.2 to 6860.8)	-2.7% (-40.4 to 59.3)	946.5 (572.7 to 1432.9)	929.5 (530.6 to 1477.7)	-1.8% (-50.5 to 95.3)
Islington	6835.1 (4624.5 to 9797.4)	7046.2 (4469.2 to 10836.9)	3.1% (-42.3 to 84.7)	5244.1 (3776.2 to 7217.0)	5287.9 (3567.8 to 7754.6)	0.8% (-39.2 to 67.5)	995.8 (605.4 to 1527.5)	1014.5 (587.7 to 1661.6)	1.9% (-49.1 to 104.4)
Kensington and Chelsea	7264.7 (4999.0 to 10234.6)	7007.4 (4439.2 to 10496.9)	-3.5% (-44.9 to 69.2)	5639.5 (4086.3 to 7739.9)	5255.6 (3557.7 to 7499.9)	-6.8% (-42.9 to 52.5)	1070.4 (664.6 to 1642.8)	1008.7 (586.7 to 1624.4)	-5.8% (-52.2 to 86.3)
Kingston upon Thames	6608.5 (4481.2 to 9441.1)	6901.4 (4366.6 to 10631.5)	4.4% (-41.5 to 87.0)	5018.9 (3621.1 to 6887.2)	5123.0 (3471.6 to 7527.2)	2.1% (-38.3 to 69.1)	957.3 (591.9 to 1476.3)	985.4 (562.9 to 1589.4)	2.9% (-48.3 to 105.6)
Lambeth	6907.8 (4664.5 to 9802.0)	6872.0 (4347.0 to 10305.7)	-0.5% (-43.7 to 76.1)	5313.3 (3802.7 to 7267.1)	5128.2 (3549.7 to 7378.0)	-3.5% (-40.7 to 57.3)	1010.4 (618.0 to 1570.4)	984.1 (567.9 to 1581.5)	-2.6% (-51.0 to 94.2)
Lewisham	6791.8 (4615.6 to 9661.1)	6638.3 (4150.0 to 9886.6)	-2.3% (-44.7 to 73.2)	5194.7 (3740.7 to 7165.3)	4906.6 (3253.5 to 6975.3)	-5.5% (-42.7 to 56.2)	989.3 (611.0 to 1530.5)	944.2 (551.8 to 1526.9)	-4.6% (-51.7 to 89.0)

Merton	6627.9 (4529.2 to 9391.6)	6594.5 (4154.2 to 9871.0)	-0.5% (-43.5 to 75.5)	5043.3 (3641.4 to 6953.8)	4858.5 (3248.3 to 6961.3)	-3.7% (-41.5 to 59.1)	961.3 (589.8 to 1485.3)	935.6 (545.9 to 1507.2)	-2.7% (-50.8 to 92.9)
Newham	6521.2 (4427.6 to 9256.0)	6775.2 (4279.8 to 10015.2)	3.9% (-40.7 to 82.6)	4942.1 (3594.5 to 6791.2)	5027.1 (3413.2 to 7205.5)	1.7% (-37.7 to 66.5)	941.4 (571.0 to 1468.5)	968.4 (552.9 to 1554.3)	2.9% (-48.7 to 106.5)
Redbridge	6440.0 (4404.3 to 9085.5)	6517.1 (4155.9 to 9944.4)	1.2% (-42.6 to 78.7)	4860.9 (3499.3 to 6723.1)	4787.0 (3281.2 to 6884.5)	-1.5% (-39.8 to 61.4)	927.4 (569.5 to 1418.3)	924.4 (538.3 to 1482.2)	-0.3% (-49.4 to 96.7)
Richmond upon Thames	6562.3 (4506.9 to 9329.2)	6556.0 (4186.5 to 9667.2)	-0.1% (-42.5 to 74.1)	4970.1 (3609.3 to 6881.7)	4812.1 (3246.5 to 6875.2)	-3.2% (-40.9 to 59.1)	948.7 (585.1 to 1467.9)	928.9 (528.7 to 1495.4)	-2.1% (-51.0 to 95.9)
Southwark	6789.8 (4678.9 to 9559.8)	6870.8 (4341.2 to 9973.0)	1.2% (-41.5 to 75.5)	5207.1 (3767.5 to 7176.1)	5126.6 (3433.4 to 7132.5)	-1.5% (-39.4 to 60.3)	988.7 (600.2 to 1545.4)	984.4 (578.8 to 1547.9)	-0.4% (-49.3 to 95.8)
Sutton	6441.0 (4359.4 to 9169.8)	6606.4 (4229.2 to 9813.1)	2.6% (-41.4 to 79.8)	4861.8 (3485.5 to 6692.7)	4855.3 (3311.1 to 6927.2)	-0.1% (-38.8 to 63.4)	928.1 (573.5 to 1420.6)	936.7 (535.8 to 1547.1)	0.9% (-49.7 to 103.2)
Tower Hamlets	6486.8 (4372.9 to 9183.4)	6932.3 (4392.8 to 10185.0)	6.9% (-38.9 to 87.2)	4917.2 (3555.6 to 6705.6)	5181.7 (3518.4 to 7293.9)	5.4% (-34.9 to 70.9)	934.0 (578.4 to 1441.5)	994.0 (570.4 to 1588.4)	6.4% (-46.3 to 111.4)
Waltham Forest	6622.5 (4520.2 to 9291.0)	6612.5 (4273.4 to 9905.1)	-0.2% (-42.5 to 74.0)	5037.6 (3641.8 to 6887.8)	4880.2 (3330.1 to 7040.3)	-3.1% (-40.7 to 58.7)	959.6 (590.7 to 1475.2)	938.4 (549.4 to 1511.7)	-2.2% (-50.4 to 93.1)
Wandsworth	7093.4 (4812.4 to 10090.4)	6941.6 (4455.8 to 10417.2)	-2.1% (-44.2 to 72.0)	5478.1 (3945.6 to 7545.4)	5187.0 (3539.4 to 7457.1)	-5.3% (-42.2 to 55.3)	1039.3 (633.8 to 1594.6)	994.0 (584.5 to 1557.5)	-4.4% (-51.0 to 86.7)
Westminster	7228.0 (4914.6 to	6960.4 (4369.0 to	-3.7% (-45.3 to 69.7)	5611.3 (4028.7 to 7790.4)	5226.7 (3546.2 to 7419.1)	-6.9% (-43.1 to 52.8)	1063.0 (651.7 to	1000.3 (581.7 to	-5.9% (-52.4 to 86.7)

	10326.8)	10266.7)					1641.4)	1608.8)	
County Durham	6362.6 (4292.2 to 8999.9)	6671.5 (4283.9 to 10016.5)	4.9% (-40.2 to 84.3)	4784.4 (3460.3 to 6572.3)	4933.2 (3359.3 to 7089.0)	3.1% (-36.9 to 69.0)	915.3 (560.3 to 1422.6)	948.0 (554.0 to 1544.3)	3.6% (-48.0 to 106.6)
Darlington	6262.6 (4245.6 to 8931.3)	6567.0 (4109.9 to 9884.0)	4.9% (-41.0 to 86.8)	4691.0 (3365.9 to 6403.9)	4826.3 (3253.5 to 6941.2)	2.9% (-37.4 to 69.5)	896.0 (544.3 to 1383.0)	930.3 (530.7 to 1499.4)	3.8% (-48.1 to 108.5)
Gateshead	6400.3 (4353.1 to 9077.7)	6561.5 (4176.7 to 9673.1)	2.5% (-41.2 to 79.1)	4818.1 (3490.5 to 6643.0)	4840.0 (3291.0 to 6960.9)	0.5% (-38.6 to 64.9)	921.3 (568.8 to 1410.3)	929.8 (546.6 to 1468.9)	0.9% (-48.2 to 96.7)
Hartlepool	6240.0 (4243.8 to 8874.8)	6615.6 (4219.8 to 9901.9)	6.0% (-39.6 to 86.5)	4677.6 (3353.0 to 6437.3)	4880.3 (3342.8 to 6955.0)	4.3% (-36.0 to 70.5)	893.0 (555.7 to 1365.7)	938.9 (554.0 to 1484.4)	5.1% (-45.8 to 104.3)
Middlesbrough	6387.5 (4315.6 to 9098.3)	6565.7 (4212.4 to 9858.6)	2.8% (-41.5 to 81.0)	4813.3 (3449.9 to 6622.2)	4855.0 (3306.7 to 6991.6)	0.9% (-38.5 to 65.8)	916.4 (565.5 to 1411.7)	933.9 (533.1 to 1462.6)	1.9% (-48.2 to 101.0)
Newcastle upon Tyne	6735.3 (4583.0 to 9580.6)	6962.8 (4436.6 to 10467.0)	3.4% (-41.2 to 82.4)	5142.9 (3710.9 to 7180.0)	5229.0 (3556.4 to 7467.2)	1.7% (-38.0 to 67.1)	980.9 (598.0 to 1482.0)	1002.3 (592.7 to 1605.7)	2.2% (-47.7 to 100.0)
North Tyneside	6260.2 (4207.0 to 8960.1)	6475.2 (4127.3 to 9607.5)	3.4% (-41.2 to 82.4)	4686.8 (3383.5 to 6489.8)	4761.5 (3239.9 to 6757.2)	1.6% (-37.7 to 66.0)	896.5 (552.2 to 1386.3)	918.1 (532.0 to 1483.6)	2.4% (-48.4 to 103.9)
Northumberland	6139.0 (4112.4 to 8832.8)	6573.0 (4119.4 to 9861.8)	7.1% (-40.0 to 91.3)	4573.2 (3266.1 to 6287.7)	4839.2 (3244.9 to 6942.5)	5.8% (-35.9 to 75.1)	875.4 (541.3 to 1348.4)	932.6 (531.5 to 1507.4)	6.5% (-46.6 to 113.0)
Redcar and Cleveland	6247.6 (4255.7 to 8825.4)	6549.7 (4073.7 to 9808.4)	4.8% (-40.8 to 85.9)	4675.8 (3394.9 to 6416.1)	4825.3 (3274.1 to 6994.7)	3.2% (-37.1 to 69.7)	896.1 (551.8 to 1387.5)	930.2 (538.6 to 1528.9)	3.8% (-48.1 to 108.1)

South Tyneside	6300.0 (4256.3 to 8988.7)	6680.7 (4179.1 to 9920.5)	6.0% (-40.1 to 88.0)	4731.3 (3431.3 to 6530.4)	4928.0 (3324.4 to 7130.1)	4.2% (-36.8 to 71.9)	904.7 (550.1 to 1398.9)	948.7 (549.9 to 1545.2)	4.9% (-47.5 to 109.9)
Stockton-on-Tees	6158.1 (4160.3 to 8711.4)	6572.2 (4117.5 to 9819.3)	6.7% (-39.6 to 89.3)	4600.0 (3342.4 to 6341.6)	4837.3 (3211.8 to 6936.0)	5.2% (-36.3 to 73.9)	880.3 (543.5 to 1361.2)	931.7 (540.4 to 1528.6)	5.8% (-46.9 to 111.7)
Sunderland	6331.6 (4260.5 to 8952.5)	6627.2 (4260.2 to 9763.3)	4.7% (-39.8 to 82.6)	4766.4 (3441.5 to 6553.4)	4907.8 (3388.4 to 6842.7)	3.0% (-35.8 to 65.4)	911.3 (562.2 to 1414.4)	943.7 (554.8 to 1509.7)	3.6% (-47.3 to 103.7)
Blackburn with Darwen	6211.6 (4233.7 to 8842.8)	6554.5 (4177.8 to 9874.6)	5.5% (-40.0 to 86.2)	4651.2 (3353.7 to 6378.0)	4815.7 (3231.6 to 6840.4)	3.5% (-36.8 to 69.9)	887.6 (551.2 to 1357.1)	926.4 (516.1 to 1487.5)	4.4% (-47.9 to 109.7)
Blackpool	6489.8 (4409.6 to 9174.3)	6653.6 (4256.9 to 9949.9)	2.5% (-41.4 to 79.9)	4909.0 (3572.2 to 6750.7)	4911.9 (3370.3 to 7033.4)	0.1% (-38.4 to 62.9)	936.0 (573.8 to 1438.4)	943.1 (565.2 to 1523.0)	0.8% (-48.5 to 97.2)
Bolton	6314.1 (4262.7 to 9042.1)	6556.9 (4154.7 to 9768.1)	3.8% (-41.1 to 83.6)	4739.4 (3403.9 to 6568.8)	4830.3 (3285.3 to 6912.3)	1.9% (-37.8 to 67.4)	906.3 (549.6 to 1382.7)	930.3 (532.1 to 1476.1)	2.7% (-48.2 to 103.8)
Bury	6245.5 (4238.8 to 8957.2)	6443.8 (4127.2 to 9835.2)	3.2% (-41.8 to 83.3)	4680.1 (3385.3 to 6394.0)	4729.2 (3241.3 to 6816.5)	1.0% (-38.0 to 65.1)	893.9 (547.5 to 1392.1)	911.6 (527.7 to 1490.8)	2.0% (-49.1 to 104.8)
Cheshire East	6242.9 (4196.2 to 8857.4)	6551.3 (4247.0 to 9762.4)	4.9% (-39.8 to 83.5)	4671.3 (3349.7 to 6450.4)	4824.5 (3308.8 to 6861.9)	3.3% (-36.6 to 68.5)	893.3 (548.7 to 1377.9)	929.8 (539.8 to 1488.6)	4.1% (-47.3 to 106.0)
Cheshire West and Chester	6319.7 (4291.3 to 9007.9)	6639.8 (4160.3 to 10010.6)	5.1% (-40.8 to 87.0)	4737.1 (3401.1 to 6575.4)	4911.4 (3330.4 to 7056.2)	3.7% (-37.0 to 71.0)	905.9 (552.5 to 1417.0)	945.0 (536.8 to 1526.3)	4.3% (-48.2 to 110.4)
Cumbria	6294.6 (4313.1 to 8913.5)	6556.2 (4161.7 to 9875.4)	4.2% (-40.8 to 83.5)	4721.0 (3418.7 to 6465.5)	4833.6 (3278.5 to 6853.5)	2.4% (-37.1 to 66.9)	902.7 (556.1 to	928.8 (541.3 to	2.9% (-47.9 to 104.0)

							1382.7)	1501.7)	
Halton	6193.3 (4194.0 to 8809.7)	6531.1 (4189.5 to 9637.8)	5.5% (-39.5 to 84.3)	4626.4 (3334.2 to 6424.4)	4804.2 (3275.6 to 6793.8)	3.8% (-36.2 to 69.4)	884.8 (537.4 to 1346.7)	923.7 (542.2 to 1435.8)	4.4% (-46.3 to 103.0)
Knowsley	6312.9 (4253.8 to 8942.0)	6540.0 (4080.0 to 9744.7)	3.6% (-41.5 to 84.0)	4742.0 (3408.1 to 6516.5)	4827.3 (3294.6 to 6955.1)	1.8% (-37.9 to 67.1)	904.7 (560.3 to 1394.2)	927.6 (528.5 to 1490.6)	2.5% (-48.5 to 104.6)
Lancashire	6296.6 (4262.8 to 9022.2)	6660.1 (4207.6 to 9876.3)	5.8% (-40.0 to 86.8)	4726.2 (3389.2 to 6468.7)	4919.0 (3303.1 to 7032.4)	4.1% (-36.6 to 71.4)	903.0 (555.7 to 1404.7)	946.6 (530.2 to 1499.5)	4.8% (-47.6 to 110.5)
Liverpool	5340.4 (3429.7 to 7927.1)	5588.7 (3363.2 to 8597.2)	4.6% (-44.1 to 96.4)	4181.8 (2856.6 to 5880.5)	4299.9 (2781.7 to 6315.7)	2.8% (-40.4 to 77.6)	780.0 (458.6 to 1227.4)	809.5 (450.0 to 1293.4)	3.8% (-49.3 to 112.6)
Manchester	6802.1 (4624.6 to 9722.9)	6876.2 (4329.8 to 10372.3)	1.1% (-43.0 to 79.8)	5207.5 (3733.5 to 7189.5)	5137.6 (3489.3 to 7444.4)	-1.3% (-40.2 to 63.0)	989.9 (602.5 to 1505.0)	984.8 (559.7 to 1581.4)	-0.5% (-50.1 to 98.8)
Oldham	6239.4 (4188.0 to 8819.5)	6532.9 (4153.7 to 9583.2)	4.7% (-40.0 to 83.3)	4676.0 (3382.8 to 6460.3)	4811.5 (3243.6 to 6809.3)	2.9% (-37.0 to 68.4)	893.7 (551.3 to 1377.6)	925.5 (536.7 to 1465.4)	3.6% (-47.3 to 103.8)
Rochdale	6210.9 (4182.1 to 8890.1)	6549.8 (4222.9 to 9740.4)	5.5% (-39.7 to 84.9)	4651.5 (3332.5 to 6433.3)	4823.7 (3283.5 to 6791.8)	3.7% (-36.3 to 69.1)	888.3 (539.7 to 1364.1)	927.3 (526.5 to 1446.5)	4.4% (-47.2 to 106.4)
Salford	6543.1 (4393.4 to 9259.3)	6586.4 (4152.3 to 9735.9)	0.7% (-42.8 to 77.3)	4961.4 (3566.4 to 6723.2)	4860.2 (3280.7 to 6854.2)	-2.0% (-39.7 to 59.5)	945.9 (572.7 to 1429.8)	936.1 (537.6 to 1515.6)	-1.0% (-50.3 to 97.5)
Sefton	6223.8 (4227.4 to 8817.6)	6525.1 (4150.5 to 9819.5)	4.8% (-40.4 to 85.1)	4660.9 (3376.3 to 6404.8)	4805.1 (3282.8 to 6944.5)	3.1% (-37.0 to 69.0)	890.4 (545.1 to 1366.6)	922.1 (558.8 to 1469.9)	3.6% (-46.6 to 101.0)

St Helens	6296.6 (4257.7 to 8939.7)	6554.3 (4209.3 to 9699.7)	4.1% (-40.3 to 82.0)	4720.7 (3409.9 to 6488.7)	4826.2 (3296.3 to 6841.9)	2.2% (-37.1 to 66.3)	901.9 (552.5 to 1383.4)	928.4 (537.4 to 1449.4)	2.9% (-47.4 to 101.5)
Stockport	6235.5 (4200.0 to 8988.8)	6439.0 (4063.4 to 9737.5)	3.3% (-42.1 to 84.4)	4670.0 (3336.4 to 6420.0)	4727.1 (3214.9 to 6718.7)	1.2% (-38.1 to 65.7)	892.4 (547.5 to 1355.1)	910.8 (524.4 to 1472.5)	2.1% (-48.5 to 103.0)
Tameside	6366.5 (4297.1 to 9075.4)	6447.5 (4103.5 to 9527.1)	1.3% (-42.2 to 77.8)	4792.9 (3449.2 to 6605.8)	4741.1 (3192.3 to 6704.3)	-1.1% (-39.5 to 62.0)	915.2 (554.3 to 1402.0)	913.3 (517.7 to 1470.2)	-0.2% (-50.2 to 100.7)
Trafford	6272.0 (4285.9 to 8889.4)	6430.9 (4115.3 to 9510.6)	2.5% (-41.1 to 78.8)	4704.8 (3384.1 to 6440.4)	4703.9 (3182.3 to 6676.6)	-0.0% (-38.7 to 63.5)	898.3 (542.8 to 1376.8)	907.4 (534.5 to 1433.8)	1.0% (-48.5 to 98.2)
Warrington	6300.7 (4266.0 to 8988.3)	6504.9 (4203.1 to 9636.8)	3.2% (-40.7 to 80.2)	4729.5 (3414.6 to 6509.0)	4796.0 (3261.4 to 6803.5)	1.4% (-37.7 to 65.4)	902.6 (554.2 to 1397.7)	923.4 (531.8 to 1464.8)	2.3% (-48.2 to 102.6)
Wigan	6358.1 (4305.2 to 9092.1)	6530.8 (4184.9 to 9723.6)	2.7% (-41.4 to 80.4)	4783.8 (3476.5 to 6582.8)	4805.5 (3312.5 to 6808.5)	0.5% (-37.8 to 62.5)	913.9 (560.4 to 1401.1)	924.6 (548.8 to 1462.5)	1.2% (-48.0 to 97.0)
Wirral	6199.7 (4283.9 to 8714.7)	6539.8 (4194.5 to 9609.1)	5.5% (-38.8 to 82.4)	4635.7 (3332.9 to 6340.4)	4806.0 (3336.5 to 6761.9)	3.7% (-35.5 to 66.9)	885.4 (539.9 to 1348.7)	925.8 (542.2 to 1457.5)	4.6% (-46.5 to 104.3)
Bracknell Forest	6283.7 (4236.5 to 8876.1)	6516.9 (4151.2 to 9671.0)	3.7% (-40.8 to 81.9)	4728.7 (3410.6 to 6476.1)	4785.3 (3291.3 to 6785.4)	1.2% (-37.5 to 64.0)	904.6 (552.9 to 1385.3)	923.6 (549.1 to 1470.0)	2.1% (-47.7 to 99.3)
Brighton and Hove	6866.4 (4662.4 to 9783.1)	6988.0 (4467.3 to 10257.7)	1.8% (-41.5 to 77.7)	5272.5 (3830.5 to 7265.1)	5246.7 (3612.3 to 7314.8)	-0.5% (-38.0 to 60.0)	1002.2 (611.9 to 1546.3)	1007.0 (594.8 to 1631.4)	0.5% (-49.1 to 98.5)
Buckinghamshire	6223.6 (4224.4 to 8930.7)	6558.8 (4192.3 to 9794.1)	5.4% (-40.0 to 85.6)	4652.8 (3360.8 to 6416.8)	4814.5 (3323.2 to 6845.2)	3.5% (-36.1 to 68.1)	891.9 (550.5 to	928.2 (561.6 to	4.1% (-46.6 to 103.0)

							1368.9)	1508.7)	
East Sussex	6198.9 (4192.2 to 8746.3)	6606.3 (4116.7 to 9837.9)	6.6% (-39.7 to 88.8)	4635.7 (3354.4 to 6347.1)	4870.5 (3288.8 to 6959.0)	5.1% (-35.7 to 72.2)	886.9 (542.1 to 1375.2)	937.8 (547.1 to 1467.7)	5.7% (-46.1 to 107.5)
Hampshire	6267.9 (4256.3 to 8928.1)	6571.3 (4232.7 to 9720.7)	4.8% (-39.8 to 83.0)	4700.9 (3399.6 to 6432.5)	4830.2 (3320.7 to 6910.3)	2.7% (-36.7 to 67.1)	901.1 (555.1 to 1389.5)	931.7 (542.1 to 1466.9)	3.4% (-47.2 to 102.6)
Isle of Wight	6170.3 (4174.6 to 8772.6)	6627.0 (4150.1 to 10006.5)	7.4% (-39.6 to 91.4)	4604.3 (3351.0 to 6331.2)	4887.5 (3304.1 to 7050.1)	6.2% (-35.3 to 74.4)	882.3 (541.6 to 1347.1)	941.3 (549.2 to 1516.6)	6.7% (-45.9 to 111.0)
Kent	6286.8 (4222.4 to 8920.9)	6590.4 (4156.4 to 9890.1)	4.8% (-40.8 to 86.1)	4712.8 (3397.8 to 6465.2)	4859.3 (3305.8 to 6929.0)	3.1% (-36.8 to 68.5)	902.2 (547.5 to 1402.8)	936.9 (536.6 to 1509.4)	3.9% (-48.1 to 108.5)
Medway	6325.0 (4265.0 to 8996.5)	6624.9 (4221.0 to 9901.5)	4.7% (-40.5 to 84.6)	4760.7 (3441.6 to 6610.3)	4893.5 (3358.7 to 6918.5)	2.8% (-36.6 to 67.0)	909.5 (559.0 to 1399.1)	940.2 (549.2 to 1474.3)	3.4% (-47.1 to 101.9)
Milton Keynes	6154.0 (4163.2 to 8758.9)	6400.0 (4095.2 to 9436.9)	4.0% (-40.4 to 82.0)	4595.6 (3321.5 to 6345.4)	4669.7 (3190.3 to 6648.6)	1.6% (-37.6 to 65.8)	879.5 (541.2 to 1343.6)	901.6 (512.4 to 1420.4)	2.5% (-48.1 to 103.1)
Oxfordshire	6540.3 (4410.3 to 9262.7)	6744.5 (4337.4 to 10009.2)	3.1% (-40.9 to 80.3)	4958.4 (3584.9 to 6809.0)	4989.5 (3422.2 to 7078.9)	0.6% (-37.9 to 63.4)	945.8 (585.4 to 1473.7)	959.6 (562.3 to 1533.3)	1.5% (-48.4 to 99.7)
Portsmouth	6775.1 (4673.4 to 9606.0)	6845.0 (4311.9 to 10184.5)	1.0% (-42.3 to 77.4)	5198.9 (3748.1 to 7165.0)	5106.9 (3422.1 to 7311.3)	-1.8% (-40.3 to 62.1)	990.8 (607.0 to 1543.8)	980.7 (577.2 to 1556.1)	-1.0% (-49.6 to 94.7)
Reading	6783.7 (4647.7 to 9545.3)	6737.6 (4226.7 to 9990.2)	-0.7% (-43.3 to 74.4)	5198.8 (3771.8 to 7183.3)	4991.4 (3364.9 to 7157.0)	-4.0% (-41.5 to 58.0)	990.0 (610.8 to 1525.8)	958.6 (552.2 to 1541.6)	-3.2% (-51.2 to 92.7)

Slough	6466.3 (4363.9 to 9150.0)	6369.1 (3985.4 to 9561.9)	-1.5% (-44.5 to 75.1)	4903.5 (3493.1 to 6719.3)	4648.1 (3133.1 to 6667.4)	-5.2% (-42.4 to 56.3)	934.3 (574.3 to 1440.5)	896.6 (504.4 to 1474.3)	-4.0% (-52.6 to 94.9)
Southampton	6692.7 (4536.3 to 9572.6)	6892.0 (4410.4 to 10187.8)	3.0% (-41.1 to 80.5)	5109.9 (3709.0 to 7065.8)	5163.4 (3552.9 to 7432.6)	1.0% (-38.0 to 65.0)	973.7 (599.2 to 1511.6)	991.4 (587.2 to 1650.4)	1.8% (-48.9 to 103.8)
Surrey	6321.4 (4280.1 to 8994.2)	6578.1 (4224.9 to 9864.4)	4.1% (-40.7 to 82.8)	4740.5 (3412.4 to 6491.4)	4835.0 (3253.8 to 7016.9)	2.0% (-38.2 to 68.7)	906.8 (560.0 to 1419.7)	930.9 (535.4 to 1436.4)	2.7% (-47.6 to 101.4)
West Berkshire	6255.7 (4234.7 to 8876.0)	6483.7 (4058.3 to 9653.9)	3.6% (-41.3 to 83.6)	4680.0 (3375.5 to 6397.1)	4744.0 (3211.9 to 6759.1)	1.4% (-37.9 to 65.9)	896.5 (548.1 to 1371.6)	915.9 (522.3 to 1459.3)	2.2% (-48.5 to 103.5)
West Sussex	6249.2 (4248.2 to 8934.2)	6516.3 (4190.2 to 9808.9)	4.3% (-40.6 to 83.4)	4682.7 (3395.6 to 6464.6)	4787.5 (3257.5 to 6929.3)	2.2% (-37.7 to 68.2)	895.7 (548.9 to 1384.9)	922.7 (528.1 to 1455.6)	3.0% (-47.9 to 104.0)
Windsor and Maidenhead	6232.7 (4199.8 to 8803.6)	6463.3 (4101.7 to 9642.2)	3.7% (-41.0 to 82.7)	4661.2 (3362.9 to 6391.8)	4742.3 (3225.6 to 6683.4)	1.7% (-37.3 to 65.3)	893.4 (542.5 to 1382.9)	915.3 (539.7 to 1447.2)	2.5% (-47.8 to 101.3)
Wokingham	6218.8 (4233.9 to 8787.7)	6443.6 (4077.4 to 9592.5)	3.6% (-40.9 to 82.2)	4647.4 (3367.8 to 6349.8)	4704.4 (3145.2 to 6736.7)	1.2% (-38.3 to 66.5)	890.4 (544.1 to 1372.2)	909.2 (520.0 to 1459.5)	2.1% (-48.8 to 104.2)
Bath and North East Somerset	6543.7 (4470.4 to 9338.4)	6889.2 (4404.8 to 10194.5)	5.3% (-39.7 to 84.1)	4954.4 (3589.6 to 6876.5)	5154.6 (3496.7 to 7284.6)	4.0% (-36.2 to 69.8)	947.1 (579.1 to 1462.5)	991.3 (577.5 to 1593.2)	4.7% (-47.1 to 107.4)
Bournemouth	6720.9 (4577.9 to 9529.6)	6844.6 (4409.1 to 10253.4)	1.8% (-41.7 to 78.3)	5118.9 (3729.8 to 7078.5)	5111.5 (3456.6 to 7297.9)	-0.1% (-38.9 to 63.6)	975.9 (605.3 to 1514.7)	981.6 (560.7 to 1569.8)	0.6% (-49.4 to 100.5)
Bristol, City of	6677.1 (4554.0 to 9533.7)	6883.5 (4417.7 to	3.1% (-41.5 to 82.1)	5091.1 (3663.6 to 7041.0)	5127.8 (3516.0 to 7442.6)	0.7% (-38.7 to 65.7)	970.3 (596.3 to	984.7 (563.0 to	1.5% (-49.3 to 103.5)

		10432.6)					1499.1)	1598.4)	
Cornwall	6151.2 (4162.1 to 8804.3)	6629.1 (4122.8 to 9945.7)	7.8% (-39.5 to 92.6)	4587.5 (3318.8 to 6322.6)	4892.7 (3311.5 to 6986.8)	6.7% (-34.8 to 74.9)	879.0 (537.6 to 1354.6)	944.9 (530.8 to 1515.1)	7.5% (-46.4 to 116.3)
Devon	6289.6 (4313.3 to 8891.5)	6610.1 (4229.7 to 9909.4)	5.1% (-39.8 to 84.2)	4724.7 (3409.0 to 6455.8)	4879.2 (3318.3 to 6903.8)	3.3% (-36.4 to 67.9)	903.7 (554.9 to 1396.6)	940.1 (538.0 to 1488.1)	4.0% (-47.4 to 106.3)
Dorset	6132.4 (4181.1 to 8711.0)	6540.9 (4207.7 to 9850.3)	6.7% (-39.1 to 87.3)	4571.1 (3299.4 to 6261.3)	4806.9 (3279.7 to 6925.6)	5.2% (-35.7 to 72.3)	874.1 (536.3 to 1336.9)	927.8 (526.7 to 1478.5)	6.2% (-46.6 to 111.3)
Gloucestershire	6338.6 (4286.9 to 9010.0)	6673.5 (4184.8 to 9951.9)	5.3% (-40.4 to 86.6)	4764.5 (3434.1 to 6571.5)	4921.8 (3337.1 to 6956.3)	3.3% (-36.6 to 68.6)	910.6 (555.7 to 1405.6)	949.3 (539.3 to 1540.7)	4.3% (-48.1 to 109.9)
North Somerset	6170.7 (4219.8 to 8786.8)	6504.1 (4185.8 to 9928.7)	5.4% (-40.2 to 86.2)	4605.8 (3355.5 to 6319.5)	4774.1 (3266.8 to 6967.1)	3.7% (-36.7 to 70.1)	882.1 (542.5 to 1353.4)	919.8 (521.8 to 1466.4)	4.3% (-47.5 to 107.7)
Plymouth	6552.6 (4440.7 to 9322.7)	6744.8 (4294.1 to 9977.4)	2.9% (-41.2 to 80.5)	4982.7 (3601.1 to 6889.7)	5015.1 (3440.5 to 7122.0)	0.7% (-38.1 to 63.9)	950.5 (580.8 to 1461.3)	962.6 (559.8 to 1515.2)	1.3% (-48.4 to 98.8)
Poole	6330.3 (4286.7 to 8980.1)	6497.1 (4081.3 to 9717.9)	2.6% (-41.9 to 81.9)	4762.4 (3421.7 to 6574.8)	4775.6 (3218.4 to 6801.9)	0.3% (-38.9 to 64.8)	910.0 (558.1 to 1378.4)	921.8 (528.5 to 1483.2)	1.3% (-48.9 to 101.2)
Somerset	6165.0 (4171.6 to 8742.4)	6536.7 (4128.4 to 9766.6)	6.0% (-39.8 to 87.5)	4605.7 (3320.4 to 6327.4)	4816.6 (3263.7 to 6892.1)	4.6% (-36.1 to 71.6)	880.3 (537.4 to 1350.2)	927.0 (538.6 to 1459.2)	5.3% (-46.3 to 106.7)
South Gloucestershire	6333.3 (4302.6 to 9155.6)	6587.6 (4208.4 to 9711.8)	4.0% (-40.6 to 82.4)	4754.9 (3442.1 to 6617.6)	4859.0 (3327.1 to 6835.6)	2.2% (-37.0 to 65.9)	909.4 (558.2 to 1395.1)	937.4 (550.7 to 1504.6)	3.1% (-47.5 to 102.9)

Swindon	6481.7 (4367.4 to 9253.5)	6563.5 (4146.1 to 9826.8)	1.3% (-42.8 to 79.5)	4891.2 (3519.8 to 6686.1)	4828.3 (3243.5 to 6919.9)	-1.3% (-39.9 to 62.5)	935.8 (571.0 to 1454.3)	932.2 (544.2 to 1482.4)	-0.4% (-49.6 to 96.8)
Torbay	6331.1 (4268.7 to 9109.2)	6520.3 (4149.8 to 9652.3)	3.0% (-41.4 to 81.5)	4756.5 (3413.8 to 6506.9)	4795.9 (3264.8 to 6857.4)	0.8% (-38.3 to 65.0)	908.3 (550.6 to 1403.7)	923.4 (522.7 to 1484.1)	1.7% (-49.4 to 104.9)
Wiltshire	6260.4 (4241.8 to 8860.8)	6575.8 (4213.2 to 9802.1)	5.0% (-39.9 to 84.0)	4697.5 (3397.2 to 6431.0)	4836.1 (3310.1 to 6939.5)	3.0% (-36.8 to 68.1)	899.3 (554.6 to 1377.9)	933.3 (540.9 to 1507.7)	3.8% (-47.6 to 106.0)
Birmingham	6472.5 (4406.3 to 9199.5)	6704.7 (4256.0 to 10034.6)	3.6% (-41.1 to 82.7)	4899.0 (3531.6 to 6736.7)	4959.3 (3381.6 to 7152.7)	1.2% (-38.2 to 66.2)	932.9 (575.5 to 1436.6)	956.0 (562.4 to 1542.7)	2.5% (-47.9 to 102.1)
Coventry	6544.8 (4418.2 to 9330.8)	6719.4 (4286.6 to 9995.7)	2.7% (-41.5 to 80.6)	4965.5 (3538.5 to 6810.4)	4985.5 (3434.7 to 7186.7)	0.4% (-38.6 to 64.4)	945.9 (581.0 to 1438.0)	958.0 (557.3 to 1571.4)	1.3% (-49.0 to 101.6)
Dudley	6355.7 (4331.6 to 9093.8)	6609.6 (4192.3 to 9902.6)	4.0% (-41.0 to 83.7)	4784.8 (3461.7 to 6581.4)	4862.4 (3264.5 to 7008.7)	1.6% (-38.3 to 67.7)	913.9 (564.2 to 1386.0)	937.9 (555.4 to 1461.1)	2.6% (-46.7 to 97.7)
Herefordshire, County of	6229.0 (4218.4 to 8809.8)	6553.1 (4156.2 to 9970.7)	5.2% (-40.6 to 86.7)	4657.8 (3380.5 to 6362.9)	4823.7 (3292.2 to 7021.6)	3.6% (-36.8 to 70.0)	892.6 (550.3 to 1370.6)	930.1 (537.1 to 1477.6)	4.2% (-47.1 to 105.6)
Sandwell	6436.1 (4330.7 to 9272.3)	6460.2 (4112.2 to 9665.1)	0.4% (-43.2 to 78.0)	4856.9 (3492.1 to 6695.7)	4743.4 (3233.4 to 6825.4)	-2.3% (-40.4 to 60.4)	926.1 (568.9 to 1432.3)	914.1 (529.5 to 1482.8)	-1.3% (-50.4 to 97.1)
Shropshire	6227.6 (4215.4 to 8818.5)	6612.1 (4142.4 to 9913.1)	6.2% (-40.0 to 88.4)	4655.5 (3342.3 to 6396.7)	4877.2 (3305.3 to 6954.0)	4.8% (-36.0 to 71.7)	892.3 (537.8 to 1352.9)	938.4 (549.9 to 1511.0)	5.2% (-46.7 to 107.9)
Solihull	6218.7 (4224.9 to 8756.2)	6511.5 (4148.0 to 9661.0)	4.7% (-40.0 to 83.3)	4647.9 (3390.9 to 6345.1)	4786.3 (3301.0 to 6901.2)	3.0% (-36.5 to 67.4)	890.2 (548.9 to	921.8 (540.6 to	3.6% (-46.4 to 100.4)

							1346.0)	1438.5)	
Staffordshire	6281.8 (4278.2 to 8887.4)	6617.8 (4228.1 to 9714.6)	5.3% (-39.3 to 83.4)	4712.4 (3402.4 to 6446.9)	4886.1 (3307.0 to 6935.5)	3.7% (-36.4 to 69.3)	901.8 (550.1 to 1390.8)	942.2 (554.0 to 1512.9)	4.5% (-47.0 to 106.1)
Stoke-on-Trent	6549.8 (4469.9 to 9252.2)	6590.2 (4229.0 to 9777.5)	0.6% (-42.2 to 75.4)	4972.5 (3582.0 to 6795.3)	4853.5 (3315.1 to 6859.1)	-2.4% (-39.8 to 58.5)	948.1 (577.9 to 1460.0)	933.1 (545.6 to 1475.5)	-1.6% (-49.9 to 93.4)
Telford and Wrekin	6257.2 (4265.9 to 8933.9)	6574.9 (4190.4 to 9766.9)	5.1% (-40.0 to 84.3)	4693.7 (3391.5 to 6468.2)	4846.7 (3299.4 to 6920.7)	3.3% (-36.7 to 68.8)	896.3 (548.2 to 1370.8)	934.1 (545.3 to 1520.7)	4.2% (-47.5 to 107.3)
Walsall	6353.5 (4284.2 to 9003.2)	6597.7 (4183.5 to 9945.6)	3.8% (-41.2 to 84.1)	4783.3 (3469.7 to 6610.9)	4860.3 (3294.4 to 7063.7)	1.6% (-38.3 to 67.7)	912.6 (564.0 to 1418.0)	935.5 (531.1 to 1505.1)	2.5% (-48.7 to 105.4)
Warwickshire	6282.9 (4252.2 to 8925.8)	6587.8 (4243.6 to 9837.8)	4.9% (-40.0 to 83.7)	4710.5 (3405.8 to 6453.7)	4862.6 (3383.6 to 6882.4)	3.2% (-35.8 to 66.2)	899.2 (554.9 to 1374.9)	935.8 (533.5 to 1499.7)	4.1% (-47.6 to 107.2)
Wolverhampton	6445.8 (4378.4 to 9102.0)	6570.7 (4174.0 to 9936.5)	1.9% (-42.2 to 80.1)	4871.8 (3497.2 to 6670.0)	4846.1 (3280.0 to 6982.3)	-0.5% (-39.4 to 63.8)	930.4 (575.2 to 1443.1)	934.7 (528.3 to 1527.5)	0.5% (-50.2 to 103.0)
Worcestershire	6174.9 (4169.9 to 8751.4)	6582.6 (4188.2 to 9873.5)	6.6% (-39.5 to 88.1)	4611.5 (3339.3 to 6317.9)	4851.4 (3328.2 to 6916.7)	5.2% (-35.2 to 71.0)	883.4 (535.7 to 1363.1)	934.6 (552.2 to 1491.2)	5.8% (-46.2 to 108.3)
Barnsley	6401.0 (4351.9 to 9064.0)	6559.1 (4187.2 to 9878.5)	2.5% (-41.7 to 80.6)	4825.3 (3510.7 to 6661.3)	4827.0 (3305.4 to 6919.6)	0.0% (-38.6 to 63.3)	920.1 (565.2 to 1406.9)	930.9 (531.0 to 1499.5)	1.2% (-49.2 to 102.1)
Bradford	6303.8 (4225.5 to 8989.1)	6513.1 (4191.6 to 9773.8)	3.3% (-41.3 to 82.2)	4737.6 (3396.1 to 6521.9)	4790.5 (3295.5 to 6869.5)	1.1% (-38.0 to 65.3)	904.8 (556.2 to 1377.0)	924.4 (550.4 to 1495.2)	2.2% (-47.7 to 100.2)

Calderdale	6297.2 (4258.3 to 8974.6)	6557.1 (4149.9 to 9914.7)	4.1% (-41.2 to 85.1)	4721.1 (3390.0 to 6494.0)	4819.7 (3303.5 to 6983.2)	2.1% (-37.7 to 67.7)	902.2 (554.6 to 1400.7)	929.0 (533.1 to 1492.2)	3.0% (-48.3 to 105.4)
Doncaster	6306.3 (4242.0 to 8960.7)	6584.5 (4251.3 to 9707.2)	4.4% (-40.0 to 81.9)	4737.7 (3443.8 to 6479.9)	4838.2 (3332.2 to 6860.9)	2.1% (-36.7 to 65.0)	904.7 (554.7 to 1386.8)	932.2 (550.0 to 1527.3)	3.0% (-47.9 to 104.6)
East Riding of Yorkshire	6141.8 (4166.4 to 8739.1)	6540.8 (4236.6 to 9777.2)	6.5% (-38.9 to 86.1)	4572.3 (3285.0 to 6265.1)	4816.0 (3345.4 to 6850.8)	5.3% (-34.8 to 70.4)	875.5 (533.1 to 1338.2)	926.6 (543.2 to 1499.9)	5.8% (-46.4 to 109.6)
Kingston upon Hull, City of	6603.6 (4497.8 to 9335.7)	6603.5 (4215.9 to 9708.8)	-0.0% (-42.5 to 74.1)	5019.7 (3628.3 to 6895.3)	4882.9 (3275.2 to 6914.2)	-2.7% (-40.5 to 59.5)	957.6 (584.8 to 1459.3)	938.3 (545.6 to 1523.6)	-2.0% (-50.6 to 95.0)
Kirklees	6371.4 (4338.3 to 9037.7)	6622.8 (4156.2 to 10108.6)	3.9% (-41.6 to 85.3)	4790.1 (3467.0 to 6611.7)	4886.2 (3309.2 to 7130.2)	2.0% (-38.2 to 68.7)	916.1 (565.7 to 1408.3)	939.4 (541.7 to 1505.6)	2.5% (-48.2 to 103.5)
Leeds	6561.5 (4452.2 to 9397.5)	6744.8 (4302.2 to 10032.9)	2.8% (-41.4 to 80.8)	4981.6 (3586.9 to 6926.7)	5009.8 (3444.3 to 7073.6)	0.6% (-38.0 to 63.4)	949.9 (581.1 to 1456.2)	962.9 (548.7 to 1519.8)	1.4% (-48.7 to 100.9)
North East Lincolnshire	6300.1 (4288.4 to 9027.3)	6555.2 (4168.3 to 9773.4)	4.1% (-40.8 to 83.2)	4728.3 (3408.5 to 6545.8)	4825.5 (3261.1 to 6936.6)	2.1% (-38.0 to 68.2)	904.2 (554.8 to 1403.2)	928.6 (544.2 to 1506.7)	2.7% (-48.2 to 104.0)
North Lincolnshire	6199.4 (4181.9 to 8800.1)	6519.3 (4130.8 to 9726.8)	5.2% (-40.3 to 85.6)	4639.3 (3353.6 to 6394.7)	4784.5 (3247.5 to 6821.8)	3.1% (-36.9 to 68.8)	886.8 (552.6 to 1365.2)	920.6 (539.1 to 1466.3)	3.8% (-46.9 to 103.4)
North Yorkshire	6244.3 (4251.2 to 8829.6)	6510.4 (4153.2 to 9723.5)	4.3% (-40.4 to 83.1)	4677.0 (3387.7 to 6447.7)	4786.5 (3227.7 to 6876.8)	2.3% (-37.7 to 68.5)	894.8 (549.8 to 1379.8)	922.9 (538.7 to 1440.5)	3.1% (-47.2 to 101.4)
Rotherham	6330.0 (4280.2 to 8973.1)	6616.2 (4253.6 to 9802.0)	4.5% (-40.0 to 82.5)	4756.0 (3453.4 to 6562.2)	4871.9 (3318.1 to 7028.2)	2.4% (-37.4 to 68.1)	909.0 (552.2 to	937.5 (567.3 to	3.1% (-46.6 to 99.2)

							1390.7)	1475.9)	
Sheffield	6668.3 (4544.7 to 9465.9)	6883.3 (4296.7 to 10273.8)	3.2% (-41.6 to 82.8)	5077.1 (3662.9 to 7006.2)	5137.4 (3491.3 to 7307.4)	1.2% (-38.0 to 65.5)	968.7 (587.7 to 1487.5)	986.4 (552.8 to 1551.0)	1.8% (-48.9 to 103.5)
Wakefield	6404.5 (4318.7 to 9080.5)	6566.1 (4251.4 to 9750.2)	2.5% (-41.1 to 79.0)	4830.7 (3485.3 to 6631.0)	4831.6 (3349.6 to 6887.6)	0.0% (-38.2 to 62.2)	921.5 (564.6 to 1411.6)	929.9 (539.5 to 1499.9)	0.9% (-49.0 to 100.5)
York	6623.1 (4492.2 to 9464.2)	6928.4 (4425.2 to 10476.9)	4.6% (-40.8 to 85.1)	5032.3 (3613.7 to 6917.6)	5180.9 (3580.8 to 7493.5)	3.0% (-36.9 to 68.4)	961.0 (598.5 to 1498.3)	995.0 (581.7 to 1614.8)	3.5% (-47.7 to 105.4)
Northern Ireland	5462.5 (3620.9 to 7854.0)	7230.6 (4376.0 to 11063.0)	32.4% (-27.7 to 142.8)	4231.3 (2968.1 to 5813.5)	5317.8 (3458.7 to 7780.9)	25.7% (-25.8 to 113.2)	798.8 (481.8 to 1220.9)	1027.9 (573.5 to 1693.9)	28.7% (-36.9 to 163.4)
Scotland	5562.1 (3929.1 to 7693.6)	6480.3 (3989.5 to 10054.6)	16.5% (-34.0 to 106.3)	4341.5 (3201.7 to 5751.1)	4877.2 (3210.5 to 7236.9)	12.3% (-31.7 to 85.2)	817.0 (505.8 to 1230.4)	931.4 (524.5 to 1513.3)	14.0% (-42.9 to 128.2)
Wales	5787.4 (3947.9 to 8117.5)	7238.8 (4483.6 to 11007.0)	25.1% (-29.5 to 122.3)	4475.9 (3187.0 to 6167.8)	5379.0 (3622.7 to 7775.5)	20.2% (-27.4 to 99.3)	848.6 (509.9 to 1285.3)	1040.0 (589.2 to 1694.5)	22.6% (-39.1 to 147.3)

UI, uncertainly interval; DALYs, disability-adjusted life-years.

Table S22 The incidence, prevalence and DALYs rate of anxiety and depressive disorders in 1990 and 2021, and changes from 1990 to 2021 in United States of America.

**Location**	**Incidence (95% UI)**			**Prevalence (95% UI)**			**DALYs (95% UI)**		
	**1990**	**2021**	**Percentage change**	**1990**	**2021**	**Percentage change**	**1990**	**2021**	**Percentage change**
Anxiety disorders									
Alabama	979.7 (655.1 to 1364.7)	1501.4 (956.5 to 2207.7)	53.3% (-12.0 to 167.4)	4831.0 (3613.8 to 6311.4)	7446.8 (5073.4 to 10458.7)	54.1% (-2.1 to 143.5)	590.9 (373.8 to 875.2)	899.1 (542.7 to 1386.1)	52.2% (-18.9 to 186.0)
Alaska	944.0 (640.6	1275.0 (786.1 to 1889.2)	35.1% (-22.8 to 136.8)	4635.7 (3475.3	6210.5 (4242.9	34.0% (-15.2 to 112.5)	565.6 (352.6	750.5 (445.1	32.7% (-30.7 to 154.7)

	to 1293.6)			to 6053.2)	to 8863.1)		to 839.9)	to 1176.7)	
Arizona	974.0 (649.8 to 1332.0)	1495.6 (934.4 to 2207.2)	53.5% (-12.3 to 169.4)	4802.3 (3576.6 to 6310.6)	7413.4 (5065.4 to 10536.7)	54.4% (-2.6 to 145.4)	583.5 (365.1 to 868.2)	894.6 (525.9 to 1383.9)	53.3% (-19.7 to 193.5)
Arkansas	973.6 (648.8 to 1365.3)	1469.9 (905.8 to 2250.0)	51.0% (-16.0 to 172.0)	4775.4 (3571.6 to 6247.9)	7236.7 (4854.6 to 10233.1)	51.5% (-4.6 to 141.4)	584.2 (361.9 to 863.5)	876.4 (484.4 to 1377.4)	50.0% (-24.0 to 196.9)
California	964.0 (652.3 to 1311.1)	1463.1 (931.9 to 2188.6)	51.8% (-12.5 to 163.7)	4857.5 (3684.9 to 6276.3)	7251.3 (4944.3 to 10047.7)	49.3% (-3.9 to 132.5)	595.0 (370.7 to 867.5)	881.6 (520.7 to 1421.8)	48.2% (-23.2 to 186.8)
Colorado	969.4 (647.4 to 1314.3)	1354.2 (836.4 to 1986.5)	39.7% (-20.0 to 144.5)	4781.1 (3584.3 to 6187.0)	6692.6 (4540.0 to 9443.3)	40.0% (-11.1 to 120.9)	581.8 (365.4 to 857.1)	806.9 (472.6 to 1244.9)	38.7% (-27.1 to 164.5)
Connecticut	987.0 (655.6 to 1432.2)	1319.0 (807.1 to 1977.8)	33.6% (-26.1 to 142.3)	4950.8 (3669.8 to 6469.1)	6577.6 (4491.6 to 9344.4)	32.9% (-16.2 to 111.1)	604.5 (375.3 to 898.3)	796.2 (466.1 to 1237.7)	31.7% (-31.4 to 153.6)
Delaware	985.9 (666.7 to 1377.1)	1437.5 (912.4 to 2129.9)	45.8% (-16.5 to 155.2)	4948.2 (3766.4 to 6393.1)	7146.9 (4857.8 to 10185.8)	44.4% (-8.1 to 127.3)	603.5 (377.2 to 886.2)	863.8 (509.1 to 1386.6)	43.1% (-25.8 to 177.2)
District of Columbia	1033.2 (682.0 to 1415.7)	1400.1 (859.8 to 2115.9)	35.5% (-24.0 to 141.9)	5357.8 (4036.7 to 6933.6)	7123.1 (4751.8 to 10034.5)	32.9% (-16.0 to 110.7)	650.2 (411.1 to 959.1)	860.9 (502.3 to 1315.6)	32.4% (-30.1 to 151.6)
Florida	971.0 (649.0 to 1337.6)	1478.4 (893.5 to 2199.0)	52.3% (-14.4 to 171.1)	4826.8 (3601.8 to 6236.1)	7339.2 (4975.6 to 10235.2)	52.1% (-3.1 to 139.4)	588.6 (364.2 to 862.6)	889.0 (524.6 to 1400.9)	51.0% (-21.3 to 190.5)
Georgia	973.6 (657.3 to 1358.0)	1460.0 (886.0 to 2197.9)	50.0% (-15.9 to 168.1)	4835.2 (3640.7 to 6237.7)	7227.6 (4867.8 to 9994.9)	49.5% (-4.4 to 134.2)	591.1 (370.6 to 871.3)	875.7 (491.7 to 1342.3)	48.2% (-23.3 to 187.2)
Hawaii	963.3 (648.5 to 1328.0)	1274.5 (803.1 to 1859.0)	32.3% (-23.7 to 130.2)	4828.2 (3621.9 to 6297.3)	6274.7 (4275.6 to 8727.9)	30.0% (-17.1 to 104.1)	589.7 (374.6 to 878.7)	763.2 (446.4 to 1182.3)	29.4% (-32.1 to 147.3)
Idaho	955.6 (648.8 to 1293.9)	1381.3 (837.1 to 2031.8)	44.6% (-17.4 to 153.5)	4597.3 (3477.8 to 5938.9)	6760.1 (4623.2 to 9613.9)	47.0% (-6.3 to 131.4)	561.6 (350.8 to 840.9)	818.3 (474.6 to 1289.0)	45.7% (-24.8 to 182.9)

Illinois	974.7 (645.9 to 1349.1)	1382.9 (860.9 to 2081.0)	41.9% (-20.2 to 152.7)	4838.0 (3616.5 to 6260.4)	6880.8 (4737.7 to 9741.6)	42.2% (-9.3 to 123.9)	591.2 (366.9 to 868.0)	832.2 (503.6 to 1328.9)	40.8% (-26.2 to 169.3)
Indiana	972.2 (658.4 to 1340.6)	1381.7 (855.2 to 2037.3)	42.1% (-18.8 to 149.3)	4809.9 (3607.9 to 6200.1)	6851.3 (4714.4 to 9834.6)	42.4% (-9.5 to 124.6)	588.1 (372.9 to 878.5)	826.2 (497.3 to 1237.7)	40.5% (-24.5 to 161.6)
Iowa	964.9 (648.7 to 1312.3)	1297.5 (813.9 to 1915.5)	34.5% (-22.7 to 134.3)	4755.6 (3548.4 to 6211.8)	6431.3 (4425.7 to 8989.4)	35.2% (-13.7 to 112.4)	580.7 (369.6 to 857.3)	779.1 (466.2 to 1185.0)	34.2% (-28.1 to 151.0)
Kansas	963.3 (641.9 to 1337.8)	1368.9 (863.5 to 2014.8)	42.1% (-18.8 to 149.2)	4760.8 (3577.3 to 6262.9)	6715.4 (4583.0 to 9428.0)	41.1% (-10.5 to 122.7)	581.0 (362.6 to 863.0)	813.9 (472.9 to 1298.2)	40.1% (-27.9 to 173.3)
Kentucky	967.6 (648.7 to 1304.6)	1510.7 (908.4 to 2229.4)	56.1% (-11.4 to 175.7)	4810.1 (3569.6 to 6242.2)	7462.8 (5035.4 to 10559.4)	55.1% (-2.1 to 146.8)	587.5 (363.9 to 870.6)	897.0 (530.7 to 1371.2)	52.7% (-19.5 to 189.8)
Louisiana	971.3 (640.0 to 1338.3)	1423.9 (872.9 to 2071.2)	46.6% (-16.9 to 159.3)	4793.0 (3594.0 to 6194.1)	7021.0 (4758.1 to 9791.0)	46.5% (-6.4 to 130.2)	585.1 (357.4 to 871.1)	849.0 (495.0 to 1324.8)	45.1% (-25.0 to 181.3)
Maine	974.5 (644.4 to 1354.7)	1300.6 (813.3 to 1916.4)	33.5% (-24.2 to 135.6)	4810.4 (3611.3 to 6282.1)	6430.5 (4350.1 to 9026.8)	33.7% (-15.2 to 111.4)	588.4 (367.6 to 872.4)	779.3 (429.4 to 1212.3)	32.5% (-32.6 to 161.0)
Maryland	990.4 (654.5 to 1369.4)	1421.2 (871.1 to 2071.2)	43.5% (-18.7 to 154.1)	4934.8 (3679.1 to 6435.5)	7033.2 (4746.2 to 9788.3)	42.5% (-9.5 to 125.2)	603.9 (372.0 to 895.2)	852.3 (497.5 to 1315.9)	41.1% (-26.4 to 171.2)
Massachusetts	997.9 (657.7 to 1356.9)	1383.5 (848.9 to 2031.7)	38.6% (-21.4 to 144.9)	5049.3 (3783.3 to 6529.0)	6908.8 (4713.3 to 9625.8)	36.8% (-12.4 to 114.6)	614.2 (389.5 to 896.6)	832.8 (490.3 to 1271.3)	35.6% (-27.9 to 155.3)
Michigan	977.3 (653.7 to 1339.7)	1429.4 (867.8 to 2108.9)	46.3% (-17.2 to 158.8)	4836.4 (3649.0 to 6254.2)	7070.2 (4774.7 to 10062.2)	46.2% (-7.5 to 131.3)	590.1 (372.0 to 869.5)	852.3 (508.0 to 1331.8)	44.4% (-23.8 to 174.6)
Minnesota	971.2 (644.5 to 1348.9)	1323.8 (814.2 to 1935.3)	36.3% (-22.8 to 141.3)	4794.4 (3600.6 to 6253.9)	6529.6 (4413.5 to 9147.2)	36.2% (-13.5 to 115.2)	586.3 (359.1 to 865.8)	791.5 (460.1 to 1219.5)	35.0% (-29.7 to 159.8)
Mississippi	971.9 (640.2 to 1367.7)	1465.4 (910.2 to 2151.7)	50.8% (-14.9 to 167.6)	4790.6 (3603.8 to 6261.6)	7218.2 (4947.1 to 9991.2)	50.7% (-3.4 to 135.6)	586.6 (362.1 to 872.8)	875.8 (524.5 to 1347.9)	49.3% (-21.3 to 183.3)

Missouri	979.6 (644.6 to 1349.0)	1407.9 (846.4 to 2094.0)	43.7% (-19.8 to 158.0)	4811.6 (3625.2 to 6266.8)	6952.9 (4599.9 to 9788.7)	44.5% (-9.1 to 130.2)	587.8 (364.3 to 864.5)	838.5 (501.8 to 1291.3)	42.6% (-24.5 to 169.8)
Montana	954.8 (645.2 to 1307.1)	1336.2 (839.9 to 2013.7)	39.9% (-20.1 to 145.4)	4610.0 (3468.3 to 5971.5)	6542.0 (4507.8 to 9296.0)	41.9% (-9.4 to 123.1)	563.4 (354.2 to 833.0)	790.4 (464.4 to 1234.7)	40.3% (-26.6 to 168.6)
Nebraska	965.2 (641.4 to 1347.4)	1306.1 (799.1 to 1970.8)	35.3% (-24.5 to 143.0)	4738.8 (3550.2 to 6146.4)	6418.8 (4374.8 to 9049.9)	35.5% (-13.8 to 113.6)	579.3 (361.1 to 847.1)	779.6 (452.6 to 1231.6)	34.6% (-30.2 to 160.4)
Nevada	972.3 (638.0 to 1344.1)	1447.6 (902.1 to 2169.0)	48.9% (-16.2 to 165.4)	4821.9 (3566.2 to 6273.3)	7168.7 (4877.3 to 9974.0)	48.7% (-5.5 to 134.5)	589.6 (367.1 to 859.1)	868.3 (509.5 to 1327.9)	47.3% (-22.2 to 179.4)
New Hampshire	977.0 (652.4 to 1318.6)	1383.0 (879.7 to 2070.8)	41.6% (-18.6 to 146.6)	4900.8 (3636.5 to 6364.9)	6849.6 (4710.5 to 9484.4)	39.8% (-10.5 to 118.7)	597.2 (371.8 to 885.6)	826.6 (489.3 to 1266.7)	38.4% (-27.0 to 162.8)
New Jersey	979.7 (639.3 to 1358.1)	1391.2 (902.7 to 2024.8)	42.0% (-18.0 to 145.8)	4874.8 (3640.4 to 6369.2)	6858.3 (4679.6 to 9590.0)	40.7% (-10.5 to 121.7)	596.6 (371.9 to 887.2)	830.2 (501.7 to 1264.8)	39.1% (-25.9 to 161.4)
New Mexico	963.8 (643.9 to 1313.5)	1444.3 (918.1 to 2123.3)	49.9% (-13.5 to 160.4)	4702.2 (3542.5 to 6117.2)	7100.4 (4864.6 to 9870.5)	51.0% (-3.2 to 136.1)	573.9 (356.8 to 845.5)	854.8 (498.4 to 1289.9)	48.9% (-21.3 to 182.4)
New York	986.0 (646.6 to 1369.2)	1411.9 (843.6 to 2092.2)	43.2% (-20.6 to 158.5)	4940.9 (3647.7 to 6431.8)	7009.8 (4686.7 to 9980.9)	41.9% (-11.2 to 127.5)	602.3 (369.6 to 880.2)	847.2 (488.7 to 1285.6)	40.7% (-26.3 to 169.3)
North Carolina	975.5 (664.6 to 1329.7)	1429.5 (898.4 to 2156.1)	46.5% (-15.9 to 156.0)	4962.7 (3853.7 to 6230.2)	7080.3 (4845.2 to 9881.5)	42.7% (-7.0 to 119.1)	608.1 (389.7 to 878.0)	859.4 (515.0 to 1340.2)	41.3% (-24.5 to 165.4)
North Dakota	965.2 (647.0 to 1330.0)	1256.5 (772.5 to 1877.3)	30.2% (-26.4 to 130.6)	4768.9 (3549.6 to 6243.8)	6192.3 (4109.8 to 8654.0)	29.8% (-18.4 to 107.3)	583.4 (367.9 to 864.0)	751.0 (437.8 to 1150.9)	28.7% (-32.3 to 145.2)
Ohio	974.2 (647.6 to 1329.6)	1418.9 (873.1 to 2128.7)	45.7% (-17.7 to 158.1)	4825.8 (3584.3 to 6256.0)	7049.9 (4781.0 to 9940.9)	46.1% (-7.5 to 131.5)	589.7 (377.5 to 859.5)	849.0 (492.2 to 1316.6)	44.0% (-24.2 to 174.1)
Oklahoma	964.6 (643.5 to 1355.7)	1434.5 (894.0 to 2096.5)	48.7% (-15.4 to 161.9)	4743.7 (3565.4 to 6179.9)	7084.7 (4832.0 to 9822.5)	49.3% (-4.4 to 133.9)	579.7 (361.2 to 853.7)	854.3 (500.9 to 1342.7)	47.4% (-23.3 to 183.6)

Oregon	806.7 (519.2 to 1125.9)	1152.2 (698.1 to 1798.5)	42.8% (-22.4 to 163.5)	3807.1 (2770.7 to 5079.5)	5402.3 (3461.6 to 8003.0)	41.9% (-15.2 to 137.8)	463.9 (282.7 to 684.4)	652.7 (365.5 to 1063.8)	40.7% (-29.7 to 182.1)
Pennsylvania	976.0 (651.4 to 1345.6)	1403.0 (886.5 to 2030.6)	43.8% (-17.0 to 149.3)	4862.7 (3629.7 to 6280.5)	6983.9 (4825.6 to 9790.2)	43.6% (-8.0 to 124.7)	593.3 (368.7 to 862.0)	840.1 (491.8 to 1272.6)	41.6% (-24.9 to 167.9)
Rhode Island	995.2 (660.1 to 1370.3)	1369.9 (819.6 to 2039.7)	37.6% (-23.1 to 146.7)	5021.7 (3776.7 to 6497.5)	6879.3 (4631.0 to 9767.5)	37.0% (-13.4 to 117.3)	612.8 (386.5 to 900.3)	831.1 (479.7 to 1306.5)	35.6% (-29.6 to 161.8)
South Carolina	978.7 (653.2 to 1342.2)	1448.6 (905.3 to 2173.7)	48.0% (-16.0 to 161.2)	4859.8 (3666.6 to 6284.4)	7191.7 (4935.2 to 9963.0)	48.0% (-4.6 to 130.6)	593.2 (375.7 to 872.3)	868.4 (524.8 to 1312.2)	46.4% (-21.1 to 171.8)
South Dakota	959.2 (652.3 to 1328.7)	1263.7 (797.0 to 1862.5)	31.7% (-24.3 to 129.7)	4683.1 (3526.1 to 6061.1)	6164.2 (4233.6 to 8668.9)	31.6% (-15.7 to 106.3)	573.6 (354.1 to 846.2)	748.3 (427.3 to 1162.1)	30.5% (-32.7 to 153.4)
Tennessee	975.2 (657.2 to 1356.2)	1424.9 (875.2 to 2153.9)	46.1% (-17.9 to 160.3)	4846.0 (3648.3 to 6269.7)	7045.4 (4732.8 to 9972.5)	45.4% (-8.1 to 130.4)	591.6 (366.4 to 869.6)	851.0 (494.9 to 1330.7)	43.8% (-25.3 to 177.4)
Texas	970.0 (646.5 to 1340.0)	1417.3 (897.9 to 2056.8)	46.1% (-15.7 to 153.8)	4798.3 (3576.4 to 6237.6)	7015.6 (4828.7 to 9755.3)	46.2% (-6.4 to 128.9)	587.0 (373.1 to 863.0)	850.7 (497.4 to 1304.5)	44.9% (-23.4 to 174.7)
Utah	963.5 (642.8 to 1342.7)	1329.0 (824.0 to 1951.9)	37.9% (-21.7 to 143.7)	4649.6 (3456.9 to 6040.7)	6536.6 (4438.7 to 9123.2)	40.6% (-10.7 to 121.7)	569.4 (352.4 to 840.7)	790.7 (458.7 to 1231.2)	38.9% (-27.9 to 168.1)
Vermont	976.2 (643.5 to 1361.8)	1462.5 (902.1 to 2161.9)	49.8% (-15.7 to 167.0)	4869.6 (3615.0 to 6261.3)	7266.6 (4944.0 to 10274.8)	49.2% (-5.2 to 135.7)	595.6 (369.5 to 877.5)	880.9 (512.9 to 1398.8)	47.9% (-23.6 to 187.4)
Virginia	984.7 (651.2 to 1366.1)	1408.6 (881.1 to 2133.8)	43.0% (-19.7 to 155.2)	4926.5 (3705.7 to 6412.0)	7026.4 (4765.4 to 9876.8)	42.6% (-9.3 to 125.0)	601.6 (379.6 to 885.1)	849.3 (482.1 to 1289.5)	41.2% (-26.2 to 170.7)
Washington	965.9 (660.6 to 1321.8)	1342.2 (828.4 to 1975.6)	39.0% (-20.1 to 142.2)	4771.2 (3557.3 to 6223.0)	6608.3 (4485.7 to 9201.8)	38.5% (-12.0 to 118.3)	580.9 (364.3 to 866.6)	797.7 (462.3 to 1261.1)	37.3% (-29.2 to 166.9)
West Virginia	964.4 (646.7 to 1327.1)	1510.2 (930.5 to 2283.5)	56.6% (-11.7 to 178.1)	4730.4 (3539.1 to 6103.8)	7472.1 (5103.1 to 10472.8)	58.0% (0.9 to 148.1)	578.3 (360.5 to 856.7)	899.3 (518.2 to 1382.8)	55.5% (-19.0 to 199.1)

Wisconsin	977.6 (652.9 to 1325.6)	1303.2 (800.8 to 1925.0)	33.3% (-24.0 to 134.2)	4811.8 (3582.2 to 6247.3)	6454.0 (4376.7 to 9037.6)	34.1% (-14.8 to 112.0)	587.3 (371.0 to 874.0)	779.1 (458.3 to 1200.6)	32.7% (-30.2 to 152.9)
Wyoming	954.6 (637.2 to 1313.6)	1368.7 (849.0 to 2033.9)	43.4% (-18.7 to 153.3)	4572.4 (3428.0 to 5891.8)	6709.8 (4540.5 to 9521.0)	46.7% (-7.0 to 132.1)	559.8 (353.6 to 827.1)	811.1 (460.8 to 1250.3)	44.9% (-24.7 to 179.8)
Depressive disorders									
Alabama	5056.5 (3632.1 to 6822.6)	10313.4 (7243.2 to 14617.9)	104.0% (27.6 to 226.8)	4075.9 (3094.7 to 5270.2)	7303.1 (5328.4 to 10111.6)	79.2% (18.1 to 172.2)	752.9 (476.7 to 1116.7)	1440.8 (878.1 to 2220.8)	91.4% (2.4 to 257.9)
Alaska	5525.7 (3903.9 to 7502.5)	9151.1 (6405.1 to 12669.3)	65.6% (3.7 to 164.5)	4317.7 (3242.1 to 5639.7)	6462.9 (4718.1 to 8680.2)	49.7% (-0.6 to 125.6)	808.8 (515.0 to 1216.2)	1268.3 (802.3 to 1954.2)	56.8% (-15.0 to 189.5)
Arizona	6034.5 (4343.4 to 8194.2)	12475.7 (8676.9 to 17305.0)	106.7% (29.8 to 229.5)	4706.2 (3568.6 to 6174.2)	8719.1 (6329.2 to 11711.5)	85.3% (22.9 to 179.8)	885.3 (568.6 to 1313.4)	1731.2 (1073.3 to 2633.4)	95.6% (6.3 to 259.8)
Arkansas	6214.3 (4486.8 to 8552.0)	12527.1 (8832.9 to 17313.9)	101.6% (27.0 to 220.1)	4799.5 (3610.2 to 6260.0)	8715.1 (6364.4 to 11732.8)	81.6% (20.6 to 173.7)	910.7 (570.7 to 1361.0)	1739.2 (1101.7 to 2667.6)	91.0% (3.4 to 253.0)
California	4159.4 (3087.7 to 5561.3)	9412.8 (6461.9 to 13216.6)	126.3% (42.5 to 259.9)	3529.7 (2761.4 to 4510.0)	6751.8 (4859.3 to 9233.4)	91.3% (28.1 to 186.7)	634.1 (405.0 to 944.8)	1332.5 (796.9 to 2024.3)	110.1% (12.4 to 293.7)
Colorado	6120.0 (4395.4 to 8346.8)	11180.5 (7883.1 to 15366.9)	82.7% (15.5 to 189.1)	4752.5 (3555.8 to 6234.6)	7862.4 (5774.1 to 10562.6)	65.4% (9.8 to 149.2)	896.6 (576.1 to 1354.6)	1556.2 (963.0 to 2342.0)	73.6% (-5.8 to 219.8)
Connecticut	3939.0 (2936.2 to 5217.9)	7353.6 (4986.5 to 10592.5)	86.7% (16.6 to 200.1)	3405.6 (2684.1 to 4324.0)	5427.7 (3945.5 to 7483.1)	59.4% (7.2 to 137.4)	604.9 (390.5 to 873.5)	1046.9 (640.5 to 1619.9)	73.1% (-6.2 to 220.3)
Delaware	5547.9 (4023.6 to 7578.1)	10910.5 (7647.8 to 15272.8)	96.7% (23.5 to 213.5)	4459.0 (3363.6 to 5835.1)	7722.7 (5604.5 to 10385.2)	73.2% (14.7 to 161.9)	828.2 (518.2 to 1243.4)	1528.2 (942.6 to 2357.0)	84.5% (-1.5 to 246.1)
District of Columbia	4876.5 (3515.3 to 6797.1)	8059.2 (5450.2 to 11539.1)	65.3% (0.5 to 172.4)	4179.3 (3184.4 to 5554.1)	5959.8 (4232.1 to 8291.5)	42.6% (-7.9 to 120.9)	752.0 (477.9 to 1130.2)	1151.8 (679.5 to 1792.3)	53.2% (-19.7 to 192.9)
Florida	6510.3 (4582.6 to 8807.2)	11574.2 (7873.7 to 16513.0)	77.8% (8.7 to 191.3)	5045.2 (3740.5 to 6603.6)	8150.7 (5755.3 to 11372.6)	61.6% (3.7 to 152.2)	956.4 (604.3 to 1421.0)	1617.0 (987.1 to 2544.3)	69.1% (-10.3 to 219.4)

Georgia	5631.7 (4028.1 to 7635.1)	10449.5 (7034.1 to 14702.8)	85.5% (14.0 to 202.6)	4475.1 (3367.0 to 5890.2)	7408.2 (5292.6 to 10129.1)	65.5% (7.9 to 154.4)	837.4 (526.7 to 1275.1)	1467.1 (891.4 to 2277.3)	75.2% (-7.6 to 232.4)
Hawaii	5125.5 (3672.9 to 6930.9)	9004.2 (6221.0 to 12656.7)	75.7% (9.3 to 182.9)	4149.1 (3149.7 to 5353.7)	6430.7 (4644.4 to 8772.8)	55.0% (2.4 to 134.9)	767.0 (487.3 to 1137.4)	1271.0 (802.6 to 1956.9)	65.7% (-9.9 to 205.3)
Idaho	5693.5 (4066.5 to 7709.9)	11222.5 (7784.4 to 15592.4)	97.1% (23.3 to 215.2)	4374.2 (3286.6 to 5687.3)	7850.7 (5690.8 to 10670.9)	79.5% (18.4 to 172.4)	826.3 (523.4 to 1236.1)	1563.5 (959.6 to 2444.7)	89.2% (0.7 to 255.7)
Illinois	4690.8 (3364.0 to 6466.3)	8909.3 (6178.9 to 12354.6)	89.9% (18.4 to 204.9)	3848.1 (2914.5 to 5087.9)	6417.1 (4637.3 to 8607.8)	66.8% (10.3 to 152.6)	704.6 (446.4 to 1058.4)	1258.4 (762.1 to 1880.2)	78.6% (-3.8 to 231.9)
Indiana	5804.0 (4147.1 to 7882.3)	10775.6 (7456.1 to 14987.5)	85.7% (15.9 to 197.5)	4571.5 (3429.0 to 5985.6)	7605.3 (5471.2 to 10405.7)	66.4% (8.8 to 154.8)	858.8 (541.2 to 1288.5)	1506.8 (924.5 to 2347.6)	75.5% (-6.8 to 230.2)
Iowa	5098.7 (3619.1 to 6955.4)	8662.4 (6102.4 to 12121.1)	69.9% (6.2 to 172.1)	4081.9 (3053.8 to 5375.0)	6225.2 (4554.9 to 8402.7)	52.5% (0.8 to 130.8)	757.4 (479.9 to 1154.6)	1220.2 (755.0 to 1896.8)	61.1% (-14.3 to 203.1)
Kansas	5589.2 (3987.2 to 7638.2)	10057.2 (6985.0 to 14118.9)	79.9% (11.8 to 189.7)	4406.1 (3284.8 to 5804.5)	7115.0 (5121.6 to 9680.0)	61.5% (5.6 to 147.5)	823.8 (523.0 to 1240.4)	1402.8 (883.2 to 2099.1)	70.3% (-7.0 to 211.8)
Kentucky	5584.7 (4001.0 to 7624.6)	11836.5 (8325.5 to 16455.0)	111.9% (33.1 to 237.7)	4417.2 (3329.0 to 5820.6)	8273.0 (5978.3 to 11074.5)	87.3% (23.9 to 183.6)	826.0 (526.7 to 1227.1)	1637.0 (1014.8 to 2531.9)	98.2% (6.8 to 268.0)
Louisiana	5067.9 (3605.9 to 6868.6)	9604.9 (6645.5 to 13493.9)	89.5% (17.8 to 205.4)	4061.3 (3053.1 to 5295.6)	6815.3 (4934.1 to 9258.5)	67.8% (10.6 to 154.9)	750.5 (472.5 to 1120.7)	1343.5 (837.2 to 2049.0)	79.0% (-3.3 to 231.5)
Maine	5538.1 (3967.2 to 7448.5)	9615.8 (6666.2 to 13612.0)	73.6% (8.1 to 179.5)	4385.9 (3312.1 to 5741.5)	6868.6 (4976.3 to 9341.9)	56.6% (3.2 to 137.9)	821.3 (517.3 to 1226.9)	1350.5 (821.2 to 2047.7)	64.4% (-11.9 to 207.1)
Maryland	3503.5 (2517.3 to 4794.7)	9418.3 (6262.2 to 13347.8)	168.8% (63.6 to 342.8)	3095.8 (2363.3 to 4036.3)	6687.6 (4686.5 to 9167.9)	116.0% (41.0 to 231.7)	541.8 (346.1 to 812.8)	1317.8 (783.3 to 2080.3)	143.2% (27.4 to 365.3)
Massachusetts	5395.9 (3891.4 to 7402.8)	9557.5 (6597.7 to 13572.4)	77.1% (9.5 to 187.3)	4397.4 (3348.1 to 5725.1)	6881.1 (4965.4 to 9400.7)	56.5% (3.1 to 137.8)	811.3 (511.7 to 1198.4)	1349.9 (817.2 to 2075.1)	66.4% (-11.1 to 211.9)

Michigan	5293.1 (3772.9 to 7192.7)	10679.8 (7434.5 to 14921.9)	101.8% (25.9 to 223.4)	4239.1 (3214.1 to 5528.7)	7574.1 (5510.1 to 10164.1)	78.7% (18.9 to 168.9)	785.1 (494.6 to 1182.8)	1491.4 (916.2 to 2235.6)	90.0% (2.6 to 252.4)
Minnesota	5574.7 (4000.9 to 7649.7)	9351.1 (6479.7 to 12998.3)	67.7% (4.6 to 169.0)	4401.5 (3336.5 to 5781.2)	6665.3 (4811.8 to 9036.5)	51.4% (-0.2 to 130.1)	824.7 (520.3 to 1239.0)	1313.9 (808.2 to 2067.2)	59.3% (-15.6 to 200.8)
Mississippi	5440.4 (3865.5 to 7485.4)	10236.1 (7046.8 to 14452.3)	88.2% (15.9 to 205.6)	4307.0 (3204.9 to 5641.7)	7228.6 (5096.2 to 10000.7)	67.8% (8.1 to 161.0)	805.7 (511.1 to 1193.1)	1431.9 (873.0 to 2192.0)	77.7% (-4.5 to 231.0)
Missouri	4082.9 (2986.3 to 5471.6)	9479.2 (6448.7 to 13502.4)	132.2% (44.1 to 274.6)	3433.3 (2636.3 to 4443.9)	6758.3 (4802.7 to 9258.0)	96.8% (29.7 to 199.3)	618.1 (402.5 to 911.6)	1328.4 (803.0 to 2075.9)	114.9% (15.0 to 303.0)
Montana	5623.5 (3980.0 to 7674.5)	10074.6 (7013.3 to 14139.4)	79.2% (11.2 to 188.7)	4338.4 (3223.0 to 5682.9)	7085.5 (5127.8 to 9616.0)	63.3% (7.2 to 149.1)	817.4 (518.8 to 1223.5)	1397.1 (863.9 to 2133.8)	70.9% (-7.9 to 217.5)
Nebraska	5061.6 (3565.9 to 6842.2)	8450.3 (5827.4 to 11892.1)	66.9% (3.3 to 170.0)	4043.3 (3017.4 to 5269.7)	6078.5 (4322.6 to 8188.2)	50.3% (-1.5 to 129.8)	750.0 (476.0 to 1121.5)	1192.7 (746.5 to 1792.8)	59.0% (-13.2 to 191.8)
Nevada	6132.0 (4410.3 to 8234.6)	11292.5 (7815.6 to 15533.4)	84.2% (16.1 to 192.5)	4768.7 (3622.2 to 6176.5)	7936.5 (5747.1 to 10542.9)	66.4% (11.2 to 149.4)	899.5 (584.0 to 1350.2)	1576.0 (977.9 to 2395.1)	75.2% (-4.7 to 222.2)
New Hampshire	5998.8 (4267.5 to 8245.7)	11085.8 (7650.2 to 15403.3)	84.8% (14.7 to 197.9)	4733.3 (3532.8 to 6229.9)	7856.6 (5668.4 to 10636.2)	66.0% (8.9 to 153.3)	889.1 (574.0 to 1337.6)	1555.8 (972.0 to 2435.9)	75.0% (-5.9 to 225.5)
New Jersey	4473.5 (3212.9 to 6090.4)	8413.2 (5701.4 to 11944.4)	88.1% (15.4 to 206.9)	3734.8 (2836.8 to 4886.5)	6075.4 (4287.0 to 8403.3)	62.7% (5.7 to 150.7)	678.0 (419.5 to 1017.8)	1186.3 (723.8 to 1847.8)	75.0% (-7.7 to 232.1)
New Mexico	6276.5 (4509.6 to 8529.0)	12769.2 (8941.7 to 17800.7)	103.4% (27.7 to 224.3)	4807.5 (3578.2 to 6283.4)	8845.1 (6421.1 to 11987.1)	84.0% (21.2 to 179.8)	912.1 (584.4 to 1373.6)	1763.0 (1091.8 to 2656.5)	93.3% (4.9 to 256.2)
New York	5927.7 (4351.0 to 7972.6)	10648.2 (7497.8 to 14766.0)	79.6% (14.3 to 183.0)	4719.3 (3595.9 to 6070.1)	7548.0 (5453.3 to 10288.2)	59.9% (6.0 to 141.6)	881.7 (569.4 to 1303.9)	1490.3 (921.5 to 2312.3)	69.0% (-8.6 to 213.4)
North Carolina	3520.0 (2549.4 to 4719.4)	8655.8 (5804.4 to 12442.5)	145.9% (50.8 to 301.4)	3116.7 (2431.4 to 3962.4)	6248.2 (4375.5 to 8716.9)	100.5% (31.7 to 205.3)	545.9 (347.6 to 813.3)	1223.8 (737.6 to 1911.7)	124.2% (18.7 to 324.6)

North Dakota	4649.8 (3350.0 to 6377.2)	7927.2 (5386.5 to 11252.0)	70.5% (4.7 to 178.1)	3796.5 (2883.0 to 4951.3)	5733.1 (4115.5 to 7884.1)	51.0% (-1.1 to 130.9)	698.6 (442.4 to 1059.4)	1117.7 (672.6 to 1763.5)	60.0% (-16.2 to 206.0)
Ohio	5332.3 (3821.2 to 7253.2)	10471.0 (7270.1 to 14535.0)	96.4% (22.9 to 213.9)	4262.6 (3214.9 to 5531.4)	7410.8 (5301.5 to 9944.8)	73.9% (14.8 to 163.7)	792.5 (500.5 to 1187.3)	1461.4 (899.1 to 2260.0)	84.4% (-1.5 to 245.4)
Oklahoma	5932.4 (4249.0 to 8037.8)	11313.7 (7865.9 to 15454.5)	90.7% (20.3 to 202.5)	4614.6 (3470.0 to 5977.0)	7907.1 (5767.0 to 10466.2)	71.4% (14.8 to 156.1)	870.4 (556.3 to 1311.2)	1565.6 (975.4 to 2368.5)	79.9% (-2.3 to 231.4)
Oregon	5682.9 (4152.3 to 7597.1)	11178.9 (7733.6 to 15720.6)	96.7% (23.5 to 214.0)	4436.2 (3376.1 to 5761.3)	7876.9 (5695.3 to 10734.4)	77.6% (17.3 to 169.1)	833.3 (528.4 to 1235.1)	1567.5 (957.8 to 2357.3)	88.1% (1.8 to 247.9)
Pennsylvania	5036.9 (3622.5 to 6846.5)	9955.1 (6808.7 to 14118.0)	97.6% (22.0 to 220.7)	4089.3 (3106.4 to 5350.4)	7083.4 (5013.9 to 9711.8)	73.2% (12.9 to 165.9)	752.4 (477.0 to 1119.8)	1387.0 (847.5 to 2124.0)	84.3% (-1.1 to 243.6)
Rhode Island	6346.5 (4567.9 to 8694.4)	11111.0 (7860.9 to 15521.4)	75.1% (10.0 to 178.8)	5029.4 (3751.9 to 6620.7)	7931.4 (5774.2 to 10835.6)	57.7% (3.5 to 140.7)	944.7 (603.1 to 1421.7)	1567.1 (974.9 to 2423.4)	65.9% (-10.8 to 208.8)
South Carolina	5645.3 (4030.8 to 7697.2)	10997.4 (7746.7 to 15292.8)	94.8% (22.3 to 210.6)	4484.0 (3351.6 to 5928.4)	7752.9 (5708.5 to 10464.2)	72.9% (14.4 to 161.4)	836.5 (535.8 to 1254.6)	1535.3 (977.0 to 2324.4)	83.5% (0.7 to 235.0)
South Dakota	4514.5 (3220.2 to 6160.8)	7456.6 (5072.0 to 10448.4)	65.2% (1.9 to 168.3)	3659.9 (2773.3 to 4734.9)	5404.5 (3893.3 to 7362.8)	47.7% (-2.6 to 124.2)	671.9 (431.7 to 1004.7)	1049.9 (647.9 to 1589.6)	56.2% (-15.2 to 188.2)
Tennessee	5735.7 (4101.8 to 7765.4)	10968.9 (7611.3 to 15330.1)	91.2% (19.5 to 206.6)	4530.4 (3414.9 to 5935.8)	7718.9 (5590.0 to 10461.9)	70.4% (12.4 to 158.8)	848.0 (530.5 to 1261.4)	1526.7 (923.9 to 2313.7)	80.0% (-3.7 to 237.1)
Texas	5100.0 (3624.6 to 6975.8)	9928.9 (6878.0 to 13829.6)	94.7% (21.0 to 213.2)	4096.0 (3092.1 to 5360.3)	7057.7 (5085.3 to 9489.6)	72.3% (13.9 to 161.2)	760.0 (478.3 to 1142.9)	1395.7 (868.1 to 2139.8)	83.6% (-1.3 to 241.8)
Utah	6363.1 (4531.3 to 8625.7)	12124.7 (8562.0 to 16904.3)	90.5% (19.8 to 203.4)	4823.6 (3605.7 to 6309.3)	8435.2 (6068.4 to 11446.1)	74.9% (14.7 to 167.0)	921.9 (580.1 to 1400.3)	1683.8 (1038.2 to 2585.4)	82.6% (-2.5 to 242.4)
Vermont	5778.4 (4135.8 to 7833.2)	11093.8 (7726.8 to 15169.2)	92.0% (21.1 to 204.5)	4575.3 (3425.1 to 6042.4)	7824.9 (5694.5 to 10592.7)	71.0% (12.6 to 159.9)	856.9 (543.0 to 1278.4)	1552.4 (974.9 to 2381.3)	81.2% (-1.9 to 234.6)

Virginia	5548.7 (3943.1 to 7445.9)	11104.8 (7769.5 to 15300.5)	100.1% (26.2 to 217.5)	4455.4 (3336.7 to 5789.2)	7849.7 (5647.8 to 10614.0)	76.2% (16.0 to 167.9)	829.2 (523.9 to 1237.1)	1556.6 (965.8 to 2396.9)	87.7% (1.0 to 249.4)
Washington	6001.0 (4258.2 to 8082.2)	10795.2 (7493.1 to 15246.6)	79.9% (11.8 to 189.9)	4671.5 (3496.0 to 6031.5)	7602.9 (5518.4 to 10393.6)	62.8% (7.2 to 147.5)	878.2 (560.7 to 1305.9)	1508.2 (897.8 to 2325.9)	71.7% (-8.9 to 224.6)
West Virginia	6042.2 (4280.9 to 8298.0)	12569.5 (8872.9 to 17304.3)	108.0% (30.6 to 231.5)	4684.3 (3463.9 to 6149.0)	8738.6 (6406.2 to 11819.2)	86.6% (23.0 to 183.1)	885.2 (559.4 to 1322.2)	1735.4 (1051.0 to 2607.4)	96.1% (5.4 to 265.0)
Wisconsin	5675.4 (4055.9 to 7695.0)	9991.3 (7000.6 to 13928.9)	76.0% (10.4 to 180.8)	4472.6 (3346.2 to 5904.9)	7097.8 (5164.3 to 9622.1)	58.7% (4.5 to 141.4)	837.5 (526.5 to 1250.4)	1402.9 (864.7 to 2159.2)	67.5% (-10.3 to 213.1)
Wyoming	5736.5 (4121.2 to 7885.4)	11030.0 (7612.2 to 15245.2)	92.3% (19.9 to 208.3)	4390.4 (3291.3 to 5713.3)	7693.3 (5614.8 to 10378.9)	75.2% (16.2 to 164.7)	831.5 (530.6 to 1249.7)	1528.4 (915.6 to 2343.2)	83.8% (-2.2 to 246.3)

UI, uncertainly interval; DALYs, disability-adjusted life-years.

Table S23 The incidence, prevalence and DALYs rate of dysthymia in 1990 and 2021, and changes from 1990 to 2021 at the global level and different regions.

Location	Incidence (95% UI)			Prevalence (95% UI)			DALYs (95% UI)		
	**1990**	**2021**	**Percentage change**	**1990**	**2021**	**Percentage change**	**1990**	**2021**	**Percentage change**
Global	171.0 (105.4 to 252.5)	180.8 (111.9 to 267.0)	5.7% (-42.5 to 94.5)	642.3 (438.3 to 899.7)	681.9 (463.7 to 957.8)	6.2% (-36.0 to 76.1)	63.7 (36.9 to 99.7)	67.7 (39.2 to 107.2)	6.2% (-47.2 to 114.1)
High SDI	199.5 (123.1 to 294.0)	191.2 (118.4 to 282.5)	-4.2% (-47.9 to 76.1)	793.1 (541.1 to 1106.5)	753.0 (511.1 to 1055.3)	-5.1% (-42.6 to 57.3)	78.7 (45.4 to 123.8)	74.5 (43.1 to 119.2)	-5.3% (-53.3 to 92.3)
High-middle SDI	160.3 (99.2 to 237.6)	156.9 (97.4 to 232.0)	-2.1% (-46.8 to 80.0)	607.5 (415.3 to 850.6)	595.4 (404.1 to 838.0)	-2.0% (-40.9 to 62.7)	60.6 (35.0 to 95.1)	59.4 (33.7 to 93.7)	-1.9% (-51.6 to 99.1)
Middle SDI	151.9 (94.8 to 223.2)	157.5 (98.8 to 230.5)	3.7% (-42.9 to 88.1)	561.8 (384.5 to 780.6)	588.1 (405.0 to 819.7)	4.7% (-36.1 to 71.6)	55.9 (32.5 to 87.5)	58.6 (34.3 to 92.2)	4.7% (-47.6 to 109.3)
Low-middle SDI	176.4 (109.2 to 259.9)	188.0 (116.9 to 275.4)	6.6% (-41.7 to 94.7)	652.2 (443.4 to 915.9)	708.8 (481.7 to 997.0)	8.7% (-34.6 to 80.7)	64.4 (37.2 to 100.9)	70.3 (40.9 to 112.0)	9.1% (-45.9 to 120.2)

Low SDI	209.8 (126.8 to 313.8)	214.2 (130.1 to 320.0)	2.1% (-45.8 to 92.0)	777.1 (517.6 to 1106.9)	799.2 (530.4 to 1139.1)	2.8% (-39.7 to 75.4)	76.4 (43.4 to 122.2)	79.0 (45.1 to 126.6)	3.4% (-49.8 to 113.2)
North Africa and Middle East	186.7 (111.1 to 283.6)	189.5 (112.1 to 289.0)	1.5% (-47.5 to 96.4)	730.6 (466.0 to 1093.6)	754.3 (477.1 to 1135.5)	3.2% (-43.5 to 88.7)	72.3 (39.9 to 118.1)	74.8 (41.2 to 124.2)	3.4% (-52.0 to 122.7)
South Asia	177.6 (111.8 to 258.1)	188.4 (119.9 to 271.8)	6.1% (-40.5 to 89.1)	652.9 (451.0 to 896.1)	708.7 (490.4 to 981.6)	8.5% (-33.0 to 76.0)	64.4 (37.8 to 100.1)	70.2 (41.6 to 110.8)	9.0% (-44.9 to 115.9)
Western Europe	209.1 (126.9 to 315.4)	202.6 (123.1 to 304.8)	-3.1% (-48.7 to 83.0)	879.0 (582.6 to 1254.3)	830.9 (546.9 to 1193.9)	-5.5% (-45.0 to 62.6)	87.1 (49.4 to 140.9)	82.3 (46.8 to 133.9)	-5.5% (-54.6 to 96.9)
Central Europe	172.9 (104.5 to 264.1)	175.4 (106.4 to 267.1)	1.4% (-46.9 to 93.7)	641.9 (416.3 to 947.1)	659.1 (432.3 to 968.2)	2.7% (-41.9 to 81.4)	63.9 (35.8 to 103.8)	65.7 (36.4 to 107.1)	2.8% (-51.4 to 117.9)
Eastern Europe	172.1 (111.4 to 250.2)	167.9 (108.7 to 244.0)	-2.5% (-44.6 to 71.7)	634.3 (441.8 to 869.5)	611.9 (426.3 to 839.1)	-3.5% (-39.9 to 54.9)	63.2 (36.5 to 99.5)	61.0 (35.7 to 96.1)	-3.5% (-52.0 to 93.7)
East Asia	140.6 (89.8 to 203.4)	128.2 (82.4 to 183.5)	-8.8% (-48.2 to 60.5)	520.1 (358.2 to 712.6)	466.1 (322.4 to 637.9)	-10.4% (-44.5 to 44.7)	52.1 (30.1 to 81.1)	46.8 (27.4 to 73.2)	-10.1% (-55.0 to 79.1)
Central Asia	174.0 (100.7 to 273.1)	173.3 (100.2 to 272.1)	-0.4% (-50.5 to 100.3)	648.5 (401.3 to 996.6)	648.5 (401.3 to 995.8)	0.0% (-47.1 to 88.8)	64.5 (34.8 to 107.1)	64.6 (35.0 to 106.0)	0.1% (-54.2 to 118.6)
Southeast Asia	144.2 (89.0 to 217.1)	147.4 (91.6 to 220.8)	2.2% (-45.0 to 89.9)	521.1 (344.9 to 752.0)	539.3 (360.3 to 772.9)	3.5% (-39.7 to 77.4)	51.8 (29.6 to 82.4)	53.8 (31.1 to 84.8)	3.9% (-48.9 to 111.0)
High-income North America	252.0 (160.5 to 362.7)	210.0 (133.4 to 303.7)	-16.7% (-53.0 to 47.9)	979.6 (682.9 to 1329.4)	799.7 (555.5 to 1090.0)	-18.4% (-48.9 to 30.6)	97.0 (57.5 to 150.0)	78.7 (46.2 to 124.2)	-18.9% (-59.0 to 60.4)
Tropical Latin America	138.1 (86.5 to 198.4)	144.9 (92.0 to 212.1)	5.0% (-41.4 to 88.0)	506.8 (352.3 to 690.8)	546.9 (379.4 to 750.8)	7.9% (-32.9 to 73.5)	50.1 (29.6 to 77.9)	54.0 (32.2 to 84.5)	7.9% (-45.1 to 112.0)
Andean Latin America	143.4 (83.5 to 223.4)	147.8 (86.2 to 229.7)	3.1% (-48.1 to 104.8)	536.2 (329.2 to 816.2)	567.3 (349.2 to 861.8)	5.8% (-43.9 to 99.3)	53.3 (28.1 to 88.9)	56.3 (30.0 to 93.8)	5.6% (-52.7 to 135.5)

Central Latin America	135.7 (82.6 to 201.1)	139.5 (84.9 to 206.6)	2.8% (-44.8 to 91.6)	505.4 (337.8 to 719.5)	531.1 (355.1 to 756.5)	5.1% (-38.1 to 78.3)	50.3 (28.9 to 80.3)	52.9 (30.3 to 84.0)	5.1% (-48.6 to 114.8)
Southern Latin America	144.7 (83.6 to 225.3)	148.0 (85.5 to 230.5)	2.3% (-48.9 to 104.7)	554.9 (345.1 to 852.9)	580.6 (361.5 to 891.8)	4.6% (-44.4 to 96.9)	55.1 (29.4 to 93.8)	57.7 (31.2 to 97.0)	4.6% (-53.1 to 133.2)
Central Sub-Saharan Africa	215.1 (124.6 to 328.4)	214.3 (124.1 to 327.2)	-0.4% (-49.4 to 96.2)	799.2 (491.5 to 1195.1)	793.3 (487.7 to 1186.7)	-0.7% (-46.7 to 84.8)	78.1 (41.9 to 129.9)	78.1 (42.5 to 129.7)	-0.0% (-54.5 to 119.2)
Eastern Sub-Saharan Africa	244.5 (147.5 to 366.7)	246.4 (149.2 to 371.0)	0.8% (-46.7 to 90.6)	912.5 (613.4 to 1303.6)	928.9 (619.5 to 1328.0)	1.8% (-40.1 to 73.2)	89.8 (50.8 to 144.1)	91.9 (52.1 to 147.0)	2.4% (-50.5 to 111.8)
Western Sub-Saharan Africa	213.7 (130.1 to 313.6)	213.0 (129.9 to 312.5)	-0.3% (-46.1 to 84.3)	783.8 (518.4 to 1113.9)	780.4 (516.3 to 1108.8)	-0.4% (-41.7 to 70.0)	77.1 (44.8 to 122.6)	77.2 (44.4 to 122.5)	0.2% (-50.6 to 103.4)
Southern Sub-Saharan Africa	215.8 (134.8 to 311.9)	216.9 (134.7 to 313.9)	0.5% (-44.3 to 81.3)	795.0 (542.9 to 1110.1)	807.2 (547.8 to 1129.0)	1.5% (-38.6 to 68.1)	78.6 (45.9 to 123.1)	79.9 (46.6 to 125.0)	1.6% (-49.0 to 102.5)
Australasia	211.1 (126.6 to 314.5)	205.4 (121.3 to 311.8)	-2.7% (-49.2 to 86.2)	843.9 (551.6 to 1229.1)	818.1 (527.4 to 1200.3)	-3.1% (-45.1 to 71.2)	83.1 (46.5 to 135.4)	80.8 (44.4 to 134.5)	-2.8% (-54.7 to 108.5)
High-income Asia Pacific	142.4 (88.2 to 205.8)	142.8 (87.9 to 206.0)	0.2% (-44.7 to 81.7)	557.3 (378.2 to 777.0)	567.4 (385.8 to 788.7)	1.8% (-38.4 to 68.2)	55.6 (32.2 to 87.2)	56.7 (33.3 to 88.5)	2.0% (-48.9 to 103.3)
Caribbean	148.0 (86.4 to 230.2)	148.3 (86.5 to 230.6)	0.2% (-49.5 to 98.9)	563.9 (347.0 to 857.5)	568.5 (349.9 to 863.8)	0.8% (-46.4 to 89.7)	55.8 (29.7 to 93.5)	56.4 (30.0 to 93.9)	1.0% (-54.6 to 124.8)
Oceania	143.1 (81.2 to 221.7)	144.5 (82.1 to 223.7)	1.0% (-49.9 to 103.7)	520.7 (316.0 to 793.3)	529.9 (321.6 to 806.7)	1.8% (-46.5 to 93.6)	51.6 (26.7 to 86.3)	52.6 (27.6 to 88.4)	2.0% (-55.0 to 130.9)

UI, uncertainly interval; DALYs, disability-adjusted life-years.

Table S24 The incidence, prevalence and DALYs rate of dysthymia in 1990 and 2021, and changes from 1990 to 2021 at 204 countries and territories around the world.

**Location**	**Incidence (95% UI)**			**Prevalence (95% UI)**			**DALYs (95% UI)**		
	**1990**	**2021**	**Percentage change**	**1990**	**2021**	**Percentage change**	**1990**	**2021**	**Percentage change**

Saint Vincent and the Grenadines	143.2 (83.4 to 222.7)	147.5 (86.0 to 229.1)	3.0% (-48.1 to 104.6)	534.5 (328.2 to 813.7)	564.0 (347.1 to 857.2)	5.5% (-44.0 to 98.9)	53.1 (27.0 to 90.4)	56.0 (29.1 to 95.0)	5.5% (-54.3 to 143.0)
Viet Nam	145.2 (82.5 to 225.0)	144.3 (82.0 to 223.4)	-0.6% (-50.7 to 100.4)	529.6 (321.5 to 807.1)	527.8 (320.3 to 803.8)	-0.3% (-47.6 to 89.7)	52.6 (27.6 to 90.6)	52.9 (27.4 to 88.5)	0.5% (-56.0 to 129.3)
United States of America	255.9 (163.4 to 366.3)	209.6 (133.8 to 303.4)	-18.1% (-53.6 to 44.9)	993.9 (697.0 to 1340.5)	796.4 (557.6 to 1076.9)	-19.9% (-49.4 to 26.9)	98.4 (58.6 to 151.7)	78.3 (46.1 to 122.5)	-20.4% (-59.4 to 56.3)
Timor-Leste	146.0 (83.0 to 225.8)	146.2 (83.2 to 226.5)	0.2% (-50.3 to 101.7)	539.0 (327.3 to 820.3)	535.4 (325.2 to 815.5)	-0.7% (-47.8 to 89.0)	53.3 (27.8 to 88.8)	53.5 (26.9 to 90.8)	0.5% (-56.3 to 131.6)
Lao People's Democratic Republic	141.2 (80.0 to 219.3)	149.0 (84.9 to 230.4)	5.6% (-47.7 to 112.9)	506.2 (307.0 to 772.5)	551.7 (335.2 to 839.6)	9.0% (-42.7 to 107.5)	50.0 (26.0 to 84.5)	55.0 (28.9 to 93.2)	10.0% (-51.7 to 150.3)
Ethiopia	239.7 (154.2 to 343.5)	239.7 (154.3 to 341.7)	0.0% (-42.8 to 74.8)	876.7 (617.7 to 1184.7)	890.5 (625.1 to 1215.8)	1.6% (-35.9 to 61.1)	86.6 (50.8 to 135.7)	88.3 (52.0 to 137.9)	2.1% (-48.6 to 102.3)
South Sudan	245.7 (143.5 to 381.8)	243.5 (142.4 to 378.5)	-0.9% (-50.0 to 96.4)	937.2 (591.7 to 1389.6)	910.6 (574.1 to 1353.9)	-2.8% (-46.6 to 76.9)	92.3 (50.2 to 151.2)	89.6 (48.3 to 146.1)	-2.9% (-55.2 to 110.2)
Suriname	147.5 (86.0 to 229.0)	146.1 (85.2 to 227.2)	-0.9% (-50.1 to 96.7)	564.1 (347.1 to 857.5)	555.6 (341.7 to 844.5)	-1.5% (-47.7 to 85.4)	55.8 (29.1 to 92.8)	54.9 (28.0 to 93.9)	-1.6% (-57.1 to 125.9)
Dominica	145.0 (84.6 to 225.2)	147.4 (86.0 to 228.7)	1.6% (-48.8 to 101.5)	545.7 (335.5 to 830.4)	560.6 (345.0 to 852.7)	2.7% (-45.5 to 93.5)	54.4 (27.2 to 93.4)	55.8 (28.5 to 94.8)	2.6% (-56.2 to 140.0)
Tuvalu	147.5 (84.0 to 228.3)	146.0 (83.1 to 225.8)	-1.1% (-50.9 to 99.2)	544.3 (330.6 to 828.8)	537.4 (326.4 to 817.9)	-1.3% (-48.1 to 87.7)	54.2 (27.6 to 93.2)	53.3 (27.5 to 89.6)	-1.6% (-57.5 to 127.4)
Republic of Moldova	172.6 (99.9 to 271.0)	177.7 (102.8 to 278.5)	3.0% (-48.7 to 106.8)	638.9 (395.2 to 982.2)	674.5 (417.9 to 1035.2)	5.6% (-44.1 to 99.4)	63.7 (33.1 to 106.4)	67.2 (35.7 to 112.9)	5.5% (-53.2 to 137.4)
Bulgaria	176.3 (102.2 to 276.5)	174.7 (101.1 to 274.1)	-0.9% (-50.6 to 98.9)	661.8 (409.8 to 1016.5)	654.5 (405.2 to 1004.9)	-1.1% (-47.6 to 86.7)	65.9 (35.1 to 110.7)	65.2 (34.5 to 108.6)	-1.0% (-55.6 to 121.0)

Germany	209.7 (121.3 to 324.5)	196.9 (115.1 to 305.7)	-6.1% (-52.7 to 86.4)	919.4 (578.7 to 1373.6)	828.8 (519.7 to 1242.5)	-9.9% (-50.9 to 65.6)	91.0 (49.8 to 151.4)	82.1 (44.2 to 139.0)	-9.8% (-59.1 to 98.9)
Romania	176.7 (102.3 to 277.0)	175.7 (101.8 to 275.7)	-0.5% (-50.5 to 99.7)	666.1 (412.5 to 1023.0)	660.2 (408.8 to 1013.7)	-0.9% (-47.5 to 87.1)	66.1 (35.4 to 111.6)	65.9 (34.3 to 111.7)	-0.4% (-55.9 to 124.9)
Lebanon	190.3 (111.2 to 292.3)	189.3 (110.5 to 290.9)	-0.5% (-49.4 to 95.7)	759.2 (467.8 to 1155.5)	757.0 (466.7 to 1152.2)	-0.3% (-47.0 to 87.6)	74.9 (39.6 to 126.4)	74.9 (39.1 to 126.3)	0.1% (-55.8 to 126.6)
China	140.8 (90.0 to 203.4)	127.8 (82.5 to 182.4)	-9.2% (-48.3 to 59.2)	520.5 (359.7 to 712.5)	463.9 (321.3 to 634.0)	-10.9% (-44.7 to 43.6)	52.1 (30.2 to 81.3)	46.6 (27.4 to 73.0)	-10.6% (-55.2 to 78.1)
Canada	215.4 (122.9 to 334.7)	213.3 (121.6 to 331.6)	-1.0% (-50.9 to 99.7)	844.1 (518.3 to 1288.6)	833.1 (511.6 to 1272.2)	-1.3% (-47.8 to 86.6)	83.5 (44.8 to 138.3)	82.4 (44.3 to 134.7)	-1.3% (-54.9 to 115.8)
Guinea-Bissau	213.0 (123.2 to 325.5)	215.5 (124.8 to 328.9)	1.1% (-48.7 to 99.3)	782.7 (481.4 to 1171.1)	800.5 (492.5 to 1196.8)	2.3% (-45.1 to 90.3)	77.3 (40.9 to 129.5)	79.3 (41.9 to 131.0)	2.5% (-54.1 to 128.5)
Philippines	141.9 (91.0 to 207.1)	145.0 (93.0 to 211.6)	2.2% (-42.5 to 81.7)	505.3 (350.5 to 697.9)	523.2 (363.0 to 721.5)	3.5% (-36.0 to 67.5)	50.2 (29.5 to 78.1)	52.1 (30.5 to 81.2)	3.8% (-47.6 to 105.8)
American Samoa	147.2 (83.7 to 227.7)	139.0 (78.8 to 216.2)	-5.5% (-53.2 to 91.0)	543.4 (330.1 to 827.4)	495.2 (300.3 to 755.8)	-8.9% (-52.2 to 73.6)	54.0 (28.6 to 90.2)	49.3 (25.2 to 85.1)	-8.6% (-60.2 to 109.8)
Burkina Faso	209.3 (120.9 to 320.3)	216.2 (125.2 to 330.0)	3.3% (-47.7 to 103.9)	760.5 (467.6 to 1138.7)	799.2 (492.1 to 1194.9)	5.1% (-43.6 to 95.7)	74.7 (39.8 to 125.1)	79.3 (41.5 to 131.9)	6.0% (-52.6 to 137.5)
Benin	212.8 (122.8 to 325.5)	216.5 (125.3 to 330.3)	1.7% (-48.5 to 100.7)	785.3 (483.5 to 1173.7)	805.4 (495.7 to 1203.8)	2.6% (-44.9 to 90.7)	77.3 (41.5 to 127.9)	80.1 (41.8 to 132.4)	3.6% (-53.3 to 130.2)
Iraq	187.0 (109.3 to 287.5)	190.0 (111.0 to 291.8)	1.6% (-48.4 to 99.8)	735.6 (453.1 to 1121.1)	758.5 (467.6 to 1154.8)	3.1% (-45.2 to 94.2)	72.6 (39.0 to 121.9)	75.1 (39.7 to 127.3)	3.5% (-53.7 to 131.9)
Antigua and Barbuda	147.7 (86.1 to 229.9)	151.1 (88.2 to 234.6)	2.3% (-48.5 to 103.0)	564.7 (347.4 to 858.2)	587.0 (361.7 to 891.0)	3.9% (-44.7 to 95.4)	56.1 (28.7 to 95.6)	58.2 (30.6 to 96.9)	3.6% (-54.5 to 135.7)

Lesotho	211.1 (121.9 to 322.9)	222.4 (129.1 to 338.7)	5.4% (-46.6 to 107.5)	766.0 (471.3 to 1146.7)	843.3 (519.5 to 1258.9)	10.1% (-40.8 to 104.8)	75.8 (40.5 to 124.9)	83.2 (44.7 to 138.9)	9.9% (-50.2 to 142.4)
Afghanistan	188.2 (110.5 to 288.9)	188.1 (109.9 to 289.0)	-0.1% (-49.1 to 96.3)	730.4 (449.5 to 1114.4)	743.1 (457.8 to 1132.1)	1.7% (-46.0 to 91.8)	71.2 (38.2 to 120.3)	73.2 (38.8 to 121.9)	2.8% (-53.9 to 128.9)
Oman	181.3 (105.3 to 279.3)	187.8 (109.0 to 288.8)	3.6% (-47.6 to 104.7)	705.2 (434.0 to 1074.2)	753.2 (464.5 to 1145.8)	6.8% (-43.3 to 101.1)	70.2 (36.9 to 120.3)	74.6 (38.6 to 126.2)	6.3% (-53.6 to 143.4)
Senegal	214.6 (124.1 to 327.6)	215.7 (125.1 to 329.2)	0.5% (-49.0 to 98.0)	790.3 (486.2 to 1182.2)	805.3 (495.2 to 1204.0)	1.9% (-45.3 to 89.7)	77.7 (41.3 to 129.2)	79.5 (42.5 to 131.2)	2.3% (-53.8 to 126.0)
Trinidad and Tobago	144.1 (84.0 to 224.3)	145.7 (84.9 to 226.6)	1.1% (-49.1 to 100.9)	542.0 (333.0 to 824.6)	551.8 (339.3 to 839.2)	1.8% (-46.0 to 91.9)	53.7 (28.1 to 89.5)	54.6 (28.6 to 91.4)	1.6% (-54.8 to 128.8)
Niger	212.5 (122.8 to 324.8)	212.3 (122.9 to 324.3)	-0.1% (-49.4 to 97.1)	778.3 (478.7 to 1164.6)	776.7 (477.5 to 1162.9)	-0.2% (-46.4 to 85.9)	76.7 (41.7 to 128.9)	77.1 (40.7 to 127.7)	0.5% (-54.6 to 122.8)
Kenya	239.0 (154.6 to 341.9)	243.9 (157.9 to 348.5)	2.0% (-41.4 to 77.7)	873.4 (617.0 to 1199.7)	903.9 (639.1 to 1240.1)	3.5% (-34.9 to 64.7)	86.8 (50.7 to 135.5)	89.8 (52.6 to 140.5)	3.5% (-47.9 to 105.8)
Costa Rica	135.3 (75.8 to 210.3)	139.6 (78.3 to 216.4)	3.2% (-49.4 to 110.4)	515.4 (313.9 to 776.0)	543.0 (331.3 to 816.5)	5.4% (-44.0 to 98.2)	51.3 (27.2 to 86.8)	53.9 (28.3 to 89.7)	5.2% (-53.2 to 136.3)
Singapore	134.8 (79.0 to 206.2)	128.4 (75.2 to 195.2)	-4.7% (-51.2 to 86.0)	545.4 (342.7 to 819.2)	510.7 (327.5 to 761.2)	-6.4% (-48.6 to 70.4)	54.2 (28.3 to 89.8)	51.0 (27.0 to 85.3)	-6.0% (-58.0 to 110.5)
Israel	209.8 (120.4 to 324.8)	211.6 (123.4 to 325.3)	0.8% (-49.2 to 100.1)	851.6 (527.6 to 1295.2)	865.3 (545.1 to 1300.8)	1.6% (-45.2 to 88.3)	84.7 (44.6 to 141.6)	85.9 (45.3 to 145.4)	1.4% (-55.0 to 128.7)
Liberia	215.7 (124.8 to 329.3)	215.8 (125.1 to 329.3)	0.0% (-49.2 to 97.0)	801.0 (493.0 to 1197.3)	801.6 (493.1 to 1198.7)	0.1% (-46.2 to 86.2)	77.6 (41.2 to 127.4)	78.3 (42.0 to 130.6)	0.9% (-54.3 to 122.7)
Comoros	246.0 (143.6 to 382.5)	253.5 (148.2 to 393.4)	3.0% (-48.0 to 104.2)	927.9 (585.3 to 1378.5)	984.2 (622.3 to 1458.7)	6.1% (-41.7 to 92.8)	92.0 (48.8 to 152.0)	97.6 (52.8 to 163.5)	6.1% (-52.0 to 134.3)

Switzerland	221.2 (126.5 to 340.5)	216.4 (124.7 to 335.9)	-2.1% (-51.1 to 95.6)	962.9 (603.9 to 1429.5)	916.9 (574.3 to 1379.1)	-4.8% (-48.2 to 75.1)	94.8 (51.3 to 155.0)	90.8 (49.1 to 151.7)	-4.3% (-56.2 to 109.4)
Kazakhstan	173.6 (100.5 to 272.6)	169.0 (97.6 to 265.8)	-2.6% (-51.6 to 96.0)	646.7 (400.2 to 993.5)	621.0 (383.8 to 954.4)	-4.0% (-49.2 to 81.5)	64.1 (34.1 to 109.5)	61.8 (32.3 to 104.3)	-3.7% (-57.5 to 118.3)
Denmark	208.5 (122.7 to 319.7)	205.8 (120.9 to 316.0)	-1.3% (-49.5 to 93.1)	886.8 (568.6 to 1318.0)	866.3 (555.0 to 1288.3)	-2.3% (-45.8 to 76.0)	88.1 (46.9 to 145.6)	85.7 (46.6 to 145.4)	-2.7% (-56.0 to 115.4)
Namibia	218.1 (126.5 to 332.5)	220.8 (128.0 to 336.4)	1.2% (-48.5 to 99.0)	813.3 (500.6 to 1215.6)	834.1 (513.6 to 1245.3)	2.6% (-44.8 to 90.6)	80.4 (42.9 to 132.6)	82.5 (43.6 to 137.1)	2.7% (-53.7 to 127.9)
Sri Lanka	146.3 (83.2 to 226.5)	147.3 (83.9 to 228.1)	0.7% (-50.0 to 102.8)	536.7 (325.9 to 817.4)	541.0 (328.6 to 823.8)	0.8% (-47.0 to 91.7)	53.5 (27.9 to 90.3)	53.8 (27.7 to 90.0)	0.6% (-55.8 to 129.1)
Botswana	215.2 (124.6 to 328.5)	222.3 (129.0 to 338.6)	3.3% (-47.5 to 103.3)	792.9 (487.9 to 1186.0)	844.9 (520.4 to 1261.0)	6.6% (-42.7 to 98.1)	78.4 (41.2 to 129.4)	83.7 (44.6 to 139.6)	6.8% (-52.0 to 137.3)
Brunei Darussalam	139.1 (80.3 to 211.6)	142.3 (82.0 to 216.1)	2.3% (-48.1 to 101.4)	548.7 (347.6 to 829.1)	573.9 (364.5 to 866.2)	4.6% (-43.0 to 91.8)	54.9 (29.5 to 91.5)	57.2 (30.1 to 94.6)	4.2% (-53.0 to 131.7)
El Salvador	133.0 (74.5 to 206.8)	138.7 (77.8 to 215.2)	4.3% (-48.9 to 112.8)	496.8 (302.1 to 749.5)	537.2 (327.5 to 808.0)	8.1% (-42.6 to 103.8)	48.9 (25.5 to 82.2)	53.5 (28.1 to 90.2)	9.5% (-51.6 to 147.7)
Russian Federation	171.2 (111.9 to 246.3)	167.0 (109.1 to 240.4)	-2.5% (-43.9 to 69.5)	629.3 (442.3 to 858.6)	606.1 (425.6 to 828.2)	-3.7% (-39.5 to 53.4)	62.7 (36.6 to 98.0)	60.4 (35.2 to 94.6)	-3.6% (-51.6 to 92.3)
Mexico	139.3 (88.5 to 199.8)	143.0 (90.7 to 205.1)	2.7% (-41.9 to 81.6)	514.6 (360.1 to 704.9)	539.2 (378.2 to 737.0)	4.8% (-34.4 to 67.4)	51.3 (30.2 to 79.7)	53.7 (31.7 to 84.0)	4.8% (-46.9 to 107.0)
Kyrgyzstan	172.5 (99.8 to 270.9)	171.7 (99.2 to 269.9)	-0.4% (-50.5 to 100.4)	638.4 (394.9 to 981.3)	639.2 (395.4 to 981.8)	0.1% (-47.0 to 89.1)	63.7 (33.2 to 107.2)	63.8 (33.2 to 108.9)	0.2% (-56.1 to 129.2)
Nicaragua	130.9 (73.3 to 203.9)	137.1 (76.8 to 212.9)	4.7% (-48.7 to 113.8)	484.7 (294.5 to 731.9)	526.2 (320.8 to 791.5)	8.5% (-42.5 to 104.7)	47.9 (25.1 to 79.6)	52.4 (27.1 to 87.7)	9.3% (-51.6 to 147.1)

Serbia	175.9 (101.9 to 276.0)	177.8 (103.1 to 278.6)	1.1% (-49.6 to 102.7)	660.7 (409.1 to 1014.5)	673.2 (417.1 to 1033.4)	1.9% (-46.0 to 92.2)	65.8 (34.9 to 110.3)	67.0 (34.6 to 111.3)	1.9% (-54.8 to 129.8)
Haiti	141.7 (82.4 to 221.0)	145.4 (84.8 to 226.4)	2.6% (-48.4 to 104.3)	525.1 (322.2 to 799.8)	549.0 (337.5 to 835.2)	4.6% (-44.6 to 97.3)	51.5 (26.4 to 87.0)	54.1 (28.2 to 91.1)	5.0% (-54.1 to 139.9)
Chile	148.5 (86.0 to 231.0)	148.5 (85.7 to 231.4)	-0.0% (-50.0 to 100.1)	580.5 (361.7 to 891.8)	585.6 (364.6 to 899.5)	0.9% (-46.4 to 89.7)	57.7 (30.2 to 97.5)	58.0 (31.2 to 97.2)	0.5% (-55.1 to 124.8)
Tonga	140.4 (79.6 to 218.1)	143.1 (81.3 to 221.9)	1.9% (-49.6 to 106.0)	504.3 (305.9 to 769.0)	519.4 (315.3 to 791.7)	3.0% (-45.9 to 96.2)	50.2 (25.9 to 84.4)	51.6 (27.3 to 88.3)	2.7% (-55.0 to 134.3)
Mozambique	246.1 (143.4 to 382.8)	249.6 (145.6 to 388.0)	1.4% (-48.9 to 101.4)	917.2 (578.0 to 1366.2)	946.2 (597.0 to 1406.8)	3.2% (-43.4 to 88.1)	89.7 (49.0 to 150.2)	92.7 (50.3 to 154.7)	3.3% (-52.8 to 126.5)
Guam	151.0 (86.2 to 232.5)	147.9 (84.3 to 228.6)	-2.1% (-51.2 to 96.6)	569.1 (345.6 to 863.5)	547.6 (332.5 to 832.9)	-3.8% (-49.3 to 82.8)	56.6 (30.2 to 96.0)	54.7 (28.8 to 93.0)	-3.4% (-57.3 to 118.4)
Brazil	137.9 (86.5 to 197.8)	144.7 (92.4 to 211.5)	5.0% (-41.2 to 87.2)	506.1 (352.6 to 689.5)	545.9 (380.4 to 748.4)	7.9% (-32.7 to 72.9)	50.0 (29.7 to 77.8)	53.9 (32.2 to 84.2)	7.8% (-45.0 to 111.2)
United States Virgin Islands	142.5 (83.1 to 221.9)	144.9 (84.6 to 225.5)	1.7% (-48.8 to 102.1)	528.3 (324.2 to 804.8)	544.4 (334.6 to 828.6)	3.1% (-45.4 to 94.5)	52.4 (27.2 to 88.3)	54.1 (28.4 to 91.2)	3.2% (-54.6 to 134.4)
Japan	138.6 (87.8 to 197.8)	138.9 (87.4 to 198.6)	0.2% (-43.4 to 77.5)	536.1 (374.1 to 726.9)	543.6 (379.7 to 735.6)	1.4% (-36.2 to 61.2)	53.5 (31.7 to 84.2)	54.3 (32.1 to 85.6)	1.5% (-48.8 to 101.4)
Saint Kitts and Nevis	144.7 (84.3 to 225.2)	153.3 (89.7 to 238.1)	5.9% (-46.6 to 110.1)	545.0 (334.9 to 829.1)	598.1 (368.9 to 908.1)	9.8% (-41.7 to 106.5)	54.0 (27.9 to 91.0)	59.5 (30.7 to 100.6)	10.3% (-51.8 to 152.7)
Kiribati	148.2 (84.3 to 229.2)	144.2 (81.9 to 223.5)	-2.7% (-51.7 to 96.2)	548.7 (333.3 to 835.2)	525.4 (318.9 to 800.6)	-4.3% (-49.7 to 82.2)	54.4 (28.7 to 90.6)	52.3 (27.3 to 88.7)	-3.8% (-57.4 to 117.6)
Greece	205.1 (120.6 to 314.9)	203.8 (119.8 to 313.2)	-0.6% (-49.2 to 94.6)	855.5 (547.4 to 1273.5)	844.8 (540.2 to 1258.0)	-1.3% (-45.3 to 78.3)	84.8 (46.2 to 143.6)	83.5 (45.4 to 141.3)	-1.6% (-55.5 to 117.7)

Jamaica	145.5 (84.9 to 226.6)	151.5 (88.4 to 235.3)	4.1% (-47.6 to 106.7)	549.0 (337.4 to 835.3)	590.0 (363.7 to 895.5)	7.5% (-42.9 to 102.2)	54.5 (28.4 to 91.7)	58.7 (31.3 to 99.3)	7.6% (-52.3 to 142.8)
Fiji	144.0 (81.8 to 223.1)	144.8 (82.3 to 224.2)	0.5% (-50.2 to 102.7)	525.2 (318.7 to 800.2)	529.5 (321.3 to 806.4)	0.8% (-47.0 to 91.9)	52.4 (27.2 to 87.4)	52.7 (27.6 to 88.3)	0.7% (-55.4 to 127.4)
Nigeria	213.0 (137.1 to 300.9)	210.4 (135.4 to 297.3)	-1.2% (-43.0 to 71.2)	774.2 (543.6 to 1059.6)	759.2 (532.7 to 1039.9)	-1.9% (-38.6 to 56.5)	76.1 (45.5 to 117.8)	75.1 (44.4 to 116.8)	-1.3% (-49.6 to 93.4)
Libya	186.7 (109.1 to 286.9)	194.5 (113.8 to 298.6)	4.2% (-47.0 to 104.7)	729.6 (449.4 to 1112.9)	791.2 (488.1 to 1203.0)	8.4% (-42.4 to 104.1)	72.3 (38.5 to 122.6)	78.4 (41.2 to 130.6)	8.5% (-51.7 to 143.5)
Armenia	176.2 (102.0 to 276.3)	173.2 (100.1 to 271.9)	-1.7% (-51.1 to 97.7)	663.2 (410.6 to 1018.8)	648.8 (401.5 to 995.9)	-2.2% (-48.2 to 84.7)	66.1 (34.6 to 113.6)	64.7 (34.1 to 112.8)	-2.2% (-57.5 to 125.4)
Cambodia	142.4 (80.8 to 221.0)	146.5 (83.3 to 226.7)	2.9% (-49.0 to 107.6)	513.6 (311.5 to 783.5)	539.1 (327.3 to 820.6)	5.0% (-44.9 to 99.8)	50.6 (26.4 to 85.4)	53.9 (27.9 to 90.3)	6.5% (-53.2 to 142.3)
Mauritius	146.8 (83.5 to 227.1)	154.4 (88.3 to 238.2)	5.2% (-47.7 to 111.2)	542.3 (329.2 to 825.3)	580.7 (353.2 to 882.7)	7.1% (-43.6 to 103.4)	53.9 (28.1 to 92.6)	57.4 (30.3 to 95.0)	6.5% (-52.9 to 140.5)
Mongolia	172.0 (99.5 to 270.2)	170.0 (98.0 to 267.3)	-1.2% (-50.9 to 99.2)	636.1 (393.4 to 977.8)	630.4 (389.8 to 968.3)	-0.9% (-47.6 to 87.3)	63.5 (33.7 to 107.9)	62.7 (33.7 to 107.1)	-1.3% (-56.1 to 122.2)
United Kingdom	209.9 (132.3 to 305.0)	203.1 (128.8 to 295.6)	-3.2% (-46.0 to 73.3)	861.8 (598.5 to 1185.4)	813.1 (564.1 to 1127.0)	-5.7% (-41.7 to 52.8)	85.2 (50.0 to 132.7)	80.3 (47.6 to 127.3)	-5.7% (-52.5 to 87.2)
Taiwan (Province of China)	132.3 (75.8 to 204.5)	140.9 (81.1 to 217.3)	6.5% (-46.7 to 112.8)	490.1 (298.9 to 749.9)	538.8 (329.4 to 822.5)	9.9% (-42.1 to 108.9)	49.0 (25.2 to 81.4)	54.0 (28.4 to 91.4)	10.2% (-51.4 to 149.6)
India	176.4 (112.6 to 253.8)	188.9 (120.8 to 270.6)	7.0% (-39.3 to 88.6)	647.8 (452.9 to 885.7)	710.5 (495.4 to 978.0)	9.7% (-31.6 to 76.1)	63.9 (37.8 to 98.4)	70.4 (42.0 to 111.0)	10.2% (-43.9 to 116.8)
Nepal	184.9 (106.0 to 285.6)	195.3 (112.3 to 300.7)	5.6% (-47.1 to 110.9)	689.8 (431.0 to 1038.1)	754.4 (472.8 to 1131.7)	9.4% (-40.8 to 101.8)	67.8 (35.8 to 114.4)	75.0 (40.2 to 126.6)	10.6% (-50.7 to 147.9)

Ecuador	143.5 (83.6 to 223.5)	147.0 (85.6 to 228.7)	2.5% (-48.5 to 103.7)	536.3 (329.3 to 816.4)	562.2 (345.9 to 854.2)	4.8% (-44.4 to 97.6)	53.5 (27.7 to 90.4)	55.7 (29.6 to 93.1)	4.2% (-53.8 to 134.7)
Cuba	155.4 (91.1 to 240.9)	150.7 (87.9 to 234.0)	-3.1% (-51.0 to 92.0)	609.2 (376.2 to 925.0)	586.4 (361.4 to 890.1)	-3.7% (-48.7 to 80.7)	60.2 (32.2 to 102.5)	58.4 (30.2 to 97.5)	-3.0% (-57.1 to 119.4)
North Macedonia	176.4 (102.2 to 276.6)	182.7 (105.9 to 286.0)	3.6% (-48.3 to 107.7)	663.6 (411.0 to 1019.1)	705.5 (437.7 to 1081.9)	6.3% (-43.6 to 100.4)	66.0 (34.6 to 112.5)	69.9 (37.4 to 117.6)	5.9% (-53.1 to 138.5)
Eswatini	214.6 (124.2 to 327.8)	219.8 (127.5 to 335.1)	2.4% (-48.0 to 101.6)	789.5 (486.0 to 1180.8)	830.4 (511.3 to 1239.9)	5.2% (-43.4 to 95.5)	78.4 (41.4 to 131.2)	82.2 (44.9 to 136.6)	4.8% (-52.5 to 131.1)
Micronesia (Federated States of)	137.2 (77.6 to 213.5)	146.3 (83.3 to 226.5)	6.7% (-47.3 to 115.6)	485.8 (294.4 to 741.9)	536.7 (325.9 to 817.2)	10.5% (-42.0 to 110.5)	48.1 (24.6 to 79.6)	53.4 (27.8 to 91.5)	11.1% (-51.5 to 154.7)
Sao Tome and Principe	209.8 (121.3 to 320.9)	217.2 (126.0 to 331.2)	3.5% (-47.5 to 104.0)	765.4 (470.1 to 1146.4)	809.0 (497.7 to 1209.7)	5.7% (-43.3 to 96.9)	76.0 (40.2 to 126.7)	80.5 (43.5 to 132.1)	5.9% (-51.9 to 132.9)
Marshall Islands	136.3 (77.0 to 212.0)	145.7 (82.9 to 225.7)	6.9% (-47.2 to 116.3)	482.9 (292.5 to 737.3)	533.3 (323.8 to 812.1)	10.4% (-42.1 to 110.5)	47.9 (24.0 to 82.0)	52.9 (26.6 to 88.0)	10.4% (-52.7 to 157.5)
France	207.6 (122.3 to 318.3)	203.4 (119.7 to 312.6)	-2.0% (-49.9 to 91.7)	873.3 (559.1 to 1299.5)	839.5 (536.4 to 1250.8)	-3.9% (-46.8 to 73.7)	86.4 (47.0 to 144.5)	83.4 (45.5 to 140.5)	-3.6% (-56.1 to 112.0)
Guatemala	128.5 (71.8 to 200.4)	138.6 (77.8 to 214.8)	7.8% (-47.3 to 120.2)	469.6 (285.1 to 709.9)	533.5 (325.3 to 802.9)	13.6% (-39.9 to 114.4)	46.3 (23.9 to 77.3)	52.7 (28.0 to 86.3)	13.9% (-49.0 to 153.9)
Zimbabwe	214.1 (124.0 to 326.9)	217.3 (125.9 to 331.5)	1.5% (-48.5 to 99.9)	787.7 (484.5 to 1178.7)	810.3 (498.8 to 1211.0)	2.9% (-44.7 to 91.4)	77.9 (41.6 to 128.2)	80.4 (43.4 to 134.0)	3.2% (-53.1 to 126.9)
Peru	144.0 (83.9 to 224.2)	148.5 (86.6 to 230.6)	3.1% (-48.1 to 104.9)	539.7 (331.5 to 821.4)	572.1 (352.3 to 869.0)	6.0% (-43.7 to 99.6)	53.5 (27.8 to 89.9)	56.8 (29.1 to 96.9)	6.2% (-53.7 to 144.0)
Cook Islands	144.4 (82.1 to 223.8)	149.3 (85.0 to 231.1)	3.4% (-48.7 to 108.3)	526.5 (319.6 to 802.1)	550.0 (334.2 to 837.9)	4.5% (-45.1 to 98.7)	52.4 (27.0 to 89.3)	54.8 (28.5 to 92.7)	4.5% (-54.5 to 139.7)

Bolivia (Plurinational State of)	141.4 (82.3 to 220.4)	147.1 (85.7 to 228.8)	4.0% (-47.7 to 106.9)	523.6 (321.2 to 797.6)	561.3 (345.3 to 853.1)	7.2% (-43.2 to 102.2)	52.0 (26.7 to 88.6)	55.5 (28.9 to 94.0)	6.8% (-53.5 to 145.2)
Indonesia	142.6 (91.5 to 208.2)	147.9 (94.8 to 215.6)	3.7% (-41.7 to 84.2)	509.0 (353.0 to 703.0)	538.2 (373.6 to 741.6)	5.8% (-34.6 to 71.0)	50.7 (29.6 to 79.3)	53.8 (31.4 to 83.8)	6.0% (-46.7 to 110.8)
Jordan	188.9 (110.4 to 290.3)	190.7 (111.5 to 292.9)	0.9% (-48.7 to 98.4)	751.2 (463.1 to 1144.2)	764.5 (471.5 to 1163.9)	1.8% (-45.9 to 91.5)	74.9 (39.0 to 126.6)	75.8 (39.4 to 126.2)	1.2% (-55.4 to 129.6)
Belize	141.5 (82.4 to 220.5)	148.0 (86.4 to 230.2)	4.6% (-47.4 to 107.8)	524.7 (321.9 to 799.1)	563.5 (346.7 to 856.9)	7.4% (-43.1 to 102.5)	52.0 (26.9 to 87.8)	56.1 (29.3 to 96.0)	7.8% (-52.9 to 146.9)
Croatia	178.2 (103.3 to 279.4)	180.3 (104.4 to 282.4)	1.1% (-49.6 to 102.9)	675.9 (418.8 to 1037.8)	691.1 (428.5 to 1060.4)	2.2% (-45.8 to 92.8)	67.3 (35.7 to 113.3)	68.9 (36.1 to 116.4)	2.3% (-54.7 to 131.2)
Netherlands	196.5 (115.6 to 302.1)	197.8 (113.9 to 306.5)	0.7% (-49.1 to 99.3)	828.5 (525.8 to 1230.4)	826.9 (519.7 to 1230.2)	-0.2% (-45.2 to 81.8)	82.3 (43.6 to 136.9)	82.1 (44.2 to 136.3)	-0.3% (-54.9 to 120.4)
Qatar	187.4 (108.8 to 288.1)	190.7 (110.1 to 293.2)	1.8% (-48.6 to 101.6)	764.9 (472.1 to 1158.4)	804.2 (497.4 to 1213.3)	5.1% (-43.8 to 96.6)	76.3 (40.3 to 128.0)	79.9 (42.3 to 133.2)	4.7% (-53.3 to 134.2)
Republic of Korea	151.1 (87.7 to 230.8)	153.0 (88.3 to 233.9)	1.2% (-48.7 to 99.8)	603.5 (377.2 to 914.9)	626.8 (392.7 to 948.1)	3.9% (-44.0 to 92.7)	60.3 (31.9 to 100.3)	62.8 (34.3 to 103.4)	4.1% (-52.5 to 128.2)
San Marino	210.2 (123.7 to 322.2)	205.6 (121.0 to 315.5)	-2.2% (-50.0 to 91.2)	897.8 (575.9 to 1334.0)	860.1 (550.5 to 1280.1)	-4.2% (-46.8 to 72.7)	89.2 (47.6 to 148.2)	85.4 (45.8 to 143.8)	-4.3% (-56.9 to 112.5)
Italy	200.6 (127.6 to 285.7)	196.6 (124.7 to 278.7)	-2.0% (-44.2 to 72.0)	810.4 (575.7 to 1104.4)	777.9 (544.7 to 1055.9)	-4.0% (-39.4 to 52.1)	80.3 (47.0 to 126.0)	77.1 (45.4 to 118.0)	-4.0% (-51.3 to 88.9)
Maldives	142.4 (80.8 to 220.9)	147.5 (84.0 to 227.3)	3.6% (-48.6 to 108.5)	515.0 (312.4 to 785.3)	551.5 (334.8 to 837.0)	7.1% (-43.7 to 103.6)	51.1 (26.8 to 86.7)	54.8 (28.7 to 90.1)	7.4% (-52.3 to 141.3)
Bermuda	153.0 (89.3 to 237.8)	147.6 (86.1 to 229.7)	-3.5% (-51.4 to 91.5)	600.6 (370.5 to 911.1)	563.6 (346.8 to 856.6)	-6.2% (-50.1 to 76.5)	60.0 (30.9 to 101.7)	56.2 (29.4 to 95.2)	-6.2% (-59.0 to 114.2)

Azerbaijan	178.4 (103.3 to 279.6)	174.5 (100.9 to 273.8)	-2.2% (-51.3 to 96.6)	675.7 (418.6 to 1038.4)	657.9 (407.3 to 1009.7)	-2.6% (-48.4 to 83.7)	67.2 (36.3 to 110.8)	65.7 (35.1 to 111.7)	-2.3% (-55.9 to 116.8)
Ukraine	172.3 (112.7 to 247.8)	167.8 (109.5 to 241.5)	-2.6% (-43.9 to 69.2)	634.3 (445.9 to 865.2)	612.2 (430.1 to 836.0)	-3.5% (-39.3 to 53.6)	63.3 (35.8 to 100.3)	61.1 (34.9 to 95.5)	-3.6% (-52.7 to 96.3)
Vanuatu	141.5 (80.3 to 219.6)	144.8 (82.3 to 224.3)	2.3% (-49.3 to 106.6)	511.4 (310.2 to 779.7)	529.5 (321.5 to 806.7)	3.6% (-45.6 to 97.2)	50.7 (26.3 to 85.7)	52.6 (28.2 to 89.3)	3.6% (-54.2 to 134.1)
Türkiye	#N/A	#N/A	#N/A	#N/A	#N/A	#N/A	69.7 (37.2 to 117.5)	73.5 (38.5 to 124.3)	5.4% (-53.2 to 137.7)
Malaysia	145.1 (82.5 to 224.8)	151.3 (86.3 to 233.5)	4.2% (-48.2 to 109.7)	530.5 (322.0 to 808.1)	566.5 (344.2 to 861.1)	6.8% (-43.8 to 103.0)	52.8 (27.4 to 90.1)	56.4 (29.1 to 93.8)	6.9% (-53.2 to 143.7)
Kuwait	193.0 (112.1 to 296.5)	193.3 (112.5 to 297.0)	0.2% (-49.2 to 97.6)	790.6 (488.0 to 1201.7)	791.3 (488.1 to 1202.3)	0.1% (-46.7 to 88.0)	78.7 (41.6 to 134.3)	78.7 (41.4 to 131.9)	0.0% (-55.8 to 125.9)
Panama	136.2 (76.4 to 211.5)	137.5 (77.0 to 213.5)	0.9% (-50.5 to 105.9)	519.3 (316.4 to 781.8)	530.1 (323.3 to 797.1)	2.1% (-45.7 to 92.0)	51.7 (26.7 to 85.8)	52.8 (27.7 to 88.4)	2.1% (-54.8 to 130.5)
Togo	213.6 (123.6 to 326.3)	215.1 (124.7 to 328.5)	0.7% (-48.9 to 98.5)	788.6 (485.1 to 1179.5)	801.6 (493.0 to 1198.6)	1.6% (-45.4 to 89.2)	77.7 (42.1 to 130.4)	79.3 (42.6 to 130.2)	2.0% (-53.6 to 123.7)
Cameroon	215.4 (124.7 to 328.9)	215.3 (124.7 to 328.7)	-0.0% (-49.3 to 96.9)	798.2 (491.4 to 1193.1)	799.7 (492.0 to 1195.8)	0.2% (-46.1 to 86.4)	78.5 (41.4 to 130.8)	79.2 (41.7 to 132.9)	0.8% (-55.1 to 126.5)
Uruguay	144.8 (83.7 to 225.5)	149.2 (86.3 to 232.1)	3.0% (-48.5 to 105.9)	556.0 (345.8 to 854.7)	586.7 (365.5 to 901.2)	5.5% (-43.9 to 98.5)	55.4 (29.7 to 95.4)	58.1 (30.9 to 98.8)	4.7% (-53.7 to 136.6)
Barbados	149.3 (87.1 to 232.1)	150.0 (87.6 to 233.0)	0.5% (-49.3 to 99.2)	574.6 (353.8 to 872.9)	578.7 (356.5 to 879.0)	0.7% (-46.4 to 89.4)	57.2 (29.3 to 97.3)	57.6 (30.1 to 96.4)	0.7% (-55.9 to 129.9)
Guyana	145.9 (85.1 to 227.2)	150.0 (87.5 to 233.2)	2.8% (-48.2 to 104.1)	551.3 (338.9 to 838.7)	580.7 (357.7 to 881.6)	5.3% (-44.0 to 98.2)	54.3 (28.1 to 92.4)	57.3 (29.8 to 97.0)	5.5% (-54.1 to 141.9)

Australia	209.0 (122.9 to 315.6)	205.9 (118.7 to 317.6)	-1.5% (-49.9 to 93.7)	839.8 (531.9 to 1246.8)	824.7 (517.7 to 1238.1)	-1.8% (-46.2 to 79.6)	82.7 (44.1 to 136.4)	81.4 (43.1 to 138.6)	-1.5% (-55.9 to 120.3)
Morocco	190.1 (111.1 to 292.0)	192.1 (112.3 to 295.0)	1.1% (-48.6 to 98.6)	755.9 (465.8 to 1151.2)	773.8 (477.1 to 1177.3)	2.4% (-45.6 to 92.6)	75.1 (40.3 to 125.7)	76.8 (40.4 to 127.7)	2.3% (-54.0 to 127.8)
United Republic of Tanzania	248.8 (145.2 to 386.9)	250.8 (146.5 to 389.7)	0.8% (-49.2 to 99.9)	940.4 (593.2 to 1398.5)	957.2 (604.2 to 1421.9)	1.8% (-44.1 to 85.3)	91.8 (49.3 to 152.7)	94.7 (51.6 to 158.5)	3.1% (-53.1 to 126.8)
Belarus	177.1 (102.6 to 277.7)	173.1 (100.1 to 271.9)	-2.3% (-51.4 to 96.5)	667.6 (413.5 to 1025.8)	647.4 (400.7 to 993.9)	-3.0% (-48.6 to 83.1)	66.6 (35.3 to 113.7)	64.5 (34.5 to 107.7)	-3.2% (-56.8 to 116.4)
Madagascar	246.9 (144.1 to 383.8)	250.3 (146.1 to 388.7)	1.4% (-48.9 to 101.0)	933.3 (588.7 to 1386.5)	955.5 (603.2 to 1418.5)	2.4% (-43.7 to 86.3)	91.6 (49.3 to 152.8)	94.3 (51.0 to 157.7)	3.0% (-53.3 to 126.9)
Eritrea	244.3 (142.6 to 379.8)	249.6 (145.8 to 387.6)	2.2% (-48.5 to 102.6)	923.4 (582.7 to 1371.1)	963.7 (608.9 to 1427.7)	4.4% (-42.6 to 89.6)	90.4 (49.0 to 152.6)	95.7 (52.7 to 160.1)	5.9% (-51.7 to 132.1)
Solomon Islands	140.6 (79.7 to 218.3)	143.2 (81.3 to 222.1)	1.9% (-49.6 to 105.9)	505.3 (306.5 to 770.7)	520.9 (316.1 to 793.7)	3.1% (-45.9 to 96.3)	50.3 (26.2 to 84.8)	52.0 (26.5 to 88.4)	3.5% (-54.9 to 138.0)
Czechia	172.9 (100.3 to 271.5)	171.9 (99.4 to 270.0)	-0.6% (-50.5 to 99.8)	638.7 (395.1 to 981.6)	637.9 (394.6 to 979.7)	-0.1% (-47.2 to 88.7)	63.5 (33.3 to 108.1)	63.4 (33.9 to 106.4)	-0.3% (-55.7 to 124.3)
Slovenia	178.2 (103.2 to 279.3)	174.7 (101.0 to 274.2)	-2.0% (-51.2 to 97.0)	676.5 (419.2 to 1038.9)	657.7 (407.2 to 1009.5)	-2.8% (-48.5 to 83.4)	67.4 (35.5 to 114.0)	65.7 (33.9 to 110.9)	-2.5% (-57.2 to 122.4)
New Zealand	221.2 (141.5 to 315.3)	202.8 (130.2 to 292.6)	-8.3% (-47.8 to 61.2)	863.2 (616.1 to 1172.3)	787.2 (557.3 to 1067.0)	-8.8% (-42.0 to 43.4)	85.0 (49.2 to 133.8)	77.5 (45.7 to 121.2)	-8.8% (-54.3 to 81.9)
Estonia	176.9 (102.5 to 277.4)	172.6 (99.8 to 271.1)	-2.4% (-51.4 to 96.1)	666.7 (412.9 to 1023.4)	643.3 (398.1 to 987.9)	-3.5% (-48.9 to 82.2)	66.4 (35.3 to 109.7)	64.0 (33.9 to 107.8)	-3.7% (-56.8 to 115.1)
Venezuela (Bolivarian Republic of)	134.6 (75.4 to 209.2)	133.3 (74.9 to 207.1)	-1.0% (-51.4 to 102.0)	509.1 (310.0 to 766.9)	496.0 (301.6 to 748.5)	-2.6% (-48.4 to 83.9)	50.6 (26.8 to 84.7)	49.5 (25.1 to 83.2)	-2.2% (-57.1 to 123.1)

Algeria	188.3 (109.9 to 289.4)	187.7 (109.3 to 288.6)	-0.3% (-49.4 to 96.5)	745.0 (458.9 to 1134.8)	744.9 (458.8 to 1134.2)	-0.0% (-46.9 to 88.3)	73.9 (39.2 to 124.7)	74.4 (39.1 to 125.3)	0.6% (-55.3 to 126.8)
Georgia	177.2 (102.7 to 277.9)	173.0 (100.0 to 271.7)	-2.3% (-51.4 to 96.4)	668.3 (413.9 to 1026.8)	649.3 (401.9 to 996.3)	-2.8% (-48.5 to 83.4)	66.7 (35.1 to 111.3)	64.6 (34.5 to 107.0)	-3.2% (-56.5 to 115.0)
Mauritania	215.6 (124.9 to 329.1)	215.5 (124.8 to 328.9)	-0.1% (-49.3 to 96.8)	800.3 (492.4 to 1196.5)	797.2 (490.5 to 1192.3)	-0.4% (-46.5 to 85.4)	79.5 (41.1 to 132.6)	79.0 (42.8 to 133.5)	-0.6% (-55.7 to 122.8)
Chad	213.5 (123.5 to 326.1)	211.9 (122.6 to 323.7)	-0.8% (-49.7 to 95.7)	784.6 (482.6 to 1173.9)	774.9 (476.4 to 1160.2)	-1.2% (-47.0 to 84.0)	77.5 (41.1 to 130.7)	76.8 (40.6 to 126.6)	-0.9% (-55.6 to 121.2)
Angola	214.2 (124.0 to 327.1)	211.8 (122.5 to 323.8)	-1.1% (-49.8 to 95.1)	793.5 (487.9 to 1187.0)	775.8 (476.9 to 1161.2)	-2.2% (-47.5 to 82.1)	77.9 (41.7 to 129.9)	76.7 (40.9 to 127.4)	-1.6% (-55.6 to 117.8)
Sierra Leone	220.6 (127.7 to 336.4)	221.4 (128.4 to 337.2)	0.4% (-49.0 to 97.4)	831.9 (512.8 to 1241.4)	835.3 (514.5 to 1247.1)	0.4% (-45.9 to 86.4)	82.1 (44.4 to 137.0)	82.5 (43.7 to 136.6)	0.4% (-54.6 to 122.3)
Pakistan	176.2 (113.4 to 252.4)	182.0 (117.2 to 260.5)	3.3% (-41.0 to 80.6)	638.6 (450.8 to 874.7)	673.2 (476.0 to 919.6)	5.4% (-33.6 to 67.3)	63.2 (36.8 to 99.0)	66.6 (39.1 to 104.6)	5.4% (-47.1 to 110.2)
Uganda	252.8 (148.4 to 386.3)	250.5 (147.1 to 382.8)	-0.9% (-49.3 to 93.5)	962.4 (607.8 to 1435.9)	951.5 (600.6 to 1419.9)	-1.1% (-45.8 to 80.4)	94.5 (51.3 to 160.0)	94.3 (50.2 to 157.6)	-0.2% (-55.1 to 121.7)
Norway	206.7 (131.5 to 295.7)	201.0 (127.7 to 287.9)	-2.8% (-44.9 to 71.6)	836.9 (591.1 to 1132.2)	800.0 (564.3 to 1083.4)	-4.4% (-39.4 to 50.8)	83.0 (49.0 to 128.6)	79.4 (46.9 to 122.2)	-4.3% (-51.2 to 87.4)
Ireland	192.2 (113.0 to 297.4)	192.6 (113.0 to 298.4)	0.2% (-49.1 to 97.6)	768.7 (487.4 to 1162.3)	779.4 (494.8 to 1176.5)	1.4% (-44.7 to 86.0)	76.1 (40.3 to 129.2)	76.9 (40.4 to 131.2)	1.0% (-55.5 to 129.6)
Andorra	207.4 (122.0 to 318.1)	204.6 (120.2 to 314.3)	-1.3% (-49.6 to 93.1)	883.0 (566.4 to 1312.0)	857.1 (548.9 to 1274.9)	-2.9% (-46.1 to 74.9)	87.7 (46.6 to 149.4)	84.8 (46.0 to 141.5)	-3.3% (-56.5 to 114.8)
United Arab Emirates	190.0 (110.3 to 292.3)	189.0 (110.2 to 290.4)	-0.6% (-49.6 to 96.3)	779.4 (480.9 to 1182.2)	753.9 (464.7 to 1147.8)	-3.3% (-48.5 to 81.9)	77.5 (41.3 to 129.8)	74.9 (40.3 to 125.6)	-3.3% (-56.5 to 114.9)

Tajikistan	172.5 (99.9 to 270.9)	173.5 (100.3 to 272.4)	0.6% (-50.0 to 102.3)	638.6 (395.0 to 982.0)	649.8 (402.2 to 997.8)	1.8% (-46.1 to 92.2)	63.8 (33.0 to 107.0)	64.6 (34.8 to 108.2)	1.3% (-54.8 to 127.0)
Bahrain	192.3 (111.7 to 295.5)	194.2 (113.2 to 298.4)	1.0% (-48.8 to 99.1)	787.2 (485.9 to 1196.5)	806.0 (497.7 to 1222.1)	2.4% (-45.4 to 92.1)	78.1 (42.0 to 130.8)	80.2 (41.8 to 134.0)	2.7% (-54.0 to 129.9)
Bhutan	186.4 (107.2 to 287.6)	195.4 (112.3 to 301.2)	4.9% (-47.4 to 109.0)	704.6 (442.0 to 1059.8)	760.9 (477.7 to 1140.7)	8.0% (-41.3 to 98.8)	70.0 (36.4 to 117.1)	75.9 (42.0 to 128.3)	8.6% (-51.1 to 141.0)
Belgium	194.6 (111.4 to 302.7)	190.4 (108.9 to 296.6)	-2.1% (-51.4 to 97.2)	816.9 (505.7 to 1229.7)	783.0 (483.8 to 1179.5)	-4.2% (-48.6 to 78.7)	80.9 (43.0 to 137.7)	77.3 (41.2 to 129.7)	-4.4% (-57.4 to 114.2)
Congo	216.4 (125.4 to 330.1)	216.9 (125.7 to 330.8)	0.2% (-49.1 to 97.2)	803.5 (494.3 to 1201.7)	807.4 (496.8 to 1207.0)	0.5% (-46.0 to 87.0)	79.2 (41.2 to 132.7)	79.7 (42.0 to 134.1)	0.7% (-55.5 to 127.5)
Paraguay	144.3 (82.4 to 228.5)	149.9 (85.8 to 236.7)	3.9% (-49.0 to 111.7)	535.5 (327.6 to 808.9)	571.1 (350.1 to 861.2)	6.6% (-43.2 to 100.4)	53.1 (27.7 to 88.6)	56.4 (29.3 to 97.0)	6.1% (-53.6 to 142.6)
Dominican Republic	146.6 (85.5 to 228.3)	149.3 (87.1 to 232.1)	1.8% (-48.7 to 102.2)	554.9 (341.2 to 844.2)	574.4 (353.7 to 872.7)	3.5% (-45.0 to 94.9)	55.0 (28.3 to 94.1)	57.1 (29.9 to 96.6)	3.7% (-54.8 to 137.7)
Samoa	139.4 (79.0 to 216.4)	141.9 (80.6 to 220.2)	1.8% (-49.6 to 105.8)	501.4 (304.1 to 764.2)	513.4 (311.4 to 782.5)	2.4% (-46.3 to 95.0)	50.0 (25.7 to 84.8)	50.9 (25.9 to 86.8)	1.7% (-56.1 to 135.9)
South Africa	216.5 (139.4 to 305.6)	215.9 (138.9 to 305.2)	-0.3% (-42.4 to 72.9)	797.8 (560.7 to 1090.2)	800.8 (563.0 to 1092.9)	0.4% (-36.9 to 59.7)	78.9 (46.8 to 122.5)	79.2 (46.7 to 122.5)	0.4% (-48.8 to 96.9)
Finland	224.4 (130.6 to 348.7)	223.0 (129.1 to 349.3)	-0.7% (-50.3 to 98.6)	950.2 (602.6 to 1417.5)	933.1 (582.3 to 1404.5)	-1.8% (-46.5 to 80.3)	94.3 (51.8 to 159.0)	92.2 (49.0 to 156.0)	-2.2% (-55.9 to 117.3)
Saudi Arabia	198.7 (113.8 to 310.8)	202.9 (117.6 to 314.0)	2.1% (-49.0 to 104.4)	790.8 (489.2 to 1202.0)	841.8 (531.7 to 1254.2)	6.4% (-42.4 to 96.5)	78.6 (41.3 to 131.8)	83.8 (44.9 to 139.0)	6.6% (-52.1 to 137.1)
Central African Republic	217.8 (126.2 to 332.3)	217.4 (126.0 to 331.6)	-0.2% (-49.3 to 96.4)	815.8 (502.3 to 1218.7)	810.2 (498.7 to 1210.9)	-0.7% (-46.6 to 84.7)	80.0 (43.1 to 132.2)	80.0 (42.0 to 132.7)	0.0% (-54.8 to 121.6)

Luxembourg	209.8 (123.0 to 321.8)	205.8 (120.8 to 316.0)	-1.9% (-49.9 to 92.2)	906.7 (582.9 to 1344.9)	870.4 (558.1 to 1293.6)	-4.0% (-46.6 to 72.7)	89.8 (48.6 to 151.5)	86.3 (47.1 to 144.9)	-3.9% (-56.5 to 111.7)
Malta	200.1 (117.7 to 307.8)	205.6 (120.7 to 315.8)	2.8% (-47.5 to 101.4)	810.7 (516.9 to 1209.6)	868.2 (556.6 to 1290.6)	7.1% (-40.8 to 93.5)	80.5 (43.4 to 136.8)	85.7 (46.0 to 142.8)	6.4% (-52.1 to 136.3)
Niue	139.2 (78.9 to 216.1)	144.2 (82.0 to 223.5)	3.6% (-48.7 to 109.3)	501.1 (304.0 to 763.9)	527.2 (320.2 to 802.9)	5.2% (-44.7 to 100.2)	50.0 (25.8 to 86.2)	52.4 (27.2 to 87.7)	4.8% (-54.3 to 140.1)
Gambia	216.3 (125.2 to 330.2)	218.6 (126.7 to 333.3)	1.0% (-48.7 to 98.9)	805.7 (495.9 to 1203.9)	817.1 (503.1 to 1221.0)	1.4% (-45.5 to 88.6)	79.7 (42.5 to 131.9)	80.8 (43.2 to 133.6)	1.3% (-54.1 to 123.3)
Myanmar	146.2 (83.1 to 226.4)	148.9 (84.8 to 230.3)	1.9% (-49.4 to 105.1)	534.9 (324.8 to 814.9)	550.1 (334.2 to 837.4)	2.8% (-45.9 to 95.6)	52.9 (27.0 to 88.9)	54.8 (29.0 to 93.3)	3.6% (-54.6 to 136.3)
Greenland	222.2 (126.1 to 344.9)	213.9 (122.0 to 332.5)	-3.7% (-52.3 to 94.4)	912.1 (561.5 to 1389.8)	834.7 (512.4 to 1274.4)	-8.5% (-51.5 to 72.9)	89.7 (48.2 to 148.5)	82.1 (44.3 to 138.9)	-8.4% (-58.6 to 102.9)
Slovakia	172.5 (99.9 to 270.9)	177.1 (102.5 to 277.7)	2.7% (-48.9 to 106.2)	638.8 (395.2 to 981.6)	671.4 (416.0 to 1030.5)	5.1% (-44.3 to 98.4)	63.8 (34.1 to 107.7)	66.9 (35.0 to 113.6)	4.9% (-53.5 to 137.2)
Bangladesh	186.5 (106.9 to 288.0)	193.1 (110.8 to 297.5)	3.5% (-48.2 to 106.7)	698.6 (436.6 to 1050.9)	739.9 (463.2 to 1110.5)	5.9% (-42.6 to 95.5)	69.2 (36.0 to 115.3)	73.4 (38.7 to 124.5)	6.1% (-53.0 to 140.0)
Uzbekistan	173.0 (100.1 to 271.6)	175.4 (101.5 to 275.2)	1.4% (-49.5 to 103.8)	641.9 (397.1 to 986.7)	661.3 (409.5 to 1015.3)	3.0% (-45.5 to 94.5)	63.7 (33.8 to 106.5)	65.8 (34.6 to 108.4)	3.3% (-53.7 to 130.3)
Monaco	212.3 (124.5 to 325.3)	206.1 (121.3 to 316.3)	-2.9% (-50.4 to 90.0)	931.4 (599.9 to 1380.2)	863.6 (552.7 to 1285.1)	-7.3% (-48.4 to 66.9)	92.5 (49.9 to 150.0)	85.6 (46.2 to 140.5)	-7.4% (-57.3 to 101.0)
Thailand	149.3 (85.1 to 230.7)	153.0 (87.3 to 236.1)	2.5% (-48.9 to 105.6)	554.0 (336.5 to 842.7)	574.4 (349.2 to 873.3)	3.7% (-45.4 to 96.9)	55.1 (28.4 to 94.7)	57.5 (30.5 to 96.1)	4.3% (-54.1 to 137.0)
Equatorial Guinea	215.9 (125.0 to 329.6)	212.5 (123.5 to 325.8)	-1.6% (-50.0 to 94.0)	799.7 (492.2 to 1195.5)	800.2 (491.3 to 1197.3)	0.1% (-46.3 to 86.4)	78.2 (41.3 to 131.0)	79.3 (42.5 to 133.8)	1.4% (-54.7 to 126.6)

Djibouti	248.2 (145.1 to 385.3)	245.5 (143.2 to 381.5)	-1.1% (-50.1 to 96.2)	954.3 (602.8 to 1413.9)	954.2 (603.2 to 1410.4)	-0.0% (-44.8 to 81.3)	94.9 (51.6 to 157.7)	95.0 (51.9 to 157.0)	0.1% (-54.1 to 118.0)
Cabo Verde	216.4 (125.3 to 330.2)	224.4 (130.3 to 341.6)	3.7% (-47.3 to 103.9)	807.3 (496.7 to 1206.5)	860.3 (530.0 to 1283.2)	6.6% (-42.6 to 98.0)	80.4 (42.8 to 134.6)	85.6 (46.0 to 142.4)	6.6% (-52.0 to 136.1)
Iceland	205.0 (120.5 to 314.8)	203.2 (119.3 to 312.4)	-0.9% (-49.4 to 94.3)	854.7 (546.9 to 1272.2)	844.1 (540.0 to 1256.3)	-1.2% (-45.3 to 78.3)	84.8 (45.1 to 142.0)	83.6 (44.6 to 141.2)	-1.4% (-55.9 to 120.5)
Seychelles	147.9 (84.2 to 228.9)	146.7 (83.5 to 226.9)	-0.8% (-50.7 to 99.6)	544.8 (330.9 to 829.4)	541.0 (328.4 to 823.1)	-0.7% (-47.8 to 88.9)	54.2 (27.7 to 91.4)	53.8 (27.8 to 91.0)	-0.8% (-56.8 to 127.9)
Democratic People's Republic of Korea	139.3 (80.1 to 215.2)	136.7 (78.5 to 211.0)	-1.9% (-50.9 to 95.9)	522.6 (320.4 to 799.4)	512.9 (313.2 to 783.8)	-1.9% (-48.3 to 86.3)	52.3 (27.2 to 87.6)	51.3 (26.6 to 85.6)	-1.8% (-56.7 to 122.6)
Nauru	143.5 (81.5 to 222.5)	144.6 (82.2 to 224.0)	0.7% (-50.1 to 103.2)	521.7 (316.6 to 795.0)	528.0 (320.5 to 804.2)	1.2% (-46.8 to 92.6)	51.9 (26.7 to 87.5)	52.6 (27.2 to 89.4)	1.5% (-55.8 to 133.2)
Montenegro	176.8 (102.5 to 277.3)	179.5 (104.1 to 281.2)	1.5% (-49.4 to 103.5)	667.0 (413.2 to 1023.7)	684.0 (423.9 to 1049.5)	2.5% (-45.6 to 93.4)	66.7 (35.0 to 111.5)	67.8 (36.0 to 113.3)	1.6% (-54.6 to 127.3)
Iran (Islamic Republic of)	188.1 (119.5 to 270.6)	192.5 (122.0 to 276.8)	2.4% (-42.3 to 81.4)	713.1 (501.1 to 972.7)	743.7 (523.9 to 1010.8)	4.3% (-34.3 to 65.5)	70.6 (41.8 to 110.1)	73.7 (43.4 to 114.3)	4.3% (-47.0 to 105.3)
Papua New Guinea	143.1 (81.2 to 221.7)	144.6 (82.2 to 223.8)	1.1% (-49.9 to 103.8)	521.1 (316.2 to 793.8)	530.9 (322.2 to 808.1)	1.9% (-46.5 to 93.9)	51.5 (26.3 to 87.1)	52.7 (27.4 to 88.4)	2.3% (-55.3 to 133.8)
Honduras	129.5 (72.5 to 201.9)	137.7 (77.3 to 213.6)	6.3% (-48.0 to 117.1)	475.3 (288.6 to 717.9)	528.2 (321.9 to 795.1)	11.1% (-41.2 to 109.7)	47.1 (24.3 to 79.3)	52.3 (27.0 to 88.4)	10.9% (-51.5 to 154.1)
Hungary	172.7 (100.0 to 271.2)	179.2 (103.9 to 280.7)	3.8% (-48.3 to 108.3)	640.6 (396.3 to 984.2)	680.9 (422.0 to 1045.2)	6.3% (-43.7 to 100.6)	63.7 (33.6 to 105.8)	67.9 (34.5 to 113.5)	6.6% (-53.0 to 141.9)
Rwanda	247.0 (144.1 to 384.0)	252.9 (147.9 to 392.6)	2.4% (-48.3 to 103.0)	931.8 (587.6 to 1384.7)	974.2 (615.5 to 1445.3)	4.6% (-42.5 to 90.2)	92.1 (49.0 to 153.4)	96.8 (52.5 to 161.2)	5.1% (-52.4 to 131.8)

Tunisia	189.8 (110.9 to 291.6)	190.4 (111.1 to 292.5)	0.3% (-49.0 to 97.3)	756.4 (466.1 to 1151.6)	762.6 (470.0 to 1160.4)	0.8% (-46.4 to 89.7)	75.1 (40.6 to 124.8)	75.6 (39.8 to 126.1)	0.7% (-54.6 to 123.4)
Grenada	142.1 (82.7 to 221.2)	151.3 (88.3 to 234.6)	6.5% (-46.4 to 111.4)	528.5 (324.4 to 804.6)	590.1 (363.9 to 895.9)	11.7% (-40.8 to 110.3)	52.3 (27.2 to 88.6)	58.6 (30.8 to 97.6)	11.9% (-50.5 to 153.0)
Colombia	129.9 (73.0 to 201.6)	134.5 (75.5 to 208.3)	3.6% (-49.1 to 110.7)	493.1 (301.8 to 737.6)	524.4 (321.4 to 782.8)	6.3% (-43.0 to 98.4)	49.1 (26.1 to 83.0)	52.2 (28.2 to 88.1)	6.3% (-52.4 to 137.1)
Gabon	218.3 (126.4 to 332.9)	222.1 (128.7 to 338.4)	1.8% (-48.3 to 100.1)	816.3 (502.5 to 1219.6)	835.8 (515.1 to 1247.7)	2.4% (-44.9 to 90.2)	80.1 (42.1 to 132.3)	82.4 (44.5 to 137.3)	2.8% (-53.5 to 127.4)
Austria	212.0 (124.6 to 324.7)	207.2 (121.8 to 317.8)	-2.3% (-50.0 to 91.1)	922.1 (593.0 to 1367.8)	880.1 (564.5 to 1307.8)	-4.6% (-46.9 to 71.6)	91.0 (49.6 to 150.2)	87.2 (47.3 to 148.7)	-4.2% (-56.5 to 110.9)
Albania	176.5 (102.3 to 276.7)	185.2 (107.5 to 289.6)	4.9% (-47.6 to 110.2)	663.2 (410.7 to 1019.1)	720.5 (447.2 to 1104.5)	8.6% (-42.4 to 104.8)	65.9 (34.6 to 110.3)	71.7 (38.2 to 120.7)	8.9% (-51.5 to 144.1)
Lithuania	180.0 (104.5 to 282.0)	180.9 (104.7 to 283.3)	0.5% (-49.9 to 101.4)	685.3 (424.8 to 1051.9)	695.7 (431.5 to 1067.2)	1.5% (-46.1 to 91.3)	68.3 (35.2 to 113.6)	69.3 (37.0 to 114.7)	1.4% (-54.7 to 126.7)
Saint Lucia	144.5 (84.2 to 225.0)	151.9 (88.7 to 235.7)	5.1% (-47.0 to 108.7)	542.6 (333.3 to 825.7)	591.1 (364.5 to 897.5)	9.0% (-42.1 to 105.1)	53.7 (27.2 to 89.6)	58.5 (29.9 to 99.1)	8.9% (-52.8 to 151.2)
Democratic Republic of the Congo	215.0 (124.5 to 328.2)	214.7 (124.4 to 327.7)	-0.2% (-49.3 to 96.6)	798.8 (491.2 to 1194.6)	796.7 (489.8 to 1191.8)	-0.3% (-46.4 to 85.7)	77.9 (41.4 to 130.5)	78.3 (42.2 to 130.9)	0.5% (-54.8 to 123.0)
Northern Mariana Islands	159.7 (91.4 to 246.1)	143.2 (81.3 to 221.9)	-10.3% (-55.4 to 80.3)	607.9 (369.9 to 923.9)	522.0 (316.7 to 795.2)	-14.1% (-54.8 to 63.3)	60.6 (32.3 to 103.4)	52.2 (26.9 to 88.7)	-14.0% (-62.2 to 96.4)
Syrian Arab Republic	185.4 (108.2 to 285.0)	193.9 (114.1 to 297.0)	4.6% (-46.7 to 105.0)	721.9 (444.4 to 1100.9)	771.0 (474.8 to 1174.0)	6.8% (-43.3 to 101.3)	71.4 (37.3 to 121.5)	76.1 (40.6 to 126.7)	6.6% (-52.6 to 139.5)
Mali	212.8 (123.0 to 325.2)	214.0 (123.9 to 326.7)	0.6% (-49.0 to 98.3)	783.8 (482.2 to 1172.1)	790.0 (485.8 to 1181.9)	0.8% (-45.9 to 87.7)	77.2 (42.0 to 127.4)	78.2 (41.5 to 132.9)	1.3% (-54.4 to 125.3)

Tokelau	142.0 (80.6 to 220.3)	140.4 (79.6 to 217.8)	-1.1% (-51.1 to 99.9)	513.5 (311.7 to 782.8)	507.8 (308.0 to 773.9)	-1.1% (-48.1 to 88.3)	51.1 (26.0 to 86.8)	50.6 (25.7 to 86.0)	-0.9% (-57.4 to 130.4)
Turkmenistan	172.9 (100.1 to 271.5)	175.4 (101.6 to 275.2)	1.5% (-49.5 to 103.8)	641.2 (396.6 to 985.5)	660.2 (408.8 to 1012.9)	3.0% (-45.5 to 94.4)	63.9 (34.0 to 108.3)	65.7 (34.8 to 108.5)	2.8% (-53.9 to 129.5)
Malawi	250.6 (146.1 to 389.4)	250.1 (146.3 to 388.5)	-0.2% (-49.7 to 97.9)	960.8 (606.8 to 1426.3)	948.8 (598.6 to 1409.7)	-1.3% (-45.7 to 79.7)	94.0 (50.5 to 155.9)	94.1 (51.9 to 157.6)	0.1% (-54.3 to 118.9)
Palestine	187.4 (109.4 to 287.9)	189.3 (110.5 to 290.8)	1.0% (-48.7 to 98.8)	737.5 (454.3 to 1123.9)	753.6 (464.4 to 1147.4)	2.2% (-45.8 to 92.4)	73.0 (38.1 to 123.6)	74.8 (39.8 to 125.2)	2.5% (-54.5 to 130.6)
Portugal	204.0 (120.1 to 313.3)	207.2 (121.9 to 317.9)	1.6% (-48.0 to 98.6)	840.7 (536.9 to 1253.0)	874.0 (559.9 to 1299.9)	4.0% (-42.4 to 87.7)	82.9 (44.6 to 138.6)	86.1 (46.7 to 144.1)	3.9% (-52.9 to 129.1)
Somalia	233.9 (136.2 to 364.3)	245.0 (143.1 to 380.7)	4.7% (-47.3 to 108.0)	860.6 (541.8 to 1278.6)	929.5 (586.5 to 1378.7)	8.0% (-40.7 to 96.6)	84.7 (45.4 to 141.6)	91.8 (50.3 to 149.9)	8.4% (-50.3 to 136.0)
Poland	166.6 (108.9 to 239.8)	172.0 (112.2 to 247.5)	3.3% (-40.6 to 79.6)	602.2 (422.7 to 823.3)	637.9 (448.6 to 869.2)	5.9% (-33.5 to 68.5)	60.1 (35.3 to 94.7)	63.6 (37.2 to 100.3)	5.9% (-47.0 to 111.9)
Bahamas	148.1 (86.4 to 230.4)	147.0 (85.8 to 228.6)	-0.8% (-50.0 to 97.0)	566.5 (348.6 to 861.0)	557.4 (342.9 to 847.9)	-1.6% (-47.7 to 85.3)	56.4 (29.0 to 96.8)	55.4 (28.2 to 93.4)	-1.8% (-57.7 to 127.3)
Bosnia and Herzegovina	179.5 (104.1 to 281.2)	181.2 (105.0 to 283.7)	1.0% (-49.6 to 102.3)	682.9 (423.3 to 1047.8)	695.4 (431.2 to 1067.1)	1.8% (-46.0 to 91.9)	68.0 (36.8 to 113.2)	69.3 (36.5 to 116.5)	1.9% (-54.2 to 126.8)
Sudan	187.5 (109.5 to 288.1)	188.6 (110.2 to 289.8)	0.6% (-48.9 to 97.8)	736.5 (453.4 to 1122.2)	747.9 (460.8 to 1139.0)	1.5% (-46.1 to 91.3)	72.9 (38.3 to 122.6)	74.3 (40.3 to 123.9)	1.9% (-54.2 to 126.3)
Yemen	179.3 (104.3 to 276.1)	185.4 (108.3 to 285.0)	3.4% (-47.6 to 103.9)	680.4 (417.9 to 1038.8)	721.1 (443.8 to 1099.8)	6.0% (-43.9 to 100.1)	67.0 (35.9 to 111.7)	70.8 (37.7 to 119.2)	5.7% (-52.5 to 135.5)
Argentina	143.0 (82.6 to 222.8)	147.8 (85.4 to 230.1)	3.3% (-48.4 to 106.8)	543.7 (337.9 to 836.0)	578.3 (360.1 to 888.4)	6.4% (-43.5 to 100.2)	54.0 (28.9 to 92.5)	57.6 (31.1 to 96.8)	6.6% (-52.3 to 137.9)

Spain	225.9 (131.2 to 347.1)	212.2 (123.6 to 331.0)	-6.1% (-52.6 to 86.6)	968.9 (605.5 to 1429.5)	870.9 (550.6 to 1302.9)	-10.1% (-50.8 to 64.1)	96.3 (50.5 to 160.7)	86.4 (46.6 to 141.9)	-10.3% (-59.4 to 98.1)
Zambia	249.9 (146.0 to 388.4)	250.2 (146.0 to 388.7)	0.1% (-49.5 to 98.5)	946.8 (597.3 to 1407.4)	954.9 (602.8 to 1418.0)	0.9% (-44.6 to 83.5)	93.1 (50.3 to 156.6)	94.1 (49.9 to 154.2)	1.1% (-54.2 to 123.1)
Latvia	180.1 (104.5 to 282.1)	172.9 (100.1 to 271.6)	-4.0% (-52.2 to 92.8)	685.5 (424.9 to 1052.3)	642.9 (397.8 to 987.5)	-6.2% (-50.3 to 77.0)	68.4 (36.0 to 114.1)	64.1 (34.1 to 109.5)	-6.2% (-58.3 to 111.5)
Egypt	187.8 (109.7 to 288.6)	188.7 (110.1 to 290.0)	0.5% (-48.9 to 97.8)	741.8 (456.9 to 1130.0)	750.9 (462.6 to 1143.1)	1.2% (-46.3 to 90.6)	73.4 (38.4 to 122.7)	74.6 (39.8 to 126.3)	1.6% (-54.8 to 128.4)
Ghana	215.4 (124.7 to 328.8)	219.6 (127.3 to 334.8)	2.0% (-48.2 to 100.7)	800.6 (492.6 to 1196.7)	828.5 (510.2 to 1236.9)	3.5% (-44.3 to 92.4)	79.0 (42.3 to 131.7)	82.2 (43.7 to 135.3)	4.0% (-52.9 to 129.8)
Côte d'Ivoire	216.8 (125.5 to 330.9)	214.7 (124.4 to 327.9)	-1.0% (-49.7 to 95.1)	811.8 (499.7 to 1212.7)	800.4 (492.2 to 1196.8)	-1.4% (-47.0 to 83.5)	79.7 (43.2 to 130.4)	78.8 (41.6 to 133.1)	-1.1% (-55.4 to 119.4)
Burundi	247.2 (144.0 to 384.4)	247.1 (144.1 to 384.3)	-0.0% (-49.7 to 98.6)	937.2 (591.3 to 1392.2)	931.7 (587.5 to 1384.7)	-0.6% (-45.4 to 81.0)	92.7 (49.5 to 153.4)	92.2 (49.5 to 151.3)	-0.5% (-54.8 to 118.3)
Palau	147.9 (84.3 to 228.6)	145.1 (82.6 to 224.2)	-1.9% (-51.2 to 97.2)	546.5 (331.9 to 831.3)	533.0 (323.3 to 810.4)	-2.5% (-48.7 to 85.4)	54.7 (28.3 to 93.3)	52.9 (26.7 to 89.0)	-3.3% (-58.1 to 123.7)
Sweden	220.2 (140.5 to 314.5)	210.6 (135.8 to 304.0)	-4.4% (-45.6 to 68.0)	900.2 (643.6 to 1209.5)	831.1 (592.3 to 1124.8)	-7.7% (-40.9 to 44.2)	89.4 (52.1 to 139.2)	82.4 (48.7 to 127.8)	-7.8% (-53.4 to 82.0)
Guinea	213.1 (123.2 to 325.8)	214.5 (124.1 to 327.6)	0.6% (-49.0 to 98.5)	785.4 (483.3 to 1174.4)	791.8 (487.3 to 1184.0)	0.8% (-45.8 to 87.6)	77.2 (41.8 to 127.7)	78.3 (41.9 to 131.3)	1.4% (-53.9 to 123.4)
Cyprus	202.8 (119.1 to 311.7)	205.1 (120.3 to 315.1)	1.2% (-48.4 to 98.4)	841.5 (538.2 to 1252.7)	867.1 (556.1 to 1288.6)	3.0% (-42.8 to 85.8)	83.5 (44.4 to 137.8)	85.9 (45.8 to 141.9)	2.8% (-53.4 to 126.9)
Puerto Rico	145.3 (84.7 to 226.1)	150.9 (88.2 to 234.4)	3.9% (-47.6 to 106.1)	547.2 (336.4 to 832.6)	583.7 (359.7 to 886.6)	6.7% (-43.3 to 100.8)	54.5 (28.0 to 90.8)	58.1 (30.3 to 96.1)	6.8% (-52.7 to 141.1)

UI, uncertainly interval; DALYs, disability-adjusted life-years.
