# Supplementary material for: Anxiety and depressive disorders in adolescent injury prevention and safety promotion: frontier and inequality mapping across 953 locations
Source: Front Public Health. 2026 Aug 11;14:1916777. doi: 10.3389/fpubh.2026.1916777 (PMC13532395; doi:10.3389/fpubh.2026.1916777)
Supplement: Supplementary file 2 [file Table_1.docx]

**Supplement 1: Methodological supplement to “Anxiety and Depressive Disorders in Adolescent Injury Prevention and Safety Promotion: Frontier and Inequality Mapping across 953 Locations”**

This supplement provides further methodological detail for “Anxiety and Depressive Disorders in Adolescent Injury Prevention and Safety Promotion: Frontier and Inequality Mapping across 953 Locations”. This study adheres to the guidelines outlined in the Guidelines for Accurate and Transparent Health Estimates Reporting (GATHER).

Table of contents

[GATHER Guidelines Checklist	3](#_Toc203070087)

[Section 1: Overview	5](#_Toc203070088)

**[Section 1.1: GBD study overview](#_Toc203070089)**	[5](#_Toc203070089)

**[Section 1.2: Results overview](#_Toc203070090)**	[5](#_Toc203070090)

**[Section 1.3: Time period of the analysis](#_Toc203070091)**	[5](#_Toc203070091)

**[Section 1.4: Sociodemographic index](#_Toc203070092)**	[5](#_Toc203070092)

**[Section 1.5: Case definition](#_Toc203070093)**	[6](#_Toc203070093)

**[Section 1.6: Burden of disease geographical hierarchy across locations](#_Toc203070094)**	[6](#_Toc203070094)

**[Section 1.7: Gross Domestic Product per capita (GDPPC) analysis](#_Toc203070095)**	[9](#_Toc203070095)

**[Section 1.8: Uncertainty estimation](#_Toc203070096)**	[10](#_Toc203070096)

[Section 2: Estimation of anxiety and depressive disorders among young people aged 10-24 years outcomes in GBD 2021	11](#_Toc203070097)

**[Section 2.1: Young people](#_Toc203070098)**	[11](#_Toc203070098)

**[Section 2.2: Input data](#_Toc203070099)**	[11](#_Toc203070099)

[Section 3: References	12](#_Toc203070100)

[Section 4: Supplement 1 Tables	14](#_Toc203070101)

**GATHER Guidelines Checklist**

| **Item #** | **Checklist** **item** | **Reported** **on** **page#** |
| --- | --- | --- |
| **Objectives** **and** **funding** | | |
| 1 | Define the indicator(s), populations (including age, sex, and geographic entities), and time period(s)for which estimates were made. | Main text (Methods) and Supplement (Supplemental Methods) |
| **2** | List the funding sources for the work. | Abstract (Funding) |
| **Data** **Inputs** | | |
| *For all data inputs from multiple sources that are synthesized as part of the study:* | | |
| **3** | Describe how the data were identified and how the data were accessed. | Main text (Methods) and Supplement (Supplemental Methods) |
| 4 | Specify the inclusion and exclusion criteria Identify all ad-hoc exclusions. | Main text (Methods and Discussion) and Supplement (Supplemental Methods) |
| 5 | Provide information on all included data sources and their main characteristics. For each data source used, report reference information or contact name/institution, population represented, data collection method, year(s) of data collection, sex and age range, diagnostic criteria or measurement method, and sample size, as relevant. | Supplement (Supplemental Methods) with additional information about these sources available at https://ghdx.healthdata.org/ |
| 6 | Identify and describe any categories of input data that have potentially important biases (e.g., based on characteristics listed in item 5) | Main Text (Discussion) |
| *For data inputs that contribute to the analysis but were not synthesized as part of the study:* | | |
| 7 | Describe and give sources for any other data inputs. | [Global Health Data Exchange (https://ghdx.healthdata.org/)](https://ghdx.healthdata.org/)) |
| *For all data inputs:* | | |
| **8** | Provide all data inputs in a file format from which data can be efficiently extracted (e.g., a spreadsheet rather than a PDF), including all relevant meta-data listed in item 5. For any data inputs that cannot be shared because of ethical or legal reasons, such as third-party ownership, provide a contact name or the name of the institution that retains the right to the data. | [Global Health Data Exchange (https://ghdx.healthdata.org/)](https://ghdx.healthdata.org/)) |
| **Data** **analysis** | | |
| **9** | Provide a conceptual overview of the data analysis method. A diagram may be helpful. | Main text (Methods) and Supplement (Supplemental Methods) |
| **10** | Provide a detailed description of all steps of the analysis, including mathematical formulae. This description should cover, as relevant, data cleaning, data pre‐processing, data adjustments and weighting of data sources, and mathematical or statistical model(s). | Main text (Methods) and Supplement (Supplemental Methods) |
| **11** | Describe how candidate models were evaluated and how the final model(s) were selected. | Main text (Methods) and Supplement (Supplemental Methods) |
| **12** | Provide the results of an evaluation of model performance, if done, as well as the results of any relevant sensitivity analysis. | Main text (Results) |
| **13** | Describe methods for calculating uncertainty of the estimates. State which sources of uncertainty were, and were not, accounted for in the uncertainty analysis. | Main text (Methods) and Supplement (Supplemental Methods) |
| **14** | State how analytic or statistical source code used to generate estimates can be accessed. | Global Health Data Exchange (https://ghdx.healthdata.org/) |
| **Results and Discussion** | | |
| **15** | Provide published estimates in a file format from which data can be efficiently extracted. | Main text (Methods, Results and Discussion) and Supplement (Supplemental Methods and Results) |
| **16** | Report a quantitative measure of the uncertainty of the estimates (e.g. uncertainty intervals). | Main text (Methods) and Supplement (Supplemental Results) |
| **17** | Interpret results in light of existing evidence. If updating a previous set of estimates, describe the reasons for changes in estimates. | Main text (Methods, Results and Discussion)  and Supplement (Supplemental Methods and Results) |
| **18** | Discuss limitations of the estimates. Include a discussion of any modelling assumptions or data limitations that affect interpretation of the estimates. | Main text (Methods and Discussion) and Supplement (Supplemental Methods) |

**This study complies with the Guidelines for Accurate and Transparent Health Estimates Reporting (GATHER) recommendations.*

**Section 1: Overview**

**Section 1.1: GBD study overview**

The Global Burden of Diseases, Injuries, and Risk Factors Study (GBD) is a collaborative research initiative aimed at estimating morbidity and mortality from a comprehensive set of diseases, injuries, and risk factors. In GBD 2021, non-fatal cases were estimated using 75,459 data sources including scientific literature, household survey data, epidemiologic surveillance data, disease registry data, and clinical informatics data^1^. The non-fatal cause list has expanded from 364 causes in GBD 2019 to 365 causes in GBD 2021, while the total number of fatal and nonfatal causes combined reached 371, including communicable, maternal, neonatal, nutritional diseases, noncommunicable diseases, and injuries.

The Global Development Goals 2021 provide comprehensive estimates of disease burden, including prevalence, morbidity, years lived with disability (YLDs), which quantify non-fatal health losses, years of life lost (YLLs), reflecting premature mortality, and disability-adjusted life years (DALYs), an integrative measure combining both fatal and non-fatal health outcomes. DALYs are derived as the summation of YLLs and YLDs. The incidence of sequelae based on cause, age, sex, site, and year is multiplied by the corresponding disability weight for each disease and injury to obtain YLD. YLL is determined by multiplying the age, sex, area, and year mortality figures by the standardized life expectancy that corresponds to the age at which the death occurs. The GBD 2021 update introduces critical methodological refinements, including the incorporation of COVID-19-related burden estimates and its secondary impacts on other diseases, the addition of 12 novel disease classifications, and enhancements in data sources. To mitigate potential biases, advanced statistical modeling techniques—such as meta-regression-Bayesian, regularization, and trimming (MR-BRT)—were employed to optimize internal consistency and robustness of the estimates.

The geographic hierarchy of GBD estimates on of five levels: beginning with subnational locations across 21 countries and territories, then aggregating 204 countries and territories into 21 regions based on epidemiologic similarity and geographic proximity. These regions are further consolidated into seven super-regions, culminating in global-level coverage^1^.

The estimation of non-fatal health outcomes in the GBD study incorporated 75,459 distinct data sources, including survey data, disease registries, notification data, hospitalized patient data, published literature and other sources. A series of data processing steps included modification of epidemiologic and clinical data to reduce identified biases to ensure comparability across time periods, regions, sexes, and age groups. MR-BRT 3 was used to identify differences and correct for comparability of epidemiological data from different sources^2^.

The method uses pairs of data obtained from different case definitions or measures (consistent with the same age, sex, location, and year) to estimate correction factors through both linear and nonlinear mixed-effects models. We applied sex-specific ratios to resolve missing data that were not categorized by sex, and we performed age-sex separation for data lacking both age and sex information.

Adjustments were made to clinical data (including inpatient admissions, outpatient visits, and medical claims). Inpatient records were corrected for readmissions, secondary diagnoses, and overlapping outpatient services to avoid duplication. The age-sex ratio of incomplete hospitalizations was estimated using the MR-BRT and adjusted by the Healthcare Access and Quality Index (HAI) to address regional differences in healthcare access. After data adjustment, DisMod-MR2.1 (disease model meta-regression; version 2.1) was used to generate internally consistent prevalence and incidence estimates based on etiology and sequelae. This Bayesian meta-regression tool also generated estimates for sites without direct epidemiologic data through stepwise calculations at different levels of the GBD geographic hierarchy.

All analytical procedures strictly adhered to the GATHER standards. This compliance ensured methodological rigor in data preparation, model specification, and uncertainty quantification, thereby guaranteeing both the reproducibility and transparency of the GBD's health metrics.

**Section 1.2: Results overview**

Supplementary tables and figures are provided in Supplementary Supplement 1 and Supplement 2.

**Section 1.3: Time period of the analysis**

We estimated numbers and rates of incidence, prevalence, and disability-adjusted life-years (DALYs) among young people aged 10-24 years for the years 1990-2021.

**Section 1.4: Sociodemographic index**

This study utilized the sociodemographic index (SDI) to explore the role of broader social, economic, and demographic conditions associated with the levels and trend of disease burden of anxiety and depressive disorders at the population level. The SDI is a composite indicator of social and economic development, constructed through a composite calculation method based on three core measures: lag-distributed income per capita, average years of schooling in ages 15 and older, and total fertility rate (TFR) for females under age 2529. It is expressed as a range of values from 0 to 1, where 0 is the lowest development and 1 the highest development30, and is categorised into five quintiles: low (<0.45), low-middle (≥0.45 to <0.61), middle (≥0.61 to <0.69), high-middle (≥0.69 to <0.80), and high (≥0.80). This classification provides an important reference for studying the level of socioeconomic development and its impact on health-related indicators in different countries or regions. Detailed information on SDI from global to local levels is provided in supplement (Supplement 1 Table S2-3).

**Section 1.5: Case definition**

Anxiety disorders are characterized by intense and persistent fear and distress, accompanied by physiological symptoms such as increased heart rate, sweating, and trembling. These disorders include panic disorder, agoraphobia, and specific phobia, as classified by DSM-IV-TR codes 300.0–300.3, 208.3, 309.21, 309.81 and ICD-10 classifications F40–F42, F43.0, F43.1, F93.0–F93.2, F93.8^3^. In contrast, the GBD stratifies depressive disorders according to DSM-IV-TR into two categories: major depressive disorders (MDDs) (DSM-IV-TR: 296.21–24, 296.31–34; ICD-10: F32.0–9, F33.0–9) and dysthymia (DSM-IV-TR: 300.4, ICD-10: F34.1), excluding mood disorders secondary to medical comorbidities or substance use^4^. MDD is defined by marked depressed mood, anhedonia, and a range of physical and cognitive symptoms. It requires at least one episode of these symptoms daily for at least 2 weeks, significantly affecting functioning^5,6^. Dysthymia presents as a chronically and persistently depressed mood for most of the day, lasting at least 2 years in adults or 1 year in children and adolescents^7^.

**Section 1.6: Burden of disease geographical hierarchy across locations**

The Supplement 1 Table S1 below is organized according to seven super-regions (headings), their respective regions (left column), and the corresponding countries within each region (right column).

Supplement 1 Table S1: Geographical hierarchy across 21 GBD regions, and 204 countries and territories

| **Central Europe, Eastern Europe, and Central Asia** | |
| --- | --- |
| Central Asia | Armenia, Azerbaijan, Georgia, Kazakhstan, Kyrgyzstan, Mongolia, Tajikistan, Turkmenistan, Uzbekistan |
| Central Europe | Albania, Bosnia and Herzegovina, Bulgaria, Croatia, Czechia, Hungary, Montenegro, North Macedonia, Poland (subnational), Romania, Serbia, Slovakia, Slovenia |
| Eastern Europe | Belarus, Estonia, Latvia, Lithuania, Republic of Moldova, Russian Federation, Ukraine |
| **High-income** | |
| Australasia | Australia, New Zealand |
| High-income Asia Pacific | Brunei Darussalam, Japan (subnational), Republic of Korea, Singapore |
| High-income North America | Canada, Greenland, United States of America (subnational) |
| Southern Latin America | Argentina, Chile, Uruguay |
| Western Europe | Andorra, Austria, Belgium, Cyprus, Denmark, Finland, France, Germany, Greece, Iceland, Ireland, Israel, Italy (subnational), Luxembourg, Malta, Monaco, Netherlands, Norway (subnational), Portugal, San Marino, Spain, Sweden (subnational), Switzerland, United Kingdom (subnational) |
| **Latin America and Caribbean** | |
| Andean Latin America | Bolivia (Plurinational State of), Ecuador, Peru |
| Caribbean | Antigua and Barbuda, Bahamas, Barbados, Belize, Bermuda, Cuba, Dominica, Dominican Republic, Grenada, Guyana, Haiti, Jamaica, Puerto Rico, Saint Kitts and Nevis, Saint Lucia, Saint Vincent and the Grenadines, Suriname, Trinidad and Tobago, United States Virgin Islands |
| Central Latin America | Colombia, Costa Rica, El Salvador, Guatemala, Honduras, Mexico (subnational), Nicaragua, Panama, Venezuela (Bolivarian Republic of) |
| Tropical Latin America | Brazil (subnational), Paraguay |
| **North Africa and Middle East** | |
| North Africa and Middle East | Afghanistan, Algeria, Bahrain, Egypt, Iran (Islamic Republic of) (subnational), Iraq, Jordan, Kuwait, Lebanon, Libya, Morocco, Oman, Palestine, Qatar, Saudi Arabia, Sudan, Syrian Arab Republic, Tunisia, Türkiye, United Arab Emirates, Yemen |
| **South Asia** | |
| South Asia | Bangladesh, Bhutan, India, Nepal, Pakistan (subnational) |
| **Southeast Asia, East Asia, and Oceania** | |
| East Asia | China (subnational), Democratic People's Republic of Korea, Taiwan (Province of China) |
| Oceania | American Samoa, Cook Islands, Fiji, Guam, Kiribati, Marshall Islands, Micronesia (Federated States of), Nauru, Niue, Northern Mariana Islands, Palau, Papua New Guinea, Samoa, Solomon Islands, Tokelau, Tonga, Tuvalu, Vanuatu |
| Southeast Asia | Cambodia, Indonesia (subnational), Lao People's Democratic Republic, Malaysia, Maldives, Mauritius, Myanmar, Philippines (subnational), Seychelles, Sri Lanka, Thailand, Timor-Leste, Viet Nam |
| **Sub-Saharan Africa** | |
| Central Sub-Saharan Africa | Angola, Central African Republic, Congo, Democratic Republic of the Congo, Equatorial Guinea, Gabon |
| Eastern Sub-Saharan Africa | Burundi, Comoros, Djibouti, Eritrea, Ethiopia (subnational), Kenya (subnational), Madagascar, Malawi, Mozambique, Rwanda, Somalia, South Sudan, Uganda, United Republic of Tanzania, Zambia |
| Southern Sub-Saharan Africa | Botswana, Eswatini, Lesotho, Namibia, South Africa (subnational), Zimbabwe |
| Western Sub-Saharan Africa | Benin, Burkina Faso, Cabo Verde, Cameroon, Chad, Côte d'Ivoire, Gambia, Ghana, Guinea, Guinea-Bissau, Liberia, Mali, Mauritania, Niger, Nigeria (subnational), Sao Tome and Principe, Senegal, Sierra Leone, Togo |

The Supplement 1 Table S2 below is organized according to 18 countries (left column), and the corresponding subnational locations within each country (right column).

Supplement 1 Table S2: Geographical hierarchy across 18 countries and 652 subnational locations

| **Country** | **Subnational locations** |
| --- | --- |
| Brazil | Acre, Amapá, Amazonas, Pará, Rondônia, Roraima, Tocantins, Alagoas, Bahia, Ceará,  Maranhão, Paraíba, Pernambuco, Piauí, Rio Grande do Norte, Sergipe, Distrito Federal,  Goiás, Mato Grosso, Mato Grosso do Sul, Paraná, Rio Grande do Sul, Santa Catarina,  Espírito Santo, Minas Gerais, Rio de Janeiro, São Paulo |
| China | Anhui, Chongqing, Beijing, Gansu, Fujian, Jiangxi, Inner Mongolia, Qinghai, Shaanxi, Macao, Jiangsu, Liaoning, Shandong, Jilin, Ningxia, Hainan, Guangdong, Hebei, Guangxi, Guizhou, Taiwan, Yunnan, Xinjiang, Zhejiang, Hong Kong, Heilongjiang, Henan, Hubei, Hunan, Tianjin, Shanghai, Tibet, Shanxi, Sichuan |
| Ethiopia | Addis Ababa, Afar, Amhara, Benishangul-Gumuz, Dire Dawa, Gambella, Harari, Oromia, Somali, Southern Nations, Nationalities, and Peoples, Tigray |
| Indonesia | Aceh, Bali, Bangka-Belitung Islands, Banten, Bengkulu, Gorontalo, Jakarta, Jambi, West Java, Central Java, East Java, West Kalimantan, South Kalimantan, Central Kalimantan, East Kalimantan, North Kalimantan, Riau Islands, Lampung, Maluku, North Maluku, West Nusa Tenggara, East Nusa Tenggara, Papua, West Papua, West Sumatra, South Sumatra, North Sumatra, Yogyakarta, Riau, North Sulawesi, Central Sulawesi, South Sulawesi, West Sulawesi, Southeast Sulawesi |
| Iran (Islamic Republic of) | Alborz, Ardebil, Bushehr, Chahar Mahaal and Bakhtiari, East Azarbayejan, Fars, Gilan, Golestan, Hamadan, Hormozgan, Ilam, Isfahan, Kerman, Kermanshah, Khorasan-e-Razavi, Khuzestan, Kohgiluyeh and Boyer-Ahmad, Kurdistan, Lorestan, Markazi, Mazandaran, North Khorasan, Qazvin, Qom, Semnan, Sistan and Baluchistan, South Khorasan, Tehran, West Azarbayejan, Yazd, Zanjan |
| Italy | Liguria, Lombardia, Piemonte, Valle d'Aosta, Emilia-Romagna, Friuli-Venezia Giulia, Provincia autonoma di Bolzano, Provincia autonoma di Trento, Veneto, Lazio, Marche, Toscana, Umbria, Abruzzo, Basilicata, Calabria, Campania, Molise, Puglia, Sardegna, Sicilia |
| Japan | Aichi, Akita, Aomori, Chiba, Ehime, Fukui, Fukuoka, Fukushima, Gifu, Gunma, Hiroshima, Hokkaidō, Hyōgo, Ibaraki, Ishikawa, Iwate, Kagawa, Kagoshima, Kanagawa, Kōchi, Kumamoto, Kyōto, Mie, Miyagi, Miyazaki, Nagano, Nagasaki, Nara, Niigata, Ōita, Okayama, Okinawa, Ōsaka, Saga, Saitama, Shiga, Shimane, Shizuoka, Tochigi, Tokushima, Tōkyō, Tottori, Toyama, Wakayama, Yamagata, Yamaguchi, Yamanashi |
| Kenya | Baringo, Bomet, Bungoma, Busia, Elgeyo-Marakwet, Embu, Garissa, Homa Bay, Isiolo, Kajiado, Kakamega, Kericho, Kiambu, Kilifi, Kirinyaga, Kisii, Kisumu, Kitui, Kwale, Laikipia, Lamu, Machakos, Makueni, Mandera, Marsabit, Meru, Migori, Mombasa, Murang'a, Nairobi, Nakuru, Nandi, Narok, Nyamira, Nyandarua, Nyeri, Samburu, Siaya, Taita Taveta, Tana River, Tharaka Nithi, Trans Nzoia, Turkana, Uasin Gishu, Vihiga, Wajir, West Pokot |
| Mexico | Aguascalientes, Baja California, Baja California Sur, Campeche, Chiapas, Chihuahua, Coahuila, Colima, Durango, Guanajuato, Guerrero, Hidalgo, Jalisco, México, Mexico City, Michoacán de Ocampo, Morelos, Nayarit, Nuevo León, Oaxaca, Puebla, Querétaro, Quintana Roo, San Luis Potosí, Sinaloa, Sonora, Tabasco, Tamaulipas, Tlaxcala, Veracruz de Ignacio de la Llave, Yucatán, Zacatecas |
| Nigeria | Abia, Adamawa, Akwa Ibom, Anambra, Bauchi, Bayelsa, Benue, Borno, Cross River, Delta, Ebonyi, Edo, Ekiti, Enugu, FCT (Abuja), Gombe, Imo, Jigawa, Kaduna, Kano, Katsina, Kebbi, Kogi, Kwara, Lagos, Nasarawa, Niger, Ogun, Ondo, Osun, Oyo, Plateau, Rivers, Sokoto, Taraba, Yobe, Zamfara |
| Norway | Agder, Innlandet, Møre og Romsdal, Nordland, Oslo, Rogaland, Troms og Finnmark, Trøndelag, Vestfold og Telemark, Vestland, Viken |
| Pakistan | Azad Jammu & Kashmir, Balochistan, Gilgit-Baltistan, Islamabad Capital Territory, Khyber Pakhtunkhwa, Punjab, Sindh |
| Philippines | Abra, Agusan Del Norte, Agusan Del Sur, Aklan, Albay, Antique, Apayao, Aurora, Basilan, Bataan, Batanes, Batangas, Benguet, Biliran, Bohol, Bukidnon, Bulacan, Cagayan, Camarines Norte, Camarines Sur, Camiguin, Capiz, Catanduanes, Cavite, Cebu, Cotabato (North Cotabato), Davao de Oro, Davao Del Norte, Davao Del Sur, Davao Occidental, Davao Oriental, Dinagat Islands, Eastern Samar, Guimaras, Ifugao, Ilocos Norte, Ilocos Sur, Iloilo, Isabela, Kalinga, La Union, Laguna, Lanao Del Norte, Lanao Del Sur, Leyte, Maguindanao, Marinduque, Masbate, Misamis Occidental, Misamis Oriental, Mountain Province, National Capital Region, Negros Occidental, Negros Oriental, Northern Samar, Nueva Ecija, Nueva Vizcaya, Occidental Mindoro, Oriental Mindoro, Palawan, Pampanga, Pangasinan, Quezon, Quirino, Rizal, Romblon, Samar(Western Samar), Sarangani, Siquijor, Sorsogon, South Cotabato, Southern Leyte, Sultan Kudarat, Sulu, Surigao Del Norte, Surigao Del Sur, Tarlac, Tawi-Tawi, Zambales, Zamboanga Del Norte, Zamboanga Del Sur, Zamboanga Sibugay |
| Poland | Dolnośląskie, Kujawsko-Pomorskie, Łódzkie, Lubelskie, Lubuskie, Małopolskie, Mazowieckie, Opolskie, Podkarpackie, Podlaskie, Pomorskie, Śląskie, Świętokrzyskie, Warmińsko-Mazurskie, Wielkopolskie, Zachodniopomorskie |
| South Africa | Eastern Cape, Free State, Gauteng, KwaZulu-Natal, Limpopo, Mpumalanga, North-West, Northern Cape, Western Cape |
| Sweden | Stockholm, Sweden except Stockholm |
| United Kingdom | Derby, Derbyshire, Leicester, Leicestershire, Lincolnshire, Northamptonshire, Nottingham, Nottinghamshire, Rutland, Bedford, Cambridgeshire, Central Bedfordshire, Essex, Hertfordshire, Luton, Norfolk, Peterborough, Southend-on-Sea, Suffolk, Thurrock, Barking and Dagenham, Barnet, Bexley, Brent, Bromley, Camden, Croydon, Ealing, Enfield, Greenwich, Hackney, Hammersmith and Fulham, Haringey, Harrow, Havering, Hillingdon, Hounslow, Islington, Kensington and Chelsea, Kingston upon Thames, Lambeth, Lewisham, Merton, Newham, Redbridge, Richmond upon Thames, Southwark, Sutton, Tower Hamlets, Waltham Forest, Wandsworth, Westminster, County Durham, Darlington, Gateshead, Hartlepool, Middlesbrough, Newcastle upon Tyne, North Tyneside, Northumberland, Redcar and Cleveland, South Tyneside, Stockton-on-Tees, Sunderland, Blackburn with Darwen, Blackpool, Bolton, Bury, Cheshire East, Cheshire West and Chester, Cumbria, Halton, Knowsley, Lancashire, Liverpool, Manchester, Oldham, Rochdale, Salford, Sefton, St Helens, Stockport, Tameside, Trafford, Warrington, Wigan, Wirral, Bracknell Forest, Brighton and Hove, Buckinghamshire, East Sussex, Hampshire, Isle of Wight, Kent, Medway, Milton Keynes, Oxfordshire, Portsmouth, Reading, Slough, Southampton, Surrey, West Berkshire, WestSussex, Windsor and Maidenhead, Wokingham, Bath and North East Somerset, Bournemouth, Bristol City of, Cornwall, Devon, Dorset, Gloucestershire, North Somerset, Plymouth, Poole, Somerset, South Gloucestershire, Swindon, Torbay, Wiltshire, Birmingham, Coventry, Dudley, Herefordshire County of, Sandwell, Shropshire, Solihull, Staffordshire, Stoke-on-Trent, Telford and Wrekin, Walsall, Warwickshire, Wolverhampton, Worcestershire, Barnsley, Bradford, Calderdale, Doncaster, East Riding of Yorkshire, Kingston upon Hull City of, Kirklees, Leeds, North East Lincolnshire, North Lincolnshire, North Yorkshire, Rotherham, Sheffield, Wakefield, York, Northern Ireland, Scotland, Wales |
| United States of America | Alabama, Alaska, Arizona, Arkansas, California, Colorado, Connecticut, Delaware, District of Columbia, Florida, Georgia, Hawaii, Idaho, Illinois, Indiana, Iowa, Kansas, Kentucky, Louisiana, Maine, Maryland, Massachusetts, Michigan, Minnesota, Mississippi, Missouri, Montana, Nebraska, Nevada, New Hampshire, New Jersey, New Mexico, New York, North Carolina, North Dakota, Ohio, Oklahoma, Oregon, Pennsylvania, Rhode Island, South Carolina, South Dakota, Tennessee, Texas, Utah, Vermont, Virginia, Washington, West Virginia, Wisconsin, Wyoming |

**Section 1.7: Gross Domestic Product per capita (GDPPC) analysis**

Per capita Gross Domestic Product (GDPPC) reflects regional economic development and population welfare, quantifying economic output normalized by demographic size^8^. For mainland China, provincial GDPPC values were obtained from official National Bureau of Statistics reports, whereas Taiwan, Hong Kong, and Macao data were extracted from World Bank repositories. Jurisdictions were stratified into tertiles according to national GDPPC distribution: the lowest quintile (below 20th percentile) designated as low-GDPPC regions, the highest quintile (above 80th percentile) classified as high-GDPPC areas, with intermediate values constituting the middle-GDPPC tier. To analyze the relationship between GDPPC and various health metrics including incidence, prevalence and DALYs, we utilized a generalized additive model with a cubic curve to account for non-linearity.

**Section 1.8: Uncertainty estimation**

Assessing uncertainty starts with evaluating the availability and variability of data across different causes, stratified by age, sex, location, and year.

**Overview of 95% Uncertainty Intervals (UIs)**

For GBD 2021, the calculation of final uncertainty intervals (UIs) employs enhanced statistical methods. Rather than relying solely on the 2.5th and 97.5th percentiles of from 1,000 ranked draws, hierarchical Bayesian models and optimized smoothing algorithms are implemented to comprehensively quantify uncertainty. These methods account for variability in input data, model specifications, and covariate dependencies, ensuring robust uncertainty estimation across all model components.

**DALYs**

Uncertainty in DALYs integrates the variability from both YLLs and YLDs, capturing fluctuations in mortality, life expectancy, disease prevalence, and disability weights. The methodology accounts for all upstream sources of uncertainty, ensuring they are represented in the final estimates. Monte Carlo simulations framework propagates these uncertainties through the calculation process, with adjustments implemented to preserve consistency with empirical epidemiological patterns.

**Incidence and Prevalence**

GBD 2021 employs DisMod-MR 2.1 to derive incidence and prevalence estimates, which integrates multiple data sources, smooths inconsistencies, and produces thousands of draws to capture uncertainty. The model accommodates variations arising from sampling errors, diagnostic criteria discrepancies, and parameter instability, ensuring that output UIs reflect both observed and imputed data uncertainty.

**Calculation of Final UIs**

The generation of final UIs in GBD 2021 combines Bayesian posterior distributions with advanced diagnostic techniques to establish credible ranges. This enhanced approach moves beyond simple draw ranking, incorporating epidemiological patterns, data reliability, and covariate dynamics to produce more accurate and meaningful uncertainty estimates. The refined methodology yields intervals that better represent true underlying uncertainty while maintaining clinical and demographic coherence.

**Section 2: Estimation of anxiety and depressive disorders among young people aged 10-24 years outcomes in GBD 2021**

**Section 2.1: Young people**

This section particularly focuses on anxiety and depressive disorders in young people aged 10-24 years. This critical developmental stage captures the complete trajectory from pubertal onset to full prefrontal cortical maturation, characterized by profound physiological changes, psychological maturation, and transitions in societal roles^13,14^, making individuals particularly susceptible to mood fluctuations. Compounding this biological vulnerability, contemporary youth face unprecedented psychosocial stressors including academic pressures, digital socialization, and economic uncertainties^15,16^. Consequently, mental health challenges, including anxiety and depressive disorders, have become increasingly prevalent within this demographic, highlighting the need for targeted research and interventions to address their unique vulnerabilities. In the presentation of partial results, the 10-24 years age range is being divided into three 5-year groups: early adolescence (10-14 years), late adolescence (15-19 years), and young adulthood (20-24 years)^17^, and prevalence, incidence and DALYs are visualised and secondary analysed separately for each age group.

**Section 2.2: Input data**

The GBD 2021 project uses a systematic approach to estimate the incidence and prevalence of anxiety and depressive disorders among young people aged 10-24 years. The study synthesizes multiple data sources from around the world, including medical records, epidemiologic surveys, and other relevant research data. Through advanced statistical modelling and data integration techniques, the GBD team analysed these data to provide reliable estimates of the burden of anxiety and depressive disorders. These estimates help to understand the prevalence of anxiety and depressive disorders at the global and regional levels and support health policy development and resource allocation.

**Section 3: References**

1.	Global incidence, prevalence, years lived with disability (YLDs), disability-adjusted life-years (DALYs), and healthy life expectancy (HALE) for 371 diseases and injuries in 204 countries and territories and 811 subnational locations, 1990–2021: a systematic analysis for the Global Burden of Disease Study 2021. *Lancet*. 2024;403(10440):2133-2161. doi:10.1016/S0140-6736(24)00757-8

2.	Zheng P, Barber R, Sorensen RJD, Murray CJL, Aravkin AY. Trimmed Constrained Mixed Effects Models: Formulations and Algorithms. Published online October 27, 2020. doi:10.48550/arXiv.1909.10700

3.	Baxter AJ, Vos T, Scott KM, Ferrari AJ, Whiteford HA. The global burden of anxiety disorders in 2010. *Psychological Medicine*. 2014;44(11):2363-2374. doi:10.1017/S0033291713003243

4.	DSM5Update_October2018.pdf. Accessed February 21, 2025. https://seragpsych.com/wordpress/wp-content/uploads/2019/02/DSM5Update_October2018.pdf

5.	Li S, Zhang X, Cai Y, Zheng L, Pang H, Lou L. Sex difference in incidence of major depressive disorder: an analysis from the Global Burden of Disease Study 2019. *Ann Gen Psychiatry*. 2023;22(1):53. doi:10.1186/s12991-023-00486-7

6.	Moitra M, Santomauro D, Collins PY, et al. The global gap in treatment coverage for major depressive disorder in 84 countries from 2000–2019: A systematic review and Bayesian meta-regression analysis. *PLOS Medicine*. 2022;19(2):e1003901. doi:10.1371/journal.pmed.1003901

7.	Thapar A, Collishaw S, Pine DS, Thapar AK. Depression in adolescence. *The Lancet*. 2012;379(9820):1056-1067. doi:10.1016/S0140-6736(11)60871-4

8.	Schumacher AE, Kyu HH, Aali A, et al. Global age-sex-specific mortality, life expectancy, and population estimates in 204 countries and territories and 811 subnational locations, 1950–2021, and the impact of the COVID-19 pandemic: a comprehensive demographic analysis for the Global Burden of Disease Study 2021. *The Lancet*. 2024;403(10440):1989-2056. doi:10.1016/S0140-6736(24)00476-8

9.	Mújica ÓJ, Moreno CM. De la retórica a la acción: medir desigualdades en salud para “no dejar a nadie atrás.” *Rev Panam Salud Publica*. 2019;43:e12. doi:10.26633/RPSP.2019.12

10.	World Health Organization. *Handbook on Health Inequality Monitoring with a Special Focus on Low- and Middle-Income Countries*. World Health Organization; 2013. Accessed February 22, 2025. https://iris.who.int/handle/10665/85345

11.	Xie Y, Bowe B, Mokdad AH, et al. Analysis of the Global Burden of Disease study highlights the global, regional, and national trends of chronic kidney disease epidemiology from 1990 to 2016. *Kidney International*. 2018;94(3):567-581. doi:10.1016/j.kint.2018.04.011

12.	Ruan R, Liu X, Zhang Y, et al. Global, Regional, and National Advances Toward the Management of Rheumatic Heart Disease Based on the Global Burden of Disease Study 2019. *Journal of the American Heart Association*. 2023;12(13):e028921. doi:10.1161/JAHA.122.028921

13.	Sawyer SM, Afifi RA, Bearinger LH, et al. Adolescence: a foundation for future health. *The Lancet*. 2012;379(9826):1630-1640. doi:10.1016/S0140-6736(12)60072-5

14.	Kapur S. ADOLESCENCE: THE STAGE OF TRANSITION. *Horizons of Holistic Education*. 2015;2:233-250.

15.	Lochner C, Mogotsi M, du Toit PL, Kaminer D, Niehaus DJ, Stein DJ. Quality of Life in Anxiety Disorders: A Comparison of Obsessive-Compulsive Disorder, Social Anxiety Disorder, and Panic Disorder. *Psychopathology*. 2003;36(5):255-262. doi:10.1159/000073451

16.	Mychailyszyn MP, Mendez JL, Kendall PC. School Functioning in Youth With and Without Anxiety Disorders: Comparisons by Diagnosis and Comorbidity. *School Psychology Review*. 2010;39(1):106-121. doi:10.1080/02796015.2010.12087793

17.	Sawyer SM, Afifi RA, Bearinger LH, et al. Adolescence: a foundation for future health. *The Lancet*. 2012;379(9826):1630-1640. doi:10.1016/S0140-6736(12)60072-5

**Section 4: Supplement 1 Tables**

Supplement 1 Table S3: Global, regional, and national SDI values and SDI quintile

| **Location** | **2021 SDI Index Value** | **SDI** **Quintile** |
| --- | --- | --- |
| Global | 0.665820975 | NA |
| Afghanistan | 0.337199998 | Low SDI |
| Albania | 0.706849791 | High-middle SDI |
| Algeria | 0.659500924 | Middle SDI |
| American Samoa | 0.723727533 | High-middle SDI |
| Andean Latin America | 0.651602456 | NA |
| Andorra | 0.869444113 | High SDI |
| Angola | 0.453721949 | Low SDI |
| Antigua and Barbuda | 0.749886887 | High-middle SDI |
| Argentina | 0.723122973 | High-middle SDI |
| Armenia | 0.701833194 | High-middle SDI |
| Australasia | 0.845514063 | NA |
| Australia | 0.844252814 | High SDI |
| Austria | 0.853837004 | High SDI |
| Azerbaijan | 0.694851274 | High-middle SDI |
| Bahamas | 0.805020668 | High-middle SDI |
| Bahrain | 0.753043204 | High-middle SDI |
| Bangladesh | 0.492420885 | Low-middle SDI |
| Barbados | 0.746748764 | High-middle SDI |
| Belarus | 0.784484711 | High-middle SDI |
| Belgium | 0.853654016 | High SDI |
| Belize | 0.610229002 | Middle SDI |
| Benin | 0.373486574 | Low SDI |
| Bermuda | 0.821365422 | High SDI |
| Bhutan | 0.473062378 | Low-middle SDI |
| Bolivia (Plurinational State of) | 0.599010799 | Low-middle SDI |
| Bosnia and Herzegovina | 0.723077893 | High-middle SDI |
| Botswana | 0.642721629 | Middle SDI |
| Brazil | 0.653043887 | Middle SDI |
| Brunei Darussalam | 0.810234367 | High SDI |
| Bulgaria | 0.768150939 | High-middle SDI |
| Burkina Faso | 0.285118402 | Low SDI |
| Burundi | 0.289374365 | Low SDI |
| Cabo Verde | 0.533534539 | Low-middle SDI |
| Cambodia | 0.473621491 | Low-middle SDI |
| Cameroon | 0.479691223 | Low-middle SDI |
| Canada | 0.87317068 | High SDI |
| Caribbean | 0.642003055 | NA |
| Central African Republic | 0.30916769 | Low SDI |
| Central Asia | 0.675163978 | NA |
| Central Europe | 0.796244448 | NA |
| Central Latin America | 0.6406851 | NA |
| Central Sub-Saharan Africa | 0.472255651 | NA |
| Chad | 0.240436019 | Low SDI |
| Chile | 0.771514716 | High-middle SDI |
| China | 0.72162976 | High-middle SDI |
| Colombia | 0.655442913 | Middle SDI |
| Comoros | 0.475978688 | Low-middle SDI |
| Congo | 0.583075236 | Low-middle SDI |
| Cook Islands | 0.779109955 | High-middle SDI |
| Costa Rica | 0.700340477 | High-middle SDI |
| Côte d'Ivoire | 0.425941883 | Low SDI |
| Croatia | 0.798341027 | High-middle SDI |
| Cuba | 0.668729864 | Middle SDI |
| Cyprus | 0.835630545 | High SDI |
| Czechia | 0.828450433 | High SDI |
| Democratic People's Republic of Korea | 0.569854634 | Low-middle SDI |
| Democratic Republic of the Congo | 0.383179849 | Low SDI |
| Denmark | 0.896424204 | High SDI |
| Djibouti | 0.487958371 | Low-middle SDI |
| Dominica | 0.746967185 | High-middle SDI |
| Dominican Republic | 0.619388201 | Middle SDI |
| East Asia | 0.725704902 | NA |
| Eastern Europe | 0.802851009 | NA |
| Eastern Sub-Saharan Africa | 0.409720983 | NA |
| Ecuador | 0.661017053 | Middle SDI |
| Egypt | 0.606787094 | Low-middle SDI |
| El Salvador | 0.563775188 | Low-middle SDI |
| Equatorial Guinea | 0.657857456 | Middle SDI |
| Eritrea | 0.403863943 | Low SDI |
| Estonia | 0.844917787 | High SDI |
| Eswatini | 0.585459713 | Low-middle SDI |
| Ethiopia | 0.358823295 | Low SDI |
| Fiji | 0.675051631 | Middle SDI |
| Finland | 0.859831368 | High SDI |
| France | 0.838364875 | High SDI |
| Gabon | 0.634691393 | Middle SDI |
| Gambia | 0.40971416 | Low SDI |
| Georgia | 0.732473604 | High-middle SDI |
| Germany | 0.902957091 | High SDI |
| Ghana | 0.56493039 | Low-middle SDI |
| Greece | 0.791854408 | High-middle SDI |
| Greenland | 0.826210336 | High SDI |
| Grenada | 0.668993028 | Middle SDI |
| Guam | 0.803982203 | High-middle SDI |
| Guatemala | 0.539972424 | Low-middle SDI |
| Guinea | 0.336401293 | Low SDI |
| Guinea-Bissau | 0.353109621 | Low SDI |
| Guyana | 0.650812335 | Middle SDI |
| Haiti | 0.448278285 | Low SDI |
| High-income Asia Pacific | 0.876767 | NA |
| High-income North America | 0.863465474 | NA |
| Honduras | 0.513037248 | Low-middle SDI |
| Hungary | 0.790754768 | High-middle SDI |
| Iceland | 0.87636168 | High SDI |
| India | 0.575401649 | Low-middle SDI |
| Indonesia | 0.656868336 | Middle SDI |
| Iran (Islamic Republic of) | 0.697207398 | High-middle SDI |
| Iraq | 0.662626231 | Middle SDI |
| Ireland | 0.87375385 | High SDI |
| Israel | 0.809011652 | High SDI |
| Italy | 0.805773534 | High SDI |
| Jamaica | 0.683263064 | Middle SDI |
| Japan | 0.871241813 | High SDI |
| Jordan | 0.725307227 | High-middle SDI |
| Kazakhstan | 0.725144495 | High-middle SDI |
| Kenya | 0.523768077 | Low-middle SDI |
| Kiribati | 0.527186583 | Low-middle SDI |
| Kuwait | 0.846651055 | High SDI |
| Kyrgyzstan | 0.603979328 | Low-middle SDI |
| Lao People's Democratic Republic | 0.489136091 | Low-middle SDI |
| Latvia | 0.830663516 | High SDI |
| Lebanon | 0.744746351 | High-middle SDI |
| Lesotho | 0.510393066 | Low-middle SDI |
| Liberia | 0.352442452 | Low SDI |
| Libya | 0.725771399 | High-middle SDI |
| Lithuania | 0.856484049 | High SDI |
| Luxembourg | 0.884428955 | High SDI |
| Madagascar | 0.400246943 | Low SDI |
| Malawi | 0.384553634 | Low SDI |
| Malaysia | 0.742523828 | High-middle SDI |
| Maldives | 0.650886627 | Middle SDI |
| Mali | 0.268579941 | Low SDI |
| Malta | 0.801585034 | High-middle SDI |
| Marshall Islands | 0.574091128 | Low-middle SDI |
| Mauritania | 0.4989451 | Low-middle SDI |
| Mauritius | 0.718260446 | High-middle SDI |
| Mexico | 0.664575304 | Middle SDI |
| Micronesia (Federated States of) | 0.587534967 | Low-middle SDI |
| Monaco | 0.908262831 | High SDI |
| Mongolia | 0.617621565 | Middle SDI |
| Montenegro | 0.795800584 | High-middle SDI |
| Morocco | 0.562698301 | Low-middle SDI |
| Mozambique | 0.326462614 | Low SDI |
| Myanmar | 0.53390084 | Low-middle SDI |
| Namibia | 0.617564872 | Middle SDI |
| Nauru | 0.625177834 | Middle SDI |
| Nepal | 0.433174635 | Low SDI |
| Netherlands | 0.888464256 | High SDI |
| New Zealand | 0.849442499 | High SDI |
| Nicaragua | 0.523958472 | Low-middle SDI |
| Niger | 0.168072774 | Low SDI |
| Nigeria | 0.503390833 | Low-middle SDI |
| Niue | 0.72622205 | High-middle SDI |
| North Africa and Middle East | 0.658224715 | NA |
| North Macedonia | 0.750629703 | High-middle SDI |
| Northern Mariana Islands | 0.771535213 | High-middle SDI |
| Norway | 0.91613281 | High SDI |
| Oceania | 0.467445126 | NA |
| Oman | 0.773391602 | High-middle SDI |
| Pakistan | 0.504028689 | Low-middle SDI |
| Palau | 0.754046931 | High-middle SDI |
| Palestine | 0.631011665 | Middle SDI |
| Panama | 0.708864828 | High-middle SDI |
| Papua New Guinea | 0.417797443 | Low SDI |
| Paraguay | 0.635718099 | Middle SDI |
| Peru | 0.662054037 | Middle SDI |
| Philippines | 0.651219329 | Middle SDI |
| Poland | 0.812042809 | High SDI |
| Portugal | 0.744151851 | High-middle SDI |
| Puerto Rico | 0.825525847 | High SDI |
| Qatar | 0.846860584 | High SDI |
| Republic of Korea | 0.886675267 | High SDI |
| Republic of Moldova | 0.732214875 | High-middle SDI |
| Romania | 0.768453864 | High-middle SDI |
| Russian Federation | 0.808536005 | High SDI |
| Rwanda | 0.435588706 | Low SDI |
| Saint Kitts and Nevis | 0.754987055 | High-middle SDI |
| Saint Lucia | 0.672509735 | Middle SDI |
| Saint Vincent and the Grenadines | 0.637195963 | Middle SDI |
| Samoa | 0.593392769 | Low-middle SDI |
| San Marino | 0.888005474 | High SDI |
| Sao Tome and Principe | 0.505413747 | Low-middle SDI |
| Saudi Arabia | 0.815143493 | High SDI |
| Senegal | 0.408054193 | Low SDI |
| Serbia | 0.792416294 | High-middle SDI |
| Seychelles | 0.730150775 | High-middle SDI |
| Sierra Leone | 0.358665881 | Low SDI |
| Singapore | 0.856097766 | High SDI |
| Slovakia | 0.81061053 | High SDI |
| Slovenia | 0.842430731 | High SDI |
| Solomon Islands | 0.429360316 | Low SDI |
| Somalia | 0.077688109 | Low SDI |
| South Africa | 0.679626598 | Middle SDI |
| South Asia | 0.557864657 | NA |
| South Sudan | 0.278371125 | Low SDI |
| Southeast Asia | 0.649777295 | NA |
| Southern Latin America | 0.735984717 | NA |
| Southern Sub-Saharan Africa | 0.642200282 | NA |
| Spain | 0.769283698 | High-middle SDI |
| Sri Lanka | 0.701534935 | High-middle SDI |
| Sudan | 0.541949735 | Low-middle SDI |
| Suriname | 0.633665739 | Middle SDI |
| Sweden | 0.886880299 | High SDI |
| Switzerland | 0.933059111 | High SDI |
| Syrian Arab Republic | 0.623004075 | Middle SDI |
| Taiwan (Province of China) | 0.874747053 | High SDI |
| Tajikistan | 0.541511187 | Low-middle SDI |
| Thailand | 0.682547933 | Middle SDI |
| Timor-Leste | 0.444667619 | Low SDI |
| Togo | 0.408533695 | Low SDI |
| Tokelau | 0.686425621 | Middle SDI |
| Tonga | 0.626349936 | Middle SDI |
| Trinidad and Tobago | 0.768763254 | High-middle SDI |
| Tropical Latin America | 0.652442394 | NA |
| Tunisia | 0.682432216 | Middle SDI |
| Türkiye | 0.712692673 | High-middle SDI |
| Turkmenistan | 0.682160776 | Middle SDI |
| Tuvalu | 0.576620529 | Low-middle SDI |
| Uganda | 0.423261181 | Low SDI |
| Ukraine | 0.760773913 | High-middle SDI |
| United Arab Emirates | 0.849317734 | High SDI |
| United Kingdom | 0.859000182 | High SDI |
| United Republic of Tanzania | 0.446568273 | Low SDI |
| United States of America | 0.862448354 | High SDI |
| United States Virgin Islands | 0.821830853 | High SDI |
| Uruguay | 0.719283445 | High-middle SDI |
| Uzbekistan | 0.662621694 | Middle SDI |
| Vanuatu | 0.473100706 | Low-middle SDI |
| Venezuela (Bolivarian Republic of) | 0.596513059 | Low-middle SDI |
| Viet Nam | 0.627933721 | Middle SDI |
| Western Europe | 0.848726316 | NA |
| Western Sub-Saharan Africa | 0.446022979 | NA |
| Yemen | 0.450376375 | Low SDI |
| Zambia | 0.505948954 | Low-middle SDI |
| Zimbabwe | 0.473819486 | Low-middle SDI |

Supplement 1 Table S4: Subnational SDI values and SDI quintiles

| **Location** | **2021 SDI Index Value** | **SDI Quintile** |
| --- | --- | --- |
| **Brazil** |  |  |
| Acre | 0.565851526 | Low-middle SDI |
| Alagoas | 0.535659976 | Low-middle SDI |
| Amazonas | 0.609484469 | Middle SDI |
| Amapá | 0.632179635 | Middle SDI |
| Bahia | 0.577394253 | Low-middle SDI |
| Ceará | 0.568665698 | Low-middle SDI |
| Distrito Federal | 0.776016643 | High-middle SDI |
| Espírito Santo | 0.670192595 | Middle SDI |
| Goiás | 0.640933873 | Middle SDI |
| Maranhão | 0.49892987 | Low-middle SDI |
| Minas Gerais | 0.652293253 | Middle SDI |
| Mato Grosso do Sul | 0.64753332 | Middle SDI |
| Mato Grosso | 0.651185759 | Middle SDI |
| Pará | 0.582134405 | Low-middle SDI |
| Paraíba | 0.563868152 | Low-middle SDI |
| Paraná | 0.674856534 | Middle SDI |
| Pernambuco | 0.586764224 | Low-middle SDI |
| Rio de Janeiro | 0.716093842 | High-middle SDI |
| Rio Grande do Norte | 0.589854818 | Low-middle SDI |
| Rondônia | 0.624854665 | Middle SDI |
| Roraima | 0.621005567 | Middle SDI |
| Rio Grande do Sul | 0.693337528 | High-middle SDI |
| Santa Catarina | 0.701340701 | High-middle SDI |
| Sergipe | 0.595840177 | Low-middle SDI |
| São Paulo | 0.713727597 | High-middle SDI |
| Tocantins | 0.608587676 | Middle SDI |
| **China** |  |  |
| Beijing | 187526 | NA |
| Tianjin | 113660 | NA |
| Hebei | 54181 | NA |
| Shanxi | 65625 | NA |
| Inner Mongolia | 88137 | NA |
| Liaoning | 64992 | NA |
| Jilin | 55148 | NA |
| Heilongjiang | 47199 | NA |
| Shanghai | 175420 | NA |
| Jiangsu | 138255 | NA |
| Zhejiang | 113839 | NA |
| Anhui | 69676 | NA |
| Fujian | 118750 | NA |
| Jiangxi | 66020 | NA |
| Shandong | 81510 | NA |
| Henan | 58587 | NA |
| Hubei | 86551 | NA |
| Hunan | 68913 | NA |
| Guangdong | 98561 | NA |
| Guangxi | 50137 | NA |
| Hainan | 63991 | NA |
| Chongqing | 87450 | NA |
| Sichuan | 64610 | NA |
| Guizhou | 50476 | NA |
| Yunnan | 57717 | NA |
| Tibet | 56831 | NA |
| Shaanxi | 76171 | NA |
| Gansu | 40976 | NA |
| Qinghai | 57036 | NA |
| Ningxia | 63461 | NA |
| Xinjiang | 62991 | NA |
| Taiwan | 455800.647 | NA |
| Hong Kong | 361832.002 | NA |
| Macao | 327912.5661 | NA |
| **Ethiopia** |  |  |
| Tigray | 0.382183506 | Low SDI |
| Afar | 0.28877341 | Low SDI |
| Amhara | 0.320453189 | Low SDI |
| Oromia | 0.335815016 | Low SDI |
| Somali | 0.268253815 | Low SDI |
| Benishangul-Gumuz | 0.320228483 | Low SDI |
| Harari | 0.538168759 | Low-middle SDI |
| Gambella | 0.458220084 | Low-middle SDI |
| Addis Ababa | 0.694729596 | High-middle SDI |
| Dire Dawa | 0.540668646 | Low-middle SDI |
| **Indonesia** |  |  |
| Aceh | 0.669551641 | Middle SDI |
| North Sumatra | 0.667025647 | Middle SDI |
| West Sumatra | 0.665763664 | Middle SDI |
| Riau | 0.722784516 | High-middle SDI |
| Jambi | 0.639563676 | Middle SDI |
| South Sumatra | 0.645536761 | Middle SDI |
| Bengkulu | 0.613670654 | Middle SDI |
| Lampung | 0.608871357 | Middle SDI |
| Bangka-Belitung Islands | 0.64425964 | Middle SDI |
| Riau Islands | 0.747754306 | High-middle SDI |
| North Kalimantan | 0.752986755 | High-middle SDI |
| Jakarta | 0.799409443 | High-middle SDI |
| West Java | 0.643676633 | Middle SDI |
| Central Java | 0.613388815 | Middle SDI |
| Yogyakarta | 0.675429917 | Middle SDI |
| East Java | 0.645517381 | Middle SDI |
| Banten | 0.640712993 | Middle SDI |
| Bali | 0.649585885 | Middle SDI |
| West Nusa Tenggara | 0.587034621 | Low-middle SDI |
| East Nusa Tenggara | 0.54945119 | Low-middle SDI |
| West Kalimantan | 0.586121672 | Low-middle SDI |
| Central Kalimantan | 0.638993825 | Middle SDI |
| South Kalimantan | 0.623206076 | Middle SDI |
| East Kalimantan | 0.759293869 | High-middle SDI |
| North Sulawesi | 0.650468306 | Middle SDI |
| Central Sulawesi | 0.616253843 | Middle SDI |
| South Sulawesi | 0.621135414 | Middle SDI |
| Southeast Sulawesi | 0.615325868 | Middle SDI |
| Gorontalo | 0.569990208 | Low-middle SDI |
| West Sulawesi | 0.57565685 | Low-middle SDI |
| Maluku | 0.580321875 | Low-middle SDI |
| North Maluku | 0.562910873 | Low-middle SDI |
| West Papua | 0.675125644 | Middle SDI |
| Papua | 0.645802883 | Middle SDI |
| **Iran (Islamic Republic of)** |  |  |
| Alborz | 0.748302022 | High-middle SDI |
| Ardebil | 0.658639771 | Middle SDI |
| East Azarbayejan | 0.668193235 | Middle SDI |
| West Azarbayejan | 0.627112463 | Middle SDI |
| Bushehr | 0.708418708 | High-middle SDI |
| Chahar Mahaal and Bakhtiari | 0.677988602 | Middle SDI |
| Fars | 0.714830769 | High-middle SDI |
| Gilan | 0.712319447 | High-middle SDI |
| Golestan | 0.65607753 | Middle SDI |
| Hamadan | 0.66673482 | Middle SDI |
| Hormozgan | 0.670331366 | Middle SDI |
| Ilam | 0.706194927 | High-middle SDI |
| Isfahan | 0.709716845 | High-middle SDI |
| Kerman | 0.668439265 | Middle SDI |
| Kermanshah | 0.674466468 | Middle SDI |
| North Khorasan | 0.651610197 | Middle SDI |
| Khorasan-e-Razavi | 0.669826641 | Middle SDI |
| South Khorasan | 0.653125383 | Middle SDI |
| Khuzestan | 0.669587913 | Middle SDI |
| Kohgiluyeh and Boyer-Ahmad | 0.693706198 | High-middle SDI |
| Kurdistan | 0.642261692 | Middle SDI |
| Lorestan | 0.669118289 | Middle SDI |
| Markazi | 0.682843822 | Middle SDI |
| Mazandaran | 0.729929903 | High-middle SDI |
| Qazvin | 0.6876536 | Middle SDI |
| Qom | 0.694075907 | High-middle SDI |
| Semnan | 0.723926385 | High-middle SDI |
| Sistan and Baluchistan | 0.551305977 | Low-middle SDI |
| Tehran | 0.776032454 | High-middle SDI |
| Yazd | 0.713344284 | High-middle SDI |
| Zanjan | 0.661729132 | Middle SDI |
| **Japan** |  |  |
| Hokkaidō | 0.843743975 | High SDI |
| Aomori | 0.828344206 | High SDI |
| Iwate | 0.834979691 | High SDI |
| Miyagi | 0.859886569 | High SDI |
| Akita | 0.832249015 | High SDI |
| Yamagata | 0.837933155 | High SDI |
| Fukushima | 0.840941474 | High SDI |
| Ibaraki | 0.85980457 | High SDI |
| Tochigi | 0.861186498 | High SDI |
| Gunma | 0.861111359 | High SDI |
| Saitama | 0.85637105 | High SDI |
| Chiba | 0.861292639 | High SDI |
| Tōkyō | 0.928810109 | High SDI |
| Kanagawa | 0.882654391 | High SDI |
| Niigata | 0.845152095 | High SDI |
| Toyama | 0.865181131 | High SDI |
| Ishikawa | 0.859378938 | High SDI |
| Fukui | 0.856142577 | High SDI |
| Yamanashi | 0.857782852 | High SDI |
| Nagano | 0.858577839 | High SDI |
| Gifu | 0.85334719 | High SDI |
| Shizuoka | 0.865450452 | High SDI |
| Aichi | 0.883048835 | High SDI |
| Mie | 0.860561189 | High SDI |
| Shiga | 0.874323368 | High SDI |
| Kyōto | 0.87601835 | High SDI |
| Ōsaka | 0.87640925 | High SDI |
| Hyōgo | 0.868170156 | High SDI |
| Nara | 0.851652195 | High SDI |
| Wakayama | 0.847037481 | High SDI |
| Tottori | 0.835956957 | High SDI |
| Shimane | 0.838672262 | High SDI |
| Okayama | 0.862560219 | High SDI |
| Hiroshima | 0.869908602 | High SDI |
| Yamaguchi | 0.856255885 | High SDI |
| Tokushima | 0.85748626 | High SDI |
| Kagawa | 0.85781836 | High SDI |
| Ehime | 0.843501706 | High SDI |
| Kōchi | 0.834906164 | High SDI |
| Fukuoka | 0.858347709 | High SDI |
| Saga | 0.834925899 | High SDI |
| Nagasaki | 0.829213881 | High SDI |
| Kumamoto | 0.834536592 | High SDI |
| Ōita | 0.849282438 | High SDI |
| Miyazaki | 0.826589859 | High SDI |
| Kagoshima | 0.832047307 | High SDI |
| Okinawa | 0.821803051 | High SDI |
| **Kenya** |  |  |
| Baringo | 0.514883215 | Low-middle SDI |
| Bomet | 0.529367852 | Low-middle SDI |
| Bungoma | 0.49672518 | Low-middle SDI |
| Busia | 0.477665024 | Low-middle SDI |
| Elgeyo-Marakwet | 0.526297038 | Low-middle SDI |
| Embu | 0.547555396 | Low-middle SDI |
| Garissa | 0.321068269 | Low SDI |
| Homa Bay | 0.493871805 | Low-middle SDI |
| Isiolo | 0.433966834 | Low SDI |
| Kajiado | 0.506969707 | Low-middle SDI |
| Kakamega | 0.509528998 | Low-middle SDI |
| Kericho | 0.523573355 | Low-middle SDI |
| Kiambu | 0.593110152 | Low-middle SDI |
| Kilifi | 0.486009157 | Low-middle SDI |
| Kirinyaga | 0.54301445 | Low-middle SDI |
| Kisii | 0.544604844 | Low-middle SDI |
| Kisumu | 0.544409324 | Low-middle SDI |
| Kitui | 0.474749457 | Low-middle SDI |
| Kwale | 0.475138205 | Low-middle SDI |
| Laikipia | 0.575711773 | Low-middle SDI |
| Lamu | 0.503786492 | Low-middle SDI |
| Machakos | 0.548035791 | Low-middle SDI |
| Makueni | 0.512568943 | Low-middle SDI |
| Mandera | 0.238263833 | Low SDI |
| Marsabit | 0.40018158 | Low SDI |
| Meru | 0.508216146 | Low-middle SDI |
| Migori | 0.472803008 | Low-middle SDI |
| Mombasa | 0.596387778 | Low-middle SDI |
| Murang'a | 0.550869493 | Low-middle SDI |
| Nairobi | 0.683722442 | Middle SDI |
| Nakuru | 0.570045398 | Low-middle SDI |
| Nandi | 0.516279712 | Low-middle SDI |
| Narok | 0.459103072 | Low-middle SDI |
| Nyamira | 0.589267078 | Low-middle SDI |
| Nyandarua | 0.576109863 | Low-middle SDI |
| Nyeri | 0.578787484 | Low-middle SDI |
| Samburu | 0.378312395 | Low SDI |
| Siaya | 0.476524395 | Low-middle SDI |
| Taita Taveta | 0.540980982 | Low-middle SDI |
| Tana River | 0.382845346 | Low SDI |
| Tharaka Nithi | 0.529680112 | Low-middle SDI |
| Trans Nzoia | 0.54850455 | Low-middle SDI |
| Turkana | 0.373434245 | Low SDI |
| Uasin Gishu | 0.567345589 | Low-middle SDI |
| Vihiga | 0.523089788 | Low-middle SDI |
| Wajir | 0.252050106 | Low SDI |
| West Pokot | 0.444120899 | Low SDI |
| **Mexico** |  |  |
| Aguascalientes | 0.681159284 | Middle SDI |
| Baja California | 0.704081972 | High-middle SDI |
| Baja California Sur | 0.709694281 | High-middle SDI |
| Campeche | 0.665363431 | Middle SDI |
| Coahuila | 0.677562875 | Middle SDI |
| Colima | 0.699116771 | High-middle SDI |
| Chiapas | 0.569940909 | Low-middle SDI |
| Chihuahua | 0.673386571 | Middle SDI |
| Mexico City | 0.759848414 | High-middle SDI |
| Durango | 0.639324841 | Middle SDI |
| Guanajuato | 0.646374308 | Middle SDI |
| Guerrero | 0.584850472 | Low-middle SDI |
| Hidalgo | 0.633478453 | Middle SDI |
| Jalisco | 0.675998373 | Middle SDI |
| México | 0.681587605 | Middle SDI |
| Michoacán de Ocampo | 0.613082024 | Middle SDI |
| Morelos | 0.670074046 | Middle SDI |
| Nayarit | 0.658178913 | Middle SDI |
| Nuevo León | 0.710141817 | High-middle SDI |
| Oaxaca | 0.589041897 | Low-middle SDI |
| Puebla | 0.622031827 | Middle SDI |
| Querétaro | 0.682558115 | Middle SDI |
| Quintana Roo | 0.681068671 | Middle SDI |
| San Luis Potosí | 0.646651514 | Middle SDI |
| Sinaloa | 0.677164972 | Middle SDI |
| Sonora | 0.710679605 | High-middle SDI |
| Tabasco | 0.648728024 | Middle SDI |
| Tamaulipas | 0.682086111 | Middle SDI |
| Tlaxcala | 0.64782243 | Middle SDI |
| Veracruz de Ignacio de la Llave | 0.628136965 | Middle SDI |
| Yucatán | 0.653050809 | Middle SDI |
| Zacatecas | 0.634773655 | Middle SDI |
| **Norway** |  |  |
| Oslo | 0.945691791 | High SDI |
| Rogaland | 0.915083733 | High SDI |
| Møre og Romsdal | 0.907426289 | High SDI |
| Nordland | 0.896942278 | High SDI |
| Trøndelag | 0.915977587 | High SDI |
| Vestland | 0.917596286 | High SDI |
| Agder | 0.907489539 | High SDI |
| Vestfold og Telemark | 0.907307419 | High SDI |
| Innlandet | 0.899754056 | High SDI |
| Viken | 0.914692607 | High SDI |
| Troms og Finnmark | 0.905163204 | High SDI |
| **Pakistan** |  |  |
| Punjab | 0.632136354 | Middle SDI |
| Azad Jammu & Kashmir | 0.54211877 | Low-middle SDI |
| Balochistan | 0.417971505 | Low SDI |
| Gilgit-Baltistan | 0.397549987 | Low SDI |
| Islamabad Capital Territory | 0.696195374 | High-middle SDI |
| Khyber Pakhtunkhwa | 0.450581146 | Low SDI |
| Sindh | 0.512574957 | Low-middle SDI |
| **South Africa** |  |  |
| Eastern Cape | 0.617204696 | Middle SDI |
| Free State | 0.677908602 | Middle SDI |
| Gauteng | 0.735426628 | High-middle SDI |
| KwaZulu-Natal | 0.660483699 | Middle SDI |
| Limpopo | 0.612287836 | Middle SDI |
| Mpumalanga | 0.646057473 | Middle SDI |
| North-West | 0.652476432 | Middle SDI |
| Northern Cape | 0.664140622 | Middle SDI |
| Western Cape | 0.718532896 | High-middle SDI |
| **Sweden** |  |  |
| Sweden except Stockholm | 0.87515115 | High SDI |
| Stockholm | 0.916622714 | High SDI |
| **United Kingdom** |  |  |
| Northern Ireland | 0.838531942 | High SDI |
| Scotland | 0.852061416 | High SDI |
| Wales | 0.834278536 | High SDI |
| Darlington | 0.837204088 | High SDI |
| Northumberland | 0.823317013 | High SDI |
| Stockton-on-Tees | 0.83098489 | High SDI |
| Newcastle upon Tyne | 0.872520663 | High SDI |
| North Tyneside | 0.836400602 | High SDI |
| Redcar and Cleveland | 0.798369621 | High-middle SDI |
| County Durham | 0.812276525 | High SDI |
| Gateshead | 0.830244183 | High SDI |
| Middlesbrough | 0.801470999 | High-middle SDI |
| South Tyneside | 0.801073026 | High-middle SDI |
| Sunderland | 0.819164125 | High SDI |
| Hartlepool | 0.798877773 | High-middle SDI |
| Cheshire East | 0.884754634 | High SDI |
| Stockport | 0.861719713 | High SDI |
| Trafford | 0.896753779 | High SDI |
| Cheshire West and Chester | 0.871177933 | High SDI |
| Sefton | 0.825841895 | High SDI |
| Lancashire | 0.841125535 | High SDI |
| Cumbria | 0.843466901 | High SDI |
| Bolton | 0.814170764 | High SDI |
| Wirral | 0.819316291 | High SDI |
| Bury | 0.830089162 | High SDI |
| St Helens | 0.8122146 | High SDI |
| Warrington | 0.878756212 | High SDI |
| Oldham | 0.798408293 | High-middle SDI |
| Rochdale | 0.802069322 | High-middle SDI |
| Wigan | 0.80685214 | High SDI |
| Halton | 0.836181349 | High SDI |
| Liverpool | 0.848437635 | High SDI |
| Tameside | 0.800546694 | High-middle SDI |
| Salford | 0.838699172 | High SDI |
| Blackburn with Darwen | 0.811503741 | High SDI |
| Knowsley | 0.813671711 | High SDI |
| Blackpool | 0.789925893 | High-middle SDI |
| Manchester | 0.881429144 | High SDI |
| North Yorkshire | 0.85619466 | High SDI |
| East Riding of Yorkshire | 0.835731941 | High SDI |
| York | 0.888738932 | High SDI |
| North East Lincolnshire | 0.804837847 | High-middle SDI |
| Calderdale | 0.837863928 | High SDI |
| North Lincolnshire | 0.825904867 | High SDI |
| Bradford | 0.815985897 | High SDI |
| Kirklees | 0.824207653 | High SDI |
| Leeds | 0.868761251 | High SDI |
| Sheffield | 0.854890971 | High SDI |
| Wakefield | 0.806811491 | High SDI |
| Rotherham | 0.804228811 | High-middle SDI |
| Doncaster | 0.795614015 | High-middle SDI |
| Kingston upon Hull, City of | 0.79993173 | High-middle SDI |
| Barnsley | 0.789261821 | High-middle SDI |
| Northamptonshire | 0.839635363 | High SDI |
| Leicestershire | 0.851888947 | High SDI |
| Lincolnshire | 0.821363757 | High SDI |
| Rutland | 0.852733752 | High SDI |
| Derby | 0.846010683 | High SDI |
| Derbyshire | 0.824238096 | High SDI |
| Nottinghamshire | 0.823007864 | High SDI |
| Nottingham | 0.85887136 | High SDI |
| Leicester | 0.8290671 | High SDI |
| Warwickshire | 0.866672661 | High SDI |
| Herefordshire, County of | 0.846310474 | High SDI |
| Solihull | 0.872079235 | High SDI |
| Shropshire | 0.843218762 | High SDI |
| Worcestershire | 0.843611304 | High SDI |
| Staffordshire | 0.829320238 | High SDI |
| Dudley | 0.804164522 | High-middle SDI |
| Coventry | 0.848618069 | High SDI |
| Telford and Wrekin | 0.827341561 | High SDI |
| Stoke-on-Trent | 0.798242518 | High-middle SDI |
| Walsall | 0.792529439 | High-middle SDI |
| Wolverhampton | 0.812389843 | High SDI |
| Birmingham | 0.838066899 | High SDI |
| Sandwell | 0.795722297 | High-middle SDI |
| Bedford | 0.858093661 | High SDI |
| Central Bedfordshire | 0.852254516 | High SDI |
| Suffolk | 0.841151028 | High SDI |
| Hertfordshire | 0.887510721 | High SDI |
| Essex | 0.845491449 | High SDI |
| Cambridgeshire | 0.888027582 | High SDI |
| Thurrock | 0.819597724 | High SDI |
| Norfolk | 0.837309678 | High SDI |
| Southend-on-Sea | 0.825801394 | High SDI |
| Peterborough | 0.837874469 | High SDI |
| Luton | 0.839717125 | High SDI |
| Richmond upon Thames | 0.932132548 | High SDI |
| Kensington and Chelsea | 0.946476699 | High SDI |
| Barnet | 0.88597606 | High SDI |
| Westminster | 0.937198627 | High SDI |
| Bromley | 0.869545327 | High SDI |
| Bexley | 0.844547626 | High SDI |
| Redbridge | 0.850265079 | High SDI |
| Merton | 0.887733957 | High SDI |
| Brent | 0.860164338 | High SDI |
| Hillingdon | 0.893138131 | High SDI |
| Havering | 0.834643869 | High SDI |
| Kingston upon Thames | 0.908698276 | High SDI |
| Sutton | 0.858421577 | High SDI |
| Harrow | 0.860015269 | High SDI |
| Enfield | 0.846461002 | High SDI |
| Croydon | 0.852066336 | High SDI |
| Hammersmith and Fulham | 0.935153171 | High SDI |
| Ealing | 0.88272888 | High SDI |
| Greenwich | 0.846080185 | High SDI |
| Wandsworth | 0.924241493 | High SDI |
| Waltham Forest | 0.840509602 | High SDI |
| Camden | 0.936474834 | High SDI |
| Lambeth | 0.916380817 | High SDI |
| Lewisham | 0.857308935 | High SDI |
| Hounslow | 0.896994582 | High SDI |
| Southwark | 0.919509828 | High SDI |
| Newham | 0.841688128 | High SDI |
| Barking and Dagenham | 0.807324954 | High SDI |
| Haringey | 0.872084552 | High SDI |
| Hackney | 0.892946278 | High SDI |
| Islington | 0.924740074 | High SDI |
| Tower Hamlets | 0.904488141 | High SDI |
| Wokingham | 0.911658755 | High SDI |
| Buckinghamshire | 0.888785243 | High SDI |
| Surrey | 0.904859403 | High SDI |
| Windsor and Maidenhead | 0.91581805 | High SDI |
| West Berkshire | 0.897757011 | High SDI |
| Hampshire | 0.87285 | High SDI |
| Bracknell Forest | 0.891002655 | High SDI |
| West Sussex | 0.864792147 | High SDI |
| Oxfordshire | 0.899475931 | High SDI |
| Reading | 0.905873593 | High SDI |
| Kent | 0.845345393 | High SDI |
| Brighton and Hove | 0.898257203 | High SDI |
| Medway | 0.820667842 | High SDI |
| East Sussex | 0.839594794 | High SDI |
| Portsmouth | 0.865875927 | High SDI |
| Isle of Wight | 0.826399628 | High SDI |
| Milton Keynes | 0.88717934 | High SDI |
| Southampton | 0.861349084 | High SDI |
| Slough | 0.878557248 | High SDI |
| South Gloucestershire | 0.884984764 | High SDI |
| Dorset | 0.851615144 | High SDI |
| Wiltshire | 0.859466242 | High SDI |
| North Somerset | 0.859017669 | High SDI |
| Devon | 0.855376103 | High SDI |
| Poole | 0.863494302 | High SDI |
| Bath and North East Somerset | 0.895435359 | High SDI |
| Gloucestershire | 0.871806302 | High SDI |
| Somerset | 0.843404311 | High SDI |
| Swindon | 0.867545906 | High SDI |
| Torbay | 0.813038477 | High SDI |
| Bristol, City of | 0.897041874 | High SDI |
| Bournemouth | 0.870464766 | High SDI |
| Cornwall | 0.839768559 | High SDI |
| Plymouth | 0.843307541 | High SDI |
| **United States of America** |  |  |
| Alabama | 0.825605142 | High SDI |
| Alaska | 0.857524599 | High SDI |
| Arizona | 0.846732871 | High SDI |
| Arkansas | 0.81770902 | High SDI |
| California | 0.87021505 | High SDI |
| Colorado | 0.876654771 | High SDI |
| Connecticut | 0.903107563 | High SDI |
| Delaware | 0.866366234 | High SDI |
| District of Columbia | 0.905964755 | High SDI |
| Florida | 0.861979282 | High SDI |
| Georgia | 0.84656427 | High SDI |
| Hawaii | 0.869546917 | High SDI |
| Idaho | 0.83651792 | High SDI |
| Illinois | 0.879187797 | High SDI |
| Indiana | 0.842859834 | High SDI |
| Iowa | 0.86405652 | High SDI |
| Kansas | 0.859383805 | High SDI |
| Kentucky | 0.821325377 | High SDI |
| Louisiana | 0.825346457 | High SDI |
| Maine | 0.865754199 | High SDI |
| Maryland | 0.889225569 | High SDI |
| Massachusetts | 0.906621364 | High SDI |
| Michigan | 0.863971128 | High SDI |
| Minnesota | 0.887013945 | High SDI |
| Mississippi | 0.810471681 | High SDI |
| Missouri | 0.848169136 | High SDI |
| Montana | 0.859107705 | High SDI |
| Nebraska | 0.865128654 | High SDI |
| Nevada | 0.847682783 | High SDI |
| New Hampshire | 0.898263681 | High SDI |
| New Jersey | 0.891922443 | High SDI |
| New Mexico | 0.831716579 | High SDI |
| New York | 0.885176868 | High SDI |
| North Carolina | 0.844445313 | High SDI |
| North Dakota | 0.873031775 | High SDI |
| Ohio | 0.850513434 | High SDI |
| Oklahoma | 0.828663242 | High SDI |
| Oregon | 0.870177689 | High SDI |
| Pennsylvania | 0.872416752 | High SDI |
| Rhode Island | 0.883893485 | High SDI |
| South Carolina | 0.83771865 | High SDI |
| South Dakota | 0.855720868 | High SDI |
| Tennessee | 0.833033239 | High SDI |
| Texas | 0.834783278 | High SDI |
| Utah | 0.854616788 | High SDI |
| Vermont | 0.8899999 | High SDI |
| Virginia | 0.880119901 | High SDI |
| Washington | 0.877584845 | High SDI |
| West Virginia | 0.817713511 | High SDI |
| Wisconsin | 0.871726096 | High SDI |
| Wyoming | 0.862148722 | High SDI |
